# Quantitative Profiling of Lysine Acetylation Reveals Dynamic Crosstalk between Receptor Tyrosine Kinases and Lysine Acetylation

**DOI:** 10.1371/journal.pone.0126242

**Published:** 2015-05-15

**Authors:** Bryan D. Bryson, Forest M. White

**Affiliations:** 1 Department of Biological Engineering, Massachusetts Institute of Technology (MIT), Cambridge, Massachusetts, United States of America; 2 David H. Koch Institute for Integrative Cancer Research, Massachusetts Institute of Technology (MIT), Cambridge, Massachusetts, United States of America; Aarhus University, DENMARK

## Abstract

Lysine acetylation has been primarily investigated in the context of transcriptional regulation, but a role for acetylation in mediating other cellular responses has emerged. Multiple studies have described global lysine acetylation profiles for particular biological states, but none to date have investigated the temporal dynamics regulating cellular response to perturbation. Reasoning that lysine acetylation may be altered in response to growth factors, we implemented quantitative mass spectrometry-based proteomics to investigate the temporal dynamics of lysine acetylation in response to growth factor stimulation in cultured carcinoma cell lines. We found that lysine acetylation changed rapidly in response to activation of several different receptor tyrosine kinases by their respective ligands. To uncover the effects of lysine acetylation dynamics on tyrosine phosphorylation signaling networks, cells were treated with an HDAC inhibitor. This short-term pharmacological inhibition of histone deacetylase activity modulated signaling networks involving phosphorylated tyrosine and thereby altered the response to receptor tyrosine kinase activation. This result highlights the interconnectivity of lysine acetylation and tyrosine phosphorylation signaling networks and suggests that HDAC inhibition may influence cellular responses by affecting both types of post-translational modifications.

## Introduction

Long thought to be primarily restricted to histone modification, lysine acetylation has recently emerged as a proteome-wide post-translational modification rivaling phosphorylation in scale and biological relevance [1–2]. High-resolution mass spectrometry has greatly expanded the lysine acetylome to include proteins involved in signaling and metabolism, in addition to previously characterized acetylated proteins such as histones [3–7]. The clinical relevance of lysine acetylation has also been demonstrated by histone deacetylase inhibitors (HDACi’s), such as vorinostat and romidepsin, being approved for the treatment of cutaneous T-cell lymphoma (CTCL) and undergoing clinical trials for other cancers [8–10]. Despite the clinical success of these compounds, the mechanism by which these inhibitors alter growth in certain cancers or cell lines is poorly understood. Although changes in histone acetylation were originally thought to drive a transcriptional program that underlies response to HDACi, the discovery of lysine acetylation sites on non-histone proteins suggests the potential for non-transcriptional mechanisms that may also contribute to the therapeutic response [11–12].

Many oncogenic phenotypes, such as increased migration and invasion, have historically been ascribed to aberrant phosphorylation signaling cascades driven by altered expression, mutation, or activation of protein kinases and phosphatases [13–16]. A plethora of systems-level studies have profiled phosphorylation signaling in cancer cells and tumors under various conditions, revealing a diverse array of temporal responses to ligand stimulation [17–18]. Paired with molecular biology, these systems-level studies have mapped complex interactions between phosphorylation sites and phenotypic outcomes. Although the connection between dysregulation of kinase signaling and cancer is well established, the role of lysine acetylation in this dynamic interplay is poorly understood. The interaction between phosphorylation and acetylation has primarily been explored at the single protein level, most commonly in studies of the histone code but also of signal transducers and activators of transcription (STAT) proteins and the tumor suppressor p53, among others [19–22]. Bioinformatic approaches to analyzing the interaction of protein phosphorylation and lysine acetylation at the network level have also been performed, but have been largely limited to cataloging the overlapping proteins identified in large-scale datasets of either modification, with minimal functional insight [23–24].

The link between oncogenic signaling from receptor tyrosine kinases and altered lysine acetylation has not been systematically explored, yet such a study may provide insight into the mechanisms by which HDACi’s act to affect phenotypic outcomes both *in vitro* and *in vivo*. To explore the effect of RTK activation on lysine acetylation at a network level, we implemented multiplex quantitative mass spectrometry based proteomics to profile temporal acetylation dynamics downstream of RTK activation in cultured carcinoma cells. Our data demonstrate that growth factor stimulation induces rapid changes in lysine acetylation across the acetylproteome, suggesting a link between tyrosine phosphorylation pathways activated by RTKs and altered lysine acetylation. These results provide insight as to how HDACi’s may modulate the tyrosine phosphorylation signaling response to RTK activation.

## Materials and Methods

### Cell Culture and Stimulation

HepG2 (ATCC # HB-8065), A549 (ATCC# CCL-185), and DLD1 (ATCC# CCL-221) cell lines were obtained from ATCC. HepG2 cells were cultured in DMEM with 10% fetal bovine serum, 2mM glutamine, 100 units/ml penicillin, and 100 mg/ml streptomycin. A549 cells were cultured in RPMI with 10% fetal bovine serum, 2mM glutamine, 100 units/ml penicillin, and 100 mg/ml streptomycin. Prior to stimulation, cells were washed with PBS and incubated in serum-free media overnight. For TSA experiments, cells were pre-treated with vehicle or 1 μM TSA for one hour prior to growth factor treatment. A549 cells were stimulated with 100 ng/mL EGF or IGF-1 for 0, 1, 5, and 30 minutes, and HepG2 cells were stimulated with 150 nM insulin for the same times. Concentrated ligand (10 μL) was added to 10mL of cell culture media for stimulation. DLD1 cells were cultured in light (K0 & R0) or heavy (K8 & R10) SILAC media for at least six doublings to achieve efficient stable isotope incorporation. Cells were serum depleted; cells cultured in heavy SILAC were then stimulated with 100 ng/mL EGF for one minute. At appropriate time points, cells were lysed in 8M urea on ice. Lysates for western blotting were prepared identically, with the exception that cells for westerns were lysed in radioimmunoprecipitation (RIPA) buffer.

### Preparation of Peptides and Protein Lysates

Protein concentration was quantified using a BCA assay. Samples for mass spectrometry were prepared as previously described [25].

### iTRAQ Labeling

iTRAQ labeling was performed as previously described (36) using 4plex iTRAQ for HepG2 analyses and 8plex iTRAQ for A549 analyses. Each timepoint was labeled with a unique iTRAQ label. Samples for a particular time course analysis were combined and concentrated to dryness in a speedvac. Peptides were then dissolved in 400 μL IP buffer (100 mM Tris, 100 mM NaCl, and 1% NP-40, pH 7.4) and the pH was adjusted to 7.4 prior to immunoprecipitation.

### Peptide Immunoprecipitation

40 μL of beads with preconjugated anti-acetyllysine antibodies (ICP0388, Immunechem) were rinsed four times and then combined with resuspended iTRAQ labeled peptides and incubated overnight at 4°C with rotation. Beads were rinsed once with IP buffer and then four times with rinse buffer (100 mM Tris, pH 7.4), and peptides were eluted into 70 μL of 100 mM glycine pH 2. Eluents were cleared using immobilized affinity metal chromatography (IMAC) [26]. Peptides were loaded onto a precolumn and were separated by reverse phase HPLC (Agilent) over a 150 minute gradient prior to electrospray into an Orbitrap Elite mass spectrometer (ThermoFisher Scientific). To correct for slight variations in protein loading, the mean iTRAQ ratios for all proteins identified in each analysis was used to normalize the data.

### Western Blotting

Equal protein amounts of lysate from each of the four time points were separated using SDS gel electrophoresis. Proteins were transferred to nitrocellulose membranes. Membranes were blocked for one hour using 5% bovine serum albumin (BSA) dissolved in Tris-Buffered saline with 0.1% Tween (TBS-T). Membranes were incubated with either a histone H3 or specific antibody against histone H3 Lys^14^ overnight at 4°C. Membranes were washed three times, five minutes each with TBS-T prior to incubation with a secondary antibody for an hour at room temperature. Membranes were again washed three times, five minutes each, prior to incubation with the enhanced chemiluminescent kit.

### Mass Spectrometry Data Analysis

Raw mass spectrometry data files were converted into the. mgf format using DTASupercharge 1.31 (http://msquant.sourceforge.net/). All resulting MS/MS spectra were searched against an NCBI 2009 *Homo sapiens* database using Mascot version: 2.1.03 (Matrix Science). Trypsin enzyme specificity was applied with a maximum of nine missed cleavages. Mass tolerance was set at 10 ppm and fragment mass tolerance was set at 0.8 Da. MS/MS spectra searches incorporated fix N-terminal iTRAQ and carbamidomethylation of cysteine while incorporating variable modifications of oxidized methionine, and acetylation of lysine or iTRAQ modification of lysine. For SILAC analyses, MS/MS spectra searches incorporated fixed carbamidomethylation of cysteine, and variable incorporation of SILAC, acetylation, and methionine oxidation. Manual validation of peptides was performed using CAMV, a MATLAB-based tool for both precursor contamination and identification [27].

### Affinity Propagation Clustering Analysis

Quantitative acetylation site profiles were clustered using affinity propagation [28]. Euclidean distance was used as the similarity metric between acetylation profiles. A BIC scoring metric was used to identify the optimal clustering as previously described [25].

### Bioinformatics Analysis

Bioinformatic annotation was performed using PTMScout [29].

### Statistical Analyses

T-tests were performed to compare the phosphorylation profiles of the cells treated with or without TSA. Temporal acetylation profiles were analyzed for statistical significance using a one-way ANOVA.

## Results

### Insulin stimulation induces rapid lysine acetylation changes in hepatocellular carcinoma cells

To investigate the temporal effects of RTK activation on protein lysine acetylation, we stimulated HepG2 hepatocellular carcinoma cells with 150 nM insulin for 0, 1, 5, and 30 minutes. At these time points, cells were lysed and lysine-acetylated peptides were quantified by mass spectrometry (MS) using isobaric tagging for relative and absolute quantification (iTRAQ). Unlike other methods to profile the acetylproteome, this method allowed for the quantification of up to eight timepoints simultaneously. Furthermore, the method developed here saw a reduction in the percent of non-specific binding (60% versus 80%) as reported elsewhere [30]. We identified 43 unique acetyllysine-containing peptides on 31 proteins (complete quantitation can be found in S1 Table and manually validated spectra for each peptide can be found in S1 Fig). Within a minute of insulin stimulation, multiple lysine acetylation sites were reduced by at least two-fold, while other sites increased by more than 1.5 fold. Acetylation sites were identified on proteins involved in transcriptional regulation, including histones and p300, and metabolism, including fatty acid synthase and glyceraldehyde-3-phosphate dehydrogenase, among others.

**Fig 1:**
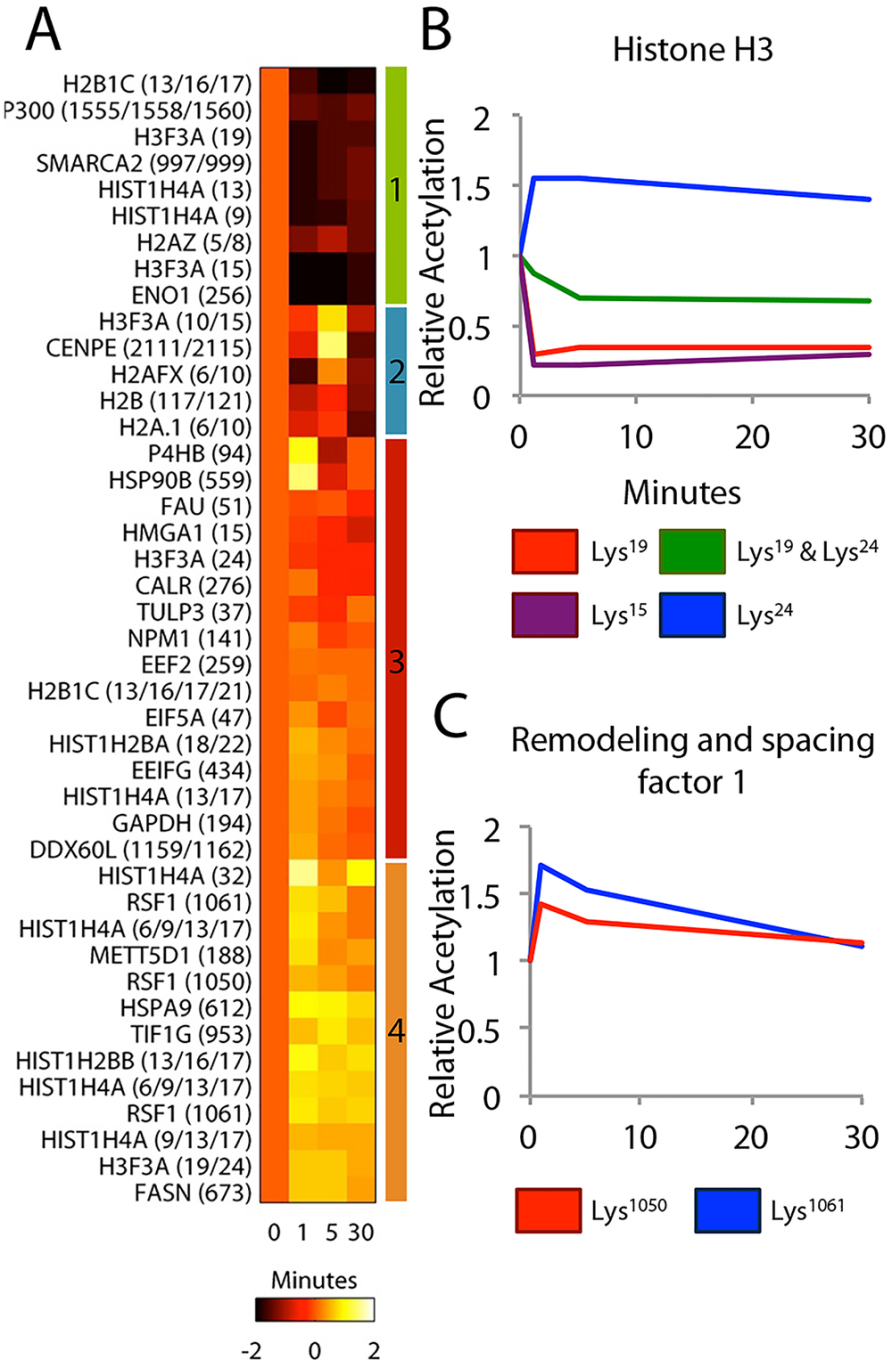
Quantitative acetyllysine profiling of HepG2 cells stimulated with insulin. **A.** Heat map representing the log_2_ fold-change relative to the unstimulated condition of the 43 acetylation sites quantified. Colored bars to the right of the heatmap indicate clusters. **B.** Acetylation dynamics on sites identified on histone H3. **C.** Acetylation dynamics on sites identified on remodeling and splicing factor 1.

To visualize the changes in lysine acetylation that had similar temporal responses, we clustered the acetylation profiles (Fig 1A). Although acetylation dynamics at various sites covered a range of responses, multiple clusters of similarly regulated acetylation sites were present. Cluster 1 is characterized by a rapid deacetylation that was sustained over the course of insulin treatment. Cluster 2 is characterized by a late decrease in acetylation; cluster 3 is relatively unchanged, and cluster 4 has a slight increase in acetylation over time. To identify potential shared pathways or protein functions within each cluster, we analyzed the biological annotations of proteins in each cluster. This analysis revealed that many of the proteins in cluster 1 are involved in transcriptional control. Although the role of many of the sites identified in this analysis remains uncharacterized, the fact that multiple sites on related proteins were regulated similarly suggests a concerted response, potentially involving activation or inhibition of a given enzyme associated with these proteins.

To better understand the regulation of acetylation dynamics by growth factor signaling, we selected proteins on which we identified multiple acetylation sites. Comparing the acetylation dynamics across multiple sites on the same protein revealed site-specific temporal dynamics. For example, histone H3 shows a diverse set of responses depending on the site modified (Fig 1B), whereas both of the acetylation sites measured on remodeling and spacing factor 1 exhibited an increase in acetylation at 1 minute after insulin stimulation followed by a decrease in acetylation (Fig 1C). Thus, lysine acetylation was rapidly and dynamically regulated in response to insulin stimulation, in a site-specific manner.

### IGF-1 and EGF stimulation induces rapid lysine acetylation changes in a NSCLC line

**Fig 2:**
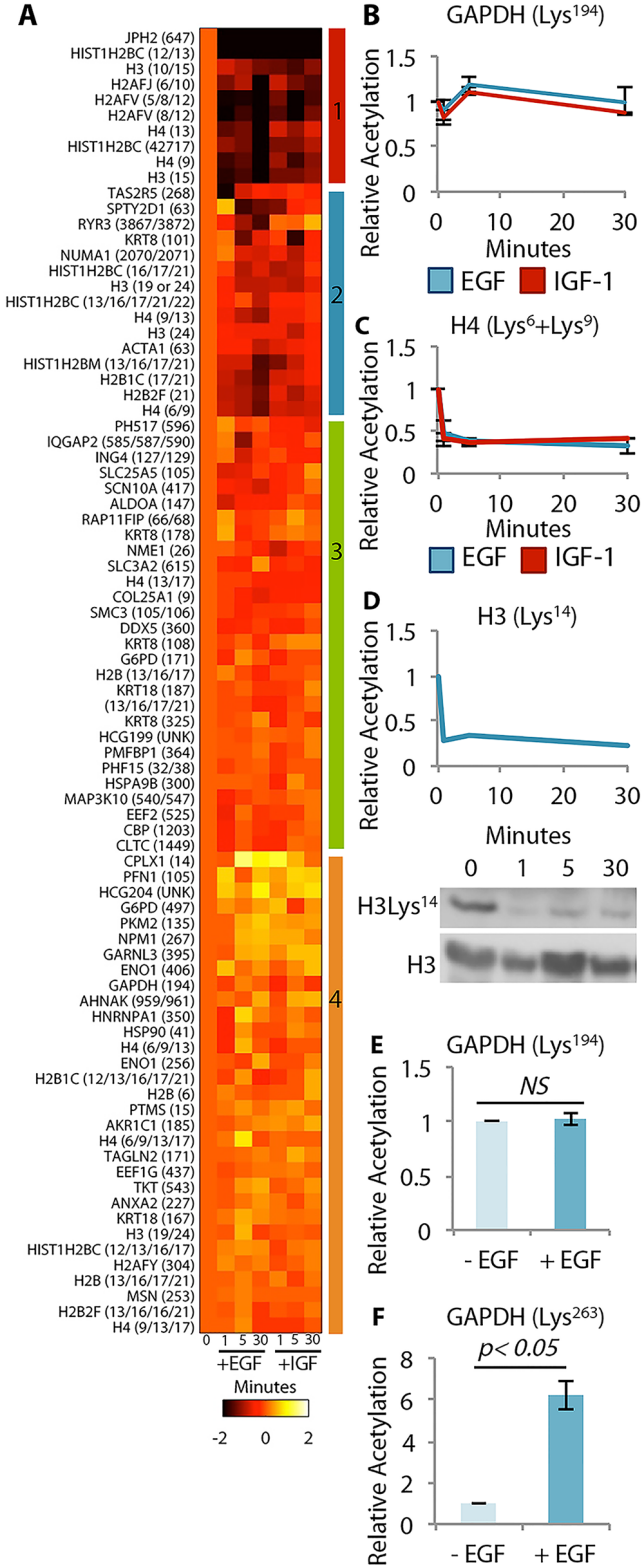
Quantitative acetyllysine profiling of A549 cells stimulated with EGF or IGF-1. **A.** Heat map of log2 fold-change relative to the unstimulated condition of the 90 acetylation sites quantified. Colored bars at the right indicate the clusters. **B.** Comparison of acetylation dynamics on GAPDH. **C.** Comparison of acetylation dynamics on histone H4, Lys^6^ and Lys^9^. **D.** EGF-stimulated Histone H3 Lys^14^ acetylation dynamics analyzed by mass spectrometry and western blotting. **E.** Acetylation dynamics quantified by SILAC on GAPDH Lys^194^. **F.** Acetylation dynamics quantified by SILAC on GAPDH Lys^263^.

To determine whether the observed rapid acetylation dynamics occurred in multiple cell lines following stimulation of different RTKs, we measured the acetylation responses of A549 non-small cell lung cancer (NSCLC) cells stimulated with either insulin-like growth factor 1 (IGF-1) or epidermal growth factor (EGF). We stimulated A549 cells with 100 ng/mL IGF-1 or EGF for 0, 1, 5, and 30 minutes and then processed the samples for simultaneous comparison of EGF- or IGF-1-stimulated lysates within the same MS analysis. This analysis resulted in the identification of 84 unique acetyllysine-containing peptides on 61 proteins (S2 Table and S2 Fig). Similar to the insulin-stimulated HepG2 cells, rapid changes in acetylation state were observed in response to either EGF or IGF-1 stimulation of A549 cells (Fig 2A).

To identify shared temporal acetylation responses to EGF or IGF-1 stimulation, we clustered the dynamic profiles. Several sites showed a rapid decrease in acetylation that was sustained across the entire length of ligand treatment, whereas other sites showed a rapid increase immediately following stimulation followed by a decrease back to basal. The acetylation status of many sites did not change in response to ligand stimulation (Fig 2A and S2 Table). Leveraging the framework utilized to describe tyrosine phosphorylation networks where different ligands can yield different tyrosine phosphorylation profiles indicating activation of RTK-specific phosphorylation networks, we investigated whether similar patterns could be observed in acetylation dynamic data. EGF and IGF-1 stimulation produced similar dynamics on GAPDH Lys^194^ (unchanged) and the doubly-acetylated histone H4 peptide Lys^6^ and Lys^9^ (rapid and sustained decrease). The commonly regulated rapid dynamics on histone H4 suggests that a shared pathway between EGF and IGF-1 signaling may drive these acetylation dynamics. Although high-confidence differential dynamics between EGF and IGF-1 stimulated samples were not identified, it is worth noting that differential acetylation dynamics between these two RTK ligands may exist, and may be revealed through additional analyses.

To assess acetylation dynamics using an alternative quantitative mass spectrometry method, we quantified the acetylation response of DLD1 colorectal adenocarcinoma cells labeled using stable isotope labeling of amino acids in culture (SILAC). Heavy isotope labeled DLD1 cells were stimulated with 100 ng/mL EGF for one minute; light isotope labeled DLD1 cells were unstimulated. These cells were then processed for MS analysis. We identified 39 unique acetyllysine-containing peptides on 23 proteins (S3 Table and S3 Fig). Rapid acetylation dynamics were detected in the DLD1 cells, confirming acetylation response to RTK stimulation (Fig 2E and 2F). Moreover, EGF treatment did not induce acetylation dynamics on GAPDH Lys^194^, recapitulating the results previously generated using iTRAQ and thereby confirming the trends observed. Additionally, we observed acetylation dynamics on a different site on GAPDH Lys^263^ where EGF stimulation induced a five-fold increase in lysine acetylation. To further confirm the trends of our mass spectrometry based analysis, we quantified histone H3 Lys^14^ acetylation dynamics in A549 cells stimulated with EGF using western blotting (Fig 2D). Western blotting and quantitative mass spectrometry revealed similar rapid deacetylation on this site. These data confirm that acetylation dynamics occur rapidly in response to RTK stimulation.

### Histone Deacetylase Inhibition Modulates Phosphotyrosine Signaling Networks

**Fig 3:**
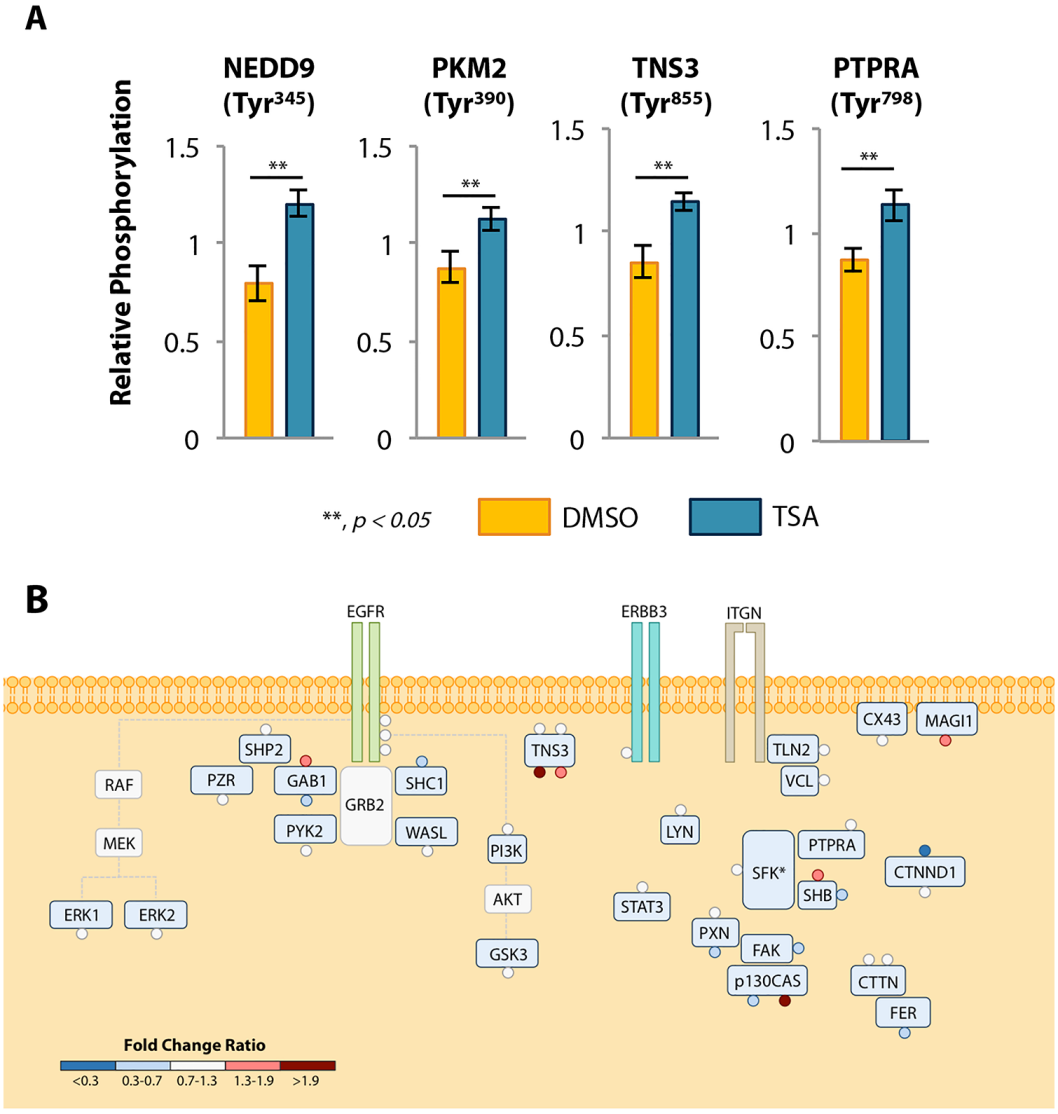
Quantitative phosphotyrosine profiling of A549 cells treated with the lysine deacetylase inhibitor, TSA, with or without EGF stimulation. **A.** The four phosphotyrosine sites significantly (*p<0*.*05*) differentially phosphorylated between A549 cells treated with TSA or DMSO. Average phosphorylation +/- standard error are shown. **B.** The fold change ratio in response to TSA treatment mapped to a visual representation of proteins involved in kinase signaling, scaffolding, and cell adhesion. Each circle represents a unique phosphorylation site identified on the protein. Sites are color coded to characterize the relative effect of TSA pre-treatment on the EGF response. Proteins are labeled by their gene name. A larger version of the figure with labeled phosphorylation sites is available in S7 Fig.

To profile the crosstalk between phosphotyrosine signaling networks and lysine acetylation at the network level, we compared the phosphotyrosine networks of A549 cells treated with the class I/II histone deacetylase inhibitor, trichostatin A (TSA). In the absence of RTK stimulation, 45 phosphotyrosine-containing peptides on 34 proteins were identified and quantified (S4 Table and S4 Fig), along with 26 acetylation sites on 13 proteins (S5 Table and S5 Fig). As expected, 1-hour inhibition of lysine deacetylases increased lysine acetylation on a subset of peptides, suggesting that short-term treatment with TSA affects deacetylases regulating histone acetylation (S5 Table). We also found four tyrosine phosphorylation sites that were statistically different between the two conditions (Fig 3A). Three of these differentially phosphorylated proteins have been implicated in cell adhesion: NEDD9, protein tyrosine phosphatase receptor A (PTPRA) and tensin-3 (TNS3). Mutation of Tyr^798^ on PTPRA has been shown to drive an increase in tyrosine phosphorylation; these effects may either be due to the direct result of reduced PTPRA activity or the altered activity of PTPRA substrates [31]. Thus, by increasing phosphorylation at this site, TSA treatment may alter PTPRA activity. The fourth phosphorylation site occurs on pyruvate kinase M2 (PKM2), a protein involved in glycolysis. While these proteins are well-characterized, aside from Tyr^798^ on PTPRA, the function of the phosphorylated tyrosine residues that showed a change in status following TSA treatment is poorly characterized in the literature. Previous studies have demonstrated that acetylation and phosphorylation can act in a coordinated fashion on the same protein, providing a potential explanation for altered tyrosine phosphorylation following short-term TSA treatment. However, the bottom-up proteomics method applied here did not identify acetylated peptides originating from these proteins (S5 Table). These data demonstrate short-term inhibition of lysine deacetylases not only perturbs lysine acetylation, but also induces changes to the phosphotyrosine signaling network.

To determine whether altered lysine acetylation affected the phosphorylation signaling network response to RTK activation, we quantified tyrosine phosphorylation in cells pre-treated with TSA for one hour and then stimulated with EGF for 5 minutes. This analysis resulted in the identification and quantification of 82 tyrosine phosphorylation sites on 64 proteins (S6 Table and S6 Fig). To quantify the magnitude of the effect of TSA on the response to EGF stimulation, we calculated the ratio of the EGF-induced fold change in phosphorylation between vehicle- and TSA-treated cells and we refer to this as the fold change ratio (FCR). In general, the response to EGF stimulation was unaltered by TSA pre-treatment for most of the phosphorylation sites. However, approximately ten percent of the sites observed either decreased or increased by at least fifty percent with respect to the FCR metric. To identify whether these altered sites fell into a particular pathway downstream of EGFR, we mapped the mean FCR of a subset of the sites to a signaling network diagram. These sites were selected to represent proteins involved in kinase signaling, scaffolding assemblies, or cell adhesion, given the earlier results demonstrating altered phosphorylation on some proteins involved with cell adhesion (Fig 3B and S7 Fig). The FCR pathway map demonstrates that short-term TSA treatment modulates the response to EGF, with some of the phosphorylation sites, such as Tyr^354^ on TNS3 and Tyr^844^ on catenin delta 1 (CTNND1), demonstrating large changes in their EGF response (FCR < 0.3 and FCR >1.9). For many other phosphorylation sites in the network, including EGFR phosphorylation sites, TSA pre-treatment did not affect the phosphorylation response to EGF stimulation. Despite a plethora of publications regarding EGFR signaling, many EGF-regulated phosphorylation sites have not been mechanistically connected to the receptor, yet may be functionally relevant. Several of the sites affected by TSA treatment fall into this group of proteins whose connection to EGFR is undefined, but phosphorylation of these proteins might be critical for regulating downstream biological response to therapy. Overall, this data demonstrates that inhibition of lysine deacetylase activity affects the tyrosine phosphorylation of specific components downstream of the receptor within the EGFR network, suggesting that acetylation may be a regulatory input into the EGF pathway.

## Discussion

To understand the potential connection between phosphotyrosine signaling and lysine acetylation, we quantified the immediate-early temporal dynamics of lysine acetylation in response to growth factor stimulation. To our surprise, many lysine acetylation sites displayed dramatic changes in their amount of modification within one minute of growth factor stimulation. Other network-level studies have demonstrated rapid changes in the phosphorylation signaling network in response to RTK activation [32–33]. While these studies were novel in terms of elucidating the speed and scale of the changes within the network, the fact that there were rapid changes in tyrosine phosphorylation following RTK activation was not unexpected. Here, using a combination of strategies including quantitative mass spectrometry and western blotting, we have elucidated a novel rapid response in lysine acetylation in response to RTK activation. Similar to tyrosine phosphorylation networks downstream of different RTKs, some of the altered acetylation sites display similar dynamics in response to either EGF or IGF-1, while other acetylation sites may be differentially regulated depending on the activated RTK. To control for ligand-independent effects such as cell stress due to turbulence that may occur during cell stimulation, a small volume of concentrated ligand was added to the large volume of media on the plate, without media exchange during stimulation. While we cannot completely rule out other ligand-independent effects on lysine acetylation dynamics, analysis of multiple unstimulated control cell lysates demonstrated very high biological reproducibility, typically <10% variance on each lysine acetylation site across 4 biological replicates, similar to previous results we have reported for tyrosine phosphorylation [34]. Analyzing specific proteins revealed that acetylation responses occur in a site-specific manner. For example, different acetylation sites on GAPDH displayed differential temporal dynamics, while other multiply acetylated proteins had sites that were either commonly or differentially regulated. This behavior suggests another similarity between tyrosine phosphorylation and lysine acetylation, as site-specific regulation of multiply phosphorylated proteins is well known. Clustering the dynamic responses of the acetylation sites has allowed us to identify sub-clusters of sites that share similar temporal profiles, suggesting that these proteins may fall within the same acetylation signaling pathways. Taken together, these data strongly suggest that lysine acetylation is a dynamic signaling process that occurs on a similar time scale to protein tyrosine phosphorylation.

Phosphotyrosine profiling of cells treated with HDAC inhibitors suggests a complex relationship between these two dynamic post-translational modifications. Our results demonstrate that short-term treatment with an HDAC inhibitor modulates the response to EGF stimulation, establishing a link between acetylation and the enzymes that regulate tyrosine phosphorylation. We first identified phosphorylation sites that were altered upon short-term TSA treatment. Three of the four proteins in which altered phosphorylation was detected upon TSA treatment are proteins implicated in cell adhesion. This result suggests that perturbation of deacetylases may influence these processes, a result that agrees with reports implicating HDACs such as HDAC6 in regulating cell motility [35]. With bi-directional interconnectivity between acetylation and phosphorylation networks, the expression and activity of various acetyltransferases and deacetylates may modulate the protein tyrosine phosphorylation signaling network, providing differential response to receptor activation in different contexts. Future analyses should endeavor to explore the effects of knocking down specific HDACs to understand the role of different classes of HDACs in regulating these responses. Moreover, knockdown of selected HDACs would reveal any off-target effects of these chemical inhibitors, which have been shown to perturb multiple aspects of cellular signaling, either through indirect or direct effects.

Lysine acetylation has long been synonymous with transcriptional control as acetylation alters chromatin structure and histone-DNA interactions. To this end, we observed rapid acetylation dynamics on multiple sites on histone proteins within one minute of EGF stimulation. How these changes might affect transcription is yet to be determined, but results from the Yarden lab have demonstrated altered expression of hundreds of genes within 20 minutes of EGF stimulation [36]. The potential connection between rapid dynamics of histone acetylation and rapid transcriptional changes will be an interesting avenue to pursue. For example, one of the sites identified as dynamic on histone H3 Lys^14^ has been identified at active sites of transcription suggesting a functional consequence of lysine acetylation on this site [37]. In addition to transcriptional regulation, a number of studies have also demonstrated that lysine acetylation occurs outside of histones and includes structural proteins as well as proteins involved in signaling and metabolism, although the functional role of lysine acetylation on many of these proteins has not been explored [3–4]. In our analysis, GAPDH acetylation was identified as altered upon stimulation in DLD1 cells, and acetylation of this protein has been shown to be influenced by metabolism and has functional consequences for nuclear translocation [38–39]. Here we demonstrate that the amount of modification of both acetylation and phosphorylation sites is rapidly altered in response to RTK stimulation, providing the potential for expeditious modulation of many cell processes following cell perturbation.

While this study has demonstrated that there exists a link between lysine acetylation and tyrosine kinases, the exact mechanism by which ligand stimulation drives acetylation dynamics remains poorly understood. Signaling pathway diagrams of the EGF signaling network rarely include enzymes involved in altering lysine acetylation, although several studies have suggested potential links between the EGF signaling network and various acetyltransferases and deacetylases. For instance, HDAC6 and EGFR have been shown to interact in a modified yeast two-hybrid assay; this same study established a negative feedback loop in which HDAC6 is phosphorylated at Tyr570 and thereby regulates EGFR endocytosis [40]. Another study showed that EGFR can be acetylated by CBP [41]. Our results suggest a requirement for a more integrated network that involves kinases, phosphatases, acetyltransferases, deacetylases and a wide array of other enzymes and scaffolding proteins. This assertion is supported by the results of our TSA experiments demonstrating that short-term inhibition of a class of lysine deacetylases can drive alterations in phosphotyrosine signaling in the basal state and in response to ligand stimulation.

This study has highlighted that tyrosine kinase activation can lead to rapid changes in lysine acetylation. This discovery suggests the existence of novel pathways that link phosphotyrosine signaling with proteins known to regulate lysine acetylation. Furthermore, the temporal dynamics experiments and subsequent experiment demonstrating feedback from HDAC inhibition to phosphotyrosine signaling highlights novel avenues in which therapeutic interventions may have a broader effect on signaling and the subsequent cellular responses. These results suggest future experiments to investigate the link between these two PTMs as well as links between other modifications. For instance, it is conceivable that rapid changes in lysine acetylation may also lead to rapid changes in other lysine post-translational modifications such as methylation and ubiquitylation, since lysine can be the site of a number of different post-translational modifications. Future studies should continue to focus both on the diversity of the perturbations that may alter these post-translational modification networks as well as the time scale over which these responses may be regulated. Further characterization of the specific function of these acetylation and phosphorylation sites will allow us to link the temporal dynamics with a particular cellular response. While the similarities between lysine acetylation and tyrosine phosphorylation dynamics are striking, these results reinforce the complex interactions at play in cellular signaling networks. Taken together, the insight into intracellular signaling gained in this study, coupled with biochemical analysis and computation, will allow us to better understand the mechanisms of a vast array of therapeutics as well as build a better portrait of the complex signaling pathways that integrate to execute and control cellular behavior.

## Supporting Information

S1 Fig Manually validated acetyllysine spectra of HepG2 cells stimulated with insulin and false discovery rate analysis. False discovery rate analyses aid in identifying a potential threshold for accepting MS/MS spectra without manual validation. Oftentimes, a particular FDR is selected and a peptide score for which that FDR is met is selected. Here, an FDR analysis was performed as a function of Mascot score. Instead of relying on an FDR analysis where the identity of true positives and true negatives are unknown, we manually validated each MS/MS spectra manually. Each page represents a manually validated MS/MS spectrum.
(PDF)

S2 Fig Manually validated acetyllysine spectra of A549 cells stimulated with EGF or IGF-1 and false discovery rate analysis. False discovery rate analyses aid in identifying a potential threshold for accepting MS/MS spectra without manual validation. Oftentimes, a particular FDR is selected and a peptide score for which that FDR is met is selected. Here, an FDR analysis was performed as a function of Mascot score. Instead of relying on an FDR analysis where the identity of true positives and true negatives are unknown, we manually validated each MS/MS spectra manually. Each page represents a manually validated MS/MS spectrum.
(PDF)

S3 Fig Manually validated acetyllysine spectra of DLD1 cells stimulated with EGF. Instead of relying on an FDR analysis where the identity of true positives and true negatives are unknown, we manually validated each MS/MS spectra manually. Each page represents a manually validated MS/MS spectrum.
(PDF)

S4 Fig Manually validated phosphotyrosine spectra of A549 cells treated with TSA. Instead of relying on an FDR analysis where the identity of true positives and true negatives are unknown, we manually validated each MS/MS spectra manually. Each page represents a manually validated MS/MS spectrum.
(PDF)

S5 Fig Manually validated acetyllysine spectra of A549 cells treated with TSA. Instead of relying on an FDR analysis where the identity of true positives and true negatives are unknown, we manually validated each MS/MS spectra manually. Each page represents a manually validated MS/MS spectrum.
(PDF)

S6 Fig Manually validated phosphotyrosine spectra of A549 cells treated with TSA with or without EGF. Instead of relying on an FDR analysis where the identity of true positives and true negatives are unknown, we manually validated each MS/MS spectra manually. Each page represents a manually validated MS/MS spectrum.
(PDF)

S7 Fig Quantitative phosphotyrosine profiling of A549 cells treated with the lysine deacetylase inhibitor, TSA, with or without EGF stimulation. The fold change ratio in response to TSA treatment mapped to a visual representation of proteins involved in kinase signaling, scaffolding, and cell adhesion. Each circle represents a unique phosphorylation site identified on the protein. The sites are color coded to characterize the relative effect of TSA pre-treatment on the EGF response. Proteins are labeled by their gene name. Sites of phosphorylation are labeled.
(TIF)

S1 Table Summary of iTRAQ quantification of manually validated acetyllysine peptides identified in the HepG2 insulin time course analyses. (XLSX)

S2 Table Summary of iTRAQ quantification of manually validated acetyllysine peptides identified in the A549 EGF and IGF time course analyses (XLSX)

S3 Table Summary of SILAC quantification of manually validated acetyllysine peptides identified in the DLD (+/-) EGF analyses. (XLSX)

S4 Table Summary of iTRAQ quantification of manually validated phosphotyrosine peptides identified in the A549 TSA analyses. (XLSX)

S5 Table Summary of iTRAQ quantification of manually validated acetyllysine peptides identified in the A549 TSA analyses. (XLSX)

S6 Table Summary of iTRAQ quantification of manually validated phosphotyrosine peptides identified in the A549 cells treated with TSA or vehicle with or without EGF. (XLSX)

## References

[1] Kouzarides T. Acetylation: a regulatory modification to rival phosphorylation? The EMBO Journal 2000; 19: 1176–1179. 10716917 10.1093/emboj/19.6.1176 PMC305658

[2] Norvell A, McMahon SB. Rise of the Rival. Science 2010: 327; 964–965. 10.1126/science.1187159 20167774

[3] Choudhary C, Kumar C, Gnad F, Nielsen ML, Rehman M, Walther TC, et al Lysine Acetylation Targets Protein Complexes and Co- Regulates Major Cellular Functions. Science 2009; 325: 834–840. 10.1126/science.1175371 19608861

[4] Kim SC, Sprung R, Chen Y, Xu Y, Ball H, Pei J, et al Substrate and functional diversity of lysine acetylation revealed by a proteomics survey. Mol Cell 2006; 23: 607–618. 16916647 10.1016/j.molcel.2006.06.026

[5] Wang Q, Zhang Y, Yang C, Xiong H, Lin Y, Yao J, et al Acetylation of Metabolic Enzymes Coordinates Carbon Source Utilization and Metabolic Flux. Science 2010; 327: 1004–1007. 10.1126/science.1179687 20167787 PMC4183141

[6] Zhang J, Sprung R, Pei J, Tan X, Kim S, Zhu H, et al Lysine Acetylation Is a Highly Abundant and Evolutionarily Conserved Modification in Escherichia Coli. Mol Cell Proteomics 2009; 8: 215–225. 10.1074/mcp.M800187-MCP200 18723842 PMC2634580

[7] Zhao S, Xu W, Jiang W, Yu W, Lin Y, Zhang T, et al Regulation of Cellular Metabolism by Protein Lysine Acetylation. Science 2010; 327: 1000–1004. 10.1126/science.1179689 20167786 PMC3232675

[8] Johnstone RW. Histone-deacetylase inhibitors: novel drugs for the treatment of cancer. Nat Rev Drug Discov 2002; 1: 287–299. 12120280 10.1038/nrd772

[9] Mann BS, Johnson JR, Cohen MH, Justice R, Pazdur R. FDA Approval Summary: Vorinostat for Treatment of Advanced Primary Cutaneous T-Cell Lymphoma. Oncologist 2007; 12: 1247–1252. 17962618 10.1634/theoncologist.12-10-1247

[10] Piekarz RL, Frye R, Turner M, Wright JJ, Allen SL, Kirschbaum MH, et al Phase II Multi-Institutional Trial of the Histone Deacetylase Inhibitor Romidepsin As Monotherapy for Patients With Cutaneous T-Cell Lymphoma. J Clin Oncol 2009; 27: 5410–5417. 10.1200/JCO.2008.21.6150 19826128 PMC2773225

[11] Khan O, La Thangue NB. HDAC inhibitors in cancer biology: emerging mechanisms and clinical applications. Immunol Cell Biol. 2012; 90: 85–94. 10.1038/icb.2011.100 22124371

[12] Xu WS, Parmigiani RB, Marks PA. Histone deacetylase inhibitors:molecular mechanisms of action. Oncogene 2007; 26: 5541–5552. 17694093 10.1038/sj.onc.1210620

[13] Blume-Jensen P, Hunter T. Oncogenic kinase signalling. Nature 2001; 411: 355–365 11357143 10.1038/35077225

[14] Downward J. Targeting RAS signalling pathways in cancer therapy. Nature Reviews Cancer 2003; 3: 11–22. 12509763 10.1038/nrc969

[15] Hanahan D, Weinberg RA. Hallmarks of Cancer: The Next Generation. Cell 2011; 144: 646–674. 10.1016/j.cell.2011.02.013 21376230

[16] Irish JM, Kotecha N, Nolan GP. Mapping normal and cancer cell signalling networks: towards single-cell proteomics. Nat Rev Cancer 2006; 6: 146–155. 16491074 10.1038/nrc1804

[17] Rikova K, Guo A, Zeng Q, Possemato A, Yu J, Haack H, et al Global Survey of Phosphotyrosine Signaling Identifies Oncogenic Kinases in Lung Cancer. Cell 2007; 131: 1190–1203. 18083107 10.1016/j.cell.2007.11.025

[18] Wolf-Yadlin A, Kumar N, Zhang Y, Hautaniemi S, Zaman M, Kim HD, et al Effects of HER2 overexpression on cell signaling networks governing proliferation and migration. Mol Syst Biol 2006; 2. 10.1038/msb4100094 PMC1682017 17016520

[19] Krämer OH, Knauer SK, Greiner G, Jandt E, Reichardt S, Guhrs KH, et al A phosphorylation-acetylation switch regulates STAT1 signaling. Genes Dev 2009; 23: 223–235. 10.1101/gad.479209 19171783 PMC2648535

[20] Li Y, Xu W, McBurney MW, Longo VD. SirT1 inhibition reduces IGF- I/IRS-2/Ras/ERK1/2 signaling and protects neurons. Cell Metab 2008; 8: 38–48. 10.1016/j.cmet.2008.05.004 18590691 PMC2822839

[21] Nie Y, Erion DM, Yuan Z, Dietrich M, Shulman GI, Horvath TL, et al STAT3 inhibition of gluconeogenesis is downregulated by SirT1. Nat Cell Biol 2009; 11: 492–500. 10.1038/ncb1857 19295512 PMC2790597

[22] Tang Y, Zhao W, Chen Y, Zhao Y, Gu W. Acetylation Is Indispensable for p53 Activation. Cell 2008; 133: 612–626. 10.1016/j.cell.2008.03.025 18485870 PMC2914560

[23] Minguez P, Parca L, Diella F, Mende DR, Kumar R, Helmer-Citterich M, et al Deciphering a global network of functionally associated post-translational modifications. Mol Syst Biol 2012; 8. 10.1038/msb.2012.31 PMC3421446 22806145

[24] van Noort V, Seebacher J, Bader S, Mohammed S, Vonkova I, Betts M, et al Cross-talk between phosphorylation and lysine acetylation in a genome-reduced bacterium. Mol Syst Biol 2012; 8. 10.1038/msb.2012.4 PMC3293634 22373819

[25] Johnson H, Del Rosario AM, Bryson BD, Schroeder MA, Sakaria JN, White FM. Molecular characterization of EGFR and EGFRvIII signaling networks in human glioblastoma tumor xenografts. Mol Cell Proteomics 2012; 11: 1724–1740. 10.1074/mcp.M112.019984 22964225 PMC3518138

[26] Zhang Y, Wolf-Yadlin A, Ross PL, Pappin DJ, Rush J, Lauffenburger DA, et al Time-resolved Mass Spectrometry of Tyrosine Phosphorylation Sites in the Epidermal Growth Factor Receptor Signaling Network Reveals Dynamic Modules. Mol Cell Proteomics 2005; 4: 1240–1250. 15951569 10.1074/mcp.M500089-MCP200

[27] Curran T, Bryson B, Riegelhaupt M, Johnson H, White FM. Computer aided manual validation of mass spectrometry-based proteomic data. Methods 2013; 61: 219–226. 10.1016/j.ymeth.2013.03.004 23500044 PMC3700613

[28] Frey BJ, Dueck D. Clustering by Passing Messages Between Data Points. Science 2007; 315: 972–976. 17218491 10.1126/science.1136800

[29] Naegle K, Gymrek M, Joughin BA, Wagner JP, Welsch RE, Yaffe MB, et al PTMScout, a Web resource for analysis of high-throughput post-translational proteomics studies. Mol Cell Proteomics 2010; 9: 2558–2570. 10.1074/mcp.M110.001206 20631208 PMC2984232

[30] Mertins P, Qiao JW, Patel J, Udeshi ND, Clauser KR, Mani DR, et al Integrated proteomic analysis of post-translational modifications by serial enrichment. Nat Methods 2013; 10: 634–637. 10.1038/nmeth.2518 23749302 PMC3943163

[31] Lammers R, Møller NP, Ullrich A. Mutant forms of the protein tyrosine phosphatase alpha show differential activities towards intracellular substrates. Biochem Biophys Res Commun 1998; 242: 32–38. 9439605 10.1006/bbrc.1997.7906

[32] Blagoev B, Ong S-E, Kratchmarova I, Mann M. Temporal analysis of phosphotyrosine-dependent signaling networks by quantitative proteomics. Nat Biotechnol 2004; 22: 1139–1145. 15314609 10.1038/nbt1005

[33] Wolf-Yadlin A, Hautaniemi S, Lauffenburger DA, White FM. Multiple reaction monitoring for robust quantitative proteomic analysis of cellular signaling networks. Proc Natl Acad Sci USA 2007; 104: 5860–5865. 17389395 10.1073/pnas.0608638104 PMC1851582

[34] Schmelzle K, Kane S, Gridley S, Lienhard G, White FM. Temporal dynamics of Tyrosine Phosphorylation in Insulin Signaling. Diabetes 2006; 55: 2171–2179. 16873679 10.2337/db06-0148

[35] Zhang X, Yuan Z, Zhang Y, Yong S, Salas-Burgos A, Koomen J, et al HDAC6 Modulates Cell Motility by Altering the Acetylation of Cortactin. Mol Cell. 2007; 27: 197–213. 17643370 10.1016/j.molcel.2007.05.033 PMC2684874

[36] Amit I, Citri A, Shay T, Lu Y, Katz M, Zhang F, et al A module of negative feedback regulators defines growth factor signaling. Nat Genet 2007; 39: 503–512. 17322878 10.1038/ng1987

[37] Koch CM, Andrews RM, Flicek P, Dillon SC, Karaöz U, Clelland GK, et al The landscape of histone modifications across 1% of the human genome in five human cells. Genome Res 2007; 17: 691–707. 17567990 10.1101/gr.5704207 PMC1891331

[38] Guan KL, Xiong Y. Regulation of intermediary metabolism by protein acetylation. Trends Biochem Sci 2011; 36: 108–116. 10.1016/j.tibs.2010.09.003 20934340 PMC3038179

[39] Ventura M, Mateo F, Serratosa J, Salaet I, Carujo S, Bachs O, et al Nuclear translocation of glyceraldehyde-3-phosphate dehydrogenase is regulated by acetylation. Int J Biochem Cell Biol. 2010; 42: 1672–1680. 10.1016/j.biocel.2010.06.014 20601085

[40] Deribe YL, Wild P, Chandrashaker A, Curak J, Schmidt MH, Kalaidzidis Y, et al Regulation of Epidermal Growth Factor Receptor Trafficking by Lysine Deacetylase HDAC6. Sci Signal 2009; 2: ra84 10.1126/scisignal.2000576 20029029

[41] Song H, Li CW, Labaff AM, Lim SO, Li LY, Kan SF, et al Acetylation of EGF receptor contributes to tumor cell resistance to histone deacetylase inhibitors. Biochem Biophys Res Commun 2011; 404: 68–73 (2011). 10.1016/j.bbrc.2010.11.064 21094134 PMC3049249

