# Supplementary material for: Quantitative Profiling of Lysine Acetylation Reveals Dynamic Crosstalk between Receptor Tyrosine Kinases and Lysine Acetylation
Source: PLoS One. 2015 May 15;10(5):e0126242. doi: 10.1371/journal.pone.0126242 (PMC4433260; doi:10.1371/journal.pone.0126242)

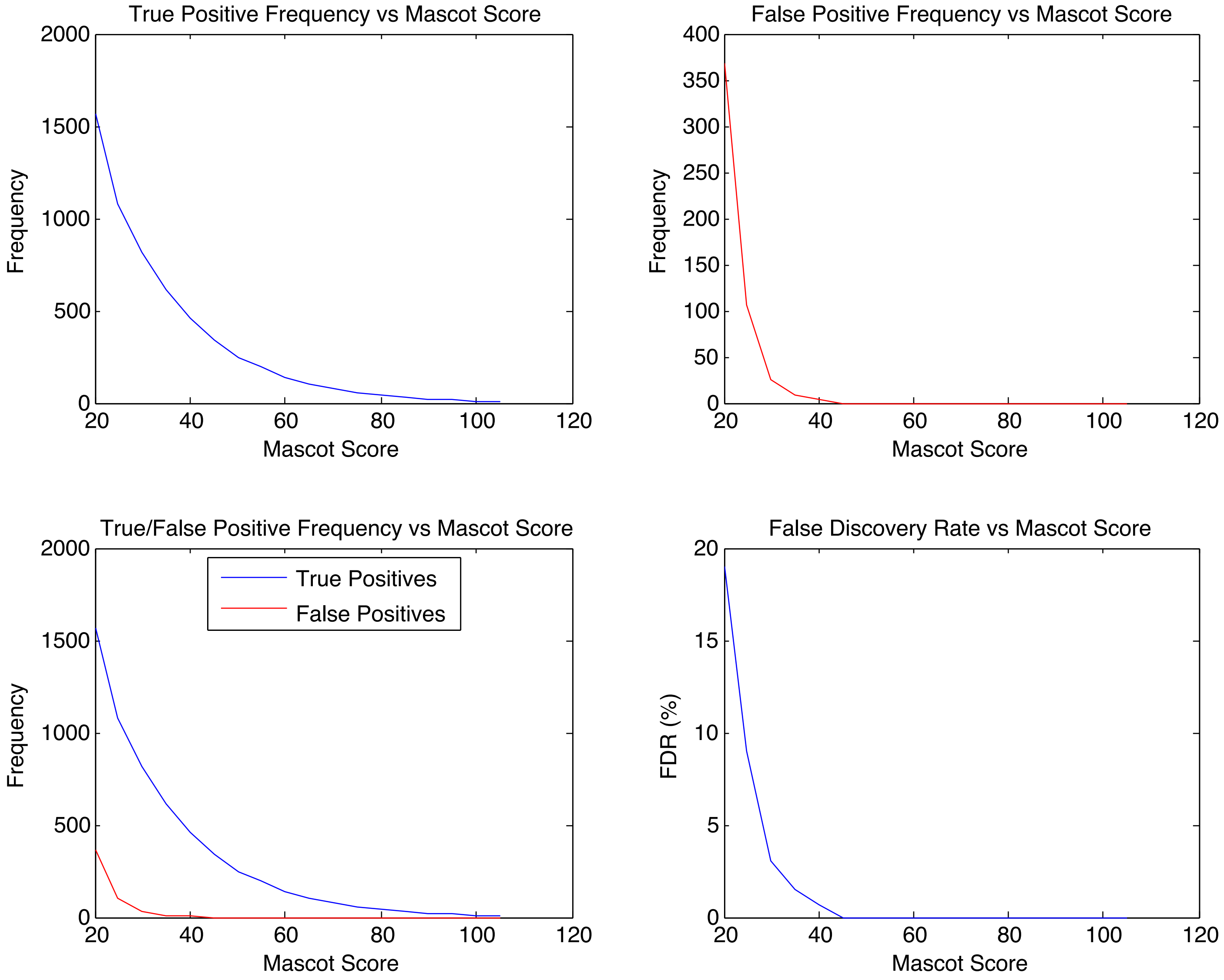

True Positive Frequency vs Mascot Score
Frequency
Mascot Score
False Positive Frequency vs Mascot Score
Frequency
Mascot Score
True/False Positive Frequency vs Mascot Score
True Positives
False Positives
Frequency
Mascot Score
False Discovery Rate vs Mascot Score
FDR (%)
Mascot Score

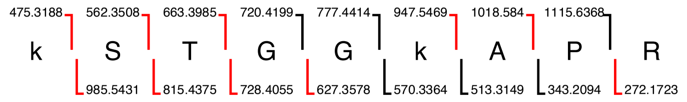

H3 histone, family 3A [Homo sapiens]
Charge State: +2
Scan Number: 4243
File Name: 120413_A549_EGFIGF_bioRepC_AcK_FT.raw

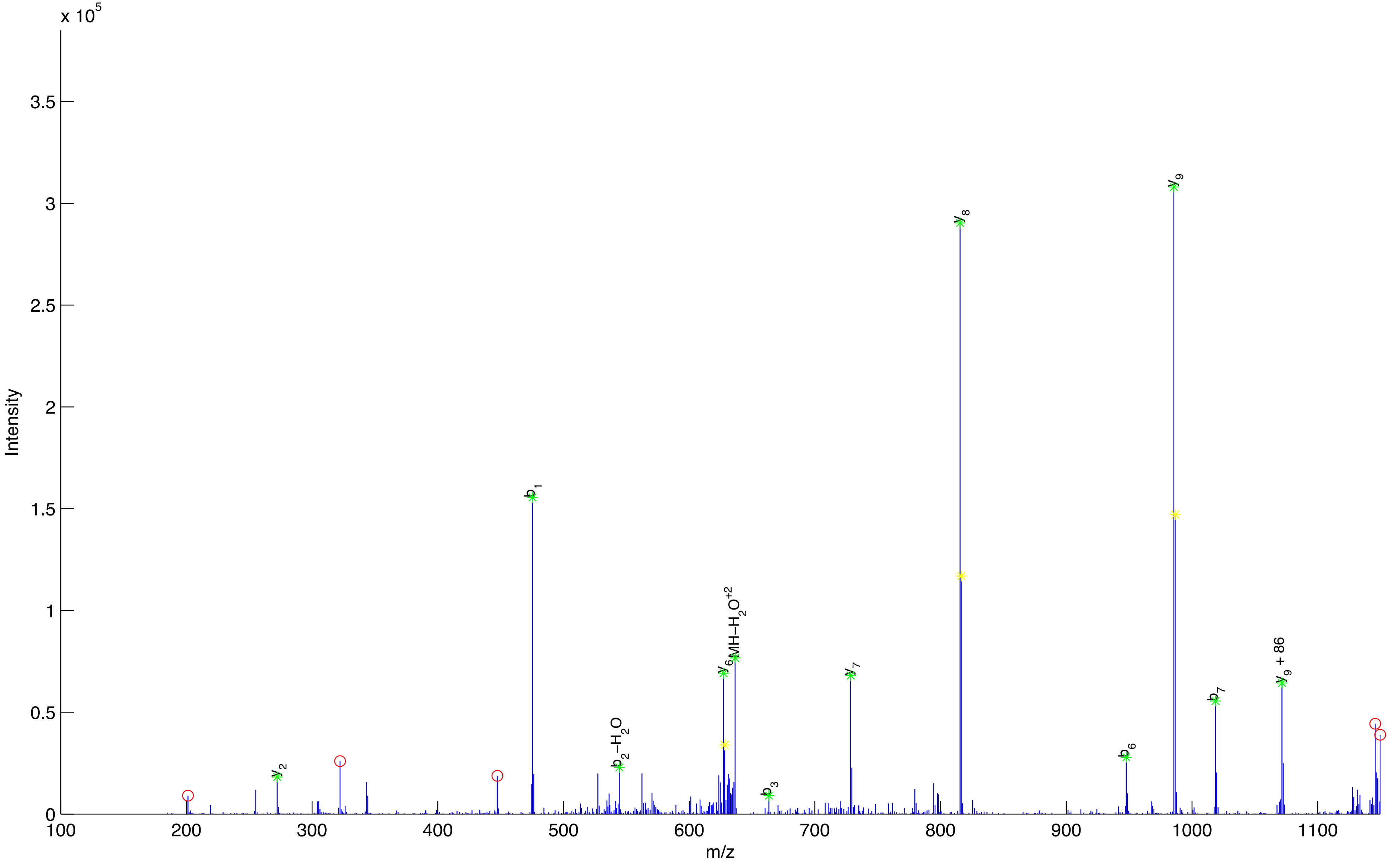

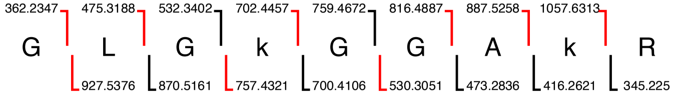

histone cluster 1, H4a [Homo sapiens]
Charge State: +2
Scan Number: 4738
File Name: 120413_A549_EGFIGF_bioRepC_AcK_FT.raw

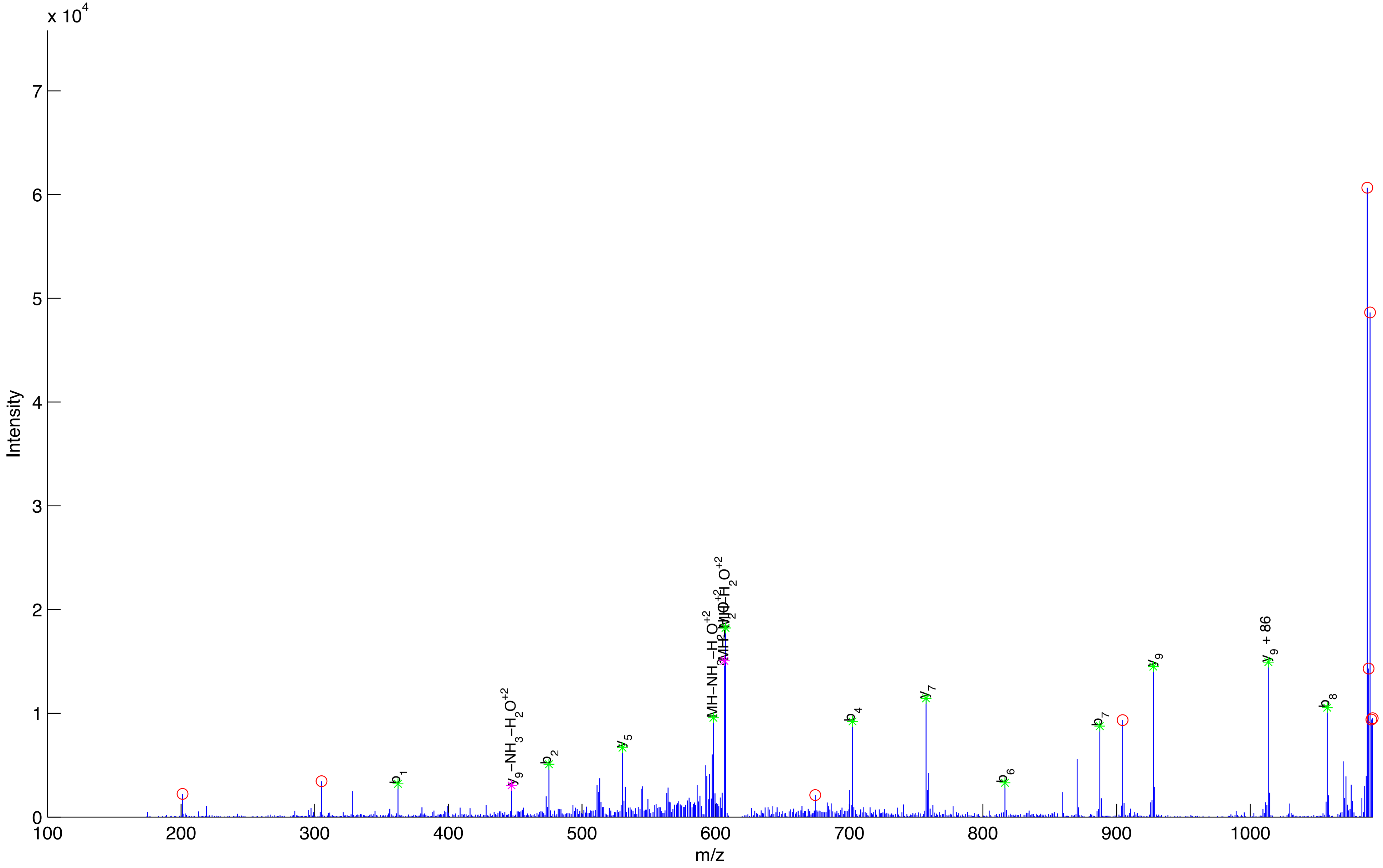

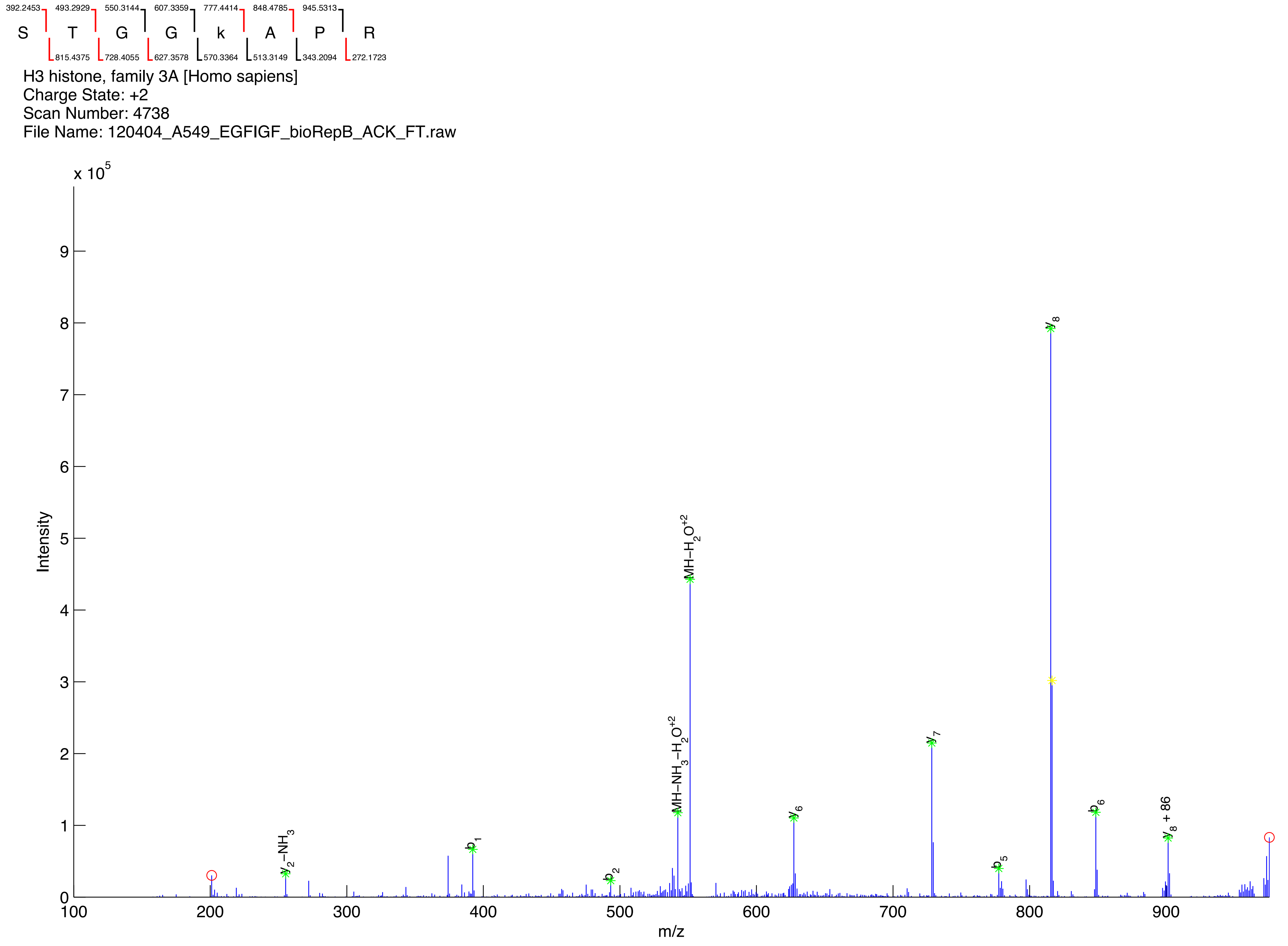

392.2453 493.2929 550.3144 607.3359 777.4414 848.4785 945.5313
S T G G k A P R
815.4375 728.4055 627.3578 570.3364 513.3149 343.2094 272.1723
H3 histone, family 3A [Homo sapiens]
Charge State: +2
Scan Number: 4738
File Name: 120404_A549_EGFIGF_bioRepB_ACK_FT.raw
x 10^5
Intensity
m/z
y2−NH3
b1
b2
MH−NH3−H2O+2
MH−H2O+2
y6
y7
b5
y8
b6
y8 + 86

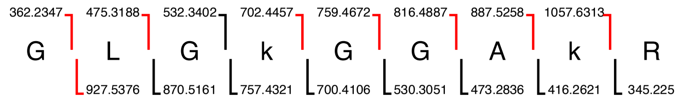

histone cluster 1, H4a [Homo sapiens]
Charge State: +1
Scan Number: 5083
File Name: 120413_A549_EGFIGF_bioRepC_AcK_FT.raw

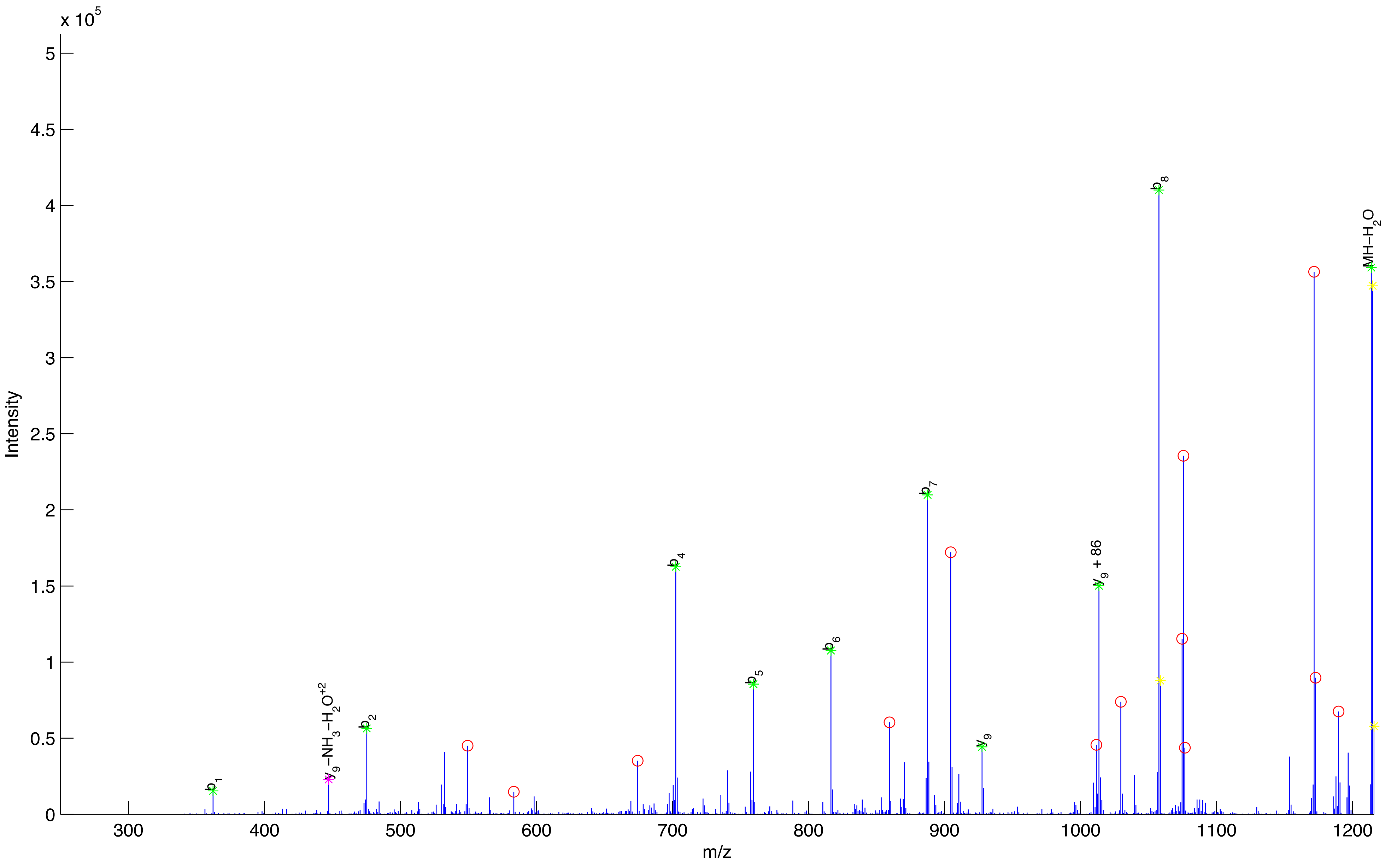

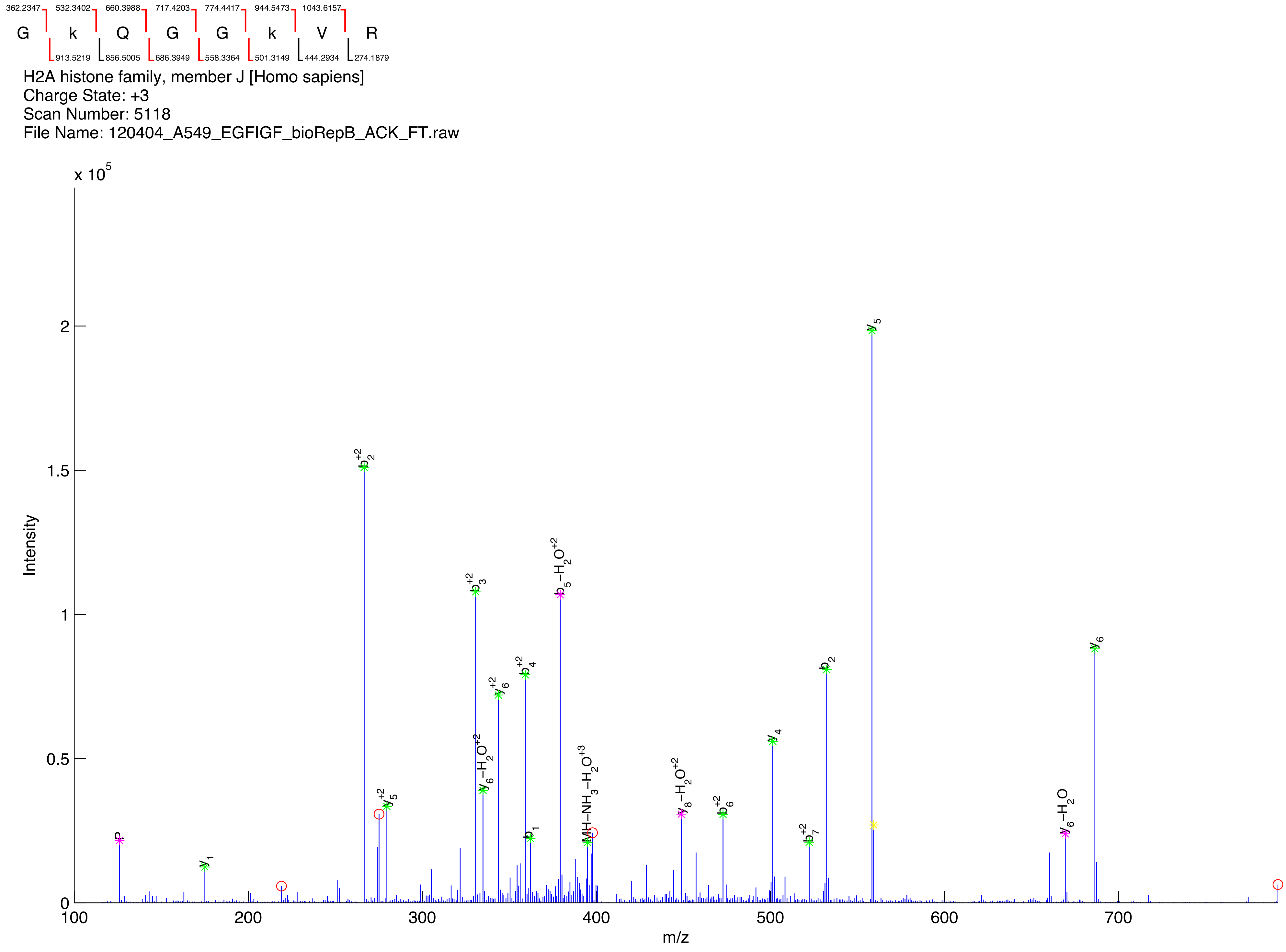
362.2347
532.3402
660.3988
717.4203
774.4417
944.5473
1043.6157
G k Q G G k V R
913.5219
856.5005
686.3949
558.3364
501.3149
444.2934
274.1879
H2A histone family, member J [Homo sapiens]
Charge State: +3
Scan Number: 5118
File Name: 120404_A549_EGFIGF_bioRepB_ACK_FT.raw
x 10^5
Intensity
m/z
0
0.5
1
1.5
2
100
200
300
400
500
600
700
P
y1
b2 +2
y5 +2
b3 +2
y6-H2O +2
y6 +2
b4 +2
b1
b5-H2O +2
MH-NH3-H2O +3
y8-H2O +2
b6 +2
y4
b7 +2
b2
y5
y6-H2O
y6

433.2718 520.3038 657.3627 827.4683 924.521 1037.6051

Q S H k P L R

907.5114 779.4528 692.4208 555.3619 385.2563 288.2036

PHD finger protein 17 long isoform [Homo sapiens]
Charge State: +3
Scan Number: 5249
File Name: 120407_A549_EGFIGF_bioRepA_ACK_FT.raw

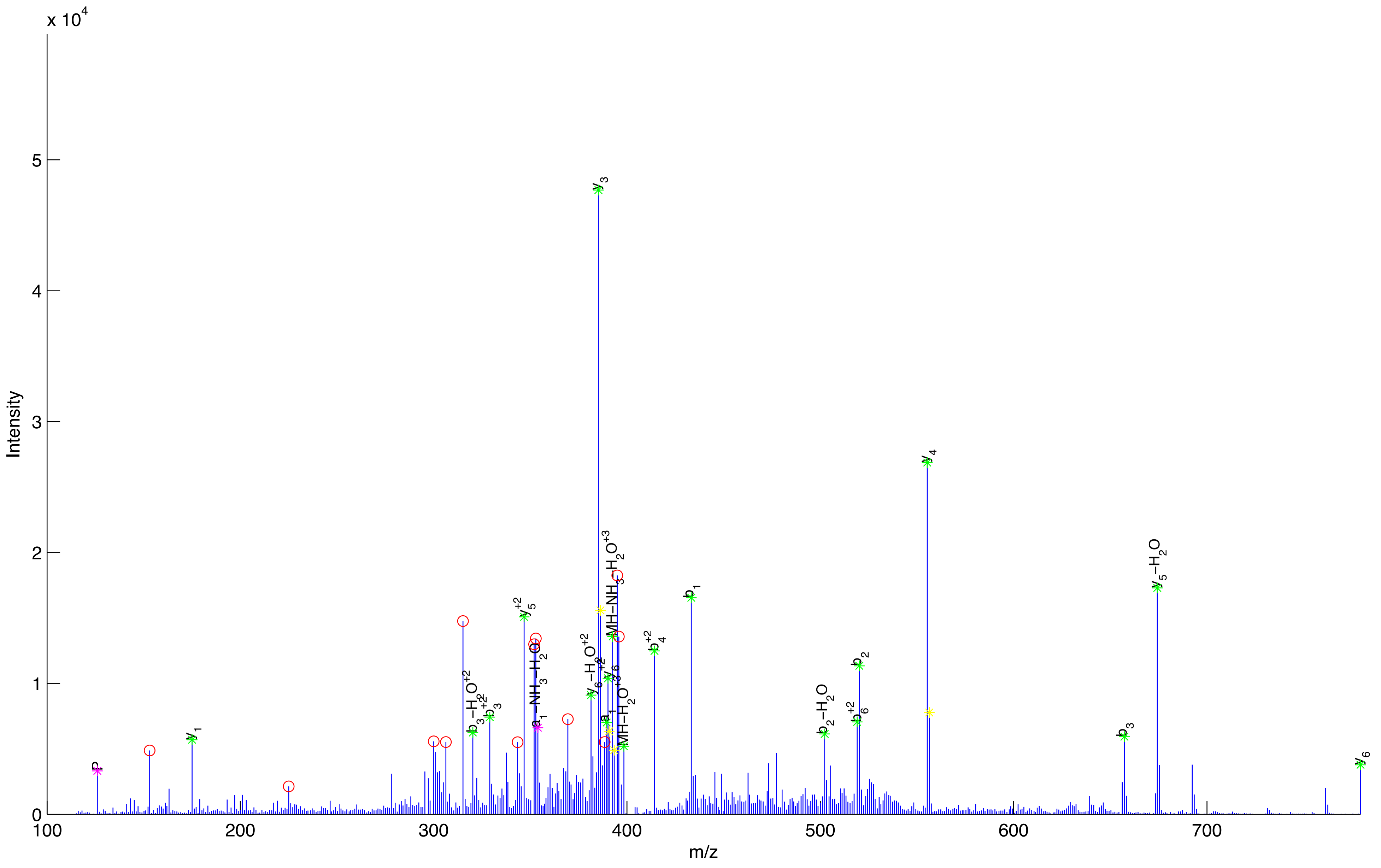

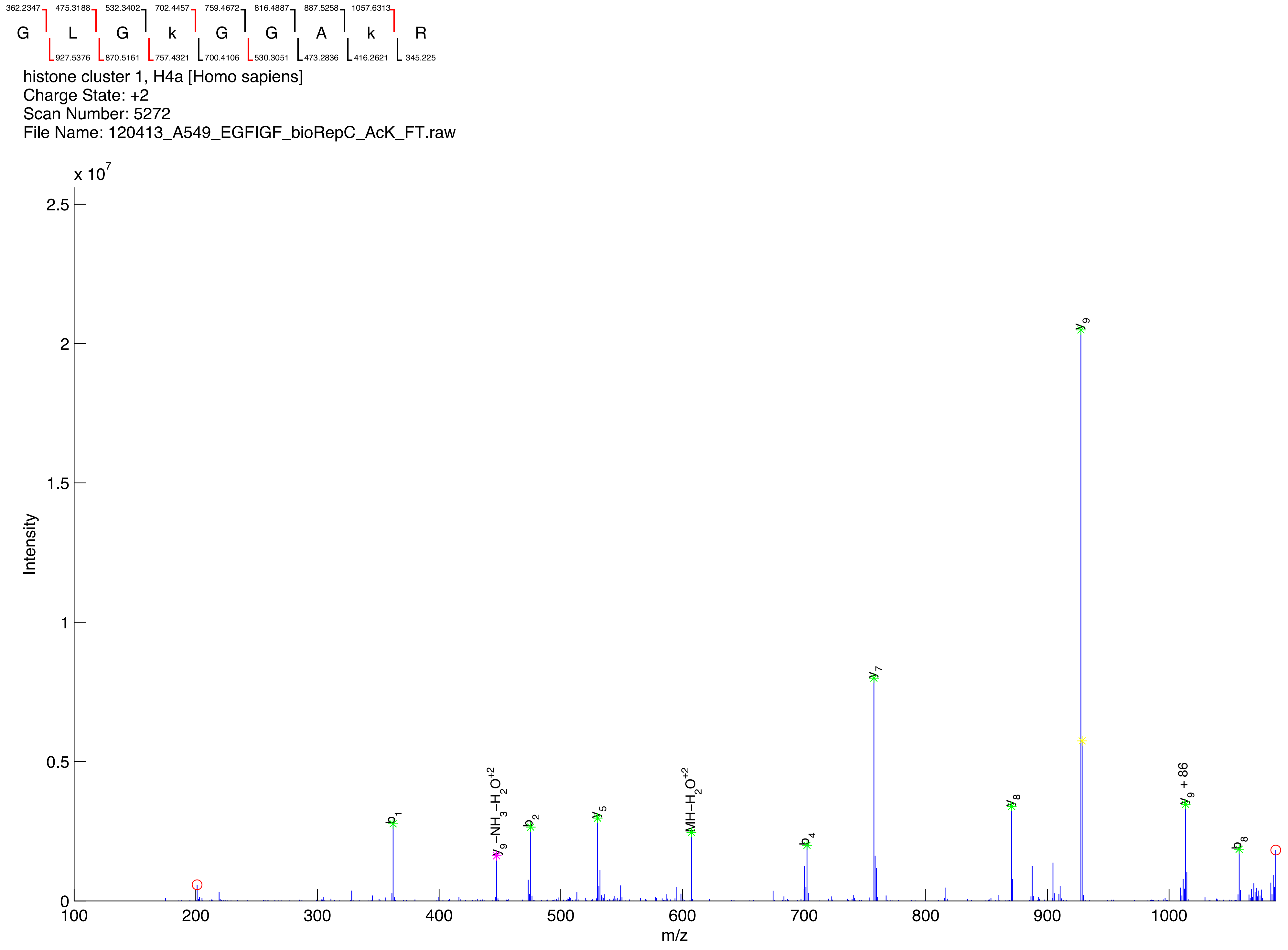
362.2347
475.3188
532.3402
702.4457
759.4672
816.4887
887.5258
1057.6313
G L G k G G A k R
927.5376
870.5161
757.4321
700.4106
530.3051
473.2836
416.2621
345.225
histone cluster 1, H4a [Homo sapiens]
Charge State: +2
Scan Number: 5272
File Name: 120413_A549_EGFIGF_bioRepC_AcK_FT.raw
x 10^7
Intensity
m/z
b1
y9−NH3−H2O+2
b2
y5
MH−H2O+2
b4
y7
y8
y9
y9 + 86
b8

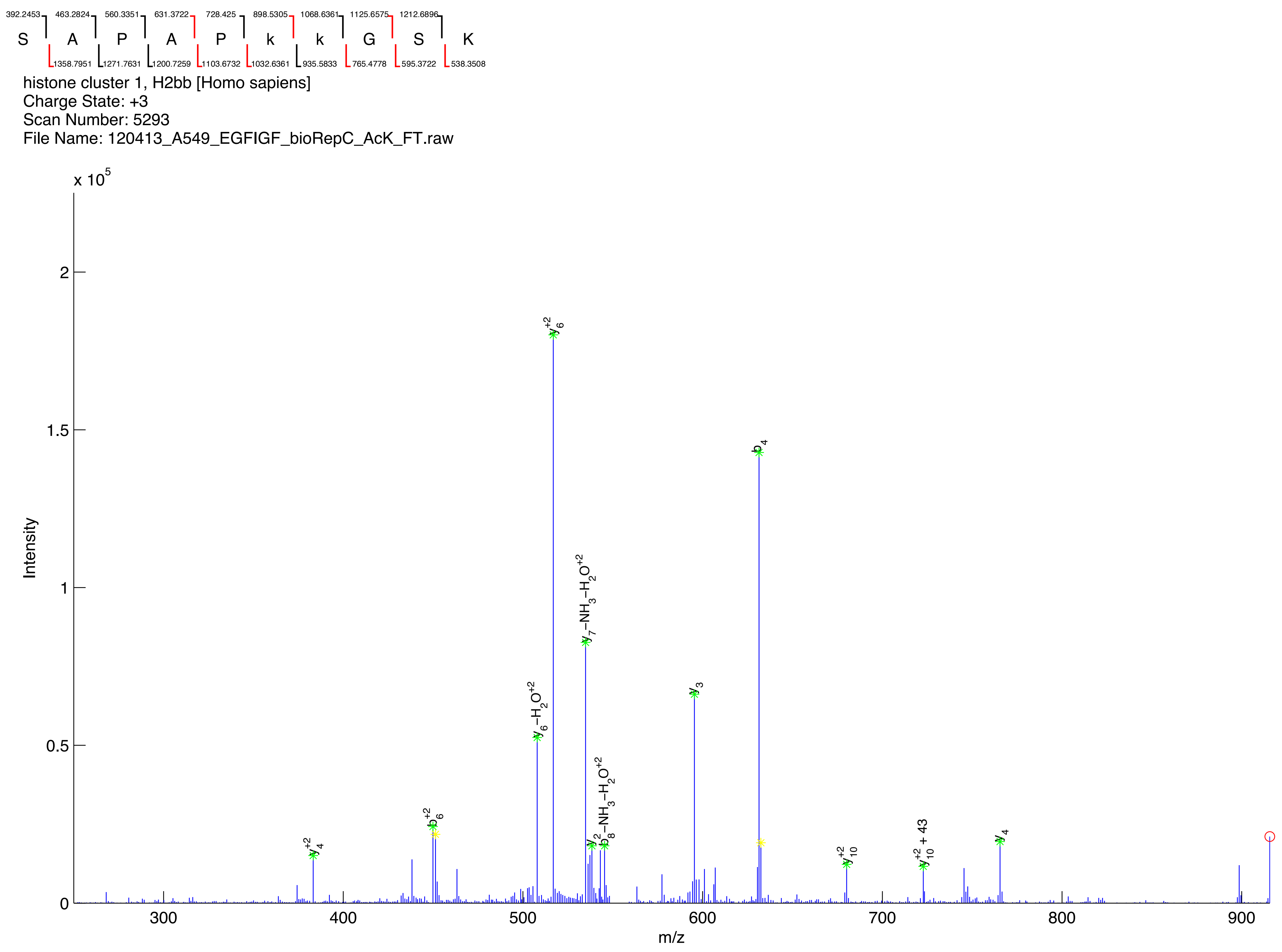
392.2453 463.2824 560.3351 631.3722 728.425 898.5305 1068.6361 1125.6575 1212.6896
S A P A P k k G S K
1358.7951 1271.7631 1200.7259 1103.6732 1032.6361 935.5833 765.4778 595.3722 538.3508
histone cluster 1, H2bb [Homo sapiens]
Charge State: +3
Scan Number: 5293
File Name: 120413_A549_EGFIGF_bioRepC_AcK_FT.raw
x 10^5
Intensity
m/z
y+2 4
b+2 6
y6−H2O+2
y+2 6
y7−NH3−H2O+2
y2
b8−NH3−H2O+2
y3
b4
y+2 10
y+2 10 + 43
y4

362.2347	475.3188	576.3664	746.472	817.5091	874.5305	945.5677	
G	L	T	k	A	G	A	K
	1091.6732	1034.6517	921.5677	820.52	650.4144	579.3773	522.3559

junctophilin 2 isoform 1 [Homo sapiens]
Charge State: +3
Scan Number: 5482
File Name: 120413_A549_EGFIGF_bioRepC_AcK_FT.raw

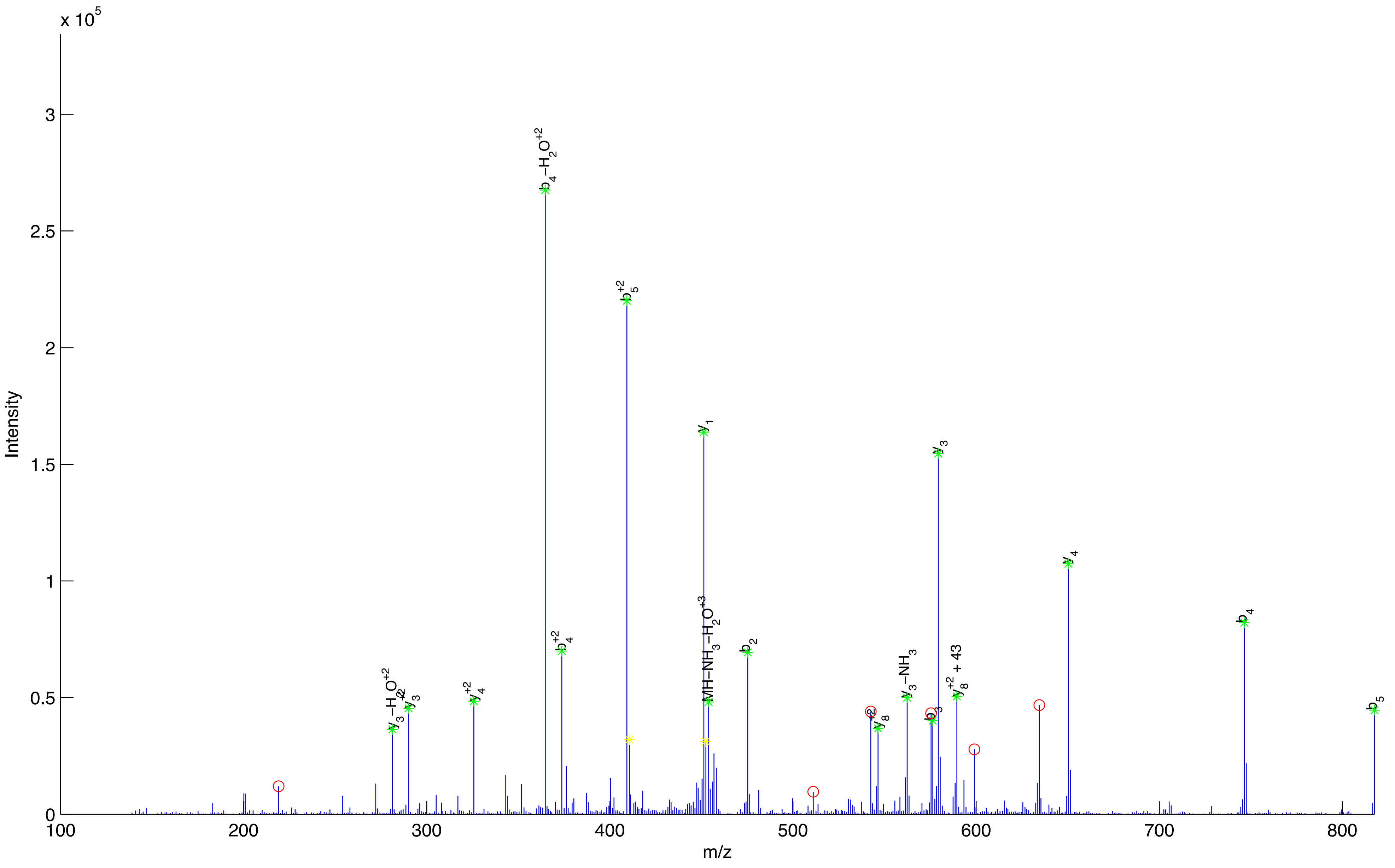

362.2347	532.3402	589.3617	646.3831	816.4887	873.5101	986.5942	1043.6157	1213.7212	1270.7427	1327.7641	1398.8012	1568.9068	
G	k	G	G	k	G	L	G	k	G	G	A	k	R
	1438.813	1381.7916	1211.686	1154.6646	1097.6431	927.5376	870.5161	757.4321	700.4106	530.3051	473.2836	416.2621	345.225

histone cluster 1, H4a [Homo sapiens]
Charge State: +
Scan Number: 5490
File Name: 120413_A549_EGFIGF_bioRepC_AcK_FT.raw

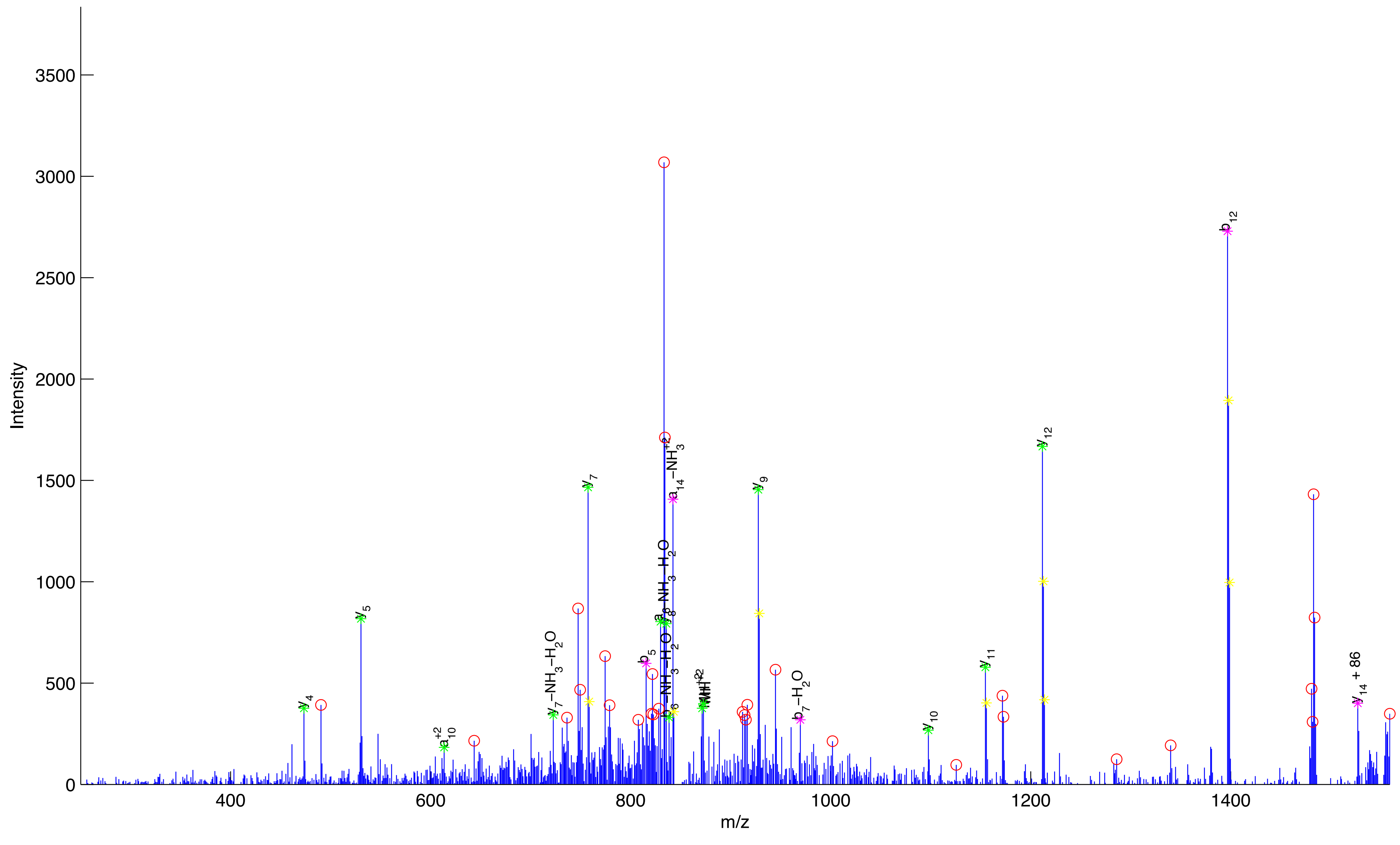

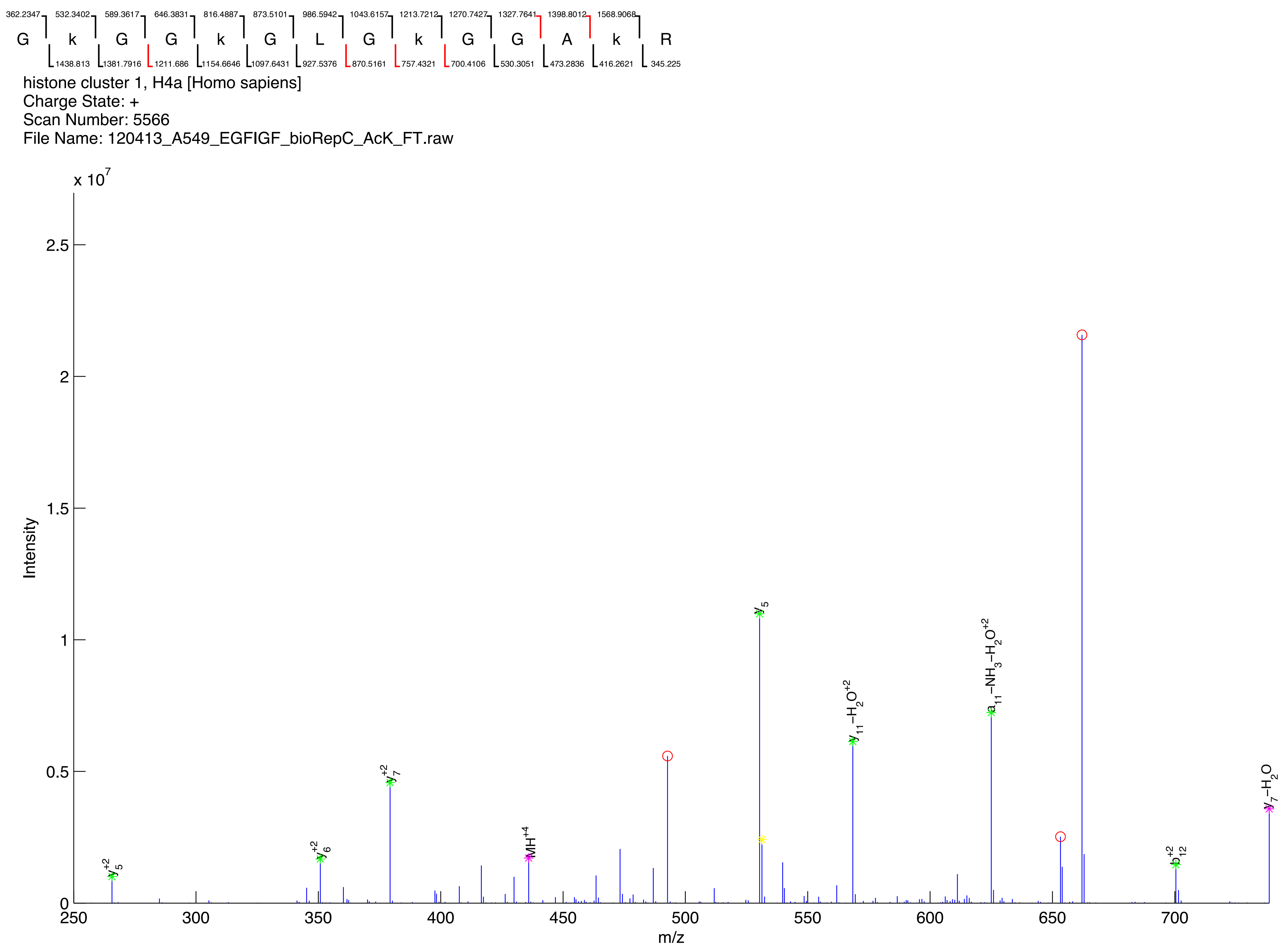

362.2347 532.3402 589.3617 646.3831 816.4887 873.5101 986.5942 1043.6157 1213.7212 1270.7427 1327.7641 1398.8012 1568.9068
G k G G k G L G k G G A k R
1438.813 1381.7916 1211.686 1154.6646 1097.6431 927.5376 870.5161 757.4321 700.4106 530.3051 473.2836 416.2621 345.225
histone cluster 1, H4a [Homo sapiens]
Charge State: +
Scan Number: 5566
File Name: 120413_A549_EGFIGF_bioRepC_AcK_FT.raw
x 10^7
Intensity
m/z
y5+2
y6+2
y7+2
MH+4
y5
y11−H2O+2
a11−NH3−H2O+2
b12+2
y7−H2O

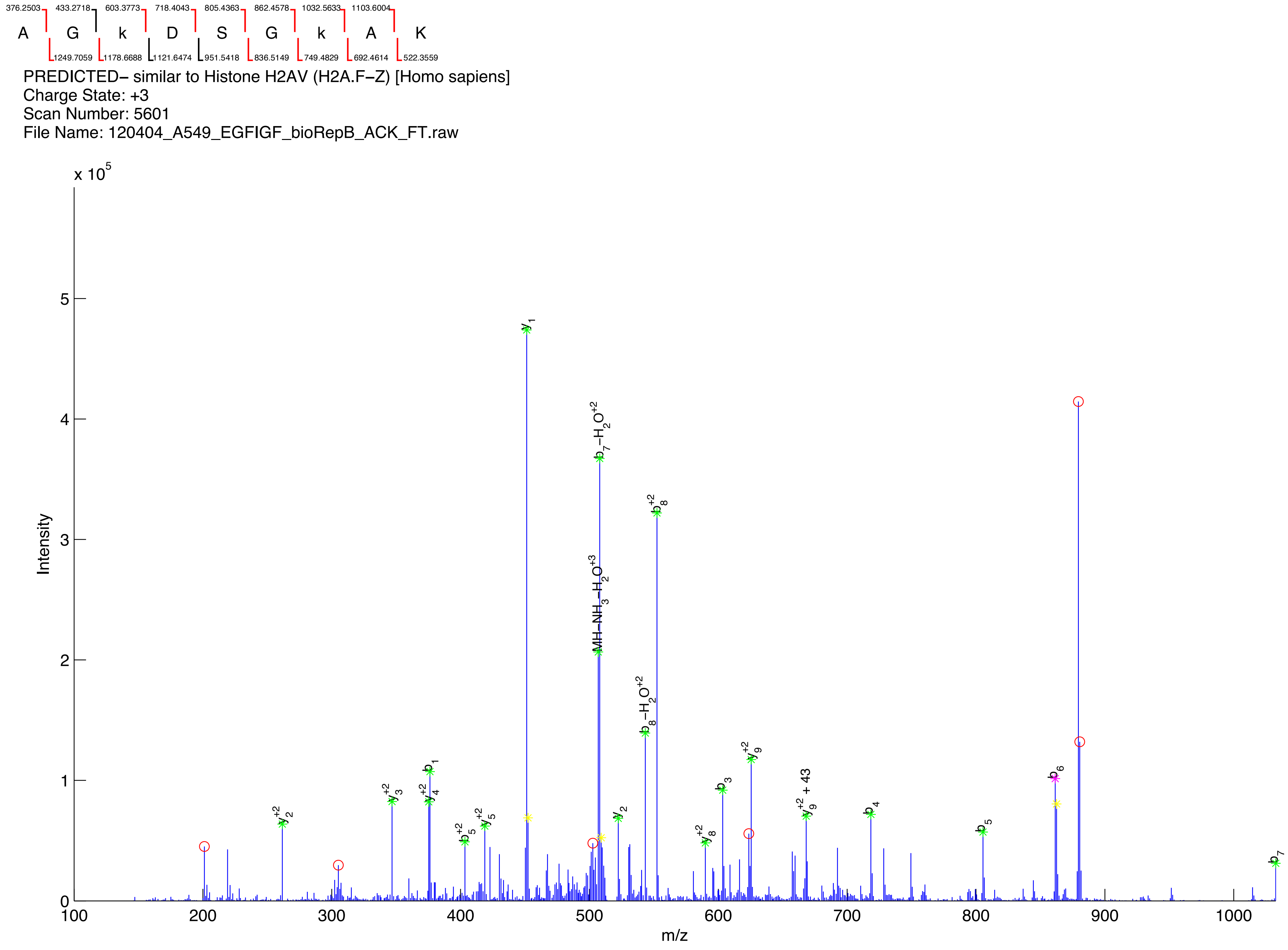
376.2503
433.2718
603.3773
718.4043
805.4363
862.4578
1032.5633
1103.6004
A G k D S G k A K
1249.7059
1178.6688
1121.6474
951.5418
836.5149
749.4829
692.4614
522.3559
PREDICTED– similar to Histone H2AV (H2A.F–Z) [Homo sapiens]
Charge State: +3
Scan Number: 5601
File Name: 120404_A549_EGFIGF_bioRepB_ACK_FT.raw
x 10^5
Intensity
m/z
y1
b7–H2O+2
b8+2
MH–NH3–H2O+3
b8–H2O+2
y9+2
b3
y9+2 + 43
b4
b5
b6
b7
y2+2
y3+2
y4+2
b1
b5+2
y5+2
y2
y8+2

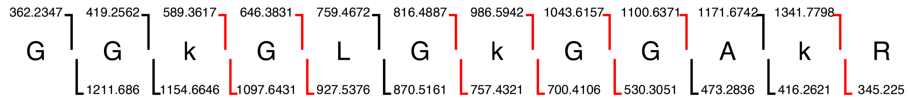

histone cluster 1, H4a [Homo sapiens]
Charge State: +3
Scan Number: 5608
File Name: 120413_A549_EGFIGF_bioRepC_AcK_FT.raw

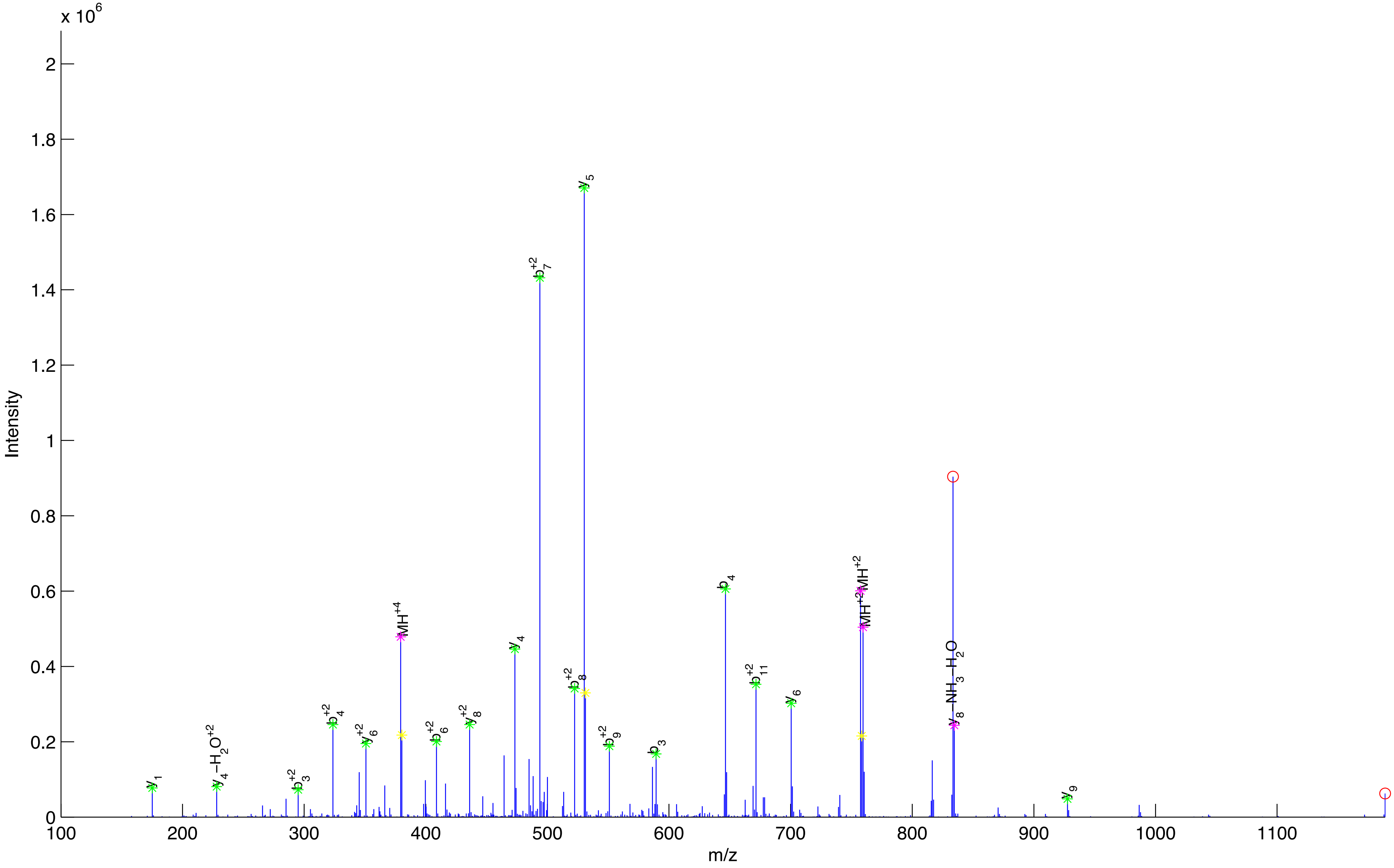

362.2347 | 532.3402 | 589.3617 | 646.3831 | 717.4203

G k G G A K

863.5258 | 806.5043 | 636.3988 | 579.3773 | 522.3559

SWI–SNF–related matrix–associated actin–dependent regulator of chromatin a2 isoform b [Homo sapiens

Charge State: +

Scan Number: 5645

File Name: 120404_A549_EGFIGF_bioRepB_ACK_FT.raw

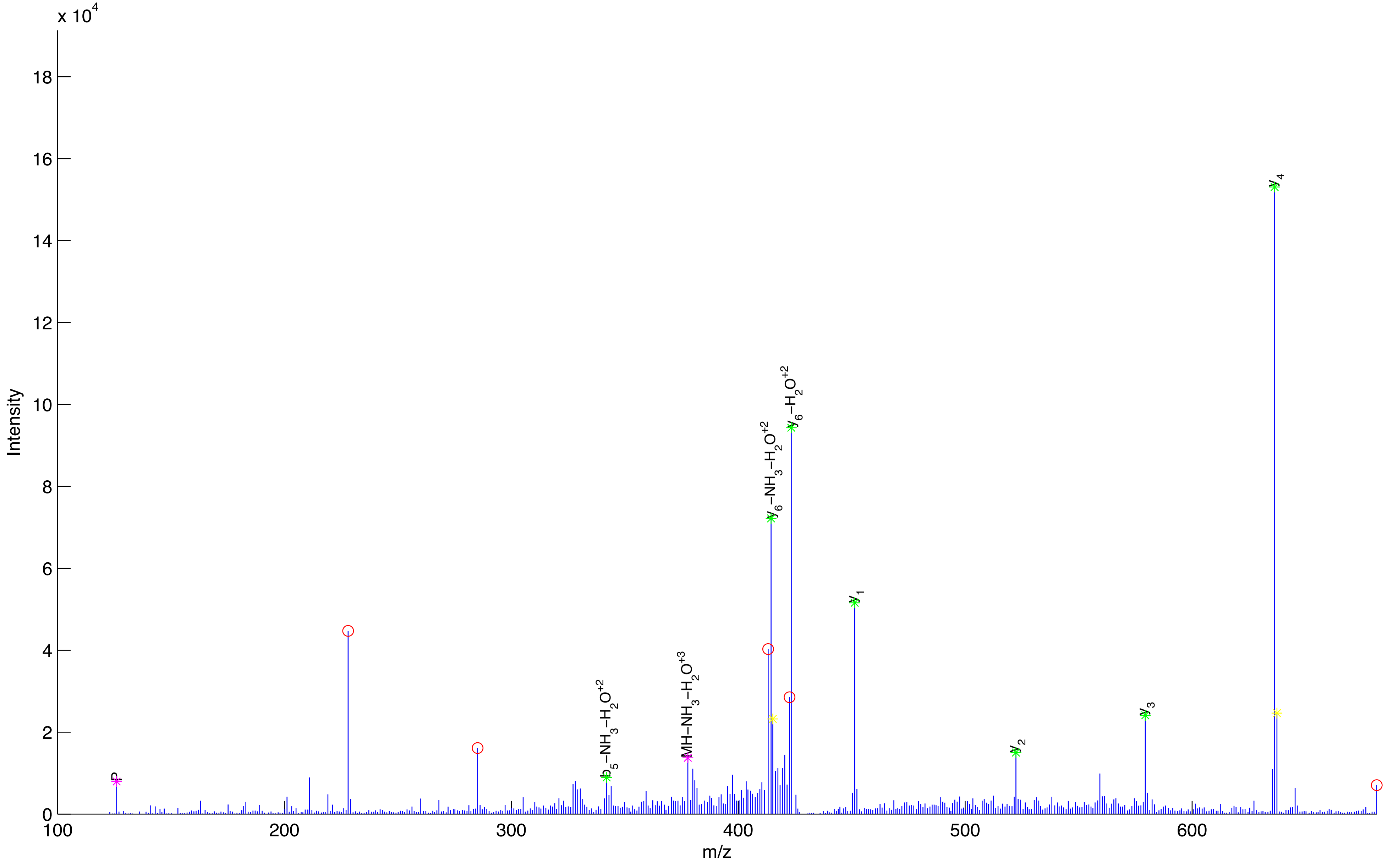

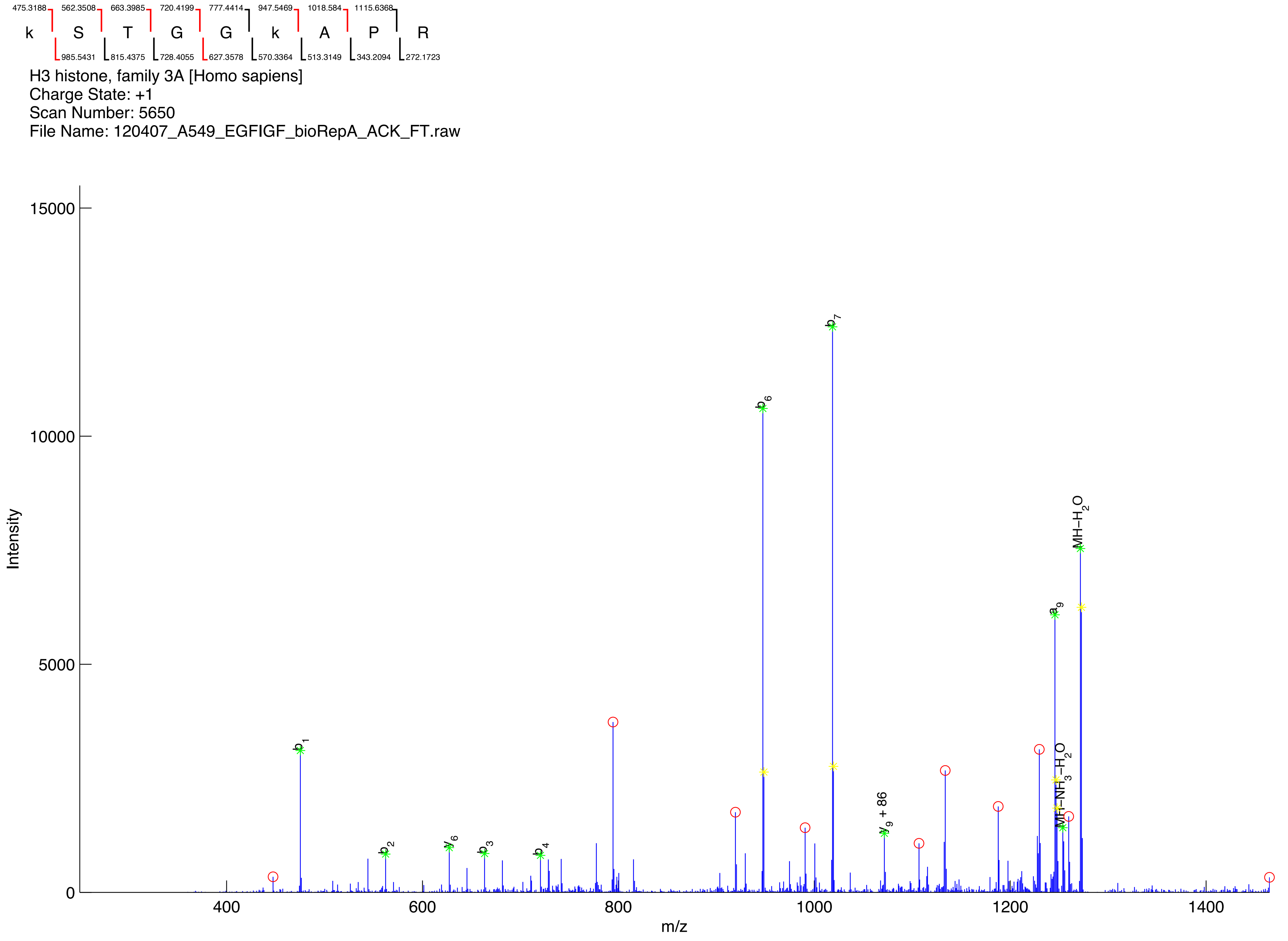
475.3188 562.3508 663.3985 720.4199 777.4414 947.5469 1018.584 1115.6368
k S T G G k A P R
985.5431 815.4375 728.4055 627.3578 570.3364 513.3149 343.2094 272.1723
H3 histone, family 3A [Homo sapiens]
Charge State: +1
Scan Number: 5650
File Name: 120407_A549_EGFIGF_bioRepA_ACK_FT.raw
Intensity
m/z
b1
b2
y6
b3
b4
b6
b7
y9 + 86
a9
MH−NH3−H2O
MH−H2O
0
5000
10000
15000
400
600
800
1000
1200
1400

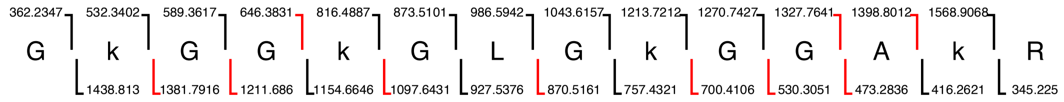

histone cluster 1, H4a [Homo sapiens]
Charge State: +
Scan Number: 5755
File Name: 120413_A549_EGFIGF_bioRepC_AcK_FT.raw

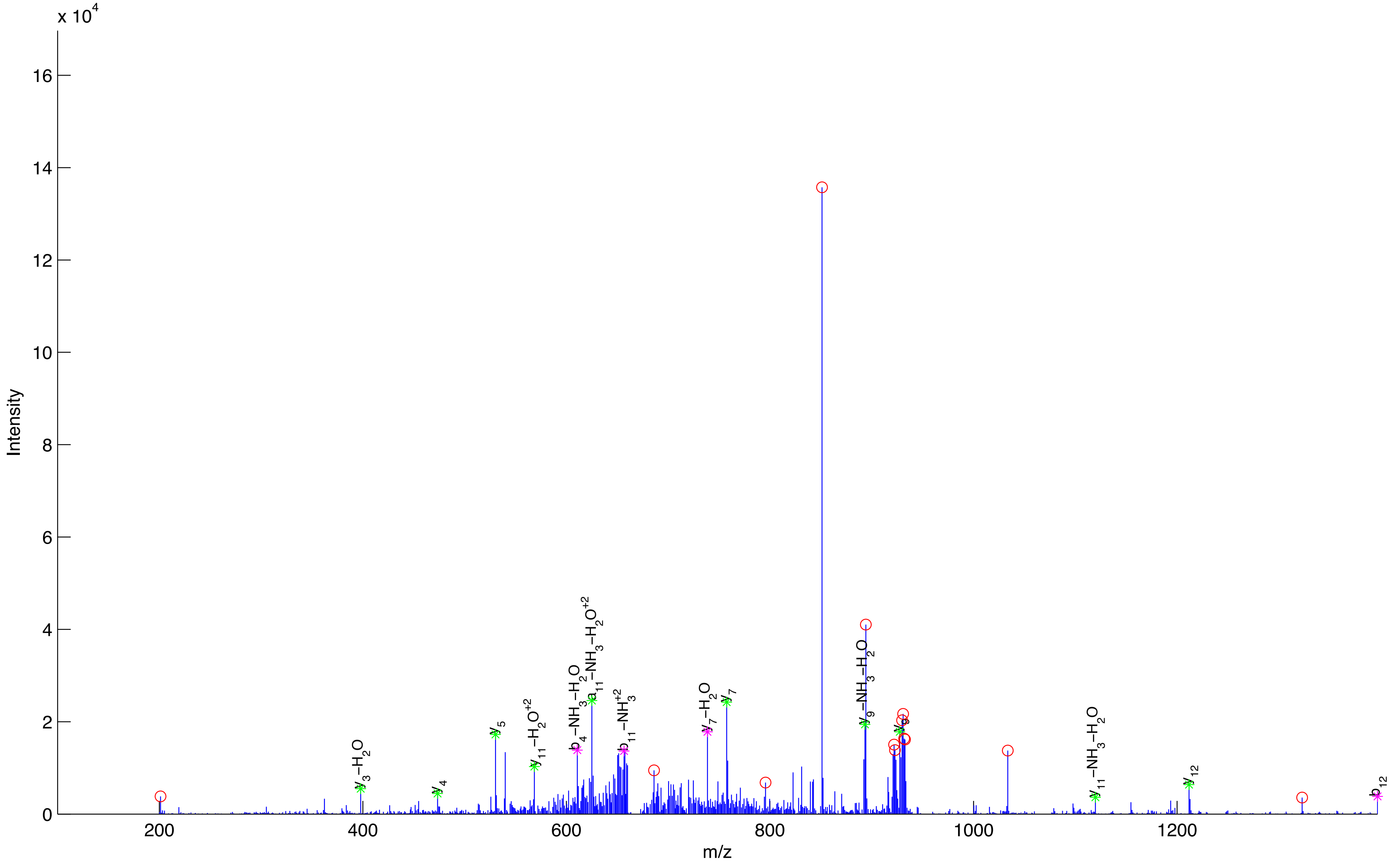

362.2347		475.3188		532.3402		702.4457		759.4672		816.4887		887.5258		1057.6313		
G		L		G		k		G		G		A		k		R
	927.5376		870.5161		757.4321		700.4106		530.3051		473.2836		416.2621		345.225	

histone cluster 1, H4a [Homo sapiens]
Charge State: +2
Scan Number: 5860
File Name: 120413_A549_EGFIGF_bioRepC_AcK_FT.raw

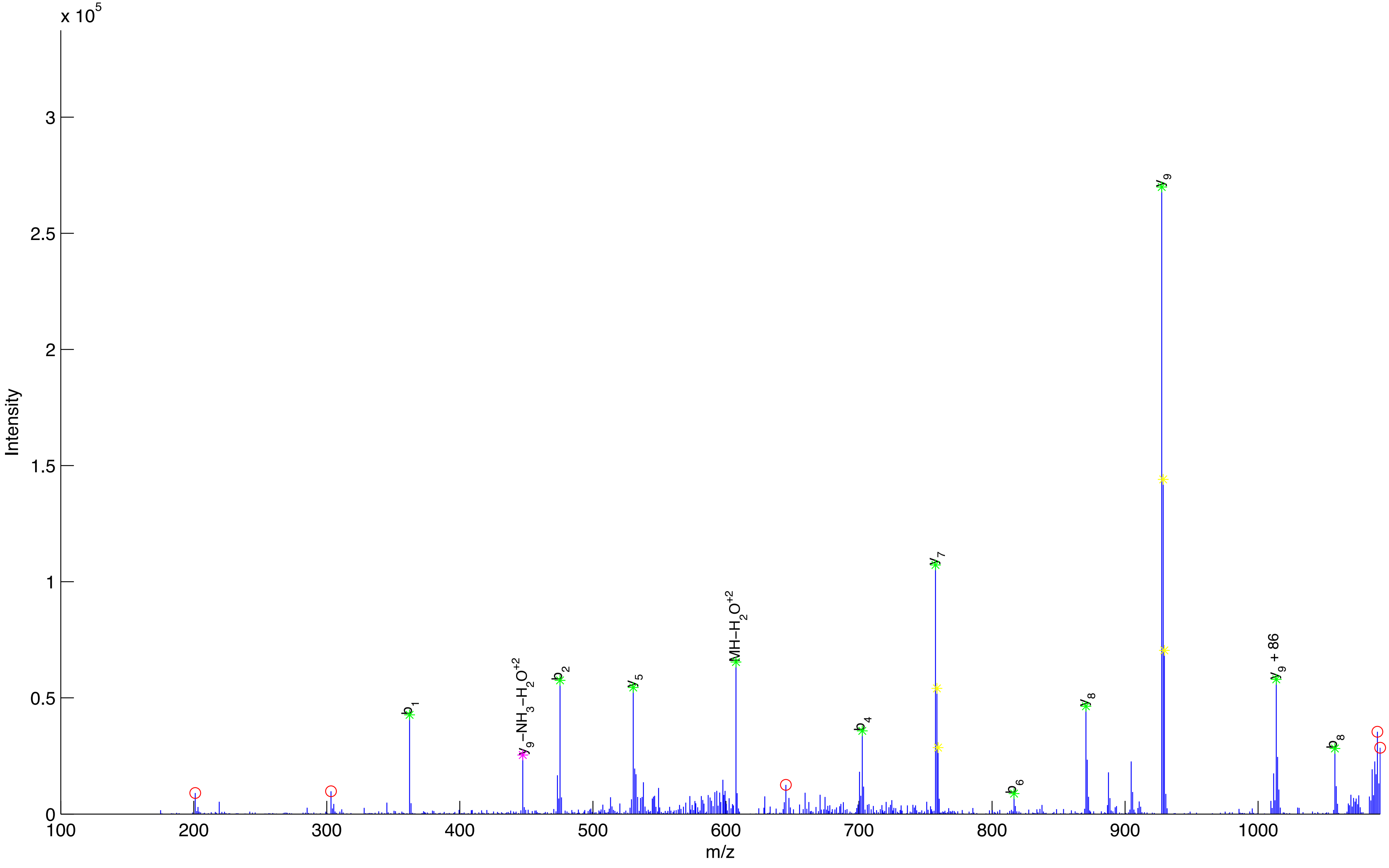

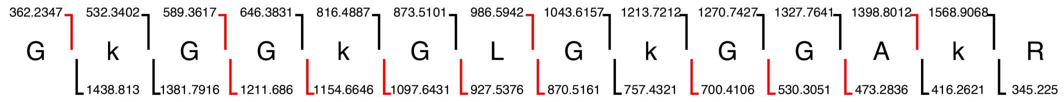

histone cluster 1, H4a [Homo sapiens]
Charge State: +
Scan Number: 5894
File Name: 120407_A549_EGFIGF_bioRepA_ACK_FT.raw

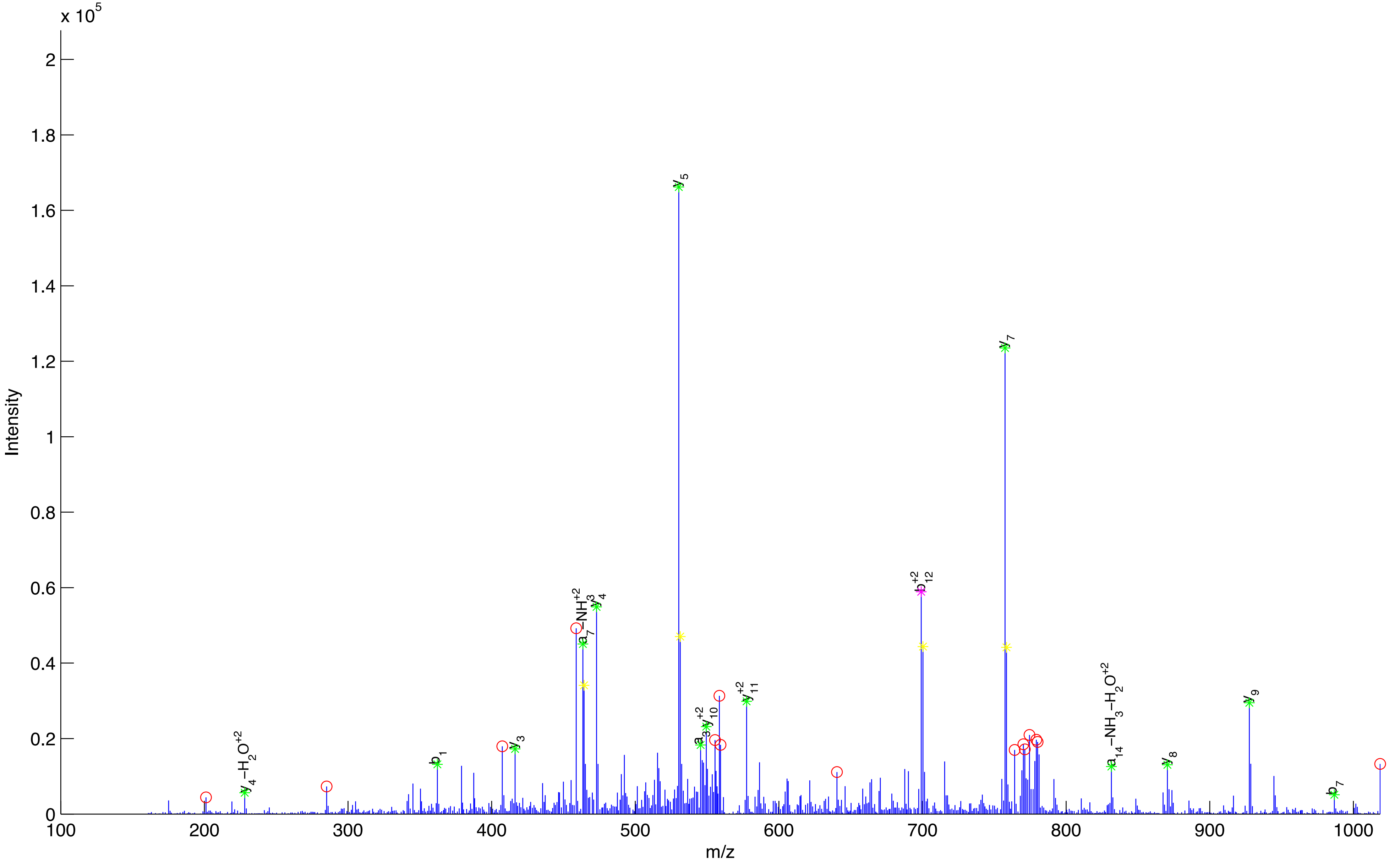

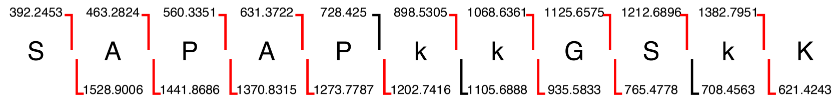

histone cluster 1, H2bb [Homo sapiens]
Charge State: +2
Scan Number: 5944
File Name: 120413_A549_EGFIGF_bioRepC_AcK_FT.raw

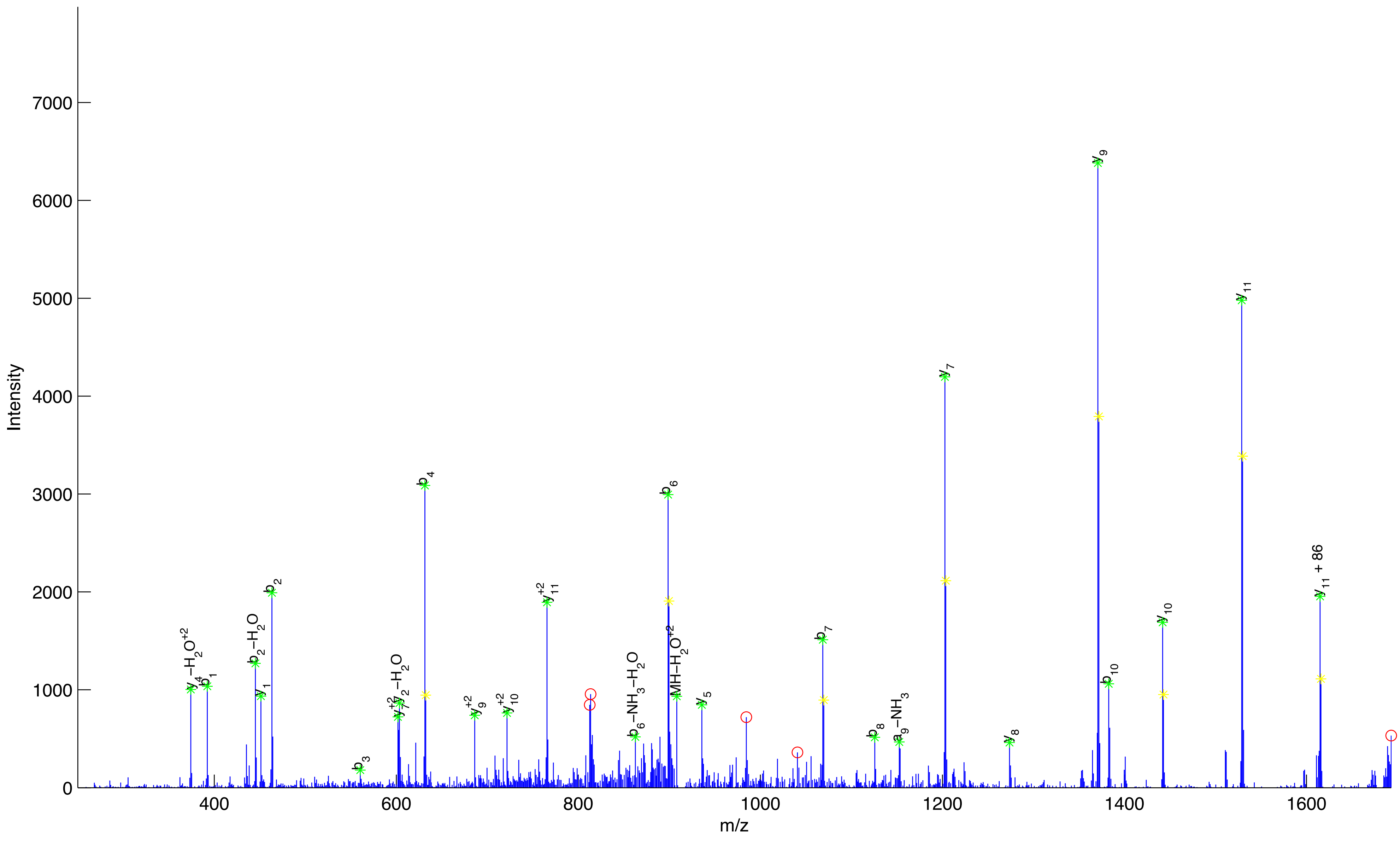

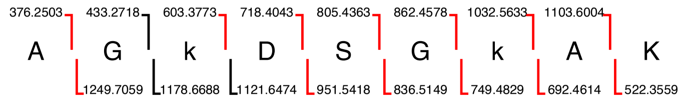

PREDICTED– similar to Histone H2AV (H2A.F–Z) [Homo sapiens]
Charge State: +3
Scan Number: 5955
File Name: 120407_A549_EGFIGF_bioRepA_ACK_FT.raw

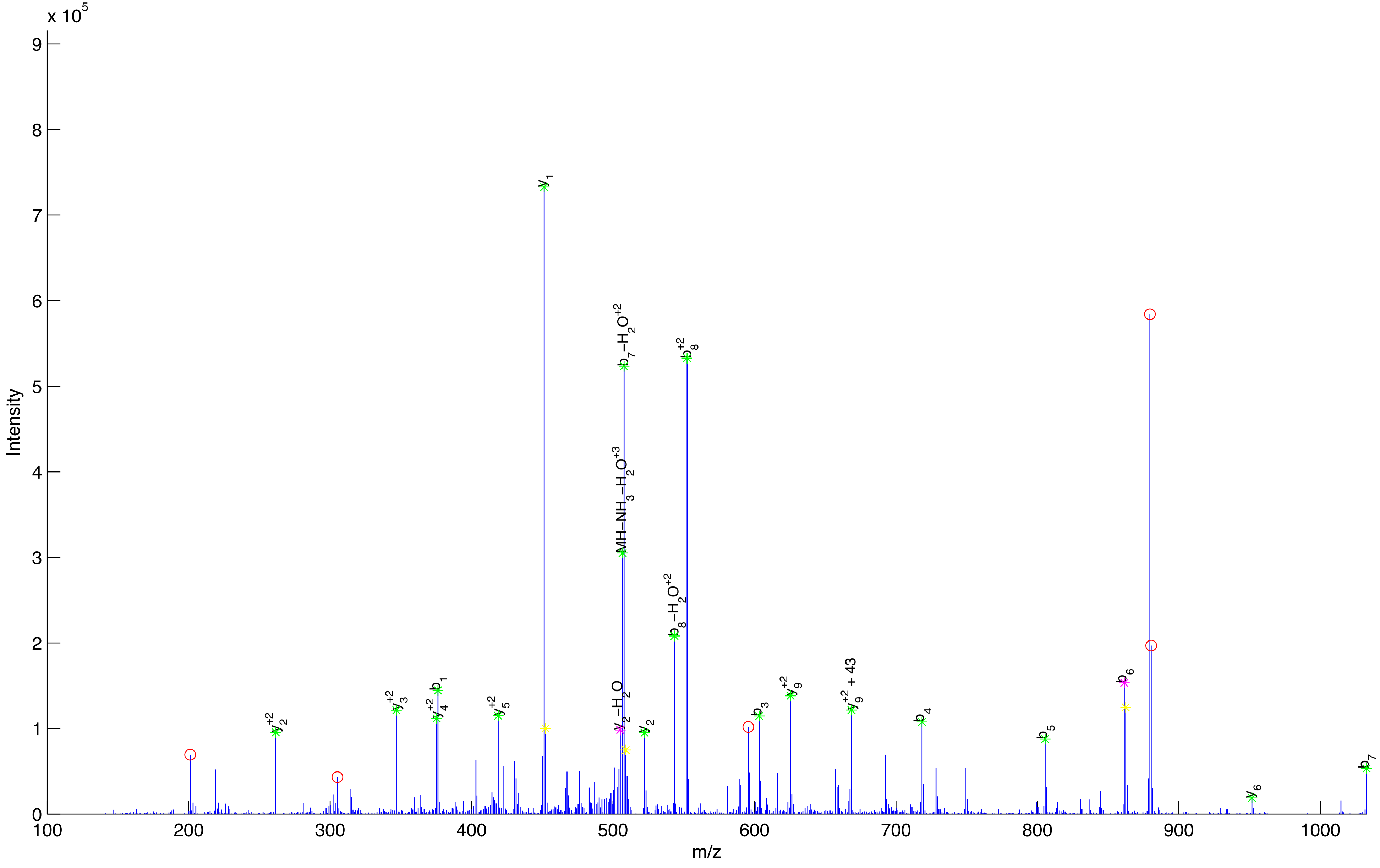

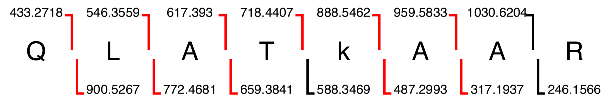

H3 histone, family 3A [Homo sapiens]
Charge State: +3
Scan Number: 6030
File Name: 120413_A549_EGFIGF_bioRepC_AcK_FT.raw

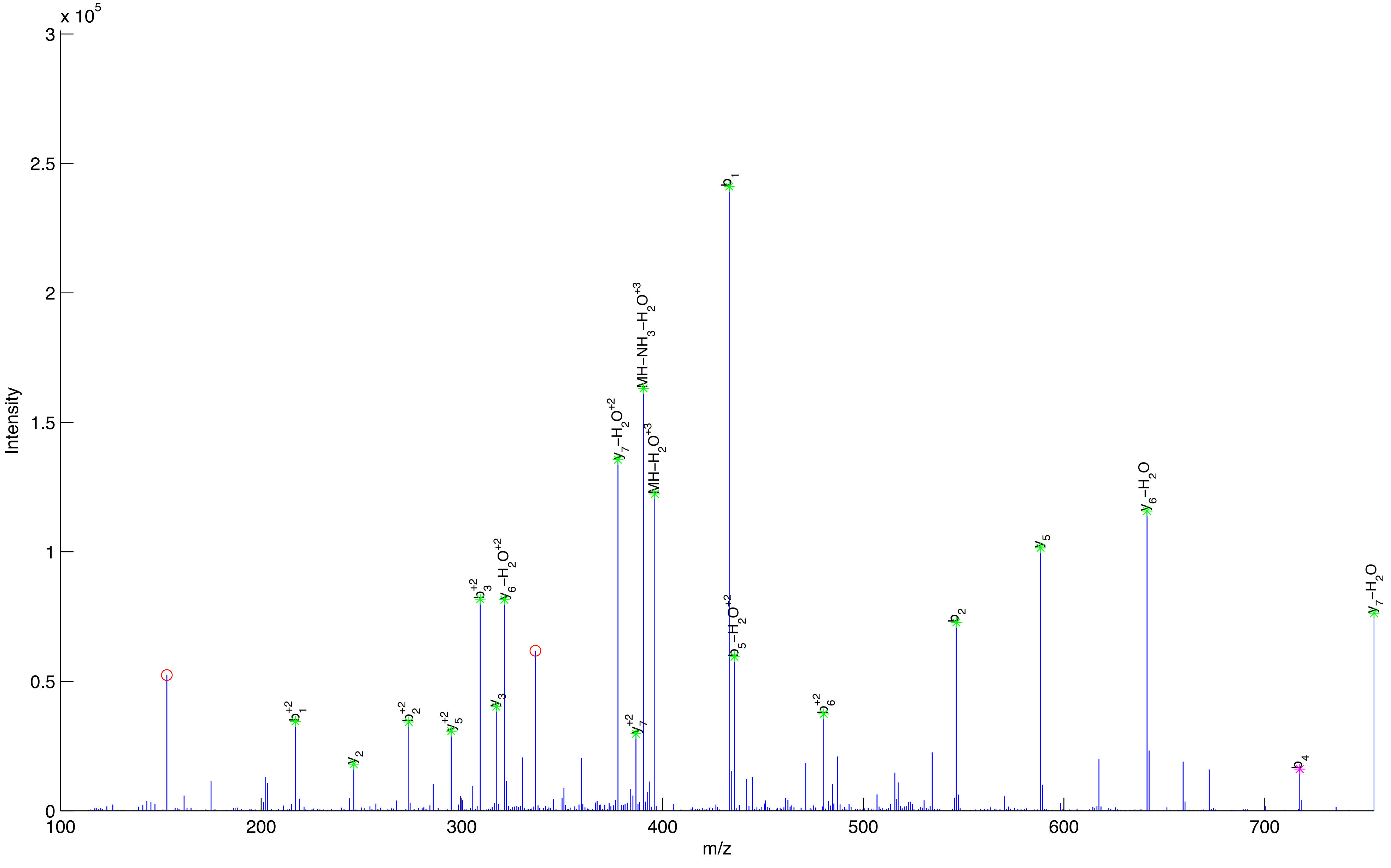

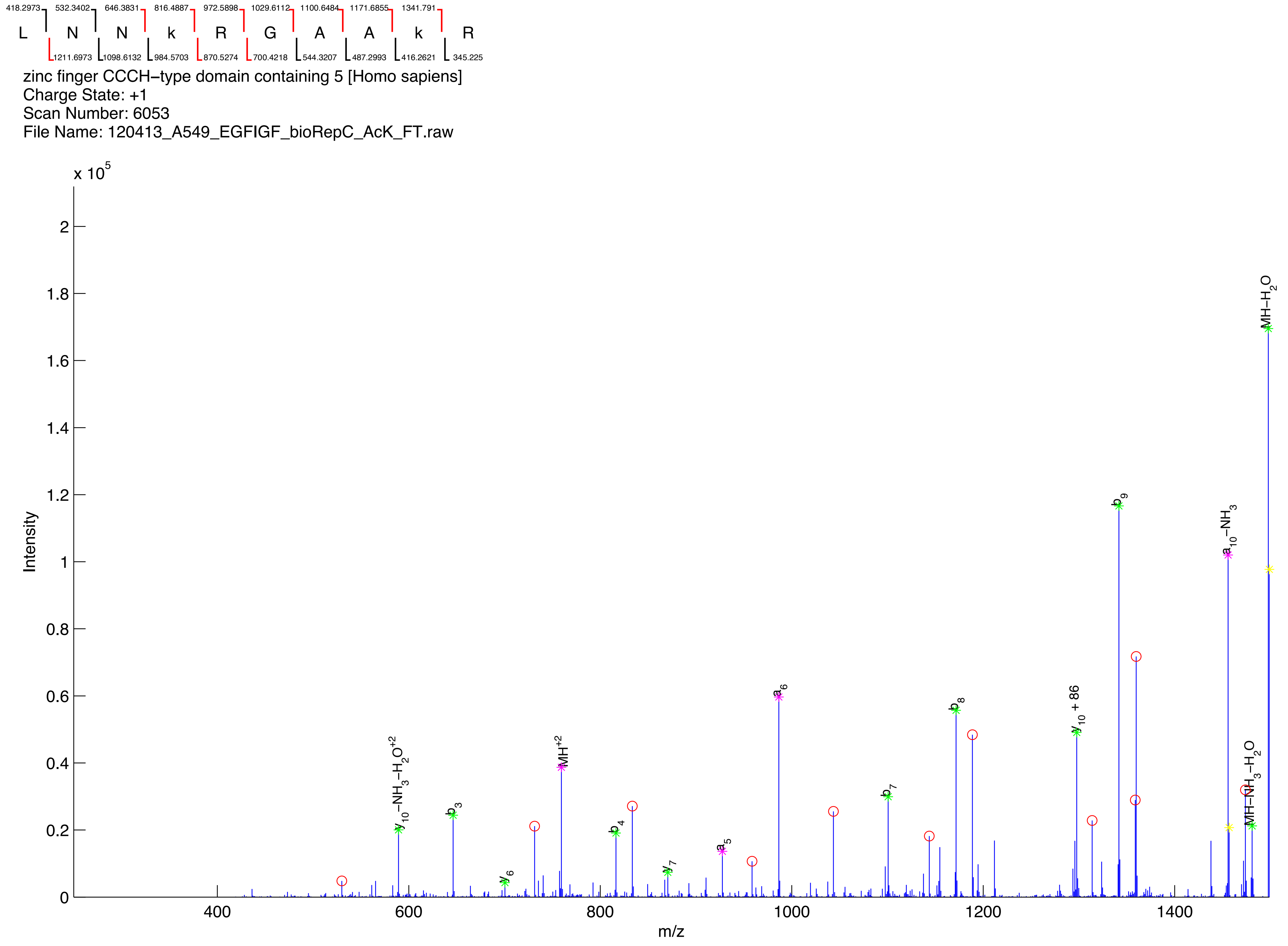
418.2973
532.3402
646.3831
816.4887
972.5898
1029.6112
1100.6484
1171.6855
1341.791
L N N k R G A A k R
1211.6973
1098.6132
984.5703
870.5274
700.4218
544.3207
487.2993
416.2621
345.225
zinc finger CCCH–type domain containing 5 [Homo sapiens]
Charge State: +1
Scan Number: 6053
File Name: 120413_A549_EGFIGF_bioRepC_AcK_FT.raw
x 10^5
Intensity
m/z
y10–NH3–H2O+2
b3
y6
MH+2
b4
y7
a5
a6
b7
b8
y10 + 86
b9
a10–NH3
MH–NH3–H2O
MH–H2O

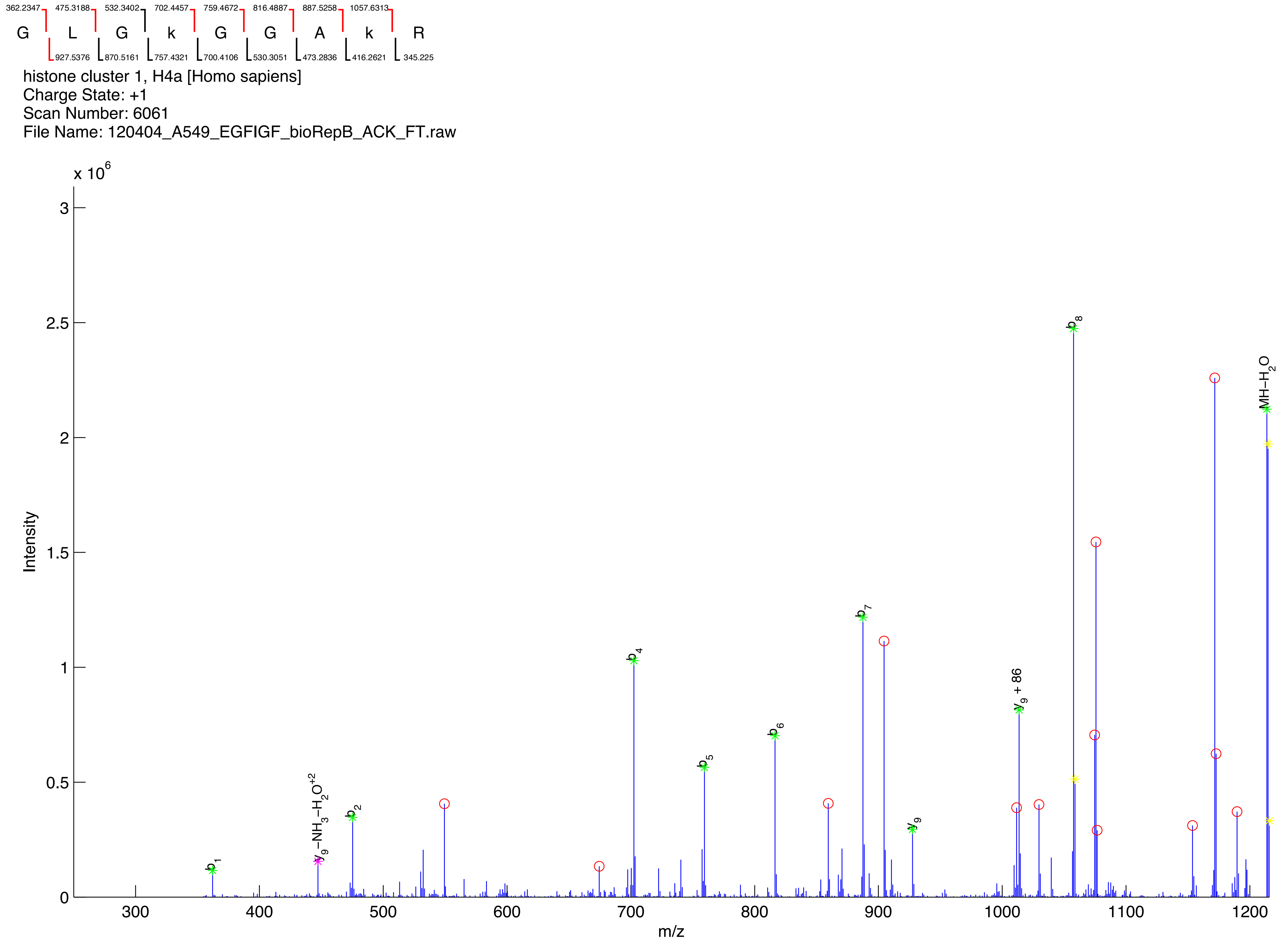

362.2347 475.3188 532.3402 702.4457 759.4672 816.4887 887.5258 1057.6313
G L G k G G A k R
927.5376 870.5161 757.4321 700.4106 530.3051 473.2836 416.2621 345.225
histone cluster 1, H4a [Homo sapiens]
Charge State: +1
Scan Number: 6061
File Name: 120404_A549_EGFIGF_bioRepB_ACK_FT.raw
x 10^6
Intensity
m/z
b1
y9−NH3−H2O+2
b2
b4
b5
b6
b7
y9
y9 + 86
b8
MH−H2O

histone cluster 1, H4a [Homo sapiens]
Charge State: +
Scan Number: 6084
File Name: 120404_A549_EGFIGF_bioRepB_ACK_FT.raw

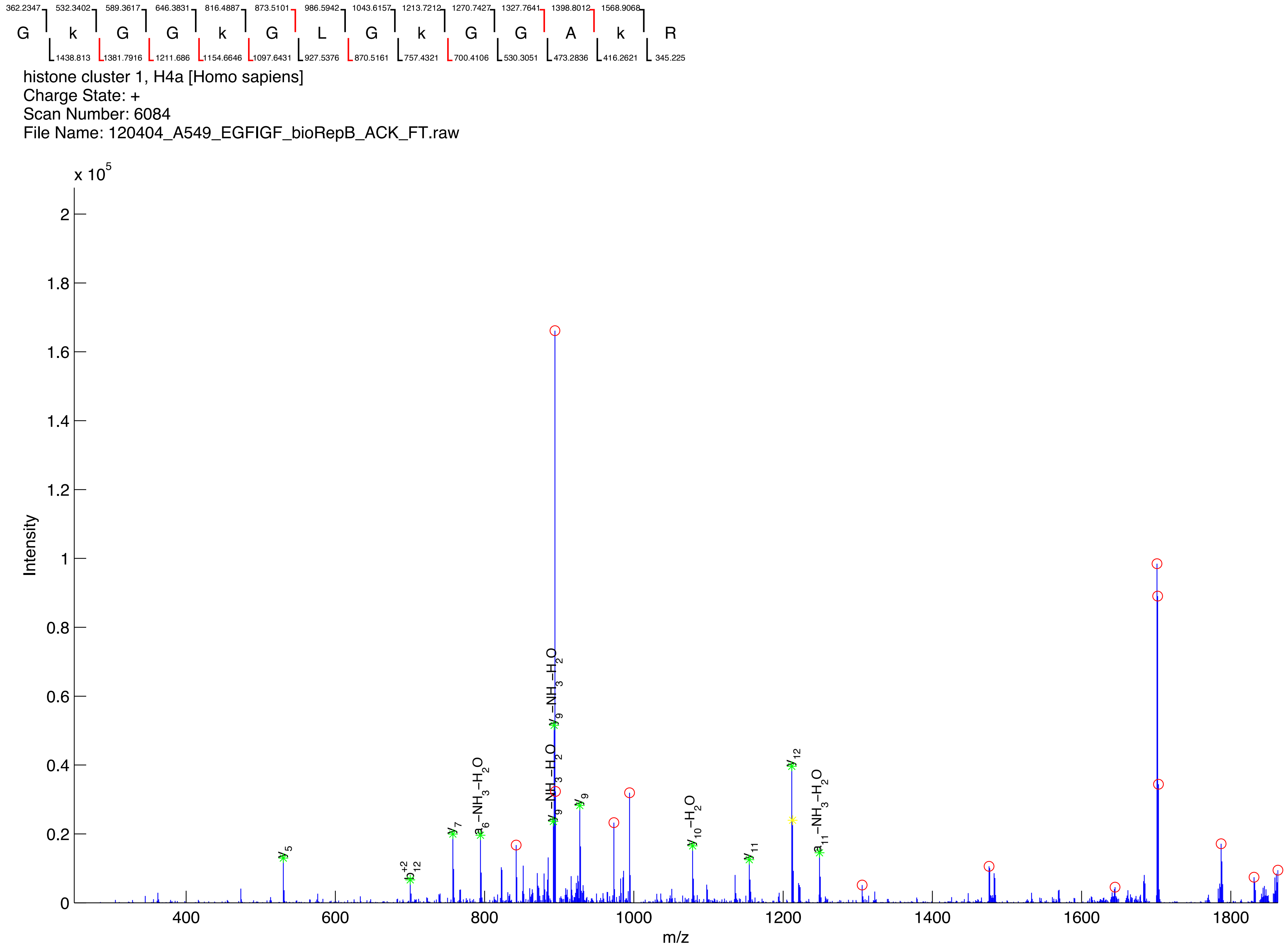

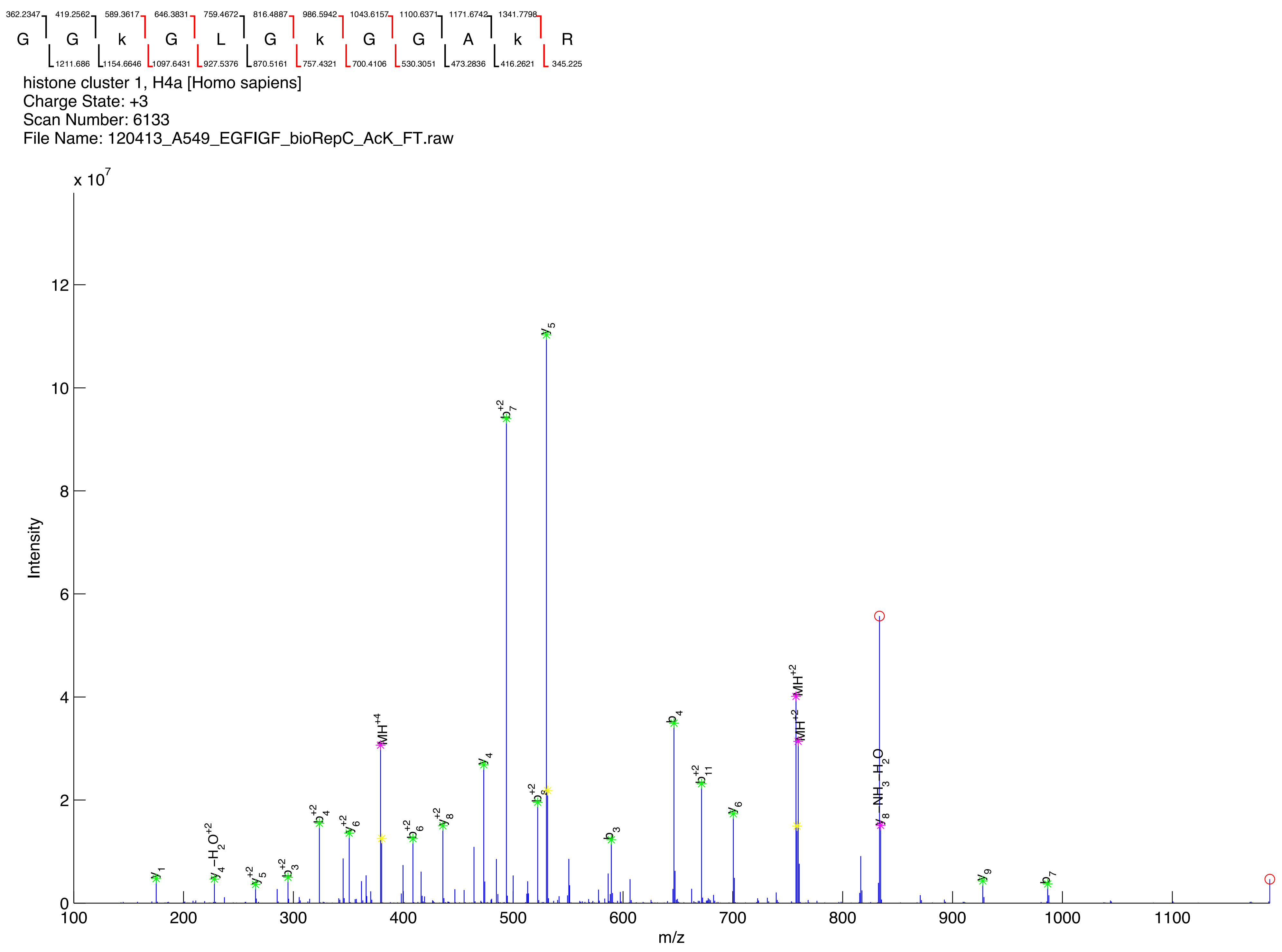

362.2347 419.2562 589.3617 646.3831 759.4672 816.4887 986.5942 1043.6157 1100.6371 1171.6742 1341.7798
G G k G L G k G G A k R
1211.686 1154.6646 1097.6431 927.5376 870.5161 757.4321 700.4106 530.3051 473.2836 416.2621 345.225
histone cluster 1, H4a [Homo sapiens]
Charge State: +3
Scan Number: 6133
File Name: 120413_A549_EGFIGF_bioRepC_AcK_FT.raw
x 10^7
Intensity
m/z
0
2
4
6
8
10
12
100
200
300
400
500
600
700
800
900
1000
1100
y1
y4-H2O+2
y+2 5
b+2 3
b+2 4
y+2 6
MH+4
b+2 6
y+2 8
y4
b+2 7
b+2 8
y5
b3
b4
b+2 11
y6
MH+2
MH+2
y8-NH3-H2O
y9
b7

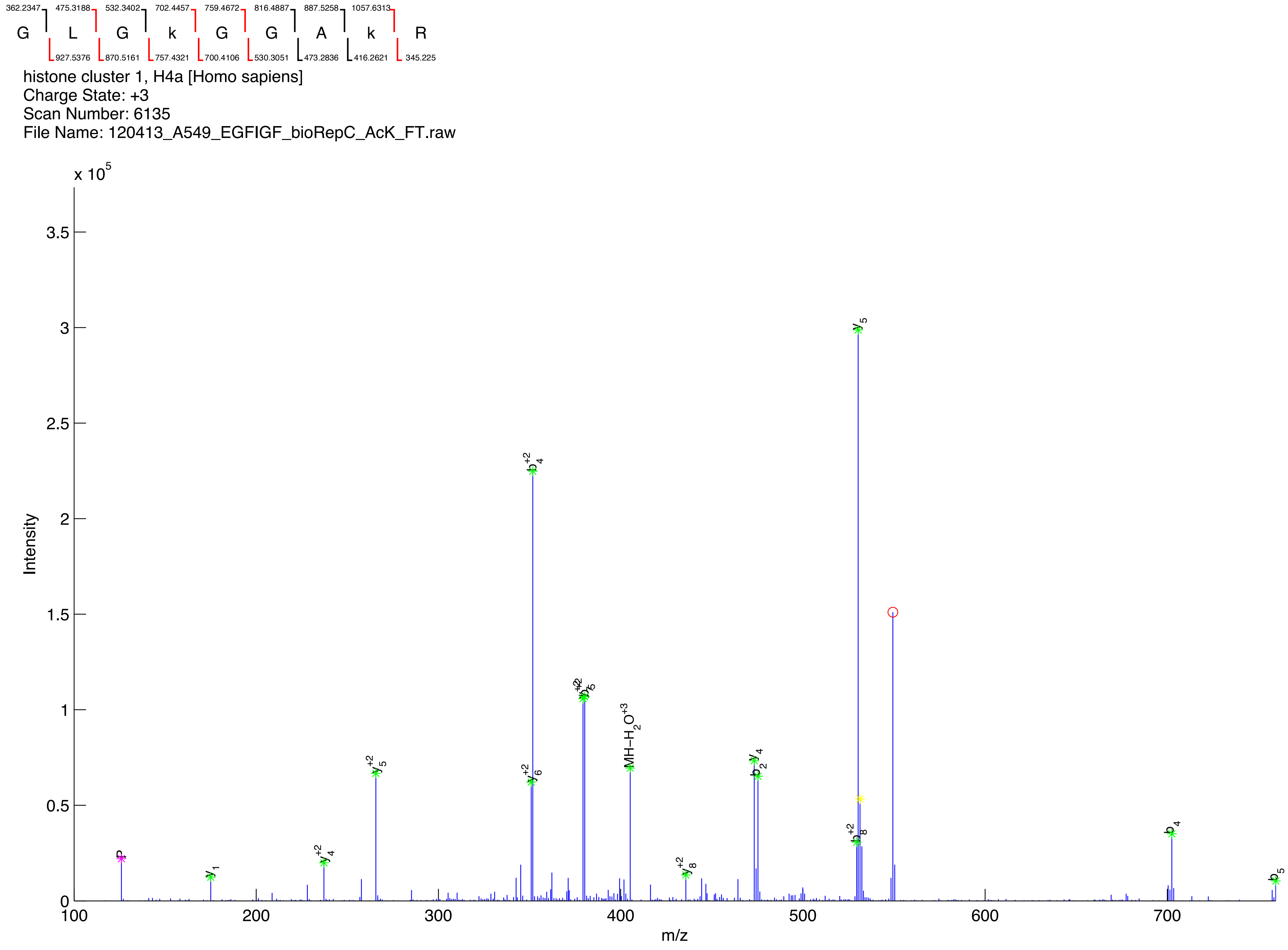
362.2347 475.3188 532.3402 702.4457 759.4672 816.4887 887.5258 1057.6313
G L G k G G A k R
927.5376 870.5161 757.4321 700.4106 530.3051 473.2836 416.2621 345.225
histone cluster 1, H4a [Homo sapiens]
Charge State: +3
Scan Number: 6135
File Name: 120413_A549_EGFIGF_bioRepC_AcK_FT.raw
x 10^5
Intensity
m/z
0
0.5
1
1.5
2
2.5
3
3.5
100
200
300
400
500
600
700
P
y1
y+2 4
y+2 5
y+2 6
b+2 4
b+2 5
MH−H2O+3
y+2 8
y4
b2
b+2 8
y5
b4
b5

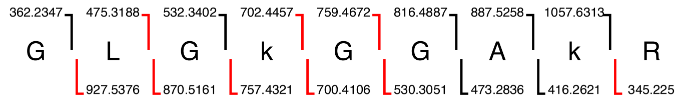

histone cluster 1, H4a [Homo sapiens]
Charge State: +3
Scan Number: 6250
File Name: 120404_A549_EGFIGF_bioRepB_ACK_FT.raw

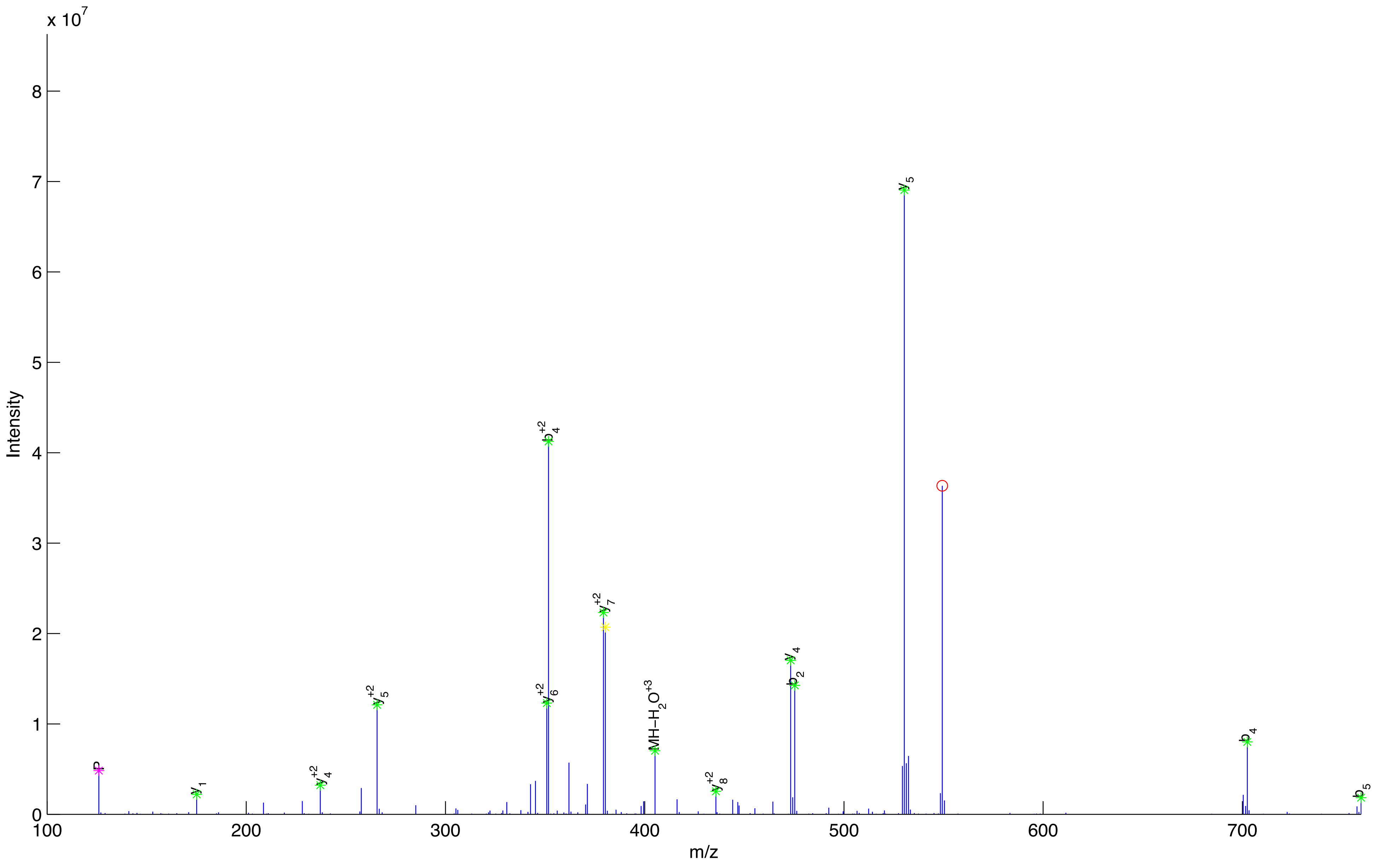

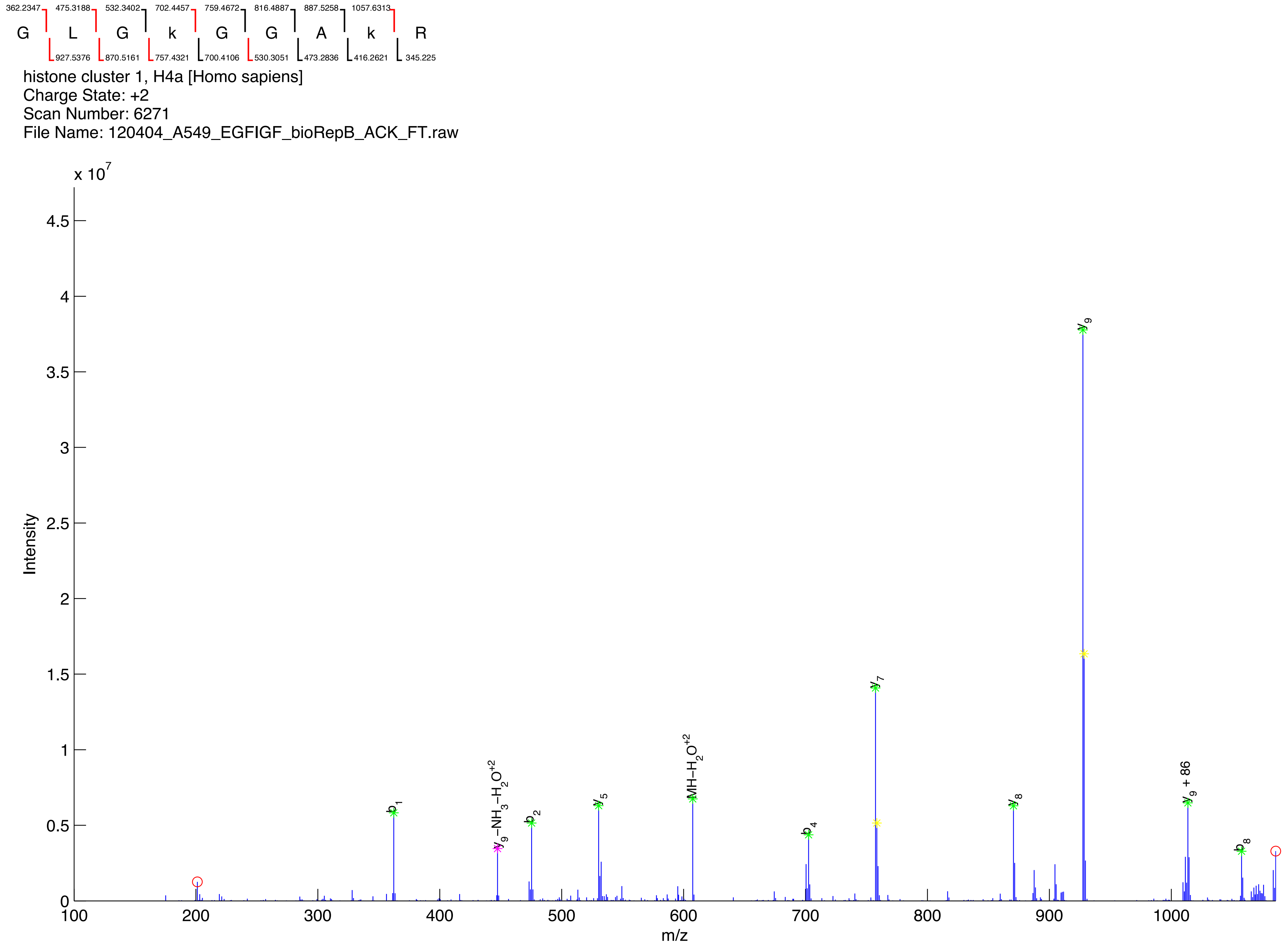
362.2347
475.3188
532.3402
702.4457
759.4672
816.4887
887.5258
1057.6313
G L G k G G A k R
927.5376
870.5161
757.4321
700.4106
530.3051
473.2836
416.2621
345.225
histone cluster 1, H4a [Homo sapiens]
Charge State: +2
Scan Number: 6271
File Name: 120404_A549_EGFIGF_bioRepB_ACK_FT.raw
x 10^7
Intensity
m/z
b1
y9−NH3−H2O+2
b2
y5
MH−H2O+2
b4
y7
y8
y9
y9 + 86
b8

376.2503	433.2718	490.2933	660.3988	731.4359	788.4574	958.5629	1073.5898	1160.6219	1217.6433	1387.7489	1458.786	
A	G	G	k	A	G	k	D	S	G	k	A	K
	1604.8915	1533.8544	1476.8329	1419.8115	1249.7059	1178.6688	1121.6474	951.5418	836.5149	749.4829	692.4614	522.3559

H2A histone family, member V isoform 1 [Homo sapiens]
Charge State: +4
Scan Number: 6317
File Name: 120404_A549_EGFIGF_bioRepB_ACK_FT.raw

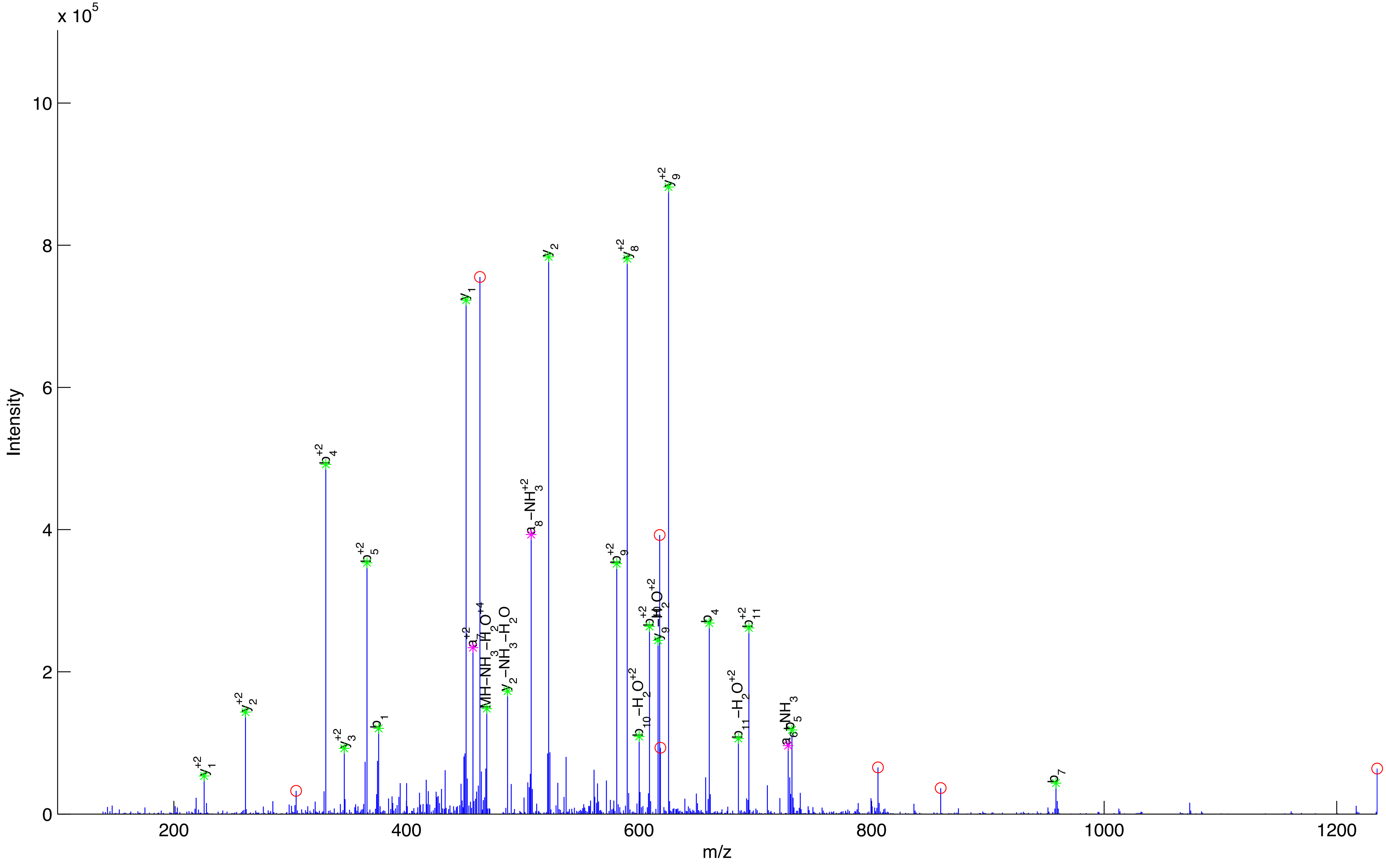

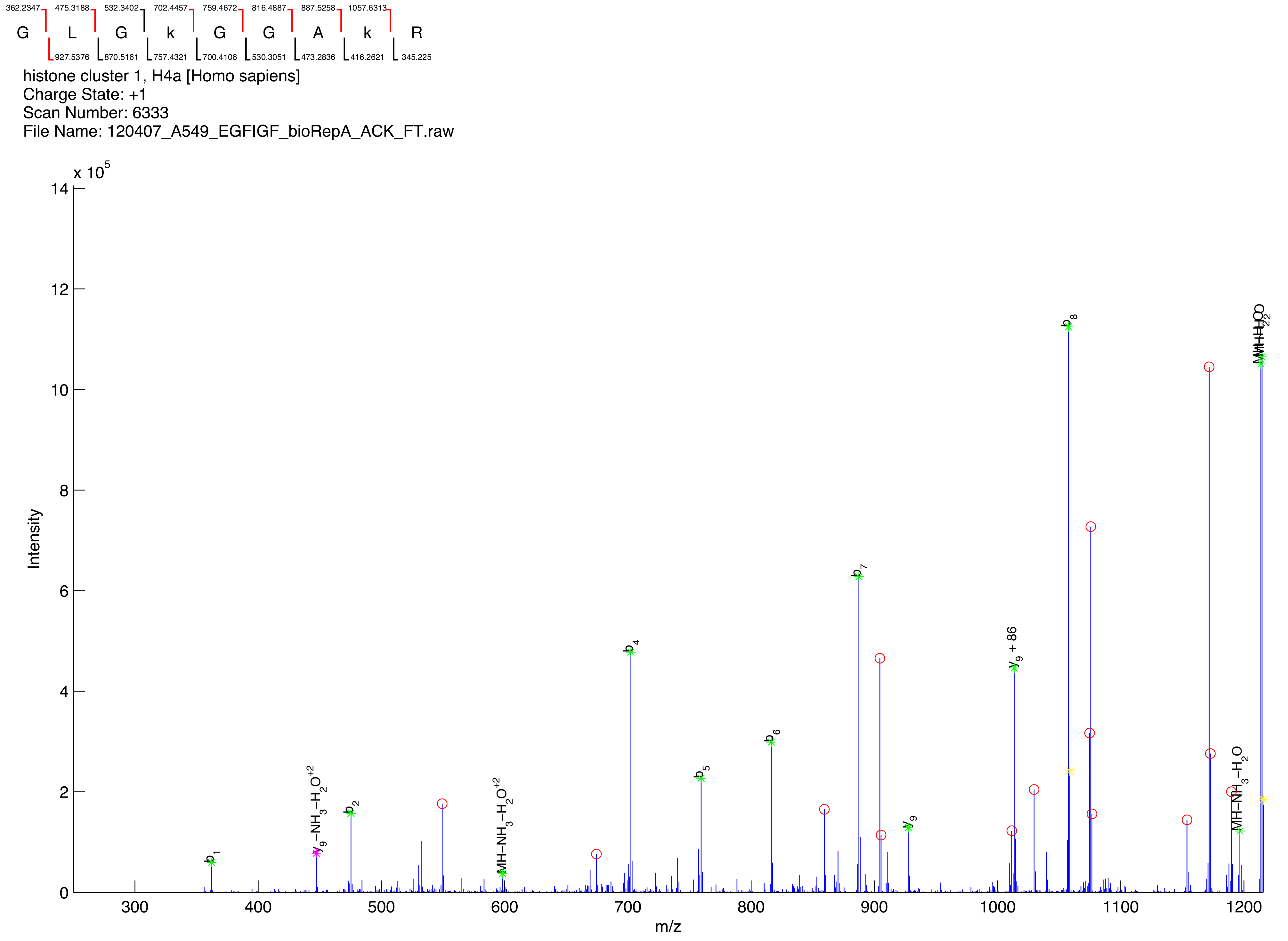
362.2347 475.3188 532.3402 702.4457 759.4672 816.4887 887.5258 1057.6313
G L G k G G A k R
927.5376 870.5161 757.4321 700.4106 530.3051 473.2836 416.2621 345.225
histone cluster 1, H4a [Homo sapiens]
Charge State: +1
Scan Number: 6333
File Name: 120407_A549_EGFIGF_bioRepA_ACK_FT.raw
x 10^5
Intensity
m/z
b1
y9−NH3−H2O+2
b2
MH−NH3−H2O+2
b4
b5
b6
b7
y9
y9 + 86
b8
MH−NH3−H2O

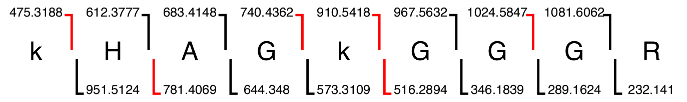

collagen, type XXV, alpha 1 isoform 2 [Homo sapiens]
Charge State: +
Scan Number: 6382
File Name: 120404_A549_EGFIGF_bioRepB_ACK_FT.raw

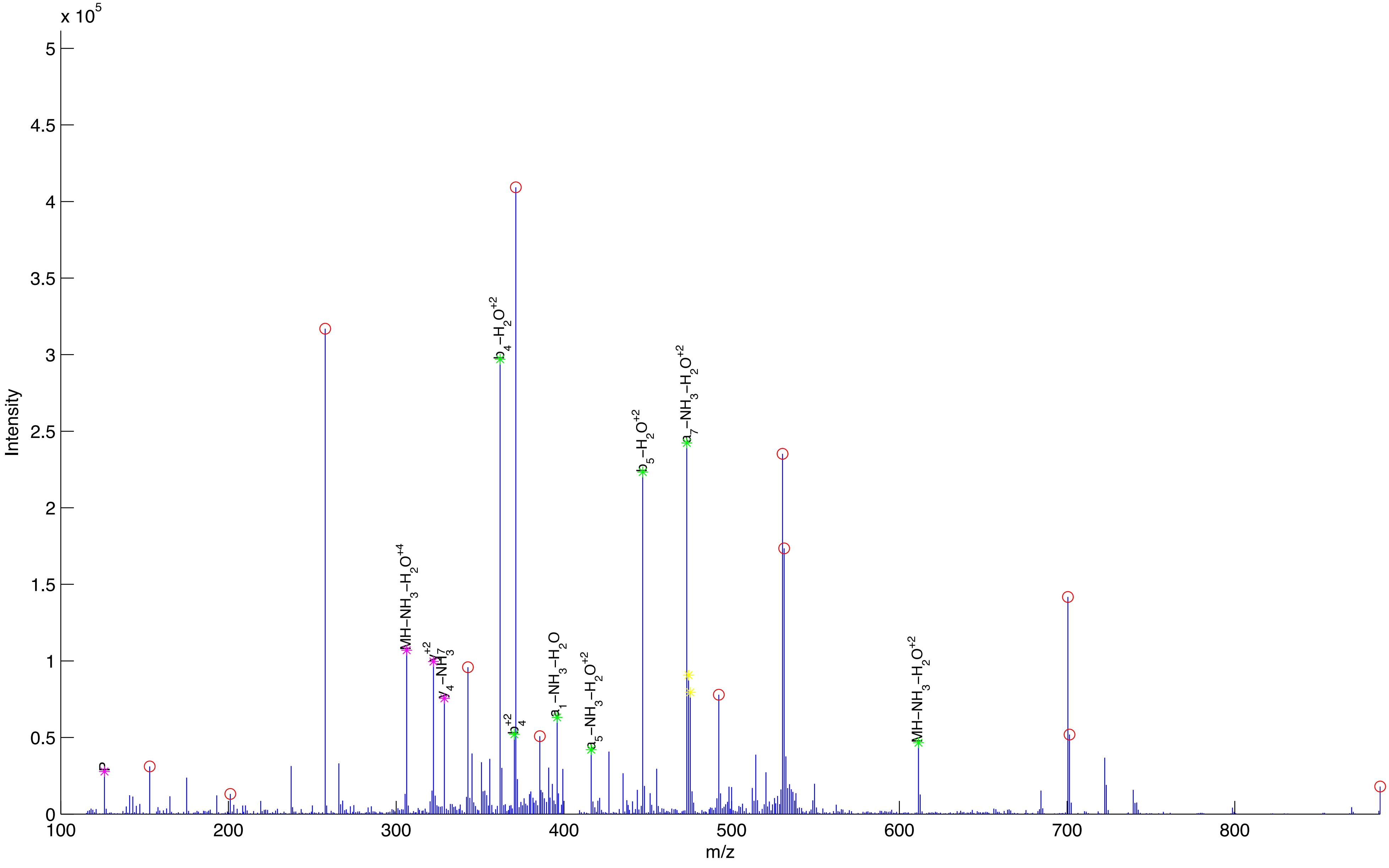

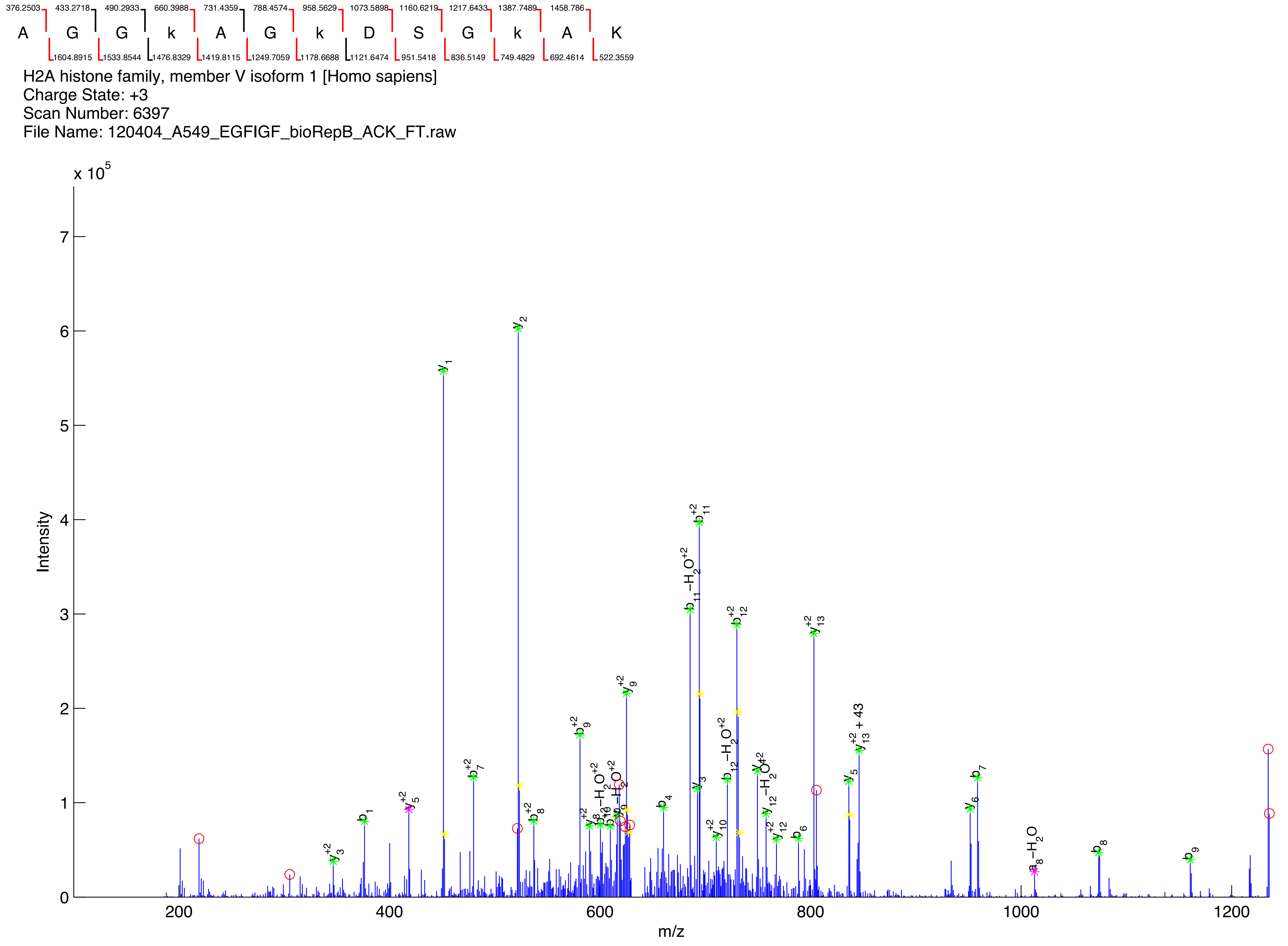

376.2503 433.2718 490.2933 660.3988 731.4359 788.4574 958.5629 1073.5898 1160.6219 1217.6433 1387.7489 1458.786
A G G k A G k D S G k A K
1604.8915 1533.8544 1476.8329 1419.8115 1249.7059 1178.6688 1121.6474 951.5418 836.5149 749.4829 692.4614 522.3559
H2A histone family, member V isoform 1 [Homo sapiens]
Charge State: +3
Scan Number: 6397
File Name: 120404_A549_EGFIGF_bioRepB_ACK_FT.raw
x 10^5
Intensity
m/z

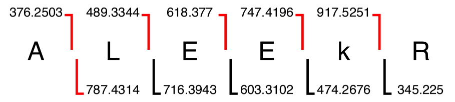

SPT2, Suppressor of Ty, domain containing 1 [Homo sapiens]
Charge State: +1
Scan Number: 6400
File Name: 120407_A549_EGFIGF_bioRepA_ACK_FT.raw

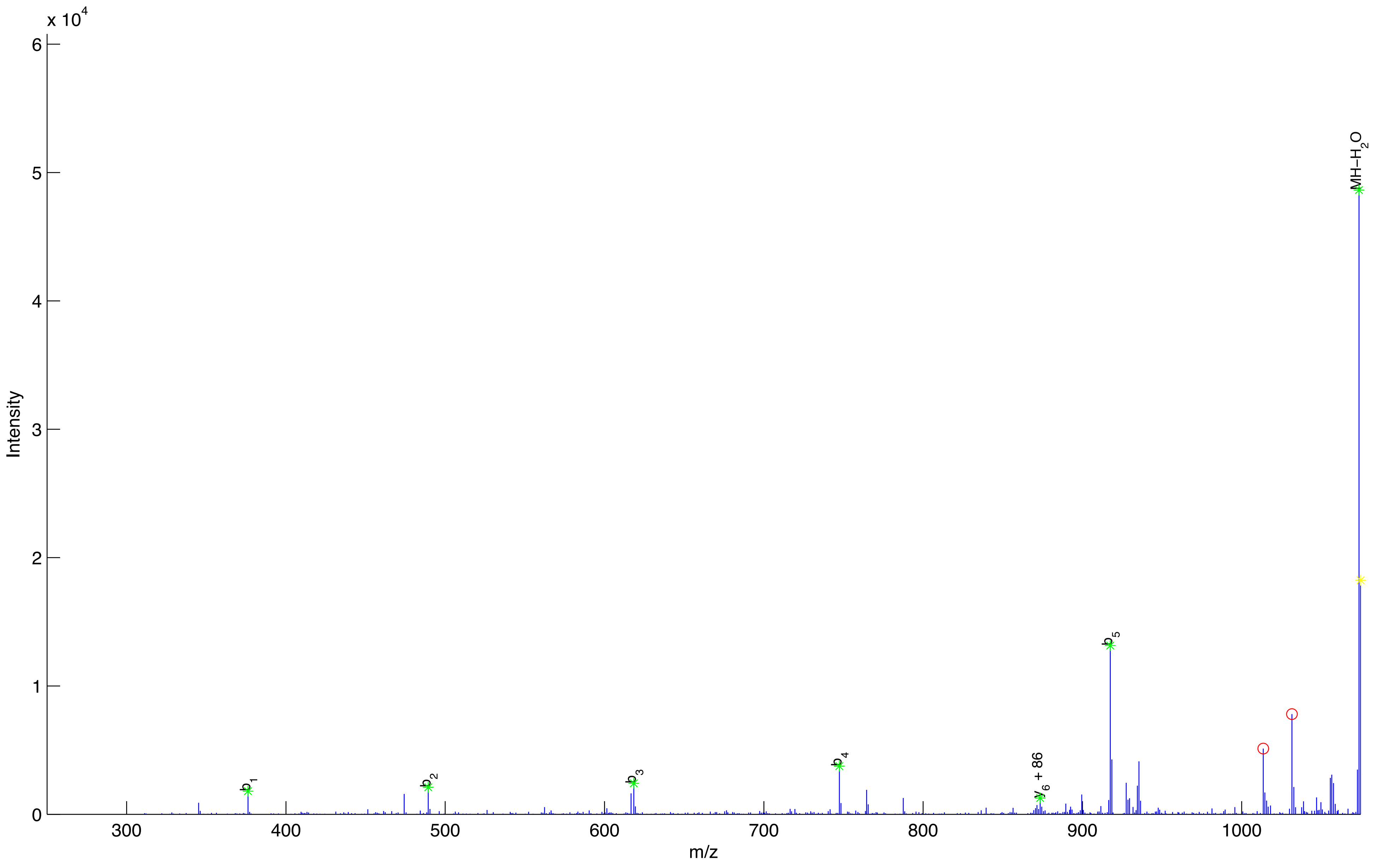

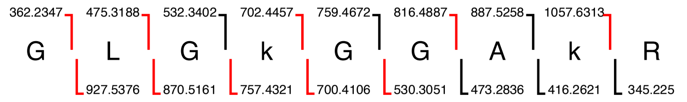

histone cluster 1, H4a [Homo sapiens]
Charge State: +2
Scan Number: 6410
File Name: 120413_A549_EGFIGF_bioRepC_AcK_FT.raw

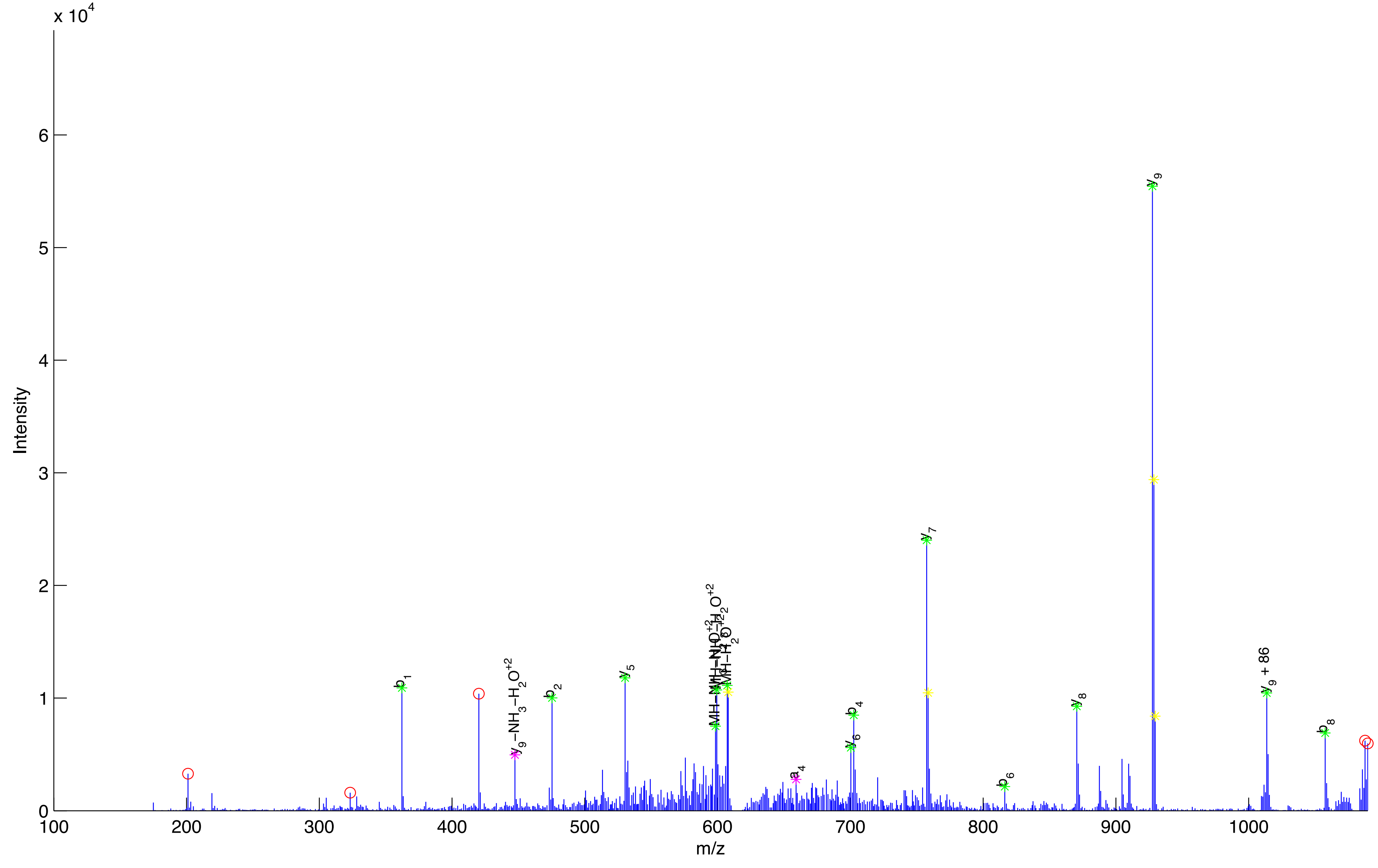

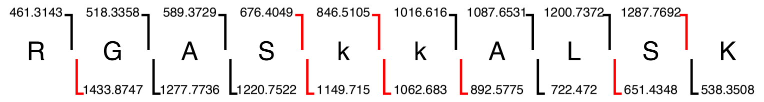

nuclear mitotic apparatus protein 1 [Homo sapiens]
Charge State: +
Scan Number: 6440
File Name: 120407_A549_EGFIGF_bioRepA_ACK_FT.raw

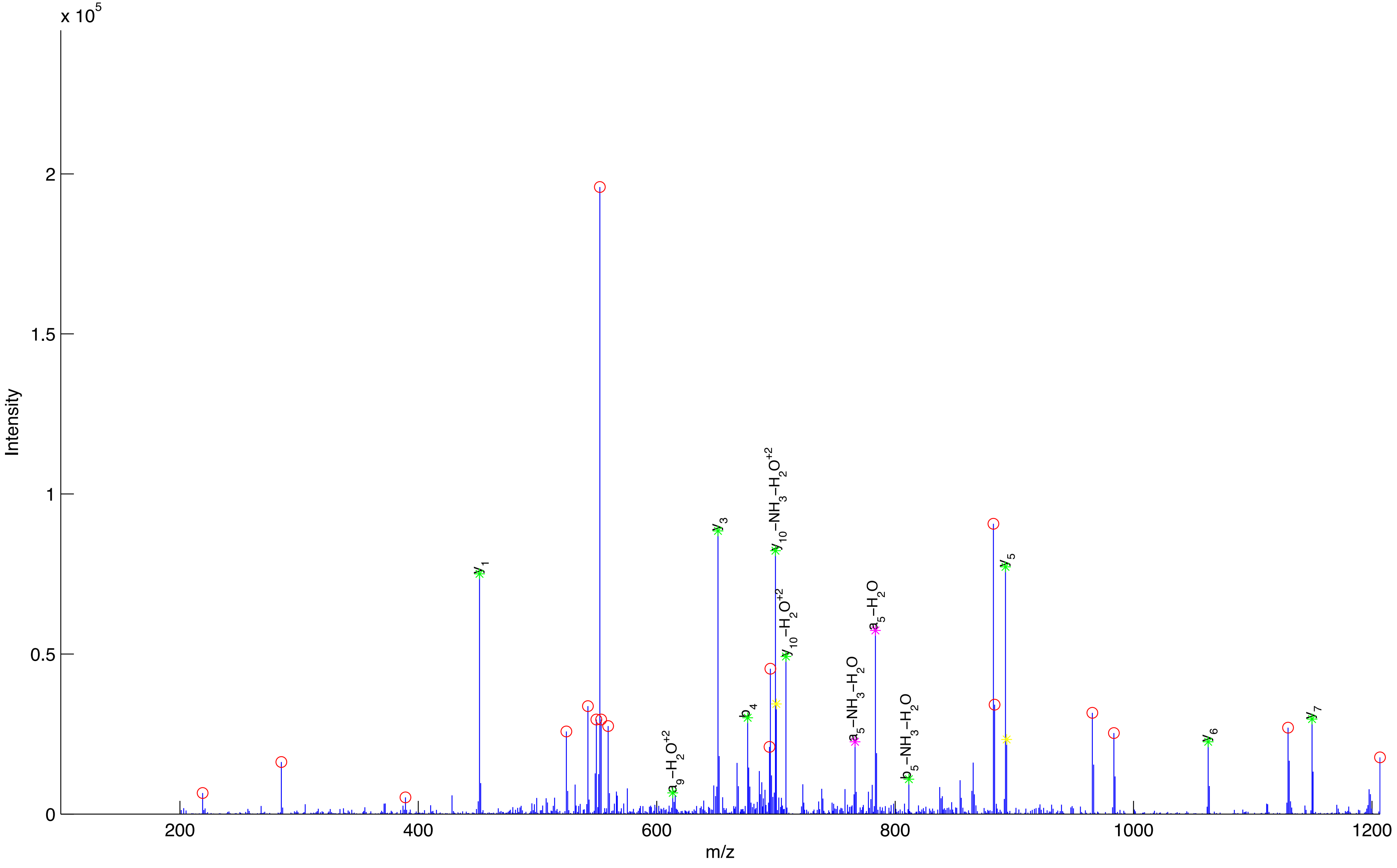

362.2347 | 475.3188 | 532.3402 | 702.4457 | 759.4672 | 816.4887 | 887.5258

G L G k G G A K

1033.6313 | 976.6099 | 863.5258 | 806.5043 | 636.3988 | 579.3773 | 522.3559

histone cluster 1, H4a [Homo sapiens]
Charge State: +3
Scan Number: 6523
File Name: 120404_A549_EGFIGF_bioRepB_ACK_FT.raw

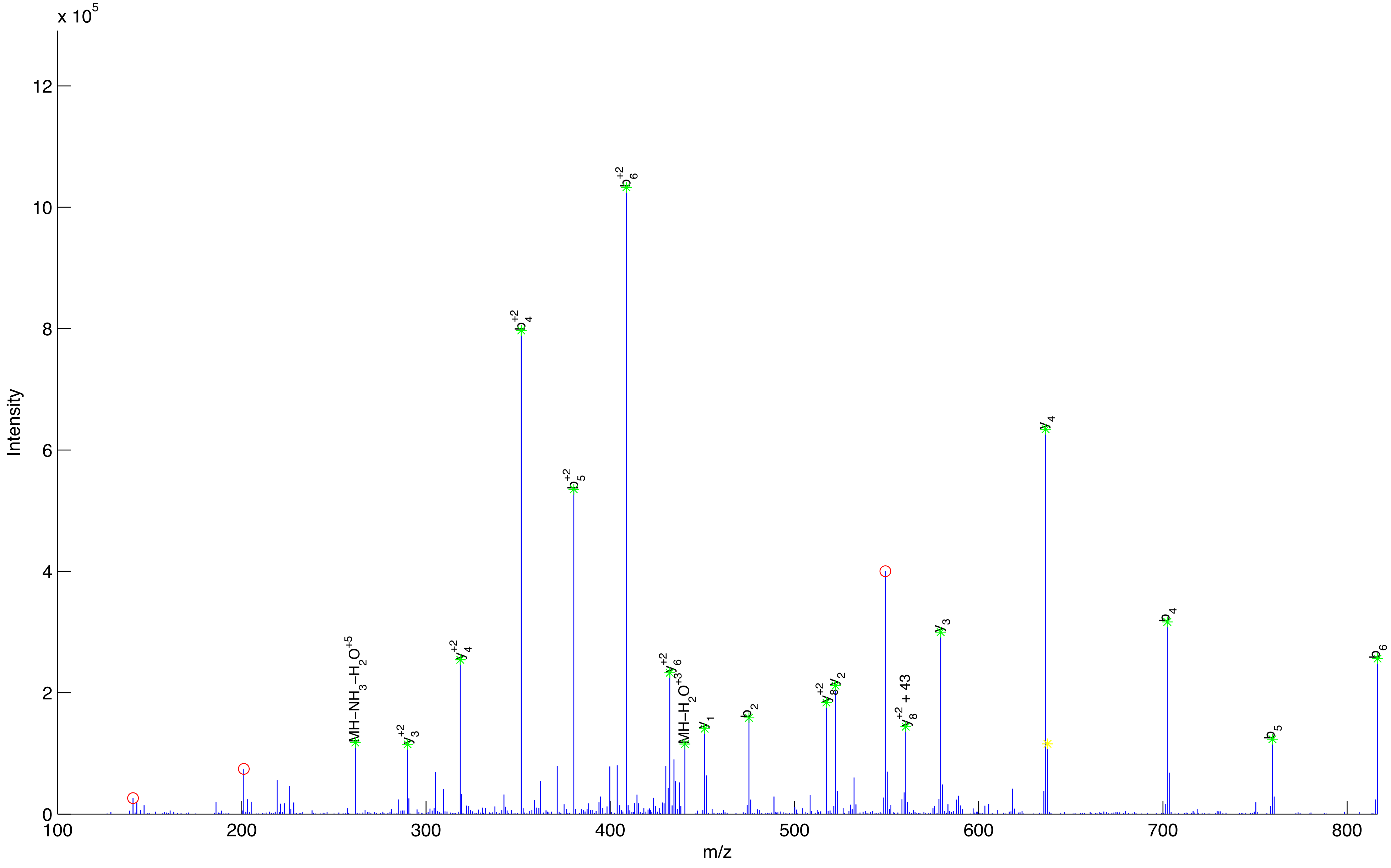

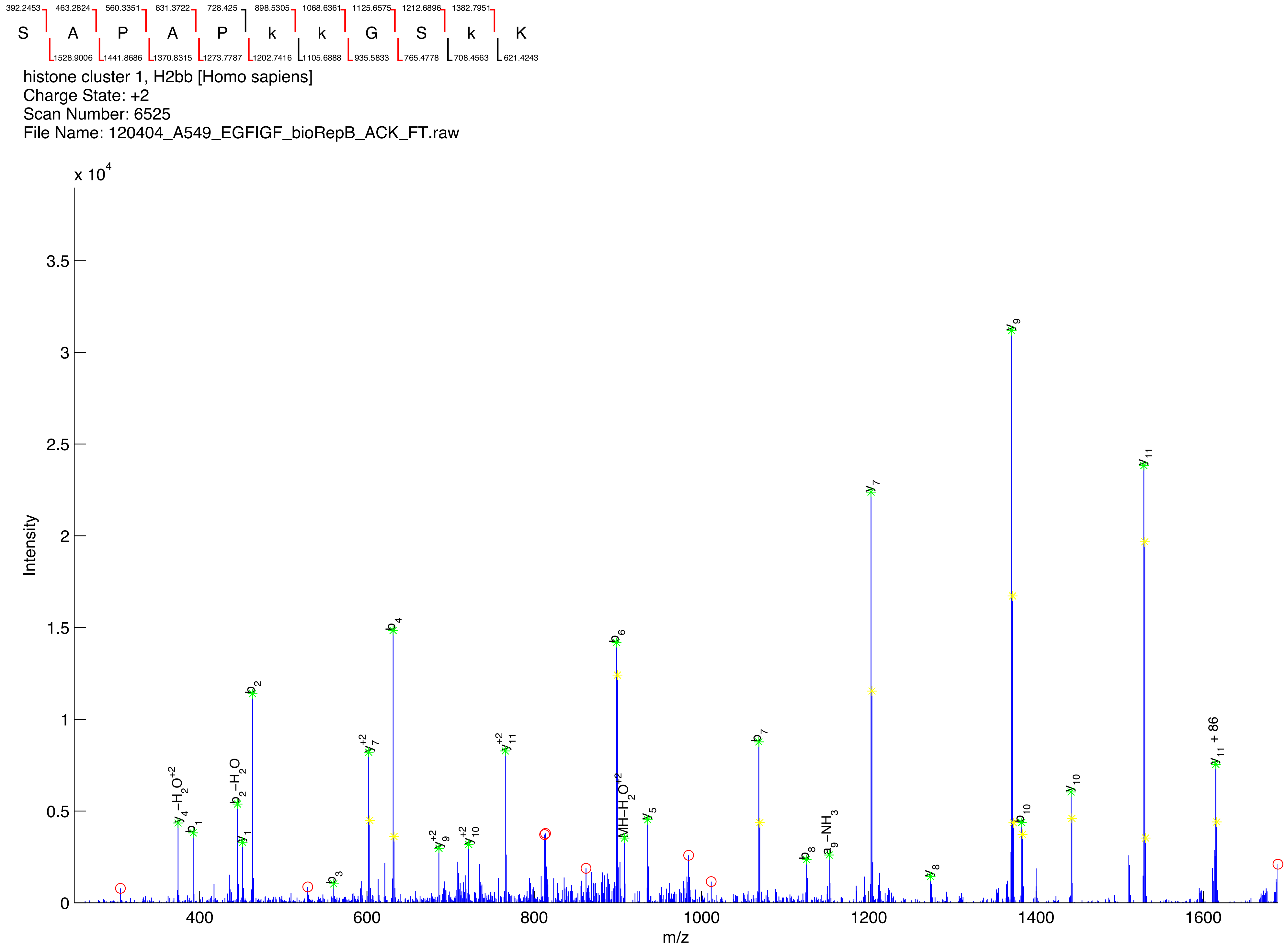

histone cluster 1, H2bb [Homo sapiens]
Charge State: +2
Scan Number: 6525
File Name: 120404_A549_EGFIGF_bioRepB_ACK_FT.raw

362.2347	532.3402	589.3617	646.3831	816.4887	873.5101	986.5942	1043.6157	1213.7212	1270.7427	1327.7641	1398.8012	1568.9068		
G	k	G	G	k	G	L	G	k	G	G	A	k	R	
	1438.813	1381.7916	1211.686	1154.6646	1097.6431	927.5376	870.5161	757.4321	700.4106	530.3051	473.2836	416.2621	345.225	

histone cluster 1, H4a [Homo sapiens]
Charge State: +
Scan Number: 6543
File Name: 120407_A549_EGFIGF_bioRepA_ACK_FT.raw

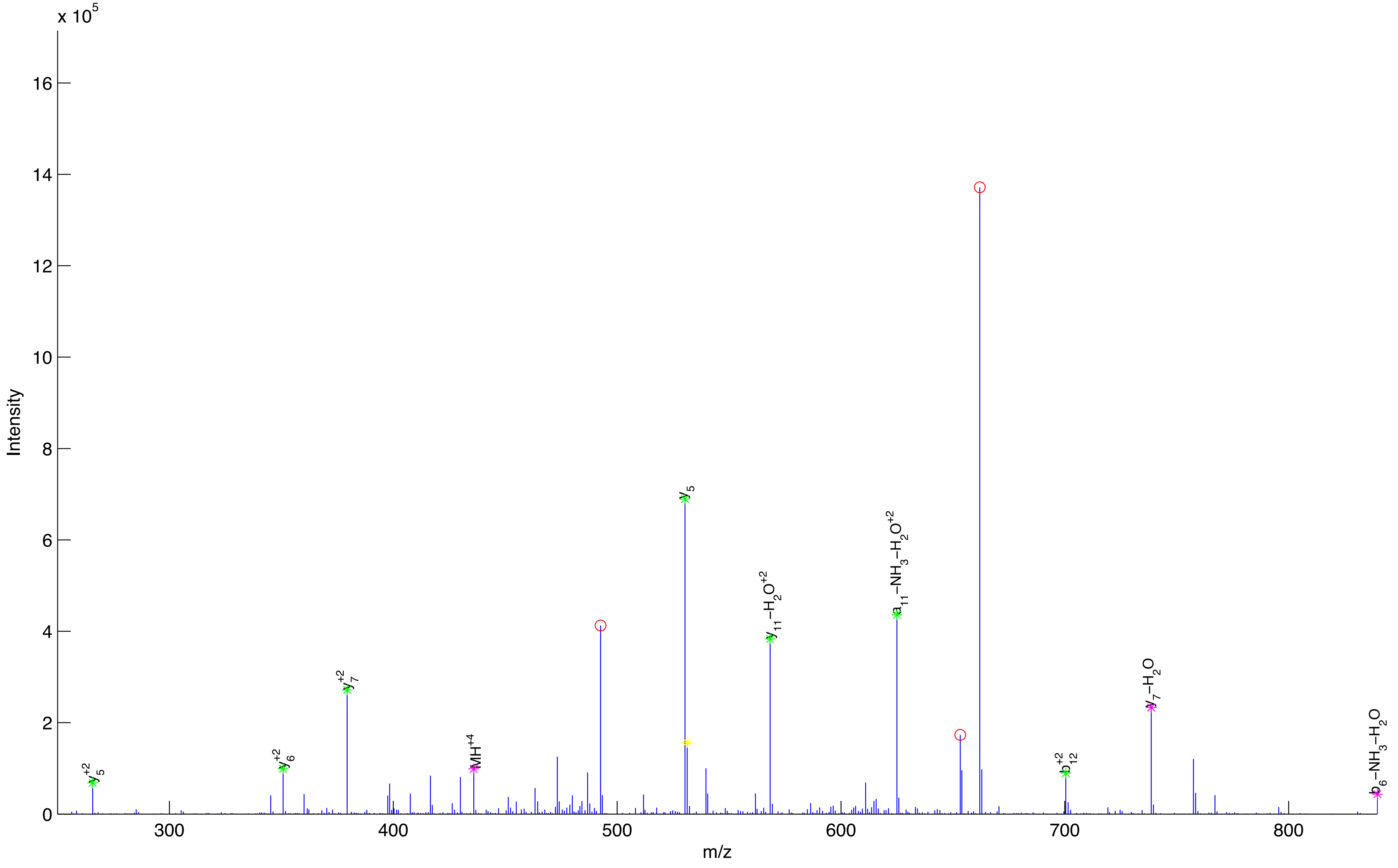

362.2347 475.3188 532.3402 702.4457 759.4672 816.4887 887.5258 1057.6313

G L G k G G A k R

927.5376 870.5161 757.4321 700.4106 530.3051 473.2836 416.2621 345.225

histone cluster 1, H4a [Homo sapiens]
Charge State: +2
Scan Number: 6564
File Name: 120407_A549_EGFIGF_bioRepA_ACK_FT.raw

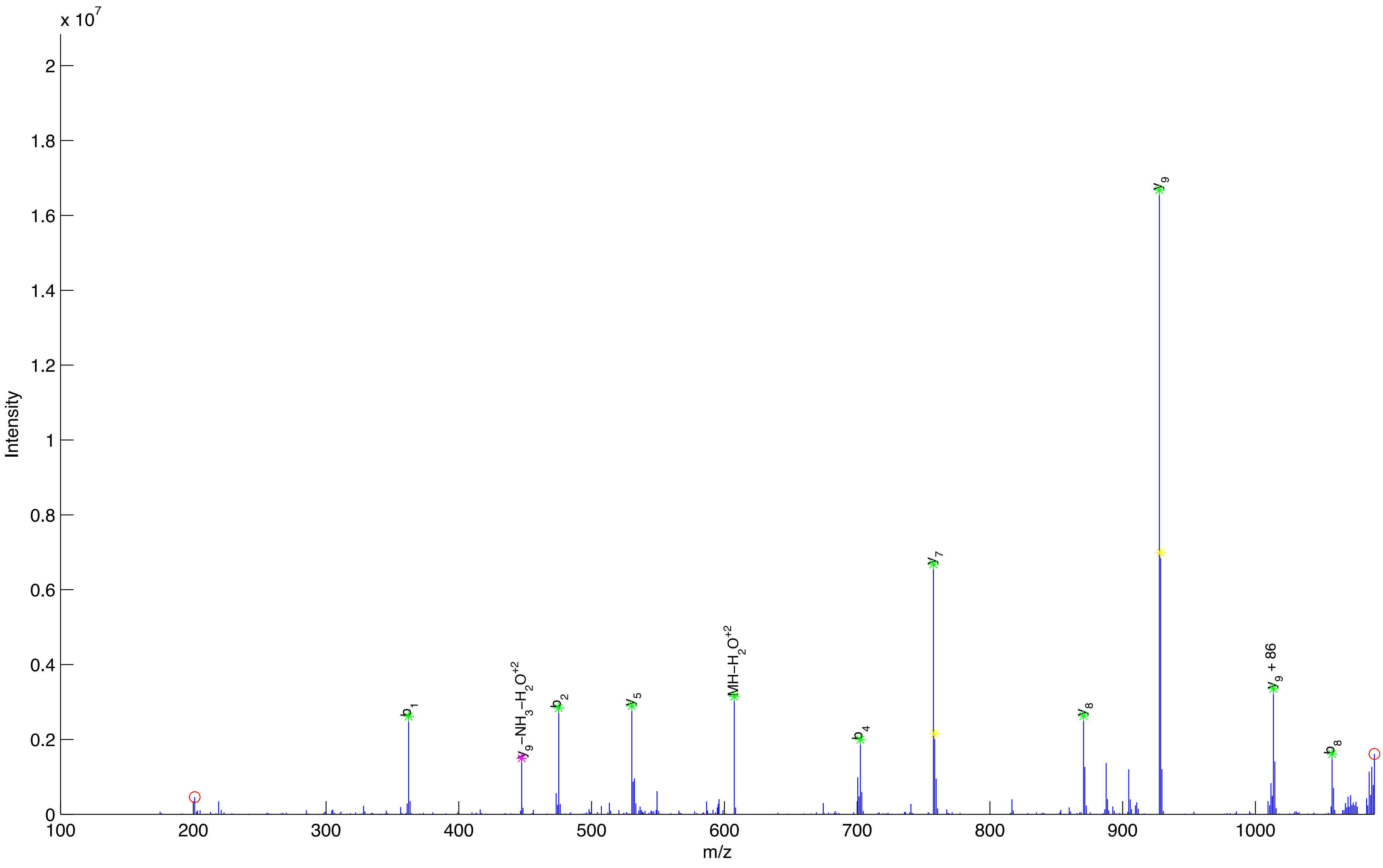

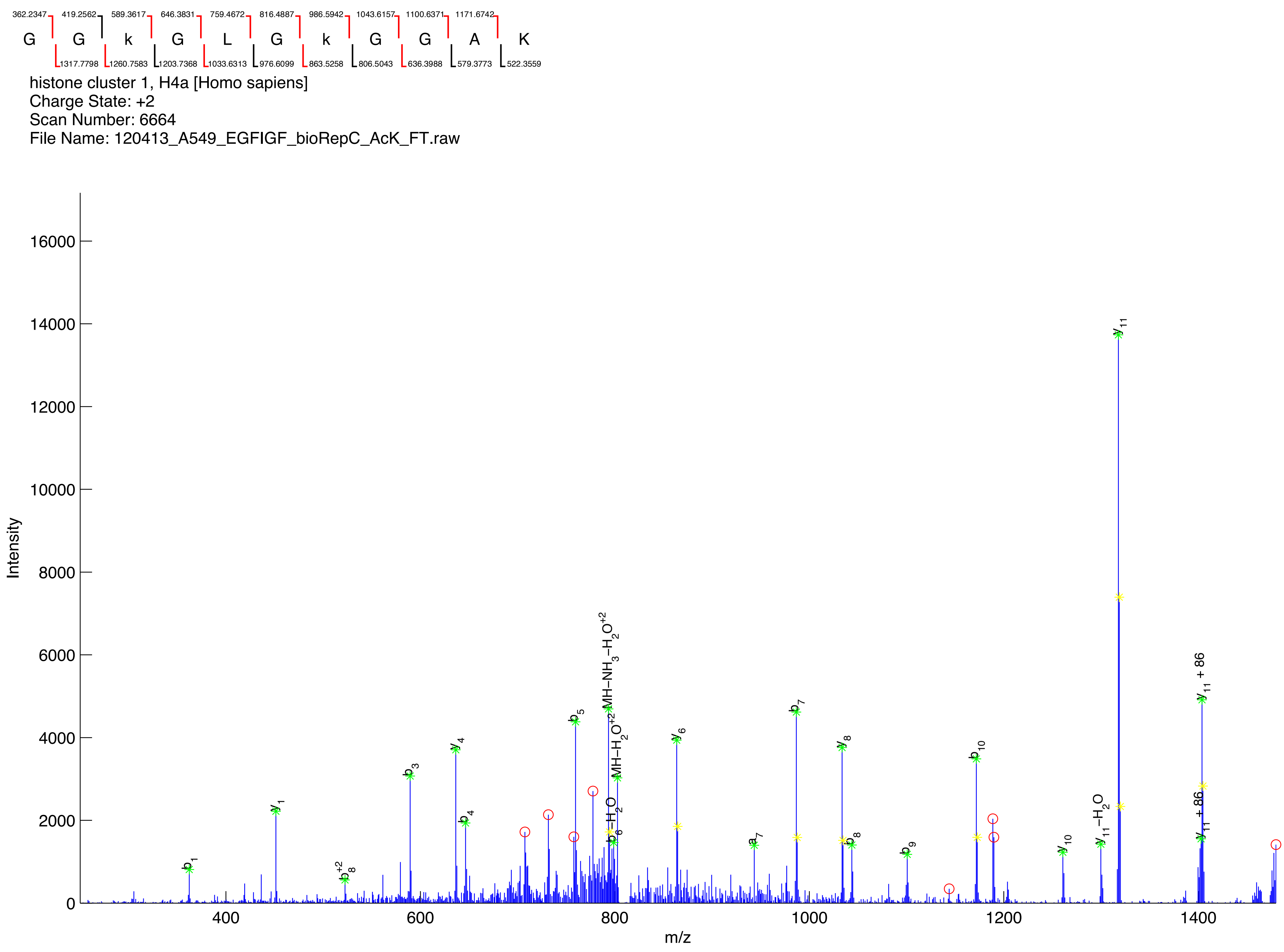

362.2347 419.2562 589.3617 646.3831 759.4672 816.4887 986.5942 1043.6157 1100.6371 1171.6742
G G k G L G k G G A K
1317.7798 1260.7583 1203.7368 1033.6313 976.6099 863.5258 806.5043 636.3988 579.3773 522.3559
histone cluster 1, H4a [Homo sapiens]
Charge State: +2
Scan Number: 6664
File Name: 120413_A549_EGFIGF_bioRepC_AcK_FT.raw
Intensity
m/z
b_1
y_1
b^{+2}_8
b_3
y_4
b_4
b_5
b_6-H_2O
$MH-H_2O^{+2}$
$MH-NH_3-H_2O^{+2}$
y_6
a_7
b_7
y_8
b_8
b_9
b_{10}
y_{10}
$y_{11}-H_2O$
y_{11}
y_{11} + 86

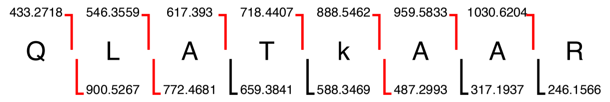

H3 histone, family 3A [Homo sapiens]
Charge State: +1
Scan Number: 6672
File Name: 120404_A549_EGFIGF_bioRepB_ACK_FT.raw

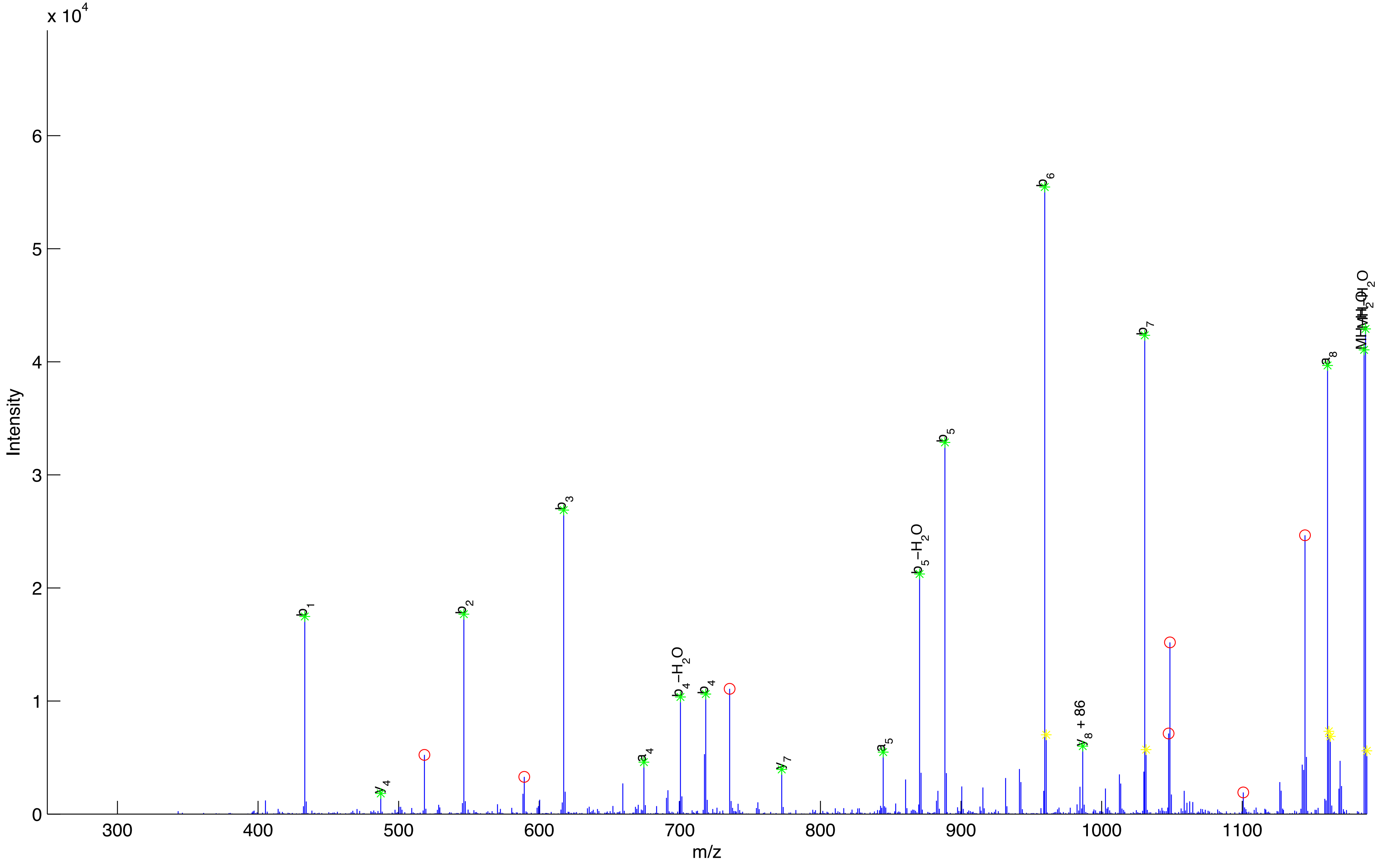

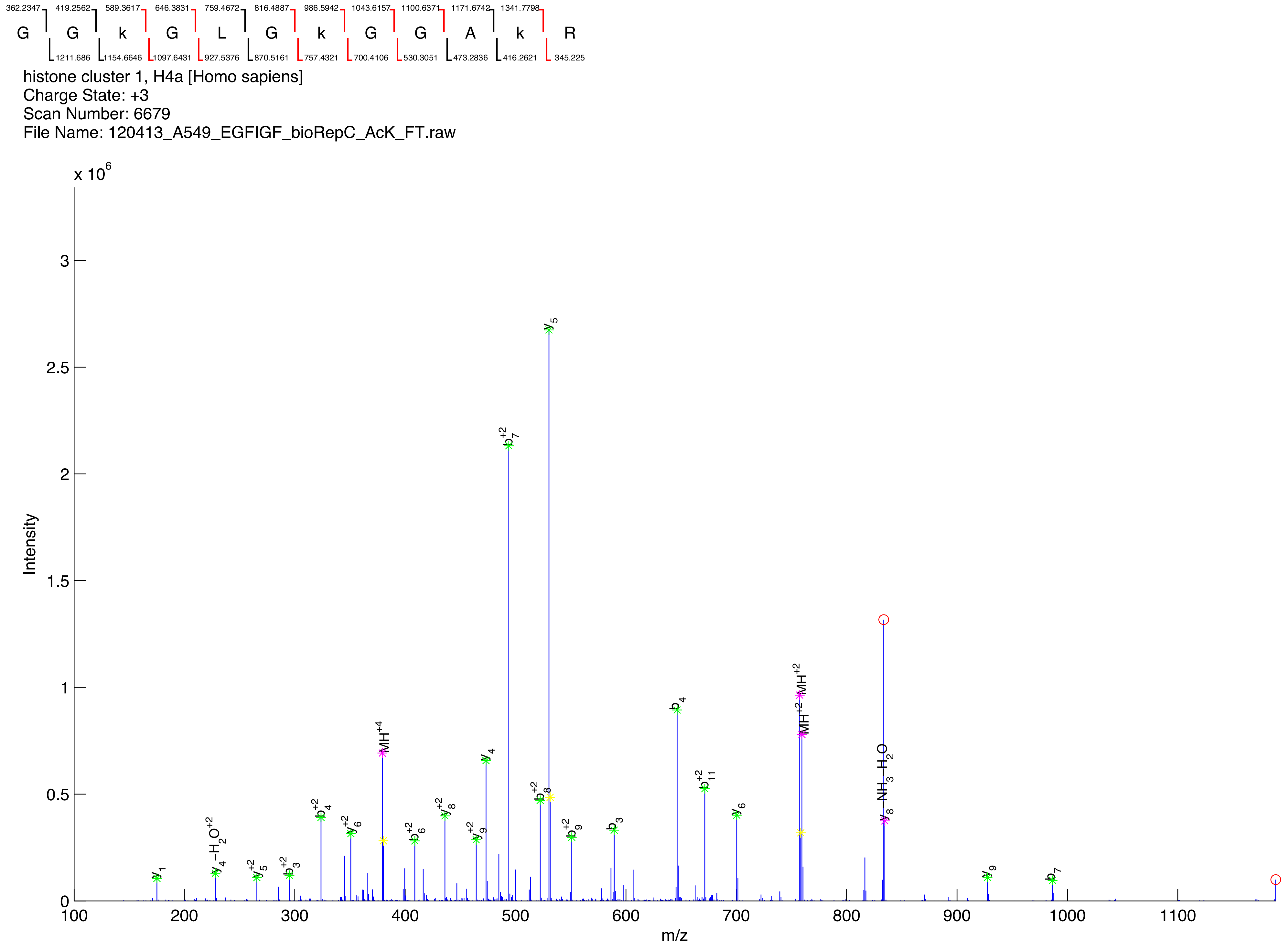
G G k G L G k G G A k R
362.2347 419.2562 589.3617 646.3831 759.4672 816.4887 986.5942 1043.6157 1100.6371 1171.6742 1341.7798
1211.686 1154.6646 1097.6431 927.5376 870.5161 757.4321 700.4106 530.3051 473.2836 416.2621 345.225
histone cluster 1, H4a [Homo sapiens]
Charge State: +3
Scan Number: 6679
File Name: 120413_A549_EGFIGF_bioRepC_AcK_FT.raw
x 10^6
Intensity
m/z

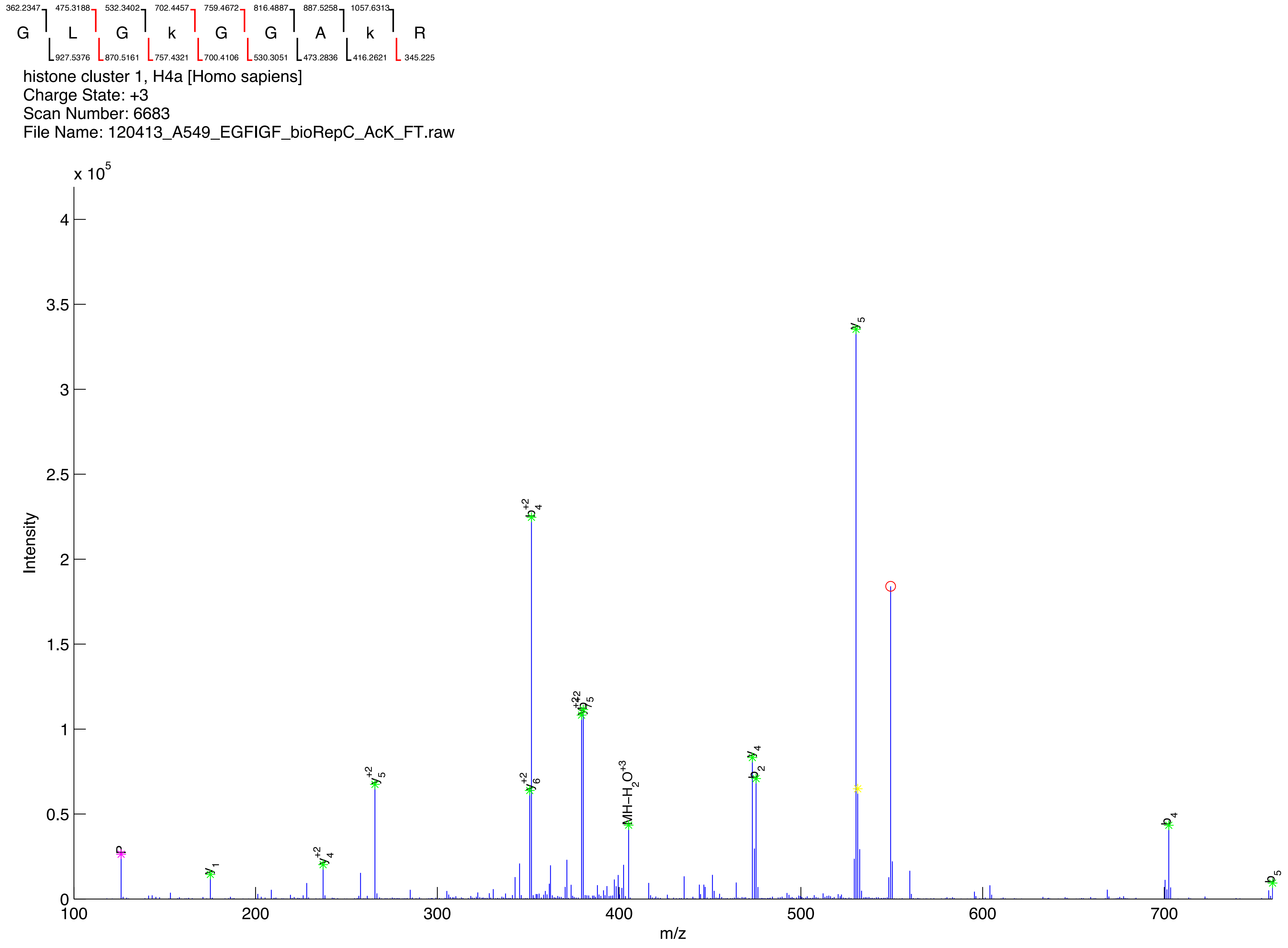
362.2347 475.3188 532.3402 702.4457 759.4672 816.4887 887.5258 1057.6313
G L G k G G A k R
927.5376 870.5161 757.4321 700.4106 530.3051 473.2836 416.2621 345.225
histone cluster 1, H4a [Homo sapiens]
Charge State: +3
Scan Number: 6683
File Name: 120413_A549_EGFIGF_bioRepC_AcK_FT.raw
x 10^5
Intensity
m/z
P
y1
y4+2
y5+2
y6+2
b4+2
b5+2
y5
MH−H2O+3
b2
y4
y5
b4
b5
0
0.5
1
1.5
2
2.5
3
3.5
4
100
200
300
400
500
600
700

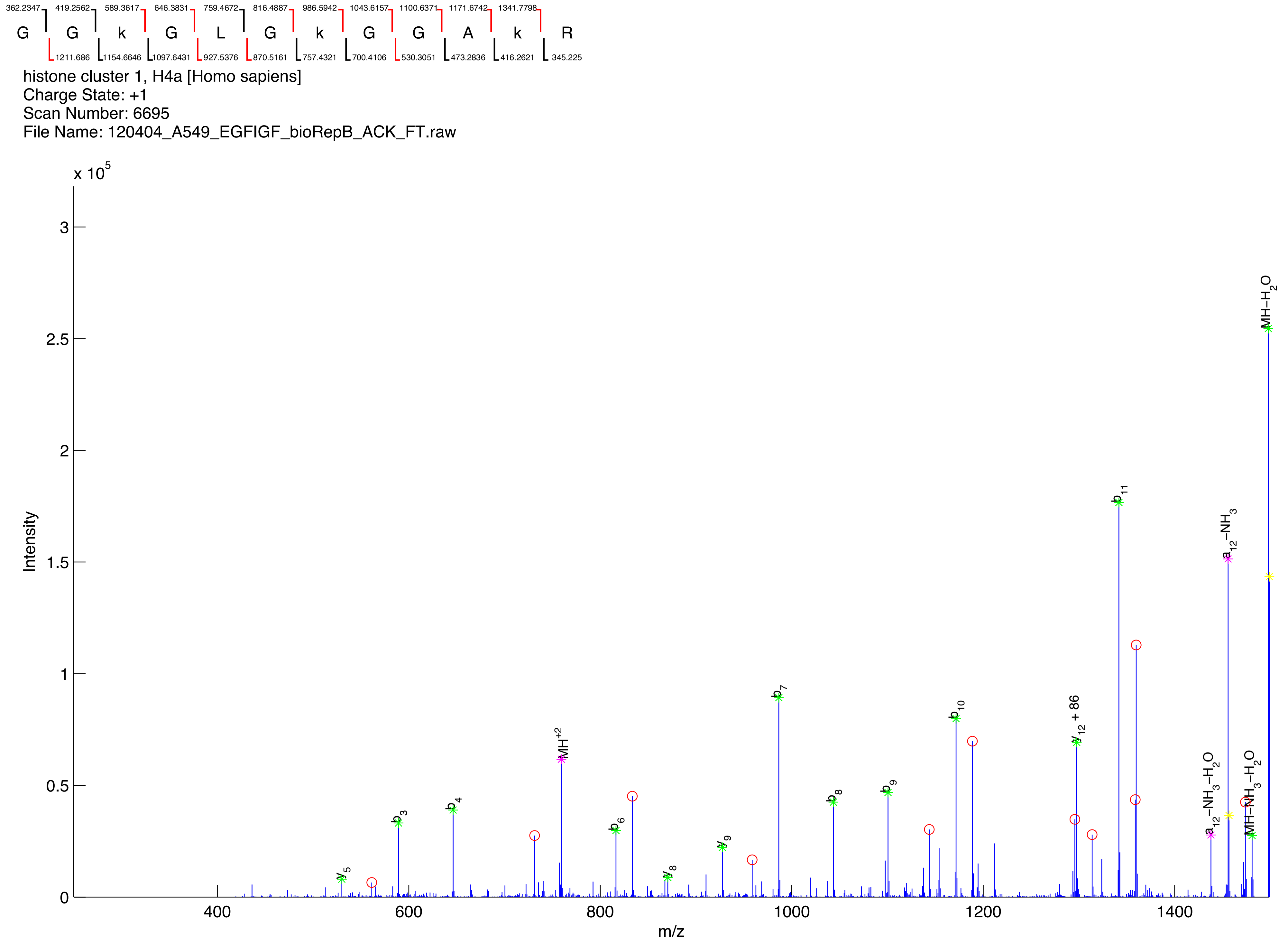
362.2347 419.2562 589.3617 646.3831 759.4672 816.4887 986.5942 1043.6157 1100.6371 1171.6742 1341.7798
G G k G L G k G G A k R
1211.686 1154.6646 1097.6431 927.5376 870.5161 757.4321 700.4106 530.3051 473.2836 416.2621 345.225
histone cluster 1, H4a [Homo sapiens]
Charge State: +1
Scan Number: 6695
File Name: 120404_A549_EGFIGF_bioRepB_ACK_FT.raw
x 10^5
Intensity
m/z
MH−H2O
b11
a12−NH3
b7
b10
y12 + 86
MH+2
b9
b8
b4
b3
b6
y9
y5
y8
a12−NH3−H2O
MH−NH3−H2O

362.2347 532.3402 589.3617 646.3831 816.4887 873.5101 986.5942 1043.6157

G k G G k G L G K

1189.7212 1132.6997 962.5942 905.5727 848.5513 678.4457 621.4243 508.3402

histone cluster 1, H4a [Homo sapiens]
Charge State: +
Scan Number: 6744
File Name: 120413_A549_EGFIGF_bioRepC_AcK_FT.raw

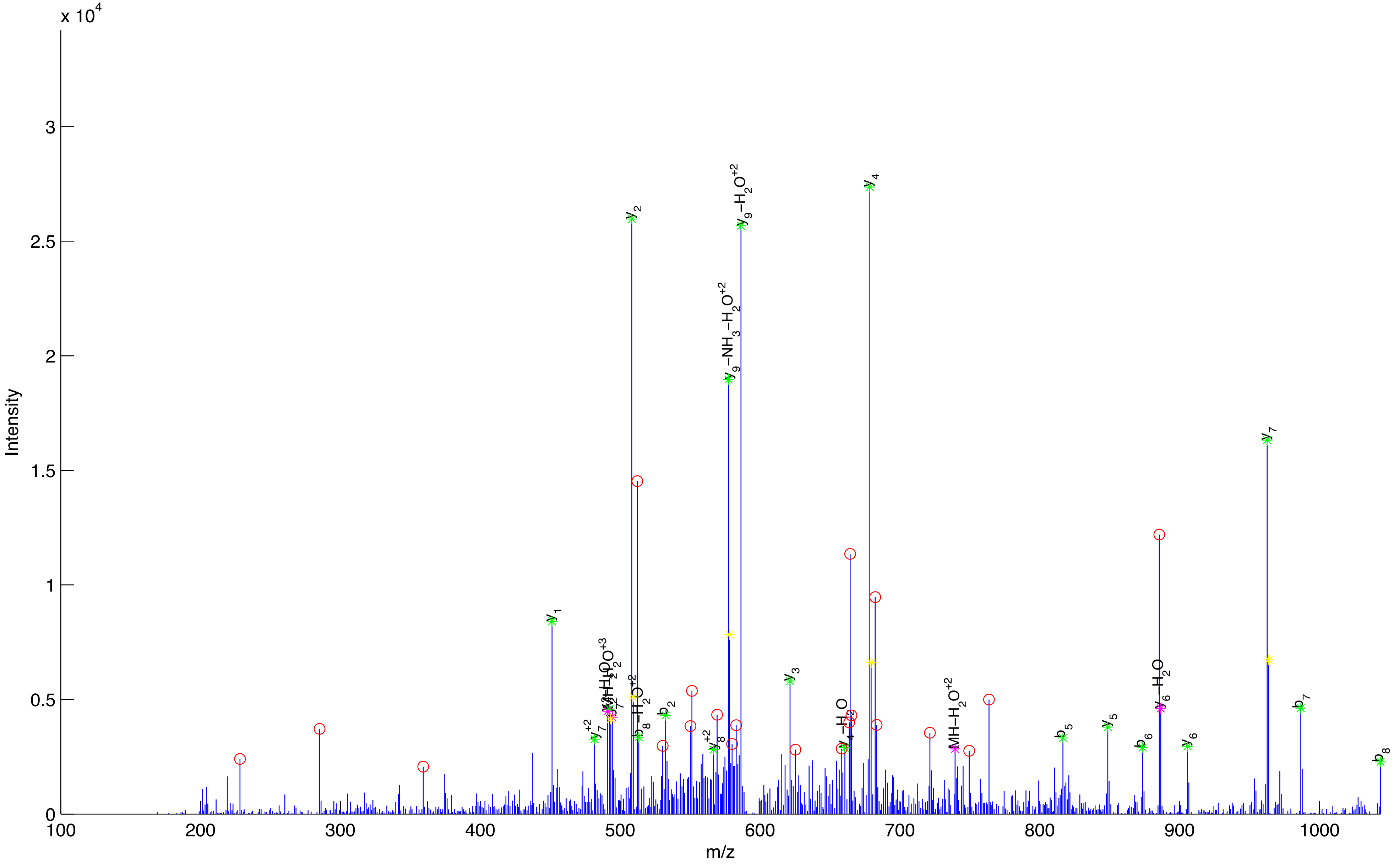

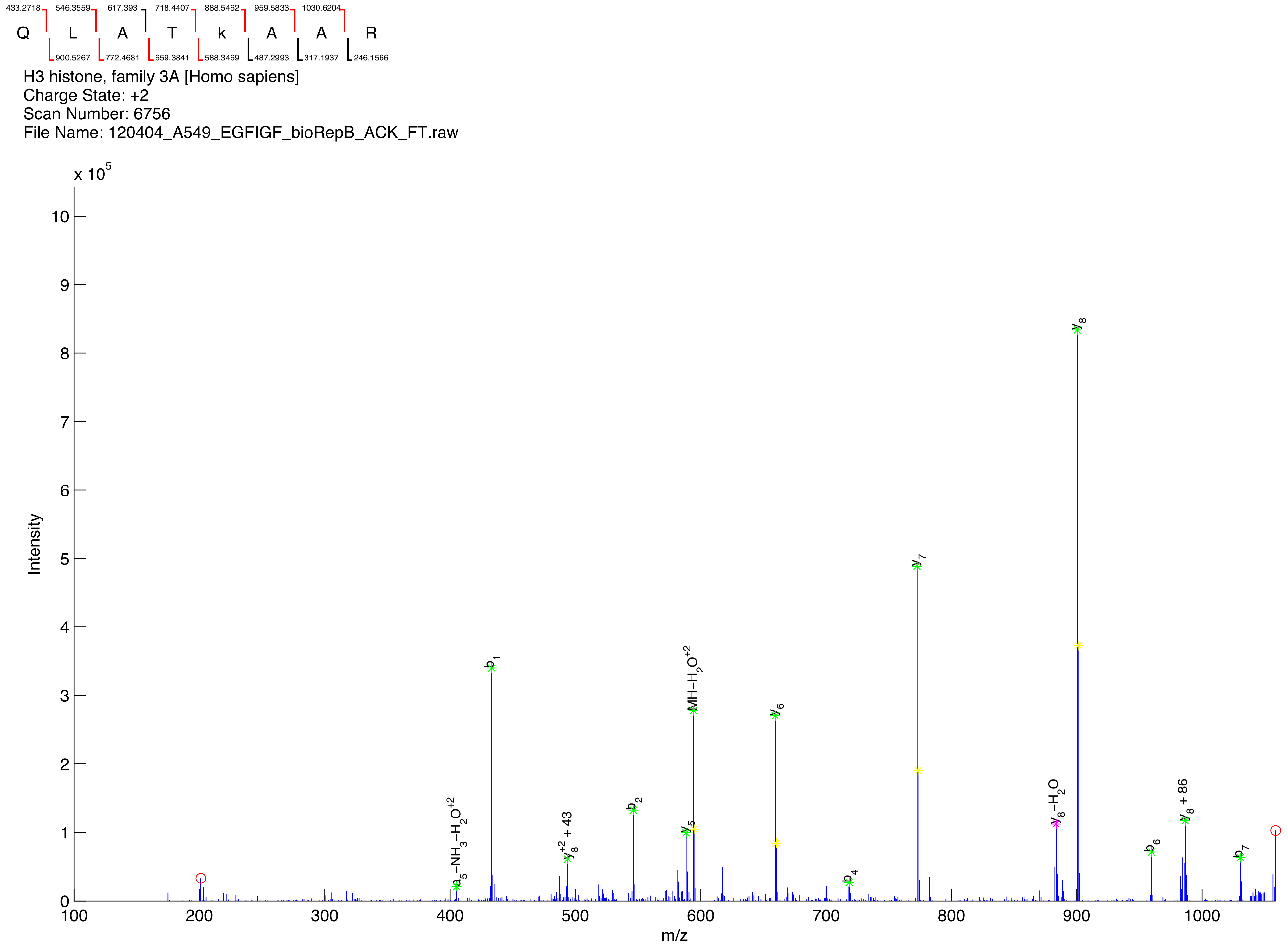

433.2718 546.3559 617.393 718.4407 888.5462 959.5833 1030.6204
Q L A T k A A R
900.5267 772.4681 659.3841 588.3469 487.2993 317.1937 246.1566
H3 histone, family 3A [Homo sapiens]
Charge State: +2
Scan Number: 6756
File Name: 120404_A549_EGFIGF_bioRepB_ACK_FT.raw
x 10^5
Intensity
m/z
a5−NH3−H2O+2
b1
y8+2 + 43
b2
y5
MH−H2O+2
y6
b4
y7
y8−H2O
y8
b6
y8 + 86
b7

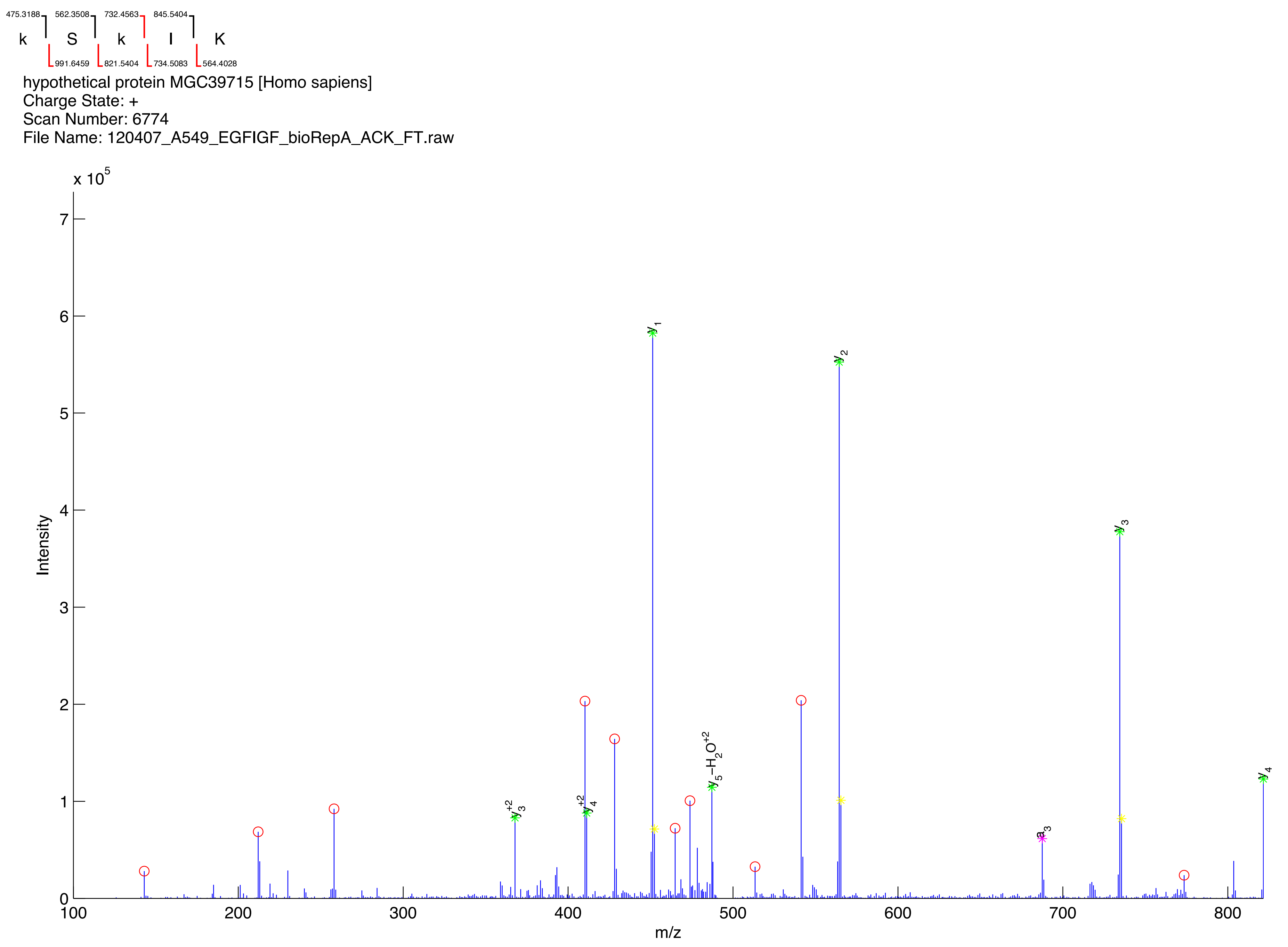

475.3188 562.3508 732.4563 845.5404
k S k I K
991.6459 821.5404 734.5083 564.4028
hypothetical protein MGC39715 [Homo sapiens]
Charge State: +
Scan Number: 6774
File Name: 120407_A549_EGFIGF_bioRepA_ACK_FT.raw
x 10^5
Intensity
m/z
y1
y2
y3
y4
y3+2
y4+2
y5-H2O+2
a3

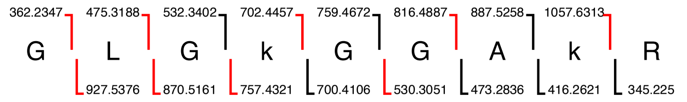

histone cluster 1, H4a [Homo sapiens]
Charge State: +2
Scan Number: 6817
File Name: 120404_A549_EGFIGF_bioRepB_ACK_FT.raw

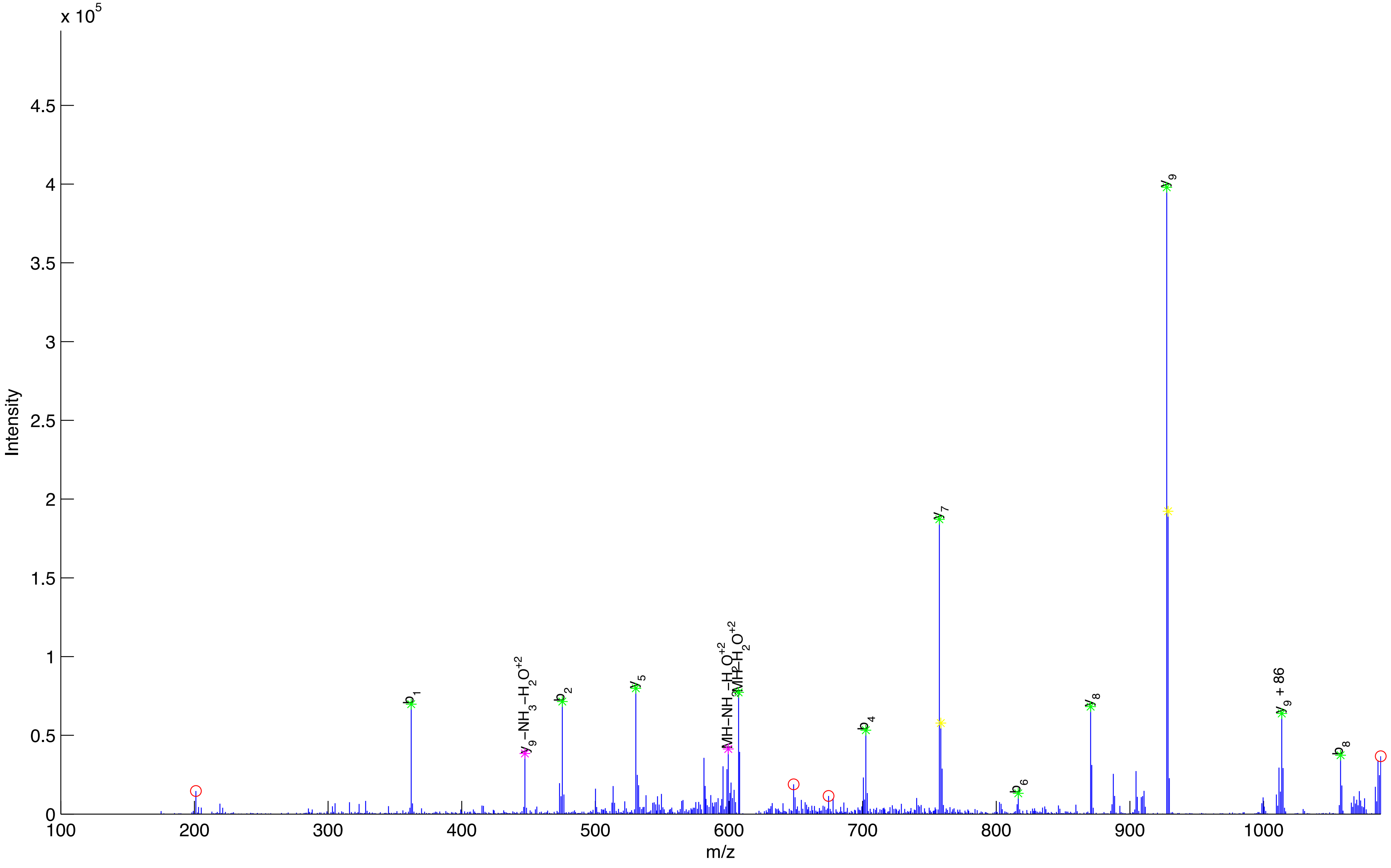

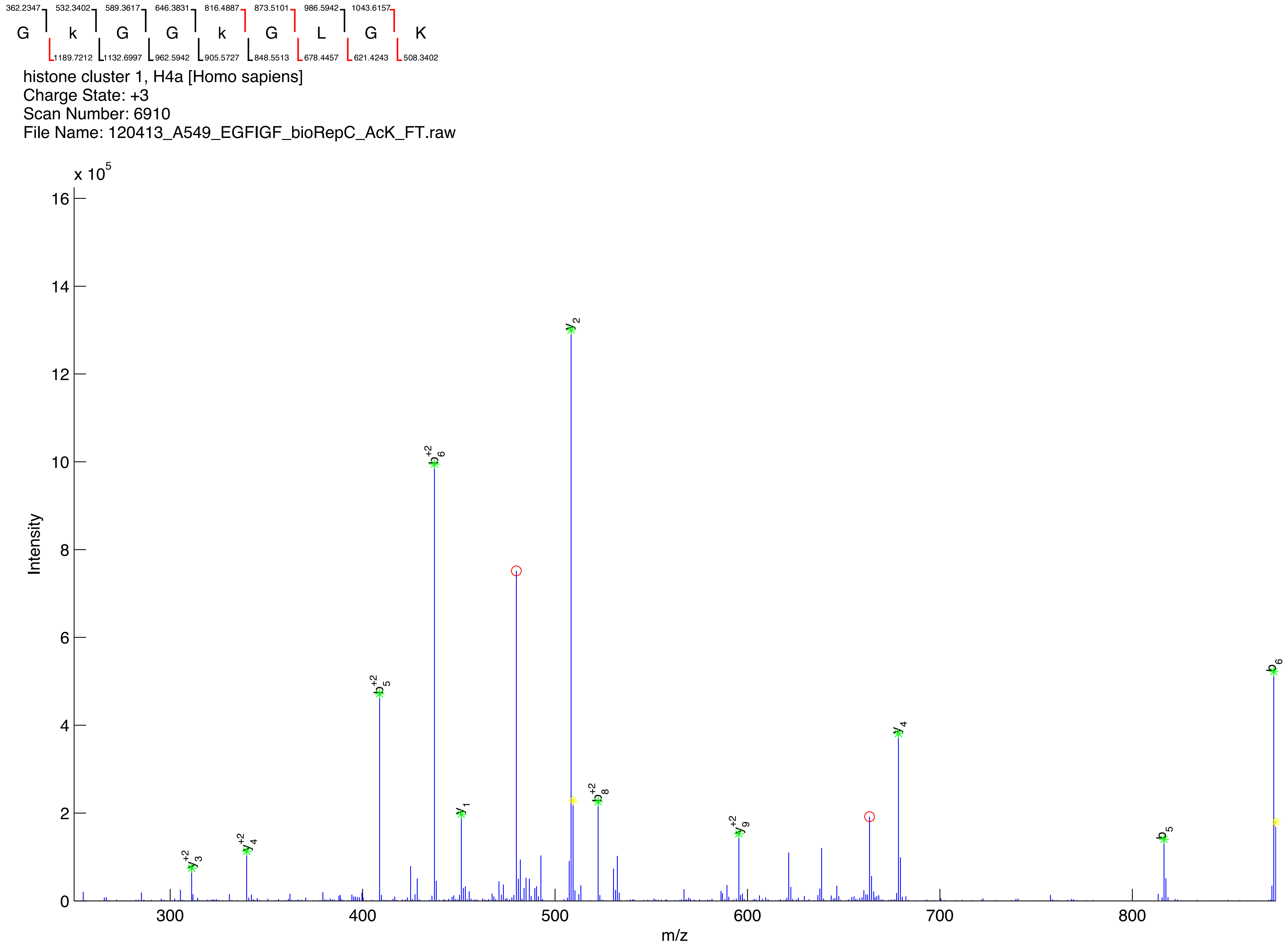
362.2347
532.3402
589.3617
646.3831
816.4887
873.5101
986.5942
1043.6157
G k G G k G L G K
1189.7212
1132.6997
962.5942
905.5727
848.5513
678.4457
621.4243
508.3402
histone cluster 1, H4a [Homo sapiens]
Charge State: +3
Scan Number: 6910
File Name: 120413_A549_EGFIGF_bioRepC_AcK_FT.raw
x 10^5
Intensity
m/z
0
2
4
6
8
10
12
14
16
300
400
500
600
700
800

362.2347	419.2562	589.3617	646.3831	759.4672	816.4887	986.5942	1043.6157	1100.6371	1171.6742	1341.7798	
G	G	k	G	L	G	k	G	G	A	k	R
	1211.686	1154.6646	1097.6431	927.5376	870.5161	757.4321	700.4106	530.3051	473.2836	416.2621	345.225

histone cluster 1, H4a [Homo sapiens]
Charge State: +2
Scan Number: 6922
File Name: 120404_A549_EGFIGF_bioRepB_ACK_FT.raw

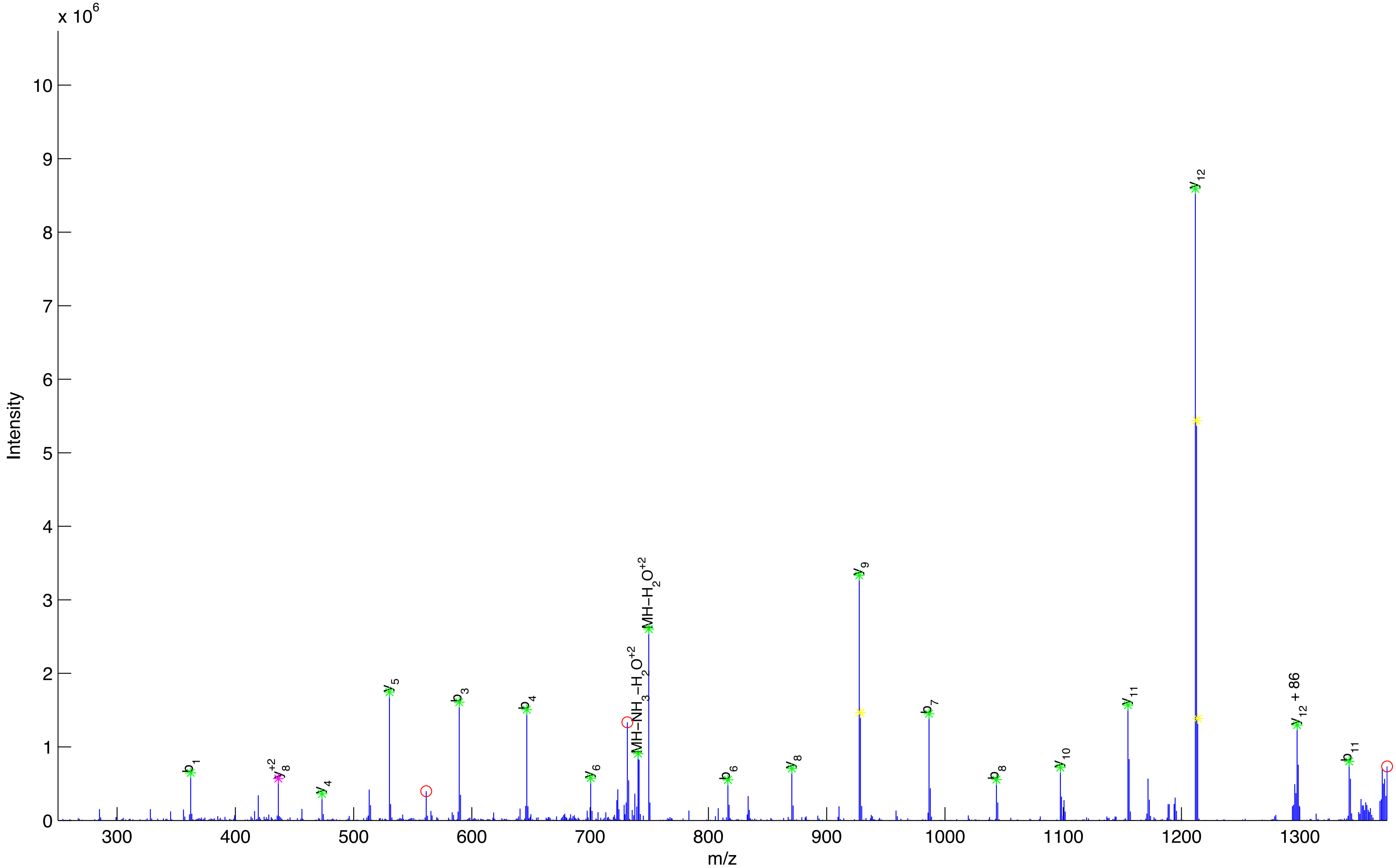

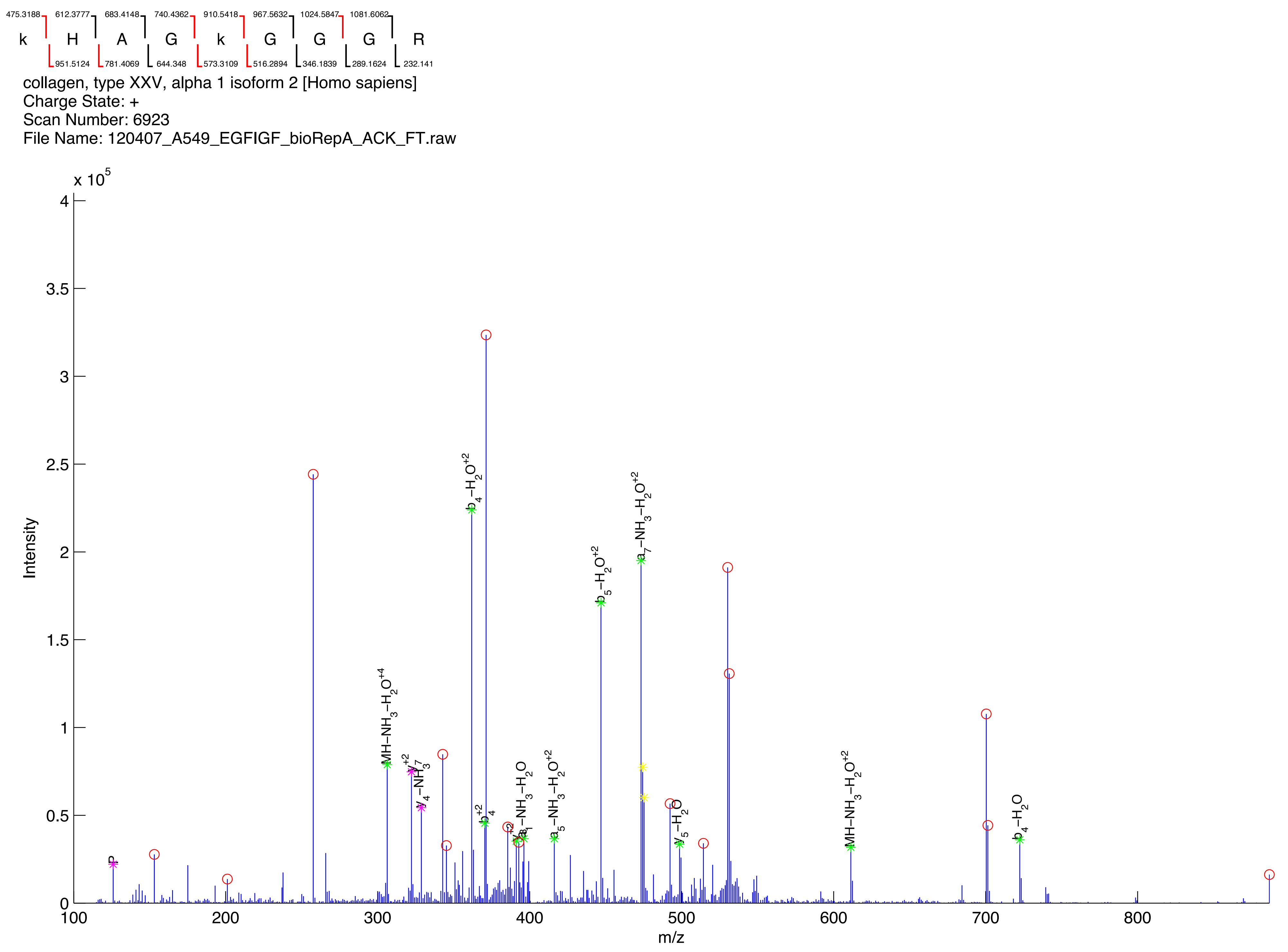
475.3188 612.3777 683.4148 740.4362 910.5418 967.5632 1024.5847 1081.6062
k H A G k G G G R
951.5124 781.4069 644.348 573.3109 516.2894 346.1839 289.1624 232.141
collagen, type XXV, alpha 1 isoform 2 [Homo sapiens]
Charge State: +
Scan Number: 6923
File Name: 120407_A549_EGFIGF_bioRepA_ACK_FT.raw
x 10^5
Intensity
m/z
MH−NH3−H2O+4
b4−H2O+2
b5−H2O+2
a7−NH3−H2O+2
a5−NH3−H2O+2
y5−H2O
MH−NH3−H2O+2
b4−H2O
y4−NH3

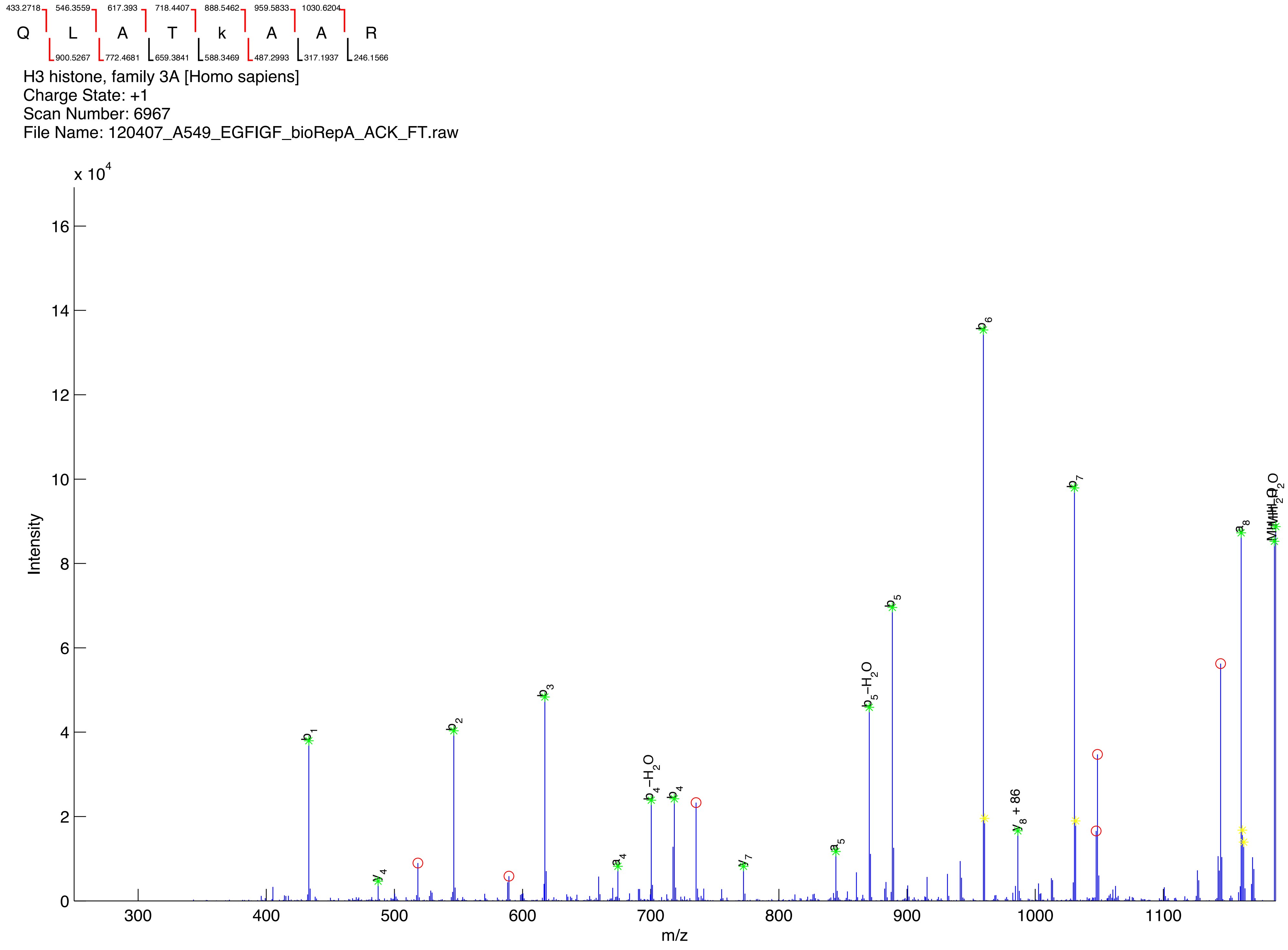
433.2718 546.3559 617.393 718.4407 888.5462 959.5833 1030.6204
Q L A T k A A R
900.5267 772.4681 659.3841 588.3469 487.2993 317.1937 246.1566
H3 histone, family 3A [Homo sapiens]
Charge State: +1
Scan Number: 6967
File Name: 120407_A549_EGFIGF_bioRepA_ACK_FT.raw
x 10^4
Intensity
m/z
b1
y4
b2
b3
a4
b4−H2O
b4
y7
a5
b5−H2O
b5
b6
y8 + 86
b7
a8

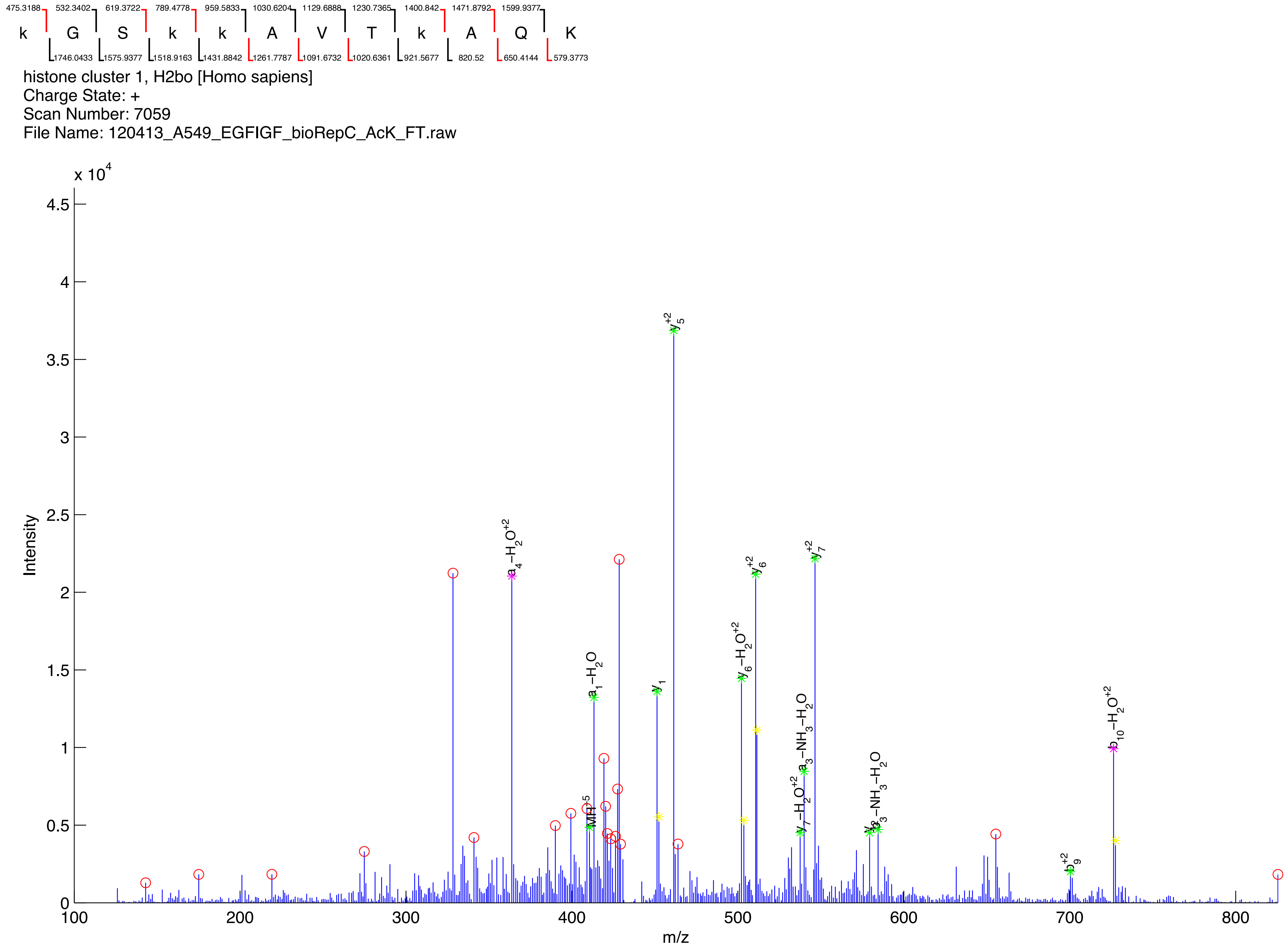
475.3188 532.3402 619.3722 789.4778 959.5833 1030.6204 1129.6888 1230.7365 1400.842 1471.8792 1599.9377
k G S k k A V T k A Q K
1746.0433 1575.9377 1518.9163 1431.8842 1261.7787 1091.6732 1020.6361 921.5677 820.52 650.4144 579.3773
histone cluster 1, H2bo [Homo sapiens]
Charge State: +
Scan Number: 7059
File Name: 120413_A549_EGFIGF_bioRepC_AcK_FT.raw
x 10^4
Intensity
m/z
0
0.5
1
1.5
2
2.5
3
3.5
4
4.5
100
200
300
400
500
600
700
800

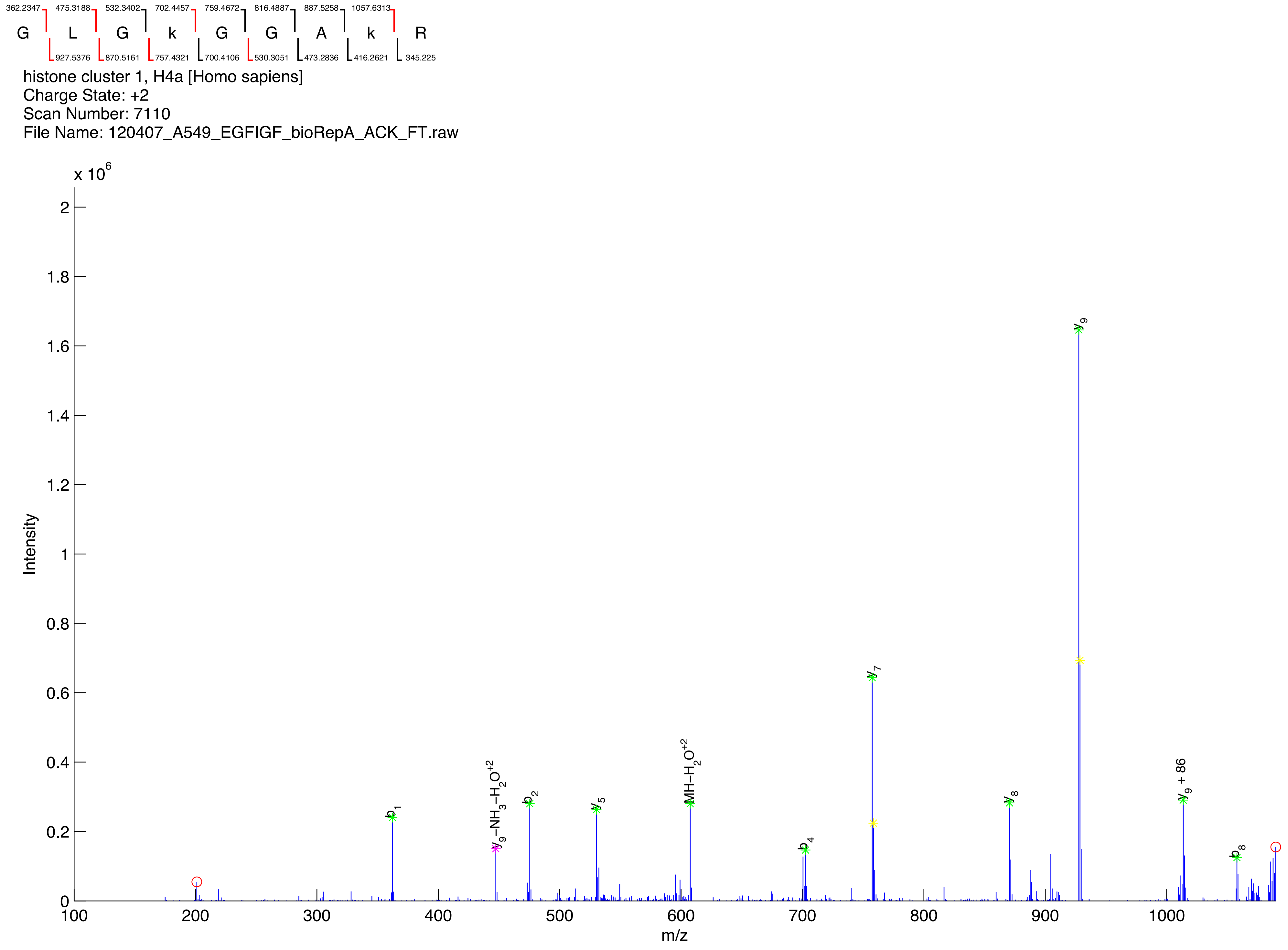

362.2347 475.3188 532.3402 702.4457 759.4672 816.4887 887.5258 1057.6313
G L G k G G A k R
927.5376 870.5161 757.4321 700.4106 530.3051 473.2836 416.2621 345.225
histone cluster 1, H4a [Homo sapiens]
Charge State: +2
Scan Number: 7110
File Name: 120407_A549_EGFIGF_bioRepA_ACK_FT.raw
x 10^6
Intensity
m/z
b1
y9−NH3−H2O+2
b2
y5
MH−H2O+2
b4
y7
y8
y9
y9 + 86
b8

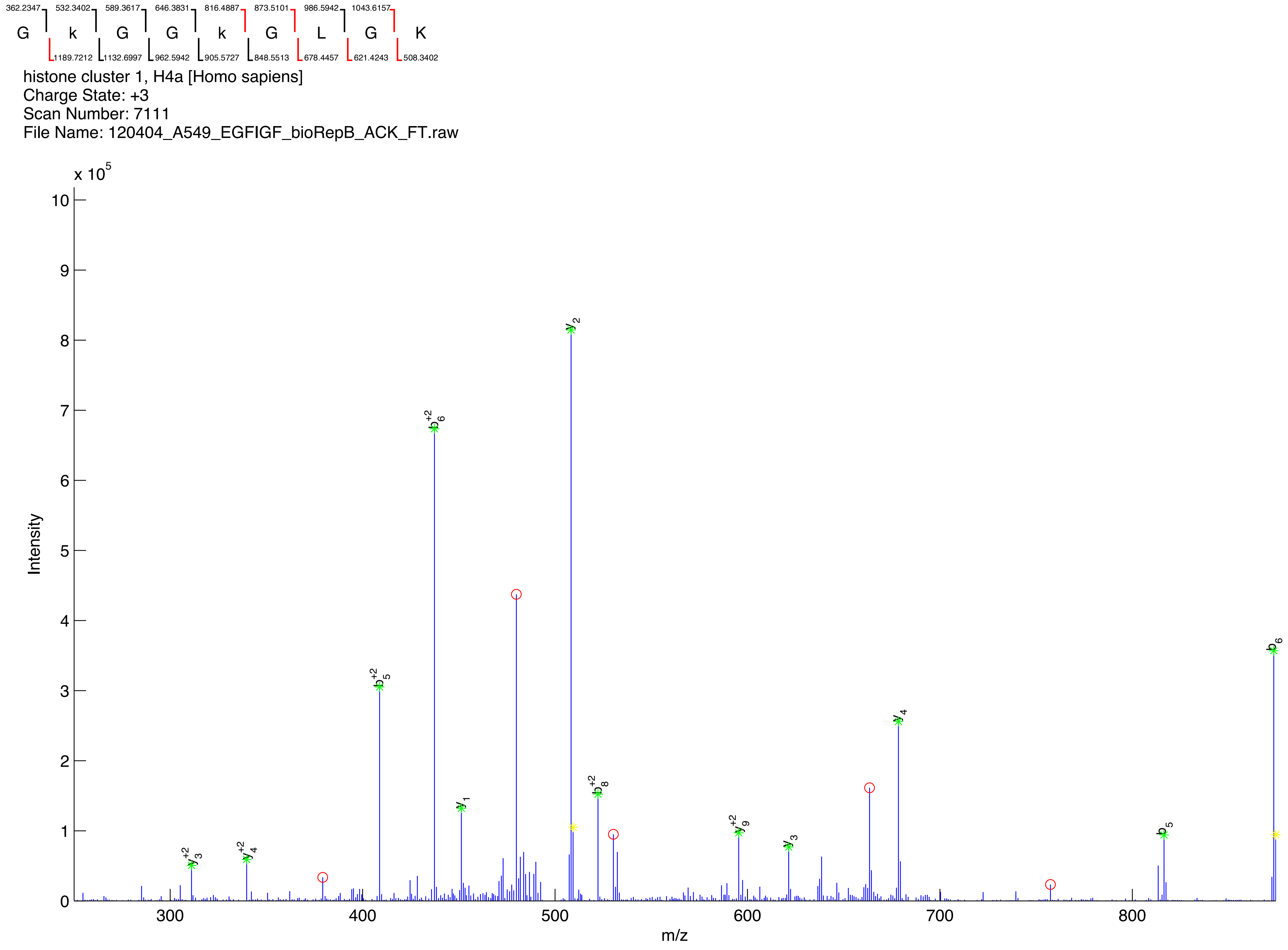
362.2347
532.3402
589.3617
646.3831
816.4887
873.5101
986.5942
1043.6157
G k G G k G L G K
1189.7212
1132.6997
962.5942
905.5727
848.5513
678.4457
621.4243
508.3402
histone cluster 1, H4a [Homo sapiens]
Charge State: +3
Scan Number: 7111
File Name: 120404_A549_EGFIGF_bioRepB_ACK_FT.raw
x 10^5
Intensity
m/z
y+2 3
y+2 4
b+2 5
b+2 6
y1
y2
b+2 8
y+2 9
y3
y4
b5
b6

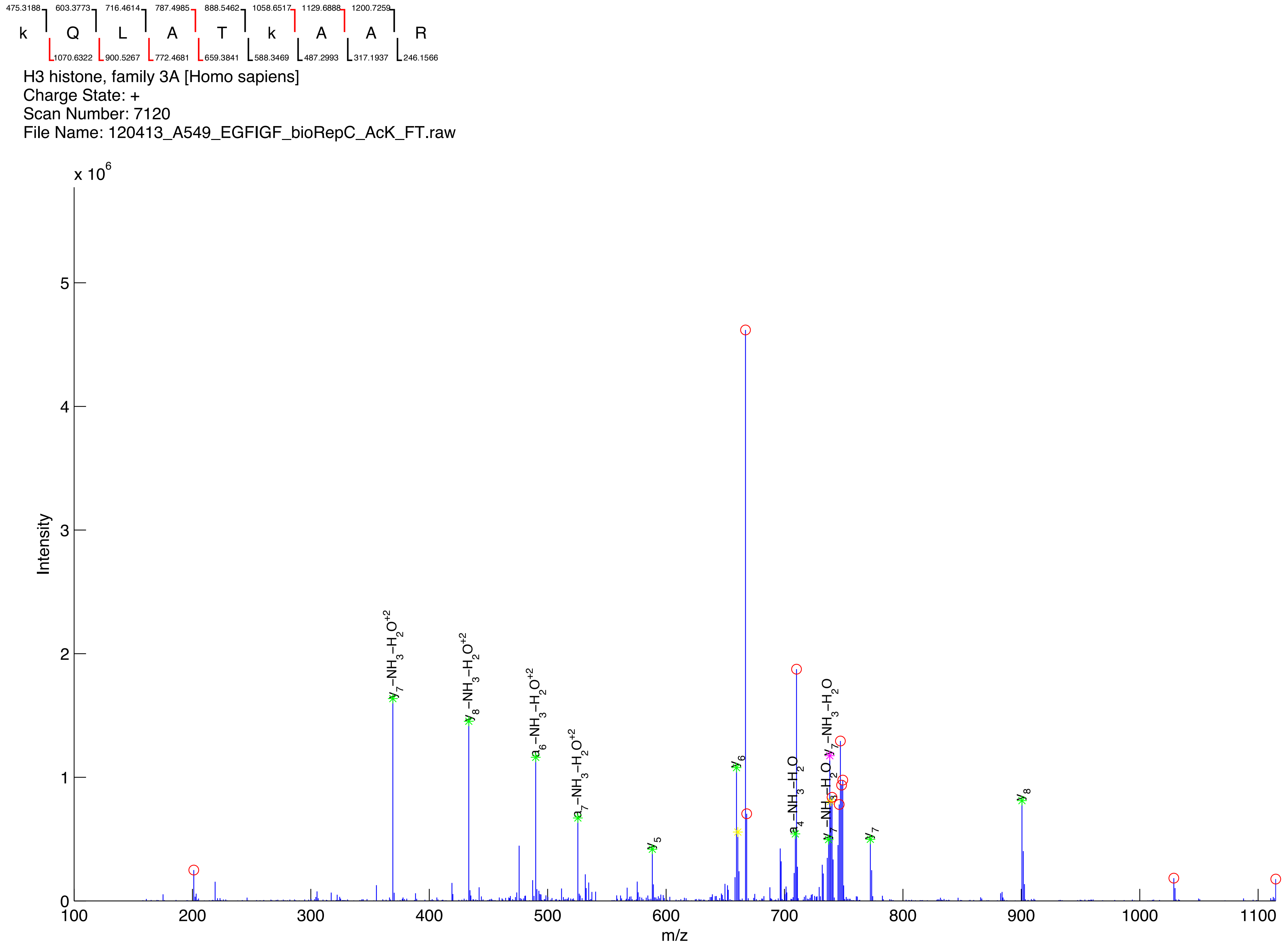

475.3188 603.3773 716.4614 787.4985 888.5462 1058.6517 1129.6888 1200.7259
k Q L A T k A A R
1070.6322 900.5267 772.4681 659.3841 588.3469 487.2993 317.1937 246.1566
H3 histone, family 3A [Homo sapiens]
Charge State: +
Scan Number: 7120
File Name: 120413_A549_EGFIGF_bioRepC_AcK_FT.raw
x 10^6
Intensity
m/z
y7−NH3−H2O+2
y8−NH3−H2O+2
a6−NH3−H2O+2
a7−NH3−H2O+2
y5
y6
a4−NH3−H2O
y7−NH3−H2O
y7
y8

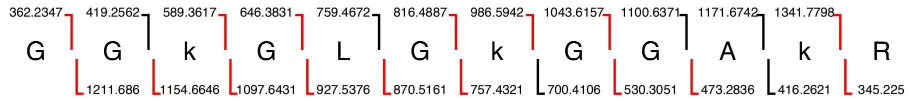

histone cluster 1, H4a [Homo sapiens]
Charge State: +2
Scan Number: 7131
File Name: 120407_A549_EGFIGF_bioRepA_ACK_FT.raw

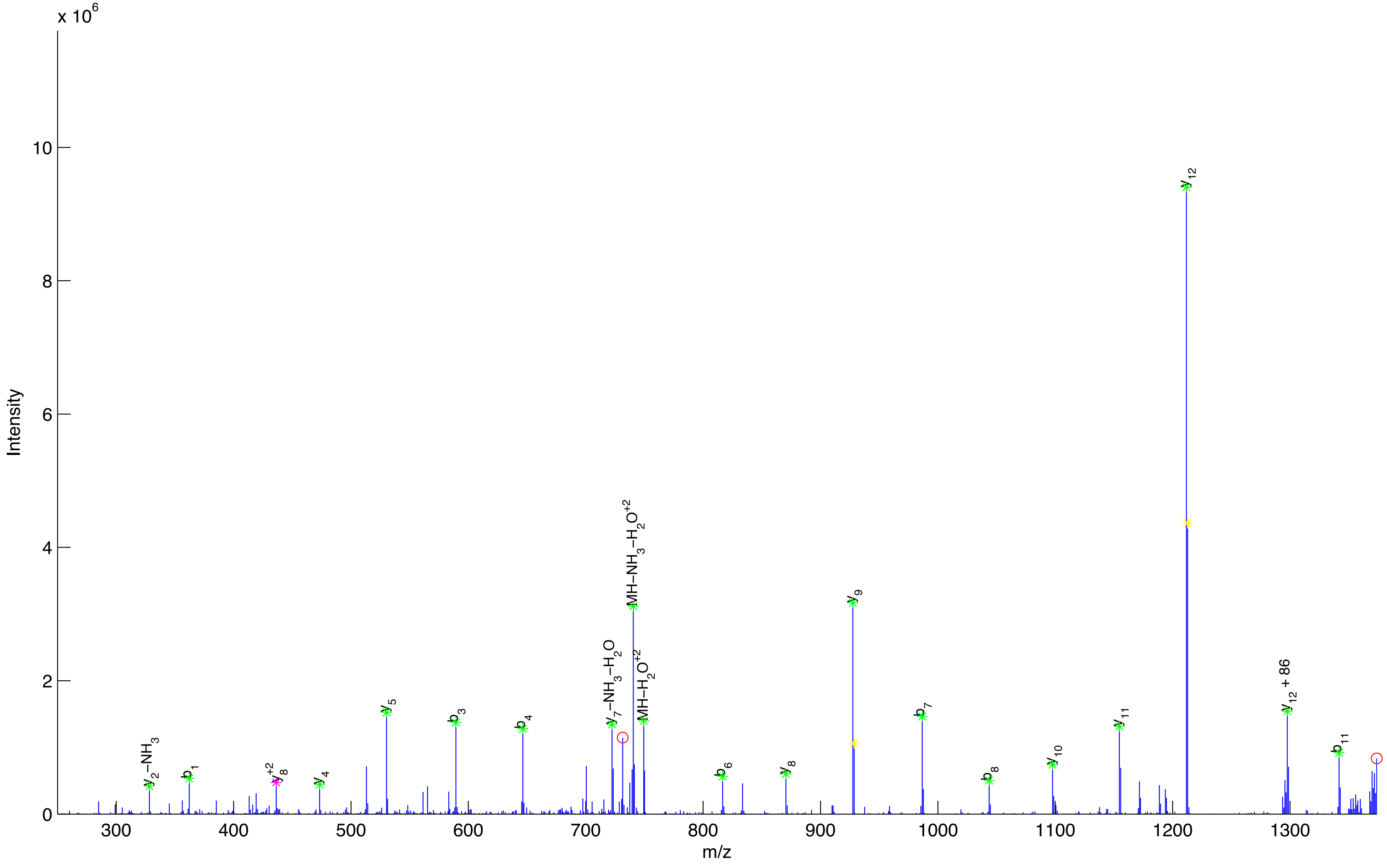

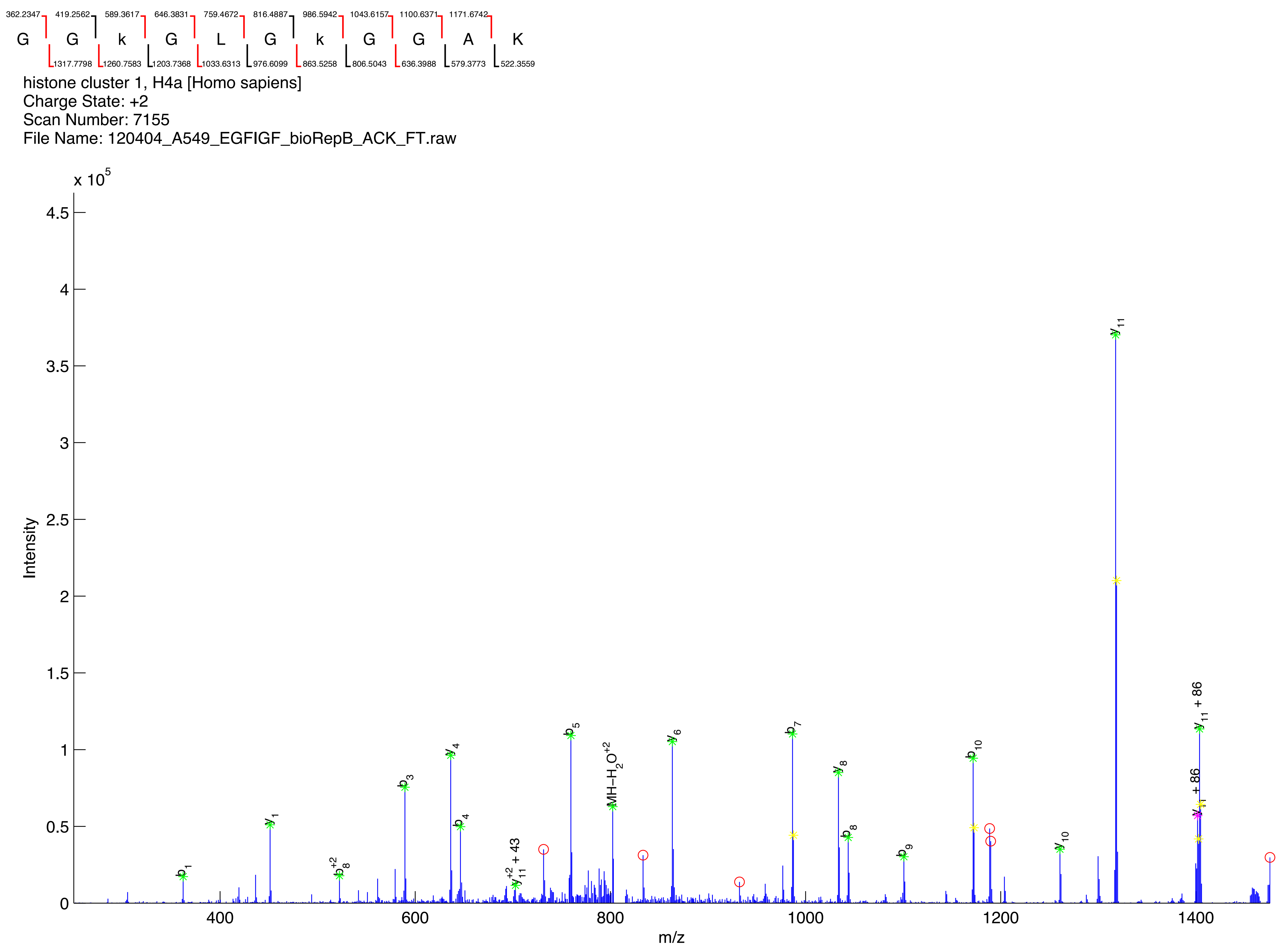

362.2347 419.2562 589.3617 646.3831 759.4672 816.4887 986.5942 1043.6157 1100.6371 1171.6742
G G k G L G k G G A K
1317.7798 1260.7583 1203.7368 1033.6313 976.6099 863.5258 806.5043 636.3988 579.3773 522.3559
histone cluster 1, H4a [Homo sapiens]
Charge State: +2
Scan Number: 7155
File Name: 120404_A549_EGFIGF_bioRepB_ACK_FT.raw
x 10^5
Intensity
m/z
MH−H2O+2
y11 + 86
y1 + 86
y11 + 43

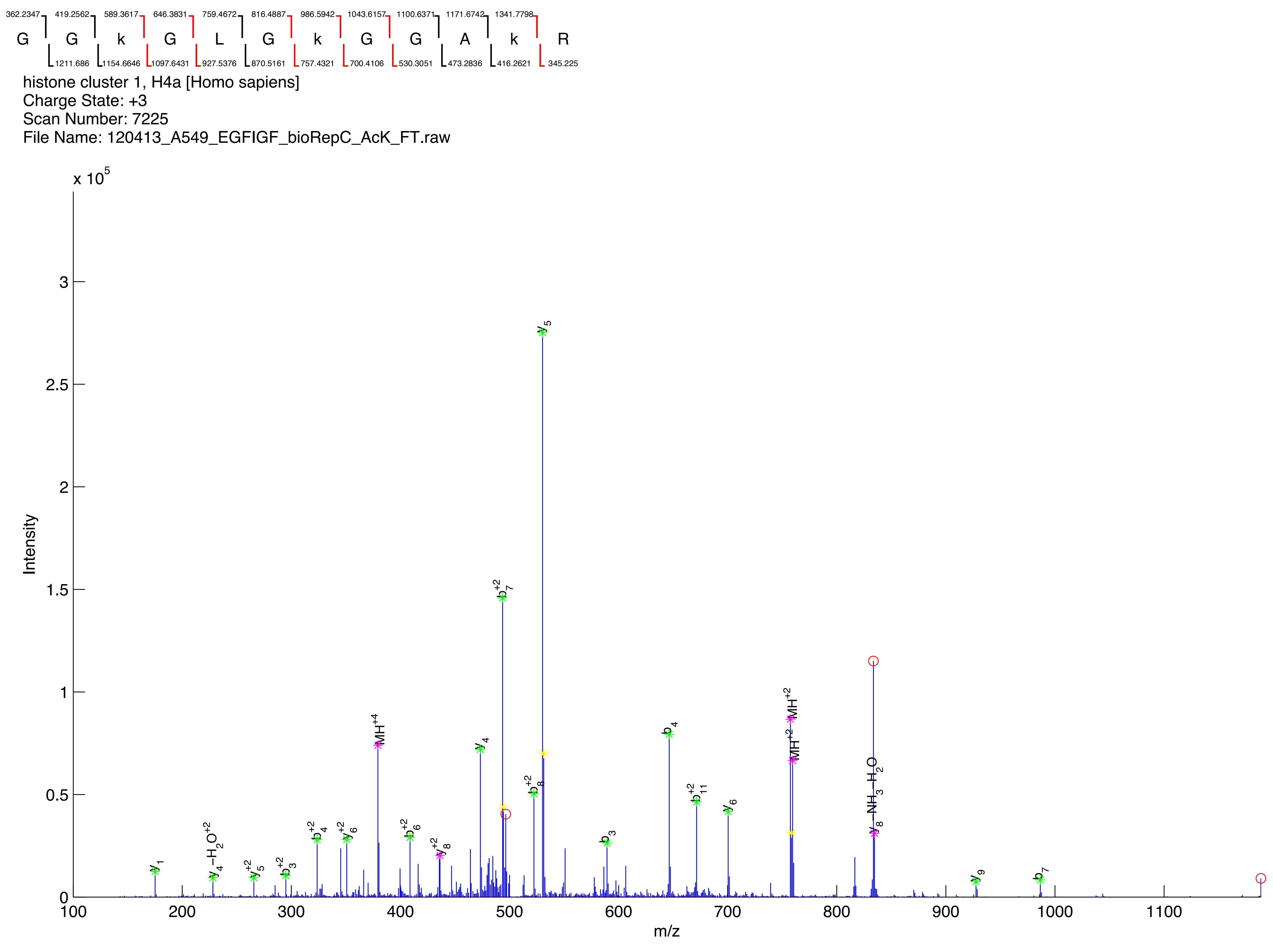
362.2347 419.2562 589.3617 646.3831 759.4672 816.4887 986.5942 1043.6157 1100.6371 1171.6742 1341.7798
G G k G L G k G G A k R
1211.686 1154.6646 1097.6431 927.5376 870.5161 757.4321 700.4106 530.3051 473.2836 416.2621 345.225
histone cluster 1, H4a [Homo sapiens]
Charge State: +3
Scan Number: 7225
File Name: 120413_A549_EGFIGF_bioRepC_AcK_FT.raw
x 10^5
Intensity
m/z
y1
y4−H2O+2
y+2 5
b+2 3
b+2 4
y+2 6
MH+4
b+2 6
y+2 8
y4
b+2 7
b+2 8
y5
b3
b4
b+2 11
y6
MH+2
MH+2
y8−NH3−H2O
y9
b7

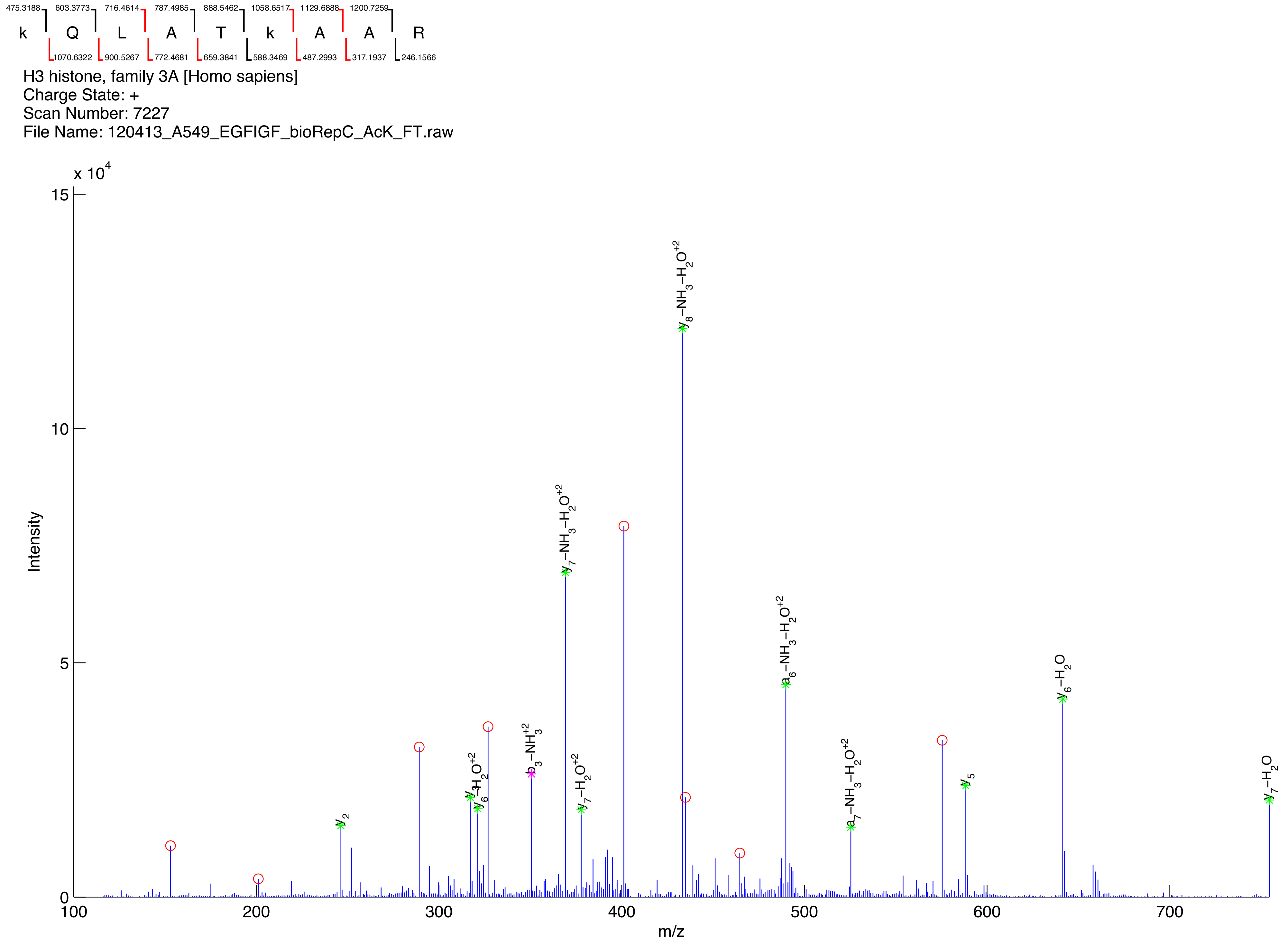
475.3188 603.3773 716.4614 787.4985 888.5462 1058.6517 1129.6888 1200.7259
k Q L A T k A A R
1070.6322 900.5267 772.4681 659.3841 588.3469 487.2993 317.1937 246.1566
H3 histone, family 3A [Homo sapiens]
Charge State: +
Scan Number: 7227
File Name: 120413_A549_EGFIGF_bioRepC_AcK_FT.raw
x 10^4
Intensity
m/z
y2
y6-H2O+2
b3-NH3+2
y7-NH3-H2O+2
y7-H2O+2
y8-NH3-H2O+2
a6-NH3-H2O+2
a7-NH3-H2O+2
y5
y6-H2O
y7-H2O

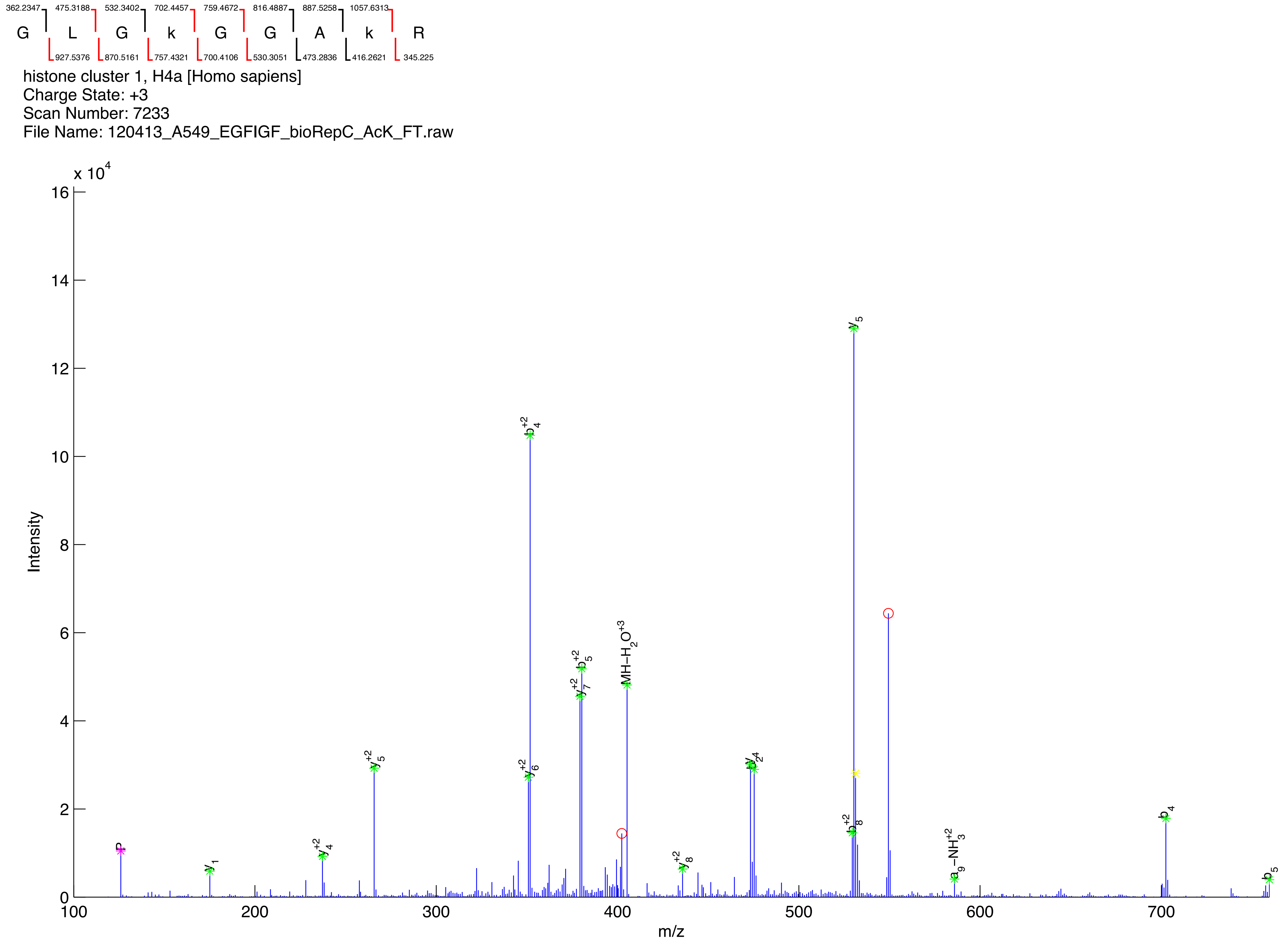

362.2347 475.3188 532.3402 702.4457 759.4672 816.4887 887.5258 1057.6313
G L G k G G A k R
927.5376 870.5161 757.4321 700.4106 530.3051 473.2836 416.2621 345.225
histone cluster 1, H4a [Homo sapiens]
Charge State: +3
Scan Number: 7233
File Name: 120413_A549_EGFIGF_bioRepC_AcK_FT.raw
Intensity
x 10^4
m/z

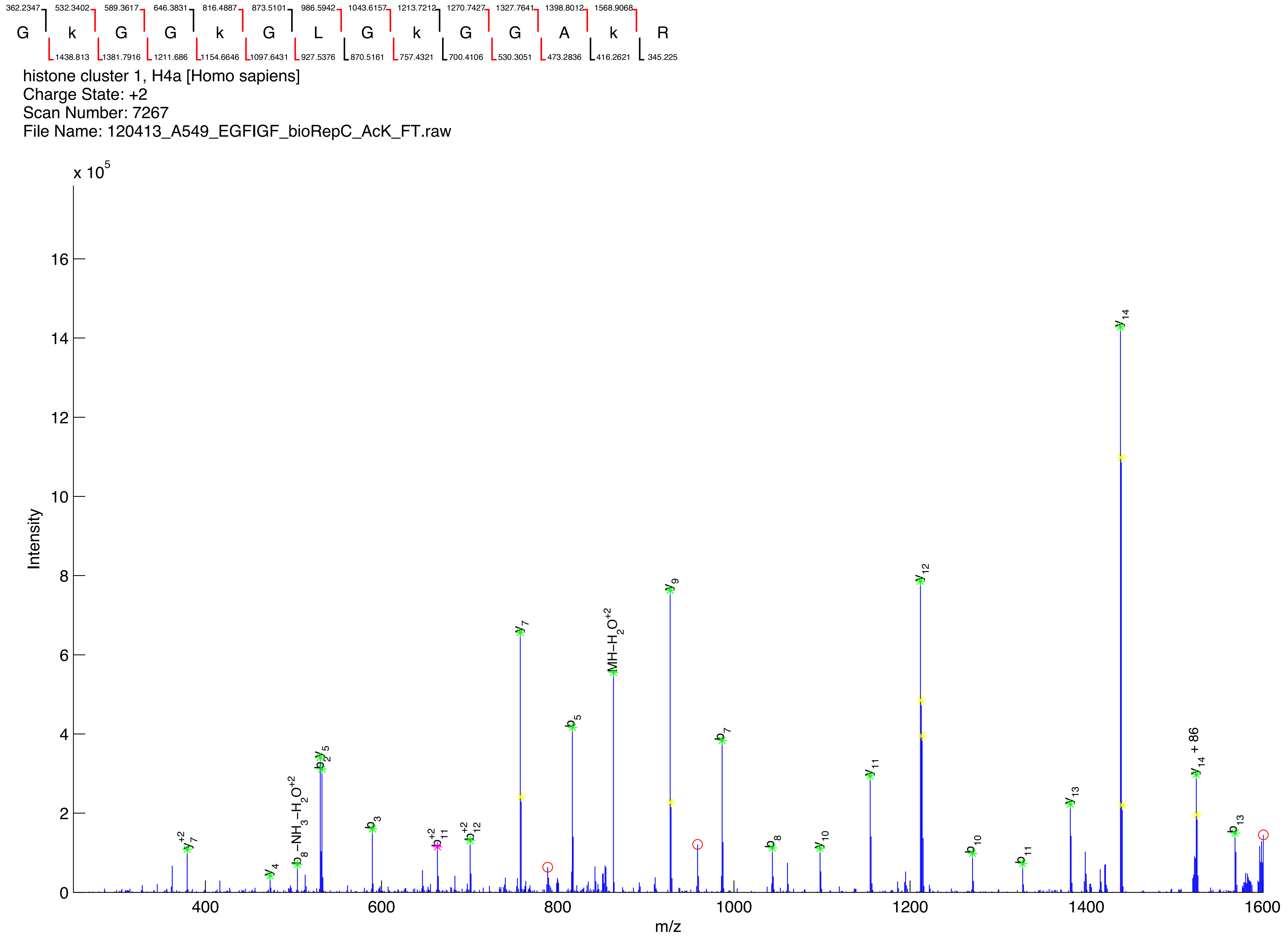
362.2347 532.3402 589.3617 646.3831 816.4887 873.5101 986.5942 1043.6157 1213.7212 1270.7427 1327.7641 1398.8012 1568.9068
G k G G k G L G k G G A k R
1438.813 1381.7916 1211.686 1154.6646 1097.6431 927.5376 870.5161 757.4321 700.4106 530.3051 473.2836 416.2621 345.225
histone cluster 1, H4a [Homo sapiens]
Charge State: +2
Scan Number: 7267
File Name: 120413_A549_EGFIGF_bioRepC_AcK_FT.raw
x 10^5
Intensity
m/z
y+2 7
y 4
b 8 −NH 3 −H 2 O+2
b y 2 5
b 3
b+2 11
b+2 12
y 7
b 5
MH−H 2 O+2
y 9
b 7
b 8
y 10
y 11
y 12
b 10
b 11
y 13
y 14
y 14 + 86
b 13

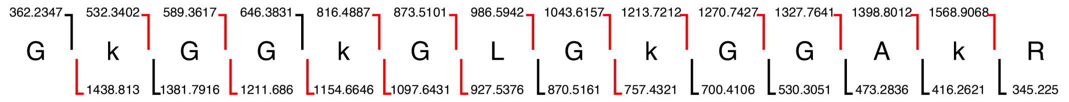

histone cluster 1, H4a [Homo sapiens]
Charge State: +1
Scan Number: 7296
File Name: 120413_A549_EGFIGF_bioRepC_AcK_FT.raw

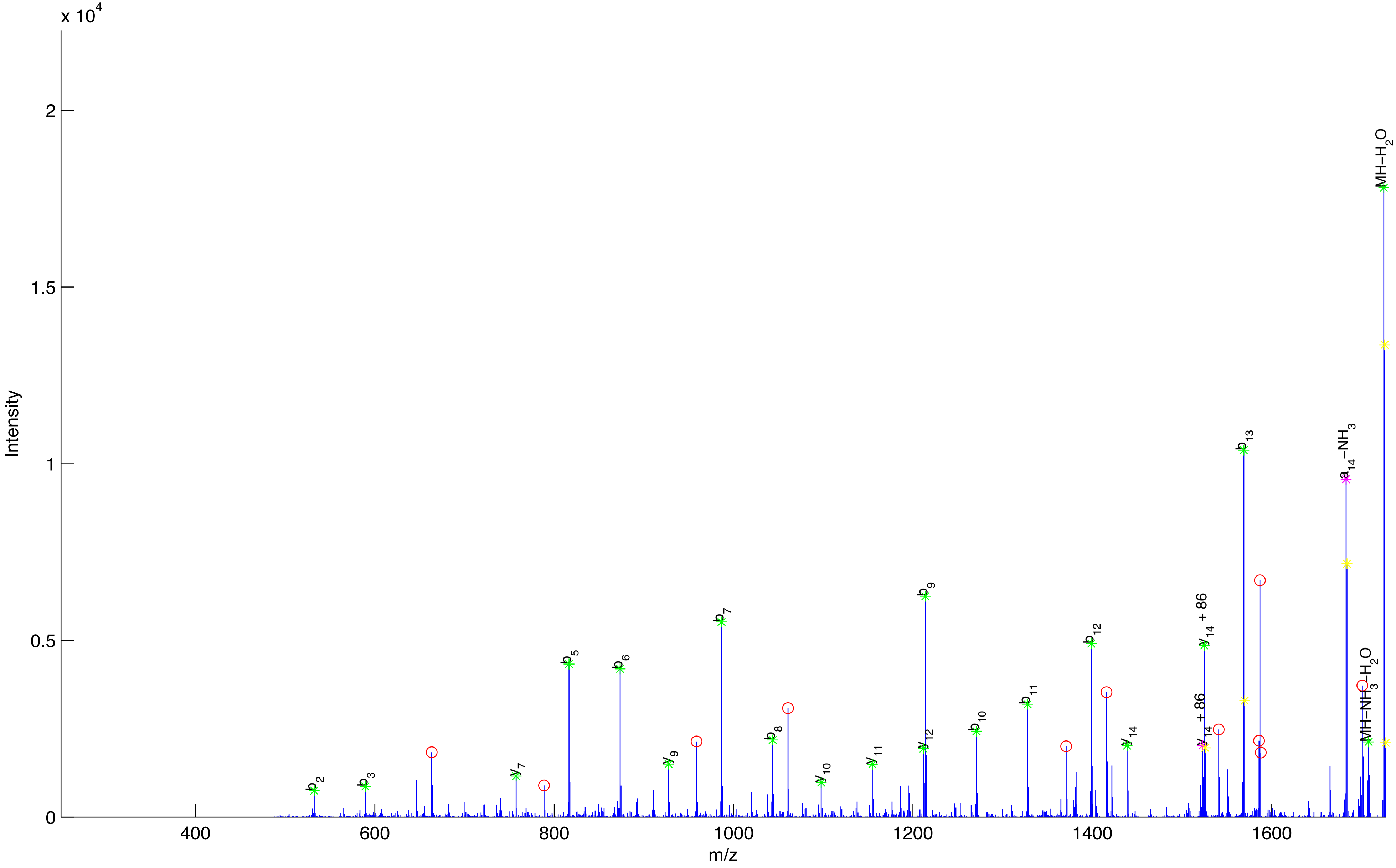

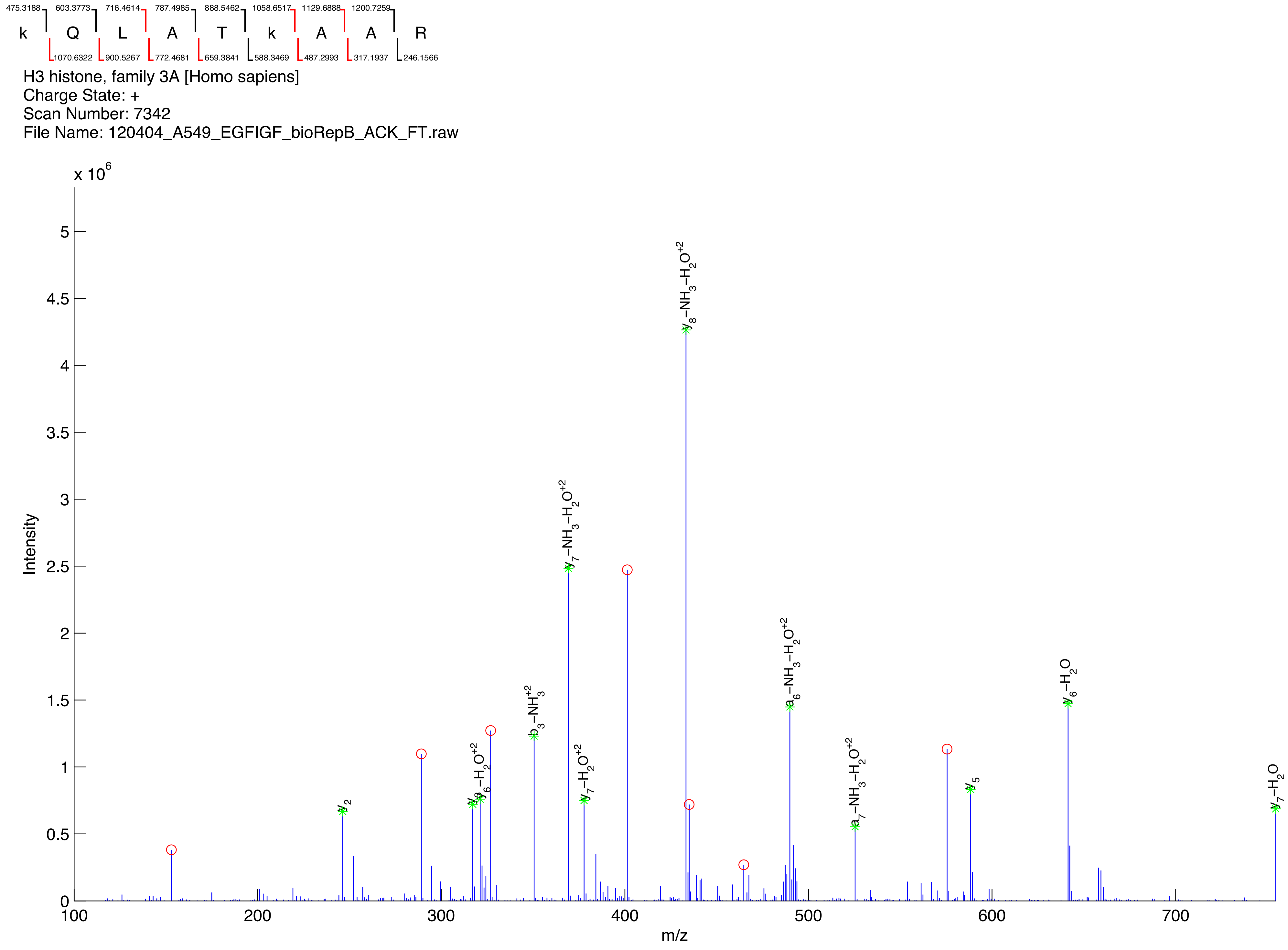
475.3188 603.3773 716.4614 787.4985 888.5462 1058.6517 1129.6888 1200.7259
k Q L A T k A A R
1070.6322 900.5267 772.4681 659.3841 588.3469 487.2993 317.1937 246.1566
H3 histone, family 3A [Homo sapiens]
Charge State: +
Scan Number: 7342
File Name: 120404_A549_EGFIGF_bioRepB_ACK_FT.raw
x 10^6
Intensity
m/z
y2
y6-H2O+2
b3-NH3+2
y7-NH3-H2O+2
y7-H2O+2
y8-NH3-H2O+2
a6-NH3-H2O+2
a7-NH3-H2O+2
y5
y6-H2O
y7-H2O

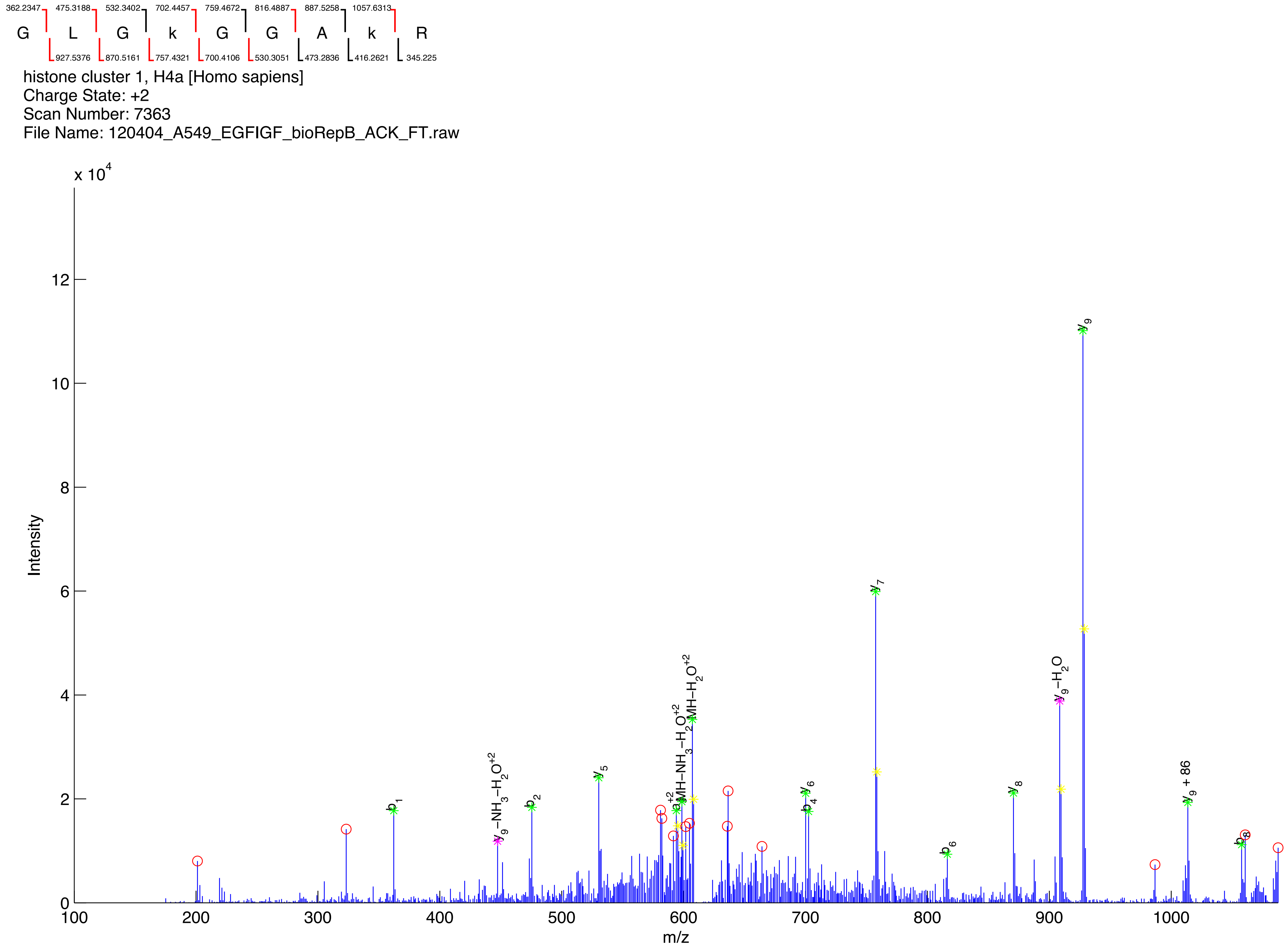
362.2347 475.3188 532.3402 702.4457 759.4672 816.4887 887.5258 1057.6313
G L G k G G A k R
927.5376 870.5161 757.4321 700.4106 530.3051 473.2836 416.2621 345.225
histone cluster 1, H4a [Homo sapiens]
Charge State: +2
Scan Number: 7363
File Name: 120404_A549_EGFIGF_bioRepB_ACK_FT.raw
x 10^4
Intensity
m/z
b1
y9−NH3−H2O+2
b2
y5
a+2
MH−NH3−H2O+2
MH−H2O+2
y6
b4
y7
b6
y8
y9−H2O
y9
y9 + 86
b8

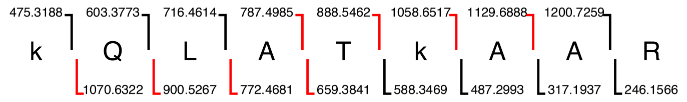

H3 histone, family 3A [Homo sapiens]
Charge State: +
Scan Number: 7405
File Name: 120404_A549_EGFIGF_bioRepB_ACK_FT.raw

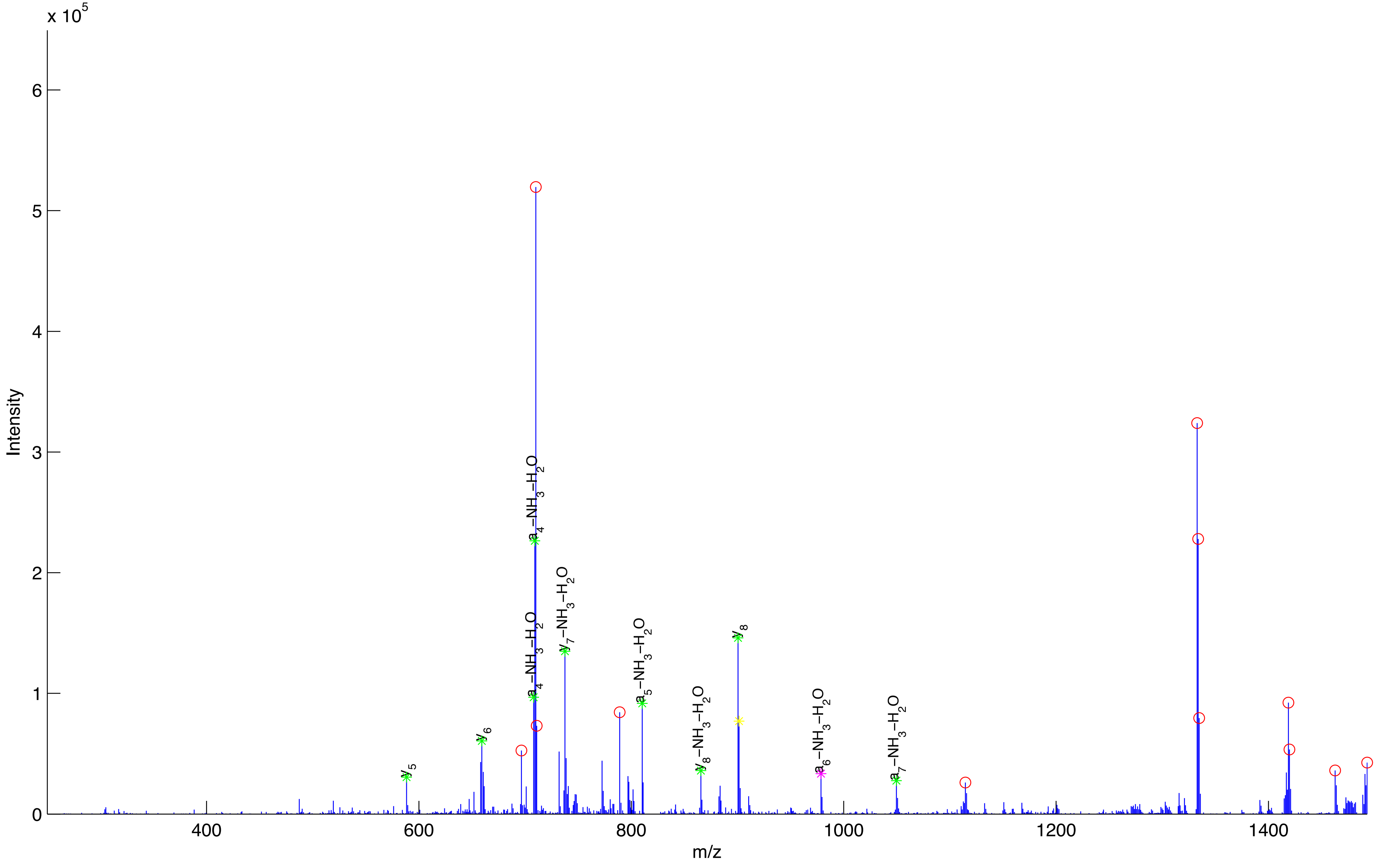

k A V T k A Q K

histone cluster 1, H2bo [Homo sapiens]
Charge State: +3
Scan Number: 7407
File Name: 120404_A549_EGFIGF_bioRepB_ACK_FT.raw

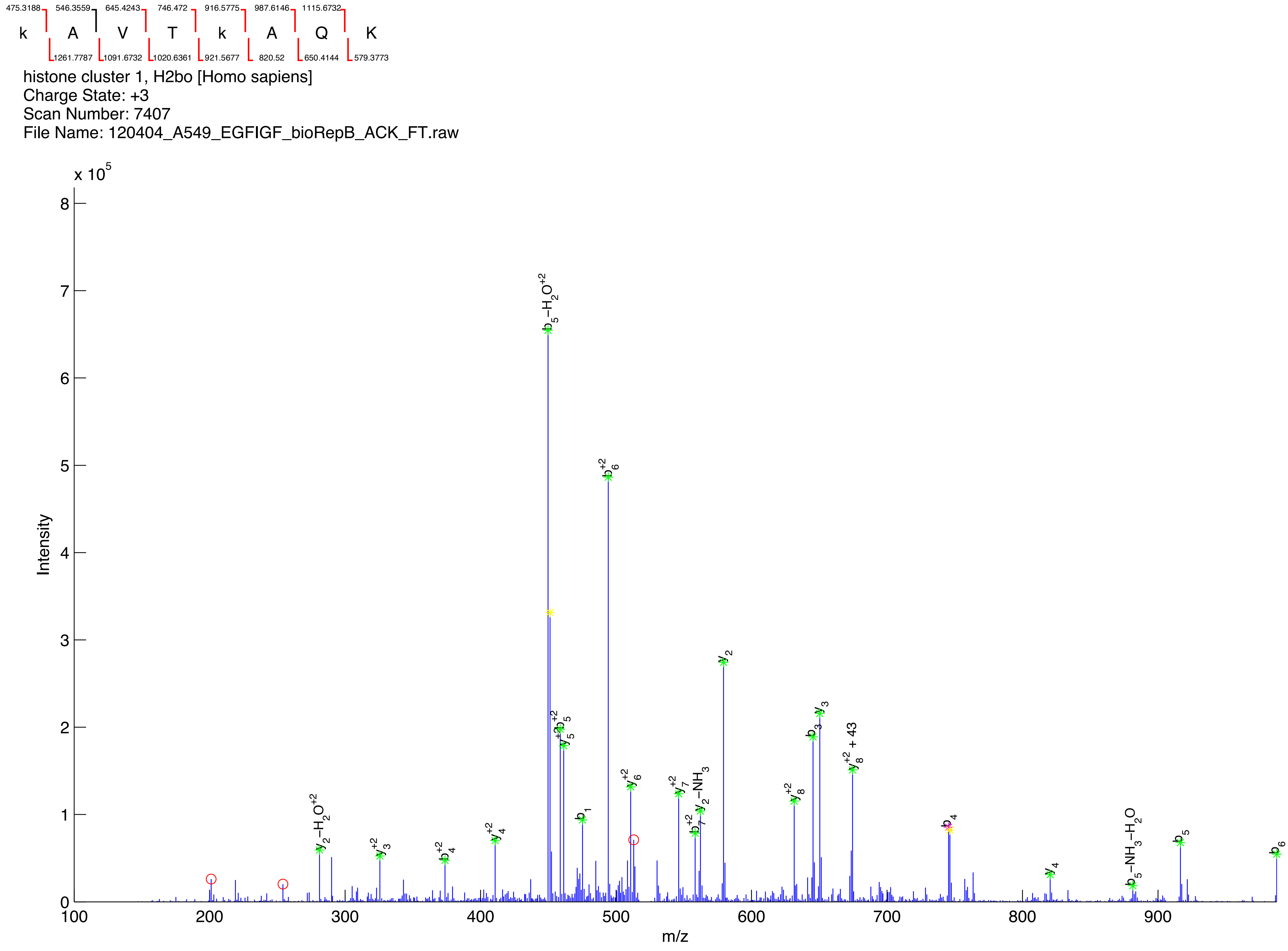

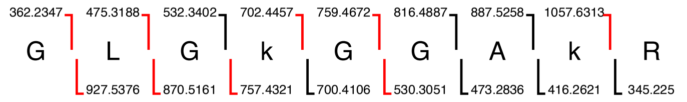

histone cluster 1, H4a [Homo sapiens]
Charge State: +2
Scan Number: 7483
File Name: 120413_A549_EGFIGF_bioRepC_AcK_FT.raw

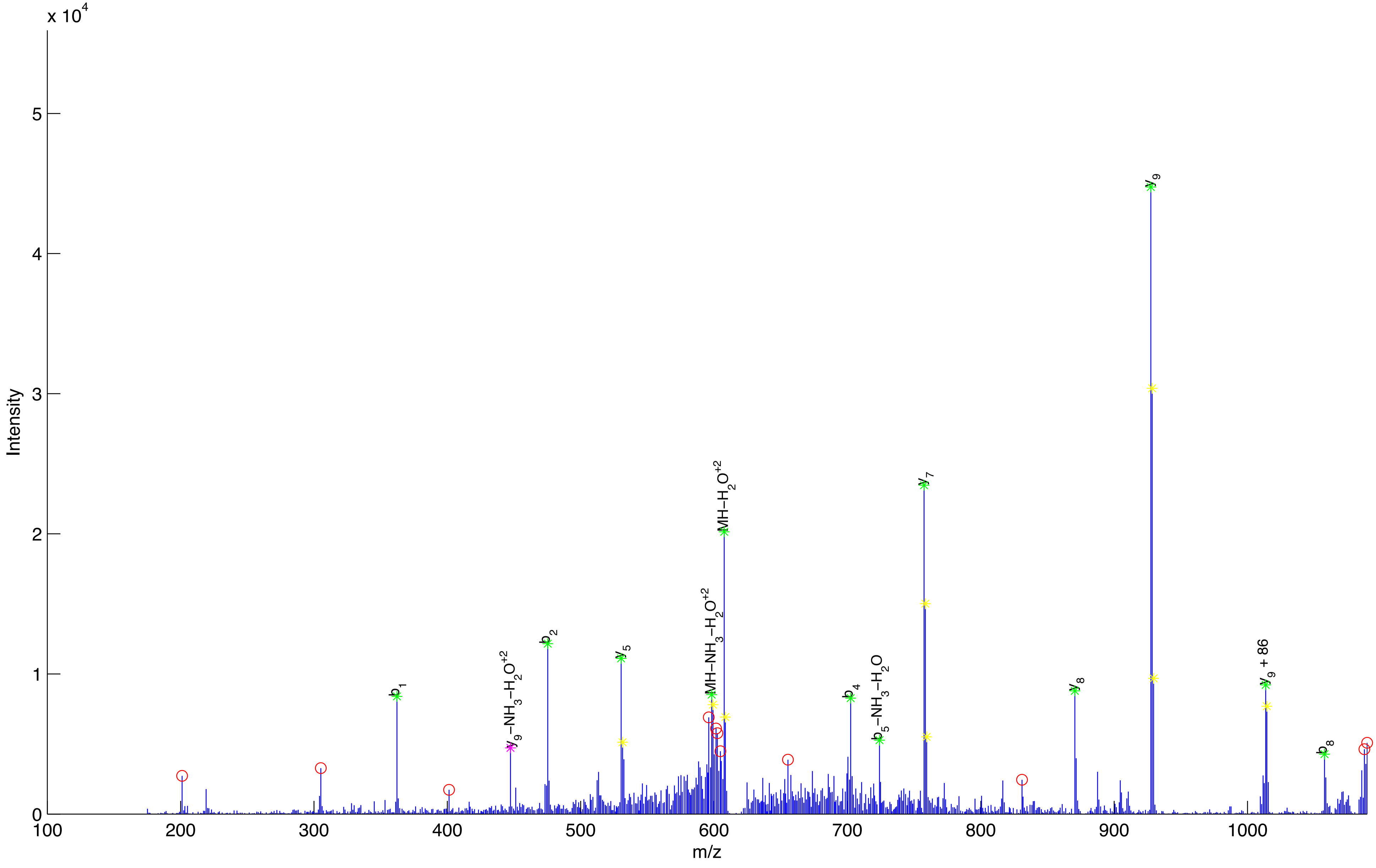

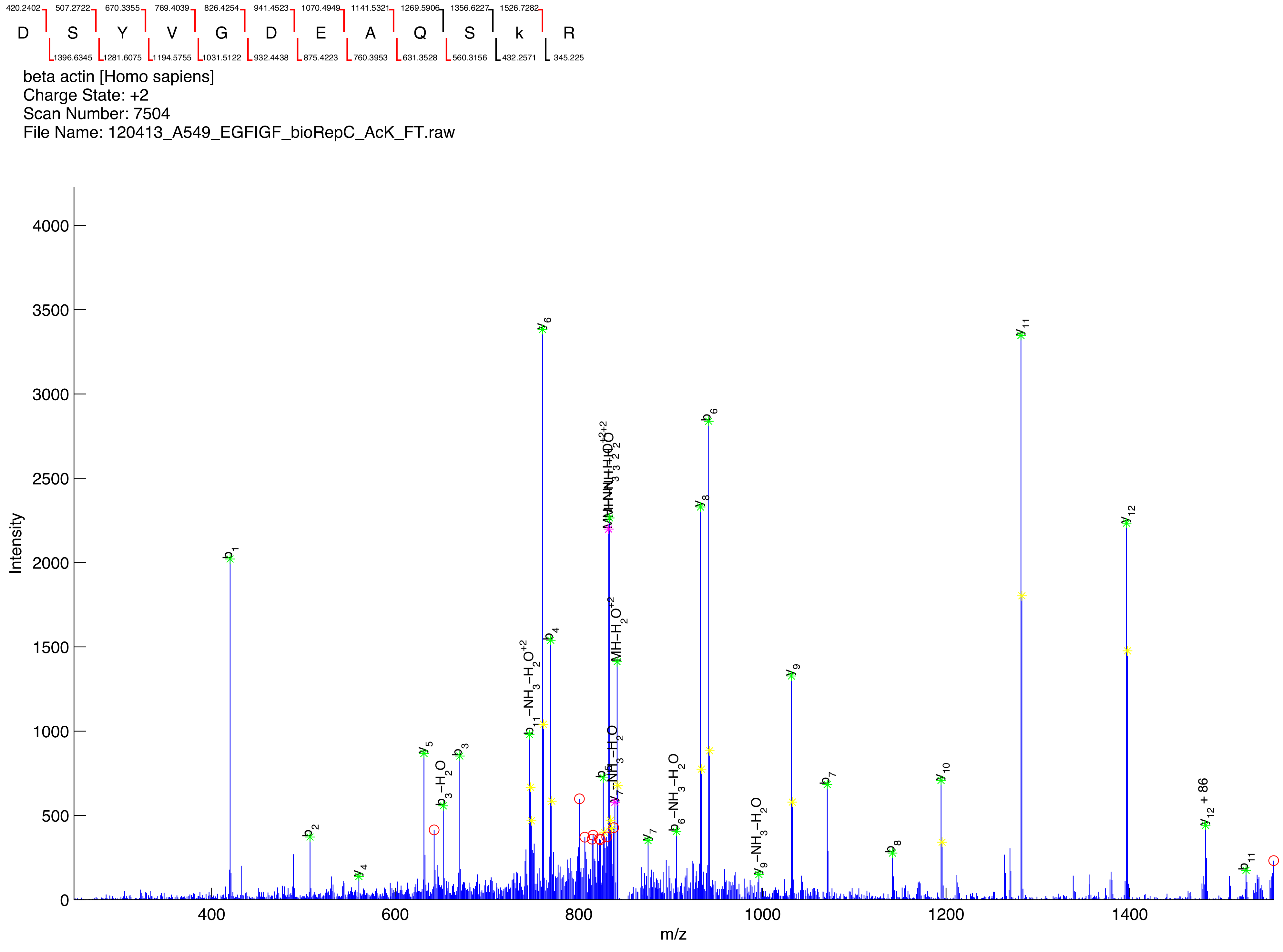
420.2402 507.2722 670.3355 769.4039 826.4254 941.4523 1070.4949 1141.5321 1269.5906 1356.6227 1526.7282
D S Y V G D E A Q S k R
1396.6345 1281.6075 1194.5755 1031.5122 932.4438 875.4223 760.3953 631.3528 560.3156 432.2571 345.225
beta actin [Homo sapiens]
Charge State: +2
Scan Number: 7504
File Name: 120413_A549_EGFIGF_bioRepC_AcK_FT.raw
Intensity
m/z
0
500
1000
1500
2000
2500
3000
3500
4000
400
600
800
1000
1200
1400
b1
b2
y4
y5
b3−H2O
b3
b11−NH3−H2O+2
y6
b4
b5
y7−NH3−H2O
MH−NH3−H2O+2
MH−H2O+2
y7
b6−NH3−H2O
y8
b6
y9−NH3−H2O
y9
b7
b8
y10
y11
y12
y12 + 86
b11

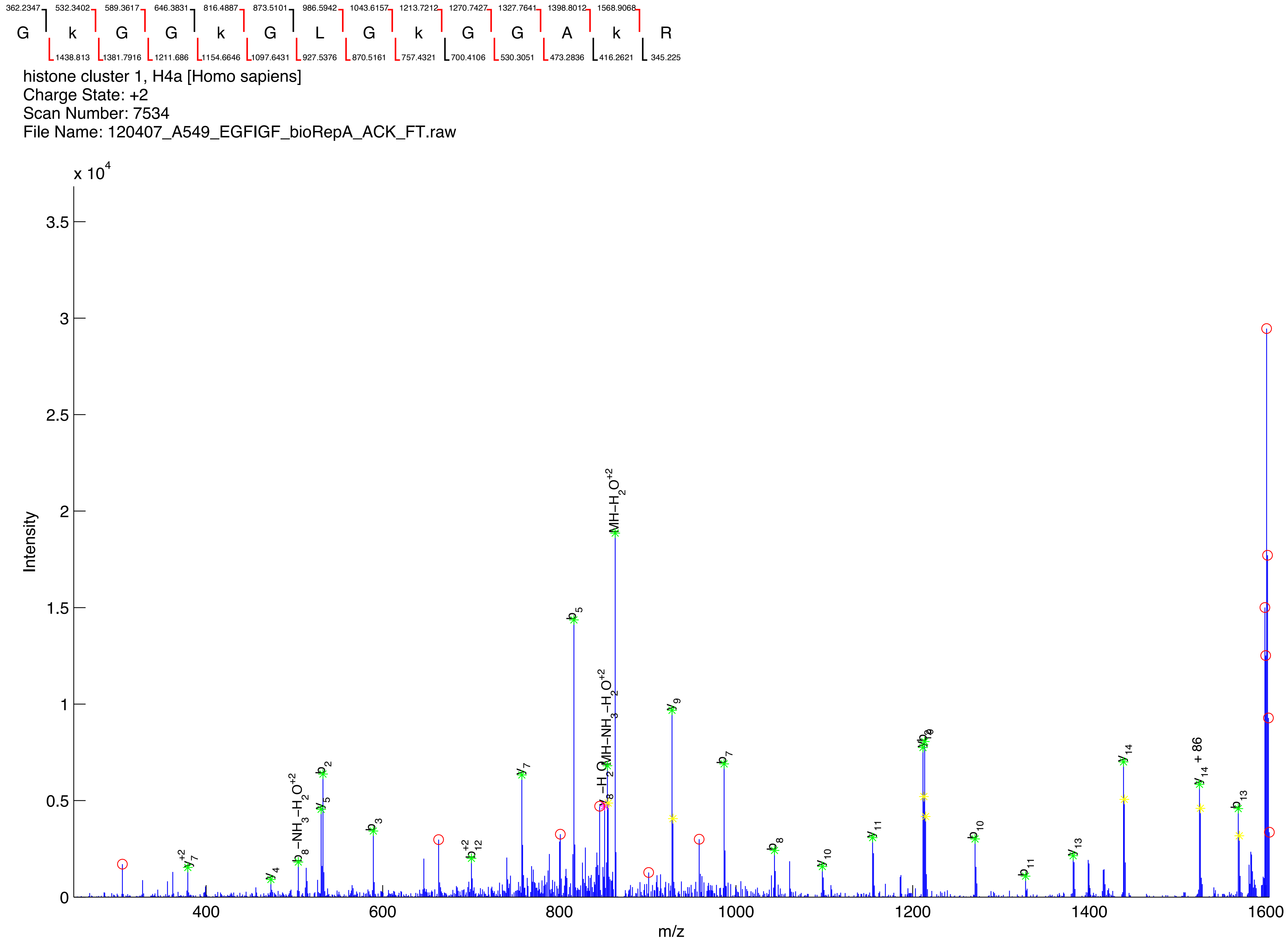
G k G G k G L G k G G A k R
362.2347 532.3402 589.3617 646.3831 816.4887 873.5101 986.5942 1043.6157 1213.7212 1270.7427 1327.7641 1398.8012 1568.9068
1438.813 1381.7916 1211.686 1154.6646 1097.6431 927.5376 870.5161 757.4321 700.4106 530.3051 473.2836 416.2621 345.225
histone cluster 1, H4a [Homo sapiens]
Charge State: +2
Scan Number: 7534
File Name: 120407_A549_EGFIGF_bioRepA_ACK_FT.raw
x 10^4
Intensity
m/z
MH−H2O+2
b5
y9
b7
y7
b2
y5
b3
y4
b12+2
y14
y14 + 86
b13
y13
b11
b10
y11
y10
b8
y8−H2O
MH−NH3−H2O+2
b8−NH3−H2O+2
y7+2

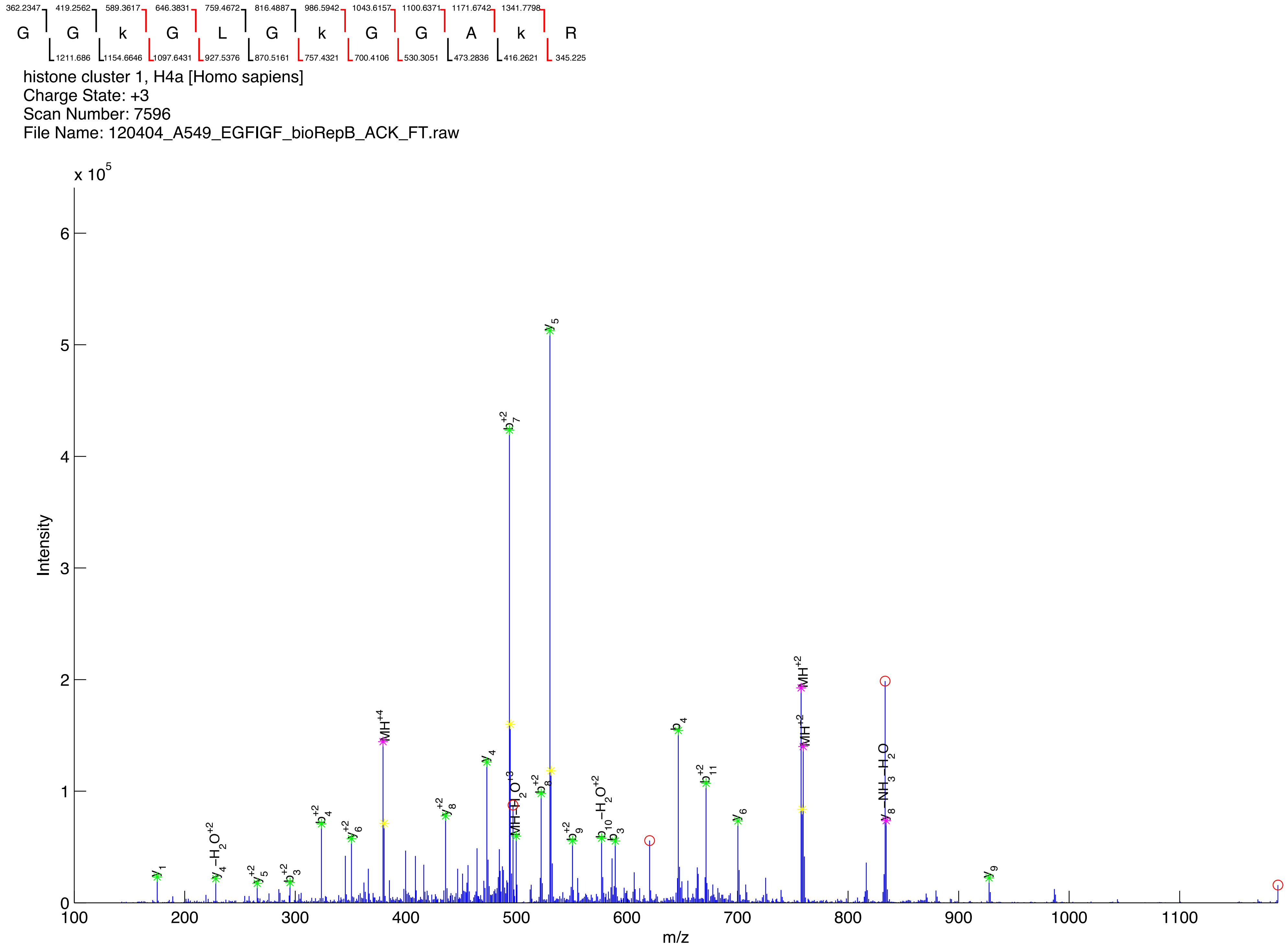

362.2347 419.2562 589.3617 646.3831 759.4672 816.4887 986.5942 1043.6157 1100.6371 1171.6742 1341.7798
G G k G L G k G G A k R
1211.686 1154.6646 1097.6431 927.5376 870.5161 757.4321 700.4106 530.3051 473.2836 416.2621 345.225
histone cluster 1, H4a [Homo sapiens]
Charge State: +3
Scan Number: 7596
File Name: 120404_A549_EGFIGF_bioRepB_ACK_FT.raw
x 10^5
Intensity
m/z

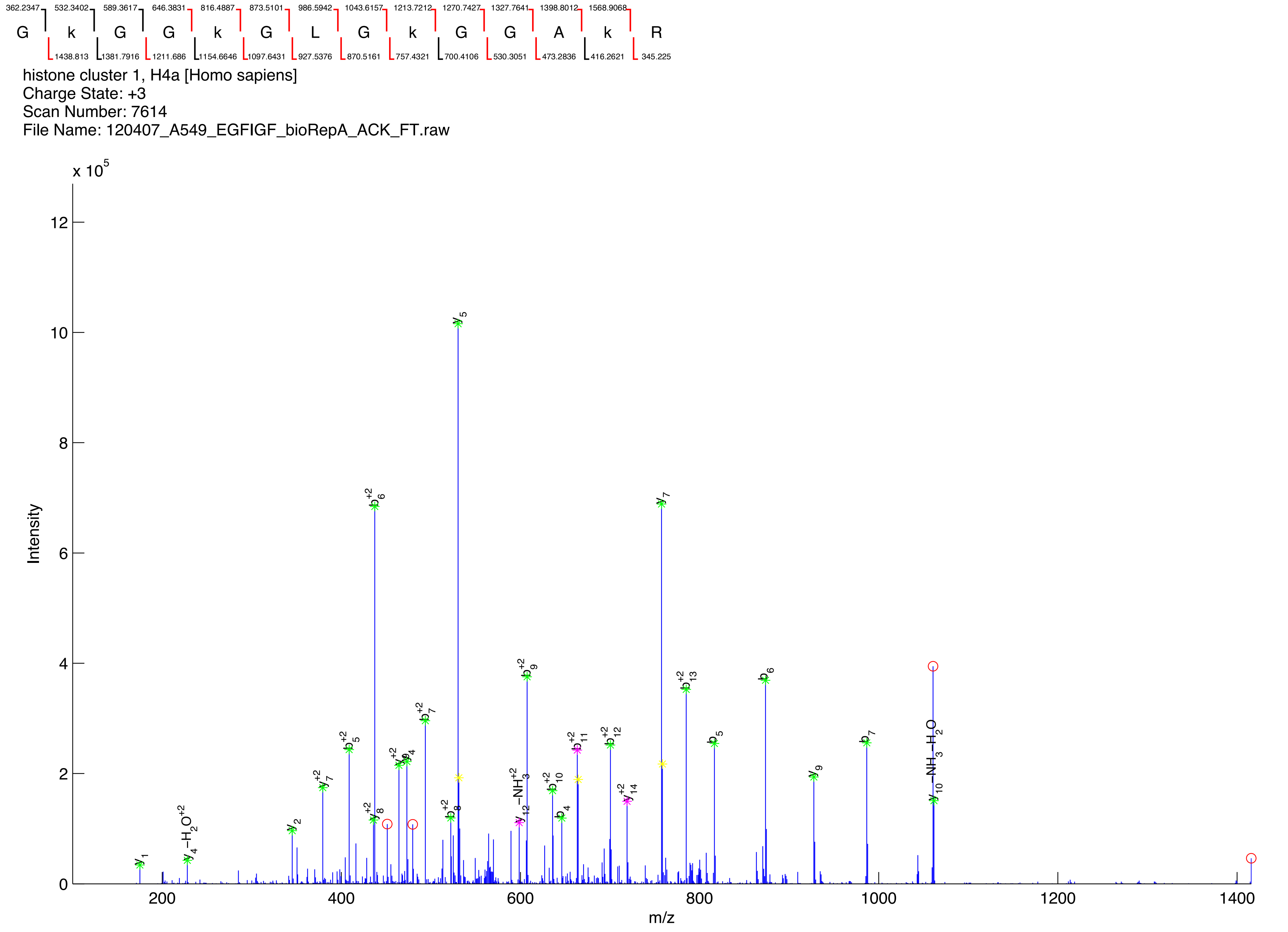
G k G G k G L G k G G A k R
362.2347 532.3402 589.3617 646.3831 816.4887 873.5101 986.5942 1043.6157 1213.7212 1270.7427 1327.7641 1398.8012 1568.9068
1438.813 1381.7916 1211.686 1154.6646 1097.6431 927.5376 870.5161 757.4321 700.4106 530.3051 473.2836 416.2621 345.225
histone cluster 1, H4a [Homo sapiens]
Charge State: +3
Scan Number: 7614
File Name: 120407_A549_EGFIGF_bioRepA_ACK_FT.raw
x 10^5
Intensity
m/z

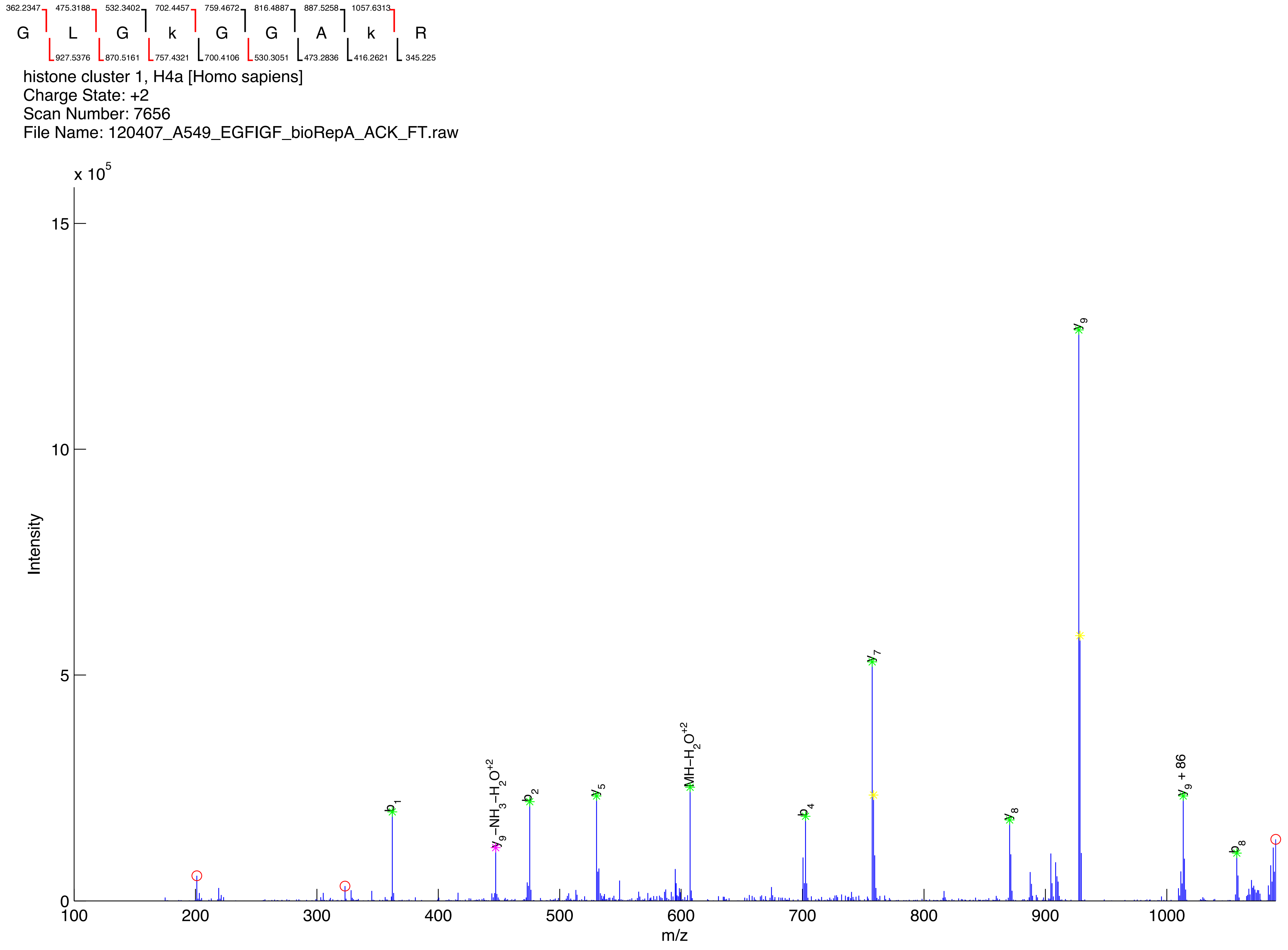
362.2347 475.3188 532.3402 702.4457 759.4672 816.4887 887.5258 1057.6313
G L G k G G A k R
927.5376 870.5161 757.4321 700.4106 530.3051 473.2836 416.2621 345.225
histone cluster 1, H4a [Homo sapiens]
Charge State: +2
Scan Number: 7656
File Name: 120407_A549_EGFIGF_bioRepA_ACK_FT.raw
x 10^5
Intensity
m/z
b1
y9−NH3−H2O+2
b2
y5
MH−H2O+2
b4
y7
y8
y9
y9 + 86
b8

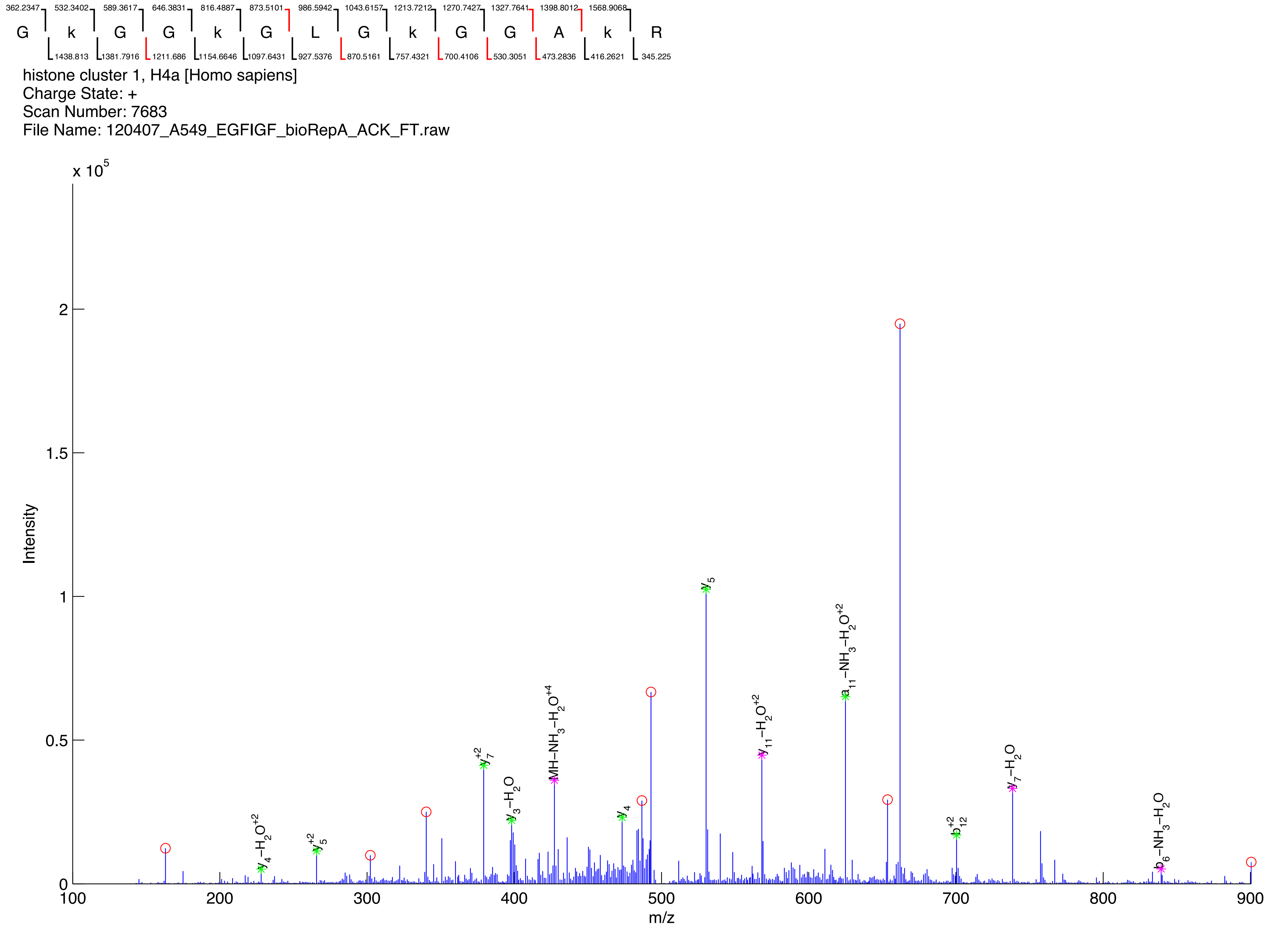
362.2347 532.3402 589.3617 646.3831 816.4887 873.5101 986.5942 1043.6157 1213.7212 1270.7427 1327.7641 1398.8012 1568.9068
G k G G k G L G k G G A k R
1438.813 1381.7916 1211.686 1154.6646 1097.6431 927.5376 870.5161 757.4321 700.4106 530.3051 473.2836 416.2621 345.225
histone cluster 1, H4a [Homo sapiens]
Charge State: +
Scan Number: 7683
File Name: 120407_A549_EGFIGF_bioRepA_ACK_FT.raw
x 10^5
Intensity
m/z
y_4−H_2O^{+2}
y^{+2}_5
y^{+2}_7
y_3−H_2O
MH−NH_3−H_2O^{+4}
y_4
y_5
y_{11}−H_2O^{+2}
a_{11}−NH_3−H_2O^{+2}
b^{+2}_{12}
y_7−H_2O
b_6−NH_3−H_2O

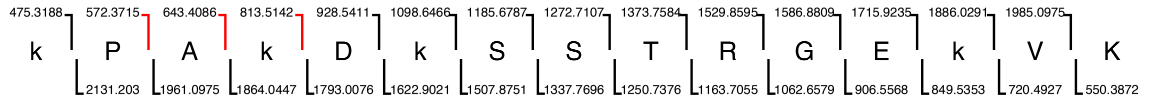

myeloid–lymphoid or mixed–lineage leukemia (trithorax homolog, Drosophila); translocated to, 1 [Hom
Charge State: +
Scan Number: 7683
File Name: 120407_A549_EGFIGF_bioRepA_ACK_FT.raw

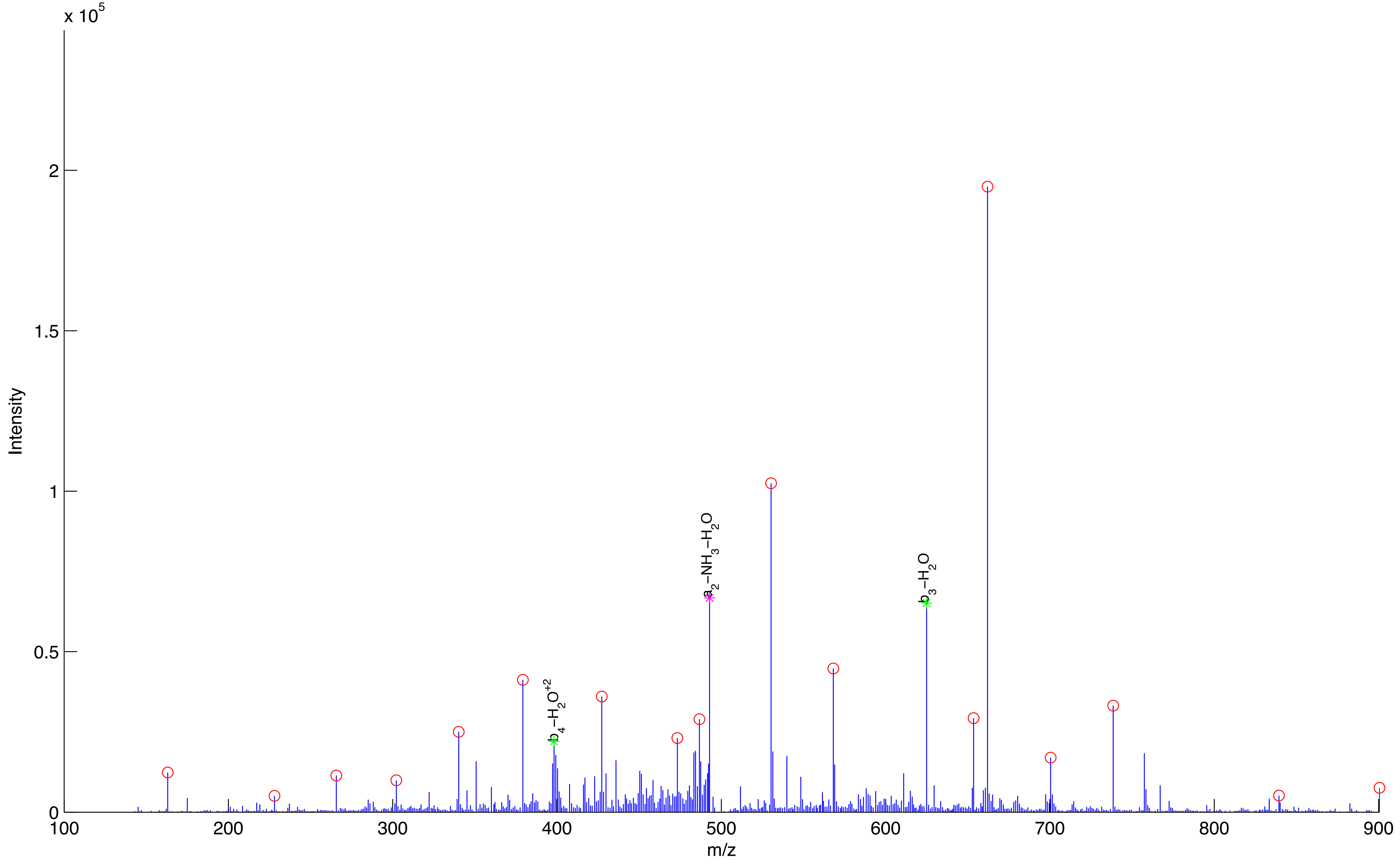

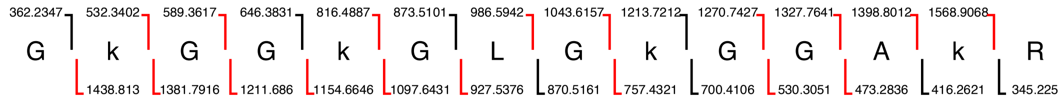

histone cluster 1, H4a [Homo sapiens]
Charge State: +2
Scan Number: 7699
File Name: 120404_A549_EGFIGF_bioRepB_ACK_FT.raw

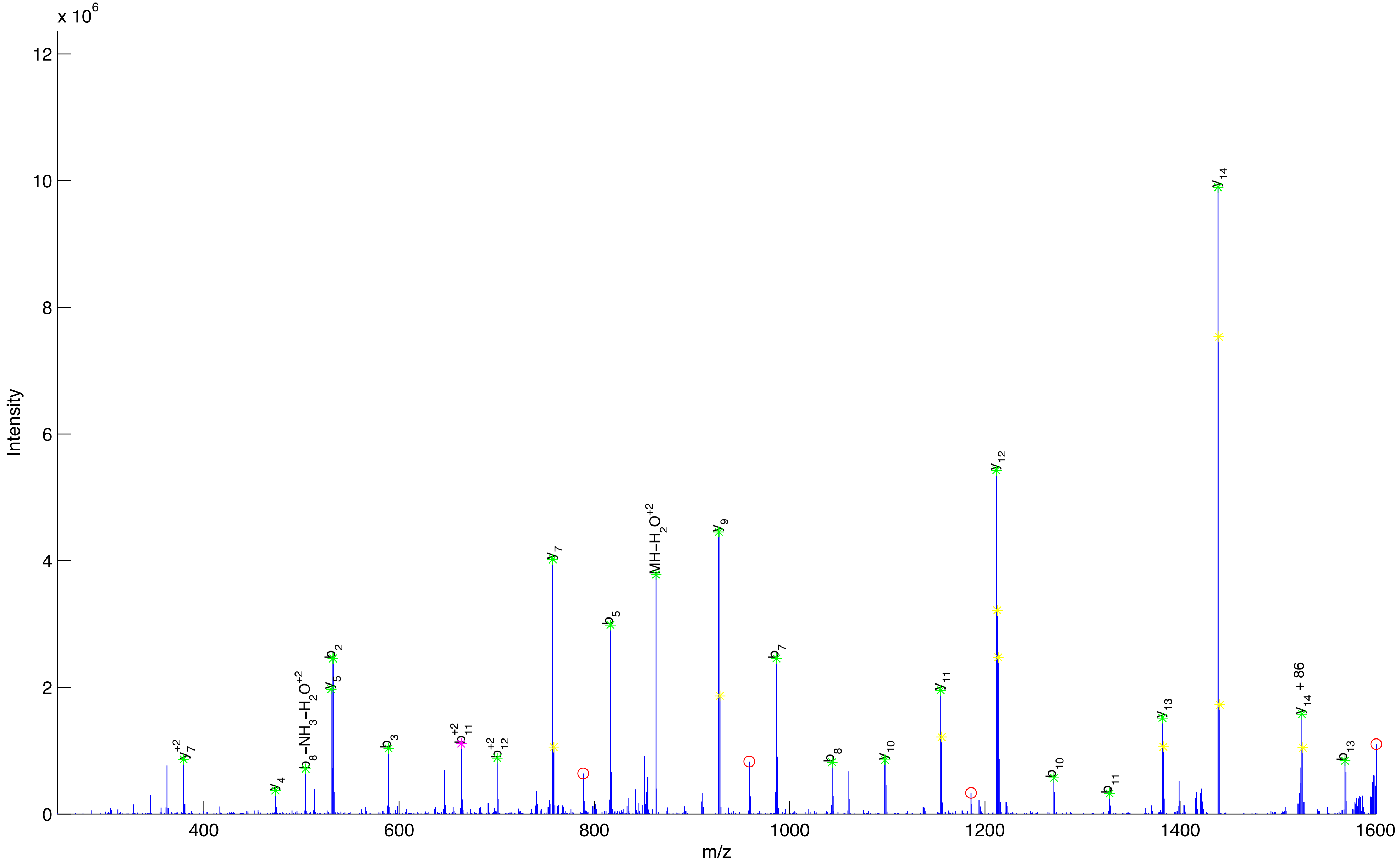

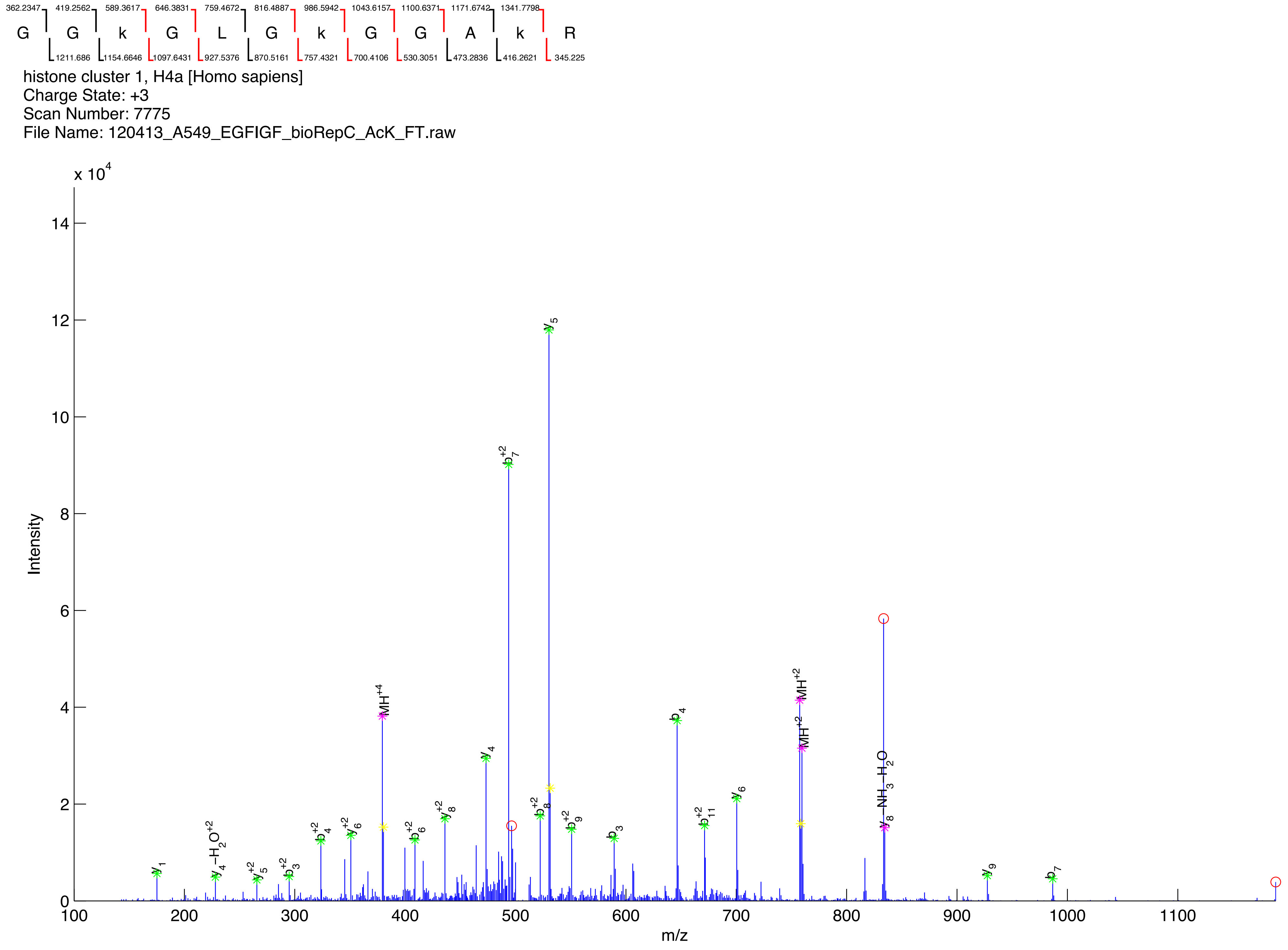
362.2347 419.2562 589.3617 646.3831 759.4672 816.4887 986.5942 1043.6157 1100.6371 1171.6742 1341.7798
G G k G L G k G G A k R
1211.686 1154.6646 1097.6431 927.5376 870.5161 757.4321 700.4106 530.3051 473.2836 416.2621 345.225
histone cluster 1, H4a [Homo sapiens]
Charge State: +3
Scan Number: 7775
File Name: 120413_A549_EGFIGF_bioRepC_AcK_FT.raw
x 10^4
Intensity
m/z

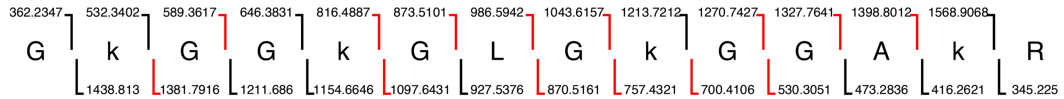

histone cluster 1, H4a [Homo sapiens]
Charge State: +4
Scan Number: 7783
File Name: 120404_A549_EGFIGF_bioRepB_ACK_FT.raw

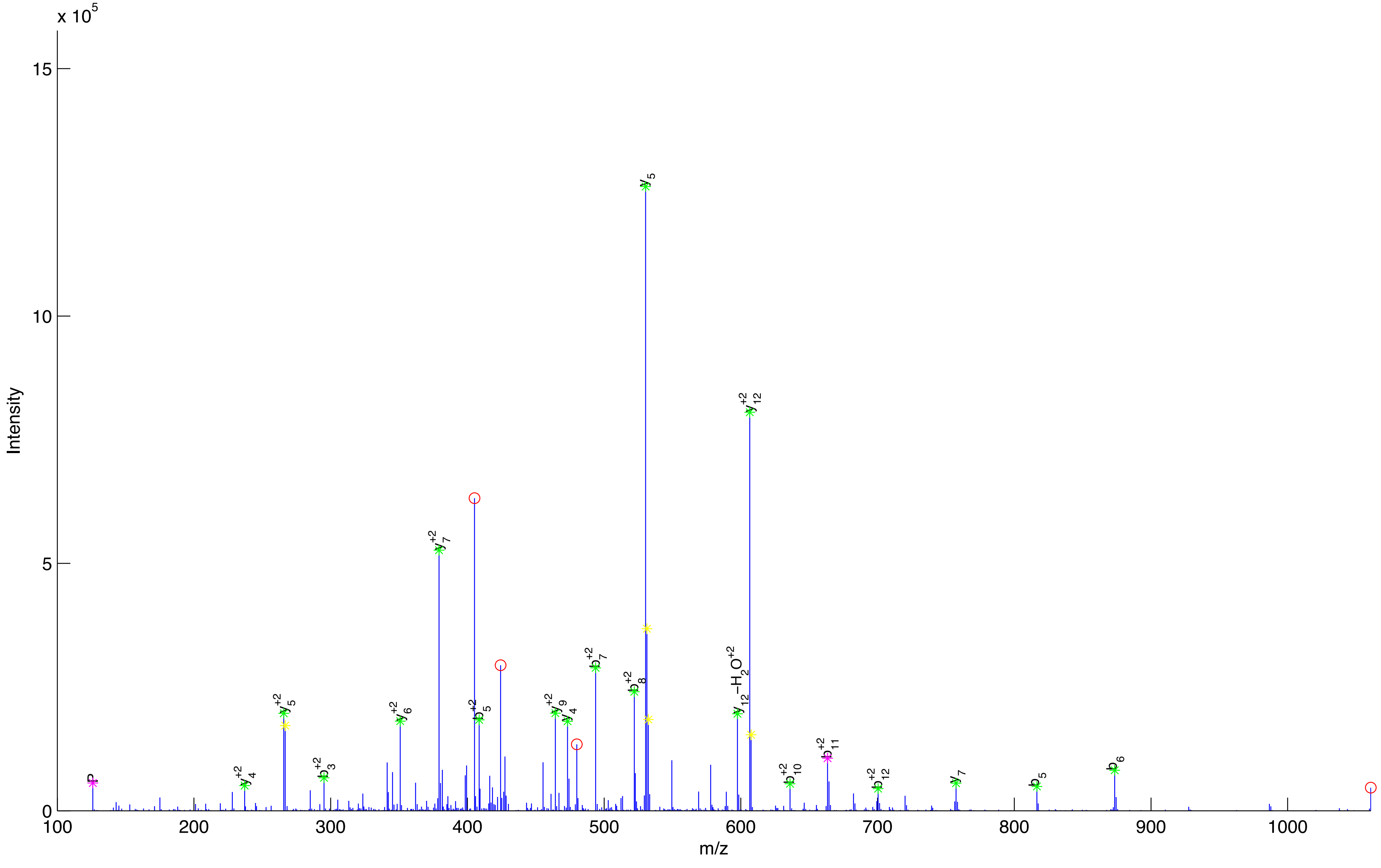

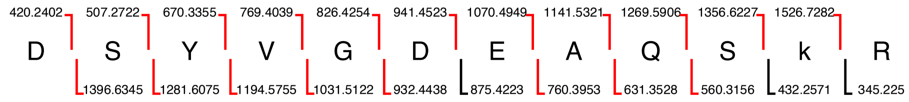

beta actin [Homo sapiens]
Charge State: +2
Scan Number: 7808
File Name: 120404_A549_EGFIGF_bioRepB_ACK_FT.raw

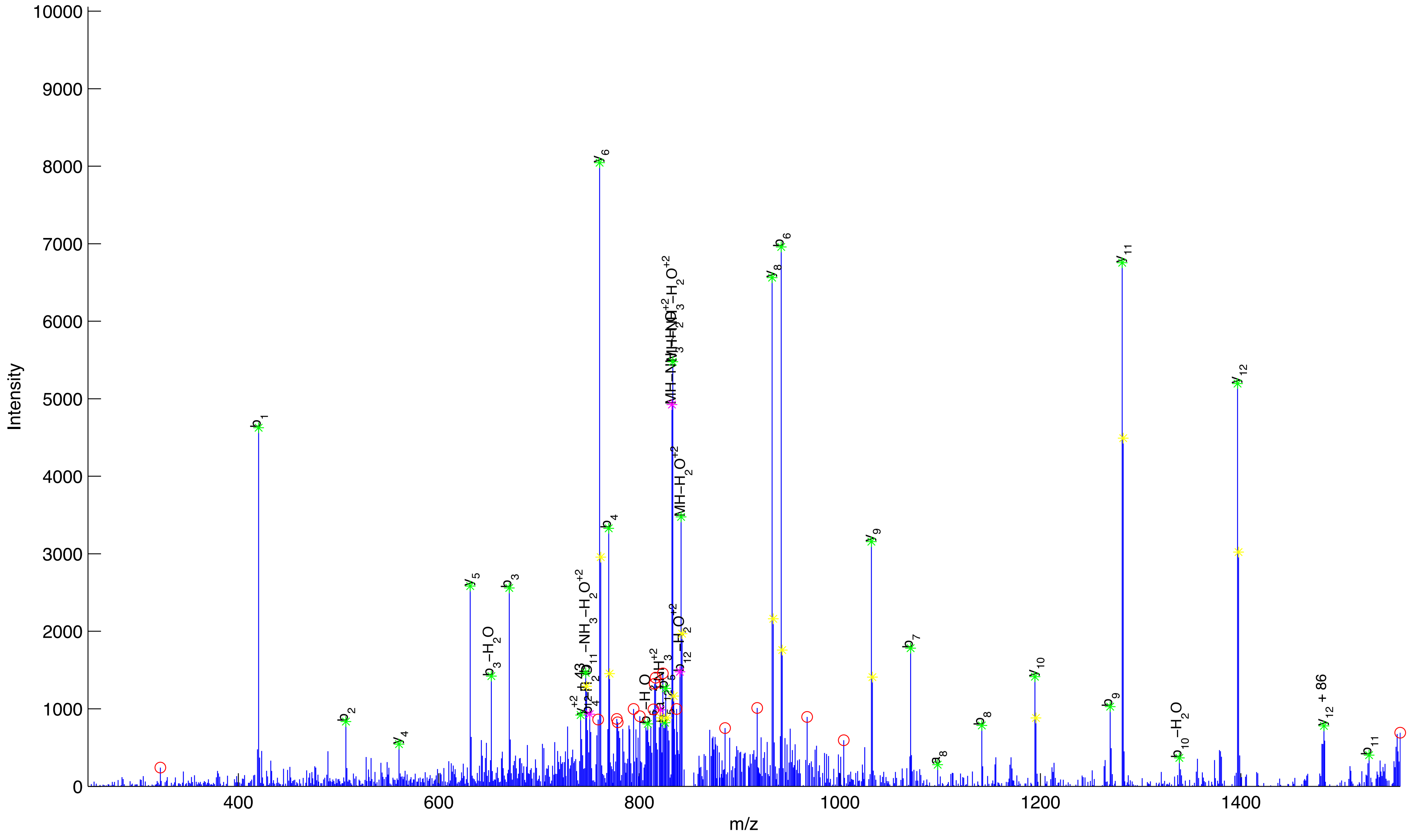

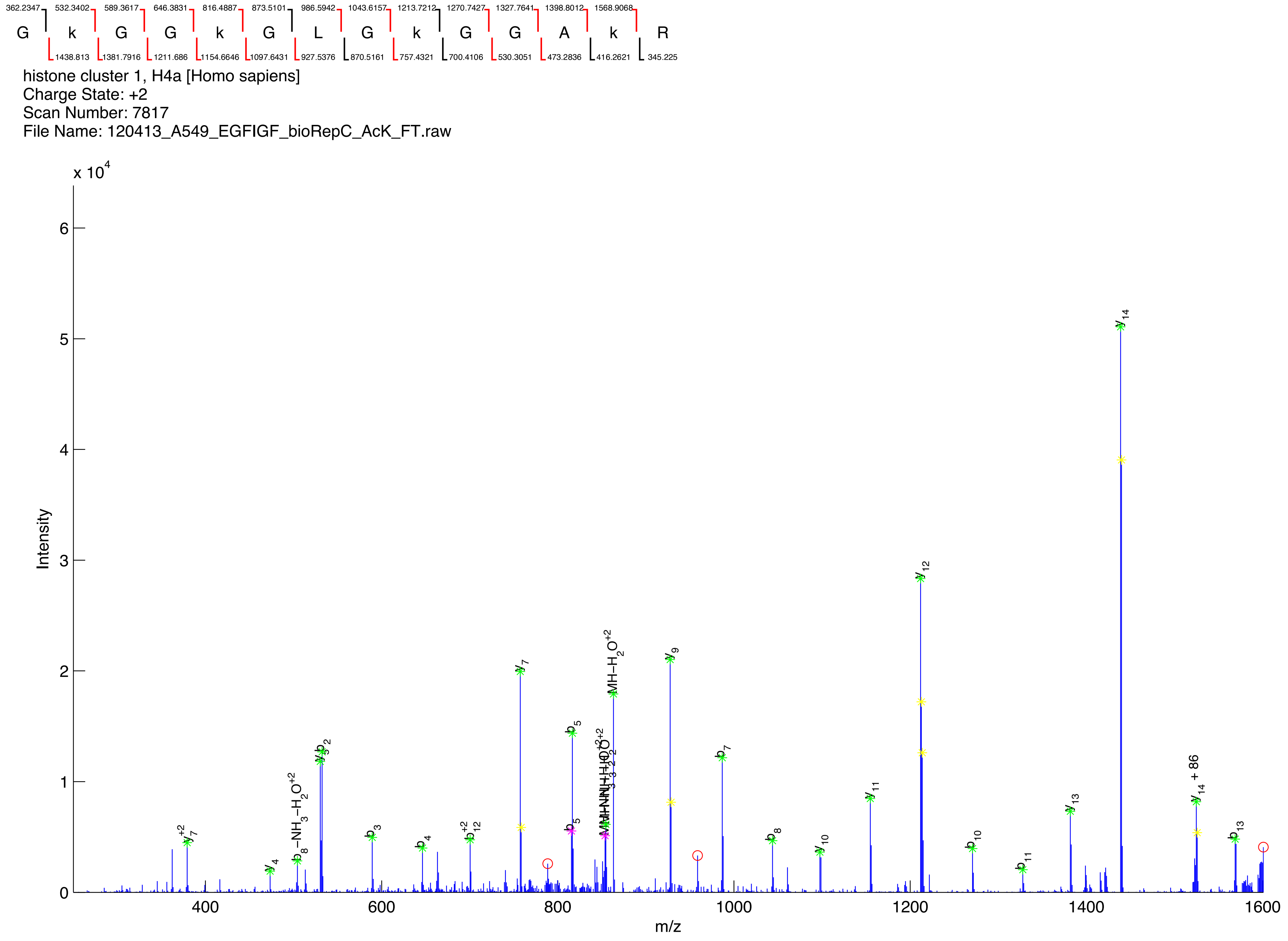

362.2347 532.3402 589.3617 646.3831 816.4887 873.5101 986.5942 1043.6157 1213.7212 1270.7427 1327.7641 1398.8012 1568.9068
G k G G k G L G k G G A k R
1438.813 1381.7916 1211.686 1154.6646 1097.6431 927.5376 870.5161 757.4321 700.4106 530.3051 473.2836 416.2621 345.225
histone cluster 1, H4a [Homo sapiens]
Charge State: +2
Scan Number: 7817
File Name: 120413_A549_EGFIGF_bioRepC_AcK_FT.raw
x 10^4
Intensity
m/z
0
1
2
3
4
5
6
400
600
800
1000
1200
1400
1600
y7+2
y4
b8−NH3−H2O+2
y3
b2
b3
b4
b12+2
y7
b5
MH−H2O+2
y9
b7
b8
y10
y11
y12
b10
b11
y13
y14
y14 + 86
b13

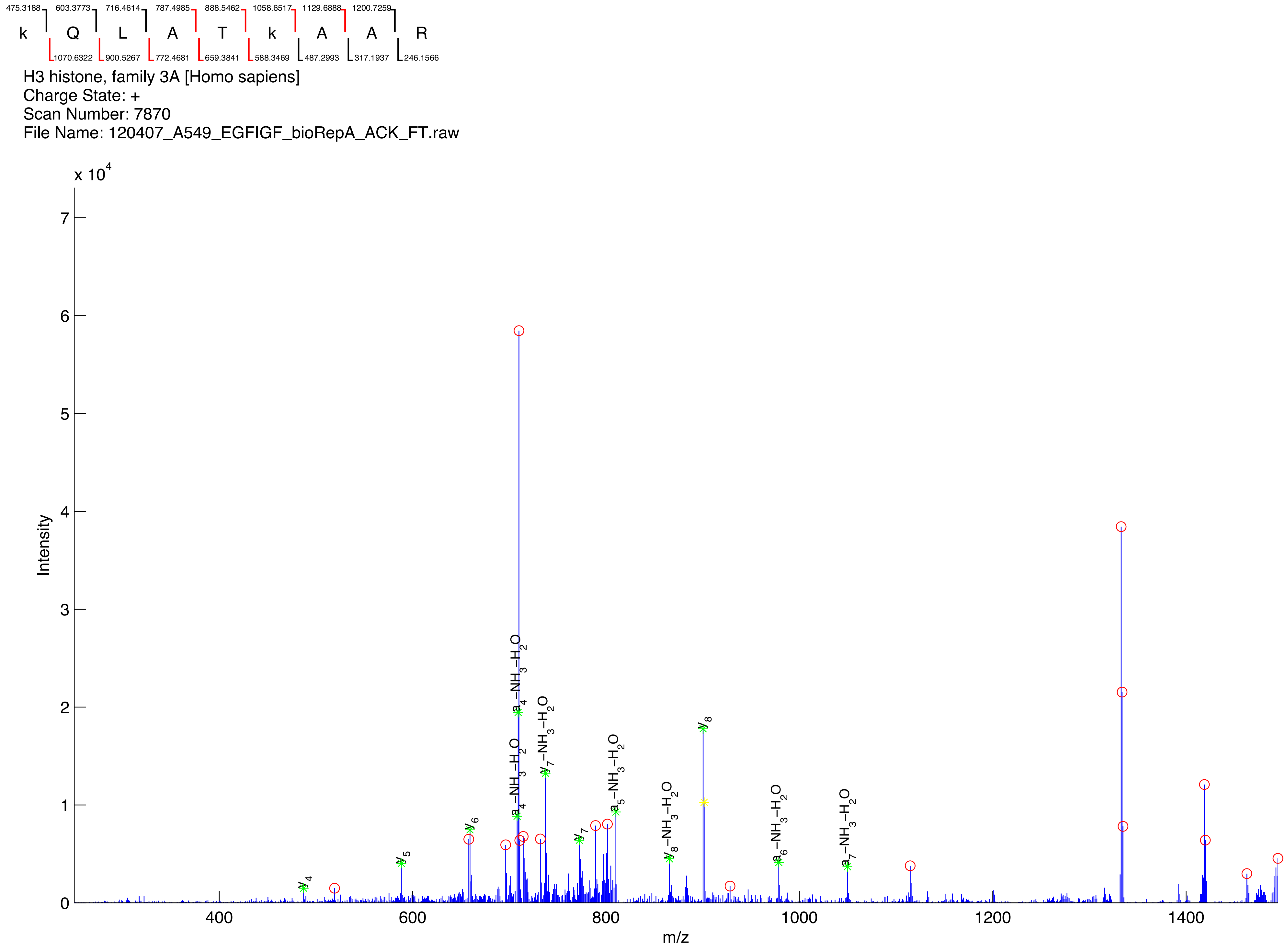

475.3188 603.3773 716.4614 787.4985 888.5462 1058.6517 1129.6888 1200.7259
k Q L A T k A A R
1070.6322 900.5267 772.4681 659.3841 588.3469 487.2993 317.1937 246.1566
H3 histone, family 3A [Homo sapiens]
Charge State: +
Scan Number: 7870
File Name: 120407_A549_EGFIGF_bioRepA_ACK_FT.raw
x 10^4
Intensity
m/z
y4
y5
y6
a4−NH3−H2O
a4−NH3−H2O
y7−NH3−H2O
y7
a5−NH3−H2O
y8−NH3−H2O
y8
a6−NH3−H2O
a7−NH3−H2O

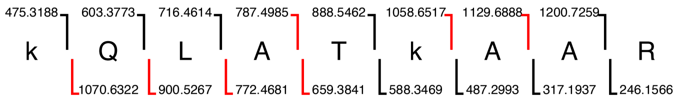

H3 histone, family 3A [Homo sapiens]
Charge State: +
Scan Number: 7908
File Name: 120407_A549_EGFIGF_bioRepA_ACK_FT.raw

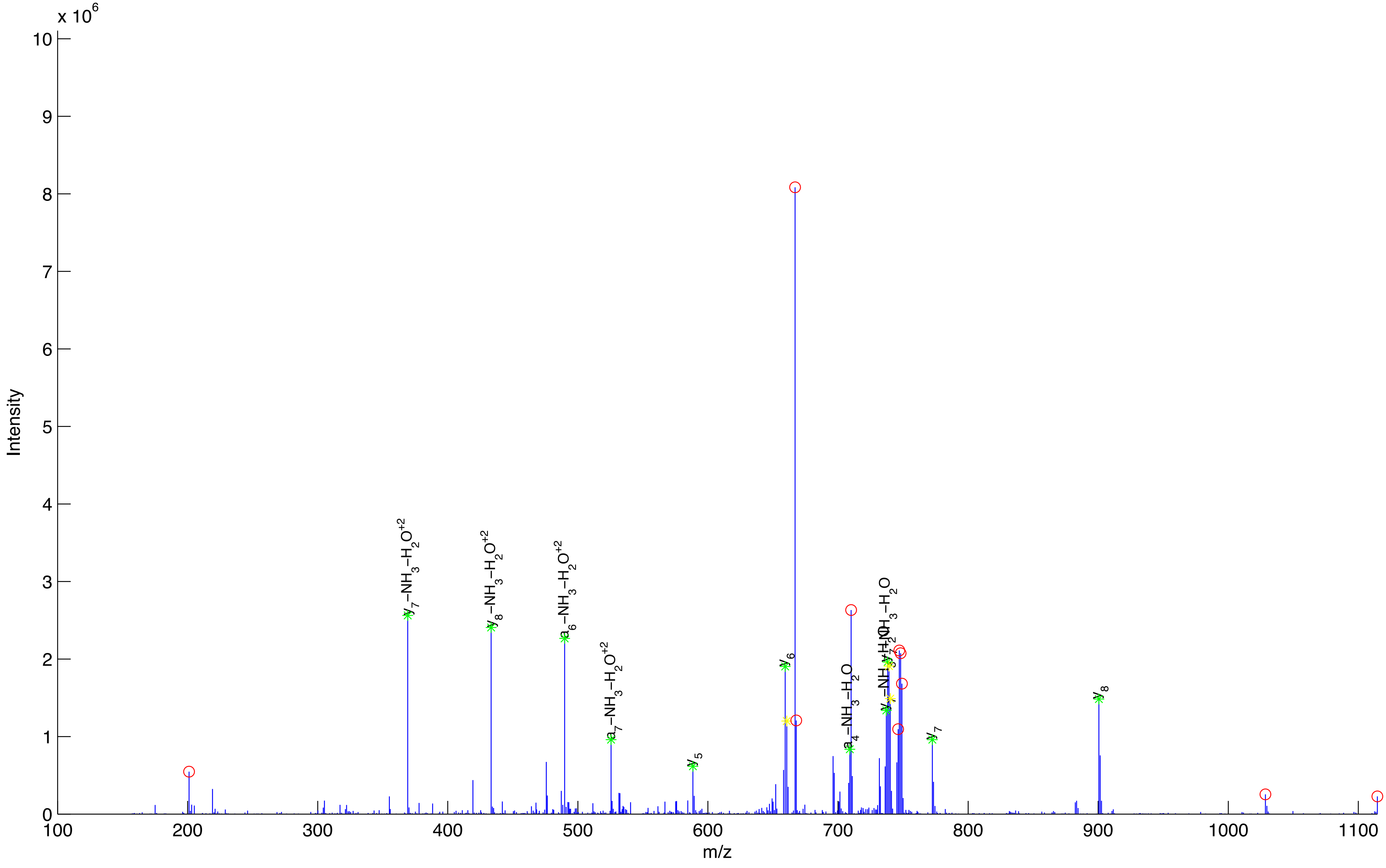

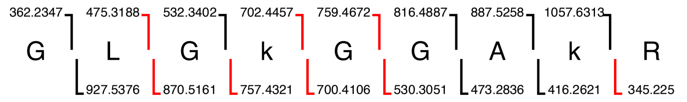

histone cluster 1, H4a [Homo sapiens]
Charge State: +3
Scan Number: 7913
File Name: 120404_A549_EGFIGF_bioRepB_ACK_FT.raw

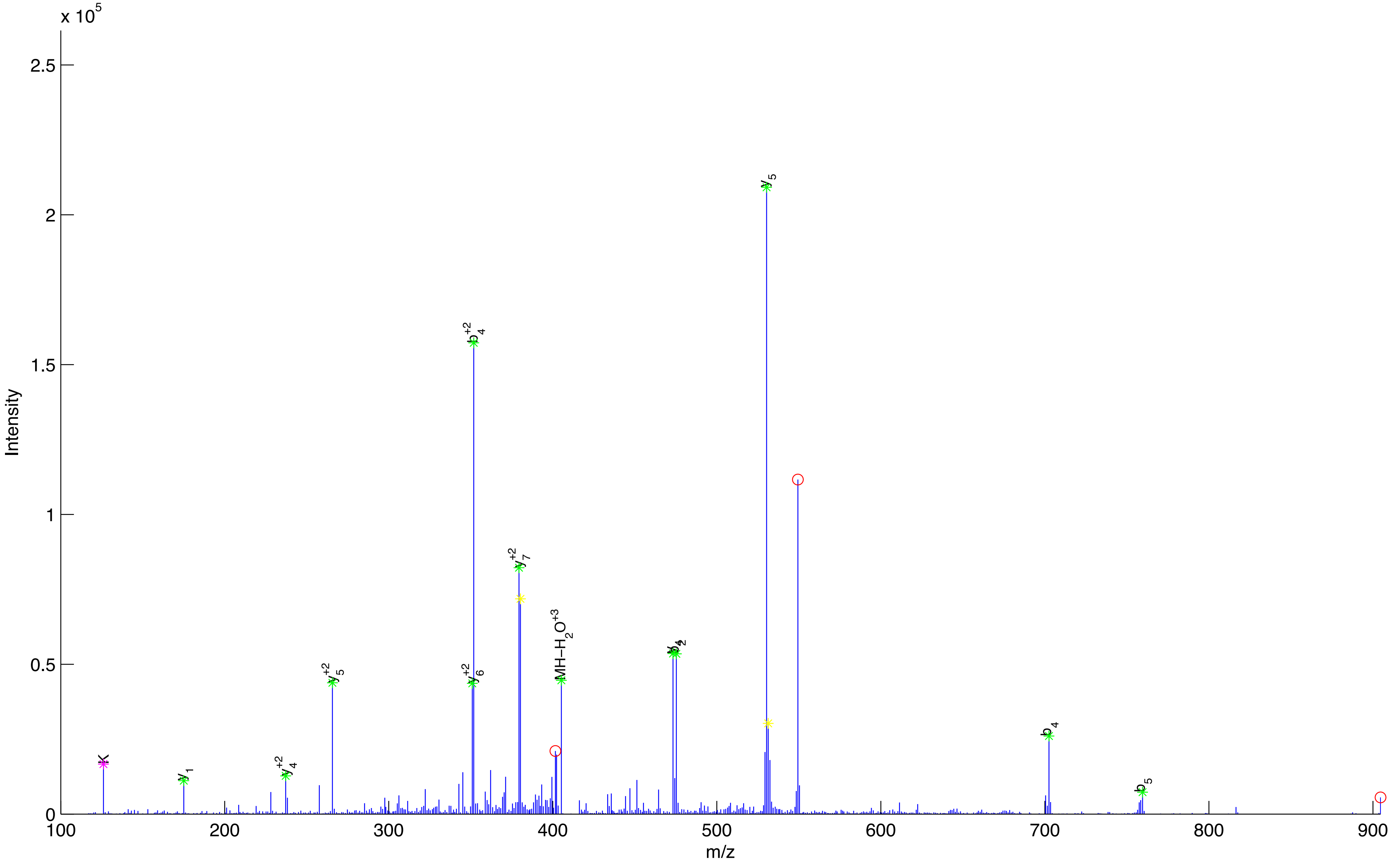

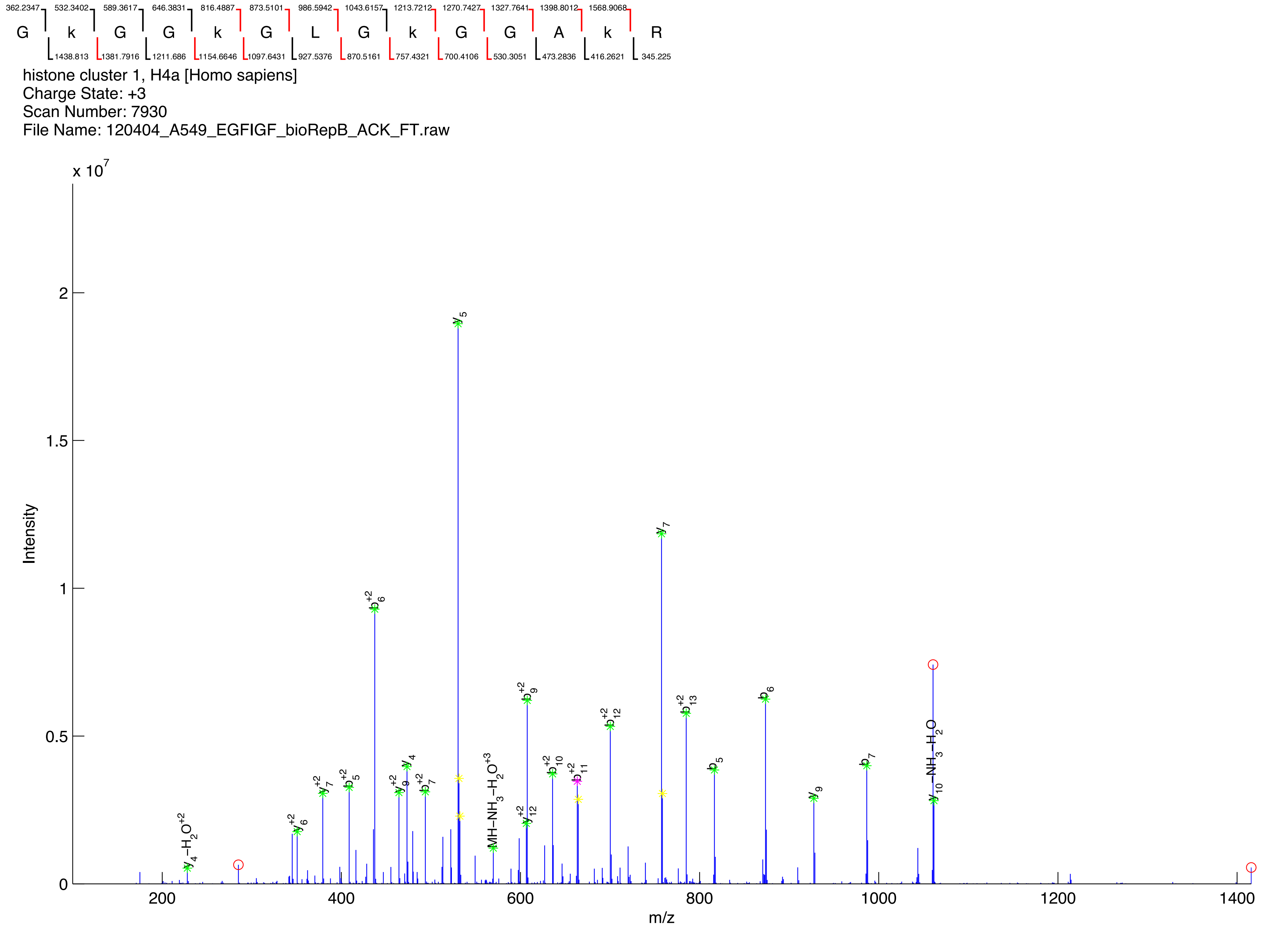

histone cluster 1, H4a [Homo sapiens]
Charge State: +3
Scan Number: 7930
File Name: 120404_A549_EGFIGF_bioRepB_ACK_FT.raw

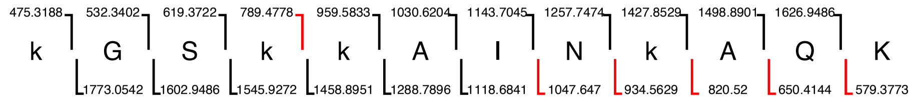

histone cluster 1, H2bm [Homo sapiens]
Charge State: +
Scan Number: 8023
File Name: 120413_A549_EGFIGF_bioRepC_AcK_FT.raw

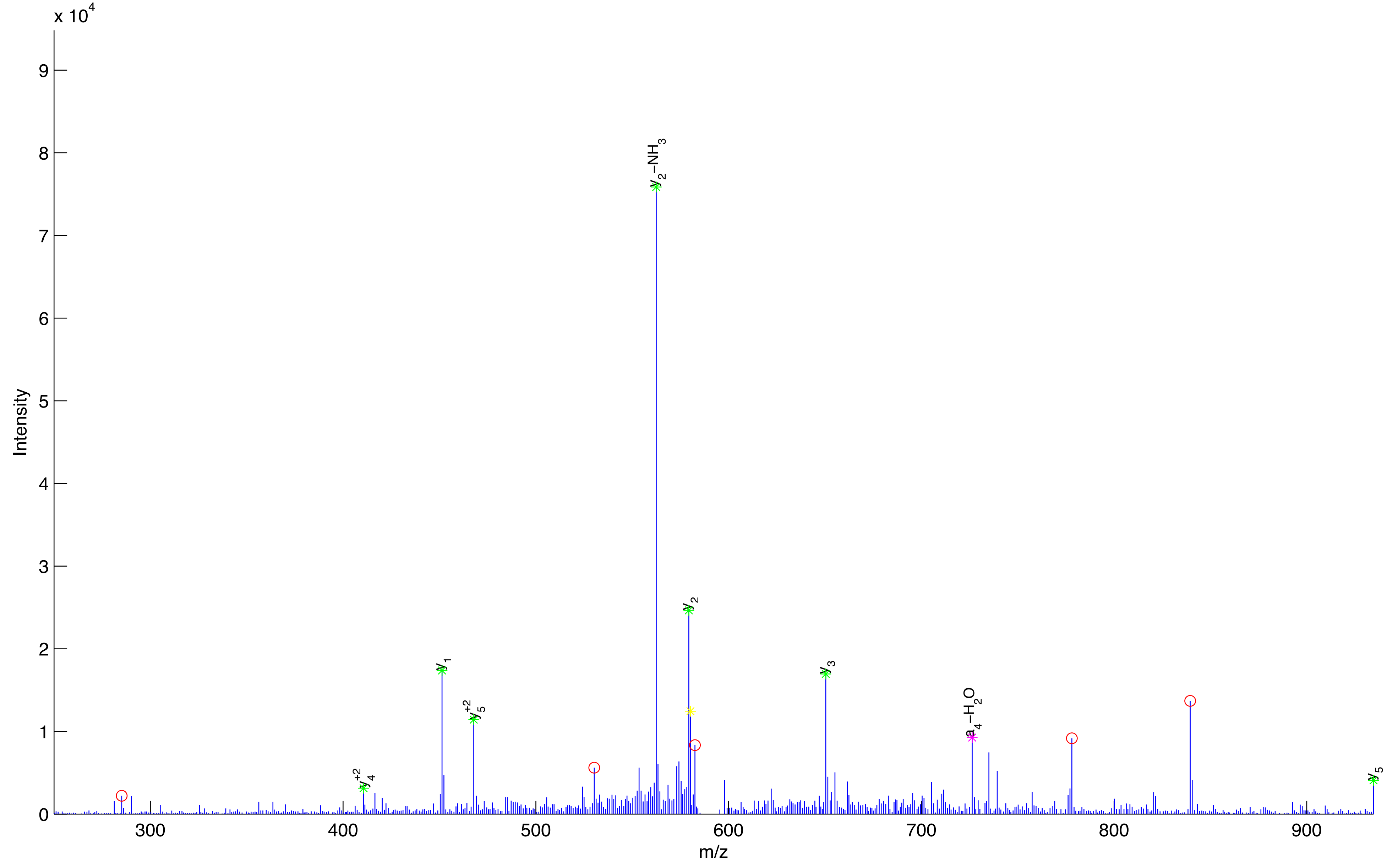

362.2347 449.2667 619.3722 789.4778 860.5149 959.5833 1060.631 1230.7365 1301.7736 1429.8322

G S k k A V T k A Q K

1575.9377 1518.9163 1431.8842 1261.7787 1091.6732 1020.6361 921.5677 820.52 650.4144 579.3773

histone cluster 1, H2bo [Homo sapiens]
Charge State: +3
Scan Number: 8044
File Name: 120413_A549_EGFIGF_bioRepC_AcK_FT.raw

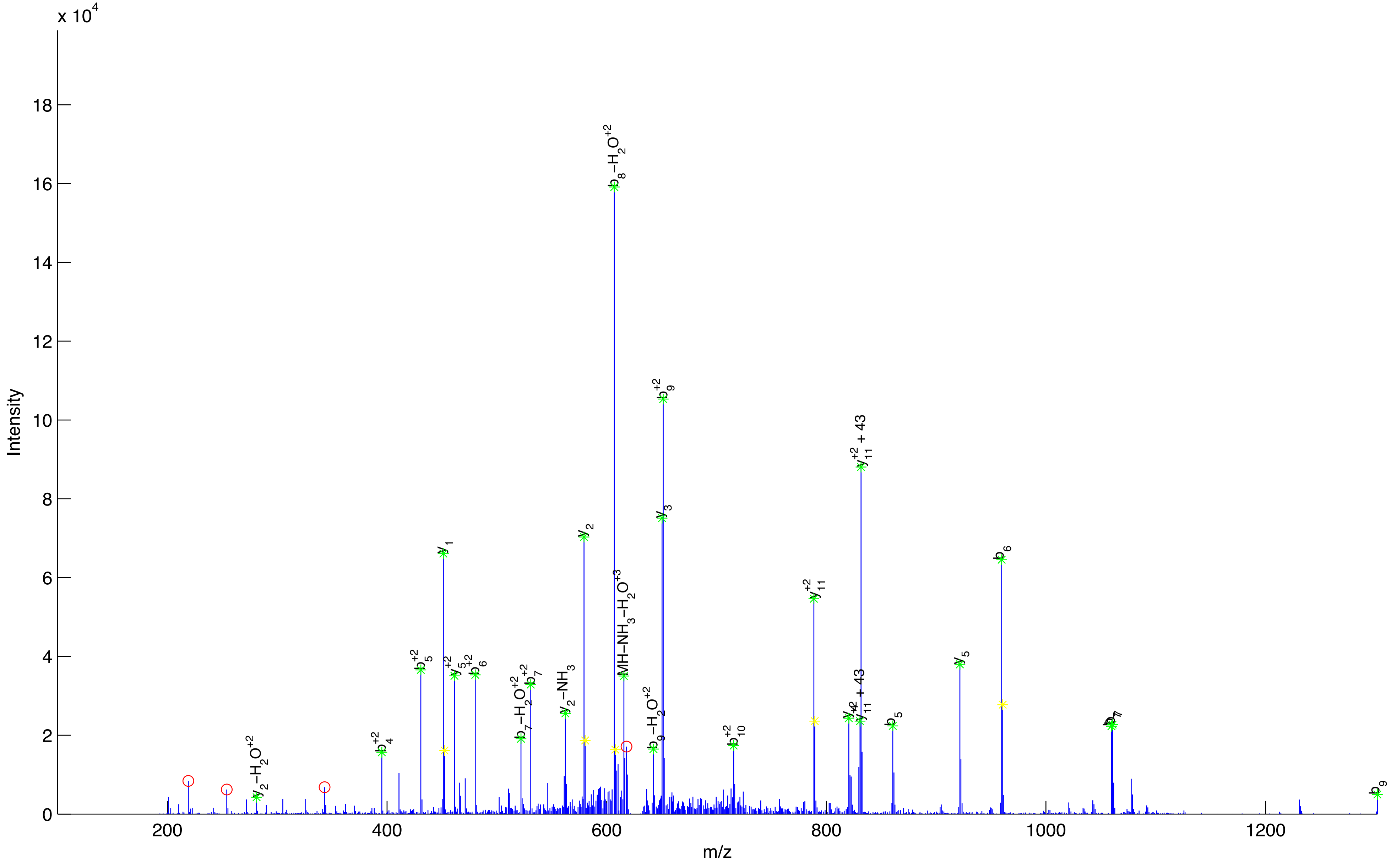

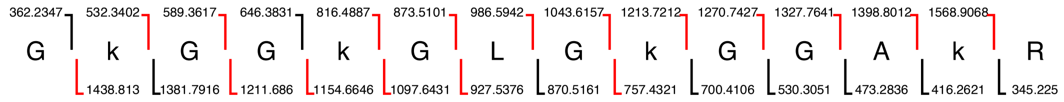

histone cluster 1, H4a [Homo sapiens]
Charge State: +1
Scan Number: 8052
File Name: 120407_A549_EGFIGF_bioRepA_ACK_FT.raw

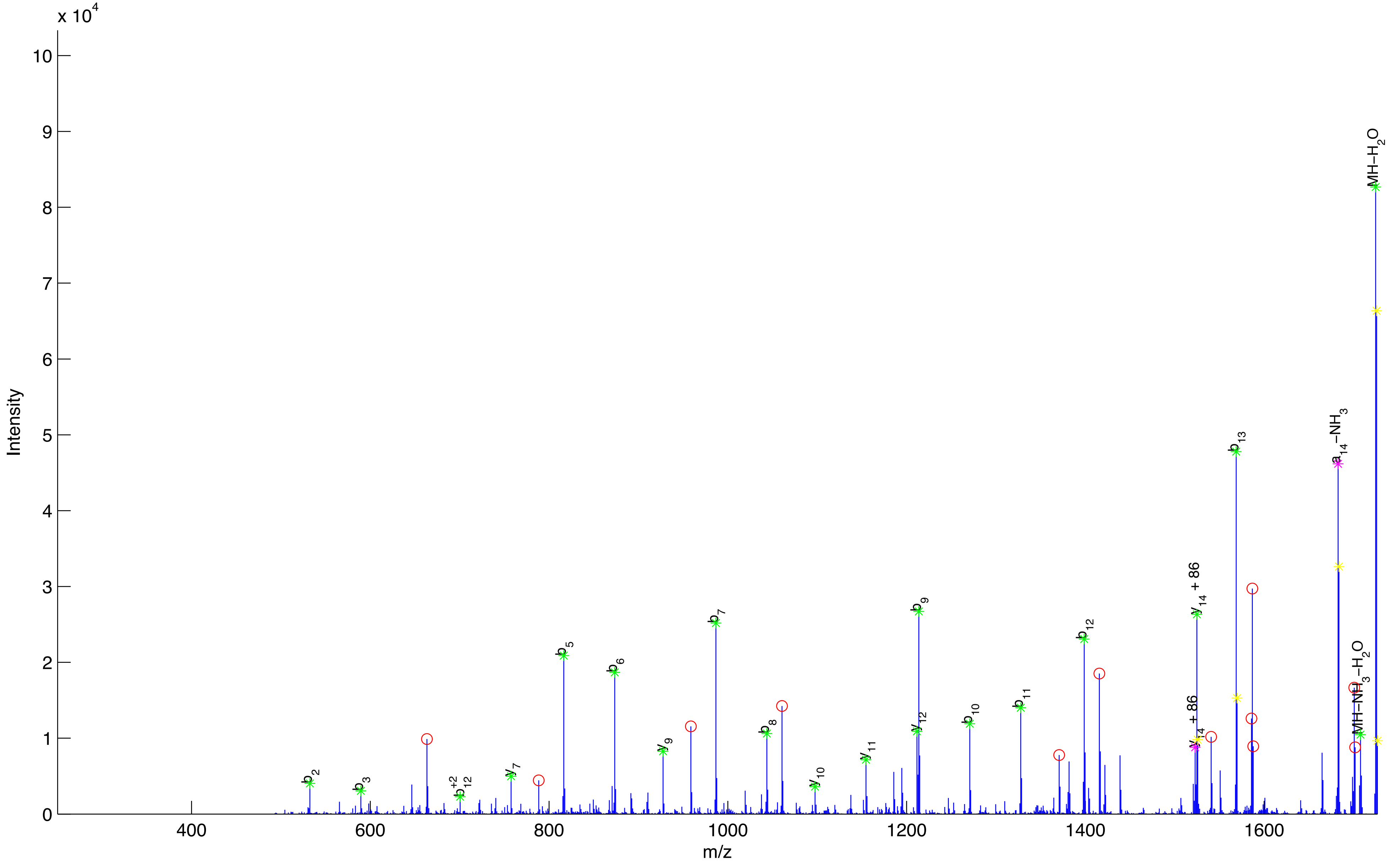

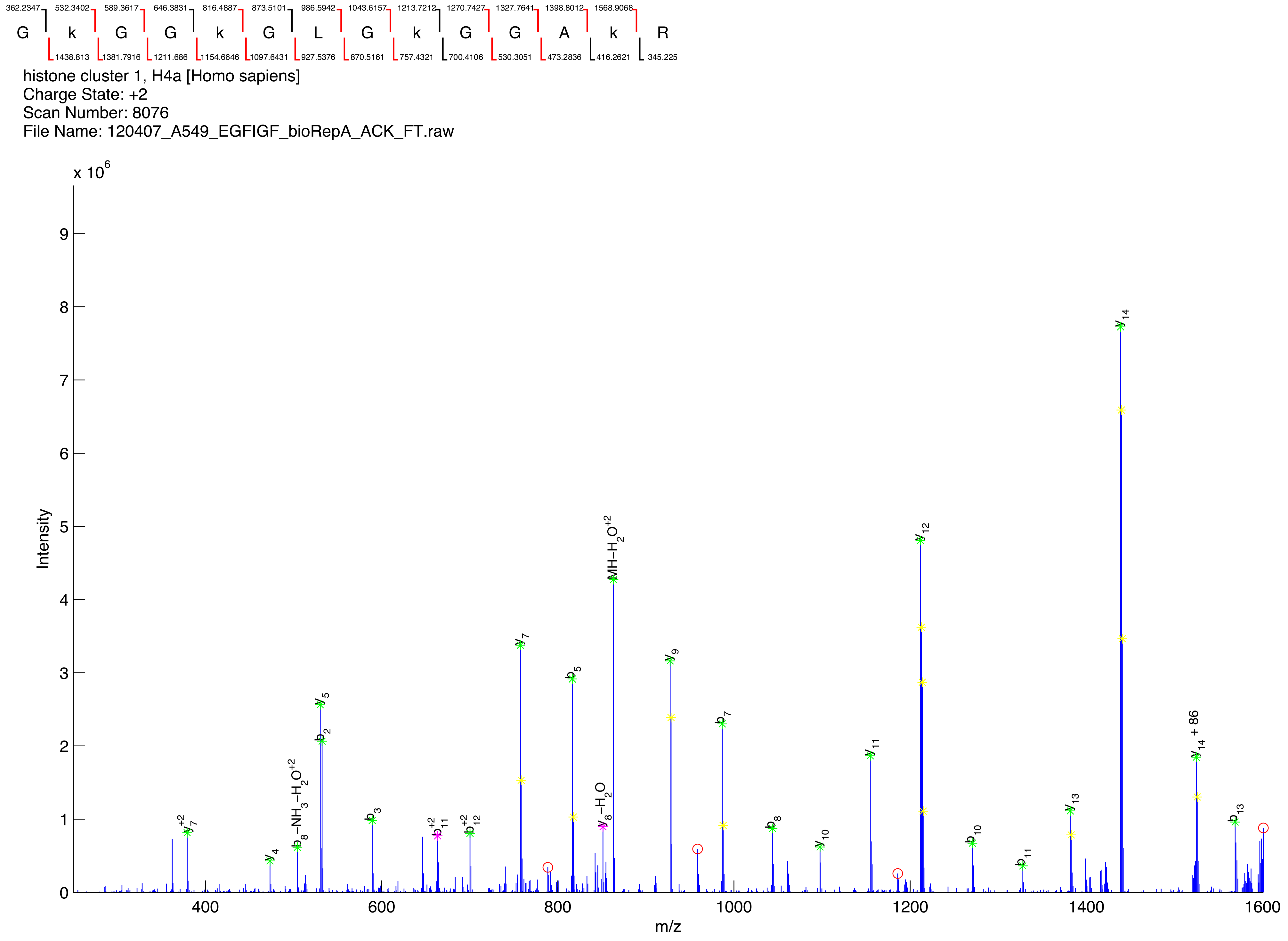
362.2347 532.3402 589.3617 646.3831 816.4887 873.5101 986.5942 1043.6157 1213.7212 1270.7427 1327.7641 1398.8012 1568.9068
G k G G k G L G k G G A k R
1438.813 1381.7916 1211.686 1154.6646 1097.6431 927.5376 870.5161 757.4321 700.4106 530.3051 473.2836 416.2621 345.225
histone cluster 1, H4a [Homo sapiens]
Charge State: +2
Scan Number: 8076
File Name: 120407_A549_EGFIGF_bioRepA_ACK_FT.raw
x 10^6
Intensity
m/z
y7 +2
y4
b8−NH3−H2O+2
y5
b2
b3
b11 +2
b12 +2
y7
b5
y8−H2O
MH−H2O+2
y9
b7
b8
y10
y11
y12
b10
b11
y13
y14
y14 + 86
b13

G	G	k	G	L	G	k	G	G	A	k	R
362.2347	419.2562	589.3617	646.3831	759.4672	816.4887	986.5942	1043.6157	1100.6371	1171.6742	1341.7798	
	1211.686	1154.6646	1097.6431	927.5376	870.5161	757.4321	700.4106	530.3051	473.2836	416.2621	345.225

histone cluster 1, H4a [Homo sapiens]
Charge State: +3
Scan Number: 8142
File Name: 120404_A549_EGFIGF_bioRepB_ACK_FT.raw

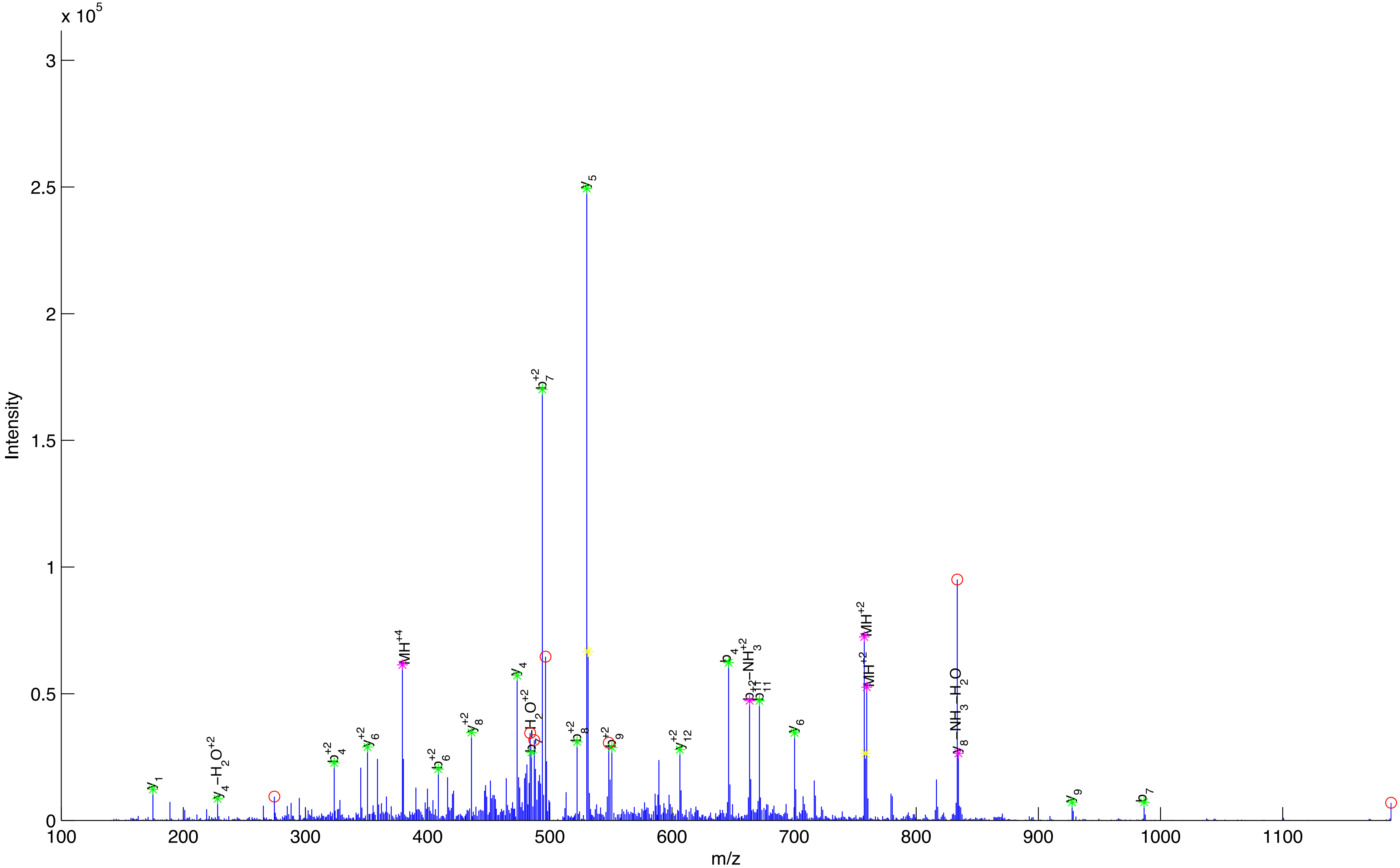

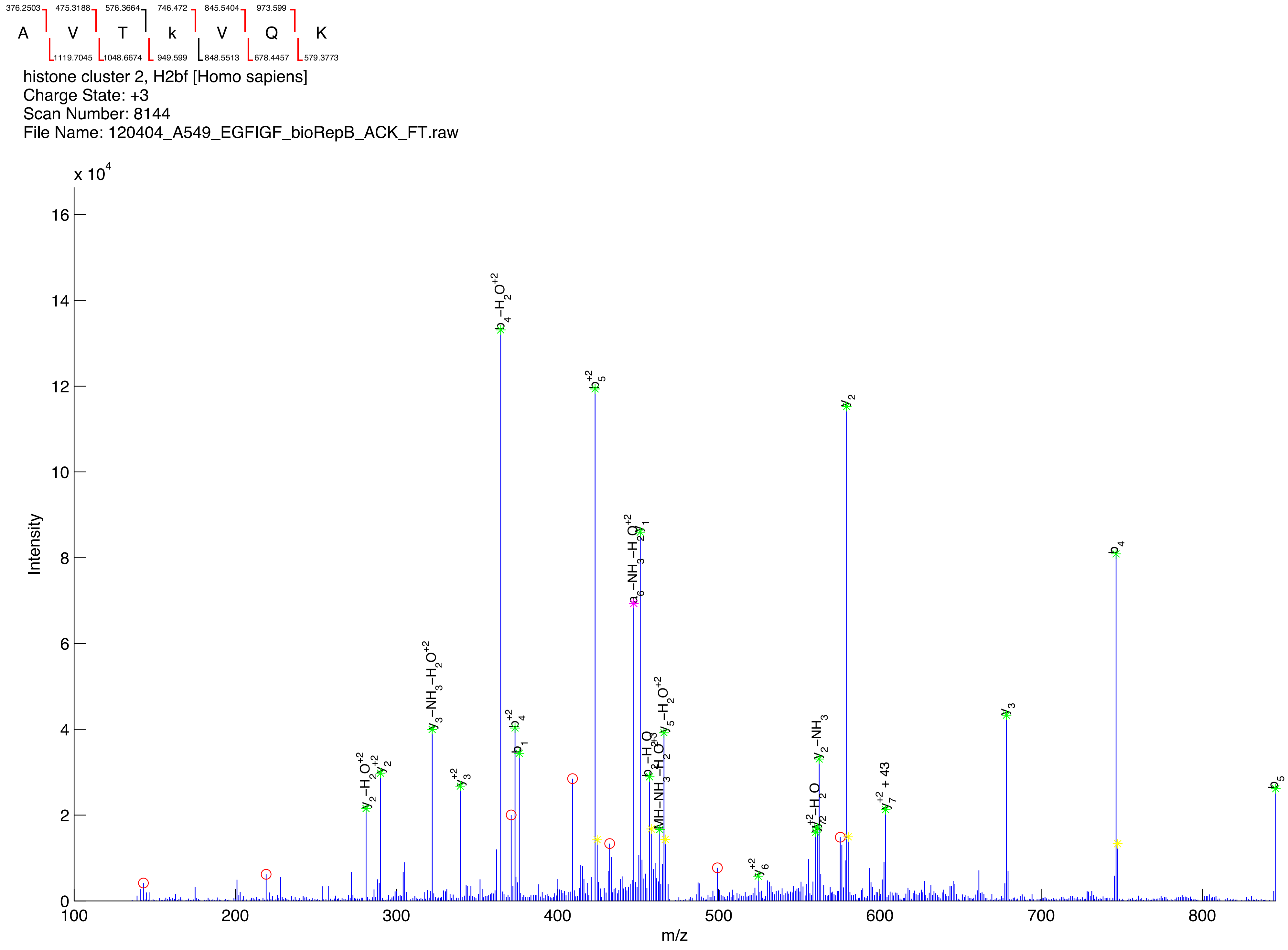

376.2503 475.3188 576.3664 746.472 845.5404 973.599
A V T k V Q K
1119.7045 1048.6674 949.599 848.5513 678.4457 579.3773
histone cluster 2, H2bf [Homo sapiens]
Charge State: +3
Scan Number: 8144
File Name: 120404_A549_EGFIGF_bioRepB_ACK_FT.raw
x 10^4
Intensity
m/z

420.2402 507.2722 670.3355 769.4039 826.4254 941.4523 1070.4949 1141.5321 1269.5906 1356.6227 1526.7282

D S Y V G D E A Q S k R

1396.6345 1281.6075 1194.5755 1031.5122 932.4438 875.4223 760.3953 631.3528 560.3156 432.2571 345.225

alpha 1 actin precursor [Homo sapiens]
Charge State: +2
Scan Number: 8155
File Name: 120407_A549_EGFIGF_bioRepA_ACK_FT.raw

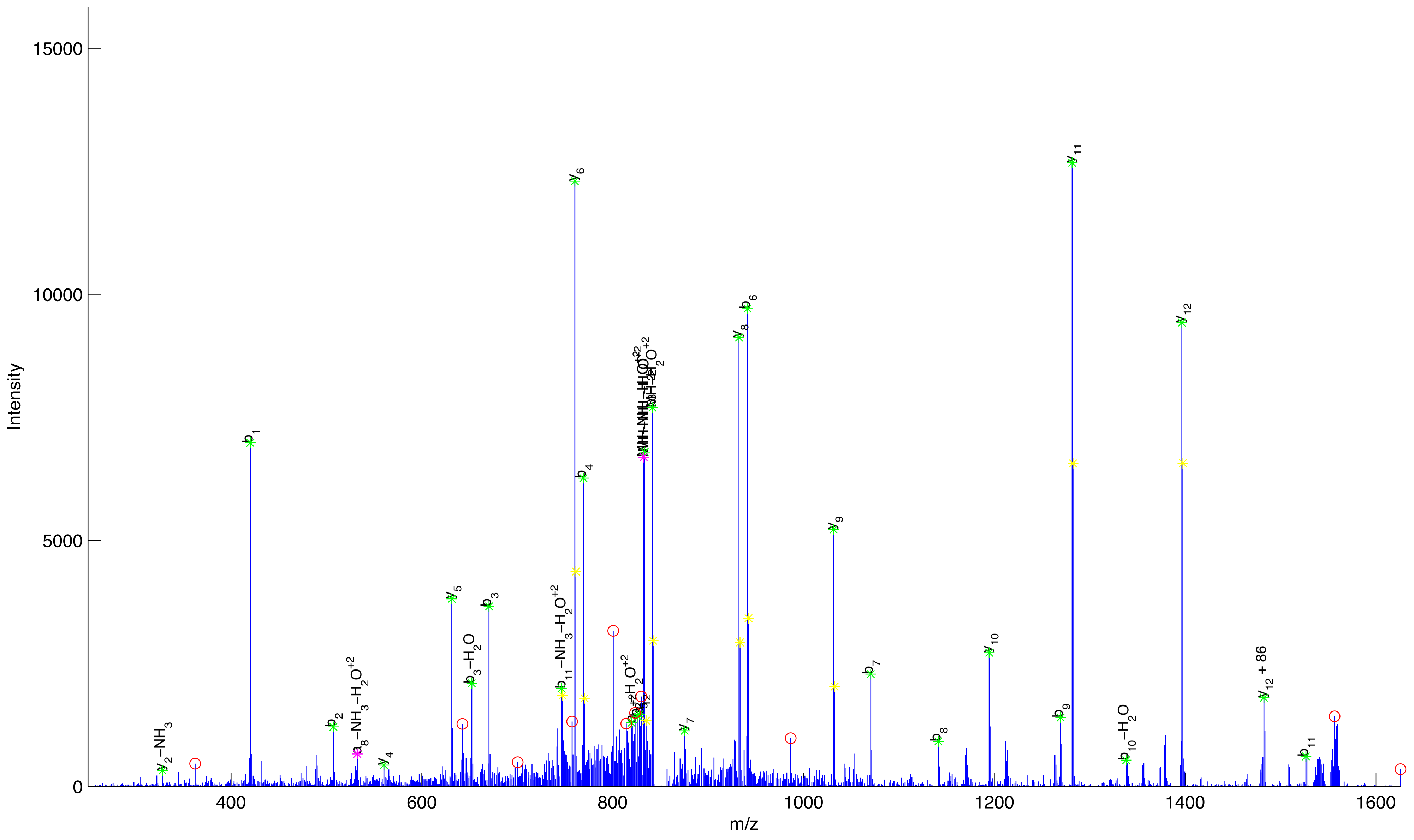

362.2347	532.3402	589.3617	646.3831	816.4887	873.5101	986.5942	1043.6157	1213.7212	1270.7427	1327.7641	1398.8012	1568.9068		
G	k	G	G	k	G	L	G	k	G	G	A	k	R	
	1438.813	1381.7916	1211.686	1154.6646	1097.6431	927.5376	870.5161	757.4321	700.4106	530.3051	473.2836	416.2621	345.225	

histone cluster 1, H4a [Homo sapiens]
Charge State: +3
Scan Number: 8160
File Name: 120407_A549_EGFIGF_bioRepA_ACK_FT.raw

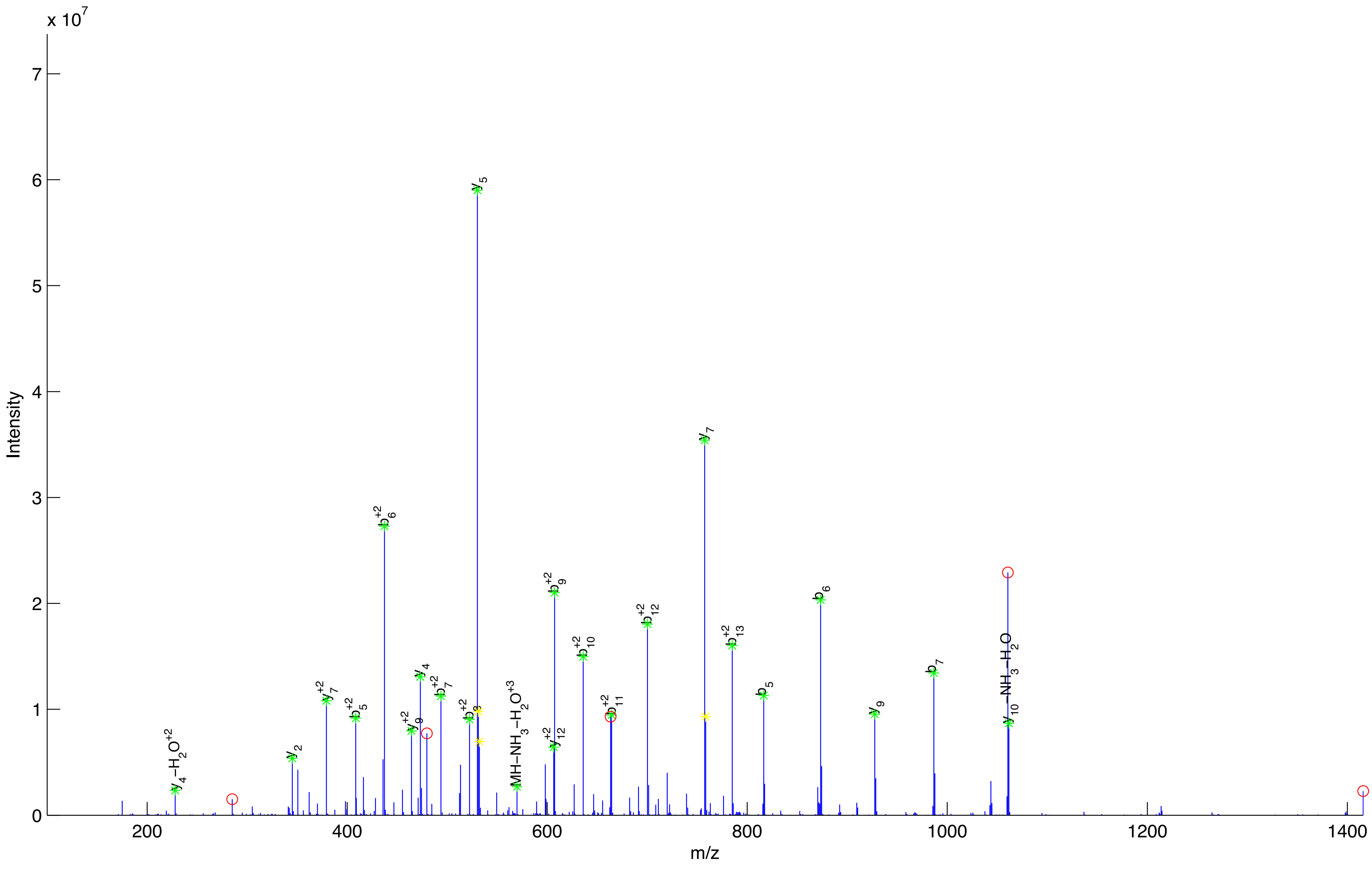

histone cluster 1, H4a [Homo sapiens]
Charge State: +2
Scan Number: 8202
File Name: 120407_A549_EGFIGF_bioRepA_ACK_FT.raw

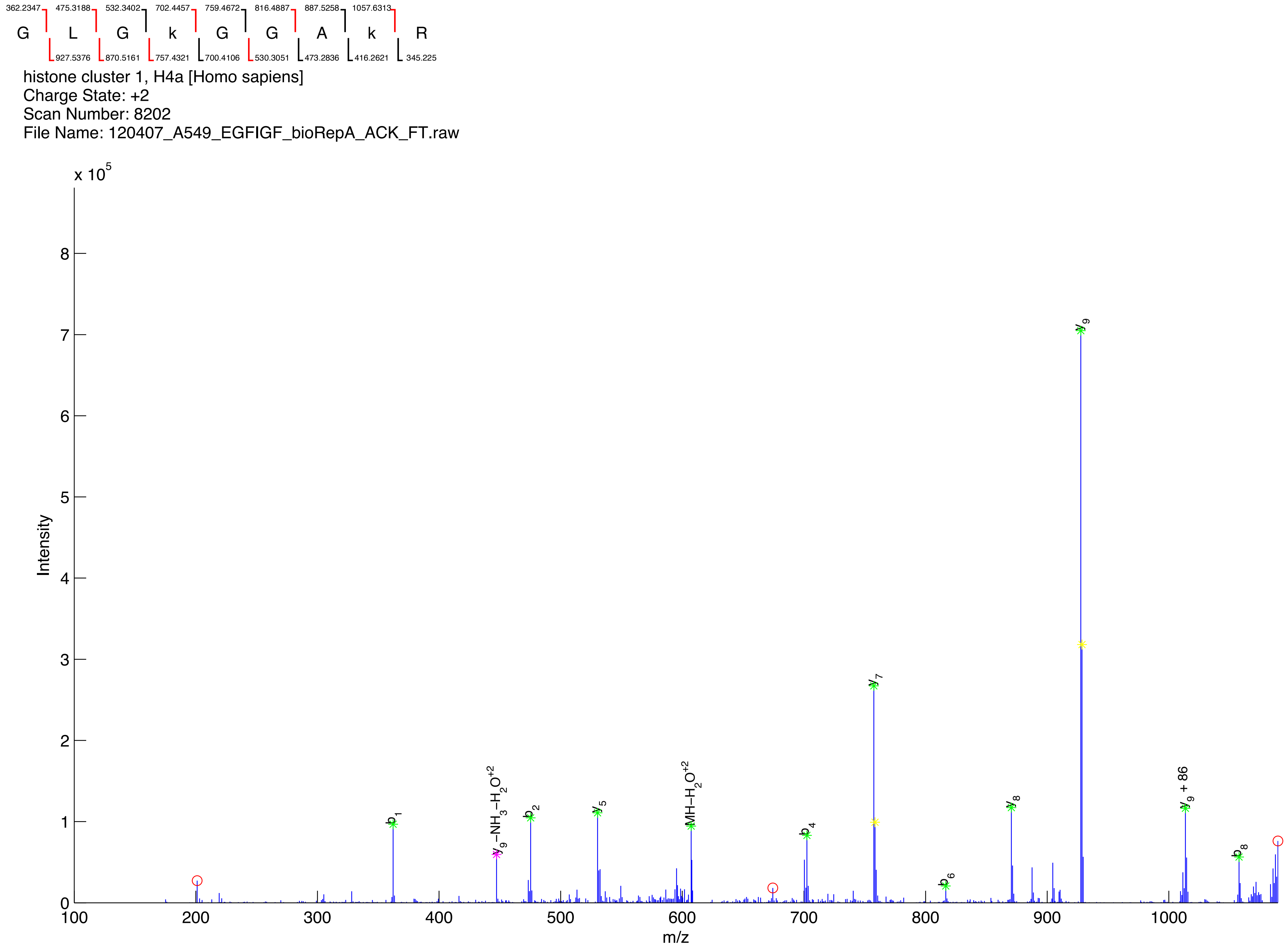

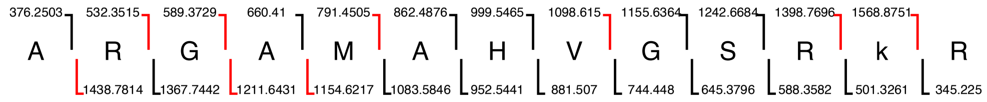

SRp25 nuclear protein isoform 4 [Homo sapiens]
Charge State: +2
Scan Number: 8245
File Name: 120404_A549_EGFIGF_bioRepB_ACK_FT.raw

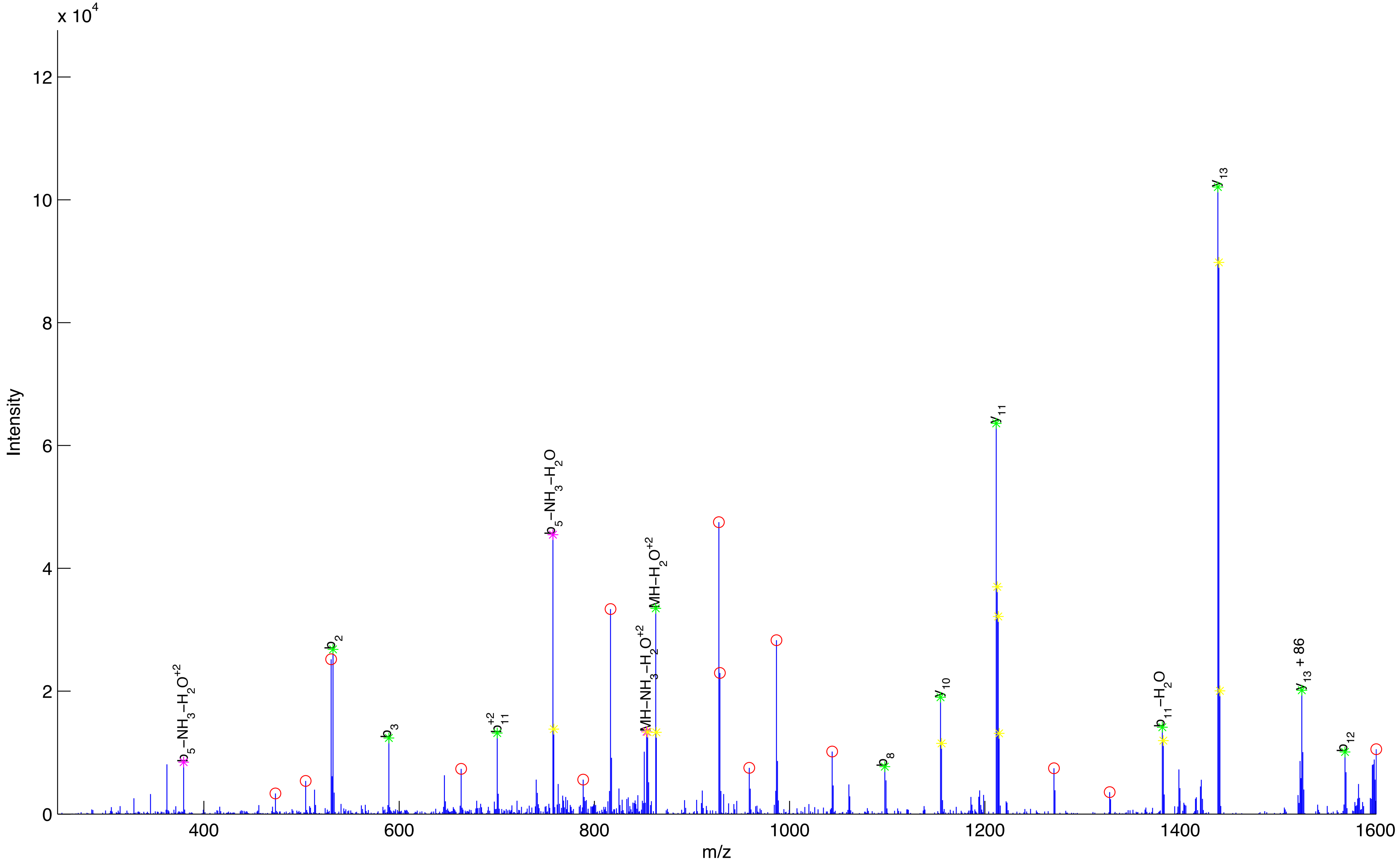

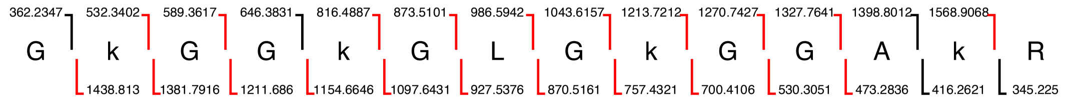

histone cluster 1, H4a [Homo sapiens]
Charge State: +2
Scan Number: 8325
File Name: 120413_A549_EGFIGF_bioRepC_AcK_FT.raw

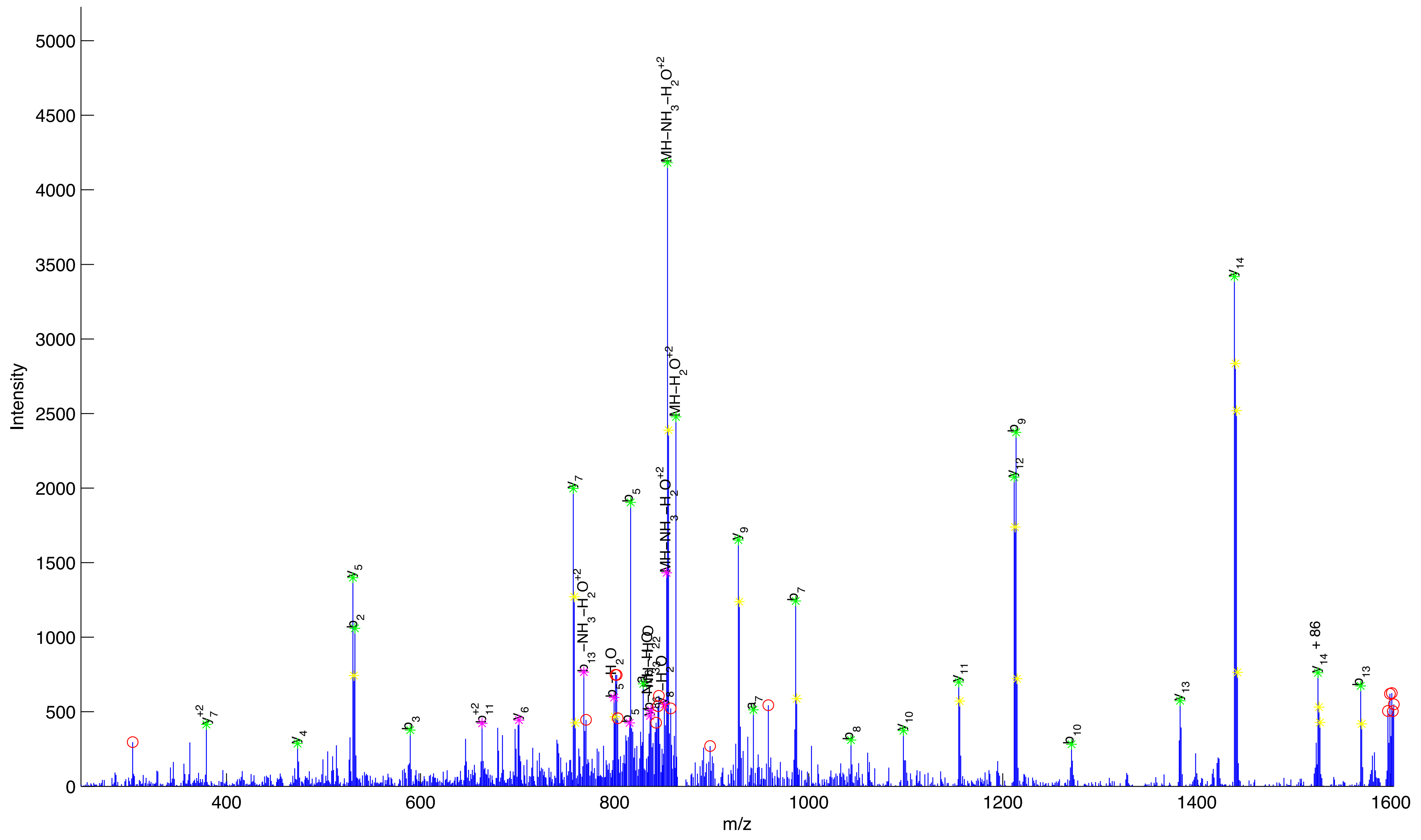

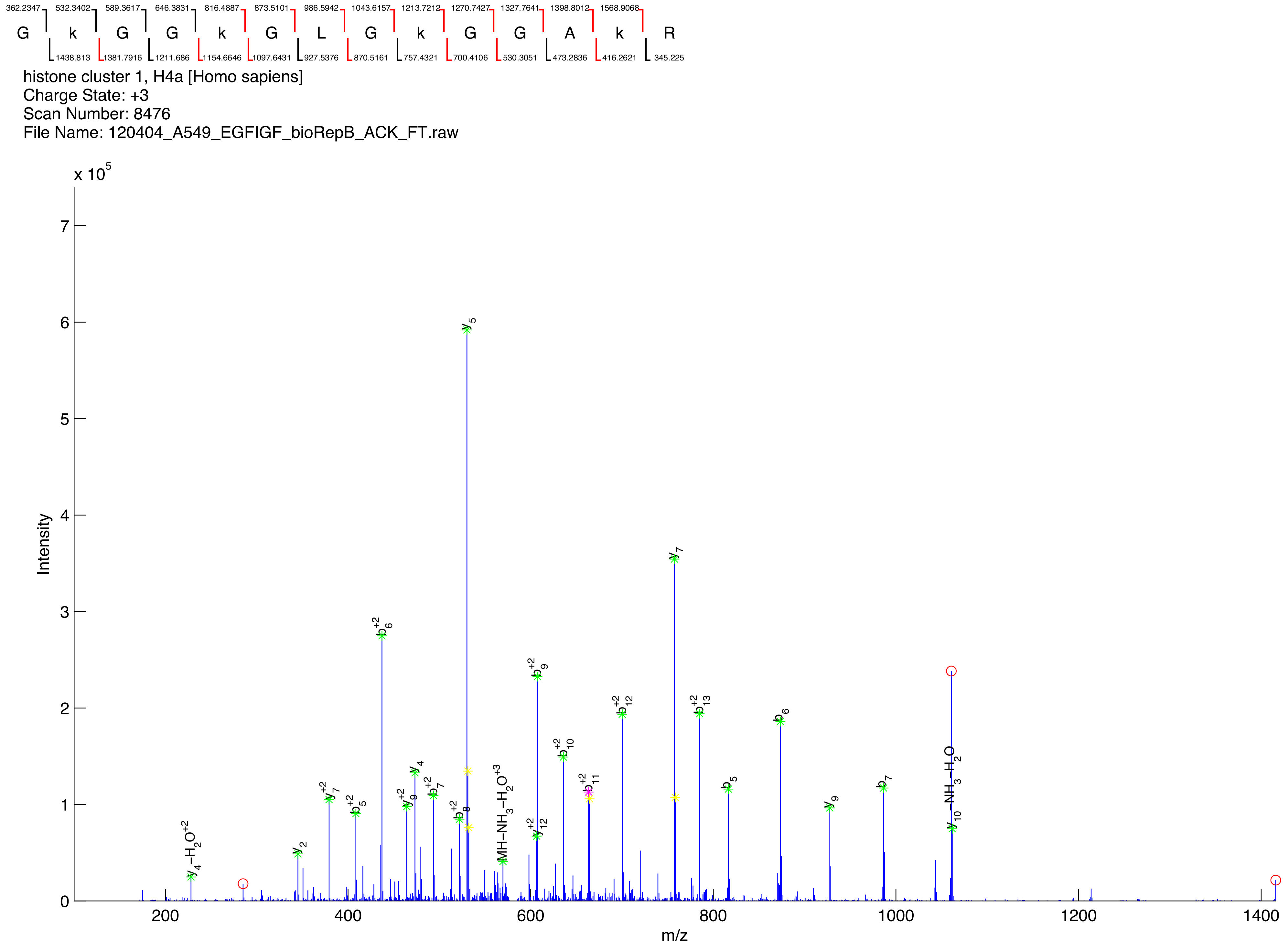

362.2347
532.3402
589.3617
646.3831
816.4887
873.5101
986.5942
1043.6157
1213.7212
1270.7427
1327.7641
1398.8012
1568.9068
G k G G k G L G k G G A k R
1438.813
1381.7916
1211.686
1154.6646
1097.6431
927.5376
870.5161
757.4321
700.4106
530.3051
473.2836
416.2621
345.225
histone cluster 1, H4a [Homo sapiens]
Charge State: +3
Scan Number: 8476
File Name: 120404_A549_EGFIGF_bioRepB_ACK_FT.raw
x 10^5
Intensity
m/z
0
1
2
3
4
5
6
7
200
400
600
800
1000
1200
1400
y4−H2O+2
y2
y7+2
b5+2
b6+2
y9+2
y4
b7+2
b8+2
y5
MH−NH3−H2O+3
y12+2
b9+2
b10+2
b11+2
b12+2
y7
b13+2
b5
b6
y9
b7
y10−NH3−H2O

434.2558 597.3191 712.3461 809.3989 924.4258 981.4473 1151.5528 1208.5743 1321.6583 1434.7424 1521.7744 1691.8799

E Y D P D G k G I I S k K

1837.9855 1708.9429 1545.8795 1430.8526 1333.7998 1218.7729 1161.7514 991.6459 934.6244 821.5404 708.4563 621.4243

ryanodine receptor 3 [Homo sapiens]
Charge State: +
Scan Number: 8492
File Name: 120404_A549_EGFIGF_bioRepB_ACK_FT.raw

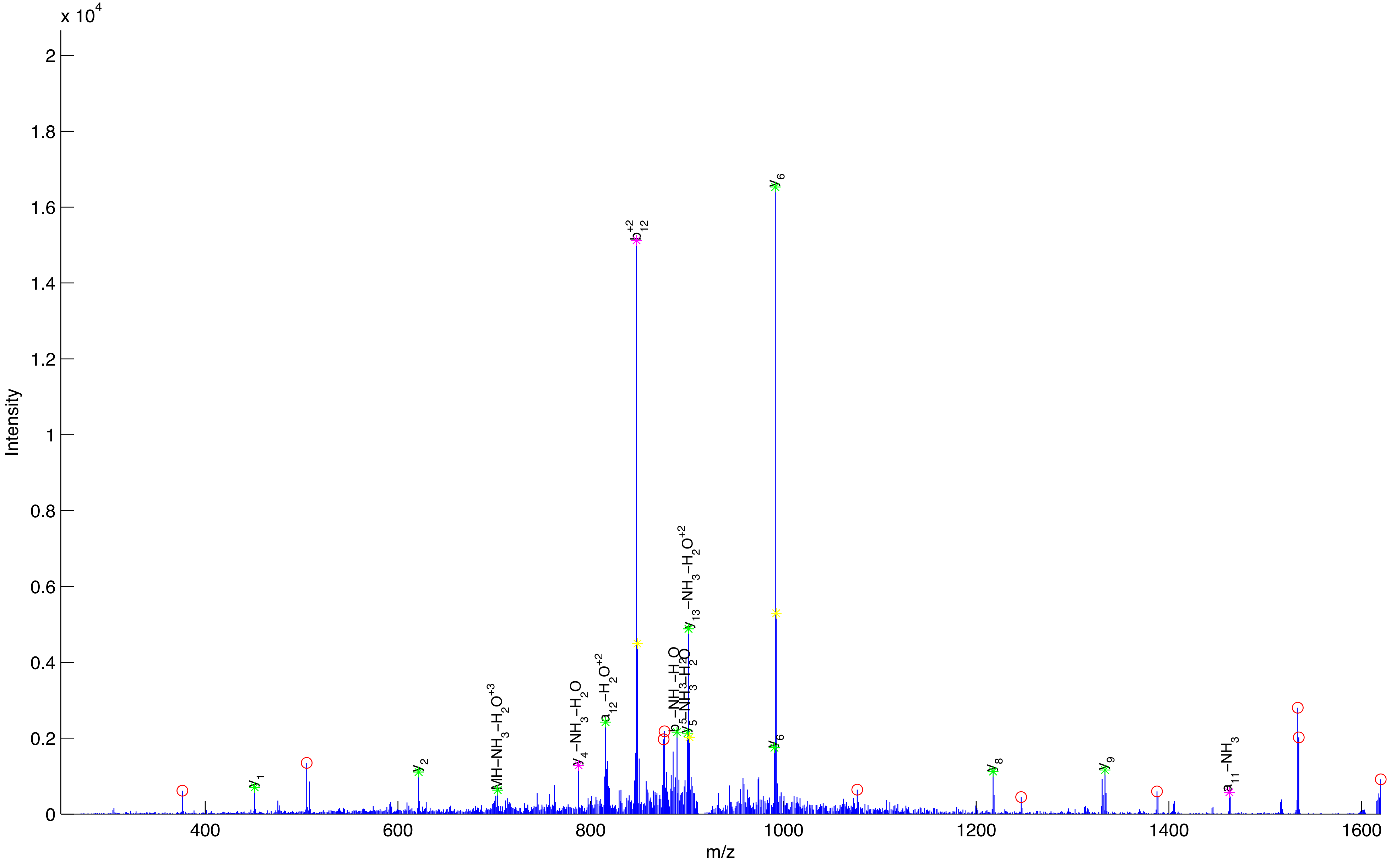

362.2347	419.2562	589.3617	646.3831	759.4672	816.4887	986.5942	1043.6157	1100.6371	1171.6742	1341.7798	
G	G	k	G	L	G	k	G	G	A	k	R
	1211.686	1154.6646	1097.6431	927.5376	870.5161	757.4321	700.4106	530.3051	473.2836	416.2621	345.225

histone cluster 1, H4a [Homo sapiens]
Charge State: +3
Scan Number: 8688
File Name: 120404_A549_EGFIGF_bioRepB_ACK_FT.raw

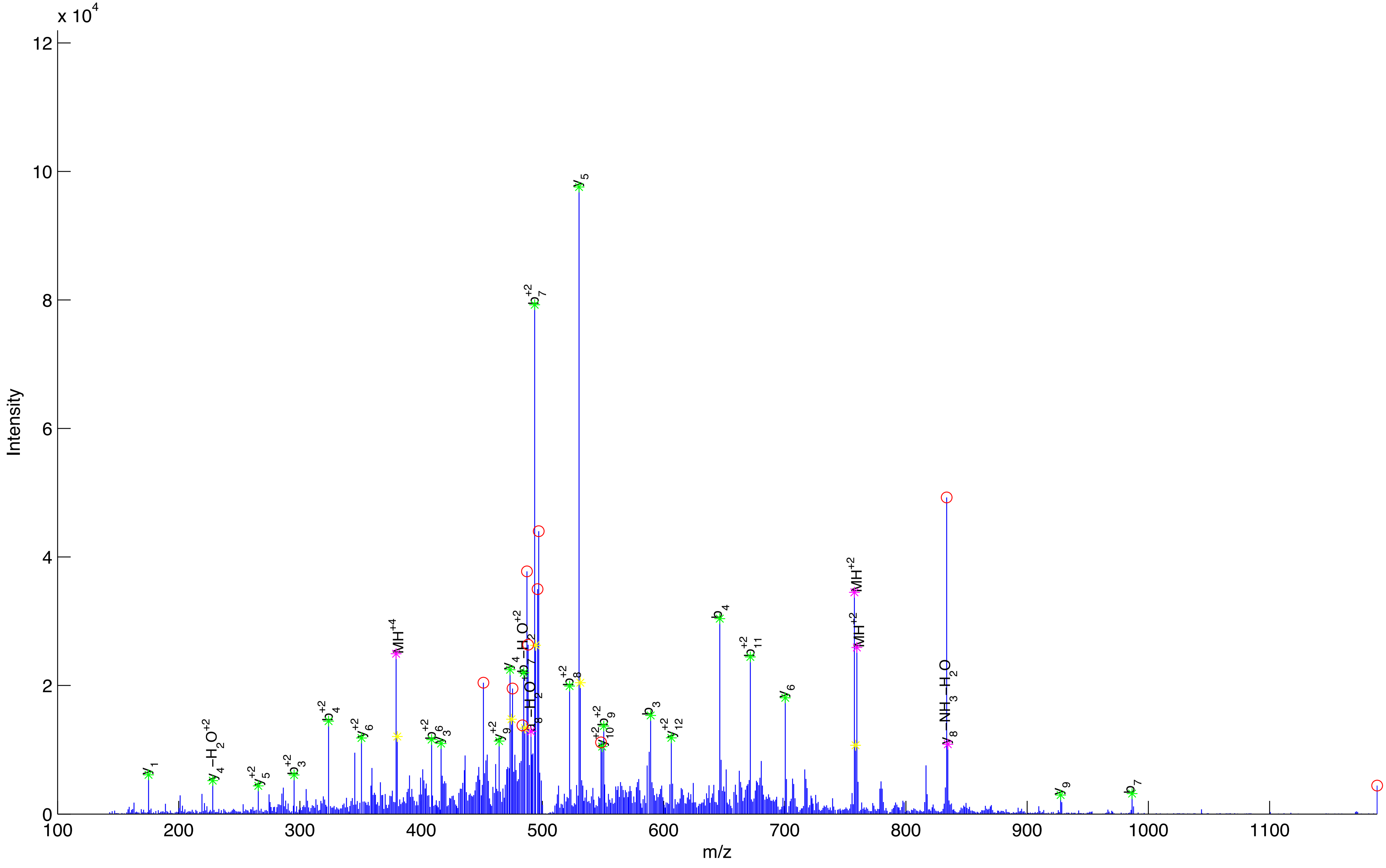

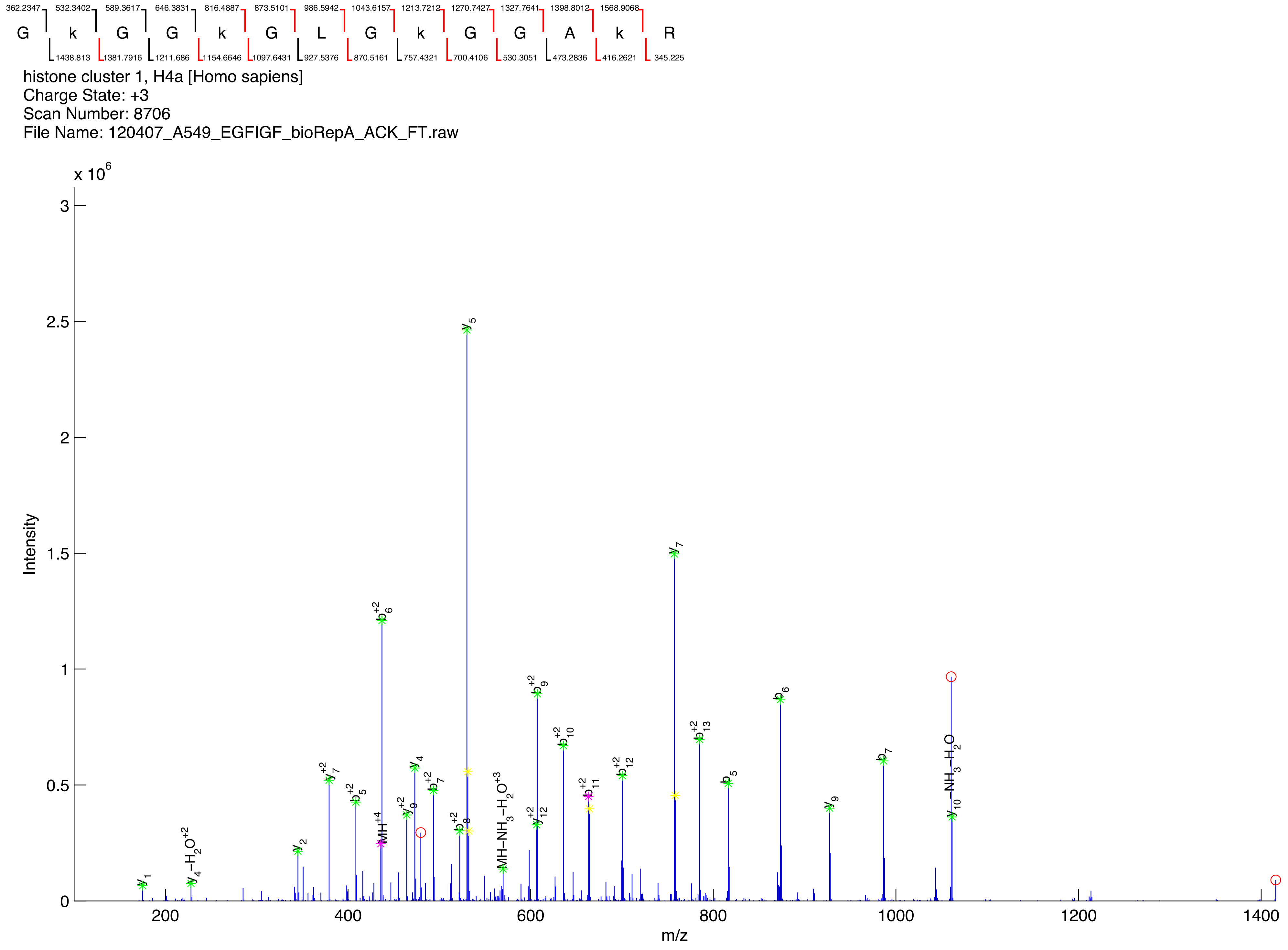

362.2347 532.3402 589.3617 646.3831 816.4887 873.5101 986.5942 1043.6157 1213.7212 1270.7427 1327.7641 1398.8012 1568.9068
G k G G k G L G k G G A k R
1438.813 1381.7916 1211.686 1154.6646 1097.6431 927.5376 870.5161 757.4321 700.4106 530.3051 473.2836 416.2621 345.225
histone cluster 1, H4a [Homo sapiens]
Charge State: +3
Scan Number: 8706
File Name: 120407_A549_EGFIGF_bioRepA_ACK_FT.raw
x 10^6
Intensity
m/z

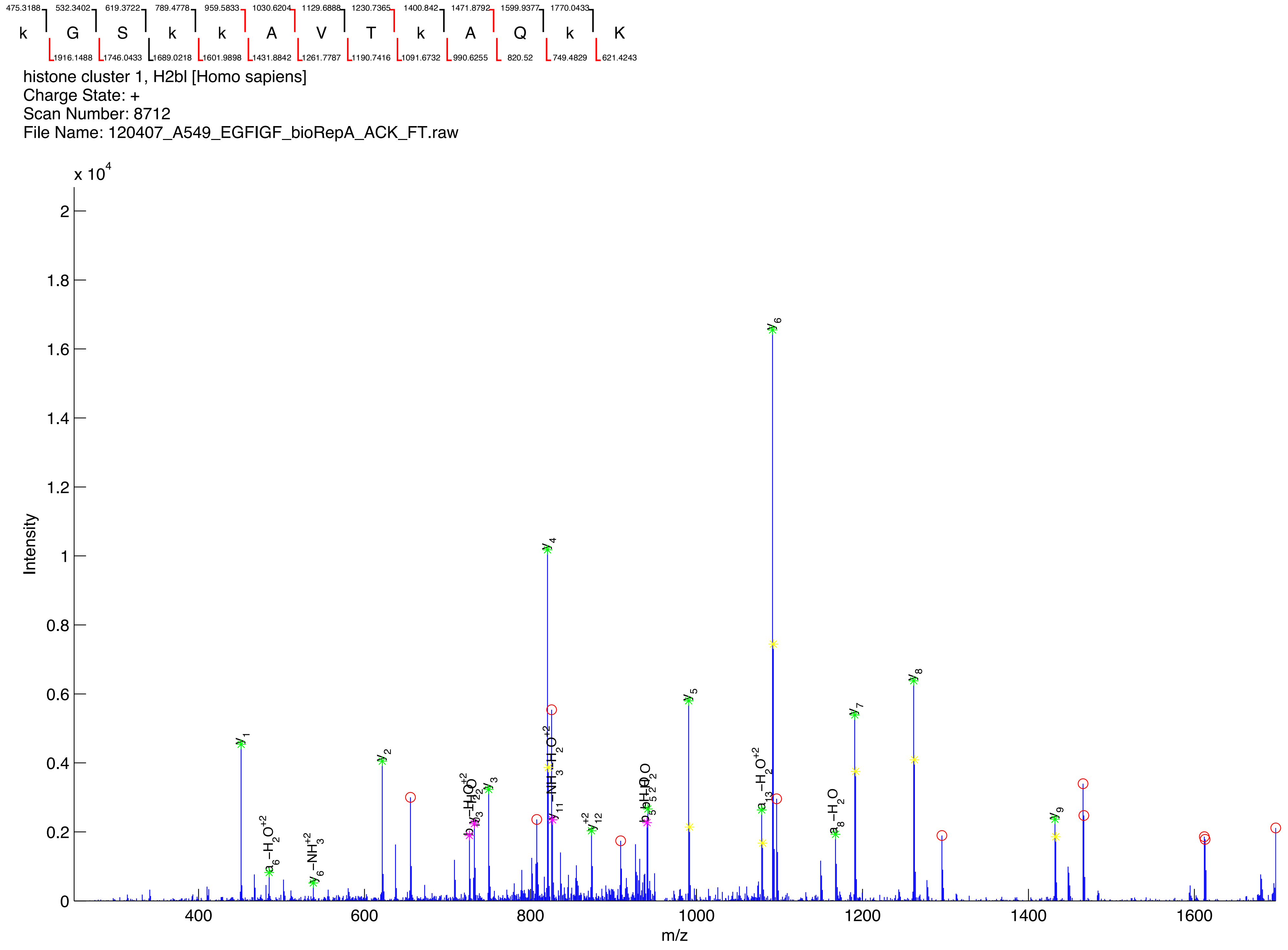

475.3188 532.3402 619.3722 789.4778 959.5833 1030.6204 1129.6888 1230.7365 1400.842 1471.8792 1599.9377 1770.0433
k G S k k A V T k A Q k K
1916.1488 1746.0433 1689.0218 1601.9898 1431.8842 1261.7787 1190.7416 1091.6732 990.6255 820.52 749.4829 621.4243
histone cluster 1, H2bl [Homo sapiens]
Charge State: +
Scan Number: 8712
File Name: 120407_A549_EGFIGF_bioRepA_ACK_FT.raw
x 10^4
Intensity
m/z

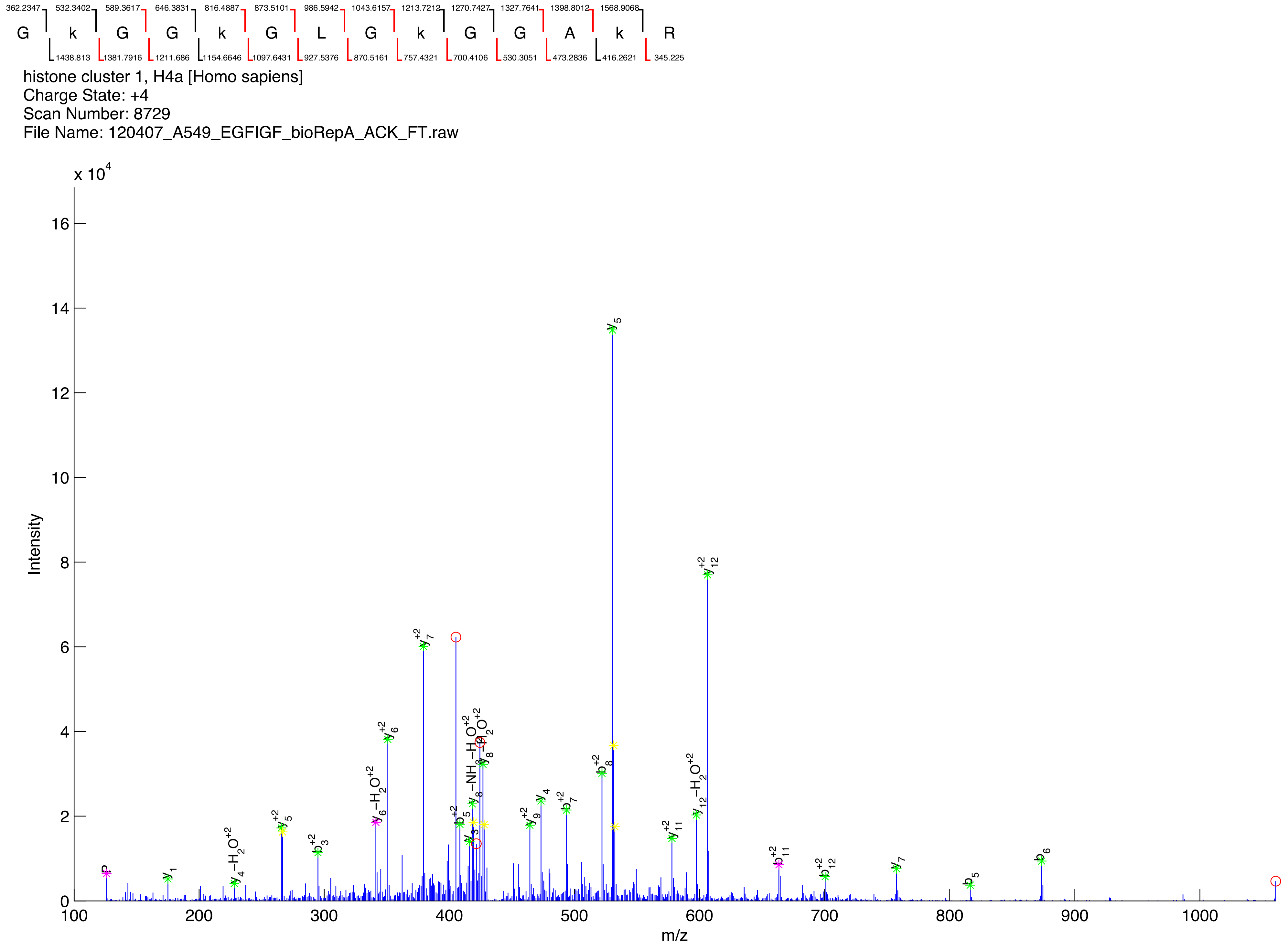

362.2347 532.3402 589.3617 646.3831 816.4887 873.5101 986.5942 1043.6157 1213.7212 1270.7427 1327.7641 1398.8012 1568.9068
G k G G k G L G k G G A k R
1438.813 1381.7916 1211.686 1154.6646 1097.6431 927.5376 870.5161 757.4321 700.4106 530.3051 473.2836 416.2621 345.225
histone cluster 1, H4a [Homo sapiens]
Charge State: +4
Scan Number: 8729
File Name: 120407_A549_EGFIGF_bioRepA_ACK_FT.raw
x 10^4
Intensity
m/z

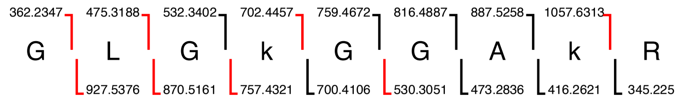

histone cluster 1, H4a [Homo sapiens]
Charge State: +2
Scan Number: 8748
File Name: 120407_A549_EGFIGF_bioRepA_ACK_FT.raw

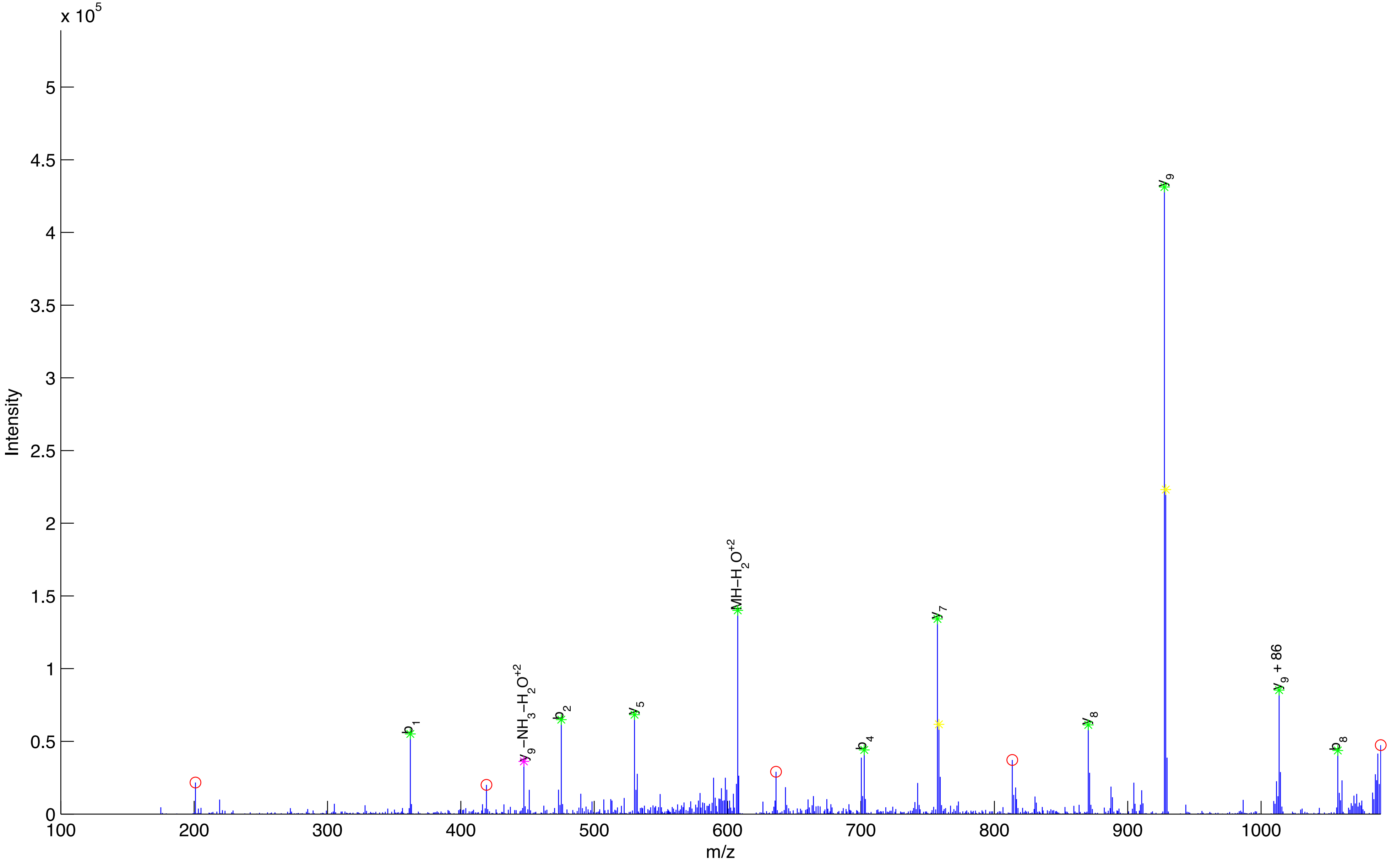

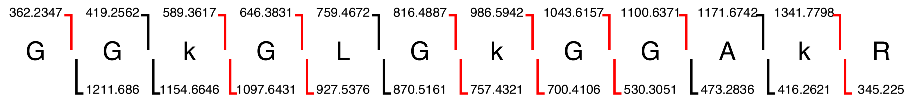

histone cluster 1, H4a [Homo sapiens]
Charge State: +3
Scan Number: 8781
File Name: 120413_A549_EGFIGF_bioRepC_AcK_FT.raw

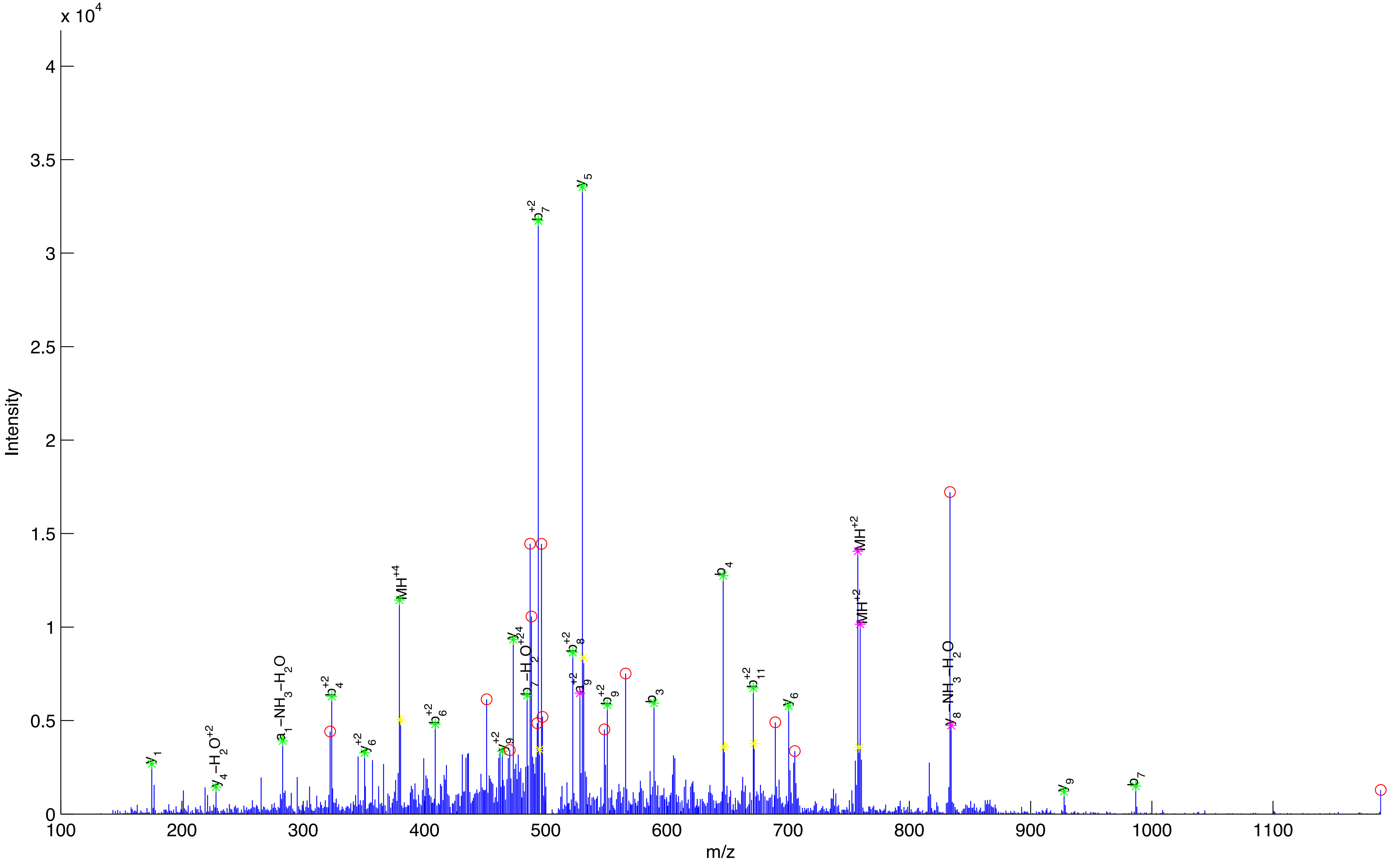

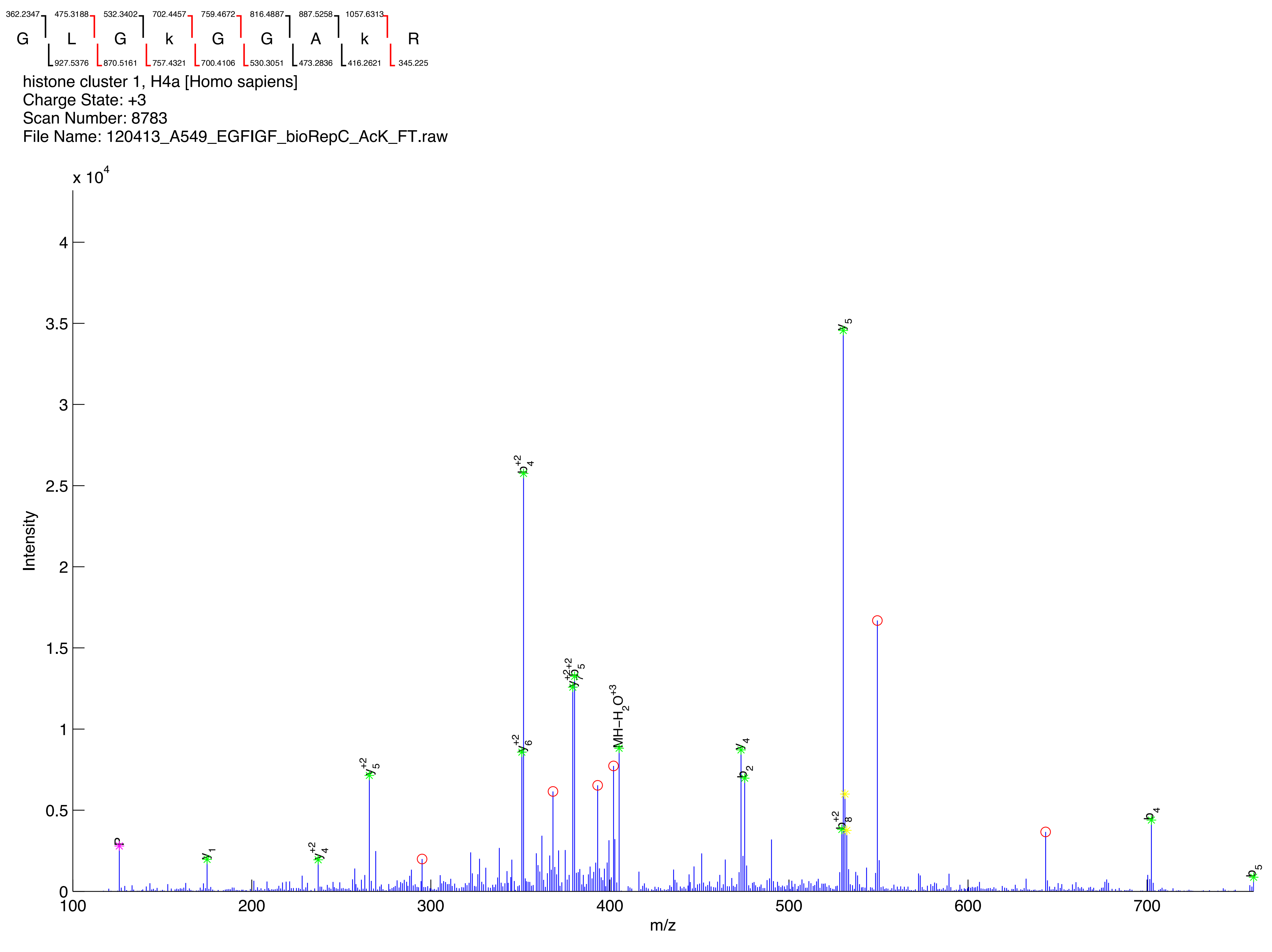

362.2347 475.3188 532.3402 702.4457 759.4672 816.4887 887.5258 1057.6313
G L G k G G A k R
927.5376 870.5161 757.4321 700.4106 530.3051 473.2836 416.2621 345.225
histone cluster 1, H4a [Homo sapiens]
Charge State: +3
Scan Number: 8783
File Name: 120413_A549_EGFIGF_bioRepC_AcK_FT.raw
x 10^4
Intensity
m/z
MH−H2O+3

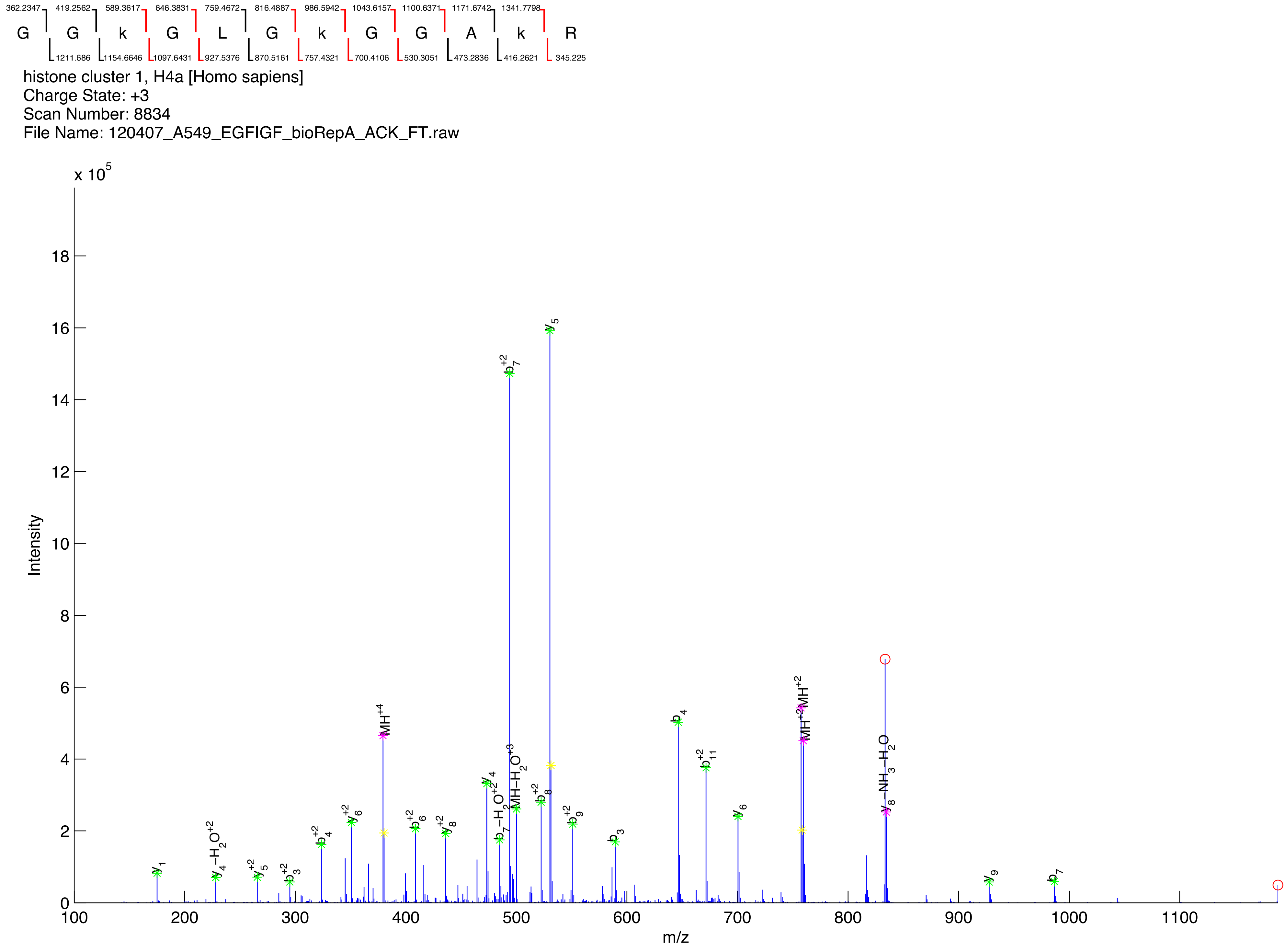
362.2347
419.2562
589.3617
646.3831
759.4672
816.4887
986.5942
1043.6157
1100.6371
1171.6742
1341.7798
G G k G L G k G G A k R
1211.686
1154.6646
1097.6431
927.5376
870.5161
757.4321
700.4106
530.3051
473.2836
416.2621
345.225
histone cluster 1, H4a [Homo sapiens]
Charge State: +3
Scan Number: 8834
File Name: 120407_A549_EGFIGF_bioRepA_ACK_FT.raw
x 10^5
Intensity
m/z
y1
y4-H2O+2
y+2 5
b+2 3
b+2 4
y+2 6
MH+4
b+2 6
y+2 8
y4
b7-H2O+2
b+2 7
MH-H2O+3
b+2 8
y5
b+2 9
b3
b4
b+2 11
y6
MH+2
MH+2
y8-NH3-H2O
y9
b7

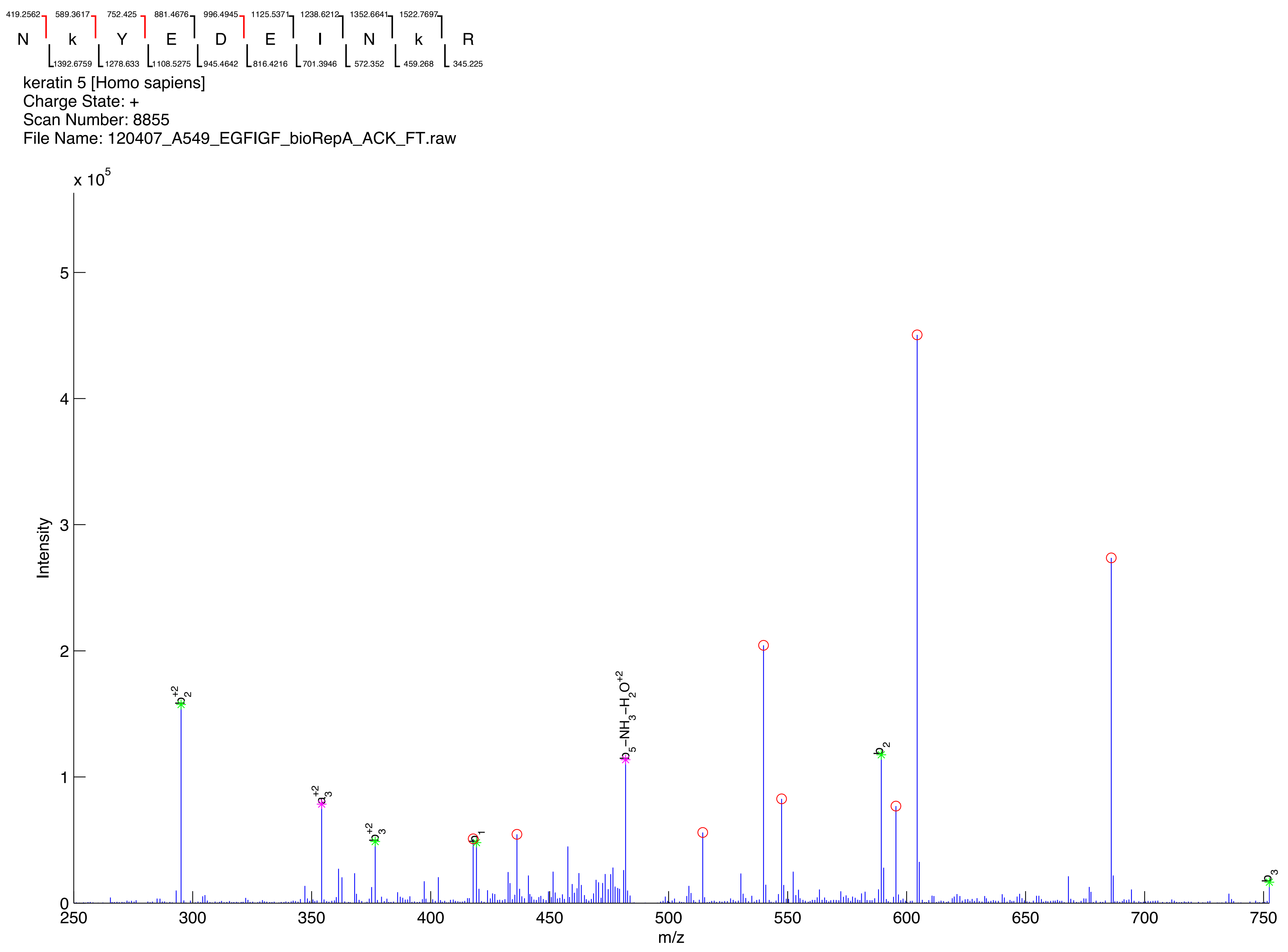
419.2562 589.3617 752.425 881.4676 996.4945 1125.5371 1238.6212 1352.6641 1522.7697
N k Y E D E I N k R
1392.6759 1278.633 1108.5275 945.4642 816.4216 701.3946 572.352 459.268 345.225
keratin 5 [Homo sapiens]
Charge State: +
Scan Number: 8855
File Name: 120407_A549_EGFIGF_bioRepA_ACK_FT.raw
x 10^5
Intensity
m/z
b2+2
a3+2
b3+2
b1
b5-NH3-H2O+2
b2
b3
0
1
2
3
4
5
250
300
350
400
450
500
550
600
650
700
750

475.3188 532.3402 619.3722 789.4778 959.5833 1030.6204 1143.7045 1244.7522

k G S k k A I T K

1390.8577 1220.7522 1163.7307 1076.6987 906.5931 736.4876 665.4505 552.3664

histone cluster 1, H2bb [Homo sapiens]
Charge State: +3
Scan Number: 8983
File Name: 120407_A549_EGFIGF_bioRepA_ACK_FT.raw

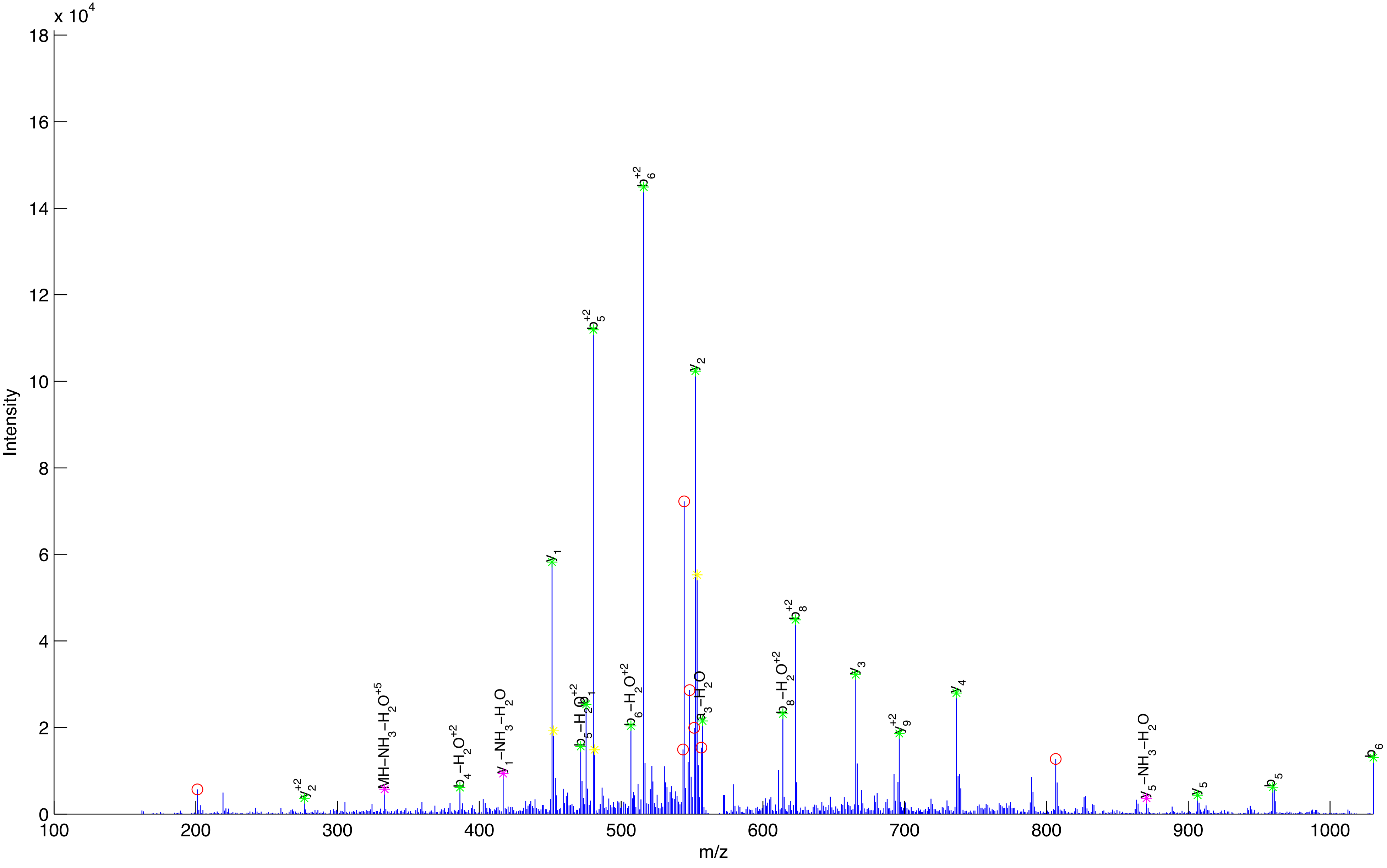

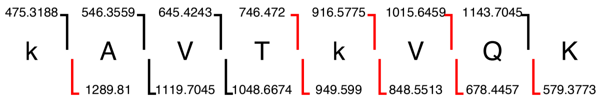

histone cluster 2, H2bf [Homo sapiens]
Charge State: +3
Scan Number: 9085
File Name: 120404_A549_EGFIGF_bioRepB_ACK_FT.raw

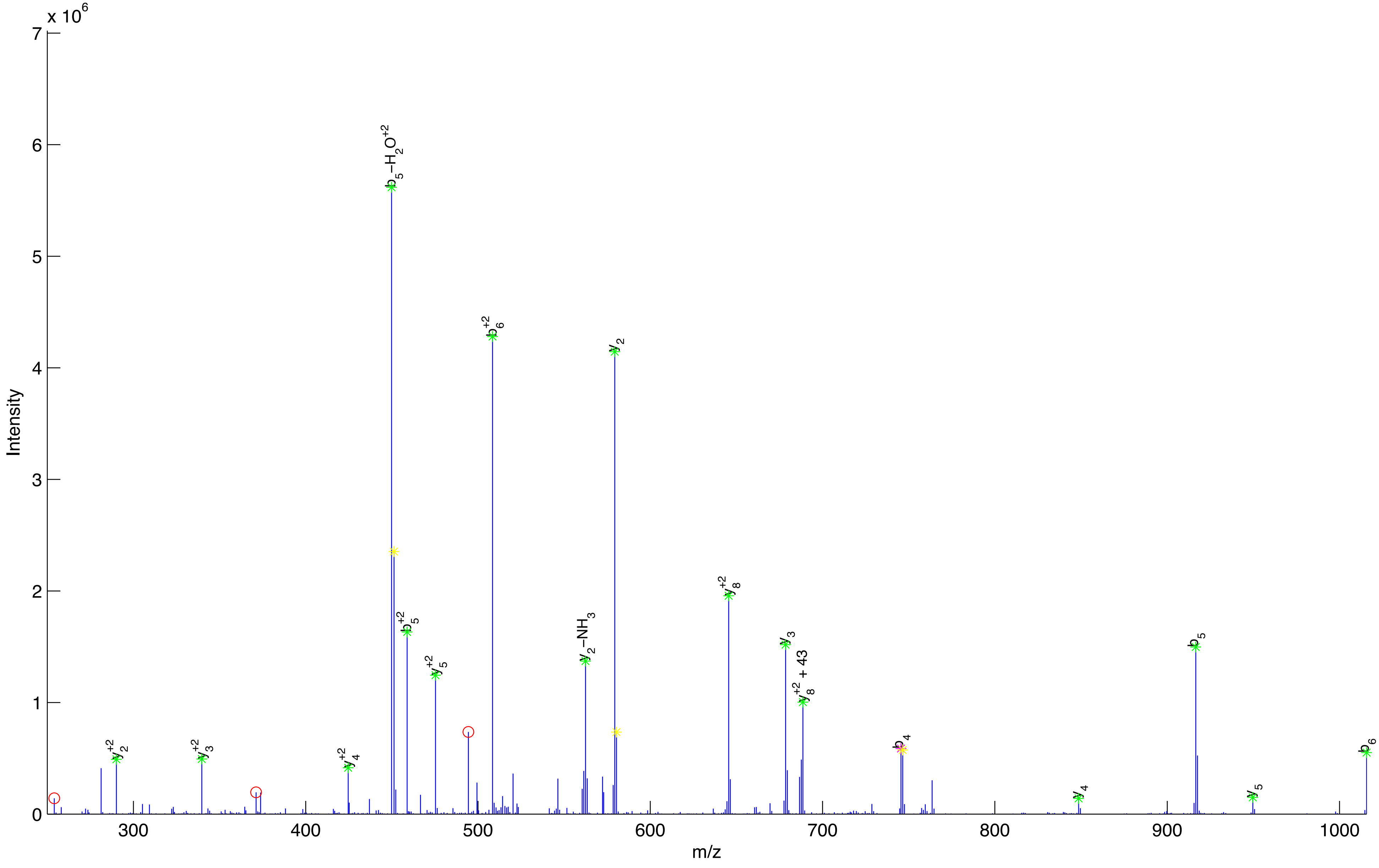

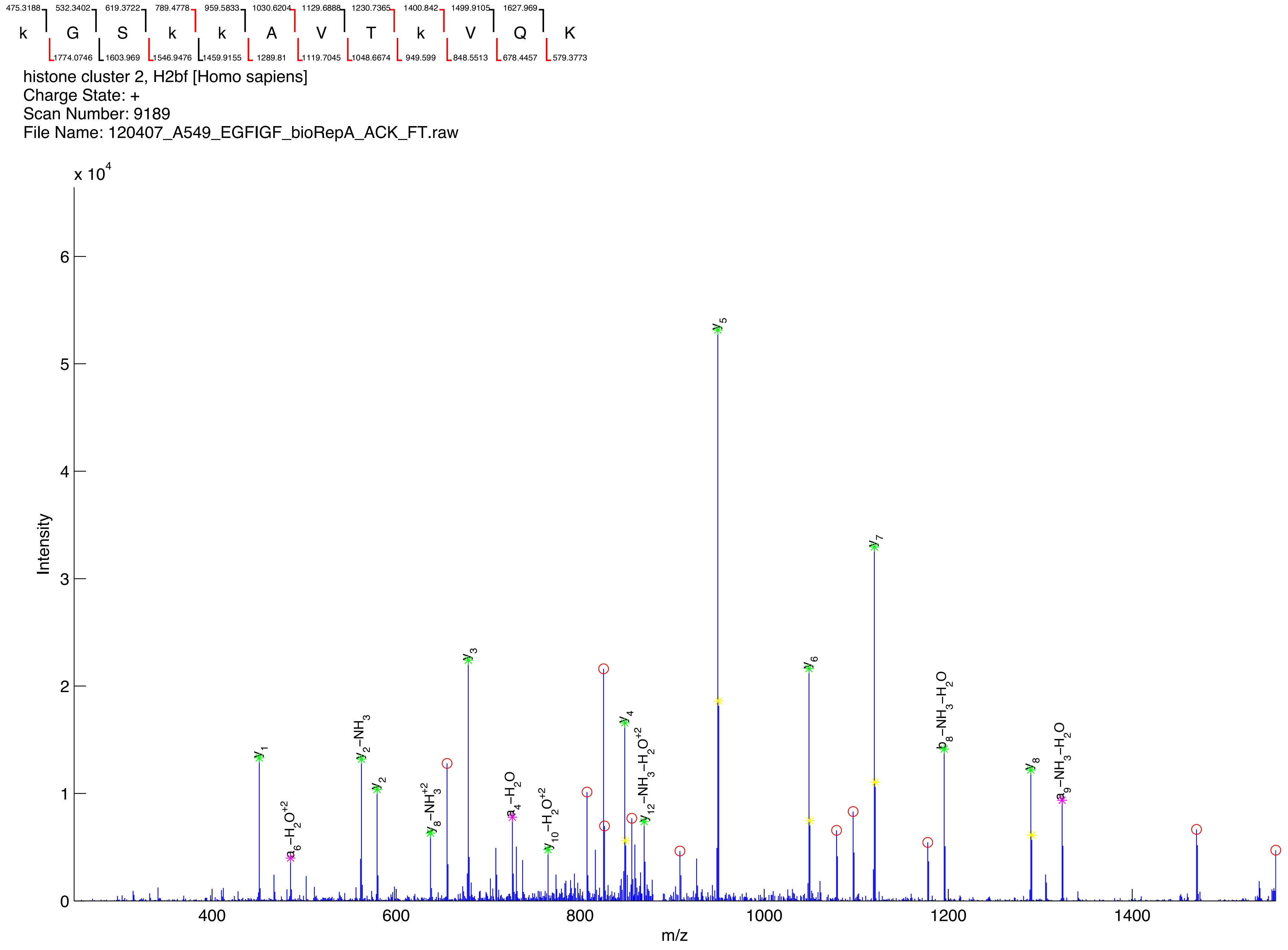

475.3188 532.3402 619.3722 789.4778 959.5833 1030.6204 1129.6888 1230.7365 1400.842 1499.9105 1627.969
k G S k k A V T k V Q K
1774.0746 1603.969 1546.9476 1459.9155 1289.81 1119.7045 1048.6674 949.599 848.5513 678.4457 579.3773
histone cluster 2, H2bf [Homo sapiens]
Charge State: +
Scan Number: 9189
File Name: 120407_A549_EGFIGF_bioRepA_ACK_FT.raw
x 10^4
Intensity
m/z
y1
a6-H2O+2
y2-NH3
y2
y8-NH3+2
y3
a4-H2O
y10-H2O+2
y4
y12-NH3-H2O+2
y5
y6
y7
b8-NH3-H2O
y8
a9-NH3-H2O

475.3188 532.3402 619.3722 789.4778 959.5833 1030.6204 1129.6888 1230.7365 1400.842 1471.8792 1599.9377

k G S k k A V T k A Q K

1746.0433 1575.9377 1518.9163 1431.8842 1261.7787 1091.6732 1020.6361 921.5677 820.52 650.4144 579.3773

histone cluster 1, H2bl [Homo sapiens]
Charge State: +2
Scan Number: 9233
File Name: 120407_A549_EGFIGF_bioRepA_ACK_FT.raw

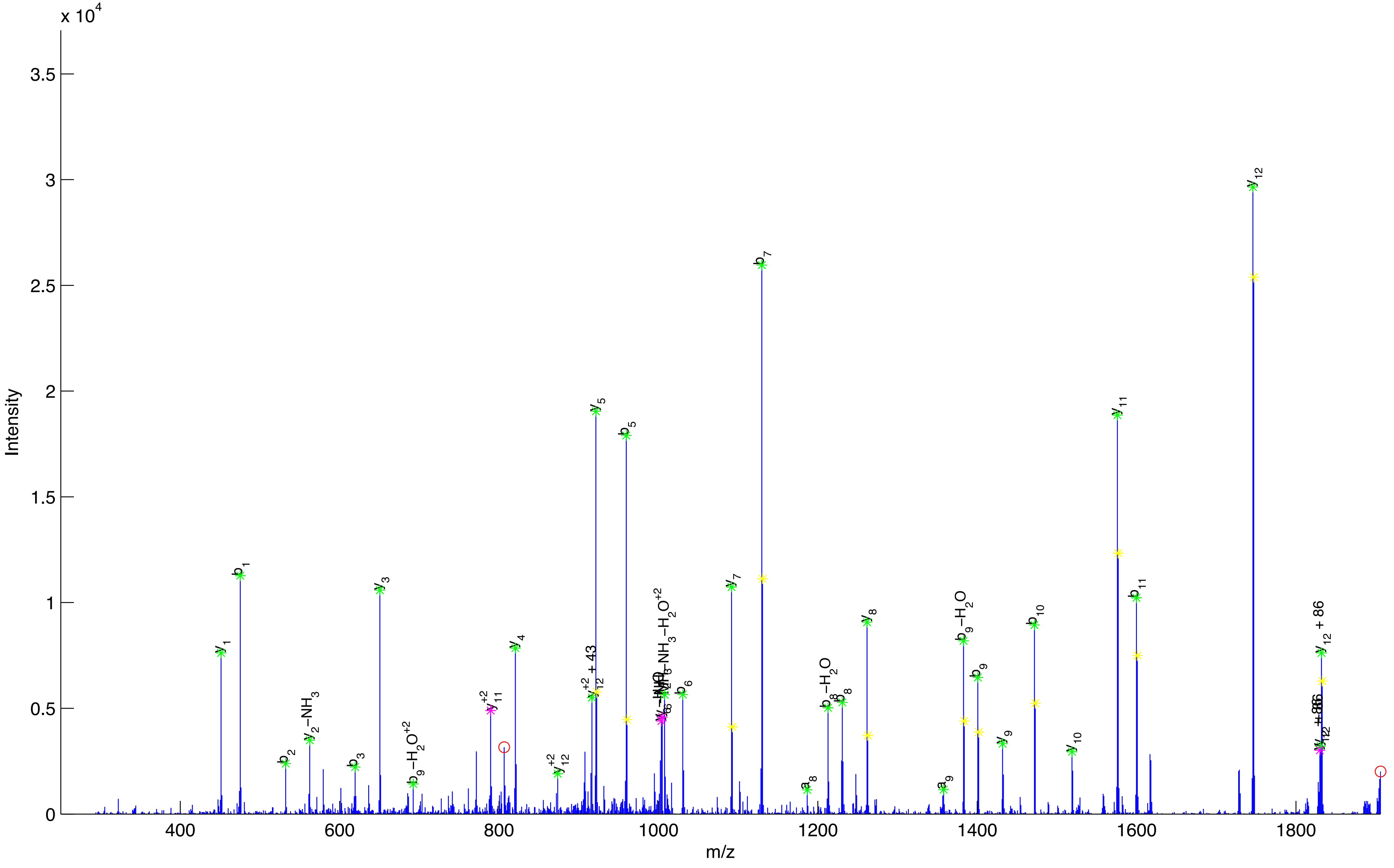

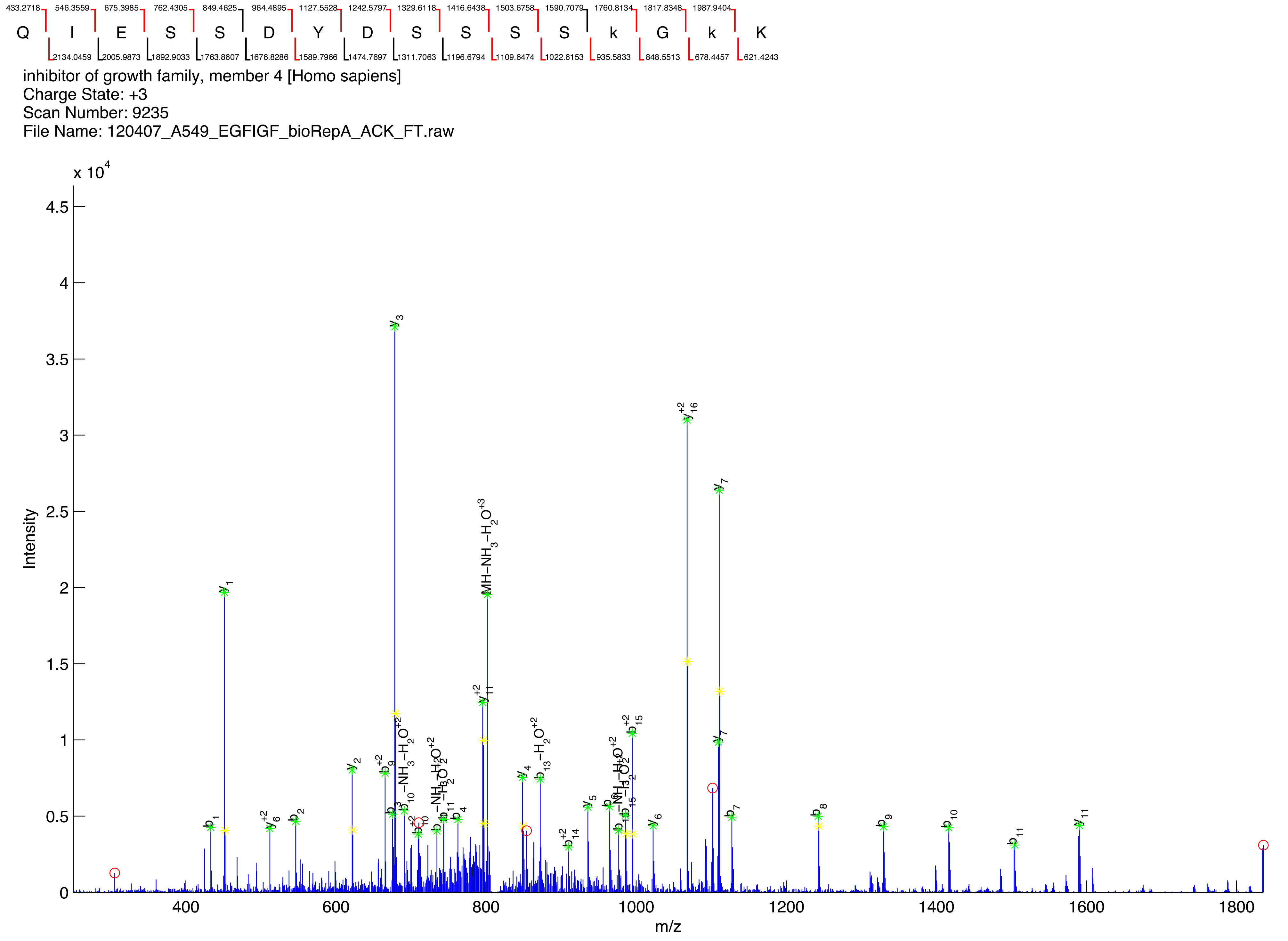

433.2718 546.3559 675.3985 762.4305 849.4625 964.4895 1127.5528 1242.5797 1329.6118 1416.6438 1503.6758 1590.7079 1760.8134 1817.8348 1987.9404
Q I E S S D Y D S S S S k G k K
2134.0459 2005.9873 1892.9033 1763.8607 1676.8286 1589.7966 1474.7697 1311.7063 1196.6794 1109.6474 1022.6153 935.5833 848.5513 678.4457 621.4243
inhibitor of growth family, member 4 [Homo sapiens]
Charge State: +3
Scan Number: 9235
File Name: 120407_A549_EGFIGF_bioRepA_ACK_FT.raw
x 10^4
Intensity
m/z

362.2347	532.3402	589.3617	646.3831	816.4887	873.5101	986.5942	1043.6157	1213.7212	1270.7427	1327.7641	1398.8012	1568.9068	
G	k	G	G	k	G	L	G	k	G	G	A	k	R
	1438.813	1381.7916	1211.686	1154.6646	1097.6431	927.5376	870.5161	757.4321	700.4106	530.3051	473.2836	416.2621	345.225

histone cluster 1, H4a [Homo sapiens]
Charge State: +3
Scan Number: 9252
File Name: 120407_A549_EGFIGF_bioRepA_ACK_FT.raw

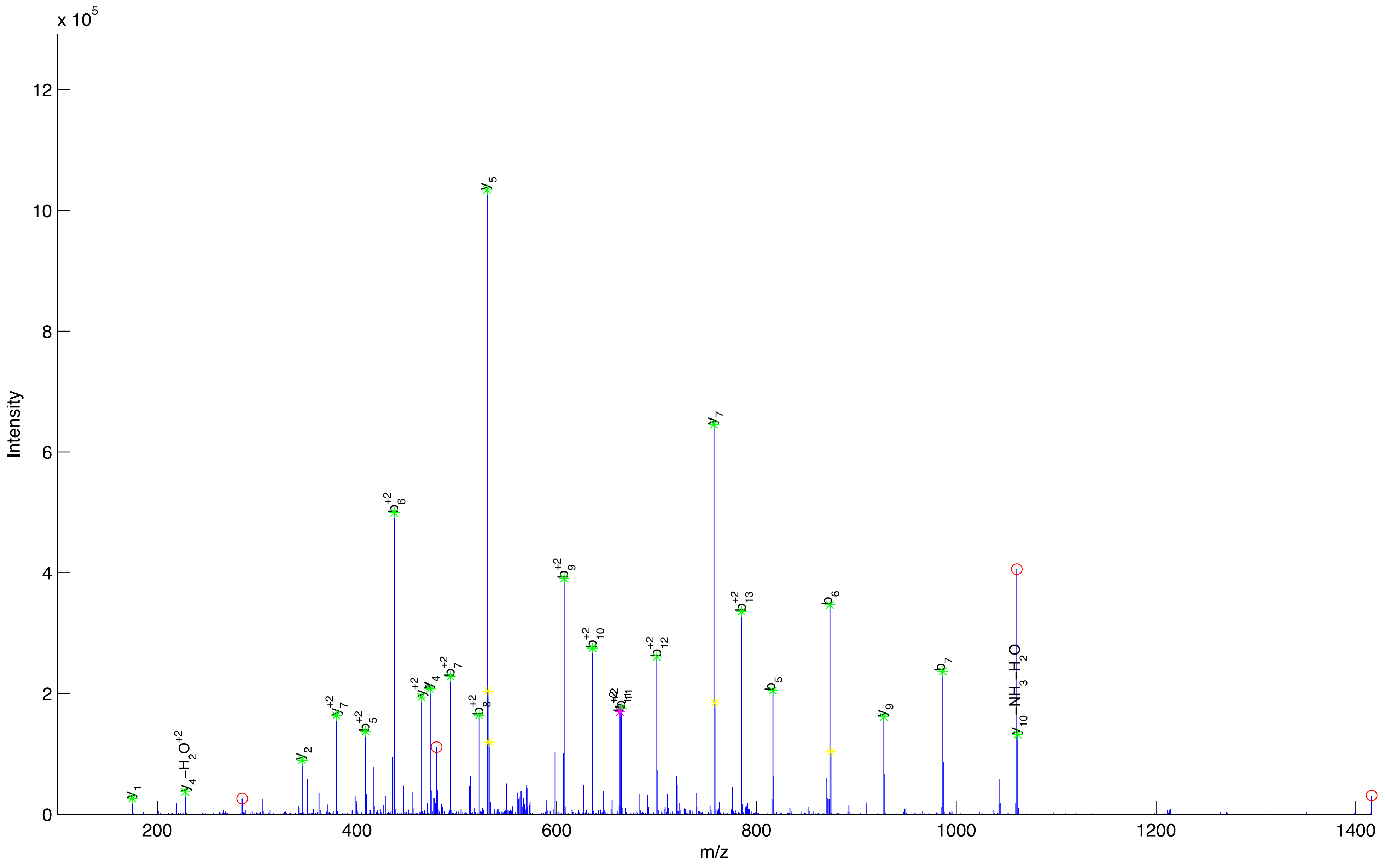

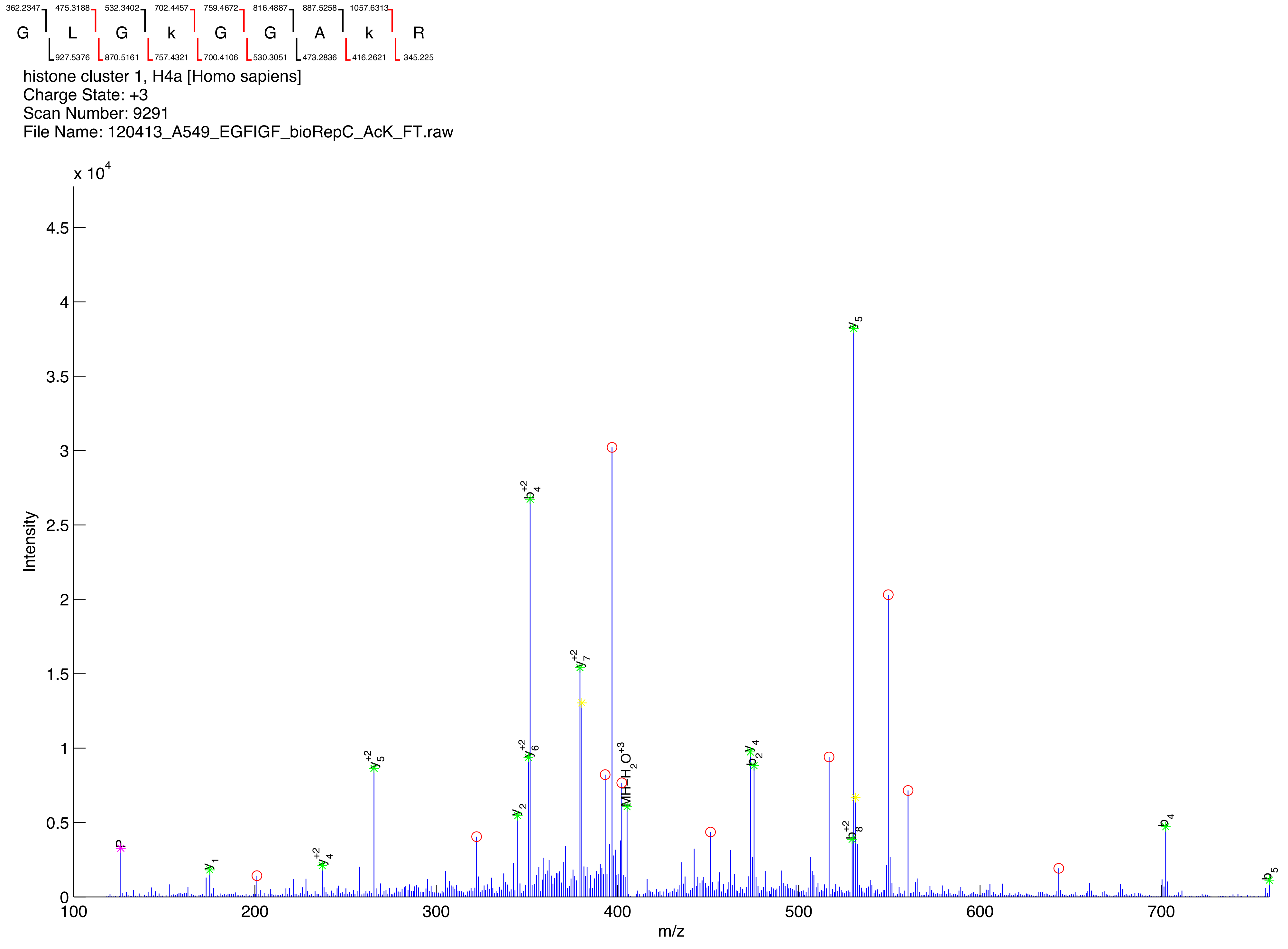

362.2347 475.3188 532.3402 702.4457 759.4672 816.4887 887.5258 1057.6313
G L G k G G A k R
927.5376 870.5161 757.4321 700.4106 530.3051 473.2836 416.2621 345.225
histone cluster 1, H4a [Homo sapiens]
Charge State: +3
Scan Number: 9291
File Name: 120413_A549_EGFIGF_bioRepC_AcK_FT.raw
x 10^4
Intensity
m/z
0
0.5
1
1.5
2
2.5
3
3.5
4
4.5
100
200
300
400
500
600
700
P
y1
y4+2
y5+2
y2
y6+2
b4+2
y7+2
MH-H2O+3
b2
y4
b8+2
y5
b4
b5

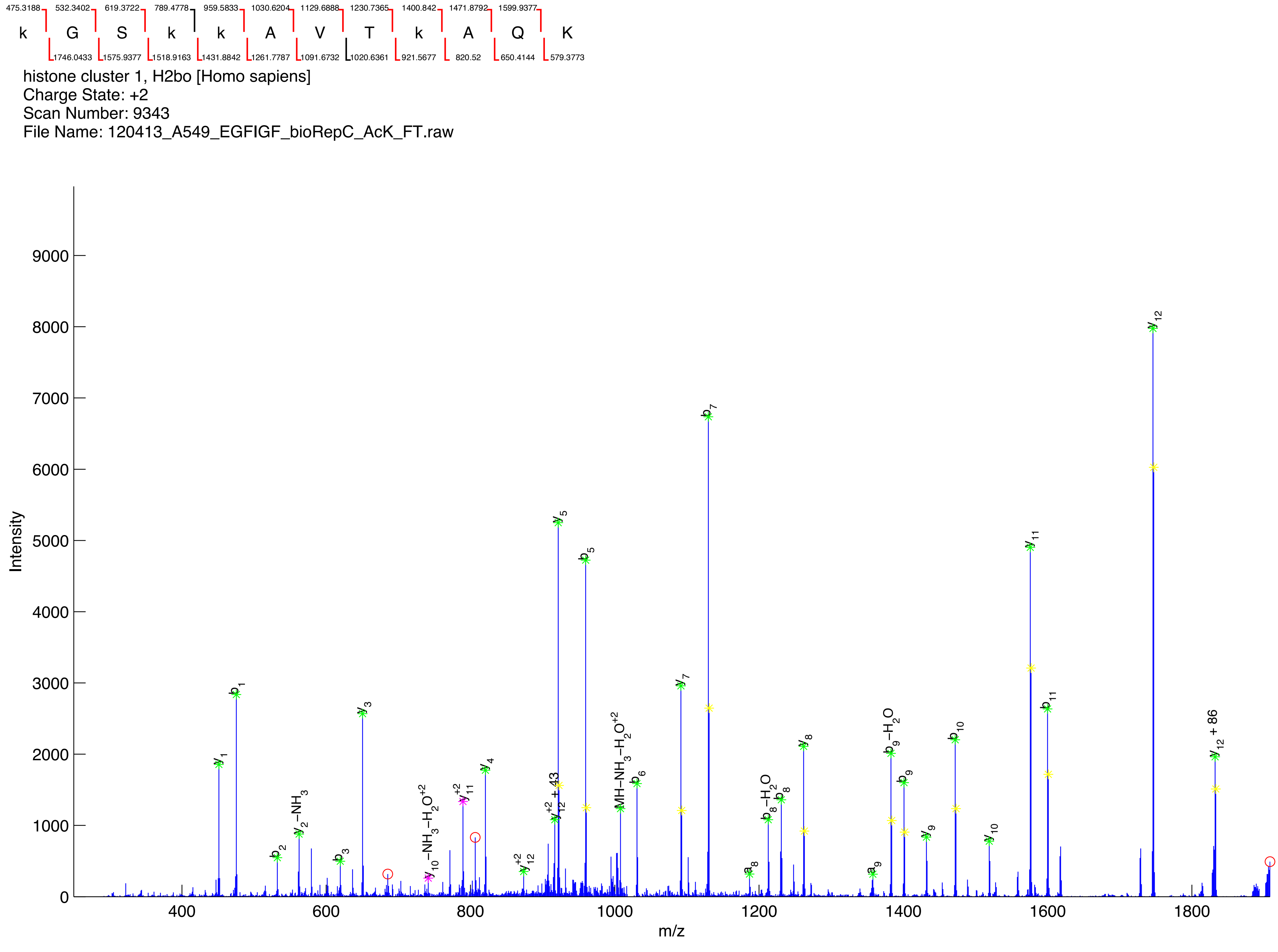

475.3188 532.3402 619.3722 789.4778 959.5833 1030.6204 1129.6888 1230.7365 1400.842 1471.8792 1599.9377
k G S k k A V T k A Q K
1746.0433 1575.9377 1518.9163 1431.8842 1261.7787 1091.6732 1020.6361 921.5677 820.52 650.4144 579.3773
histone cluster 1, H2bo [Homo sapiens]
Charge State: +2
Scan Number: 9343
File Name: 120413_A549_EGFIGF_bioRepC_AcK_FT.raw
Intensity
m/z

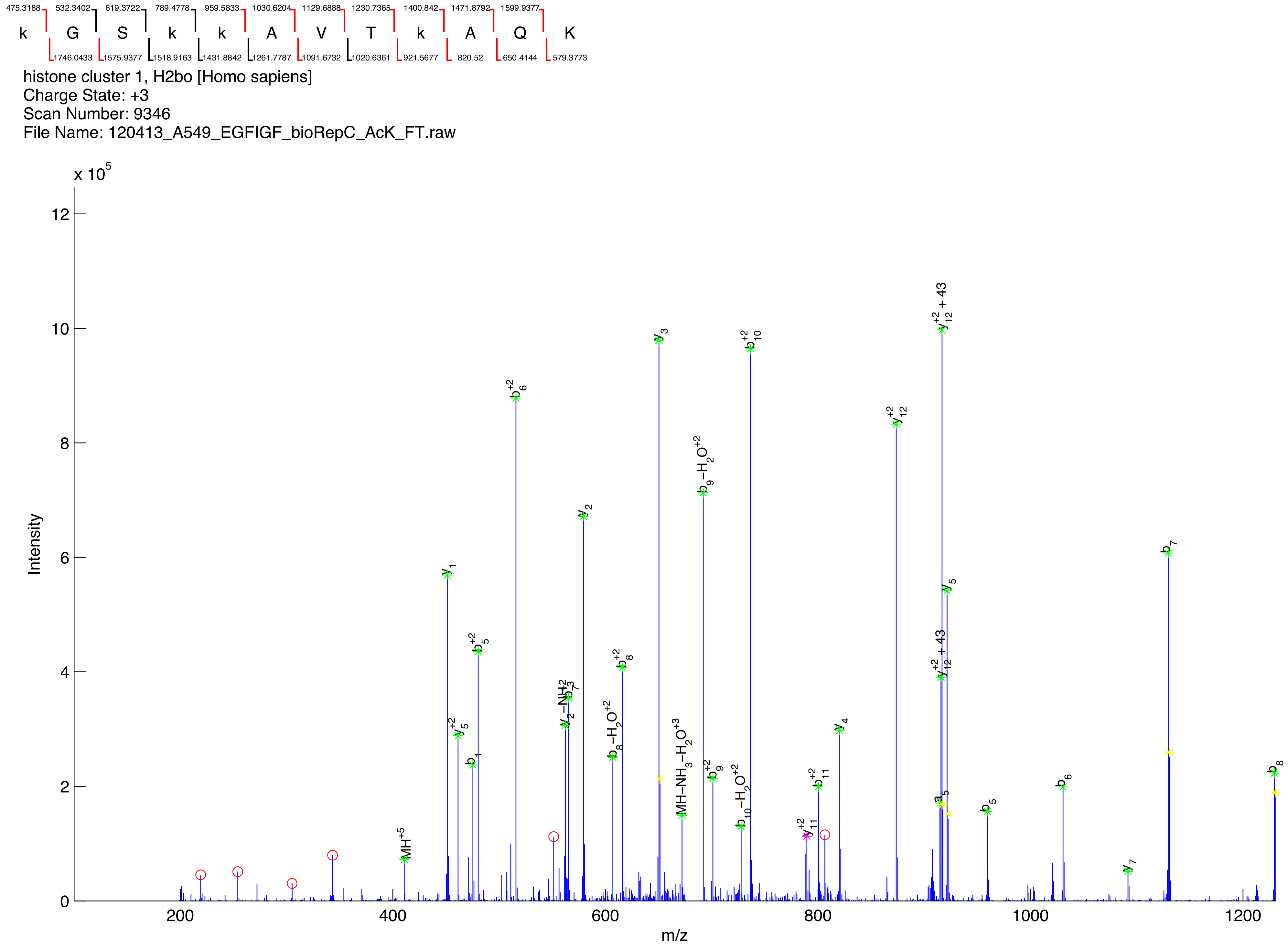

475.3188 532.3402 619.3722 789.4778 959.5833 1030.6204 1129.6888 1230.7365 1400.842 1471.8792 1599.9377
k G S k k A V T k A Q K
1746.0433 1575.9377 1518.9163 1431.8842 1261.7787 1091.6732 1020.6361 921.5677 820.52 650.4144 579.3773
histone cluster 1, H2bo [Homo sapiens]
Charge State: +3
Scan Number: 9346
File Name: 120413_A549_EGFIGF_bioRepC_AcK_FT.raw
x 10^5
Intensity
m/z

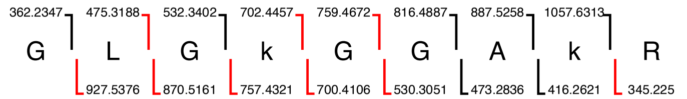

histone cluster 1, H4a [Homo sapiens]
Charge State: +3
Scan Number: 9380
File Name: 120407_A549_EGFIGF_bioRepA_ACK_FT.raw

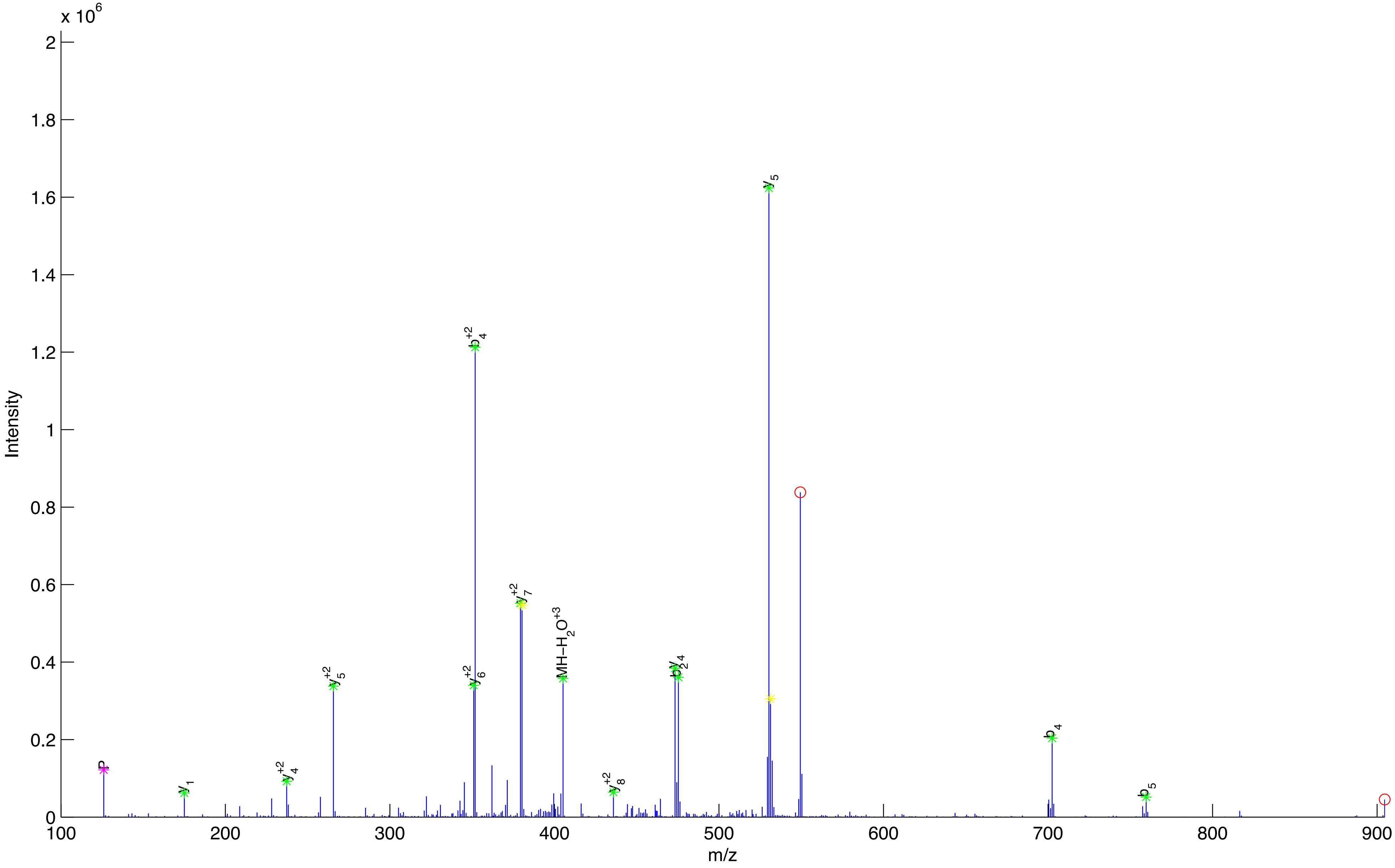

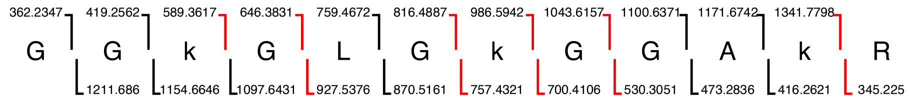

histone cluster 1, H4a [Homo sapiens]
Charge State: +3
Scan Number: 9382
File Name: 120407_A549_EGFIGF_bioRepA_ACK_FT.raw

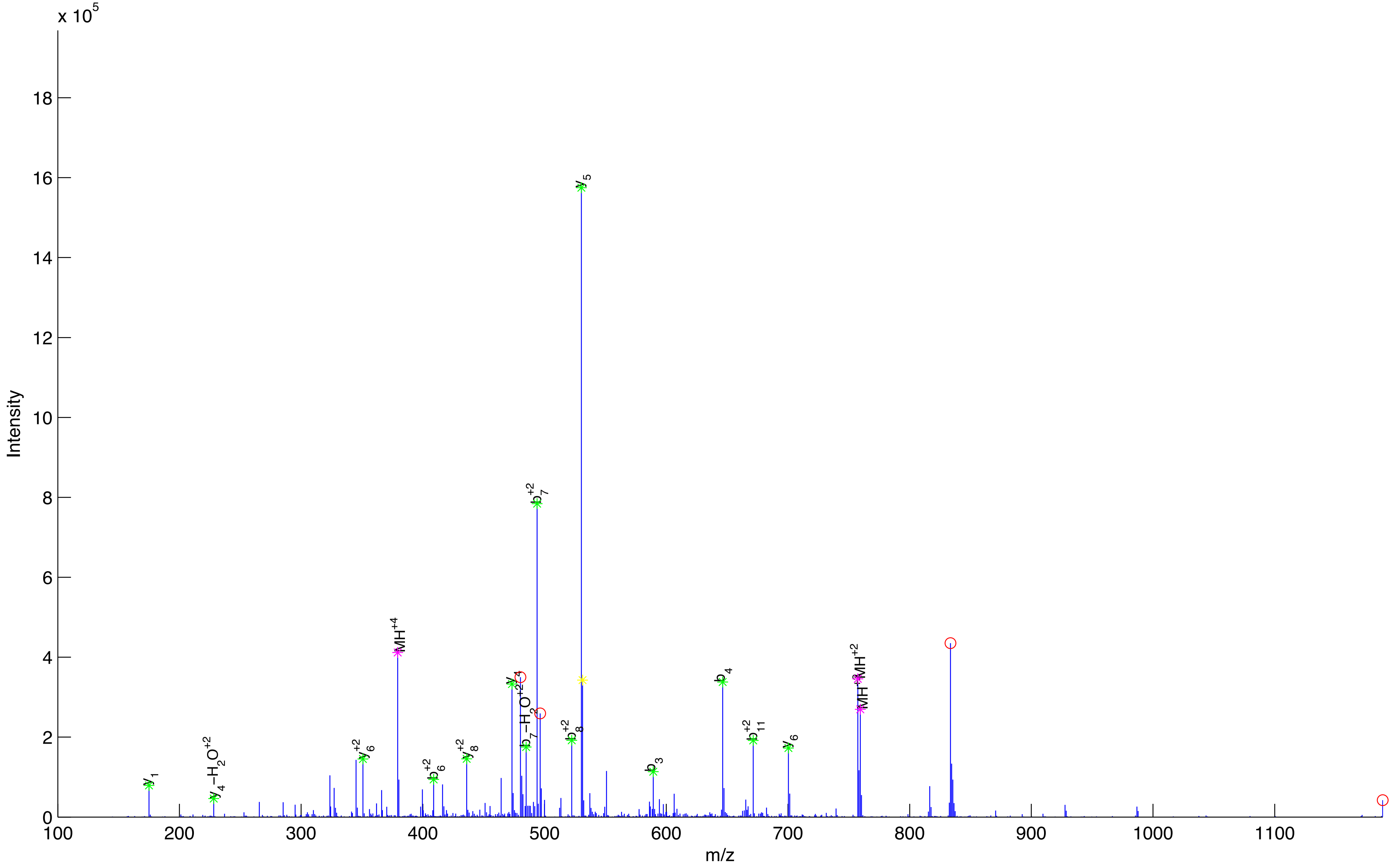

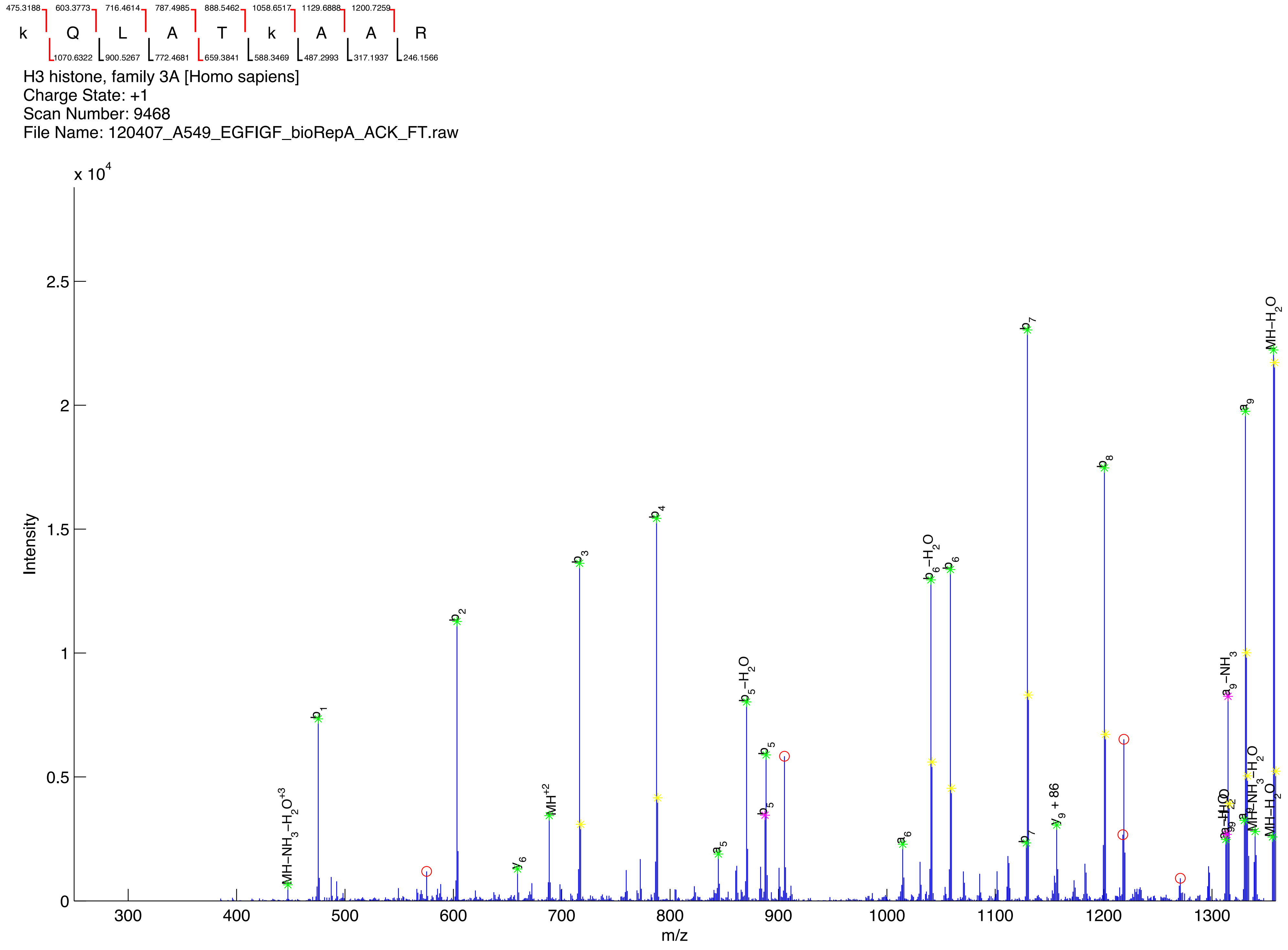

H3 histone, family 3A [Homo sapiens]
Charge State: +1
Scan Number: 9468
File Name: 120407_A549_EGFIGF_bioRepA_ACK_FT.raw

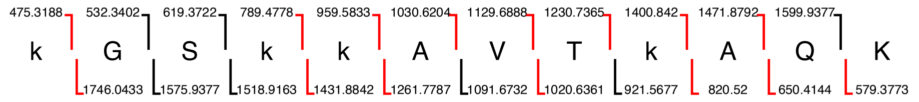

histone cluster 1, H2bo [Homo sapiens]
Charge State: +4
Scan Number: 9556
File Name: 120413_A549_EGFIGF_bioRepC_AcK_FT.raw

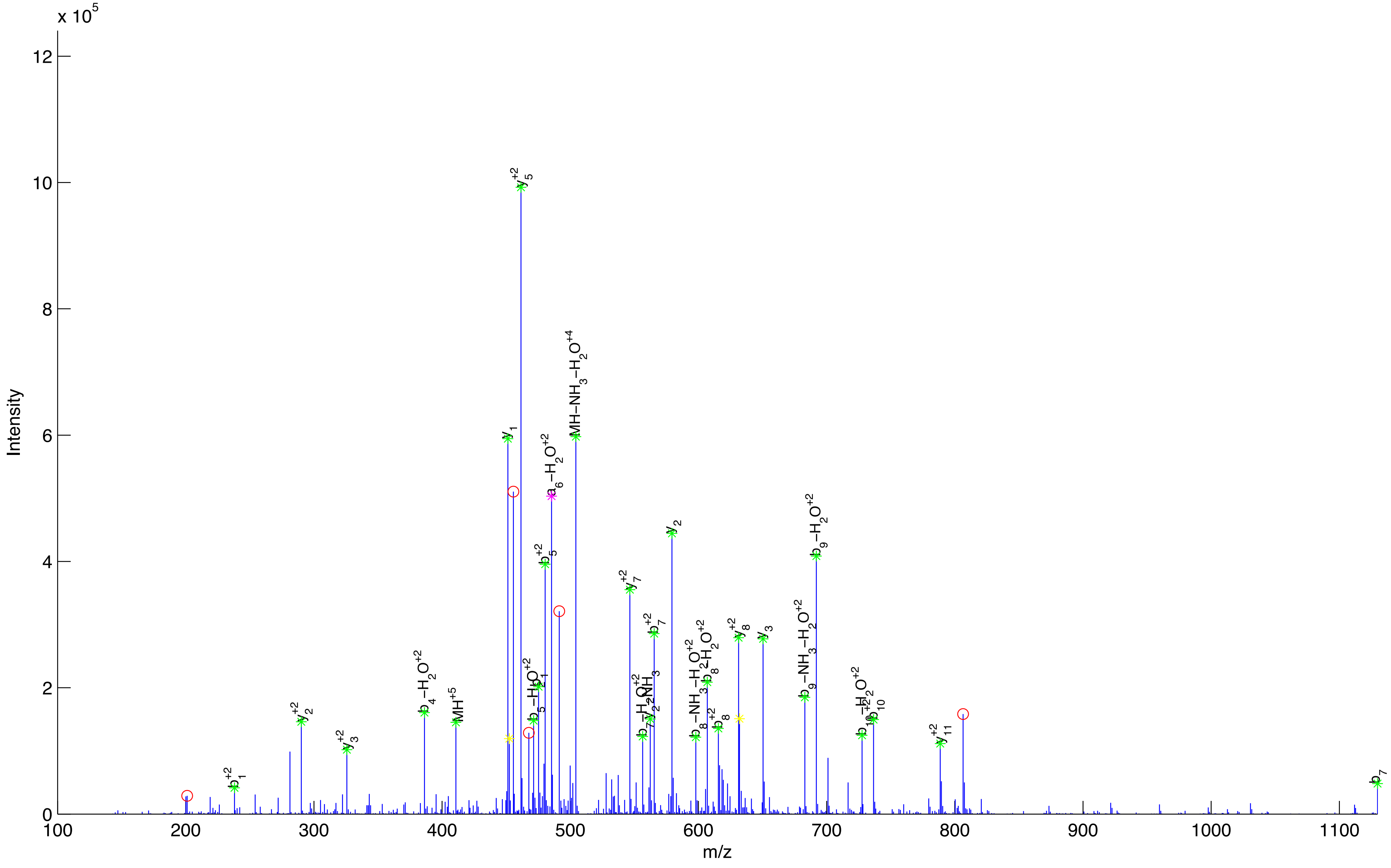

H3 histone, family 3A [Homo sapiens]
Charge State: +2
Scan Number: 9577
File Name: 120413_A549_EGFIGF_bioRepC_AcK_FT.raw

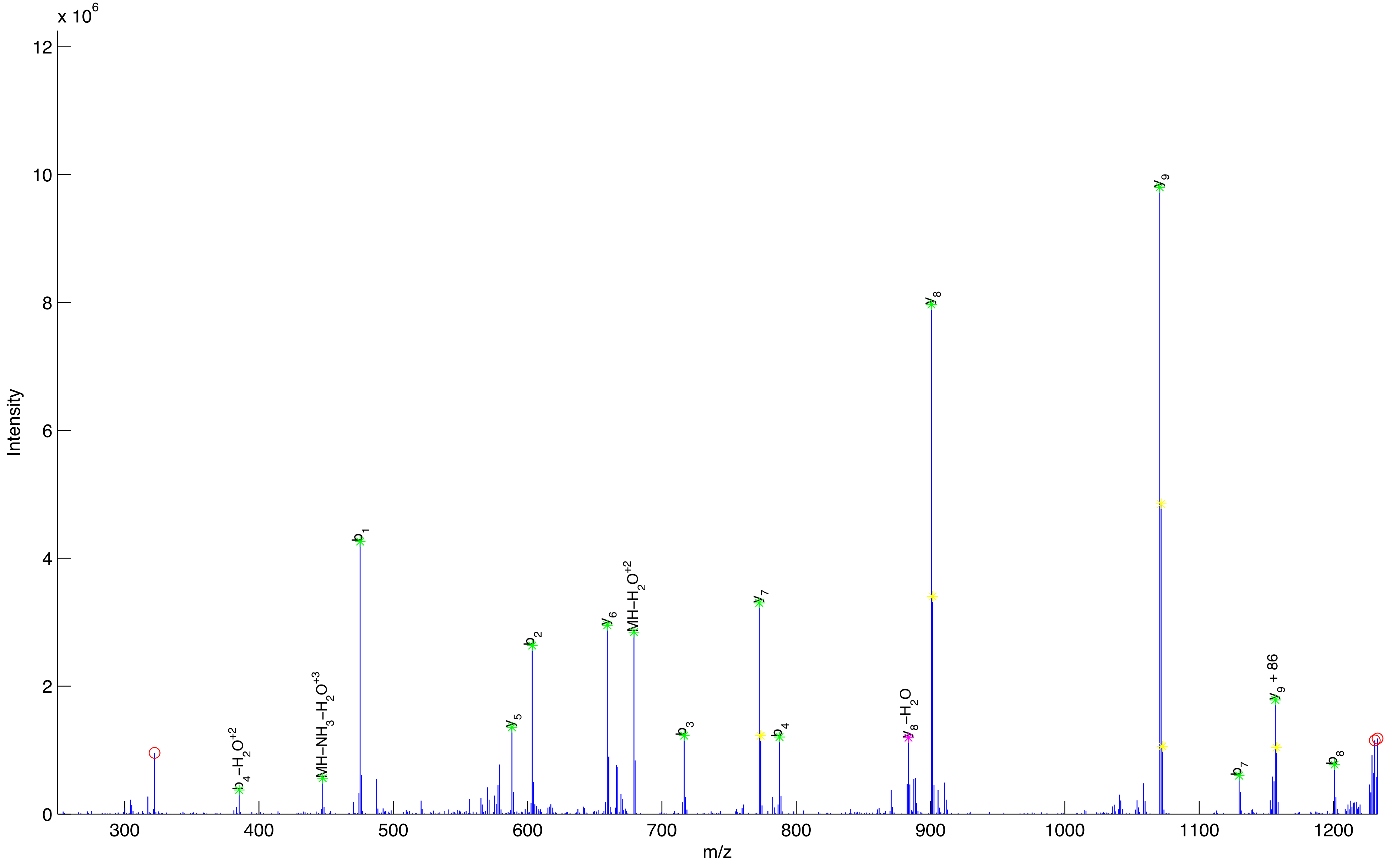

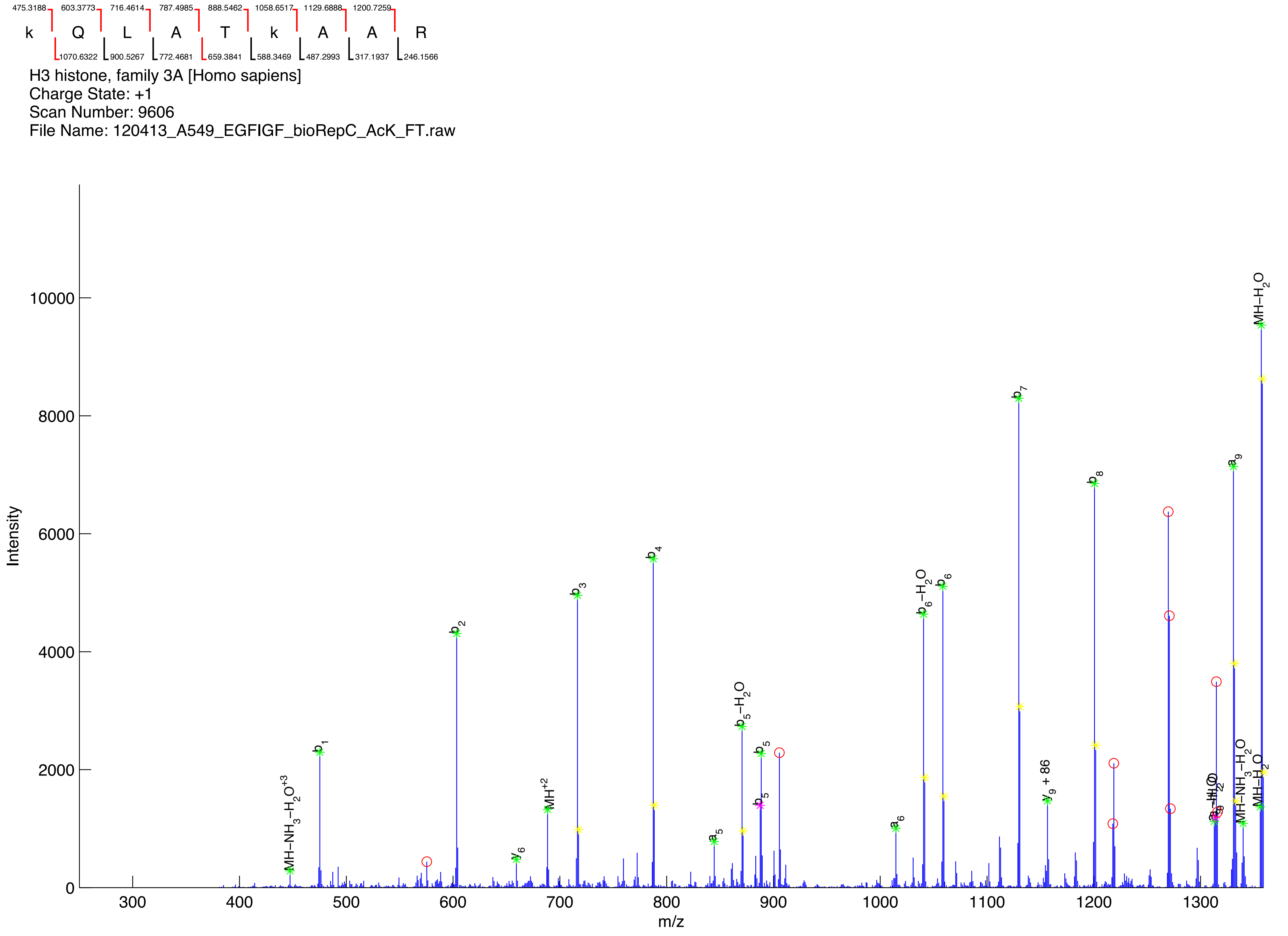
475.3188 603.3773 716.4614 787.4985 888.5462 1058.6517 1129.6888 1200.7259
k Q L A T k A A R
1070.6322 900.5267 772.4681 659.3841 588.3469 487.2993 317.1937 246.1566
H3 histone, family 3A [Homo sapiens]
Charge State: +1
Scan Number: 9606
File Name: 120413_A549_EGFIGF_bioRepC_AcK_FT.raw
Intensity
m/z
MH−NH3−H2O+3
b1
b2
y6
MH+2
b3
b4
a5
b5−H2O
b5
a6
b6−H2O
b6
b7
y9 + 86
b8
a9
MH−NH3−H2O
MH−H2O

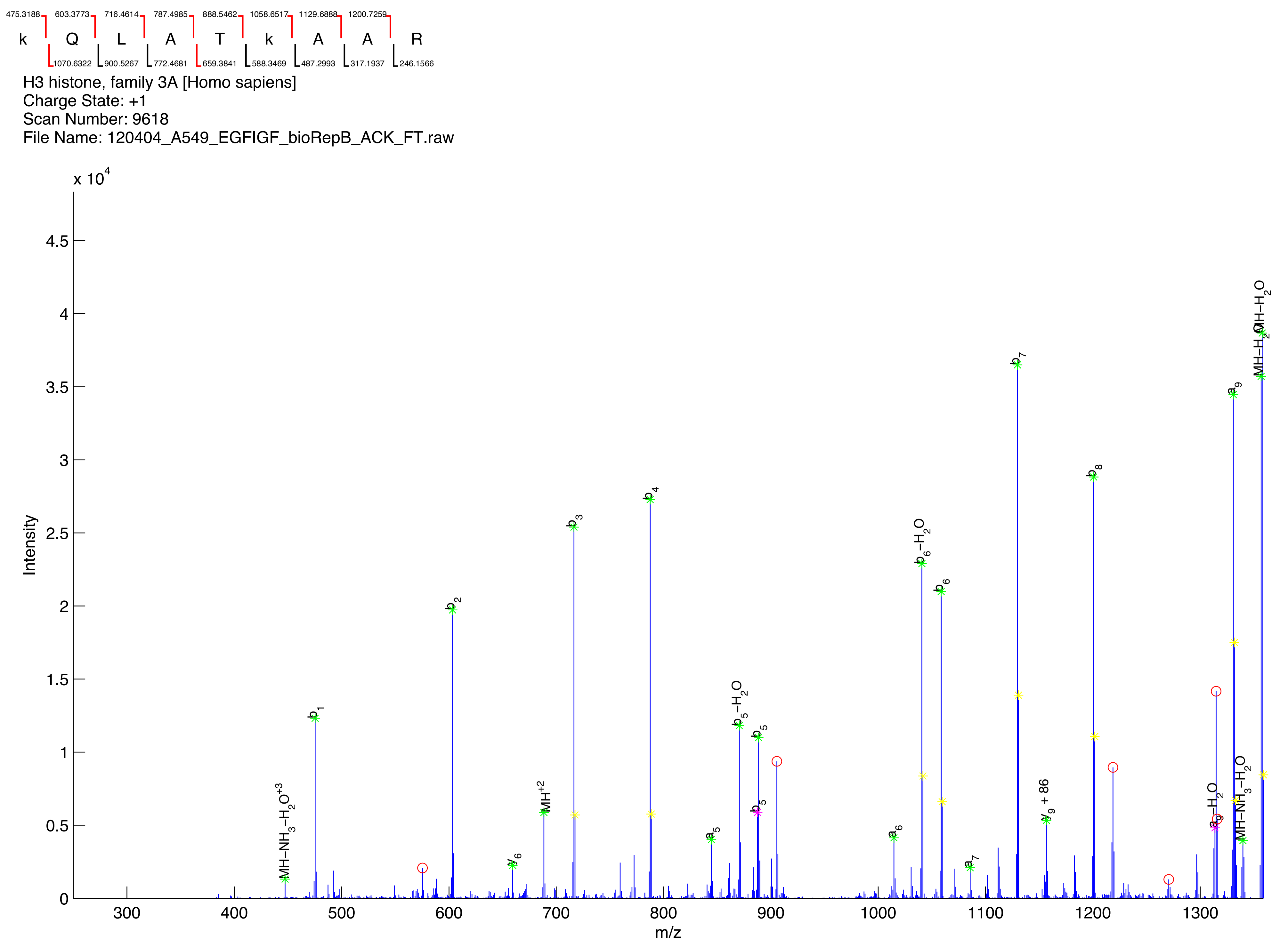
475.3188 603.3773 716.4614 787.4985 888.5462 1058.6517 1129.6888 1200.7259
k Q L A T k A A R
1070.6322 900.5267 772.4681 659.3841 588.3469 487.2993 317.1937 246.1566
H3 histone, family 3A [Homo sapiens]
Charge State: +1
Scan Number: 9618
File Name: 120404_A549_EGFIGF_bioRepB_ACK_FT.raw
x 10^4
Intensity
m/z
MH−NH3−H2O+3
b1
b2
y6
MH+2
b3
b4
a5
b5−H2O
b5
b5
a6
b6−H2O
b6
a7
b7
y9 + 86
b8
a9−H2O
a9
a9
MH−NH3−H2O
MH−H2O
MH−H2O

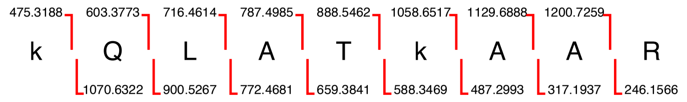

H3 histone, family 3A [Homo sapiens]
Charge State: +3
Scan Number: 9652
File Name: 120404_A549_EGFIGF_bioRepB_ACK_FT.raw

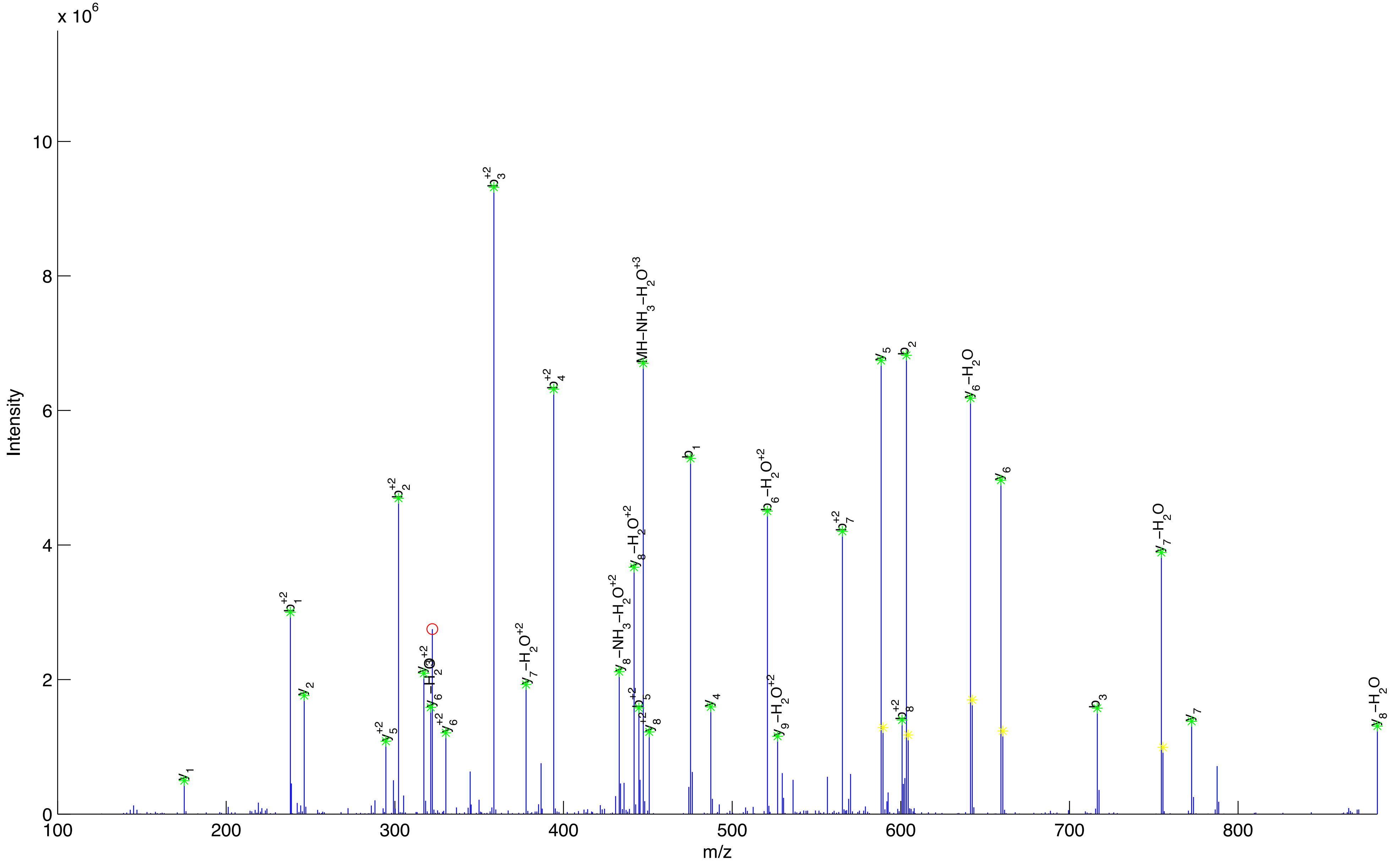

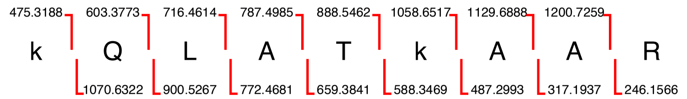

H3 histone, family 3A [Homo sapiens]
Charge State: +3
Scan Number: 9693
File Name: 120407_A549_EGFIGF_bioRepA_ACK_FT.raw

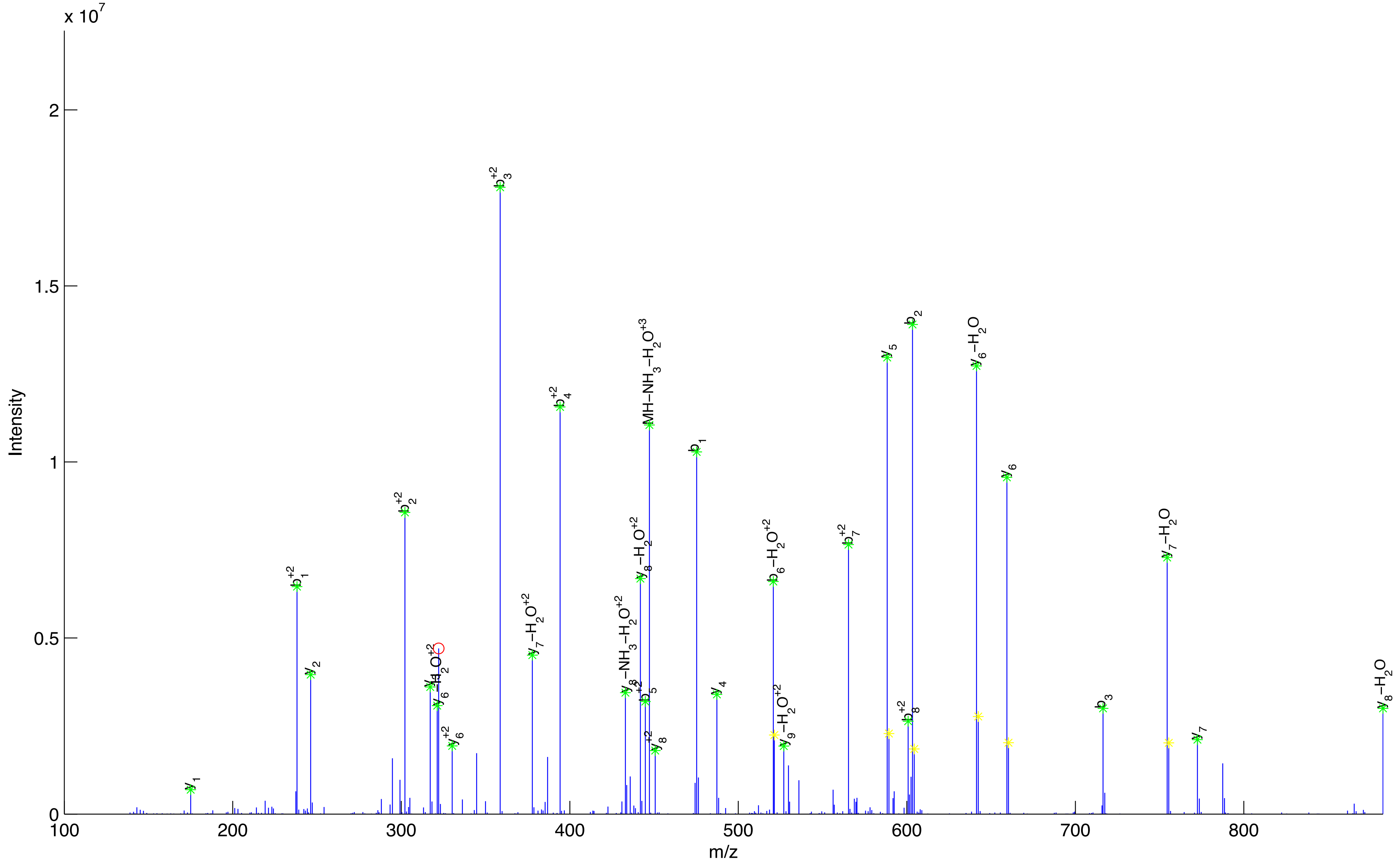

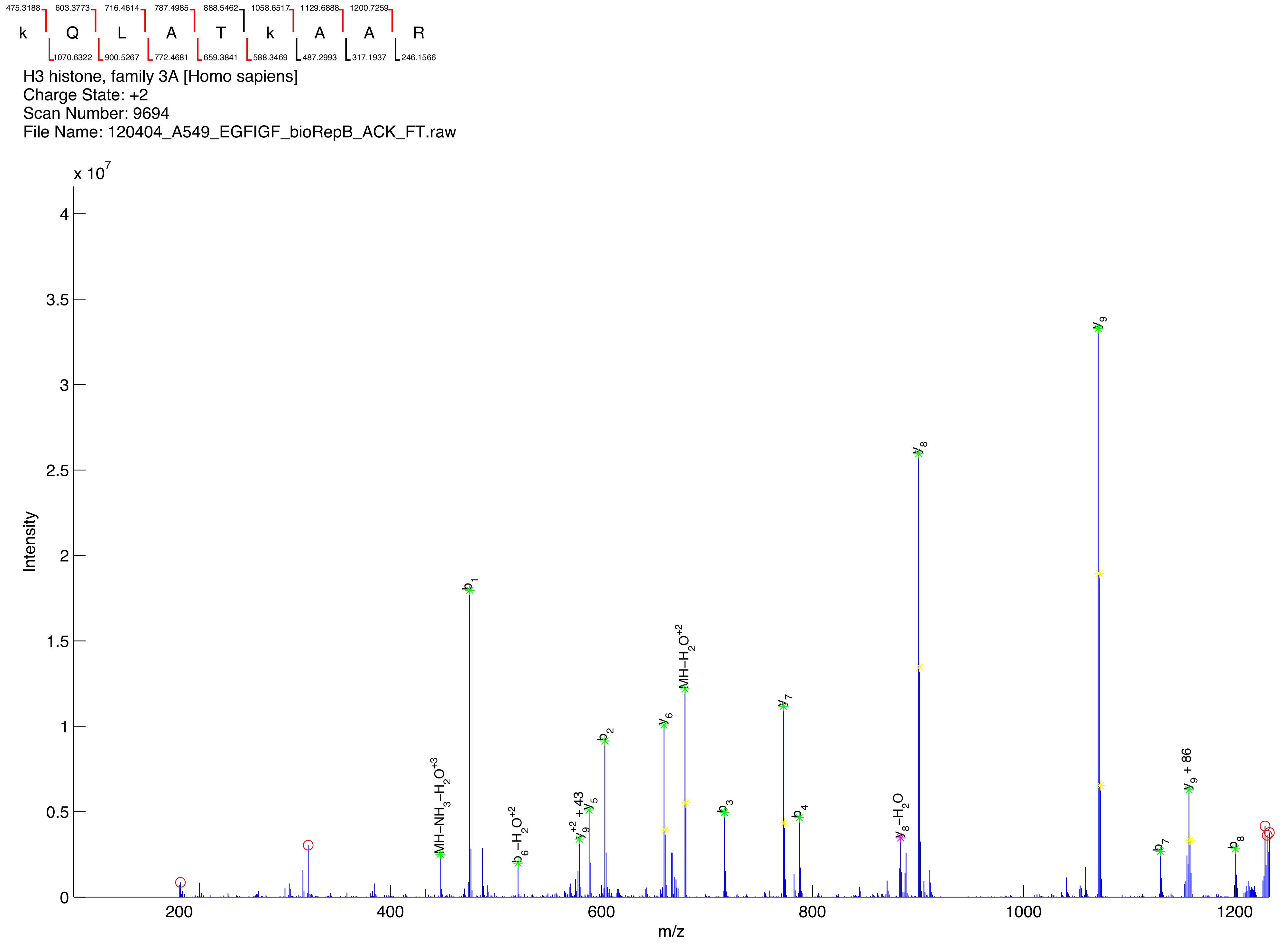

475.3188 603.3773 716.4614 787.4985 888.5462 1058.6517 1129.6888 1200.7259
k Q L A T k A A R
1070.6322 900.5267 772.4681 659.3841 588.3469 487.2993 317.1937 246.1566
H3 histone, family 3A [Homo sapiens]
Charge State: +2
Scan Number: 9694
File Name: 120404_A549_EGFIGF_bioRepB_ACK_FT.raw
x 10^7
Intensity
m/z
MH-NH3-H2O+3
b1
b6-H2O+2
y9+2 + 43
y5
b2
y6
MH-H2O+2
b3
y7
b4
y8-H2O
y8
y9
b7
y9 + 86
b8

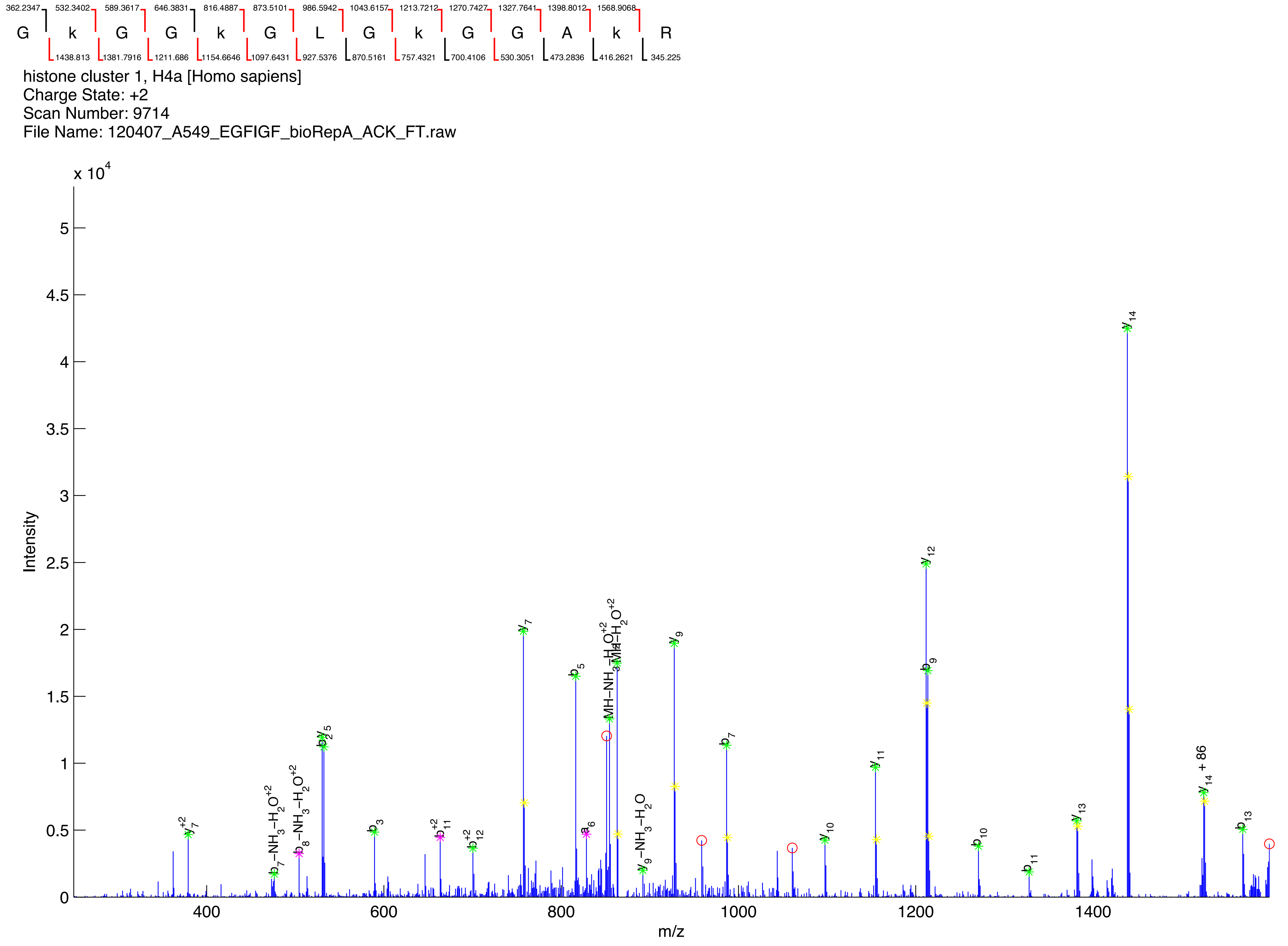
362.2347 532.3402 589.3617 646.3831 816.4887 873.5101 986.5942 1043.6157 1213.7212 1270.7427 1327.7641 1398.8012 1568.9068
G k G G k G L G k G G A k R
1438.813 1381.7916 1211.686 1154.6646 1097.6431 927.5376 870.5161 757.4321 700.4106 530.3051 473.2836 416.2621 345.225
histone cluster 1, H4a [Homo sapiens]
Charge State: +2
Scan Number: 9714
File Name: 120407_A549_EGFIGF_bioRepA_ACK_FT.raw
Intensity
x 10^4
m/z

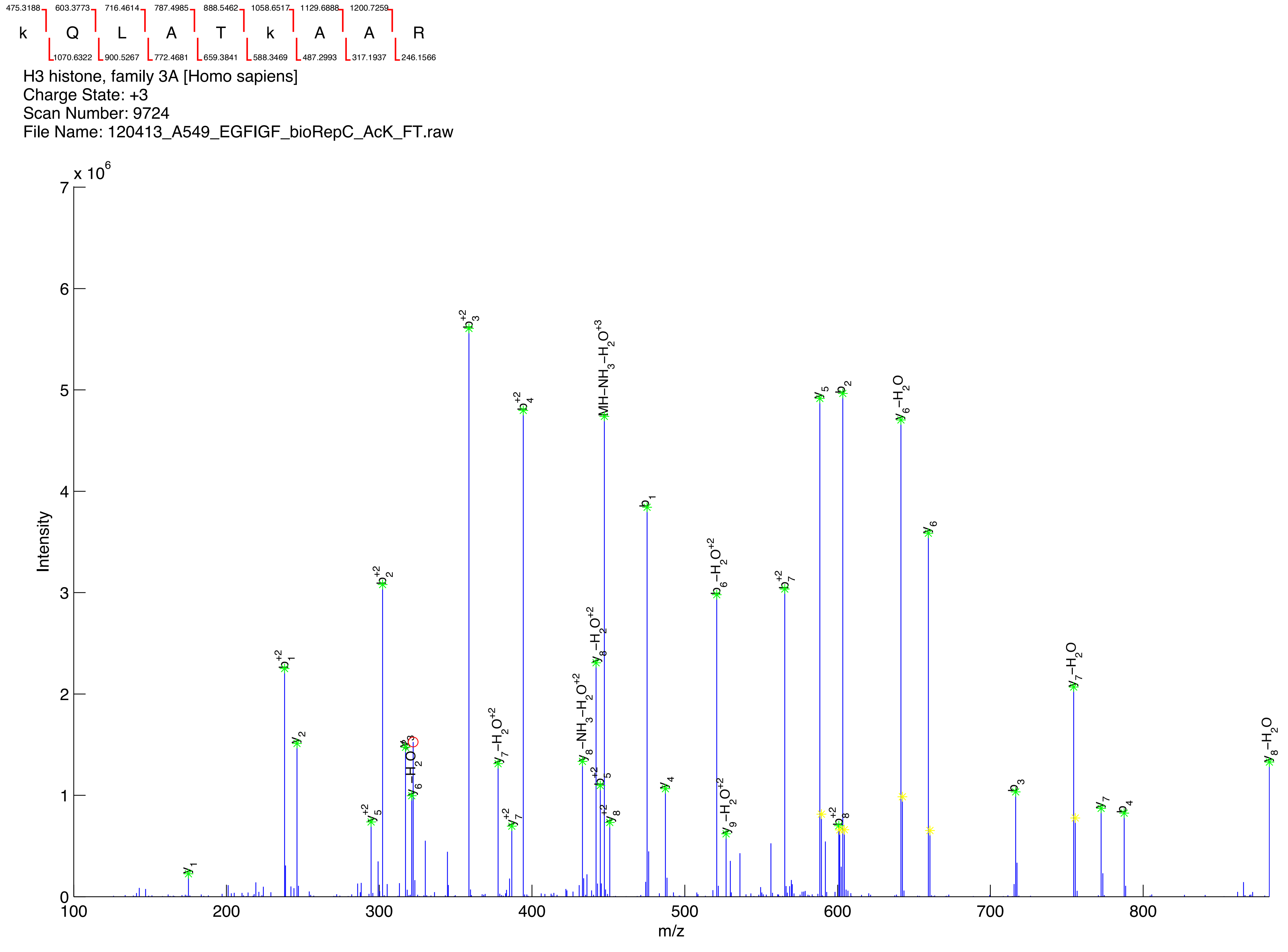

k Q L A T k A A R
475.3188 603.3773 716.4614 787.4985 888.5462 1058.6517 1129.6888 1200.7259
1070.6322 900.5267 772.4681 659.3841 588.3469 487.2993 317.1937 246.1566
H3 histone, family 3A [Homo sapiens]
Charge State: +3
Scan Number: 9724
File Name: 120413_A549_EGFIGF_bioRepC_AcK_FT.raw
Intensity
x 10^6
m/z

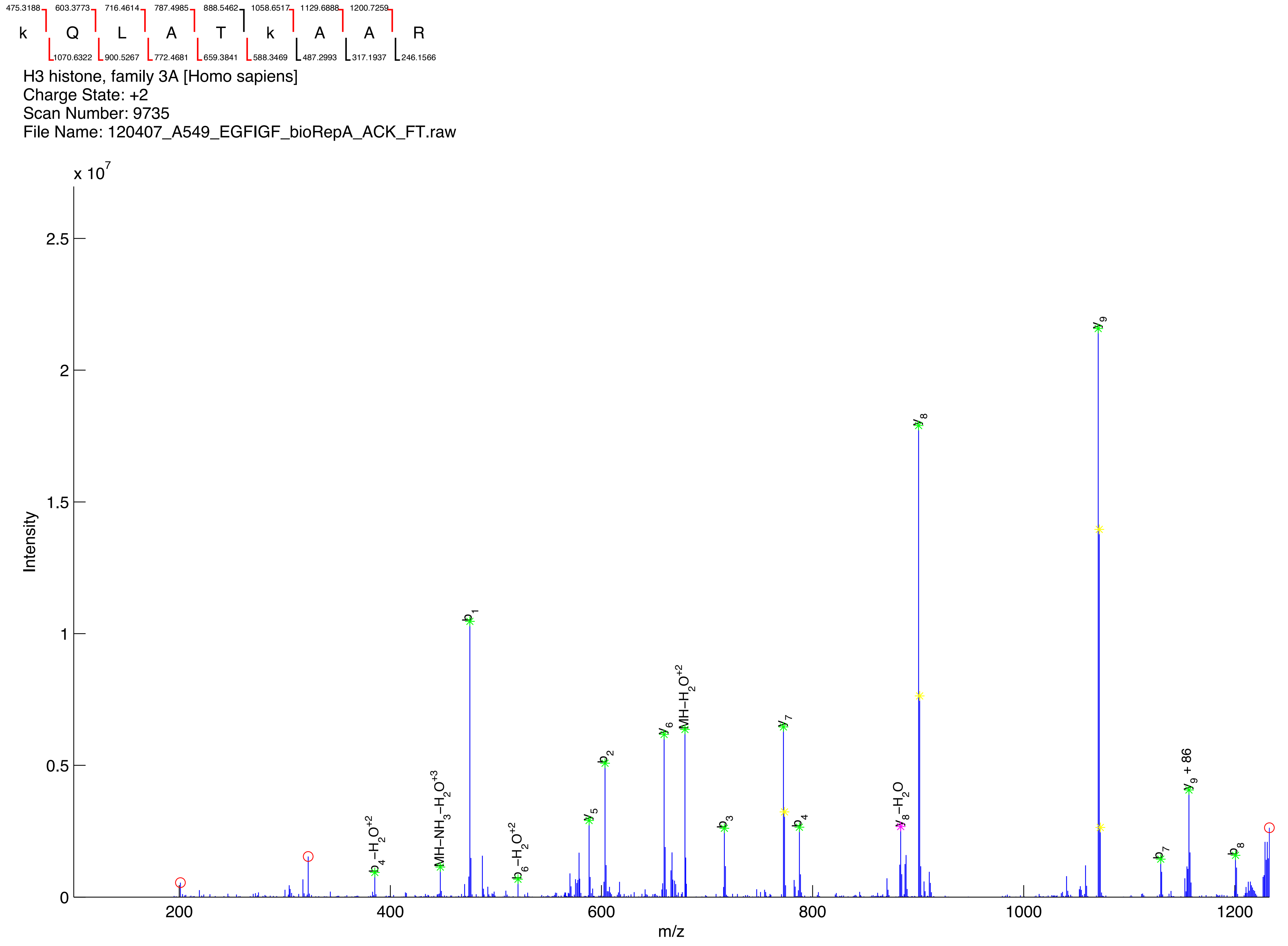

475.3188 603.3773 716.4614 787.4985 888.5462 1058.6517 1129.6888 1200.7259
k Q L A T k A A R
1070.6322 900.5267 772.4681 659.3841 588.3469 487.2993 317.1937 246.1566
H3 histone, family 3A [Homo sapiens]
Charge State: +2
Scan Number: 9735
File Name: 120407_A549_EGFIGF_bioRepA_ACK_FT.raw
x 10^7
Intensity
m/z
b4−H2O+2
MH−NH3−H2O+3
b1
b6−H2O+2
y5
b2
y6
MH−H2O+2
b3
y7
b4
y8−H2O
y8
y9
b7
y9 + 86
b8

475.3188	532.3402	619.3722	789.4778	959.5833	1030.6204	1129.6888	1230.7365	1400.842	1471.8792	1599.9377	
k	G	S	k	k	A	V	T	k	A	Q	K
1746.0433	1575.9377	1518.9163	1431.8842	1261.7787	1091.6732	1020.6361	921.5677	820.52	650.4144	579.3773	

histone cluster 1, H2bl [Homo sapiens]
Charge State: +3
Scan Number: 9737
File Name: 120407_A549_EGFIGF_bioRepA_ACK_FT.raw

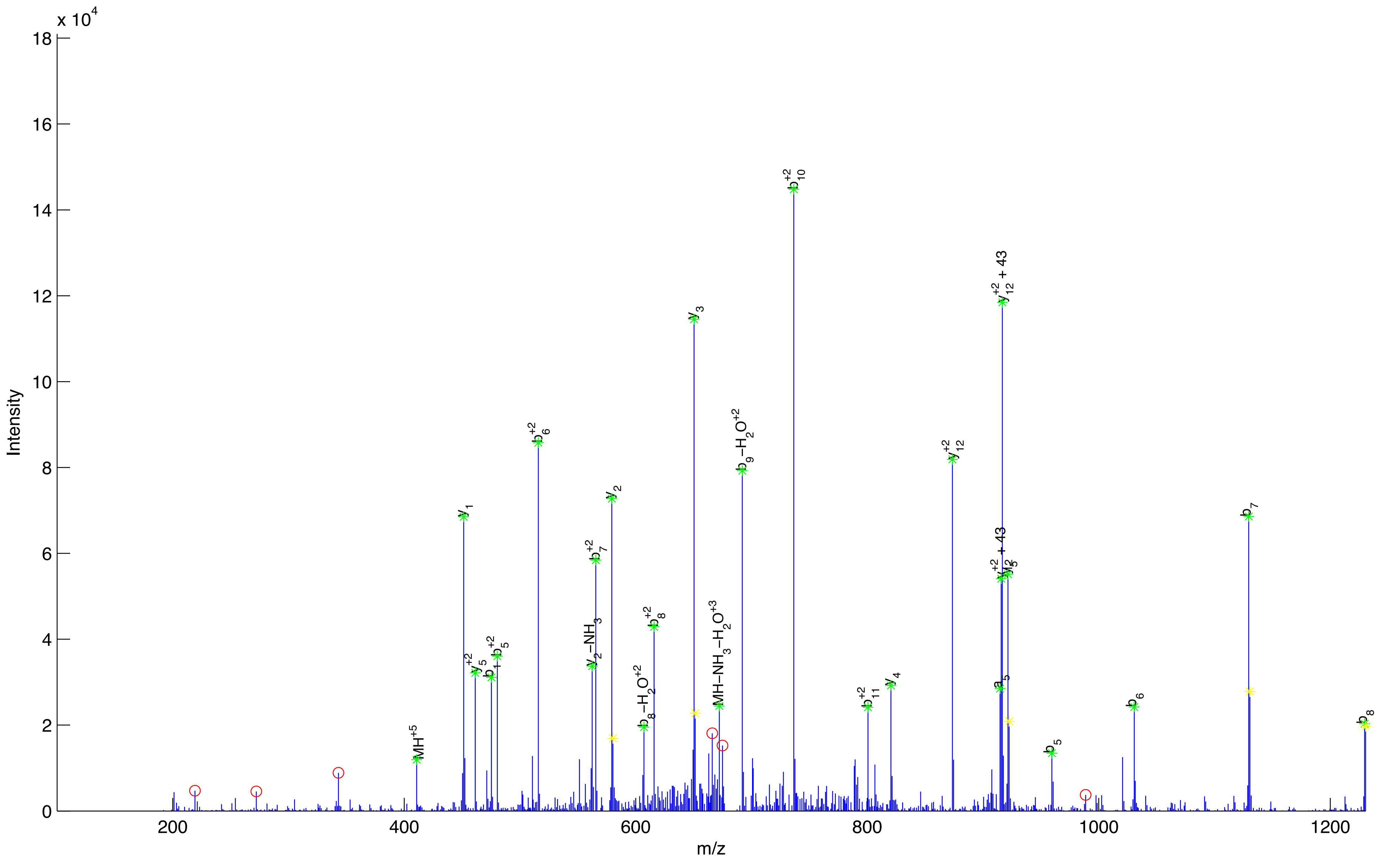

G k G G k G L G k G G A k R

b ions: 362.2347, 532.3402, 589.3617, 646.3831, 816.4887, 873.5101, 986.5942, 1043.6157, 1213.7212, 1270.7427, 1327.7641, 1398.8012, 1568.9068

y ions: 1438.813, 1381.7916, 1211.686, 1154.6646, 1097.6431, 927.5376, 870.5161, 757.4321, 700.4106, 530.3051, 473.2836, 416.2621, 345.225

histone cluster 1, H4a [Homo sapiens]
Charge State: +3
Scan Number: 9798
File Name: 120407_A549_EGFIGF_bioRepA_ACK_FT.raw

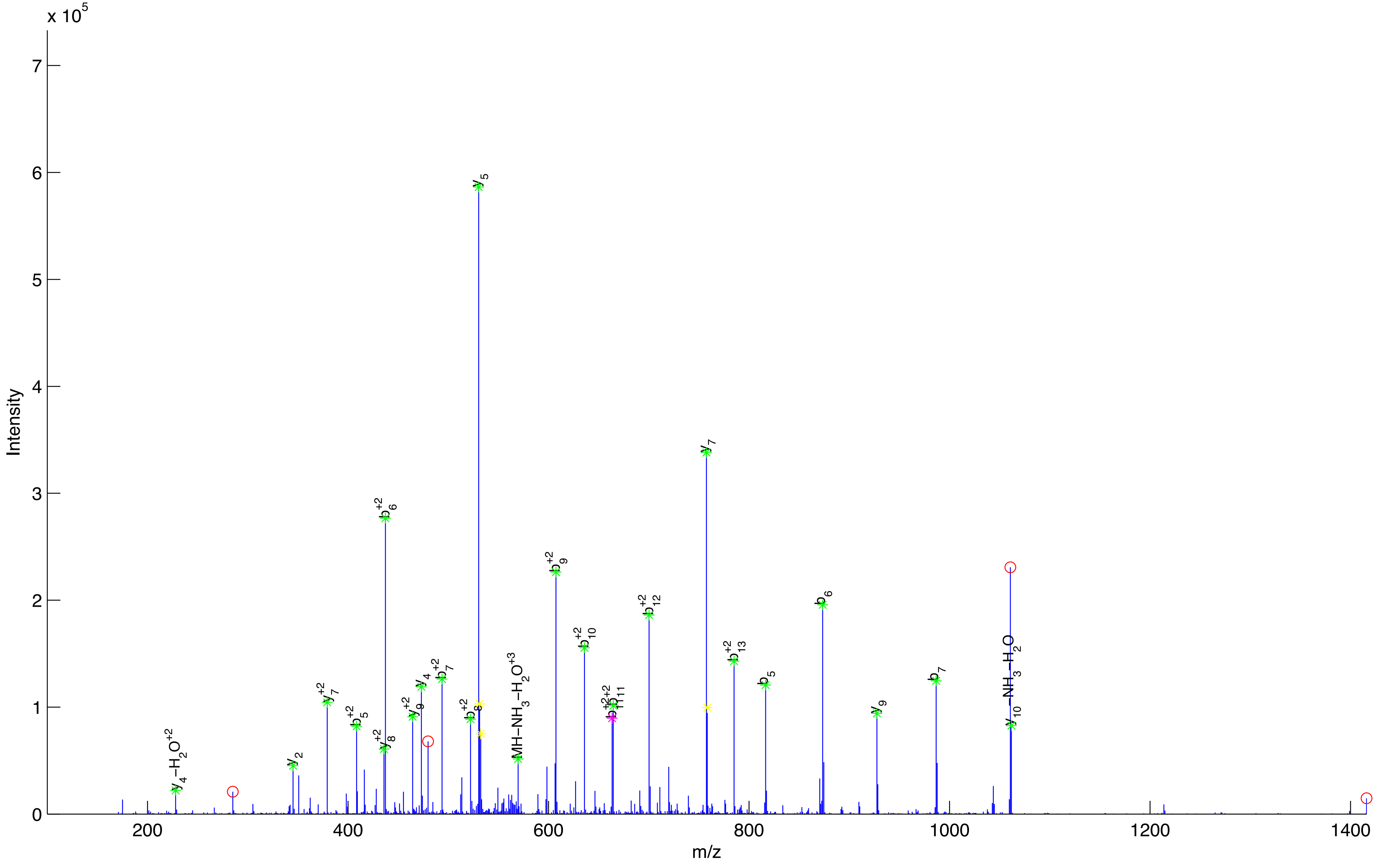

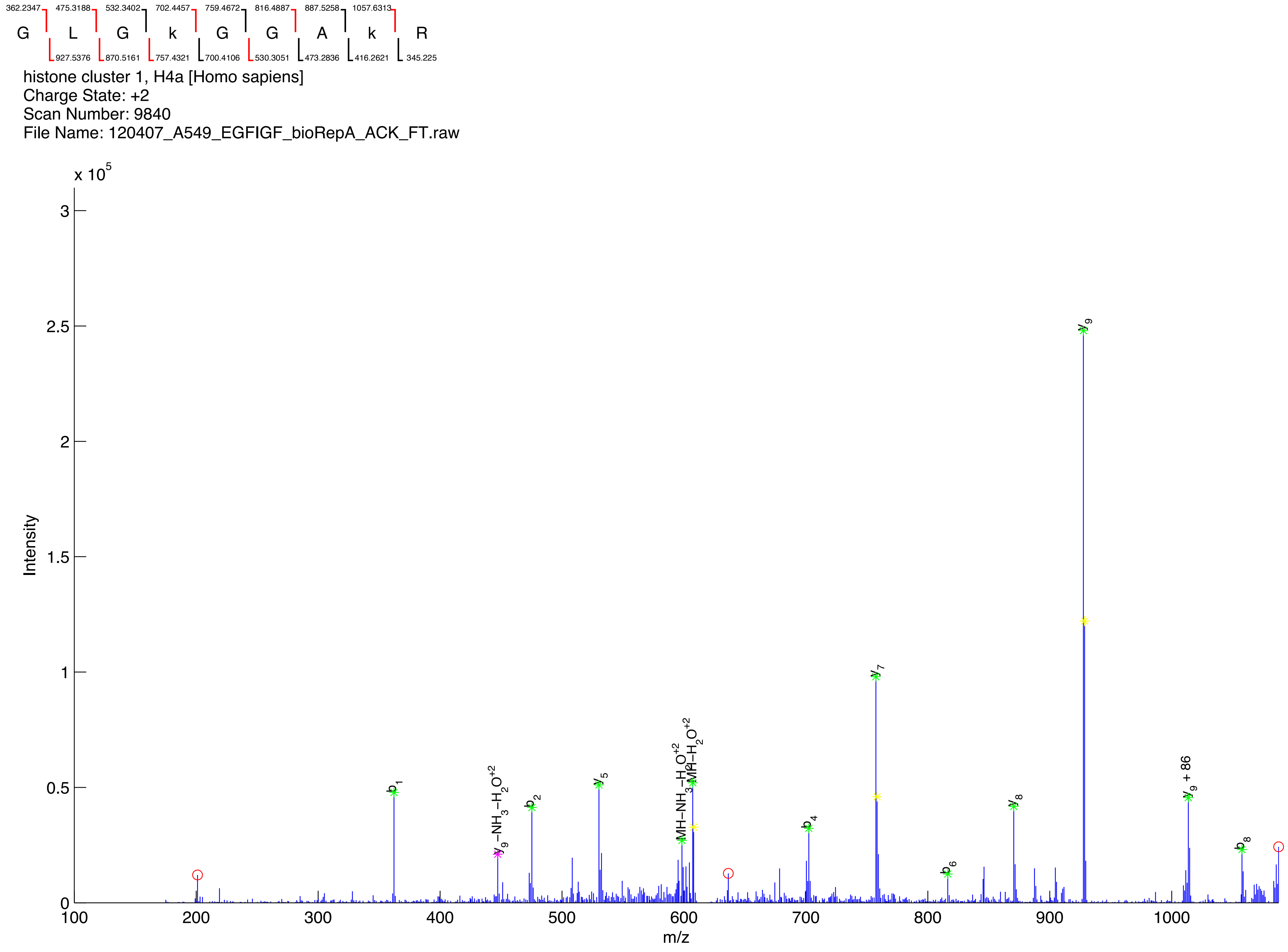
362.2347
475.3188
532.3402
702.4457
759.4672
816.4887
887.5258
1057.6313
G L G k G G A k R
927.5376
870.5161
757.4321
700.4106
530.3051
473.2836
416.2621
345.225
histone cluster 1, H4a [Homo sapiens]
Charge State: +2
Scan Number: 9840
File Name: 120407_A549_EGFIGF_bioRepA_ACK_FT.raw
x 10^5
Intensity
m/z
b1
y9−NH3−H2O+2
b2
y5
MH−NH3−H2O+2
MH−H2O+2
b4
y7
b6
y8
y9
y9 + 86
b8

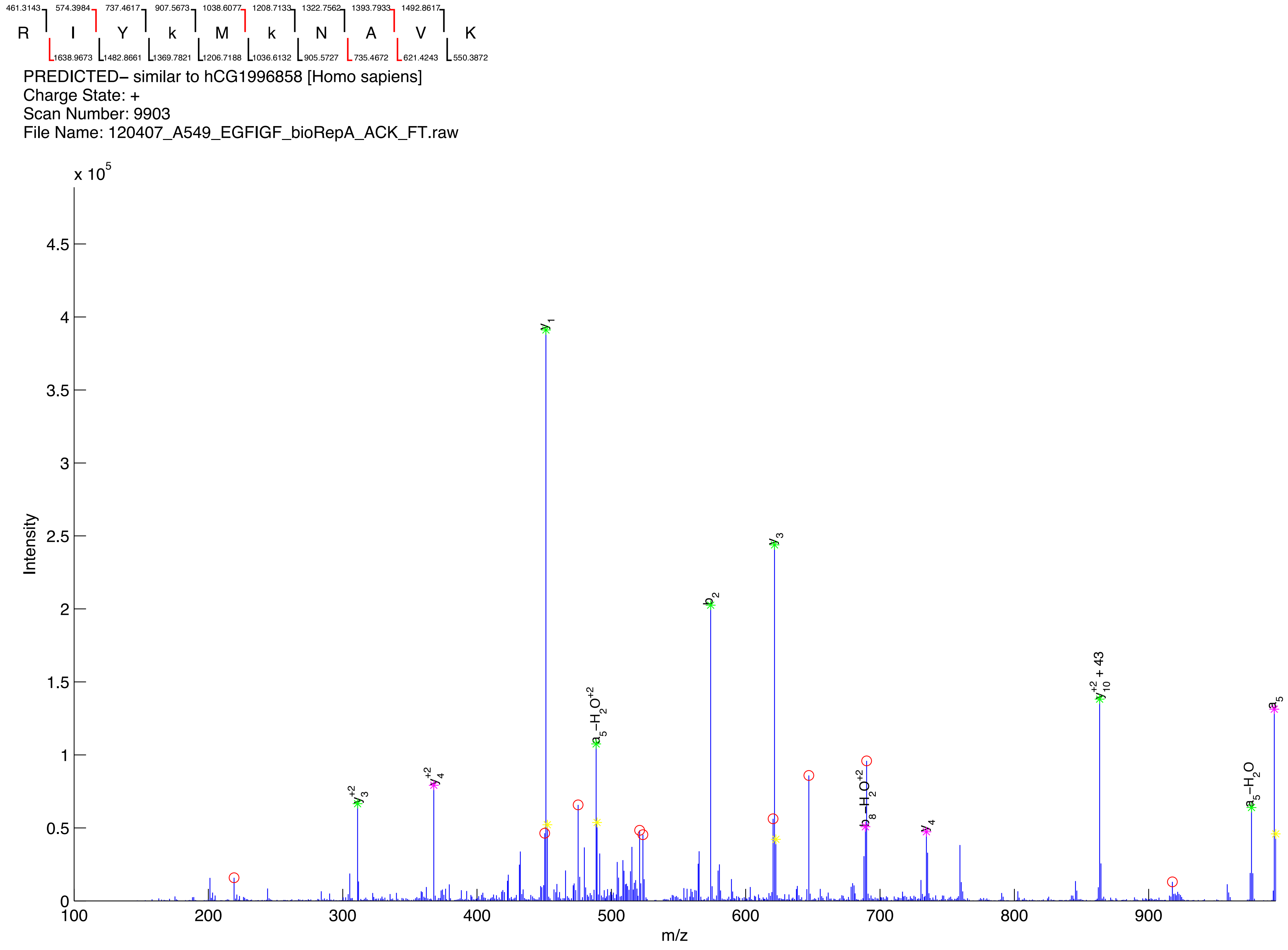
461.3143 574.3984 737.4617 907.5673 1038.6077 1208.7133 1322.7562 1393.7933 1492.8617
R I Y k M k N A V K
1638.9673 1482.8661 1369.7821 1206.7188 1036.6132 905.5727 735.4672 621.4243 550.3872
PREDICTED– similar to hCG1996858 [Homo sapiens]
Charge State: +
Scan Number: 9903
File Name: 120407_A549_EGFIGF_bioRepA_ACK_FT.raw
x 10^5
Intensity
m/z
y1
y3
b2
a5−H2O+2
y+2 3
y+2 4
b8−H2O+2
y4
y+2 10 + 43
a5−H2O
a5

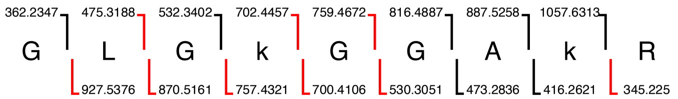

histone cluster 1, H4a [Homo sapiens]
Charge State: +3
Scan Number: 9926
File Name: 120407_A549_EGFIGF_bioRepA_ACK_FT.raw

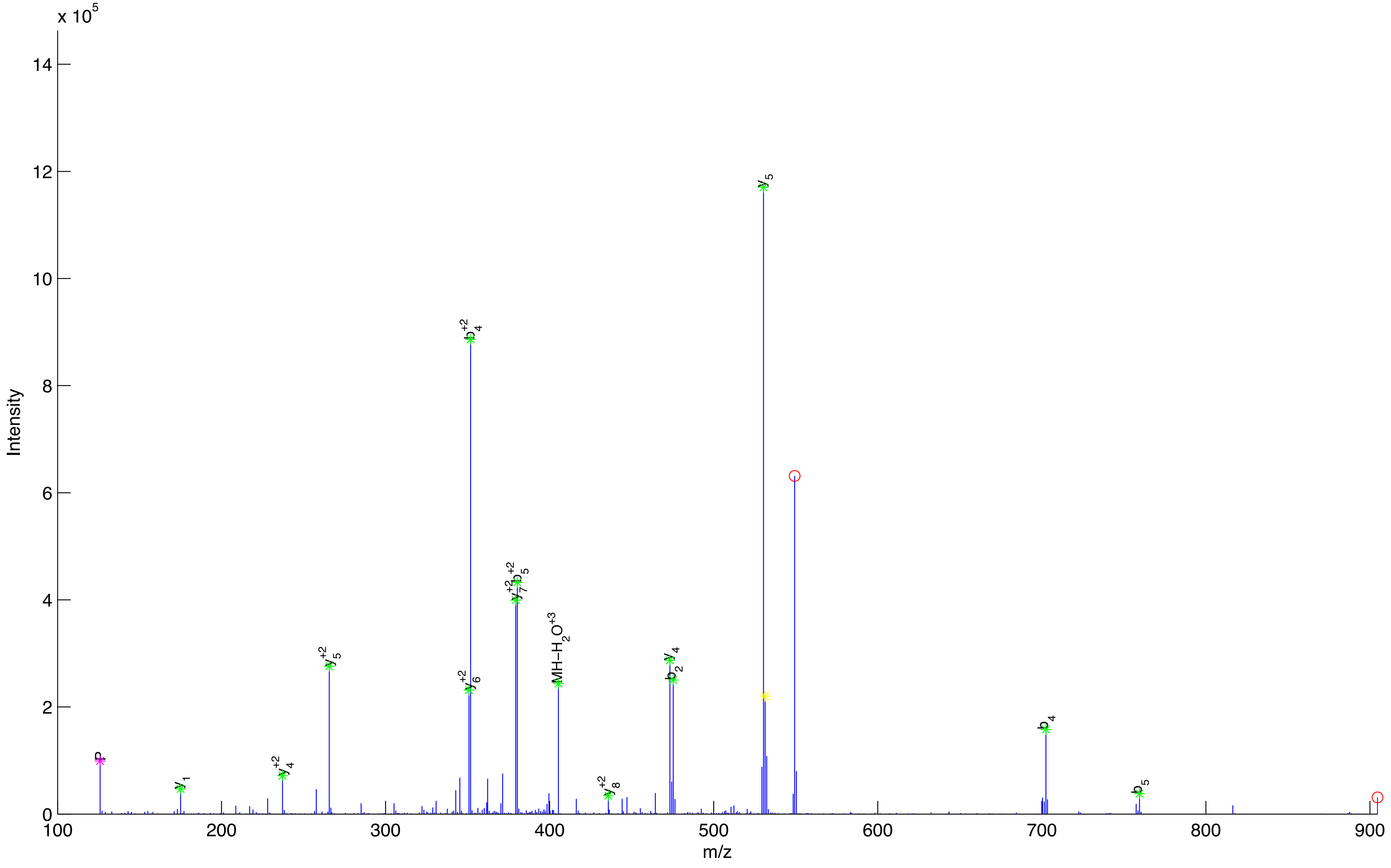

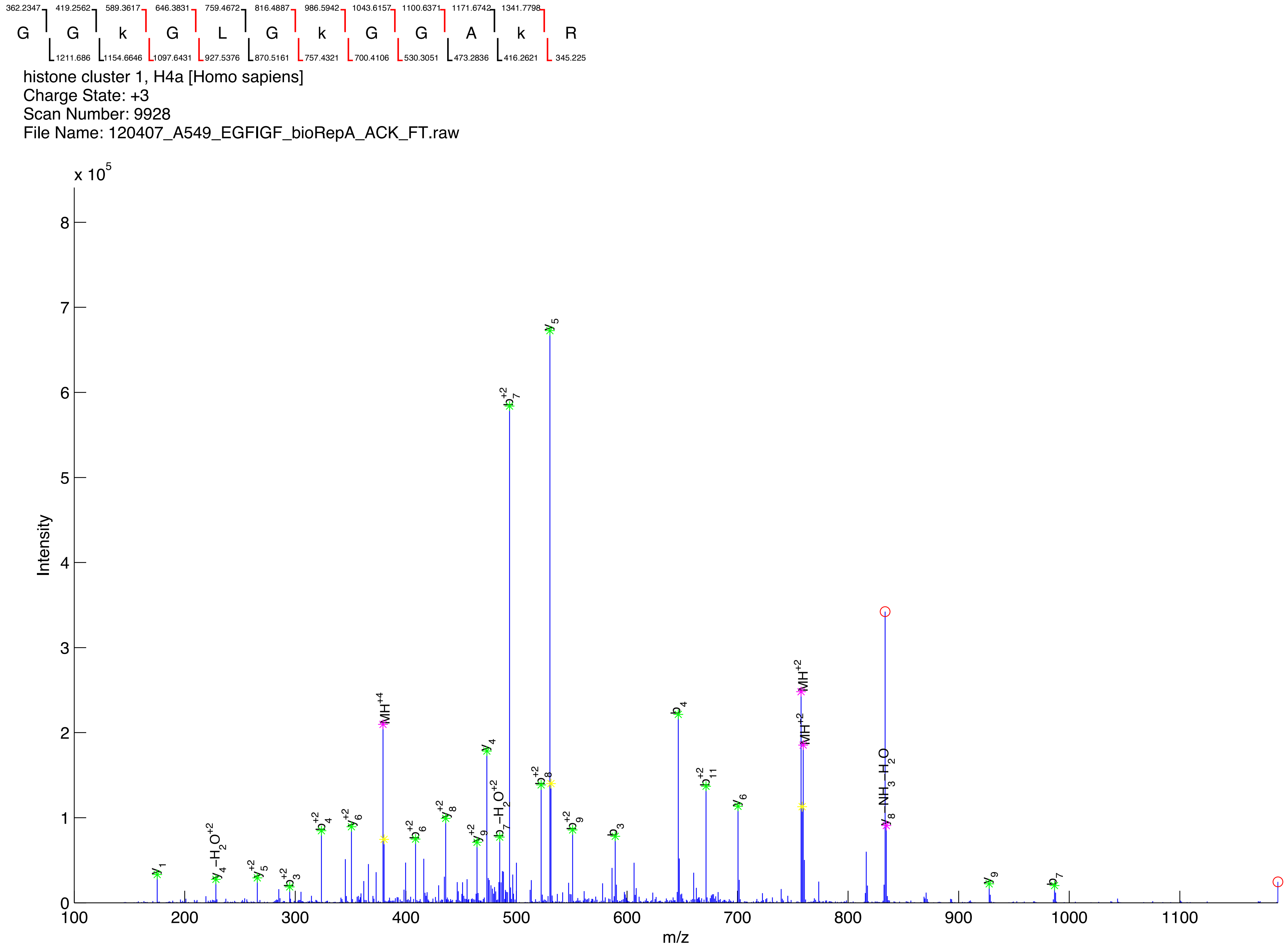

362.2347 419.2562 589.3617 646.3831 759.4672 816.4887 986.5942 1043.6157 1100.6371 1171.6742 1341.7798
G G k G L G k G G A k R
1211.686 1154.6646 1097.6431 927.5376 870.5161 757.4321 700.4106 530.3051 473.2836 416.2621 345.225
histone cluster 1, H4a [Homo sapiens]
Charge State: +3
Scan Number: 9928
File Name: 120407_A549_EGFIGF_bioRepA_ACK_FT.raw
x 10^5
Intensity
m/z

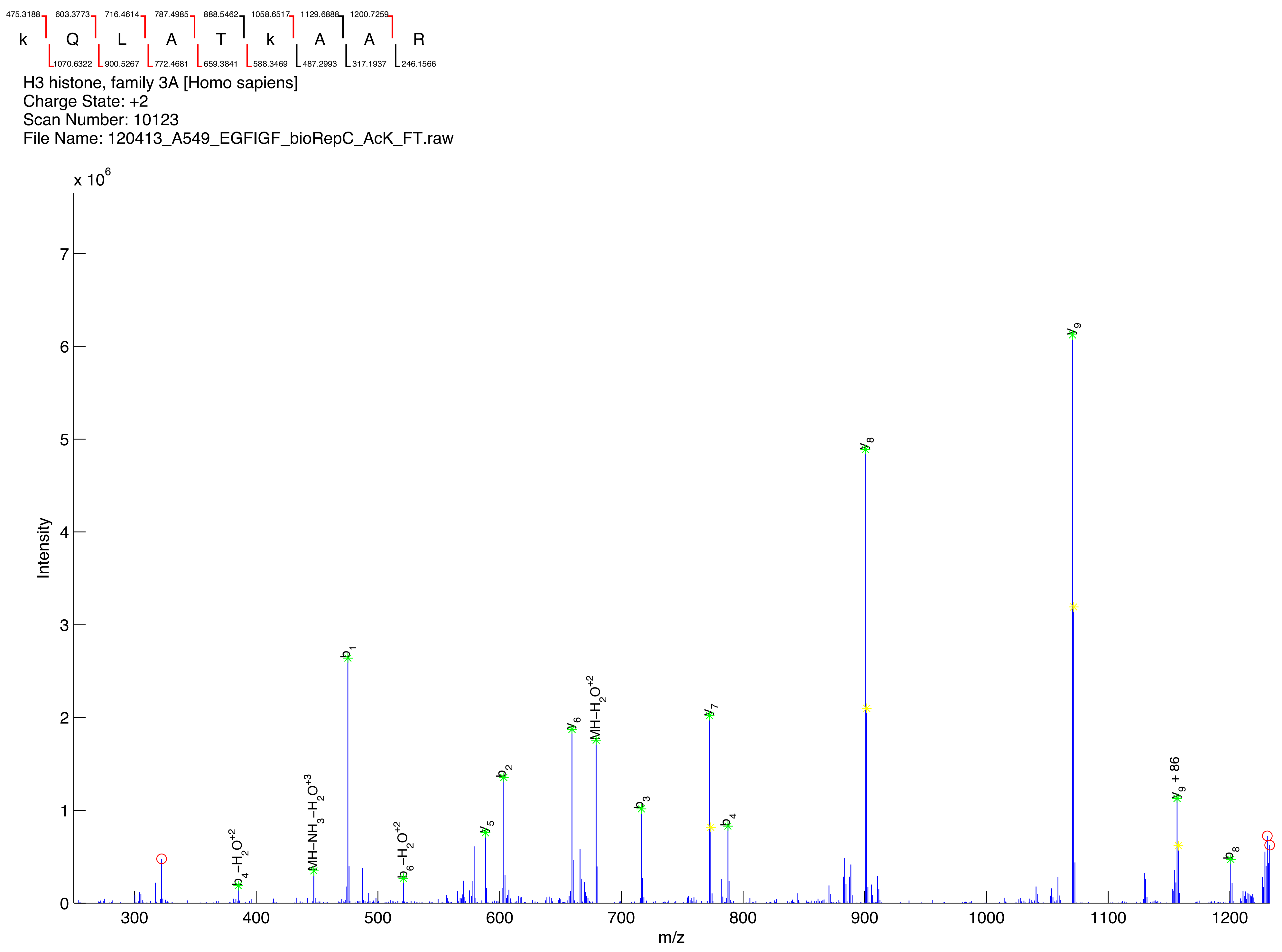

475.3188 603.3773 716.4614 787.4985 888.5462 1058.6517 1129.6888 1200.7259
k Q L A T k A A R
1070.6322 900.5267 772.4681 659.3841 588.3469 487.2993 317.1937 246.1566
H3 histone, family 3A [Homo sapiens]
Charge State: +2
Scan Number: 10123
File Name: 120413_A549_EGFIGF_bioRepC_AcK_FT.raw
x 10^6
Intensity
m/z
b4−H2O+2
MH−NH3−H2O+3
b1
b6−H2O+2
y5
b2
y6
MH−H2O+2
b3
y7
b4
y8
y9
y9 + 86
b8

475.3188 k 622.3872 F 750.4457 Q 879.4883 E 950.5255 A 1063.6095 L 1192.6521 E 1323.6926 M 1436.7767 L 1592.8778 R K

1738.9833 1568.8778 1421.8094 1293.7508 1164.7082 1093.6711 980.587 851.5444 720.5039 607.4199

sodium channel, voltage−gated, type X, alpha [Homo sapiens]
Charge State: +
Scan Number: 10124
File Name: 120404_A549_EGFIGF_bioRepB_ACK_FT.raw

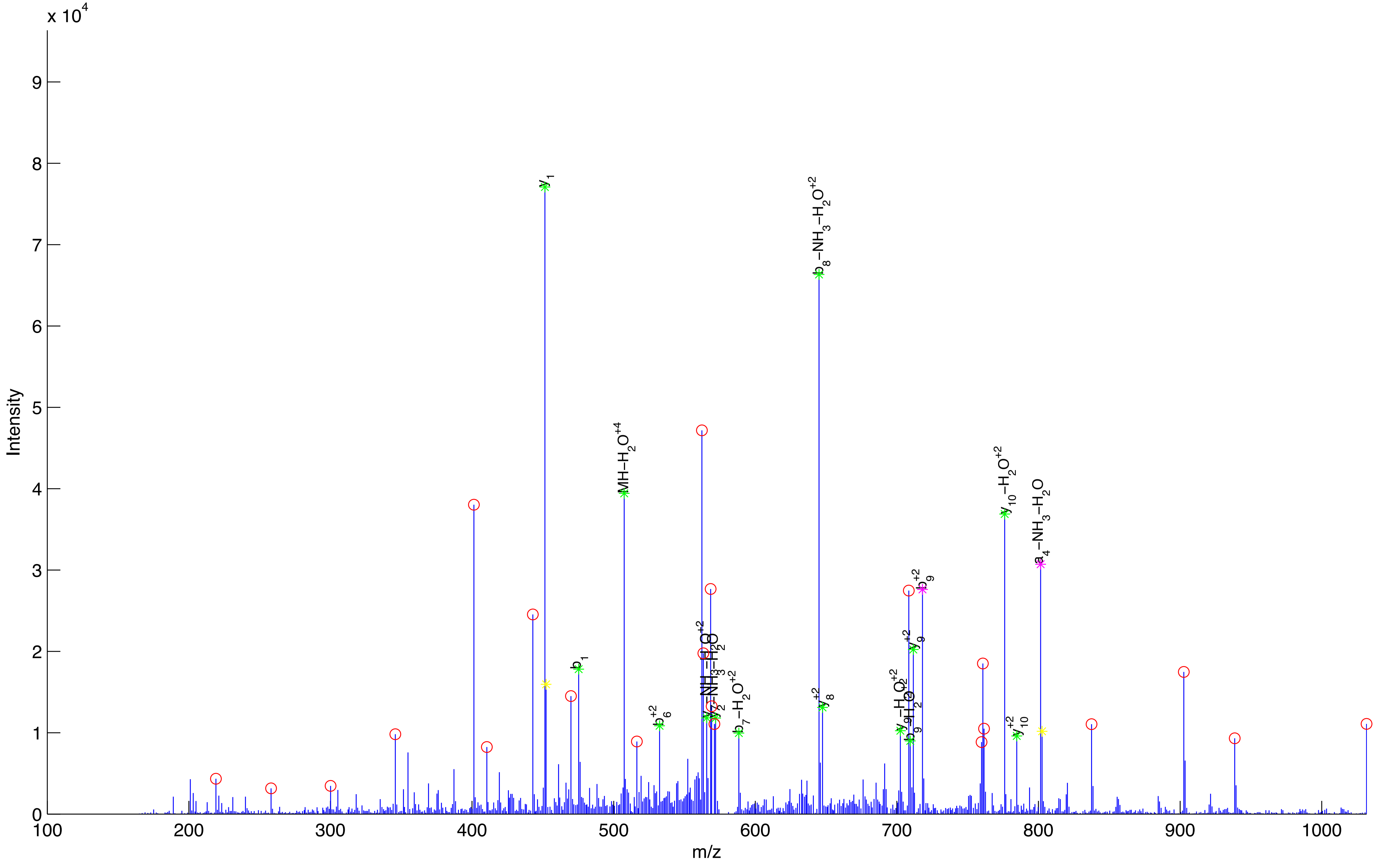

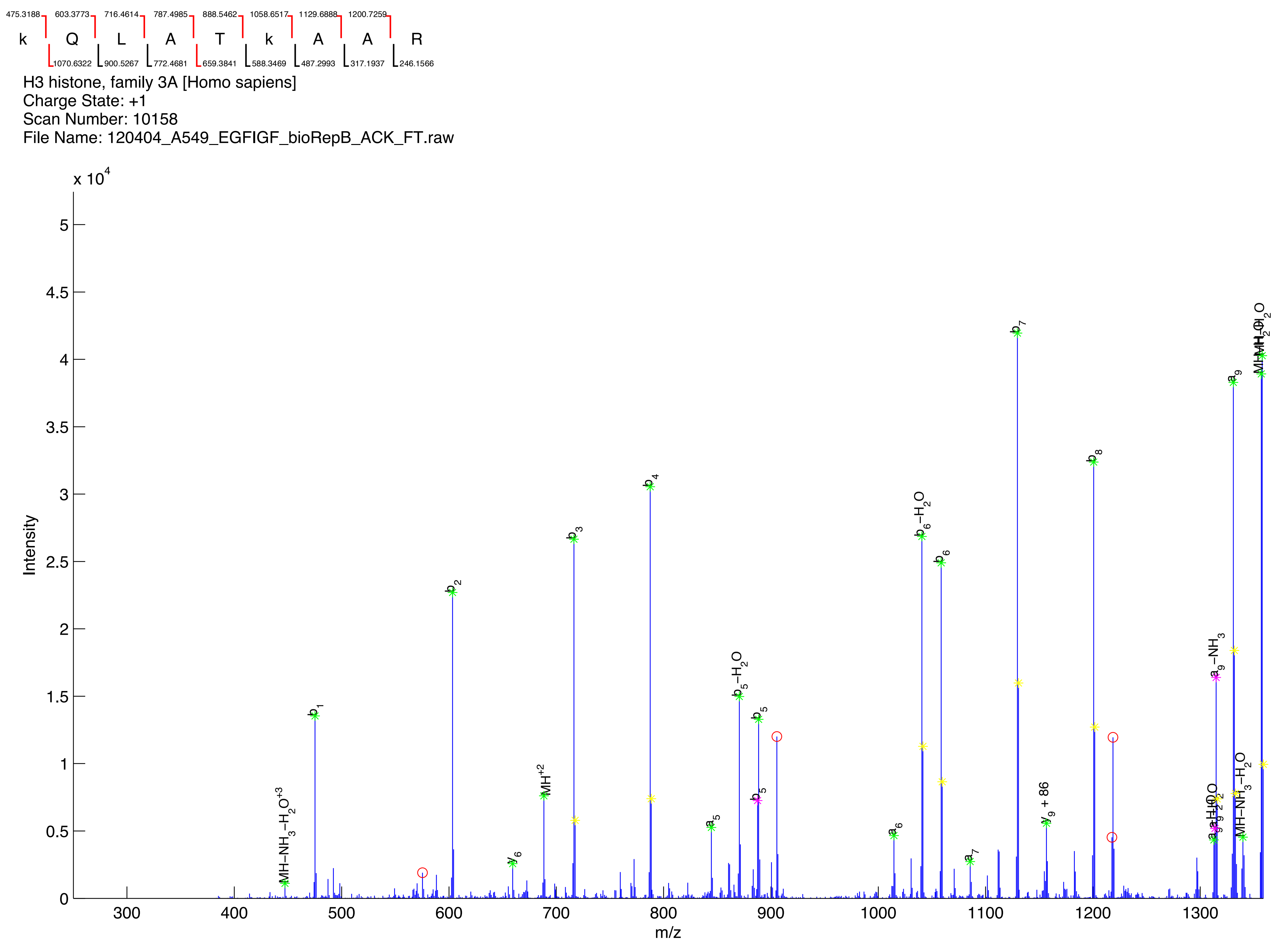
475.3188 603.3773 716.4614 787.4985 888.5462 1058.6517 1129.6888 1200.7259
k Q L A T k A A R
1070.6322 900.5267 772.4681 659.3841 588.3469 487.2993 317.1937 246.1566
H3 histone, family 3A [Homo sapiens]
Charge State: +1
Scan Number: 10158
File Name: 120404_A549_EGFIGF_bioRepB_ACK_FT.raw
x 10^4
Intensity
m/z
b1
b2
b3
b4
b5
b5−H2O
a5
a6
b6
b6−H2O
a7
b7
y9 + 86
b8
a9
a9−NH3
MH−NH3−H2O
MH+2
y6
MH−NH3−H2O+3

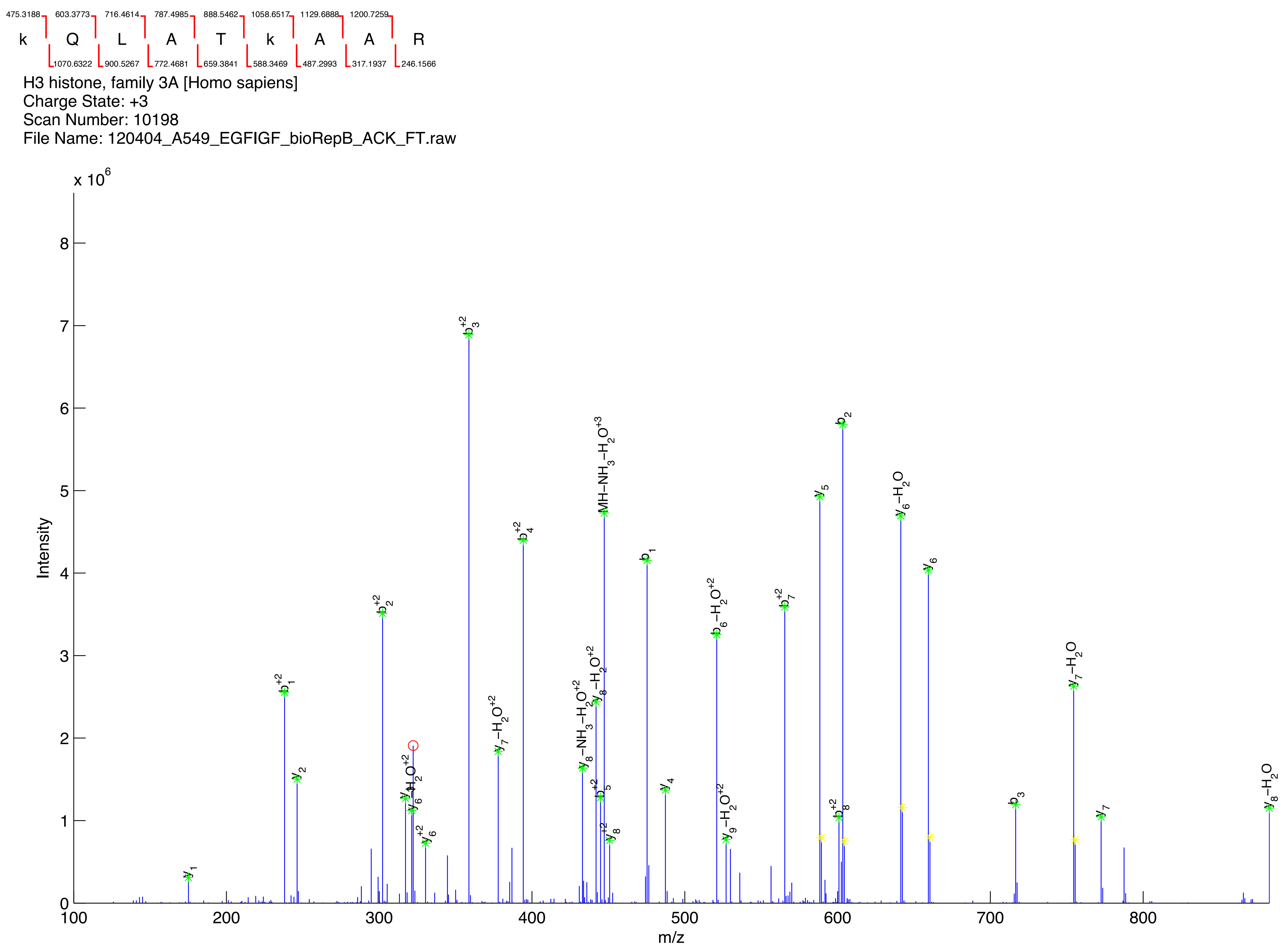
475.3188 603.3773 716.4614 787.4985 888.5462 1058.6517 1129.6888 1200.7259
k Q L A T k A A R
1070.6322 900.5267 772.4681 659.3841 588.3469 487.2993 317.1937 246.1566
H3 histone, family 3A [Homo sapiens]
Charge State: +3
Scan Number: 10198
File Name: 120404_A549_EGFIGF_bioRepB_ACK_FT.raw
x 10^6
Intensity
m/z

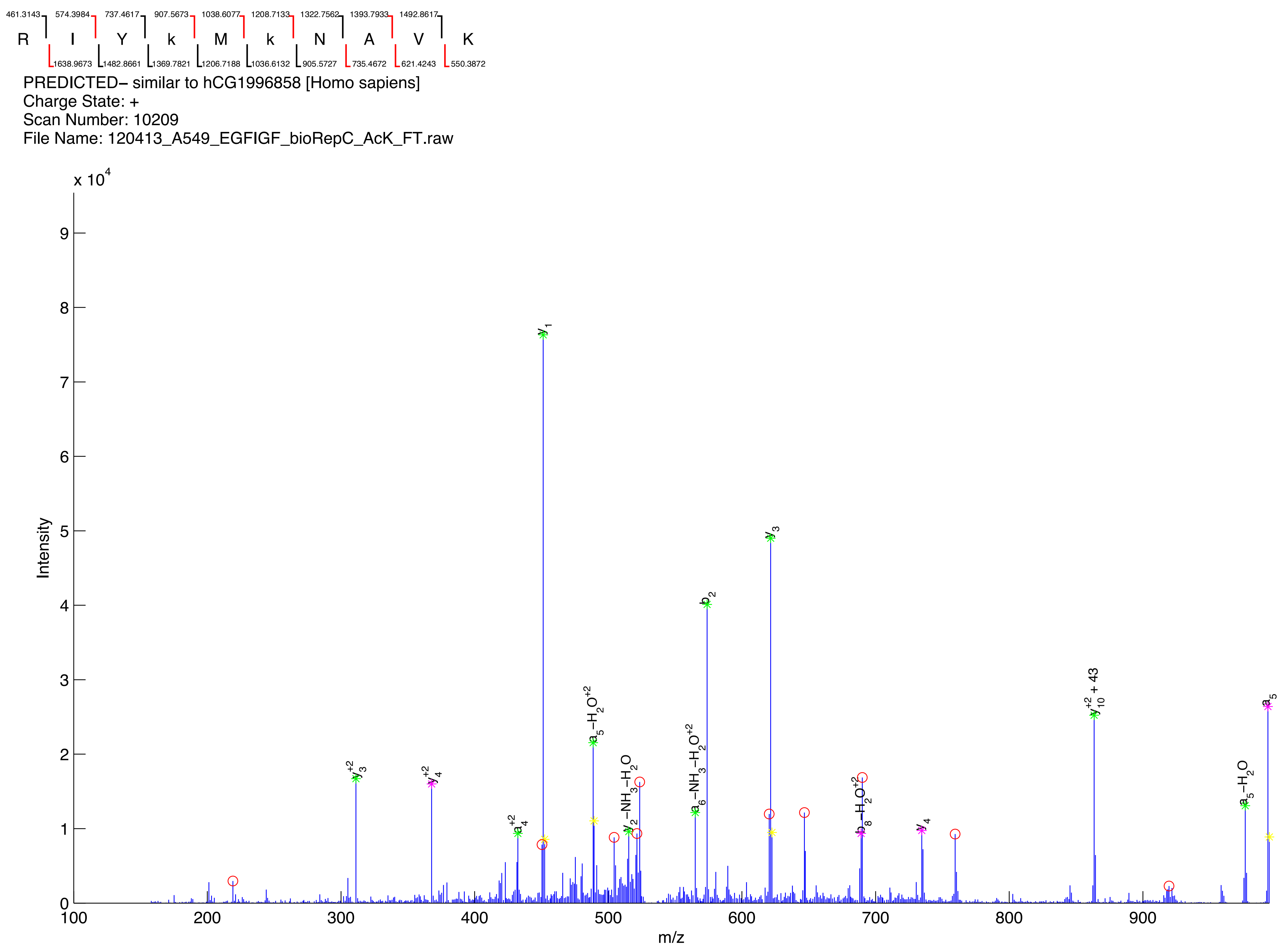

461.3143 574.3984 737.4617 907.5673 1038.6077 1208.7133 1322.7562 1393.7933 1492.8617
R I Y k M k N A V K
1638.9673 1482.8661 1369.7821 1206.7188 1036.6132 905.5727 735.4672 621.4243 550.3872
PREDICTED– similar to hCG1996858 [Homo sapiens]
Charge State: +
Scan Number: 10209
File Name: 120413_A549_EGFIGF_bioRepC_AcK_FT.raw
x 10^4
Intensity
m/z
y1
y3
b2
a5
y10+2 + 43
a5–H2O
a5–H2O+2
a6–NH3–H2O+2
y2–NH3–H2O
b8–H2O+2
y3+2
y4+2
a4+2
y4

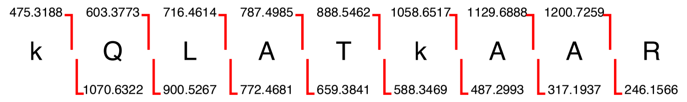

H3 histone, family 3A [Homo sapiens]
Charge State: +3
Scan Number: 10239
File Name: 120407_A549_EGFIGF_bioRepA_ACK_FT.raw

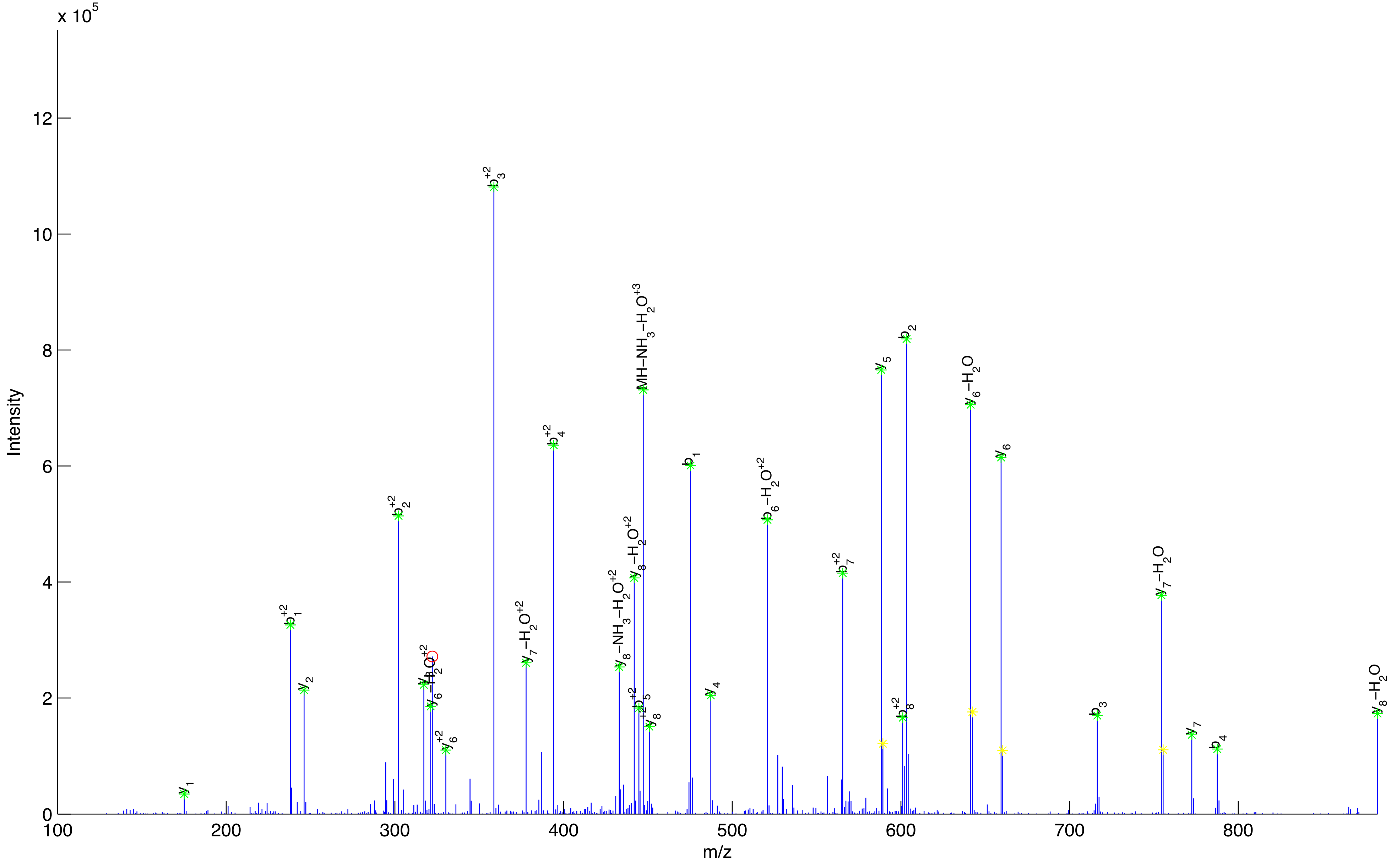

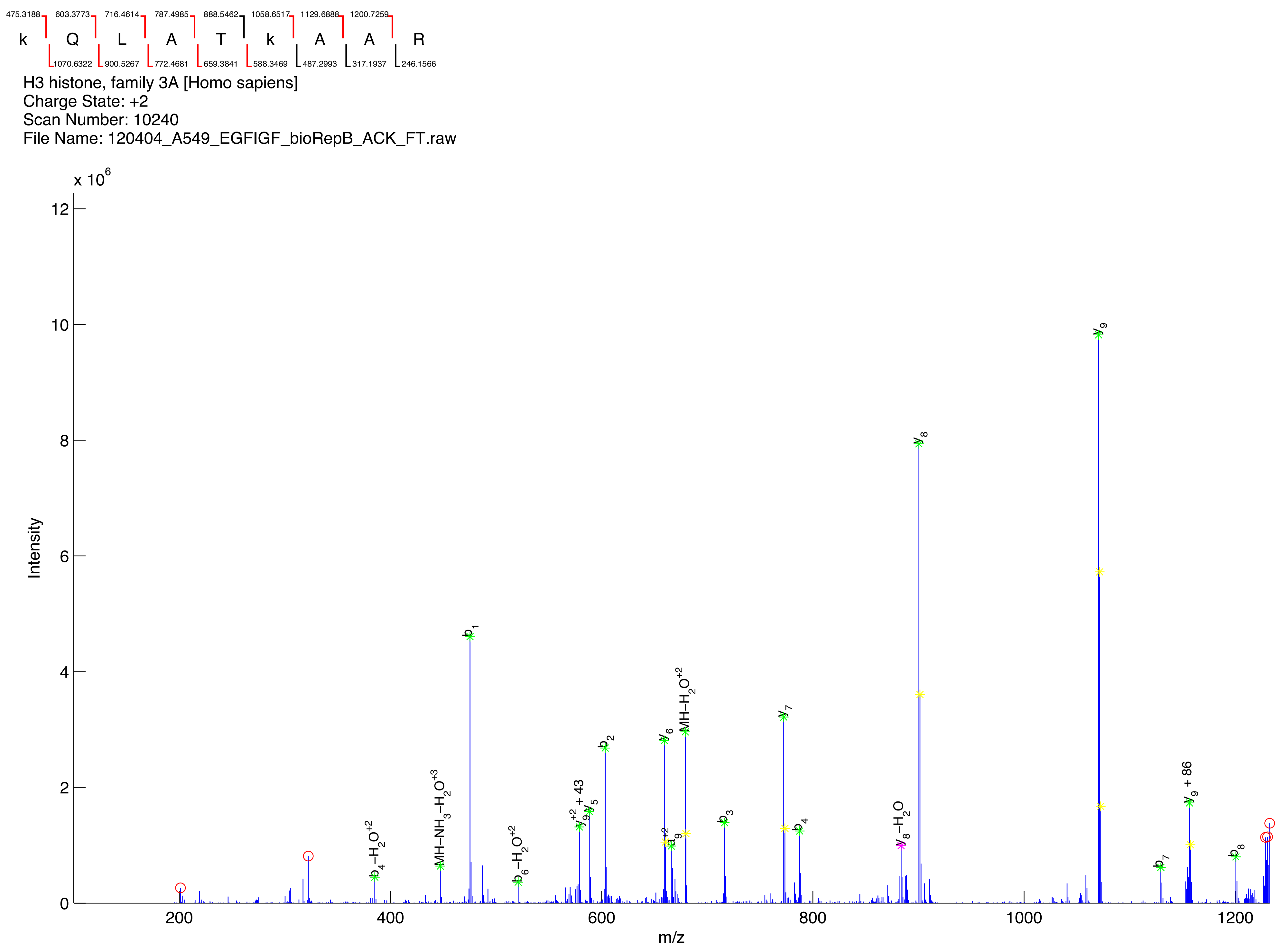
475.3188 603.3773 716.4614 787.4985 888.5462 1058.6517 1129.6888 1200.7259
k Q L A T k A A R
1070.6322 900.5267 772.4681 659.3841 588.3469 487.2993 317.1937 246.1566
H3 histone, family 3A [Homo sapiens]
Charge State: +2
Scan Number: 10240
File Name: 120404_A549_EGFIGF_bioRepB_ACK_FT.raw
x 10^6
Intensity
m/z
b1
b2
b3
b4
b7
b8
y5
y6
y7
y8
y9
b4−H2O+2
MH−NH3−H2O+3
b6−H2O+2
y9+2 + 43
a9+2
MH−H2O+2
y8−H2O
y9 + 86

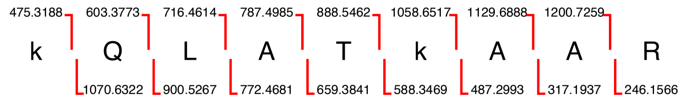

H3 histone, family 3A [Homo sapiens]
Charge State: +3
Scan Number: 10249
File Name: 120413_A549_EGFIGF_bioRepC_AcK_FT.raw

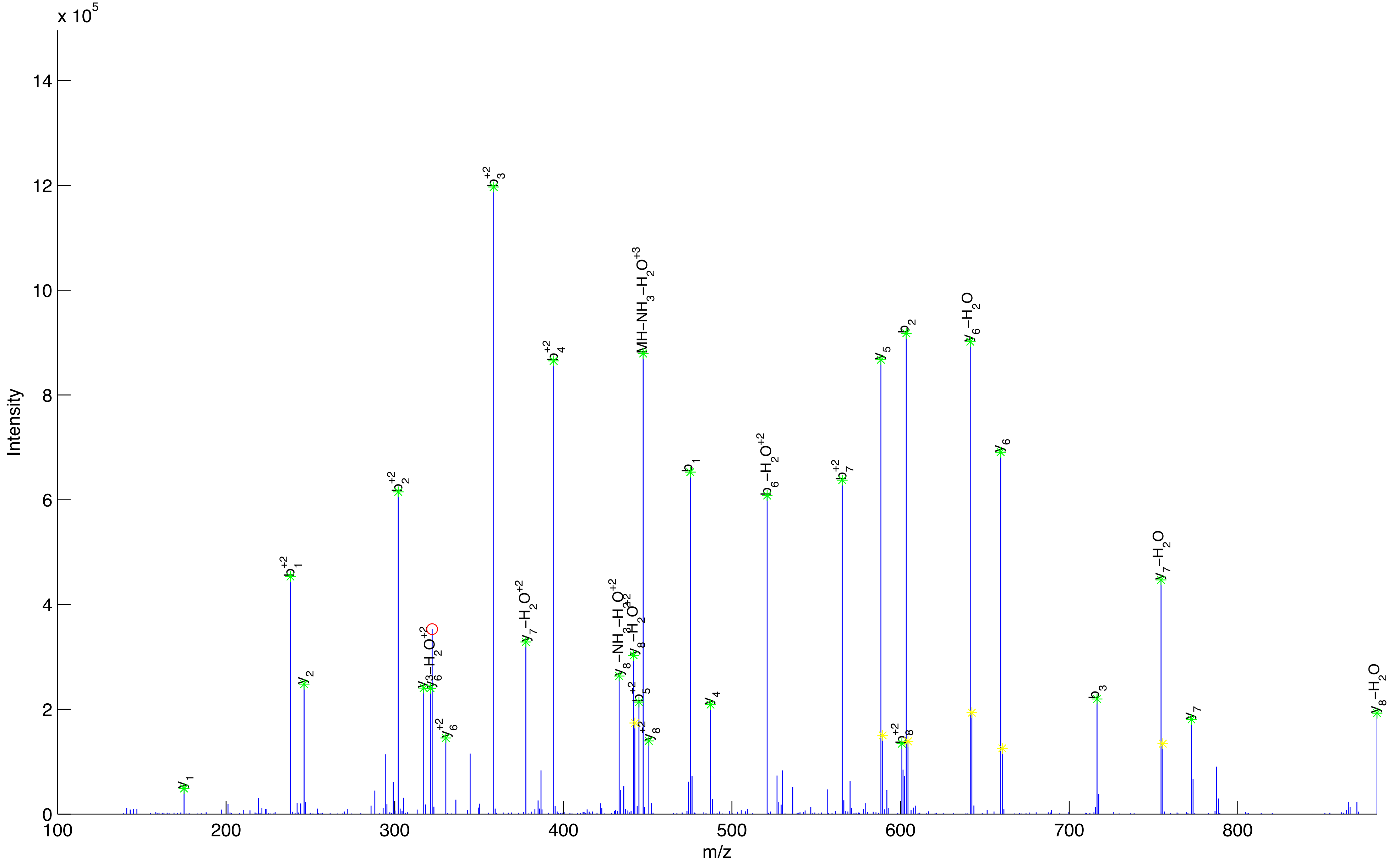

362.2347	532.3402	589.3617	646.3831	816.4887	873.5101	986.5942	1043.6157	1213.7212	1270.7427	1327.7641	1398.8012	1568.9068	
G	k	G	G	k	G	L	G	k	G	G	A	k	R
	1438.813	1381.7916	1211.686	1154.6646	1097.6431	927.5376	870.5161	757.4321	700.4106	530.3051	473.2836	416.2621	345.225

histone cluster 1, H4a [Homo sapiens]
Charge State: +2
Scan Number: 10260
File Name: 120407_A549_EGFIGF_bioRepA_ACK_FT.raw

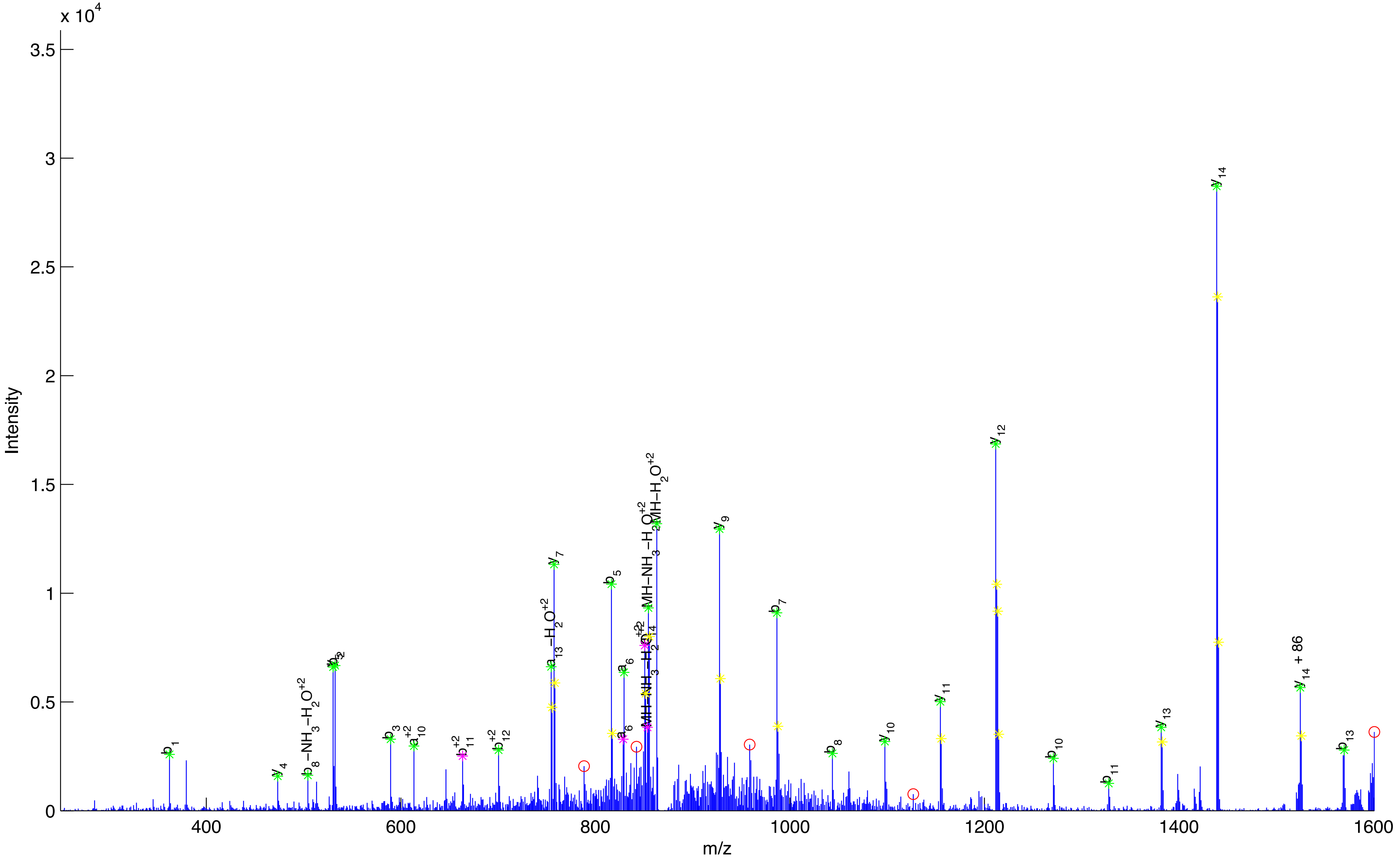

475.3188 603.3773 716.4614 787.4985 888.5462 1058.6517 1129.6888 1200.7259

k Q L A T k A A R

1070.6322 900.5267 772.4681 659.3841 588.3469 487.2993 317.1937 246.1566

H3 histone, family 3A [Homo sapiens]
Charge State: +2
Scan Number: 10281
File Name: 120407_A549_EGFIGF_bioRepA_ACK_FT.raw

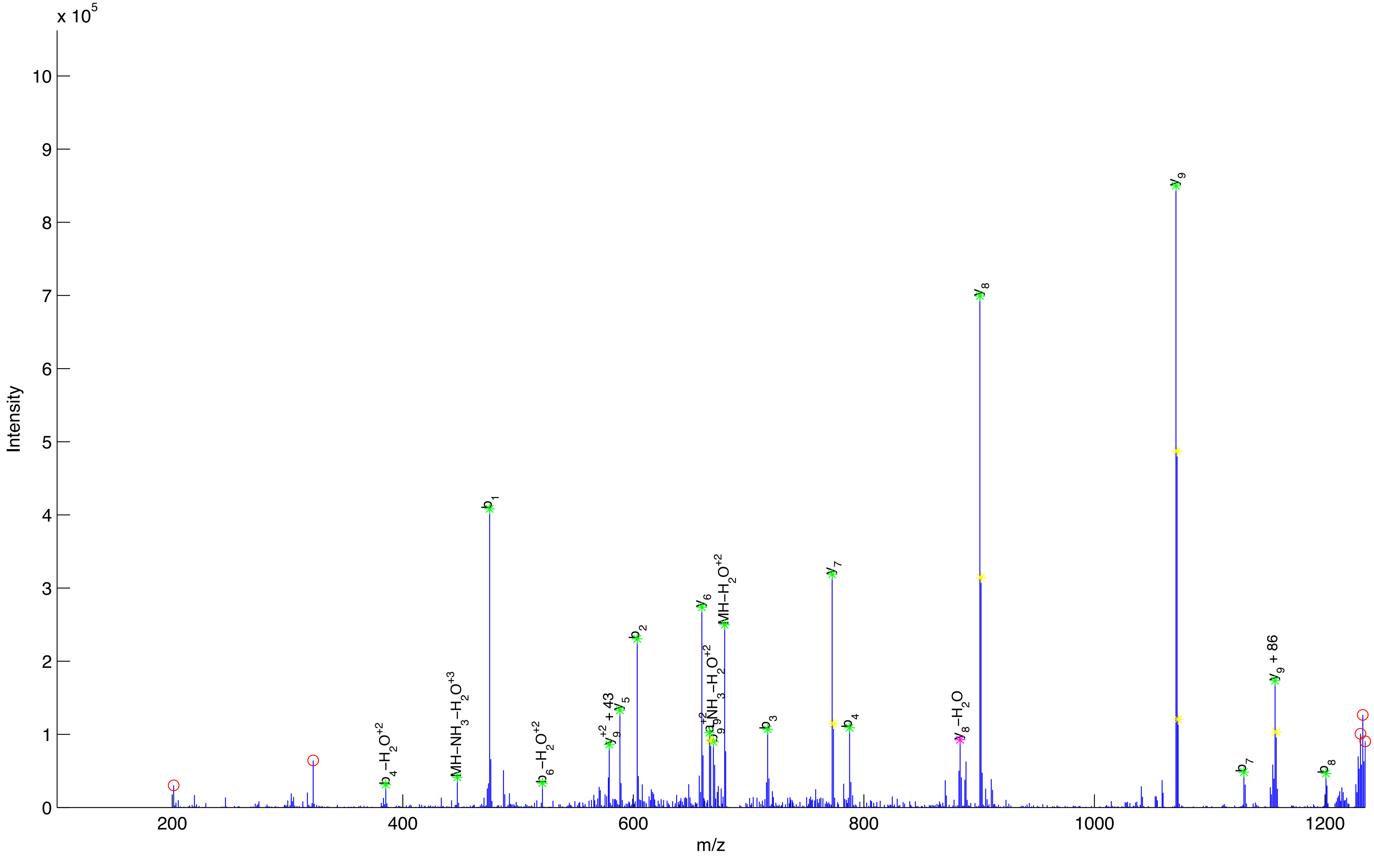

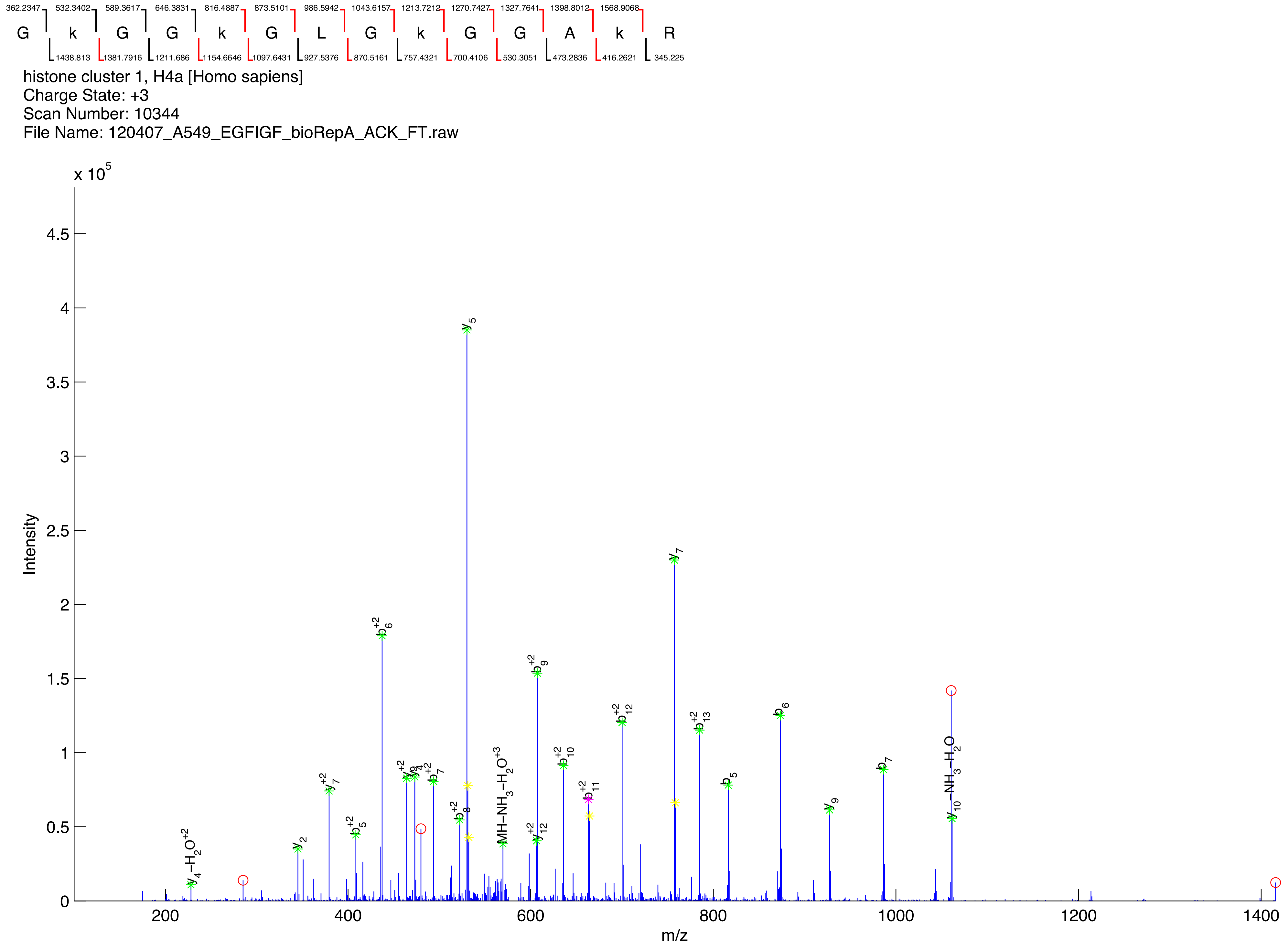

362.2347 532.3402 589.3617 646.3831 816.4887 873.5101 986.5942 1043.6157 1213.7212 1270.7427 1327.7641 1398.8012 1568.9068
G k G G k G L G k G G A k R
1438.813 1381.7916 1211.686 1154.6646 1097.6431 927.5376 870.5161 757.4321 700.4106 530.3051 473.2836 416.2621 345.225
histone cluster 1, H4a [Homo sapiens]
Charge State: +3
Scan Number: 10344
File Name: 120407_A549_EGFIGF_bioRepA_ACK_FT.raw
x 10^5
Intensity
m/z

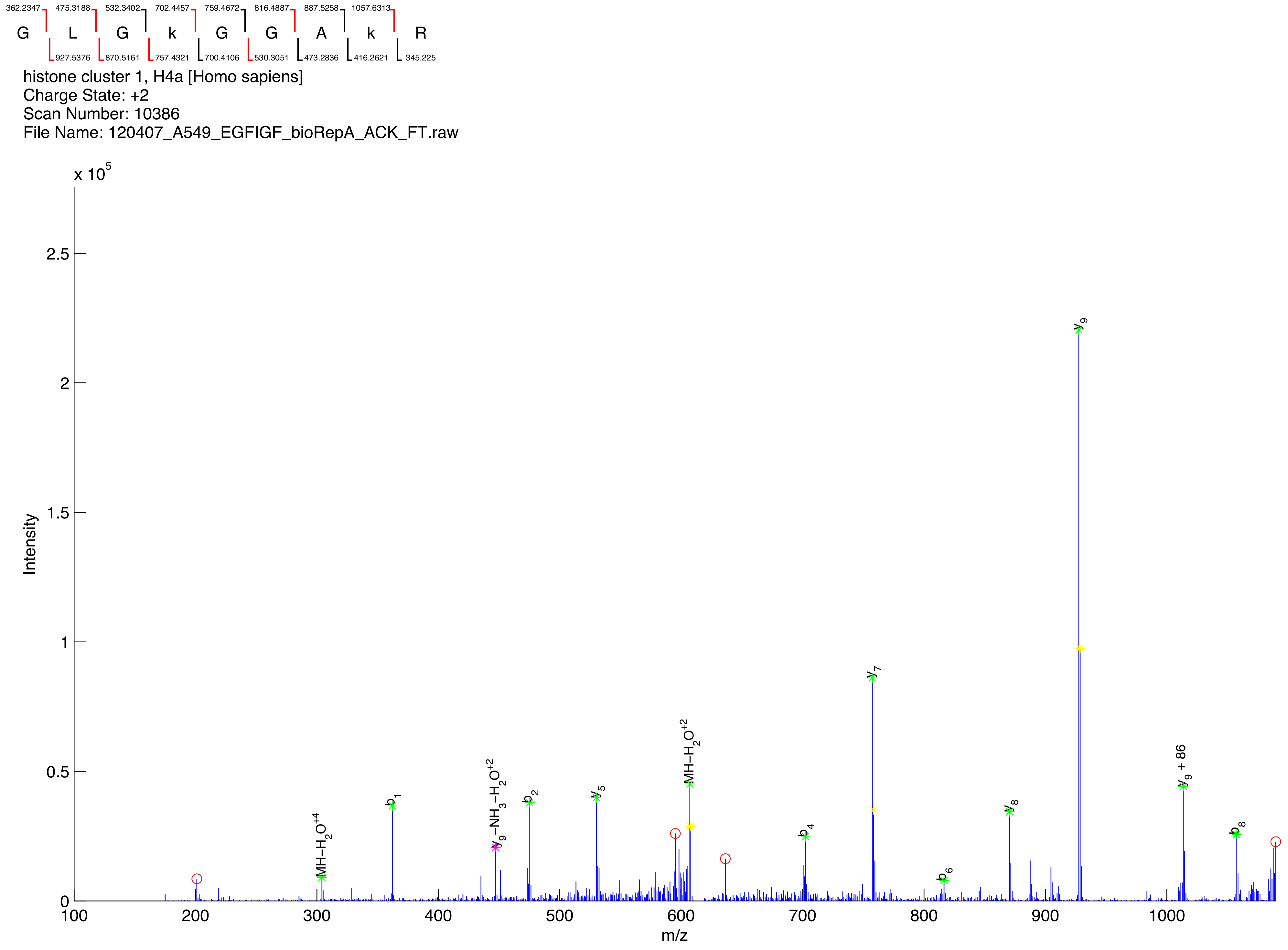

362.2347 475.3188 532.3402 702.4457 759.4672 816.4887 887.5258 1057.6313
G L G k G G A k R
927.5376 870.5161 757.4321 700.4106 530.3051 473.2836 416.2621 345.225
histone cluster 1, H4a [Homo sapiens]
Charge State: +2
Scan Number: 10386
File Name: 120407_A549_EGFIGF_bioRepA_ACK_FT.raw
x 10^5
Intensity
m/z
MH−H2O+4
b1
y9−NH3−H2O+2
b2
y5
MH−H2O+2
b4
y7
b6
y8
y9
y9 + 86
b8

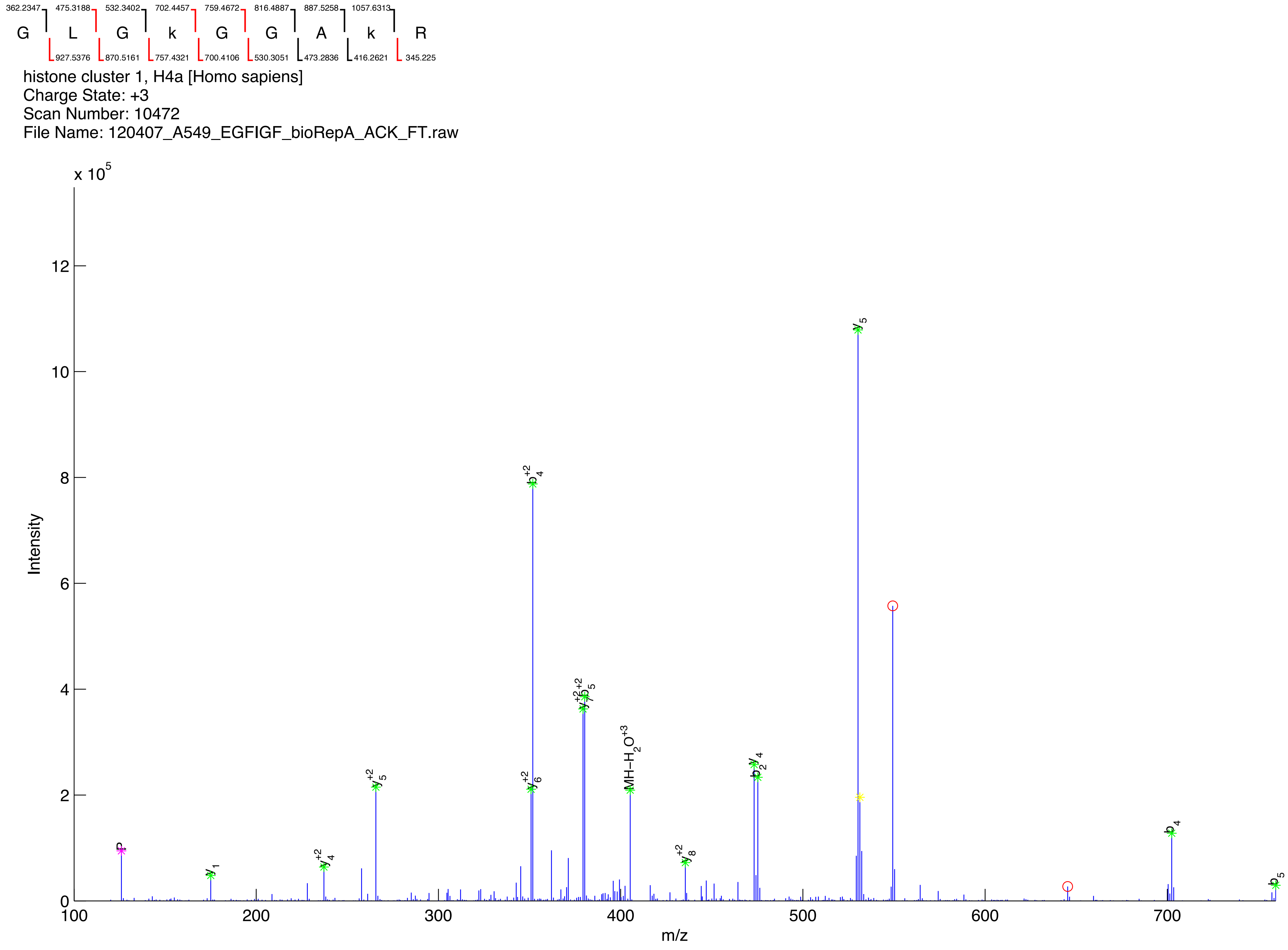
362.2347 475.3188 532.3402 702.4457 759.4672 816.4887 887.5258 1057.6313
G L G k G G A k R
927.5376 870.5161 757.4321 700.4106 530.3051 473.2836 416.2621 345.225
histone cluster 1, H4a [Homo sapiens]
Charge State: +3
Scan Number: 10472
File Name: 120407_A549_EGFIGF_bioRepA_ACK_FT.raw
x 10^5
Intensity
m/z

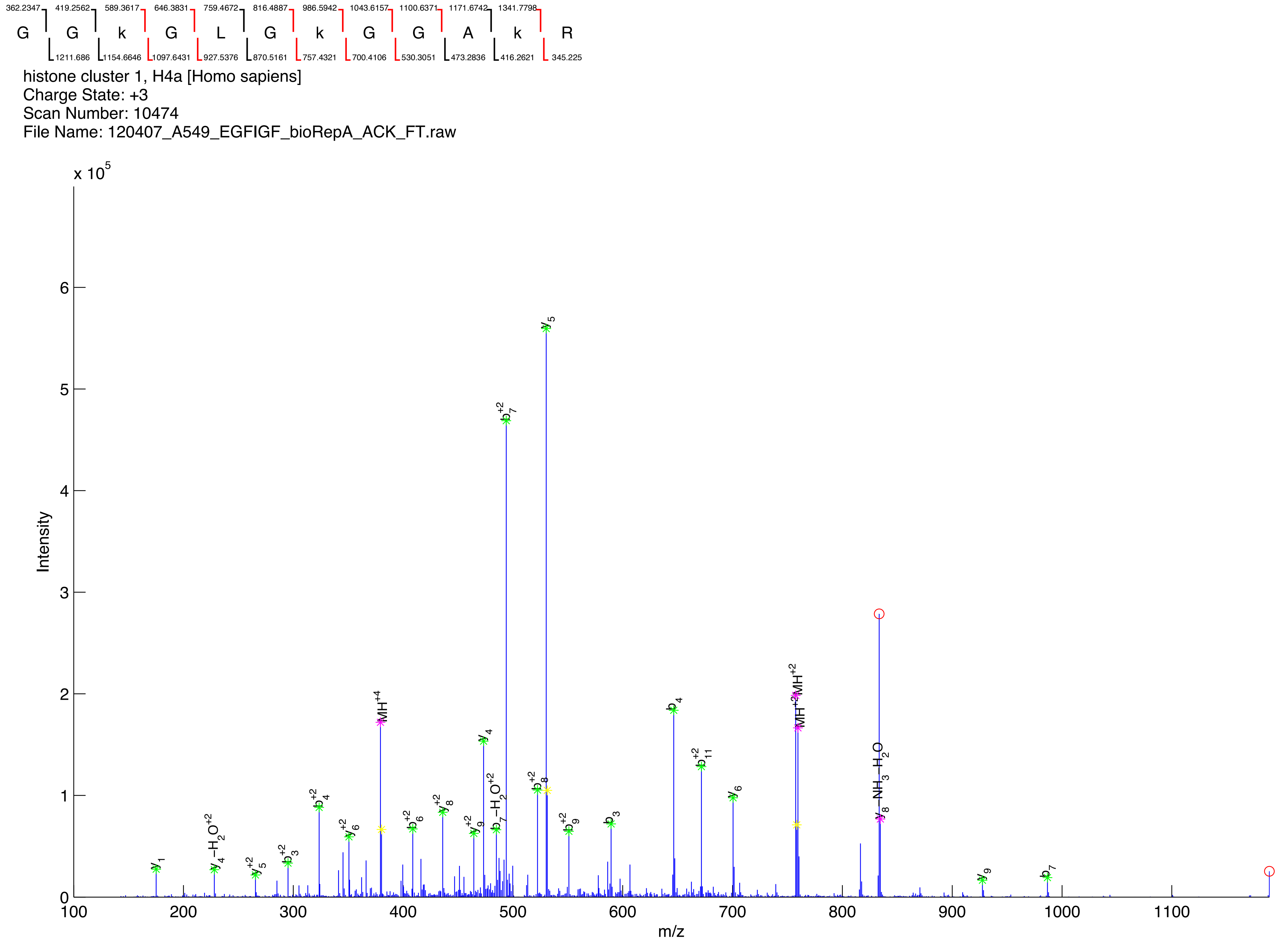

362.2347 419.2562 589.3617 646.3831 759.4672 816.4887 986.5942 1043.6157 1100.6371 1171.6742 1341.7798
G G k G L G k G G A k R
1211.686 1154.6646 1097.6431 927.5376 870.5161 757.4321 700.4106 530.3051 473.2836 416.2621 345.225
histone cluster 1, H4a [Homo sapiens]
Charge State: +3
Scan Number: 10474
File Name: 120407_A549_EGFIGF_bioRepA_ACK_FT.raw
x 10^5
Intensity
m/z

475.3188	532.3402	619.3722	789.4778	959.5833	1030.6204	1129.6888	1230.7365	1400.842	1499.9105	1627.969	
k	G	S	k	k	A	V	T	k	V	Q	K
	1774.0746	1603.969	1546.9476	1459.9155	1289.81	1119.7045	1048.6674	949.599	848.5513	678.4457	579.3773

histone cluster 2, H2bf [Homo sapiens]
Charge State: +4
Scan Number: 10575
File Name: 120407_A549_EGFIGF_bioRepA_ACK_FT.raw

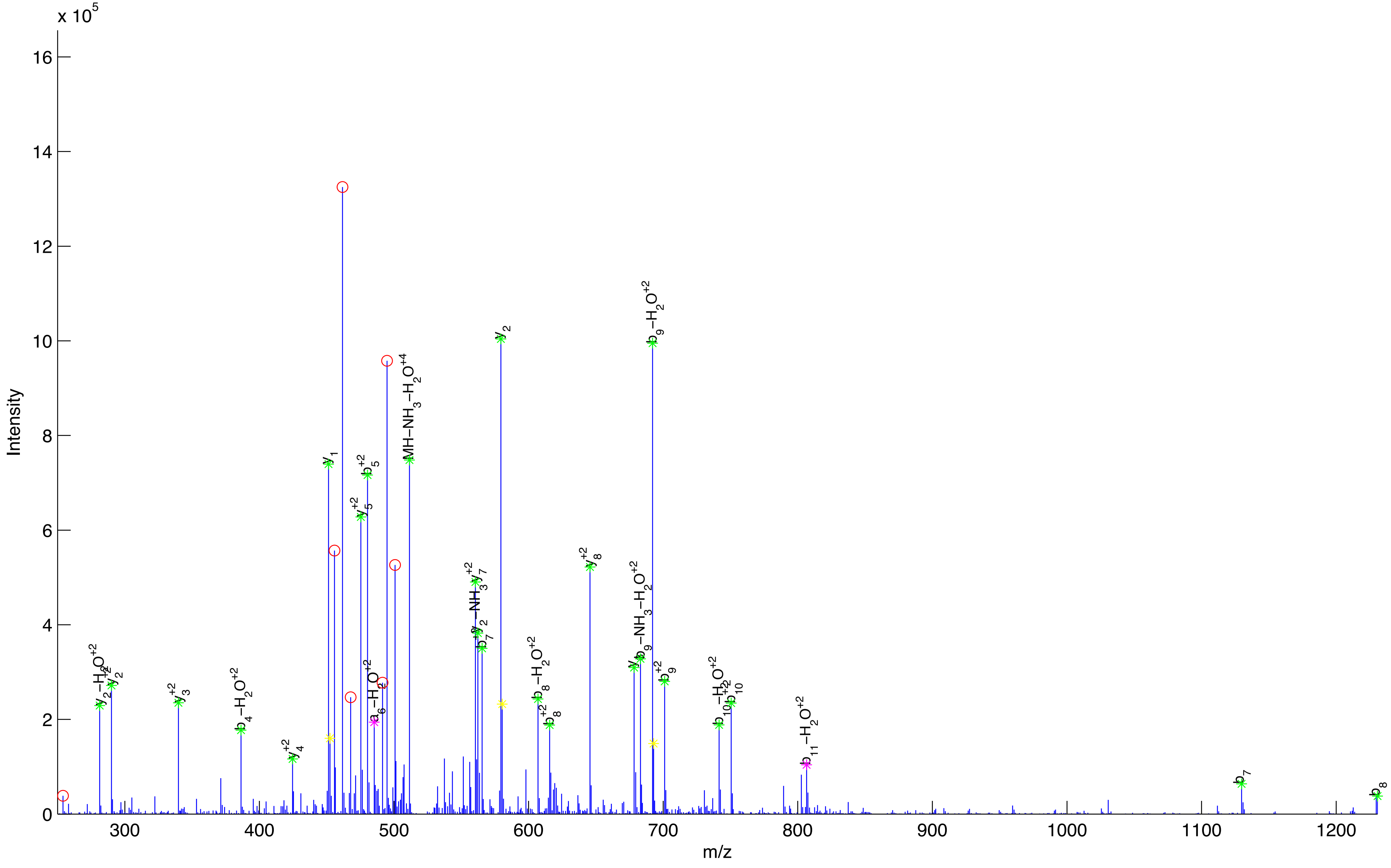

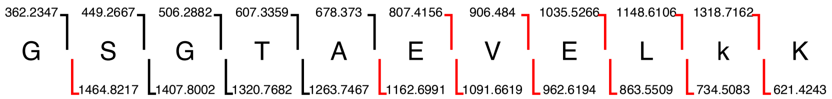

PREDICTED– similar to pyruvate kinase, muscle [Homo sapiens]
Charge State: +3
Scan Number: 10599
File Name: 120404_A549_EGFIGF_bioRepB_ACK_FT.raw

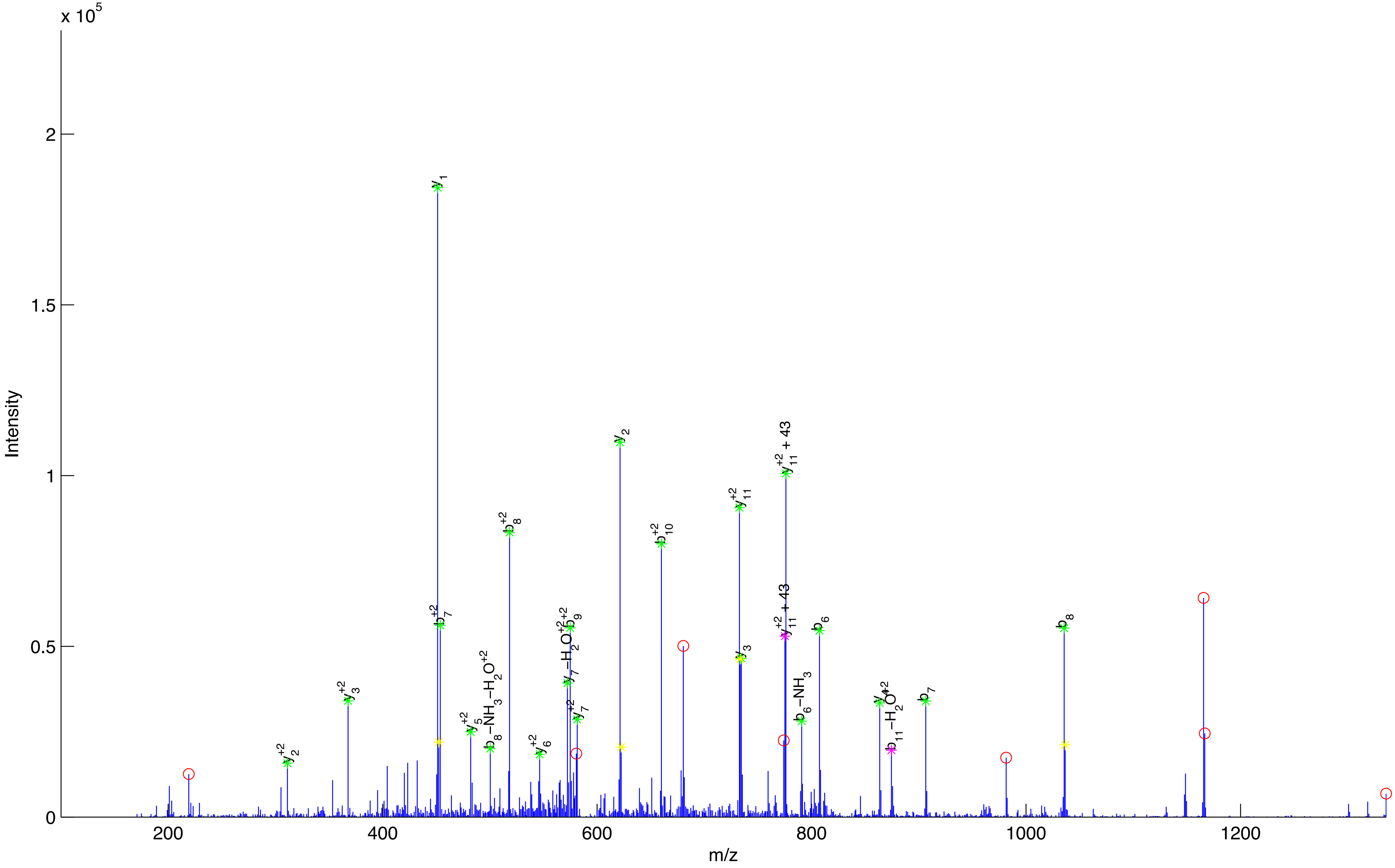

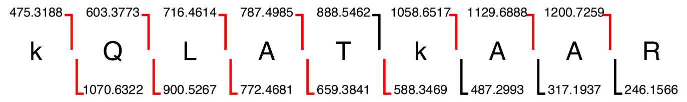

H3 histone, family 3A [Homo sapiens]
Charge State: +2
Scan Number: 10627
File Name: 120413_A549_EGFIGF_bioRepC_AcK_FT.raw

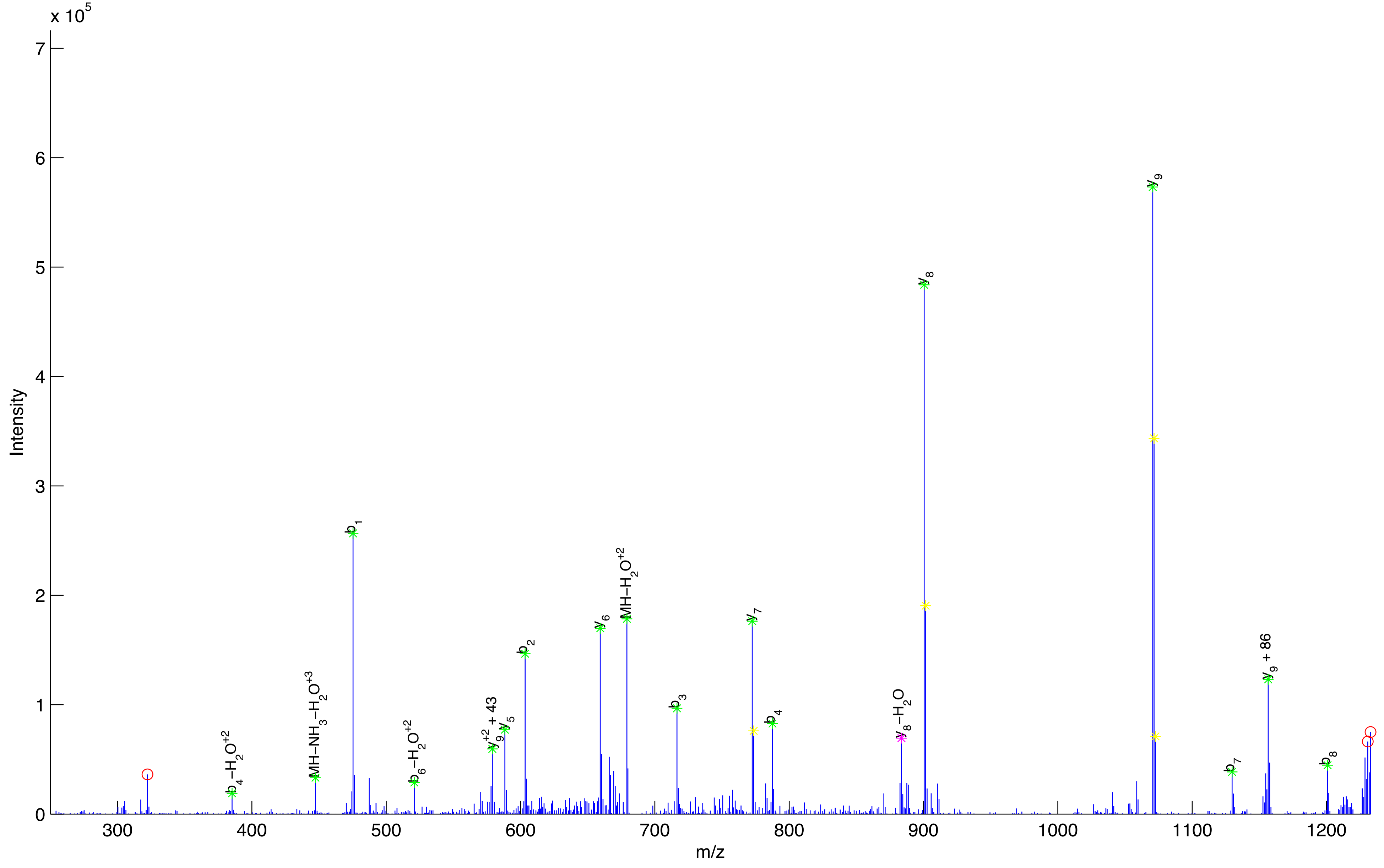

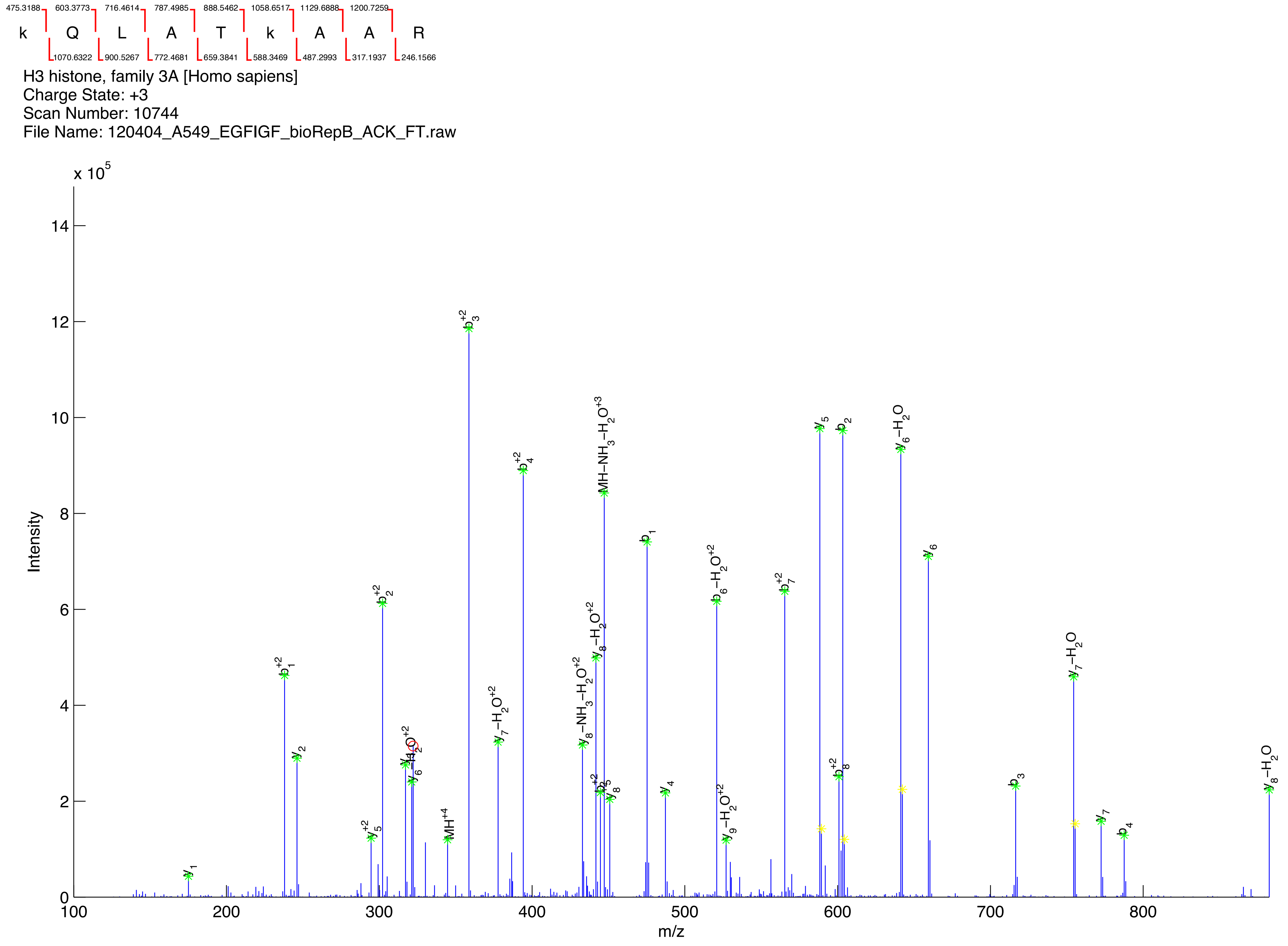
475.3188 603.3773 716.4614 787.4985 888.5462 1058.6517 1129.6888 1200.7259
k Q L A T k A A R
1070.6322 900.5267 772.4681 659.3841 588.3469 487.2993 317.1937 246.1566
H3 histone, family 3A [Homo sapiens]
Charge State: +3
Scan Number: 10744
File Name: 120404_A549_EGFIGF_bioRepB_ACK_FT.raw
x 10^5
Intensity
m/z

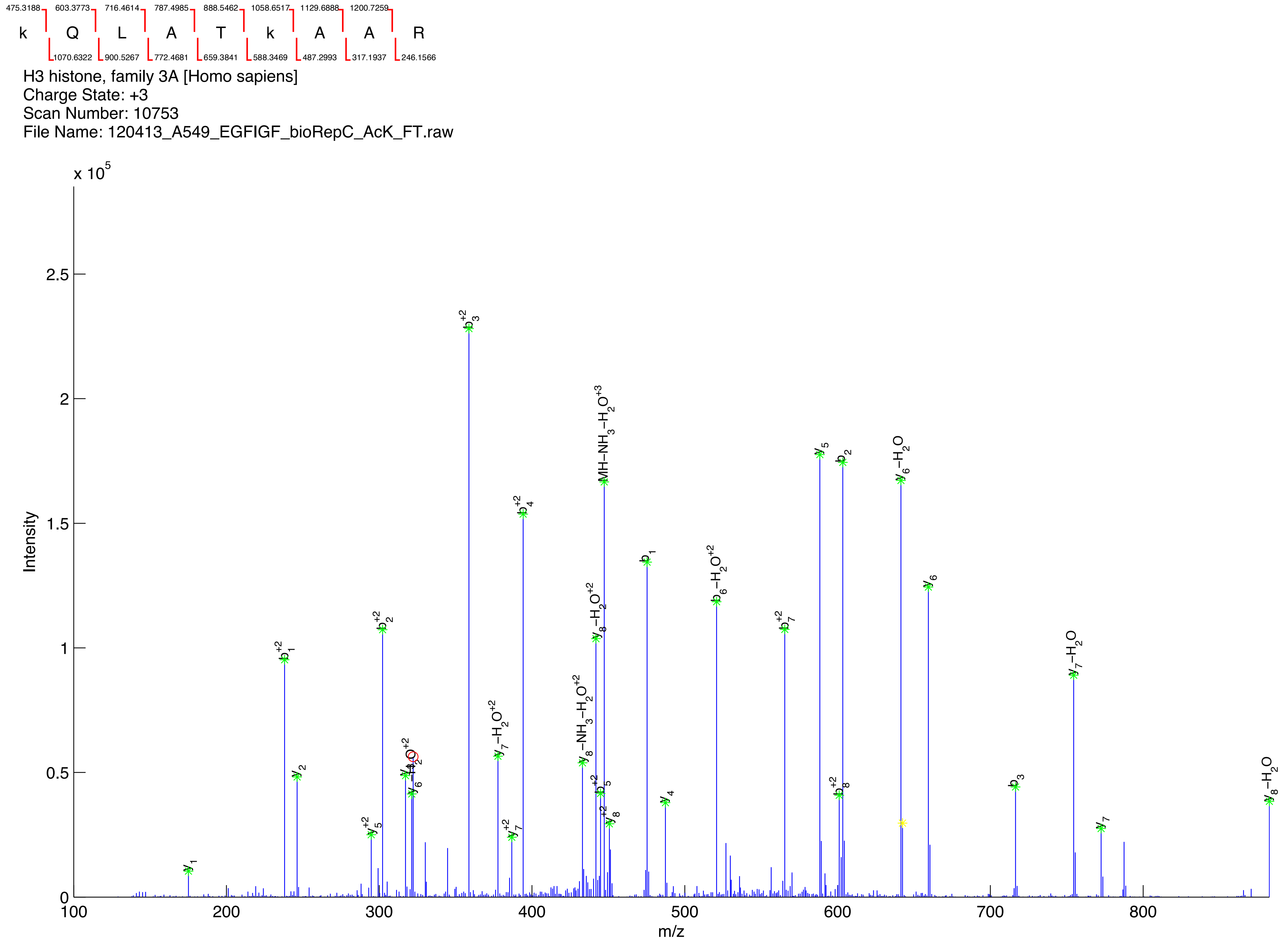
475.3188
603.3773
716.4614
787.4985
888.5462
1058.6517
1129.6888
1200.7259
k Q L A T k A A R
1070.6322
900.5267
772.4681
659.3841
588.3469
487.2993
317.1937
246.1566
H3 histone, family 3A [Homo sapiens]
Charge State: +3
Scan Number: 10753
File Name: 120413_A549_EGFIGF_bioRepC_AcK_FT.raw
x 10^5
Intensity
m/z
MH−NH3−H2O+3

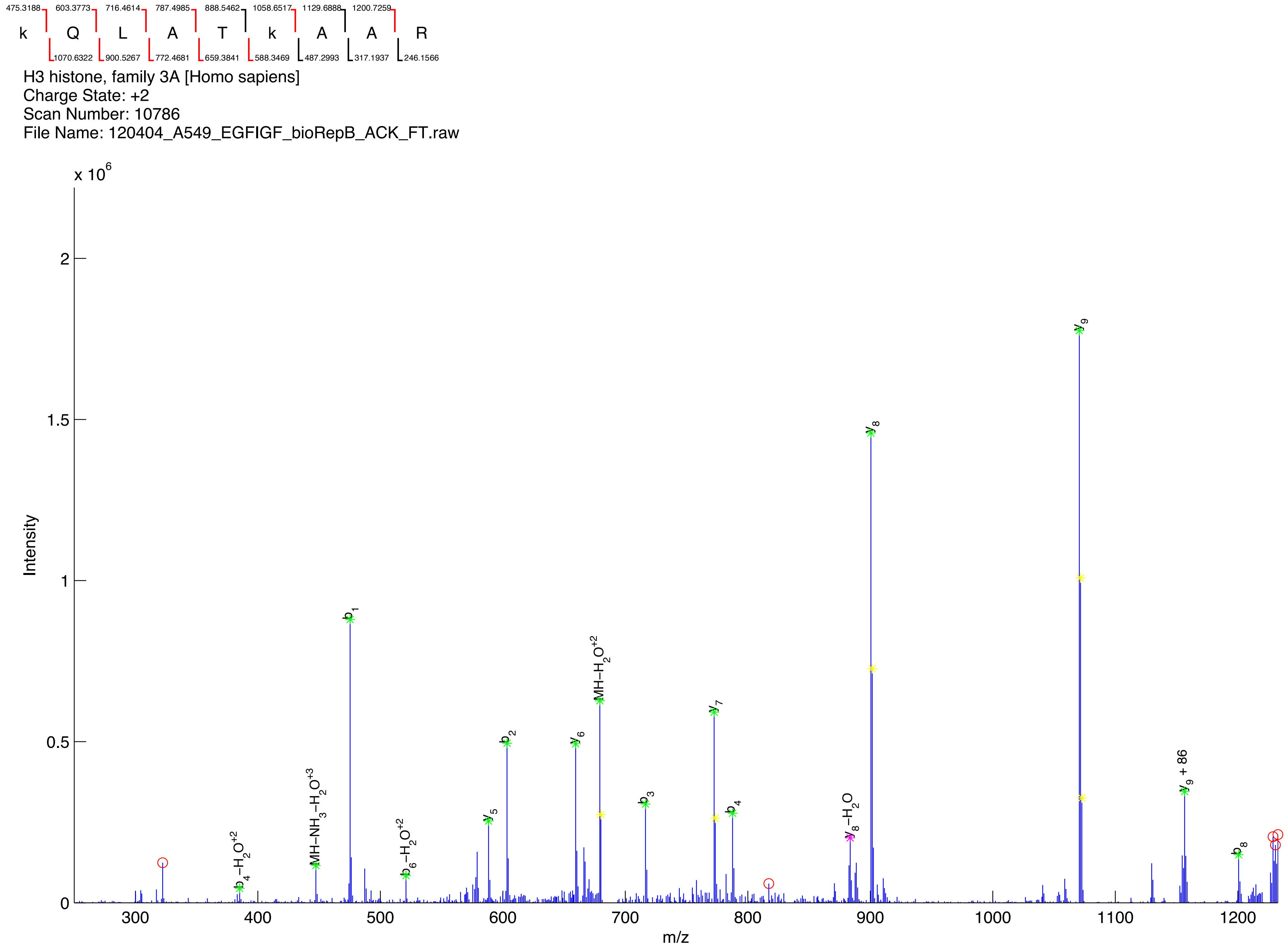

475.3188 603.3773 716.4614 787.4985 888.5462 1058.6517 1129.6888 1200.7259
k Q L A T k A A R
1070.6322 900.5267 772.4681 659.3841 588.3469 487.2993 317.1937 246.1566
H3 histone, family 3A [Homo sapiens]
Charge State: +2
Scan Number: 10786
File Name: 120404_A549_EGFIGF_bioRepB_ACK_FT.raw
x 10^6
Intensity
m/z
b1
b2
b3
b4
b8
y5
y6
y7
y8
y9
y9 + 86
y8−H2O
b4−H2O+2
b6−H2O+2
MH−H2O+2
MH−NH3−H2O+3

475.3188	603.3773	716.4614	787.4985	888.5462	1058.6517	1129.6888	1200.7259	
k	Q	L	A	T	k	A	A	R
	1070.6322	900.5267	772.4681	659.3841	588.3469	487.2993	317.1937	246.1566

H3 histone, family 3A [Homo sapiens]
Charge State: +3
Scan Number: 10787
File Name: 120407_A549_EGFIGF_bioRepA_ACK_FT.raw

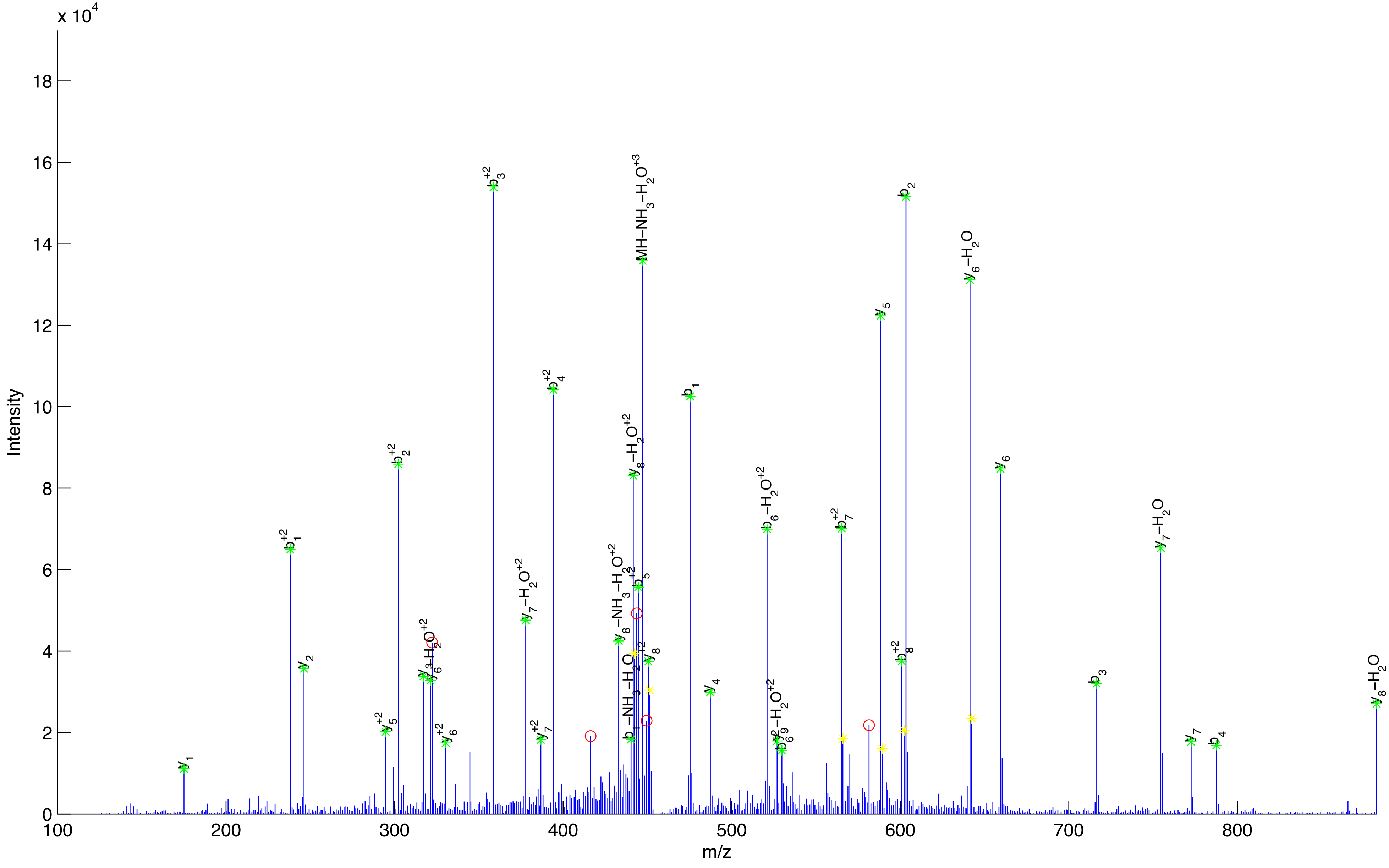

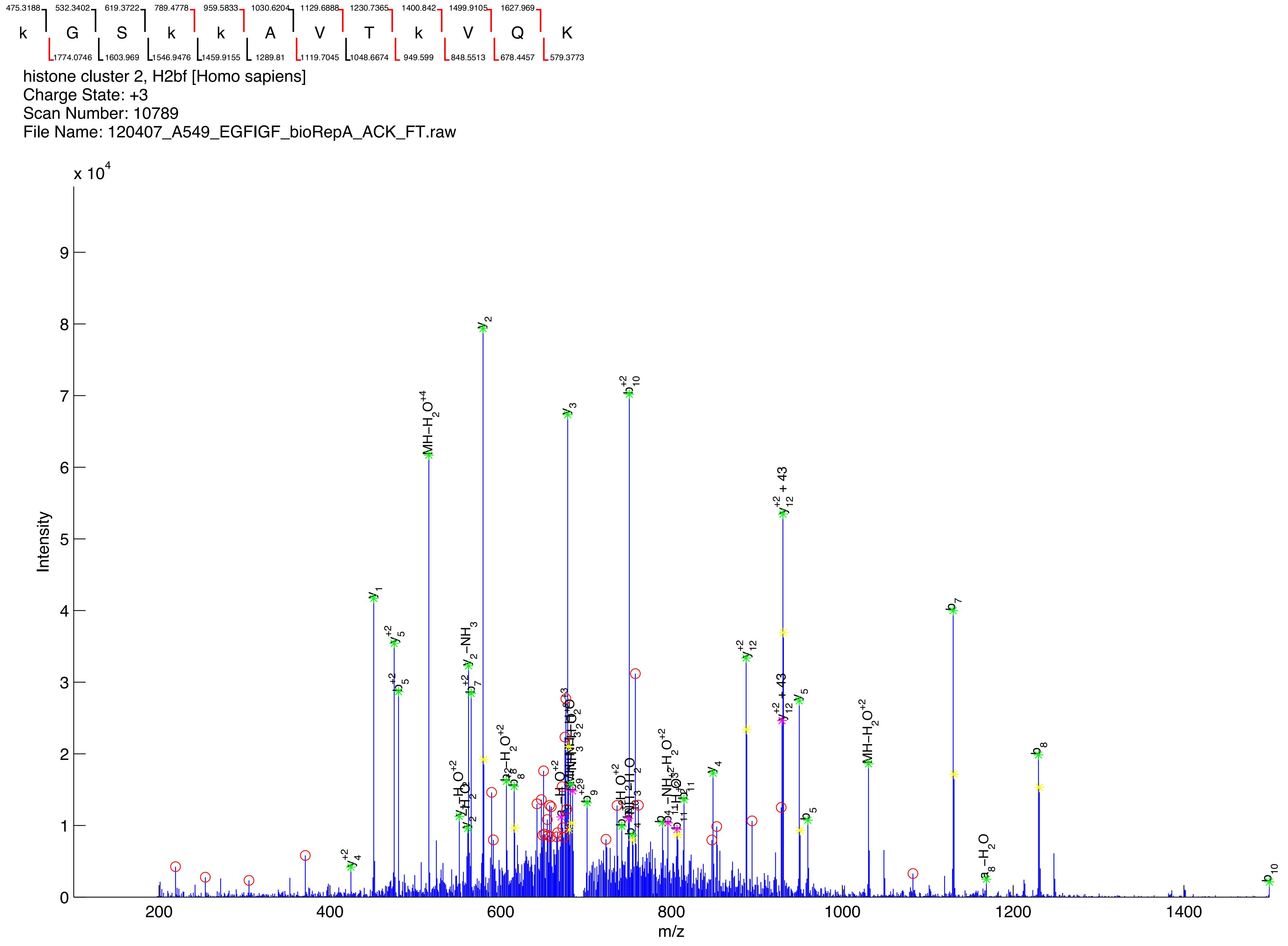
475.3188 532.3402 619.3722 789.4778 959.5833 1030.6204 1129.6888 1230.7365 1400.842 1499.9105 1627.969
k G S k k A V T k V Q K
1774.0746 1603.969 1546.9476 1459.9155 1289.81 1119.7045 1048.6674 949.599 848.5513 678.4457 579.3773
histone cluster 2, H2bf [Homo sapiens]
Charge State: +3
Scan Number: 10789
File Name: 120407_A549_EGFIGF_bioRepA_ACK_FT.raw
x 10^4
Intensity
m/z
0
1
2
3
4
5
6
7
8
9
200
400
600
800
1000
1200
1400
y_1
y_2
y_3
y_4
y_5
b_5
b_7
b_8
b_9
b_10
MH−H_2O^+4
MH−H_2O^+2
a_8−H_2O
y^+2_12
y^+2_12 + 43

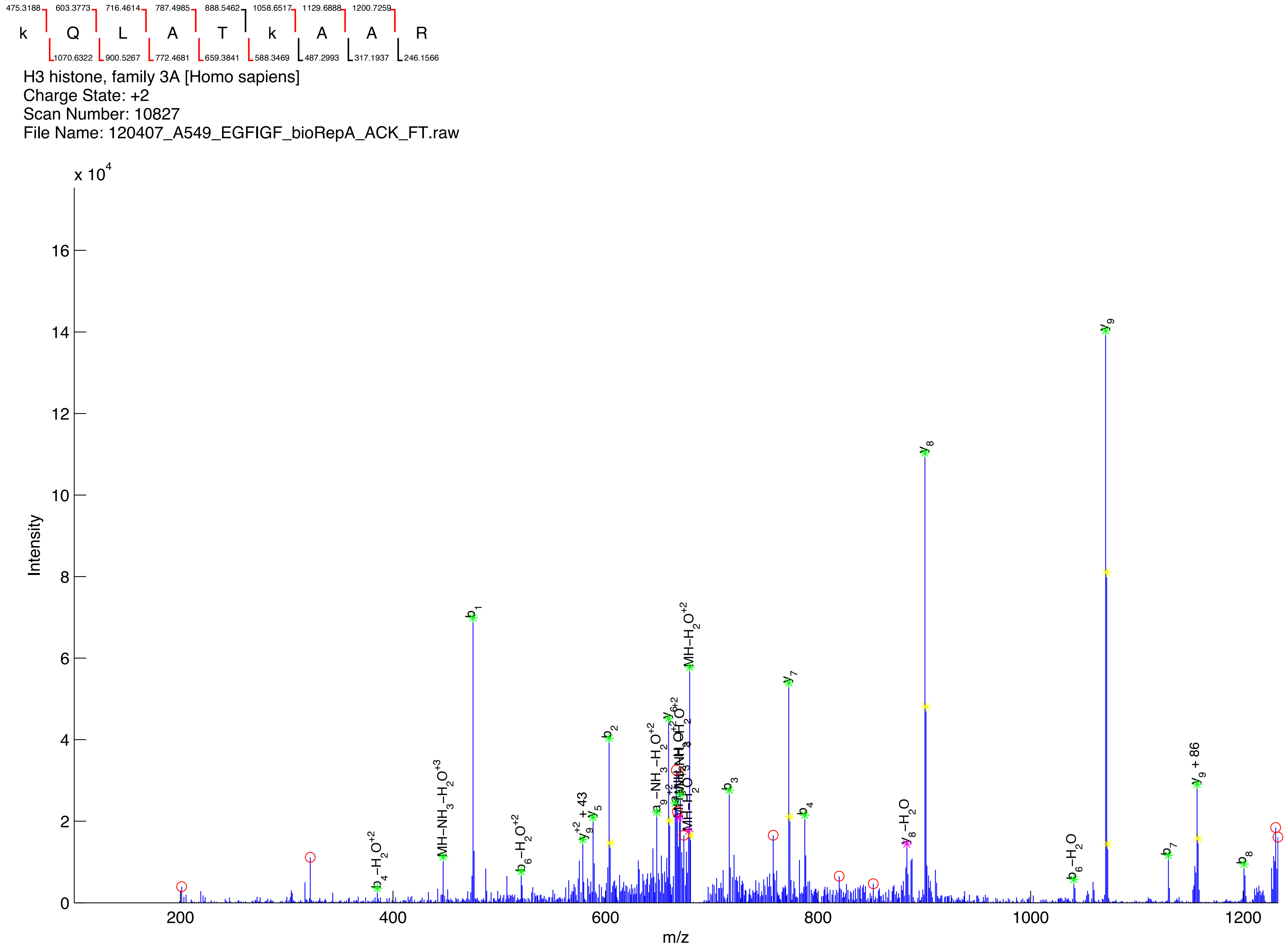

475.3188 603.3773 716.4614 787.4985 888.5462 1058.6517 1129.6888 1200.7259
k Q L A T k A A R
1070.6322 900.5267 772.4681 659.3841 588.3469 487.2993 317.1937 246.1566
H3 histone, family 3A [Homo sapiens]
Charge State: +2
Scan Number: 10827
File Name: 120407_A549_EGFIGF_bioRepA_ACK_FT.raw
x 10^4
Intensity
m/z
b1
b2
b3
b4
b7
b8
y7
y8
y9
y9 + 86
y5
MH-H2O+2
b6-H2O
y8-H2O

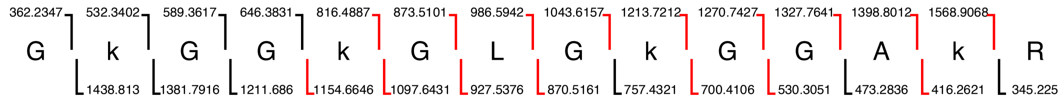

histone cluster 1, H4a [Homo sapiens]
Charge State: +3
Scan Number: 10890
File Name: 120407_A549_EGFIGF_bioRepA_ACK_FT.raw

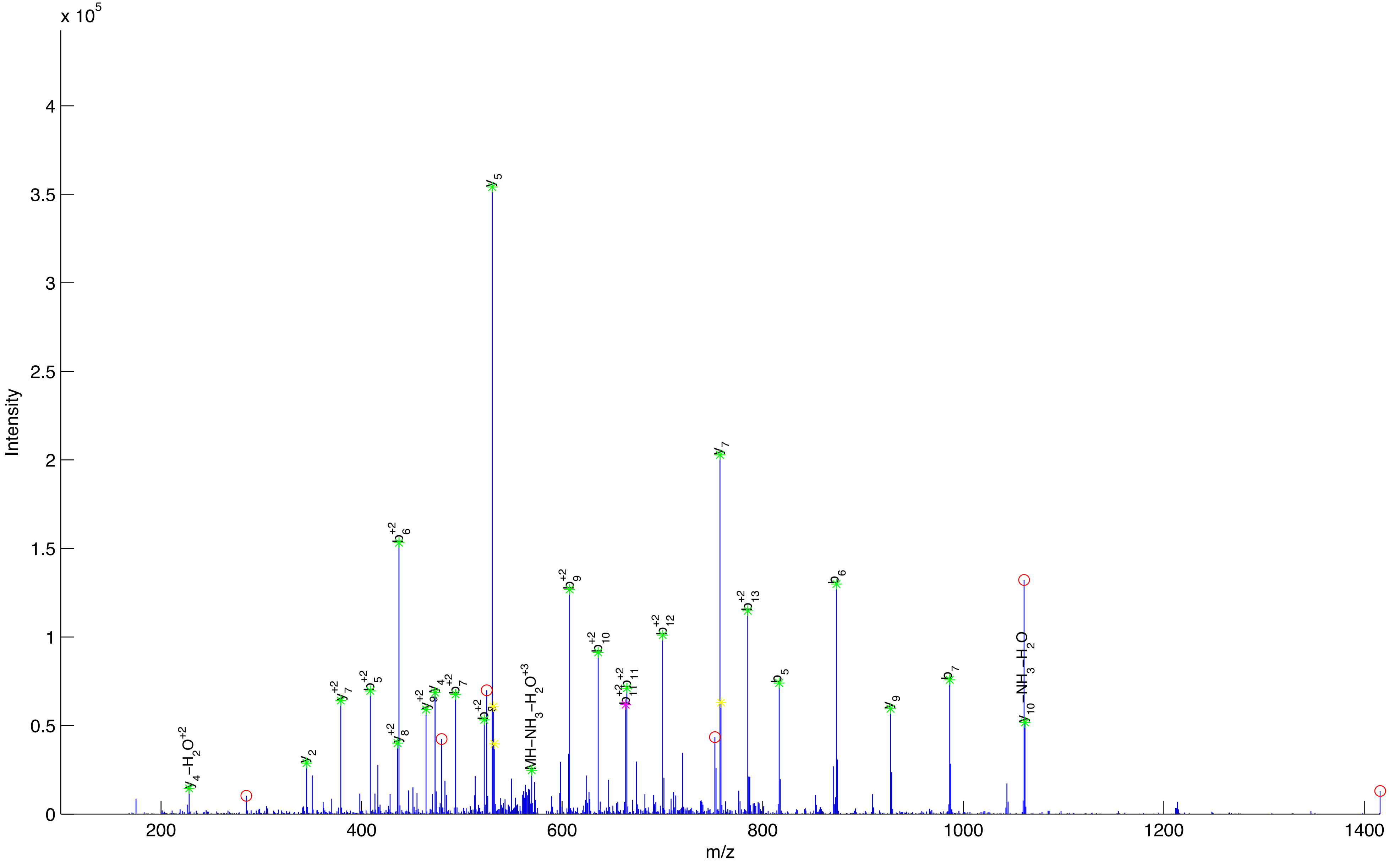

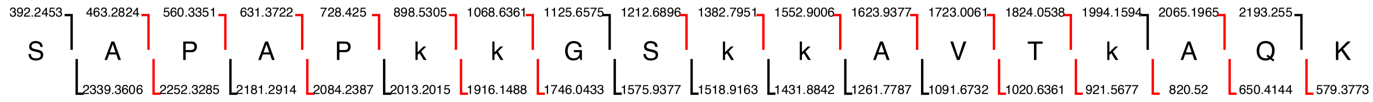

histone cluster 1, H2bl [Homo sapiens]
Charge State: +4
Scan Number: 10932
File Name: 120407_A549_EGFIGF_bioRepA_ACK_FT.raw

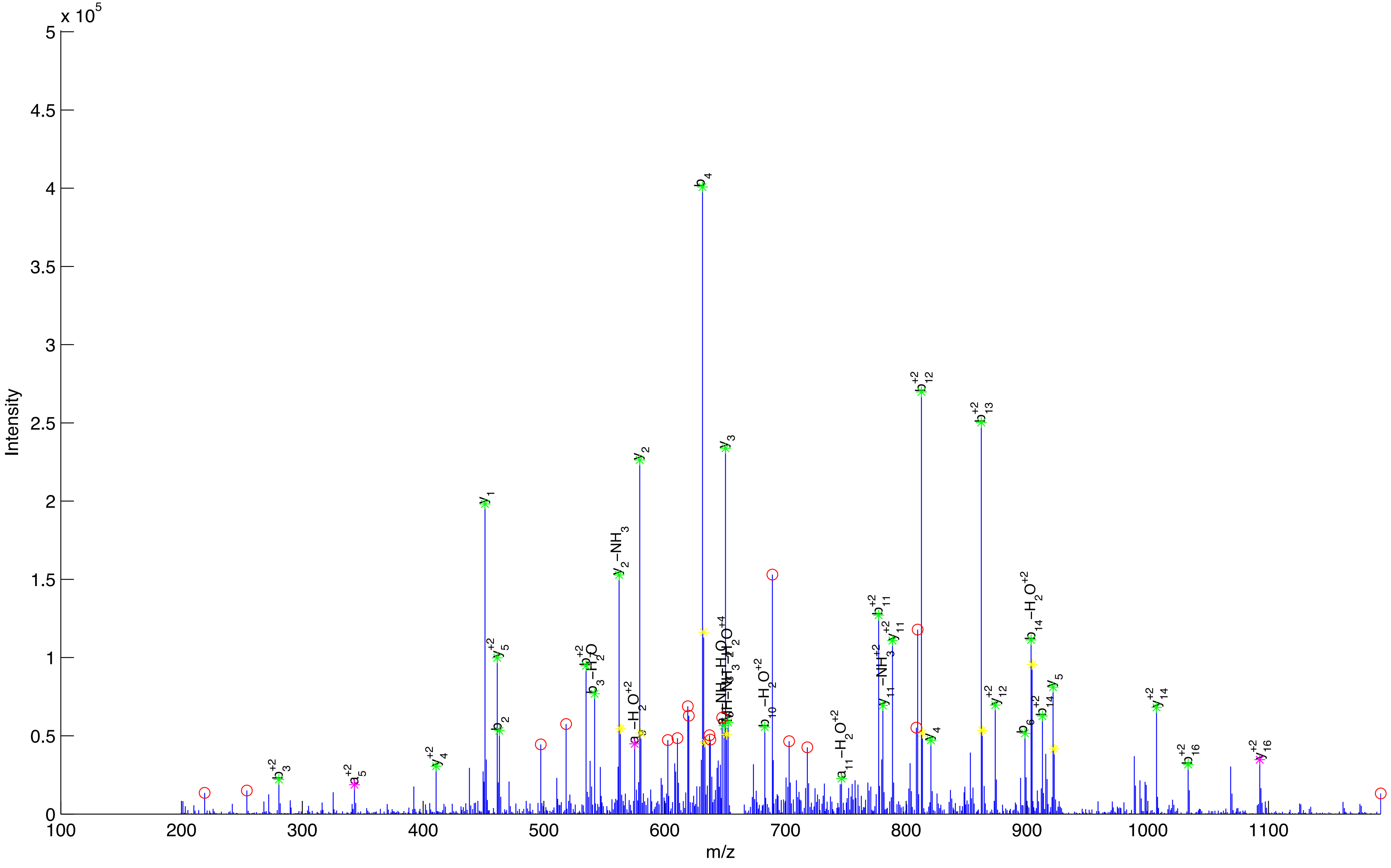

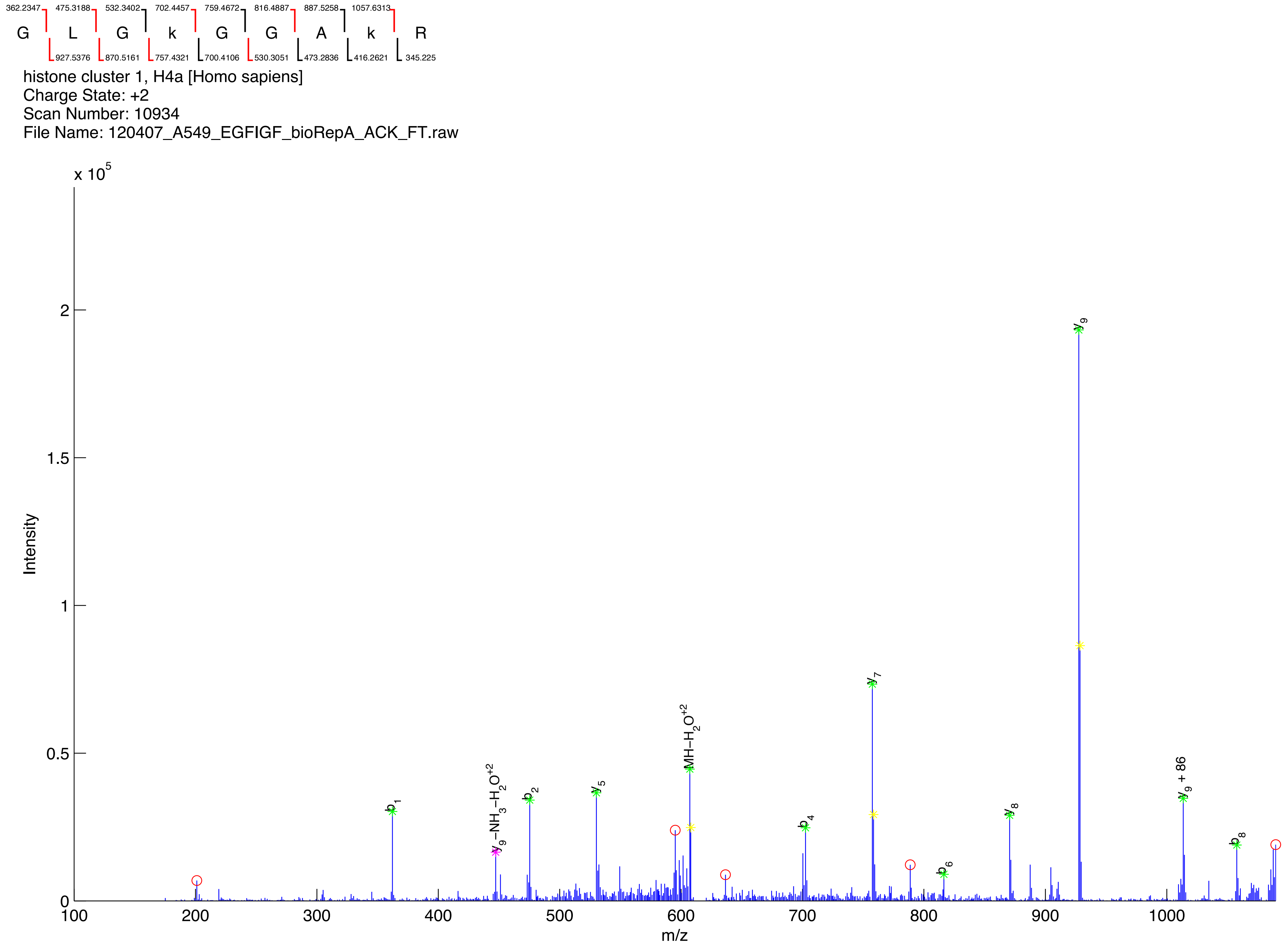
362.2347 475.3188 532.3402 702.4457 759.4672 816.4887 887.5258 1057.6313
G L G k G G A k R
927.5376 870.5161 757.4321 700.4106 530.3051 473.2836 416.2621 345.225
histone cluster 1, H4a [Homo sapiens]
Charge State: +2
Scan Number: 10934
File Name: 120407_A549_EGFIGF_bioRepA_ACK_FT.raw
x 10^5
Intensity
m/z
b1
y9−NH3−H2O+2
b2
y5
MH−H2O+2
b4
y7
b6
y8
y9
y9 + 86
b8

362.2347 419.2562 589.3617 646.3831 759.4672 816.4887 986.5942 1043.6157 1100.6371 1171.6742 1341.7798

G G k G L G k G G A k R

1211.686 1154.6646 1097.6431 927.5376 870.5161 757.4321 700.4106 530.3051 473.2836 416.2621 345.225

histone cluster 1, H4a [Homo sapiens]
Charge State: +3
Scan Number: 11018
File Name: 120407_A549_EGFIGF_bioRepA_ACK_FT.raw

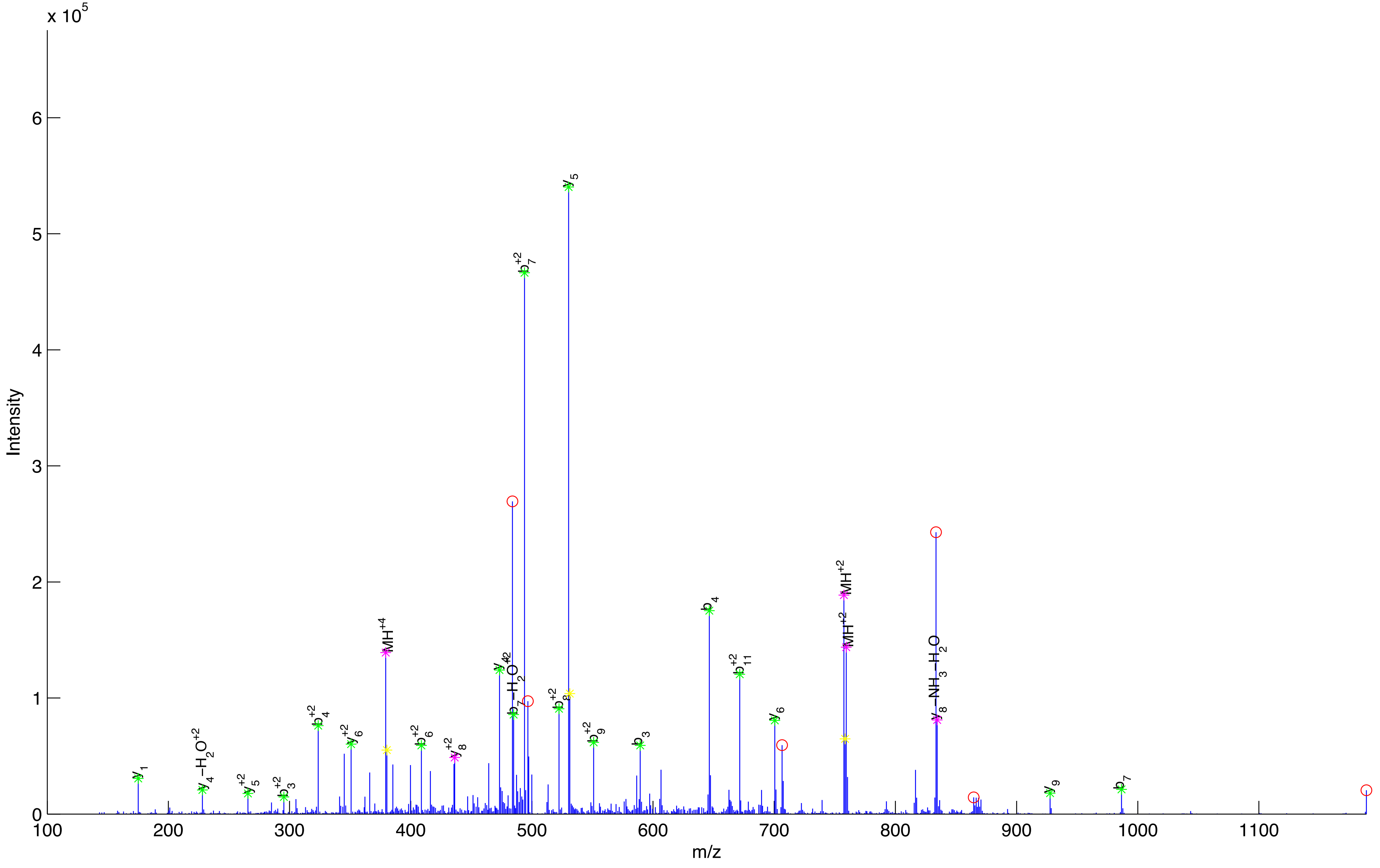

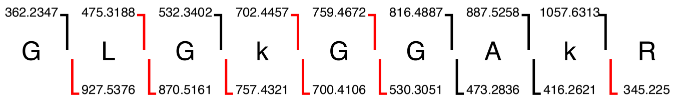

histone cluster 1, H4a [Homo sapiens]
Charge State: +3
Scan Number: 11020
File Name: 120407_A549_EGFIGF_bioRepA_ACK_FT.raw

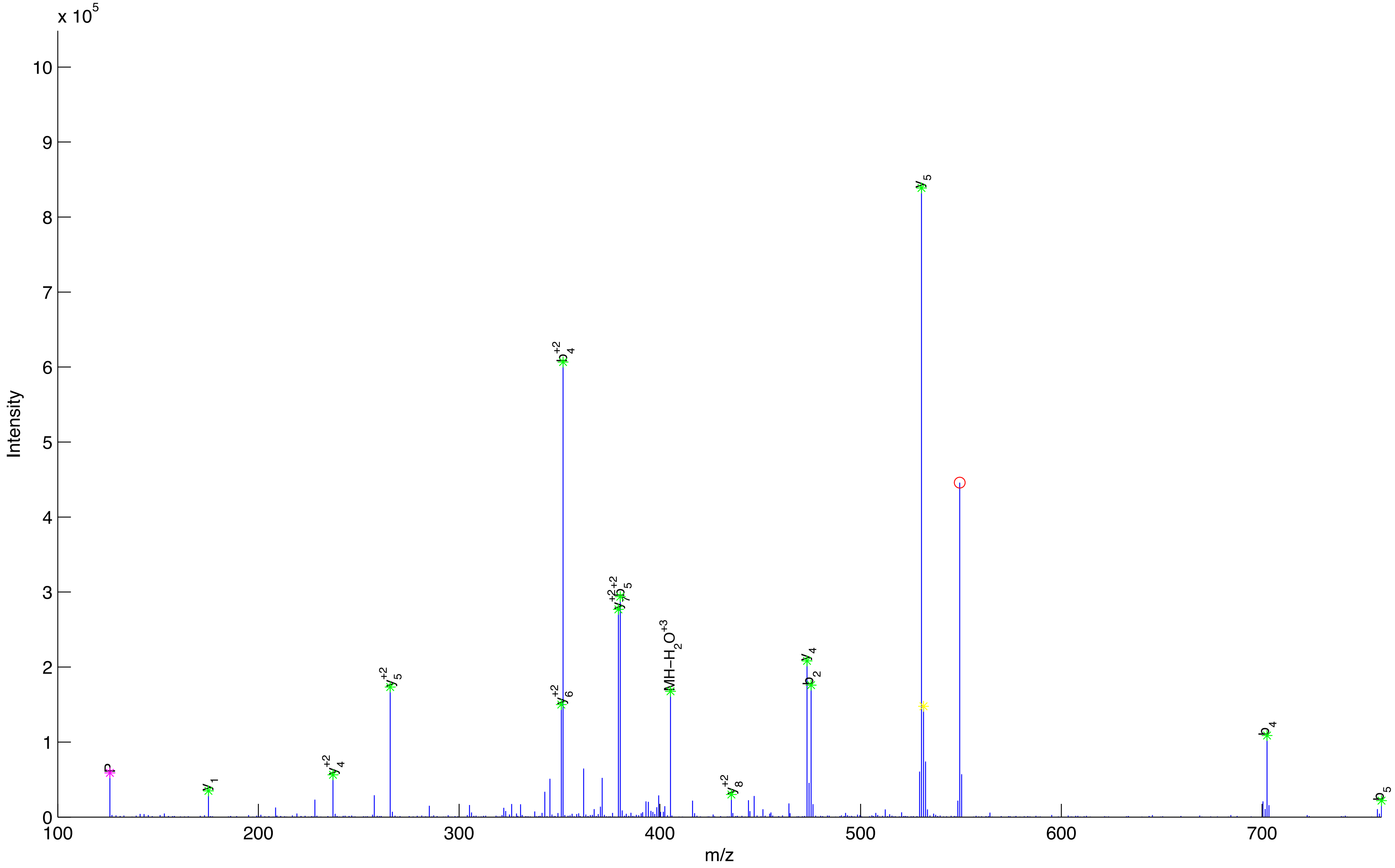

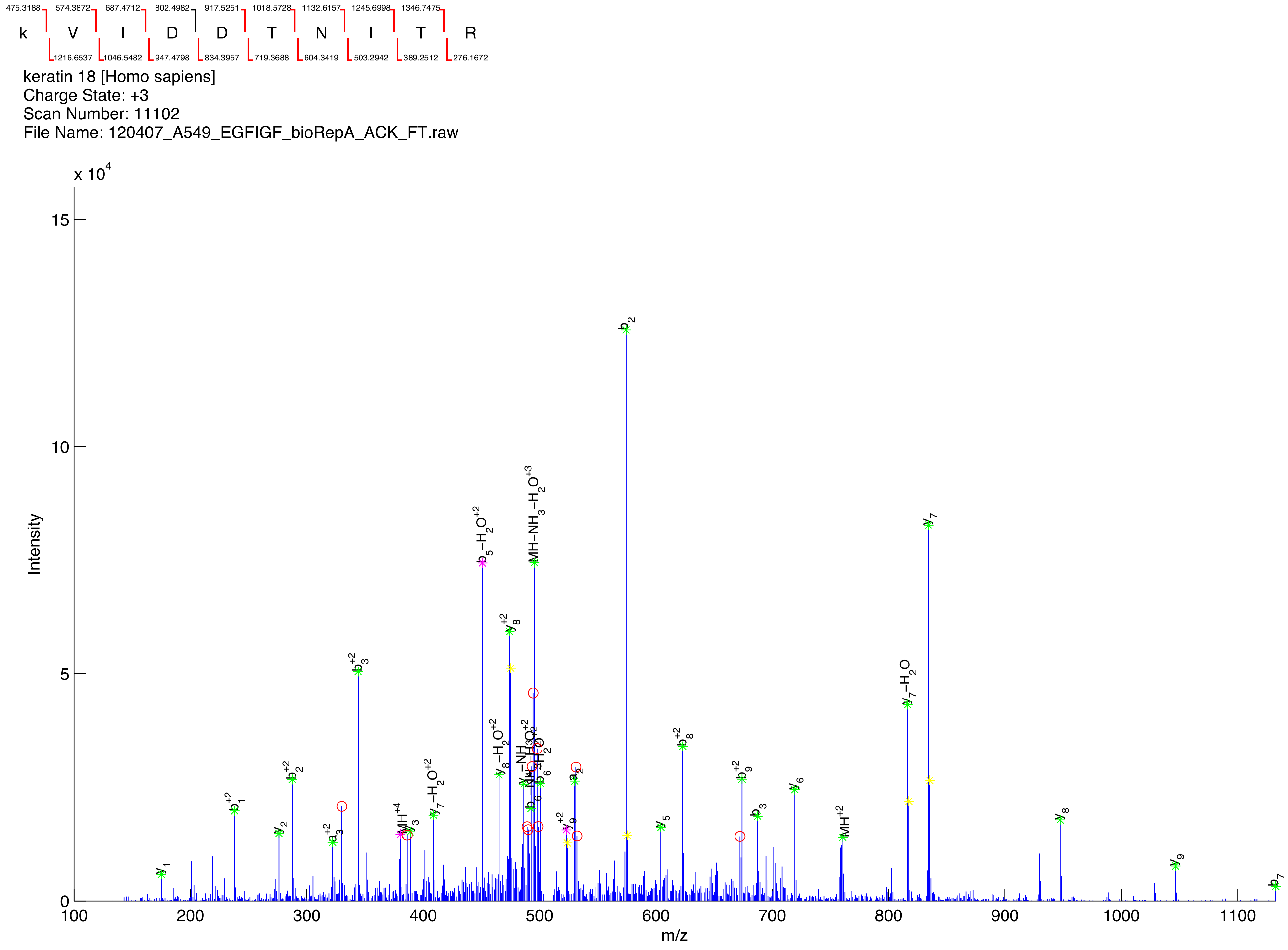

475.3188 574.3872 687.4712 802.4982 917.5251 1018.5728 1132.6157 1245.6998 1346.7475
k V I D D T N I T R
1216.6537 1046.5482 947.4798 834.3957 719.3688 604.3419 503.2942 389.2512 276.1672
keratin 18 [Homo sapiens]
Charge State: +3
Scan Number: 11102
File Name: 120407_A549_EGFIGF_bioRepA_ACK_FT.raw
x 10^4
Intensity
m/z

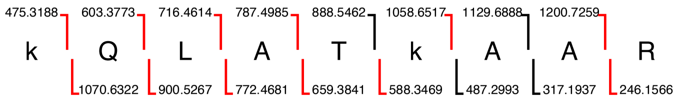

H3 histone, family 3A [Homo sapiens]
Charge State: +2
Scan Number: 11154
File Name: 120413_A549_EGFIGF_bioRepC_AcK_FT.raw

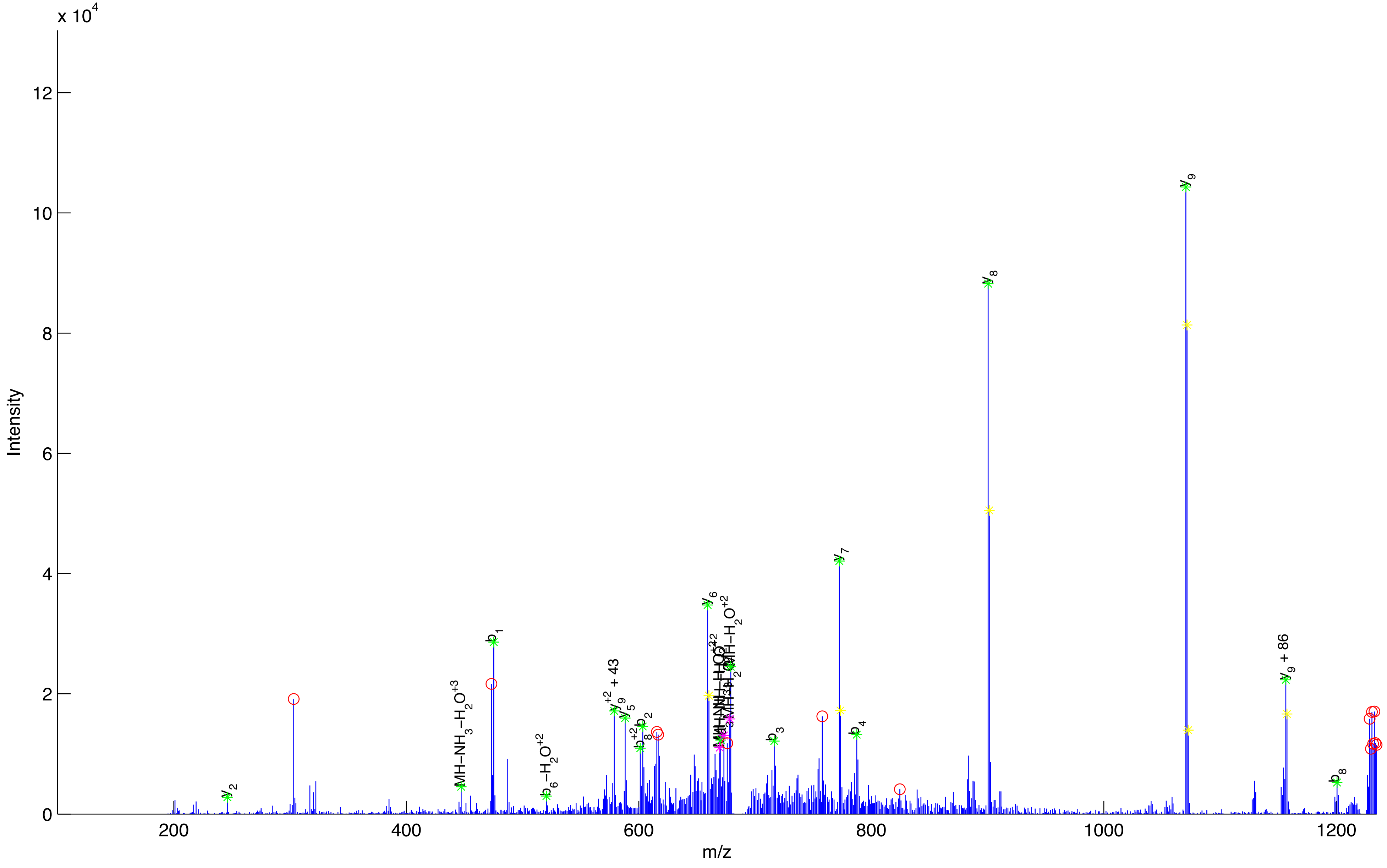

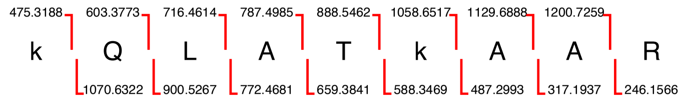

H3 histone, family 3A [Homo sapiens]
Charge State: +3
Scan Number: 11284
File Name: 120413_A549_EGFIGF_bioRepC_AcK_FT.raw

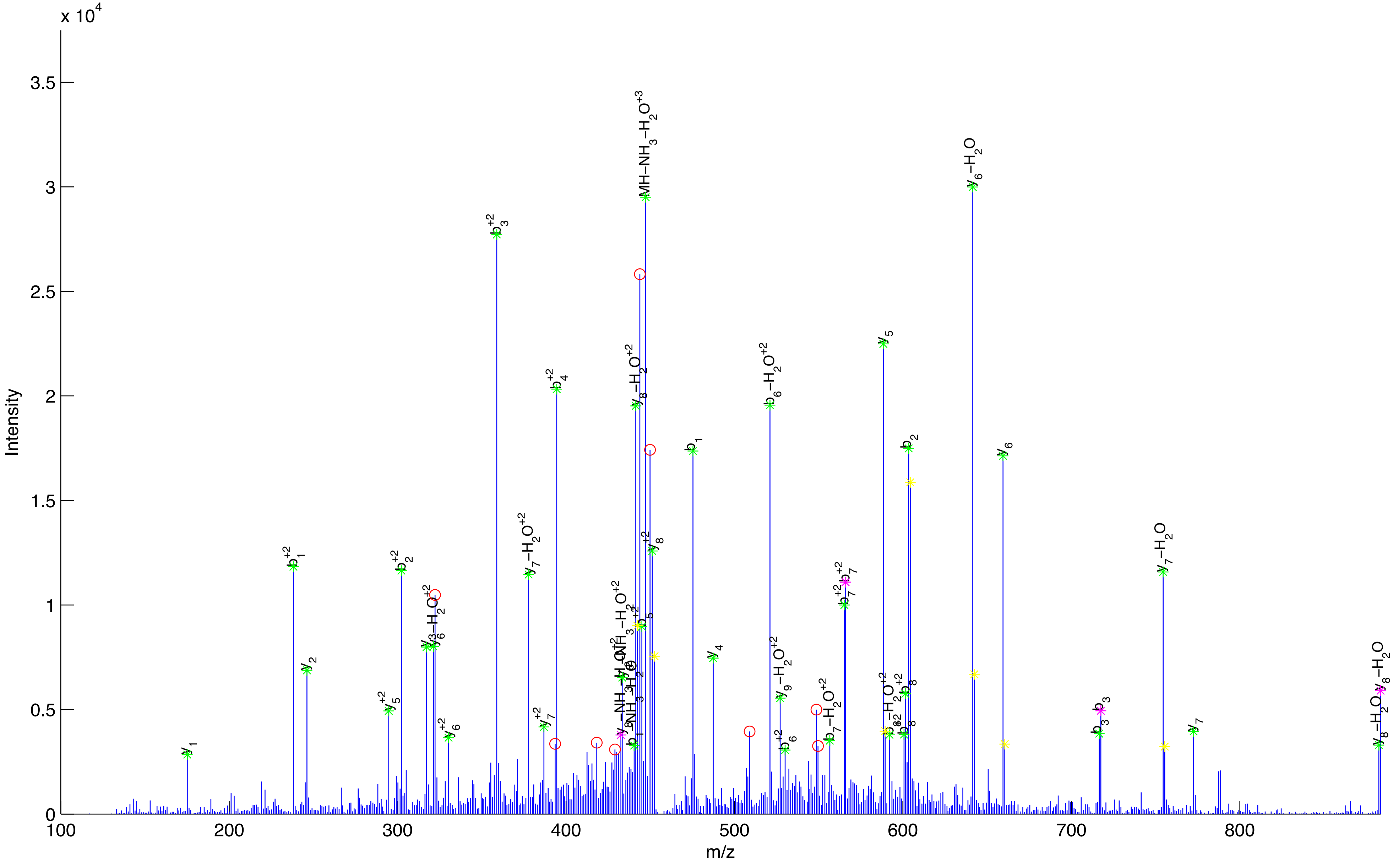

k Q L A T k A A R

b ions: 475.3188, 603.3773, 716.4614, 787.4985, 888.5462, 1058.6517, 1129.6888, 1200.7259

y ions: 1070.6322, 900.5267, 772.4681, 659.3841, 588.3469, 487.2993, 317.1937, 246.1566

H3 histone, family 3A [Homo sapiens]
Charge State: +3
Scan Number: 11290
File Name: 120404_A549_EGFIGF_bioRepB_ACK_FT.raw

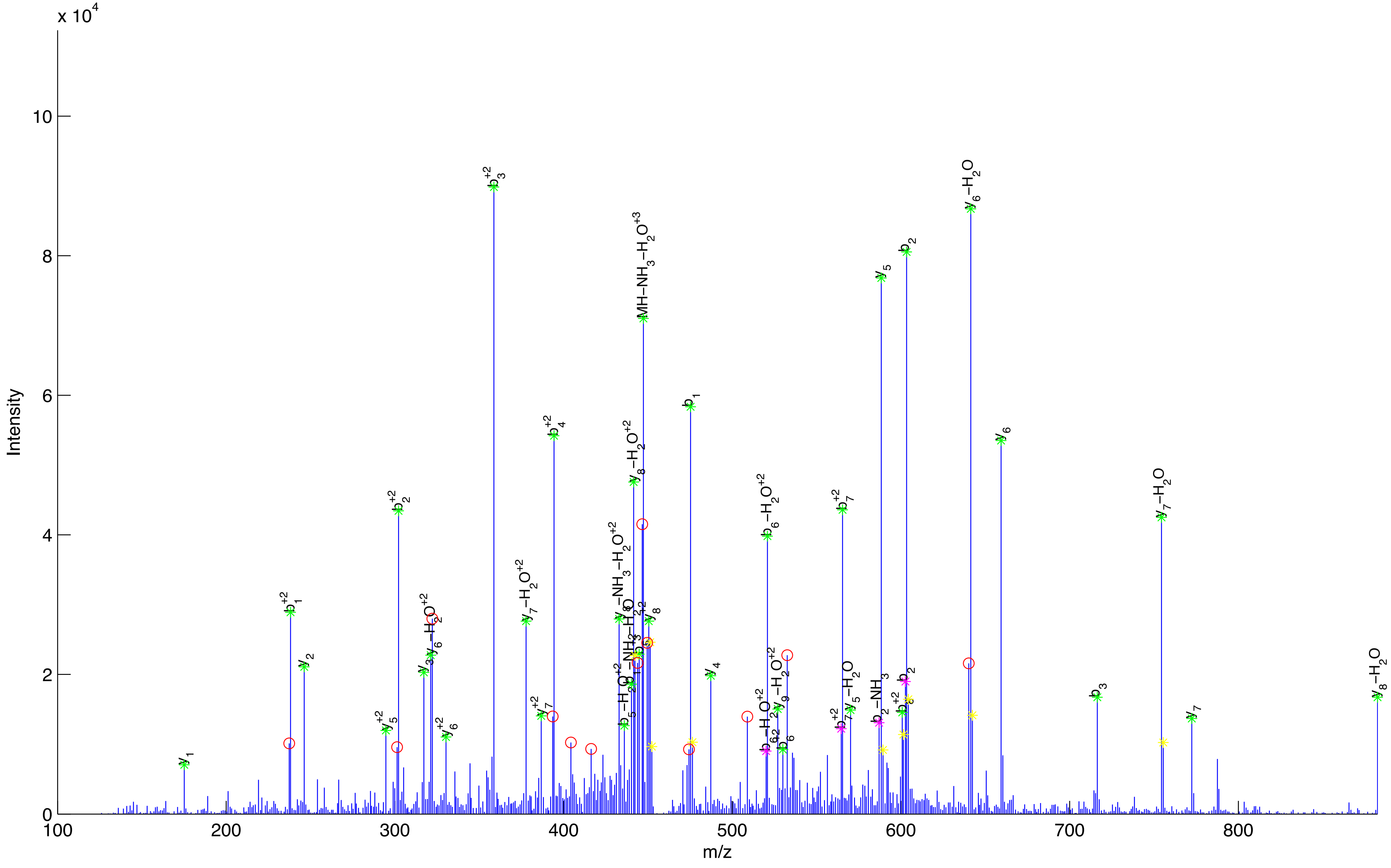

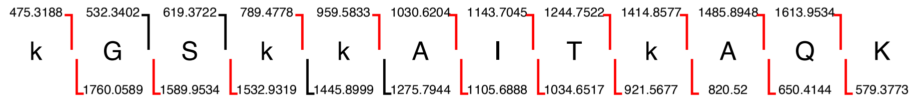

histone cluster 1, H2bb [Homo sapiens]
Charge State: +3
Scan Number: 11332
File Name: 120404_A549_EGFIGF_bioRepB_ACK_FT.raw

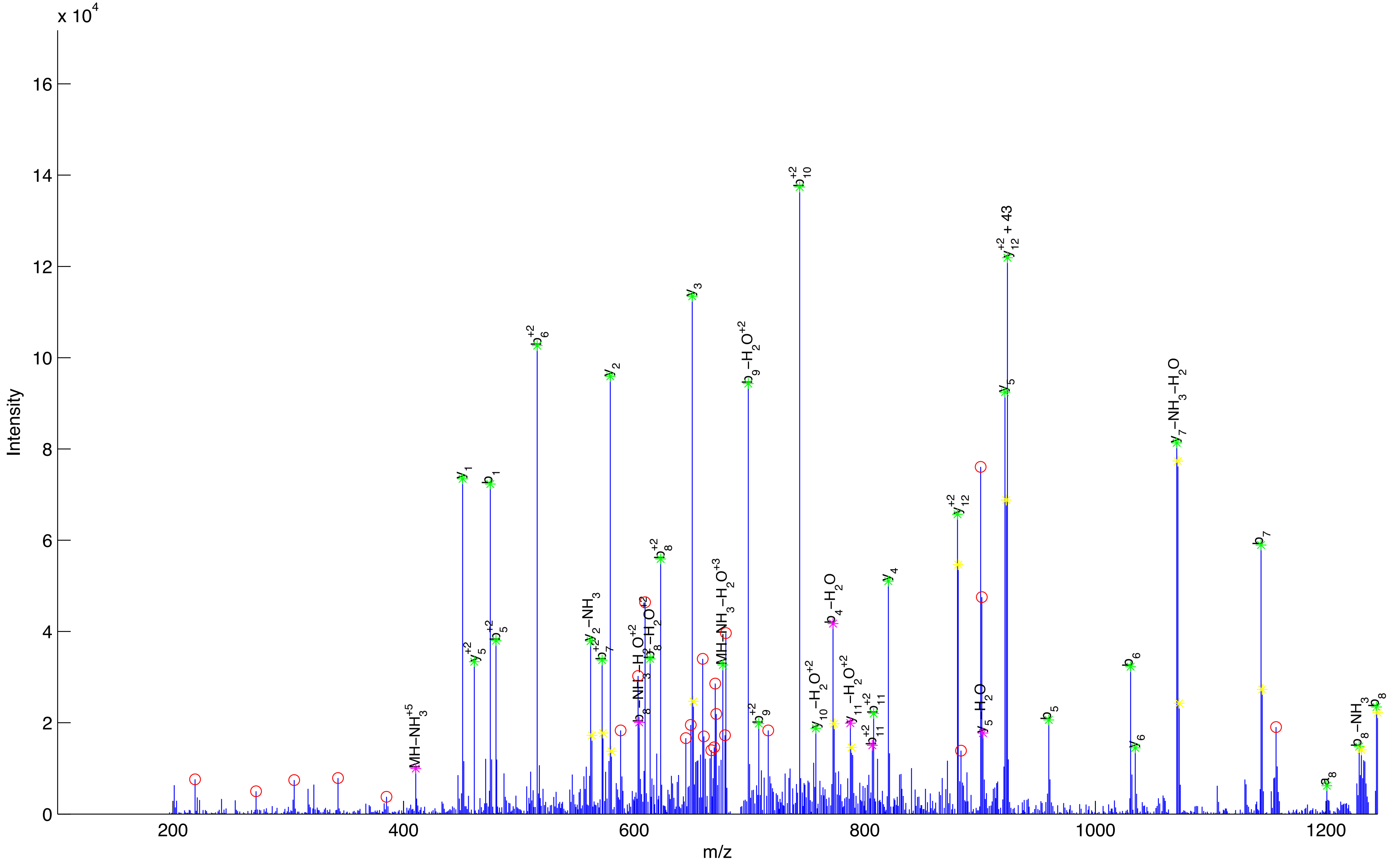

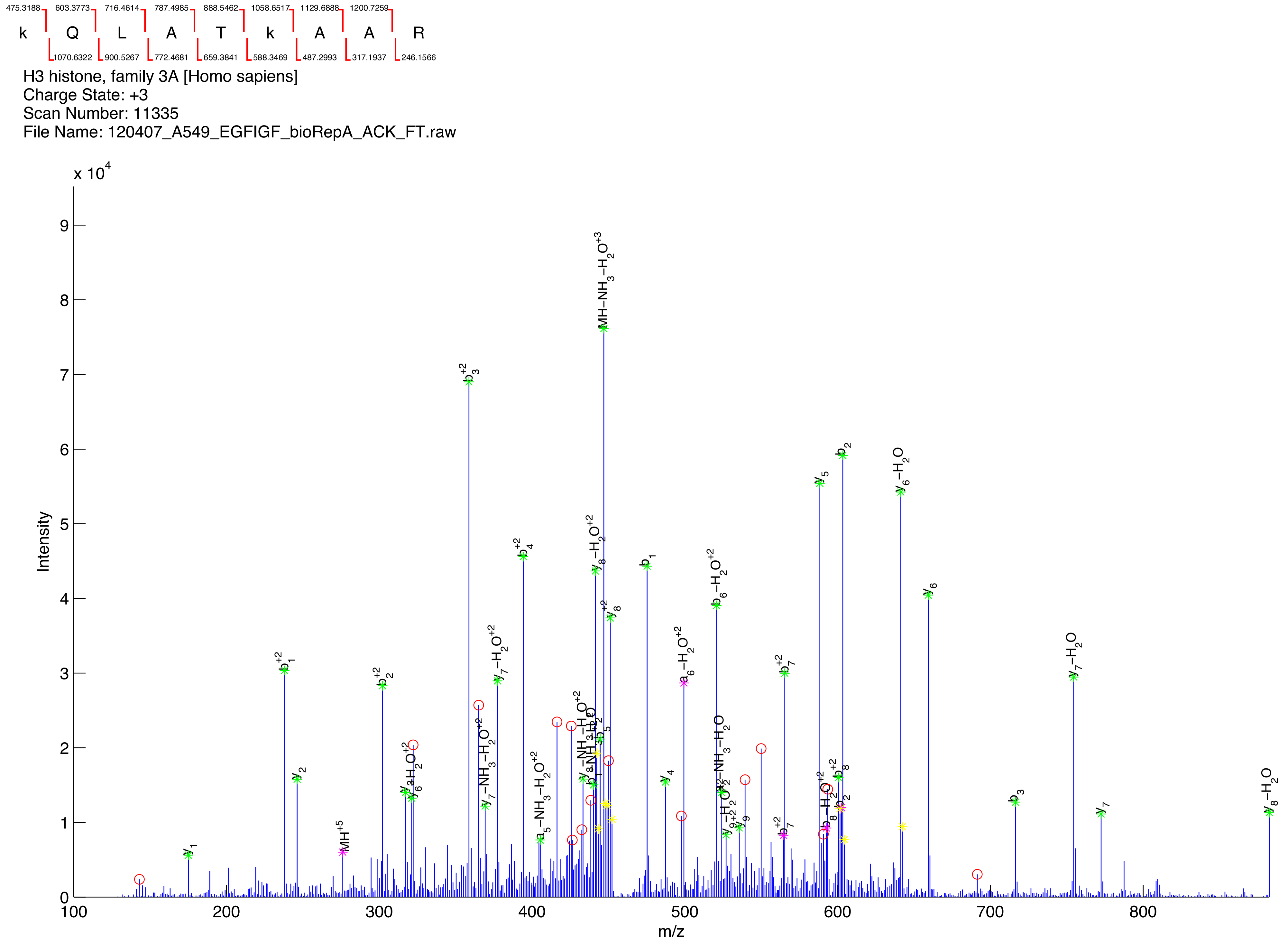
475.3188 603.3773 716.4614 787.4985 888.5462 1058.6517 1129.6888 1200.7259
k Q L A T k A A R
1070.6322 900.5267 772.4681 659.3841 588.3469 487.2993 317.1937 246.1566
H3 histone, family 3A [Homo sapiens]
Charge State: +3
Scan Number: 11335
File Name: 120407_A549_EGFIGF_bioRepA_ACK_FT.raw
x 10^4
Intensity
m/z

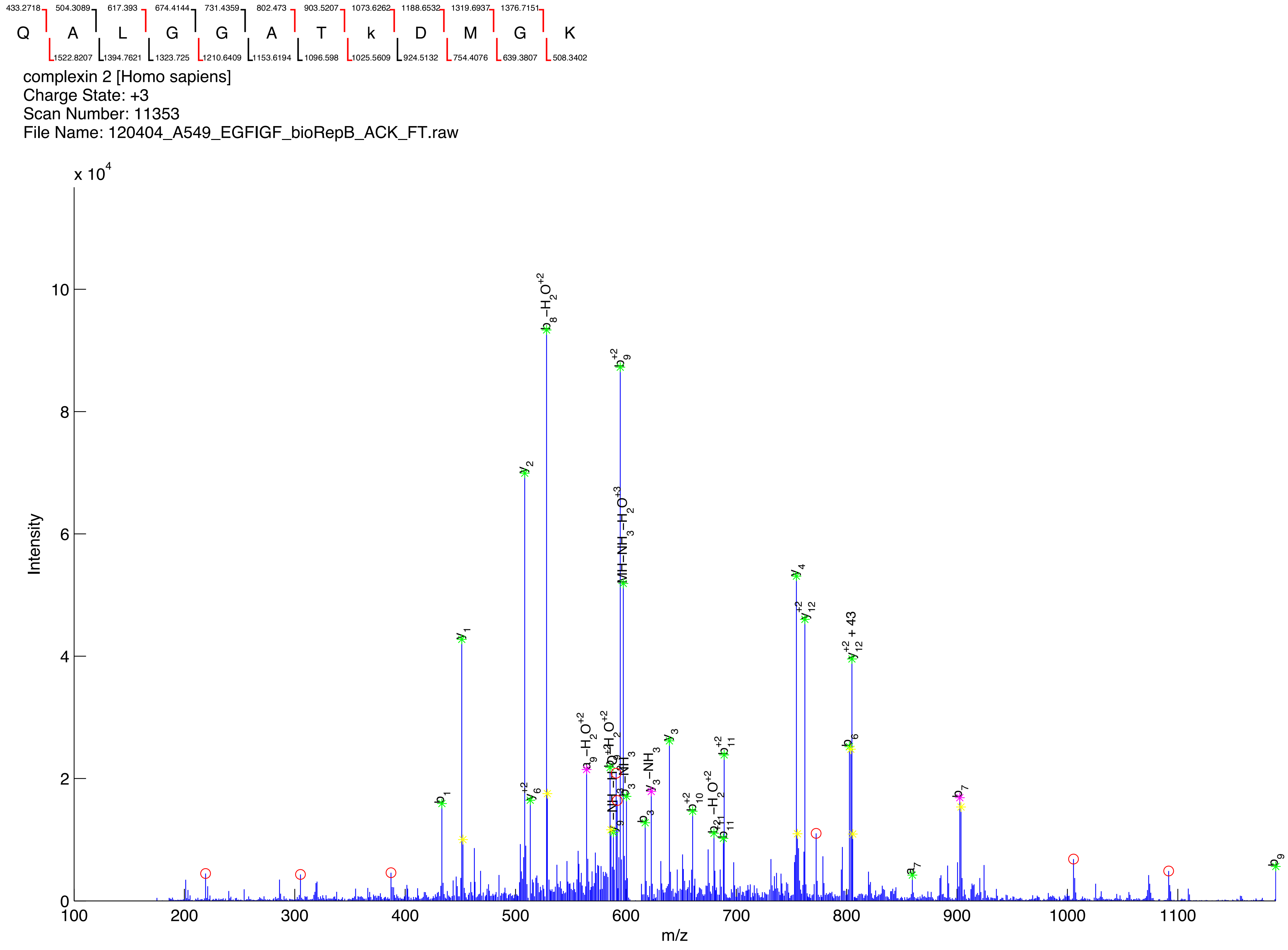

433.2718 504.3089 617.393 674.4144 731.4359 802.473 903.5207 1073.6262 1188.6532 1319.6937 1376.7151
Q A L G G A T k D M G K
1522.8207 1394.7621 1323.725 1210.6409 1153.6194 1096.598 1025.5609 924.5132 754.4076 639.3807 508.3402
complexin 2 [Homo sapiens]
Charge State: +3
Scan Number: 11353
File Name: 120404_A549_EGFIGF_bioRepB_ACK_FT.raw
x 10^4
Intensity
m/z

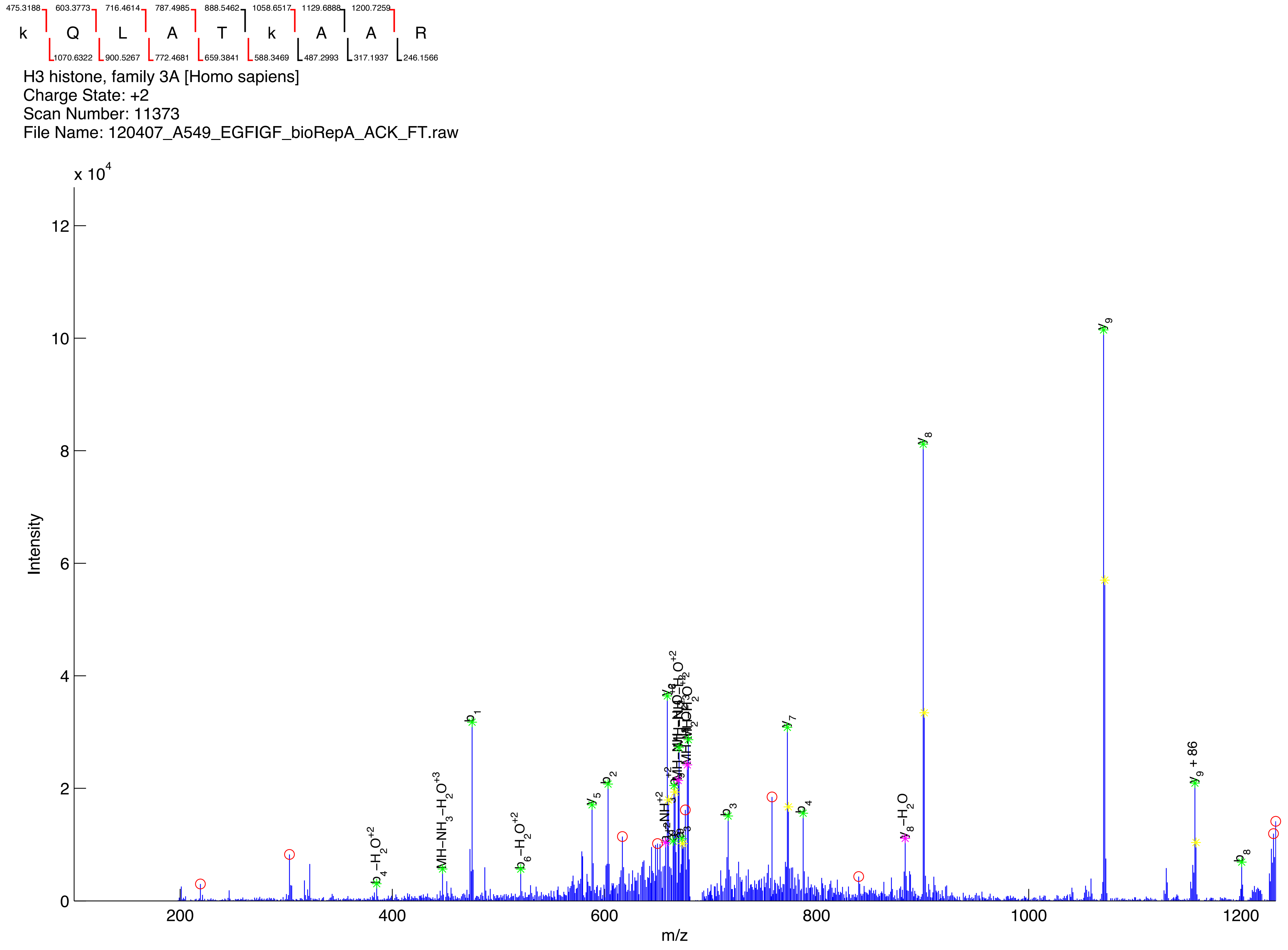

475.3188
603.3773
716.4614
787.4985
888.5462
1058.6517
1129.6888
1200.7259
k Q L A T k A A R
1070.6322
900.5267
772.4681
659.3841
588.3469
487.2993
317.1937
246.1566
H3 histone, family 3A [Homo sapiens]
Charge State: +2
Scan Number: 11373
File Name: 120407_A549_EGFIGF_bioRepA_ACK_FT.raw
x 10^4
Intensity
m/z
0
2
4
6
8
10
12
200
400
600
800
1000
1200
b4-H2O+2
MH-NH3-H2O+3
b1
b6-H2O+2
y5
b2
b3
y7
b4
y8-H2O
y8
y9
y9 + 86
b8

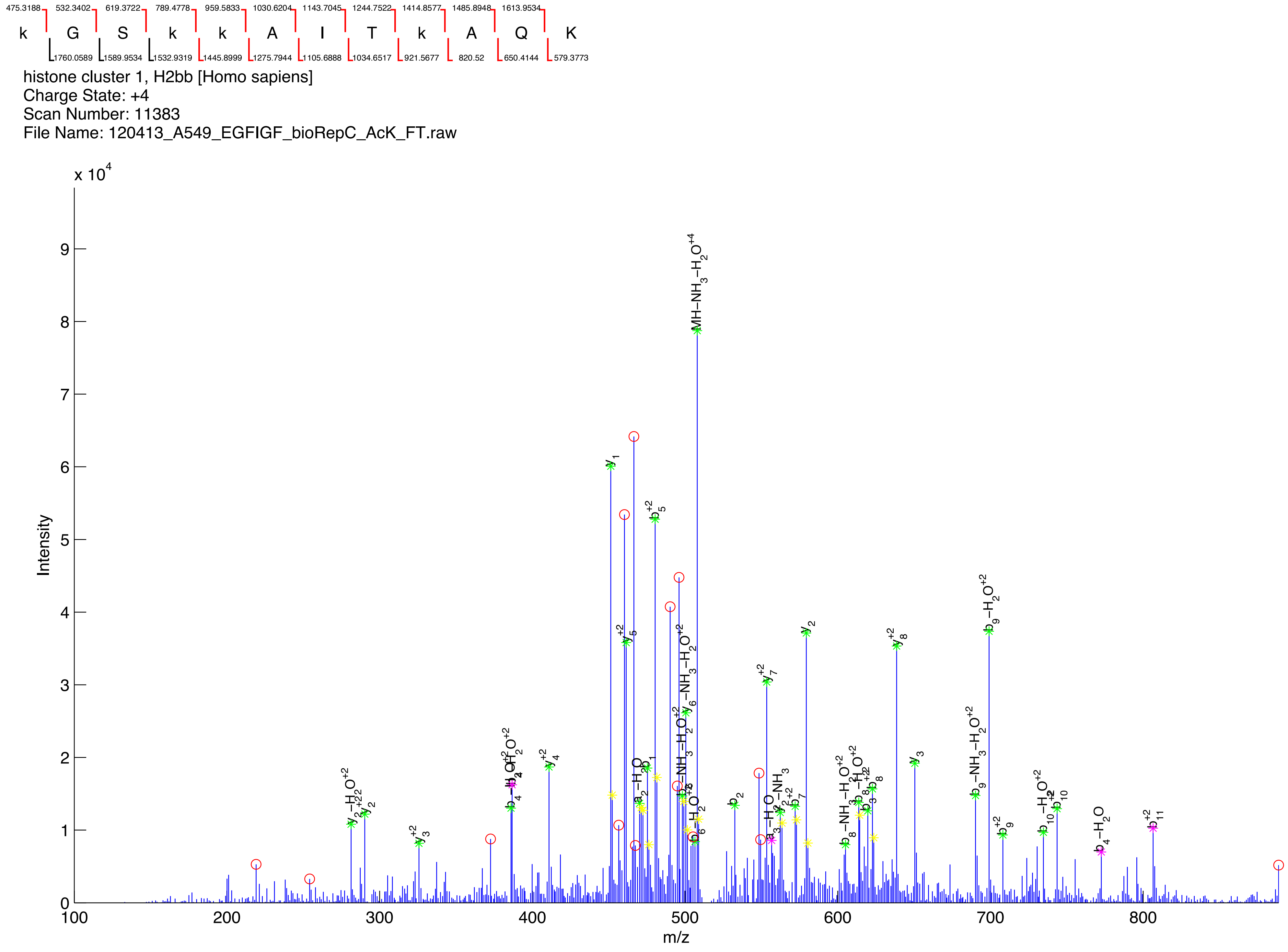

k G S k k A I T k A Q K
475.3188 532.3402 619.3722 789.4778 959.5833 1030.6204 1143.7045 1244.7522 1414.8577 1485.8948 1613.9534
1760.0589 1589.9534 1532.9319 1445.8999 1275.7944 1105.6888 1034.6517 921.5677 820.52 650.4144 579.3773
histone cluster 1, H2bb [Homo sapiens]
Charge State: +4
Scan Number: 11383
File Name: 120413_A549_EGFIGF_bioRepC_AcK_FT.raw
x 10^4
Intensity
m/z

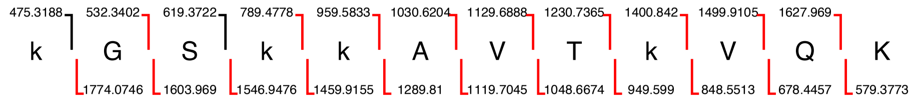

histone cluster 2, H2bf [Homo sapiens]
Charge State: +2
Scan Number: 11416
File Name: 120404_A549_EGFIGF_bioRepB_ACK_FT.raw

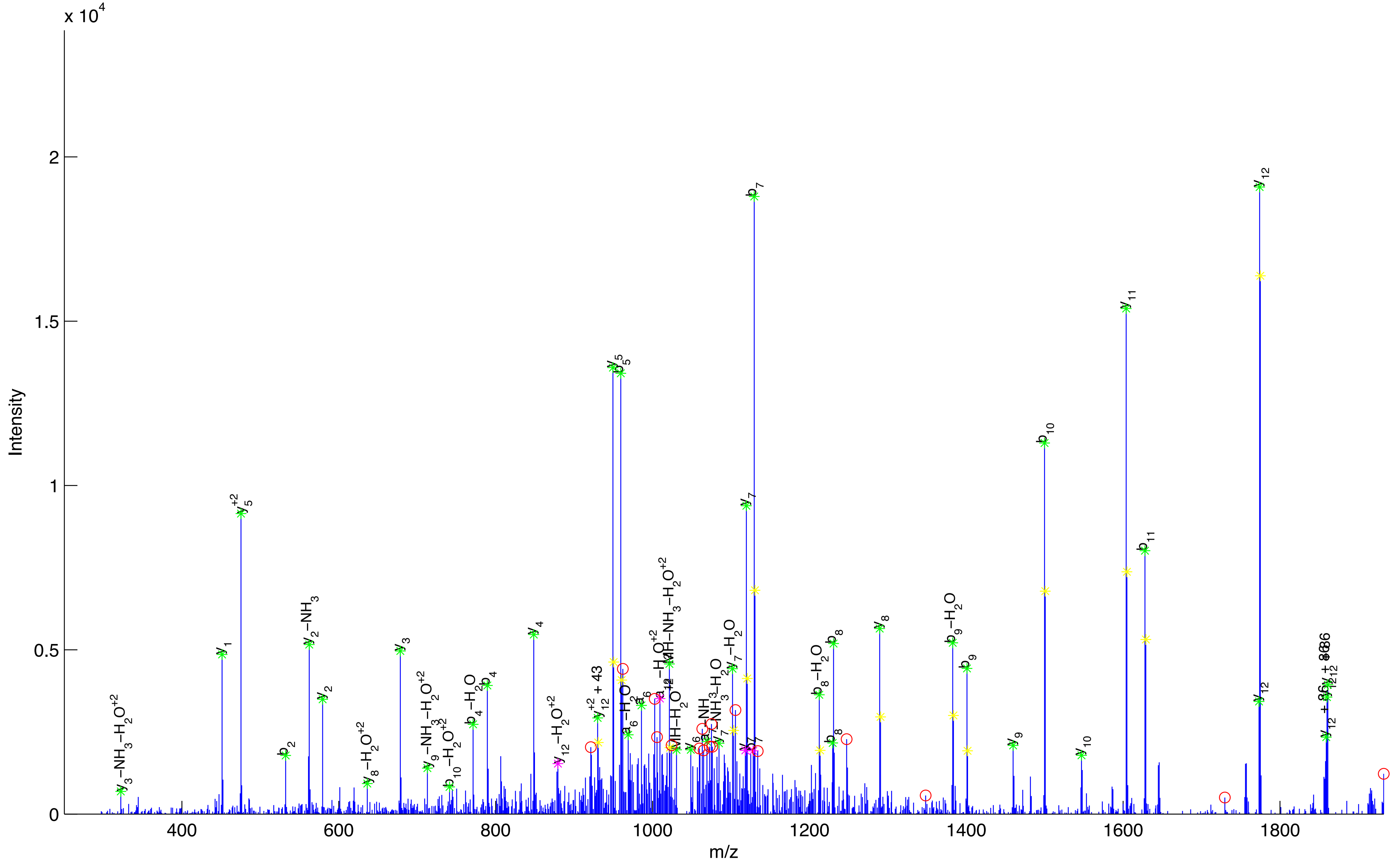

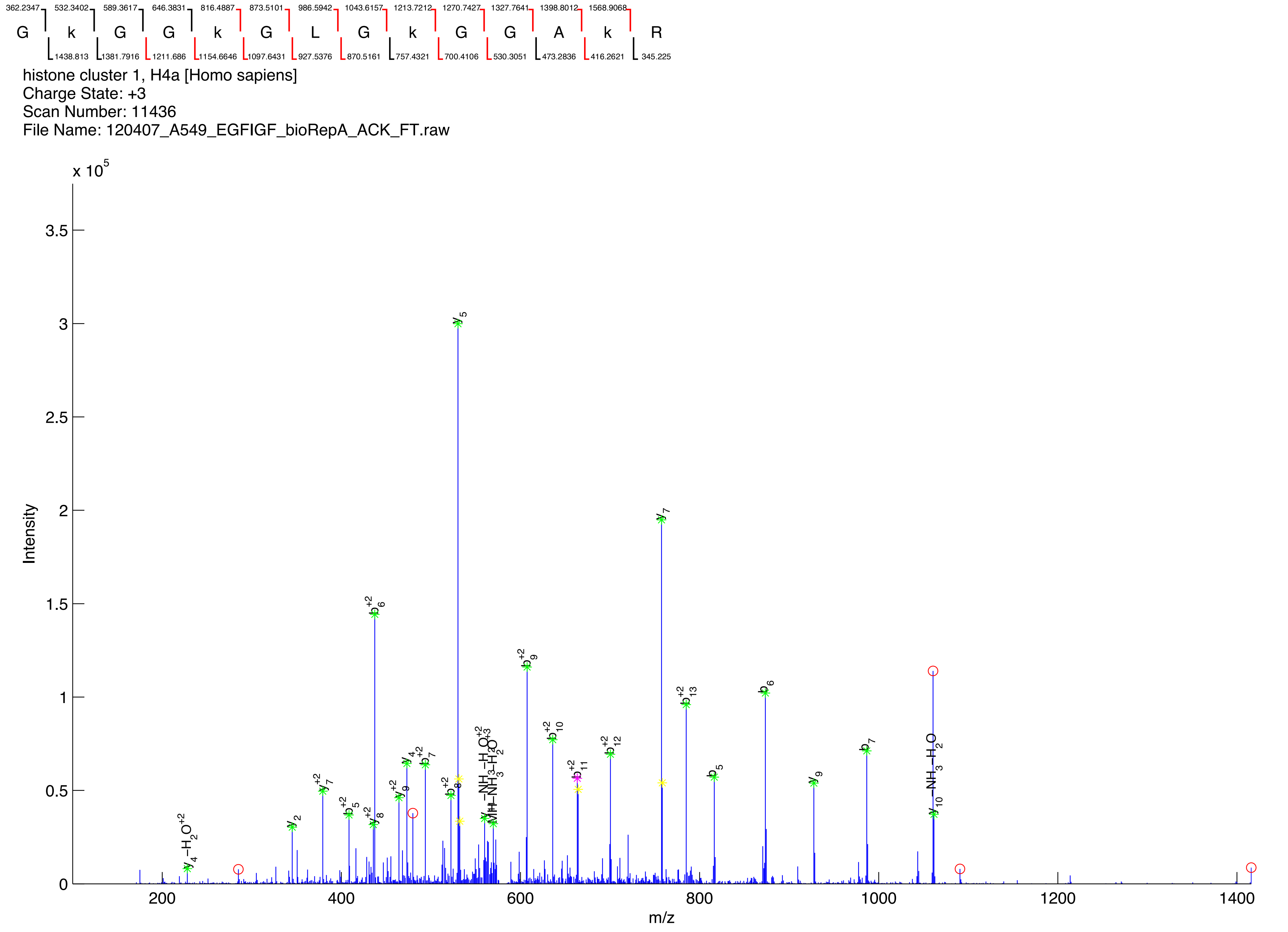

362.2347 532.3402 589.3617 646.3831 816.4887 873.5101 986.5942 1043.6157 1213.7212 1270.7427 1327.7641 1398.8012 1568.9068
G k G G k G L G k G G A k R
1438.813 1381.7916 1211.686 1154.6646 1097.6431 927.5376 870.5161 757.4321 700.4106 530.3051 473.2836 416.2621 345.225
histone cluster 1, H4a [Homo sapiens]
Charge State: +3
Scan Number: 11436
File Name: 120407_A549_EGFIGF_bioRepA_ACK_FT.raw
x 10^5
Intensity
m/z

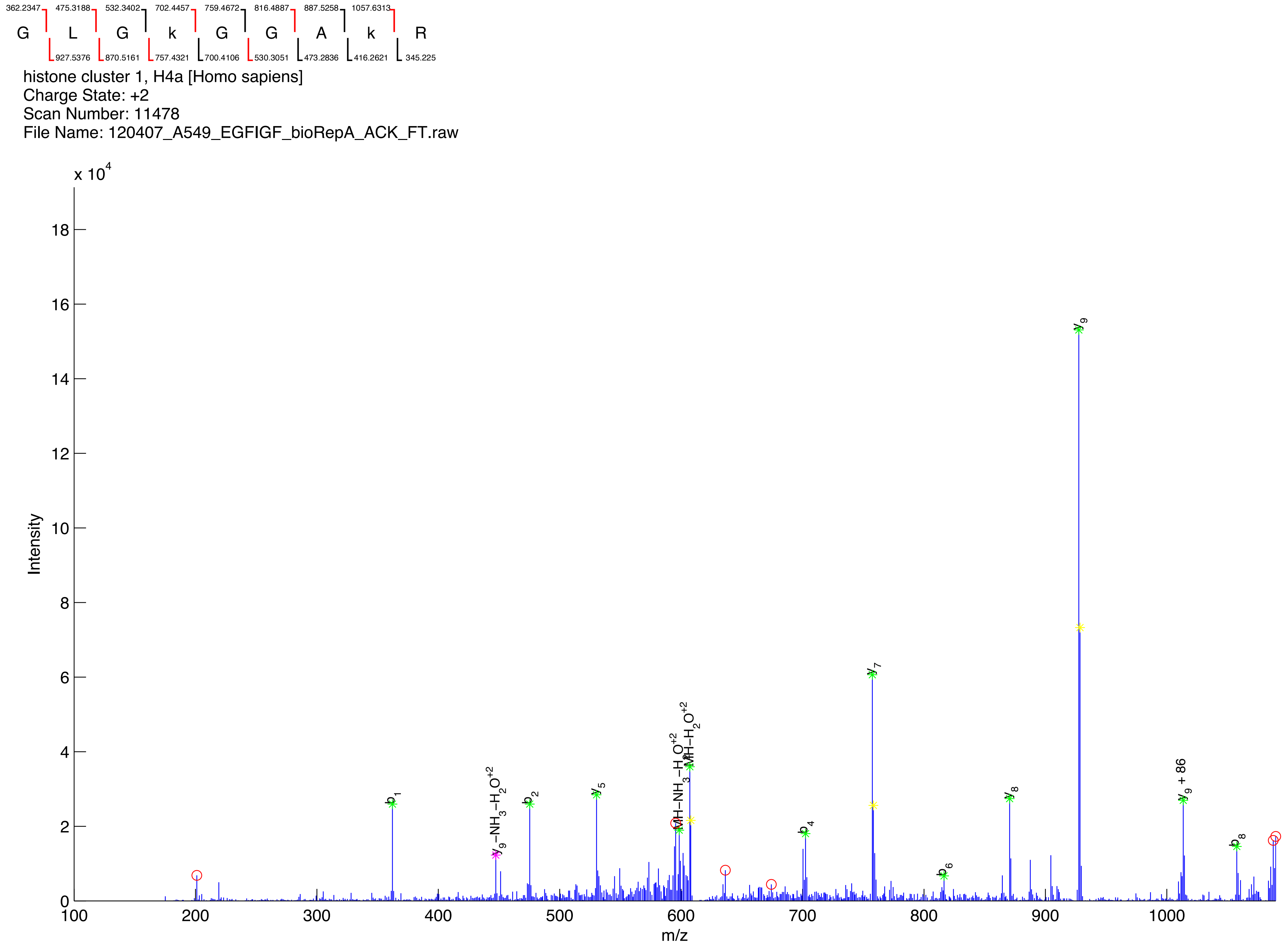

362.2347 475.3188 532.3402 702.4457 759.4672 816.4887 887.5258 1057.6313
G L G k G G A k R
927.5376 870.5161 757.4321 700.4106 530.3051 473.2836 416.2621 345.225
histone cluster 1, H4a [Homo sapiens]
Charge State: +2
Scan Number: 11478
File Name: 120407_A549_EGFIGF_bioRepA_ACK_FT.raw
x 10^4
Intensity
m/z
b1
y9−NH3−H2O+2
b2
y5
MH−NH3−H2O+2
MH−H2O+2
b4
y7
b6
y8
y9
y9 + 86
b8

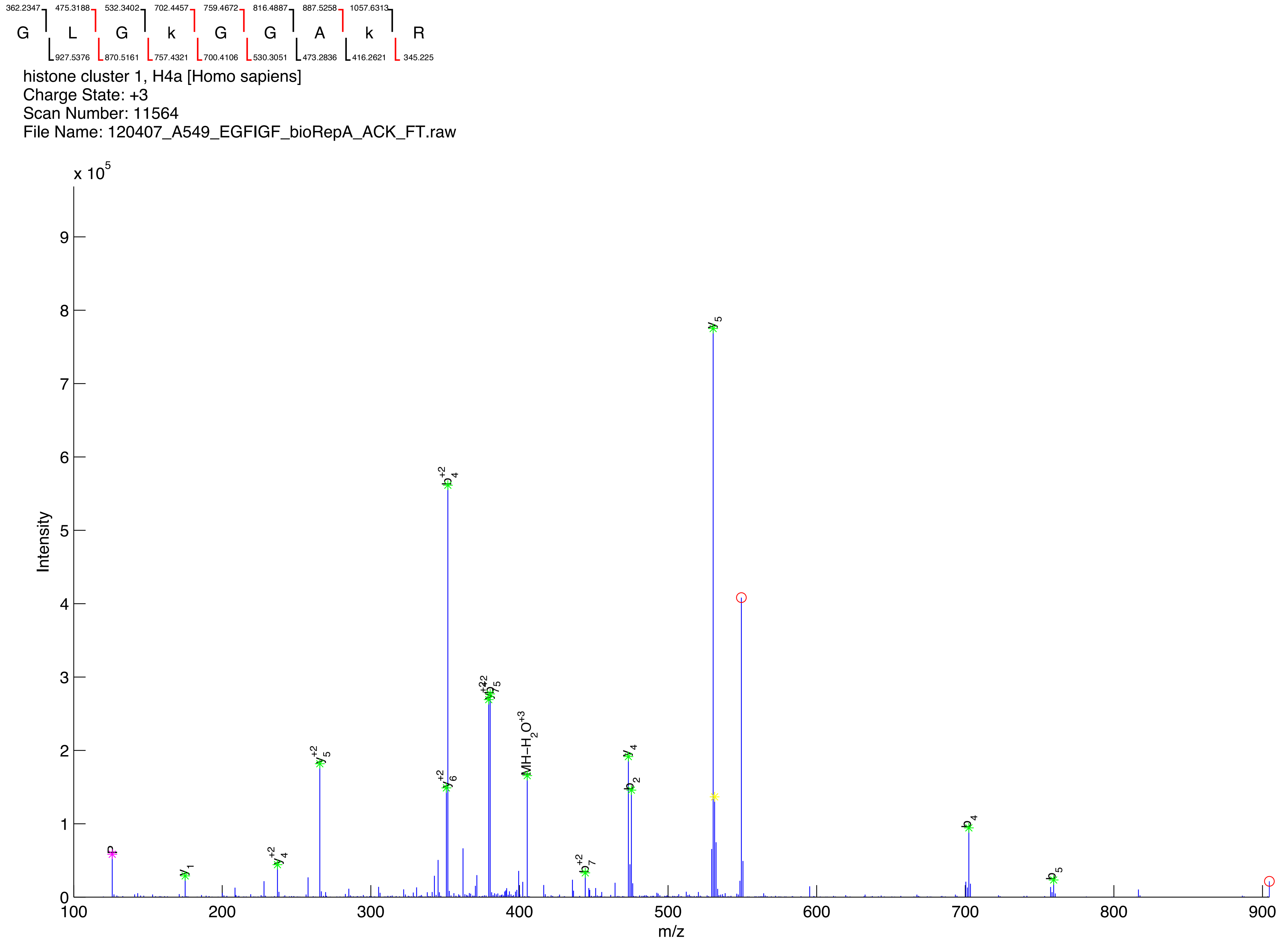

362.2347 475.3188 532.3402 702.4457 759.4672 816.4887 887.5258 1057.6313
G L G k G G A k R
927.5376 870.5161 757.4321 700.4106 530.3051 473.2836 416.2621 345.225
histone cluster 1, H4a [Homo sapiens]
Charge State: +3
Scan Number: 11564
File Name: 120407_A549_EGFIGF_bioRepA_ACK_FT.raw
x 10^5
Intensity
m/z
P
y1
y4+2
y5+2
y6+2
b4+2
y5
MH-H2O+3
b7+2
y4
b2
b4
b5

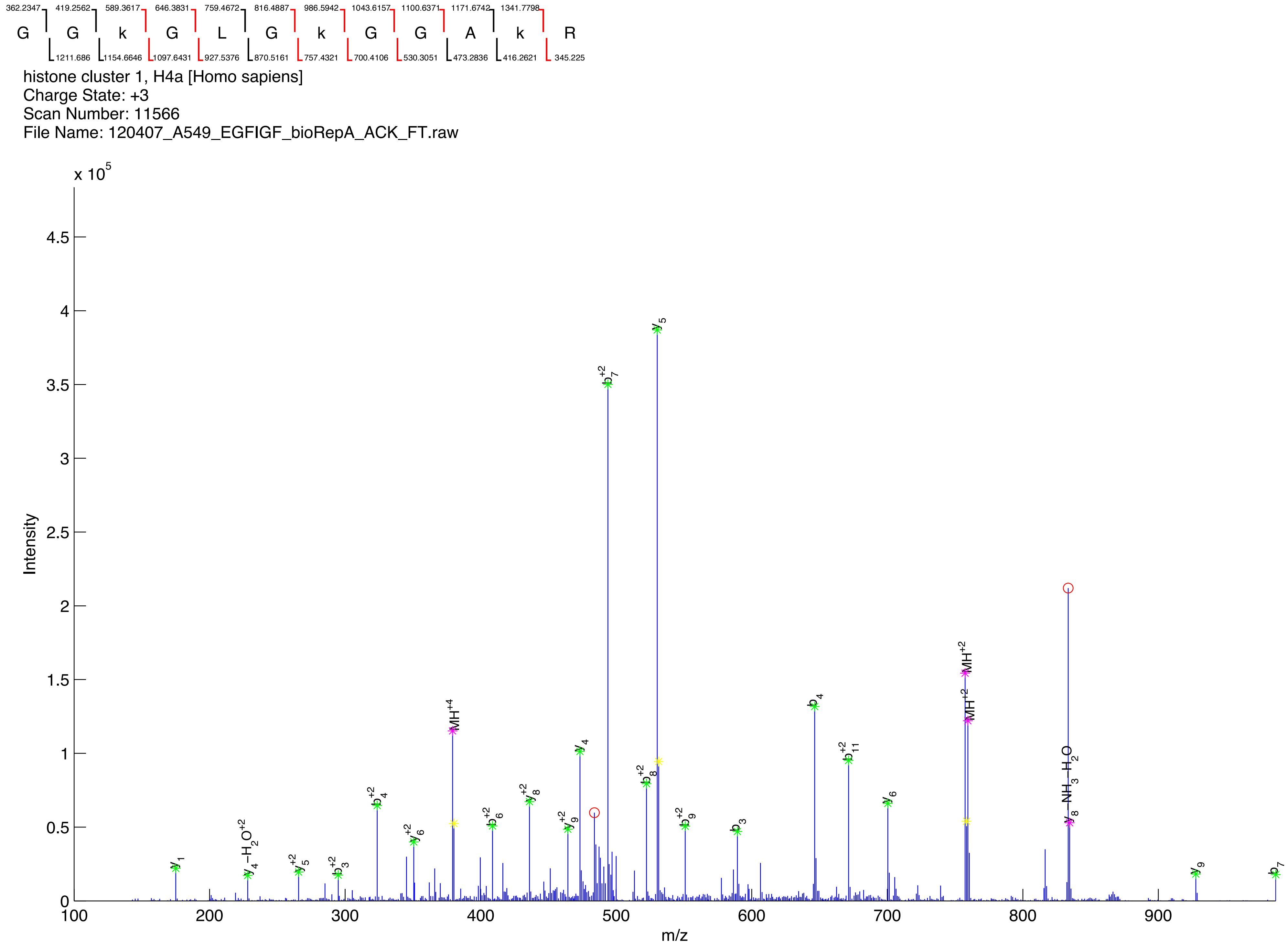
362.2347 419.2562 589.3617 646.3831 759.4672 816.4887 986.5942 1043.6157 1100.6371 1171.6742 1341.7798
G G k G L G k G G A k R
1211.686 1154.6646 1097.6431 927.5376 870.5161 757.4321 700.4106 530.3051 473.2836 416.2621 345.225
histone cluster 1, H4a [Homo sapiens]
Charge State: +3
Scan Number: 11566
File Name: 120407_A549_EGFIGF_bioRepA_ACK_FT.raw
x 10^5
Intensity
m/z

475.3188 532.3402 619.3722 789.4778 959.5833 1030.6204 1129.6888 1230.7365 1400.842 1499.9105 1627.969

k G S k k A V T k V Q K

1774.0746 1603.969 1546.9476 1459.9155 1289.81 1119.7045 1048.6674 949.599 848.5513 678.4457 579.3773

histone cluster 2, H2bf [Homo sapiens]
Charge State: +4
Scan Number: 11572
File Name: 120413_A549_EGFIGF_bioRepC_AcK_FT.raw

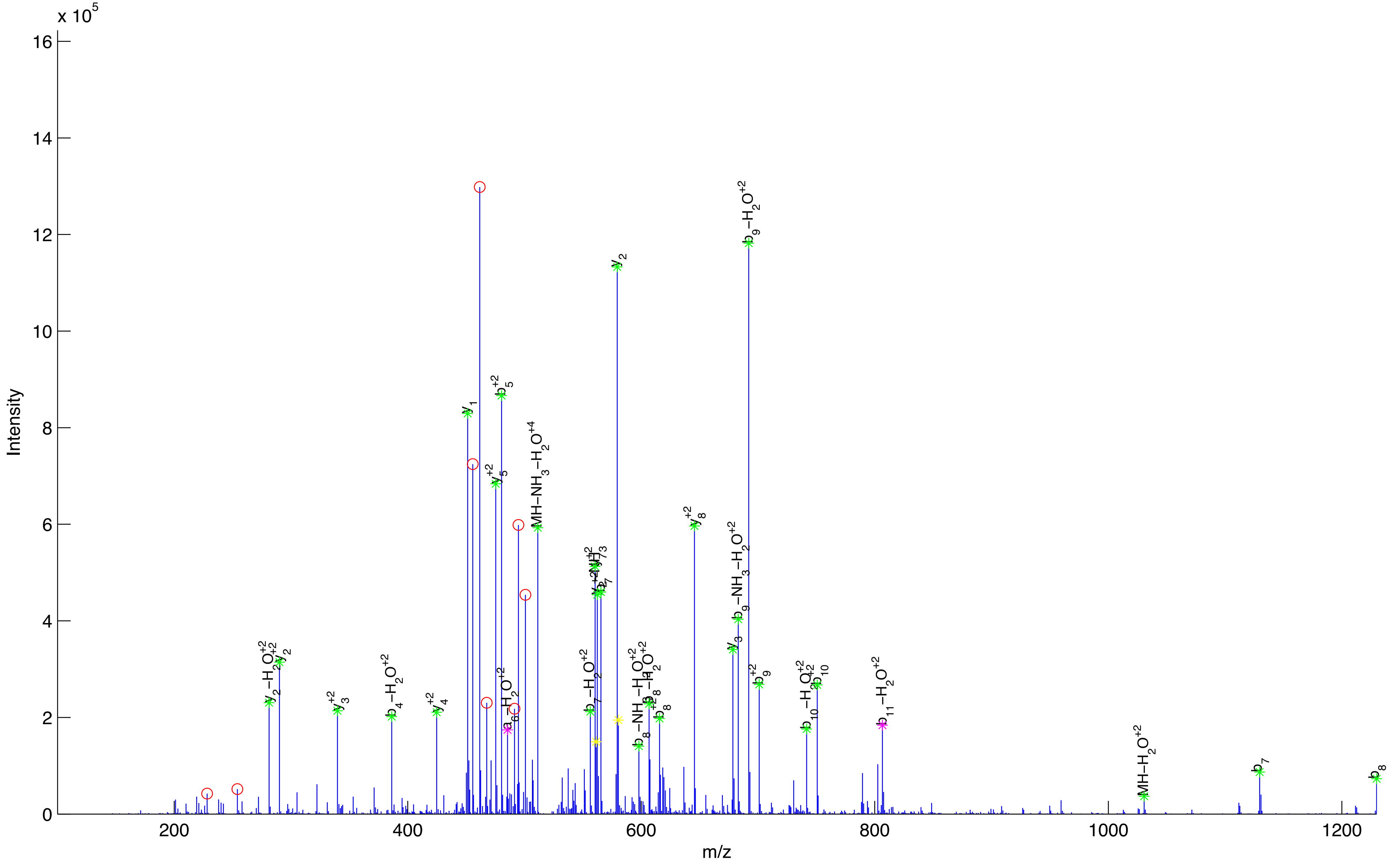

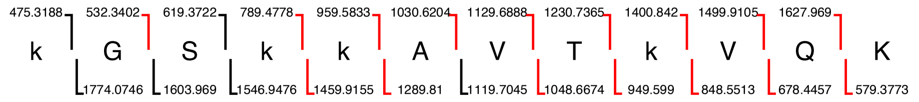

histone cluster 2, H2bf [Homo sapiens]
Charge State: +4
Scan Number: 11584
File Name: 120404_A549_EGFIGF_bioRepB_ACK_FT.raw

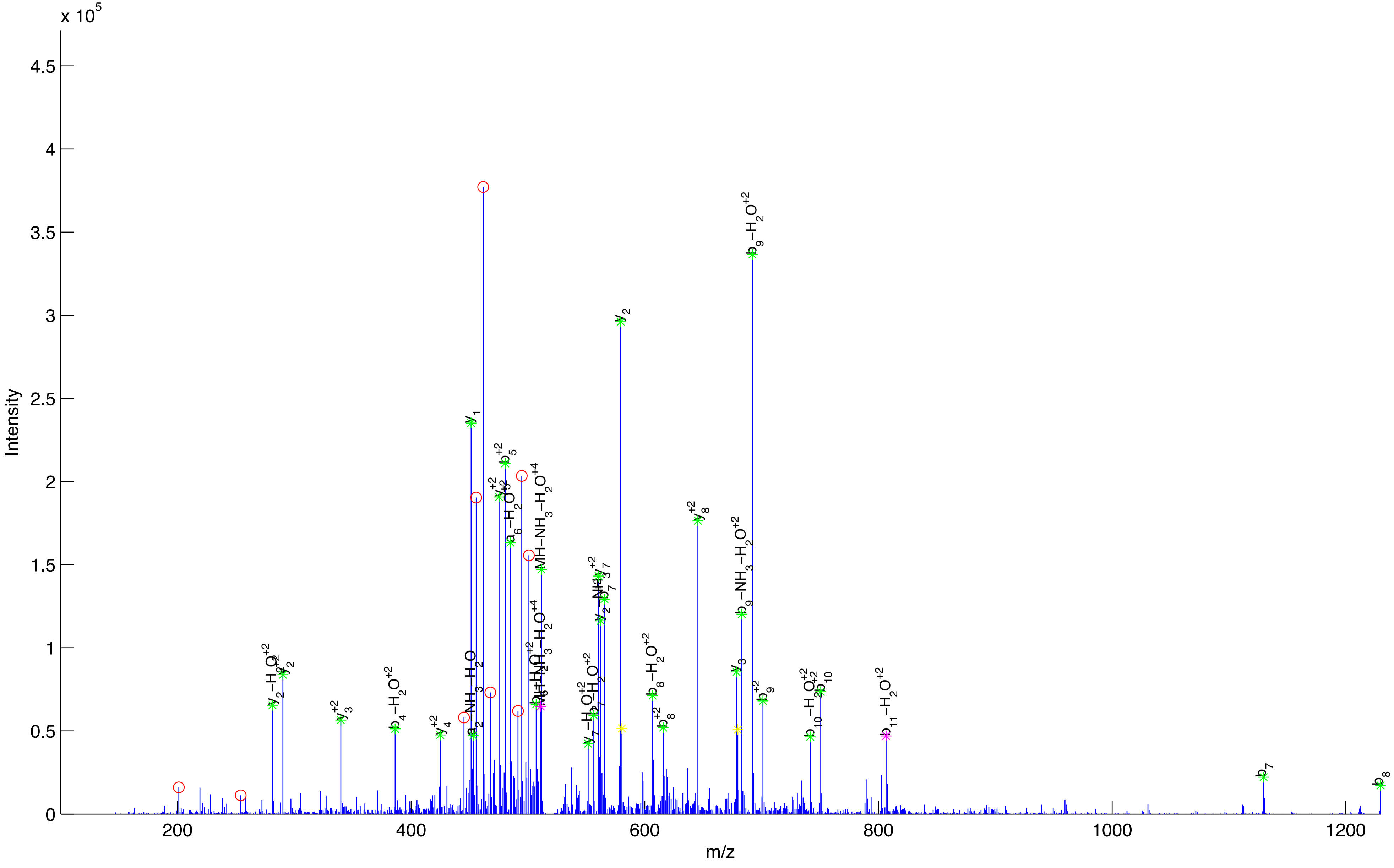

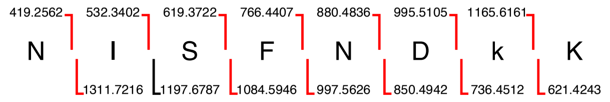

moesin [Homo sapiens]
Charge State: +3
Scan Number: 11712
File Name: 120404_A549_EGFIGF_bioRepB_ACK_FT.raw

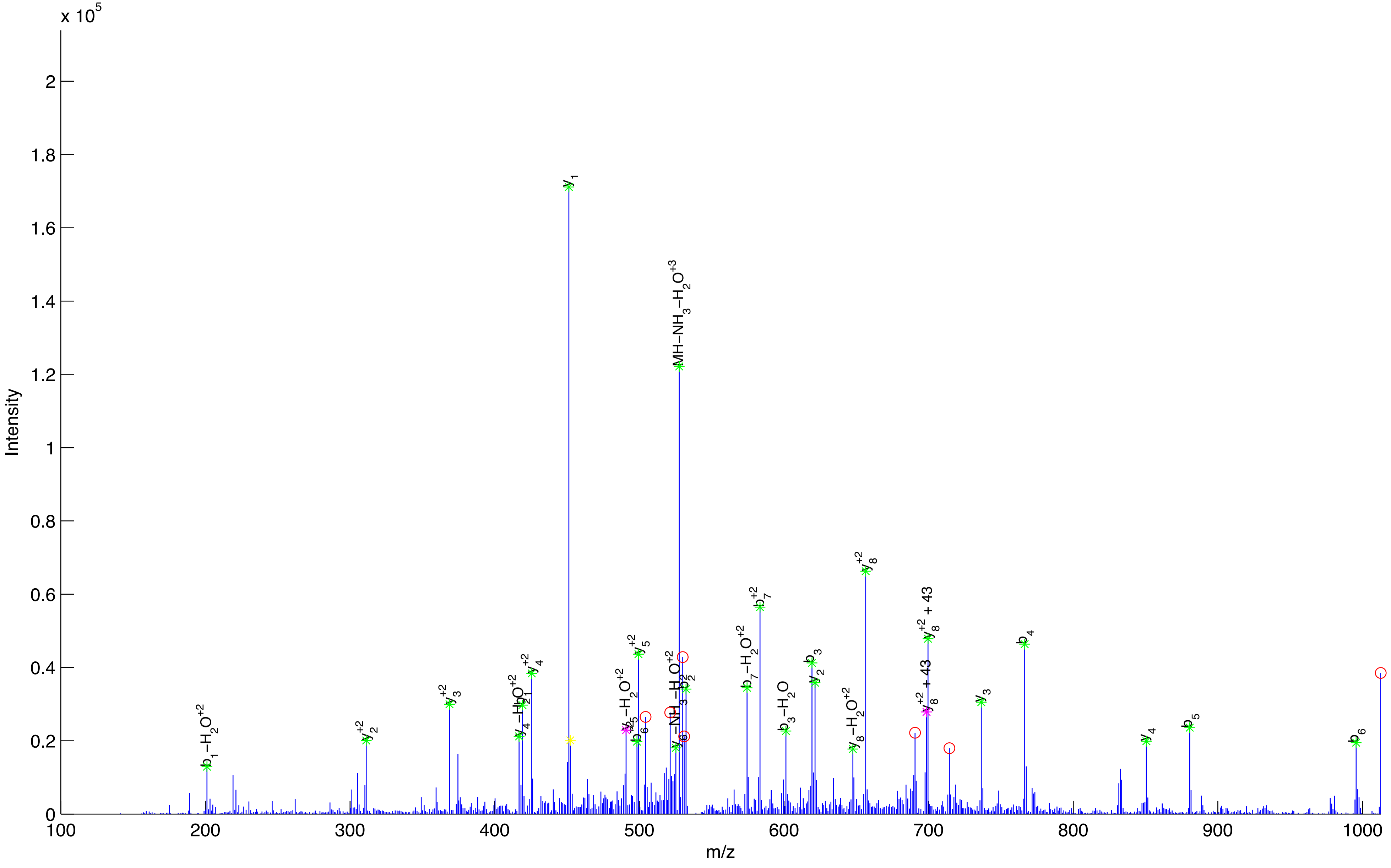

362.2347	532.3402	589.3617	646.3831	816.4887	873.5101	986.5942	1043.6157	1213.7212	1270.7427	1327.7641	1398.8012	1568.9068	
G	k	G	G	k	G	L	G	k	G	G	A	k	R
	1438.813	1381.7916	1211.686	1154.6646	1097.6431	927.5376	870.5161	757.4321	700.4106	530.3051	473.2836	416.2621	345.225

histone cluster 1, H4a [Homo sapiens]
Charge State: +3
Scan Number: 11982
File Name: 120407_A549_EGFIGF_bioRepA_ACK_FT.raw

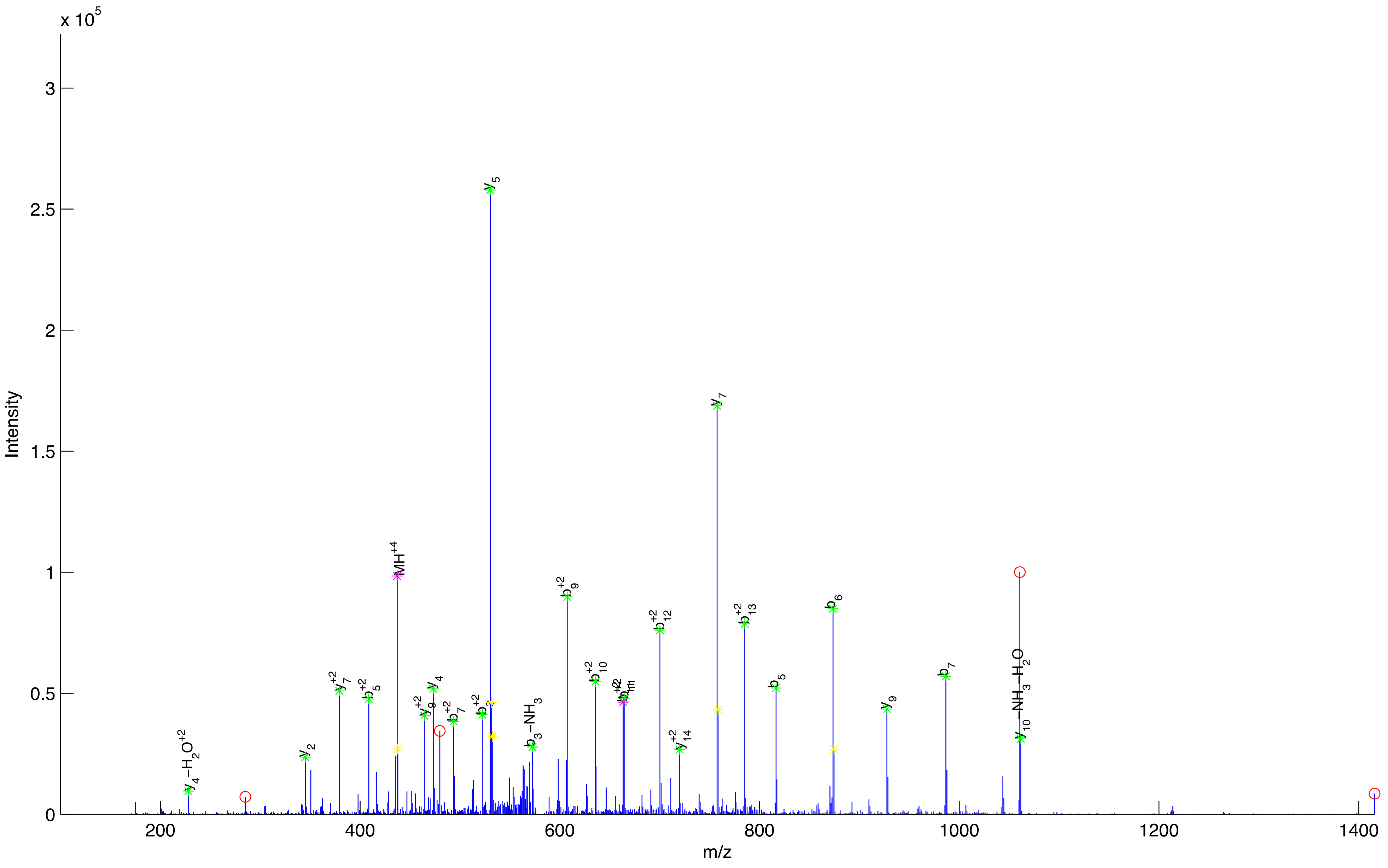

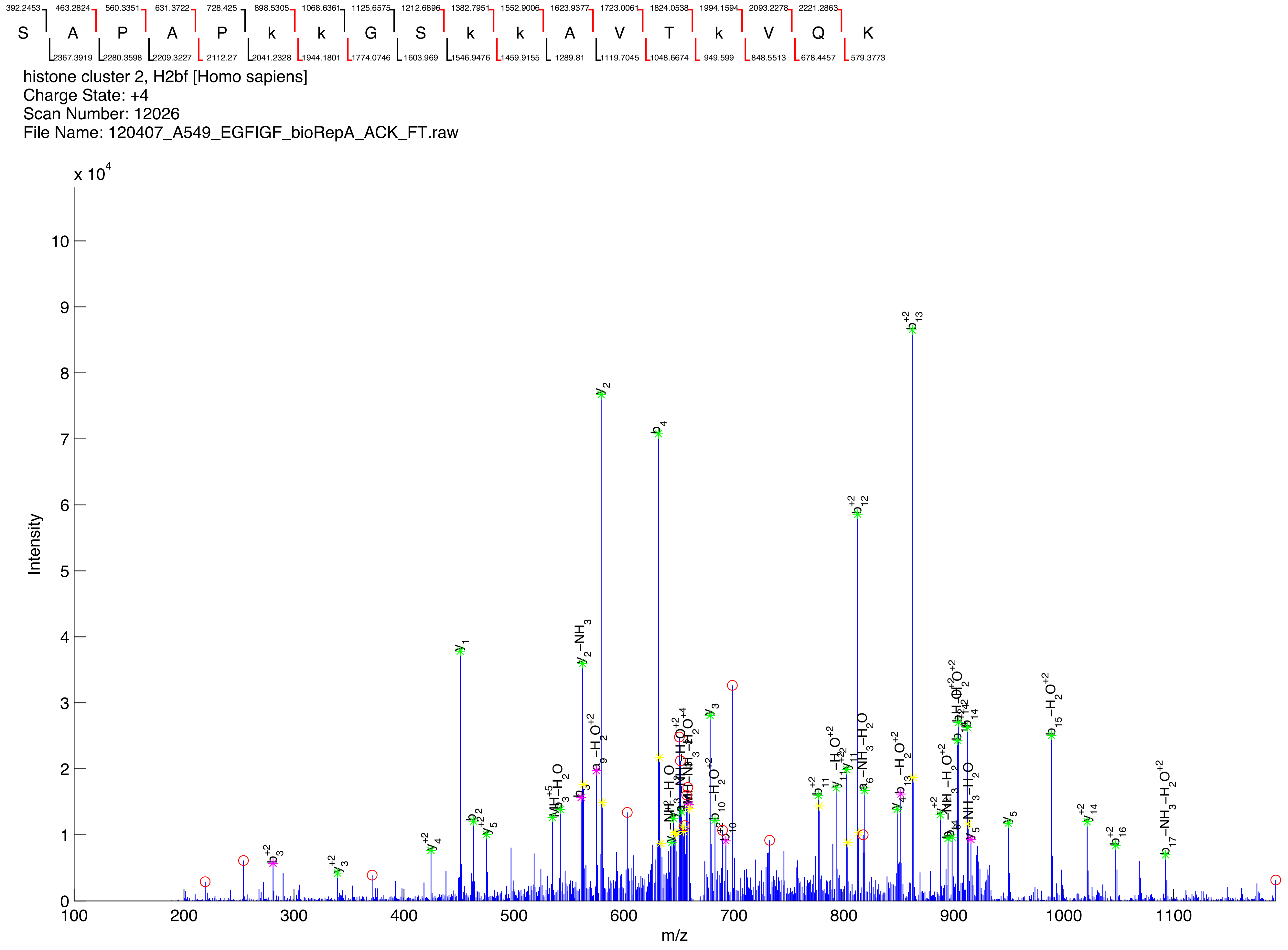

392.2453 463.2824 560.3351 631.3722 728.425 898.5305 1068.6361 1125.6575 1212.6896 1382.7951 1552.9006 1623.9377 1723.0061 1824.0538 1994.1594 2093.2278 2221.2863
S A P A P k k G S k k A V T k V Q K
2367.3919 2280.3598 2209.3227 2112.27 2041.2328 1944.1801 1774.0746 1603.969 1546.9476 1459.9155 1289.81 1119.7045 1048.6674 949.599 848.5513 678.4457 579.3773
histone cluster 2, H2bf [Homo sapiens]
Charge State: +4
Scan Number: 12026
File Name: 120407_A549_EGFIGF_bioRepA_ACK_FT.raw
x 10^4
Intensity
m/z

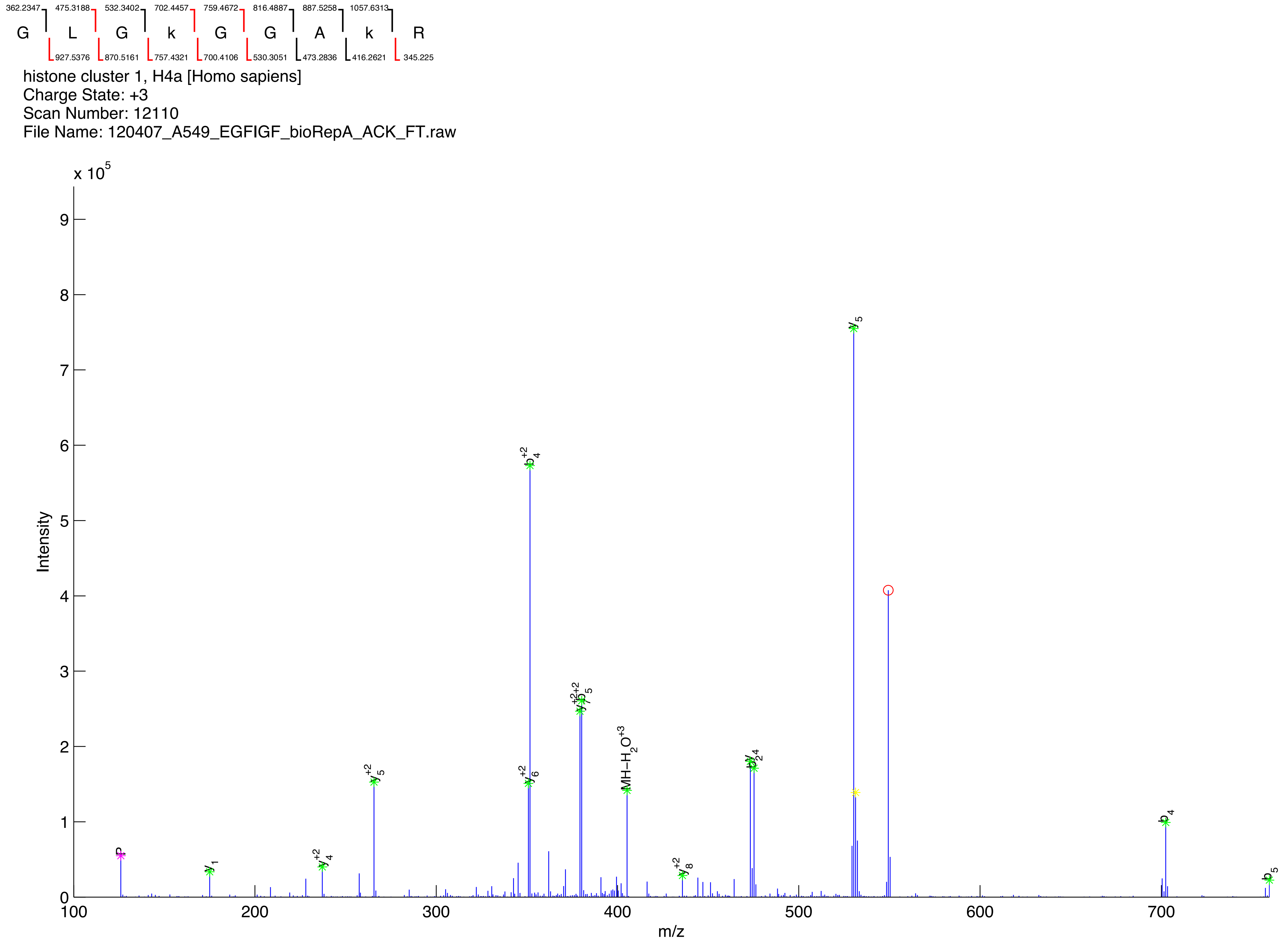
362.2347
475.3188
532.3402
702.4457
759.4672
816.4887
887.5258
1057.6313
G L G k G G A k R
927.5376
870.5161
757.4321
700.4106
530.3051
473.2836
416.2621
345.225
histone cluster 1, H4a [Homo sapiens]
Charge State: +3
Scan Number: 12110
File Name: 120407_A549_EGFIGF_bioRepA_ACK_FT.raw
x 10^5
Intensity
m/z

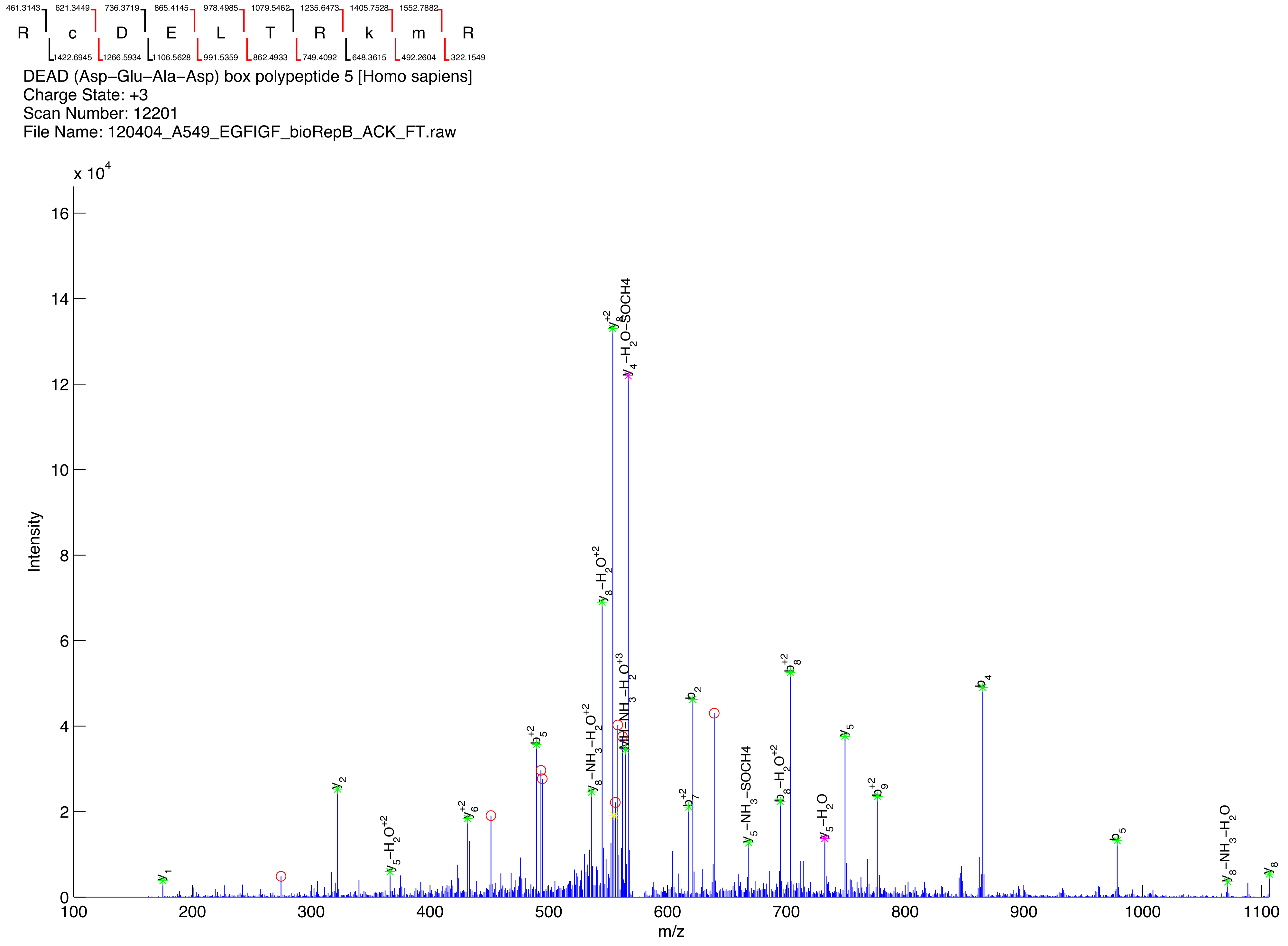
461.3143 621.3449 736.3719 865.4145 978.4985 1079.5462 1235.6473 1405.7528 1552.7882
R c D E L T R k m R
1422.6945 1266.5934 1106.5628 991.5359 862.4933 749.4092 648.3615 492.2604 322.1549
DEAD (Asp−Glu−Ala−Asp) box polypeptide 5 [Homo sapiens]
Charge State: +3
Scan Number: 12201
File Name: 120404_A549_EGFIGF_bioRepB_ACK_FT.raw
x 10^4
Intensity
m/z
y1
y2
y5−H2O+2
y6+2
b5+2
y8−NH3−H2O+2
y8−H2O+2
y8+2
MH−NH3−H2O+3
y4−H2O−SOCH4
b7+2
b2
y5−NH3−SOCH4
b8−H2O+2
b8+2
y5−H2O
y5
b9+2
b4
b5
y8−NH3−H2O
y8

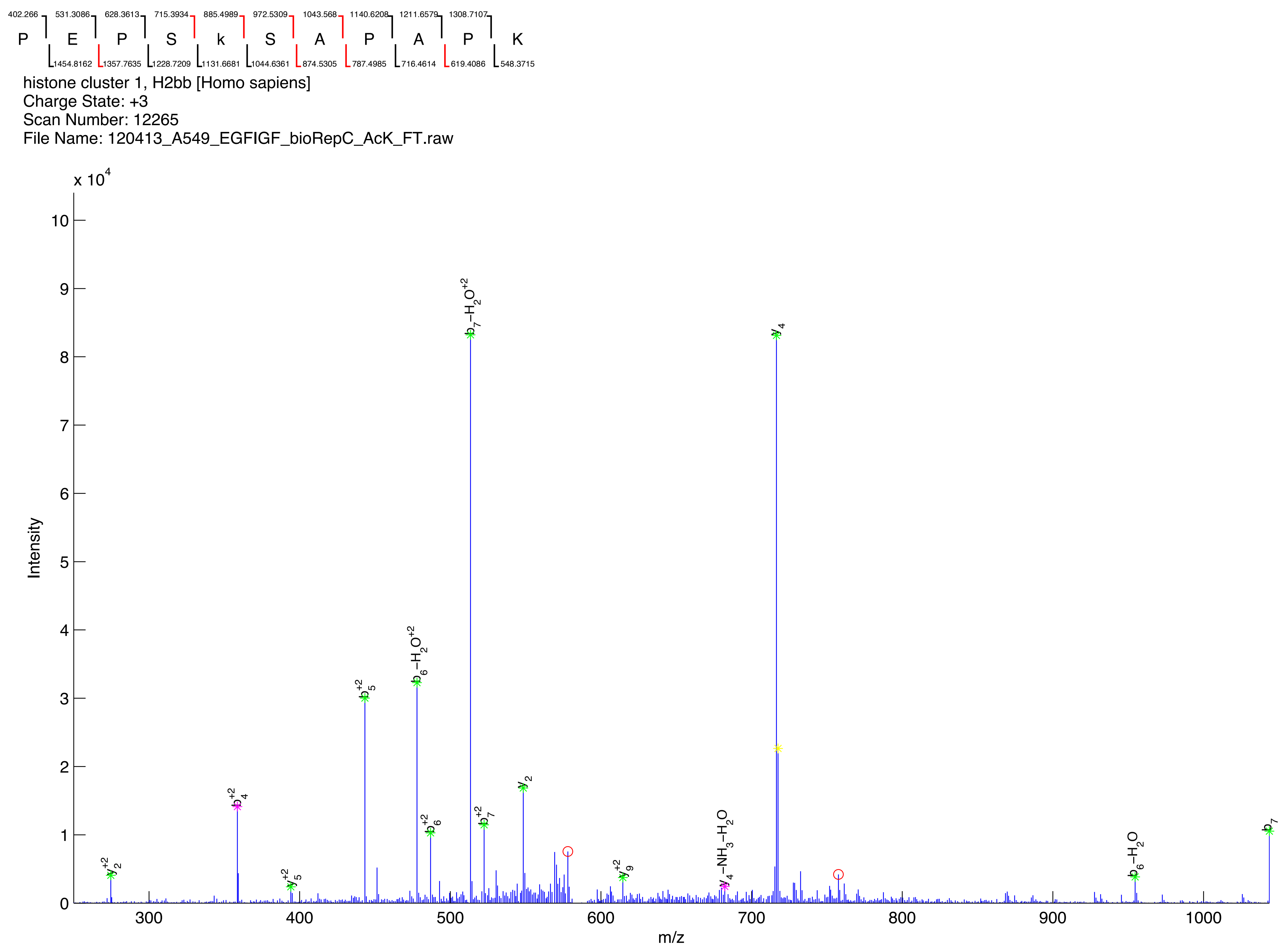

402.266 531.3086 628.3613 715.3934 885.4989 972.5309 1043.568 1140.6208 1211.6579 1308.7107
P E P S k S A P A P K
1454.8162 1357.7635 1228.7209 1131.6681 1044.6361 874.5305 787.4985 716.4614 619.4086 548.3715
histone cluster 1, H2bb [Homo sapiens]
Charge State: +3
Scan Number: 12265
File Name: 120413_A549_EGFIGF_bioRepC_AcK_FT.raw
x 10^4
Intensity
m/z

H3 histone, family 3A [Homo sapiens]
Charge State: +2
Scan Number: 12465
File Name: 120407_A549_EGFIGF_bioRepA_ACK_FT.raw

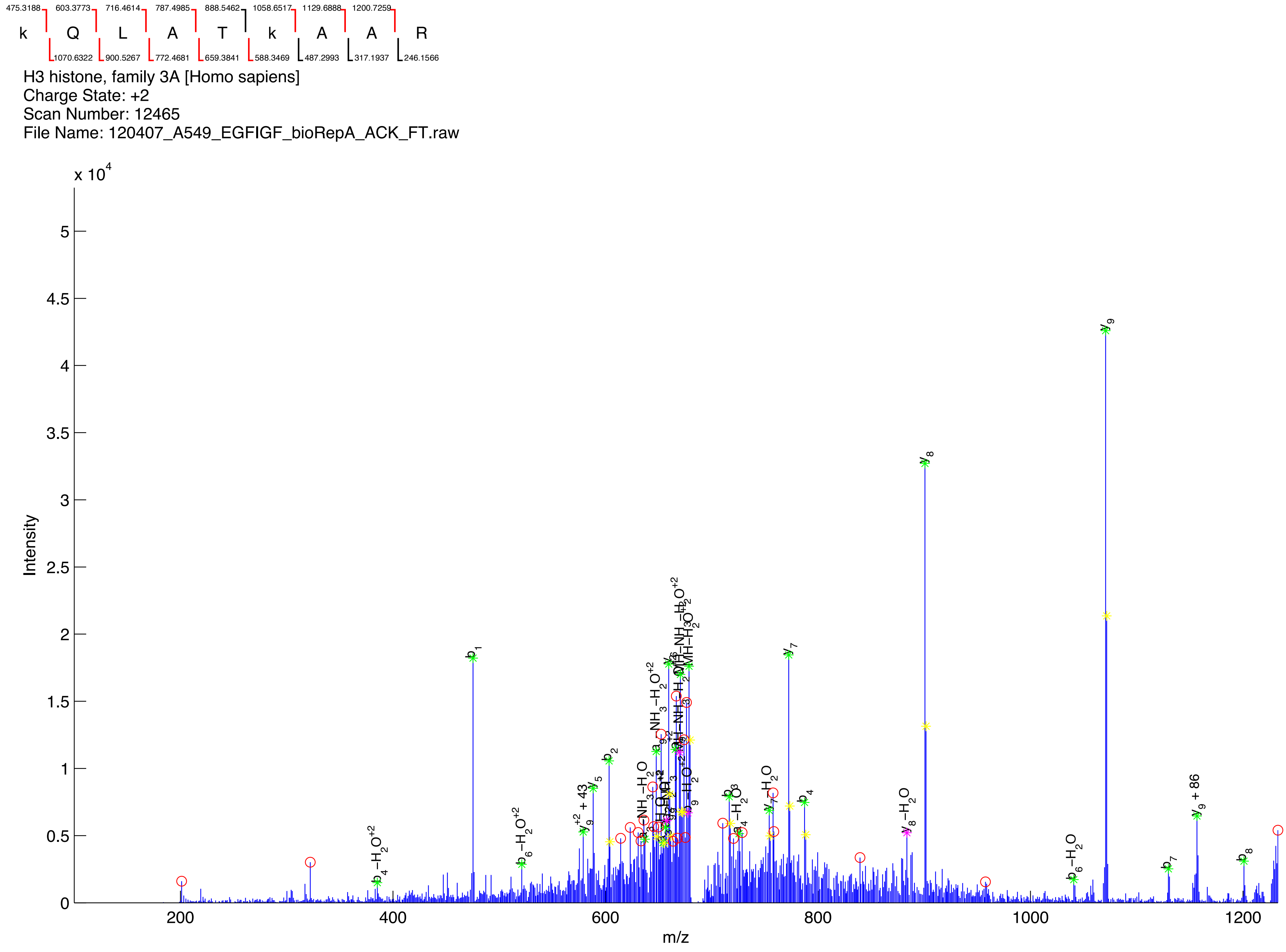

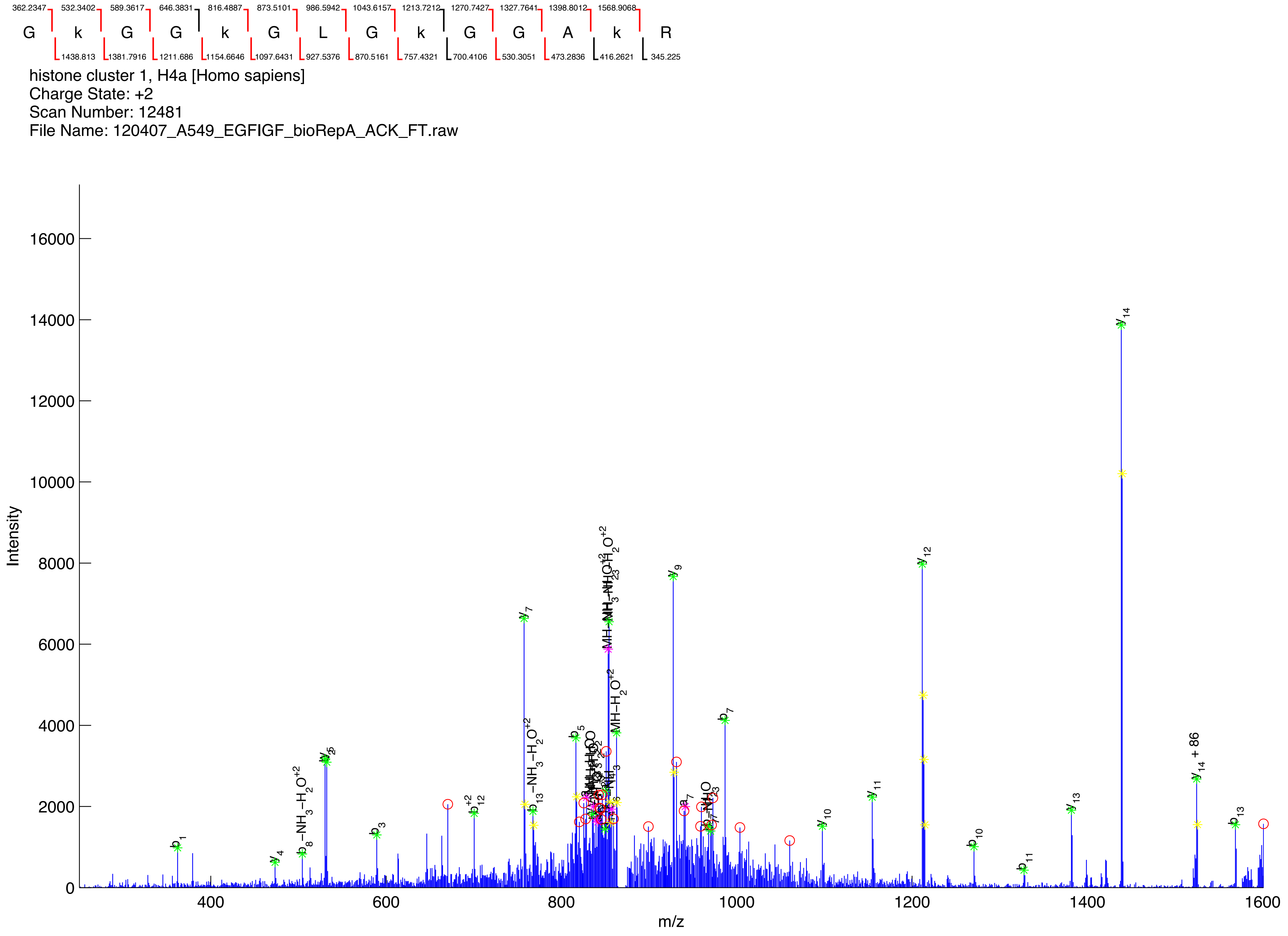

362.2347
532.3402
589.3617
646.3831
816.4887
873.5101
986.5942
1043.6157
1213.7212
1270.7427
1327.7641
1398.8012
1568.9068
G k G G k G L G k G G A k R
1438.813
1381.7916
1211.686
1154.6646
1097.6431
927.5376
870.5161
757.4321
700.4106
530.3051
473.2836
416.2621
345.225
histone cluster 1, H4a [Homo sapiens]
Charge State: +2
Scan Number: 12481
File Name: 120407_A549_EGFIGF_bioRepA_ACK_FT.raw
Intensity
m/z
0
2000
4000
6000
8000
10000
12000
14000
16000
400
600
800
1000
1200
1400
1600
b1
y4
b8-NH3-H2O+2
y5
b3
b12+2
y7
b13-NH3-H2O+2
b5
MH-H2O+2
y9
a7
b7
y10
y11
y12
b10
b11
y13
y14
y14 + 86
b13

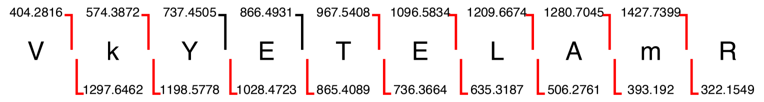

keratin 18 [Homo sapiens]
Charge State: +3
Scan Number: 12490
File Name: 120407_A549_EGFIGF_bioRepA_ACK_FT.raw

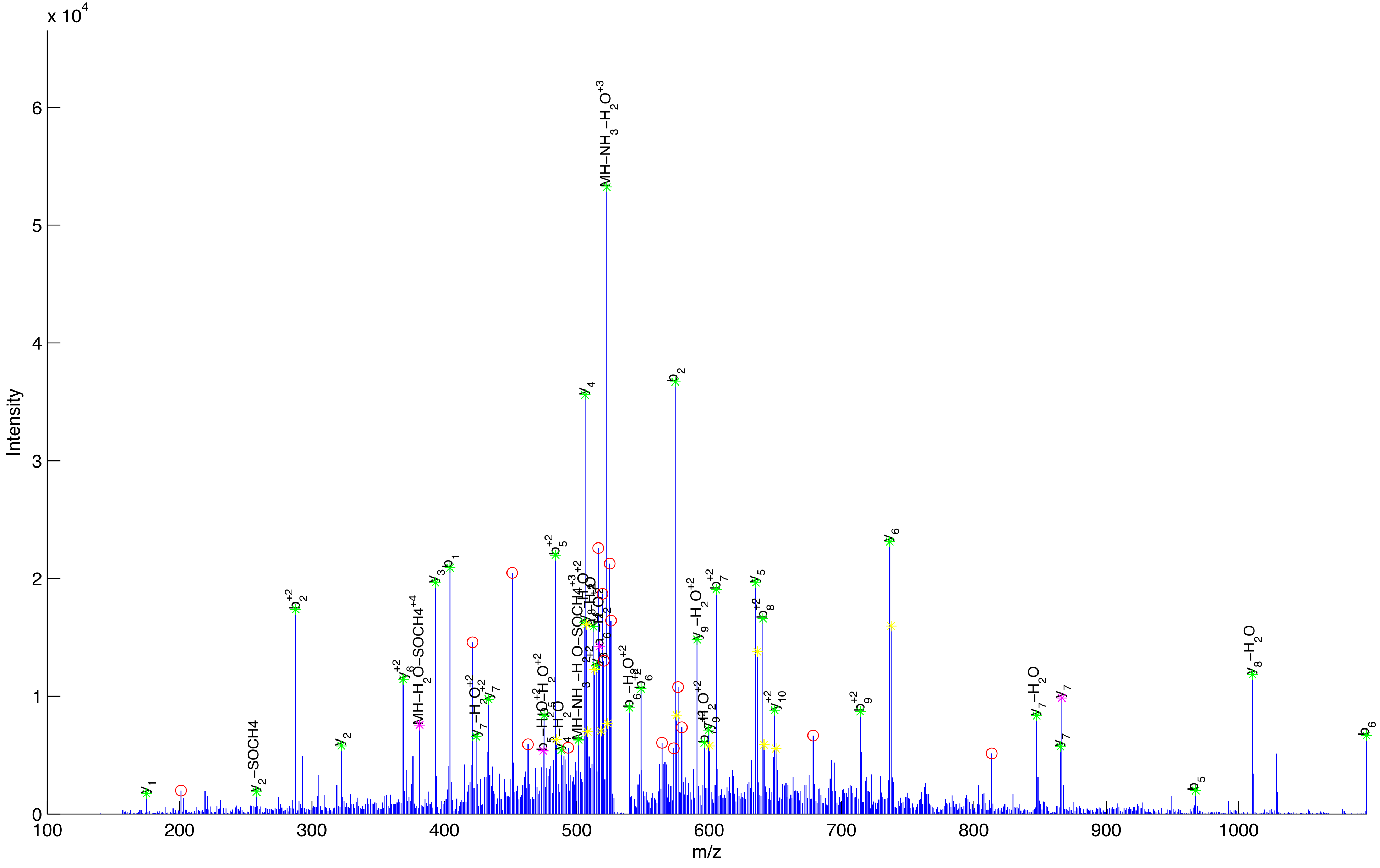

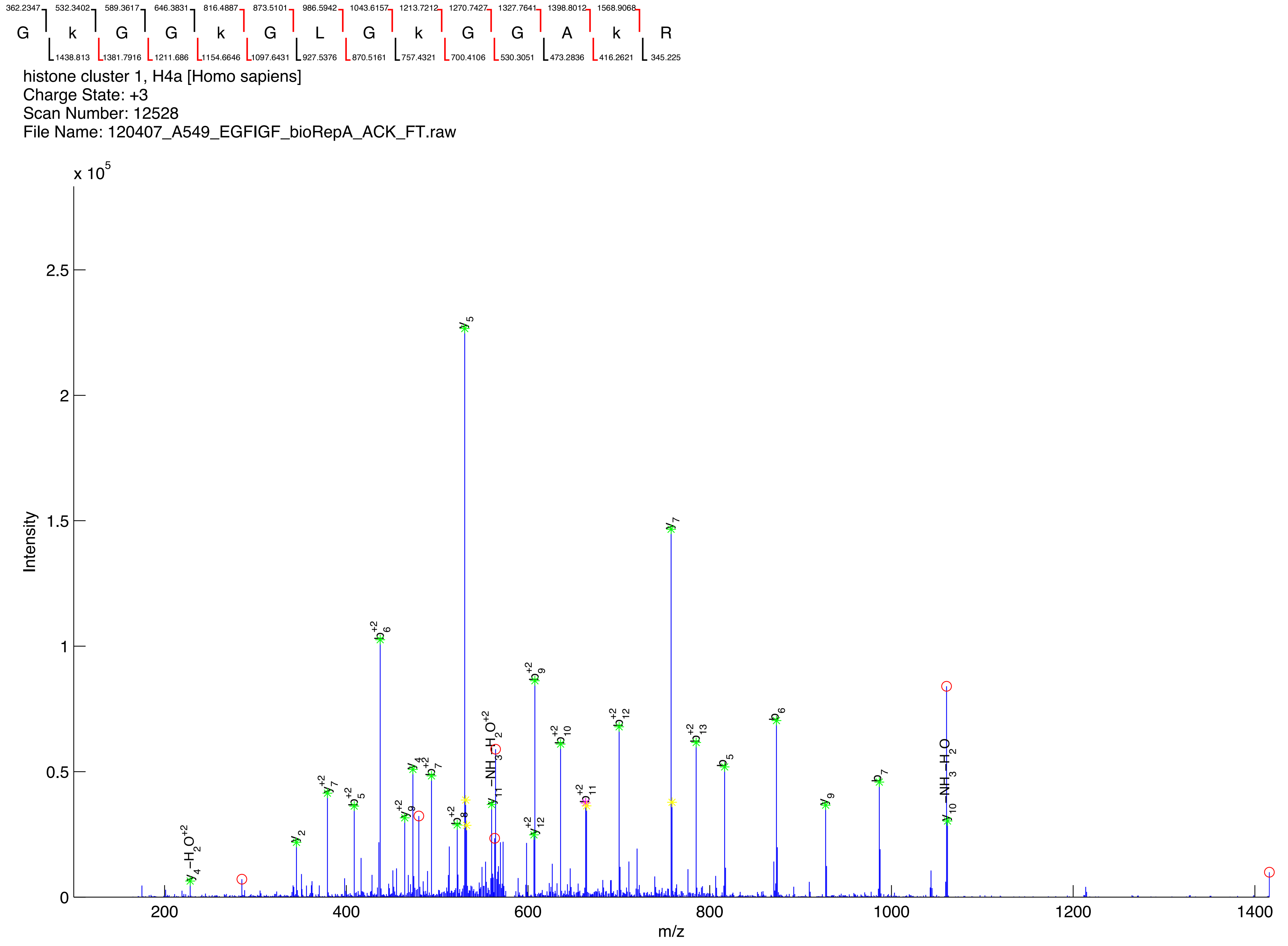
362.2347 532.3402 589.3617 646.3831 816.4887 873.5101 986.5942 1043.6157 1213.7212 1270.7427 1327.7641 1398.8012 1568.9068
G k G G k G L G k G G A k R
1438.813 1381.7916 1211.686 1154.6646 1097.6431 927.5376 870.5161 757.4321 700.4106 530.3051 473.2836 416.2621 345.225
histone cluster 1, H4a [Homo sapiens]
Charge State: +3
Scan Number: 12528
File Name: 120407_A549_EGFIGF_bioRepA_ACK_FT.raw
x 10^5
Intensity
m/z
0
0.5
1
1.5
2
2.5
200
400
600
800
1000
1200
1400

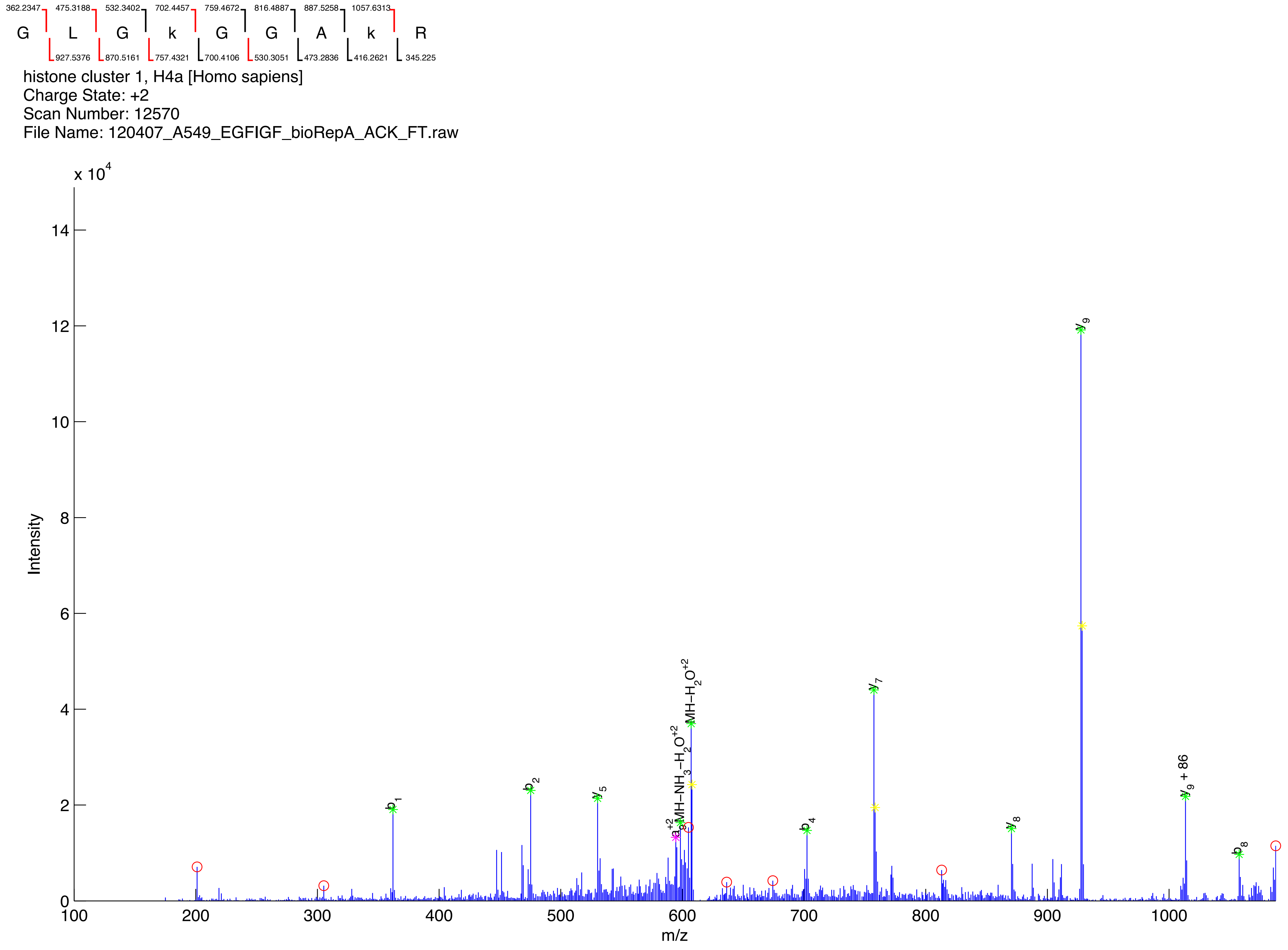

362.2347 475.3188 532.3402 702.4457 759.4672 816.4887 887.5258 1057.6313
G L G k G G A k R
927.5376 870.5161 757.4321 700.4106 530.3051 473.2836 416.2621 345.225
histone cluster 1, H4a [Homo sapiens]
Charge State: +2
Scan Number: 12570
File Name: 120407_A549_EGFIGF_bioRepA_ACK_FT.raw
x 10^4
Intensity
m/z
b1
b2
y5
MH−NH3−H2O+2
MH−H2O+2
b4
y7
y8
y9
y9 + 86
b8

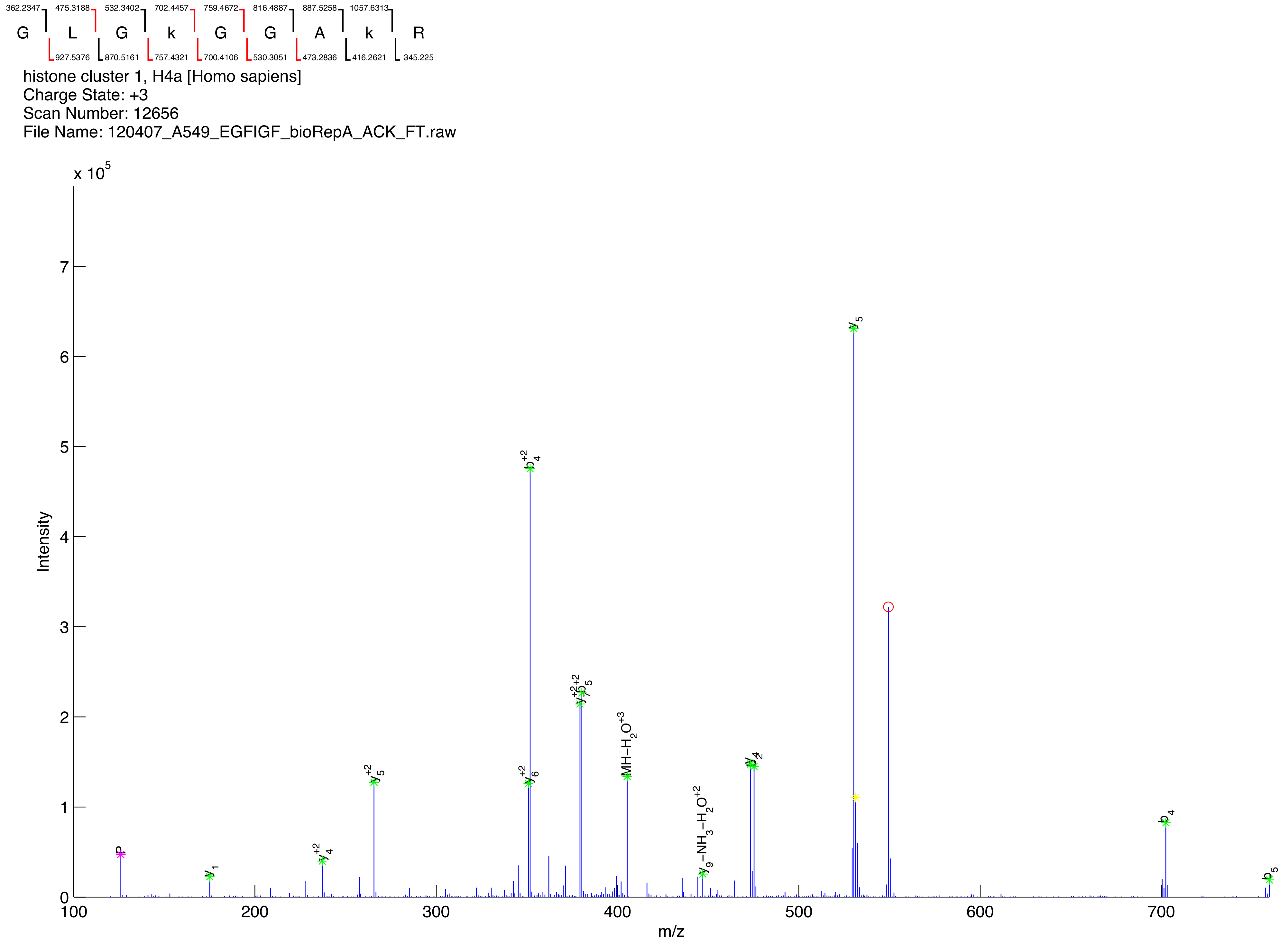

362.2347 475.3188 532.3402 702.4457 759.4672 816.4887 887.5258 1057.6313
G L G k G G A k R
927.5376 870.5161 757.4321 700.4106 530.3051 473.2836 416.2621 345.225
histone cluster 1, H4a [Homo sapiens]
Charge State: +3
Scan Number: 12656
File Name: 120407_A549_EGFIGF_bioRepA_ACK_FT.raw
x 10^5
Intensity
m/z

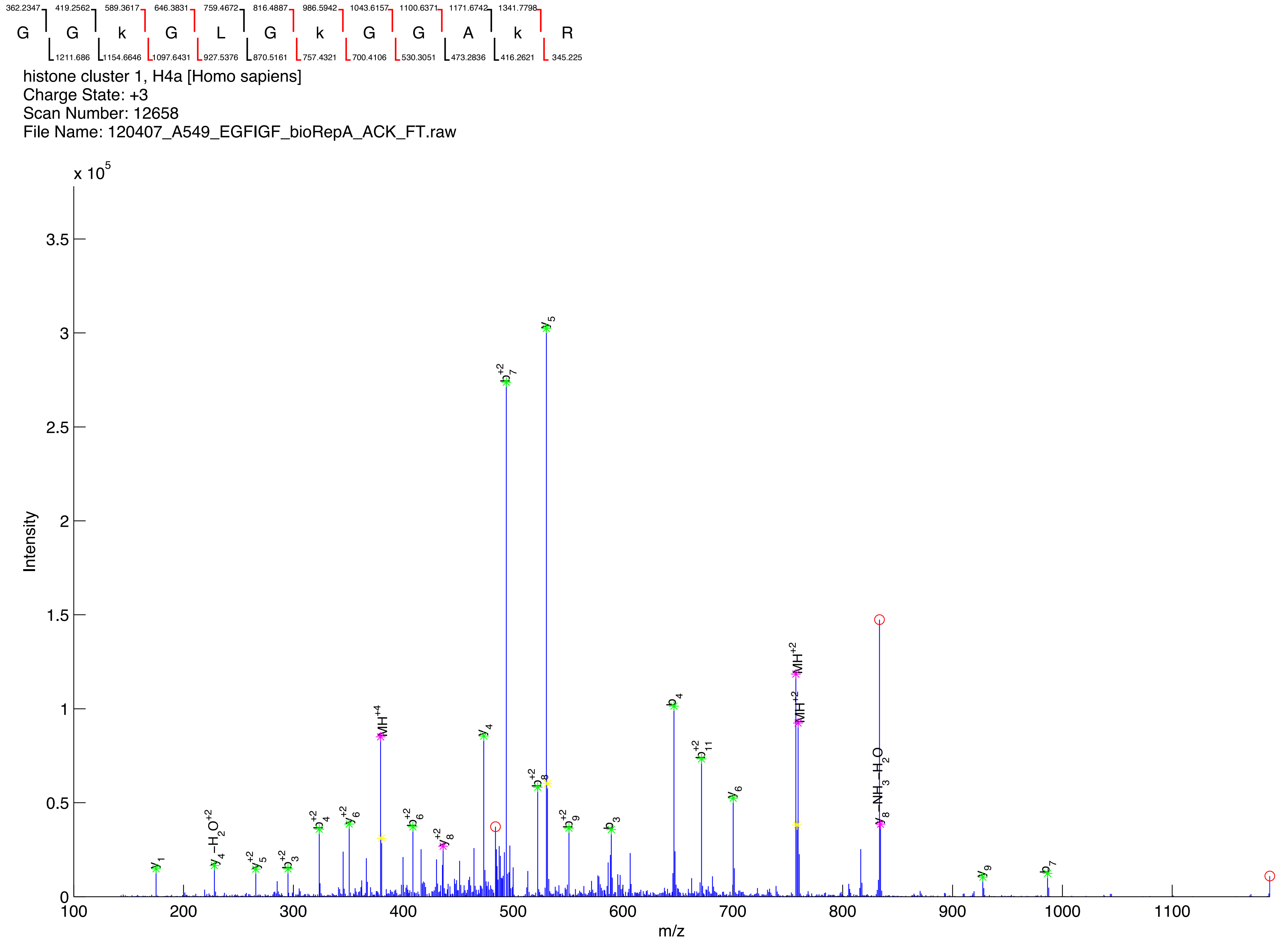

362.2347 419.2562 589.3617 646.3831 759.4672 816.4887 986.5942 1043.6157 1100.6371 1171.6742 1341.7798
G G k G L G k G G A k R
1211.686 1154.6646 1097.6431 927.5376 870.5161 757.4321 700.4106 530.3051 473.2836 416.2621 345.225
histone cluster 1, H4a [Homo sapiens]
Charge State: +3
Scan Number: 12658
File Name: 120407_A549_EGFIGF_bioRepA_ACK_FT.raw
x 10^5
Intensity
m/z

PREDICTED– similar to heterogeneous nuclear ribonucleoprotein A1 [Homo sapiens]
Charge State: +2
Scan Number: 12680
File Name: 120407_A549_EGFIGF_bioRepA_ACK_FT.raw

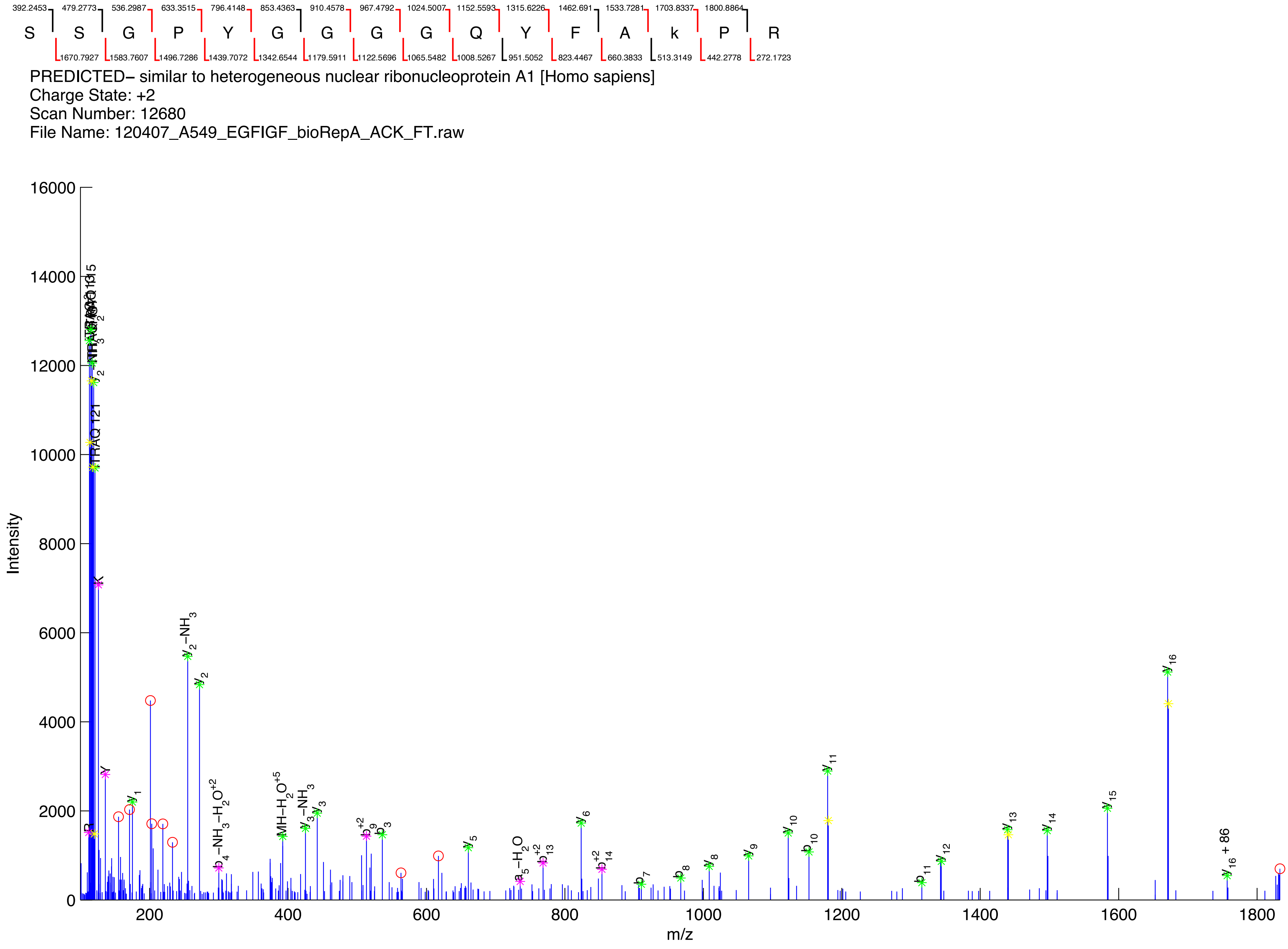

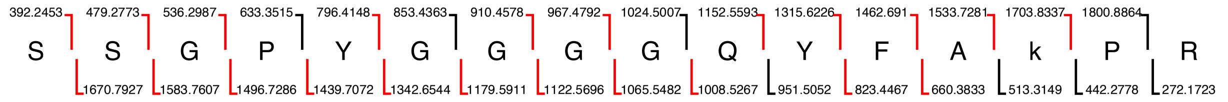

PREDICTED– similar to heterogeneous nuclear ribonucleoprotein A1 [Homo sapiens]
Charge State: +2
Scan Number: 12681
File Name: 120407_A549_EGFIGF_bioRepA_ACK_FT.raw

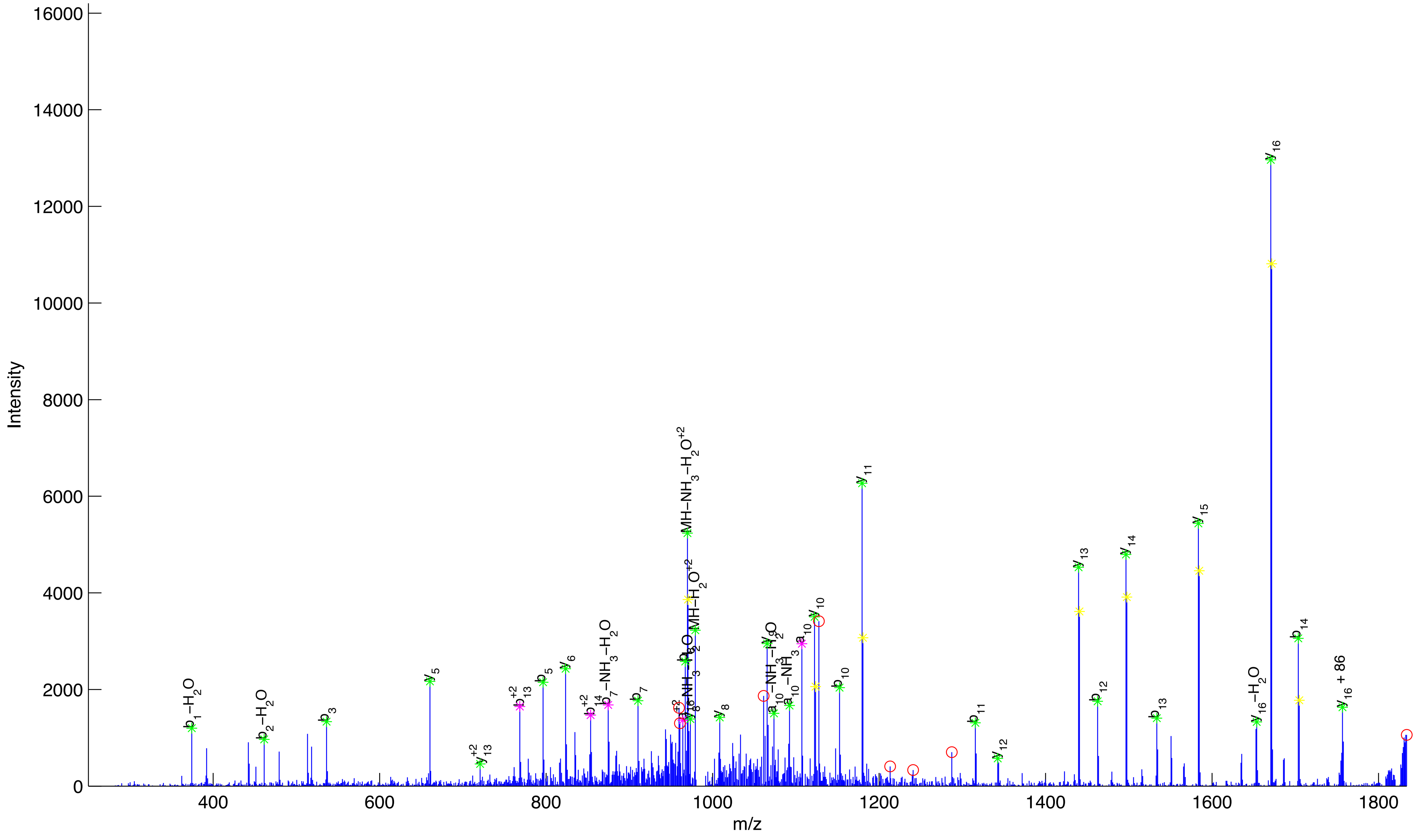

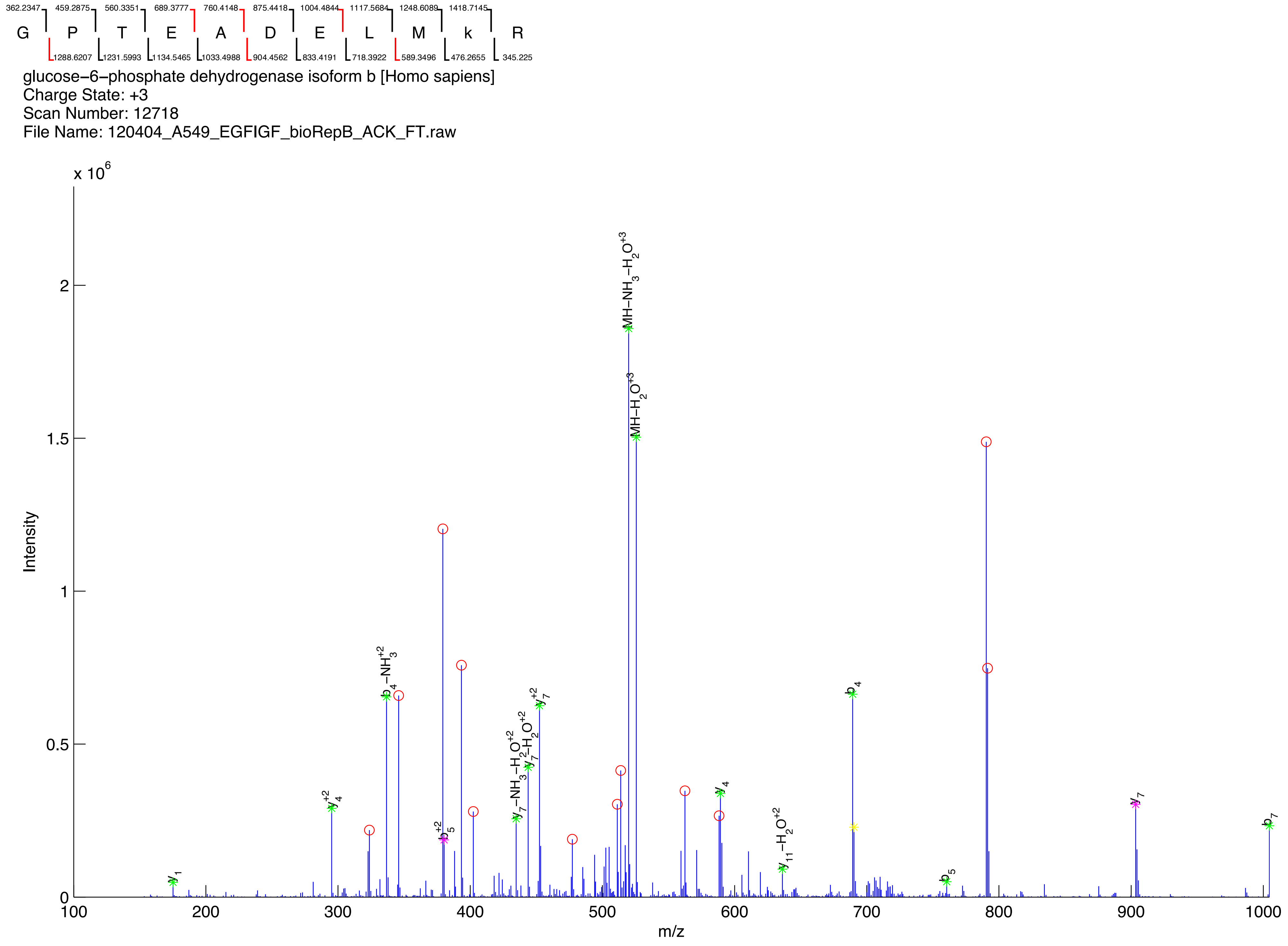

362.2347 459.2875 560.3351 689.3777 760.4148 875.4418 1004.4844 1117.5684 1248.6089 1418.7145
G P T E A D E L M k R
1288.6207 1231.5993 1134.5465 1033.4988 904.4562 833.4191 718.3922 589.3496 476.2655 345.225
glucose–6–phosphate dehydrogenase isoform b [Homo sapiens]
Charge State: +3
Scan Number: 12718
File Name: 120404_A549_EGFIGF_bioRepB_ACK_FT.raw
x 10^6
Intensity
m/z
MH−NH3−H2O+3
MH−H2O+3
b4−NH3+2
y4+2
b5+2
y7−NH3−H2O+2
y7−H2O+2
y7+2
y4
y11−H2O+2
b4
b5
y7
b7
y1

H2A histone family, member Y isoform 2 [Homo sapiens]
Charge State: +3
Scan Number: 12745
File Name: 120404_A549_EGFIGF_bioRepB_ACK_FT.raw

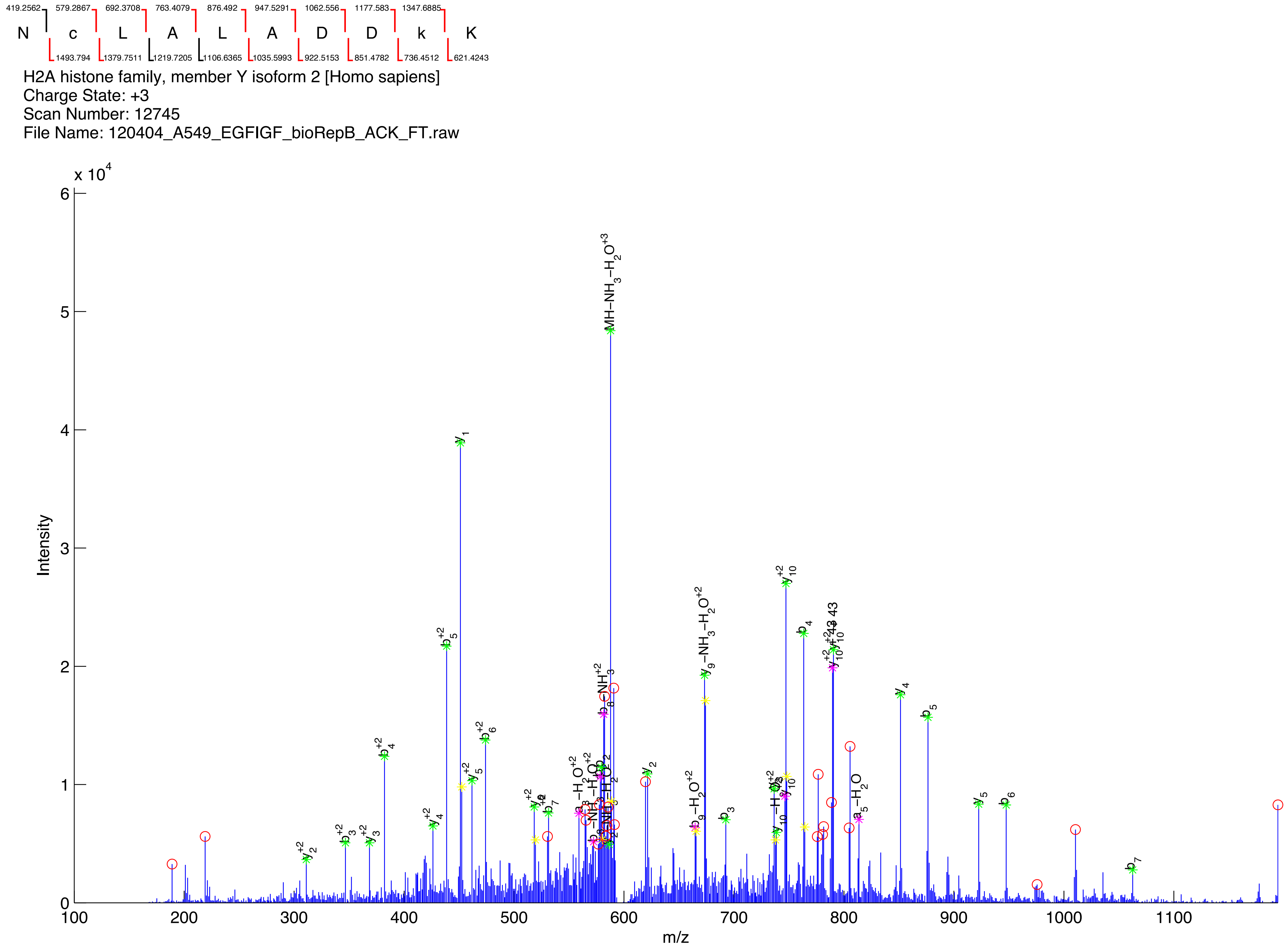

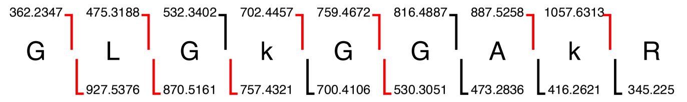

histone cluster 1, H4a [Homo sapiens]
Charge State: +2
Scan Number: 13116
File Name: 120407_A549_EGFIGF_bioRepA_ACK_FT.raw

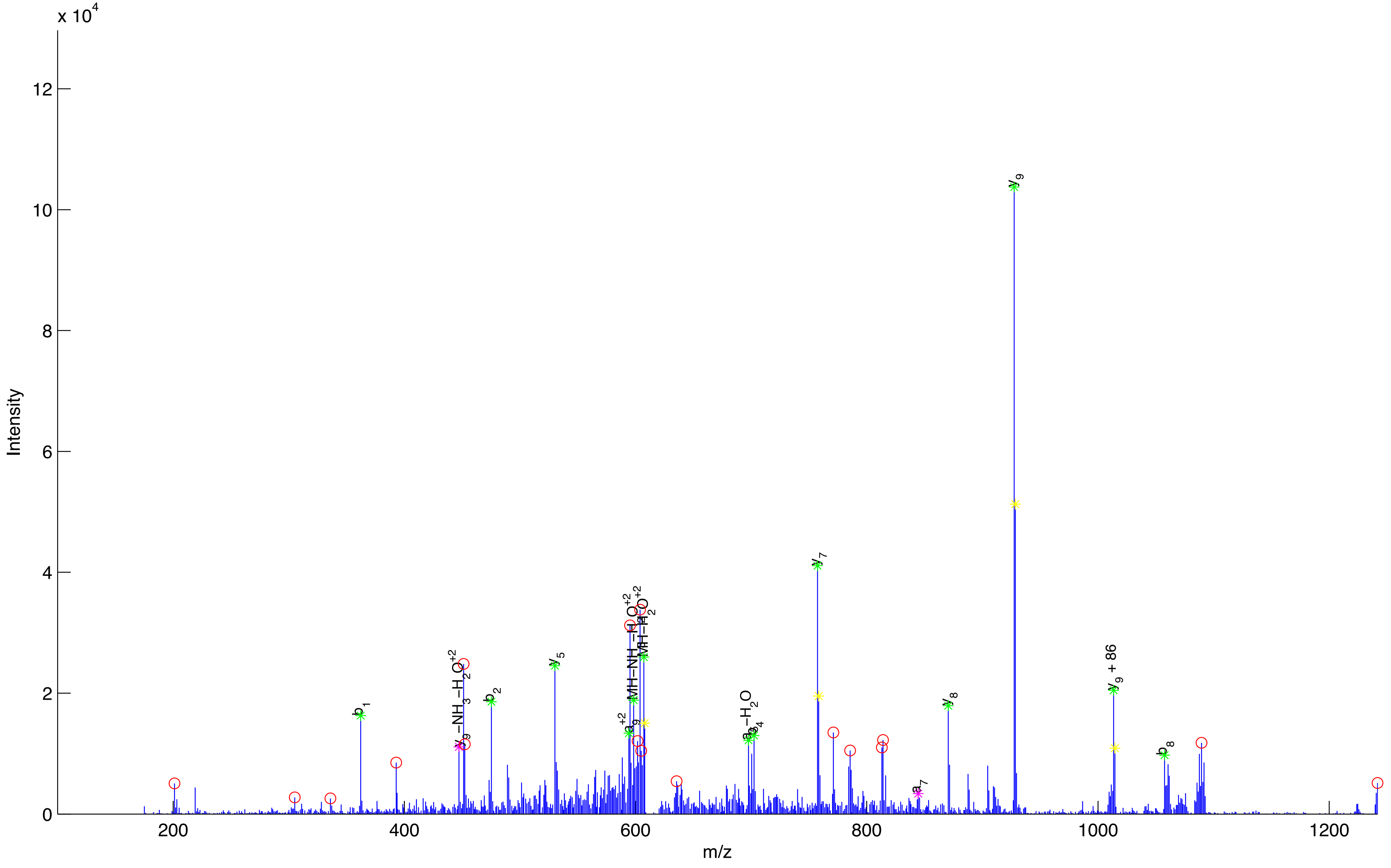

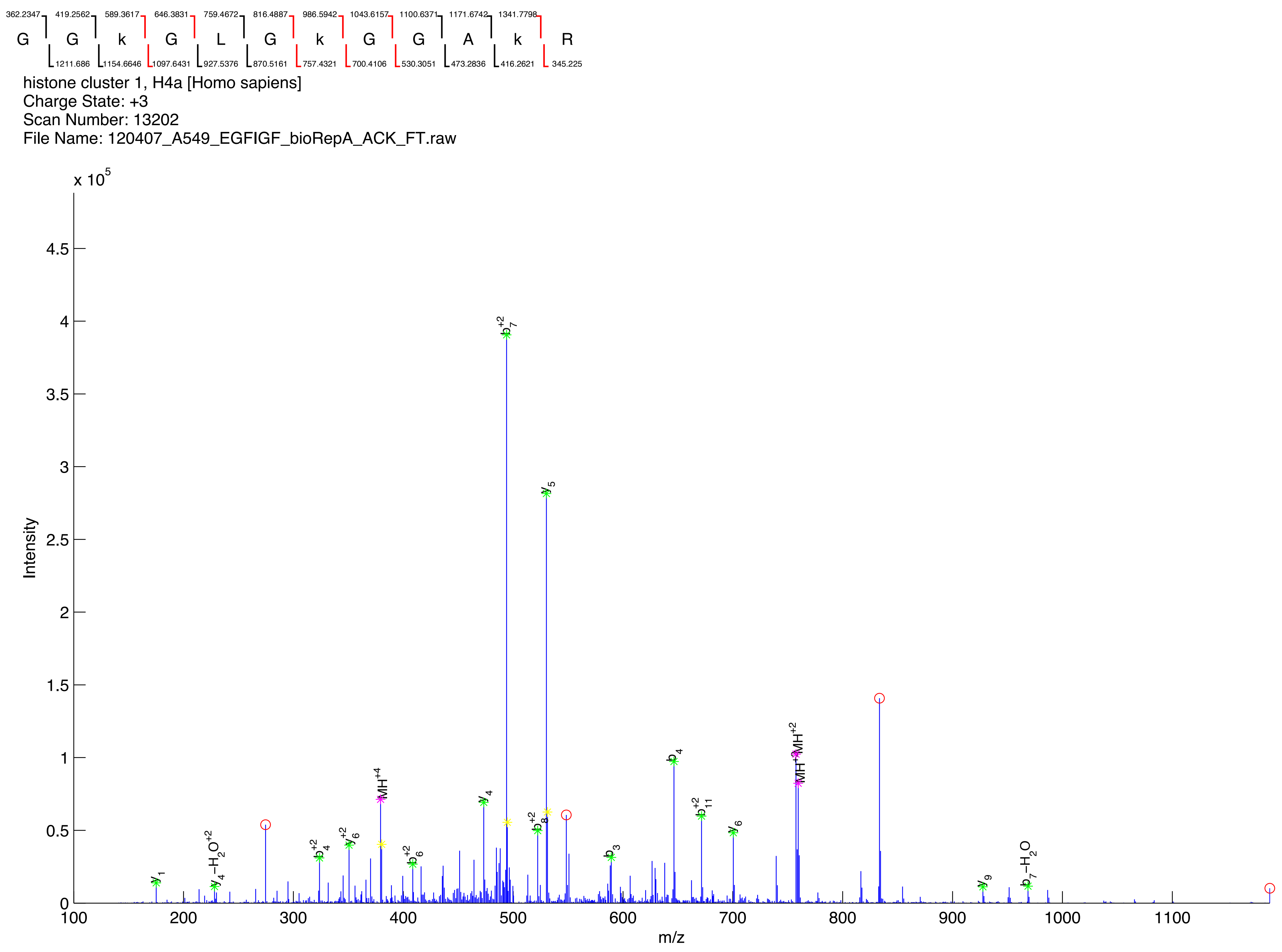

362.2347 419.2562 589.3617 646.3831 759.4672 816.4887 986.5942 1043.6157 1100.6371 1171.6742 1341.7798
G G k G L G k G G A k R
1211.686 1154.6646 1097.6431 927.5376 870.5161 757.4321 700.4106 530.3051 473.2836 416.2621 345.225
histone cluster 1, H4a [Homo sapiens]
Charge State: +3
Scan Number: 13202
File Name: 120407_A549_EGFIGF_bioRepA_ACK_FT.raw
x 10^5
Intensity
m/z
0
0.5
1
1.5
2
2.5
3
3.5
4
4.5
100
200
300
400
500
600
700
800
900
1000
1100
y1
y4−H2O+2
b4+2
y6+2
MH+4
b6+2
y4
b7+2
b8+2
y5
b3
b4
b11+2
y6
MH+3
MH+2
y9
b7−H2O

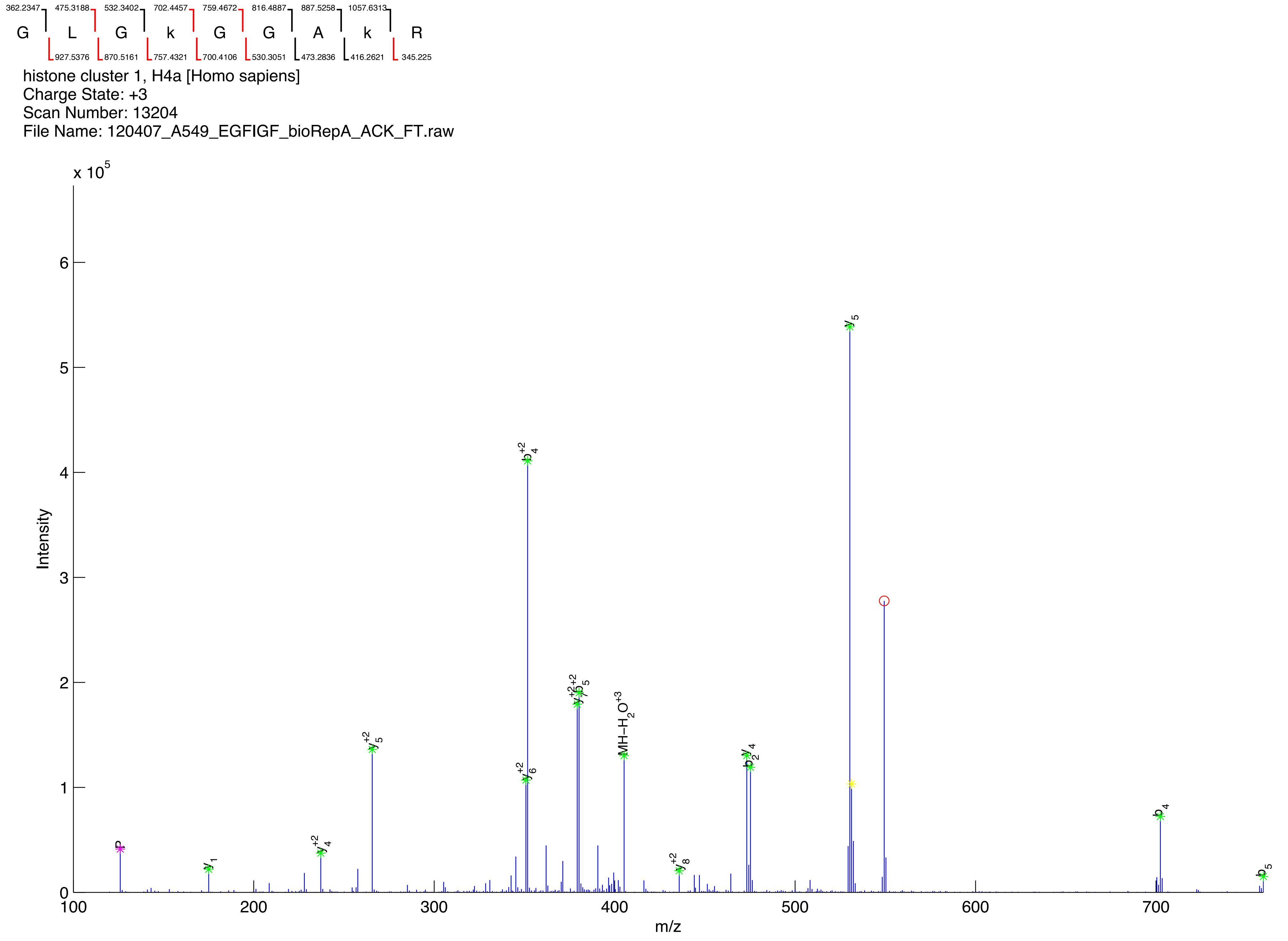

362.2347 475.3188 532.3402 702.4457 759.4672 816.4887 887.5258 1057.6313
G L G k G G A k R
927.5376 870.5161 757.4321 700.4106 530.3051 473.2836 416.2621 345.225
histone cluster 1, H4a [Homo sapiens]
Charge State: +3
Scan Number: 13204
File Name: 120407_A549_EGFIGF_bioRepA_ACK_FT.raw
x 10^5
Intensity
m/z

419.2562		589.3617		718.4043		831.4883		978.5568		1091.6408		
N		k		E		I		F		L		R
	961.5471		847.5042		677.3986		548.356		435.272		288.2036	

tumor rejection antigen (gp96) 1 [Homo sapiens]
Charge State: +2
Scan Number: 13307
File Name: 120407_A549_EGFIGF_bioRepA_ACK_FT.raw

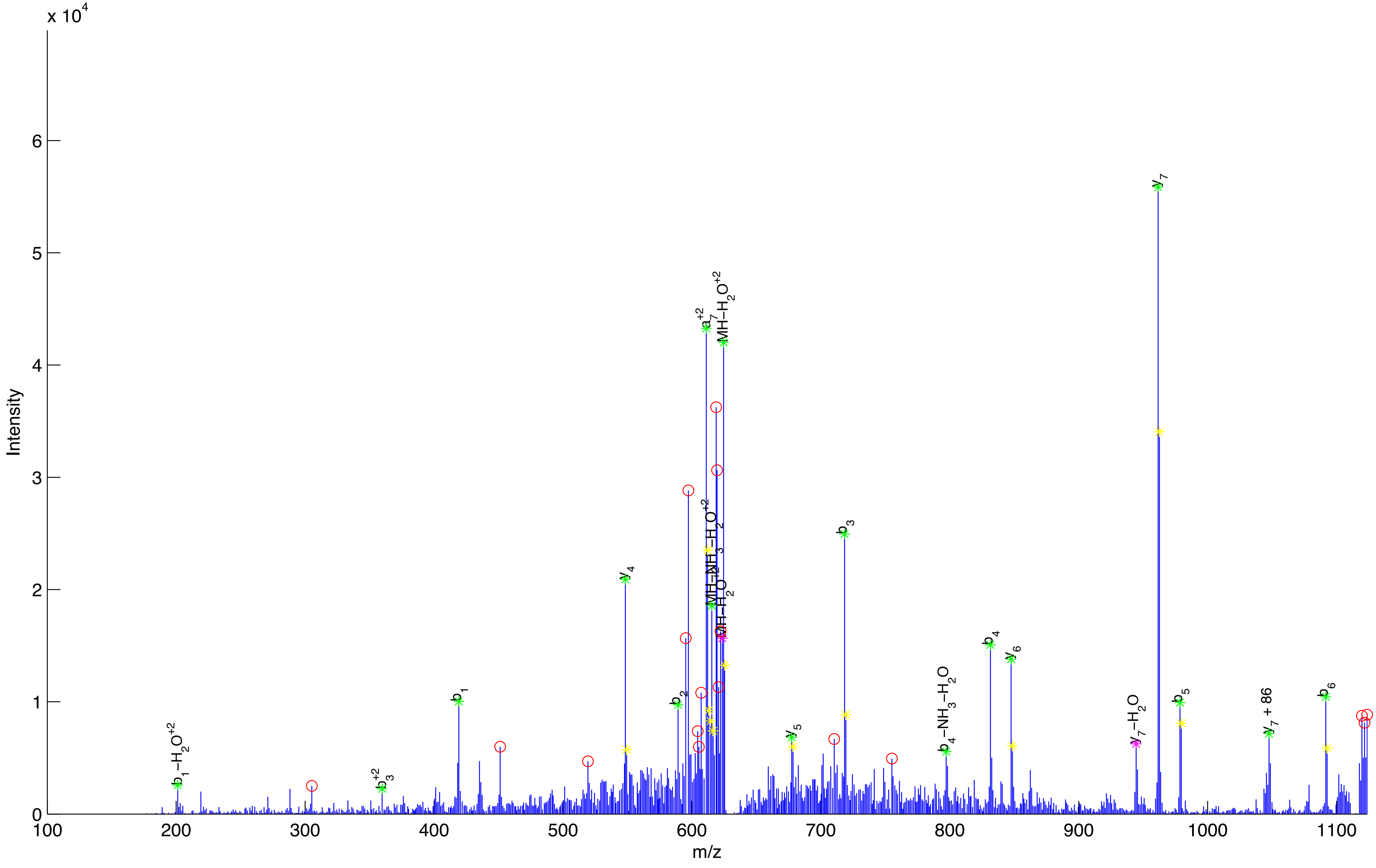

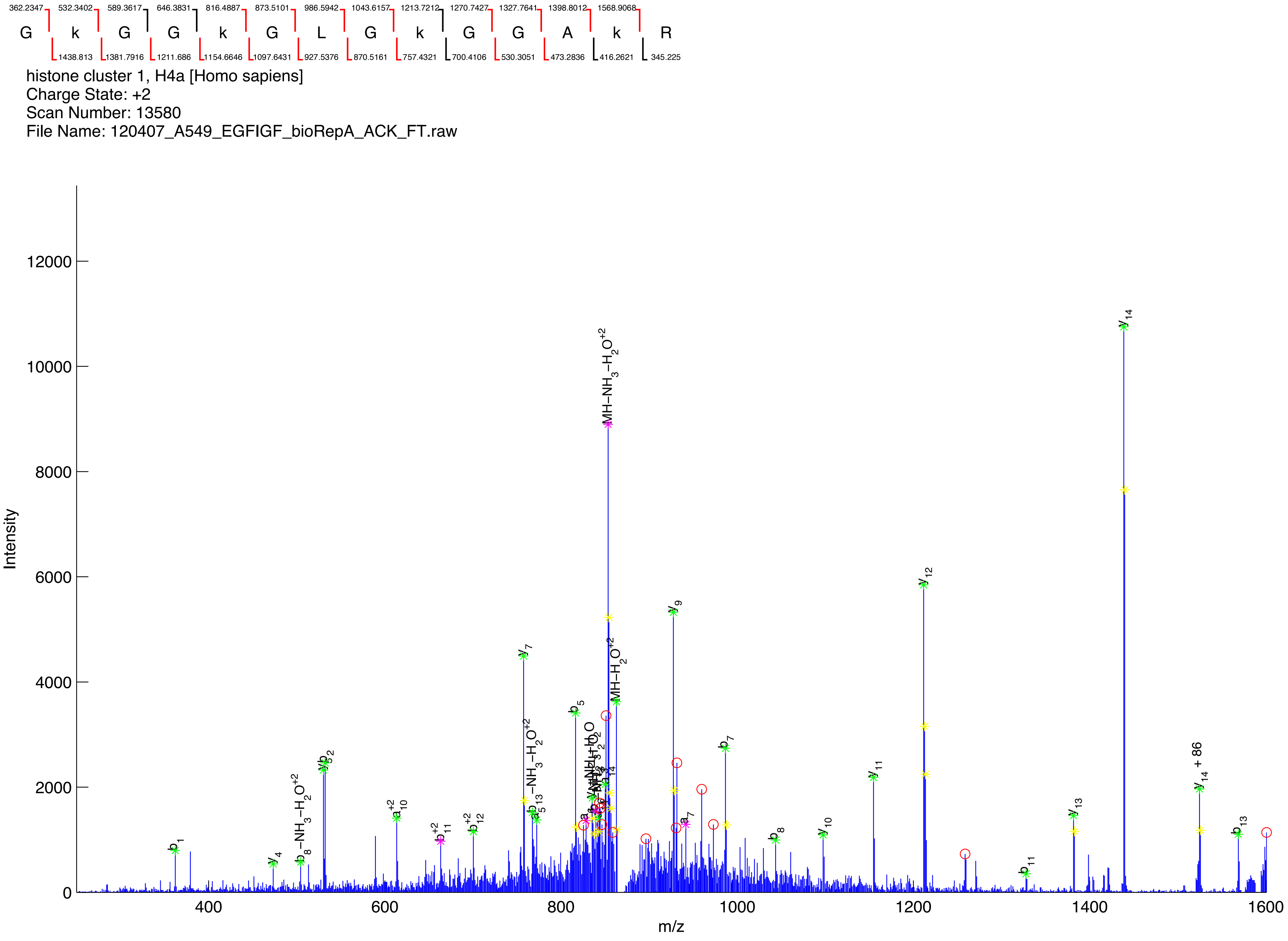
362.2347 532.3402 589.3617 646.3831 816.4887 873.5101 986.5942 1043.6157 1213.7212 1270.7427 1327.7641 1398.8012 1568.9068
G k G G k G L G k G G A k R
1438.813 1381.7916 1211.686 1154.6646 1097.6431 927.5376 870.5161 757.4321 700.4106 530.3051 473.2836 416.2621 345.225
histone cluster 1, H4a [Homo sapiens]
Charge State: +2
Scan Number: 13580
File Name: 120407_A549_EGFIGF_bioRepA_ACK_FT.raw
Intensity
m/z
b_1
y_4
b_8−NH_3−H_2O^{+2}
a^{+2}_{10}
b^{+2}_{11}
b^{+2}_{12}
y_7
b_{13}−NH_3−H_2O^{+2}
b_5
MH−NH_3−H_2O^{+2}
MH−H_2O^{+2}
y_9
a_7
b_7
b_8
y_{10}
y_{11}
y_{12}
b_{11}
y_{13}
y_{14}
y_{14} + 86
b_{13}

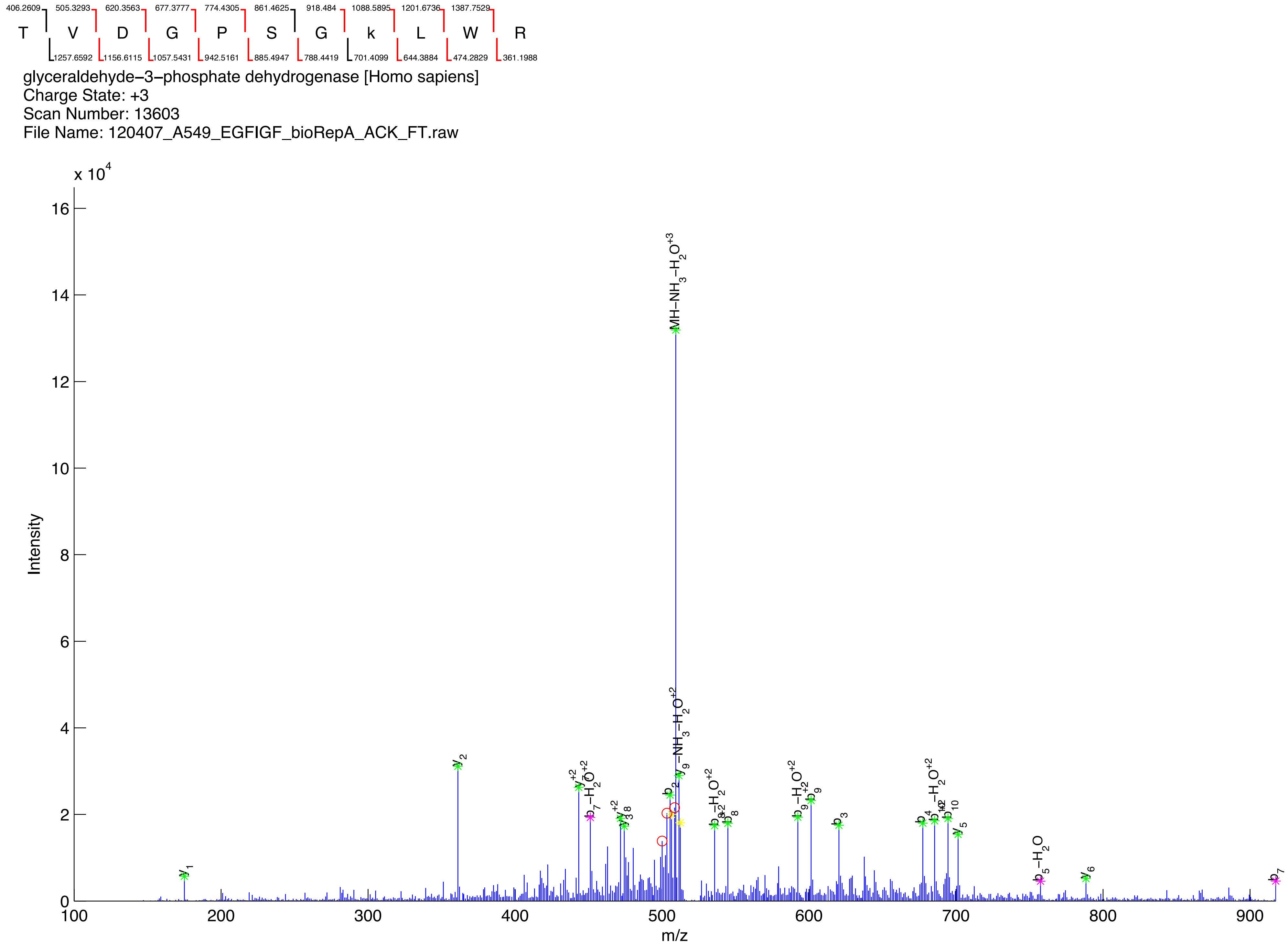
406.2609 505.3293 620.3563 677.3777 774.4305 861.4625 918.484 1088.5895 1201.6736 1387.7529
T V D G P S G k L W R
1257.6592 1156.6115 1057.5431 942.5161 885.4947 788.4419 701.4099 644.3884 474.2829 361.1988
glyceraldehyde−3−phosphate dehydrogenase [Homo sapiens]
Charge State: +3
Scan Number: 13603
File Name: 120407_A549_EGFIGF_bioRepA_ACK_FT.raw
x 10^4
Intensity
m/z
MH−NH3−H2O+3
y9−NH3−H2O+2
y1
y2
y8
y5
y6
b3
b7
b8
b9
b10
b5−H2O

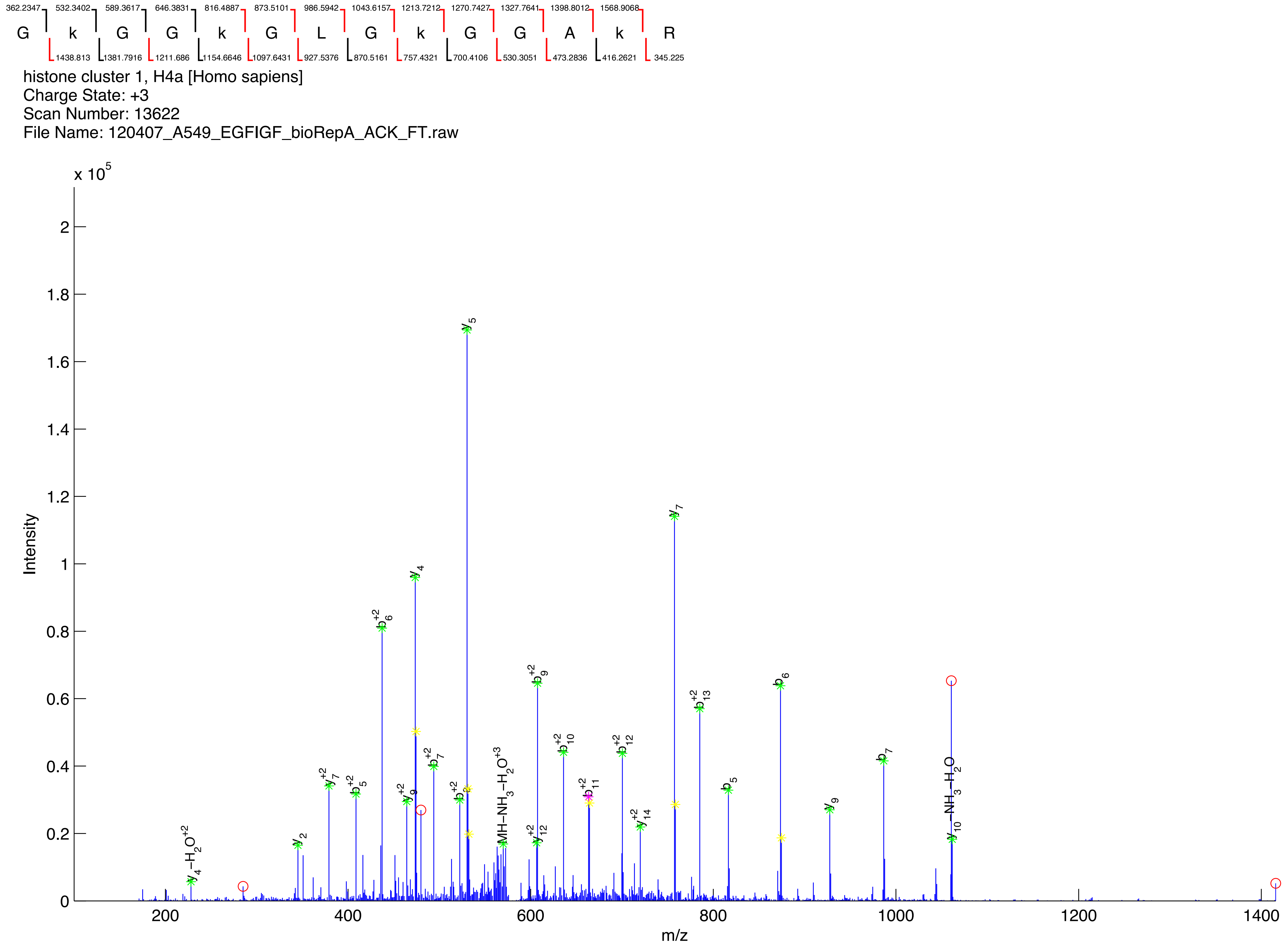

G k G G k G L G k G G A k R
362.2347 532.3402 589.3617 646.3831 816.4887 873.5101 986.5942 1043.6157 1213.7212 1270.7427 1327.7641 1398.8012 1568.9068
1438.813 1381.7916 1211.686 1154.6646 1097.6431 927.5376 870.5161 757.4321 700.4106 530.3051 473.2836 416.2621 345.225
histone cluster 1, H4a [Homo sapiens]
Charge State: +3
Scan Number: 13622
File Name: 120407_A549_EGFIGF_bioRepA_ACK_FT.raw
x 10^5
Intensity
m/z

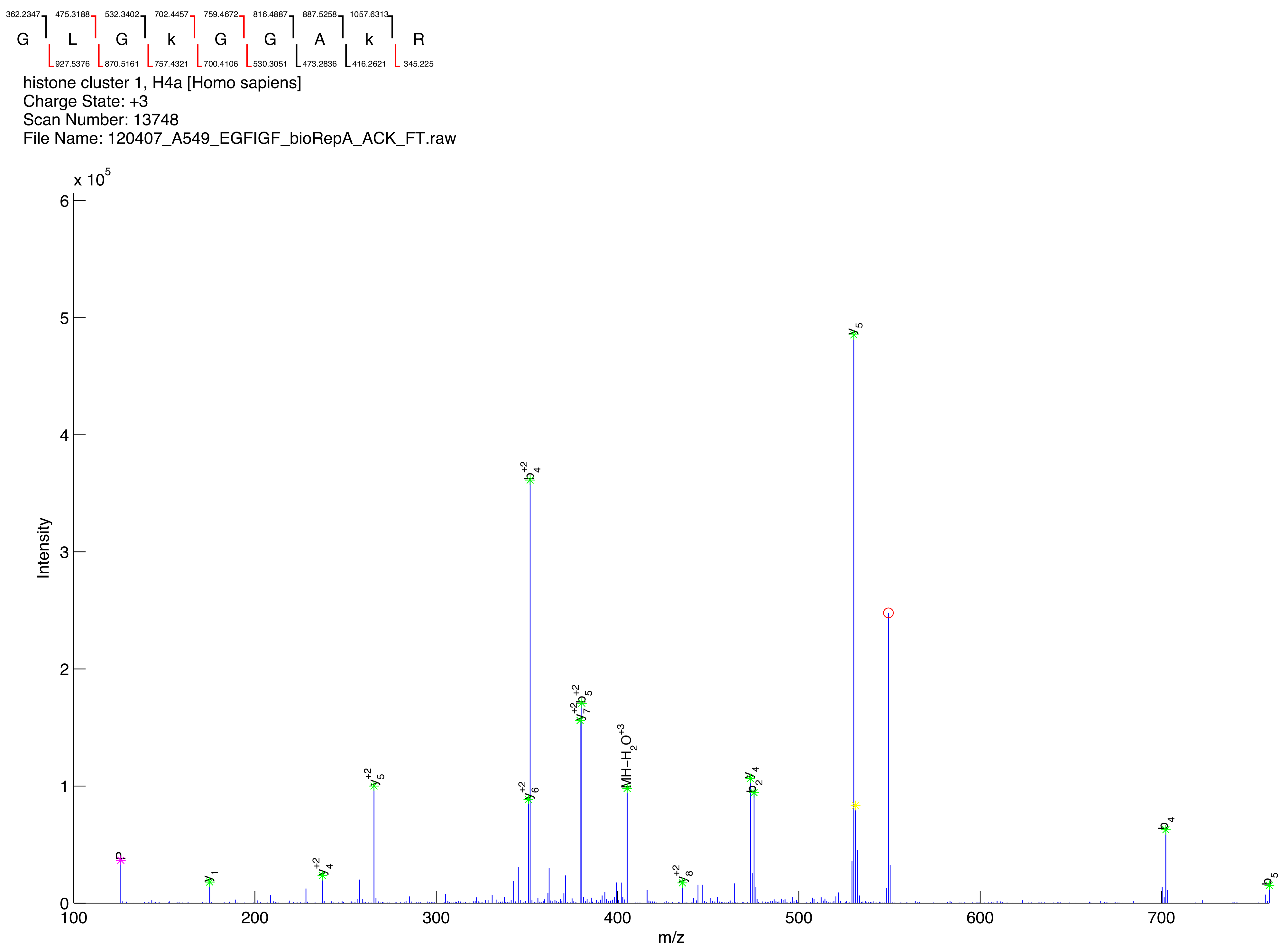

362.2347 475.3188 532.3402 702.4457 759.4672 816.4887 887.5258 1057.6313
G L G k G G A k R
927.5376 870.5161 757.4321 700.4106 530.3051 473.2836 416.2621 345.225
histone cluster 1, H4a [Homo sapiens]
Charge State: +3
Scan Number: 13748
File Name: 120407_A549_EGFIGF_bioRepA_ACK_FT.raw
x 10^5
Intensity
m/z
P
y_1
y_4^{+2}
y_5^{+2}
y_6^{+2}
b_4^{+2}
y_7^{+2}
b_5^{+2}
$MH-H_2O^{+3}$
y_8^{+2}
y_4
b_2
y_5
b_4
b_5

362.2347	532.3402	589.3617	646.3831	816.4887	873.5101	986.5942	1043.6157	1213.7212	1270.7427	1327.7641	1398.8012	1568.9068		
G	k	G	G	k	G	L	G	k	G	G	A	k	R	
	1438.813	1381.7916	1211.686	1154.6646	1097.6431	927.5376	870.5161	757.4321	700.4106	530.3051	473.2836	416.2621	345.225	

histone cluster 1, H4a [Homo sapiens]
Charge State: +3
Scan Number: 14168
File Name: 120407_A549_EGFIGF_bioRepA_ACK_FT.raw

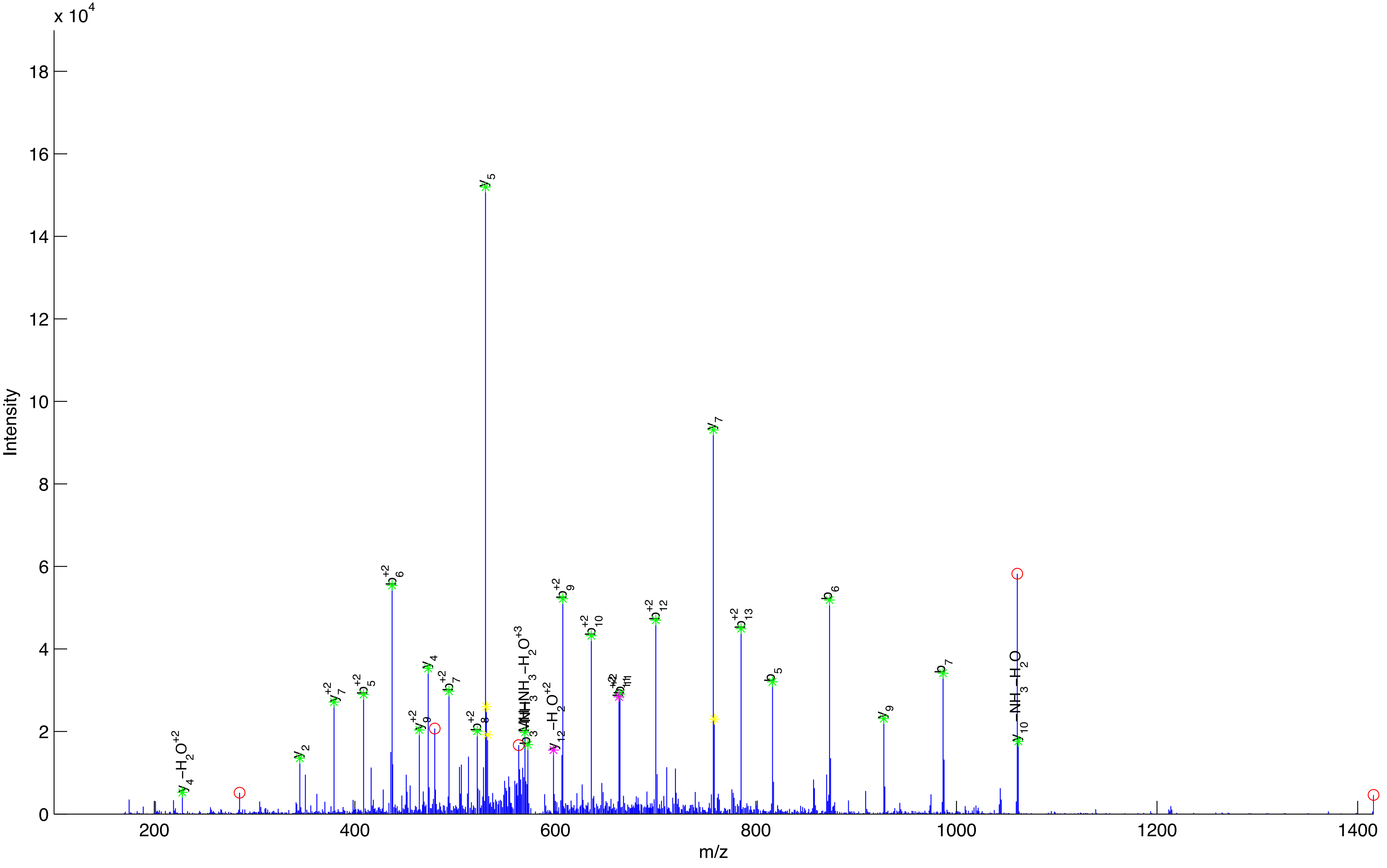

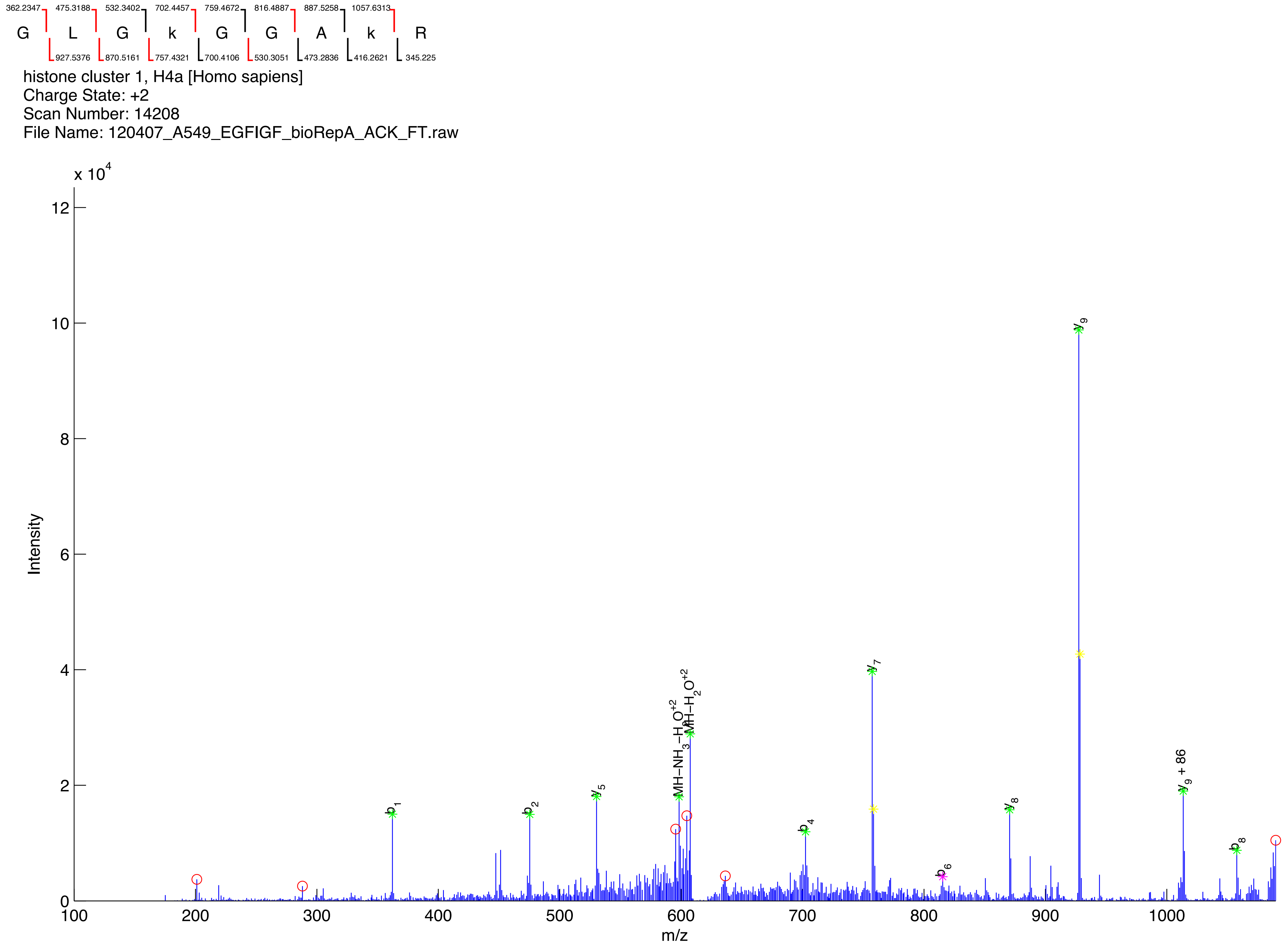
362.2347
475.3188
532.3402
702.4457
759.4672
816.4887
887.5258
1057.6313
G L G k G G A k R
927.5376
870.5161
757.4321
700.4106
530.3051
473.2836
416.2621
345.225
histone cluster 1, H4a [Homo sapiens]
Charge State: +2
Scan Number: 14208
File Name: 120407_A549_EGFIGF_bioRepA_ACK_FT.raw
x 10^4
Intensity
m/z
b1
b2
y5
MH−NH3−H2O+2
MH−H2O+2
b4
y7
b6
y8
y9
y9 + 86
b8

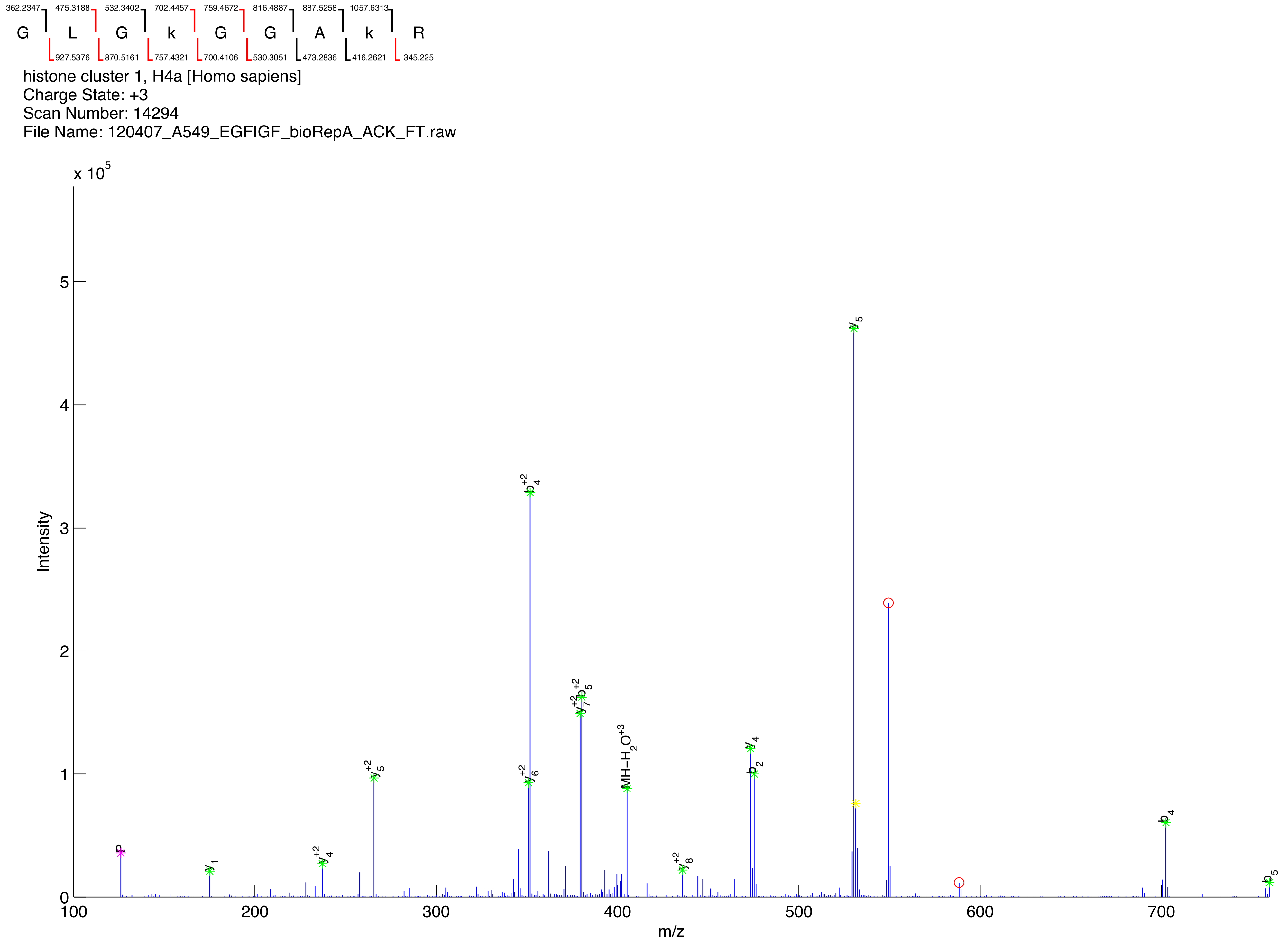
362.2347
475.3188
532.3402
702.4457
759.4672
816.4887
887.5258
1057.6313
G L G k G G A k R
927.5376
870.5161
757.4321
700.4106
530.3051
473.2836
416.2621
345.225
histone cluster 1, H4a [Homo sapiens]
Charge State: +3
Scan Number: 14294
File Name: 120407_A549_EGFIGF_bioRepA_ACK_FT.raw
x 10^5
Intensity
m/z
MH−H2O+3

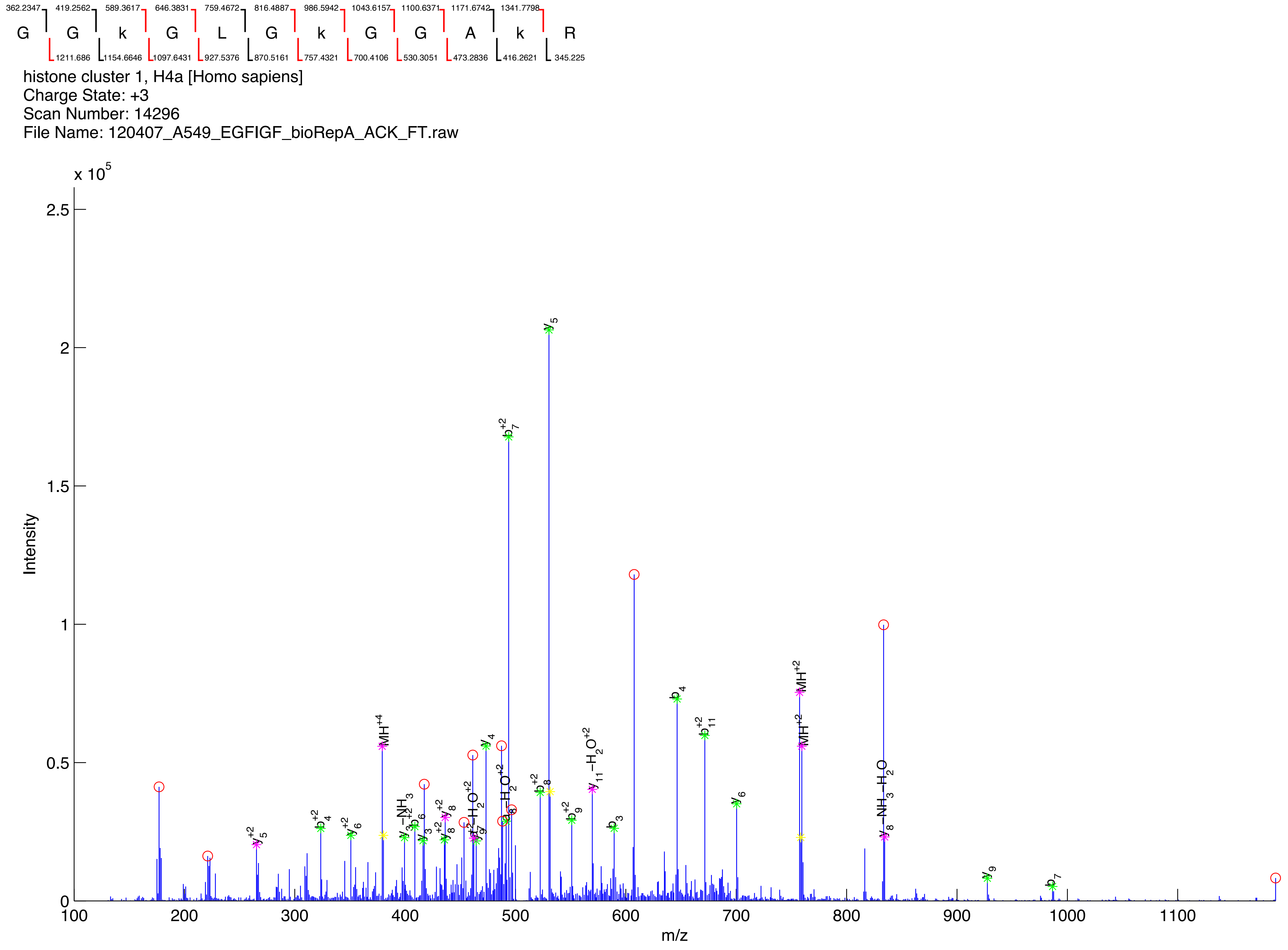

362.2347 419.2562 589.3617 646.3831 759.4672 816.4887 986.5942 1043.6157 1100.6371 1171.6742 1341.7798
G G k G L G k G G A k R
1211.686 1154.6646 1097.6431 927.5376 870.5161 757.4321 700.4106 530.3051 473.2836 416.2621 345.225
histone cluster 1, H4a [Homo sapiens]
Charge State: +3
Scan Number: 14296
File Name: 120407_A549_EGFIGF_bioRepA_ACK_FT.raw
x 10^5
Intensity
m/z

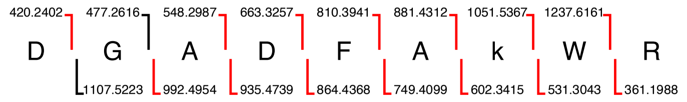

aldolase A [Homo sapiens]
Charge State: +3
Scan Number: 14325
File Name: 120407_A549_EGFIGF_bioRepA_ACK_FT.raw

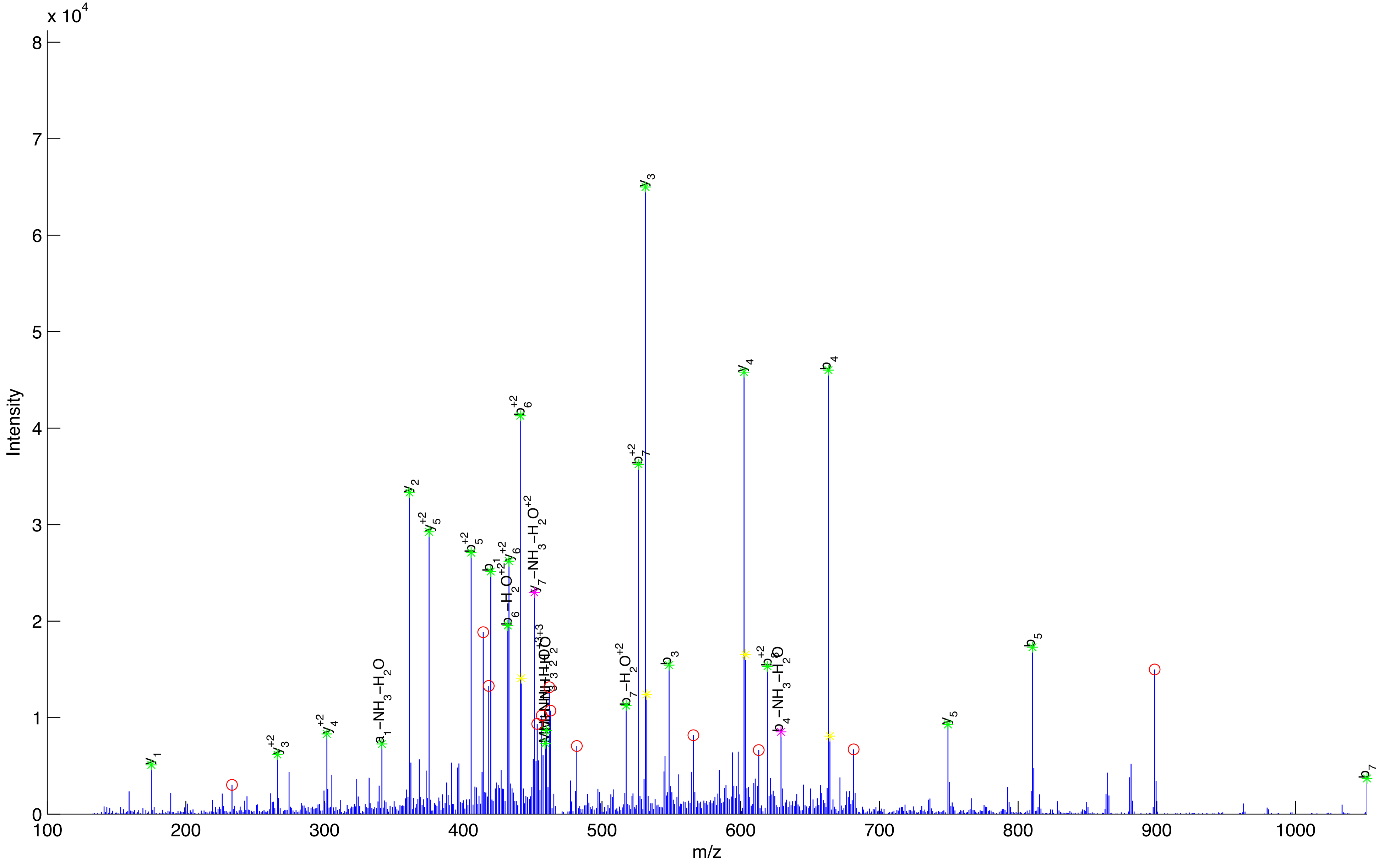

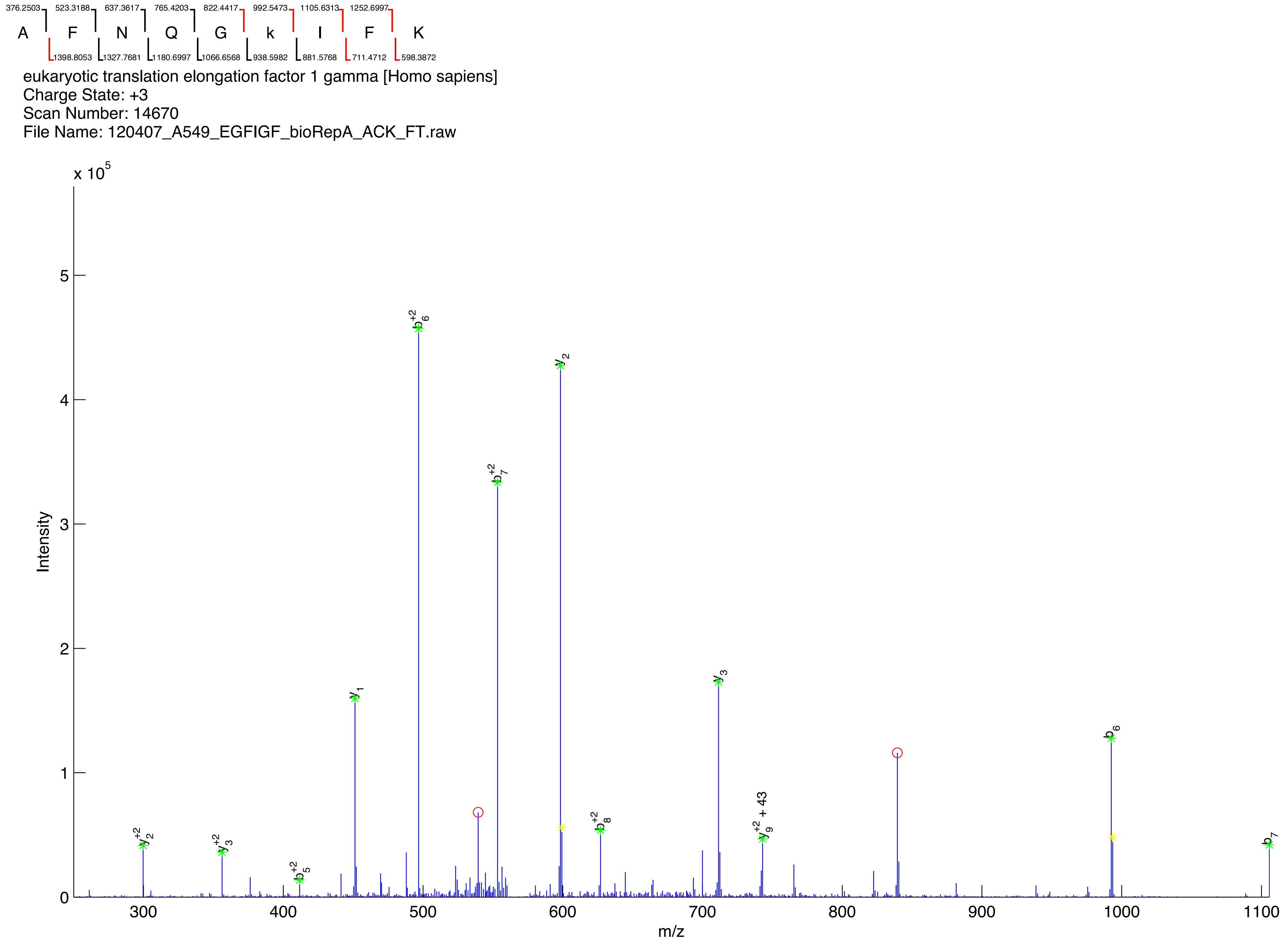
376.2503 523.3188 637.3617 765.4203 822.4417 992.5473 1105.6313 1252.6997
A F N Q G k I F K
1398.8053 1327.7681 1180.6997 1066.6568 938.5982 881.5768 711.4712 598.3872
eukaryotic translation elongation factor 1 gamma [Homo sapiens]
Charge State: +3
Scan Number: 14670
File Name: 120407_A549_EGFIGF_bioRepA_ACK_FT.raw
x 10^5
Intensity
m/z
0
1
2
3
4
5
300
400
500
600
700
800
900
1000
1100
y+2 2
y+2 3
b+2 5
y1
b+2 6
b+2 7
y2
b+2 8
y3
y+2 9 + 43
b6
b7

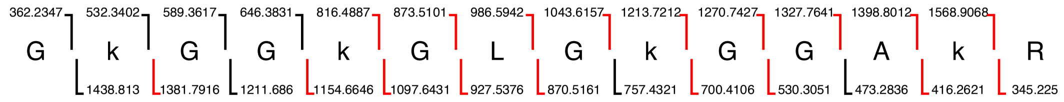

histone cluster 1, H4a [Homo sapiens]
Charge State: +3
Scan Number: 14714
File Name: 120407_A549_EGFIGF_bioRepA_ACK_FT.raw

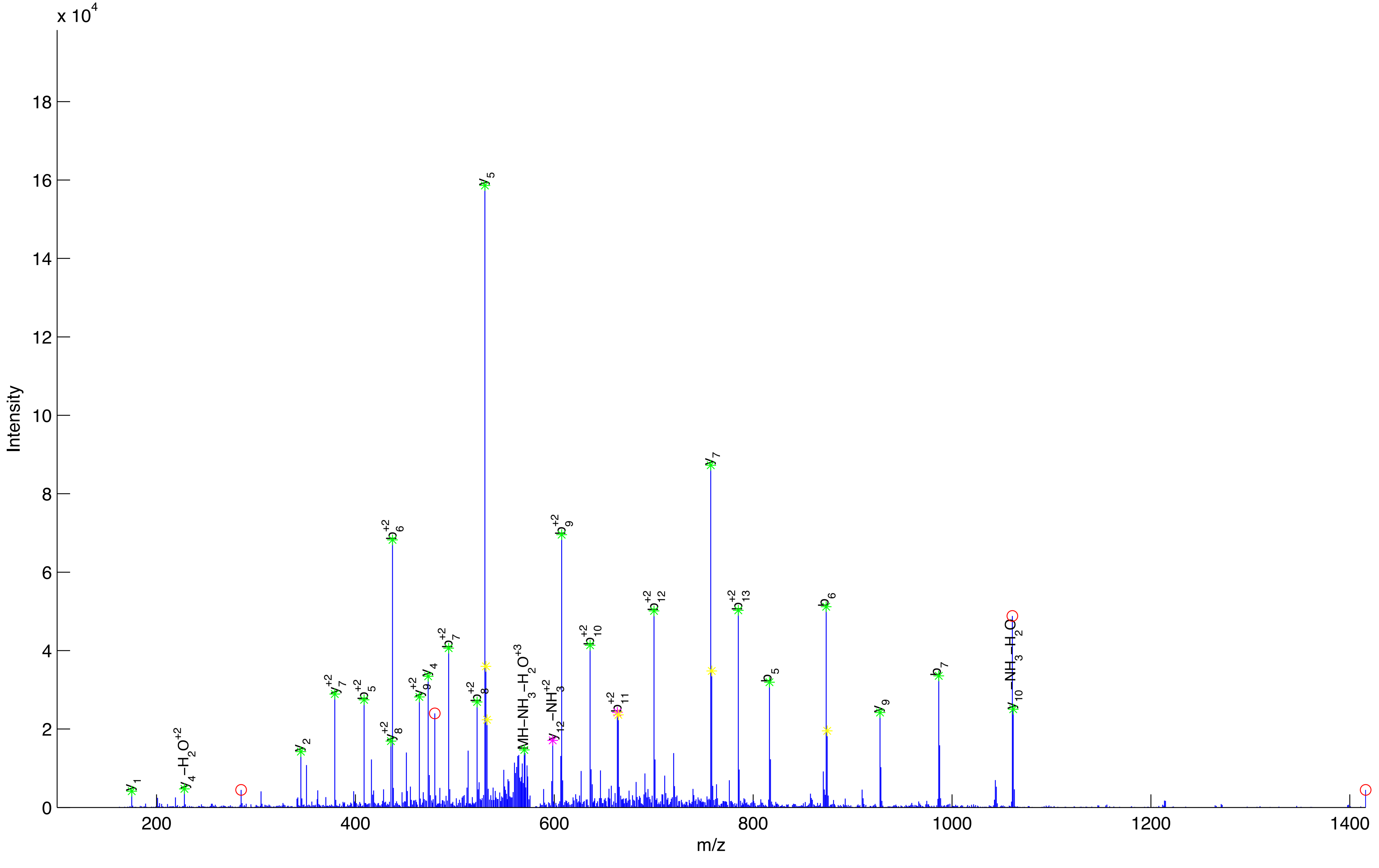

keratin 18 [Homo sapiens]
Charge State: +3
Scan Number: 14797
File Name: 120404_A549_EGFIGF_bioRepB_ACK_FT.raw

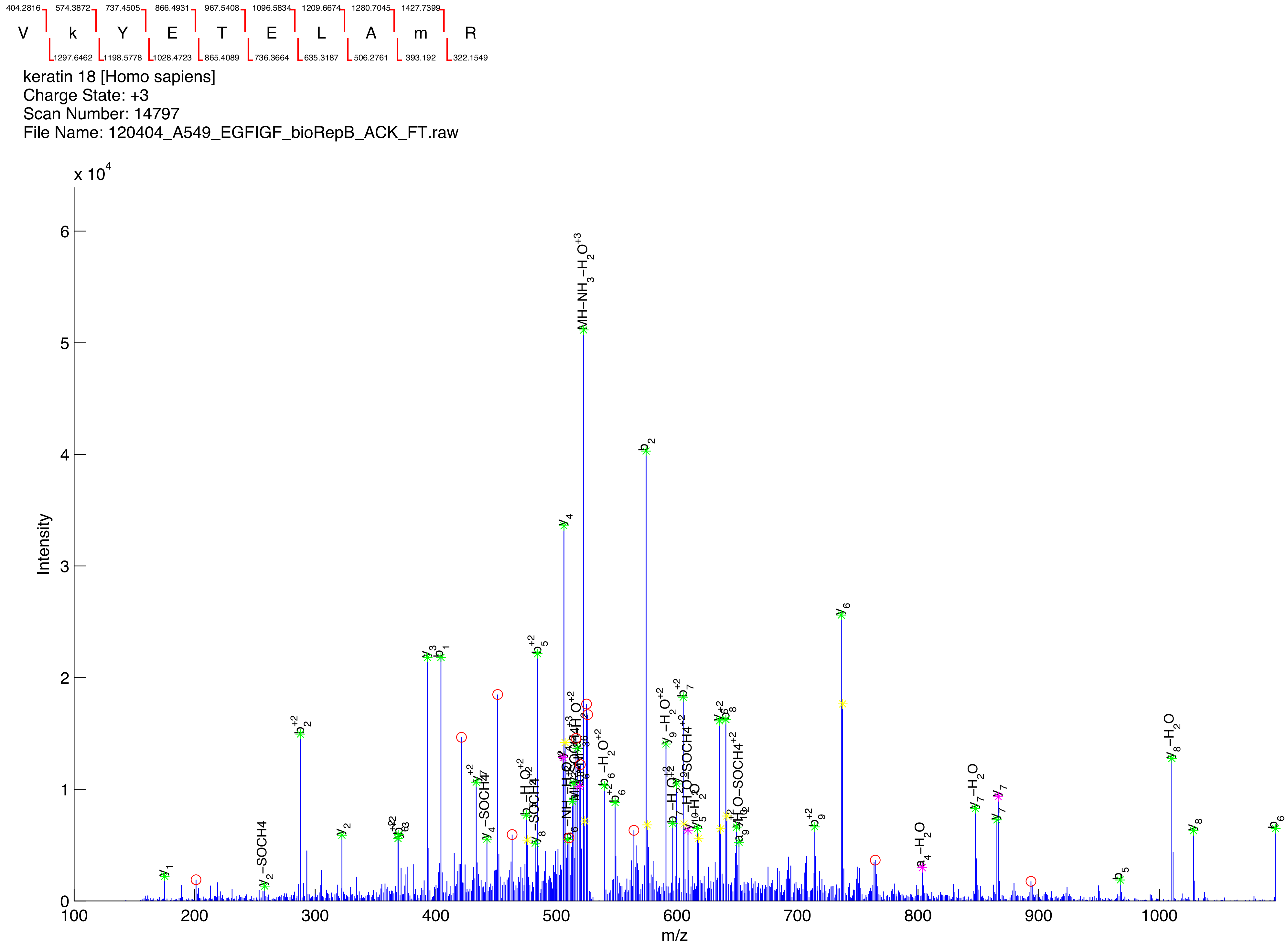

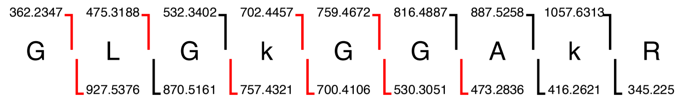

histone cluster 1, H4a [Homo sapiens]
Charge State: +3
Scan Number: 14840
File Name: 120407_A549_EGFIGF_bioRepA_ACK_FT.raw

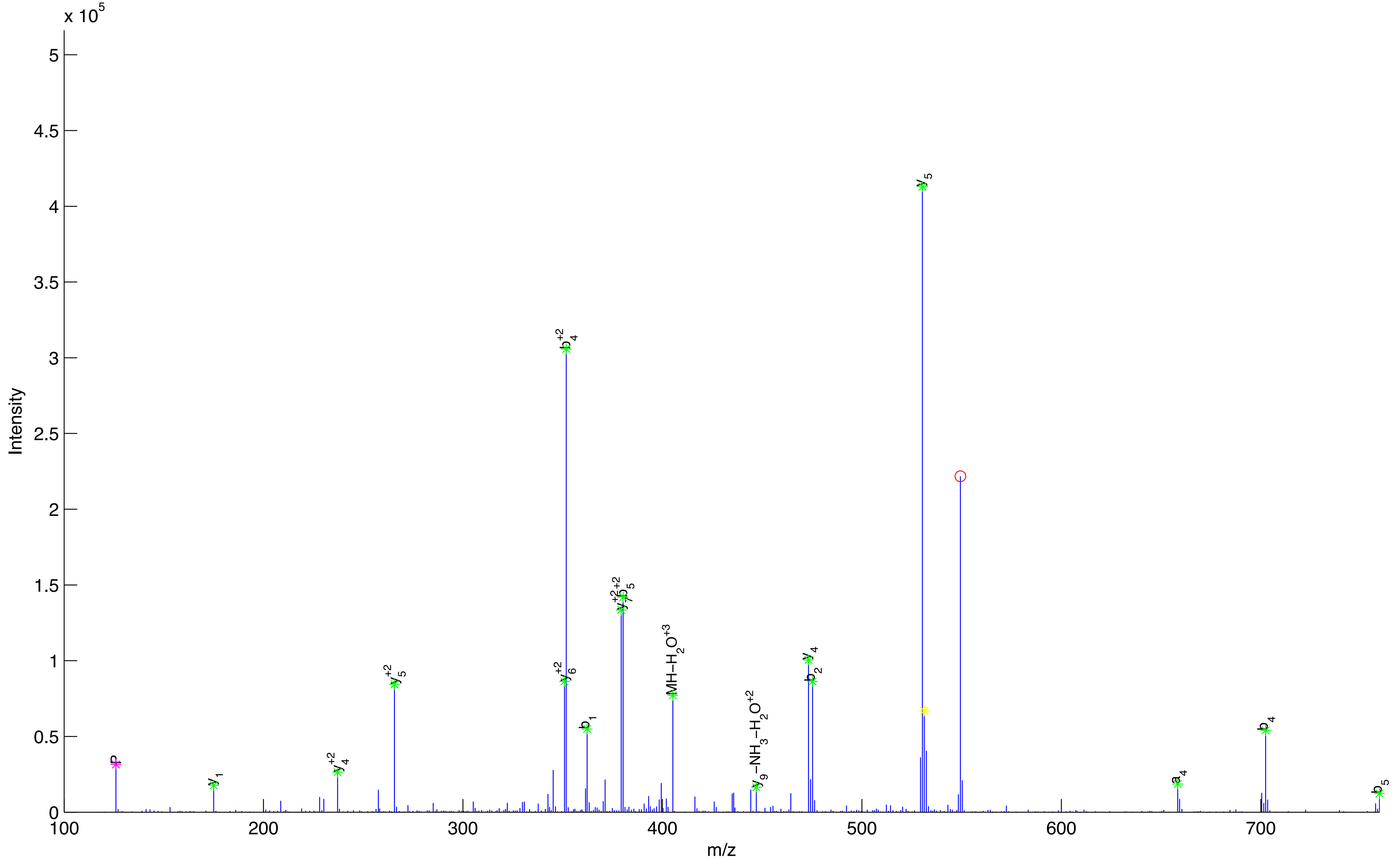

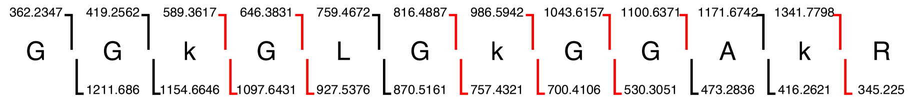

histone cluster 1, H4a [Homo sapiens]
Charge State: +3
Scan Number: 14842
File Name: 120407_A549_EGFIGF_bioRepA_ACK_FT.raw

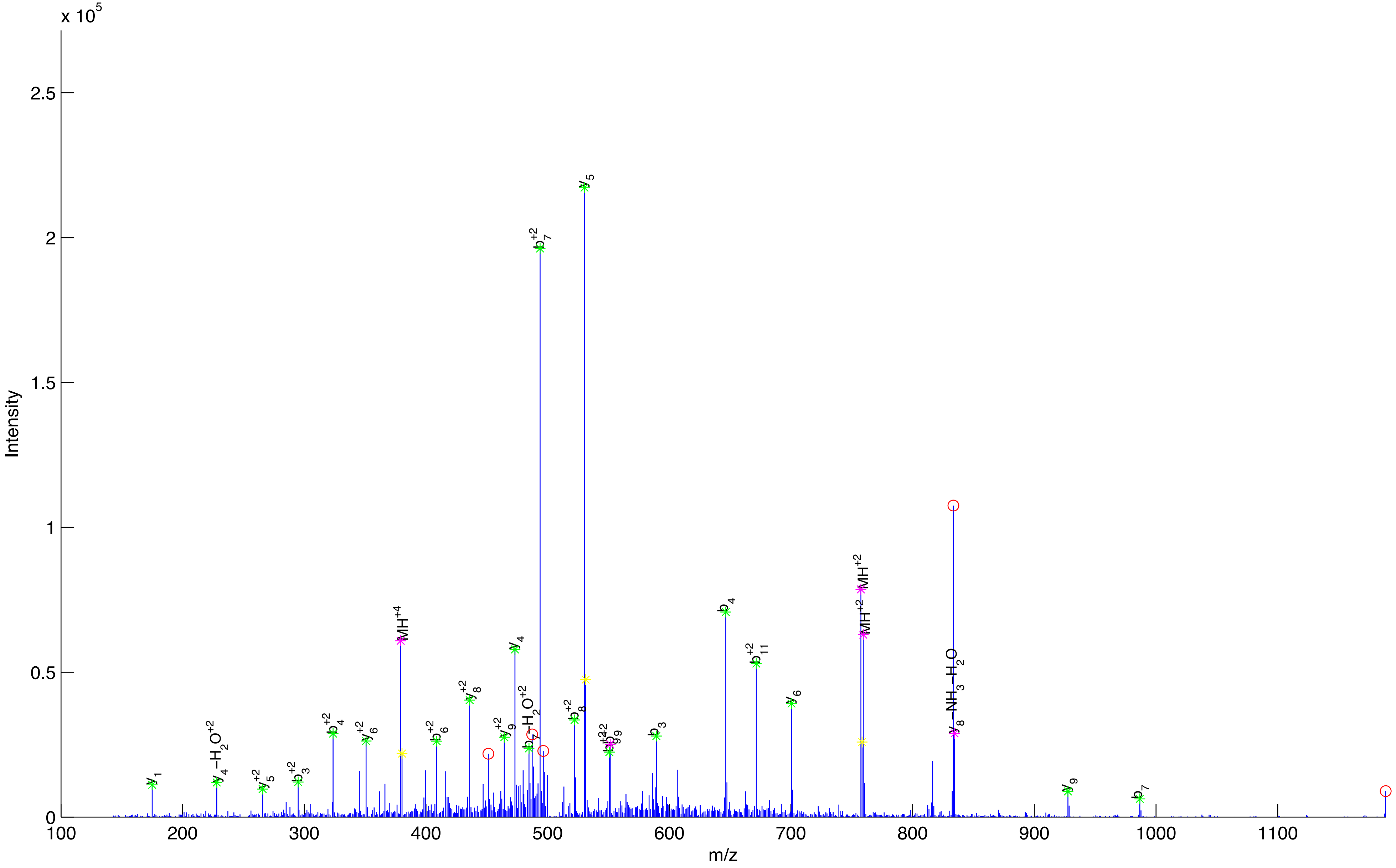

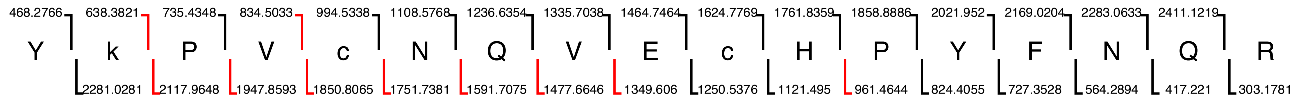

aldo–keto reductase family 1, member C1 [Homo sapiens]
Charge State: +4
Scan Number: 14976
File Name: 120413_A549_EGFIGF_bioRepC_AcK_FT.raw

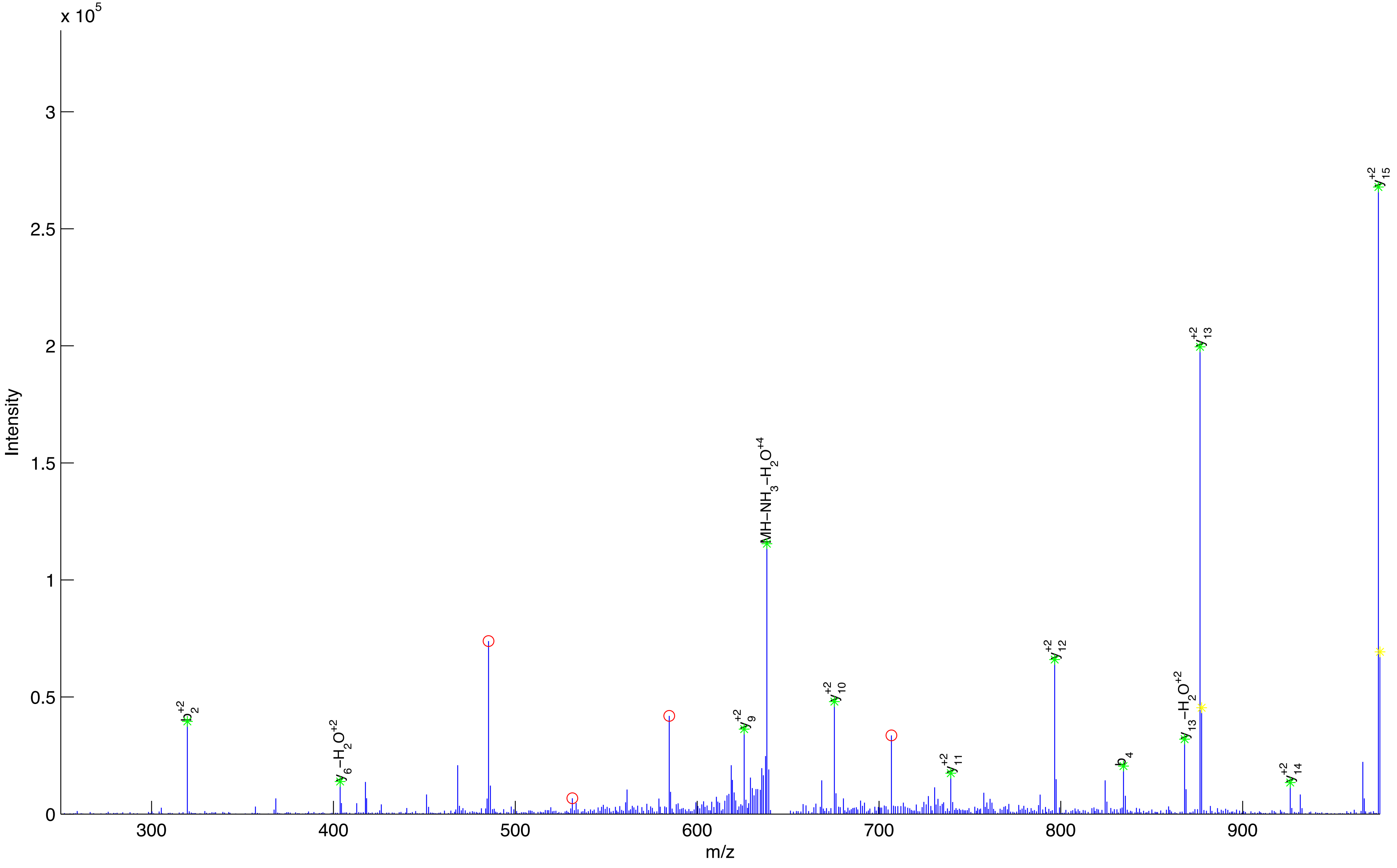

362.2347	532.3402	589.3617	646.3831	816.4887	873.5101	986.5942	1043.6157	1213.7212	1270.7427	1327.7641	1398.8012	1568.9068	
G	k	G	G	k	G	L	G	k	G	G	A	k	R
	1438.813	1381.7916	1211.686	1154.6646	1097.6431	927.5376	870.5161	757.4321	700.4106	530.3051	473.2836	416.2621	345.225

histone cluster 1, H4a [Homo sapiens]
Charge State: +3
Scan Number: 15258
File Name: 120407_A549_EGFIGF_bioRepA_ACK_FT.raw

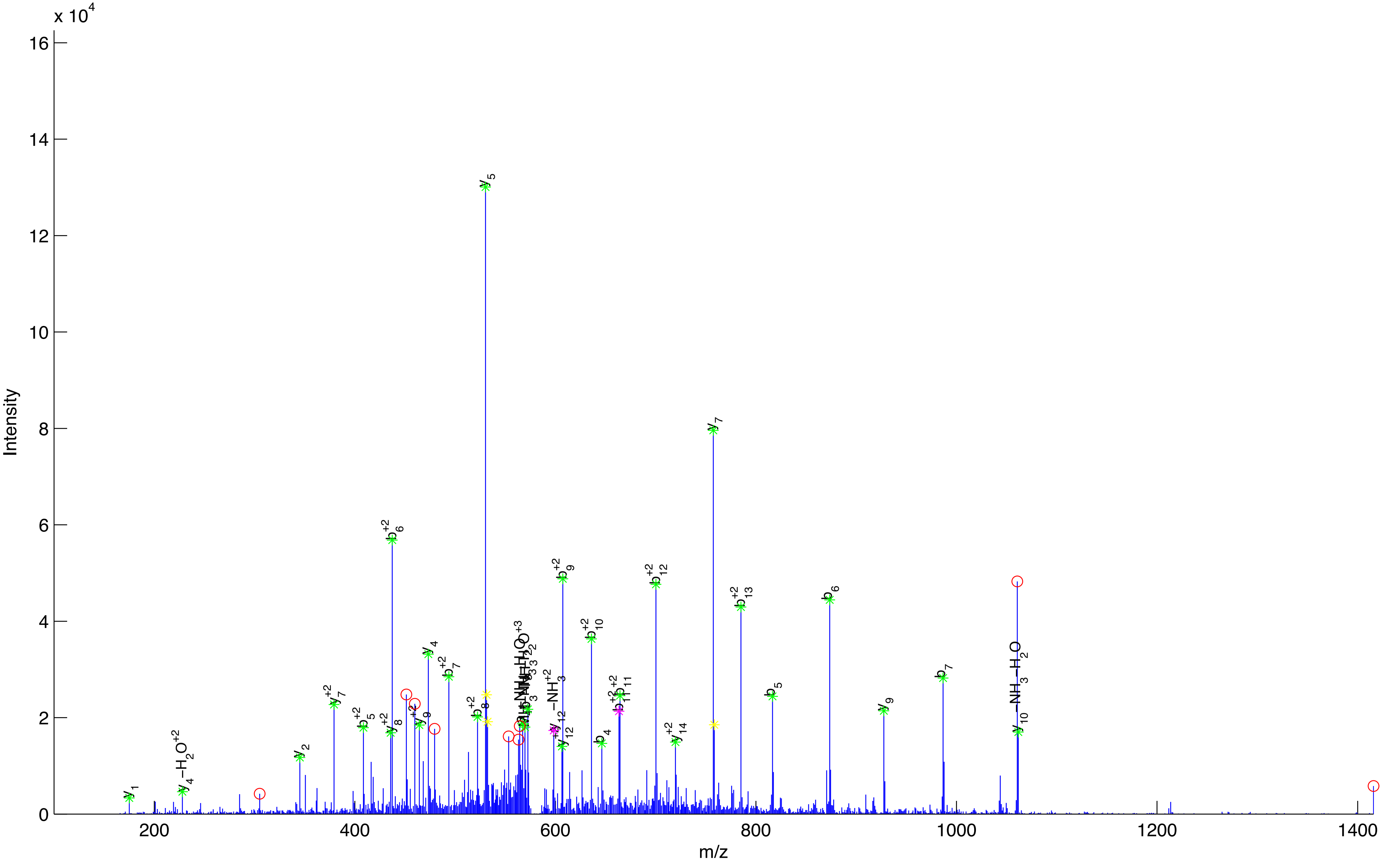

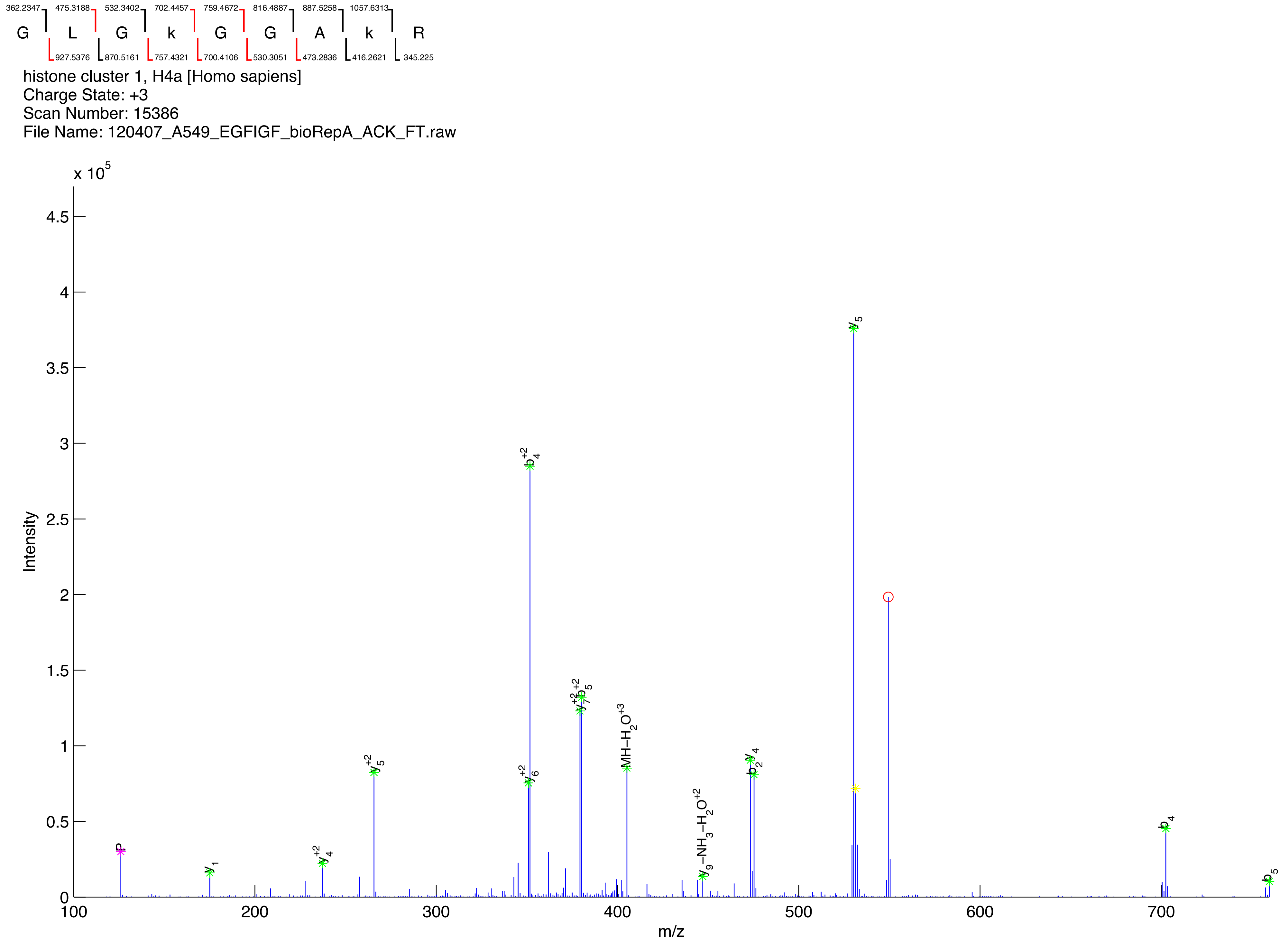
362.2347 475.3188 532.3402 702.4457 759.4672 816.4887 887.5258 1057.6313
G L G k G G A k R
927.5376 870.5161 757.4321 700.4106 530.3051 473.2836 416.2621 345.225
histone cluster 1, H4a [Homo sapiens]
Charge State: +3
Scan Number: 15386
File Name: 120407_A549_EGFIGF_bioRepA_ACK_FT.raw
x 10^5
Intensity
m/z
P
y1
y4+2
y5+2
y6+2
b4+2
y7+2
b5+2
MH−H2O+3
y9−NH3−H2O+2
b2
y4
y5
b4
b5

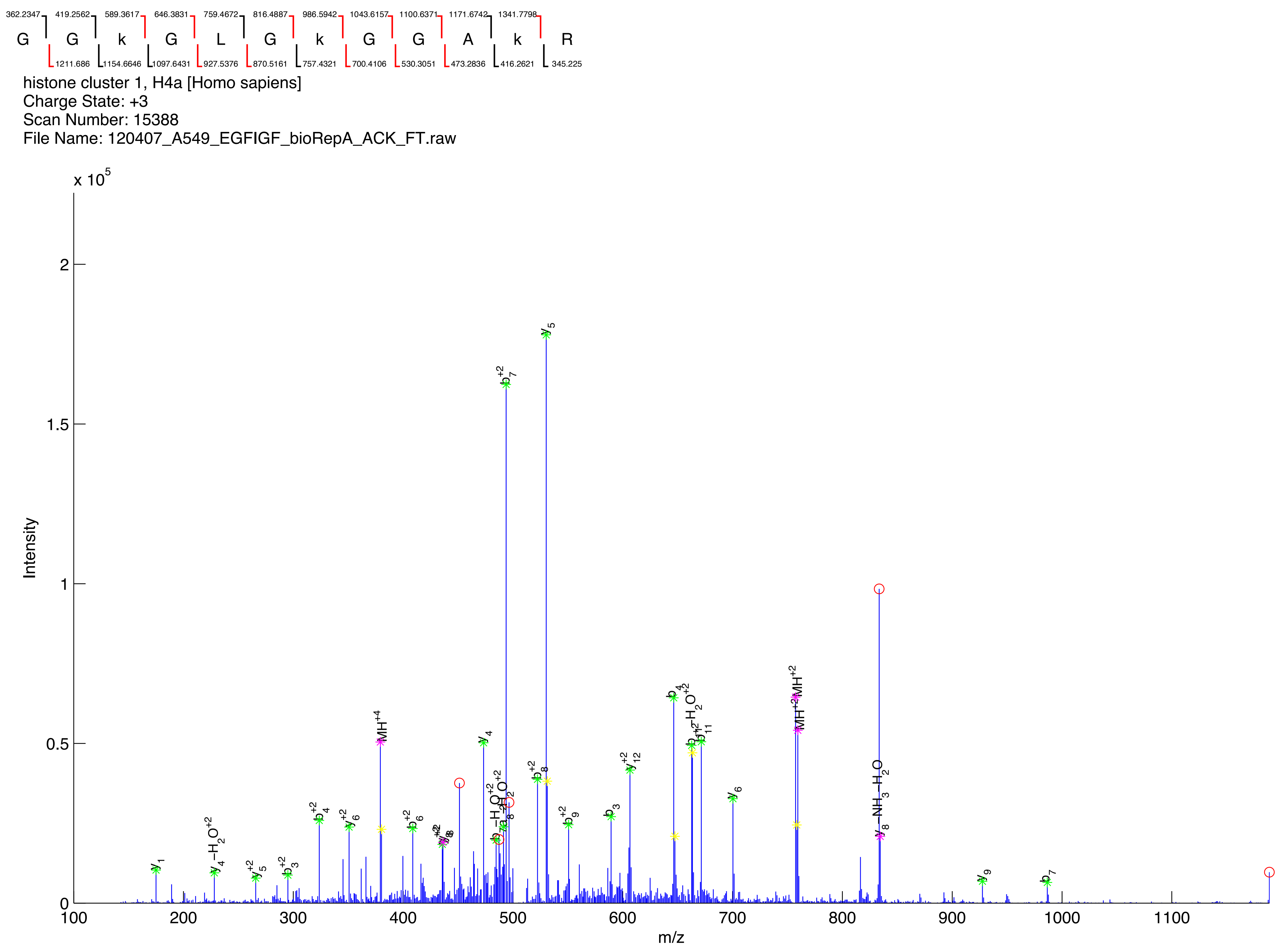
362.2347
419.2562
589.3617
646.3831
759.4672
816.4887
986.5942
1043.6157
1100.6371
1171.6742
1341.7798
G G k G L G k G G A k R
1211.686
1154.6646
1097.6431
927.5376
870.5161
757.4321
700.4106
530.3051
473.2836
416.2621
345.225
histone cluster 1, H4a [Homo sapiens]
Charge State: +3
Scan Number: 15388
File Name: 120407_A549_EGFIGF_bioRepA_ACK_FT.raw
x 10^5
Intensity
m/z

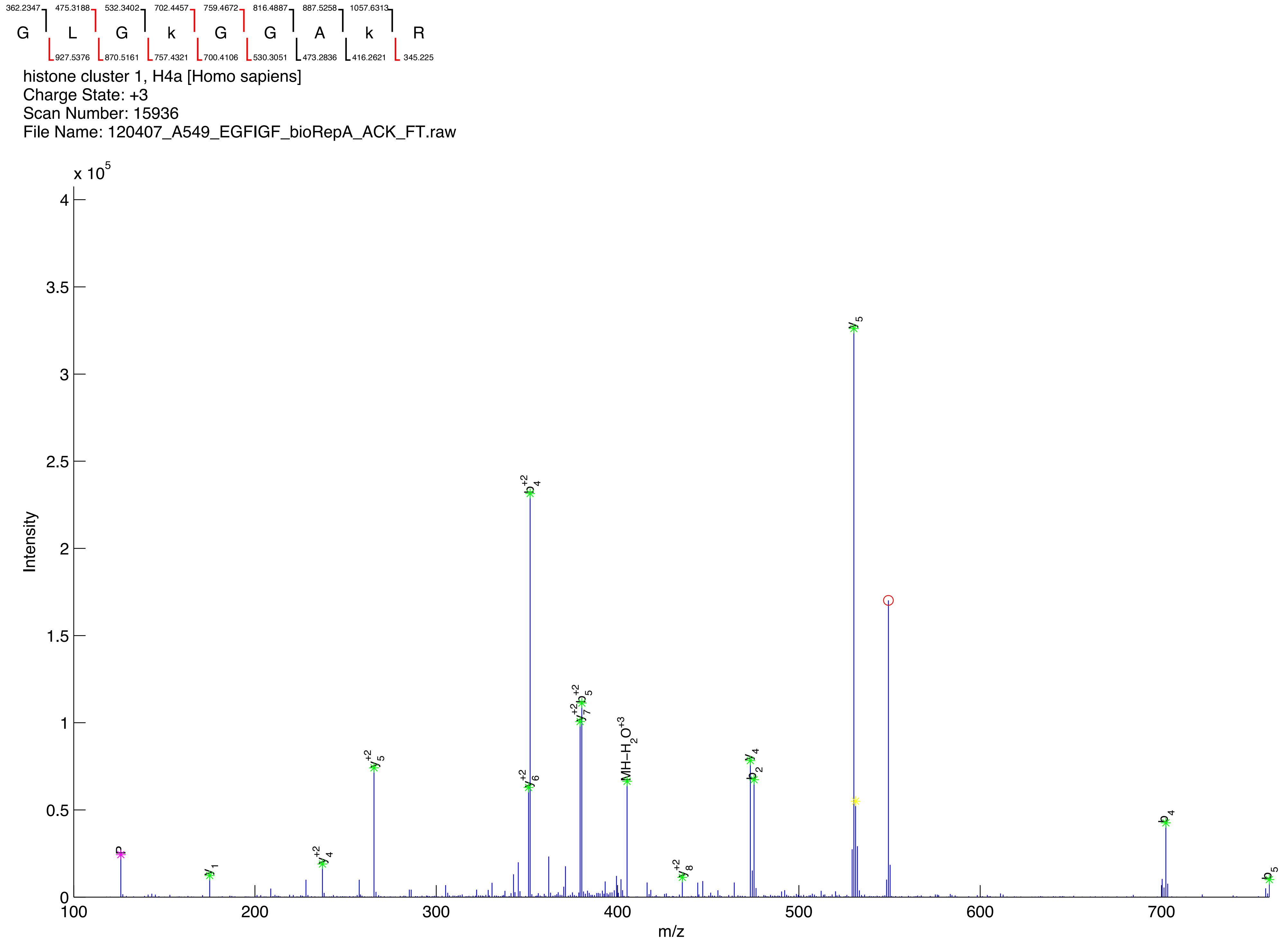
362.2347 475.3188 532.3402 702.4457 759.4672 816.4887 887.5258 1057.6313
G L G k G G A k R
927.5376 870.5161 757.4321 700.4106 530.3051 473.2836 416.2621 345.225
histone cluster 1, H4a [Homo sapiens]
Charge State: +3
Scan Number: 15936
File Name: 120407_A549_EGFIGF_bioRepA_ACK_FT.raw
x 10^5
Intensity
m/z
MH−H2O+3

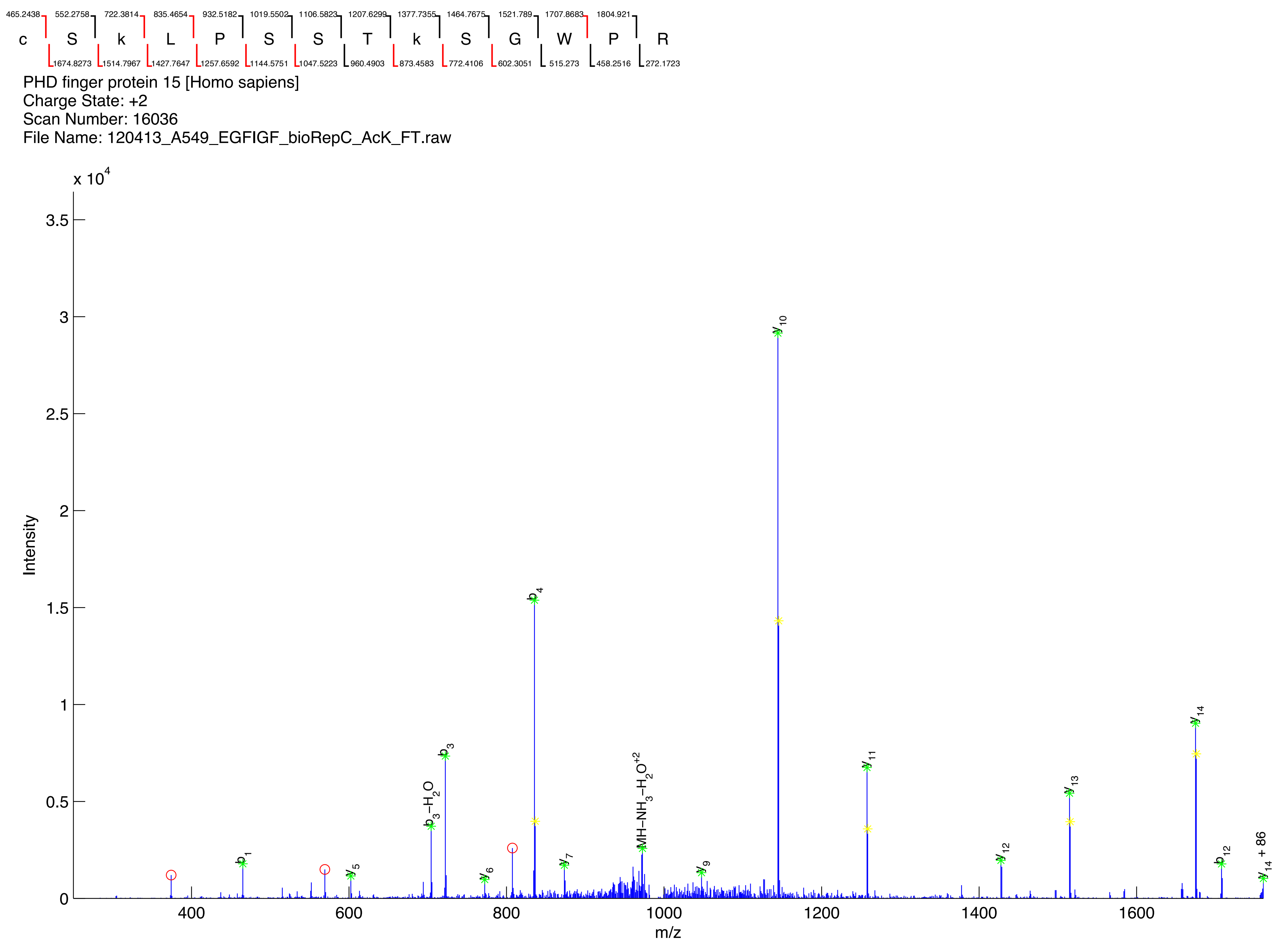

465.2438 552.2758 722.3814 835.4654 932.5182 1019.5502 1106.5823 1207.6299 1377.7355 1464.7675 1521.789 1707.8683 1804.921
c S k L P S S T k S G W P R
1674.8273 1514.7967 1427.7647 1257.6592 1144.5751 1047.5223 960.4903 873.4583 772.4106 602.3051 515.273 458.2516 272.1723
PHD finger protein 15 [Homo sapiens]
Charge State: +2
Scan Number: 16036
File Name: 120413_A549_EGFIGF_bioRepC_AcK_FT.raw
x 10^4
Intensity
m/z
0
0.5
1
1.5
2
2.5
3
3.5
400
600
800
1000
1200
1400
1600
b1
y5
b3−H2O
b3
y6
b4
y7
MH−NH3−H2O+2
y9
y10
y11
y12
y13
y14
b12
y14 + 86

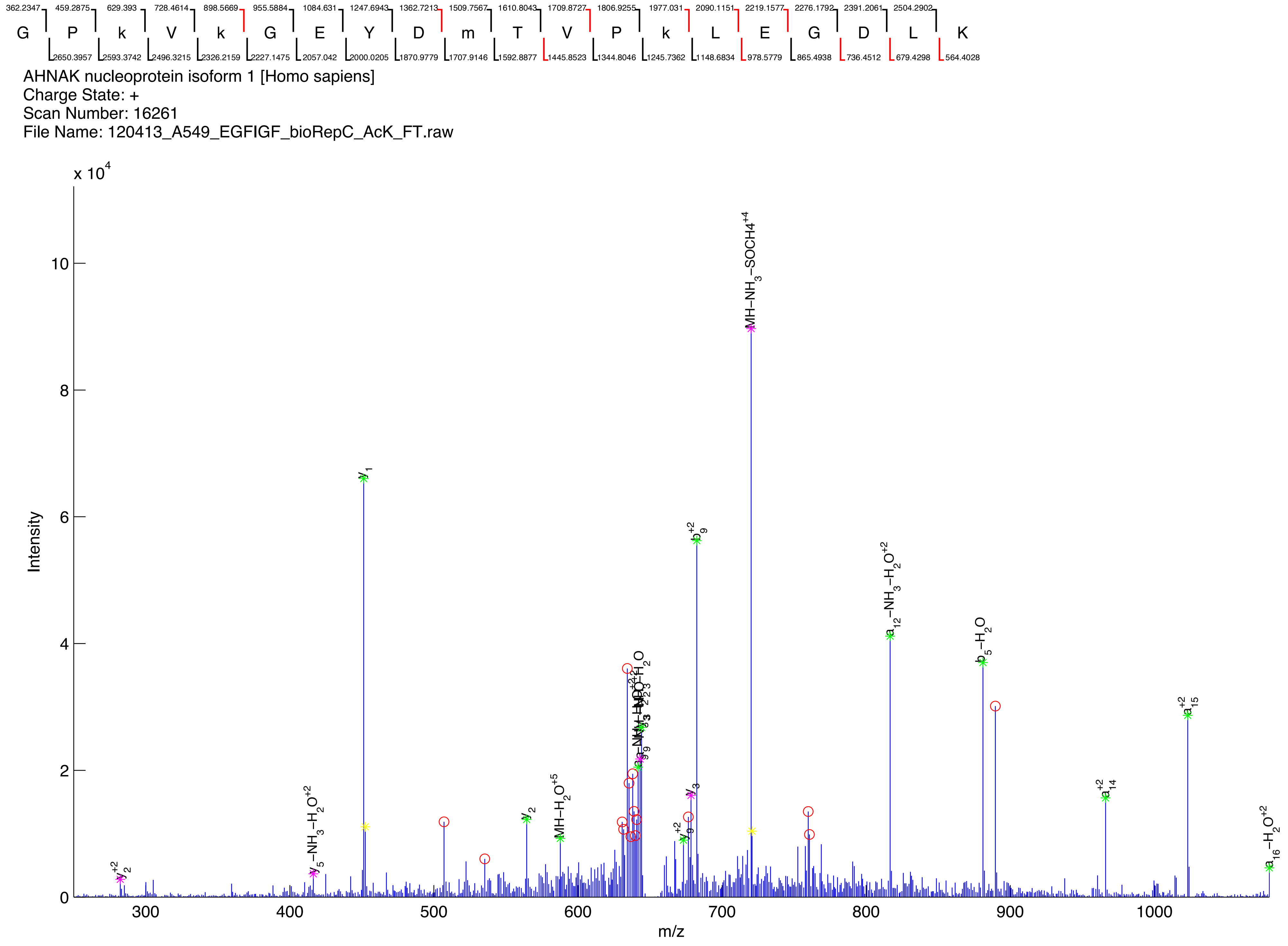
362.2347 459.2875 629.393 728.4614 898.5669 955.5884 1084.631 1247.6943 1362.7213 1509.7567 1610.8043 1709.8727 1806.9255 1977.031 2090.1151 2219.1577 2276.1792 2391.2061 2504.2902
G P k V k G E Y D m T V P k L E G D L K
2650.3957 2593.3742 2496.3215 2326.2159 2227.1475 2057.042 2000.0205 1870.9779 1707.9146 1592.8877 1445.8523 1344.8046 1245.7362 1148.6834 978.5779 865.4938 736.4512 679.4298 564.4028
AHNAK nucleoprotein isoform 1 [Homo sapiens]
Charge State: +
Scan Number: 16261
File Name: 120413_A549_EGFIGF_bioRepC_AcK_FT.raw
x 10^4
Intensity
m/z
MH−NH3−SOCH4+4
y1
b9+2
a12−NH3−H2O+2
b5−H2O
a15+2
a14+2
a16−H2O+2
y5−NH3−H2O+2
MH−H2O+5
y2
y2+2
y3
y9+2

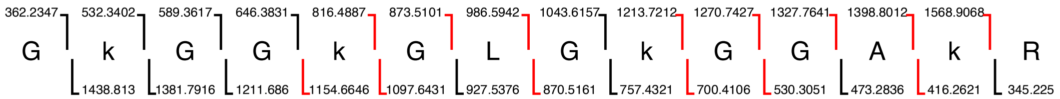

histone cluster 1, H4a [Homo sapiens]
Charge State: +3
Scan Number: 16350
File Name: 120407_A549_EGFIGF_bioRepA_ACK_FT.raw

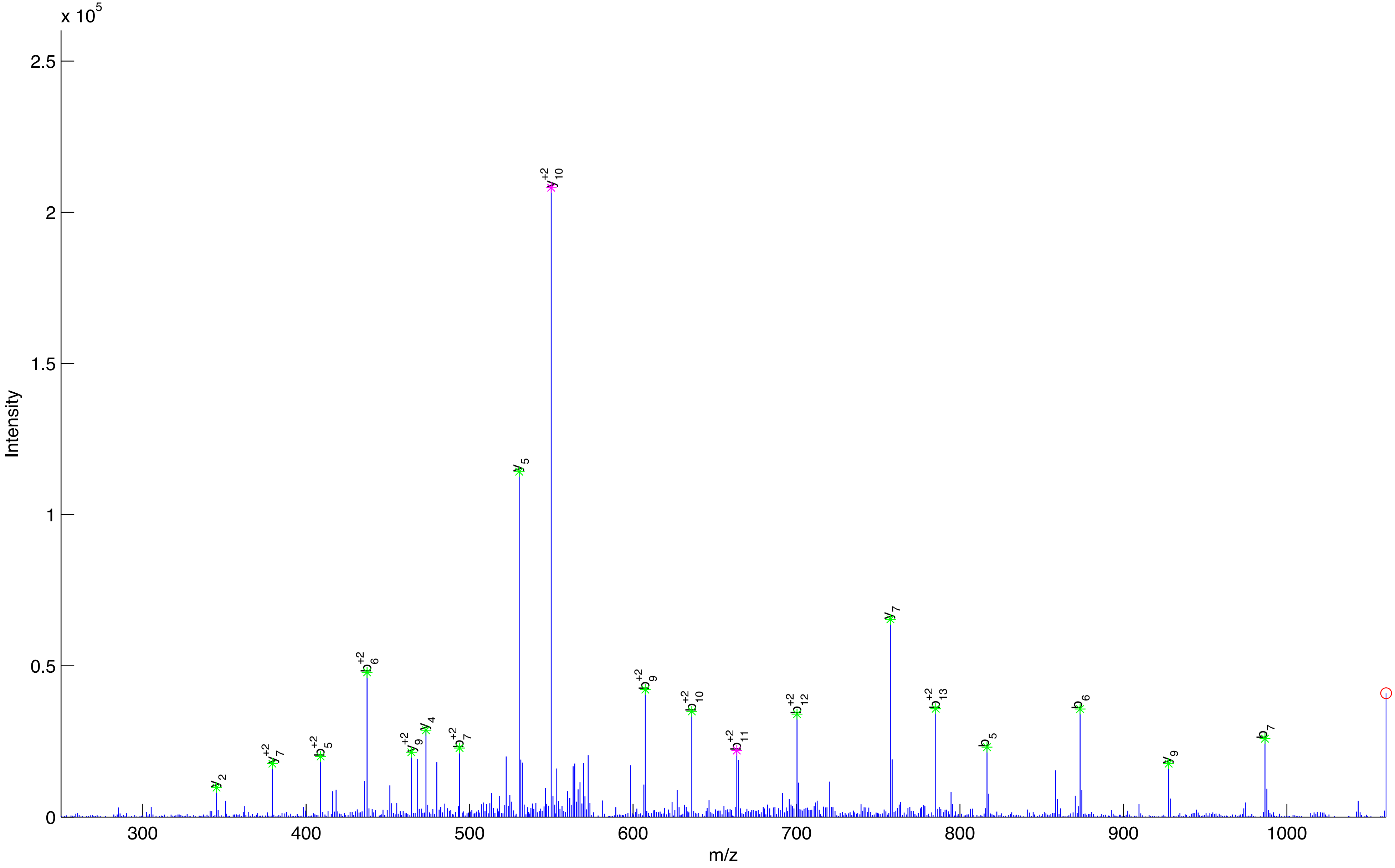

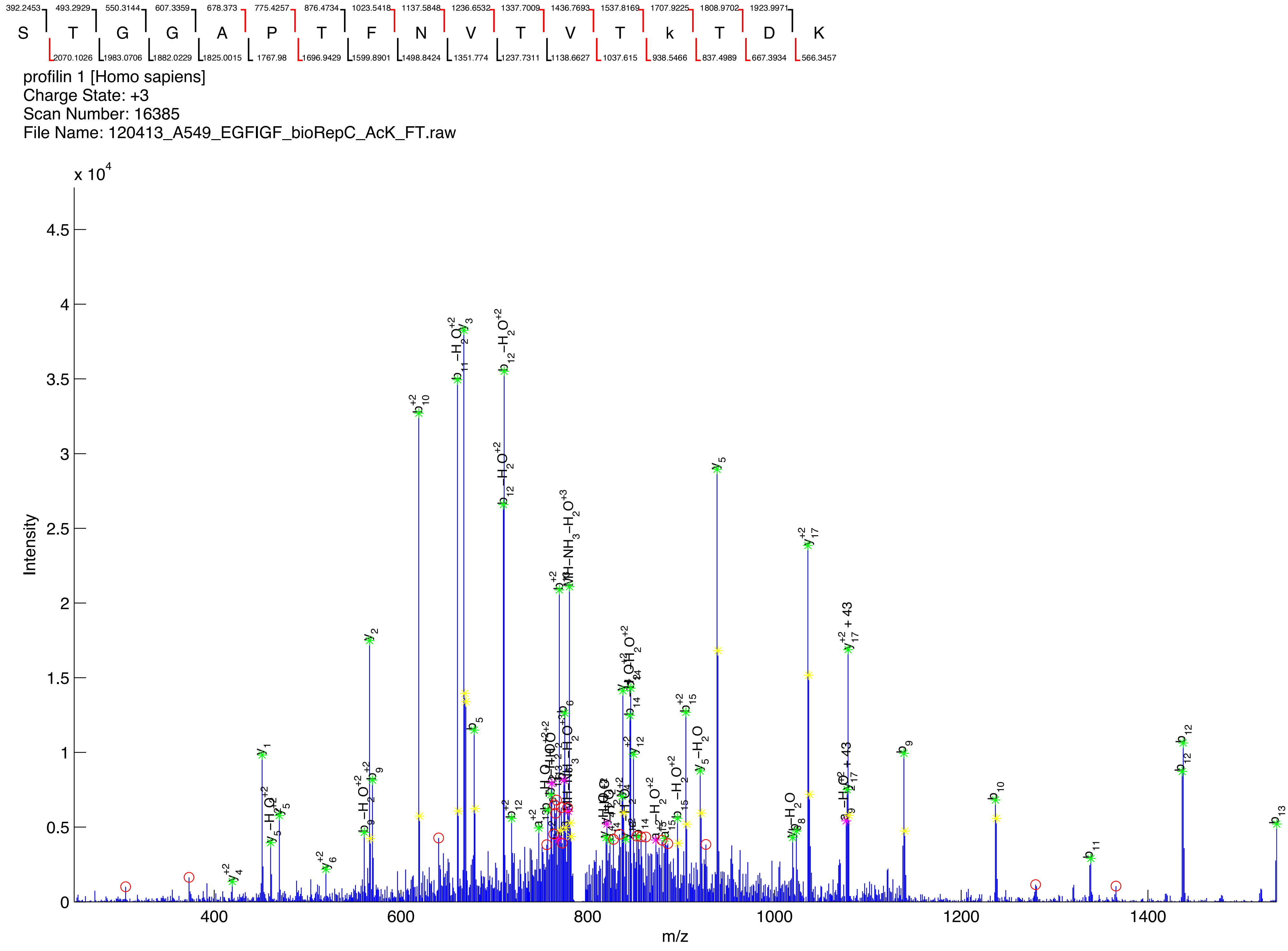
392.2453 493.2929 550.3144 607.3359 678.373 775.4257 876.4734 1023.5418 1137.5848 1236.6532 1337.7009 1436.7693 1537.8169 1707.9225 1808.9702 1923.9971
S T G G A P T F N V T V T k T D K
2070.1026 1983.0706 1882.0229 1825.0015 1767.98 1696.9429 1599.8901 1498.8424 1351.774 1237.7311 1138.6627 1037.615 938.5466 837.4989 667.3934 566.3457
profilin 1 [Homo sapiens]
Charge State: +3
Scan Number: 16385
File Name: 120413_A549_EGFIGF_bioRepC_AcK_FT.raw
Intensity
m/z

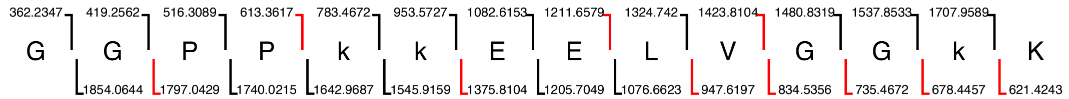

mitogen−activated protein kinase kinase kinase 10 [Homo sapiens]
Charge State: +
Scan Number: 16438
File Name: 120407_A549_EGFIGF_bioRepA_ACK_FT.raw

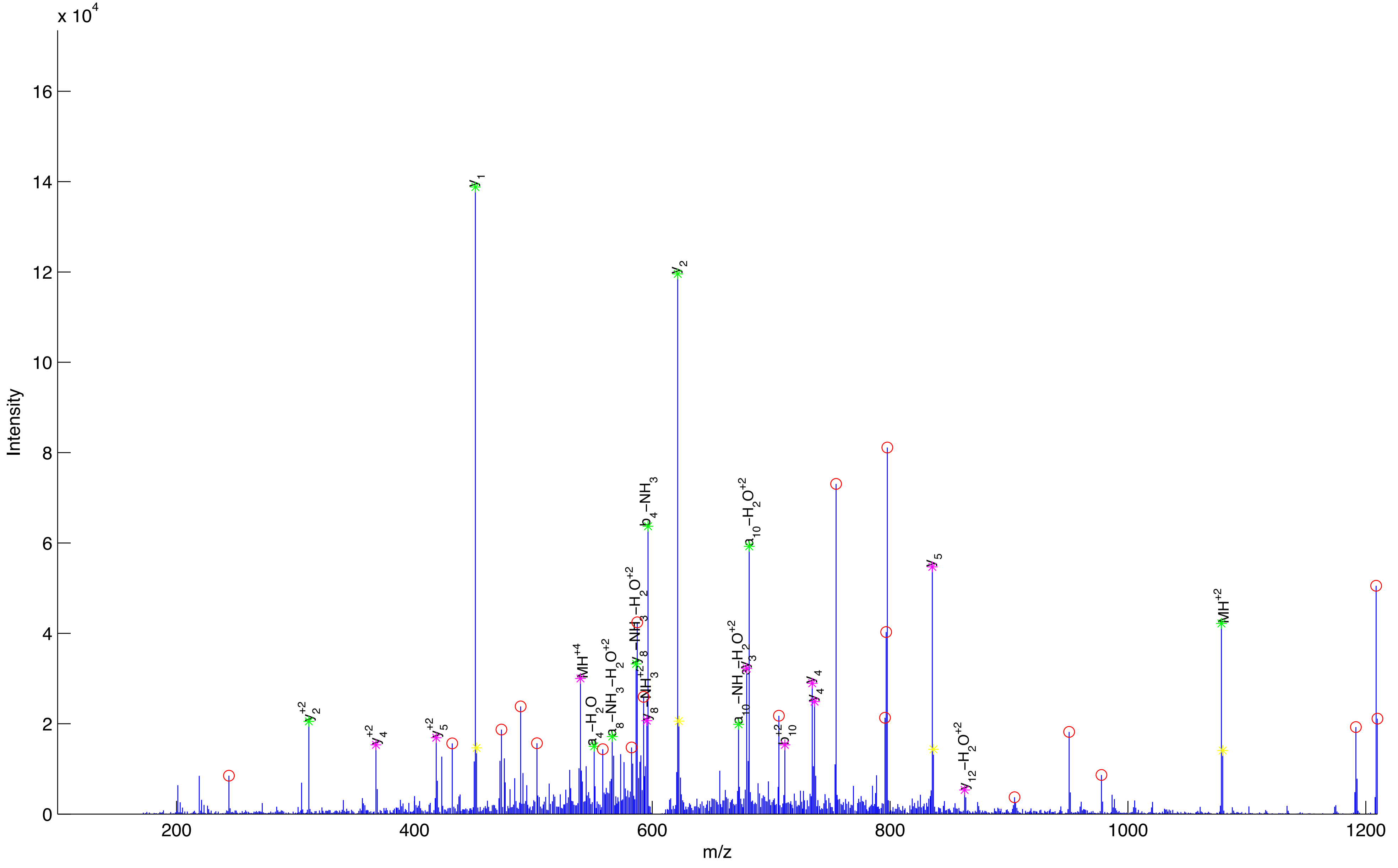

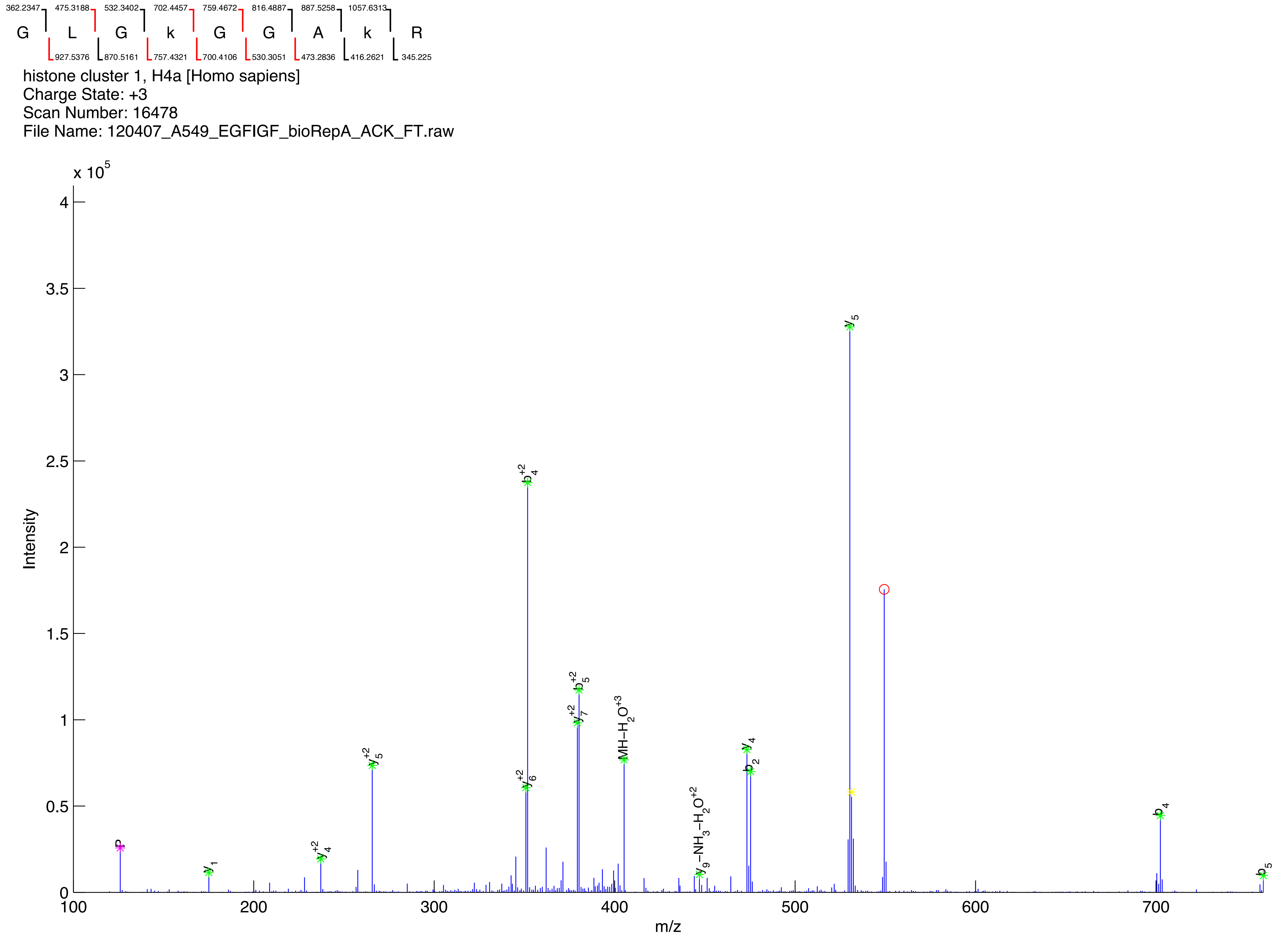
362.2347 475.3188 532.3402 702.4457 759.4672 816.4887 887.5258 1057.6313
G L G k G G A k R
927.5376 870.5161 757.4321 700.4106 530.3051 473.2836 416.2621 345.225
histone cluster 1, H4a [Homo sapiens]
Charge State: +3
Scan Number: 16478
File Name: 120407_A549_EGFIGF_bioRepA_ACK_FT.raw
x 10^5
Intensity
m/z

362.2347	419.2562	589.3617	646.3831	759.4672	816.4887	986.5942	1043.6157	1100.6371	1171.6742	1341.7798	
G	G	k	G	L	G	k	G	G	A	k	R
	1211.686	1154.6646	1097.6431	927.5376	870.5161	757.4321	700.4106	530.3051	473.2836	416.2621	345.225

histone cluster 1, H4a [Homo sapiens]
Charge State: +3
Scan Number: 16480
File Name: 120407_A549_EGFIGF_bioRepA_ACK_FT.raw

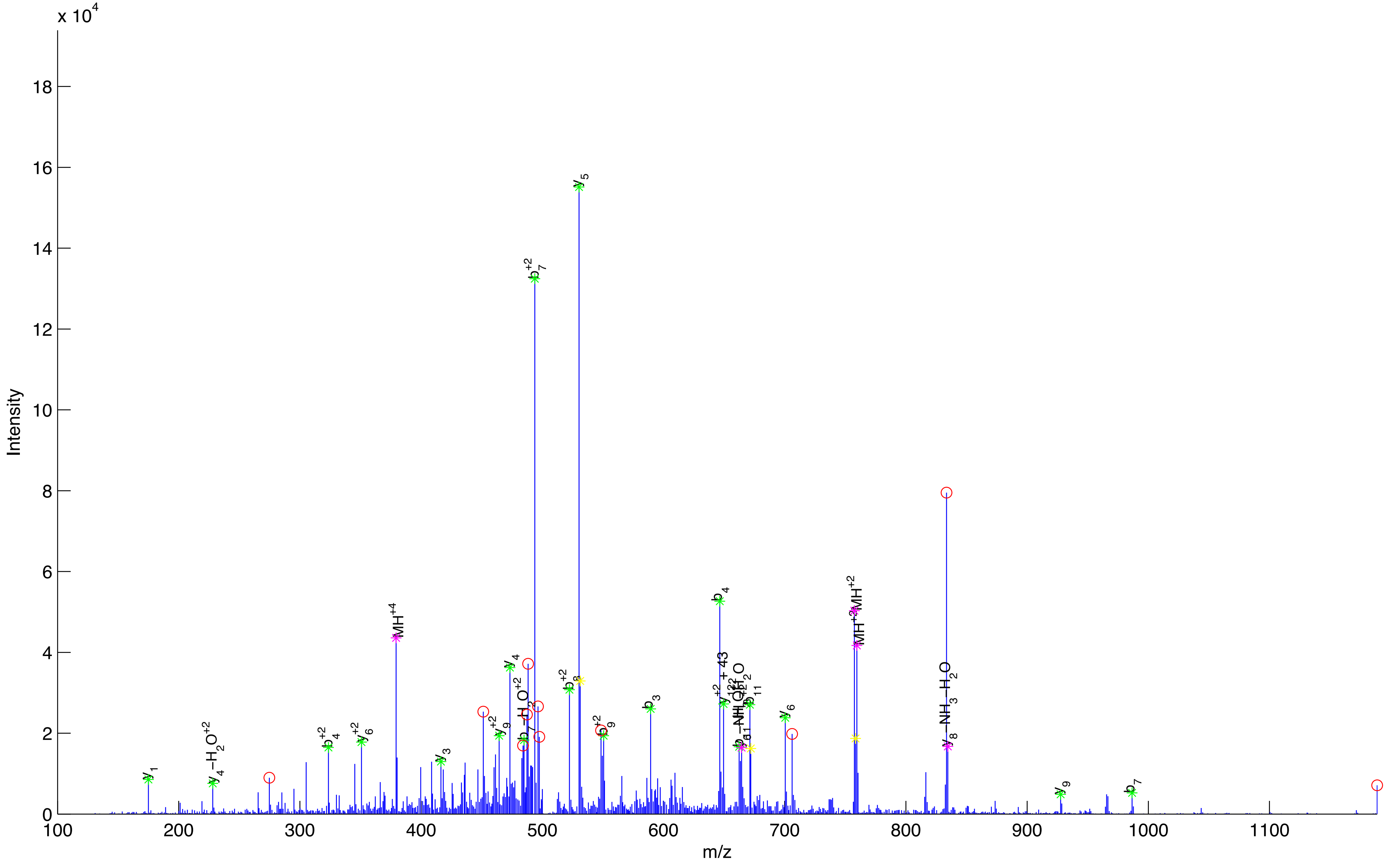

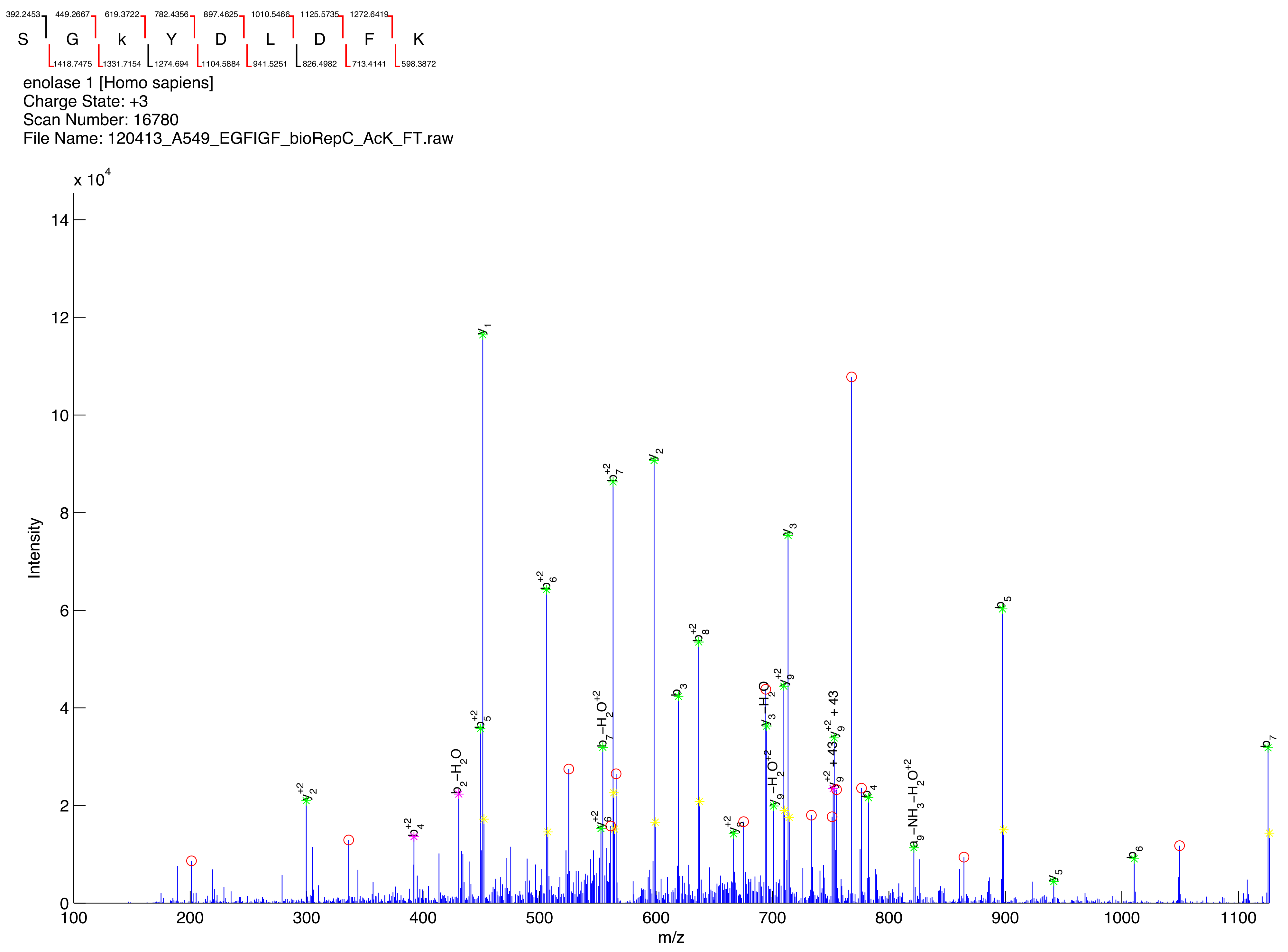
392.2453 449.2667 619.3722 782.4356 897.4625 1010.5466 1125.5735 1272.6419
S G k Y D L D F K
1418.7475 1331.7154 1274.694 1104.5884 941.5251 826.4982 713.4141 598.3872
enolase 1 [Homo sapiens]
Charge State: +3
Scan Number: 16780
File Name: 120413_A549_EGFIGF_bioRepC_AcK_FT.raw
x 10^4
Intensity
m/z

418.2973 531.3814 630.4498 759.4924 929.5979 1026.6507 1173.7191 1230.7405

I I V E k P F G R

1100.6468 987.5627 874.4787 775.4103 646.3677 476.2621 379.2094 232.141

glucose–6–phosphate dehydrogenase isoform b [Homo sapiens]
Charge State: +3
Scan Number: 16791
File Name: 120407_A549_EGFIGF_bioRepA_ACK_FT.raw

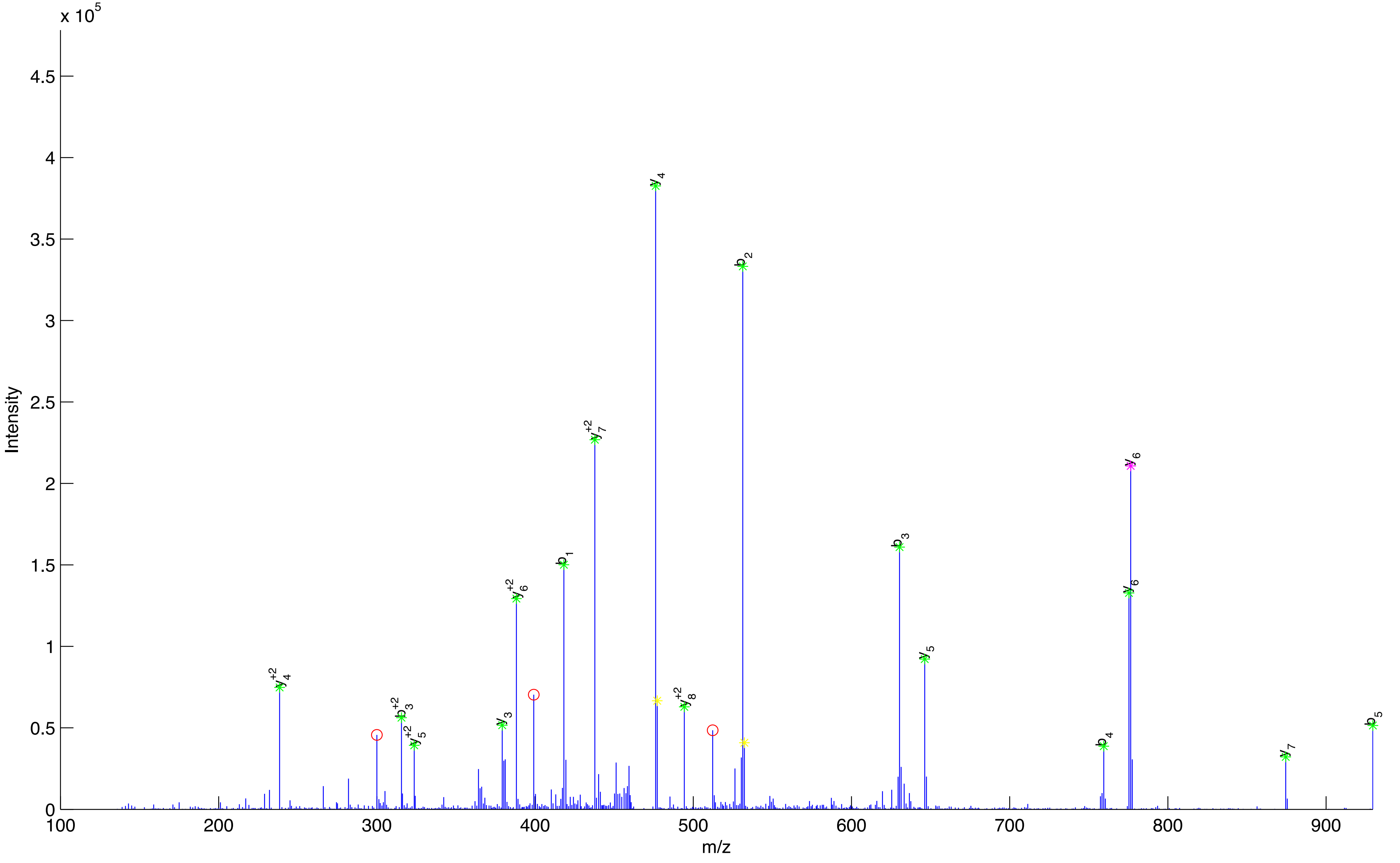

GTPase activating Rap−RanGAP domain−like 3 [Homo sapiens]
Charge State: +3
Scan Number: 16938
File Name: 120407_A549_EGFIGF_bioRepA_ACK_FT.raw

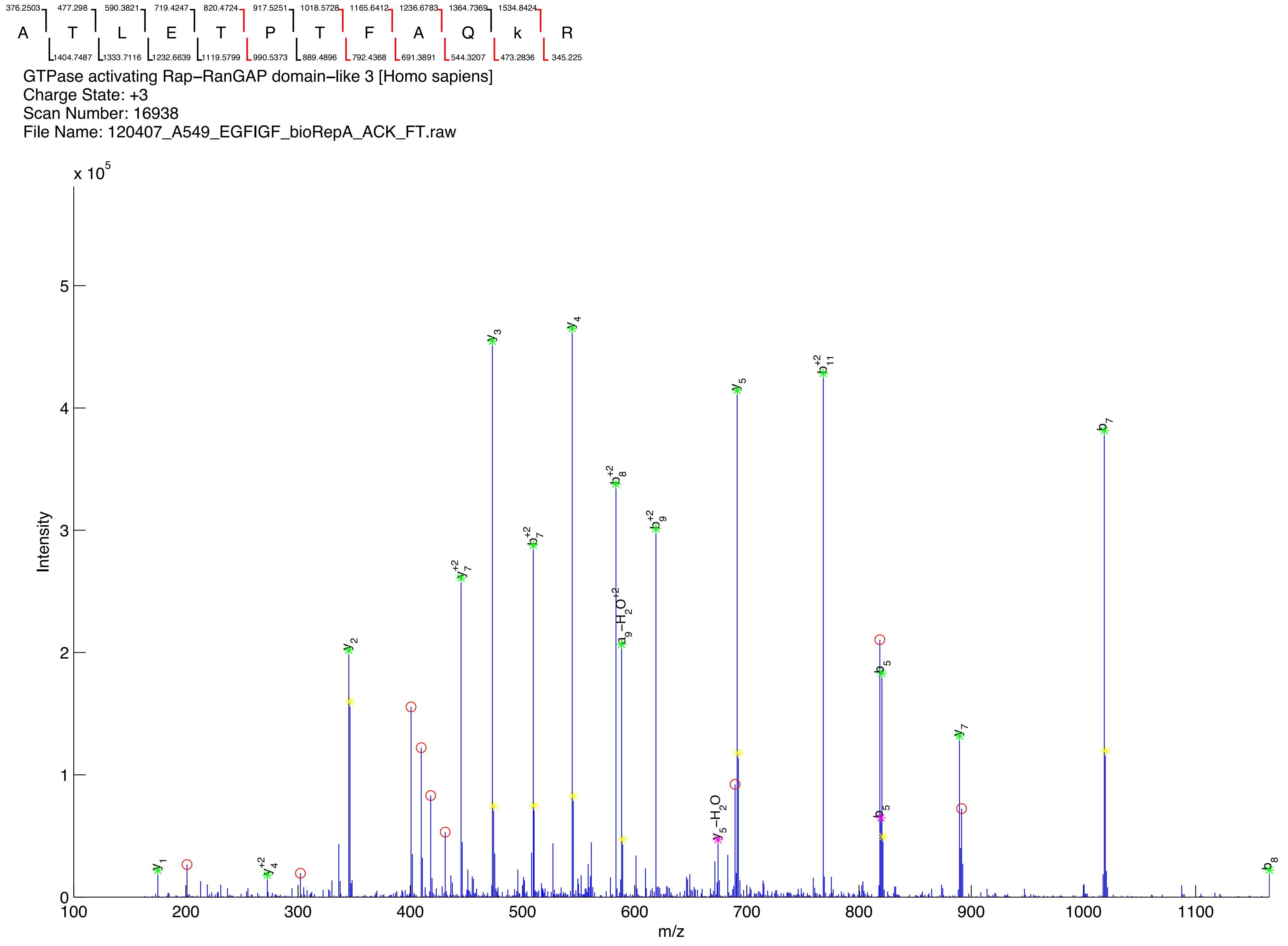

406.2609 505.3293 620.3563 677.3777 774.4305 861.4625 918.484 1088.5895 1201.6736 1387.7529

T V D G P S G k L W R

1257.6592 1156.6115 1057.5431 942.5161 885.4947 788.4419 701.4099 644.3884 474.2829 361.1988

glyceraldehyde−3−phosphate dehydrogenase [Homo sapiens]
Charge State: +3
Scan Number: 17004
File Name: 120404_A549_EGFIGF_bioRepB_ACK_FT.raw

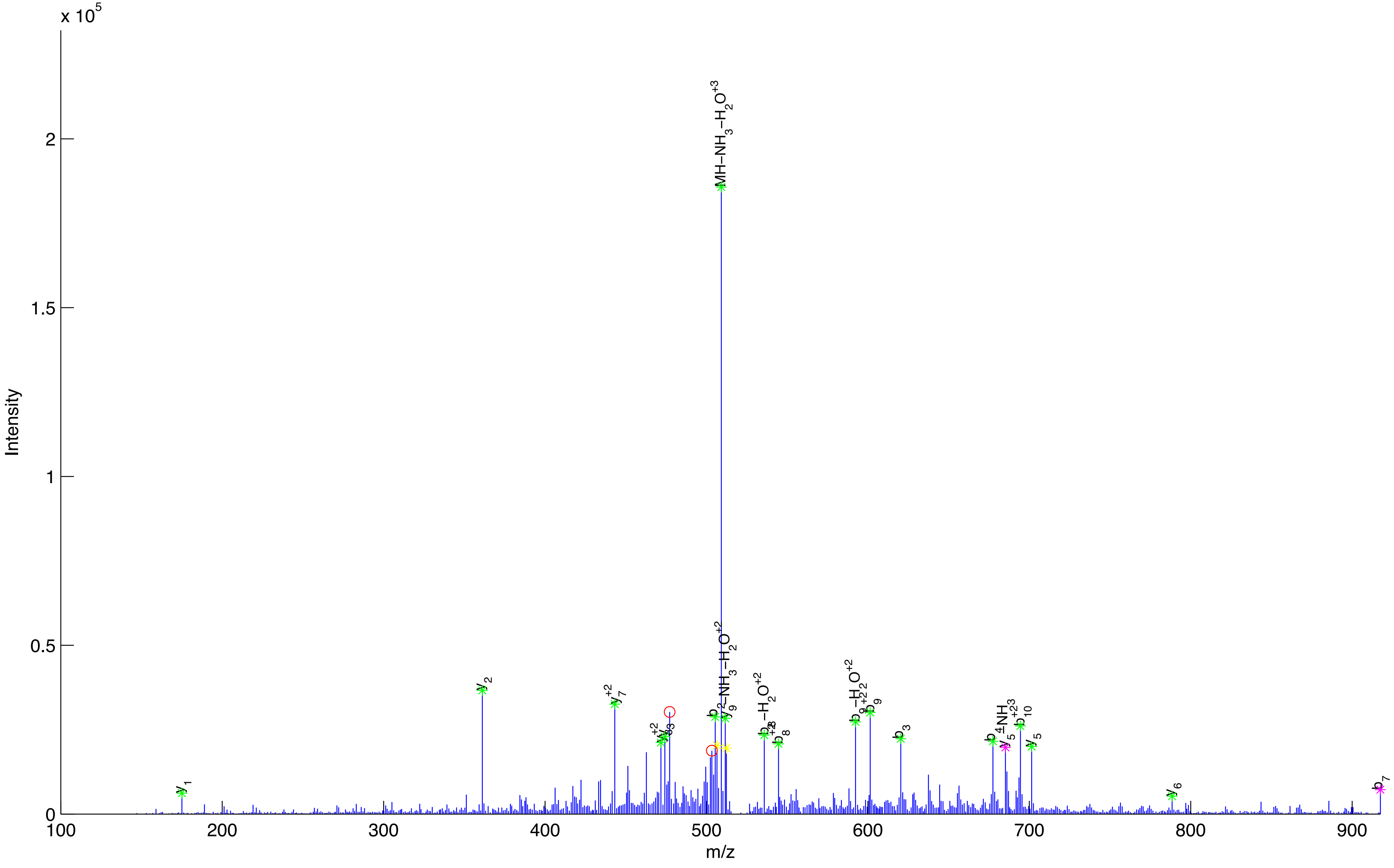

362.2347 475.3188 532.3402 702.4457 759.4672 816.4887 887.5258 1057.6313

G L G k G G A k R

927.5376 870.5161 757.4321 700.4106 530.3051 473.2836 416.2621 345.225

histone cluster 1, H4a [Homo sapiens]
Charge State: +3
Scan Number: 17022
File Name: 120407_A549_EGFIGF_bioRepA_ACK_FT.raw

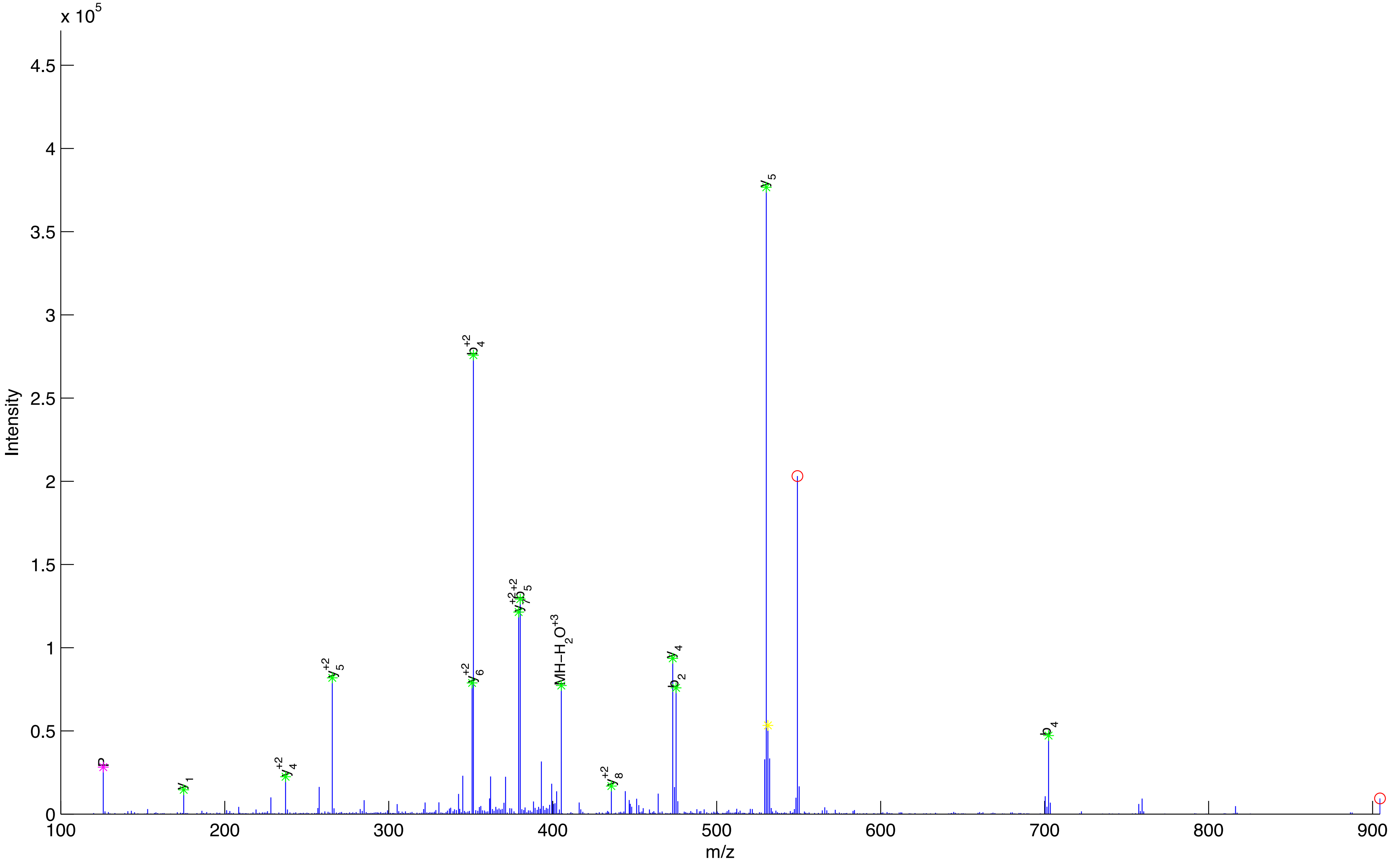

362.2347	419.2562	589.3617	646.3831	759.4672	816.4887	986.5942	1043.6157	1100.6371	1171.6742	1341.7798	
G	G	k	G	L	G	k	G	G	A	k	R
	1211.686	1154.6646	1097.6431	927.5376	870.5161	757.4321	700.4106	530.3051	473.2836	416.2621	345.225

histone cluster 1, H4a [Homo sapiens]
Charge State: +3
Scan Number: 17024
File Name: 120407_A549_EGFIGF_bioRepA_ACK_FT.raw

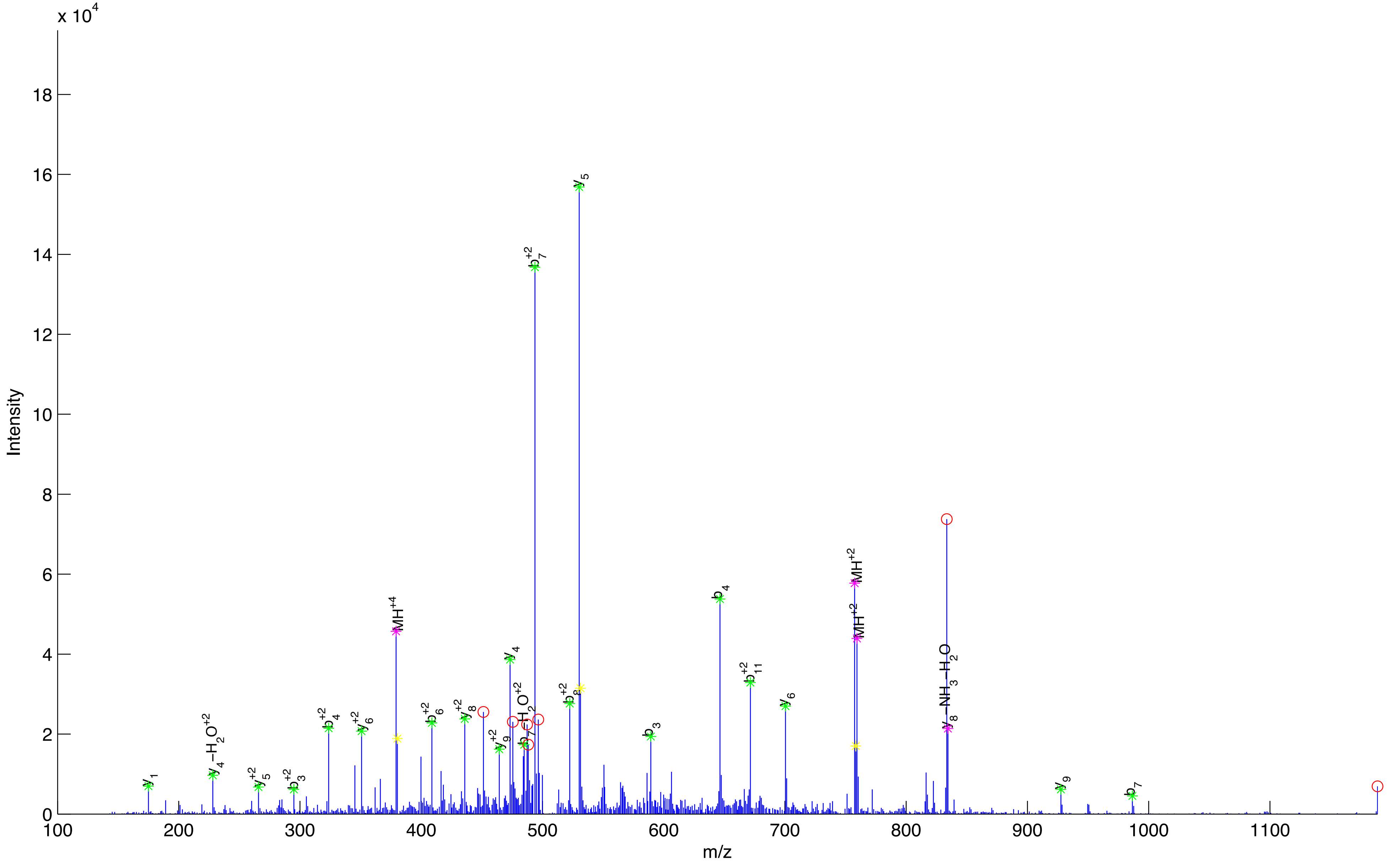

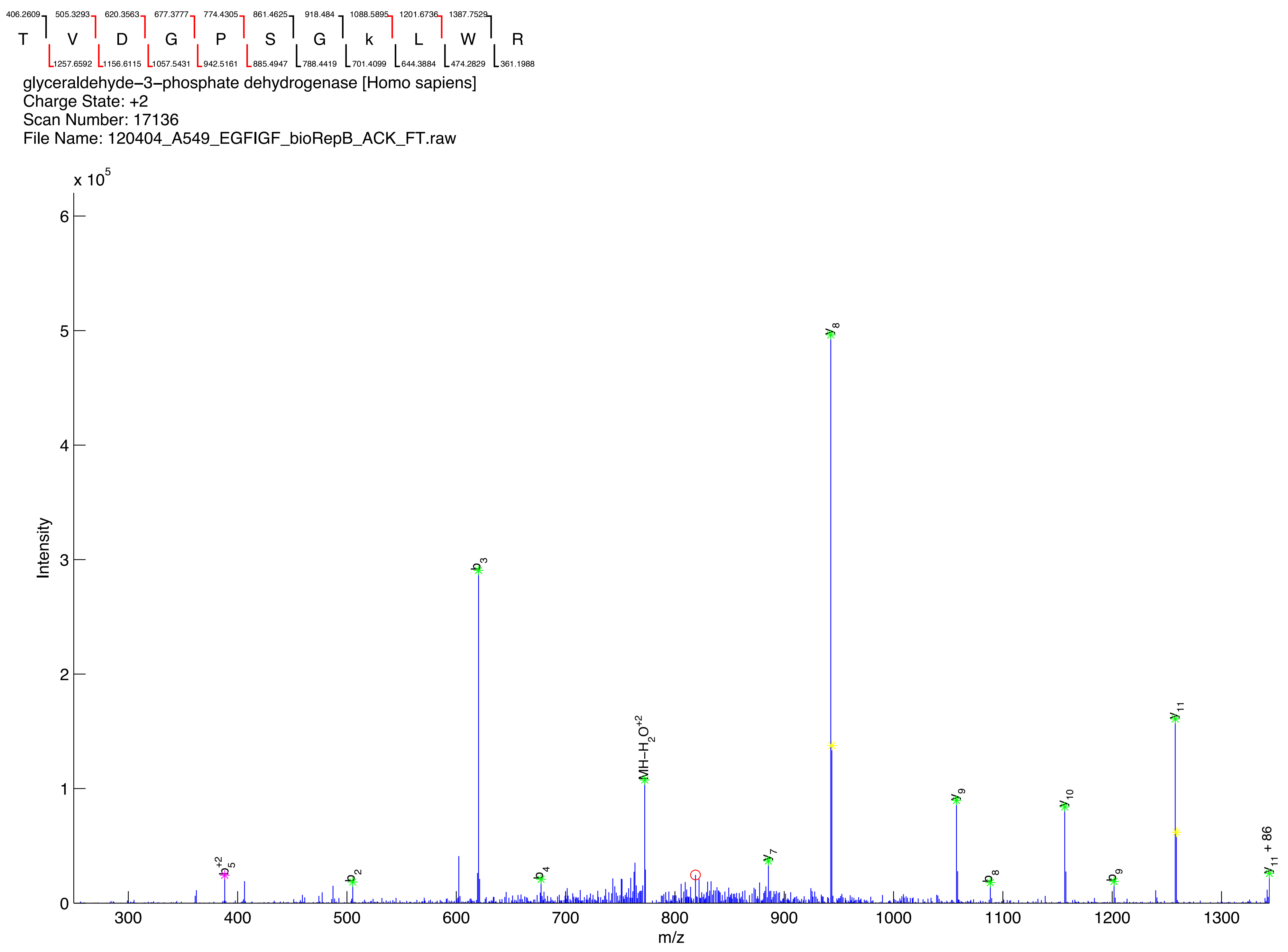
406.2609 505.3293 620.3563 677.3777 774.4305 861.4625 918.484 1088.5895 1201.6736 1387.7529
T V D G P S G k L W R
1257.6592 1156.6115 1057.5431 942.5161 885.4947 788.4419 701.4099 644.3884 474.2829 361.1988
glyceraldehyde−3−phosphate dehydrogenase [Homo sapiens]
Charge State: +2
Scan Number: 17136
File Name: 120404_A549_EGFIGF_bioRepB_ACK_FT.raw
x 10^5
Intensity
m/z
b+2 5
b2
b3
b4
MH−H2O+2
y7
y8
y9
b8
y10
b9
y11
y11 + 86

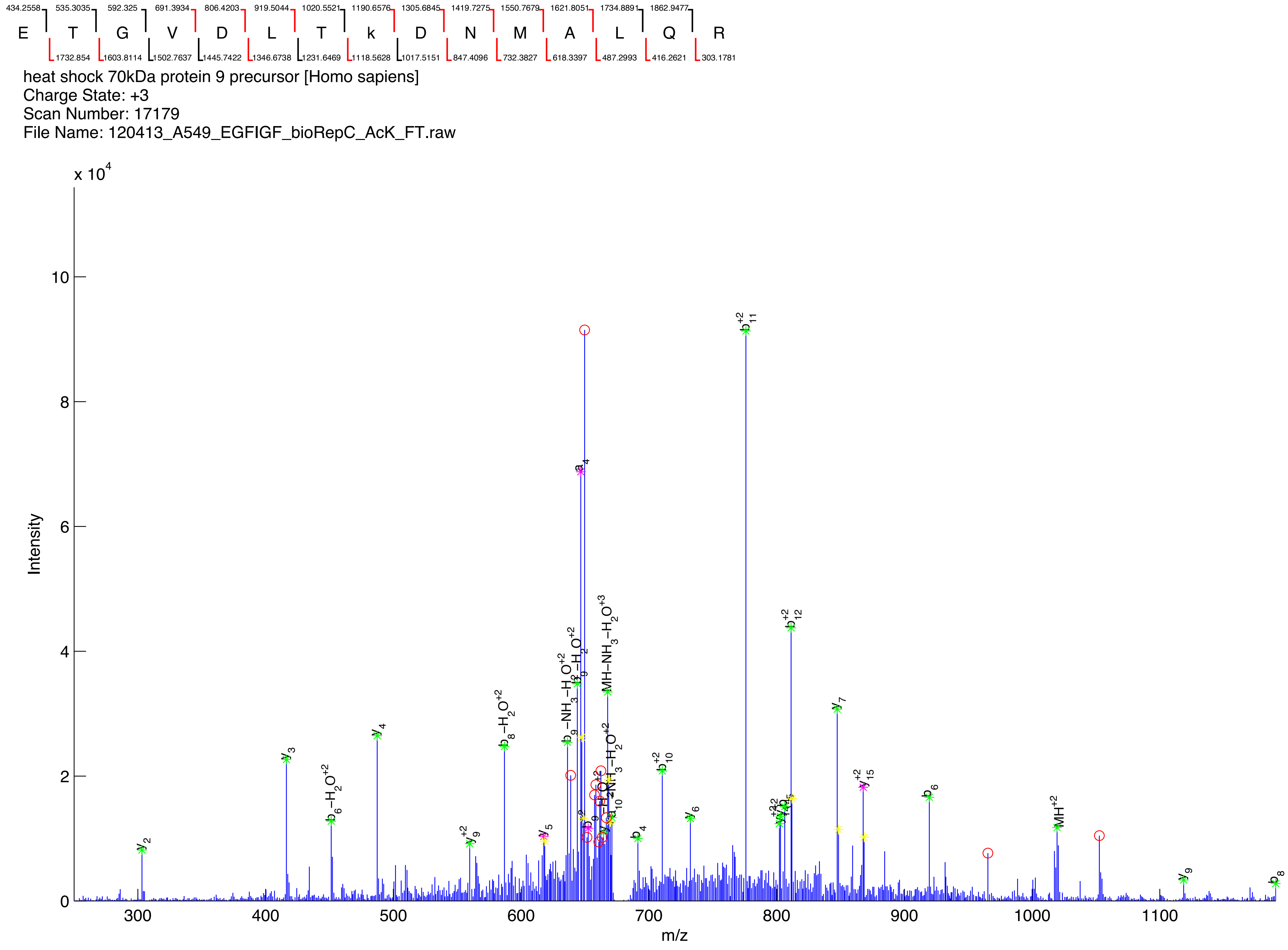

434.2558 535.3035 592.325 691.3934 806.4203 919.5044 1020.5521 1190.6576 1305.6845 1419.7275 1550.7679 1621.8051 1734.8891 1862.9477
E T G V D L T k D N M A L Q R
1732.854 1603.8114 1502.7637 1445.7422 1346.6738 1231.6469 1118.5628 1017.5151 847.4096 732.3827 618.3397 487.2993 416.2621 303.1781
heat shock 70kDa protein 9 precursor [Homo sapiens]
Charge State: +3
Scan Number: 17179
File Name: 120413_A549_EGFIGF_bioRepC_AcK_FT.raw
x 10^4
Intensity
m/z

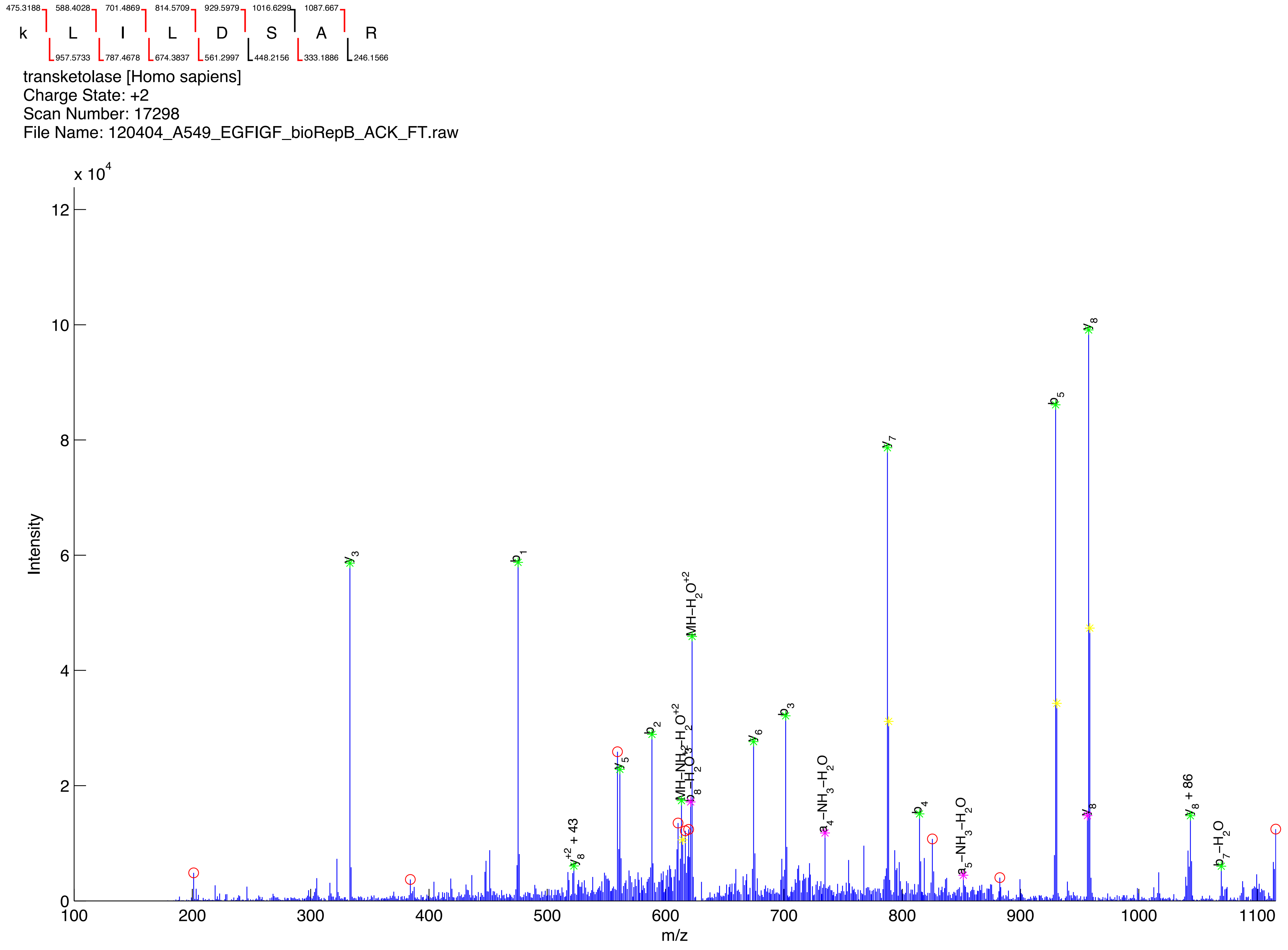
475.3188 588.4028 701.4869 814.5709 929.5979 1016.6299 1087.667
k L I L D S A R
957.5733 787.4678 674.3837 561.2997 448.2156 333.1886 246.1566
transketolase [Homo sapiens]
Charge State: +2
Scan Number: 17298
File Name: 120404_A549_EGFIGF_bioRepB_ACK_FT.raw
x 10^4
Intensity
m/z
y3
b1
y8+2 + 43
y5
b2
MH−NH3−H2O+2
b8−H2O
MH−H2O+2
y6
b3
a4−NH3−H2O
y7
b4
a5−NH3−H2O
b5
y8
y8
y8 + 86
b7−H2O

solute carrier family 25, member 5 [Homo sapiens]
Charge State: +3
Scan Number: 17418
File Name: 120413_A549_EGFIGF_bioRepC_AcK_FT.raw

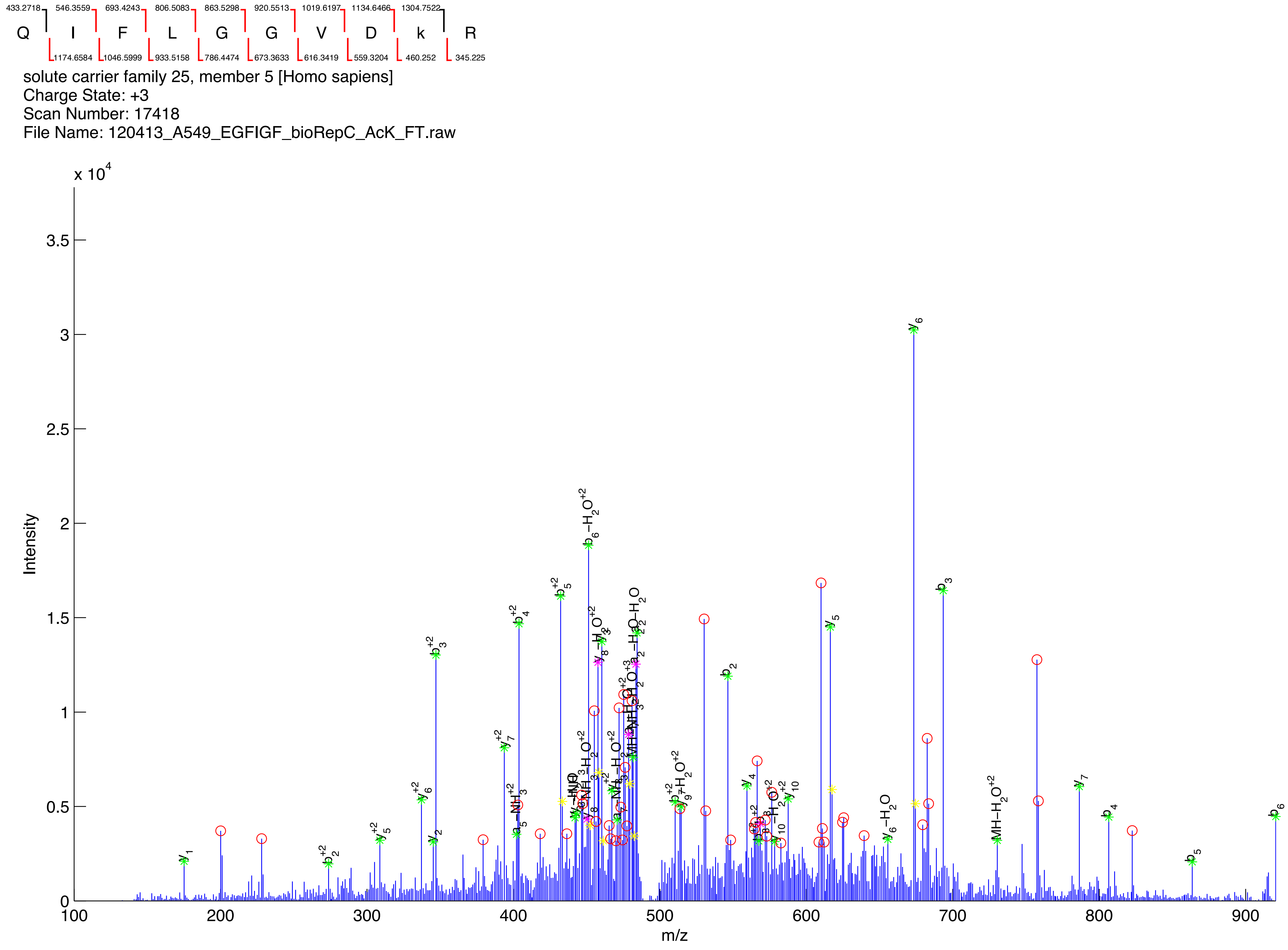

362.2347 532.3402 589.3617 646.3831 816.4887 873.5101 986.5942 1043.6157 1213.7212 1270.7427 1327.7641 1398.8012 1568.9068

G k G G k G L G k G G A k R

1438.813 1381.7916 1211.686 1154.6646 1097.6431 927.5376 870.5161 757.4321 700.4106 530.3051 473.2836 416.2621 345.225

histone cluster 1, H4a [Homo sapiens]
Charge State: +3
Scan Number: 17442
File Name: 120407_A549_EGFIGF_bioRepA_ACK_FT.raw

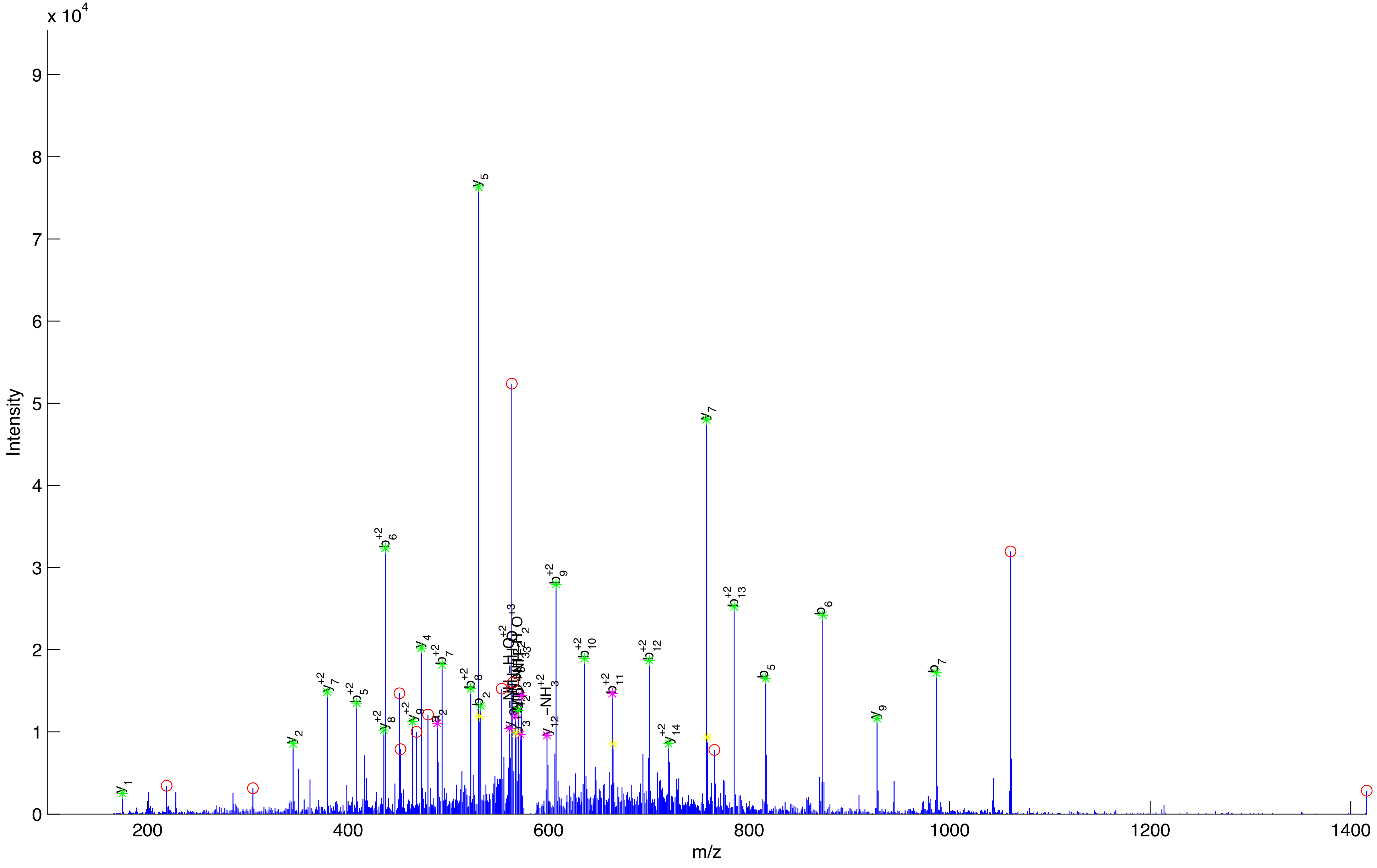

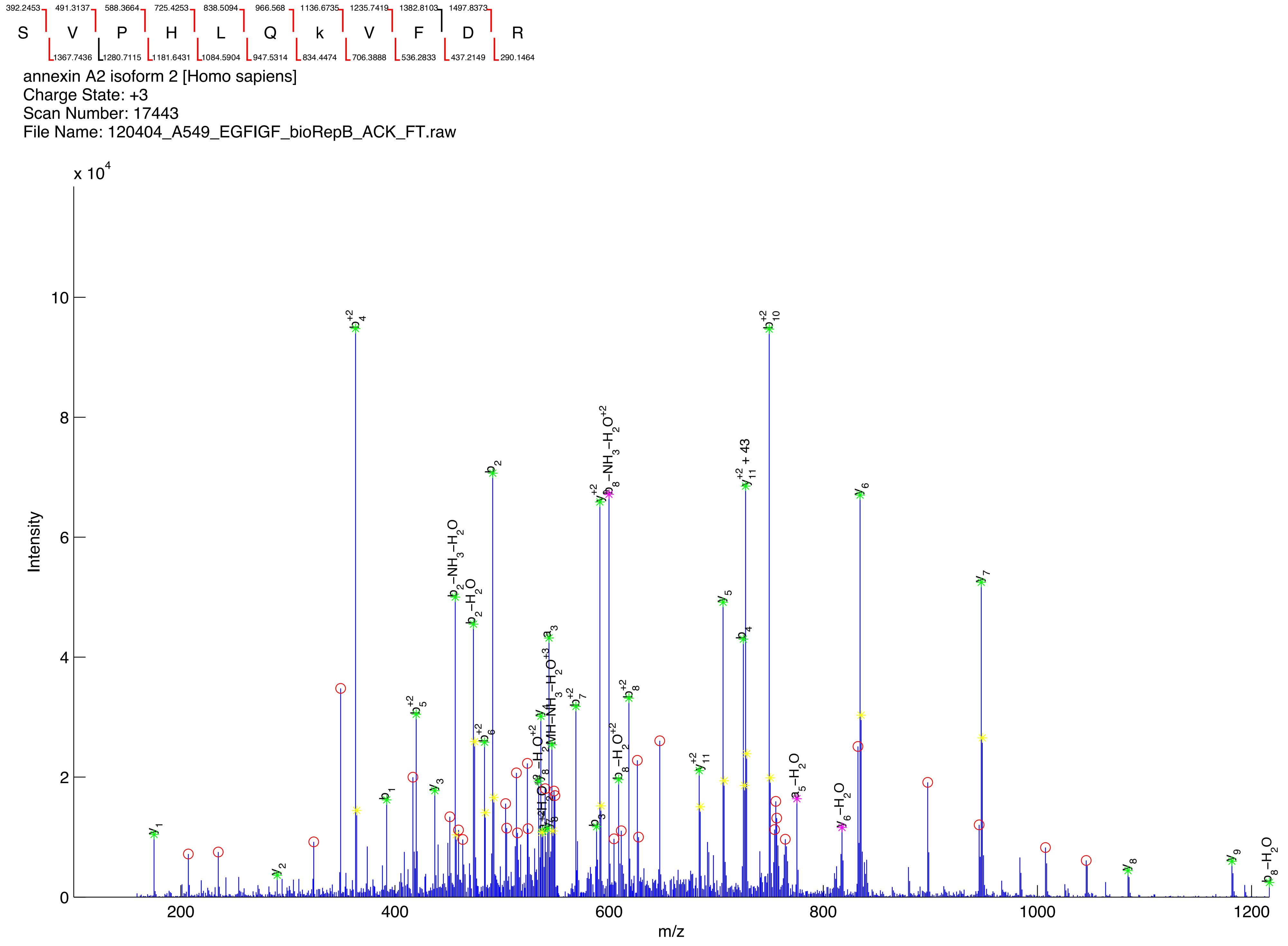
392.2453 491.3137 588.3664 725.4253 838.5094 966.568 1136.6735 1235.7419 1382.8103 1497.8373
S V P H L Q k V F D R
1367.7436 1280.7115 1181.6431 1084.5904 947.5314 834.4474 706.3888 536.2833 437.2149 290.1464
annexin A2 isoform 2 [Homo sapiens]
Charge State: +3
Scan Number: 17443
File Name: 120404_A549_EGFIGF_bioRepB_ACK_FT.raw
x 10^4
Intensity
m/z

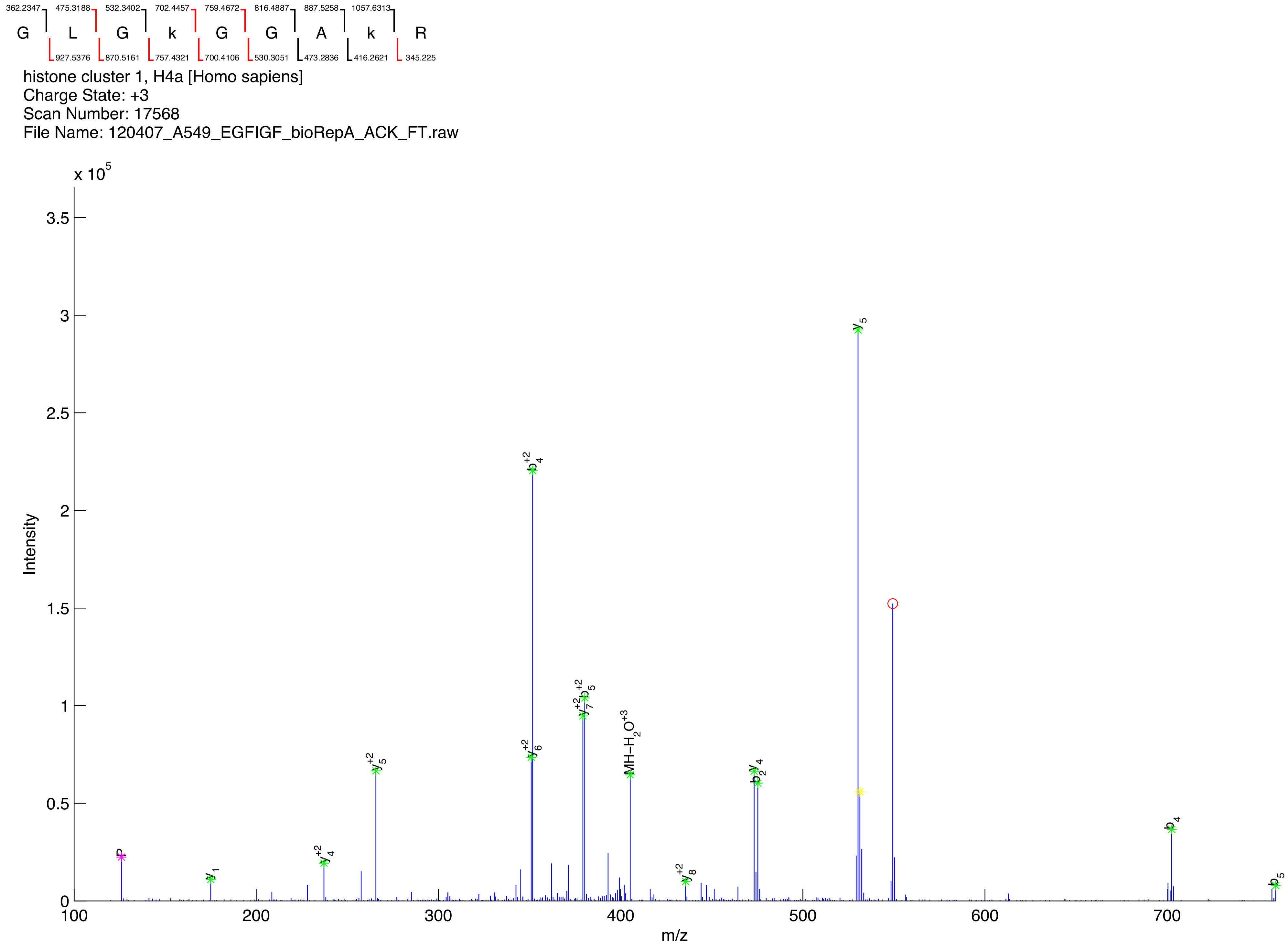
362.2347 475.3188 532.3402 702.4457 759.4672 816.4887 887.5258 1057.6313
G L G k G G A k R
927.5376 870.5161 757.4321 700.4106 530.3051 473.2836 416.2621 345.225
histone cluster 1, H4a [Homo sapiens]
Charge State: +3
Scan Number: 17568
File Name: 120407_A549_EGFIGF_bioRepA_ACK_FT.raw
x 10^5
Intensity
m/z
MH-H2O+3
0
0.5
1
1.5
2
2.5
3
3.5
100
200
300
400
500
600
700

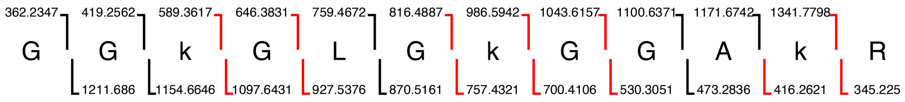

histone cluster 1, H4a [Homo sapiens]
Charge State: +3
Scan Number: 17570
File Name: 120407_A549_EGFIGF_bioRepA_ACK_FT.raw

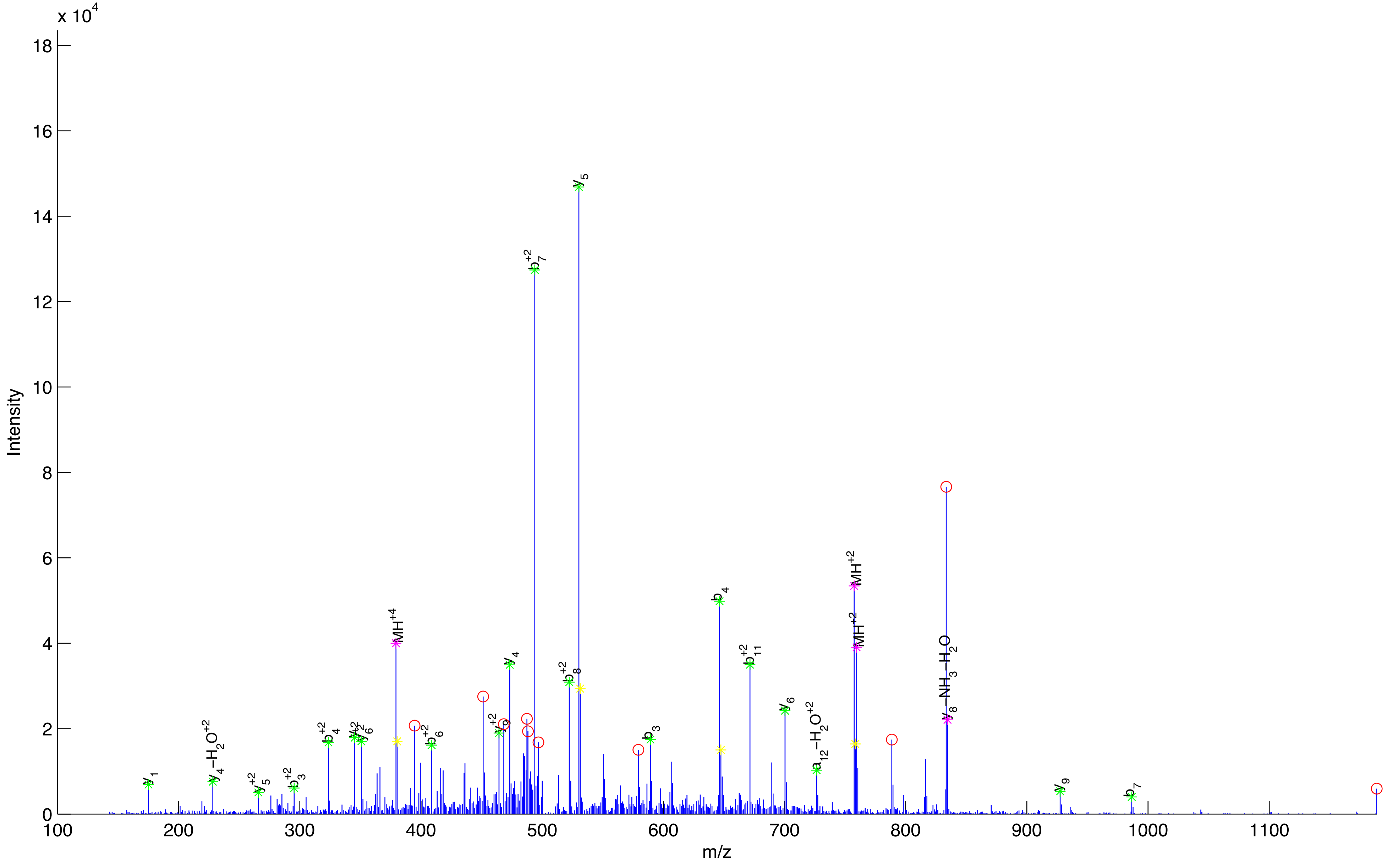

419.2562	516.3089	587.346	702.373	815.457	912.5098	1082.6153	1195.6994	1294.7678	1423.8104	1480.8319	1593.9159	1764.0215	
N	P	A	D	L	P	k	L	V	E	G	L	k	R
	1633.9277	1519.8848	1422.832	1351.7949	1236.768	1123.6839	1026.6312	856.5256	743.4416	644.3732	515.3306	458.3091	345.225

eukaryotic translation elongation factor 2 [Homo sapiens]
Charge State: +
Scan Number: 17971
File Name: 120407_A549_EGFIGF_bioRepA_ACK_FT.raw

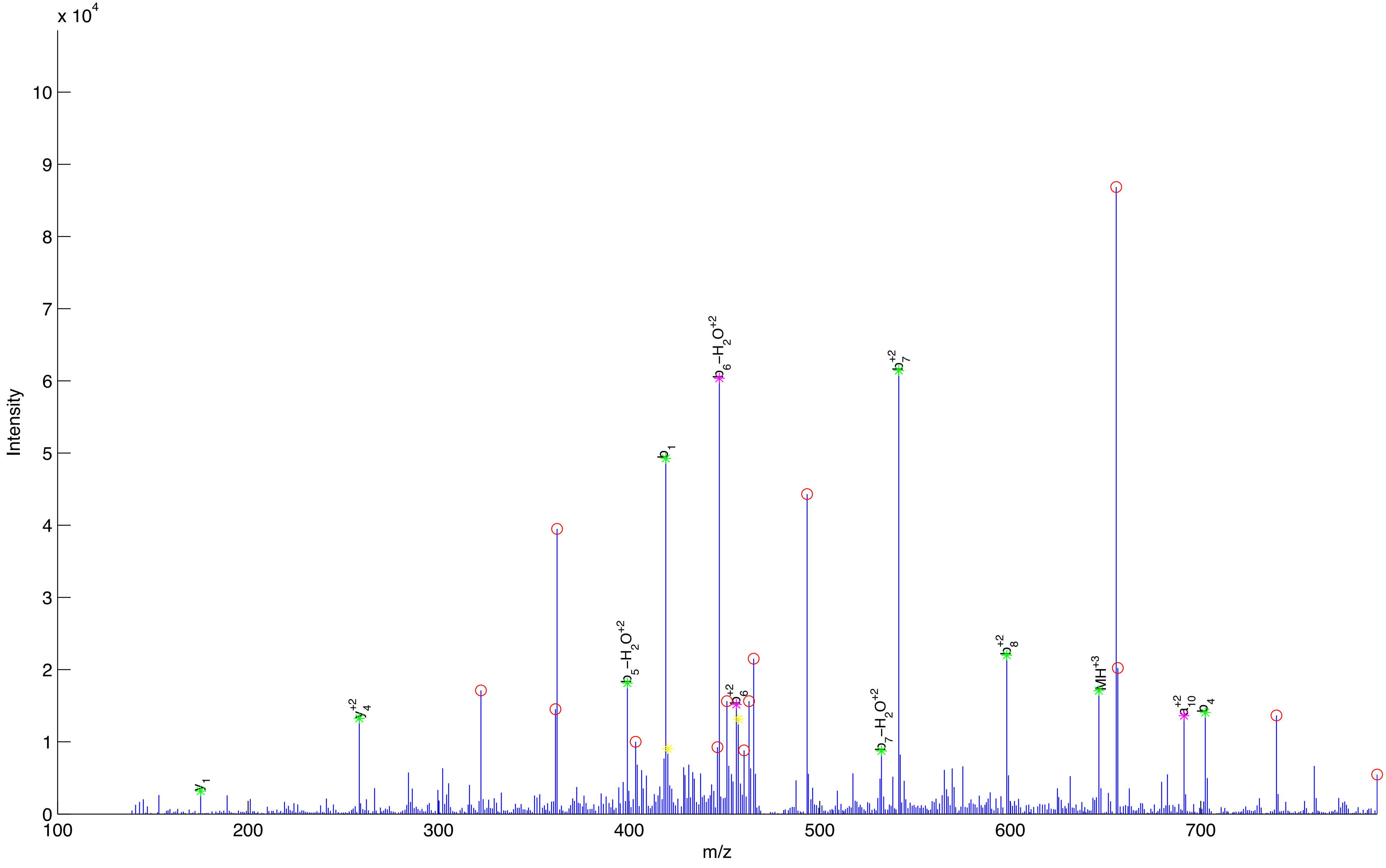

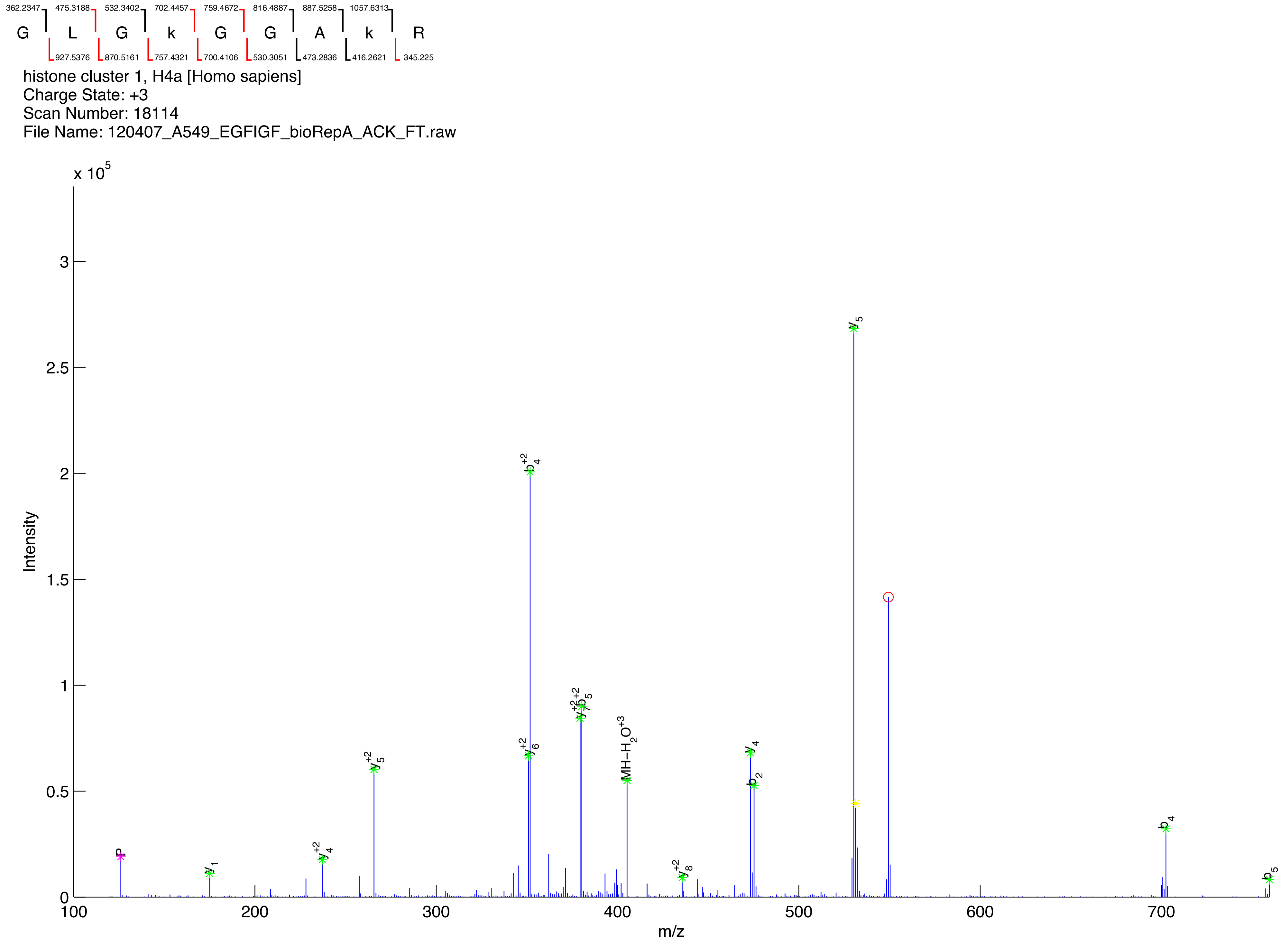
362.2347 475.3188 532.3402 702.4457 759.4672 816.4887 887.5258 1057.6313
G L G k G G A k R
927.5376 870.5161 757.4321 700.4106 530.3051 473.2836 416.2621 345.225
histone cluster 1, H4a [Homo sapiens]
Charge State: +3
Scan Number: 18114
File Name: 120407_A549_EGFIGF_bioRepA_ACK_FT.raw
x 10^5
Intensity
m/z

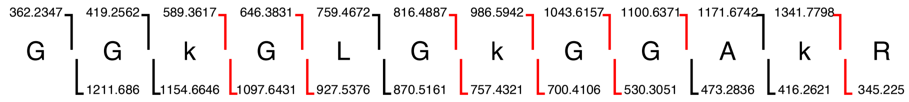

histone cluster 1, H4a [Homo sapiens]
Charge State: +3
Scan Number: 18116
File Name: 120407_A549_EGFIGF_bioRepA_ACK_FT.raw

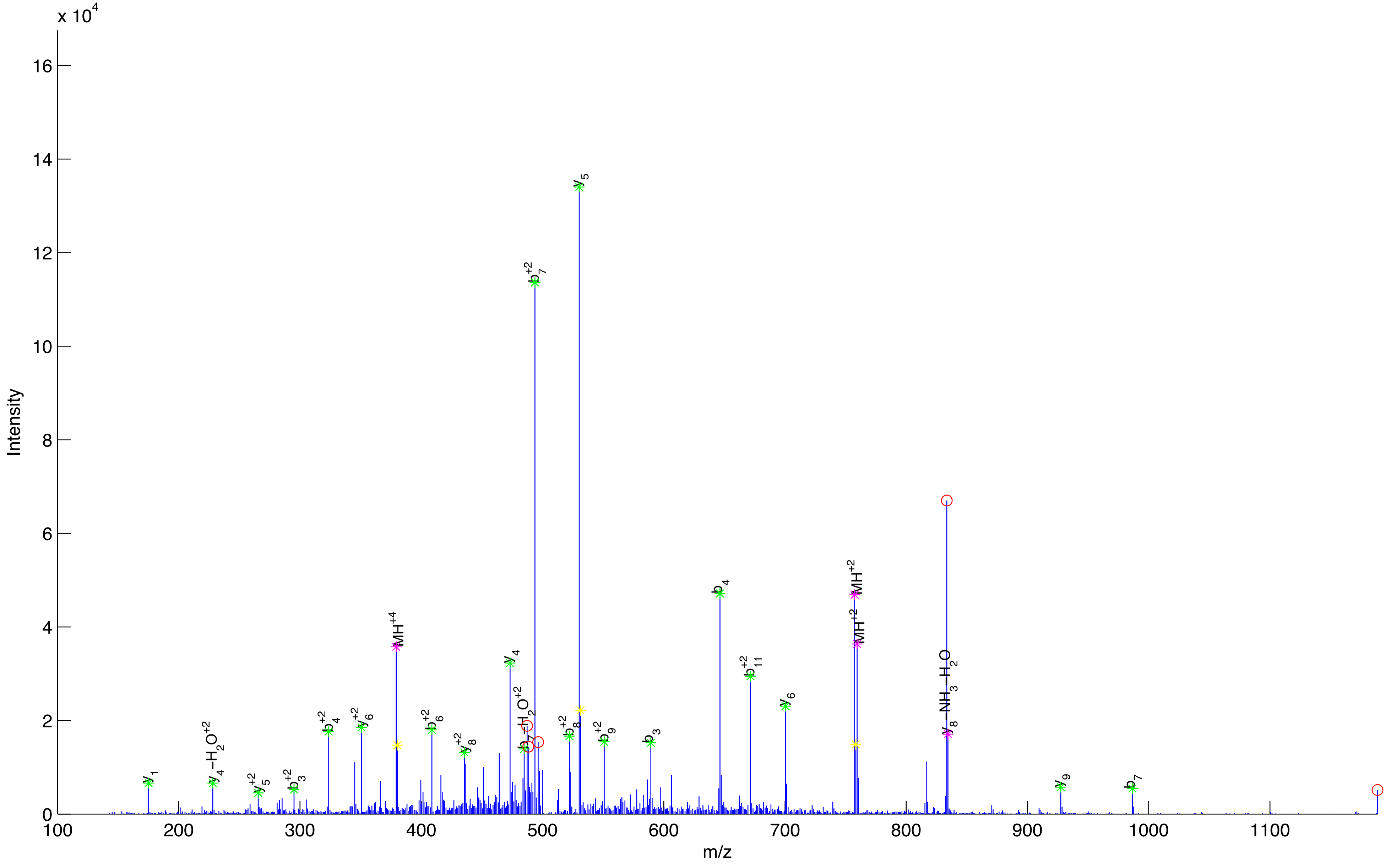

S V P T S T V F Y P S D G V A T E K

transketolase [Homo sapiens]
Charge State: +
Scan Number: 18164
File Name: 120407_A549_EGFIGF_bioRepA_ACK_FT.raw

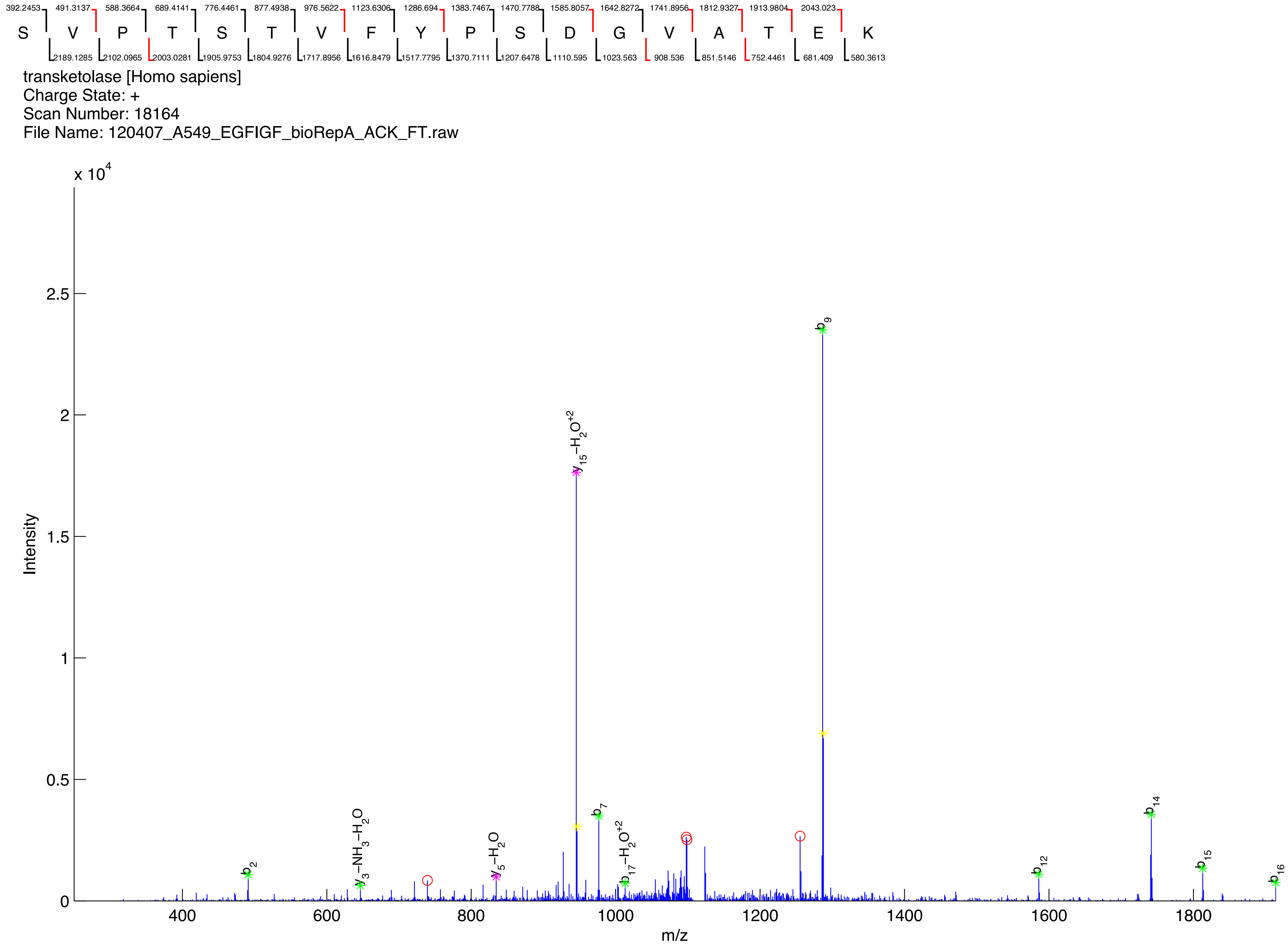

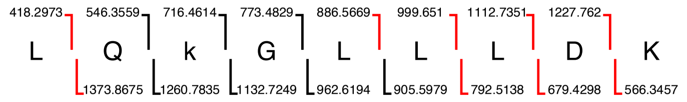

polyamine modulated factor 1 binding protein 1 [Homo sapiens]
Charge State: +
Scan Number: 18523
File Name: 120413_A549_EGFIGF_bioRepC_AcK_FT.raw

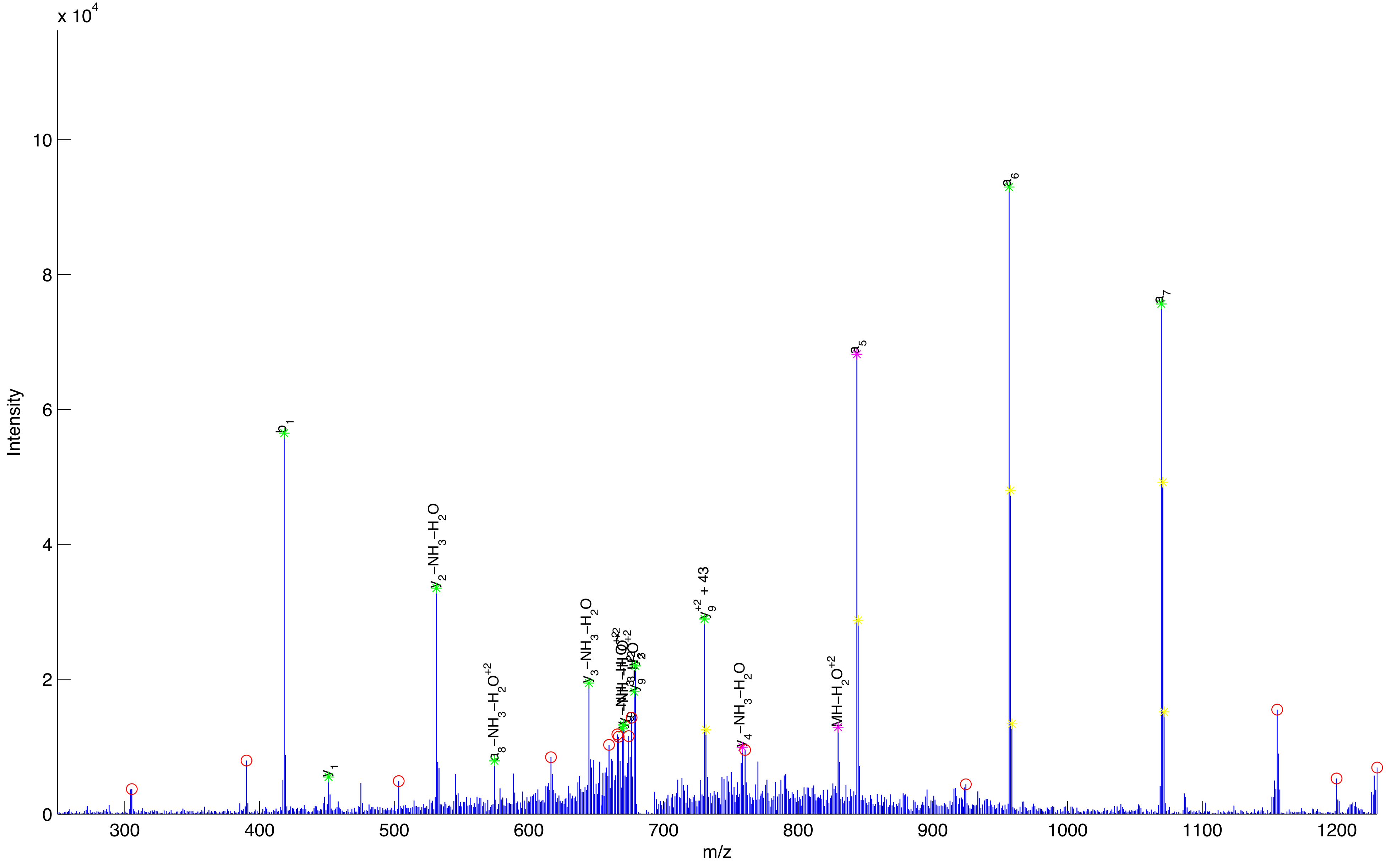

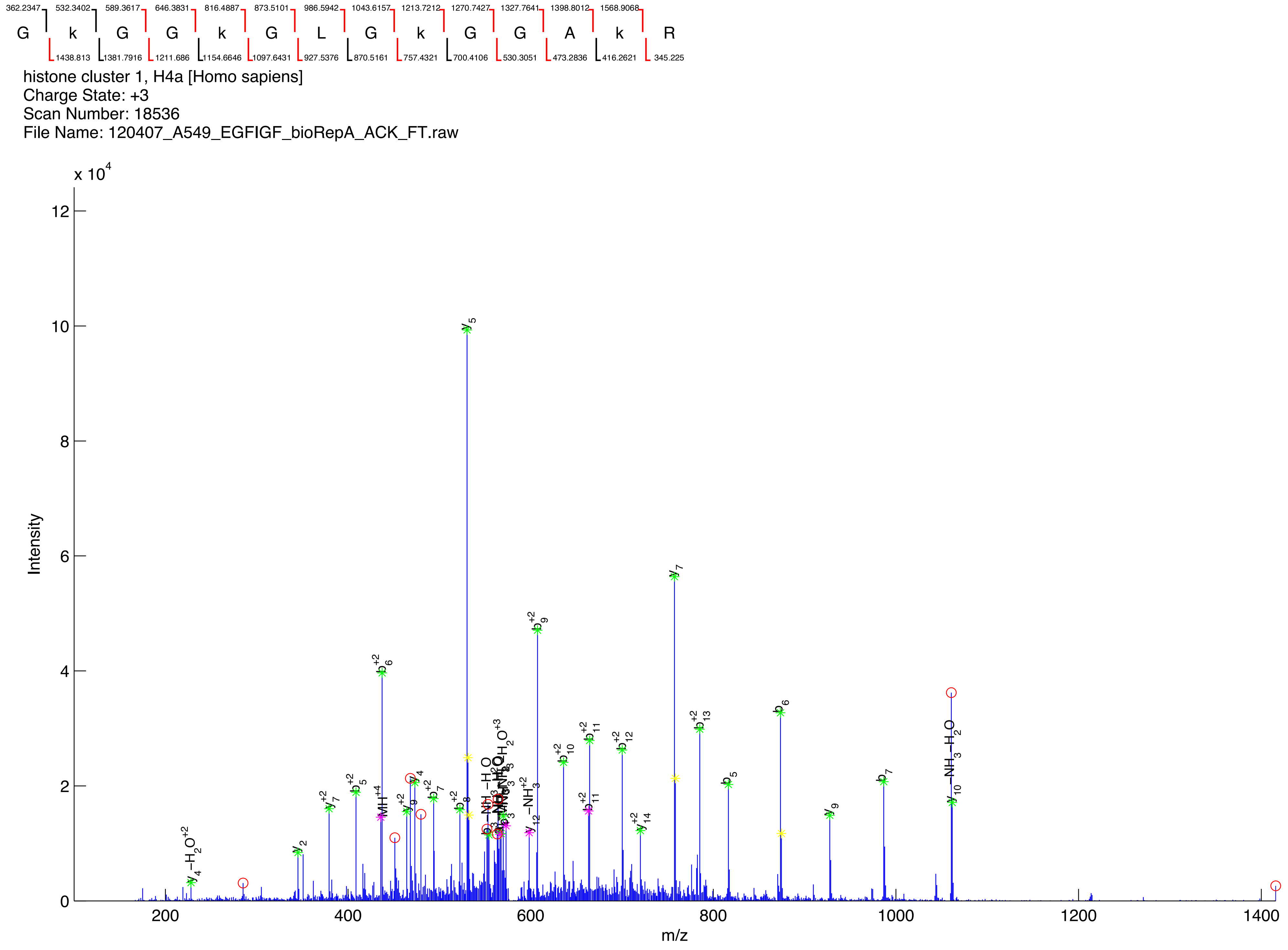

362.2347 532.3402 589.3617 646.3831 816.4887 873.5101 986.5942 1043.6157 1213.7212 1270.7427 1327.7641 1398.8012 1568.9068
G k G G k G L G k G G A k R
1438.813 1381.7916 1211.686 1154.6646 1097.6431 927.5376 870.5161 757.4321 700.4106 530.3051 473.2836 416.2621 345.225
histone cluster 1, H4a [Homo sapiens]
Charge State: +3
Scan Number: 18536
File Name: 120407_A549_EGFIGF_bioRepA_ACK_FT.raw
x 10^4
Intensity
m/z

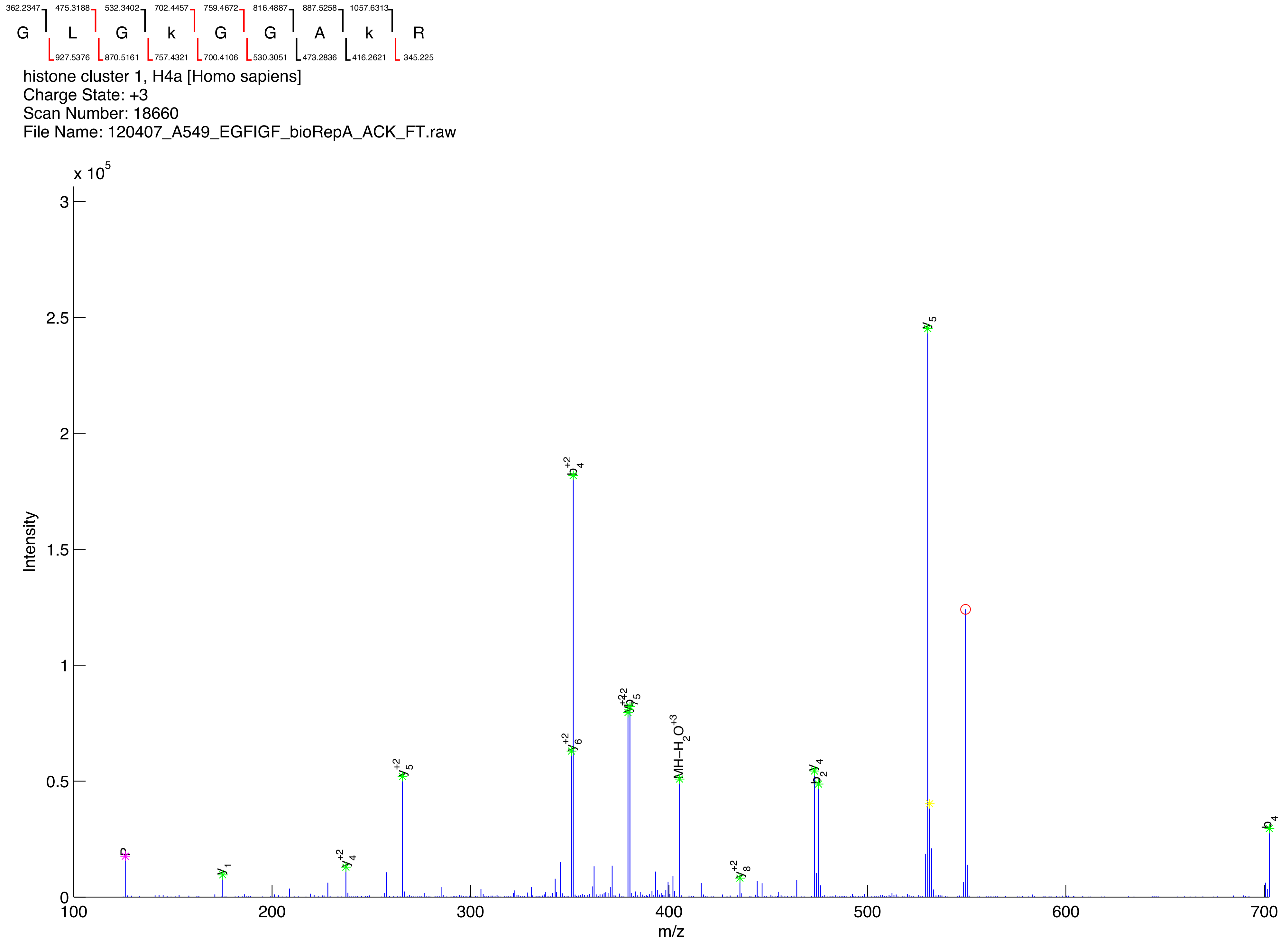
362.2347
475.3188
532.3402
702.4457
759.4672
816.4887
887.5258
1057.6313
G L G k G G A k R
927.5376
870.5161
757.4321
700.4106
530.3051
473.2836
416.2621
345.225
histone cluster 1, H4a [Homo sapiens]
Charge State: +3
Scan Number: 18660
File Name: 120407_A549_EGFIGF_bioRepA_ACK_FT.raw
x 10^5
Intensity
m/z
0
0.5
1
1.5
2
2.5
3
100
200
300
400
500
600
700

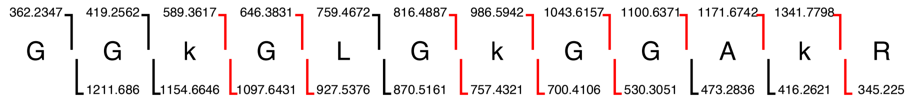

histone cluster 1, H4a [Homo sapiens]
Charge State: +3
Scan Number: 18664
File Name: 120407_A549_EGFIGF_bioRepA_ACK_FT.raw

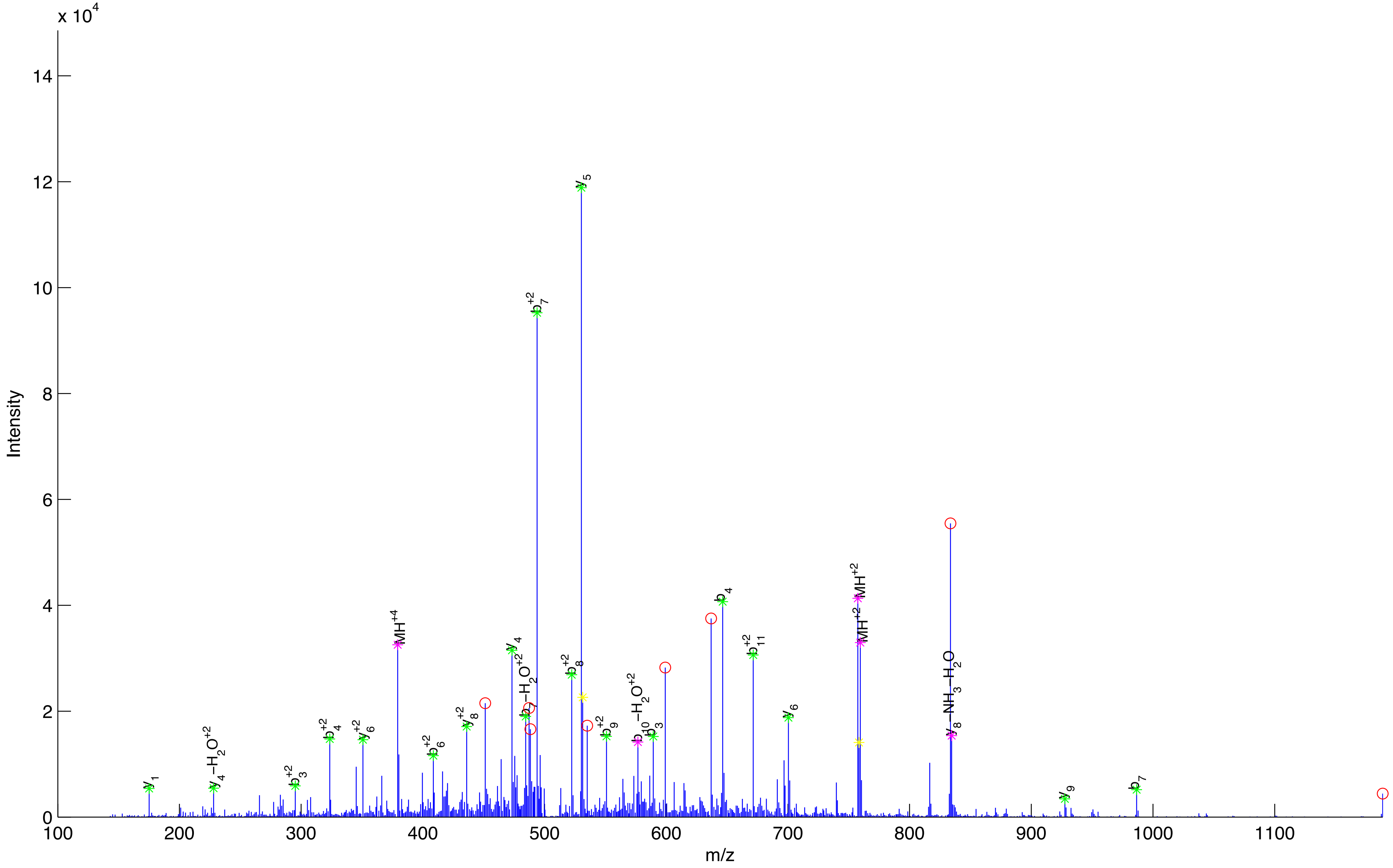

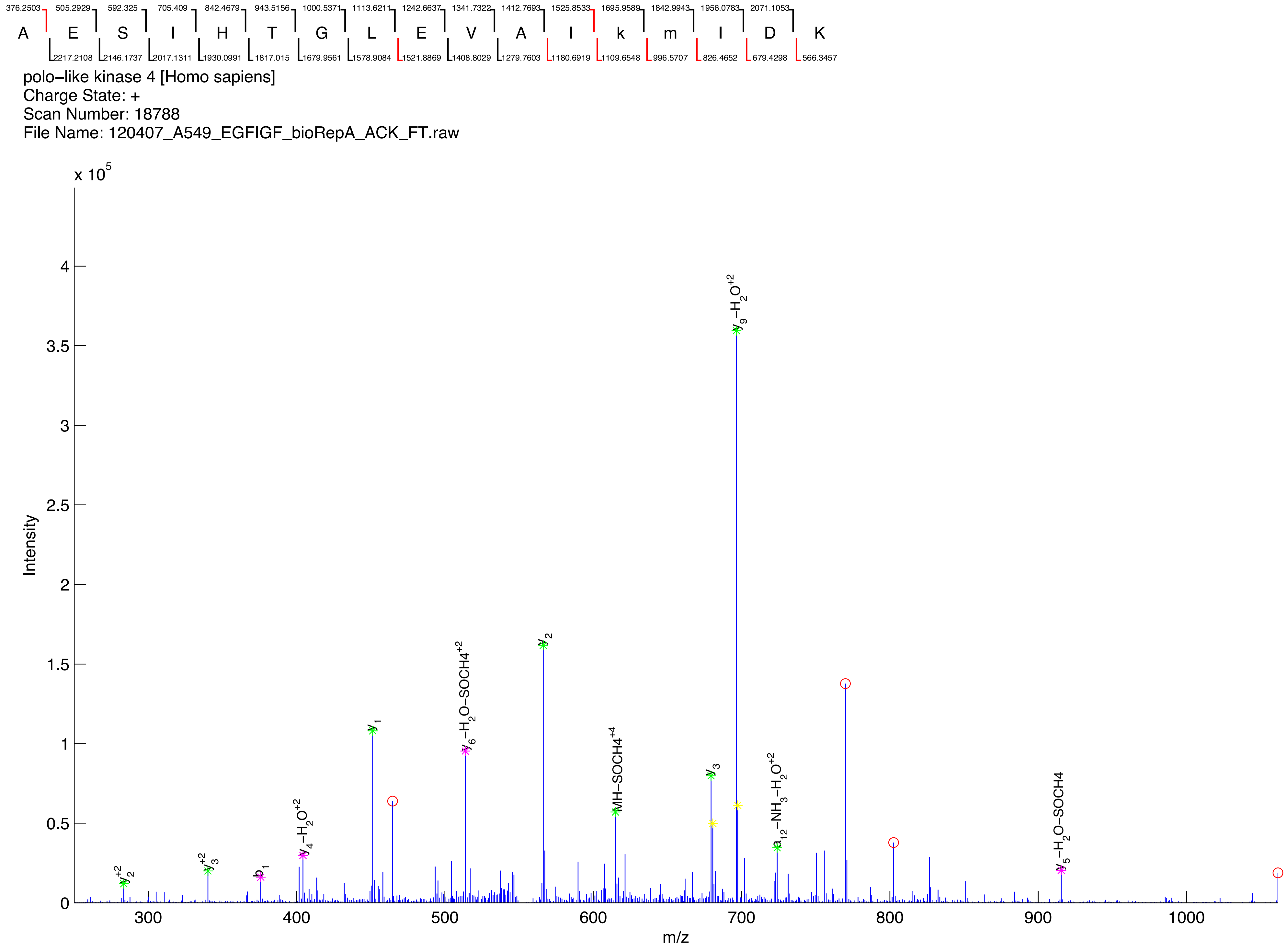

376.2503 505.2929 592.325 705.409 842.4679 943.5156 1000.5371 1113.6211 1242.6637 1341.7322 1412.7693 1525.8533 1695.9589 1842.9943 1956.0783 2071.1053
A E S I H T G L E V A I k m I D K
2217.2108 2146.1737 2017.1311 1930.0991 1817.015 1679.9561 1578.9084 1521.8869 1408.8029 1279.7603 1180.6919 1109.6548 996.5707 826.4652 679.4298 566.3457
polo−like kinase 4 [Homo sapiens]
Charge State: +
Scan Number: 18788
File Name: 120407_A549_EGFIGF_bioRepA_ACK_FT.raw
x 10^5
Intensity
m/z
y_2^+2
y_3^+2
b_1
y_4−H_2O^+2
y_1
y_6−H_2O−SOCH4^+2
y_2
MH−SOCH4^+4
y_3
y_9−H_2O^+2
a_12−NH_3−H_2O^+2
y_5−H_2O−SOCH4

404.2816	517.3657	574.3872	645.4243	815.5298	985.6353	1100.6623	1228.7209	1391.7842	1538.8526	1651.9367	1766.9636	
V	I	G	A	k	k	D	Q	Y	F	L	D	K
	1913.0691	1814.0007	1700.9167	1643.8952	1572.8581	1402.7526	1232.647	1117.6201	989.5615	826.4982	679.4298	566.3457

structural maintenance of chromosomes 3 [Homo sapiens]
Charge State: +4
Scan Number: 18788
File Name: 120407_A549_EGFIGF_bioRepA_ACK_FT.raw

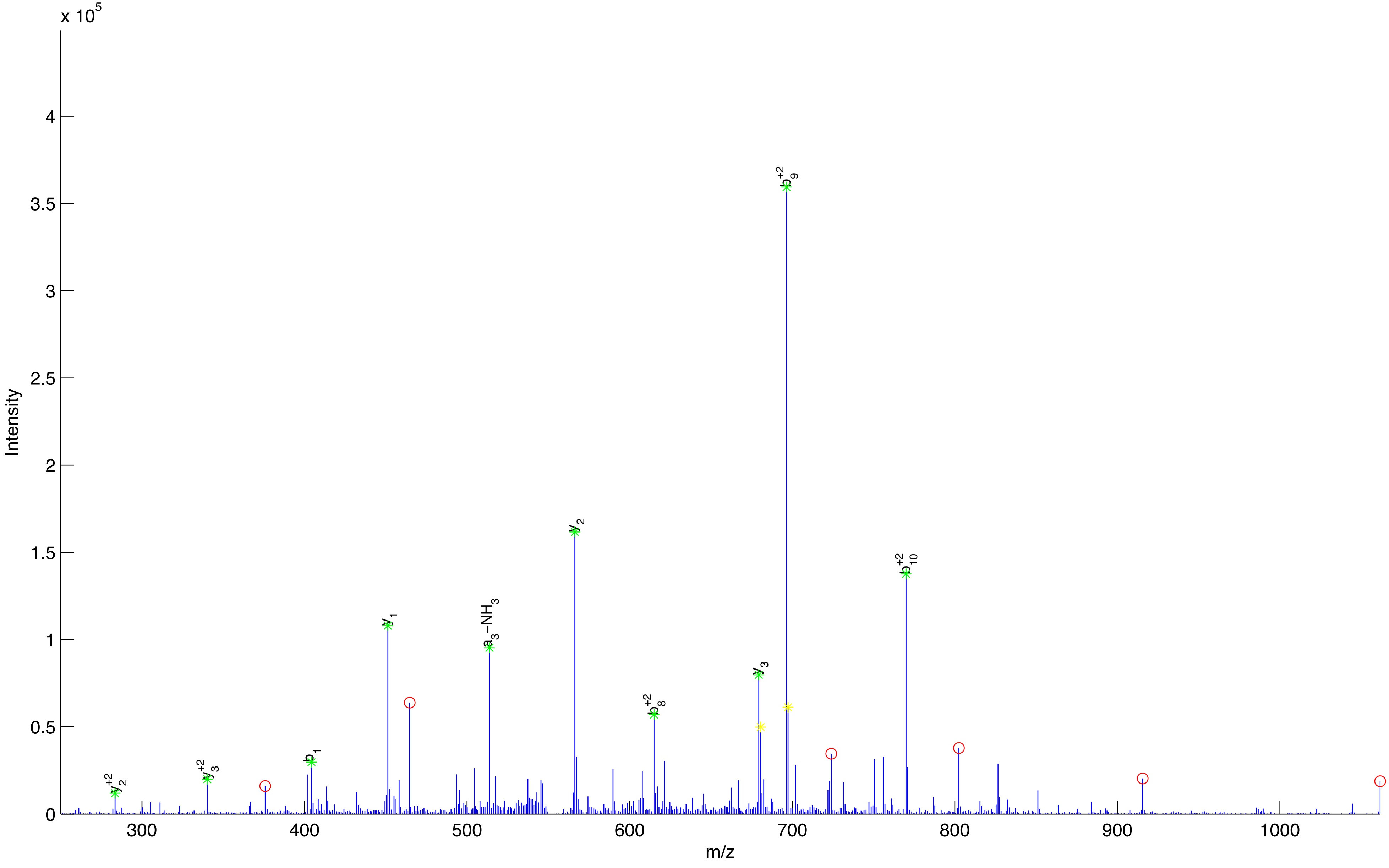

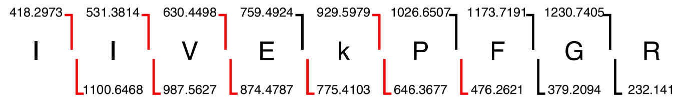

glucose−6−phosphate dehydrogenase isoform b [Homo sapiens]
Charge State: +3
Scan Number: 18882
File Name: 120413_A549_EGFIGF_bioRepC_AcK_FT.raw

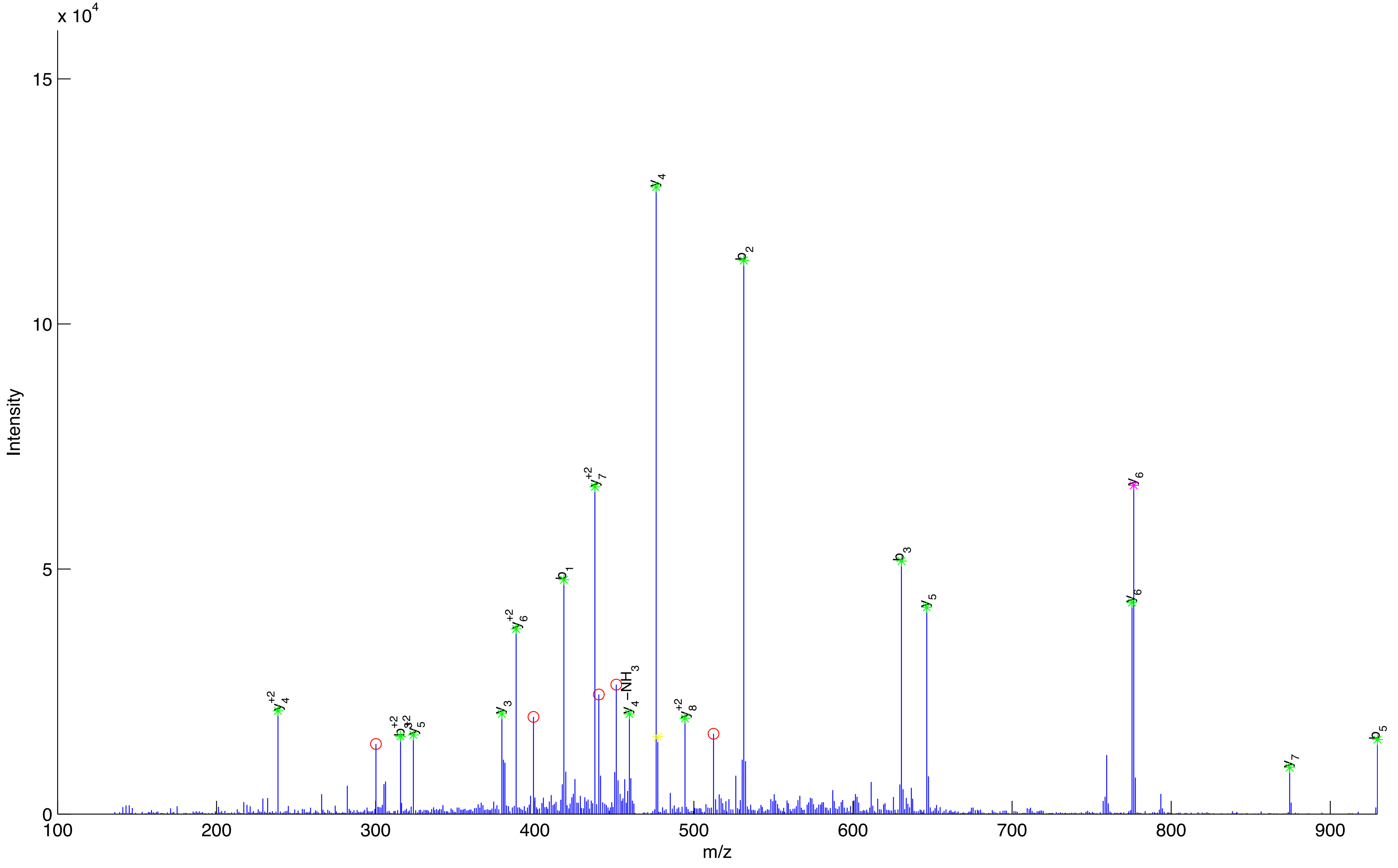

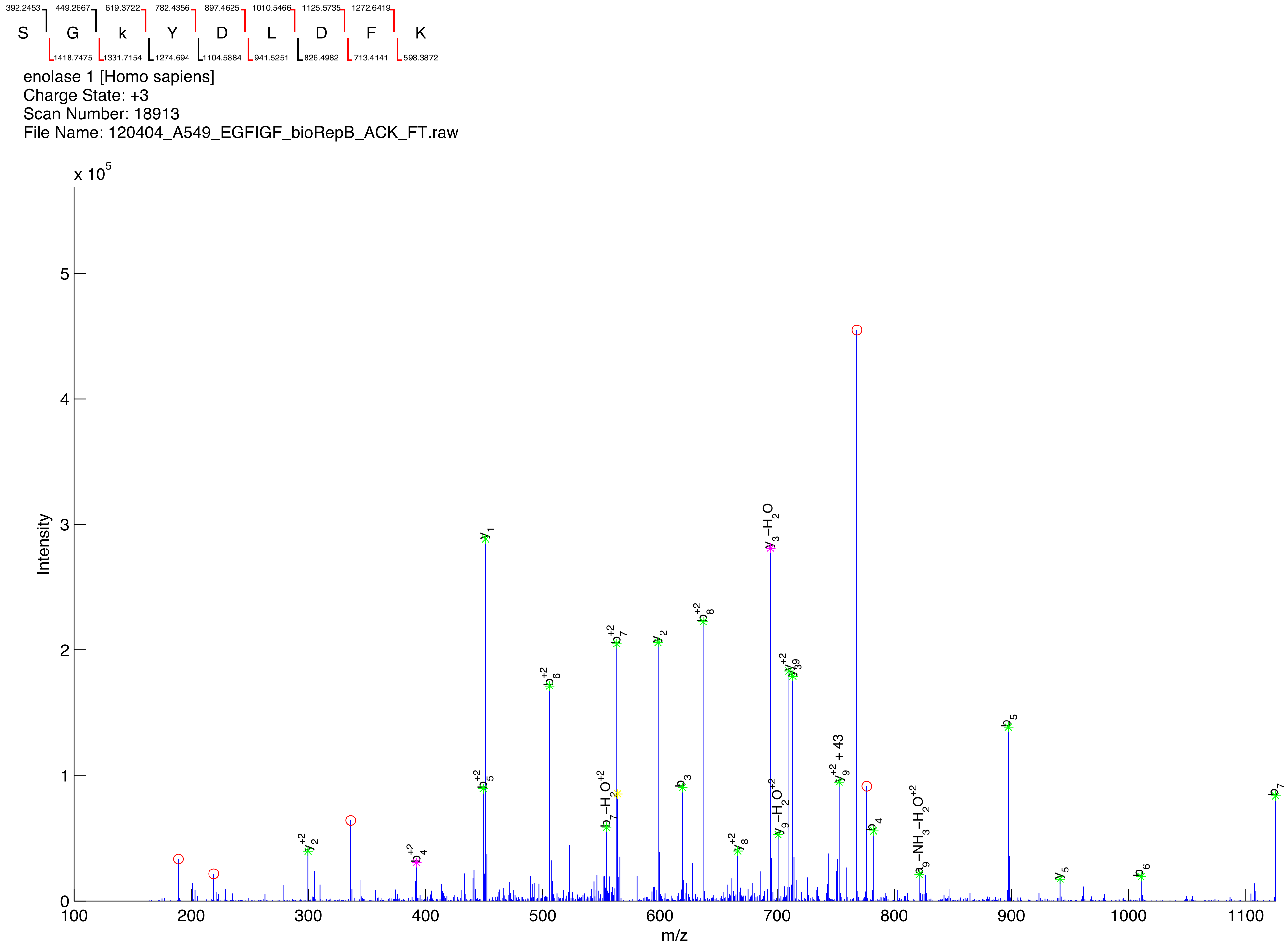
392.2453 449.2667 619.3722 782.4356 897.4625 1010.5466 1125.5735 1272.6419
S G k Y D L D F K
1418.7475 1331.7154 1274.694 1104.5884 941.5251 826.4982 713.4141 598.3872
enolase 1 [Homo sapiens]
Charge State: +3
Scan Number: 18913
File Name: 120404_A549_EGFIGF_bioRepB_ACK_FT.raw
x 10^5
Intensity
m/z

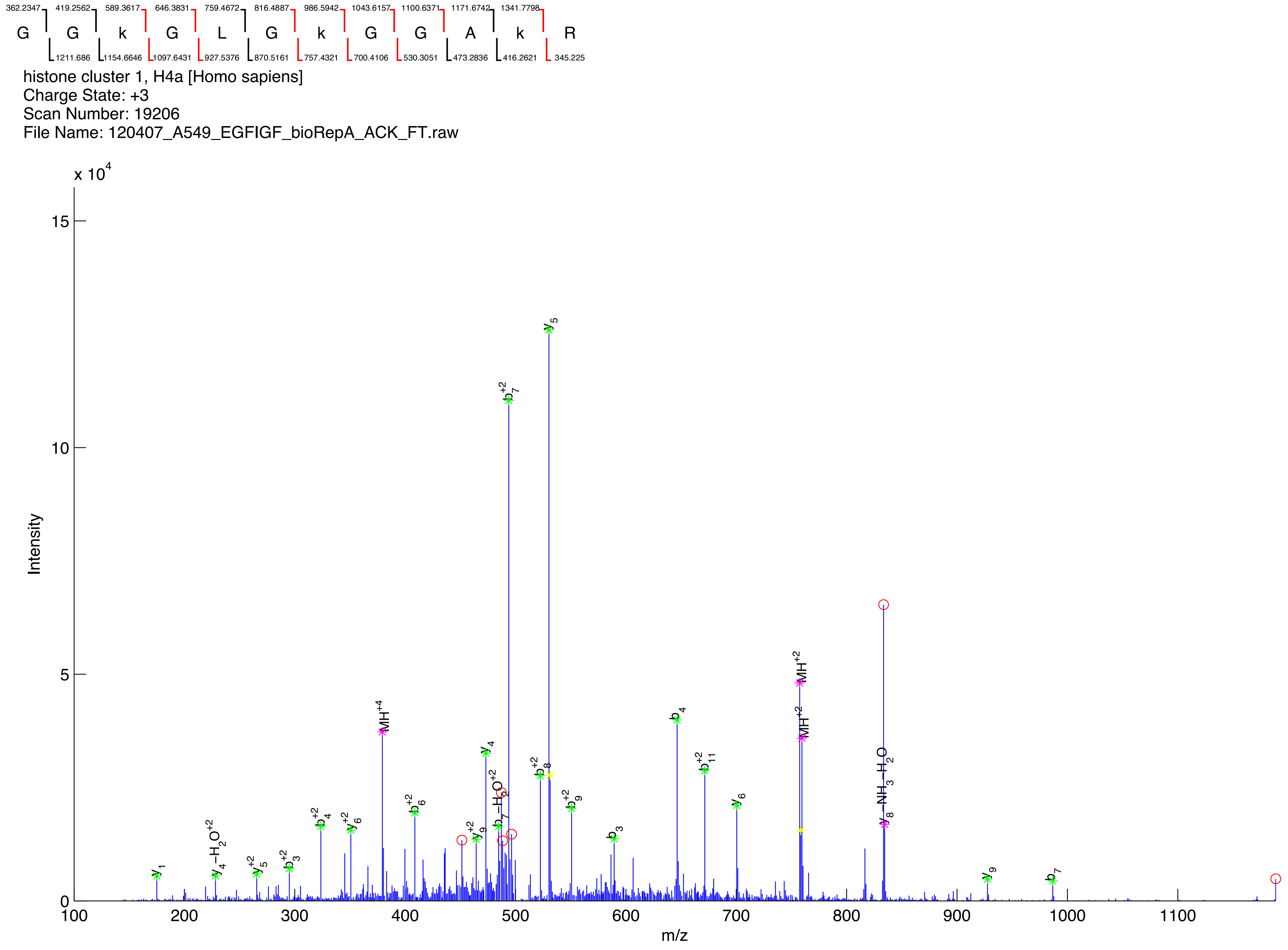

362.2347 419.2562 589.3617 646.3831 759.4672 816.4887 986.5942 1043.6157 1100.6371 1171.6742 1341.7798
G G k G L G k G G A k R
1211.686 1154.6646 1097.6431 927.5376 870.5161 757.4321 700.4106 530.3051 473.2836 416.2621 345.225
histone cluster 1, H4a [Homo sapiens]
Charge State: +3
Scan Number: 19206
File Name: 120407_A549_EGFIGF_bioRepA_ACK_FT.raw
x 10^4
Intensity
m/z

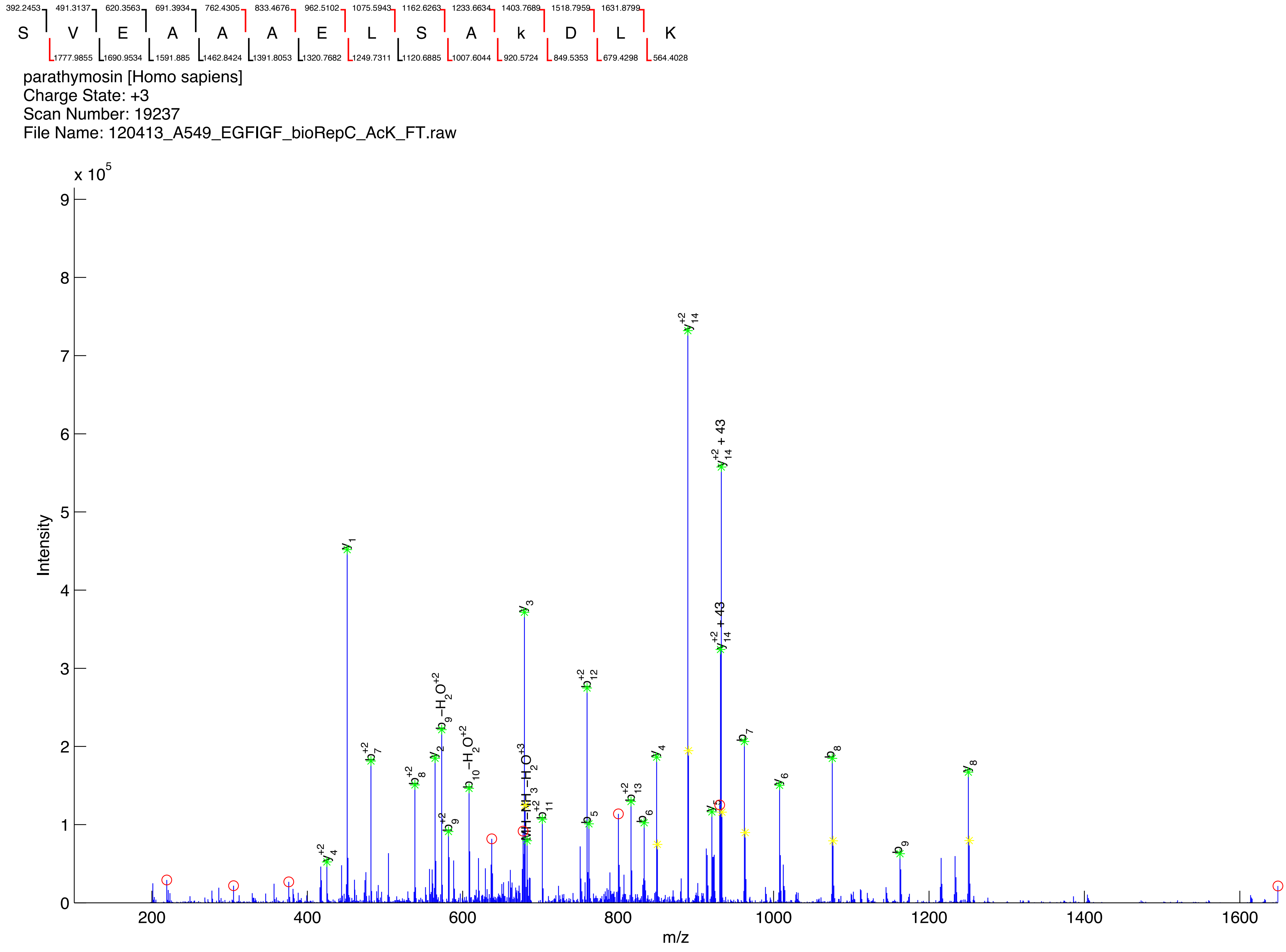

392.2453 491.3137 620.3563 691.3934 762.4305 833.4676 962.5102 1075.5943 1162.6263 1233.6634 1403.7689 1518.7959 1631.8799
S V E A A A E L S A k D L K
1777.9855 1690.9534 1591.885 1462.8424 1391.8053 1320.7682 1249.7311 1120.6885 1007.6044 920.5724 849.5353 679.4298 564.4028
parathymosin [Homo sapiens]
Charge State: +3
Scan Number: 19237
File Name: 120413_A549_EGFIGF_bioRepC_AcK_FT.raw
x 10^5
Intensity
m/z

k Y E F S P Q T L c c Y G k Q L c T I P R

475.3188 638.3821 767.4247 914.4931 1001.5251 1098.5779 1226.6365 1327.6841 1440.7682 1600.7988 1760.8294 1923.8927 1980.9142 2151.0197 2279.0783 2392.1623 2552.1929 2653.2406 2766.3247 2863.3774

2733.2837 2563.1782 2400.1149 2271.0723 2124.0038 2036.9718 1939.9191 1811.8605 1710.8128 1597.7287 1437.6981 1277.6676 1114.6042 1057.5828 887.4772 759.4187 646.3346 486.304 385.2563 272.1723

CREB binding protein isoform b [Homo sapiens]
Charge State: +
Scan Number: 19338
File Name: 120407_A549_EGFIGF_bioRepA_ACK_FT.raw

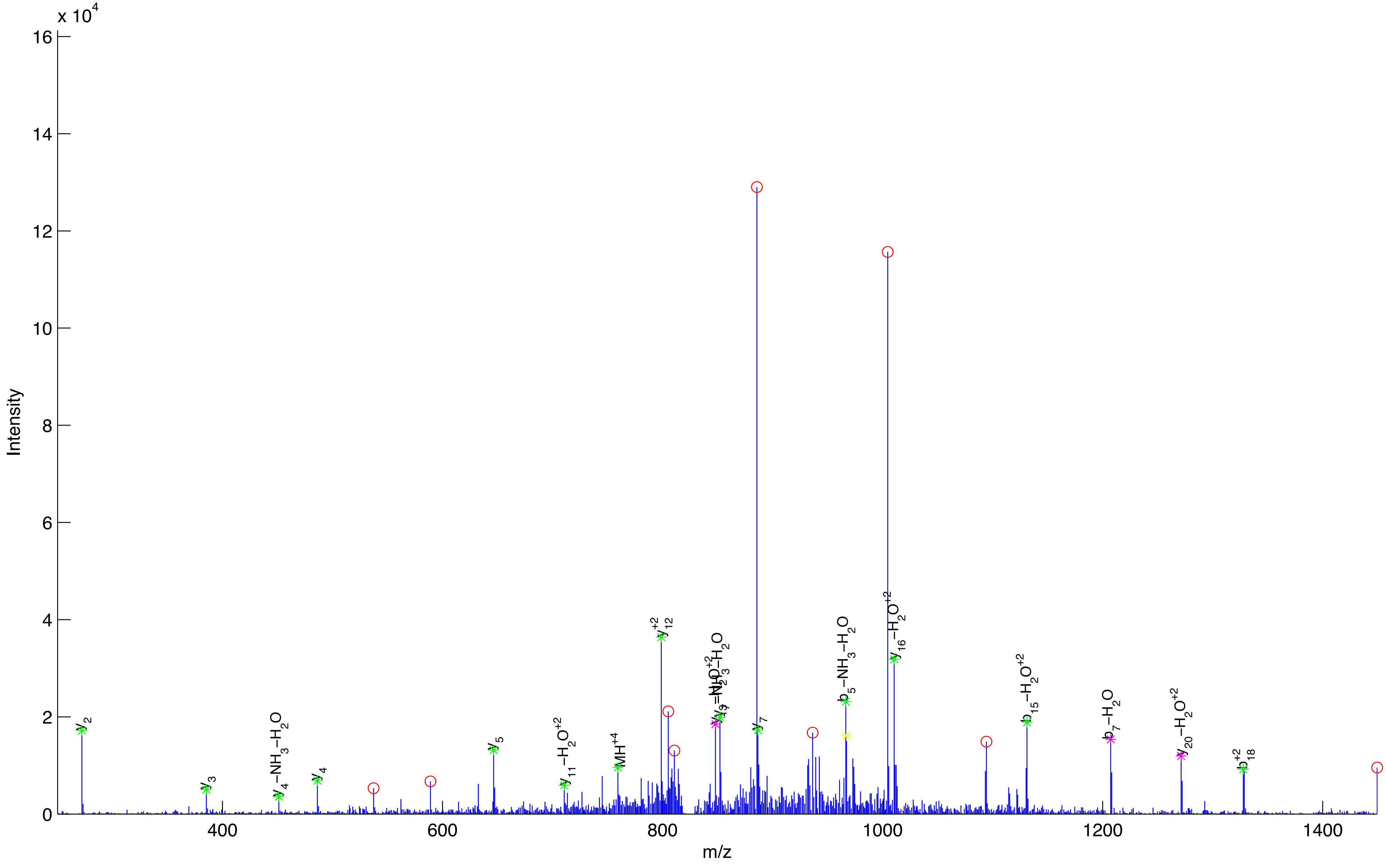

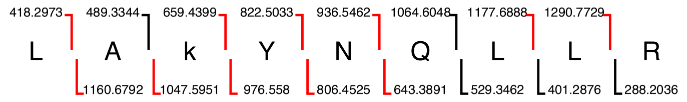

enolase 1 [Homo sapiens]
Charge State: +2
Scan Number: 19720
File Name: 120413_A549_EGFIGF_bioRepC_AcK_FT.raw

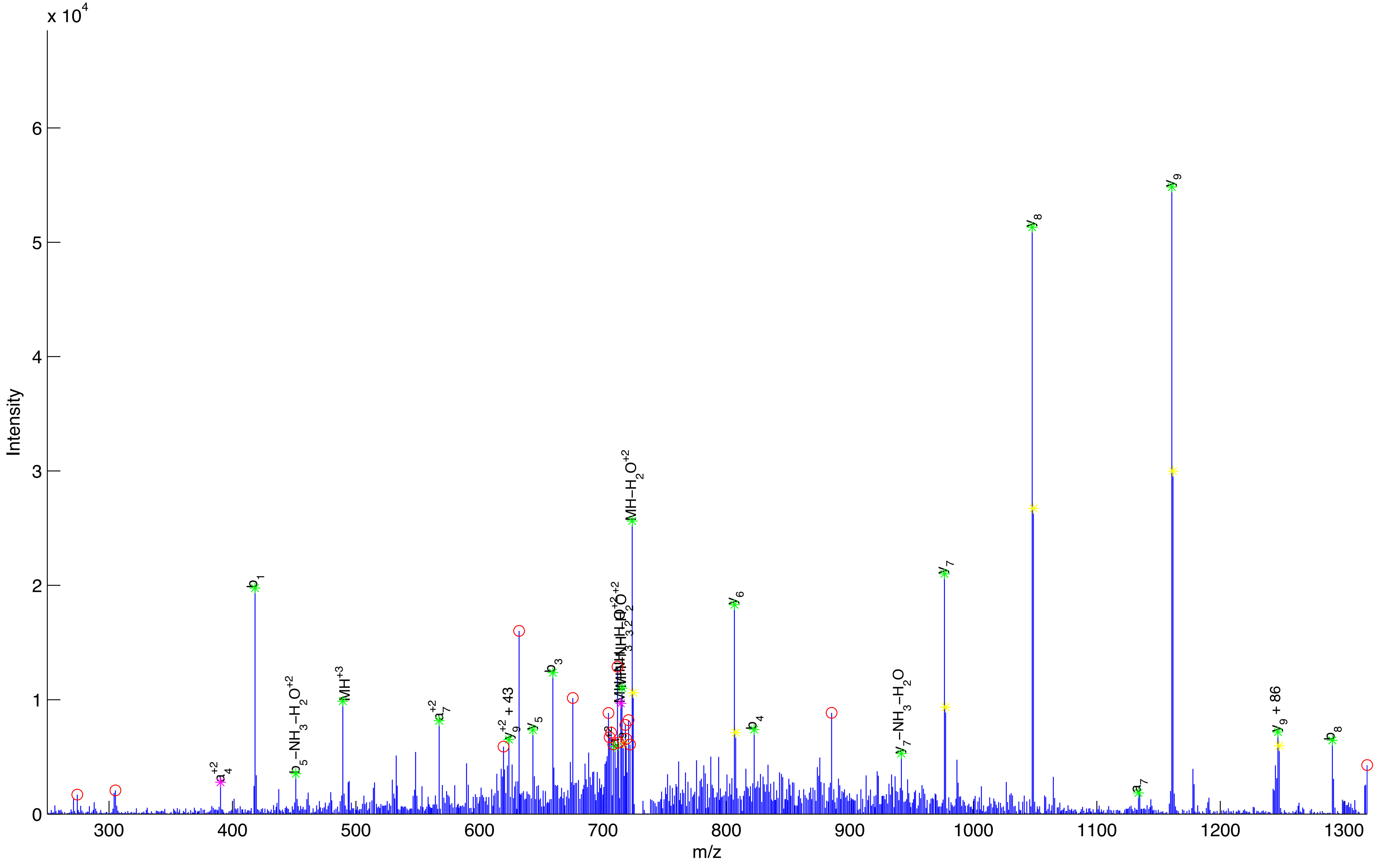

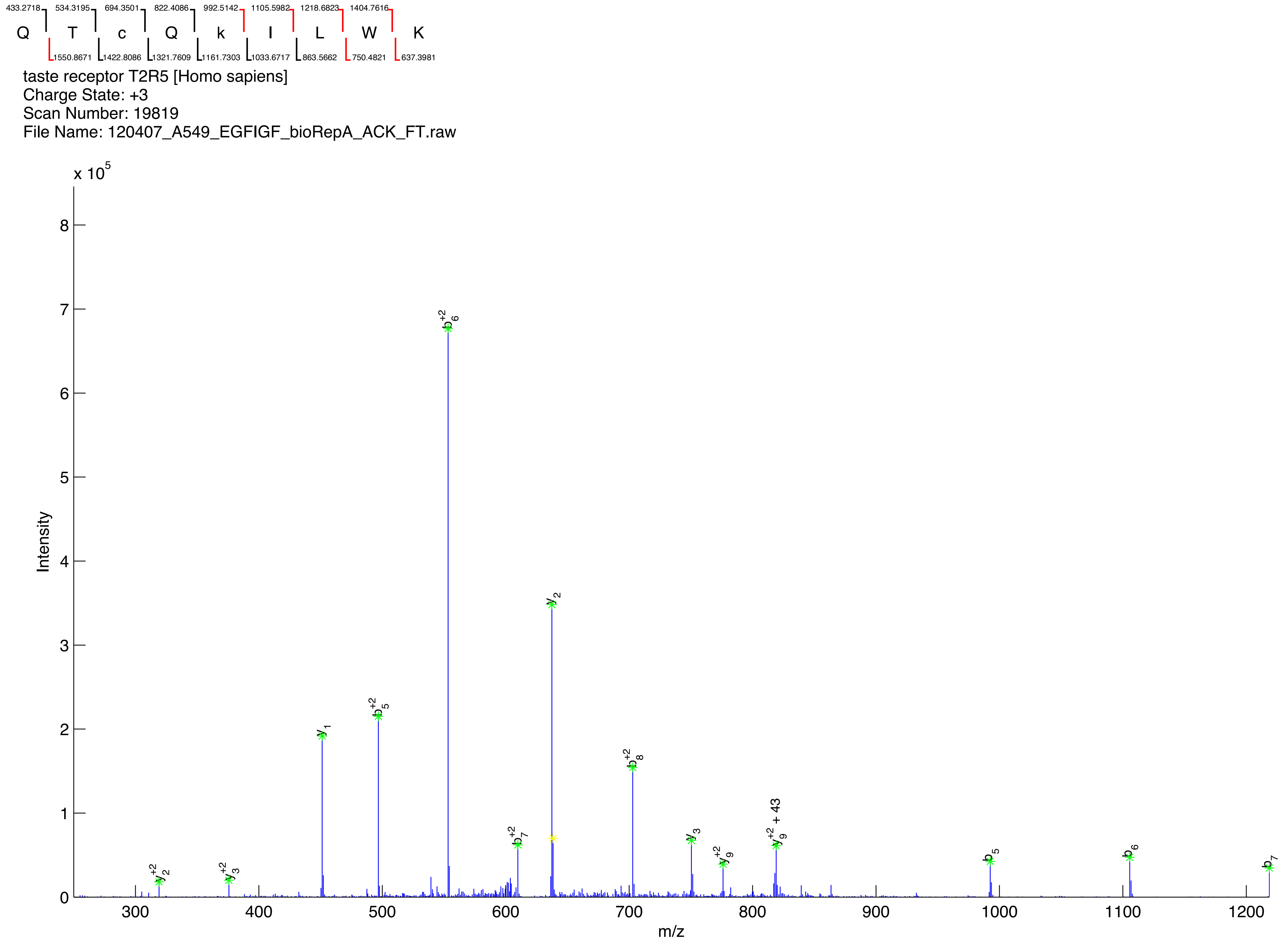
433.2718 534.3195 694.3501 822.4086 992.5142 1105.5982 1218.6823 1404.7616
Q T c Q k I L W K
1550.8671 1422.8086 1321.7609 1161.7303 1033.6717 863.5662 750.4821 637.3981
taste receptor T2R5 [Homo sapiens]
Charge State: +3
Scan Number: 19819
File Name: 120407_A549_EGFIGF_bioRepA_ACK_FT.raw
x 10^5
Intensity
m/z
y+2 2
y+2 3
y 1
b+2 5
b+2 6
b+2 7
y 2
b+2 8
y 3
y+2 9
y+2 9 + 43
b 5
b 6
b 7

468.2766	631.3399	746.3668	817.4039	930.488	1029.5564	1199.6619	1270.6991	1440.8046	1569.8472	1682.9312	1853.0368	1940.0688	2069.1114	
Y	Y	D	A	L	V	k	A	k	E	L	k	S	E	R
	1939.0177	1775.9543	1612.891	1497.8641	1426.827	1313.7429	1214.6745	1044.569	973.5318	803.4263	674.3837	561.2997	391.1941	304.1621

IQ motif containing GTPase activating protein 2 [Homo sapiens]
Charge State: +3
Scan Number: 20098
File Name: 120413_A549_EGFIGF_bioRepC_AcK_FT.raw

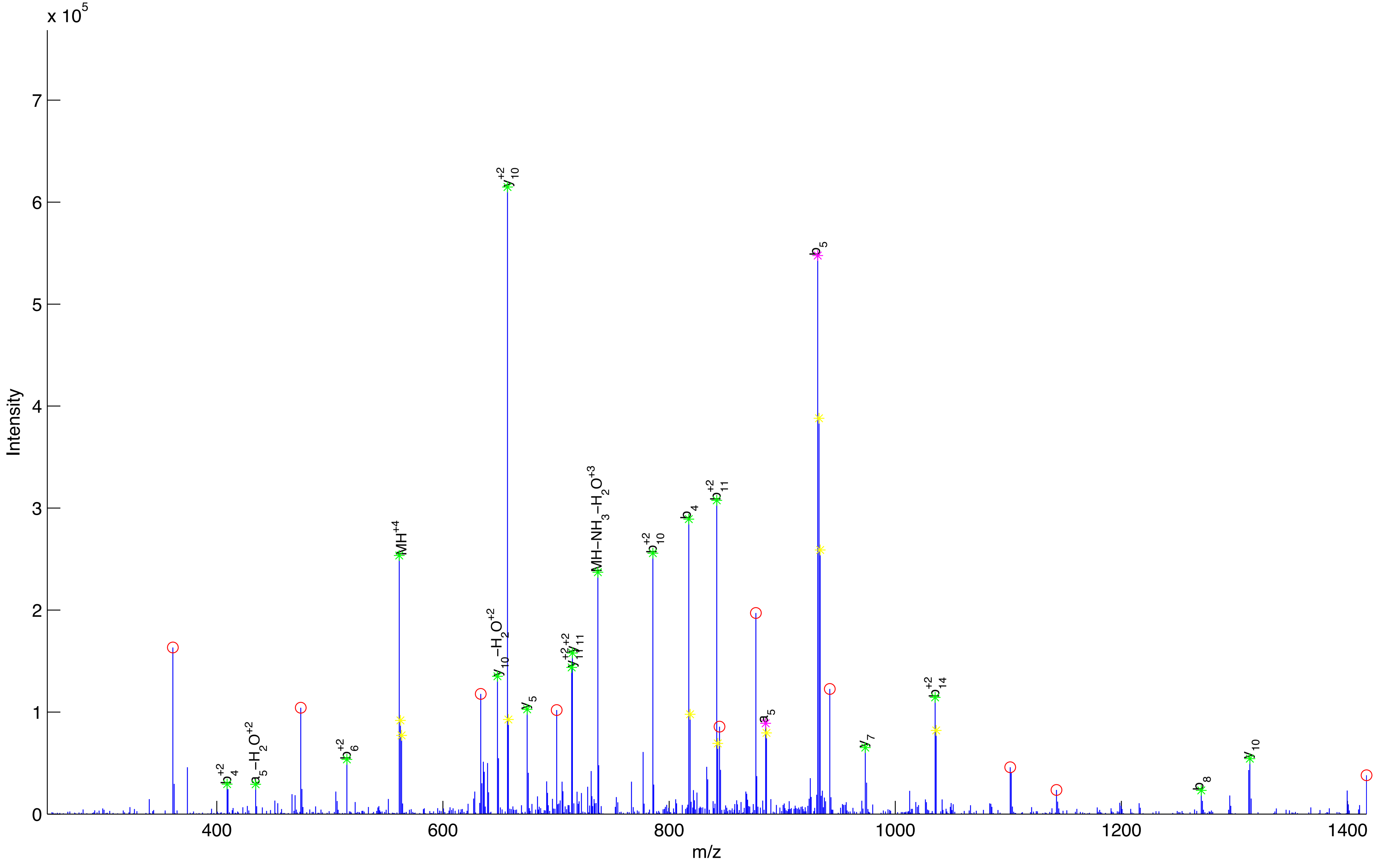

non−metastatic cells 2, protein (NM23B) expressed in [Homo sapiens]
Charge State: +3
Scan Number: 20716
File Name: 120404_A549_EGFIGF_bioRepB_ACK_FT.raw

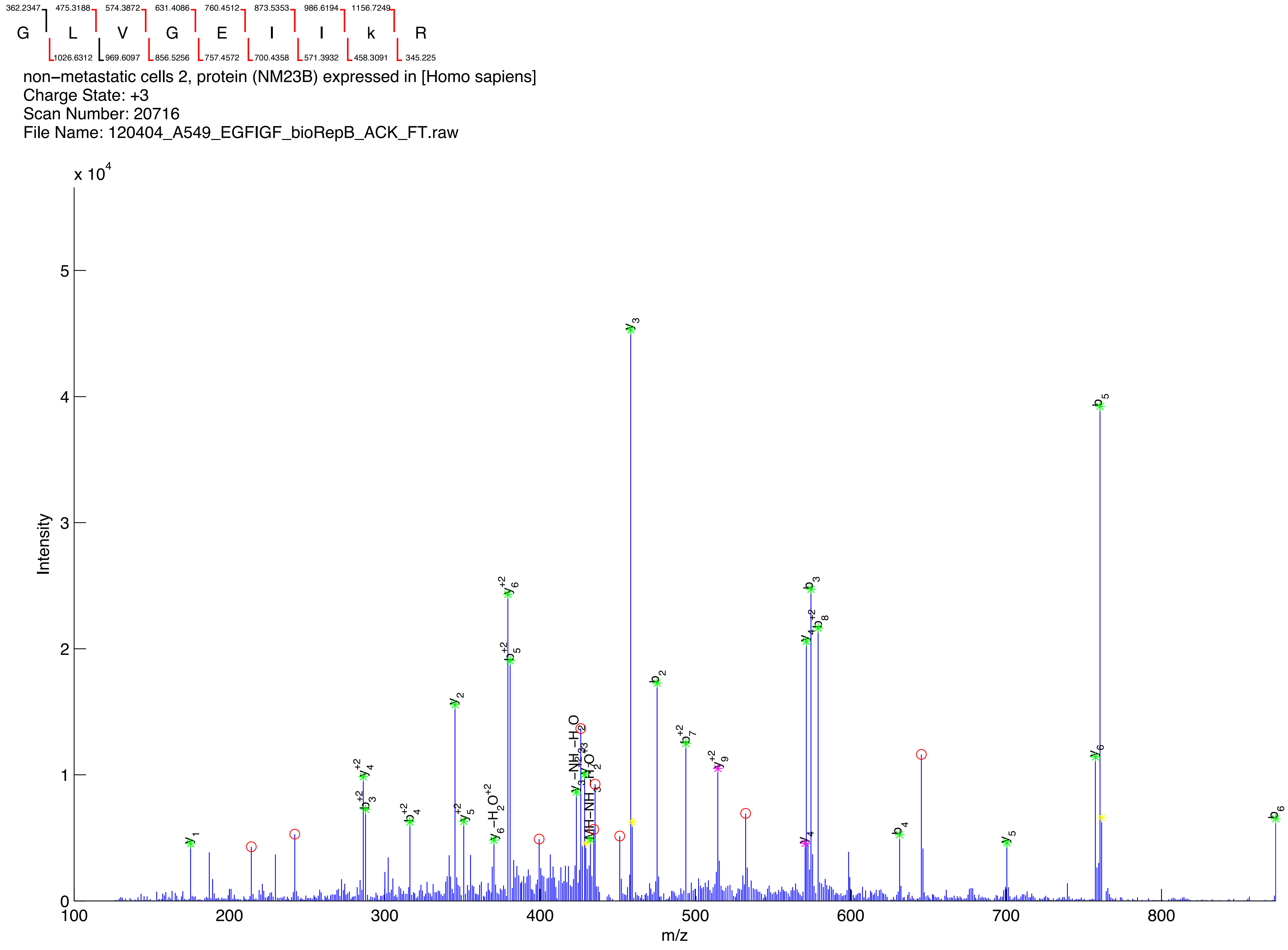

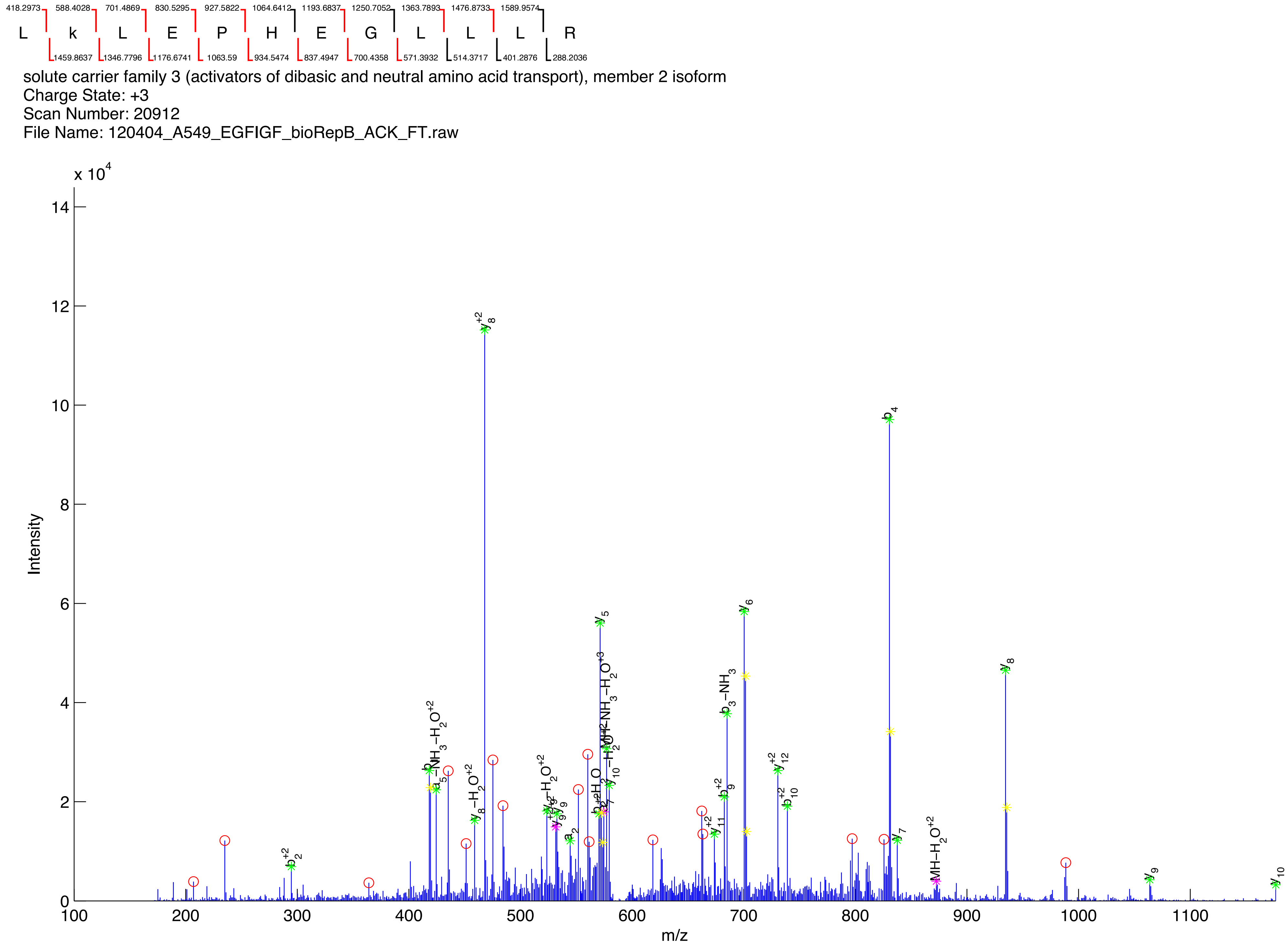

418.2973 588.4028 701.4869 830.5295 927.5822 1064.6412 1193.6837 1250.7052 1363.7893 1476.8733 1589.9574
L k L E P H E G L L L R
1459.8637 1346.7796 1176.6741 1063.59 934.5474 837.4947 700.4358 571.3932 514.3717 401.2876 288.2036
solute carrier family 3 (activators of dibasic and neutral amino acid transport), member 2 isoform
Charge State: +3
Scan Number: 20912
File Name: 120404_A549_EGFIGF_bioRepB_ACK_FT.raw
x 10^4
Intensity
m/z

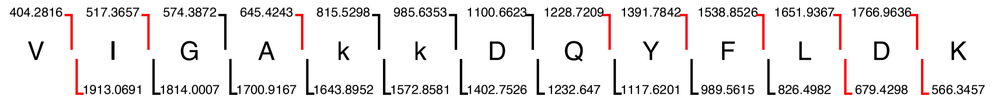

structural maintenance of chromosomes 3 [Homo sapiens]
Charge State: +3
Scan Number: 21043
File Name: 120413_A549_EGFIGF_bioRepC_AcK_FT.raw

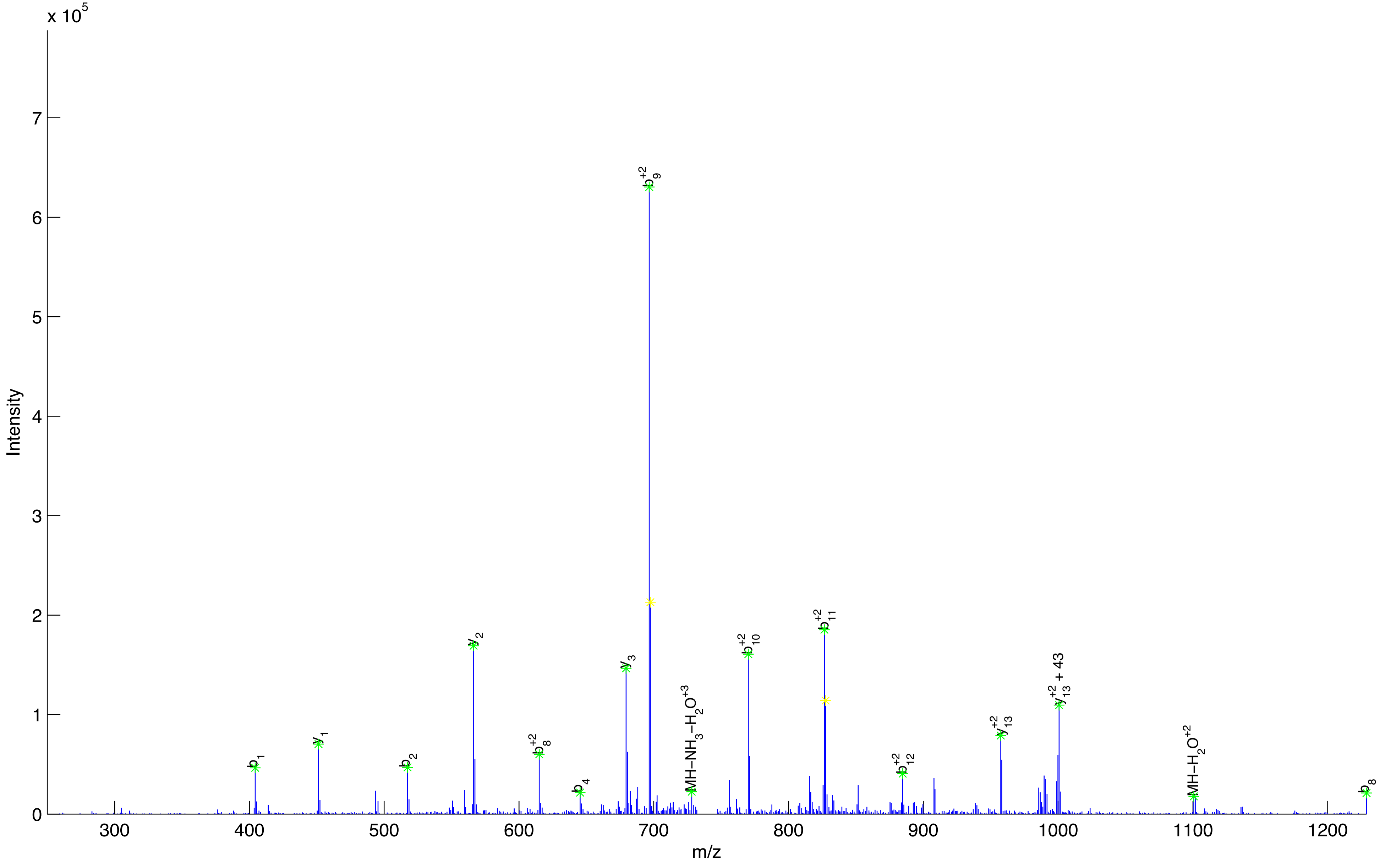

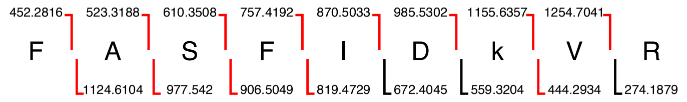

keratin 6 irs4 [Homo sapiens]
Charge State: +2
Scan Number: 21100
File Name: 120407_A549_EGFIGF_bioRepA_ACK_FT.raw

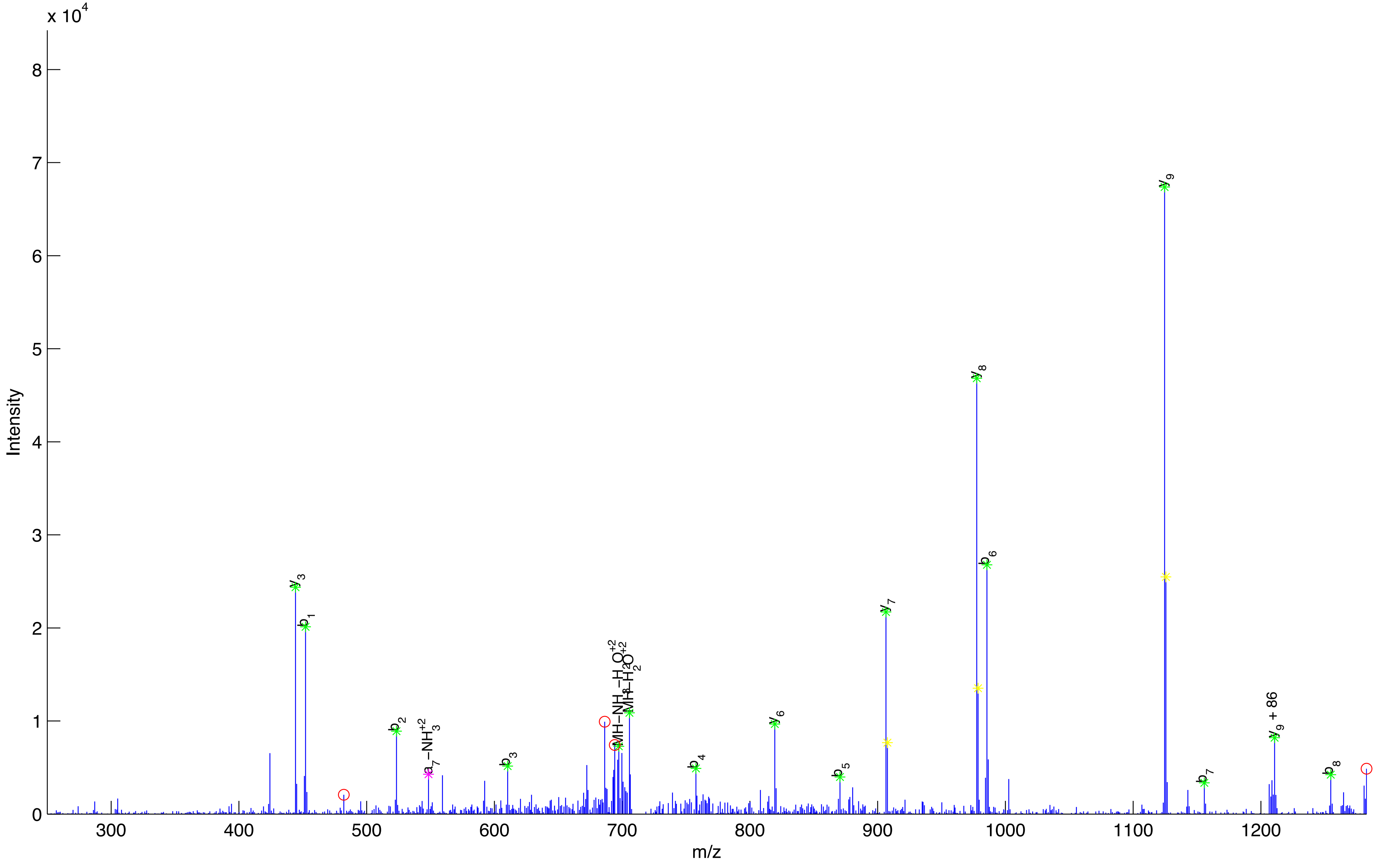

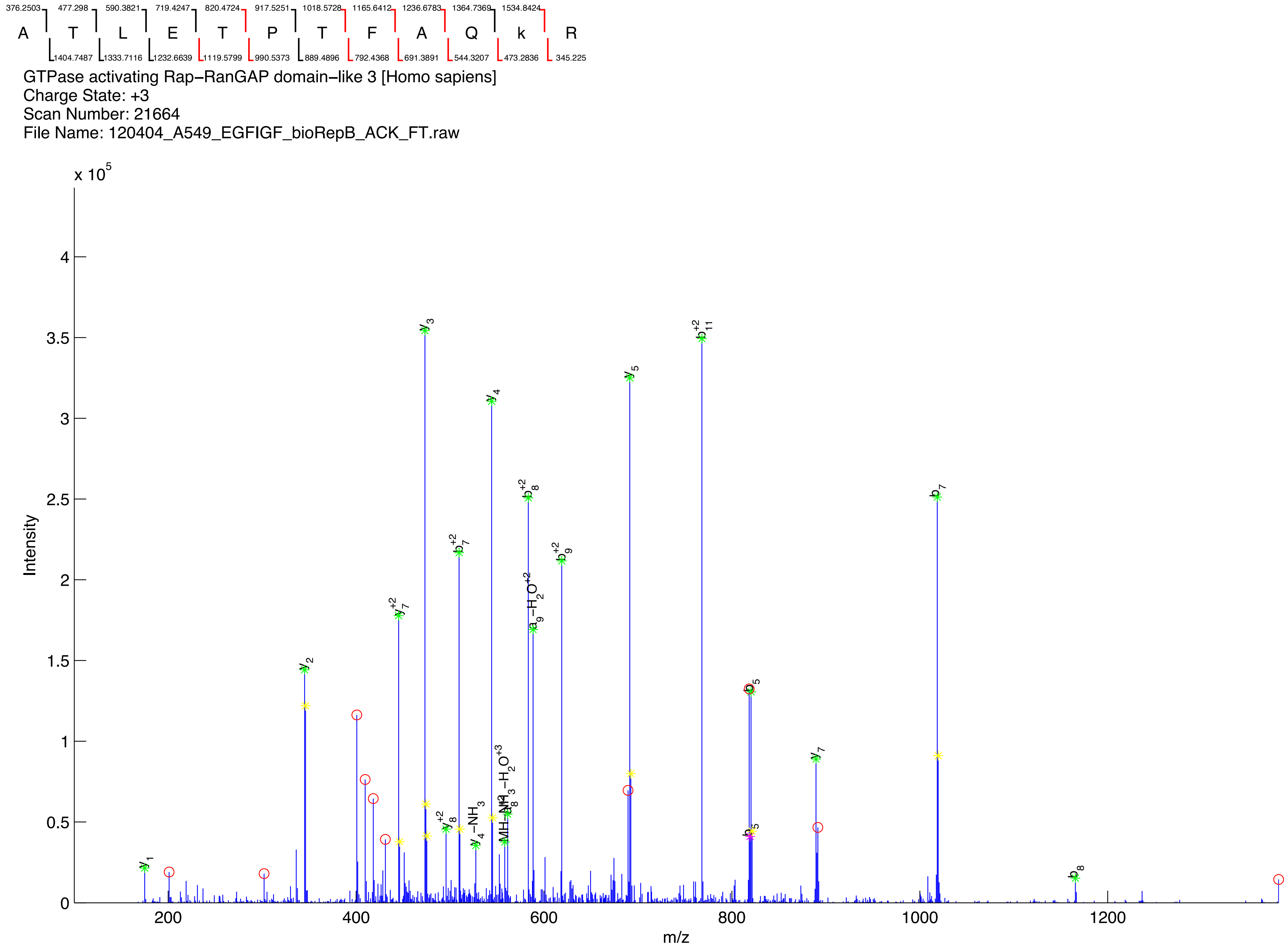

376.2503 477.298 590.3821 719.4247 820.4724 917.5251 1018.5728 1165.6412 1236.6783 1364.7369 1534.8424
A T L E T P T F A Q k R
1404.7487 1333.7116 1232.6639 1119.5799 990.5373 889.4896 792.4368 691.3891 544.3207 473.2836 345.225
GTPase activating Rap–RanGAP domain–like 3 [Homo sapiens]
Charge State: +3
Scan Number: 21664
File Name: 120404_A549_EGFIGF_bioRepB_ACK_FT.raw
x 10^5
Intensity
m/z

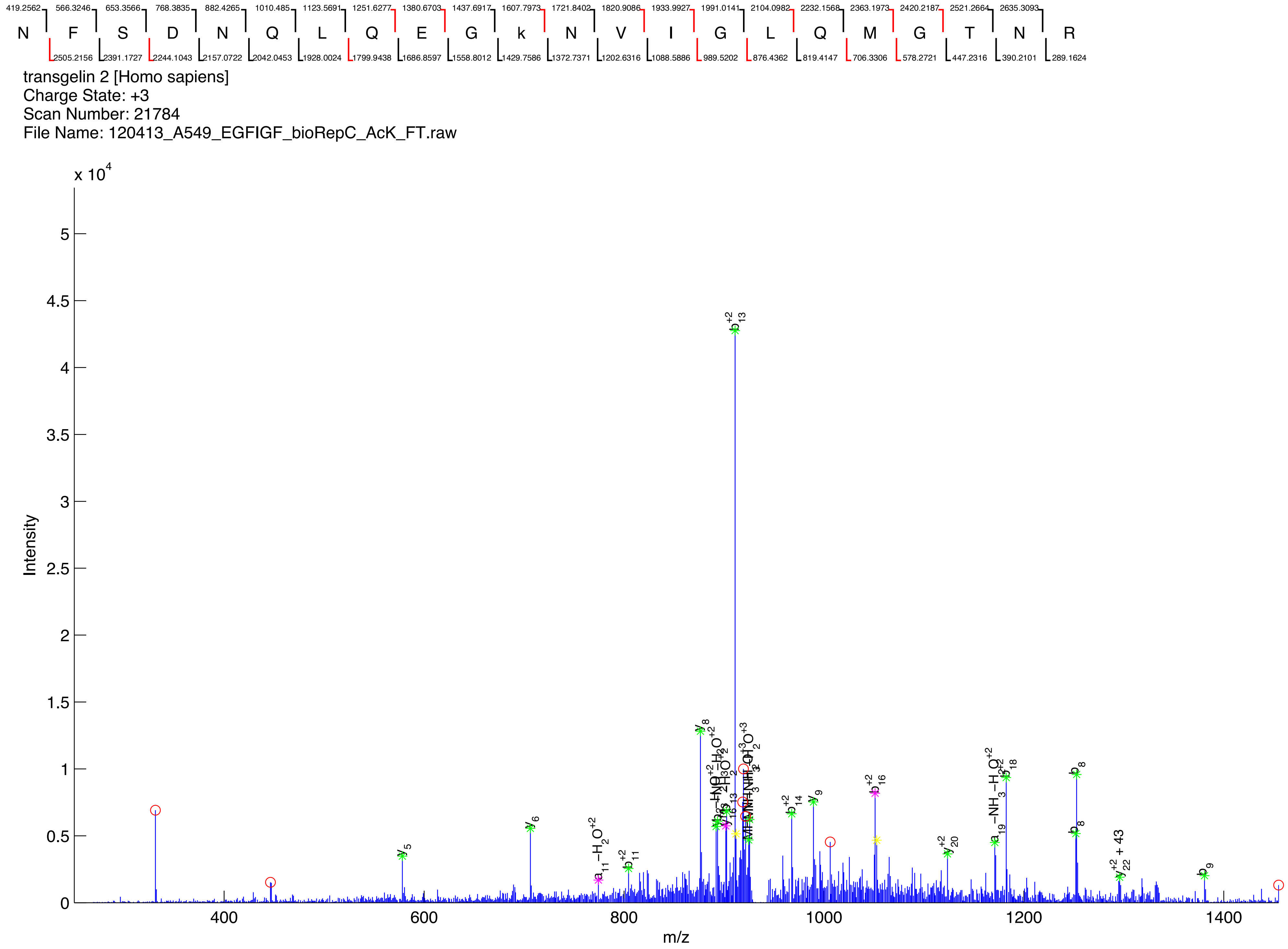
419.2562 566.3246 653.3566 768.3835 882.4265 1010.485 1123.5691 1251.6277 1380.6703 1437.6917 1607.7973 1721.8402 1820.9086 1933.9927 1991.0141 2104.0982 2232.1568 2363.1973 2420.2187 2521.2664 2635.3093
N F S D N Q L Q E G k N V I G L Q M G T N R
2505.2156 2391.1727 2244.1043 2157.0722 2042.0453 1928.0024 1799.9438 1686.8597 1558.8012 1429.7586 1372.7371 1202.6316 1088.5886 989.5202 876.4362 819.4147 706.3306 578.2721 447.2316 390.2101 289.1624
transgelin 2 [Homo sapiens]
Charge State: +3
Scan Number: 21784
File Name: 120413_A549_EGFIGF_bioRepC_AcK_FT.raw
x 10^4
Intensity
m/z
y5
y6
b+2 11
y8
b+2 13
b+2 14
y9
b+2 16
y+2 20
b8
b9

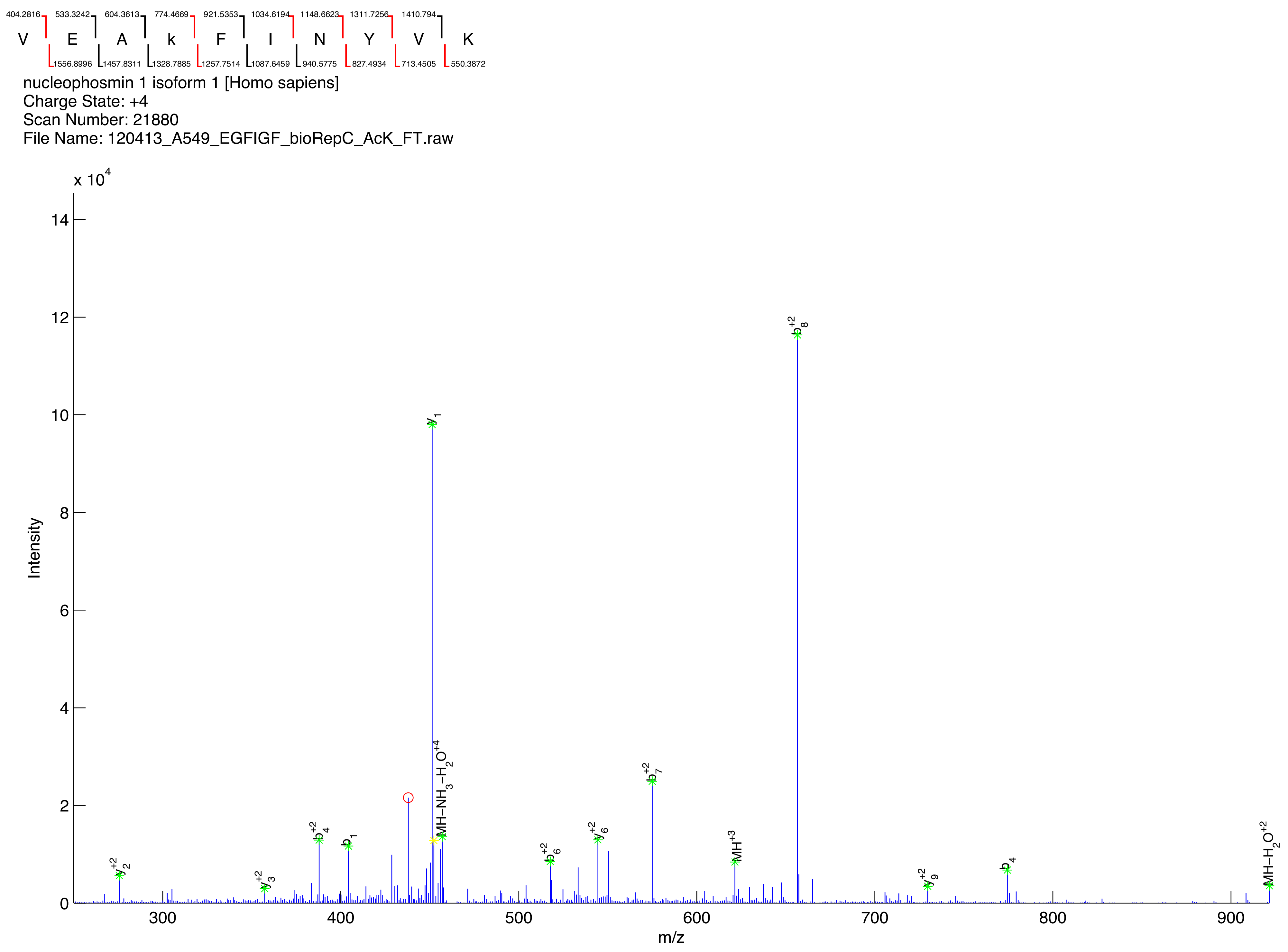

404.2816 533.3242 604.3613 774.4669 921.5353 1034.6194 1148.6623 1311.7256 1410.794
V E A k F I N Y V K
1556.8996 1457.8311 1328.7885 1257.7514 1087.6459 940.5775 827.4934 713.4505 550.3872
nucleophosmin 1 isoform 1 [Homo sapiens]
Charge State: +4
Scan Number: 21880
File Name: 120413_A549_EGFIGF_bioRepC_AcK_FT.raw
x 10^4
Intensity
m/z
y_2^{+2}
y_3^{+2}
b_4^{+2}
b_1
y_1
$MH-NH_3-H_2O^{+4}$
b_6^{+2}
y_6^{+2}
b_7^{+2}
MH^{+3}
b_8^{+2}
y_9^{+2}
b_4
$MH-H_2O^{+2}$

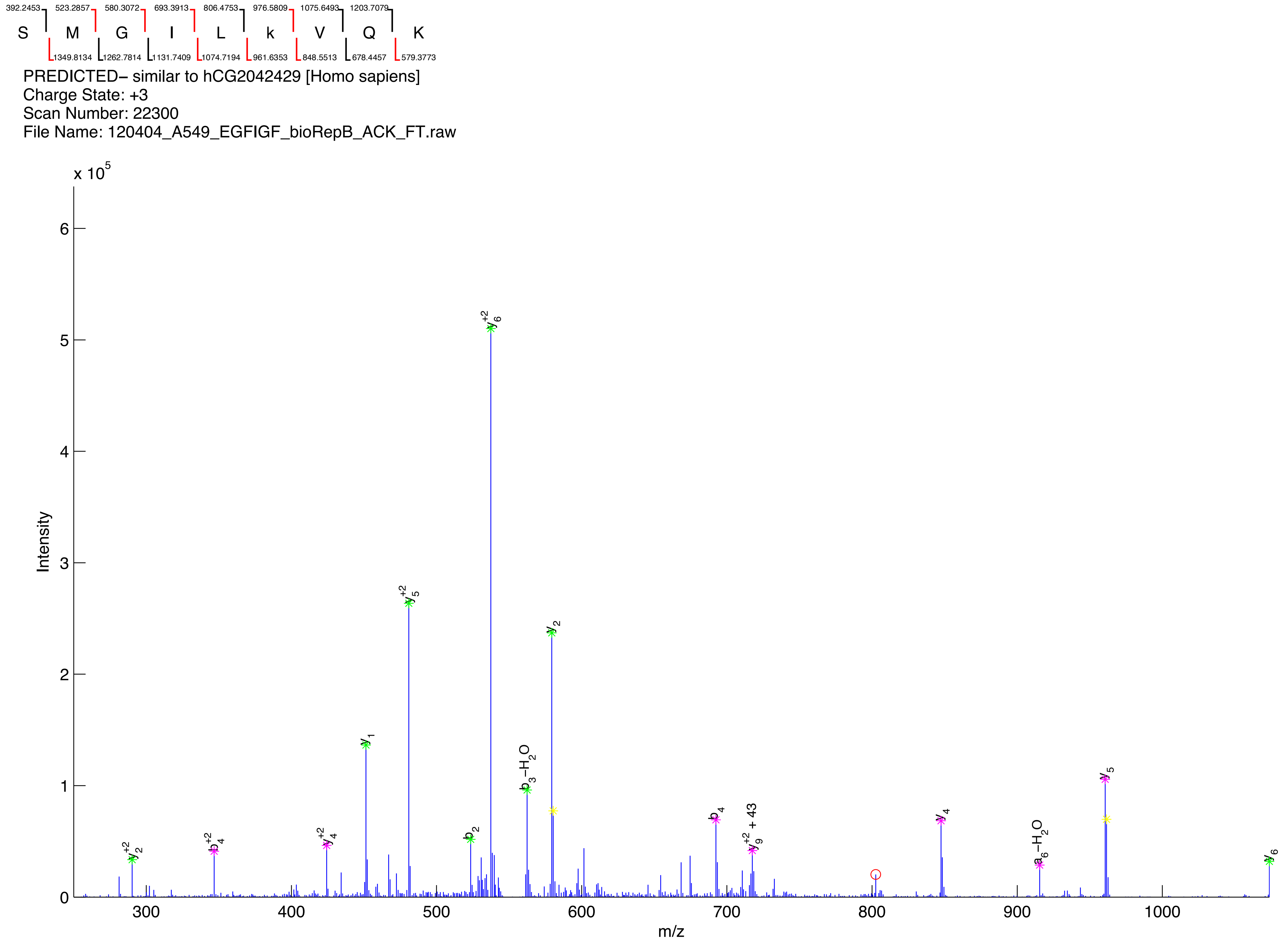
392.2453 523.2857 580.3072 693.3913 806.4753 976.5809 1075.6493 1203.7079
S M G I L k V Q K
1349.8134 1262.7814 1131.7409 1074.7194 961.6353 848.5513 678.4457 579.3773
PREDICTED– similar to hCG2042429 [Homo sapiens]
Charge State: +3
Scan Number: 22300
File Name: 120404_A549_EGFIGF_bioRepB_ACK_FT.raw
x 10^5
Intensity
m/z
y+2 2
b+2 4
y+2 4
y1
y+2 5
b2
y+2 6
b3−H2O
y2
b4
y+2 9 + 43
y4
a6−H2O
y5
y6

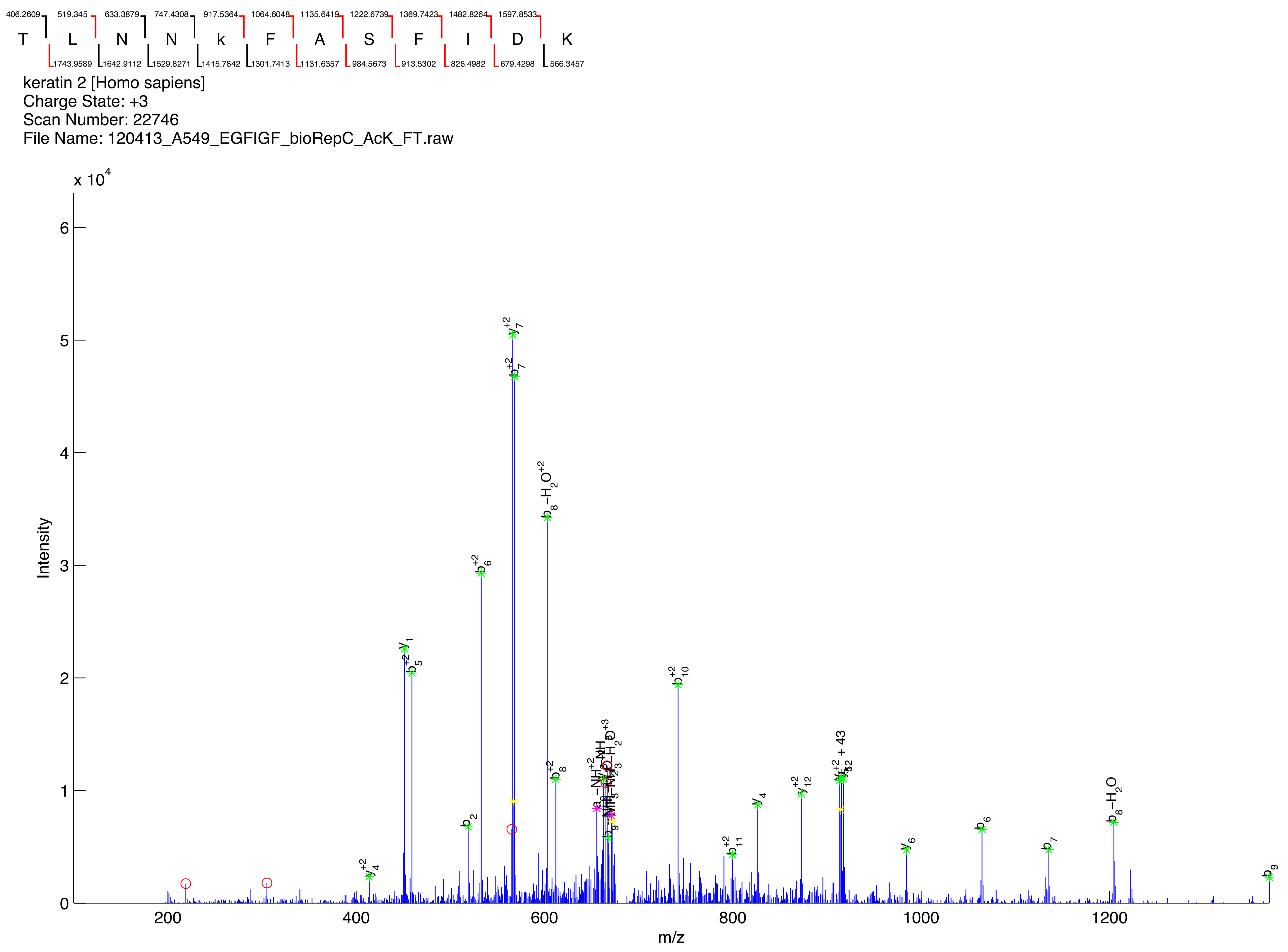

406.2609 519.345 633.3879 747.4308 917.5364 1064.6048 1135.6419 1222.6739 1369.7423 1482.8264 1597.8533
T L N N k F A S F I D K
1743.9589 1642.9112 1529.8271 1415.7842 1301.7413 1131.6357 984.5673 913.5302 826.4982 679.4298 566.3457
keratin 2 [Homo sapiens]
Charge State: +3
Scan Number: 22746
File Name: 120413_A549_EGFIGF_bioRepC_AcK_FT.raw
x 10^4
Intensity
m/z

433.2718 546.3559 643.4086 756.4927 855.5611 1025.6666 1122.7194 1285.7827 1398.8668

Q L P L V k P Y L R

1268.7731 1140.7145 1027.6304 930.5777 817.4936 718.4252 548.3197 451.2669 288.2036

clathrin heavy chain 1 [Homo sapiens]
Charge State: +3
Scan Number: 23936
File Name: 120404_A549_EGFIGF_bioRepB_ACK_FT.raw

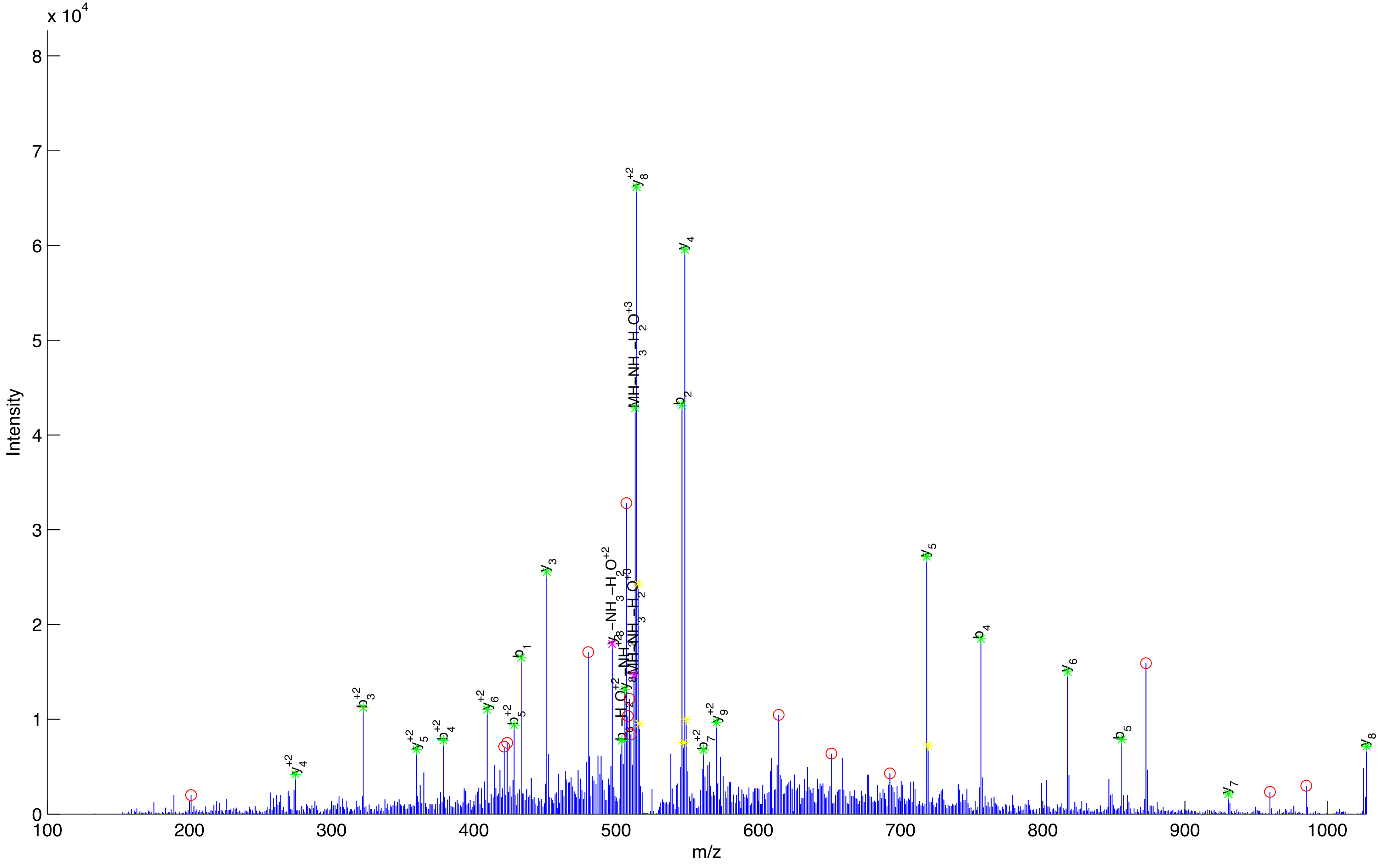

362.2347	419.2562	516.3089	573.3304	660.3624	731.3995	830.4679	917.5	1014.5527	1177.6161	1274.6688	1375.7165	1522.7849	1636.8278	1733.8806	1820.9126	1907.9447	2022.9716	2122.04	2193.0771	2264.1143	2377.1983	2514.2572	
G	G	P	G	S	A	V	S	P	Y	P	T	F	N	P	S	S	D	V	A	A	L	H	K
	2660.3628	2603.3413	2546.3198	2449.2671	2392.2456	2305.2136	2234.1765	2135.108	2048.076	1951.0233	1787.9599	1690.9072	1589.8595	1442.7911	1328.7481	1231.6954	1144.6633	1057.6313	942.6044	843.536	772.4988	701.4617	588.3777

annexin I [Homo sapiens]
Charge State: +
Scan Number: 24360
File Name: 120404_A549_EGFIGF_bioRepB_ACK_FT.raw

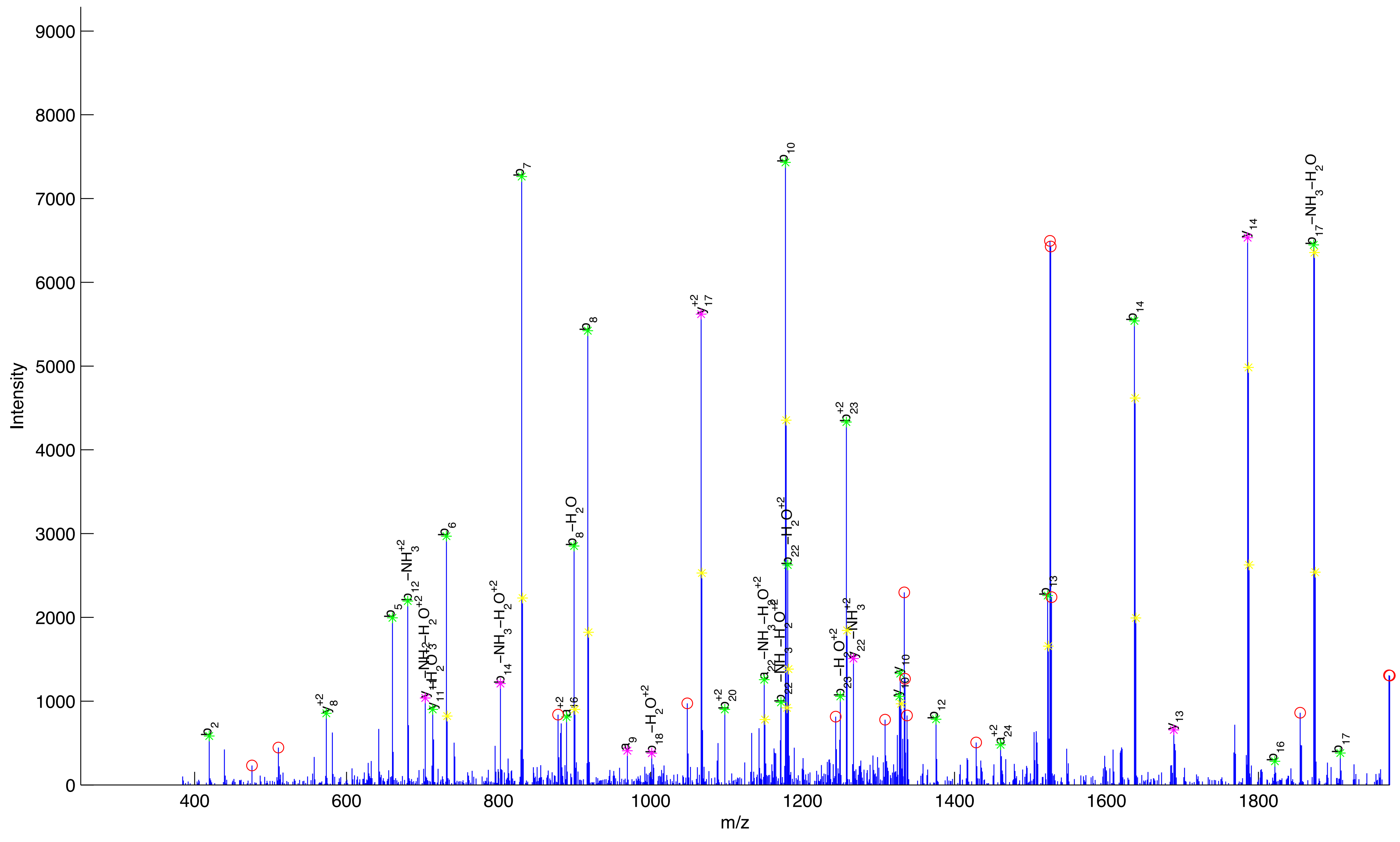

V	I	G	A	k	k	D	Q	Y	F	L	D	K
404.2816	517.3657	574.3872	645.4243	815.5298	985.6353	1100.6623	1228.7209	1391.7842	1538.8526	1651.9367	1766.9636	
	1913.0691	1814.0007	1700.9167	1643.8952	1572.8581	1402.7526	1232.647	1117.6201	989.5615	826.4982	679.4298	566.3457

structural maintenance of chromosomes 3 [Homo sapiens]
Charge State: +2
Scan Number: 24452
File Name: 120404_A549_EGFIGF_bioRepB_ACK_FT.raw

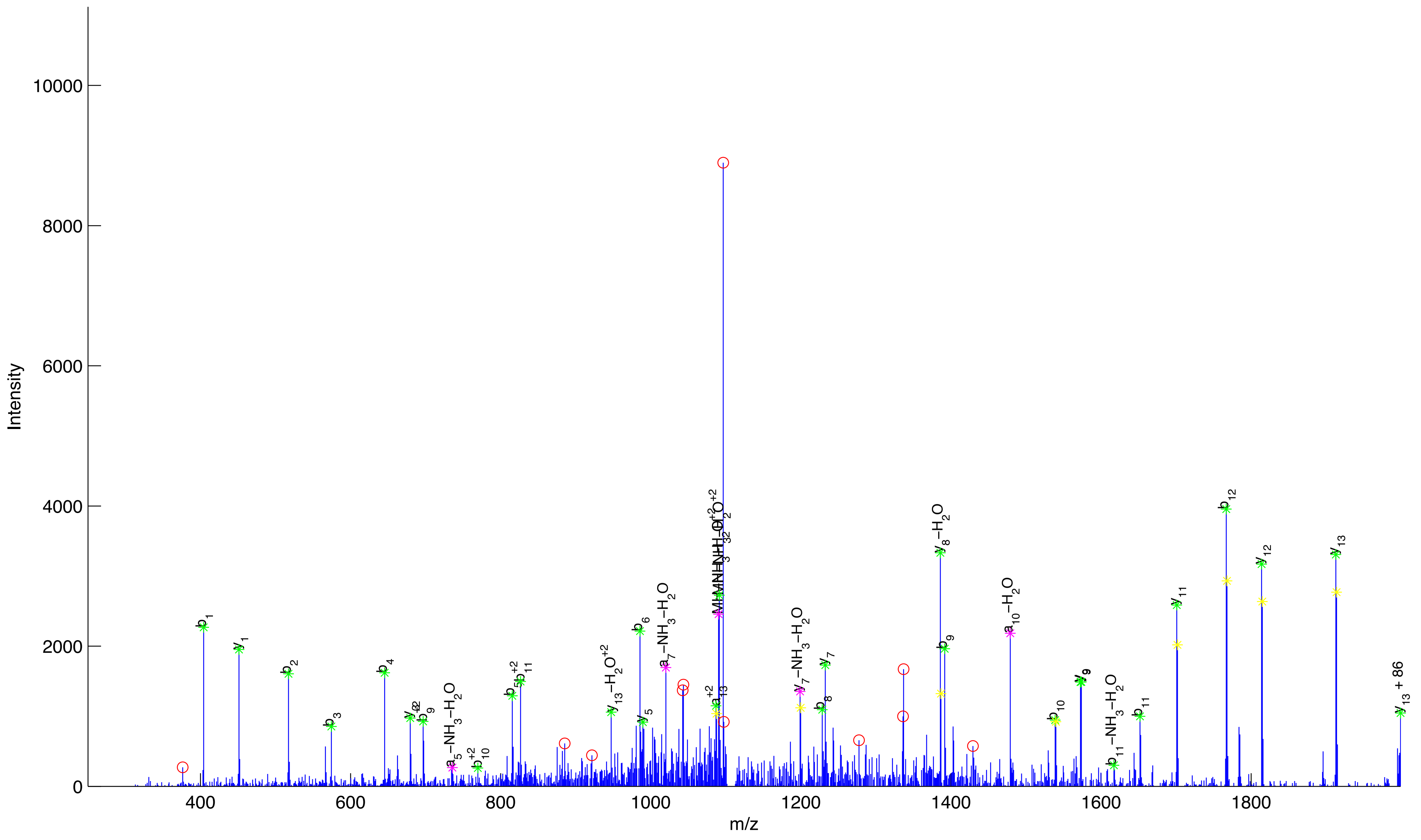

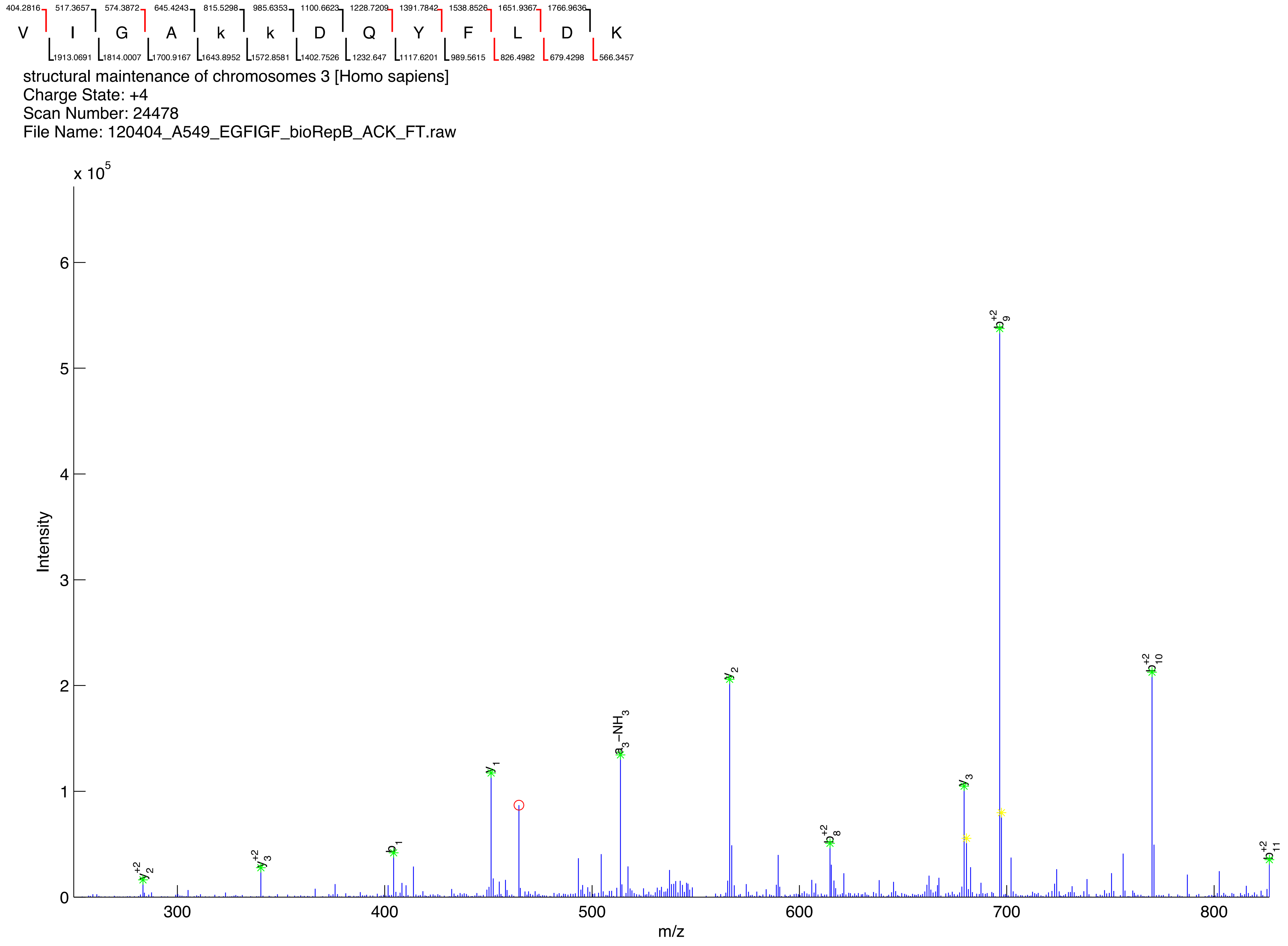
404.2816 517.3657 574.3872 645.4243 815.5298 985.6353 1100.6623 1228.7209 1391.7842 1538.8526 1651.9367 1766.9636
V I G A k k D Q Y F L D K
1913.0691 1814.0007 1700.9167 1643.8952 1572.8581 1402.7526 1232.647 1117.6201 989.5615 826.4982 679.4298 566.3457
structural maintenance of chromosomes 3 [Homo sapiens]
Charge State: +4
Scan Number: 24478
File Name: 120404_A549_EGFIGF_bioRepB_ACK_FT.raw
x 10^5
Intensity
m/z
y+2 2
y+2 3
b1
y1
a3−NH3
y2
b+2 8
y3
b+2 9
b+2 10
b+2 11

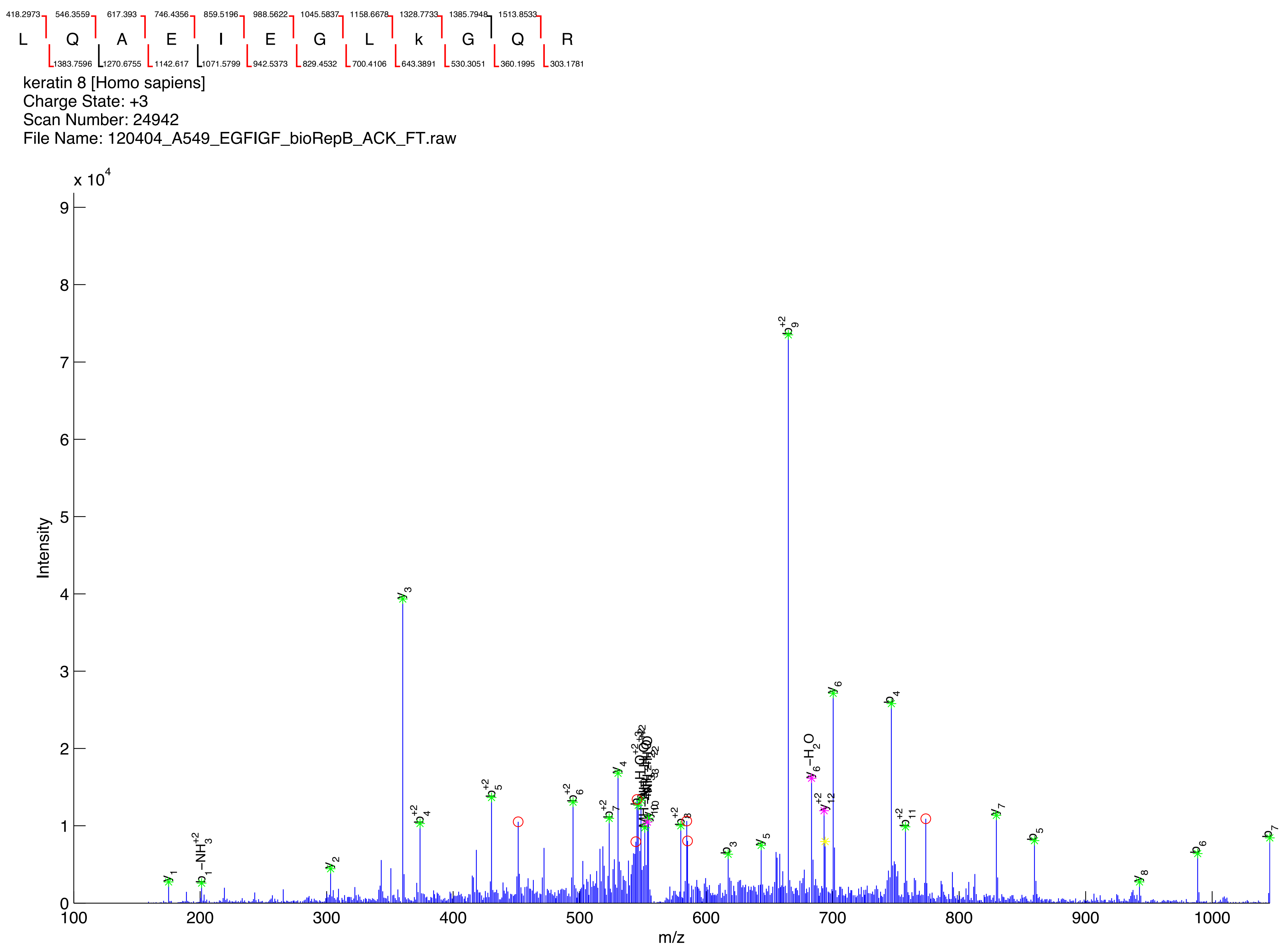

418.2973 546.3559 617.393 746.4356 859.5196 988.5622 1045.5837 1158.6678 1328.7733 1385.7948 1513.8533
L Q A E I E G L k G Q R
1383.7596 1270.6755 1142.617 1071.5799 942.5373 829.4532 700.4106 643.3891 530.3051 360.1995 303.1781
keratin 8 [Homo sapiens]
Charge State: +3
Scan Number: 24942
File Name: 120404_A549_EGFIGF_bioRepB_ACK_FT.raw
x 10^4
Intensity
m/z

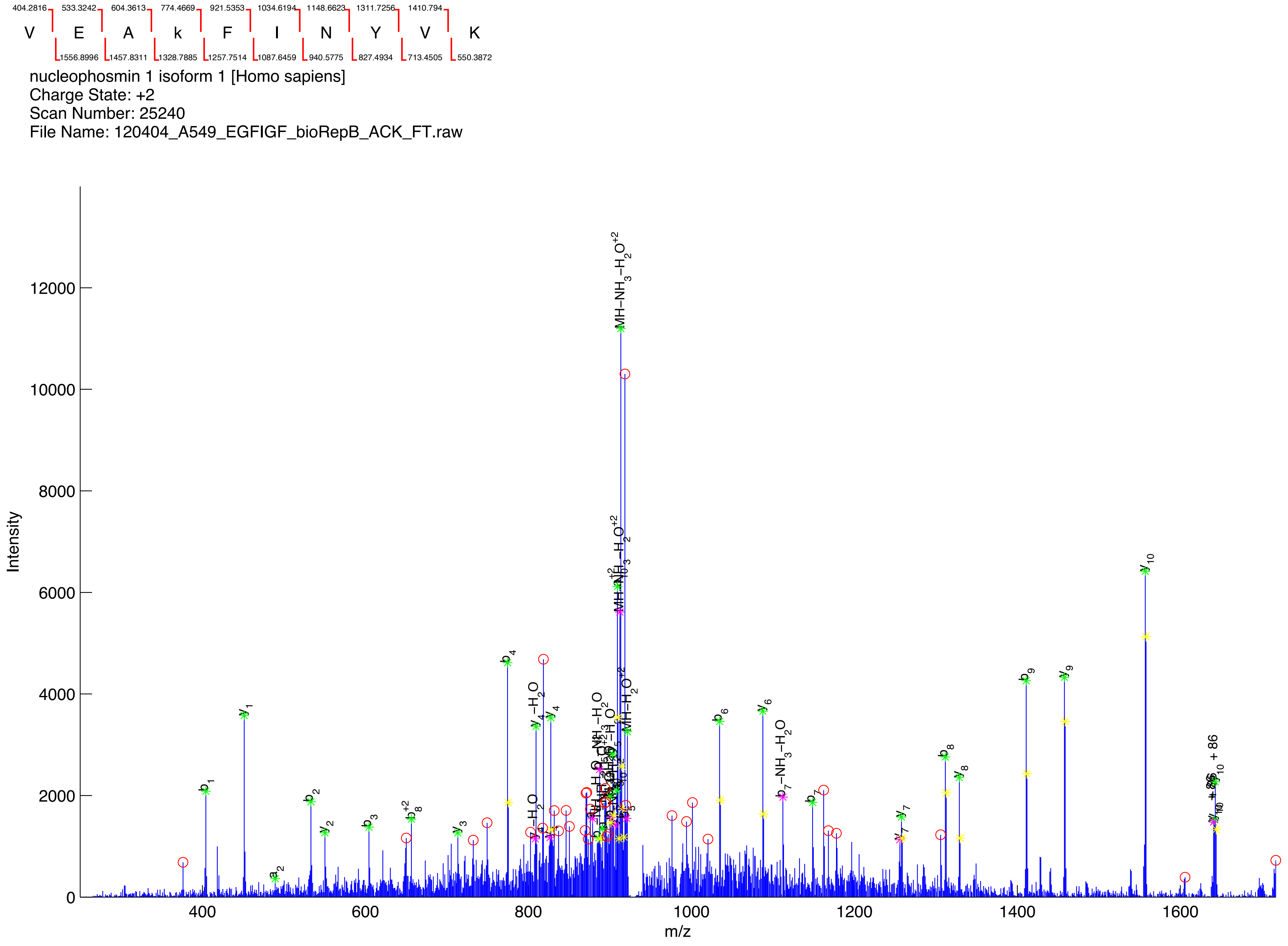

404.2816 533.3242 604.3613 774.4669 921.5353 1034.6194 1148.6623 1311.7256 1410.794
V E A k F I N Y V K
1556.8996 1457.8311 1328.7885 1257.7514 1087.6459 940.5775 827.4934 713.4505 550.3872
nucleophosmin 1 isoform 1 [Homo sapiens]
Charge State: +2
Scan Number: 25240
File Name: 120404_A549_EGFIGF_bioRepB_ACK_FT.raw
Intensity
m/z

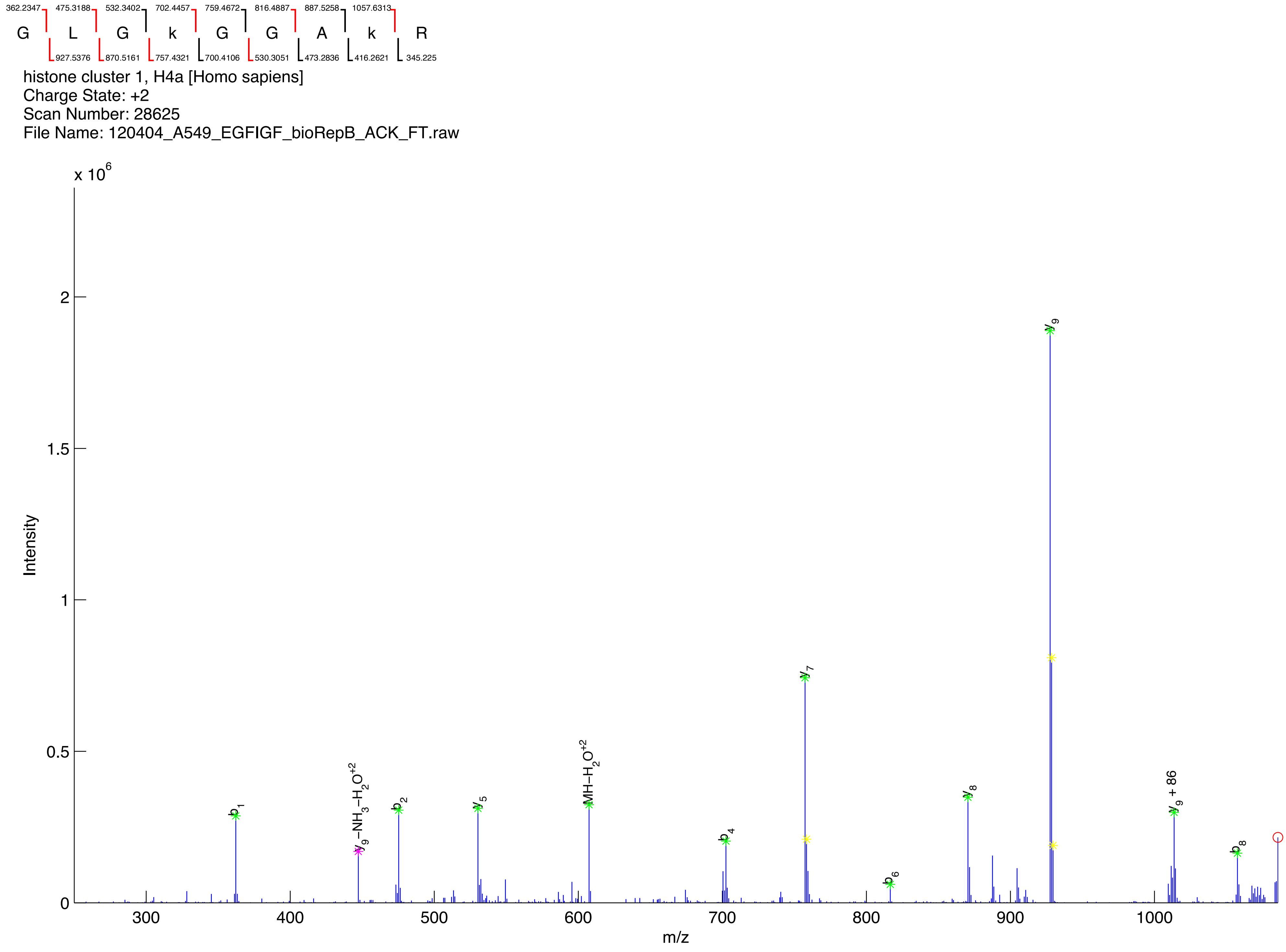
362.2347 475.3188 532.3402 702.4457 759.4672 816.4887 887.5258 1057.6313
G L G k G G A k R
927.5376 870.5161 757.4321 700.4106 530.3051 473.2836 416.2621 345.225
histone cluster 1, H4a [Homo sapiens]
Charge State: +2
Scan Number: 28625
File Name: 120404_A549_EGFIGF_bioRepB_ACK_FT.raw
x 10^6
Intensity
m/z
b1
y9−NH3−H2O+2
b2
y5
MH−H2O+2
b4
y7
b6
y8
y9
y9 + 86
b8

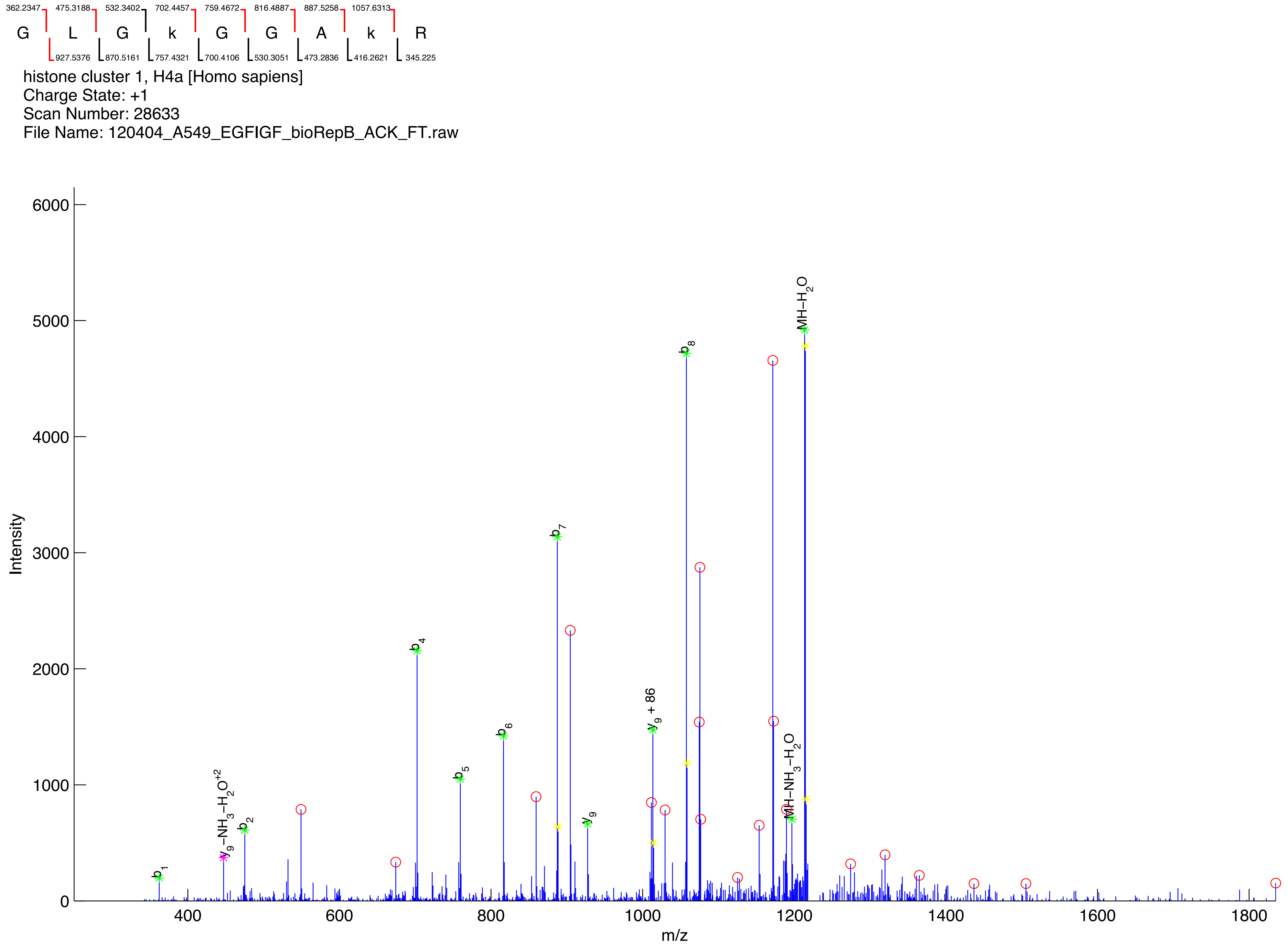
362.2347 475.3188 532.3402 702.4457 759.4672 816.4887 887.5258 1057.6313
G L G k G G A k R
927.5376 870.5161 757.4321 700.4106 530.3051 473.2836 416.2621 345.225
histone cluster 1, H4a [Homo sapiens]
Charge State: +1
Scan Number: 28633
File Name: 120404_A549_EGFIGF_bioRepB_ACK_FT.raw
Intensity
m/z
b1
y9−NH3−H2O+2
b2
b4
b5
b6
b7
y9
y9 + 86
b8
MH−NH3−H2O
MH−H2O
0
1000
2000
3000
4000
5000
6000
400
600
800
1000
1200
1400
1600
1800

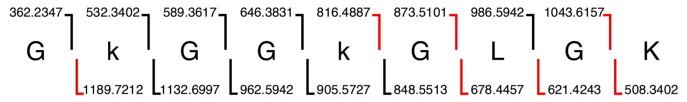

histone cluster 1, H4a [Homo sapiens]
Charge State: +3
Scan Number: 28648
File Name: 120404_A549_EGFIGF_bioRepB_ACK_FT.raw

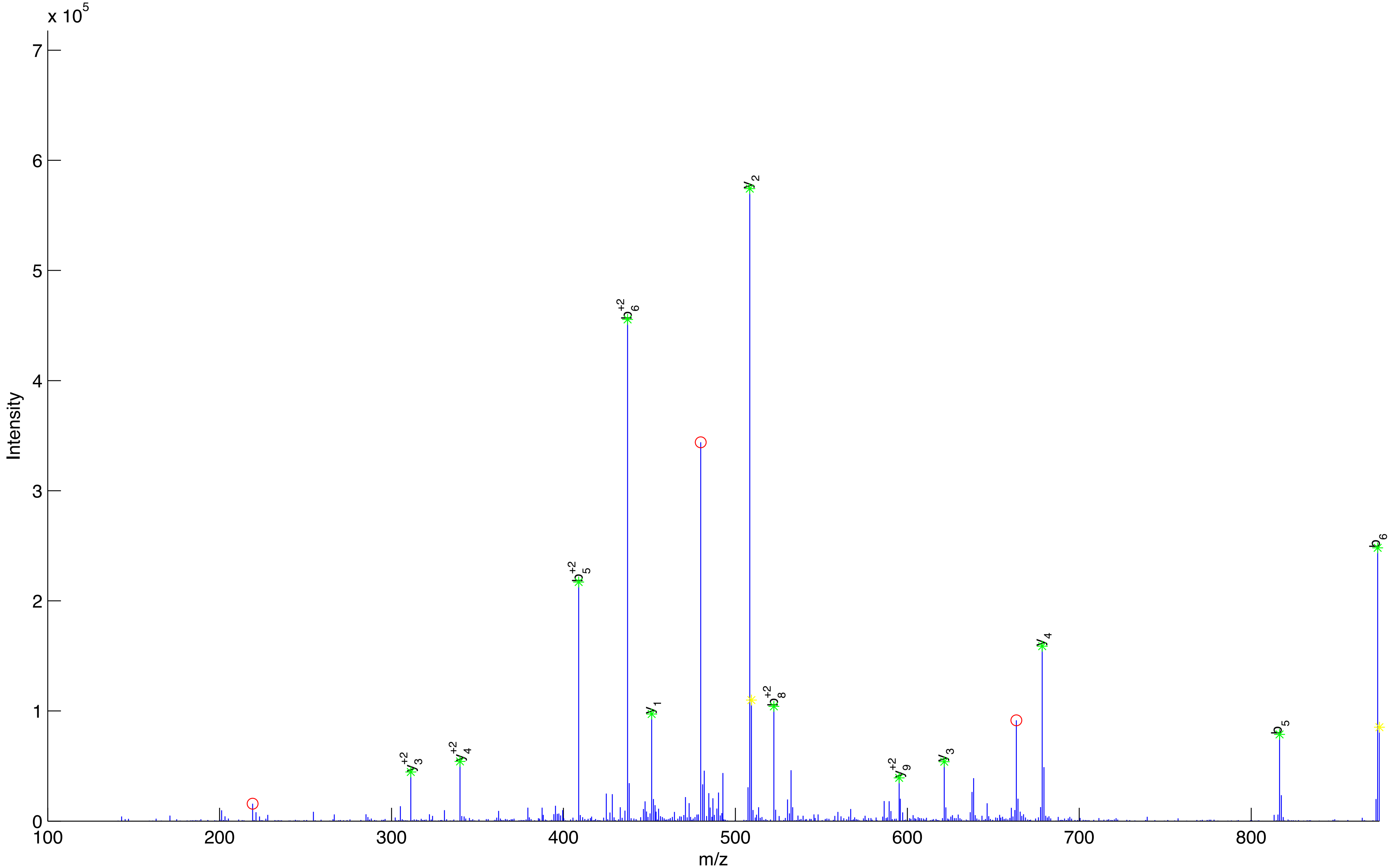

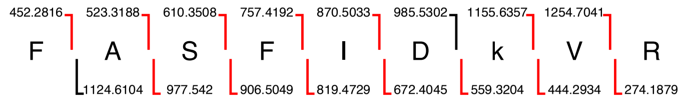

keratin 72 [Homo sapiens]
Charge State: +3
Scan Number: 29360
File Name: 120404_A549_EGFIGF_bioRepB_ACK_FT.raw

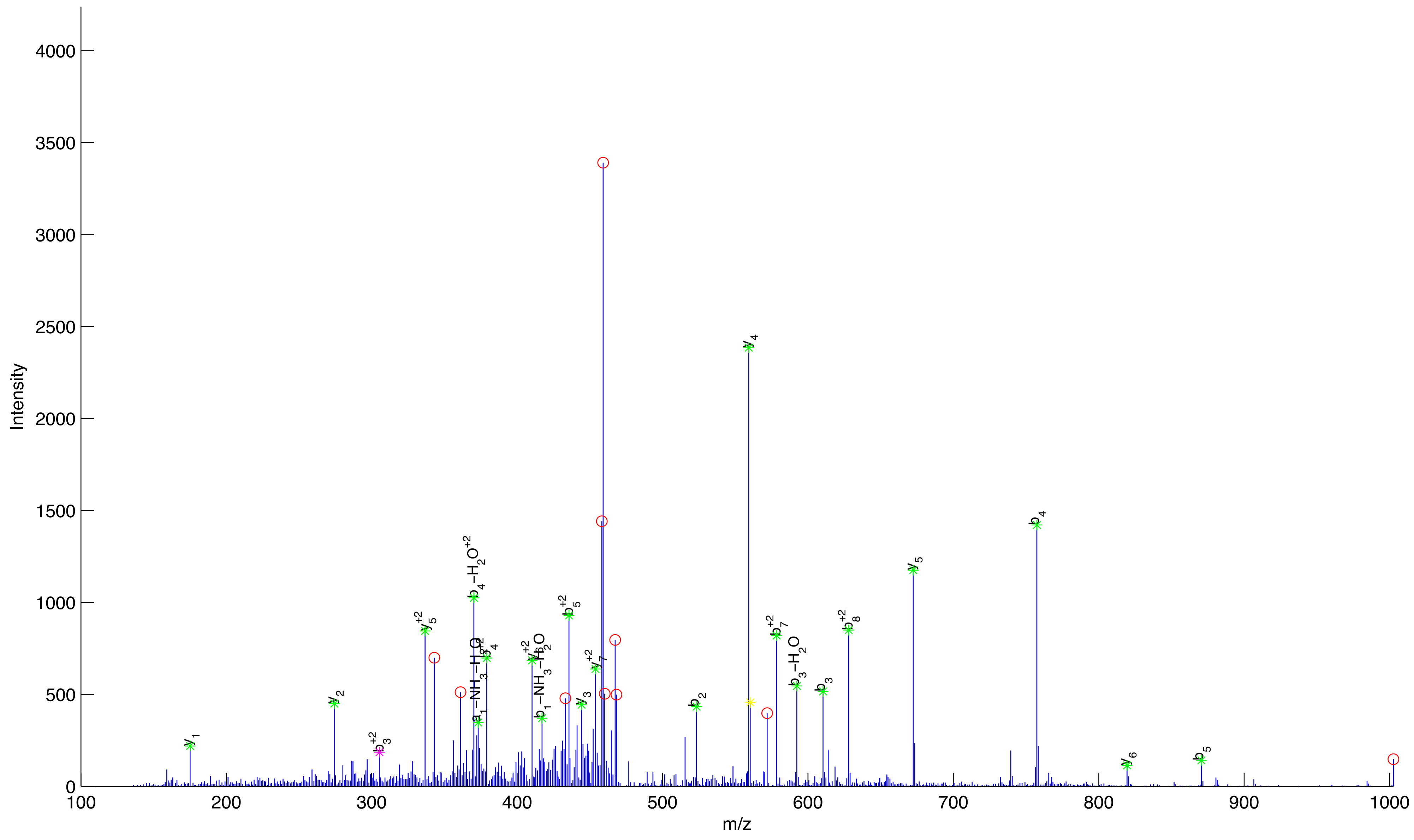

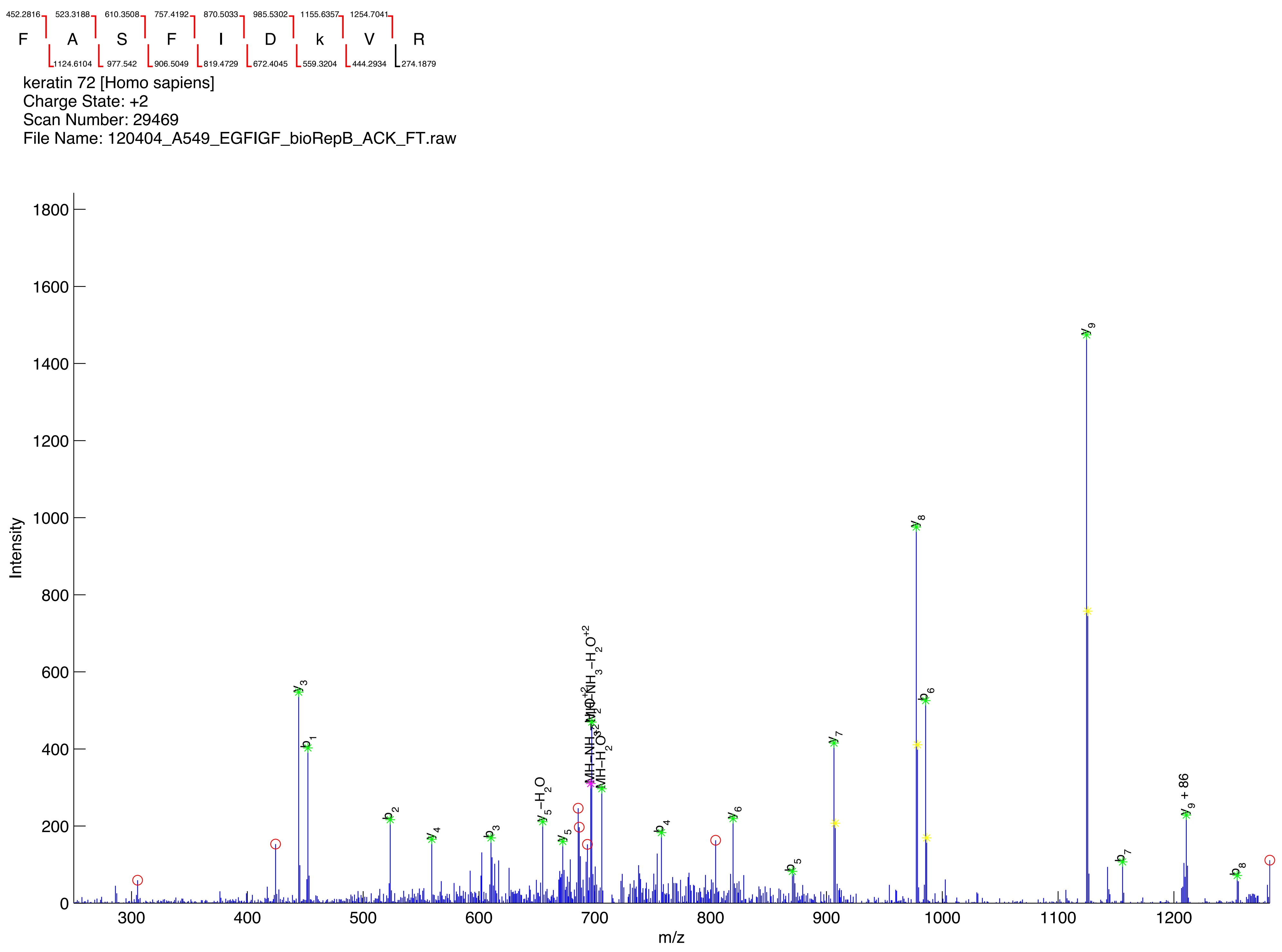
452.2816
523.3188
610.3508
757.4192
870.5033
985.5302
1155.6357
1254.7041
F A S F I D k V R
1124.6104
977.542
906.5049
819.4729
672.4045
559.3204
444.2934
274.1879
keratin 72 [Homo sapiens]
Charge State: +2
Scan Number: 29469
File Name: 120404_A549_EGFIGF_bioRepB_ACK_FT.raw
Intensity
m/z
y3
b1
b2
y4
b3
y5−H2O
y5
MH−NH3−H2O+2
MH−H2O+2
MH−H2O
b4
y6
b5
y7
y8
b6
y9
b7
y9 + 86
b8

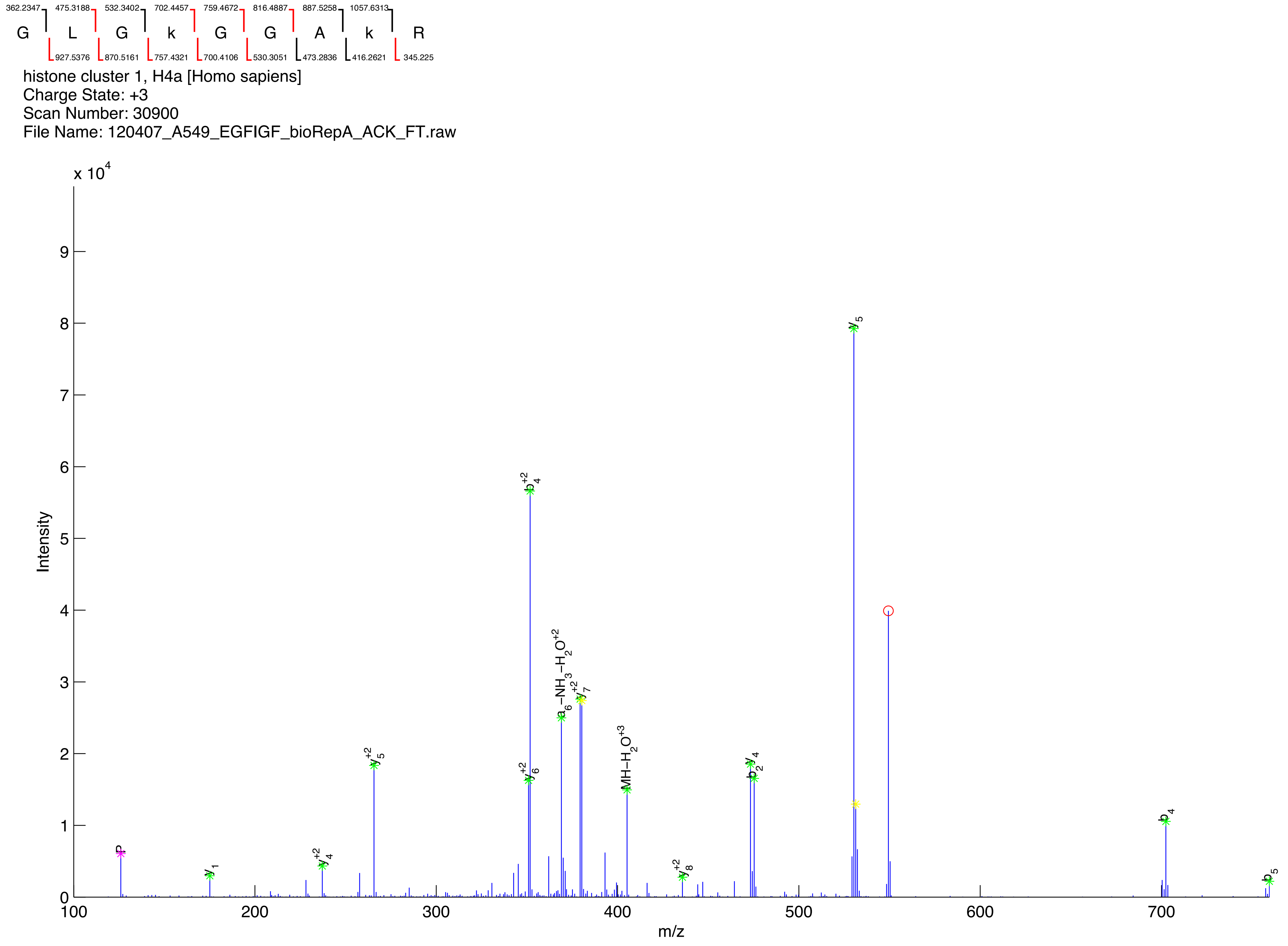
362.2347 475.3188 532.3402 702.4457 759.4672 816.4887 887.5258 1057.6313
G L G k G G A k R
927.5376 870.5161 757.4321 700.4106 530.3051 473.2836 416.2621 345.225
histone cluster 1, H4a [Homo sapiens]
Charge State: +3
Scan Number: 30900
File Name: 120407_A549_EGFIGF_bioRepA_ACK_FT.raw
x 10^4
Intensity
m/z

362.2347 419.2562 589.3617 646.3831 759.4672 816.4887 986.5942 1043.6157 1100.6371 1171.6742 1341.7798

G G k G L G k G G A k R

1211.686 1154.6646 1097.6431 927.5376 870.5161 757.4321 700.4106 530.3051 473.2836 416.2621 345.225

histone cluster 1, H4a [Homo sapiens]
Charge State: +3
Scan Number: 30984
File Name: 120407_A549_EGFIGF_bioRepA_ACK_FT.raw

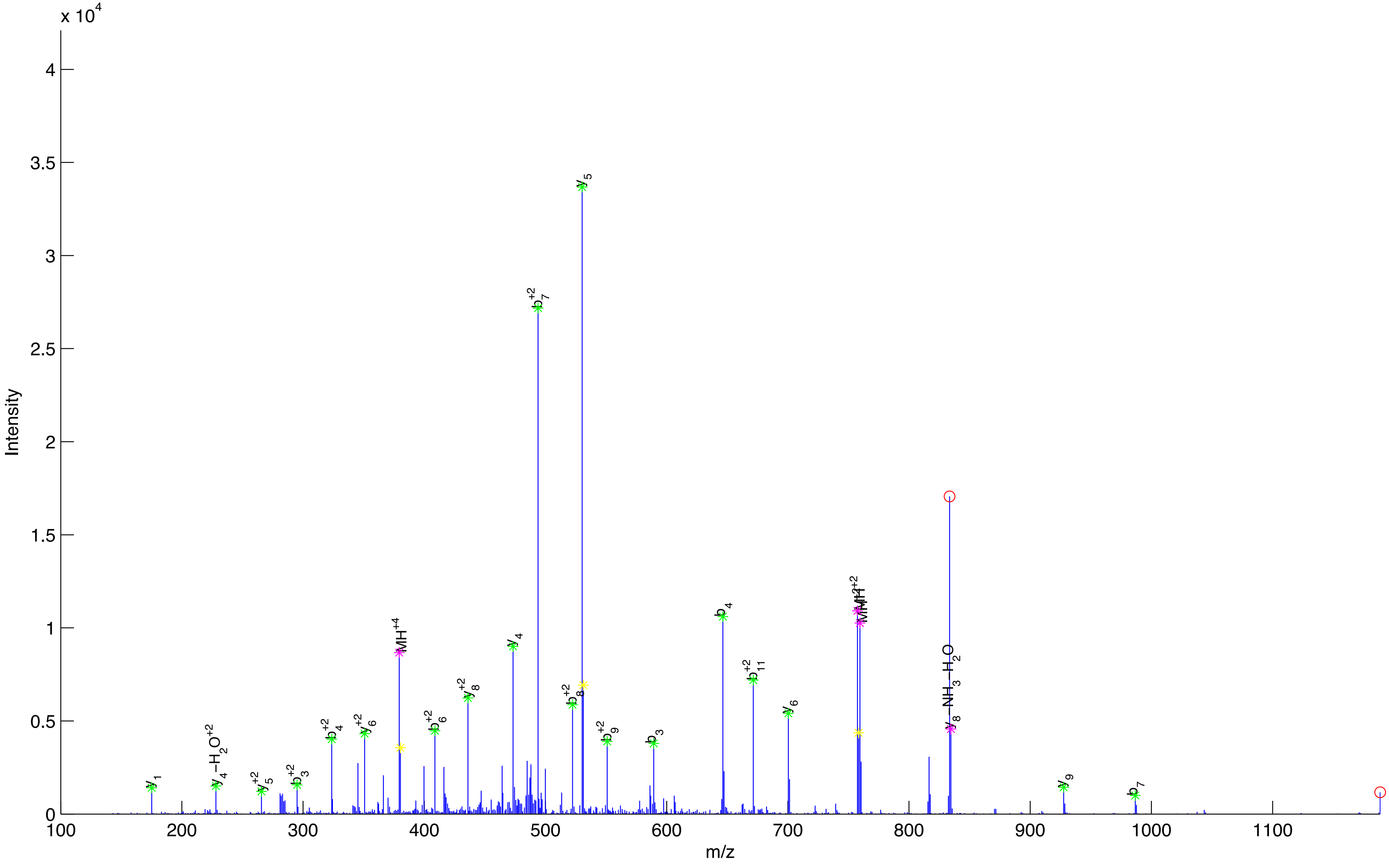

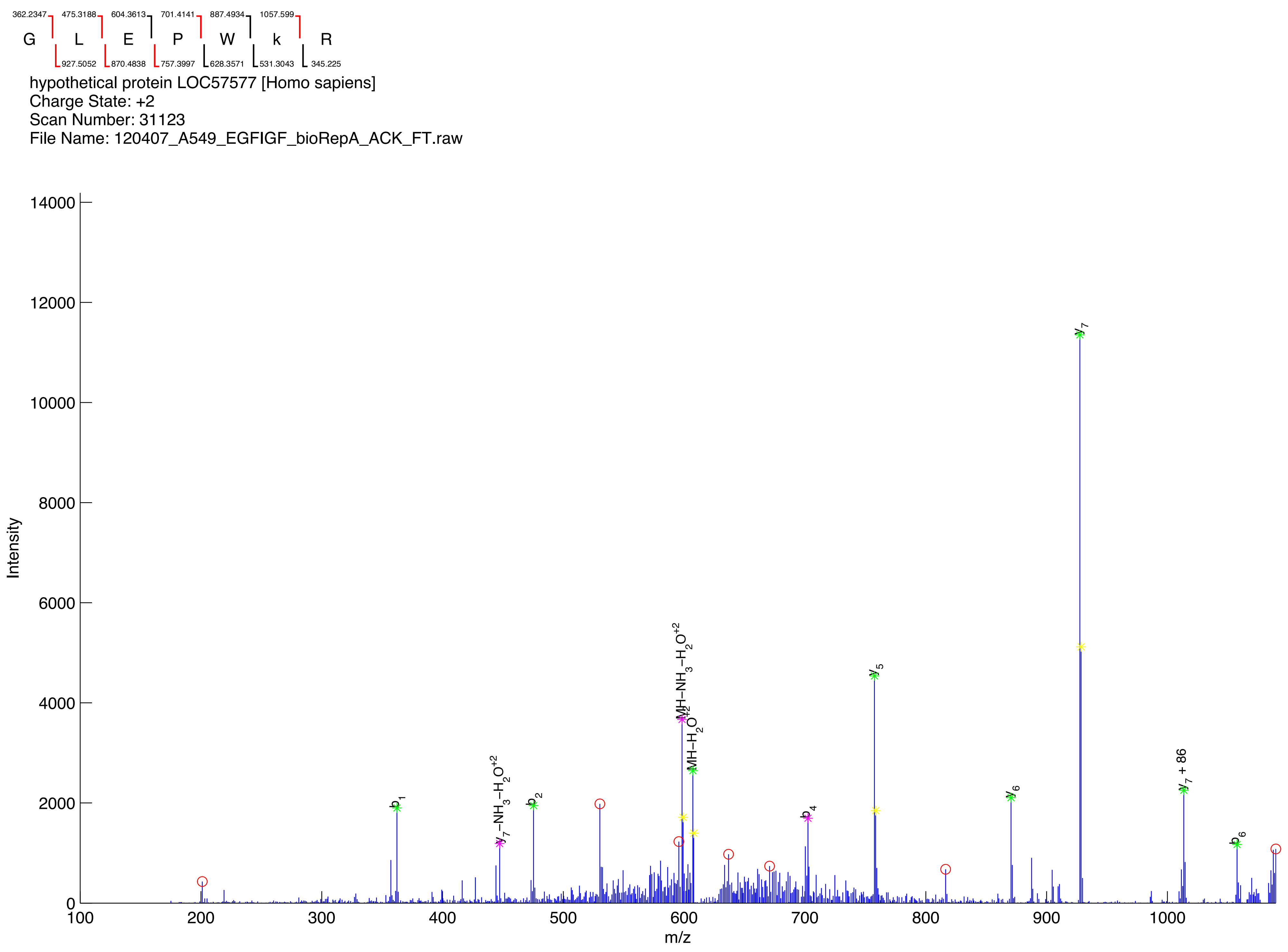
362.2347
475.3188
604.3613
701.4141
887.4934
1057.599
G L E P W k R
927.5052
870.4838
757.3997
628.3571
531.3043
345.225
hypothetical protein LOC57577 [Homo sapiens]
Charge State: +2
Scan Number: 31123
File Name: 120407_A549_EGFIGF_bioRepA_ACK_FT.raw
Intensity
m/z
b_1
y_7−NH_3−H_2O^{+2}
b_2
MH−NH_3−H_2O^{+2}
MH−H_2O^{+2}
b_4
y_5
y_6
y_7
y_7 + 86
b_6

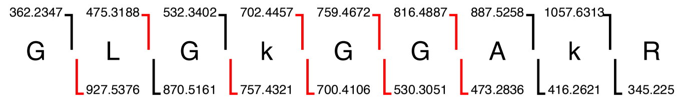

histone cluster 1, H4a [Homo sapiens]
Charge State: +3
Scan Number: 31365
File Name: 120407_A549_EGFIGF_bioRepA_ACK_FT.raw

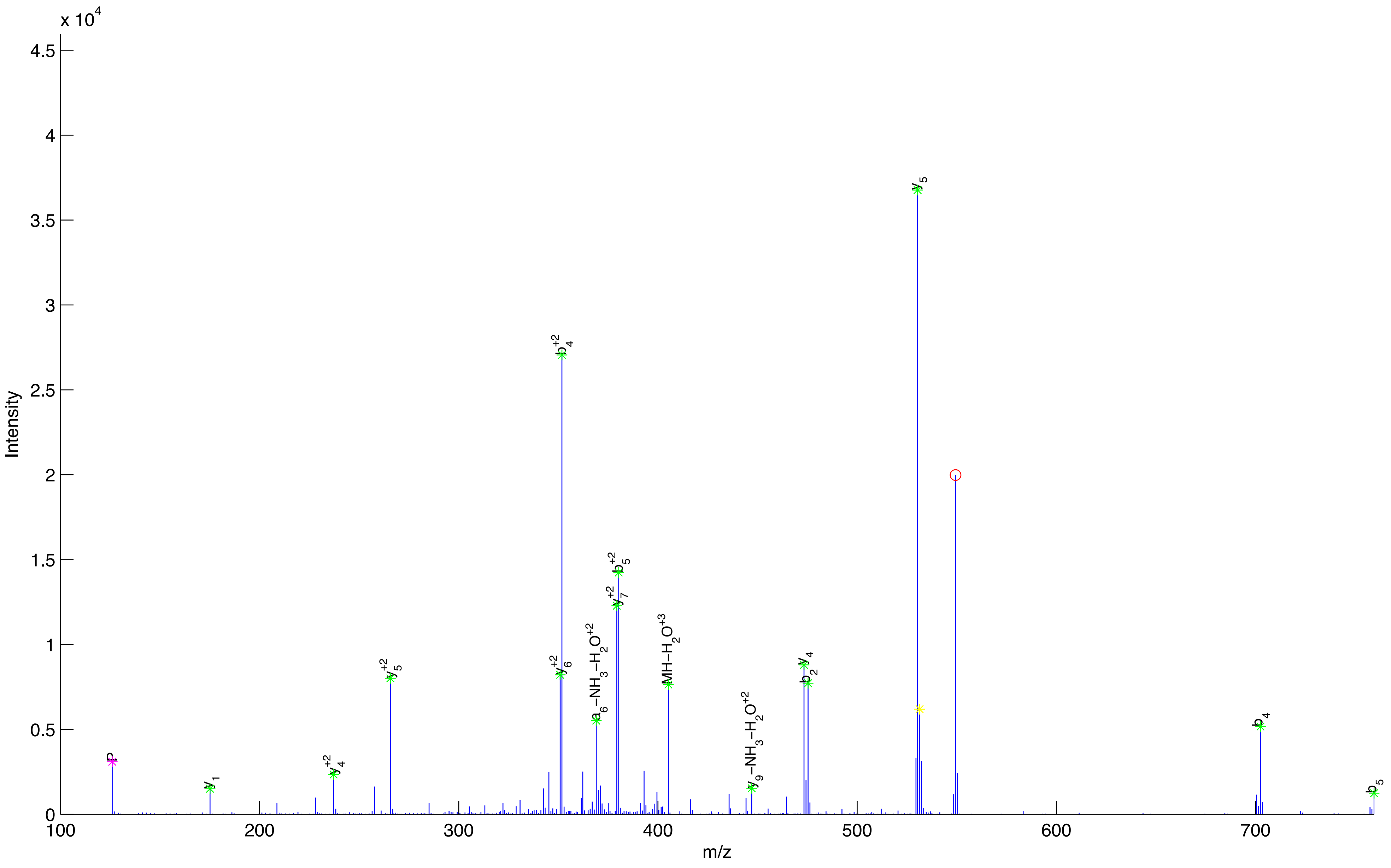

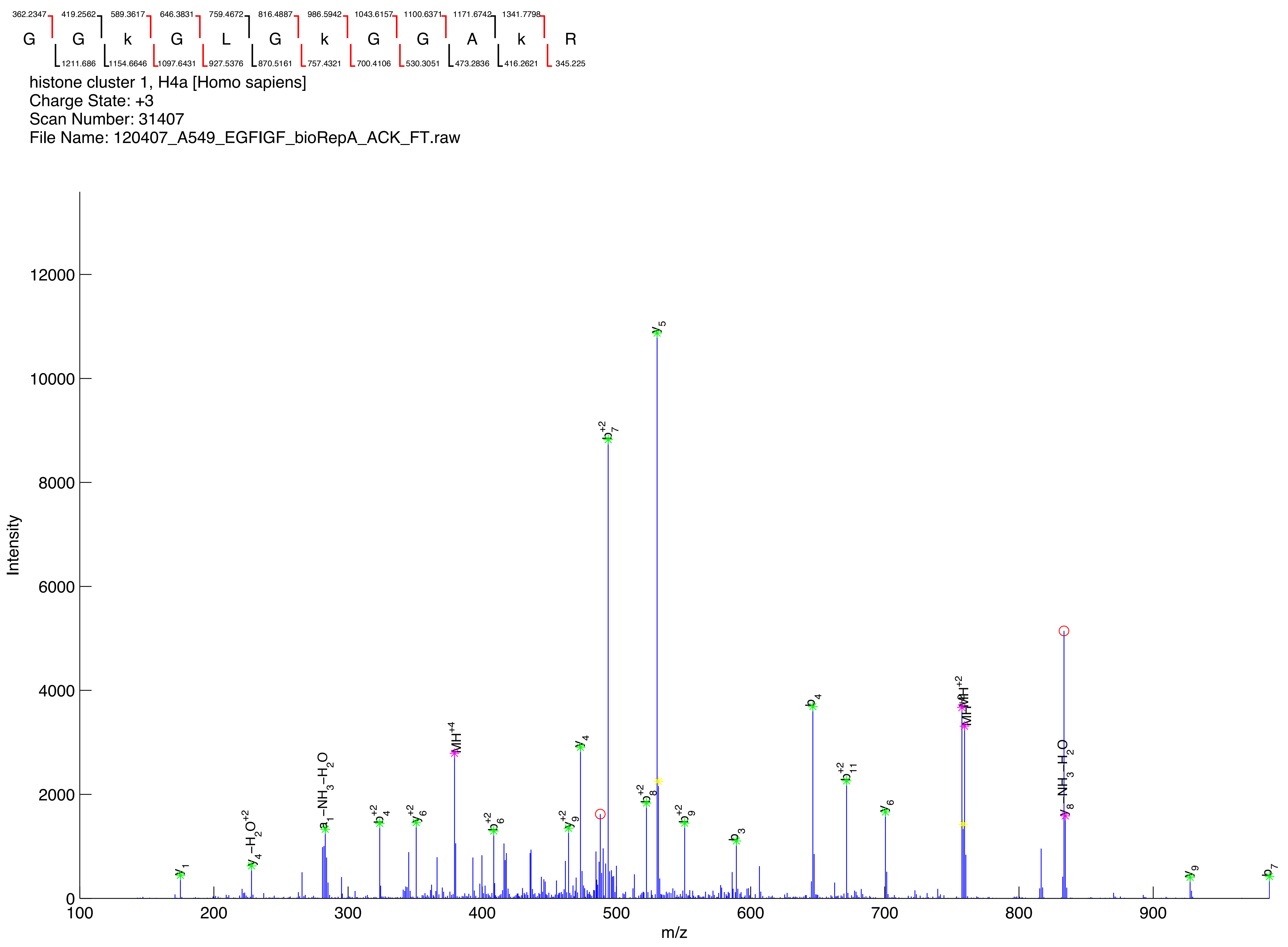

362.2347 419.2562 589.3617 646.3831 759.4672 816.4887 986.5942 1043.6157 1100.6371 1171.6742 1341.7798
G G k G L G k G G A k R
1211.686 1154.6646 1097.6431 927.5376 870.5161 757.4321 700.4106 530.3051 473.2836 416.2621 345.225
histone cluster 1, H4a [Homo sapiens]
Charge State: +3
Scan Number: 31407
File Name: 120407_A549_EGFIGF_bioRepA_ACK_FT.raw
Intensity
m/z

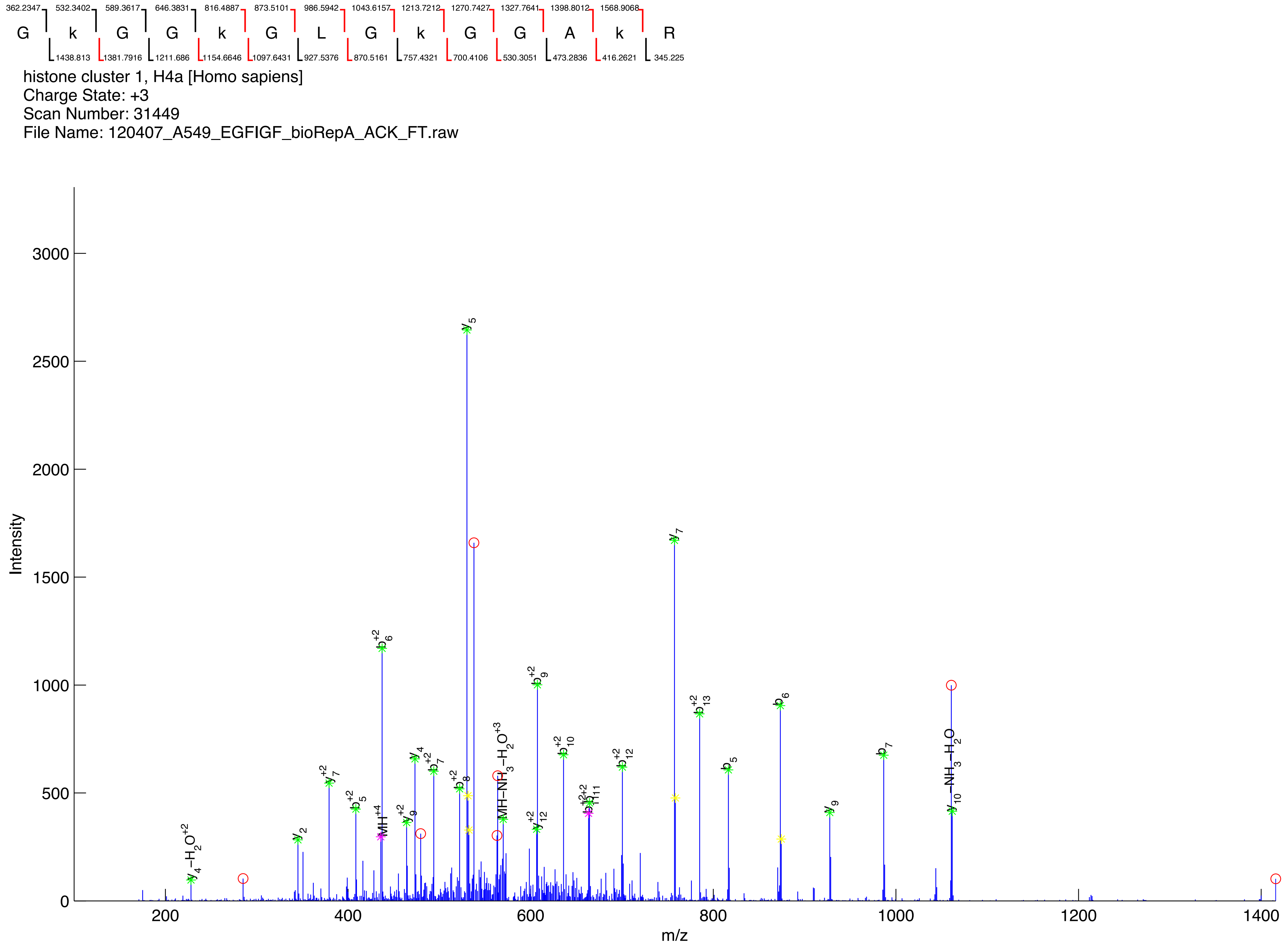

362.2347 532.3402 589.3617 646.3831 816.4887 873.5101 986.5942 1043.6157 1213.7212 1270.7427 1327.7641 1398.8012 1568.9068
G k G G k G L G k G G A k R
1438.813 1381.7916 1211.686 1154.6646 1097.6431 927.5376 870.5161 757.4321 700.4106 530.3051 473.2836 416.2621 345.225
histone cluster 1, H4a [Homo sapiens]
Charge State: +3
Scan Number: 31449
File Name: 120407_A549_EGFIGF_bioRepA_ACK_FT.raw
Intensity
m/z

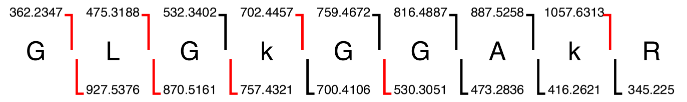

histone cluster 1, H4a [Homo sapiens]
Charge State: +2
Scan Number: 31512
File Name: 120407_A549_EGFIGF_bioRepA_ACK_FT.raw

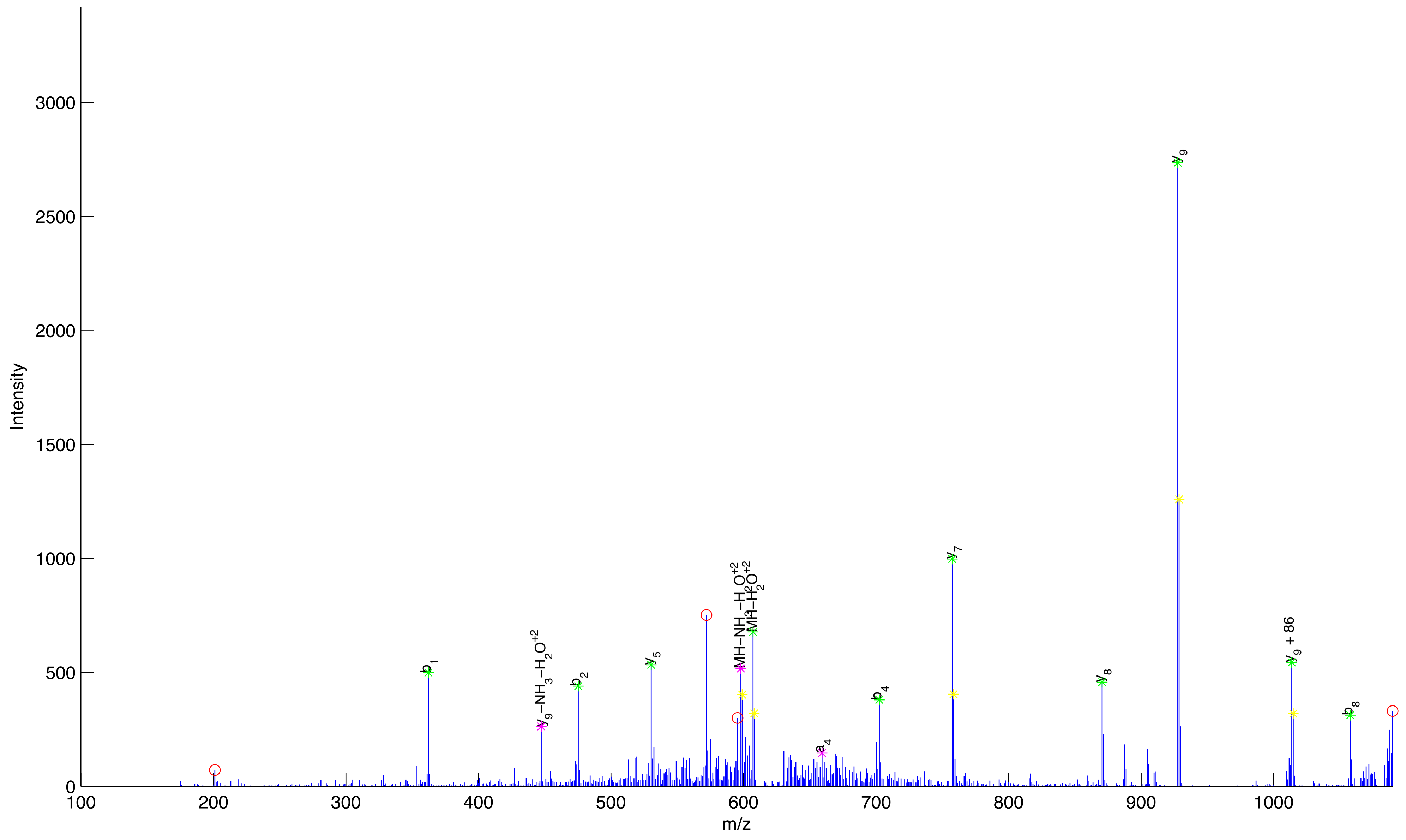

Supplement: S2 Fig — False discovery rate analyses aid in identifying a potential threshold for accepting MS/MS spectra without manual validation. Oftentimes, a particular FDR is selected and a peptide score for which that FDR is met is selected. Here, an FDR analysis was performed as a function of Mascot score. Instead of relying on an FDR analysis where the identity of true positives and true negatives are unknown, we manually validated each MS/MS spectra manually. Each page represents a manually validated MS/MS spectrum. (PDF) [file pone.0126242.s002.pdf]
