# Supplementary material for: Quantitative Profiling of Lysine Acetylation Reveals Dynamic Crosstalk between Receptor Tyrosine Kinases and Lysine Acetylation
Source: PLoS One. 2015 May 15;10(5):e0126242. doi: 10.1371/journal.pone.0126242 (PMC4433260; doi:10.1371/journal.pone.0126242)

Y G k D A T N V G D E G G F A P N I L E N K

Alpha–enolase
Charge State: +2
Scan Number: 12184
File Name: 130605_Ack_IP_1.raw

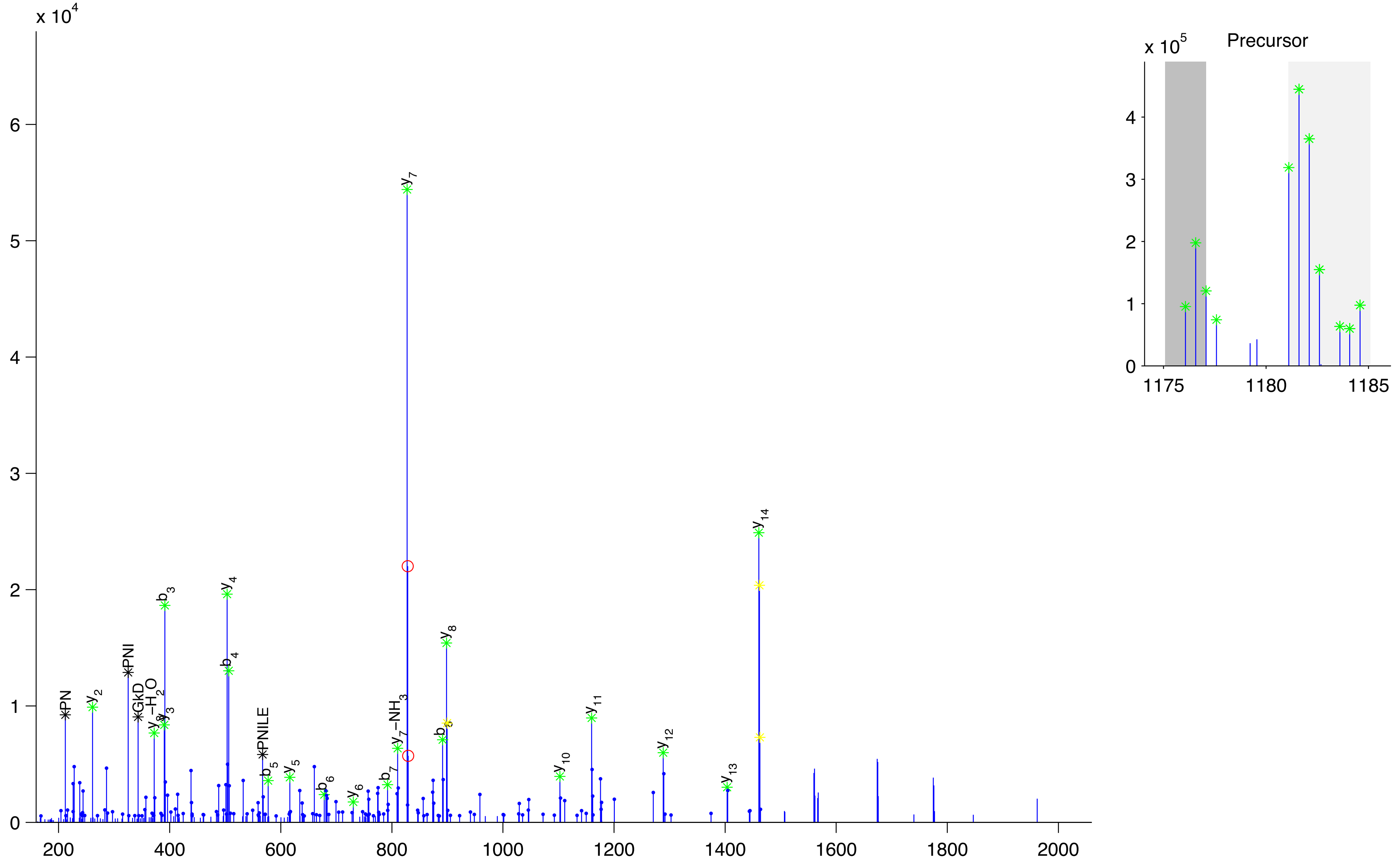

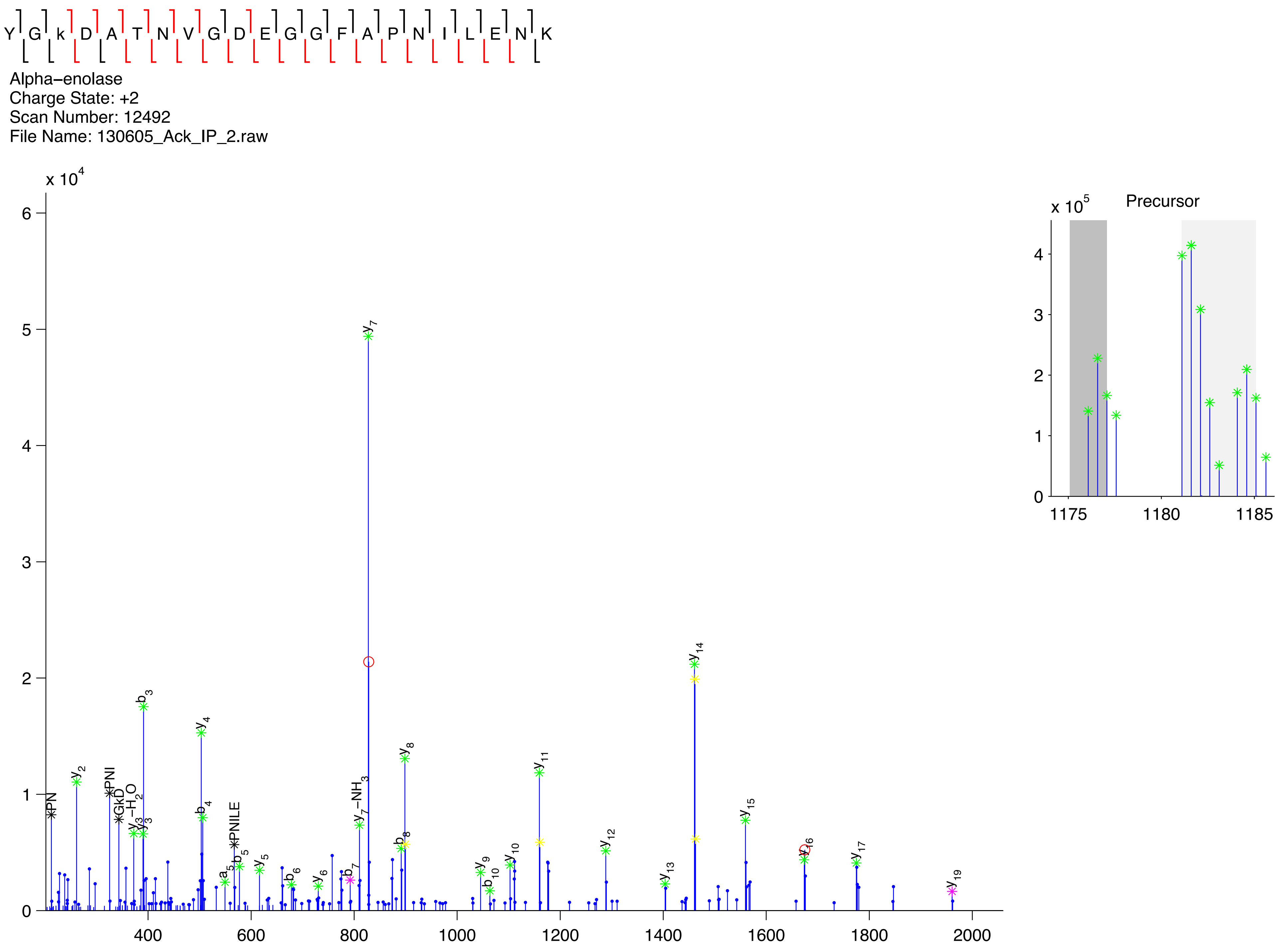
Y G k D A T N V G D E G G F A P N I L E N K
Alpha–enolase
Charge State: +2
Scan Number: 12492
File Name: 130605_Ack_IP_2.raw
x 10^4
Precursor
x 10^5

A F N Q G k I F K

Elongation factor 1−gamma
Charge State: +2
Scan Number: 8462
File Name: 130605_Ack_IP_1.raw

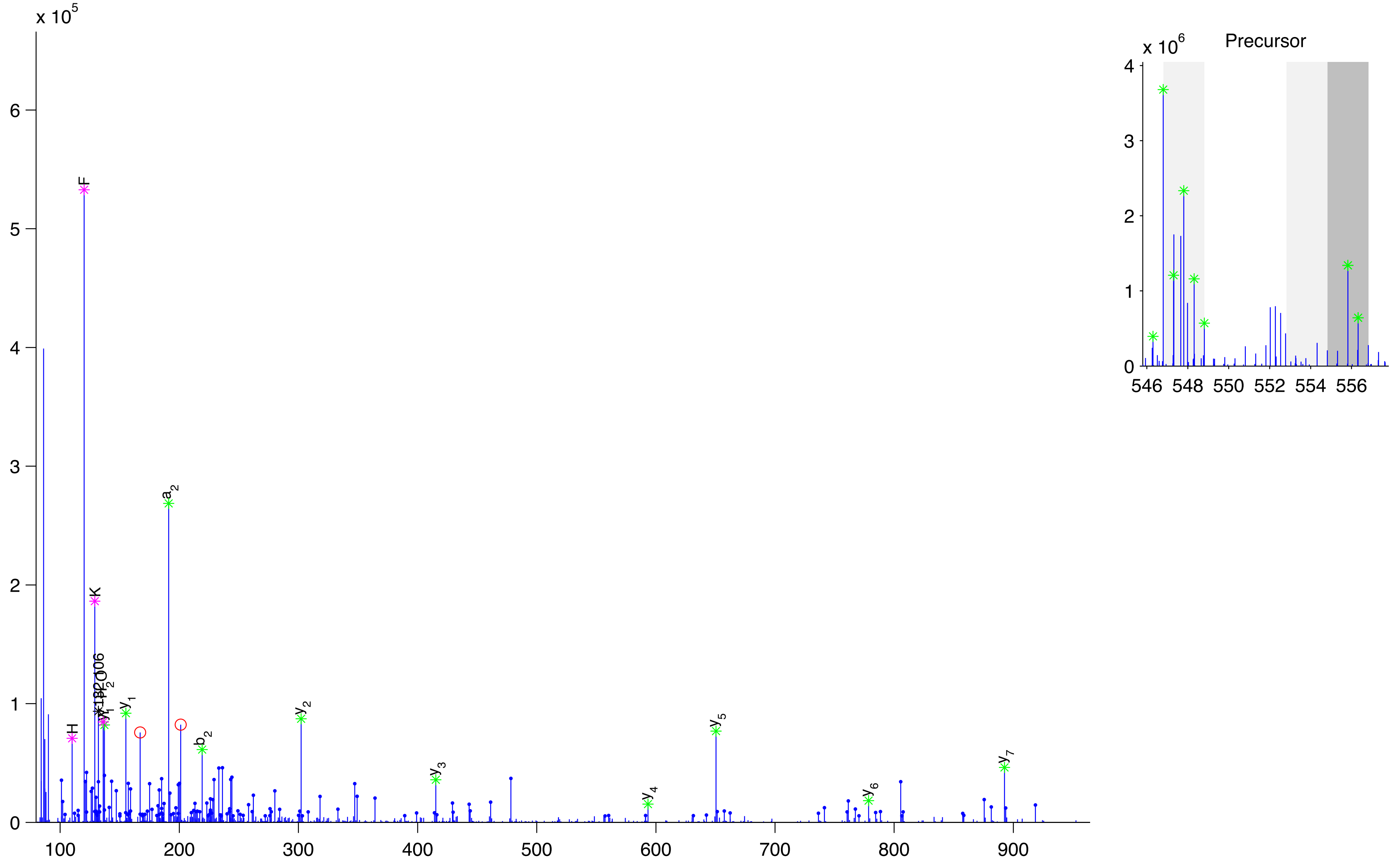

V V k Q A S E G P L K

Glyceraldehyde–3–phosphate dehydrogenase
Charge State: +2
Scan Number: 4431
File Name: 130605_Ack_IP_1.raw

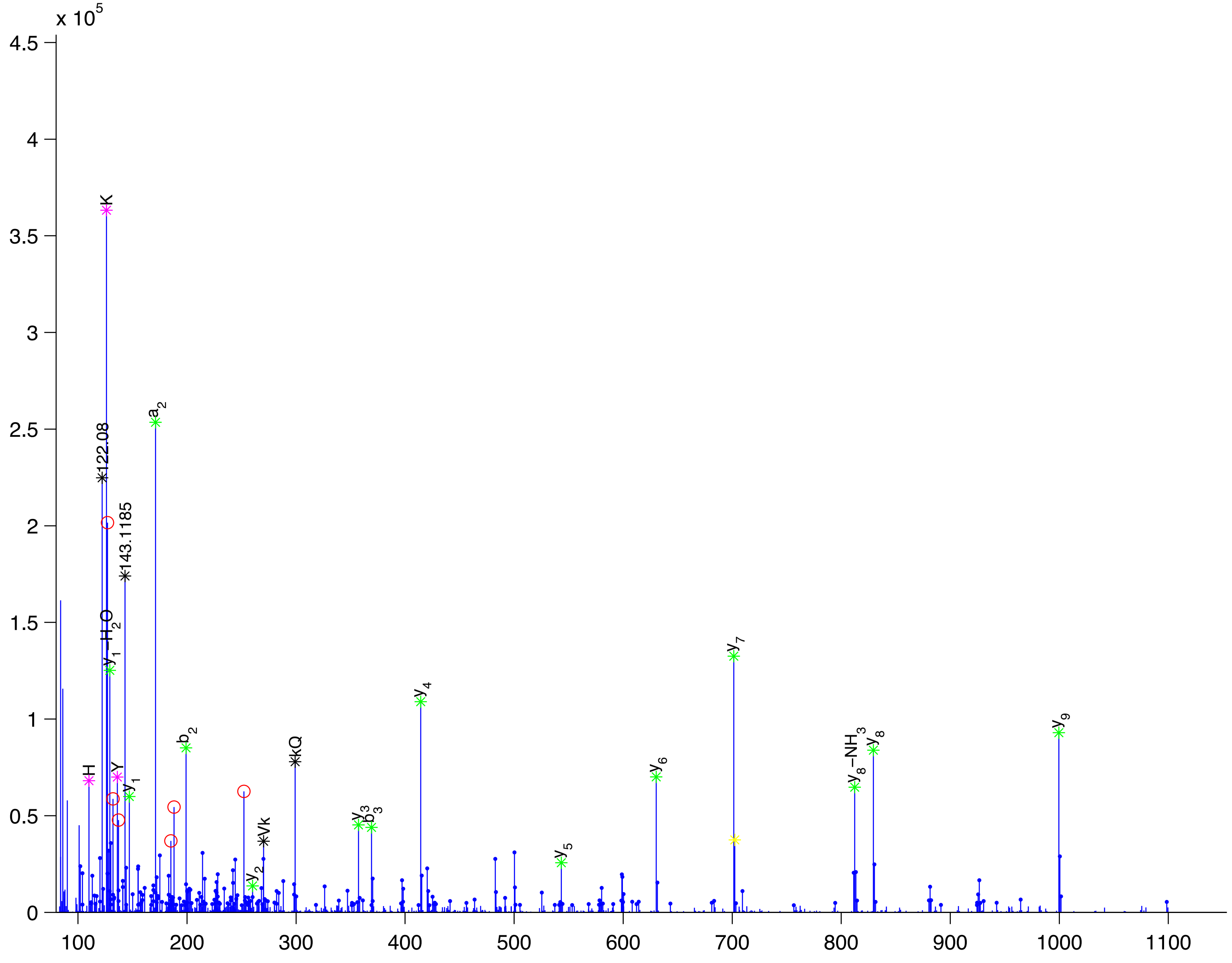

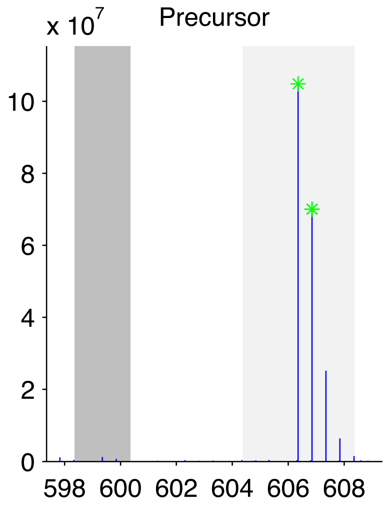

V V k Q A S E G P L K

Glyceraldehyde−3−phosphate dehydrogenase
Charge State: +2
Scan Number: 4658
File Name: 130605_Ack_IP_2.raw

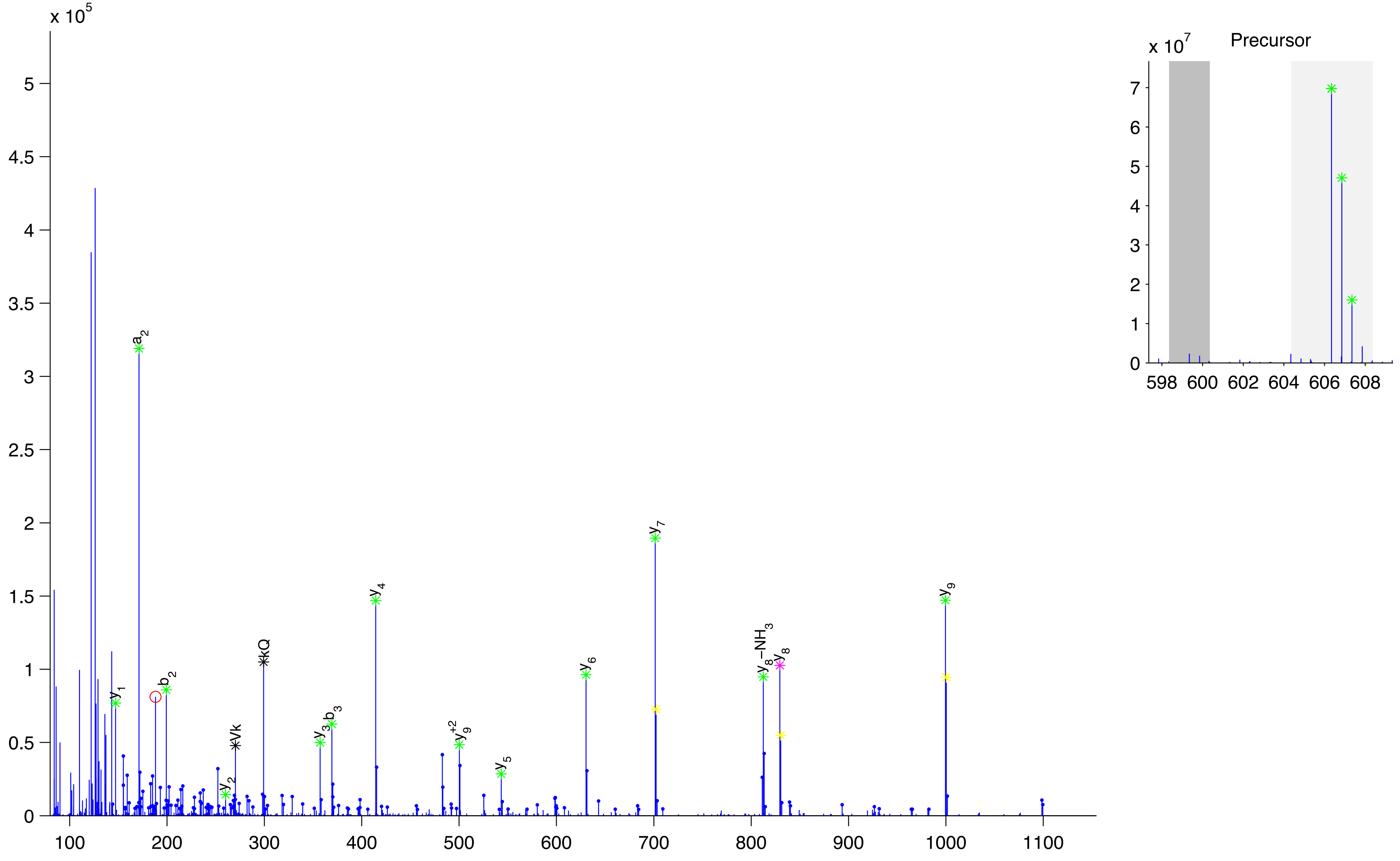

V V k Q A S E G P L K

Glyceraldehyde−3−phosphate dehydrogenase
Charge State: +2
Scan Number: 4717
File Name: 130605_Ack_IP_3.raw

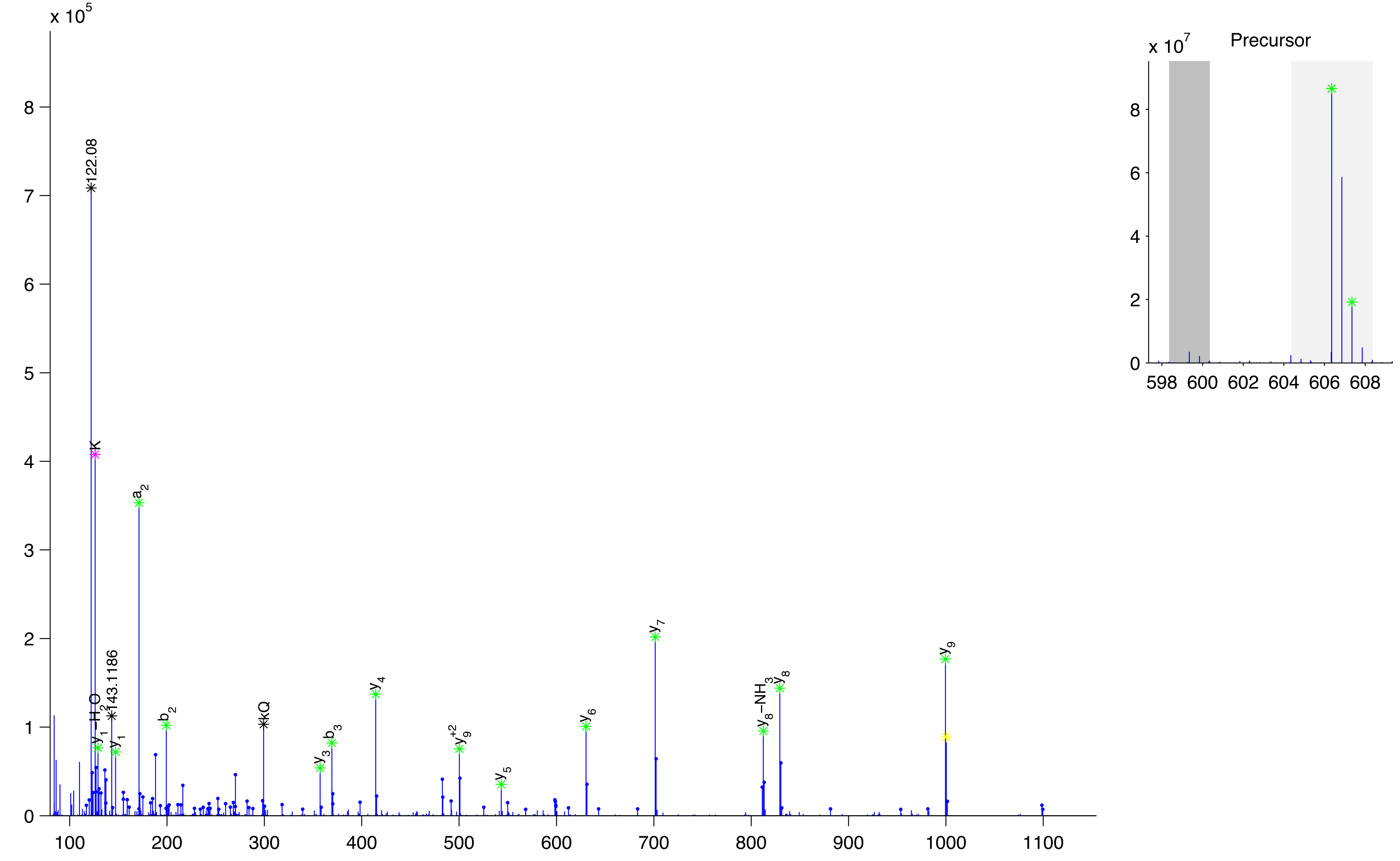

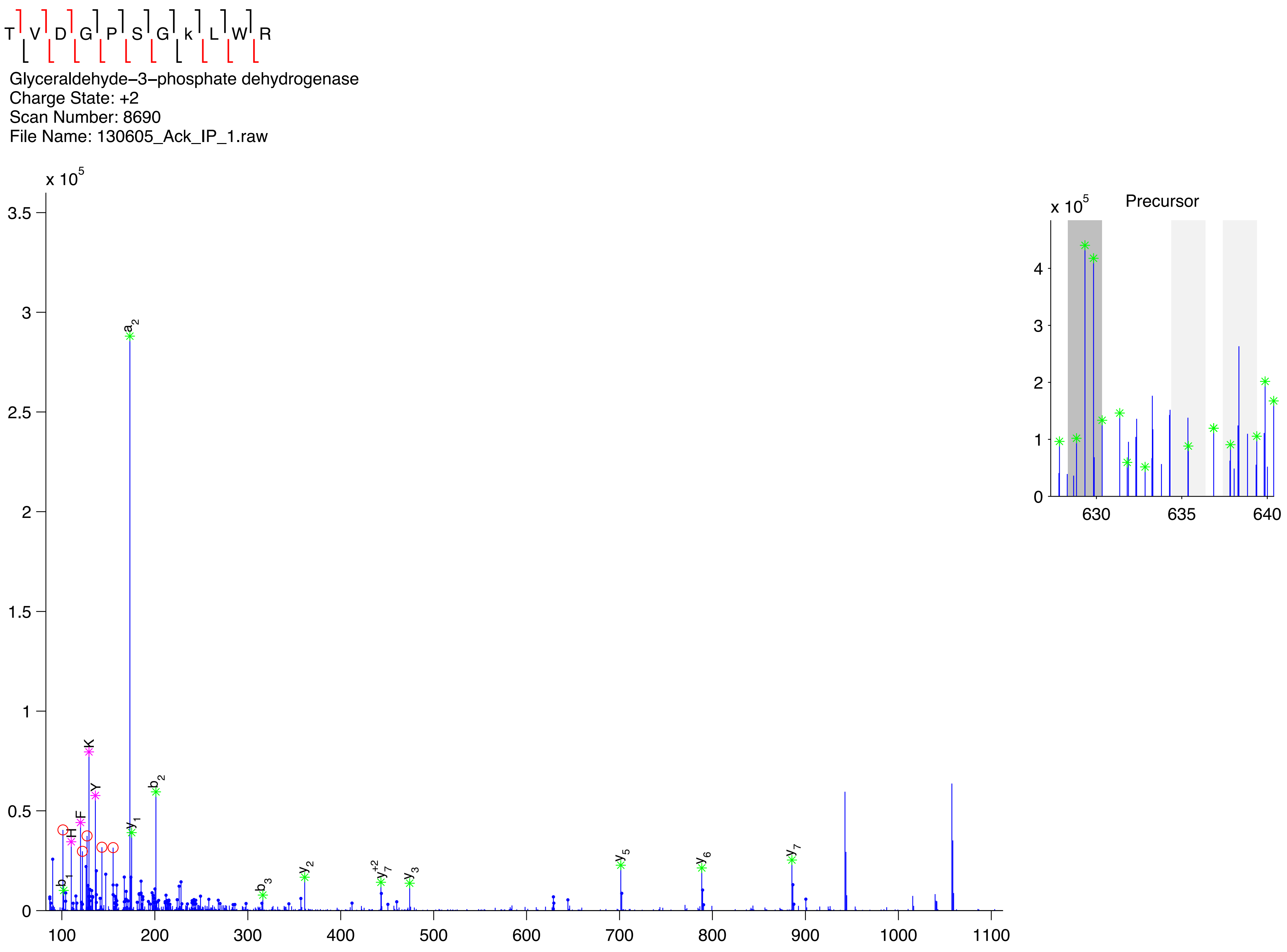

T V D G P S G k L W R
Glyceraldehyde–3–phosphate dehydrogenase
Charge State: +2
Scan Number: 8690
File Name: 130605_Ack_IP_1.raw
x 10^5
a2
b2
y1
K
Y
F
H
b1
b3
y2
y7+2
y3
y5
y6
y7
Precursor

T V D G P S G k L W R

Glyceraldehyde−3−phosphate dehydrogenase
Charge State: +2
Scan Number: 8733
File Name: 130605_Ack_IP_1.raw

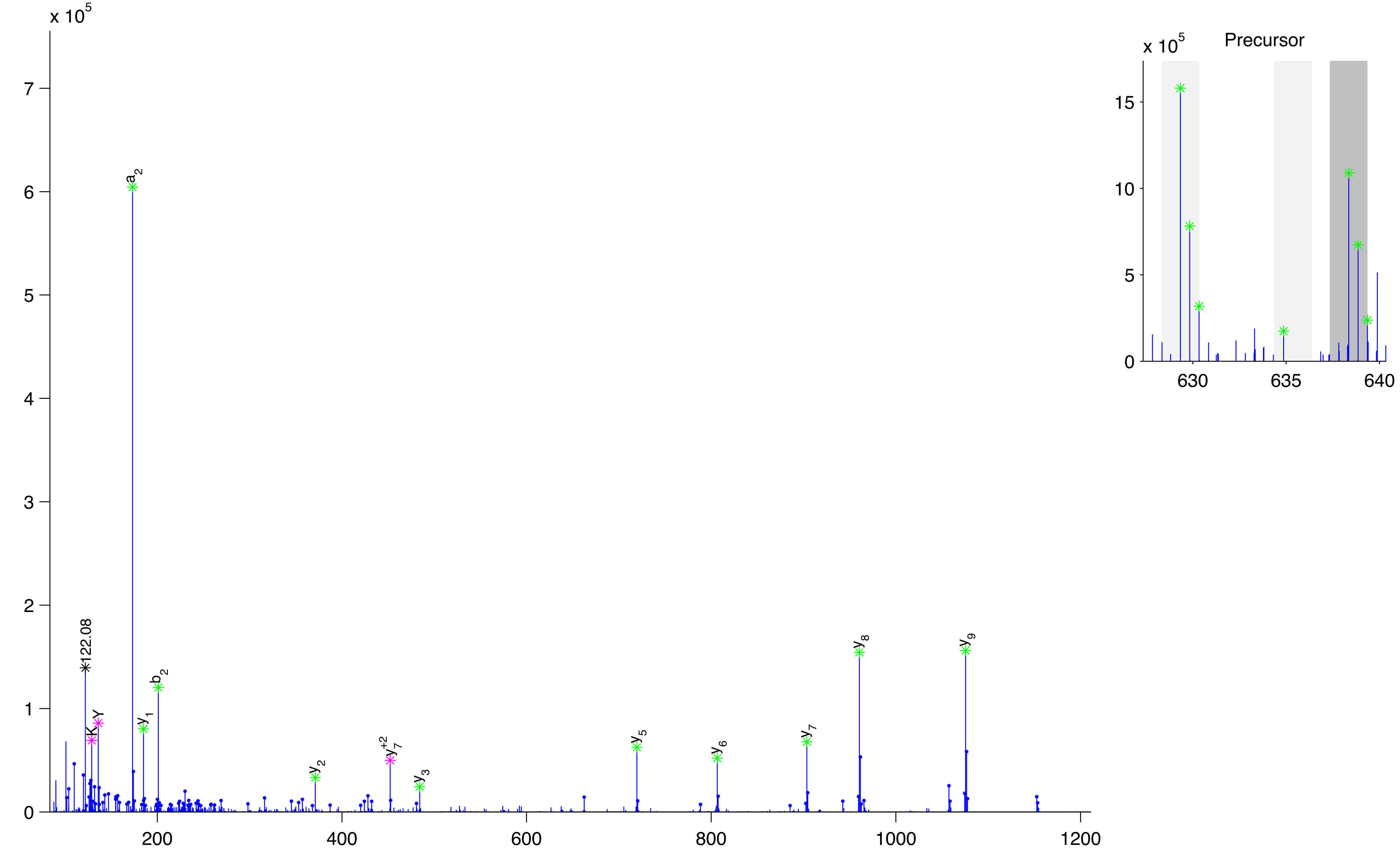

T V D G P S G k L W R

Glyceraldehyde−3−phosphate dehydrogenase
Charge State: +2
Scan Number: 8821
File Name: 130605_Ack_IP_1.raw

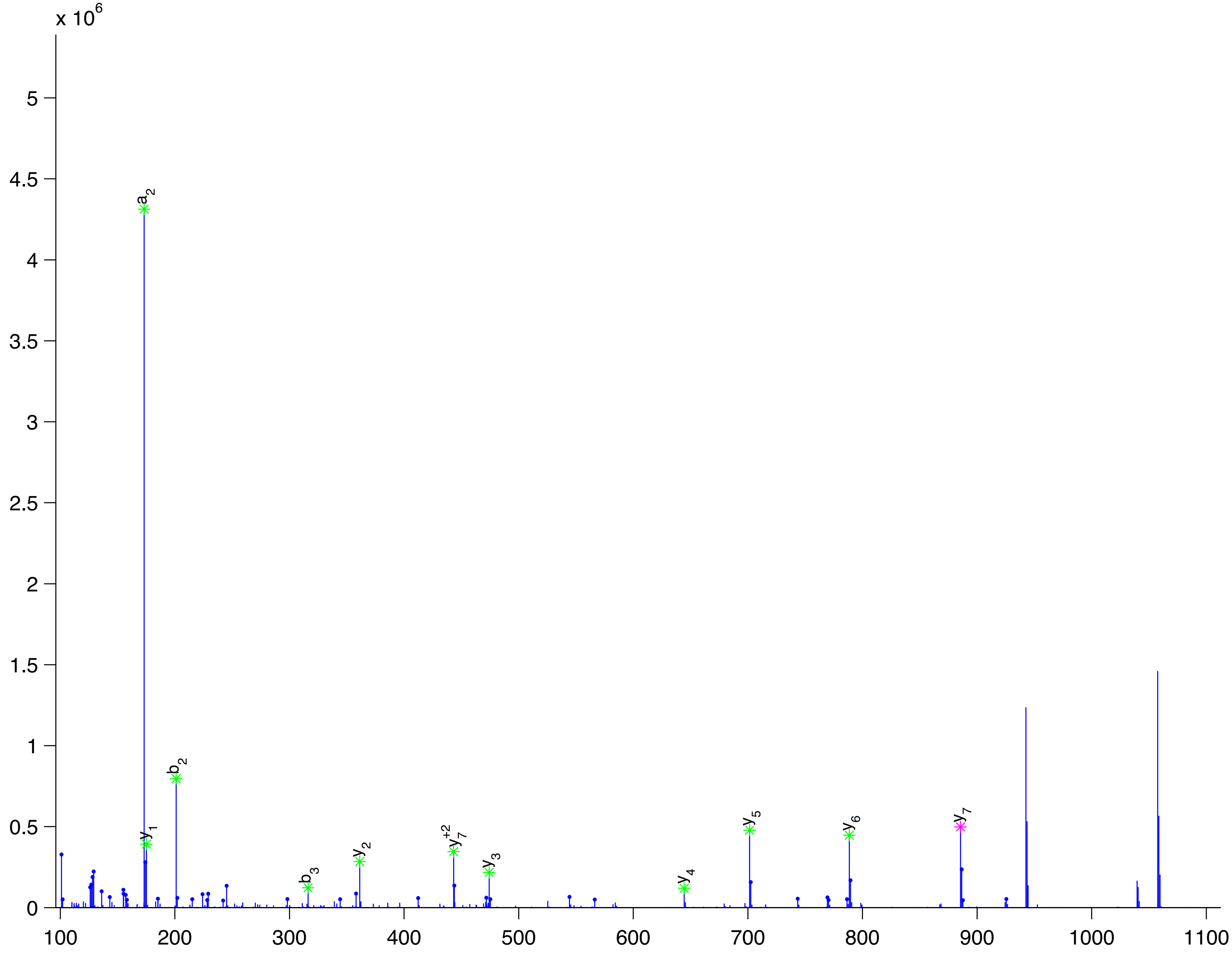

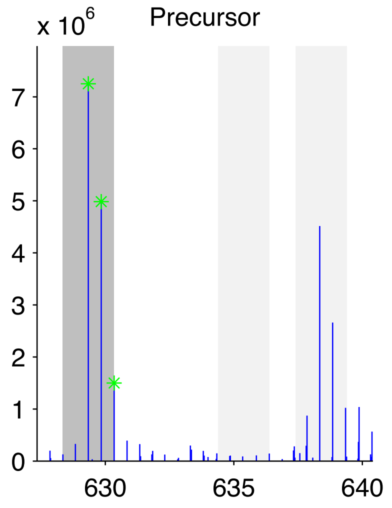

T V D G P S G k L W R

Glyceraldehyde−3−phosphate dehydrogenase
Charge State: +2
Scan Number: 8857
File Name: 130605_Ack_IP_1.raw

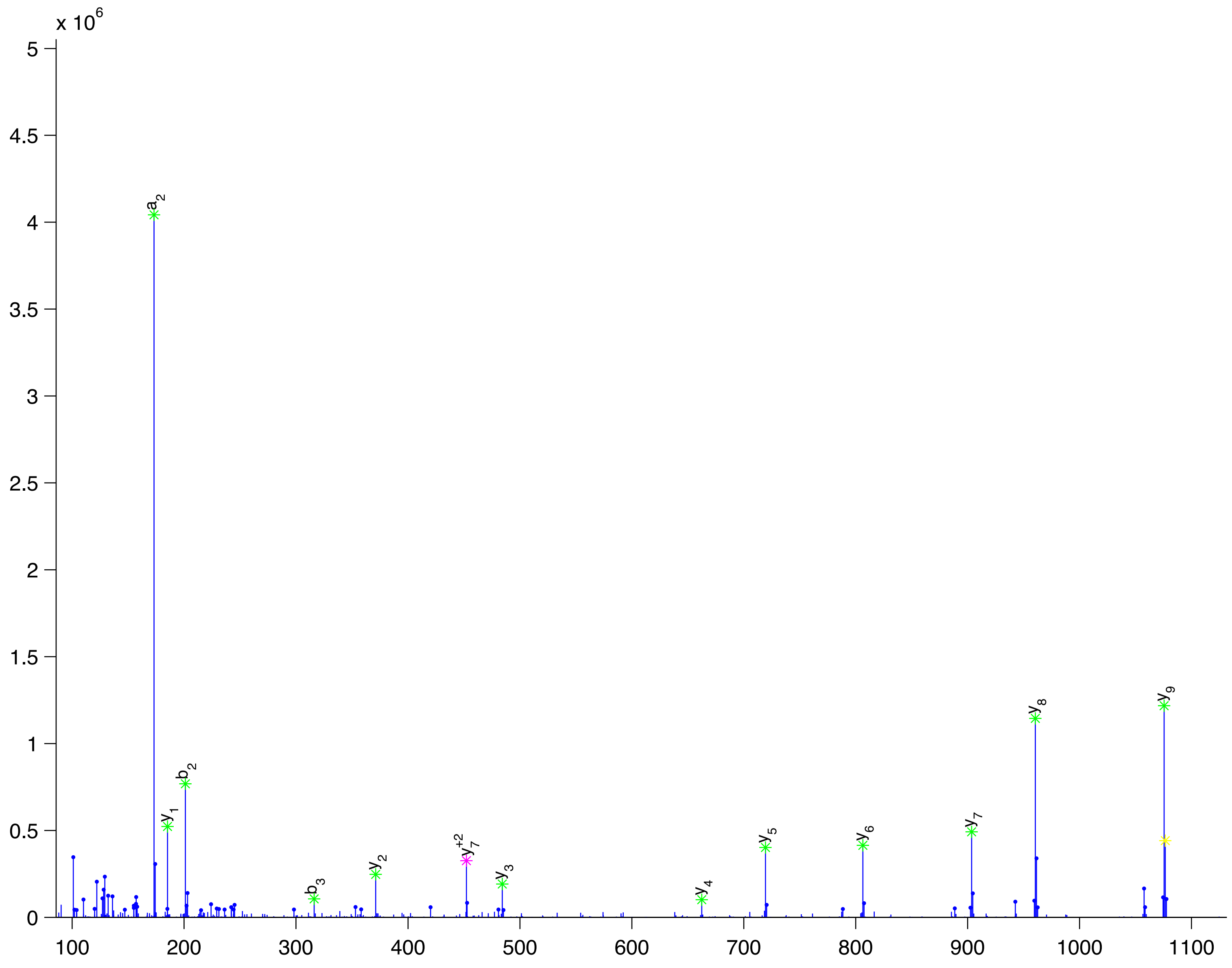

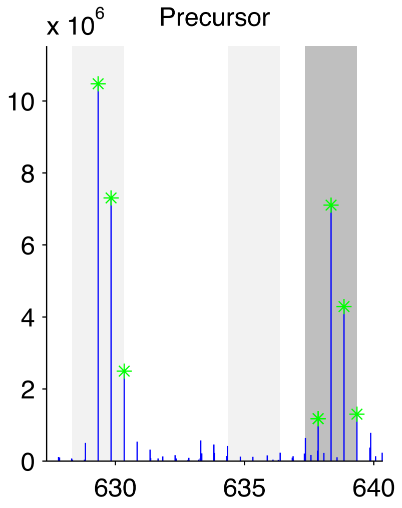

T V D G P S G k L W R

Glyceraldehyde−3−phosphate dehydrogenase
Charge State: +2
Scan Number: 8937
File Name: 130605_Ack_IP_1.raw

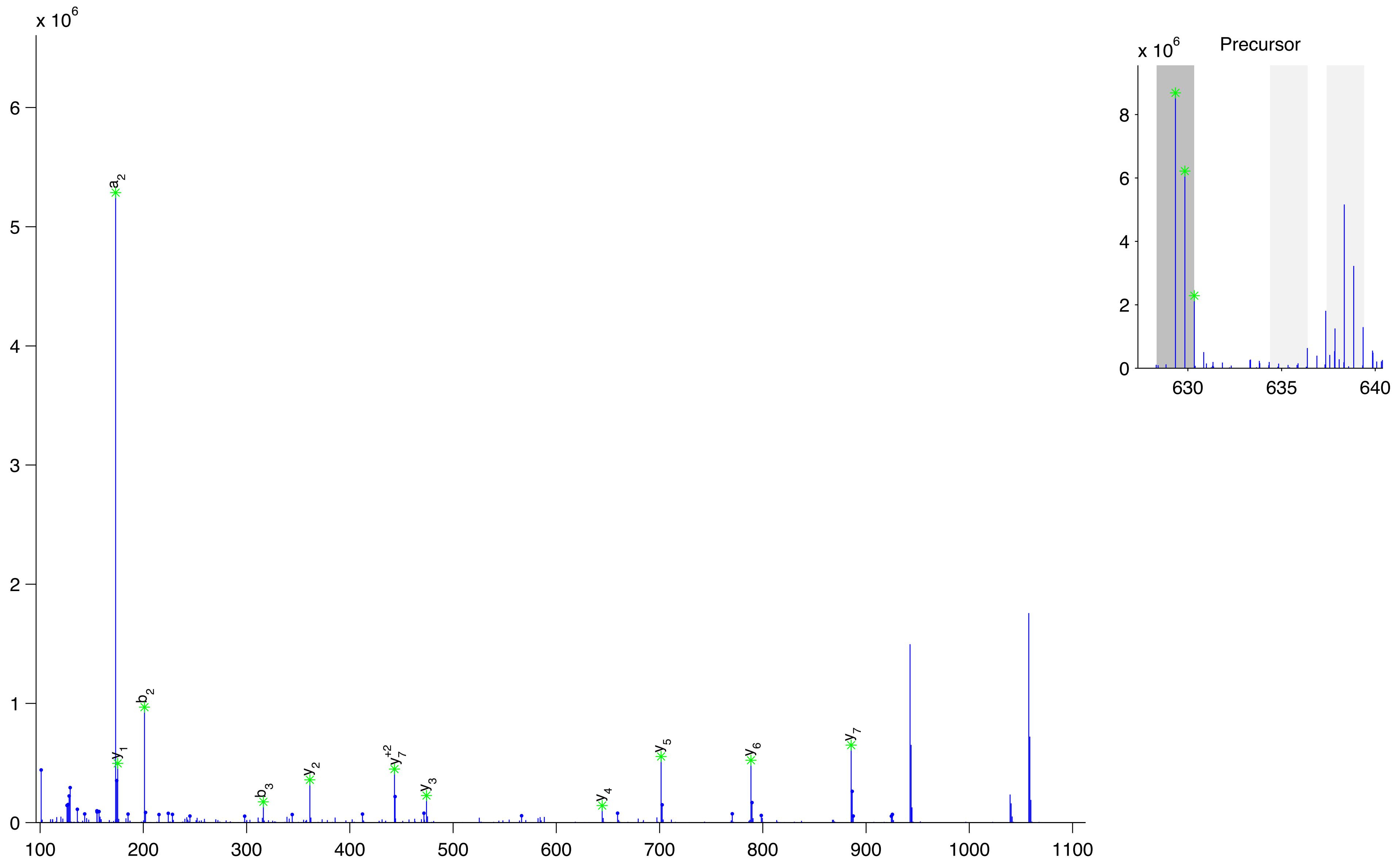

T V D G P S G k L W R

Glyceraldehyde–3–phosphate dehydrogenase
Charge State: +2
Scan Number: 9008
File Name: 130605_Ack_IP_2.raw

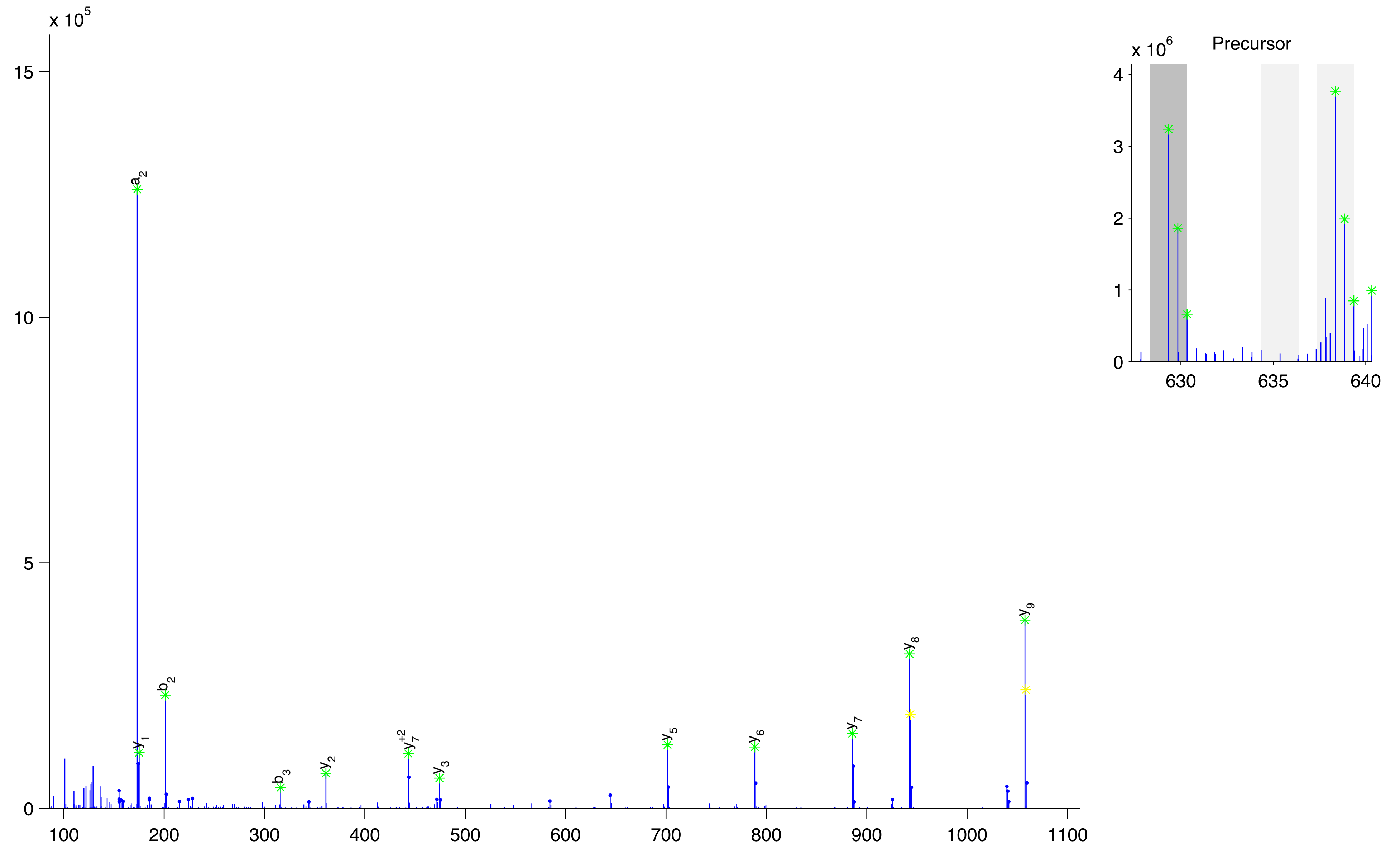

T V D G P S G k L W R

Glyceraldehyde–3–phosphate dehydrogenase
Charge State: +2
Scan Number: 9101
File Name: 130605_Ack_IP_3.raw

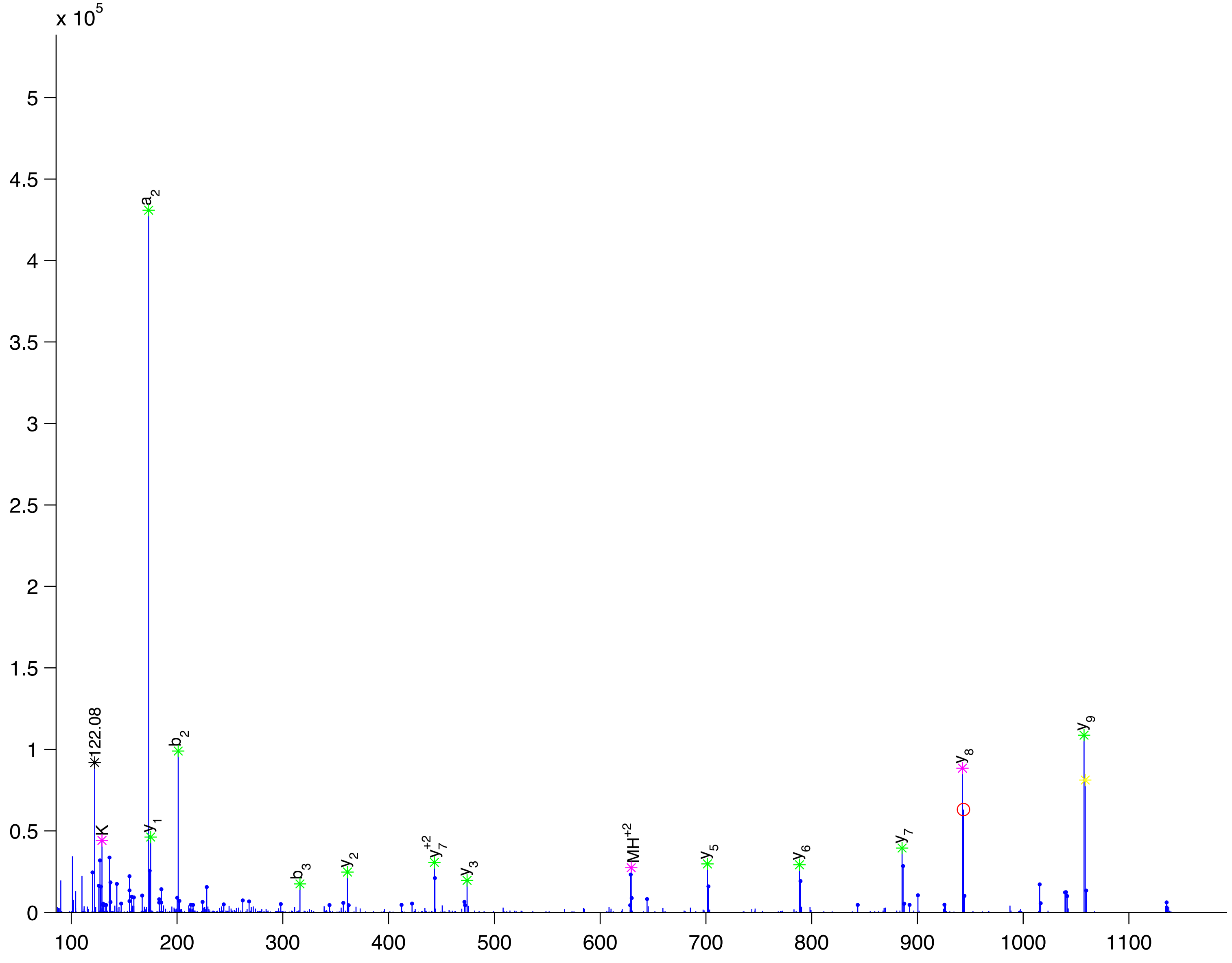

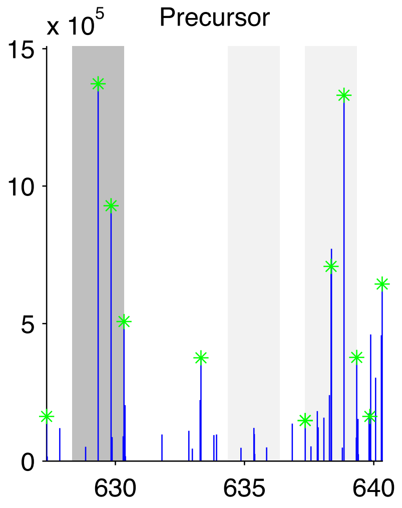

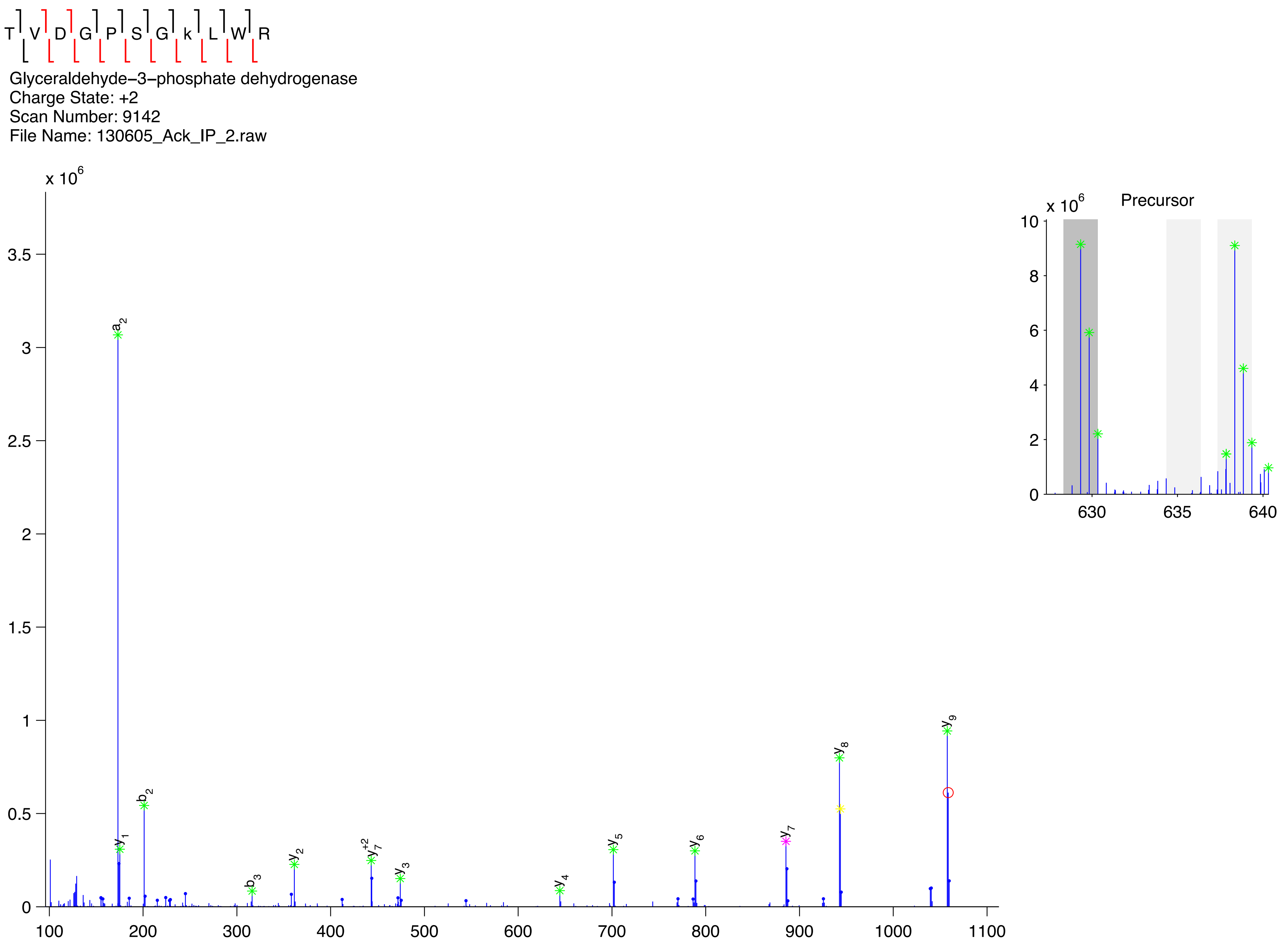

T V D G P S G k L W R
Glyceraldehyde−3−phosphate dehydrogenase
Charge State: +2
Scan Number: 9142
File Name: 130605_Ack_IP_2.raw
x 10^6
a2
b2
y1
b3
y2
y7+2
y3
y4
y5
y6
y7
y8
y9
Precursor

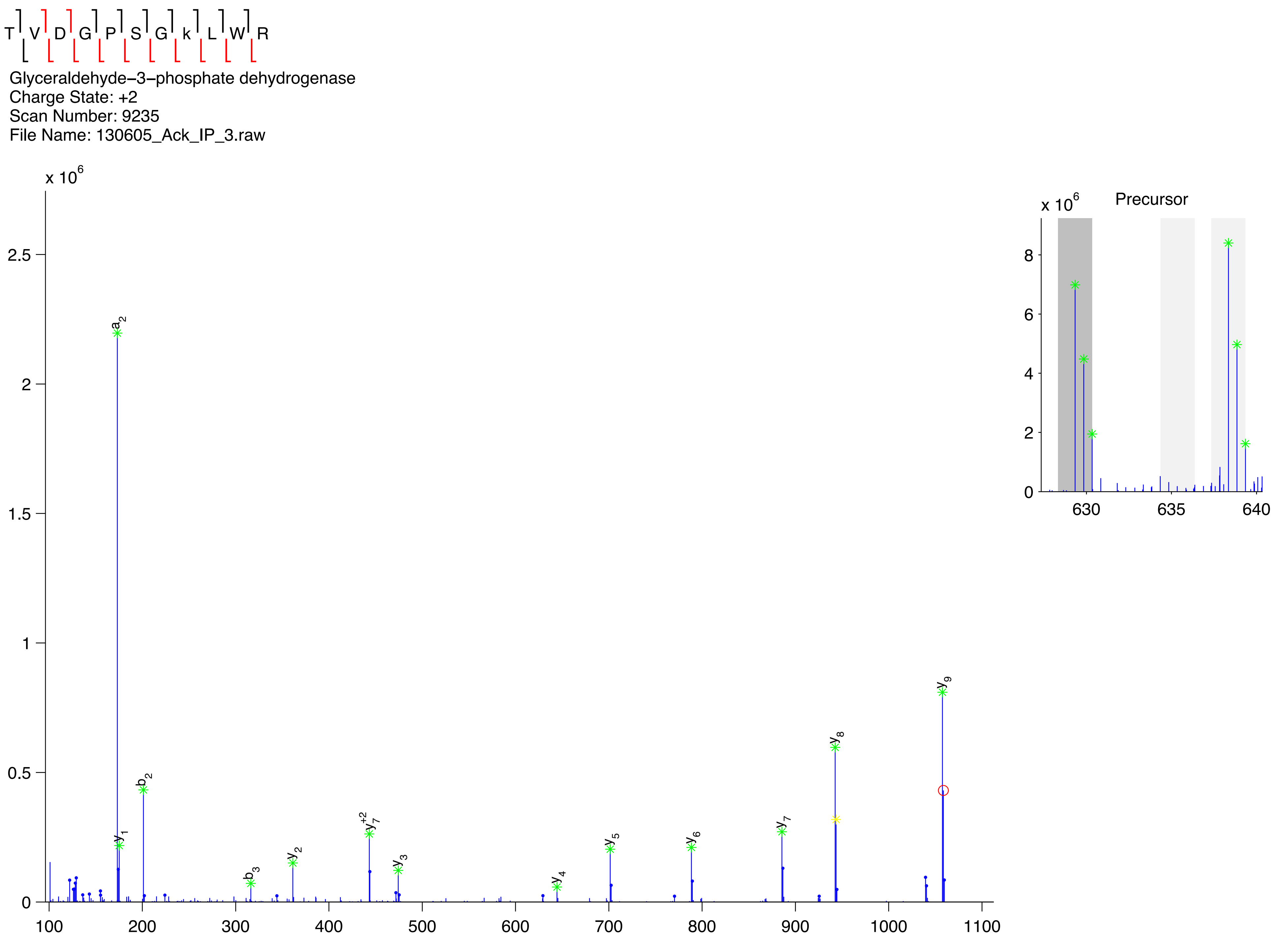
TVDGPSGkLWR
Glyceraldehyde–3–phosphate dehydrogenase
Charge State: +2
Scan Number: 9235
File Name: 130605_Ack_IP_3.raw
x 10^6
a2
b2
y1
b3
y2
y7+2
y3
y4
y5
y6
y7
y8
y9
Precursor

T V D G P S G k L W R

Glyceraldehyde−3−phosphate dehydrogenase
Charge State: +2
Scan Number: 9241
File Name: 130605_Ack_IP_3.raw

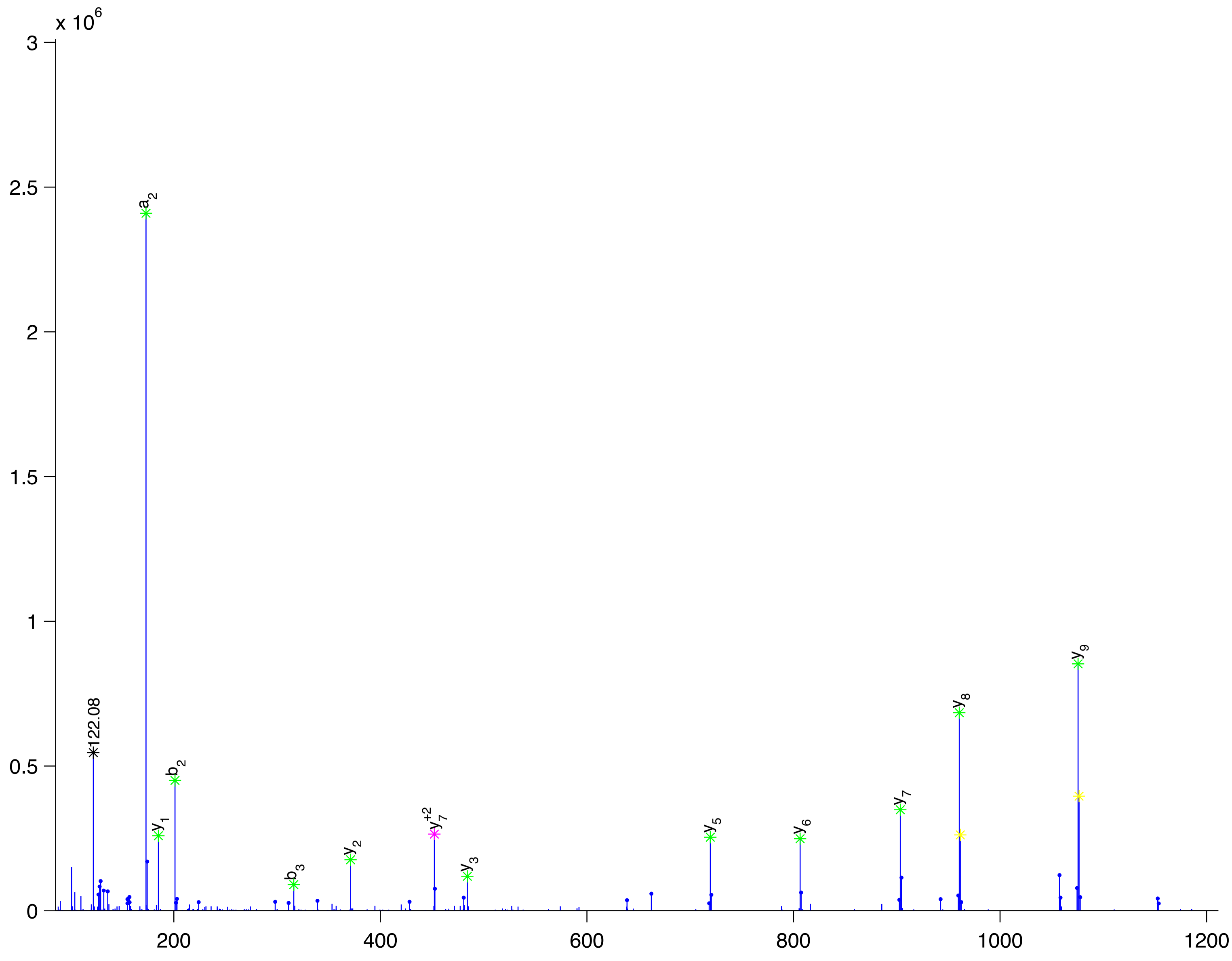

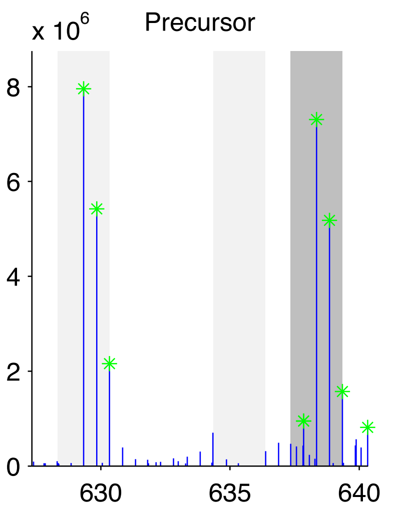

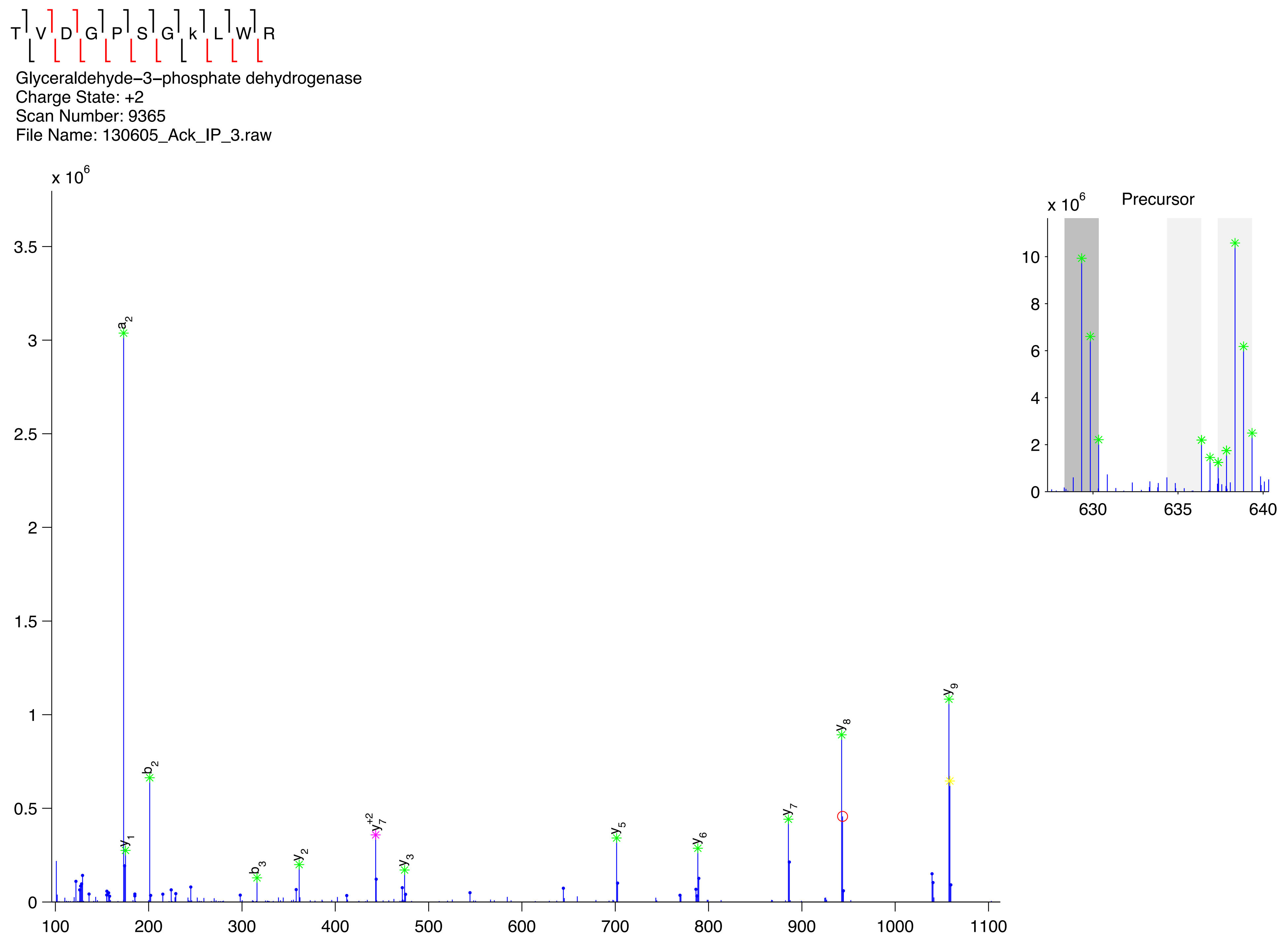

T V D G P S G k L W R
Glyceraldehyde−3−phosphate dehydrogenase
Charge State: +2
Scan Number: 9365
File Name: 130605_Ack_IP_3.raw
x 10^6
a2
b2
y1
b3
y2
y7+2
y3
y5
y6
y7
y8
y9
Precursor

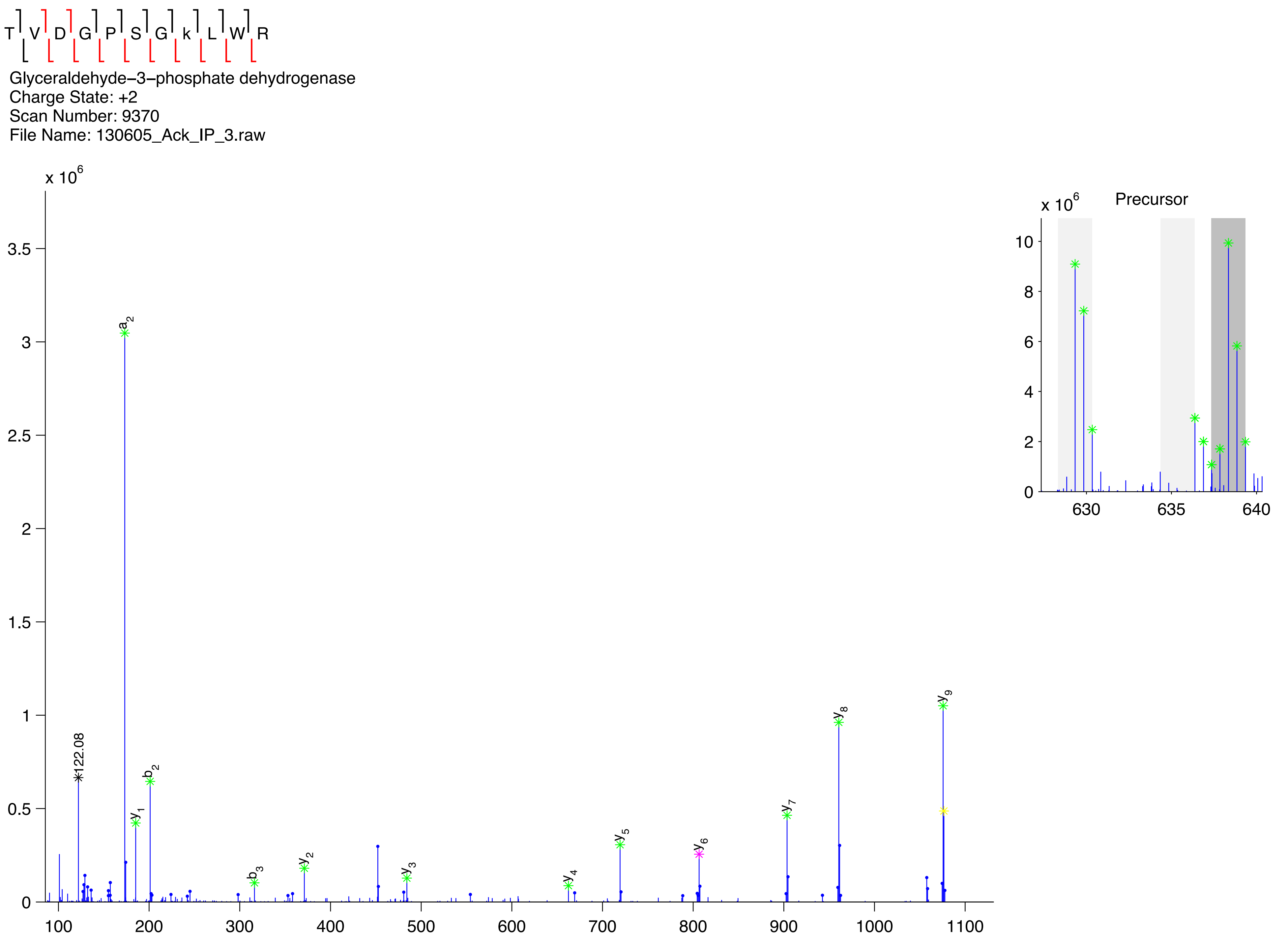
T V D G P S G k L W R
Glyceraldehyde–3–phosphate dehydrogenase
Charge State: +2
Scan Number: 9370
File Name: 130605_Ack_IP_3.raw
x 10^6
0
0.5
1
1.5
2
2.5
3
3.5
100
200
300
400
500
600
700
800
900
1000
1100
122.08
a2
b2
y1
b3
y2
y3
y4
y5
y6
y7
y8
y9
Precursor
x 10^6
0
2
4
6
8
10
630
635
640

V I P E L N G k L T G M A F R

Glyceraldehyde−3−phosphate dehydrogenase
Charge State: +2
Scan Number: 15099
File Name: 130605_Ack_IP_1.raw

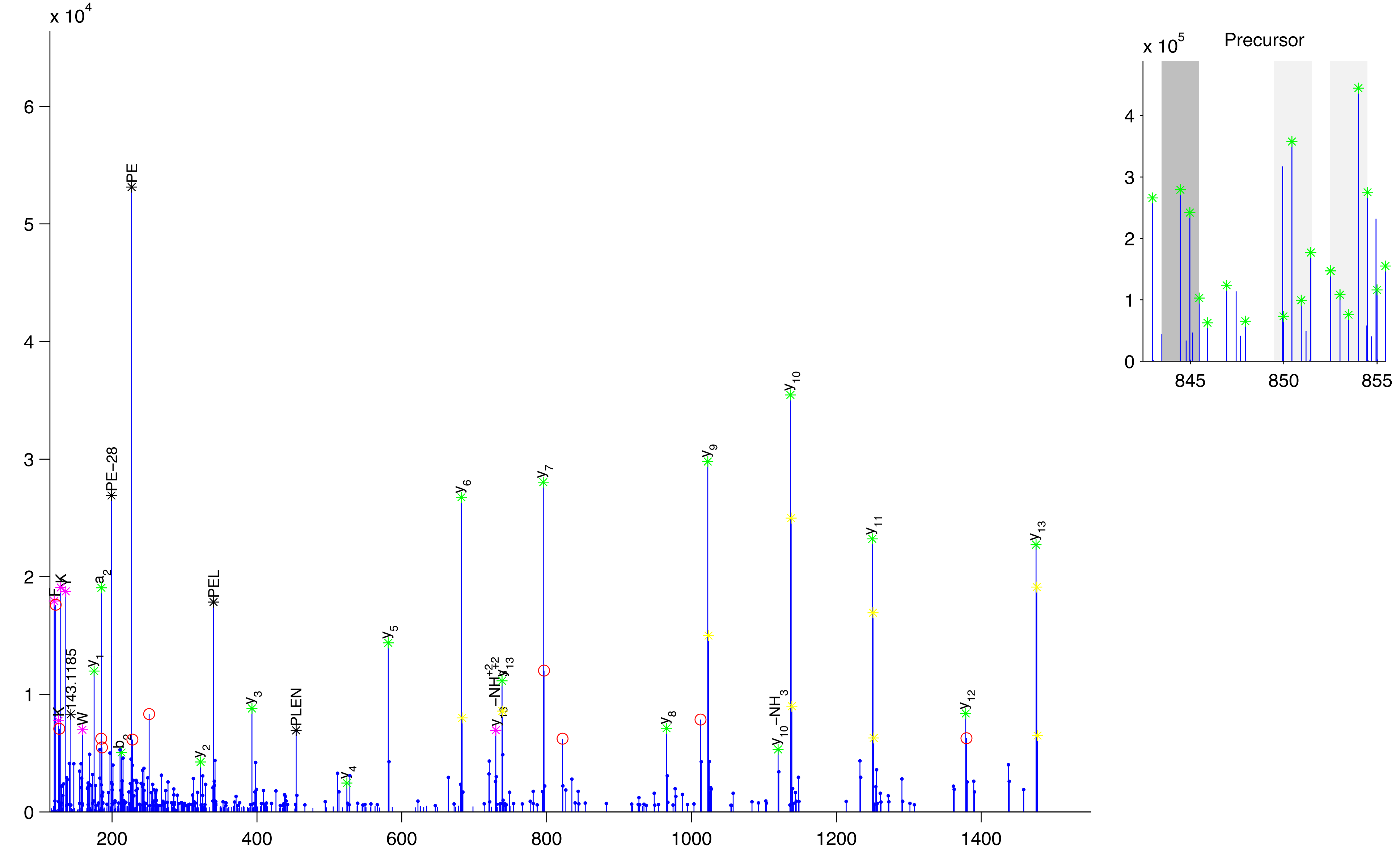

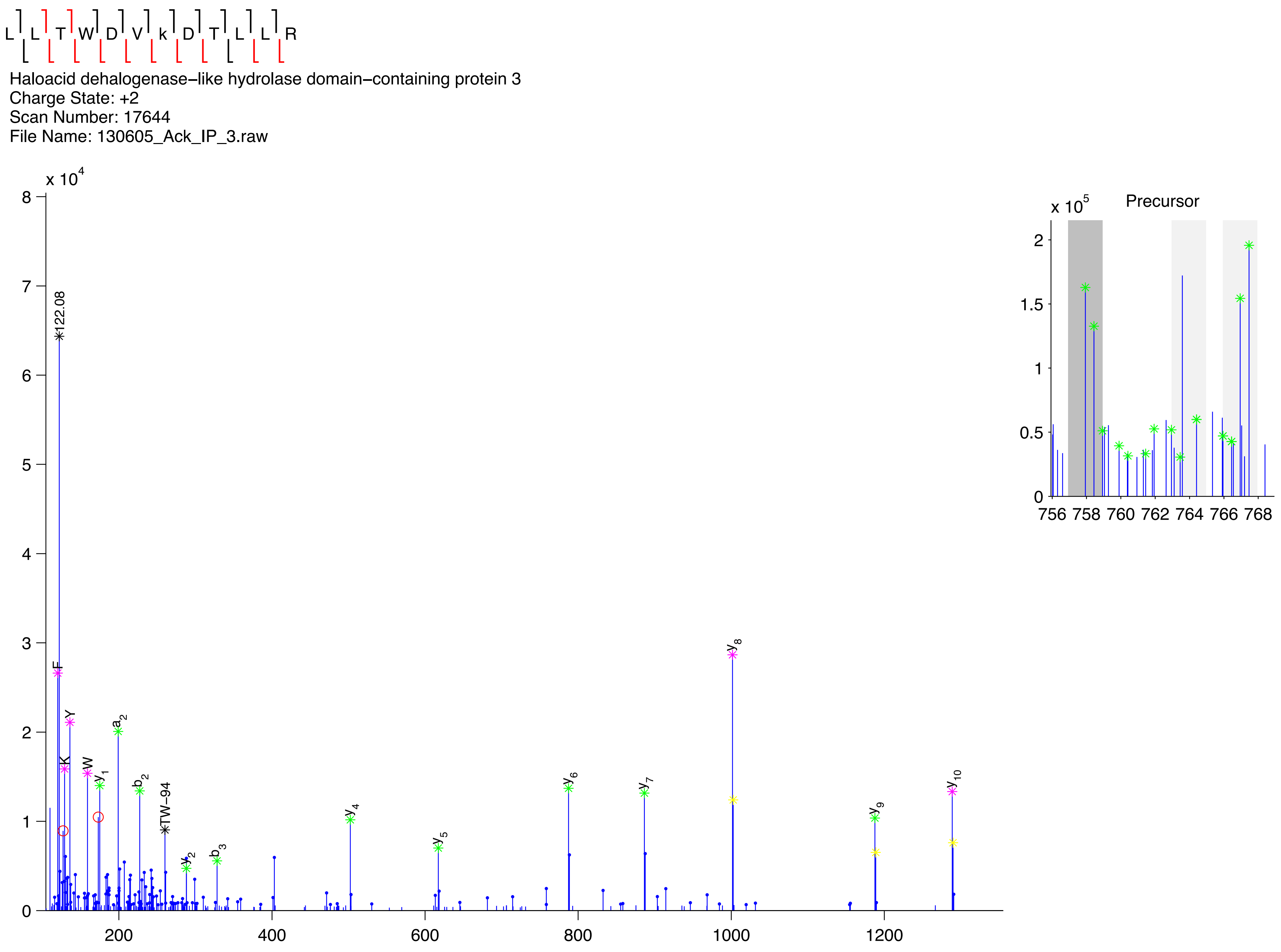

L L T W D V k D T L L R
Haloacid dehalogenase–like hydrolase domain–containing protein 3
Charge State: +2
Scan Number: 17644
File Name: 130605_Ack_IP_3.raw
x 10^4
122.08
F
Y
K
W
y1
a2
b2
TW–94
y2
b3
y4
y5
y6
y7
y8
y9
y10
Precursor
x 10^5

S S G P Y G G G G Q Y F A k P R

Heterogeneous nuclear ribonucleoprotein A1
Charge State: +2
Scan Number: 8508
File Name: 130605_Ack_IP_1.raw

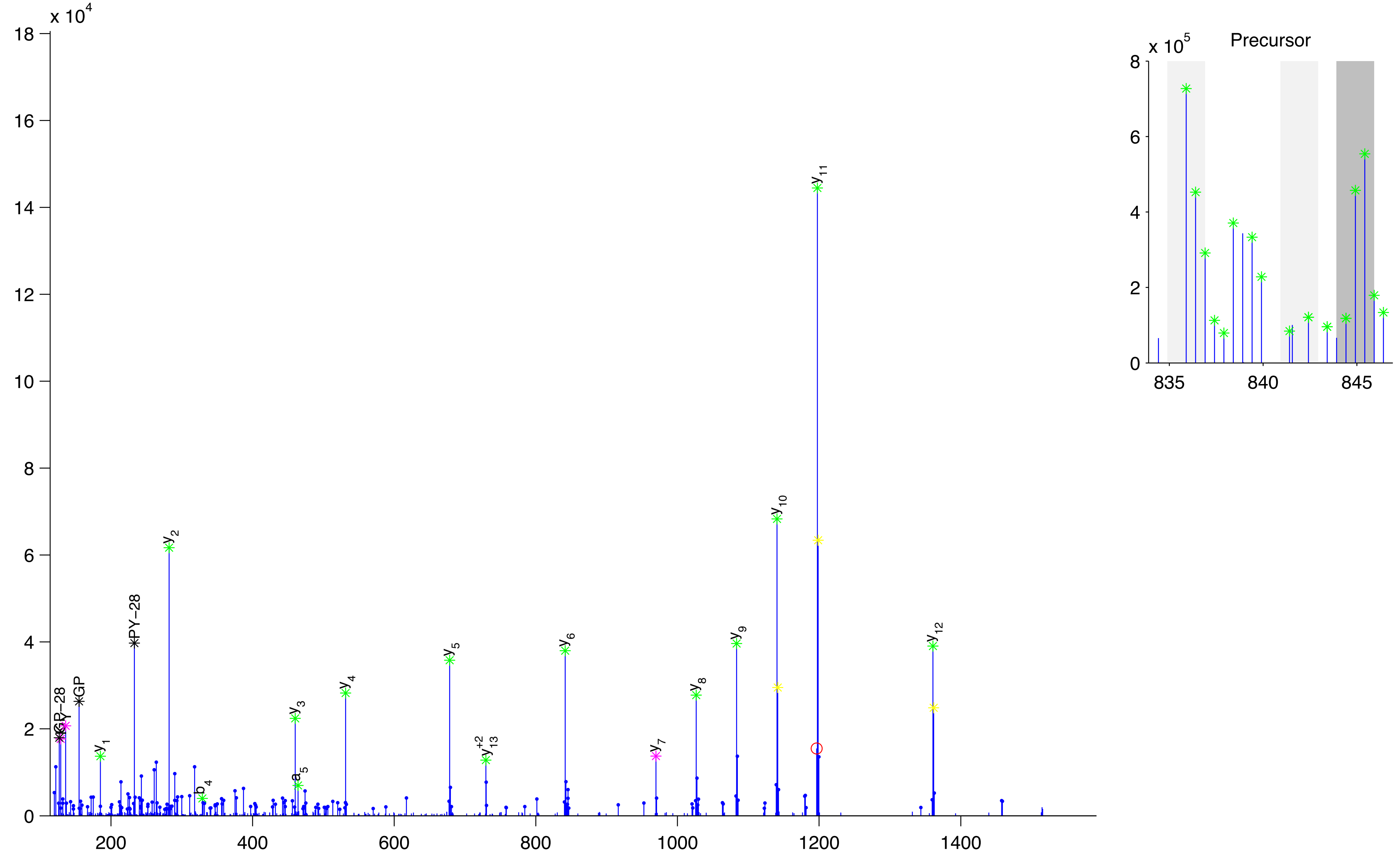

S S G P Y G G G G Q Y F A k P R

Heterogeneous nuclear ribonucleoprotein A1
Charge State: +2
Scan Number: 8531
File Name: 130605_Ack_IP_1.raw

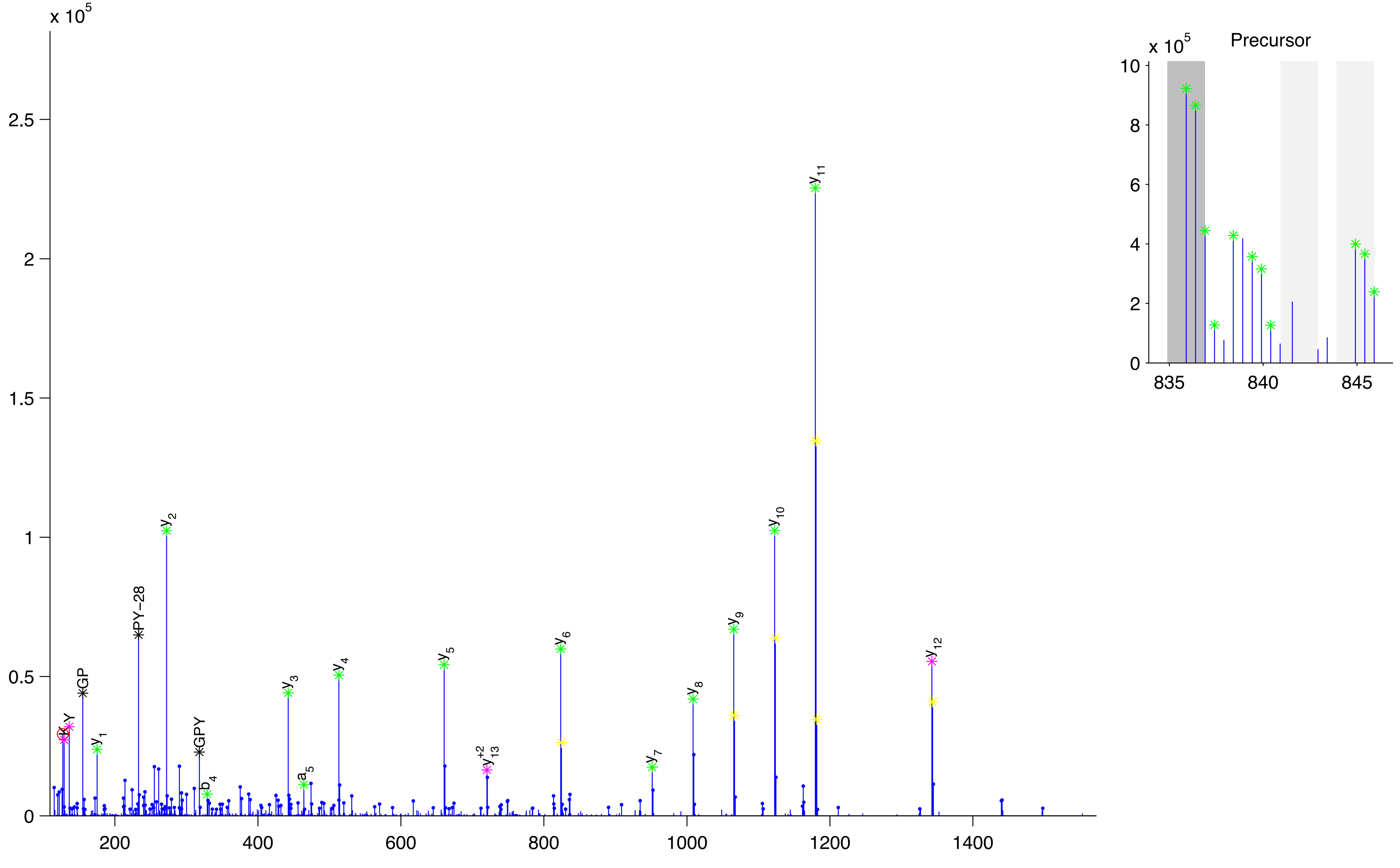

S S G P Y G G G G Q Y F A k P R

Heterogeneous nuclear ribonucleoprotein A1
Charge State: +2
Scan Number: 8622
File Name: 130605_Ack_IP_1.raw

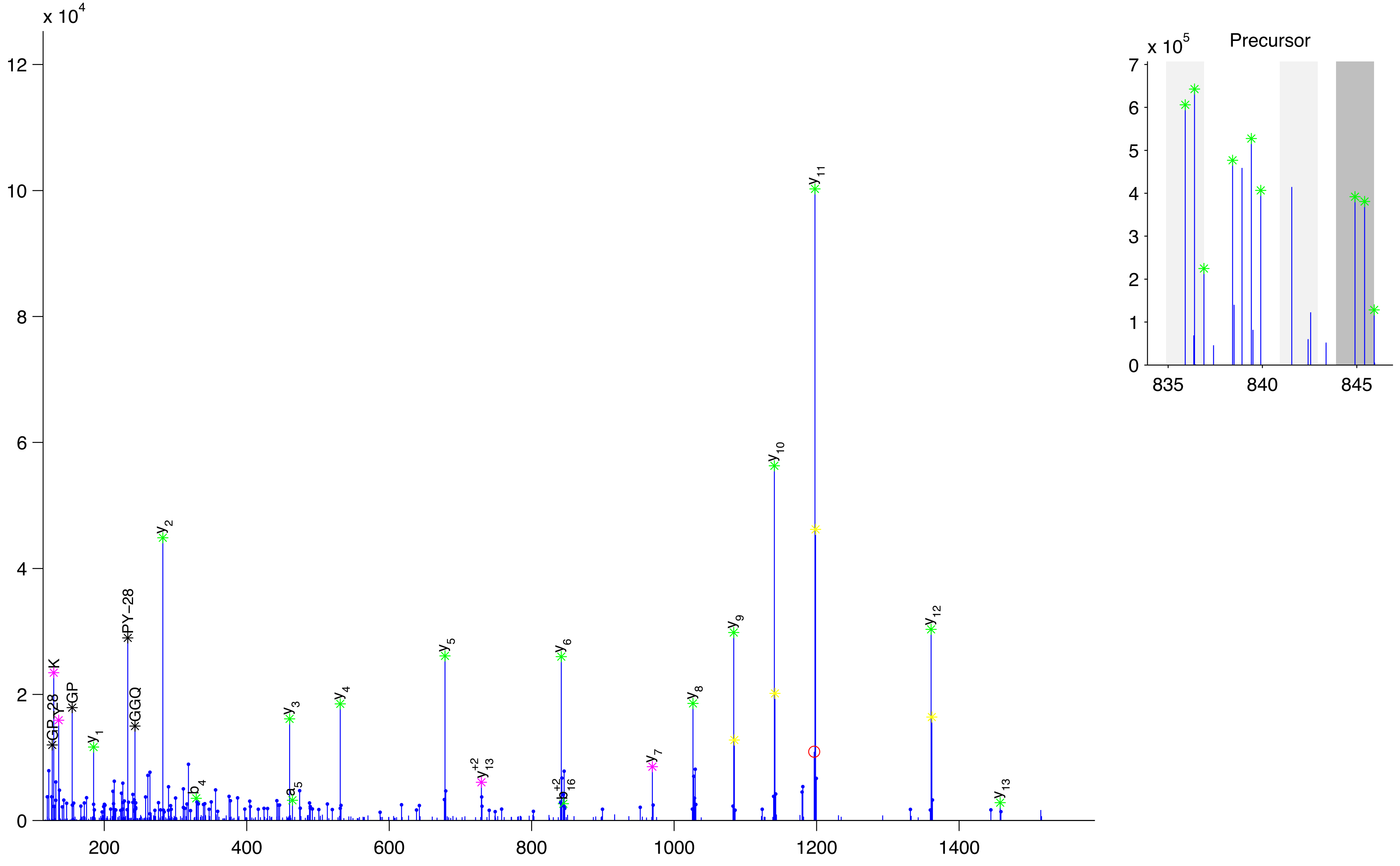

S S G P Y G G G G Q Y F A k P R

Heterogeneous nuclear ribonucleoprotein A1
Charge State: +2
Scan Number: 8643
File Name: 130605_Ack_IP_1.raw

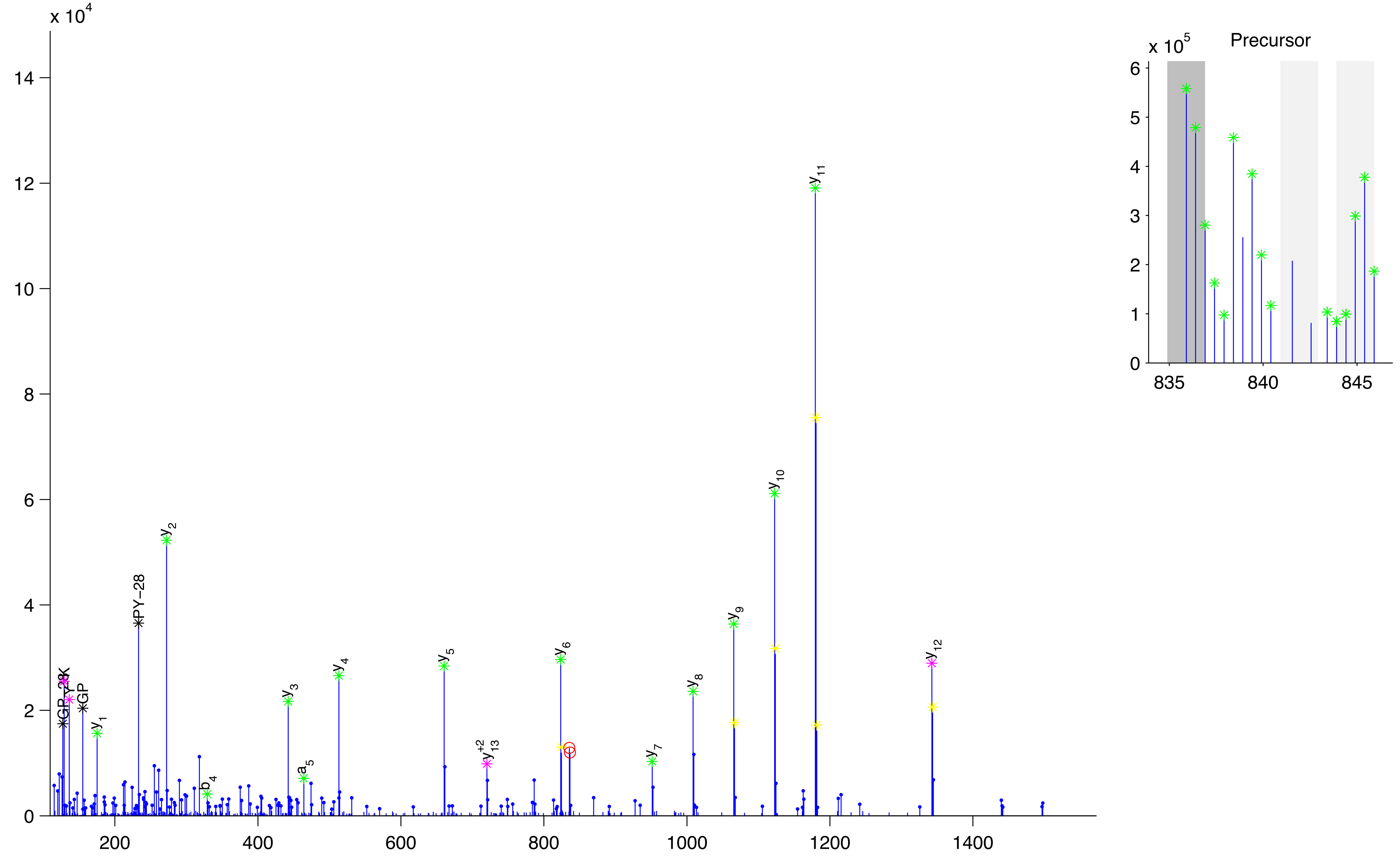

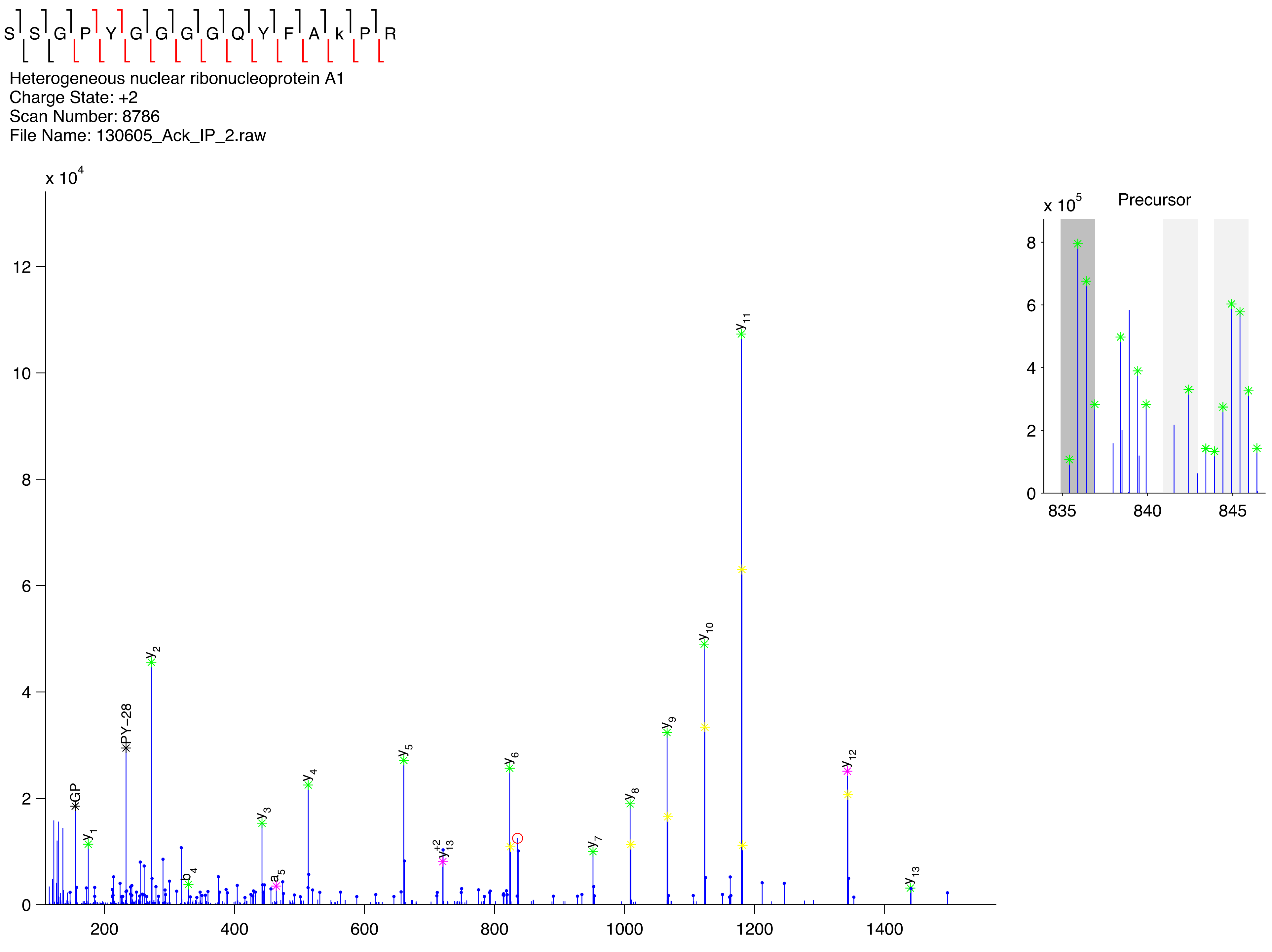
S S G P Y G G G G Q Y F A k P R
Heterogeneous nuclear ribonucleoprotein A1
Charge State: +2
Scan Number: 8786
File Name: 130605_Ack_IP_2.raw
x 10^4
GP
PY−28
y1
y2
b4
y3
a5
y4
y5
y13 +2
y6
y7
y8
y9
y10
y11
y12
y13
x 10^5
Precursor

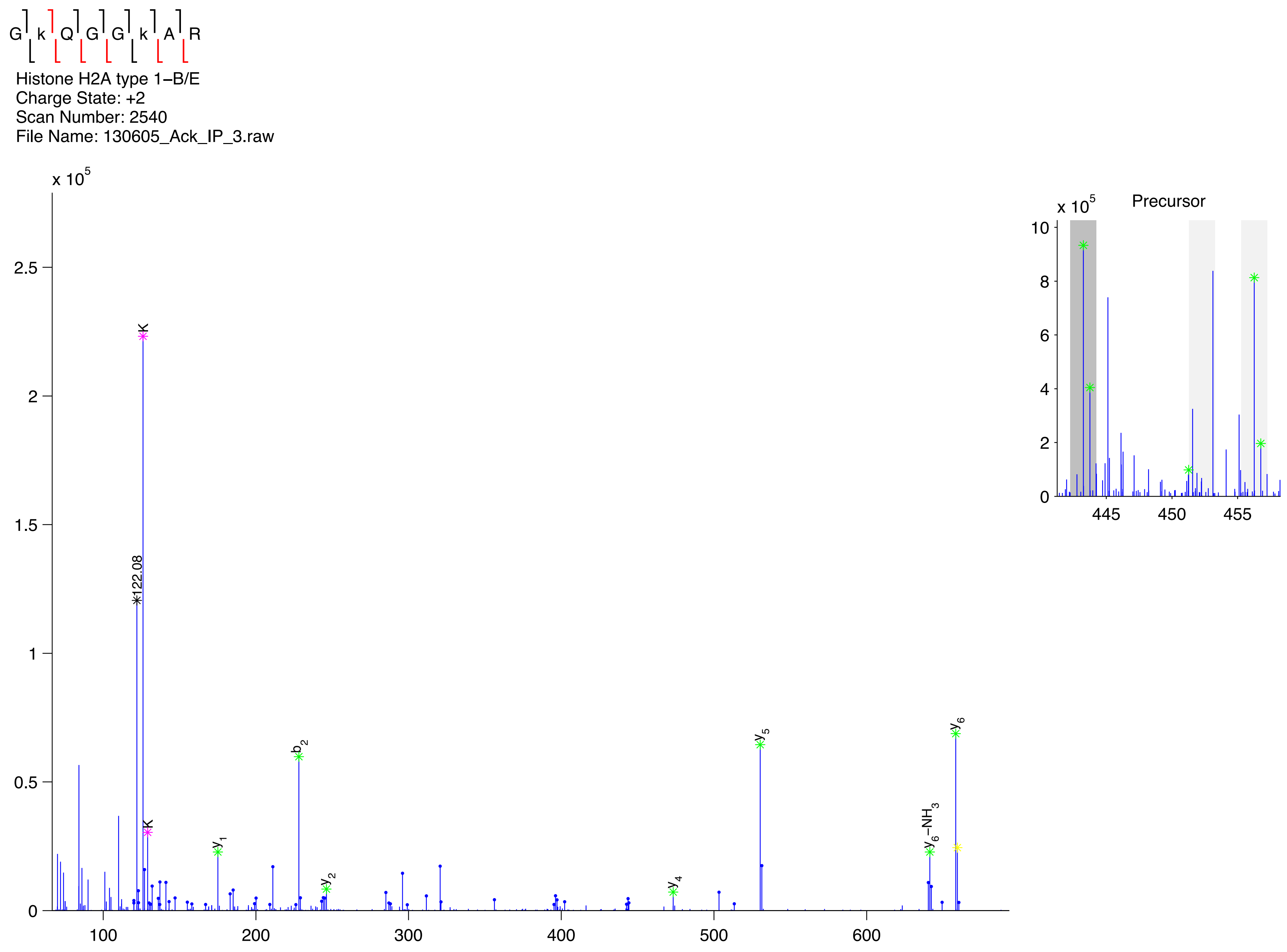

G k Q G G k A R
Histone H2A type 1–B/E
Charge State: +2
Scan Number: 2540
File Name: 130605_Ack_IP_3.raw
x 10^5
122.08
K
K
y1
b2
y2
y4
y5
y6–NH3
y6
Precursor

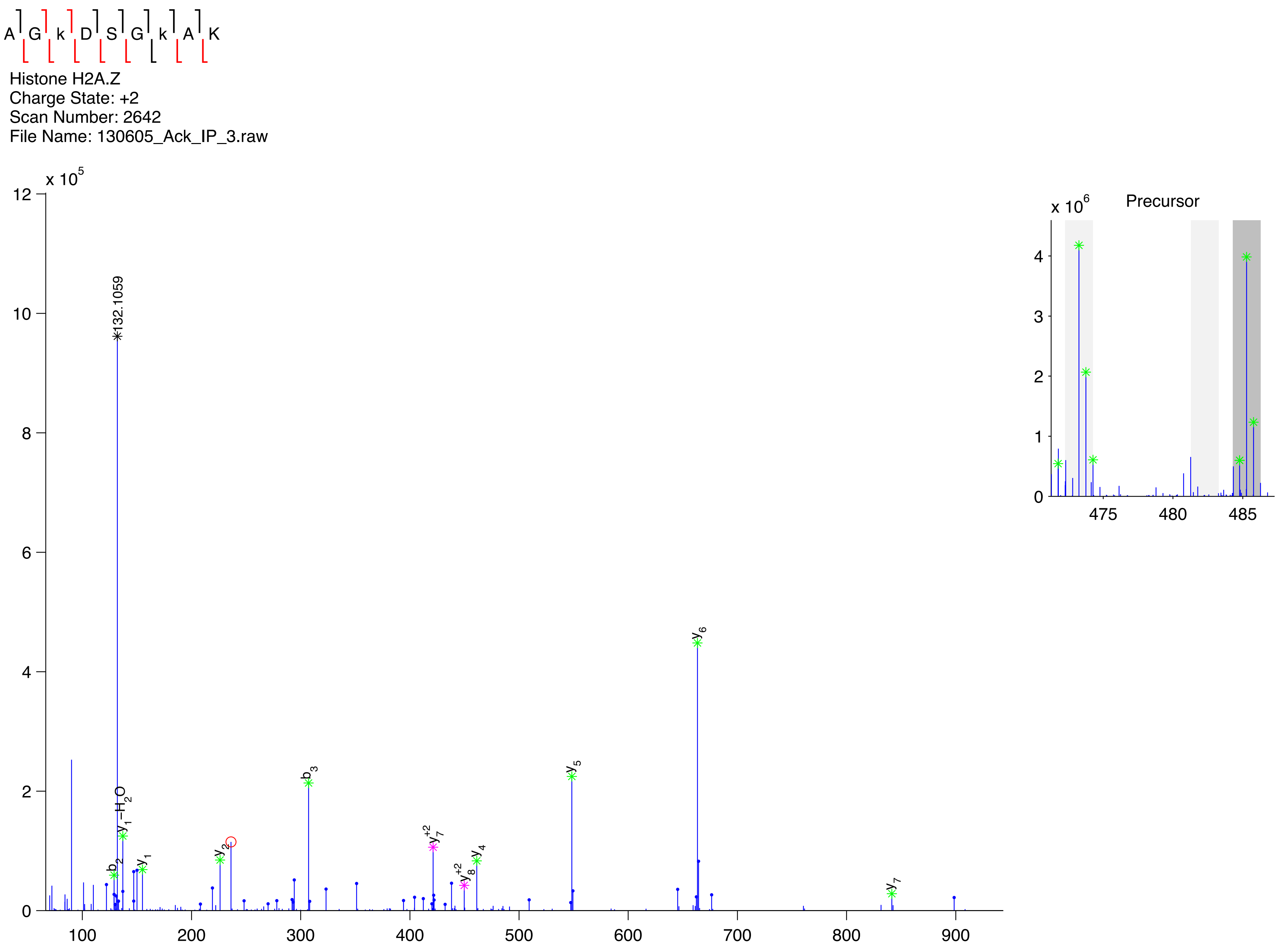

A G k D S G k A K
Histone H2A.Z
Charge State: +2
Scan Number: 2642
File Name: 130605_Ack_IP_3.raw
x 10^5
132.1059
b2
y1−H2O
y1
y2
b3
y7+2
y8+2
y4
y5
y6
y7
x 10^6
Precursor

A G G k A G k D S G K

Histone H2A.Z
Charge State: +2
Scan Number: 2645
File Name: 130605_Ack_IP_2.raw

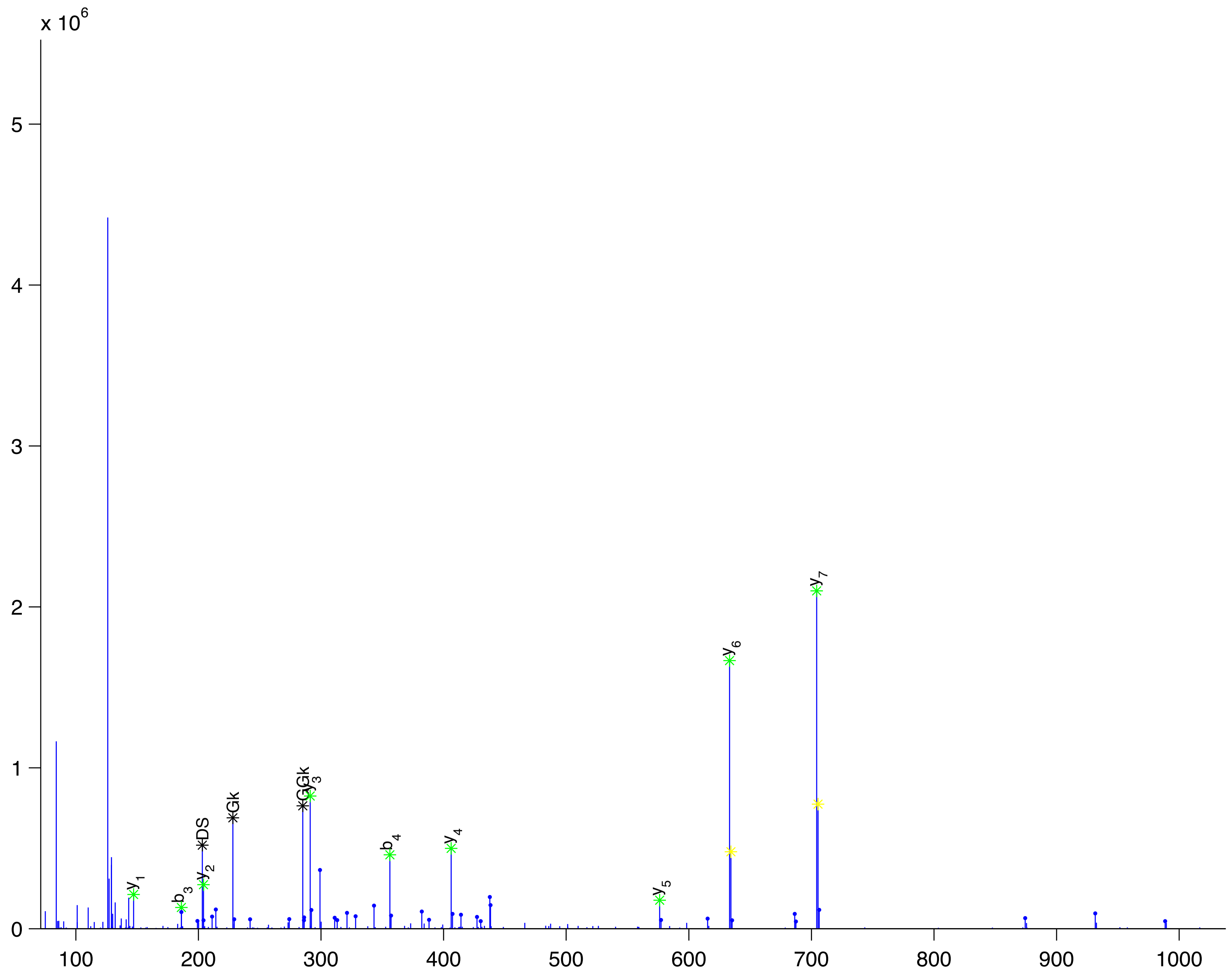

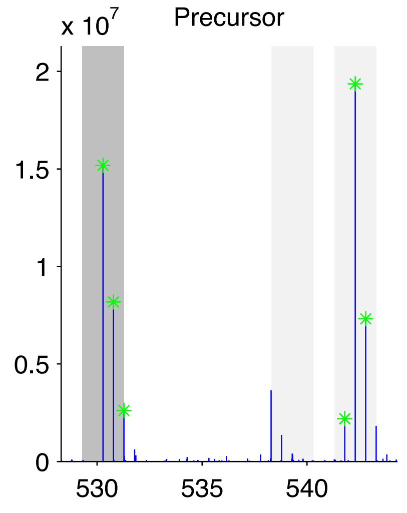

A G G k A G k D S G K

Histone H2A.Z
Charge State: +2
Scan Number: 2652
File Name: 130605_Ack_IP_1.raw

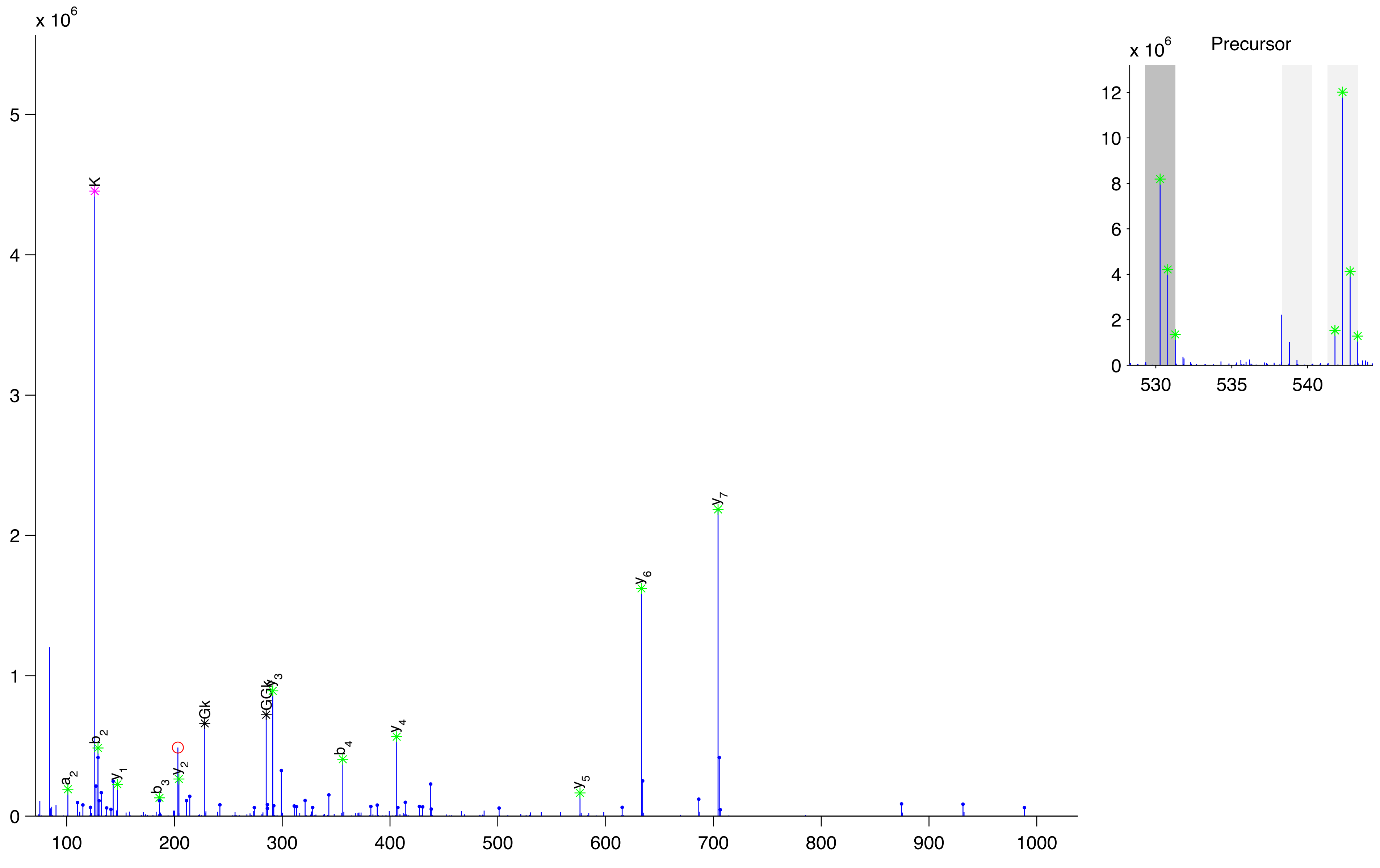

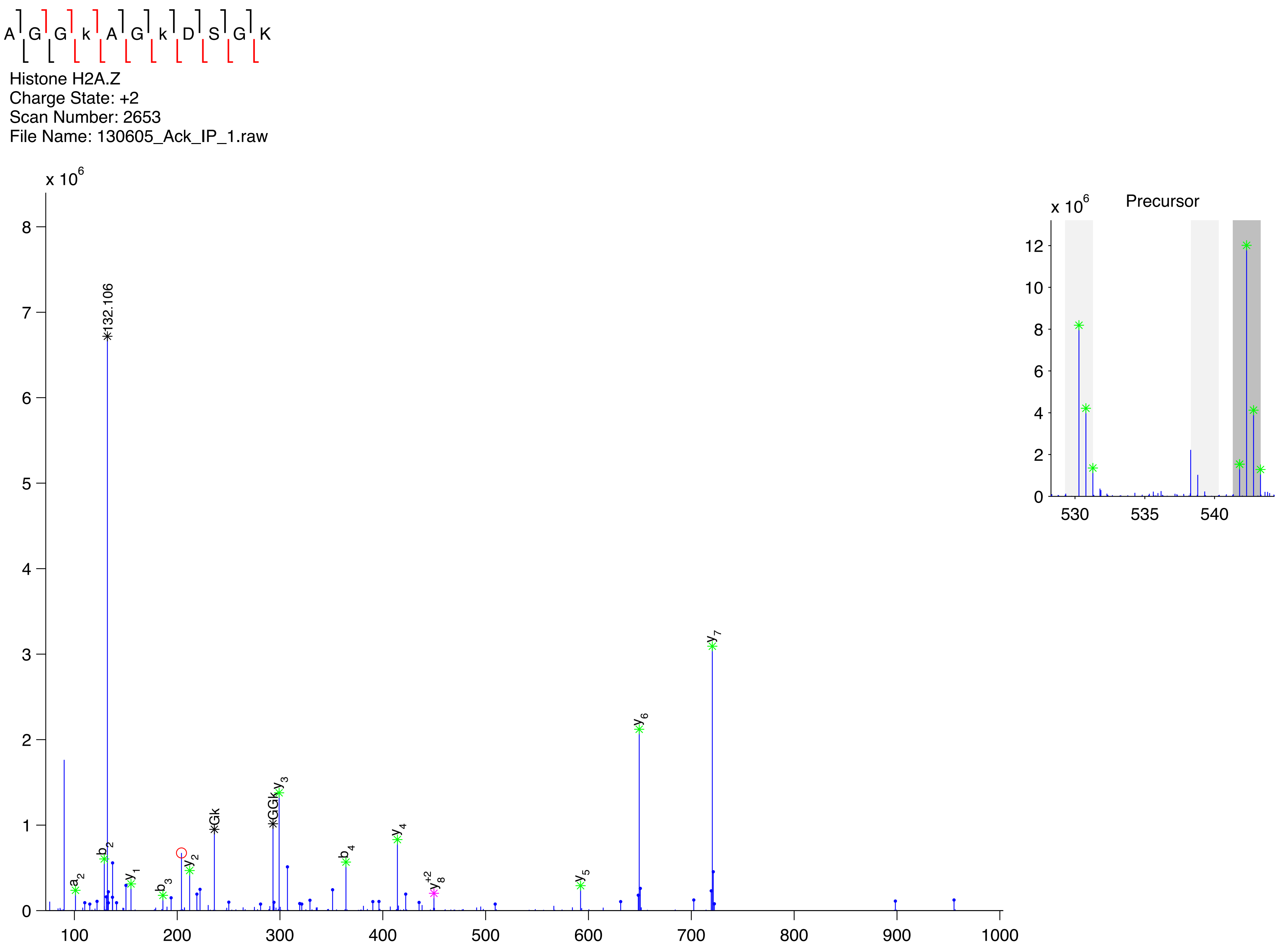

A G G k A G k D S G K
Histone H2A.Z
Charge State: +2
Scan Number: 2653
File Name: 130605_Ack_IP_1.raw
x 10^6
132.106
a2
b2
y1
b3
y2
Gk
GGk
y3
b4
y4
y8+2
y5
y6
y7
Precursor

A G G k A G k D S G K

Histone H2A.Z
Charge State: +2
Scan Number: 2683
File Name: 130605_Ack_IP_3.raw

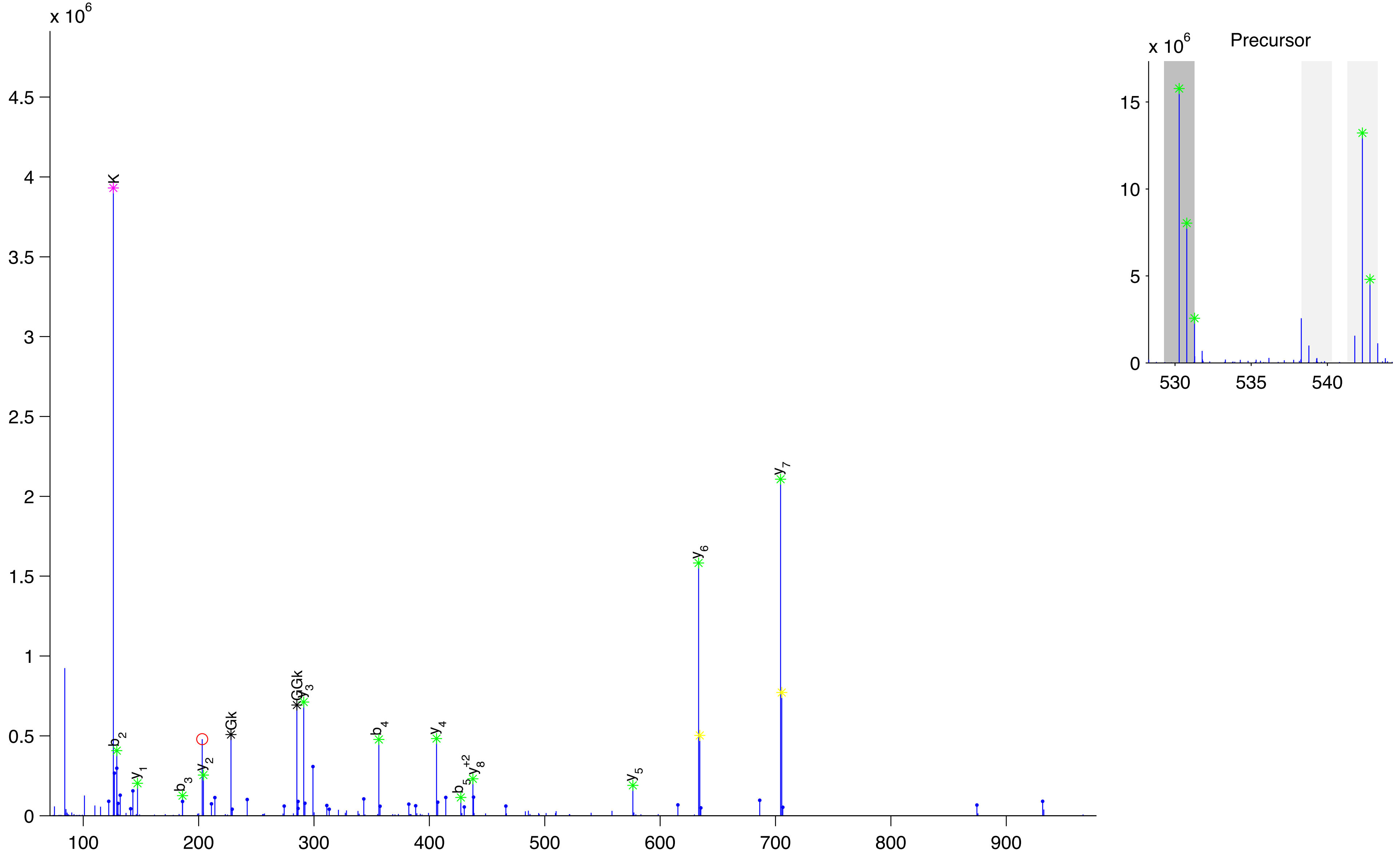

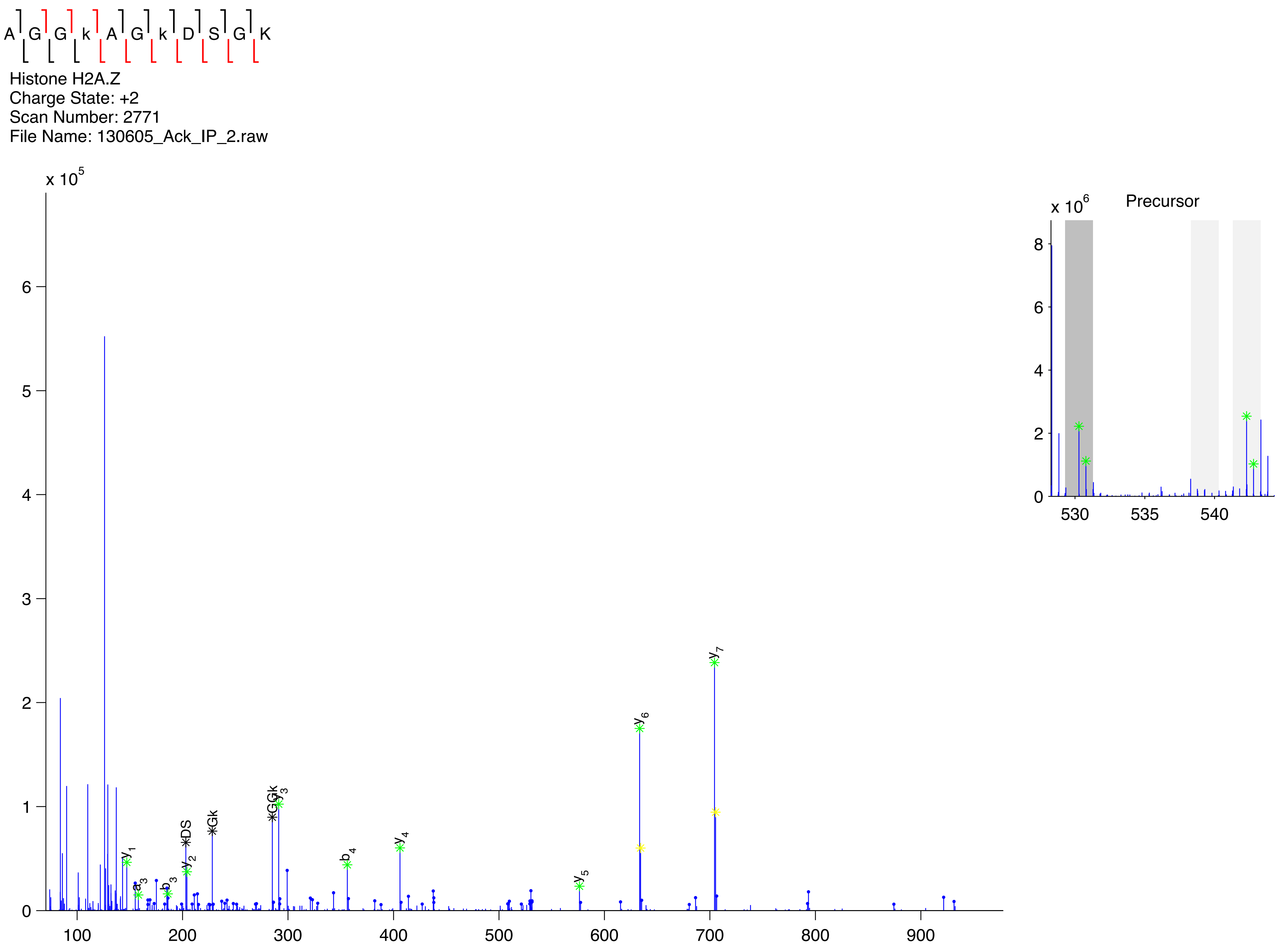
A G G k A G k D S G K
Histone H2A.Z
Charge State: +2
Scan Number: 2771
File Name: 130605_Ack_IP_2.raw
x 10^5
y1
a3
b3
y2
DS
Gk
GGk
y3
b4
y4
y5
y6
y7
x 10^6
Precursor

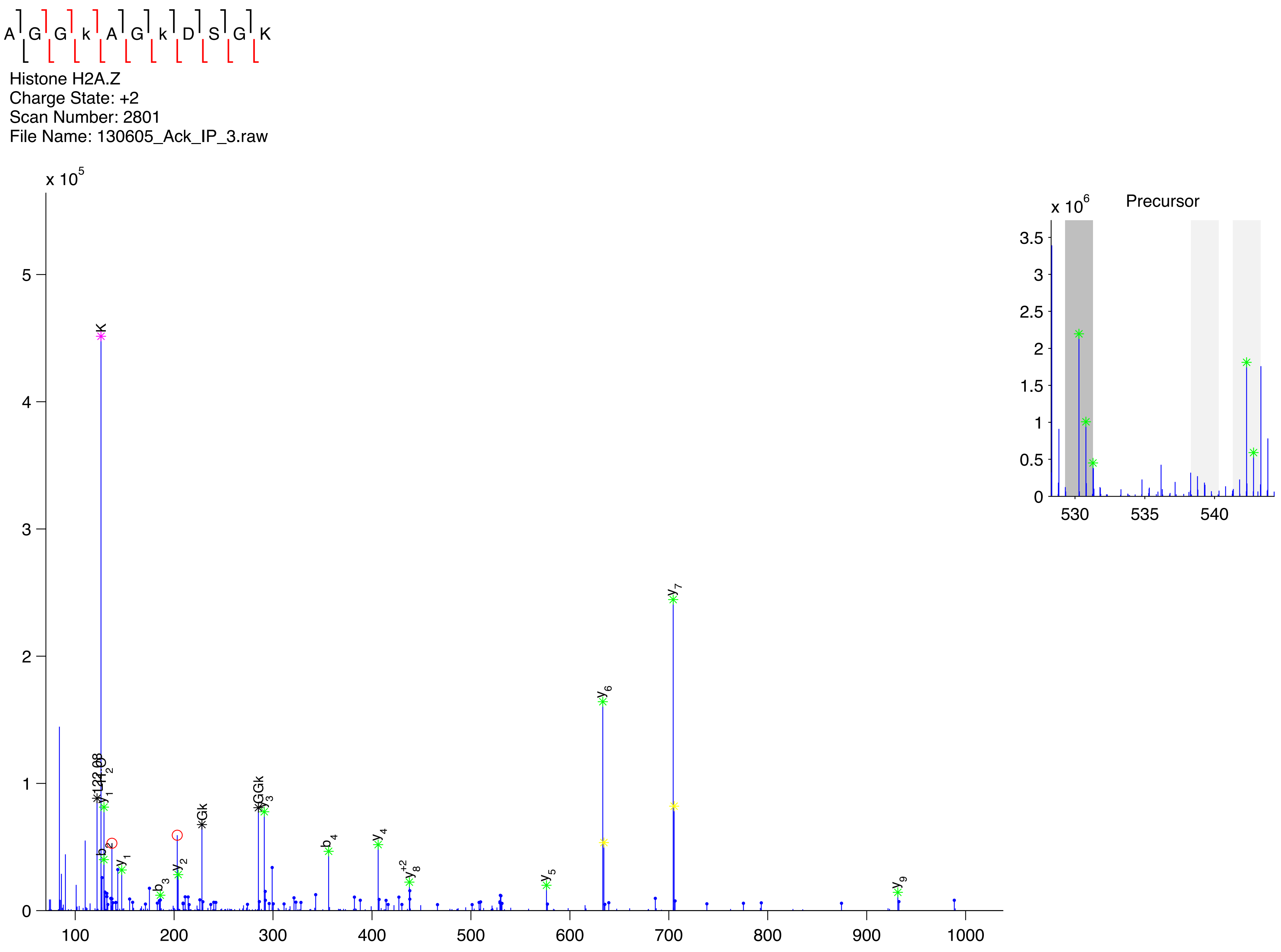
A G G k A G k D S G K
Histone H2A.Z
Charge State: +2
Scan Number: 2801
File Name: 130605_Ack_IP_3.raw
x 10^5
K
122.08
y1-H2O
b2
y1
b3
y2
Gk
GGk
y3
b4
y4
y8+2
y5
y6
y7
y9
x 10^6
Precursor

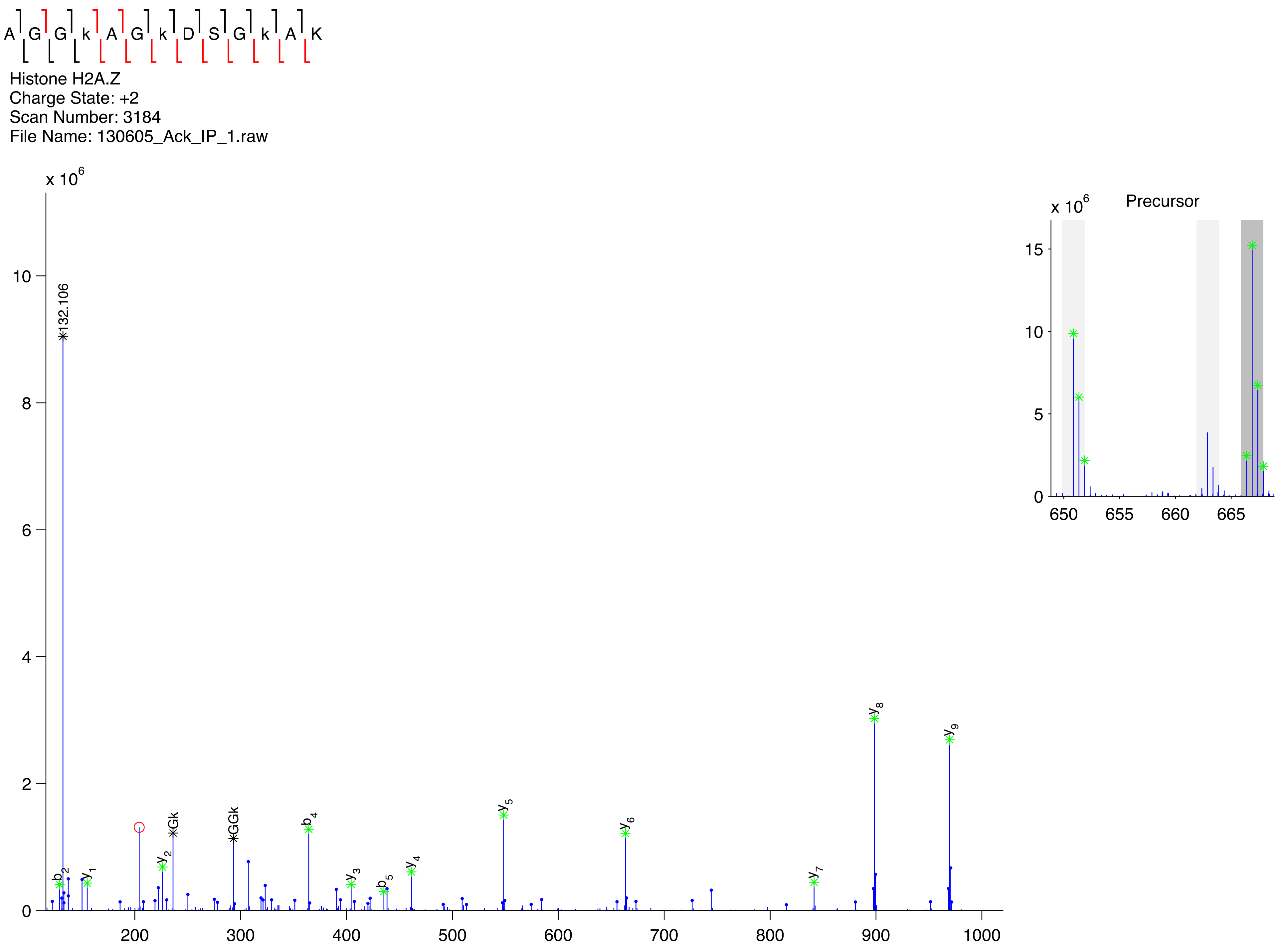
A G G k A G k D S G k A K
Histone H2A.Z
Charge State: +2
Scan Number: 3184
File Name: 130605_Ack_IP_1.raw
x 10^6
132.106
Gk
GGk
b2
y1
y2
b4
y3
b5
y4
y5
y6
y7
y8
y9
Precursor
x 10^6

A G G k A G k D S G k A K

Histone H2A.Z
Charge State: +2
Scan Number: 3196
File Name: 130605_Ack_IP_1.raw

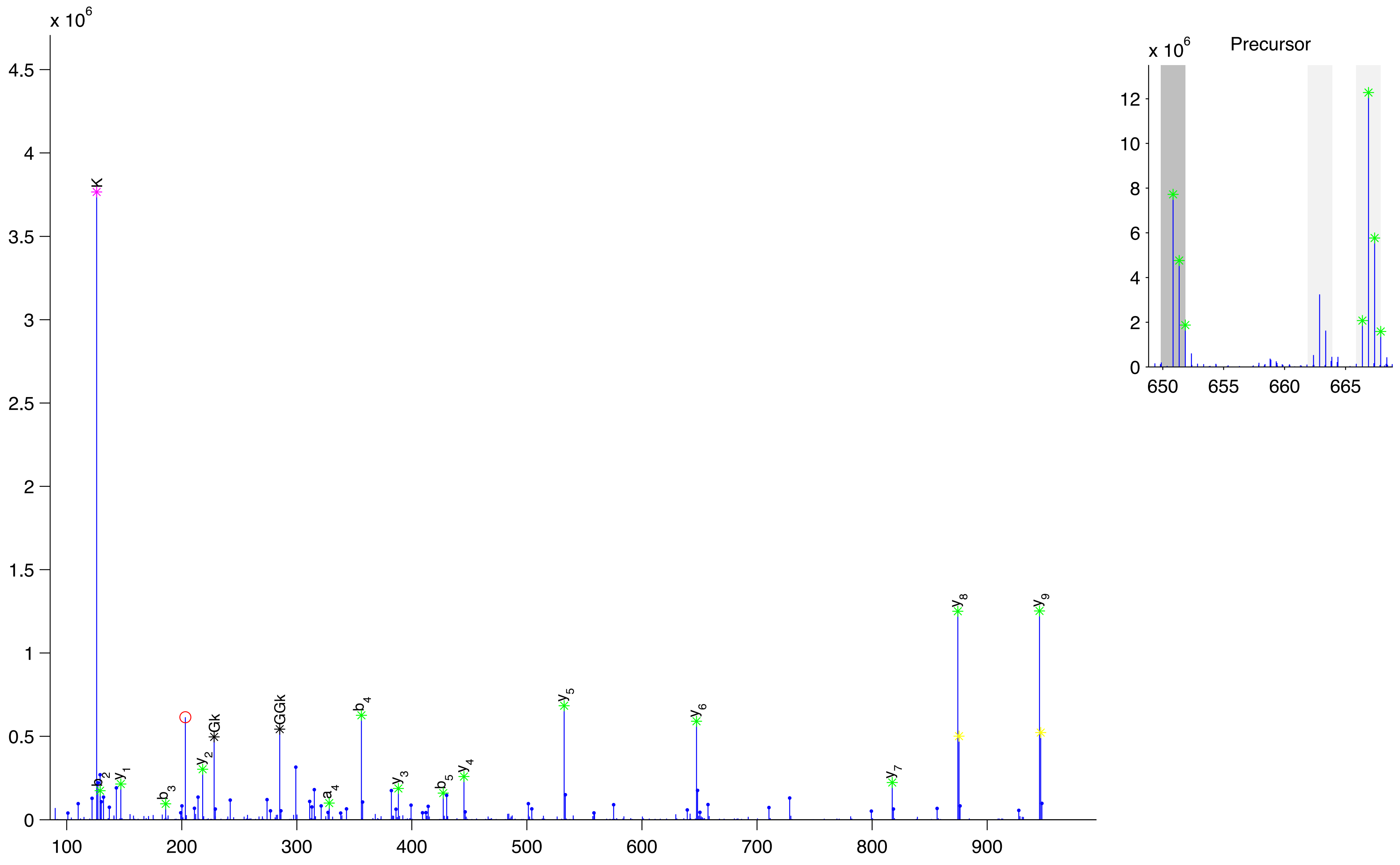

A G G k A G k D S G k A K

Histone H2A.Z
Charge State: +2
Scan Number: 3241
File Name: 130605_Ack_IP_3.raw

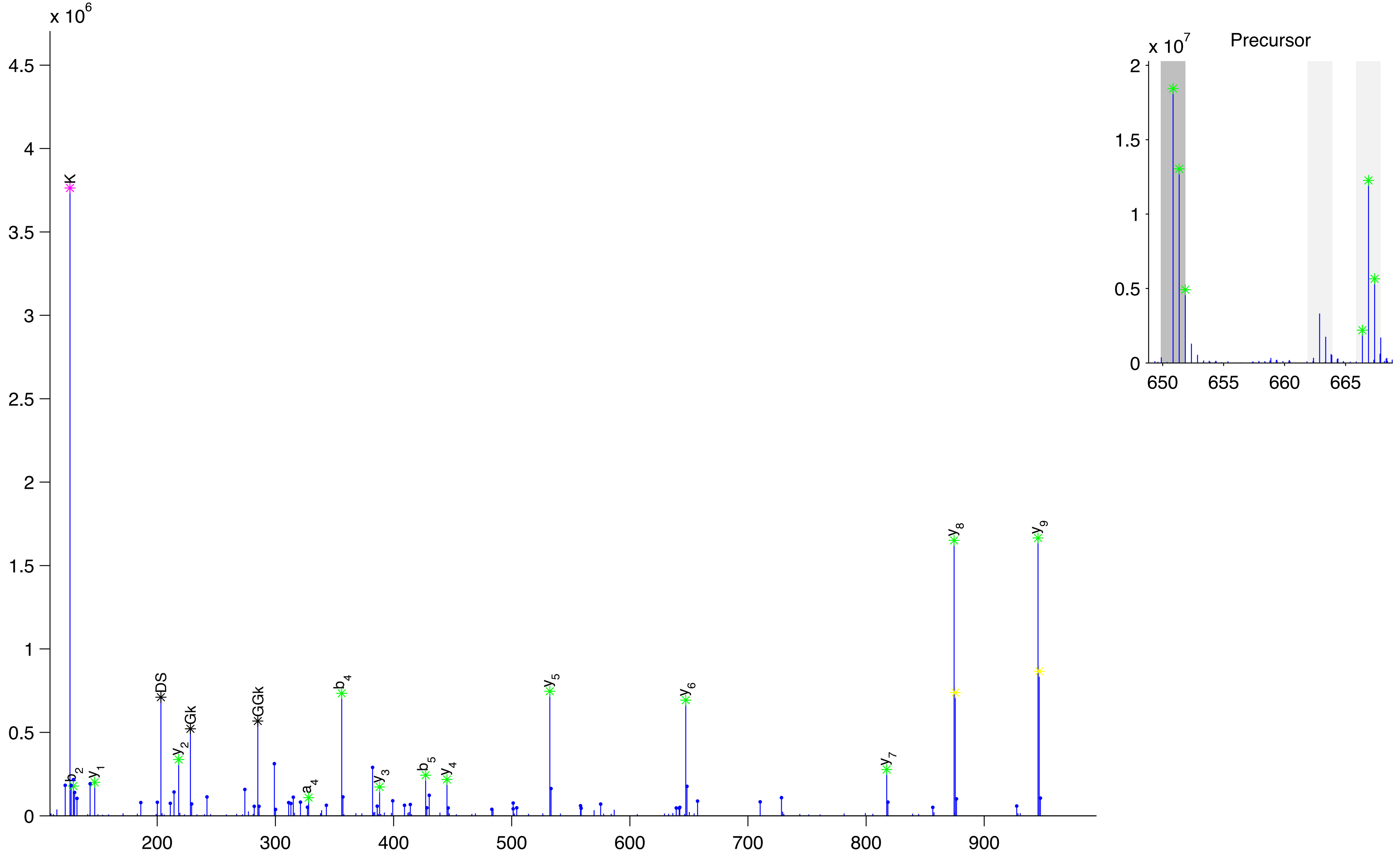

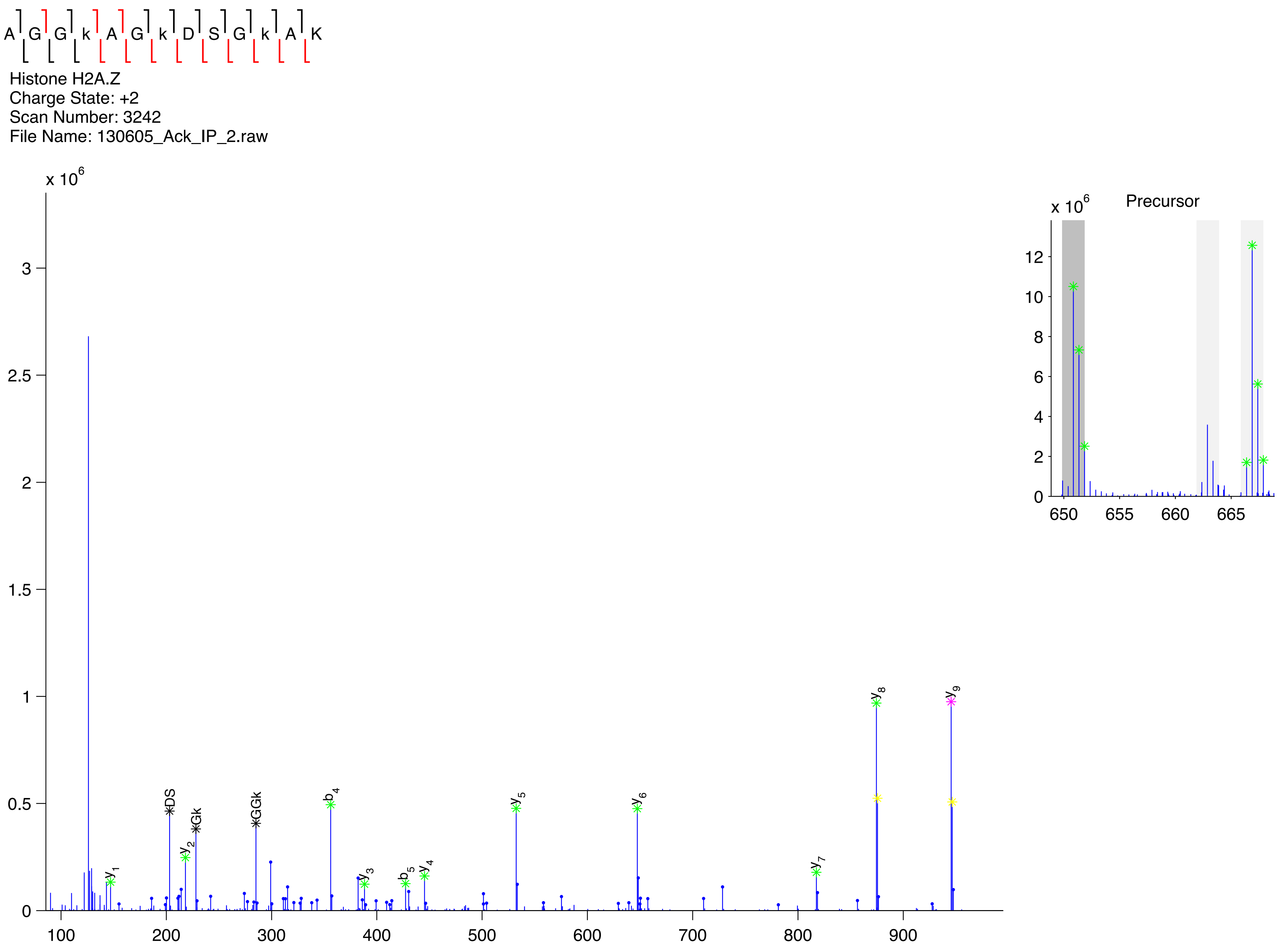
A G G k A G k D S G k A K
Histone H2A.Z
Charge State: +2
Scan Number: 3242
File Name: 130605_Ack_IP_2.raw
x 10^6
0
0.5
1
1.5
2
2.5
3
100
200
300
400
500
600
700
800
900
y1
DS
y2
Gk
GGk
b4
y3
b5
y4
y5
y6
y7
y8
y9
Precursor
x 10^6
0
2
4
6
8
10
12
650
655
660
665

A G G k A G k D S G k A K

Histone H2A.Z
Charge State: +2
Scan Number: 3330
File Name: 130605_Ack_IP_1.raw

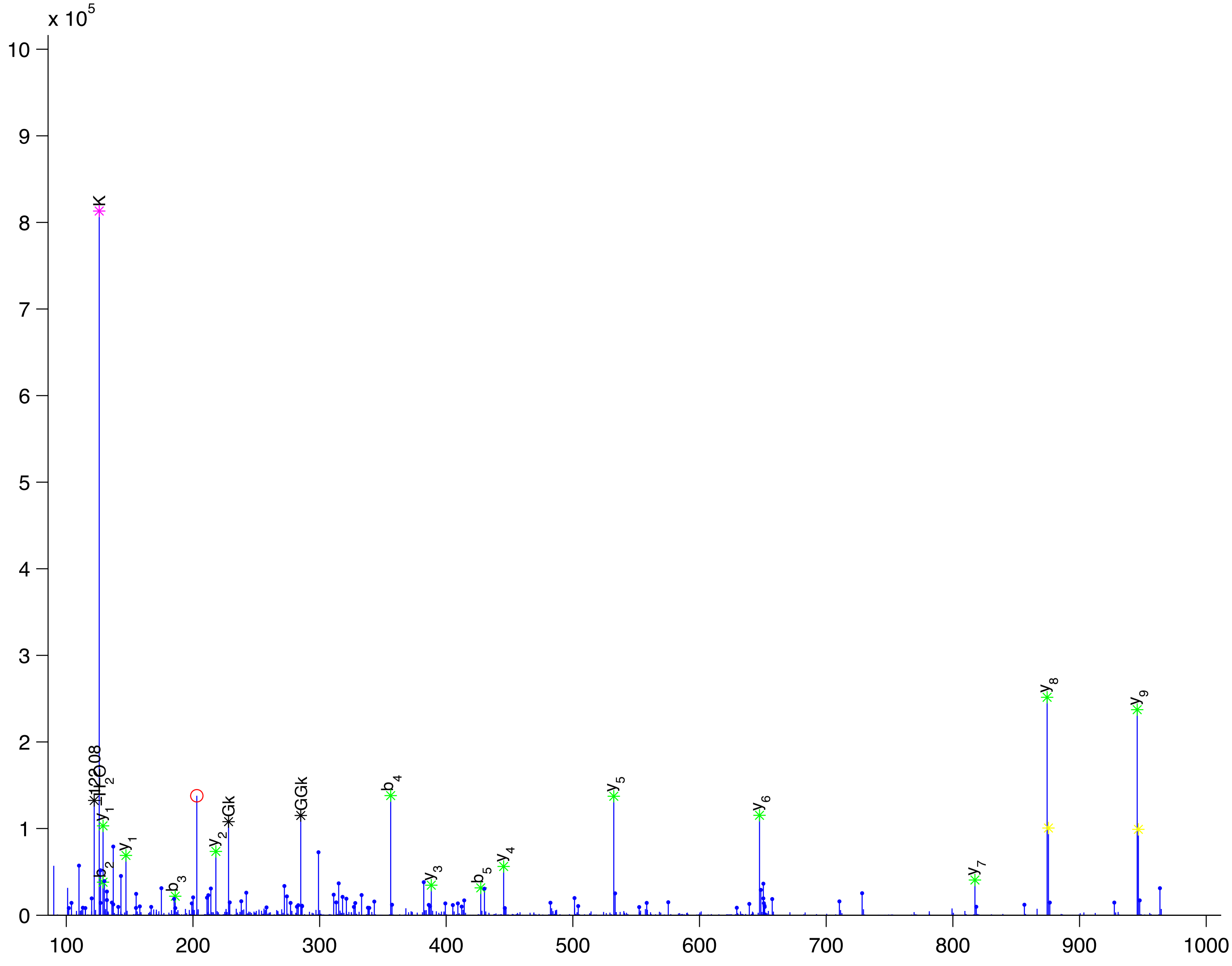

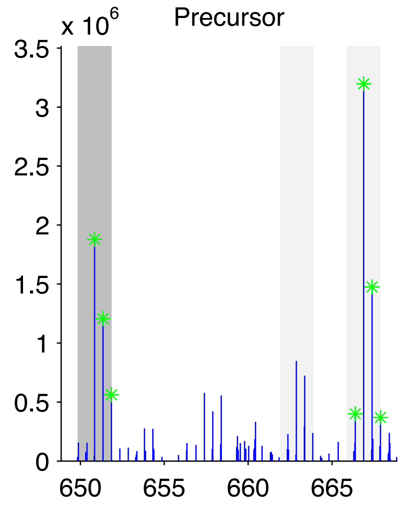

A G G k A G k D S G k A K

Histone H2A.Z
Charge State: +2
Scan Number: 3370
File Name: 130605_Ack_IP_1.raw

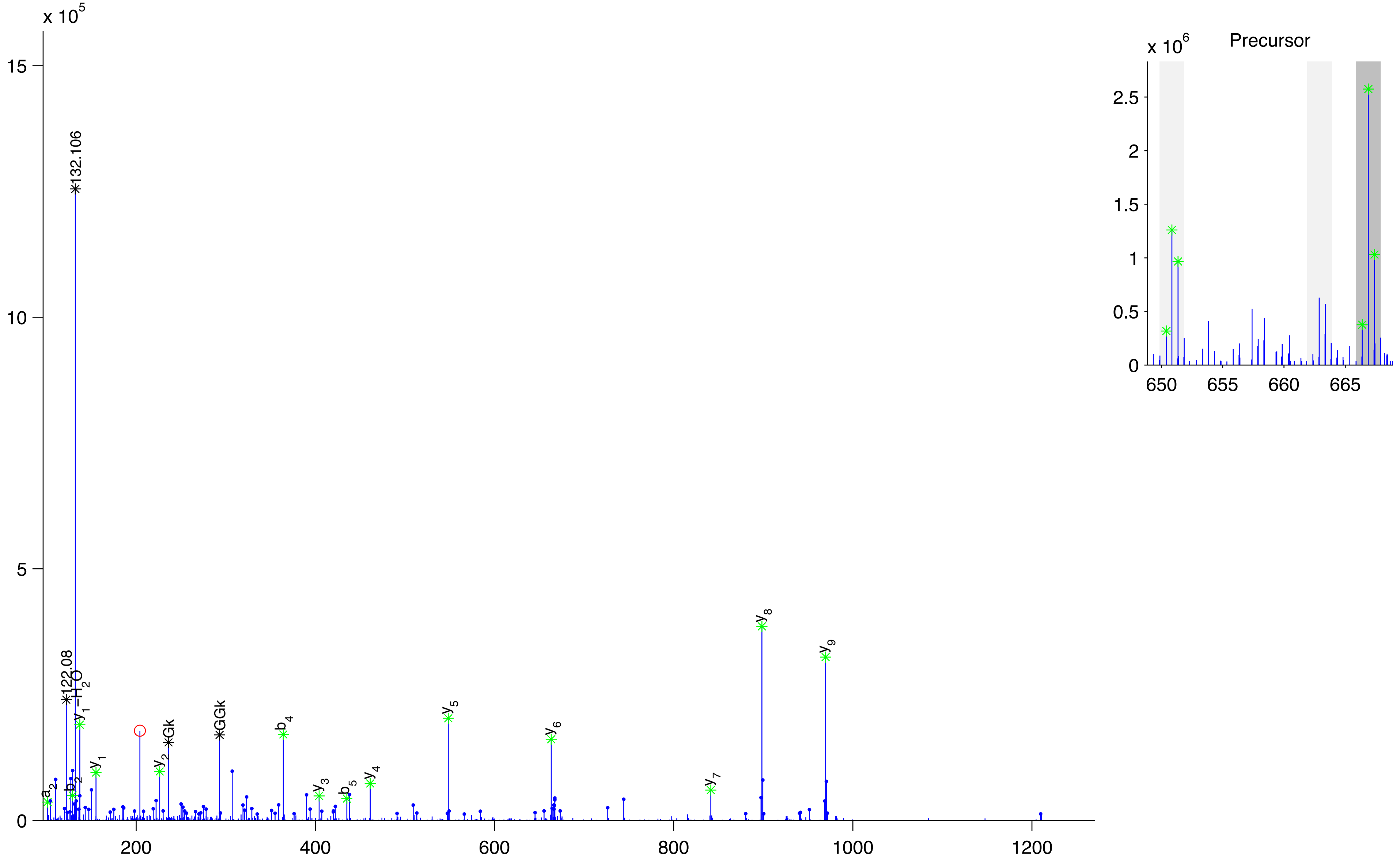

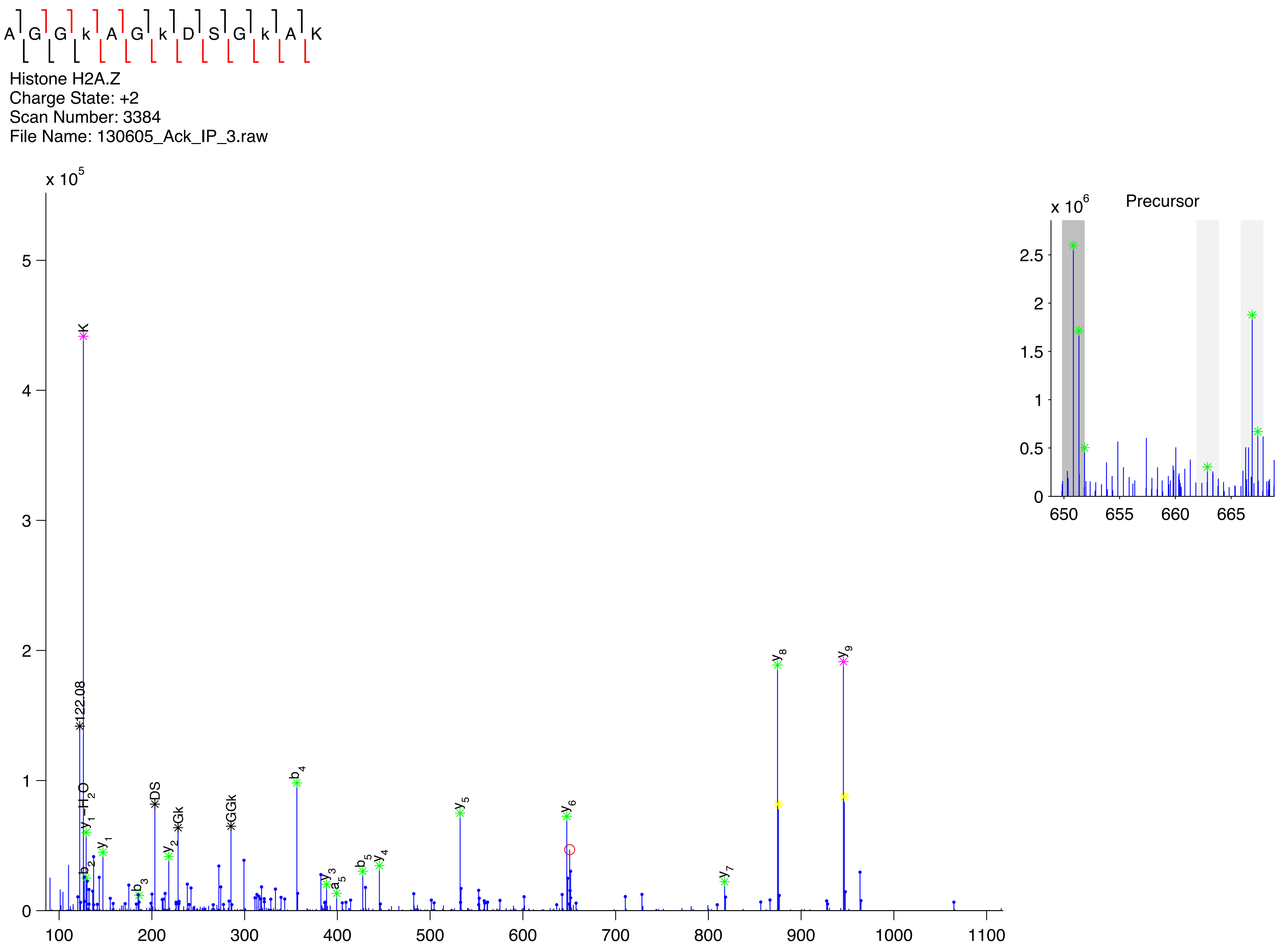

A G G k A G k D S G k A K
Histone H2A.Z
Charge State: +2
Scan Number: 3384
File Name: 130605_Ack_IP_3.raw
x 10^5
K
122.08
DS
Gk
GGk
b2
y1-H2O
y1
b3
y2
b4
y3
a5
b5
y4
y5
y6
y7
y8
y9
x 10^6
Precursor

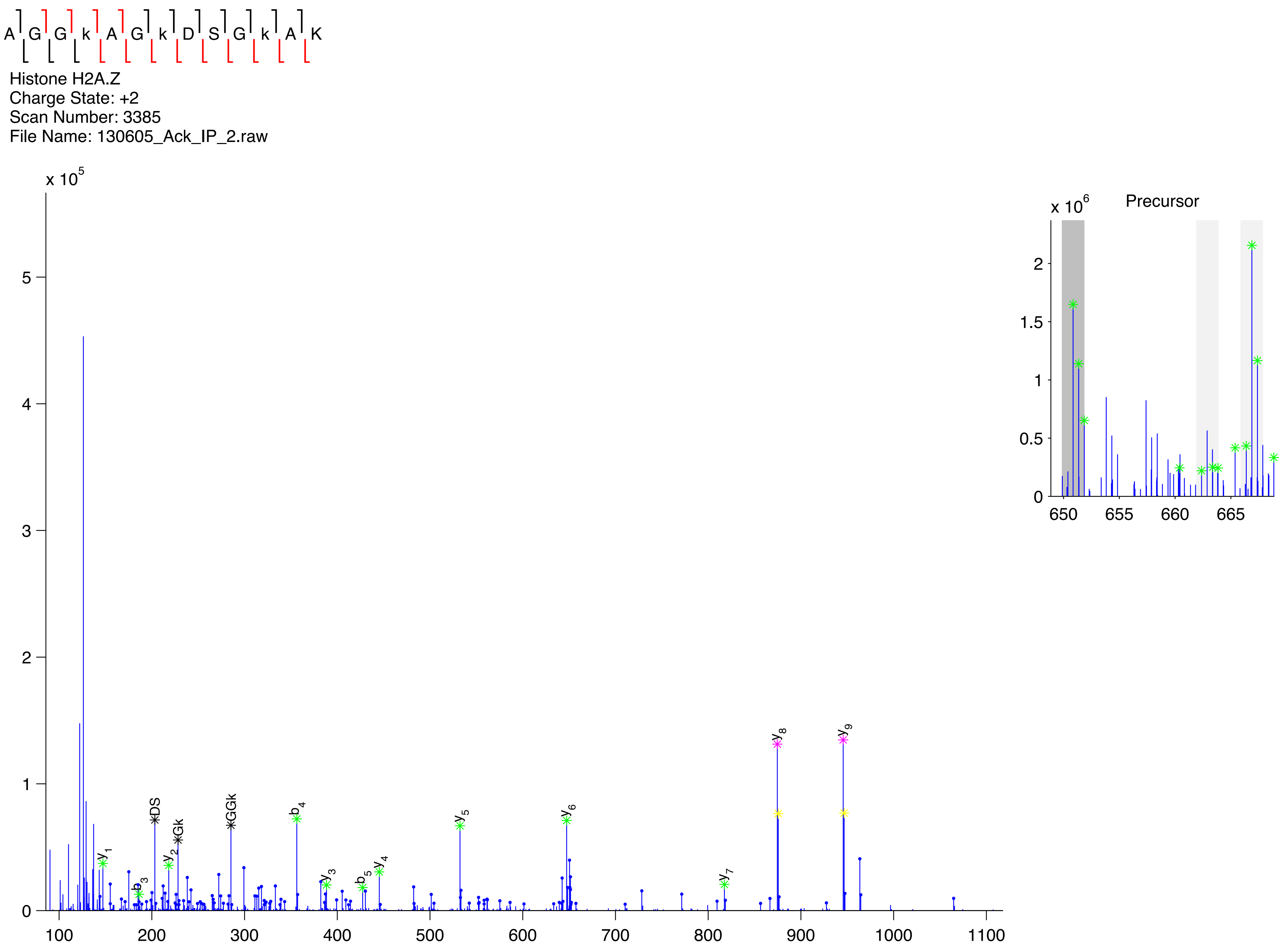
A G G k A G k D S G k A K
Histone H2A.Z
Charge State: +2
Scan Number: 3385
File Name: 130605_Ack_IP_2.raw
x 10^5
y1
b3
DS
y2
Gk
GGk
b4
y3
b5
y4
y5
y6
y7
y8
y9
x 10^6
Precursor

k G S k k A V T K

Histone H2B type 1–L
Charge State: +2
Scan Number: 3353
File Name: 130605_Ack_IP_2.raw

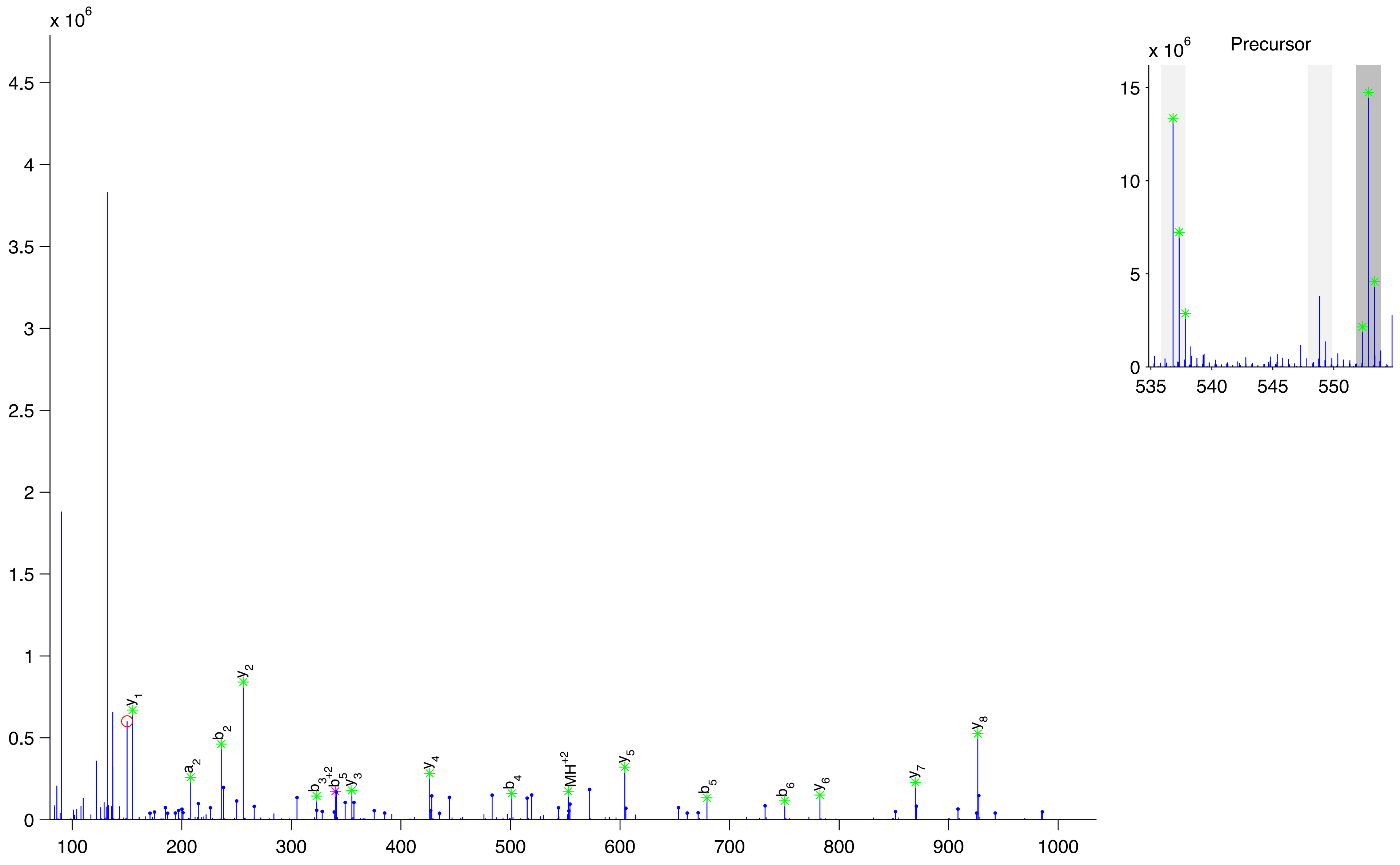

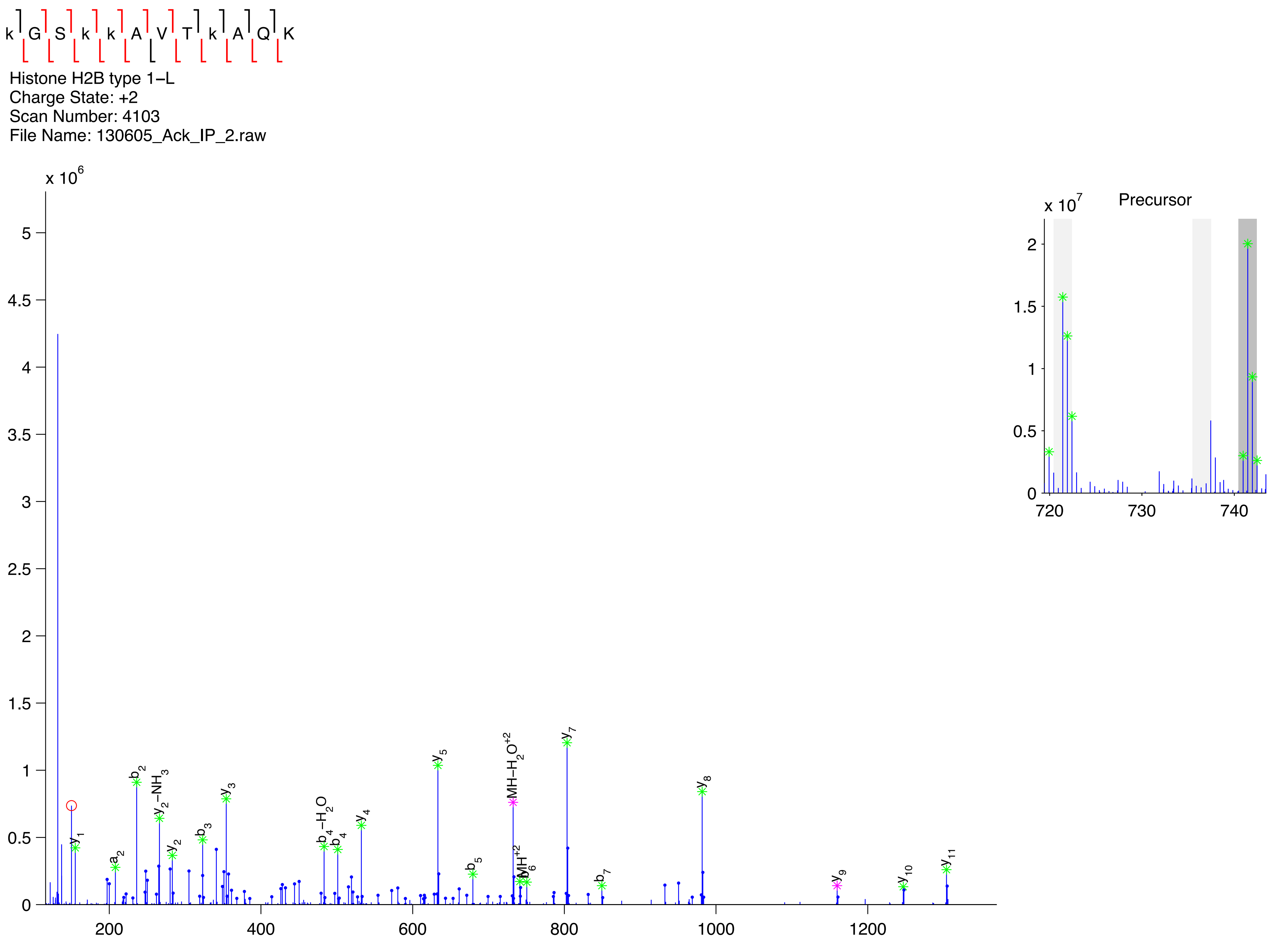

k G S k k A V T k A Q K
Histone H2B type 1–L
Charge State: +2
Scan Number: 4103
File Name: 130605_Ack_IP_2.raw
x 10^6
y1
a2
b2
y2–NH3
y2
b3
y3
b4–H2O
b4
y4
y5
b5
MH–H2O+2
MH+2
b6
y7
b7
y8
y9
y10
y11
x 10^7
Precursor

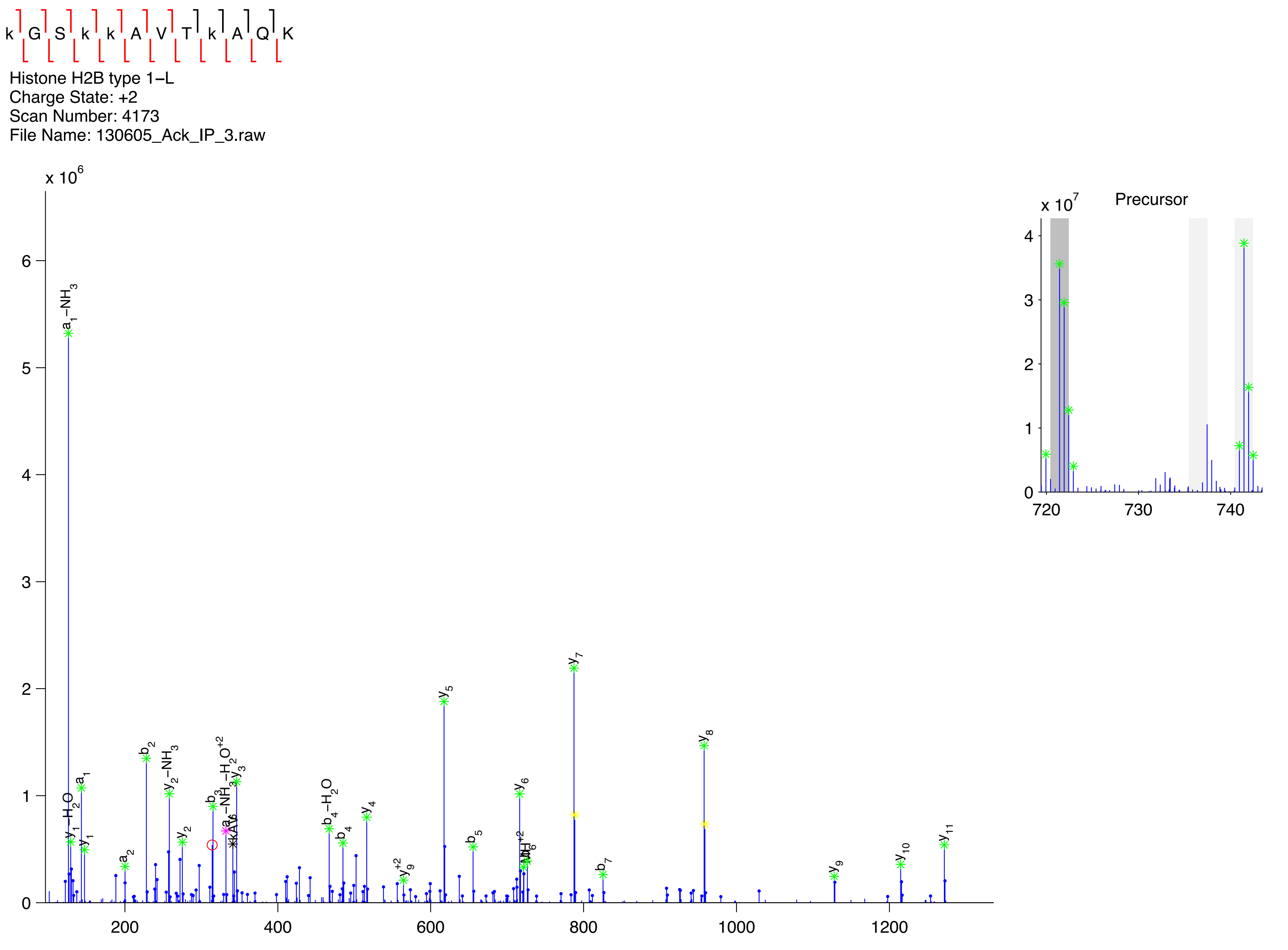

k G S k k A V T k A Q K
Histone H2B type 1–L
Charge State: +2
Scan Number: 4173
File Name: 130605_Ack_IP_3.raw
x 10^6
a1–NH3
a1
y1–H2O
y1
a2
b2
y2–NH3
y2
b3
a4–NH3–H2O+2
y3
b4–H2O
b4
y4
y9+2
y5
b5
y6
b7
y7
y8
y9
y10
y11
Precursor
x 10^7

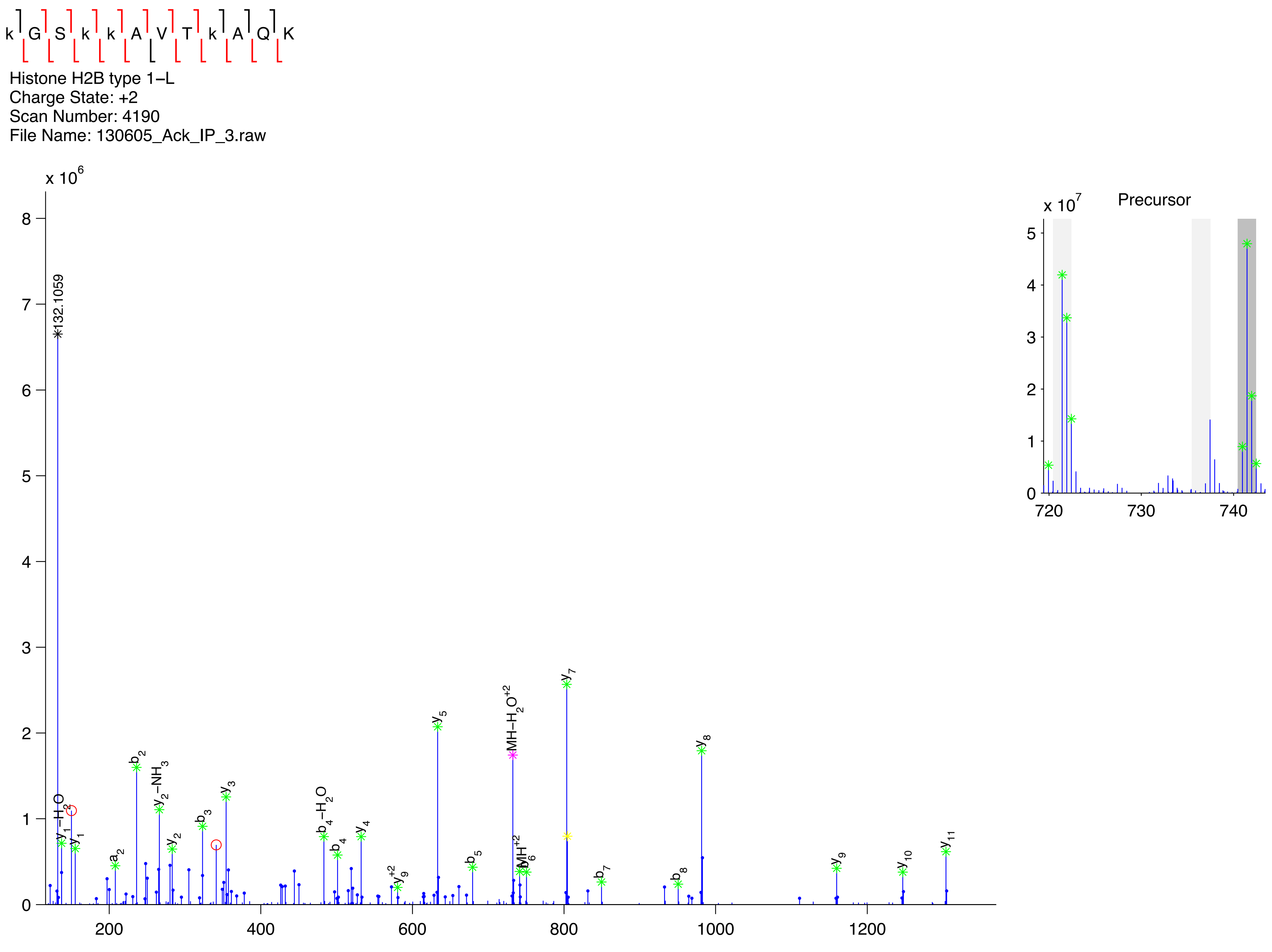

k G S k k A V T k A Q K
Histone H2B type 1–L
Charge State: +2
Scan Number: 4190
File Name: 130605_Ack_IP_3.raw
x 10^6
132.1059
Precursor
x 10^7

k G S k k A V T k A Q K

Histone H2B type 1–L
Charge State: +2
Scan Number: 4212
File Name: 130605_Ack_IP_2.raw

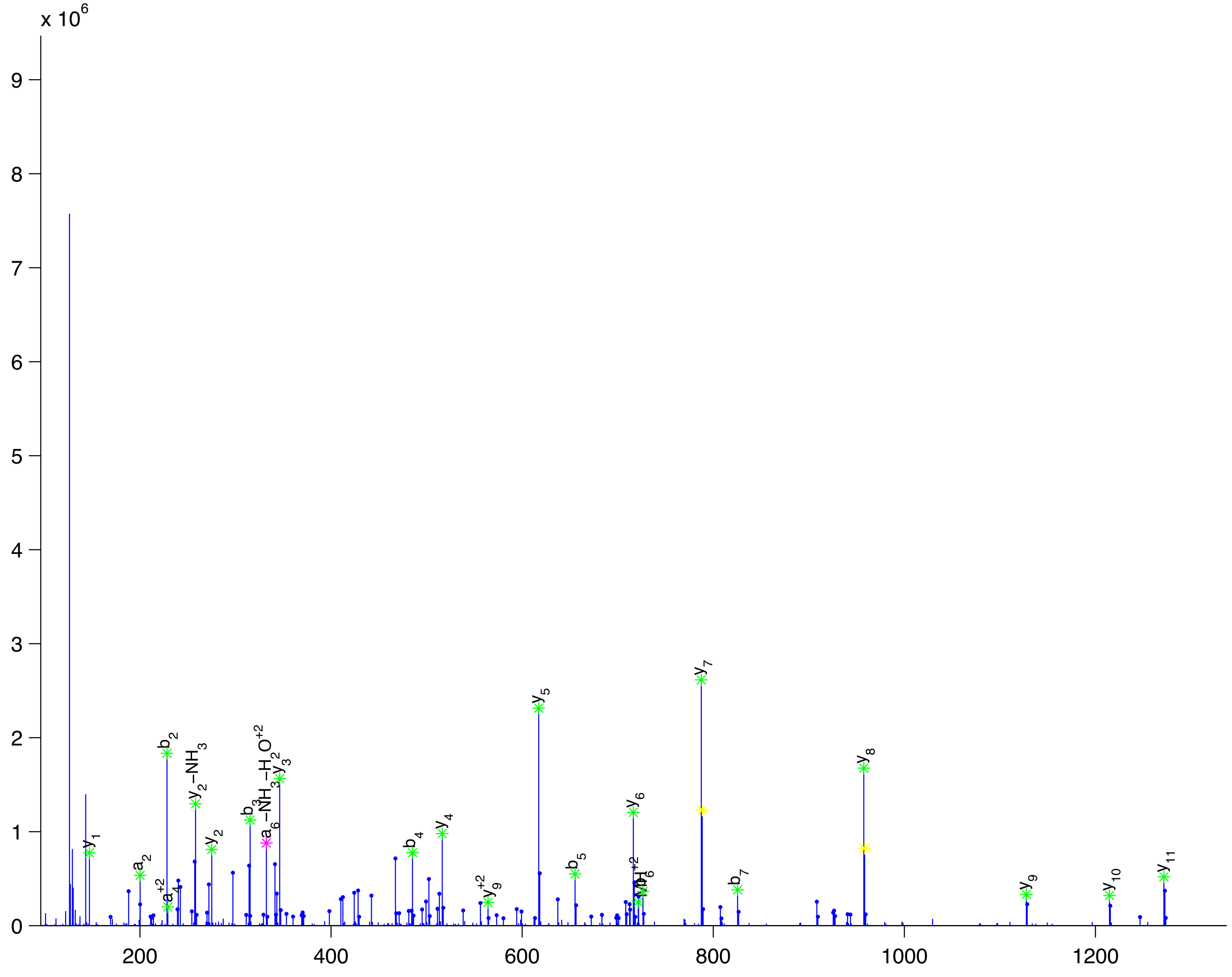

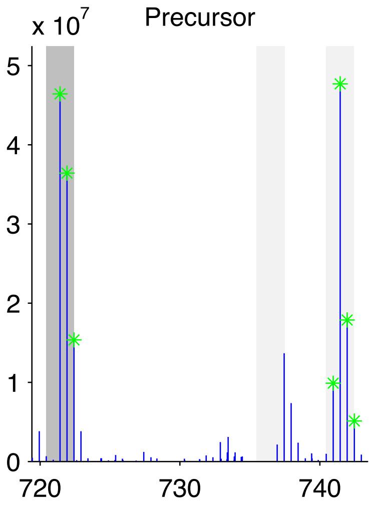

k G S k k A V T k A Q K

Histone H2B type 1–L
Charge State: +2
Scan Number: 4265
File Name: 130605_Ack_IP_2.raw

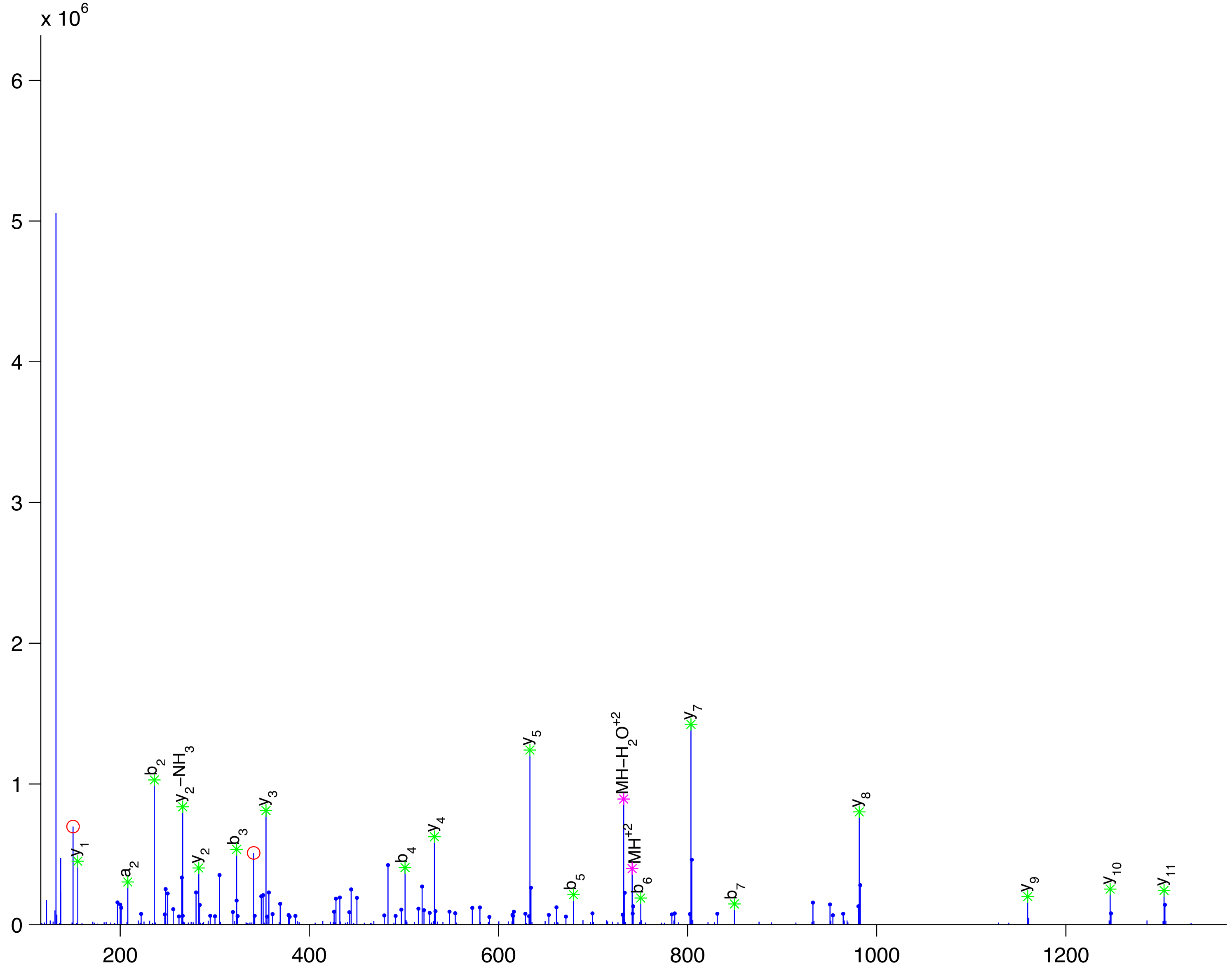

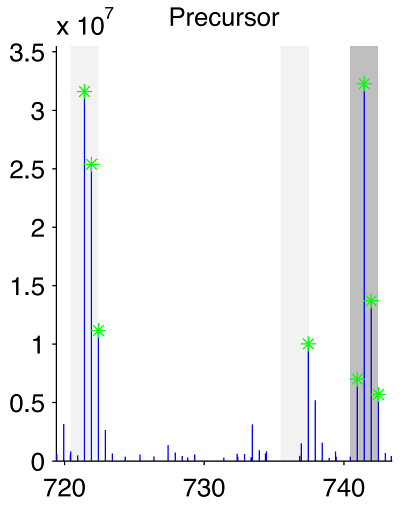

k G S k k A V T k A Q K

Histone H2B type 1–L
Charge State: +2
Scan Number: 4338
File Name: 130605_Ack_IP_3.raw

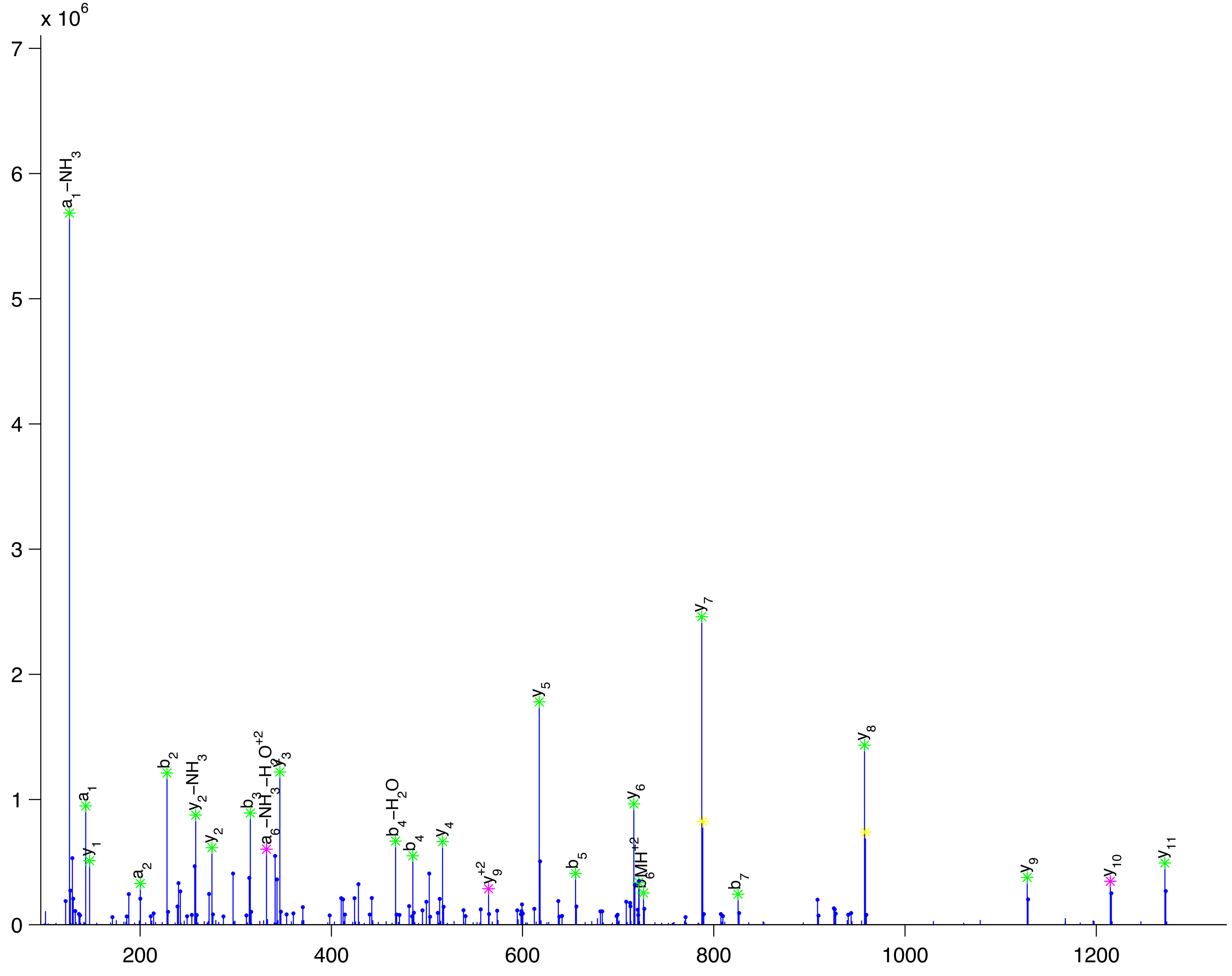

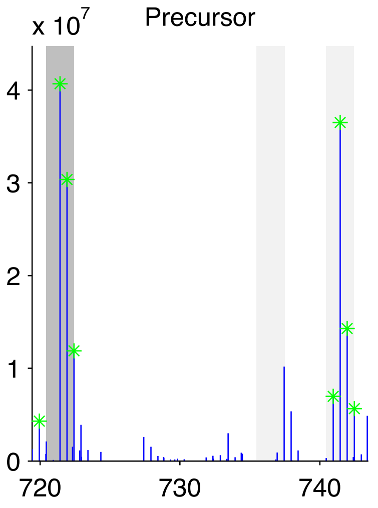

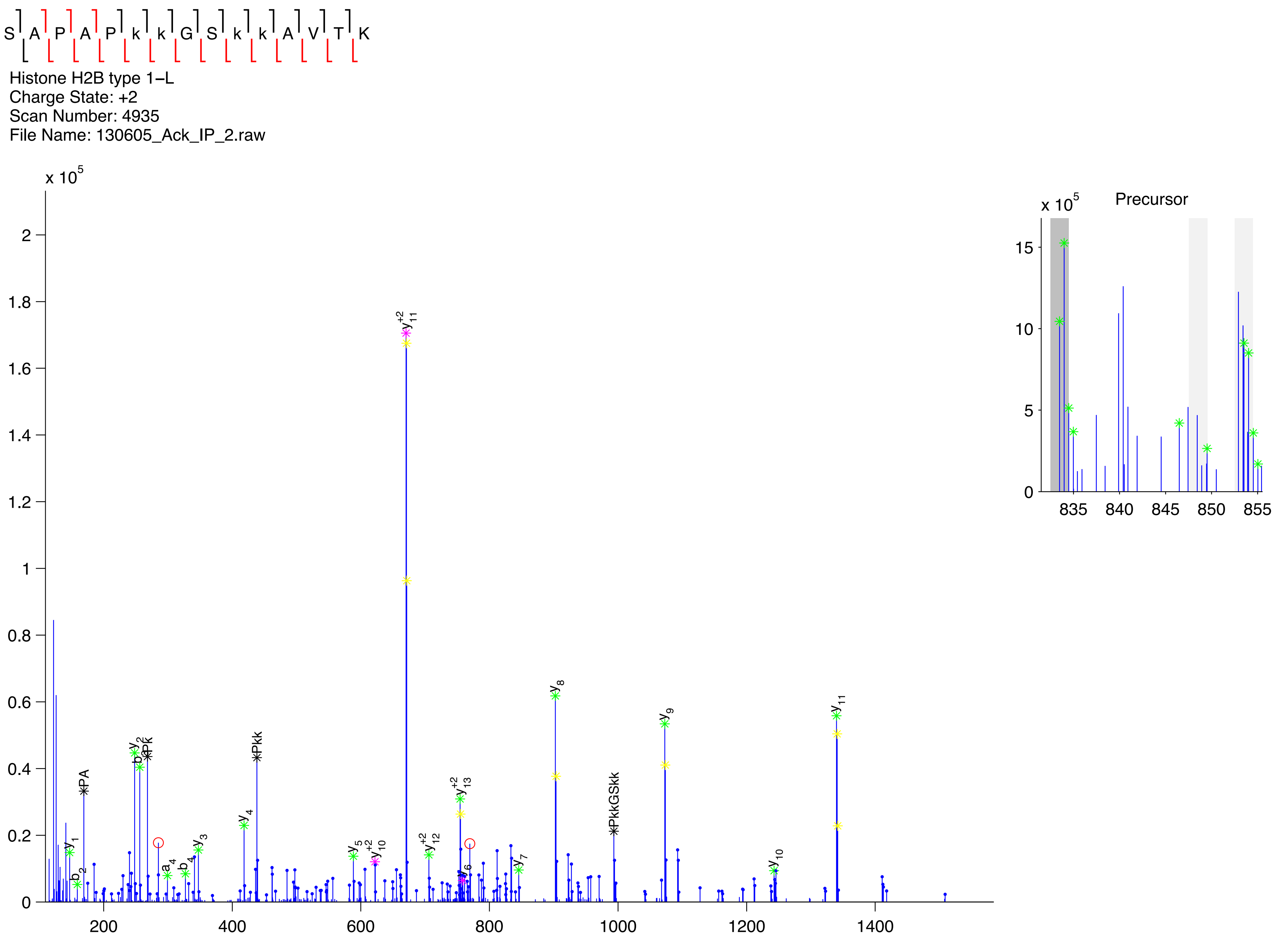

S A P A P k k G S k k A V T K
Histone H2B type 1–L
Charge State: +2
Scan Number: 4935
File Name: 130605_Ack_IP_2.raw
x 10^5
y1
b2
PA
y2
b2
Pk
a4
b4
y3
y4
Pkk
y5
y+2 10
y+2 11
y+2 12
y+2 13
y6
y7
y8
PkkGSkk
y9
y10
y11
Precursor

S A P A P k k G S k k A V T K

Histone H2B type 1–L
Charge State: +2
Scan Number: 5188
File Name: 130605_Ack_IP_3.raw

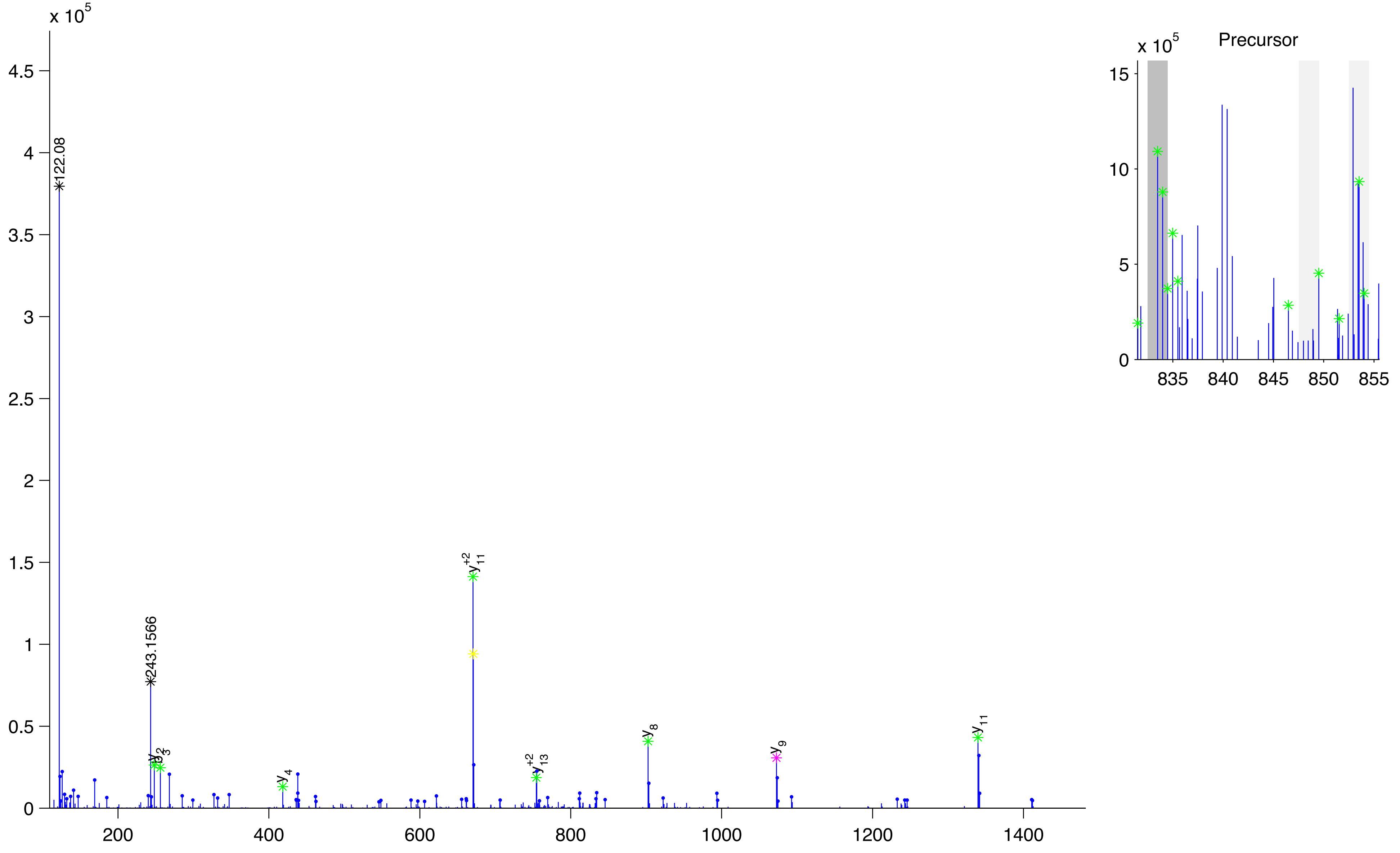

k A V T k A Q K

Histone H2B type 1–O
Charge State: +2
Scan Number: 3002
File Name: 130605_Ack_IP_1.raw

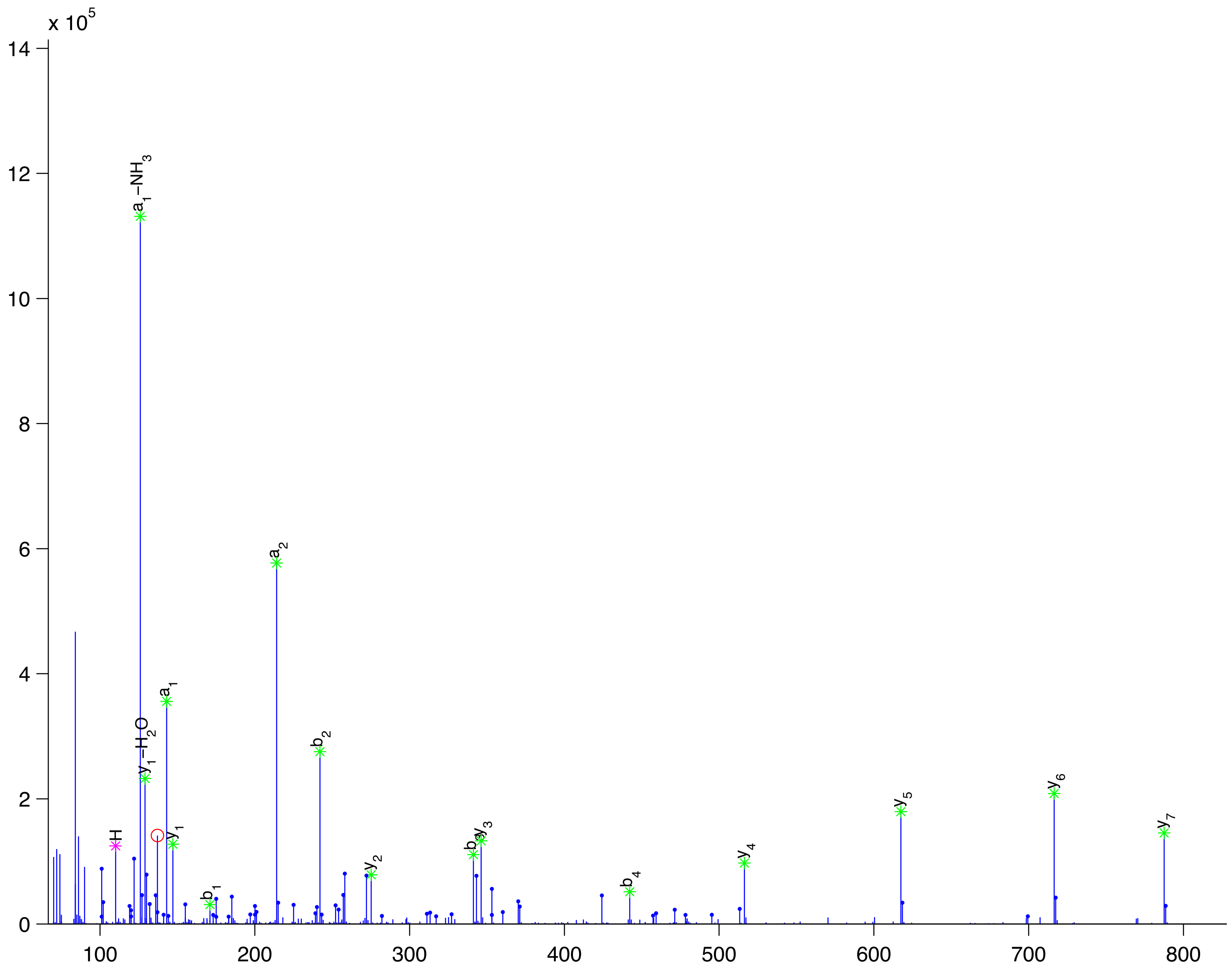

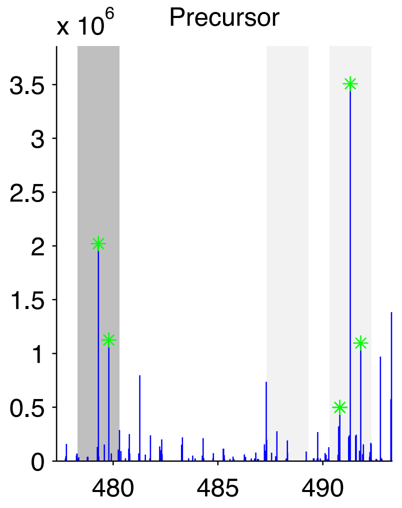

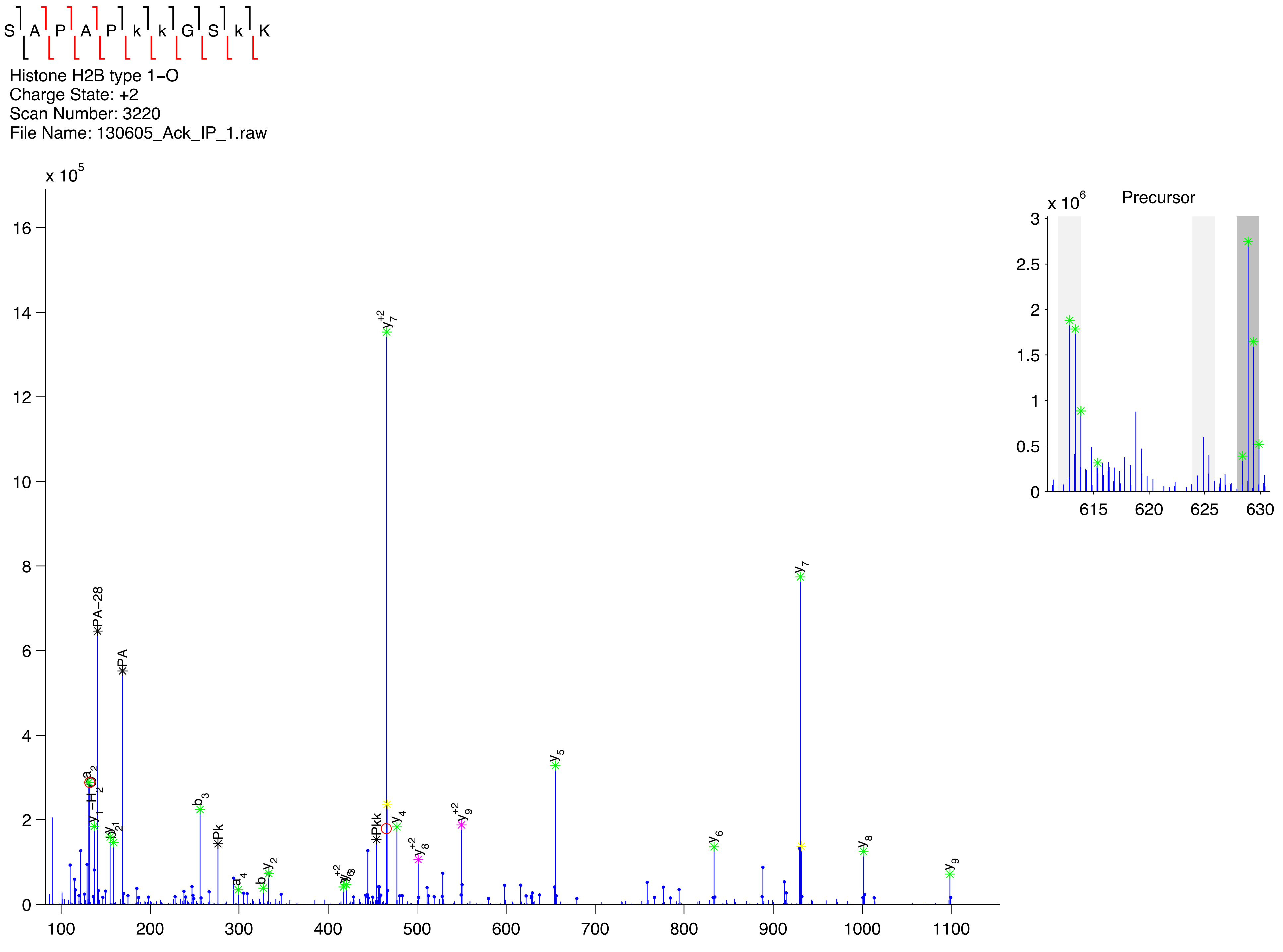

S A P A P k k G S k K
Histone H2B type 1–O
Charge State: +2
Scan Number: 3220
File Name: 130605_Ack_IP_1.raw
x 10^5
PA–28
PA
Pk
Pkk
a2
y1–H2
y2
b2
b3
a4
b
y2
y7 +2
y4
y8 +2
y9 +2
y5
y6
y7
y8
y9
x 10^6
Precursor

S A P A P k k G S k K

Histone H2B type 1–O
Charge State: +2
Scan Number: 3222
File Name: 130605_Ack_IP_1.raw

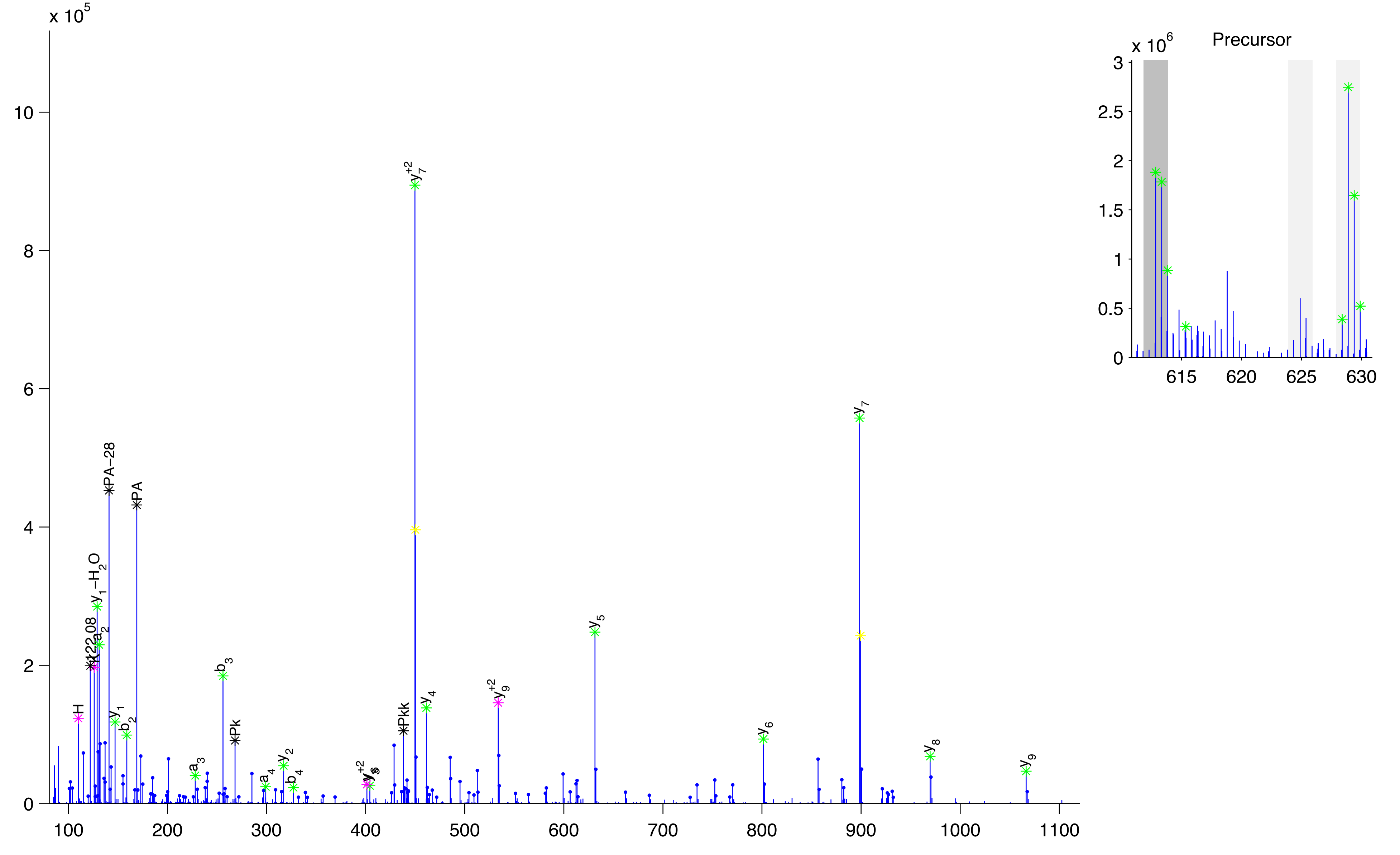

P D P A k S A P A P K

Histone H2B type 1–O
Charge State: +2
Scan Number: 3420
File Name: 130605_Ack_IP_1.raw

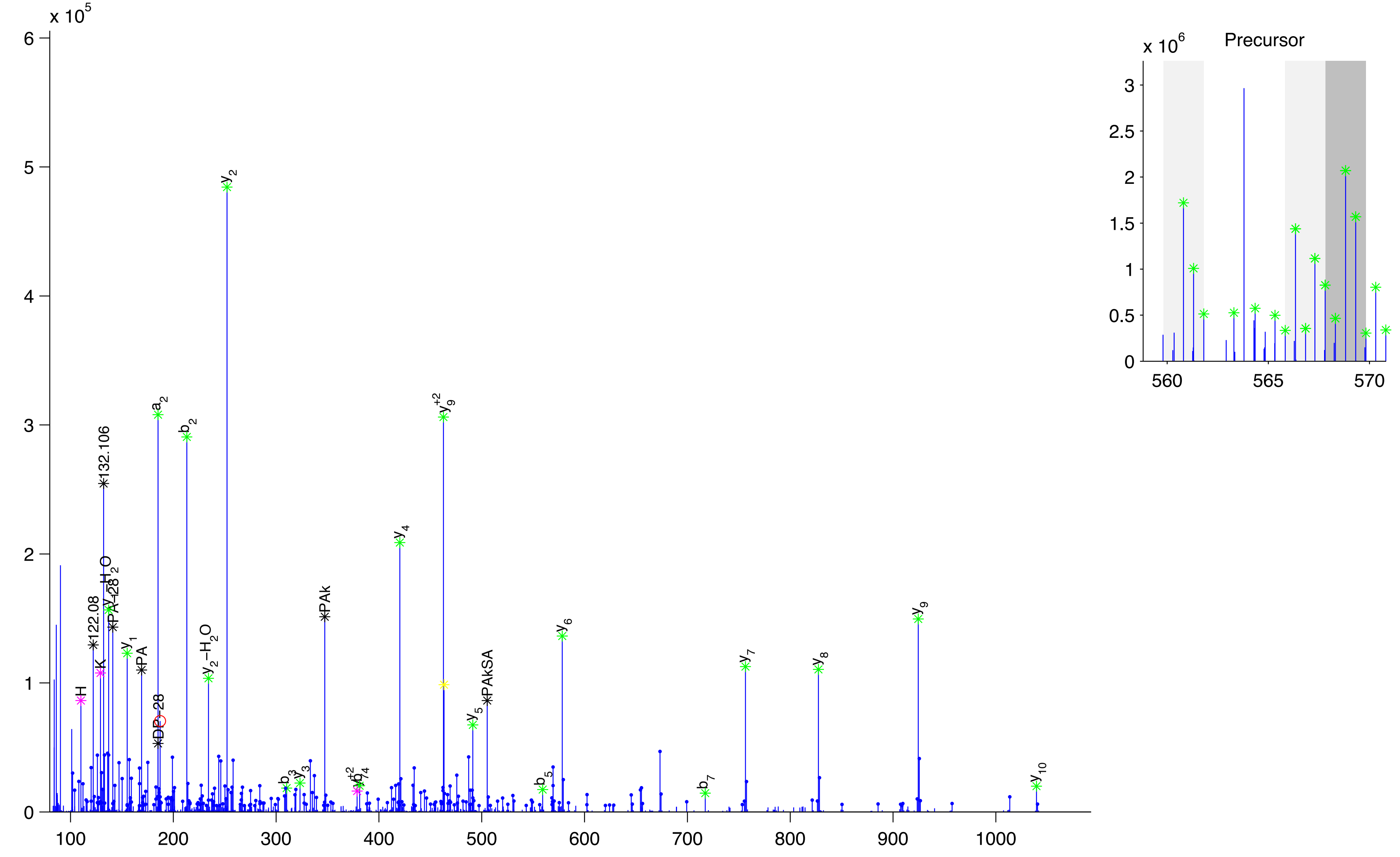

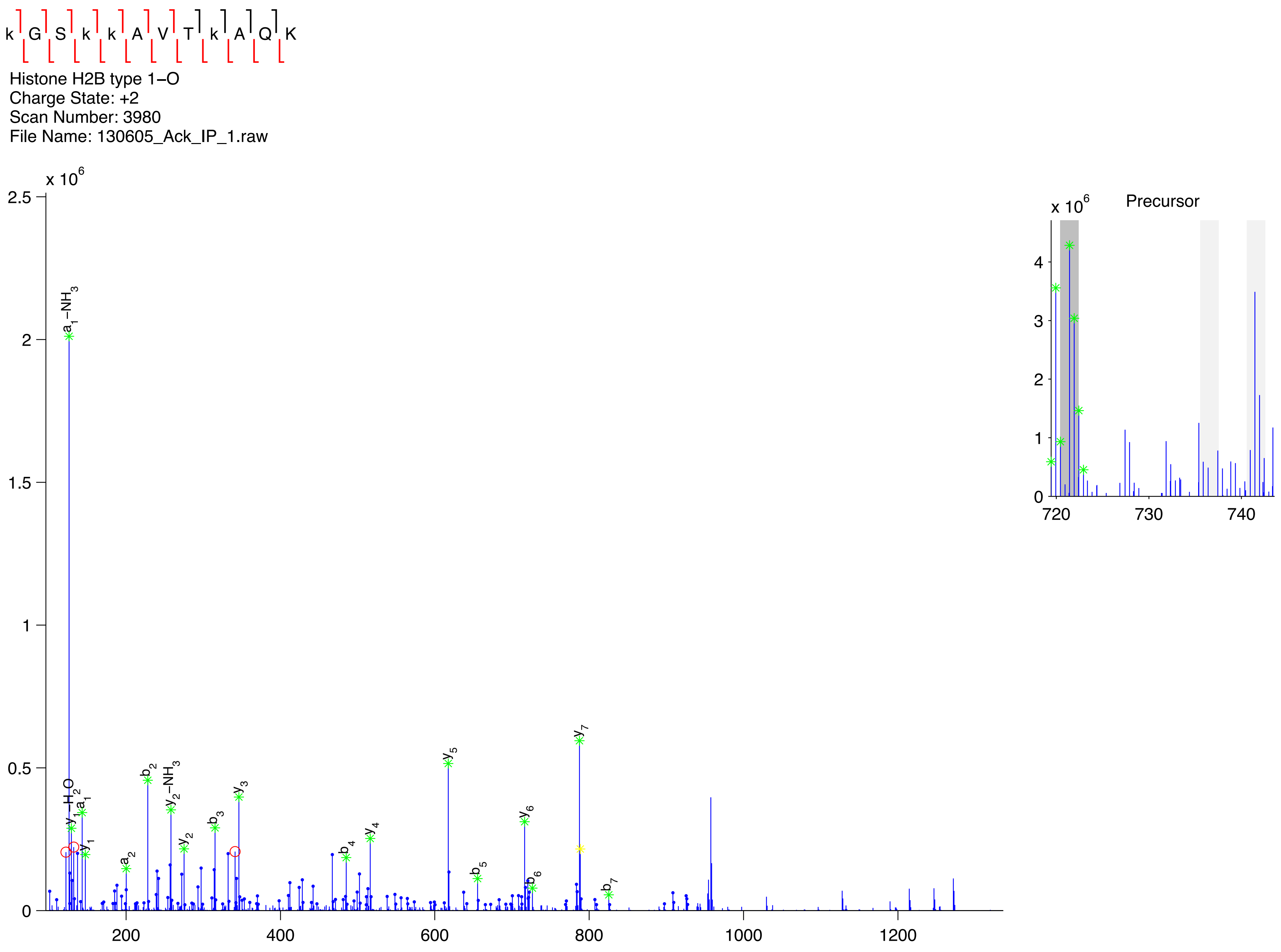
k G S k k A V T k A Q K
Histone H2B type 1–O
Charge State: +2
Scan Number: 3980
File Name: 130605_Ack_IP_1.raw
x 10^6
a1–NH3
y1–H2O
a1
y1
a2
b2
y2–NH3
y2
b3
y3
b4
y4
y5
b5
y6
b6
y7
b7
Precursor
x 10^6

k G S k k A V T k A Q K

Histone H2B type 1–O
Charge State: +2
Scan Number: 4121
File Name: 130605_Ack_IP_1.raw

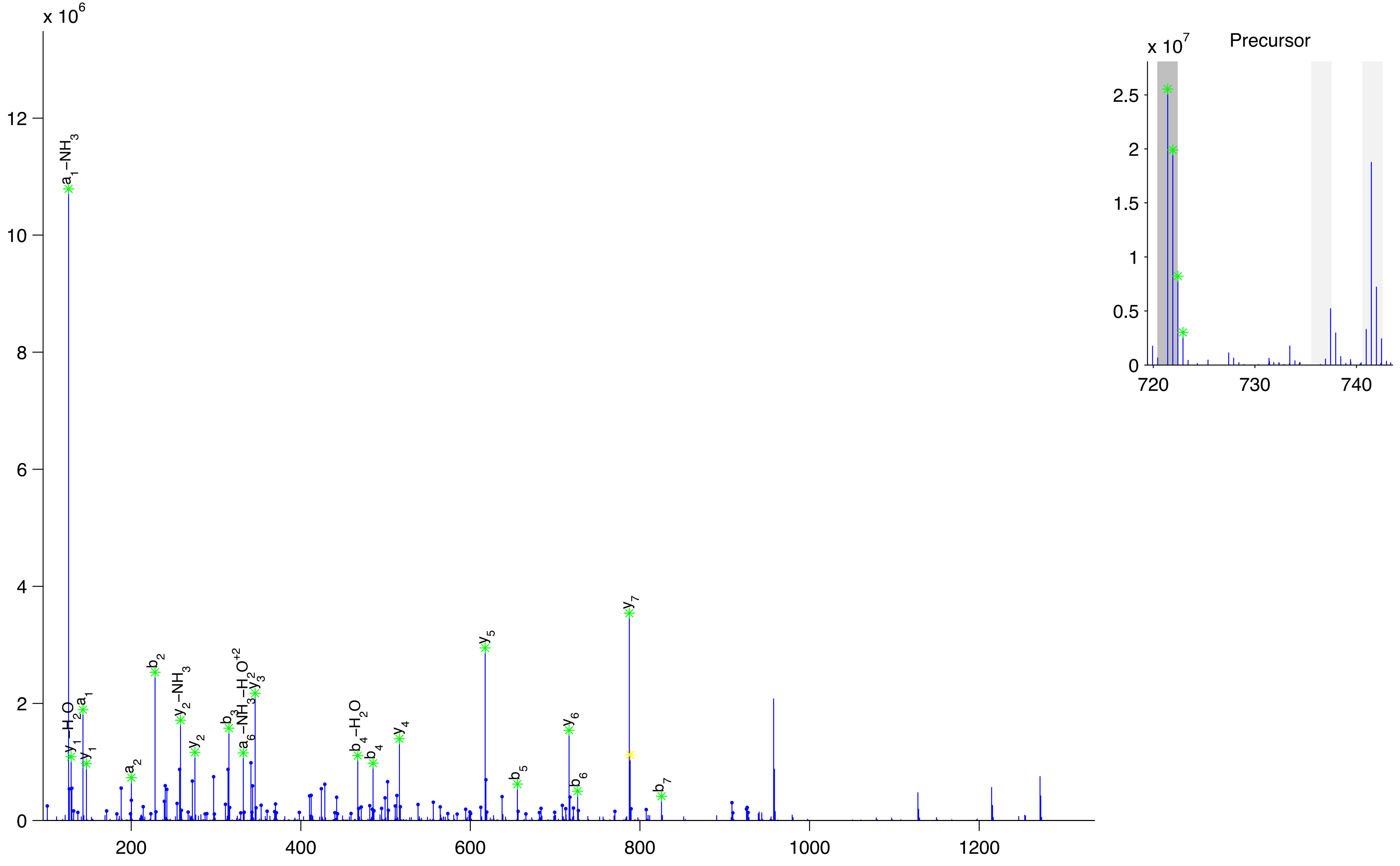

S A P A P k k G S k k A V T K

Histone H2B type 1–O
Charge State: +2
Scan Number: 4768
File Name: 130605_Ack_IP_1.raw

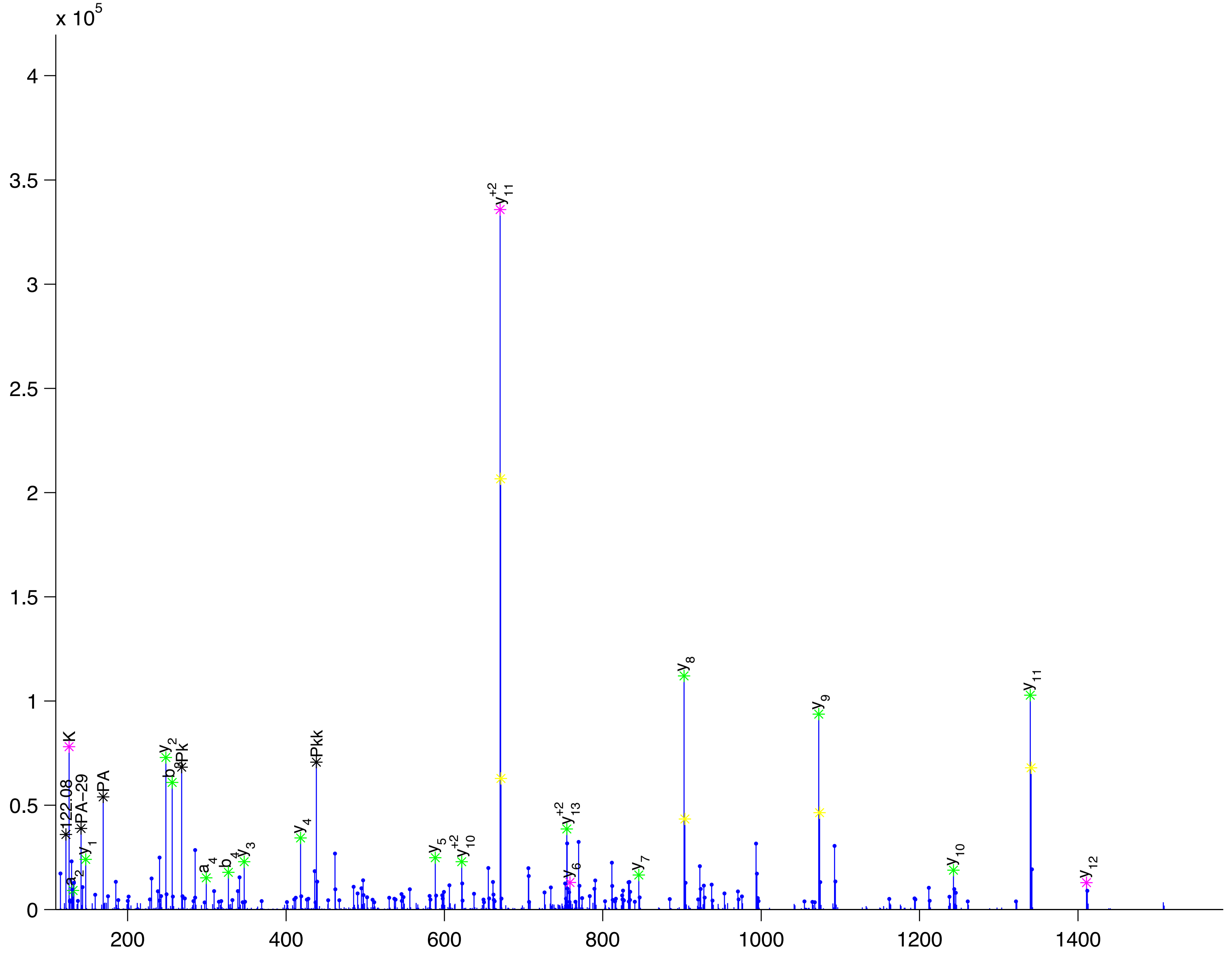

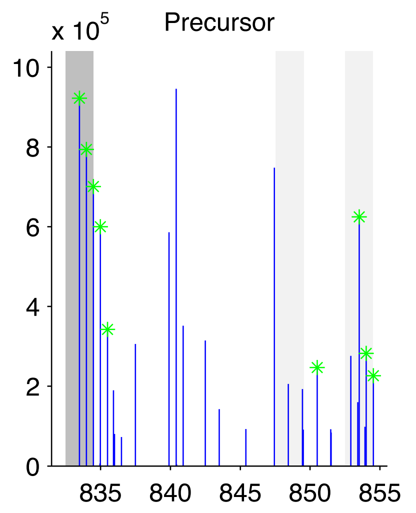

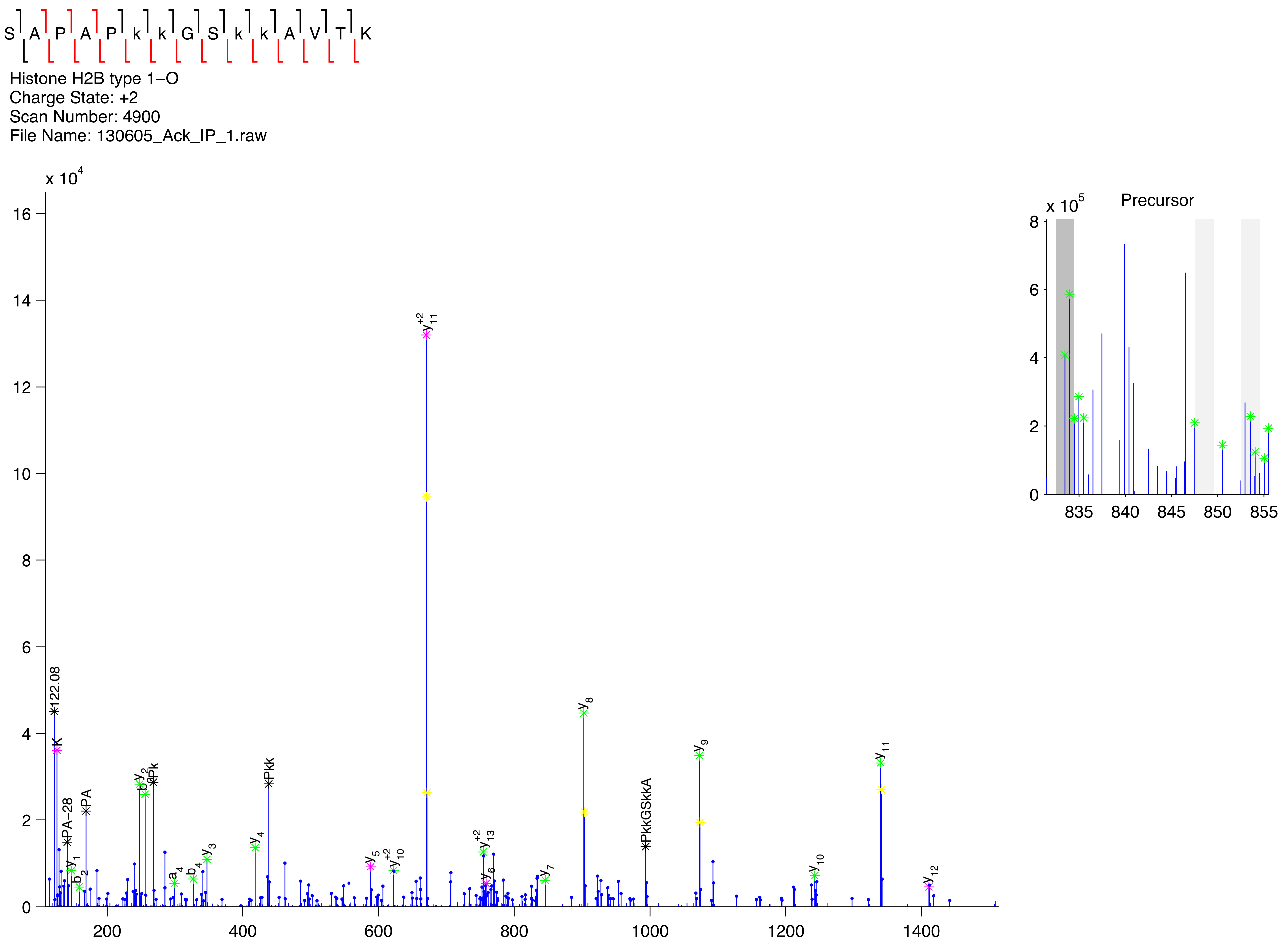

S A P A P k k G S k k A V T K
Histone H2B type 1–O
Charge State: +2
Scan Number: 4900
File Name: 130605_Ack_IP_1.raw
x 10^4
122.08
K
PA–28
y1
b2
PA
y2
b2
Pk
a4
b4
y3
y4
Pkk
y5
y10 +2
y11 +2
y13 +2
y6
y7
y8
PkkGSkkA
y9
y10
y11
y12
Precursor
x 10^5

S A P A P k k G S k k A V T k A Q K

Histone H2B type 1–O
Charge State: +2
Scan Number: 6066
File Name: 130605_Ack_IP_1.raw

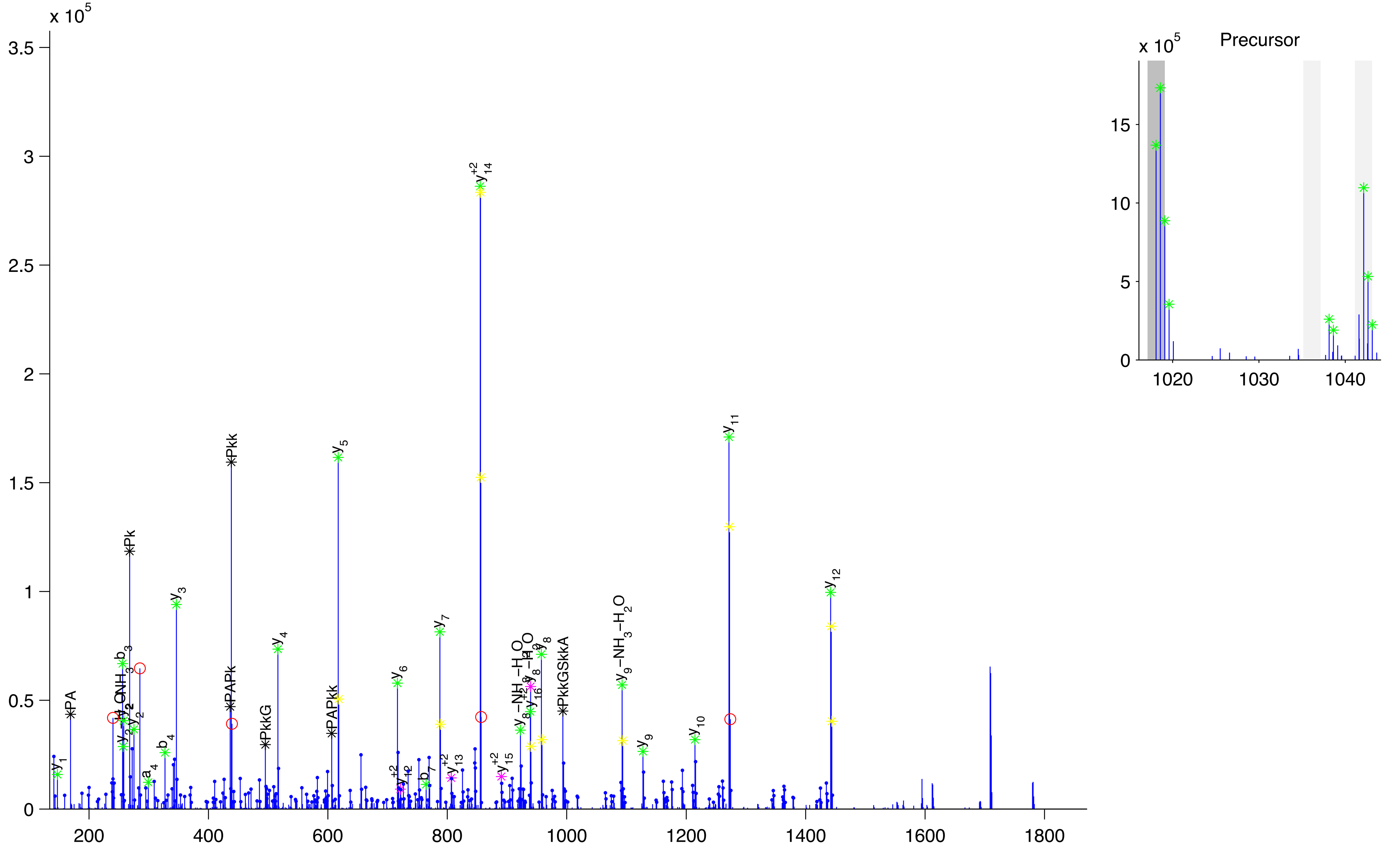

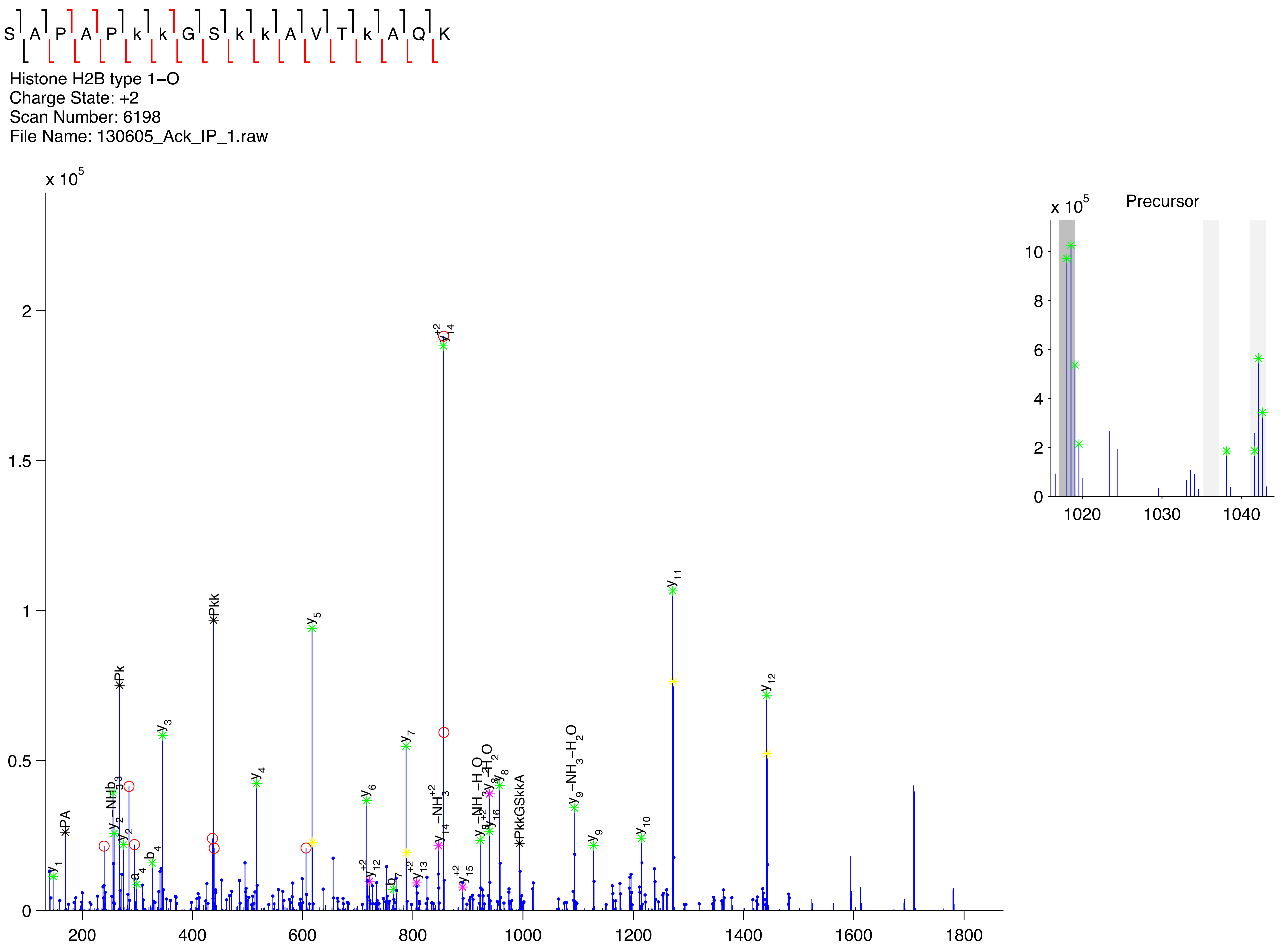
S A P A P k k G S k k A V T k A Q K
Histone H2B type 1–O
Charge State: +2
Scan Number: 6198
File Name: 130605_Ack_IP_1.raw
x 10^5
Precursor

L L L P G E L A k H A V S E G T K

Histone H2B type 1–O
Charge State: +3
Scan Number: 12656
File Name: 130605_Ack_IP_1.raw

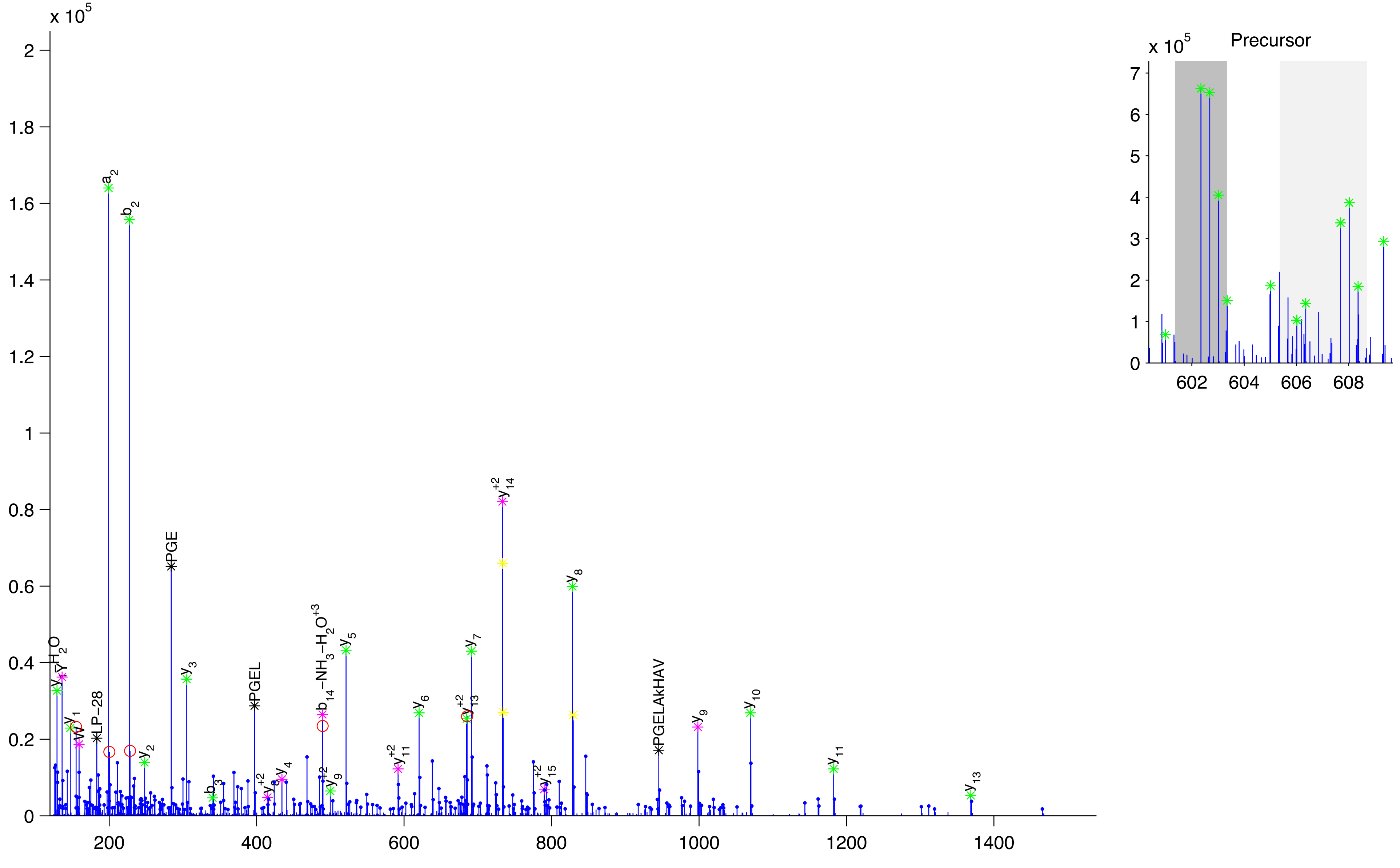

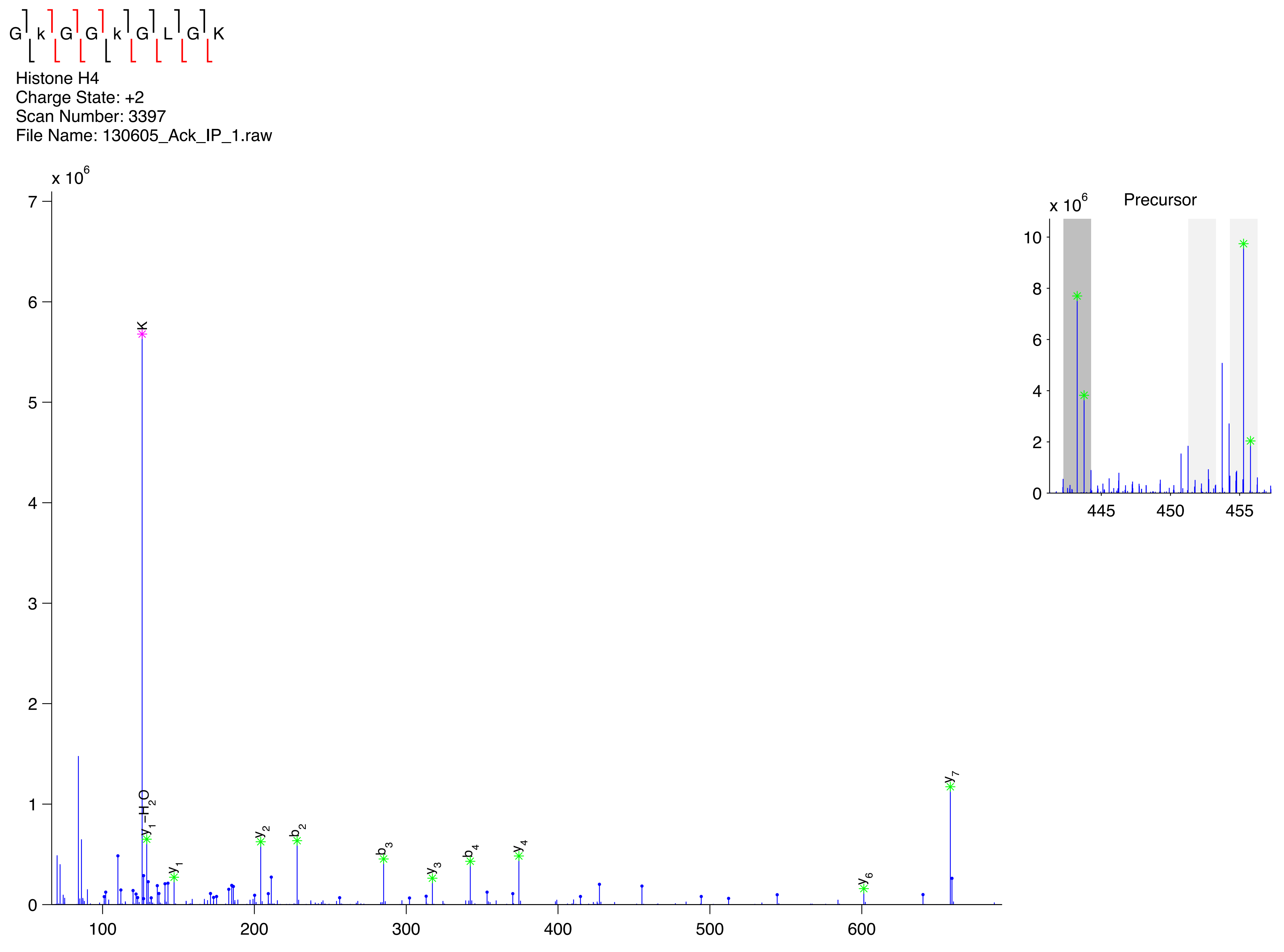

G k G G k G L G K
Histone H4
Charge State: +2
Scan Number: 3397
File Name: 130605_Ack_IP_1.raw
x 10^6
K
y1-H2O
y1
y2
b2
b3
y3
b4
y4
y6
y7
Precursor

G k G G k G L G K
Histone H4
Charge State: +2
Scan Number: 3416
File Name: 130605_Ack_IP_2.raw

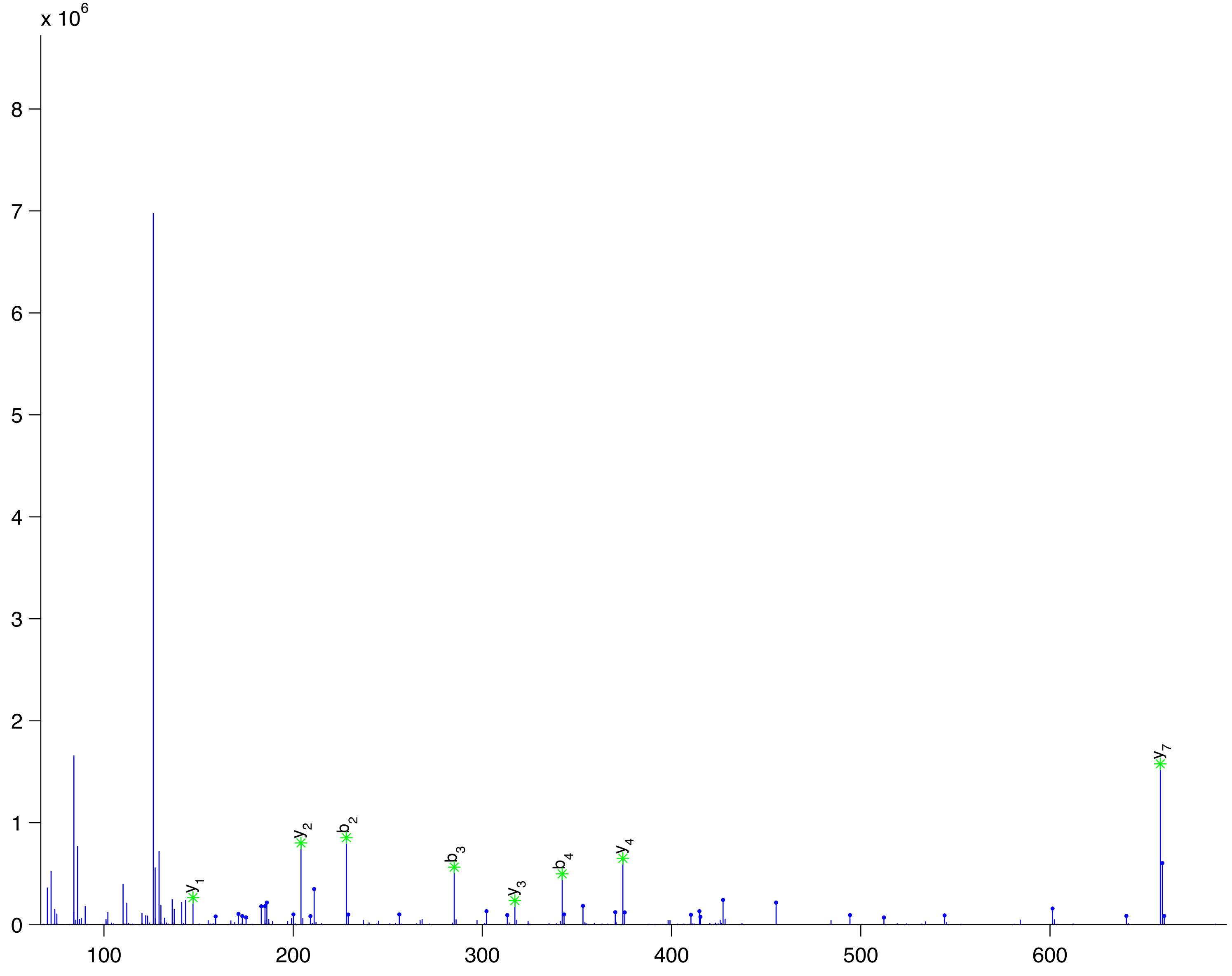

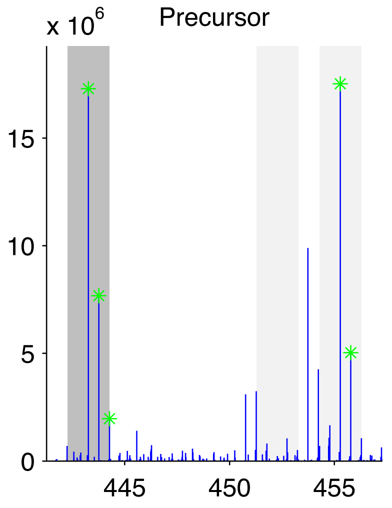

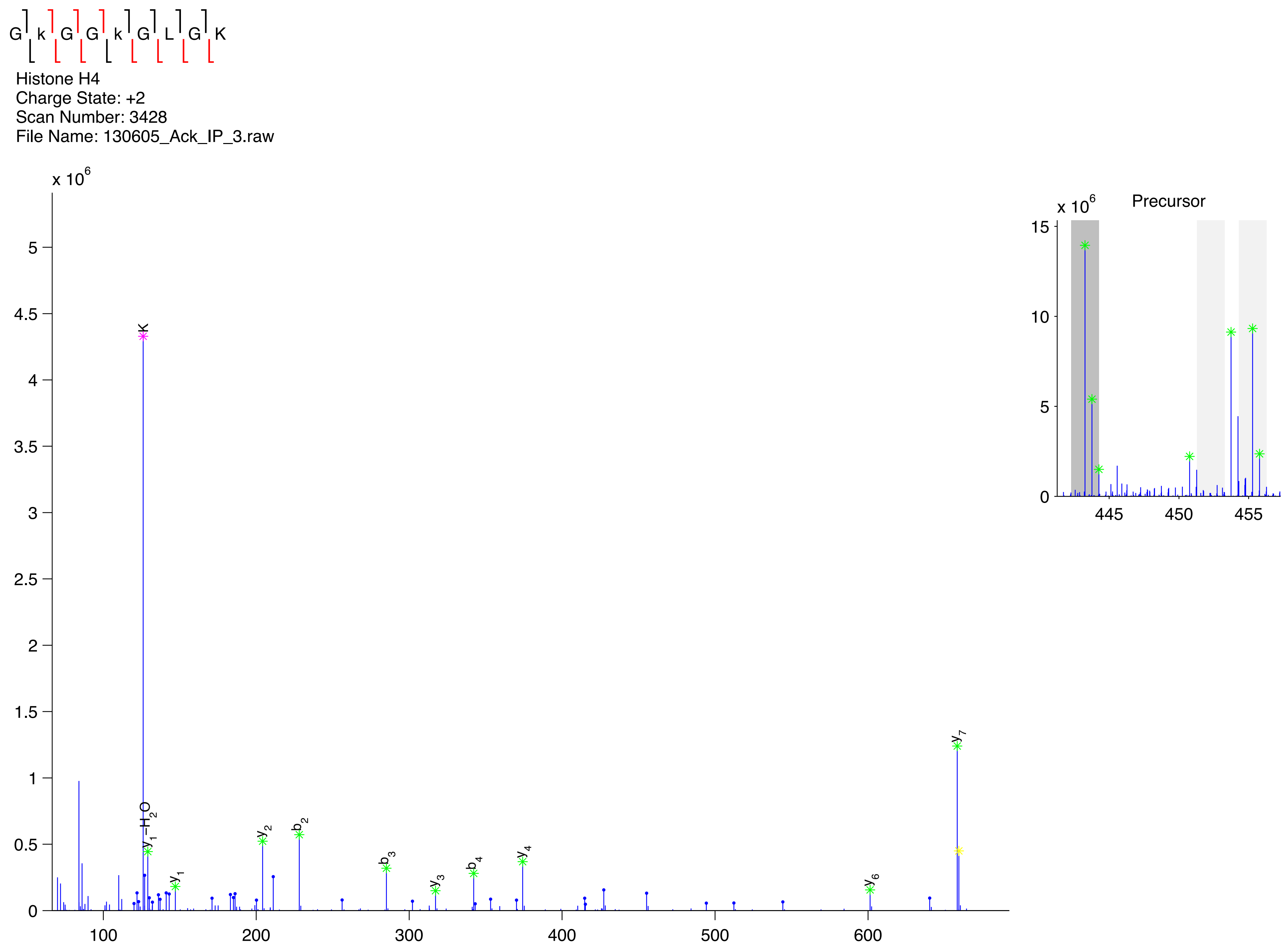

G k G G k G L G K
Histone H4
Charge State: +2
Scan Number: 3428
File Name: 130605_Ack_IP_3.raw
x 10^6
K
y1−H2O
y1
y2
b2
b3
y3
b4
y4
y6
y7
Precursor

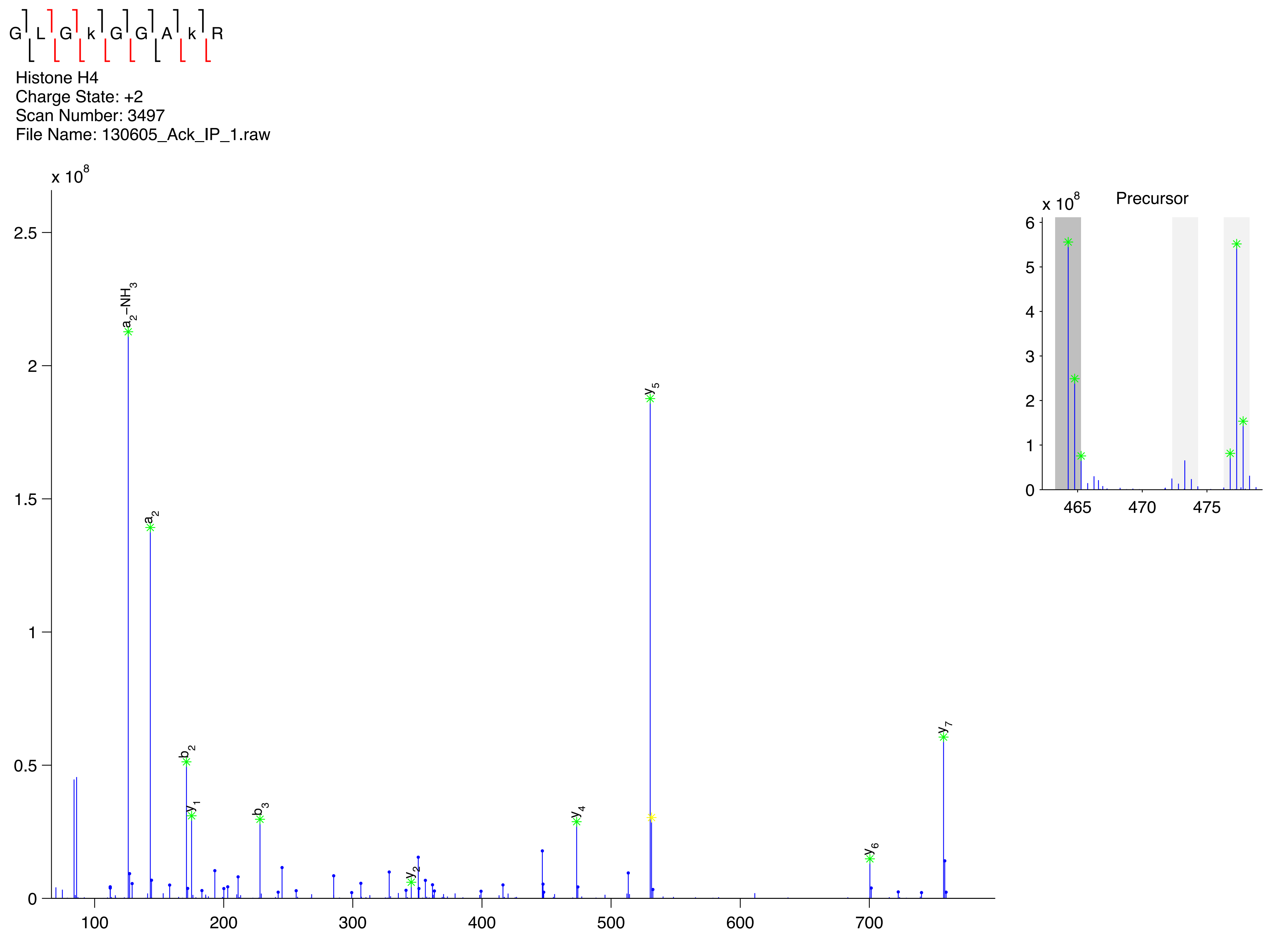
G L G k G G A k R
Histone H4
Charge State: +2
Scan Number: 3497
File Name: 130605_Ack_IP_1.raw
x 10^8
a2–NH3
a2
b2
y1
b3
y2
y4
y5
y6
y7
Precursor

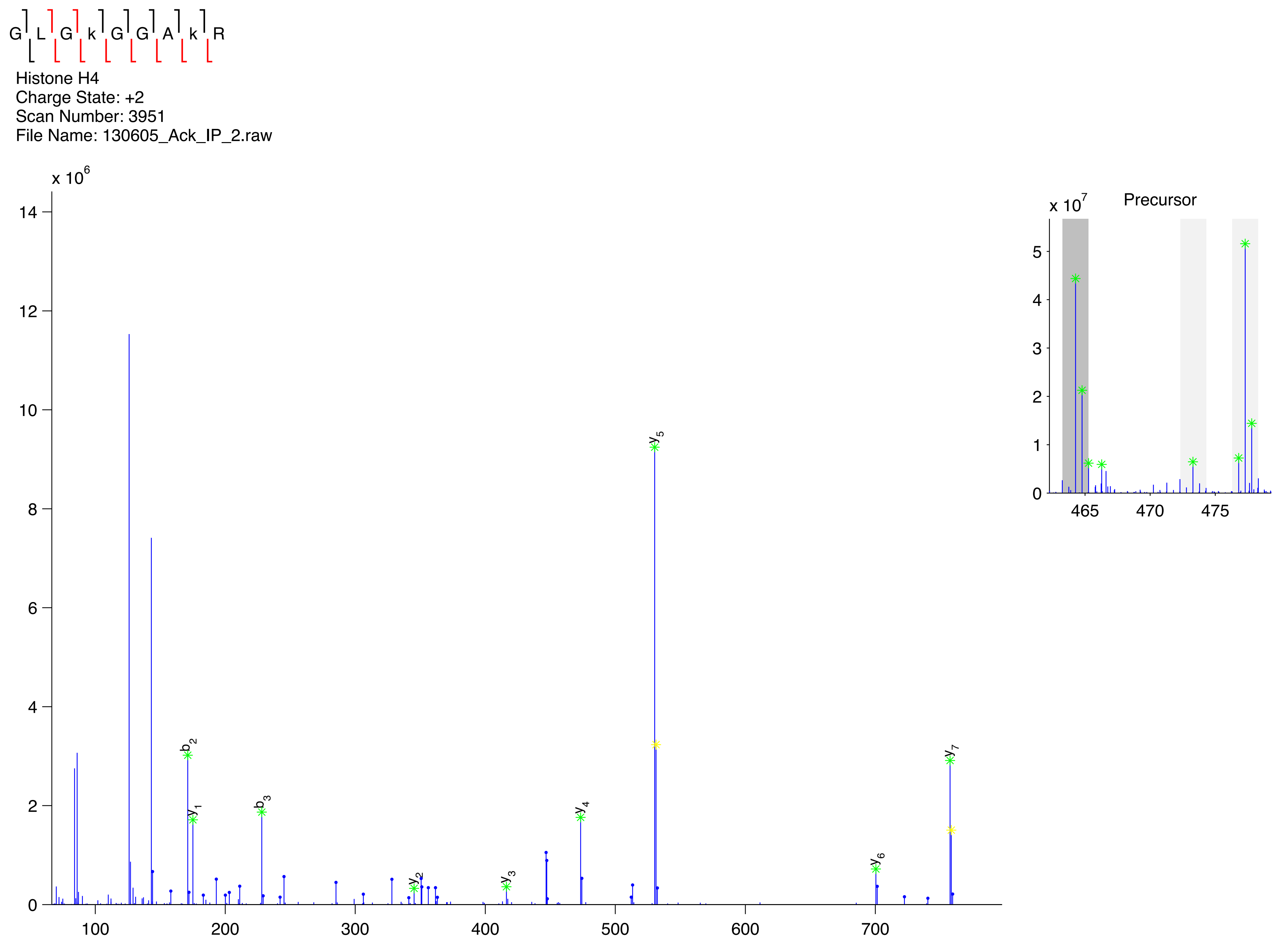
G L G k G G A k R
Histone H4
Charge State: +2
Scan Number: 3951
File Name: 130605_Ack_IP_2.raw
x 10^6
b2
y1
b3
y2
y3
y4
y5
y6
y7
x 10^7
Precursor

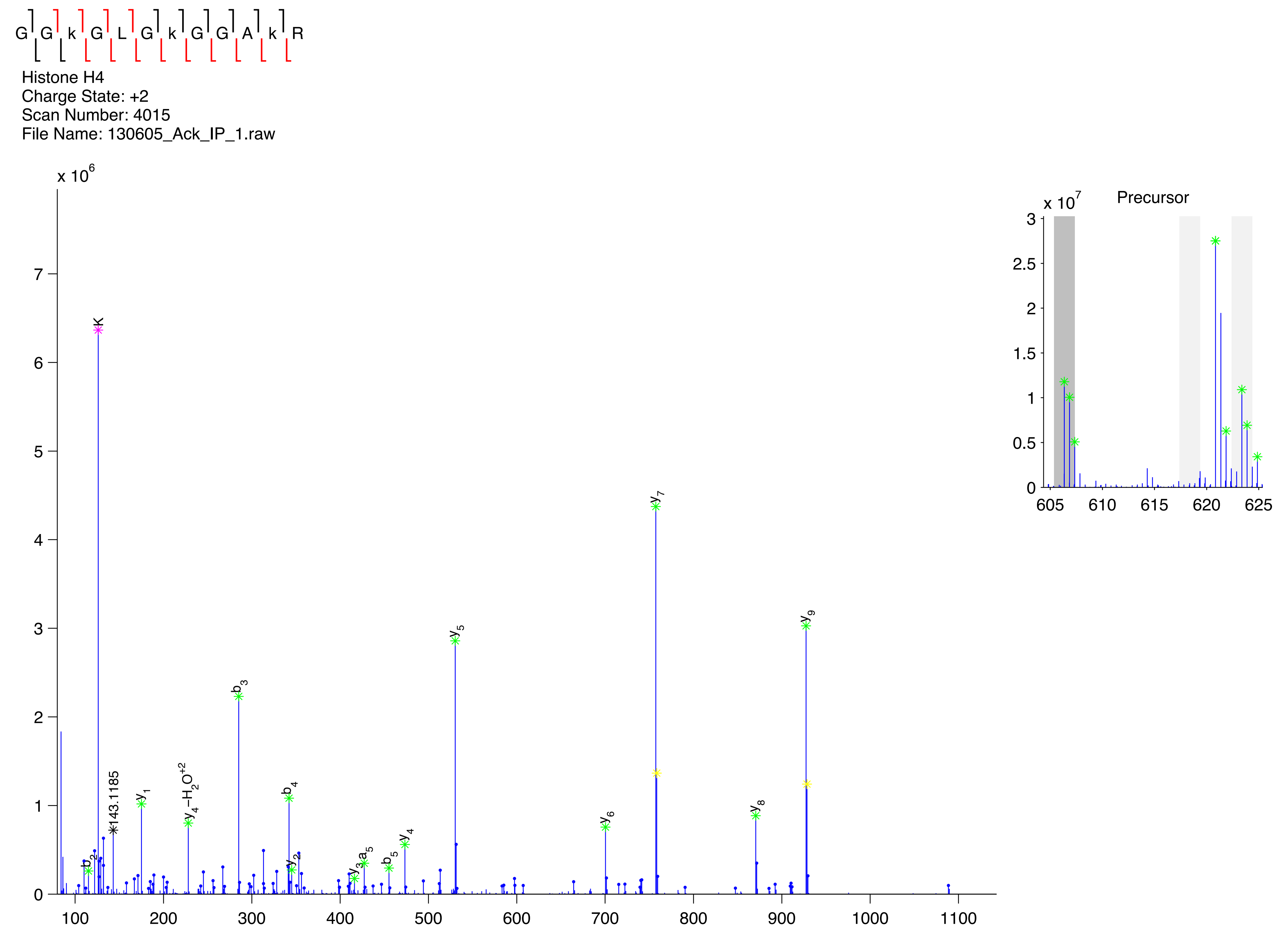
G G k G L G k G G A k R
Histone H4
Charge State: +2
Scan Number: 4015
File Name: 130605_Ack_IP_1.raw
x 10^6
K
143.1185
b2
y1
y4–H2O+2
b3
y2
b4
y3
a5
b5
y4
y5
y6
y7
y8
y9
x 10^7
Precursor

GkGGkGLGkGGAK

Histone H4
Charge State: +2
Scan Number: 4094
File Name: 130605_Ack_IP_2.raw

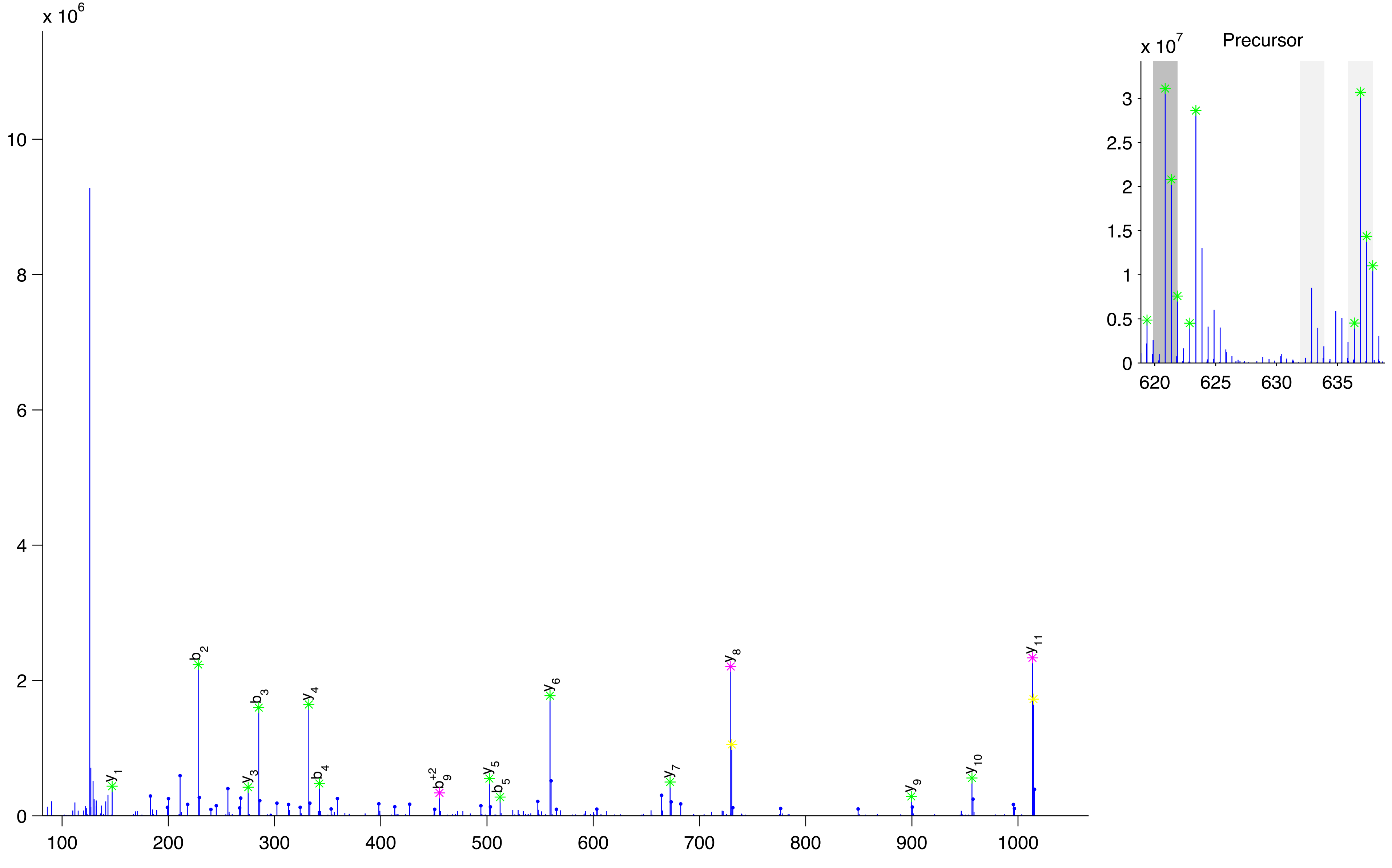

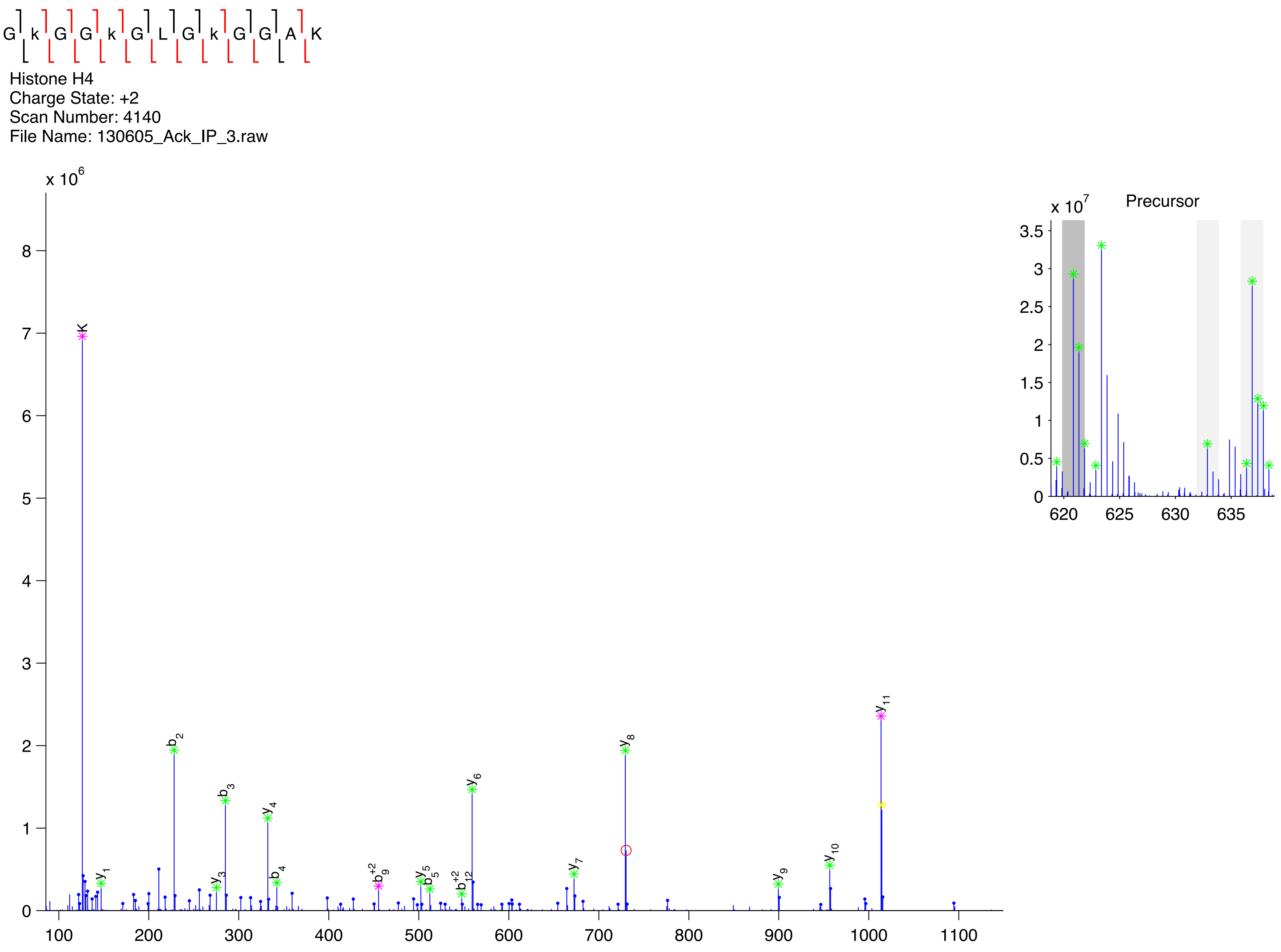
G k G G k G L G k G G A K
Histone H4
Charge State: +2
Scan Number: 4140
File Name: 130605_Ack_IP_3.raw
x 10^6
K
y1
b2
y3
b3
y4
b4
b9 +2
y5
b5
b12 +2
y6
y7
y8
y9
y10
y11
0
1
2
3
4
5
6
7
8
100
200
300
400
500
600
700
800
900
1000
1100
x 10^7
Precursor
0
0.5
1
1.5
2
2.5
3
3.5
620
625
630
635

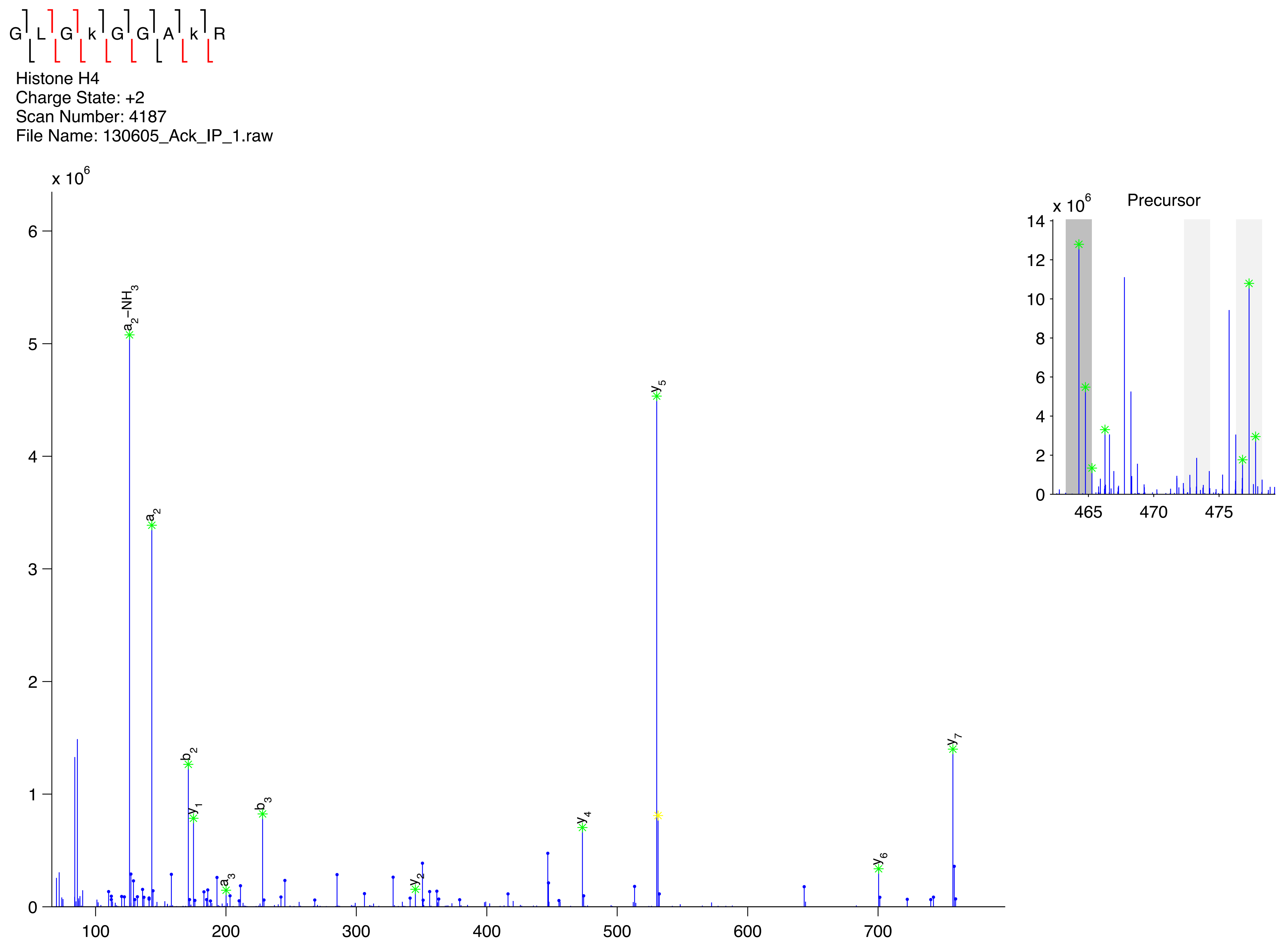
G L G k G G A k R
Histone H4
Charge State: +2
Scan Number: 4187
File Name: 130605_Ack_IP_1.raw
x 10^6
a_2–NH_3
a_2
b_2
y_1
a_3
b_3
y_2
y_4
y_5
y_6
y_7
Precursor
x 10^6

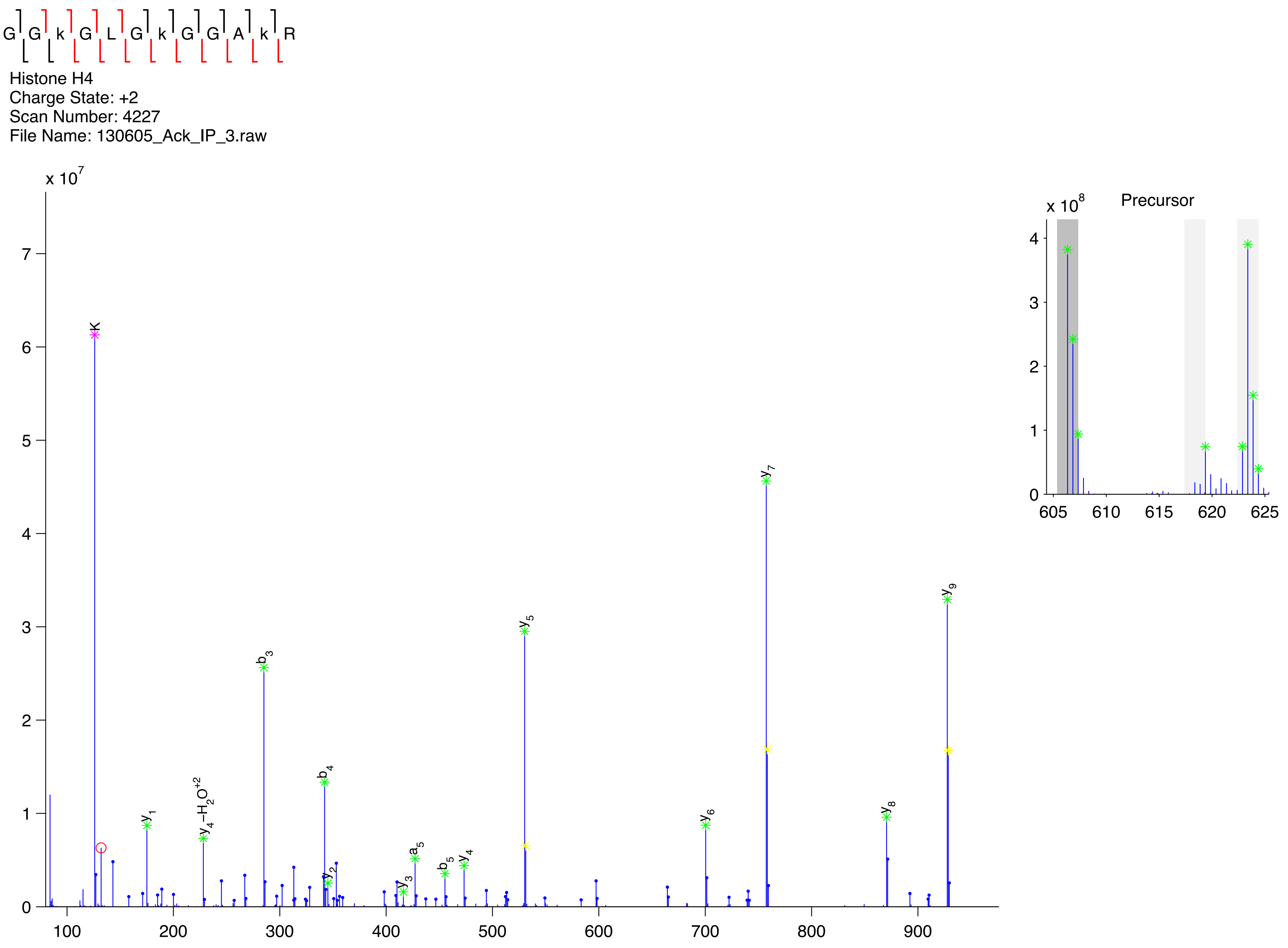
G G k G L G k G G A k R
Histone H4
Charge State: +2
Scan Number: 4227
File Name: 130605_Ack_IP_3.raw
x 10^7
K
y1
y4–H2O+2
b3
b4
y2
y3
a5
b5
y4
y5
y6
y7
y8
y9
x 10^8
Precursor

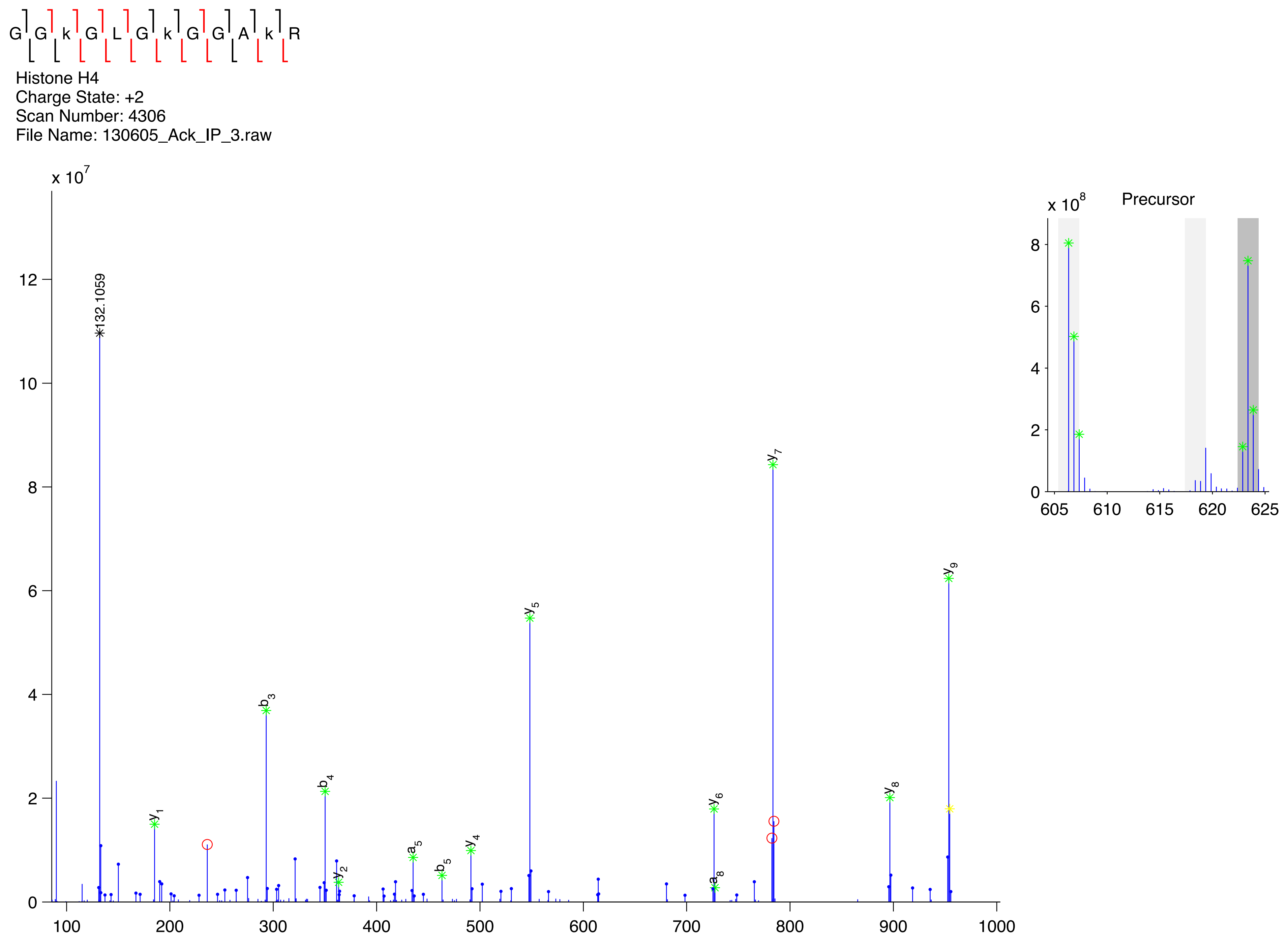

G G k G L G k G G A k R
Histone H4
Charge State: +2
Scan Number: 4306
File Name: 130605_Ack_IP_3.raw
x 10^7
132.1059
y1
b3
b4
y2
a5
b5
y4
y5
y6
a8
y7
y8
y9
x 10^8
Precursor

G G k G L G k G G A k R

Histone H4
Charge State: +2
Scan Number: 4390
File Name: 130605_Ack_IP_3.raw

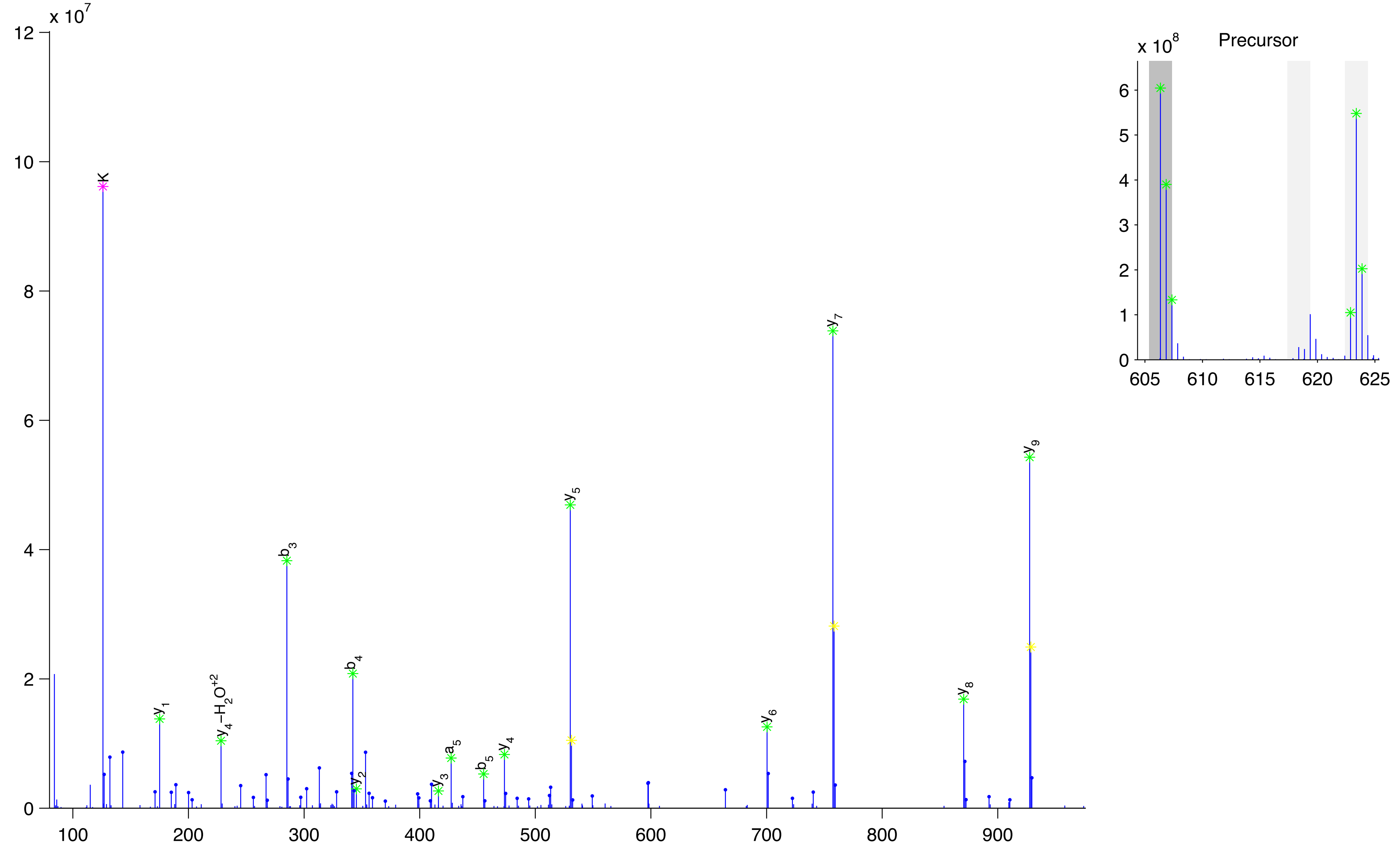

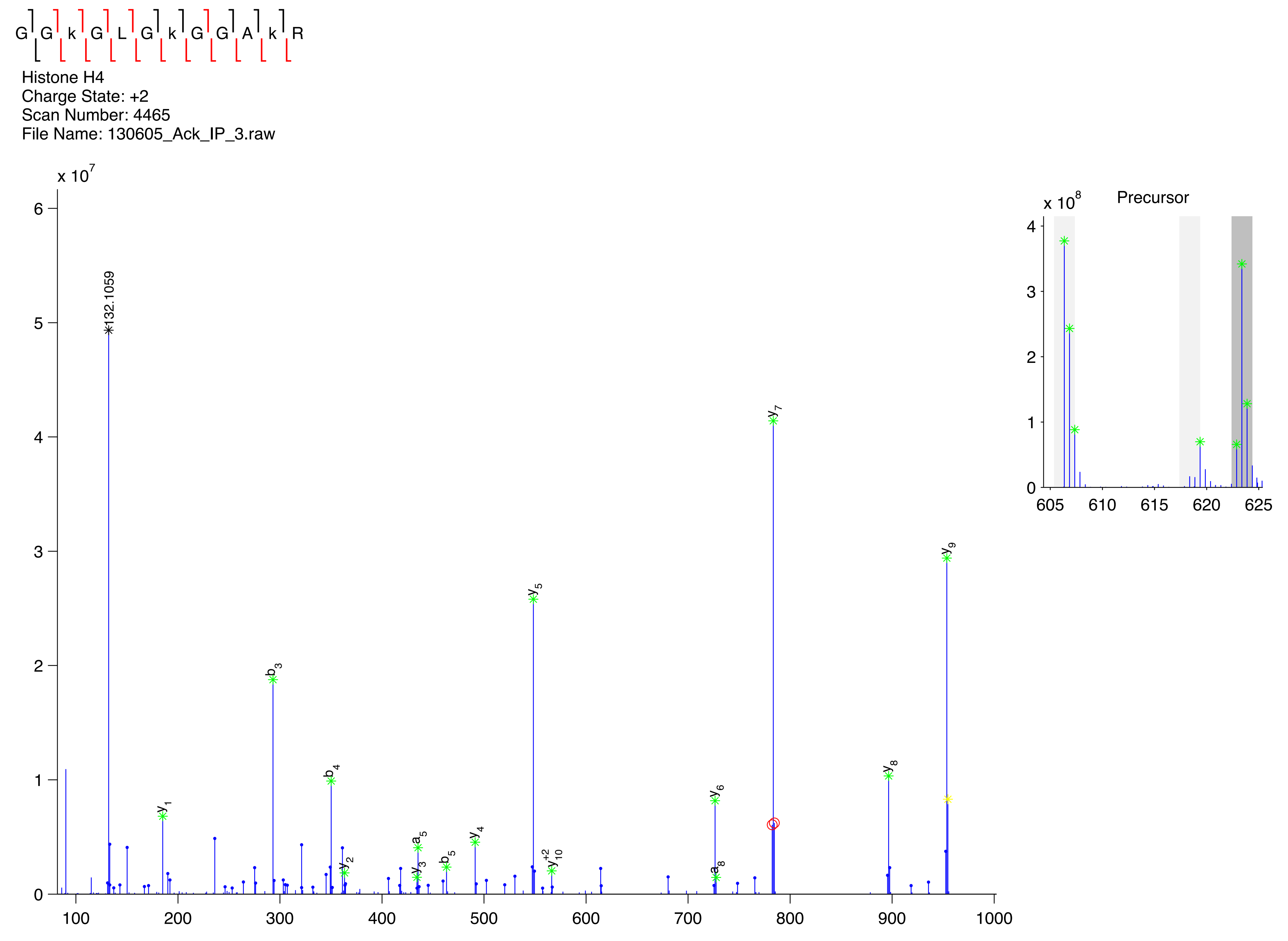

G G k G L G k G G A k R
Histone H4
Charge State: +2
Scan Number: 4465
File Name: 130605_Ack_IP_3.raw
x 10^7
132.1059
y1
b3
b4
y2
y3
a5
b5
y4
y5
y10 +2
a8
y6
y7
y8
y9
x 10^8
Precursor

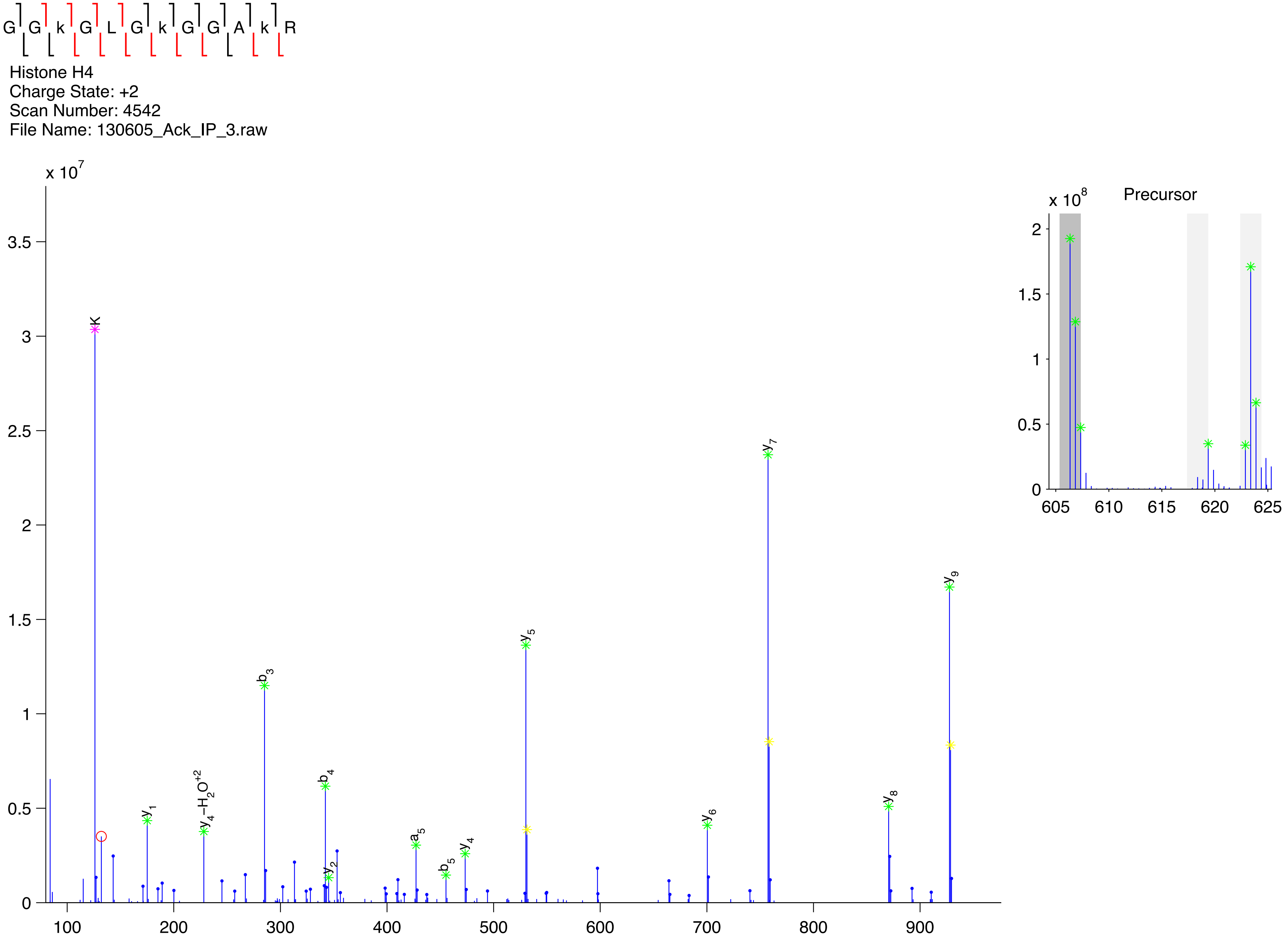
G G k G L G k G G A k R
Histone H4
Charge State: +2
Scan Number: 4542
File Name: 130605_Ack_IP_3.raw
x 10^7
K
y1
y4–H2O+2
b3
b4
y2
a5
b5
y4
y5
y6
y7
y8
y9
x 10^8
Precursor

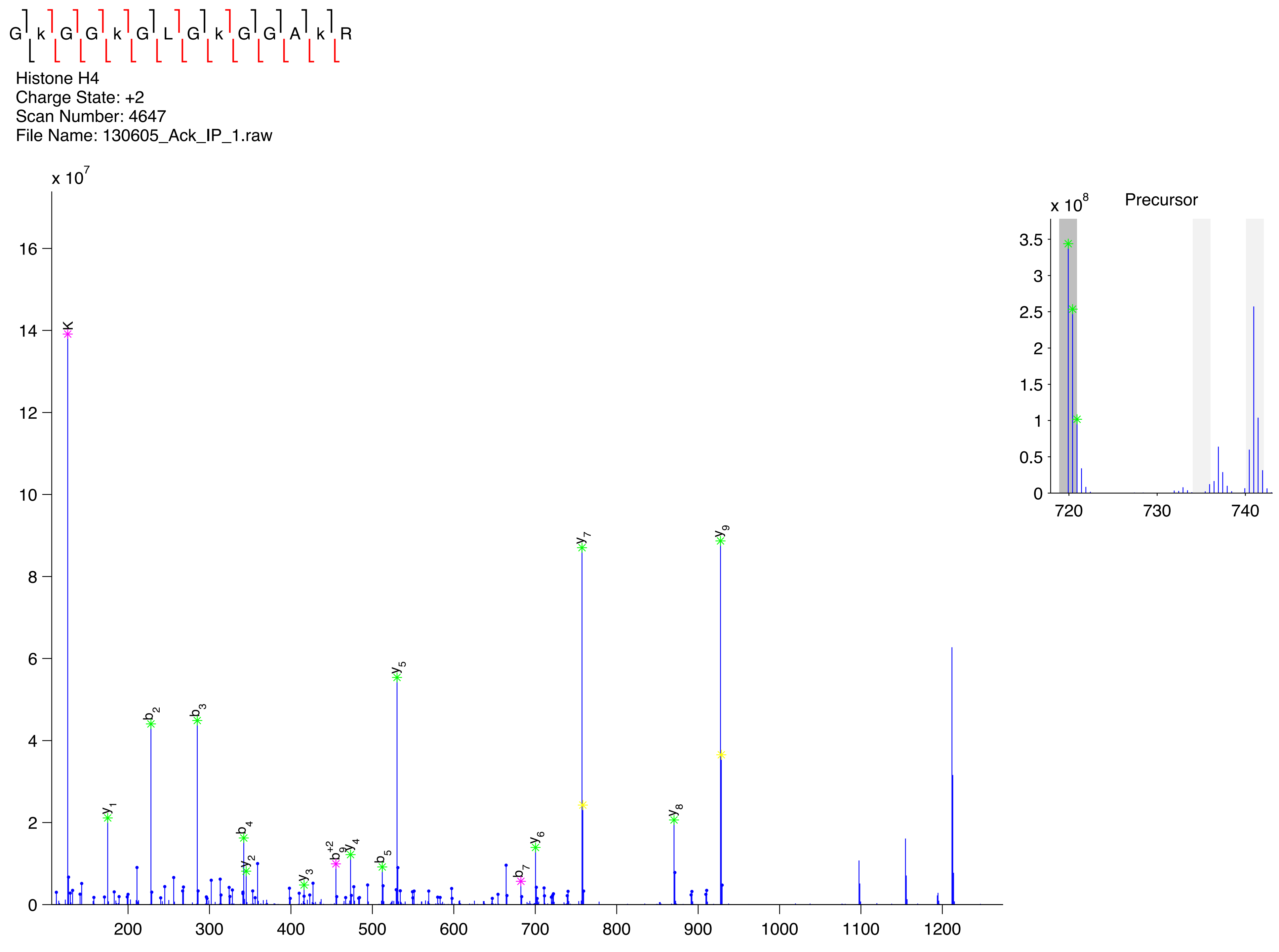

G k G G k G L G k G G A k R
Histone H4
Charge State: +2
Scan Number: 4647
File Name: 130605_Ack_IP_1.raw
x 10^7
K
y1
b2
b3
b4
y2
y3
b9 +2
y4
b5
y5
b7
y6
y7
y8
y9
x 10^8
Precursor

G G k G L G k G G A k R

Histone H4
Charge State: +2
Scan Number: 4689
File Name: 130605_Ack_IP_3.raw

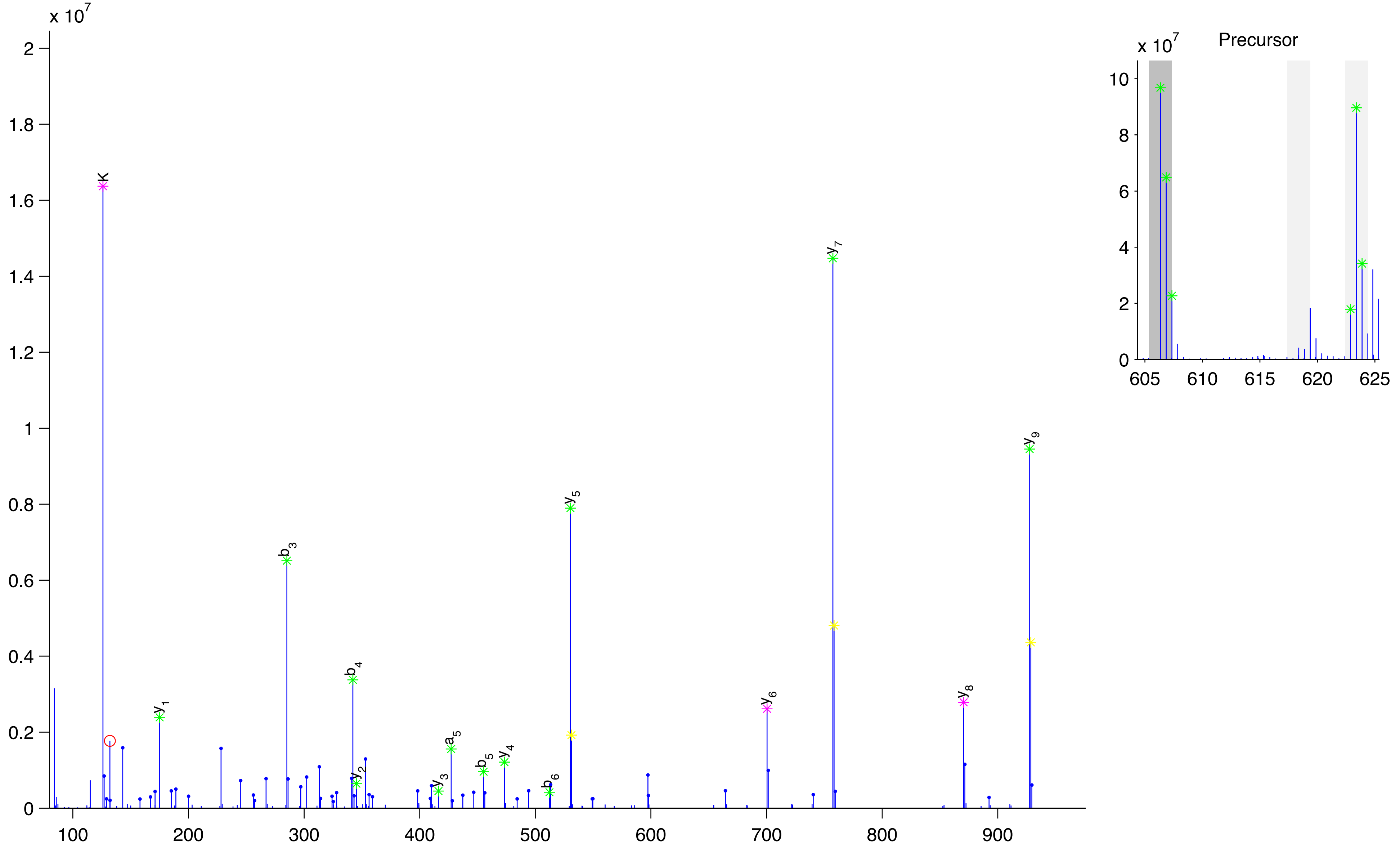

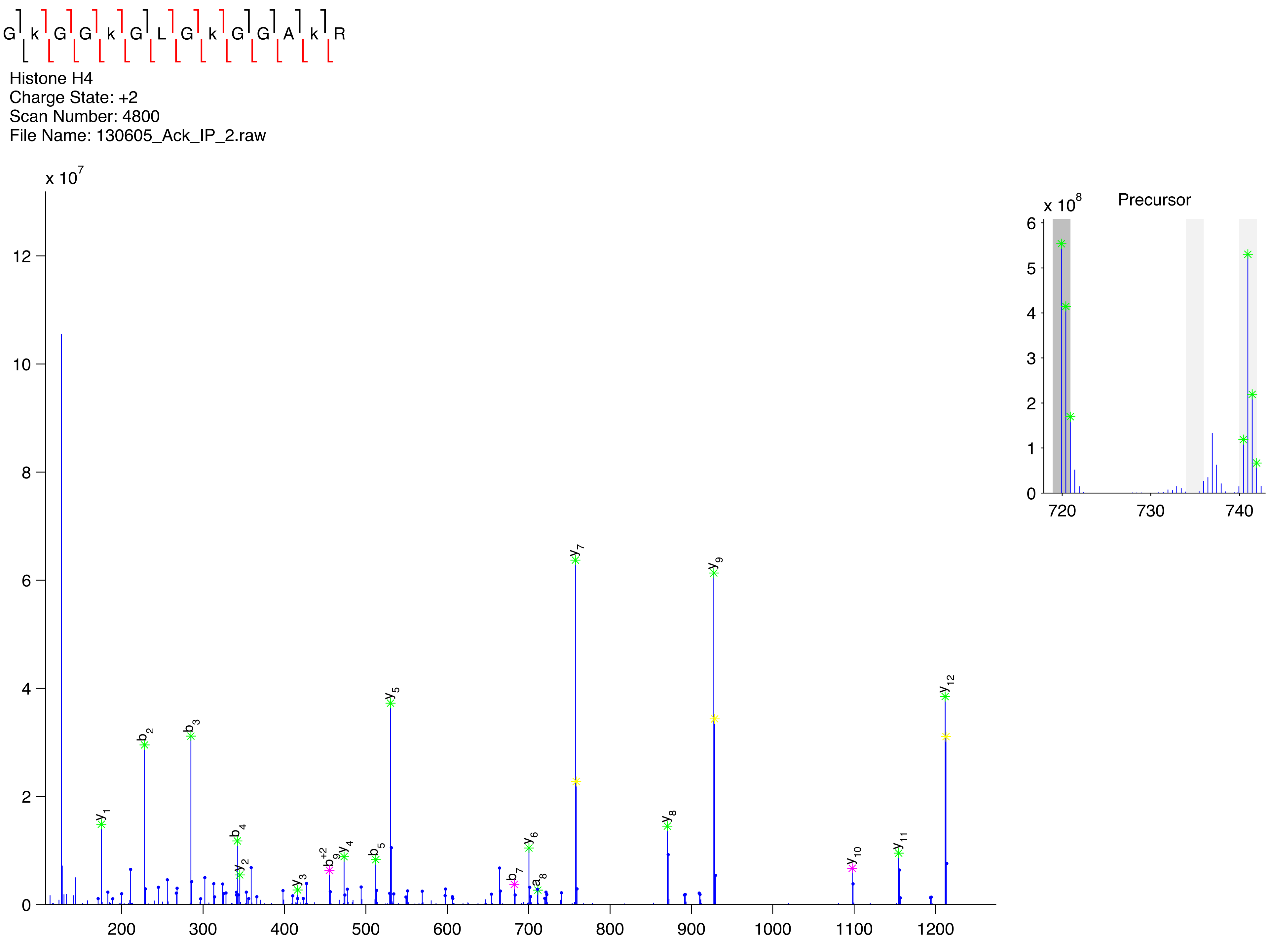

G k G G k G L G k G G A k R
Histone H4
Charge State: +2
Scan Number: 4800
File Name: 130605_Ack_IP_2.raw
x 10^7
y1
b2
b3
b4
y2
y3
b9+2
y4
b5
y5
b7
y6
a8
y7
y8
y9
y10
y11
y12
x 10^8
Precursor

G G k G L G k G G A k R

Histone H4
Charge State: +2
Scan Number: 5083
File Name: 130605_Ack_IP_3.raw

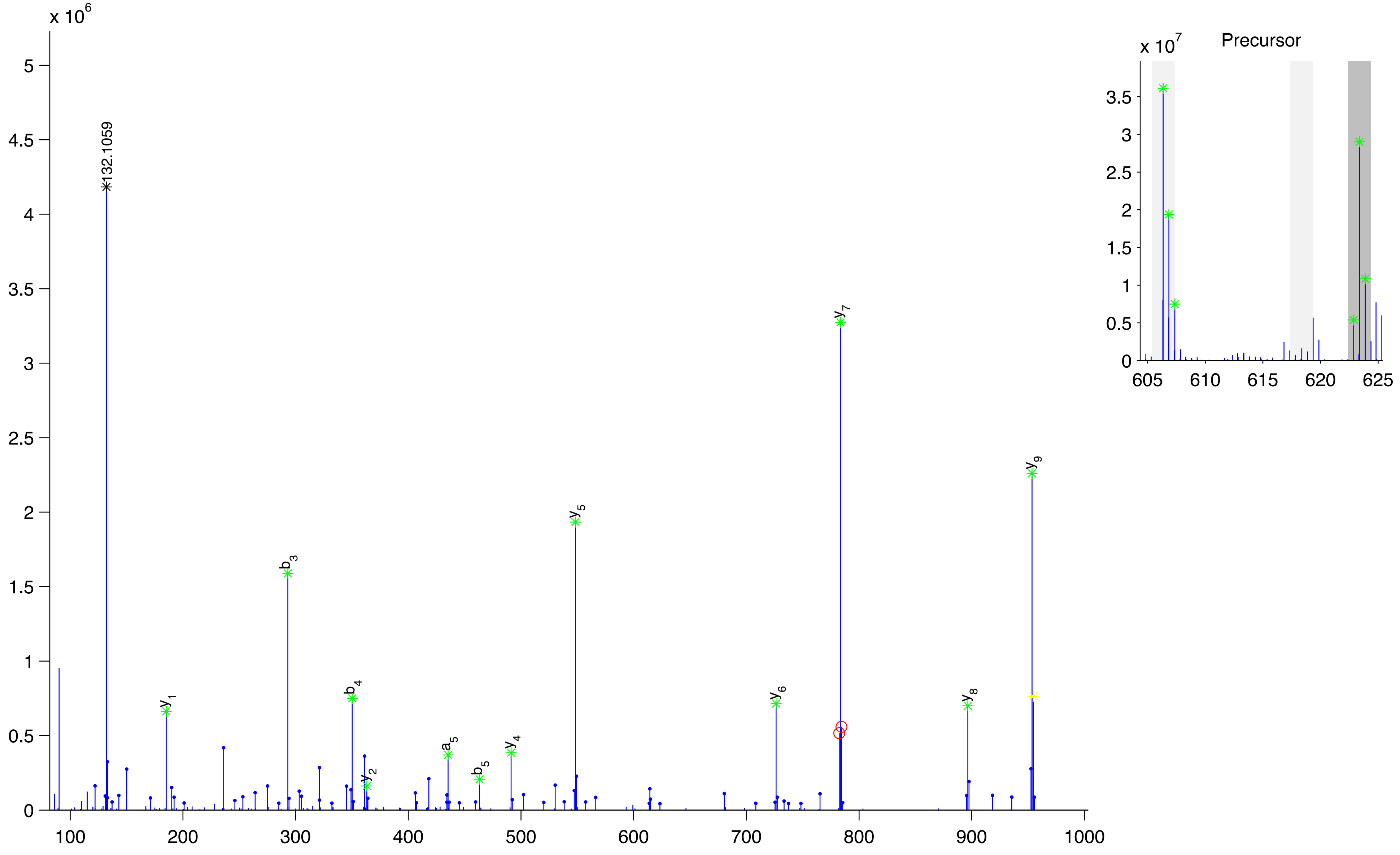

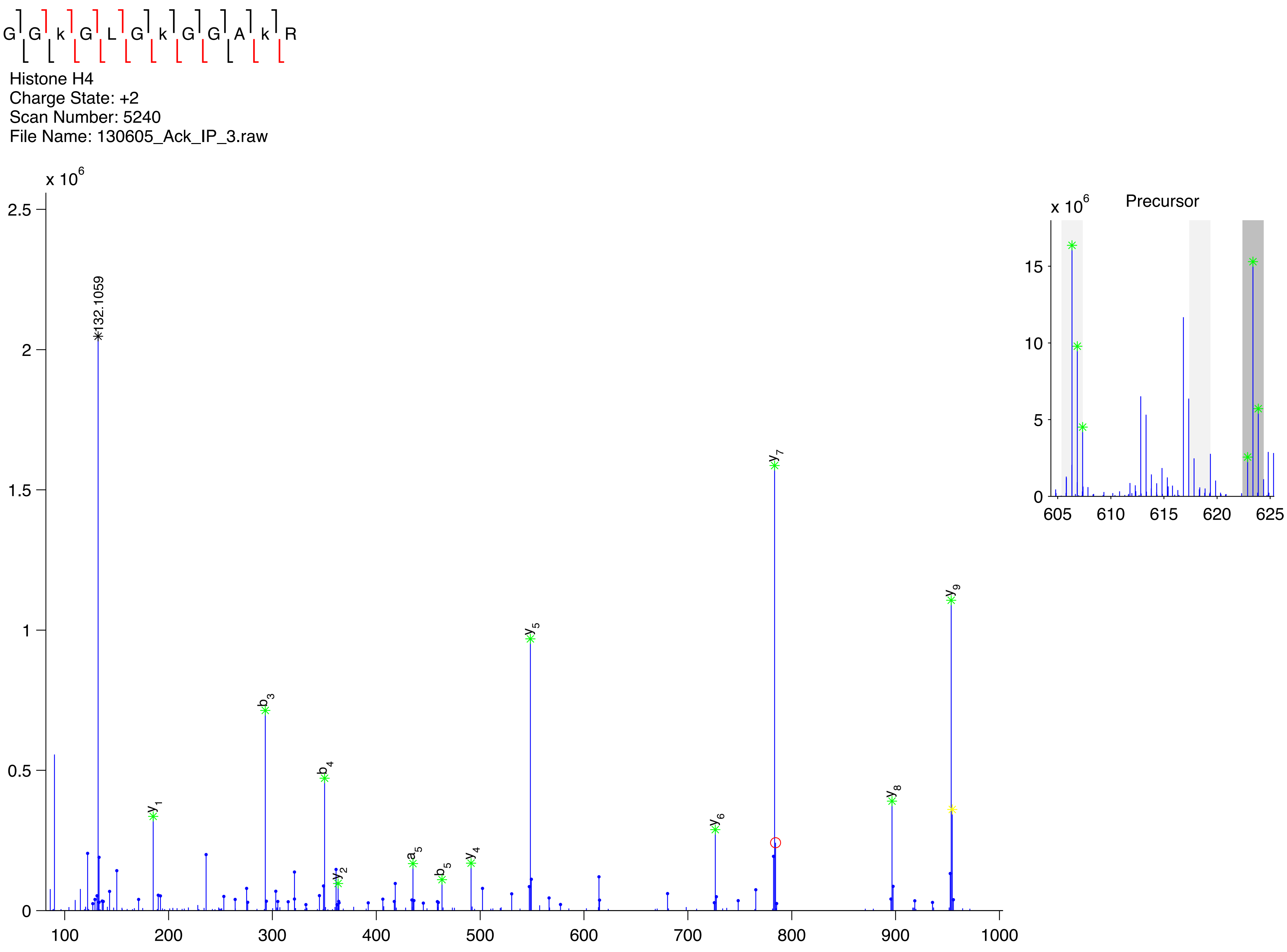
G G k G L G k G G A k R
Histone H4
Charge State: +2
Scan Number: 5240
File Name: 130605_Ack_IP_3.raw
x 10^6
132.1059
y1
b3
b4
y2
a5
b5
y4
y5
y6
y7
y8
y9
Precursor
x 10^6

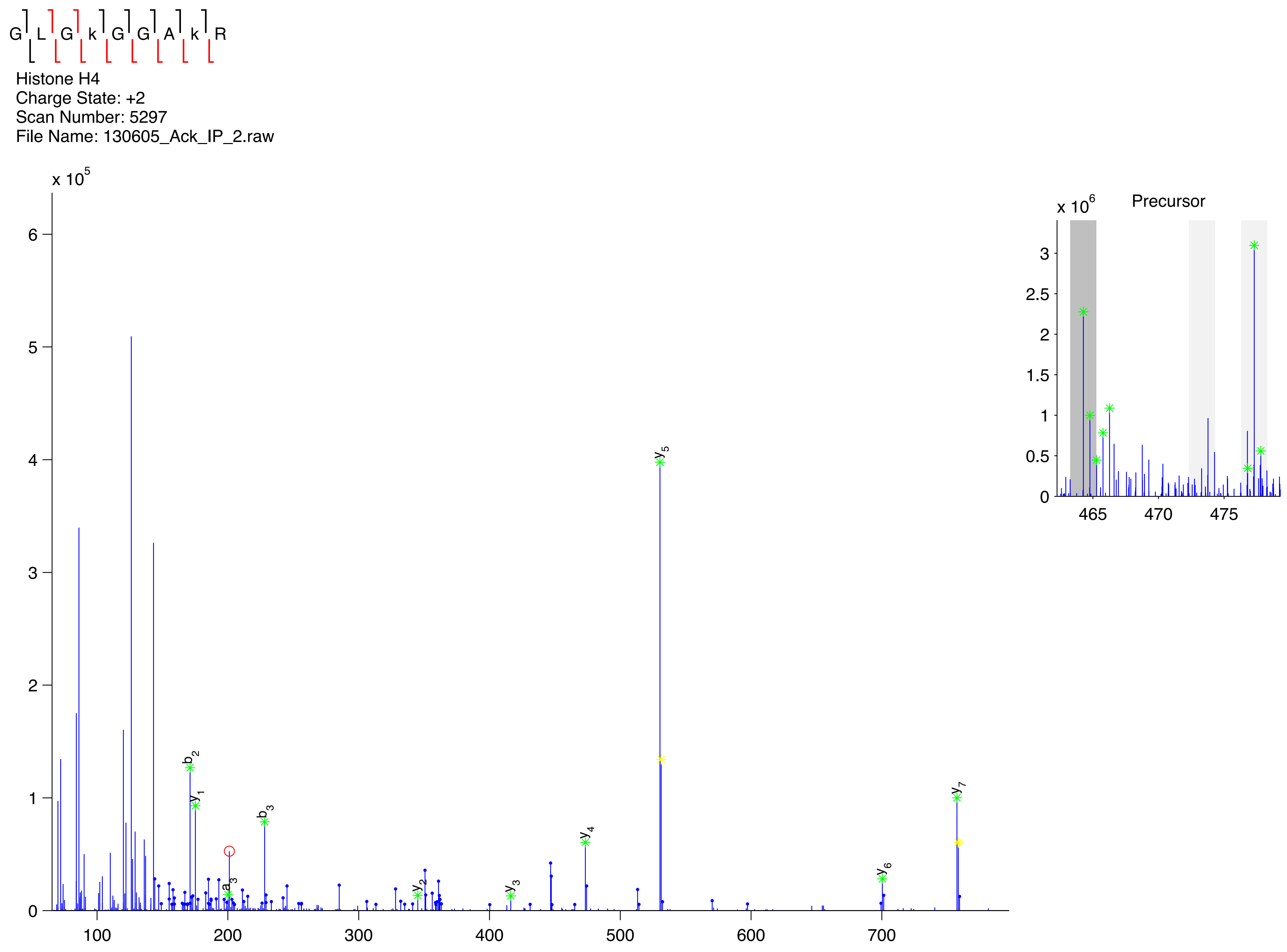
G L G k G G A k R
Histone H4
Charge State: +2
Scan Number: 5297
File Name: 130605_Ack_IP_2.raw
x 10^5
b2
y1
a3
b3
y2
y3
y4
y5
y6
y7
x 10^6
Precursor

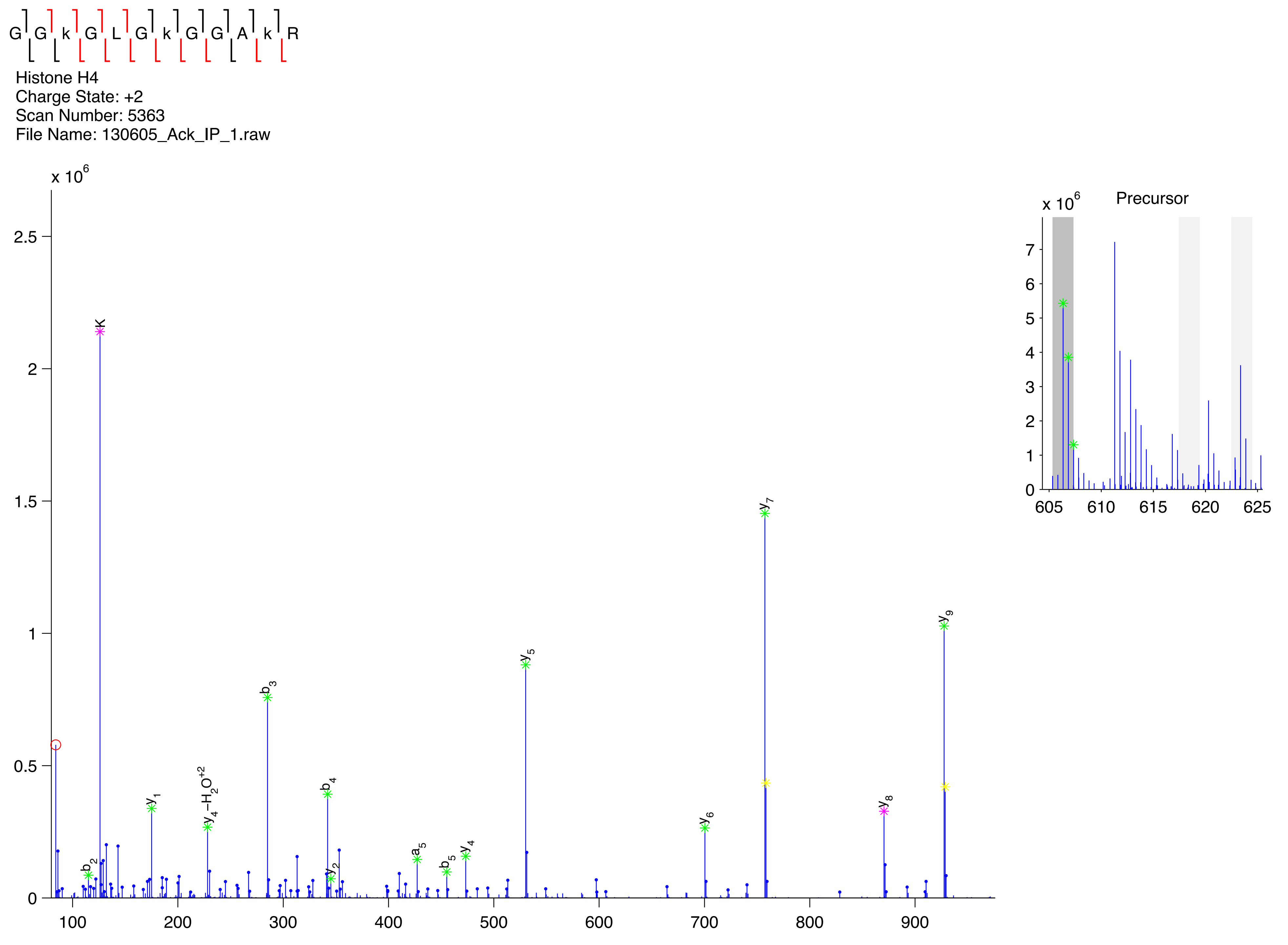

G G k G L G k G G A k R
Histone H4
Charge State: +2
Scan Number: 5363
File Name: 130605_Ack_IP_1.raw
x 10^6
K
b2
y1
y4−H2O+2
b3
b4
y2
a5
b5
y4
y5
y6
y7
y8
y9
Precursor
x 10^6

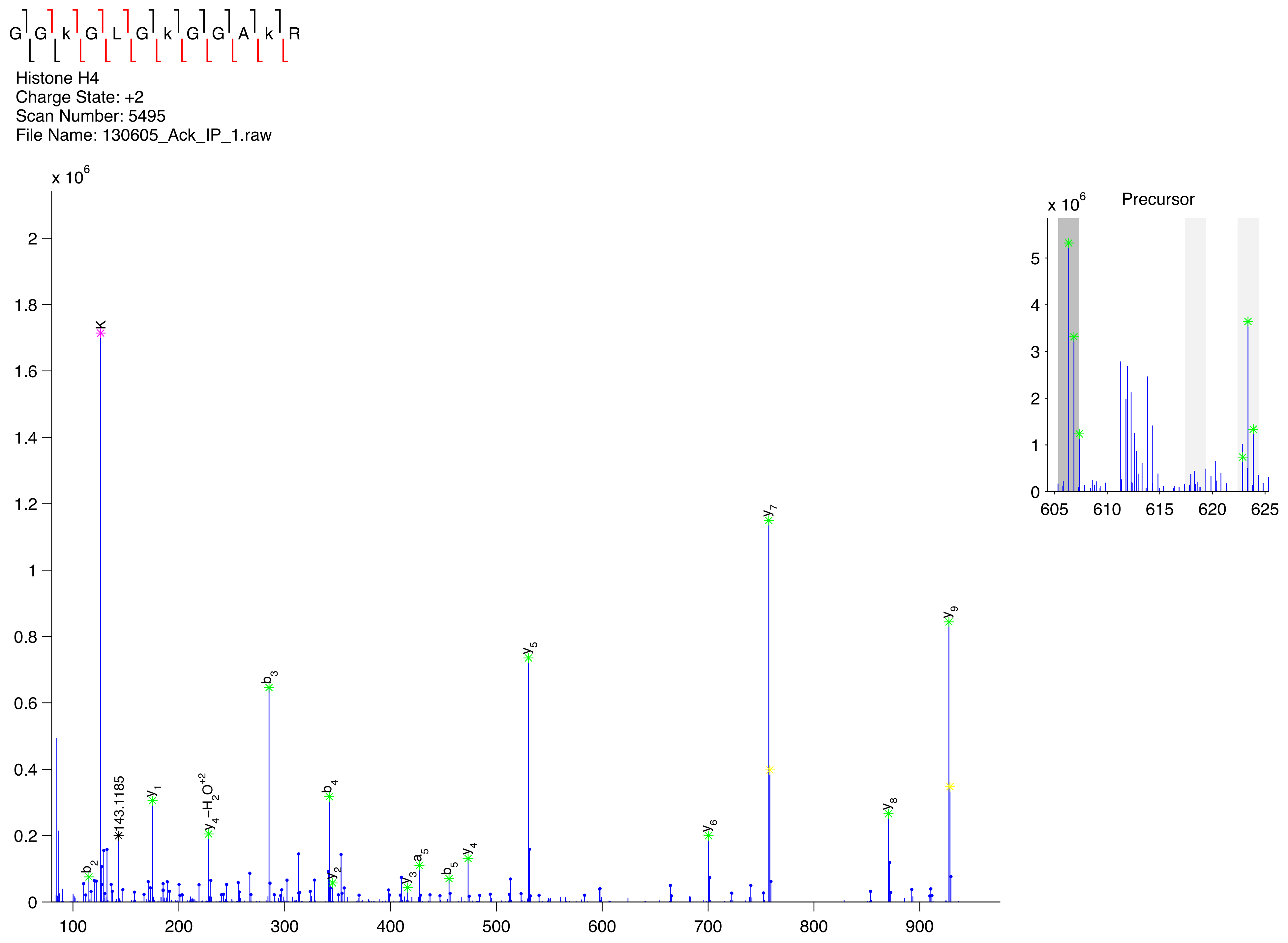
G G k G L G k G G A k R
Histone H4
Charge State: +2
Scan Number: 5495
File Name: 130605_Ack_IP_1.raw
x 10^6
K
143.1185
b2
y1
y4-H2O+2
b3
b4
y2
y3
a5
b5
y4
y5
y6
y7
y8
y9
Precursor
x 10^6
0
0.2
0.4
0.6
0.8
1
1.2
1.4
1.6
1.8
2
100
200
300
400
500
600
700
800
900
0
1
2
3
4
5
605
610
615
620
625

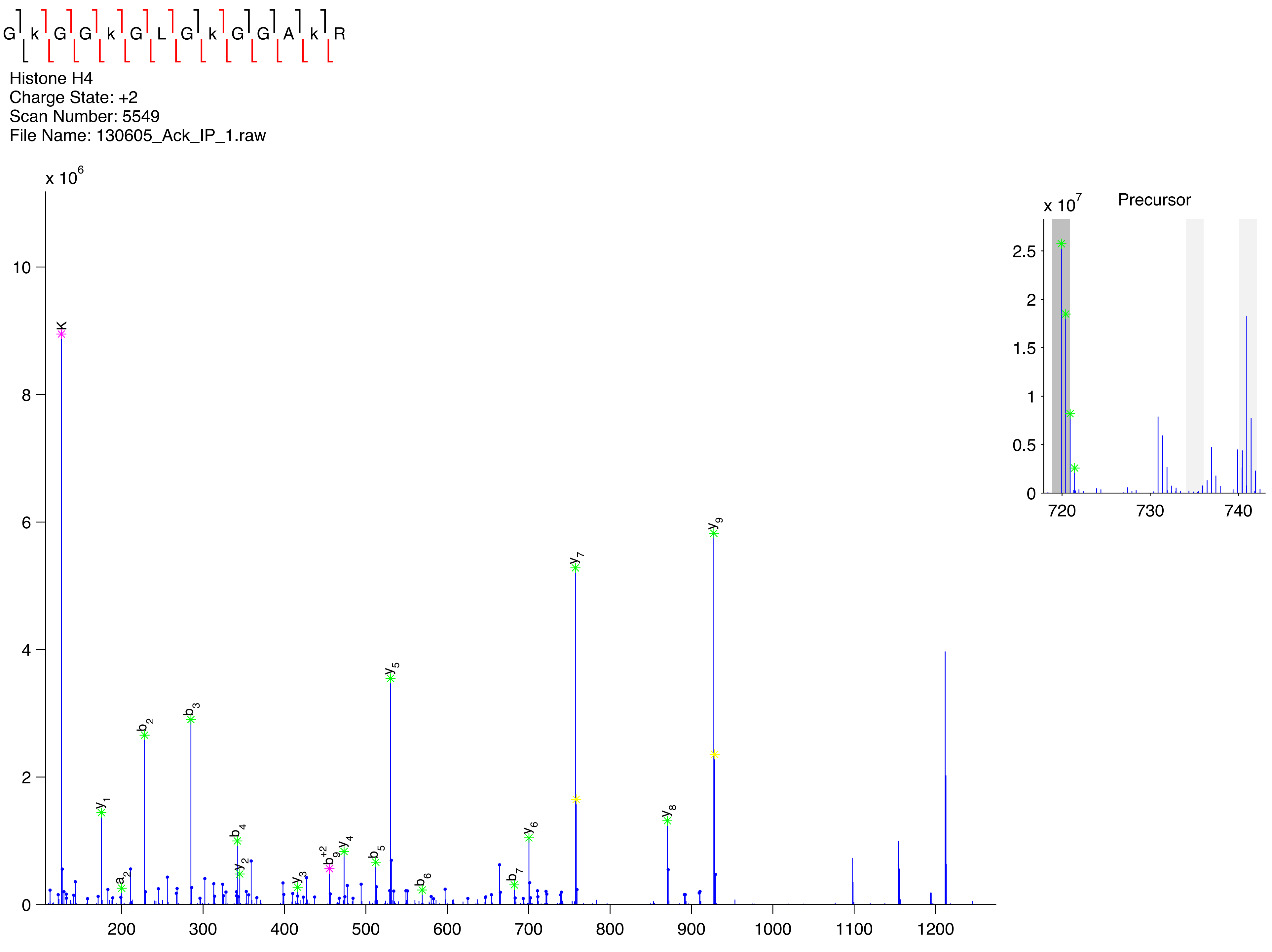
G k G G k G L G k G G A k R
Histone H4
Charge State: +2
Scan Number: 5549
File Name: 130605_Ack_IP_1.raw
x 10^6
K
y1
a2
b2
b3
b4
y2
y3
b9 +2
y4
b5
y5
b6
b7
y6
y7
y8
y9
Precursor
x 10^7

G G k G L G k G G A k R

Histone H4
Charge State: +2
Scan Number: 5565
File Name: 130605_Ack_IP_3.raw

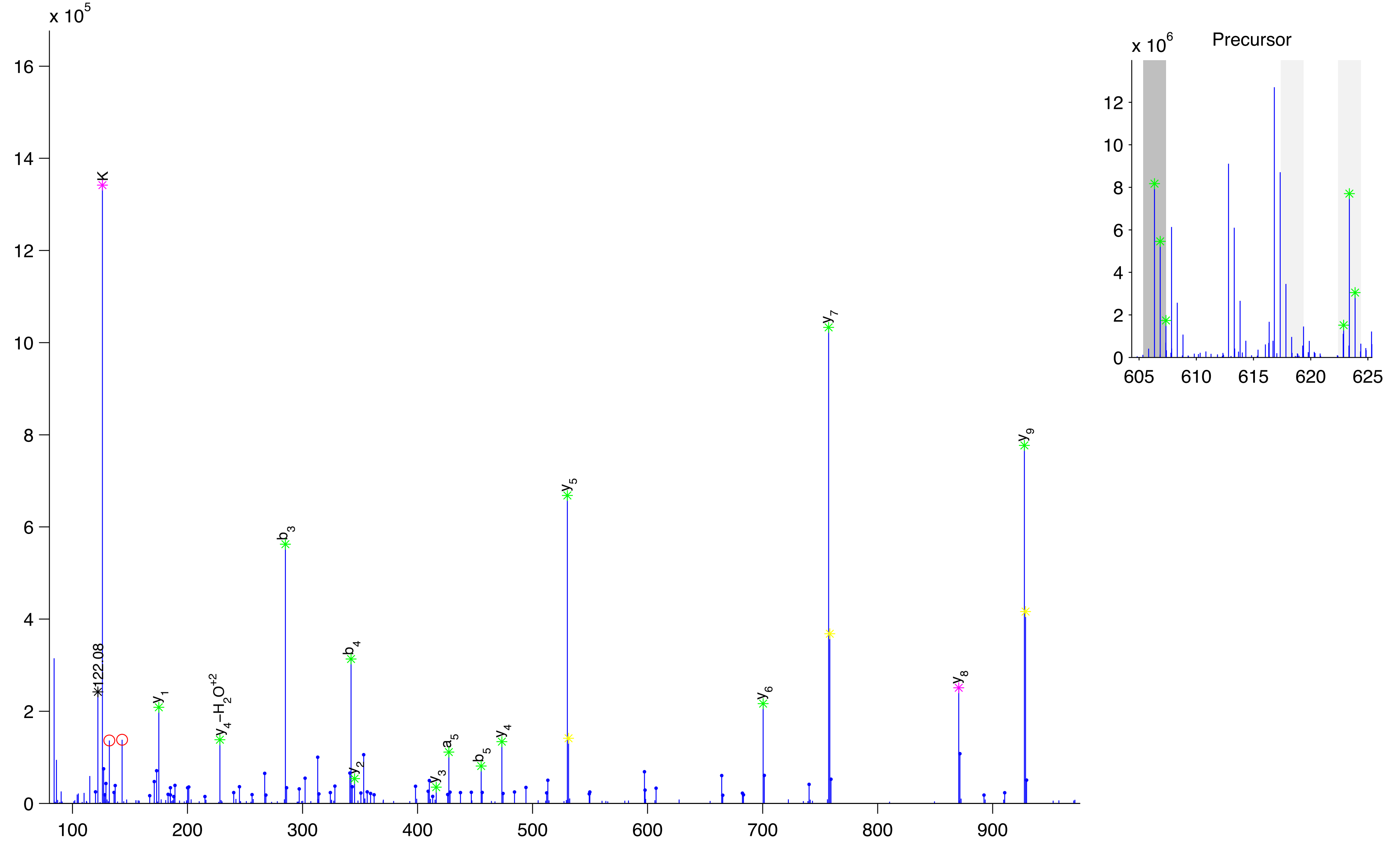

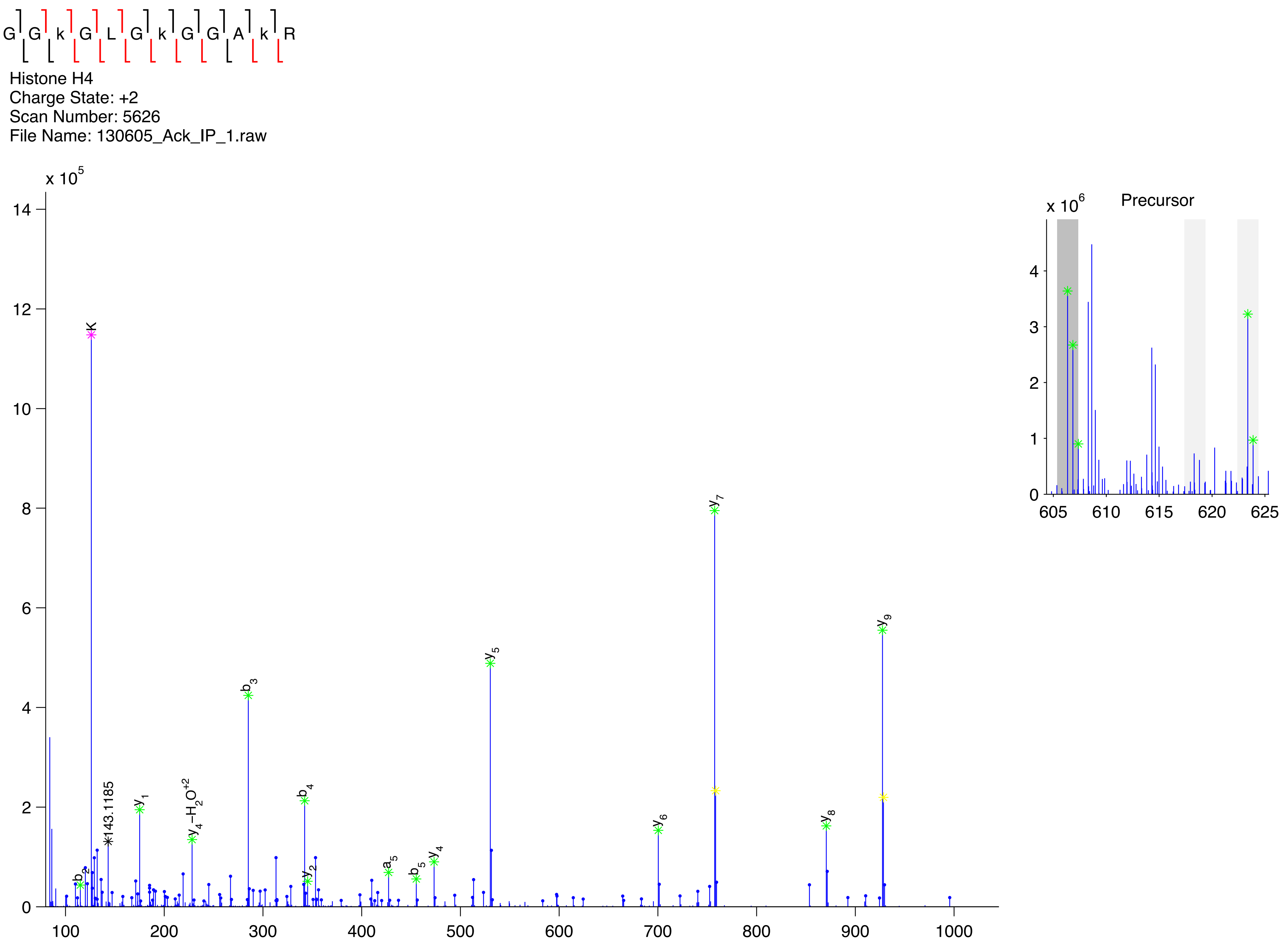

G G k G L G k G G A k R
Histone H4
Charge State: +2
Scan Number: 5626
File Name: 130605_Ack_IP_1.raw
x 10^5
K
143.1185
b2
b3
b4
b5
a5
y1
y2
y4
y4–H2O+2
y5
y6
y7
y8
y9
Precursor
x 10^6

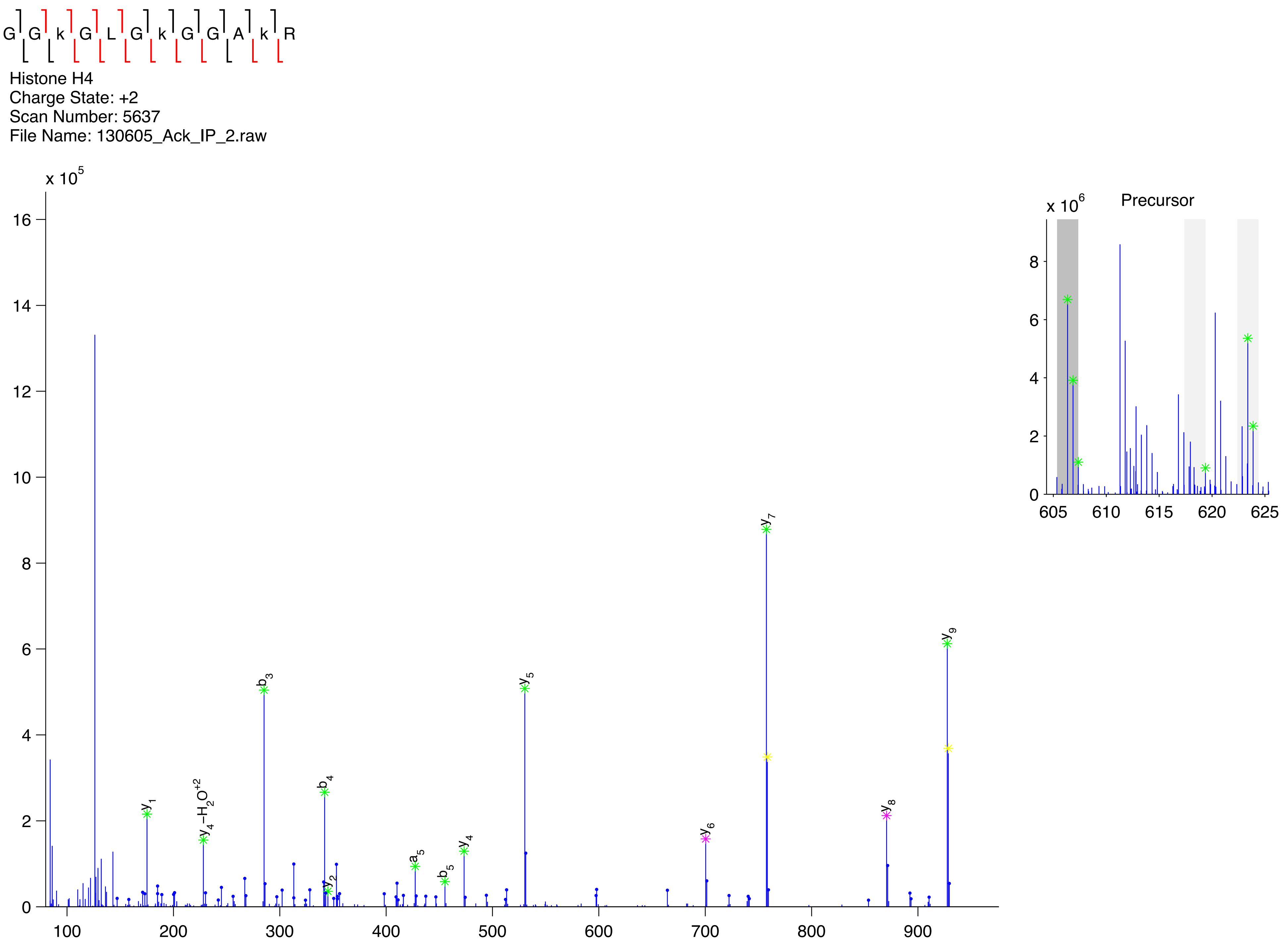
G G k G L G k G G A k R
Histone H4
Charge State: +2
Scan Number: 5637
File Name: 130605_Ack_IP_2.raw
x 10^5
y1
y4–H2O+2
b3
b4
y2
a5
b5
y4
y5
y6
y7
y8
y9
x 10^6
Precursor

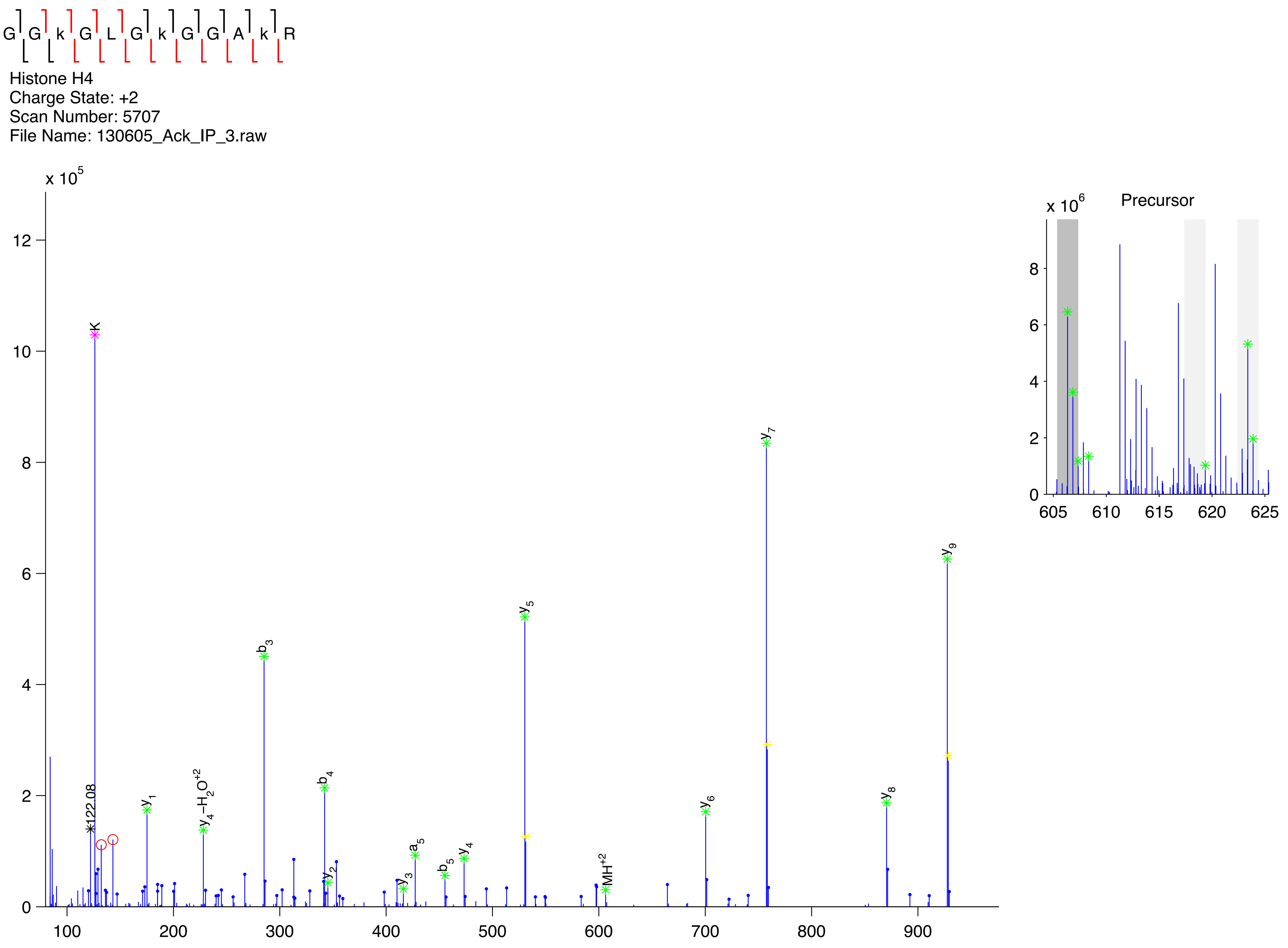
G G k G L G k G G A k R
Histone H4
Charge State: +2
Scan Number: 5707
File Name: 130605_Ack_IP_3.raw
x 10^5
K
122.08
y1
y4−H2O+2
b3
b4
y2
y3
a5
b5
y4
y5
MH+2
y6
y7
y8
y9
x 10^6
Precursor

G G k G L G k G G A k R

Histone H4
Charge State: +2
Scan Number: 5850
File Name: 130605_Ack_IP_3.raw

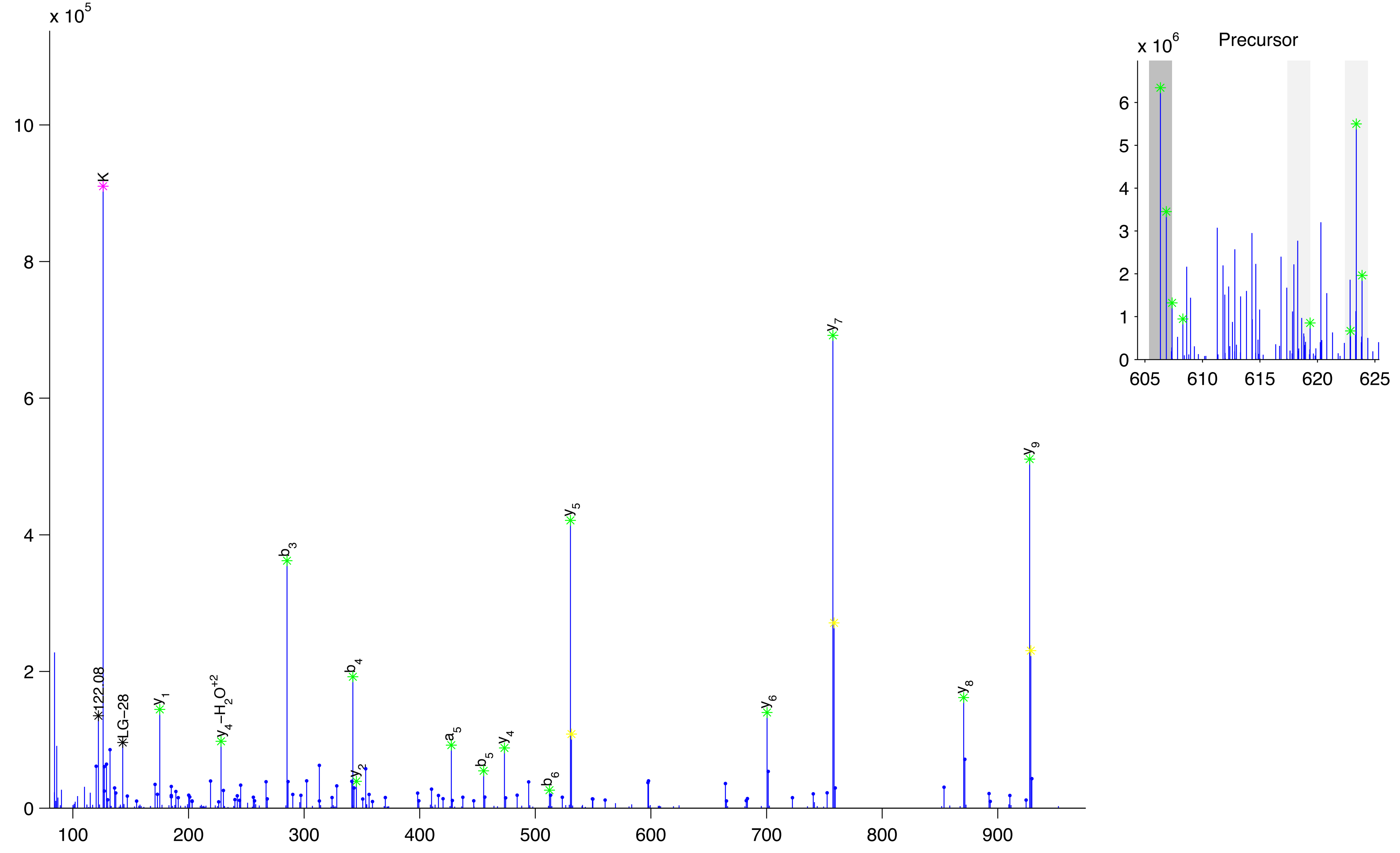

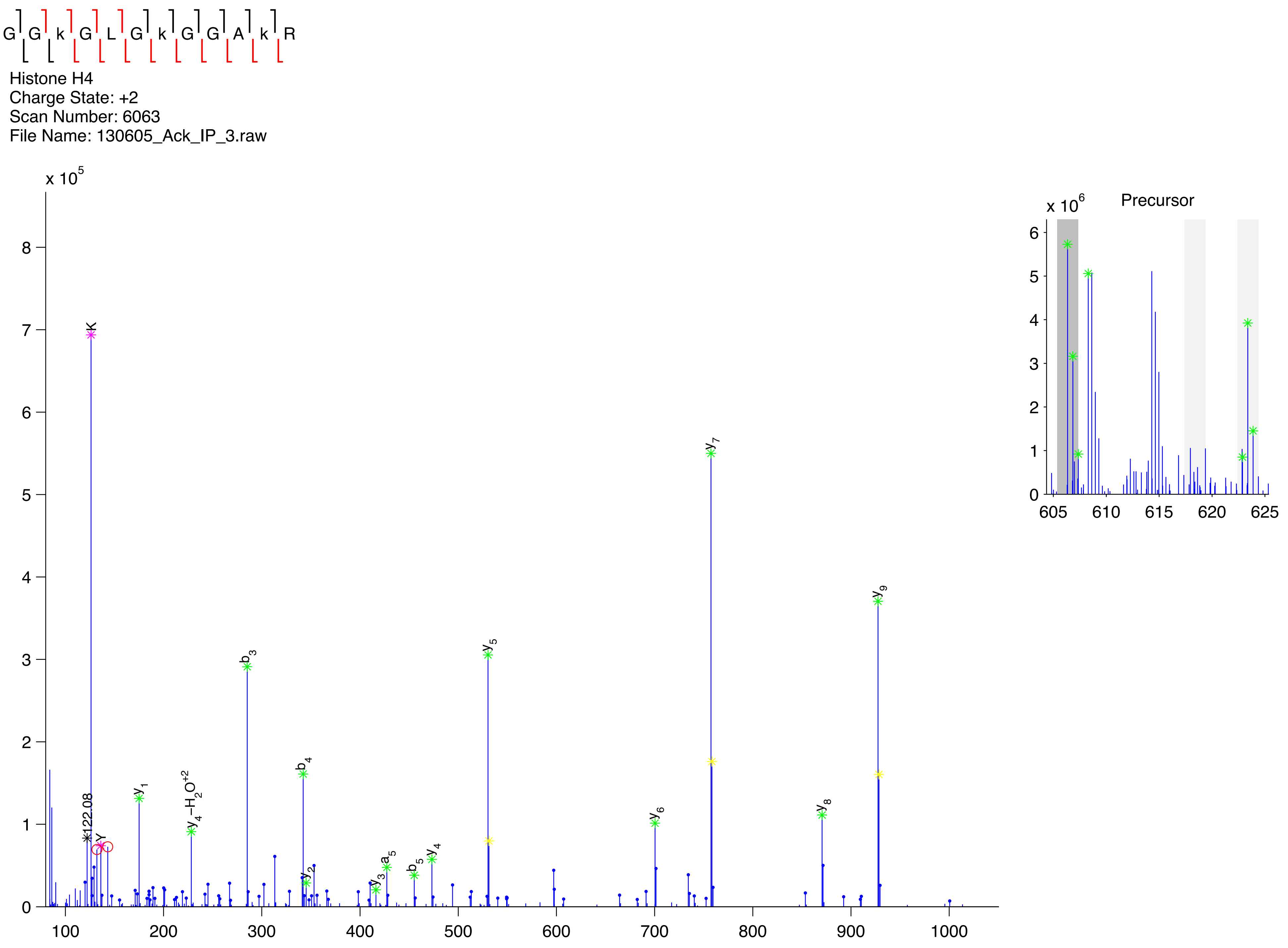
G G k G L G k G G A k R
Histone H4
Charge State: +2
Scan Number: 6063
File Name: 130605_Ack_IP_3.raw
x 10^5
K
*122.08
Y
y1
y4-H2O+2
b3
b4
y2
y3
a5
b5
y4
y5
y6
y7
y8
y9
x 10^6
Precursor

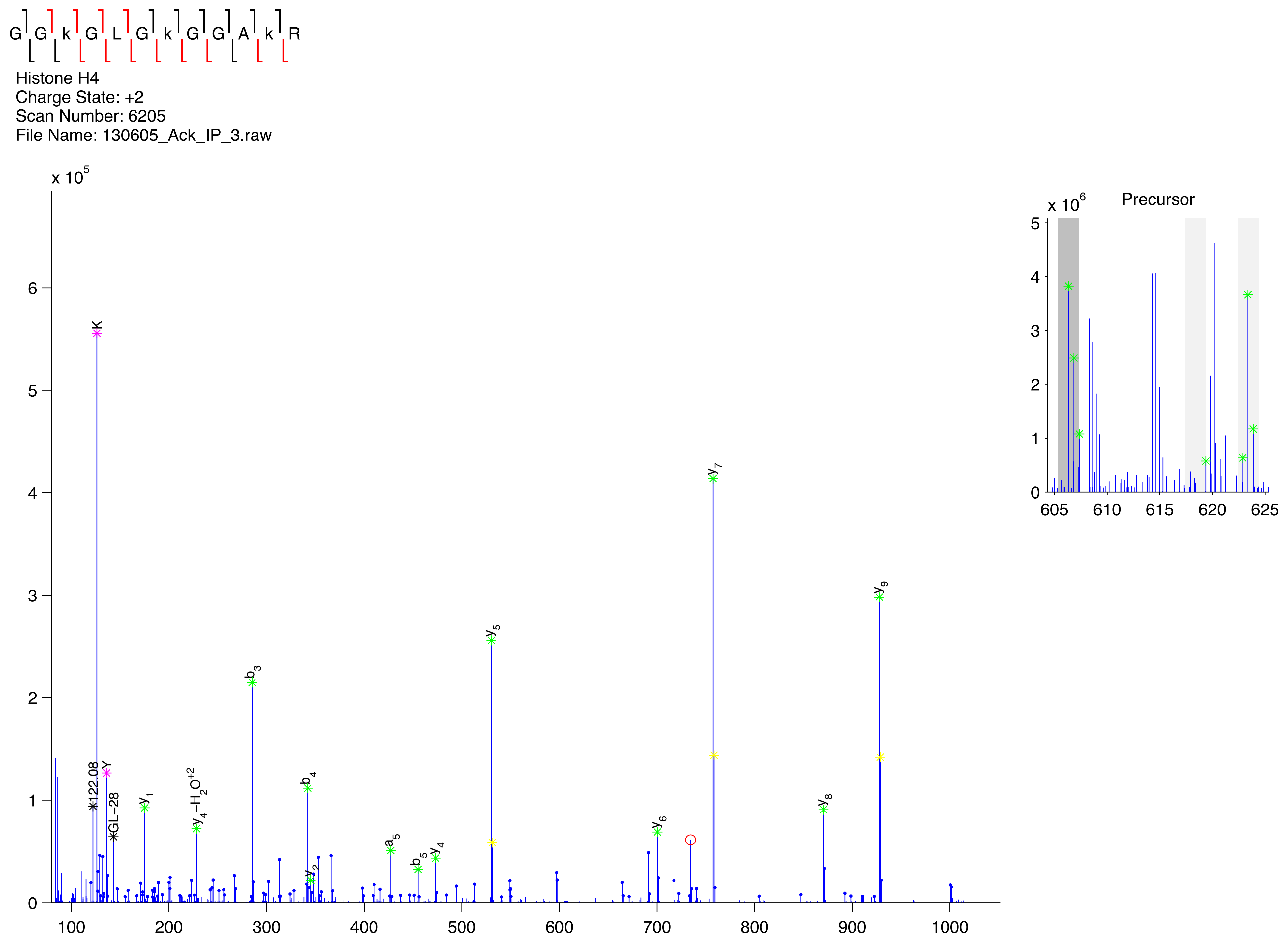

G G k G L G k G G A k R
Histone H4
Charge State: +2
Scan Number: 6205
File Name: 130605_Ack_IP_3.raw
x 10^5
K
Y
122.08
GL−28
y1
y4−H2O+2
b3
b4
y2
a5
b5
y4
y5
y6
y7
y8
y9
x 10^6
Precursor

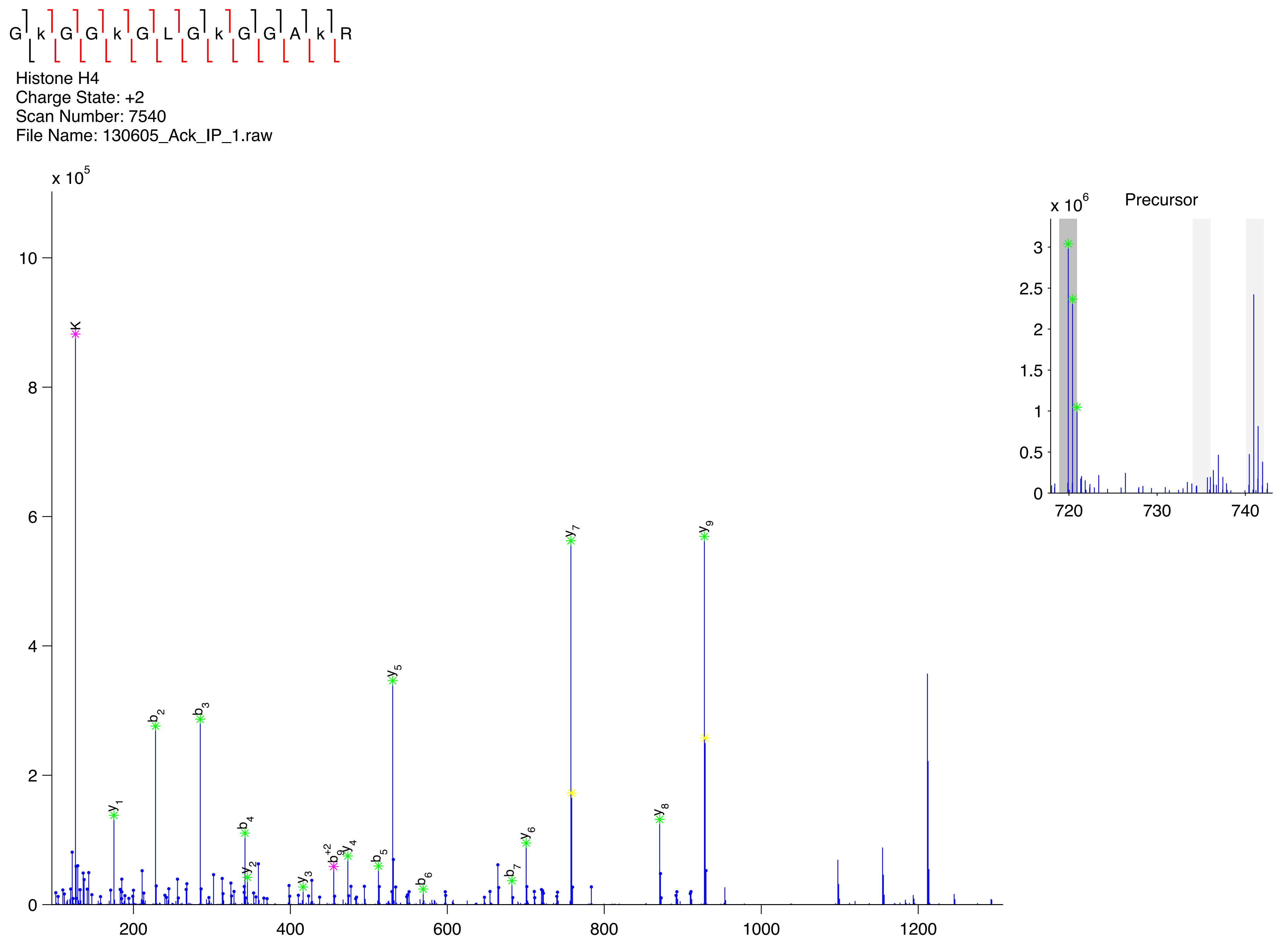

G k G G k G L G k G G A k R
Histone H4
Charge State: +2
Scan Number: 7540
File Name: 130605_Ack_IP_1.raw
x 10^5
K
y1
b2
b3
b4
y2
y3
b9+2
y4
b5
y5
b6
b7
y6
y7
y8
y9
0
2
4
6
8
10
200
400
600
800
1000
1200
x 10^6
Precursor
0
0.5
1
1.5
2
2.5
3
720
730
740

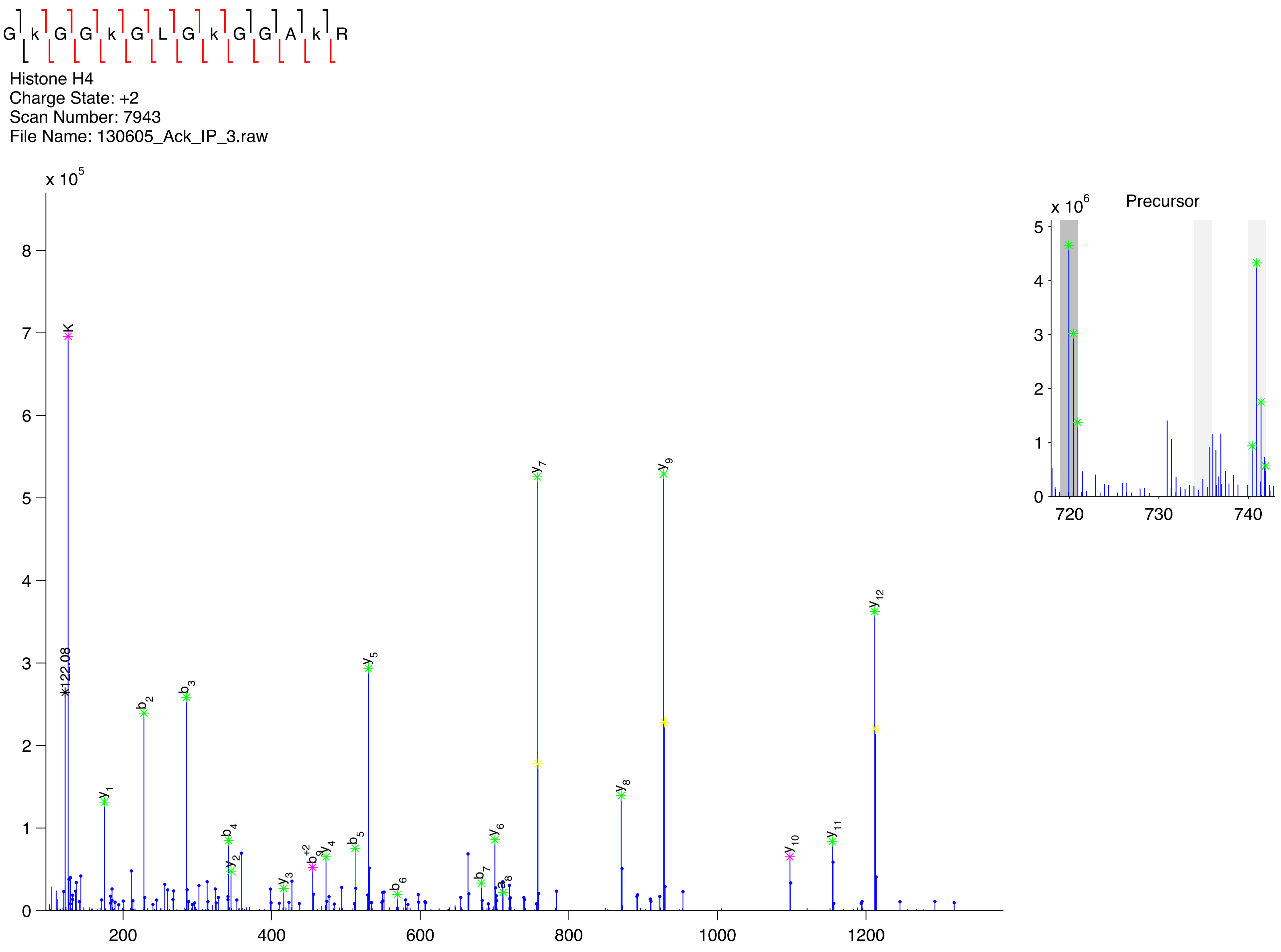

G k G G k G L G k G G A k R
Histone H4
Charge State: +2
Scan Number: 7943
File Name: 130605_Ack_IP_3.raw
x 10^5
K
122.08
y1
b2
b3
b4
y2
y3
b9+2
y4
b5
y5
b6
b7
y6
a8
y7
y8
y9
y10
y11
y12
0
1
2
3
4
5
6
7
8
200
400
600
800
1000
1200
x 10^6
Precursor
720
730
740

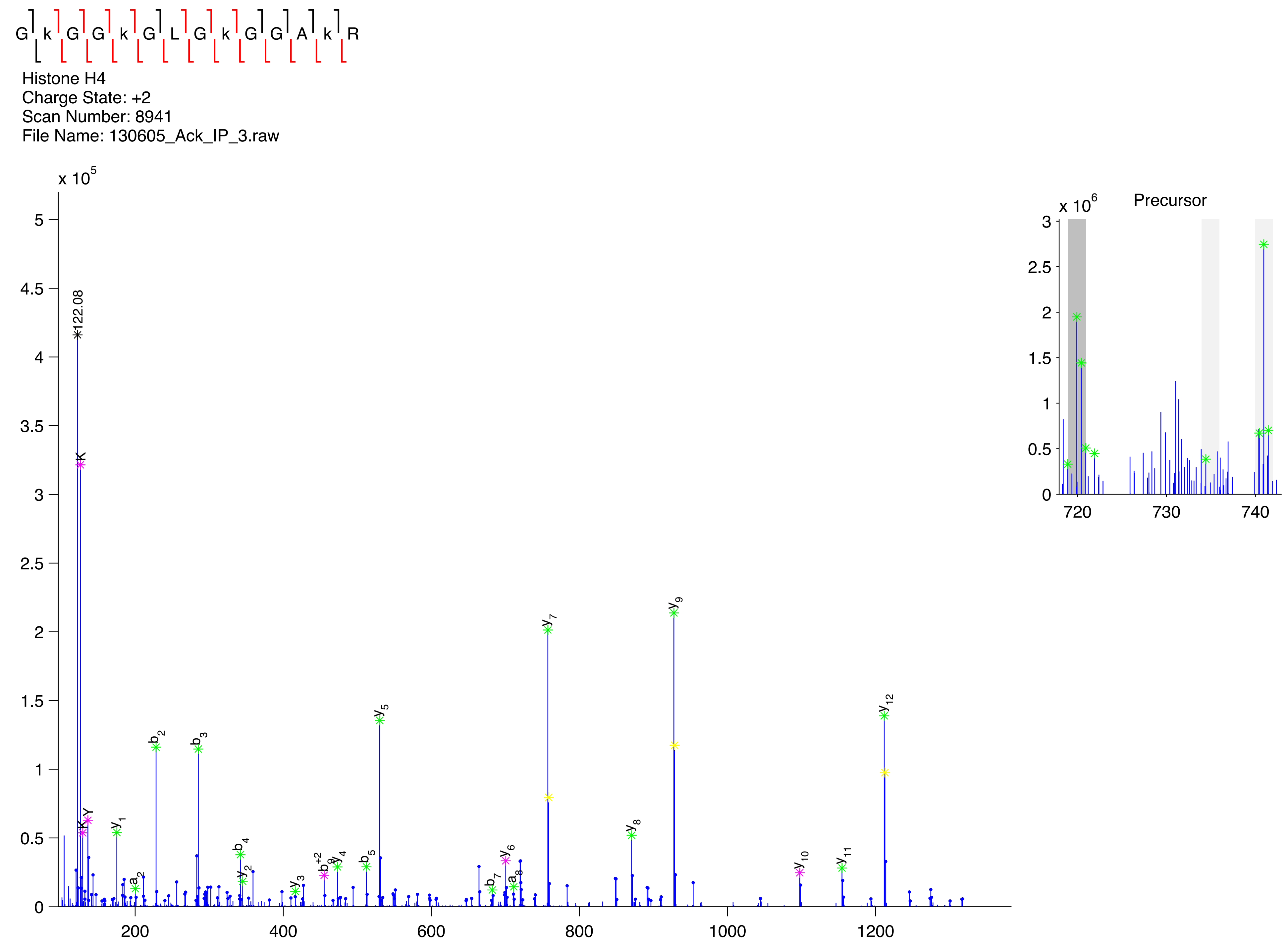
G k G G k G L G k G G A k R
Histone H4
Charge State: +2
Scan Number: 8941
File Name: 130605_Ack_IP_3.raw
x 10^5
122.08
K
K
Y
y1
a2
b2
b3
b4
y2
y3
b9+2
y4
b5
y5
b7
y6
a8
y7
y8
y9
y10
y11
y12
x 10^6
Precursor

G k G G k G L G k G G A k R

Histone H4
Charge State: +2
Scan Number: 29169
File Name: 130605_Ack_IP_3.raw

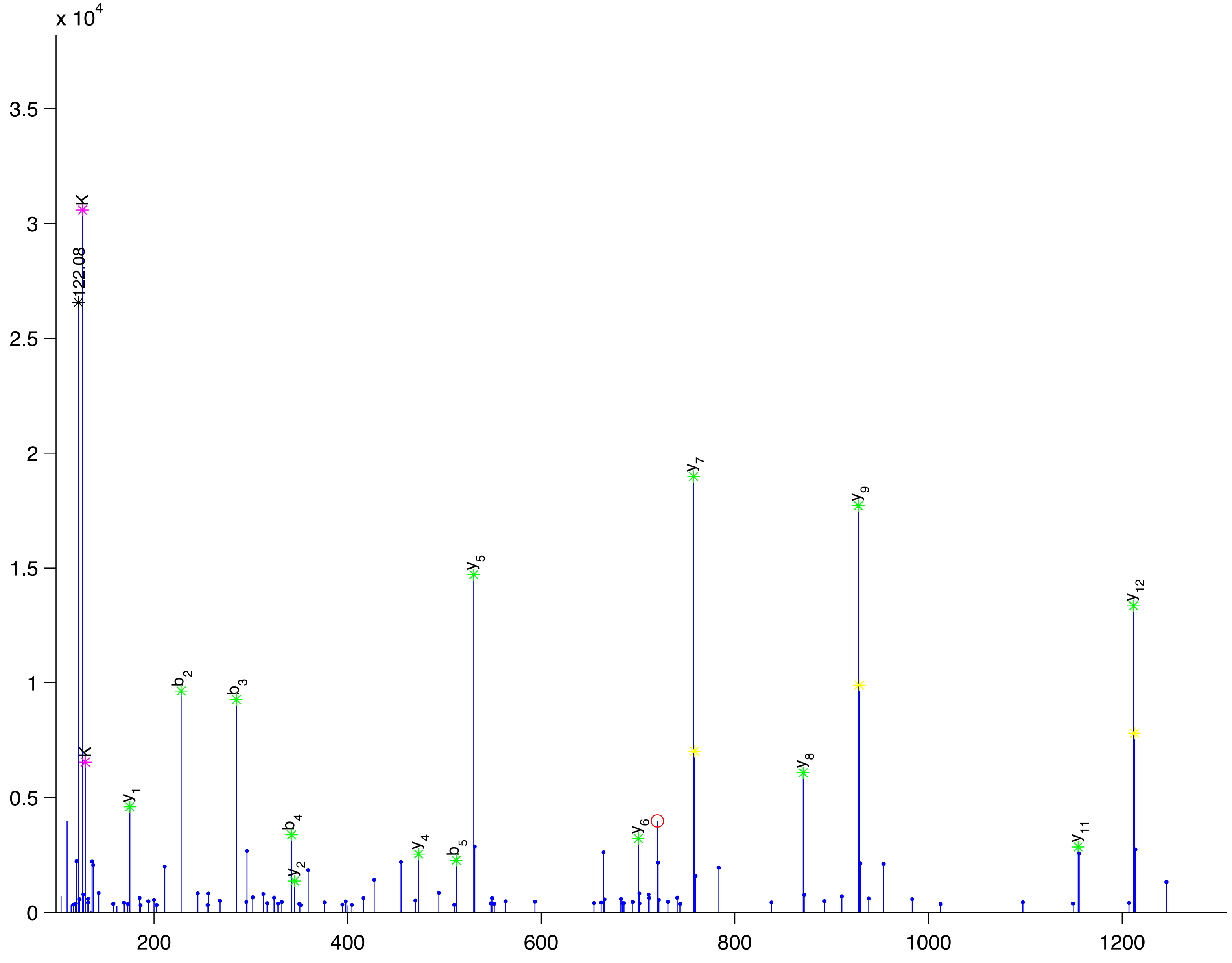

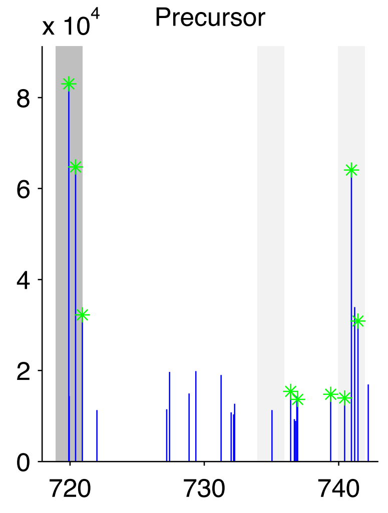

kSTGGkAPR

Histone H3.3
Charge State: +2
Scan Number: 2909
File Name: 130605_Ack_IP_1.raw

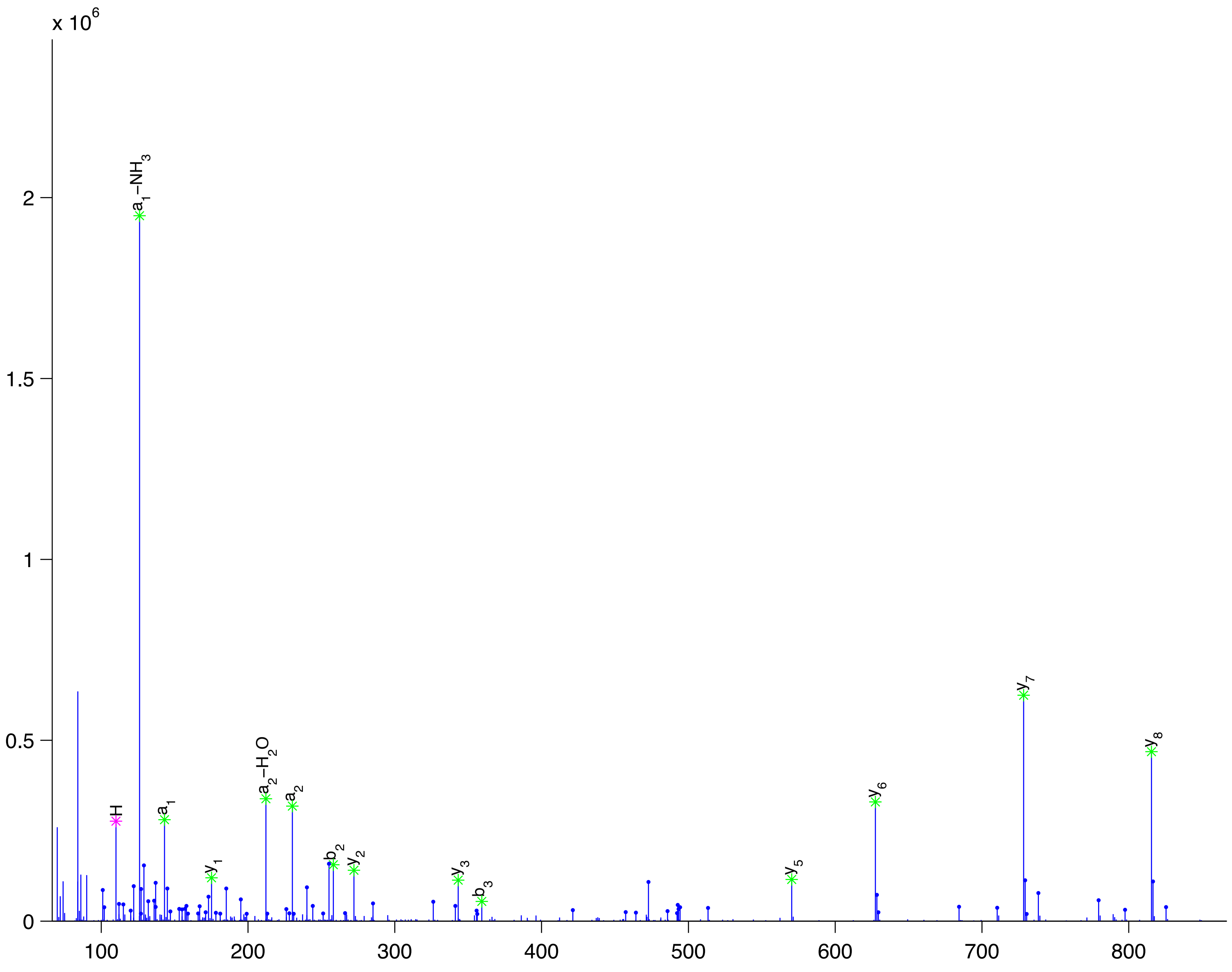

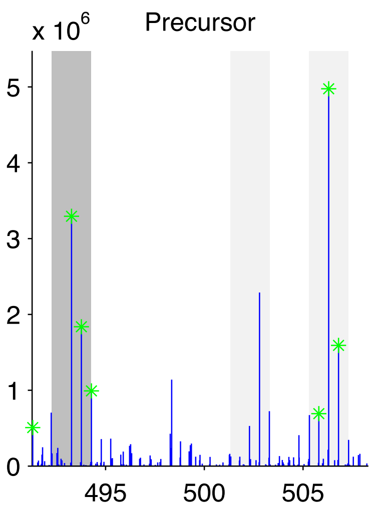

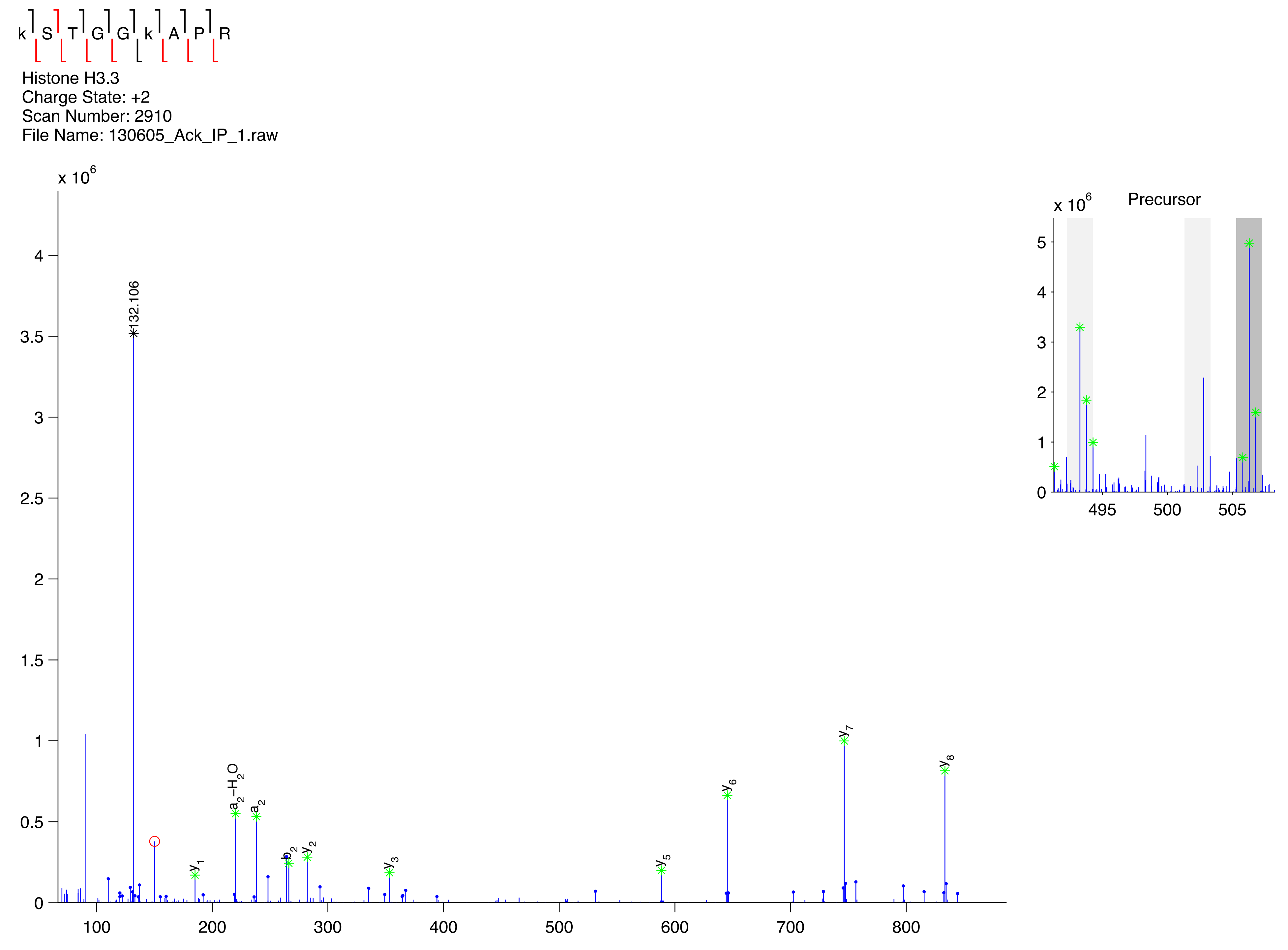

k S T G G k A P R
Histone H3.3
Charge State: +2
Scan Number: 2910
File Name: 130605_Ack_IP_1.raw
x 10^6
132.106
a2−H2O
a2
b2
y1
y2
y3
y5
y6
y7
y8
Precursor

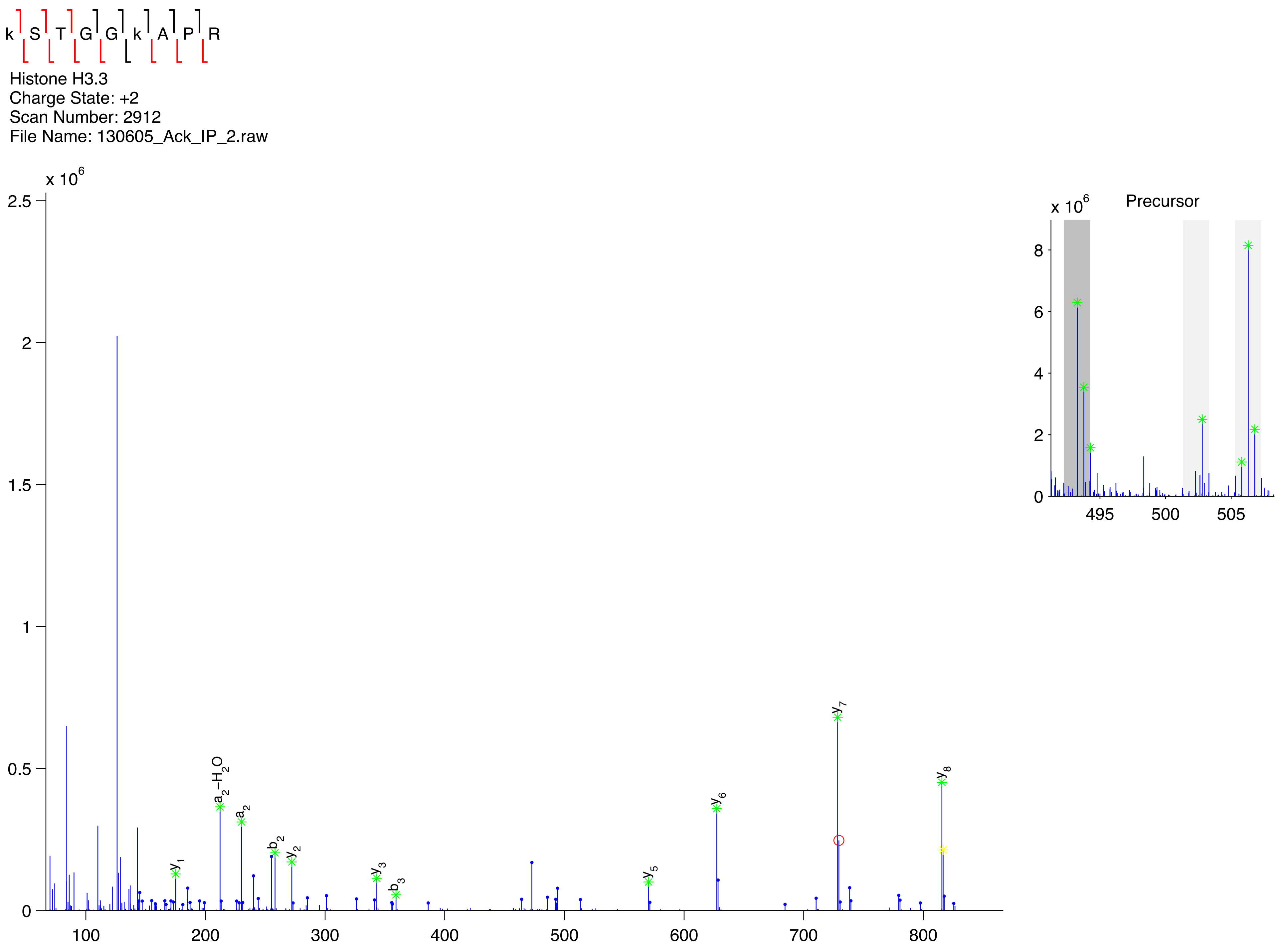
k S T G G k A P R
Histone H3.3
Charge State: +2
Scan Number: 2912
File Name: 130605_Ack_IP_2.raw
x 10^6
0
0.5
1
1.5
2
2.5
100
200
300
400
500
600
700
800
y1
a2–H2O
a2
b2
y2
y3
b3
y5
y6
y7
y8
Precursor
x 10^6
0
2
4
6
8
495
500
505

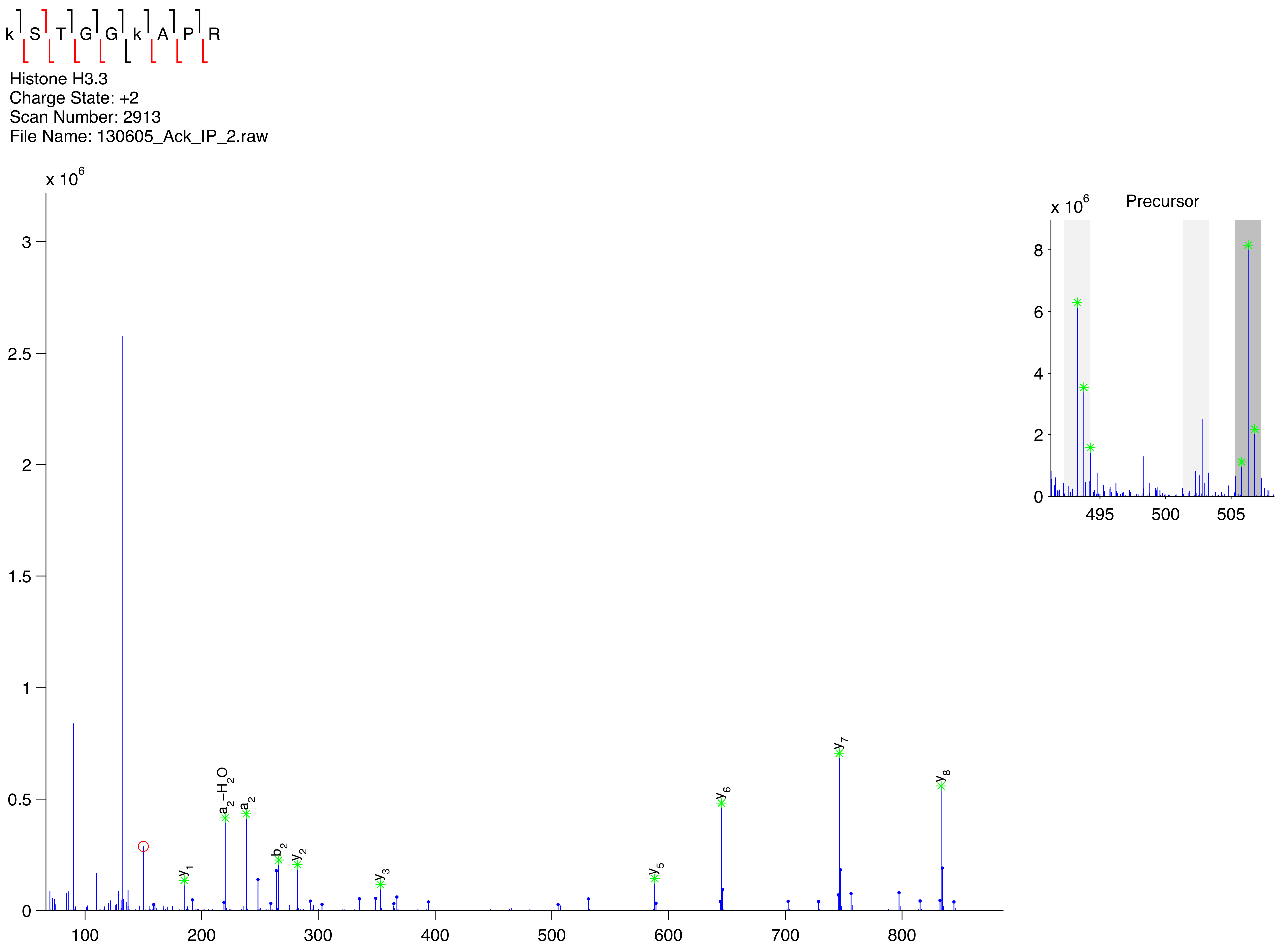

k S T G G k A P R
Histone H3.3
Charge State: +2
Scan Number: 2913
File Name: 130605_Ack_IP_2.raw
x 10^6
3
2.5
2
1.5
1
0.5
0
100
200
300
400
500
600
700
800
y1
a2−H2O
a2
b2
y2
y3
y5
y6
y7
y8
Precursor
x 10^6
8
6
4
2
0
495
500
505

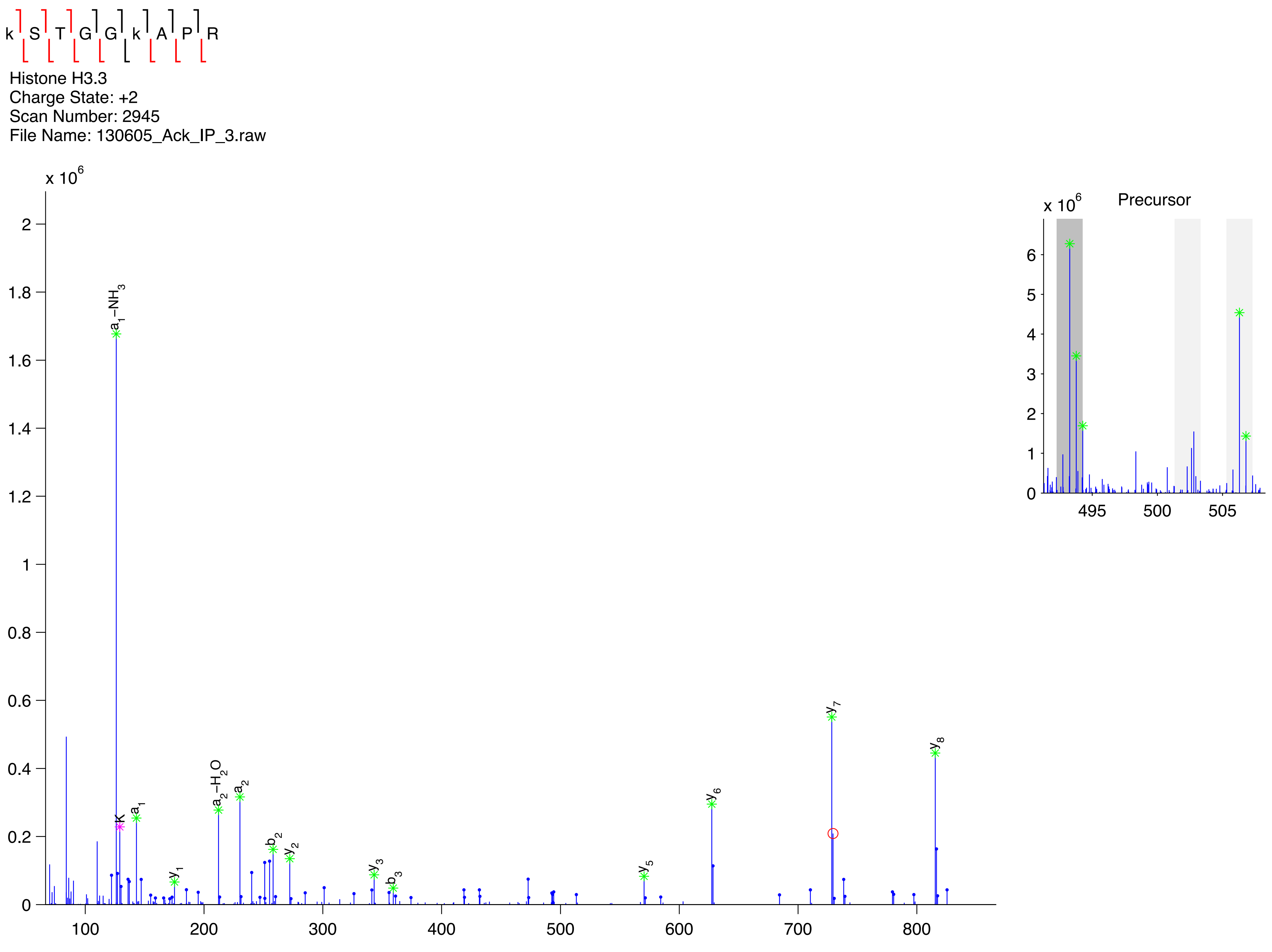

k S T G G k A P R
Histone H3.3
Charge State: +2
Scan Number: 2945
File Name: 130605_Ack_IP_3.raw
x 10^6
a1–NH3
K
a1
y1
a2–H2O
a2
b2
y2
y3
b3
y5
y6
y7
y8
Precursor

k S T G G k A P R

Histone H3.3
Charge State: +2
Scan Number: 2949
File Name: 130605_Ack_IP_3.raw

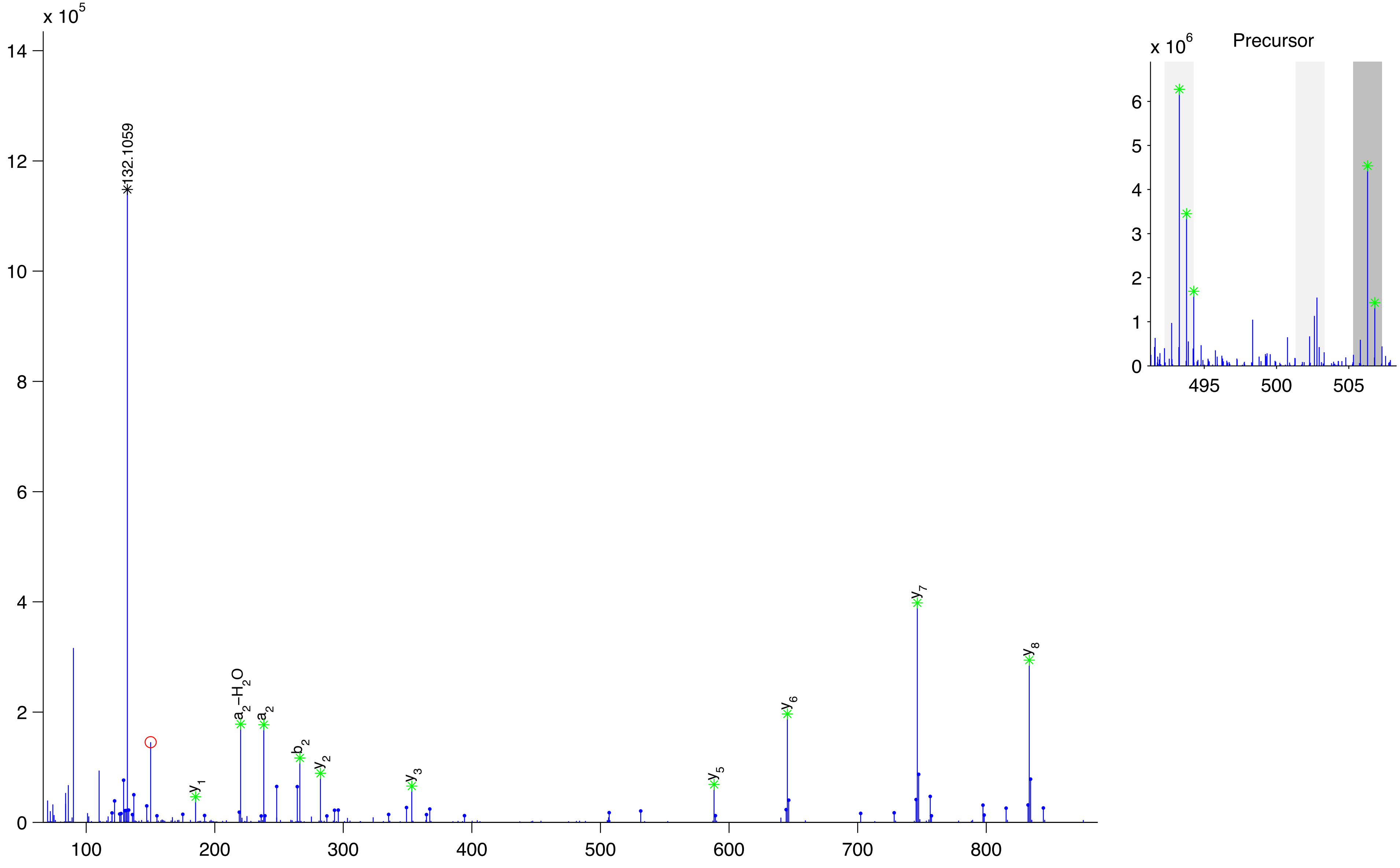

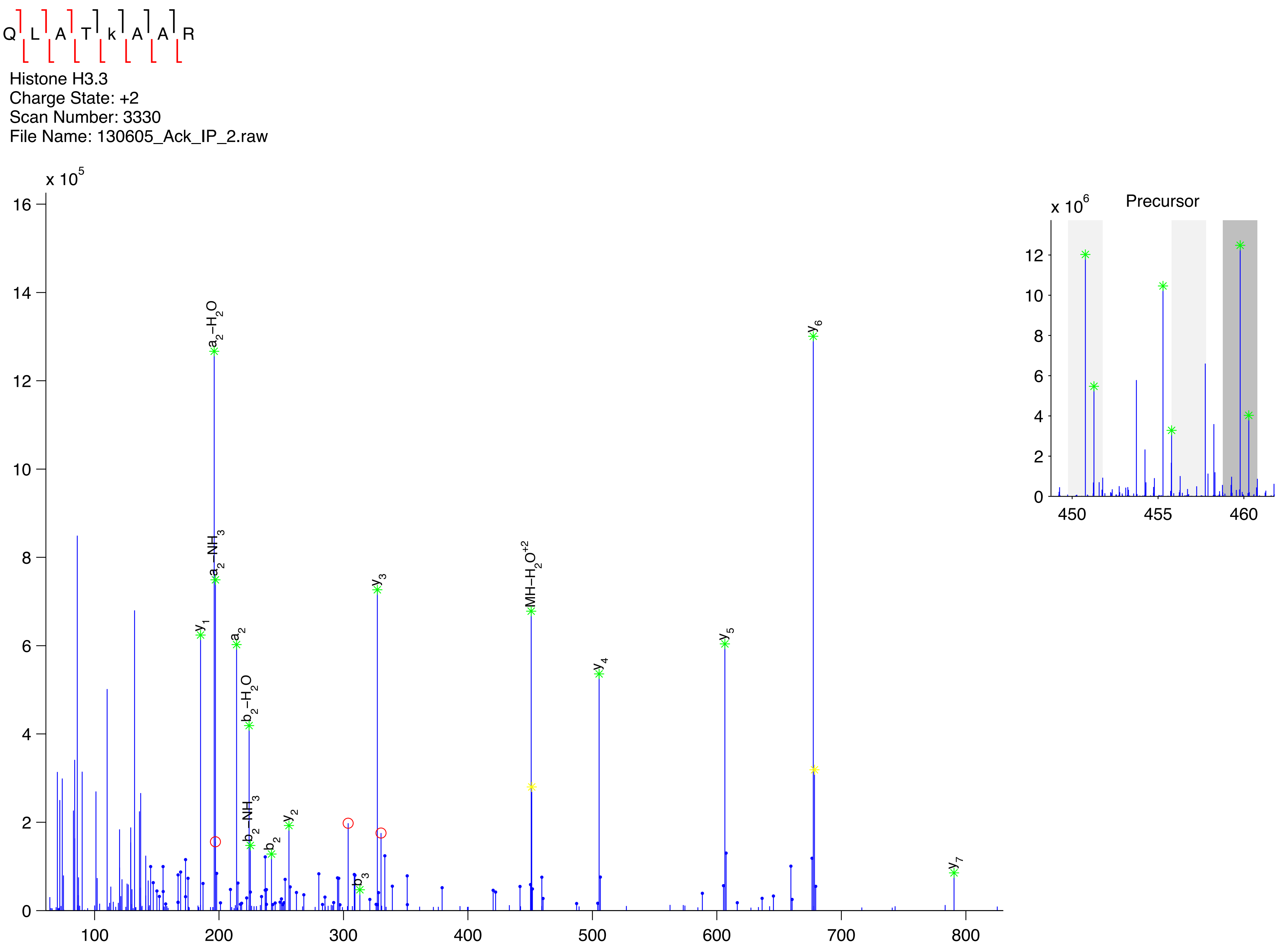

Q L A T k A A R
Histone H3.3
Charge State: +2
Scan Number: 3330
File Name: 130605_Ack_IP_2.raw
x 10^5
y1
a2−H2O
a2−NH3
a2
b2−H2O
b2−NH3
b2
y2
b3
y3
MH−H2O+2
y4
y5
y6
y7
Precursor
x 10^6

Q L A T k A A R

Histone H3.3
Charge State: +2
Scan Number: 3363
File Name: 130605_Ack_IP_3.raw

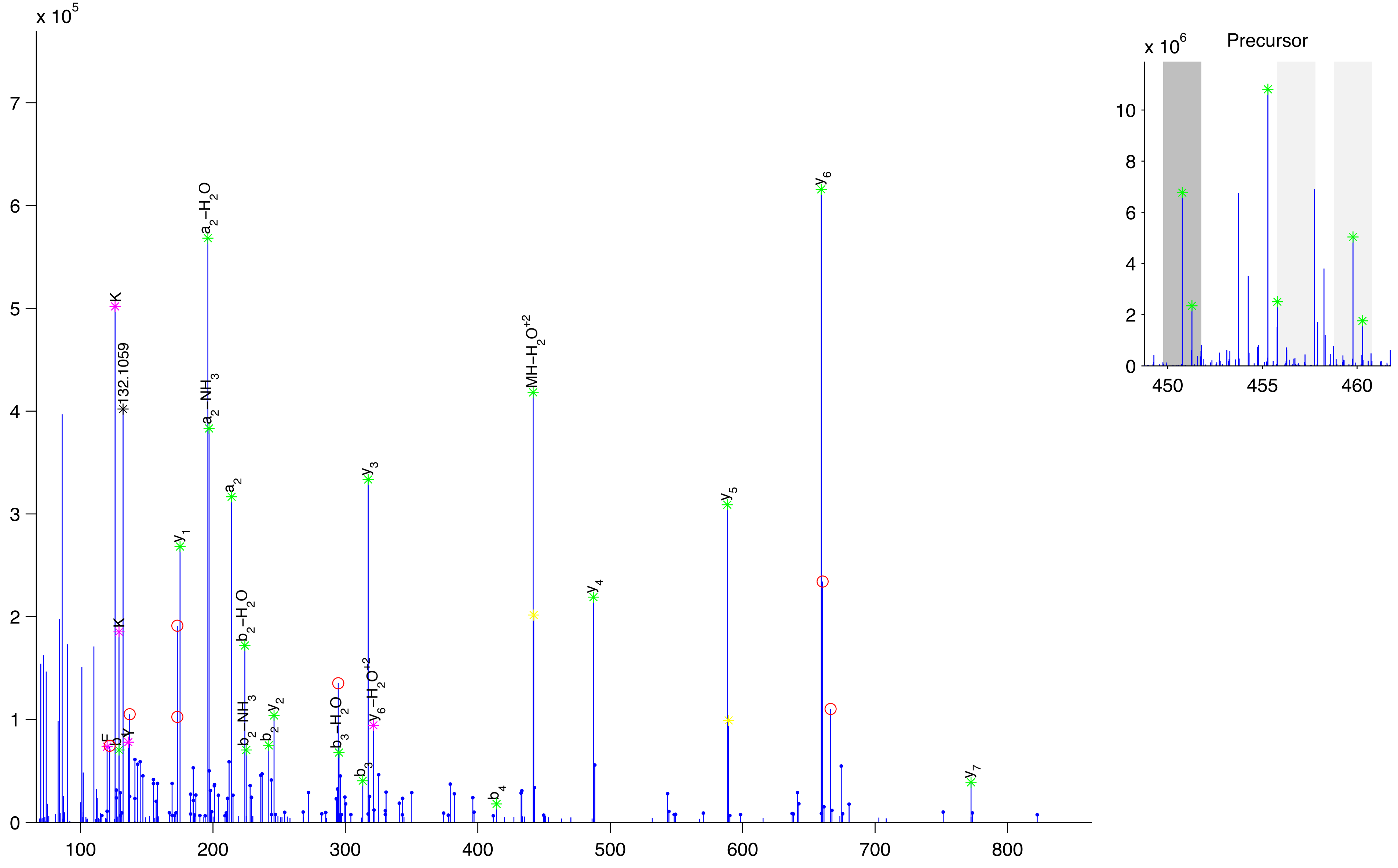

k Q L A T k A A R

Histone H3.3
Charge State: +2
Scan Number: 3879
File Name: 130605_Ack_IP_2.raw

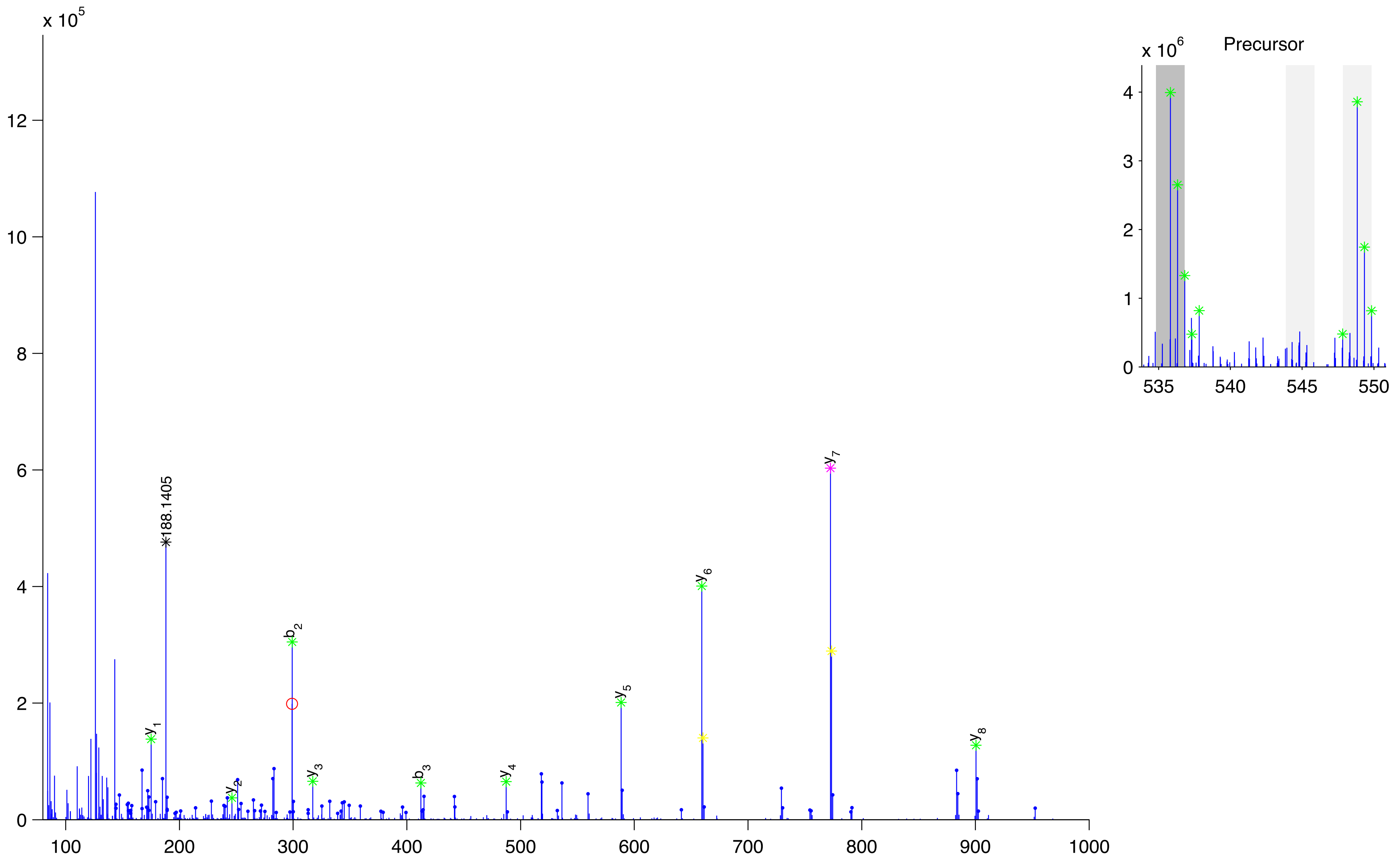

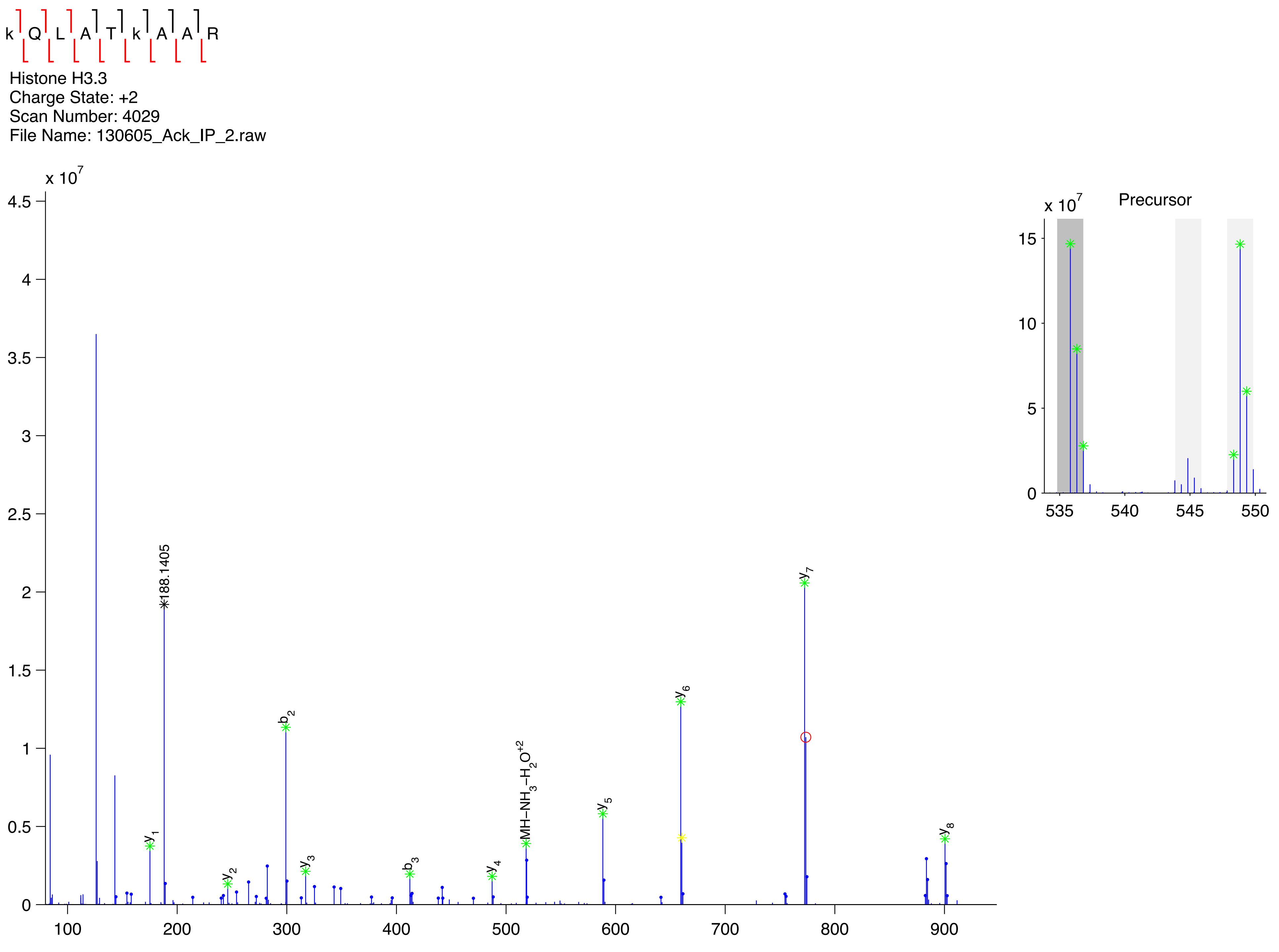
k Q L A T k A A R
Histone H3.3
Charge State: +2
Scan Number: 4029
File Name: 130605_Ack_IP_2.raw
x 10^7
188.1405
y1
y2
b2
y3
b3
y4
MH−NH3−H2O+2
y5
y6
y7
y8
Precursor
x 10^7

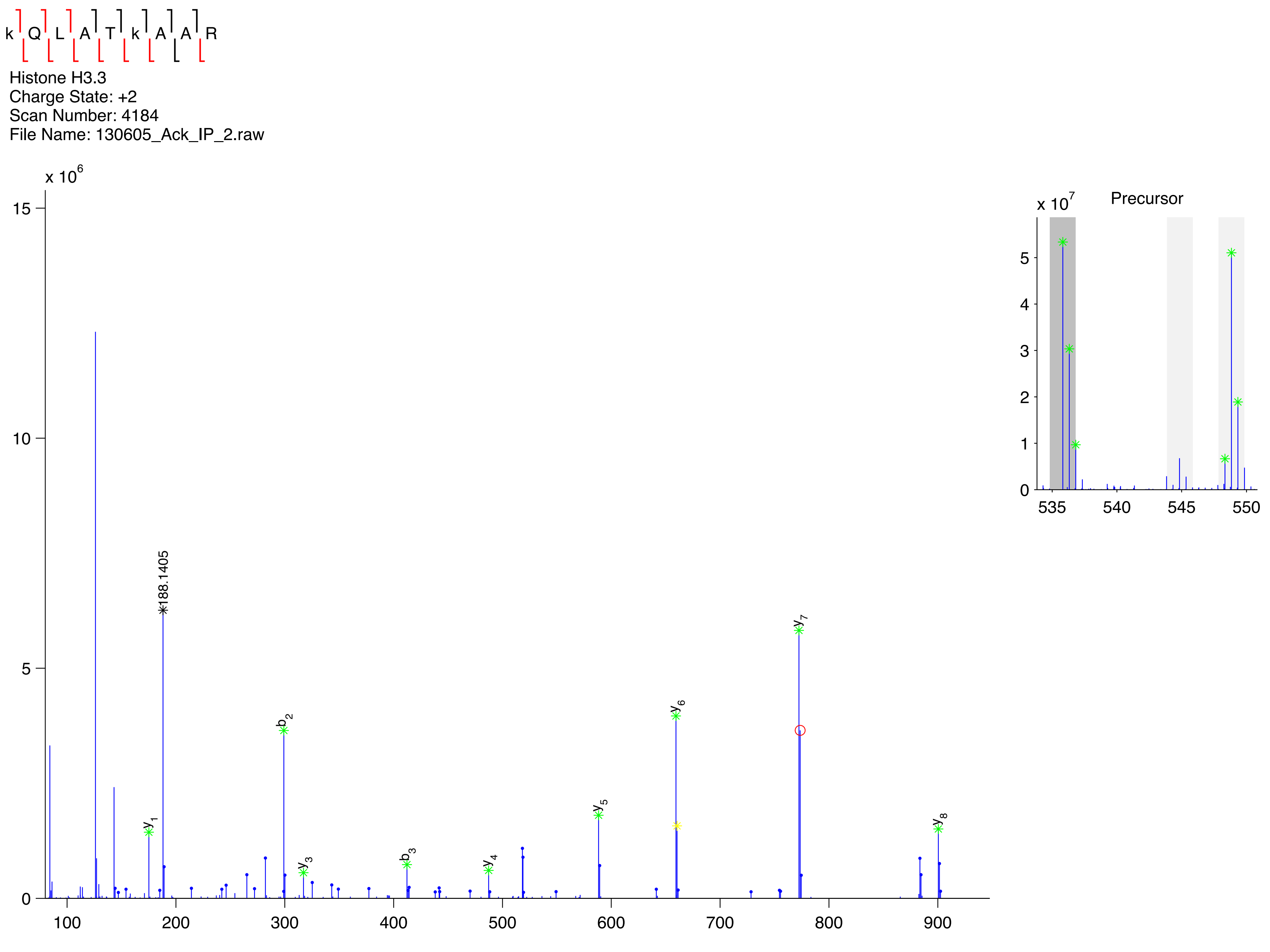
k Q L A T k A A R
Histone H3.3
Charge State: +2
Scan Number: 4184
File Name: 130605_Ack_IP_2.raw
x 10^6
188.1405
y1
b2
y3
b3
y4
y5
y6
y7
y8
Precursor
x 10^7

D G L Q N E k N I V S T P V K

Hydroxymethylglutaryl–CoA lyase, mitochondrial
Charge State: +2
Scan Number: 9223
File Name: 130605_Ack_IP_1.raw

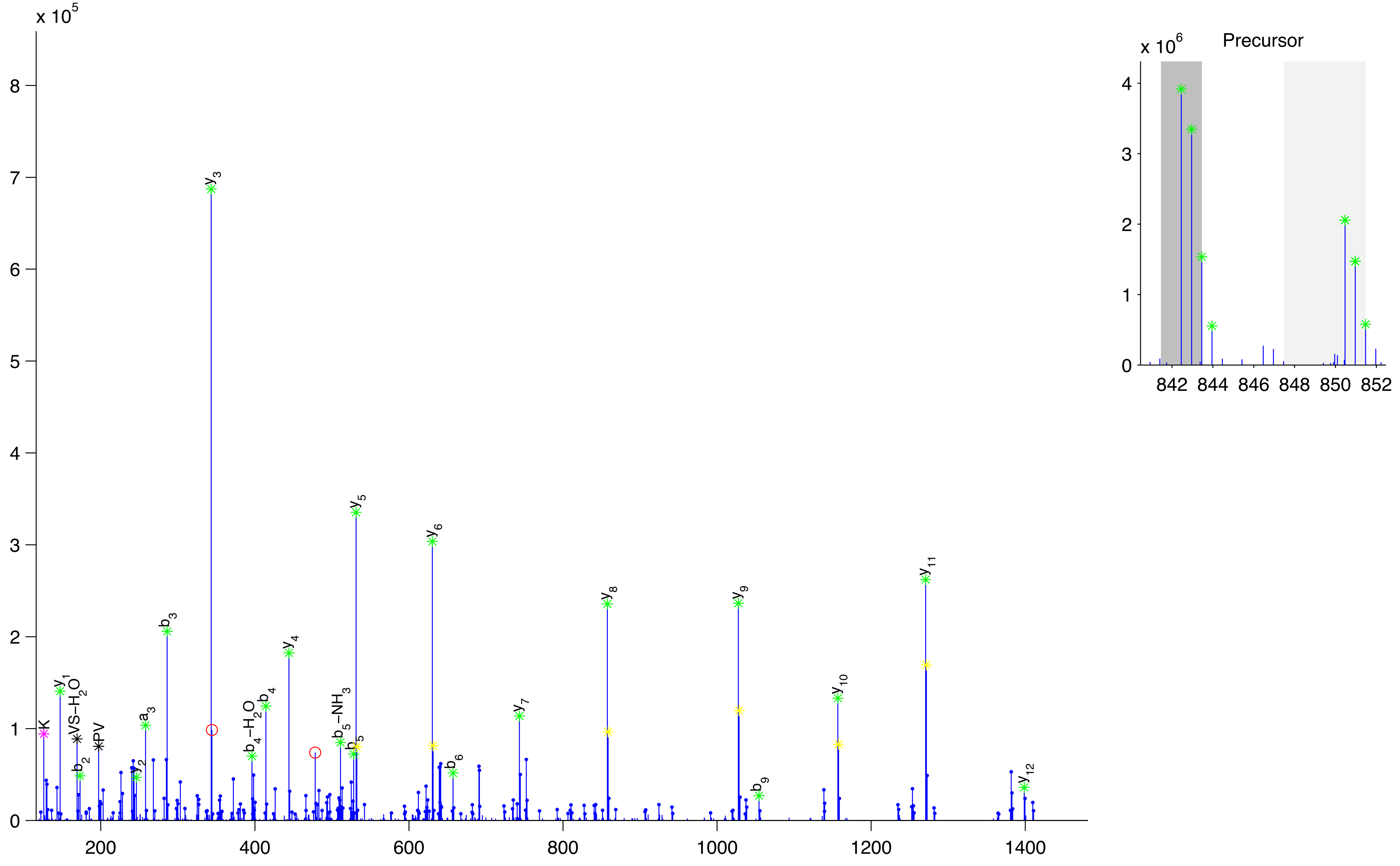

D G L Q N E k N I V S T P V K

Hydroxymethylglutaryl−CoA lyase, mitochondrial
Charge State: +2
Scan Number: 9509
File Name: 130605_Ack_IP_2.raw

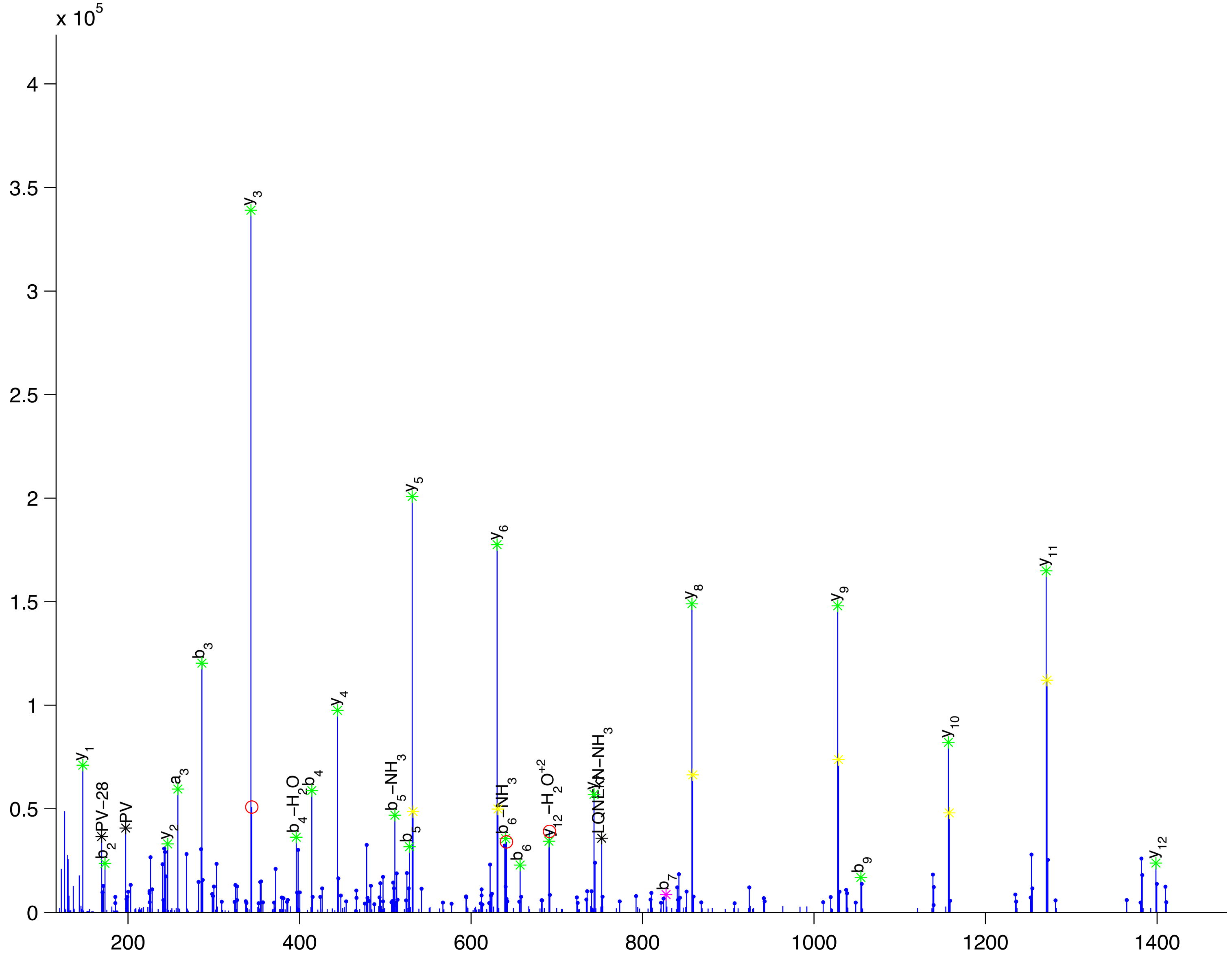

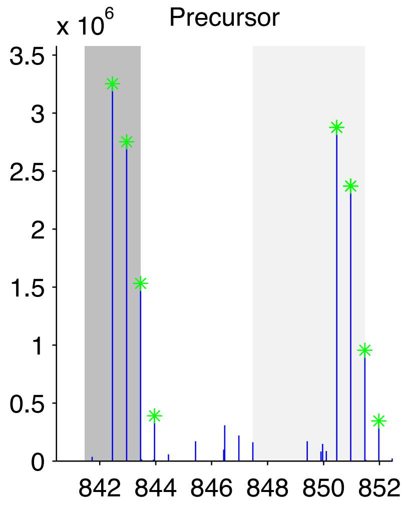

D G L Q N E k N I V S T P V K

Hydroxymethylglutaryl–CoA lyase, mitochondrial
Charge State: +2
Scan Number: 9644
File Name: 130605_Ack_IP_3.raw

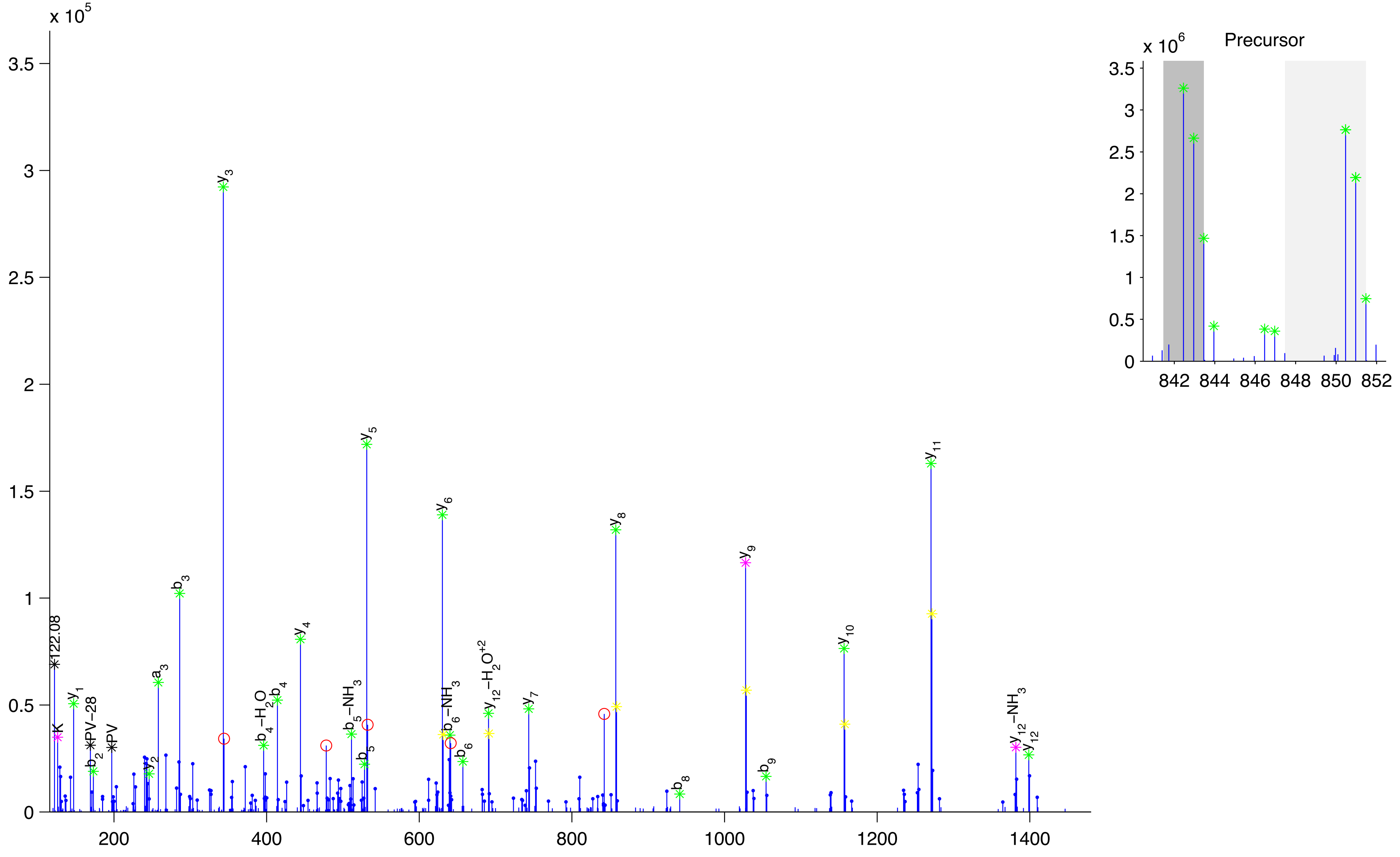

V V S E T N D T k V L R

Keratin, type I cytoskeletal 18
Charge State: +2
Scan Number: 5528
File Name: 130605_Ack_IP_1.raw

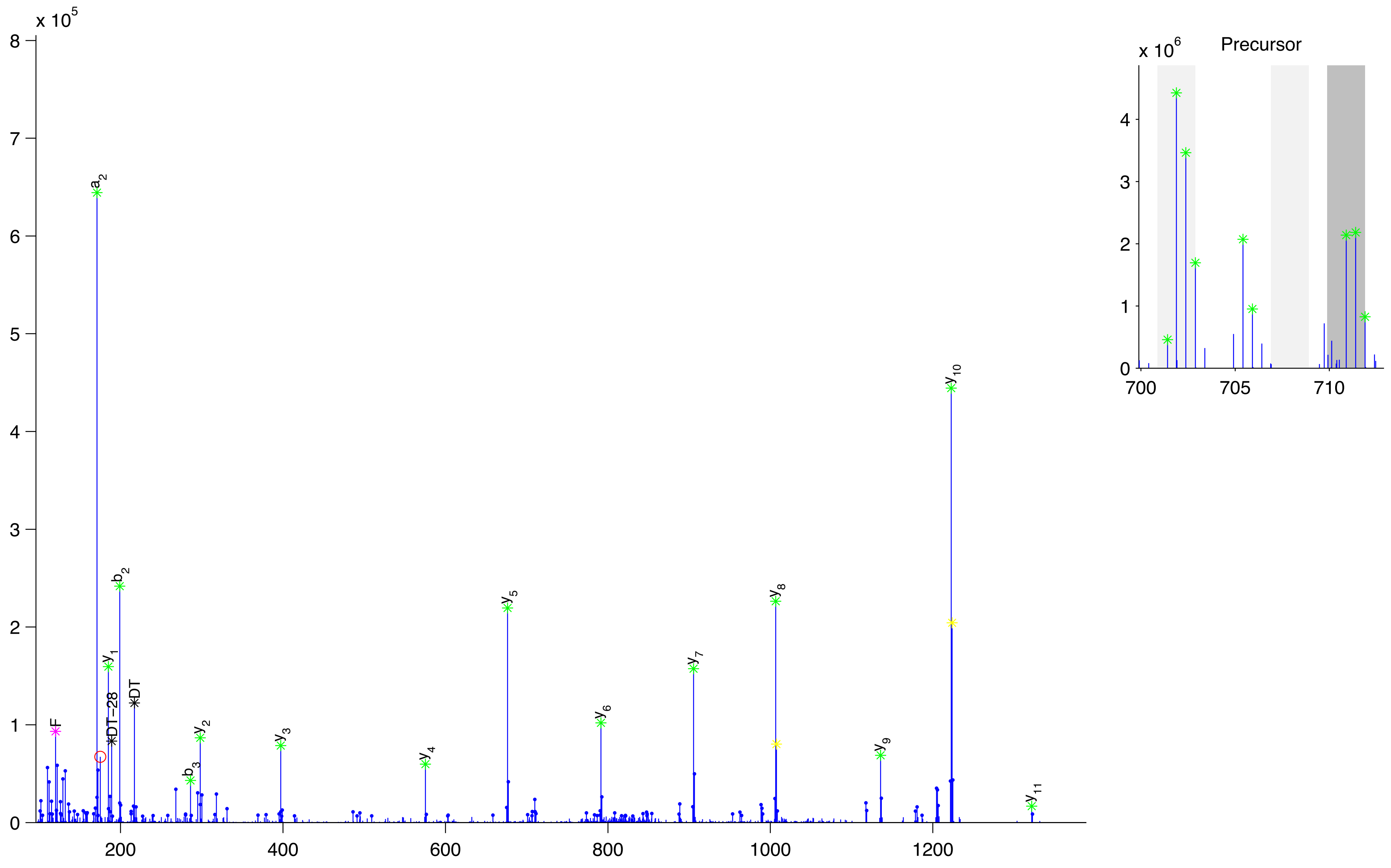

V V S E T N D T k V L R

Keratin, type I cytoskeletal 18
Charge State: +2
Scan Number: 5582
File Name: 130605_Ack_IP_1.raw

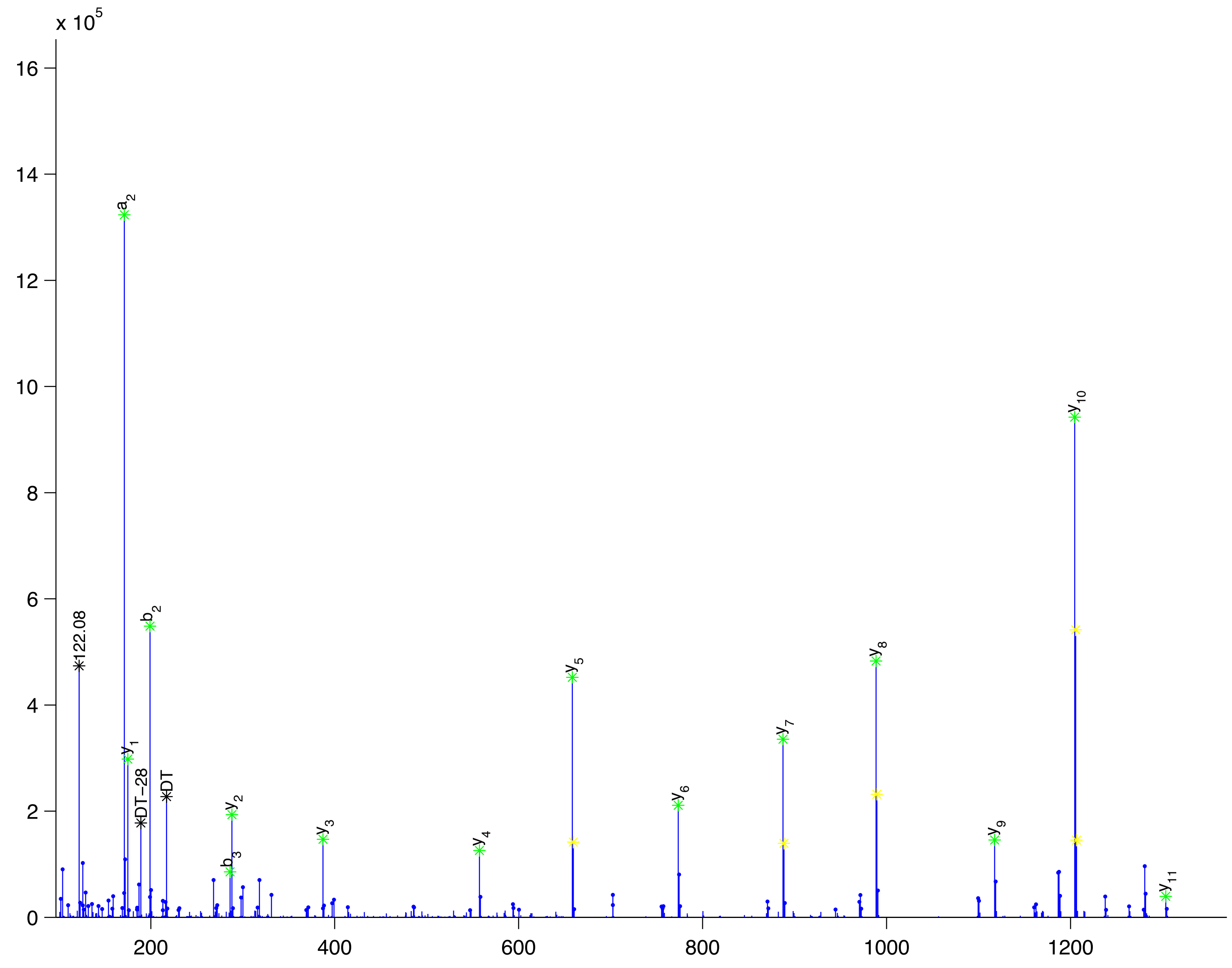

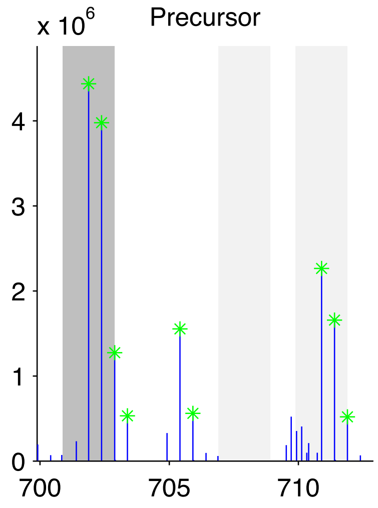

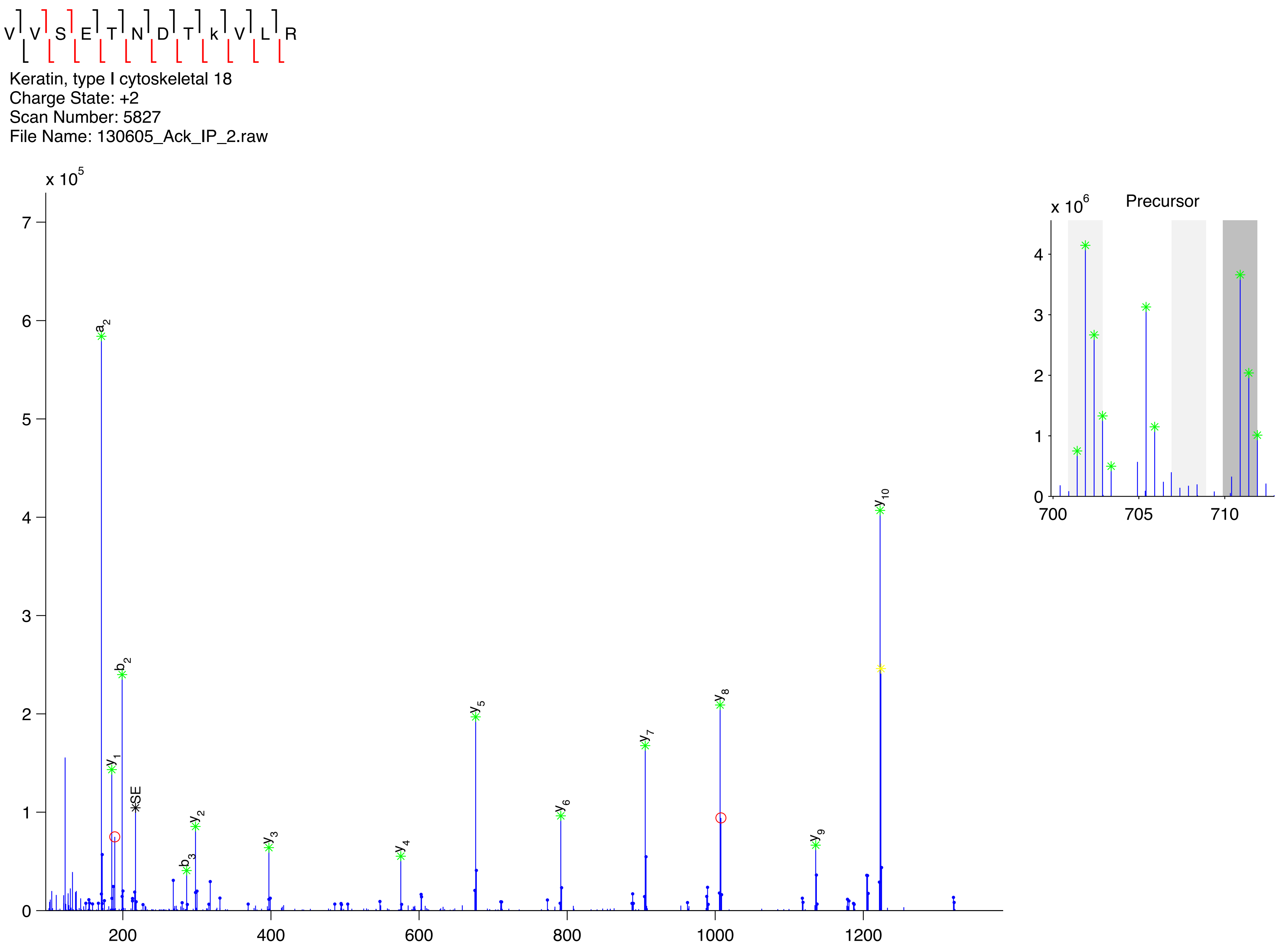
V V S E T N D T k V L R
Keratin, type I cytoskeletal 18
Charge State: +2
Scan Number: 5827
File Name: 130605_Ack_IP_2.raw
x 10^5
a2
b2
y1
SE
b3
y2
y3
y4
y5
y6
y7
y8
y9
y10
x 10^6
Precursor

V V S E T N D T k V L R

Keratin, type I cytoskeletal 18
Charge State: +2
Scan Number: 5950
File Name: 130605_Ack_IP_3.raw

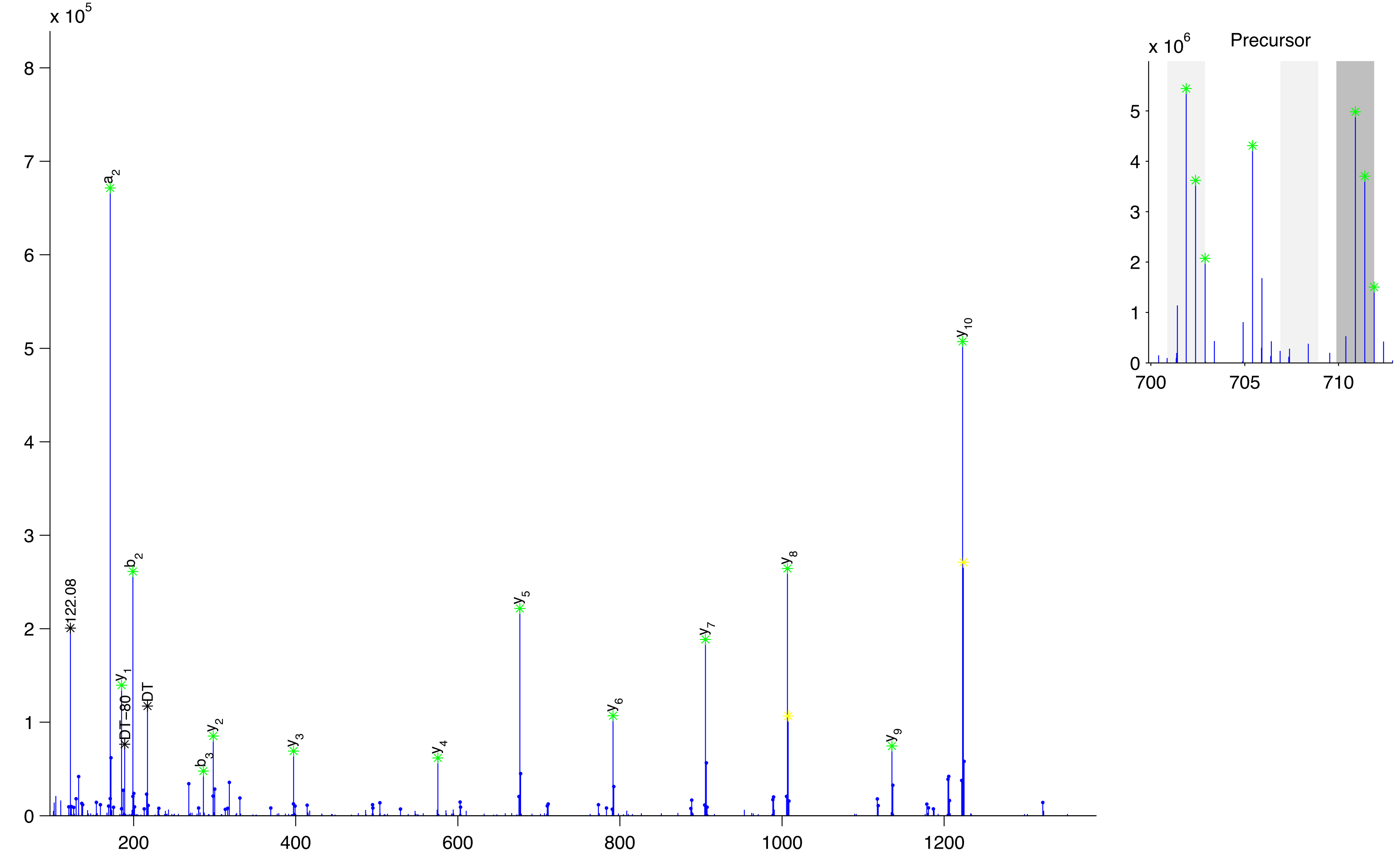

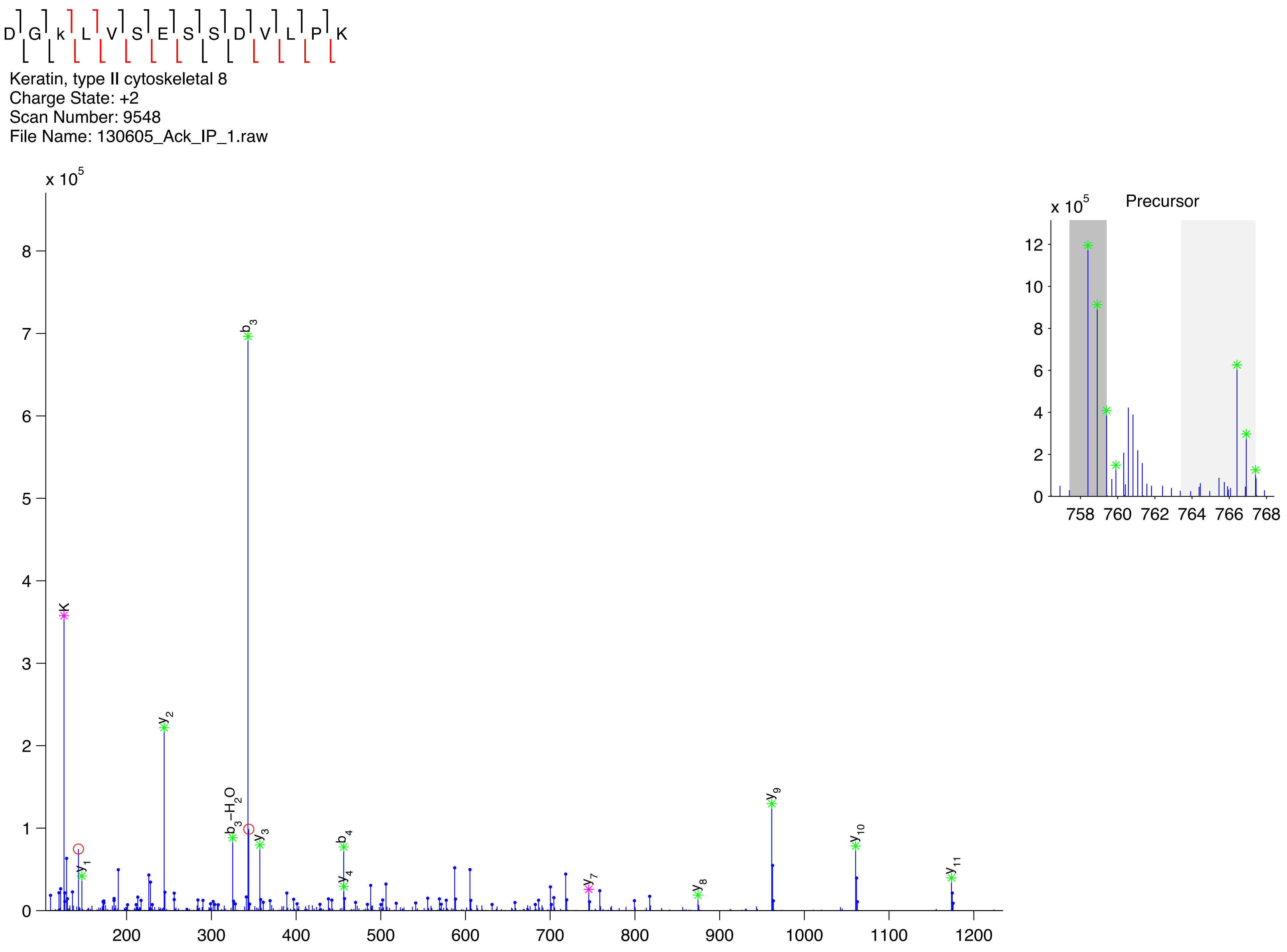

D G k L V S E S S D V L P K
Keratin, type II cytoskeletal 8
Charge State: +2
Scan Number: 9548
File Name: 130605_Ack_IP_1.raw
x 10^5
K
y1
y2
b3-H2O
b3
y3
y4
b4
y7
y8
y9
y10
y11
Precursor

D G k L V S E S S D V L P K

Keratin, type II cytoskeletal 8
Charge State: +2
Scan Number: 9652
File Name: 130605_Ack_IP_1.raw

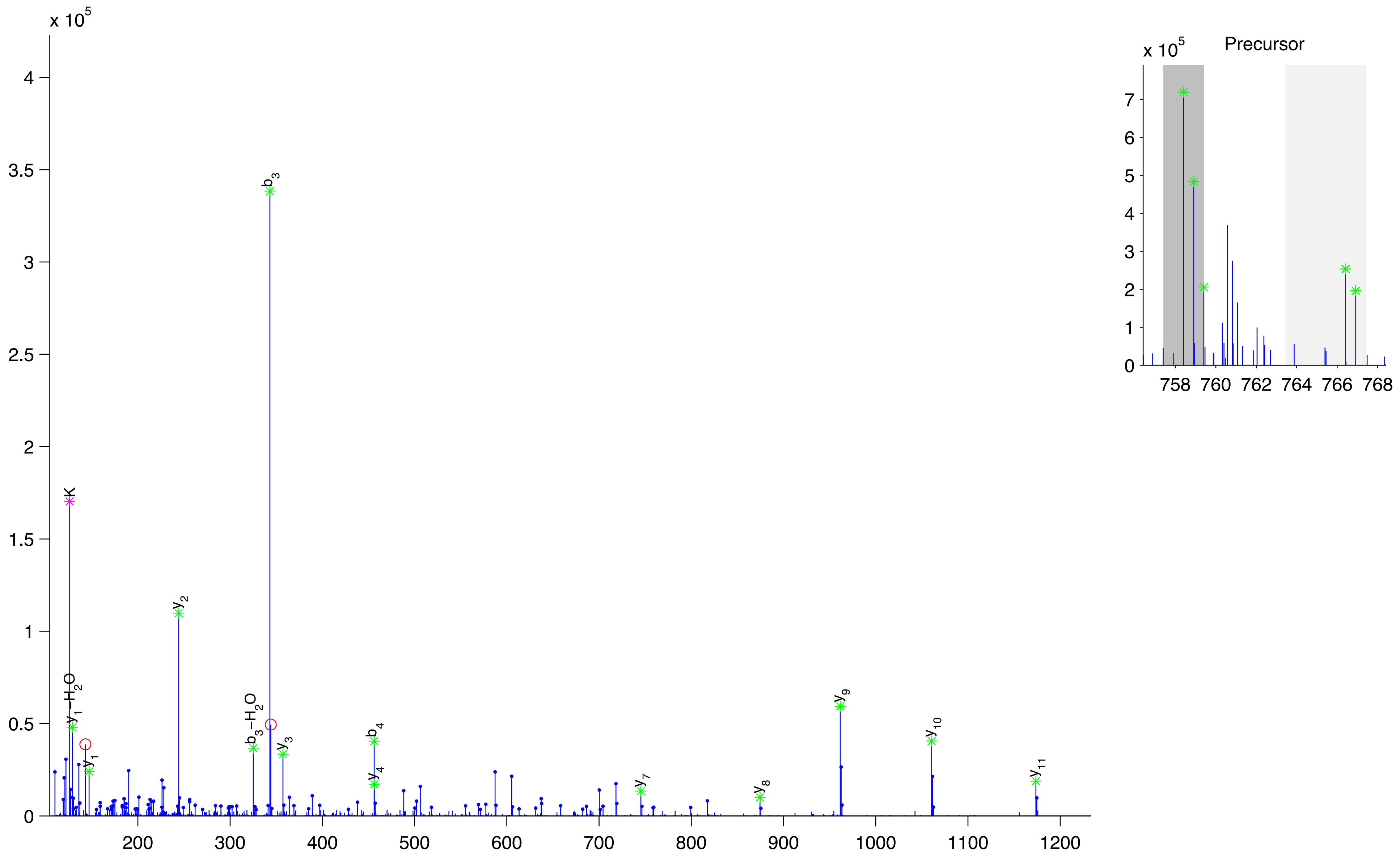

W S L L Q Q Q k T A R

Keratin, type II cytoskeletal 8
Charge State: +2
Scan Number: 9785
File Name: 130605_Ack_IP_1.raw

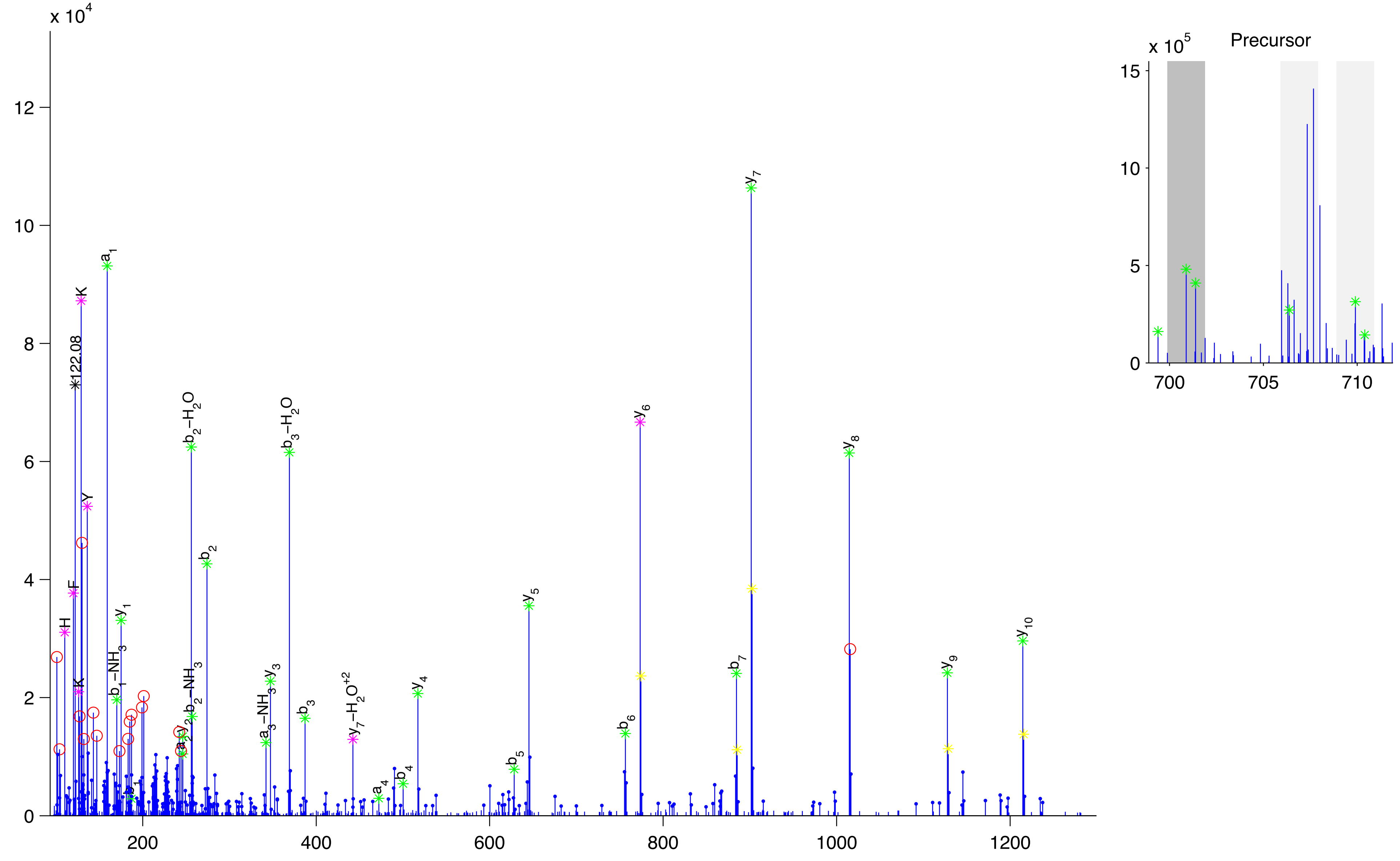

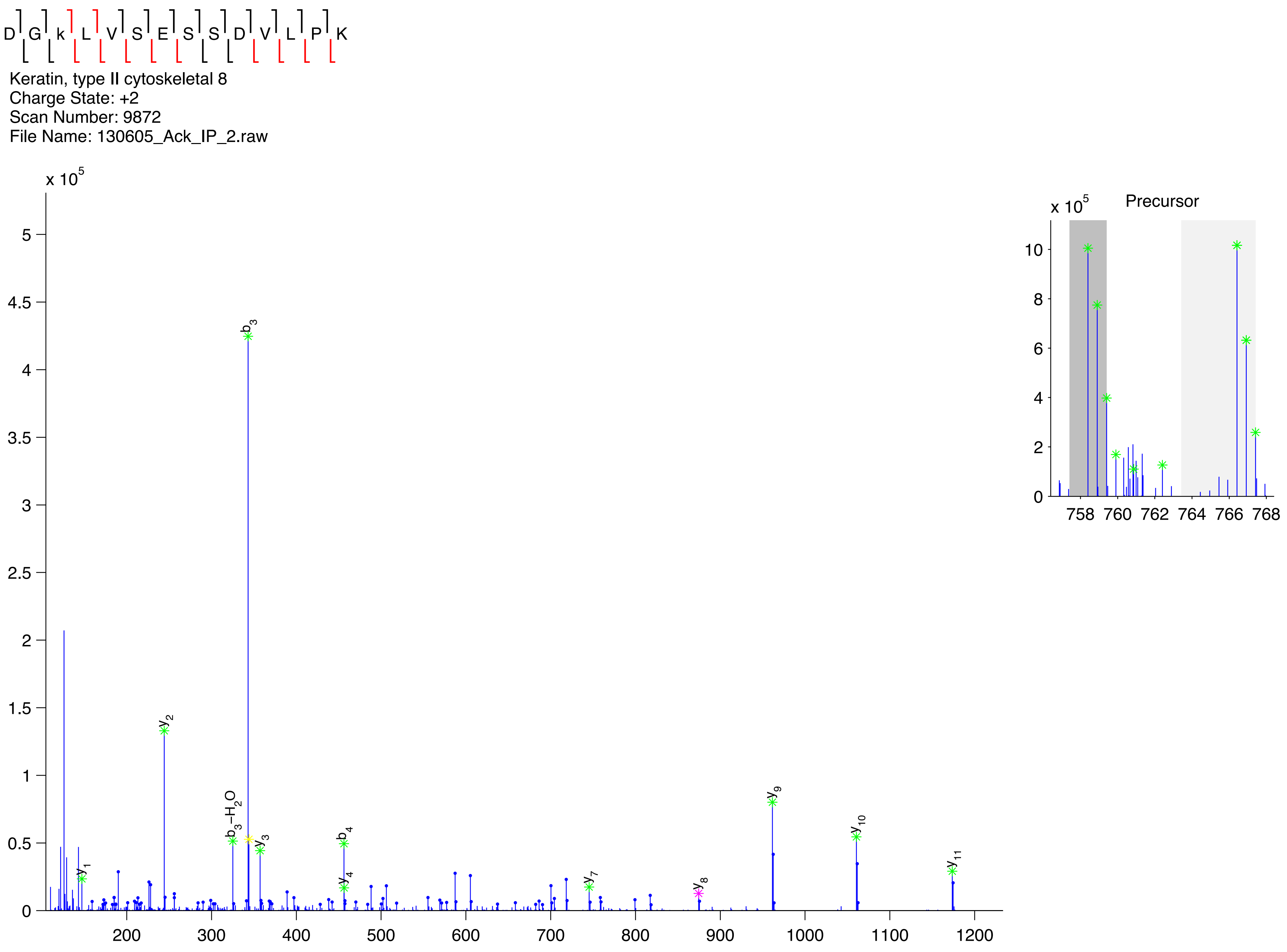

D G k L V S E S S D V L P K
Keratin, type II cytoskeletal 8
Charge State: +2
Scan Number: 9872
File Name: 130605_Ack_IP_2.raw
x 10^5
y1
y2
b3−H2O
b3
y3
y4
b4
y7
y8
y9
y10
y11
Precursor

D G k L V S E S S D V L P K

Keratin, type II cytoskeletal 8
Charge State: +2
Scan Number: 10043
File Name: 130605_Ack_IP_3.raw

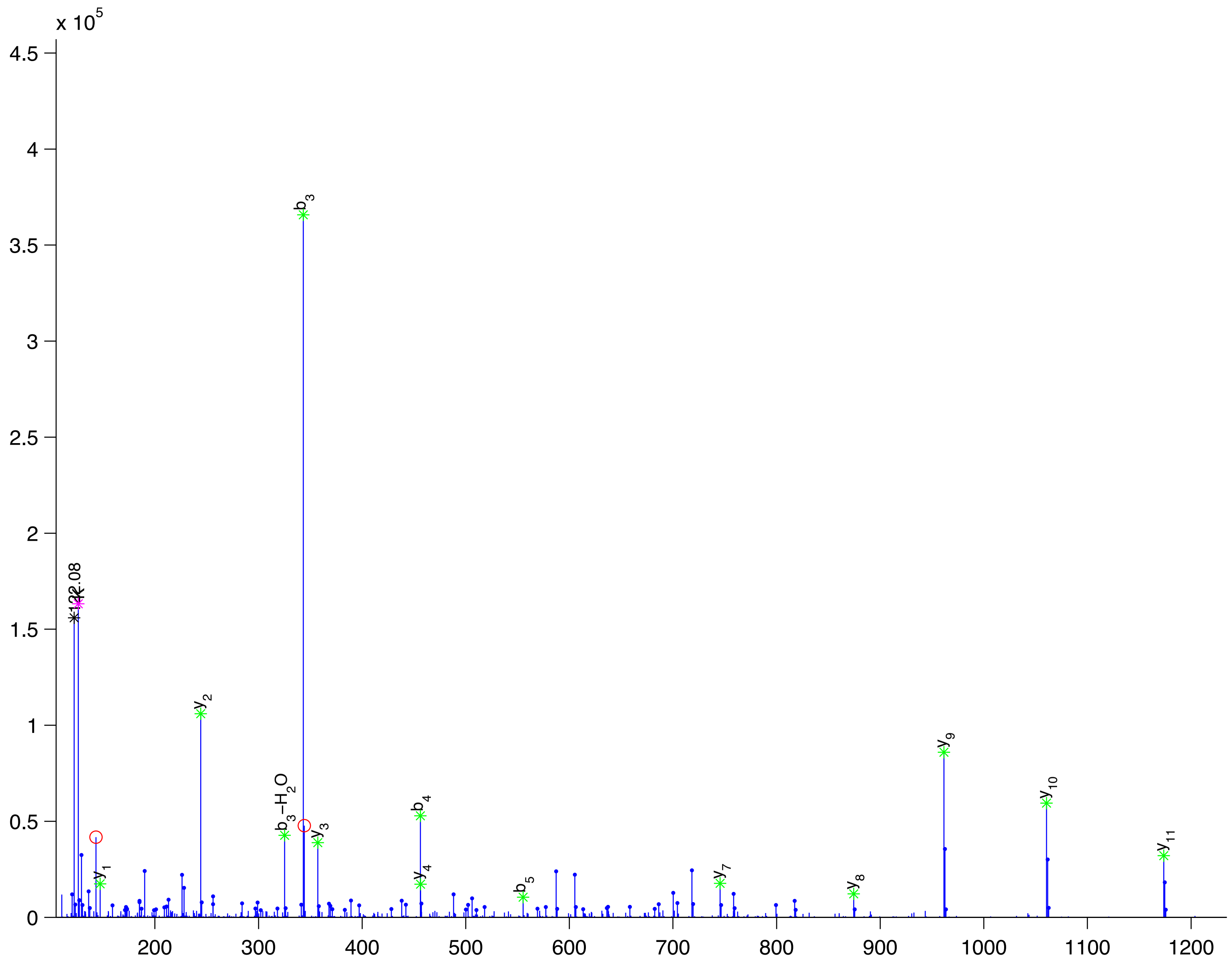

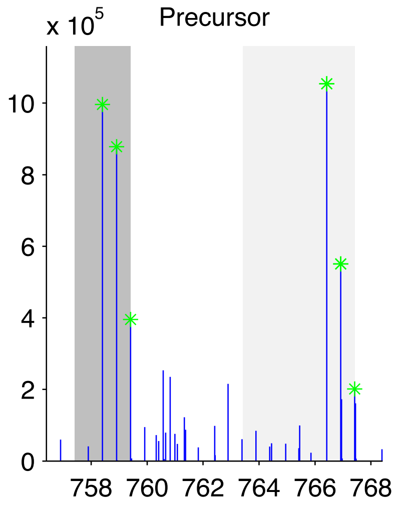

W S L L Q Q Q k T A R

Keratin, type II cytoskeletal 8
Charge State: +2
Scan Number: 10128
File Name: 130605_Ack_IP_2.raw

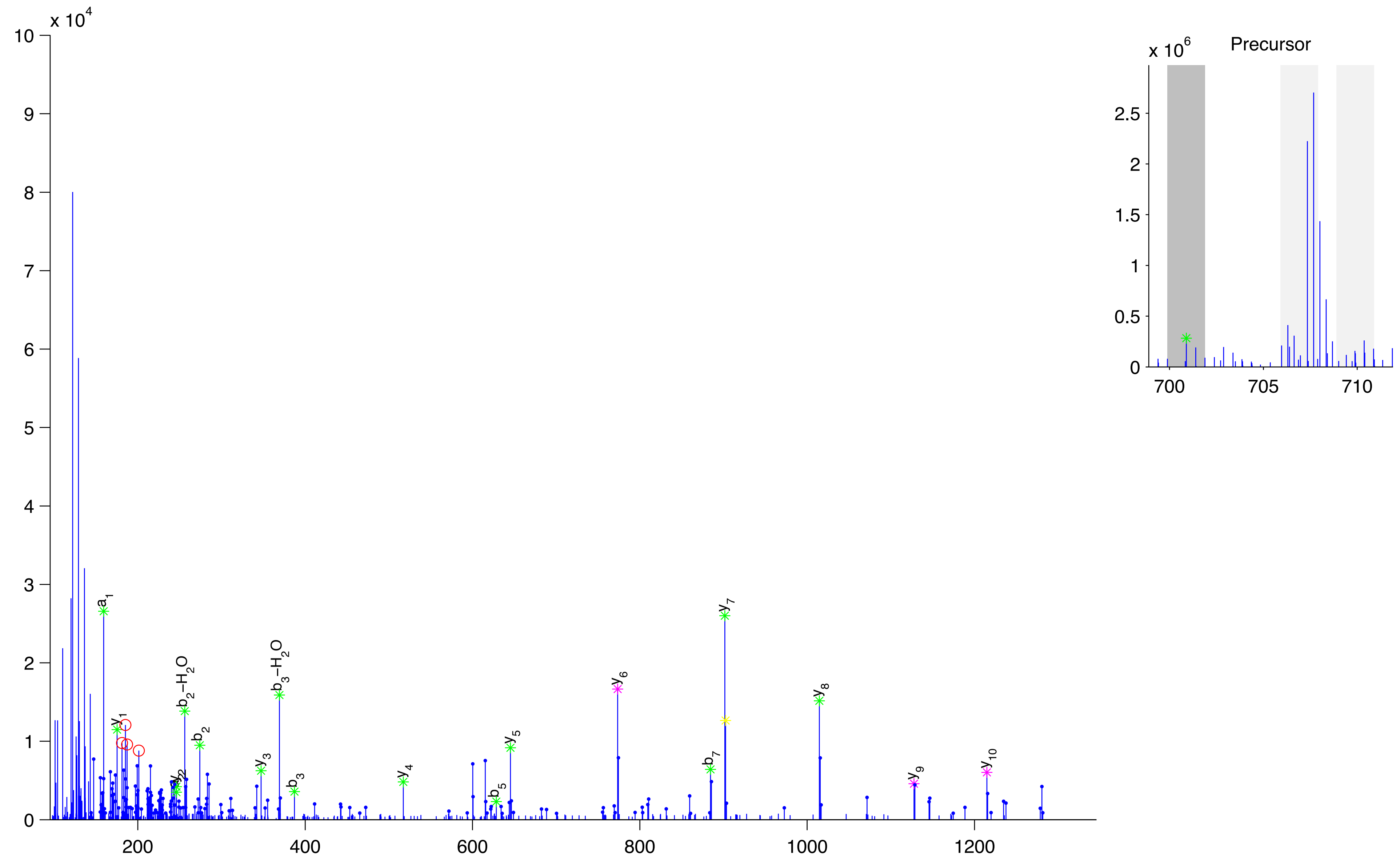

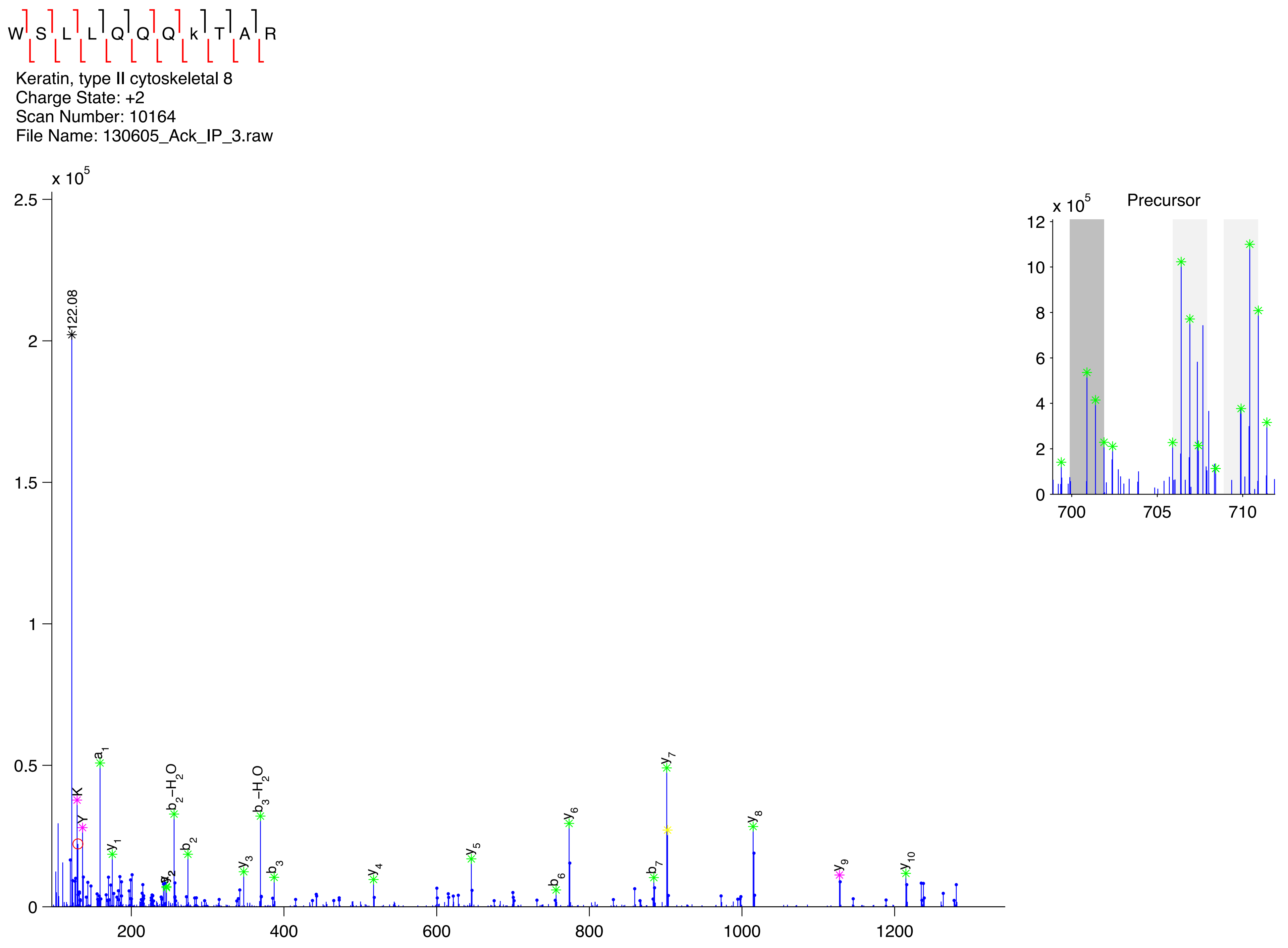
W S L L Q Q Q k T A R
Keratin, type II cytoskeletal 8
Charge State: +2
Scan Number: 10164
File Name: 130605_Ack_IP_3.raw
x 10^5
122.08
K
Y
a1
y1
b2-H2O
b2
y3
b3-H2O
b3
y4
y5
b6
y6
b7
y7
y8
y9
y10
Precursor

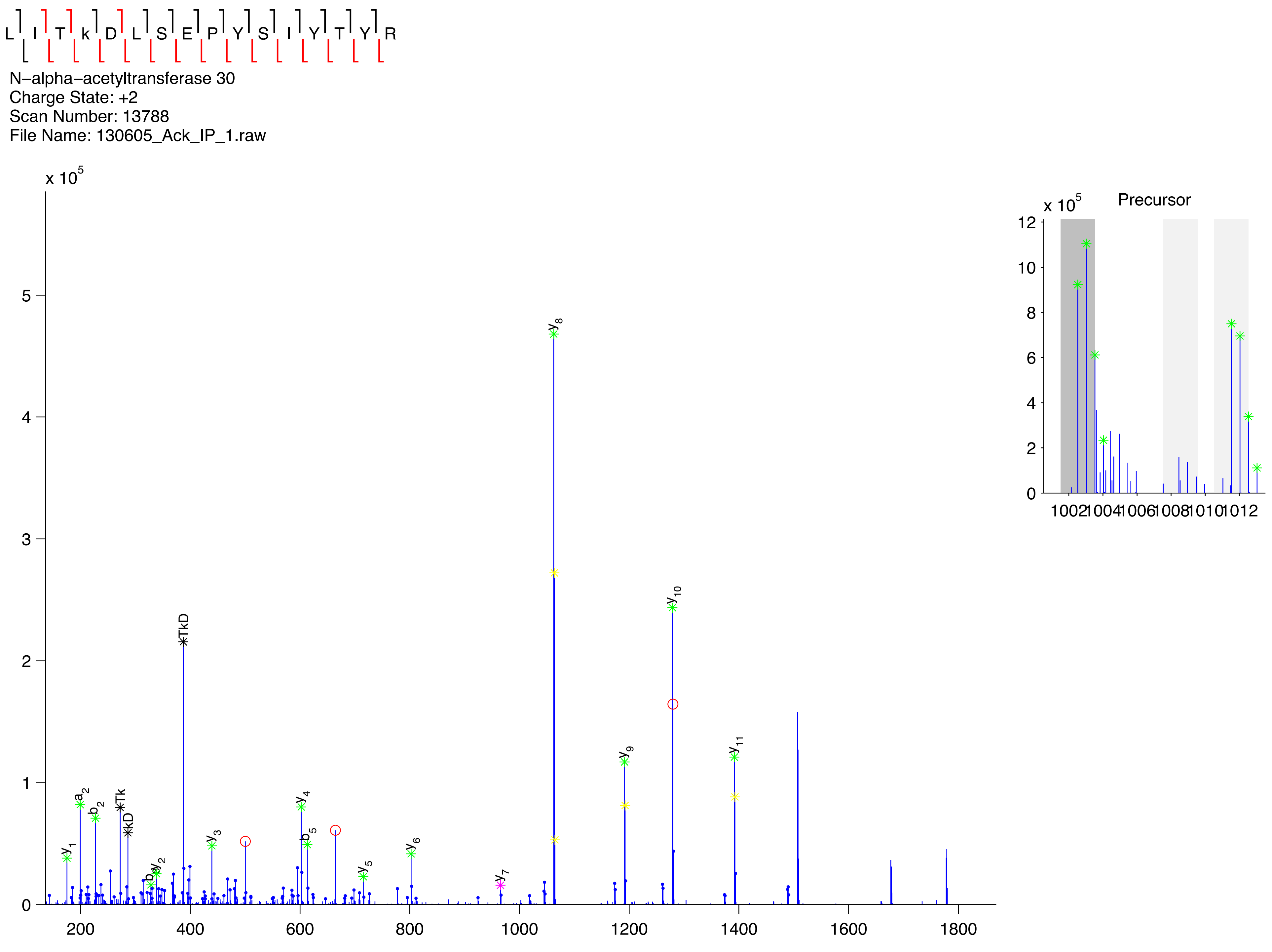

L I T k D L S E P Y S I Y T Y R
N−alpha−acetyltransferase 30
Charge State: +2
Scan Number: 13788
File Name: 130605_Ack_IP_1.raw
x 10^5
y1
a2
b2
Tk
kD
b3
y2
TkD
y3
y4
b5
y5
y6
y7
y8
y9
y10
y11
Precursor

L I T k D L S E P Y S I Y T Y R

N–alpha–acetyltransferase 30
Charge State: +2
Scan Number: 14010
File Name: 130605_Ack_IP_2.raw

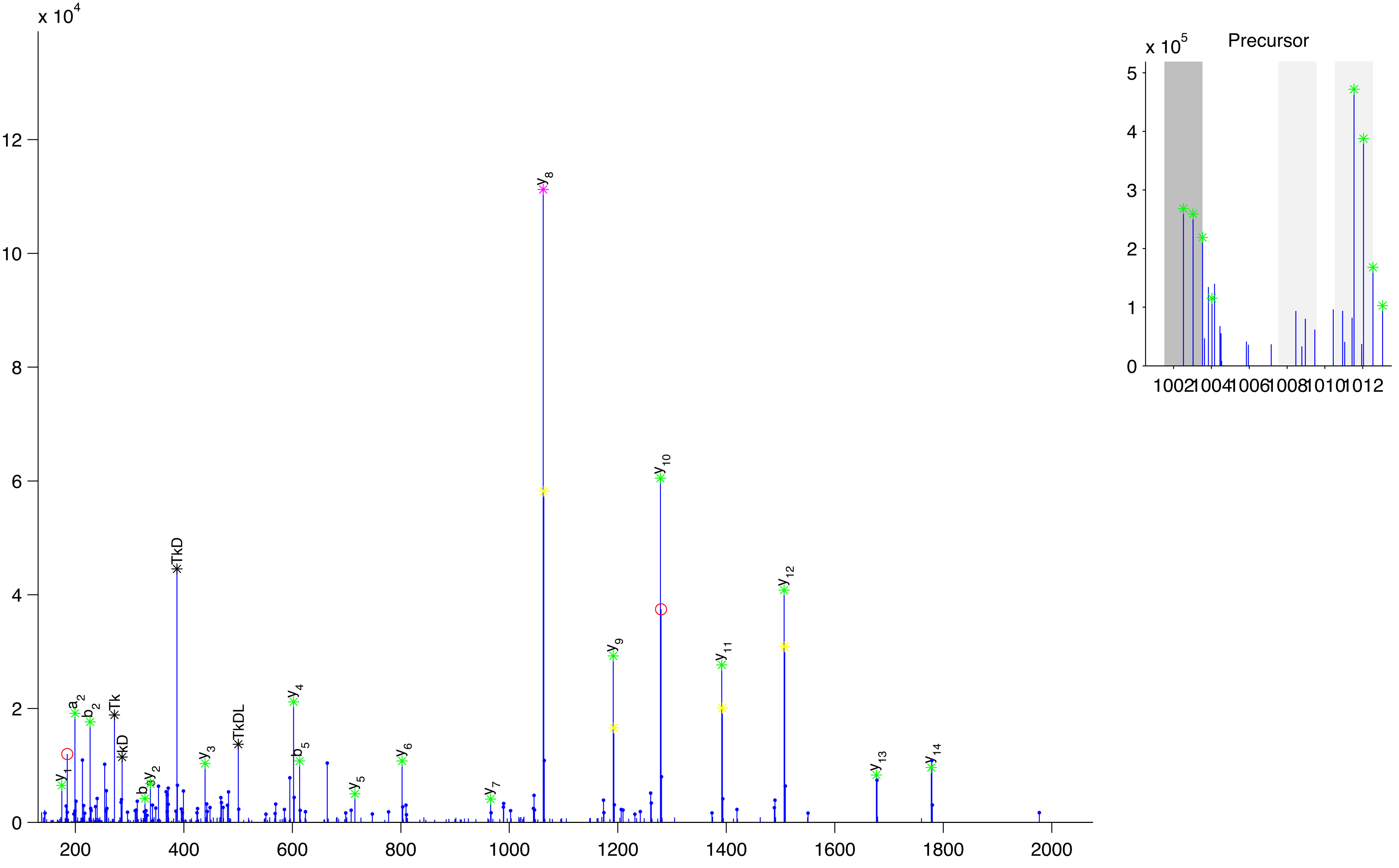

L N Q V I F P V S Y N D k F Y K

N−alpha−acetyltransferase 50
Charge State: +2
Scan Number: 14847
File Name: 130605_Ack_IP_1.raw

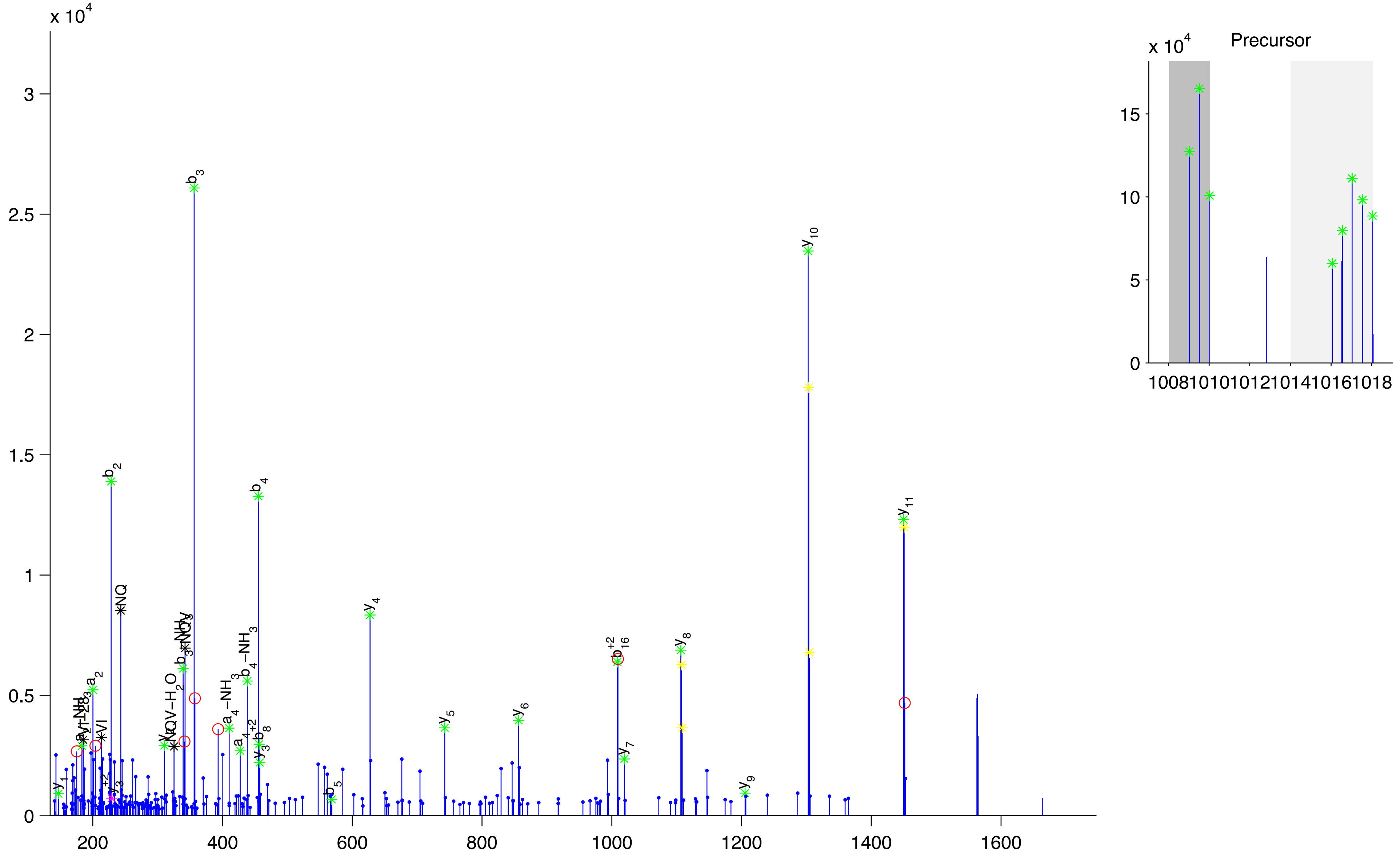

V E A k F I N Y V K

Nucleophosmin
Charge State: +2
Scan Number: 10764
File Name: 130605_Ack_IP_1.raw

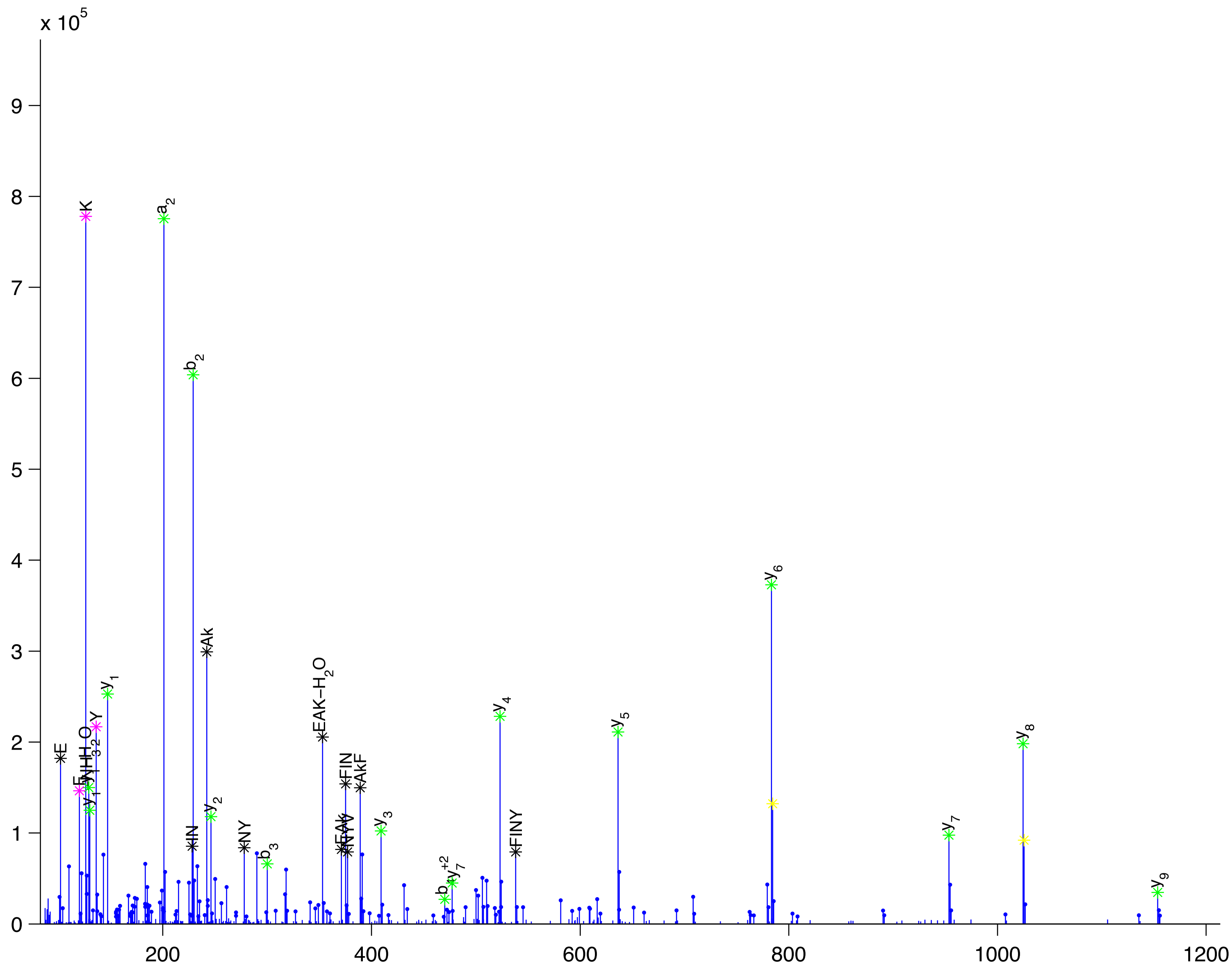

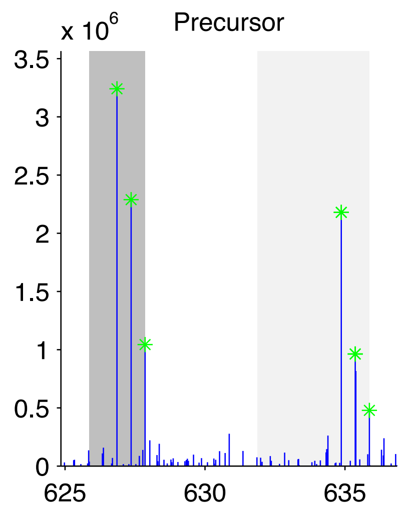

V E A k F I N Y V K

Nucleophosmin
Charge State: +2
Scan Number: 11001
File Name: 130605_Ack_IP_2.raw

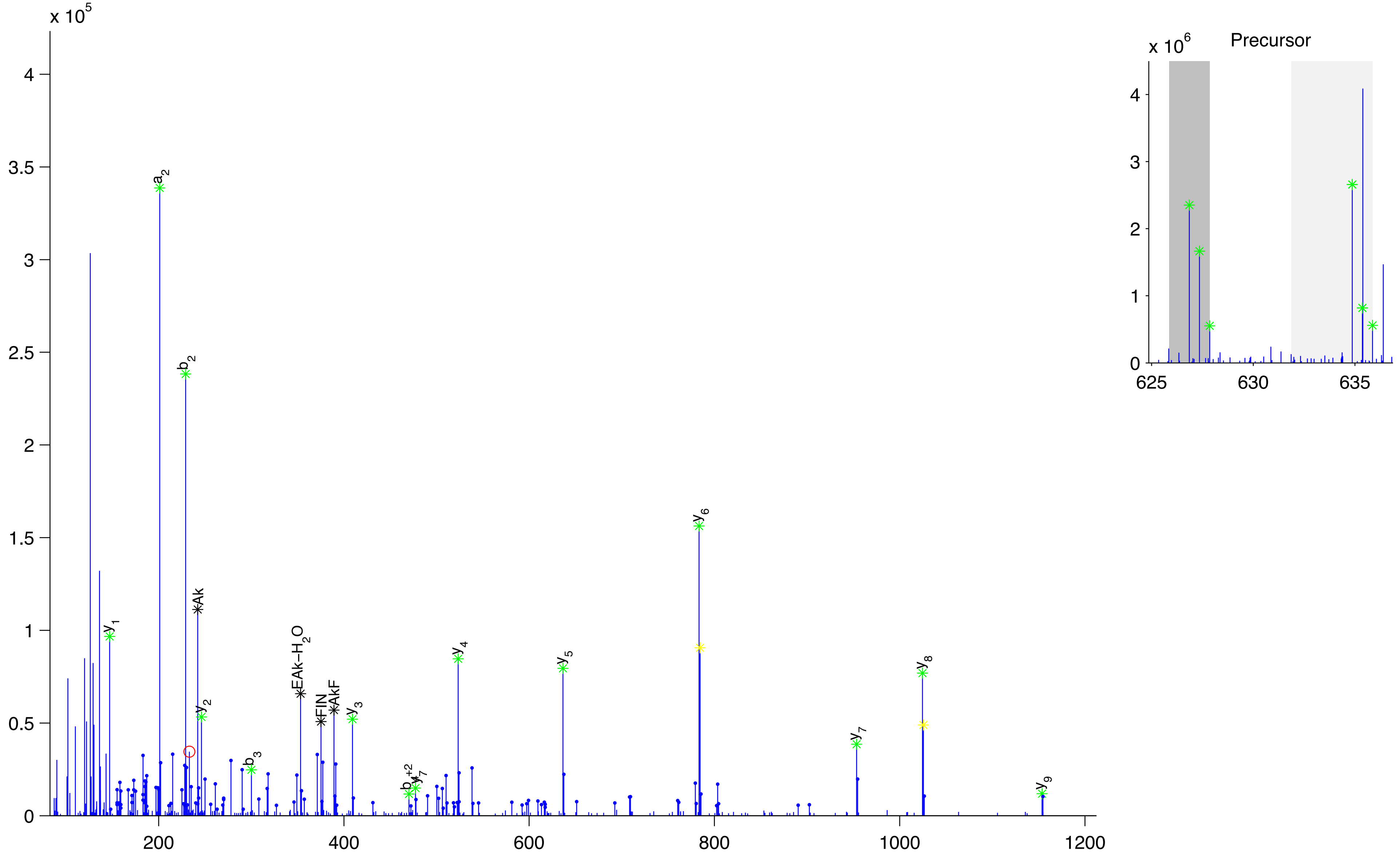

V E A k F I N Y V K

Nucleophosmin
Charge State: +2
Scan Number: 11187
File Name: 130605_Ack_IP_3.raw

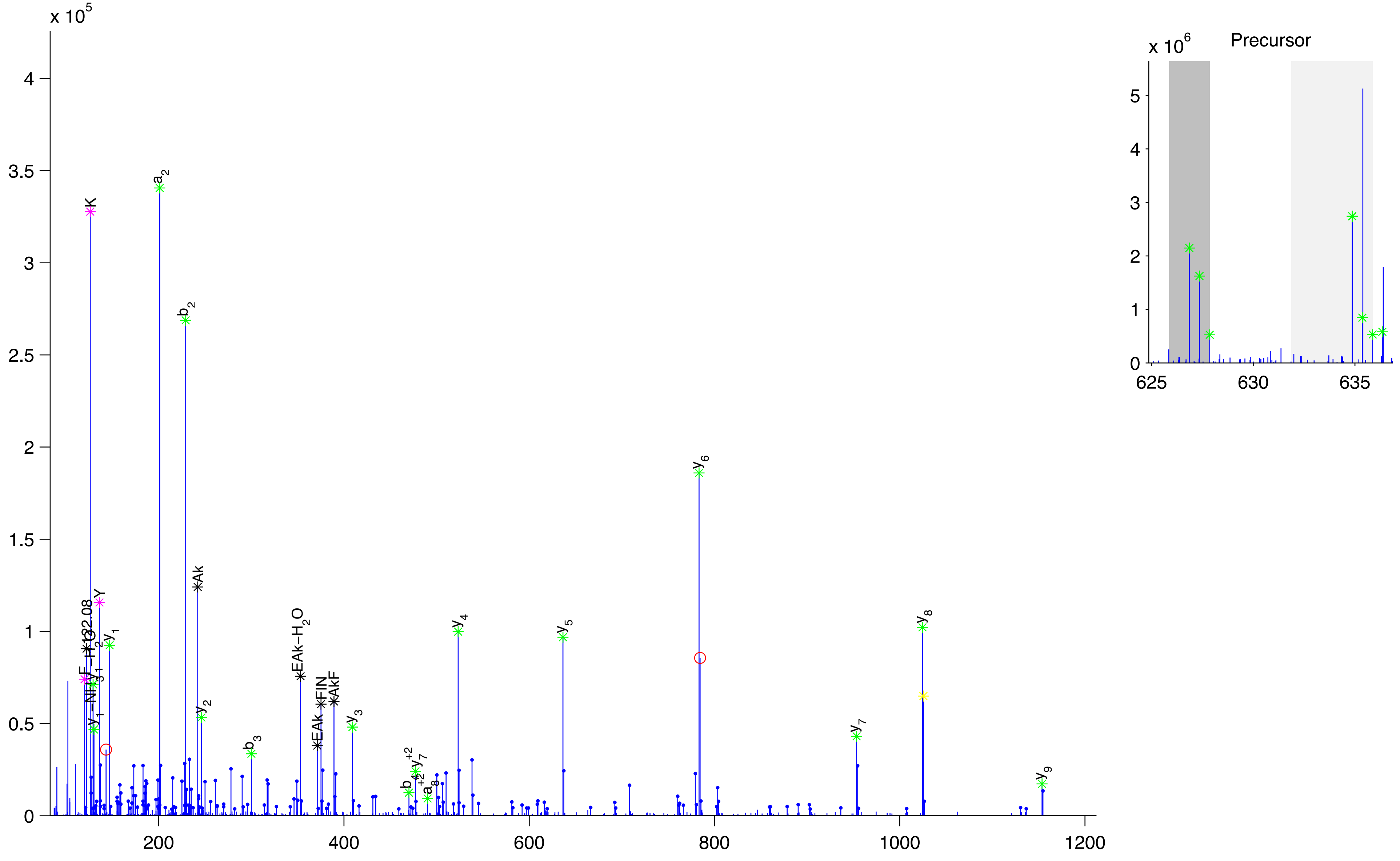

A L S T G E k G F G Y K

Peptidyl–prolyl cis–trans isomerase A
Charge State: +2
Scan Number: 7291
File Name: 130605_Ack_IP_1.raw

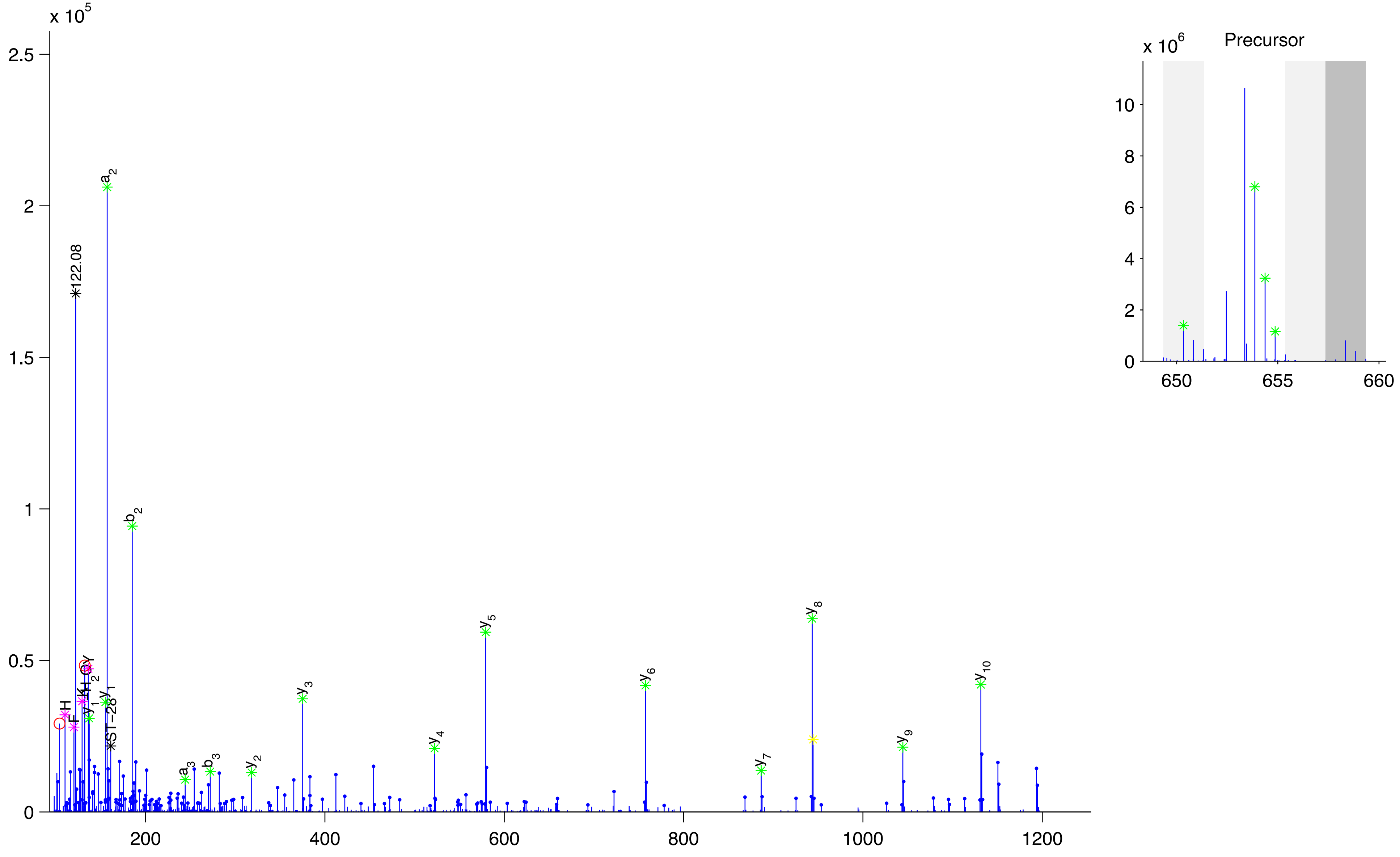

A L S T G E k G F G Y K

Peptidyl–prolyl cis–trans isomerase A
Charge State: +2
Scan Number: 7541
File Name: 130605_Ack_IP_2.raw

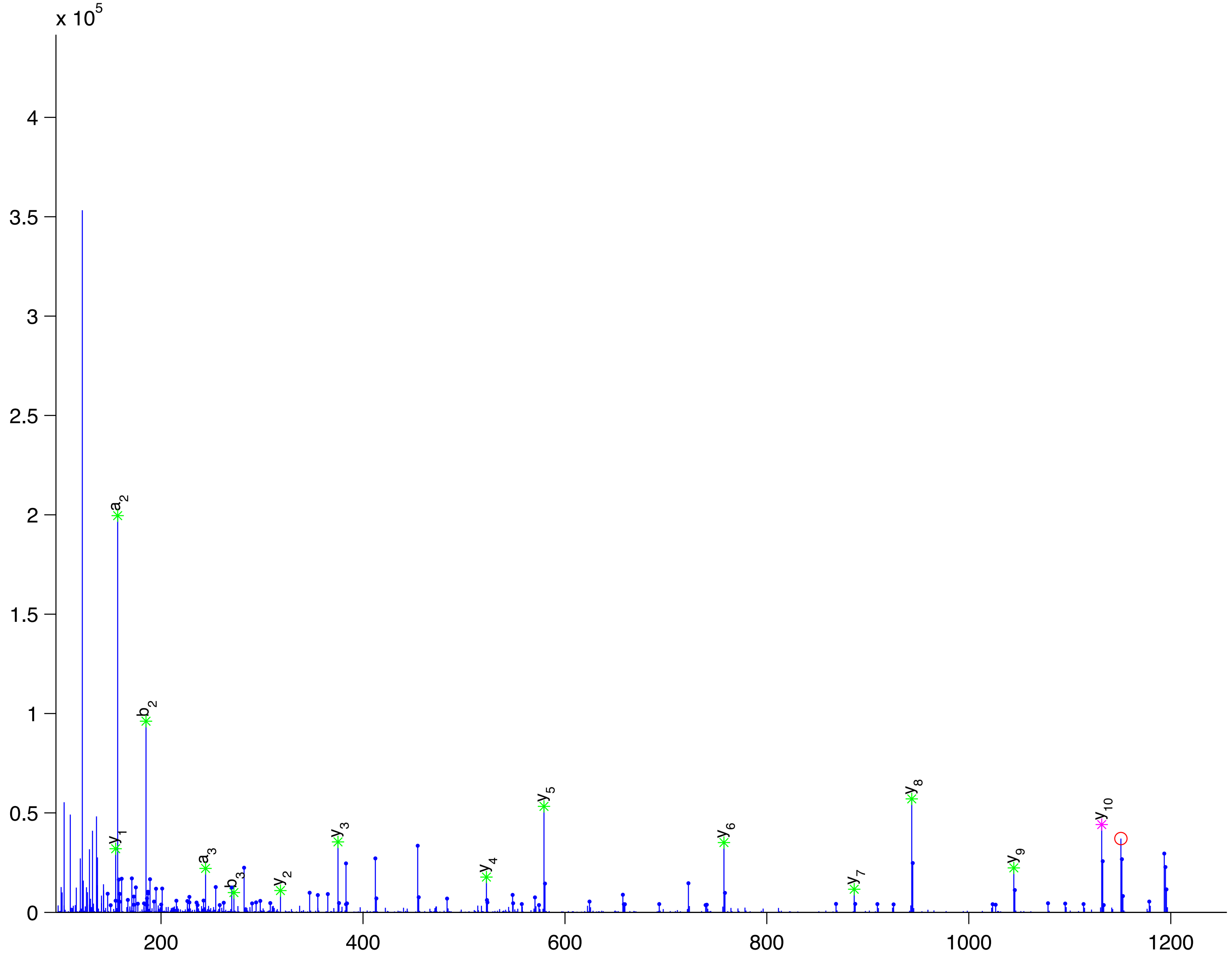

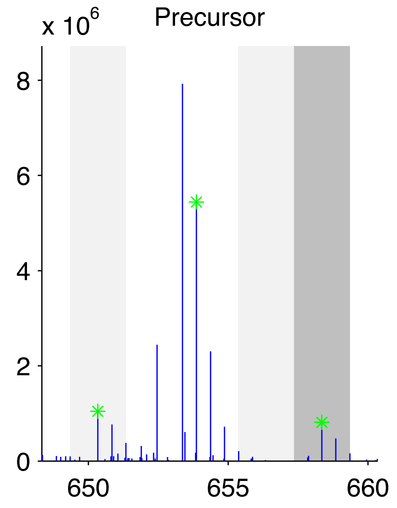

A L S T G E k G F G Y K

Peptidyl–prolyl cis–trans isomerase A
Charge State: +2
Scan Number: 7654
File Name: 130605_Ack_IP_3.raw

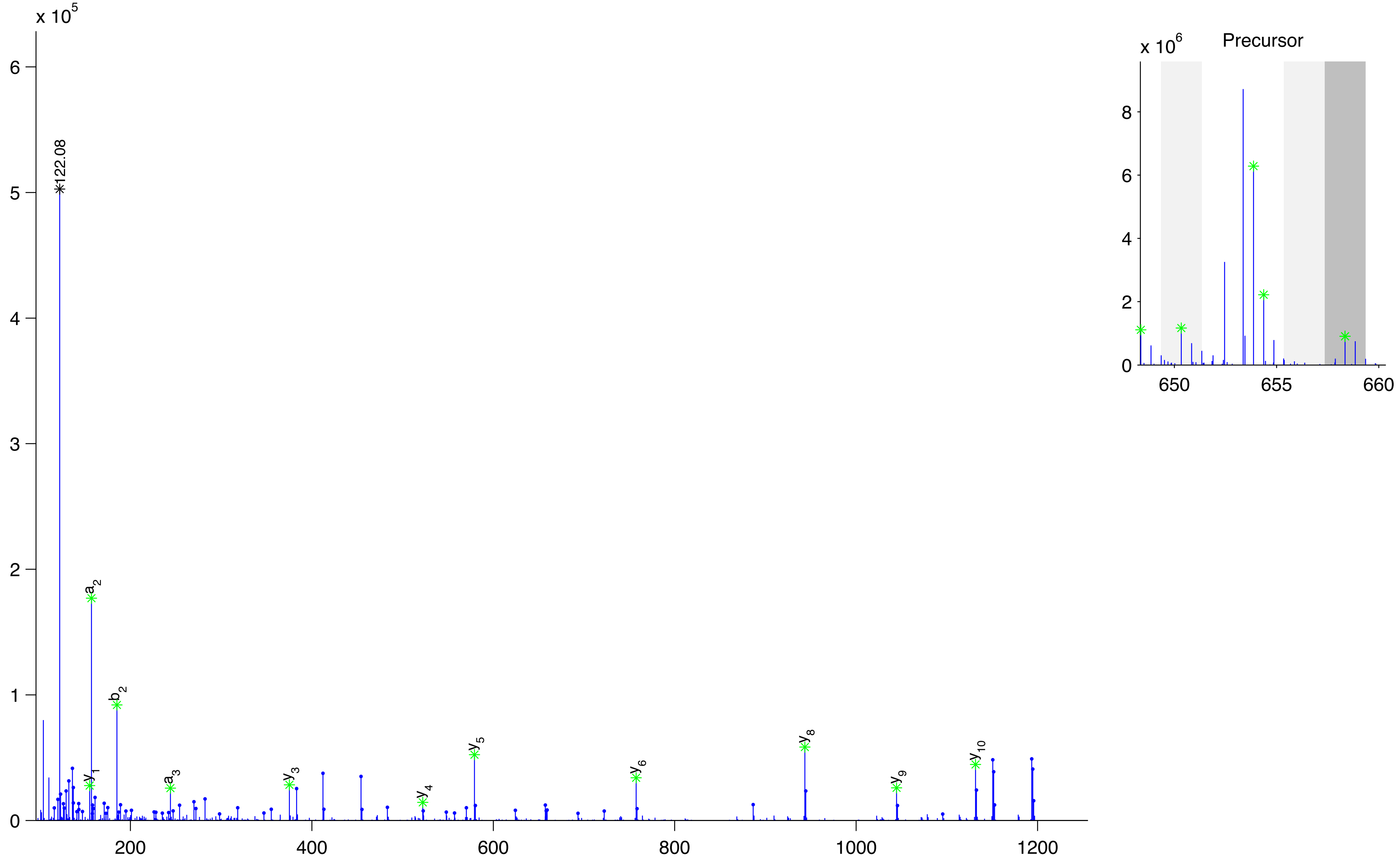

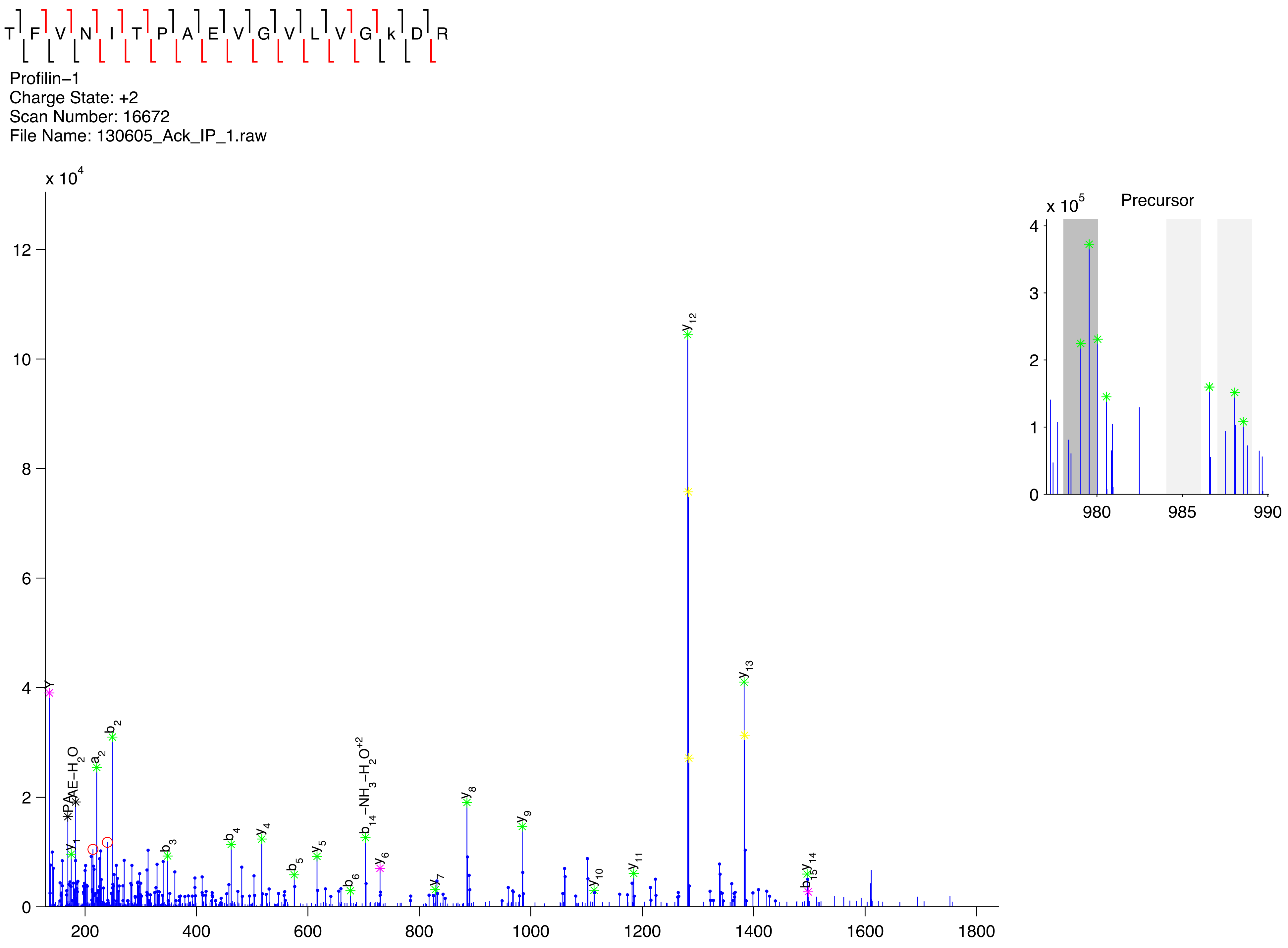
T F V N I T P A E V G V L V G k D R
Profilin−1
Charge State: +2
Scan Number: 16672
File Name: 130605_Ack_IP_1.raw
x 10^4
Y
PA
AE−H2O
y1
a2
b2
b3
b4
y4
b5
y5
b6
b14−NH3−H2O+2
y6
y7
y8
y9
y10
y11
y12
y13
b15
y14
x 10^5
Precursor
980
985
990

c S k L P S S T k S G W P R

Protein Jade−2
Charge State: +2
Scan Number: 8841
File Name: 130605_Ack_IP_1.raw

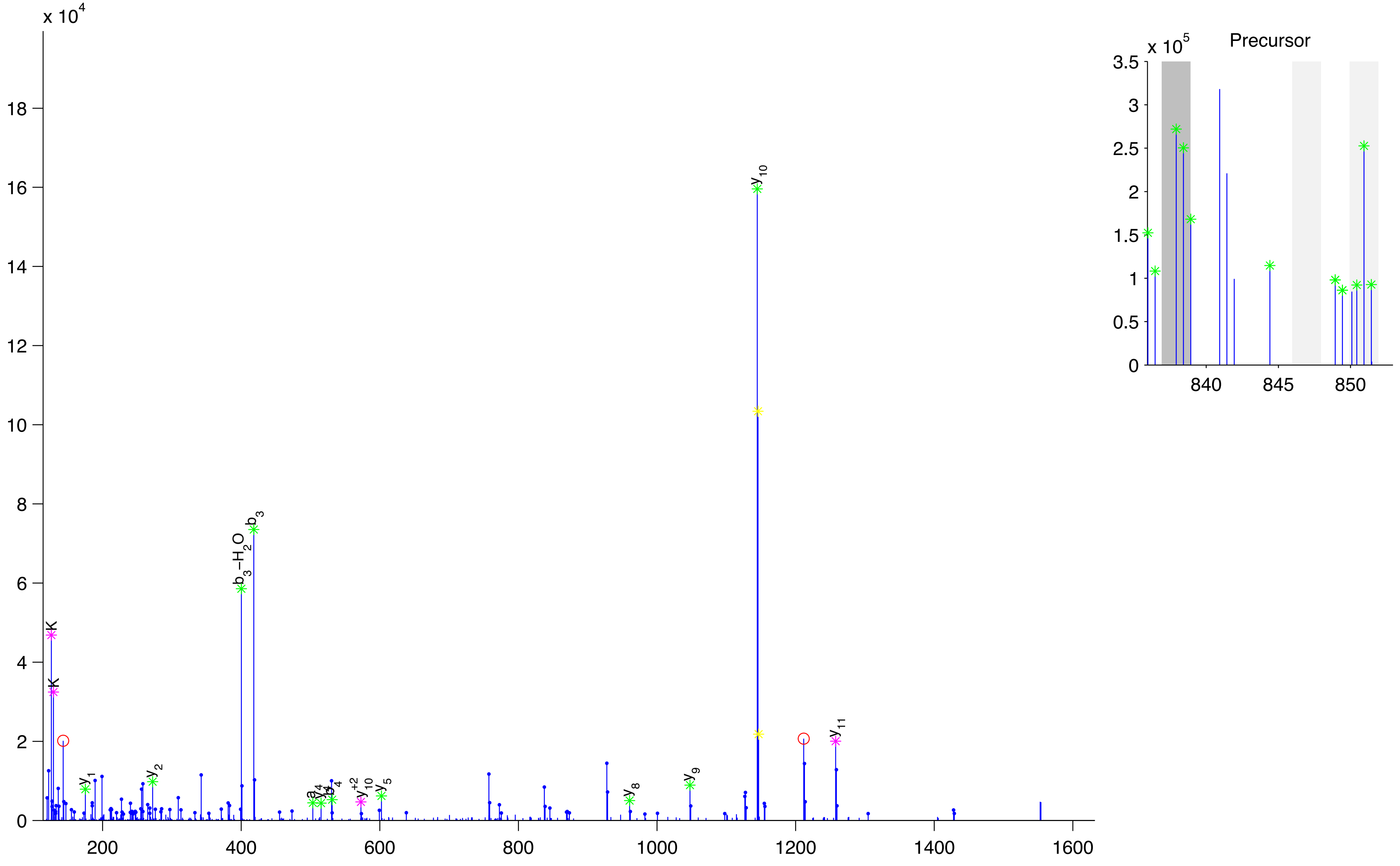

c S k L P S S T k S G W P R

Protein Jade−2
Charge State: +2
Scan Number: 8960
File Name: 130605_Ack_IP_1.raw

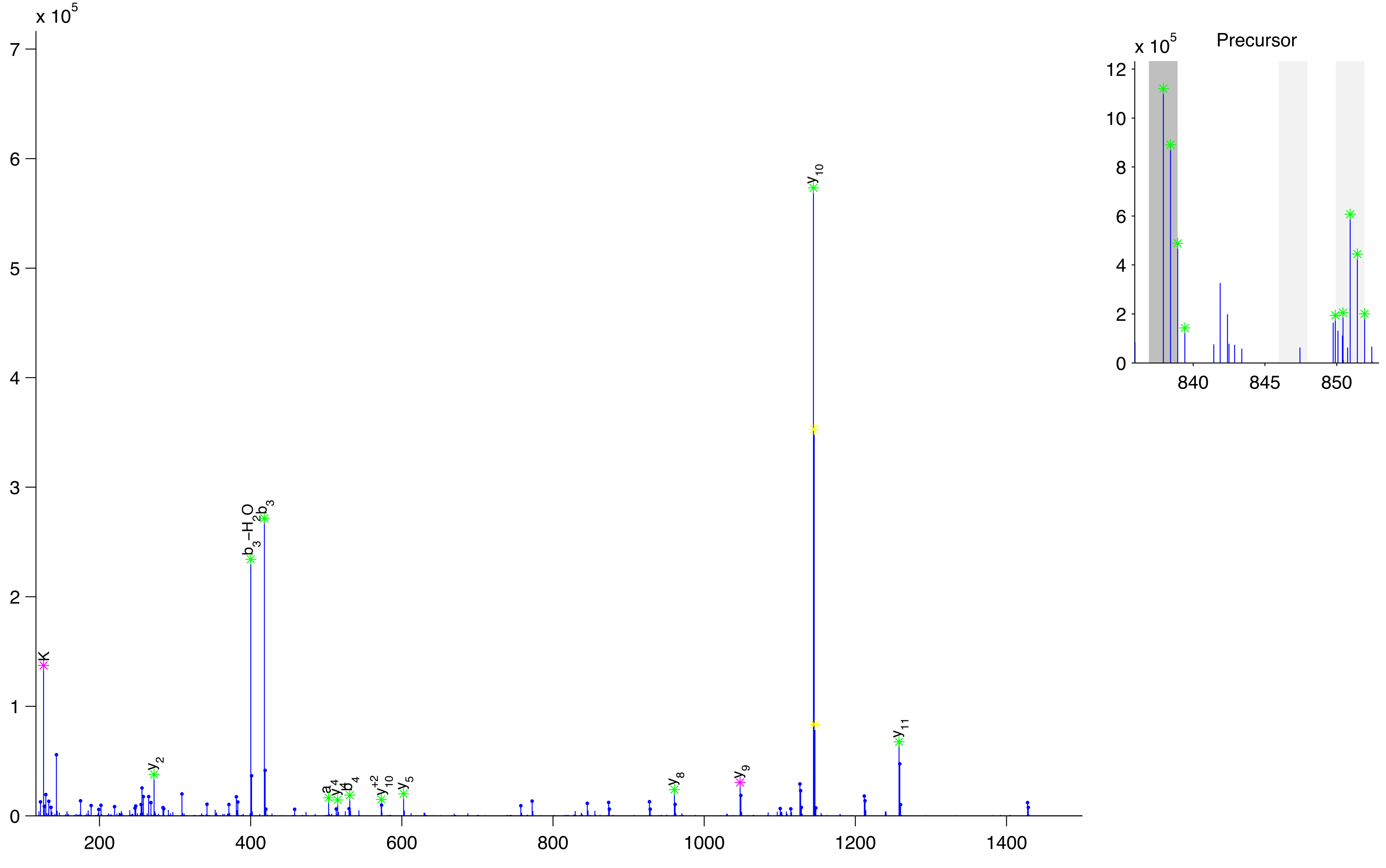

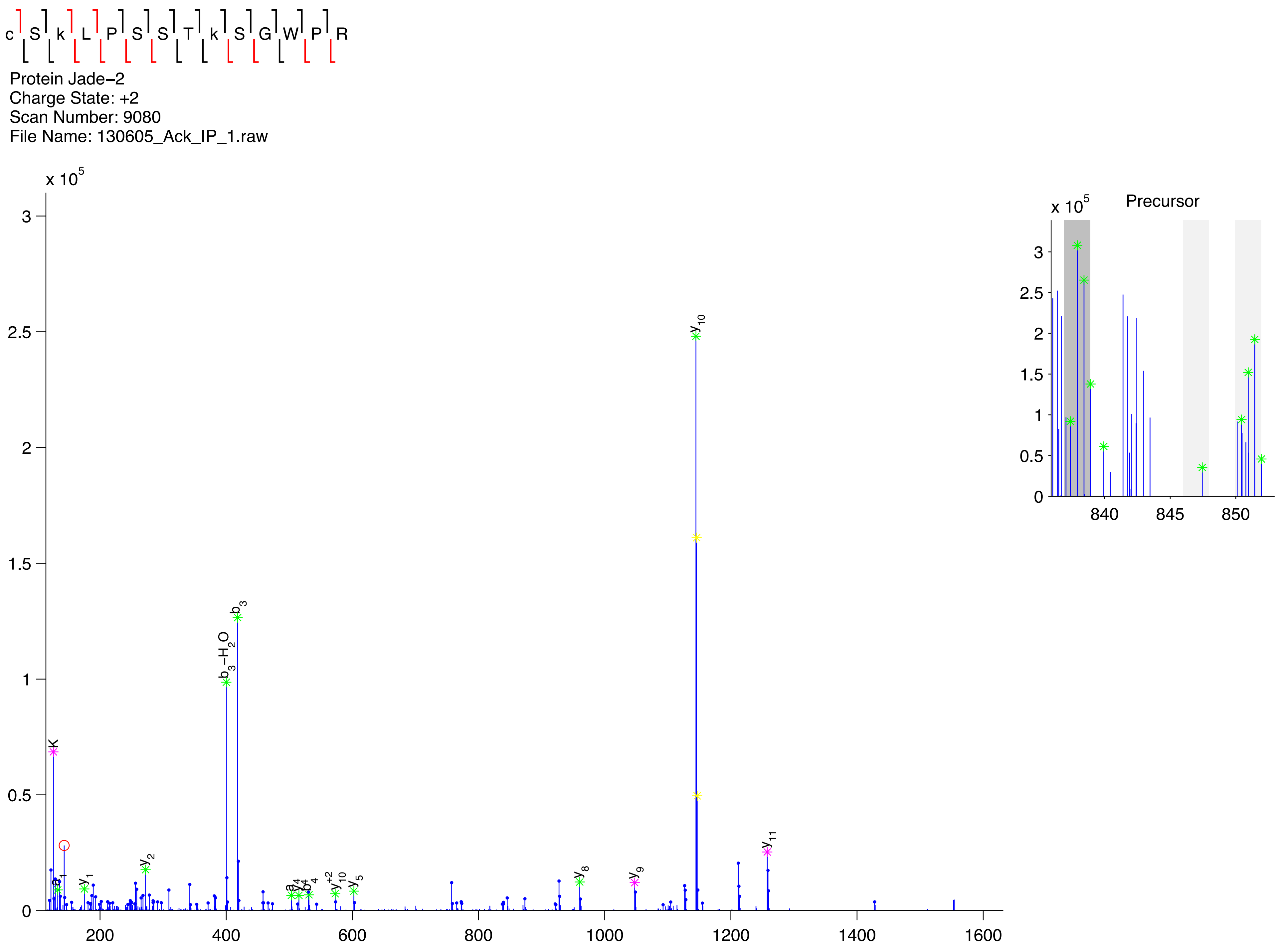
c S k L P S S T k S G W P R
Protein Jade−2
Charge State: +2
Scan Number: 9080
File Name: 130605_Ack_IP_1.raw
x 10^5
K
a_1
y_1
y_2
b_3−H_2O
b_3
a
y_4
b_4
y^{+2}_{10}
y_5
y_8
y_9
y_{10}
y_{11}
0
0.5
1
1.5
2
2.5
3
200
400
600
800
1000
1200
1400
1600
Precursor
840
845
850

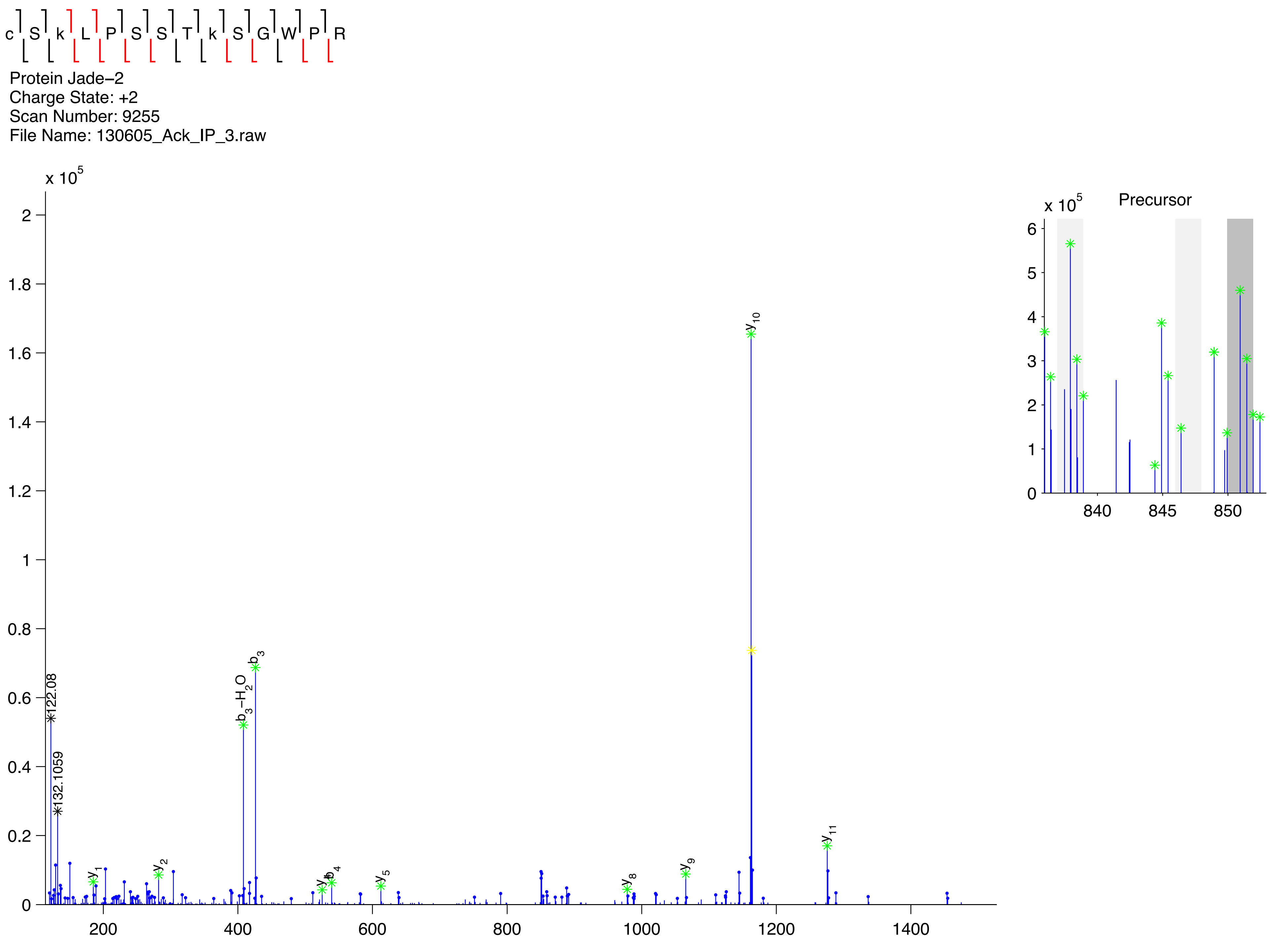

c S k L P S S T k S G W P R
Protein Jade−2
Charge State: +2
Scan Number: 9255
File Name: 130605_Ack_IP_3.raw
x 10^5
122.08
132.1059
y1
y2
b3−H2O
b3
b4
y5
y8
y9
y10
y11
Precursor

c S k L P S S T k S G W P R

Protein Jade−2
Charge State: +2
Scan Number: 9278
File Name: 130605_Ack_IP_2.raw

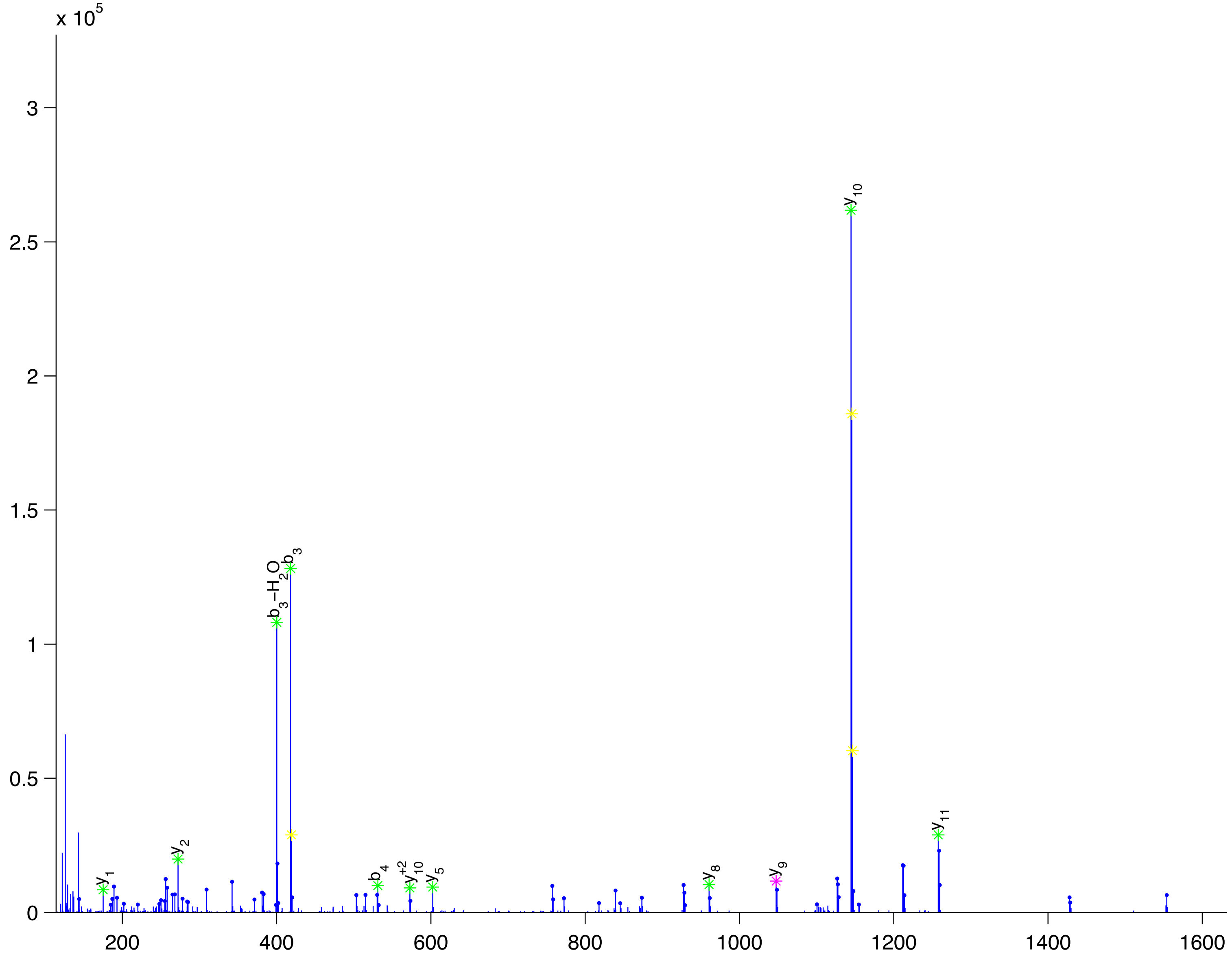

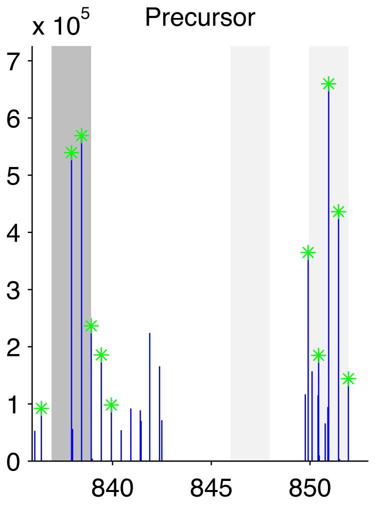

c S k L P S S T k S G W P R

Protein Jade−2
Charge State: +2
Scan Number: 9389
File Name: 130605_Ack_IP_3.raw

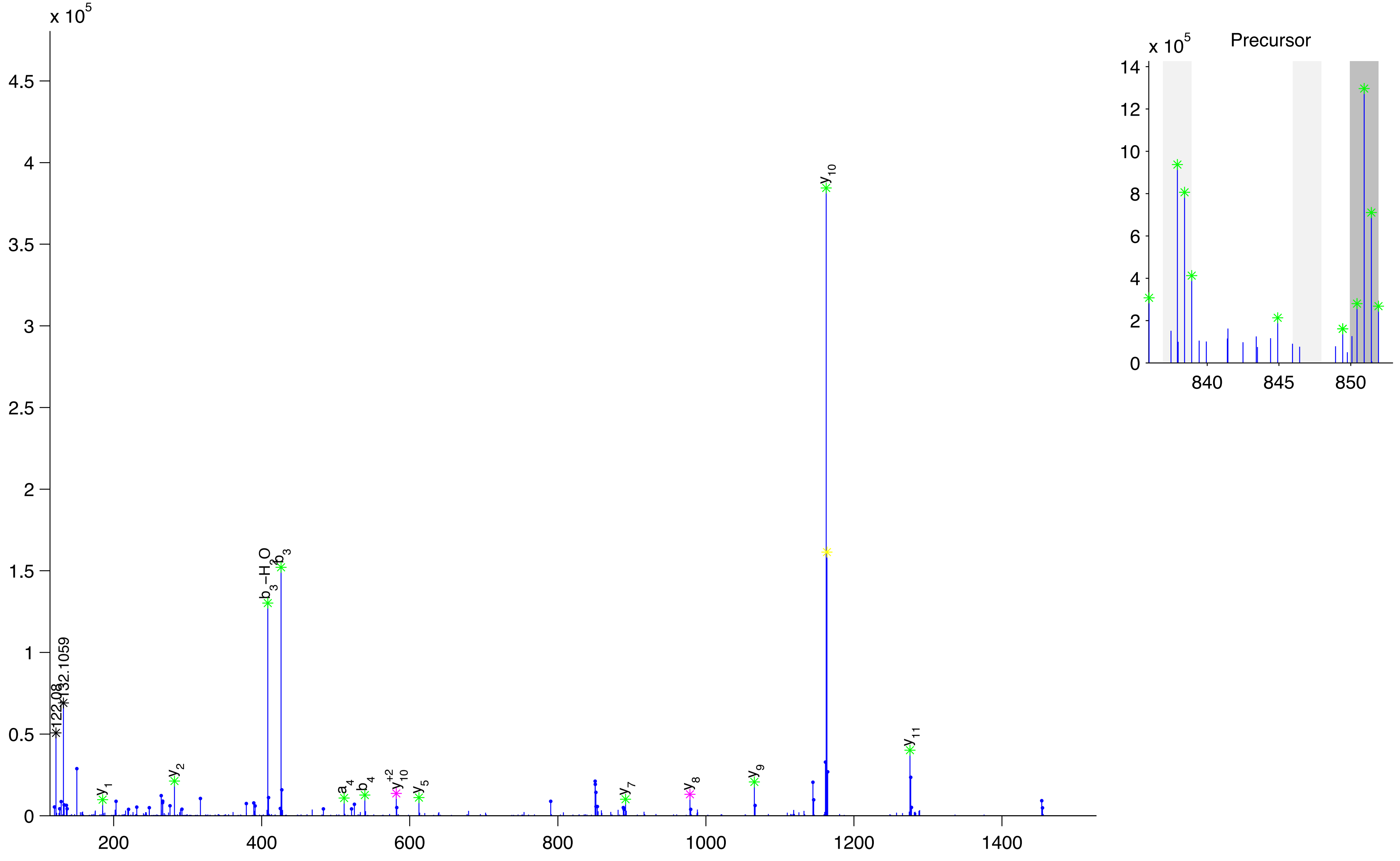

c A S L Q k F G E R

Serum albumin
Charge State: +2
Scan Number: 6498
File Name: 130605_Ack_IP_1.raw

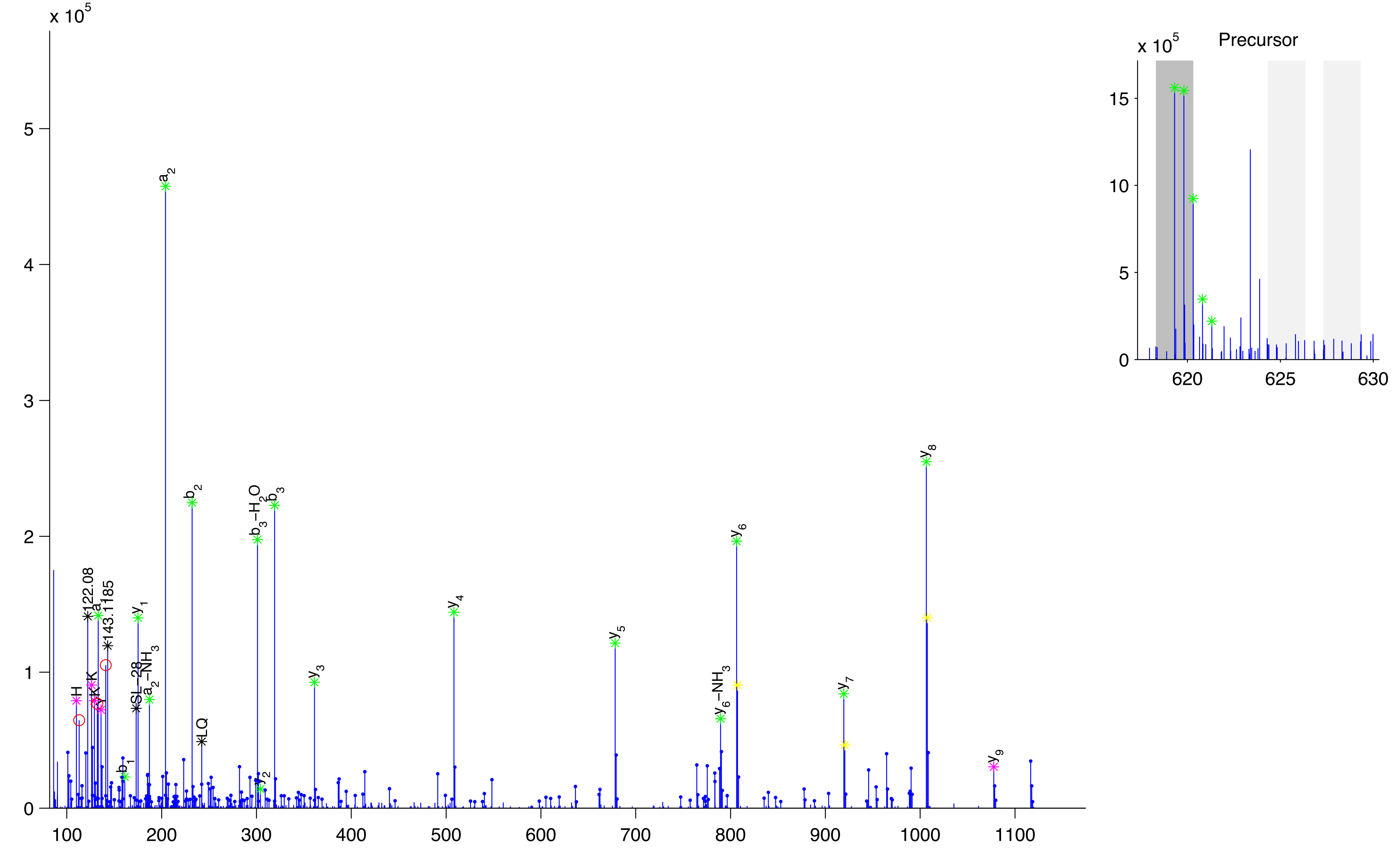

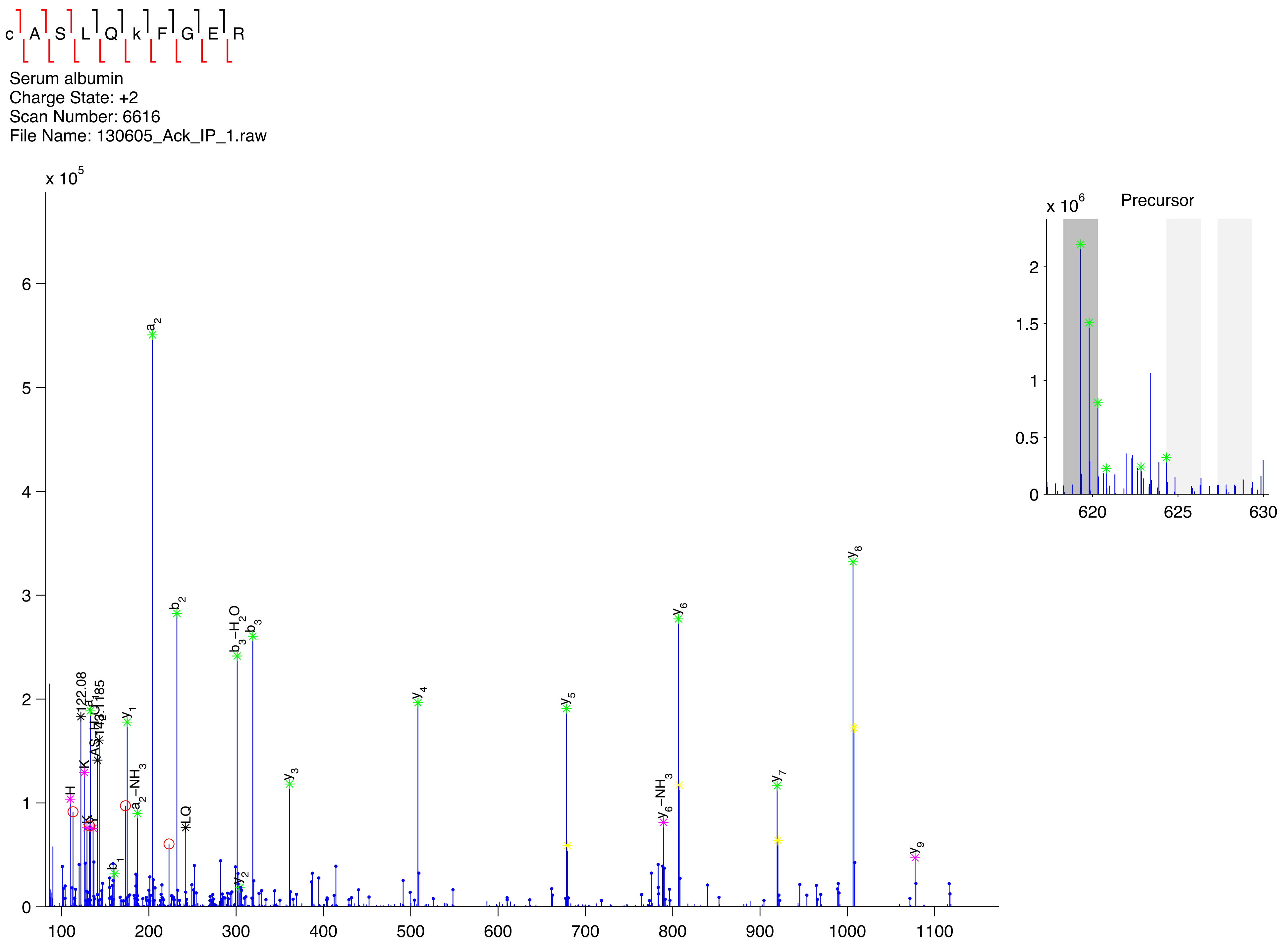

c A S L Q k F G E R
Serum albumin
Charge State: +2
Scan Number: 6616
File Name: 130605_Ack_IP_1.raw
x 10^5
a2
b2
b3-H2O
b3
y1
y2
y3
y4
y5
y6
y6-NH3
y7
y8
y9
b1
a2-NH3
H
K
LQ
122.08
AS-H2O
142.1185
x 10^6
Precursor

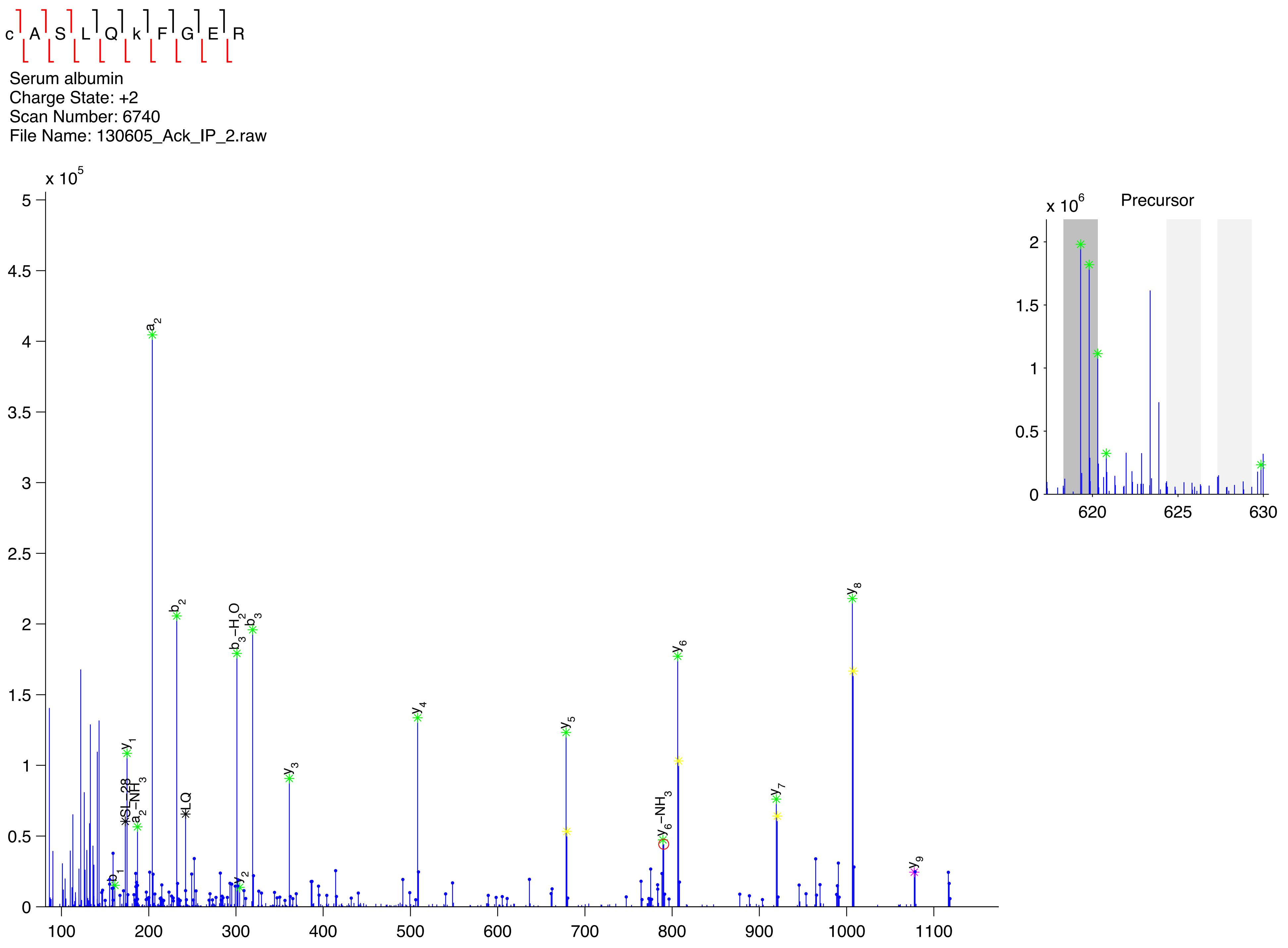
c A S L Q k F G E R
Serum albumin
Charge State: +2
Scan Number: 6740
File Name: 130605_Ack_IP_2.raw
x 10^5
a2
b2
b3−H2O
b3
y1
SL−28
a2−NH3
LQ
b1
y2
y3
y4
y5
y6−NH3
y6
y7
y8
y9
x 10^6
Precursor

c A S L Q k F G E R

Serum albumin
Charge State: +2
Scan Number: 7030
File Name: 130605_Ack_IP_3.raw

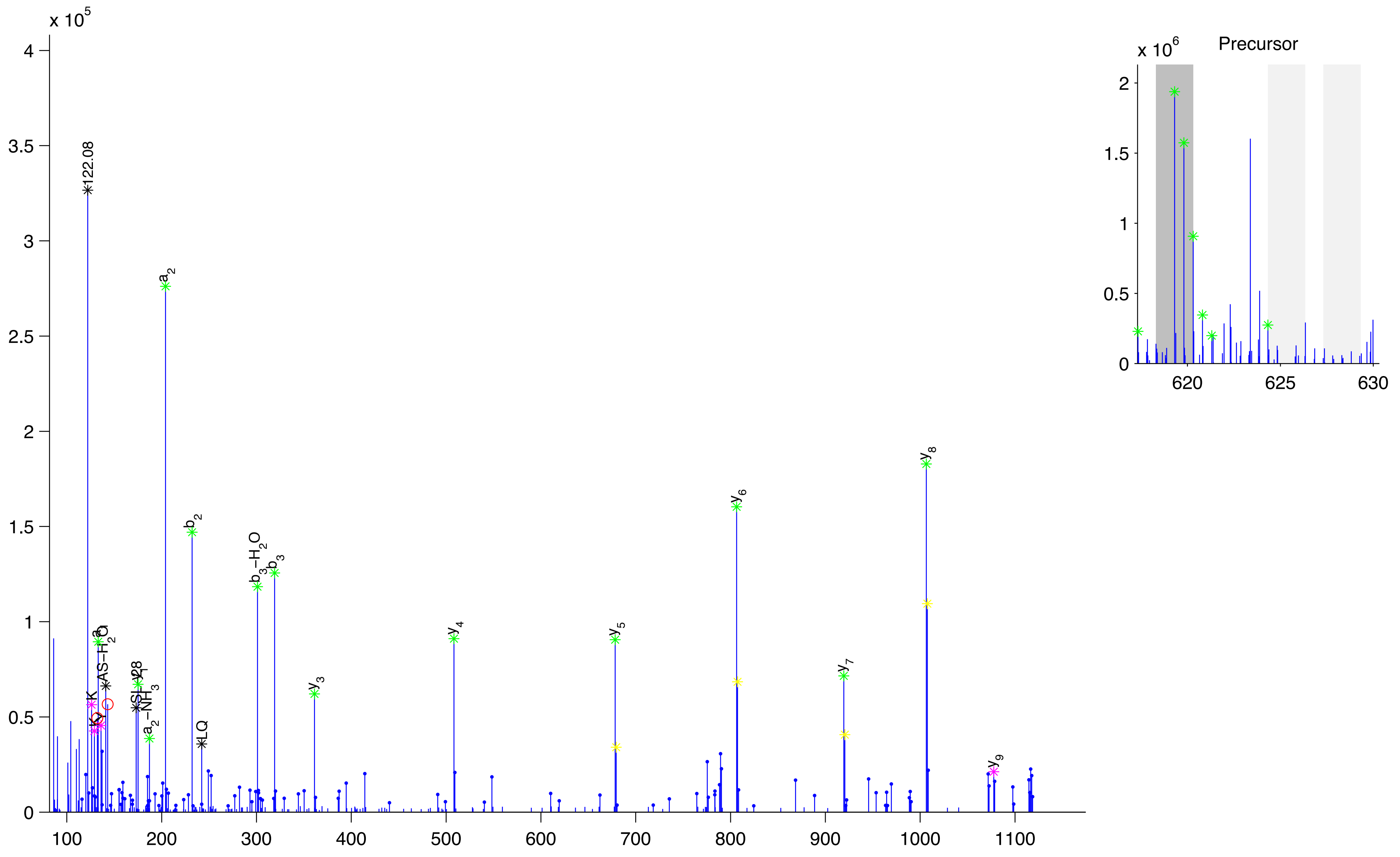

V I G A k k D Q Y F L D K

Structural maintenance of chromosomes protein 3
Charge State: +2
Scan Number: 11449
File Name: 130605_Ack_IP_3.raw

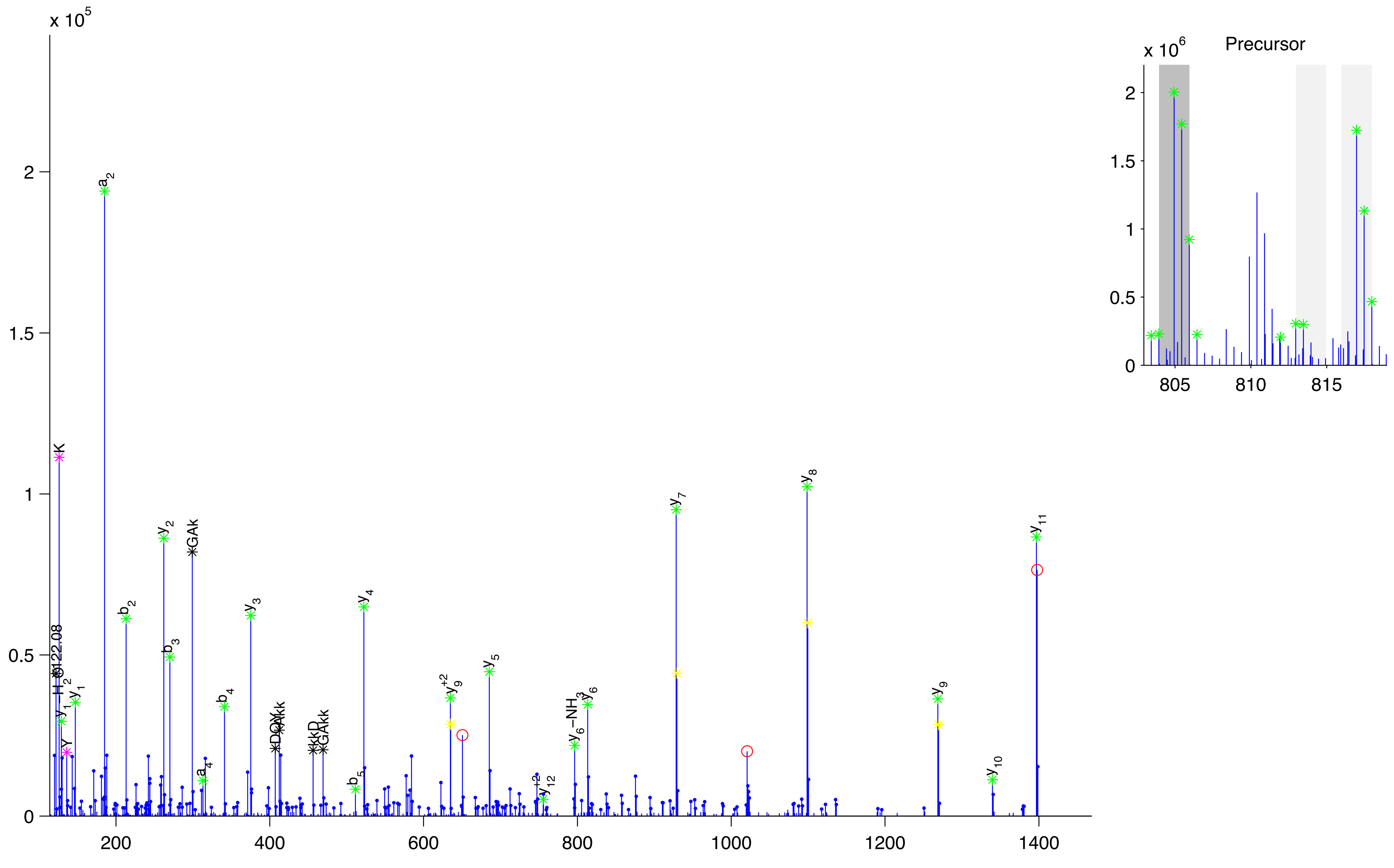

G k G k G Q k R

Vascular endothelial growth factor A
Charge State: +2
Scan Number: 4452
File Name: 130605_Ack_IP_3.raw

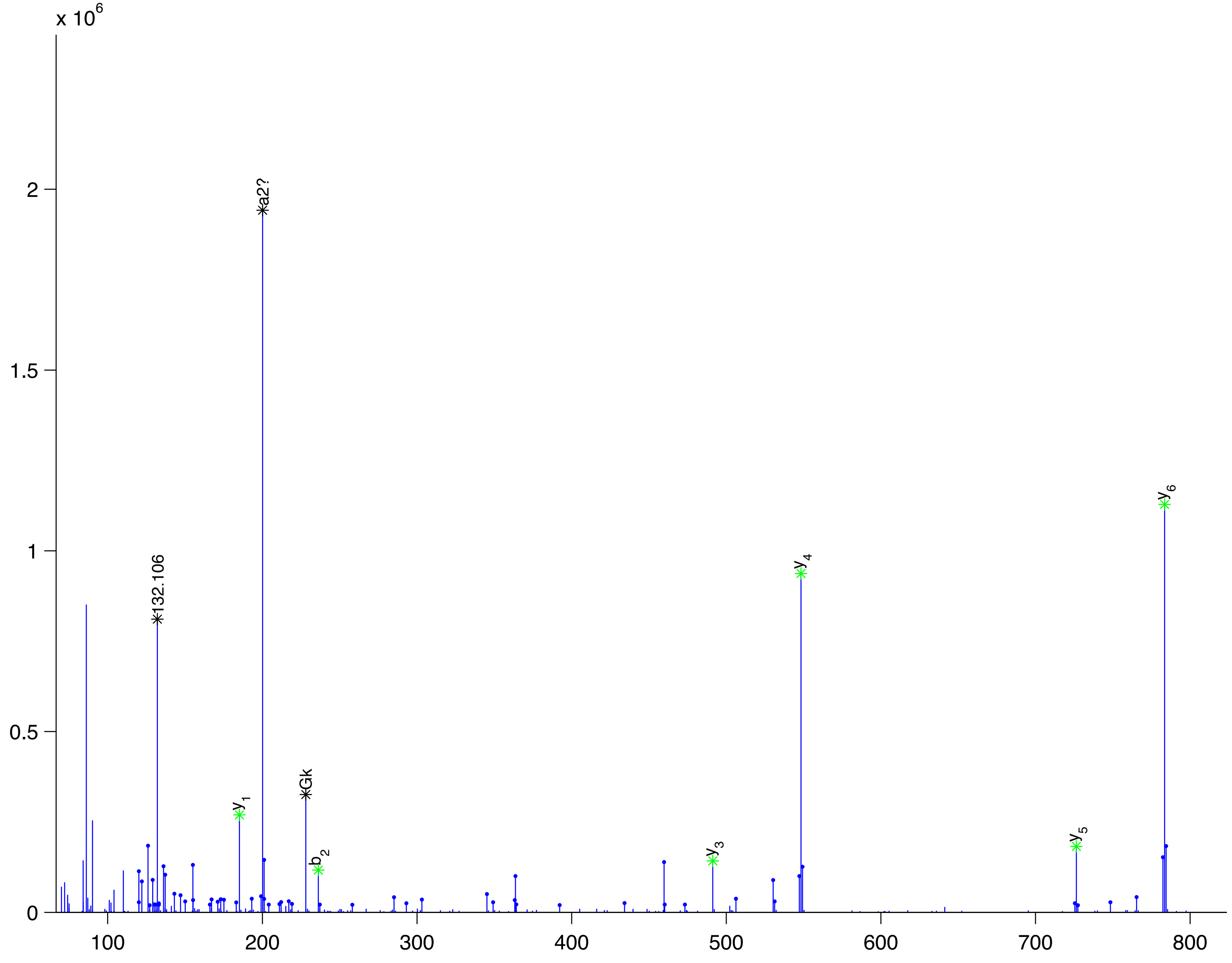

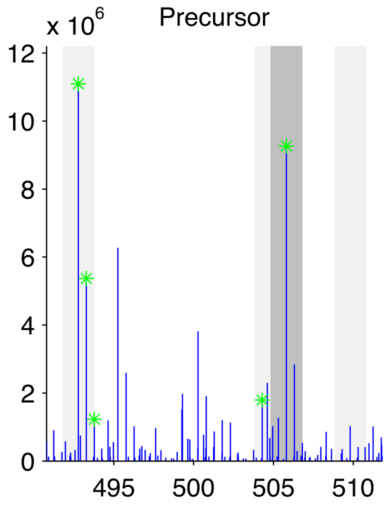

Supplement: S3 Fig — Instead of relying on an FDR analysis where the identity of true positives and true negatives are unknown, we manually validated each MS/MS spectra manually. Each page represents a manually validated MS/MS spectrum. (PDF) [file pone.0126242.s003.pdf]
