# Supplementary material for: Quantitative Profiling of Lysine Acetylation Reveals Dynamic Crosstalk between Receptor Tyrosine Kinases and Lysine Acetylation
Source: PLoS One. 2015 May 15;10(5):e0126242. doi: 10.1371/journal.pone.0126242 (PMC4433260; doi:10.1371/journal.pone.0126242)

I F V E E S I y D E F V R R

aldehyde dehydrogenase 1A1 [Homo sapiens]
Charge State: +3
Scan Number: 20576
File Name: 120429_A549_TSA_pY.raw

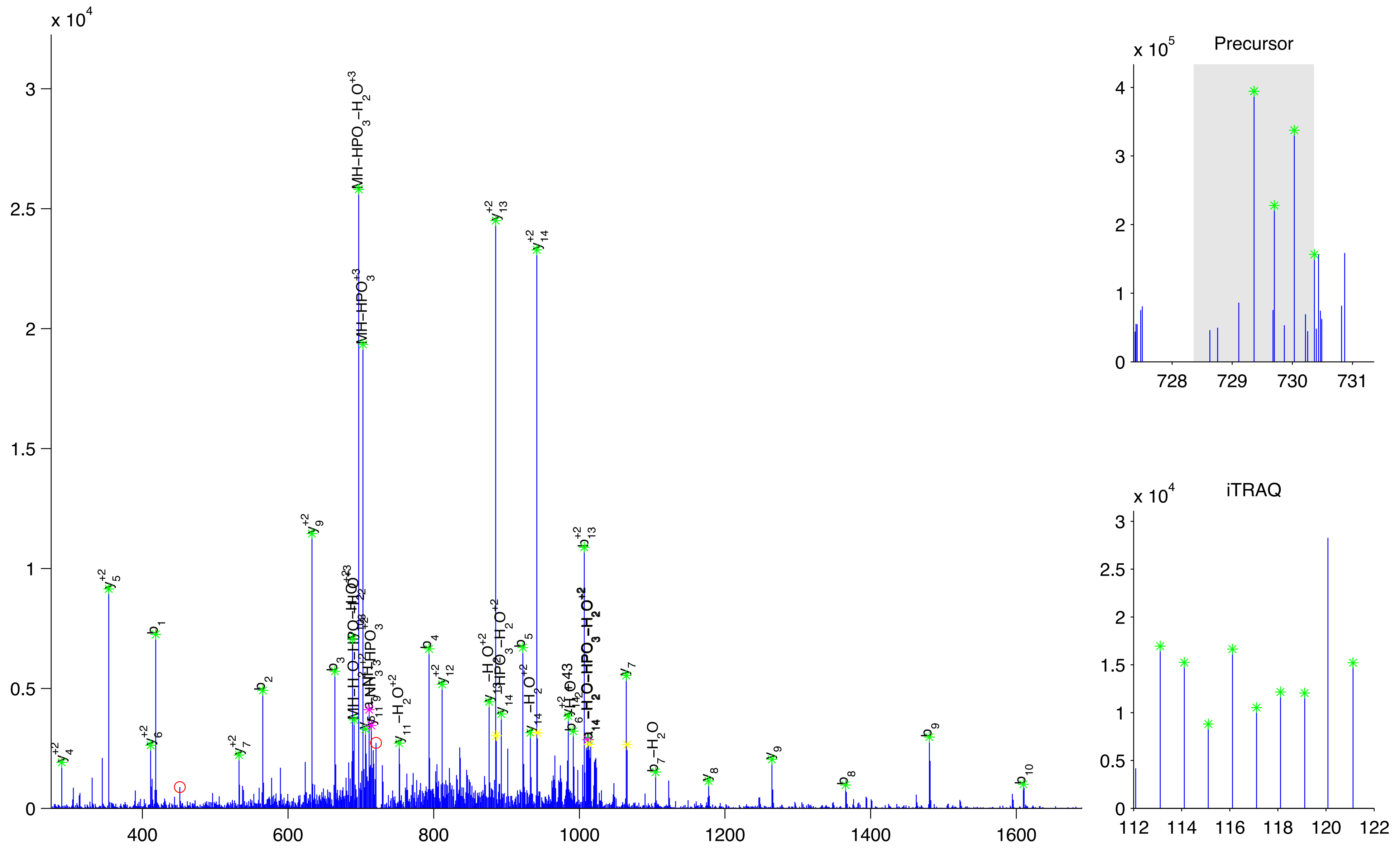

L S L E G D H S T P P S A y G S V K

annexin A2 isoform 2 [Homo sapiens]
Charge State: +3
Scan Number: 10066
File Name: 120429_A549_TSA_pY.raw

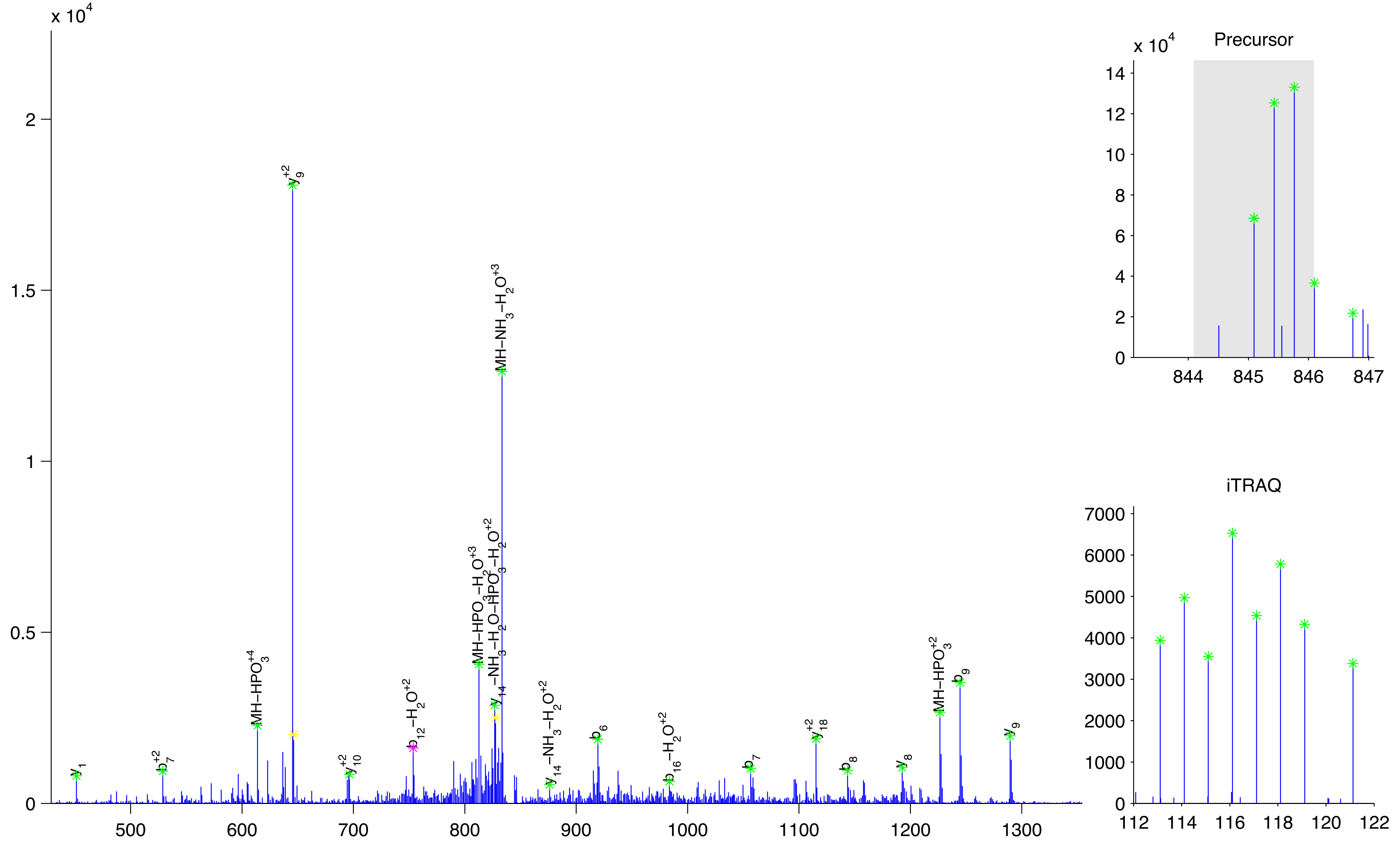

S Y S P y D M L E S I R

annexin A2 isoform 2 [Homo sapiens]
Charge State: +3
Scan Number: 19257
File Name: 120429_A549_TSA_pY.raw

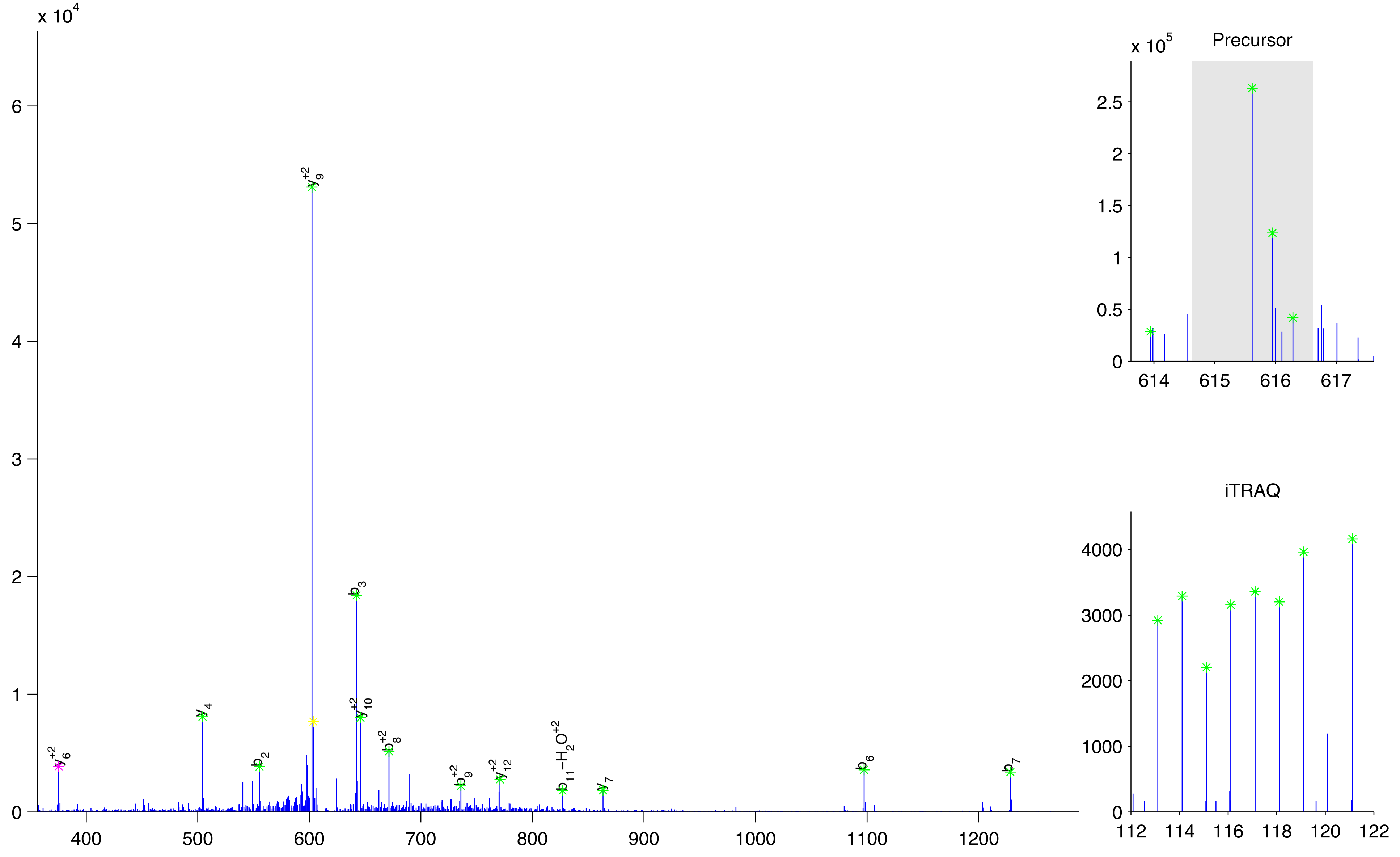

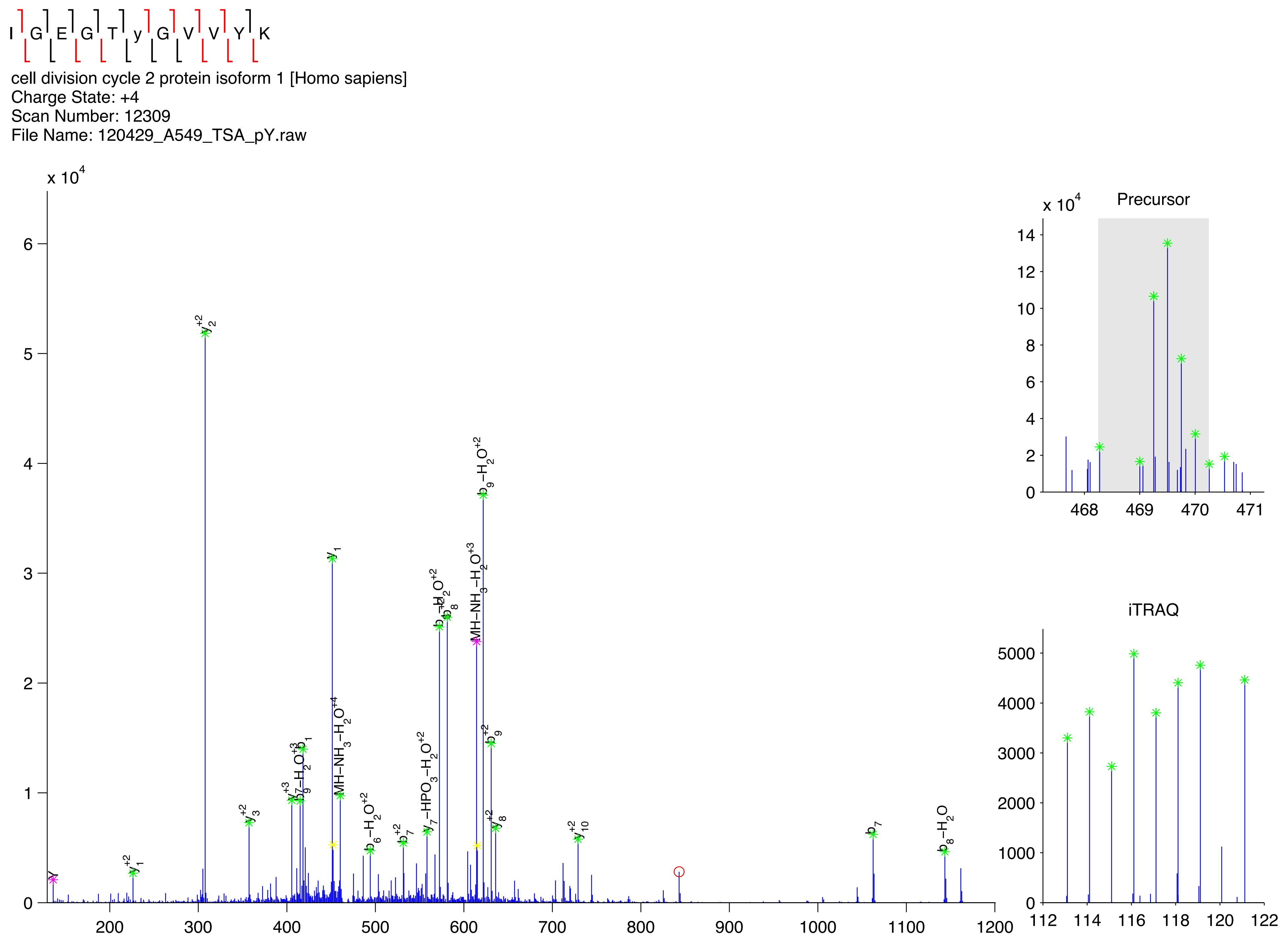

I G E G T y G V V Y K
cell division cycle 2 protein isoform 1 [Homo sapiens]
Charge State: +4
Scan Number: 12309
File Name: 120429_A549_TSA_pY.raw
x 10^4
Precursor
iTRAQ

I G E G T y G V V Y K

cell division cycle 2 protein isoform 1 [Homo sapiens]
Charge State: +2
Scan Number: 12344
File Name: 120429_A549_TSA_pY.raw

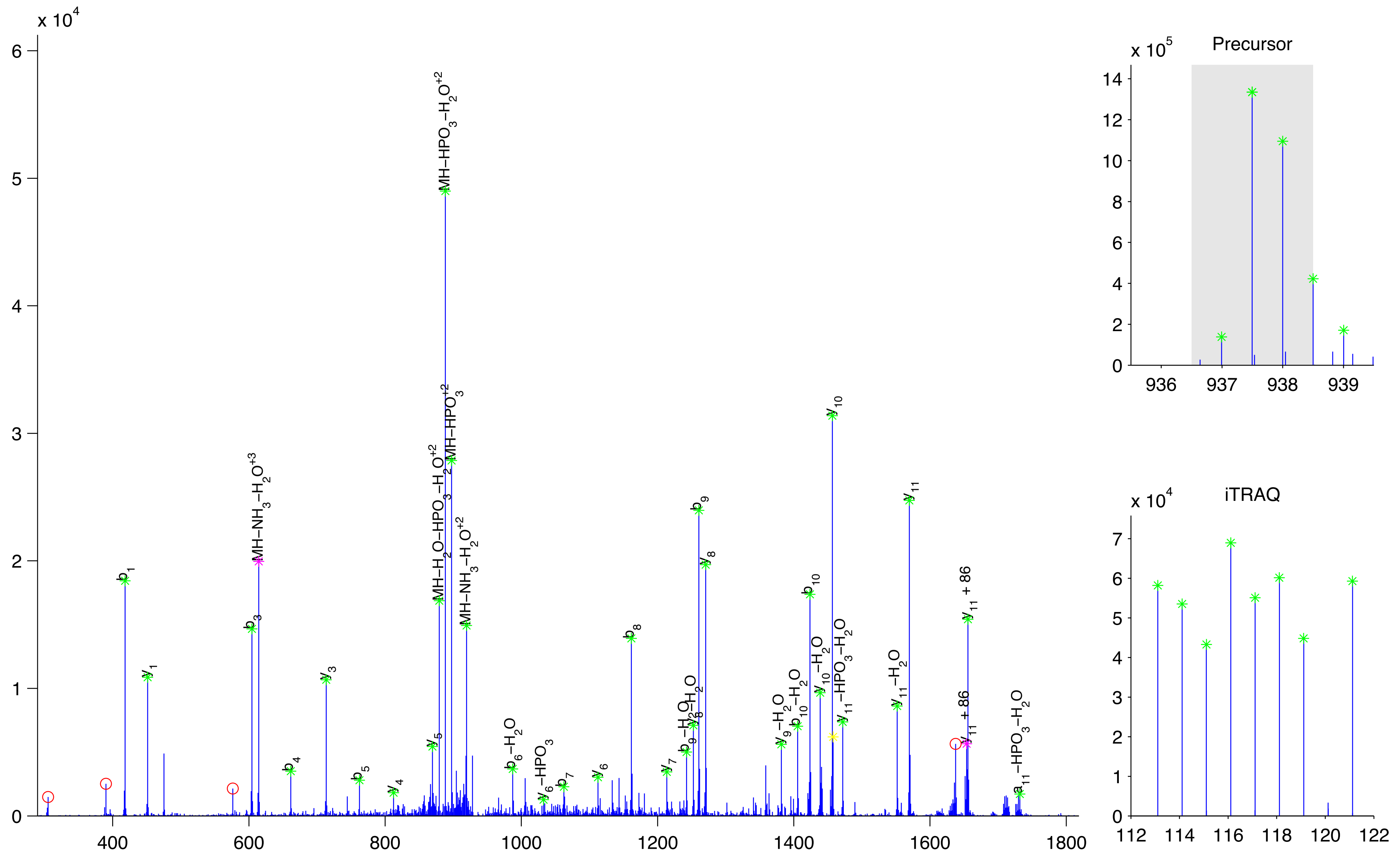

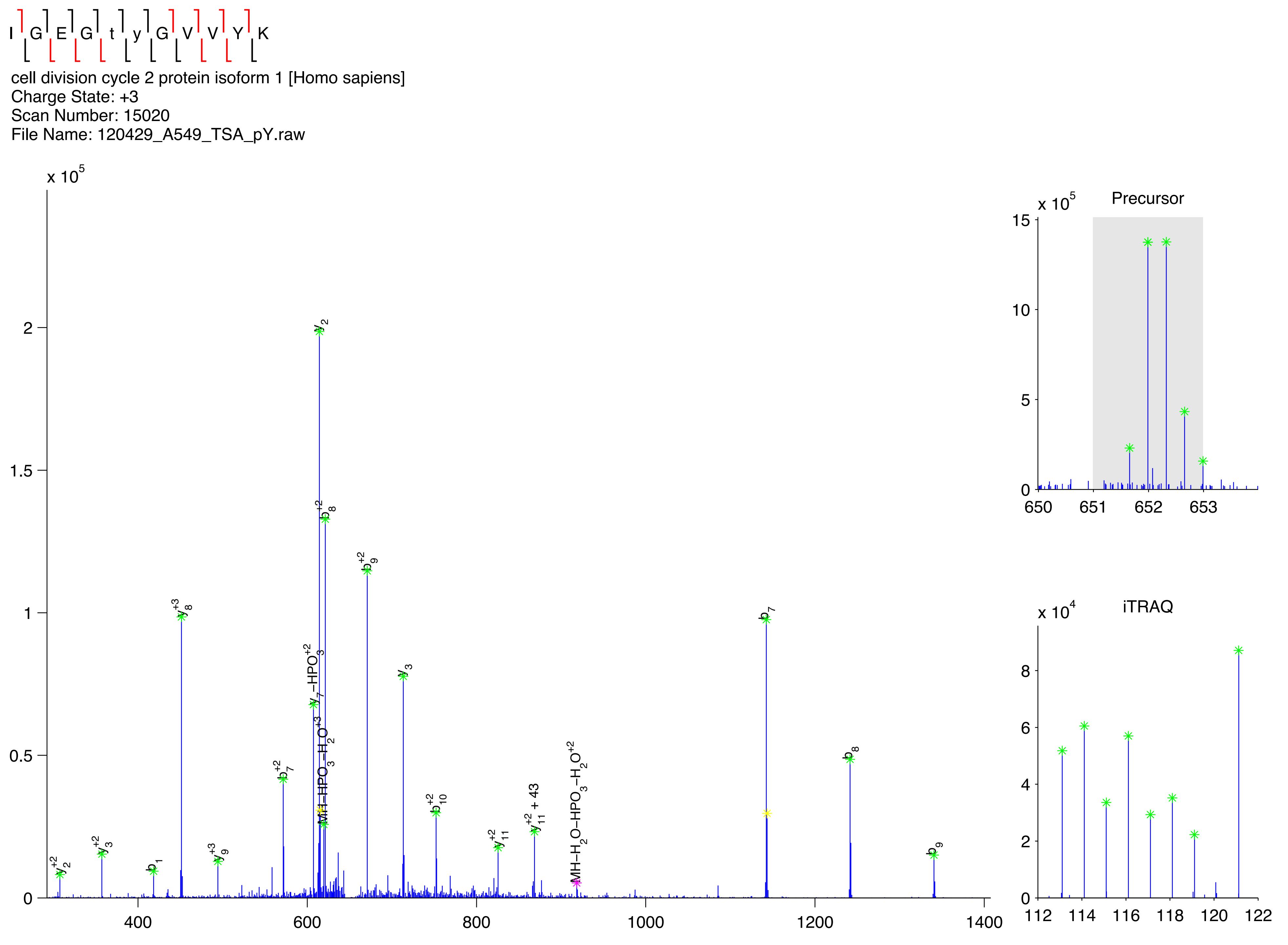
I G E G t y G V V Y K
cell division cycle 2 protein isoform 1 [Homo sapiens]
Charge State: +3
Scan Number: 15020
File Name: 120429_A549_TSA_pY.raw
x 10^5
0
0.5
1
1.5
2
400
600
800
1000
1200
1400
y+2 2
y+2 3
b1
y+3 8
y+3 9
b+2 7
y7 –HPO+2 3
MH–HPO3–H2O+3
y2
b+2 8
b+2 9
y3
b+2 10
y+2 11
y+2 11 + 43
MH–H2O–HPO3–H2O+2
b7
b8
b9
Precursor
x 10^5
0
5
10
15
650
651
652
653
iTRAQ
x 10^4
0
2
4
6
8
112
114
116
118
120
122

I G E G t y G V V Y K

cell division cycle 2 protein isoform 1 [Homo sapiens]
Charge State: +3
Scan Number: 15503
File Name: 120429_A549_TSA_pY.raw

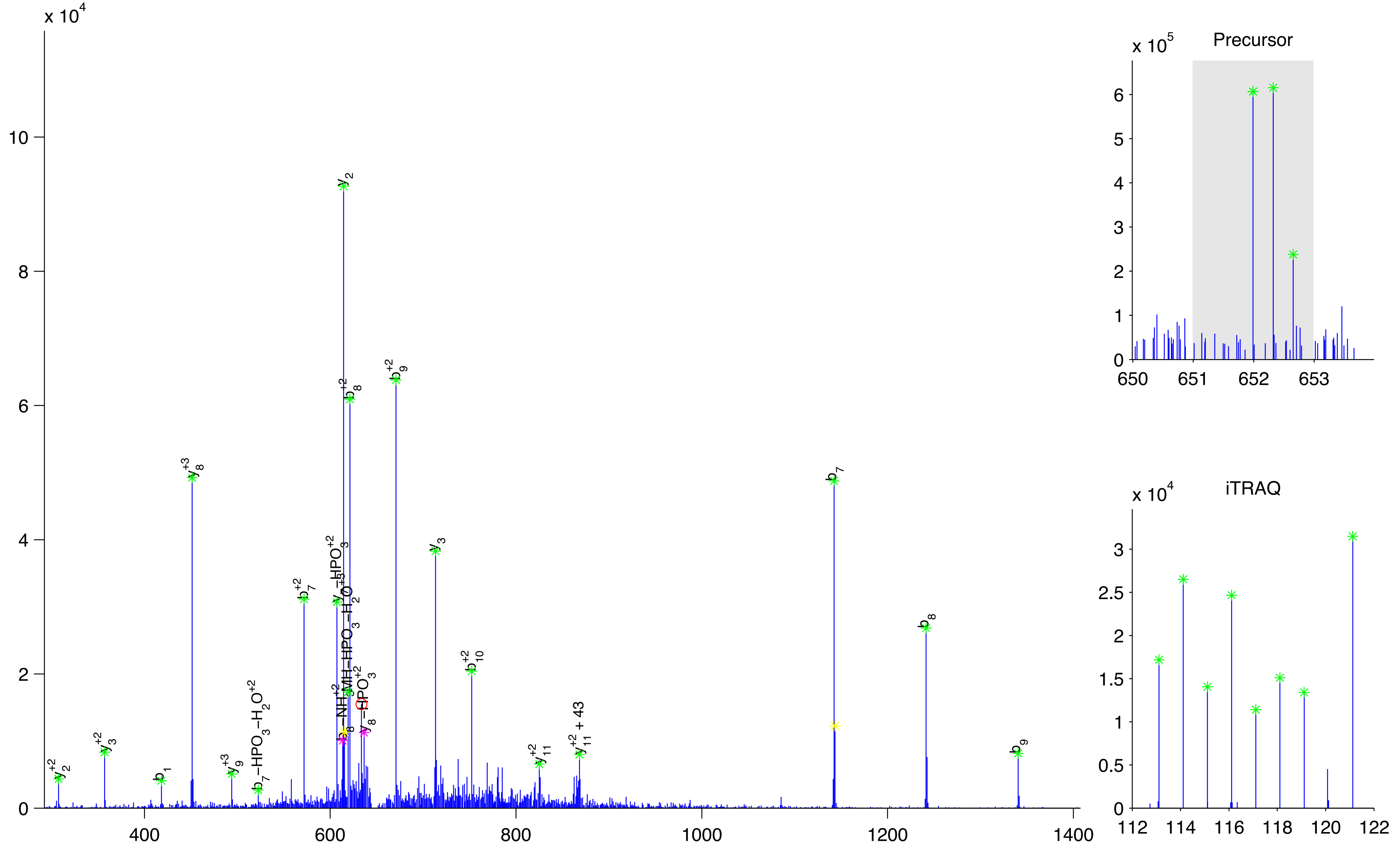

L P S S P V y E D A A S F K

cortactin isoform a [Homo sapiens]
Charge State: +3
Scan Number: 14516
File Name: 120429_A549_TSA_pY.raw

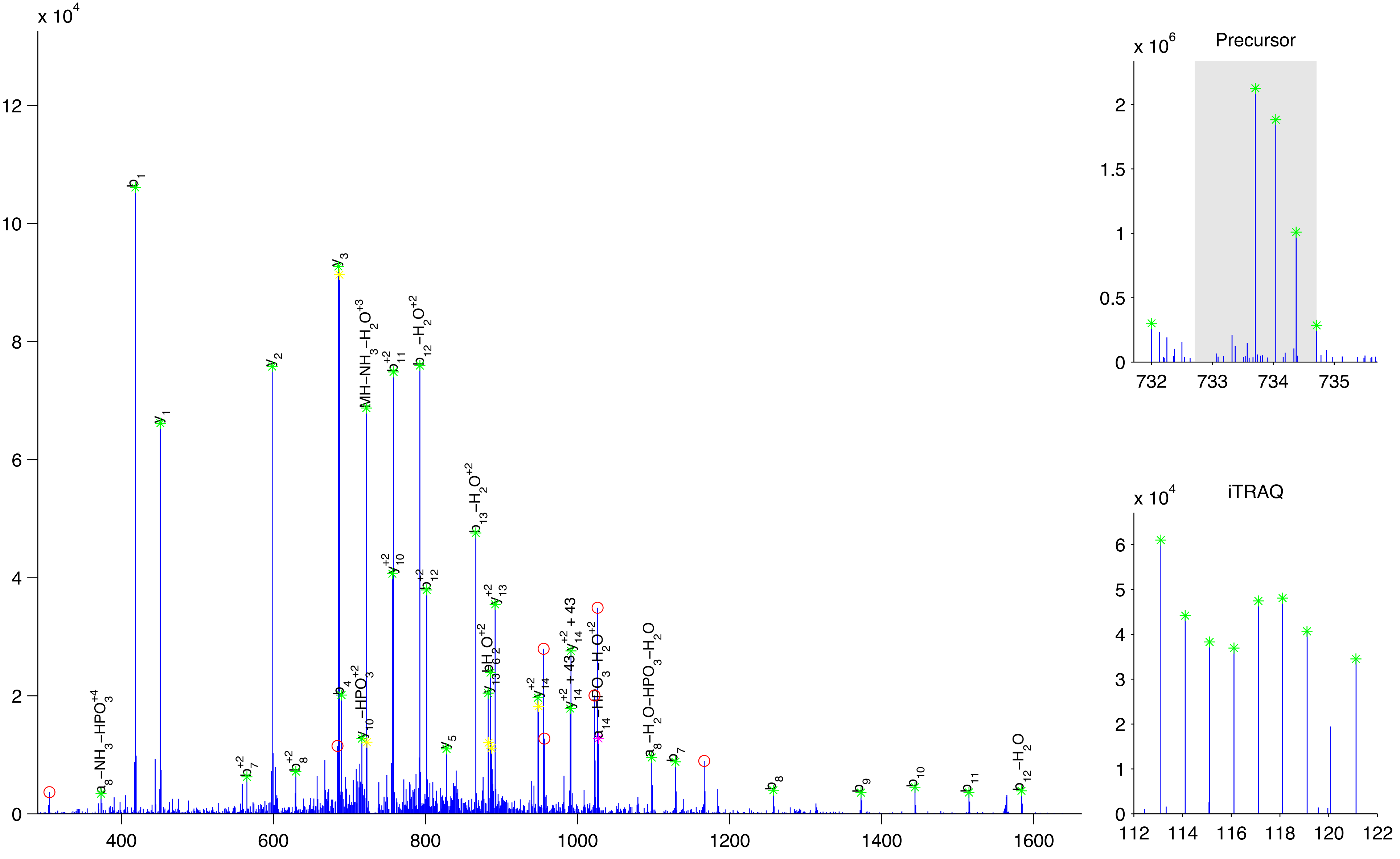

A G K P G L P A P D E L V y Q V P Q S T Q E V S G A G R

discoidin, CUB and LCCL domain containing 2 [Homo sapiens]
Charge State: +4
Scan Number: 14482
File Name: 120429_A549_TSA_pY.raw

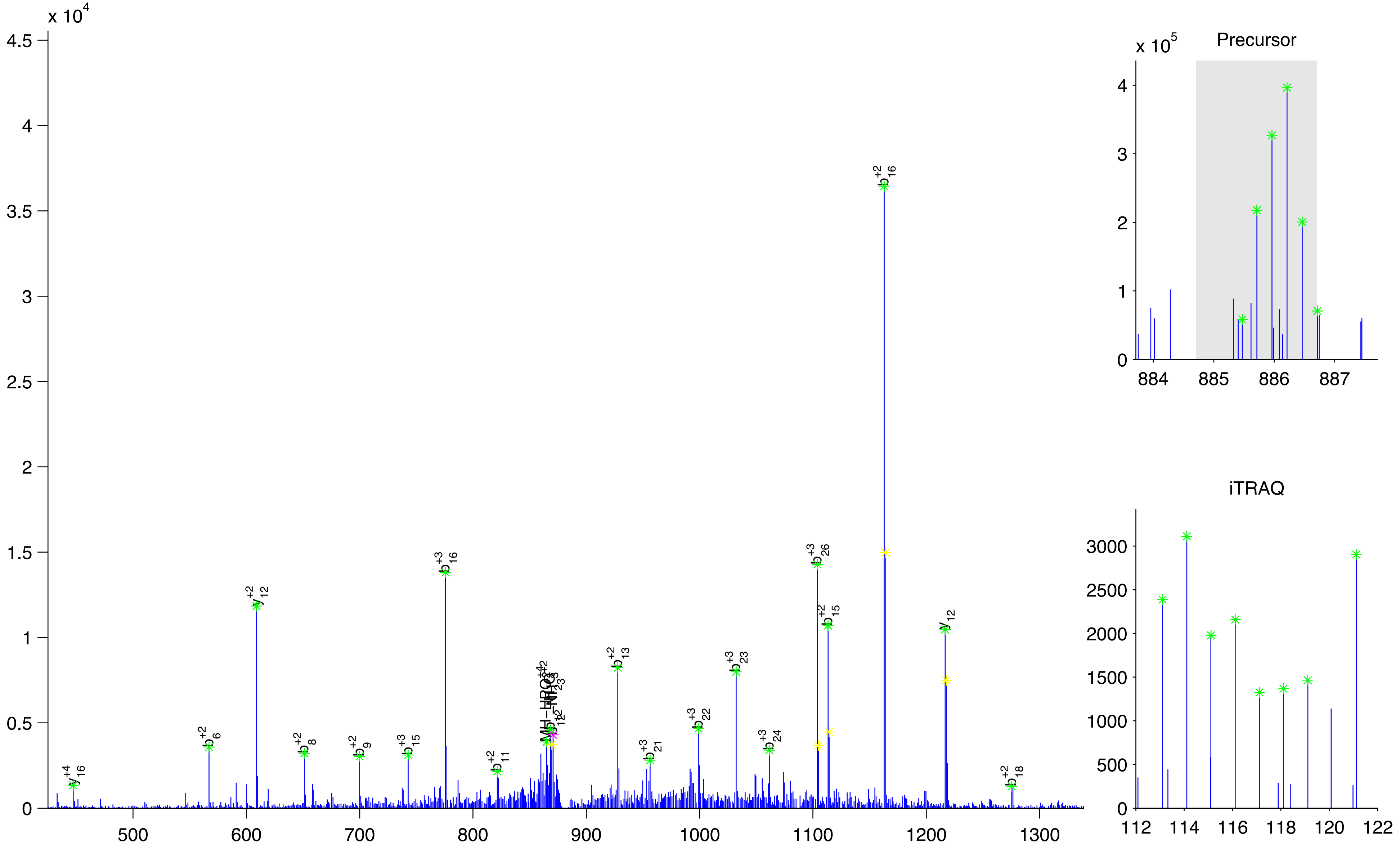

I Y Q y I Q S R

dual–specificity tyrosine–(Y)–phosphorylation regulated kinase 1A isoform 3 [Homo sapiens]
Charge State: +3
Scan Number: 13466
File Name: 120429_A549_TSA_pY.raw

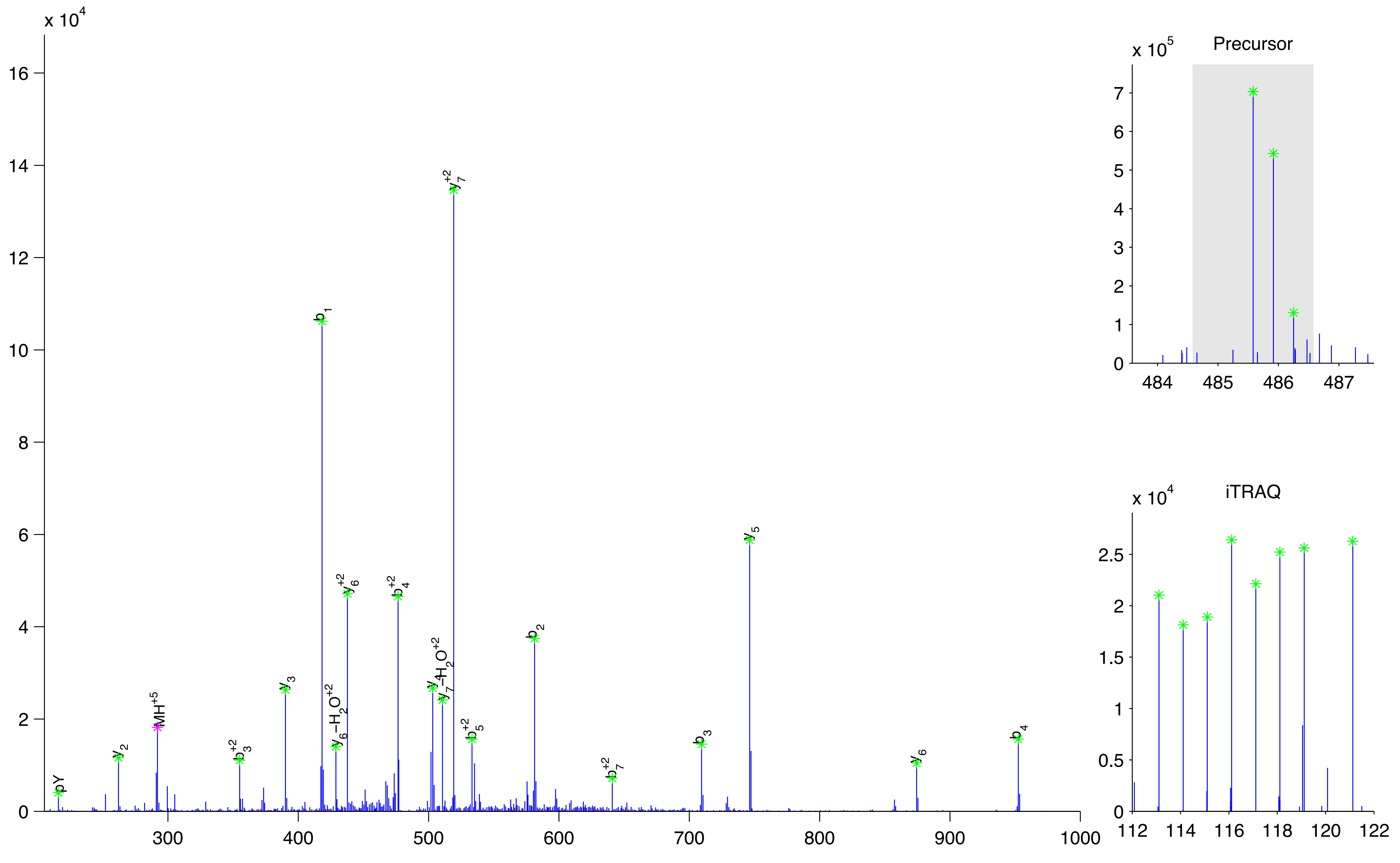

I Y Q y I Q S R

dual–specificity tyrosine–(Y)–phosphorylation regulated kinase 1B isoform b [Homo sapiens]
Charge State: +3
Scan Number: 13466
File Name: 120429_A549_TSA_pY.raw

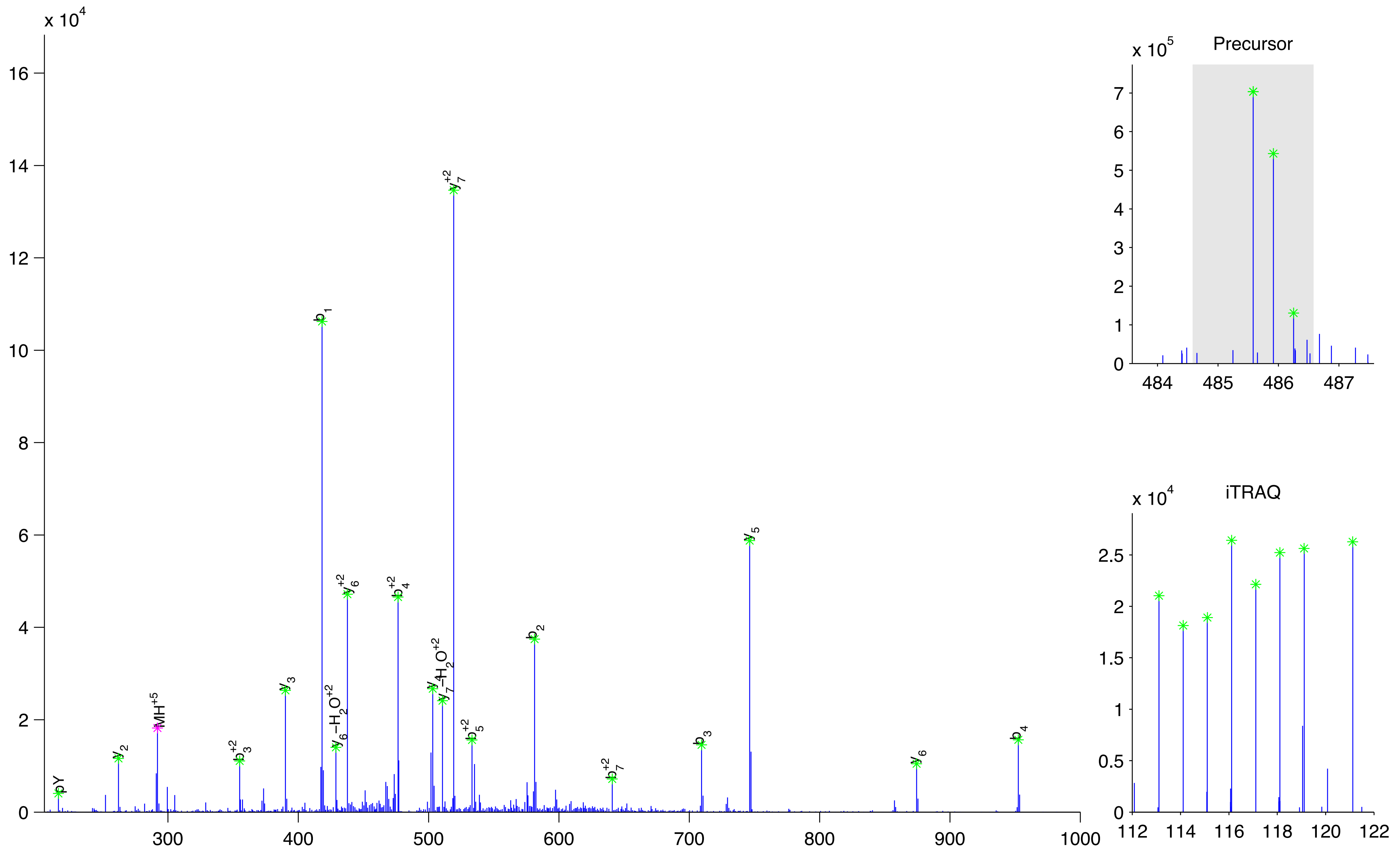

V L E D D P E A T y T T S G G K I P I R

ephrin receptor EphA2 [Homo sapiens]
Charge State: +4
Scan Number: 13086
File Name: 120429_A549_TSA_pY.raw

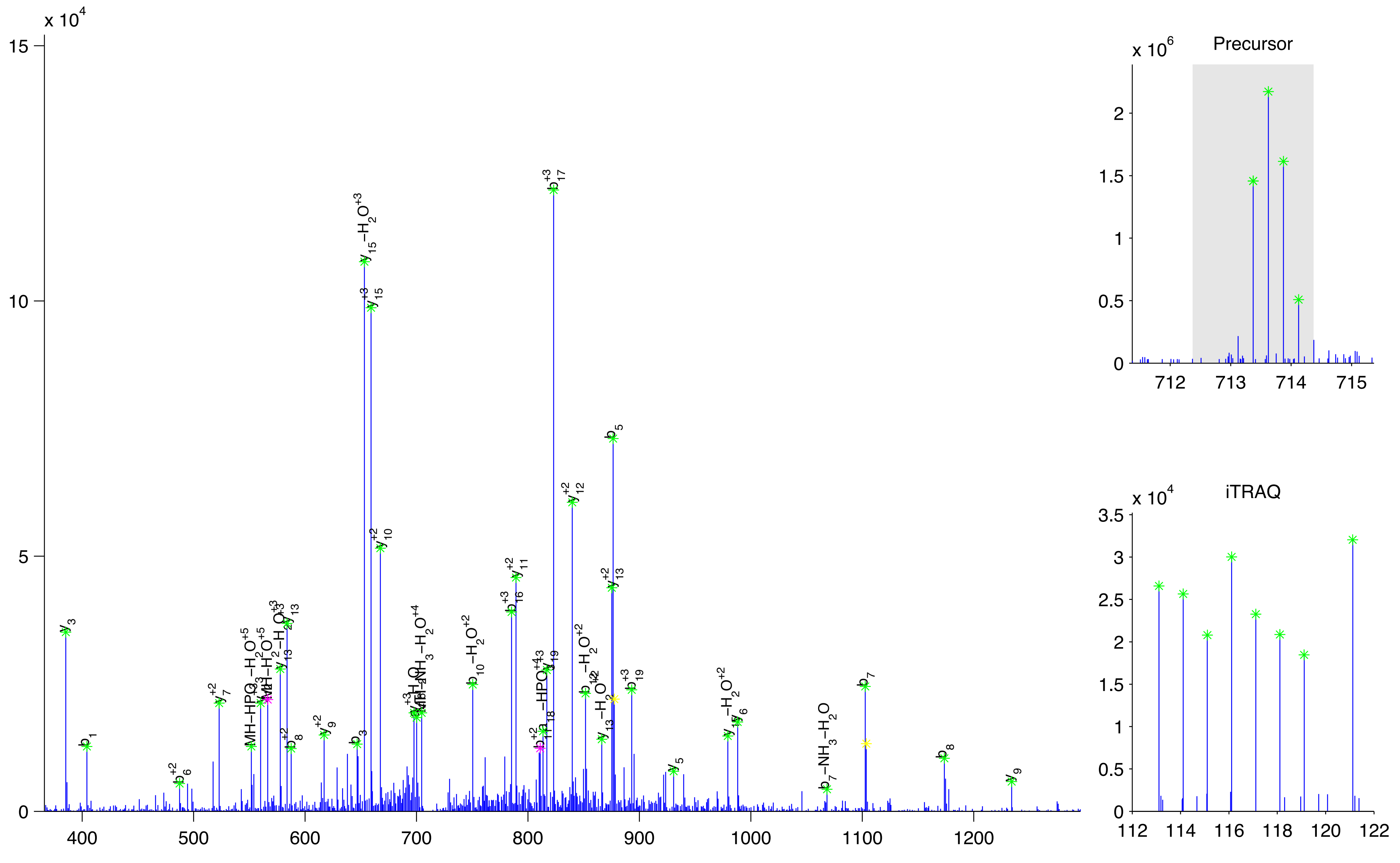

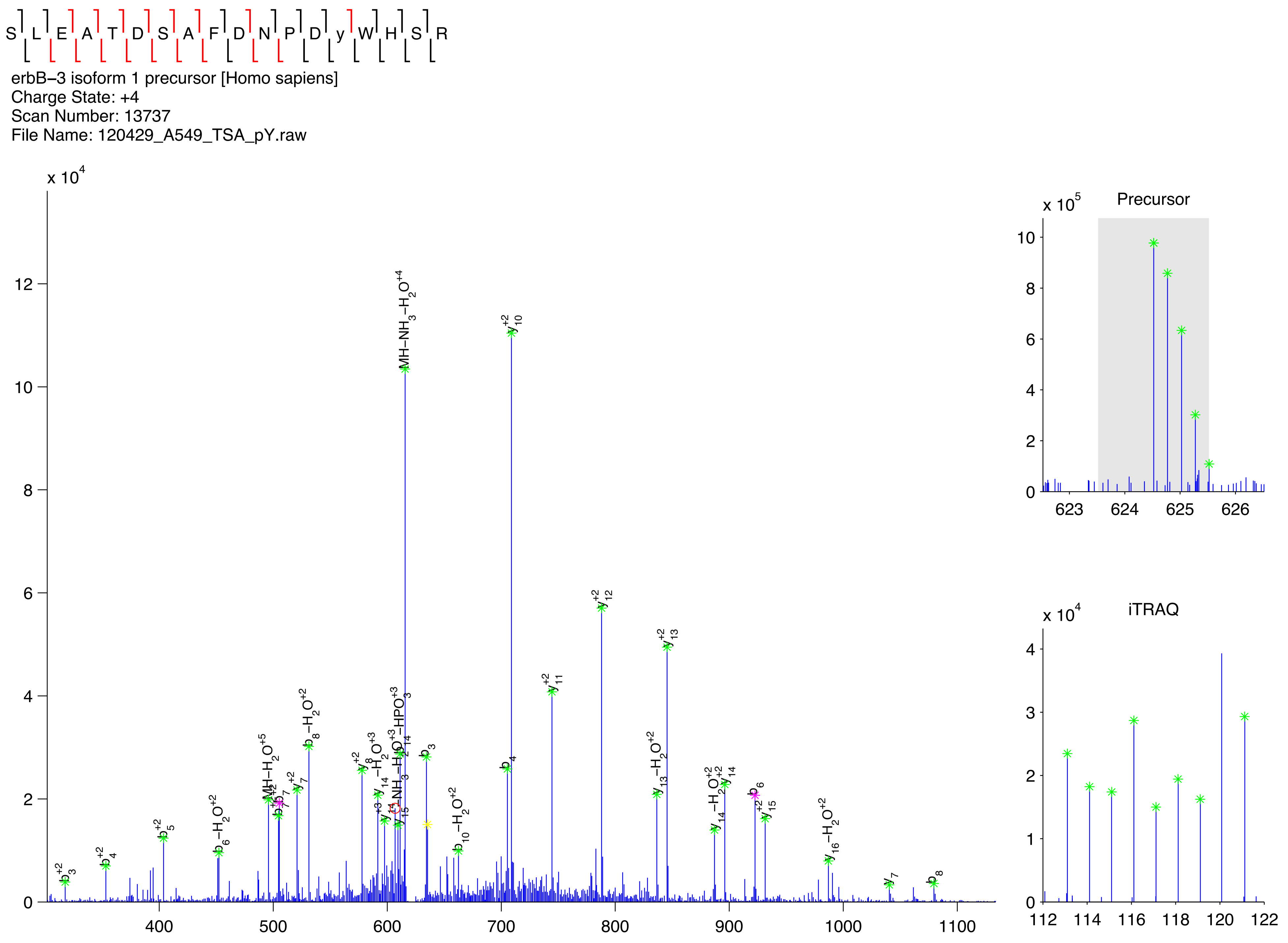
S L E A T D S A F D N P D y W H S R
erbB–3 isoform 1 precursor [Homo sapiens]
Charge State: +4
Scan Number: 13737
File Name: 120429_A549_TSA_pY.raw
x 10^4
Precursor
x 10^5
iTRAQ
x 10^4

R A Q I P E G D Y L S y R

ERBB2 interacting protein isoform 7 [Homo sapiens]
Charge State: +3
Scan Number: 9873
File Name: 120429_A549_TSA_pY.raw

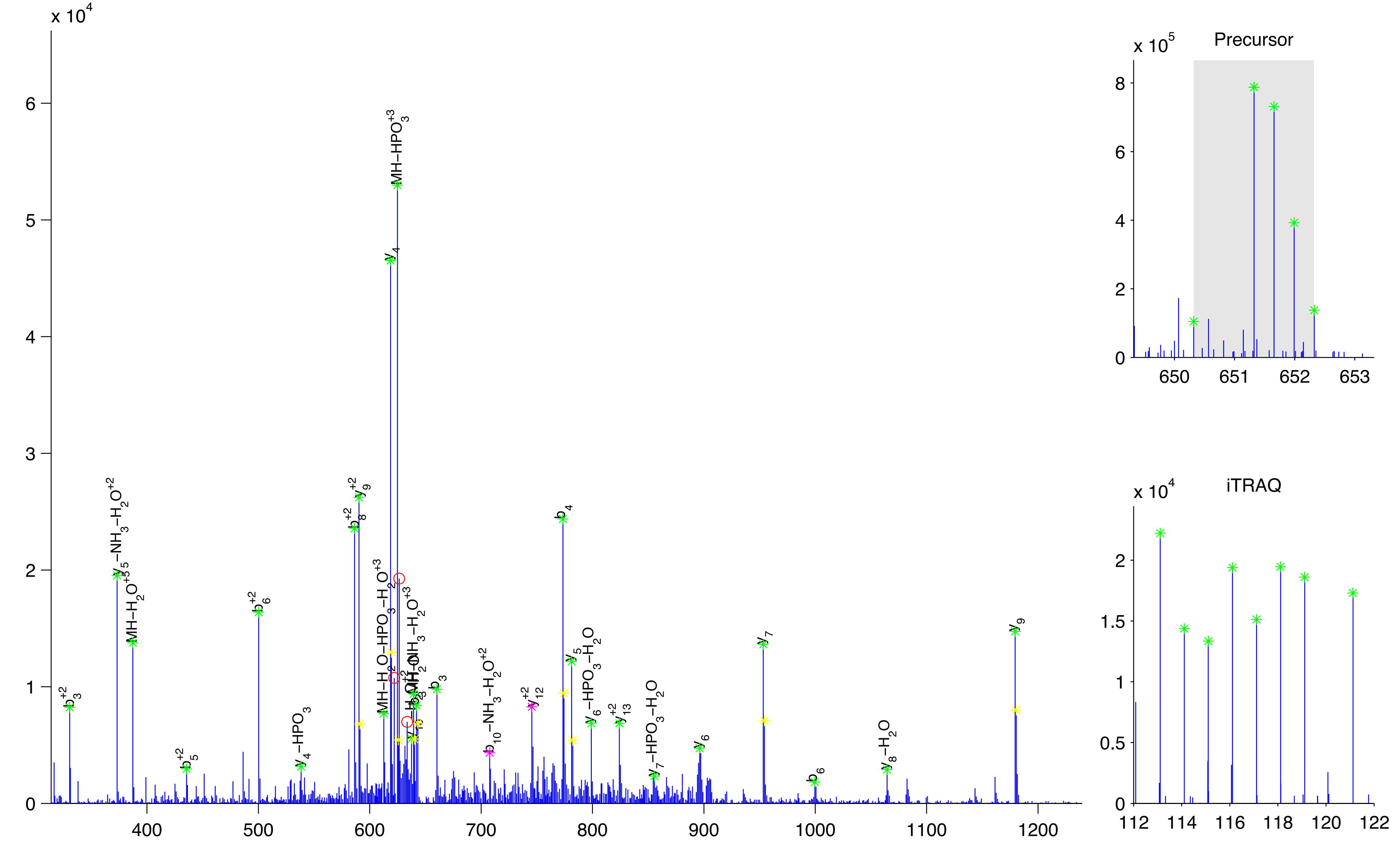

A Q I P E G D y L S Y R

ERBB2 interacting protein isoform 7 [Homo sapiens]
Charge State: +3
Scan Number: 12708
File Name: 120429_A549_TSA_pY.raw

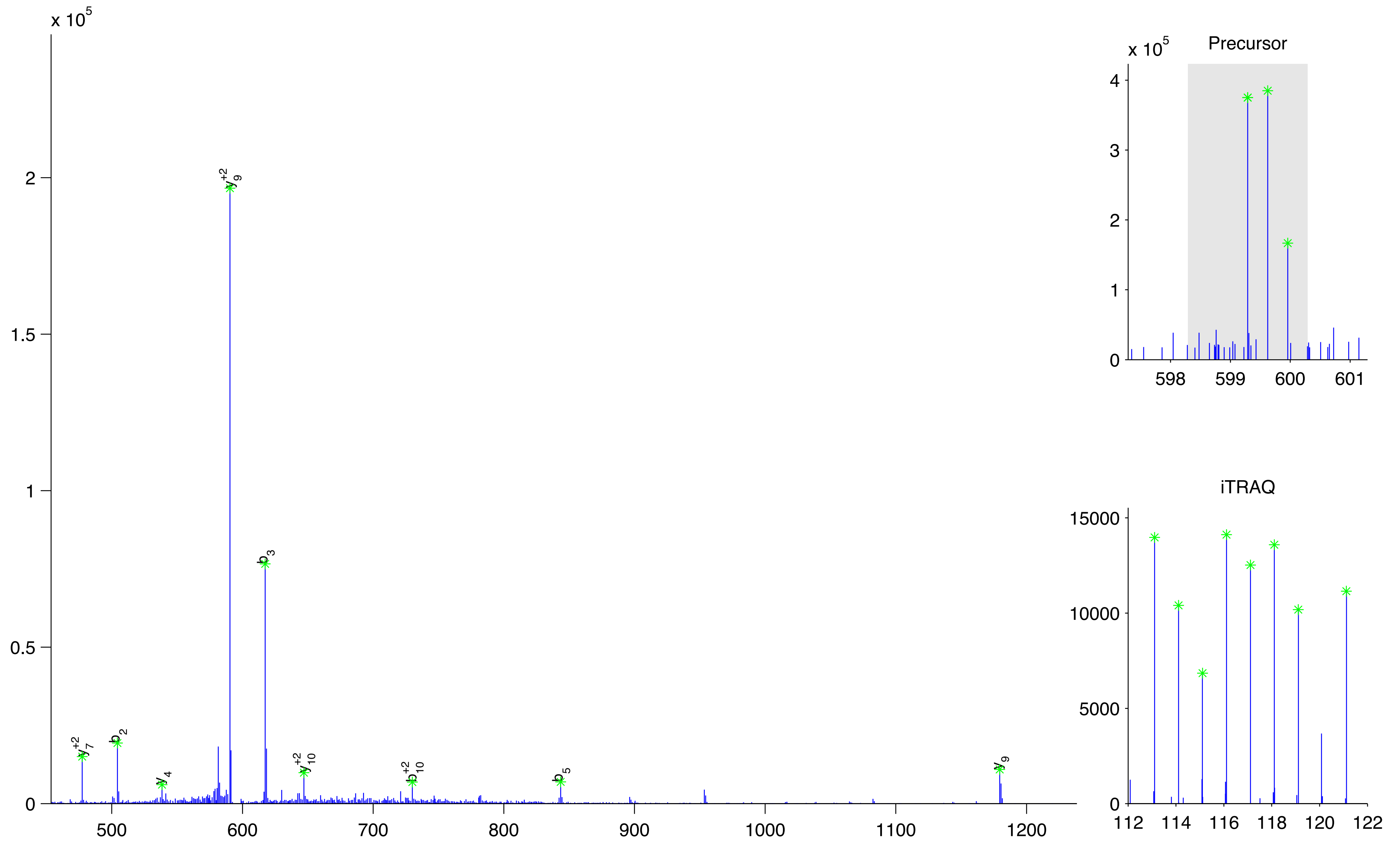

E L E R V D S G y R

glutaryl–Coenzyme A dehydrogenase isoform a precursor [Homo sapiens]
Charge State: +3
Scan Number: 10209
File Name: 120429_A549_TSA_pY.raw

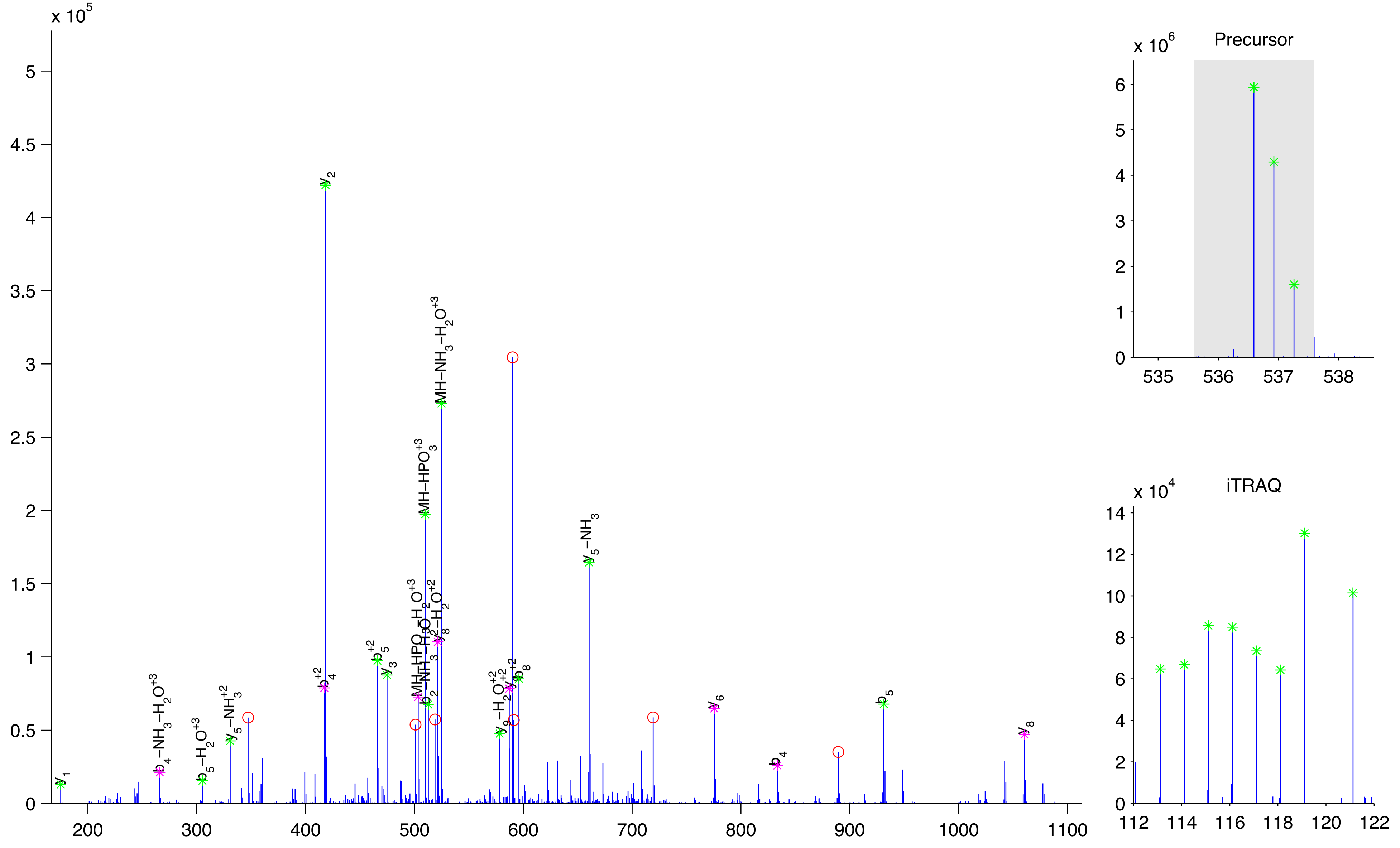

G E P N V S y I c S R

glycogen synthase kinase 3 alpha [Homo sapiens]
Charge State: +2
Scan Number: 9033
File Name: 120429_A549_TSA_pY.raw

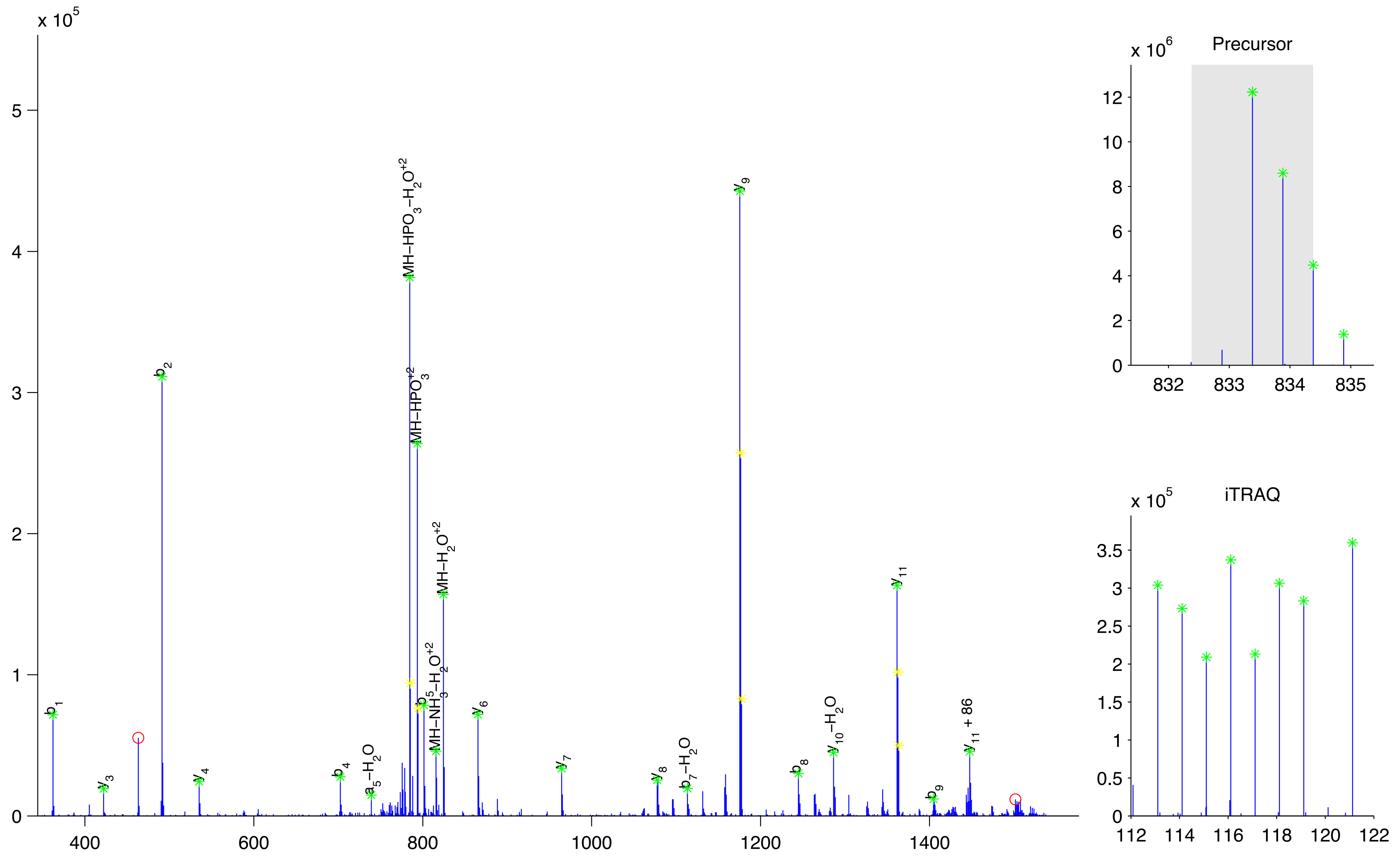

G E P N V S y I c S R

glycogen synthase kinase 3 alpha [Homo sapiens]
Charge State: +3
Scan Number: 9180
File Name: 120429_A549_TSA_pY.raw

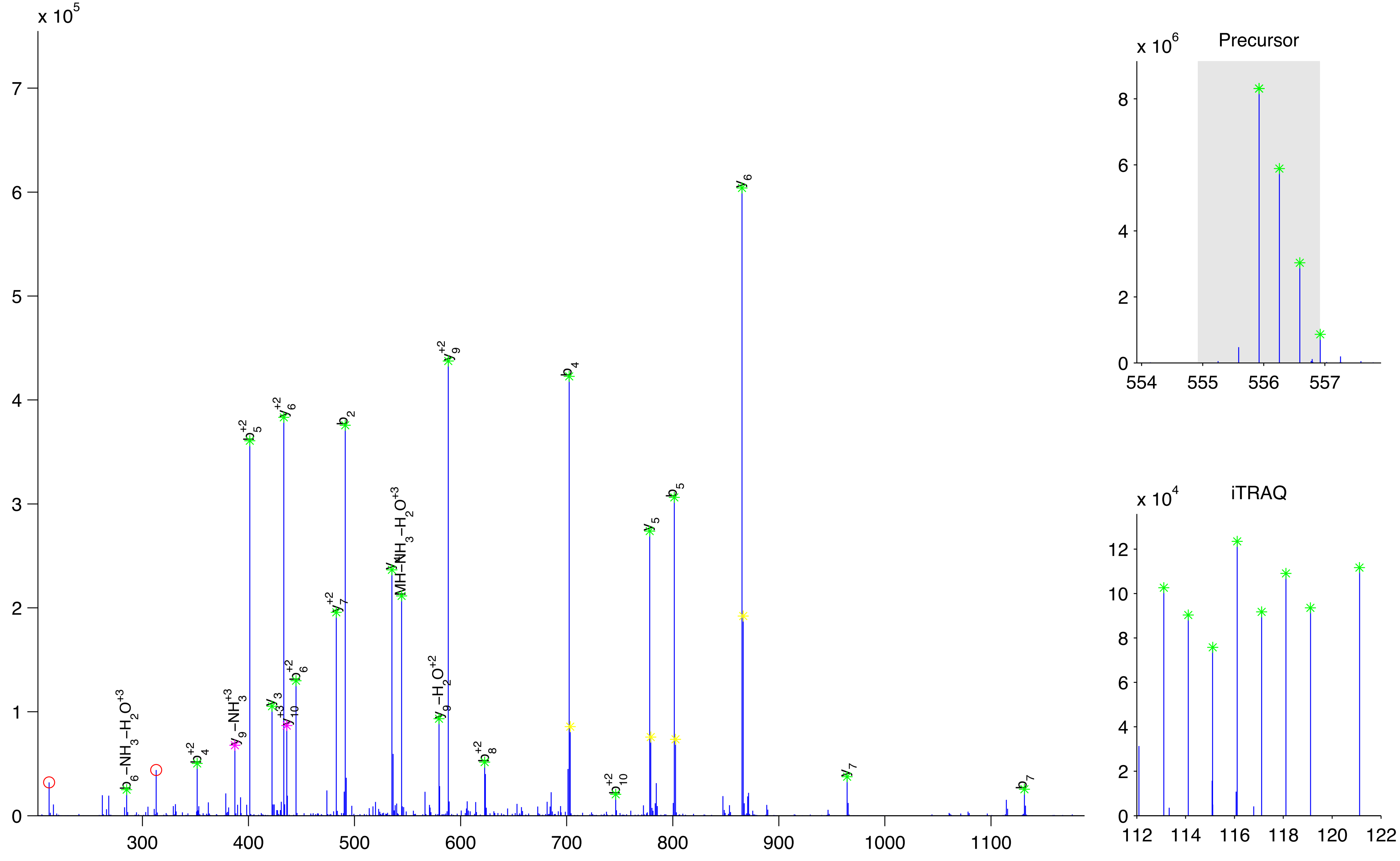

G E P N V S y I c S R

glycogen synthase kinase 3 alpha [Homo sapiens]
Charge State: +2
Scan Number: 9453
File Name: 120429_A549_TSA_pY.raw

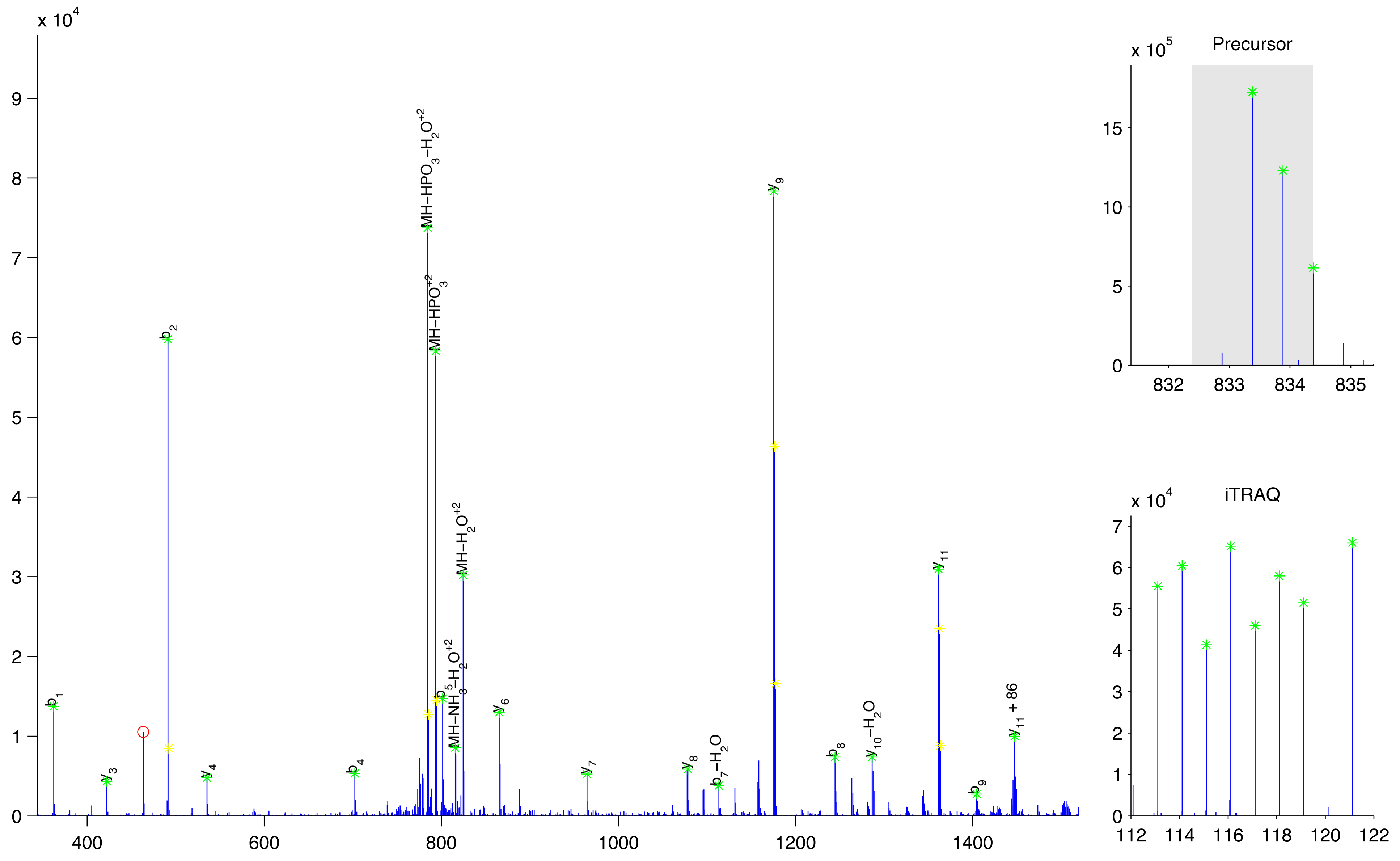

G E P N V S y I c S R

glycogen synthase kinase 3 beta [Homo sapiens]
Charge State: +2
Scan Number: 9033
File Name: 120429_A549_TSA_pY.raw

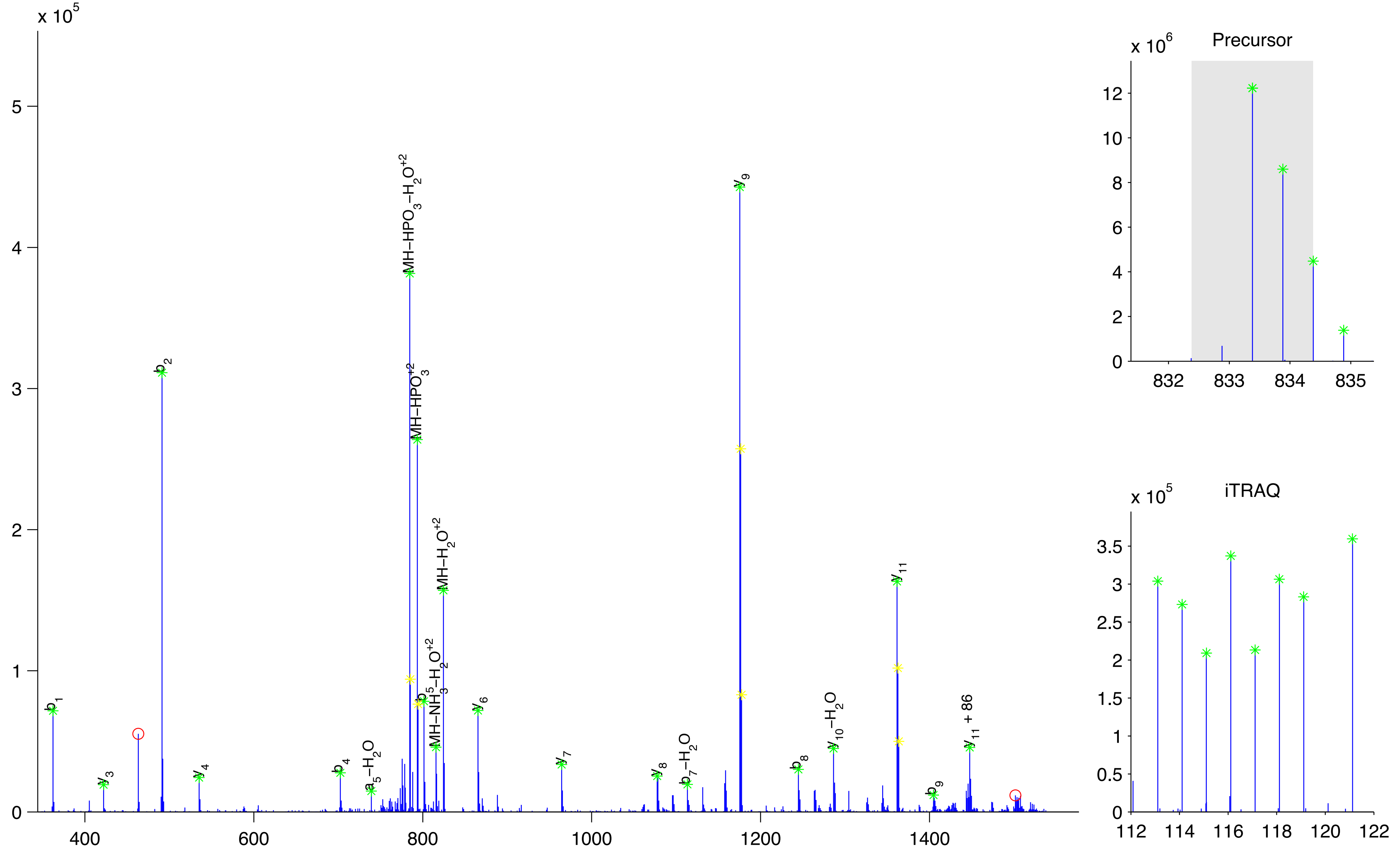

G E P N V S y I c S R

glycogen synthase kinase 3 beta [Homo sapiens]
Charge State: +3
Scan Number: 9180
File Name: 120429_A549_TSA_pY.raw

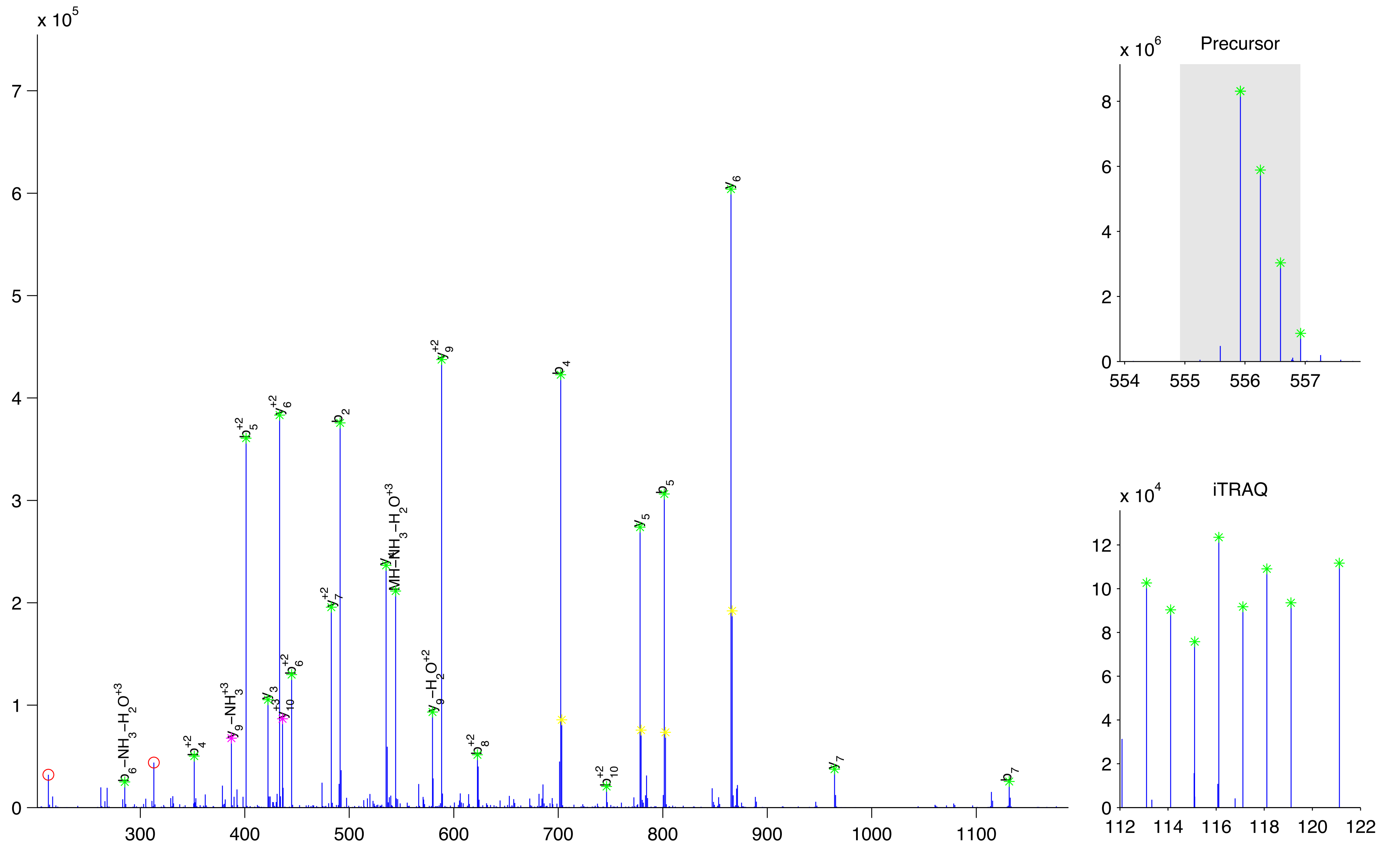

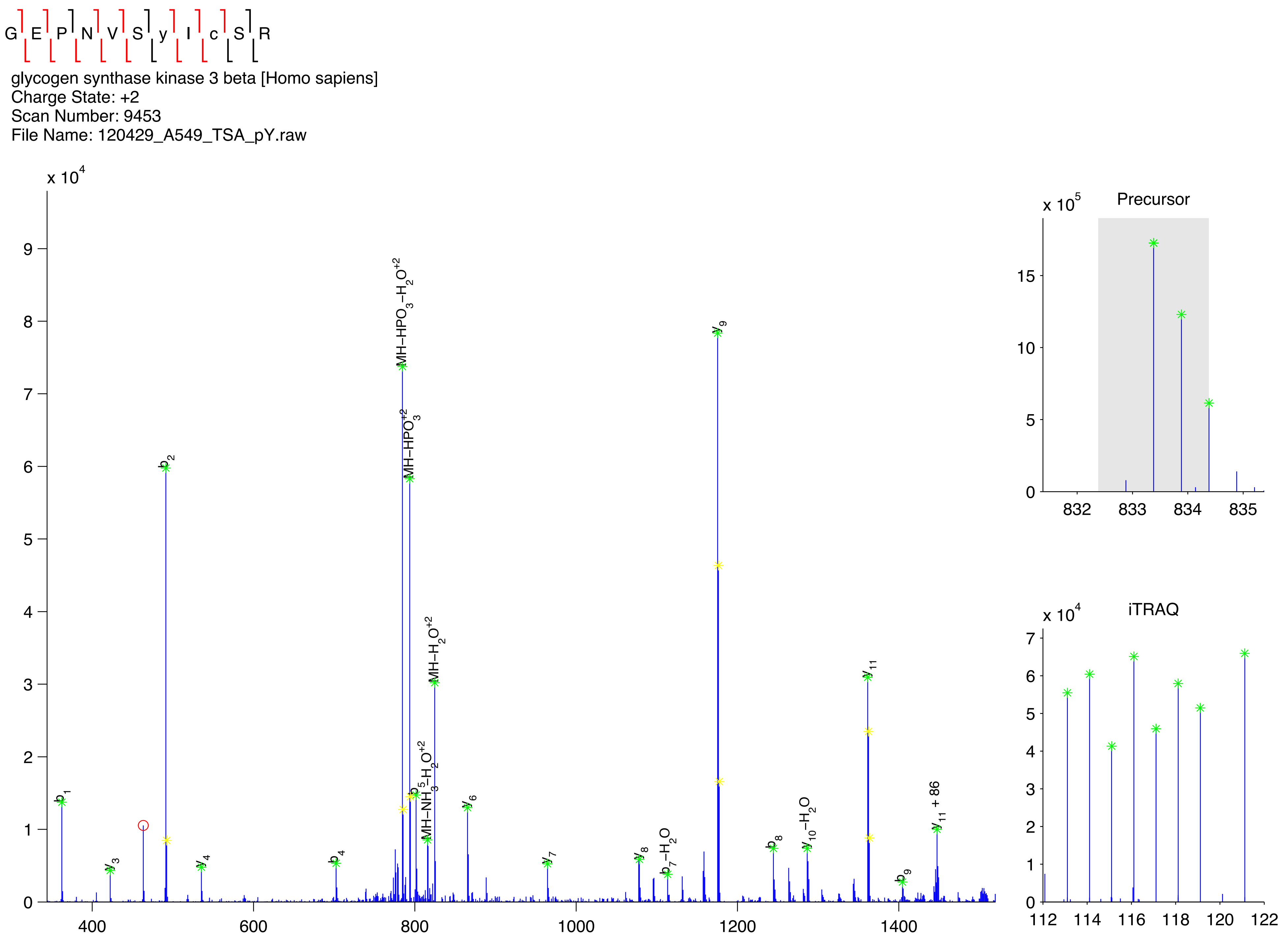
G E P N V S y I c S R
glycogen synthase kinase 3 beta [Homo sapiens]
Charge State: +2
Scan Number: 9453
File Name: 120429_A549_TSA_pY.raw
x 10^4
b_1
y_3
b_2
y_4
b_4
MH−HPO$_3$−H$_2$O^{+2}
MH−HPO$_3^{+2}$
b_5
MH−NH$_3$−H$_2$O^{+2}
MH−H$_2$O^{+2}
y_6
y_7
y_8
b_7−H$_2$O
y_9
b_8
y_{10}−H$_2$O
y_{11}
b_9
y_{11} + 86
Precursor
x 10^5
iTRAQ
x 10^4

y G s H D G s S T V A D G K K K P R K

jumonji, AT rich interactive domain 2 protein [Homo sapiens]
Charge State: +3
Scan Number: 7439
File Name: 120429_A549_TSA_pY.raw

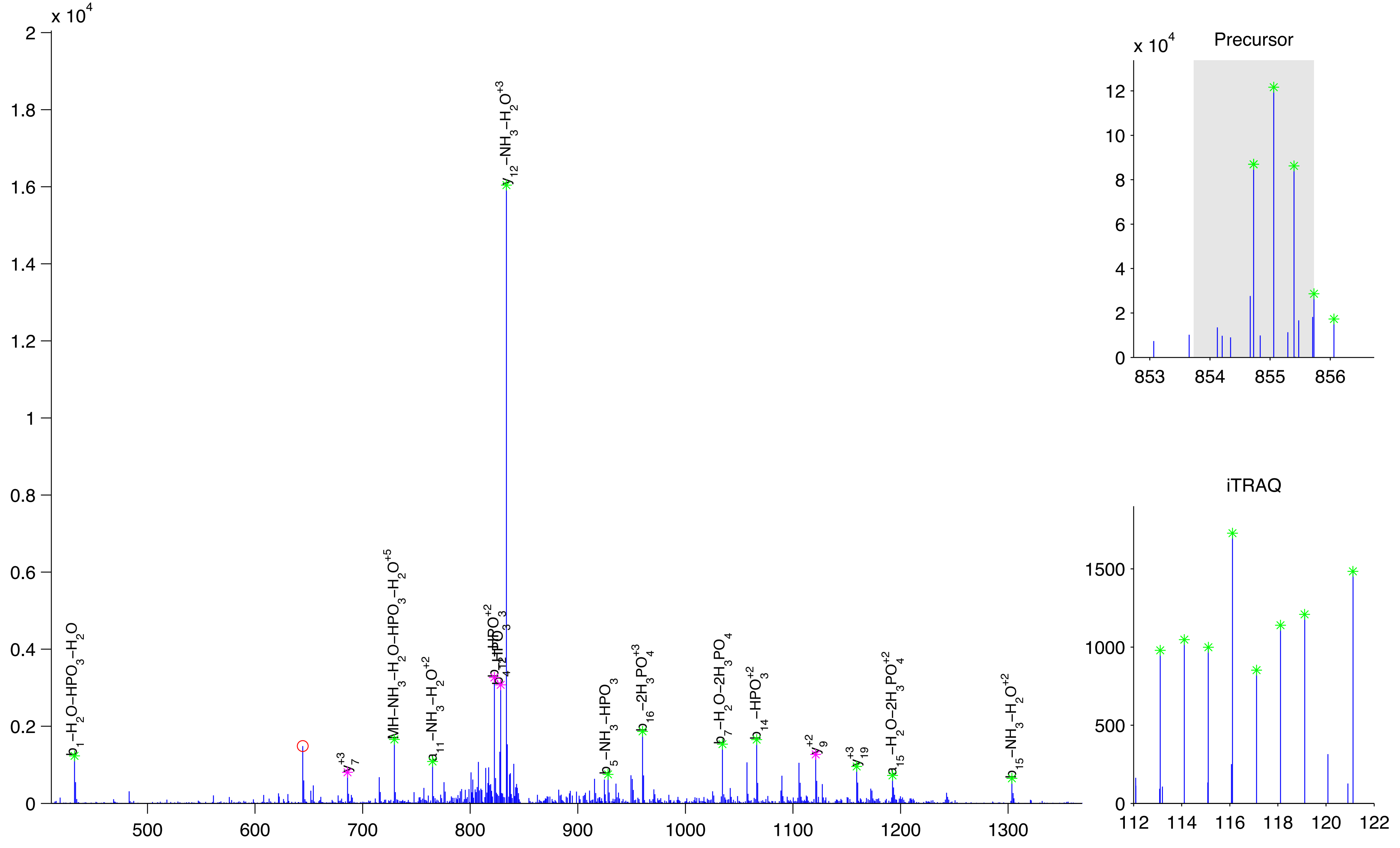

N D S D P T y G Q Q G H P N T W K

LIM domain containing preferred translocation partner in lipoma [Homo sapiens]
Charge State: +3
Scan Number: 6767
File Name: 120429_A549_TSA_pY.raw

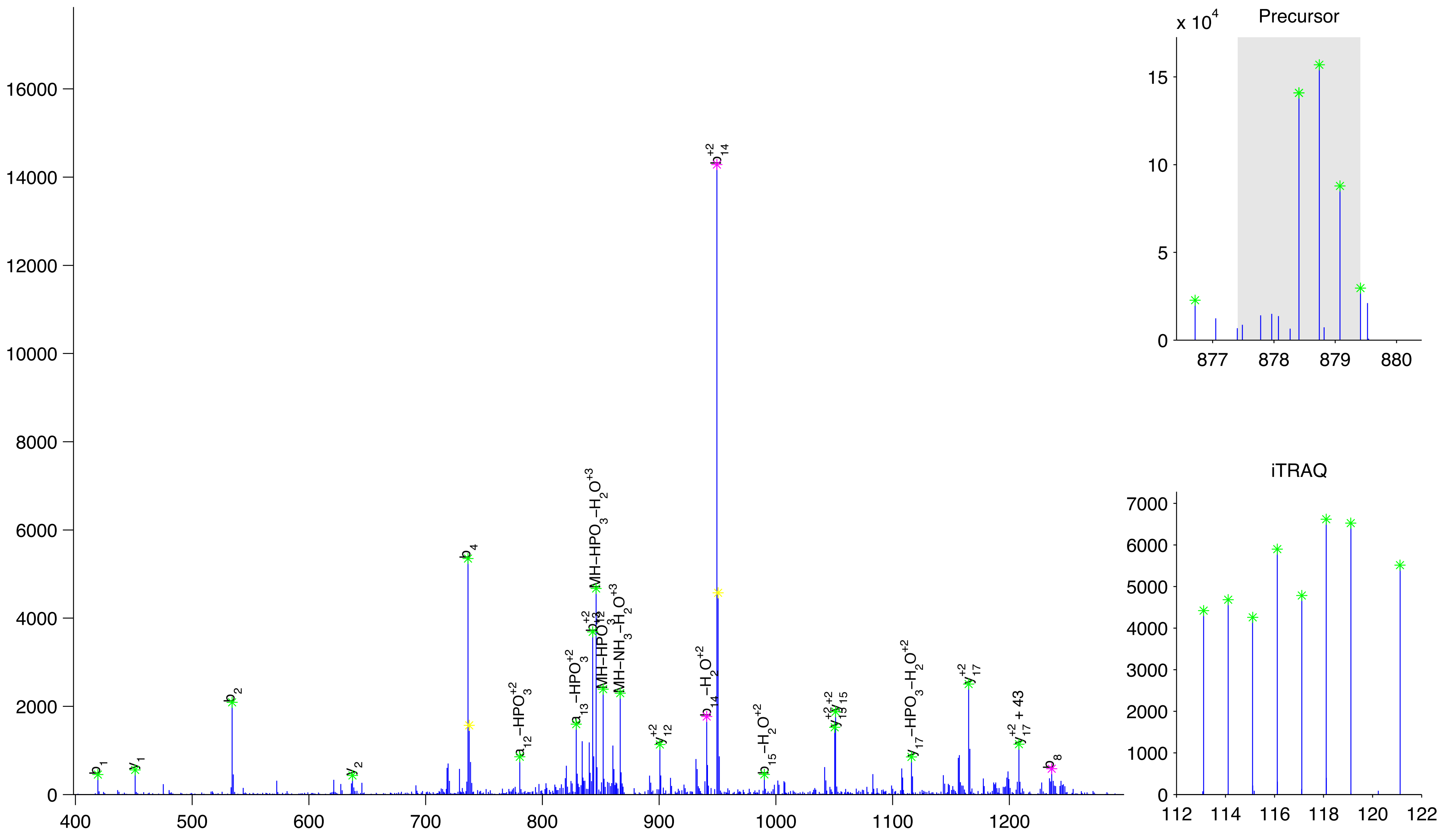

Y Y E G Y Y A A G P G y G G R

LIM domain containing preferred translocation partner in lipoma [Homo sapiens]
Charge State: +2
Scan Number: 12264
File Name: 120429_A549_TSA_pY.raw

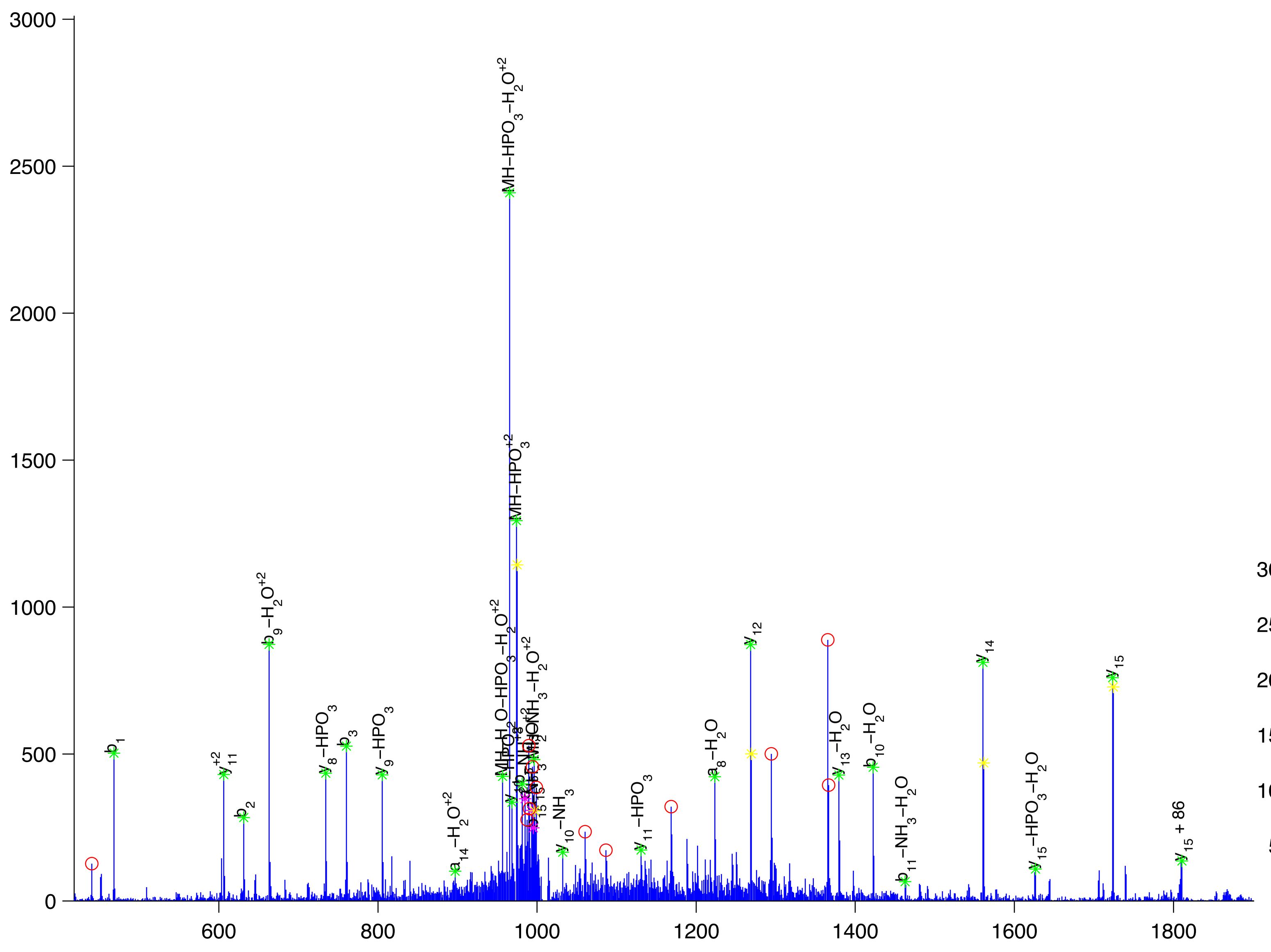

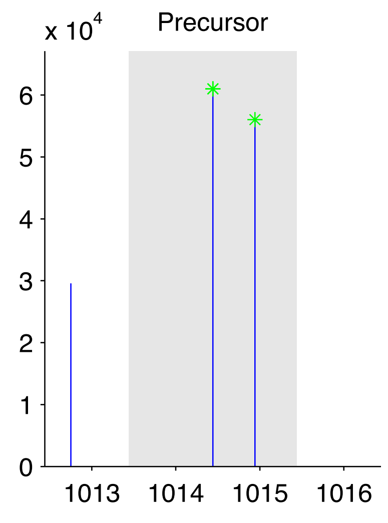

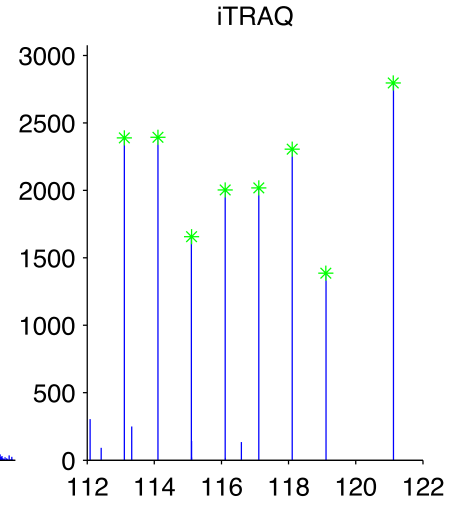

V A D P D H D H T G F L T E y V A T R

mitogen−activated protein kinase 1 [Homo sapiens]
Charge State: +3
Scan Number: 12057
File Name: 120429_A549_TSA_pY.raw

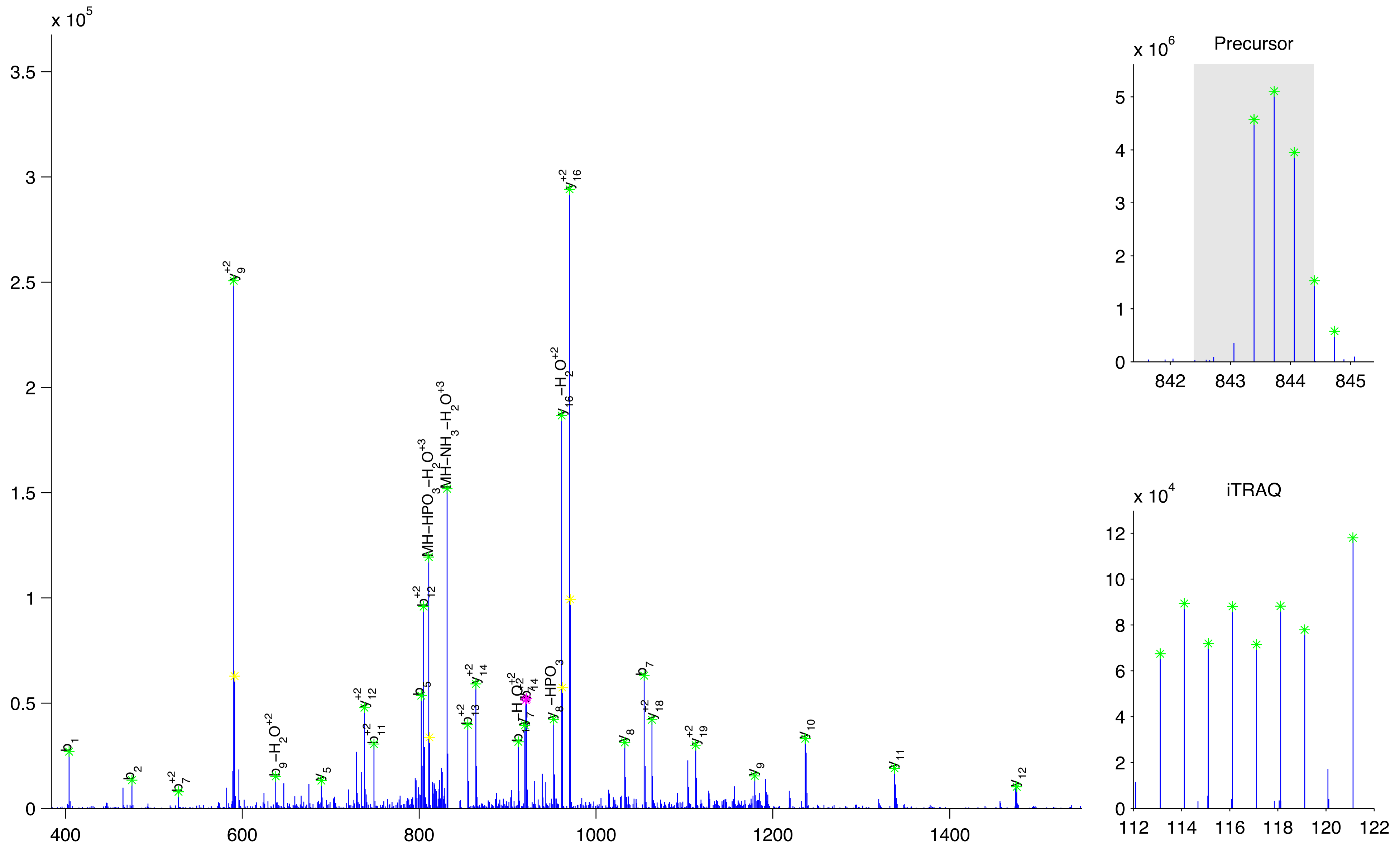

V A D P D H D H T G F L T E y V A T R

mitogen–activated protein kinase 1 [Homo sapiens]
Charge State: +4
Scan Number: 12225
File Name: 120429_A549_TSA_pY.raw

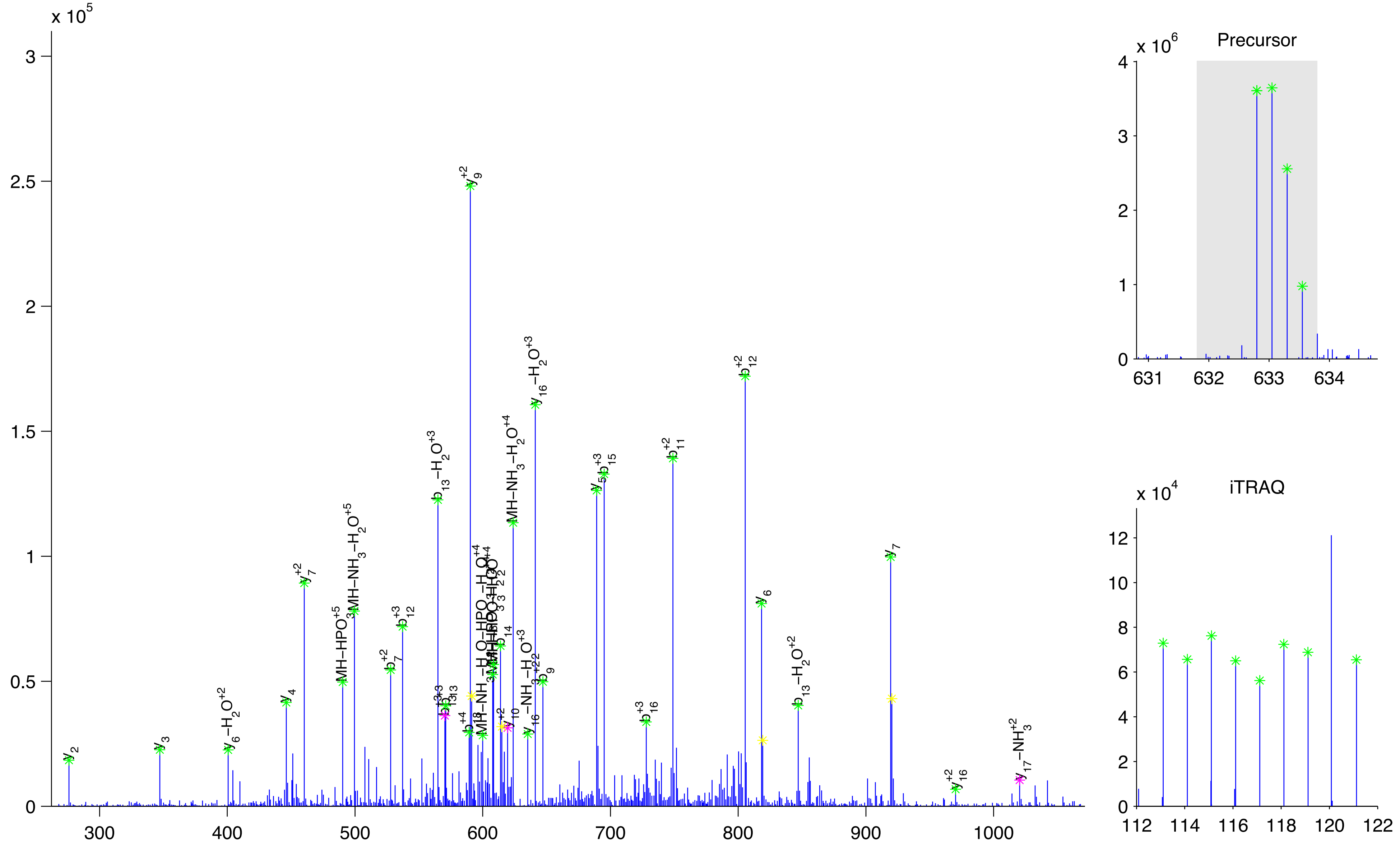

I A D P E H D H T G F L T E y V A t R

mitogen–activated protein kinase 3 isoform 2 [Homo sapiens]
Charge State: +4
Scan Number: 13988
File Name: 120429_A549_TSA_pY.raw

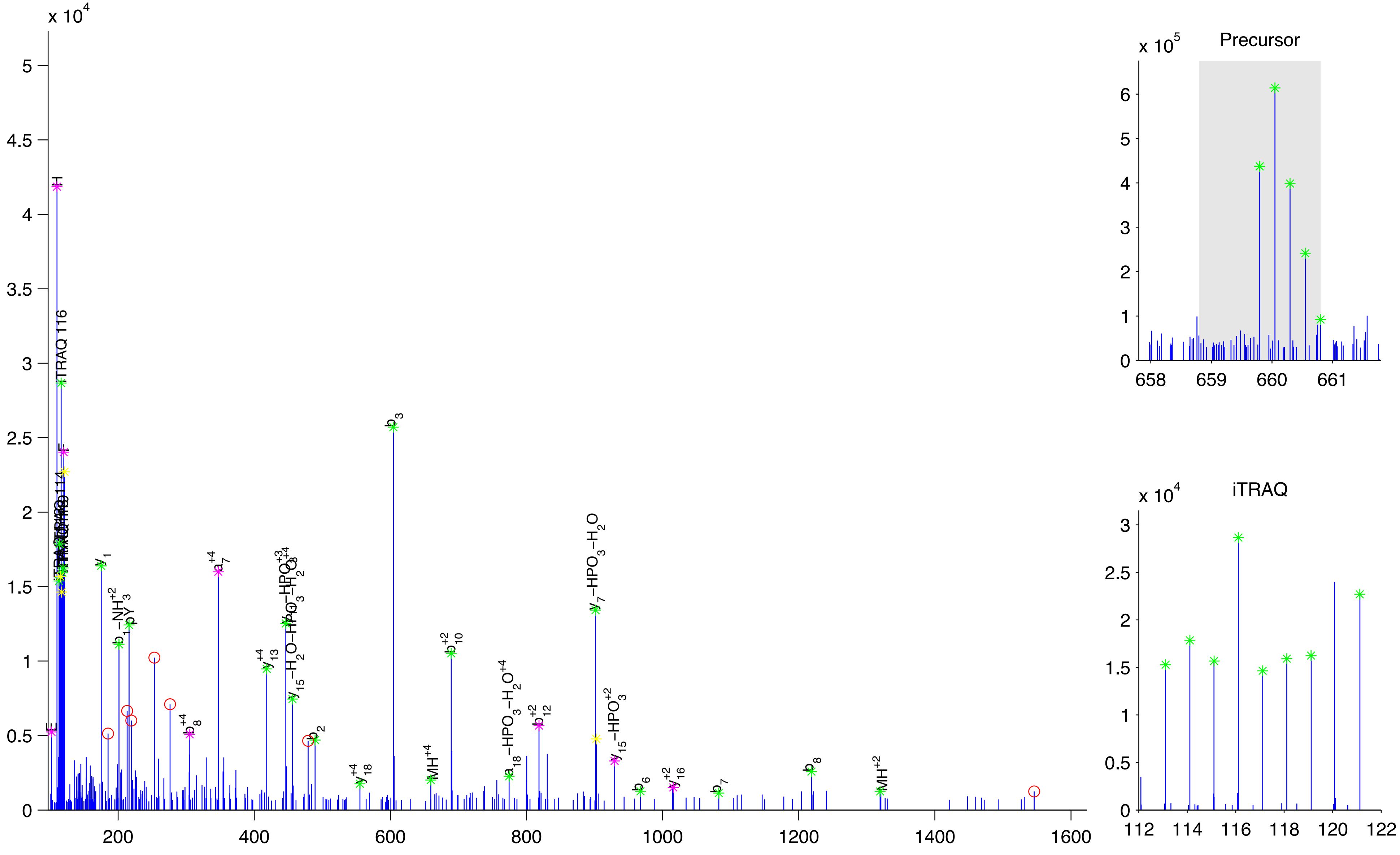

H T D D E M T G y V A T R

mitogen–activated protein kinase 14 isoform 1 [Homo sapiens]
Charge State: +2
Scan Number: 5788
File Name: 120429_A549_TSA_pY.raw

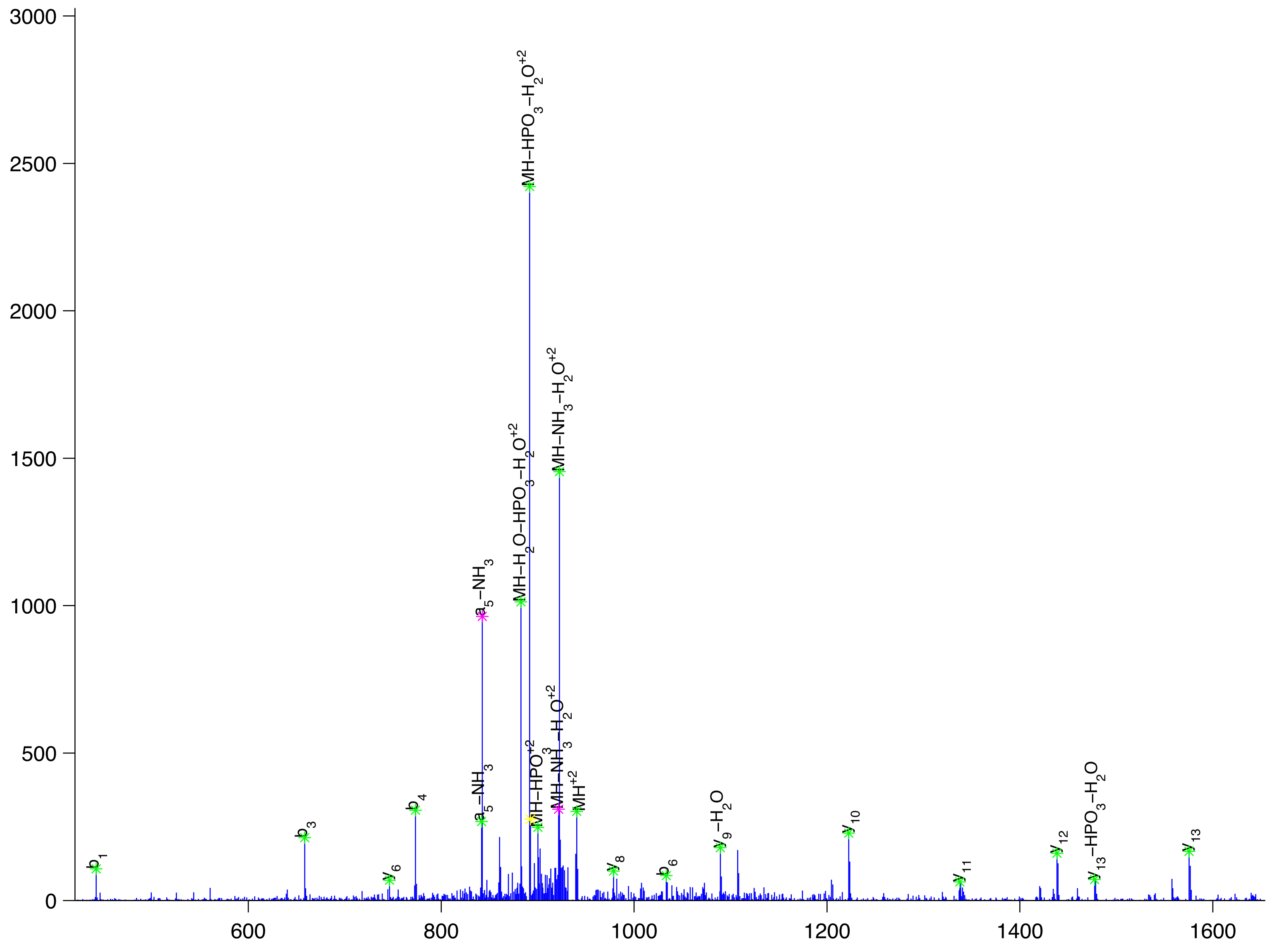

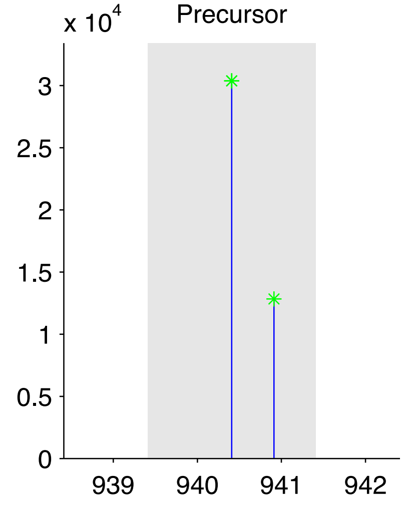

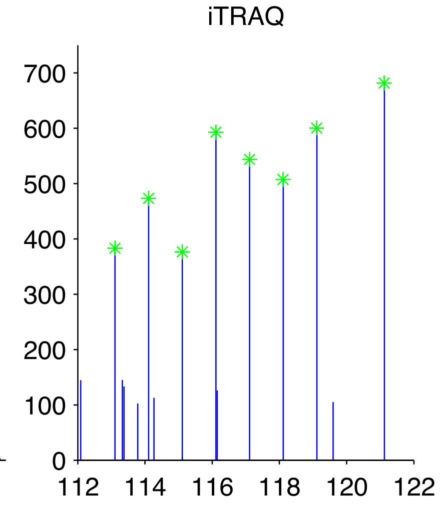

H T D D E M T G y V A T R

mitogen−activated protein kinase 14 isoform 1 [Homo sapiens]
Charge State: +3
Scan Number: 5903
File Name: 120429_A549_TSA_pY.raw

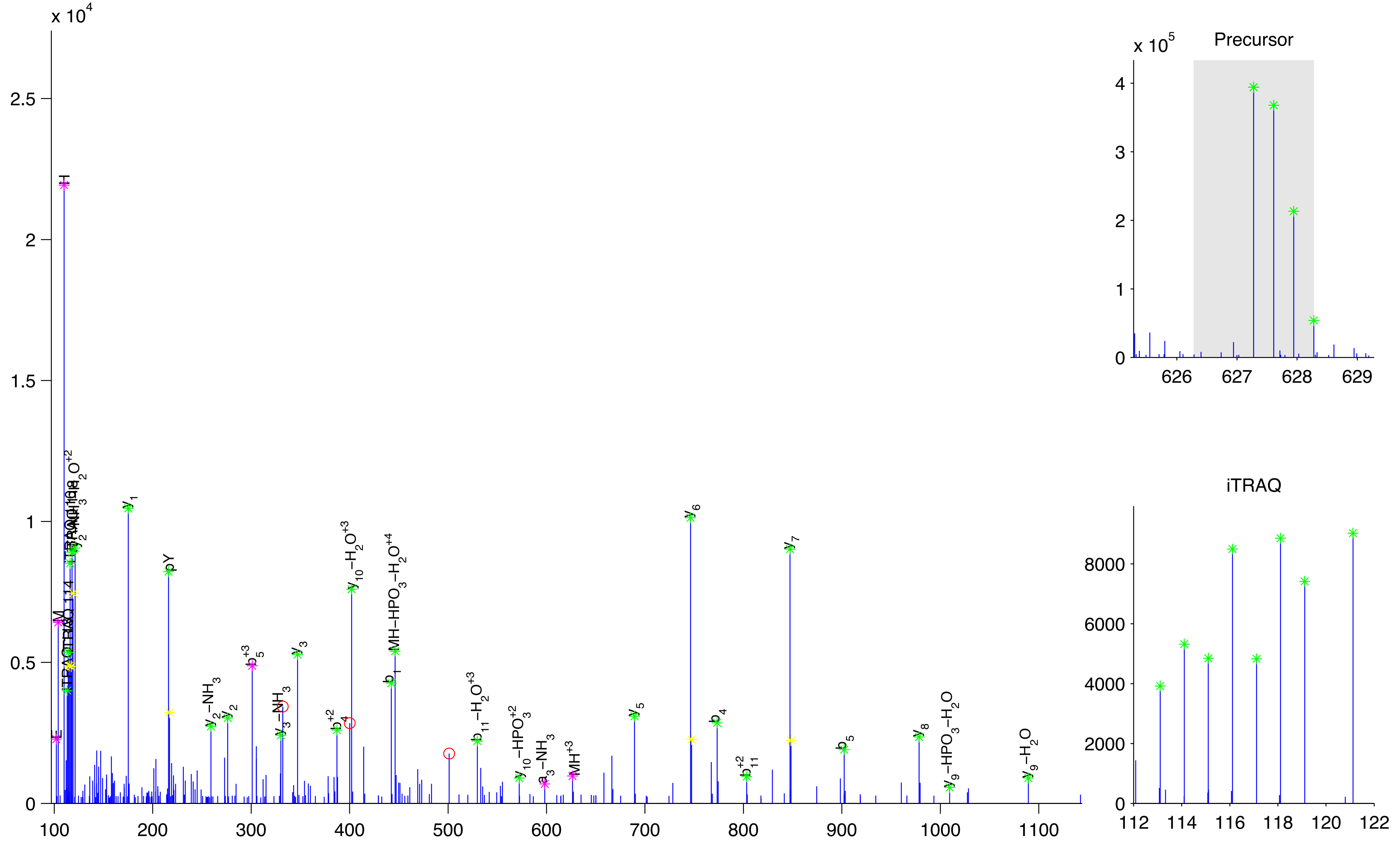

mitogen-activated protein kinase 14 isoform 1 [Homo sapiens]
Charge State: +3
Scan Number: 5904
File Name: 120429_A549_TSA_pY.raw

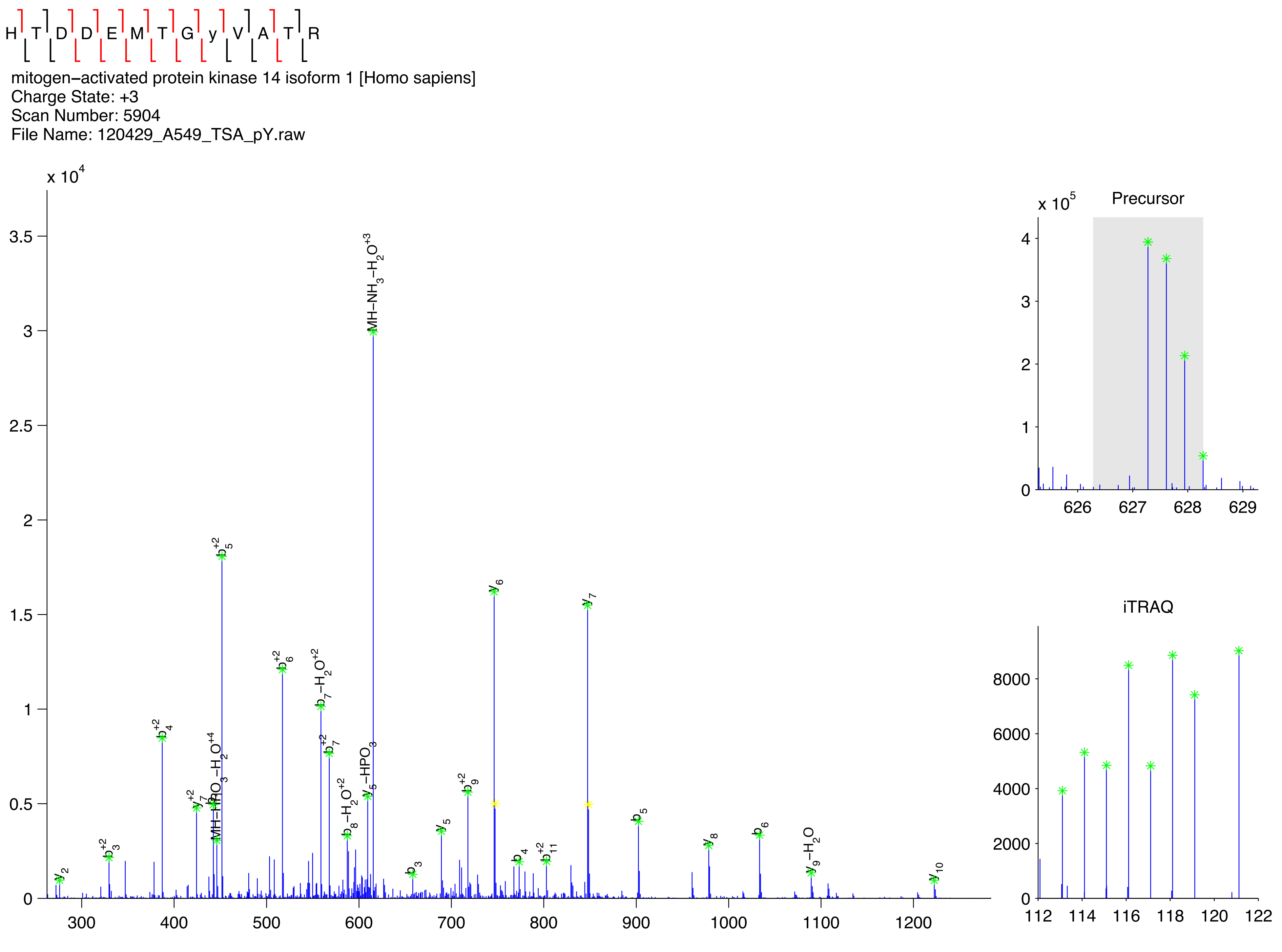

H T D D E M T G y V A T R

mitogen–activated protein kinase 14 isoform 1 [Homo sapiens]
Charge State: +3
Scan Number: 6701
File Name: 120429_A549_TSA_pY.raw

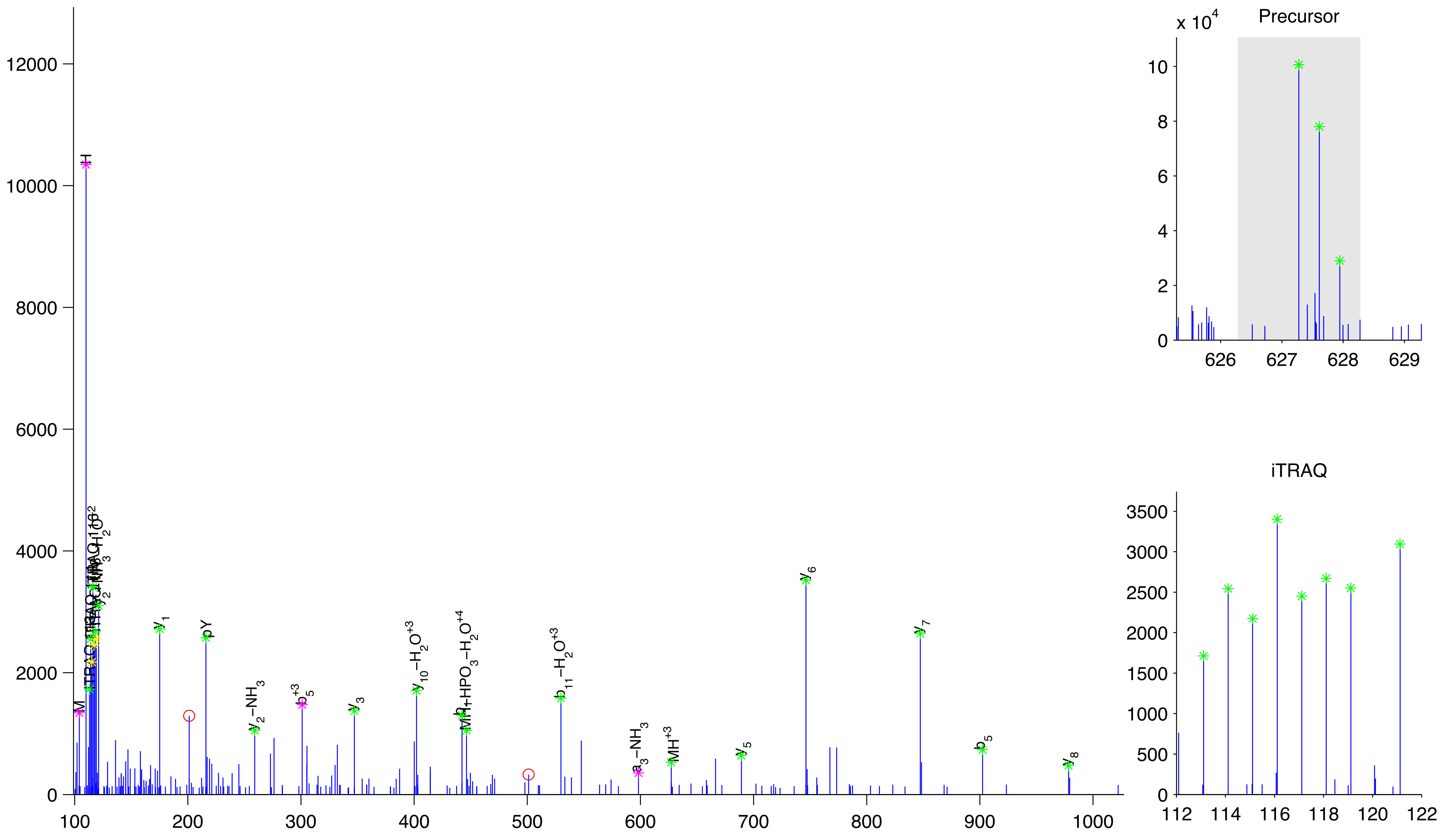

H T D D E M T G y V A T R

mitogen−activated protein kinase 14 isoform 1 [Homo sapiens]
Charge State: +3
Scan Number: 6702
File Name: 120429_A549_TSA_pY.raw

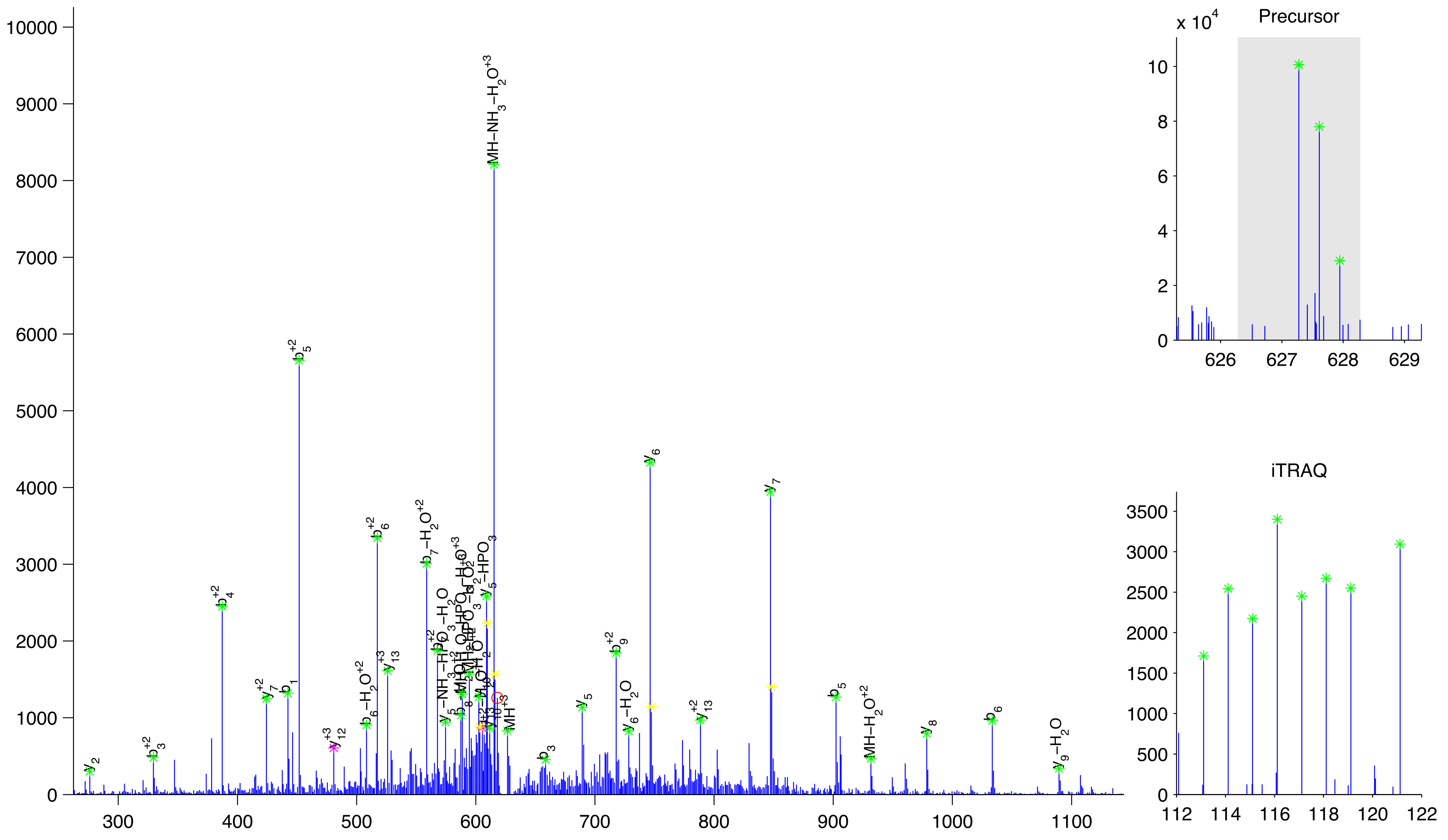

S E S V V y A D I R

myelin protein zero−like 1 isoform a [Homo sapiens]
Charge State: +3
Scan Number: 11723
File Name: 120429_A549_TSA_pY.raw

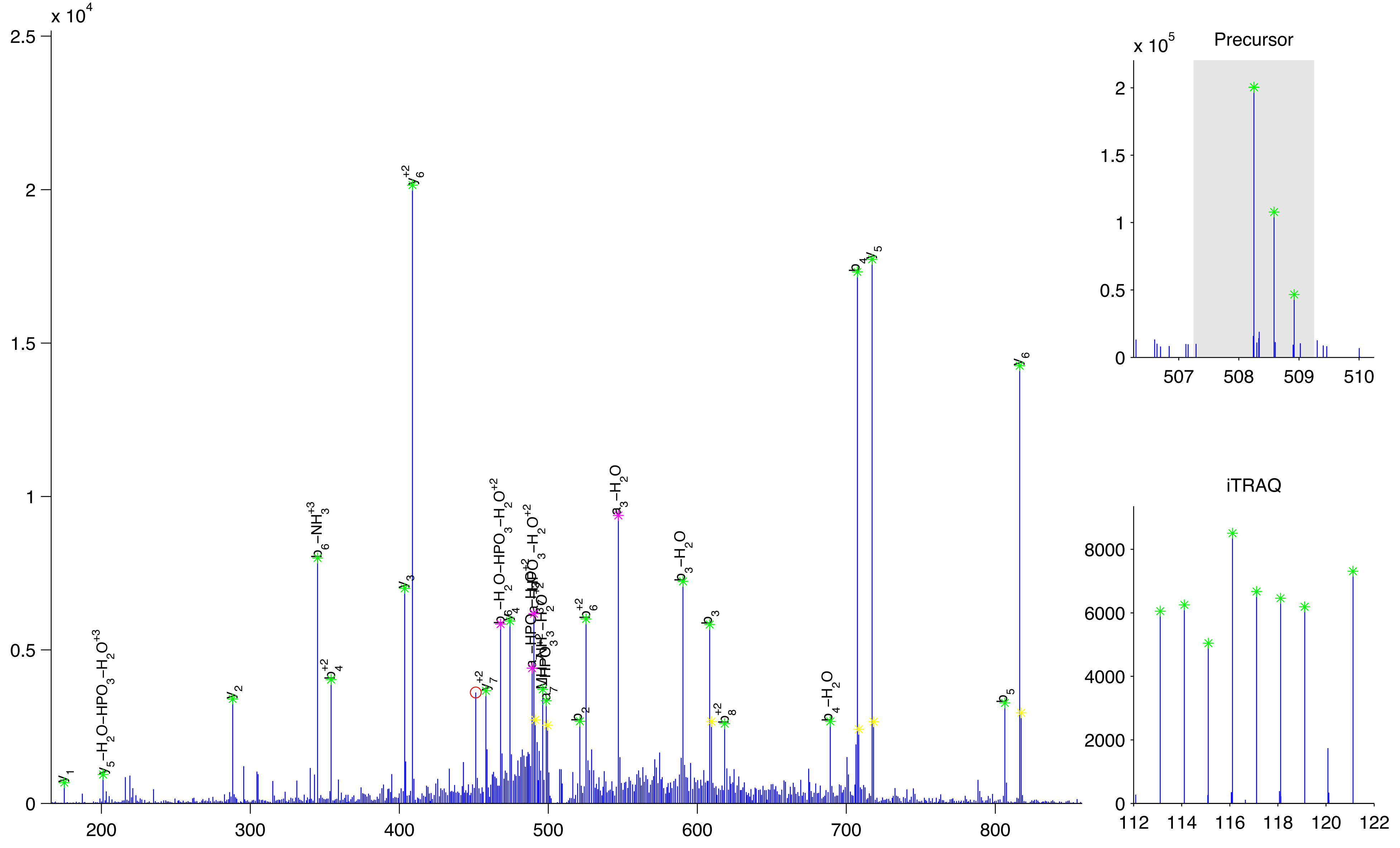

S E S V V y A D I R

myelin protein zero–like 1 isoform a [Homo sapiens]
Charge State: +2
Scan Number: 11784
File Name: 120429_A549_TSA_pY.raw

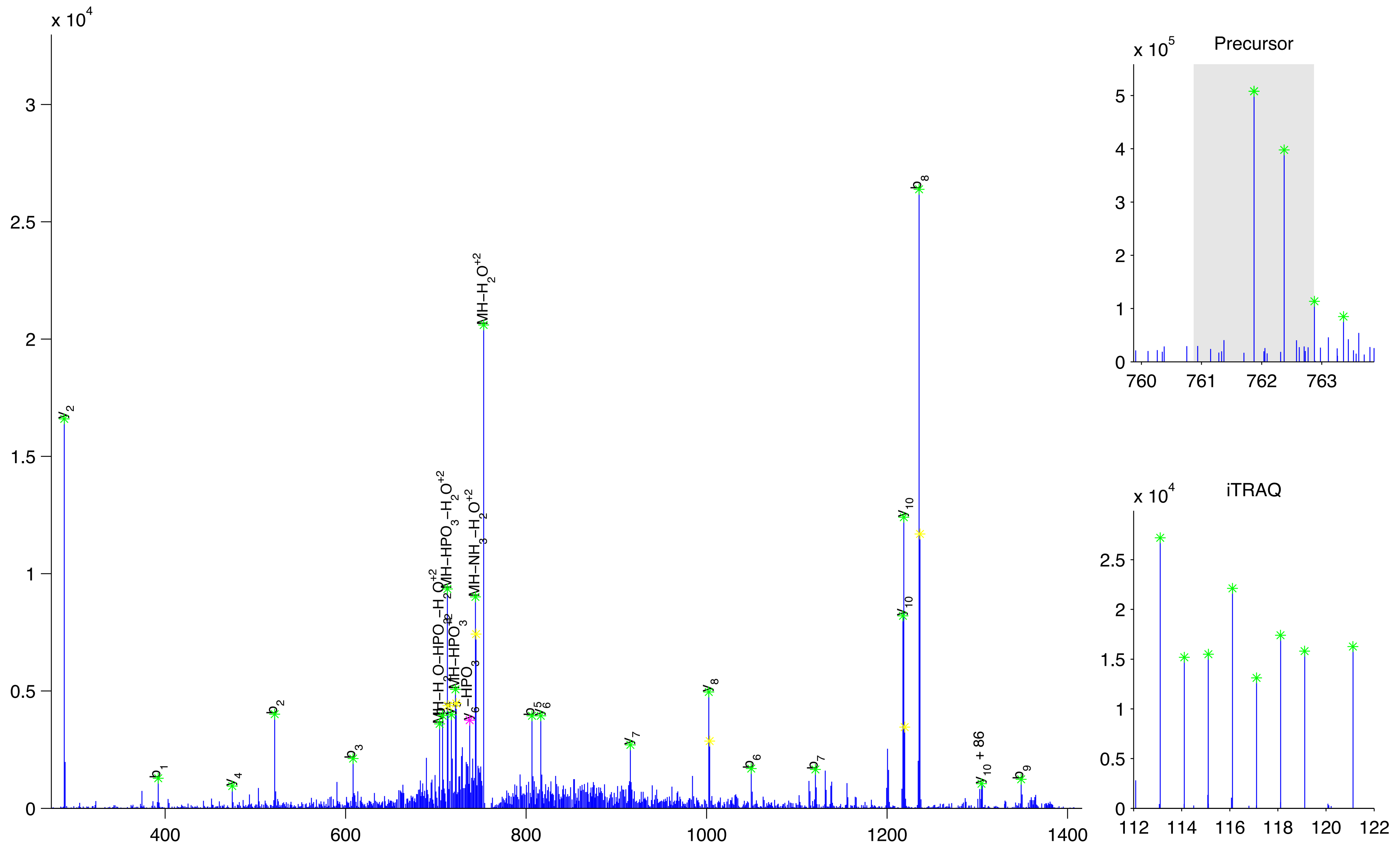

G I V V y T G D R

Na+/K+ –ATPase alpha 1 subunit isoform a proprotein [Homo sapiens]
Charge State: +2
Scan Number: 9578
File Name: 120429_A549_TSA_pY.raw

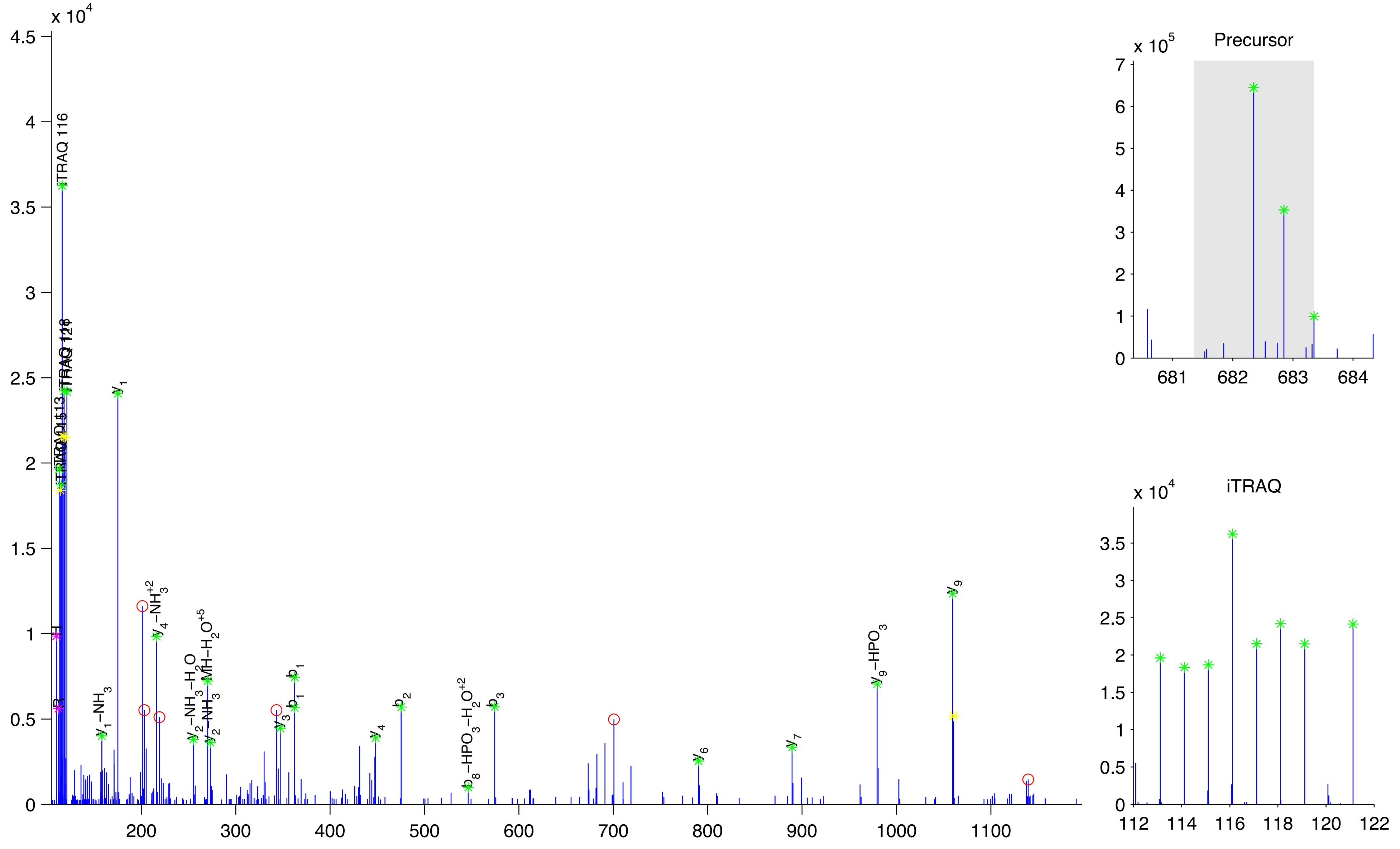

T G H G Y V Y E y P S R

neural precursor cell expressed, developmentally down−regulated 9 isoform 1 [Homo sapiens]
Charge State: +3
Scan Number: 6323
File Name: 120429_A549_TSA_pY.raw

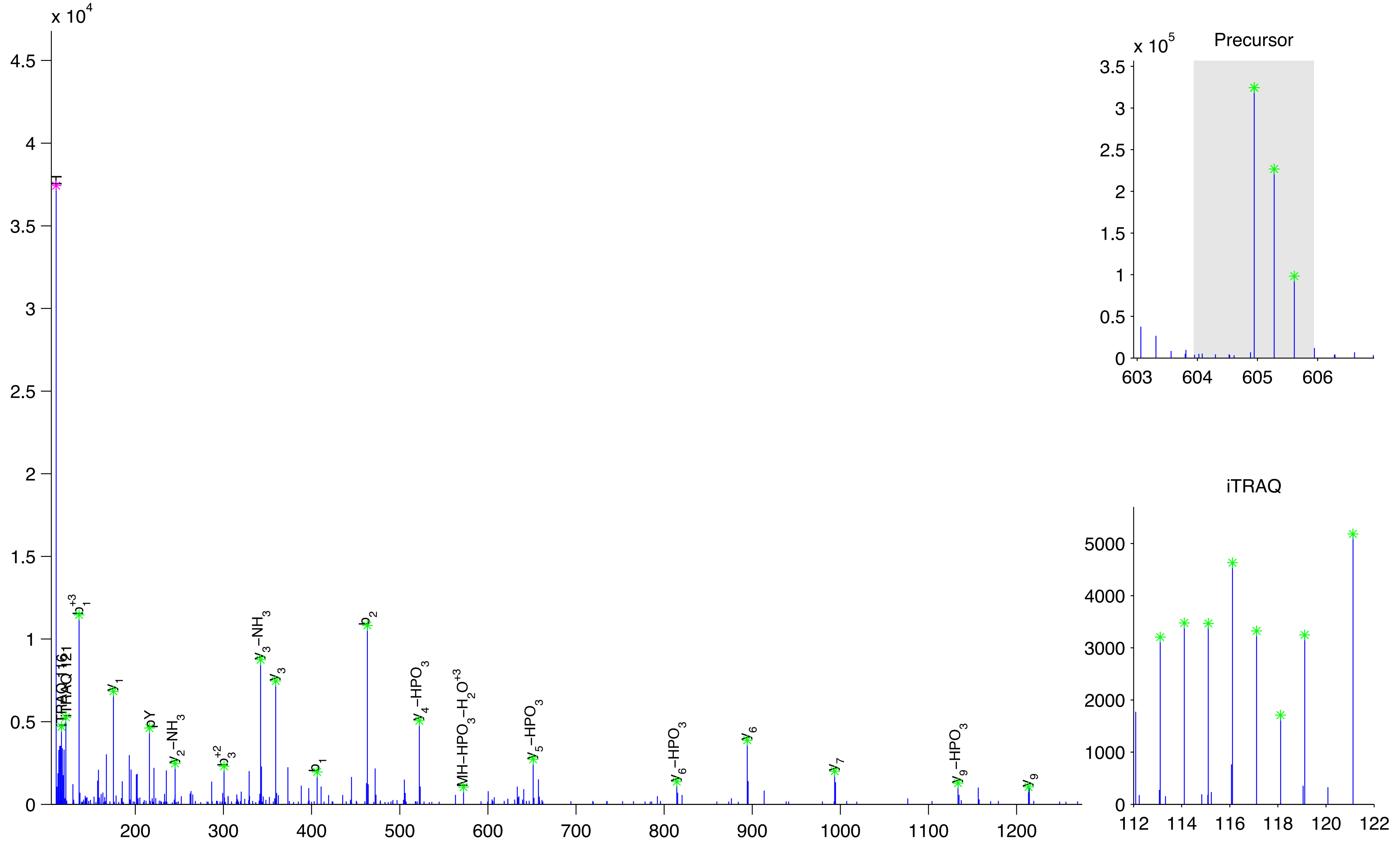

A N P Q E R D G V y D V P L H N P P D A K

neural precursor cell expressed, developmentally down−regulated 9 isoform 1 [Homo sapiens]
Charge State: +5
Scan Number: 8067
File Name: 120429_A549_TSA_pY.raw

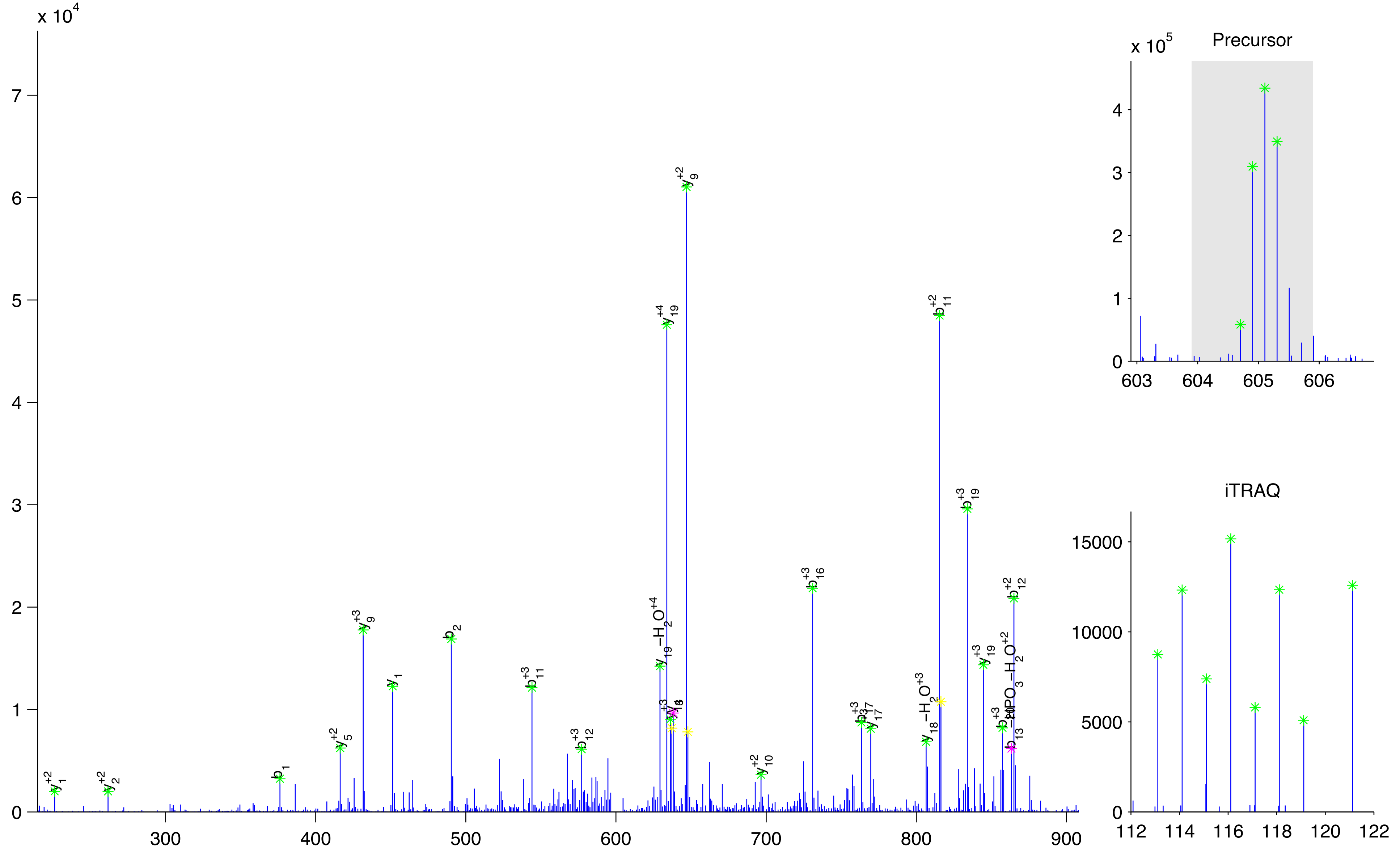

A N P Q E R D G V y D V P L H N P P D A K

neural precursor cell expressed, developmentally down−regulated 9 isoform 1 [Homo sapiens]
Charge State: +5
Scan Number: 8487
File Name: 120429_A549_TSA_pY.raw

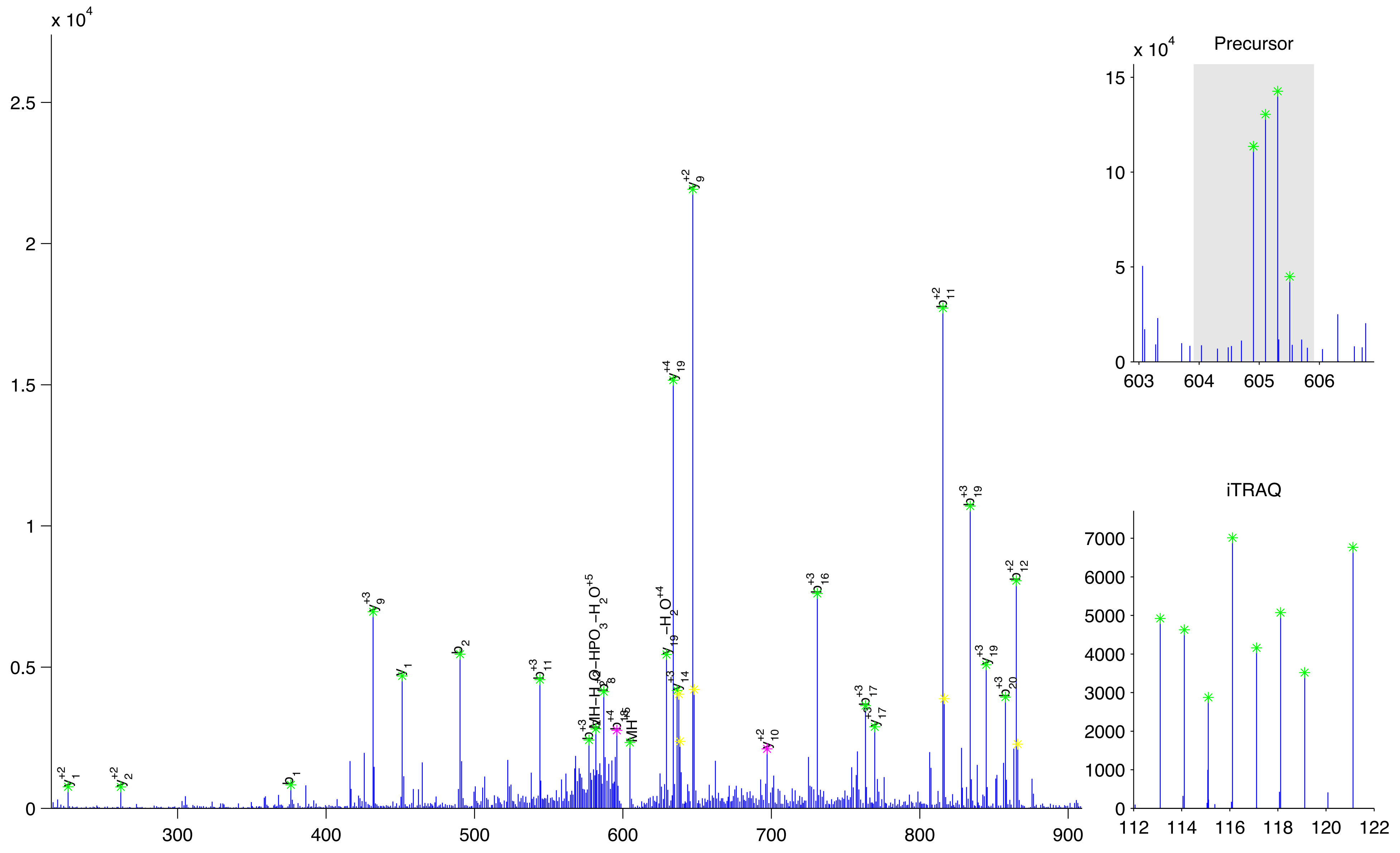

A N P Q E R D G V y D V P L H N P P D A K

neural precursor cell expressed, developmentally down−regulated 9 isoform 1 [Homo sapiens]
Charge State: +4
Scan Number: 8722
File Name: 120429_A549_TSA_pY.raw

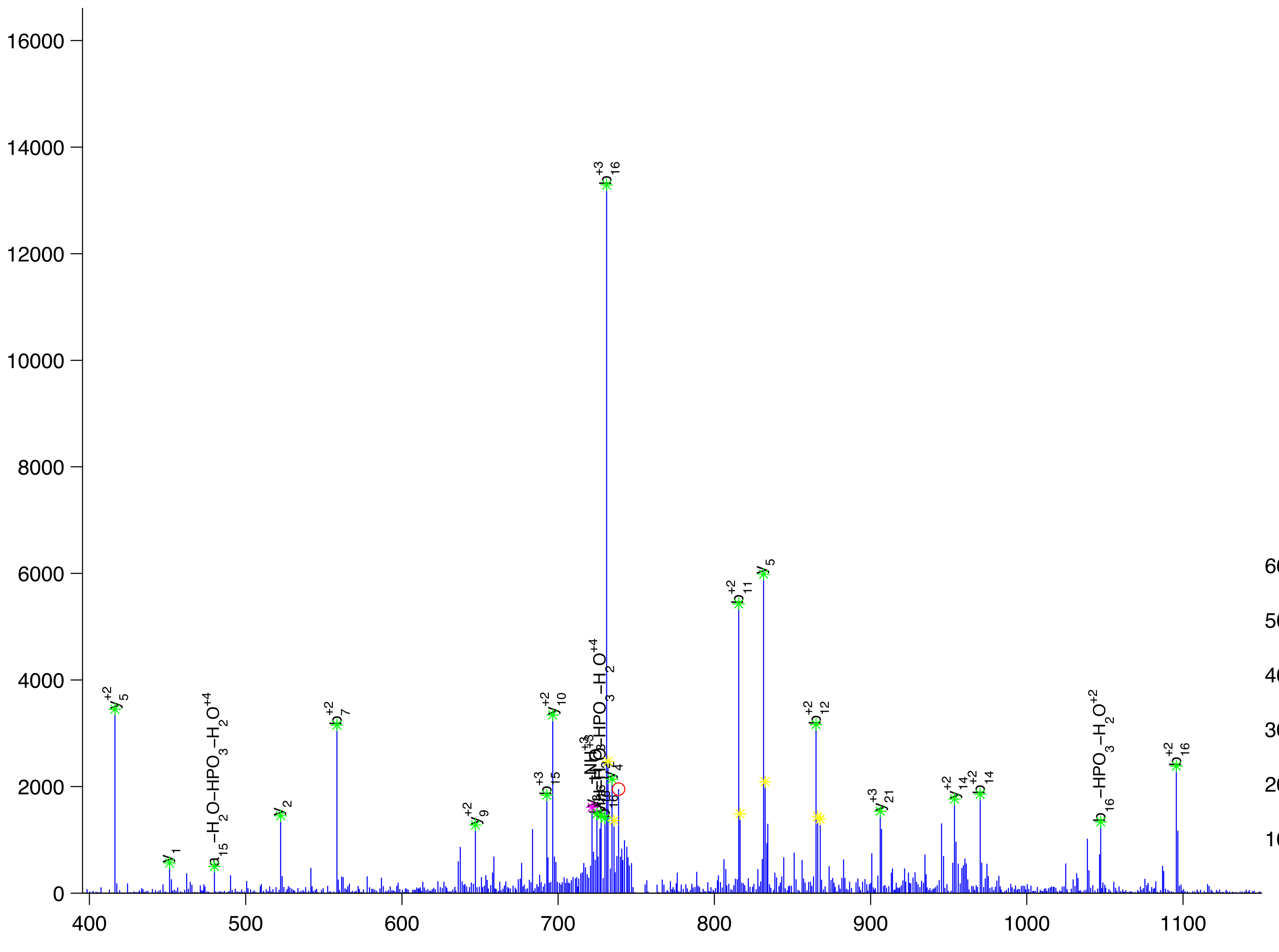

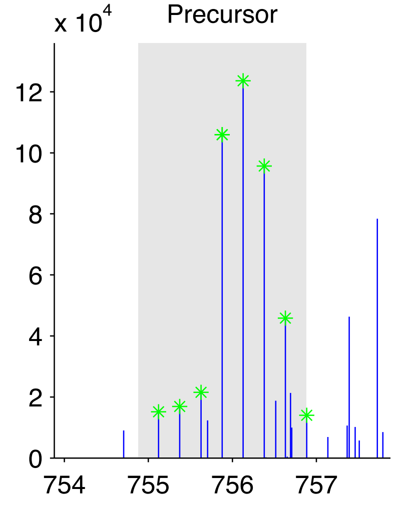

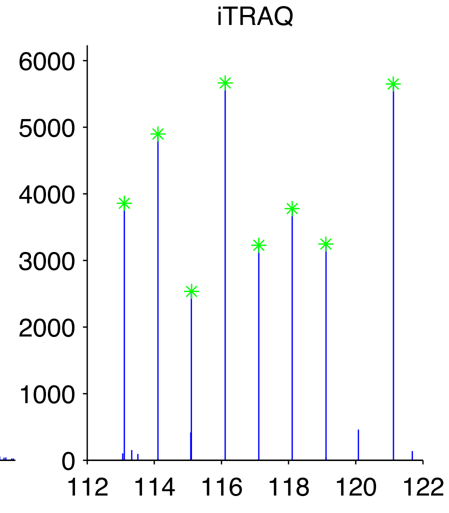

D G V y D V P L H N P P D A K

neural precursor cell expressed, developmentally down−regulated 9 isoform 1 [Homo sapiens]
Charge State: +4
Scan Number: 9663
File Name: 120429_A549_TSA_pY.raw

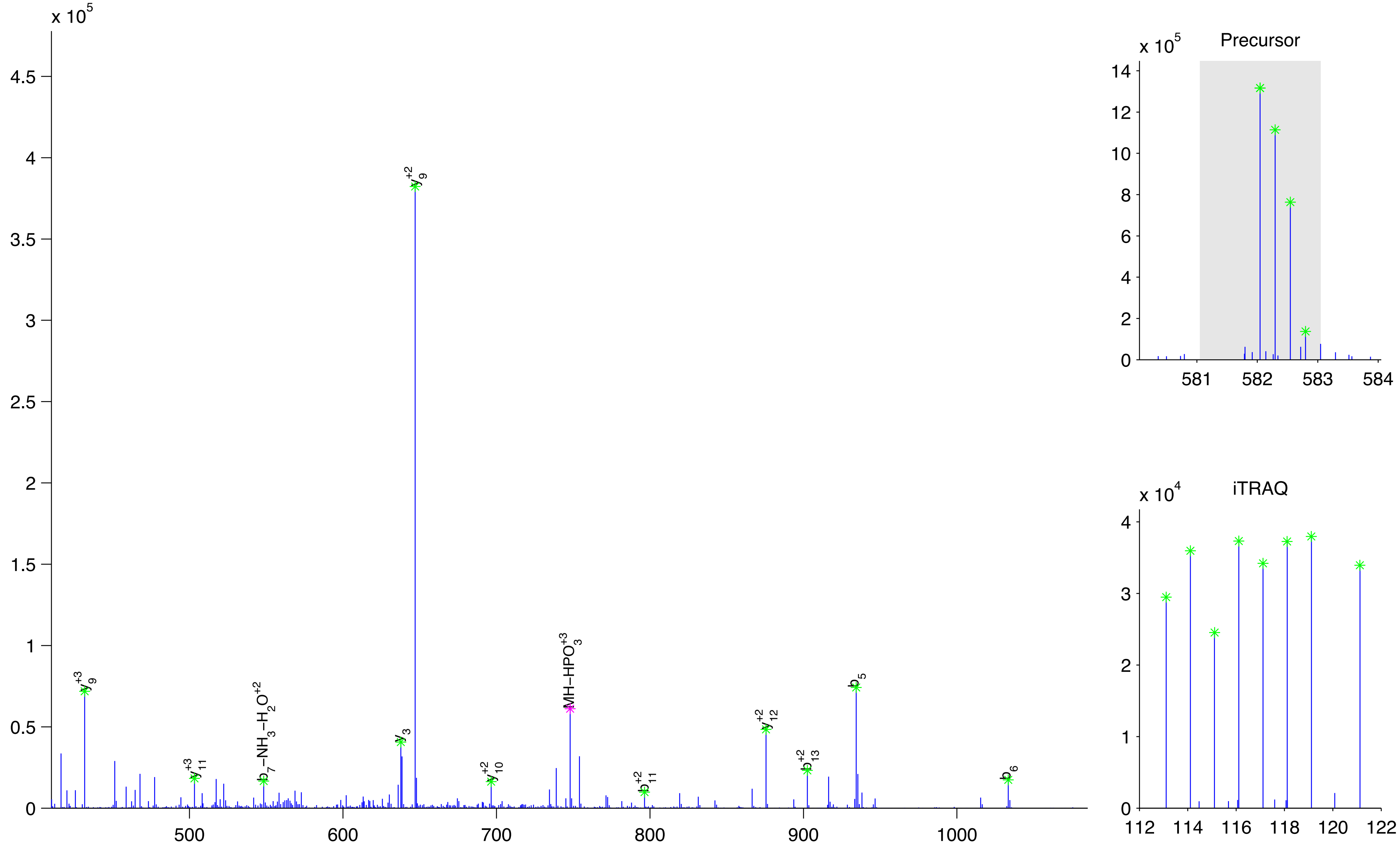

D G V y D V P L H N P P D A K

neural precursor cell expressed, developmentally down−regulated 9 isoform 1 [Homo sapiens]
Charge State: +3
Scan Number: 9789
File Name: 120429_A549_TSA_pY.raw

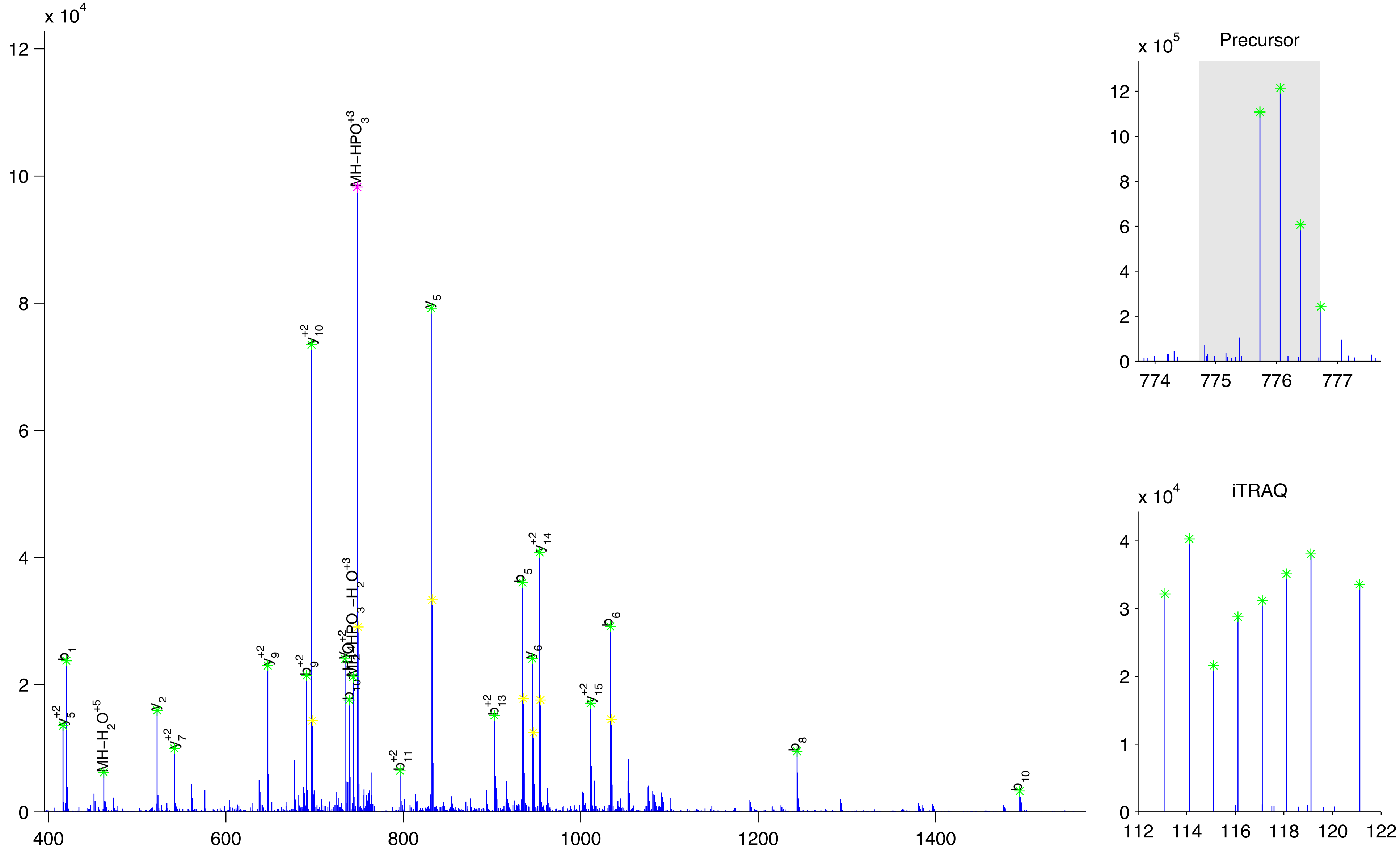

E K D y D F P P P M R

neural precursor cell expressed, developmentally down−regulated 9 isoform 1 [Homo sapiens]
Charge State: +3
Scan Number: 10356
File Name: 120429_A549_TSA_pY.raw

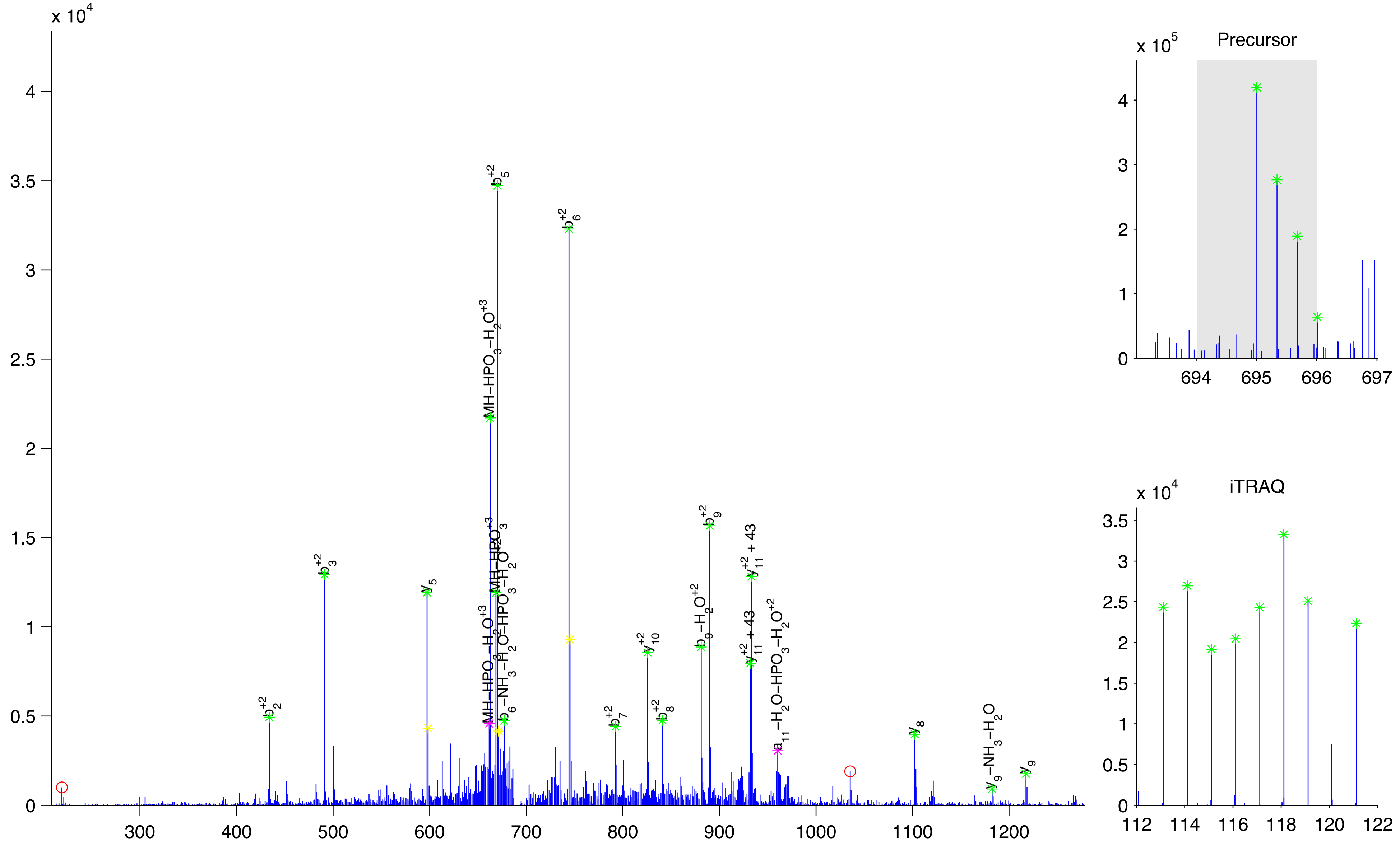

E R D y A E I Q D F H R

partitioning−defective protein 3 homolog [Homo sapiens]
Charge State: +3
Scan Number: 7105
File Name: 120429_A549_TSA_pY.raw

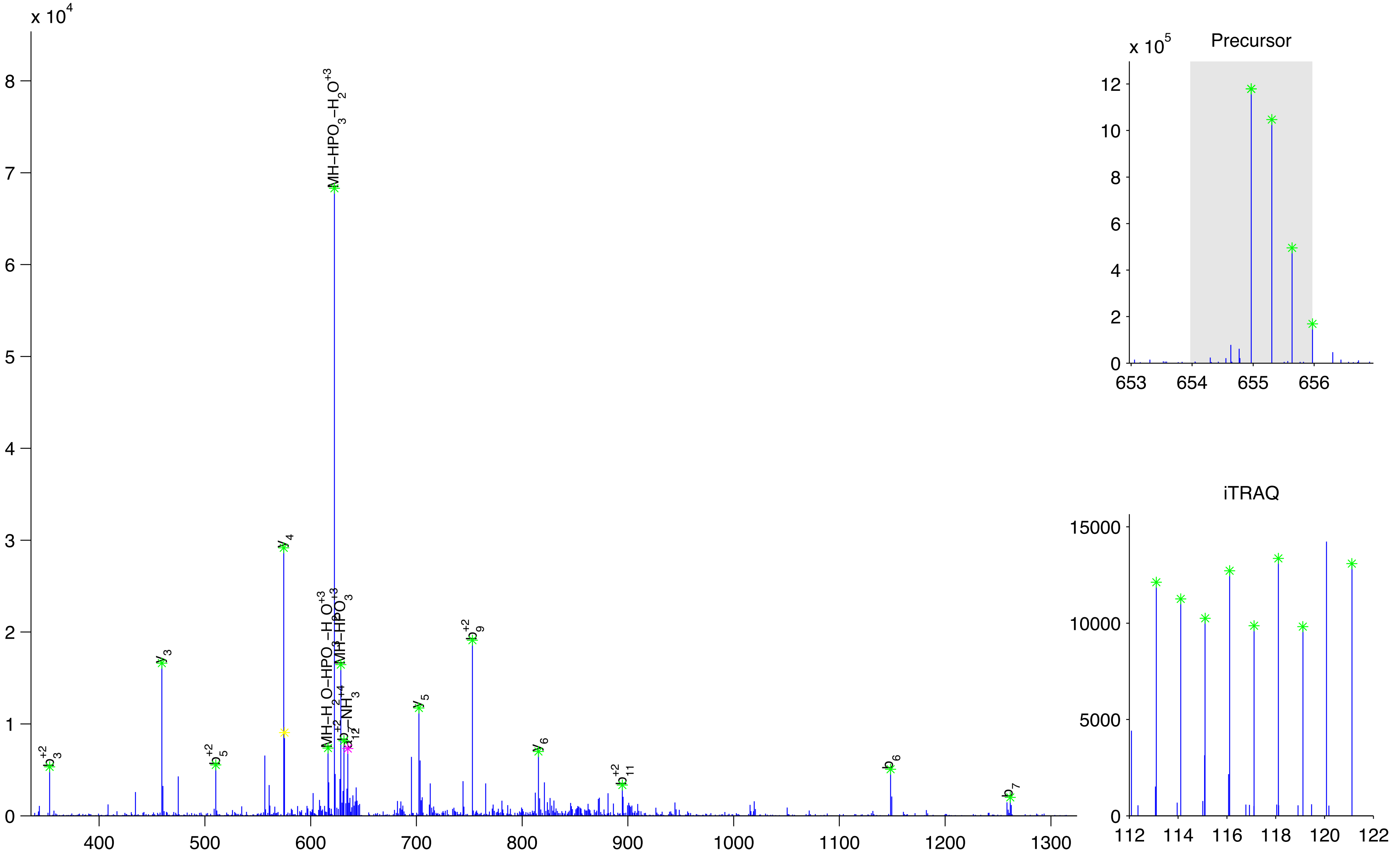

V G E E E H V y S F P N K

paxillin [Homo sapiens]
Charge State: +3
Scan Number: 8571
File Name: 120429_A549_TSA_pY.raw

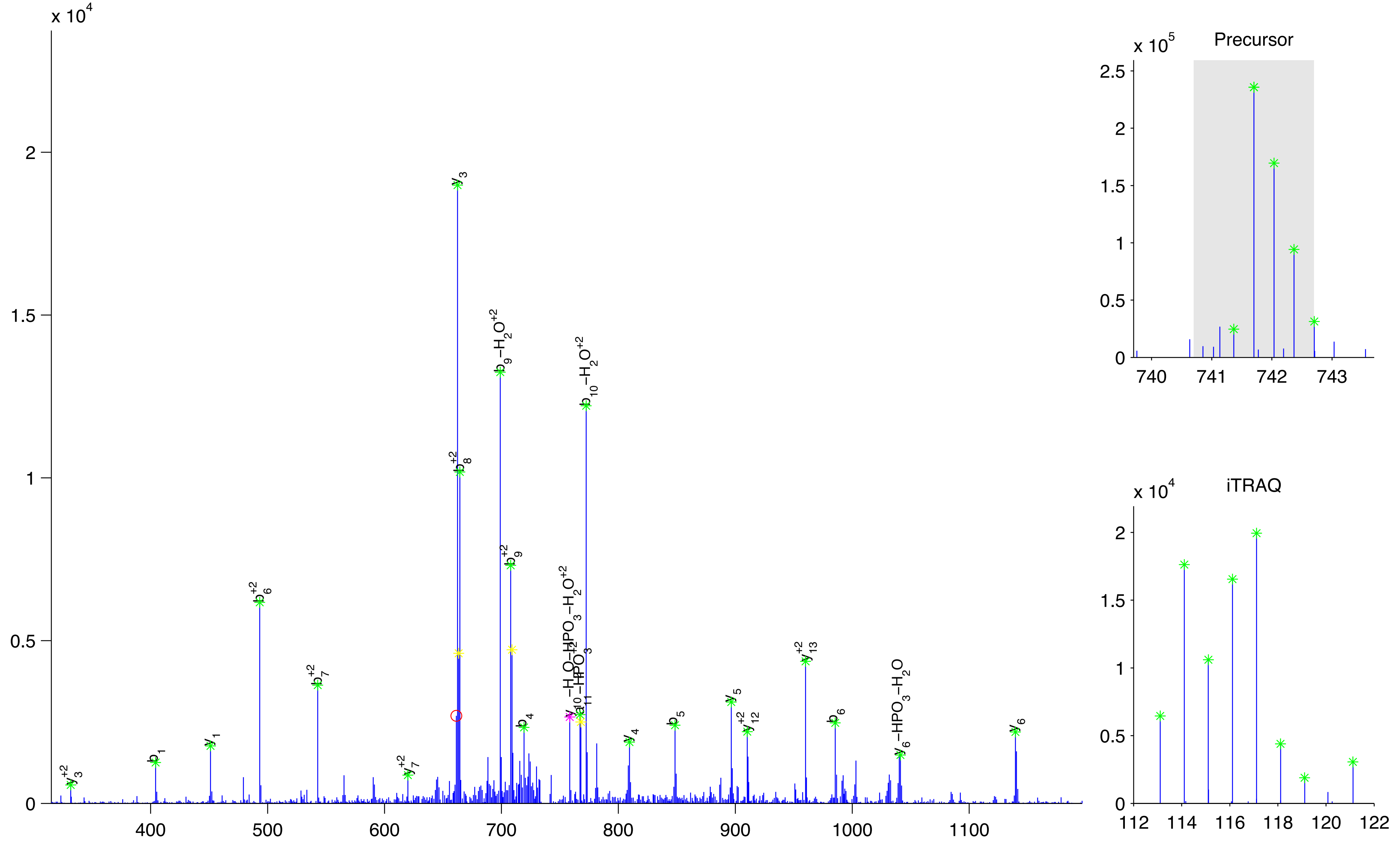

V G E E E H V y S F P N K

paxillin [Homo sapiens]
Charge State: +3
Scan Number: 9432
File Name: 120429_A549_TSA_pY.raw

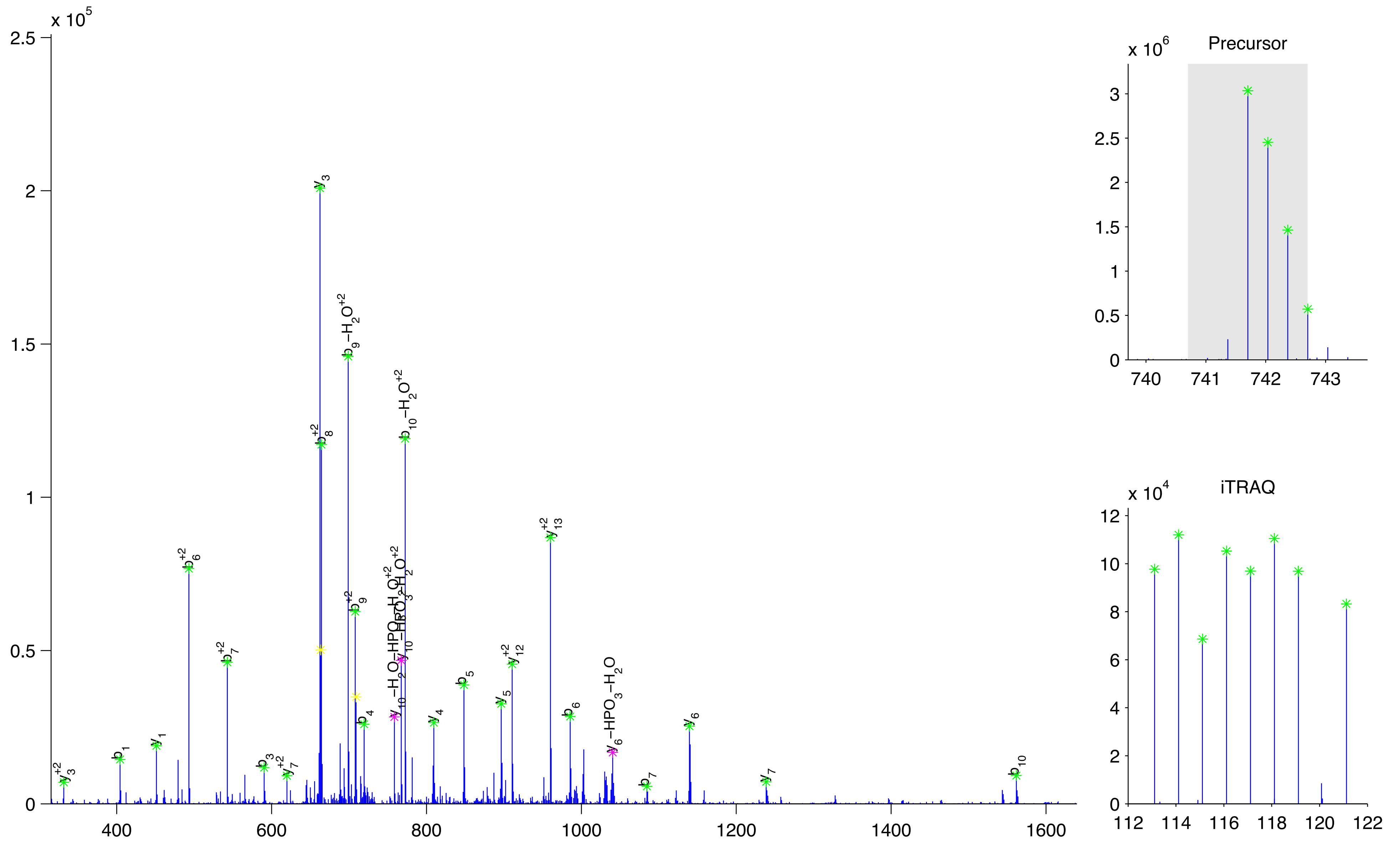

F I H Q Q P Q S S S P V y G s S A K

paxillin [Homo sapiens]
Charge State: +4
Scan Number: 9747
File Name: 120429_A549_TSA_pY.raw

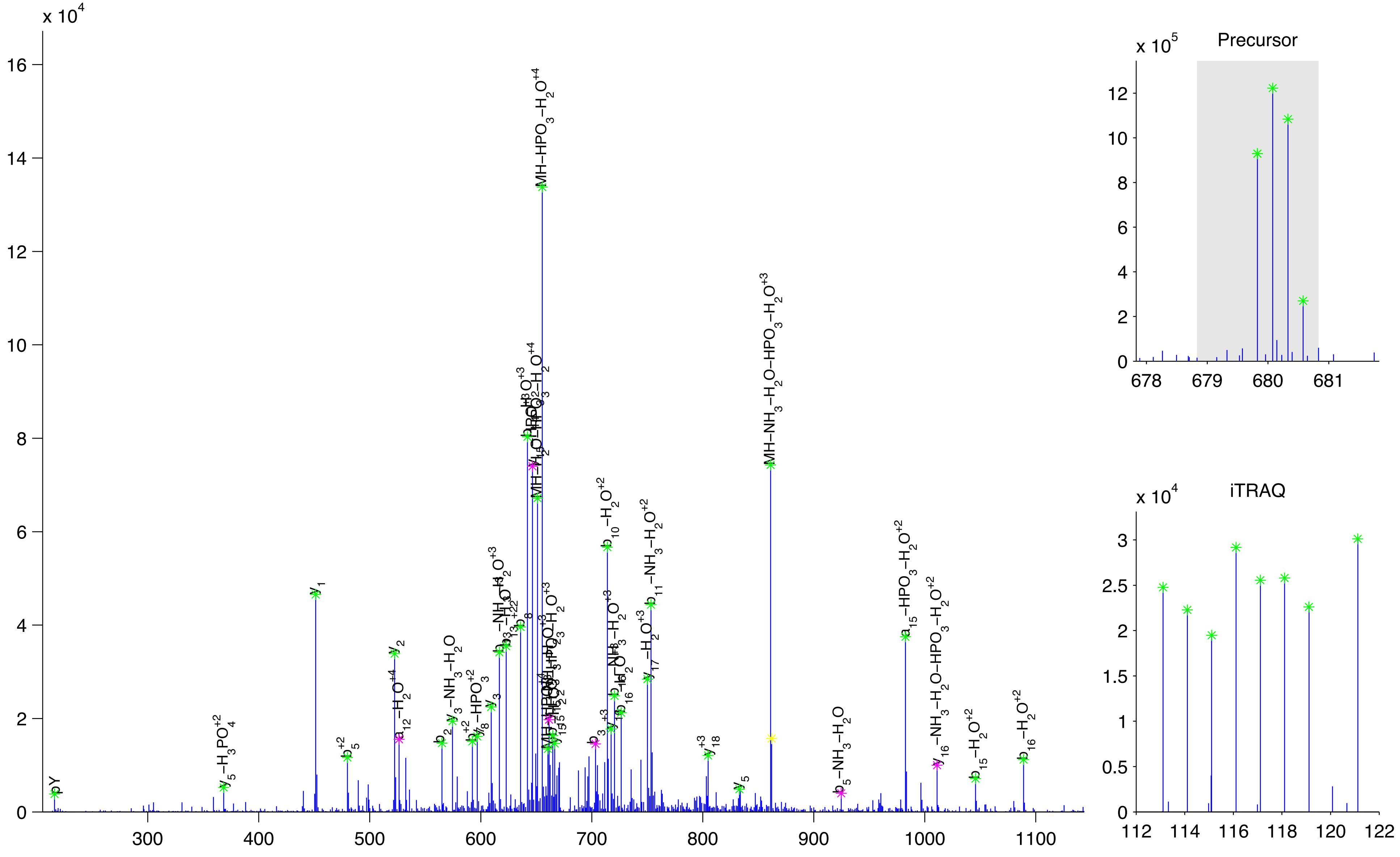

F I H Q Q P Q S S S P V y G S s A K

paxillin [Homo sapiens]
Charge State: +3
Scan Number: 9749
File Name: 120429_A549_TSA_pY.raw

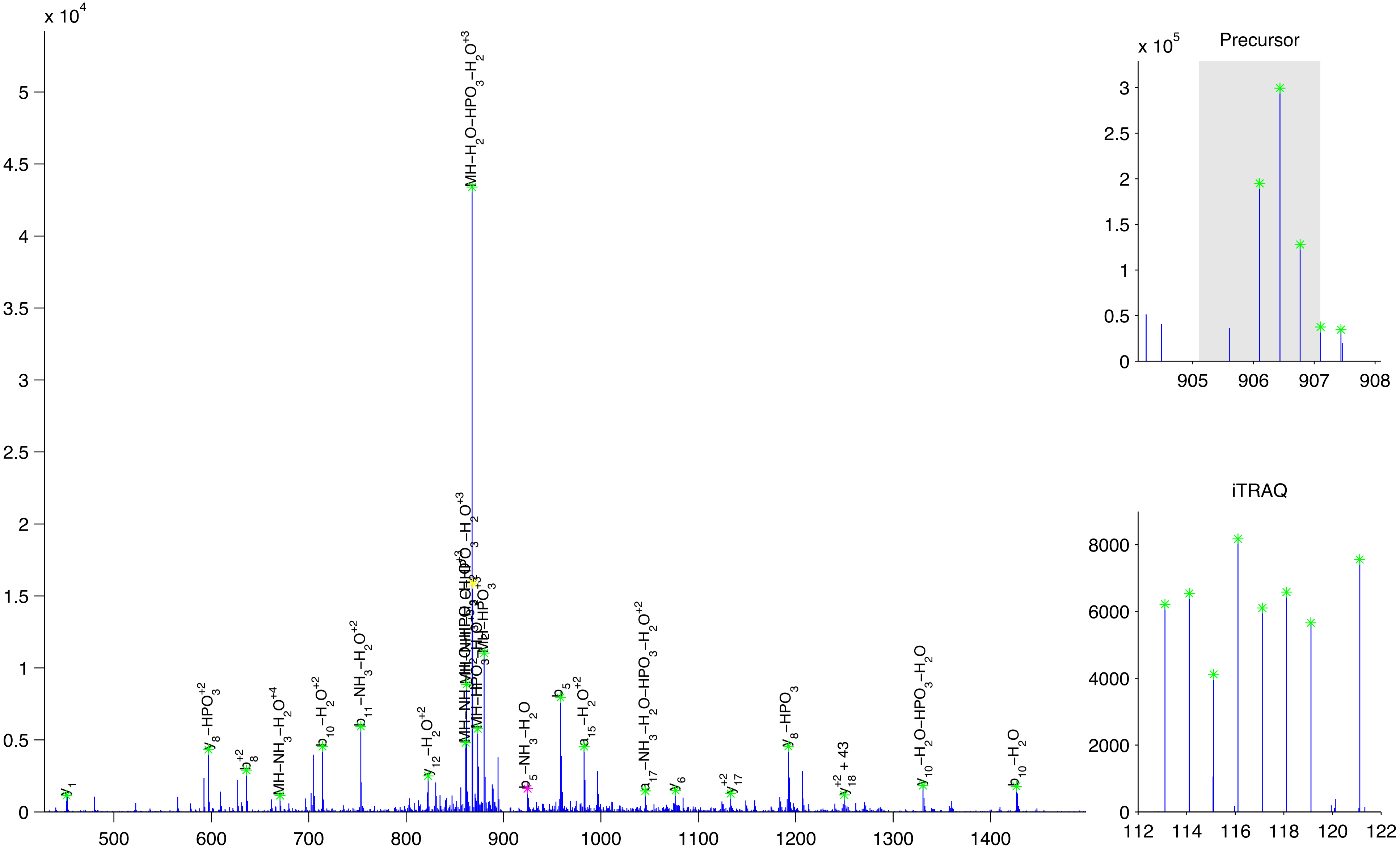

V G E E E H V y S F P N K

paxillin [Homo sapiens]
Charge State: +3
Scan Number: 9894
File Name: 120429_A549_TSA_pY.raw

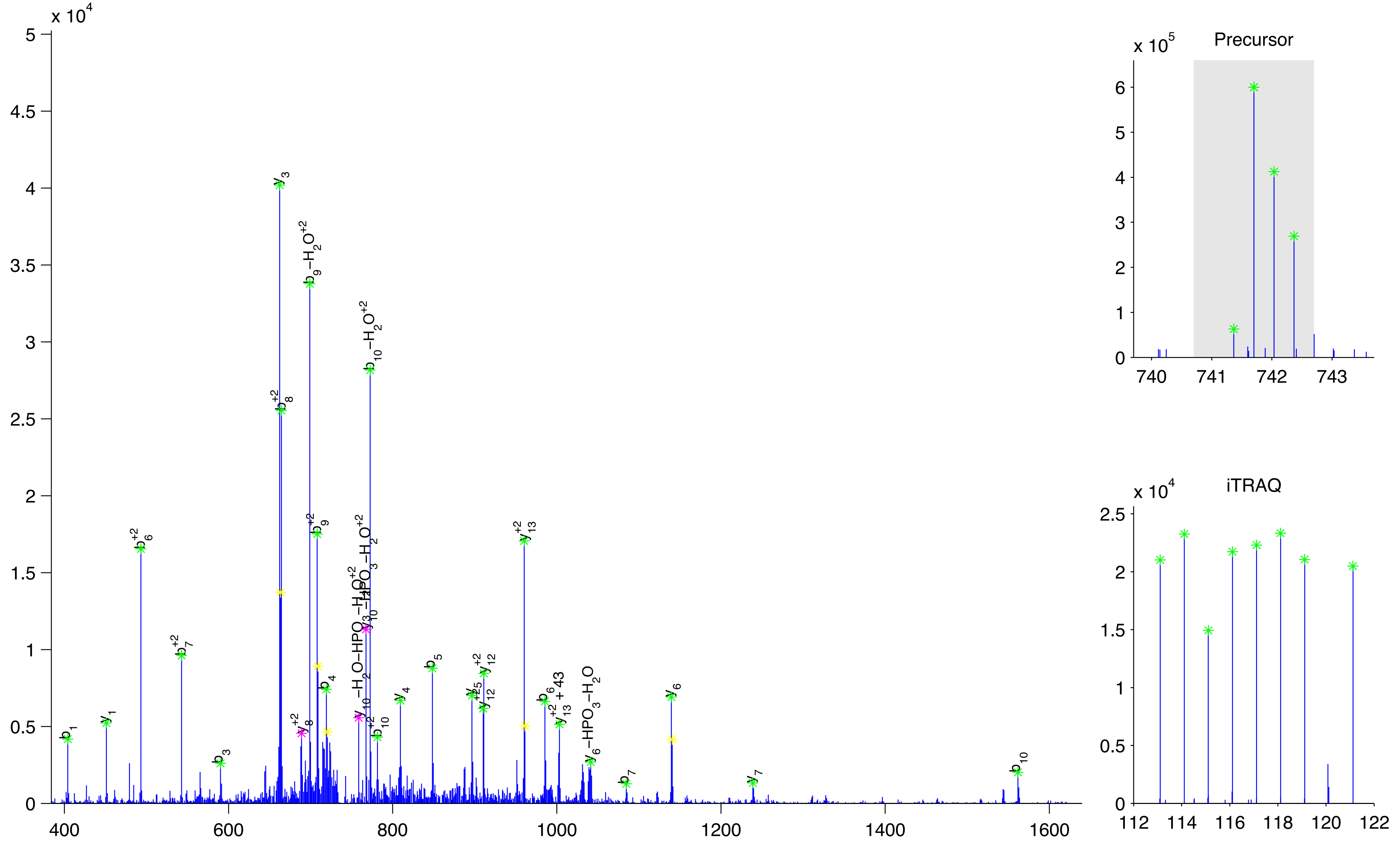

S R E y D Q L Y E E Y T R

phosphoinositide-3-kinase, regulatory subunit 2 (beta) [Homo sapiens]
Charge State: +3
Scan Number: 11175
File Name: 120429_A549_TSA_pY.raw

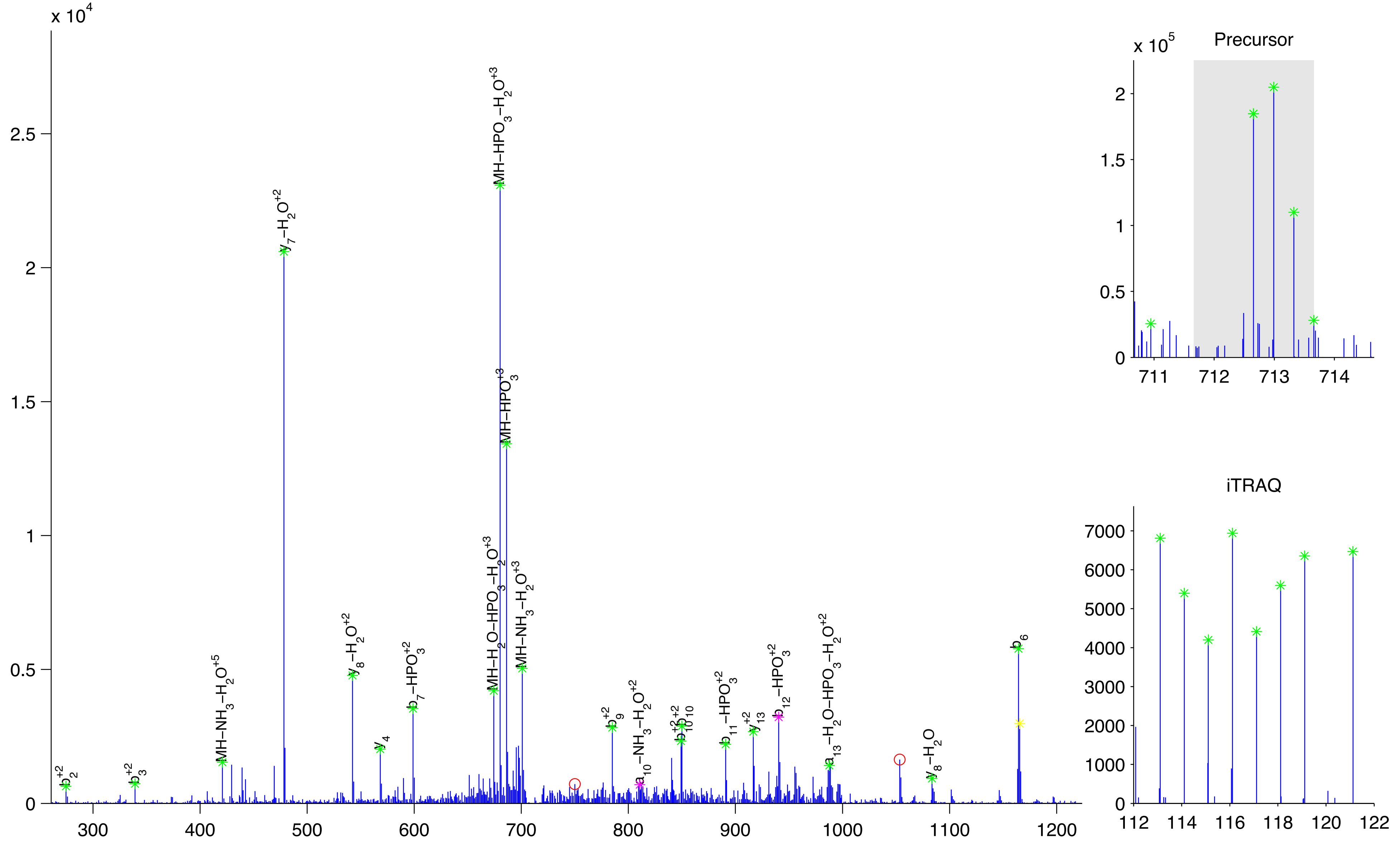

E A T Q P E P I y A E S T K R

pragmin [Homo sapiens]
Charge State: +4
Scan Number: 6492
File Name: 120429_A549_TSA_pY.raw

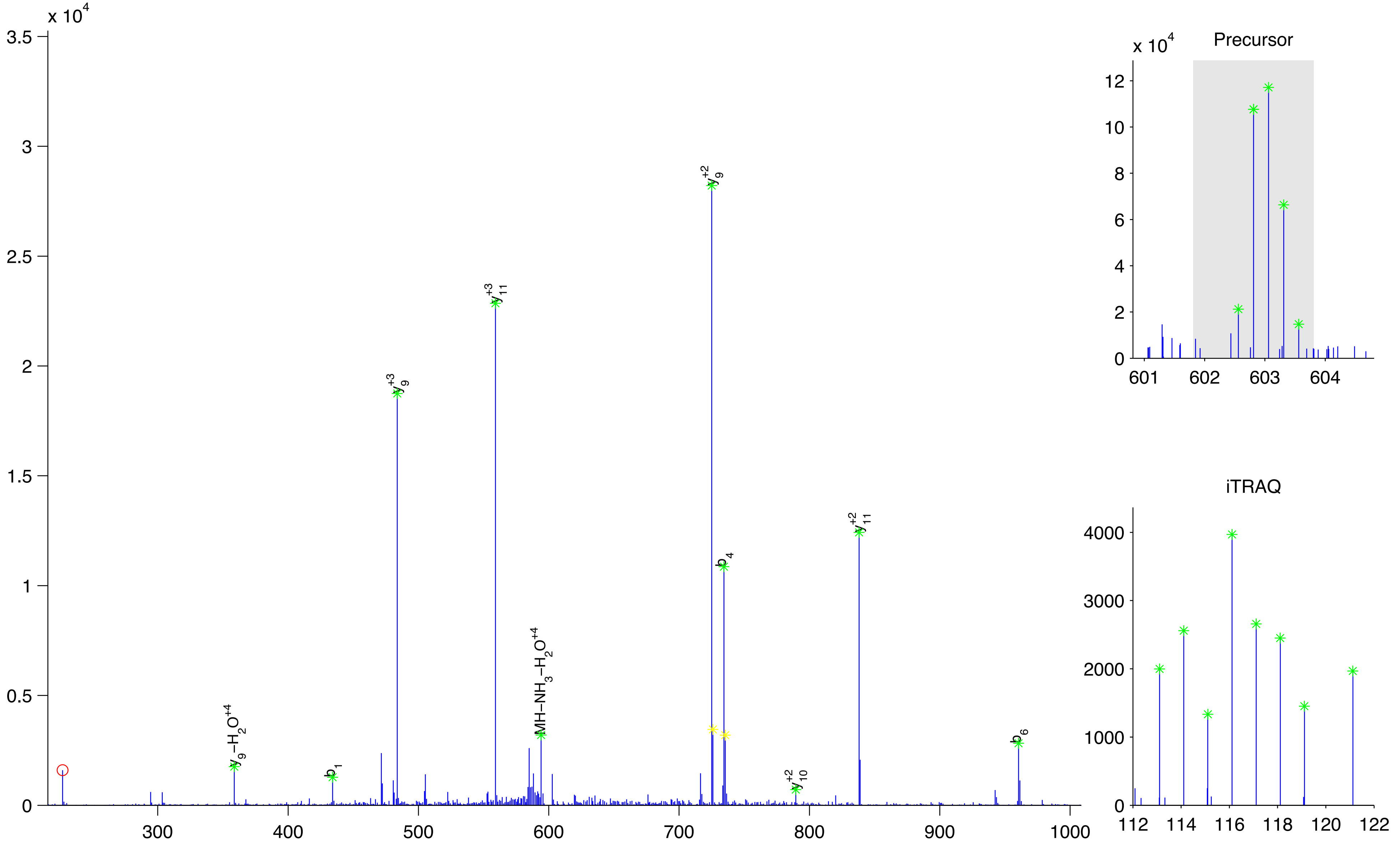

E A T Q P E P I y A E S T K R

pragmin [Homo sapiens]
Charge State: +3
Scan Number: 6534
File Name: 120429_A549_TSA_pY.raw

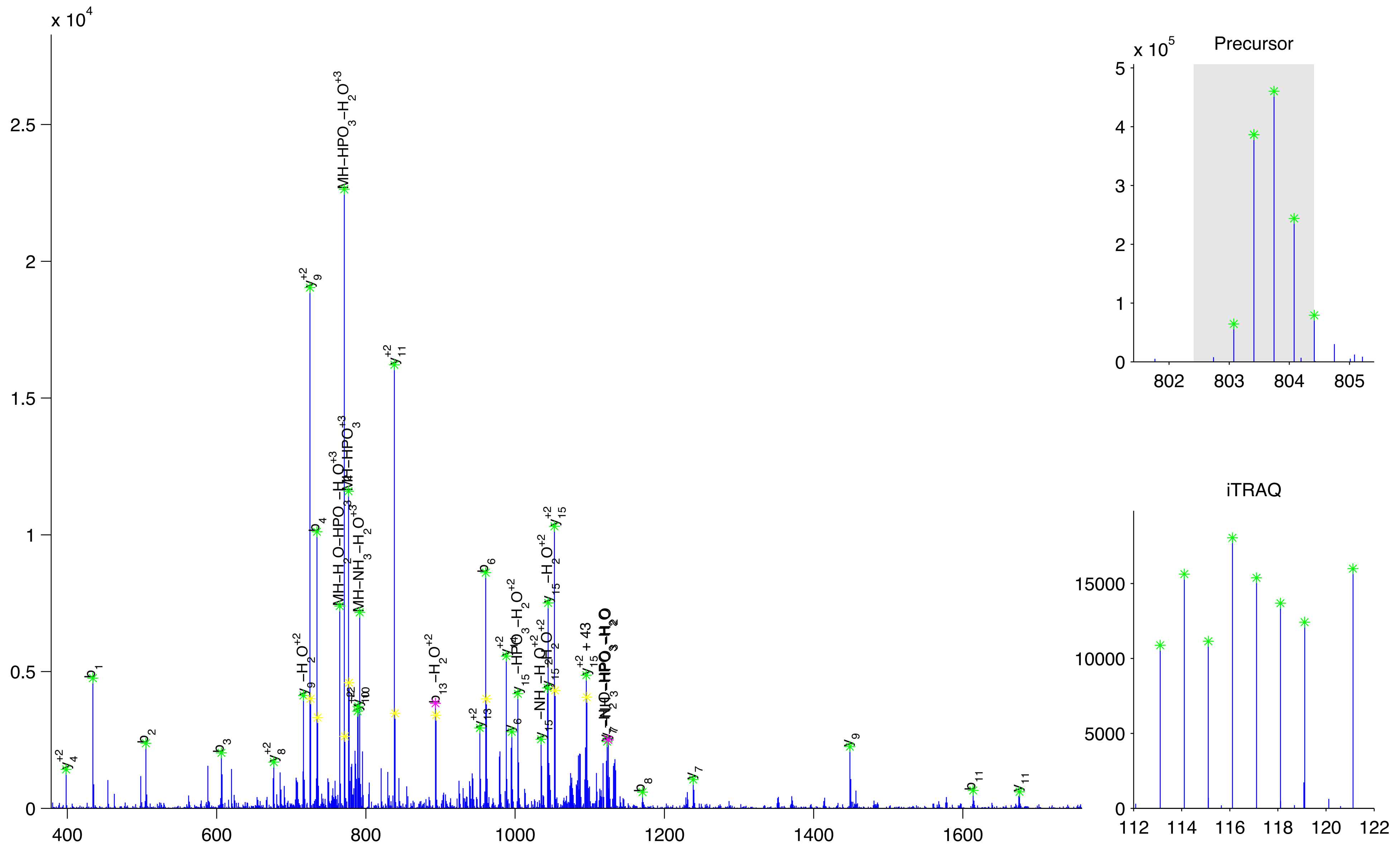

E A T Q P E P I y A E S T K R

pragmin [Homo sapiens]
Charge State: +3
Scan Number: 6933
File Name: 120429_A549_TSA_pY.raw

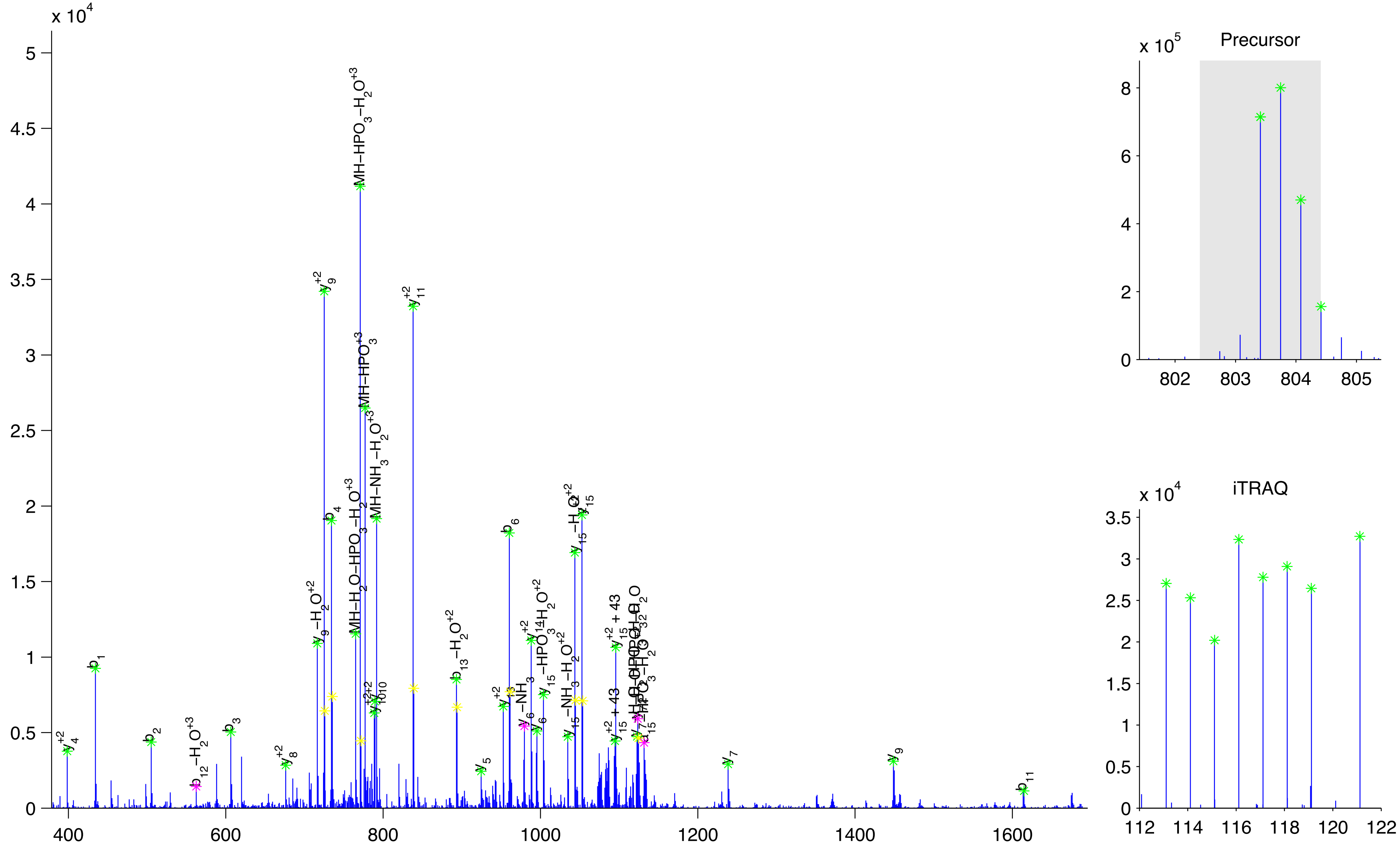

E A T Q P E P I y A E S T K R

pragmin [Homo sapiens]
Charge State: +4
Scan Number: 7710
File Name: 120429_A549_TSA_pY.raw

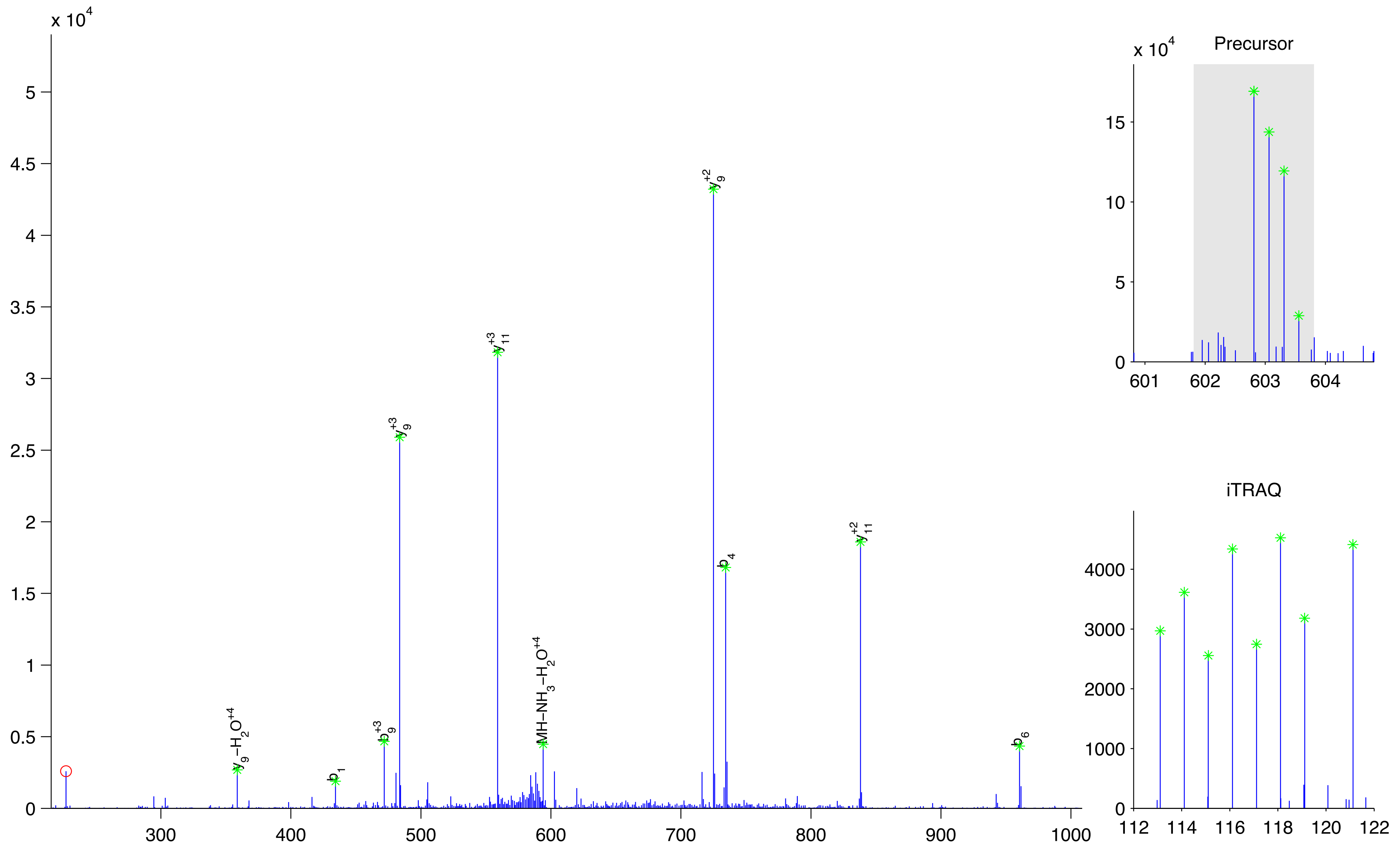

E A T Q P E P I y A E S T K R

pragmin [Homo sapiens]
Charge State: +4
Scan Number: 8132
File Name: 120429_A549_TSA_pY.raw

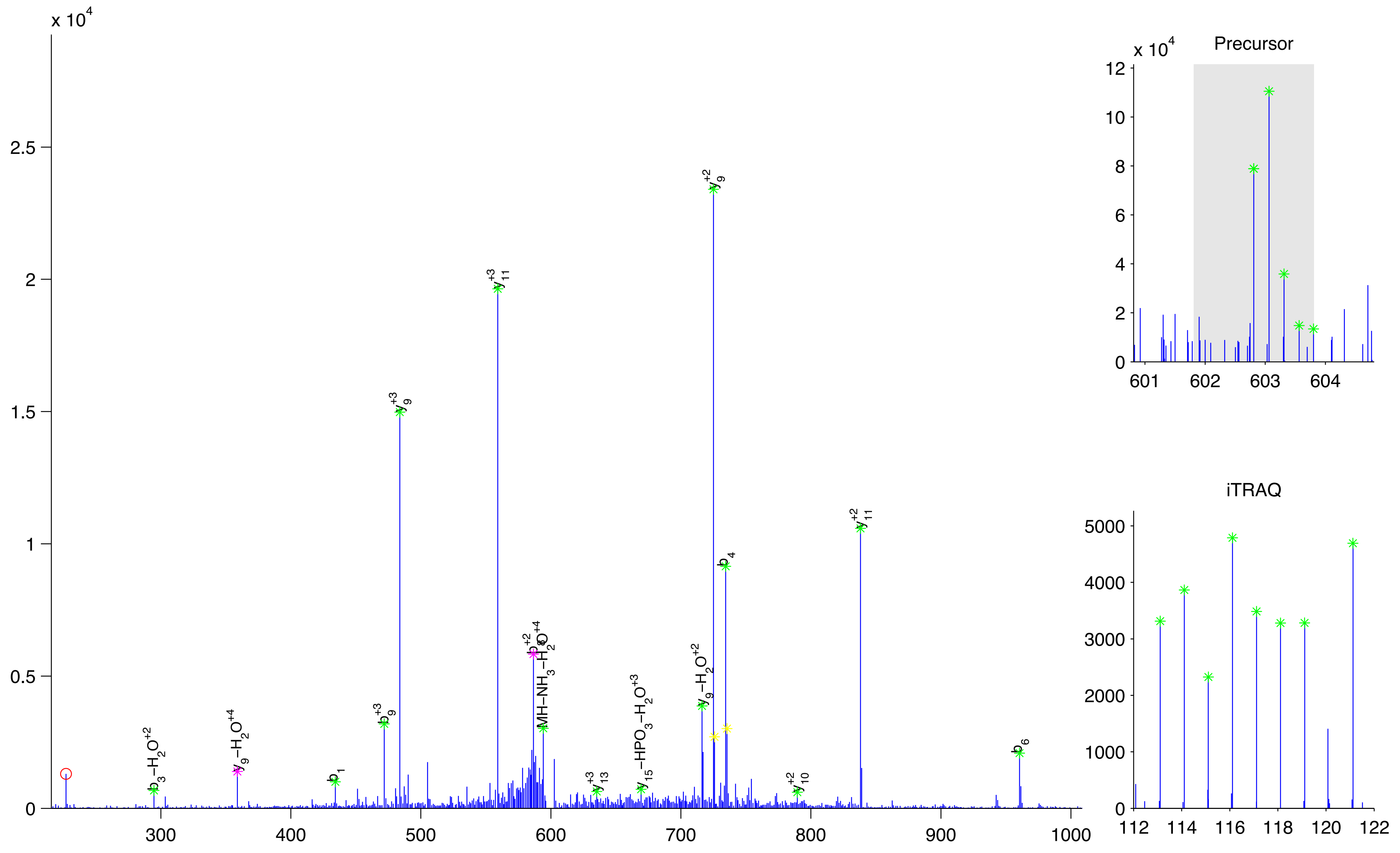

E A T Q P E P I y A E S T K

pragmin [Homo sapiens]
Charge State: +2
Scan Number: 8237
File Name: 120429_A549_TSA_pY.raw

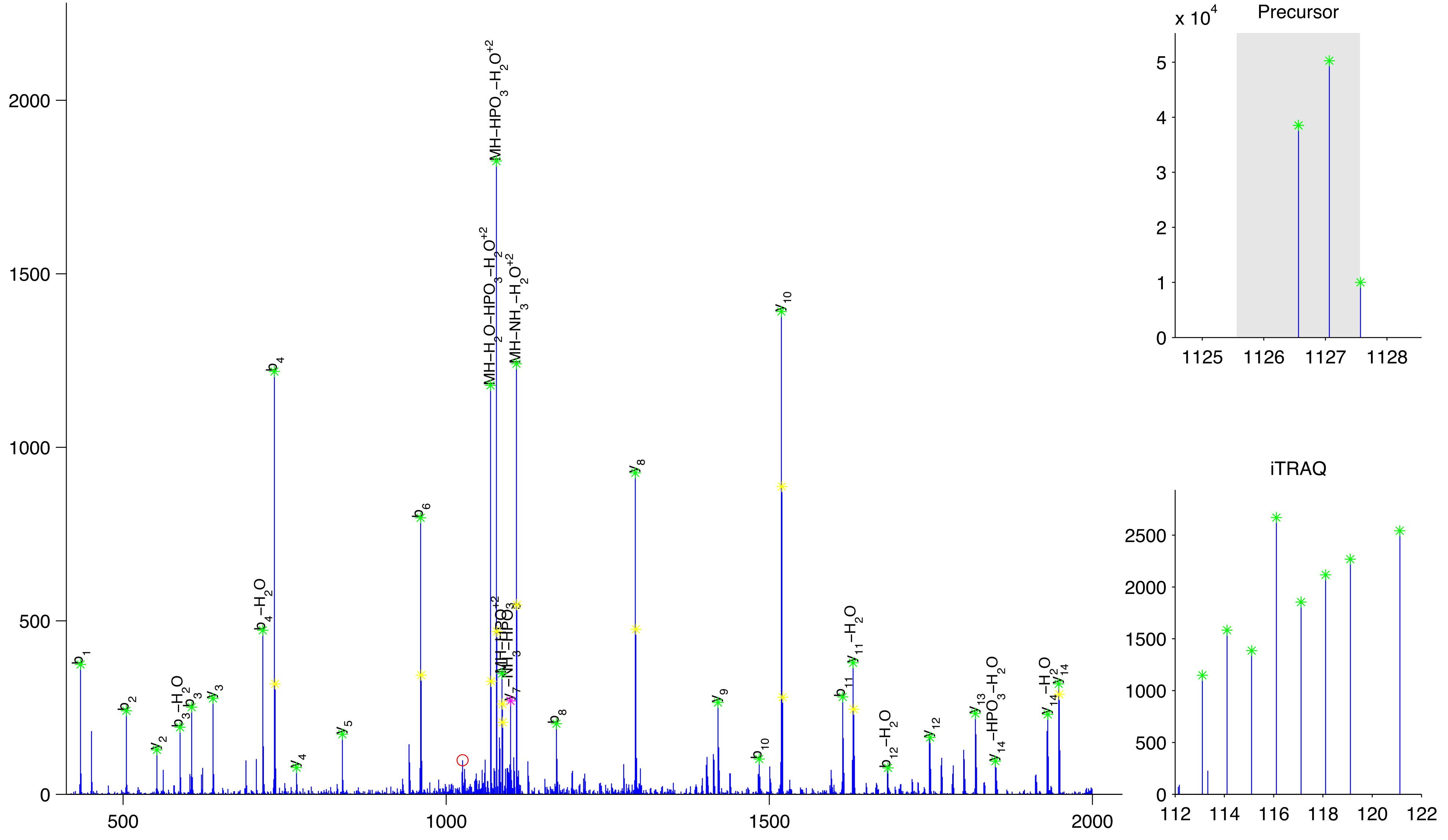

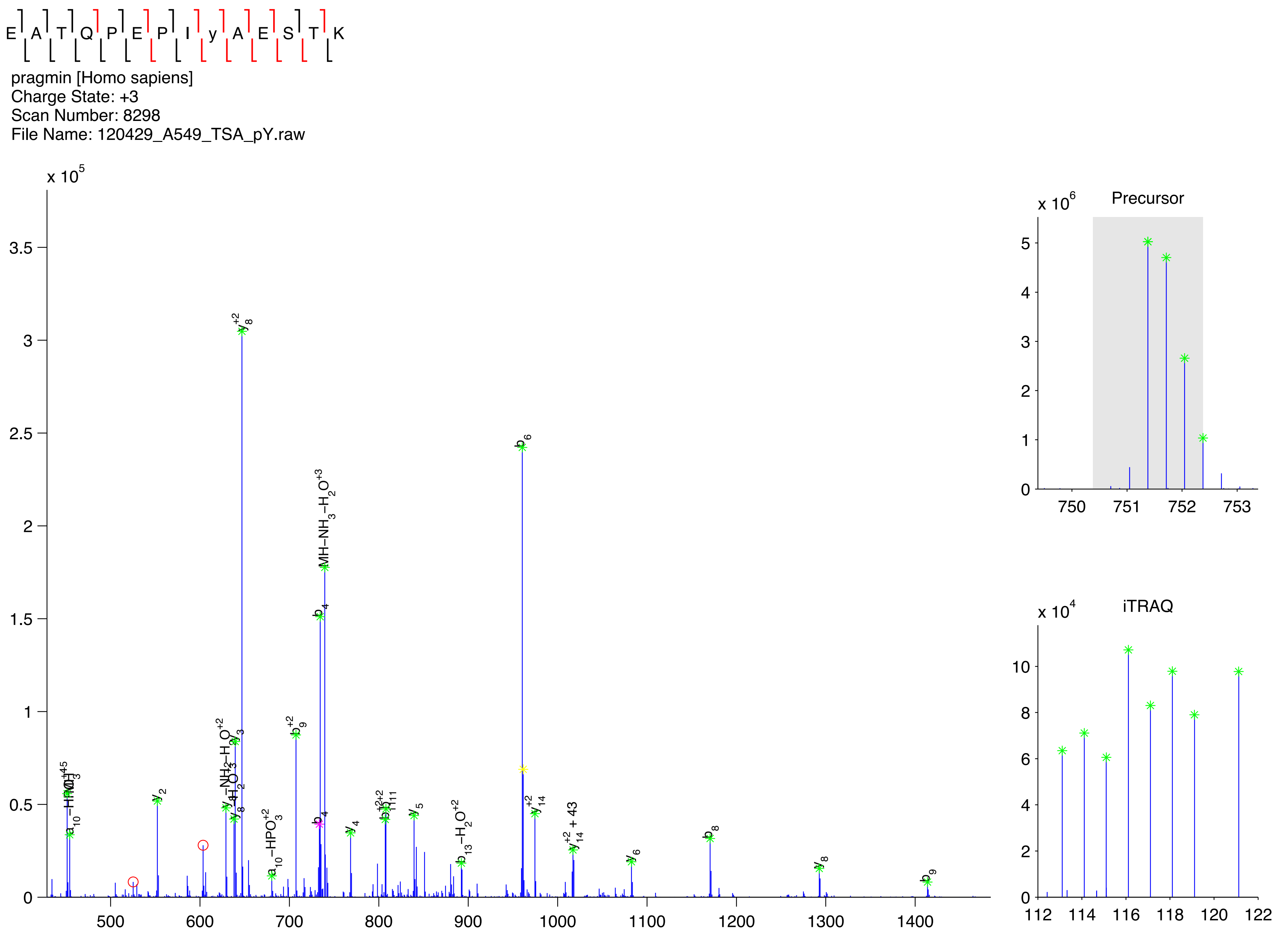
E A T Q P E P I y A E S T K
pragmin [Homo sapiens]
Charge State: +3
Scan Number: 8298
File Name: 120429_A549_TSA_pY.raw
Precursor
iTRAQ

E A T Q P E P I y A E S T K

pragmin [Homo sapiens]
Charge State: +4
Scan Number: 8424
File Name: 120429_A549_TSA_pY.raw

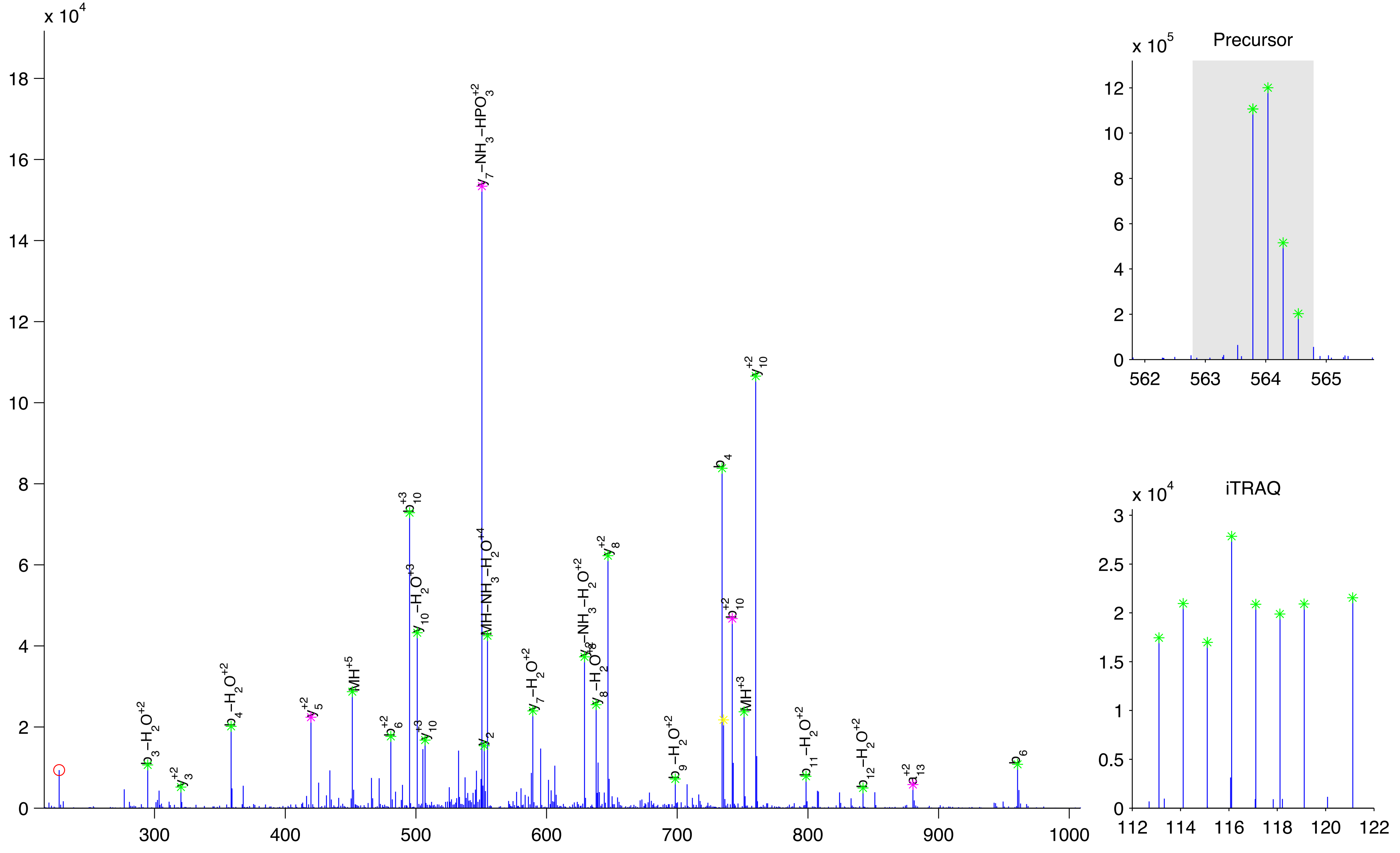

E A T Q P E P I y A E S T K

pragmin [Homo sapiens]
Charge State: +3
Scan Number: 8718
File Name: 120429_A549_TSA_pY.raw

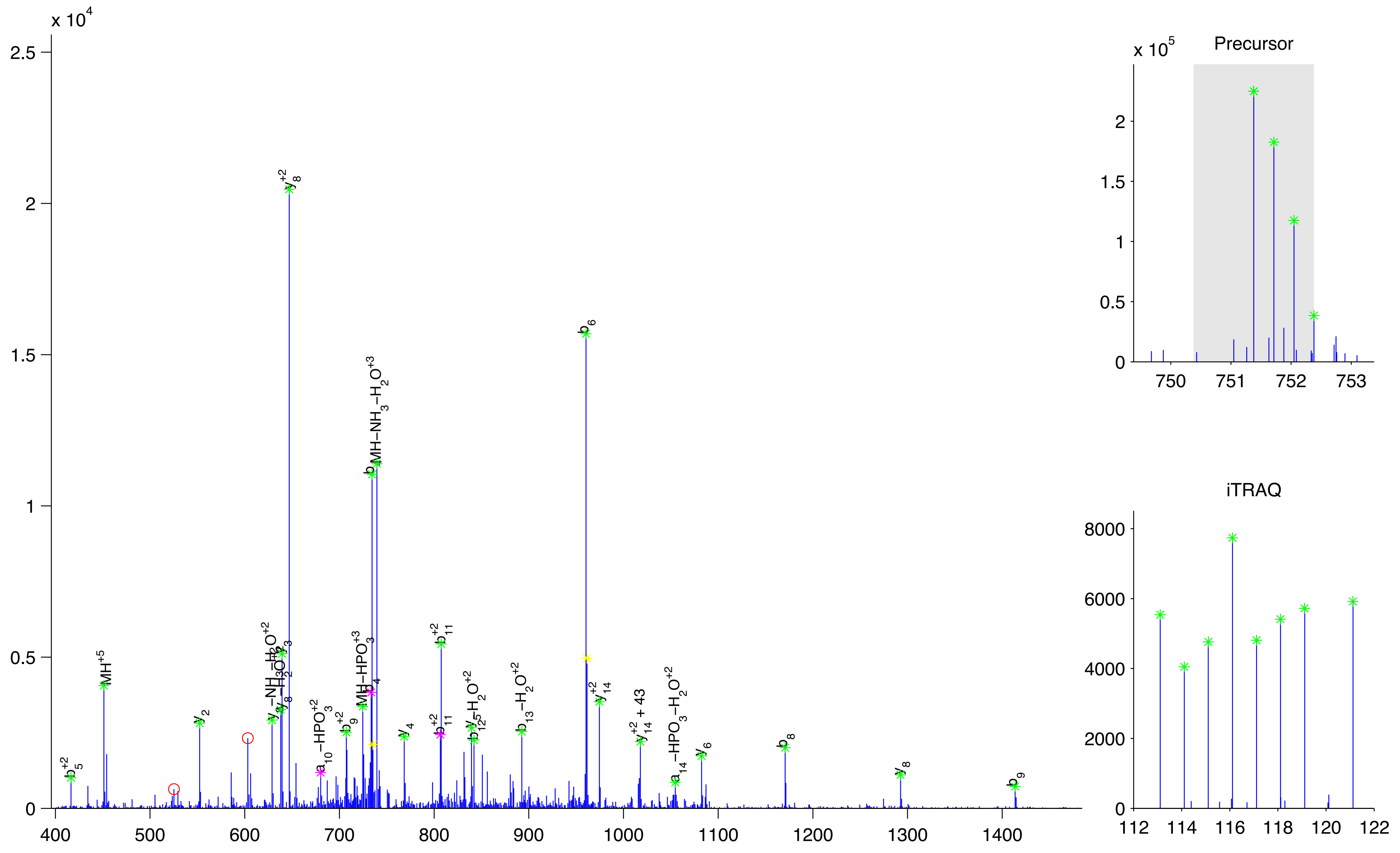

V V Q E Y I D A F S D y A N F K

protein tyrosine phosphatase, receptor type, A isoform 1 precursor [Homo sapiens]
Charge State: +4
Scan Number: 23502
File Name: 120429_A549_TSA_pY.raw

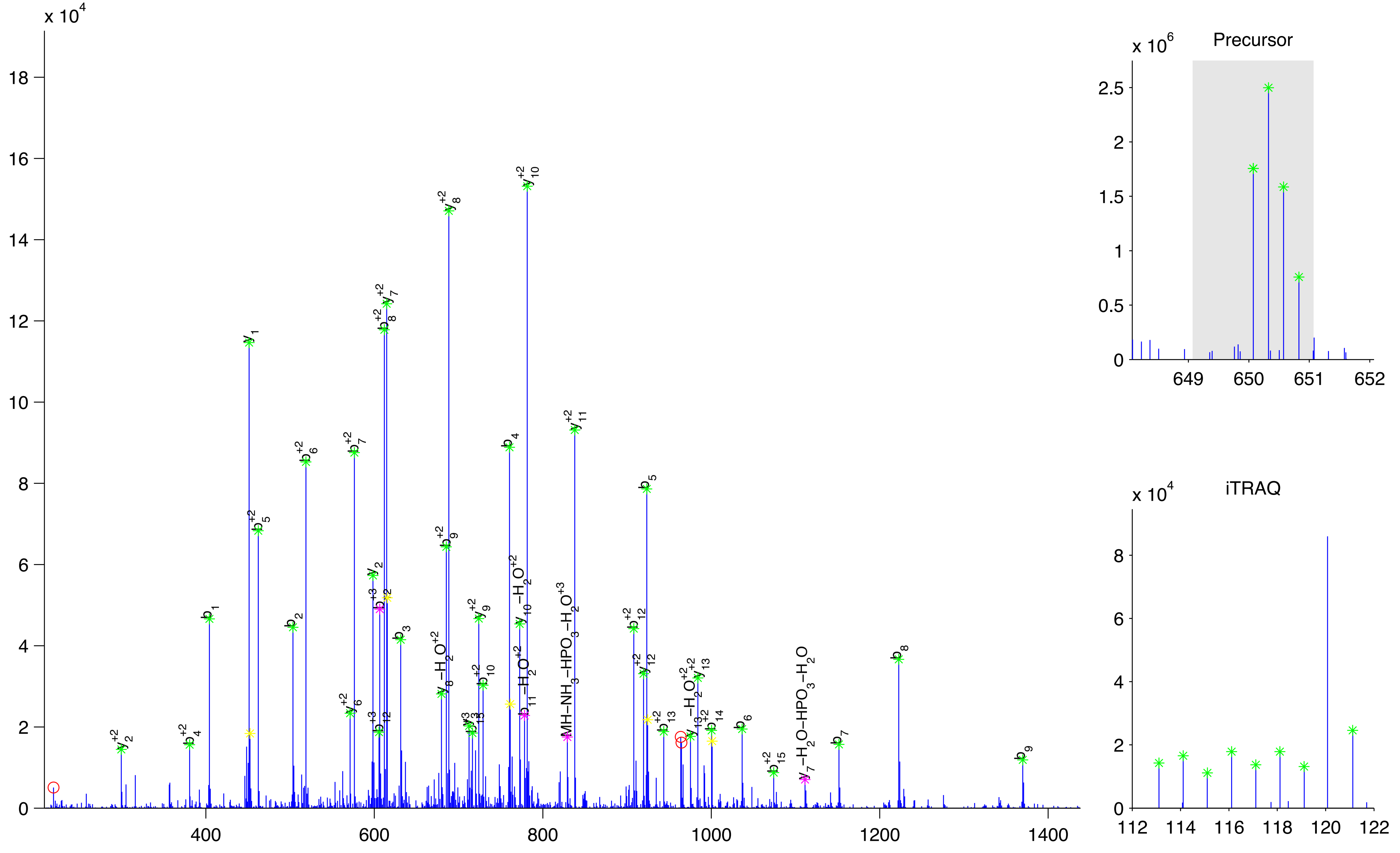

L I E D N E y T A R

protein−tyrosine kinase fyn isoform a [Homo sapiens]
Charge State: +3
Scan Number: 10209
File Name: 120429_A549_TSA_pY.raw

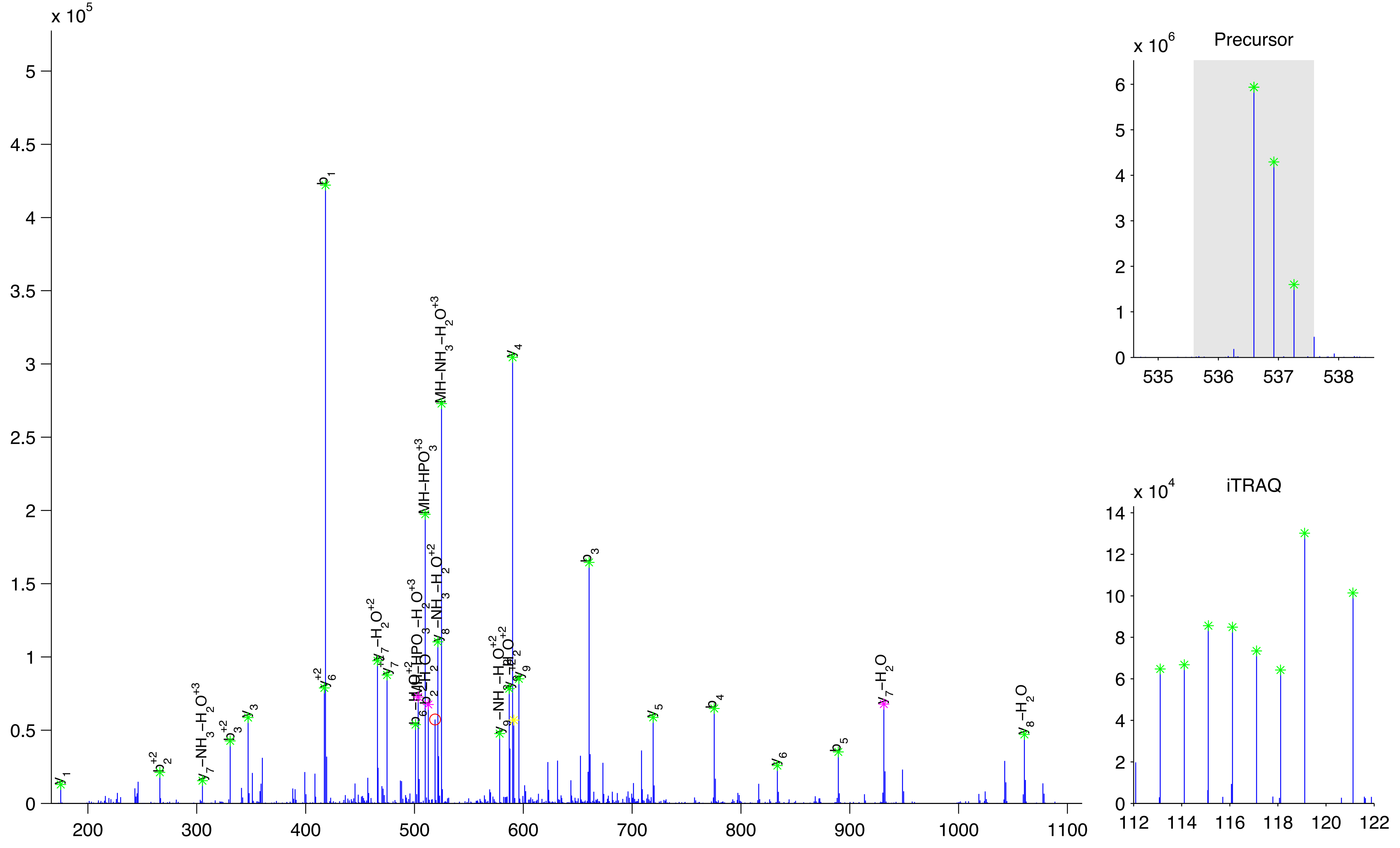

L I E D N E y T A R

protein–tyrosine kinase fyn isoform a [Homo sapiens]
Charge State: +2
Scan Number: 10442
File Name: 120429_A549_TSA_pY.raw

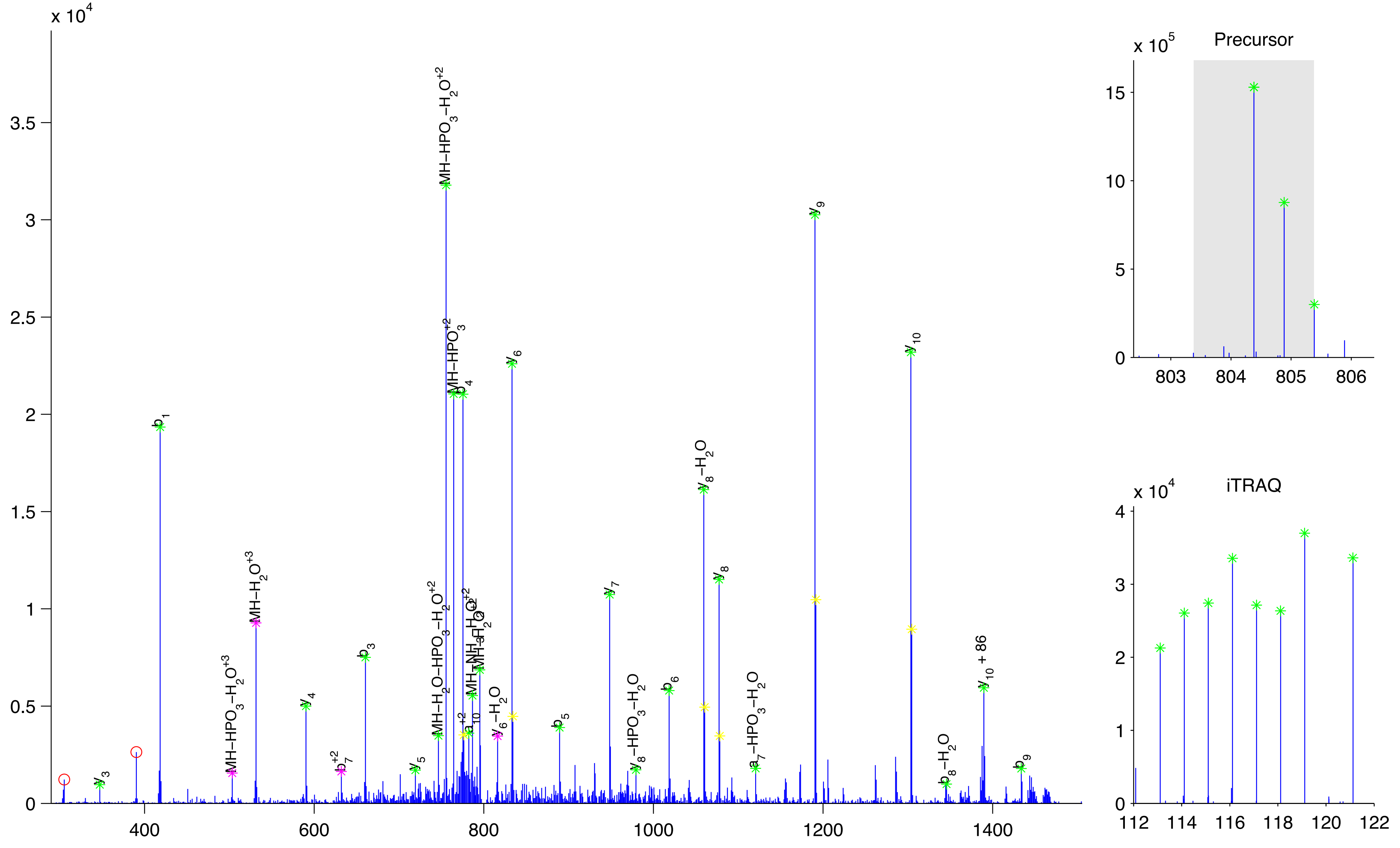

L I E D N E y T A R

protein–tyrosine kinase fyn isoform a [Homo sapiens]
Charge State: +3
Scan Number: 10650
File Name: 120429_A549_TSA_pY.raw

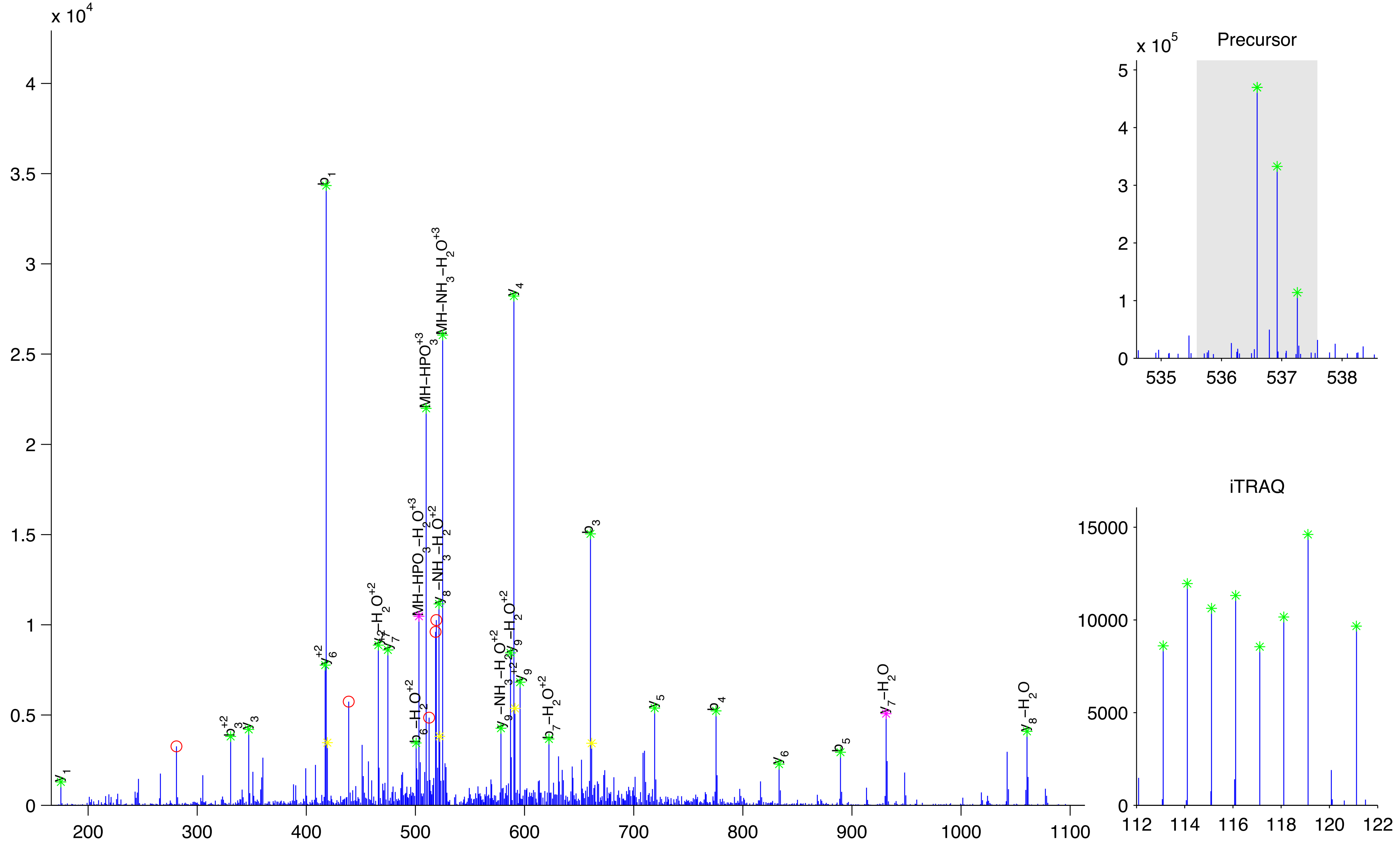

Y m E D S T Y y K

PTK2 protein tyrosine kinase 2 isoform a [Homo sapiens]
Charge State: +2
Scan Number: 7882
File Name: 120429_A549_TSA_pY.raw

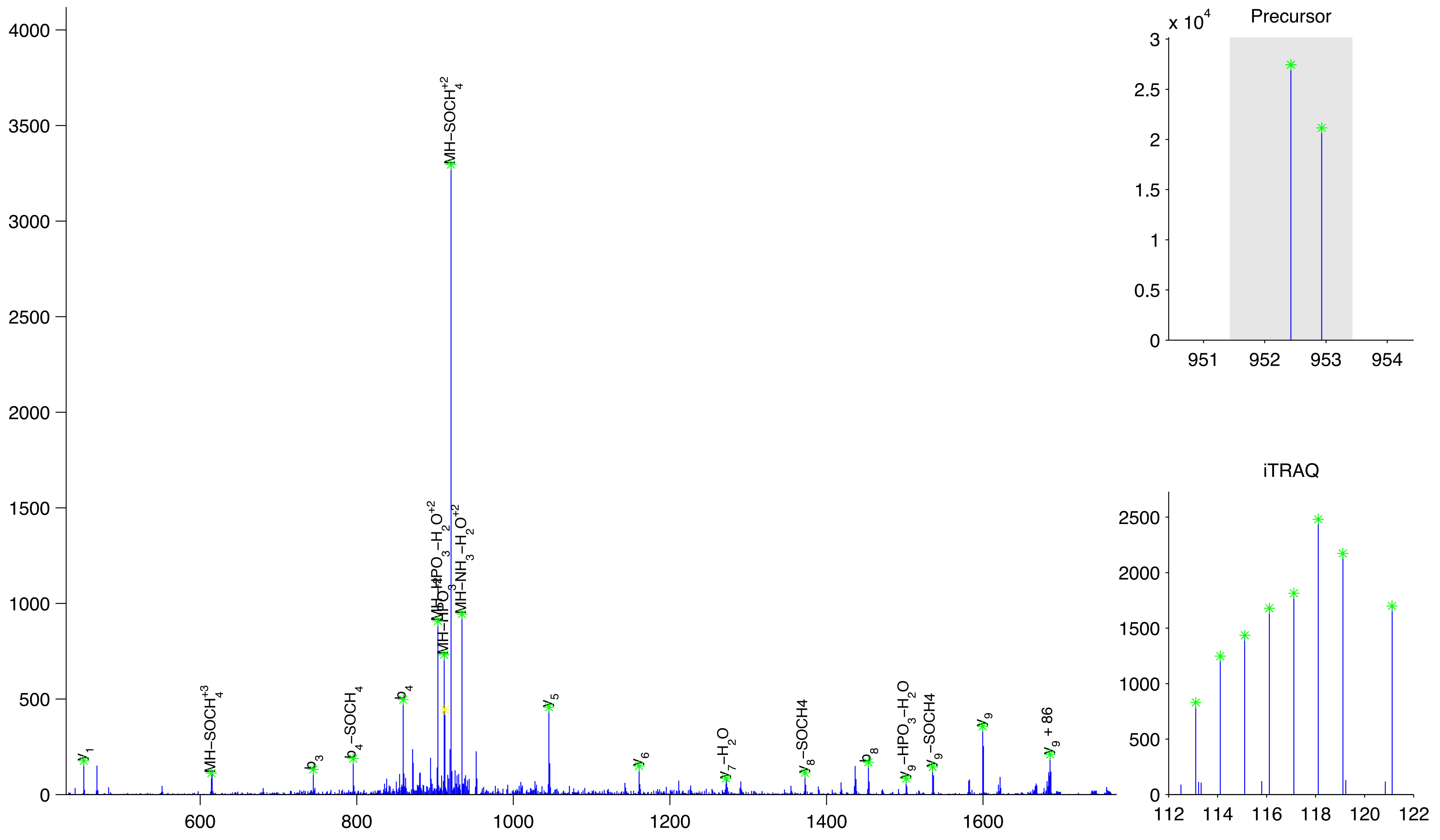

y m E D S T Y Y K

PTK2 protein tyrosine kinase 2 isoform a [Homo sapiens]
Charge State: +3
Scan Number: 8025
File Name: 120429_A549_TSA_pY.raw

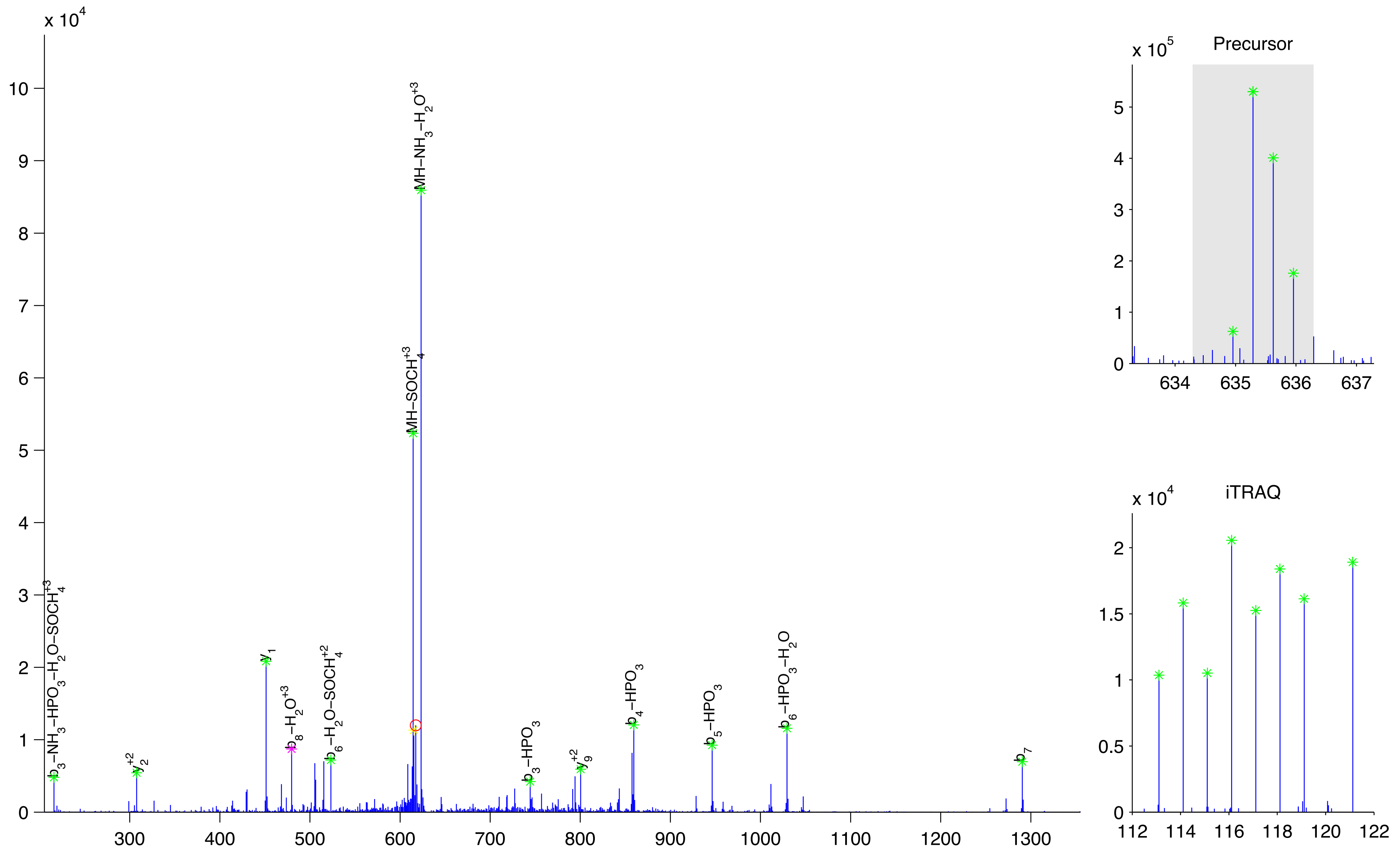

E A E A A I y H L Q L F E E L R R

pyruvate kinase, muscle isoform 1 [Homo sapiens]
Charge State: +4
Scan Number: 17989
File Name: 120429_A549_TSA_pY.raw

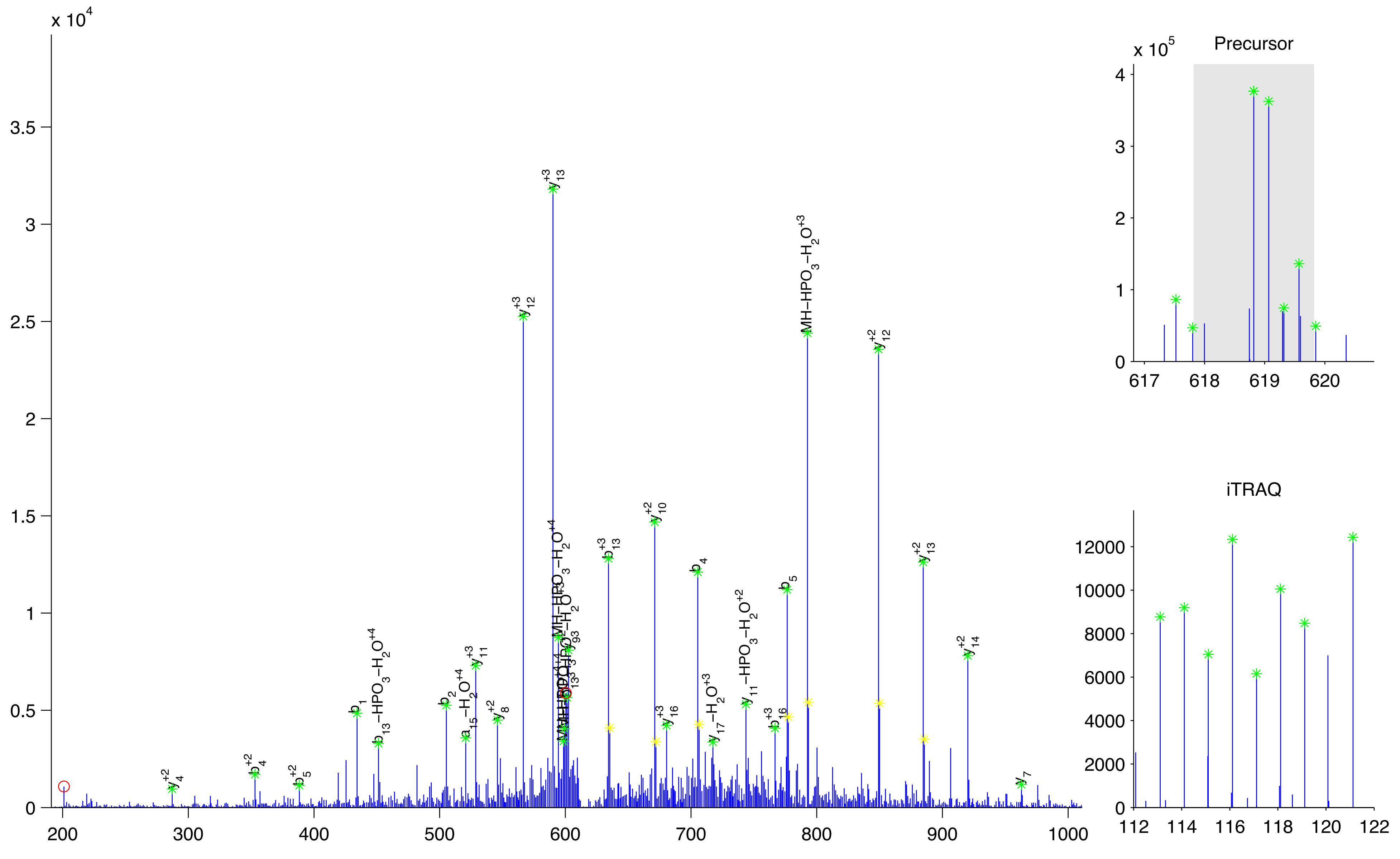

G I F P V L c K D P V Q E A W A E D V D L R

pyruvate kinase, muscle isoform 1 [Homo sapiens]
Charge State: +4
Scan Number: 18924
File Name: 120429_A549_TSA_pY.raw

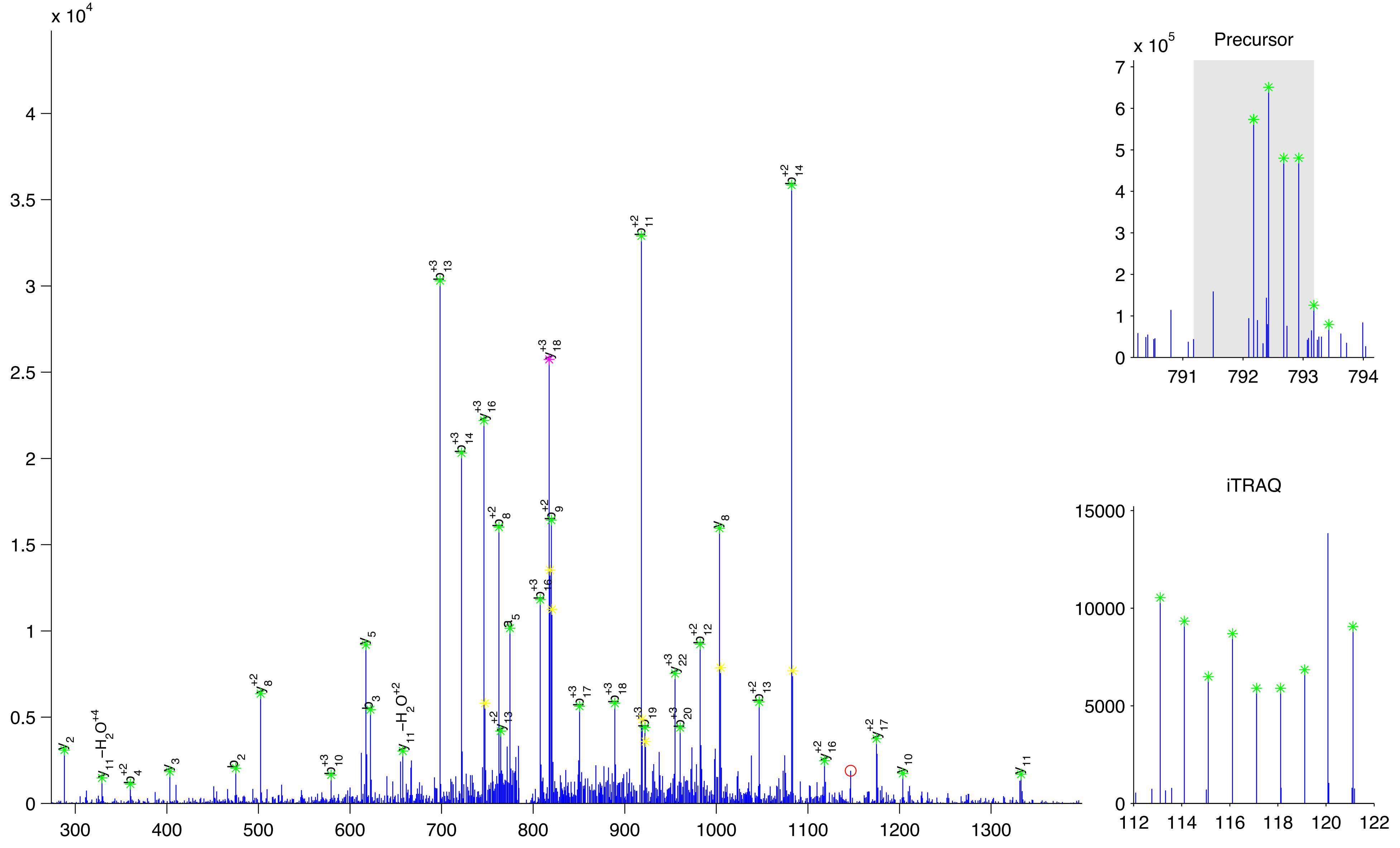

L c D F G S A S H V A D N D I T P y L V S R

serine/threonine−protein kinase PRP4K [Homo sapiens]
Charge State: +3
Scan Number: 16550
File Name: 120429_A549_TSA_pY.raw

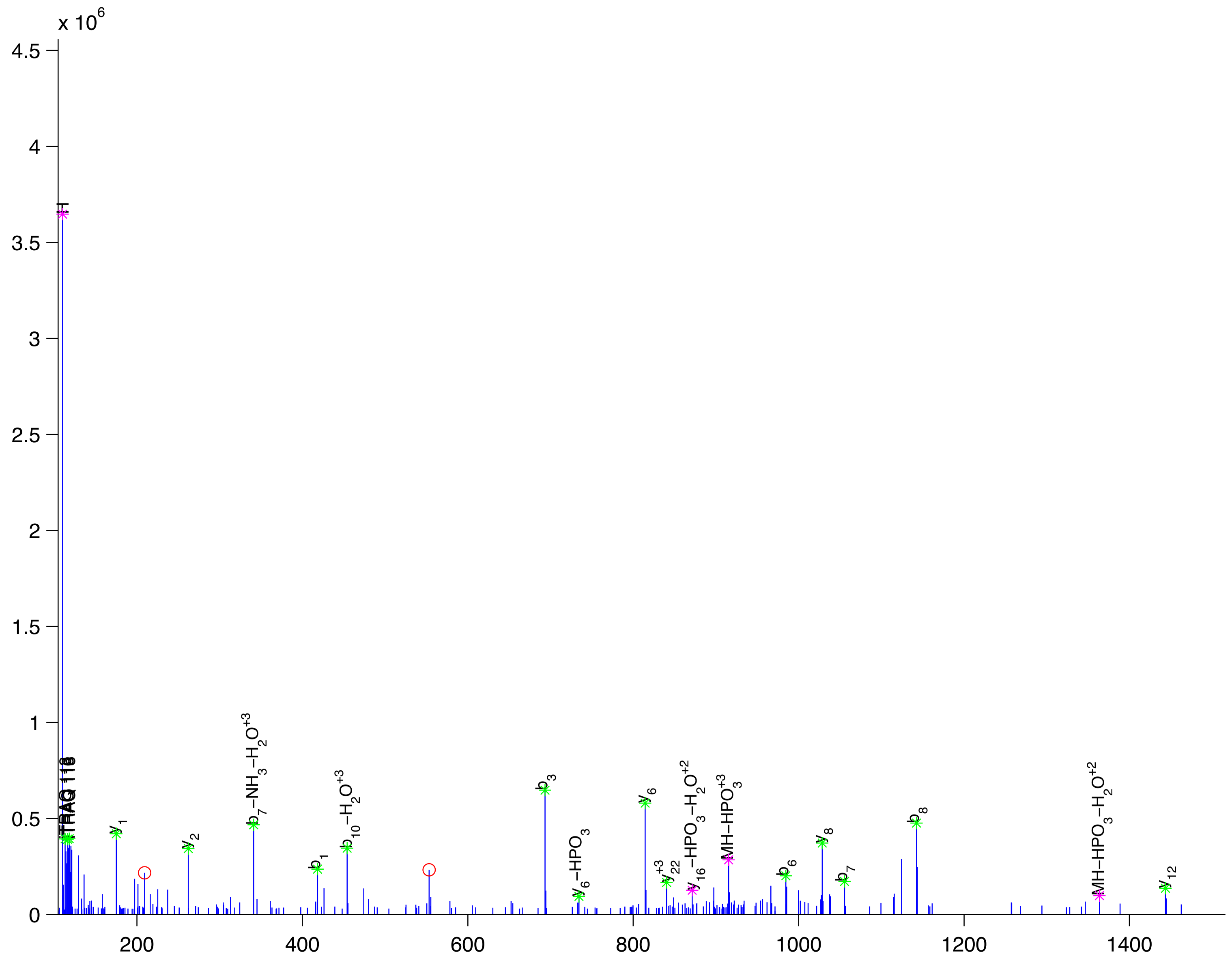

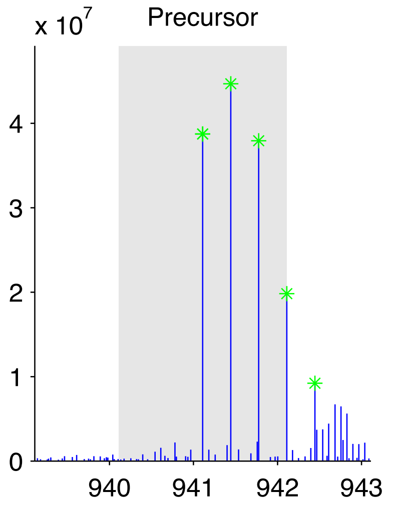

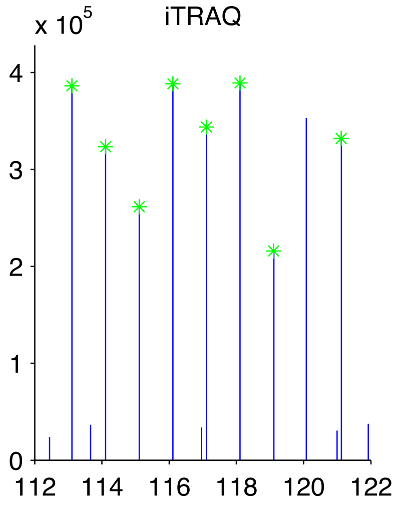

L c D F G S A S H V A D N D I T P y L V S R

serine/threonine–protein kinase PRP4K [Homo sapiens]
Charge State: +3
Scan Number: 16551
File Name: 120429_A549_TSA_pY.raw

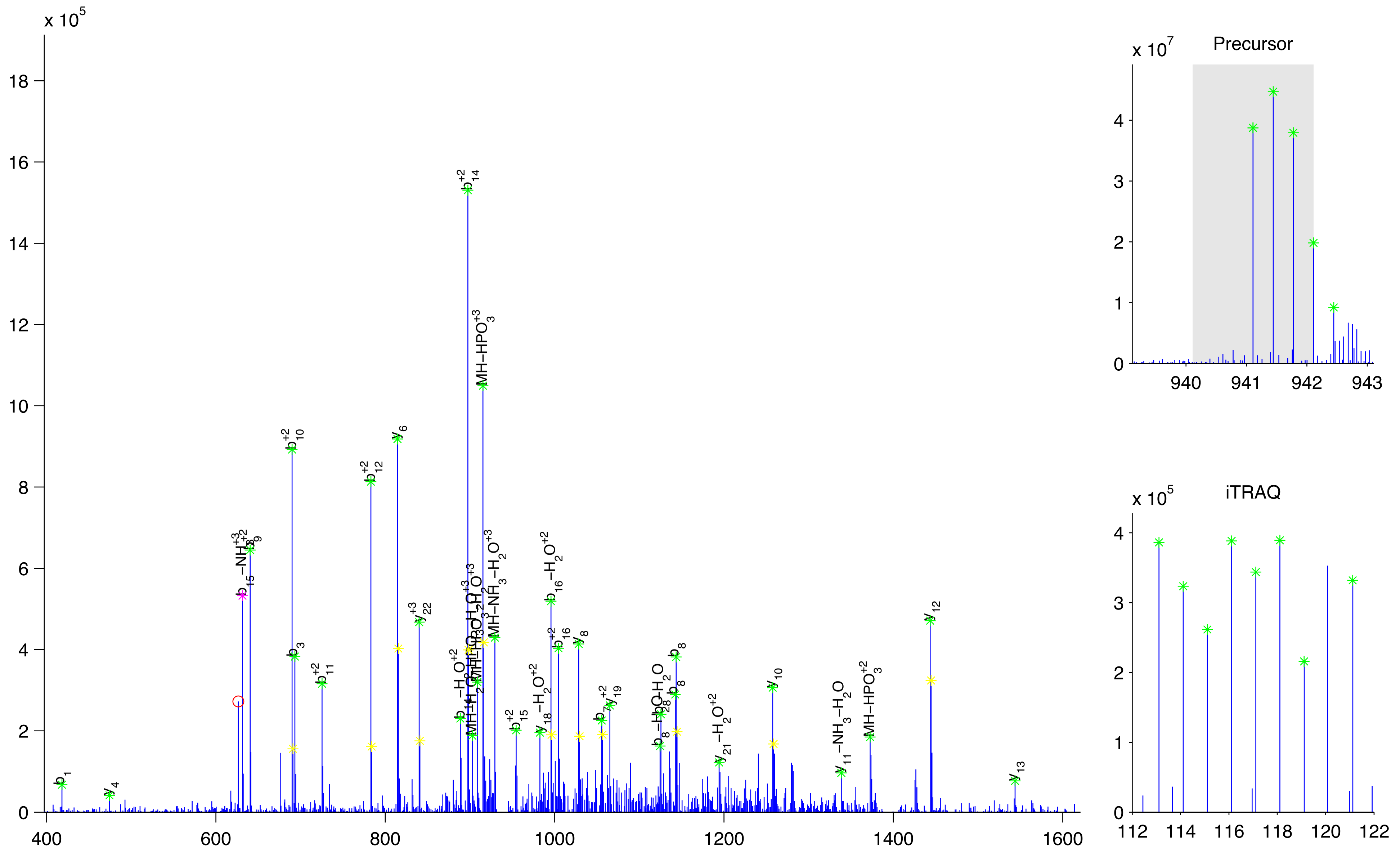

W m y k G R L L s L G M k G R A R G T A P K

synapse defective 1, Rho GTPase, homolog 2 [Homo sapiens]
Charge State: +5
Scan Number: 10578
File Name: 120429_A549_TSA_pY.raw

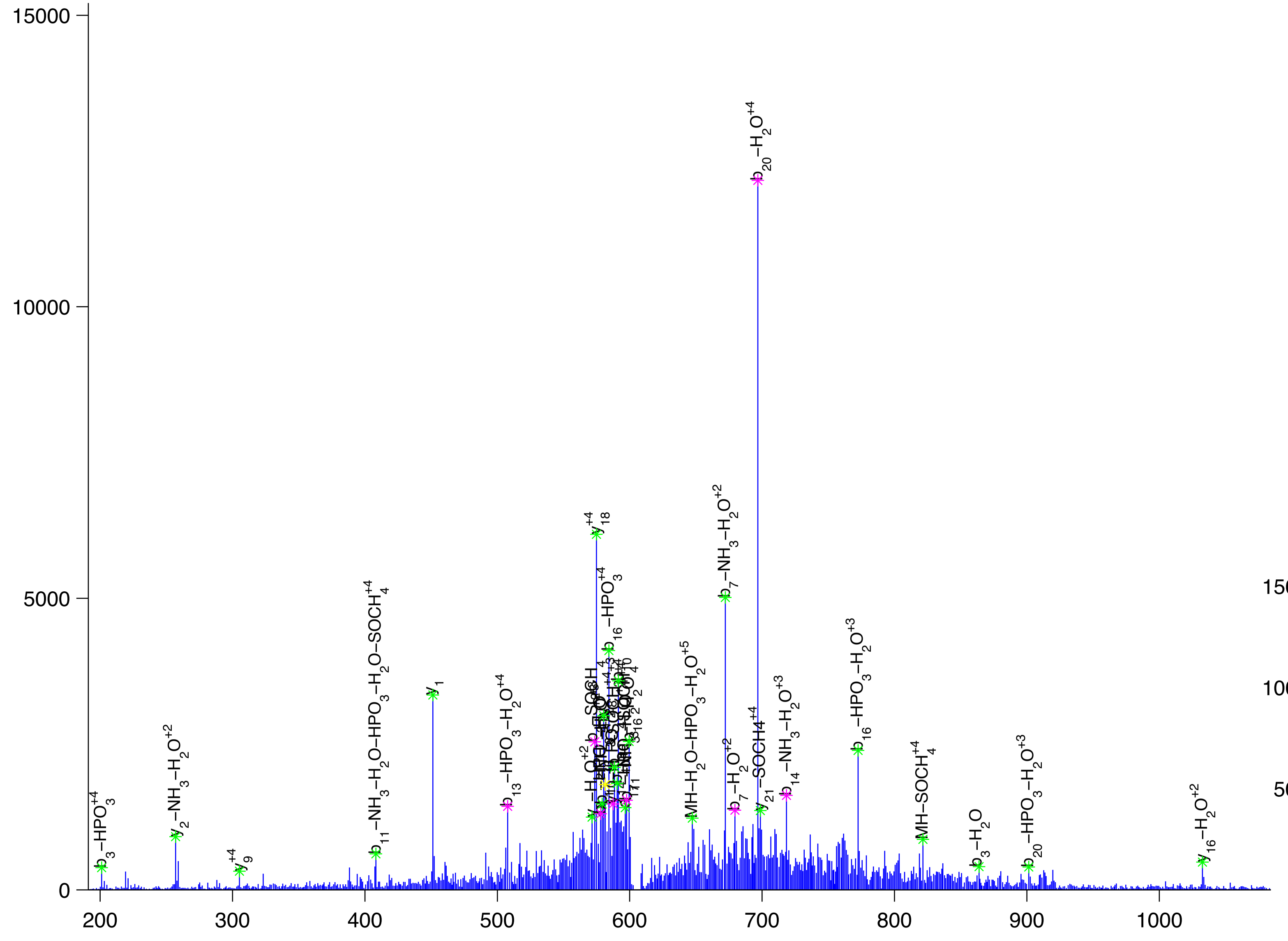

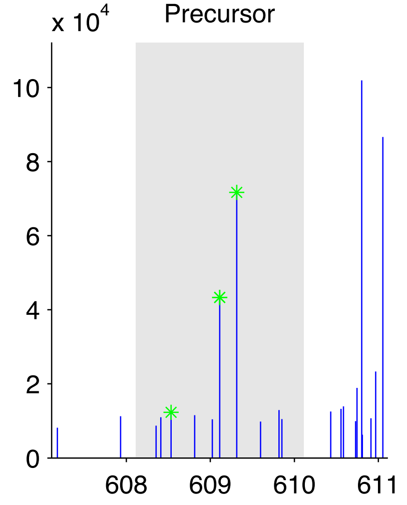

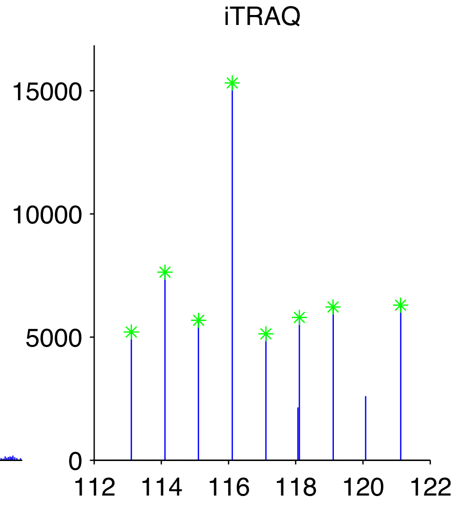

Q Q Q m V V A H Q y S F A P D G E A R

tensin 3 [Homo sapiens]
Charge State: +3
Scan Number: 7439
File Name: 120429_A549_TSA_pY.raw

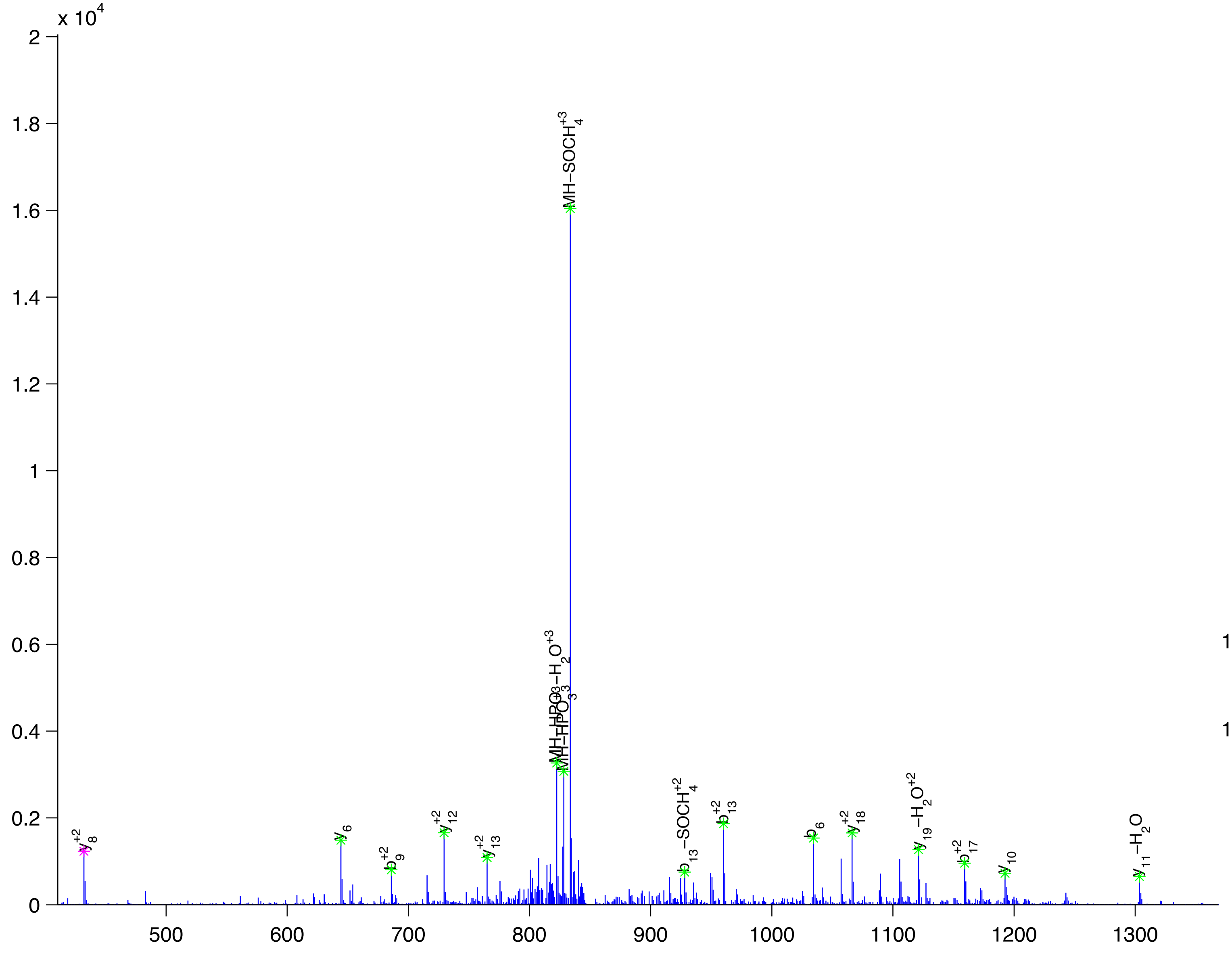

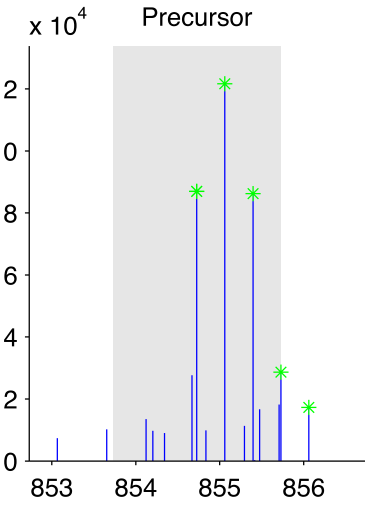

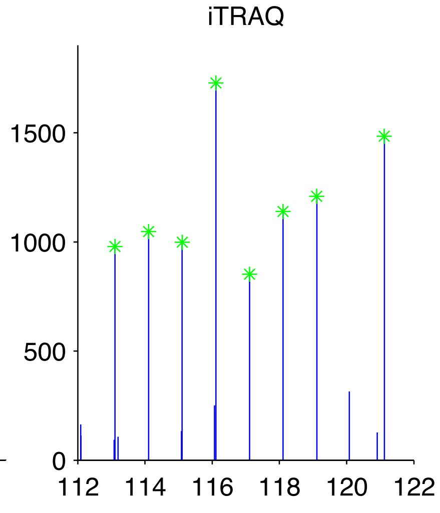

K L S L G Q y D N D A G G Q L P F S K

tensin 3 [Homo sapiens]
Charge State: +5
Scan Number: 13947
File Name: 120429_A549_TSA_pY.raw

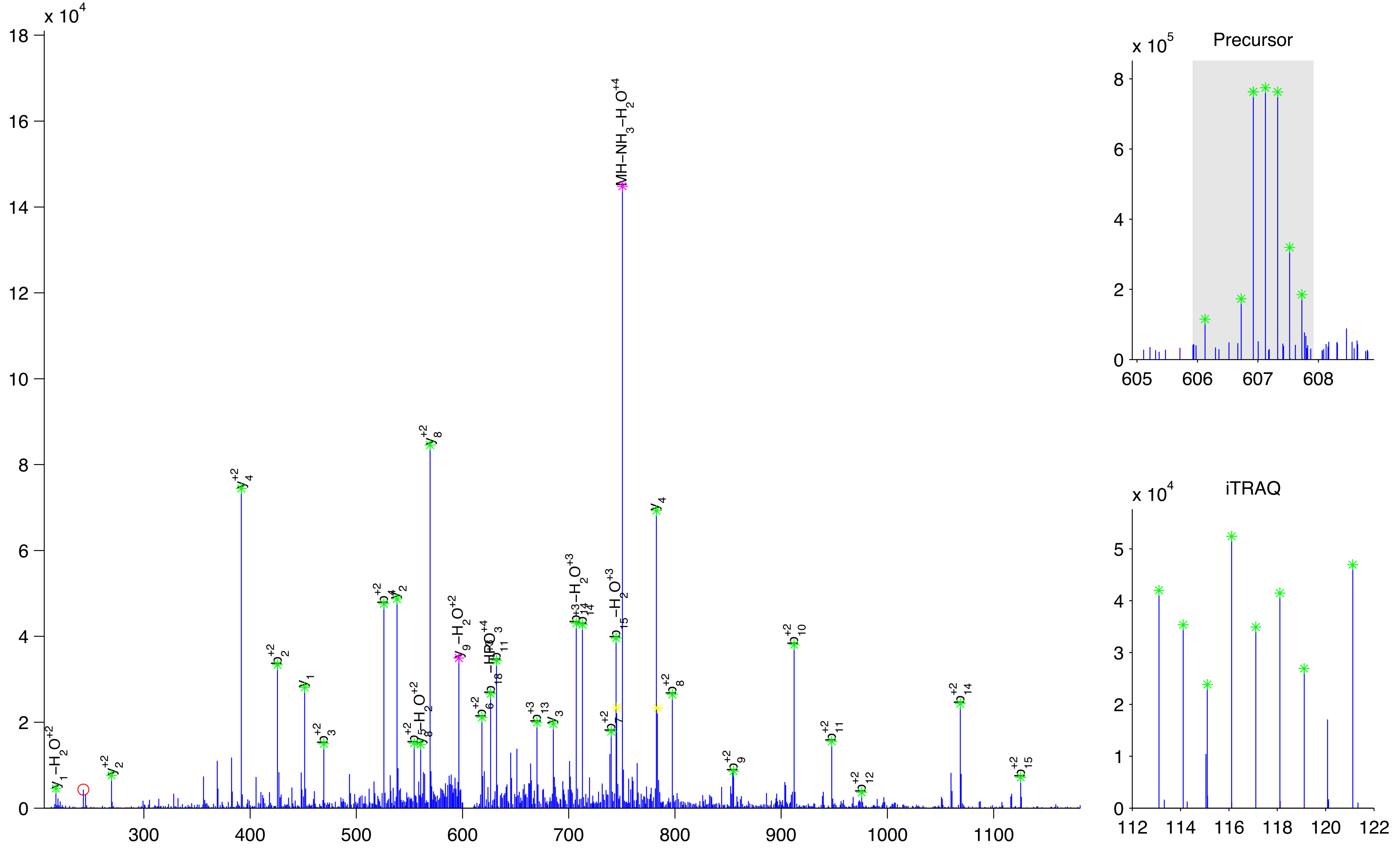

K L S L G Q y D N D A G G Q L P F S K

tensin 3 [Homo sapiens]
Charge State: +4
Scan Number: 14094
File Name: 120429_A549_TSA_pY.raw

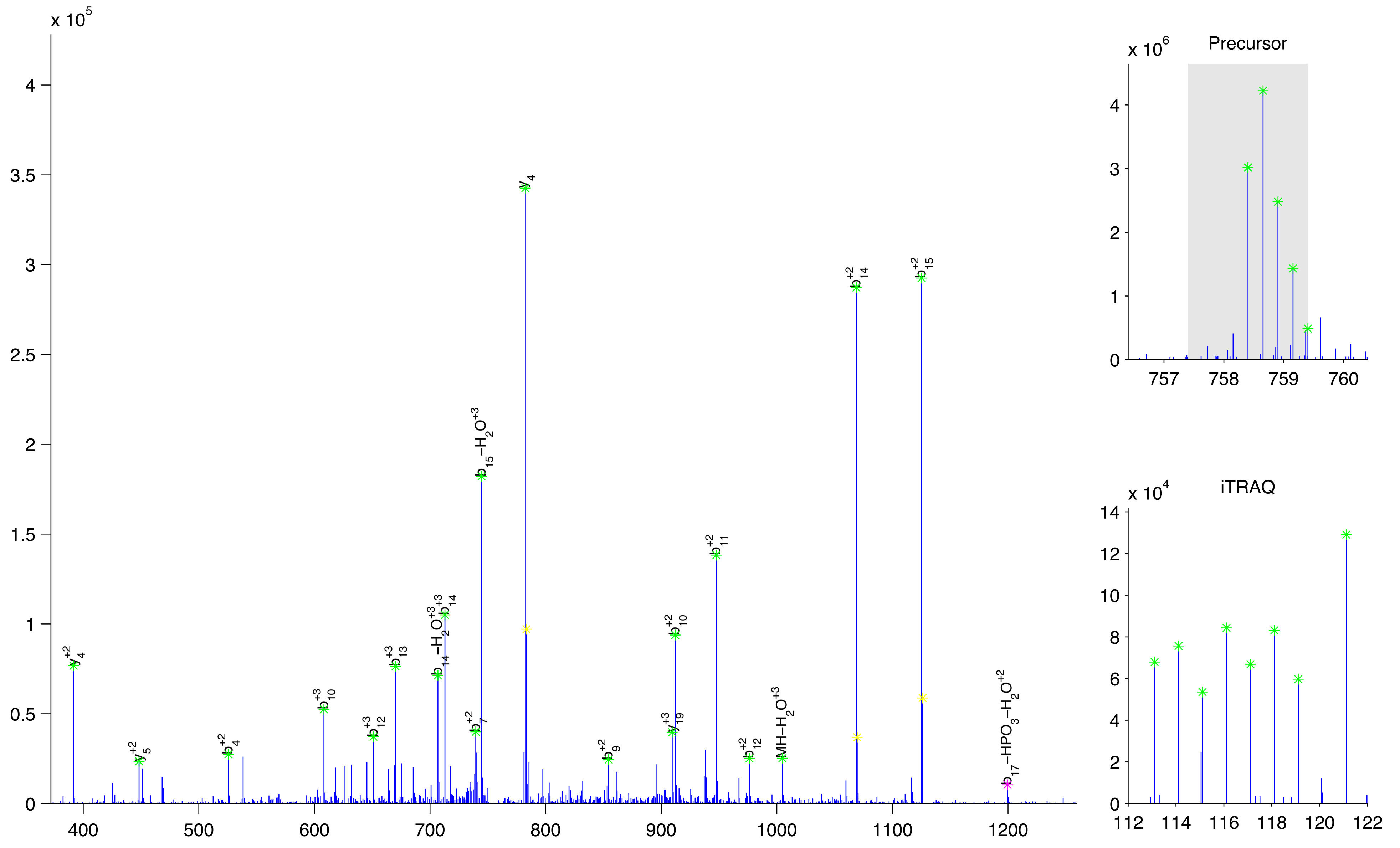

E S M c S T P A F P V S P E T P y V K

tensin 3 [Homo sapiens]
Charge State: +4
Scan Number: 14661
File Name: 120429_A549_TSA_pY.raw

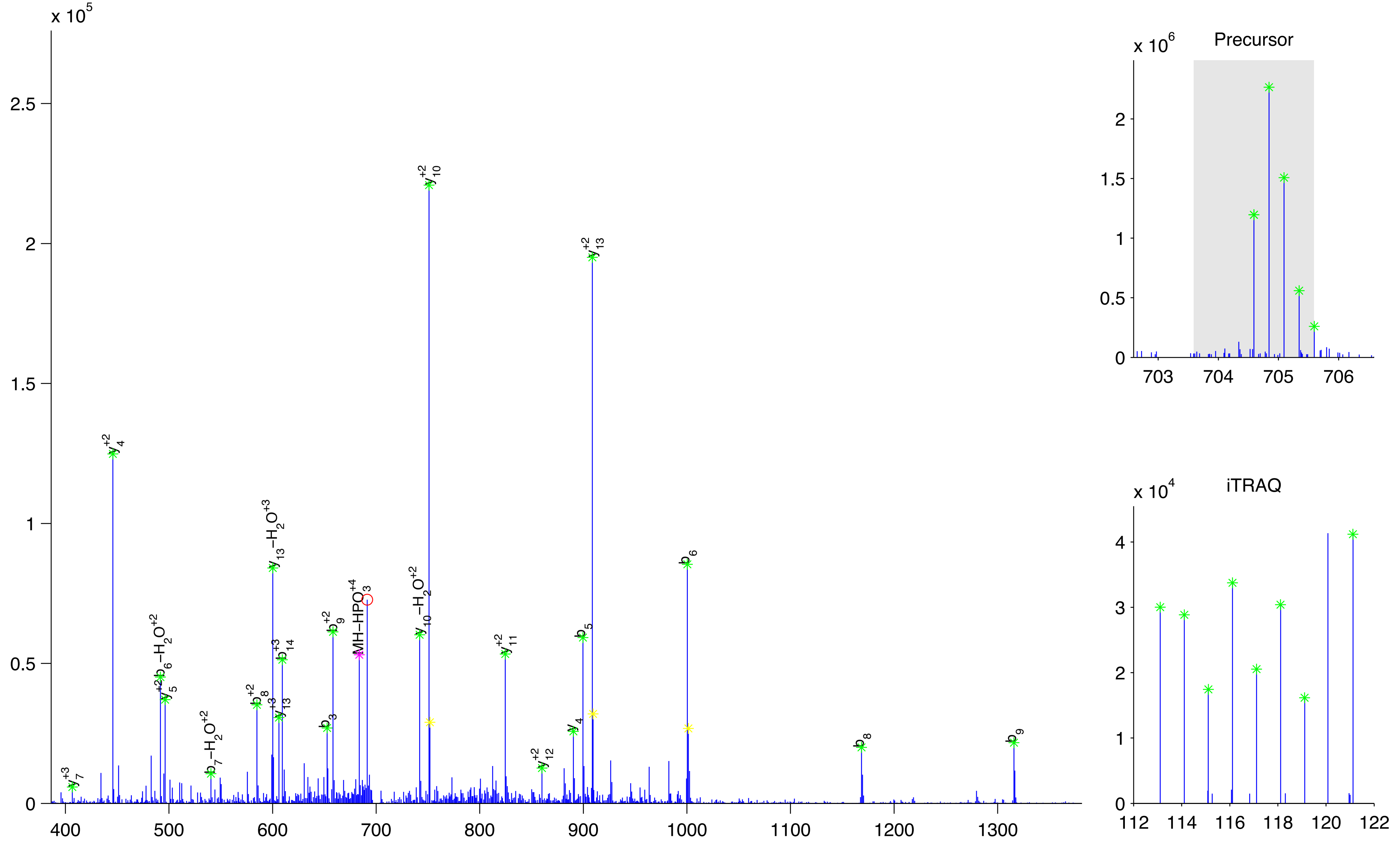

E S M c S T P A F P V S P E T P y V K

tensin 3 [Homo sapiens]
Charge State: +3
Scan Number: 14766
File Name: 120429_A549_TSA_pY.raw

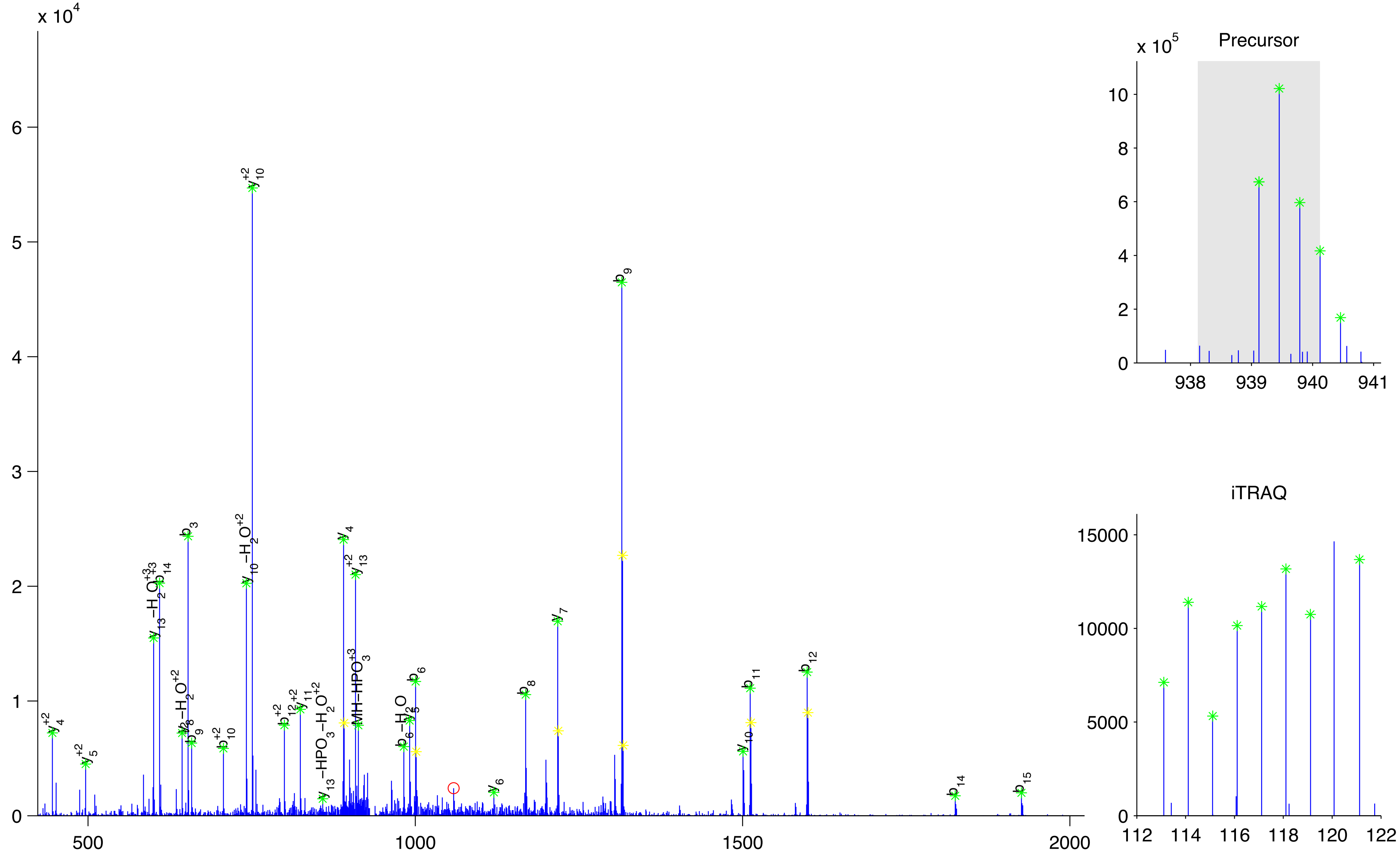

L S L G Q y D N D A G G Q L P F S K

tensin 3 [Homo sapiens]
Charge State: +4
Scan Number: 16641
File Name: 120429_A549_TSA_pY.raw

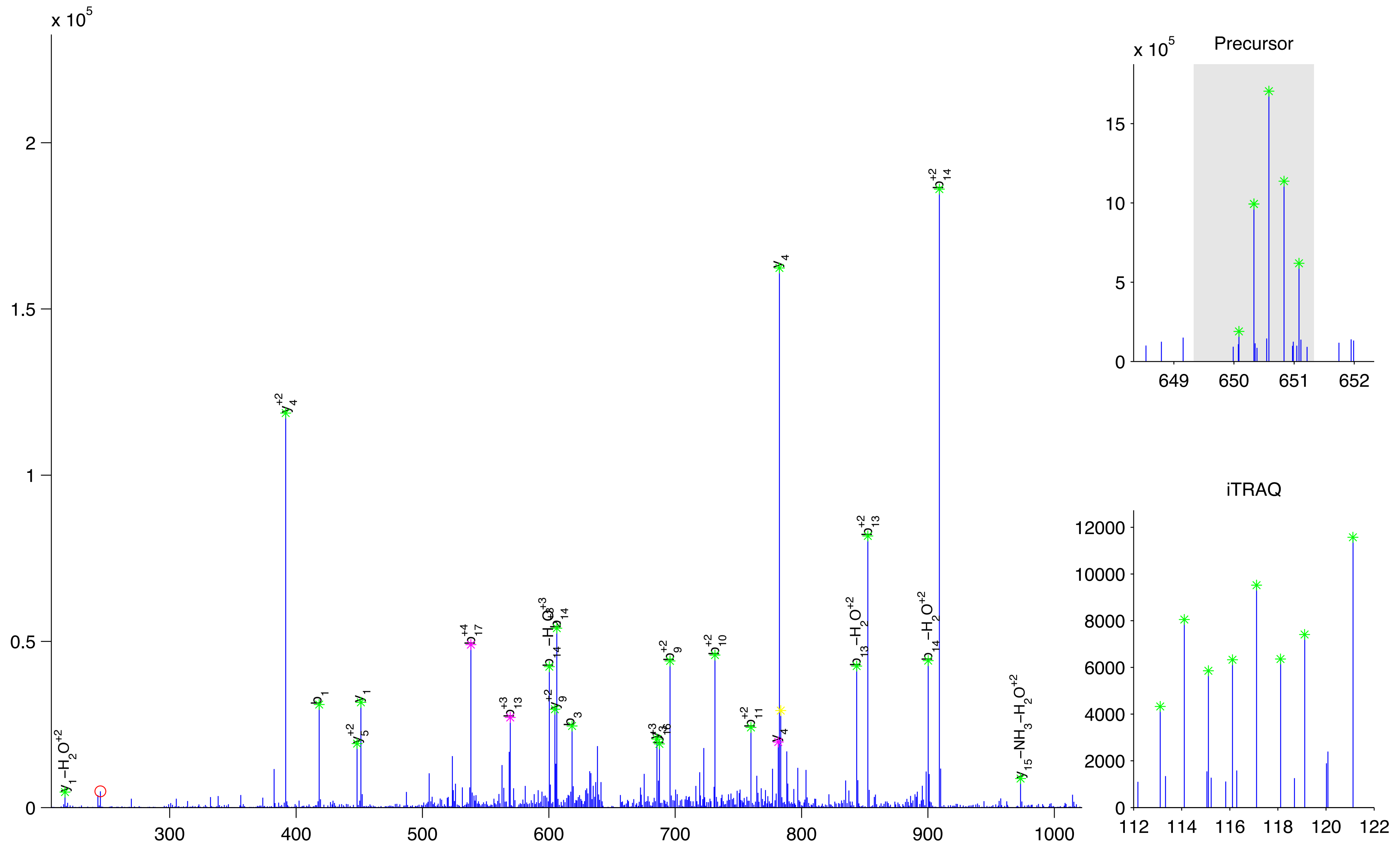

A G V D y A P N L P P F P S P A D V K

tensin 3 [Homo sapiens]
Charge State: +4
Scan Number: 17189
File Name: 120429_A549_TSA_pY.raw

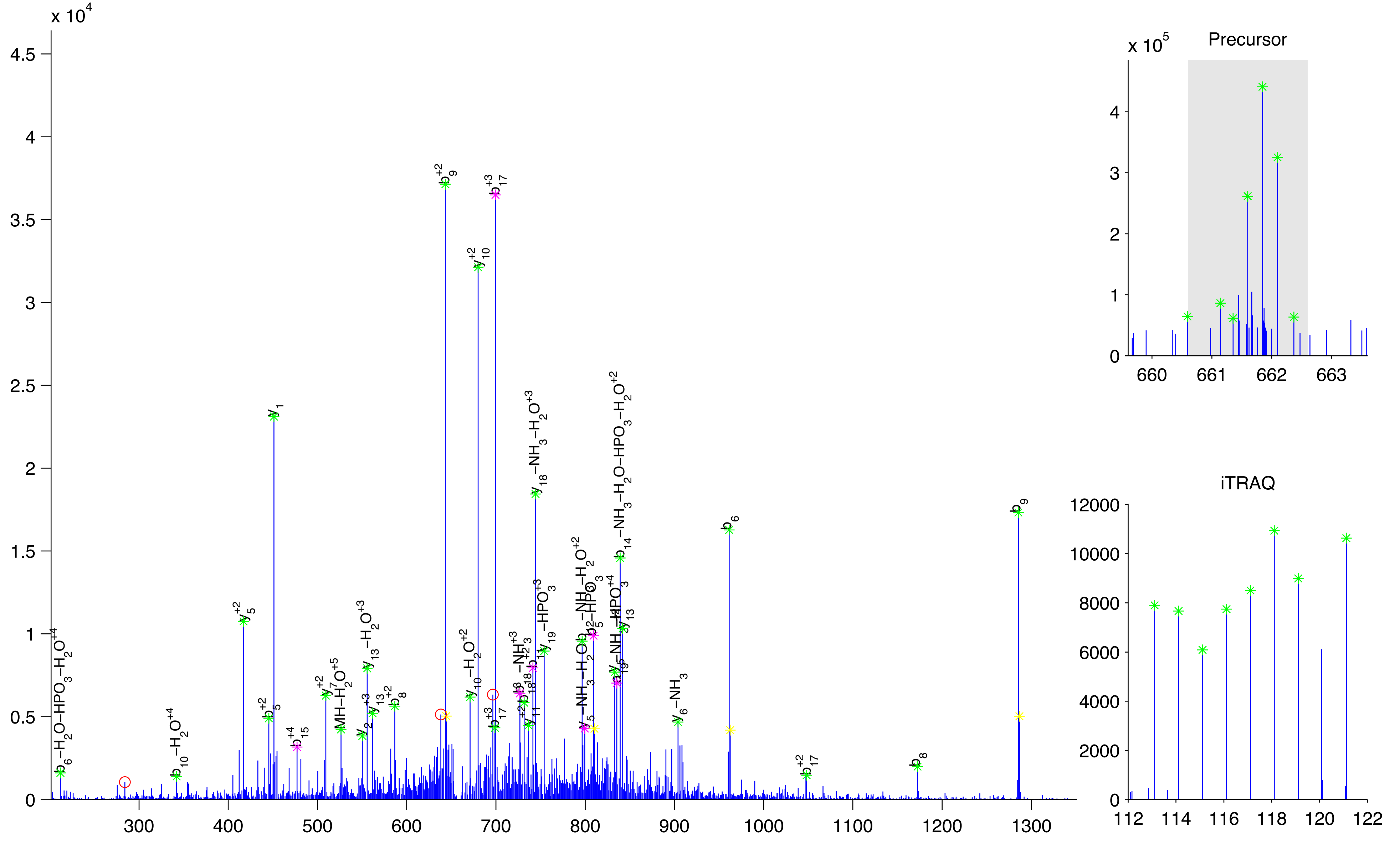

K P T y D P V S E D Q D P L S S D F K R

tyrosine kinase, non−receptor, 2 isoform 1 [Homo sapiens]
Charge State: +4
Scan Number: 11658
File Name: 120429_A549_TSA_pY.raw

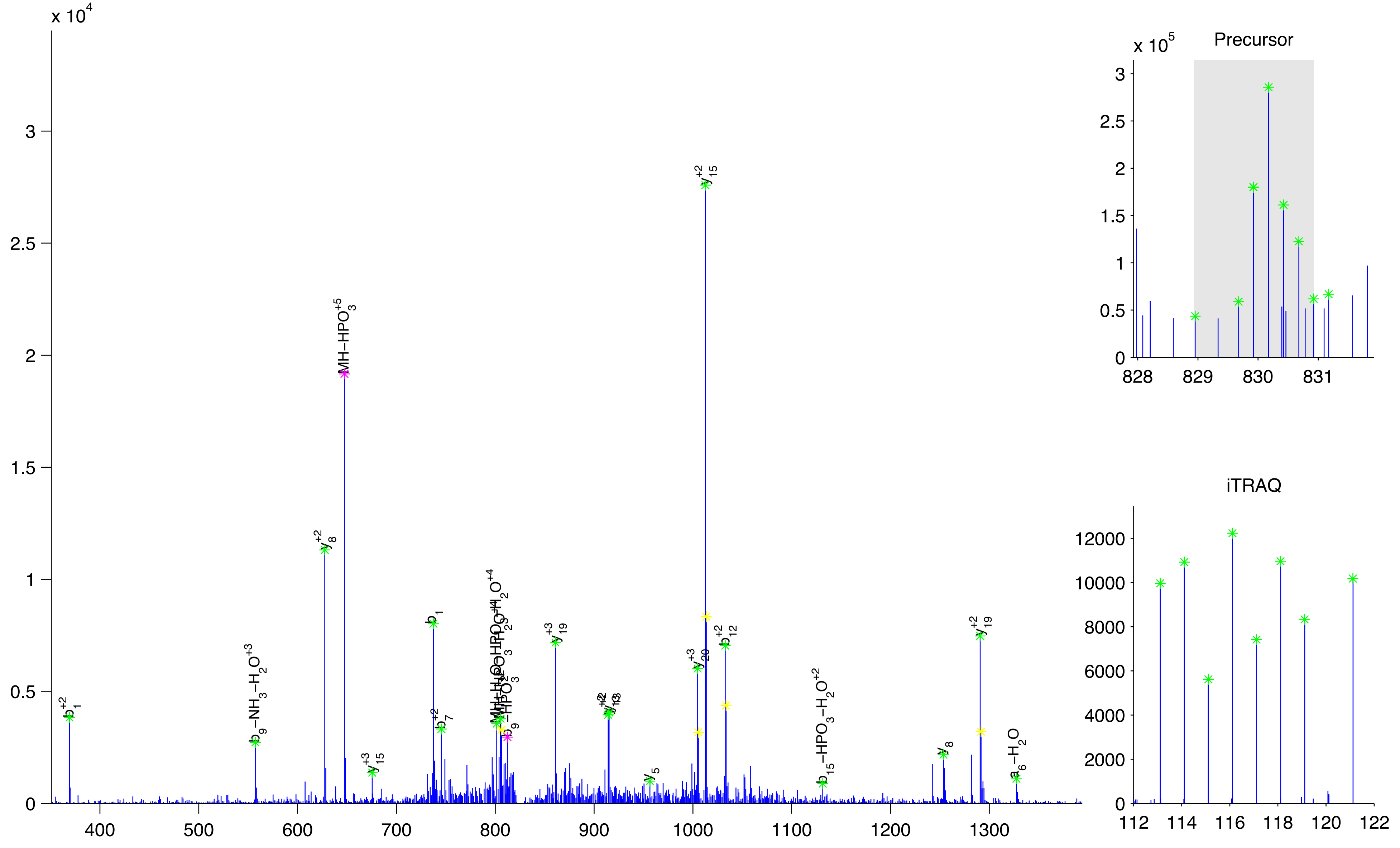

S F L D S G y R

vinculin isoform meta−VCL [Homo sapiens]
Charge State: +3
Scan Number: 11128
File Name: 120429_A549_TSA_pY.raw

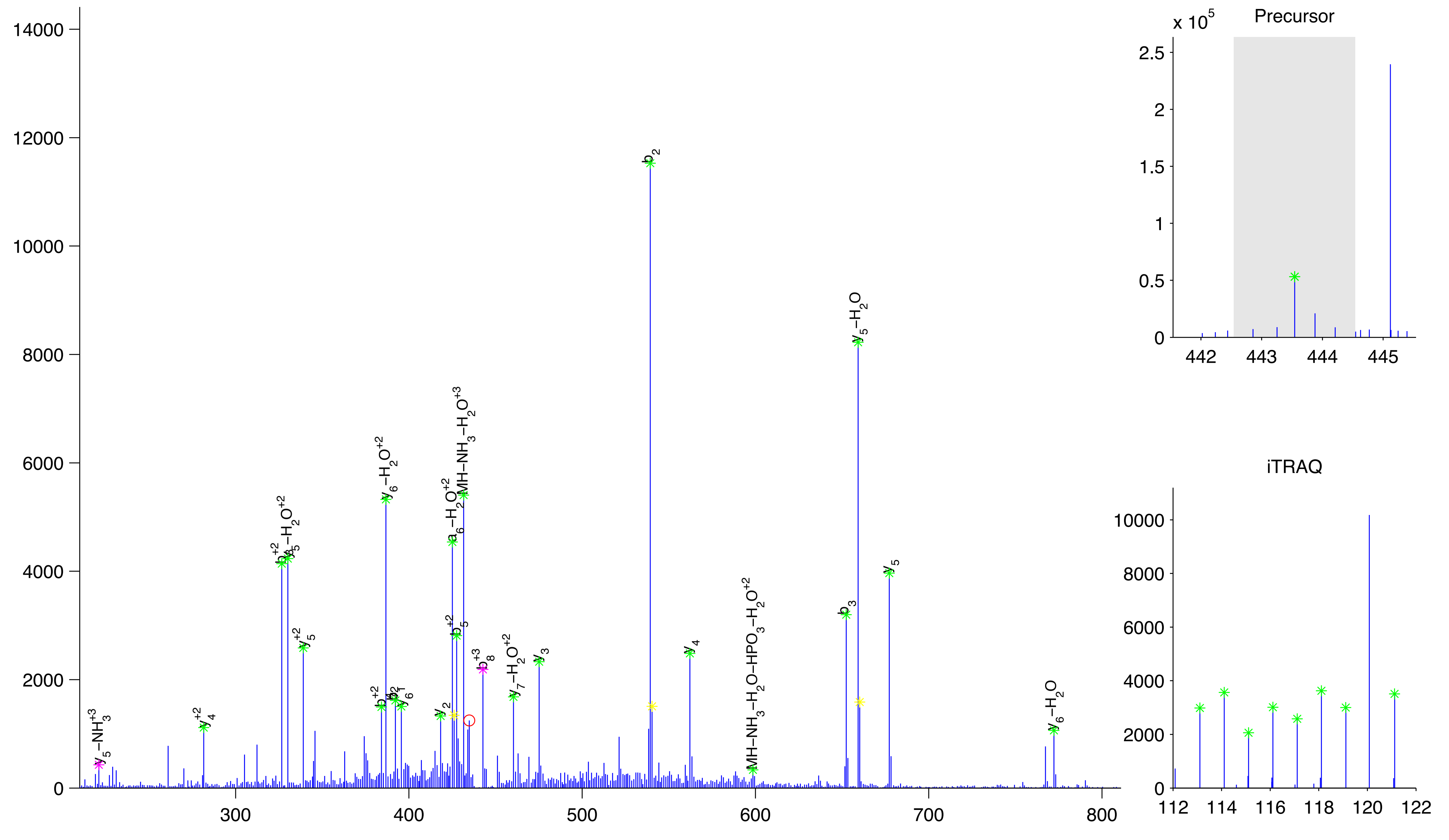

S F L D S G y R

vinculin isoform VCL [Homo sapiens]
Charge State: +2
Scan Number: 11049
File Name: 120429_A549_TSA_pY.raw

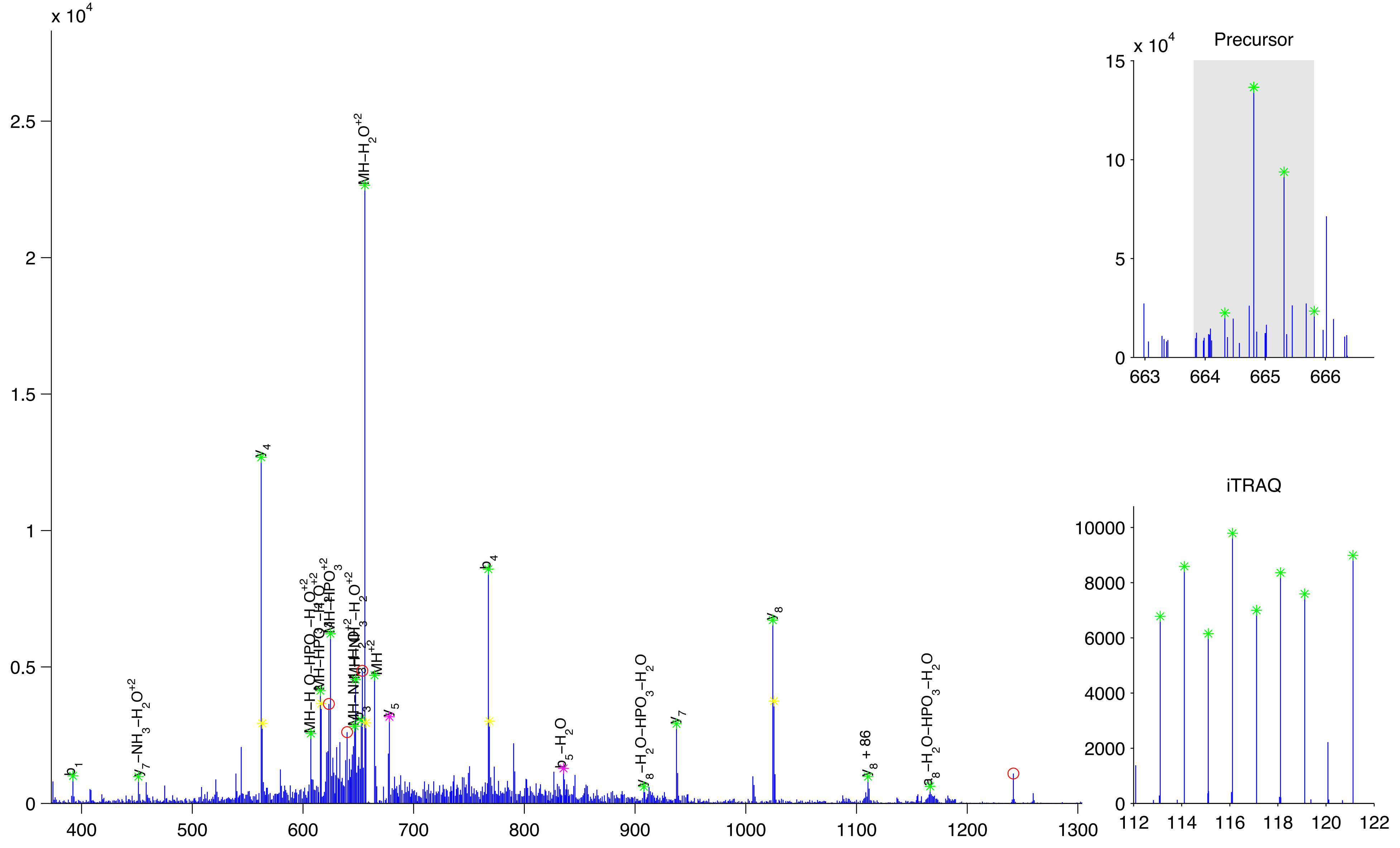

S F L D S G y R

vinculin isoform VCL [Homo sapiens]
Charge State: +3
Scan Number: 11128
File Name: 120429_A549_TSA_pY.raw

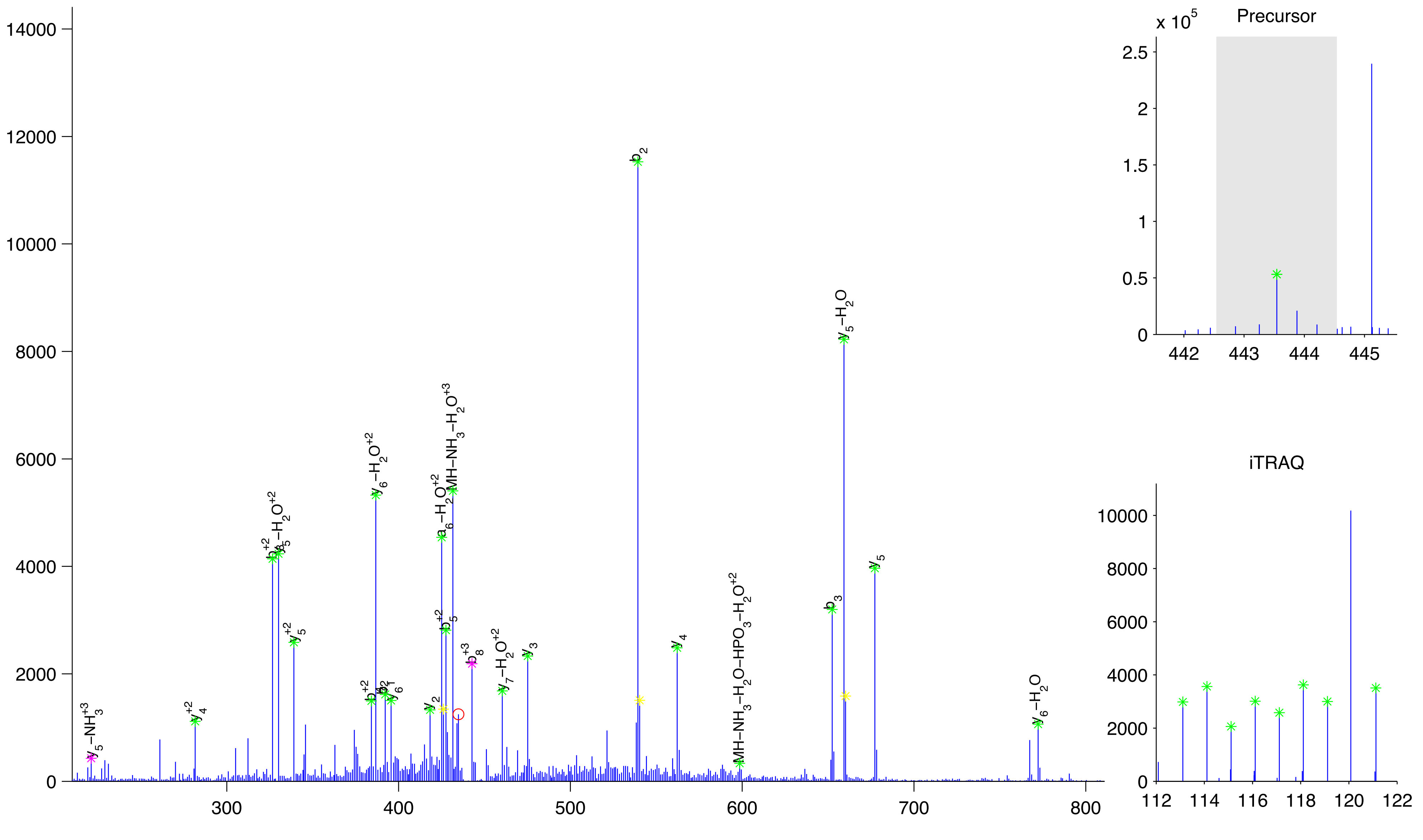

L I E D N E y T A R

viral oncogene yes−1 homolog 1 [Homo sapiens]
Charge State: +3
Scan Number: 10209
File Name: 120429_A549_TSA_pY.raw

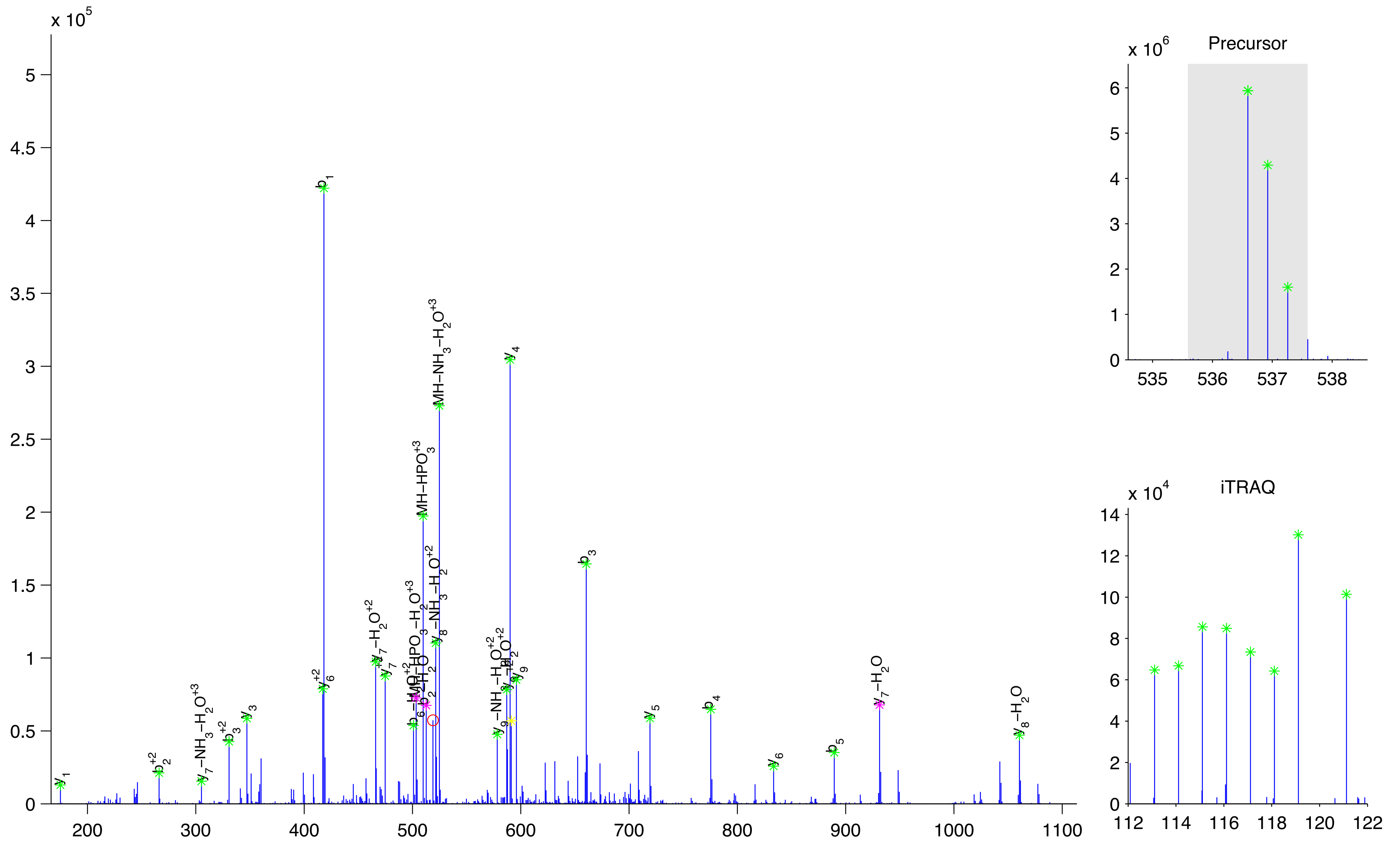

L I E D N E y T A R

viral oncogene yes−1 homolog 1 [Homo sapiens]
Charge State: +2
Scan Number: 10442
File Name: 120429_A549_TSA_pY.raw

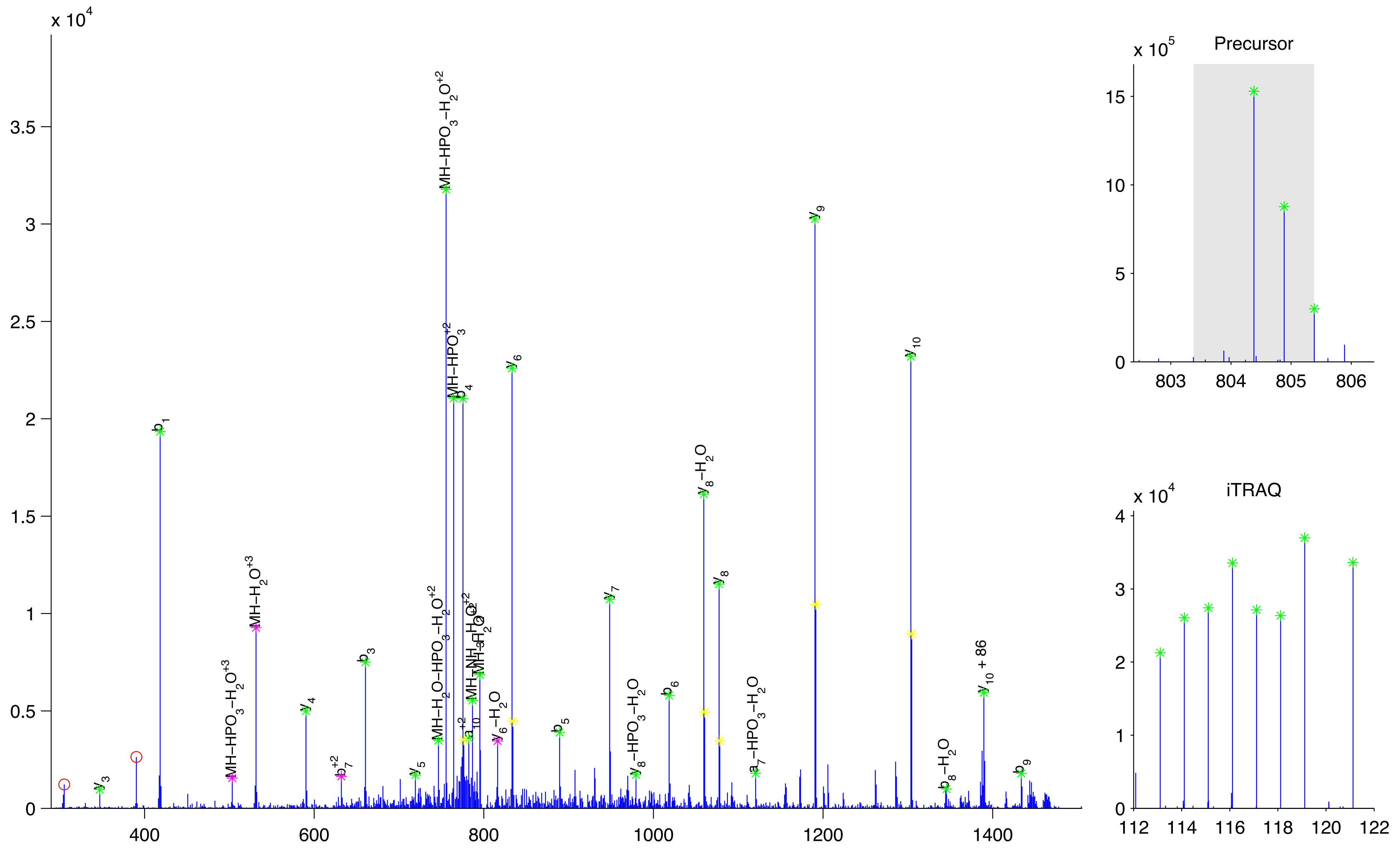

L I E D N E y T A R

viral oncogene yes−1 homolog 1 [Homo sapiens]
Charge State: +3
Scan Number: 10650
File Name: 120429_A549_TSA_pY.raw

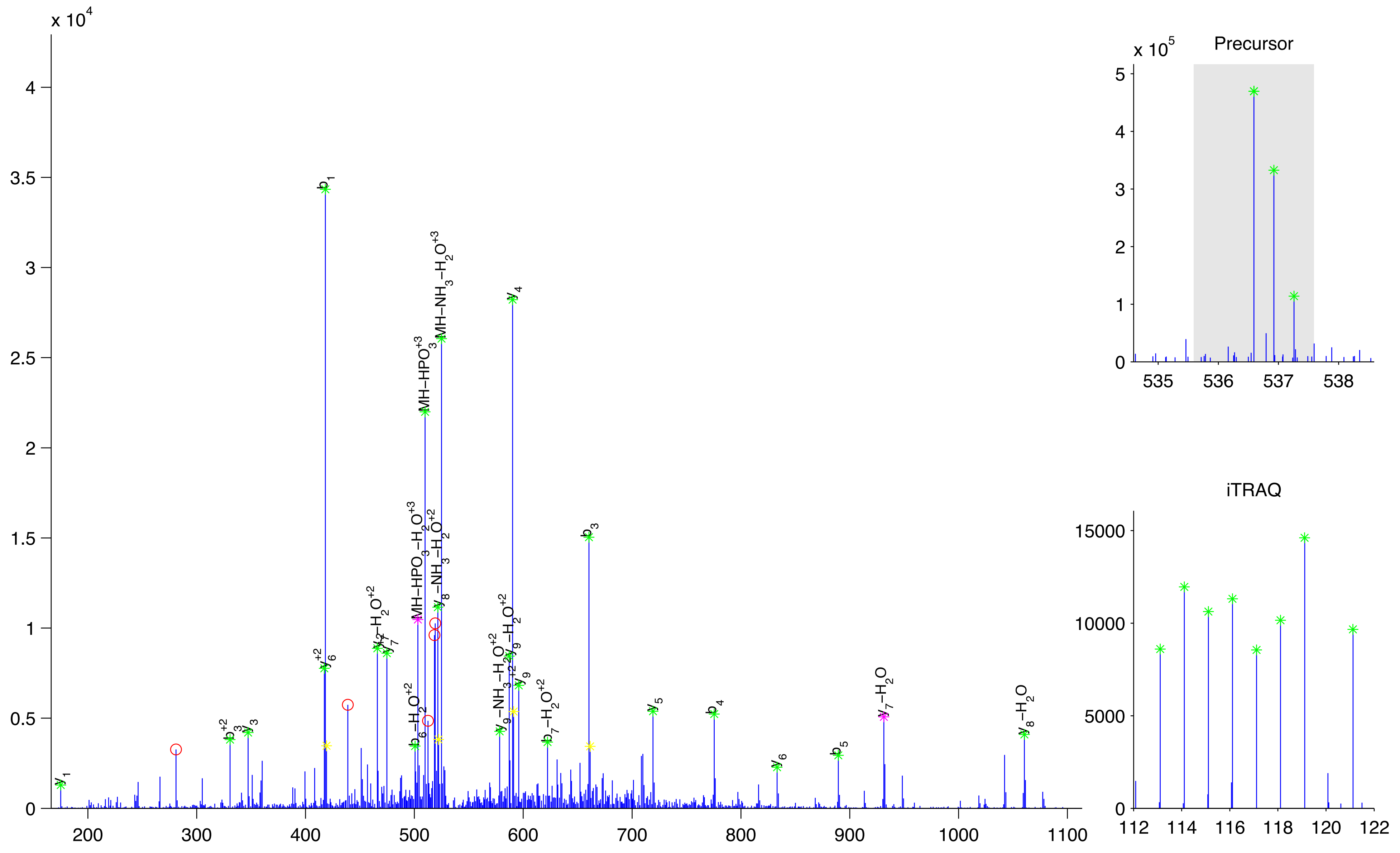

V I y D F I E K

Wiskott–Aldrich syndrome gene–like protein [Homo sapiens]
Charge State: +3
Scan Number: 20389
File Name: 120429_A549_TSA_pY.raw

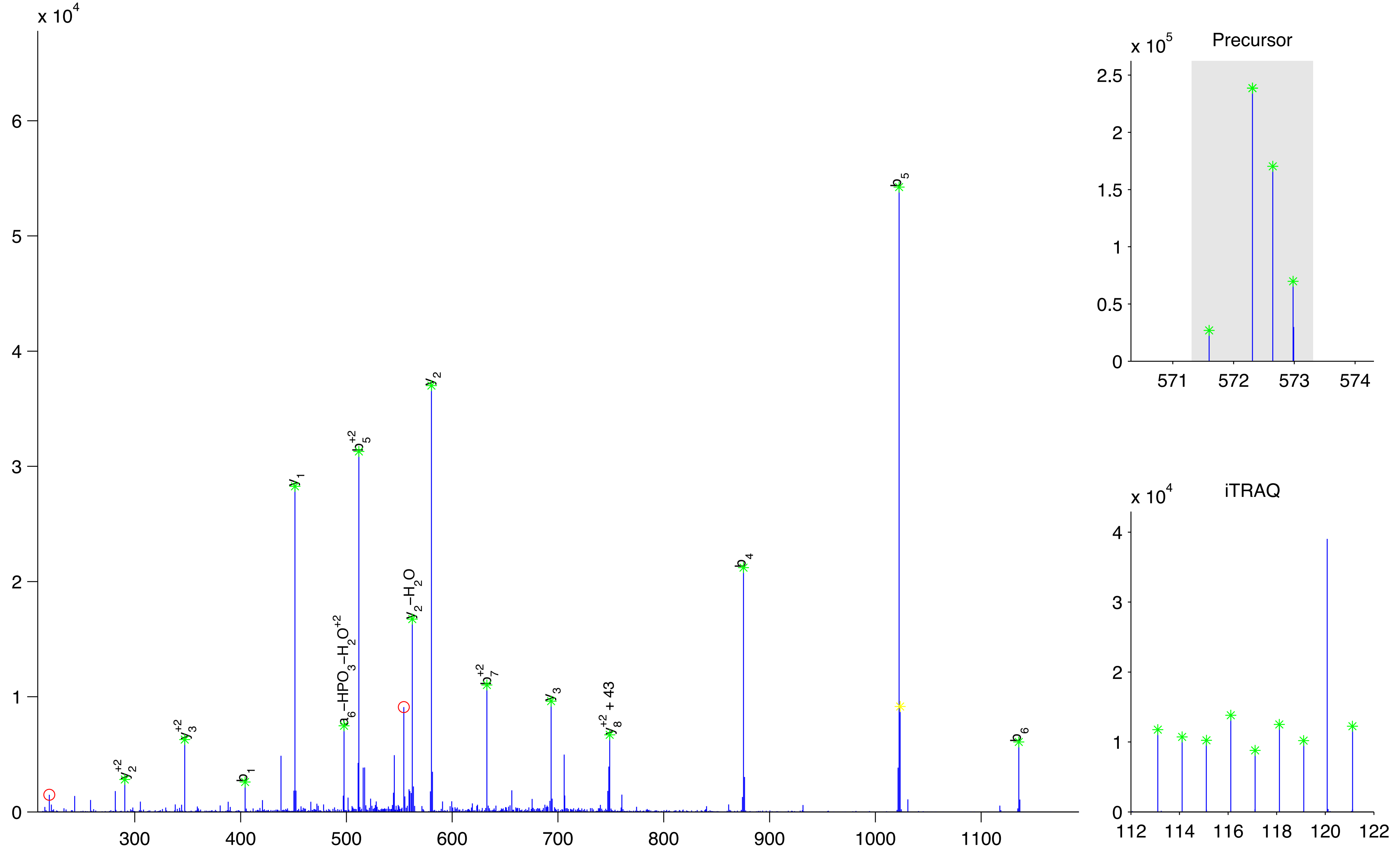

V I y D F I E K

Wiskott–Aldrich syndrome gene–like protein [Homo sapiens]
Charge State: +3
Scan Number: 20919
File Name: 120429_A549_TSA_pY.raw

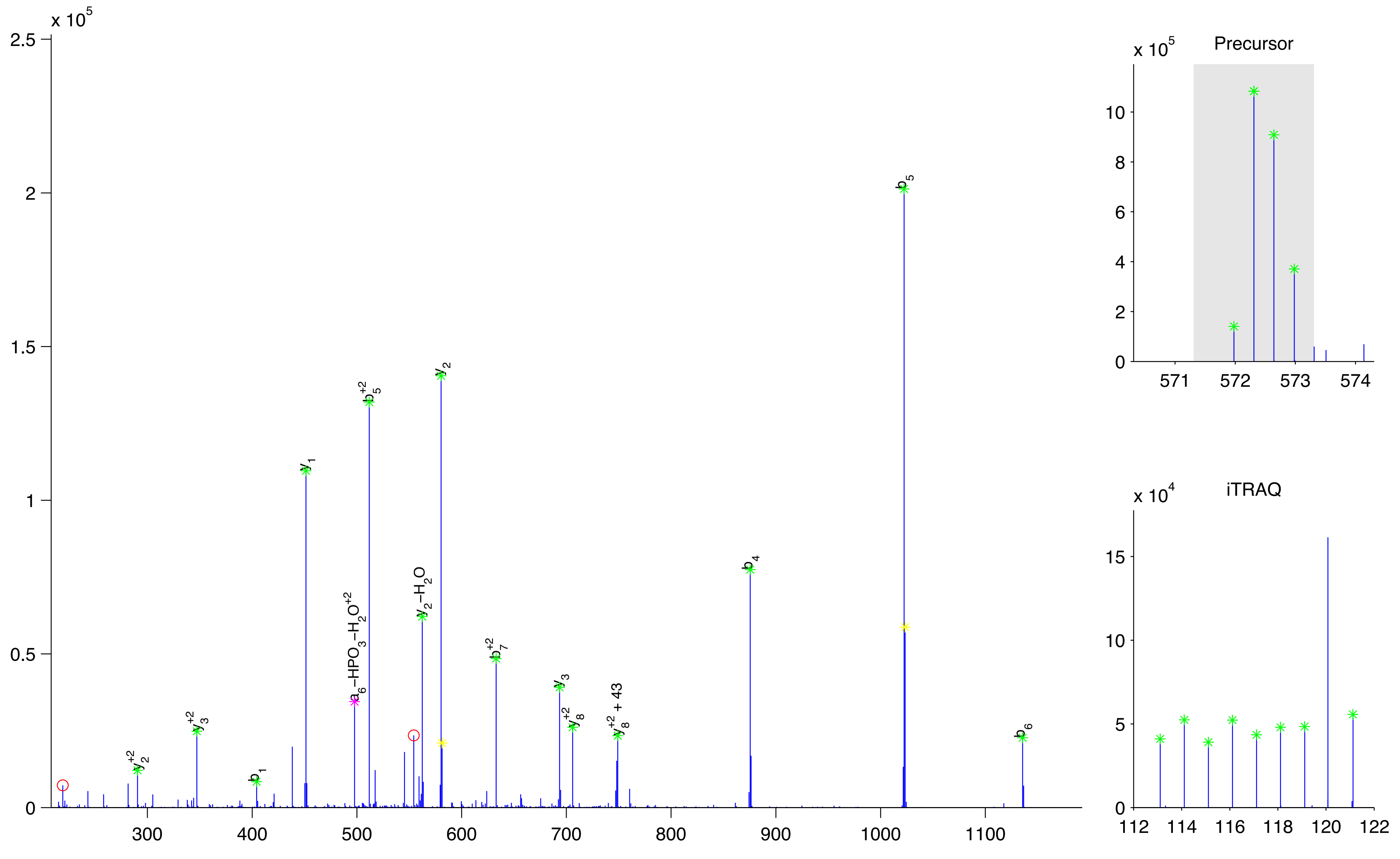

Supplement: S4 Fig — Instead of relying on an FDR analysis where the identity of true positives and true negatives are unknown, we manually validated each MS/MS spectra manually. Each page represents a manually validated MS/MS spectrum. (PDF) [file pone.0126242.s004.pdf]
