# Supplementary material for: Quantitative Profiling of Lysine Acetylation Reveals Dynamic Crosstalk between Receptor Tyrosine Kinases and Lysine Acetylation
Source: PLoS One. 2015 May 15;10(5):e0126242. doi: 10.1371/journal.pone.0126242 (PMC4433260; doi:10.1371/journal.pone.0126242)

D G L Q N E k N I V S T P V K

3–hydroxymethyl–3–methylglutaryl–Coenzyme A lyase (hydroxymethylglutaricaciduria) [Homo sapiens]
Charge State: +3
Scan Number: 20983
File Name: 120501_A549_TSA_AcK.raw

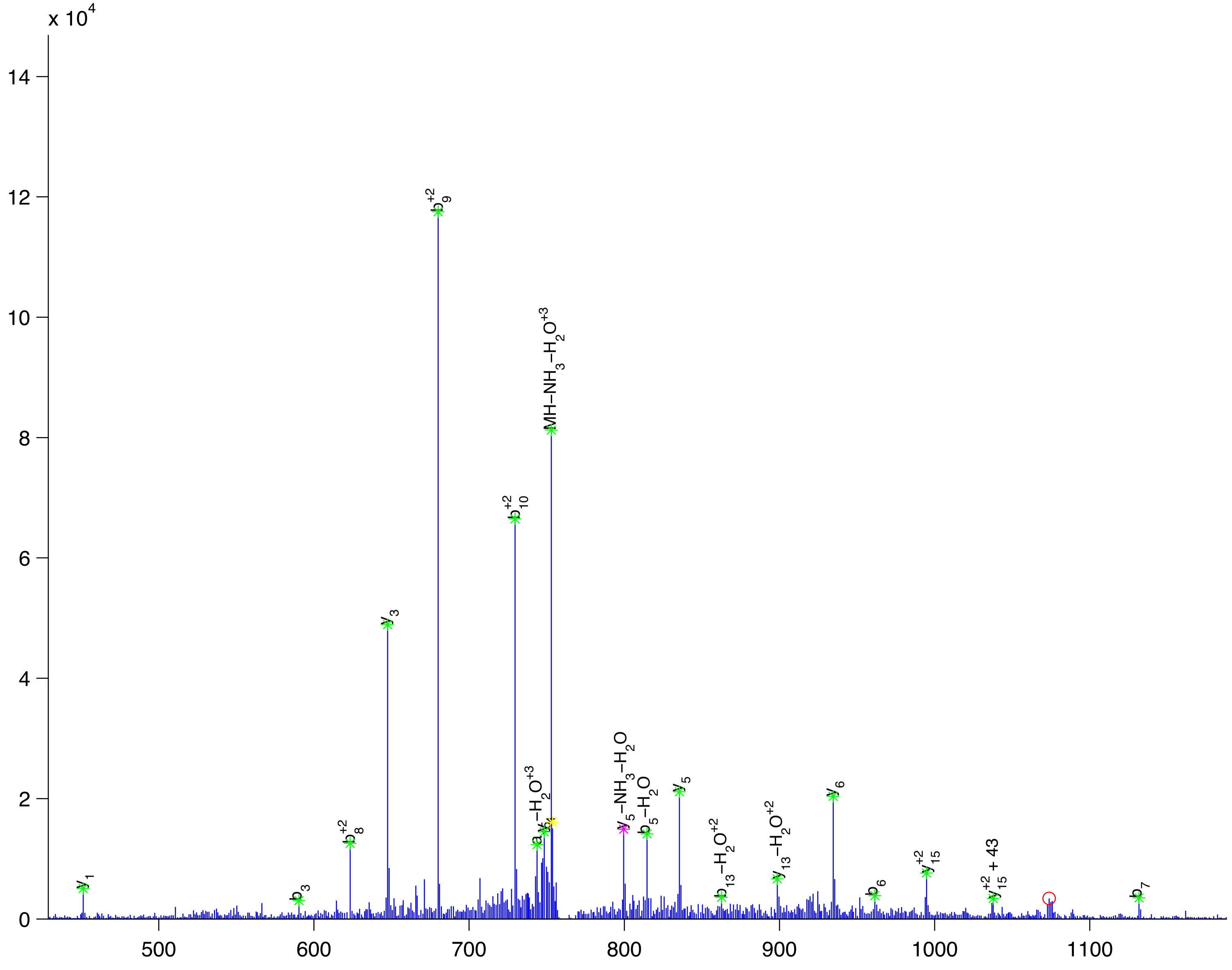

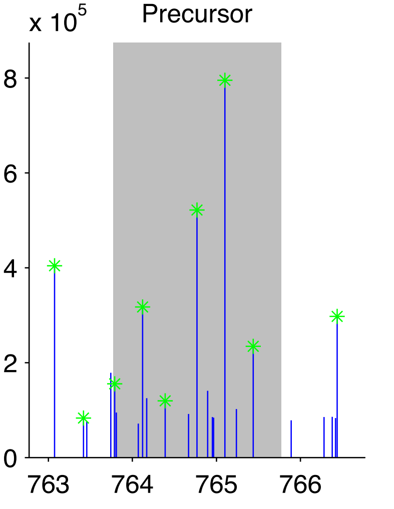

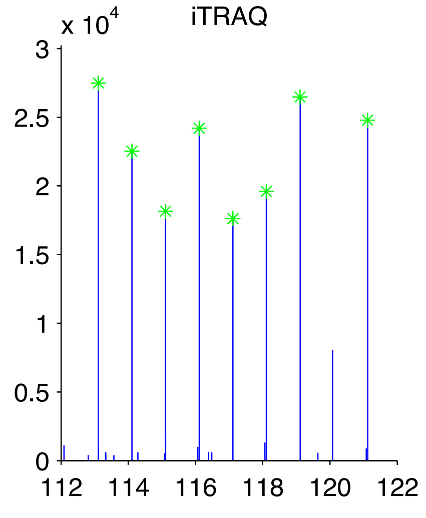

k k N N k k T N k N k S S I S R

CREB binding protein isoform b [Homo sapiens]
Charge State: +3
Scan Number: 11059
File Name: 120501_A549_TSA_AcK.raw

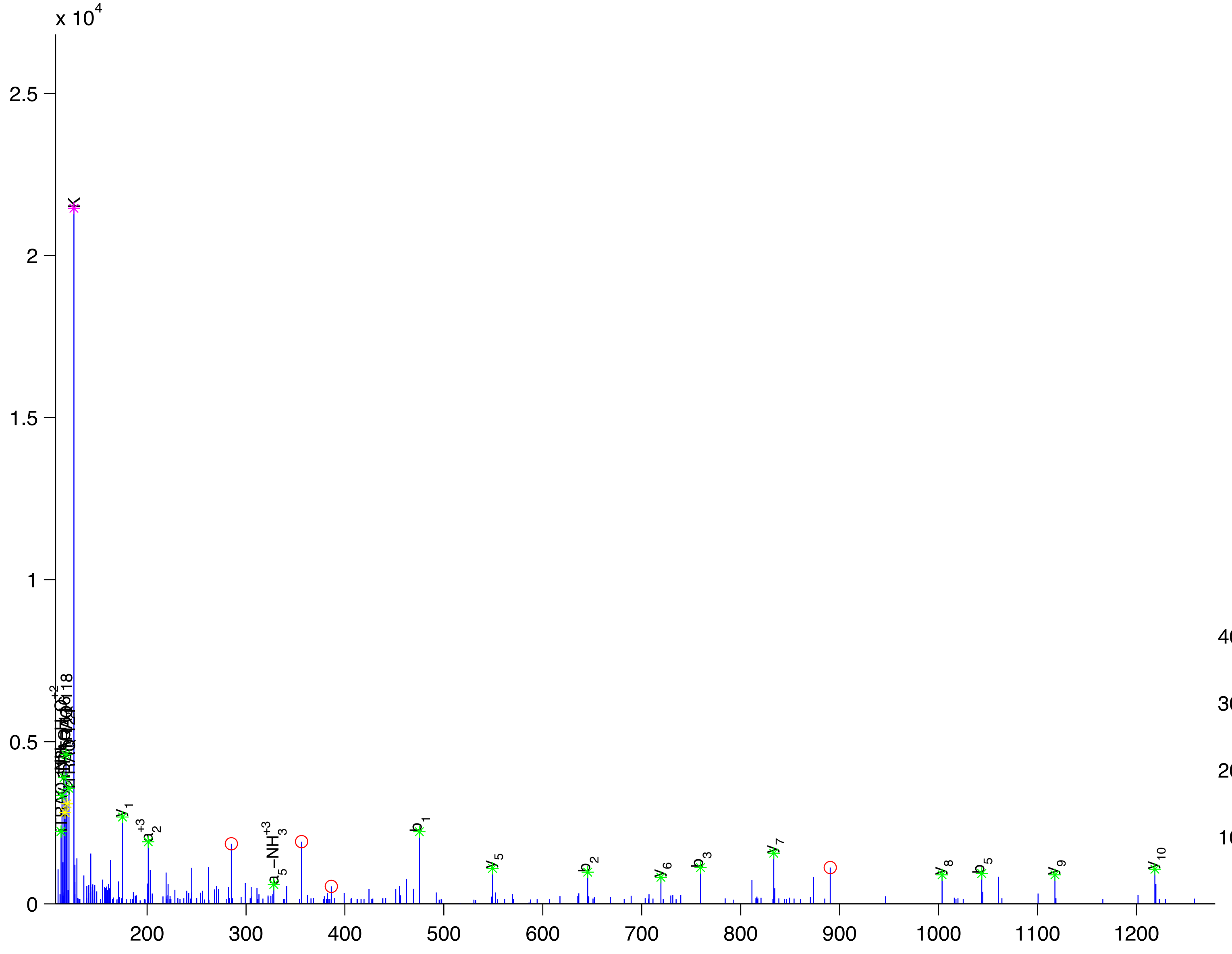

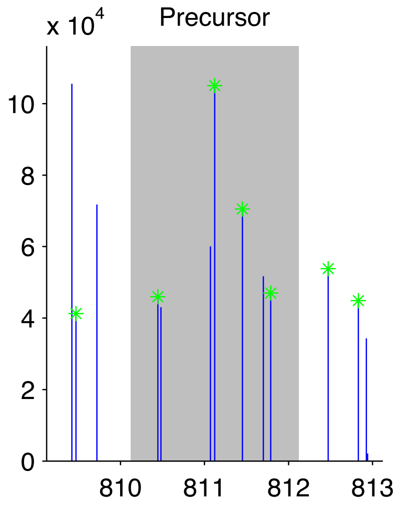

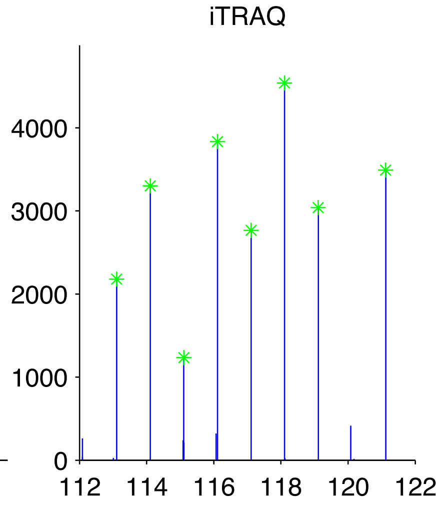

T V D G P S G k L W R

glyceraldehyde–3–phosphate dehydrogenase [Homo sapiens]
Charge State: +2
Scan Number: 20998
File Name: 120501_A549_TSA_AcK.raw

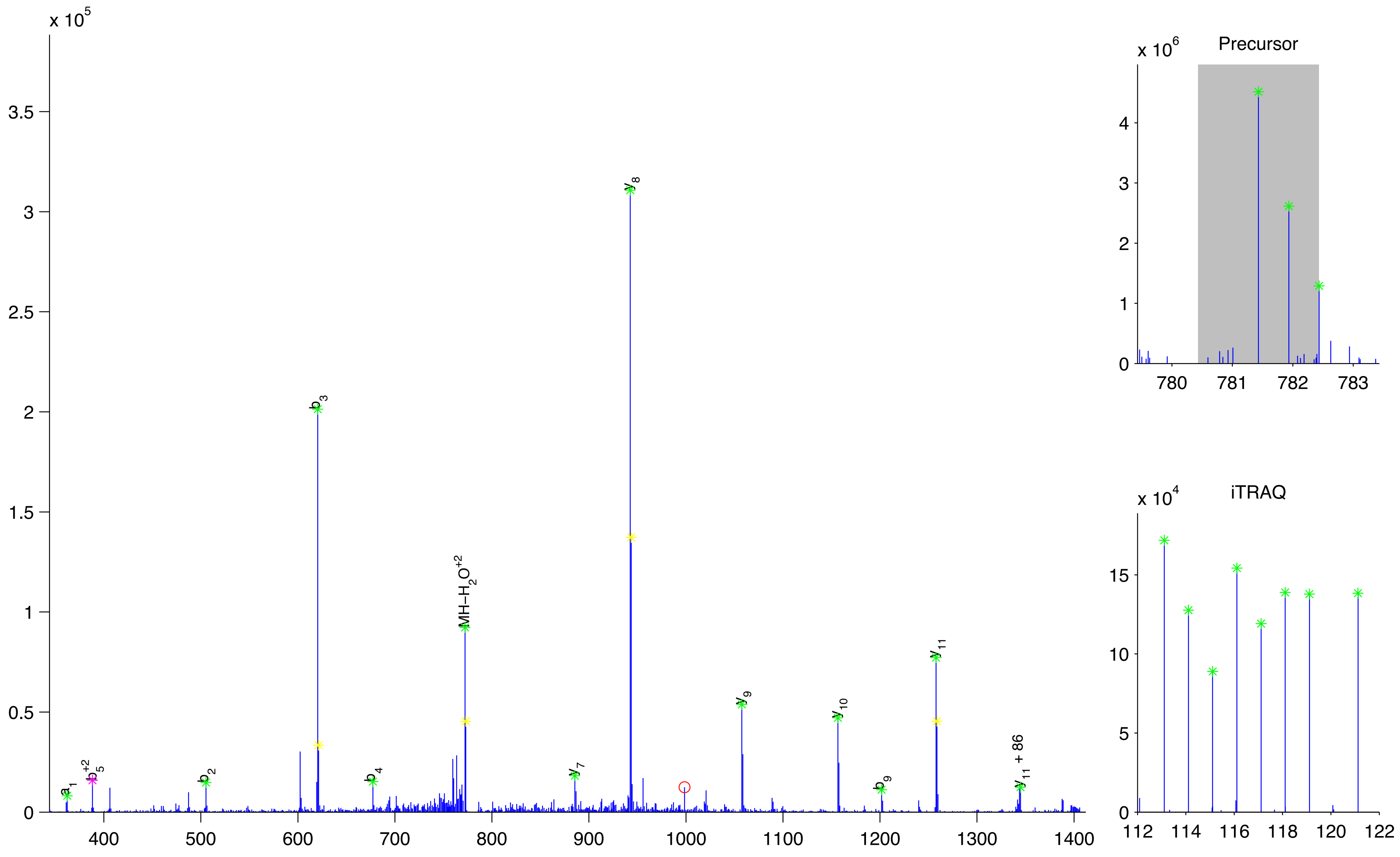

A G G k A G k D S G k A K

H2A histone family, member V isoform 1 [Homo sapiens]
Charge State: +3
Scan Number: 7033
File Name: 120501_A549_TSA_AcK.raw

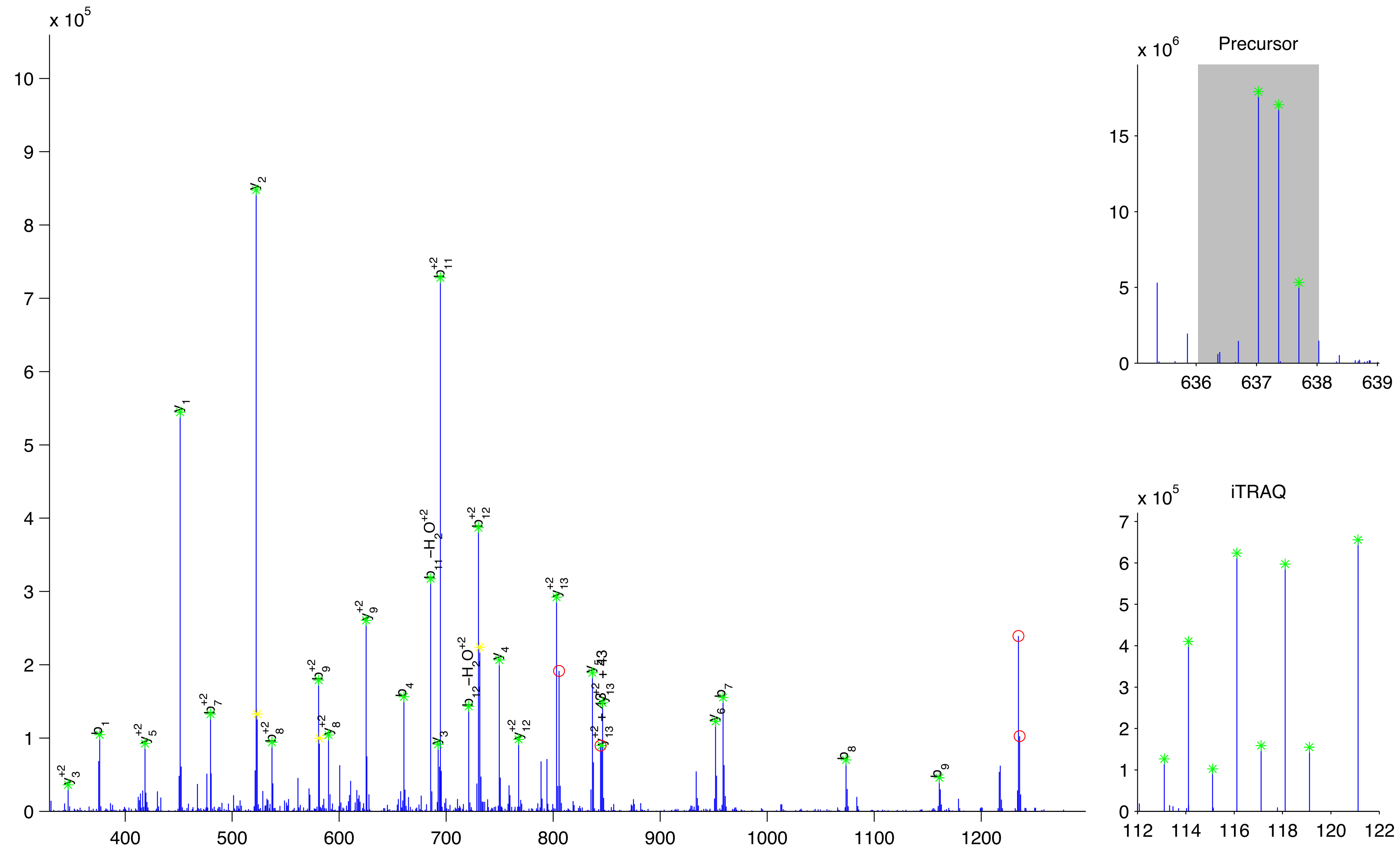

A G G k A G k D S G k A K

H2A histone family, member V isoform 1 [Homo sapiens]
Charge State: +2
Scan Number: 7289
File Name: 120501_A549_TSA_AcK.raw

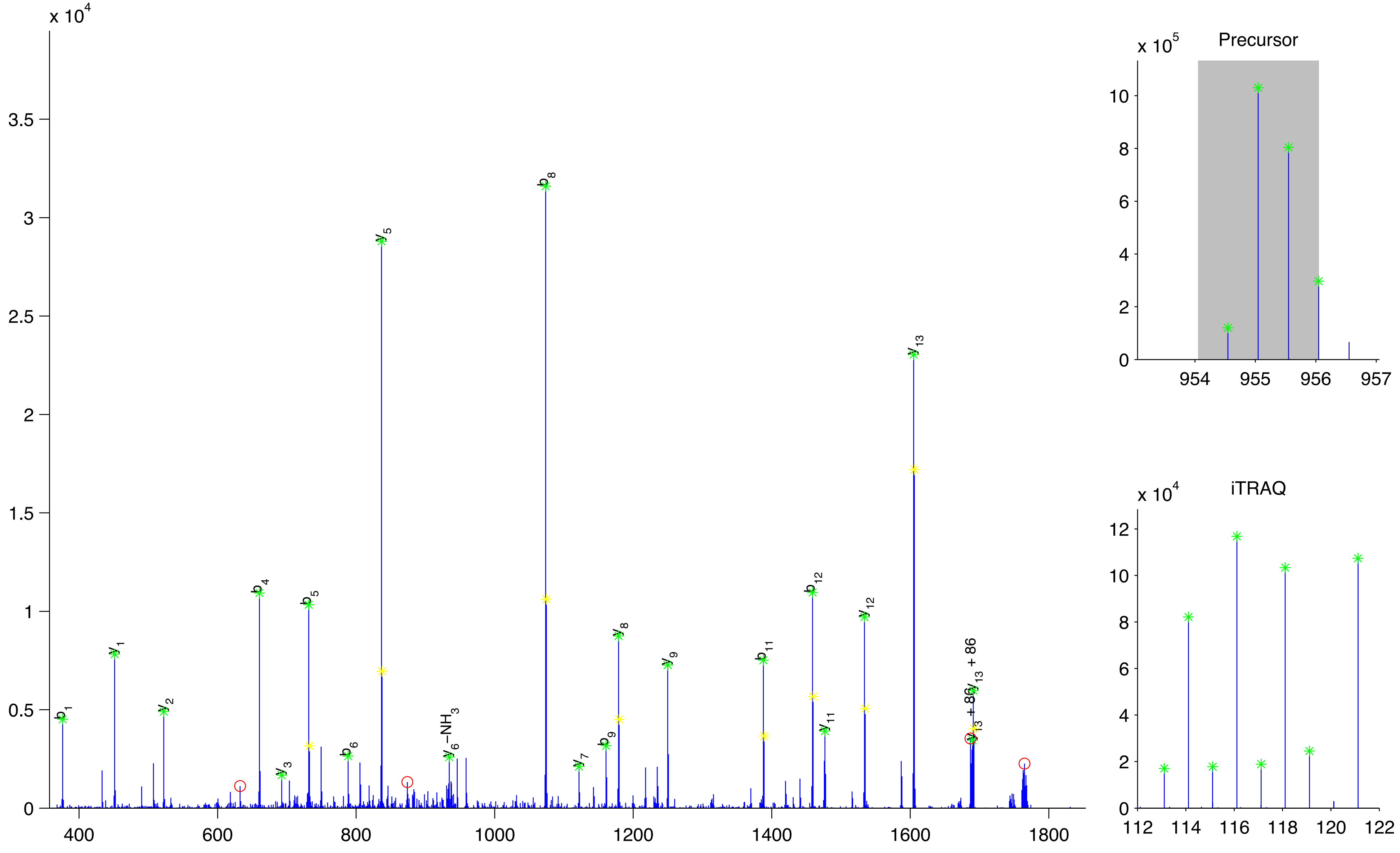

A G G k A G k D S G k A K

H2A histone family, member V isoform 1 [Homo sapiens]
Charge State: +4
Scan Number: 7432
File Name: 120501_A549_TSA_AcK.raw

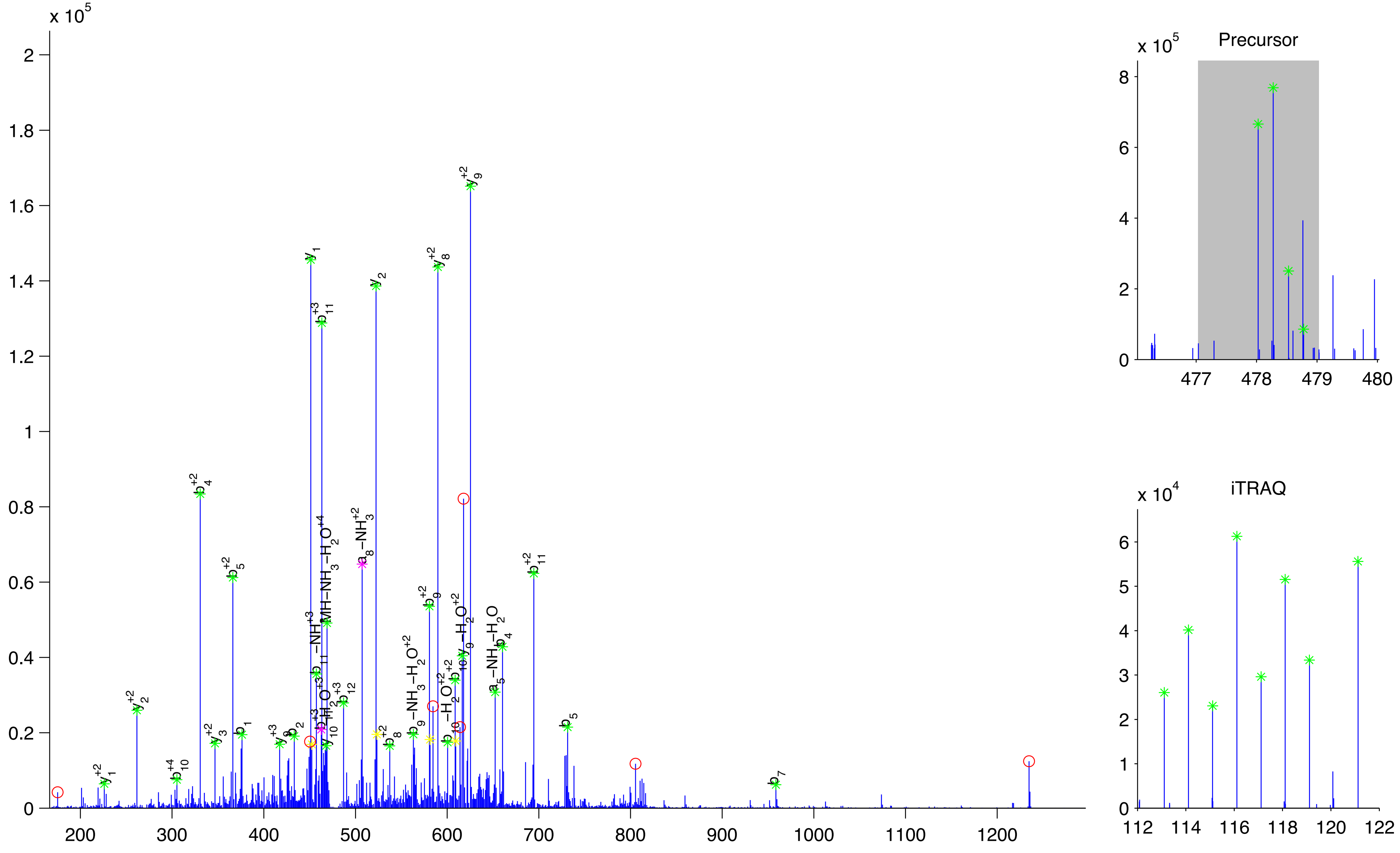

A G G k A G k D S G k A K

H2A histone family, member V isoform 1 [Homo sapiens]
Charge State: +3
Scan Number: 7581
File Name: 120501_A549_TSA_AcK.raw

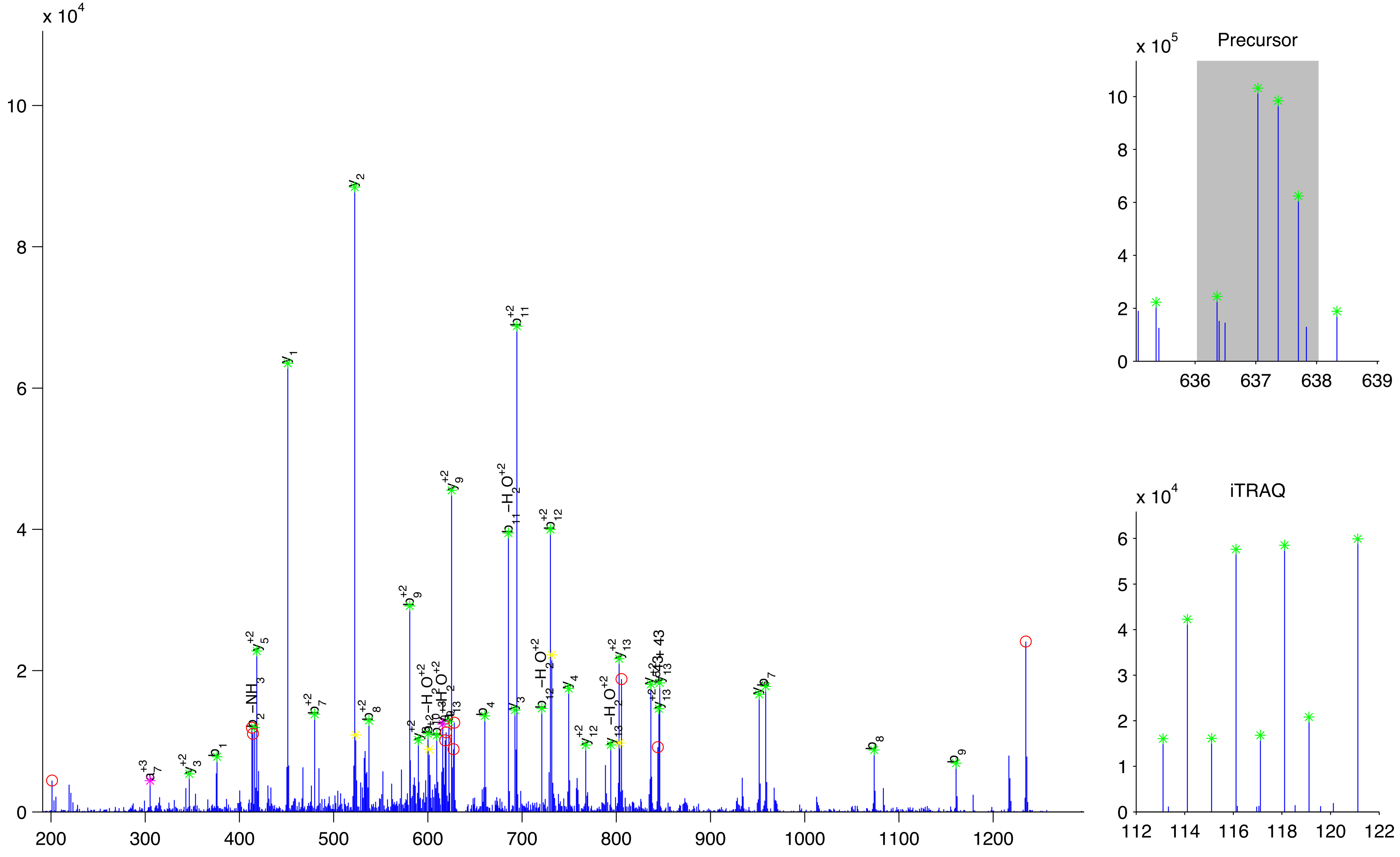

A G G k A G k D S G k A K

H2A histone family, member V isoform 1 [Homo sapiens]
Charge State: +3
Scan Number: 8129
File Name: 120501_A549_TSA_AcK.raw

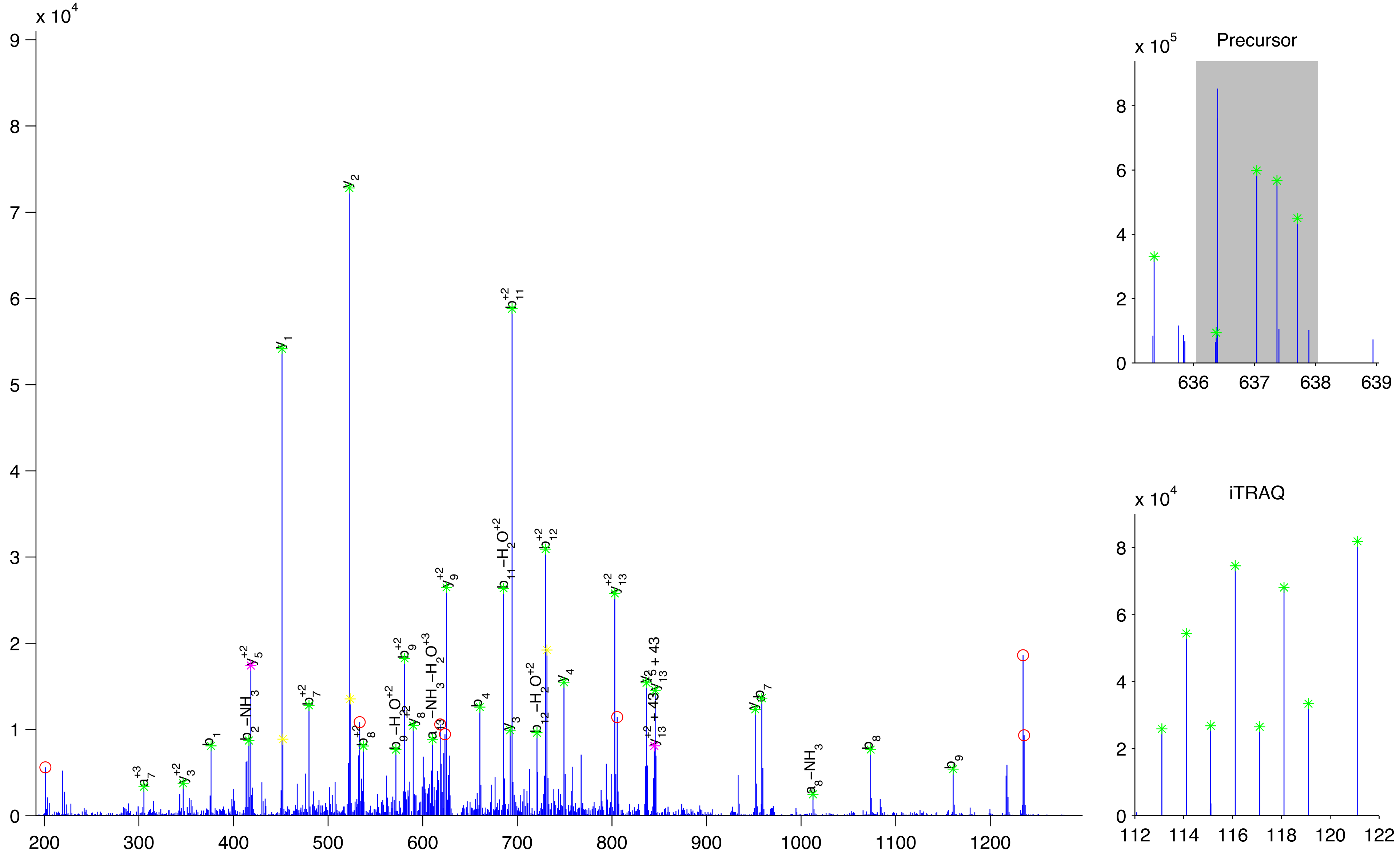

A G G k A G k D S G k A K

H2A histone family, member Z [Homo sapiens]
Charge State: +3
Scan Number: 7033
File Name: 120501_A549_TSA_AcK.raw

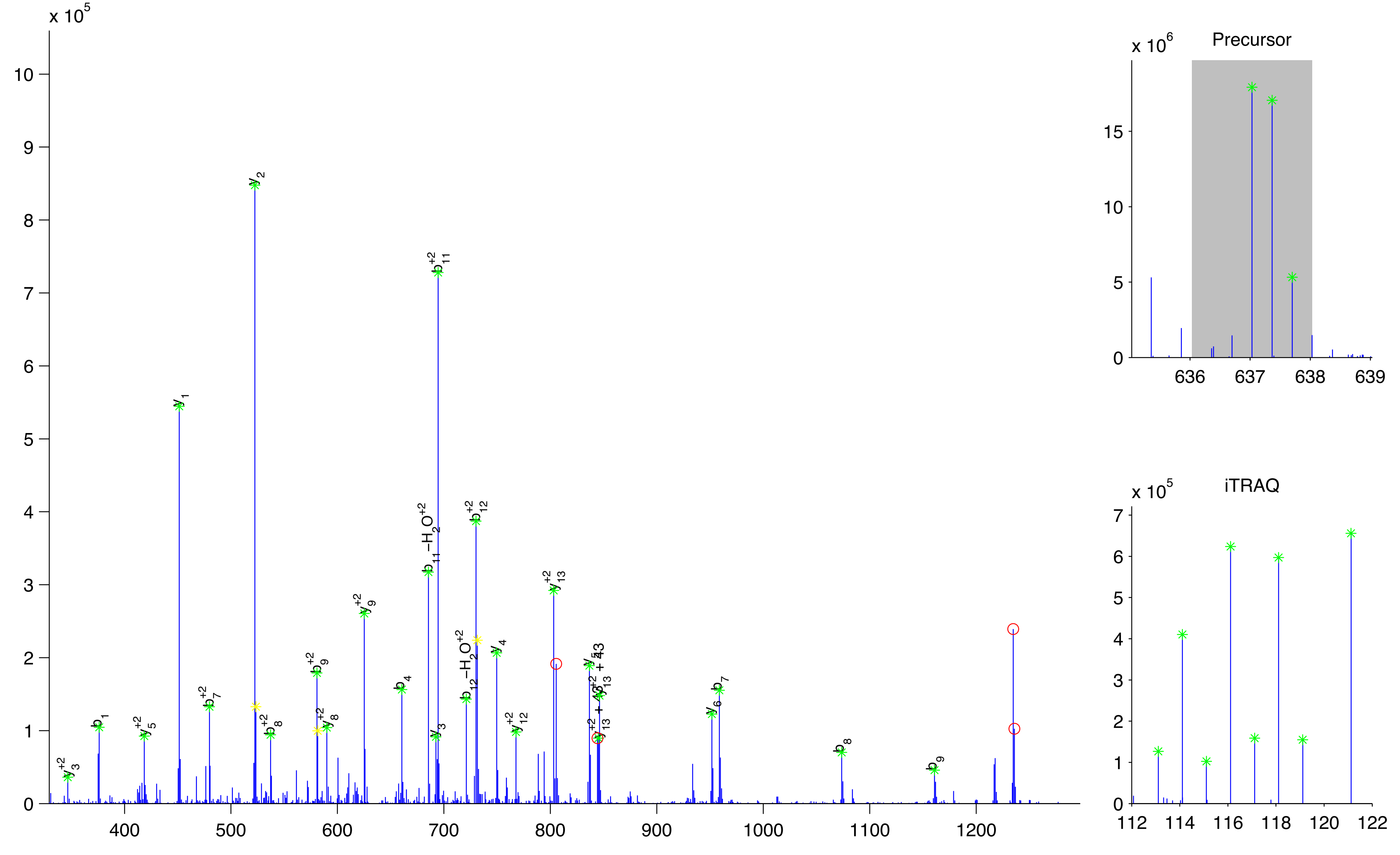

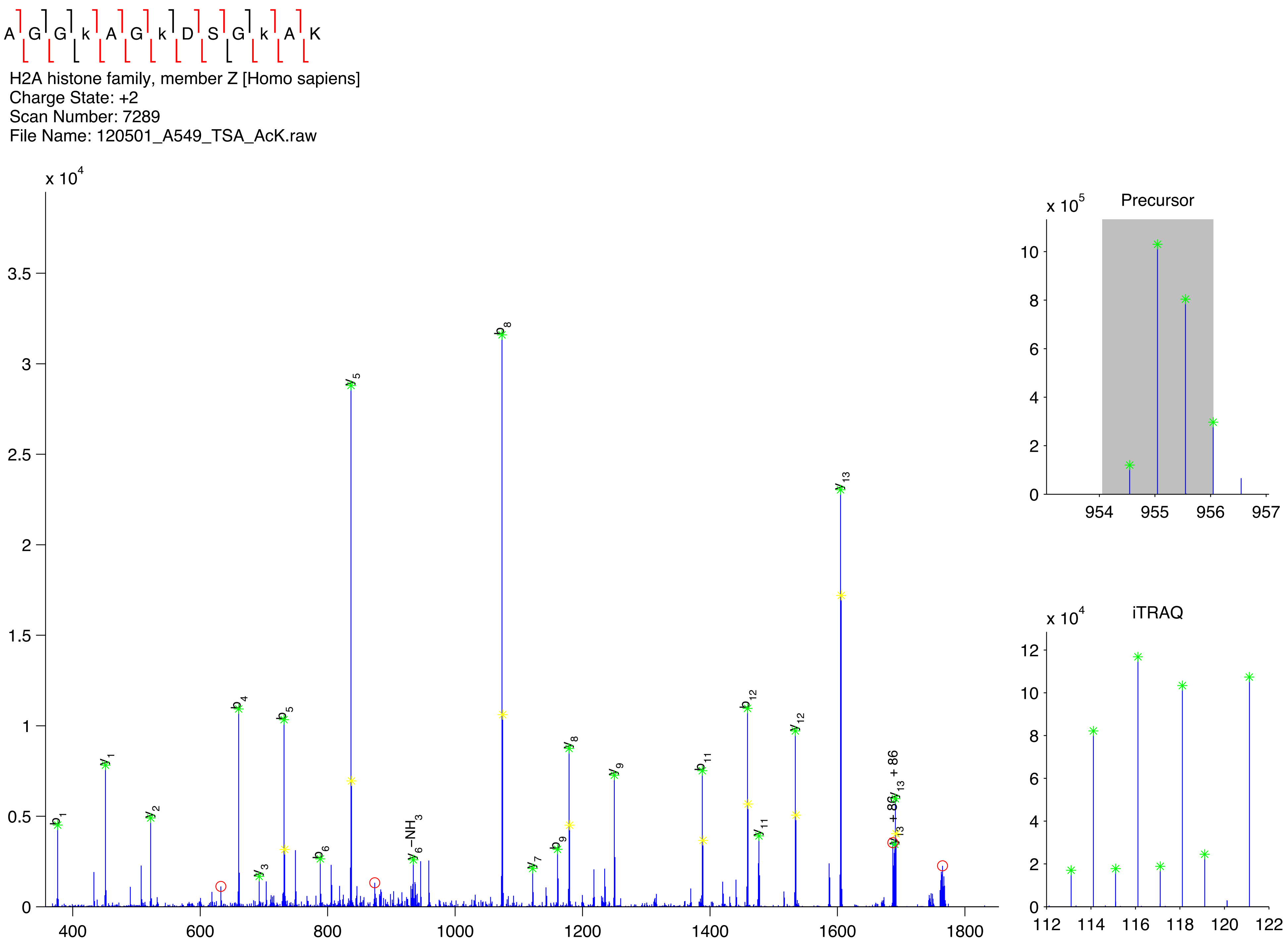

A G G k A G k D S G k A K
H2A histone family, member Z [Homo sapiens]
Charge State: +2
Scan Number: 7289
File Name: 120501_A549_TSA_AcK.raw
x 10^4
Precursor
x 10^5
iTRAQ
x 10^4

A G G k A G k D S G k A K

H2A histone family, member Z [Homo sapiens]
Charge State: +4
Scan Number: 7432
File Name: 120501_A549_TSA_AcK.raw

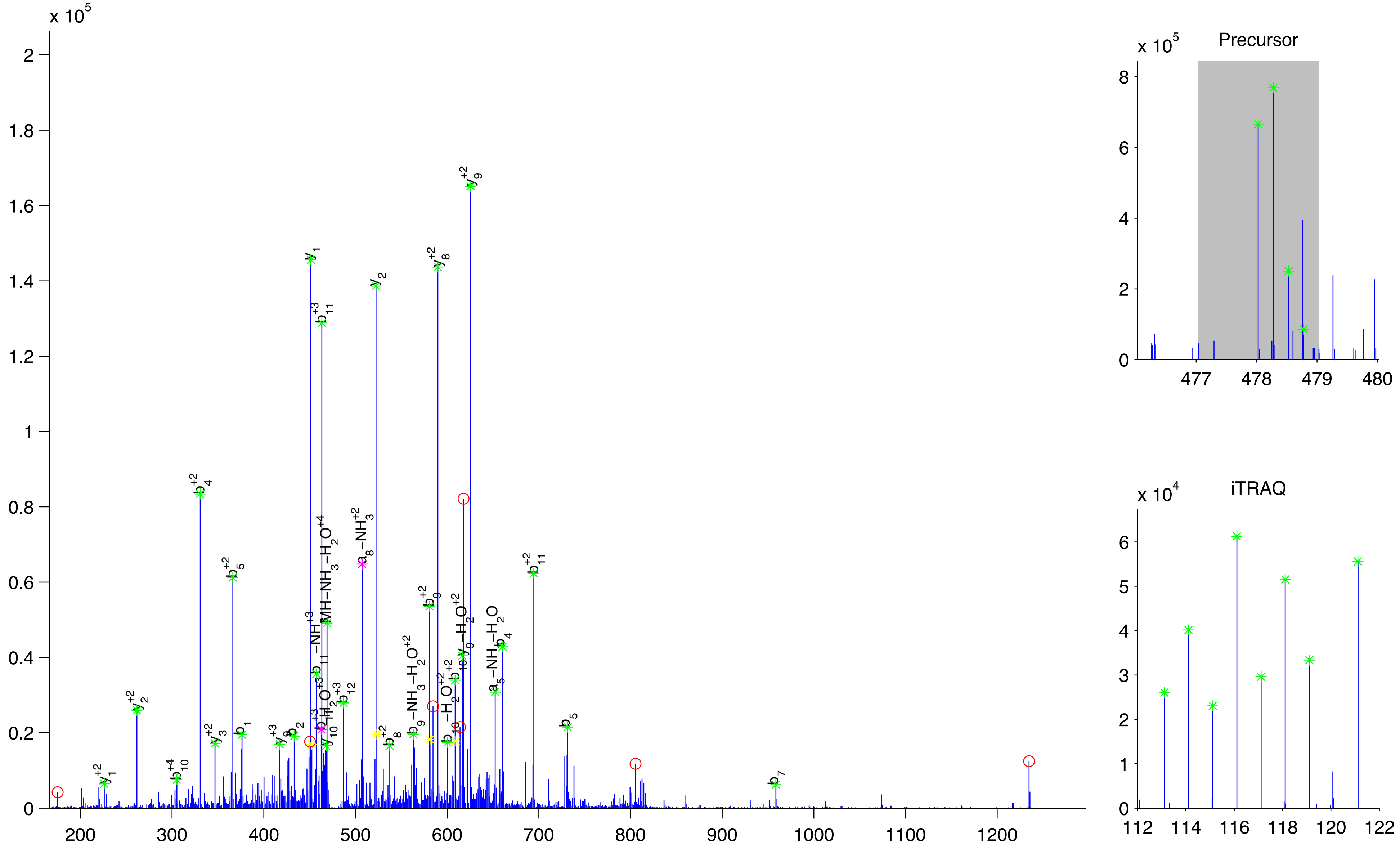

A G G k A G k D S G k A K

H2A histone family, member Z [Homo sapiens]
Charge State: +3
Scan Number: 7581
File Name: 120501_A549_TSA_AcK.raw

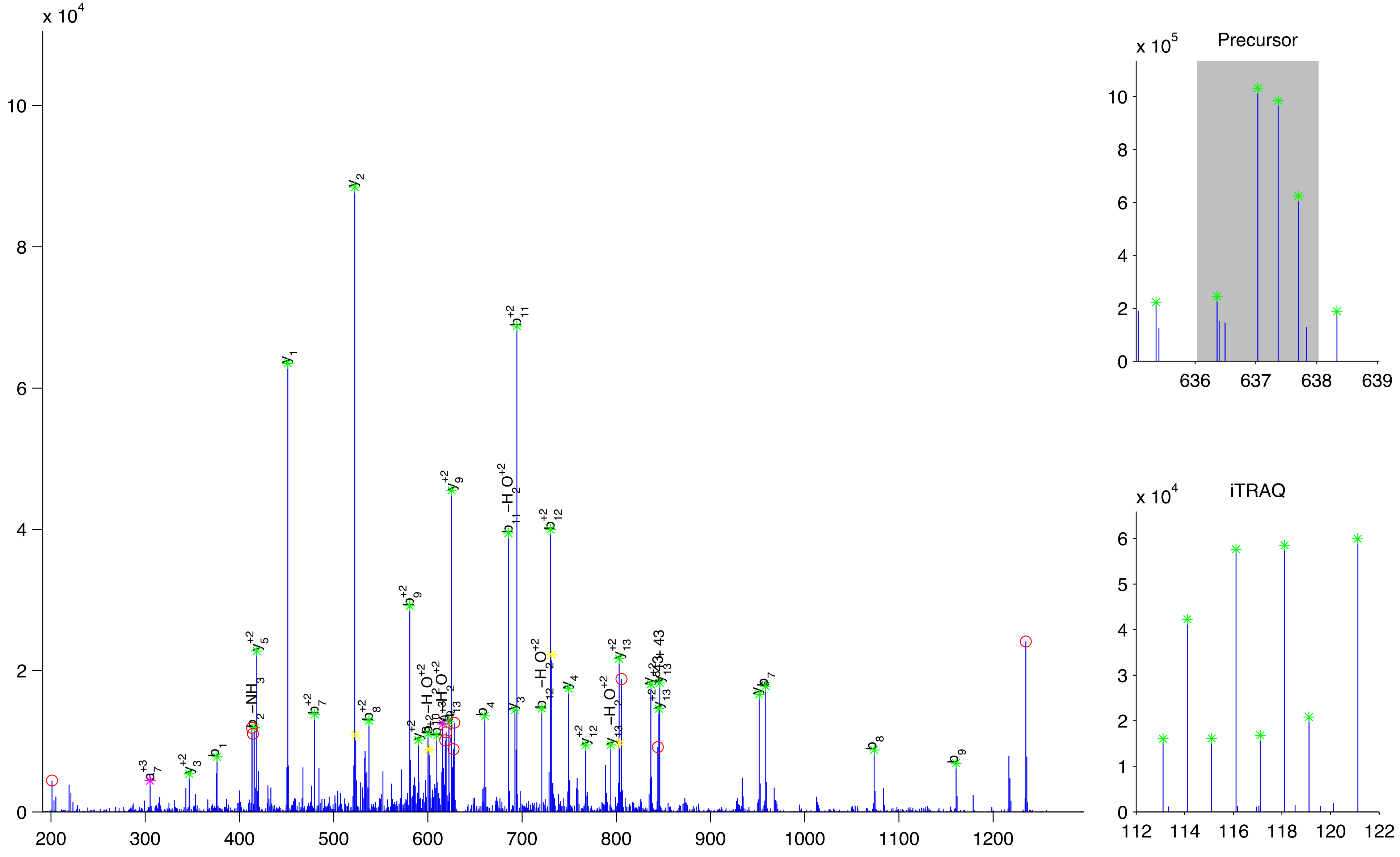

A G G k A G k D S G k A K

H2A histone family, member Z [Homo sapiens]
Charge State: +3
Scan Number: 8129
File Name: 120501_A549_TSA_AcK.raw

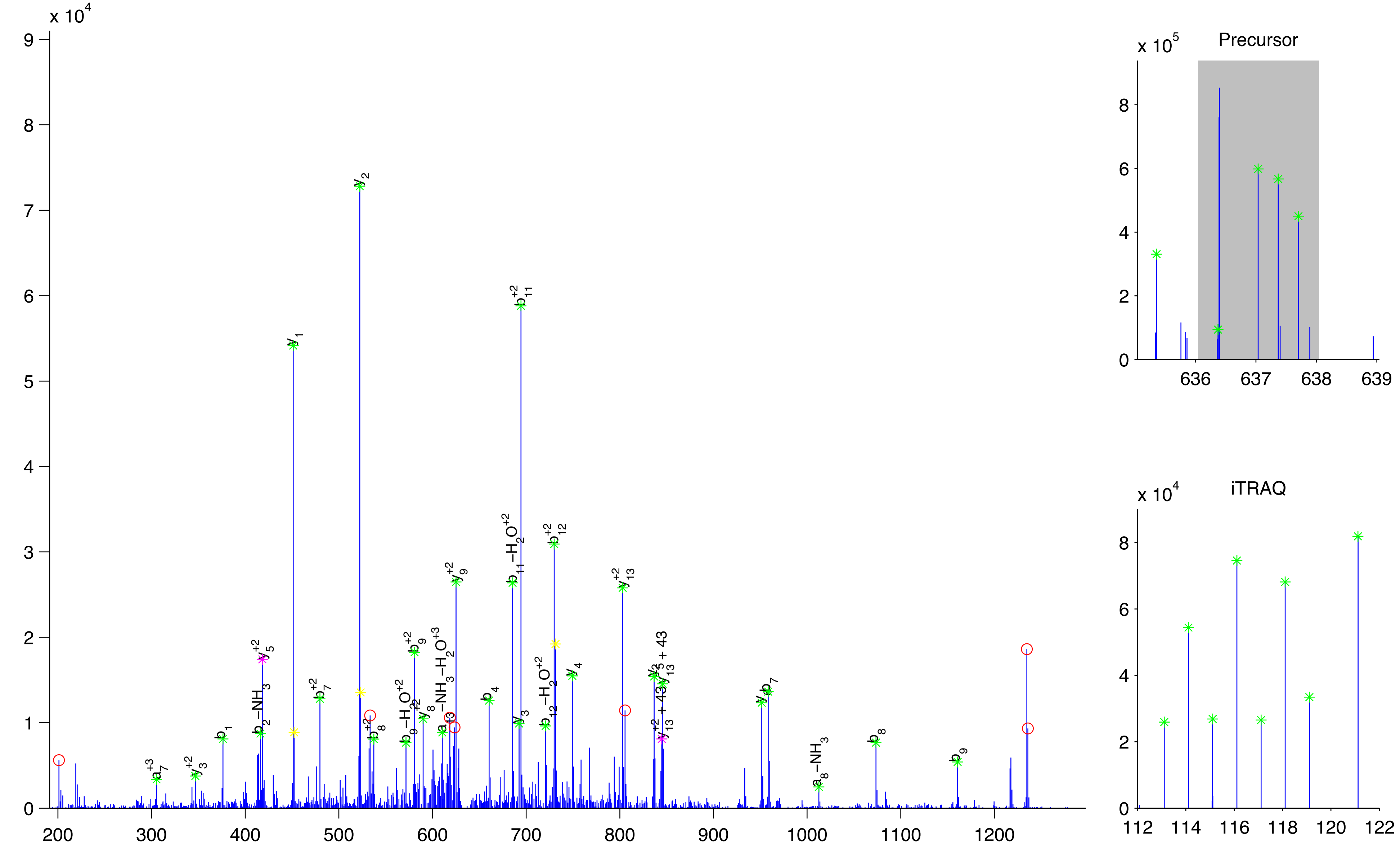

Q L A T k A A R

H3 histone, family 3A [Homo sapiens]
Charge State: +2
Scan Number: 7579
File Name: 120501_A549_TSA_AcK.raw

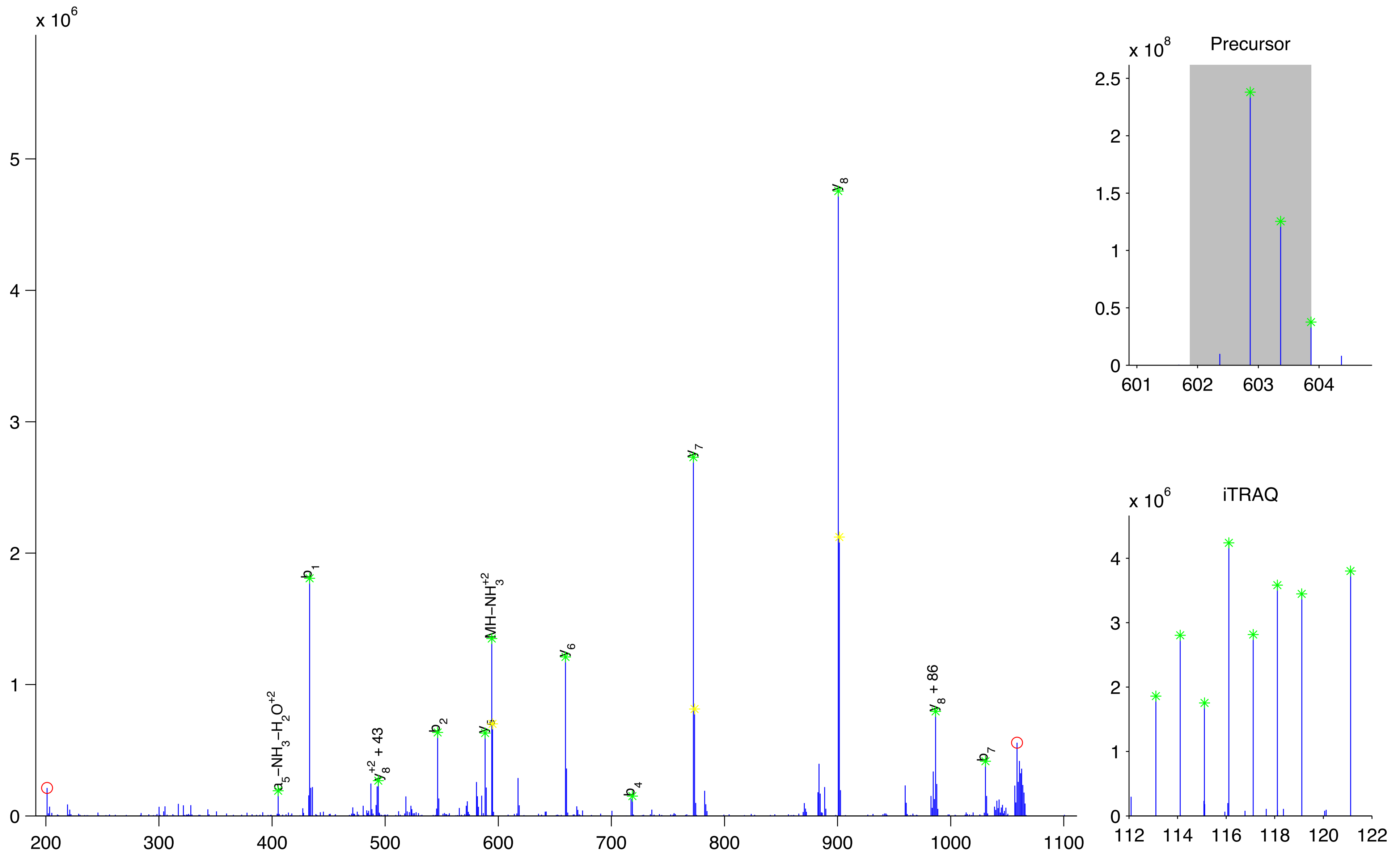

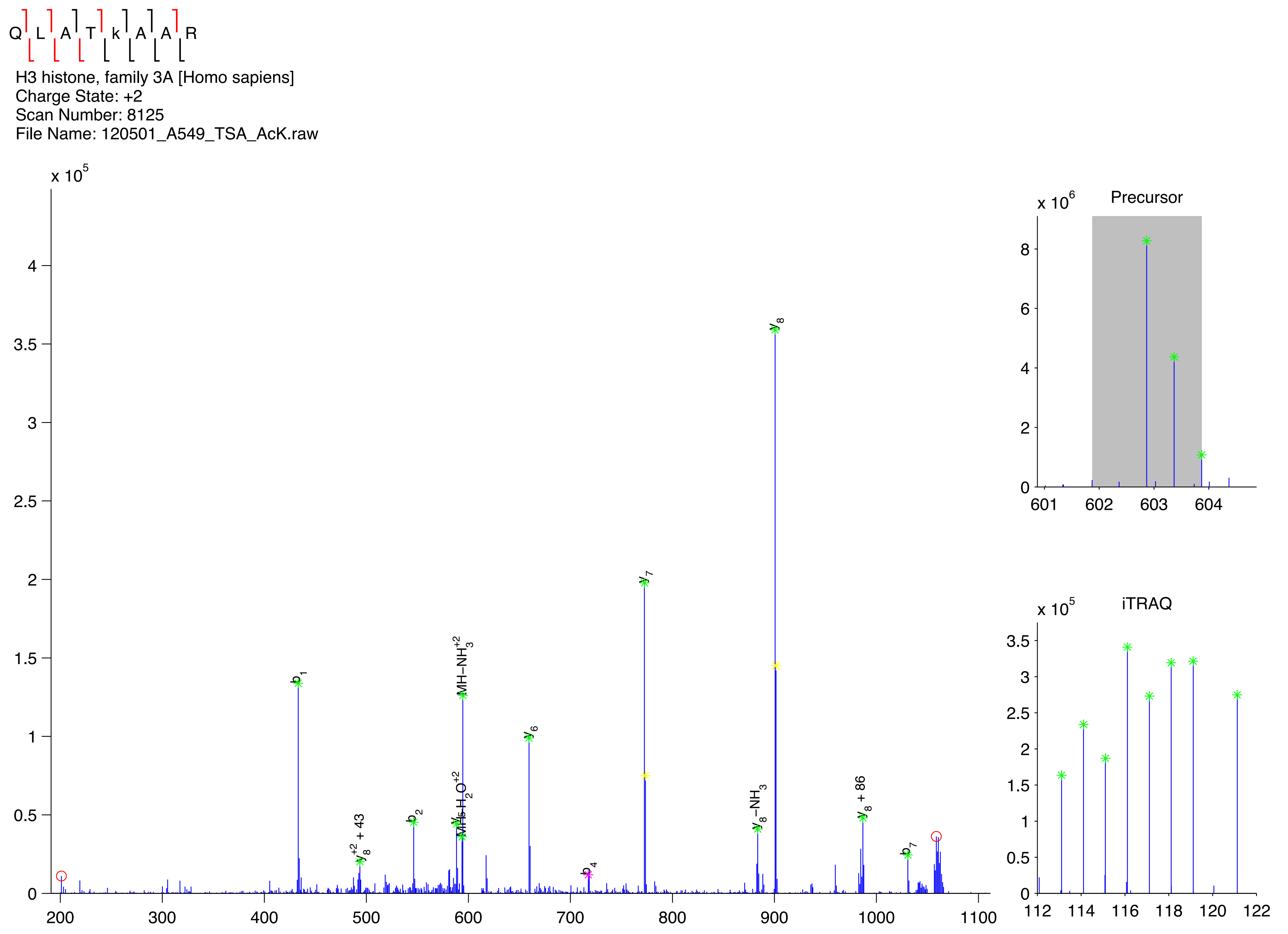

Q L A T k A A R
H3 histone, family 3A [Homo sapiens]
Charge State: +2
Scan Number: 8125
File Name: 120501_A549_TSA_AcK.raw
x 10^5
b1
y8+2 + 43
b2
y5
MH-H2O+2
MH-NH3+2
y6
b4
y7
y8-NH3
y8
y8 + 86
b7
Precursor
x 10^6
iTRAQ
x 10^5

k Q L A T k A A R

H3 histone, family 3A [Homo sapiens]
Charge State: +3
Scan Number: 12451
File Name: 120501_A549_TSA_AcK.raw

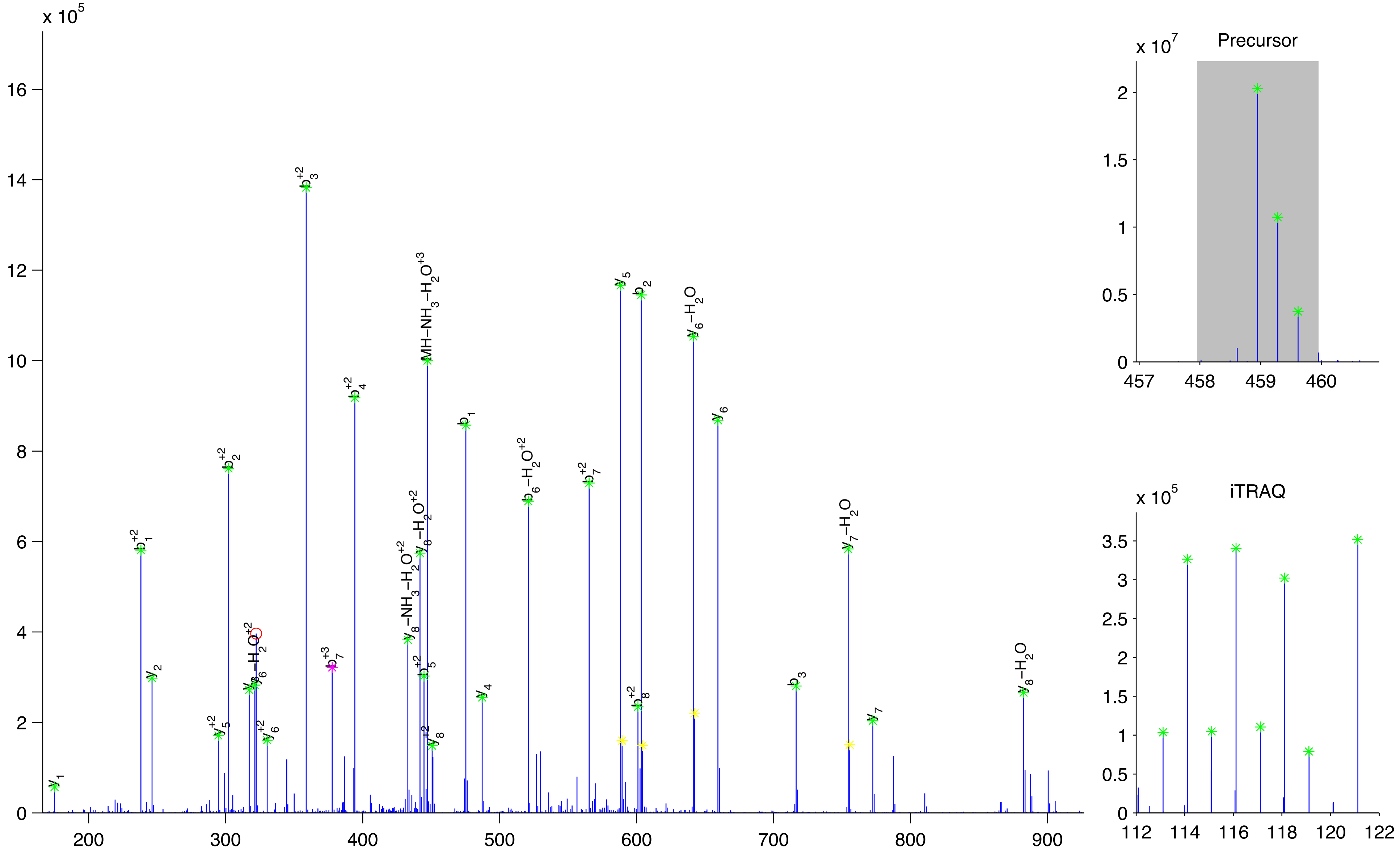

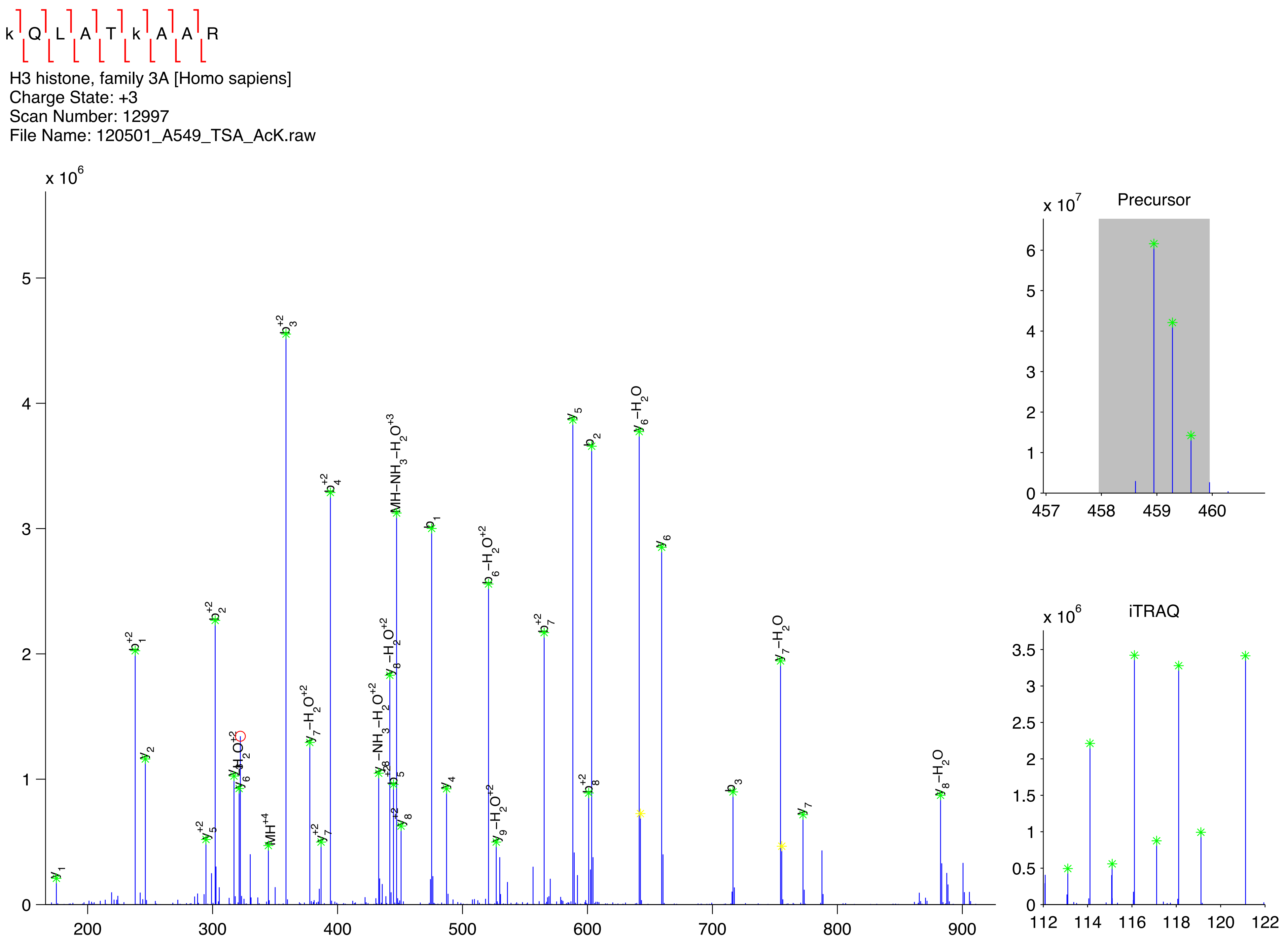
k Q L A T k A A R
H3 histone, family 3A [Homo sapiens]
Charge State: +3
Scan Number: 12997
File Name: 120501_A549_TSA_AcK.raw
x 10^6
Precursor
x 10^7
iTRAQ
x 10^6
MH–NH3–H2O+3
MH+4
y6–H2O
y7–H2O
y8–H2O
b6–H2O+2
y9–H2O+2
y7–H2O+2
y8–H2O+2

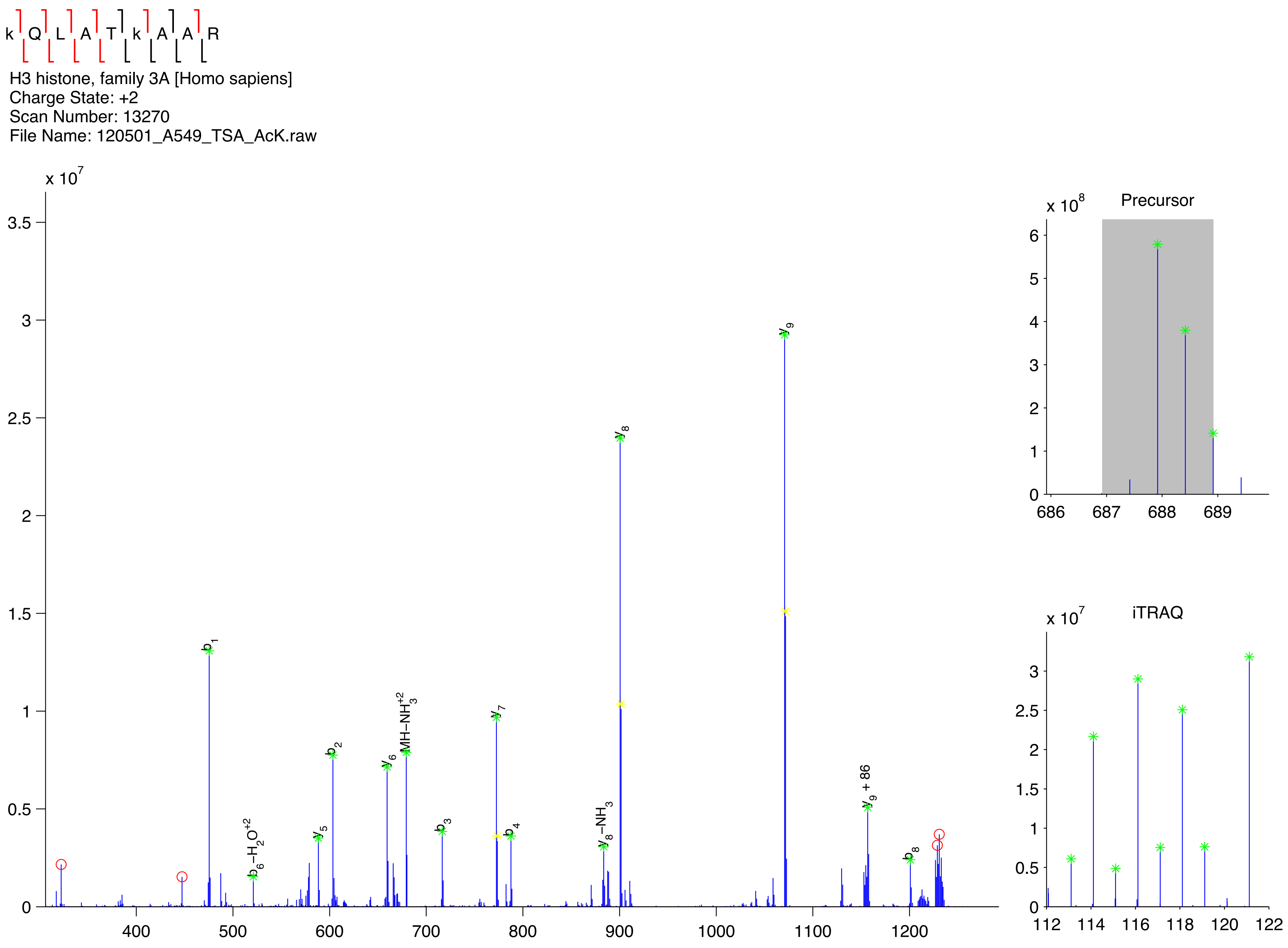

k Q L A T k A A R
H3 histone, family 3A [Homo sapiens]
Charge State: +2
Scan Number: 13270
File Name: 120501_A549_TSA_AcK.raw
x 10^7
b1
b6−H2O+2
y5
b2
y6
MH−NH3+2
b3
y7
b4
y8−NH3
y8
y9
y9 + 86
b8
Precursor
x 10^8
iTRAQ
x 10^7

k Q L A T k A A R

H3 histone, family 3A [Homo sapiens]
Charge State: +1
Scan Number: 13523
File Name: 120501_A549_TSA_AcK.raw

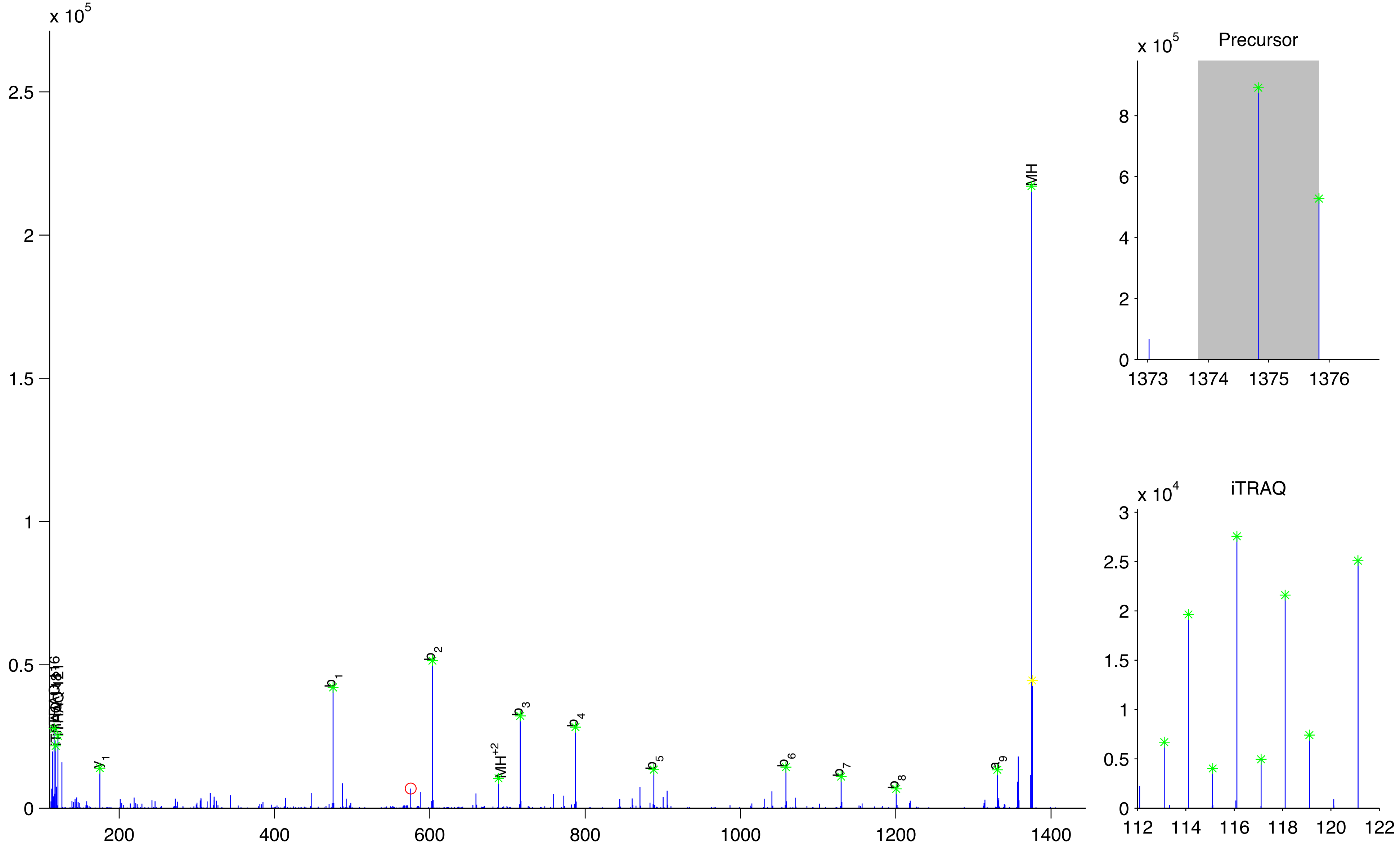

k Q L A T k A A R

H3 histone, family 3A [Homo sapiens]
Charge State: +3
Scan Number: 13543
File Name: 120501_A549_TSA_AcK.raw

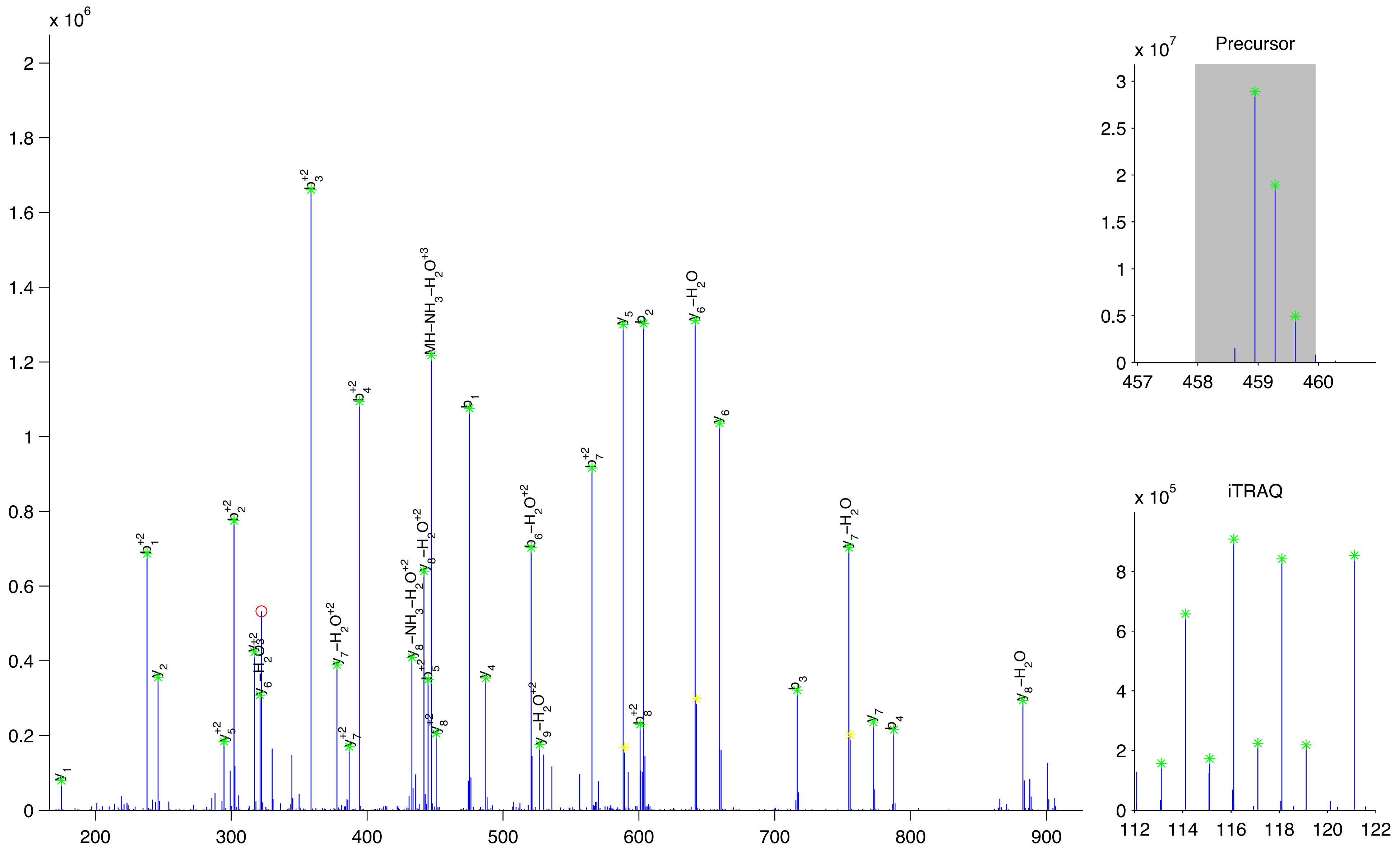

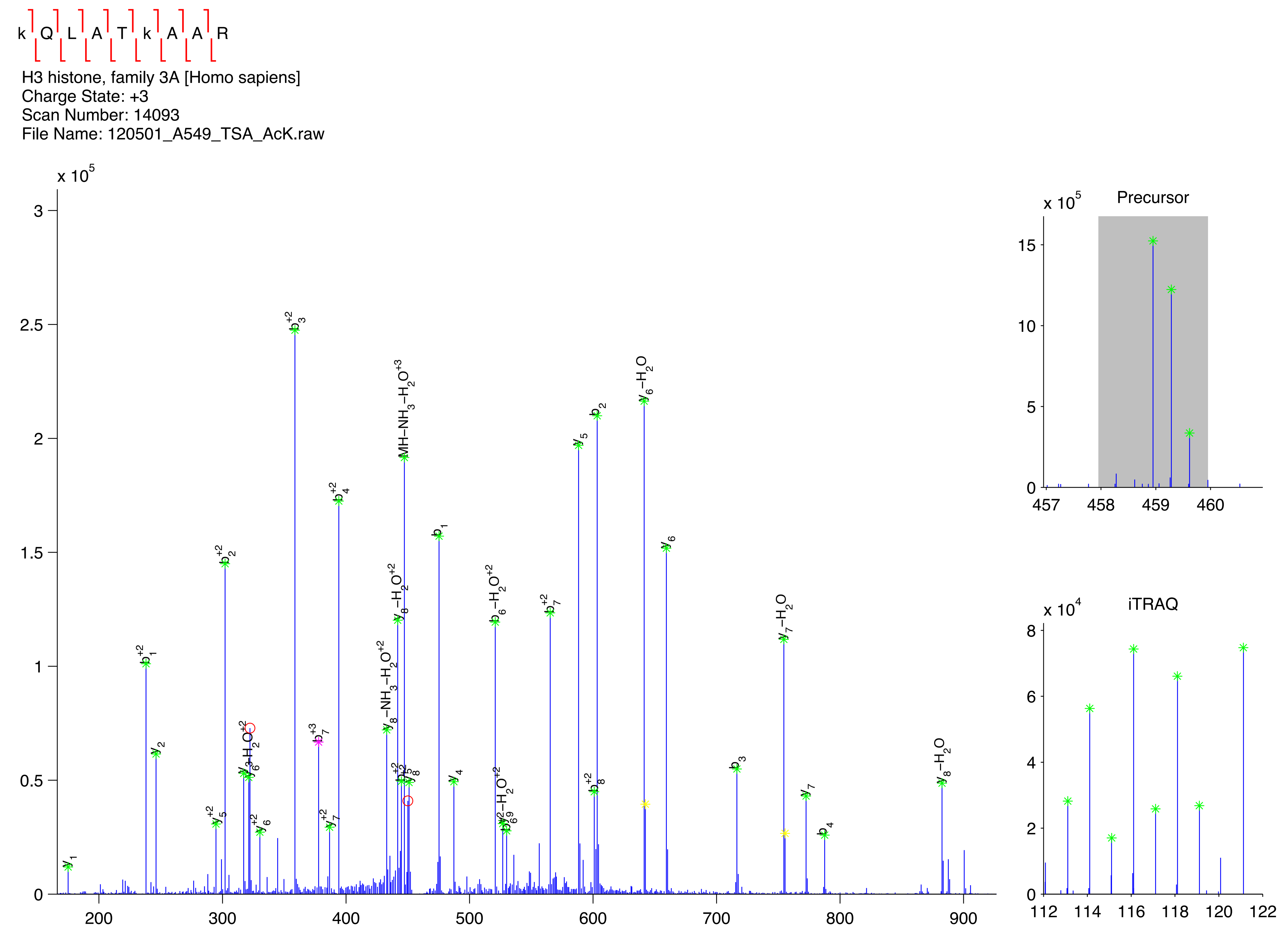
k Q L A T k A A R
H3 histone, family 3A [Homo sapiens]
Charge State: +3
Scan Number: 14093
File Name: 120501_A549_TSA_AcK.raw
Precursor
iTRAQ

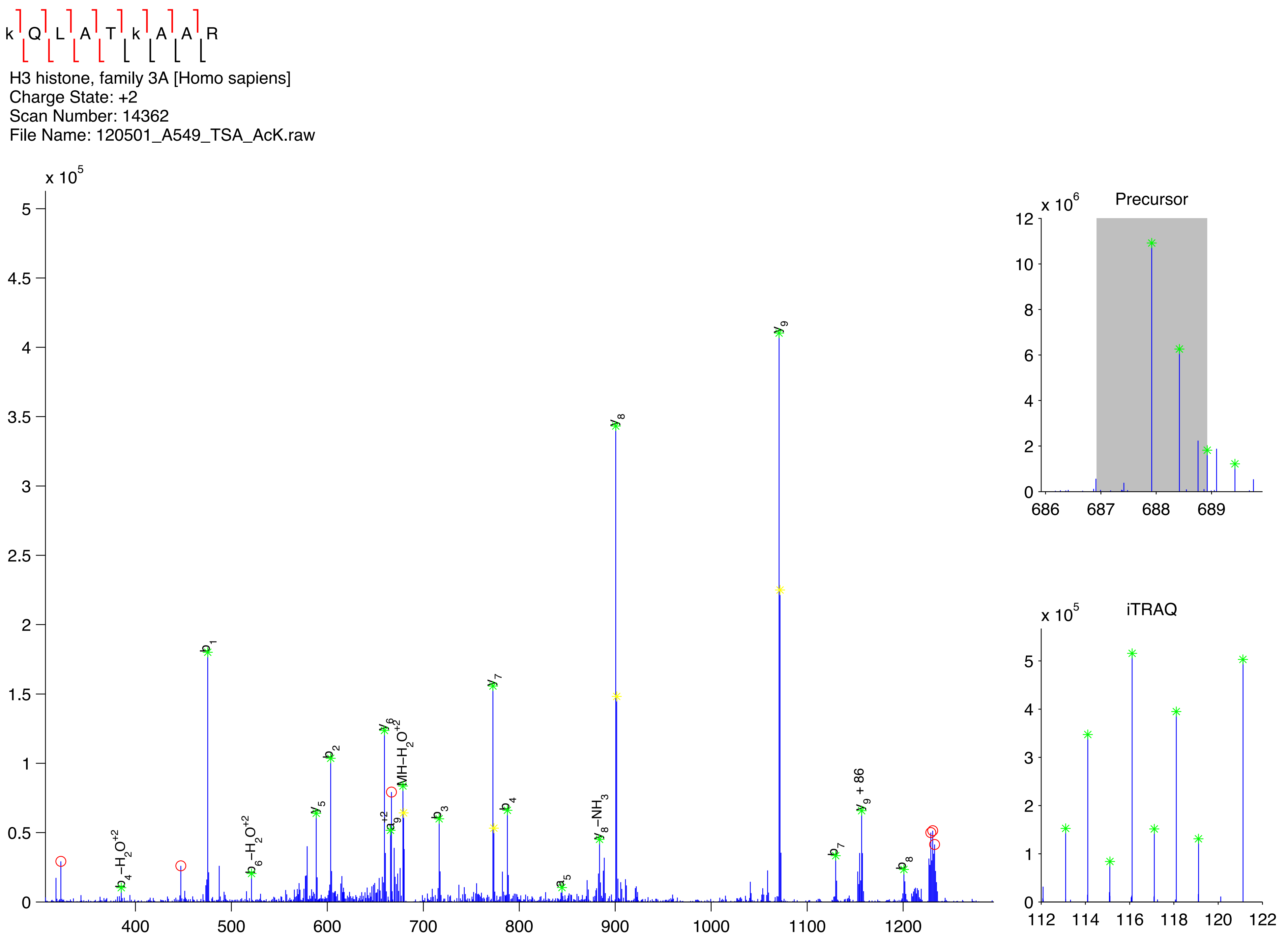

k Q L A T k A A R
H3 histone, family 3A [Homo sapiens]
Charge State: +2
Scan Number: 14362
File Name: 120501_A549_TSA_AcK.raw
x 10^5
b_4–H_2O^{+2}
b_1
b_6–H_2O^{+2}
y_5
b_2
y_6
a_9^{+2}
MH–H_2O^{+2}
b_3
y_7
b_4
a_5
y_8–NH_3
y_8
y_9
b_7
y_9 + 86
b_8
Precursor
x 10^6
iTRAQ
x 10^5

k Q L A T k A A R

H3 histone, family 3A [Homo sapiens]
Charge State: +2
Scan Number: 35519
File Name: 120501_A549_TSA_AcK.raw

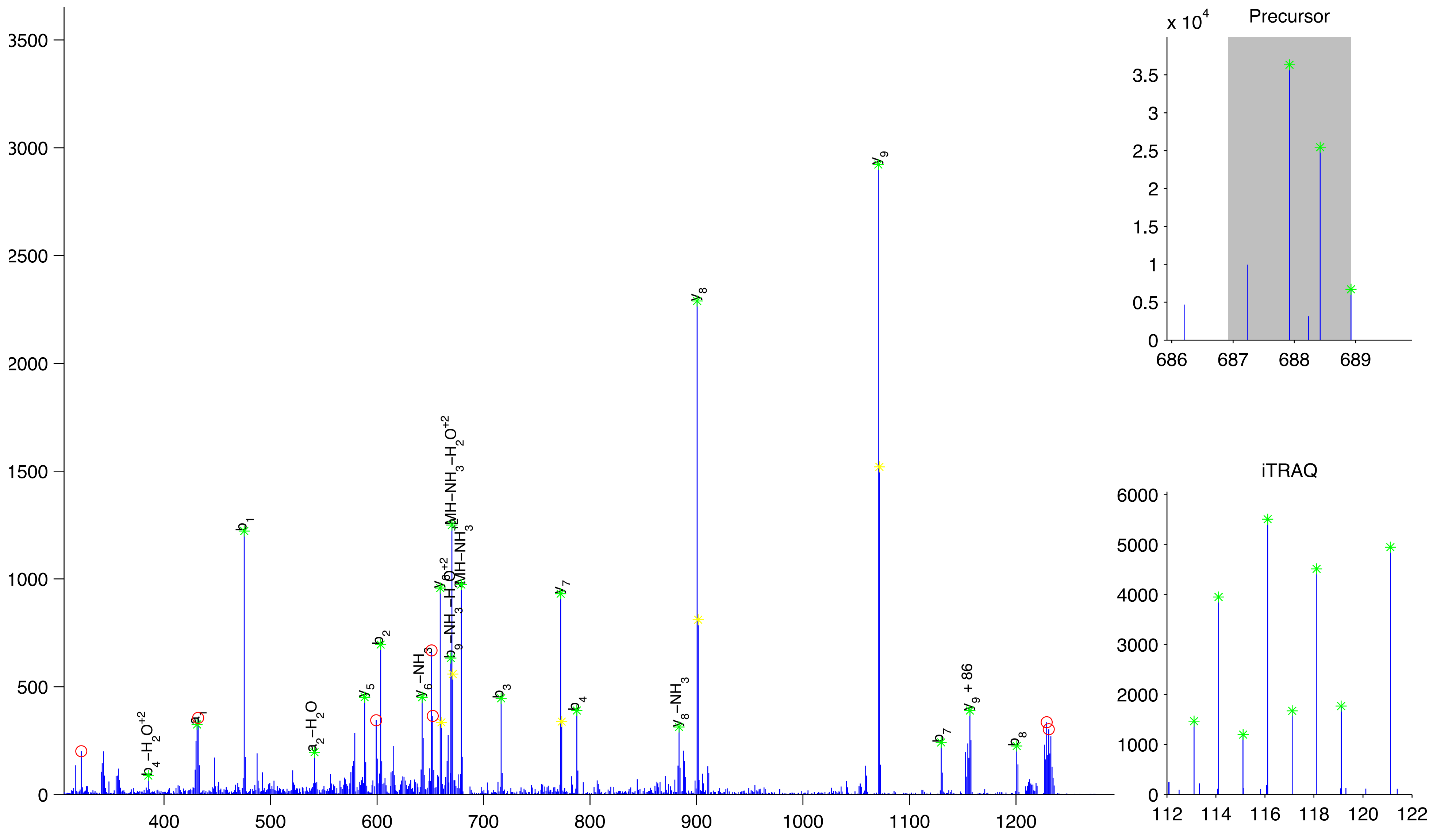

k S T G G k A P R

H3 histone, family 3A [Homo sapiens]
Charge State: +2
Scan Number: 35908
File Name: 120501_A549_TSA_AcK.raw

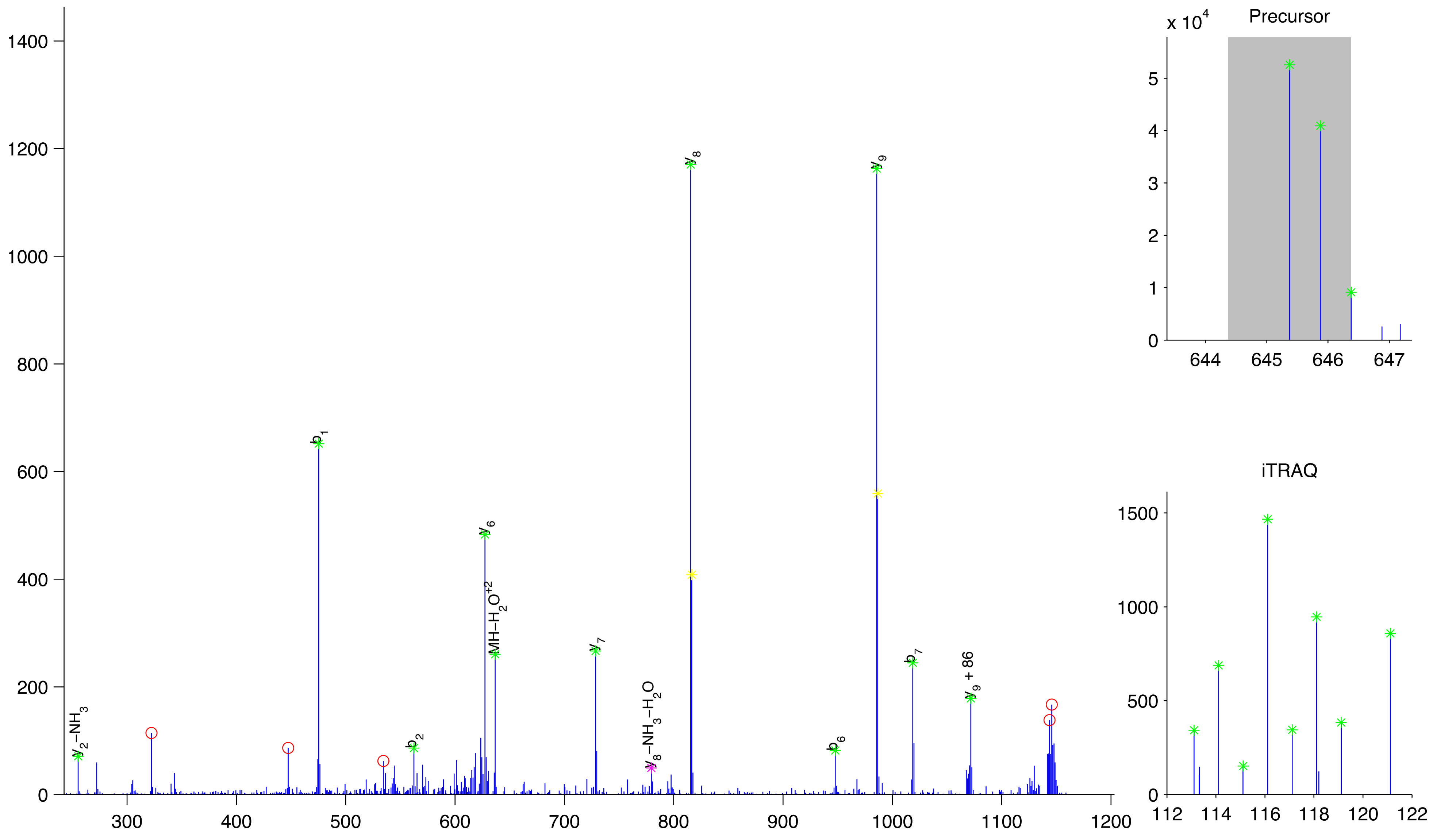

S A P A P k k G S k K

histone cluster 1, H2bb [Homo sapiens]
Charge State: +2
Scan Number: 7436
File Name: 120501_A549_TSA_AcK.raw

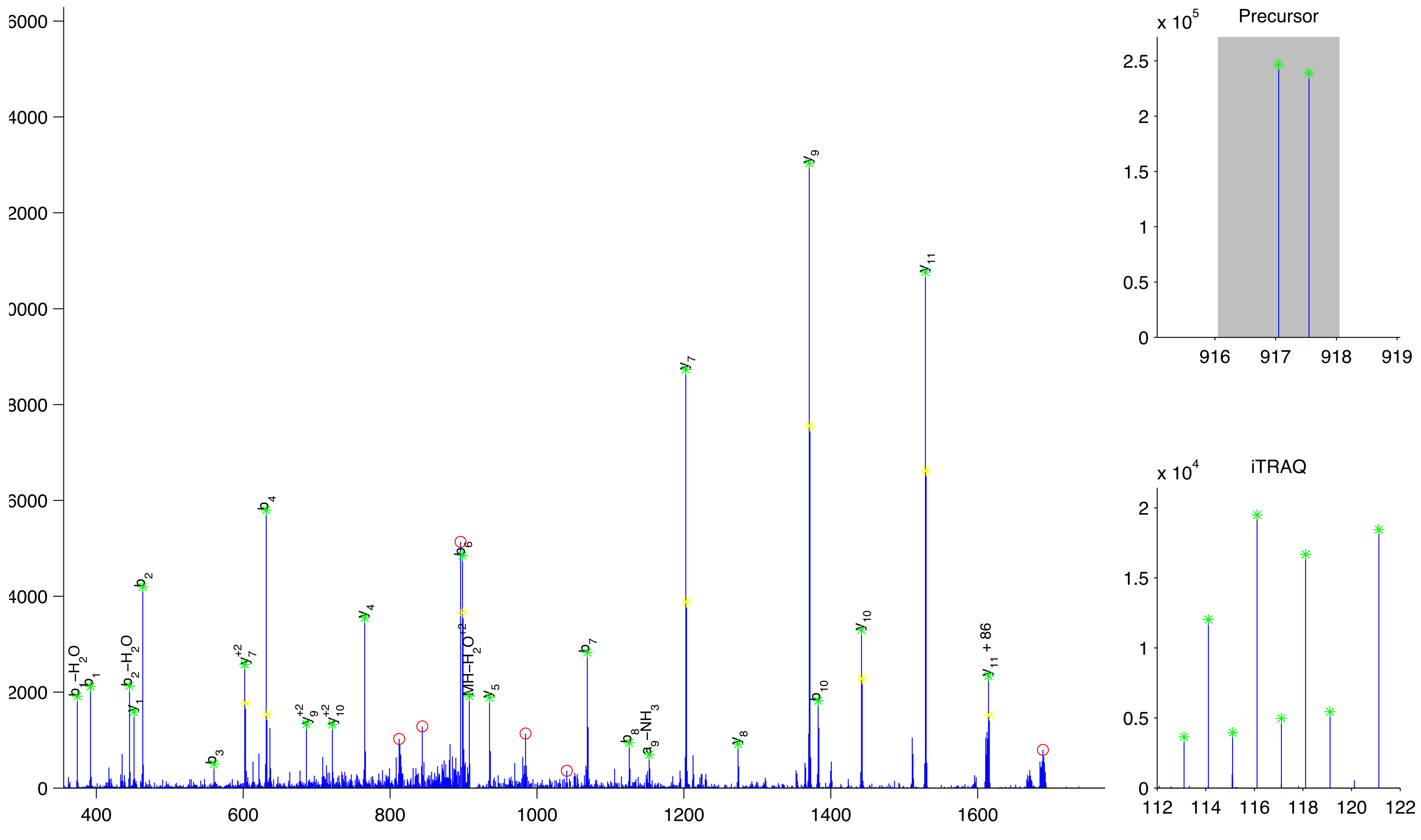

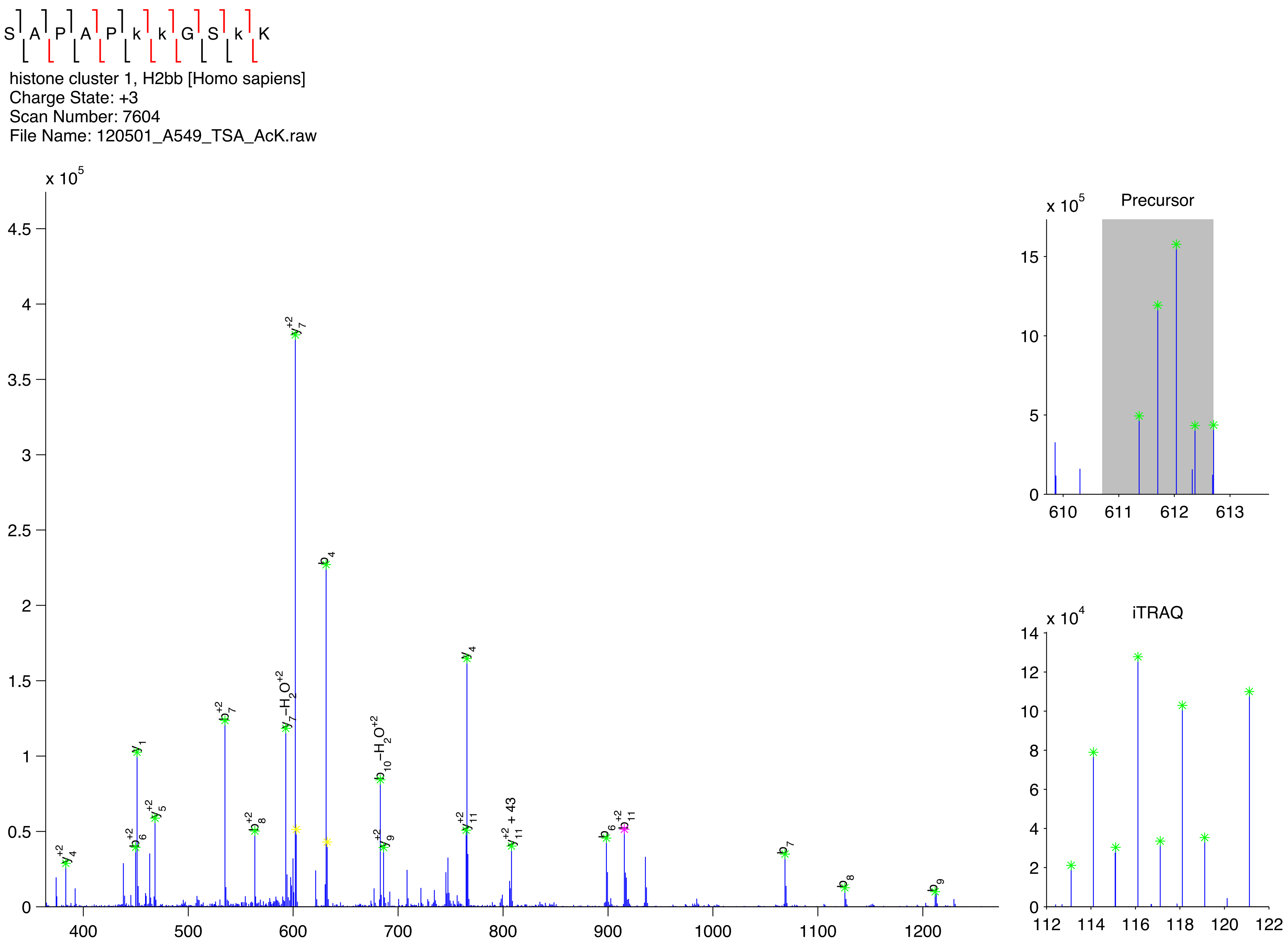

S A P A P k k G S k K
histone cluster 1, H2bb [Homo sapiens]
Charge State: +3
Scan Number: 7604
File Name: 120501_A549_TSA_AcK.raw
x 10^5
Precursor
iTRAQ
x 10^4

S A P A P k k G S k K

histone cluster 1, H2bb [Homo sapiens]
Charge State: +2
Scan Number: 8028
File Name: 120501_A549_TSA_AcK.raw

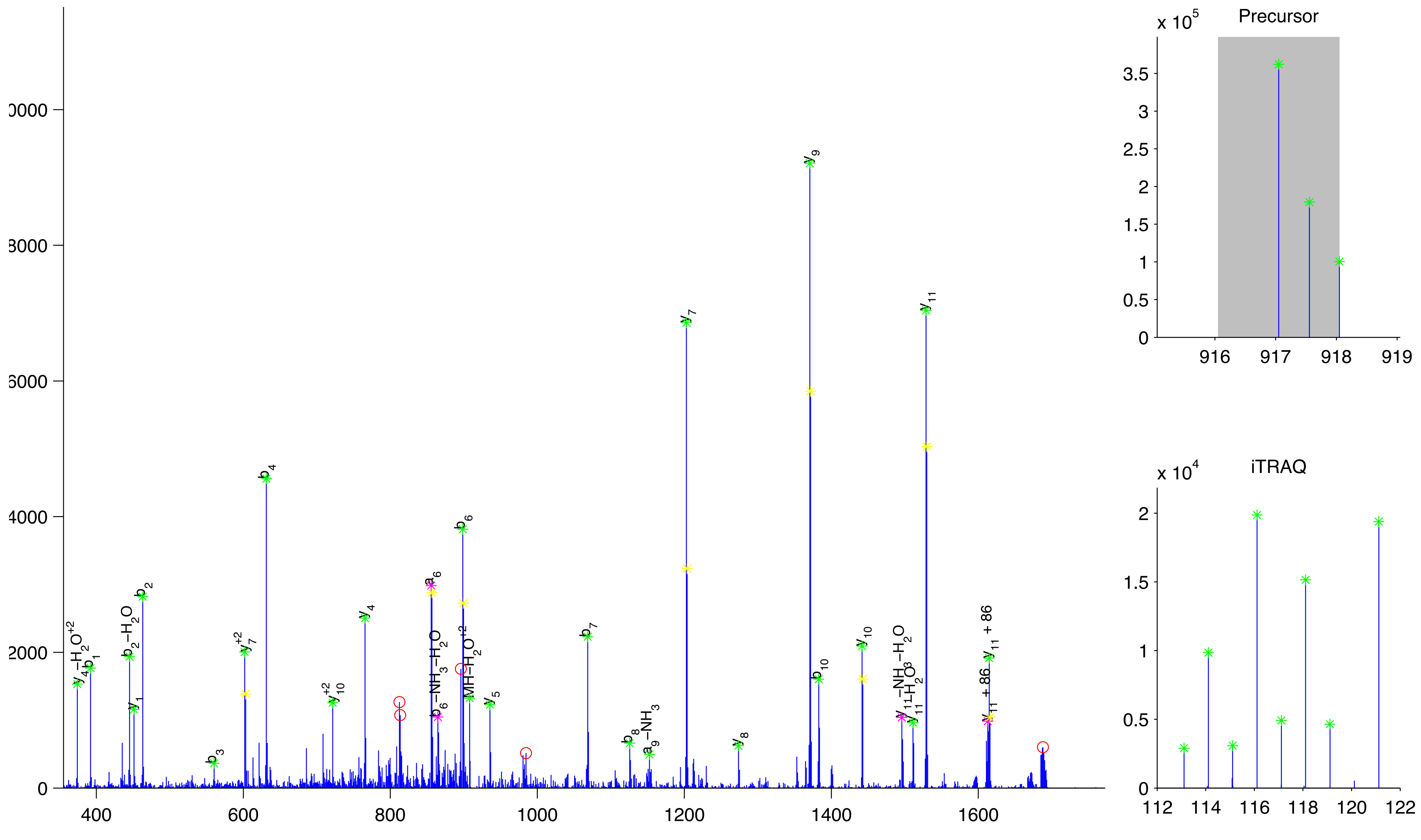

k G S k k A I T K

histone cluster 1, H2bb [Homo sapiens]
Charge State: +3
Scan Number: 12001
File Name: 120501_A549_TSA_AcK.raw

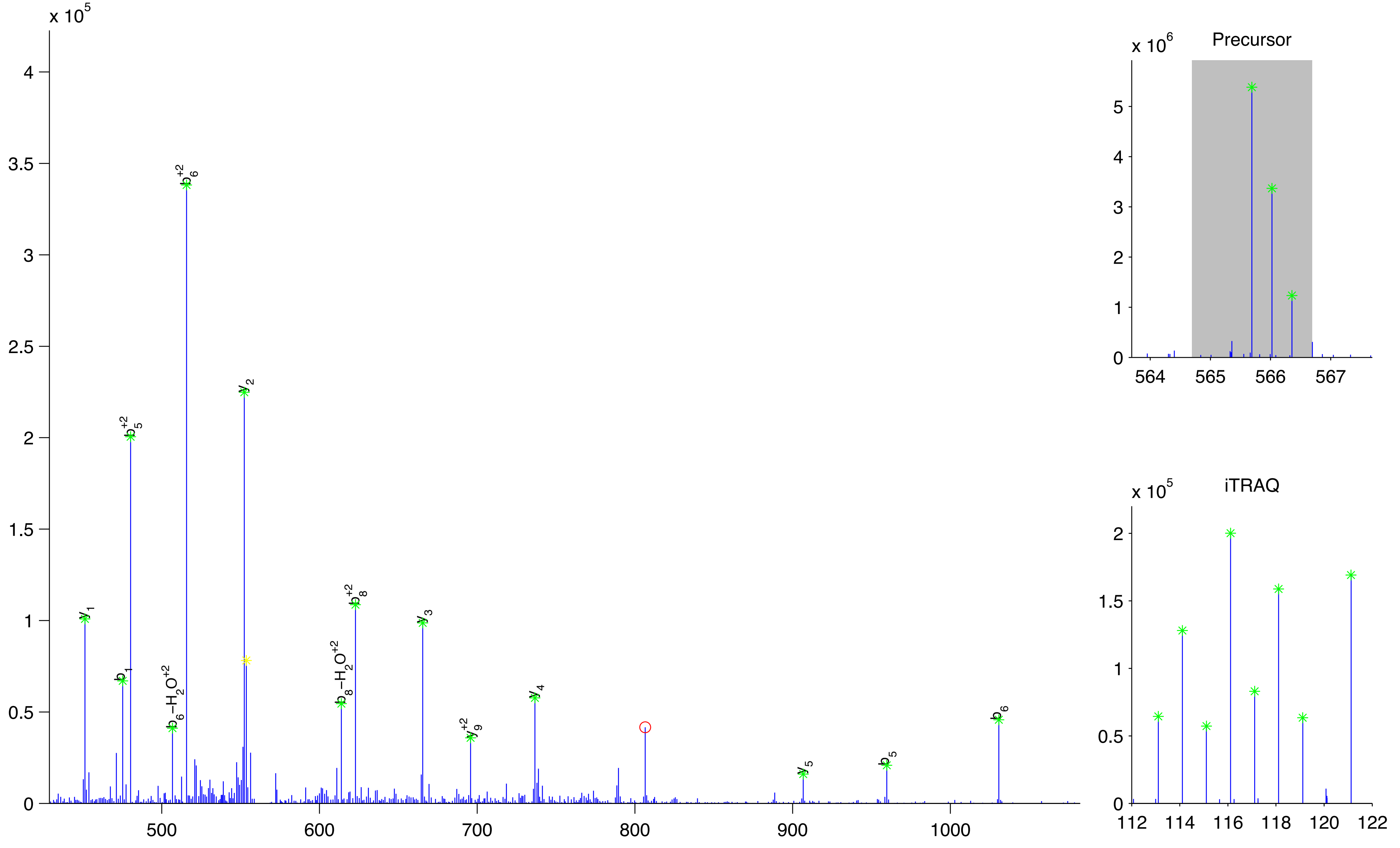

S A P A P k k G S k K

histone cluster 1, H2bg [Homo sapiens]
Charge State: +2
Scan Number: 7436
File Name: 120501_A549_TSA_AcK.raw

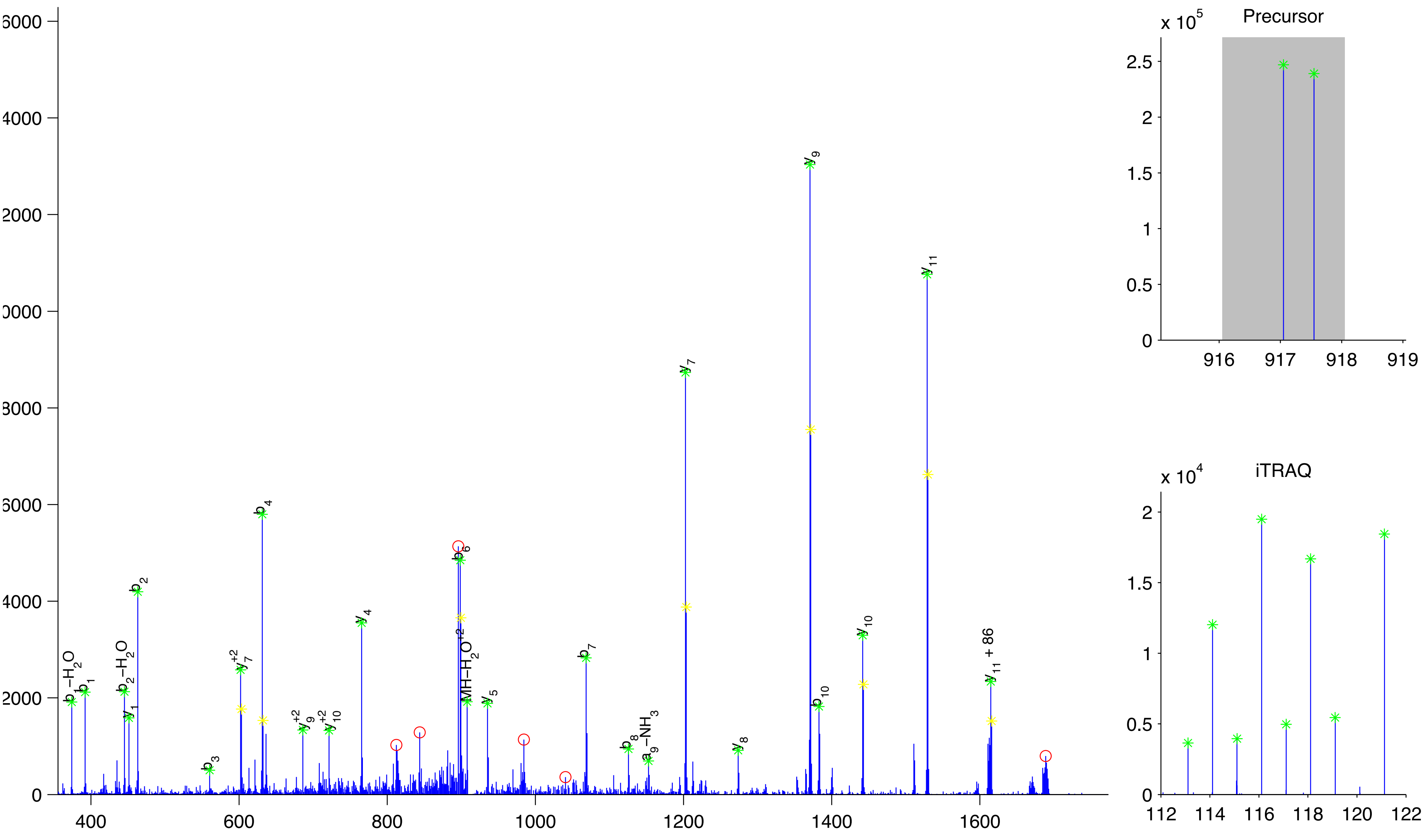

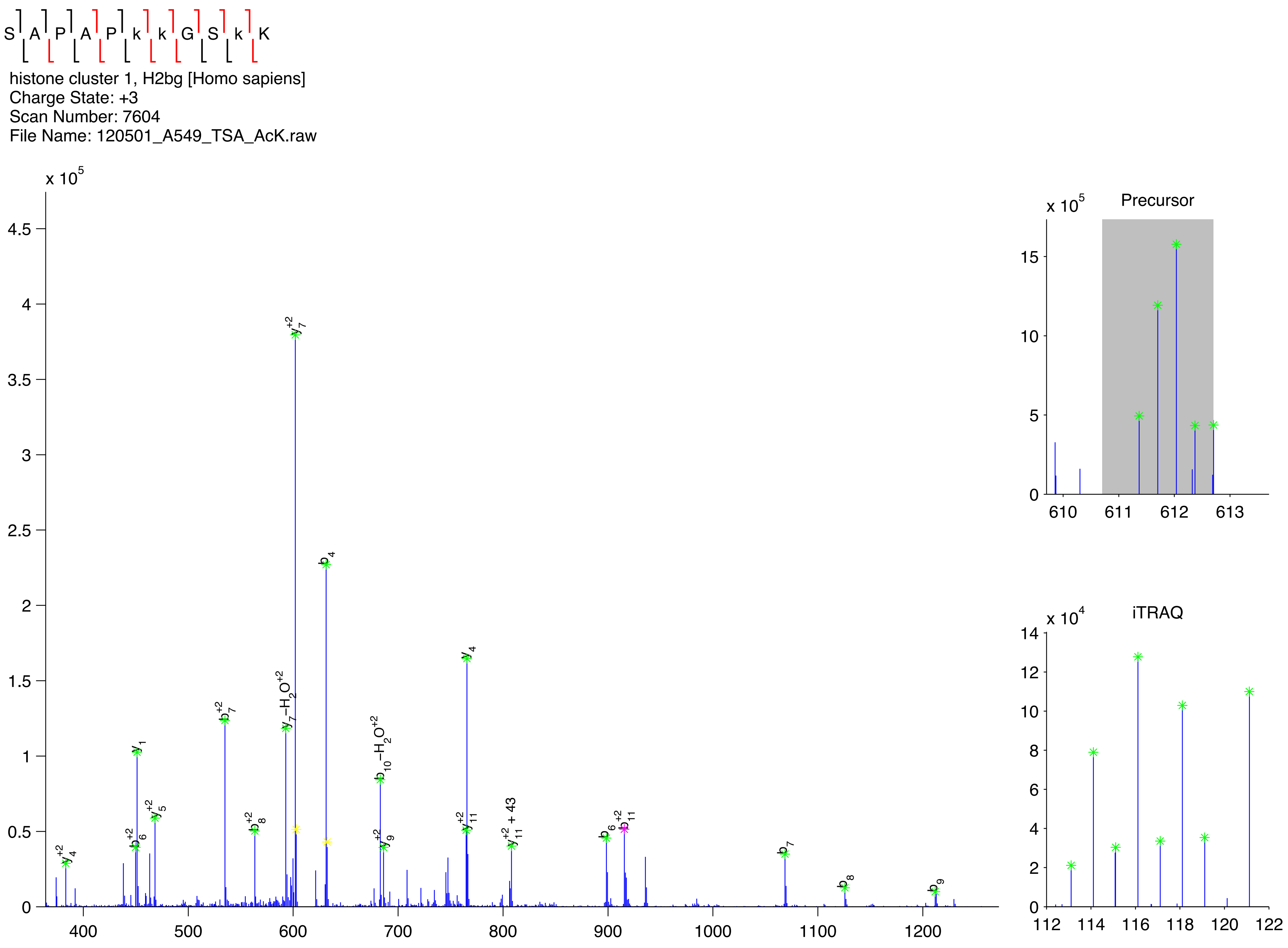

S A P A P k k G S k K
histone cluster 1, H2bg [Homo sapiens]
Charge State: +3
Scan Number: 7604
File Name: 120501_A549_TSA_AcK.raw
x 10^5
Precursor
iTRAQ
x 10^4

S A P A P k k G S k K

histone cluster 1, H2bg [Homo sapiens]
Charge State: +2
Scan Number: 8028
File Name: 120501_A549_TSA_AcK.raw

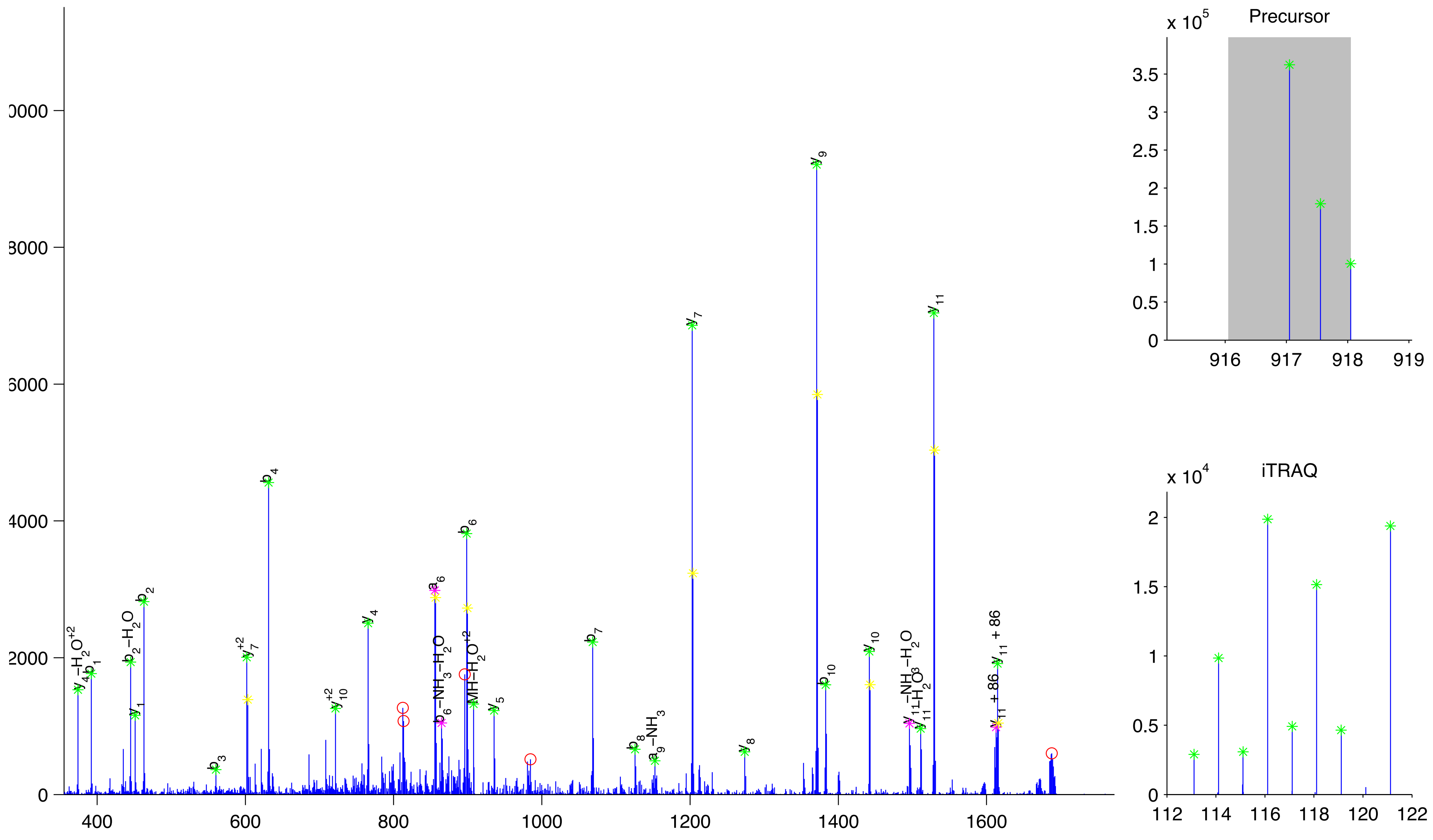

k G S k k A V T K

histone cluster 1, H2bg [Homo sapiens]
Charge State: +2
Scan Number: 9872
File Name: 120501_A549_TSA_AcK.raw

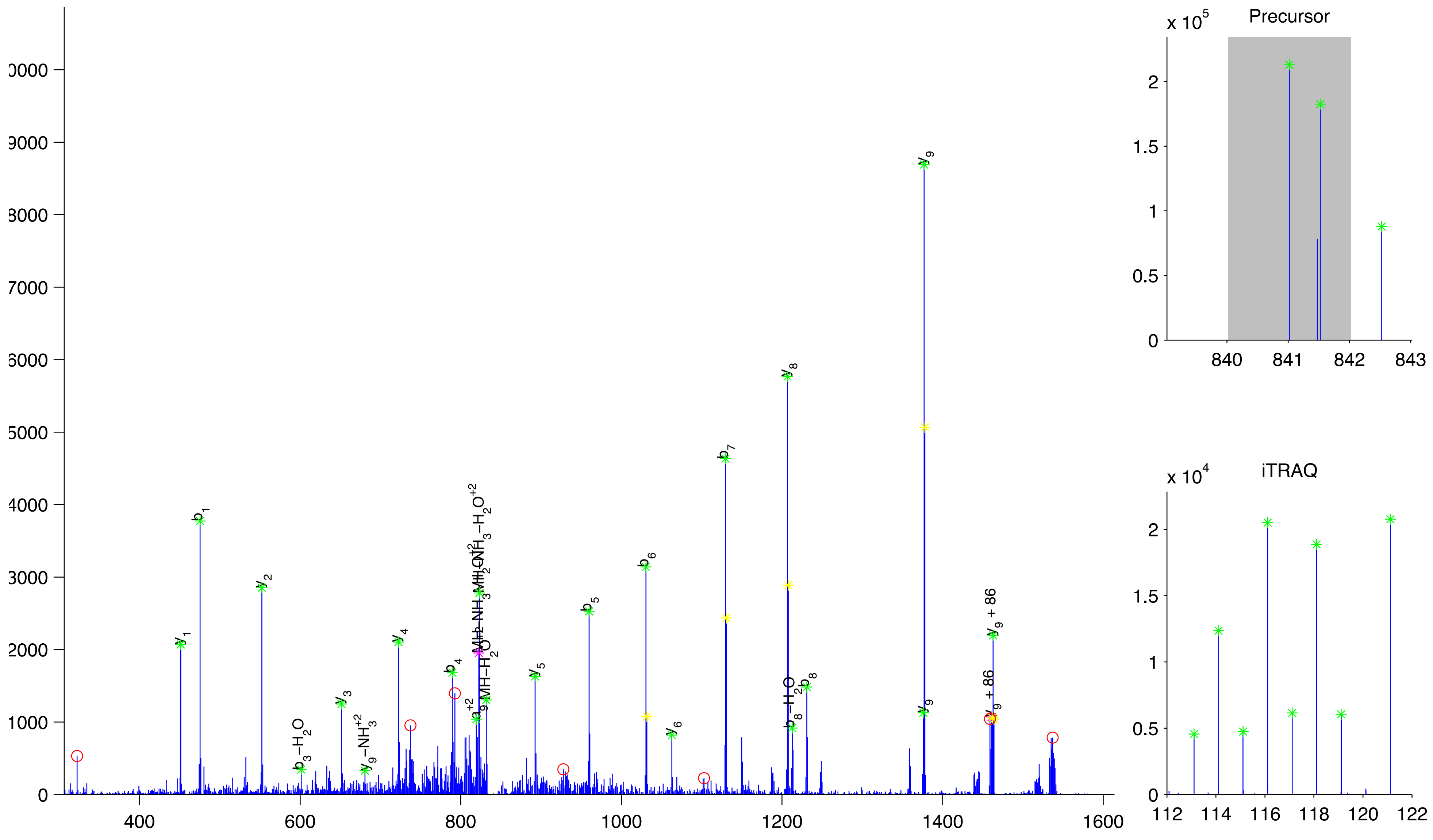

k A V T k A Q k K

histone cluster 1, H2bg [Homo sapiens]
Charge State: +3
Scan Number: 10731
File Name: 120501_A549_TSA_AcK.raw

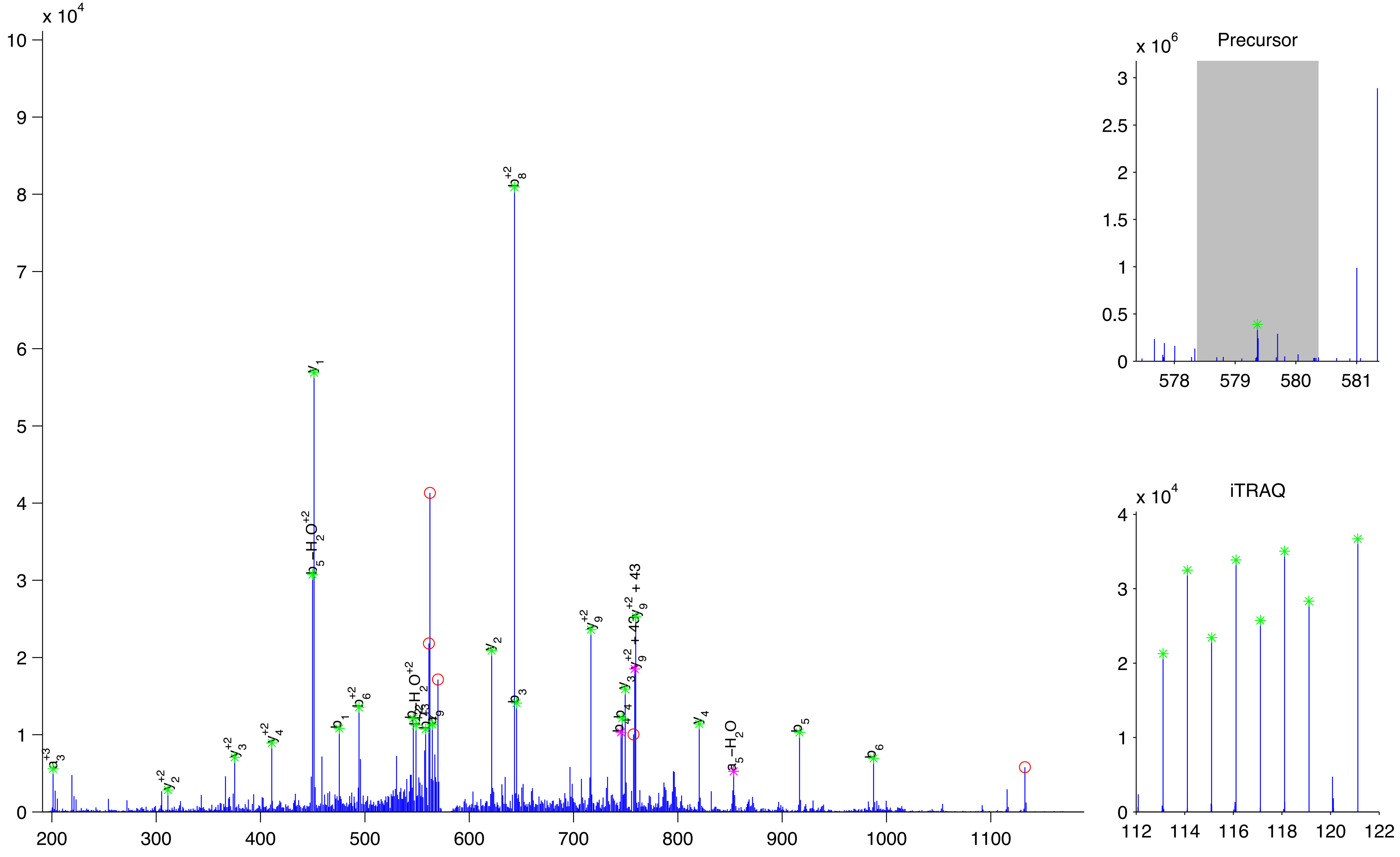

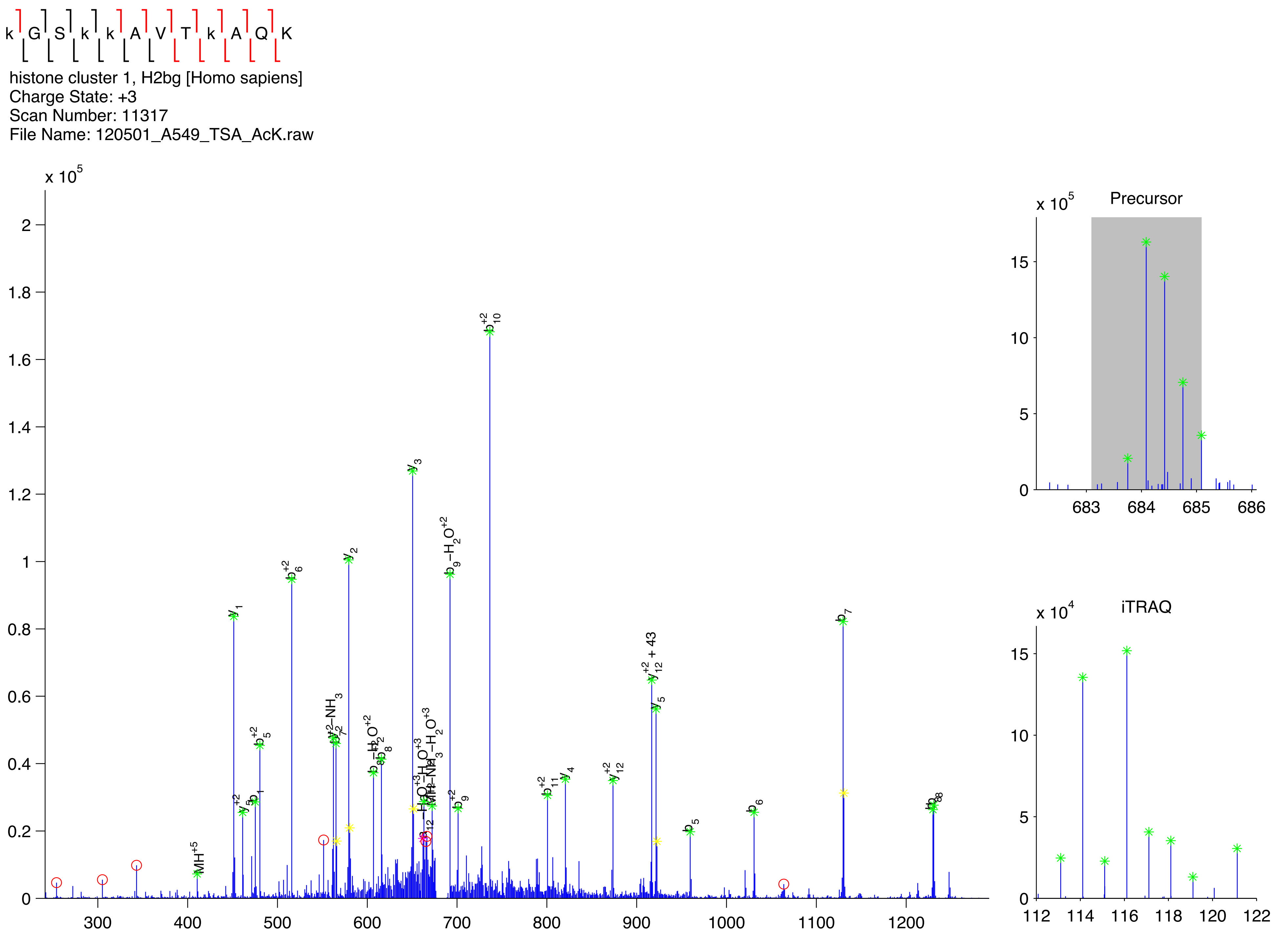

k G S k k A V T k A Q K
histone cluster 1, H2bg [Homo sapiens]
Charge State: +3
Scan Number: 11317
File Name: 120501_A549_TSA_AcK.raw
x 10^5
Precursor
iTRAQ
x 10^4

k G S k k A V T k A Q K

histone cluster 1, H2bg [Homo sapiens]
Charge State: +2
Scan Number: 11978
File Name: 120501_A549_TSA_AcK.raw

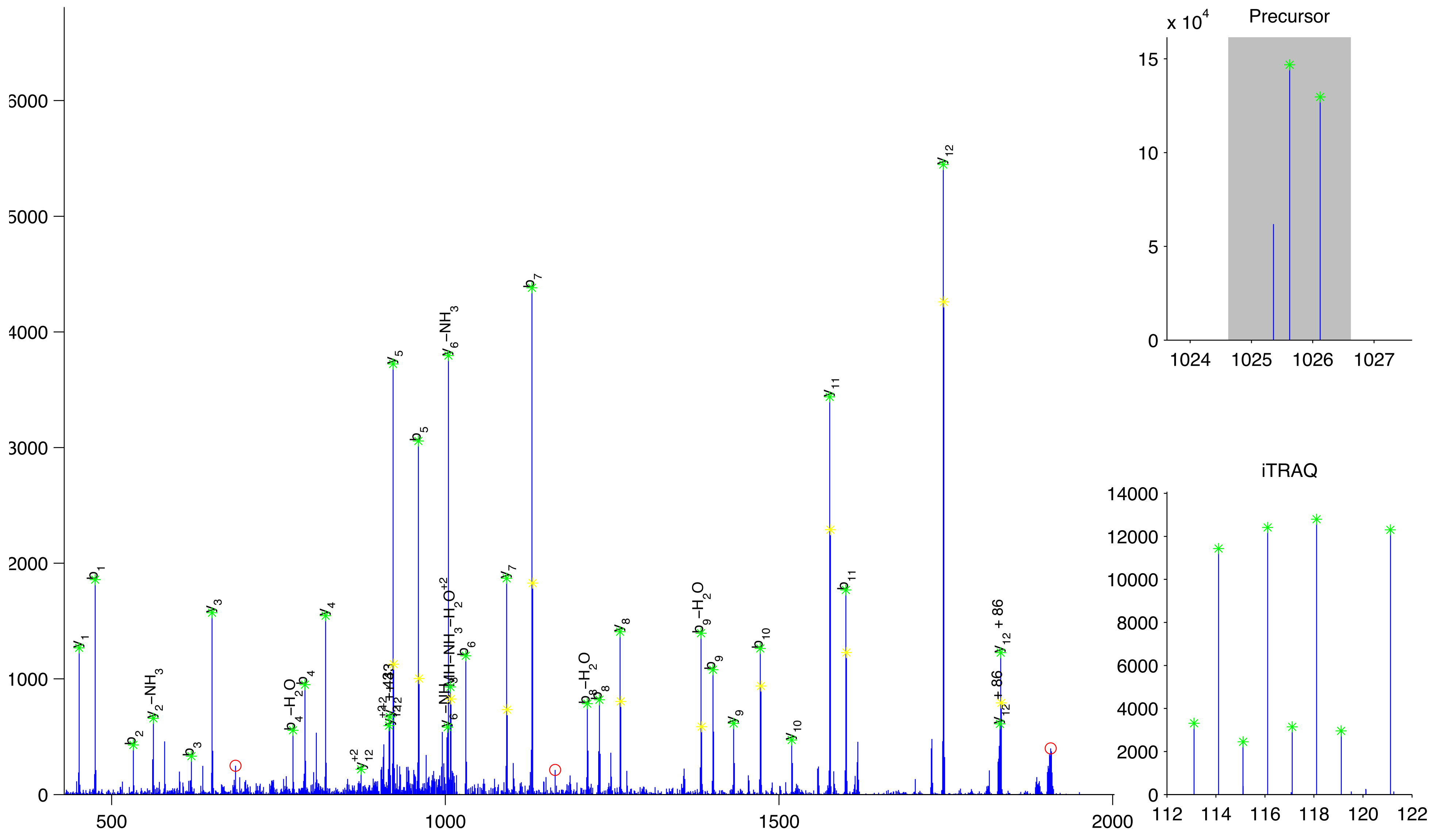

k G S k k A V T k A Q K

histone cluster 1, H2bg [Homo sapiens]
Charge State: +3
Scan Number: 12388
File Name: 120501_A549_TSA_AcK.raw

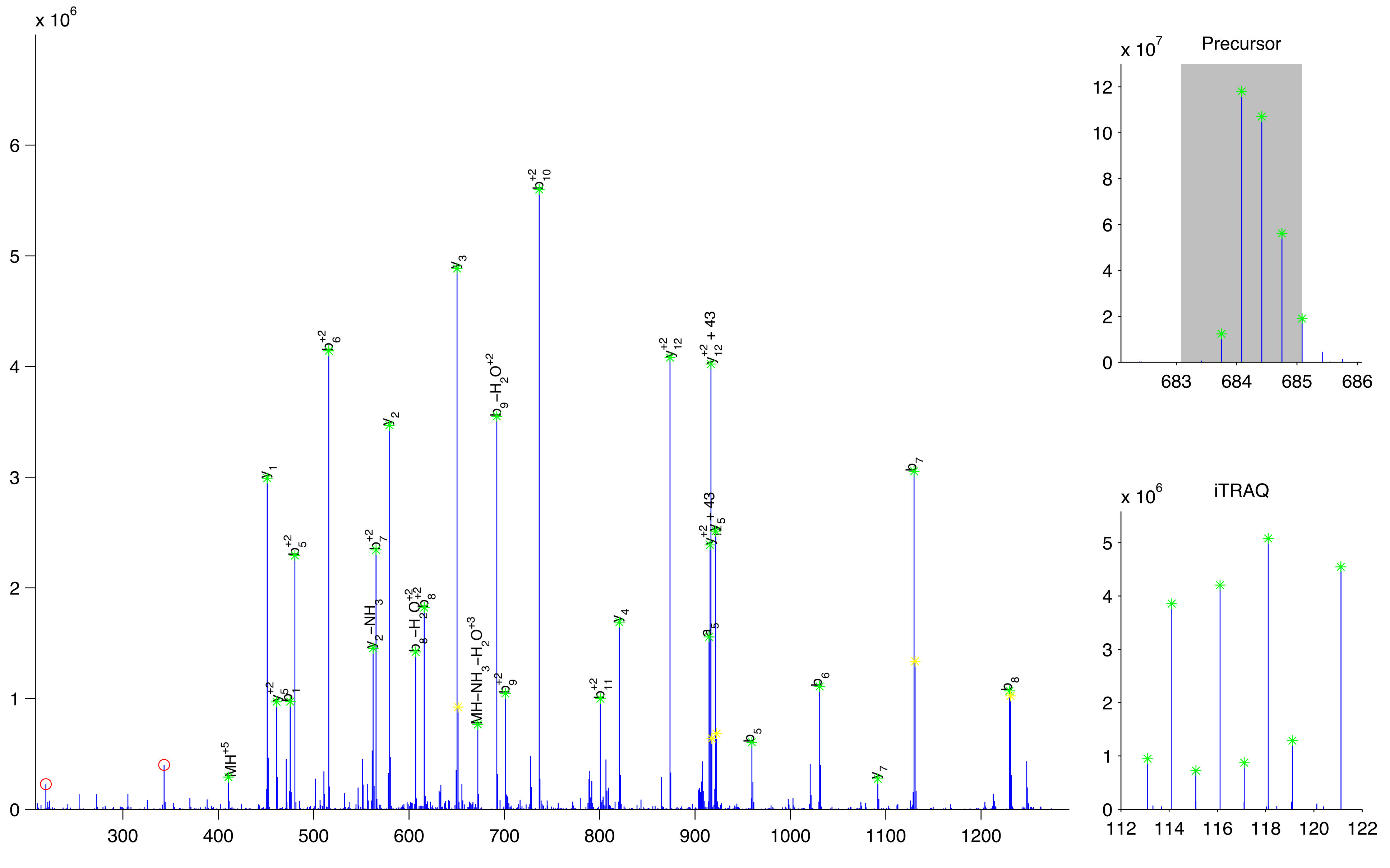

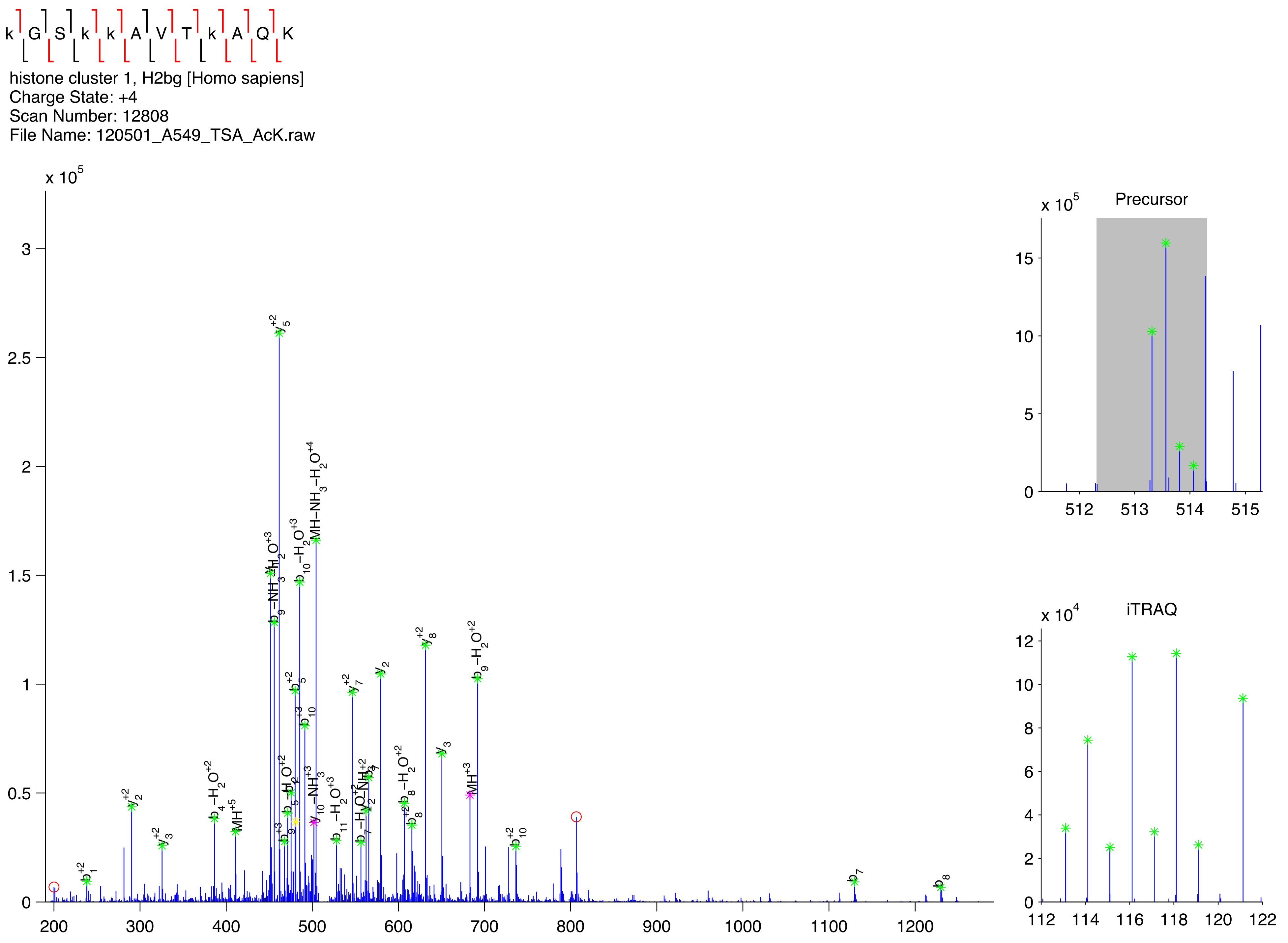
k G S k k A V T k A Q K
histone cluster 1, H2bg [Homo sapiens]
Charge State: +4
Scan Number: 12808
File Name: 120501_A549_TSA_AcK.raw
Precursor
iTRAQ

k G S k k A V T k A Q K

histone cluster 1, H2bg [Homo sapiens]
Charge State: +3
Scan Number: 12934
File Name: 120501_A549_TSA_AcK.raw

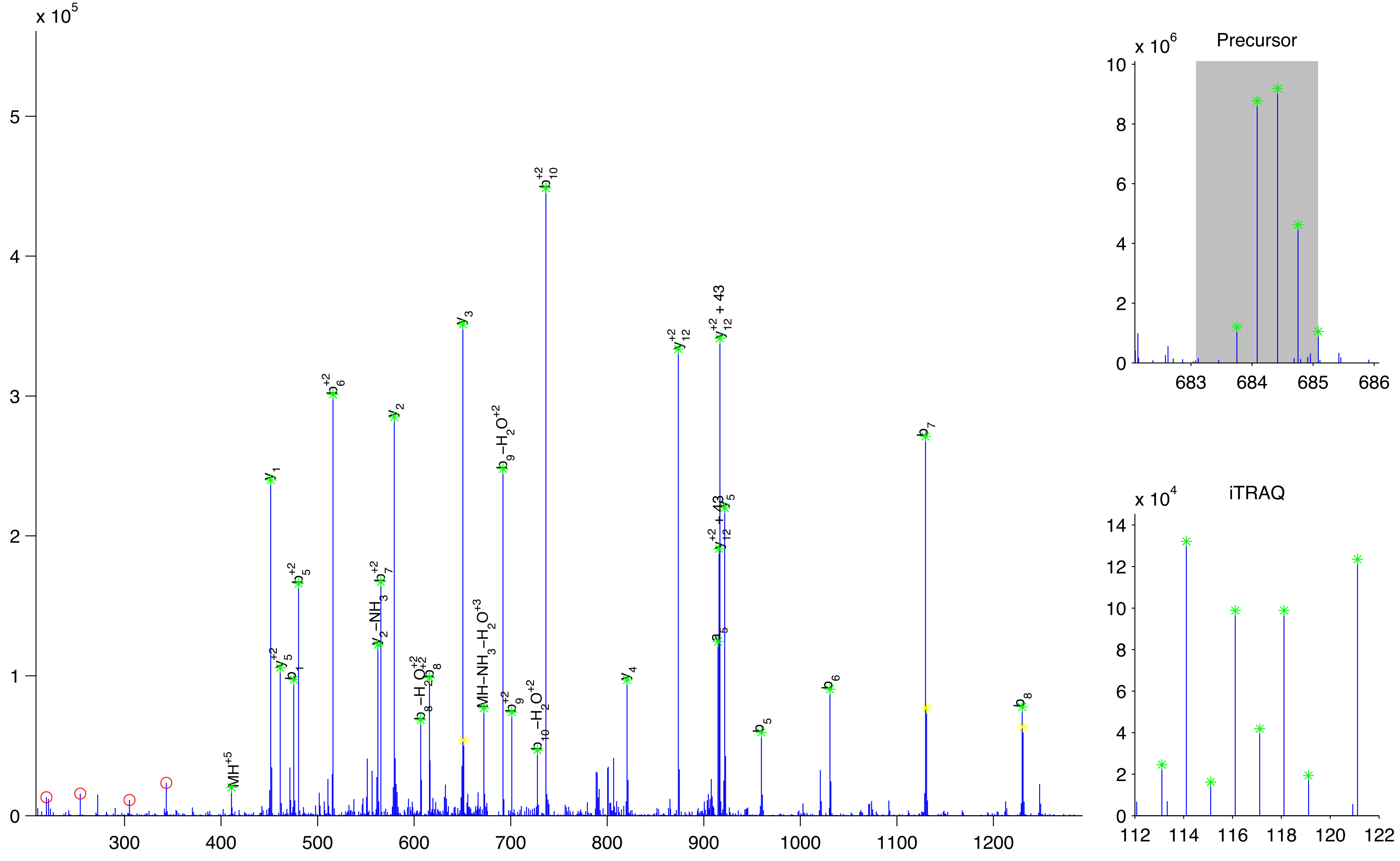

k G S k k A V T k A Q K

histone cluster 1, H2bg [Homo sapiens]
Charge State: +4
Scan Number: 13354
File Name: 120501_A549_TSA_AcK.raw

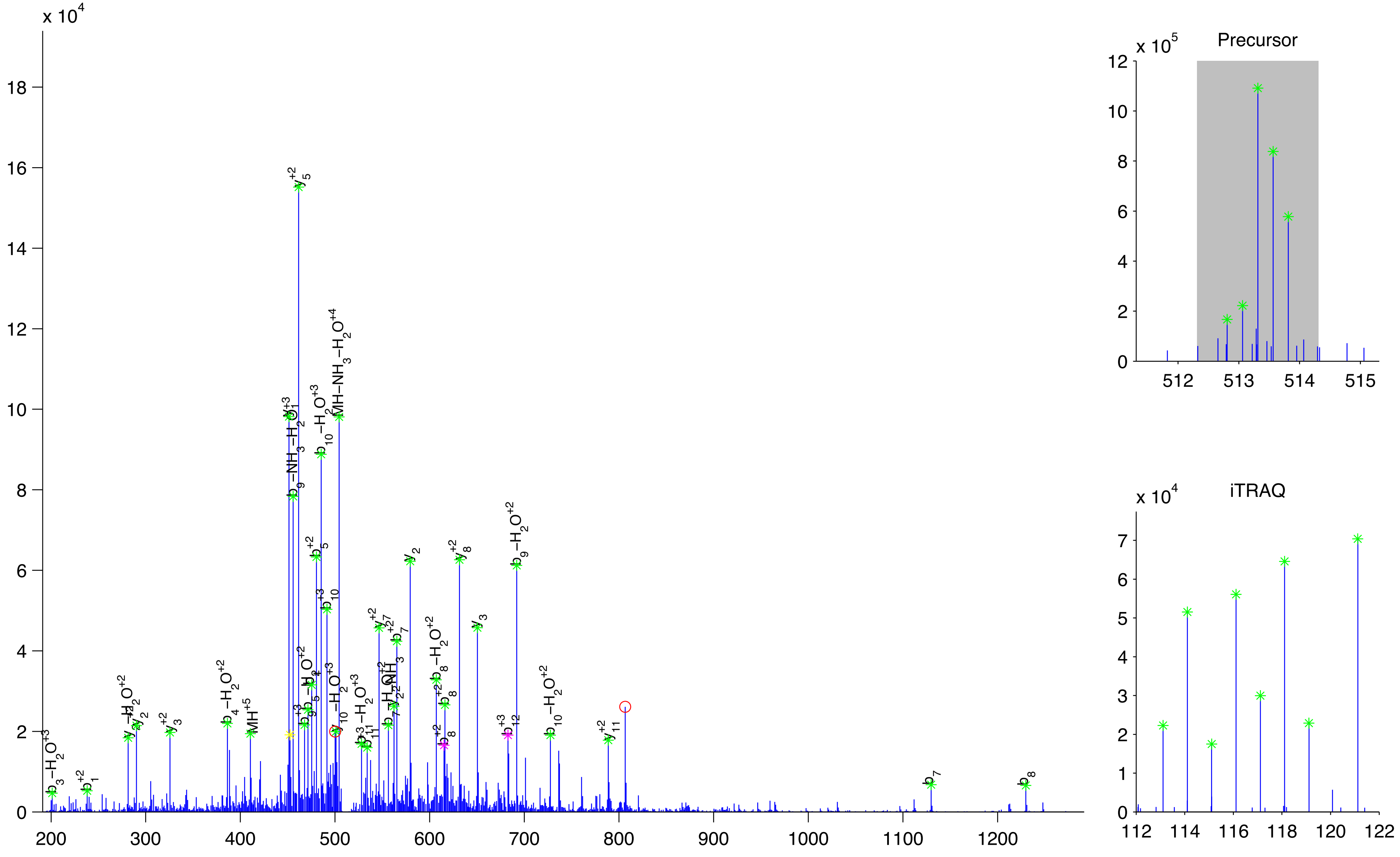

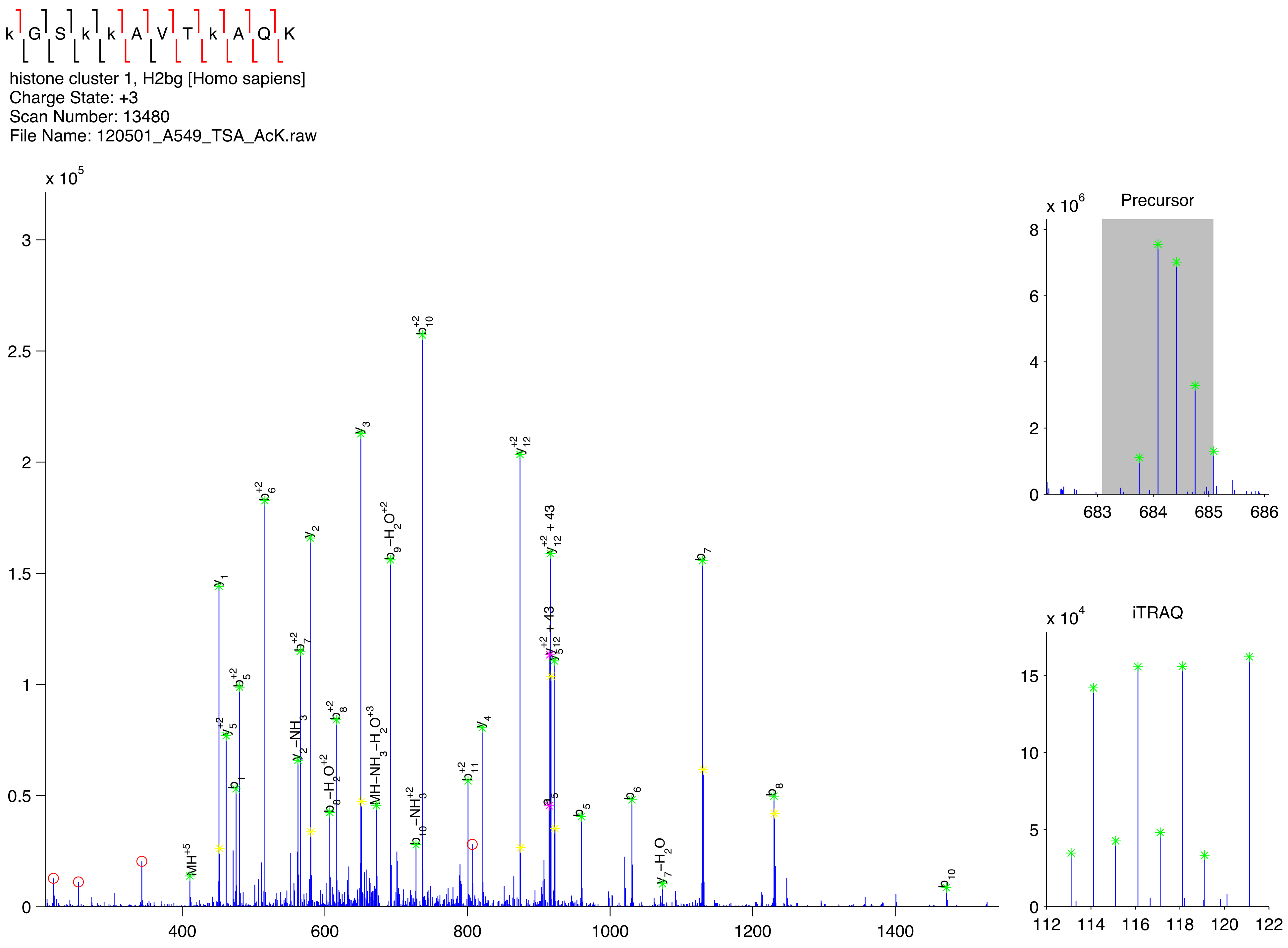
k G S k k A V T k A Q K
histone cluster 1, H2bg [Homo sapiens]
Charge State: +3
Scan Number: 13480
File Name: 120501_A549_TSA_AcK.raw
x 10^5
Precursor
x 10^6
iTRAQ
x 10^4

k G S k k A V T k A Q K

histone cluster 1, H2bg [Homo sapiens]
Charge State: +2
Scan Number: 13637
File Name: 120501_A549_TSA_AcK.raw

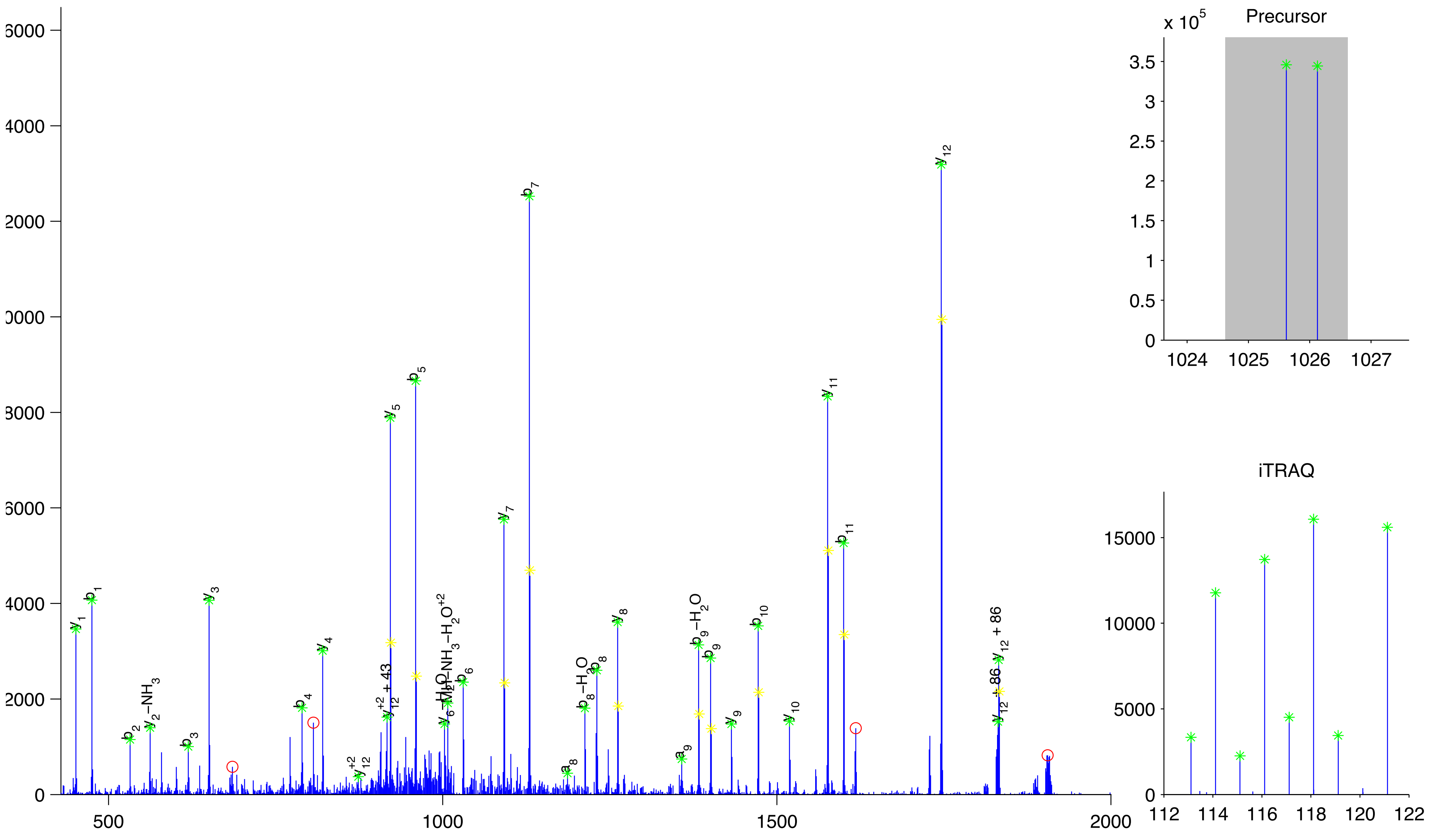

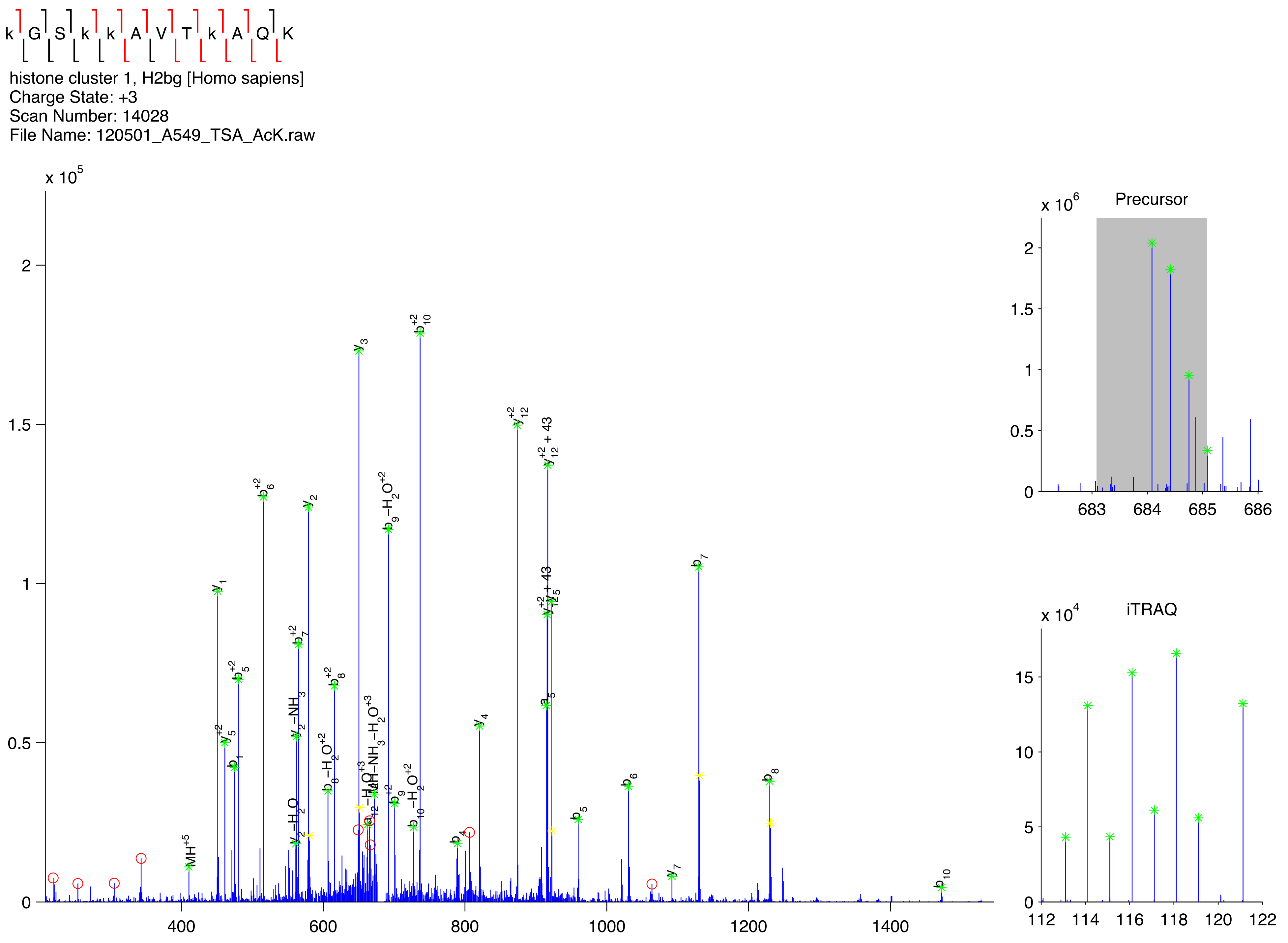

k G S k k A V T k A Q K
histone cluster 1, H2bg [Homo sapiens]
Charge State: +3
Scan Number: 14028
File Name: 120501_A549_TSA_AcK.raw
x 10^5
Precursor
x 10^6
iTRAQ
x 10^4

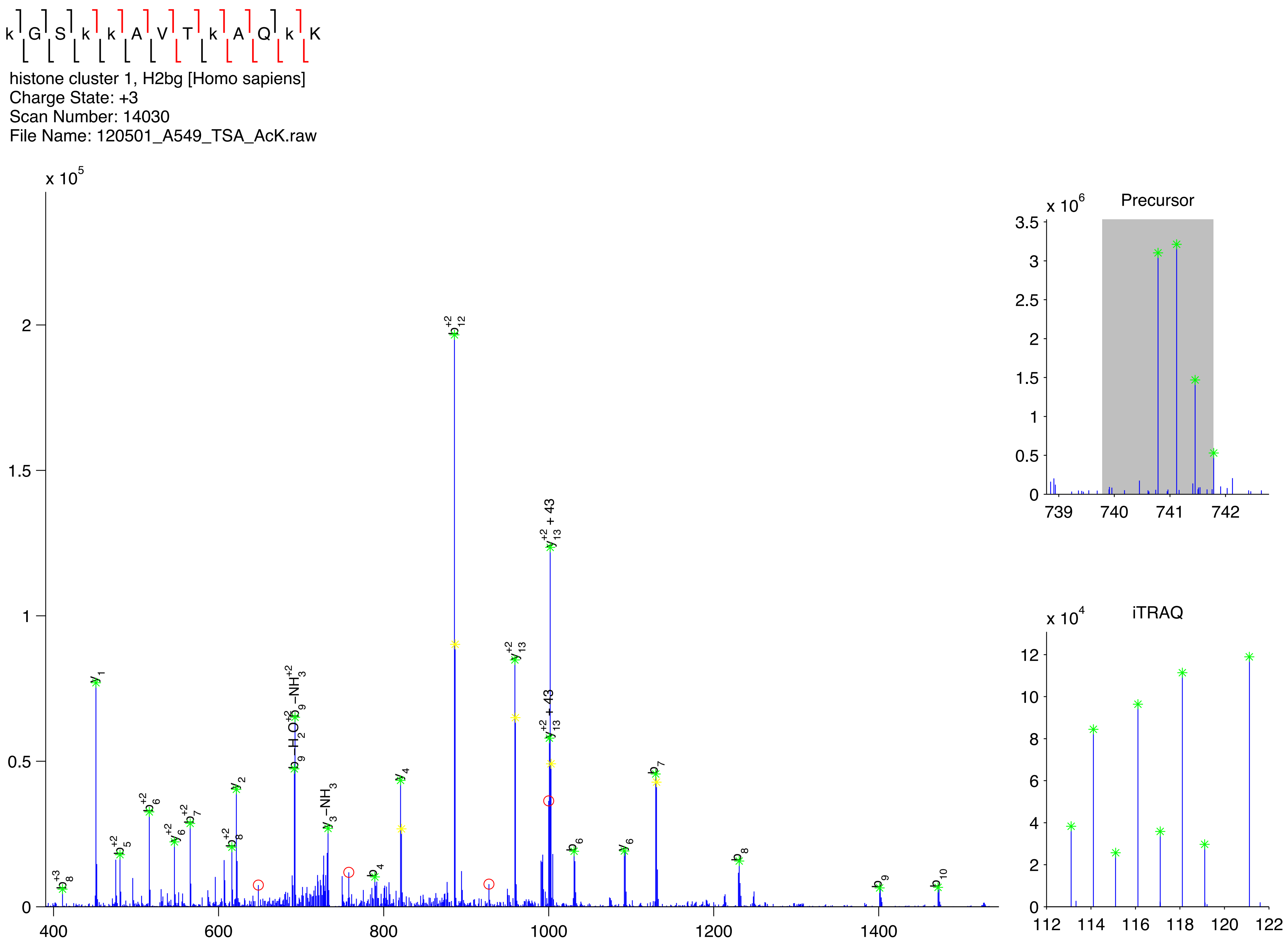

k G S k k A V T k A Q k K
histone cluster 1, H2bg [Homo sapiens]
Charge State: +3
Scan Number: 14030
File Name: 120501_A549_TSA_AcK.raw
x 10^5
Precursor
x 10^6
iTRAQ
x 10^4

k G S k k A V T k A Q K

histone cluster 1, H2bg [Homo sapiens]
Charge State: +3
Scan Number: 14574
File Name: 120501_A549_TSA_AcK.raw

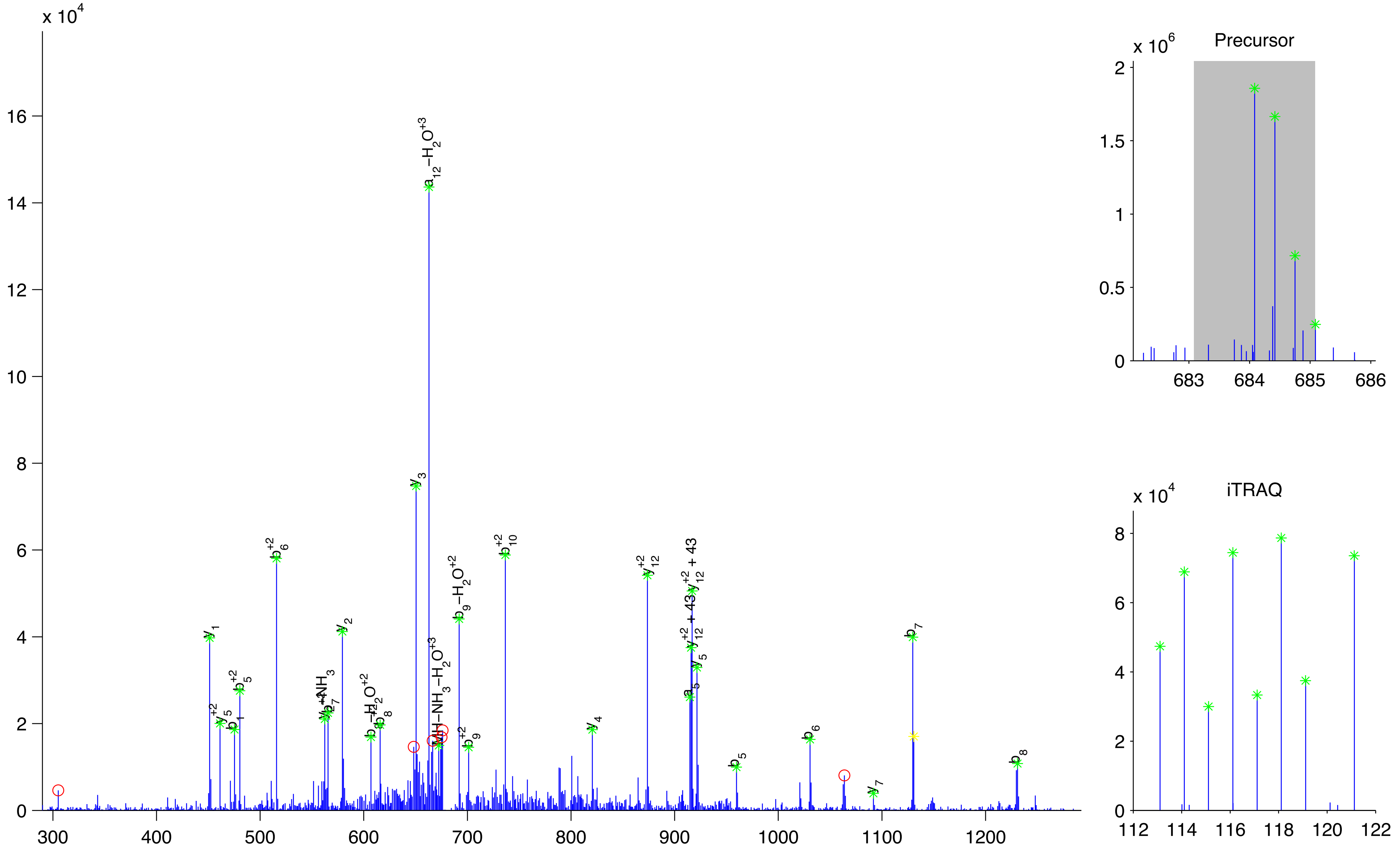

S A P A P k k G S k k A V T k A Q K

histone cluster 1, H2bg [Homo sapiens]
Charge State: +4
Scan Number: 15265
File Name: 120501_A549_TSA_AcK.raw

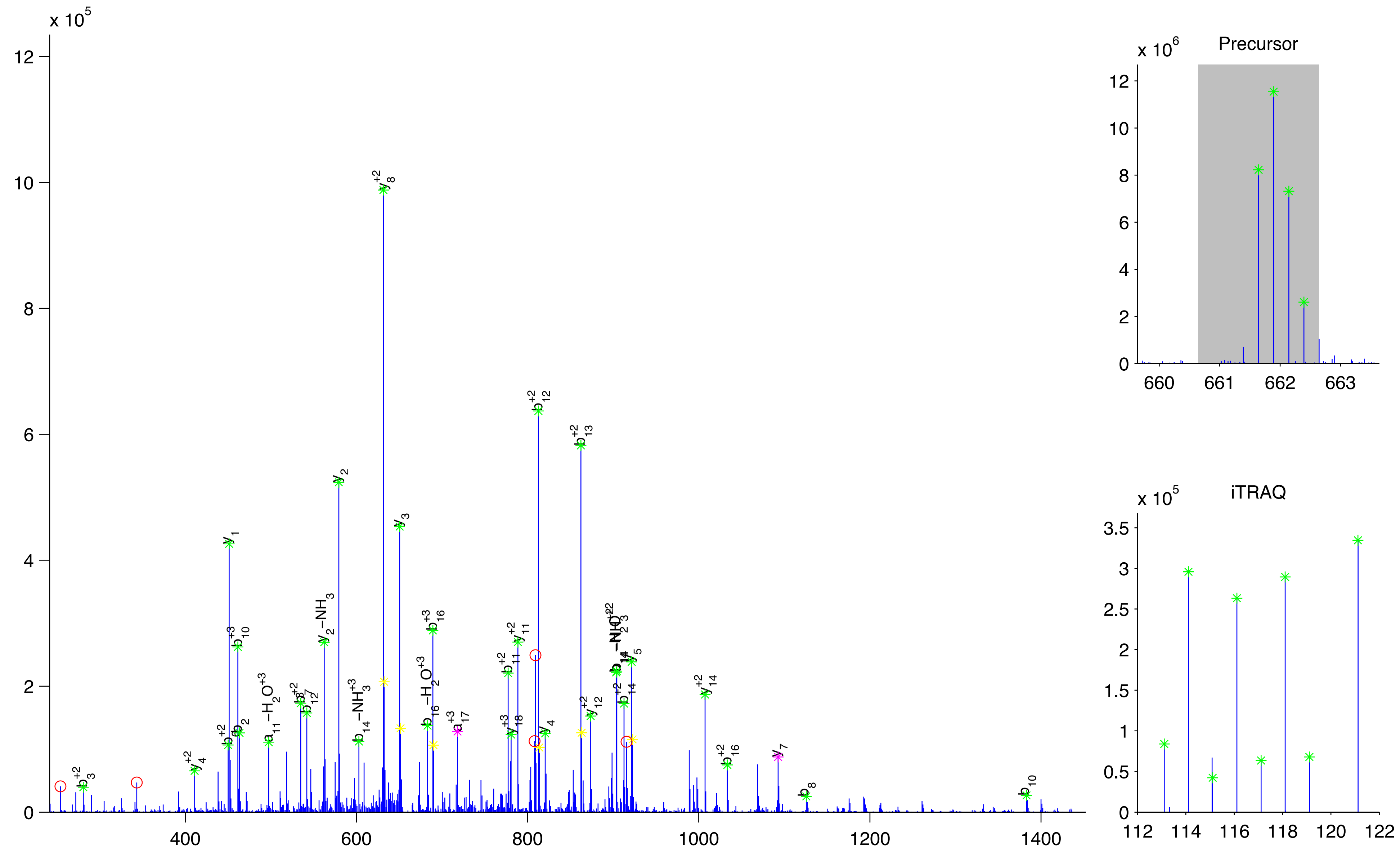

S A P A P k k G S k k A V T k A Q K

histone cluster 1, H2bg [Homo sapiens]
Charge State: +3
Scan Number: 15559
File Name: 120501_A549_TSA_AcK.raw

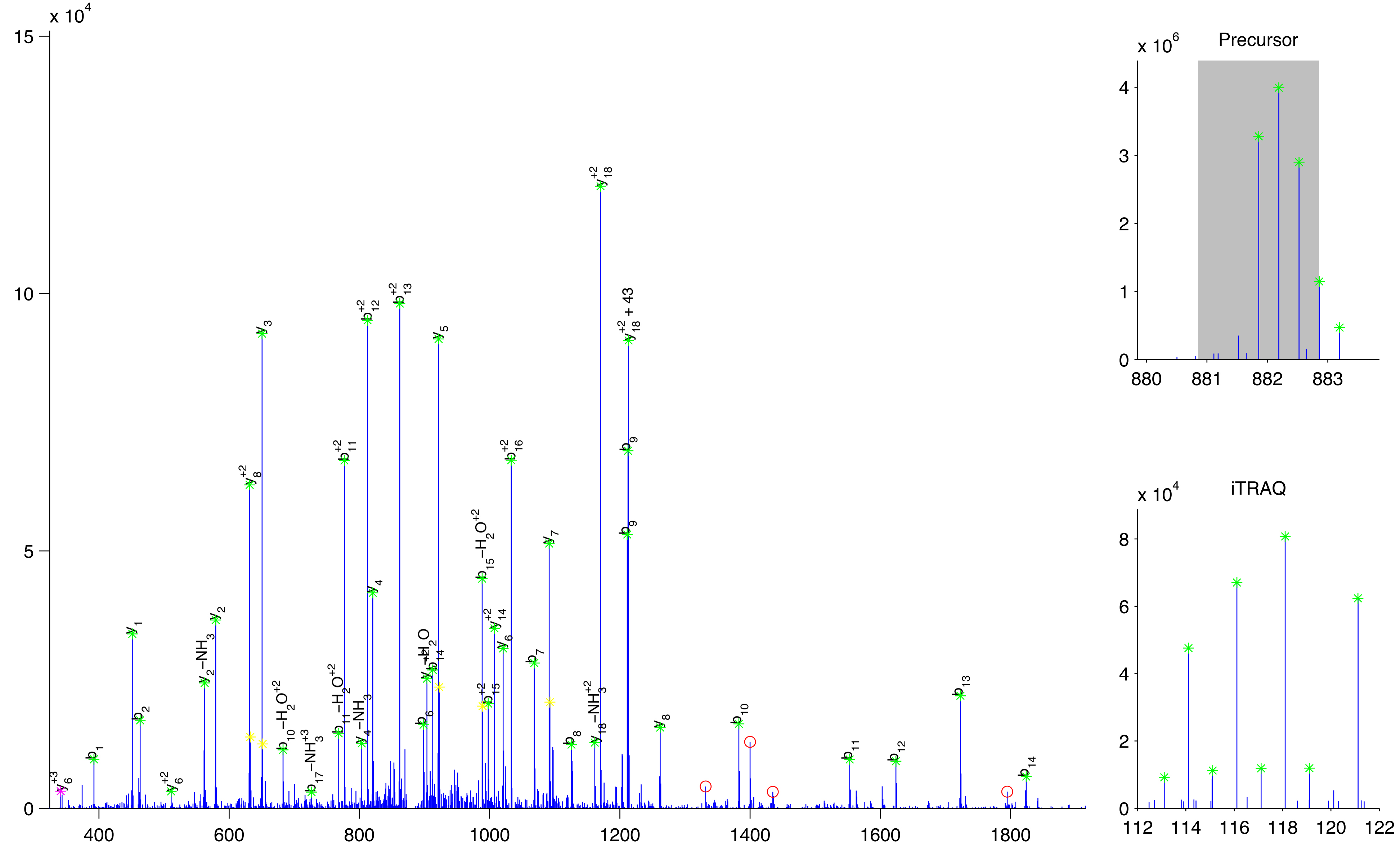

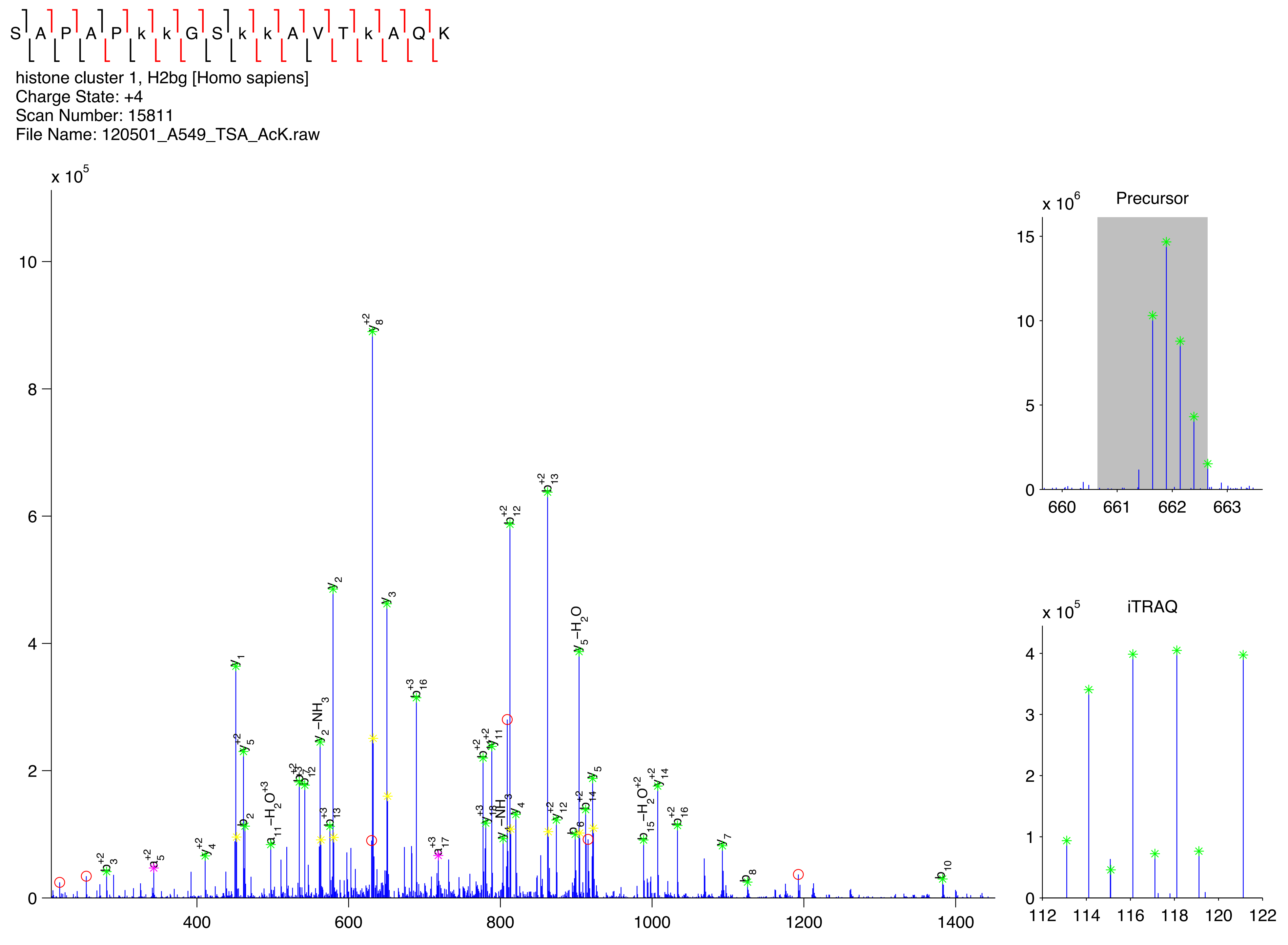
S A P A P k k G S k k A V T k A Q K
histone cluster 1, H2bg [Homo sapiens]
Charge State: +4
Scan Number: 15811
File Name: 120501_A549_TSA_AcK.raw
x 10^5
Precursor
x 10^6
iTRAQ
x 10^5

S A P A P k k G S k k A V T k A Q K

histone cluster 1, H2bg [Homo sapiens]
Charge State: +4
Scan Number: 16359
File Name: 120501_A549_TSA_AcK.raw

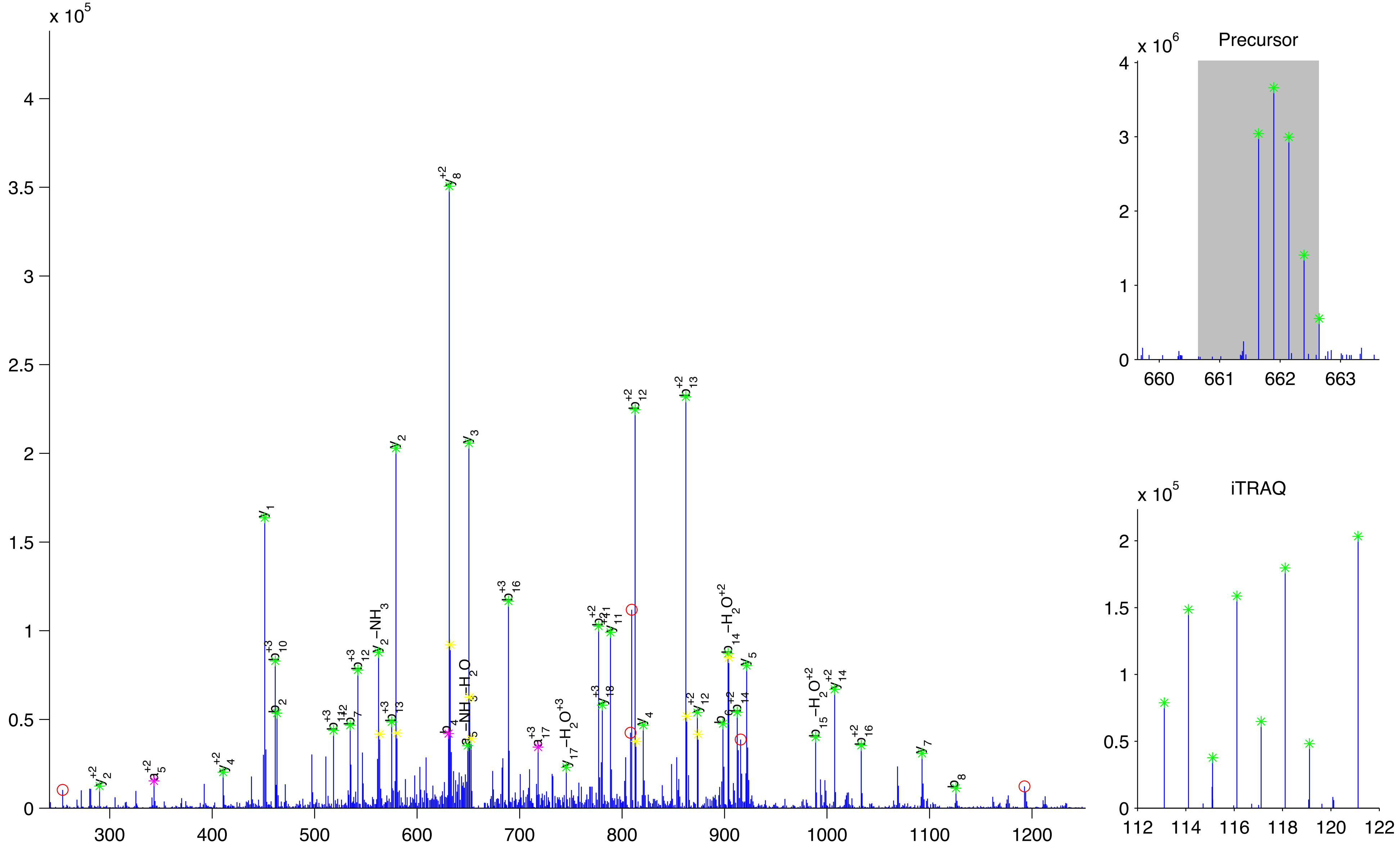

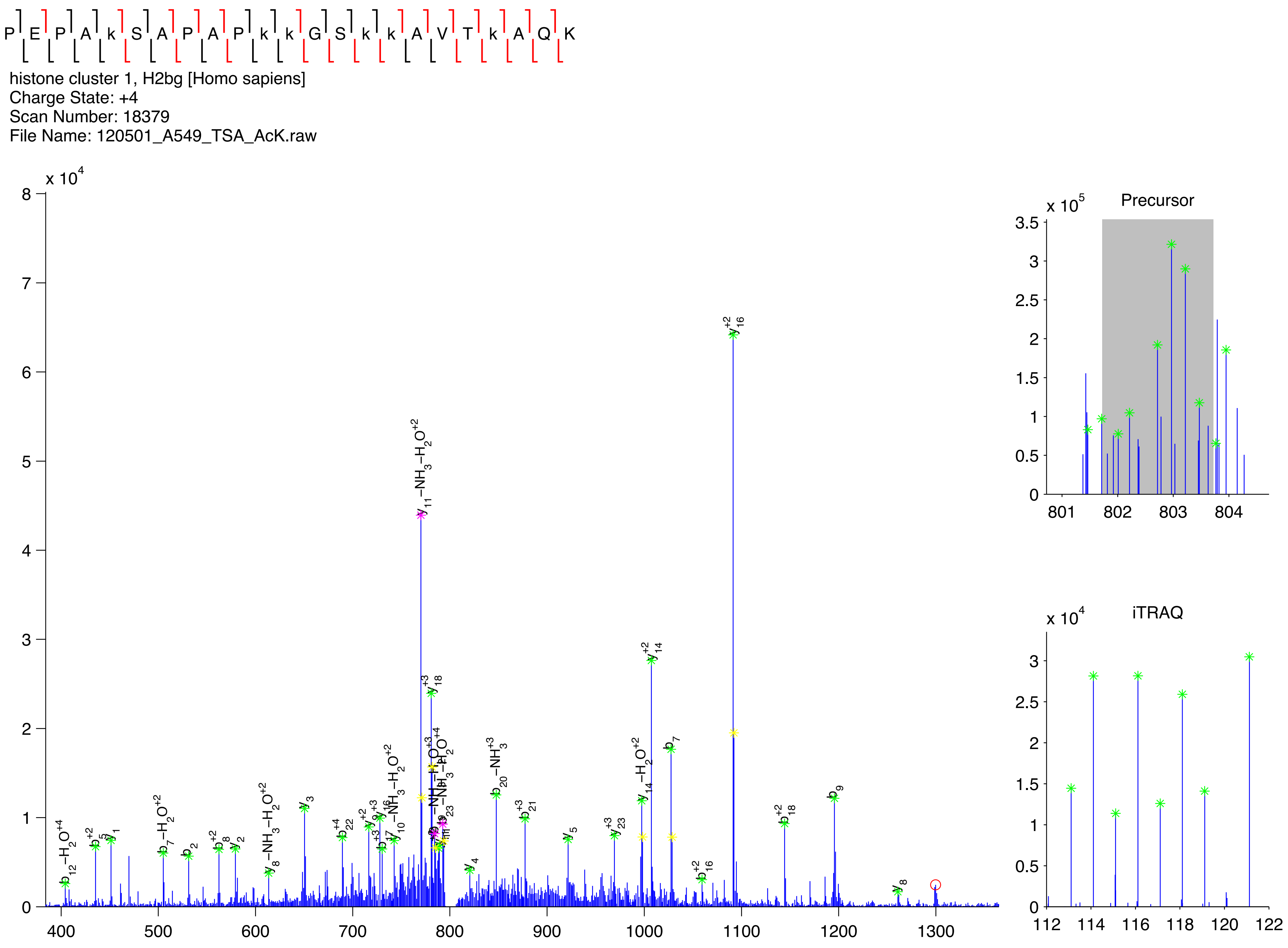

P E P A k S A P A P k k G S k k A V T k A Q K
histone cluster 1, H2bg [Homo sapiens]
Charge State: +4
Scan Number: 18379
File Name: 120501_A549_TSA_AcK.raw
x 10^4
Precursor
x 10^5
iTRAQ
x 10^4

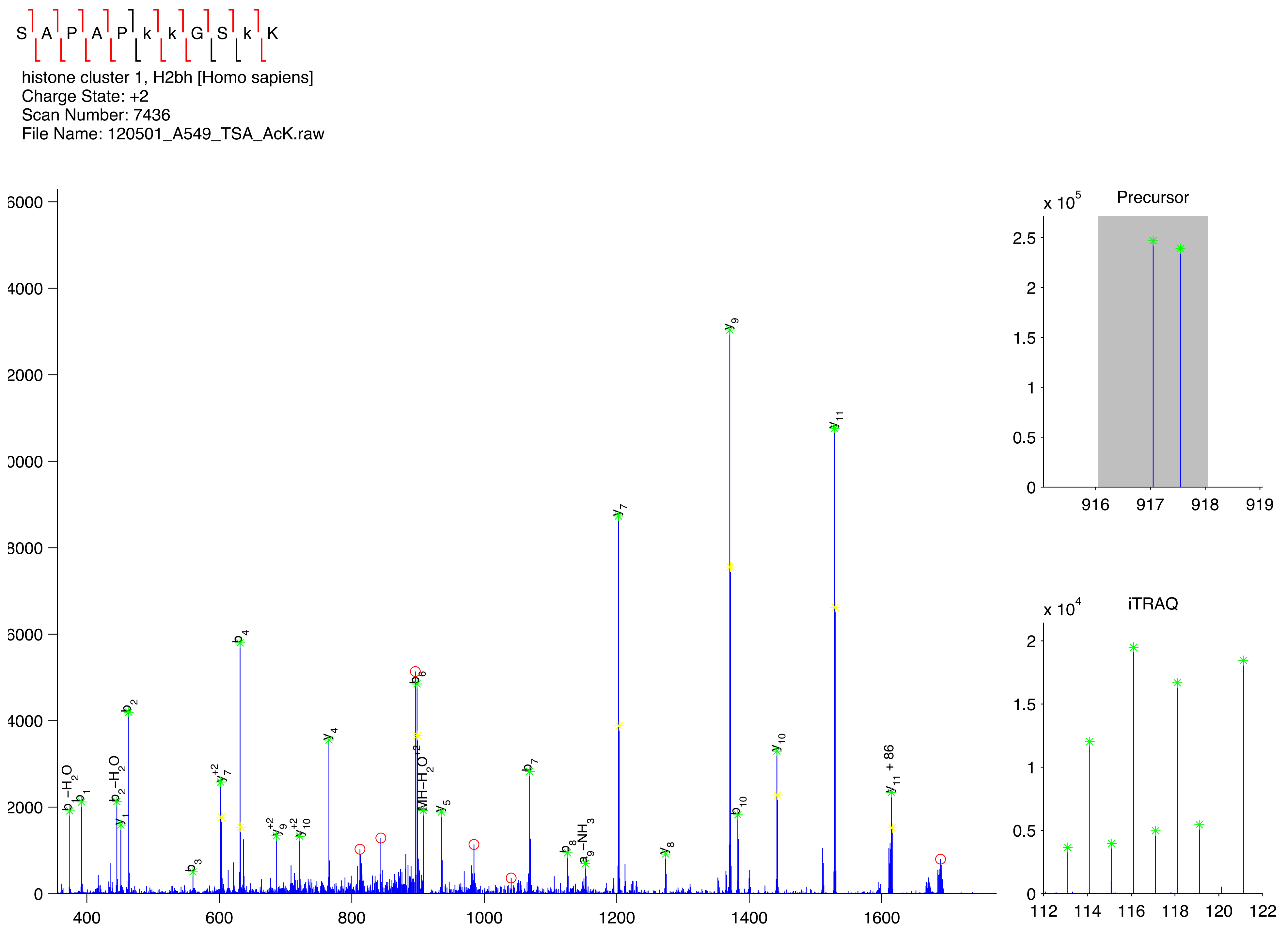
S A P A P k k G S k K
histone cluster 1, H2bh [Homo sapiens]
Charge State: +2
Scan Number: 7436
File Name: 120501_A549_TSA_AcK.raw
Precursor
x 10^5
iTRAQ
x 10^4

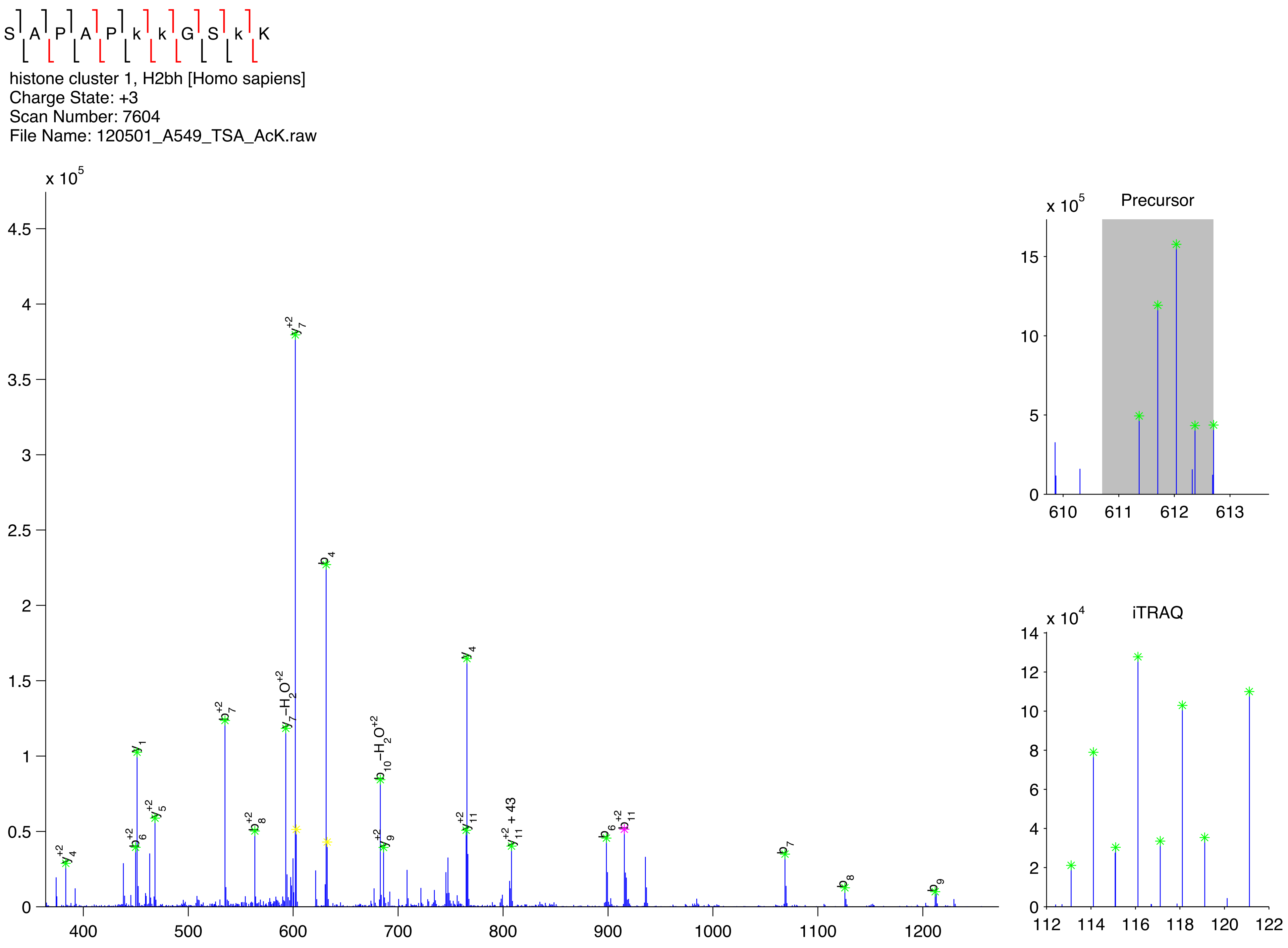
S A P A P k k G S k K
histone cluster 1, H2bh [Homo sapiens]
Charge State: +3
Scan Number: 7604
File Name: 120501_A549_TSA_AcK.raw
x 10^5
Precursor
iTRAQ
x 10^4
y4+2
b6+2
y1
y5+2
b7+2
b8+2
y7−H2O+2
y7+2
b4
b10−H2O+2
y9+2
y11+2
y4
y11+2 + 43
b6
b11+2
b7
b8
b9

S A P A P k k G S k K

histone cluster 1, H2bh [Homo sapiens]
Charge State: +2
Scan Number: 8028
File Name: 120501_A549_TSA_AcK.raw

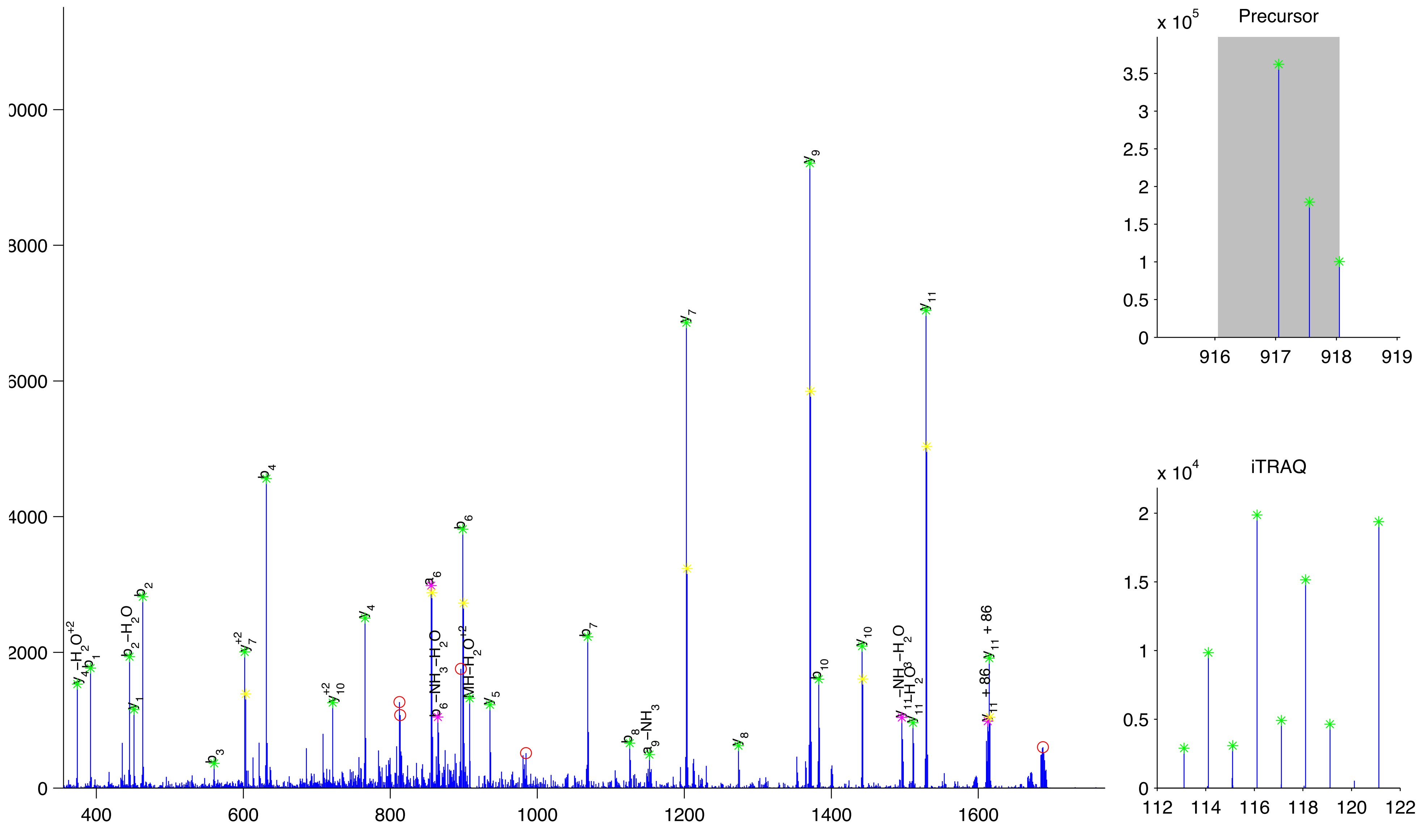

k G S k k A V T K

histone cluster 1, H2bh [Homo sapiens]
Charge State: +3
Scan Number: 9217
File Name: 120501_A549_TSA_AcK.raw

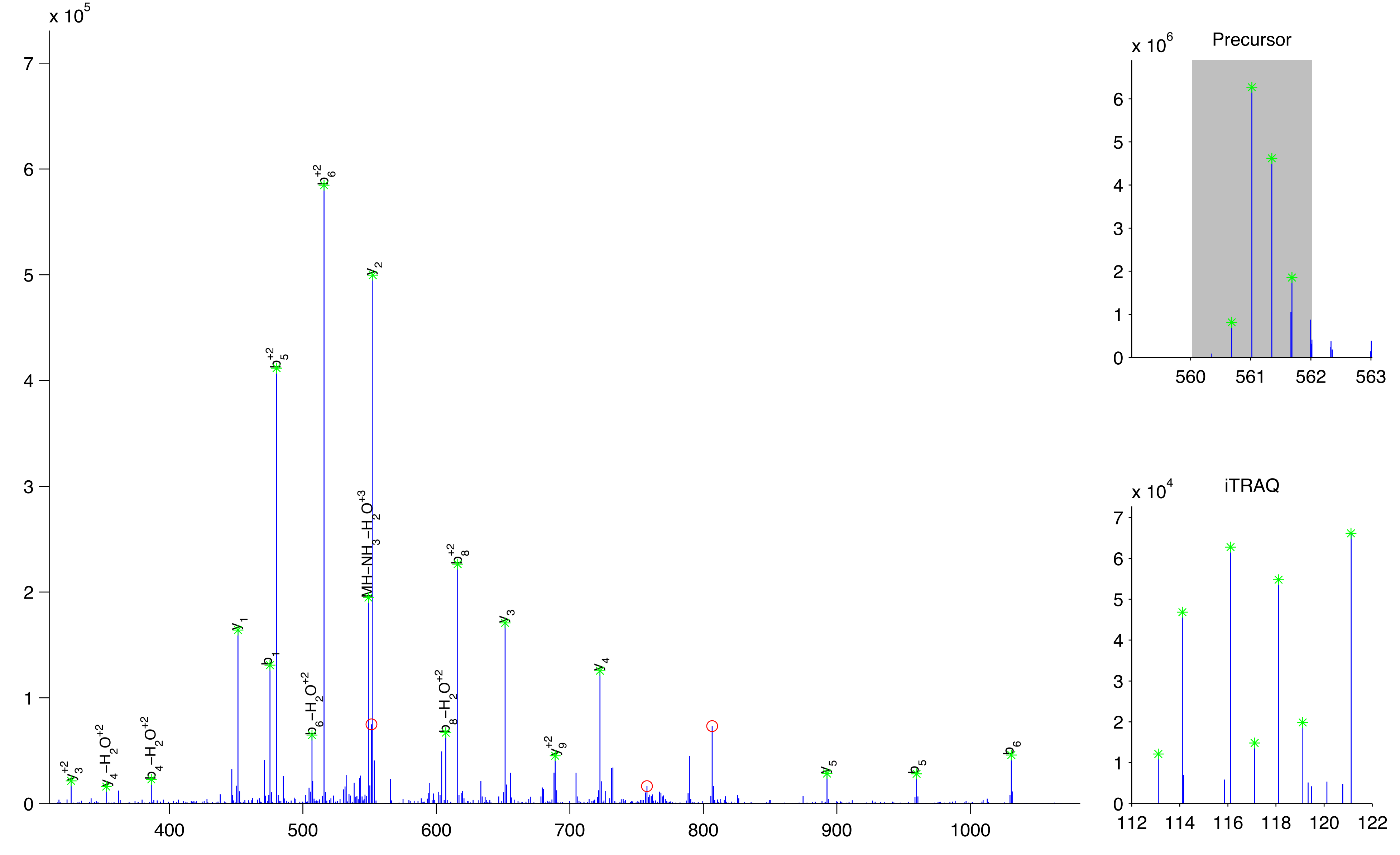

k G S k k A V T K

histone cluster 1, H2bh [Homo sapiens]
Charge State: +2
Scan Number: 9872
File Name: 120501_A549_TSA_AcK.raw

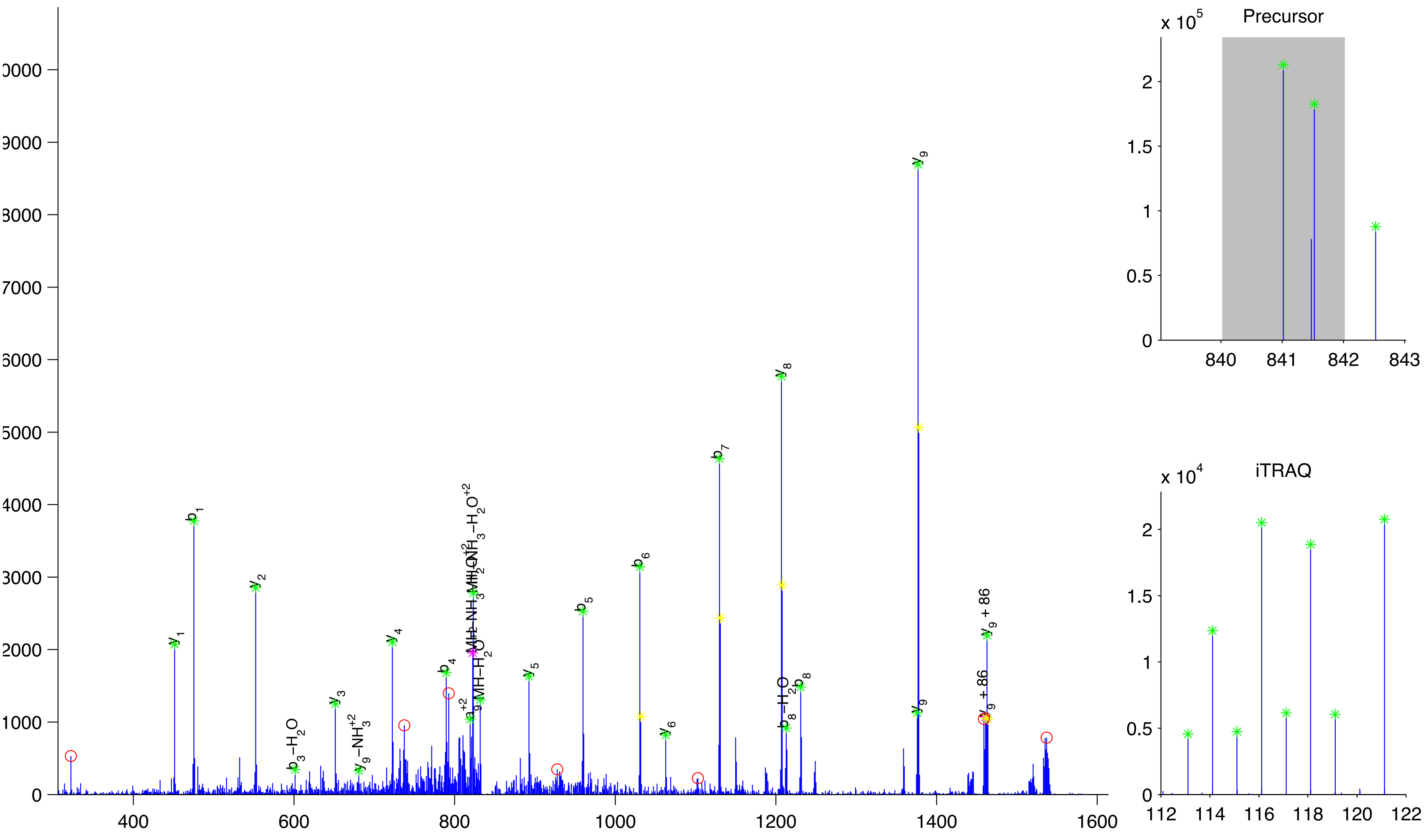

k A V T k A Q k K

histone cluster 1, H2bh [Homo sapiens]
Charge State: +3
Scan Number: 10731
File Name: 120501_A549_TSA_AcK.raw

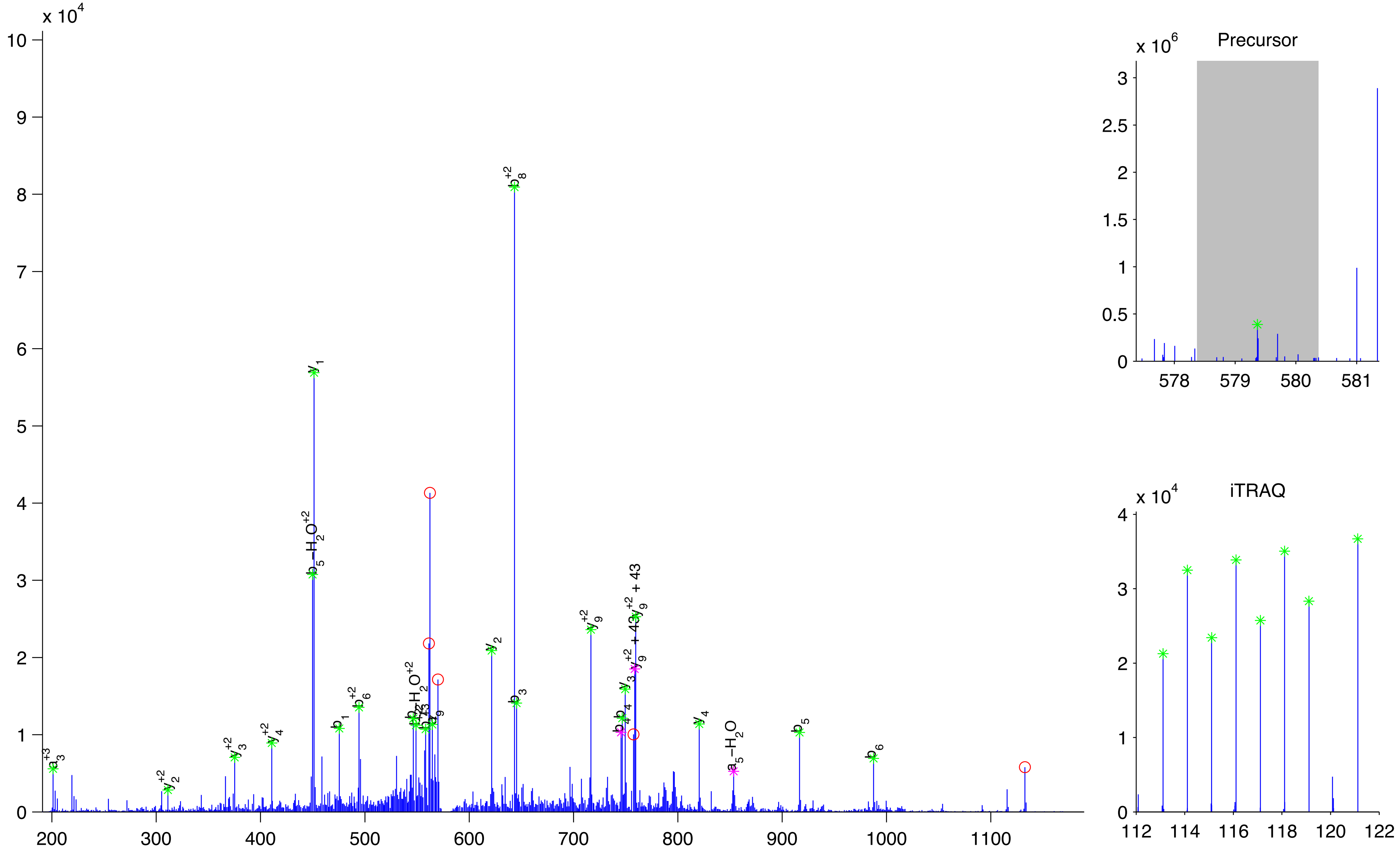

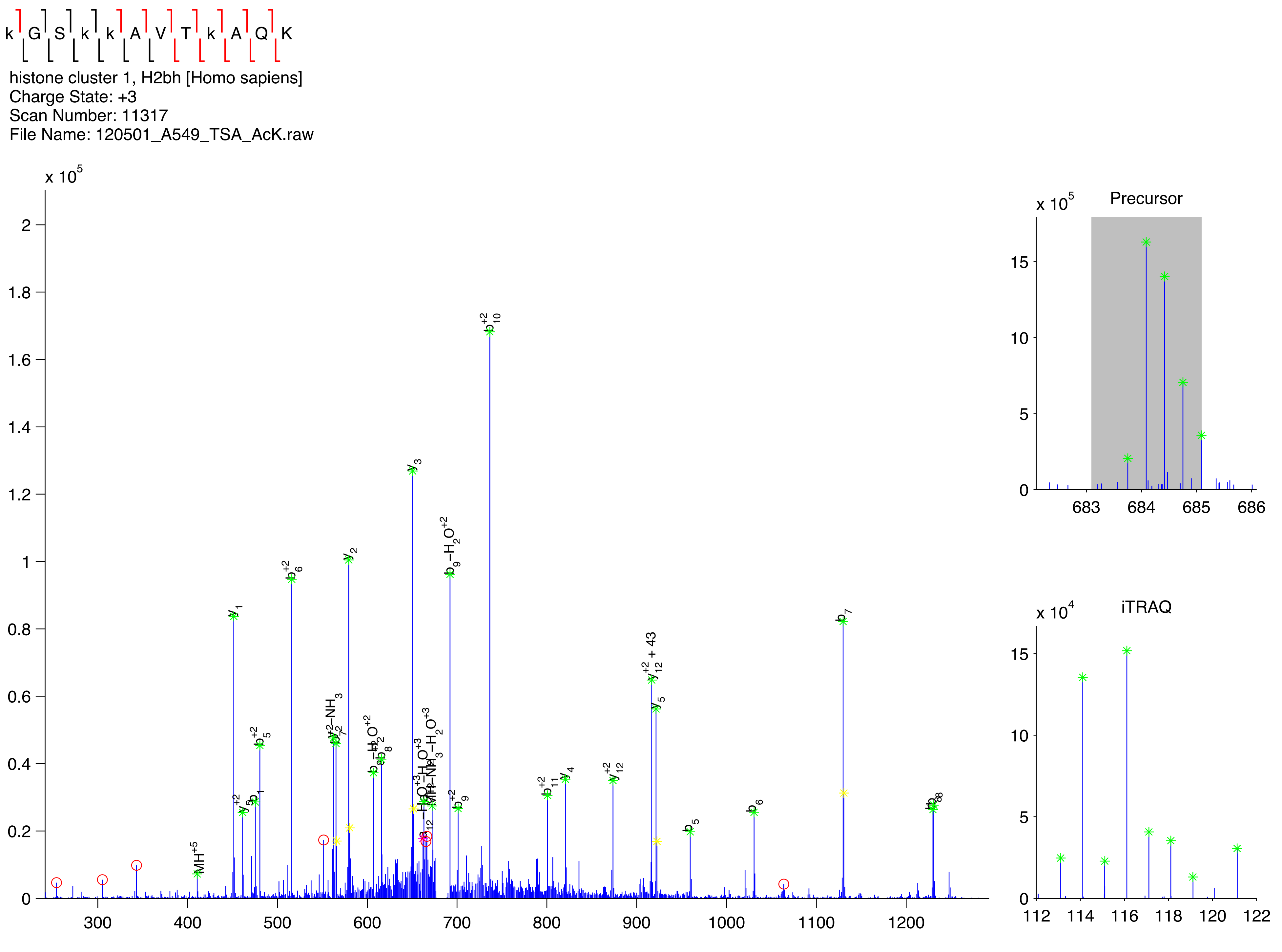
k G S k k A V T k A Q K
histone cluster 1, H2bh [Homo sapiens]
Charge State: +3
Scan Number: 11317
File Name: 120501_A549_TSA_AcK.raw
x 10^5
Precursor
iTRAQ
x 10^4

k G S k k A V T k A Q K

histone cluster 1, H2bh [Homo sapiens]
Charge State: +2
Scan Number: 11978
File Name: 120501_A549_TSA_AcK.raw

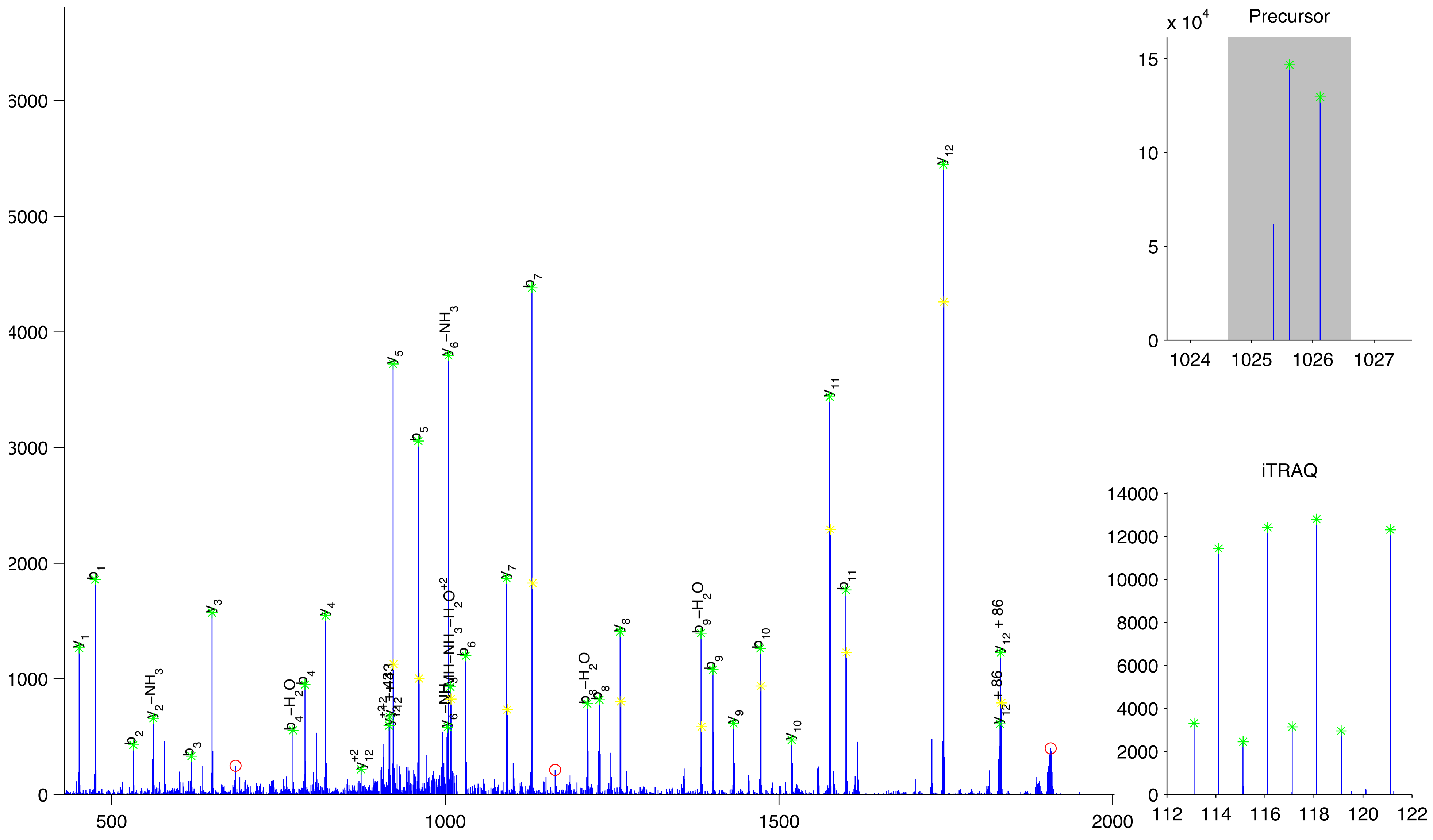

k G S k k A V T k A Q K

histone cluster 1, H2bh [Homo sapiens]
Charge State: +3
Scan Number: 12388
File Name: 120501_A549_TSA_AcK.raw

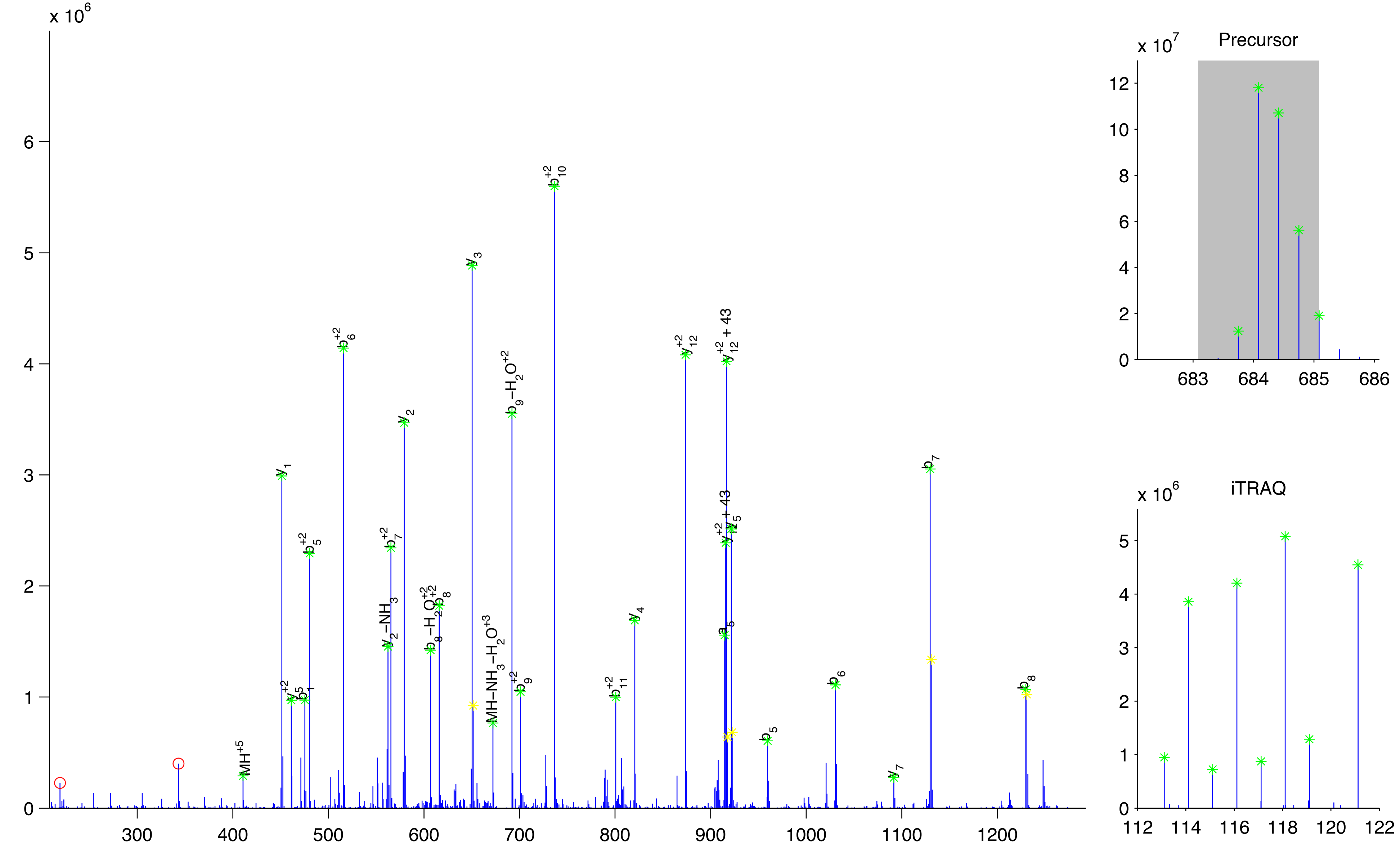

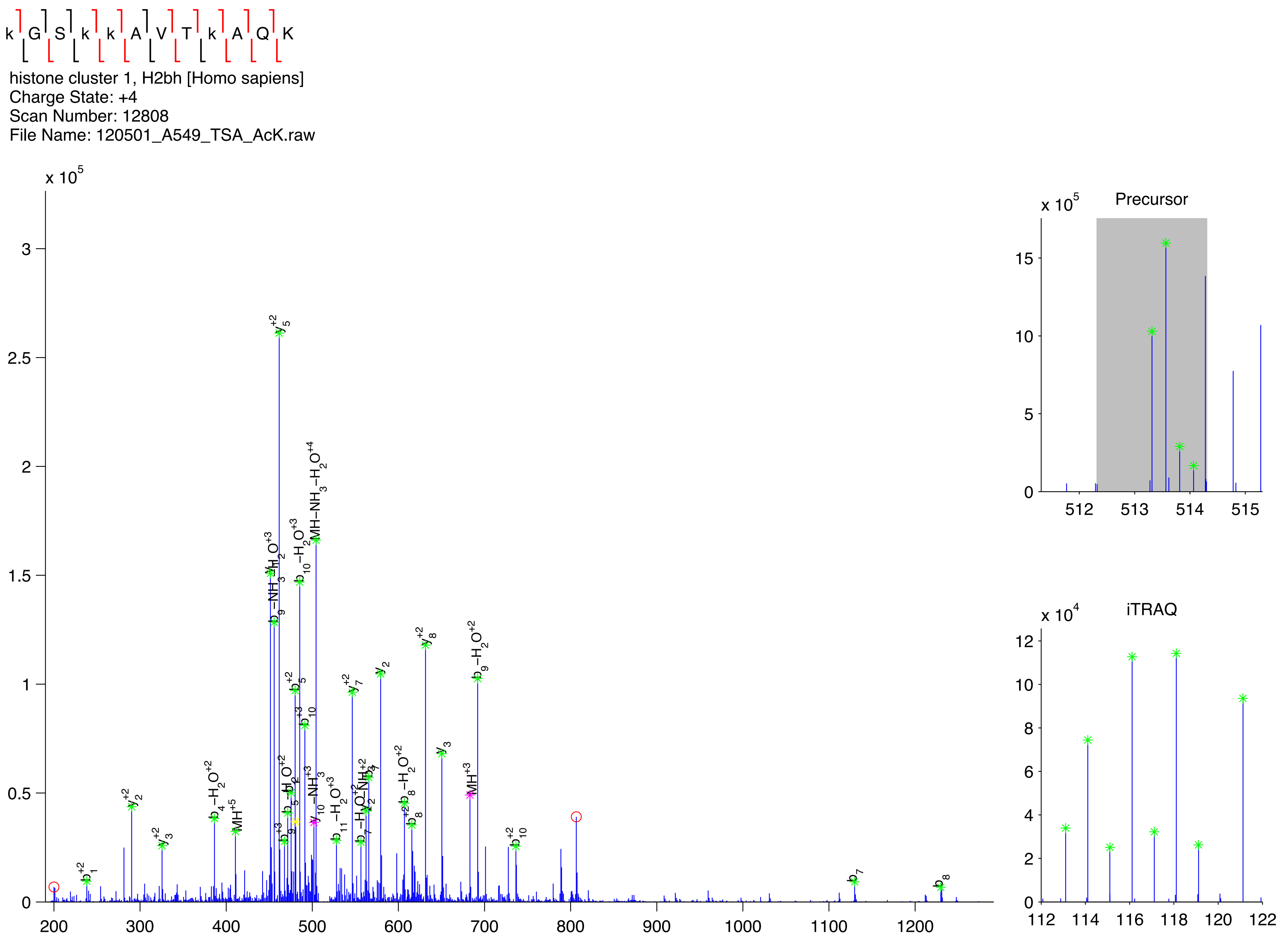

k G S k k A V T k A Q K
histone cluster 1, H2bh [Homo sapiens]
Charge State: +4
Scan Number: 12808
File Name: 120501_A549_TSA_AcK.raw
x 10^5
Precursor
iTRAQ
x 10^4

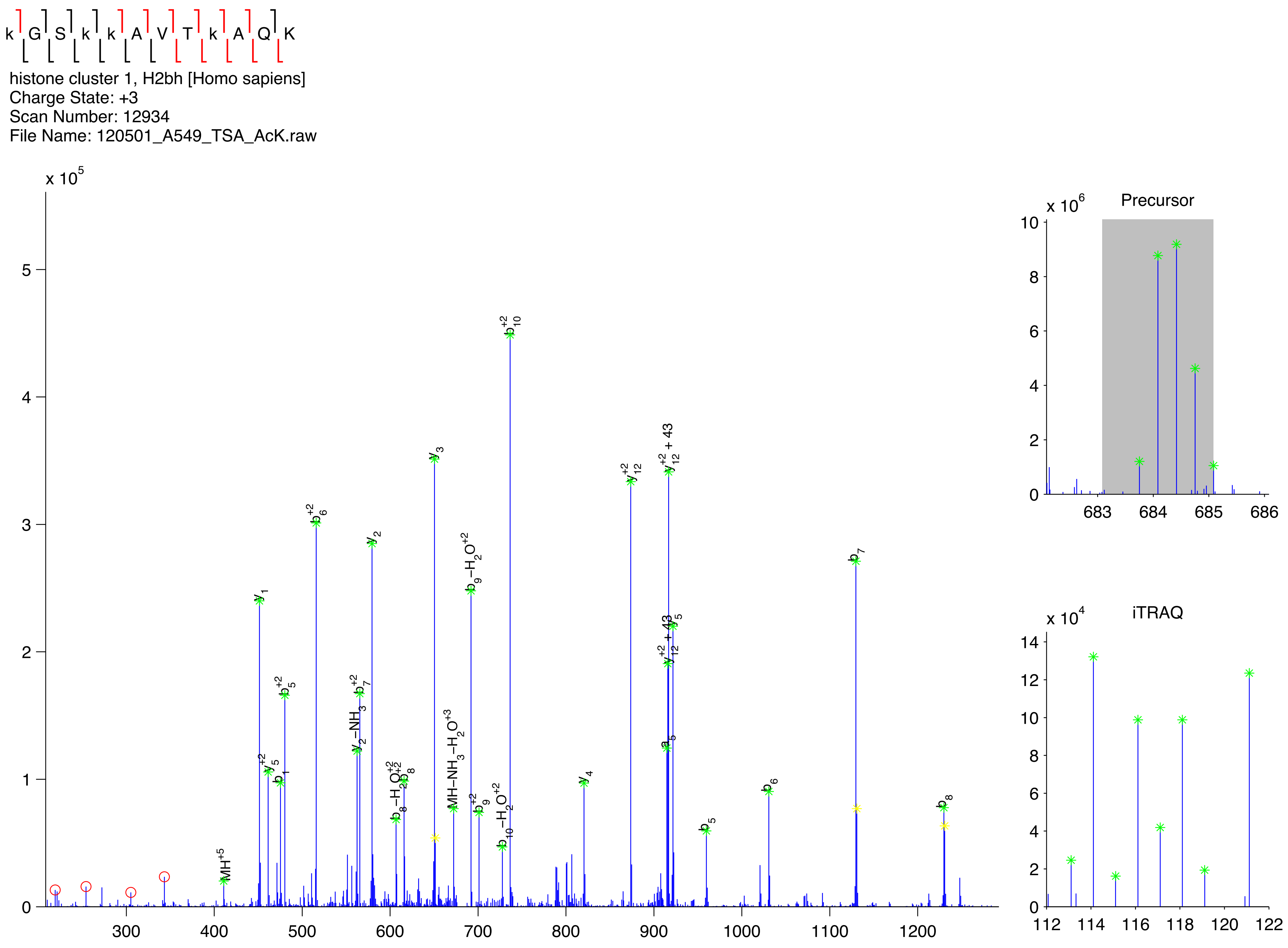

k G S k k A V T k A Q K
histone cluster 1, H2bh [Homo sapiens]
Charge State: +3
Scan Number: 12934
File Name: 120501_A549_TSA_AcK.raw
x 10^5
MH+5
y1
y5+2
b1
b5+2
b6+2
y2−NH3
b7+2
y2
b8−H2O+2
b8+2
y3
MH−NH3−H2O+3
b9−H2O+2
b9+2
b10−H2O+2
b10+2
y4
y12+2
a5
y12+2 + 43
y5
b5
b6
b7
b8
Precursor
x 10^6
iTRAQ
x 10^4

k G S k k A V T k A Q K

histone cluster 1, H2bh [Homo sapiens]
Charge State: +4
Scan Number: 13354
File Name: 120501_A549_TSA_AcK.raw

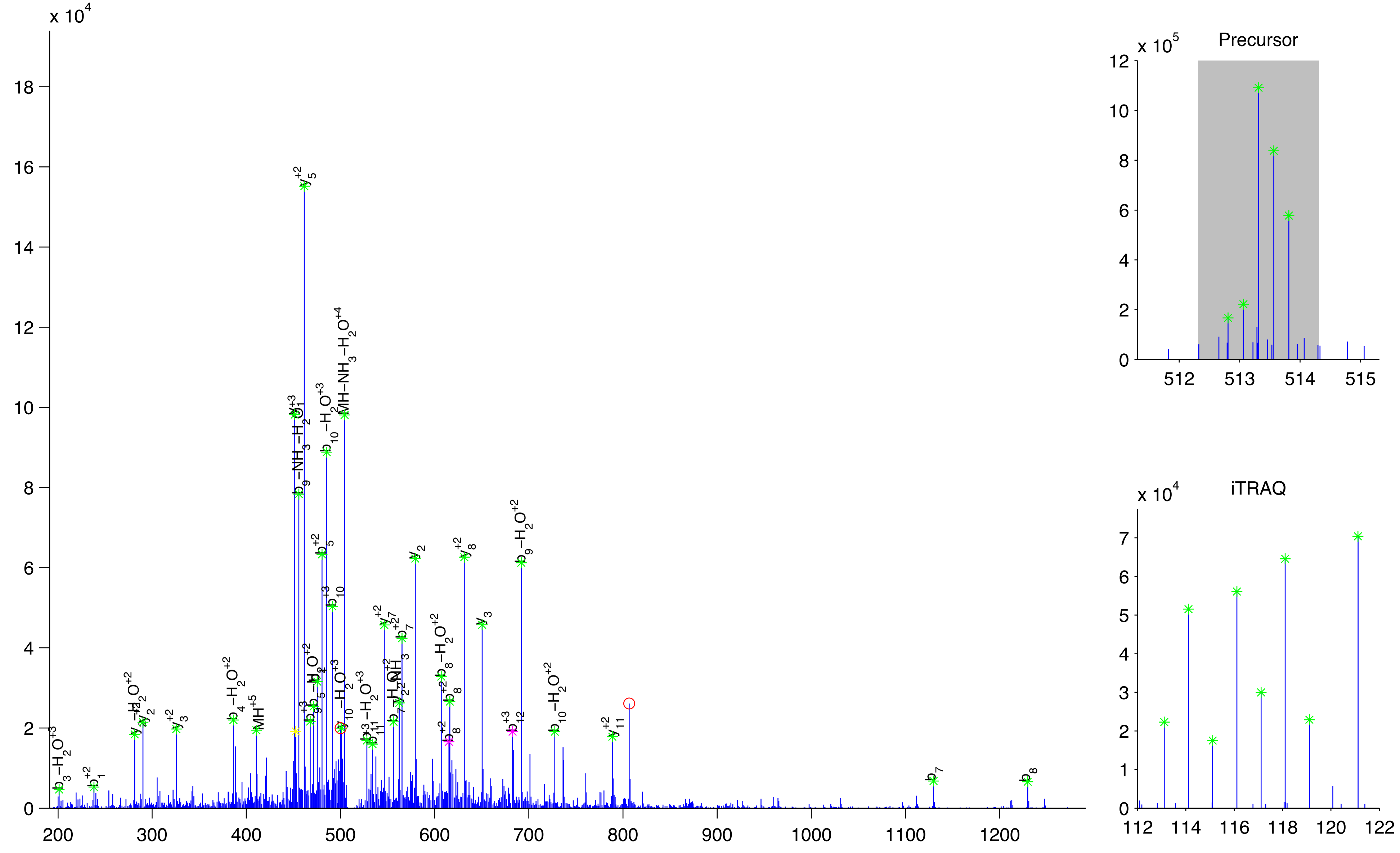

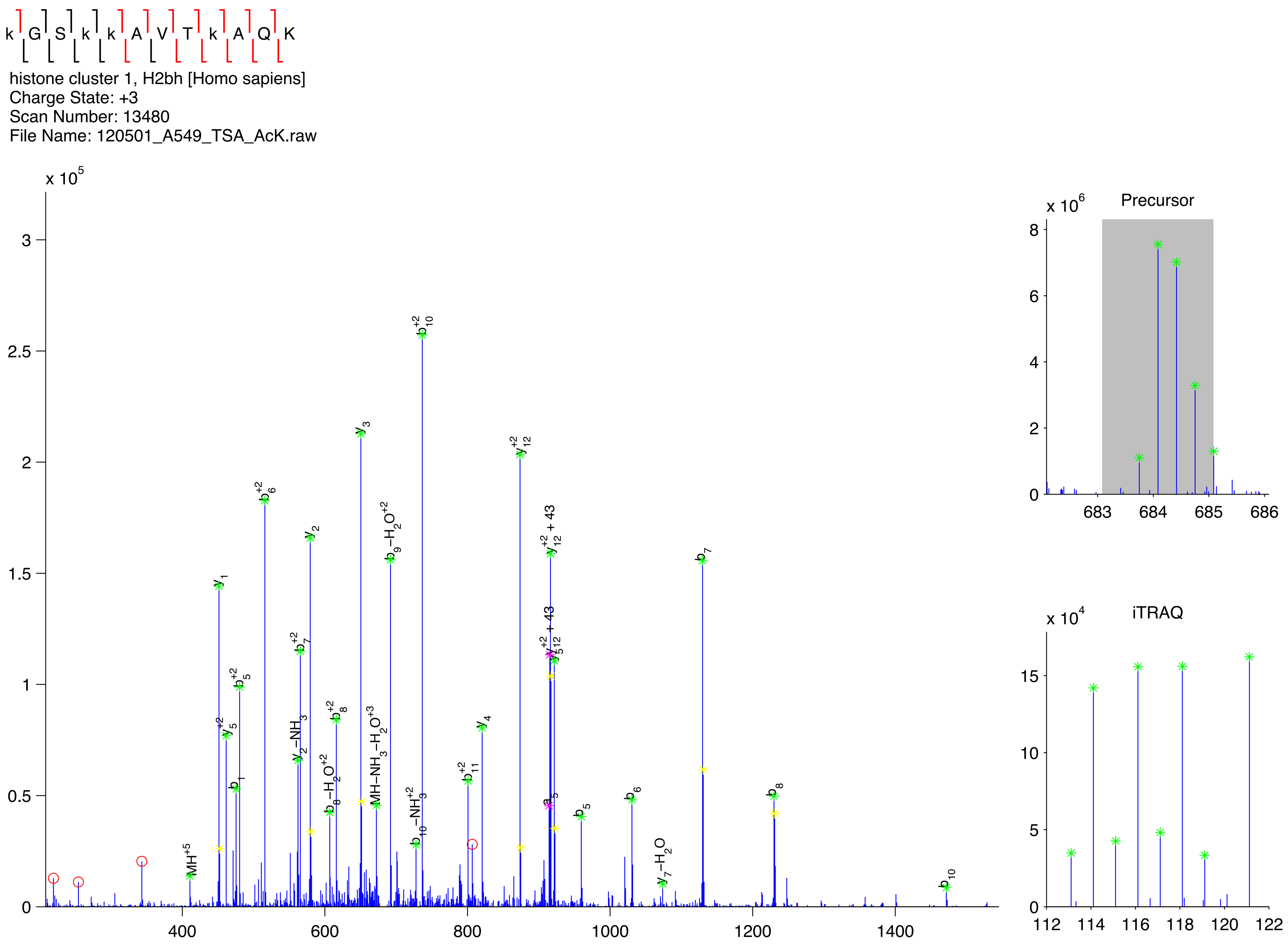

k G S k k A V T k A Q K
histone cluster 1, H2bh [Homo sapiens]
Charge State: +3
Scan Number: 13480
File Name: 120501_A549_TSA_AcK.raw
x 10^5
Precursor
x 10^6
iTRAQ
x 10^4

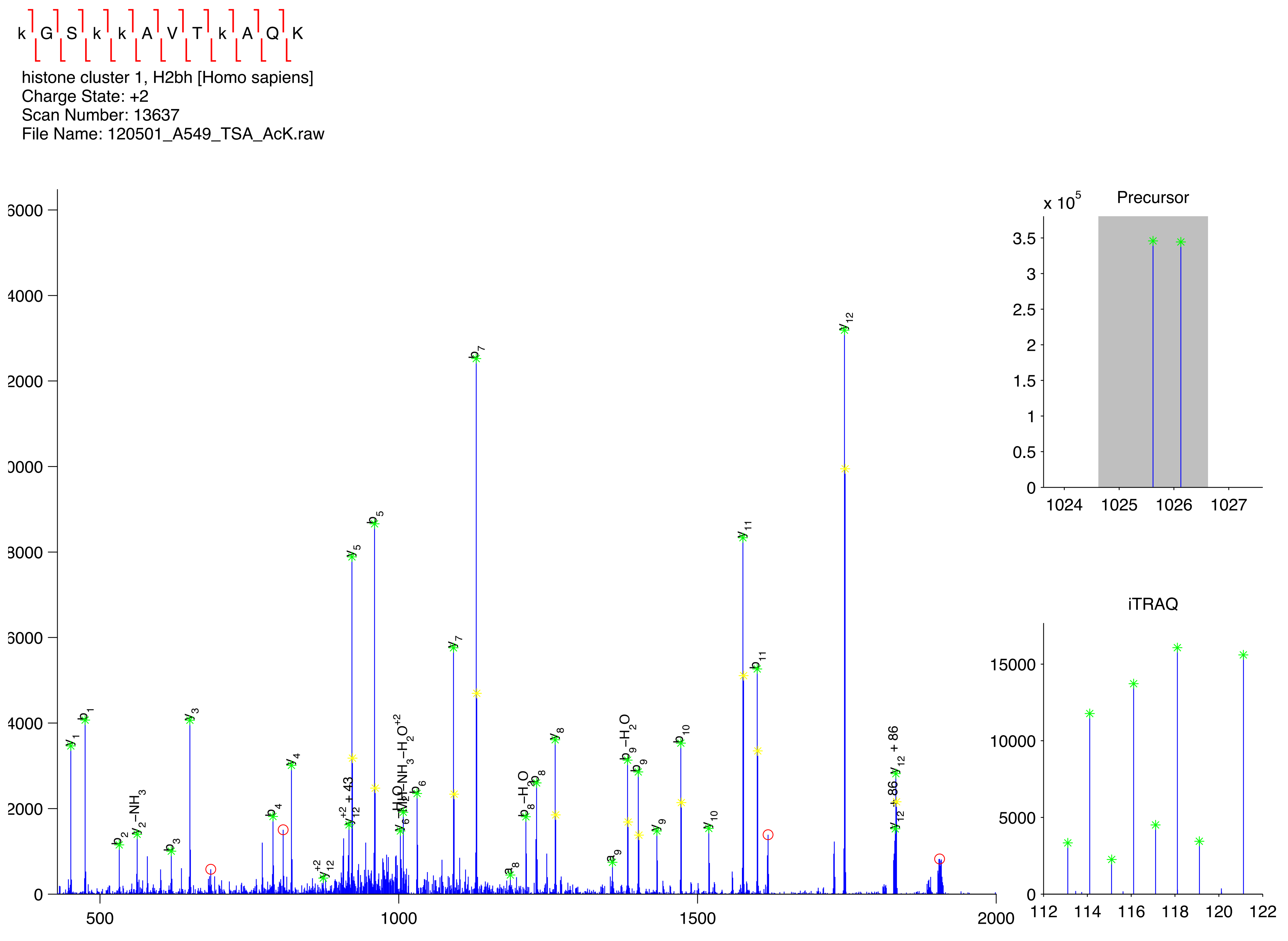

k G S k k A V T k A Q K
histone cluster 1, H2bh [Homo sapiens]
Charge State: +2
Scan Number: 13637
File Name: 120501_A549_TSA_AcK.raw
Precursor
iTRAQ

k G S k k A V T k A Q K

histone cluster 1, H2bh [Homo sapiens]
Charge State: +3
Scan Number: 14028
File Name: 120501_A549_TSA_AcK.raw

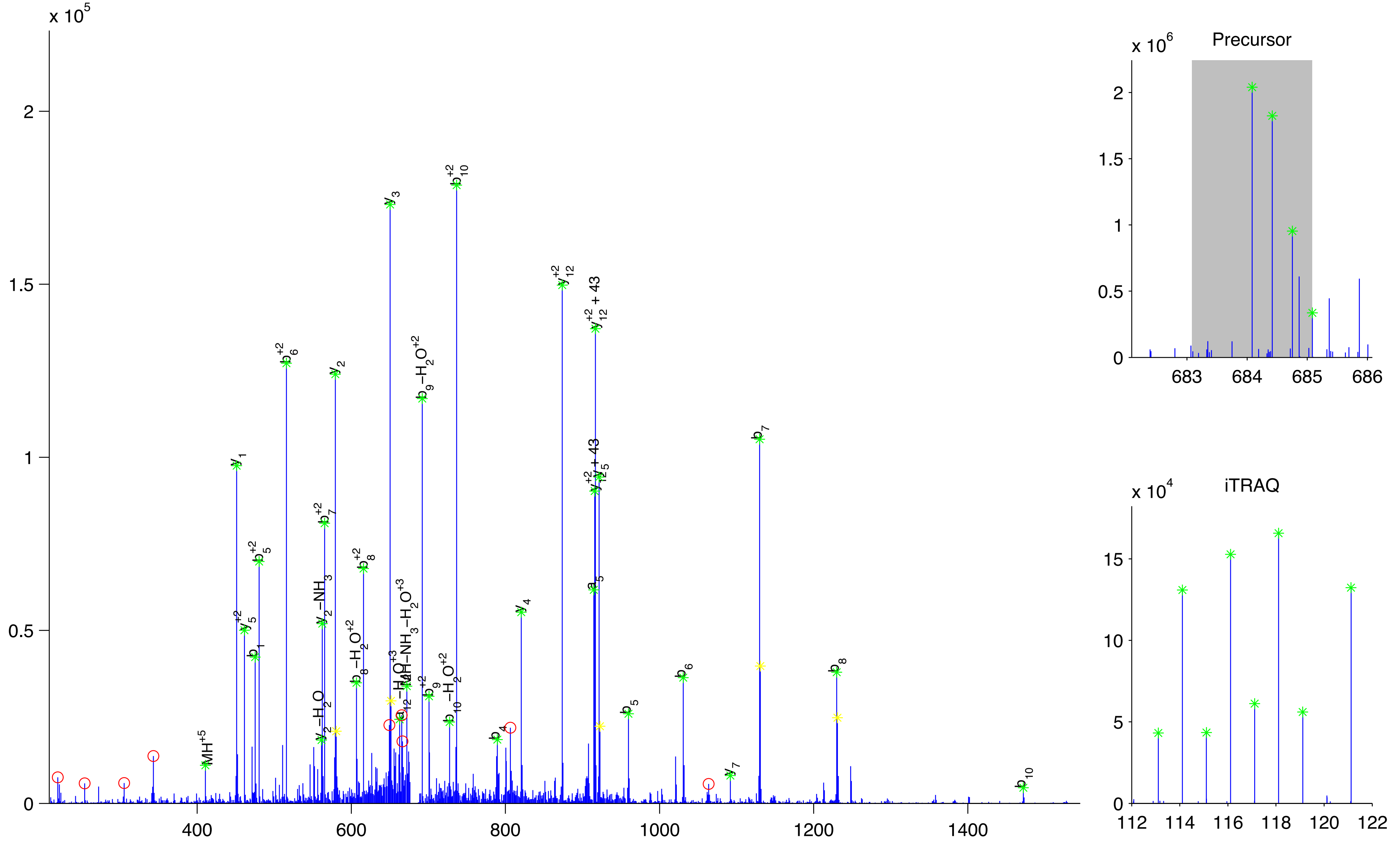

kGSkkAVTkAQkK
histone cluster 1, H2bh [Homo sapiens]
Charge State: +3
Scan Number: 14030
File Name: 120501_A549_TSA_AcK.raw

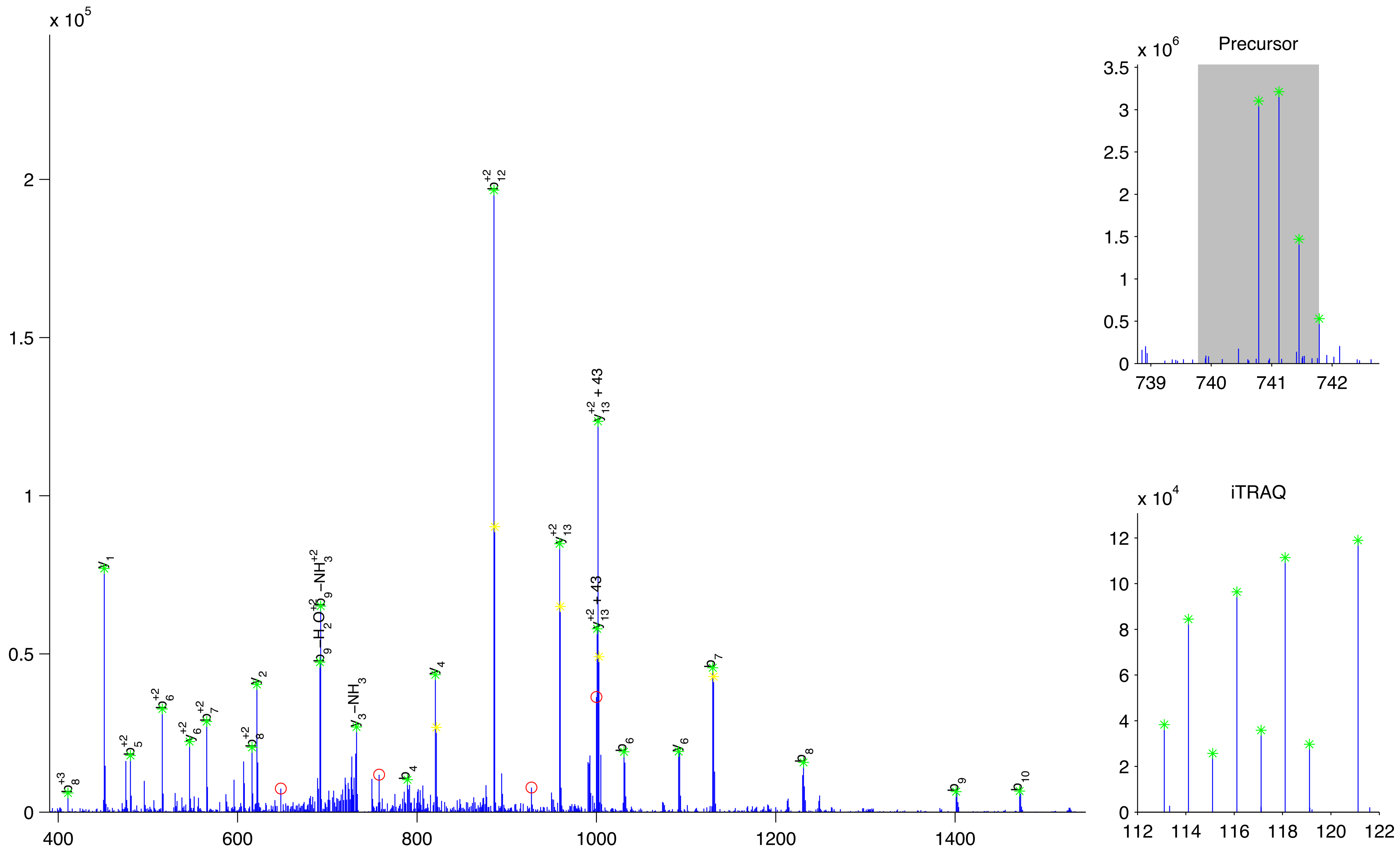

k G S k k A V T k A Q K

histone cluster 1, H2bh [Homo sapiens]
Charge State: +3
Scan Number: 14574
File Name: 120501_A549_TSA_AcK.raw

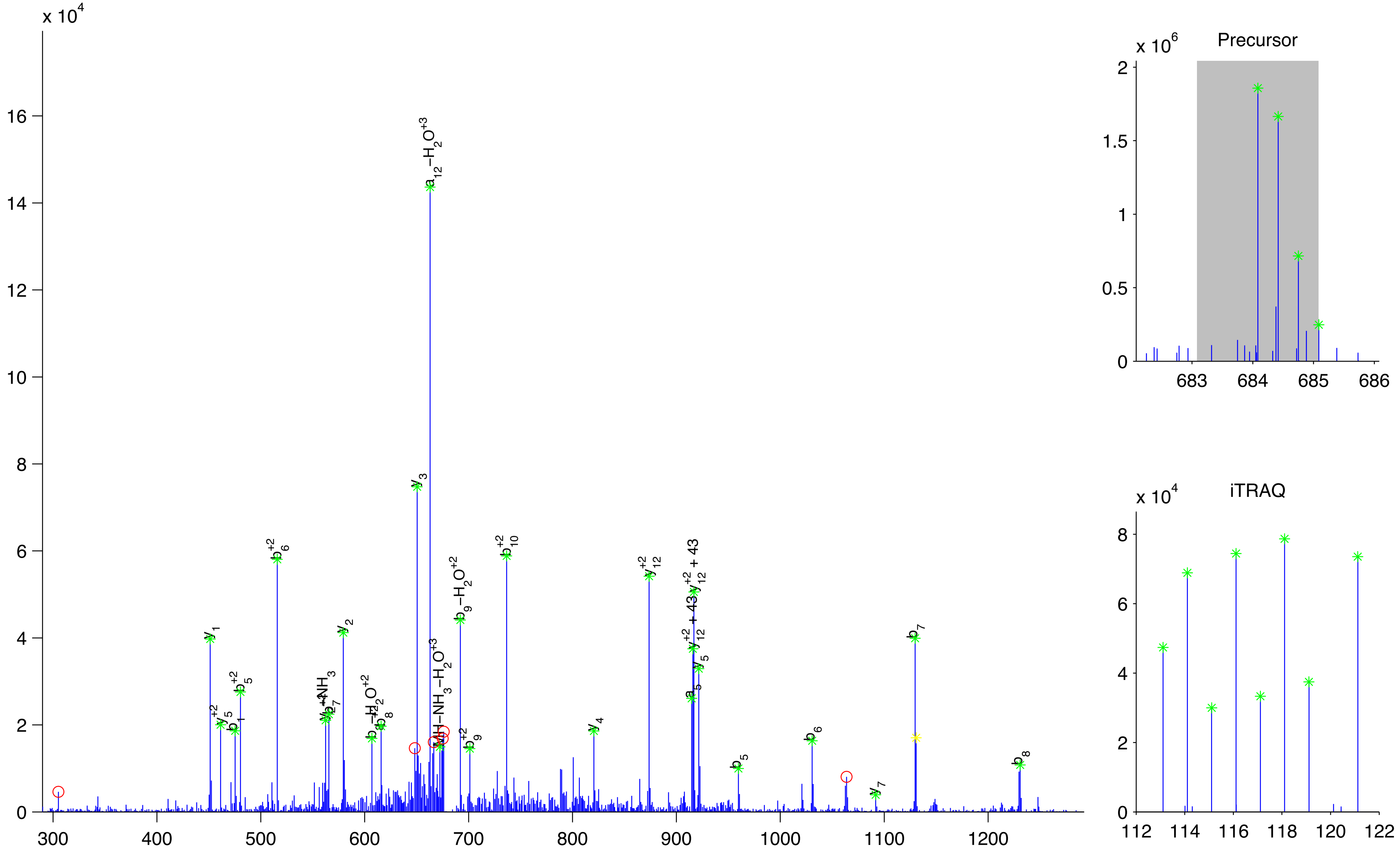

S A P A P k k G S k k A V T k A Q K

histone cluster 1, H2bh [Homo sapiens]
Charge State: +4
Scan Number: 15265
File Name: 120501_A549_TSA_AcK.raw

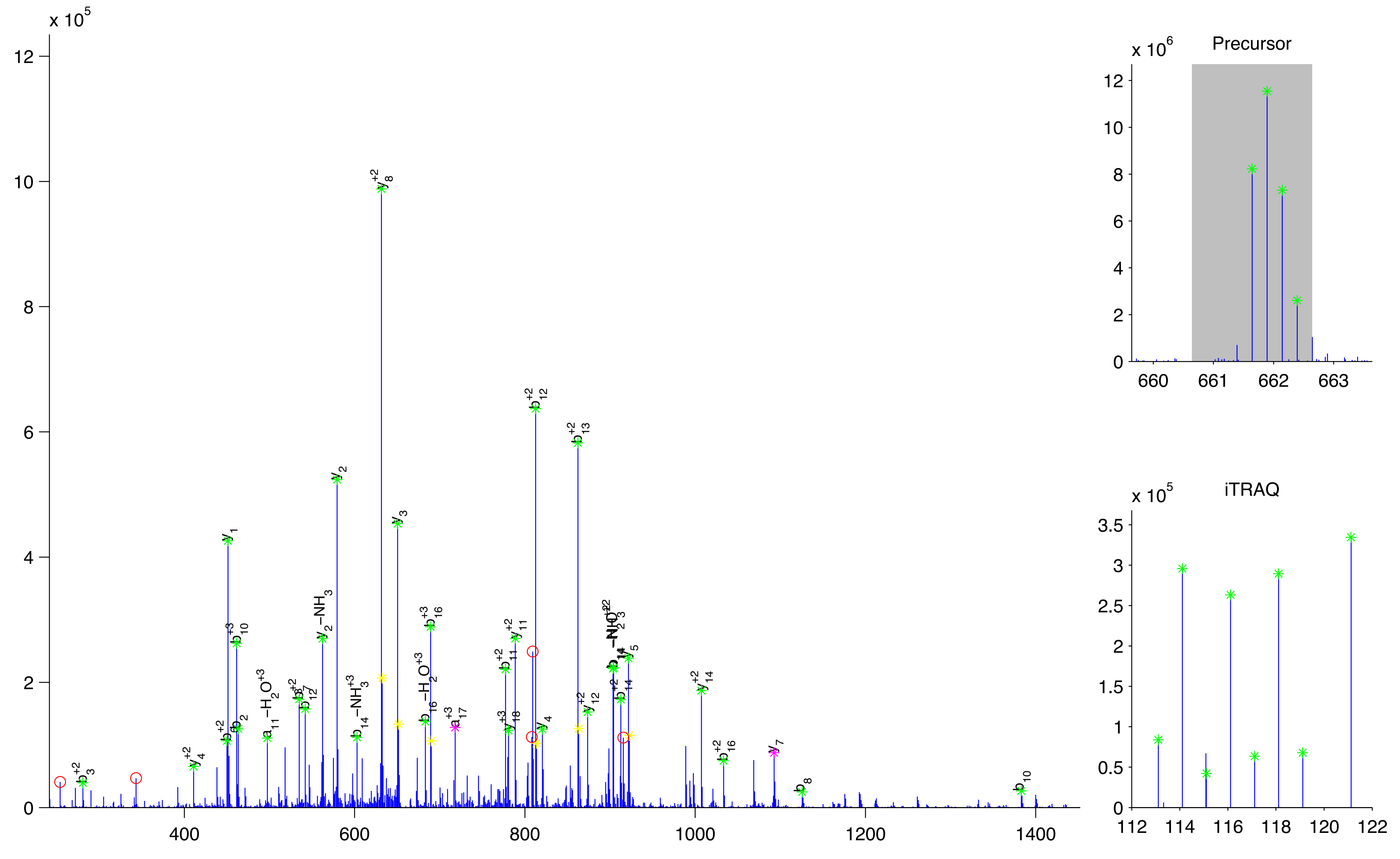

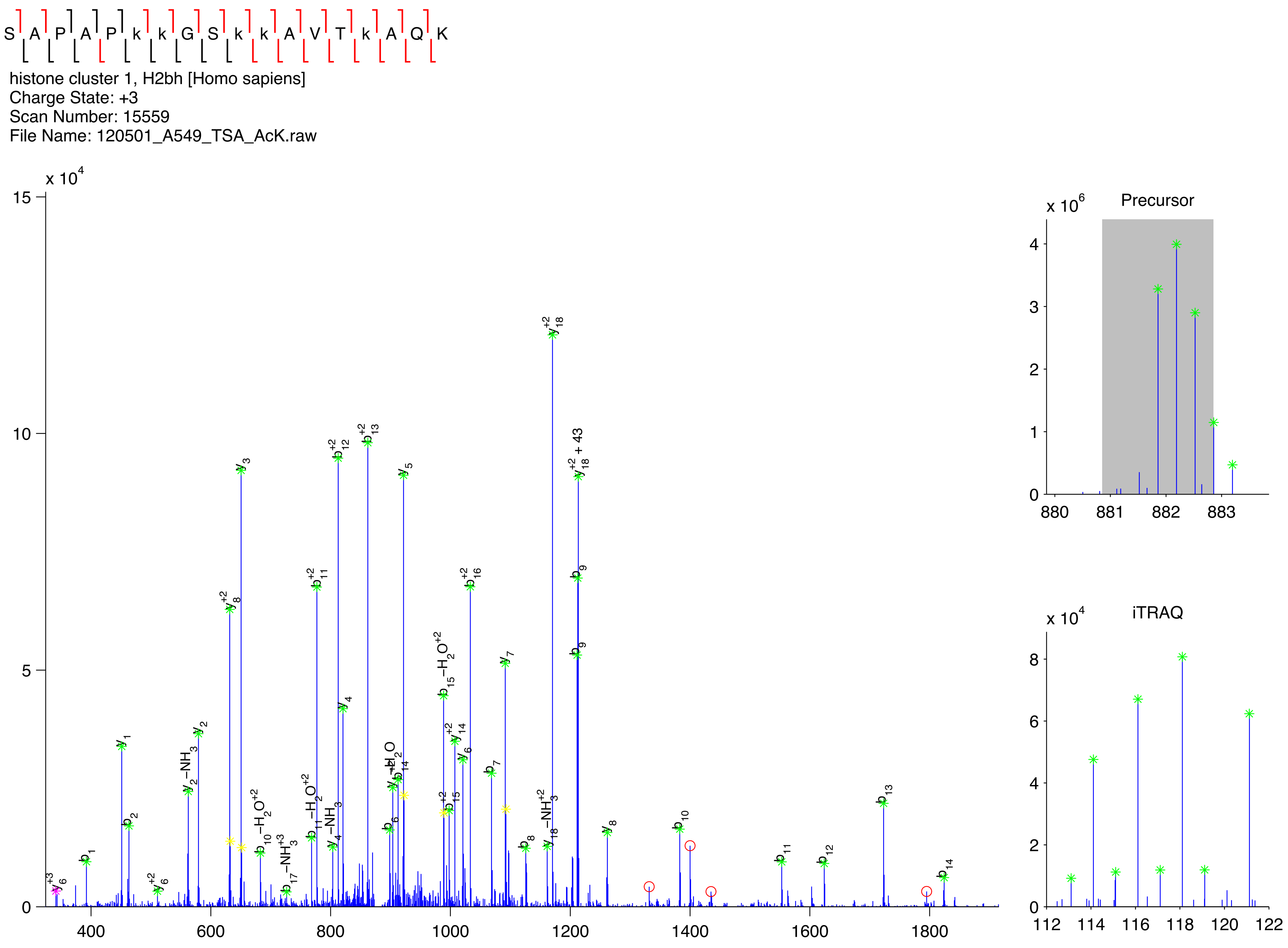

S A P A P k k G S k k A V T k A Q K
histone cluster 1, H2bh [Homo sapiens]
Charge State: +3
Scan Number: 15559
File Name: 120501_A549_TSA_AcK.raw
x 10^4
Precursor
x 10^6
iTRAQ

S A P A P k k G S k k A V T k A Q K

histone cluster 1, H2bh [Homo sapiens]
Charge State: +4
Scan Number: 15811
File Name: 120501_A549_TSA_AcK.raw

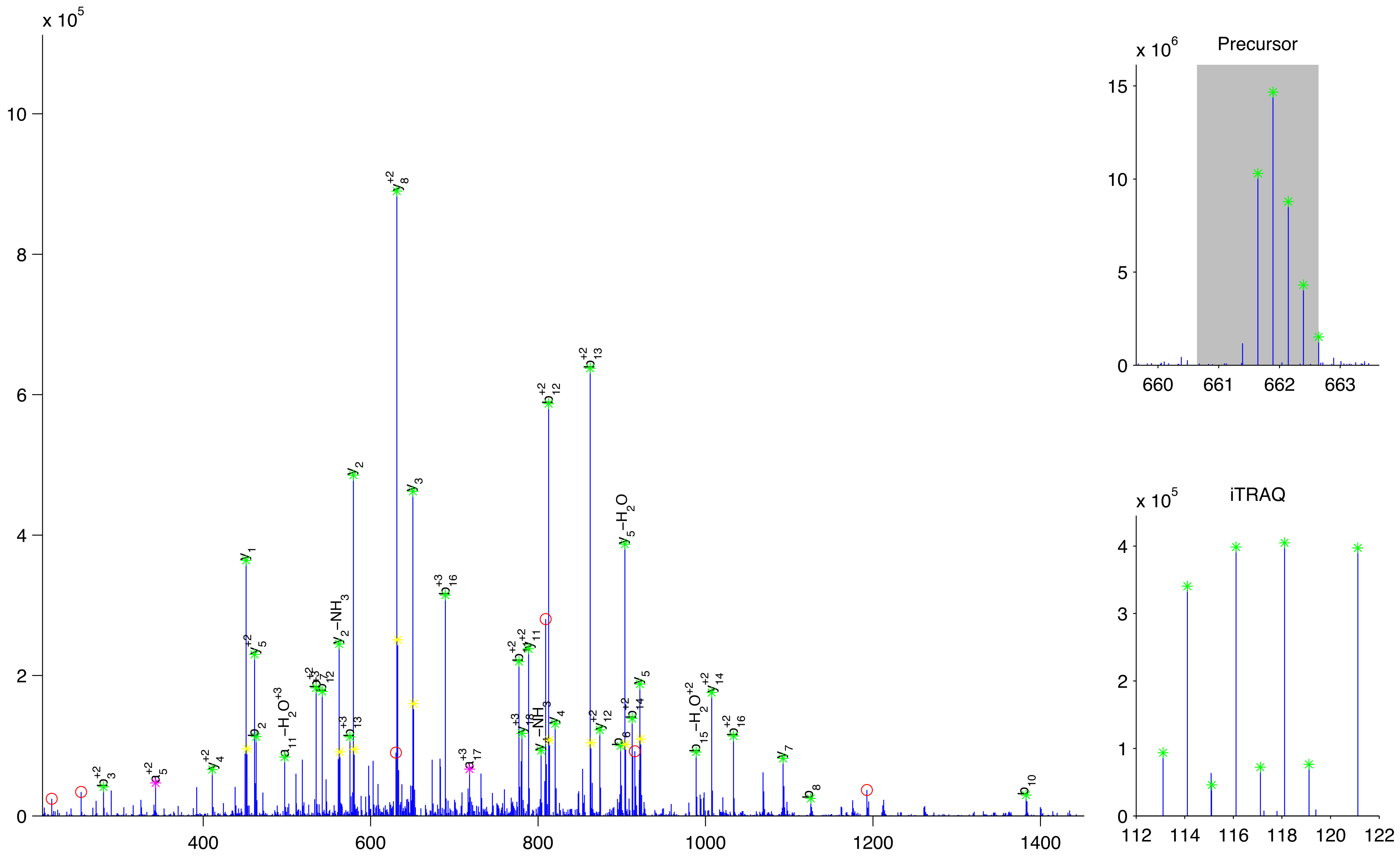

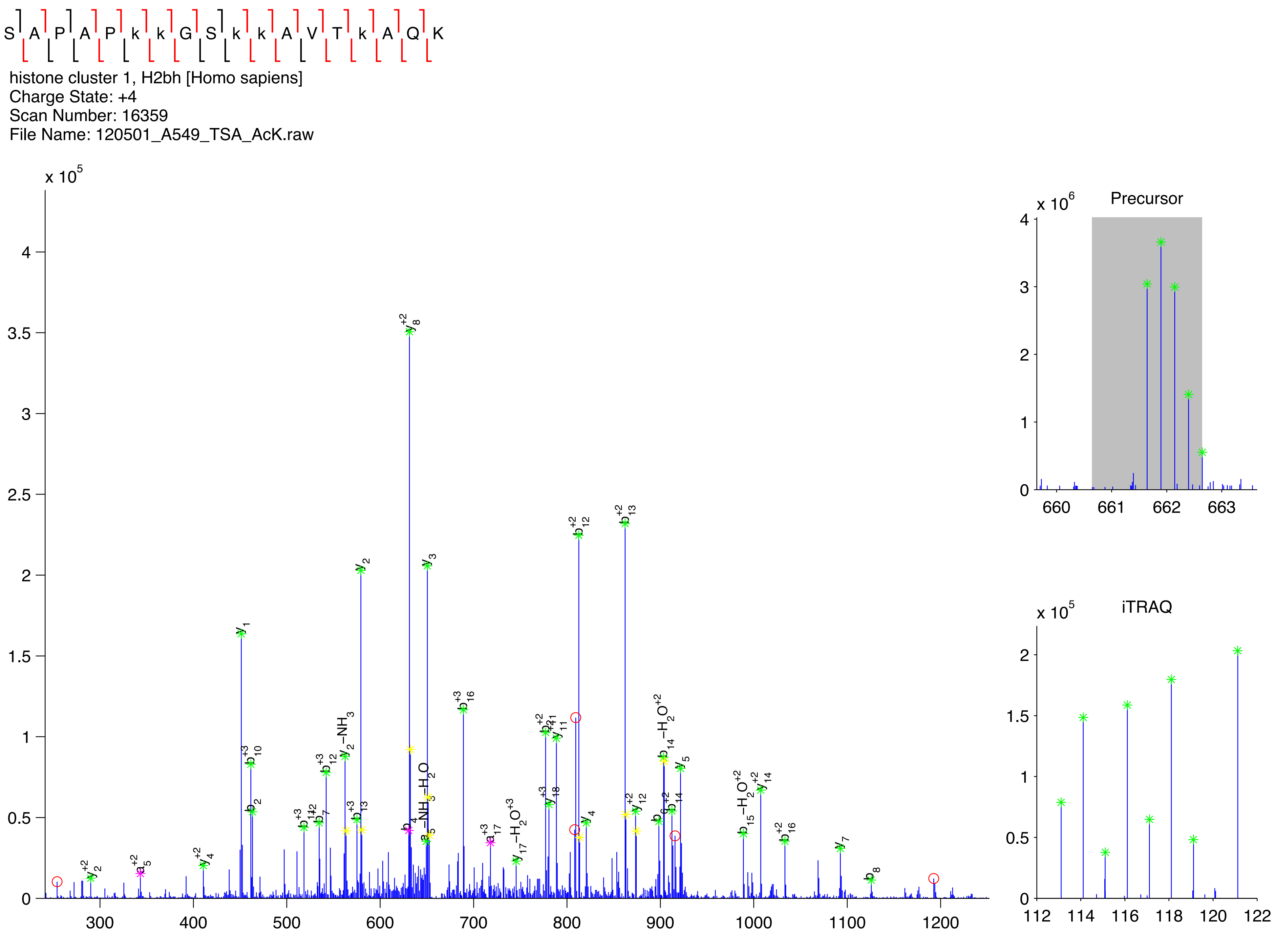
S A P A P k k G S k k A V T k A Q K
histone cluster 1, H2bh [Homo sapiens]
Charge State: +4
Scan Number: 16359
File Name: 120501_A549_TSA_AcK.raw
x 10^5
Precursor
x 10^6
iTRAQ
x 10^5

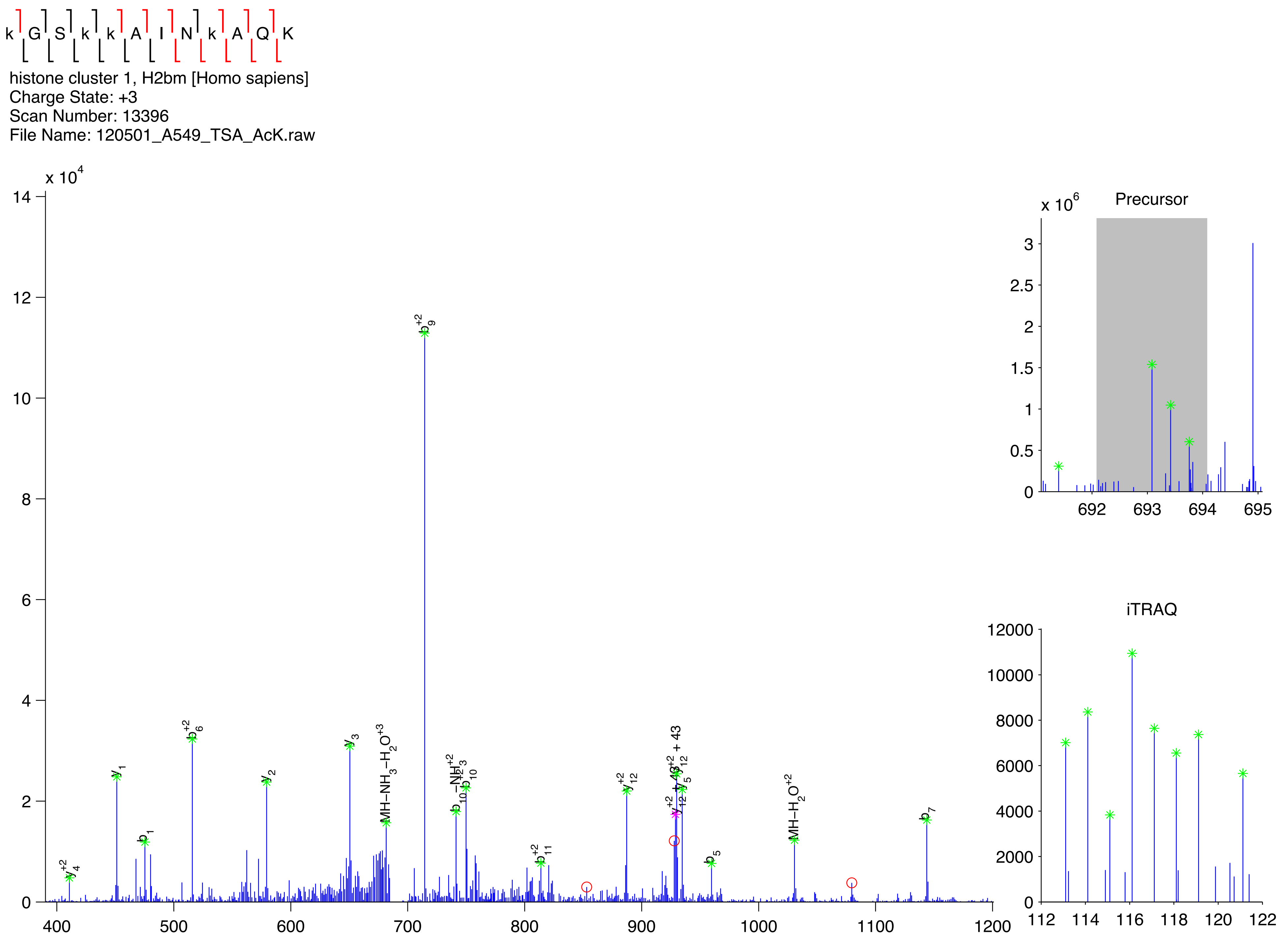
k G S k k A I N k A Q K
histone cluster 1, H2bm [Homo sapiens]
Charge State: +3
Scan Number: 13396
File Name: 120501_A549_TSA_AcK.raw
x 10^4
Precursor
x 10^6
iTRAQ
MH–NH3–H2O+3
MH–H2O+2
b9+2
b6+2
y1
b1
y2
y3
y4+2
b10–NH3+2
b10+2
b11+2
y12+2
y12+2 + 43
y5
b5
b7

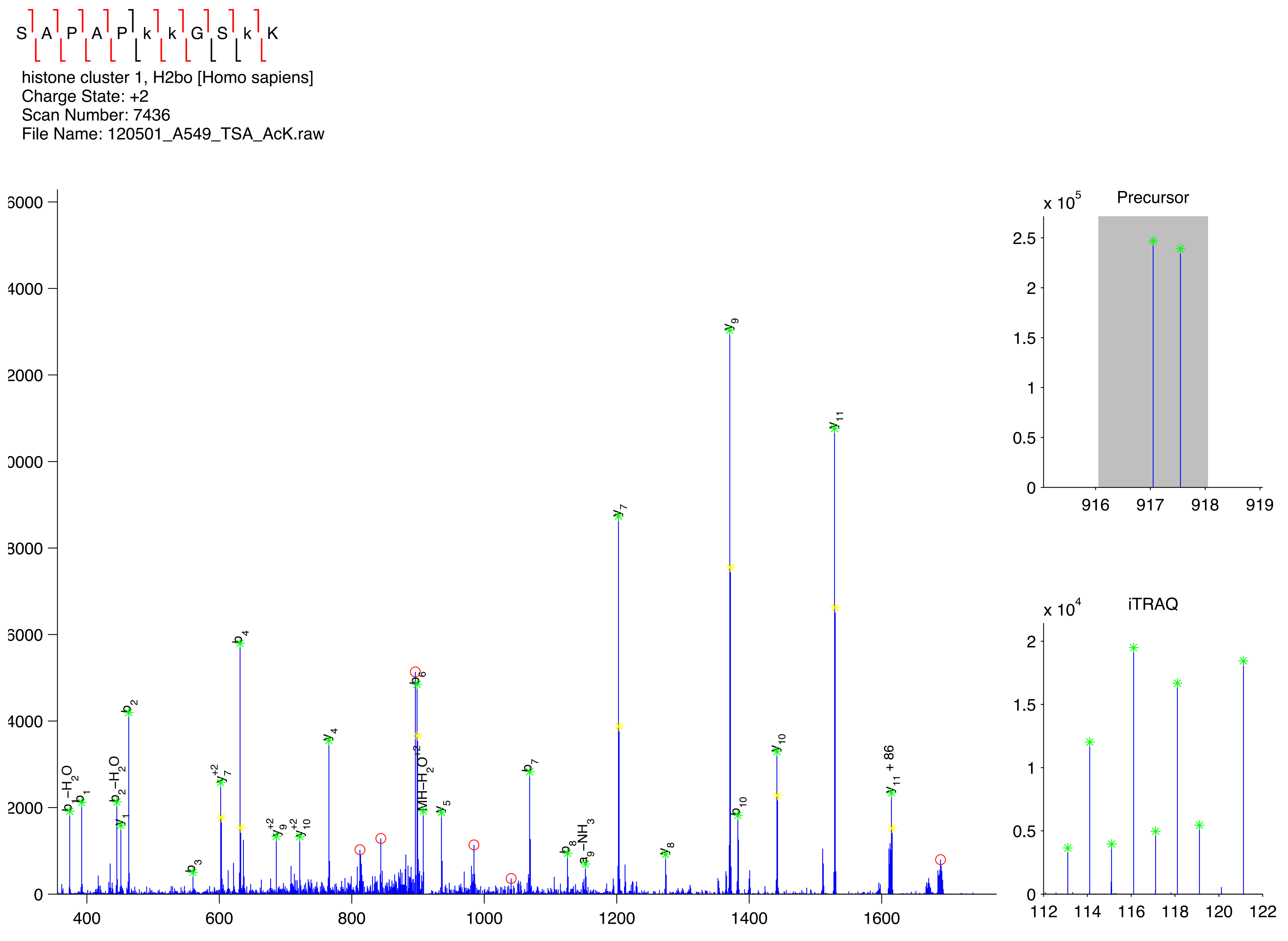
S A P A P k k G S k K
histone cluster 1, H2bo [Homo sapiens]
Charge State: +2
Scan Number: 7436
File Name: 120501_A549_TSA_AcK.raw
Precursor
iTRAQ

S A P A P k k G S k K

histone cluster 1, H2bo [Homo sapiens]
Charge State: +3
Scan Number: 7604
File Name: 120501_A549_TSA_AcK.raw

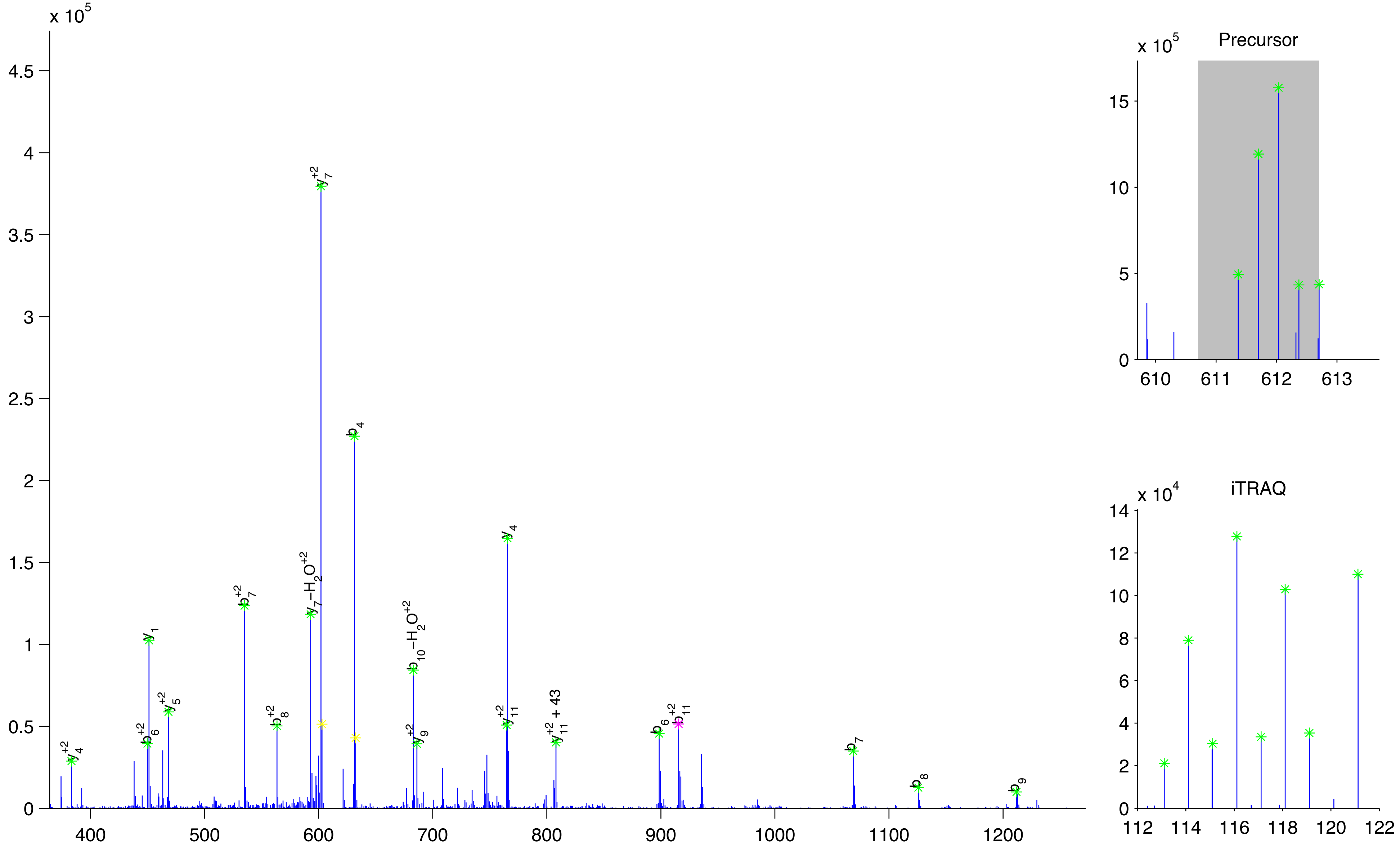

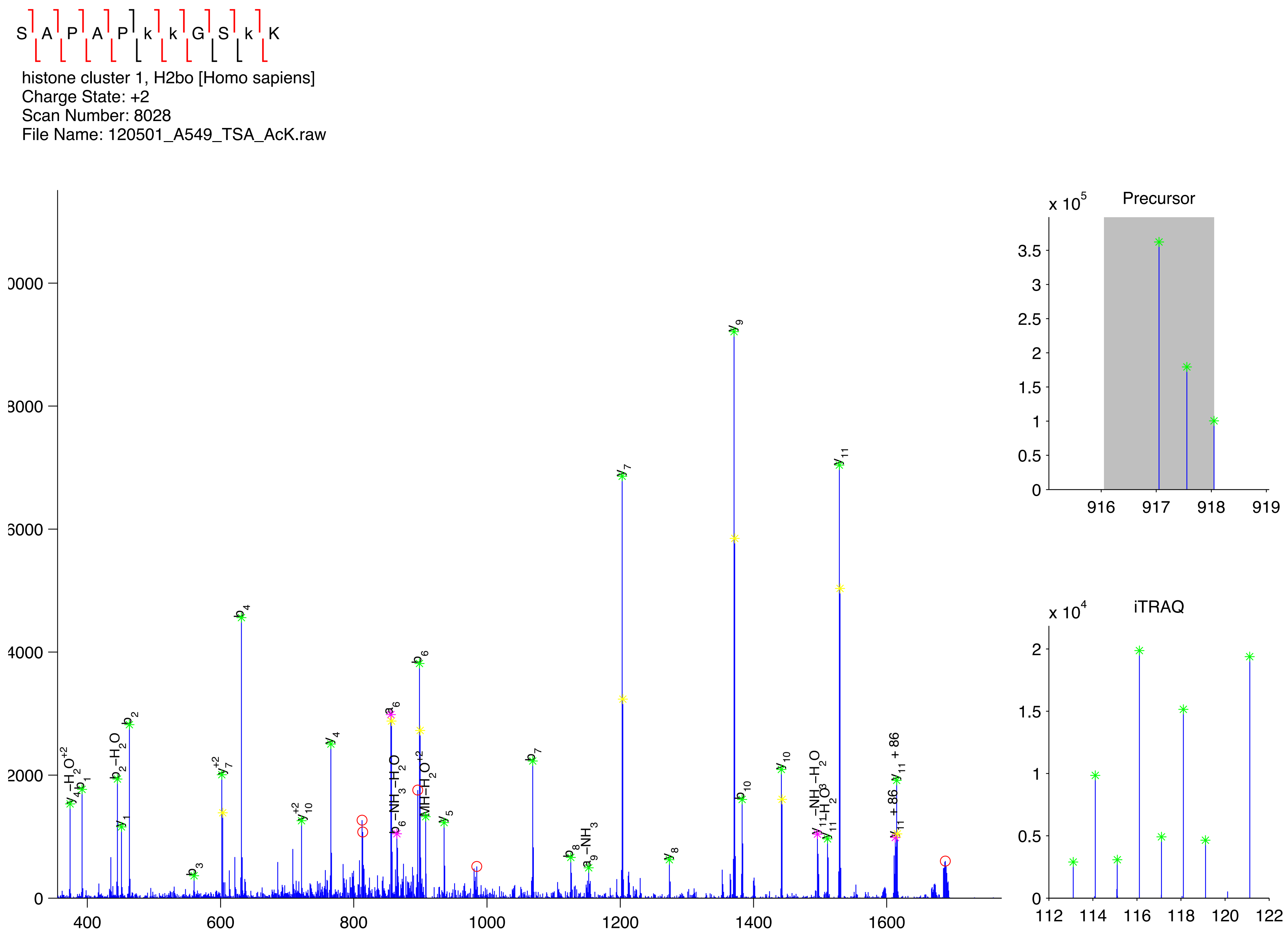
S A P A P k k G S k K
histone cluster 1, H2bo [Homo sapiens]
Charge State: +2
Scan Number: 8028
File Name: 120501_A549_TSA_AcK.raw
Precursor
x 10^5
iTRAQ
x 10^4

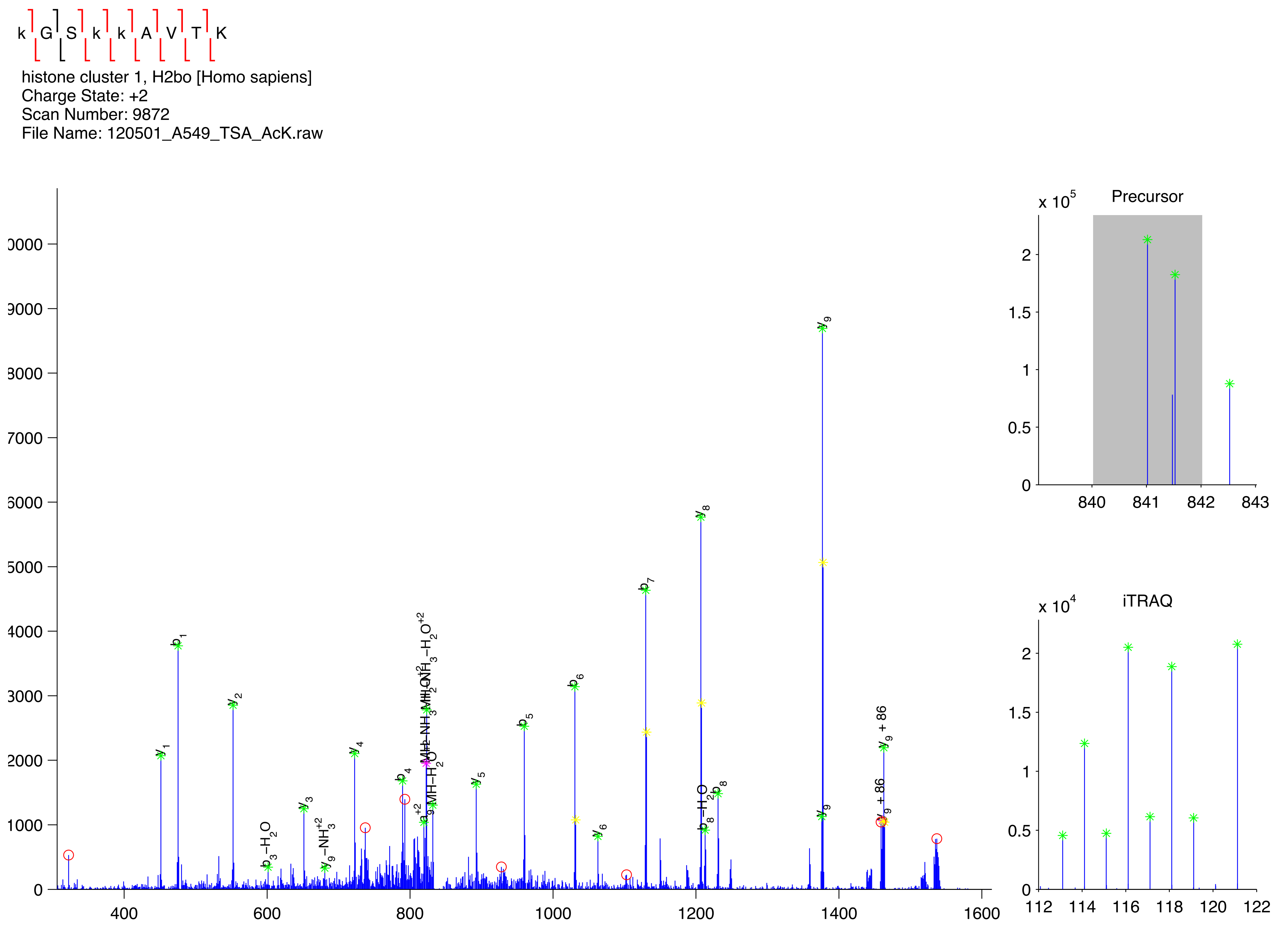
k G S k k A V T K
histone cluster 1, H2bo [Homo sapiens]
Charge State: +2
Scan Number: 9872
File Name: 120501_A549_TSA_AcK.raw
Precursor
iTRAQ

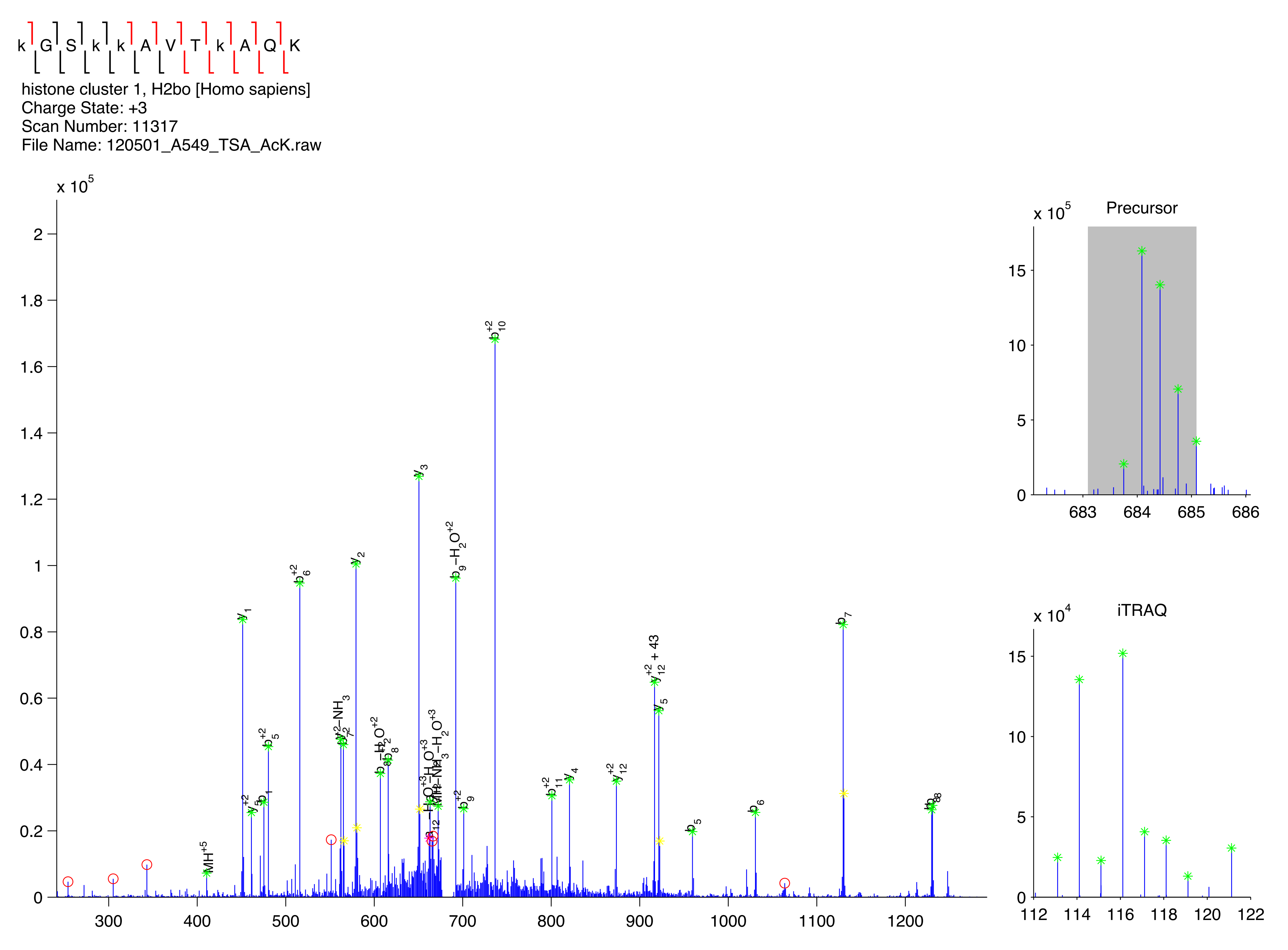

k G S k k A V T k A Q K
histone cluster 1, H2bo [Homo sapiens]
Charge State: +3
Scan Number: 11317
File Name: 120501_A549_TSA_AcK.raw
x 10^5
Precursor
iTRAQ
x 10^4

k G S k k A V T k A Q K

histone cluster 1, H2bo [Homo sapiens]
Charge State: +2
Scan Number: 11978
File Name: 120501_A549_TSA_AcK.raw

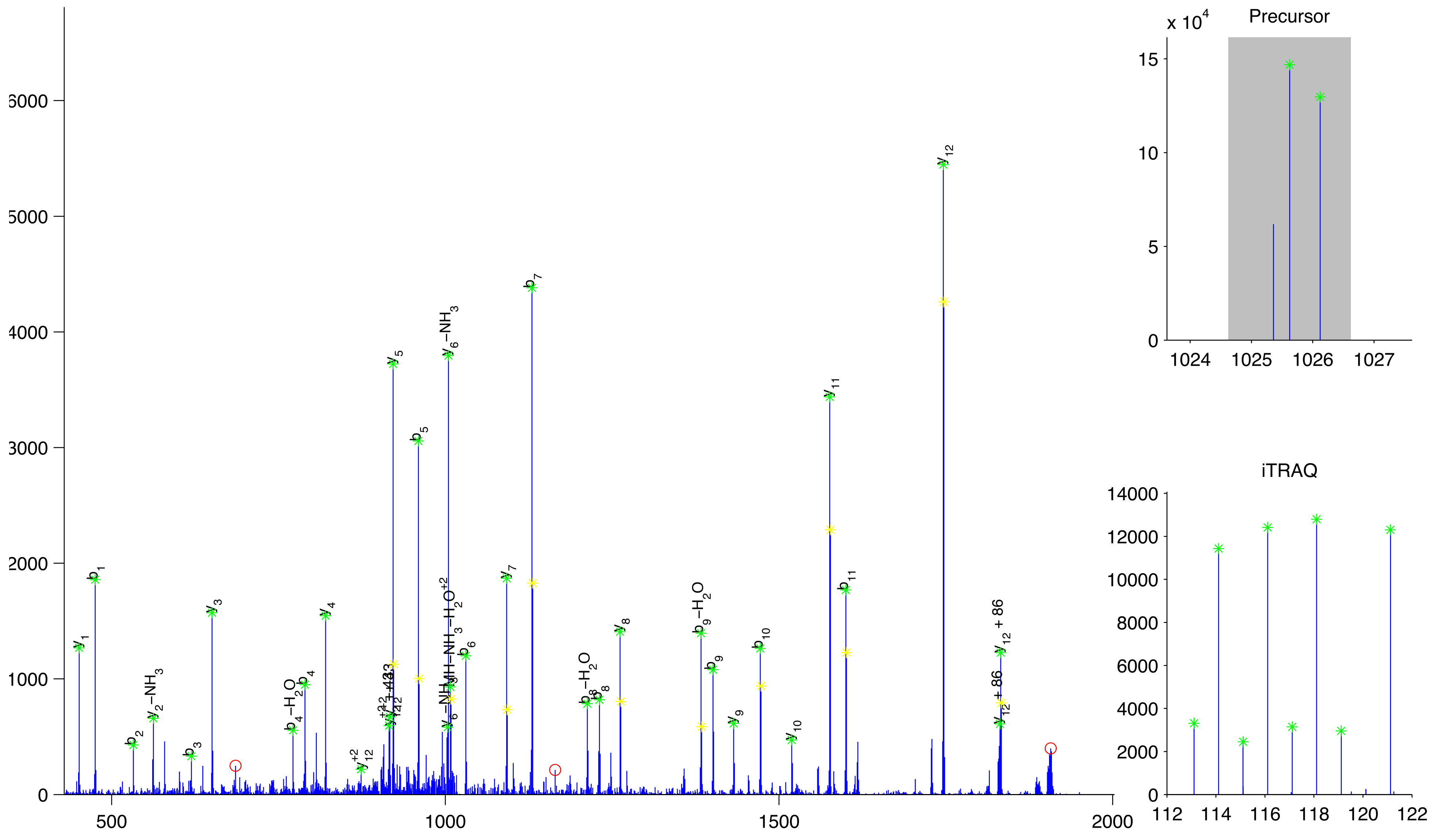

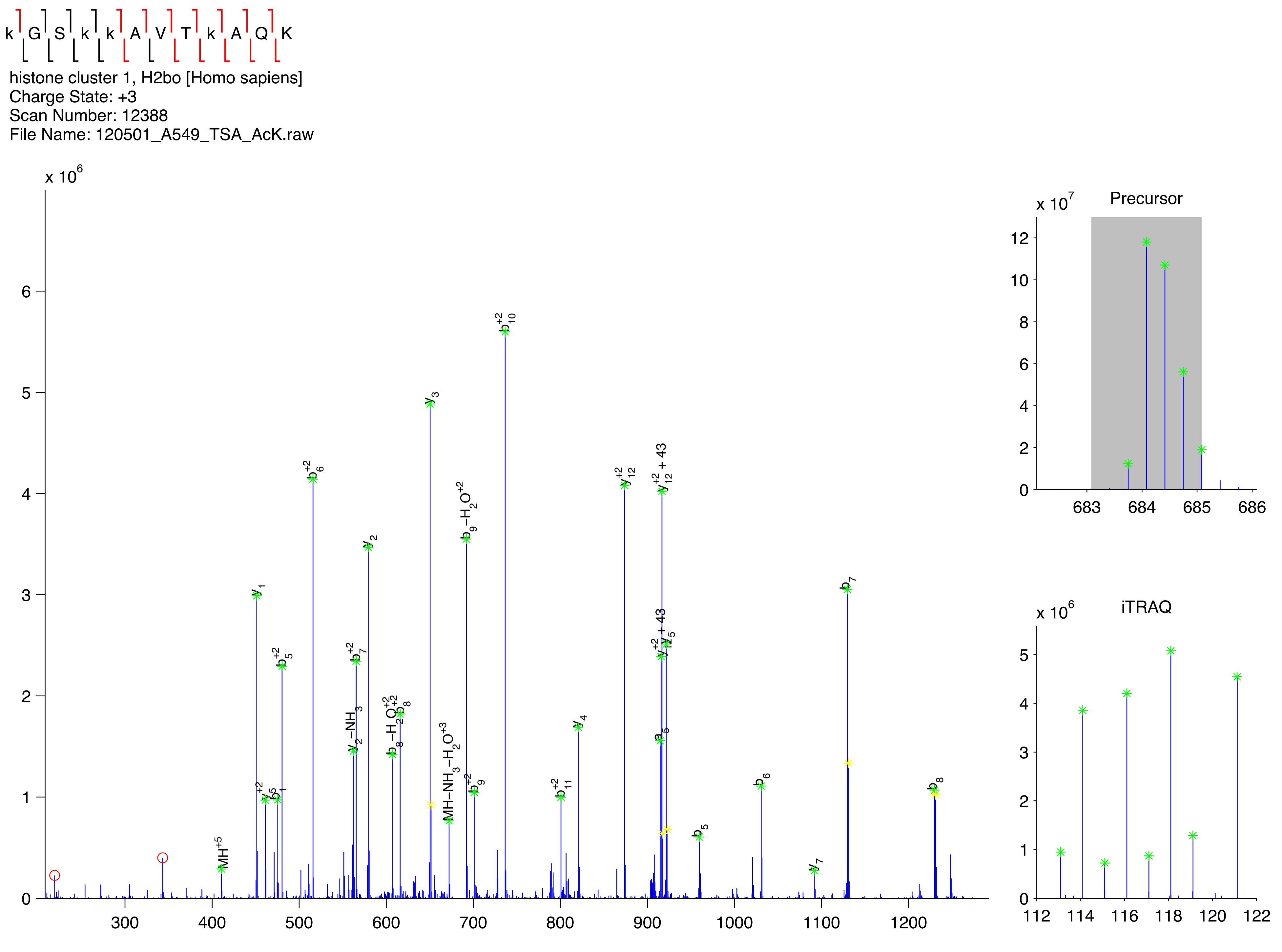

k G S k k A V T k A Q K
histone cluster 1, H2bo [Homo sapiens]
Charge State: +3
Scan Number: 12388
File Name: 120501_A549_TSA_AcK.raw
x 10^6
Precursor
x 10^7
iTRAQ
x 10^6

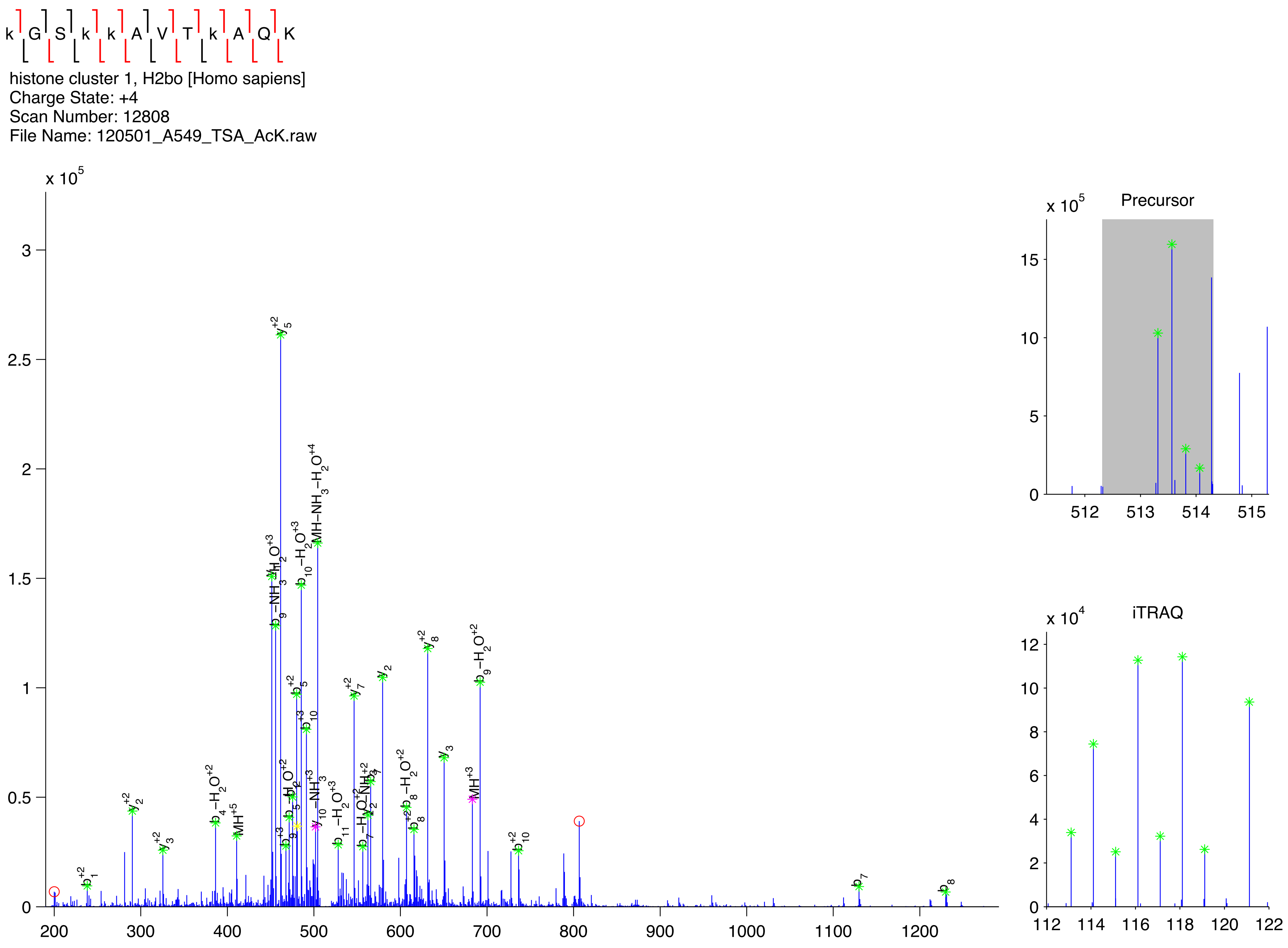

kGSkkAVTkAQK
histone cluster 1, H2bo [Homo sapiens]
Charge State: +4
Scan Number: 12808
File Name: 120501_A549_TSA_AcK.raw
x 10^5
Precursor
iTRAQ
x 10^4

k G S k k A V T k A Q K

histone cluster 1, H2bo [Homo sapiens]
Charge State: +3
Scan Number: 12934
File Name: 120501_A549_TSA_AcK.raw

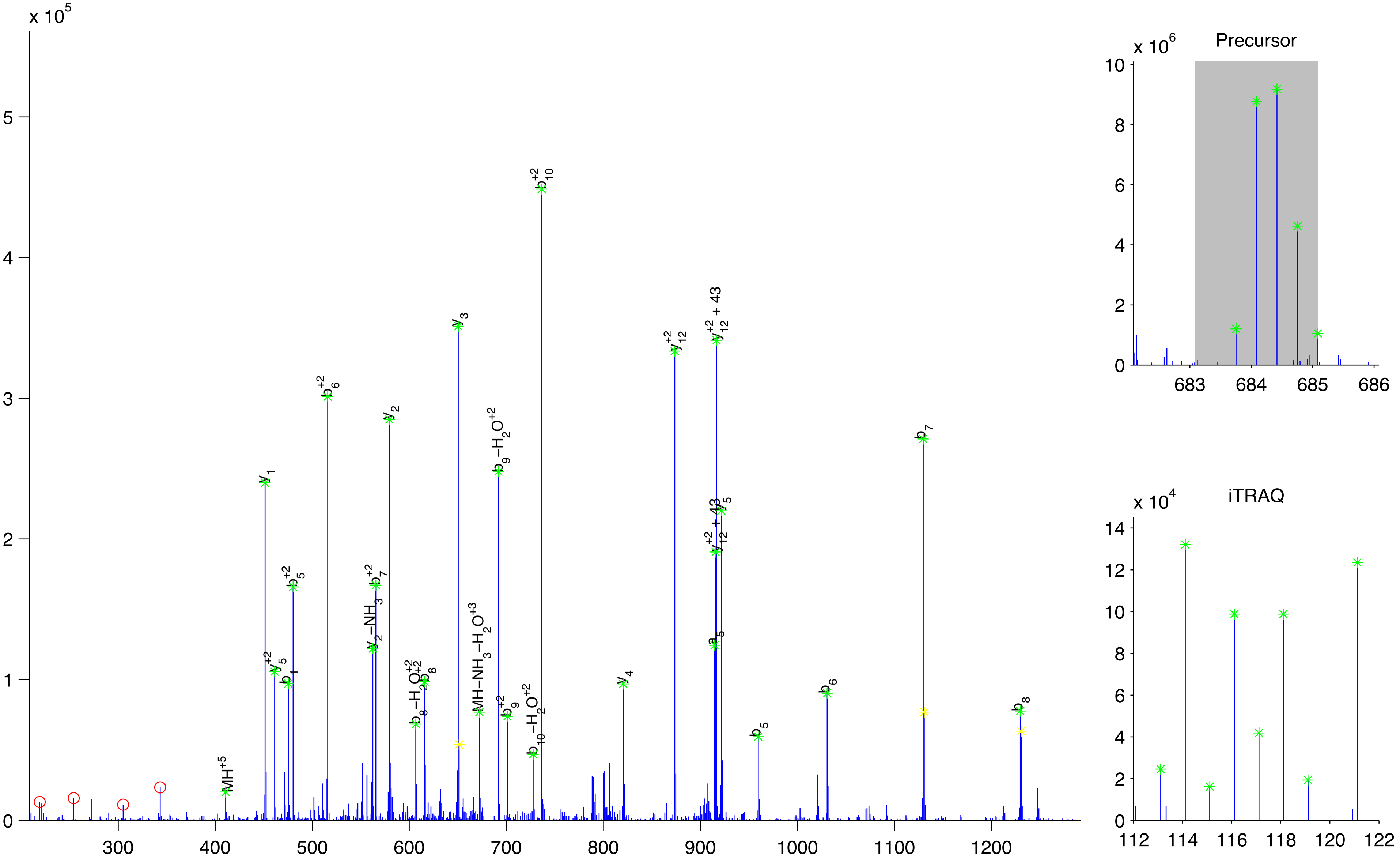

k G S k k A V T k A Q K

histone cluster 1, H2bo [Homo sapiens]
Charge State: +4
Scan Number: 13354
File Name: 120501_A549_TSA_AcK.raw

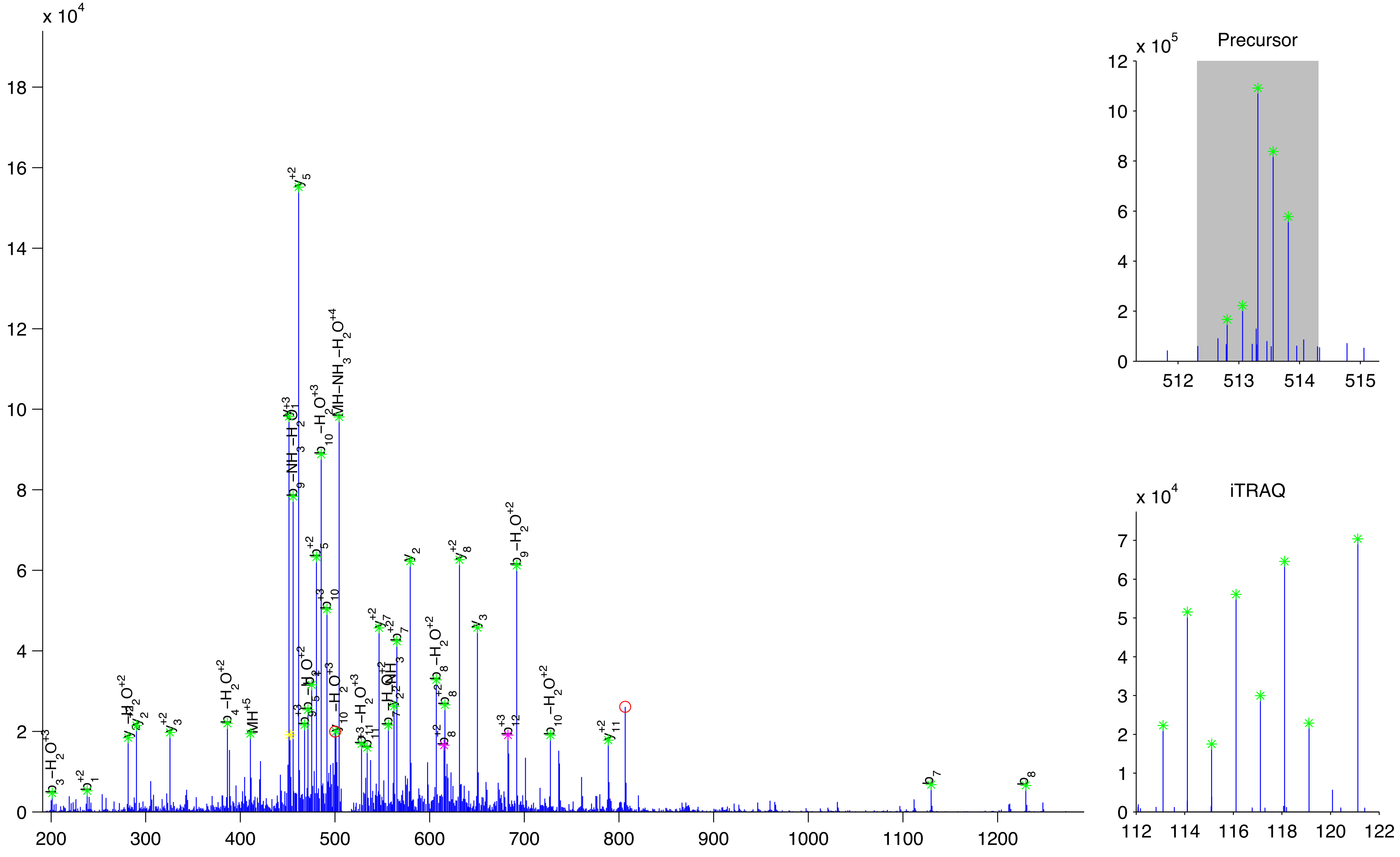

k G S k k A V T k A Q K

histone cluster 1, H2bo [Homo sapiens]
Charge State: +3
Scan Number: 13480
File Name: 120501_A549_TSA_AcK.raw

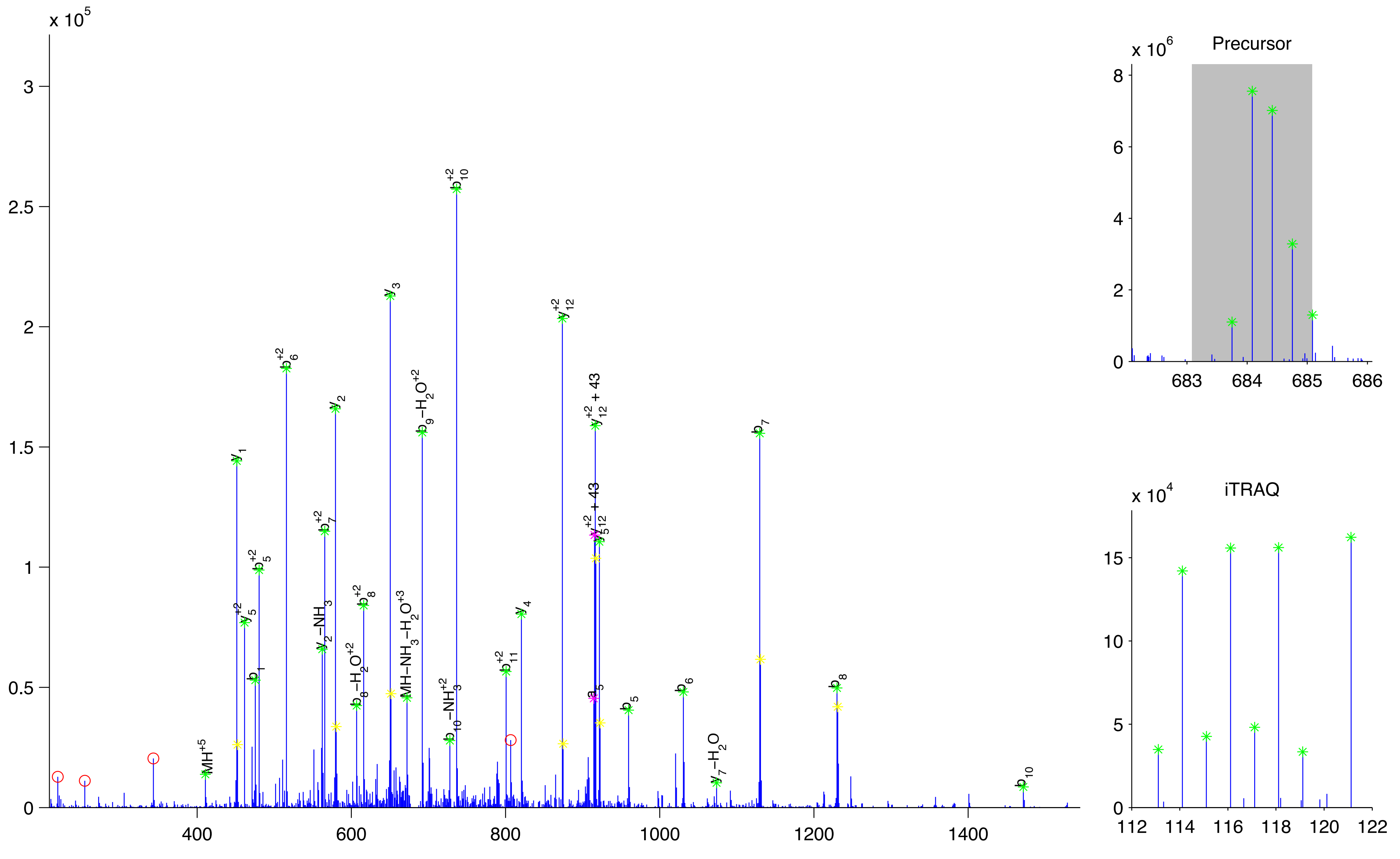

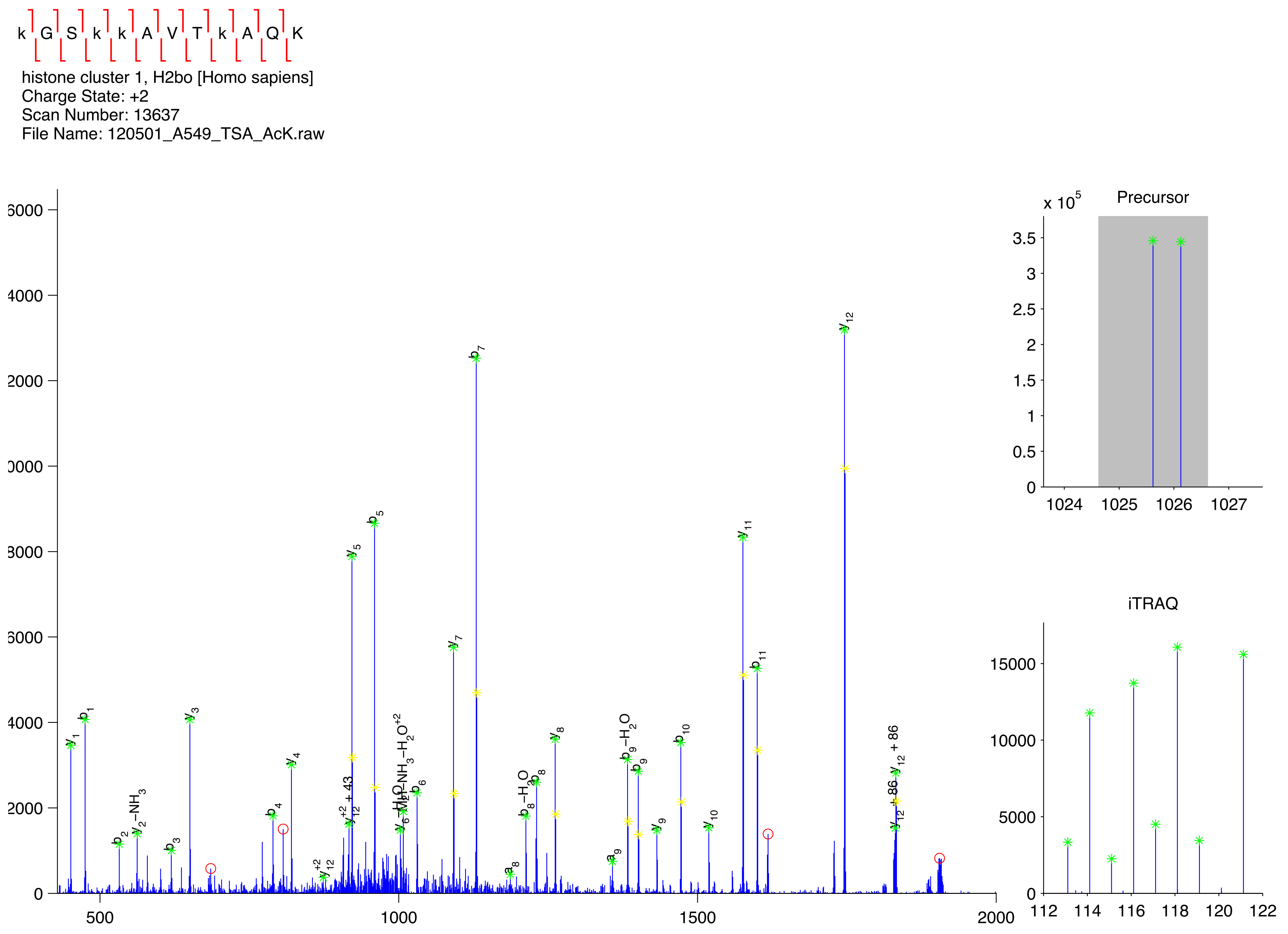
k G S k k A V T k A Q K
histone cluster 1, H2bo [Homo sapiens]
Charge State: +2
Scan Number: 13637
File Name: 120501_A549_TSA_AcK.raw
Precursor
x 10^5
iTRAQ

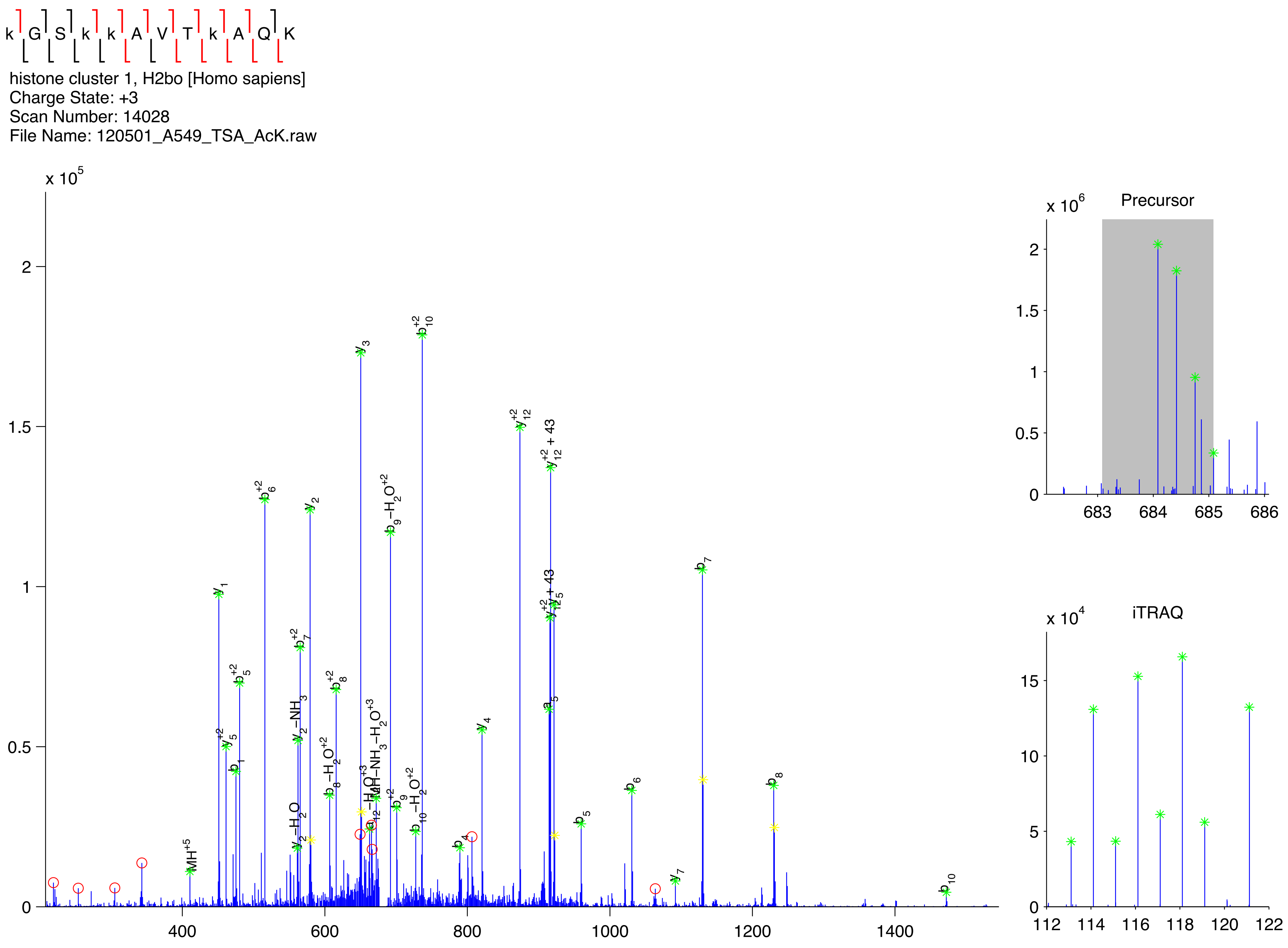

k G S k k A V T k A Q K
histone cluster 1, H2bo [Homo sapiens]
Charge State: +3
Scan Number: 14028
File Name: 120501_A549_TSA_AcK.raw
x 10^5
Precursor
x 10^6
iTRAQ
x 10^4

k G S k k A V T k A Q k K

histone cluster 1, H2bo [Homo sapiens]
Charge State: +3
Scan Number: 14030
File Name: 120501_A549_TSA_AcK.raw

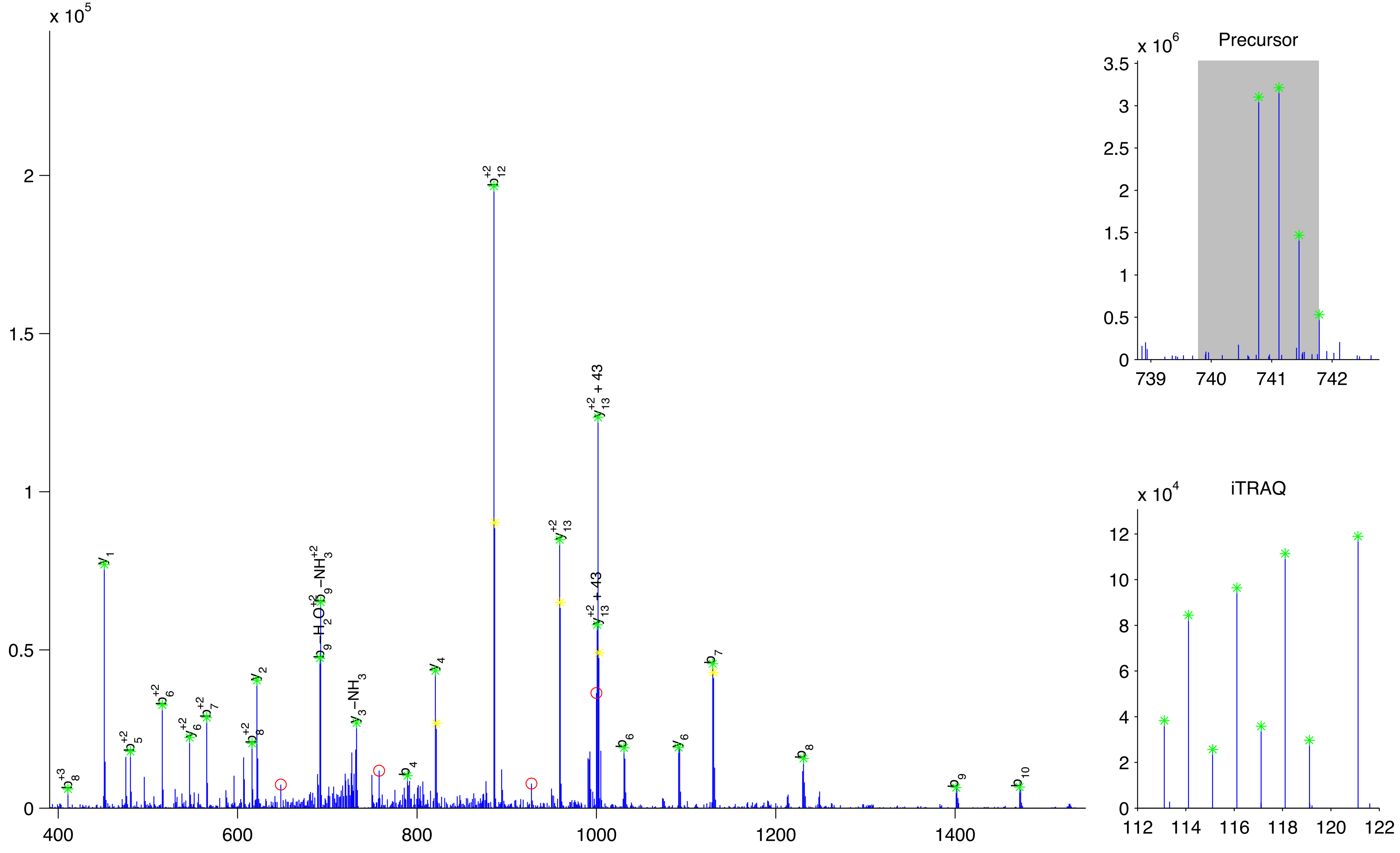

k G S k k A V T k A Q K

histone cluster 1, H2bo [Homo sapiens]
Charge State: +3
Scan Number: 14574
File Name: 120501_A549_TSA_AcK.raw

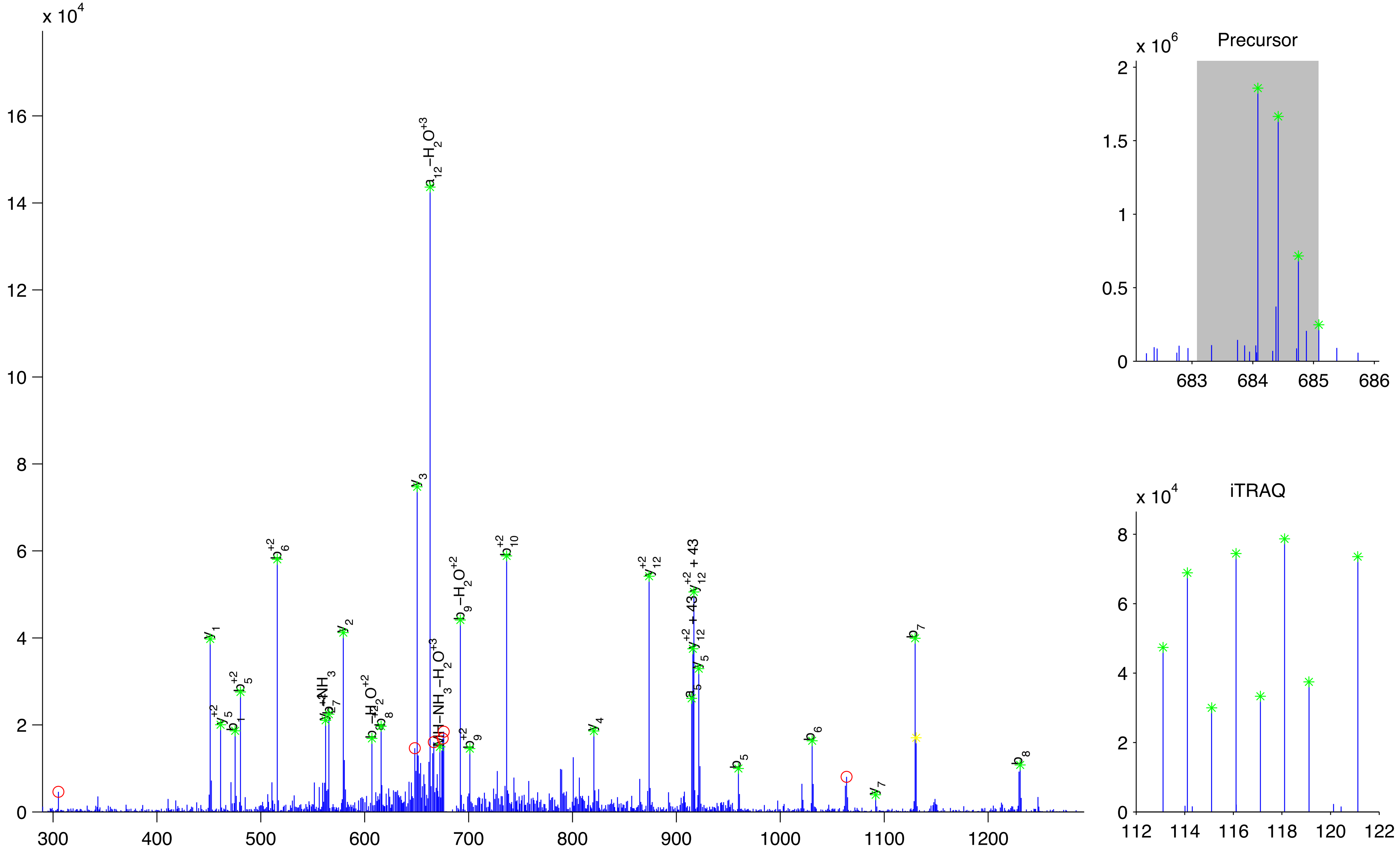

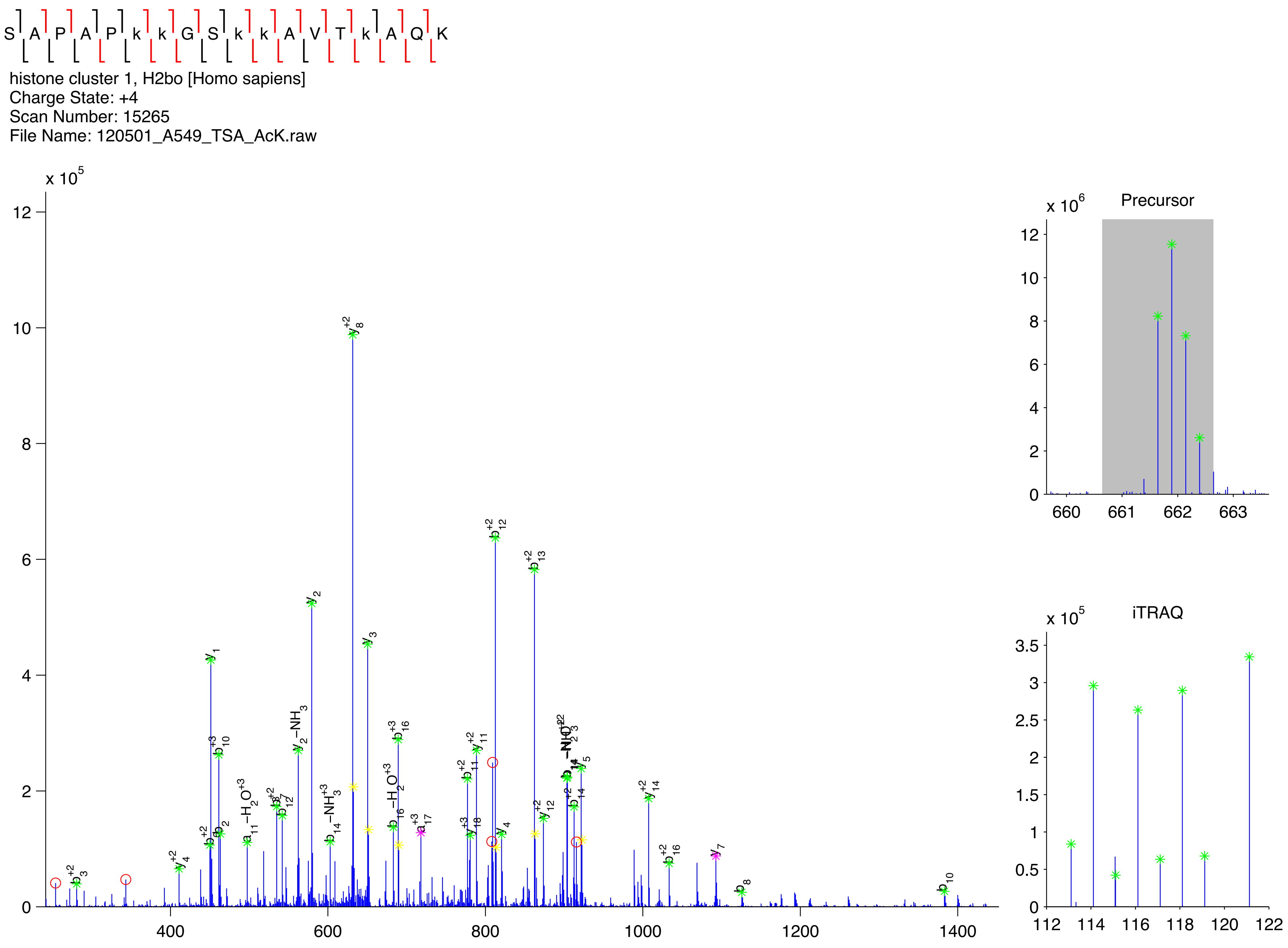
S A P A P k k G S k k A V T k A Q K
histone cluster 1, H2bo [Homo sapiens]
Charge State: +4
Scan Number: 15265
File Name: 120501_A549_TSA_AcK.raw
x 10^5
Precursor
x 10^6
iTRAQ
x 10^5

S A P A P k k G S k k A V T k A Q K

histone cluster 1, H2bo [Homo sapiens]
Charge State: +3
Scan Number: 15559
File Name: 120501_A549_TSA_AcK.raw

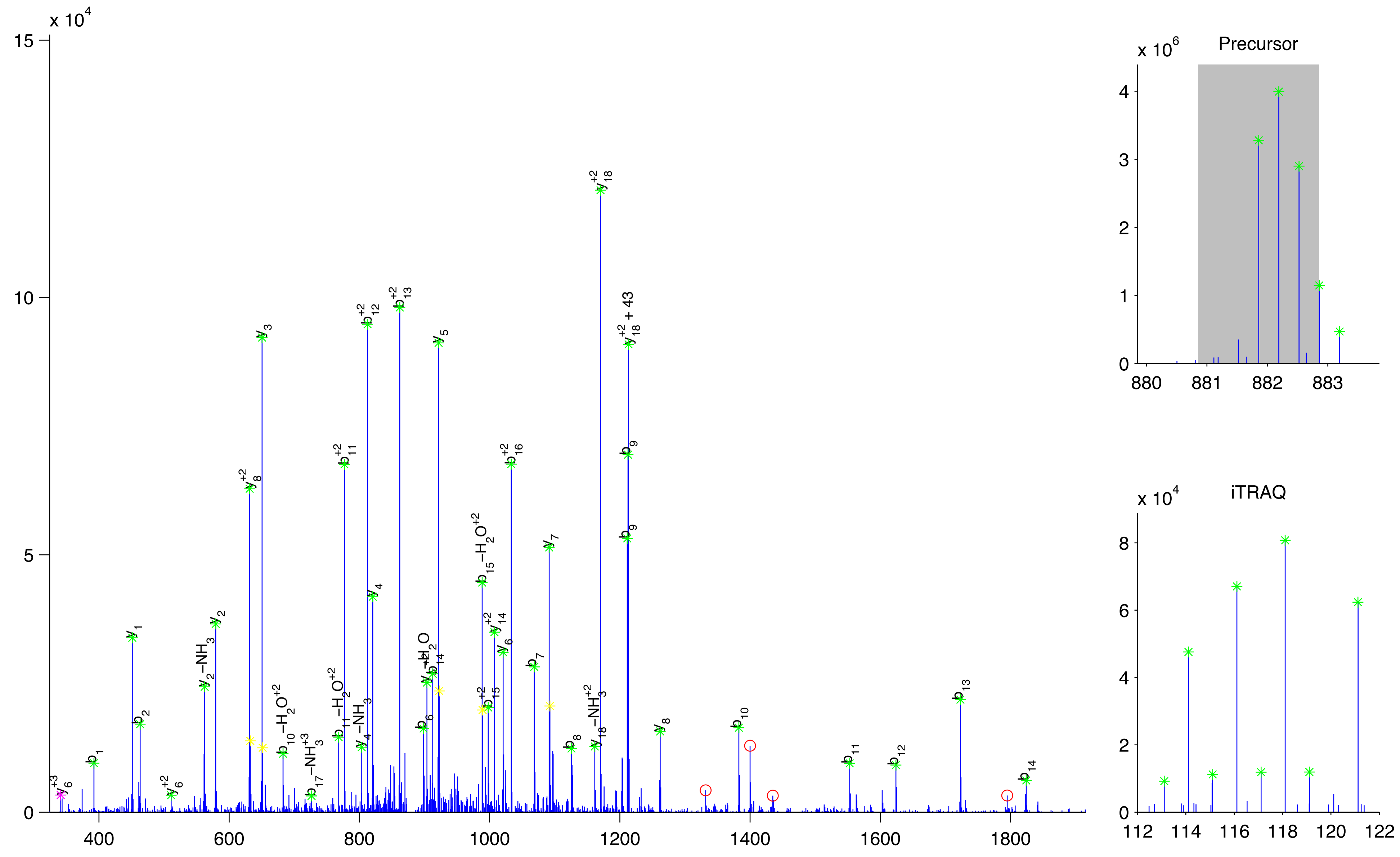

S A P A P k k G S k k A V T k A Q K

histone cluster 1, H2bo [Homo sapiens]
Charge State: +4
Scan Number: 15811
File Name: 120501_A549_TSA_AcK.raw

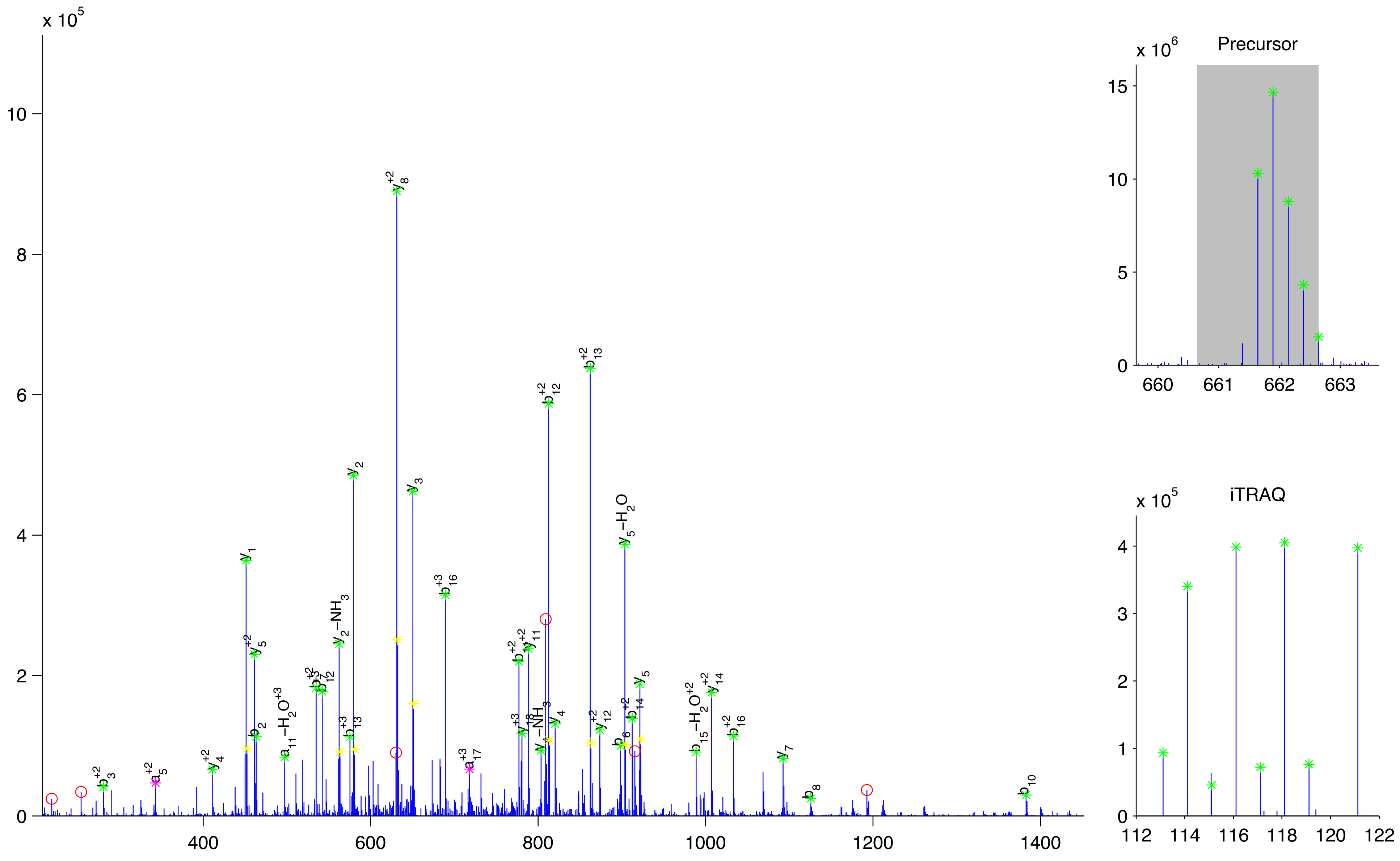

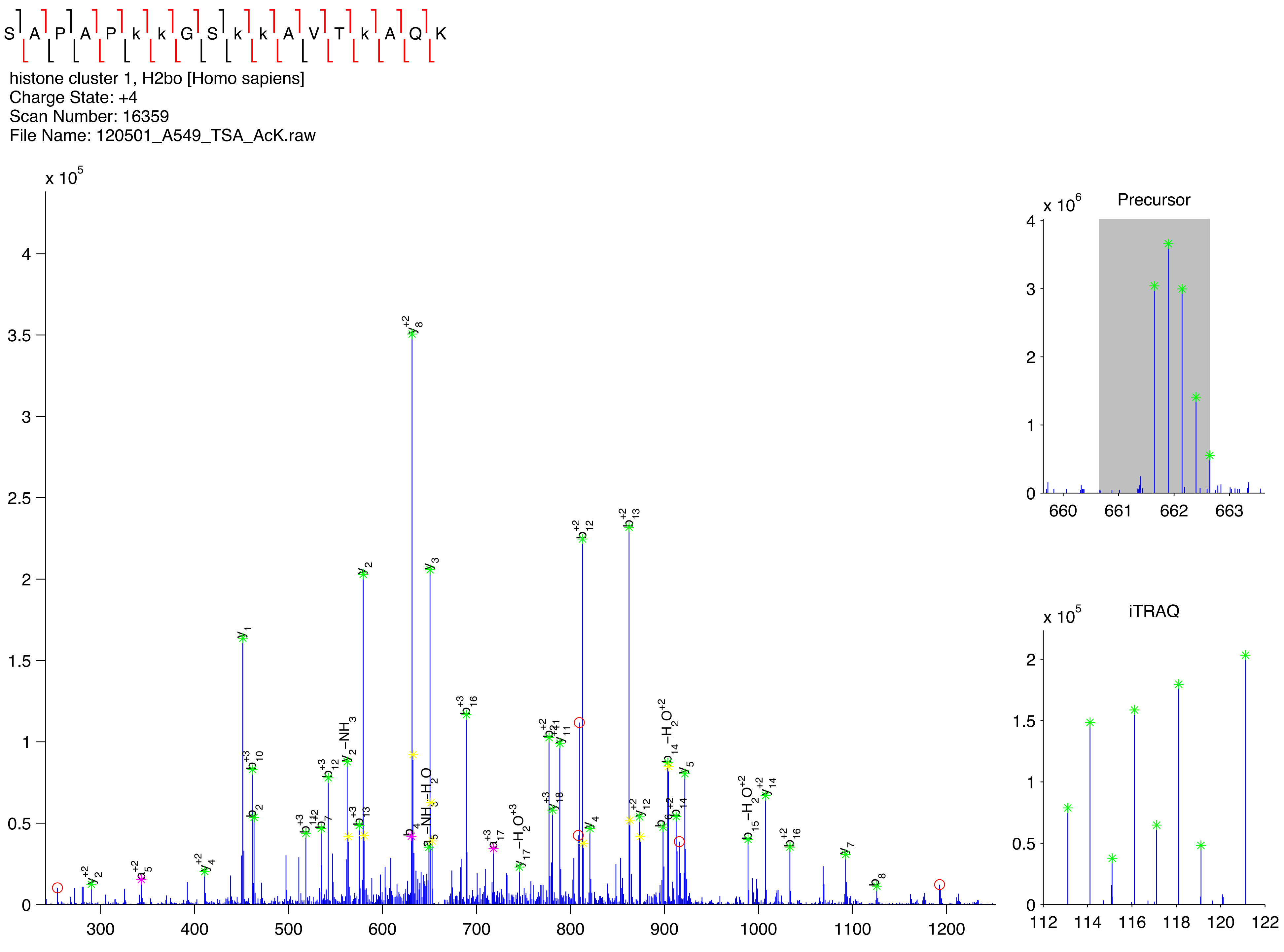
S A P A P k k G S k k A V T k A Q K
histone cluster 1, H2bo [Homo sapiens]
Charge State: +4
Scan Number: 16359
File Name: 120501_A549_TSA_AcK.raw
x 10^5
Precursor
x 10^6
iTRAQ
x 10^5

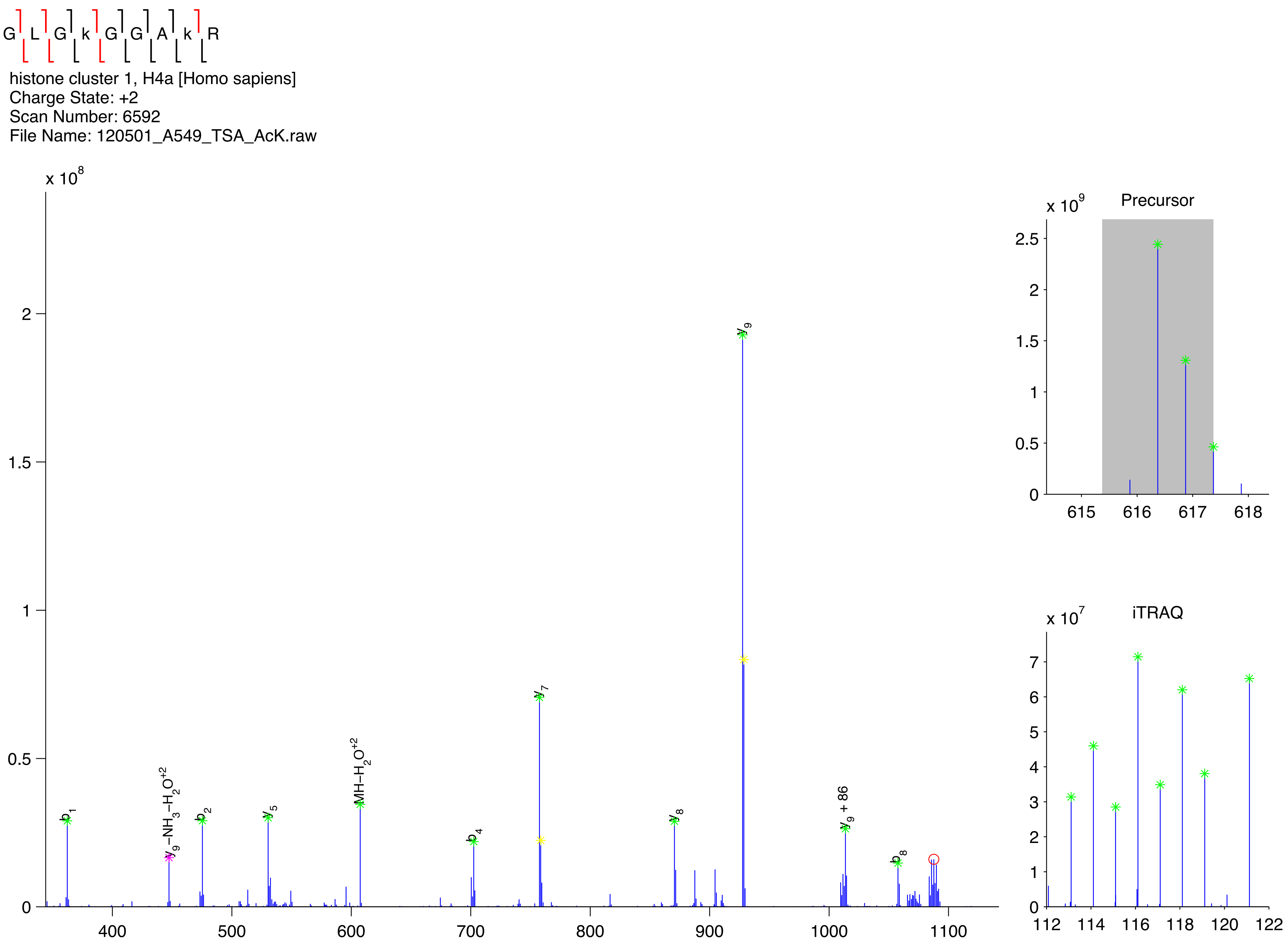

G L G k G G A k R
histone cluster 1, H4a [Homo sapiens]
Charge State: +2
Scan Number: 6592
File Name: 120501_A549_TSA_AcK.raw
x 10^8
b1
y9−NH3−H2O+2
b2
y5
MH−H2O+2
b4
y7
y8
y9
y9 + 86
b8
Precursor
x 10^9
iTRAQ
x 10^7

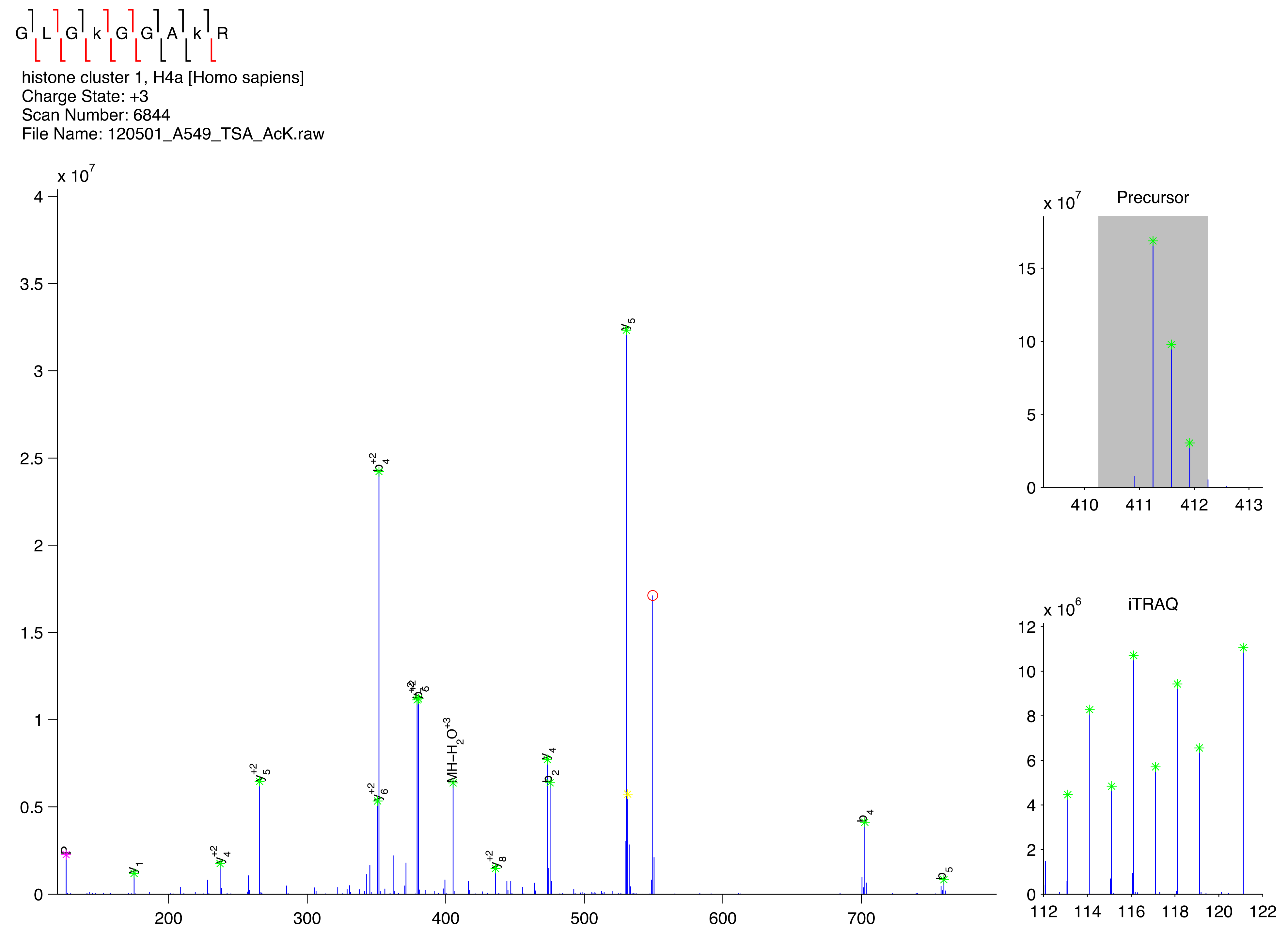

G L G k G G A k R
histone cluster 1, H4a [Homo sapiens]
Charge State: +3
Scan Number: 6844
File Name: 120501_A549_TSA_AcK.raw
x 10^7
P
y1
y4+2
y5+2
y6+2
b4+2
MH-H2O+3
y8+2
b2
y4
y5
b4
b5
Precursor
iTRAQ
x 10^6

G L G k G G A K

histone cluster 1, H4a [Homo sapiens]
Charge State: +3
Scan Number: 7159
File Name: 120501_A549_TSA_AcK.raw

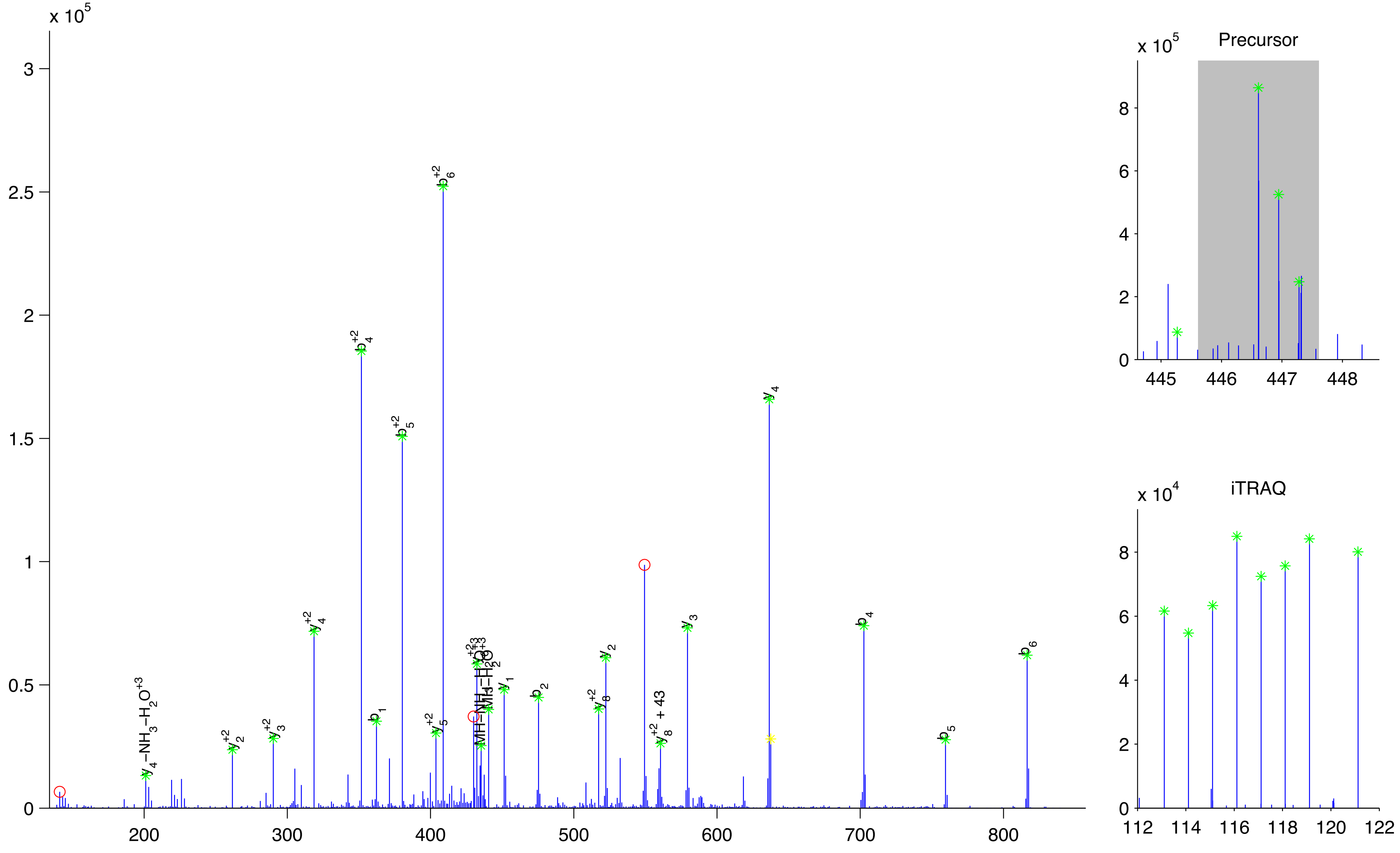

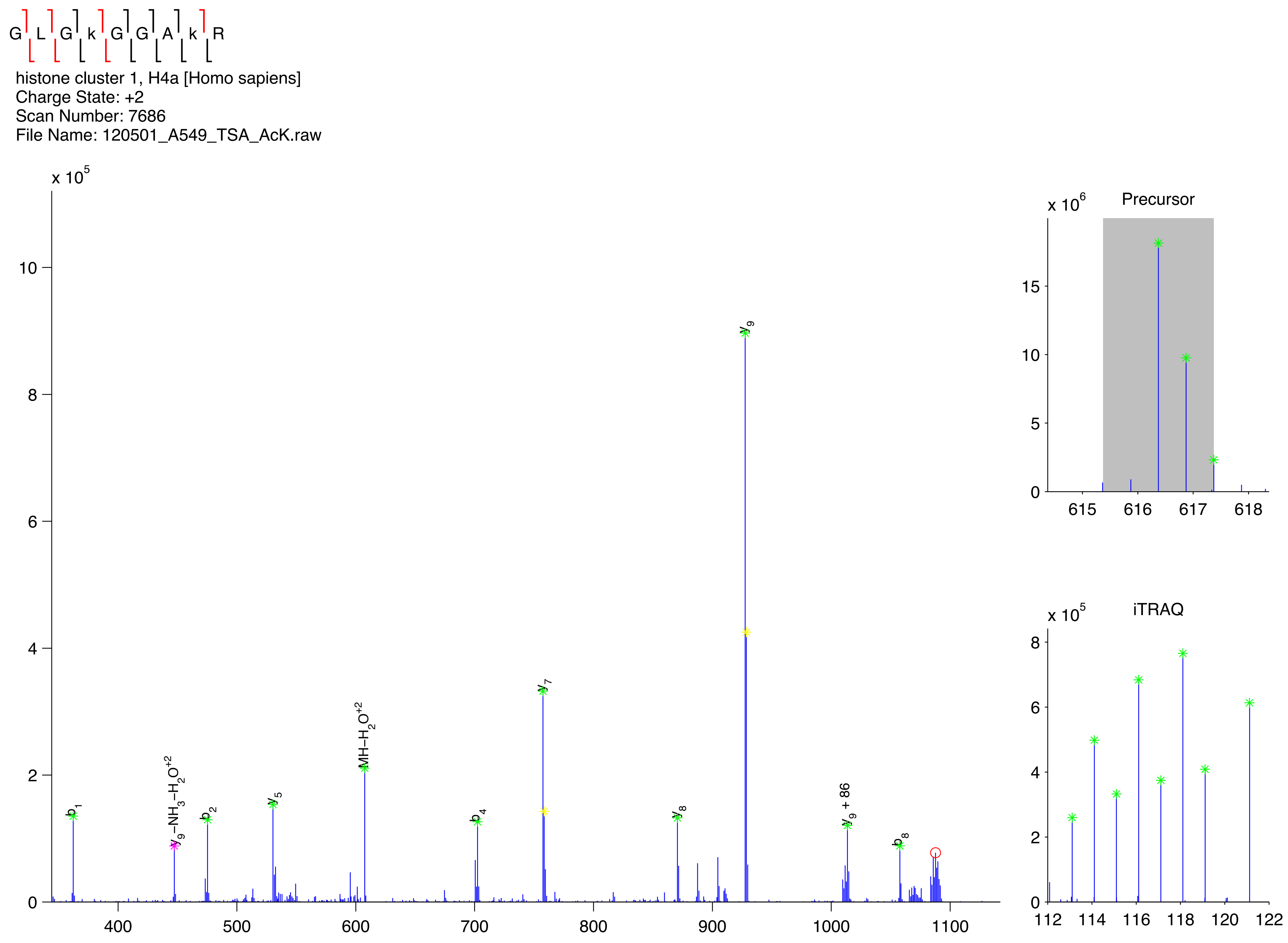

G L G k G G A k R
histone cluster 1, H4a [Homo sapiens]
Charge State: +2
Scan Number: 7686
File Name: 120501_A549_TSA_AcK.raw
x 10^5
b1
y9–NH3–H2O+2
b2
y5
MH–H2O+2
b4
y7
y8
y9
y9 + 86
b8
400
500
600
700
800
900
1000
1100
0
2
4
6
8
10
Precursor
x 10^6
615
616
617
618
0
5
10
15
iTRAQ
x 10^5
112
114
116
118
120
122
0
2
4
6
8

G G k G L G k G G A k R

histone cluster 1, H4a [Homo sapiens]
Charge State: +2
Scan Number: 7831
File Name: 120501_A549_TSA_AcK.raw

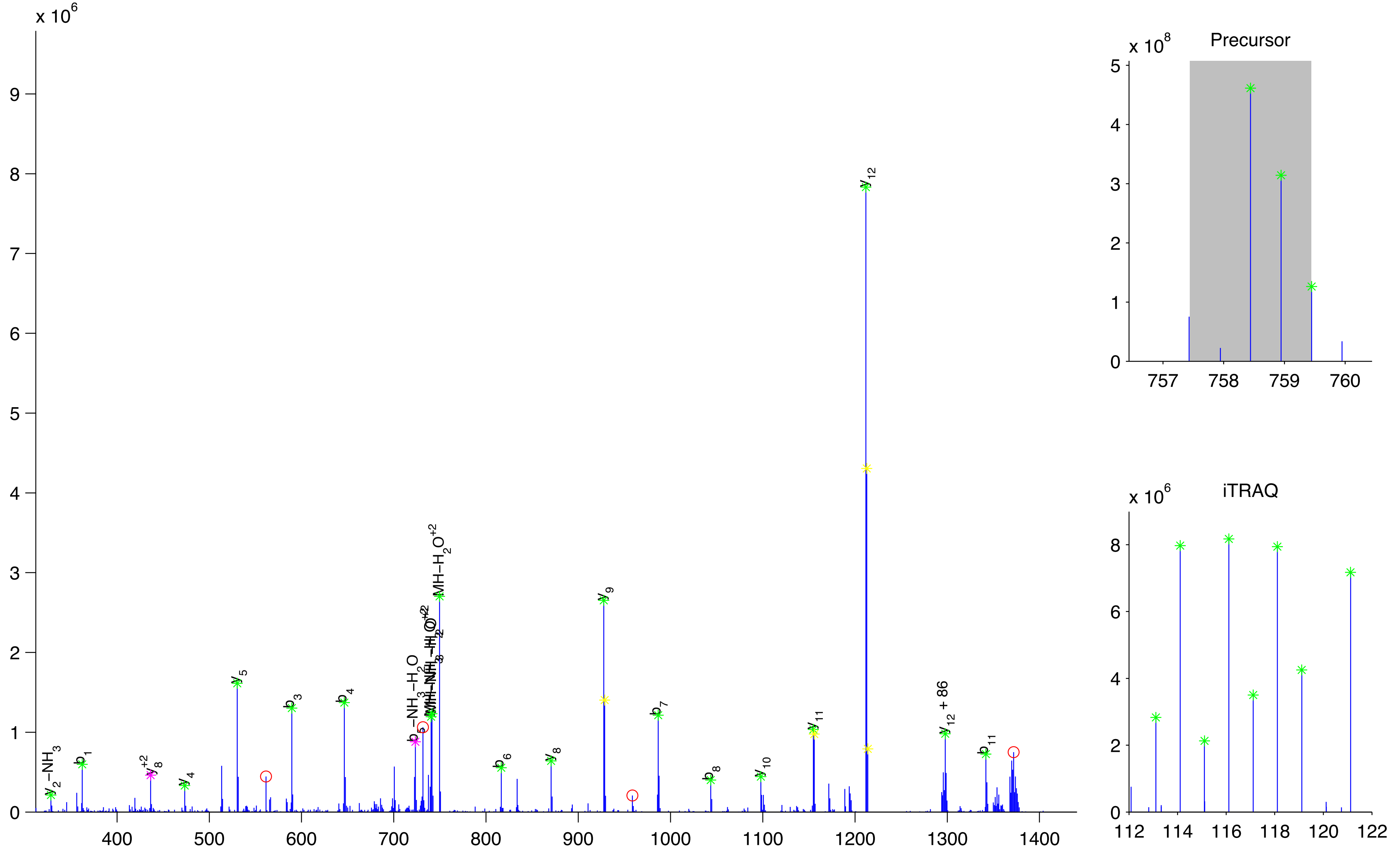

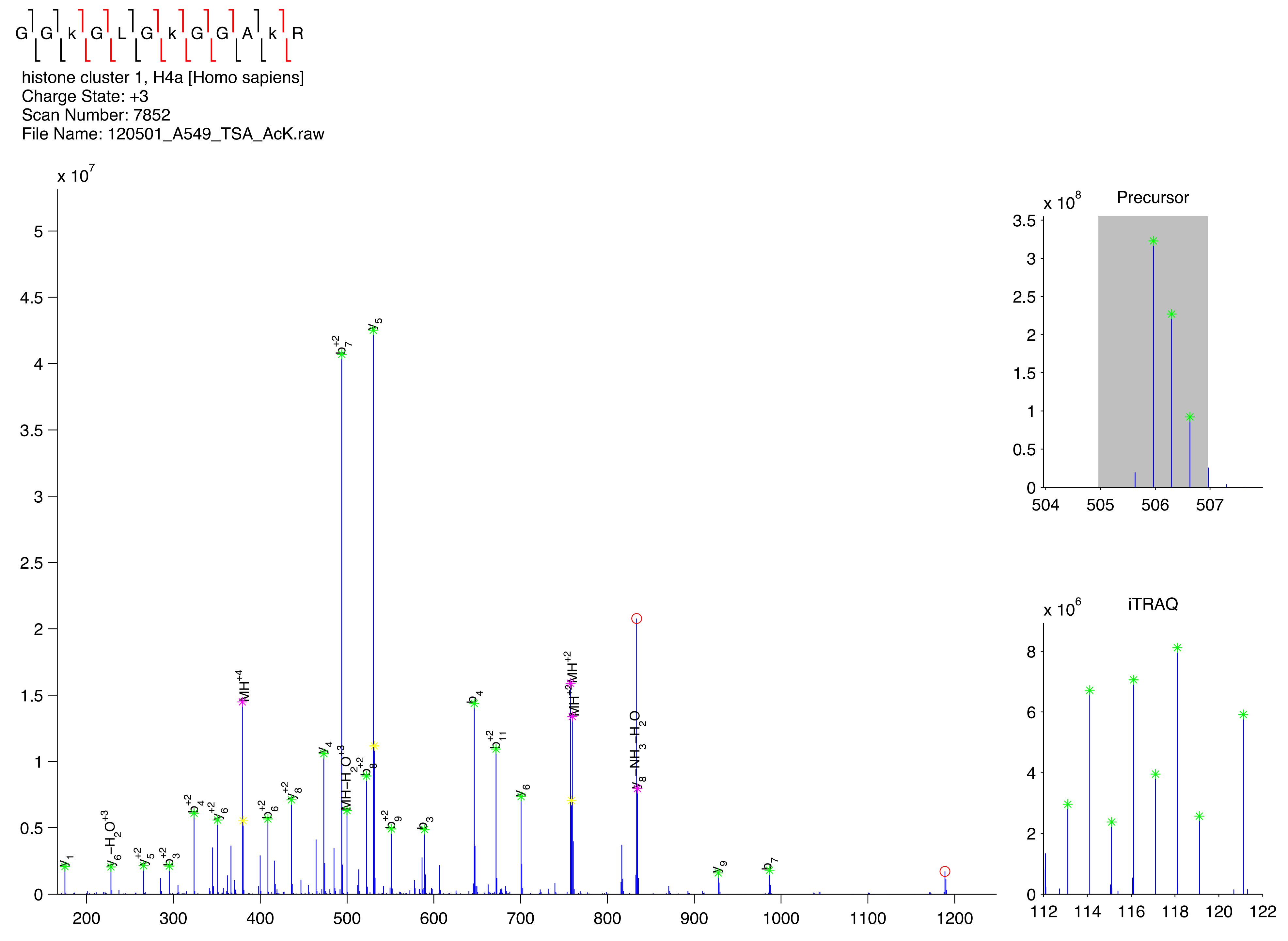

G G k G L G k G G A k R
histone cluster 1, H4a [Homo sapiens]
Charge State: +3
Scan Number: 7852
File Name: 120501_A549_TSA_AcK.raw
x 10^7
y1
y6−H2O+3
y5+2
b3+2
b4+2
y6+2
MH+4
b6+2
y8+2
y4
b7+2
MH−H2O+3
b8+2
y5
b9+2
b3
b4
b11+2
y6
MH+2
MH+2
y8−NH3−H2O
y9
b7
Precursor
x 10^8
iTRAQ
x 10^6

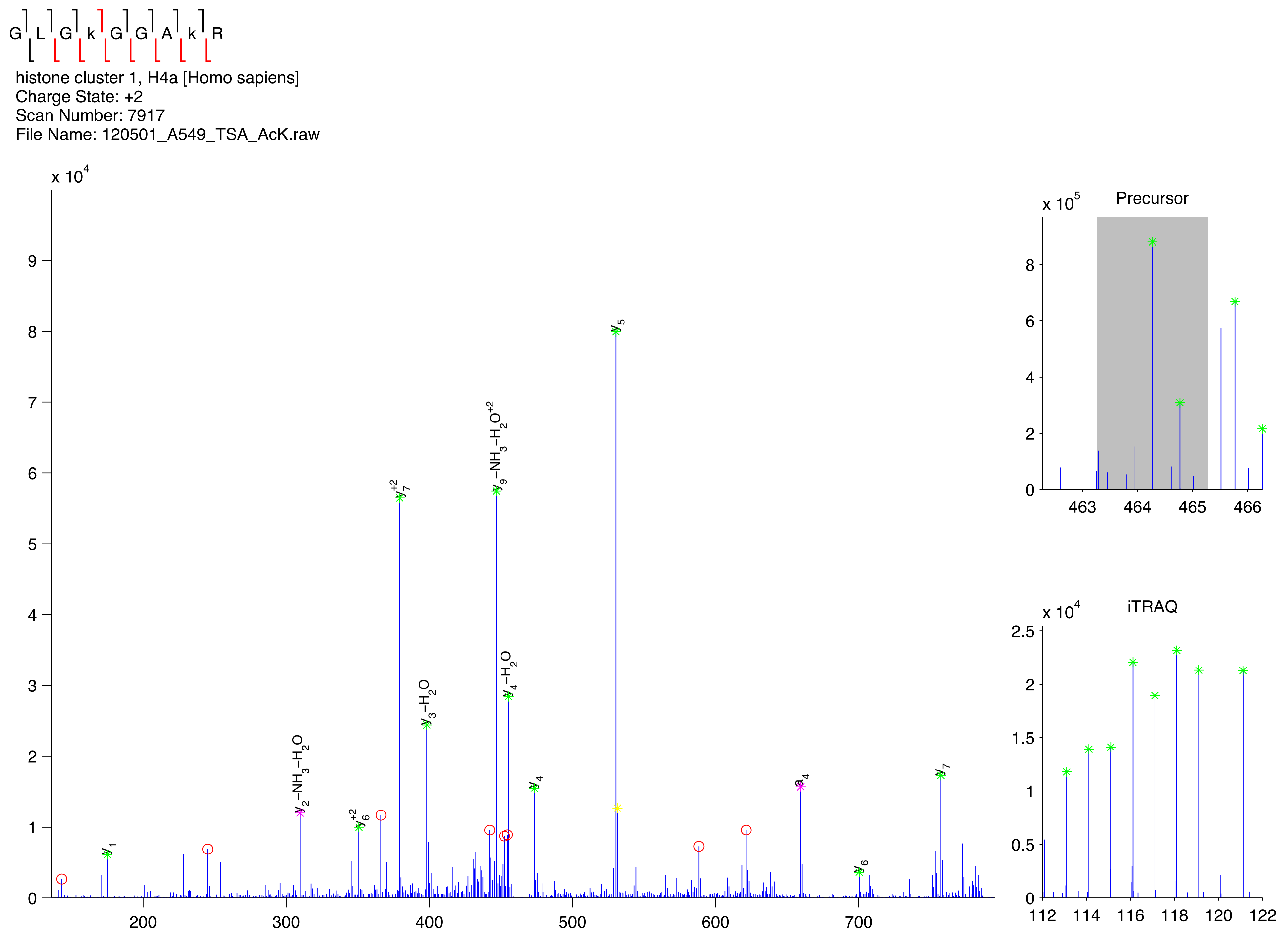
G L G k G G A k R
histone cluster 1, H4a [Homo sapiens]
Charge State: +2
Scan Number: 7917
File Name: 120501_A549_TSA_AcK.raw
x 10^4
y1
y2–NH3–H2O
y6+2
y7+2
y3–H2O
y9–NH3–H2O+2
y4–H2O
y4
y5
a4
y6
y7
Precursor
x 10^5
iTRAQ
x 10^4

G L G k G G A k R

histone cluster 1, H4a [Homo sapiens]
Charge State: +3
Scan Number: 7936
File Name: 120501_A549_TSA_AcK.raw

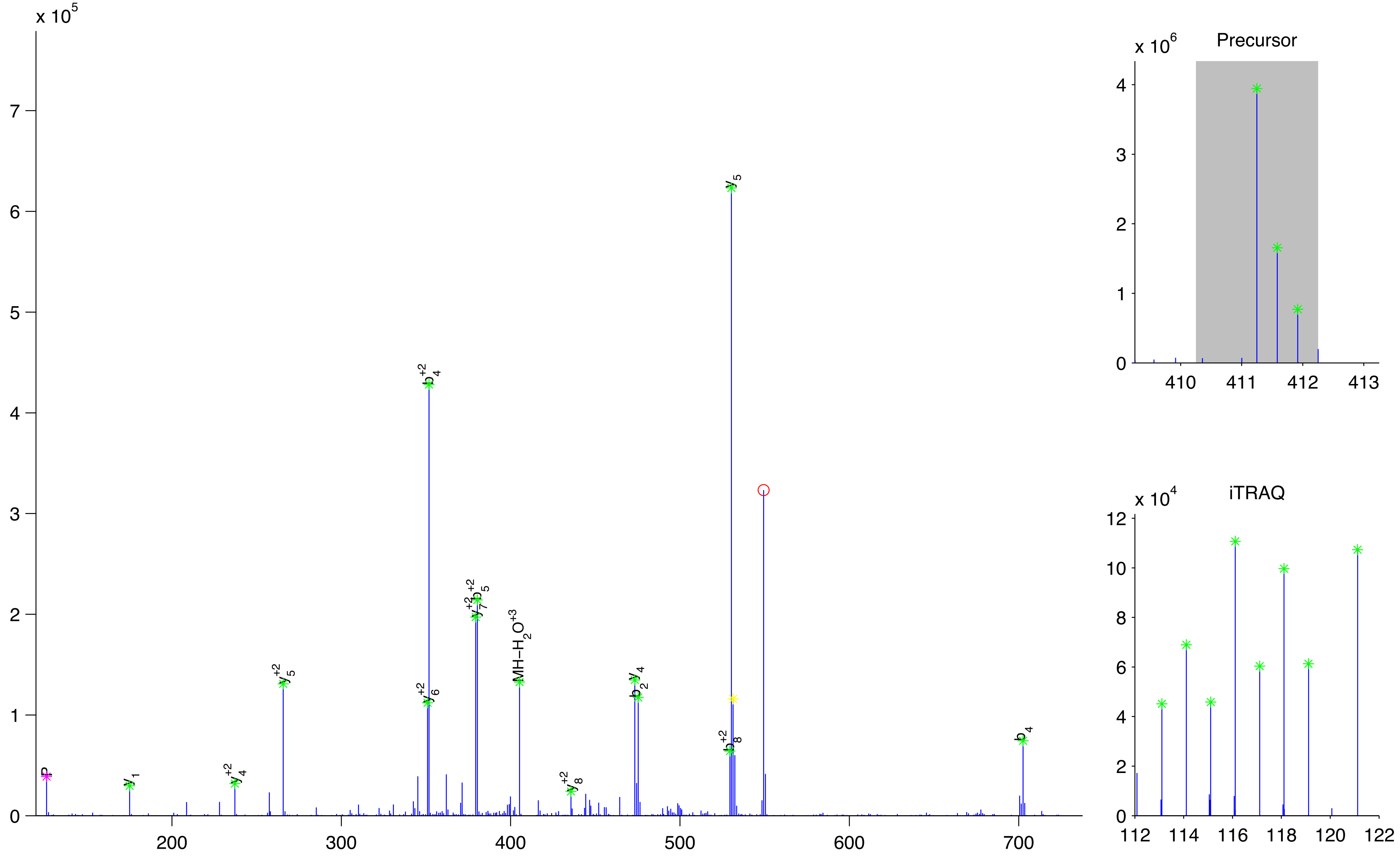

G G k G L G k G G A k R

histone cluster 1, H4a [Homo sapiens]
Charge State: +2
Scan Number: 8001
File Name: 120501_A549_TSA_AcK.raw

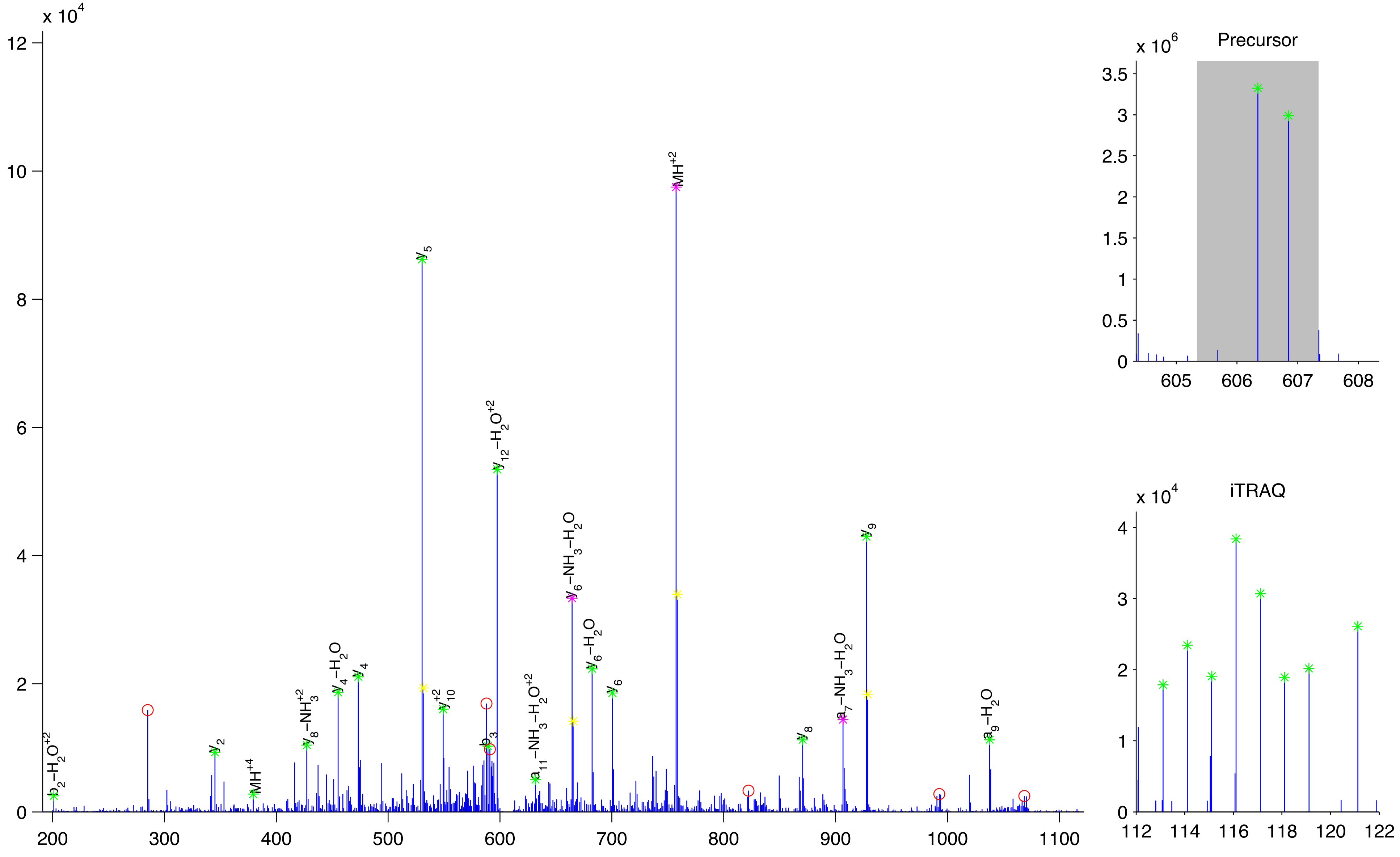

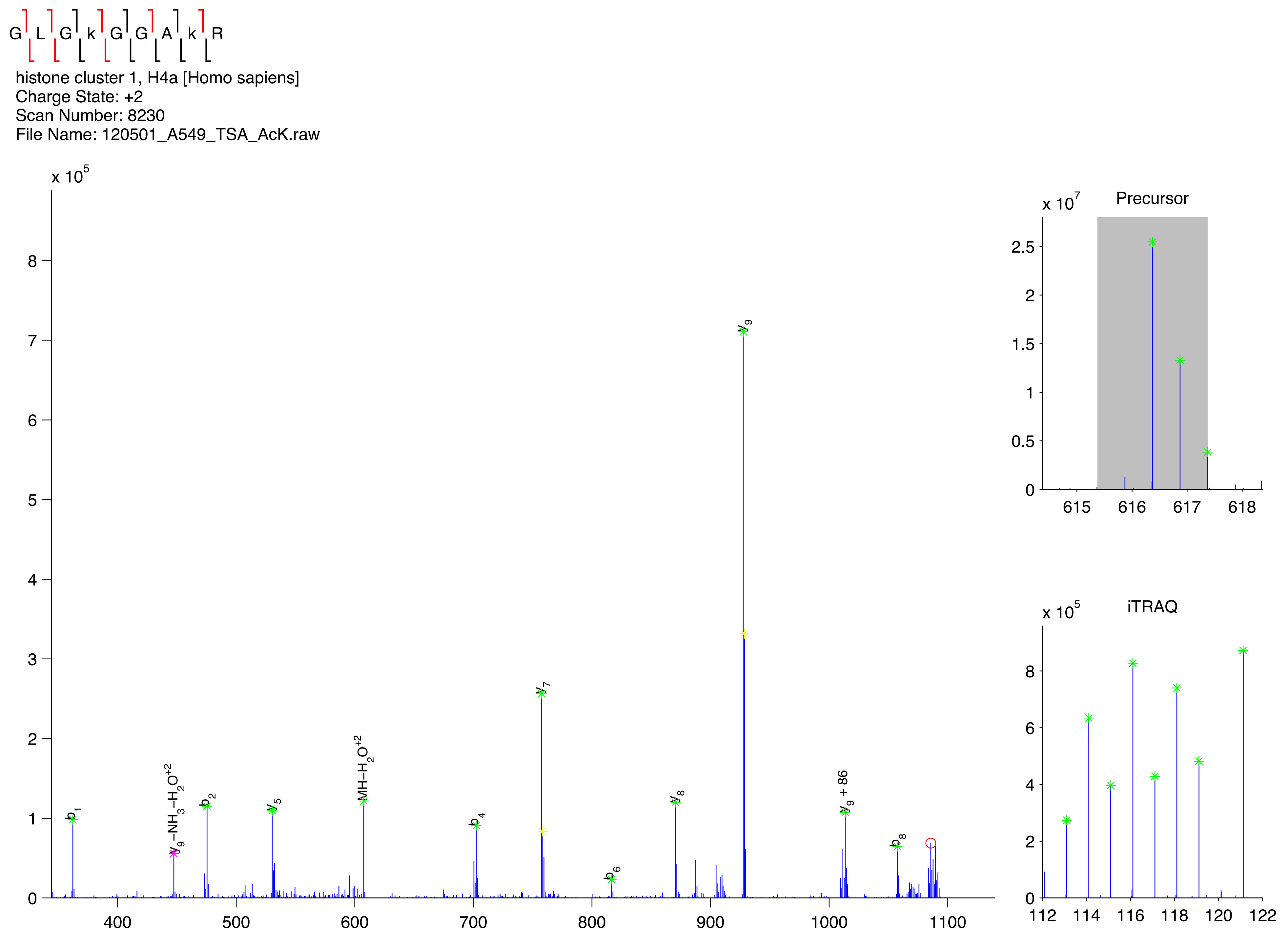

G L G k G G A k R
histone cluster 1, H4a [Homo sapiens]
Charge State: +2
Scan Number: 8230
File Name: 120501_A549_TSA_AcK.raw
x 10^5
b1
y9–NH3–H2O+2
b2
y5
MH–H2O+2
b4
y7
b6
y8
y9
y9 + 86
b8
Precursor
x 10^7
iTRAQ
x 10^5

G k G G k G L G k G G A k R

histone cluster 1, H4a [Homo sapiens]
Charge State: +3
Scan Number: 8272
File Name: 120501_A549_TSA_AcK.raw

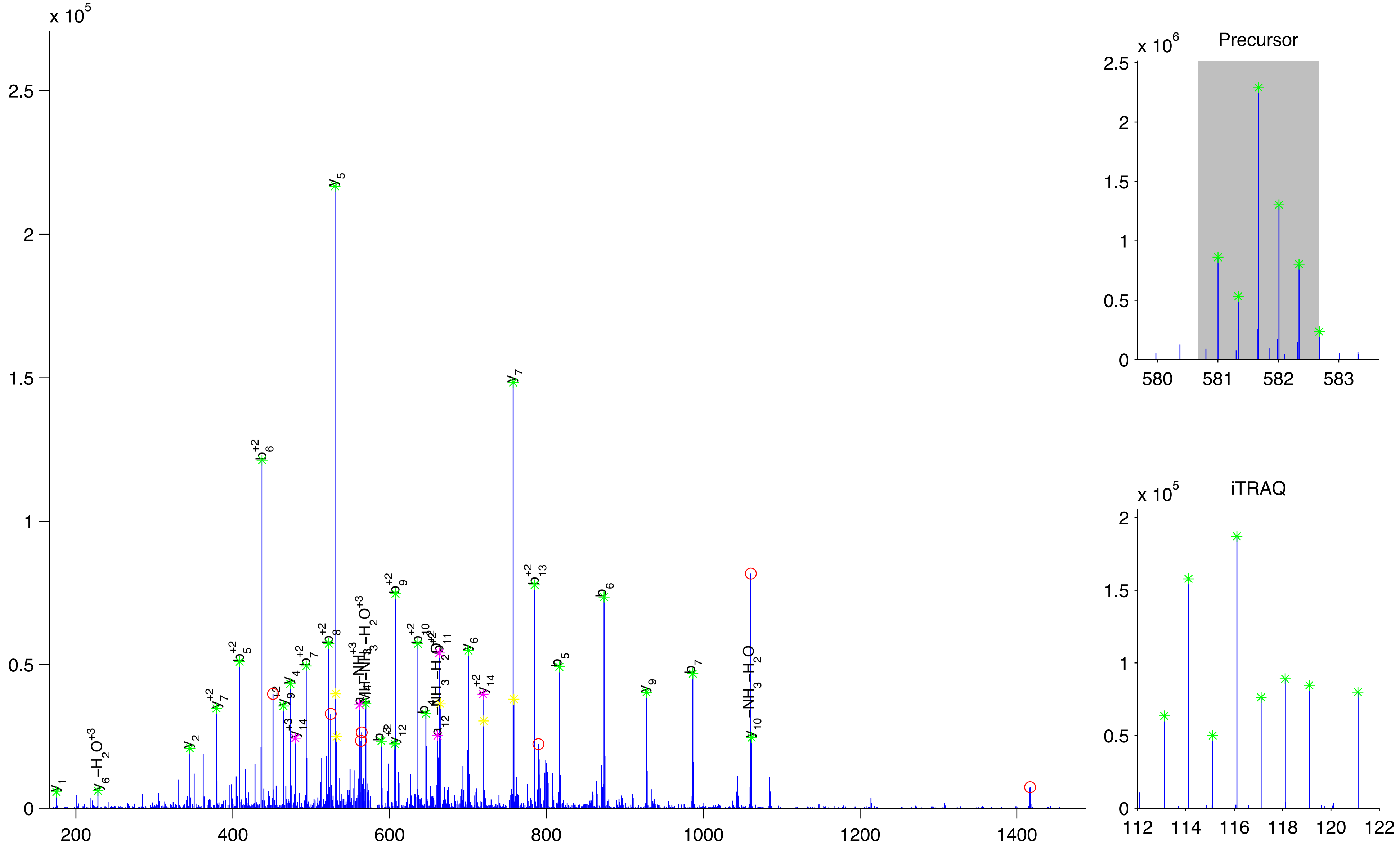

G G k G L G k G G A k R

histone cluster 1, H4a [Homo sapiens]
Charge State: +3
Scan Number: 8398
File Name: 120501_A549_TSA_AcK.raw

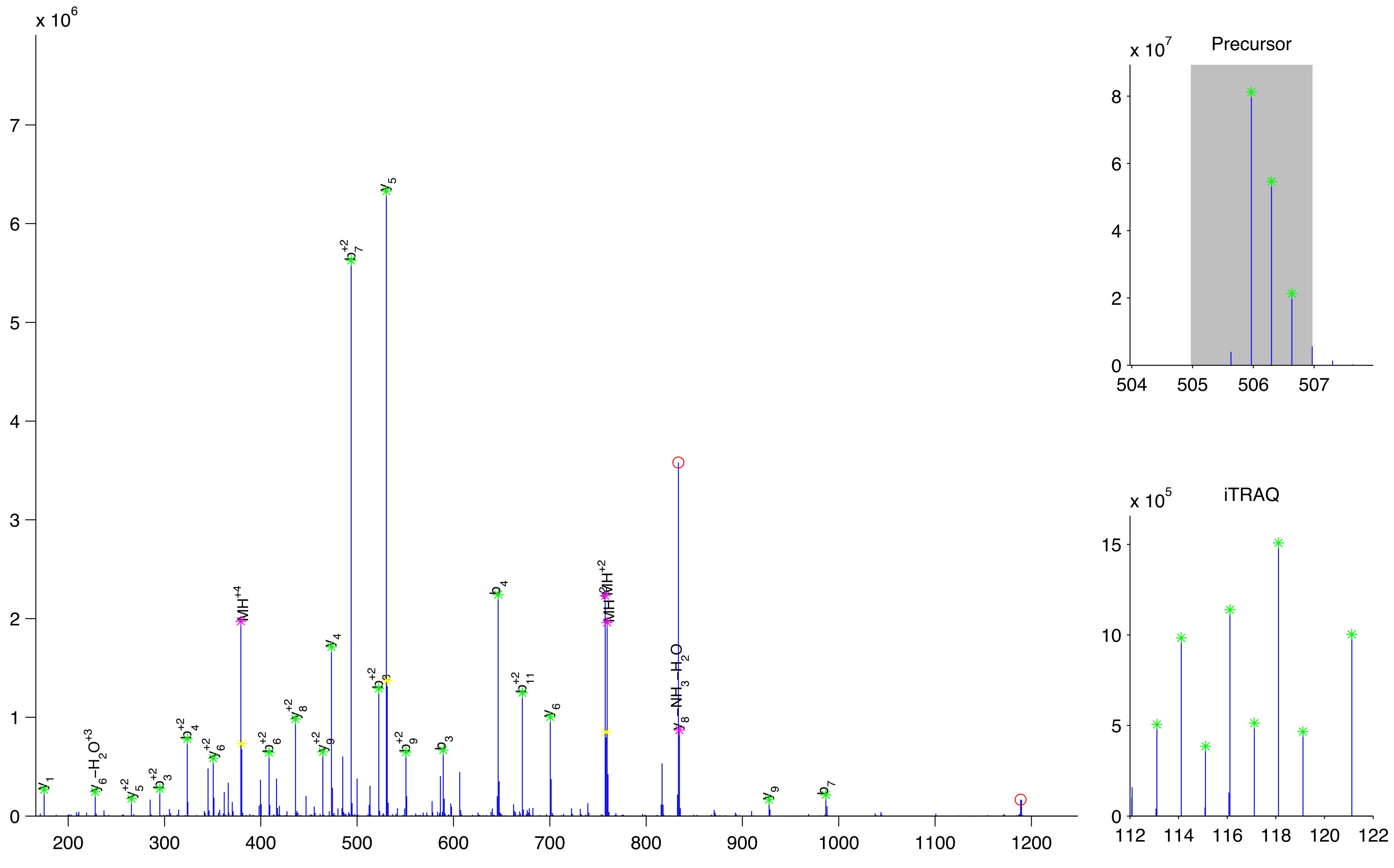

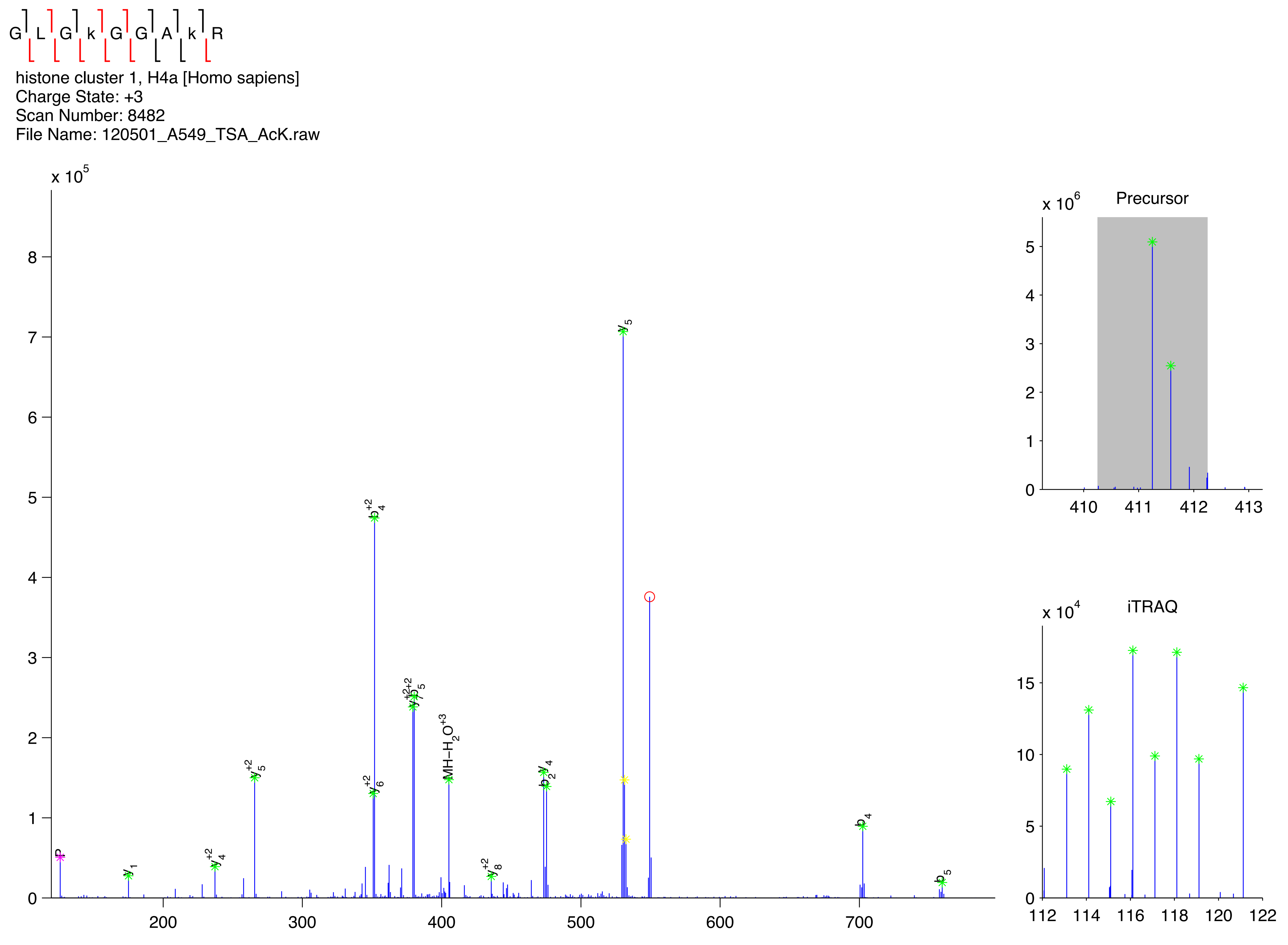

G L G k G G A k R
histone cluster 1, H4a [Homo sapiens]
Charge State: +3
Scan Number: 8482
File Name: 120501_A549_TSA_AcK.raw
x 10^5
y5
b4+2
y7+2 b5+2
MH−H2O+3
b2 y4
y5+2
y6+2
y4+2
y1
y8+2
b4
b5
p
Precursor
x 10^6
iTRAQ
x 10^4

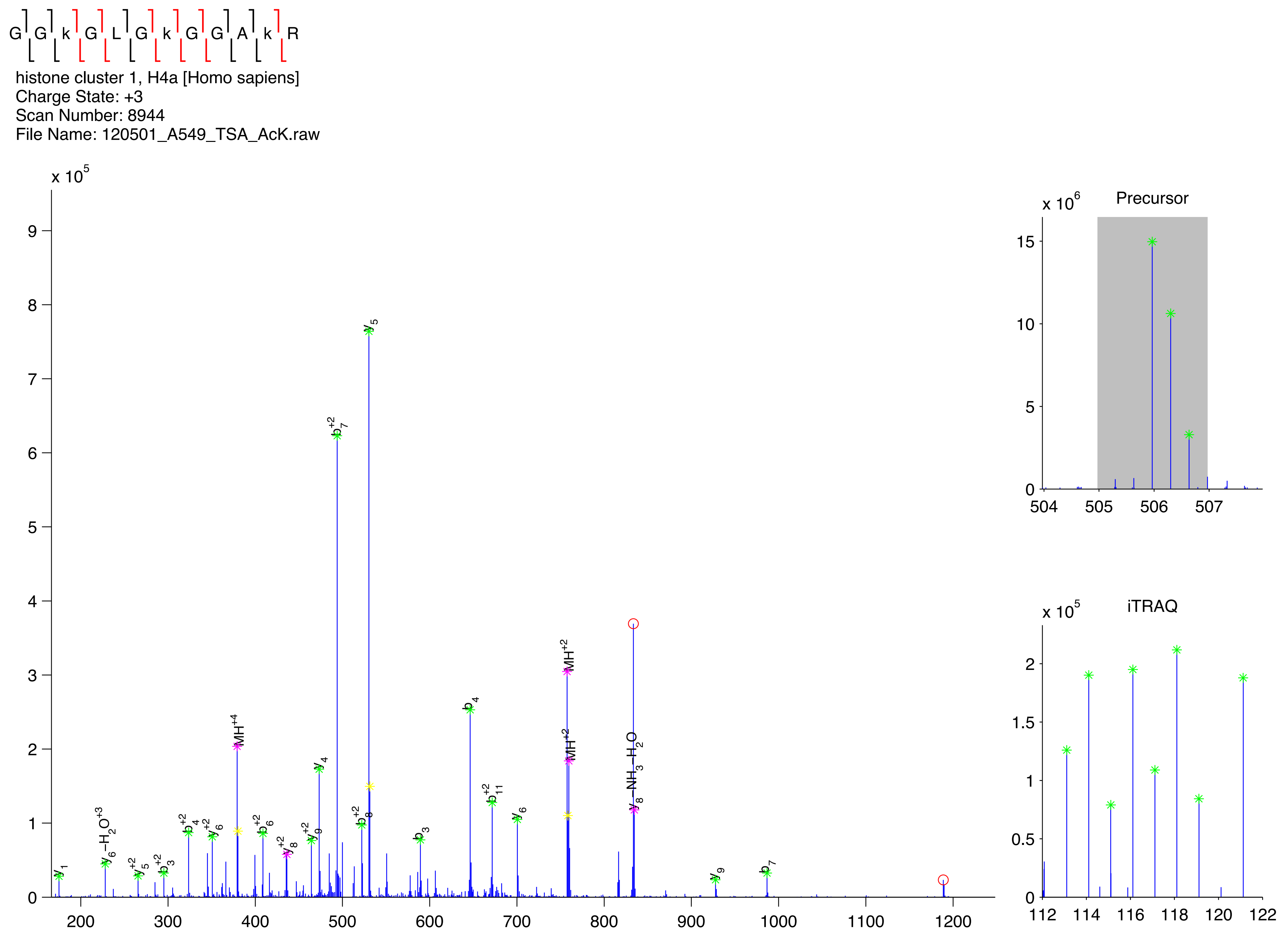

G G k G L G k G G A k R
histone cluster 1, H4a [Homo sapiens]
Charge State: +3
Scan Number: 8944
File Name: 120501_A549_TSA_AcK.raw
x 10^5
y1
y6–H2O+3
y5+2
b3+2
b4+2
y6+2
MH+4
b6+2
y8+2
y9+2
y4
b7+2
b8+2
y5
b3
b4
b11+2
y6
MH+2
MH+2
y8–NH3–H2O
y9
b7
Precursor
x 10^6
iTRAQ
x 10^5

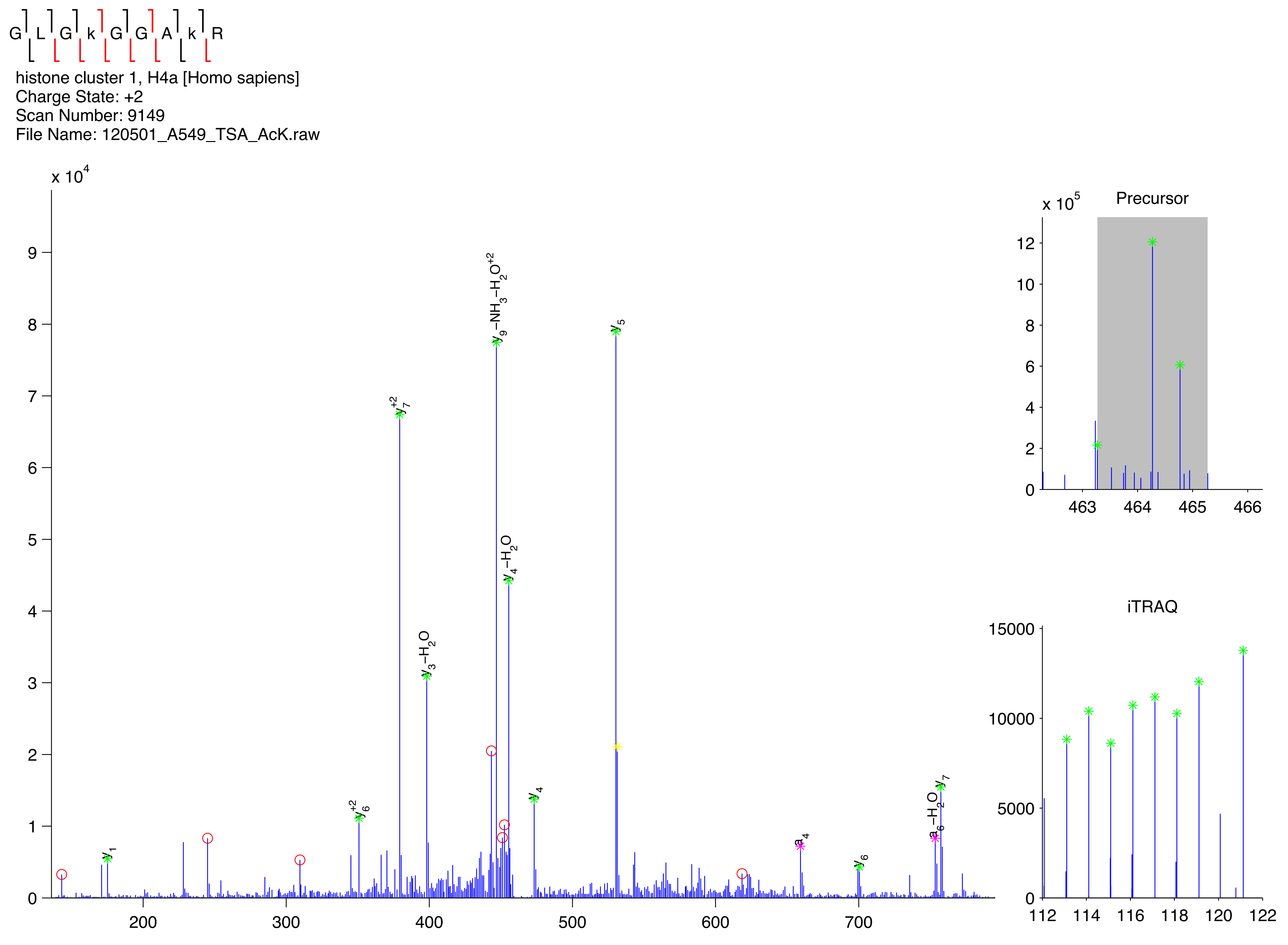
G L G k G G A k R
histone cluster 1, H4a [Homo sapiens]
Charge State: +2
Scan Number: 9149
File Name: 120501_A549_TSA_AcK.raw
x 10^4
y1
y6+2
y7+2
y3−H2O
y9−NH3−H2O+2
y4−H2O
y4
y5
a4
y6
a6−H2O
y7
x 10^5
Precursor
iTRAQ

G L G k G G A k R

histone cluster 1, H4a [Homo sapiens]
Charge State: +2
Scan Number: 9322
File Name: 120501_A549_TSA_AcK.raw

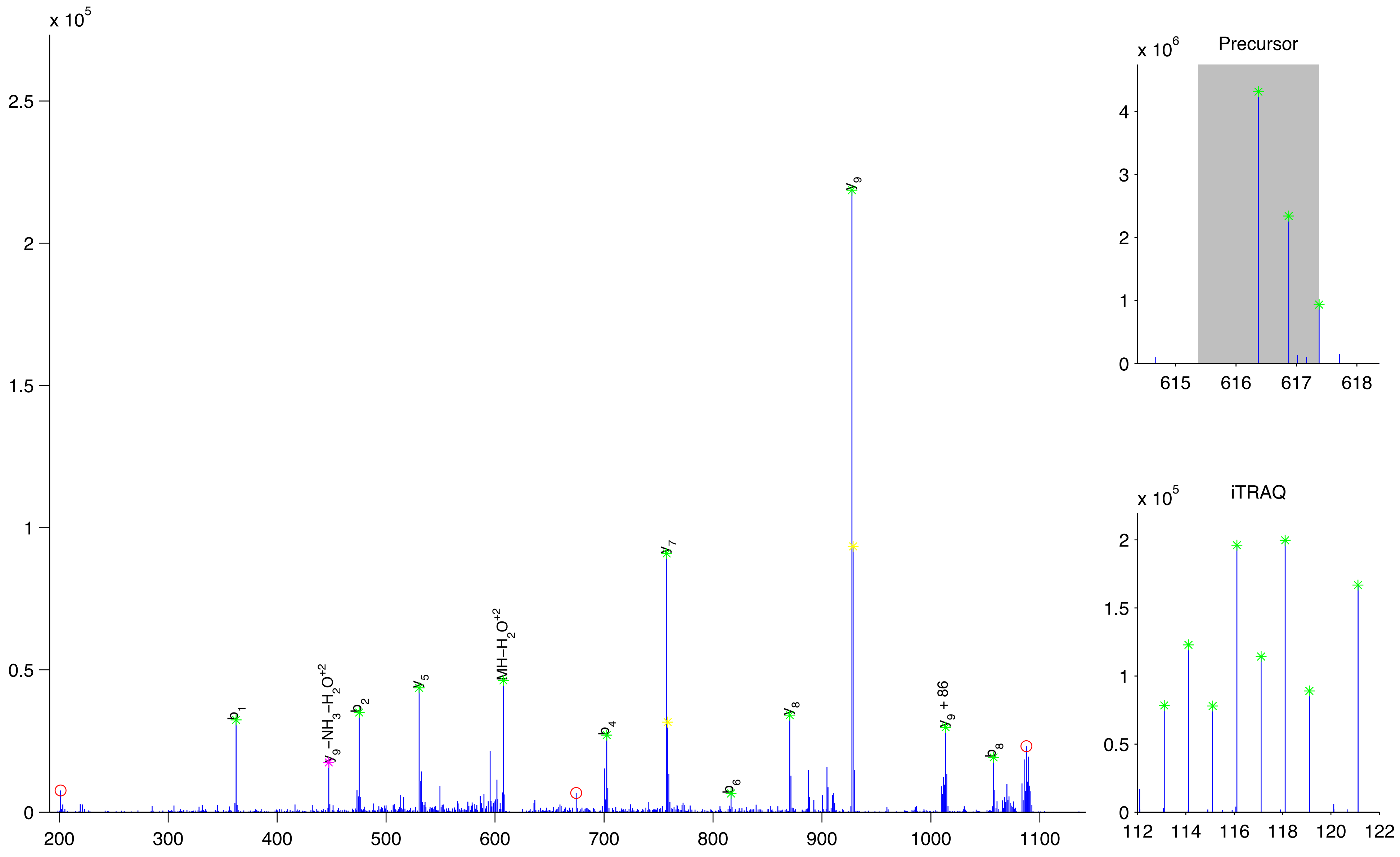

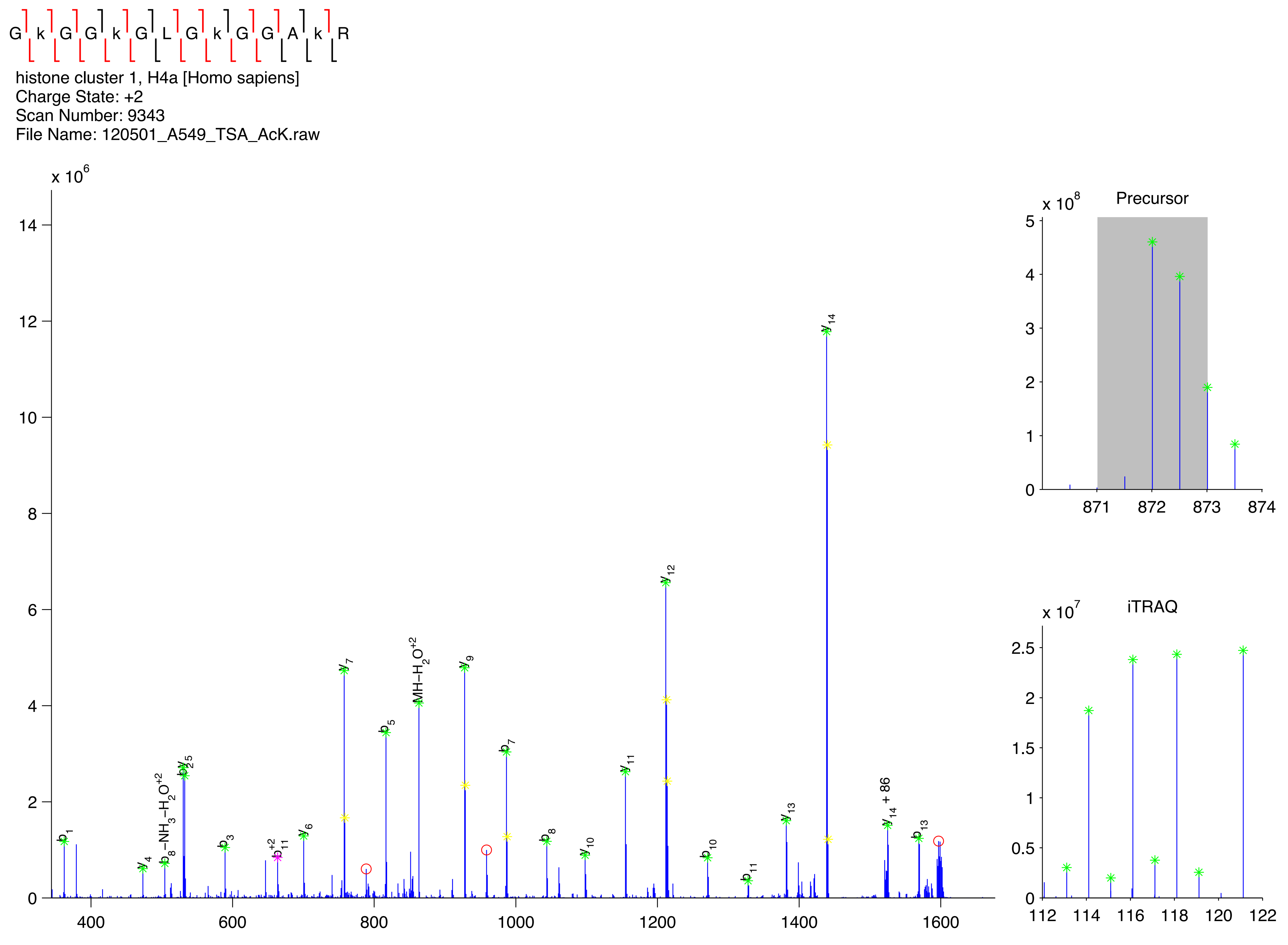
G k G G k G L G k G G A k R
histone cluster 1, H4a [Homo sapiens]
Charge State: +2
Scan Number: 9343
File Name: 120501_A549_TSA_AcK.raw
x 10^6
Precursor
x 10^8
iTRAQ
x 10^7

G k G G k G L G k G G A k R

histone cluster 1, H4a [Homo sapiens]
Charge State: +3
Scan Number: 9364
File Name: 120501_A549_TSA_AcK.raw

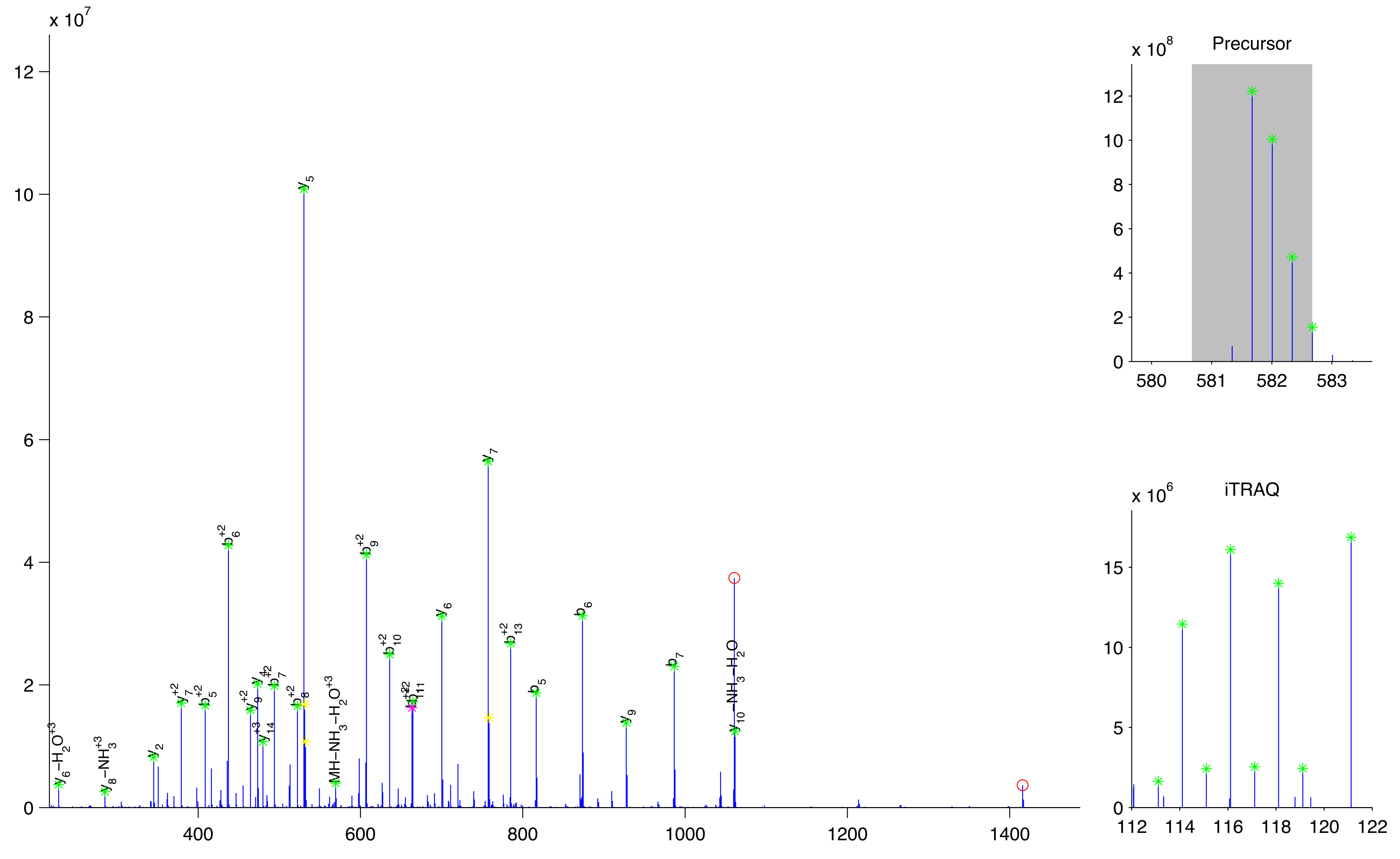

G L G k G G A k R

histone cluster 1, H4a [Homo sapiens]
Charge State: +3
Scan Number: 9578
File Name: 120501_A549_TSA_AcK.raw

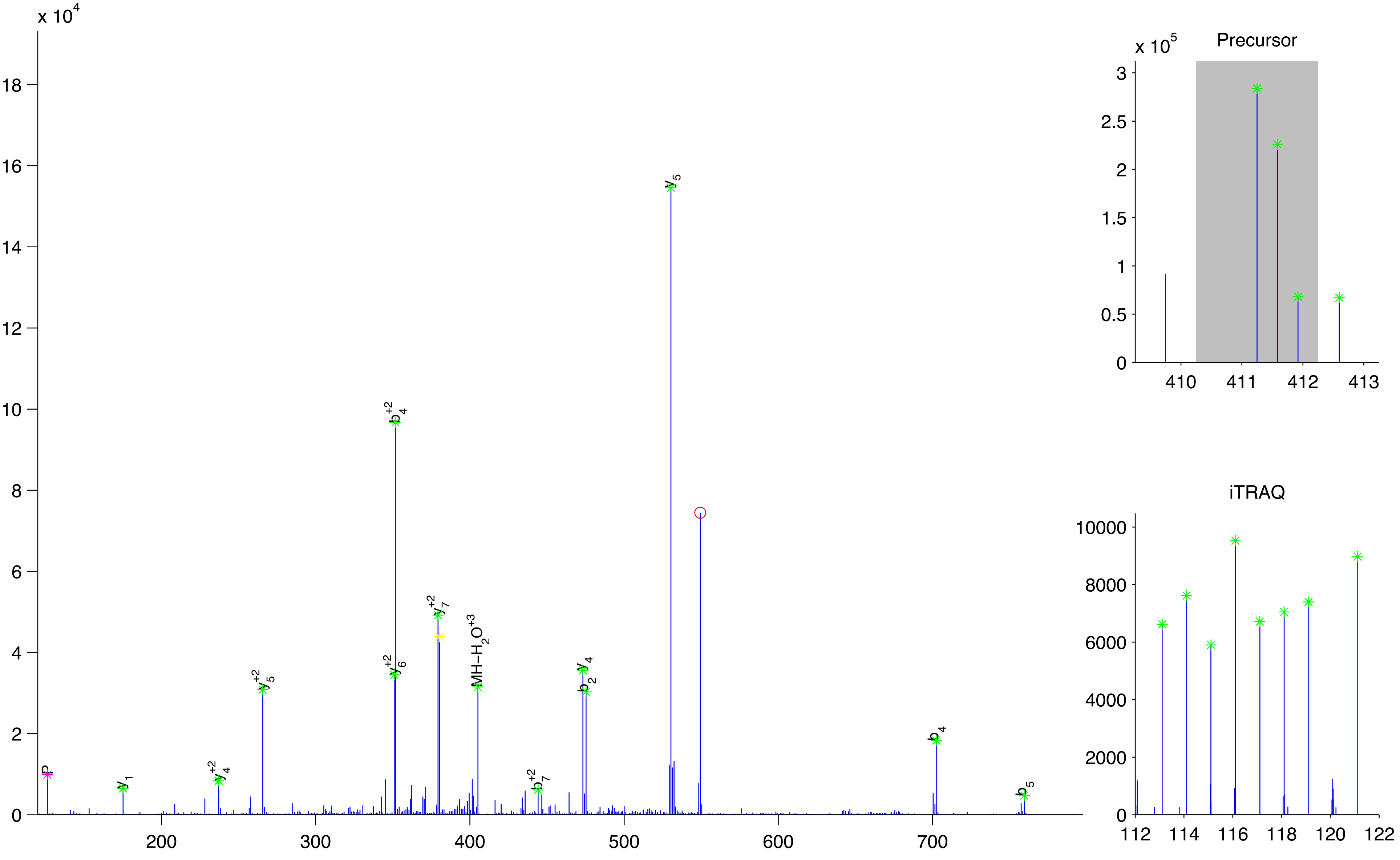

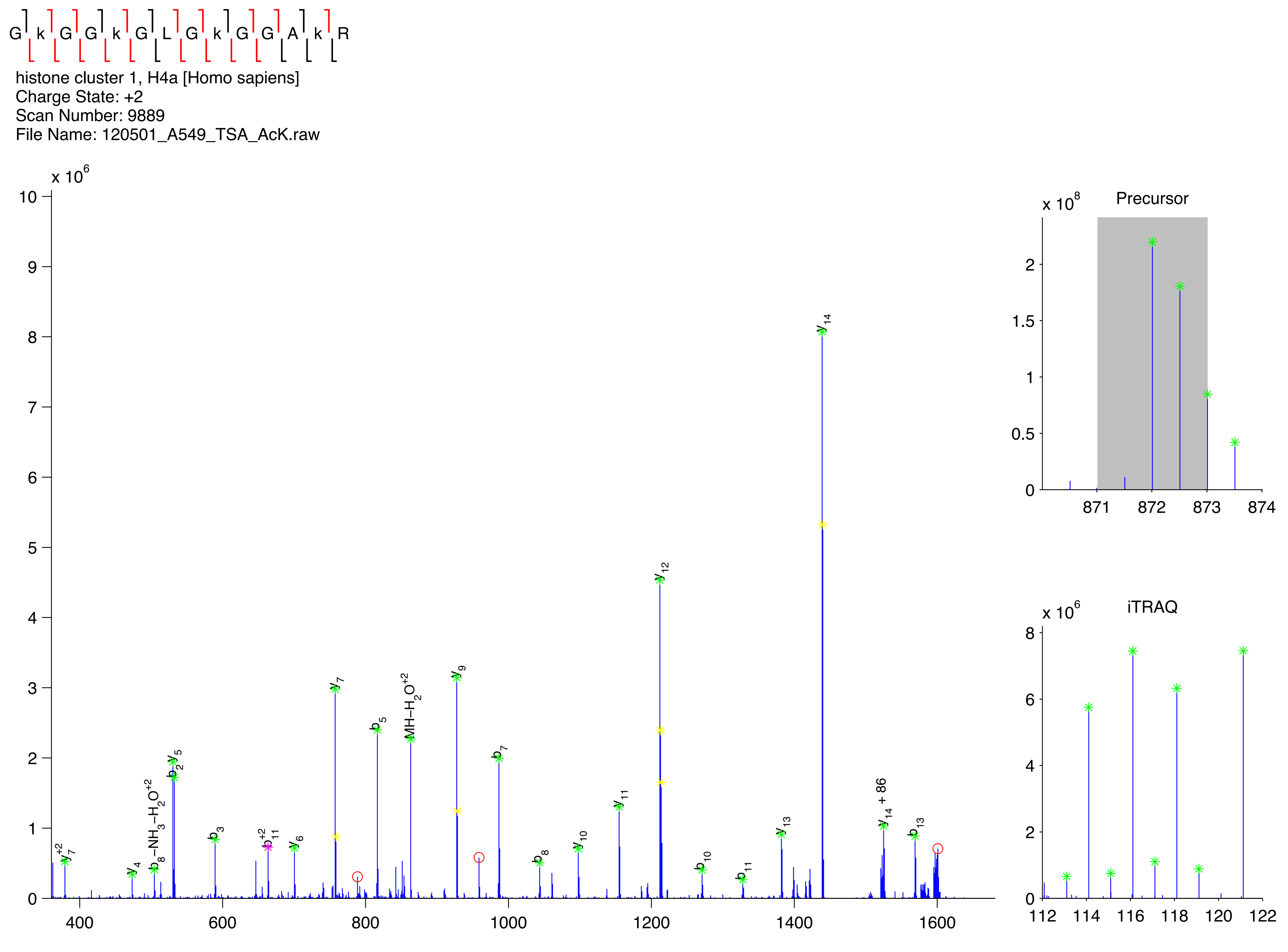

G k G G k G L G k G G A k R
histone cluster 1, H4a [Homo sapiens]
Charge State: +2
Scan Number: 9889
File Name: 120501_A549_TSA_AcK.raw
x 10^6
Precursor
x 10^8
iTRAQ
x 10^6

G k G G k G L G k G G A k R

histone cluster 1, H4a [Homo sapiens]
Charge State: +3
Scan Number: 9910
File Name: 120501_A549_TSA_AcK.raw

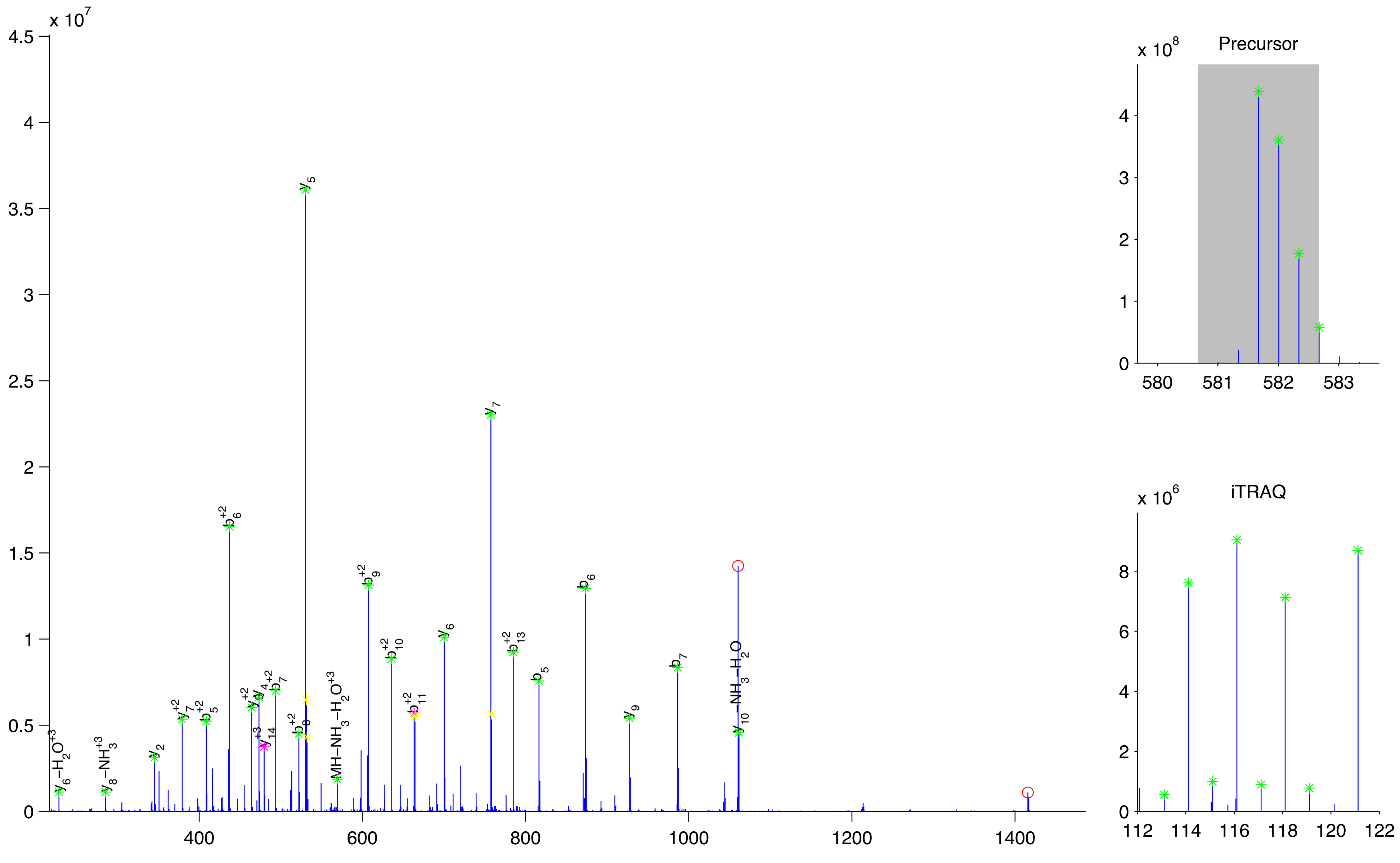

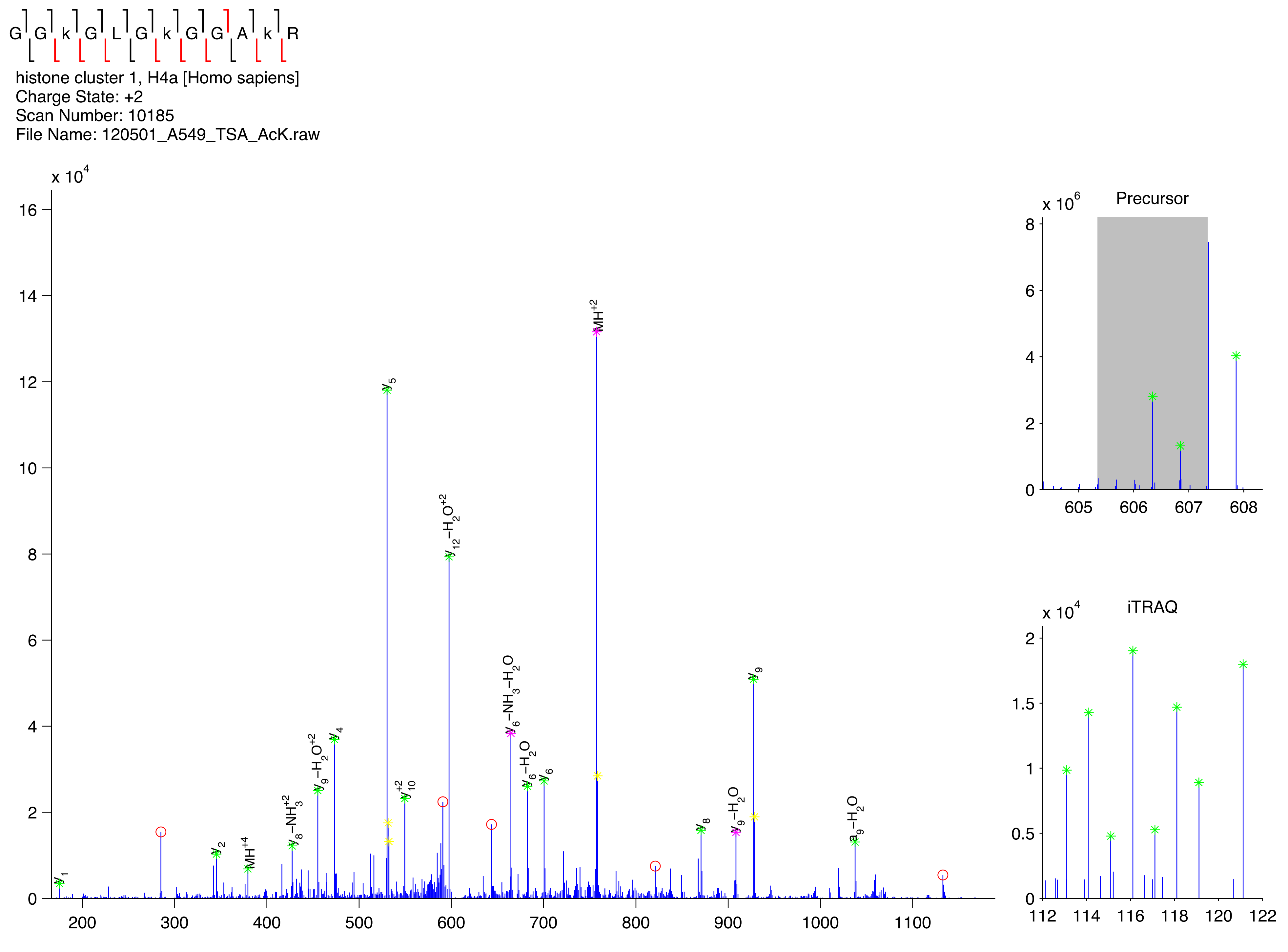

G G k G L G k G G A k R
histone cluster 1, H4a [Homo sapiens]
Charge State: +2
Scan Number: 10185
File Name: 120501_A549_TSA_AcK.raw
x 10^4
y1
y2
MH+4
y8−NH3+2
y9−H2O+2
y4
y5
y10+2
y12−H2O+2
y6−NH3−H2O
y6−H2O
y6
MH+2
y8
y9−H2O
y9
a9−H2O
Precursor
x 10^6
iTRAQ
x 10^4

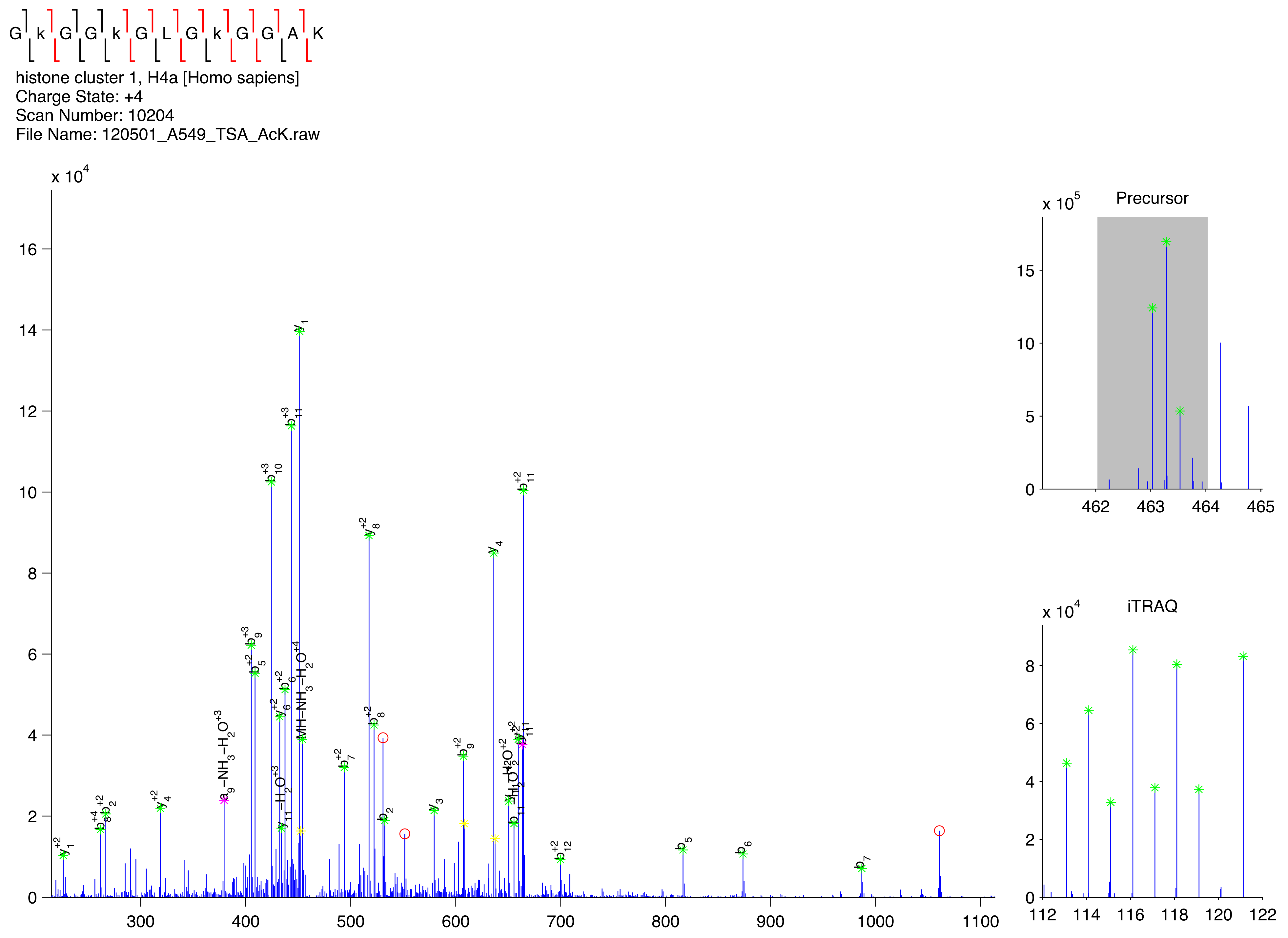
G k G G k G L G k G G A K
histone cluster 1, H4a [Homo sapiens]
Charge State: +4
Scan Number: 10204
File Name: 120501_A549_TSA_AcK.raw
Precursor
iTRAQ

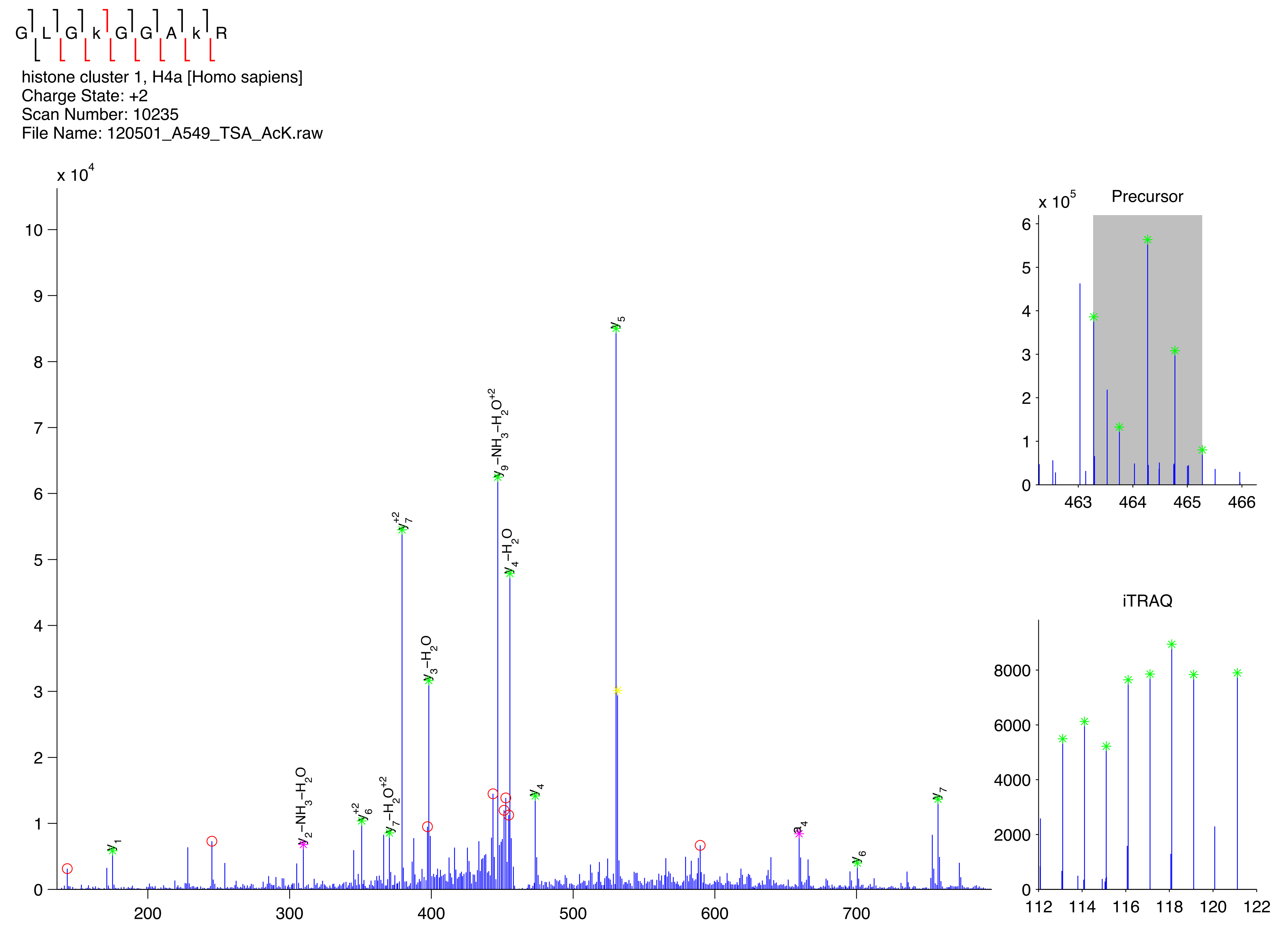

G L G k G G A k R
histone cluster 1, H4a [Homo sapiens]
Charge State: +2
Scan Number: 10235
File Name: 120501_A549_TSA_AcK.raw
x 10^4
y1
y2-NH3-H2O
y6+2
y7-H2O+2
y7+2
y3-H2O
y9-NH3-H2O+2
y4-H2O
y4
y5
a4
y6
y7
x 10^5
Precursor
iTRAQ

G k G G k G L G k G G A k R

histone cluster 1, H4a [Homo sapiens]
Charge State: +2
Scan Number: 10435
File Name: 120501_A549_TSA_AcK.raw

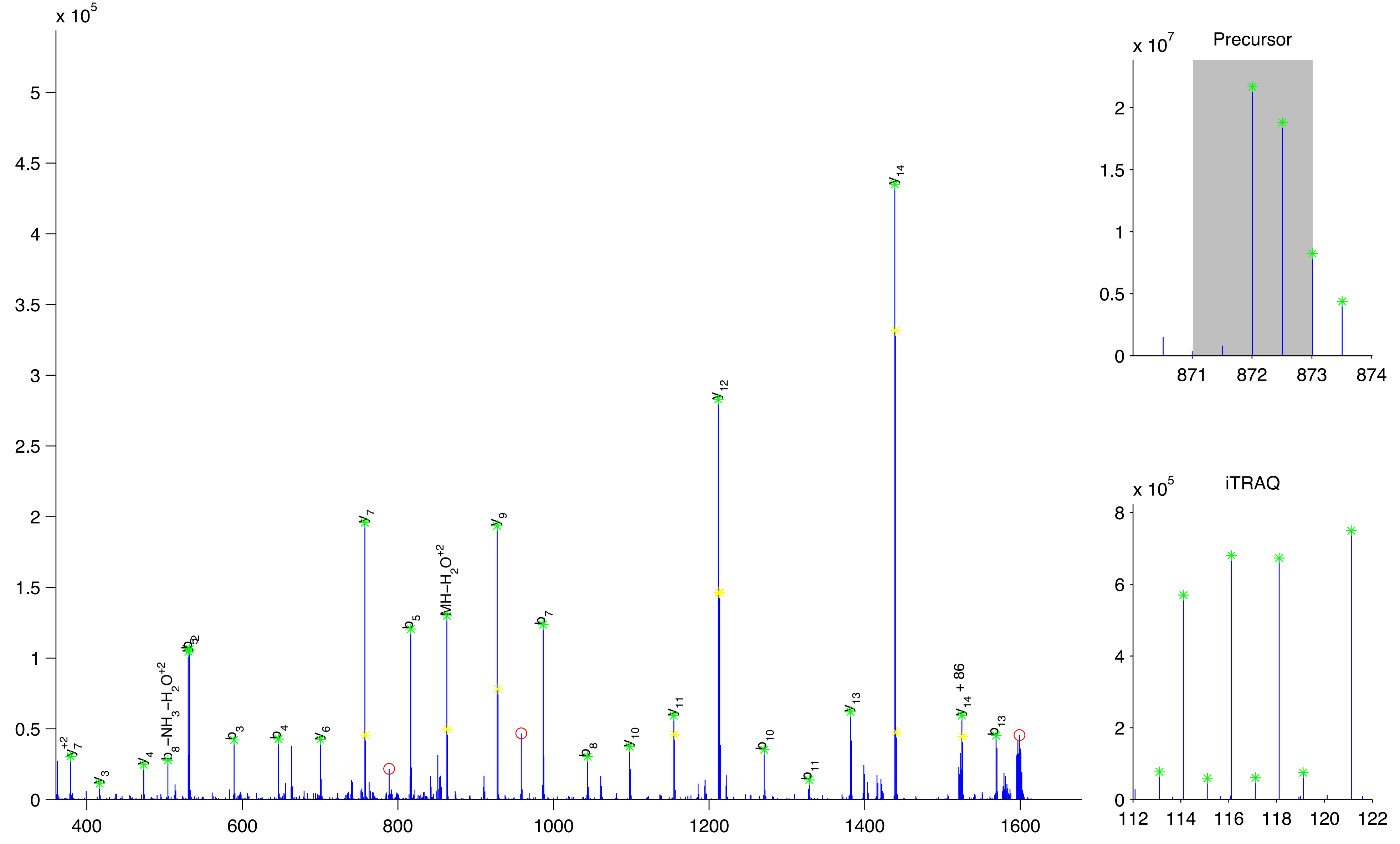

G k G G k G L G k G G A k R

histone cluster 1, H4a [Homo sapiens]
Charge State: +3
Scan Number: 10456
File Name: 120501_A549_TSA_AcK.raw

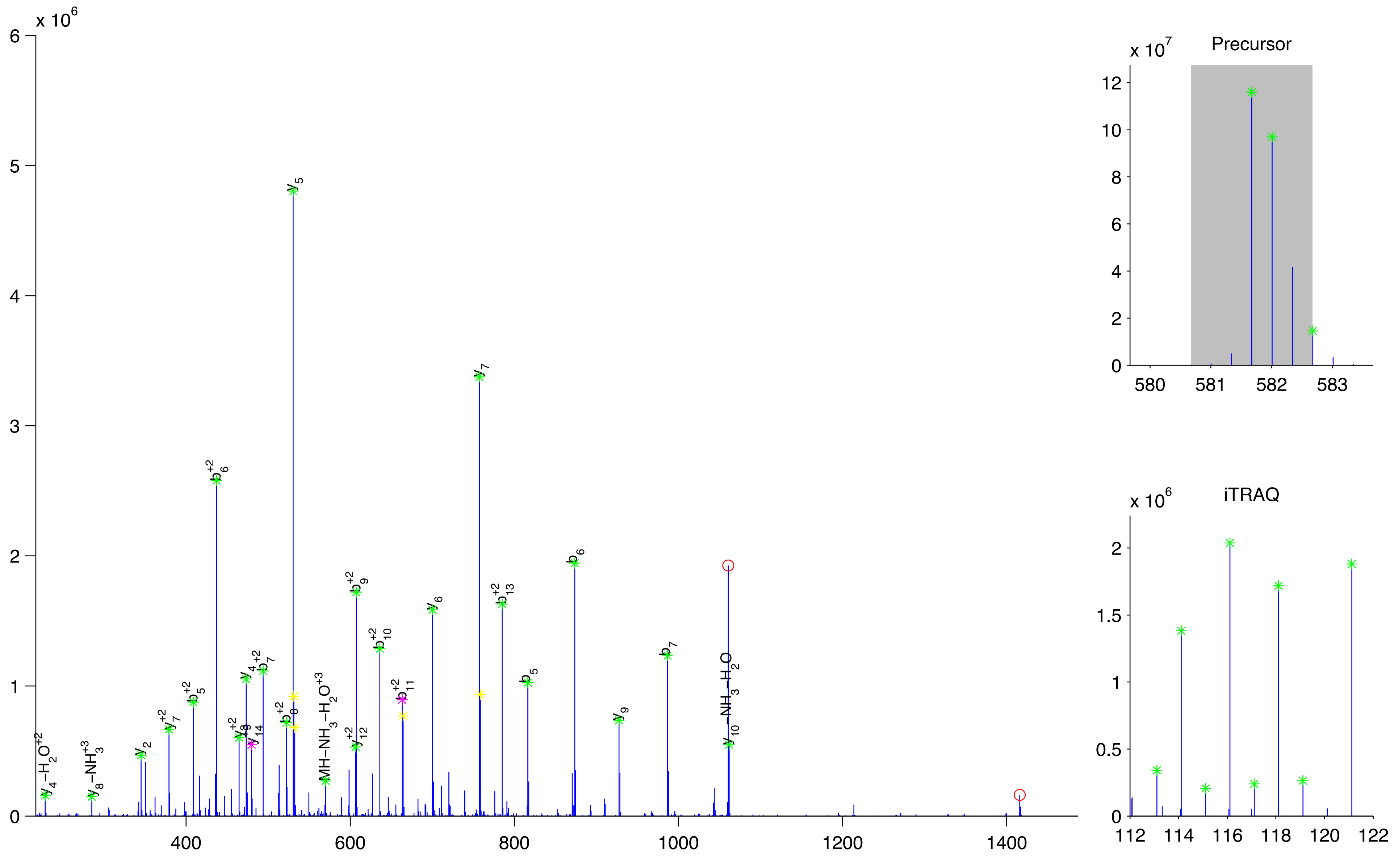

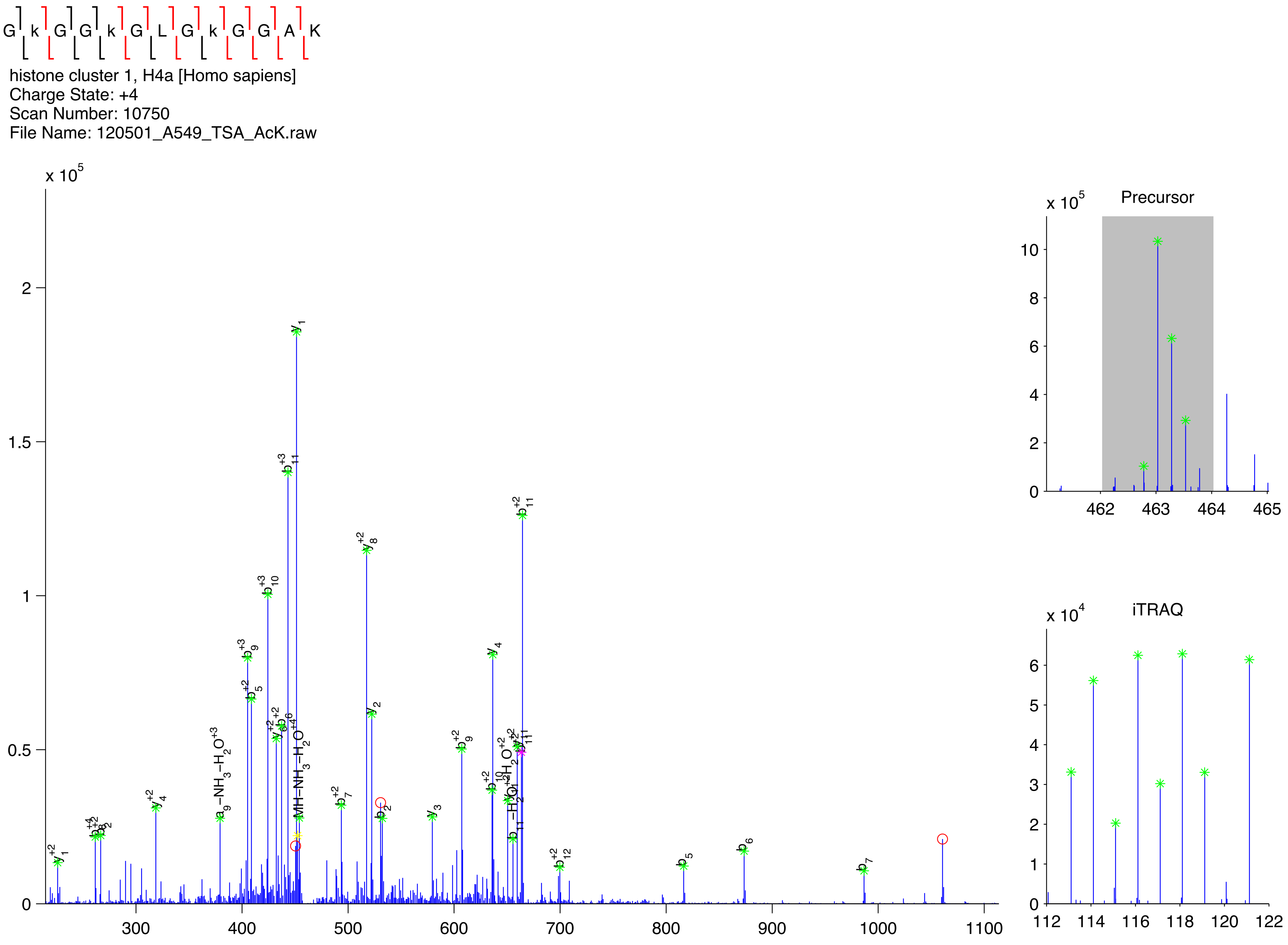
G k G G k G L G k G G A K
histone cluster 1, H4a [Homo sapiens]
Charge State: +4
Scan Number: 10750
File Name: 120501_A549_TSA_AcK.raw
Precursor
iTRAQ

G k G G k G L G k G G A K

histone cluster 1, H4a [Homo sapiens]
Charge State: +2
Scan Number: 10771
File Name: 120501_A549_TSA_AcK.raw

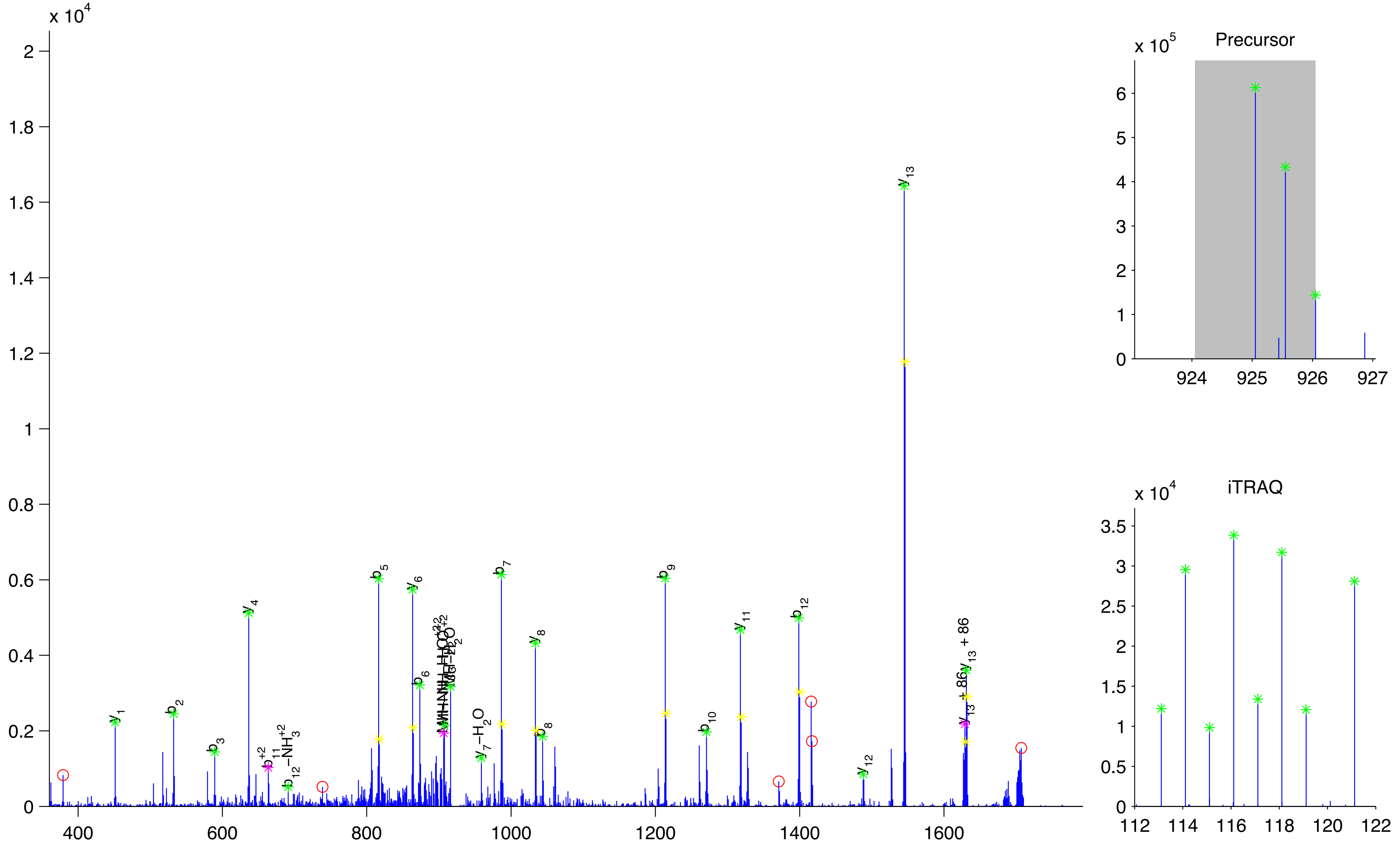

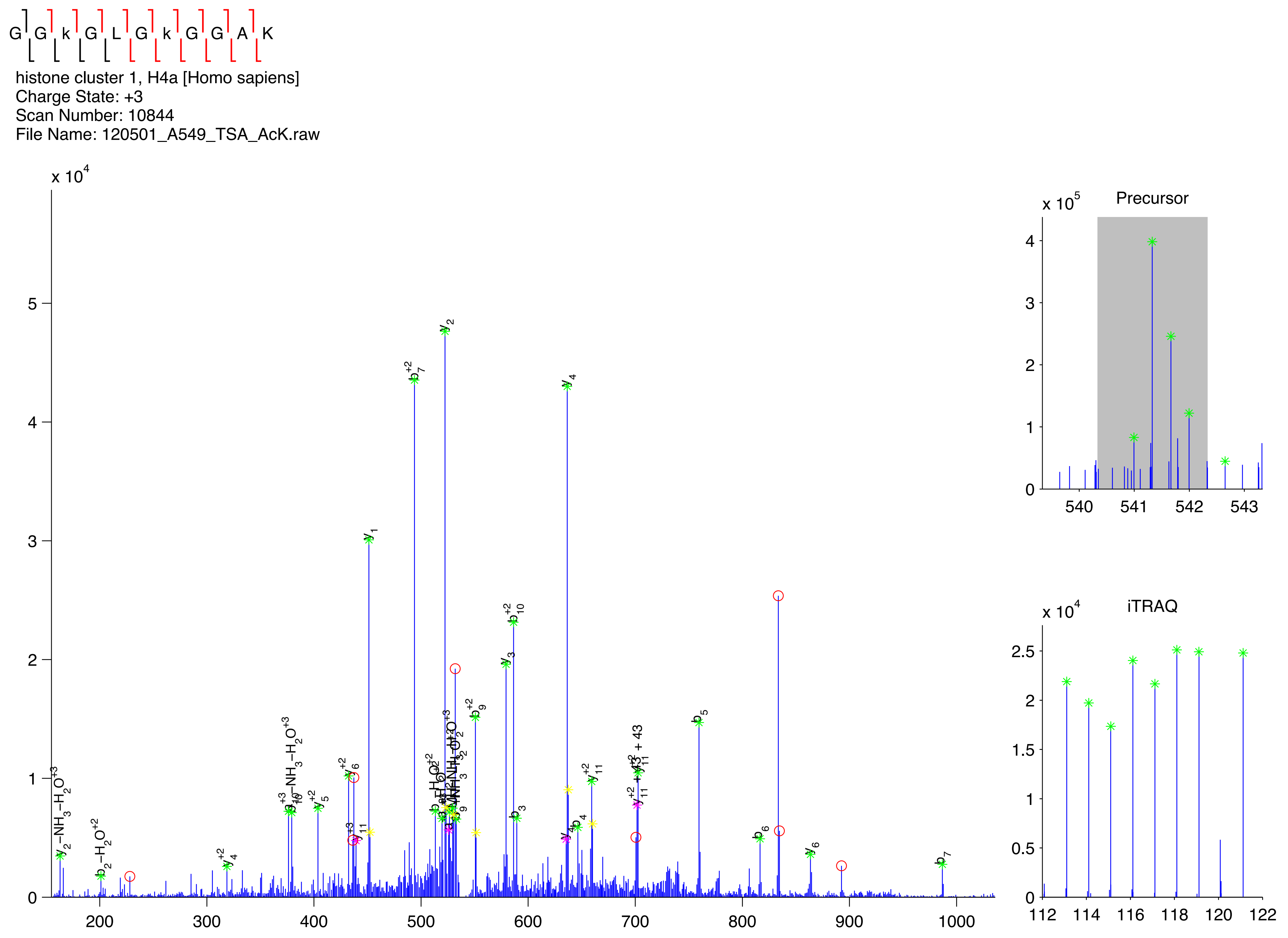

G G k G L G k G G A K
histone cluster 1, H4a [Homo sapiens]
Charge State: +3
Scan Number: 10844
File Name: 120501_A549_TSA_AcK.raw
x 10^4
Precursor
x 10^5
iTRAQ
x 10^4

G k G G k G L G k G G A k R

histone cluster 1, H4a [Homo sapiens]
Charge State: +3
Scan Number: 10980
File Name: 120501_A549_TSA_AcK.raw

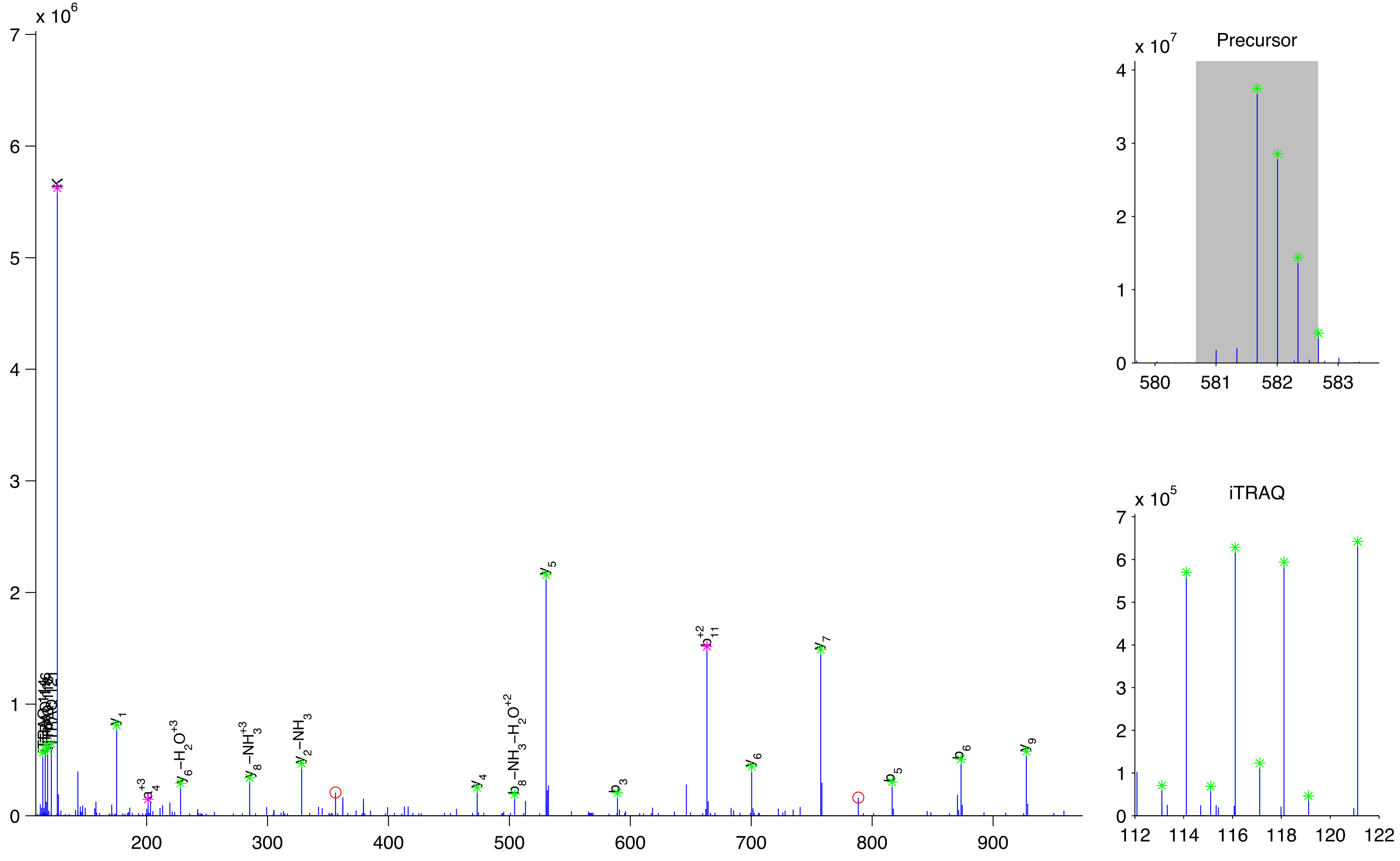

G k G G k G L G k G G A k R

histone cluster 1, H4a [Homo sapiens]
Charge State: +3
Scan Number: 10981
File Name: 120501_A549_TSA_AcK.raw

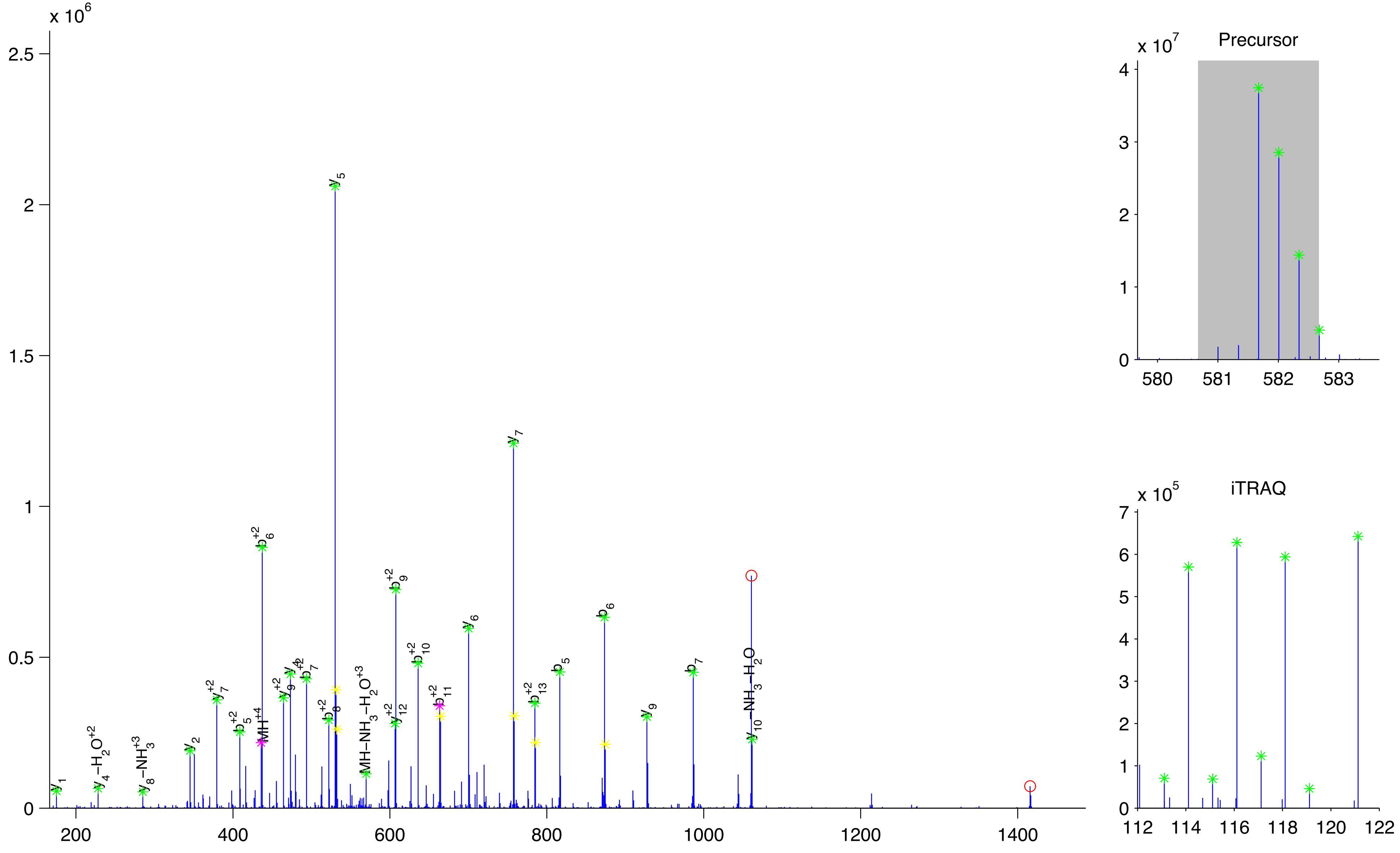

G k G G k G L G k G G A k R

histone cluster 1, H4a [Homo sapiens]
Charge State: +2
Scan Number: 10983
File Name: 120501_A549_TSA_AcK.raw

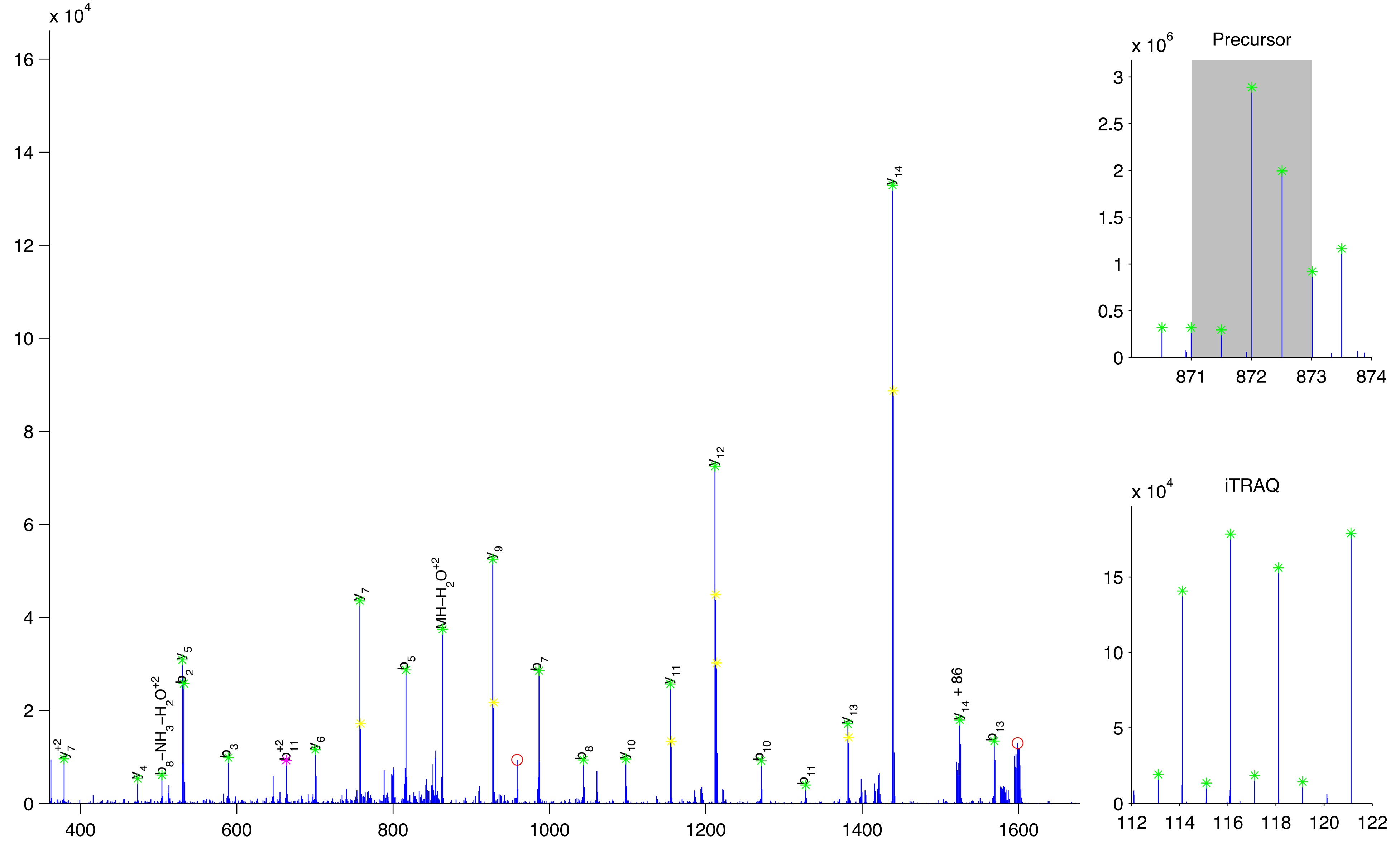

G k G G k G L G k G G A K

histone cluster 1, H4a [Homo sapiens]
Charge State: +4
Scan Number: 11277
File Name: 120501_A549_TSA_AcK.raw

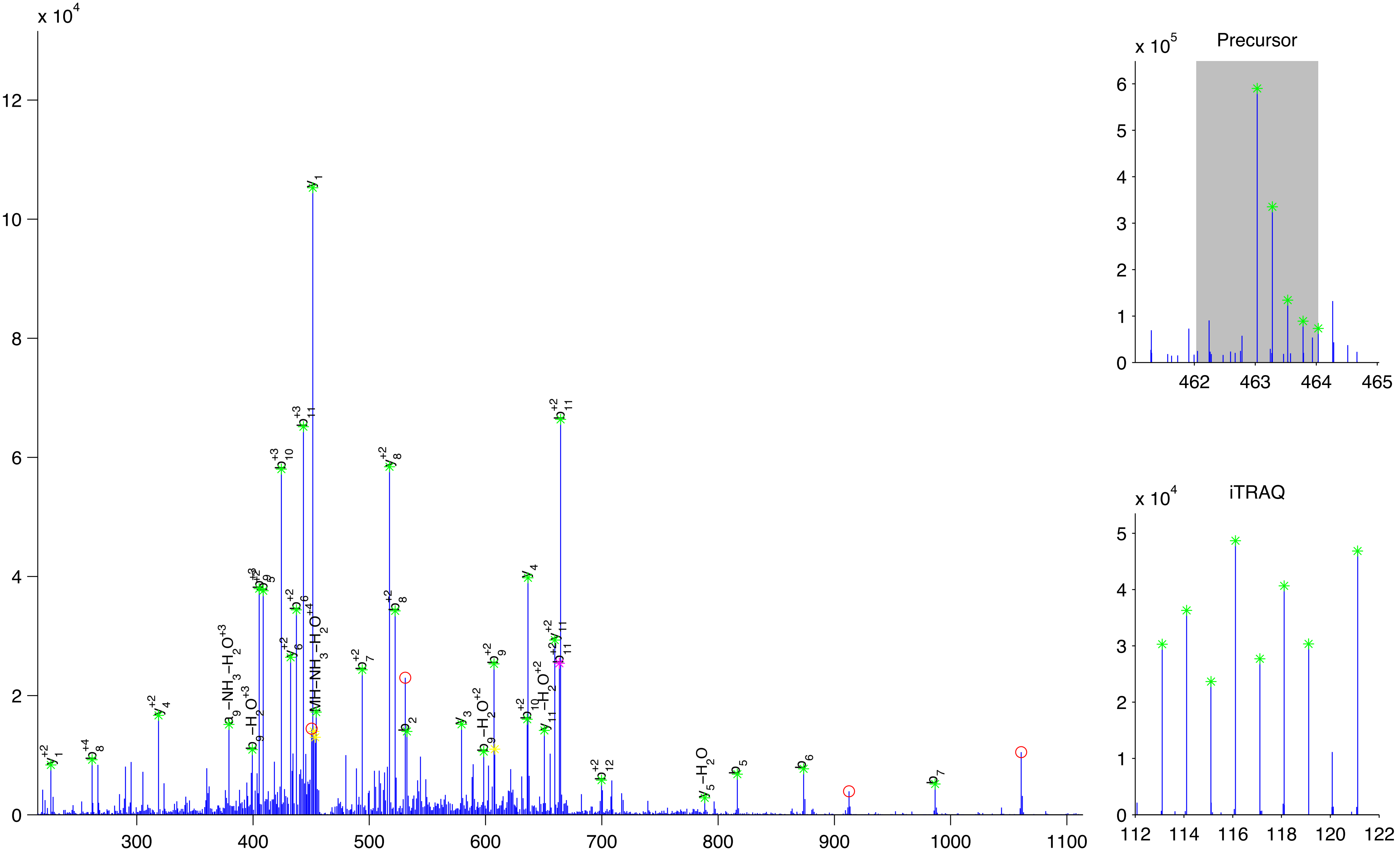

G k G G k G L G k G G A K

histone cluster 1, H4a [Homo sapiens]
Charge State: +2
Scan Number: 11300
File Name: 120501_A549_TSA_AcK.raw

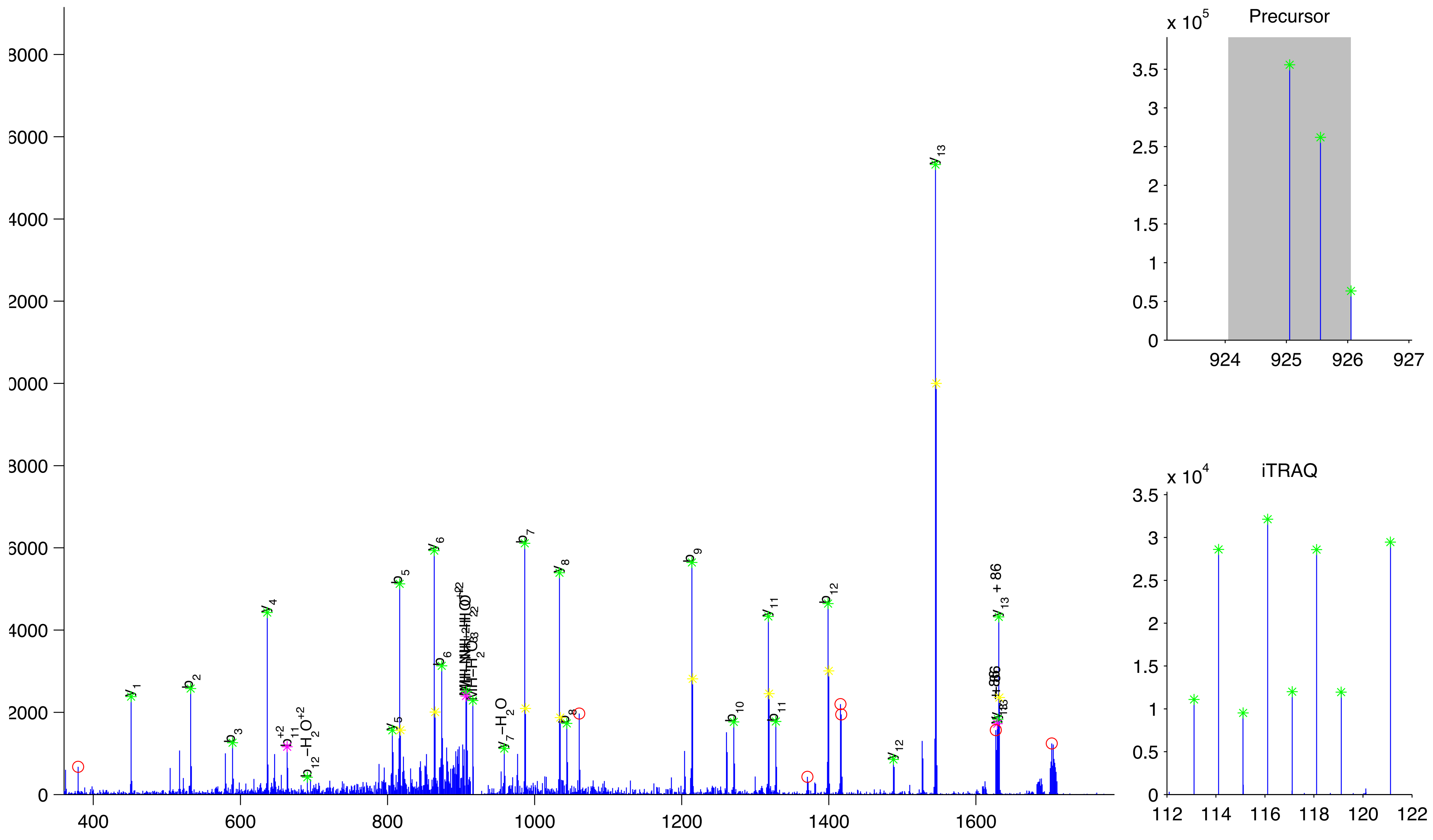

G k G G k G L G k G G A k R

histone cluster 1, H4a [Homo sapiens]
Charge State: +3
Scan Number: 11506
File Name: 120501_A549_TSA_AcK.raw

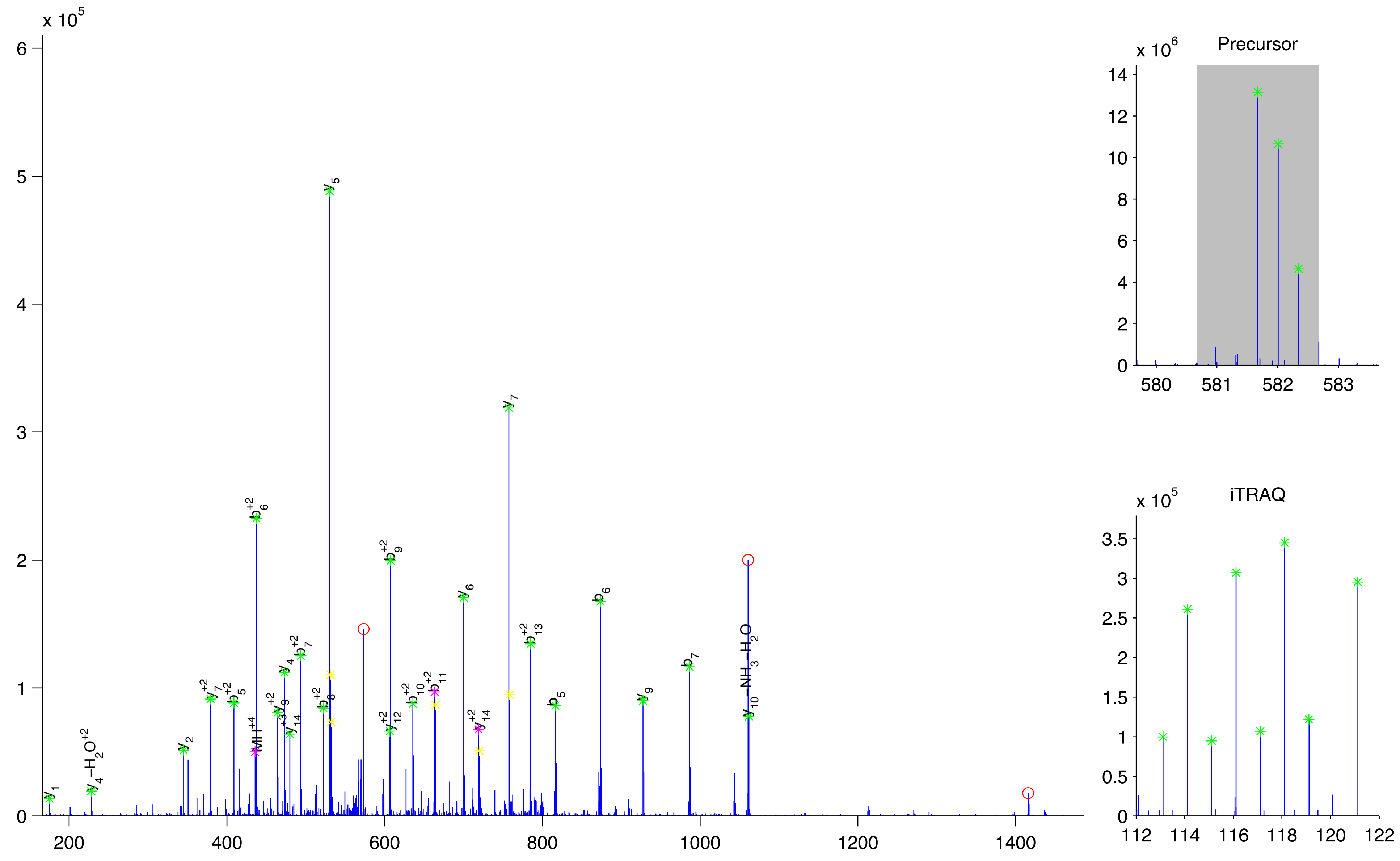

G k G G k G L G k G G A k R

histone cluster 1, H4a [Homo sapiens]
Charge State: +3
Scan Number: 12052
File Name: 120501_A549_TSA_AcK.raw

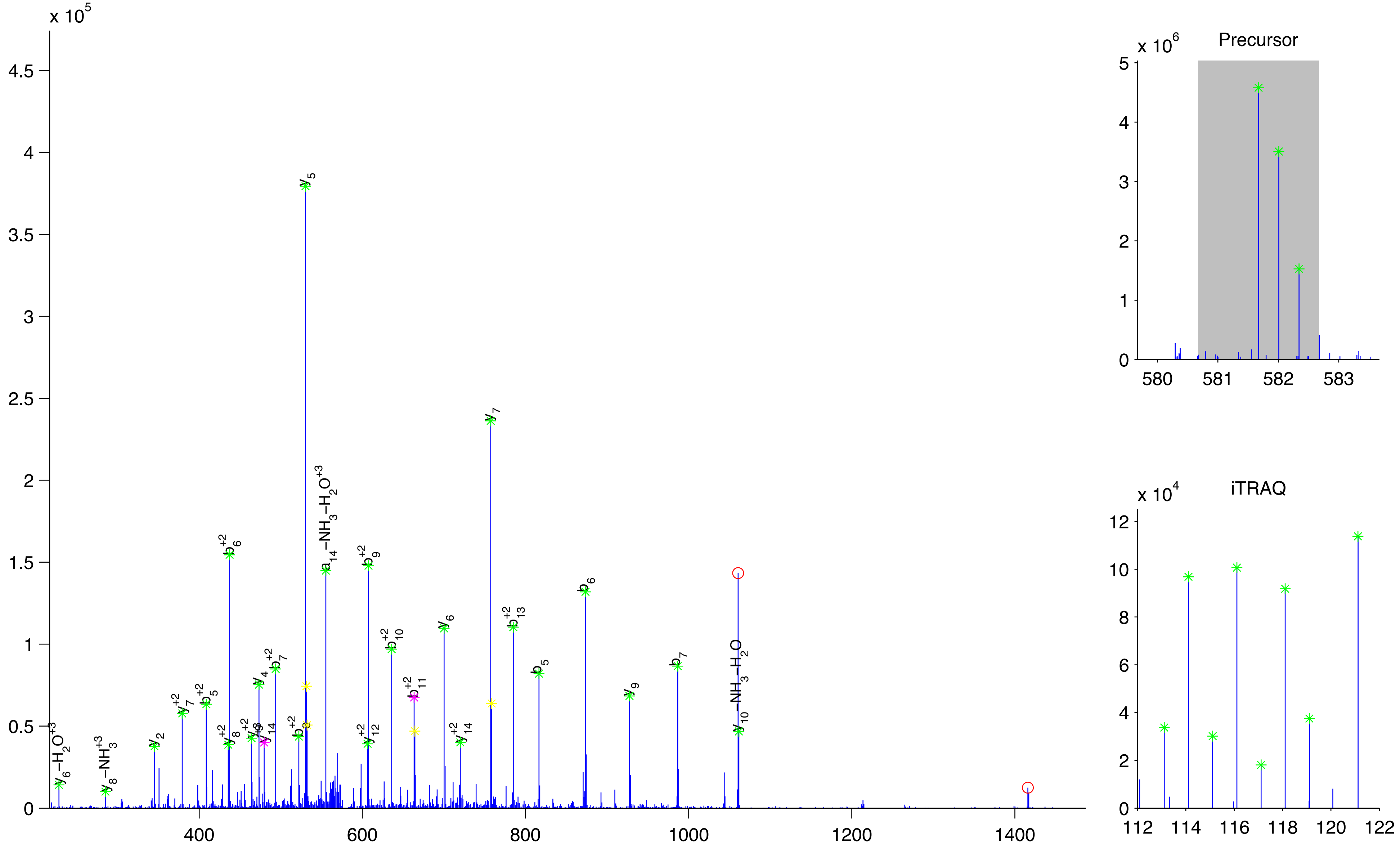

G k G G k G L G k G G A k R

histone cluster 1, H4a [Homo sapiens]
Charge State: +2
Scan Number: 12056
File Name: 120501_A549_TSA_AcK.raw

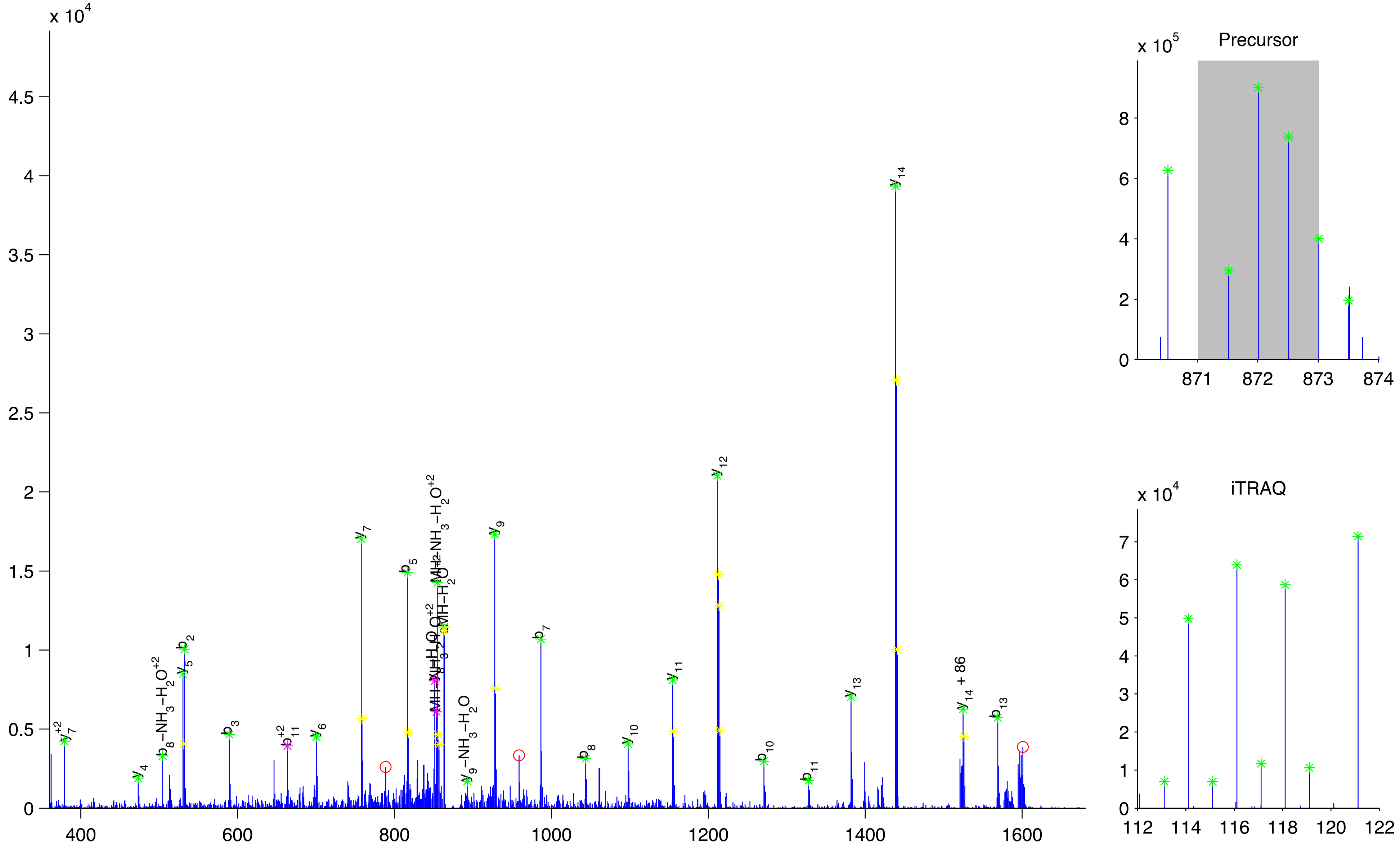

G k G G k G L G k G G A k R

histone cluster 1, H4a [Homo sapiens]
Charge State: +3
Scan Number: 12600
File Name: 120501_A549_TSA_AcK.raw

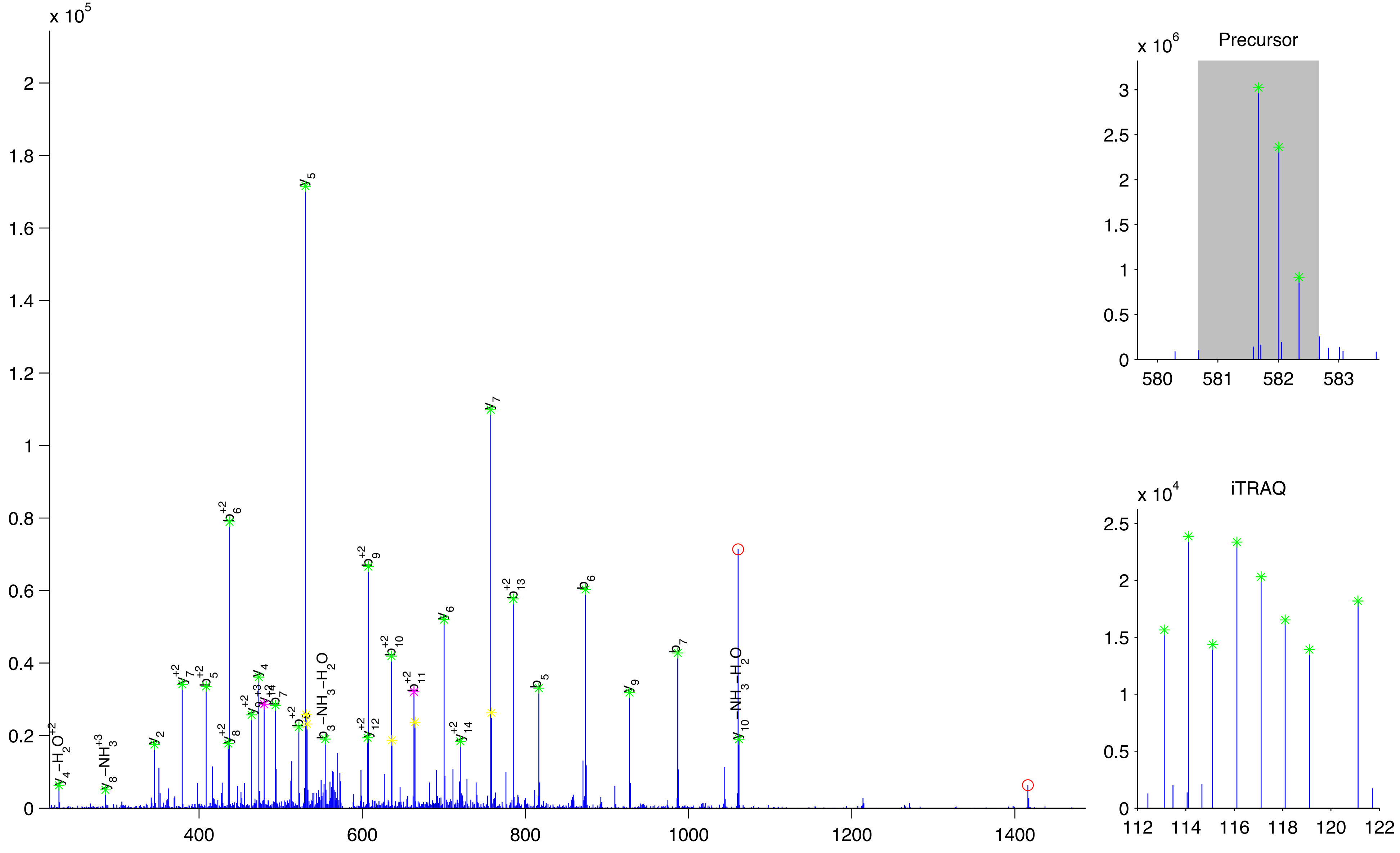

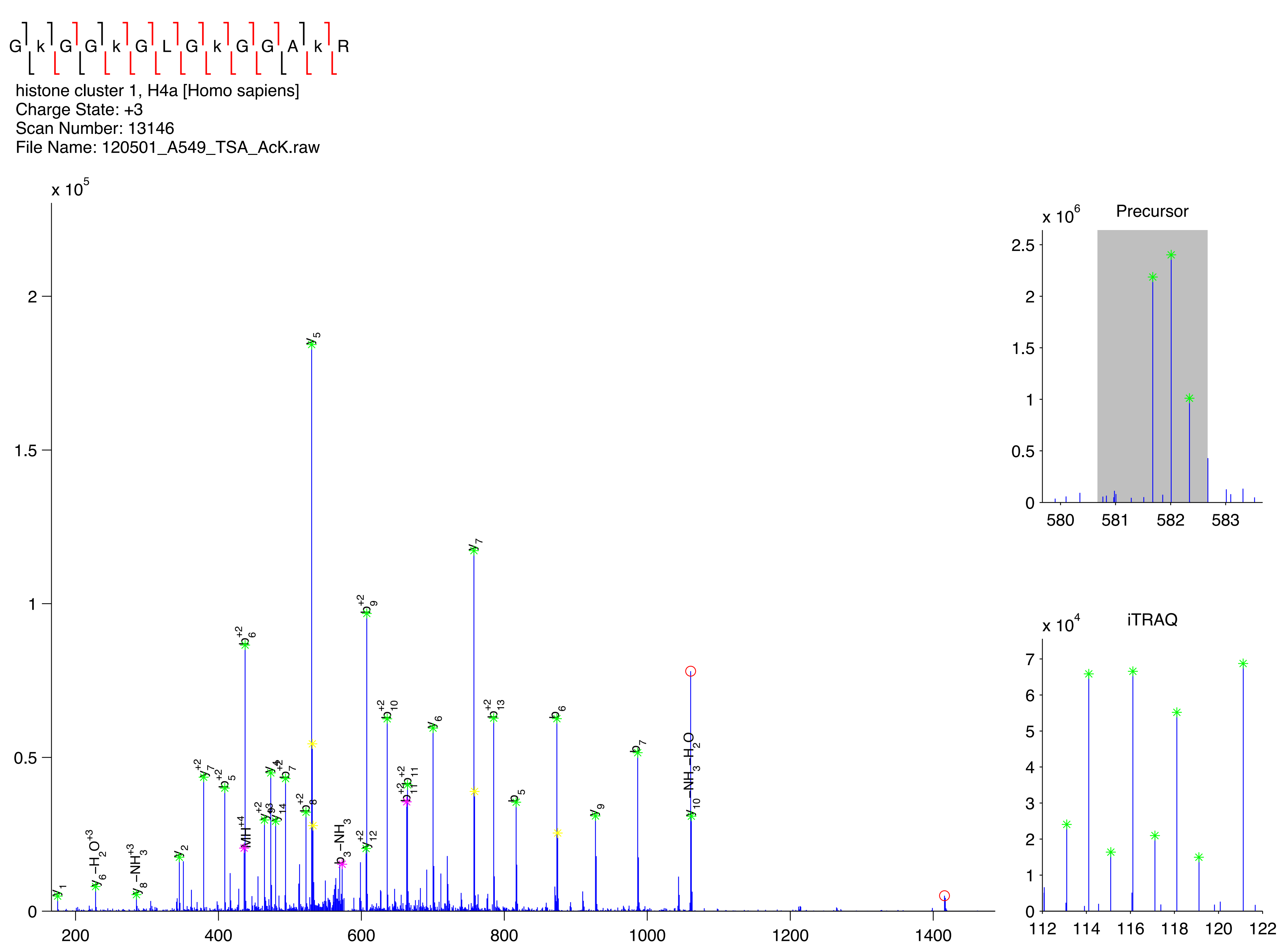
G k G G k G L G k G G A k R
histone cluster 1, H4a [Homo sapiens]
Charge State: +3
Scan Number: 13146
File Name: 120501_A549_TSA_AcK.raw
x 10^5
Precursor
x 10^6
iTRAQ
x 10^4

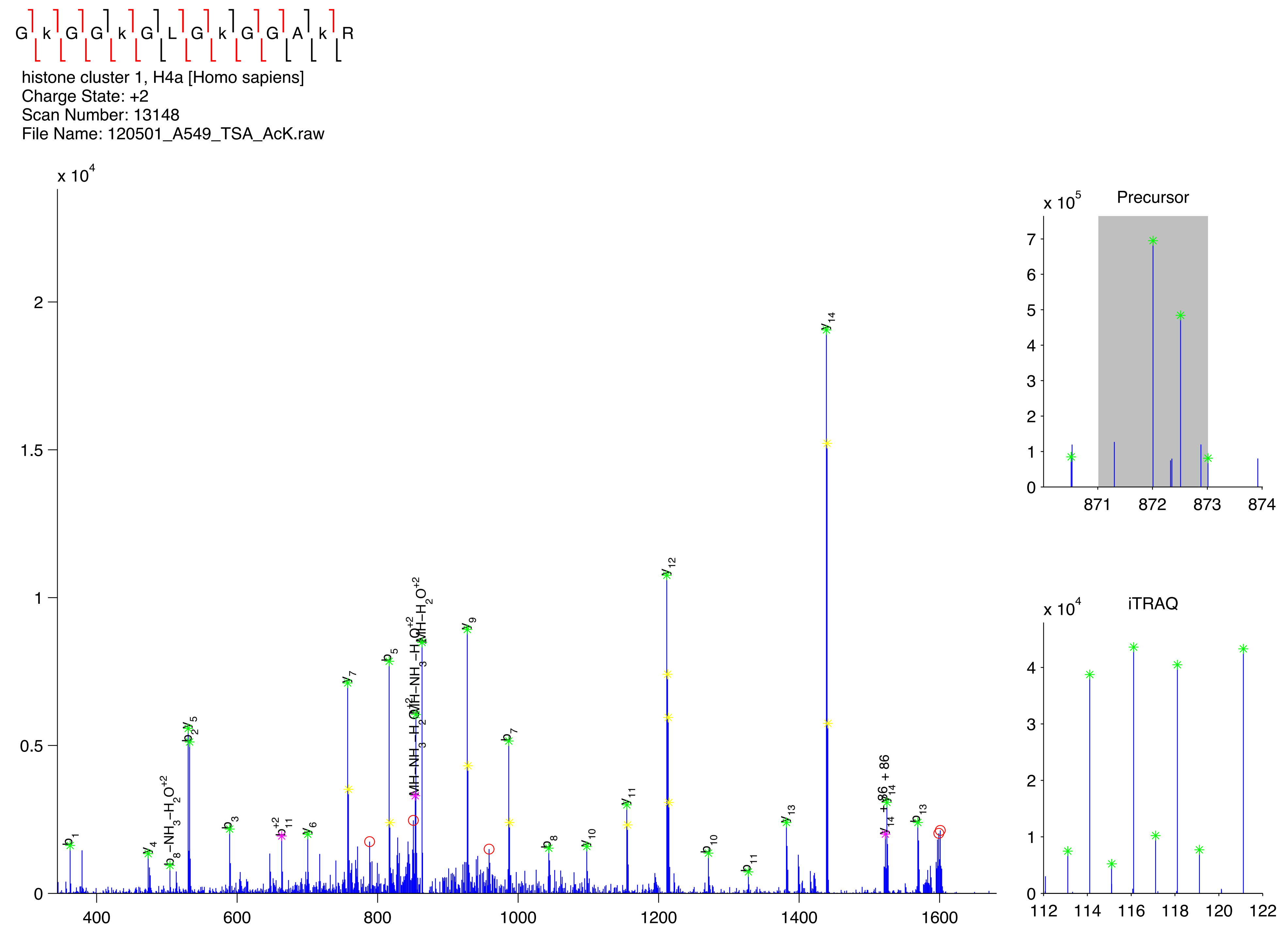

G k G G k G L G k G G A k R
histone cluster 1, H4a [Homo sapiens]
Charge State: +2
Scan Number: 13148
File Name: 120501_A549_TSA_AcK.raw
x 10^4
Precursor
x 10^5
iTRAQ
x 10^4

G k G G k G L G k G G A k R

histone cluster 1, H4a [Homo sapiens]
Charge State: +3
Scan Number: 13690
File Name: 120501_A549_TSA_AcK.raw

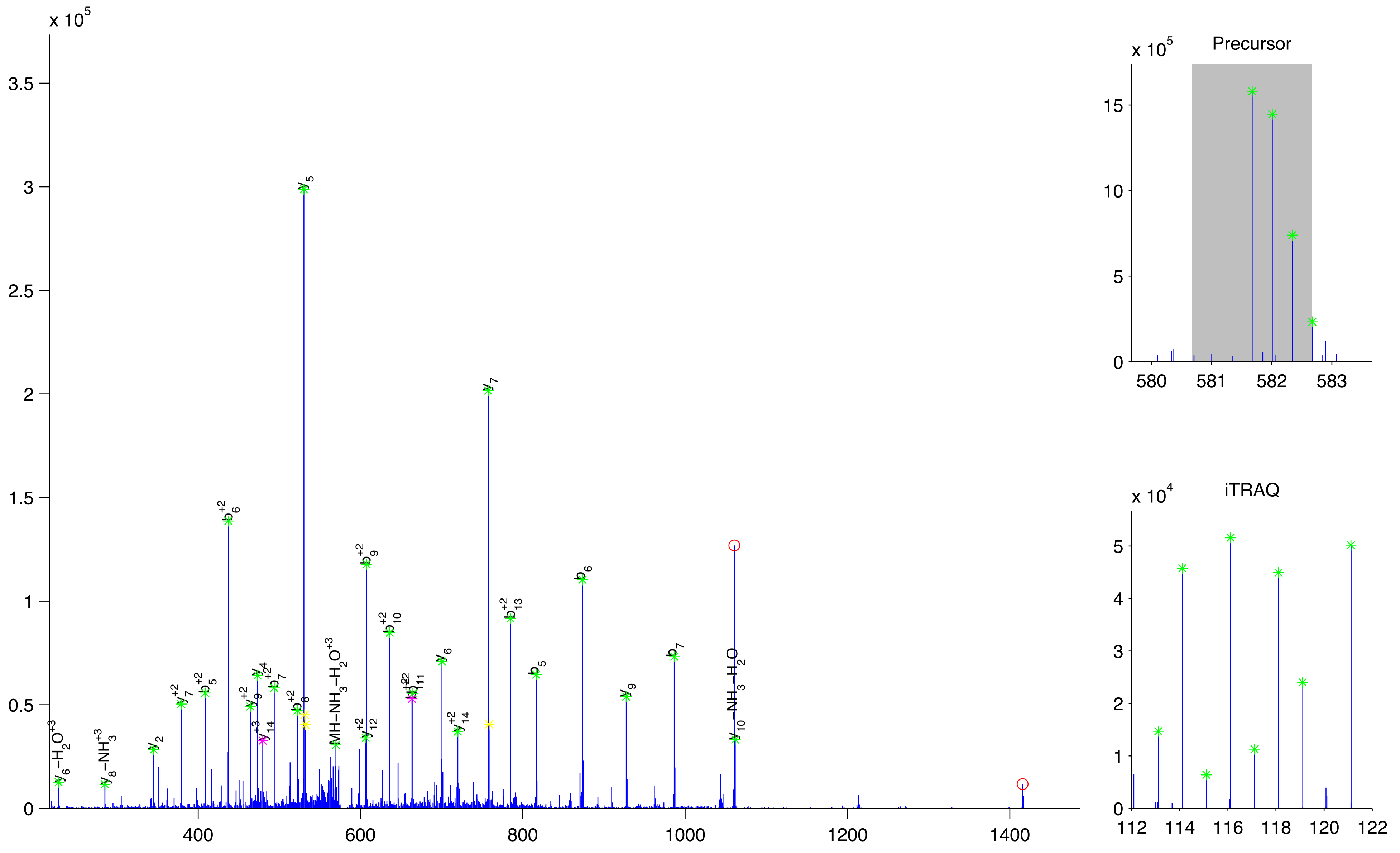

G L G k G G A k R

histone cluster 1, H4a [Homo sapiens]
Charge State: +3
Scan Number: 14013
File Name: 120501_A549_TSA_AcK.raw

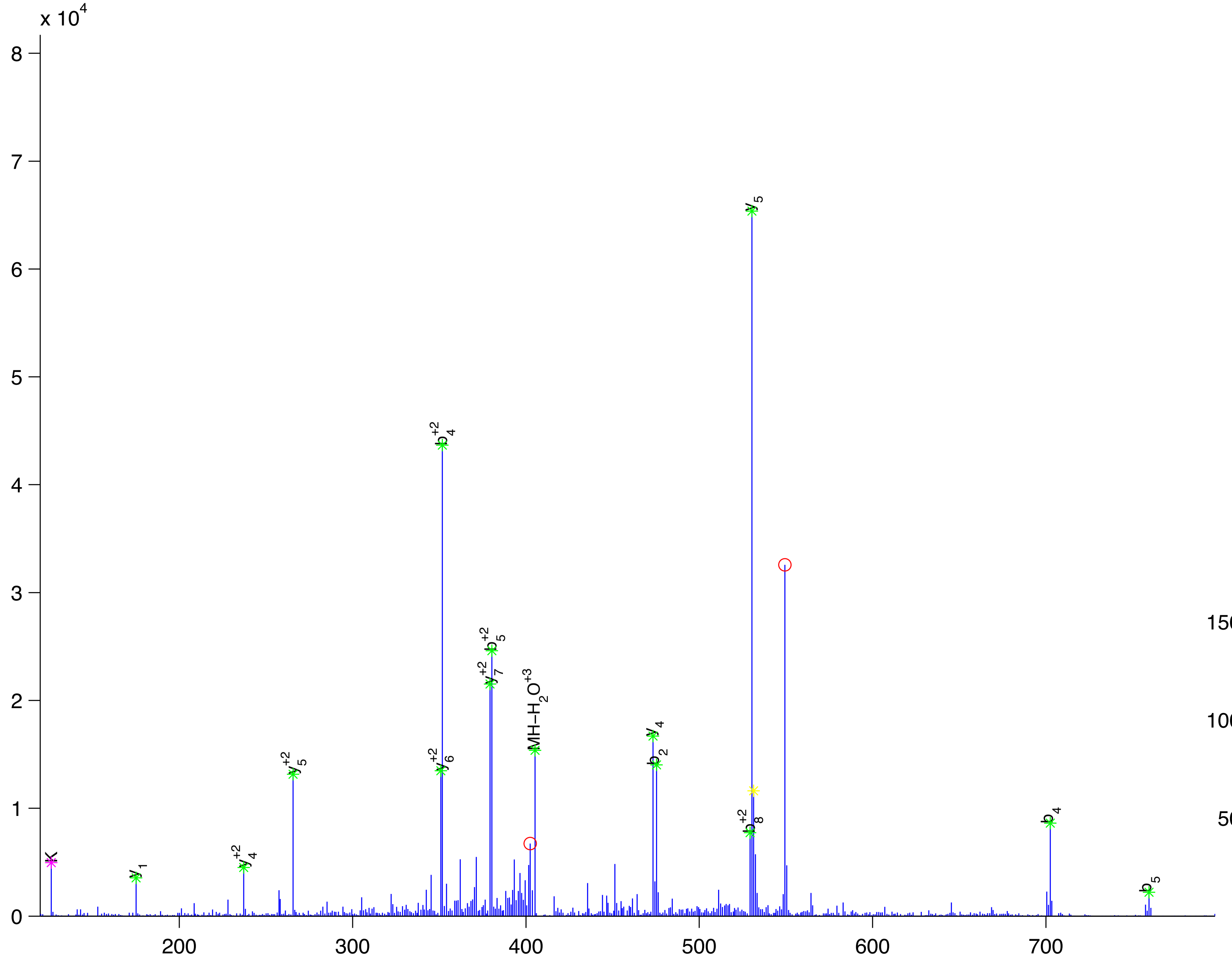

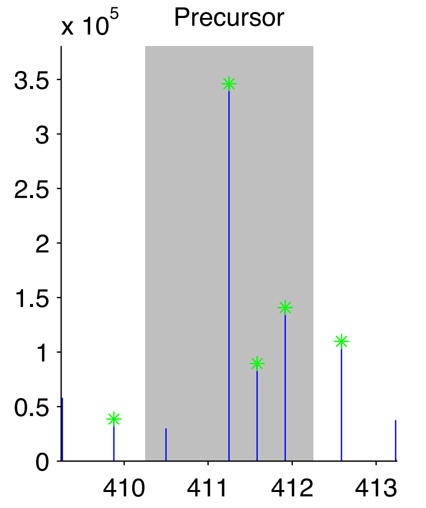

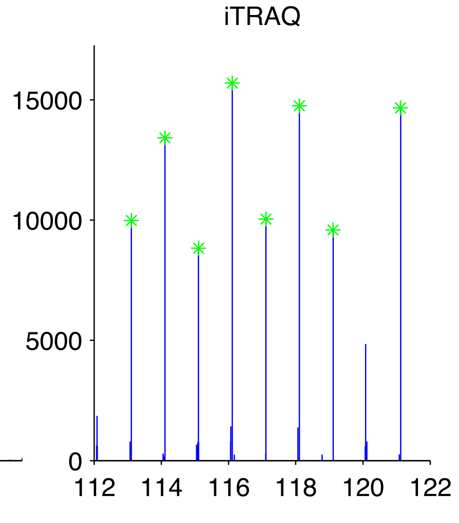

G k G G k G L G k G G A k R

histone cluster 1, H4a [Homo sapiens]
Charge State: +3
Scan Number: 14236
File Name: 120501_A549_TSA_AcK.raw

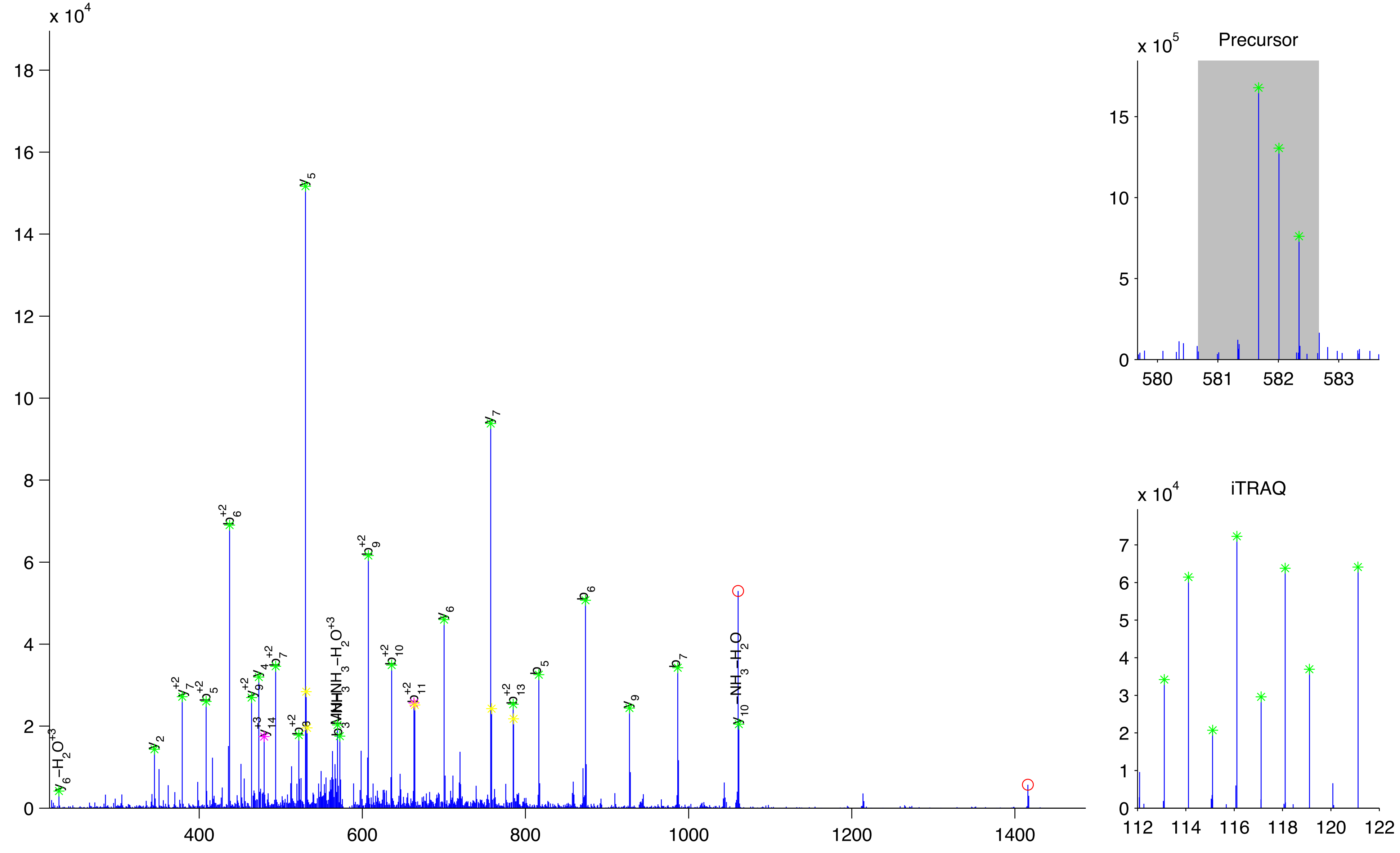

G k G G k G L G k G G A k R

histone cluster 1, H4a [Homo sapiens]
Charge State: +3
Scan Number: 15328
File Name: 120501_A549_TSA_AcK.raw

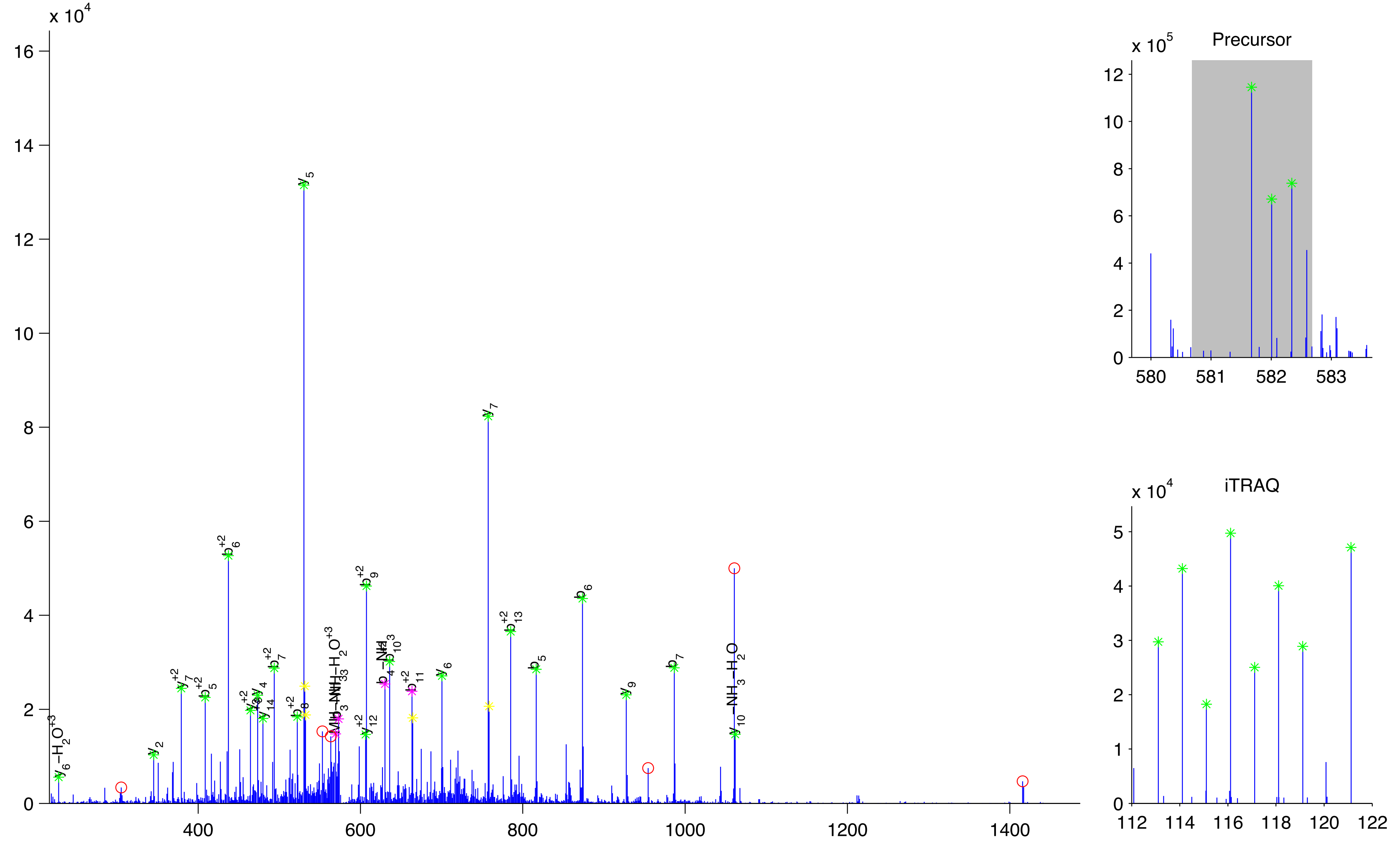

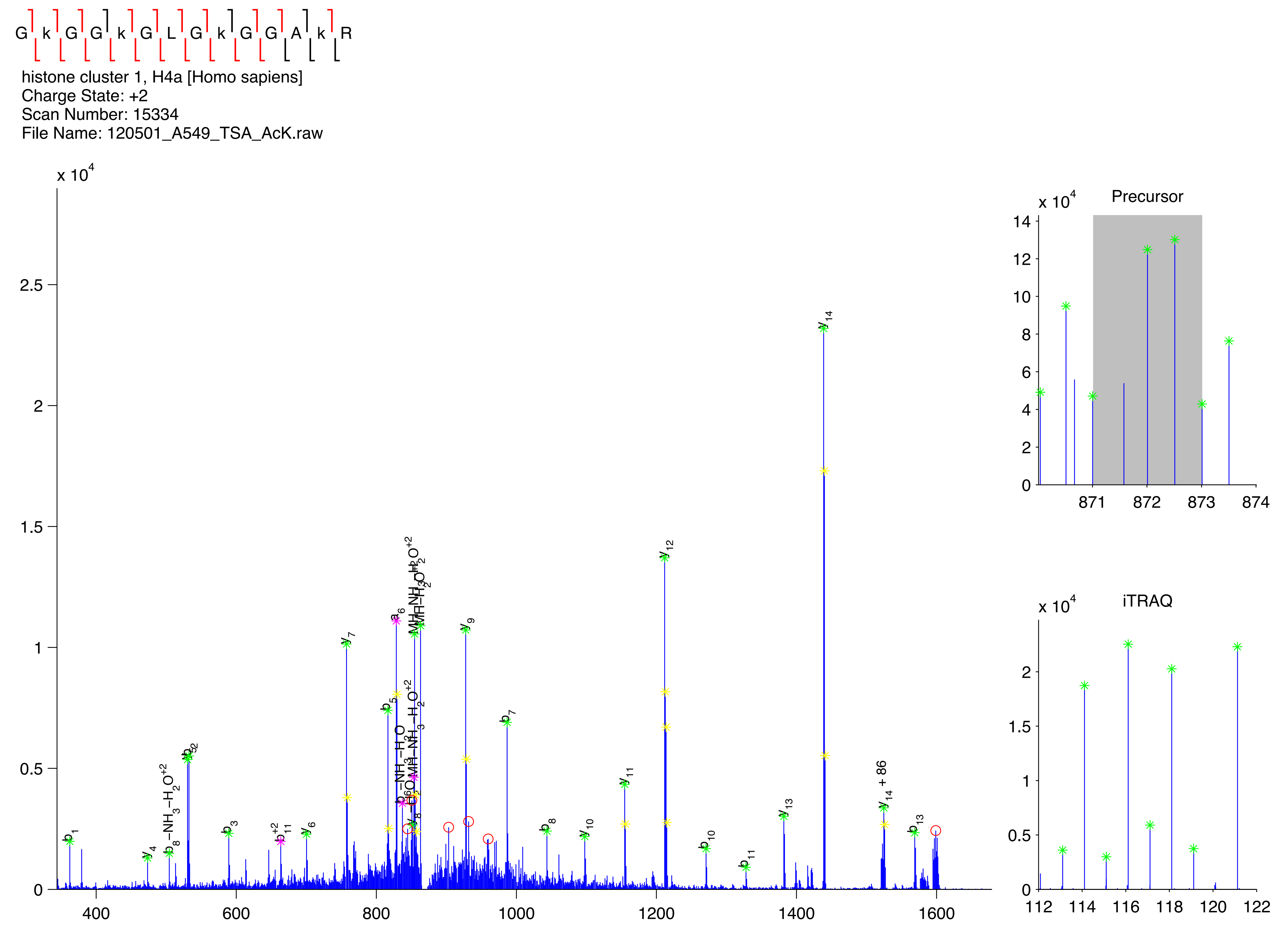
G k G G k G L G k G G A k R
histone cluster 1, H4a [Homo sapiens]
Charge State: +2
Scan Number: 15334
File Name: 120501_A549_TSA_AcK.raw
x 10^4
Precursor
iTRAQ
b1
y4
b8–NH3–H2O+2
b3
b11+2
y6
y7
b5
a6
y9
b7
b8
y10
y11
y12
b10
b11
y13
y14
y14 + 86
b13

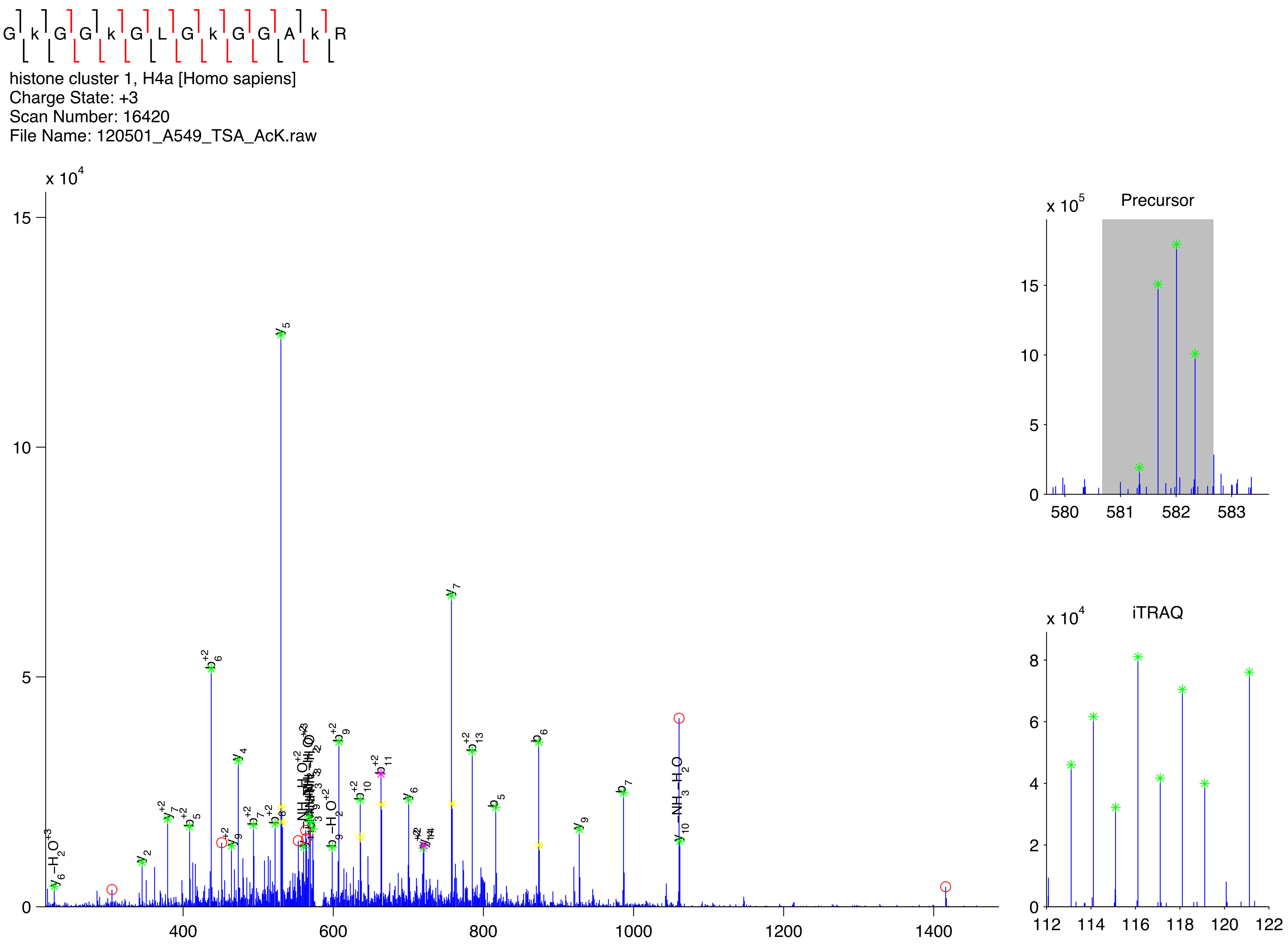

G k G G k G L G k G G A k R
histone cluster 1, H4a [Homo sapiens]
Charge State: +3
Scan Number: 16420
File Name: 120501_A549_TSA_AcK.raw
x 10^4
Precursor
x 10^5
iTRAQ
x 10^4

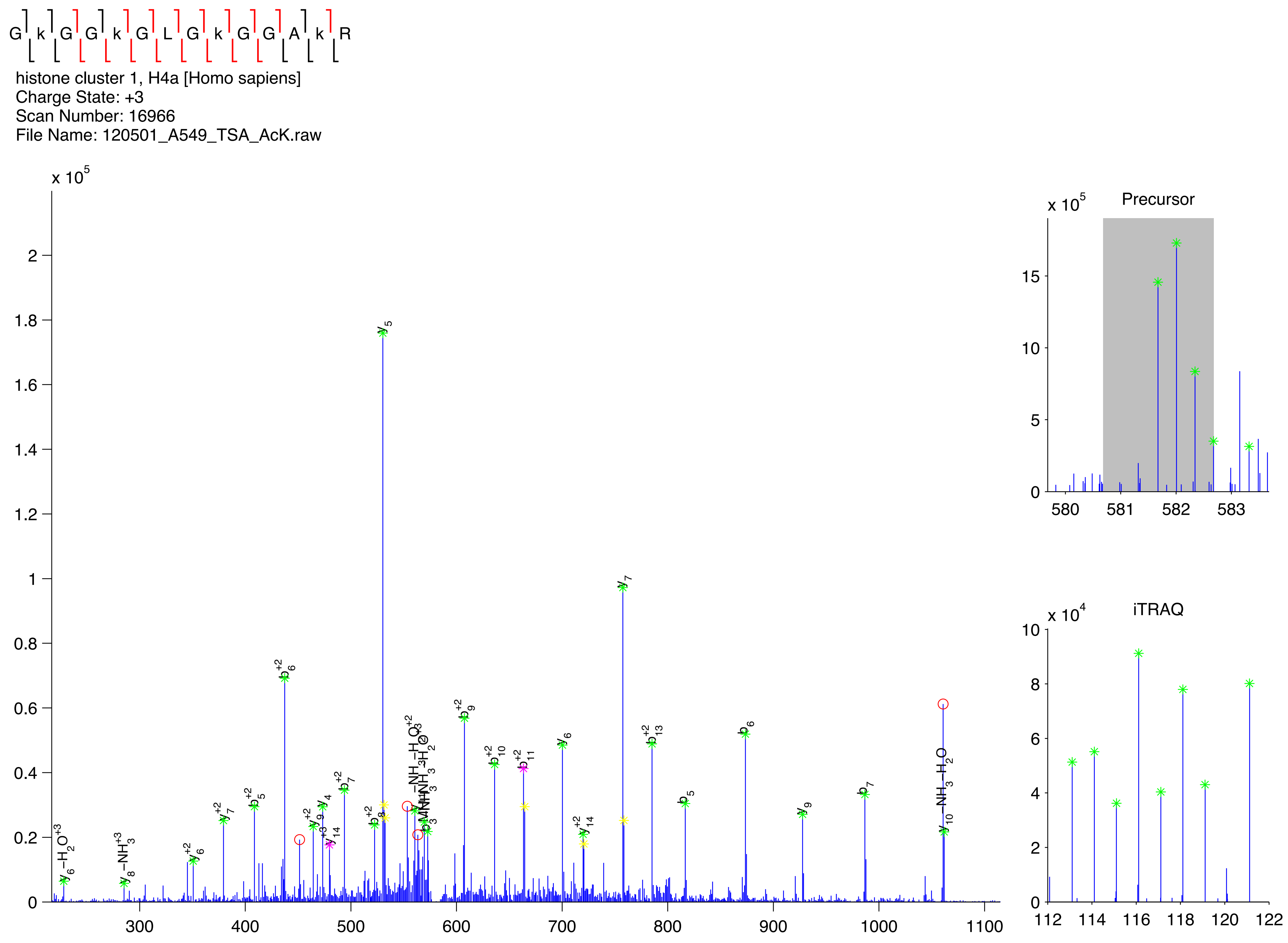

G k G G k G L G k G G A k R
histone cluster 1, H4a [Homo sapiens]
Charge State: +3
Scan Number: 16966
File Name: 120501_A549_TSA_AcK.raw
x 10^5
Precursor
iTRAQ
x 10^4

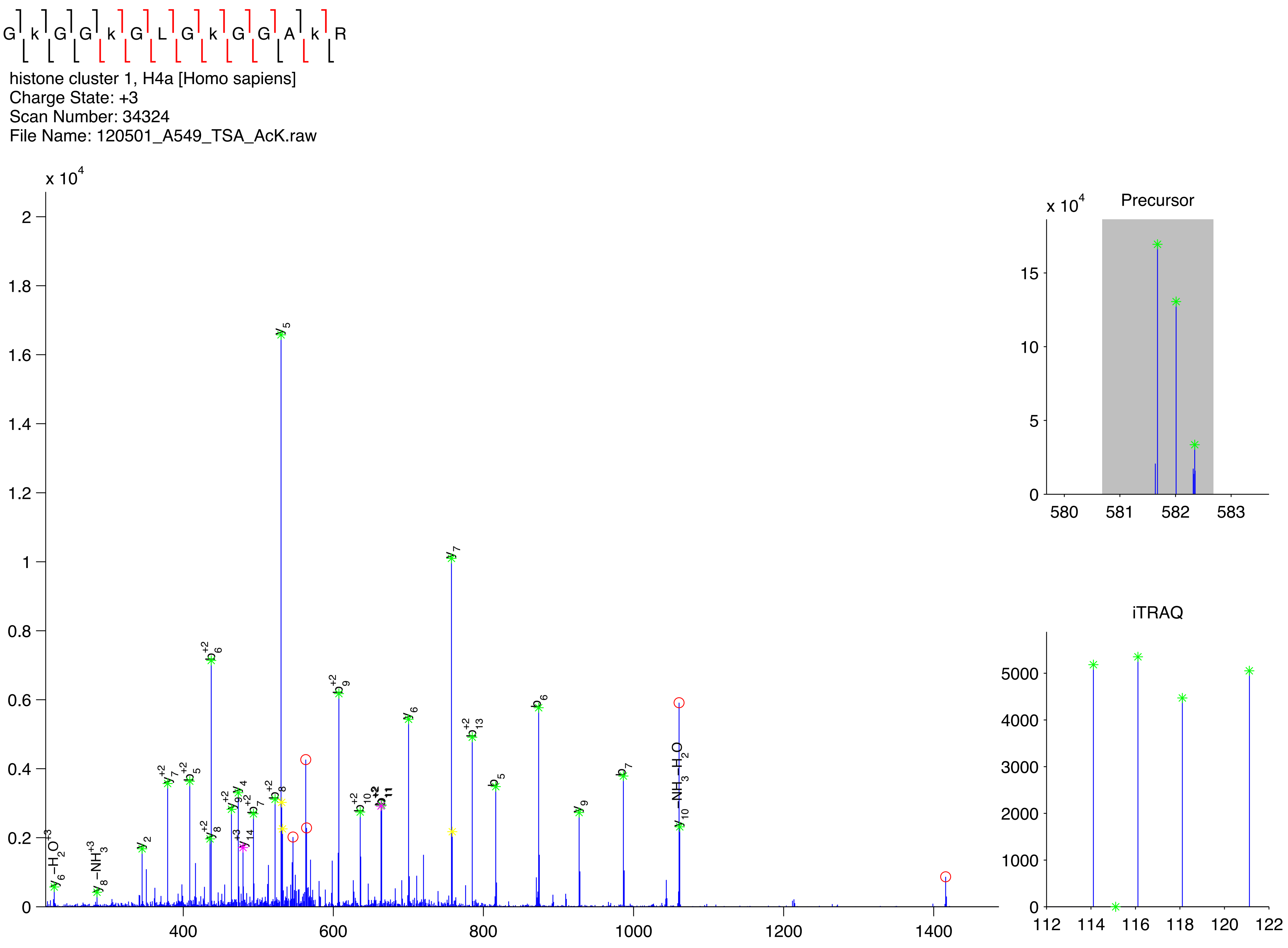

G k G G k G L G k G G A k R
histone cluster 1, H4a [Homo sapiens]
Charge State: +3
Scan Number: 34324
File Name: 120501_A549_TSA_AcK.raw
x 10^4
Precursor
iTRAQ

G k G G k G L G k G G A k R

histone cluster 1, H4a [Homo sapiens]
Charge State: +3
Scan Number: 34860
File Name: 120501_A549_TSA_AcK.raw

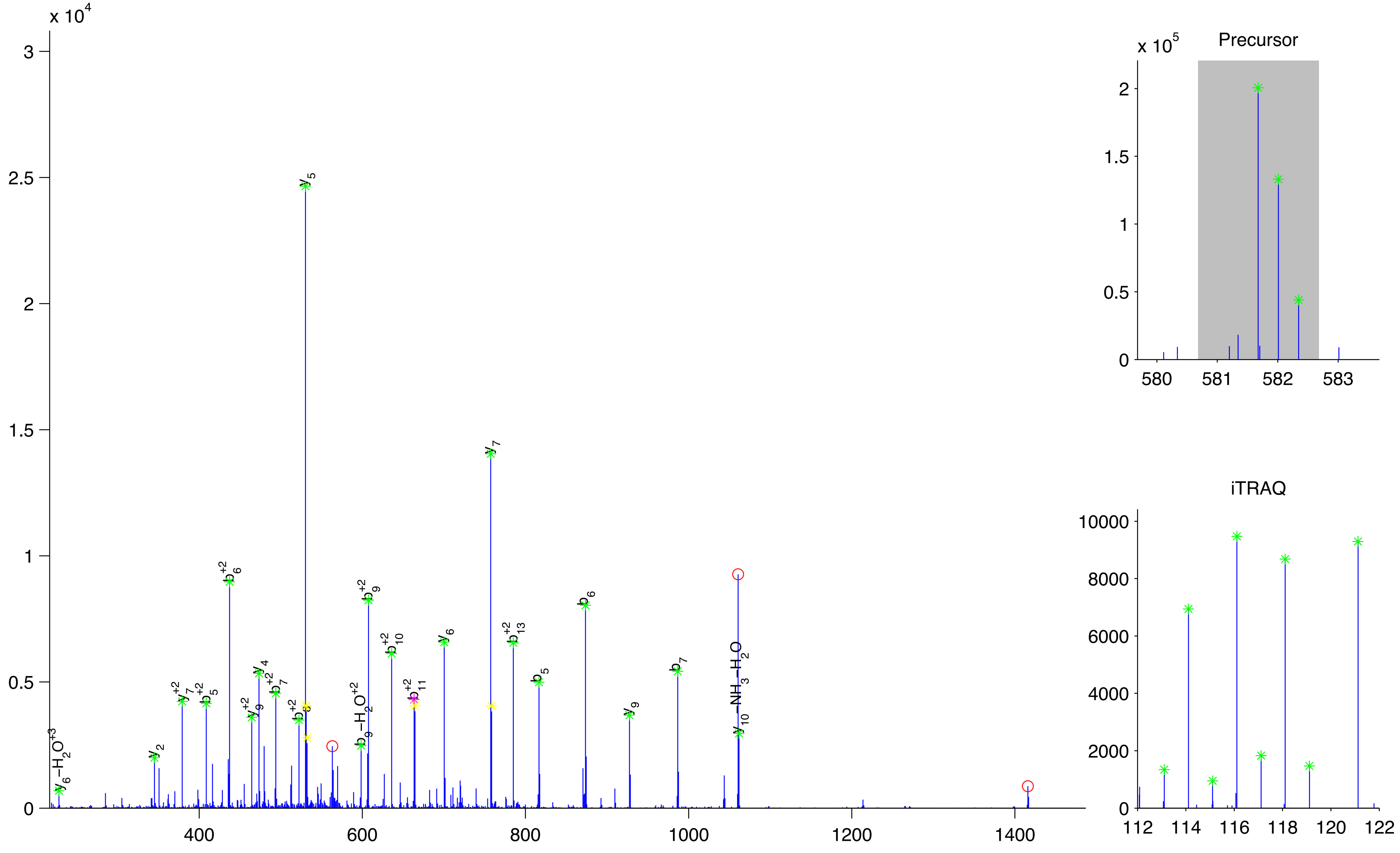

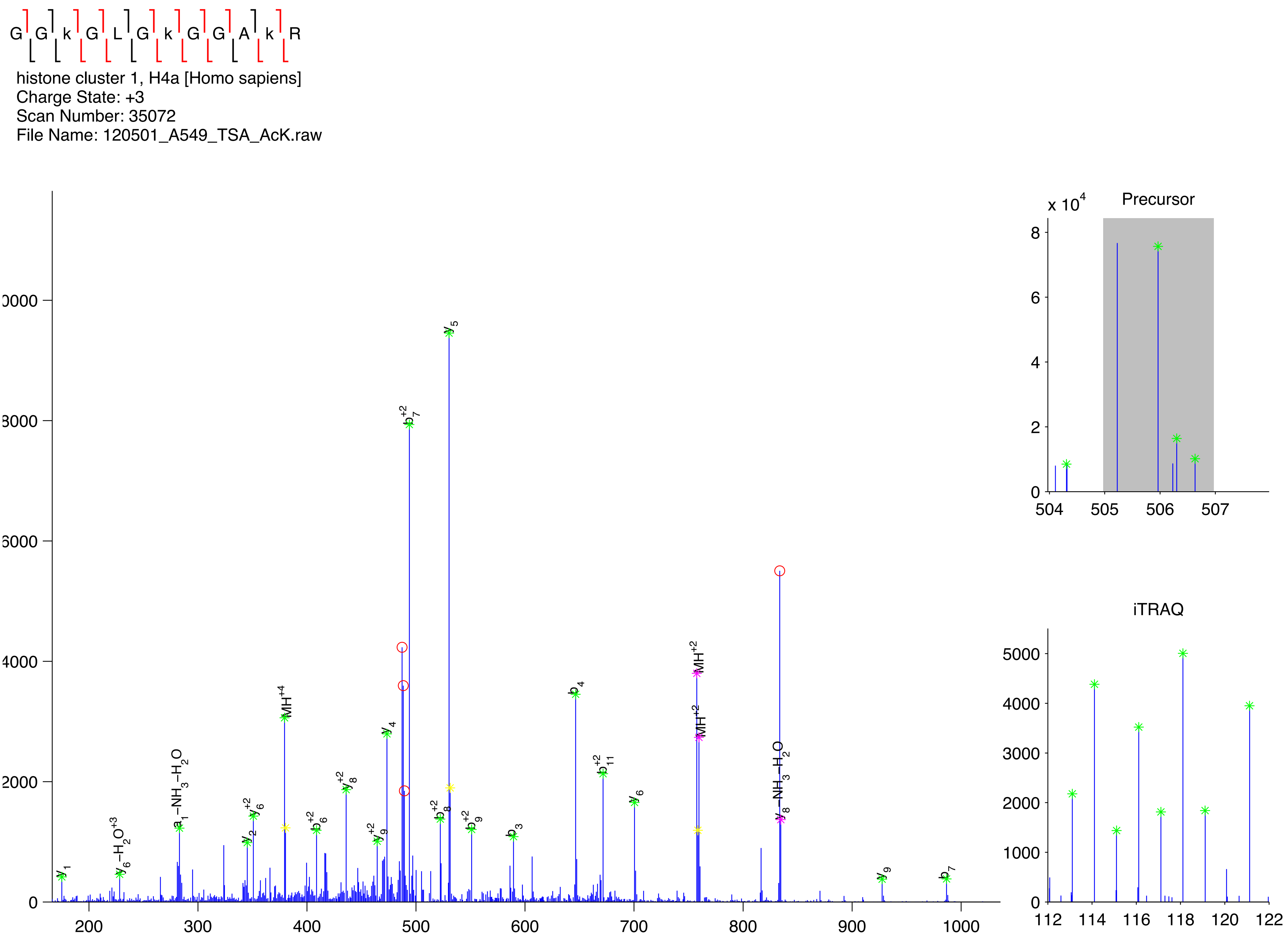

G G k G L G k G G A k R
histone cluster 1, H4a [Homo sapiens]
Charge State: +3
Scan Number: 35072
File Name: 120501_A549_TSA_AcK.raw
y1
y6–H2O+3
a1–NH3–H2O
y2
y6+2
MH+4
b6+2
y8+2
y9+2
y4
b7+2
b8+2
y5
b9+2
b3
b4
b11+2
y6
MH+2
MH+2
y8–NH3–H2O
y9
b7
200
300
400
500
600
700
800
900
1000
0
2000
4000
Precursor
x 10^4
0
2
4
6
8
504
505
506
507
iTRAQ
0
1000
2000
3000
4000
5000
112
114
116
118
120
122

G k G G k G L G k G G A k R
histone cluster 1, H4a [Homo sapiens]
Charge State: +3
Scan Number: 35341
File Name: 120501_A549_TSA_AcK.raw

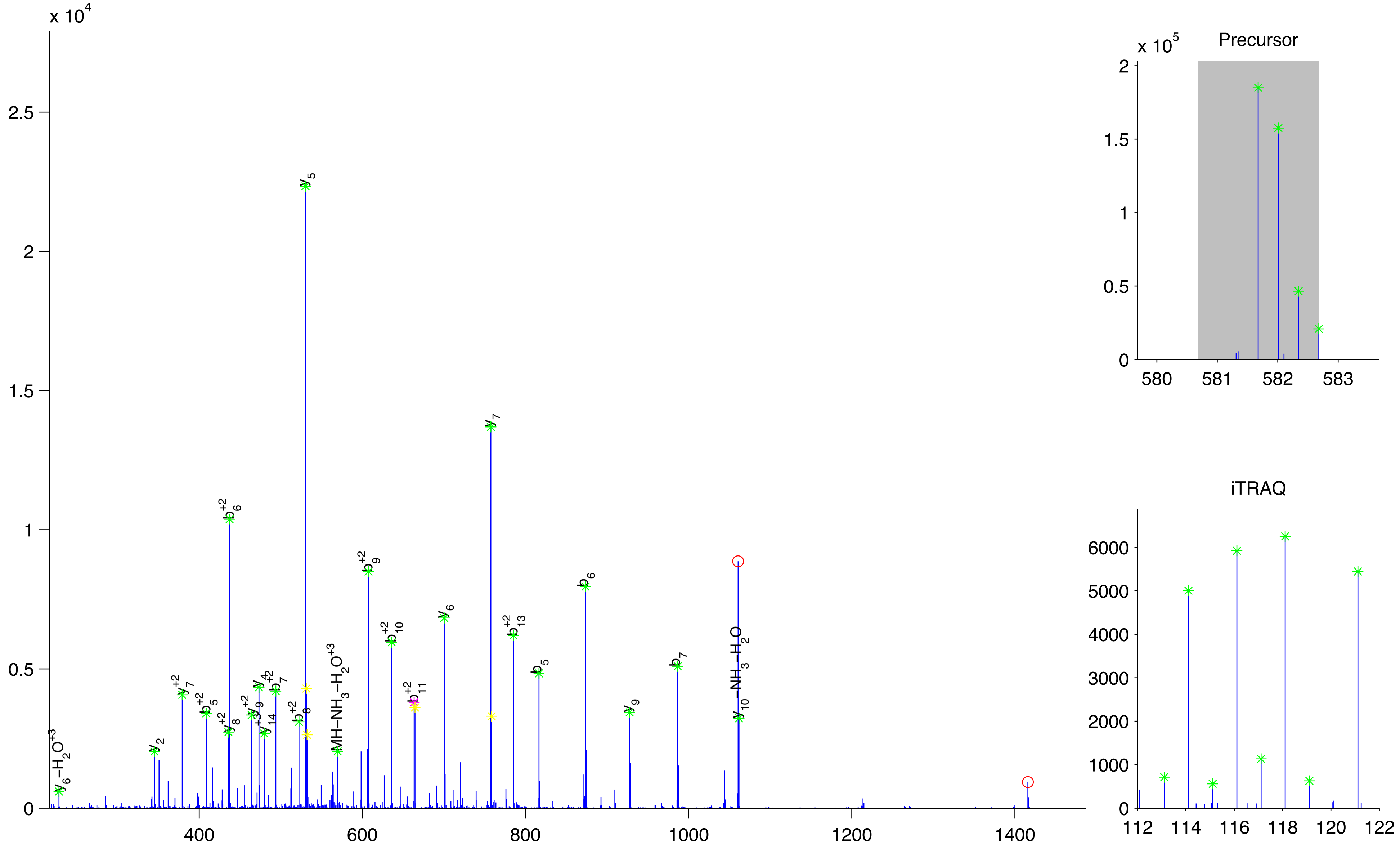

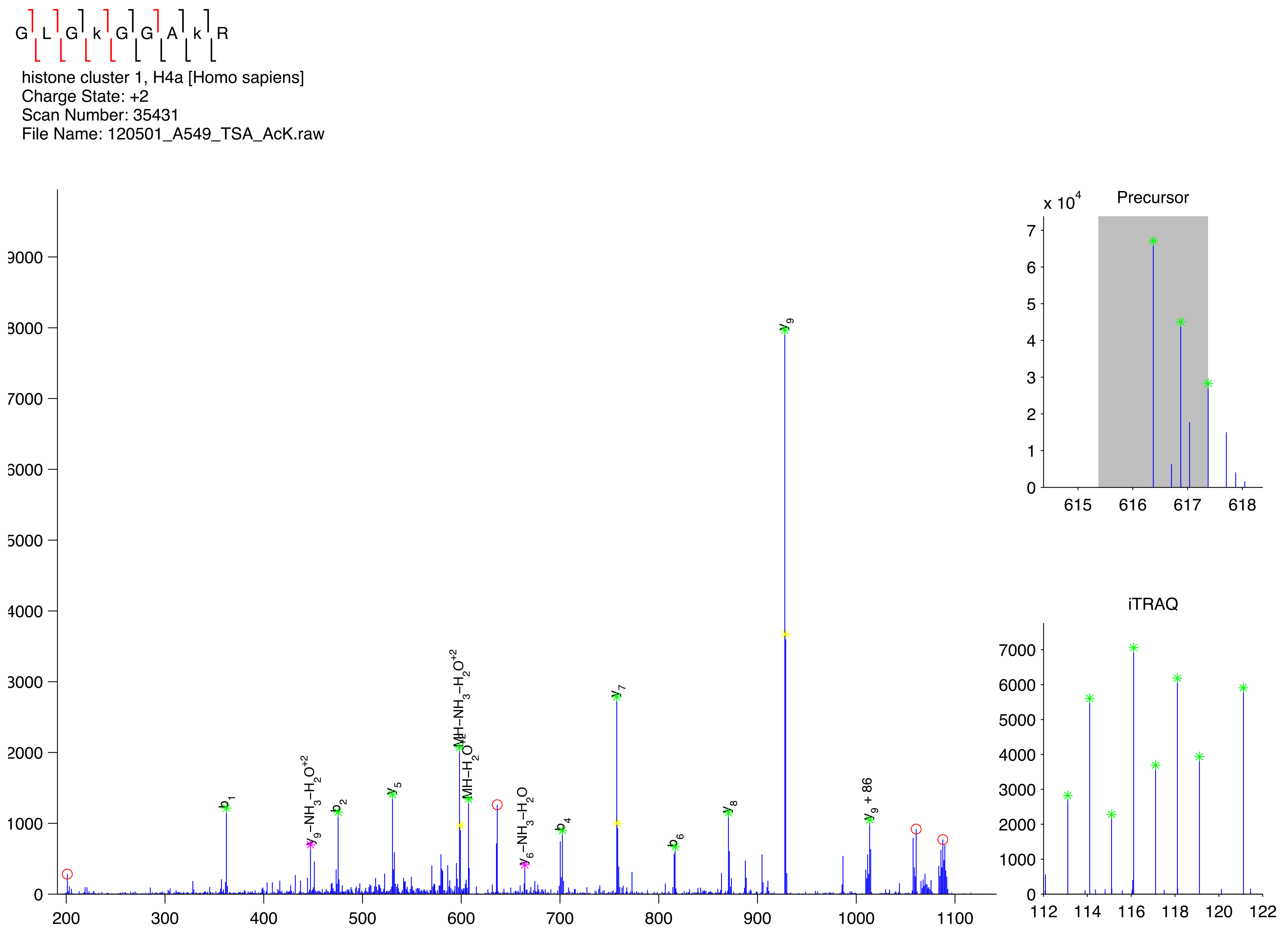

G L G k G G A k R
histone cluster 1, H4a [Homo sapiens]
Charge State: +2
Scan Number: 35431
File Name: 120501_A549_TSA_AcK.raw
Precursor
x 10^4
iTRAQ
b1
y9-NH3-H2O+2
b2
y5
MH-NH3-H2O+2
MH-H2O
y6-NH3-H2O
b4
y7
b6
y8
y9
y9 + 86

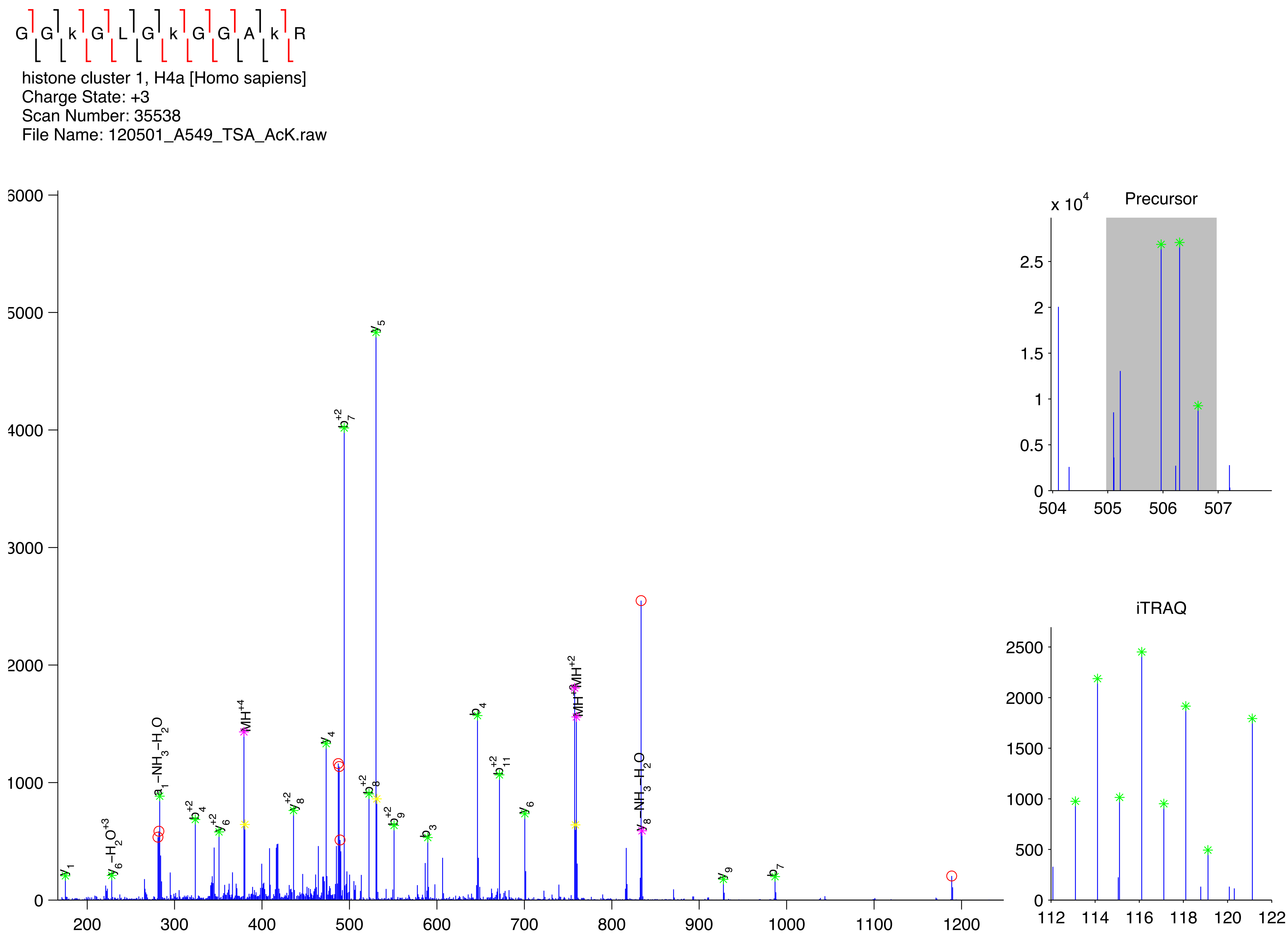

G G k G L G k G G A k R
histone cluster 1, H4a [Homo sapiens]
Charge State: +3
Scan Number: 35538
File Name: 120501_A549_TSA_AcK.raw
Precursor
x 10^4
iTRAQ

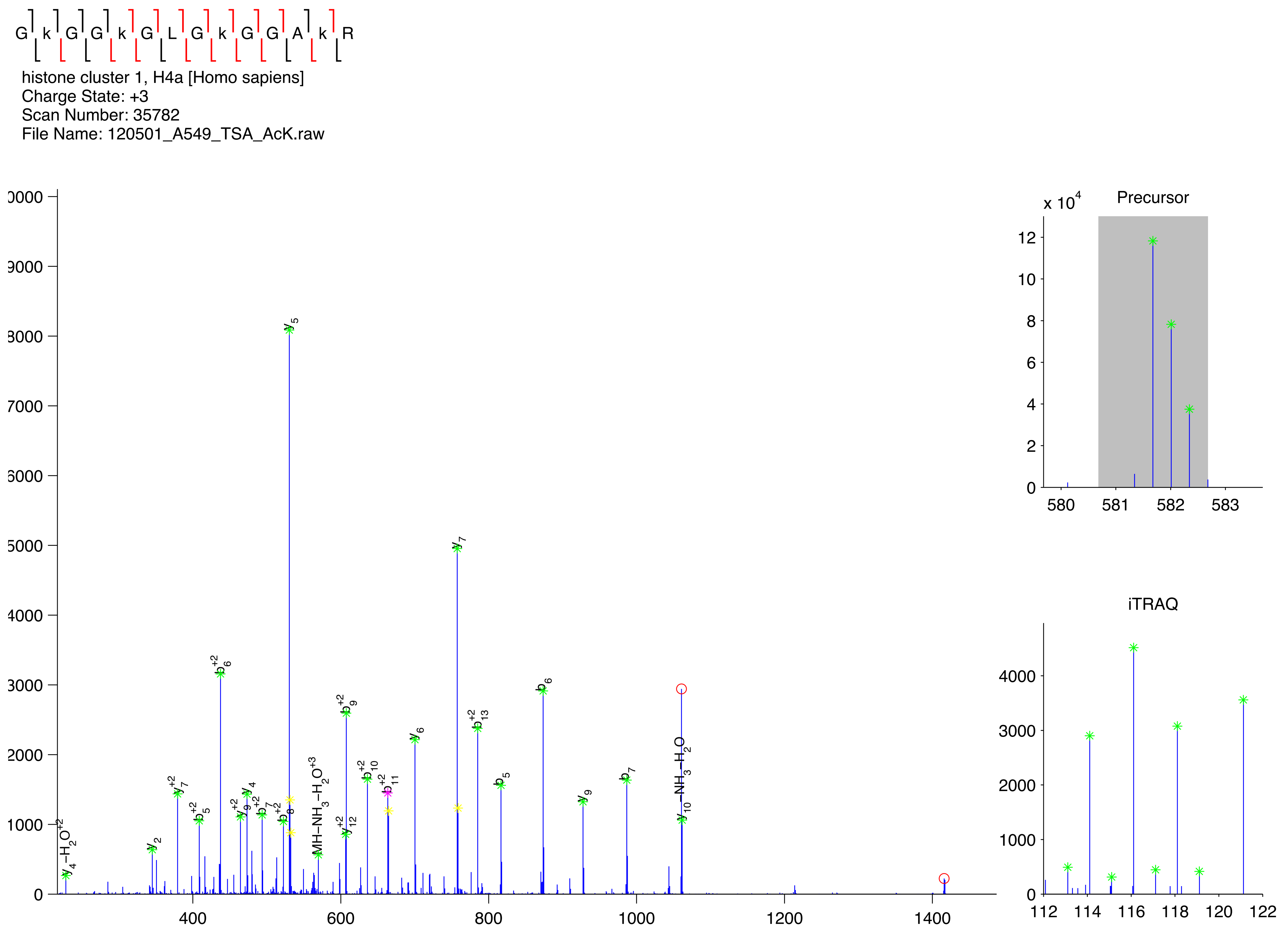
G k G G k G L G k G G A k R
histone cluster 1, H4a [Homo sapiens]
Charge State: +3
Scan Number: 35782
File Name: 120501_A549_TSA_AcK.raw
Precursor
iTRAQ

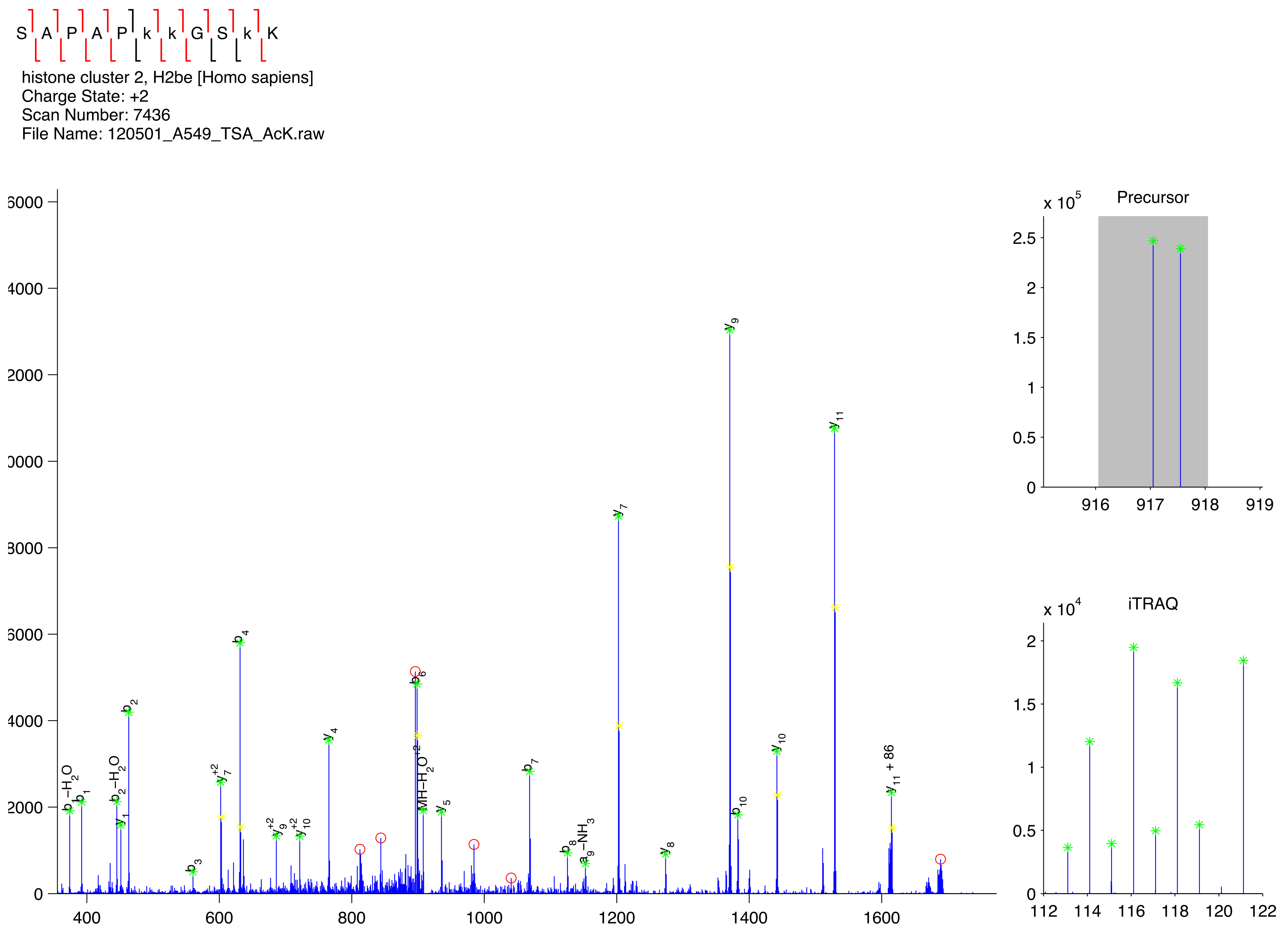

S A P A P k k G S k K
histone cluster 2, H2be [Homo sapiens]
Charge State: +2
Scan Number: 7436
File Name: 120501_A549_TSA_AcK.raw
Precursor
iTRAQ

S A P A P k k G S k K

histone cluster 2, H2be [Homo sapiens]
Charge State: +3
Scan Number: 7604
File Name: 120501_A549_TSA_AcK.raw

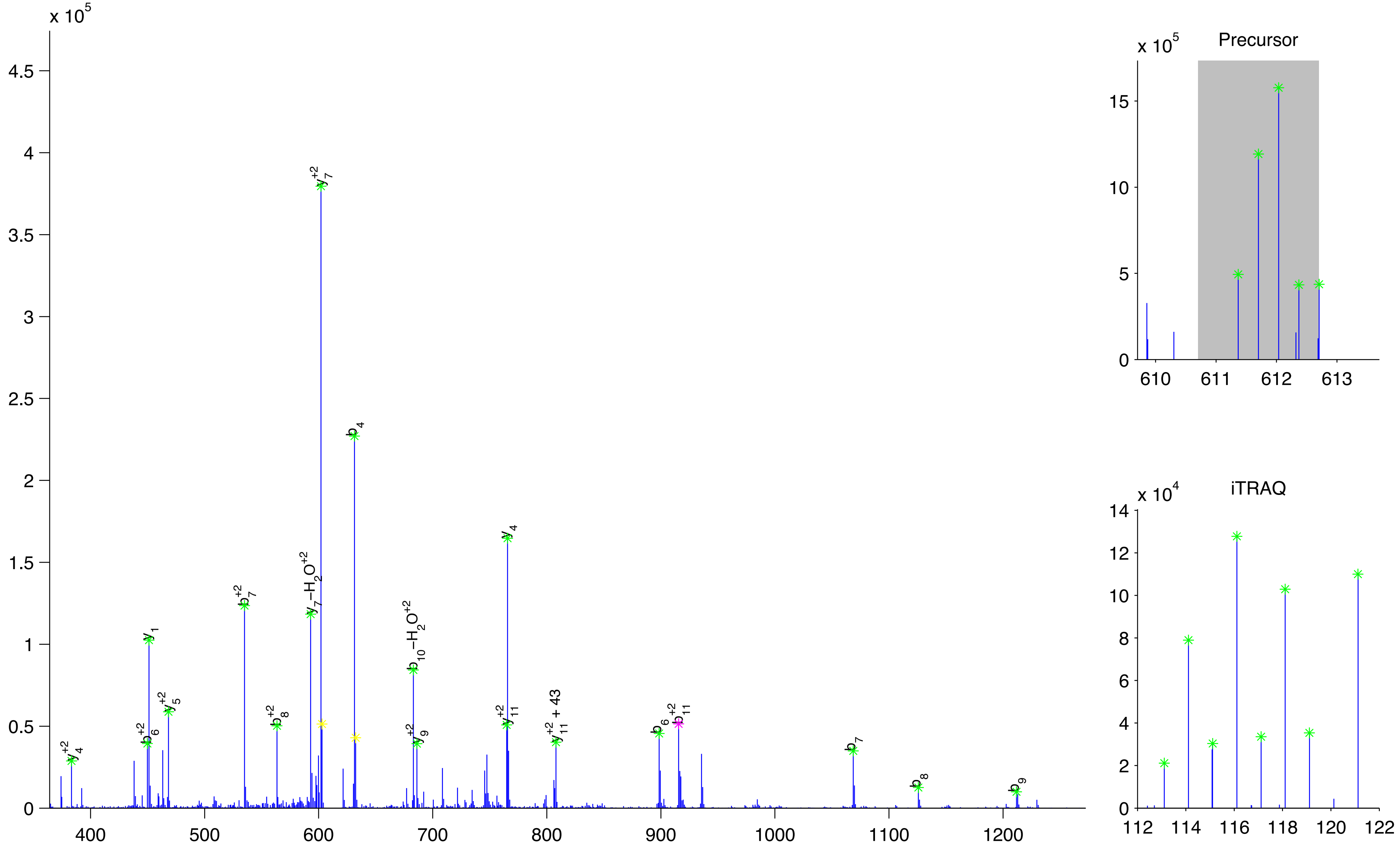

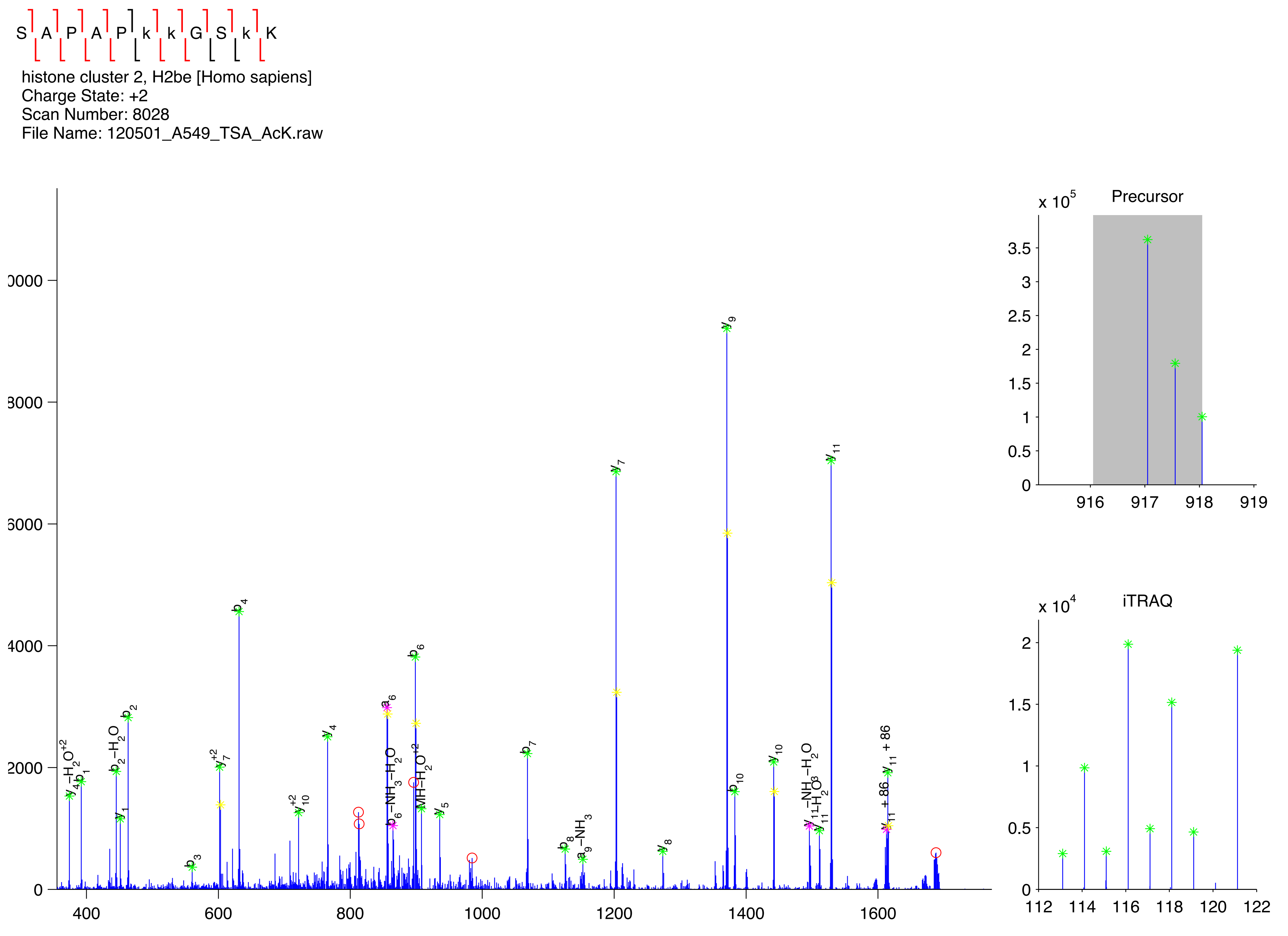
S A P A P k k G S k K
histone cluster 2, H2be [Homo sapiens]
Charge State: +2
Scan Number: 8028
File Name: 120501_A549_TSA_AcK.raw
Precursor
iTRAQ
x 10^5
x 10^4

k G S k k A V T K

histone cluster 2, H2be [Homo sapiens]
Charge State: +3
Scan Number: 9217
File Name: 120501_A549_TSA_AcK.raw

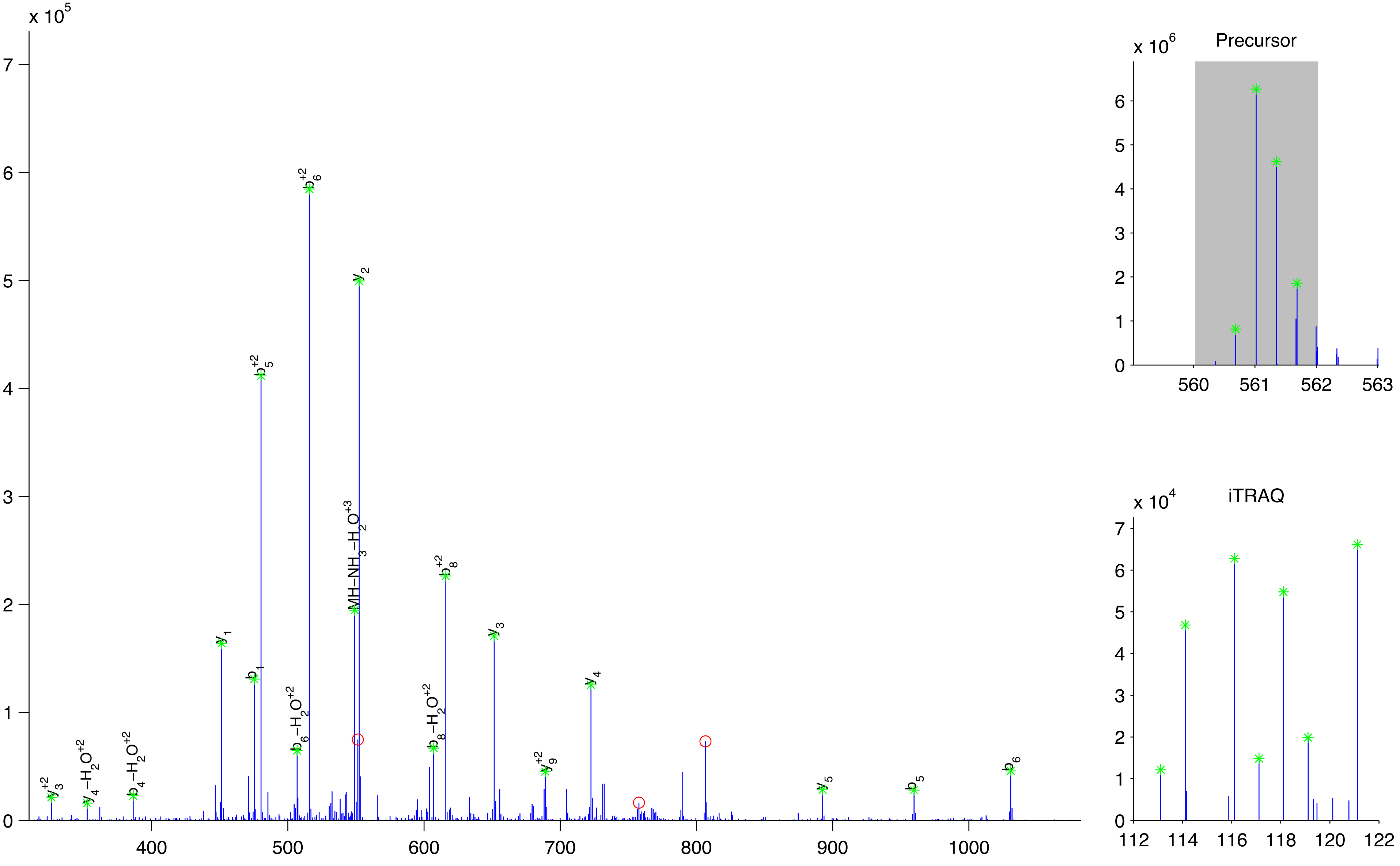

k G S k k A V T K

histone cluster 2, H2be [Homo sapiens]
Charge State: +2
Scan Number: 9872
File Name: 120501_A549_TSA_AcK.raw

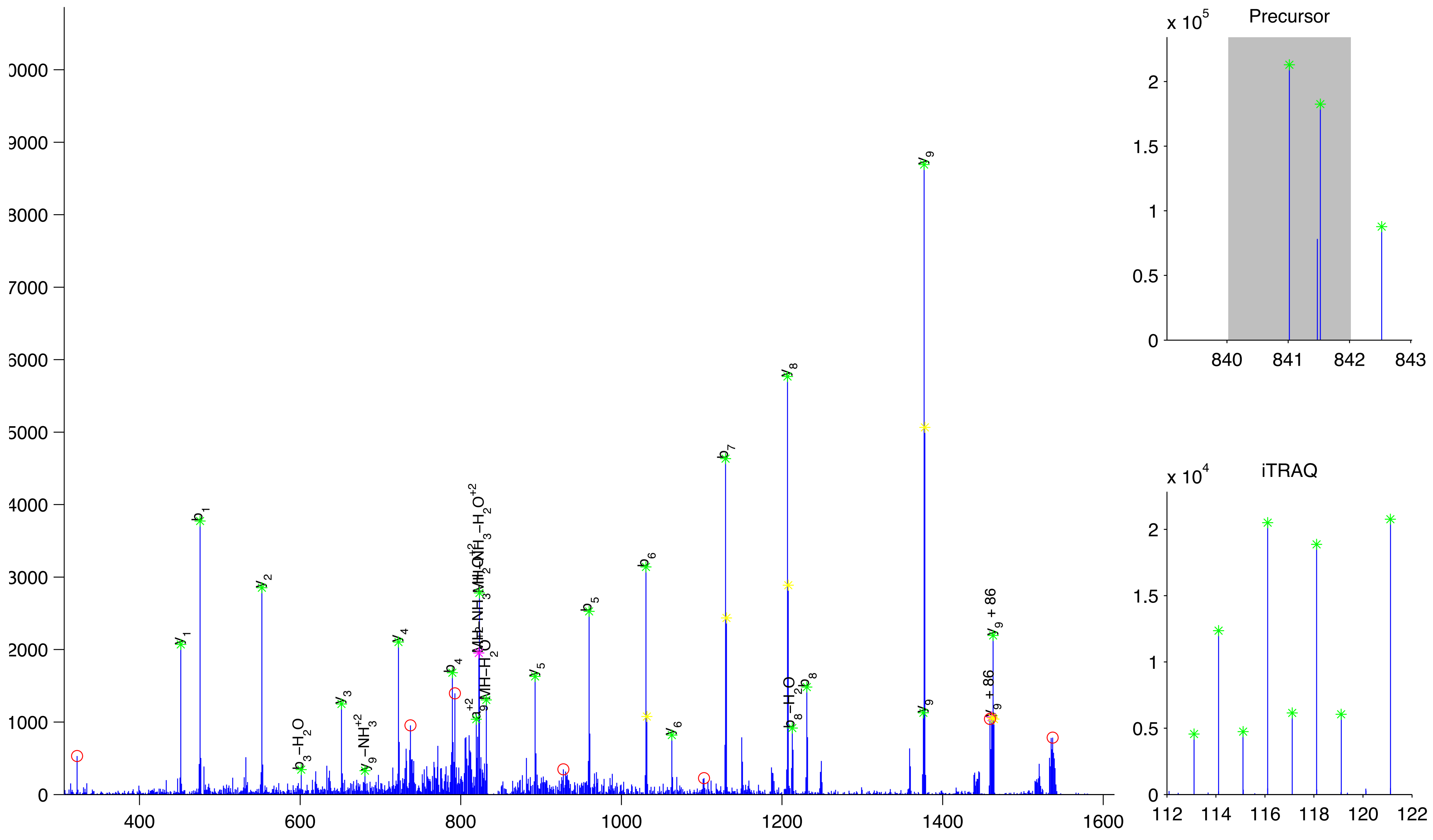

k A V T k A Q k K

histone cluster 2, H2be [Homo sapiens]
Charge State: +3
Scan Number: 10731
File Name: 120501_A549_TSA_AcK.raw

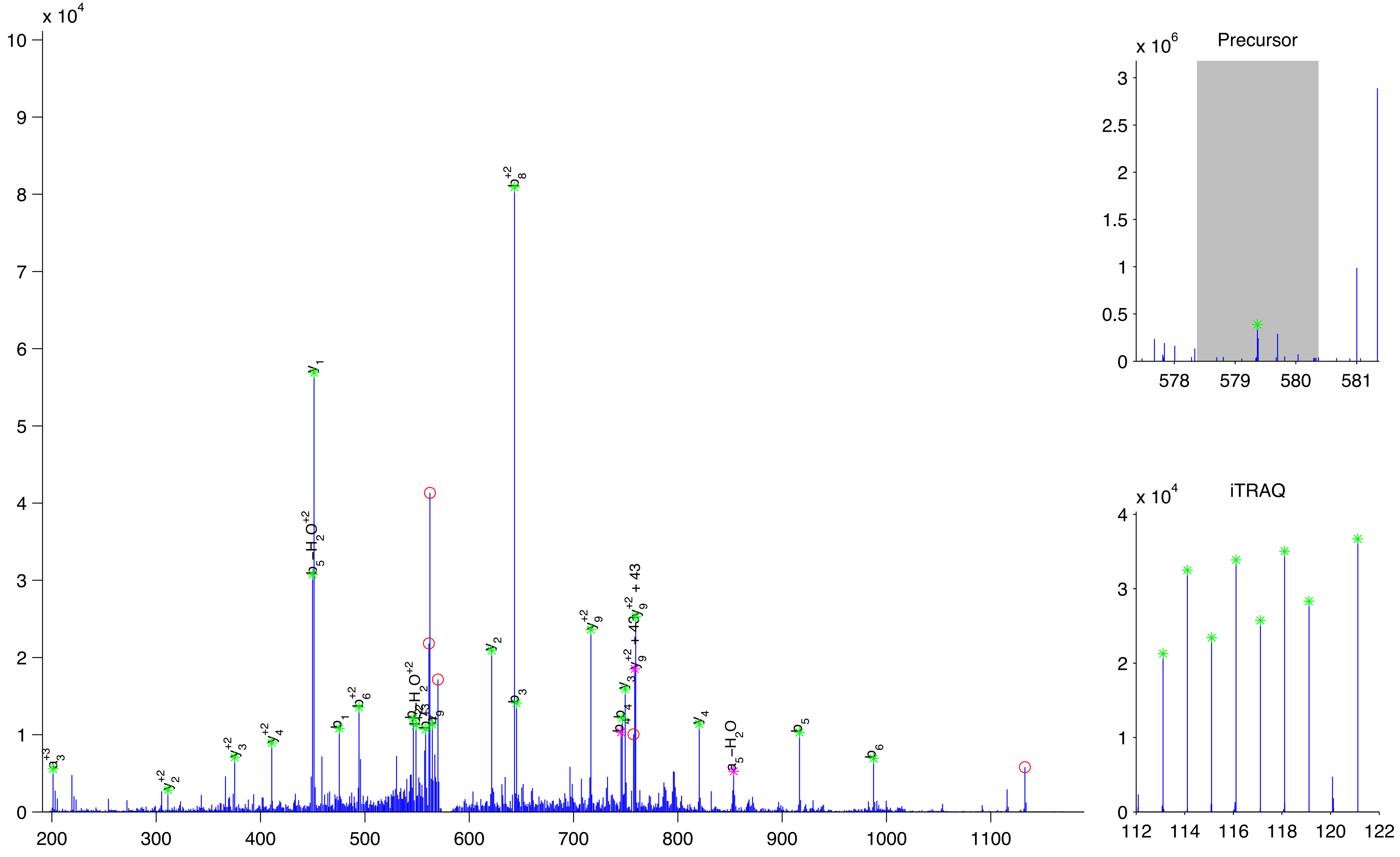

k G S k k A V T k A Q K

histone cluster 2, H2be [Homo sapiens]
Charge State: +3
Scan Number: 11317
File Name: 120501_A549_TSA_AcK.raw

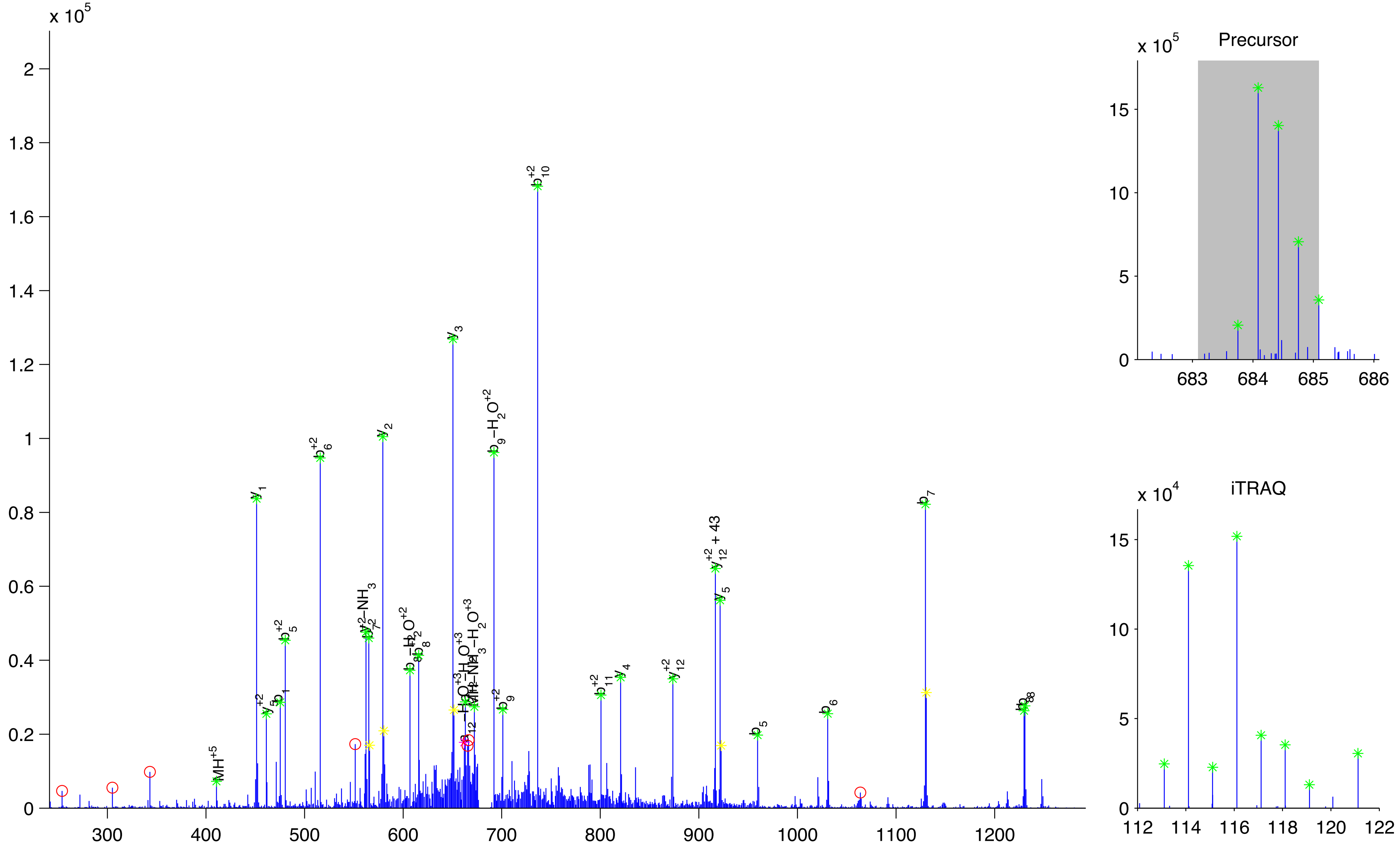

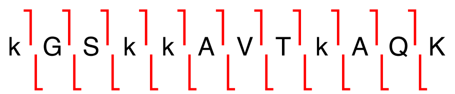

histone cluster 2, H2be [Homo sapiens]
Charge State: +2
Scan Number: 11978
File Name: 120501_A549_TSA_AcK.raw

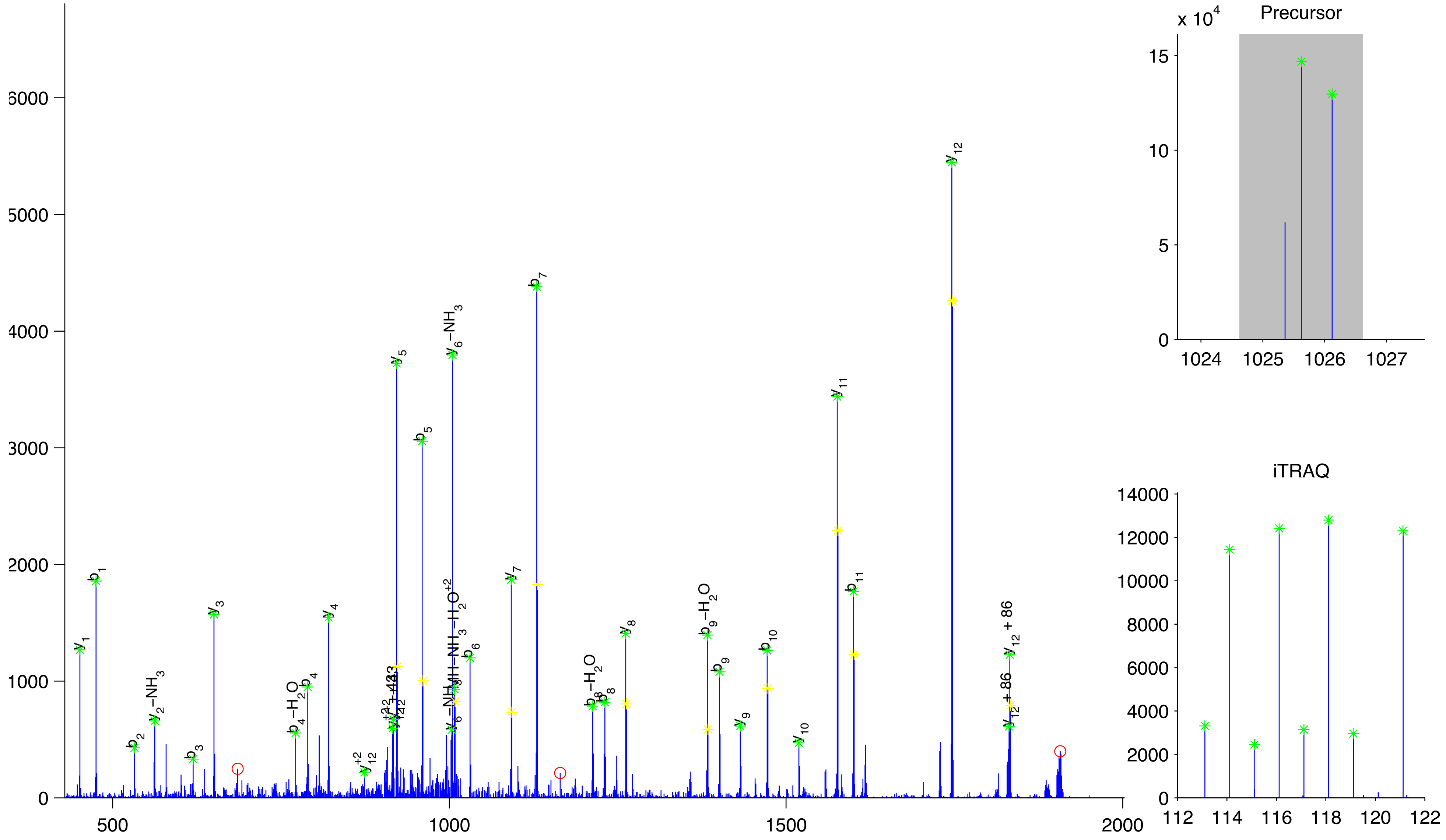

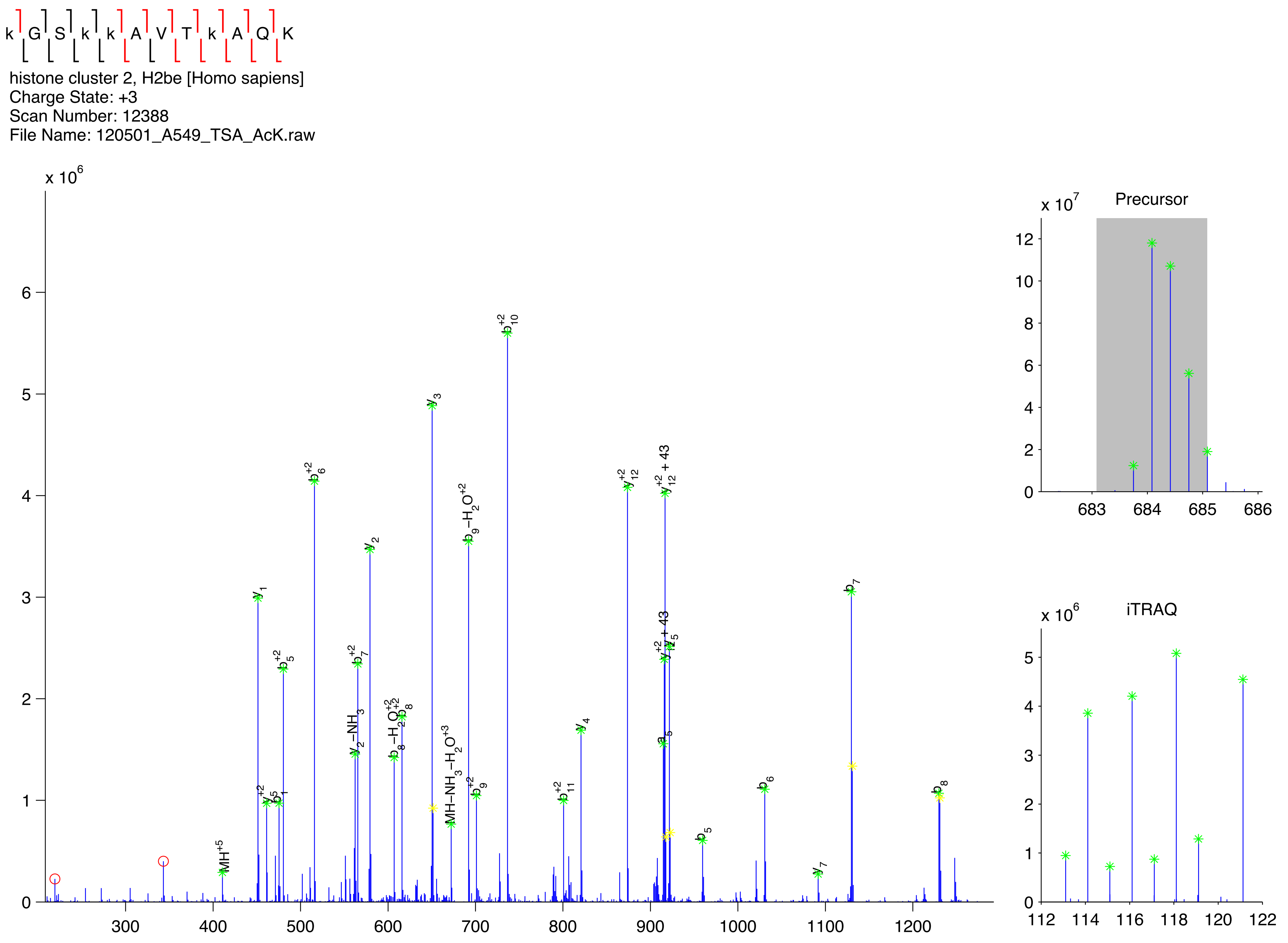
k G S k k A V T k A Q K
histone cluster 2, H2be [Homo sapiens]
Charge State: +3
Scan Number: 12388
File Name: 120501_A549_TSA_AcK.raw
x 10^6
MH+5
y1
b5+2
b6+2
y2-NH3
b7+2
y2
b8-H2O+2
b8+2
y3
MH-NH3-H2O+3
b9-H2O+2
b9+2
b10+2
b11+2
y4
y12+2
a5
y12+2 + 43
b5
b6
y7
b7
b8
Precursor
x 10^7
iTRAQ
x 10^6

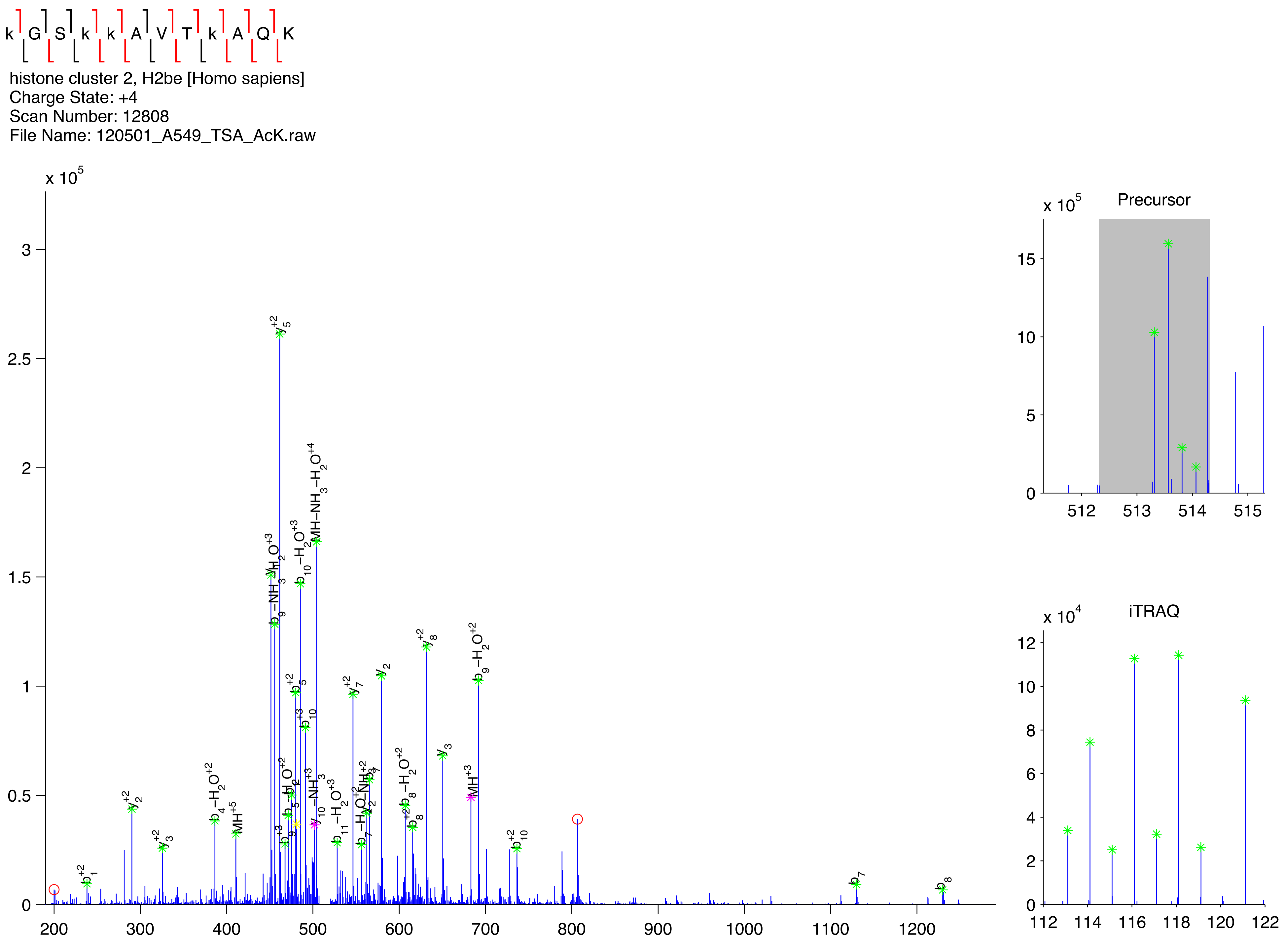

k G S k k A V T k A Q K
histone cluster 2, H2be [Homo sapiens]
Charge State: +4
Scan Number: 12808
File Name: 120501_A549_TSA_AcK.raw
x 10^5
Precursor
iTRAQ
x 10^4

k G S k k A V T k A Q K

histone cluster 2, H2be [Homo sapiens]
Charge State: +3
Scan Number: 12934
File Name: 120501_A549_TSA_AcK.raw

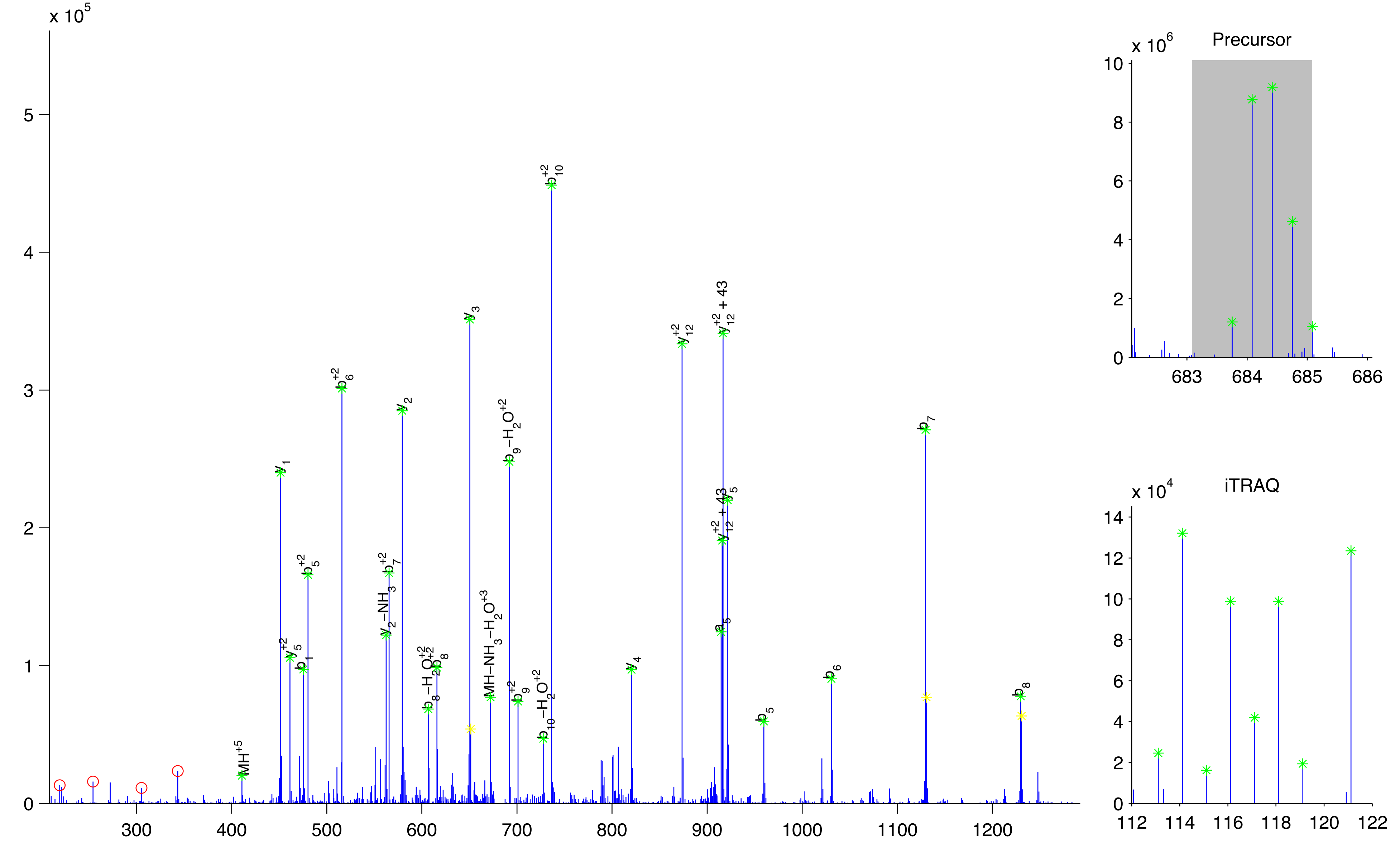

k G S k k A V T k A Q K

histone cluster 2, H2be [Homo sapiens]
Charge State: +4
Scan Number: 13354
File Name: 120501_A549_TSA_AcK.raw

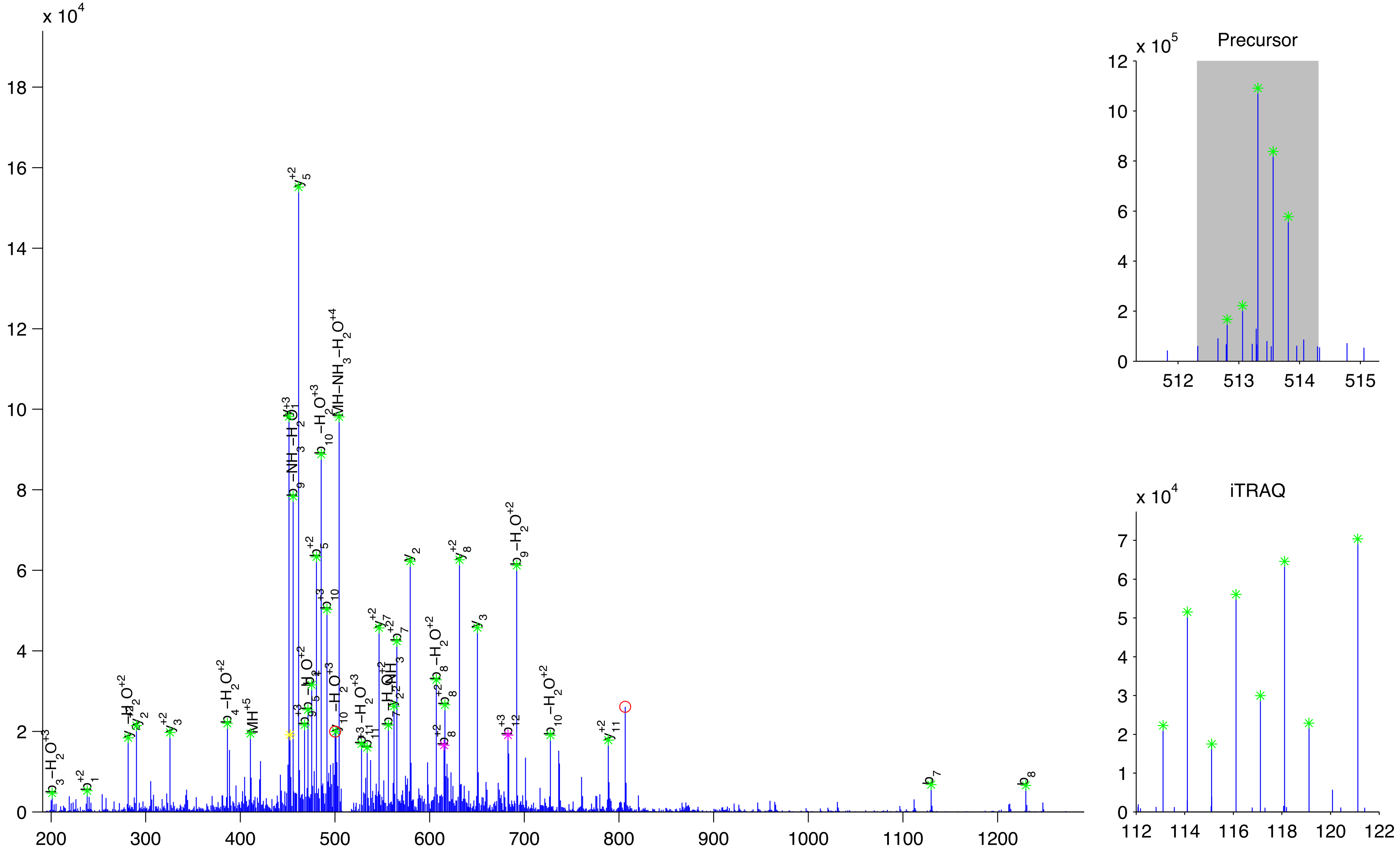

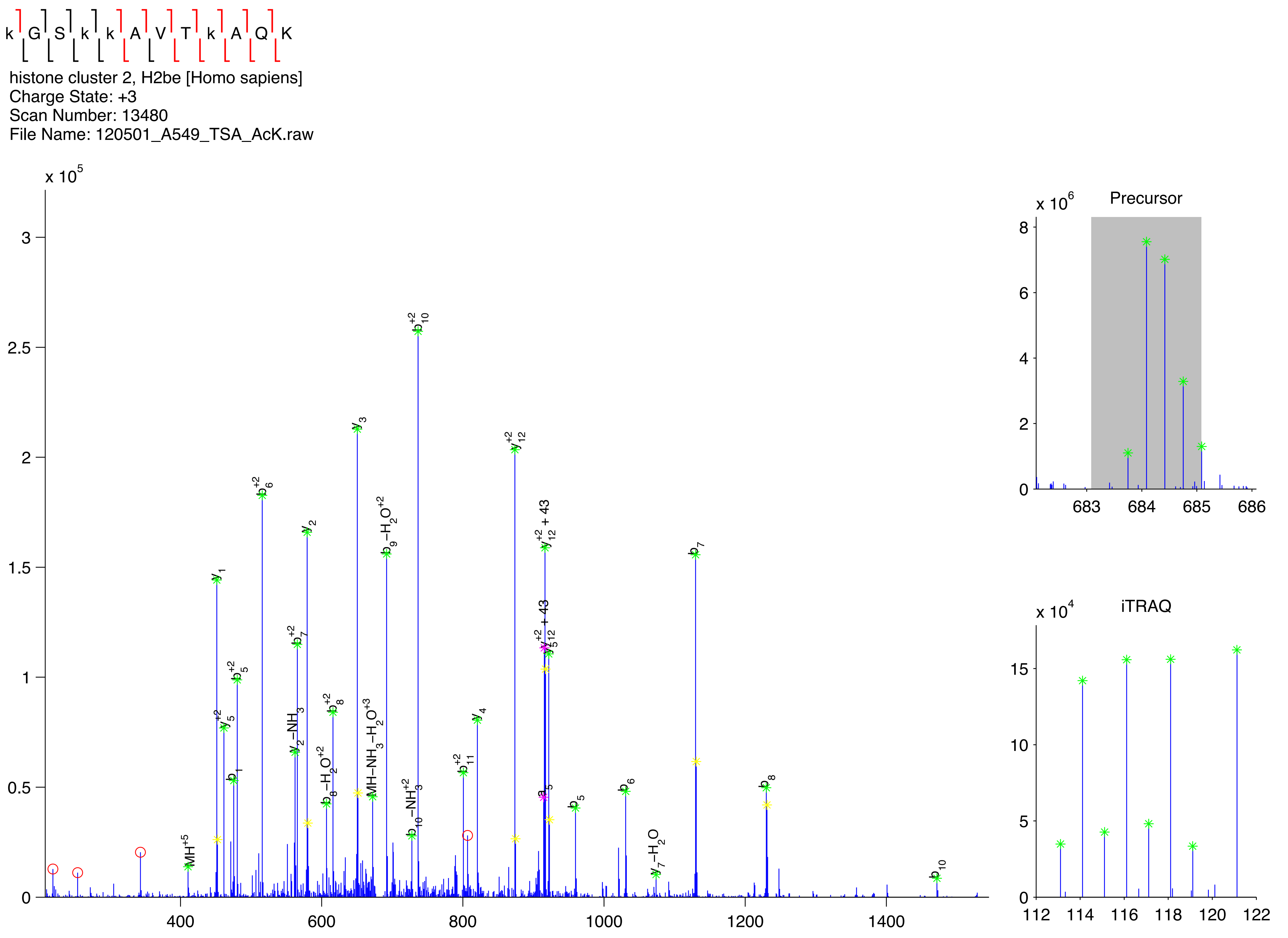

k G S k k A V T k A Q K
histone cluster 2, H2be [Homo sapiens]
Charge State: +3
Scan Number: 13480
File Name: 120501_A549_TSA_AcK.raw
x 10^5
Precursor
x 10^6
iTRAQ
x 10^4

k G S k k A V T k A Q K

histone cluster 2, H2be [Homo sapiens]
Charge State: +2
Scan Number: 13637
File Name: 120501_A549_TSA_AcK.raw

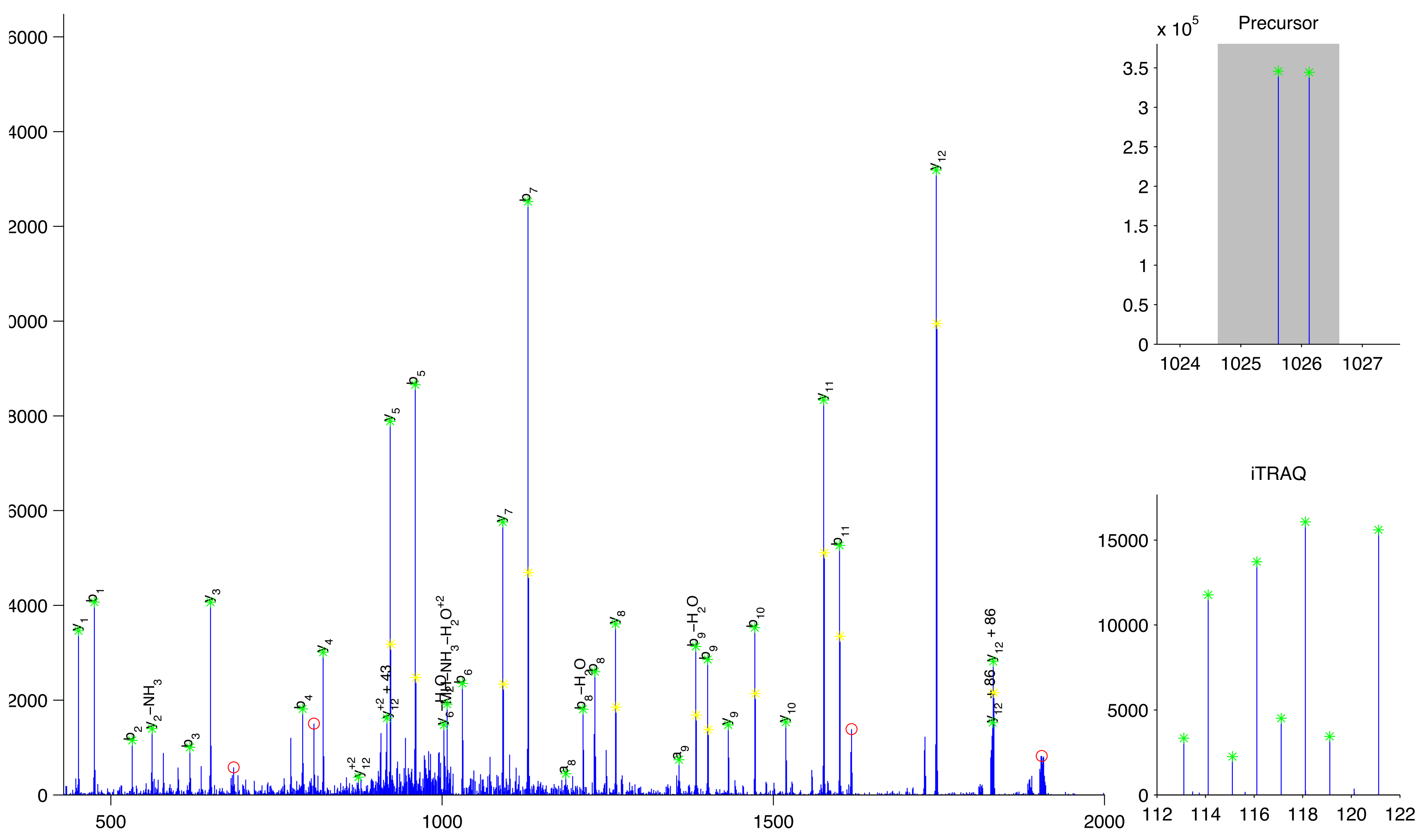

k G S k k A V T k A Q K

histone cluster 2, H2be [Homo sapiens]
Charge State: +3
Scan Number: 14028
File Name: 120501_A549_TSA_AcK.raw

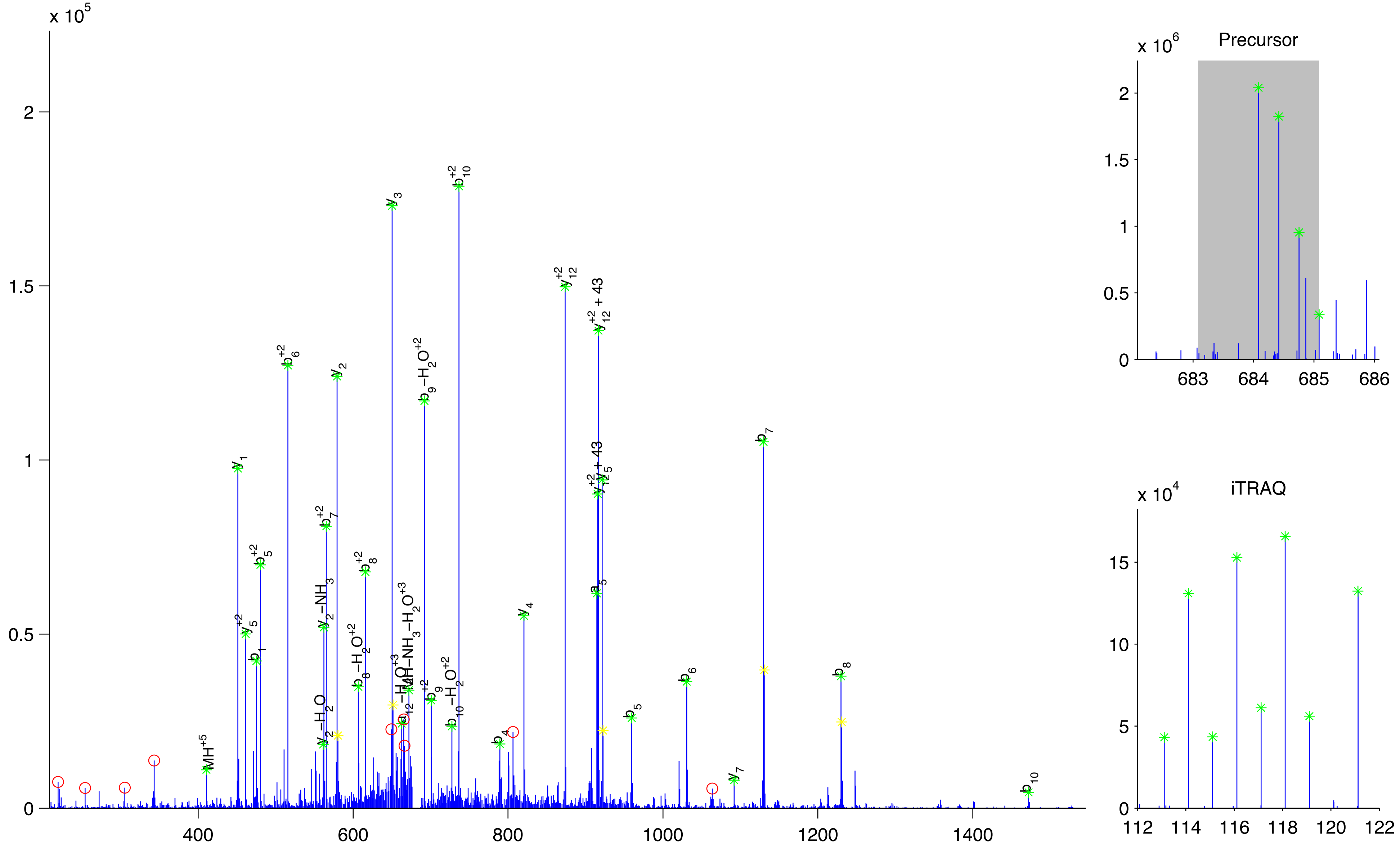

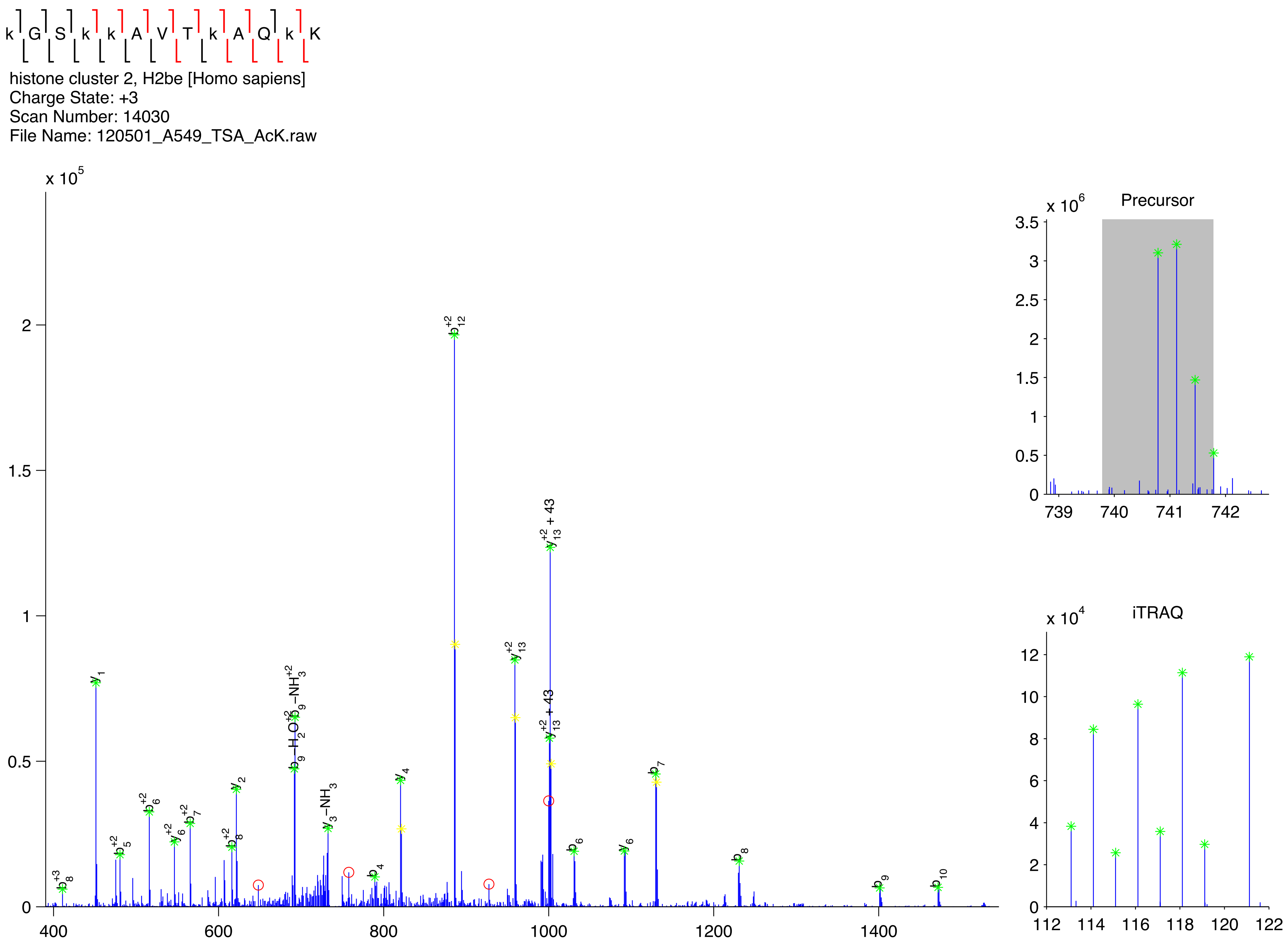
k G S k k A V T k A Q k K
histone cluster 2, H2be [Homo sapiens]
Charge State: +3
Scan Number: 14030
File Name: 120501_A549_TSA_AcK.raw
x 10^5
Precursor
x 10^6
iTRAQ
x 10^4

k G S k k A V T k A Q K

histone cluster 2, H2be [Homo sapiens]
Charge State: +3
Scan Number: 14574
File Name: 120501_A549_TSA_AcK.raw

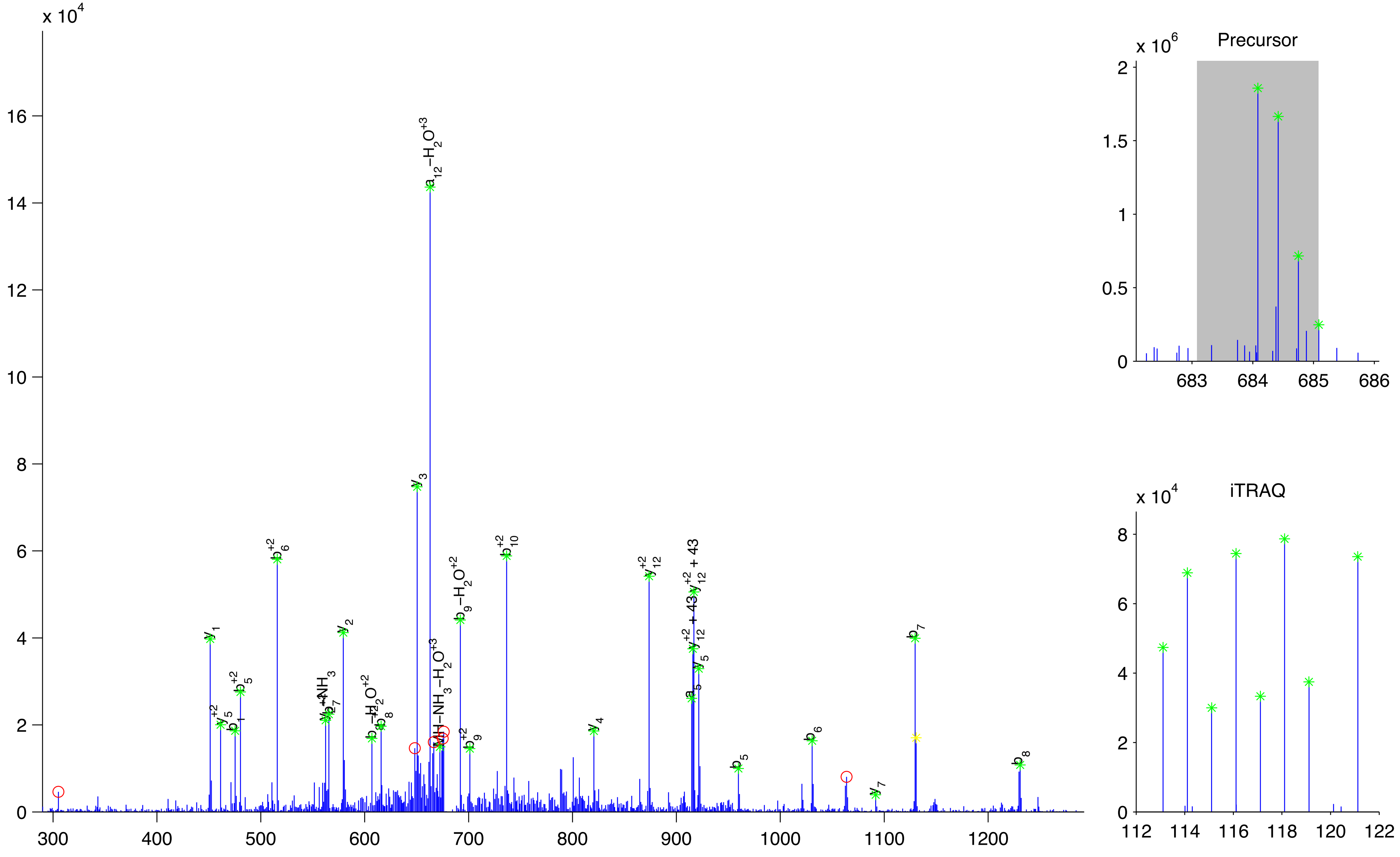

S A P A P k k G S k k A V T k A Q K

histone cluster 2, H2be [Homo sapiens]
Charge State: +4
Scan Number: 15265
File Name: 120501_A549_TSA_AcK.raw

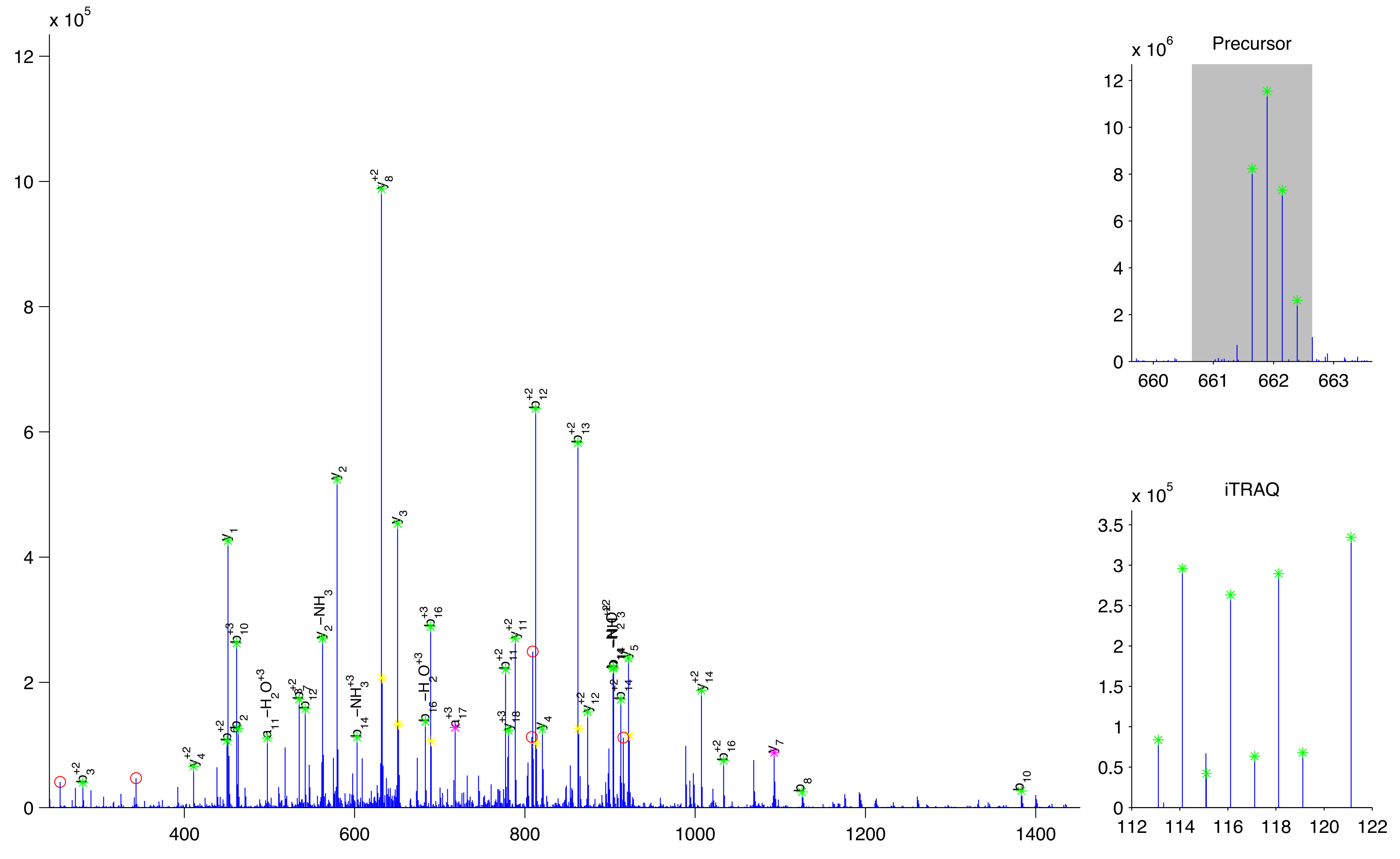

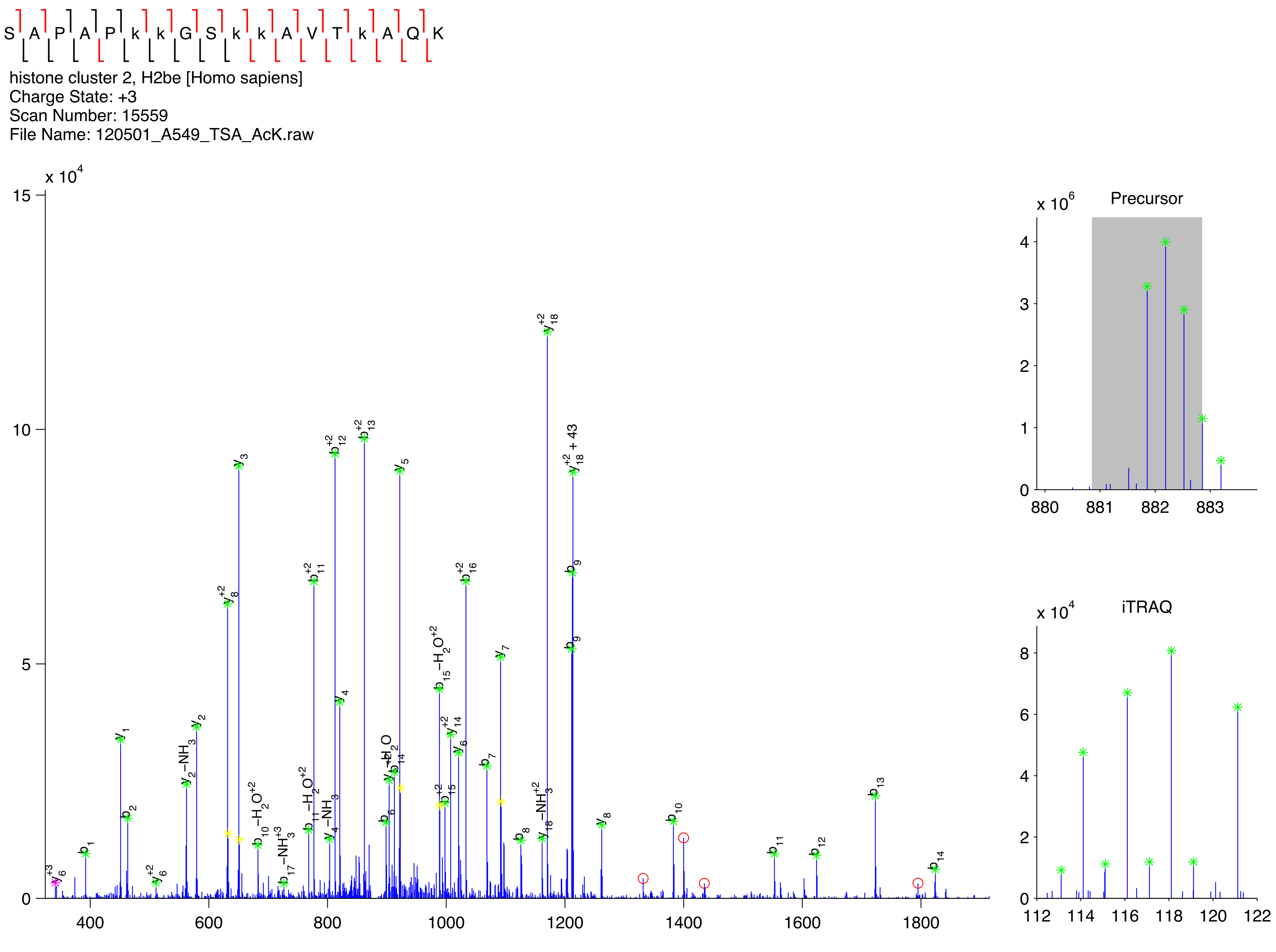
S A P A P k k G S k k A V T k A Q K
histone cluster 2, H2be [Homo sapiens]
Charge State: +3
Scan Number: 15559
File Name: 120501_A549_TSA_AcK.raw
x 10^4
Precursor
x 10^6
iTRAQ
x 10^4

S A P A P k k G S k k A V T k A Q K

histone cluster 2, H2be [Homo sapiens]
Charge State: +4
Scan Number: 15811
File Name: 120501_A549_TSA_AcK.raw

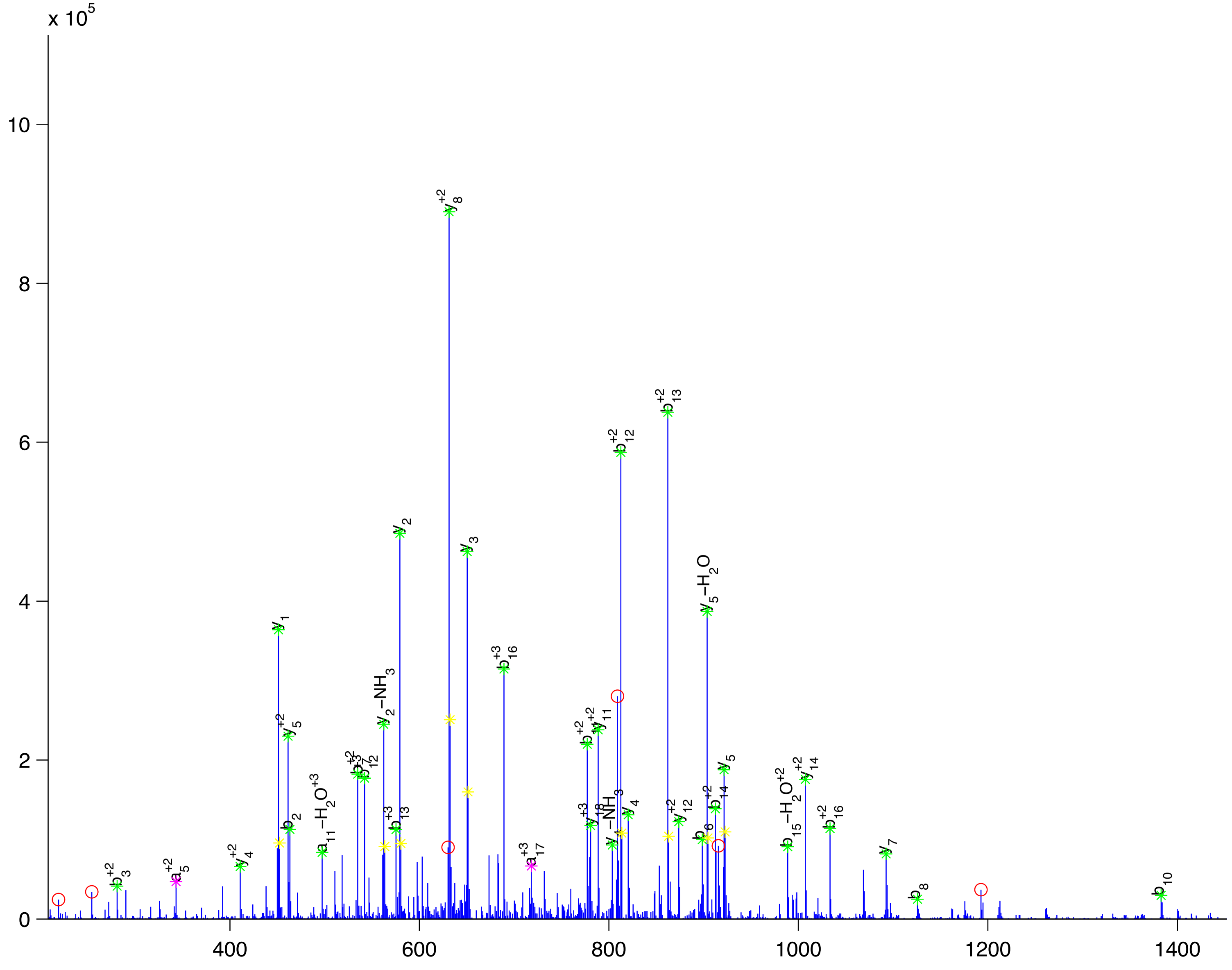

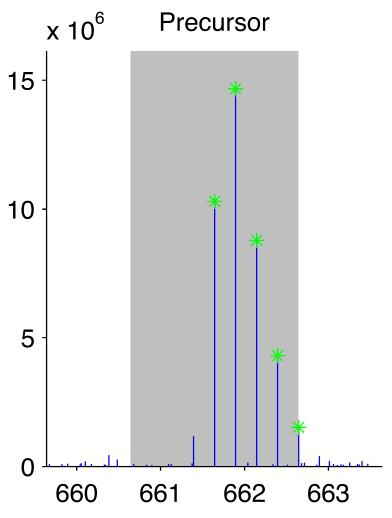

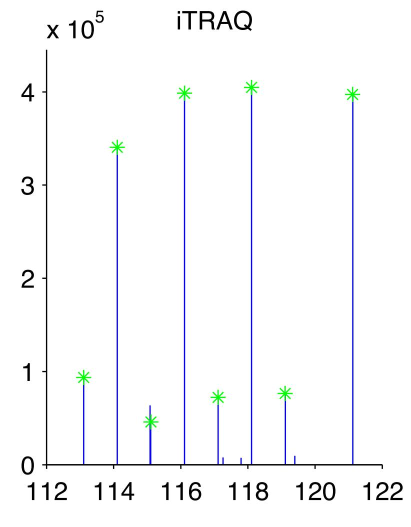

S A P A P k k G S k k A V T k A Q K

histone cluster 2, H2be [Homo sapiens]
Charge State: +4
Scan Number: 16359
File Name: 120501_A549_TSA_AcK.raw

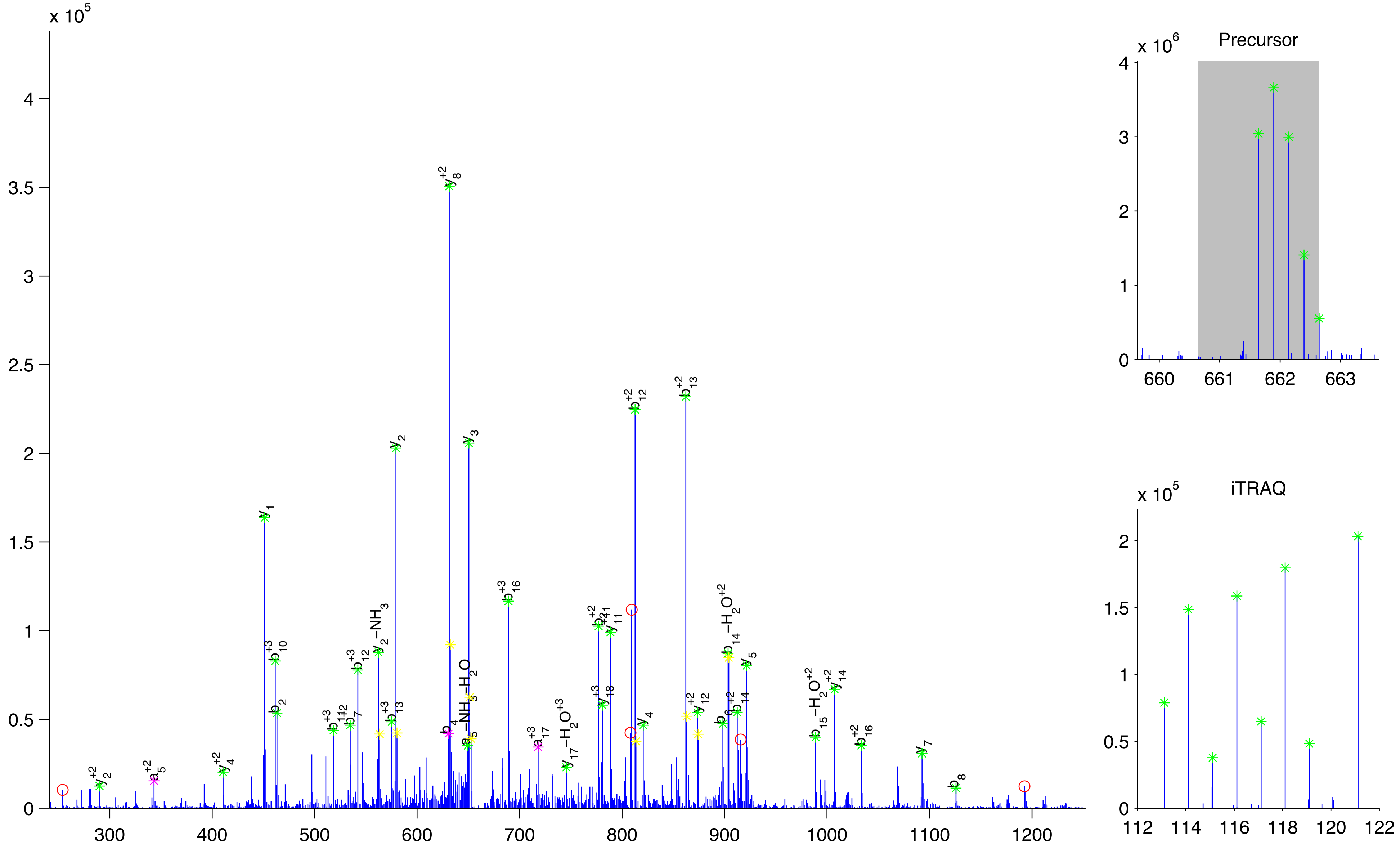

P E P A k S A P A P k k G S k k A V T k A Q K

histone cluster 2, H2be [Homo sapiens]
Charge State: +4
Scan Number: 18379
File Name: 120501_A549_TSA_AcK.raw

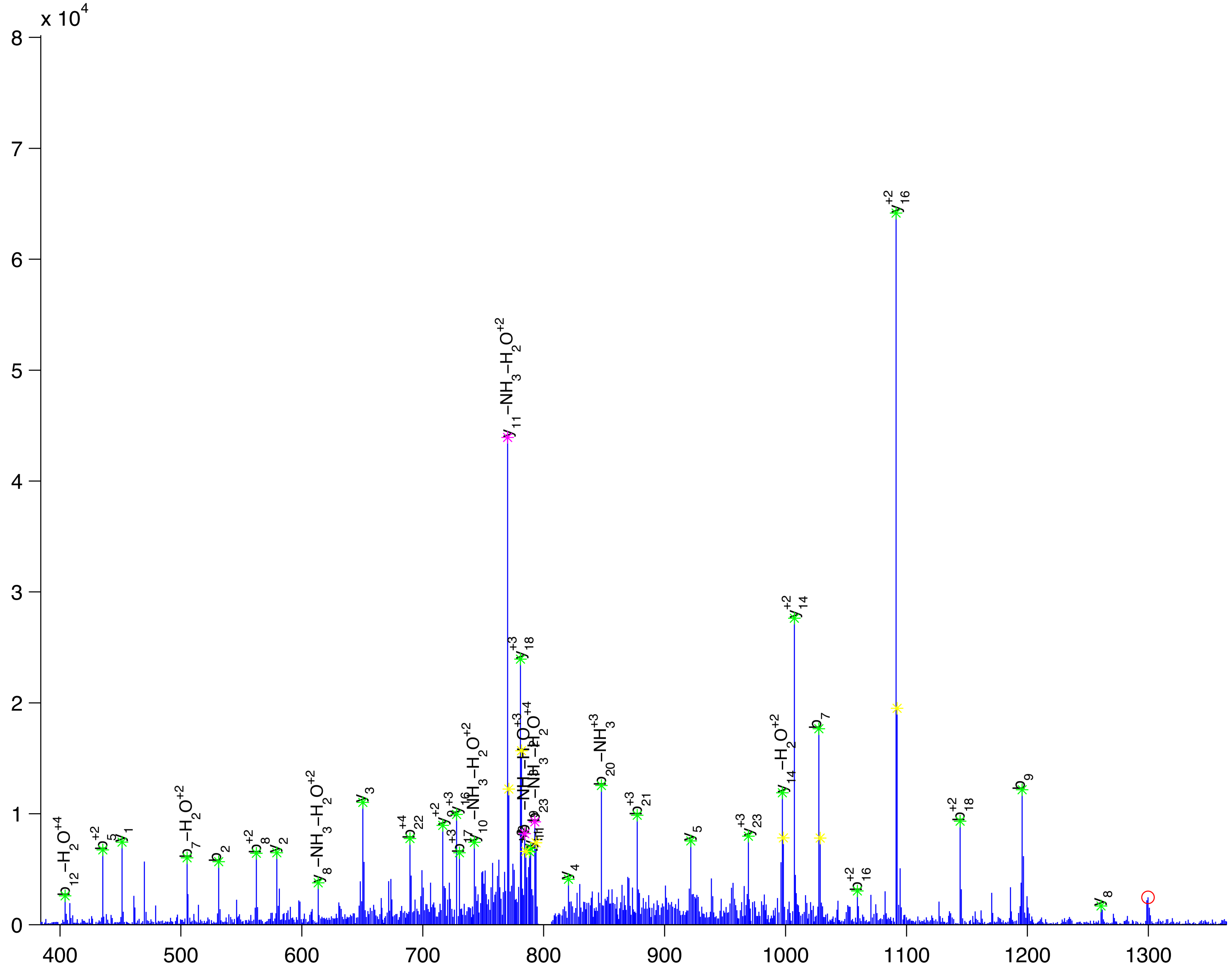

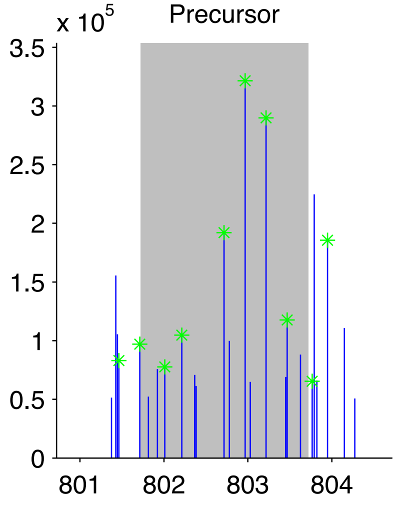

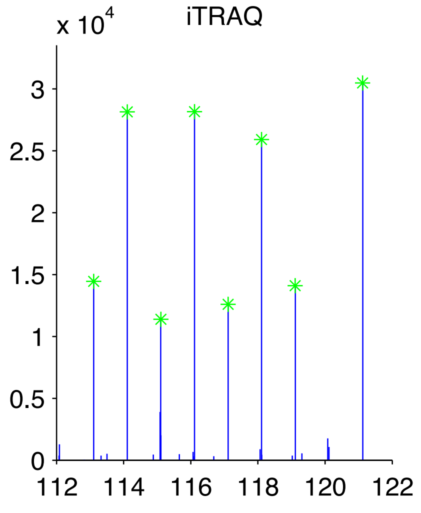

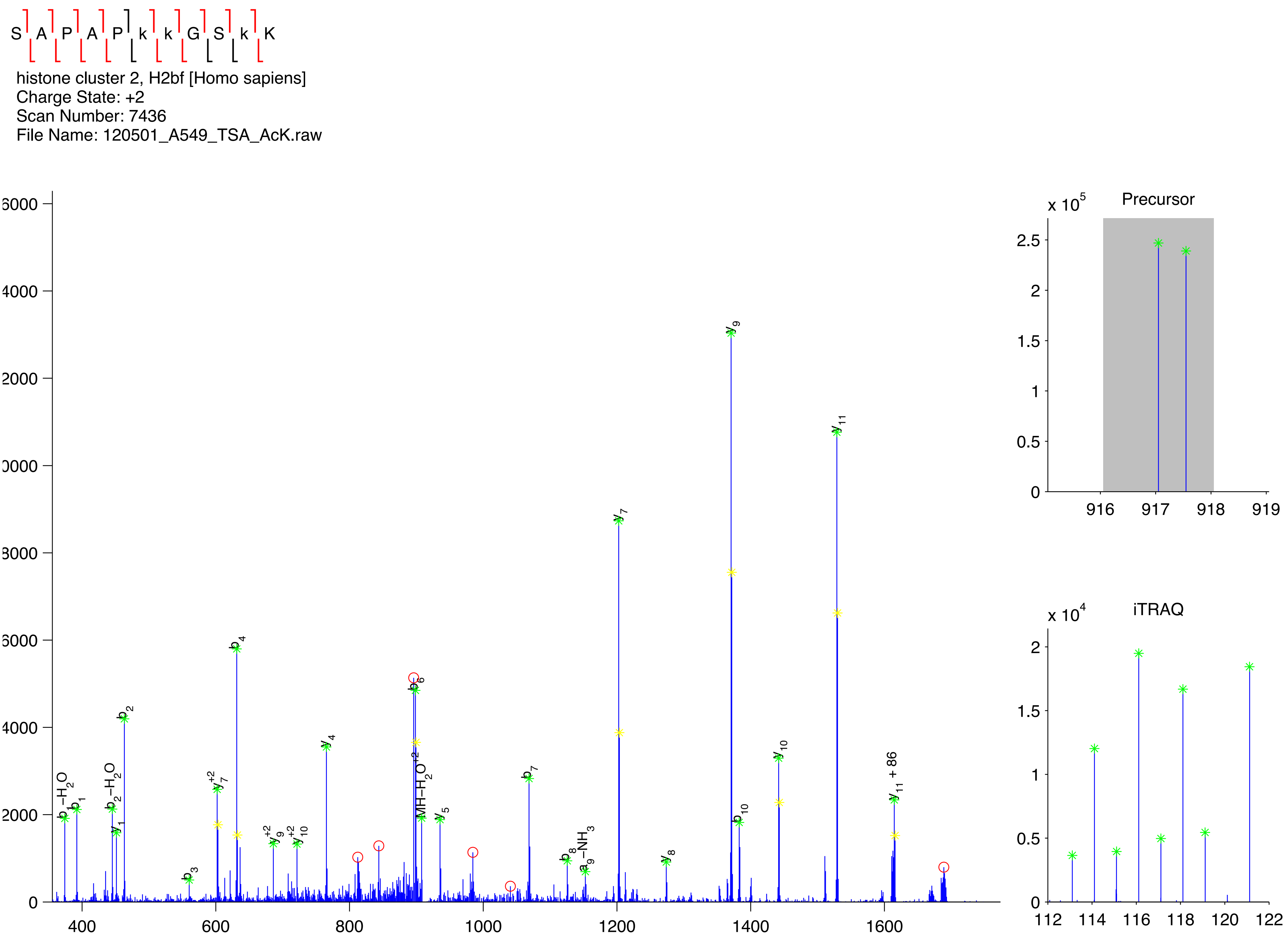
S A P A P k k G S k K
histone cluster 2, H2bf [Homo sapiens]
Charge State: +2
Scan Number: 7436
File Name: 120501_A549_TSA_AcK.raw
b1−H2O
b1
b2−H2O
y1
b2
b3
y7+2
b4
y9+2
y10+2
y4
b6
MH−H2O+2
y5
b7
b8
a9−NH3
y7
y8
y9
b10
y10
y11
y11 + 86
400
600
800
1000
1200
1400
1600
Precursor
x 10^5
916
917
918
919
iTRAQ
x 10^4
112
114
116
118
120
122

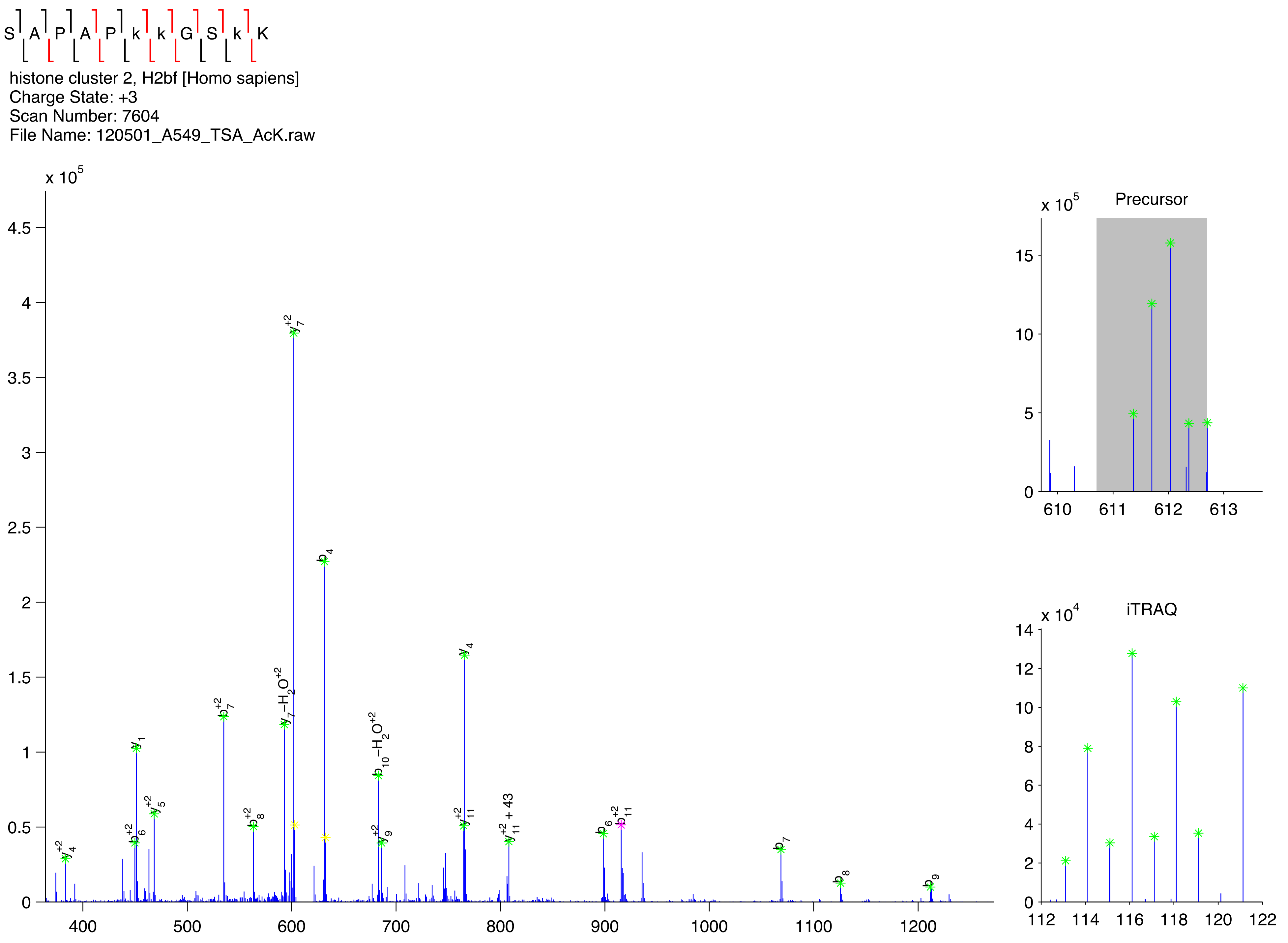
S A P A P k k G S k K
histone cluster 2, H2bf [Homo sapiens]
Charge State: +3
Scan Number: 7604
File Name: 120501_A549_TSA_AcK.raw
x 10^5
Precursor
iTRAQ
x 10^4

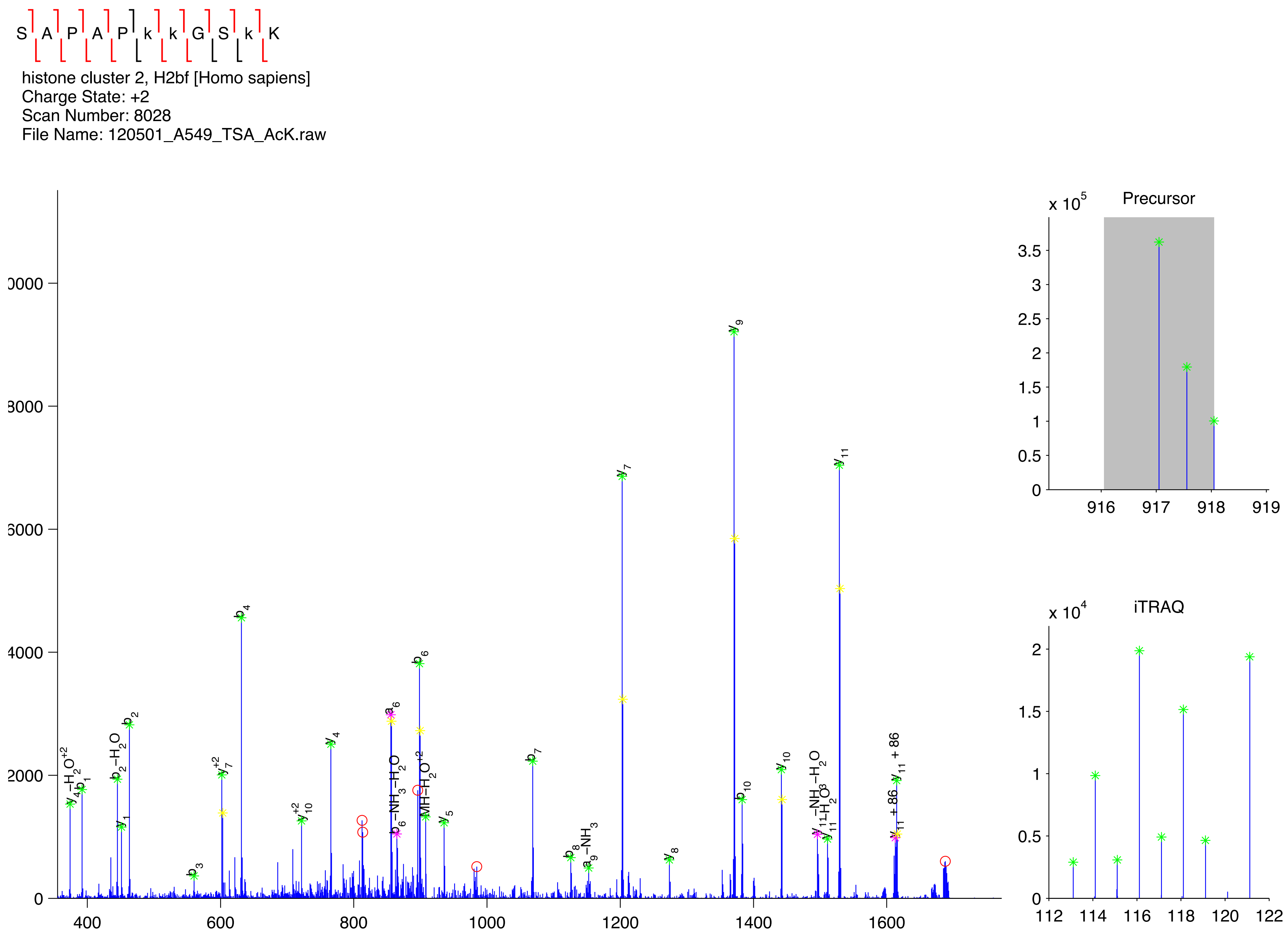

S A P A P k k G S k K
histone cluster 2, H2bf [Homo sapiens]
Charge State: +2
Scan Number: 8028
File Name: 120501_A549_TSA_AcK.raw
Precursor
x 10^5
iTRAQ
x 10^4

k G S k k A V T K
histone cluster 2, H2bf [Homo sapiens]
Charge State: +2
Scan Number: 9872
File Name: 120501_A549_TSA_AcK.raw

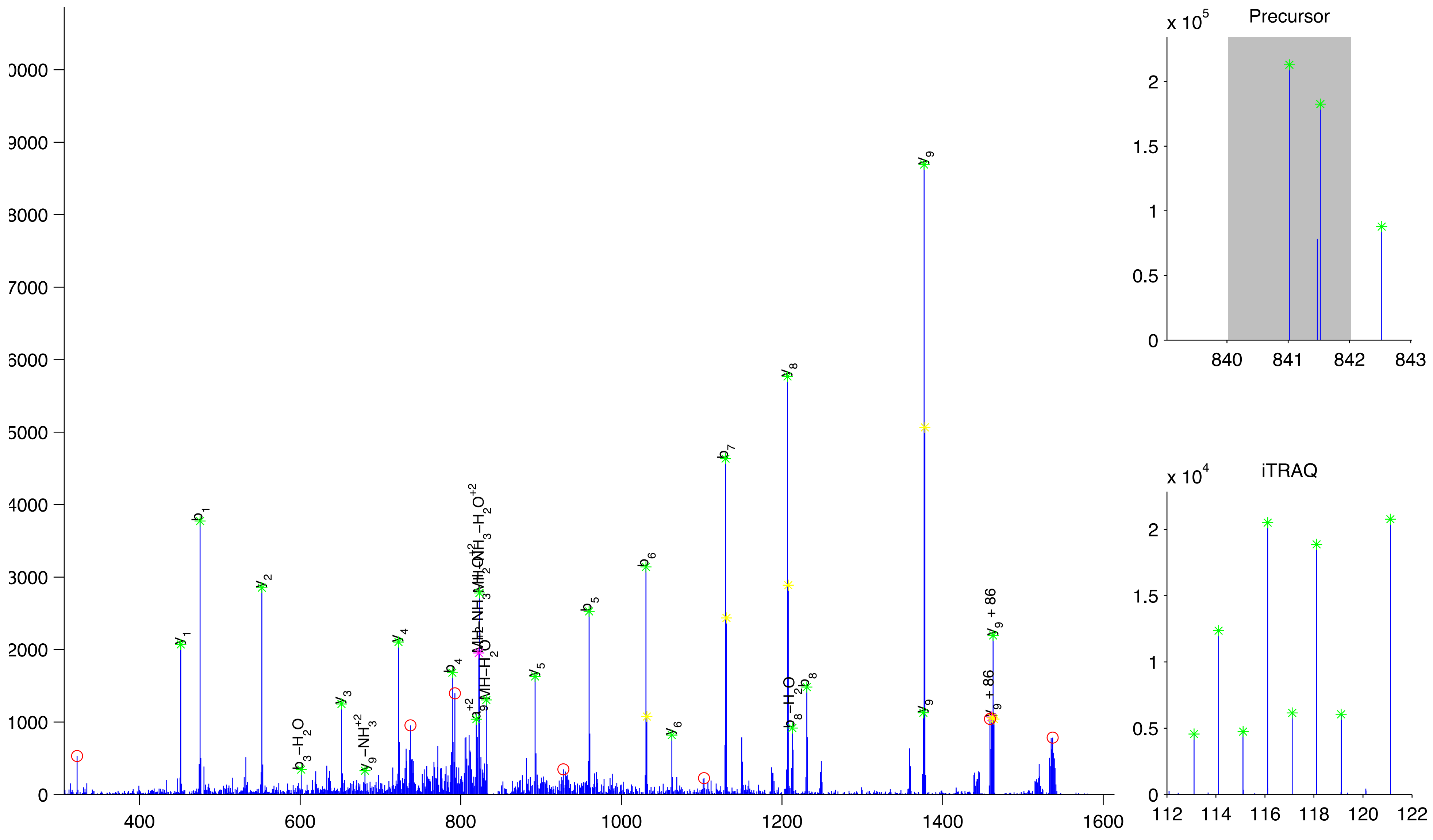

k A V T k V Q K

histone cluster 2, H2bf [Homo sapiens]
Charge State: +2
Scan Number: 11758
File Name: 120501_A549_TSA_AcK.raw

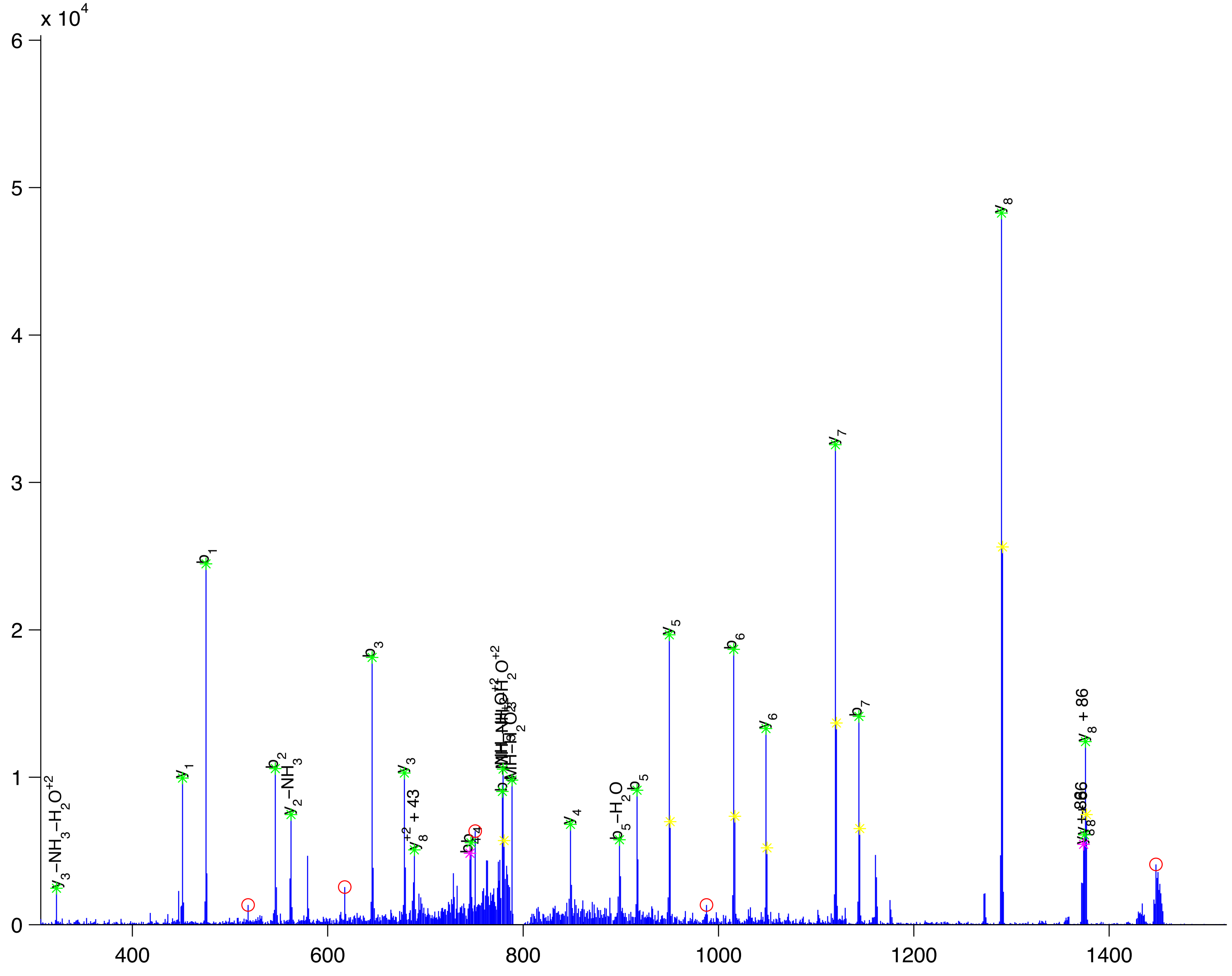

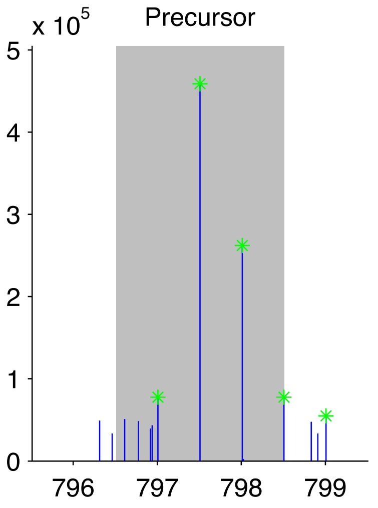

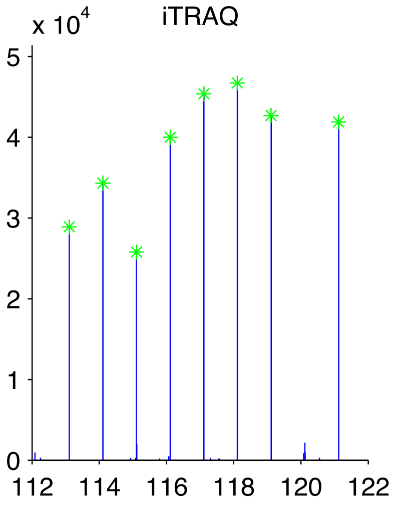

k G S k k A V T k V Q K

histone cluster 2, H2bf [Homo sapiens]
Charge State: +3
Scan Number: 14488
File Name: 120501_A549_TSA_AcK.raw

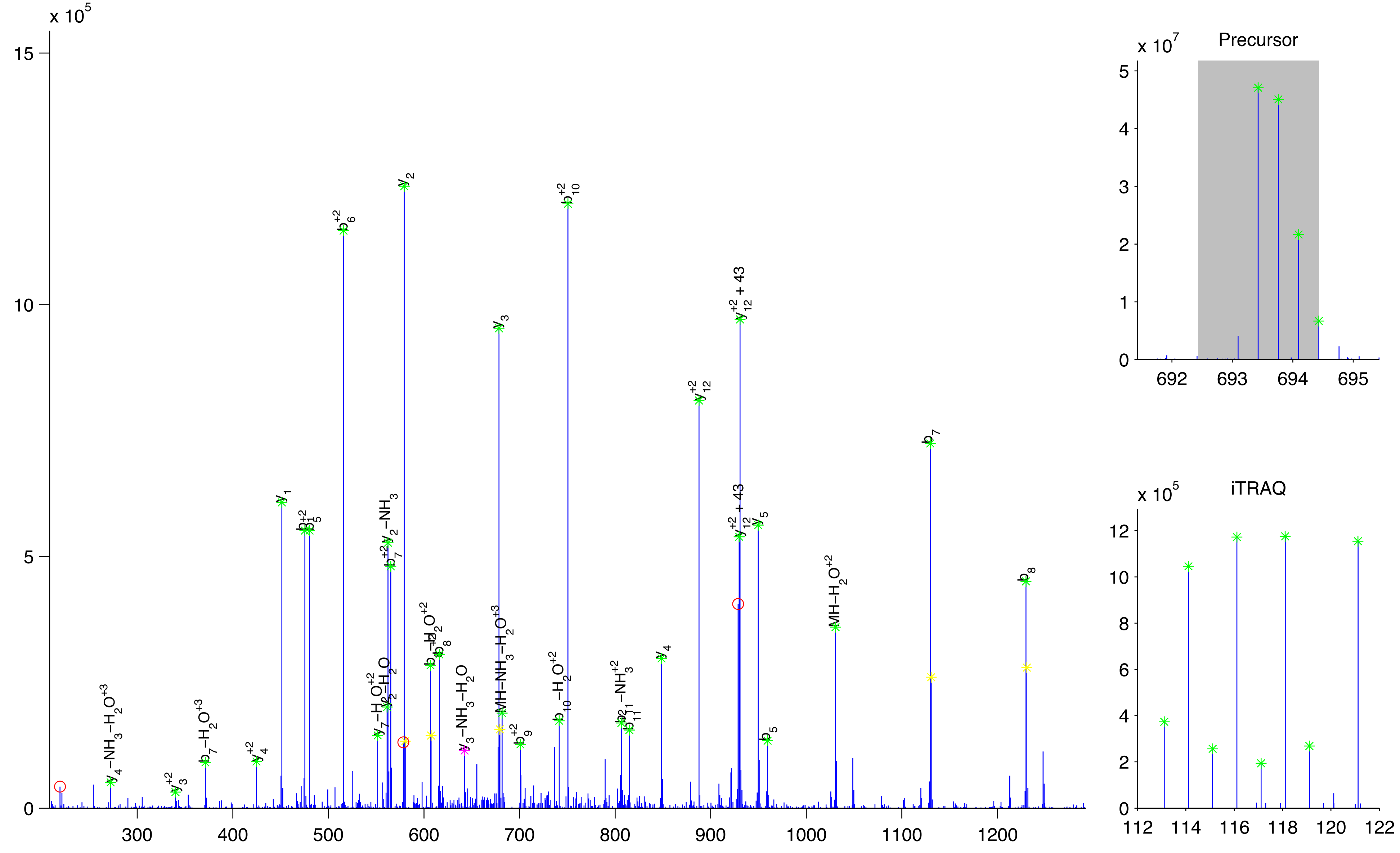

k G S k k A V T k V Q K

histone cluster 2, H2bf [Homo sapiens]
Charge State: +4
Scan Number: 14803
File Name: 120501_A549_TSA_AcK.raw

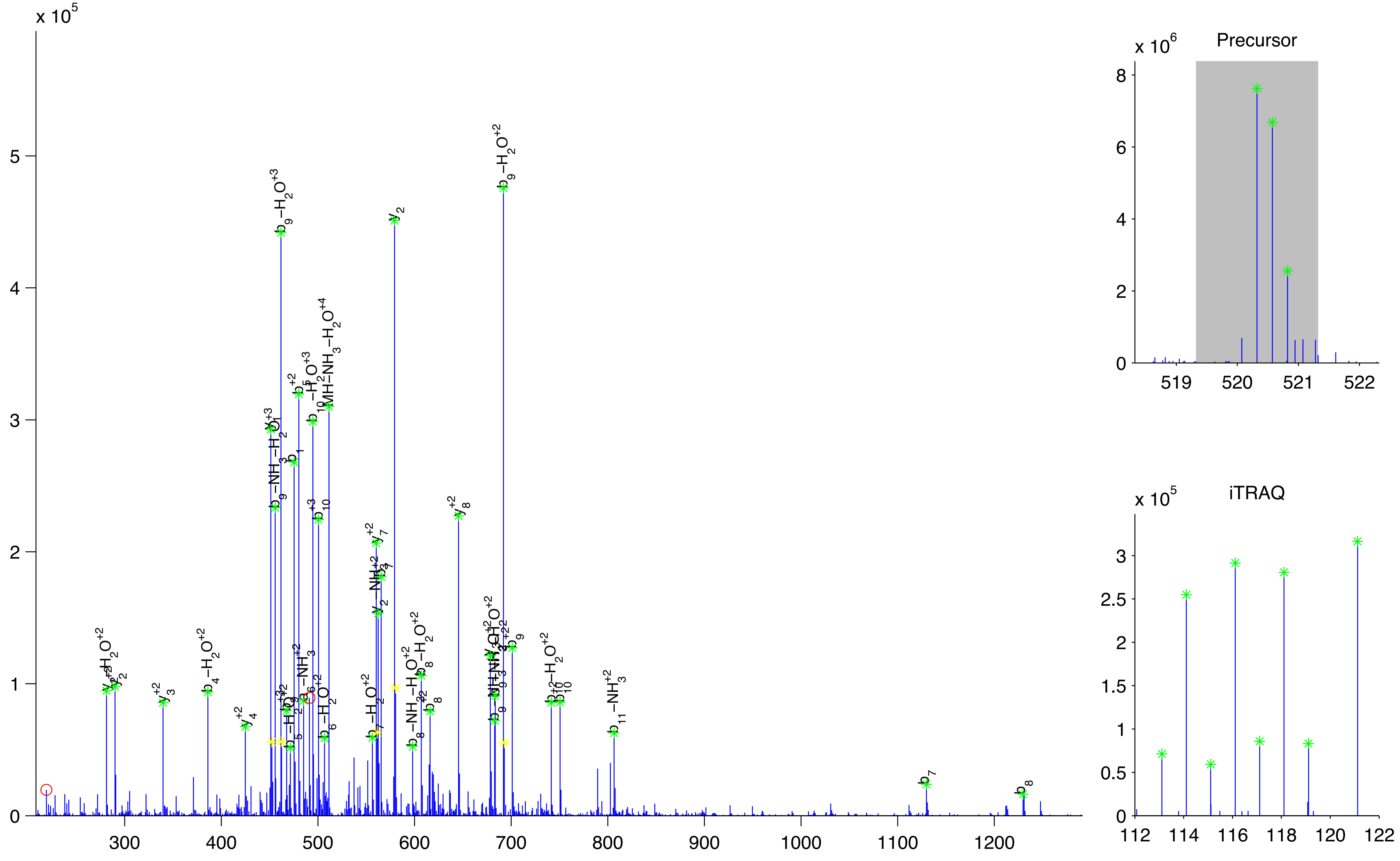

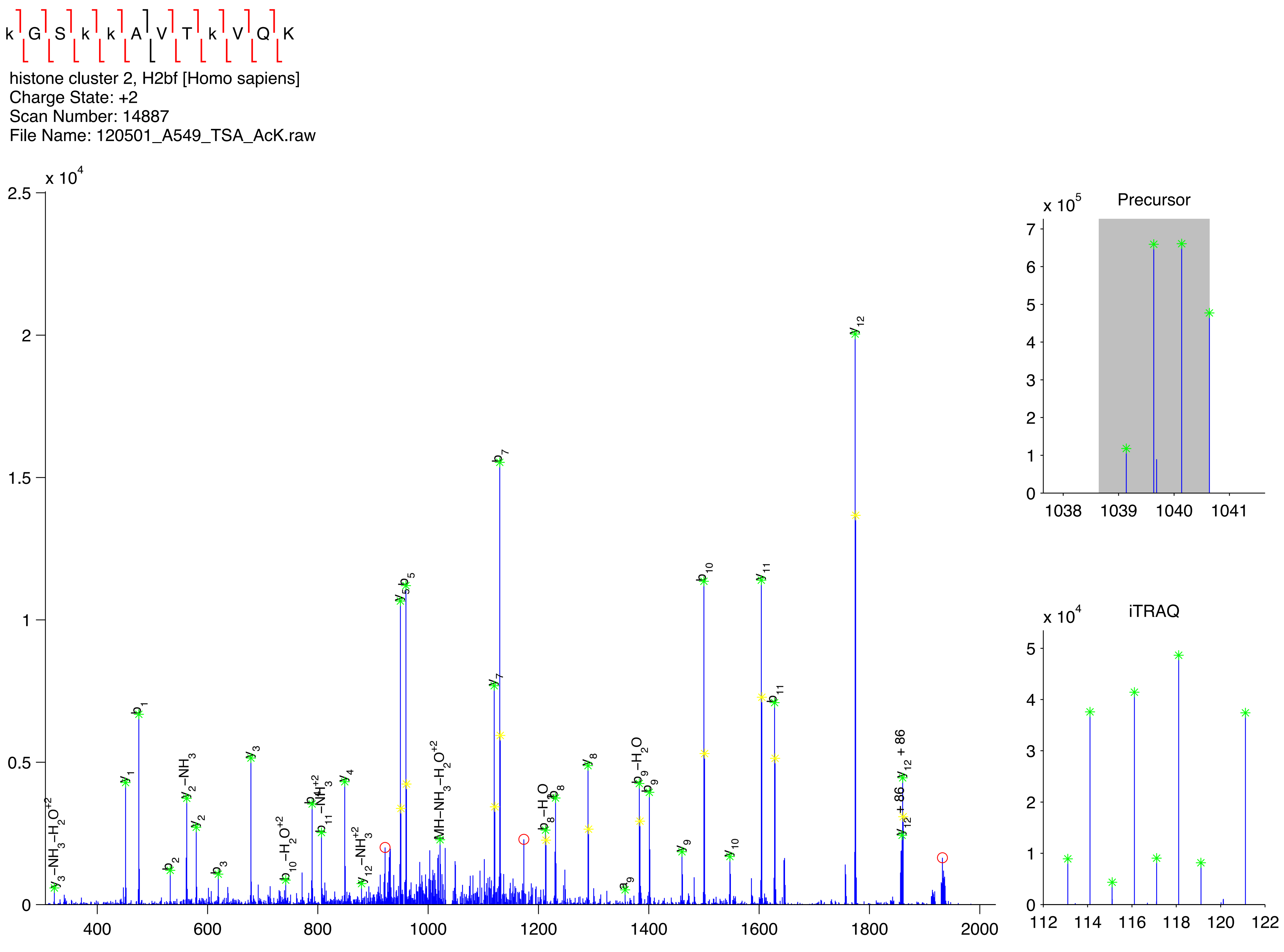
k G S k k A V T k V Q K
histone cluster 2, H2bf [Homo sapiens]
Charge State: +2
Scan Number: 14887
File Name: 120501_A549_TSA_AcK.raw
x 10^4
y3−NH3−H2O+2
y1
b1
b2
y2−NH3
y2
b3
y3
b10−H2O+2
b4+2
b11−NH3
y4
y12−NH3+2
y5
b5
MH−NH3−H2O+2
y7
b7
b8−H2O
b8
y8
a9
b9−H2O
b9
y9
b10
y10
y11
b11
y12
y12 + 86
y12 + 86
x 10^5
Precursor
iTRAQ

k G S k k A V T k V Q K

histone cluster 2, H2bf [Homo sapiens]
Charge State: +3
Scan Number: 15034
File Name: 120501_A549_TSA_AcK.raw

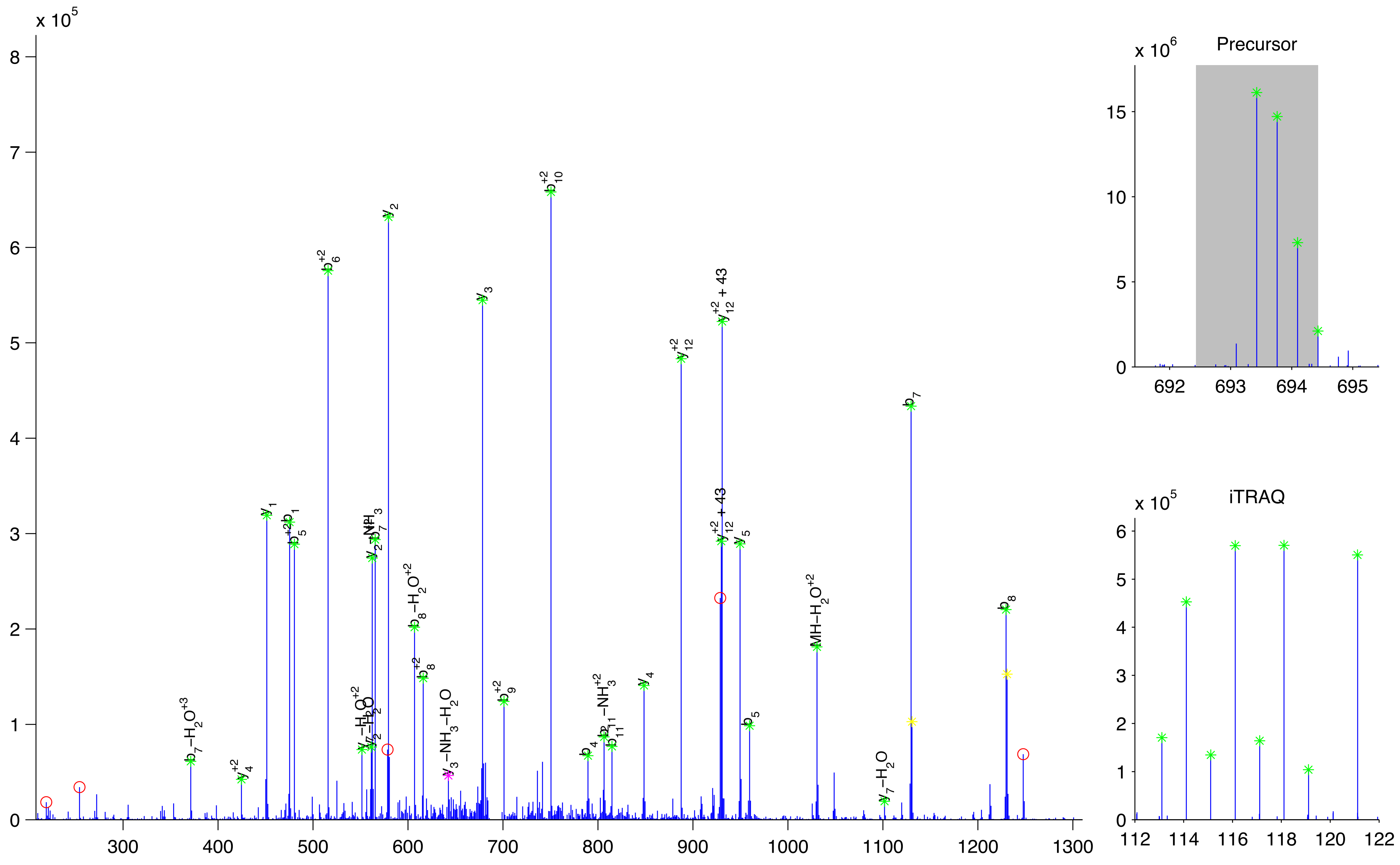

k G S k k A V T k V Q K

histone cluster 2, H2bf [Homo sapiens]
Charge State: +3
Scan Number: 15580
File Name: 120501_A549_TSA_AcK.raw

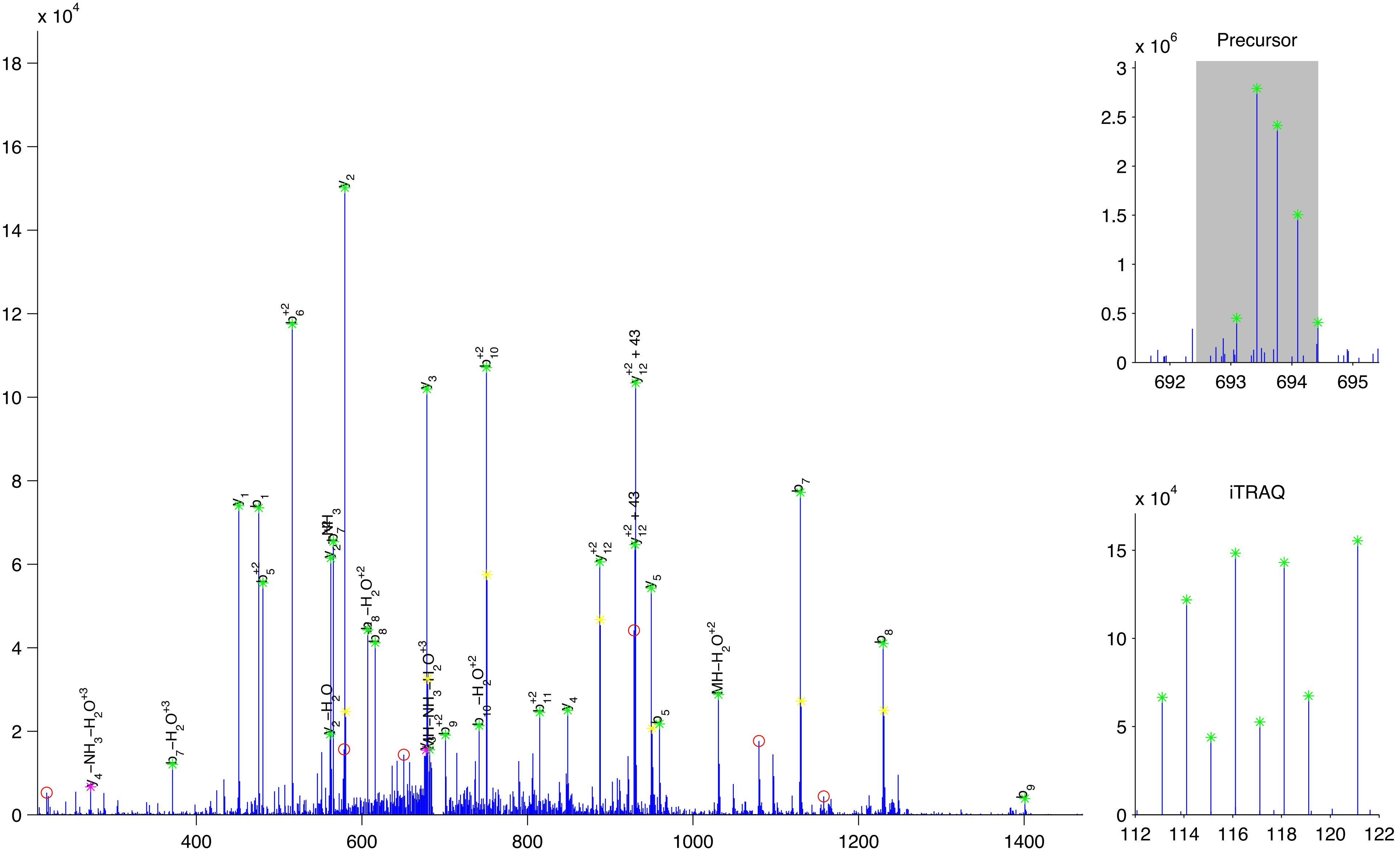

S A P A P k k G S k k A V T k V Q K

histone cluster 2, H2bf [Homo sapiens]
Charge State: +4
Scan Number: 17470
File Name: 120501_A549_TSA_AcK.raw

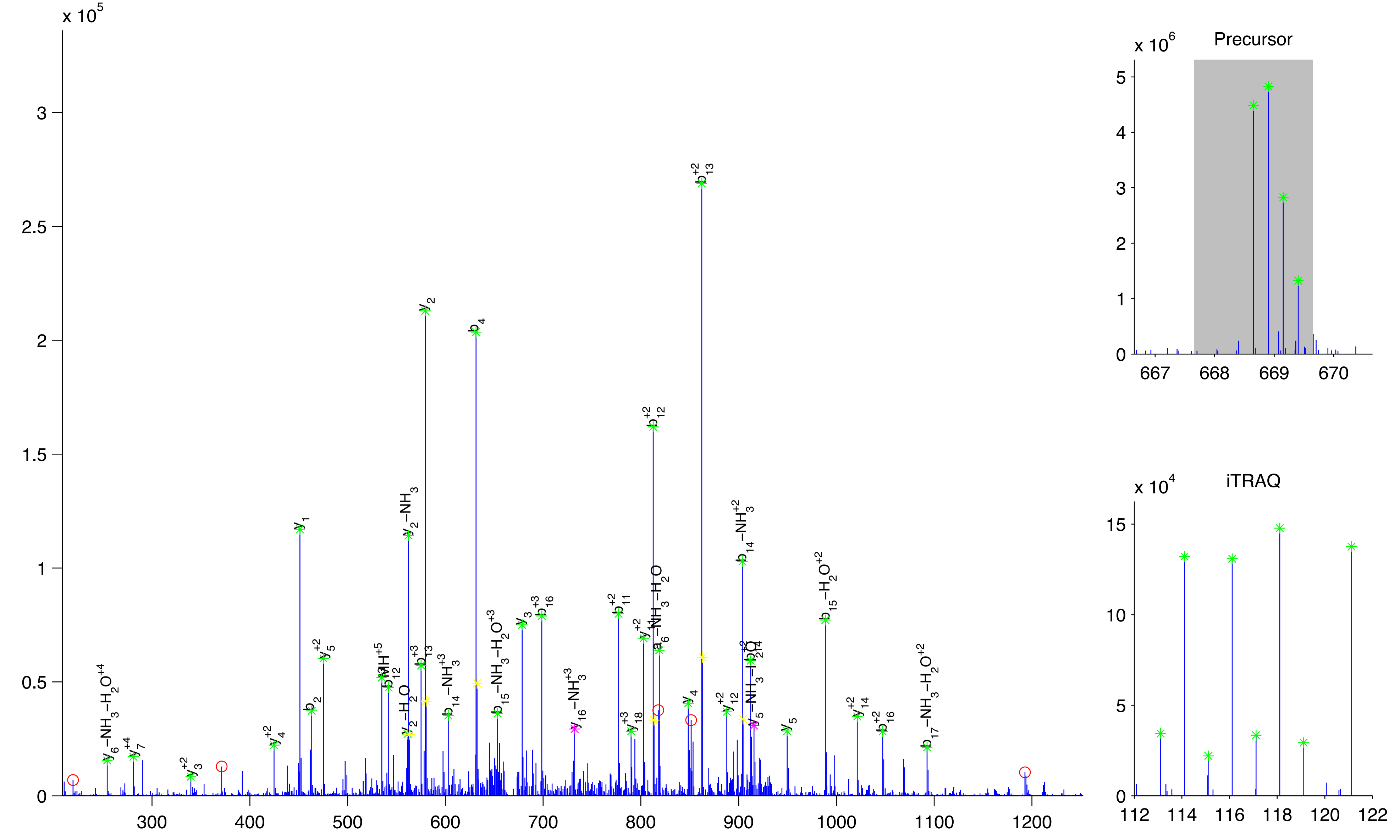

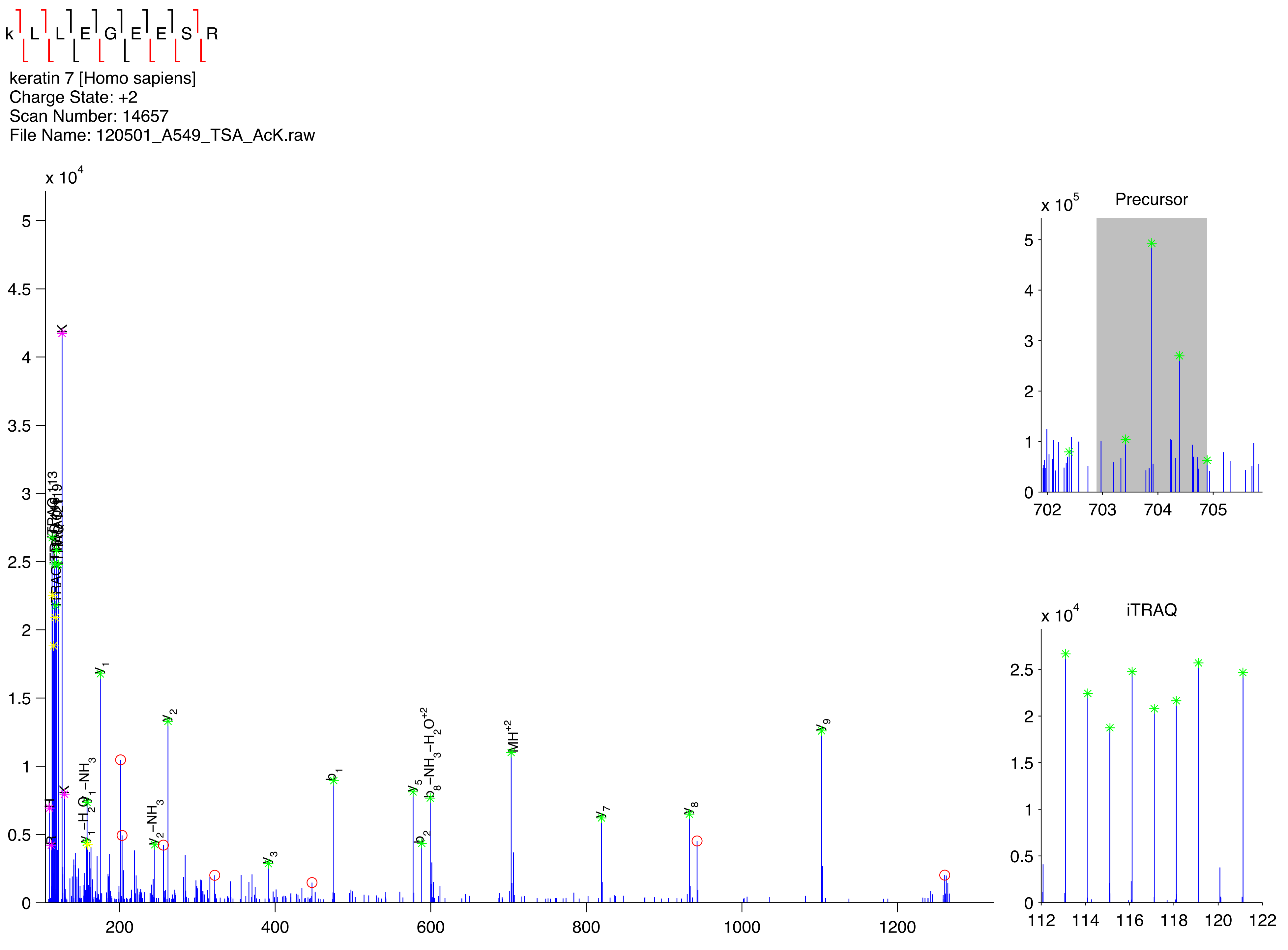
k L L E G E E S R
keratin 7 [Homo sapiens]
Charge State: +2
Scan Number: 14657
File Name: 120501_A549_TSA_AcK.raw
x 10^4
K
H
R
y1
y1-H2O
y1-NH3
y2-NH3
y2
y3
b1
y5
b2
b8-NH3-H2O+2
MH+2
y7
y8
y9
Precursor
x 10^5
iTRAQ
x 10^4

k G k G G A K

SWI/SNF–related matrix–associated actin–dependent regulator of chromatin a2 isoform b [Homo sapiens

Charge State: +3

Scan Number: 7159

File Name: 120501_A549_TSA_AcK.raw

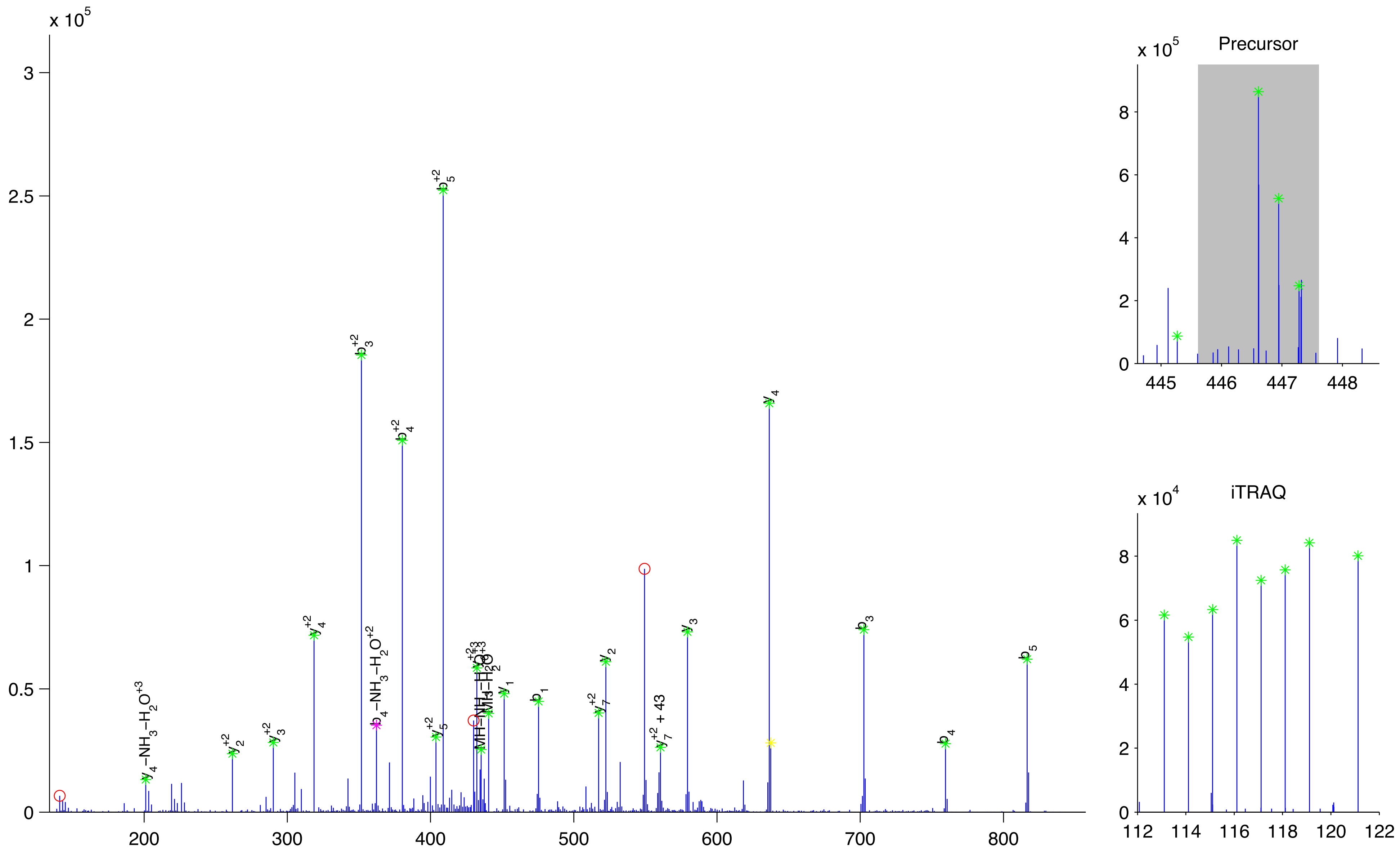

Supplement: S5 Fig — Instead of relying on an FDR analysis where the identity of true positives and true negatives are unknown, we manually validated each MS/MS spectra manually. Each page represents a manually validated MS/MS spectrum. (PDF) [file pone.0126242.s005.pdf]
