# Supplementary material for: Quantitative Profiling of Lysine Acetylation Reveals Dynamic Crosstalk between Receptor Tyrosine Kinases and Lysine Acetylation
Source: PLoS One. 2015 May 15;10(5):e0126242. doi: 10.1371/journal.pone.0126242 (PMC4433260; doi:10.1371/journal.pone.0126242)

E L G E y G F H E Y T E V K

aldehyde dehydrogenase 1A1 [Homo sapiens]
Charge State: +3
Scan Number: 22414
File Name: 120518_A549_EGFTSA_pY.raw

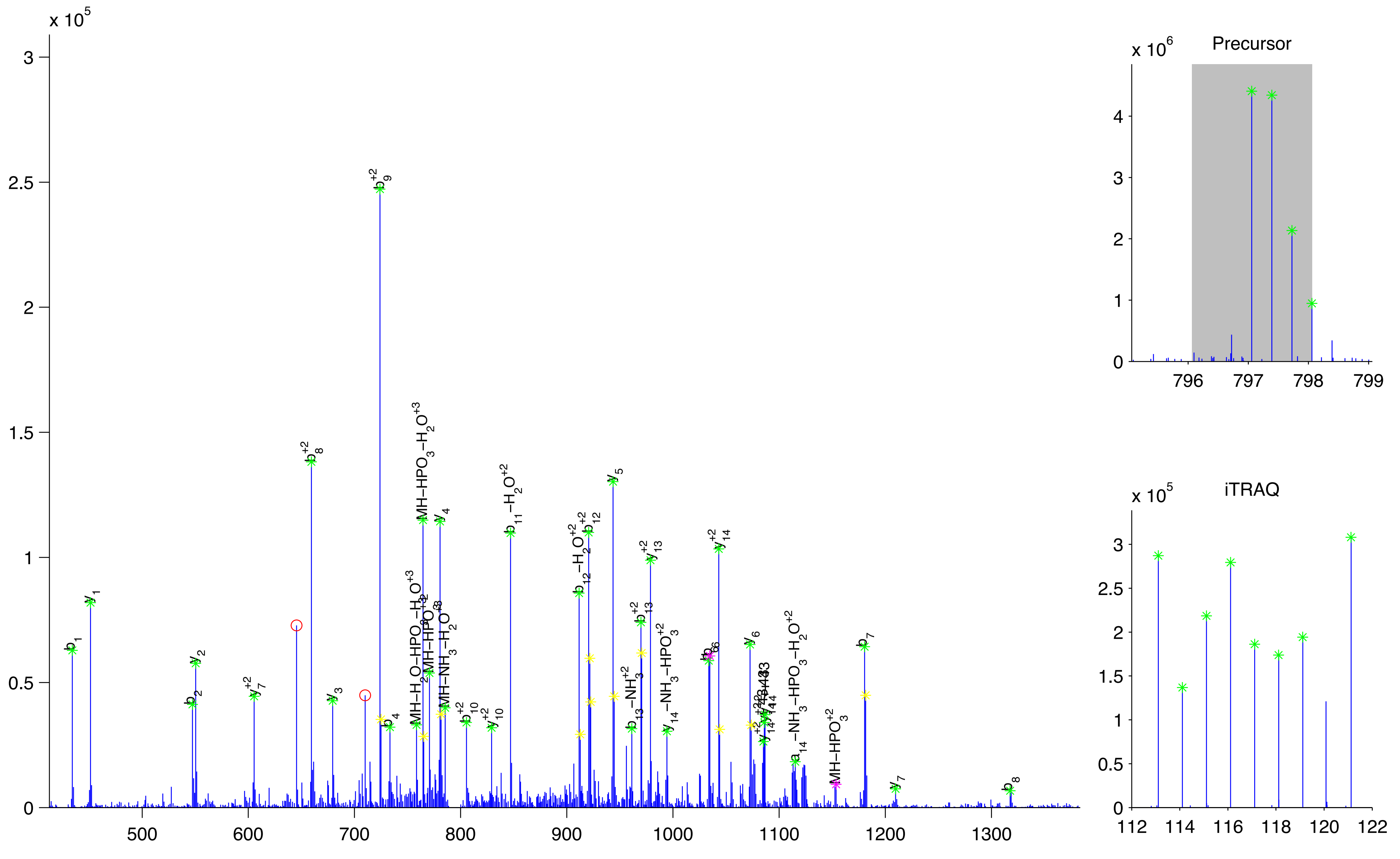

H I D c A Y V y Q N E H E V G E A I Q E K

aldo–keto reductase family 1, member B10 [Homo sapiens]
Charge State: +4
Scan Number: 10606
File Name: 120527_A549_TSAEGF_pY34_el.raw

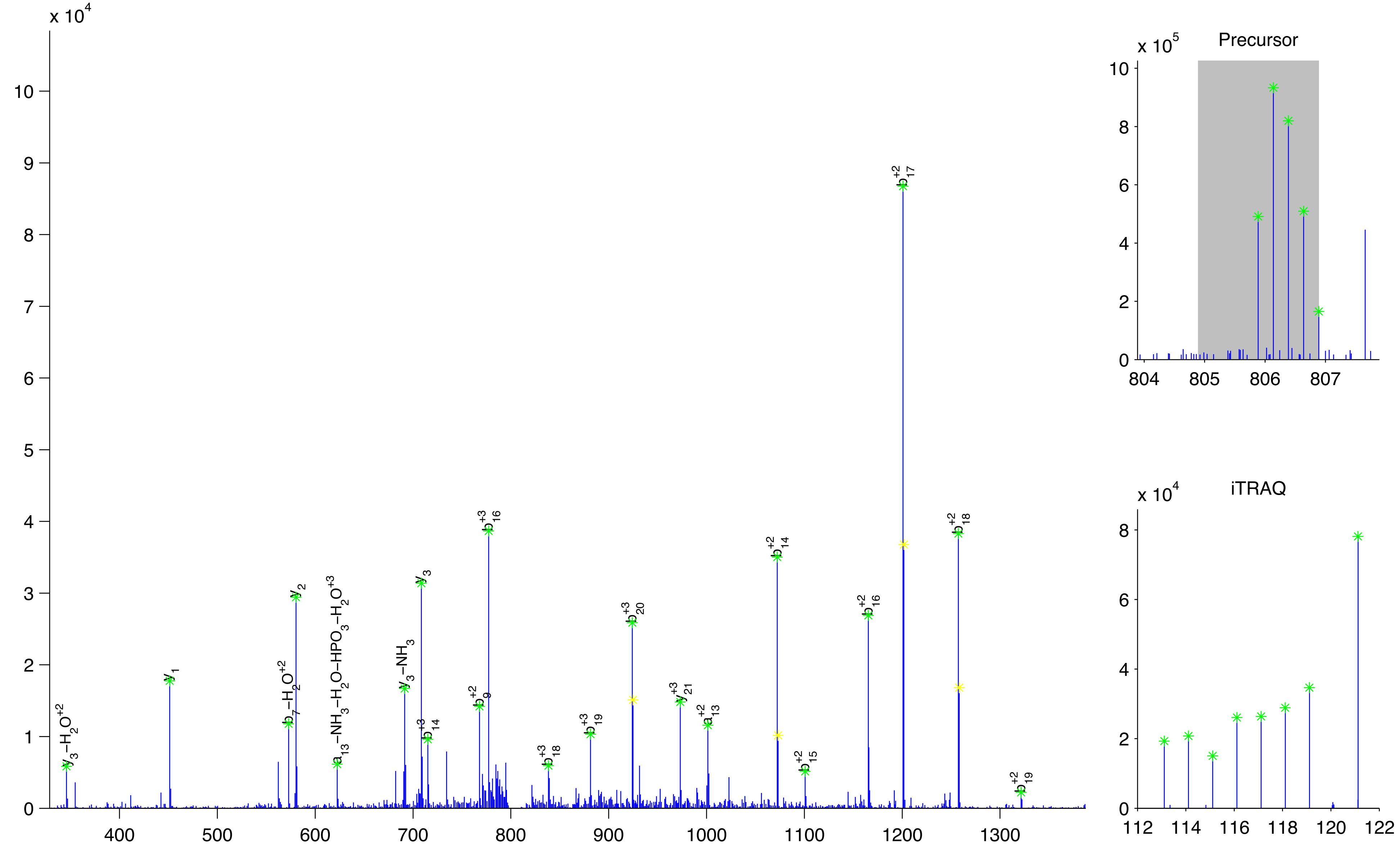

S Y S P y D M L E S I R

annexin A2 isoform 2 [Homo sapiens]
Charge State: +3
Scan Number: 16553
File Name: 120527_A549_TSAEGF_pY34_el.raw

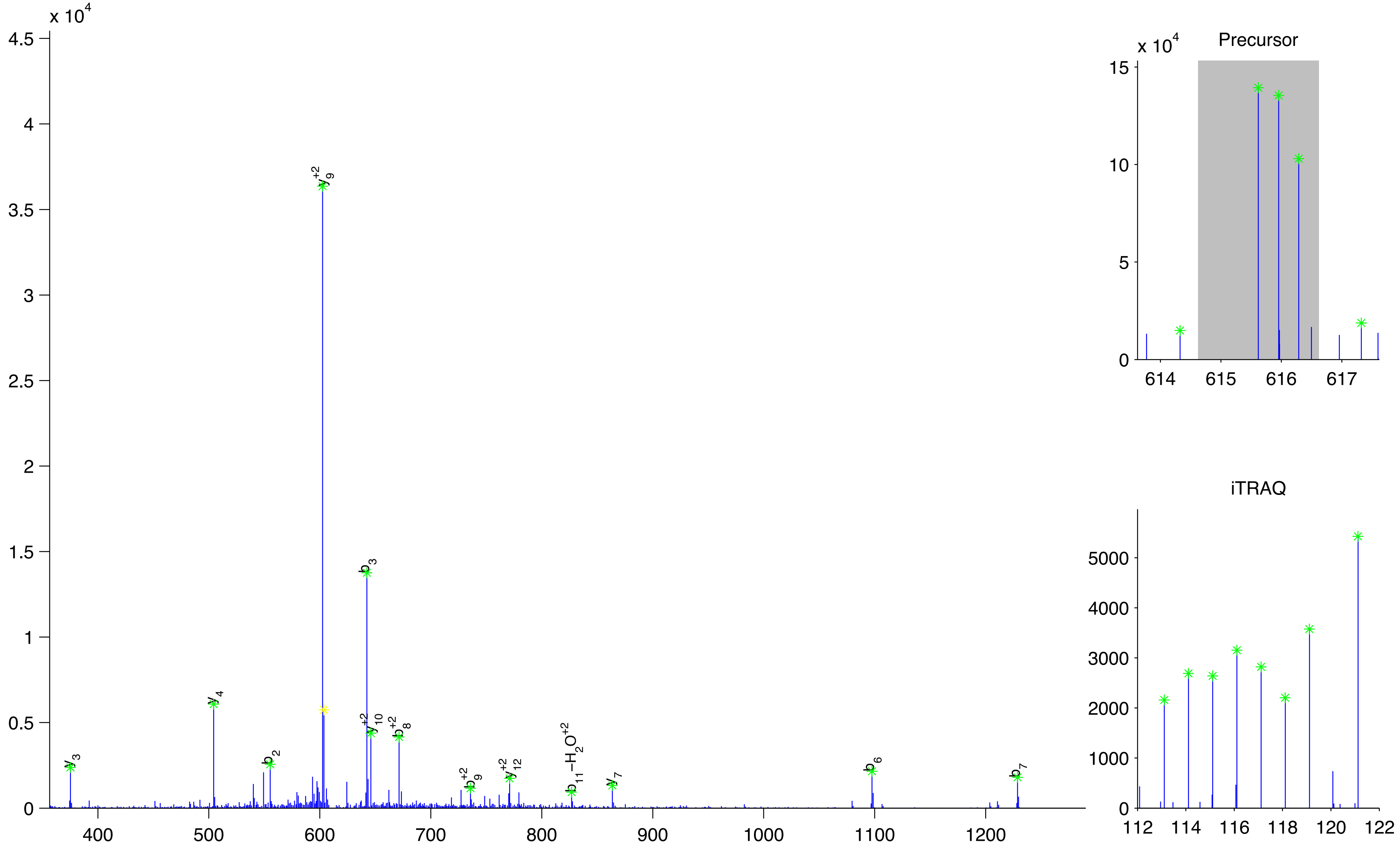

VGQGYVYEAAQPEQDEyDIPR

breast cancer anti–estrogen resistance 1 [Homo sapiens]
Charge State: +2
Scan Number: 12143
File Name: 120527_A549_TSAEGF_pY34_el.raw

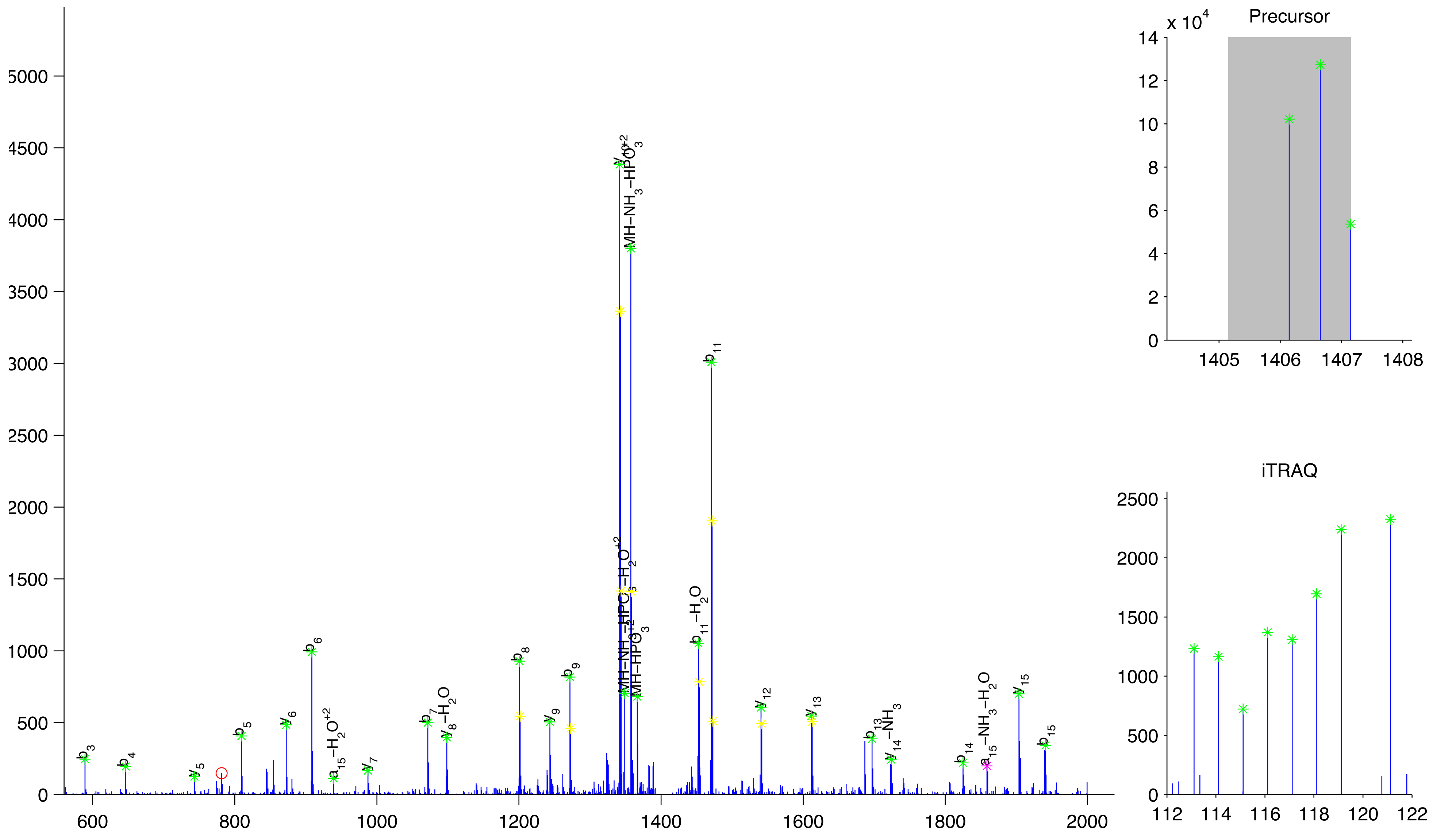

V L P P E V A D G G V V D S G V y A V P P P A E R

breast cancer anti−estrogen resistance 1 [Homo sapiens]
Charge State: +3
Scan Number: 14802
File Name: 120527_A549_TSAEGF_pY34_el.raw

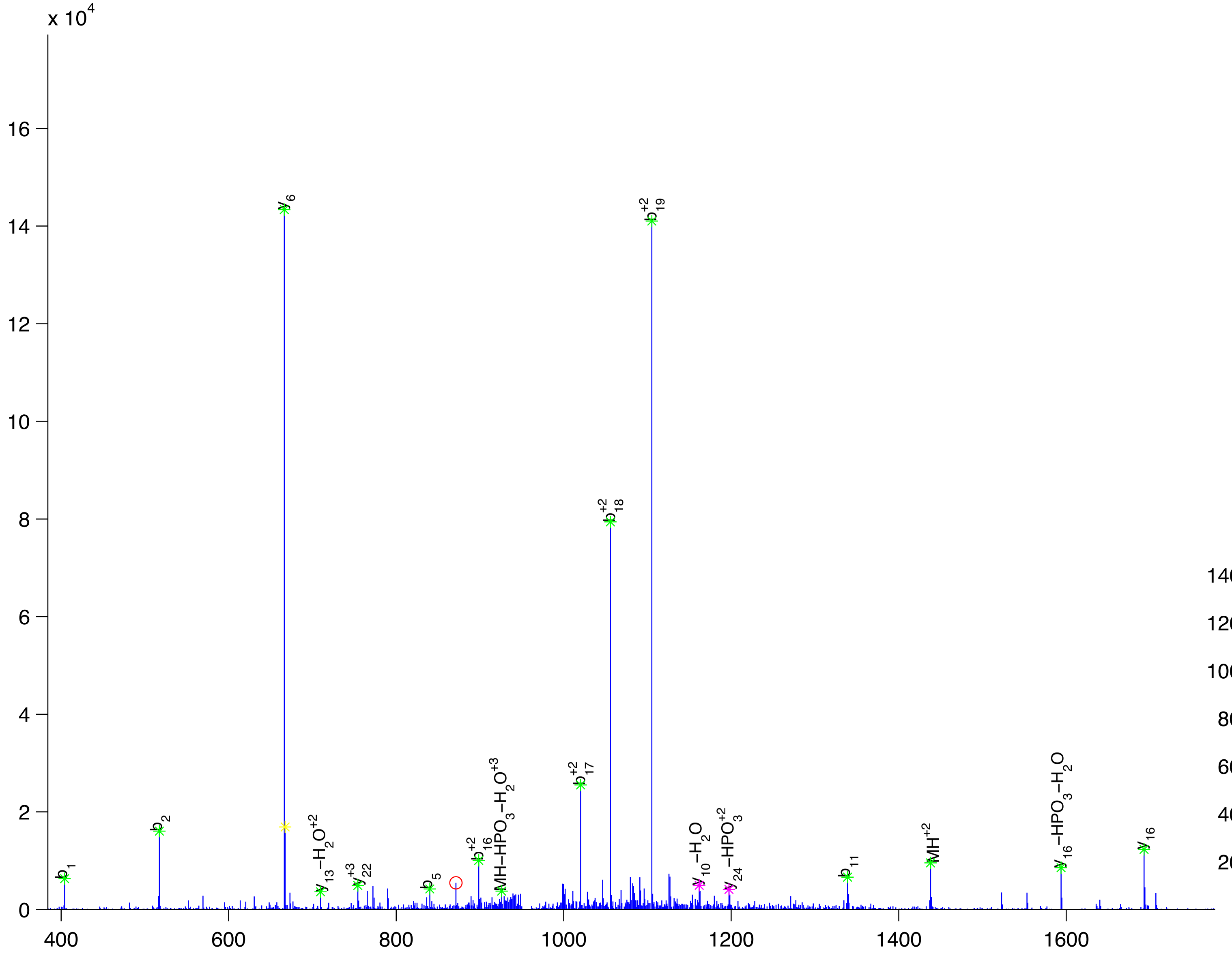

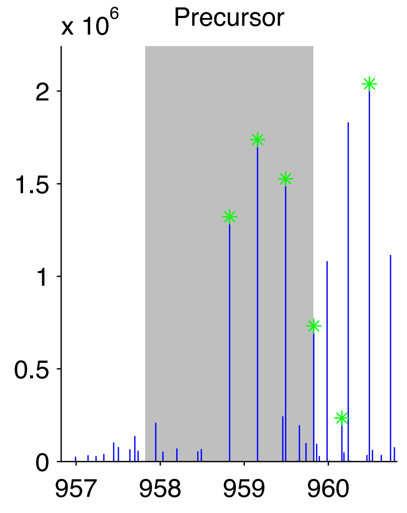

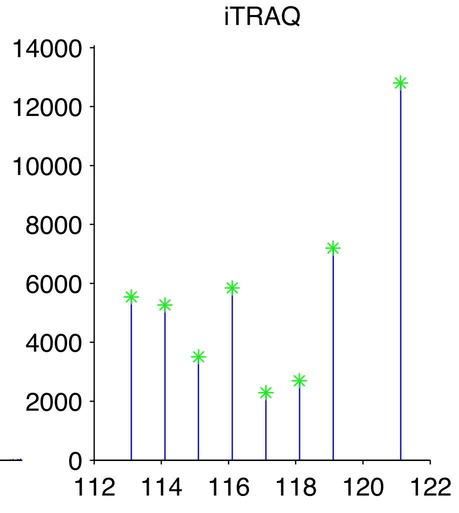

V G Q G Y V y E A A Q P E Q D E Y D I P R

breast cancer anti−estrogen resistance 1 [Homo sapiens]
Charge State: +3
Scan Number: 23611
File Name: 120518_A549_EGFTSA_pY.raw

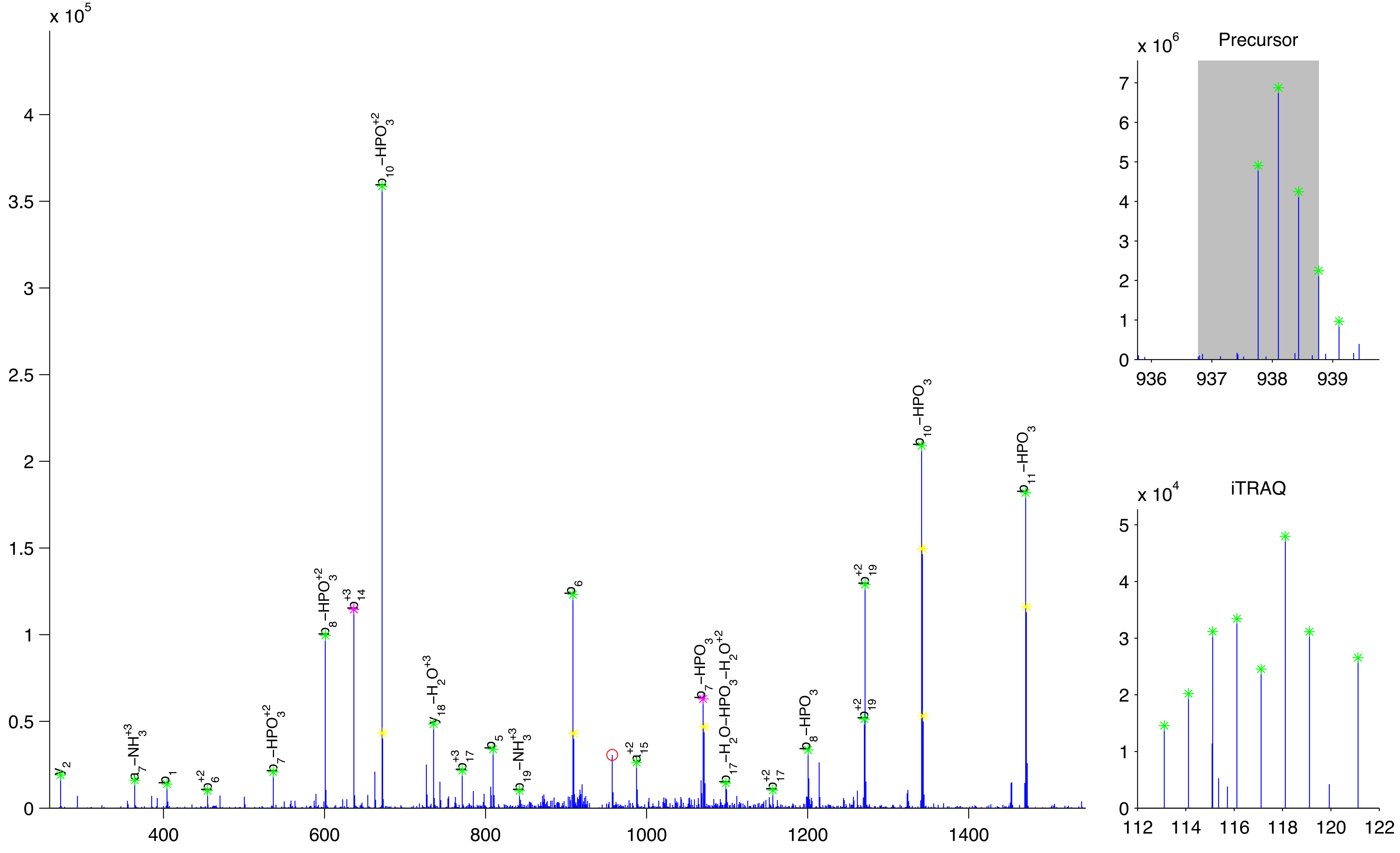

L R Q H H D E y E D E I R

carboxypeptidase D precursor [Homo sapiens]
Charge State: +4
Scan Number: 3256
File Name: 120527_A549_TSAEGF_pY34_el.raw

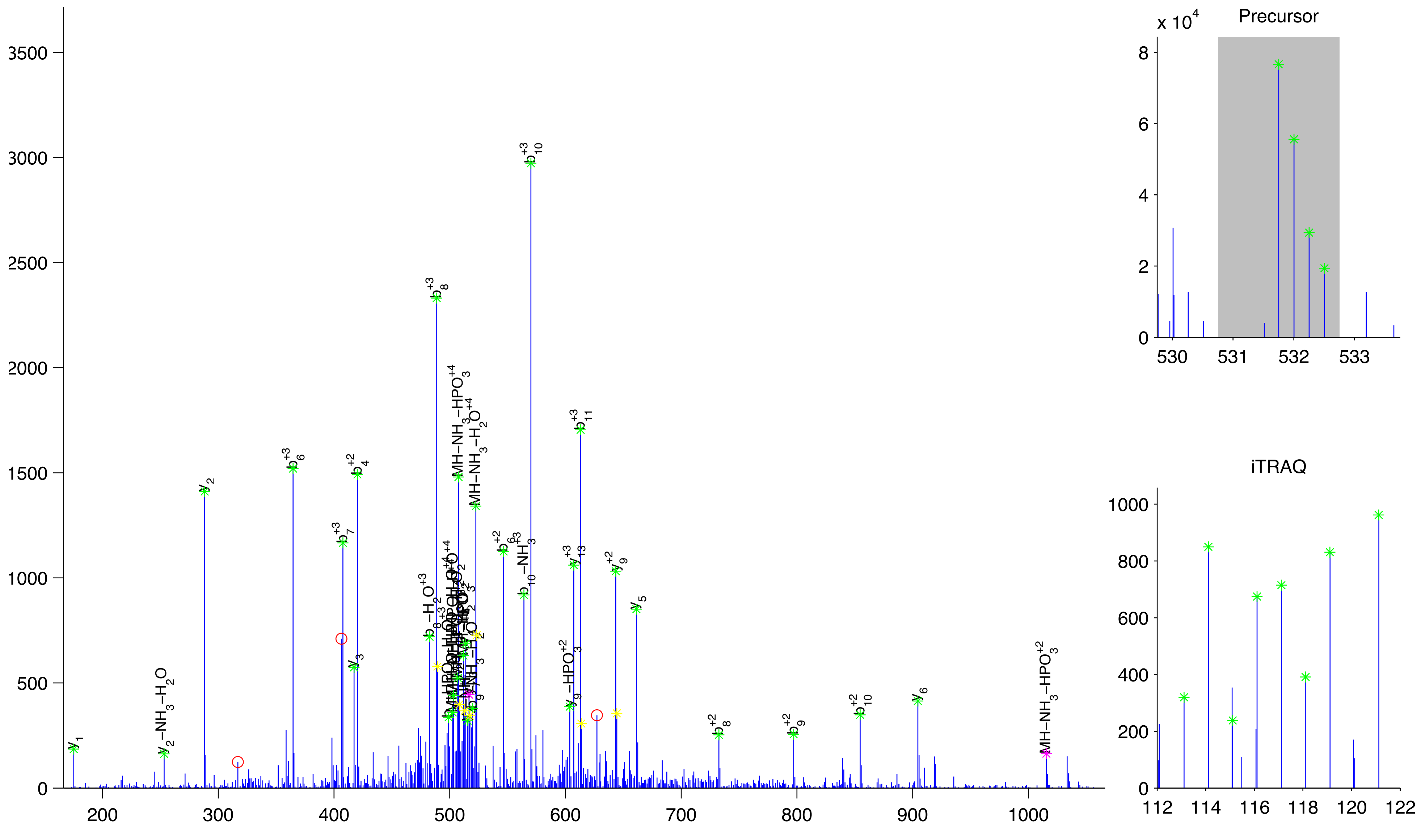

S L D N N y S T P N E R

catenin, delta 1 isoform 1A [Homo sapiens]
Charge State: +2
Scan Number: 4222
File Name: 120527_A549_TSAEGF_pY34_el.raw

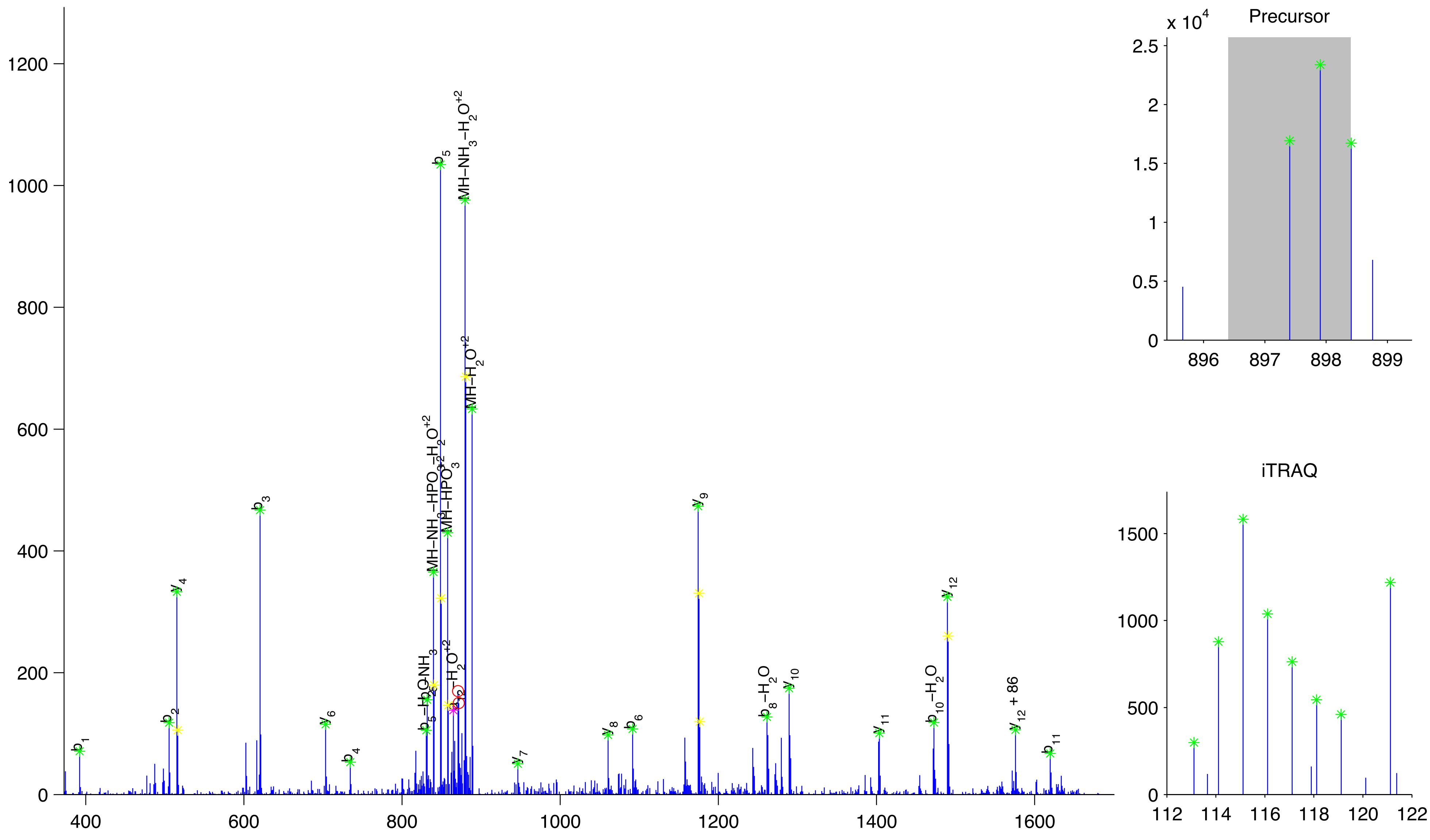

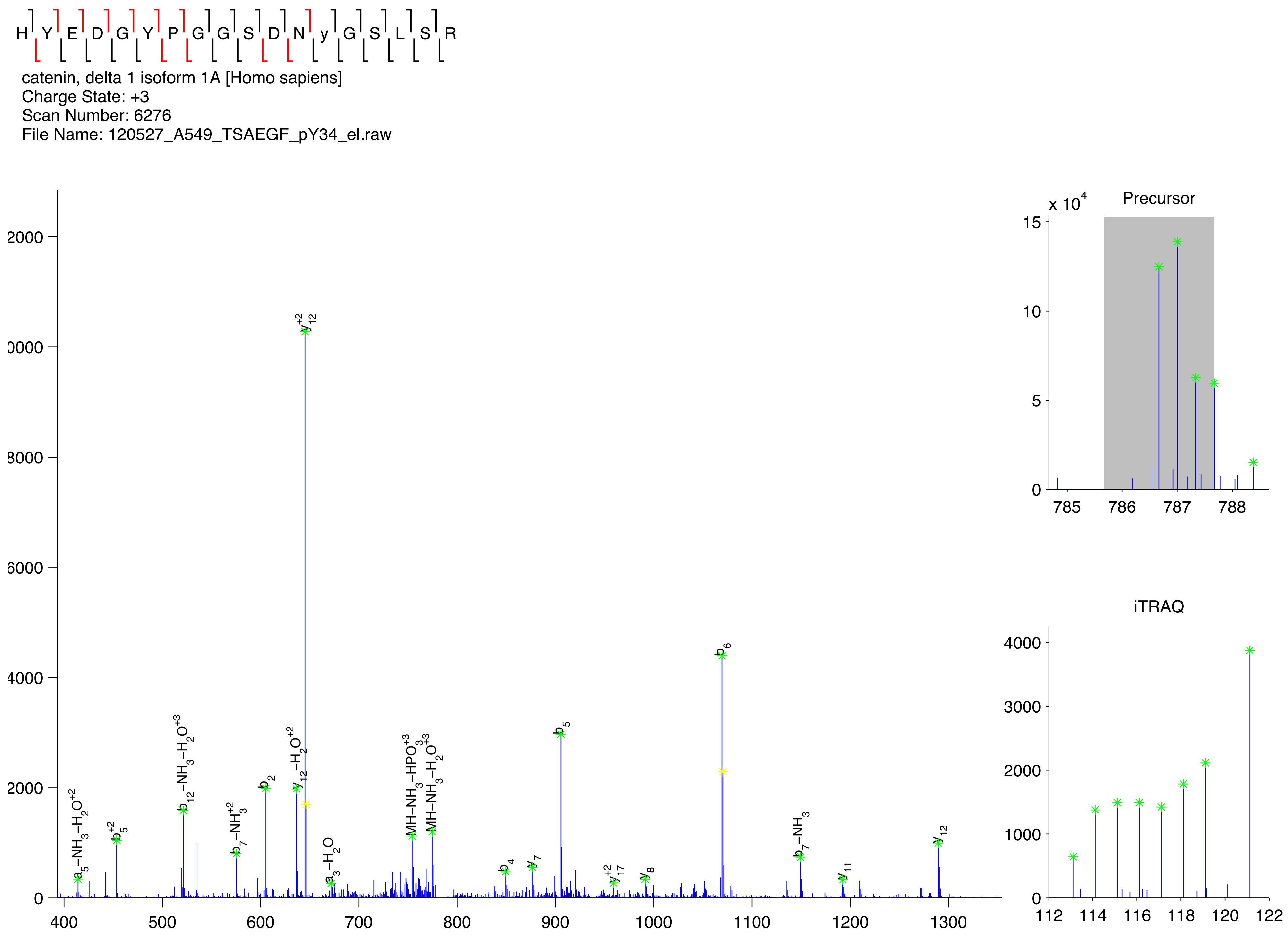
H Y E D G Y P G G S D N y G S L S R
catenin, delta 1 isoform 1A [Homo sapiens]
Charge State: +3
Scan Number: 6276
File Name: 120527_A549_TSAEGF_pY34_el.raw
Precursor
x 10^4
iTRAQ

I G E G T y G V V Y K

cell division cycle 2 protein isoform 1 [Homo sapiens]
Charge State: +4
Scan Number: 10442
File Name: 120527_A549_TSAEGF_pY34_el.raw

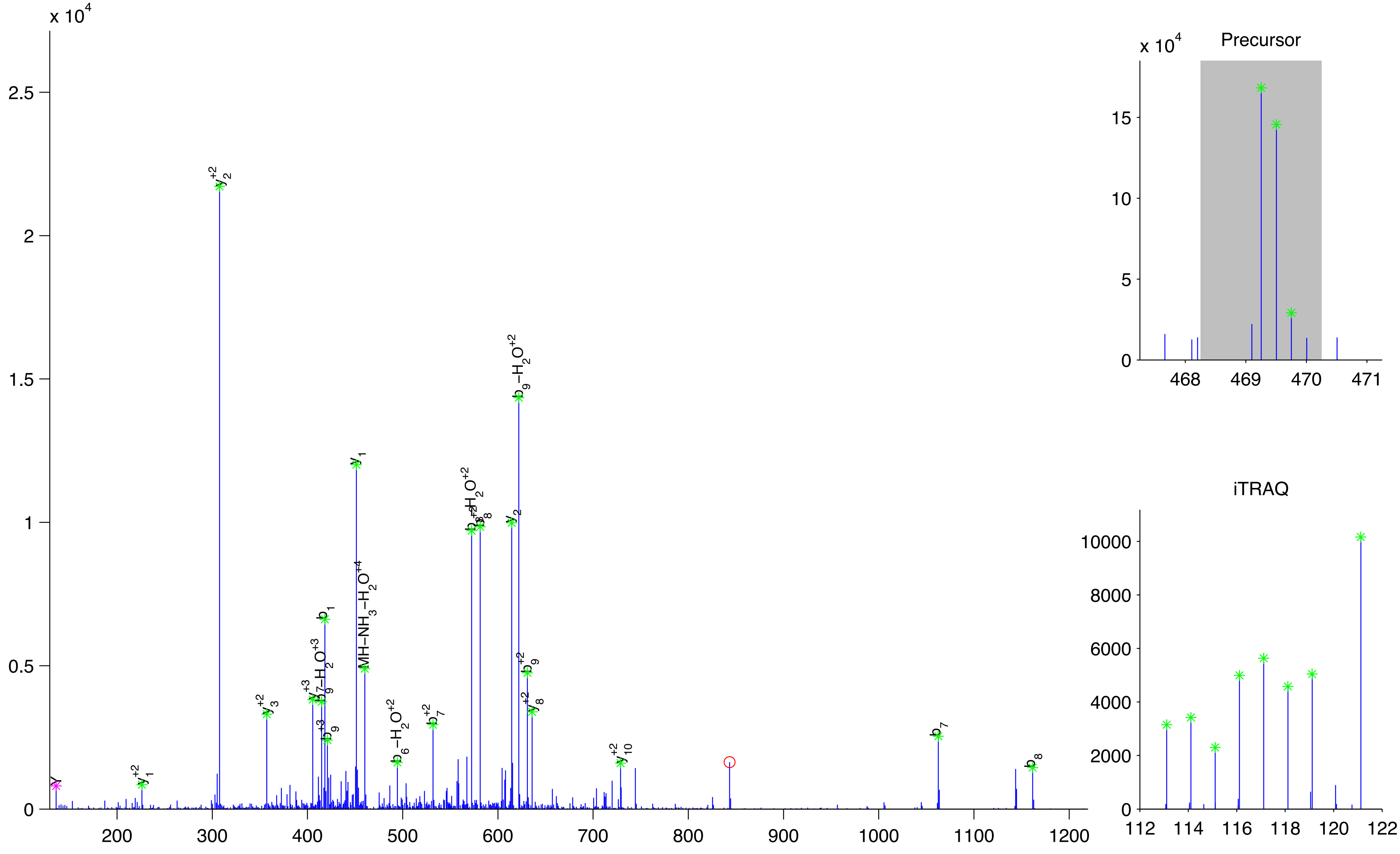

I G E G T y G V V Y K

cell division cycle 2 protein isoform 1 [Homo sapiens]
Charge State: +3
Scan Number: 18529
File Name: 120518_A549_EGFTSA_pY.raw

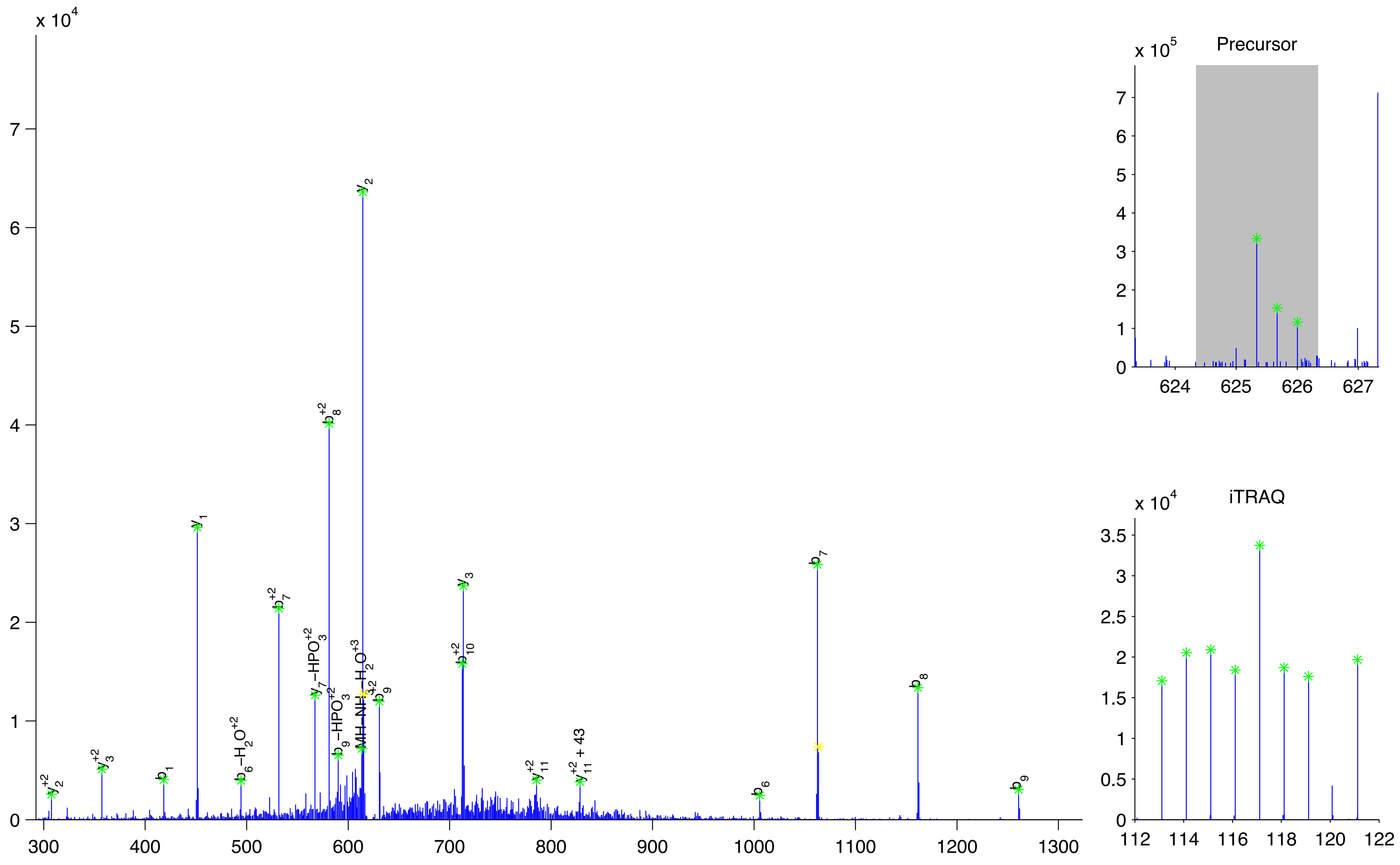

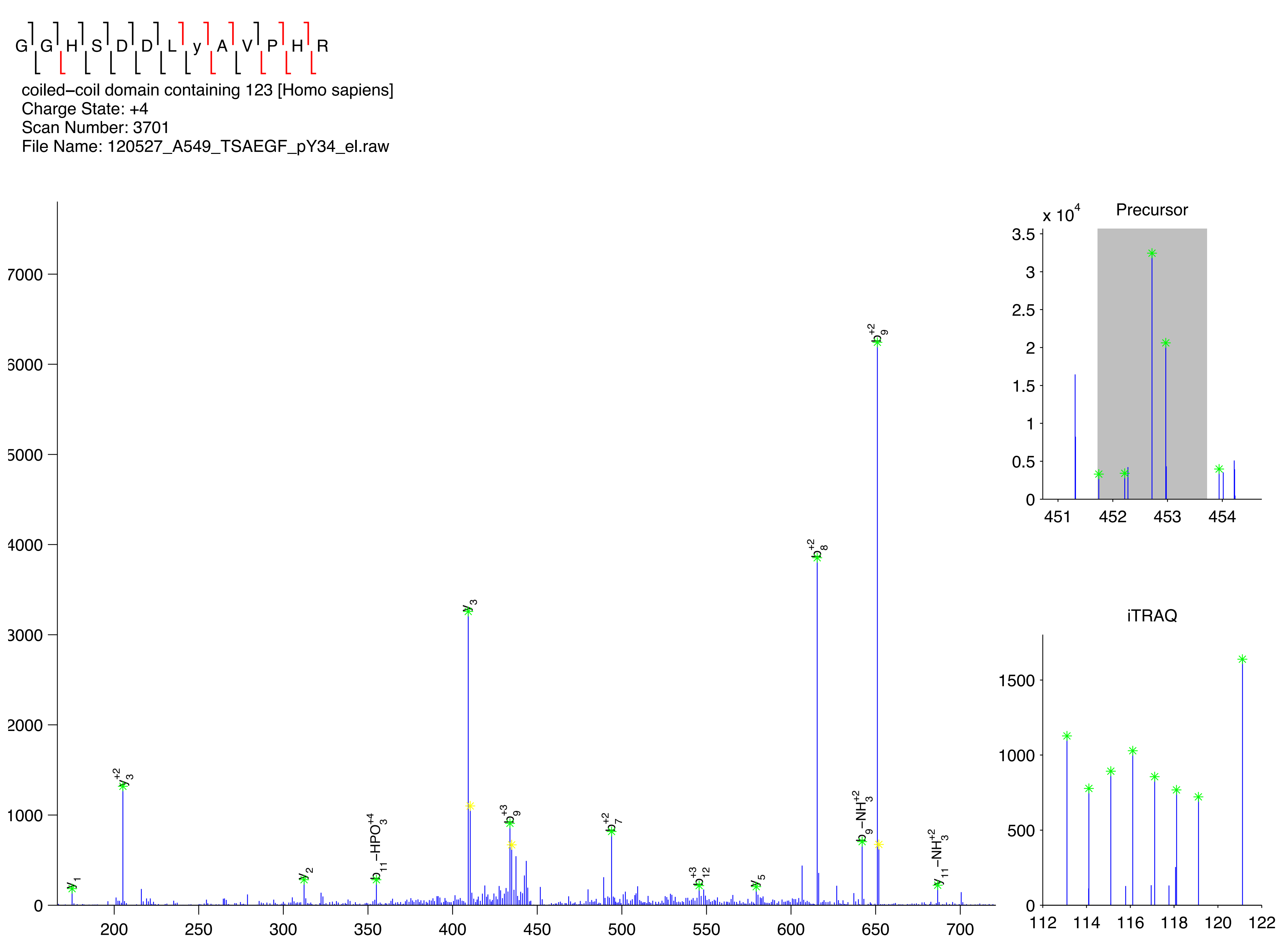
G G H S D D L y A V P H R
coiled–coil domain containing 123 [Homo sapiens]
Charge State: +4
Scan Number: 3701
File Name: 120527_A549_TSAEGF_pY34_el.raw
y_1
y_3^{+2}
y_2
$b_{11}-HPO_3^{+4}$
y_3
b_9^{+3}
b_7^{+2}
b_{12}^{+3}
y_5
b_8^{+2}
$b_9-NH_3^{+2}$
b_9^{+2}
$y_{11}-NH_3^{+2}$
Precursor
x 10^4
iTRAQ

Q A S E Q N W A N y S A E Q N R

connexin 43 [Homo sapiens]
Charge State: +3
Scan Number: 13049
File Name: 120518_A549_EGFTSA_pY.raw

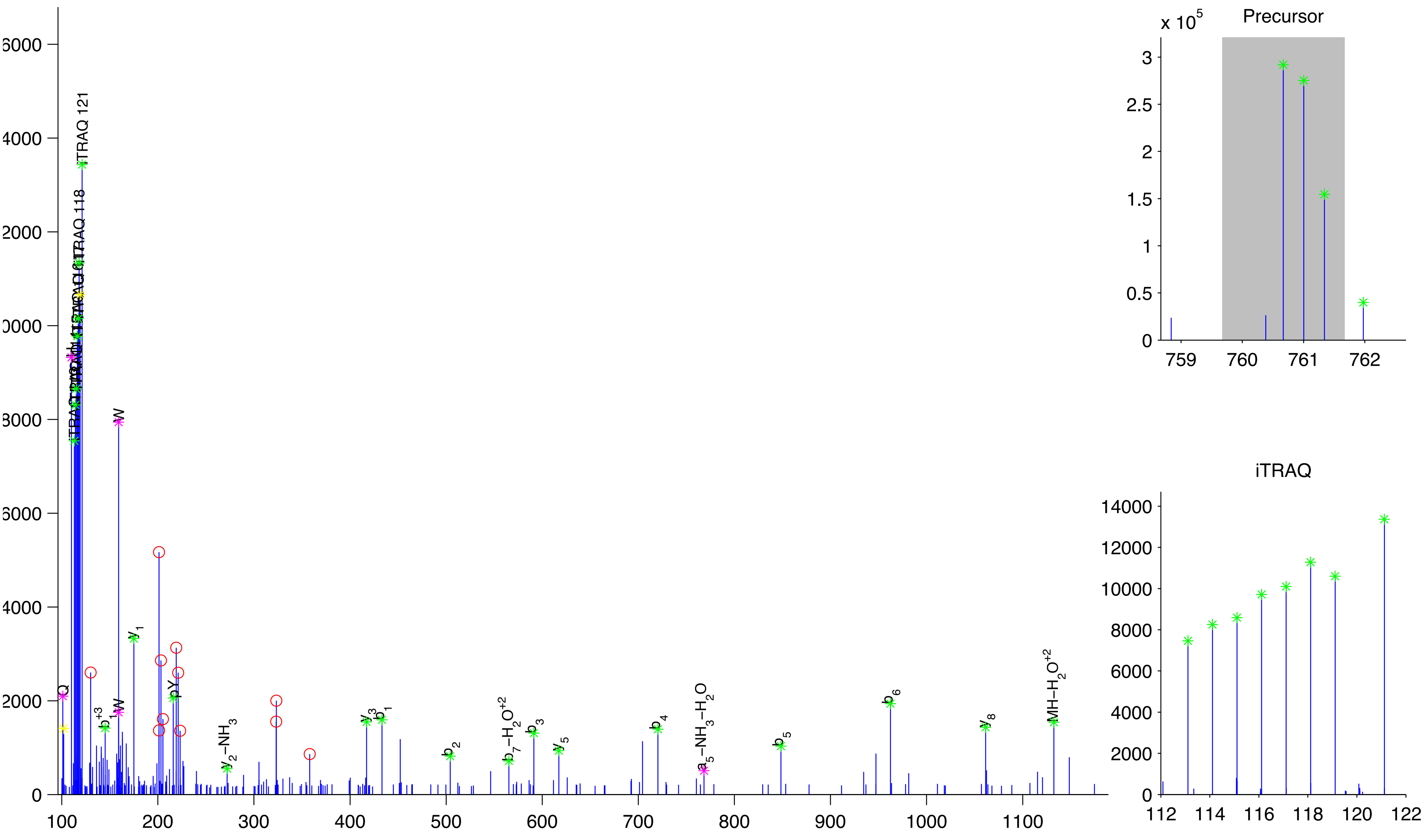

T Q T P P V S P A P Q P T E E R L P S S P V y E D A A S F K

cortactin isoform a [Homo sapiens]
Charge State: +4
Scan Number: 11442
File Name: 120527_A549_TSAEGF_pY34_el.raw

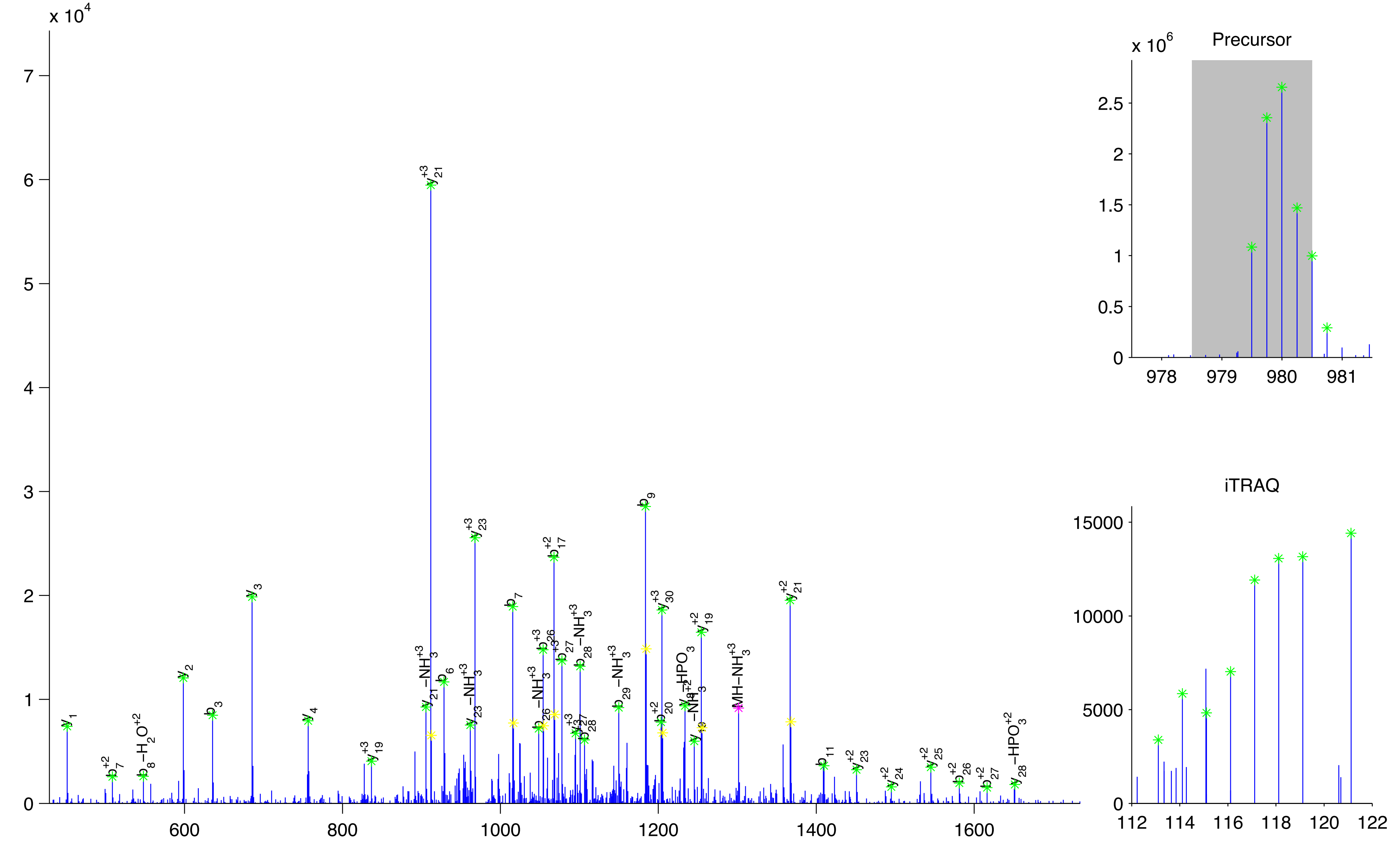

G P V S G T E P E P V Y S M E A A D y R

cortactin isoform a [Homo sapiens]
Charge State: +2
Scan Number: 12069
File Name: 120527_A549_TSAEGF_pY34_el.raw

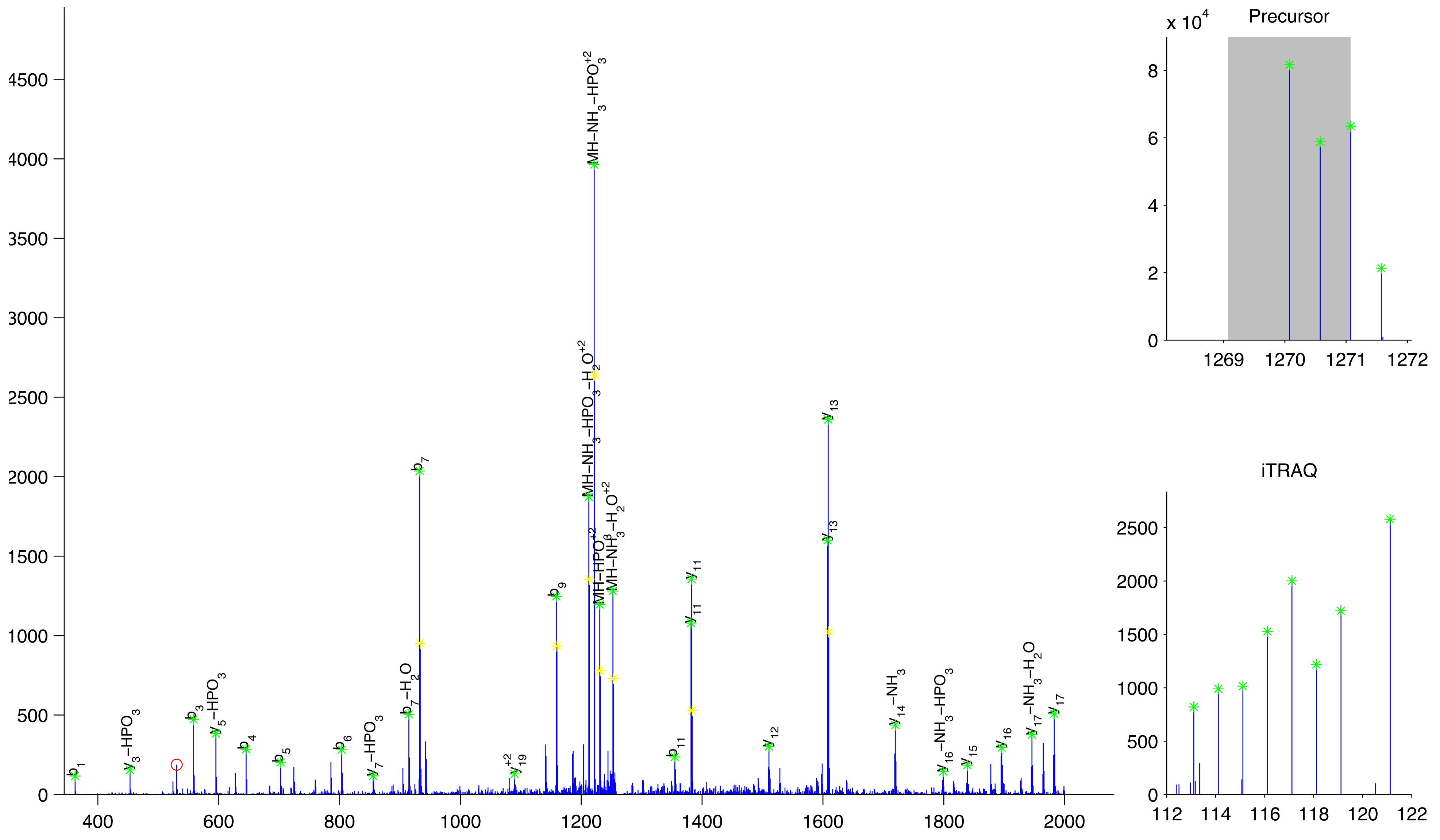

G P V S G T E P E P V Y S M E A A D y R

cortactin isoform a [Homo sapiens]
Charge State: +3
Scan Number: 12240
File Name: 120527_A549_TSAEGF_pY34_el.raw

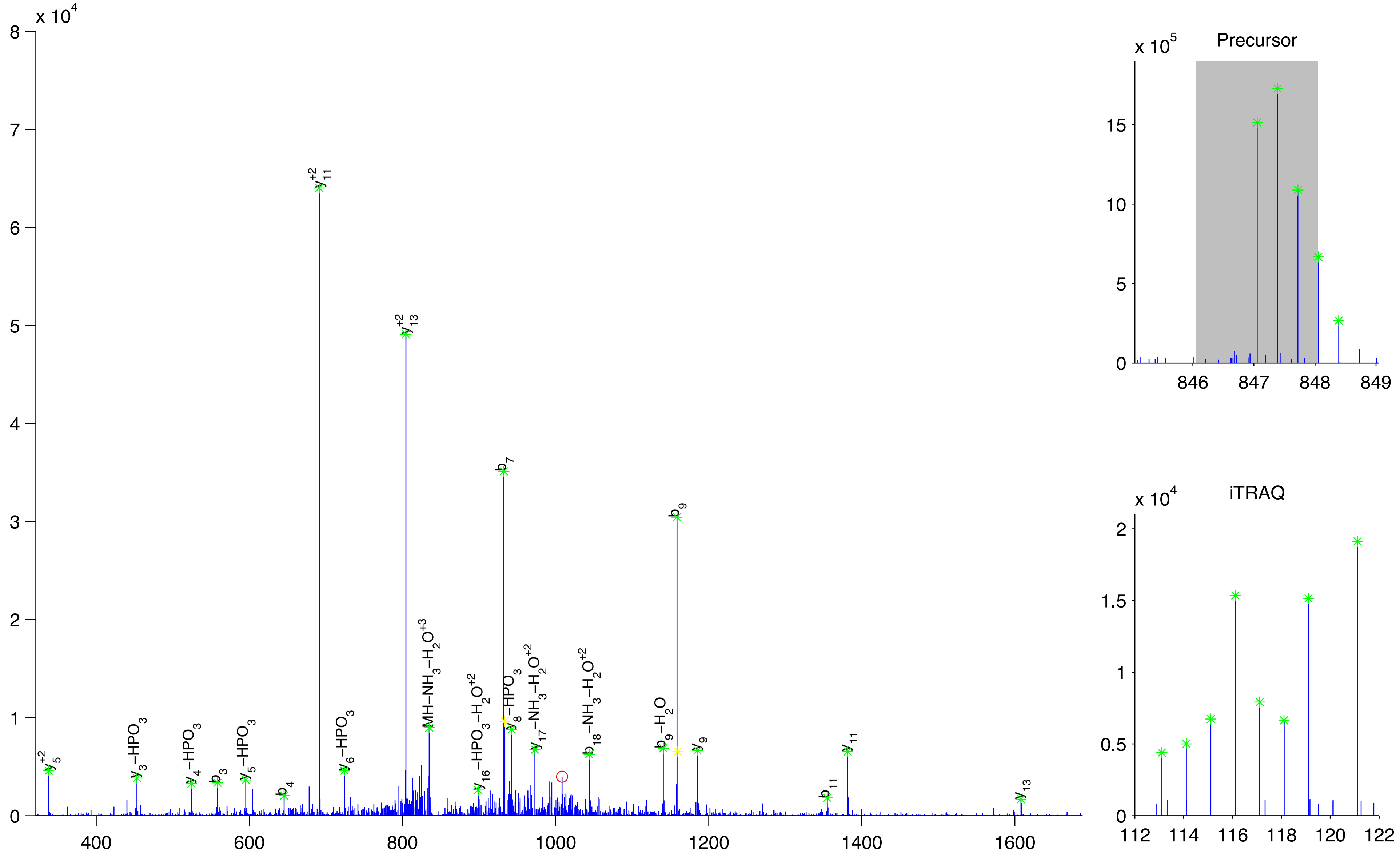

G P V S G T E P E P V y S m E A A D Y R

cortactin isoform a [Homo sapiens]
Charge State: +3
Scan Number: 16639
File Name: 120518_A549_EGFTSA_pY.raw

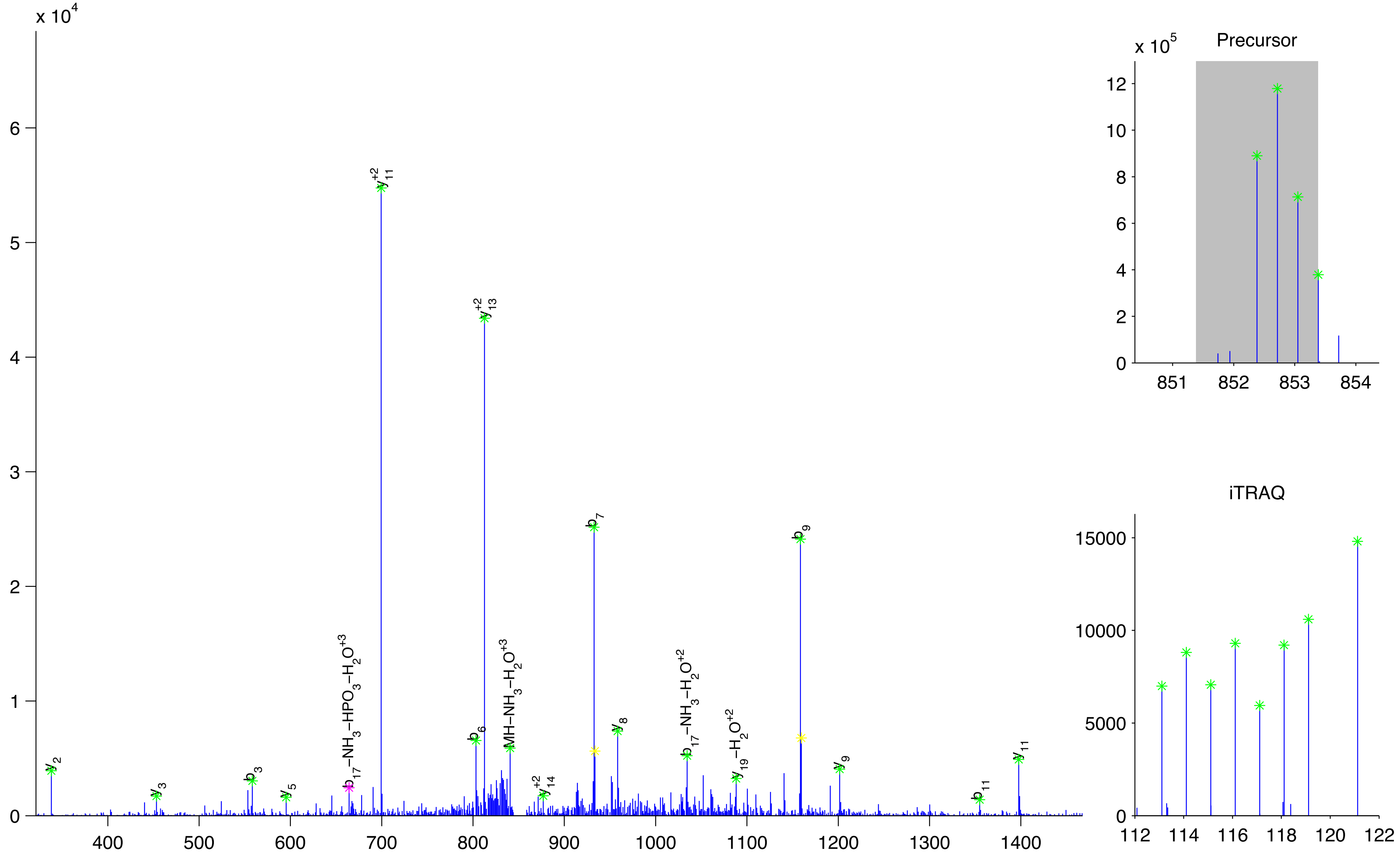

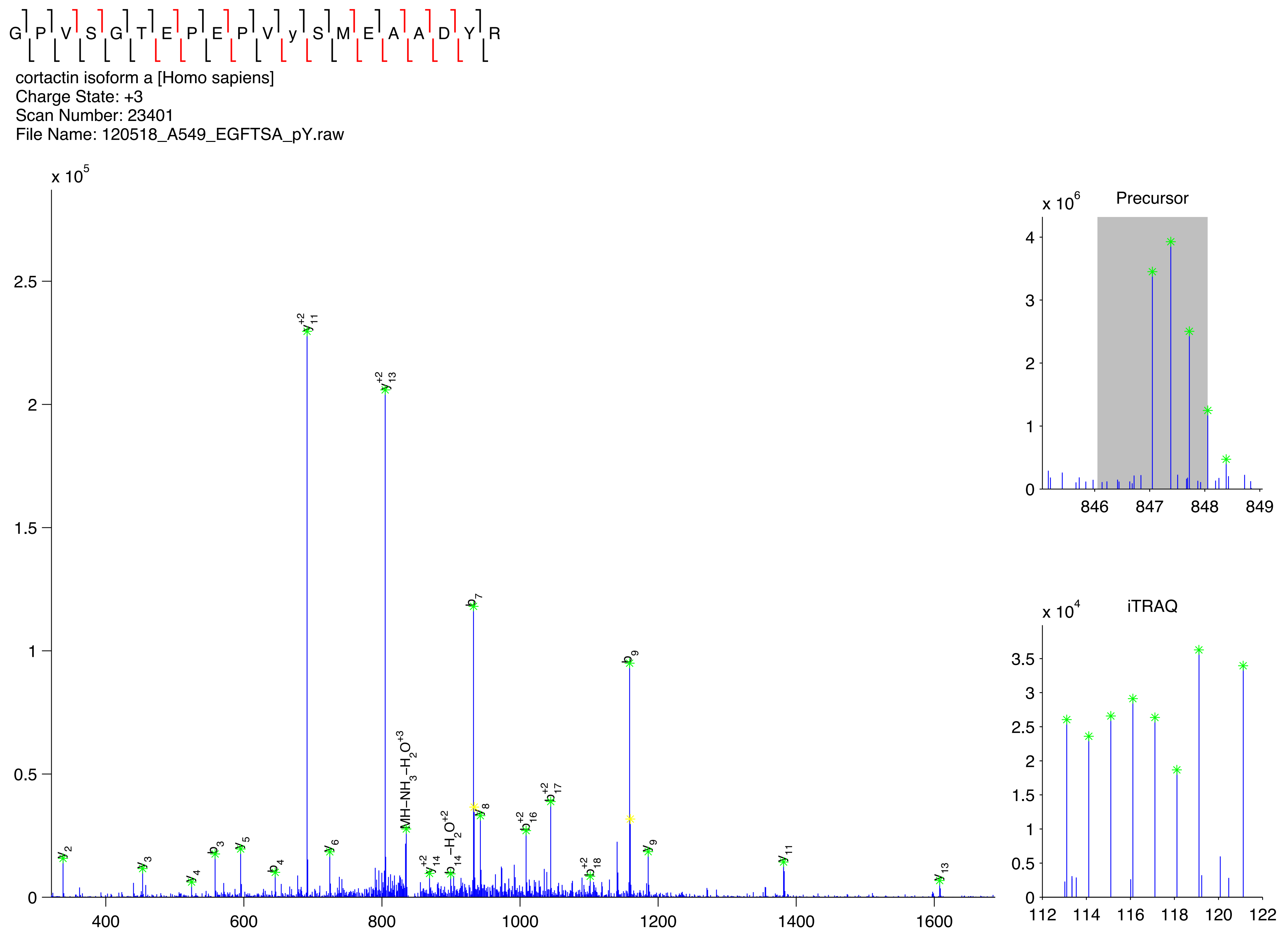
G P V S G T E P E P V y S M E A A D Y R
cortactin isoform a [Homo sapiens]
Charge State: +3
Scan Number: 23401
File Name: 120518_A549_EGFTSA_pY.raw
x 10^5
y2 y3 y4 b3 y5 b4 y11+2 y6 y13+2 MH−NH3−H2O+3 y14+2 b14−H2O+2 b7 y8 b16+2 b17+2 b18+2 b9 y9 y11 y13
x 10^6
Precursor
x 10^4
iTRAQ

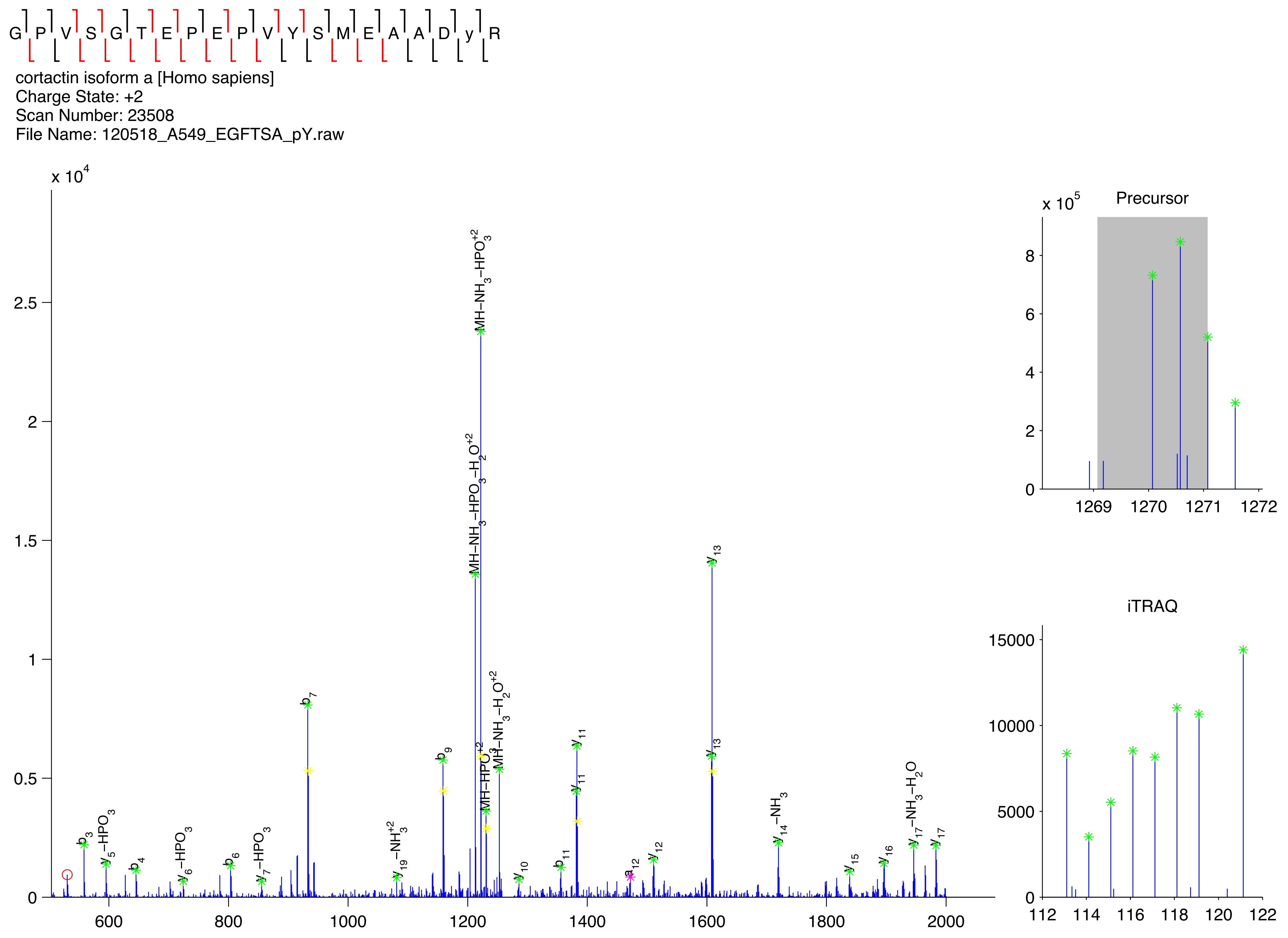
G P V S G T E P E P V Y S M E A A D y R
cortactin isoform a [Homo sapiens]
Charge State: +2
Scan Number: 23508
File Name: 120518_A549_EGFTSA_pY.raw
x 10^4
b3
y5–HPO3
b4
y6–HPO3
b6
y7–HPO3
b7
y19–NH3+2
b9
MH–NH3–HPO3–H2O+2
MH–NH3–HPO3+2
MH–HPO3+2
MH–NH3–H2O+2
y10
b11
y11
y11
a12
y12
y13
y13
y14–NH3
y15
y16
y17–NH3–H2O
y17
x 10^5
Precursor
iTRAQ

T Q T P P V S P A P Q P T E E R L P S S P V y E D A A S F K

cortactin isoform a [Homo sapiens]
Charge State: +4
Scan Number: 23590
File Name: 120518_A549_EGFTSA_pY.raw

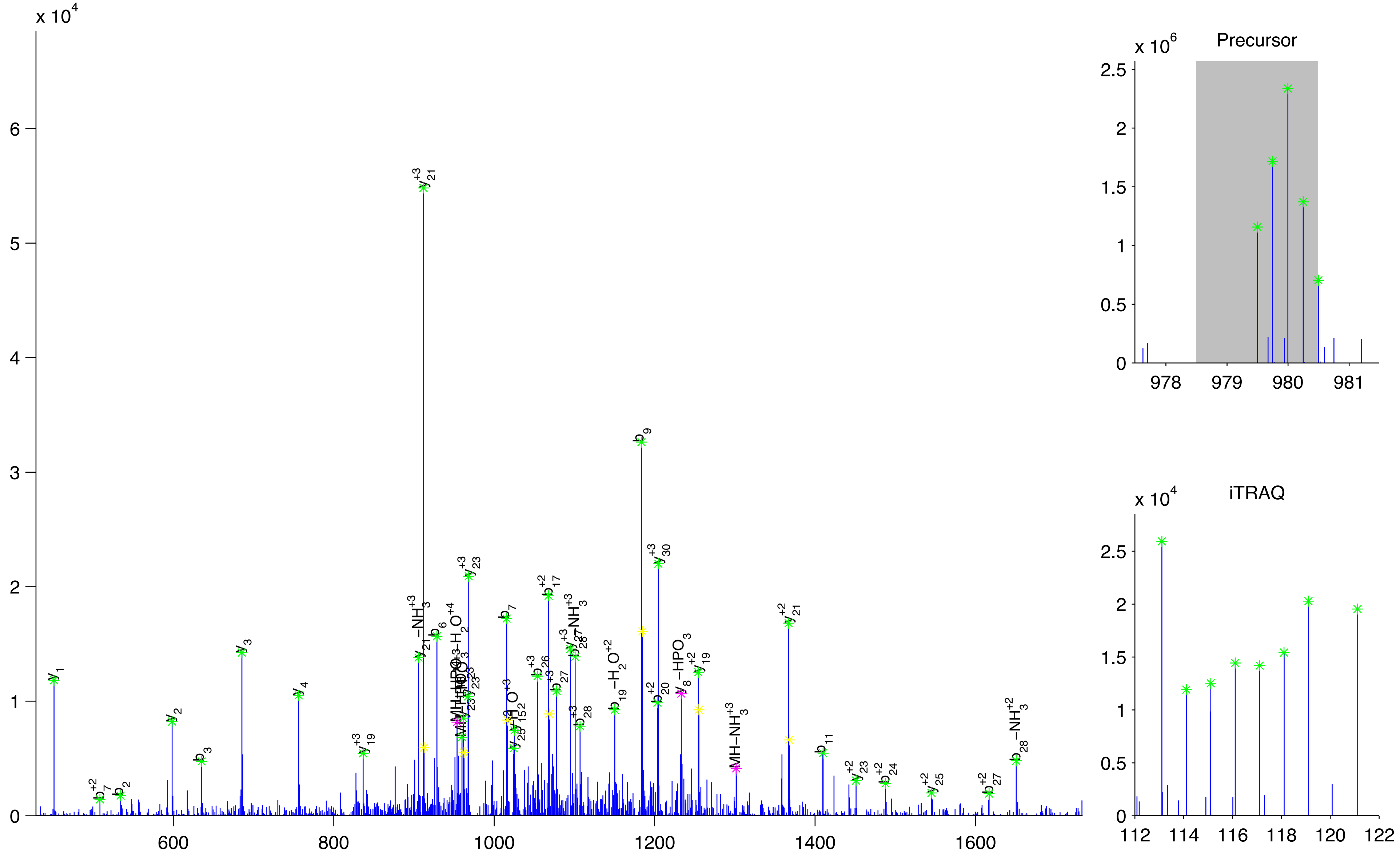

N L S E G N N A N Y T E y V A T R

cyclin–dependent kinase–like 5 [Homo sapiens]
Charge State: +2
Scan Number: 8922
File Name: 120527_A549_TSAEGF_pY34_el.raw

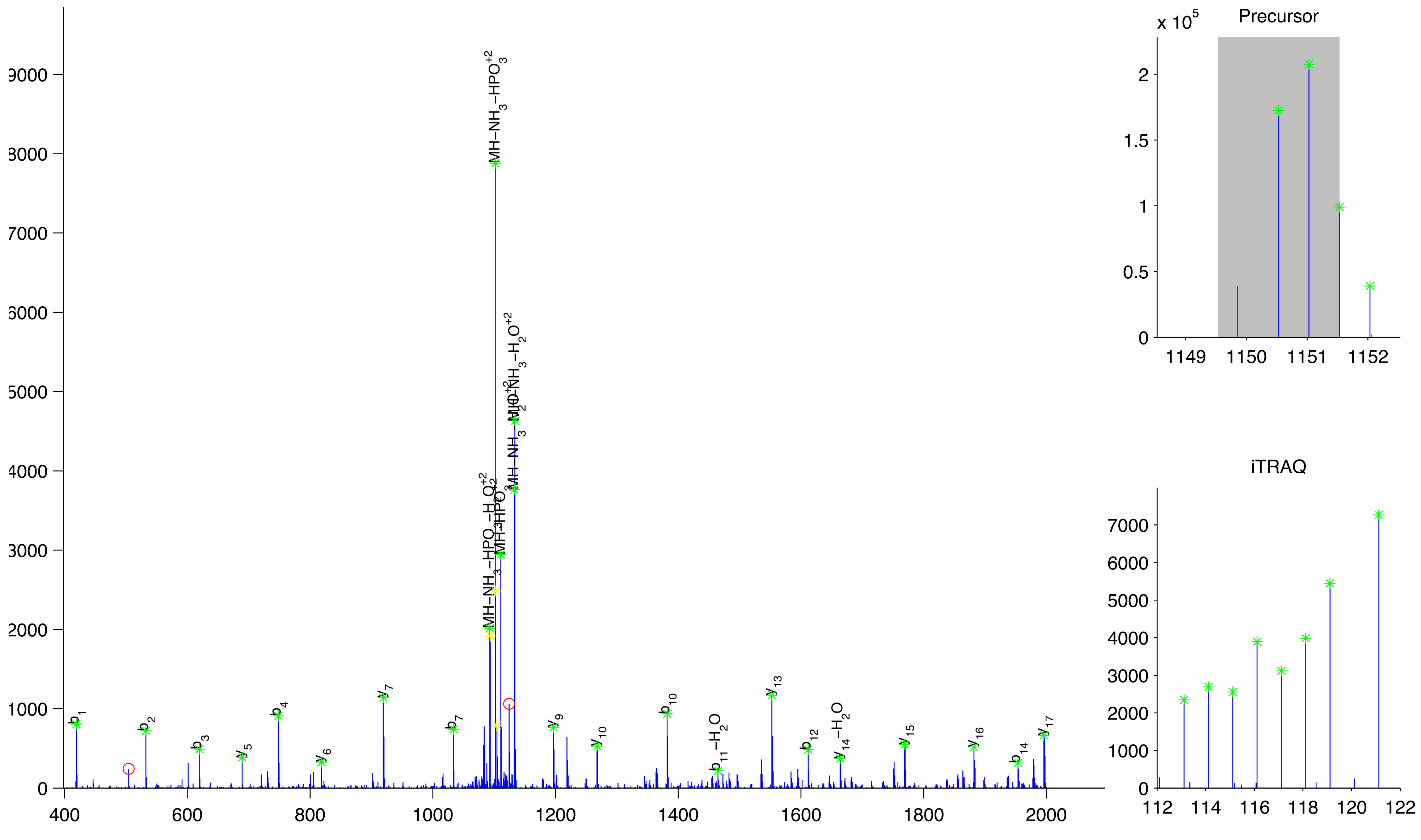

N L S E G N N A N Y T E y V A T R

cyclin–dependent kinase–like 5 [Homo sapiens]
Charge State: +2
Scan Number: 16017
File Name: 120518_A549_EGFTSA_pY.raw

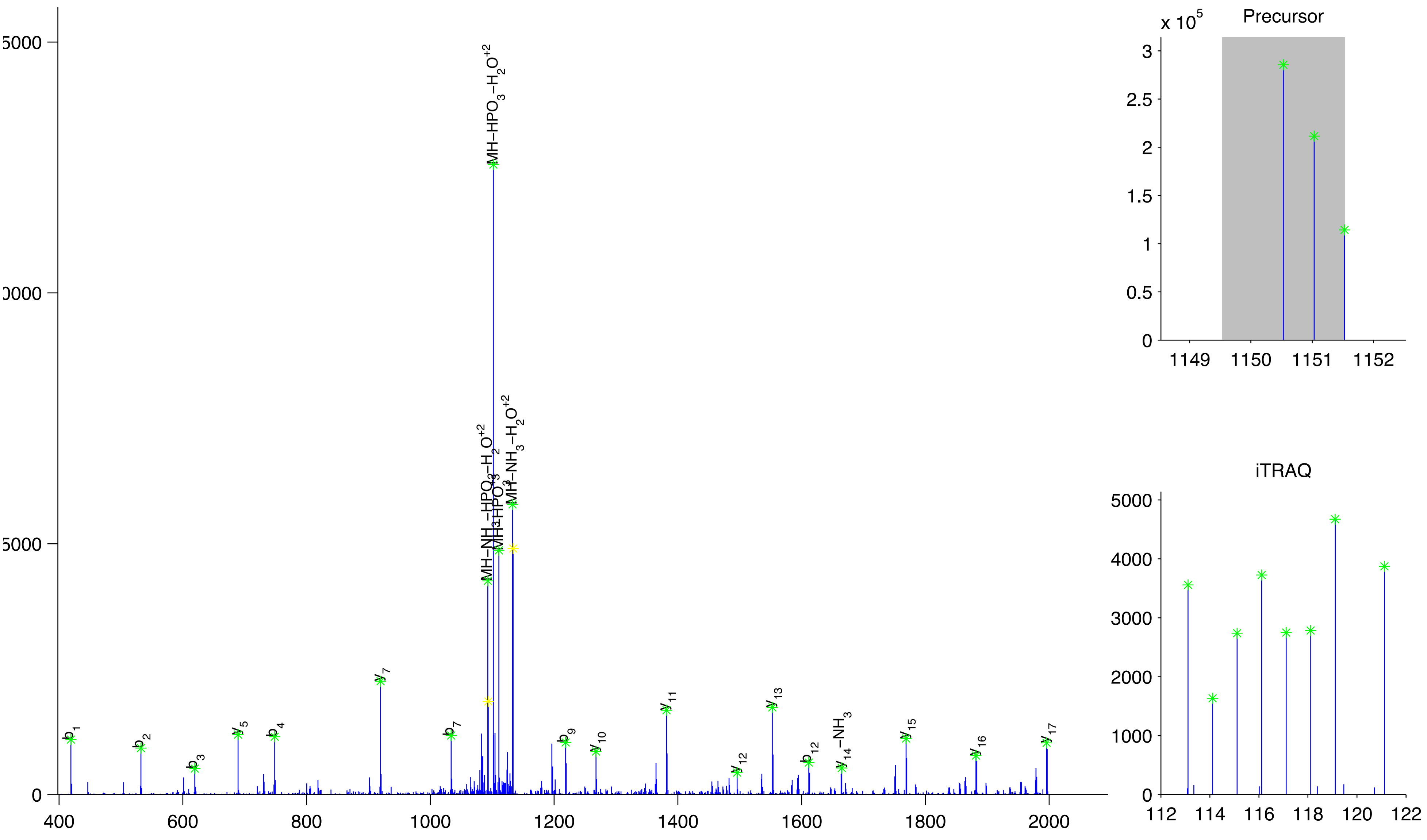

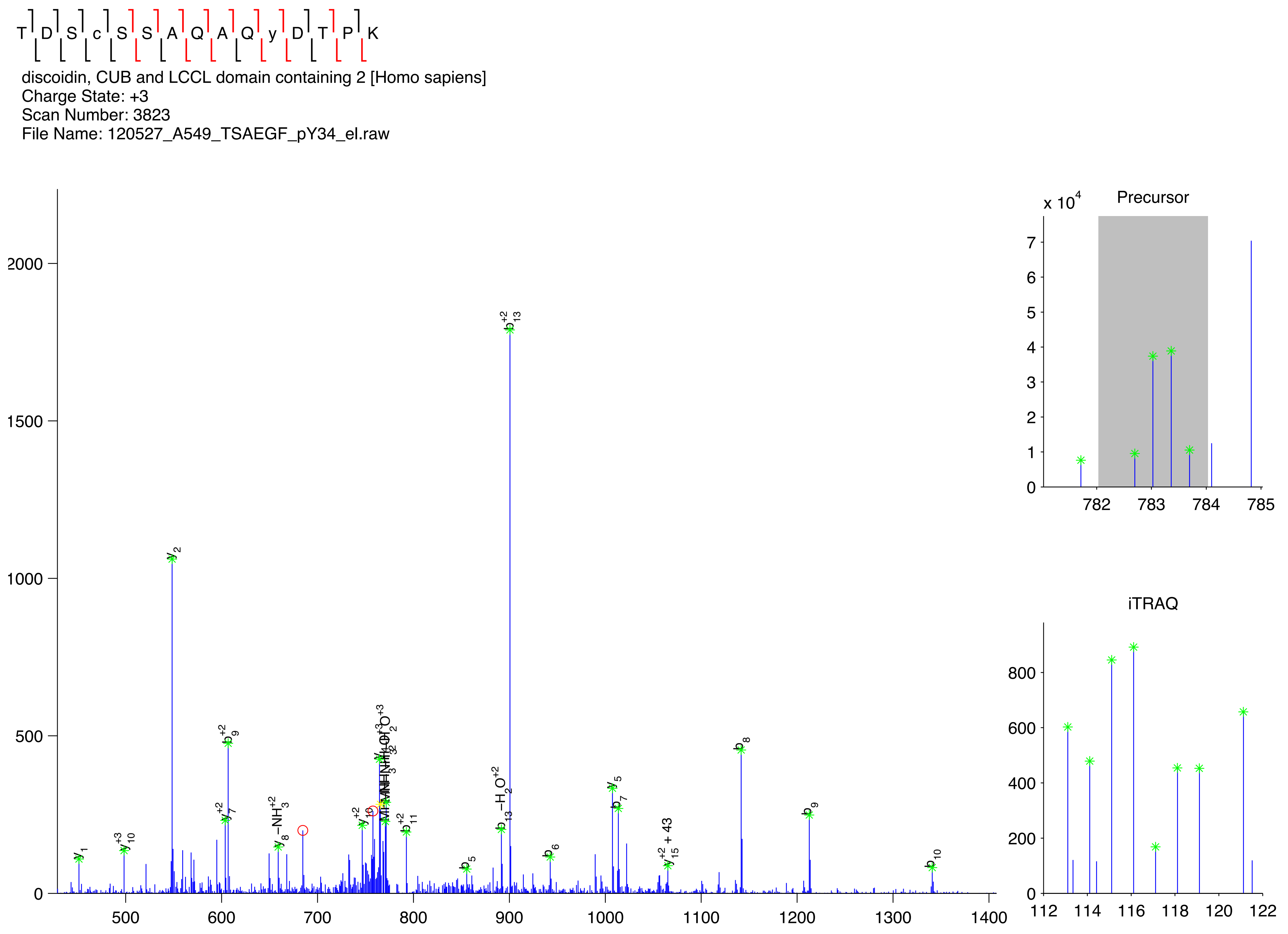
T D S c S S A Q A Q y D T P K
discoidin, CUB and LCCL domain containing 2 [Homo sapiens]
Charge State: +3
Scan Number: 3823
File Name: 120527_A549_TSAEGF_pY34_el.raw
Precursor
x 10^4
iTRAQ

I Y Q y I Q S R

dual–specificity tyrosine–(Y)–phosphorylation regulated kinase 1A isoform 3 [Homo sapiens]
Charge State: +3
Scan Number: 11366
File Name: 120527_A549_TSAEGF_pY34_el.raw

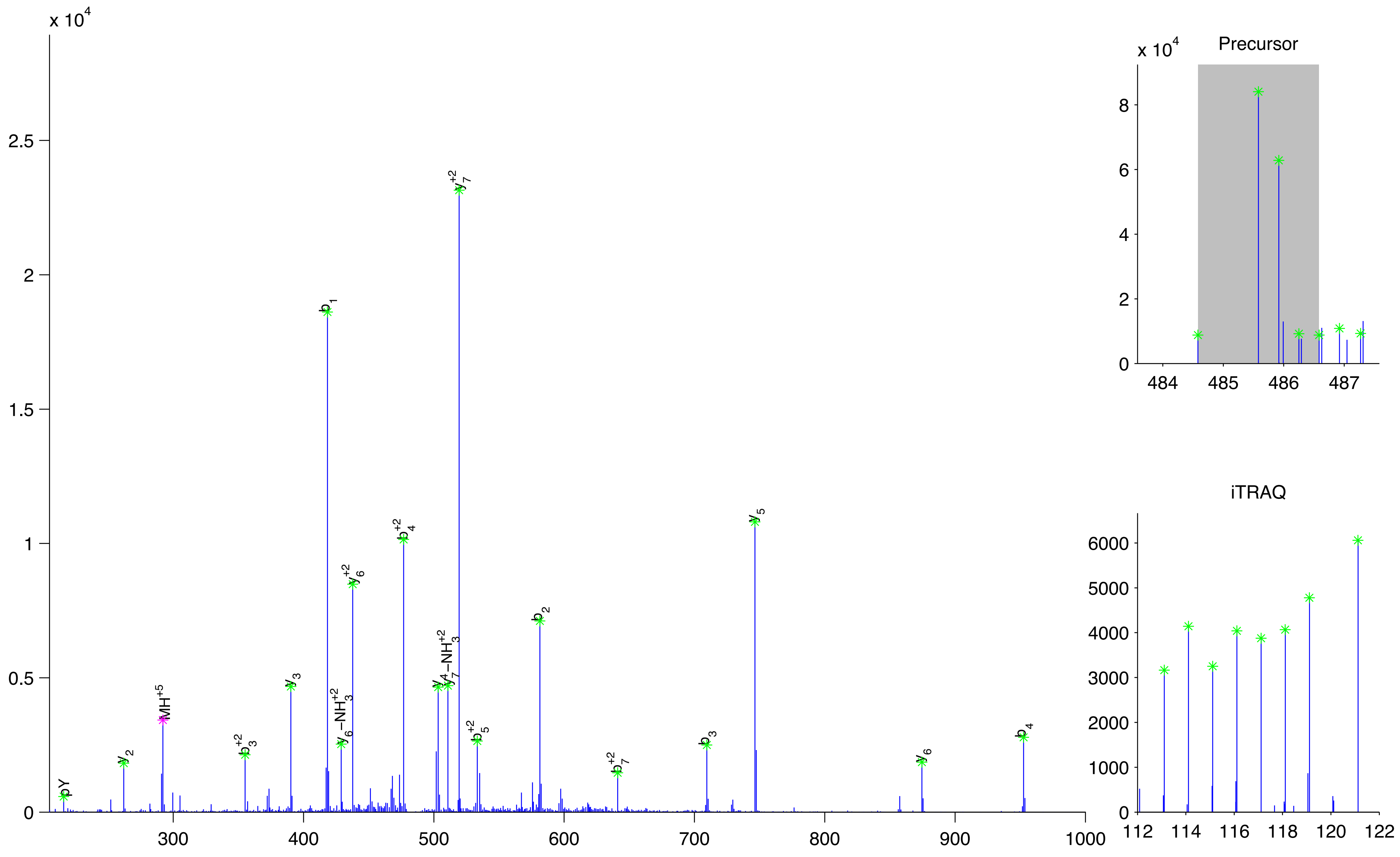

I Y Q y I Q S R

dual–specificity tyrosine–(Y)–phosphorylation regulated kinase 1B isoform a [Homo sapiens]
Charge State: +2
Scan Number: 21910
File Name: 120518_A549_EGFTSA_pY.raw

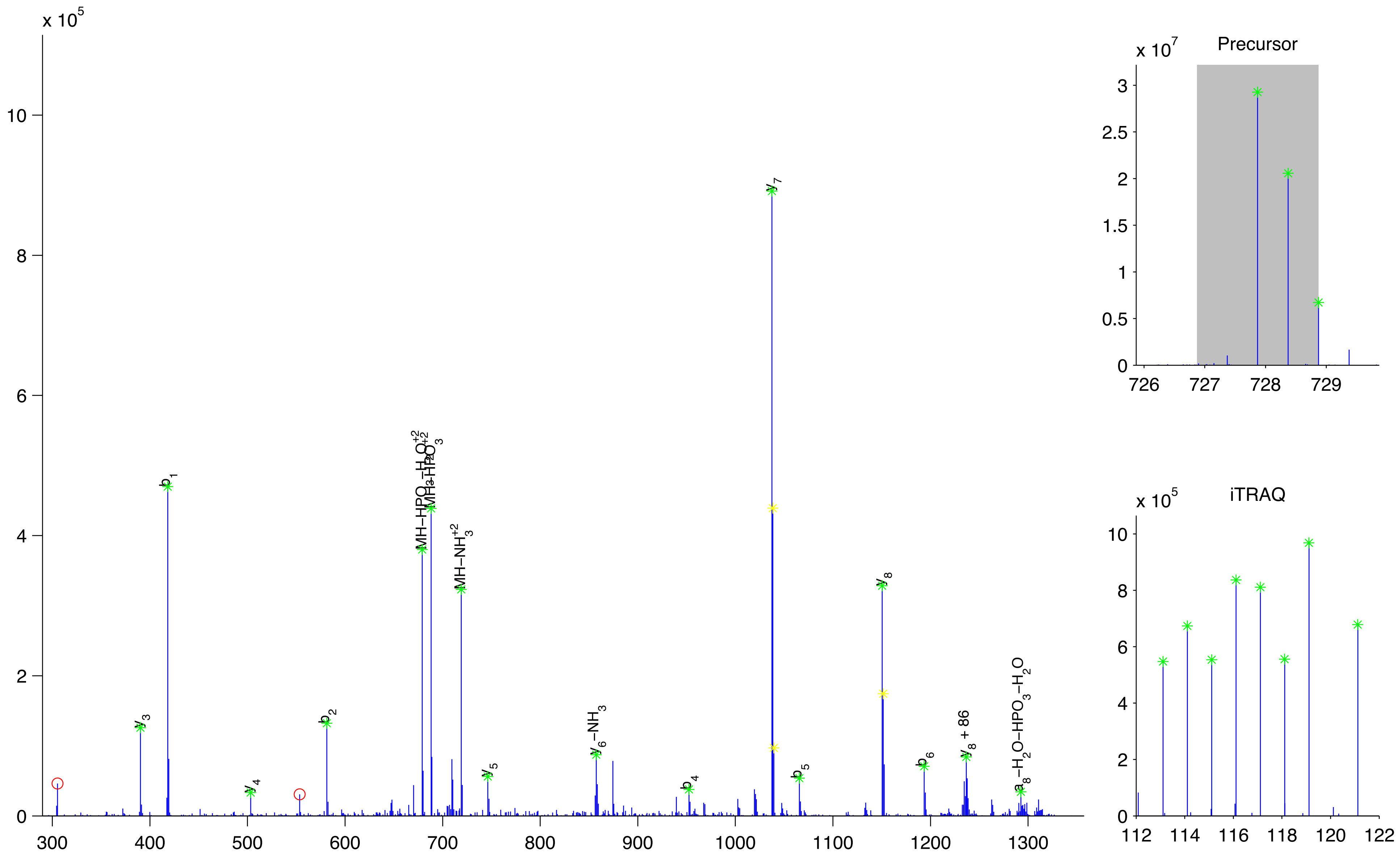

V L E D D P E A T Y T T s G G K

ephrin receptor EphA2 [Homo sapiens]
Charge State: +2
Scan Number: 18041
File Name: 120518_A549_EGFTSA_pY.raw

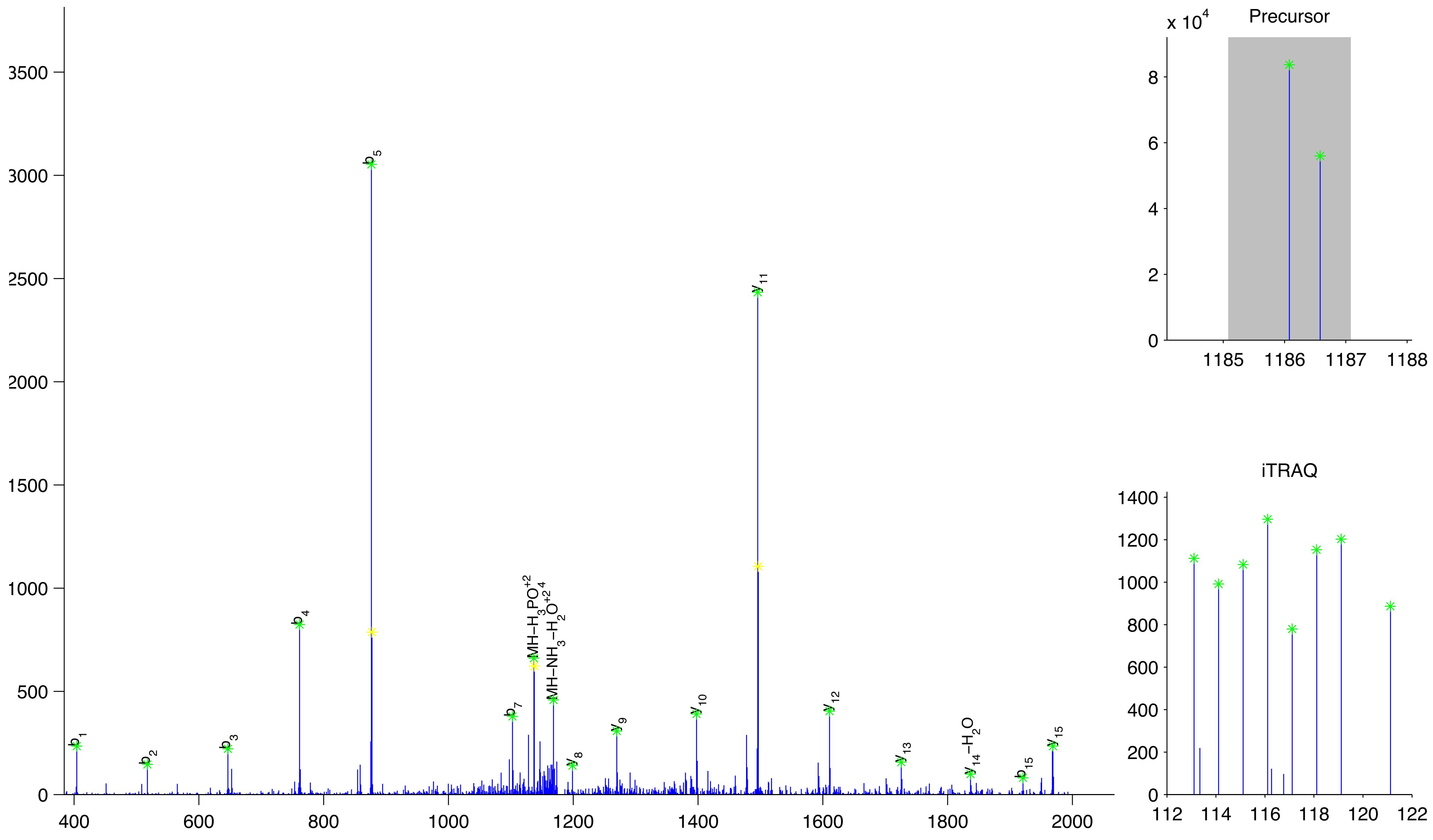

V L E D D P E A T y T T S G G K

ephrin receptor EphA2 [Homo sapiens]
Charge State: +3
Scan Number: 18279
File Name: 120518_A549_EGFTSA_pY.raw

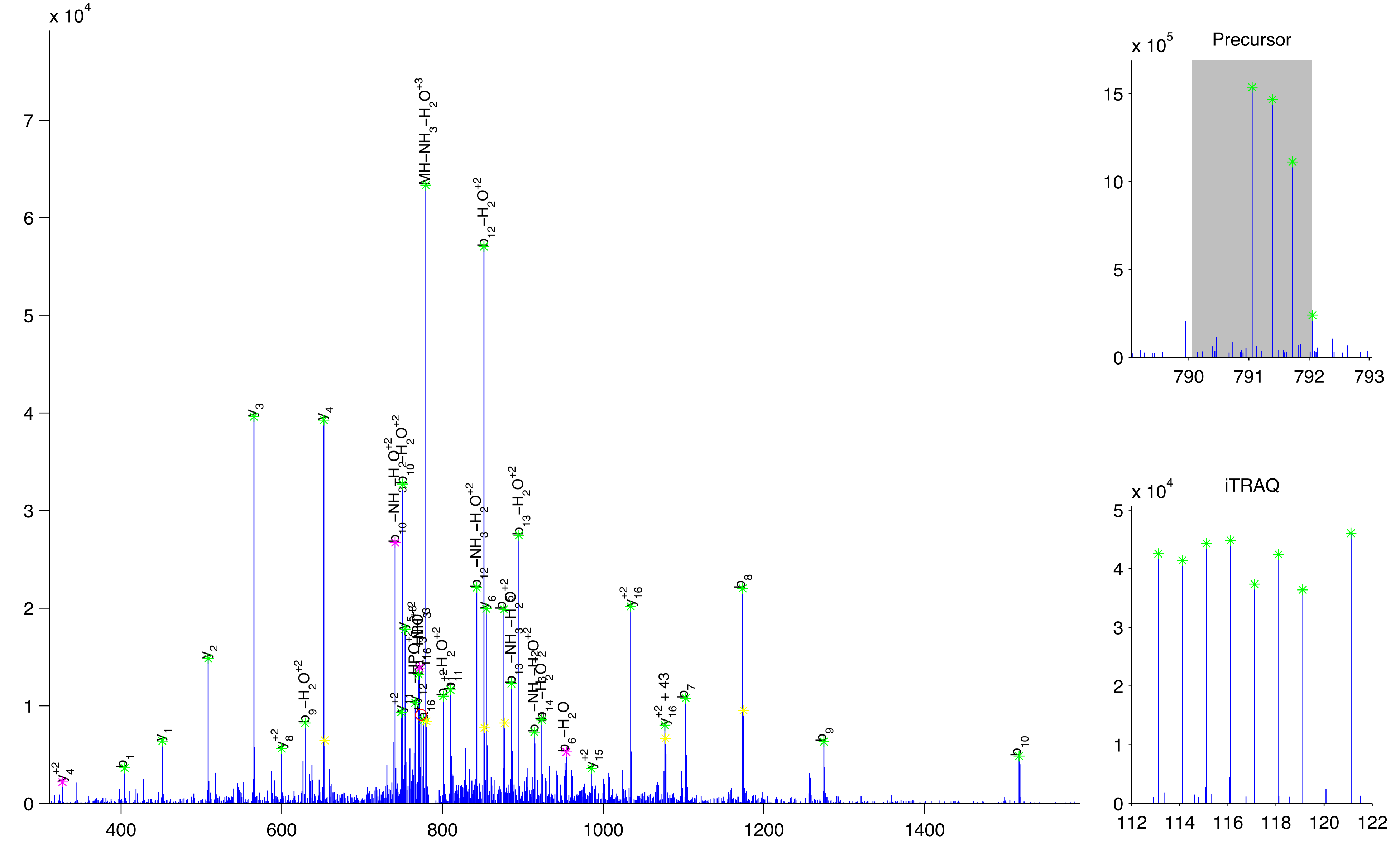

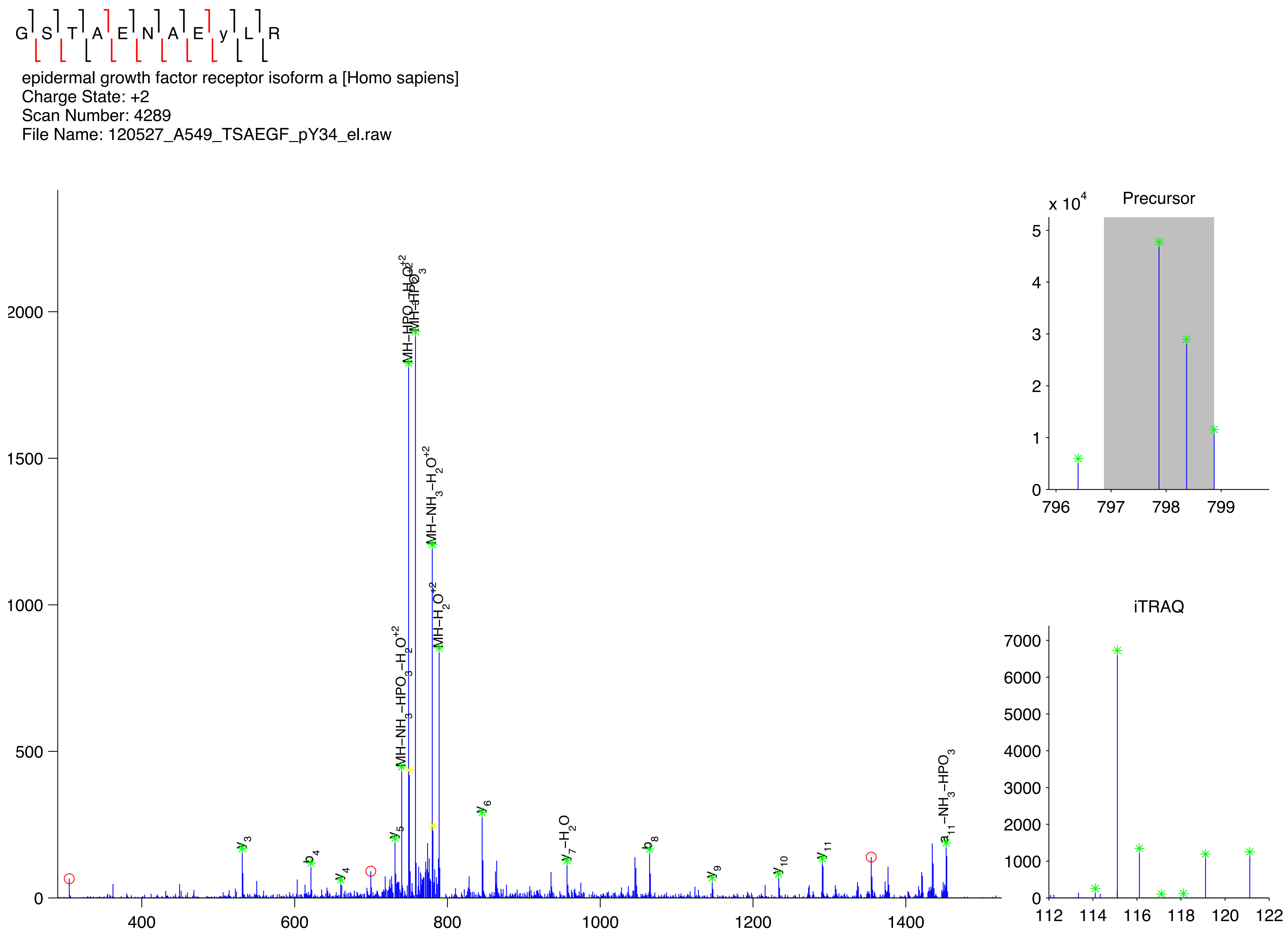

G S T A E N A E y L R
epidermal growth factor receptor isoform a [Homo sapiens]
Charge State: +2
Scan Number: 4289
File Name: 120527_A549_TSAEGF_pY34_el.raw
Precursor
x 10^4
iTRAQ
MH–HPO3–H2O+2
MH–HPO3
MH–NH3–H2O+2
MH–H2O+2
MH–NH3–HPO3–H2O+2
y3
b4
y4
y5
y6
y7–H2O
b8
y9
y10
y11
a11–NH3–HPO3

G S T A E N A E y L R

epidermal growth factor receptor isoform a [Homo sapiens]
Charge State: +3
Scan Number: 4997
File Name: 120527_A549_TSAEGF_pY34_el.raw

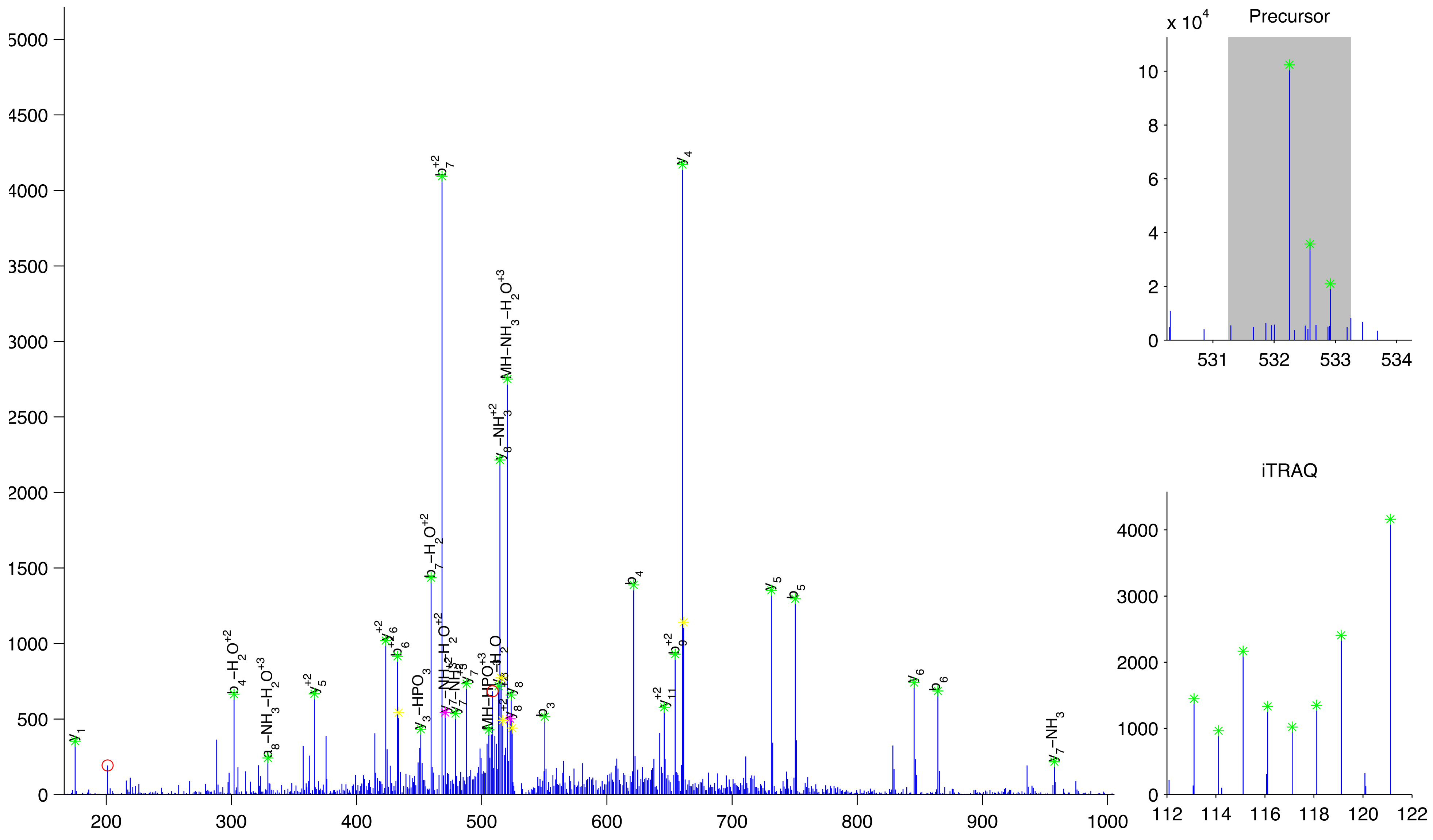

G S T A E N A E y L R

epidermal growth factor receptor isoform a [Homo sapiens]
Charge State: +2
Scan Number: 10532
File Name: 120518_A549_EGFTSA_pY.raw

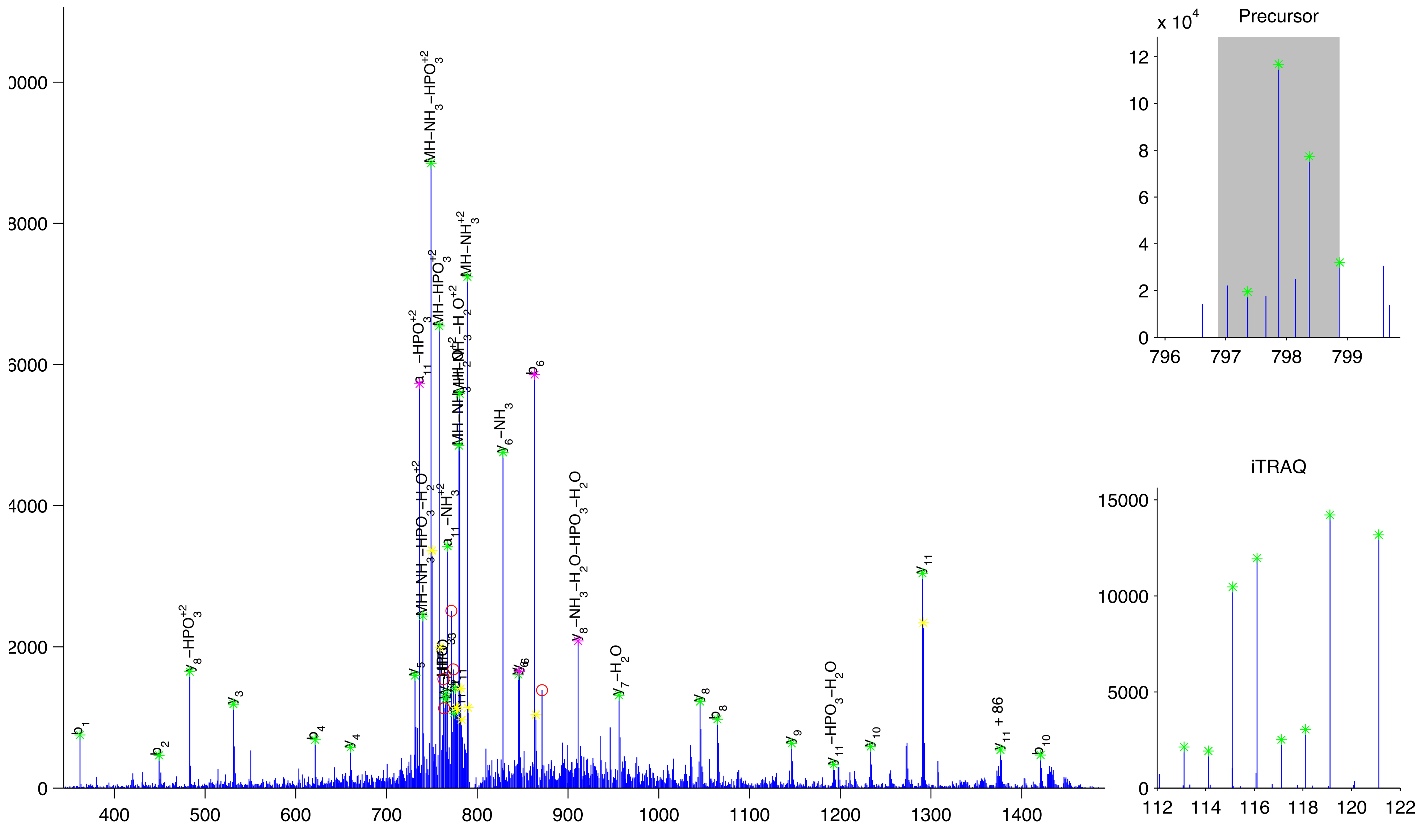

R P A G S V Q N P V y H N Q P L N P A P S R

epidermal growth factor receptor isoform a [Homo sapiens]
Charge State: +4
Scan Number: 11346
File Name: 120518_A549_EGFTSA_pY.raw

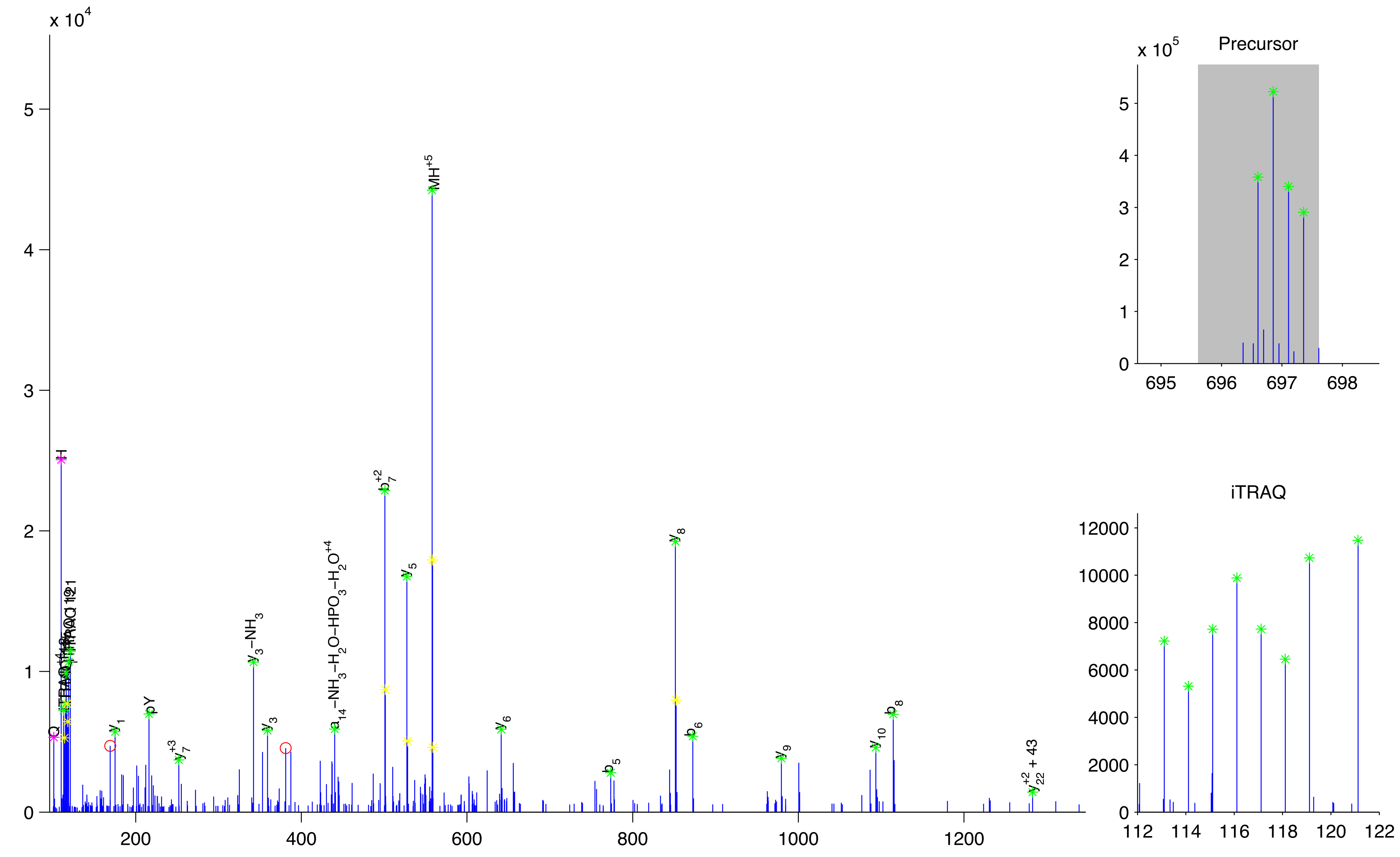

G S H Q I S L D N P D y Q Q D F F P K

epidermal growth factor receptor isoform a [Homo sapiens]
Charge State: +4
Scan Number: 12366
File Name: 120527_A549_TSAEGF_pY34_el.raw

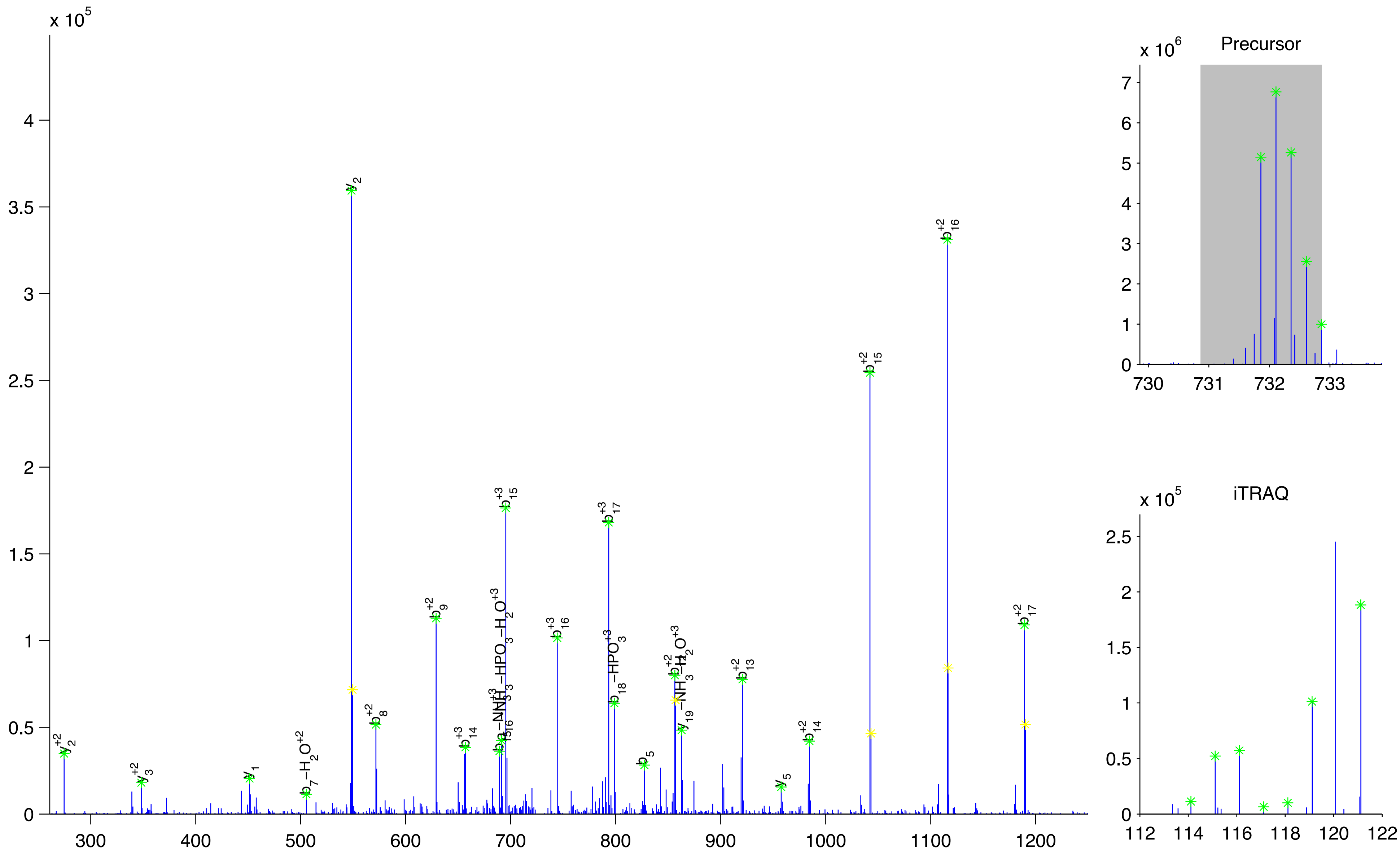

G S H Q I S L D N P D y Q Q D F F P K

epidermal growth factor receptor isoform a [Homo sapiens]
Charge State: +3
Scan Number: 12408
File Name: 120527_A549_TSAEGF_pY34_el.raw

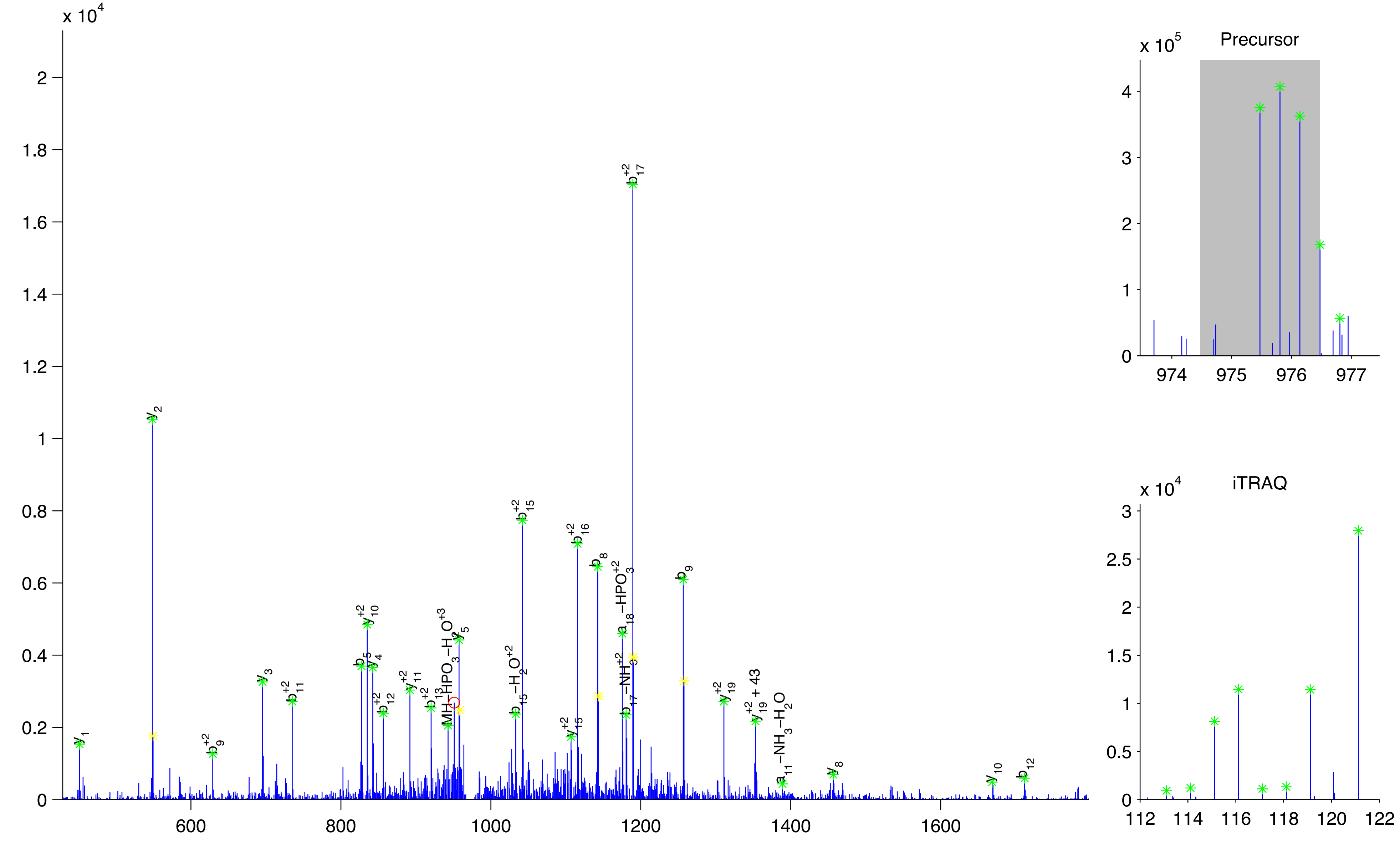

G S H Q I S L D N P D y Q Q D F F P K

epidermal growth factor receptor isoform a [Homo sapiens]
Charge State: +3
Scan Number: 24411
File Name: 120518_A549_EGFTSA_pY.raw

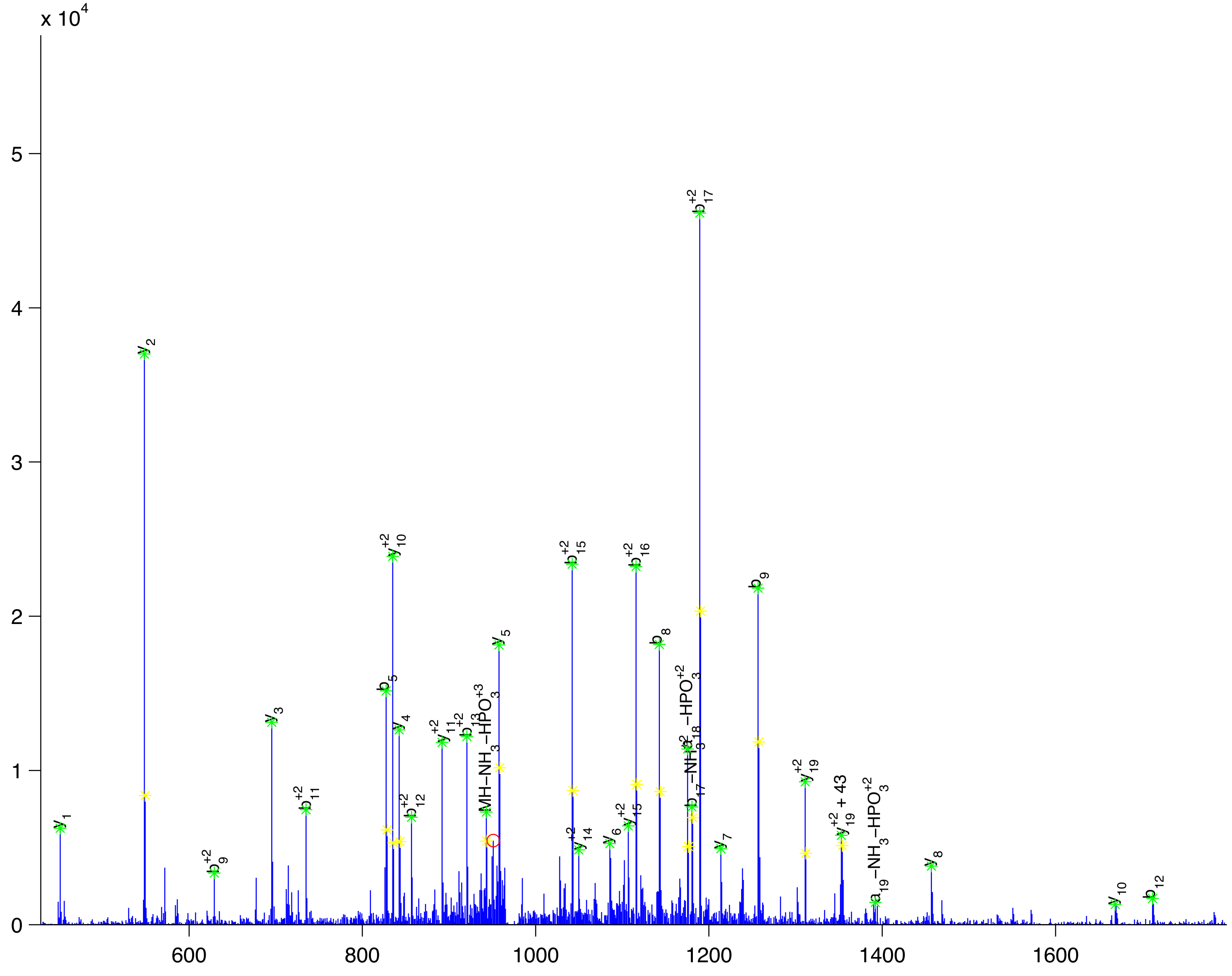

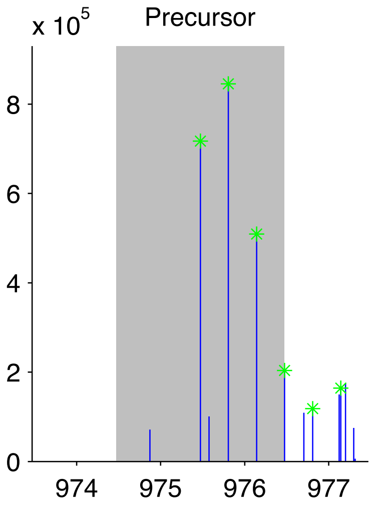

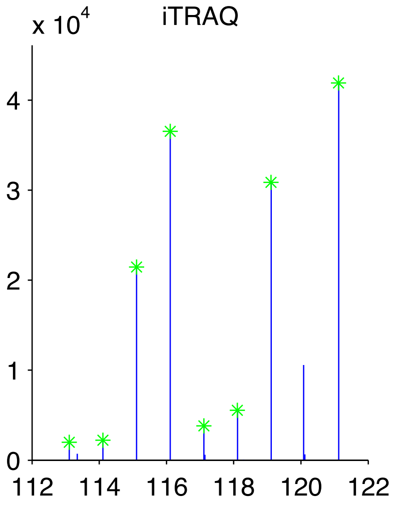

G S H Q I S L D N P D y Q Q D F F P K

epidermal growth factor receptor isoform a [Homo sapiens]
Charge State: +4
Scan Number: 24430
File Name: 120518_A549_EGFTSA_pY.raw

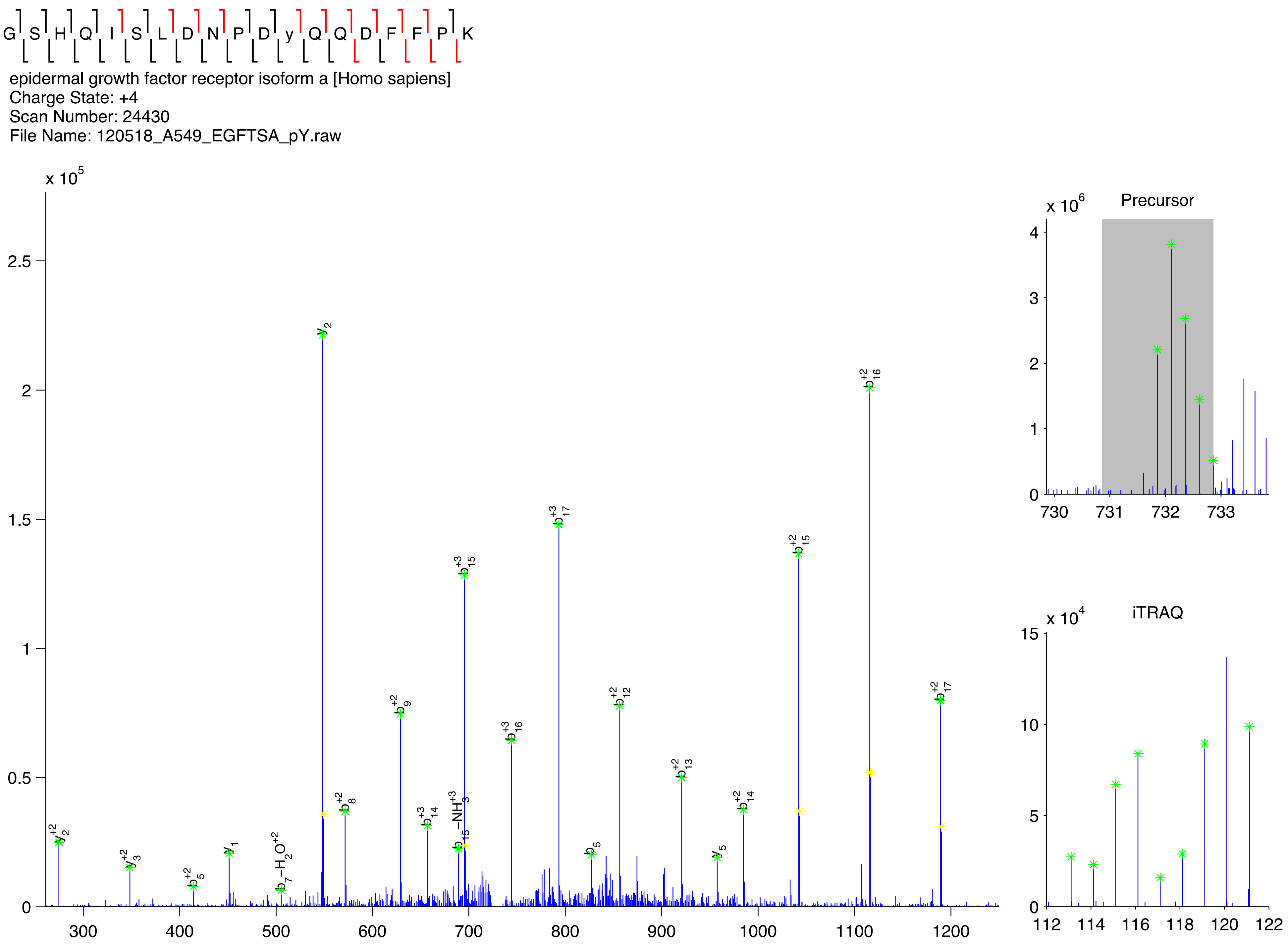

S L E A T D S A F D N P D y W H S R

erbB–3 isoform 1 precursor [Homo sapiens]
Charge State: +3
Scan Number: 23421
File Name: 120518_A549_EGFTSA_pY.raw

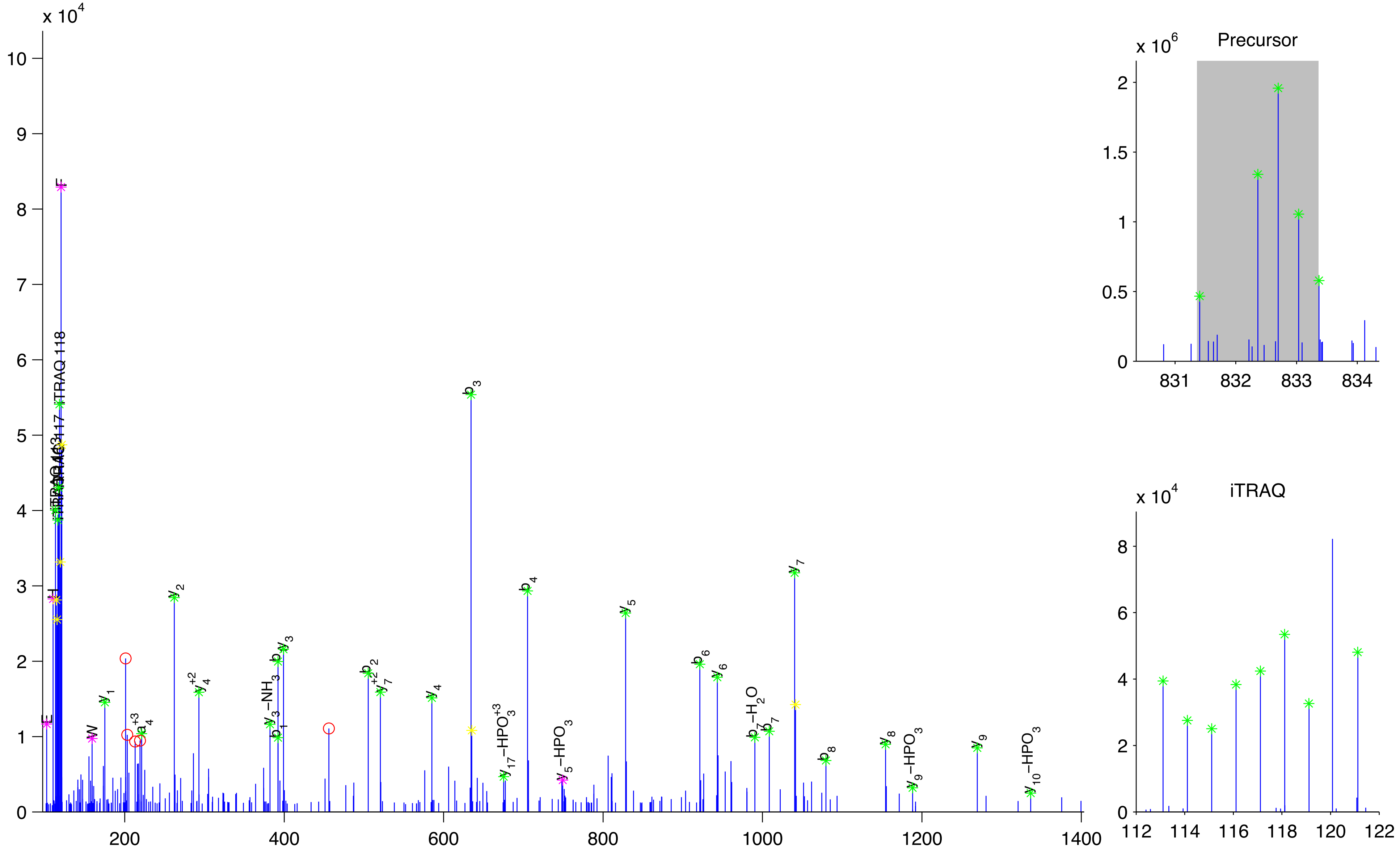

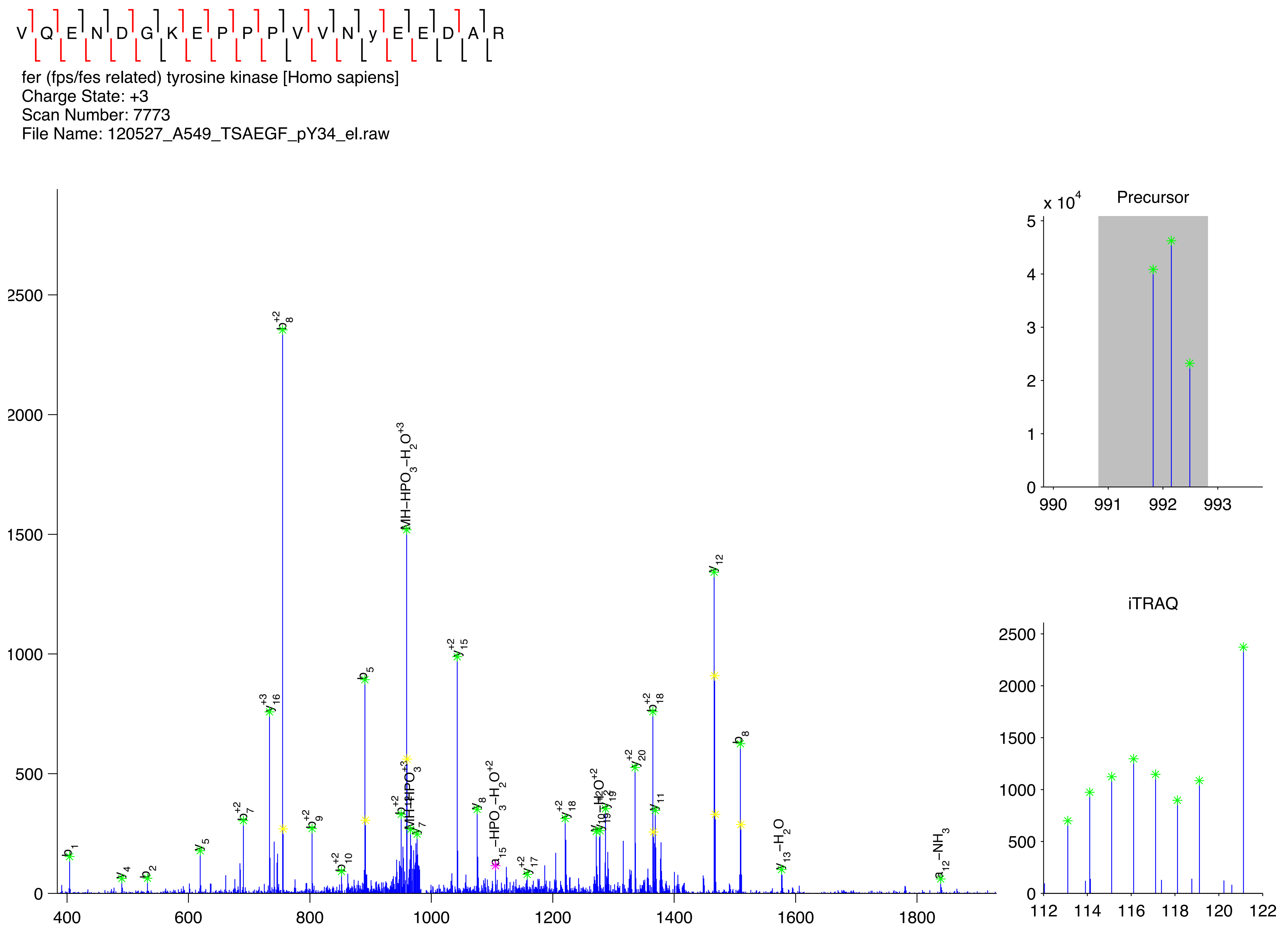
V Q E N D G K E P P P V V N y E E D A R
fer (fps/fes related) tyrosine kinase [Homo sapiens]
Charge State: +3
Scan Number: 7773
File Name: 120527_A549_TSAEGF_pY34_el.raw
Precursor
x 10^4
iTRAQ

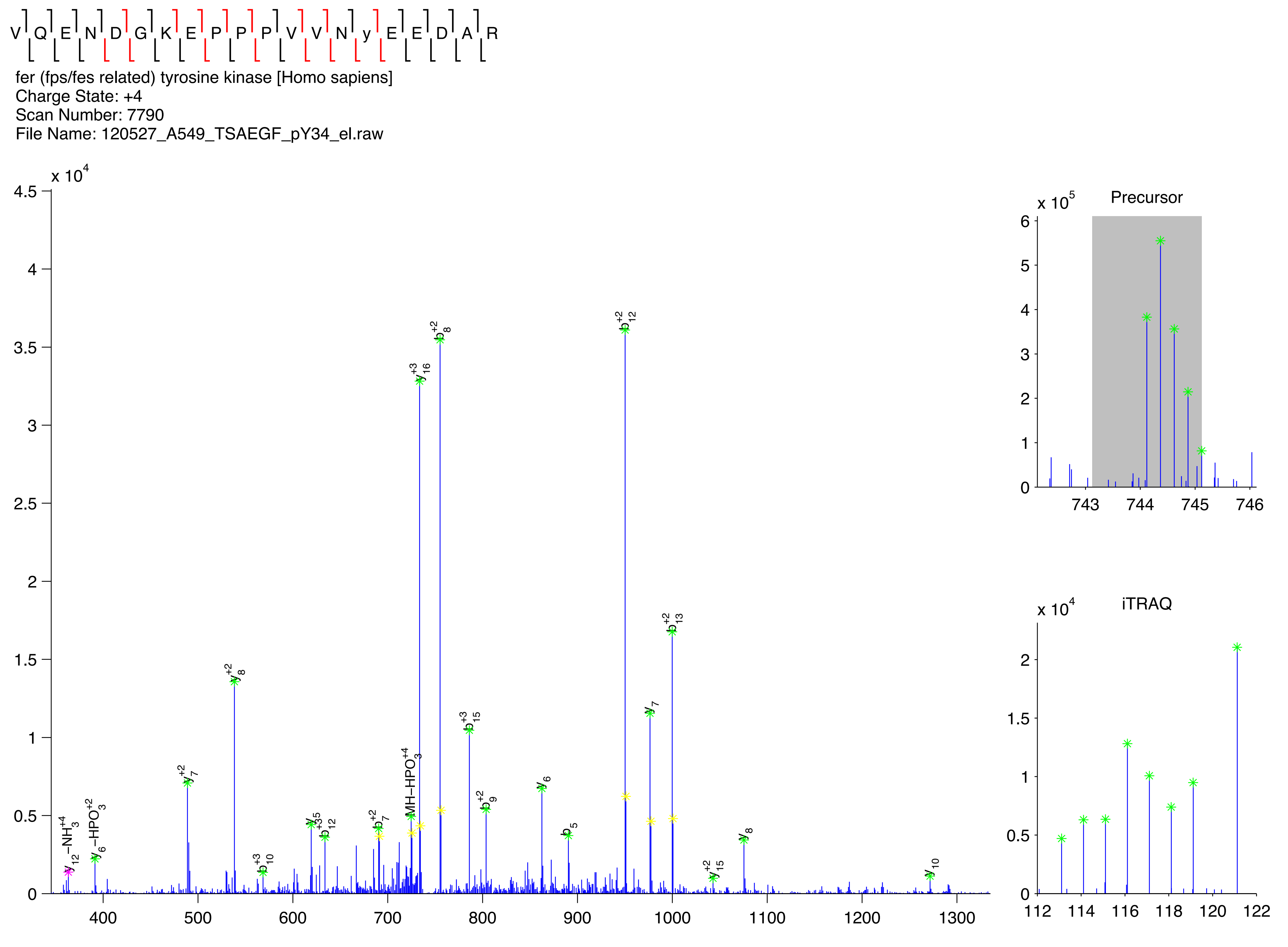

V Q E N D G K E P P P V V N y E E D A R
fer (fps/fes related) tyrosine kinase [Homo sapiens]
Charge State: +4
Scan Number: 7790
File Name: 120527_A549_TSAEGF_pY34_el.raw
x 10^4
Precursor
x 10^5
iTRAQ
x 10^4

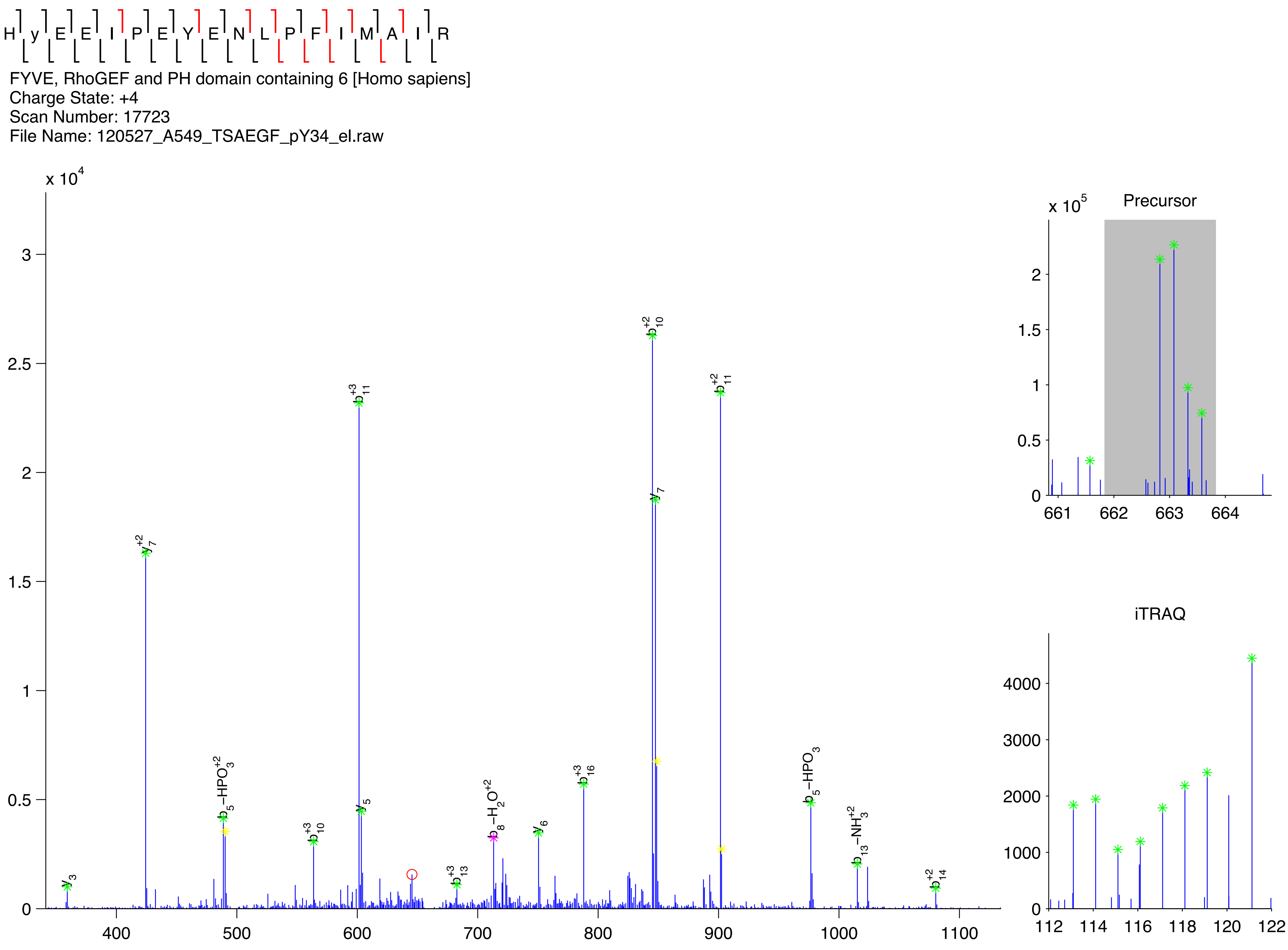

H y E E I P E Y E N L P F I M A I R
FYVE, RhoGEF and PH domain containing 6 [Homo sapiens]
Charge State: +4
Scan Number: 17723
File Name: 120527_A549_TSAEGF_pY34_el.raw
x 10^4
y3
y7+2
b5-HPO3+2
b10+3
b11+3
y5
b13+3
b8-H2O+2
y6
b16+3
b10+2
y7
b11+2
b5-HPO3
b13-NH3+2
b14+2
x 10^5
Precursor
iTRAQ

H Y E E I P E y E N L P F I M A I R

FYVE, RhoGEF and PH domain containing 6 [Homo sapiens]
Charge State: +3
Scan Number: 17784
File Name: 120527_A549_TSAEGF_pY34_el.raw

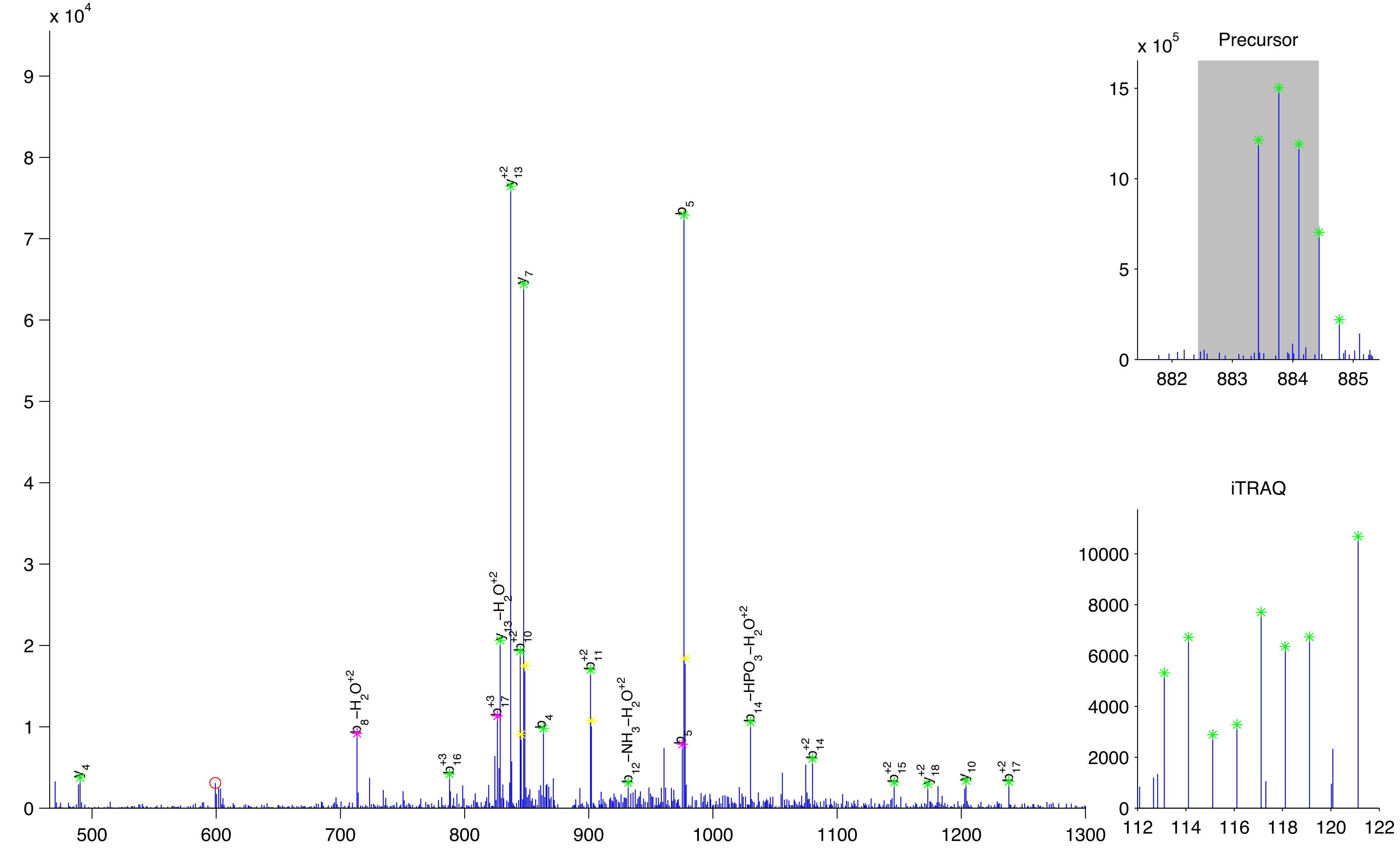

N E E E N I y S V P H D S T Q G K

glucocorticoid receptor DNA binding factor 1 [Homo sapiens]
Charge State: +3
Scan Number: 6213
File Name: 120527_A549_TSAEGF_pY34_el.raw

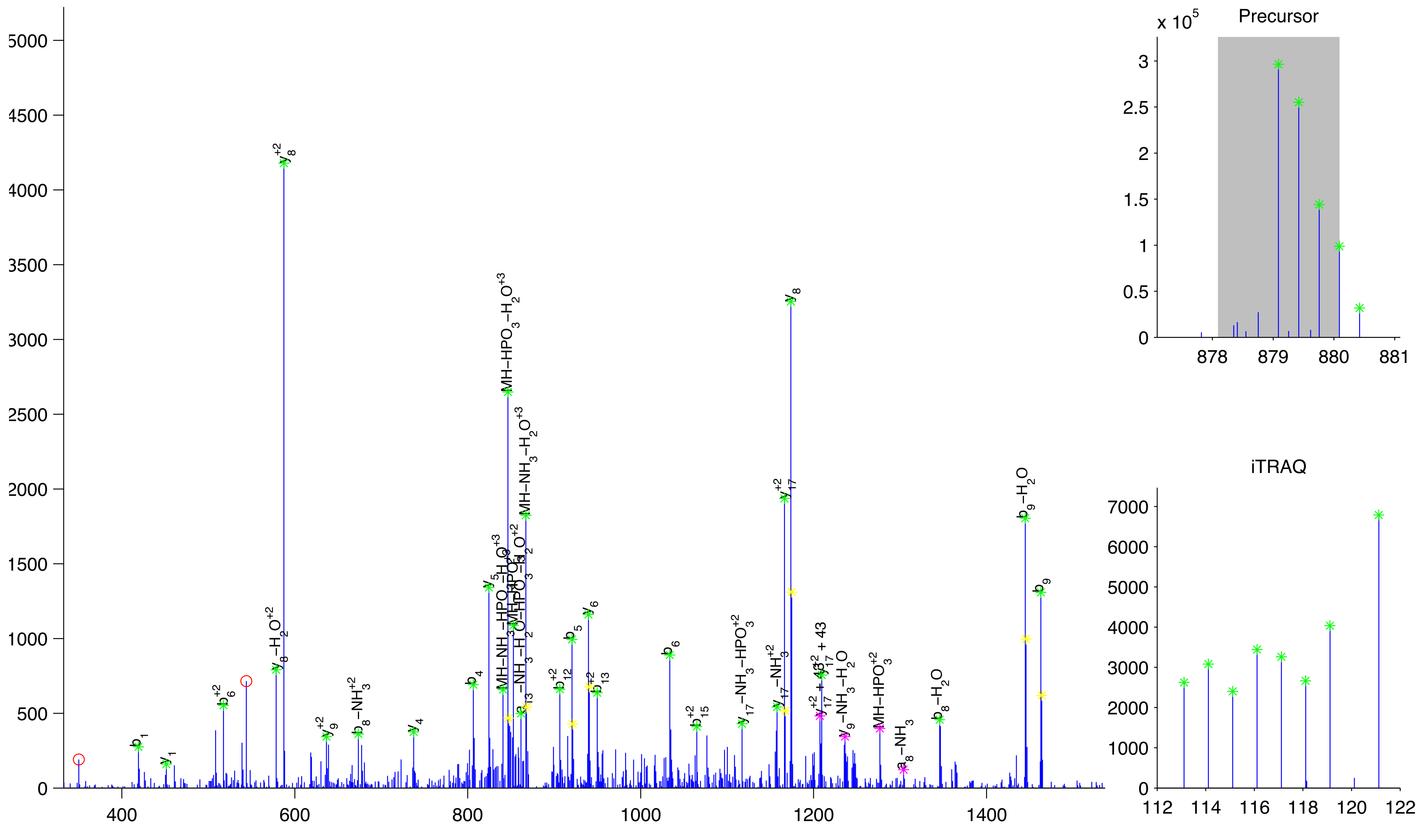

N E E E N I y S V P H D S T Q G K

glucocorticoid receptor DNA binding factor 1 [Homo sapiens]
Charge State: +4
Scan Number: 6236
File Name: 120527_A549_TSAEGF_pY34_el.raw

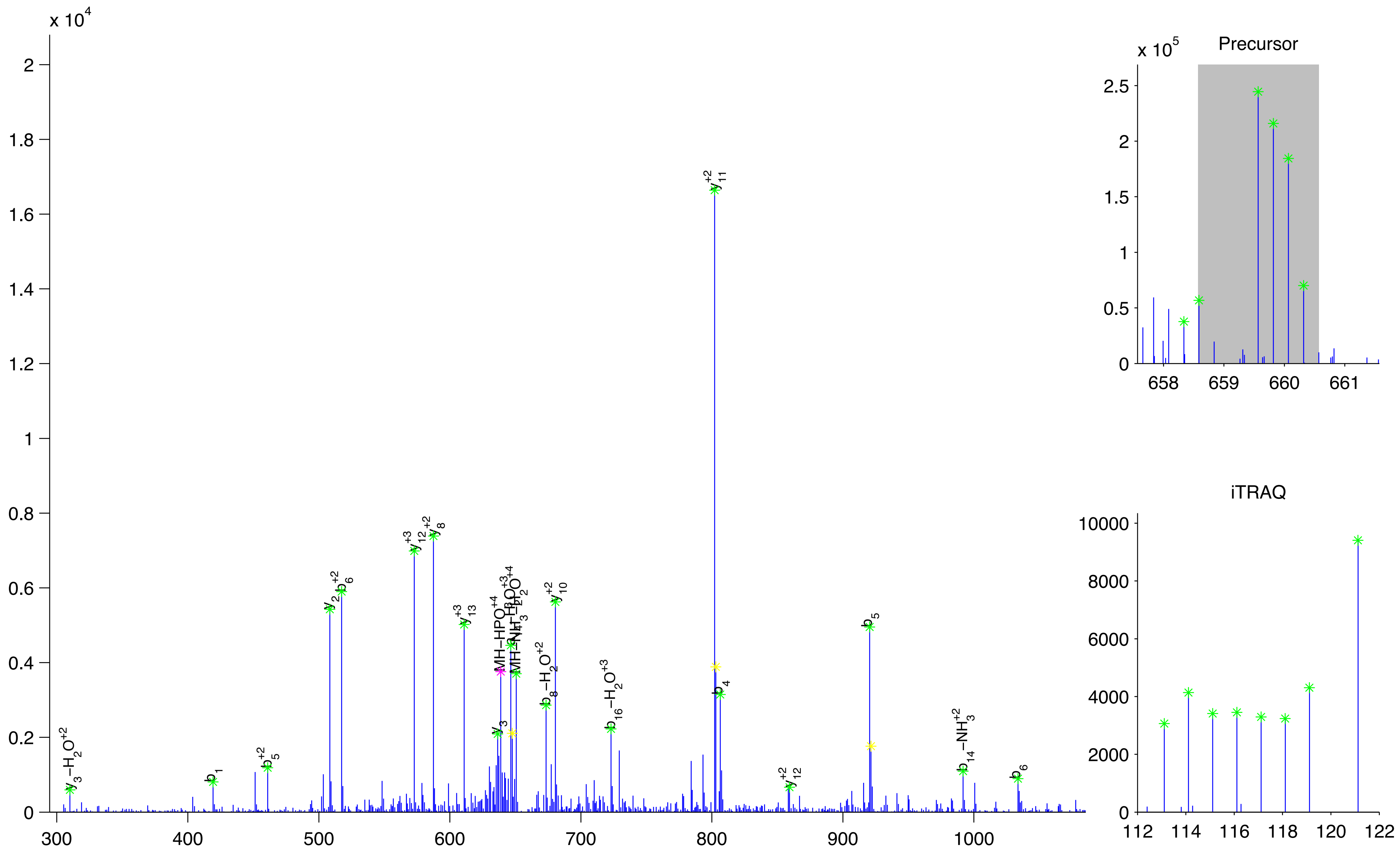

S V S S S P W L P Q D G F D P S D y A E P M D A V V K P R

glucocorticoid receptor DNA binding factor 1 [Homo sapiens]
Charge State: +4
Scan Number: 28609
File Name: 120518_A549_EGFTSA_pY.raw

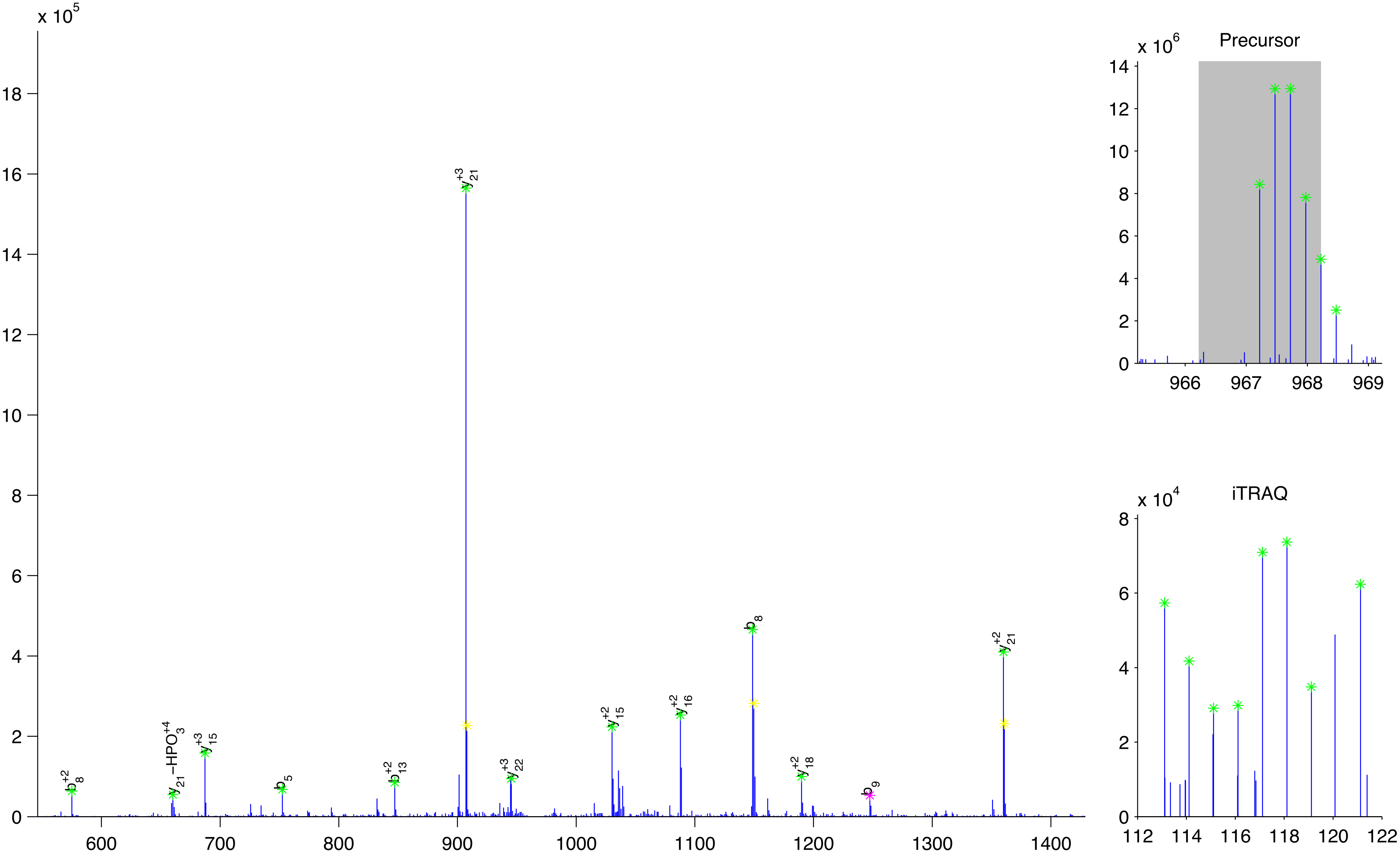

N S Y V A G Q y D D A A S Y Q R

glucose−6−phosphate dehydrogenase isoform b [Homo sapiens]
Charge State: +2
Scan Number: 7433
File Name: 120527_A549_TSAEGF_pY34_el.raw

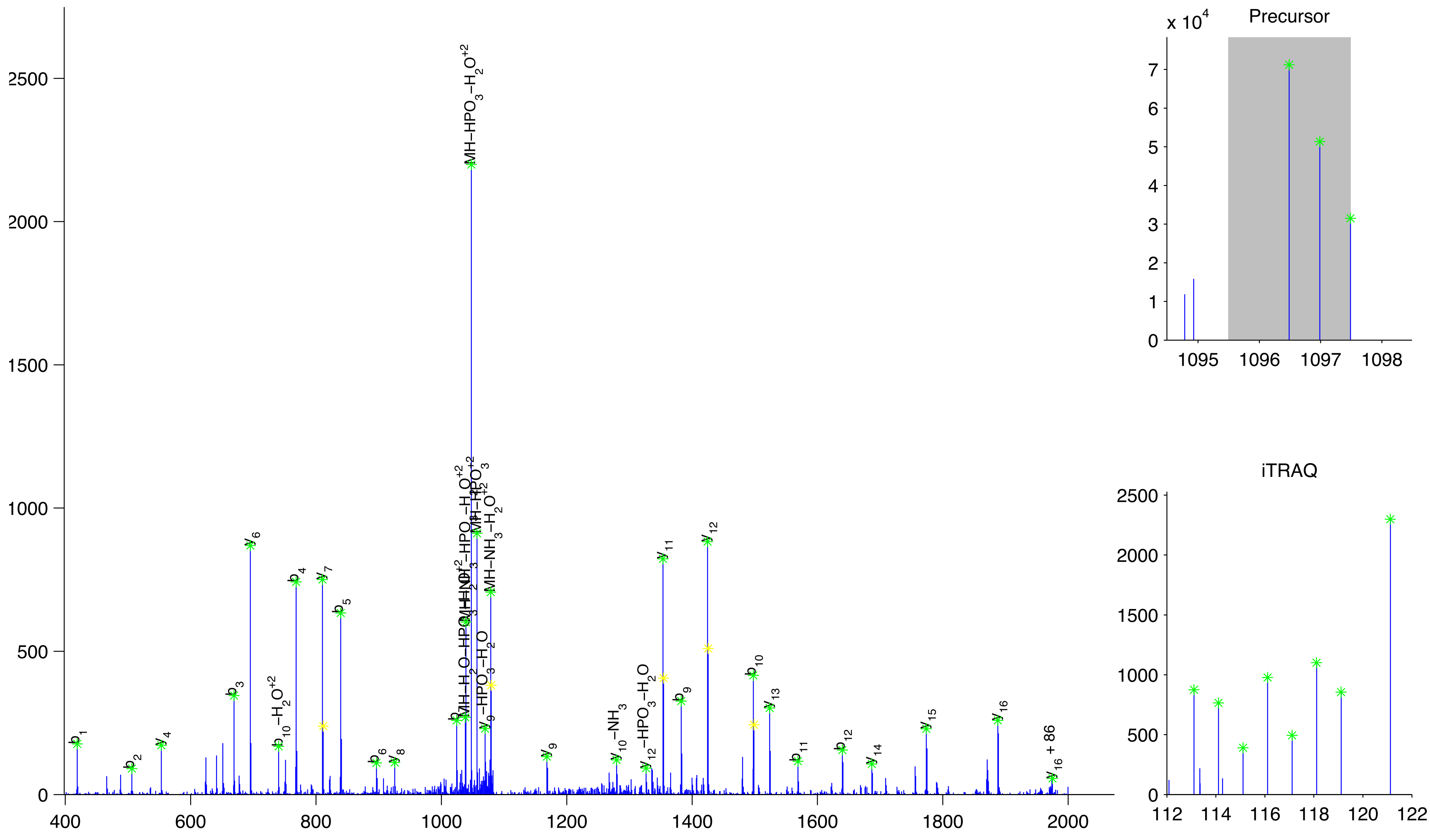

V G F Q y E G T Y K

glucose−6−phosphate dehydrogenase isoform b [Homo sapiens]
Charge State: +3
Scan Number: 18592
File Name: 120518_A549_EGFTSA_pY.raw

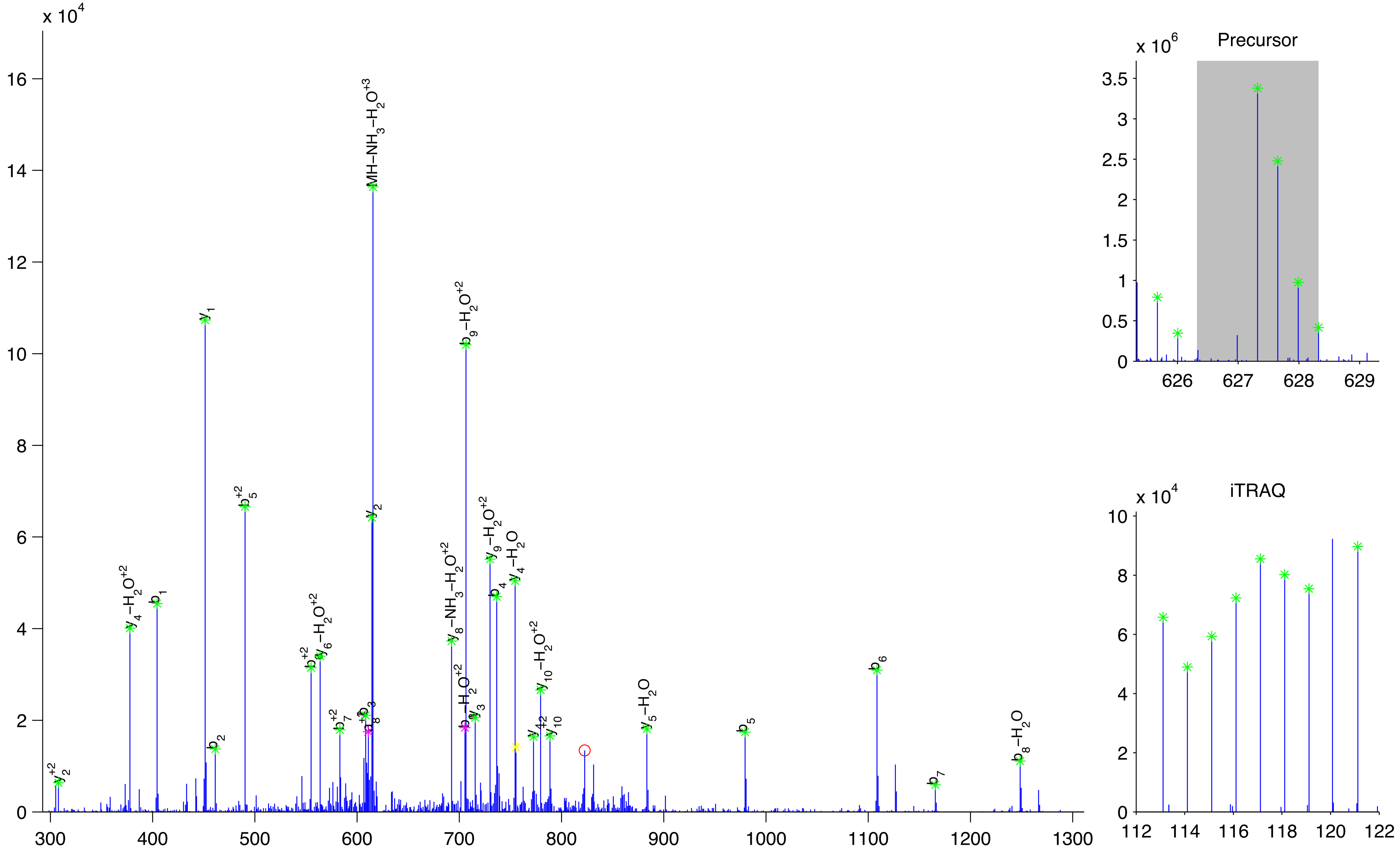

V G F Q Y E G T y K

glucose−6−phosphate dehydrogenase isoform b [Homo sapiens]
Charge State: +3
Scan Number: 19054
File Name: 120518_A549_EGFTSA_pY.raw

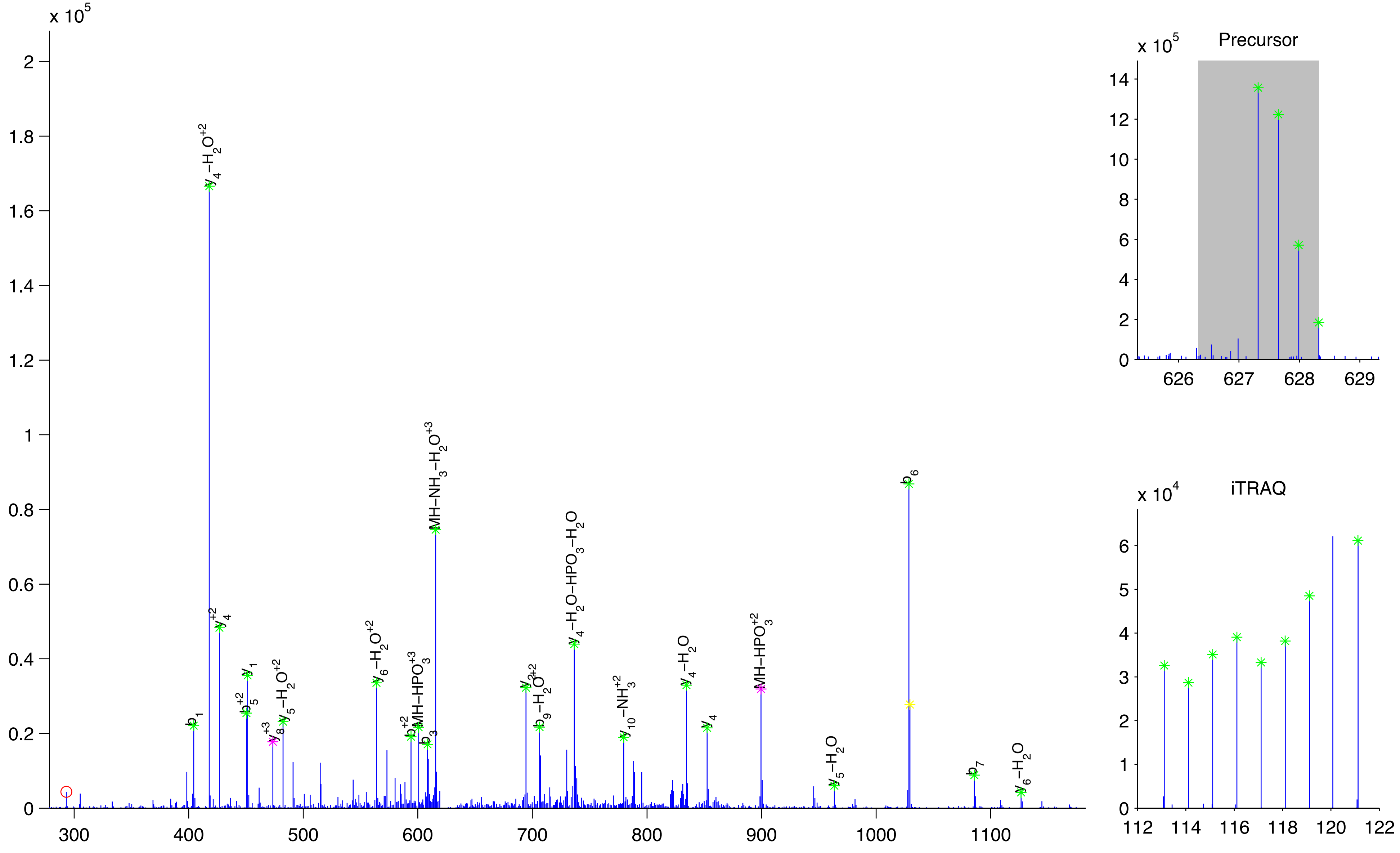

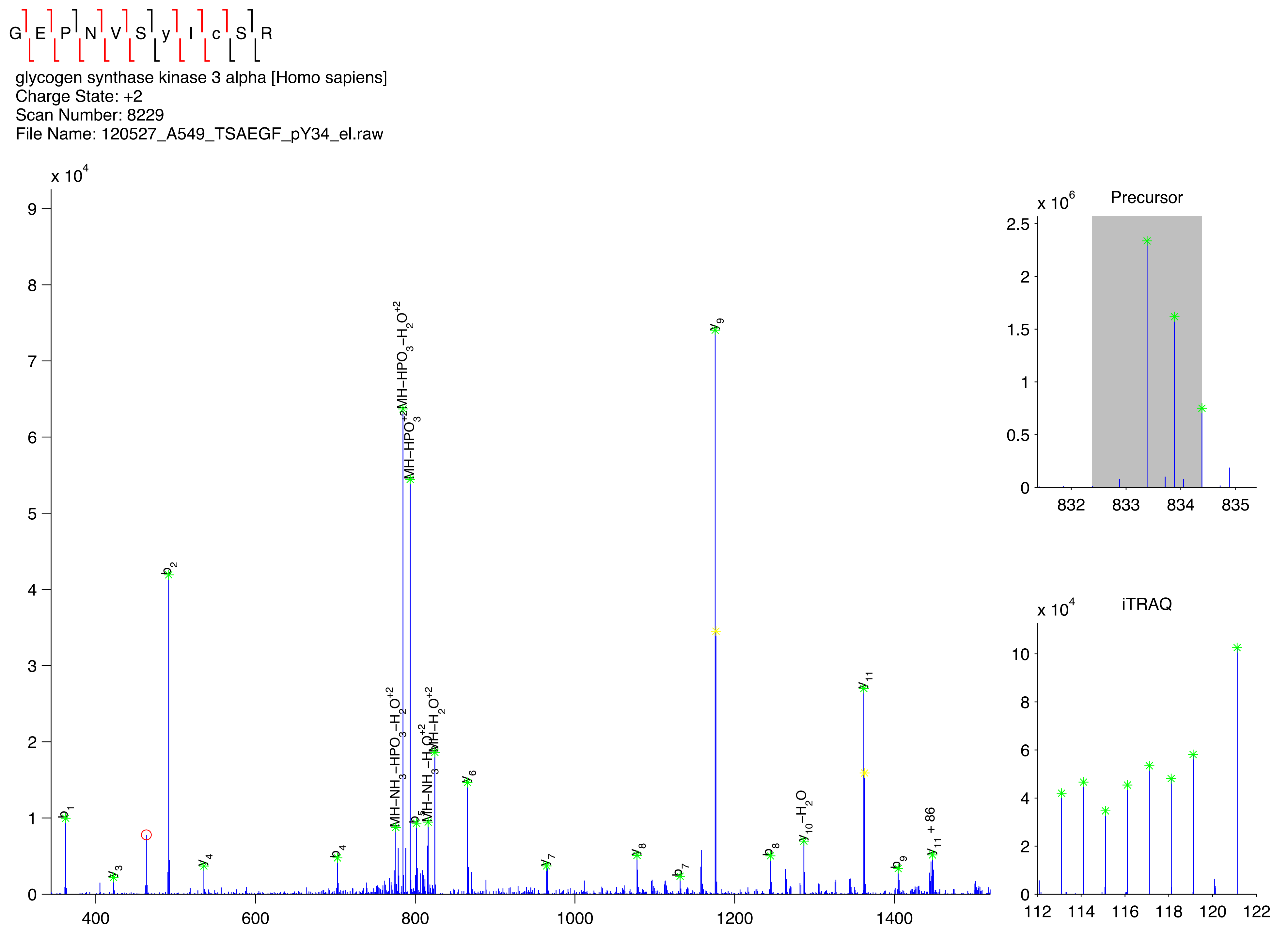
G E P N V S y I c S R
glycogen synthase kinase 3 alpha [Homo sapiens]
Charge State: +2
Scan Number: 8229
File Name: 120527_A549_TSAEGF_pY34_el.raw
x 10^4
b1
y3
b2
y4
b4
MH−NH3−HPO3−H2O+2
MH−HPO3−H2O+2
MH−HPO3+2
b5
MH−NH3−H2O+2
MH−H2O+2
y6
y7
y8
b7
y9
b8
y10−H2O
y11
b9
y11 + 86
Precursor
x 10^6
iTRAQ
x 10^4

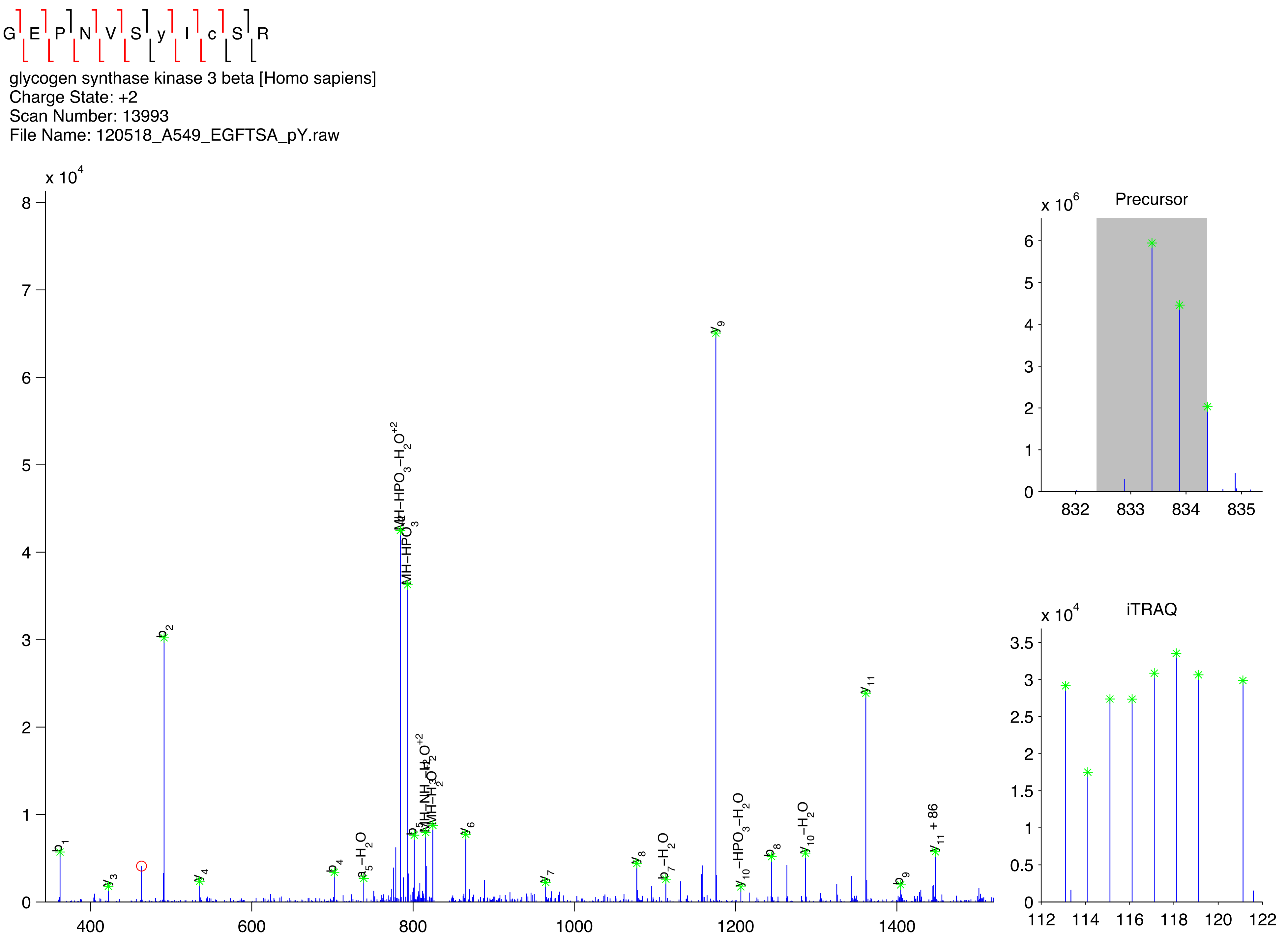

G E P N V S y I c S R
glycogen synthase kinase 3 beta [Homo sapiens]
Charge State: +2
Scan Number: 13993
File Name: 120518_A549_EGFTSA_pY.raw
x 10^4
b1
y3
b2
y4
b4
a5-H2O
MH-HPO3-H2O+2
MH-HPO3
b5
MH-NH3-H2O+2
MH-H2O
y6
y7
y8
b7-H2O
y9
y10-HPO3-H2O
b8
y10-H2O
y11
b9
y11 + 86
Precursor
x 10^6
iTRAQ
x 10^4

G E P N V S y I c S R

glycogen synthase kinase 3 beta [Homo sapiens]
Charge State: +3
Scan Number: 14287
File Name: 120518_A549_EGFTSA_pY.raw

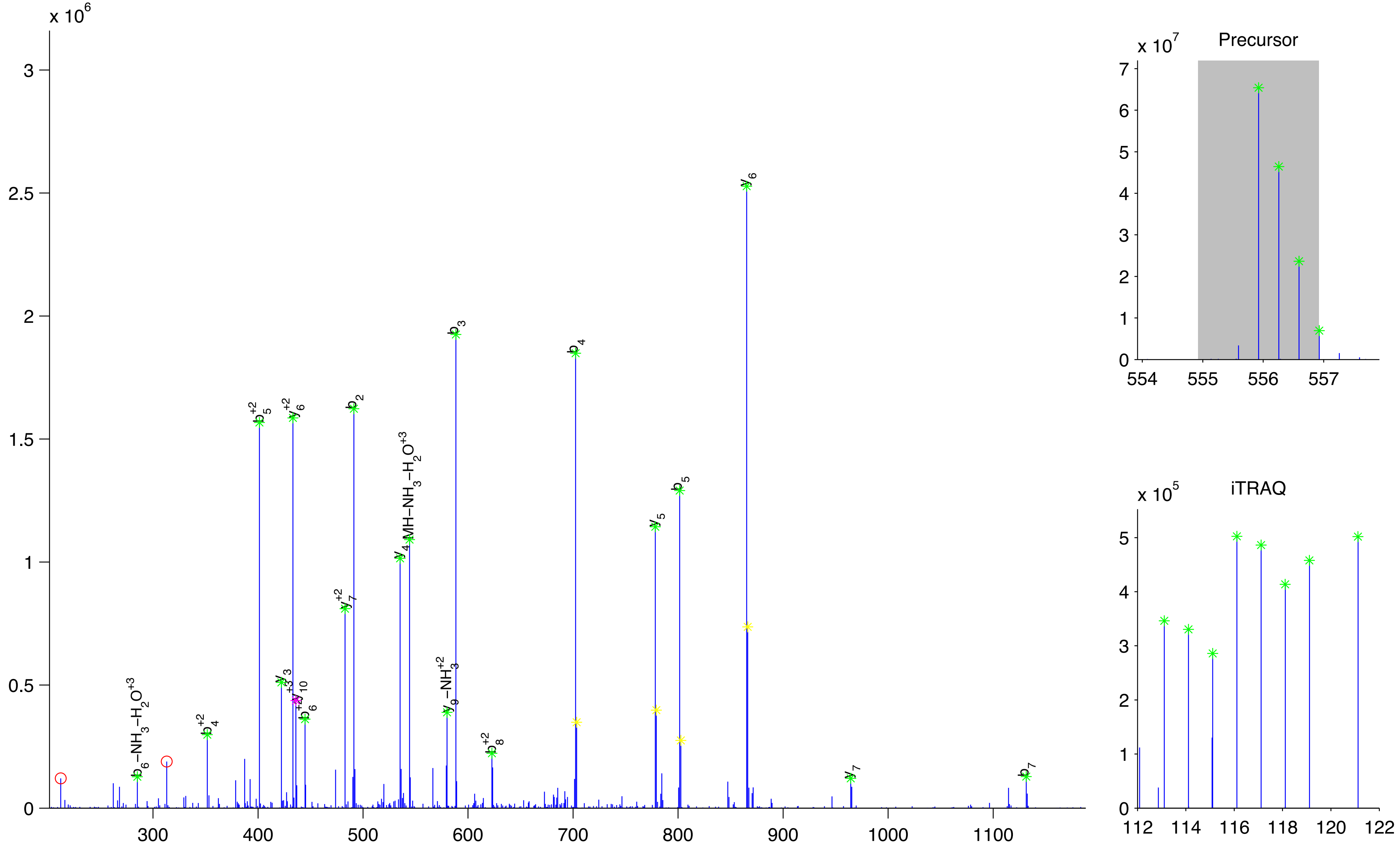

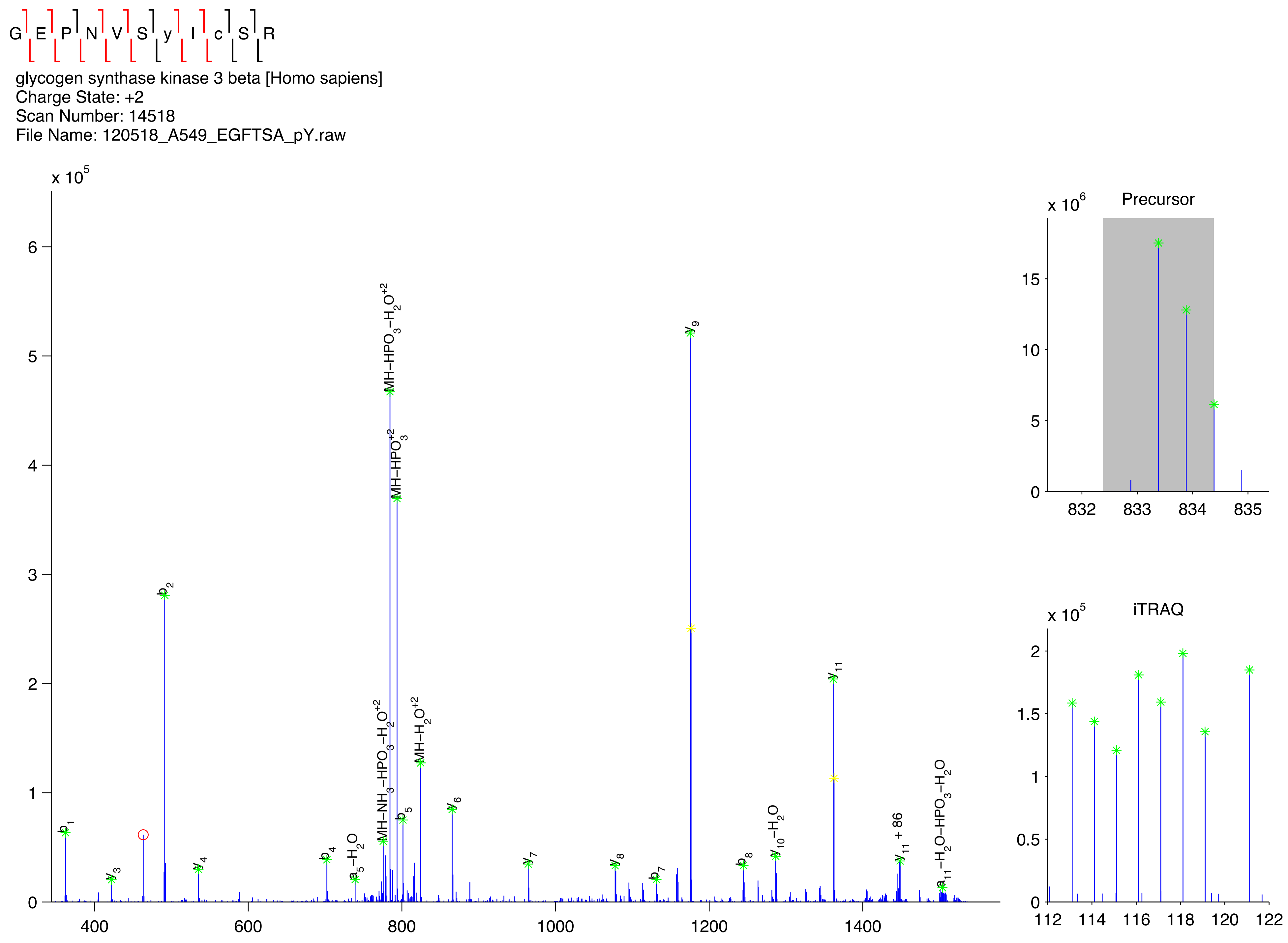

G E P N V S y I c S R
glycogen synthase kinase 3 beta [Homo sapiens]
Charge State: +2
Scan Number: 14518
File Name: 120518_A549_EGFTSA_pY.raw
x 10^5
b1
y3
b2
y4
b4
a5−H2O
MH−NH3−HPO3−H2O+2
MH−HPO3−H2O+2
MH−HPO3+2
b5
MH−H2O+2
y6
y7
y8
b7
y9
b8
y10−H2O
y11
y11 + 86
a11−H2O−HPO3−H2O
Precursor
x 10^6
iTRAQ
x 10^5

S S G S G S S V A D E R V D y V V V D Q Q K

GRB2−associated binding protein 1 isoform a [Homo sapiens]
Charge State: +4
Scan Number: 8774
File Name: 120527_A549_TSAEGF_pY34_el.raw

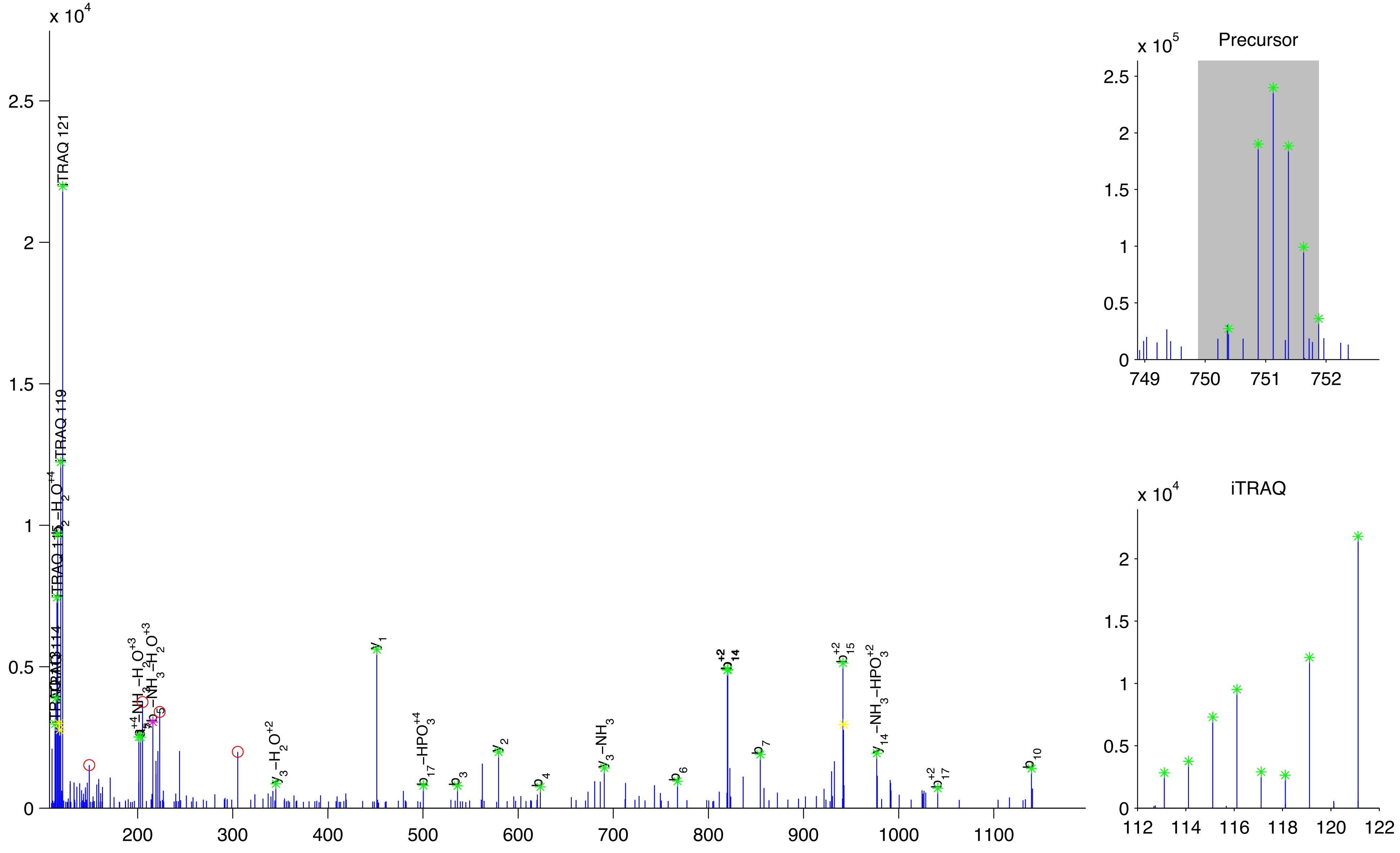

D A S S Q D c y D I P R

GRB2−associated binding protein 1 isoform a [Homo sapiens]
Charge State: +2
Scan Number: 11137
File Name: 120518_A549_EGFTSA_pY.raw

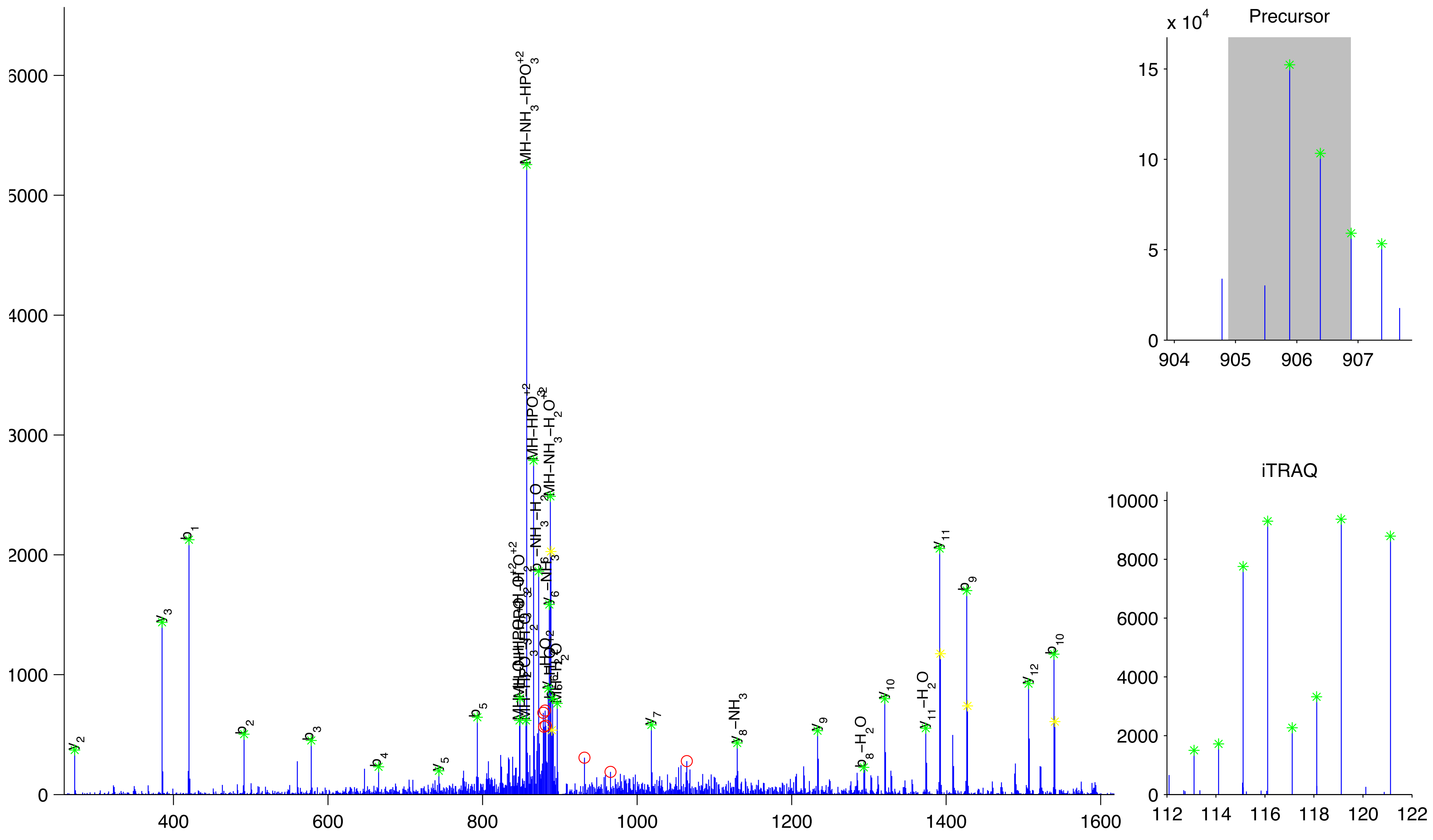

A E P M P S A S S A P P A S S L y S S P V N S S A P L A E D I D P E L A R

hepatocyte growth factor–regulated tyrosine kinase substrate [Homo sapiens]
Charge State: +3
Scan Number: 16083
File Name: 120527_A549_TSAEGF_pY34_el.raw

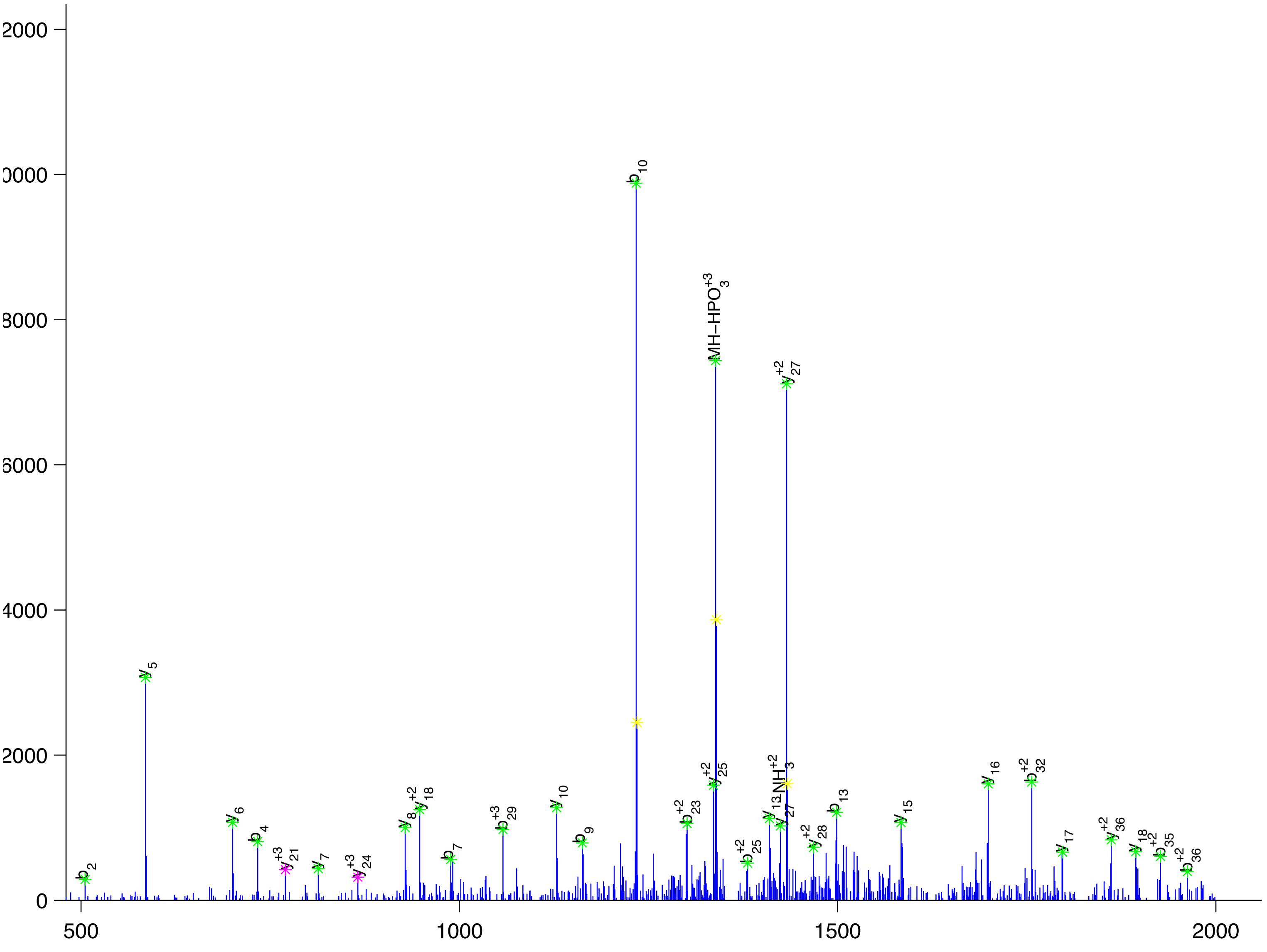

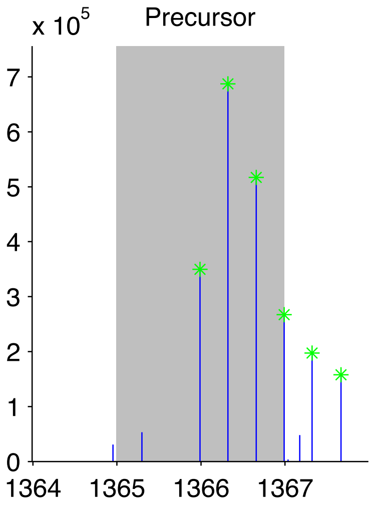

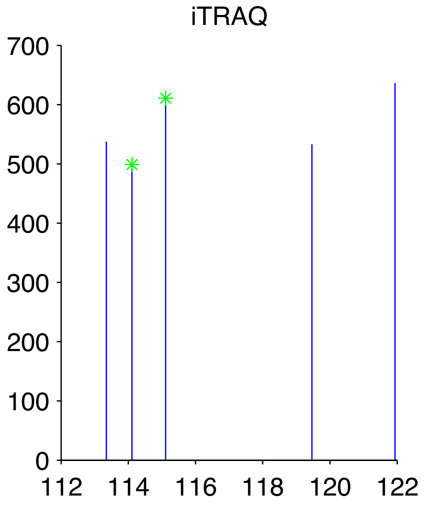

T V c S T y L Q S R

homeodomain interacting protein kinase 3 isoform 1 [Homo sapiens]
Charge State: +2
Scan Number: 5774
File Name: 120527_A549_TSAEGF_pY34_el.raw

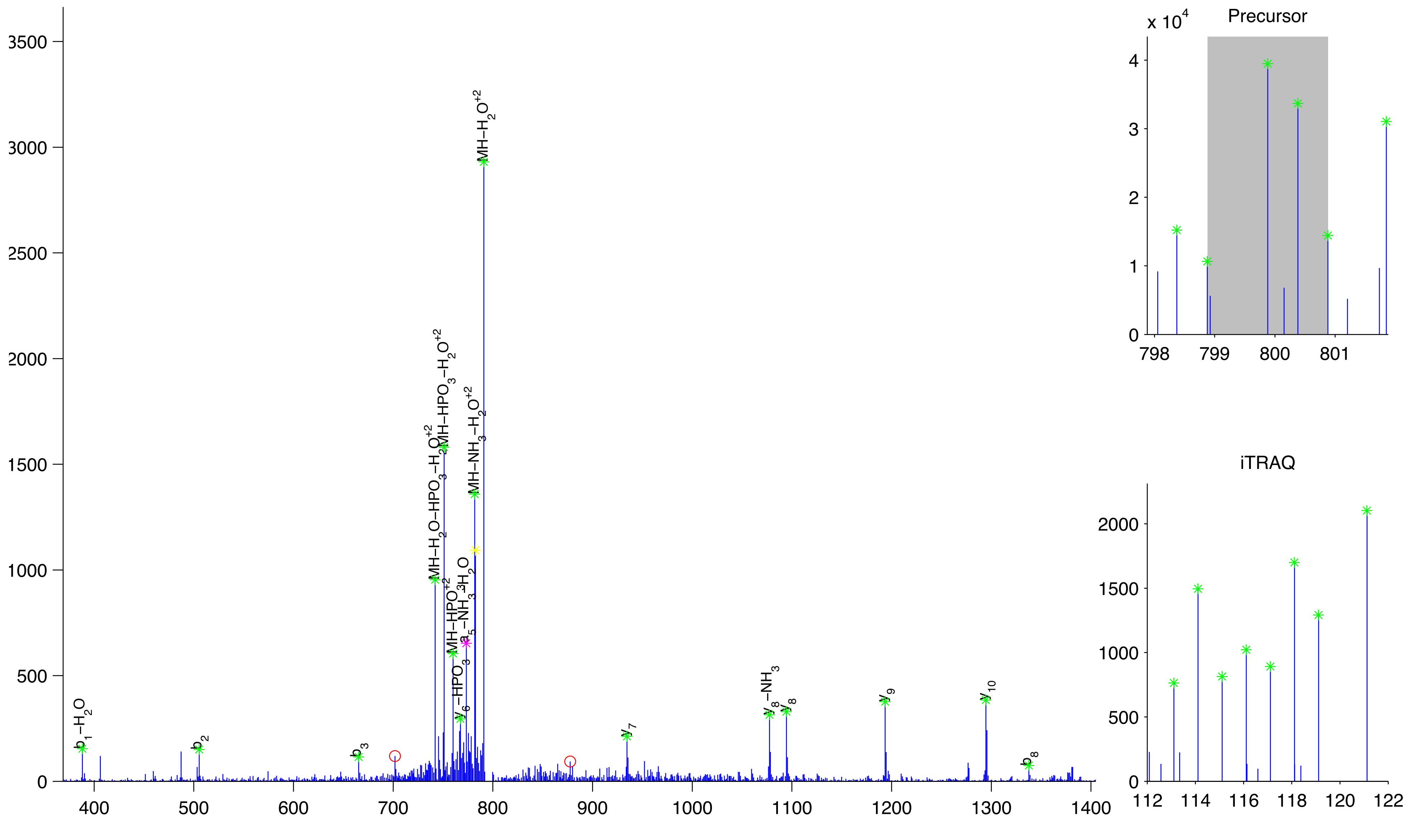

T L S E V D y A P A G P A R

inositol polyphosphate phosphatase–like 1 [Homo sapiens]
Charge State: +2
Scan Number: 9321
File Name: 120527_A549_TSAEGF_pY34_el.raw

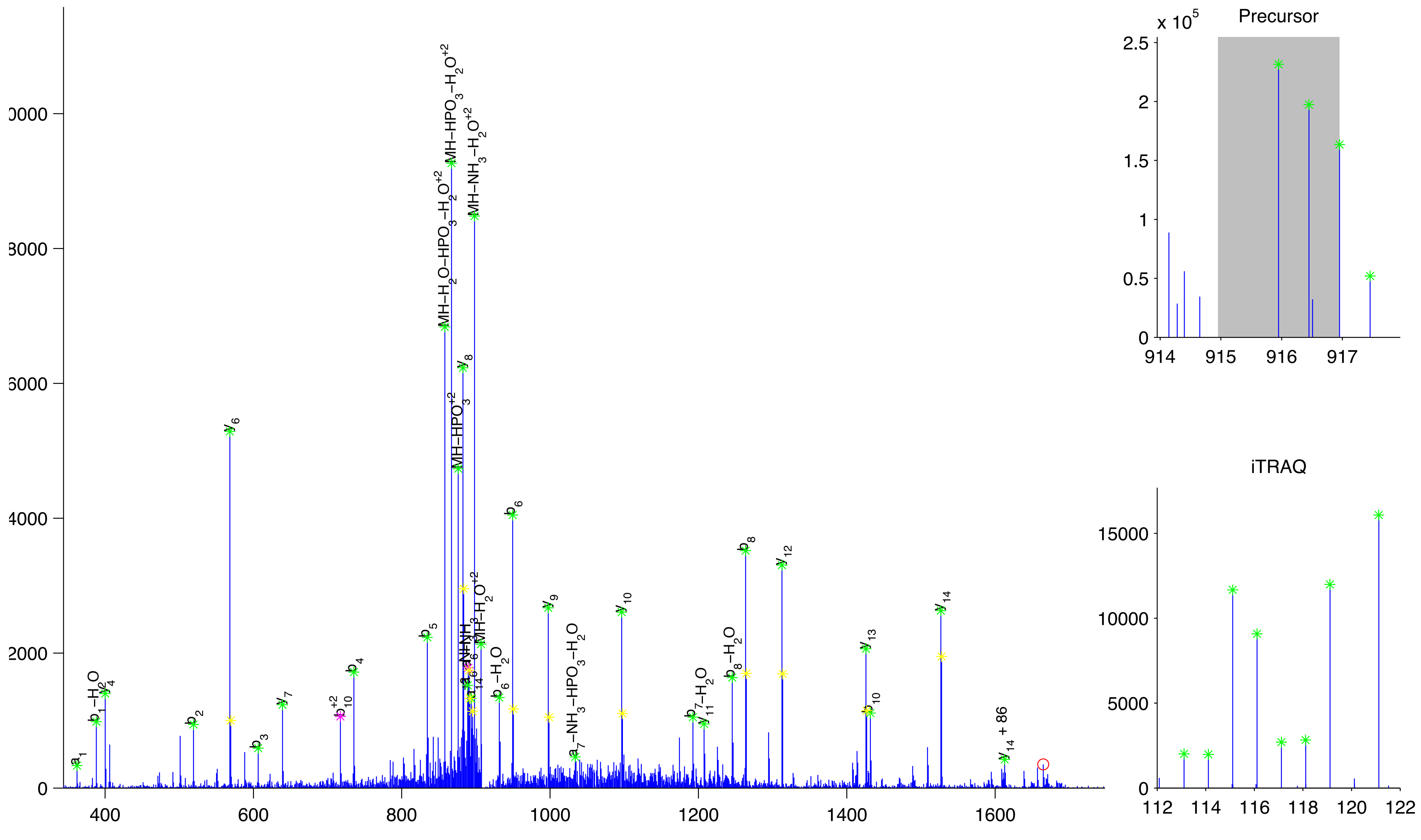

R E E P E A L y A A V N K

intersectin 2 isoform 3 [Homo sapiens]
Charge State: +3
Scan Number: 17985
File Name: 120518_A549_EGFTSA_pY.raw

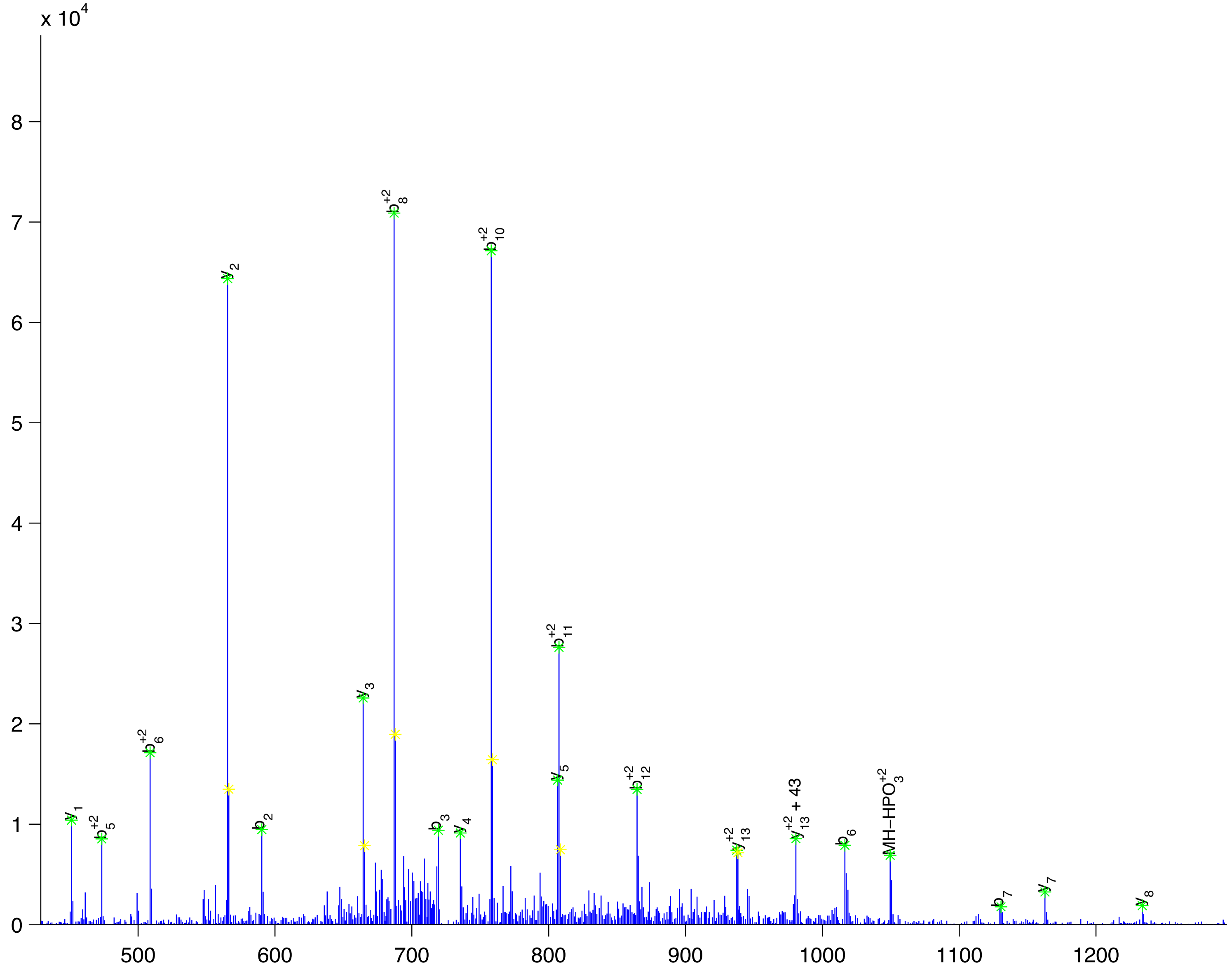

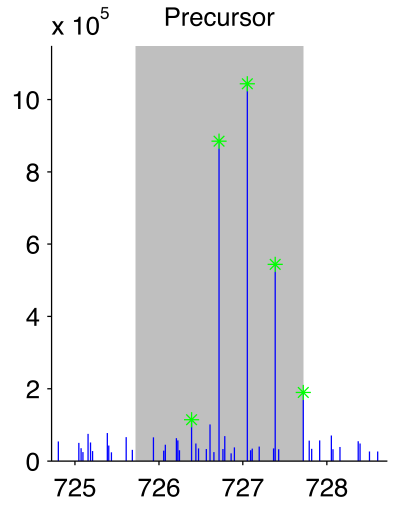

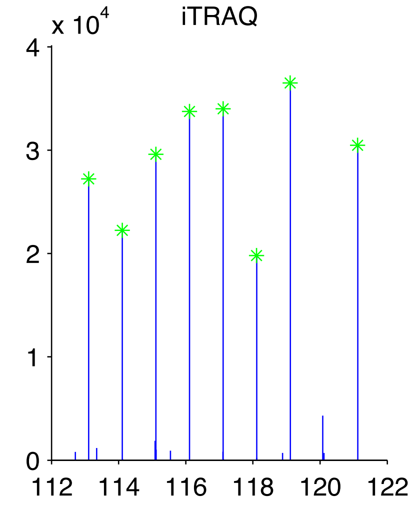

H V A A G T Q Q P y T D G V R

junction plakoglobin [Homo sapiens]
Charge State: +3
Scan Number: 3357
File Name: 120527_A549_TSAEGF_pY34_el.raw

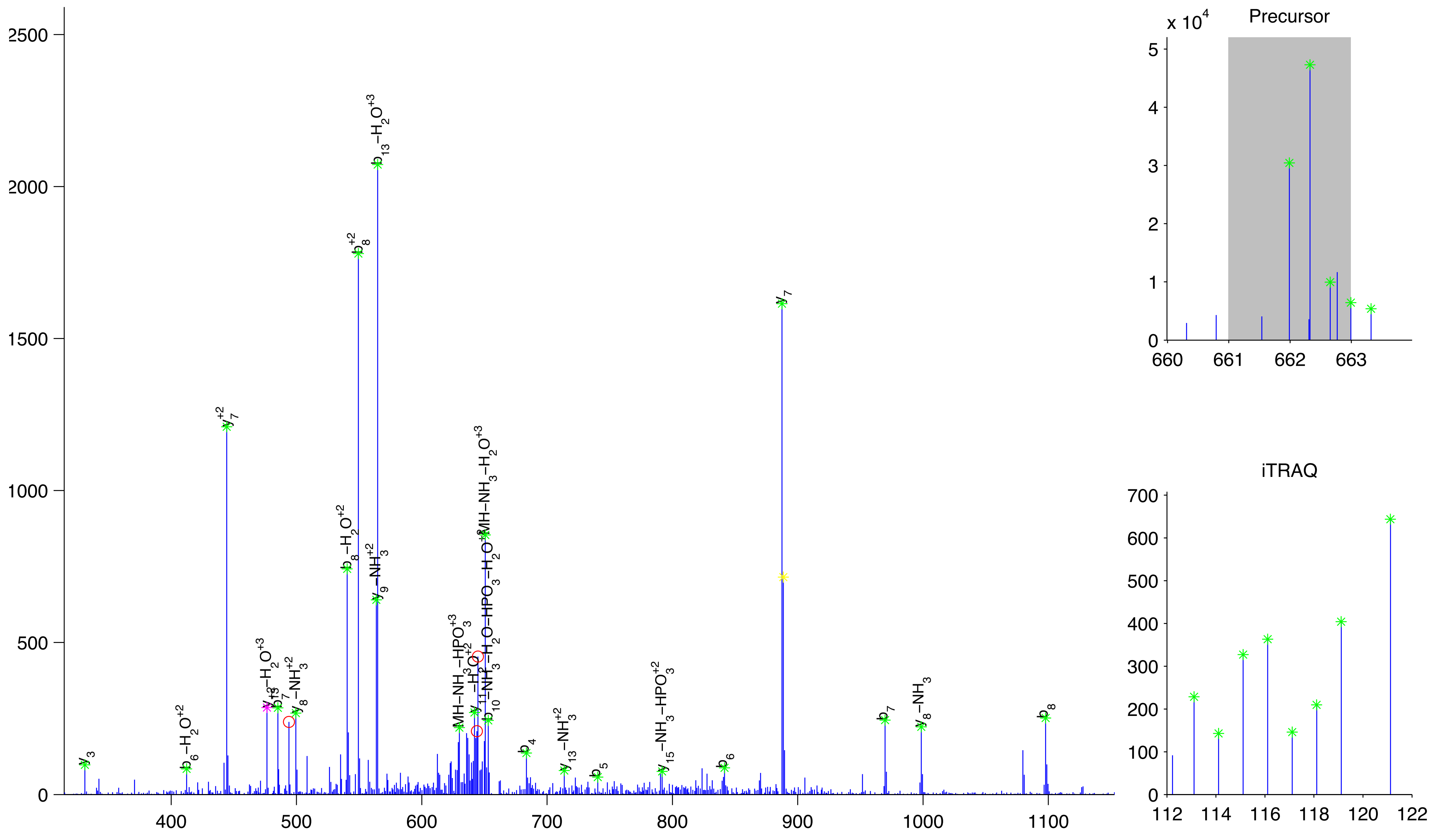

H V A A G T Q Q P y T D G V R

junction plakoglobin [Homo sapiens]
Charge State: +3
Scan Number: 7831
File Name: 120518_A549_EGFTSA_pY.raw

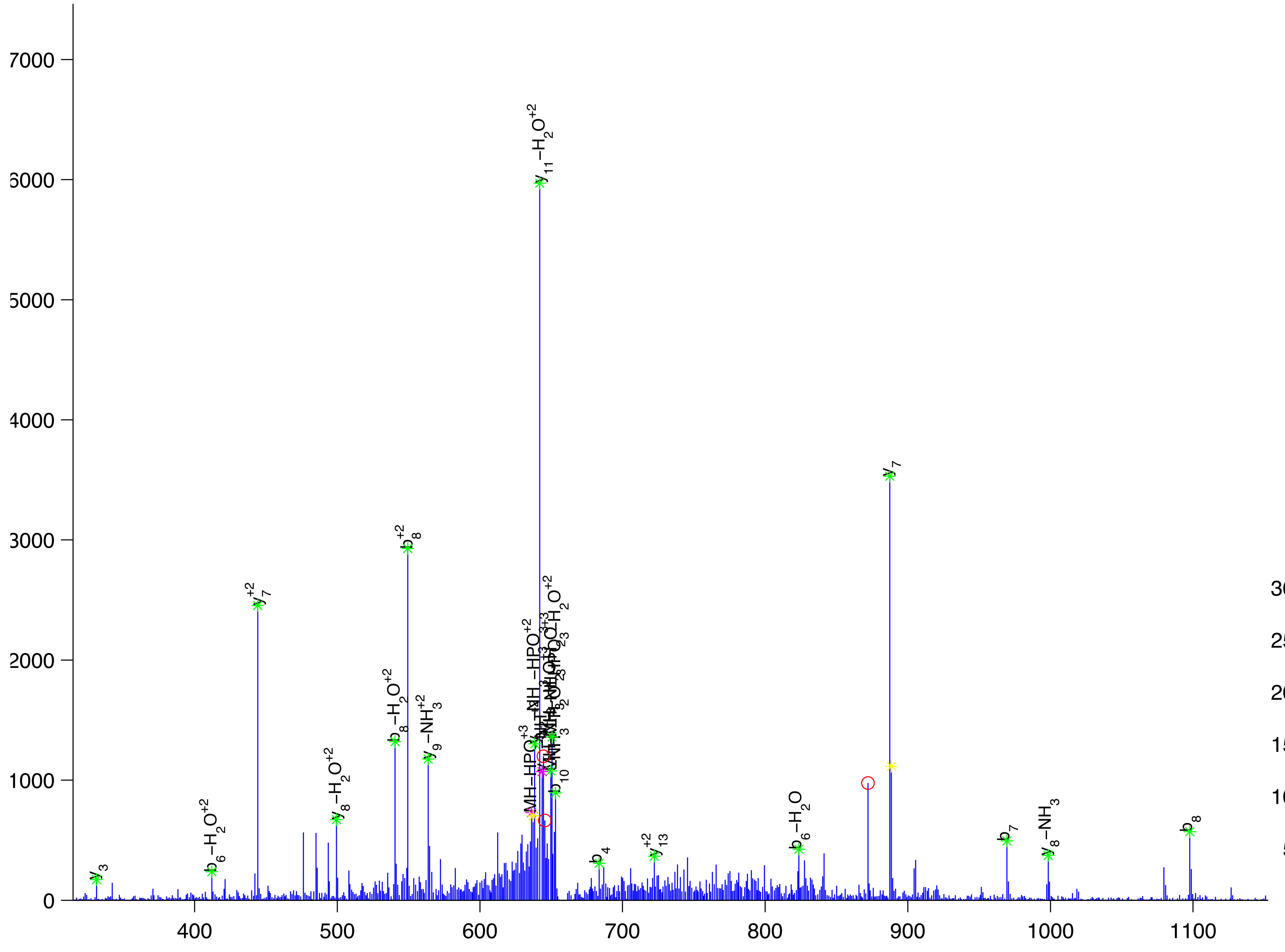

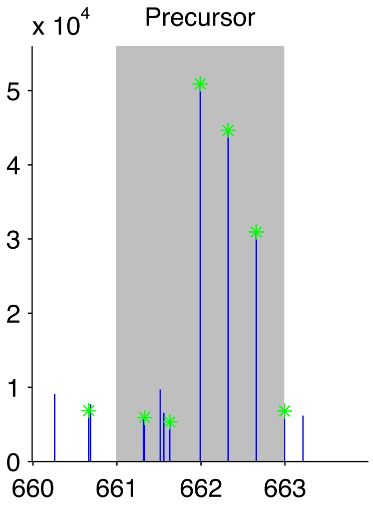

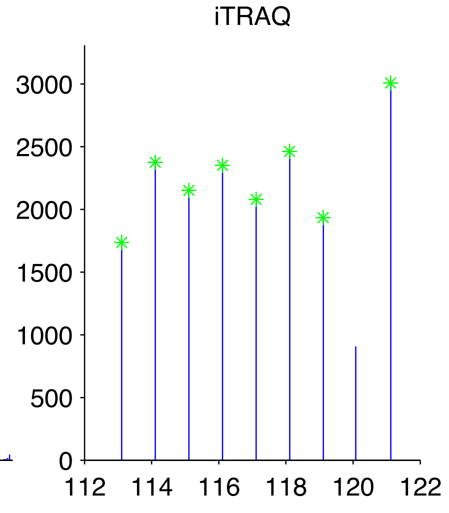

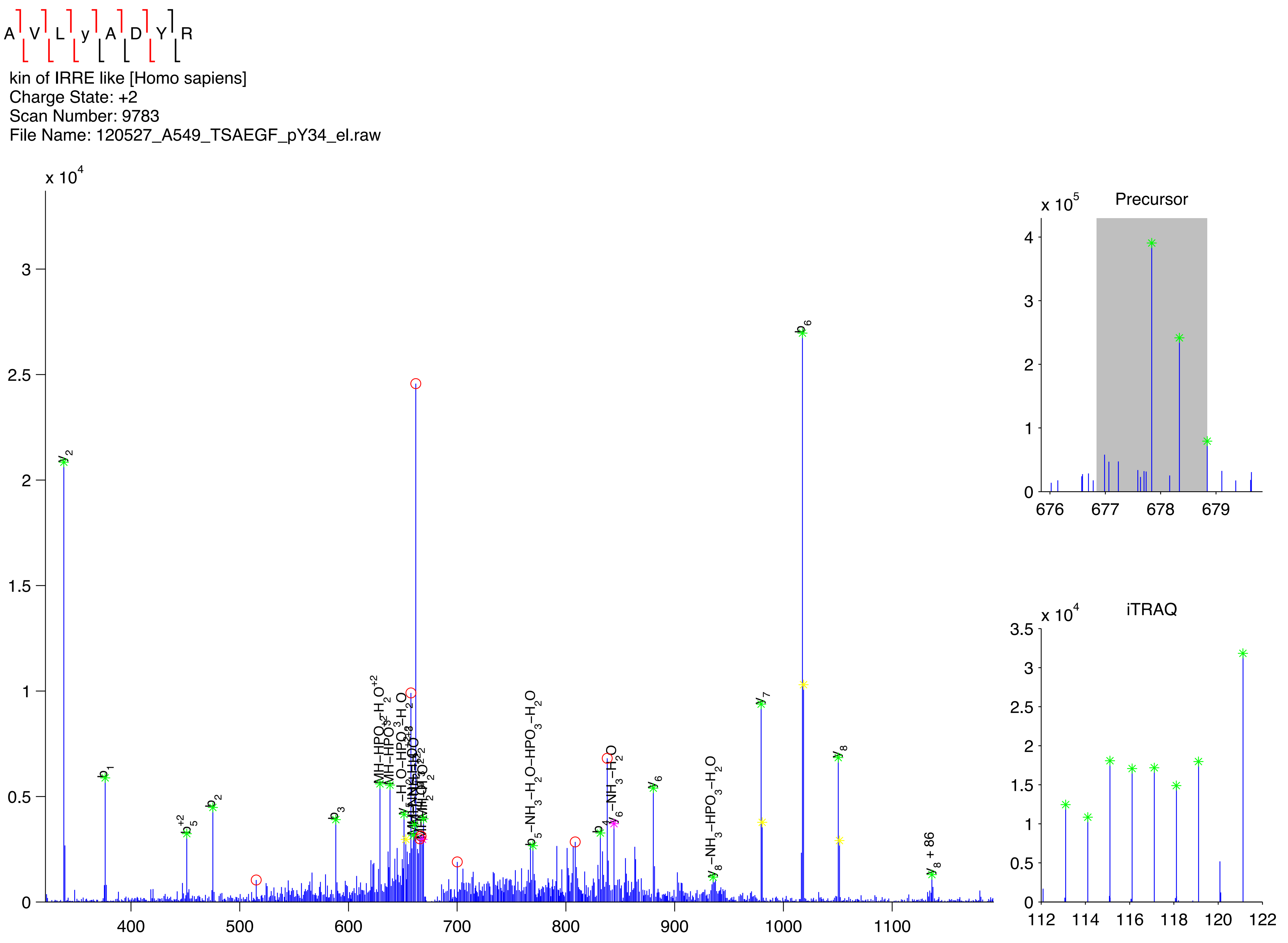
A V L y A D Y R
kin of IRRE like [Homo sapiens]
Charge State: +2
Scan Number: 9783
File Name: 120527_A549_TSAEGF_pY34_el.raw
x 10^4
y2
b1
b5+2
b2
b3
b6
y7
y8
y6
b4
y6−NH3−H2O
b5−NH3−H2O−HPO3−H2O
y8−NH3−HPO3−H2O
y8 + 86
MH−HPO3−H2O+2
Precursor
x 10^5
iTRAQ
x 10^4

N D S D P T y G Q Q G H P N T W K R

LIM domain containing preferred translocation partner in lipoma [Homo sapiens]
Charge State: +4
Scan Number: 3892
File Name: 120527_A549_TSAEGF_pY34_el.raw

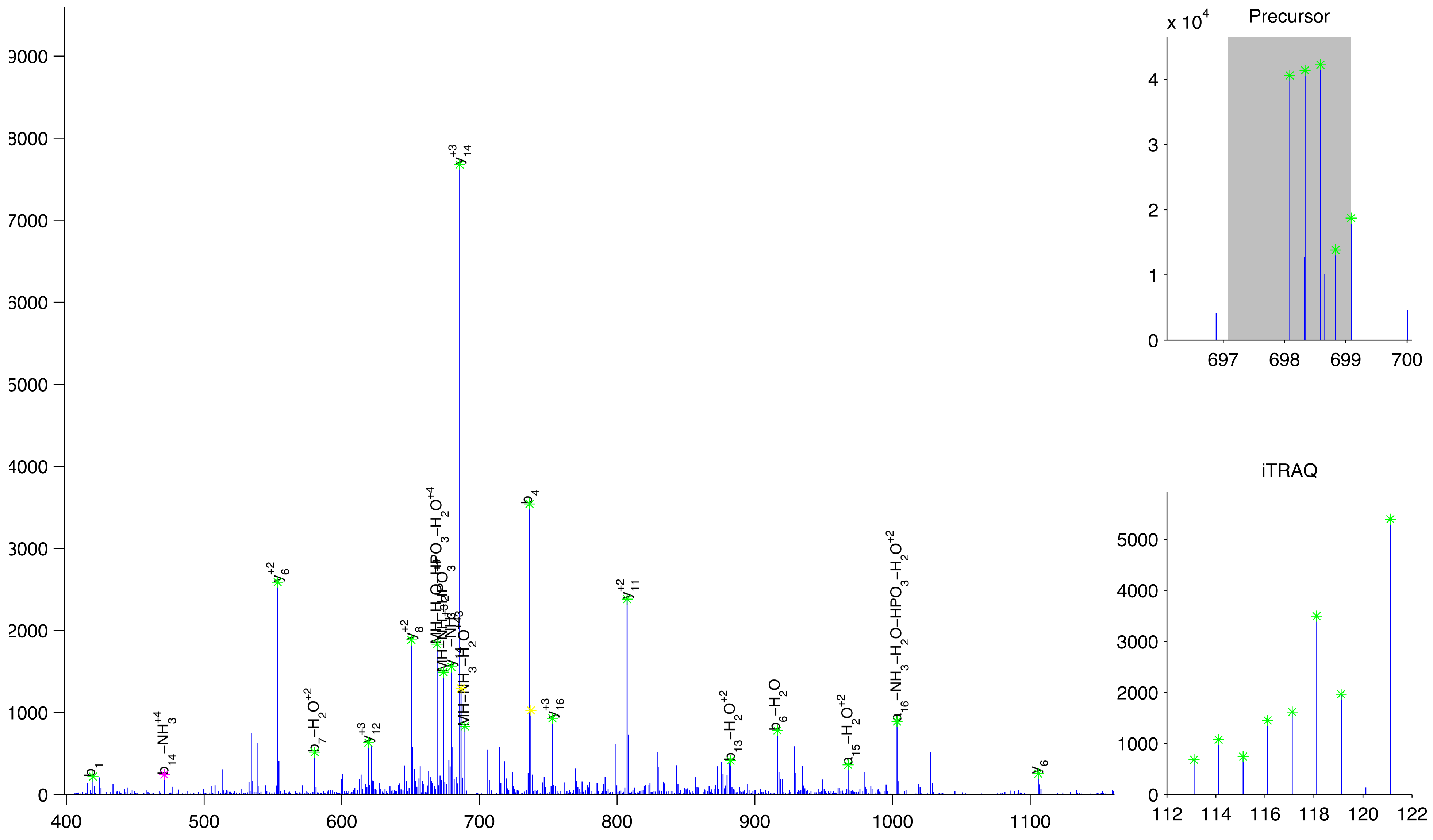

NDSDPTyGQQGHPNTWK

LIM domain containing preferred translocation partner in lipoma [Homo sapiens]
Charge State: +3
Scan Number: 5299
File Name: 120527_A549_TSAEGF_pY34_el.raw

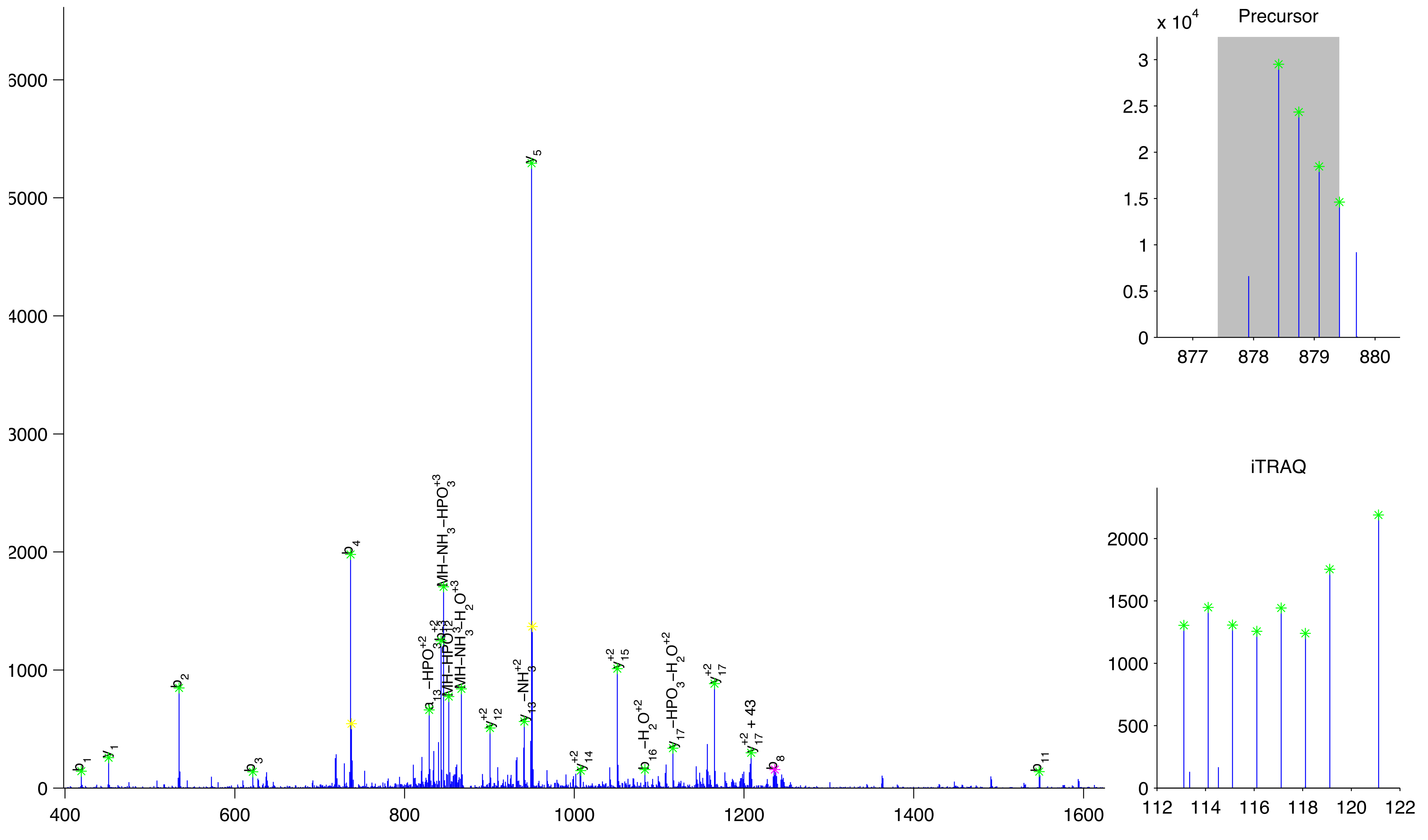

Y S Y Y D E S Q G E I y R

mediator of cell motility 1 [Homo sapiens]
Charge State: +2
Scan Number: 18352
File Name: 120518_A549_EGFTSA_pY.raw

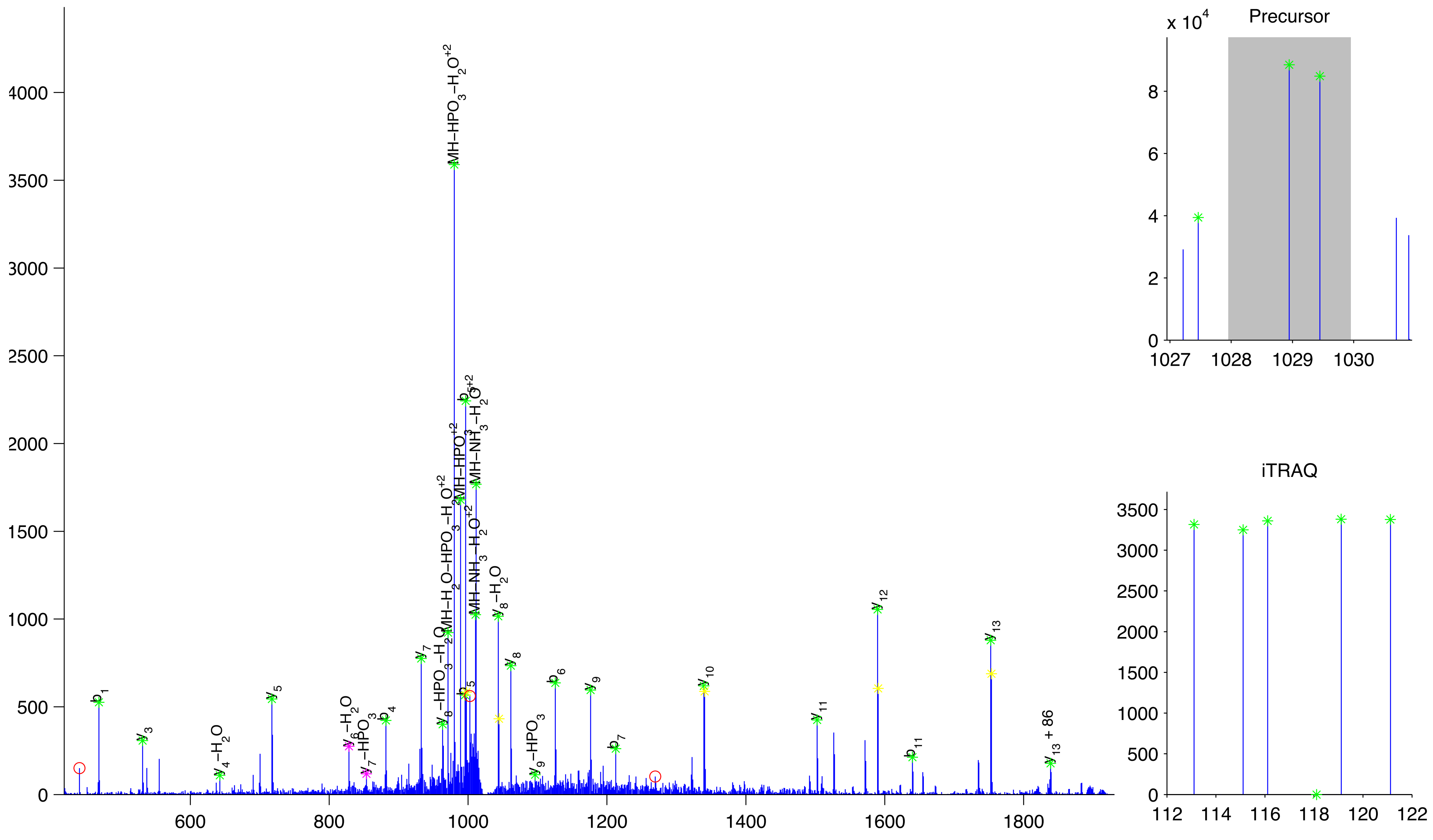

I E D P V y G I Y Y V D H I N R

membrane associated guanylate kinase, WW and PDZ domain containing 1 isoform b [Homo sapiens]
Charge State: +3
Scan Number: 16146
File Name: 120527_A549_TSAEGF_pY34_el.raw

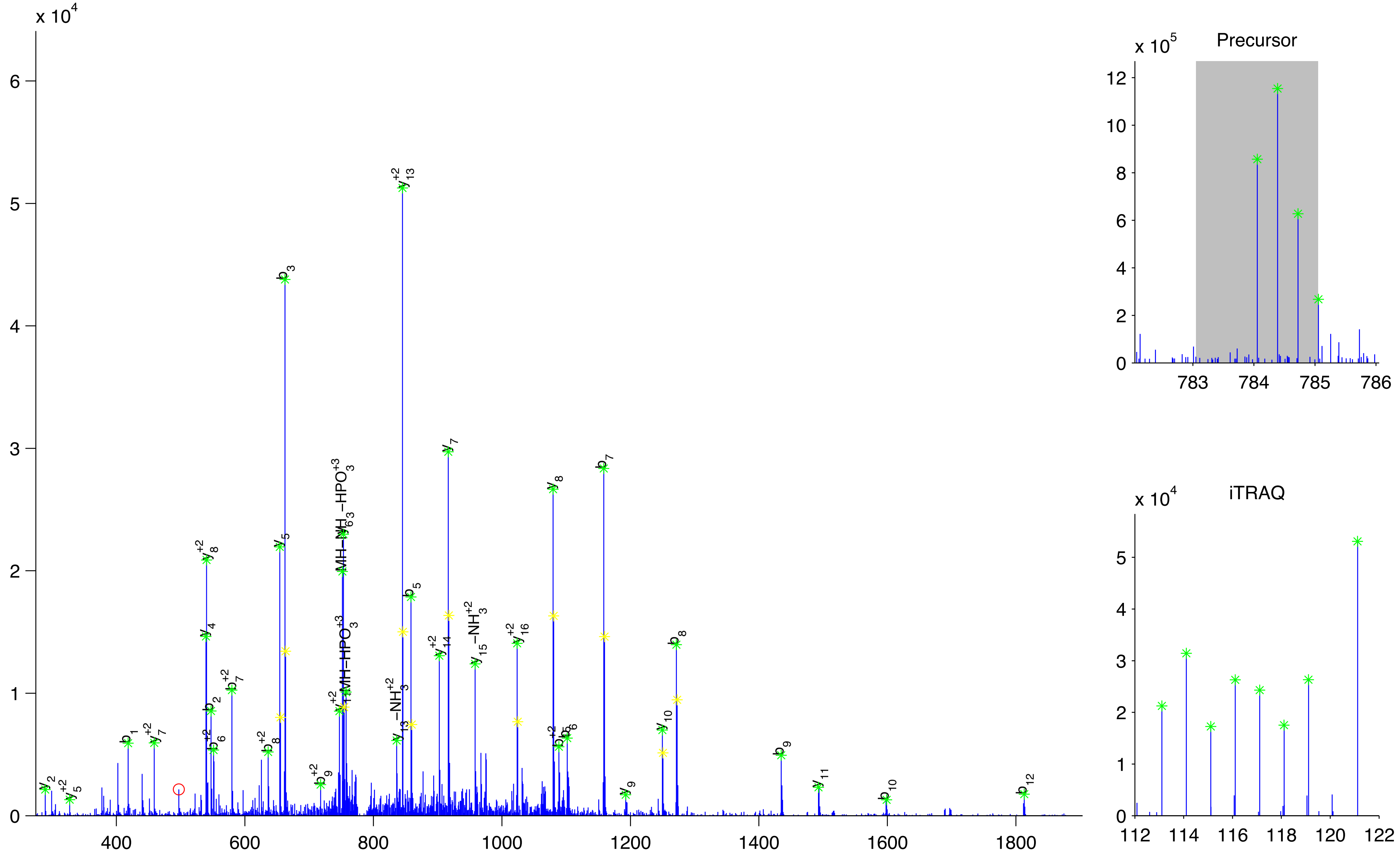

V A D P D H D H T G F L T E y V A T R

mitogen–activated protein kinase 1 [Homo sapiens]
Charge State: +3
Scan Number: 10497
File Name: 120527_A549_TSAEGF_pY34_el.raw

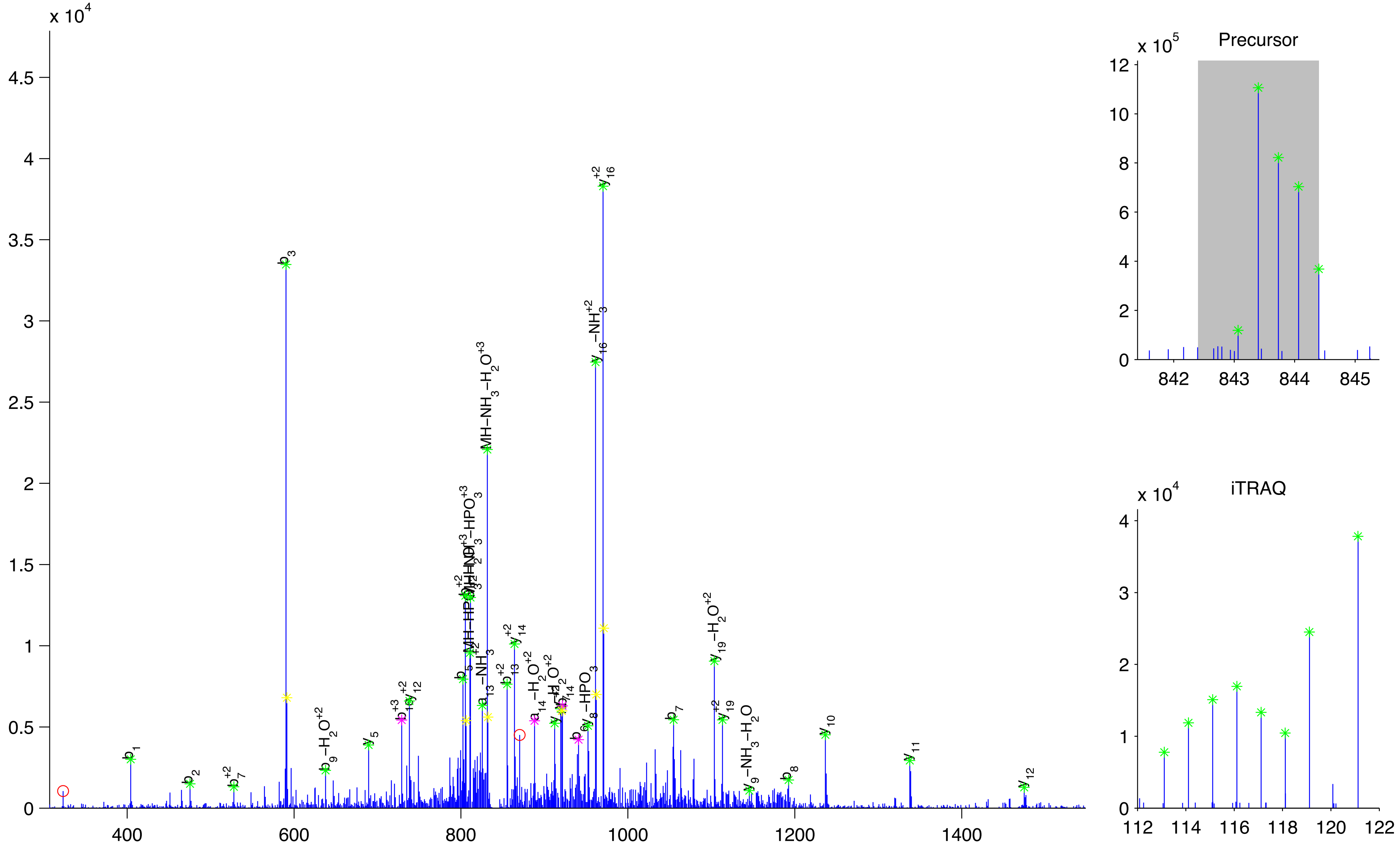

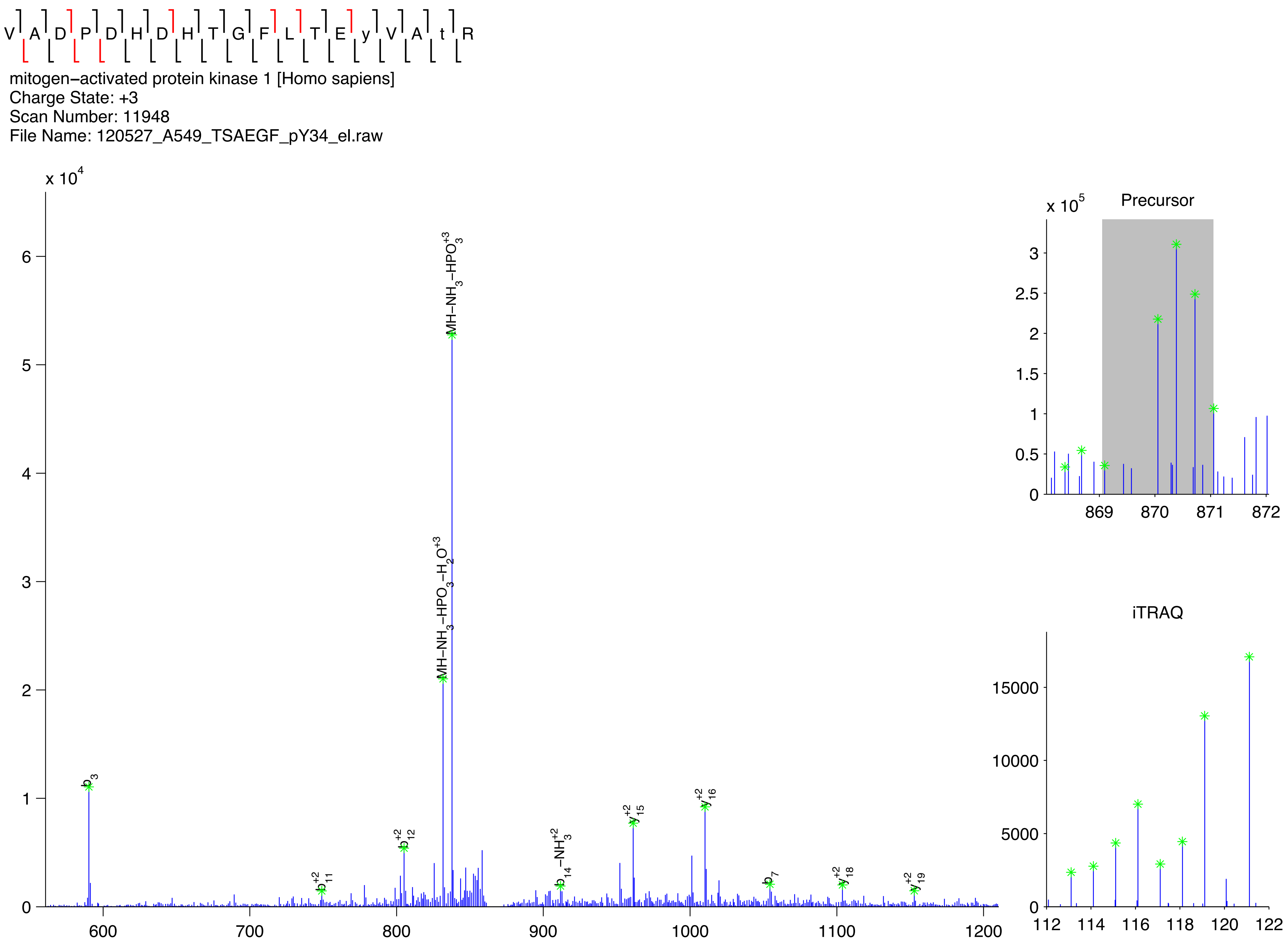

V A D P D H D H T G F L T E y V A t R
mitogen−activated protein kinase 1 [Homo sapiens]
Charge State: +3
Scan Number: 11948
File Name: 120527_A549_TSAEGF_pY34_el.raw
x 10^4
b3
b11 +2
b12 +2
MH−NH3−HPO3−H2O+3
MH−NH3−HPO3+3
b14−NH3 +2
y15 +2
y16 +2
b7
y18 +2
y19 +2
Precursor
x 10^5
iTRAQ

V A D P D H D H T G F L T E y V A T R

mitogen–activated protein kinase 1 [Homo sapiens]
Charge State: +4
Scan Number: 20608
File Name: 120518_A549_EGFTSA_pY.raw

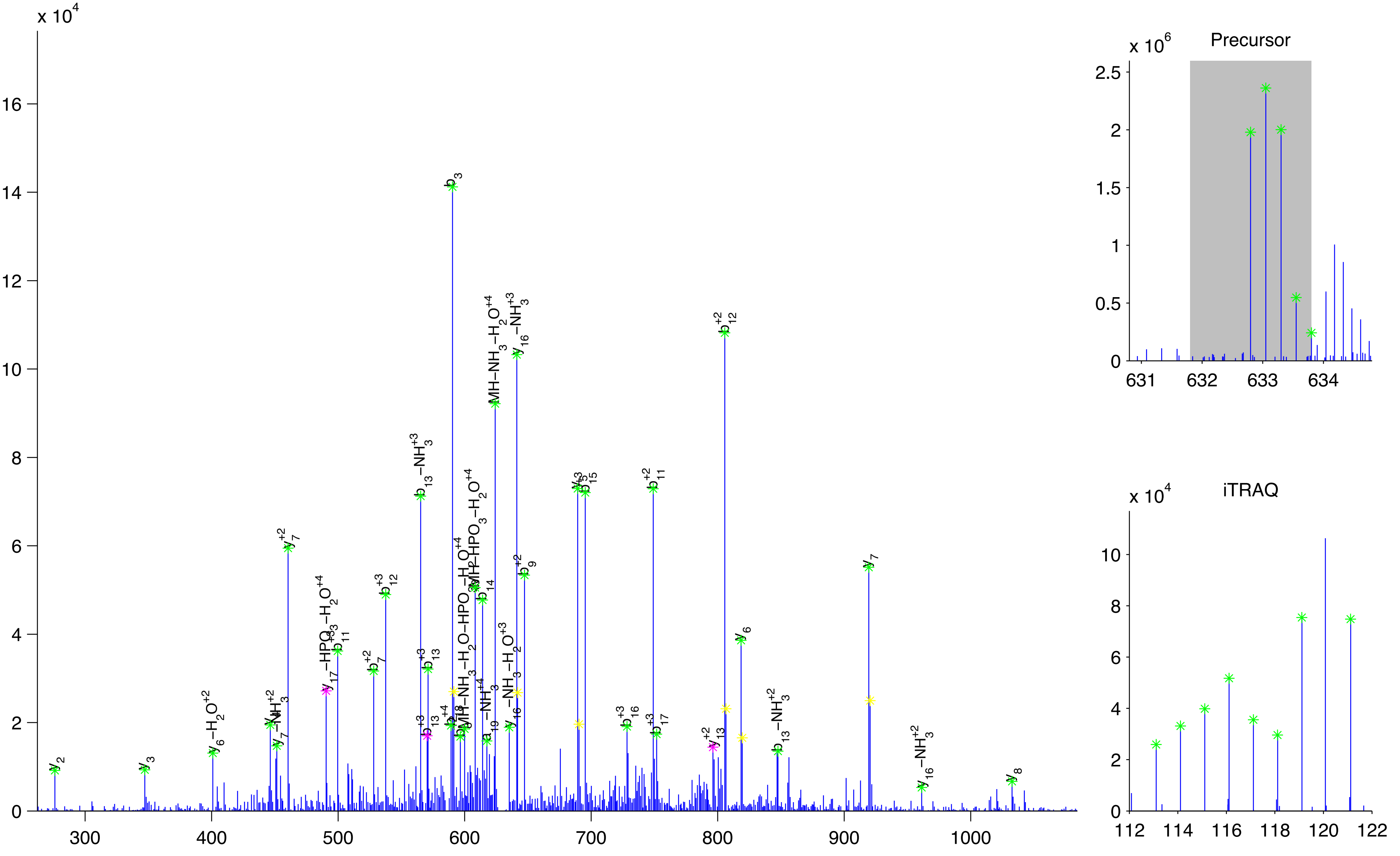

I A D P E H D H T G F L T E y V A T R

mitogen−activated protein kinase 3 isoform 2 [Homo sapiens]
Charge State: +3
Scan Number: 11022
File Name: 120527_A549_TSAEGF_pY34_el.raw

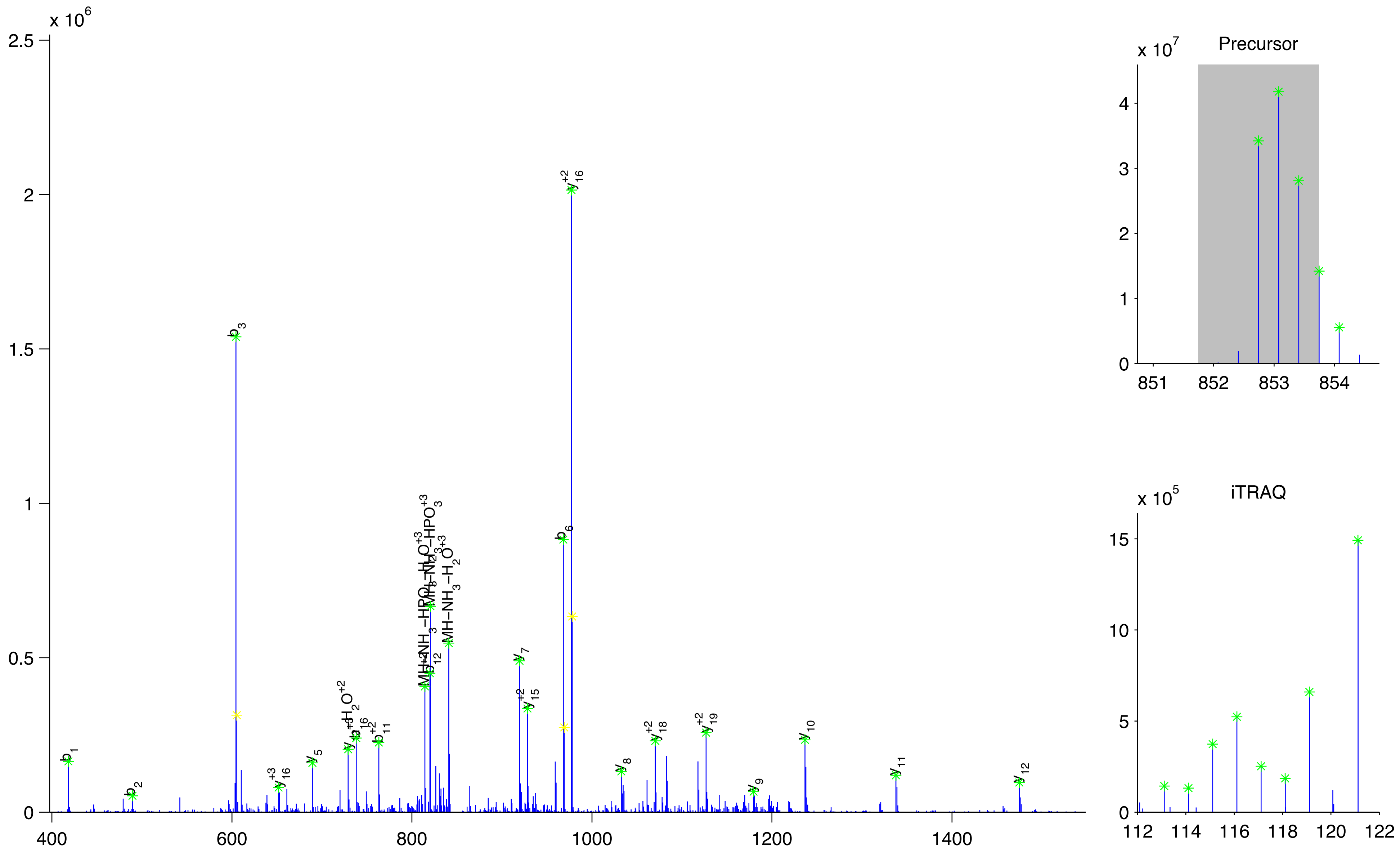

I A D P E H D H T G F L T E y V A T R

mitogen−activated protein kinase 3 isoform 2 [Homo sapiens]
Charge State: +4
Scan Number: 22078
File Name: 120518_A549_EGFTSA_pY.raw

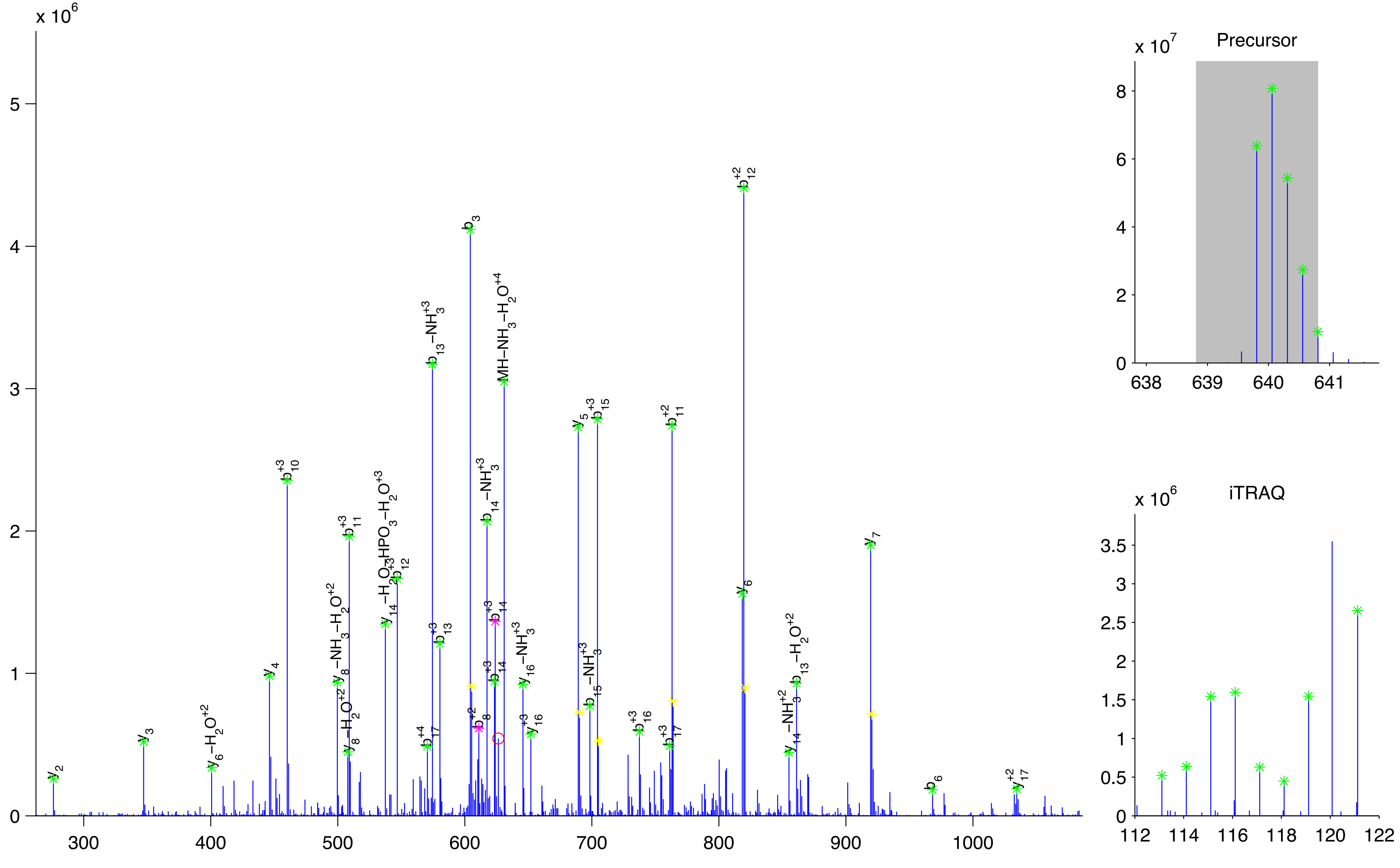

I A D P E H D H T G F L T E y V A T R

mitogen–activated protein kinase 3 isoform 2 [Homo sapiens]
Charge State: +3
Scan Number: 22099
File Name: 120518_A549_EGFTSA_pY.raw

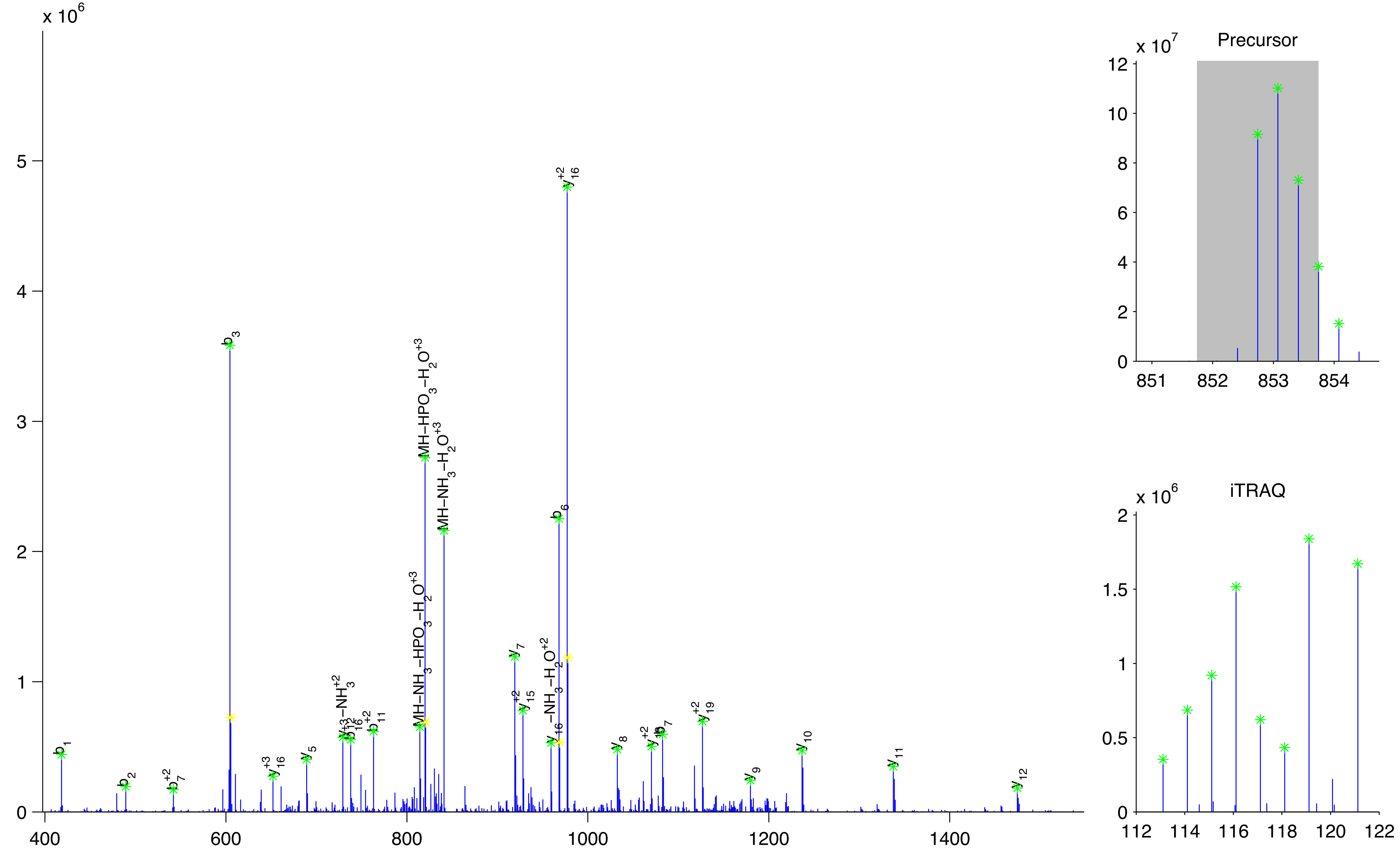

H T D D E M T G y V A T R

mitogen–activated protein kinase 14 isoform 1 [Homo sapiens]
Charge State: +3
Scan Number: 9226
File Name: 120518_A549_EGFTSA_pY.raw

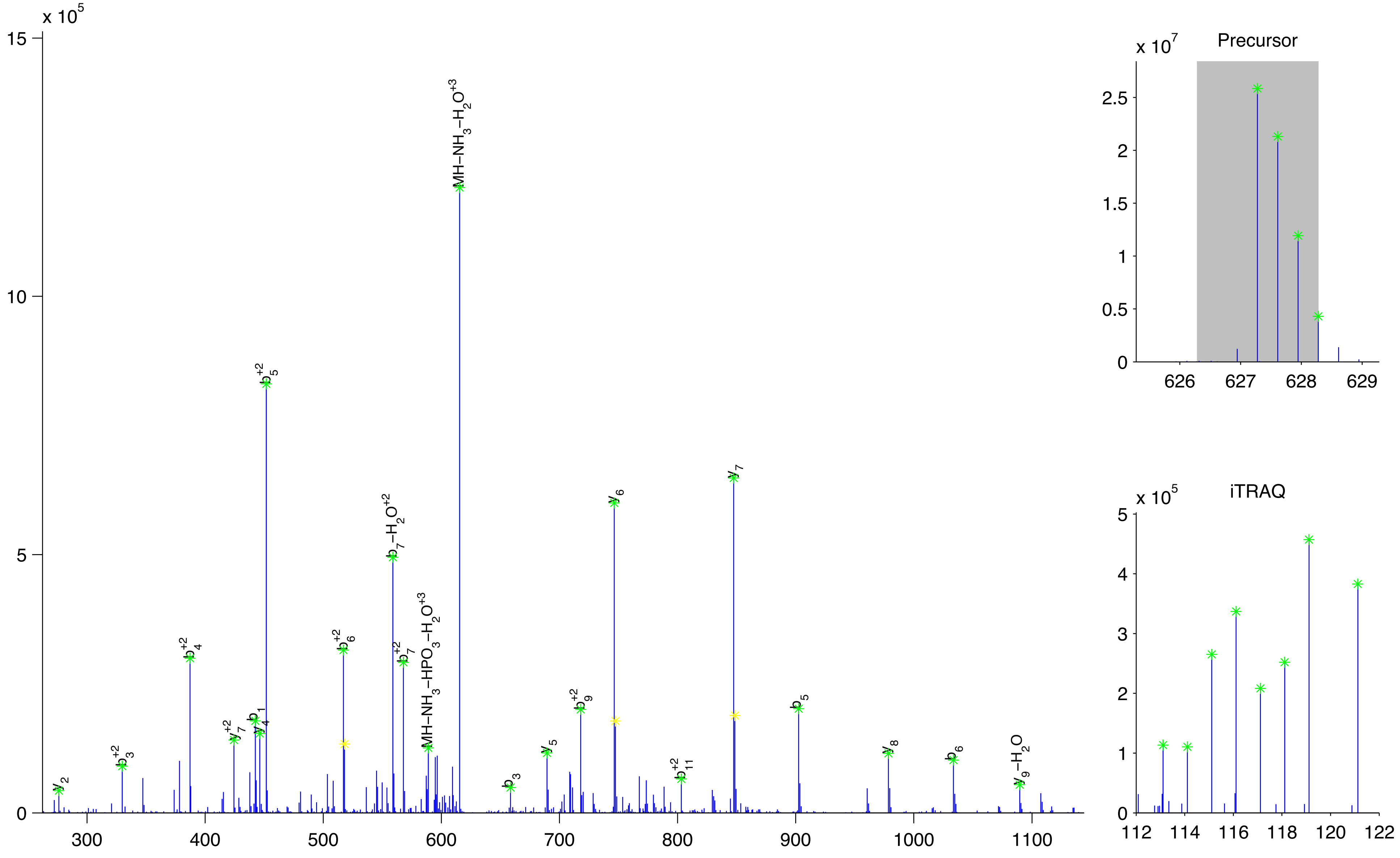

S E S V V y A D I R

myelin protein zero–like 1 isoform a [Homo sapiens]
Charge State: +2
Scan Number: 16685
File Name: 120518_A549_EGFTSA_pY.raw

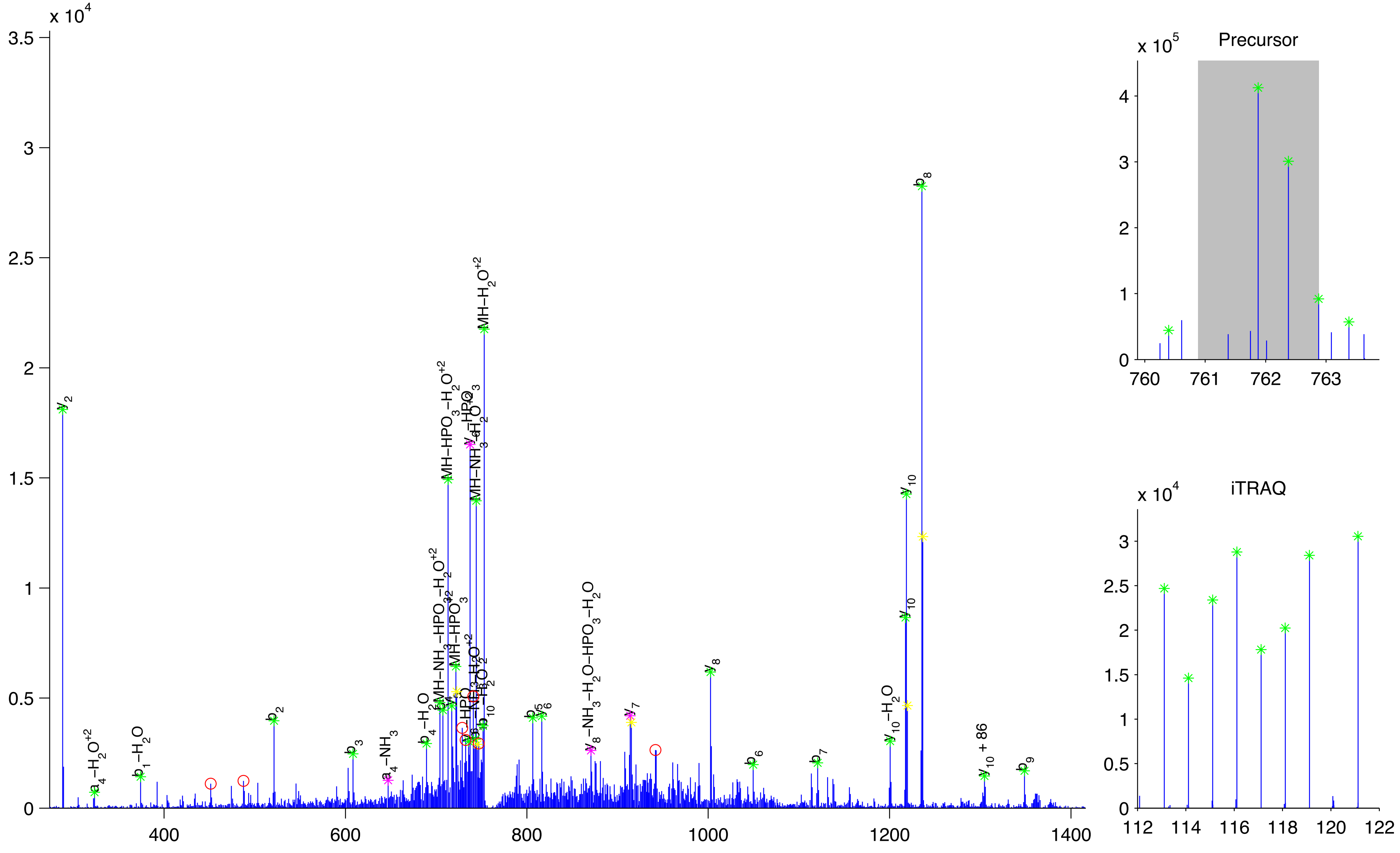

S E S V V y A D I R

myelin protein zero–like 1 isoform a [Homo sapiens]
Charge State: +2
Scan Number: 17206
File Name: 120518_A549_EGFTSA_pY.raw

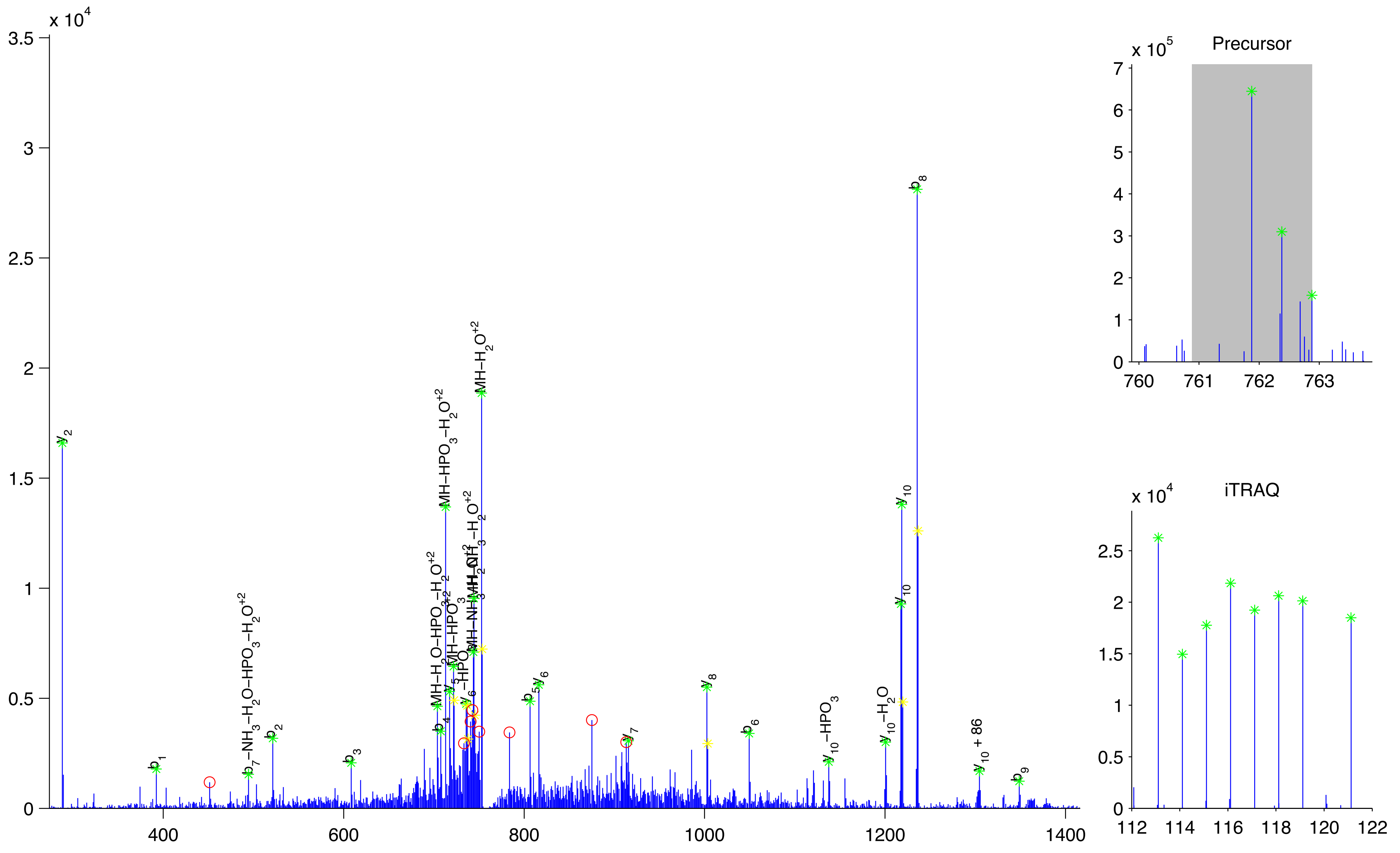

G I V V y T G D R

Na+/K+ –ATPase alpha 1 subunit isoform a proprotein [Homo sapiens]
Charge State: +3
Scan Number: 7882
File Name: 120527_A549_TSAEGF_pY34_el.raw

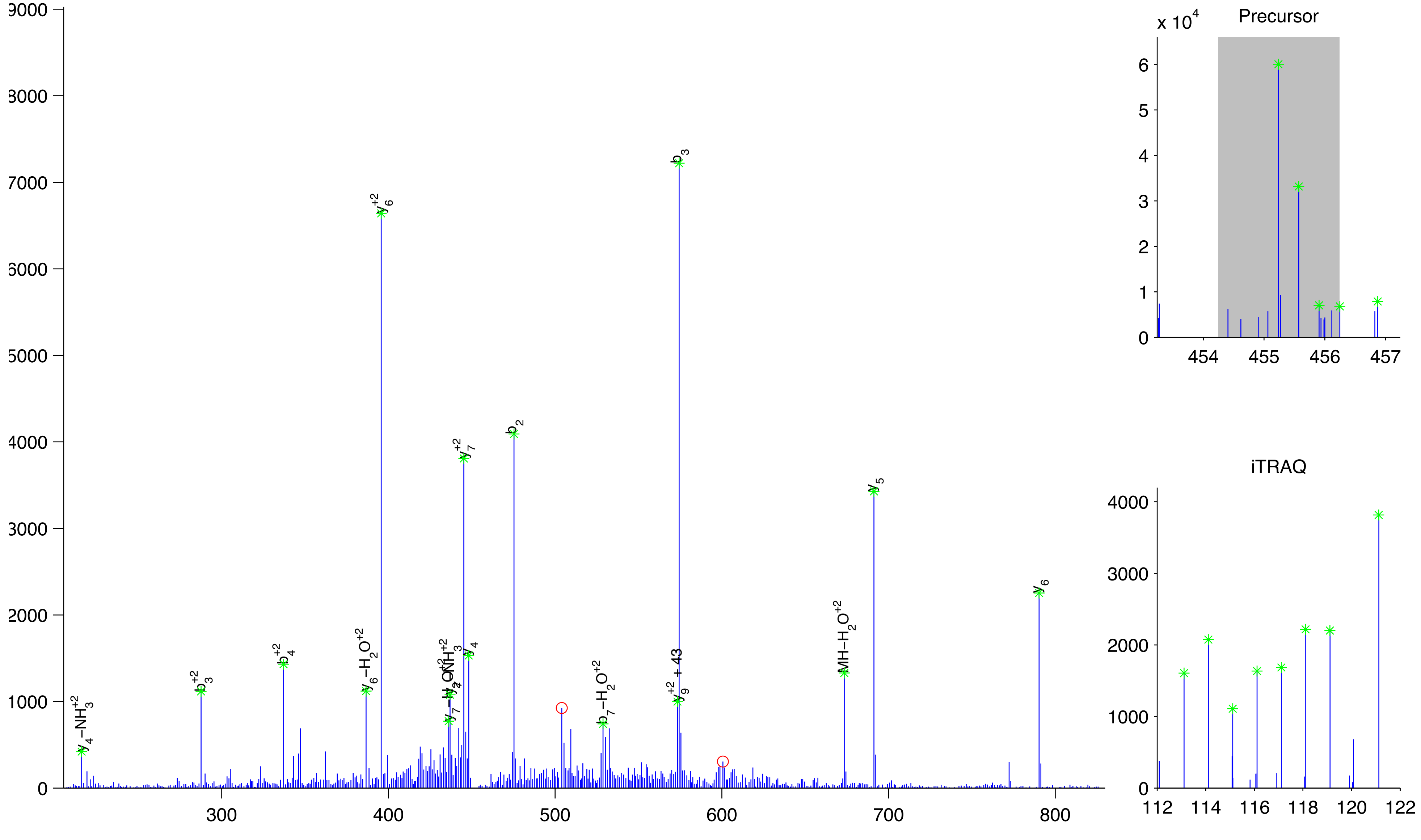

G I V V y T G D R

Na+/K+ −ATPase alpha 1 subunit isoform a proprotein [Homo sapiens]
Charge State: +2
Scan Number: 13883
File Name: 120518_A549_EGFTSA_pY.raw

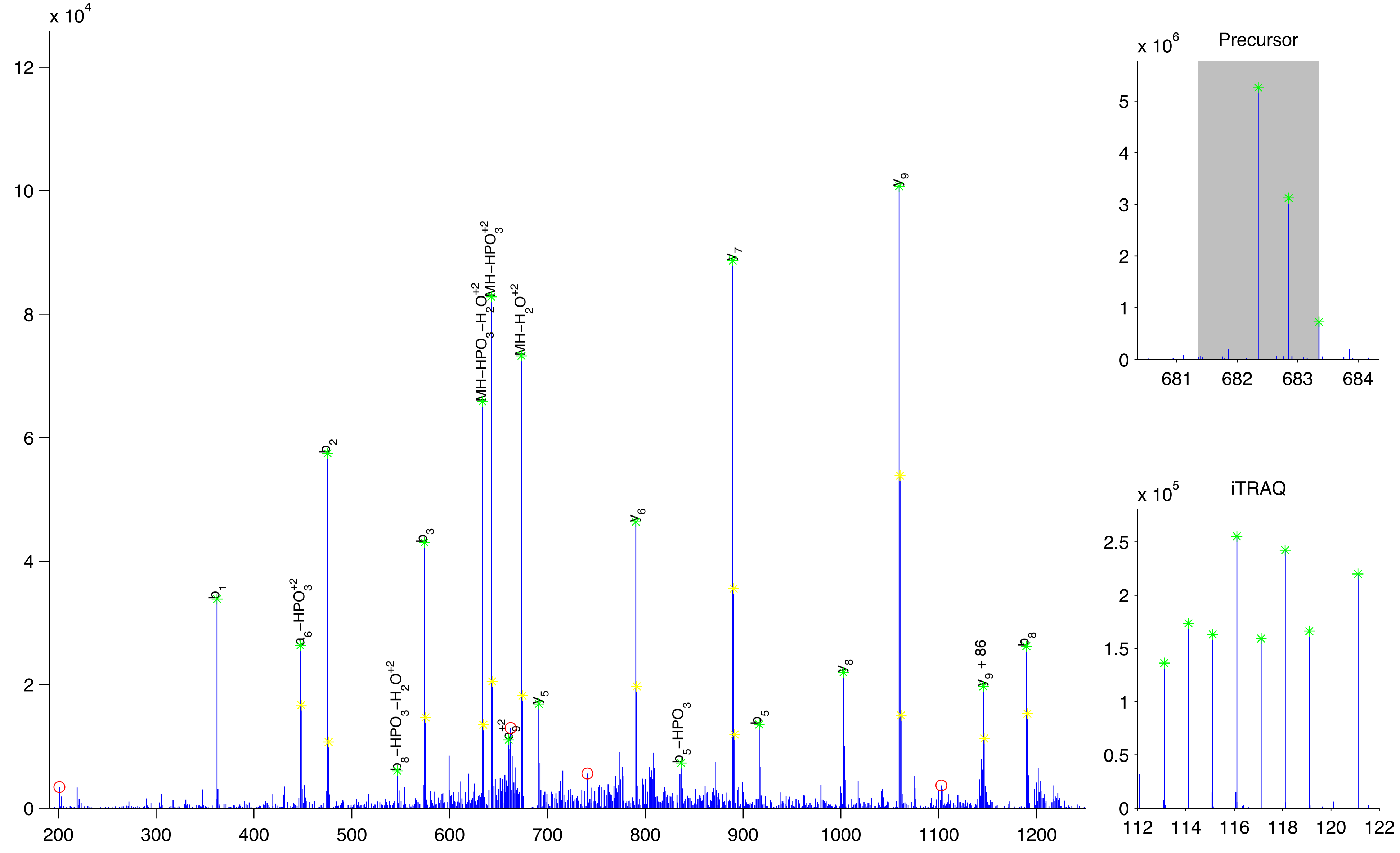

A N P Q E R D G V y D V P L H N P P D A K

neural precursor cell expressed, developmentally down−regulated 9 isoform 1 [Homo sapiens]
Charge State: +4
Scan Number: 7305
File Name: 120527_A549_TSAEGF_pY34_el.raw

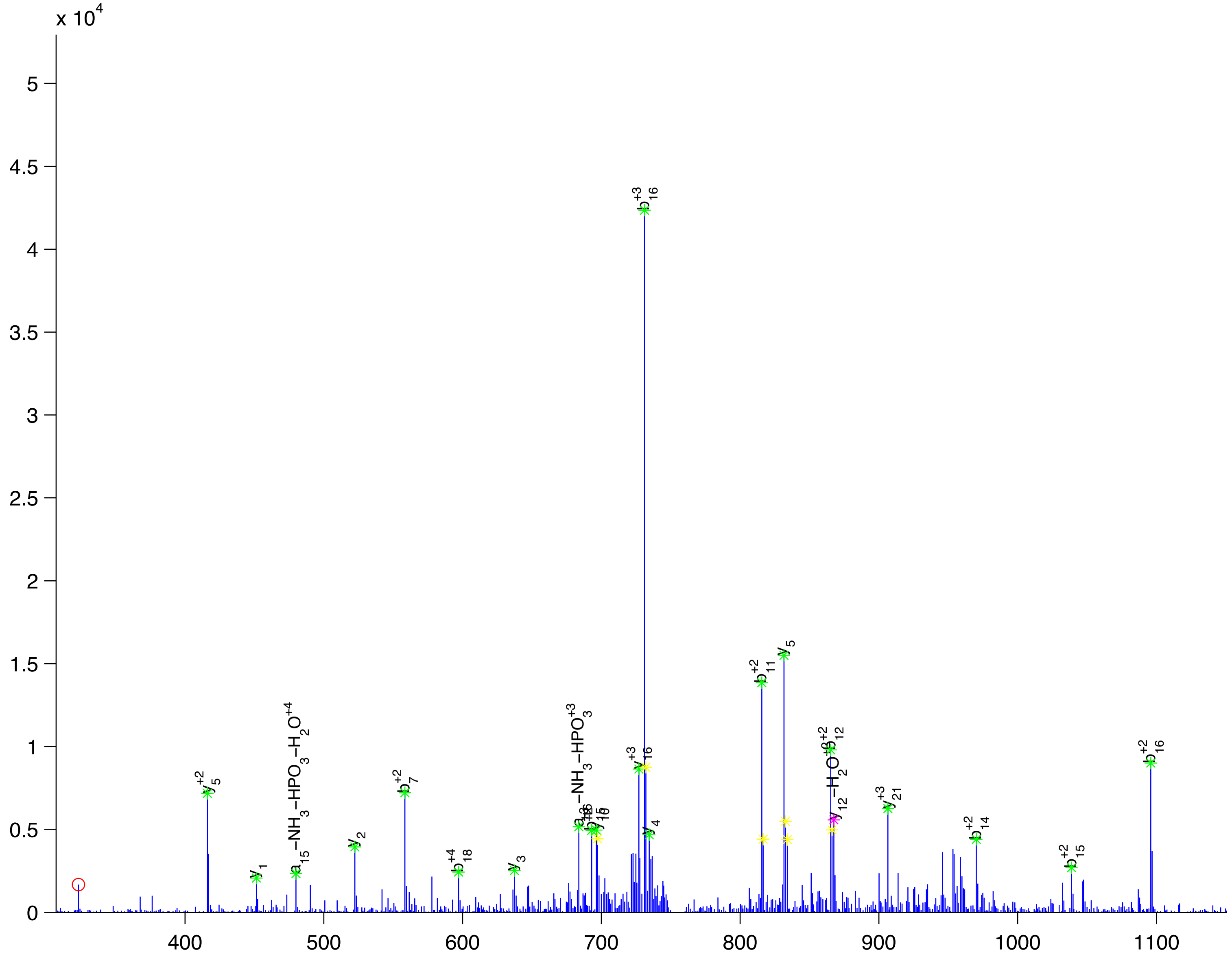

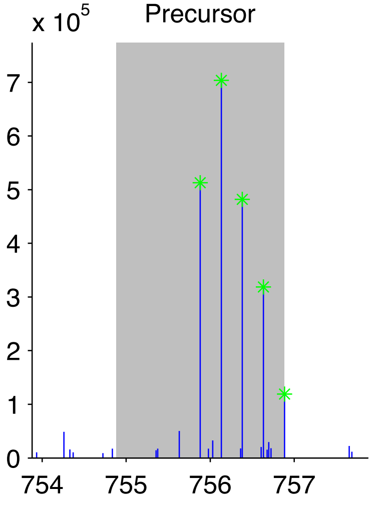

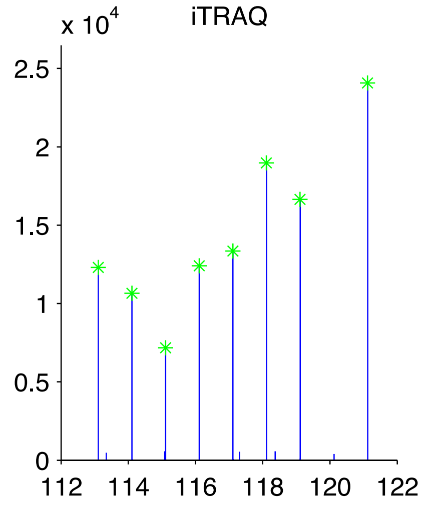

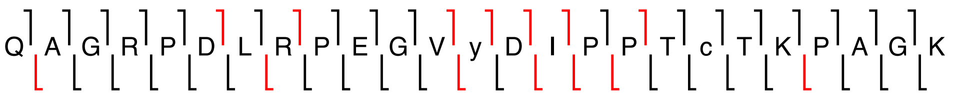

neural precursor cell expressed, developmentally down−regulated 9 isoform 1 [Homo sapiens]
Charge State: +5
Scan Number: 8250
File Name: 120527_A549_TSAEGF_pY34_el.raw

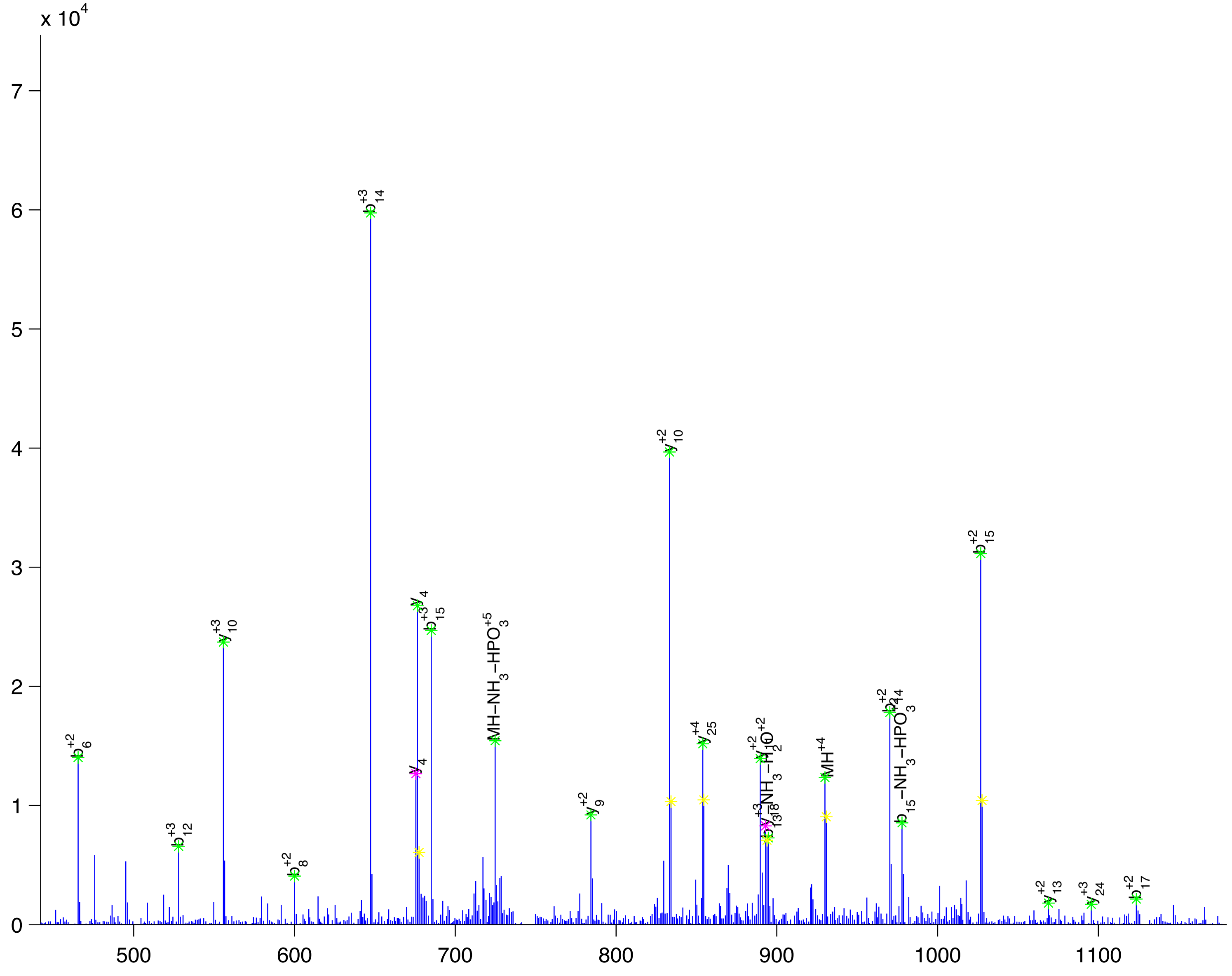

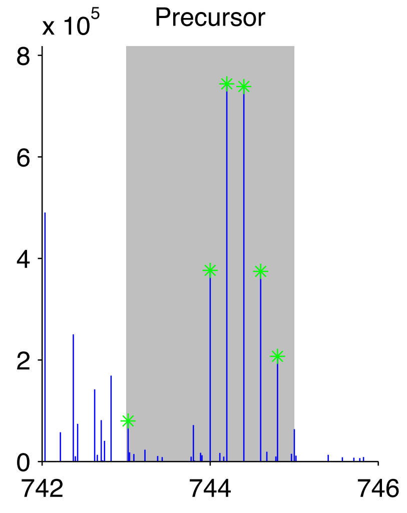

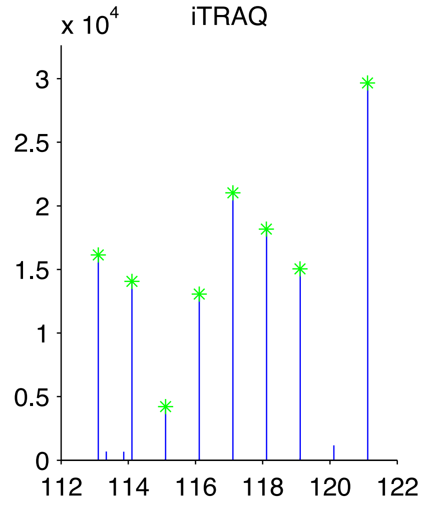

D G V y D V P L H N P P D A K

neural precursor cell expressed, developmentally down−regulated 9 isoform 1 [Homo sapiens]
Charge State: +4
Scan Number: 8502
File Name: 120527_A549_TSAEGF_pY34_el.raw

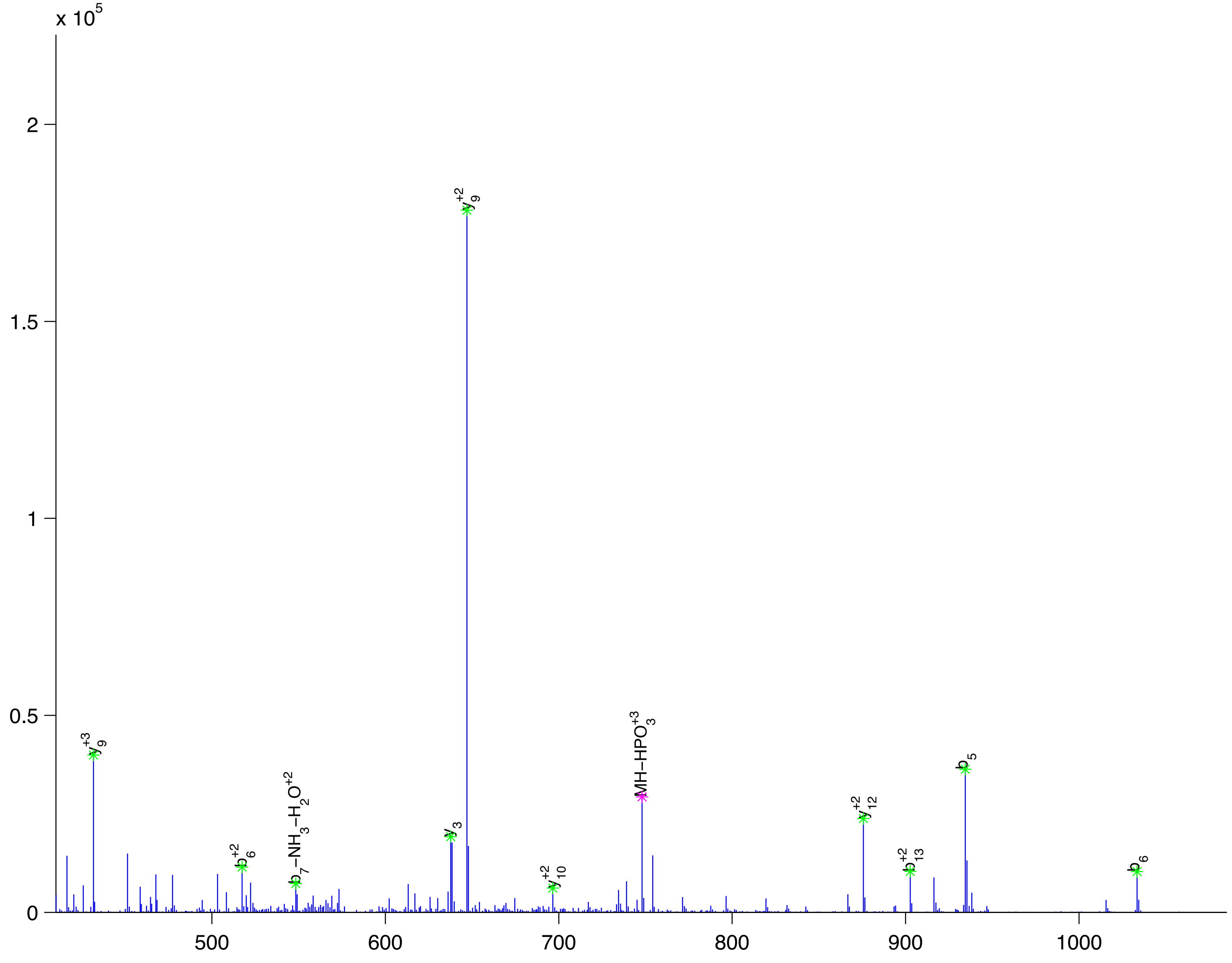

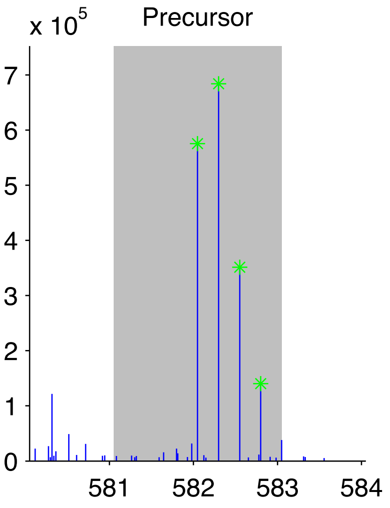

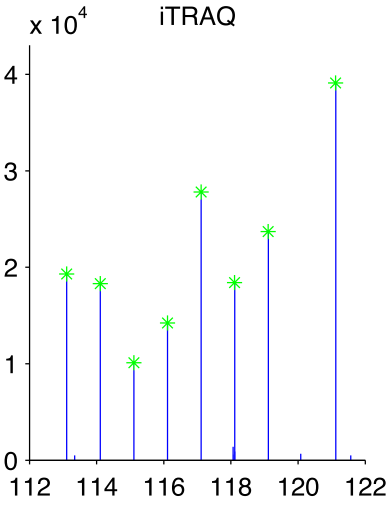

A N P Q E R D G V y D V P L H N P P D A K

neural precursor cell expressed, developmentally down−regulated 9 isoform 1 [Homo sapiens]
Charge State: +3
Scan Number: 14020
File Name: 120518_A549_EGFTSA_pY.raw

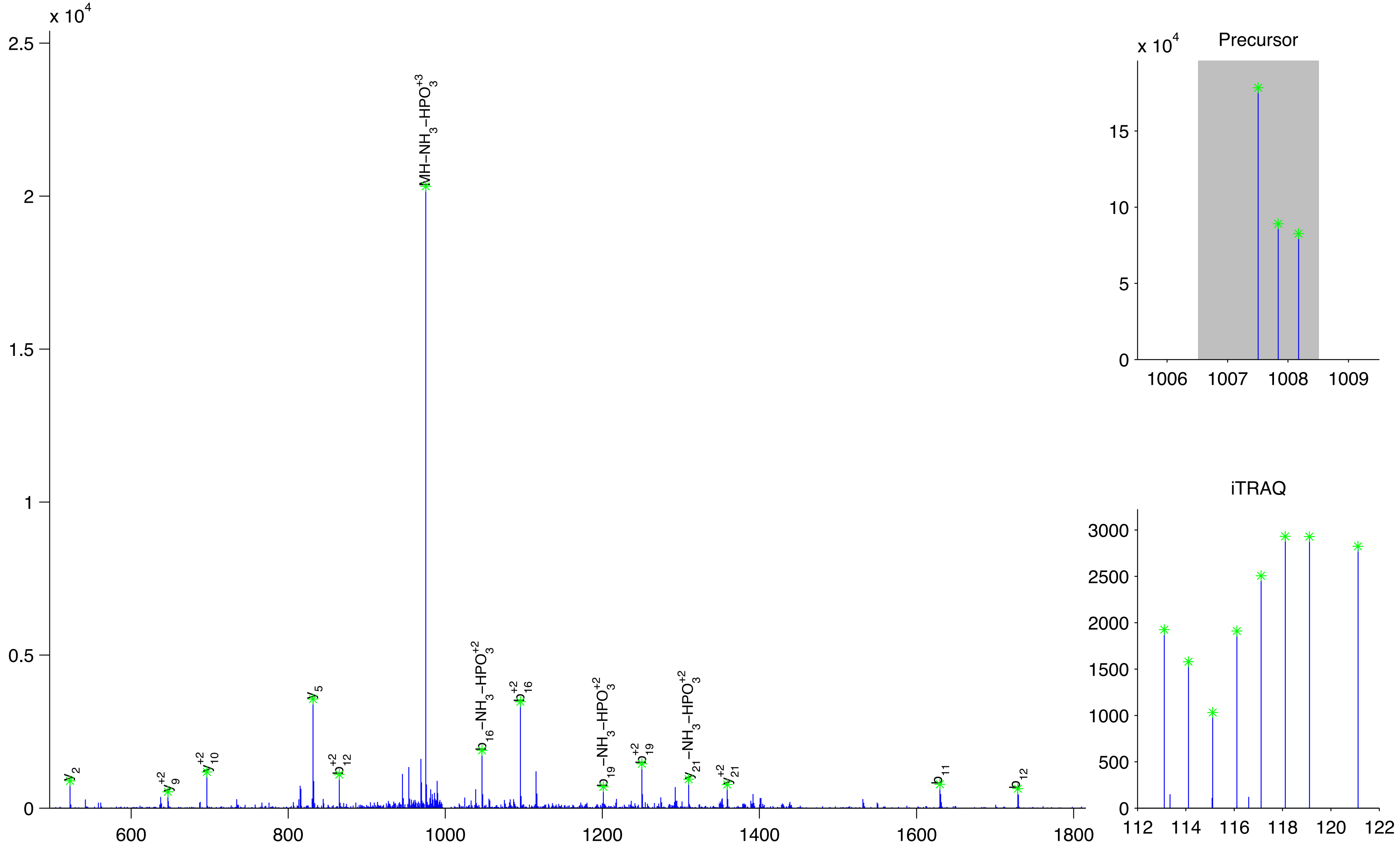

A N P Q E R D G V y D V P L H N P P D A K

neural precursor cell expressed, developmentally down−regulated 9 isoform 1 [Homo sapiens]
Charge State: +4
Scan Number: 14291
File Name: 120518_A549_EGFTSA_pY.raw

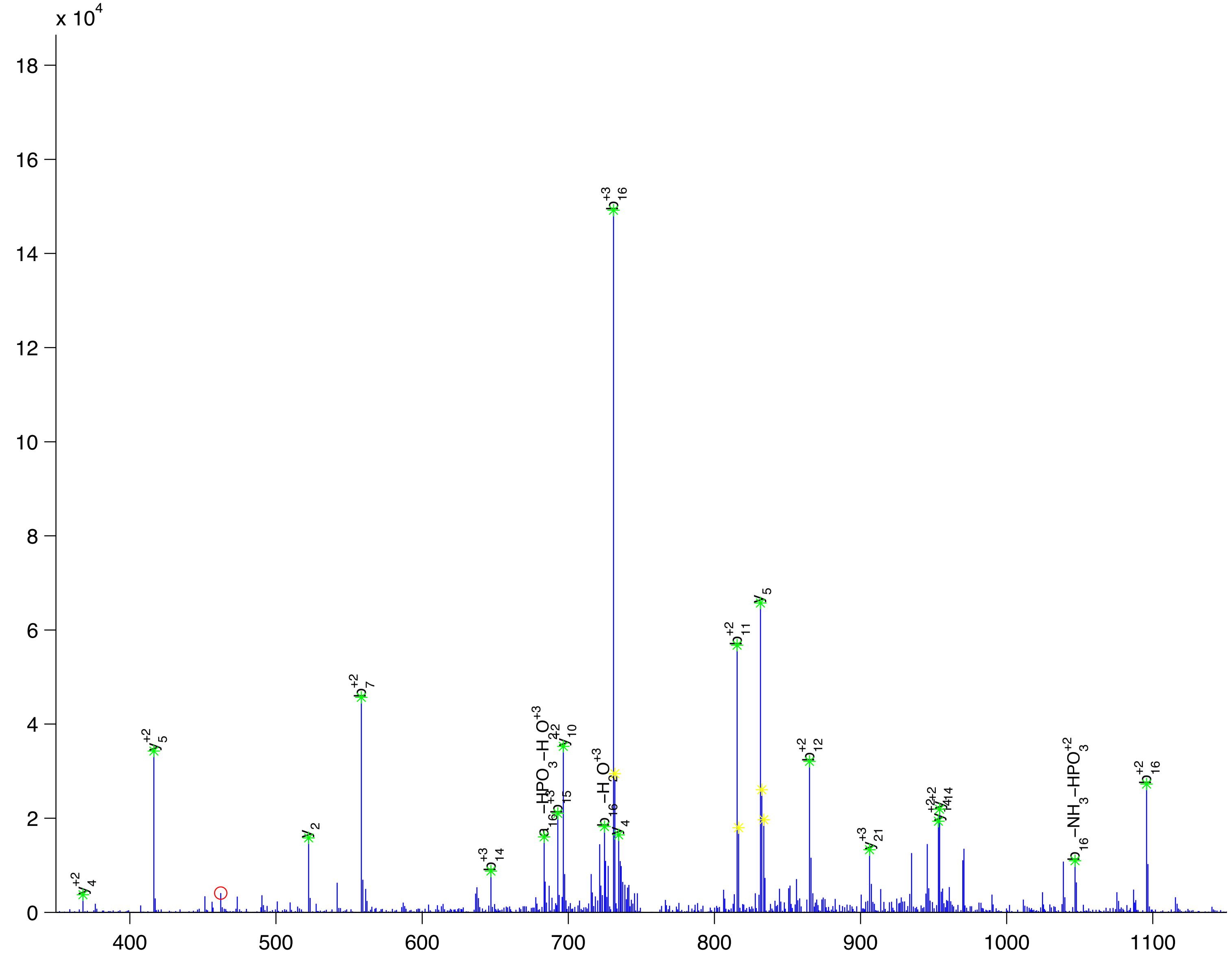

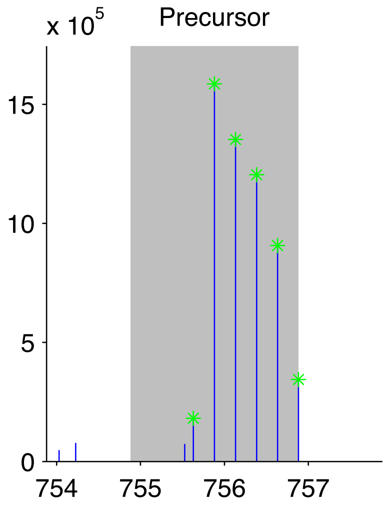

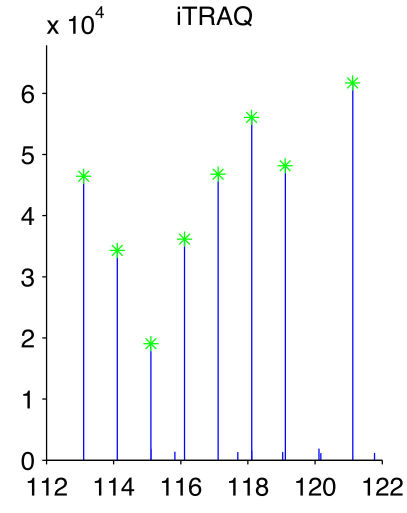

Q A G R P D L R P E G V y D I P P T c T K P A G K

neural precursor cell expressed, developmentally down−regulated 9 isoform 1 [Homo sapiens]
Charge State: +5
Scan Number: 15148
File Name: 120518_A549_EGFTSA_pY.raw

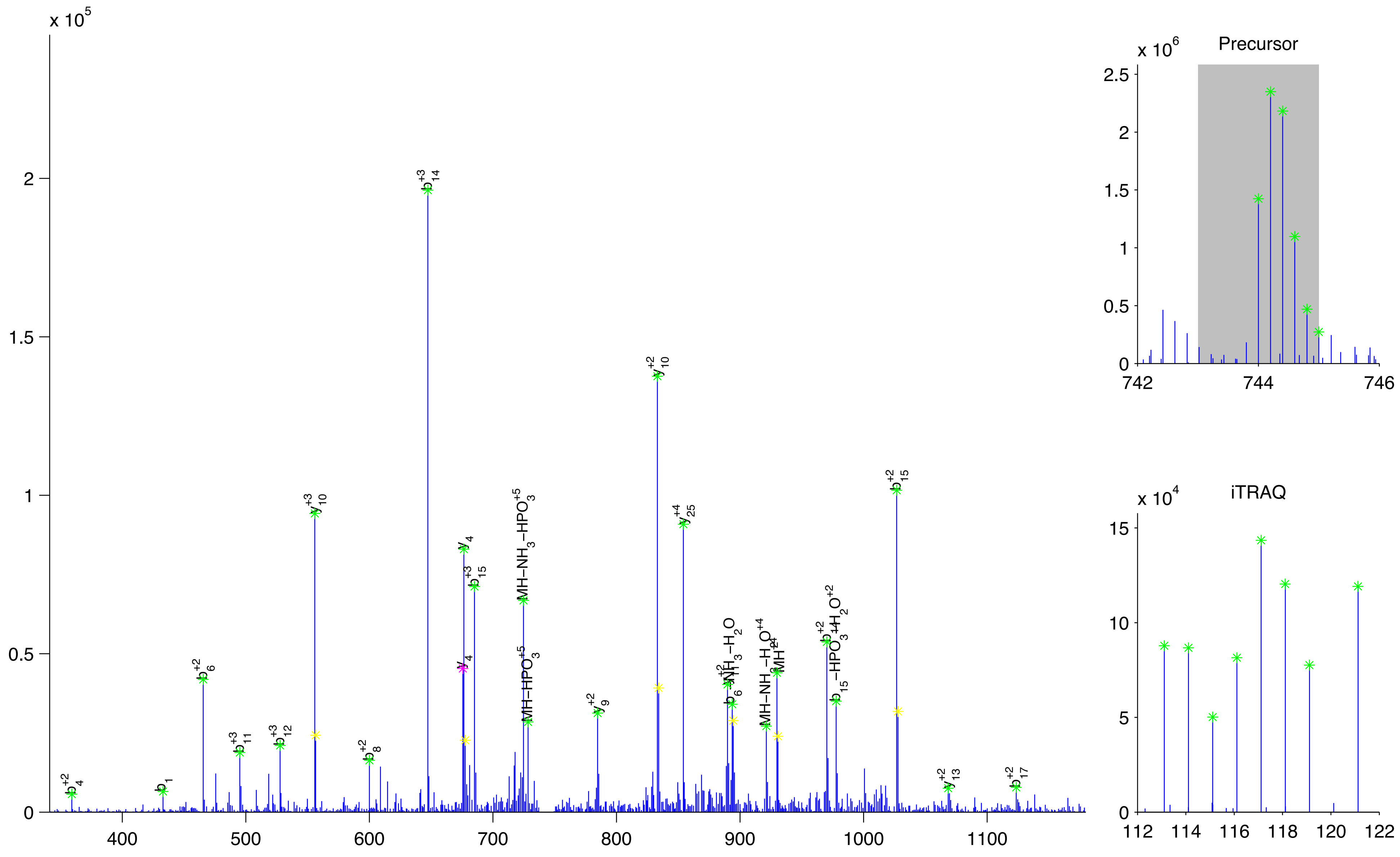

Q A G R P D L R P E G V y D I P P T c T K P A G K

neural precursor cell expressed, developmentally down–regulated 9 isoform 1 [Homo sapiens]
Charge State: +4
Scan Number: 15158
File Name: 120518_A549_EGFTSA_pY.raw

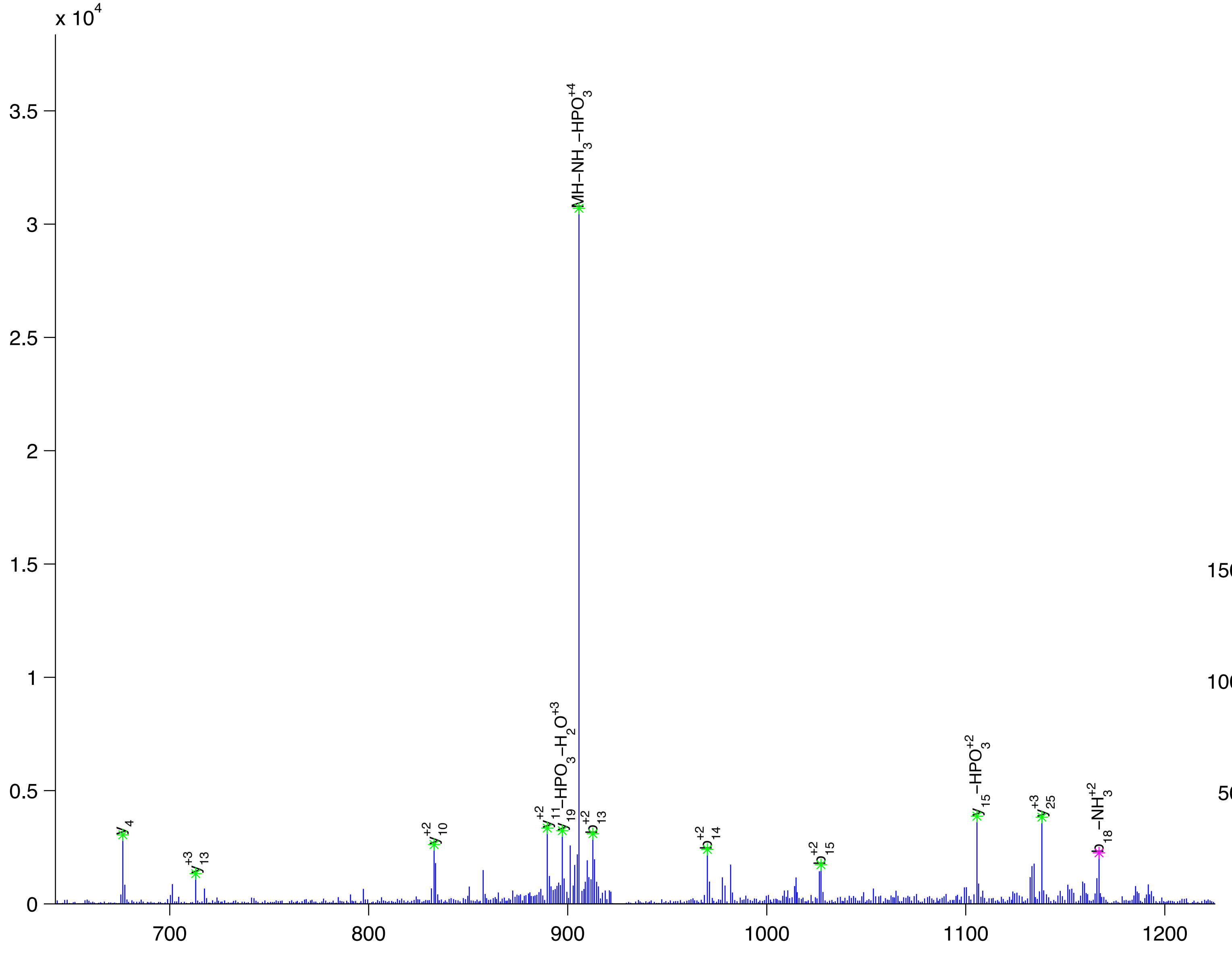

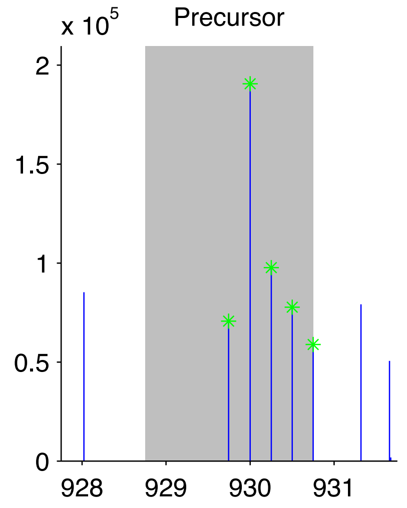

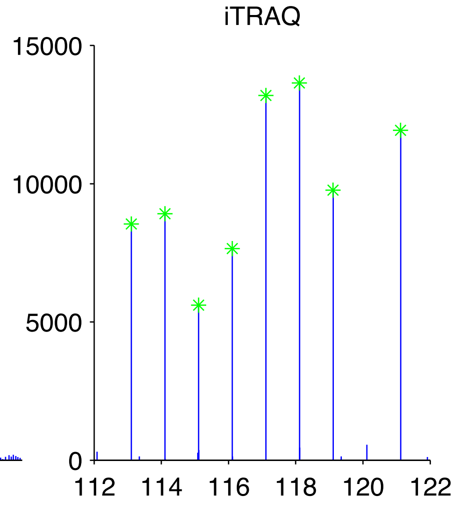

N A I K V P I V I N P N A y D N L A I Y K

NKF3 kinase family member [Homo sapiens]
Charge State: +4
Scan Number: 15497
File Name: 120527_A549_TSAEGF_pY34_el.raw

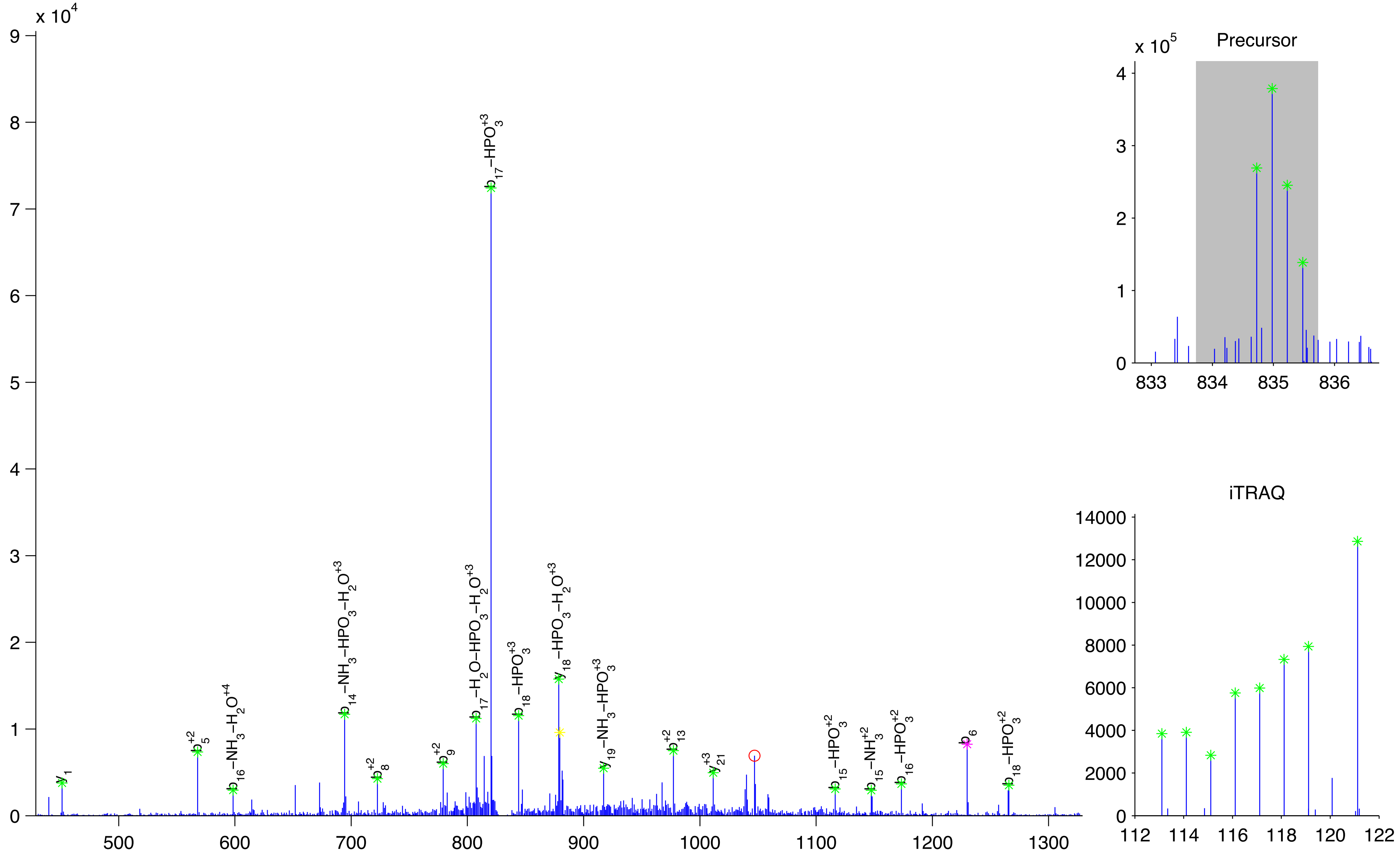

V P I V I N P N A Y D N L A I y K

NKF3 kinase family member [Homo sapiens]
Charge State: +4
Scan Number: 29236
File Name: 120518_A549_EGFTSA_pY.raw

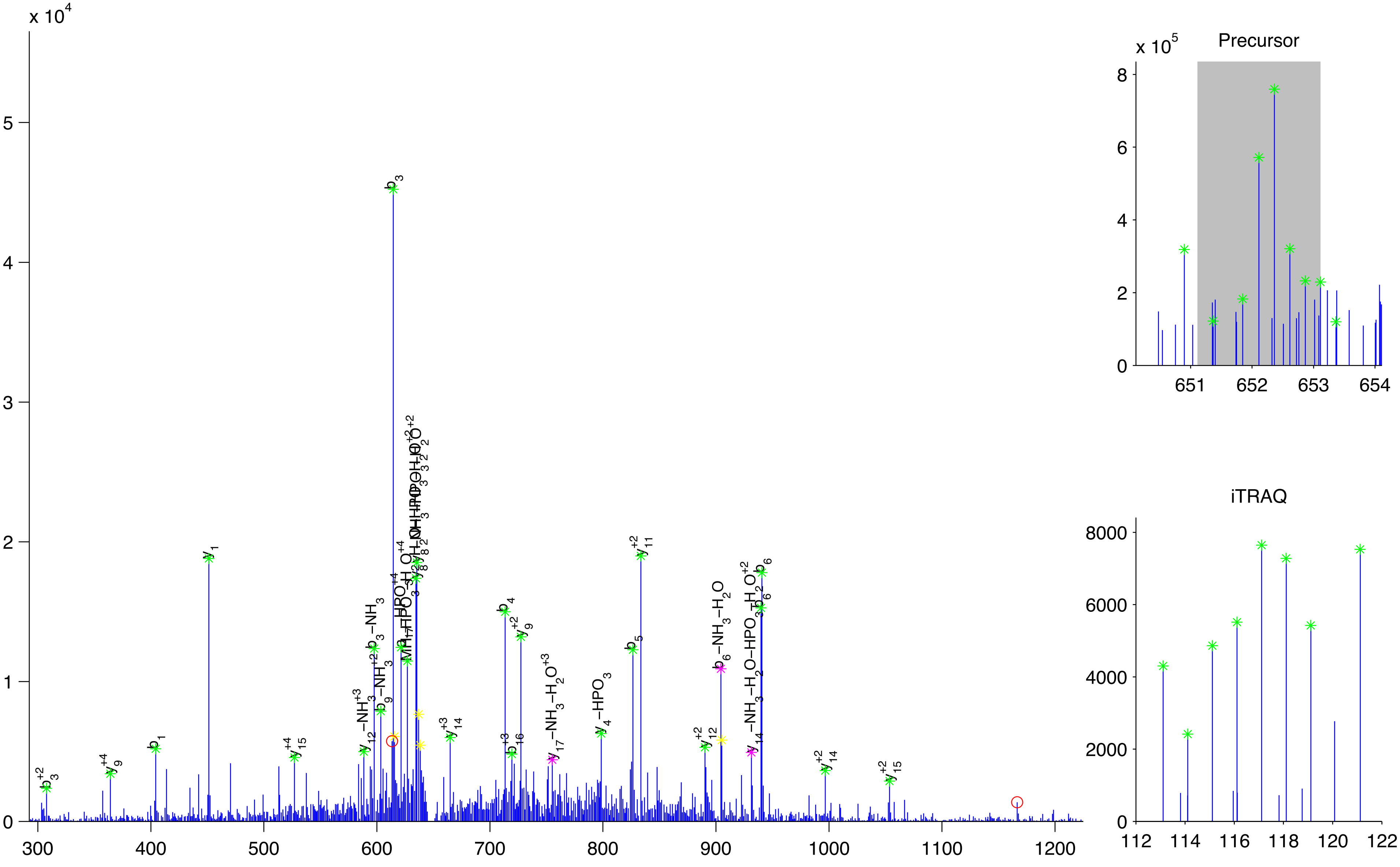

E R D y A E I Q D F H R

partitioning–defective protein 3 homolog [Homo sapiens]
Charge State: +3
Scan Number: 5856
File Name: 120527_A549_TSAEGF_pY34_el.raw

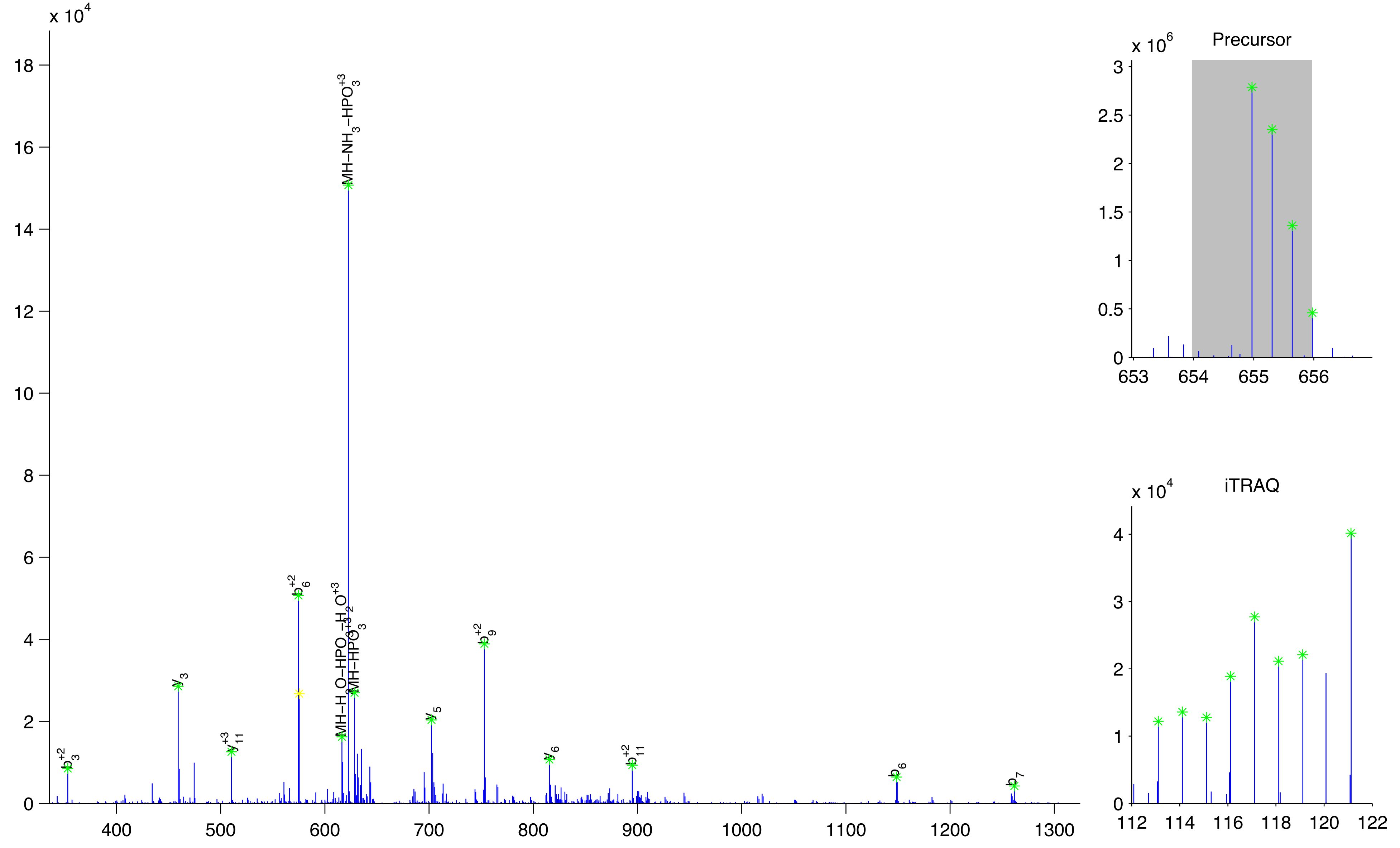

E R D y A E I Q D F H R

partitioning−defective protein 3 homolog [Homo sapiens]
Charge State: +3
Scan Number: 6255
File Name: 120527_A549_TSAEGF_pY34_el.raw

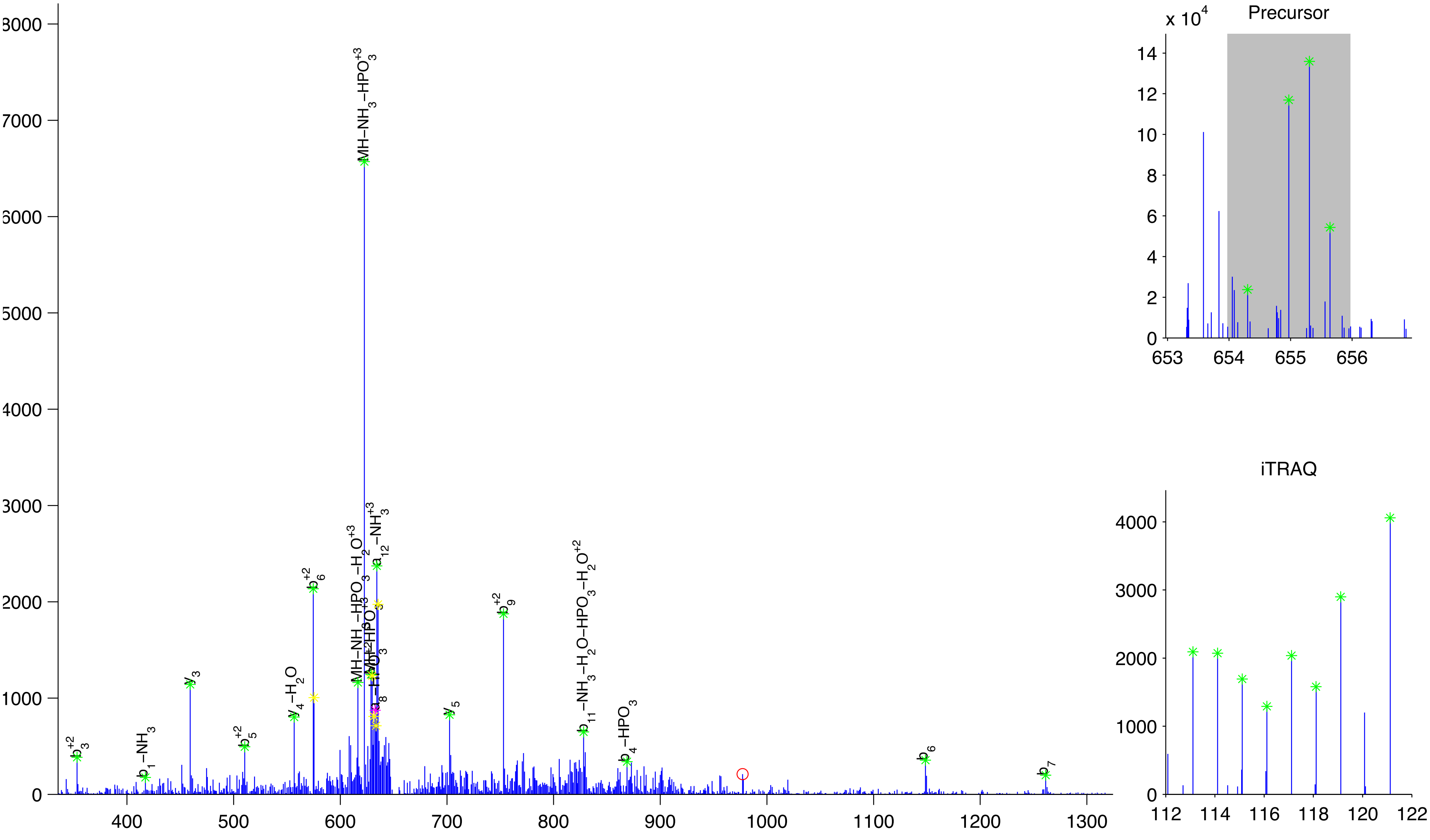

E R D y A E I Q D F H R

partitioning−defective protein 3 homolog [Homo sapiens]
Charge State: +3
Scan Number: 12292
File Name: 120518_A549_EGFTSA_pY.raw

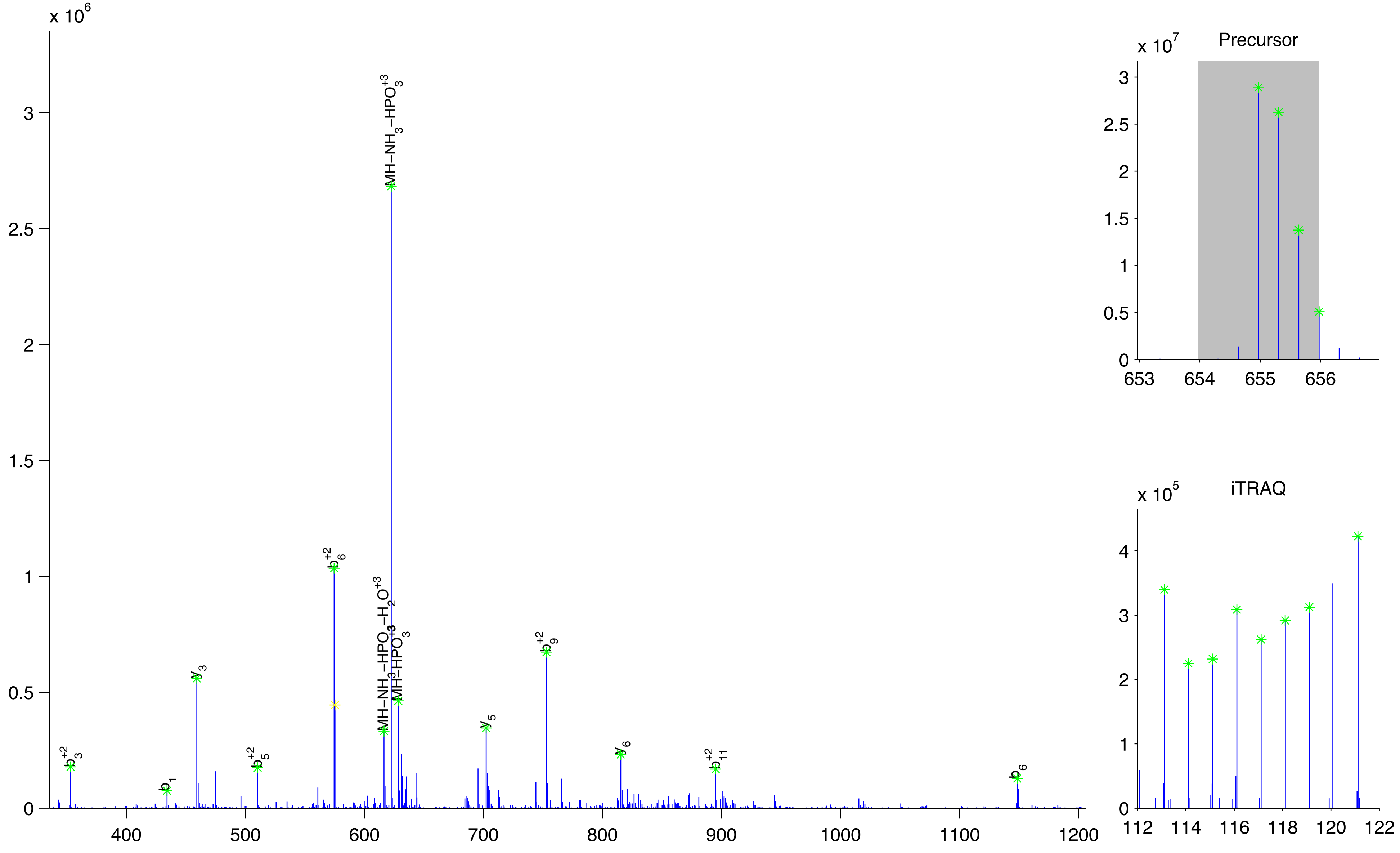

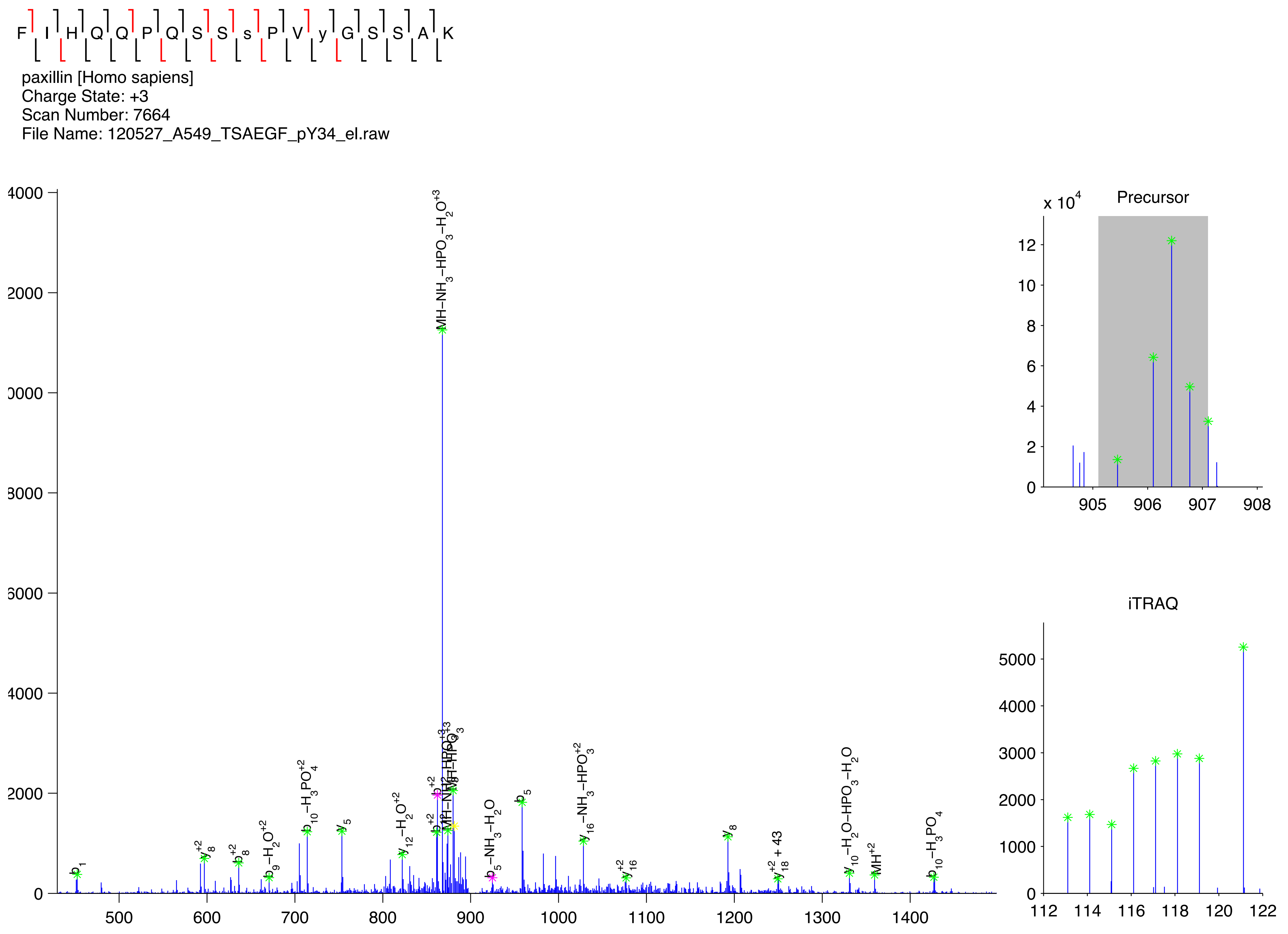

F I H Q Q P Q S S s P V y G S S A K
paxillin [Homo sapiens]
Charge State: +3
Scan Number: 7664
File Name: 120527_A549_TSAEGF_pY34_el.raw
MH−NH3−HPO3−H2O+3
b1
y8+2
b8+2
b9−H2O+2
b10−H3PO4+2
y5
y12−H2O+2
b5−NH3−H2O
b5
y16−NH3−HPO3+2
y16+2
y8
y18+2 + 43
y10−H2O−HPO3−H2O
MH+2
b10−H3PO4
Precursor
x 10^4
iTRAQ

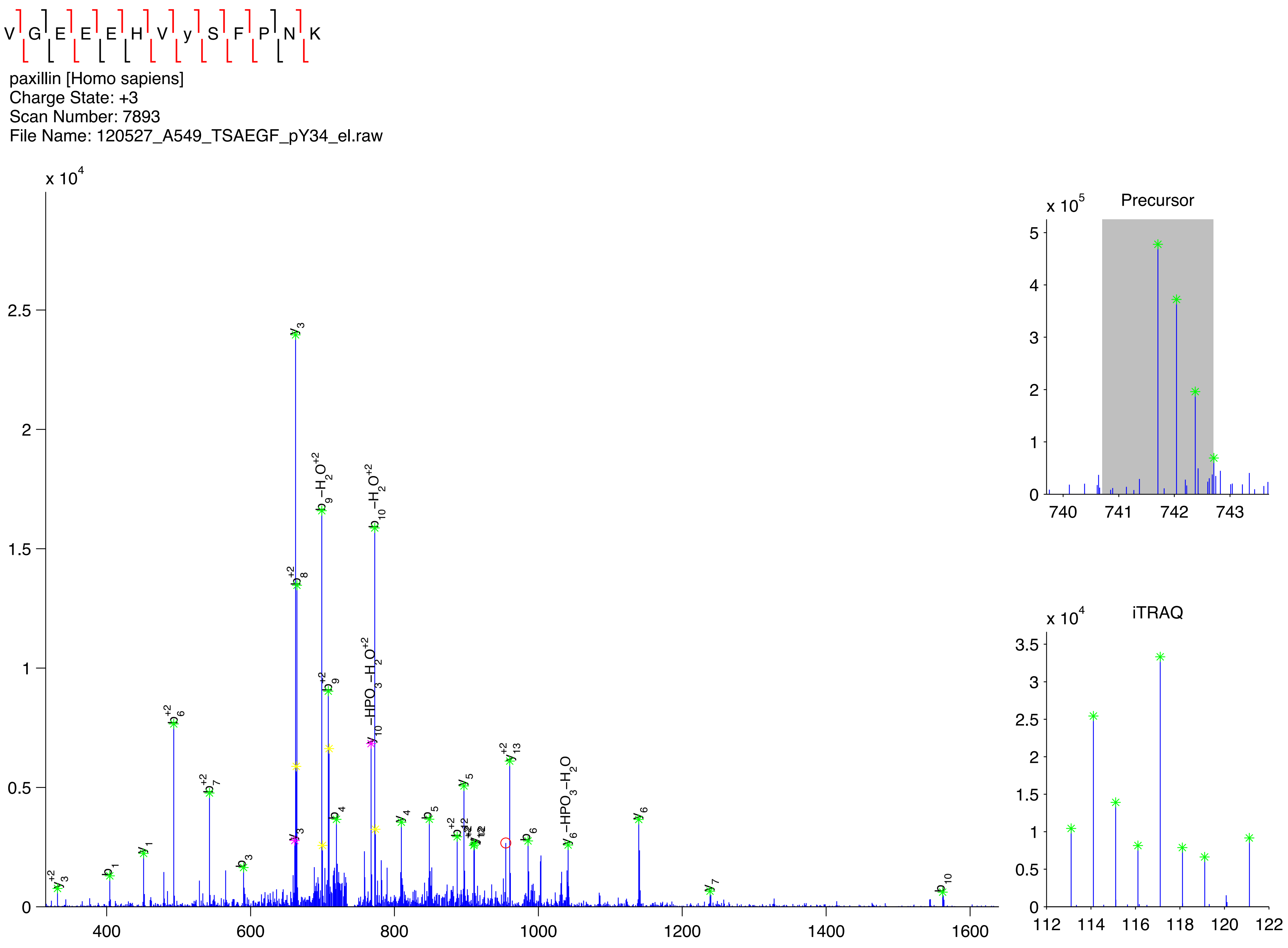
V G E E E H V y S F P N K
paxillin [Homo sapiens]
Charge State: +3
Scan Number: 7893
File Name: 120527_A549_TSAEGF_pY34_el.raw
x 10^4
Precursor
x 10^5
iTRAQ
x 10^4

V G E E H V y S F P N K

paxillin [Homo sapiens]
Charge State: +3
Scan Number: 8355
File Name: 120527_A549_TSAEGF_pY34_el.raw

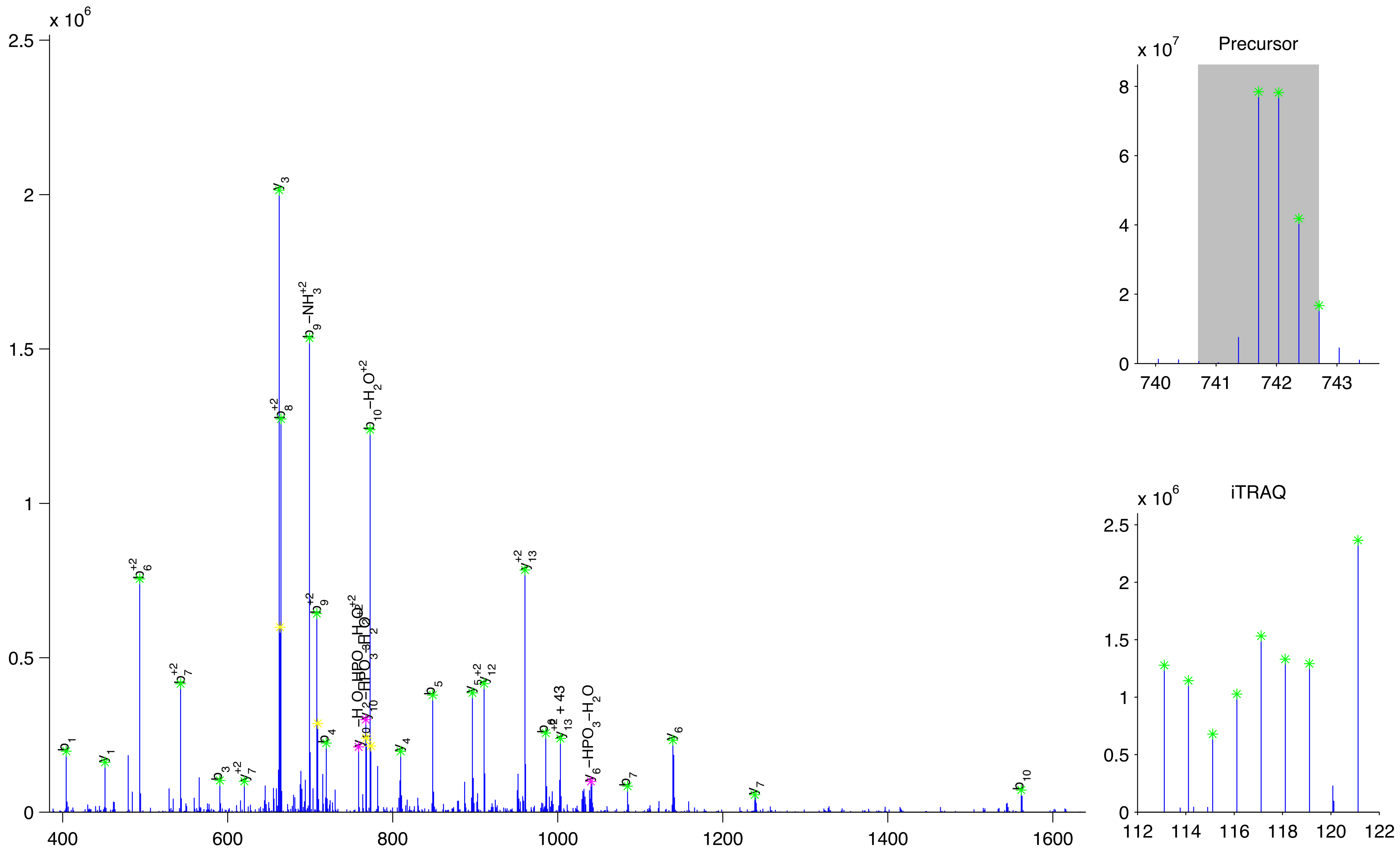

V G E E E H V y S F P N K Q K

paxillin [Homo sapiens]
Charge State: +3
Scan Number: 13644
File Name: 120518_A549_EGFTSA_pY.raw

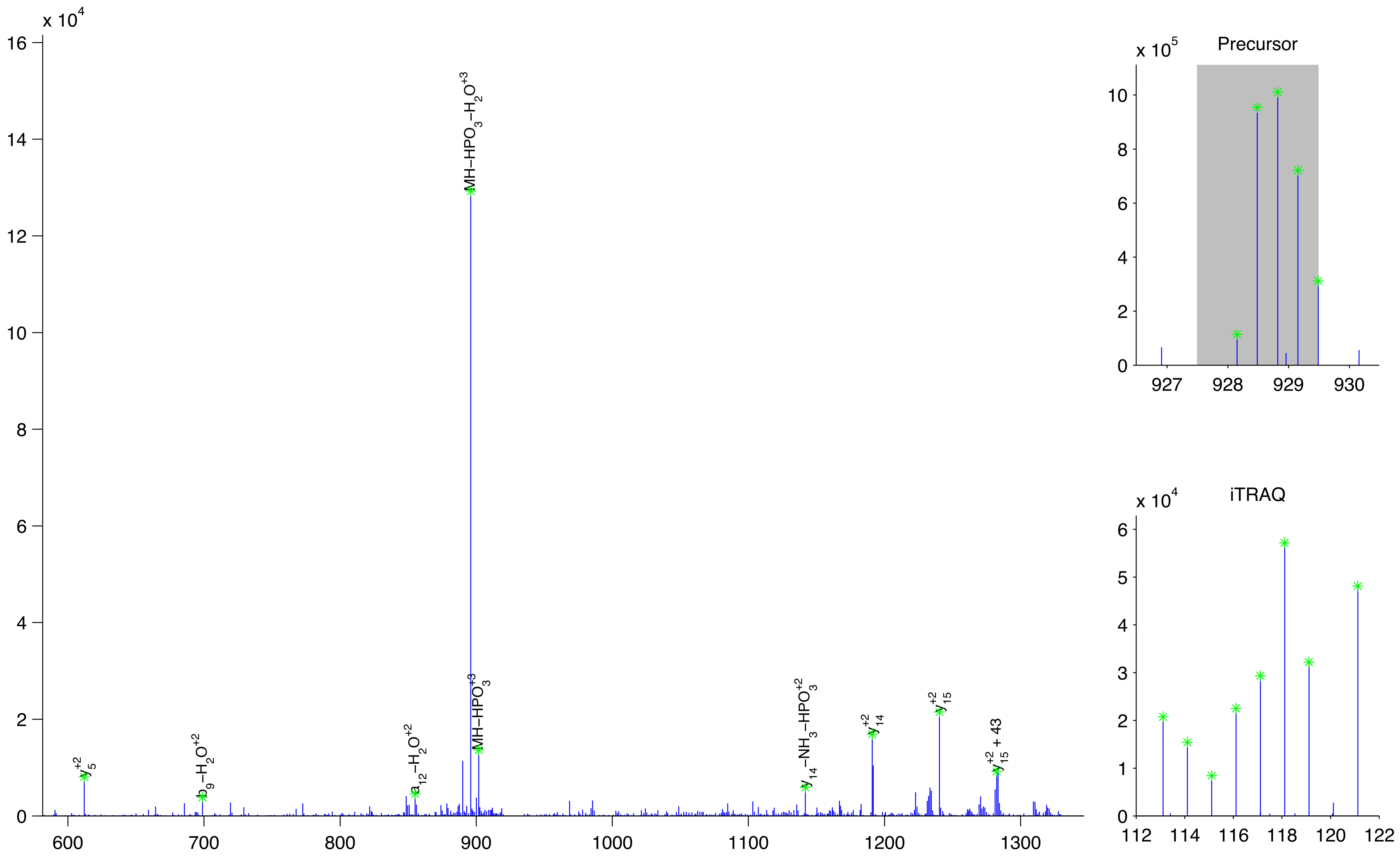

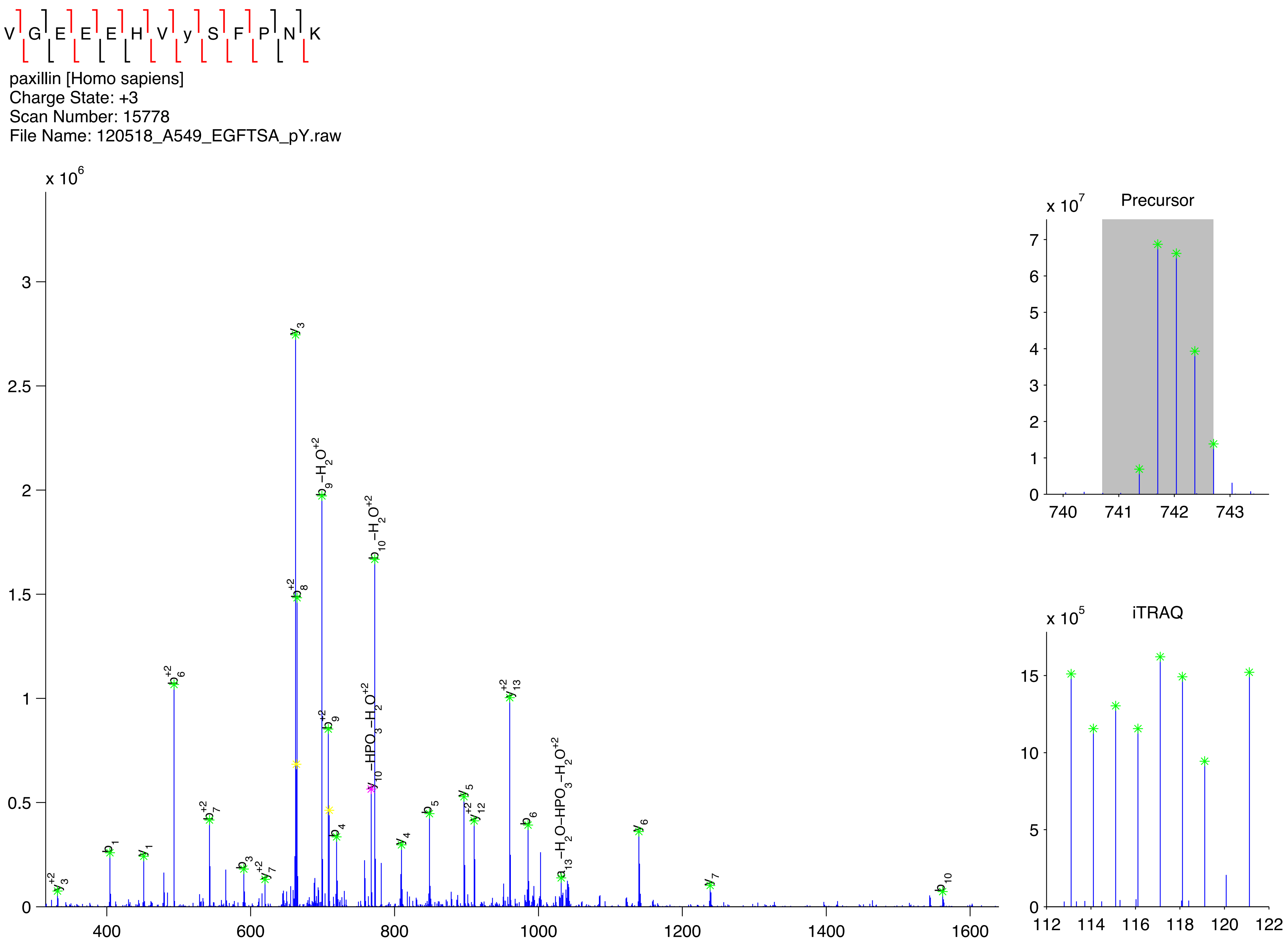

V G E E E H V y S F P N K
paxillin [Homo sapiens]
Charge State: +3
Scan Number: 15778
File Name: 120518_A549_EGFTSA_pY.raw
x 10^6
y3 b9−H2O+2 b10−H2O+2 b8+2 b6+2 y13+2 b9+2 y10−HPO3−H2O+2 a13−H2O−HPO3−H2O+2 b7+2 y5 y12+2 b5 y6 b6 y4 b4 b1 y1 b3 y7+2 y3+2 y7 b10
Precursor
x 10^7
iTRAQ
x 10^5

L L L E L N A V Q H N P P G F P A D E A N S S P P L P G A L S P L y G V P E T N S P L G G K

paxillin [Homo sapiens]
Charge State: +5
Scan Number: 31717
File Name: 120518_A549_EGFTSA_pY.raw

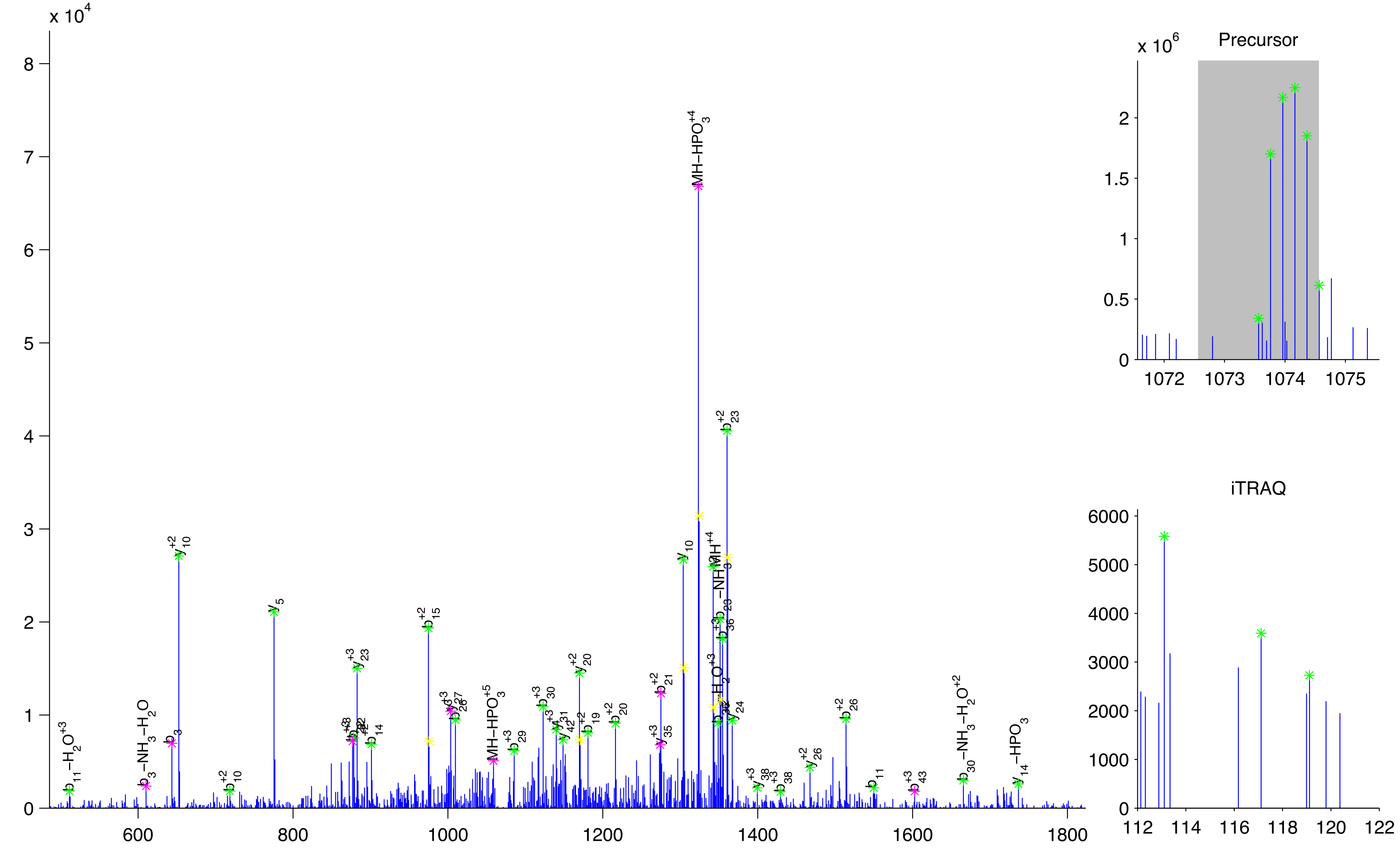

S R E Y D Q L y E E Y T R

phosphoinositide−3−kinase, regulatory subunit 2 (beta) [Homo sapiens]
Charge State: +3
Scan Number: 18865
File Name: 120518_A549_EGFTSA_pY.raw

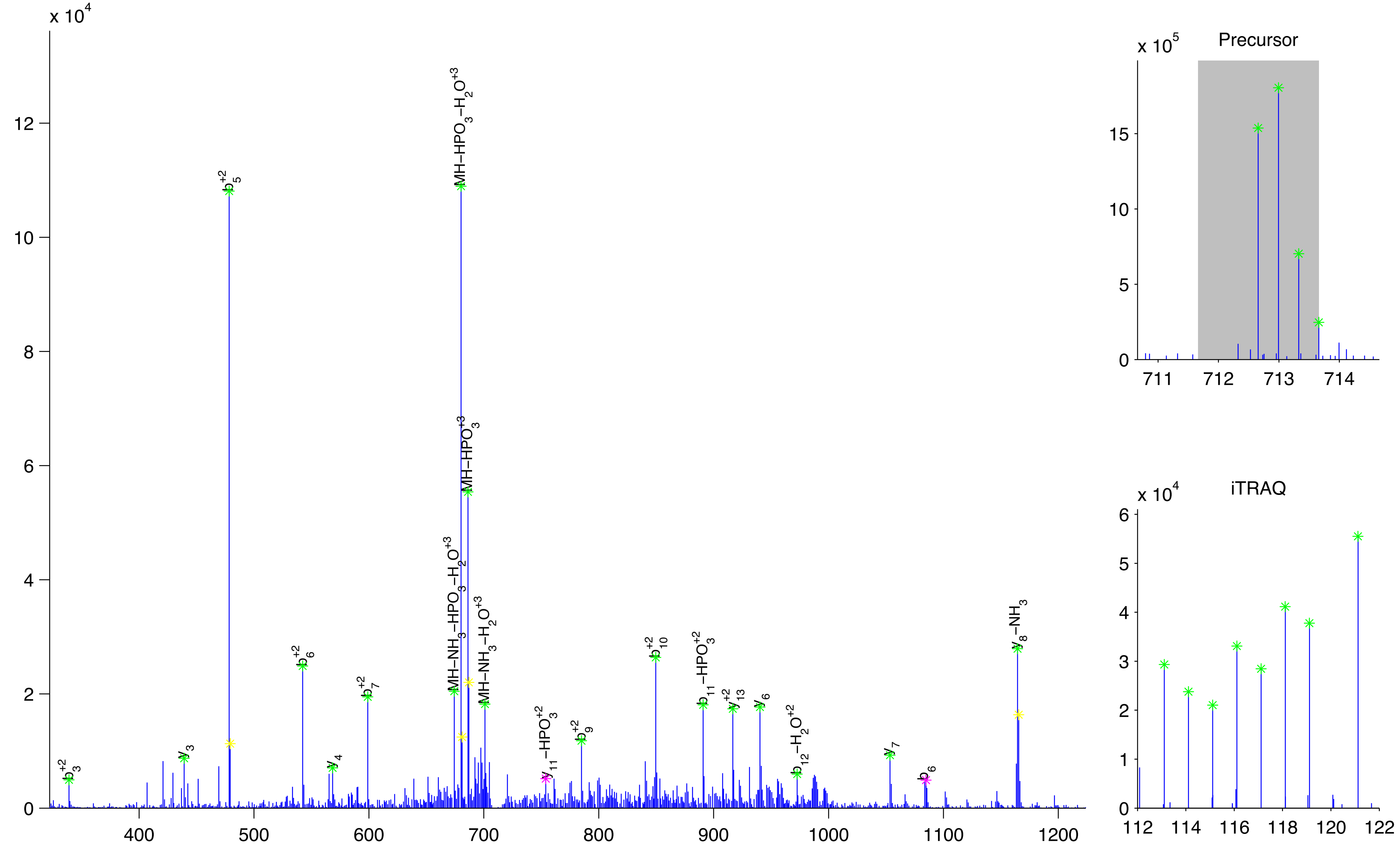

A E E P G G H A y E T P R E E I A R

phosphoinositol 3–phosphate–binding protein–3 [Homo sapiens]
Charge State: +3
Scan Number: 9070
File Name: 120518_A549_EGFTSA_pY.raw

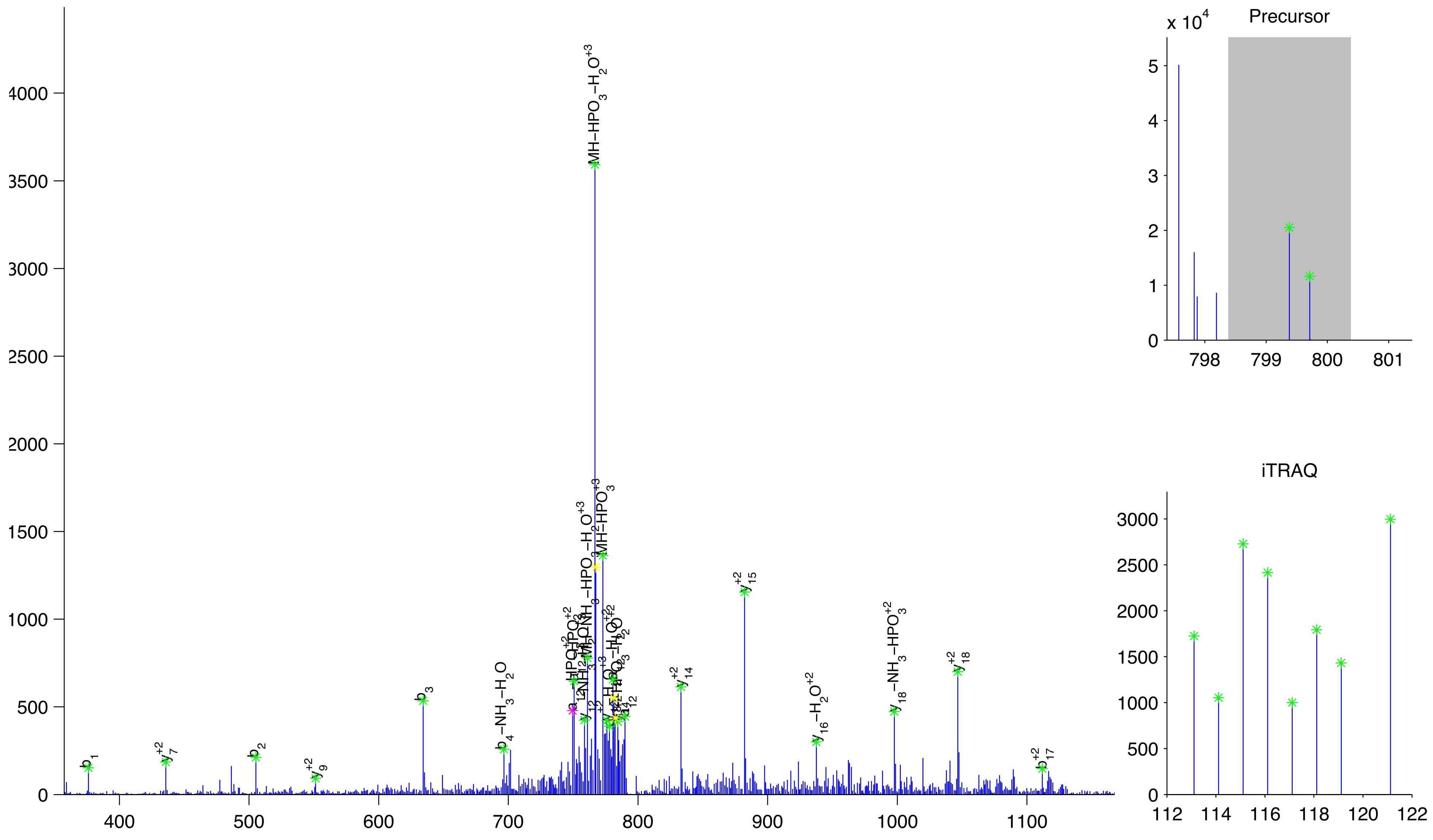

plakophilin 4 isoform a [Homo sapiens]
Charge State: +3
Scan Number: 22707
File Name: 120518_A549_EGFTSA_pY.raw

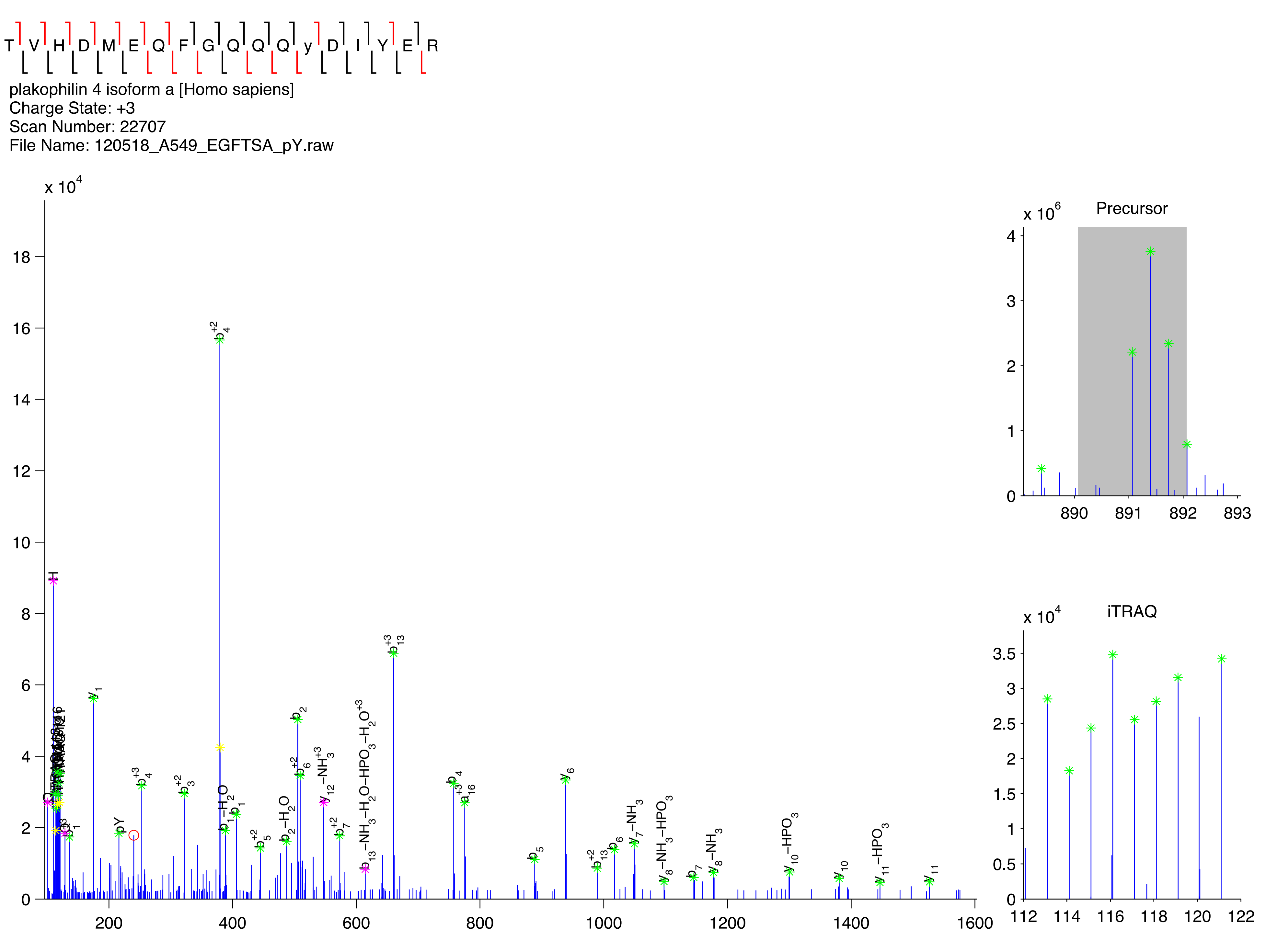

A R Q E E L y S E L Q A R

plectin 1 isoform 3 [Homo sapiens]
Charge State: +3
Scan Number: 14875
File Name: 120518_A549_EGFTSA_pY.raw

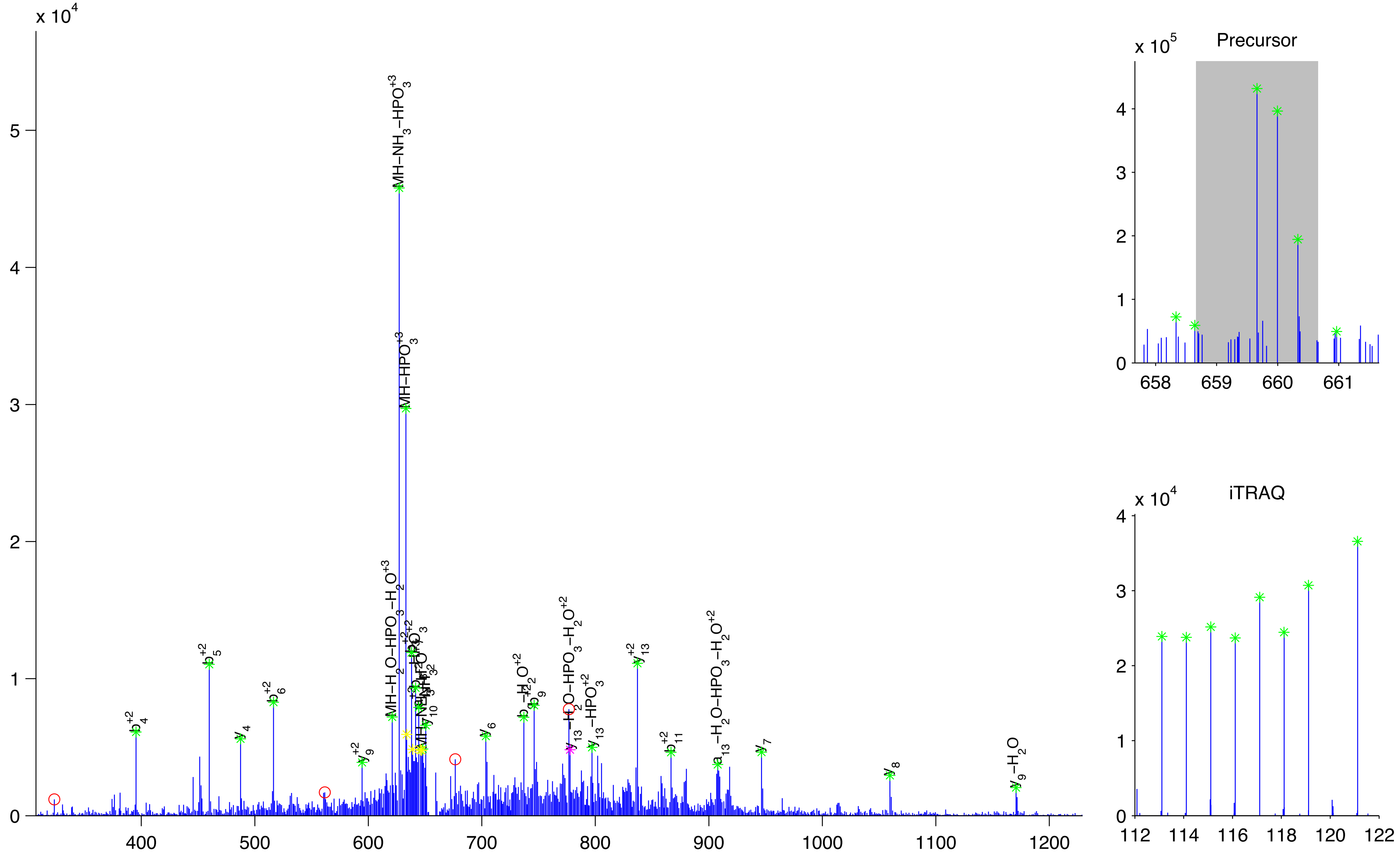

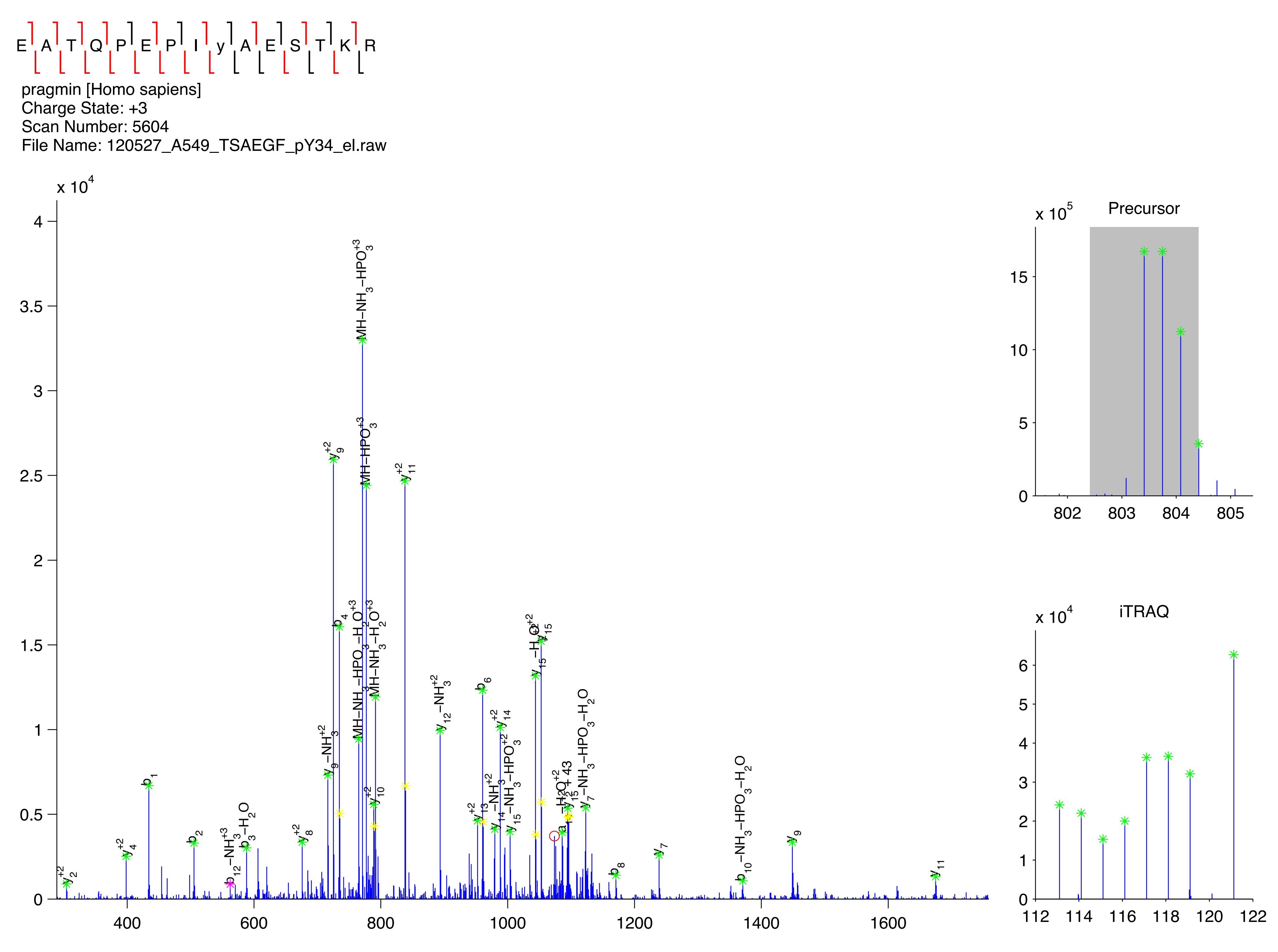

E A T Q P E P I y A E S T K R
pragmin [Homo sapiens]
Charge State: +3
Scan Number: 5604
File Name: 120527_A549_TSAEGF_pY34_el.raw
x 10^4
Precursor
x 10^5
iTRAQ
x 10^4

E A T Q P E P I y A E S T K

pragmin [Homo sapiens]
Charge State: +3
Scan Number: 6446
File Name: 120527_A549_TSAEGF_pY34_el.raw

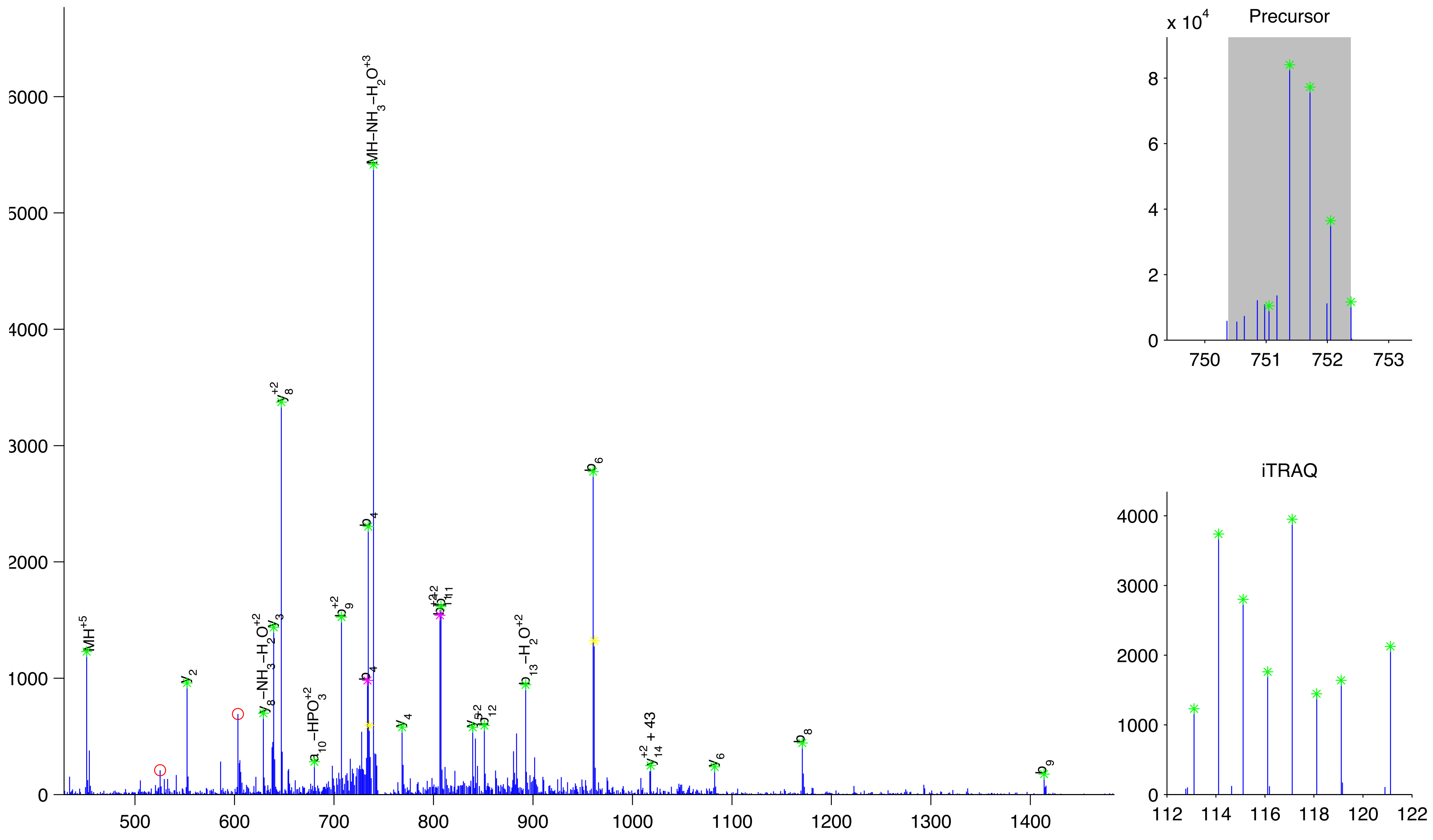

E A T Q P E P I y A E S T K

pragmin [Homo sapiens]
Charge State: +2
Scan Number: 7309
File Name: 120527_A549_TSAEGF_pY34_el.raw

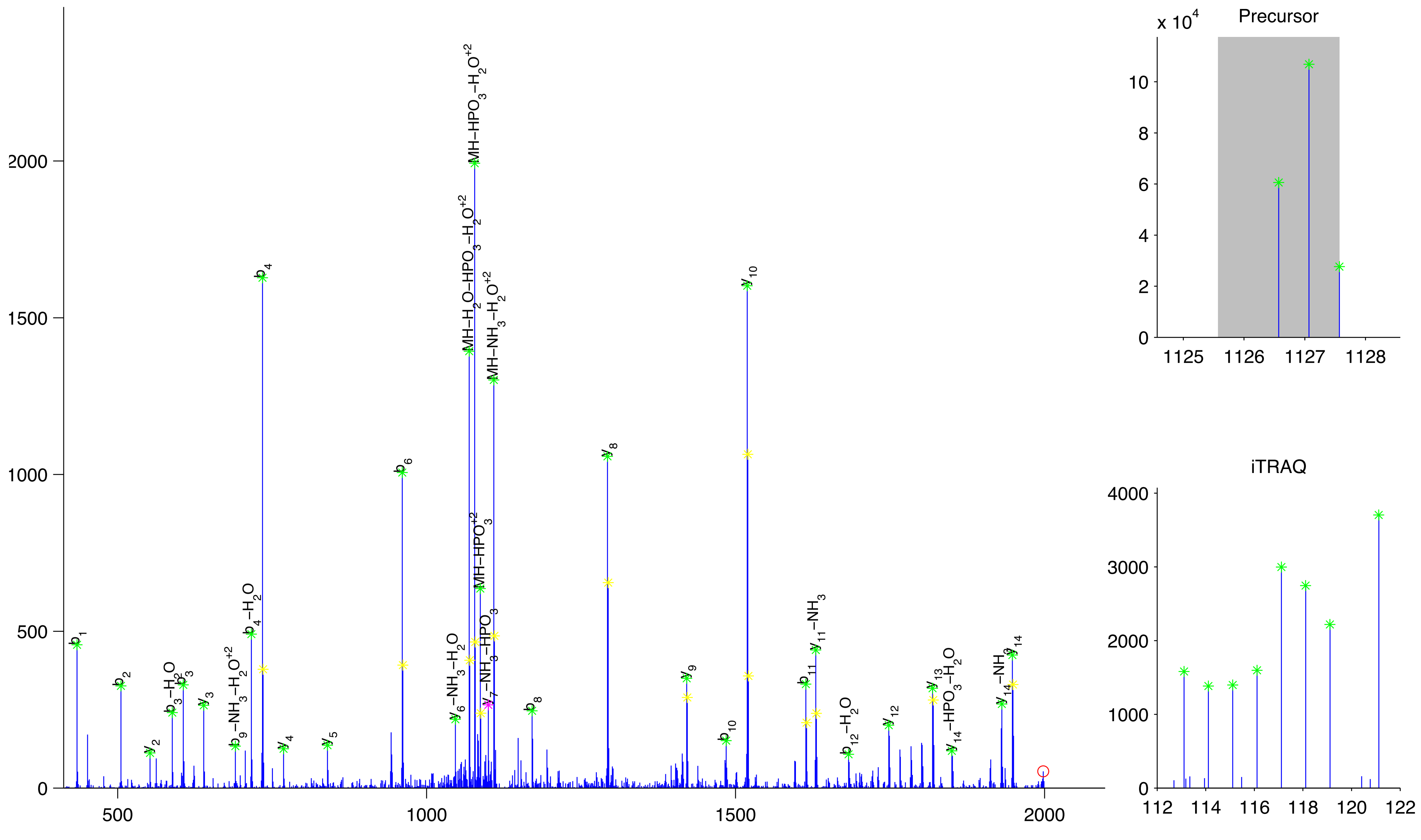

E A T Q P E P I y A E S T K

pragmin [Homo sapiens]
Charge State: +3
Scan Number: 7389
File Name: 120527_A549_TSAEGF_pY34_el.raw

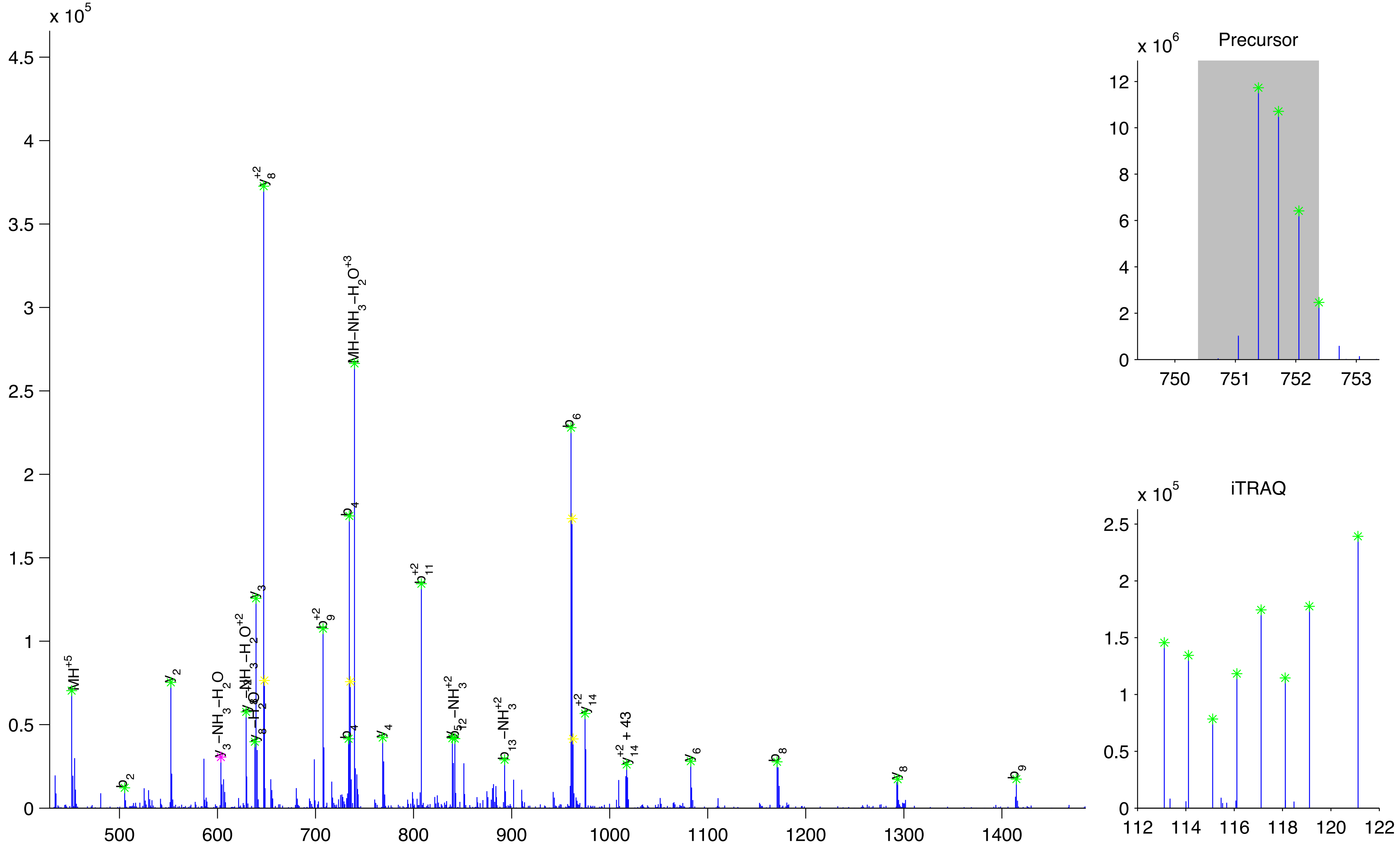

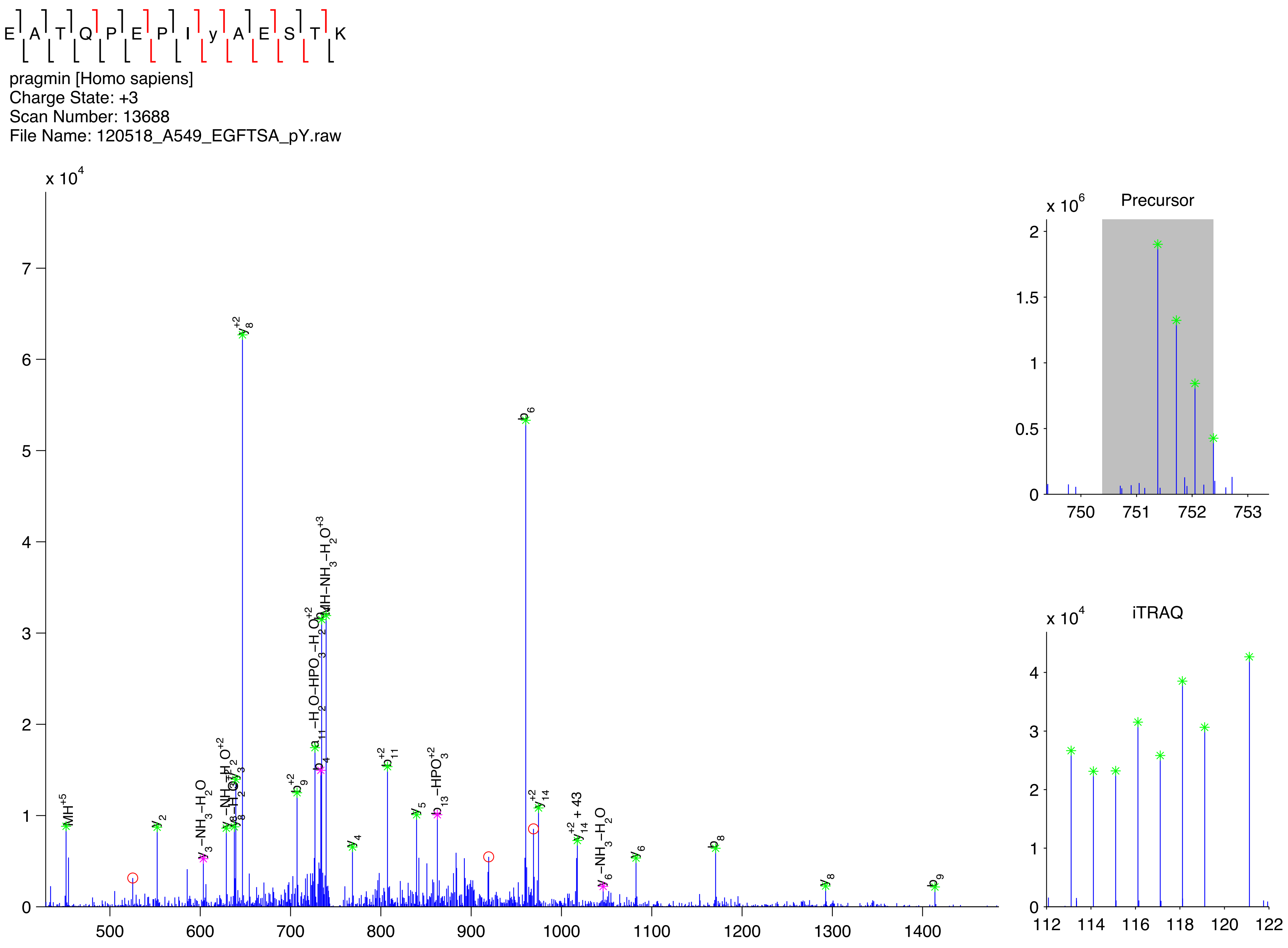
E A T Q P E P I y A E S T K
pragmin [Homo sapiens]
Charge State: +3
Scan Number: 13688
File Name: 120518_A549_EGFTSA_pY.raw
Precursor
iTRAQ

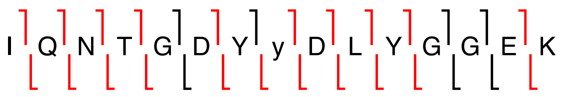

protein tyrosine phosphatase, non−receptor type 11 [Homo sapiens]
Charge State: +2
Scan Number: 22651
File Name: 120518_A549_EGFTSA_pY.raw

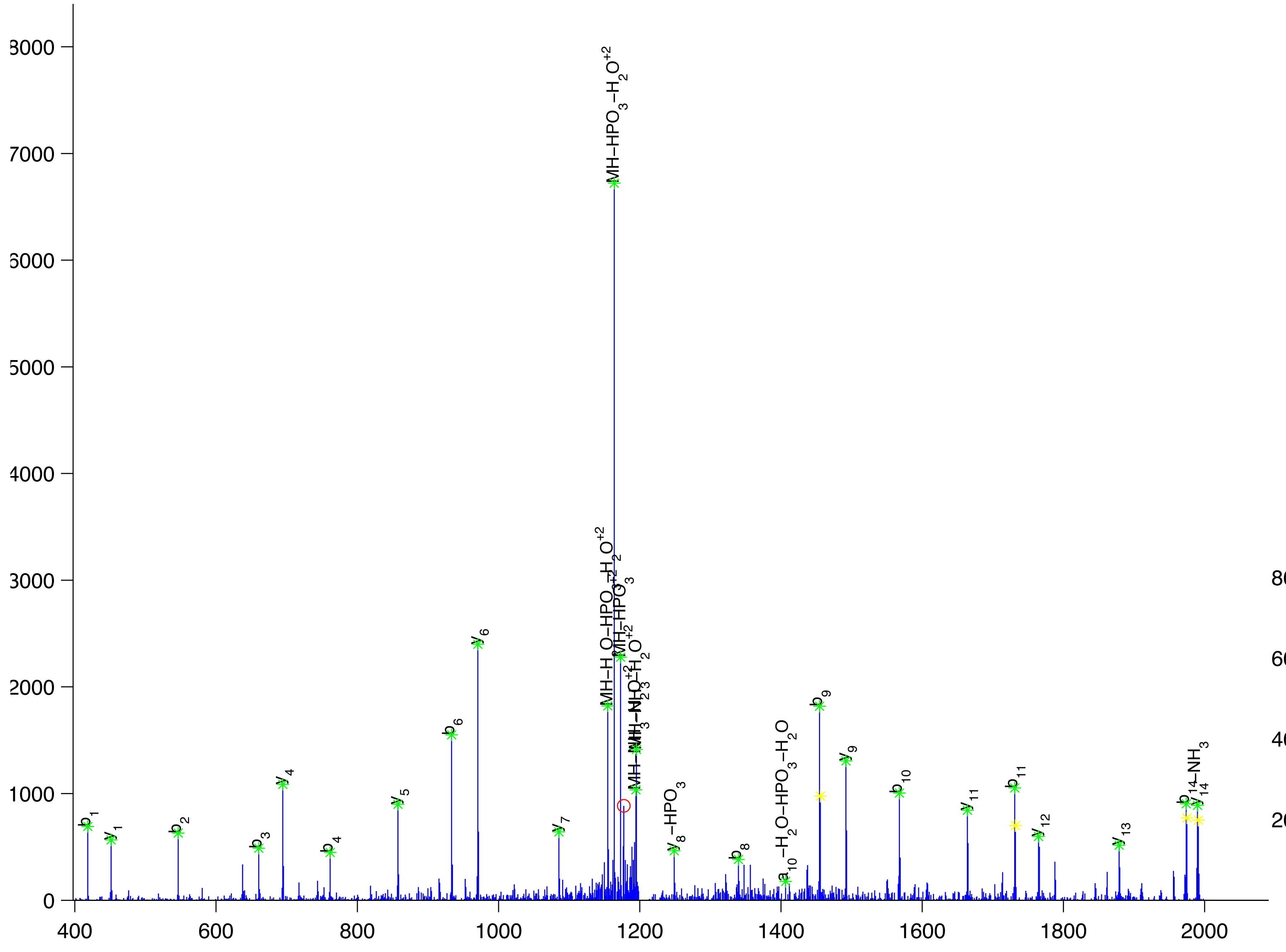

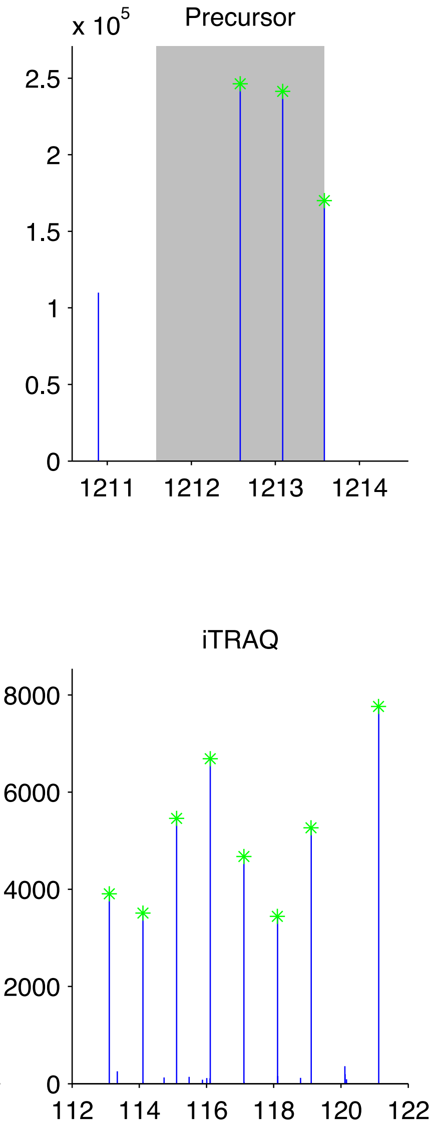

V V Q E Y I D A F S D y A N F K

protein tyrosine phosphatase, receptor type, A isoform 1 precursor [Homo sapiens]
Charge State: +4
Scan Number: 21816
File Name: 120527_A549_TSAEGF_pY34_el.raw

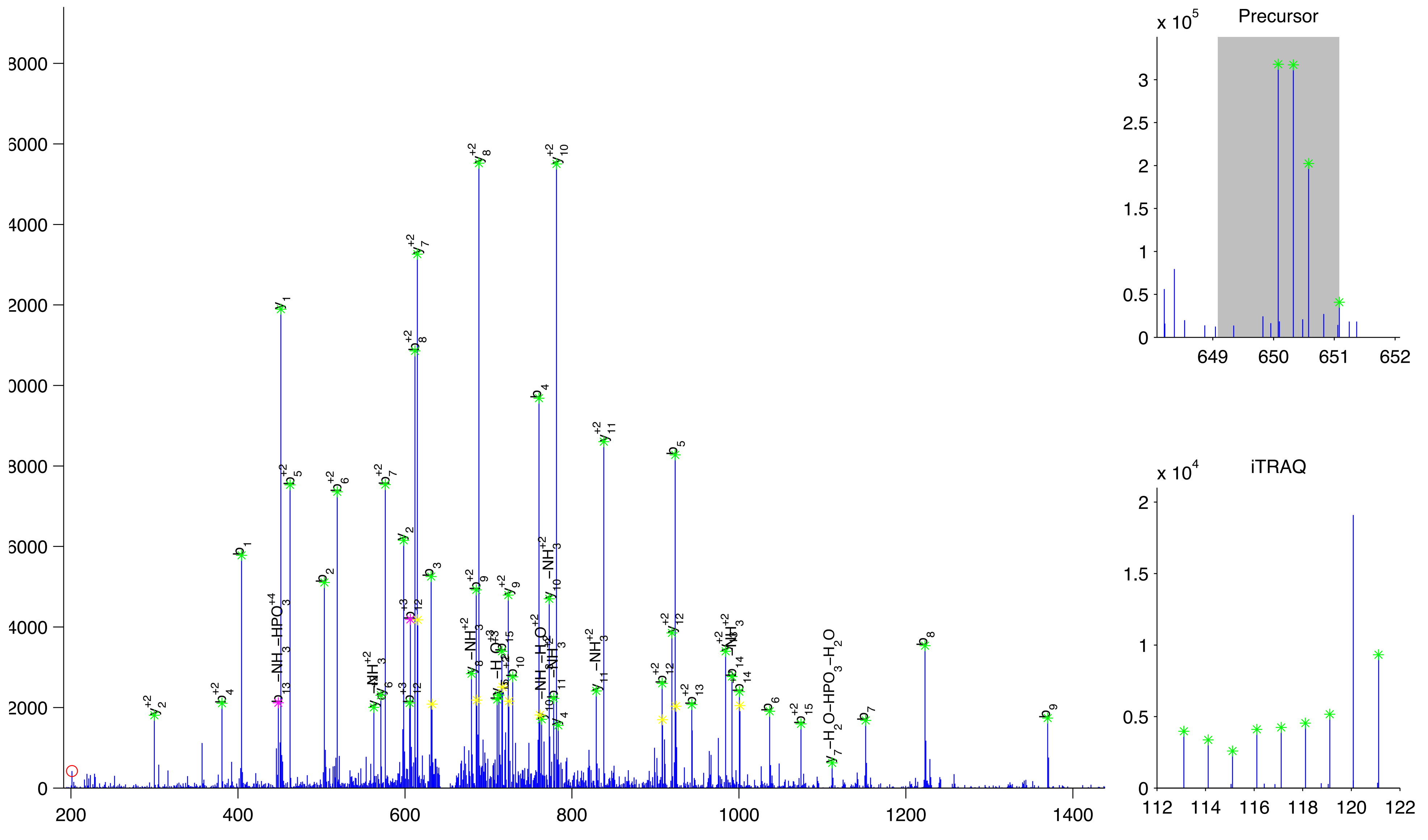

L I E D N E y T A R

protein–tyrosine kinase fyn isoform a [Homo sapiens]
Charge State: +2
Scan Number: 15211
File Name: 120518_A549_EGFTSA_pY.raw

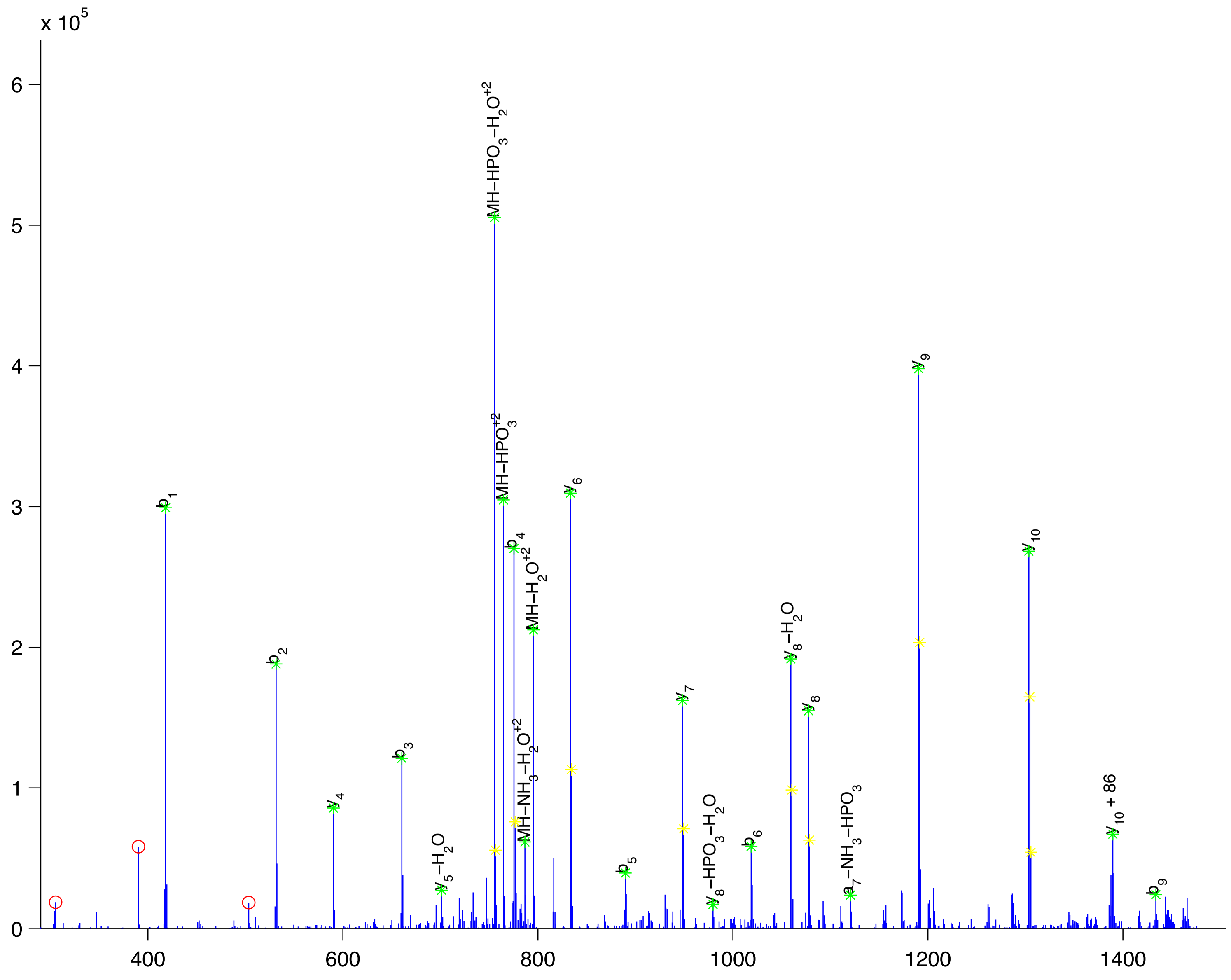

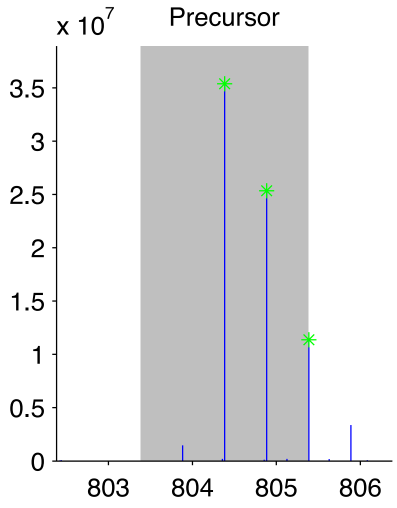

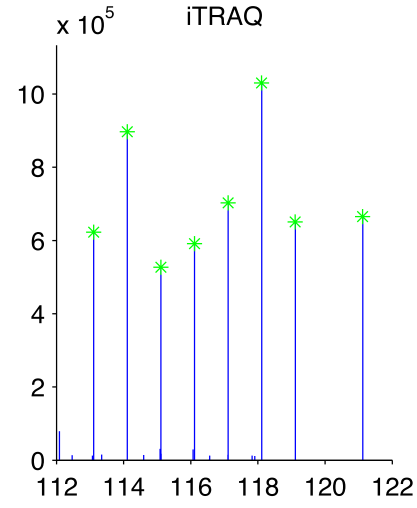

Y m E D S T Y y K

PTK2 protein tyrosine kinase 2 isoform a [Homo sapiens]
Charge State: +3
Scan Number: 6843
File Name: 120527_A549_TSAEGF_pY34_el.raw

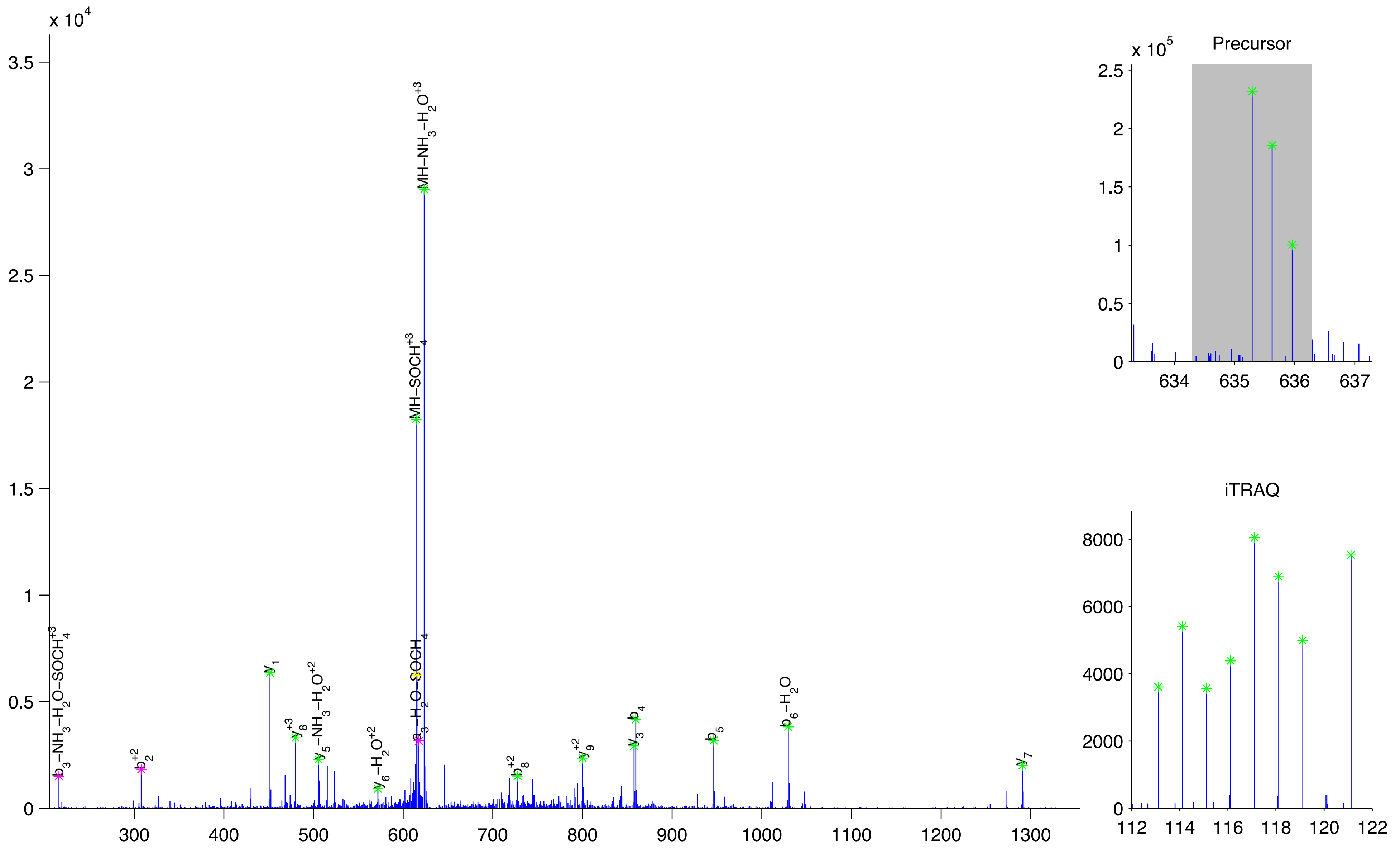

y I E D E D Y Y K A S V T R

PTK2B protein tyrosine kinase 2 beta isoform a [Homo sapiens]
Charge State: +3
Scan Number: 17857
File Name: 120518_A549_EGFTSA_pY.raw

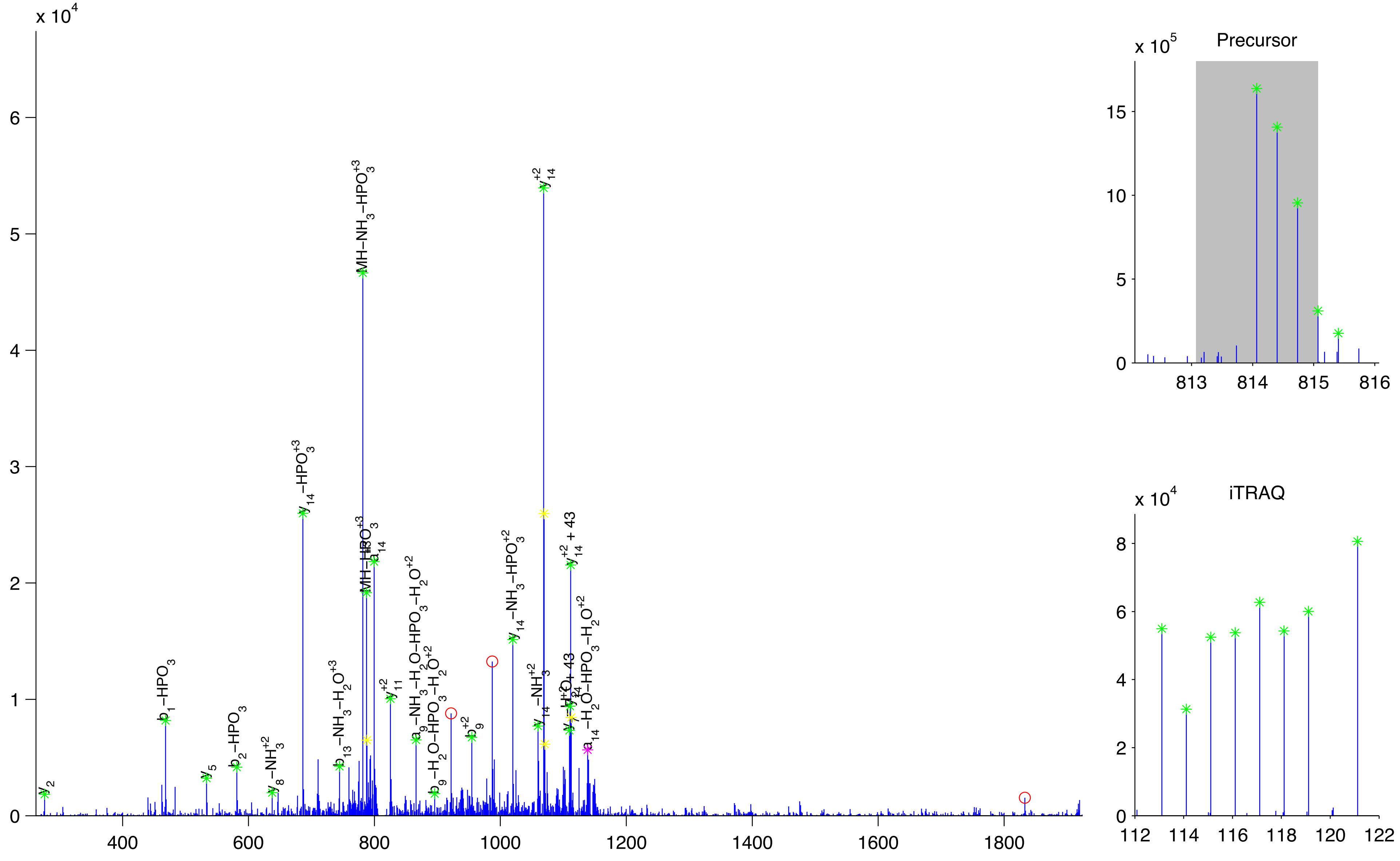

A t T P P N Q G R P D S P V y A N L Q E L K

Rho GTPase activating protein 12 [Homo sapiens]
Charge State: +4
Scan Number: 11673
File Name: 120527_A549_TSAEGF_pY34_el.raw

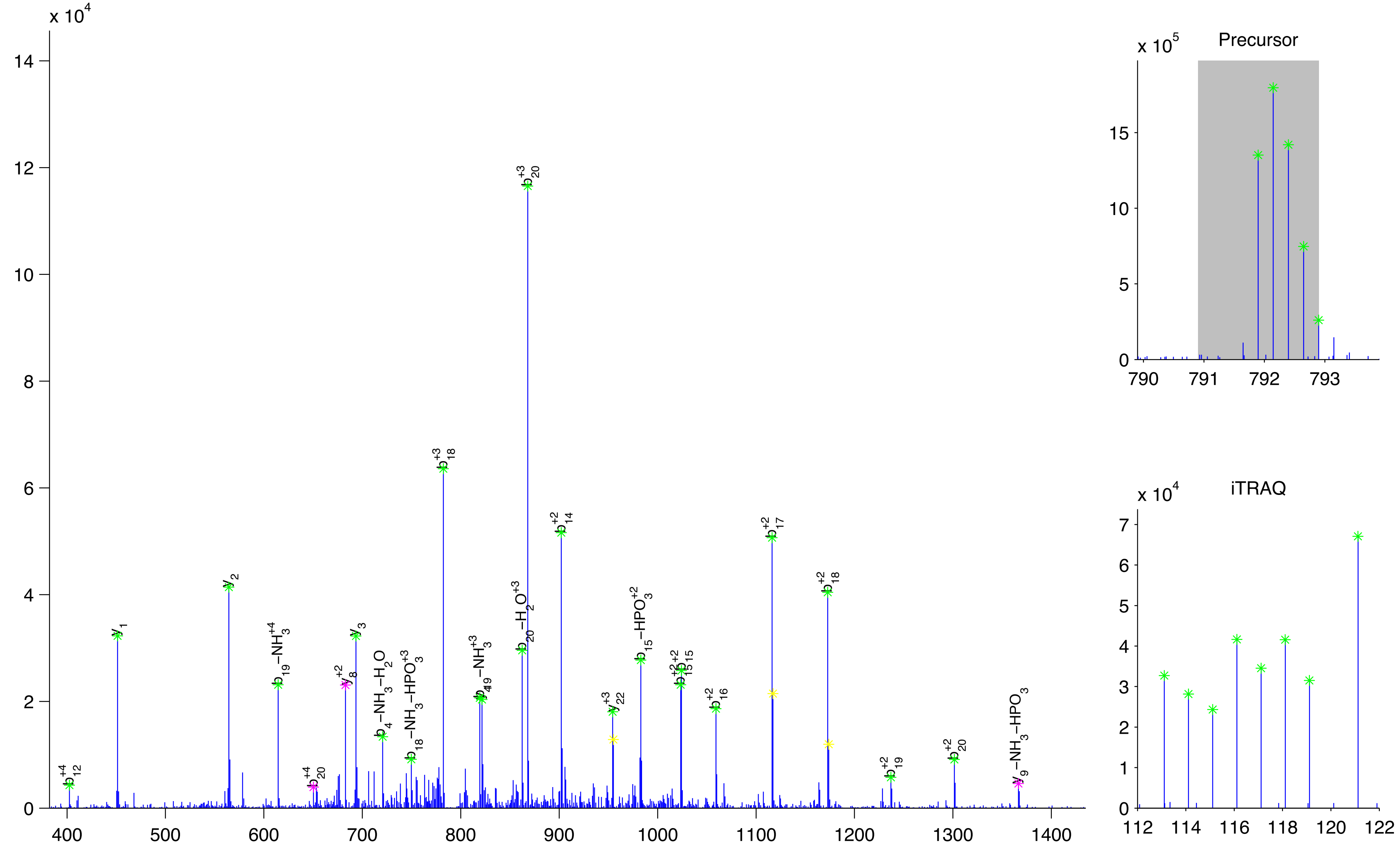

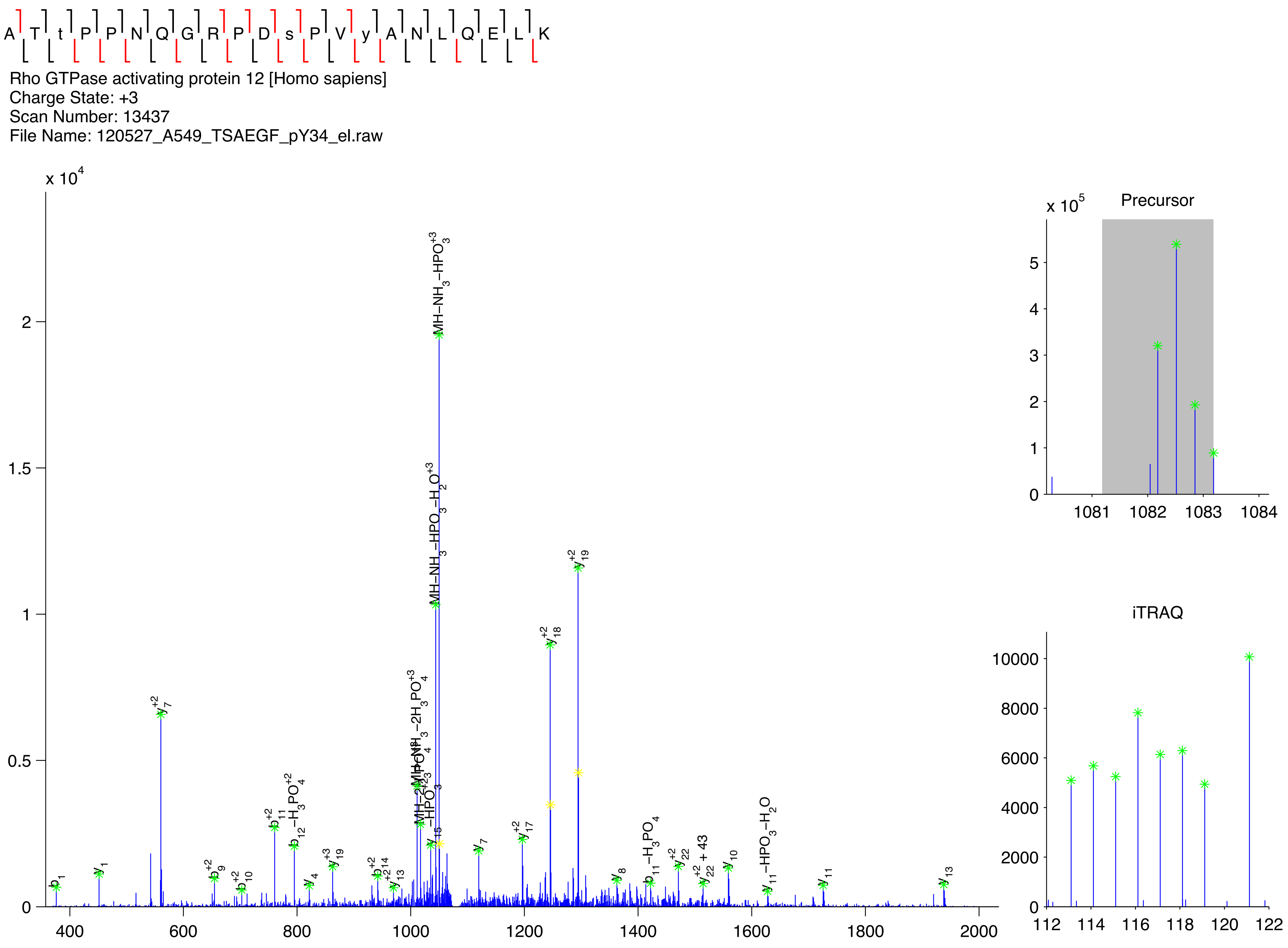

A T t P P N Q G R P D s P V y A N L Q E L K
Rho GTPase activating protein 12 [Homo sapiens]
Charge State: +3
Scan Number: 13437
File Name: 120527_A549_TSAEGF_pY34_el.raw
x 10^4
MH−NH3−HPO3 +3
MH−NH3−HPO3−H2O +3
y19 +2
y18 +2
y7 +2
b1
y1
b9 +2
b10 +2
b11 +2
b12−H3PO4 +2
y4
y19 +3
b14 +2
y13 +2
y15
y7
y17 +2
y8
b11−H3PO4
y22 +2
y22 +2 + 43
y10
y11−HPO3−H2O
y11
y13
Precursor
x 10^5
iTRAQ

A T T P P N Q G R P D s P V y A N L Q E L K

Rho GTPase activating protein 12 [Homo sapiens]
Charge State: +3
Scan Number: 23398
File Name: 120518_A549_EGFTSA_pY.raw

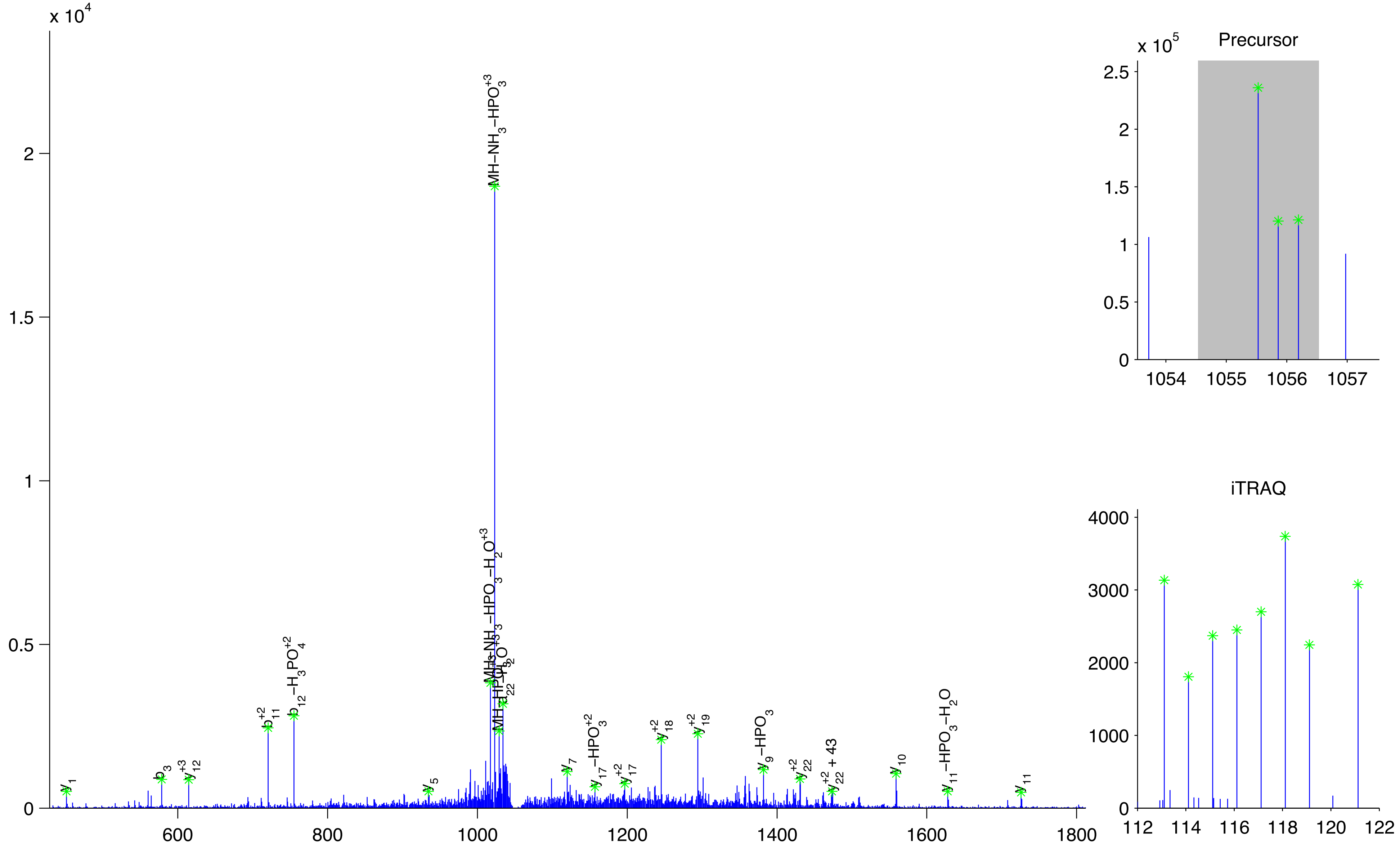

A T t P P N Q G R P D s P V y A N L Q E L K

Rho GTPase activating protein 12 [Homo sapiens]
Charge State: +4
Scan Number: 25627
File Name: 120518_A549_EGFTSA_pY.raw

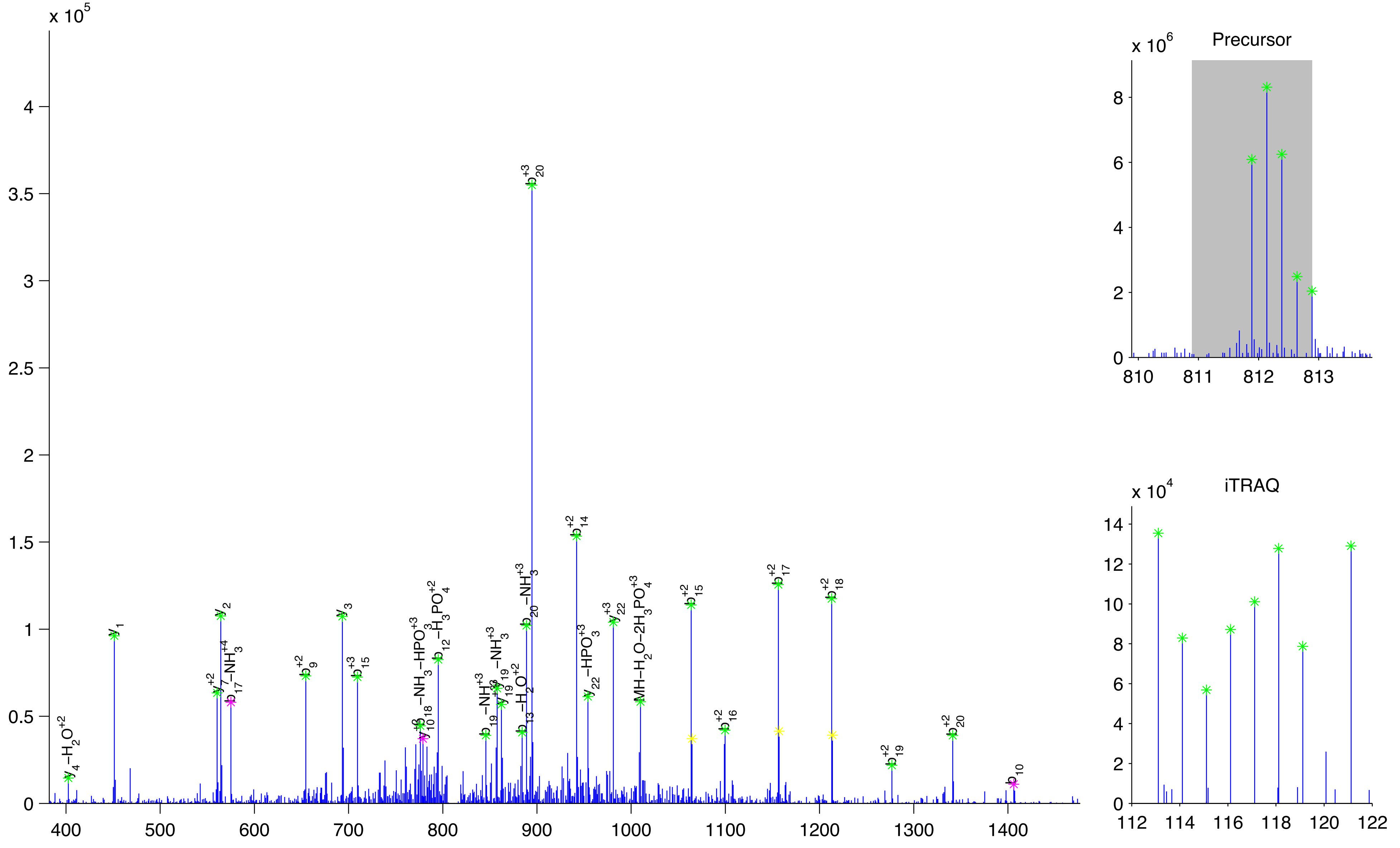

A T t P P N Q G R P D s P V y A N L Q E L K

Rho GTPase activating protein 12 [Homo sapiens]
Charge State: +3
Scan Number: 25711
File Name: 120518_A549_EGFTSA_pY.raw

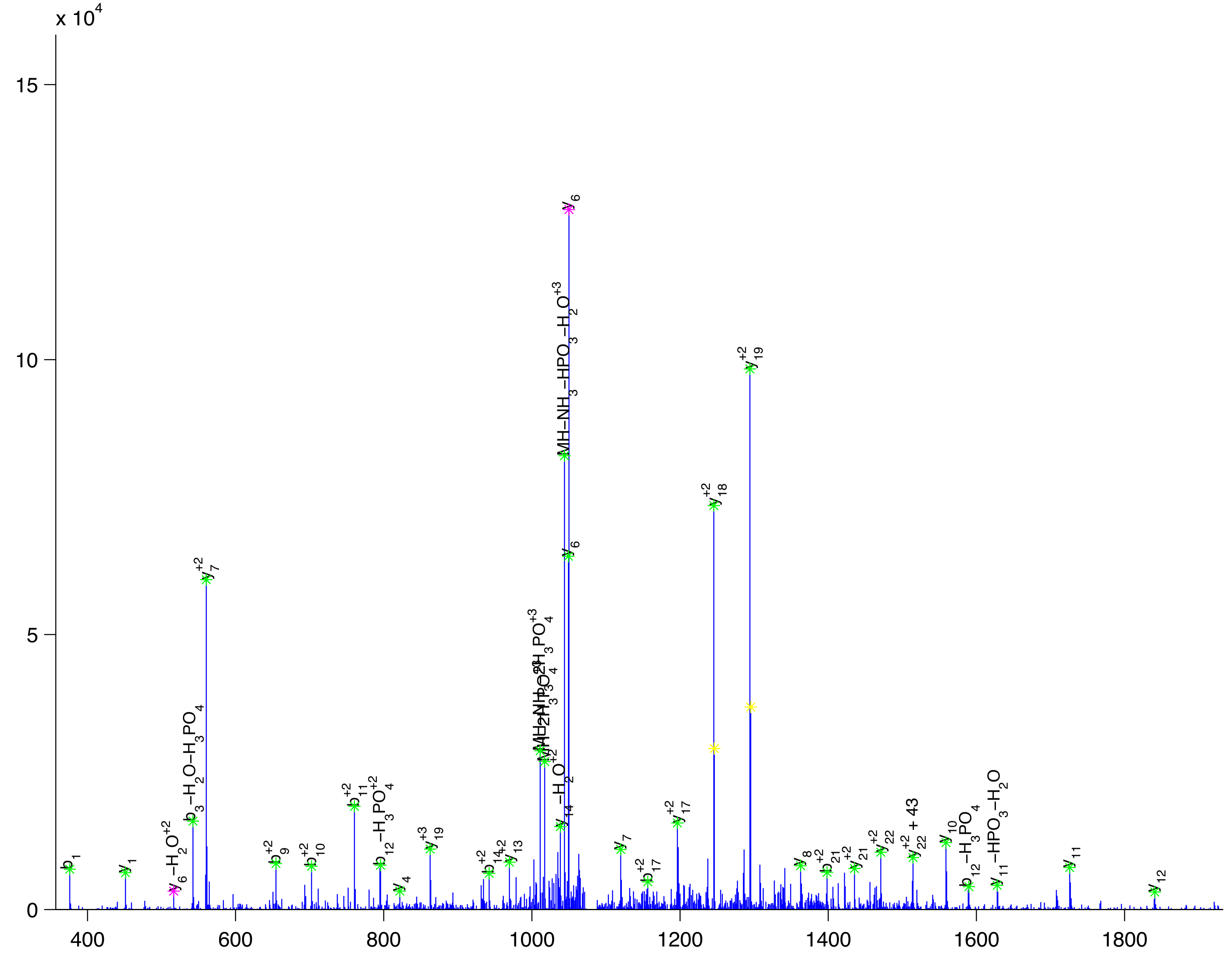

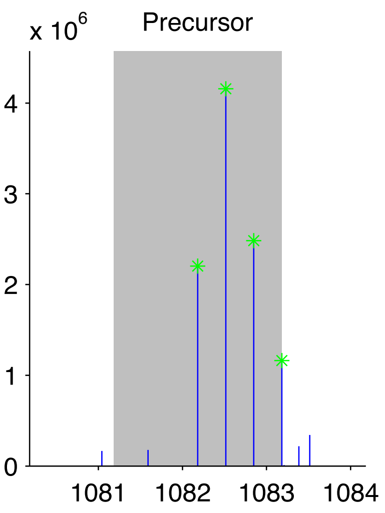

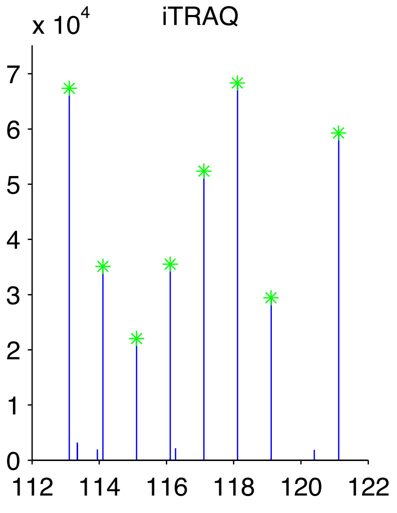

R P D P D S D E D E D y E R E R

RNA binding motif protein 17 [Homo sapiens]
Charge State: +4
Scan Number: 3415
File Name: 120527_A549_TSAEGF_pY34_el.raw

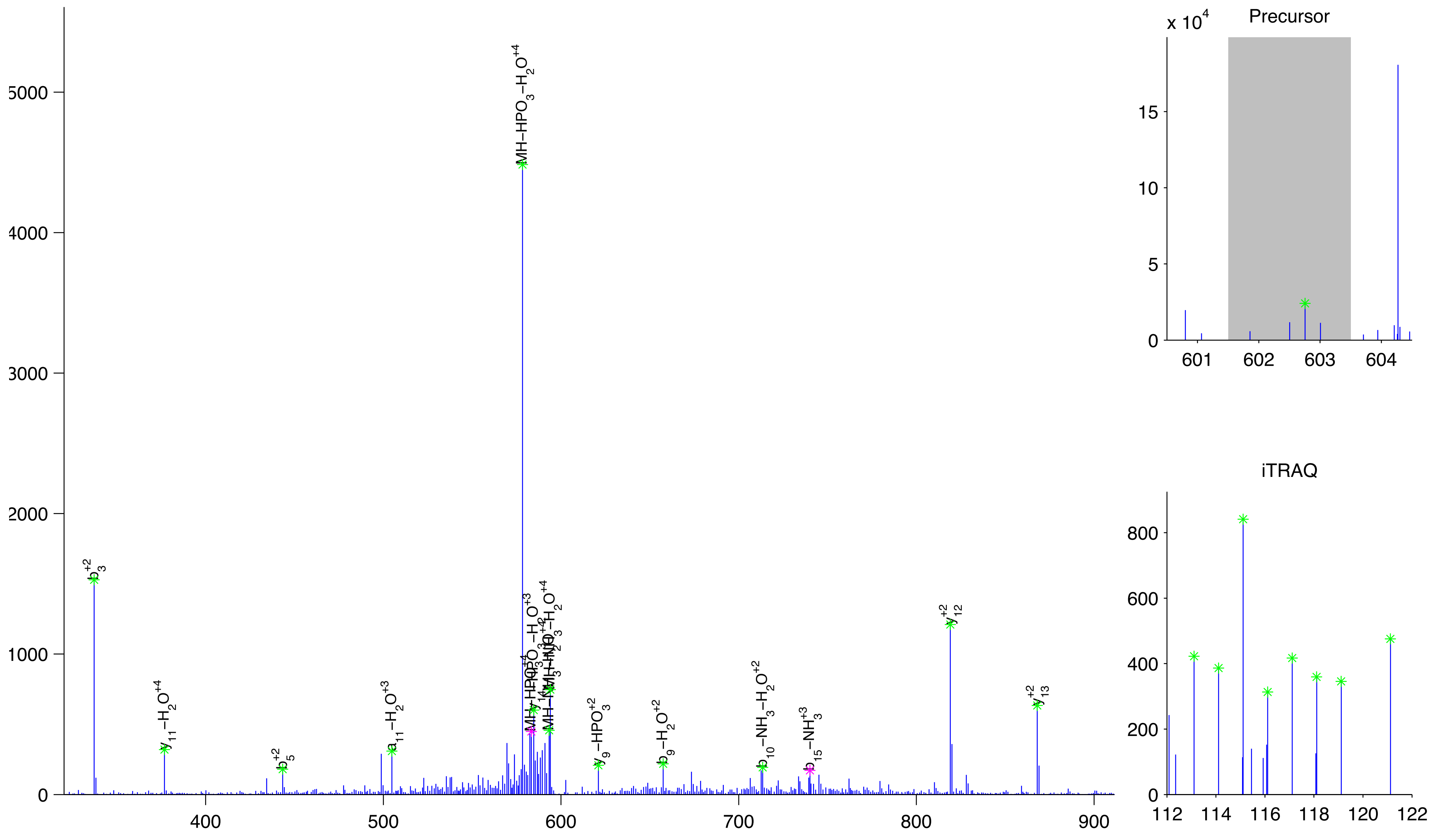

L c D F G S A S H V A D N D I T P y L V S R
serine/threonine−protein kinase PRP4K [Homo sapiens]
Charge State: +3
Scan Number: 14382
File Name: 120527_A549_TSAEGF_pY34_el.raw

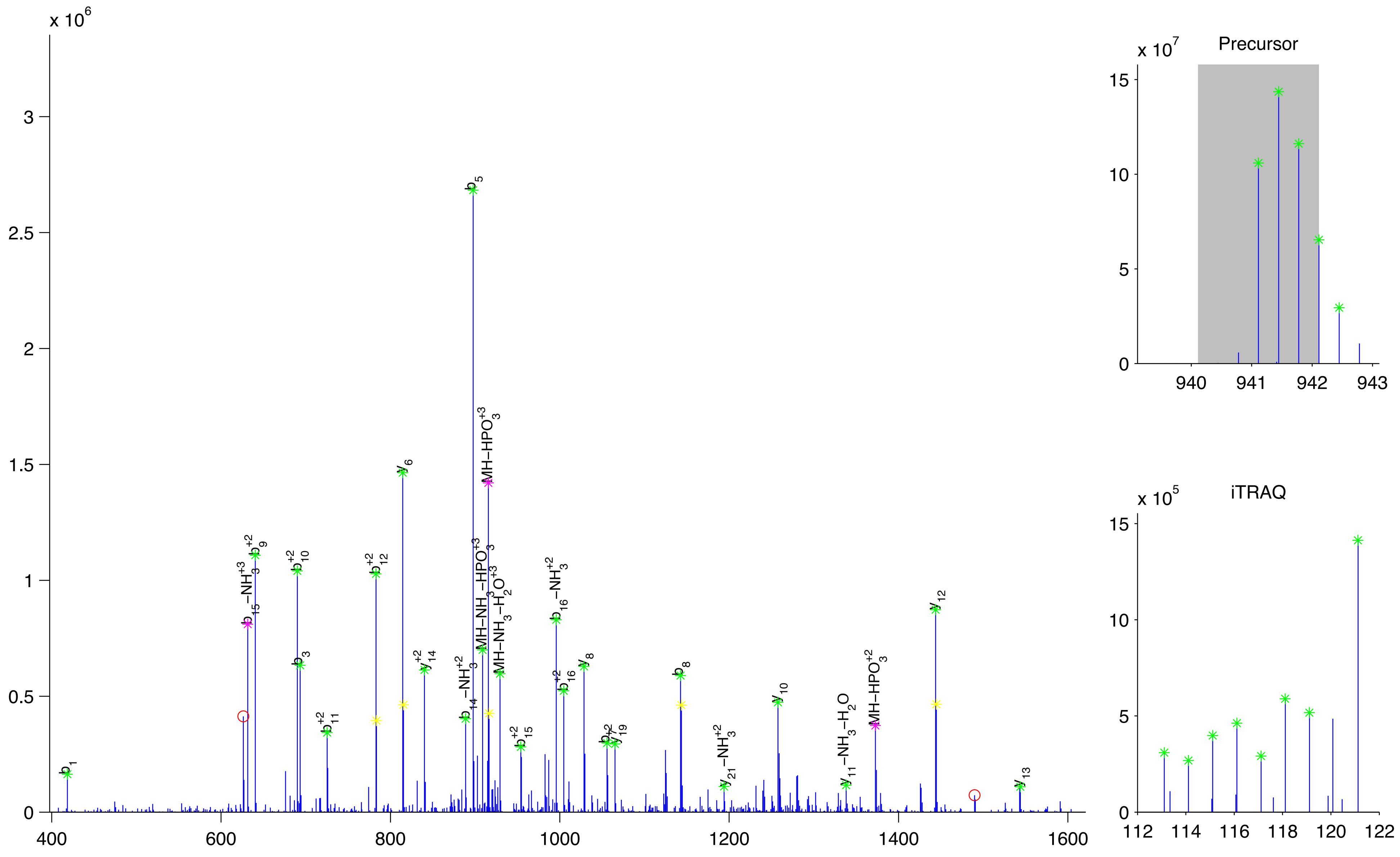

E L F D D P S y V N V Q N L D K

SHC (Src homology 2 domain containing) transforming protein 1 isoform p52Shc [Homo sapiens]
Charge State: +2
Scan Number: 14510
File Name: 120527_A549_TSAEGF_pY34_el.raw

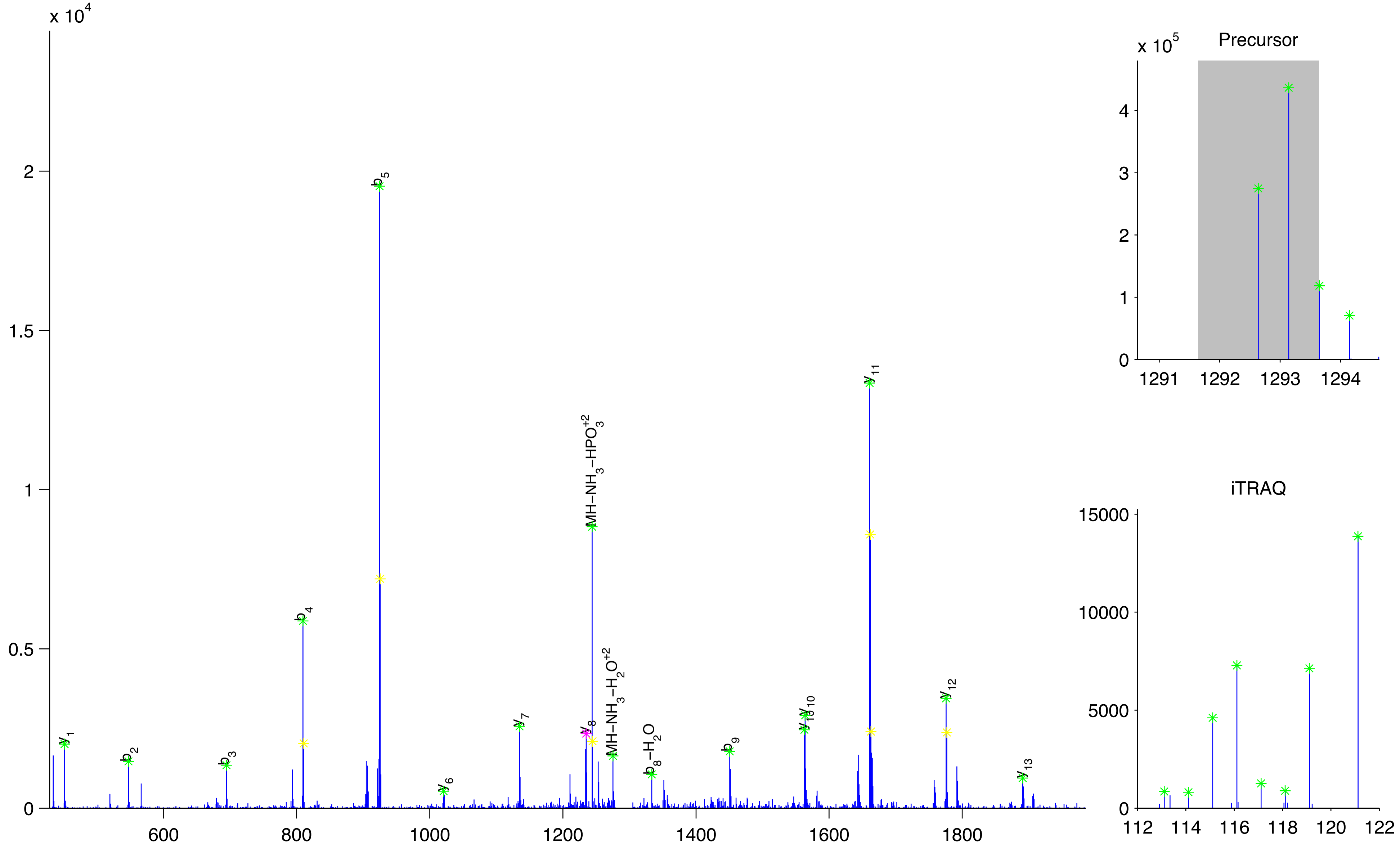

E L F D D P S y V N V Q N L D K

SHC (Src homology 2 domain containing) transforming protein 1 isoform p52Shc [Homo sapiens]
Charge State: +4
Scan Number: 14571
File Name: 120527_A549_TSAEGF_pY34_el.raw

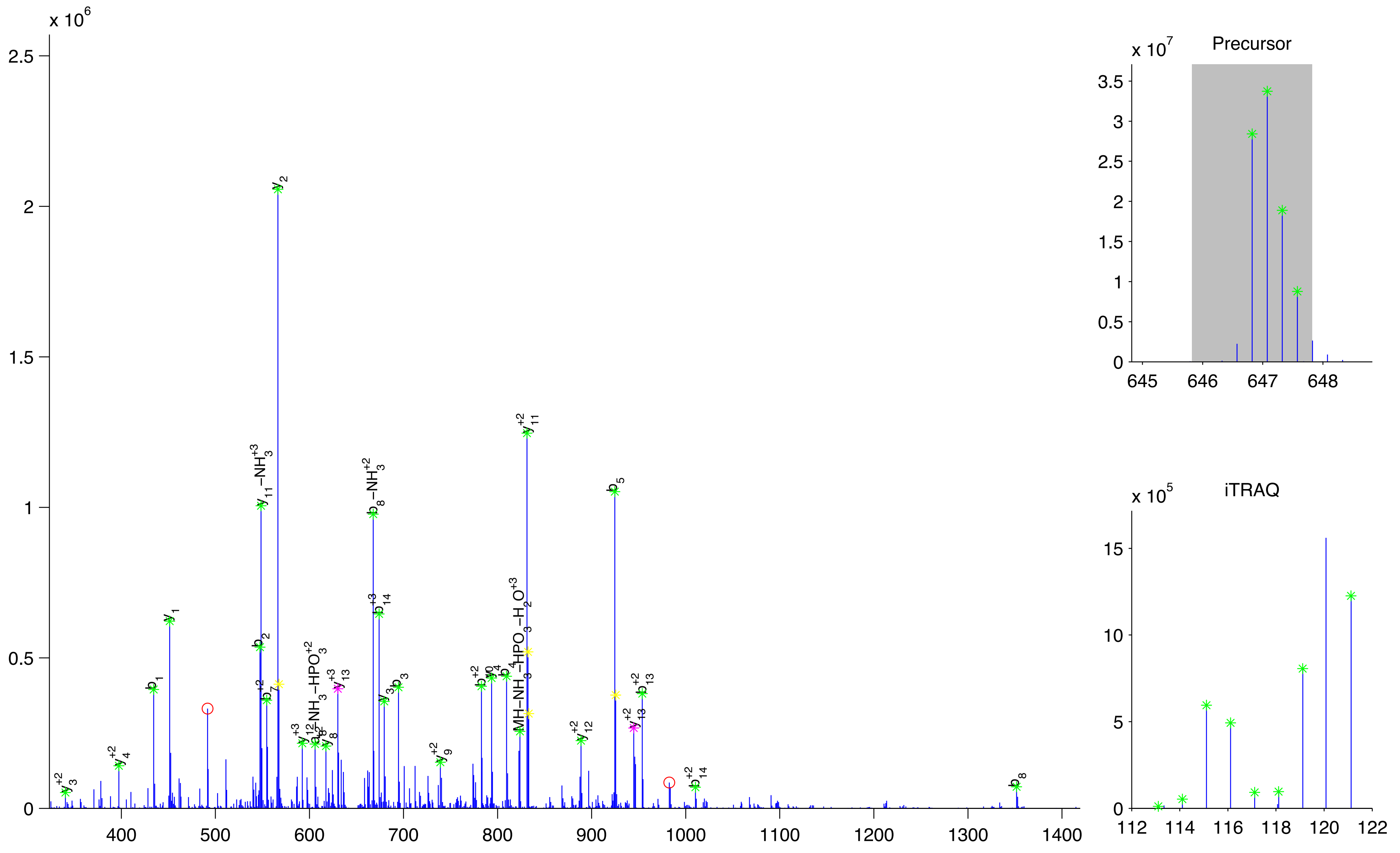

E L F D D P S y V N V Q N L D K

SHC (Src homology 2 domain containing) transforming protein 1 isoform p52Shc [Homo sapiens]
Charge State: +4
Scan Number: 26971
File Name: 120518_A549_EGFTSA_pY.raw

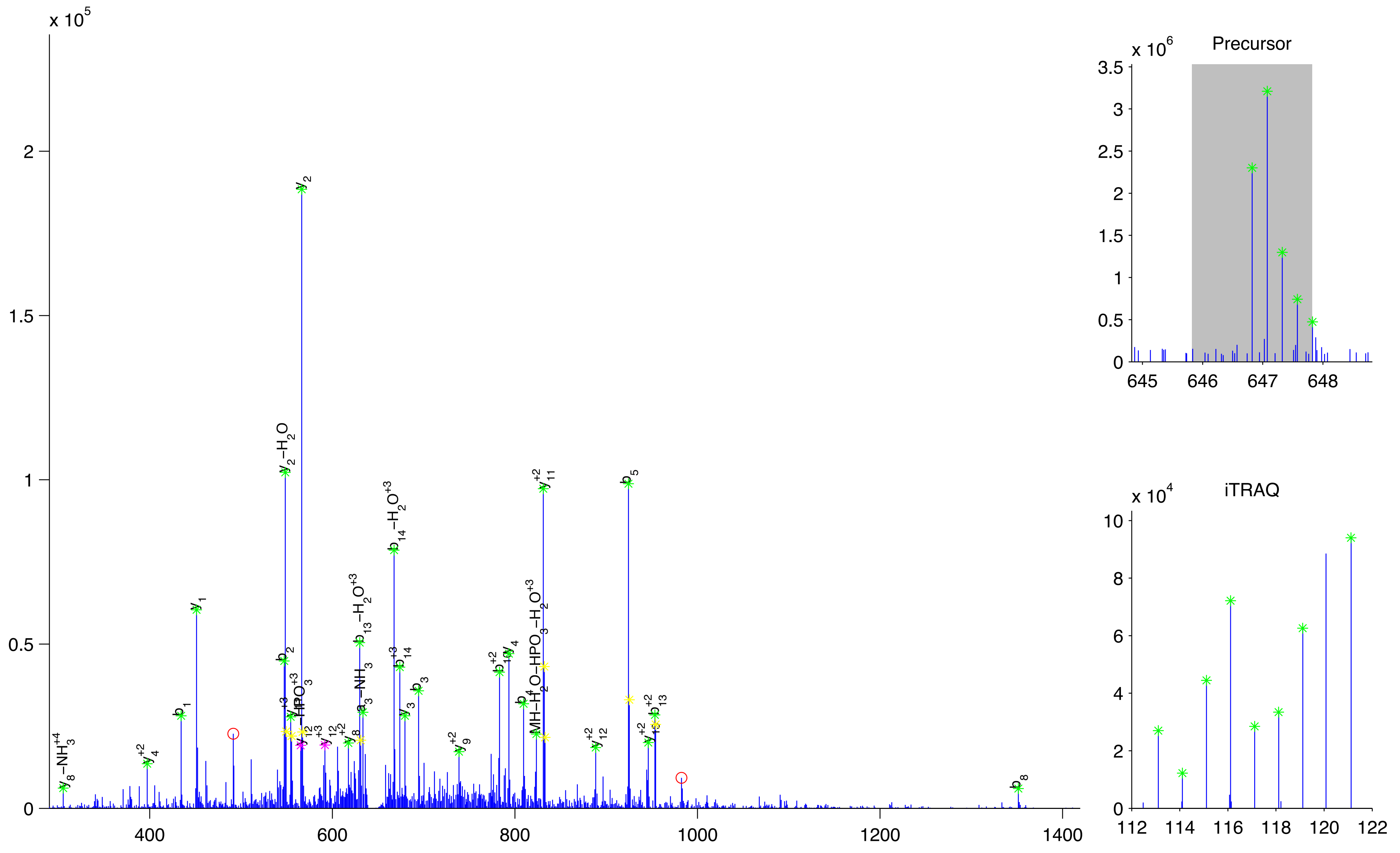

E L F D D P S y V N V Q N L D K

SHC (Src homology 2 domain containing) transforming protein 1 isoform p52Shc [Homo sapiens]
Charge State: +2
Scan Number: 26973
File Name: 120518_A549_EGFTSA_pY.raw

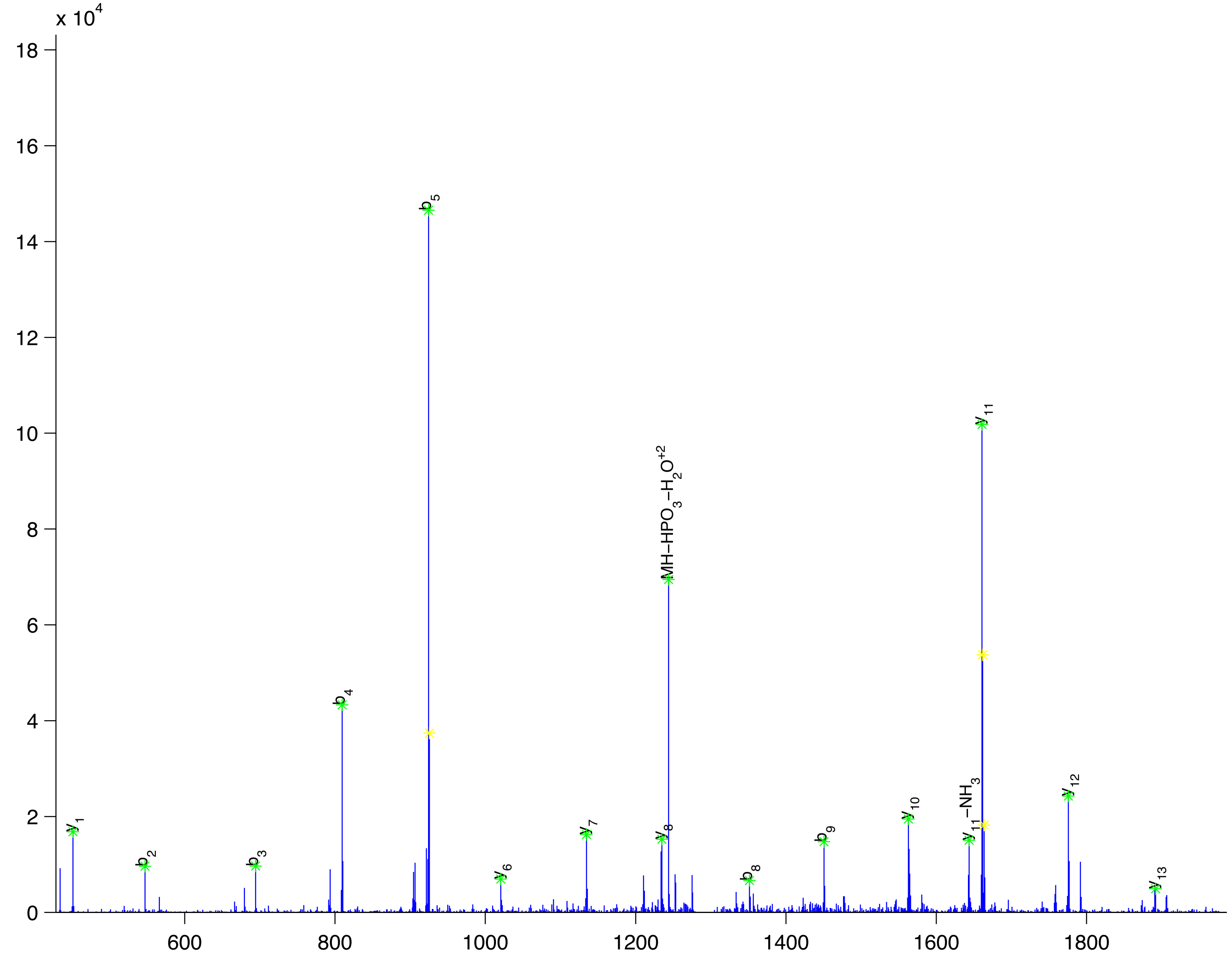

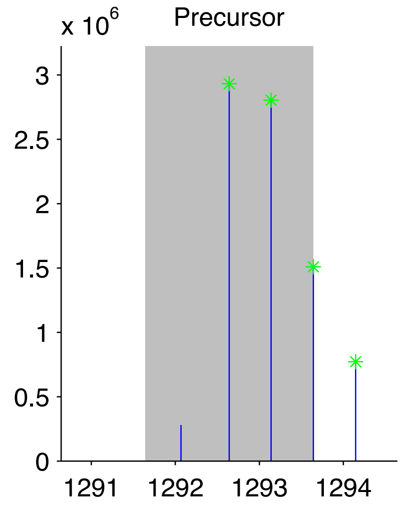

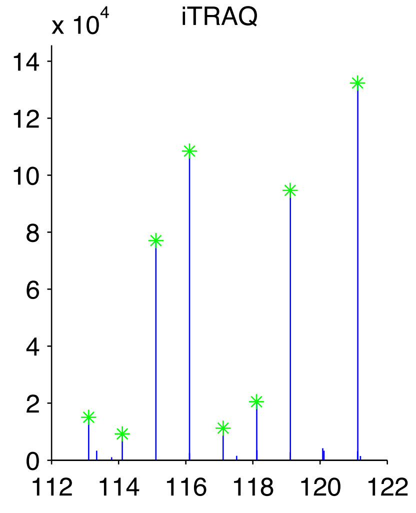

E L F D D P S y V N V Q N L D K

SHC (Src homology 2 domain containing) transforming protein 1 isoform p52Shc [Homo sapiens]
Charge State: +3
Scan Number: 27013
File Name: 120518_A549_EGFTSA_pY.raw

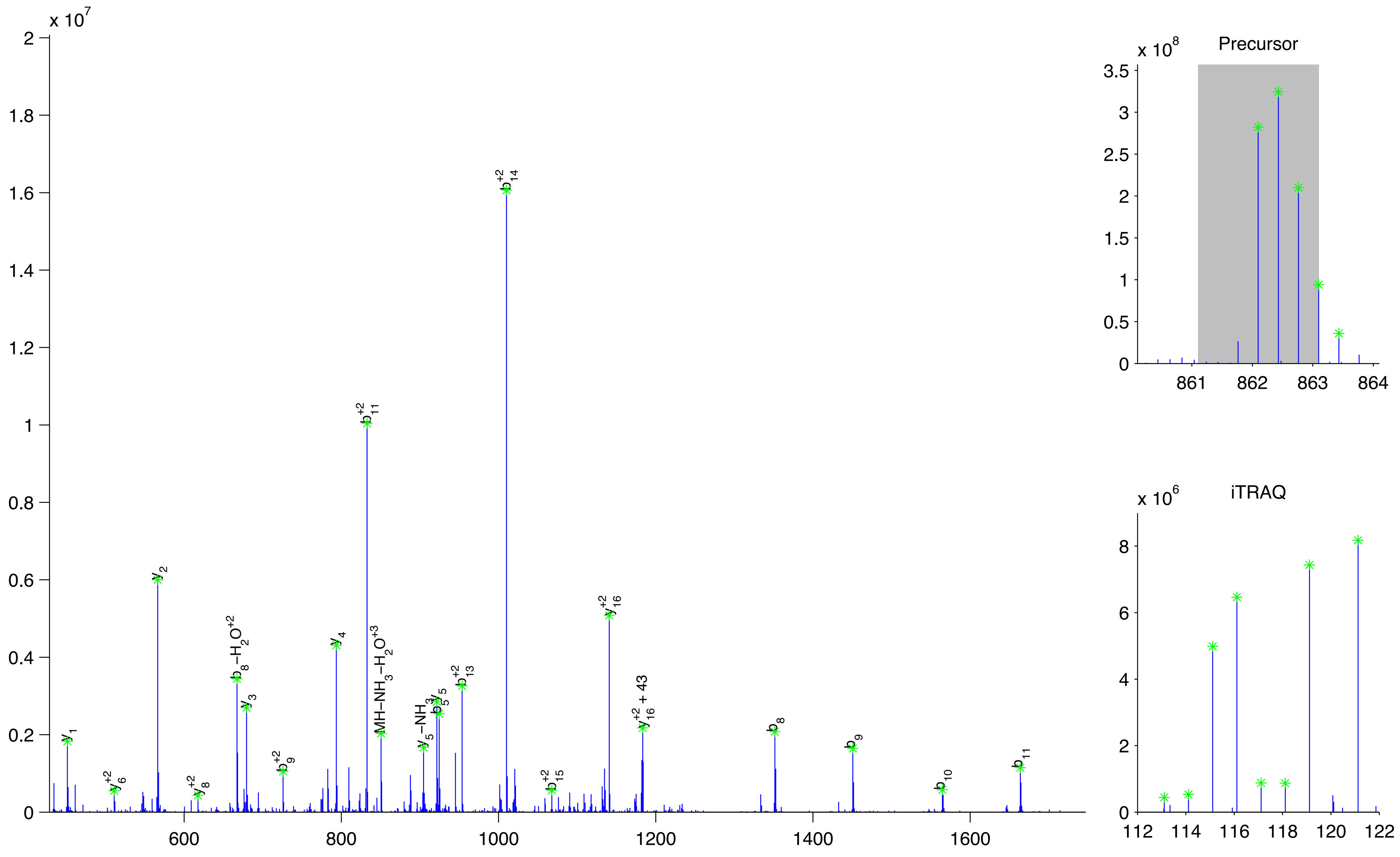

y c R P E S Q E H P E A D P G S A A P Y L K

signal transducer and activator of transcription 3 isoform 1 [Homo sapiens]
Charge State: +3
Scan Number: 6969
File Name: 120527_A549_TSAEGF_pY34_el.raw

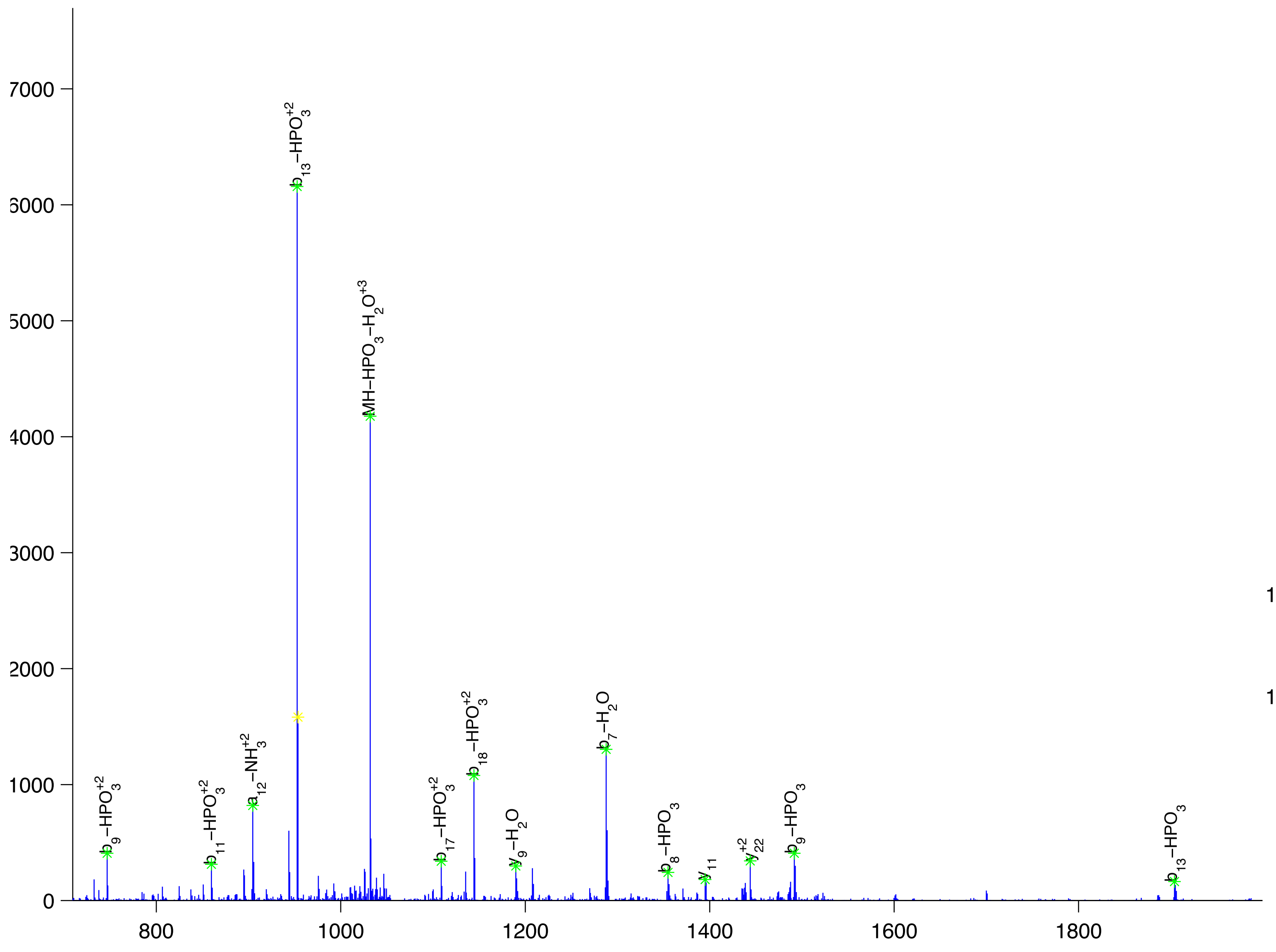

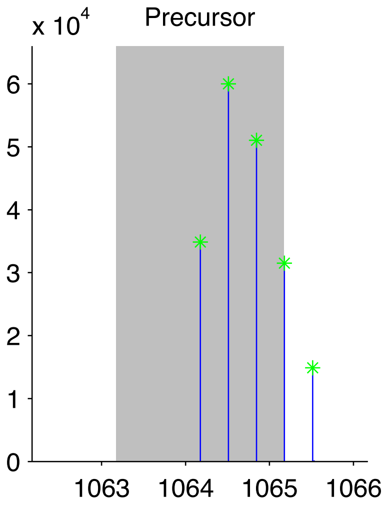

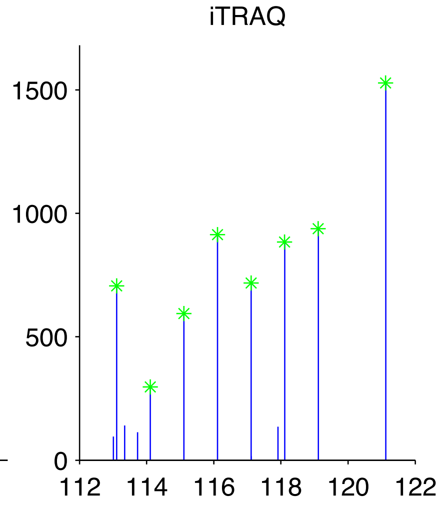

y c R P E S Q E H P E A D P G S A A P Y L K

signal transducer and activator of transcription 3 isoform 1 [Homo sapiens]
Charge State: +3
Scan Number: 13365
File Name: 120518_A549_EGFTSA_pY.raw

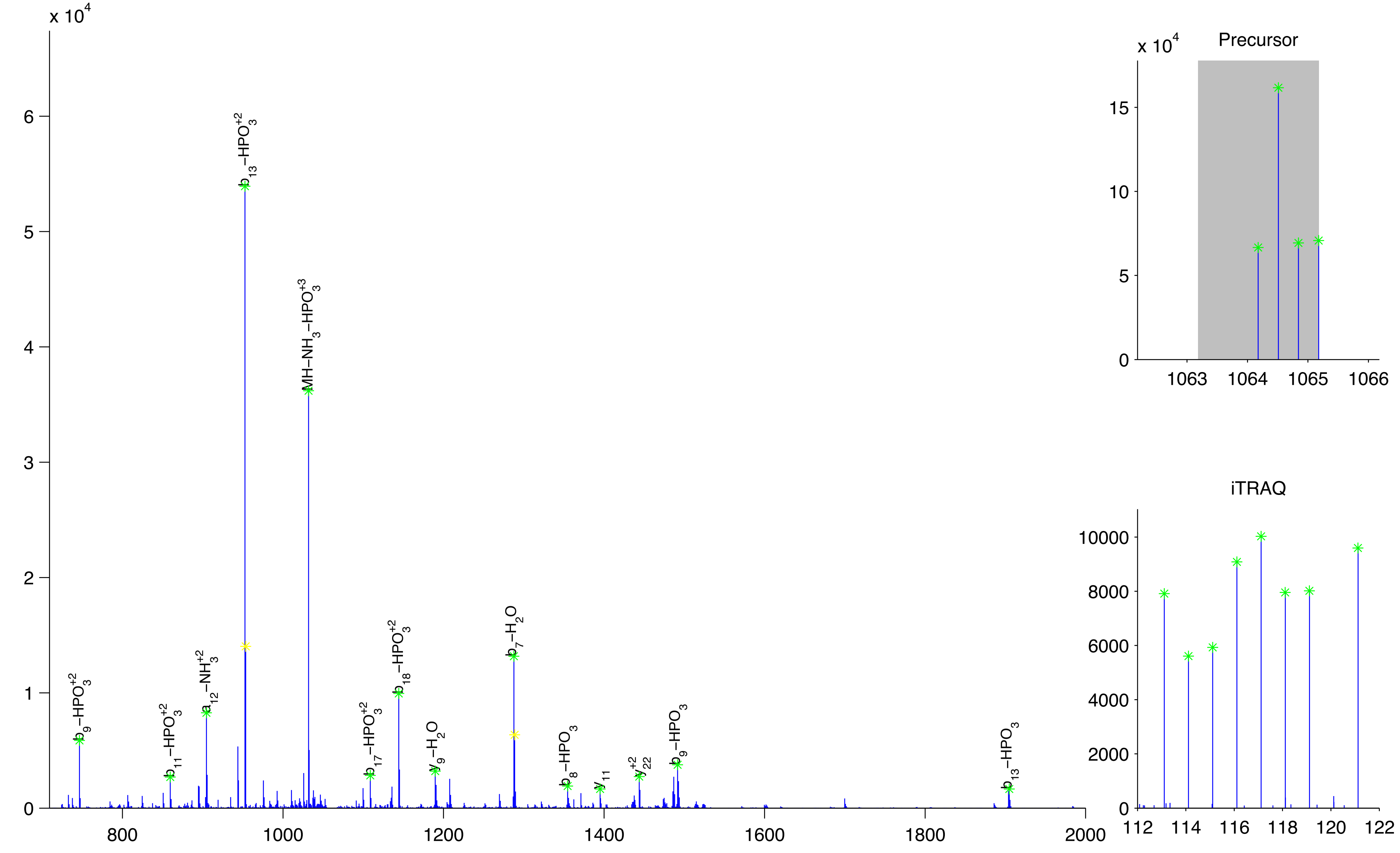

Y c R P E S Q E H P E A D P G A A P y L K

signal transducer and activator of transcription 3 isoform 2 [Homo sapiens]
Charge State: +4
Scan Number: 6801
File Name: 120527_A549_TSAEGF_pY34_el.raw

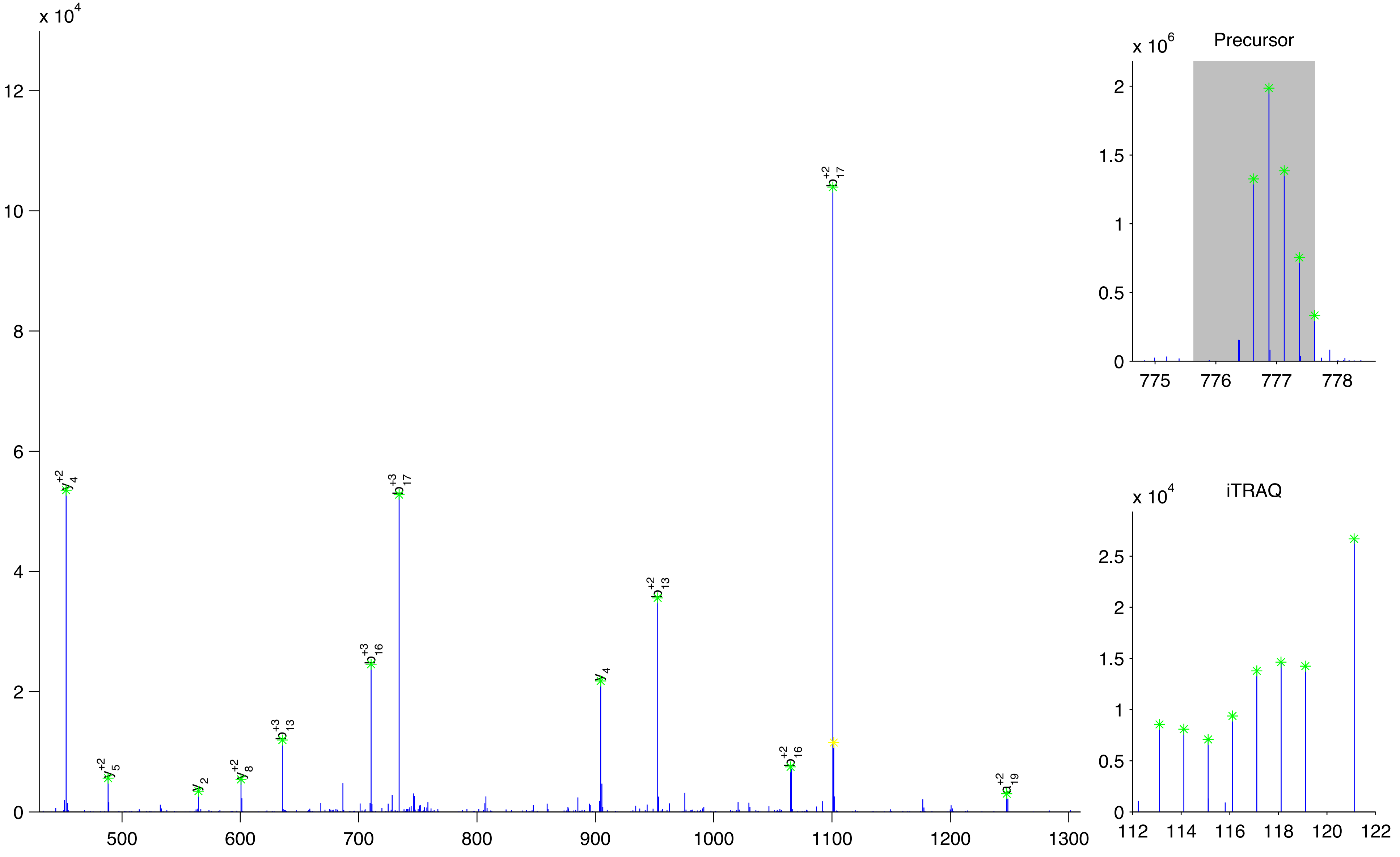

y c R P E S Q E H P E A D P G A A P Y L K T K

signal transducer and activator of transcription 3 isoform 2 [Homo sapiens]
Charge State: +4
Scan Number: 12371
File Name: 120518_A549_EGFTSA_pY.raw

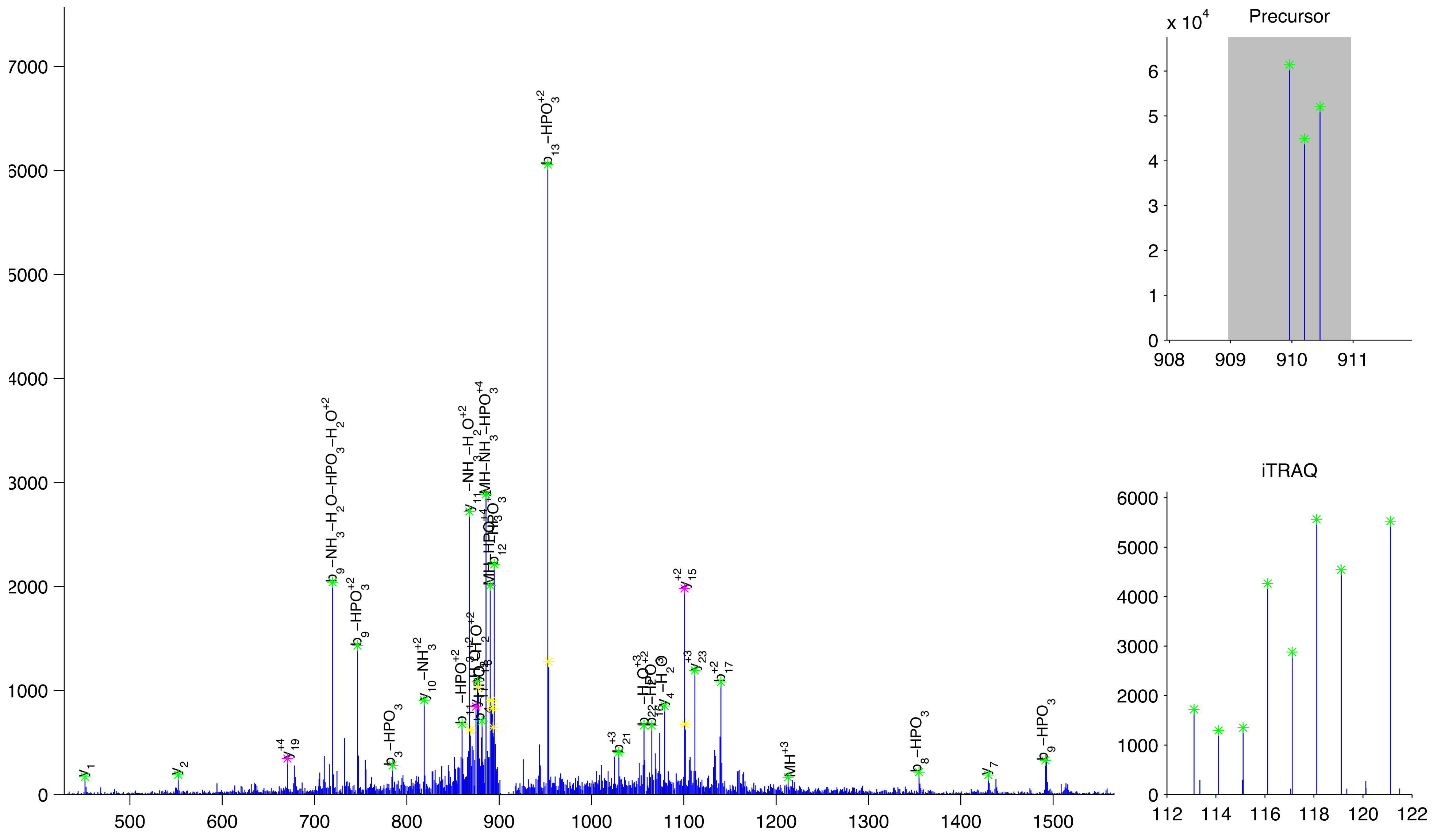

S H y A D V D P E N Q N F L L E S N L G K K

solute carrier family 38, member 2 [Homo sapiens]
Charge State: +5
Scan Number: 11276
File Name: 120527_A549_TSAEGF_pY34_el.raw

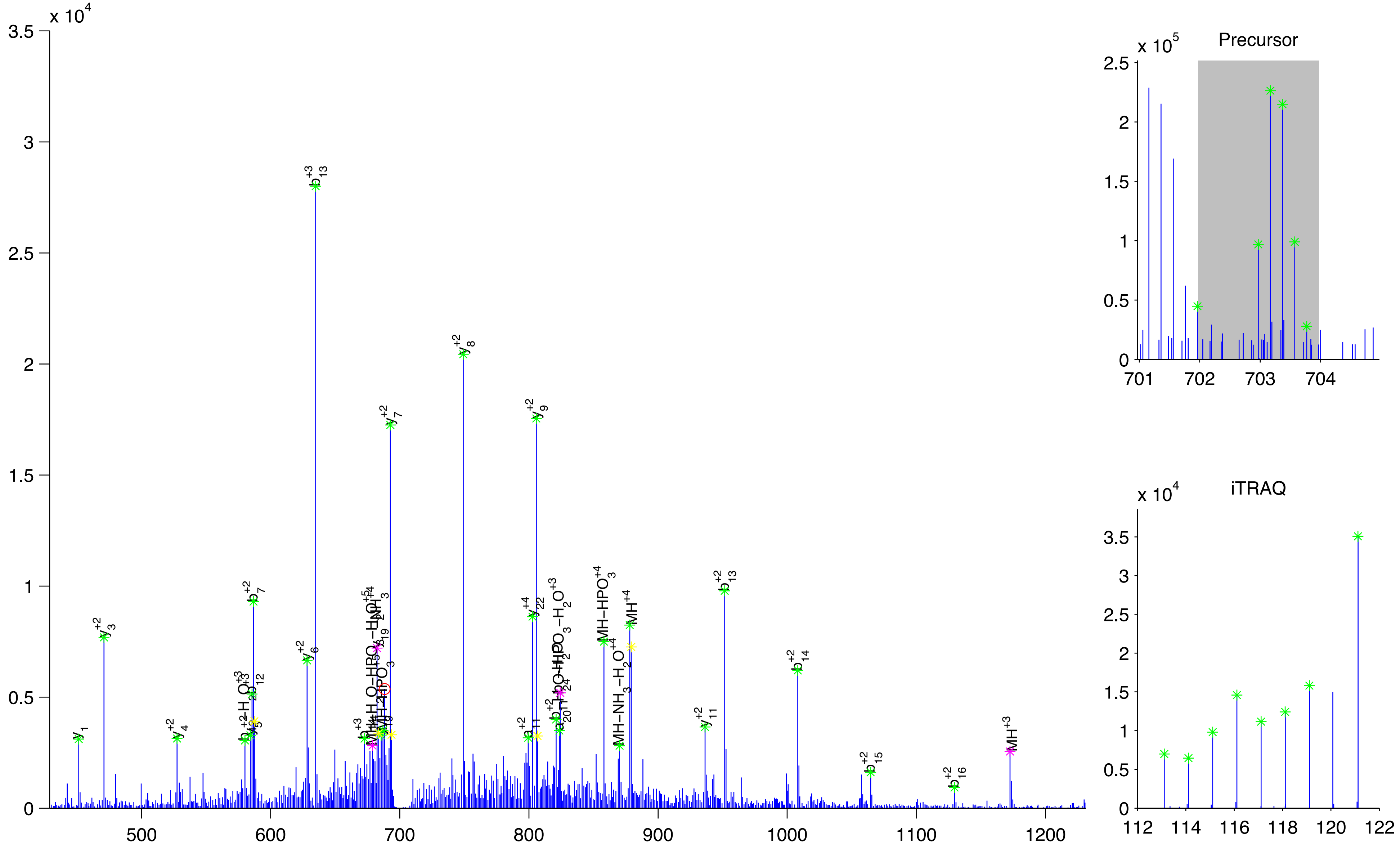

S H y A D V D P E N Q N F L L E S N L G K

solute carrier family 38, member 2 [Homo sapiens]
Charge State: +4
Scan Number: 12891
File Name: 120527_A549_TSAEGF_pY34_el.raw

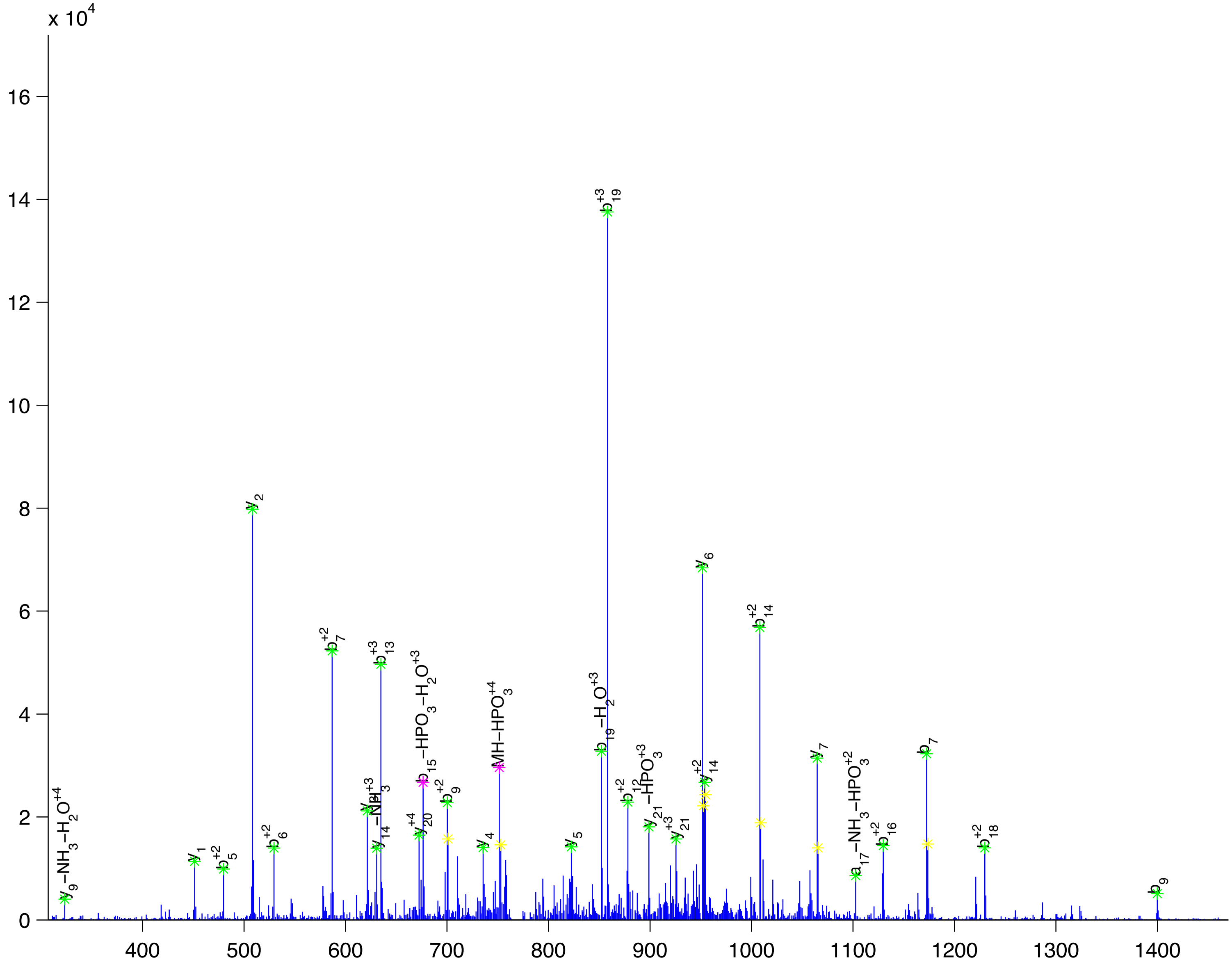

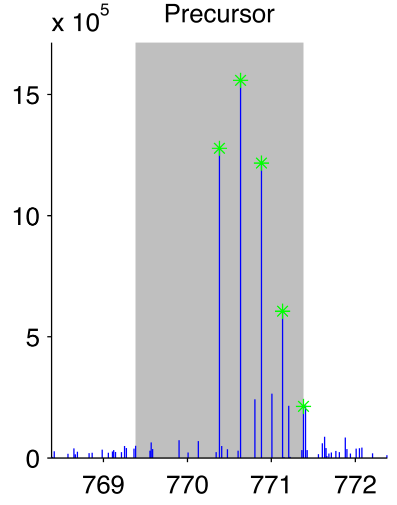

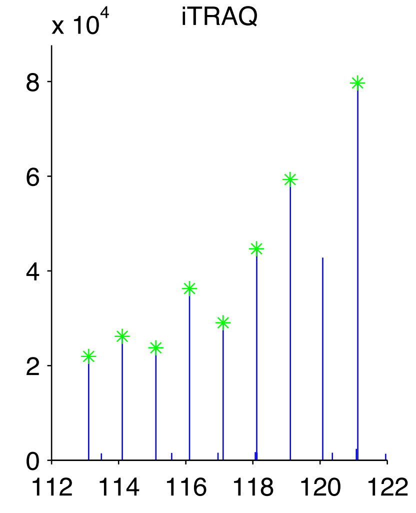

S H y A D V D P E N Q N F L L E S N L G K

solute carrier family 38, member 2 [Homo sapiens]
Charge State: +3
Scan Number: 25608
File Name: 120518_A549_EGFTSA_pY.raw

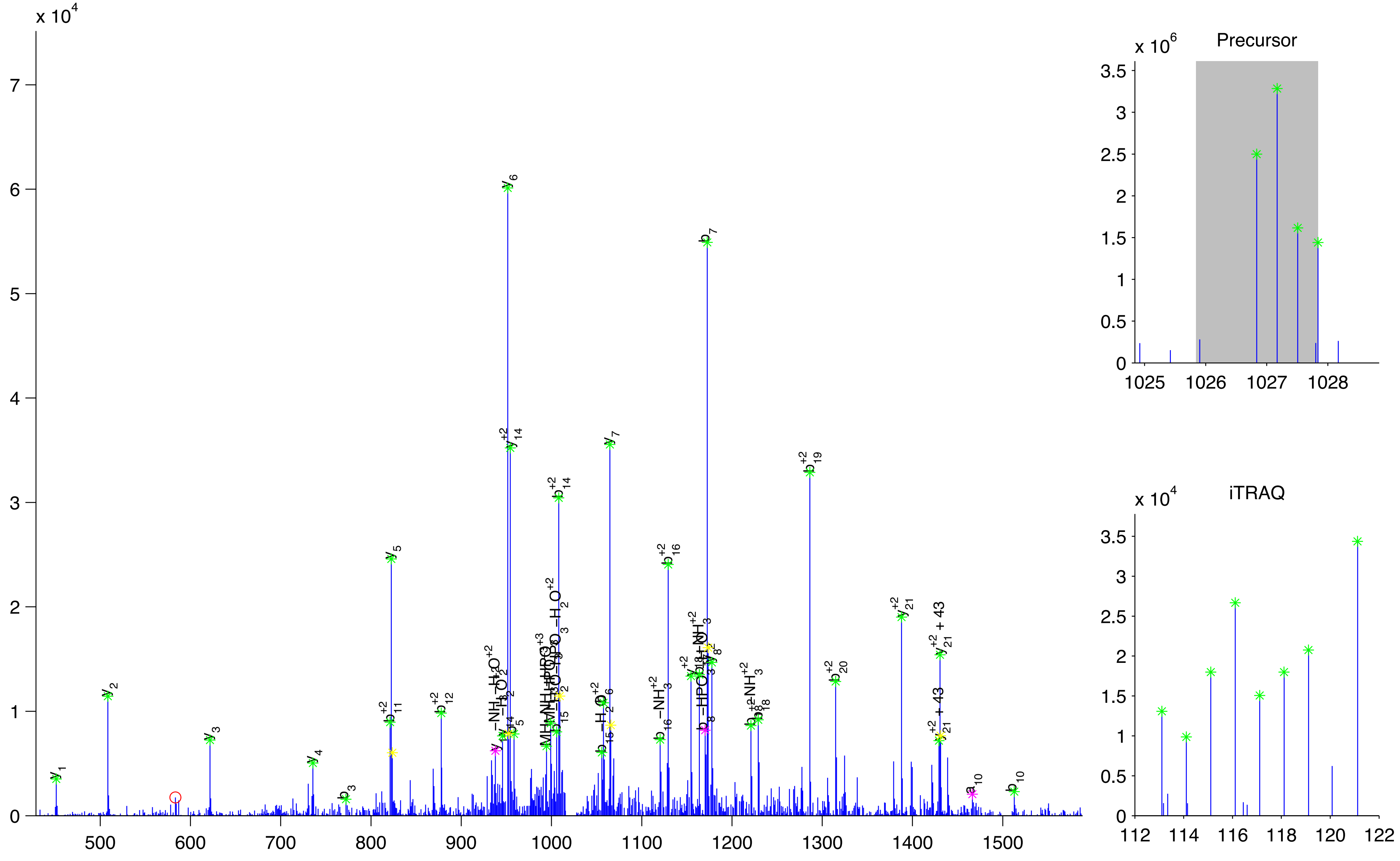

A G K G E S A G Y m E P y E A Q R

Src homology 2 domain containing adaptor protein B [Homo sapiens]
Charge State: +3
Scan Number: 3963
File Name: 120527_A549_TSAEGF_pY34_el.raw

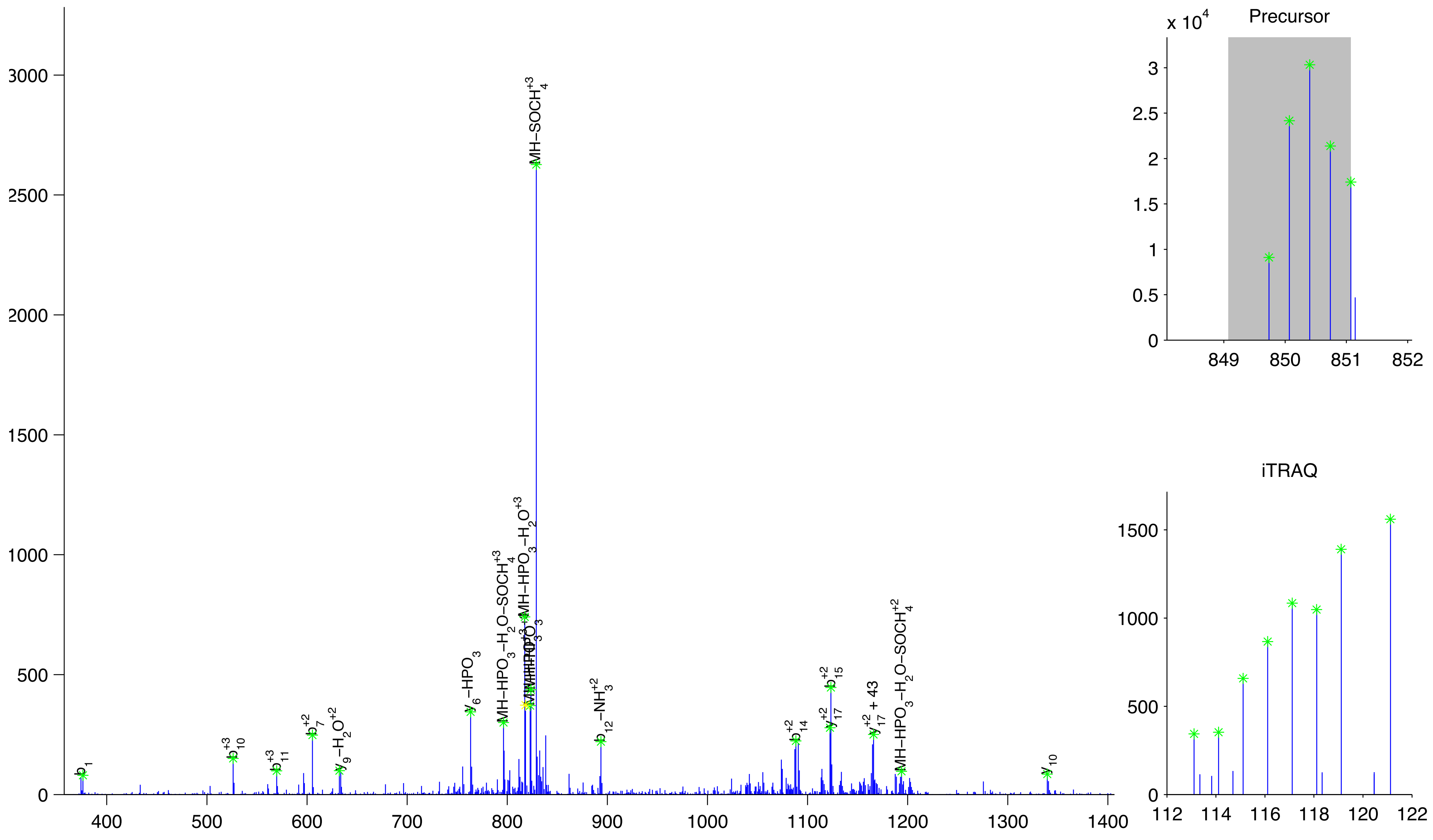

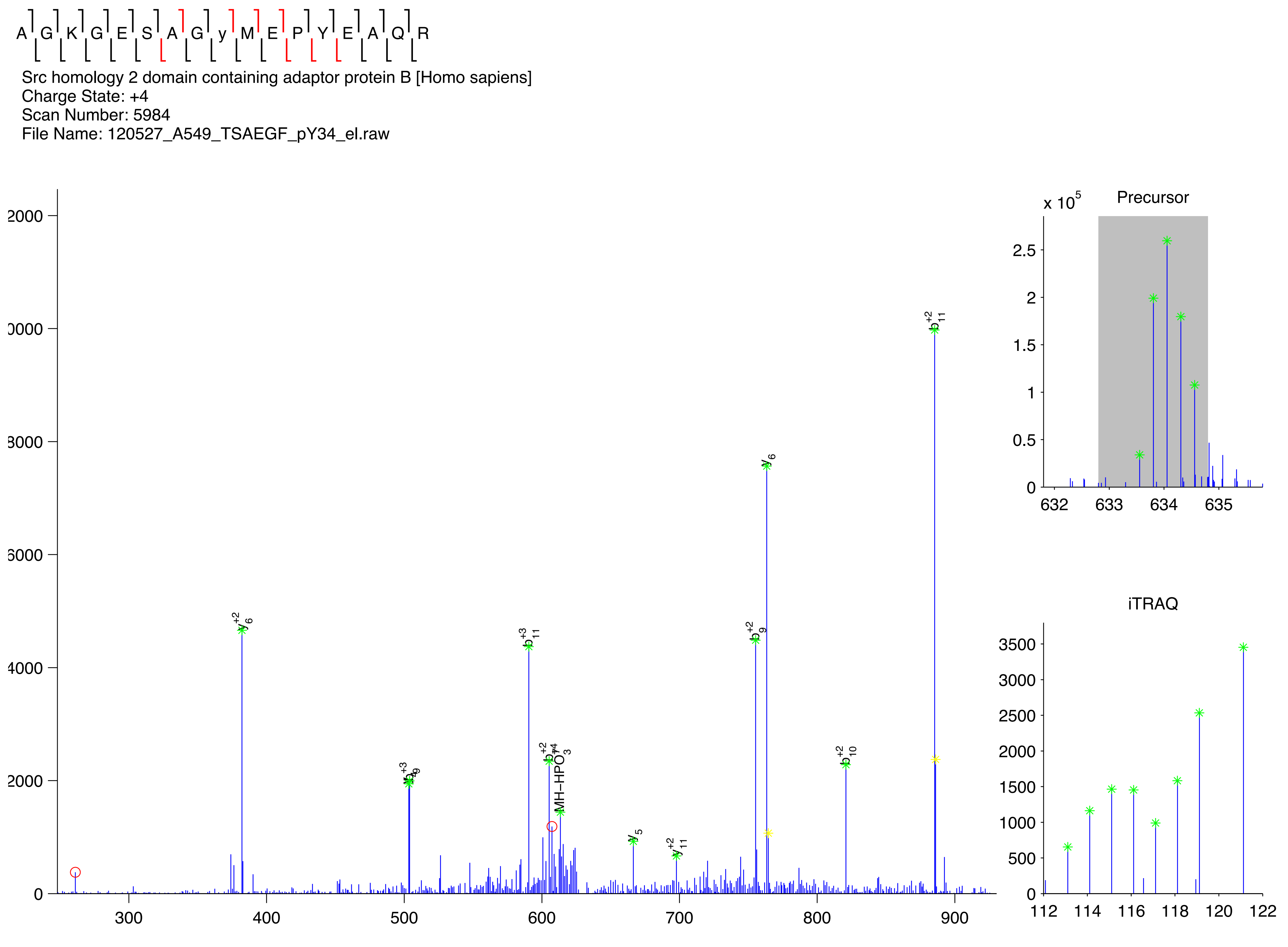

A G K G E S A G y M E P Y E A Q R
Src homology 2 domain containing adaptor protein B [Homo sapiens]
Charge State: +4
Scan Number: 5984
File Name: 120527_A549_TSAEGF_pY34_el.raw
Precursor
x 10^5
iTRAQ
MH−HPO3

D K V T I A D D y S D P F D A K

Src homology 2 domain containing adaptor protein B [Homo sapiens]
Charge State: +4
Scan Number: 11066
File Name: 120527_A549_TSAEGF_pY34_el.raw

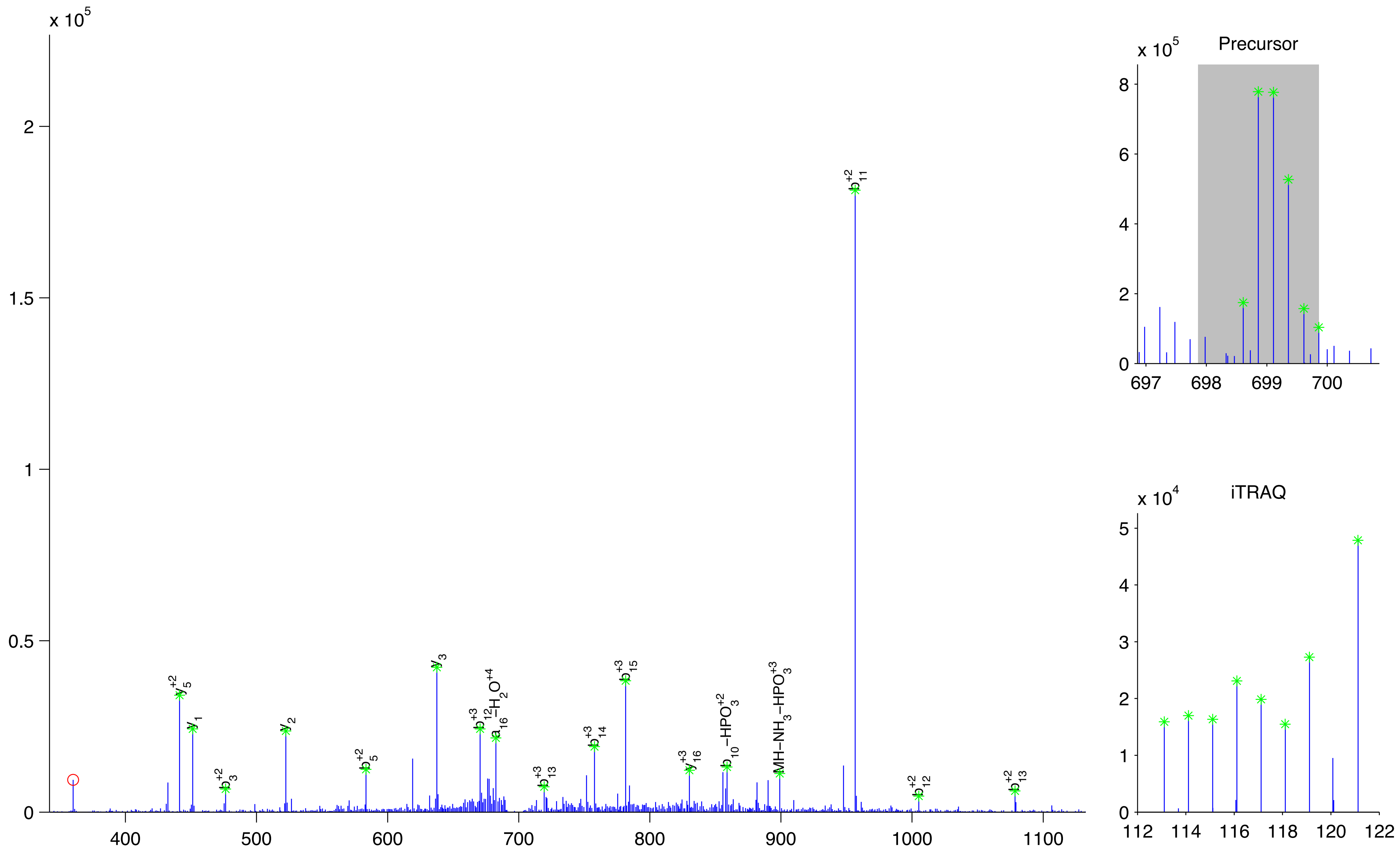

V T I A D D y S D P F D A K

Src homology 2 domain containing adaptor protein B [Homo sapiens]
Charge State: +3
Scan Number: 13405
File Name: 120527_A549_TSAEGF_pY34_el.raw

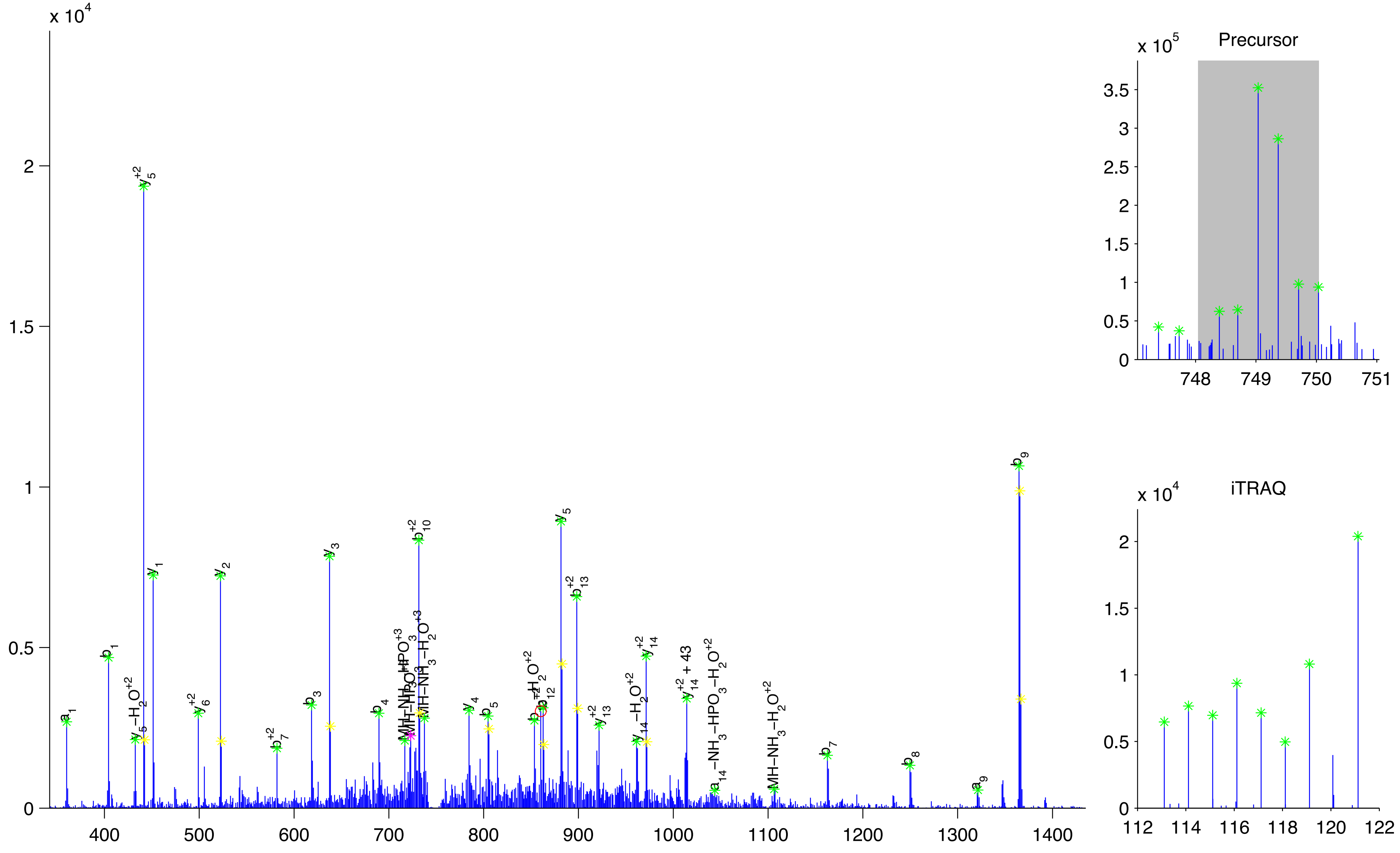

D K V T I A D D y S D P F D A K

Src homology 2 domain containing adaptor protein B [Homo sapiens]
Charge State: +4
Scan Number: 22477
File Name: 120518_A549_EGFTSA_pY.raw

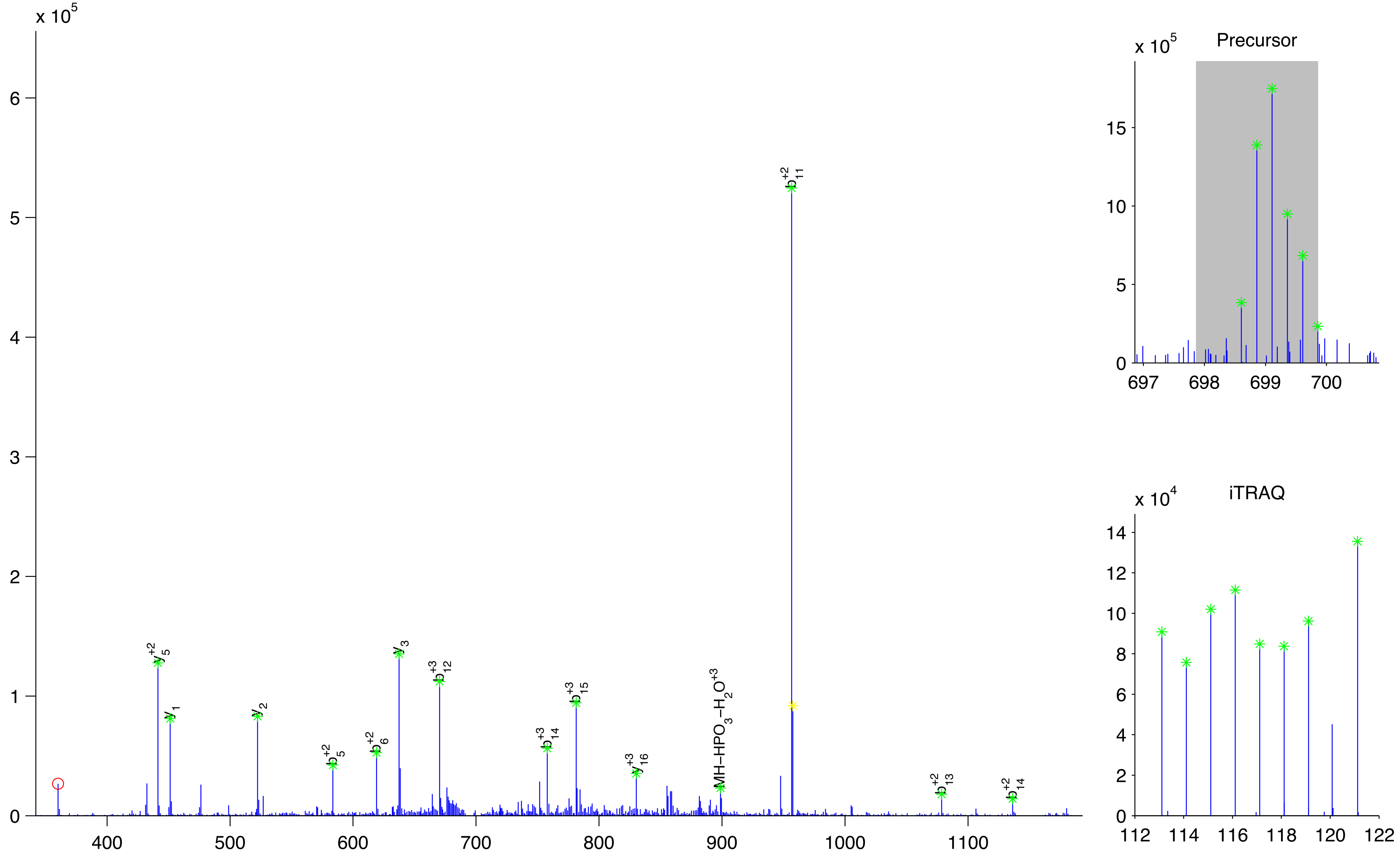

L P Q D D D R P A D E y D Q P W E W N R

Src homology 2 domain containing adaptor protein B [Homo sapiens]
Charge State: +3
Scan Number: 24913
File Name: 120518_A549_EGFTSA_pY.raw

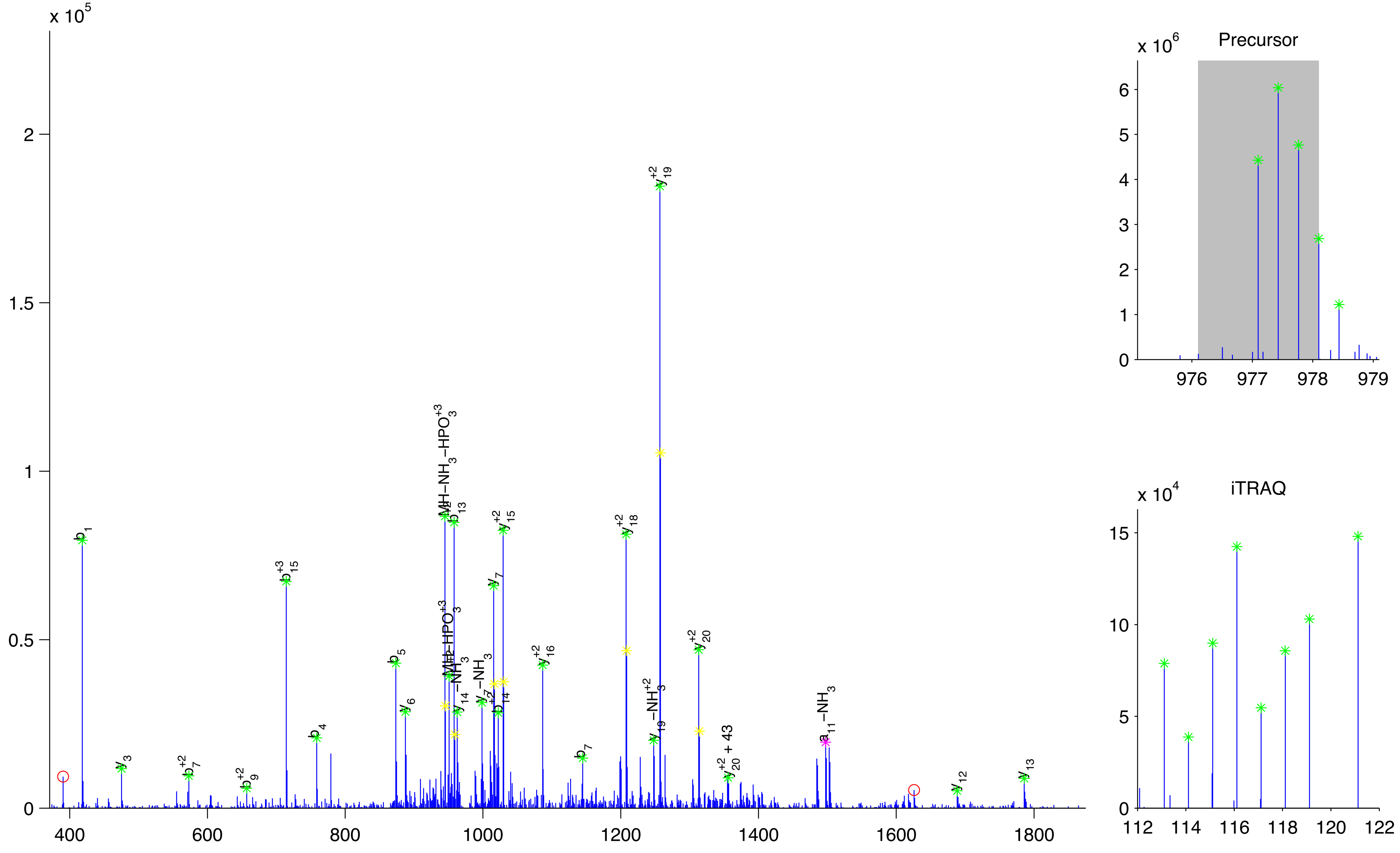

E c D y S I D G I N R

talin 2 [Homo sapiens]
Charge State: +2
Scan Number: 14245
File Name: 120518_A549_EGFTSA_pY.raw

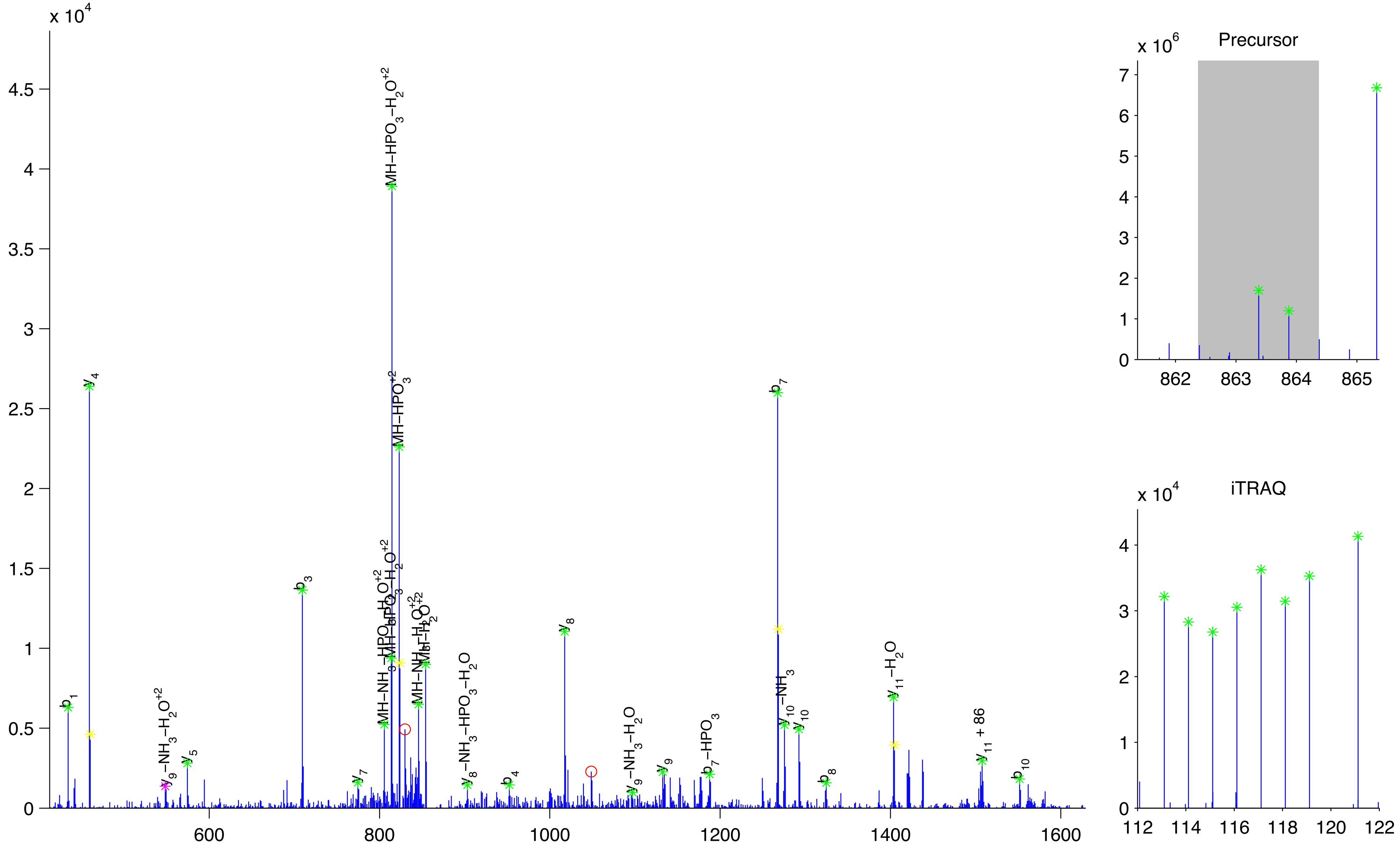

H V A Y G G y S T P E D R

tensin [Homo sapiens]
Charge State: +3
Scan Number: 8659
File Name: 120518_A549_EGFTSA_pY.raw

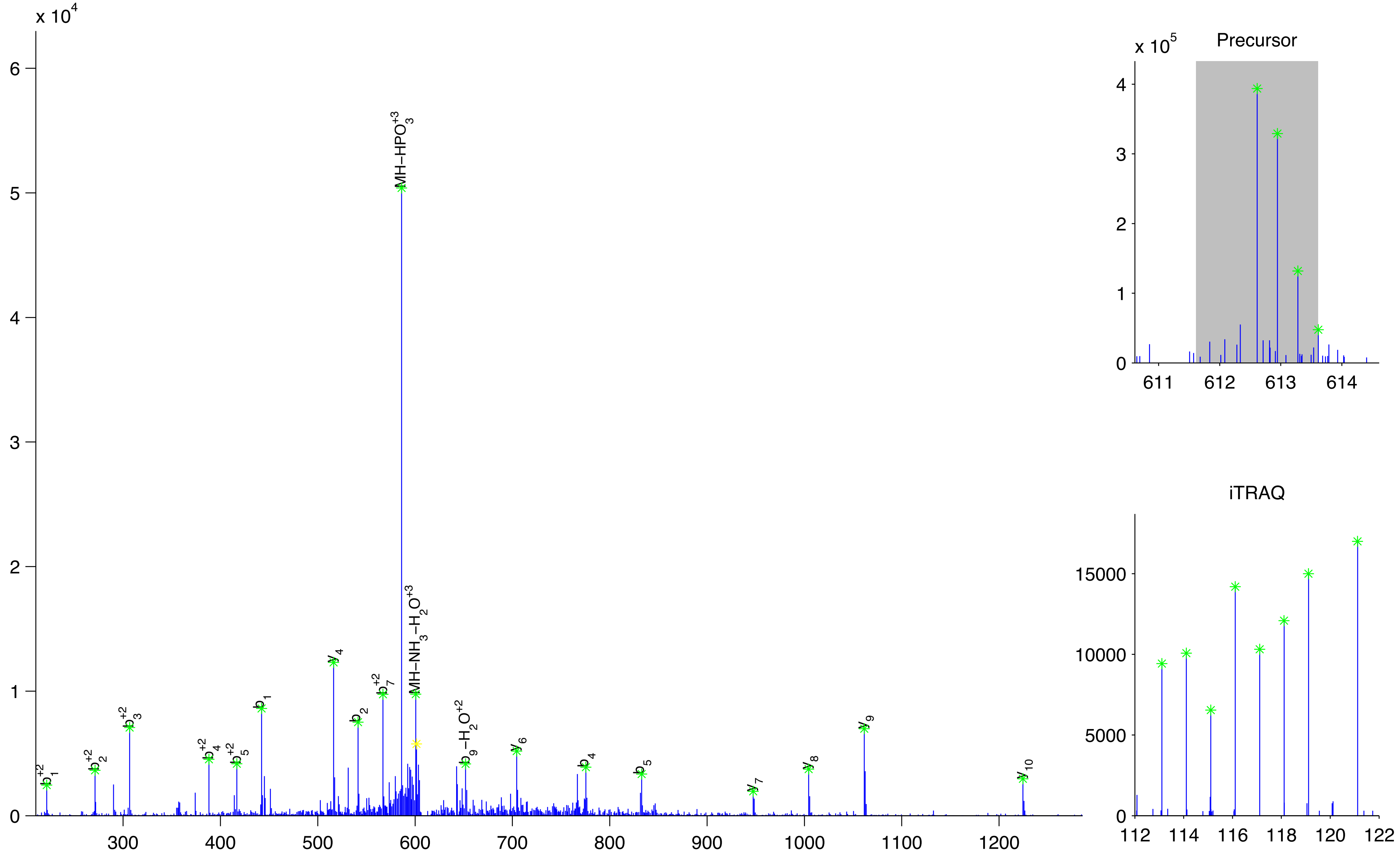

H V A Y G G y S T P E D R

tensin [Homo sapiens]
Charge State: +2
Scan Number: 9024
File Name: 120518_A549_EGFTSA_pY.raw

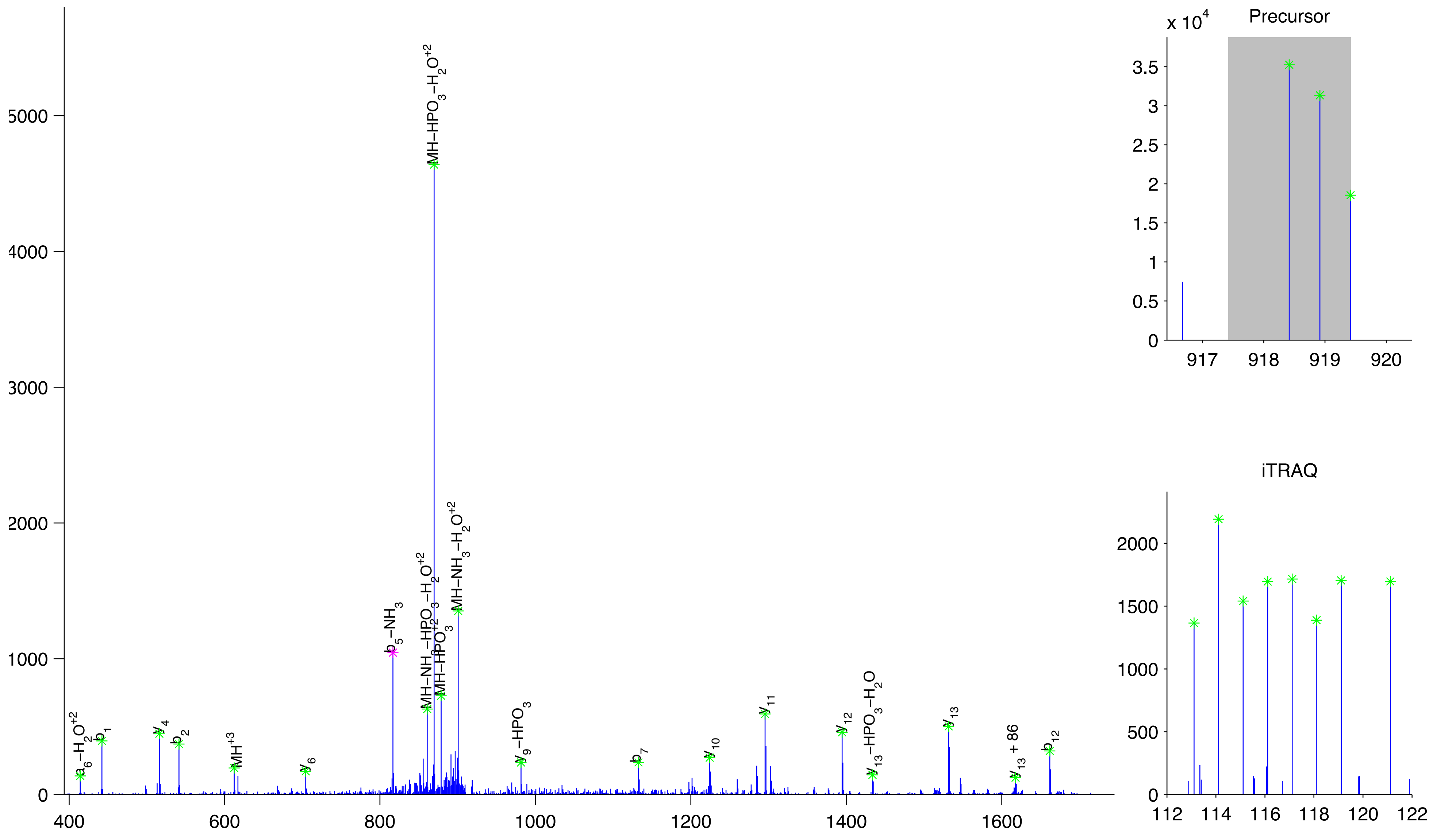

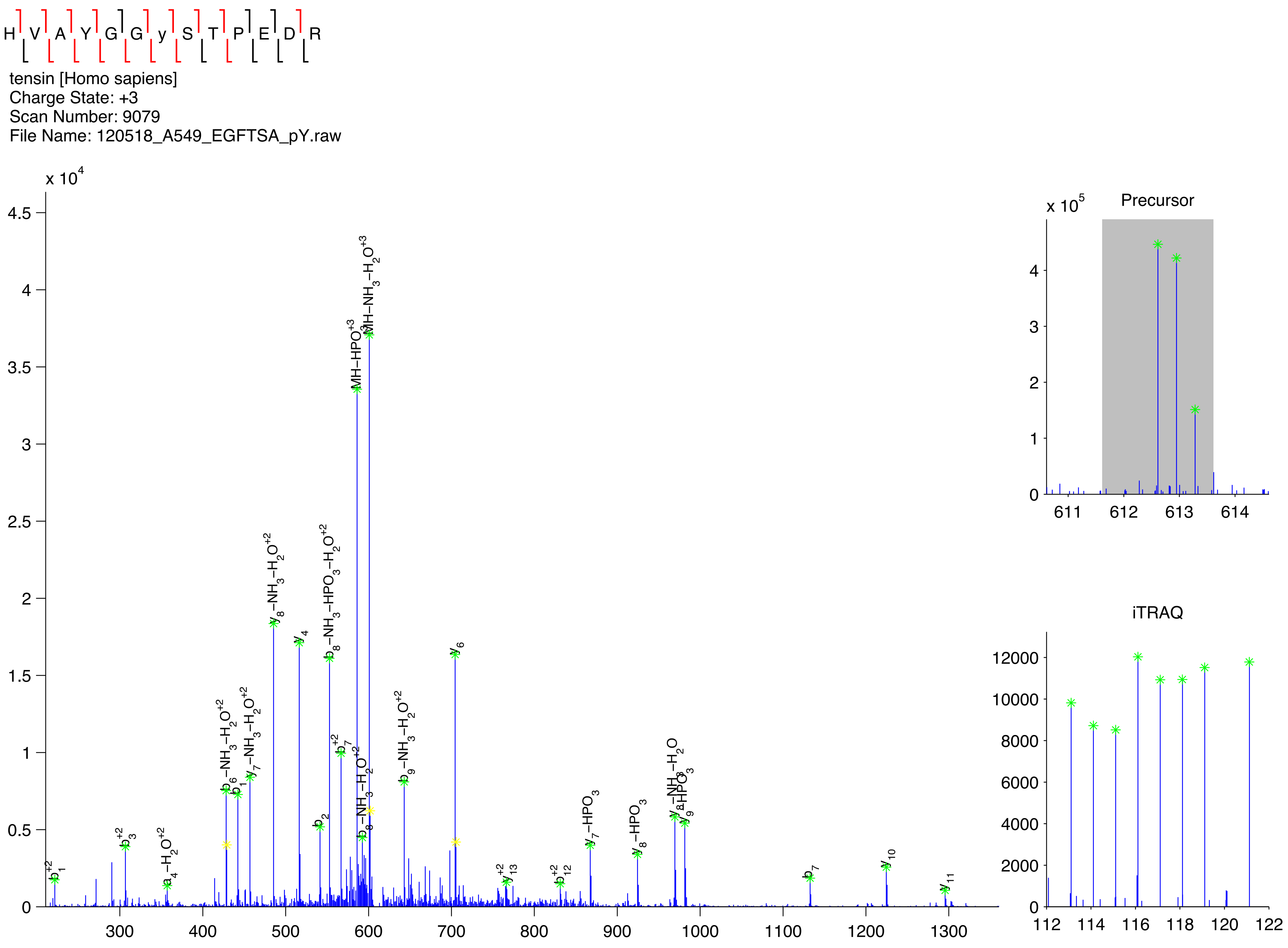
H V A Y G G y S T P E D R
tensin [Homo sapiens]
Charge State: +3
Scan Number: 9079
File Name: 120518_A549_EGFTSA_pY.raw
x 10^4
MH−HPO3+3
MH−NH3−H2O+3
y8−NH3−H2O+2
y4
b8−NH3−HPO3−H2O+2
y6
b7+2
b9−NH3−H2O+2
b6−NH3−H2O+2
y7−NH3−H2O+2
b2
b8−NH3−H2O+2
y7−HPO3
y8−HPO3
y8−NH3−H2O
y9−HPO3
b1+2
b3+2
a4−H2O+2
y13+2
b12+2
b7
y10
y11
Precursor
x 10^5
iTRAQ

K L S L G Q y D N D A G G Q L P F S K

tensin 3 [Homo sapiens]
Charge State: +4
Scan Number: 11988
File Name: 120527_A549_TSAEGF_pY34_el.raw

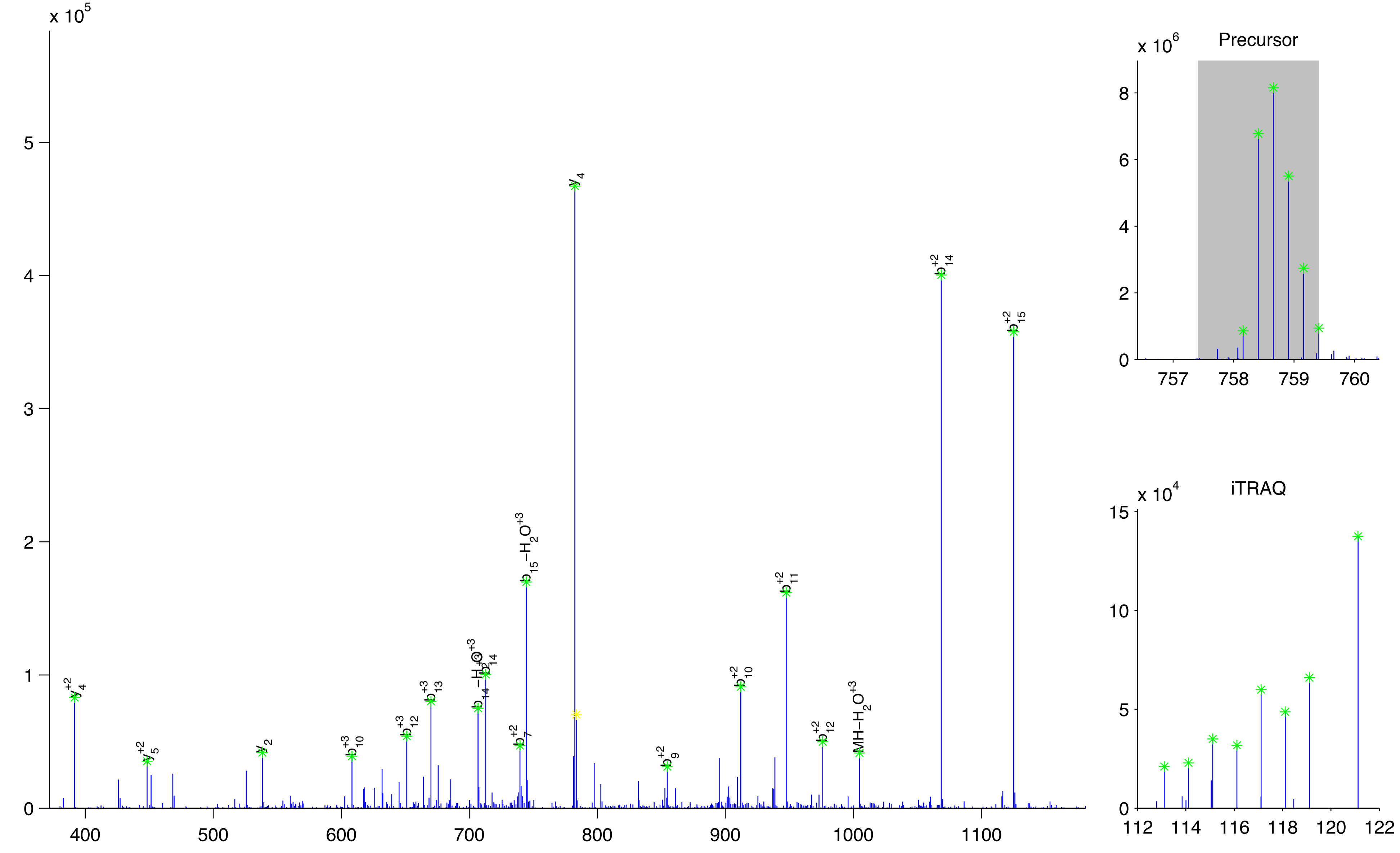

K L S L G Q y D N D A G G Q L P F S K

tensin 3 [Homo sapiens]
Charge State: +5
Scan Number: 12030
File Name: 120527_A549_TSAEGF_pY34_el.raw

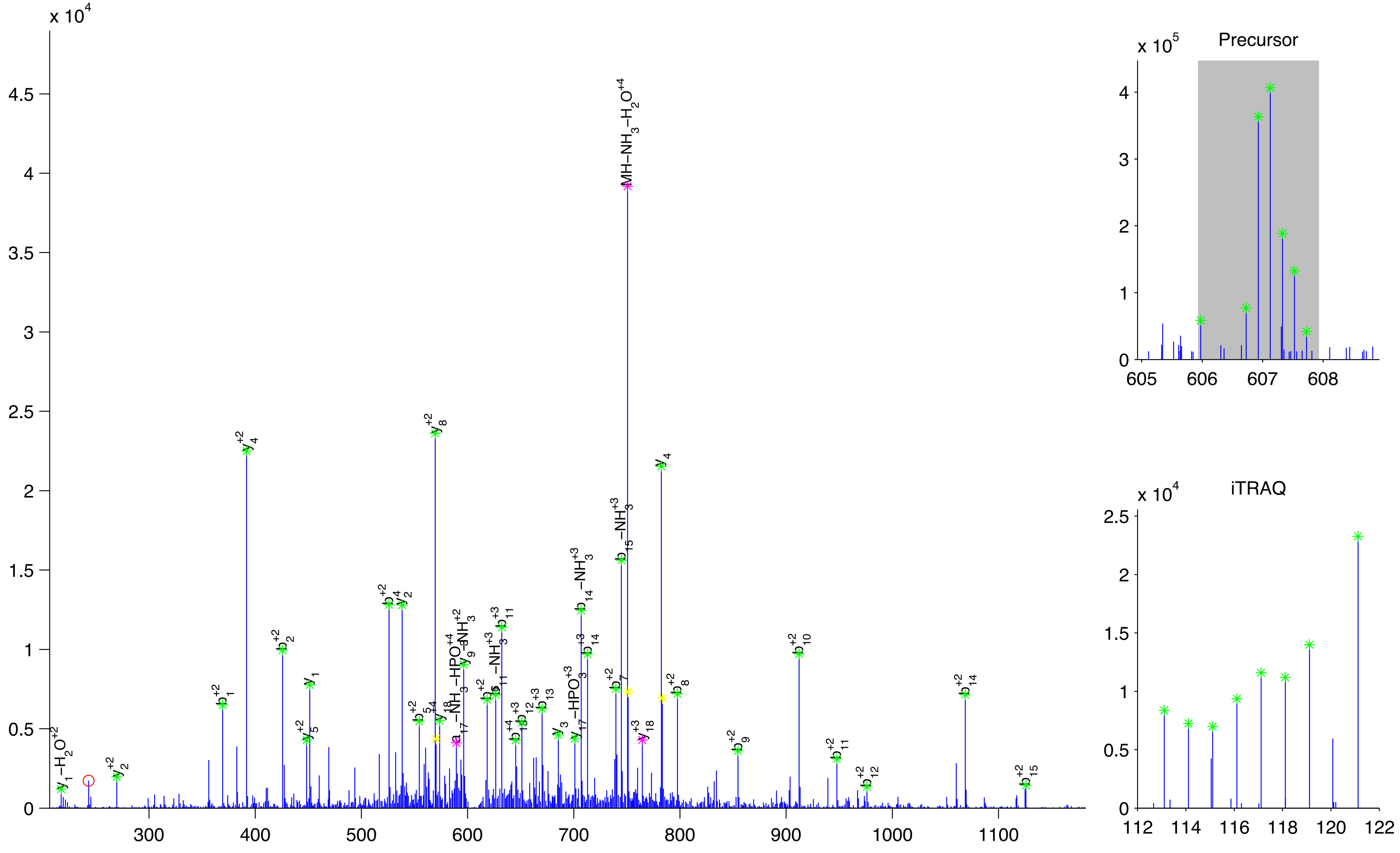

Q Q Q m V V A H Q y S F A P D G E A R

tensin 3 [Homo sapiens]
Charge State: +3
Scan Number: 12678
File Name: 120518_A549_EGFTSA_pY.raw

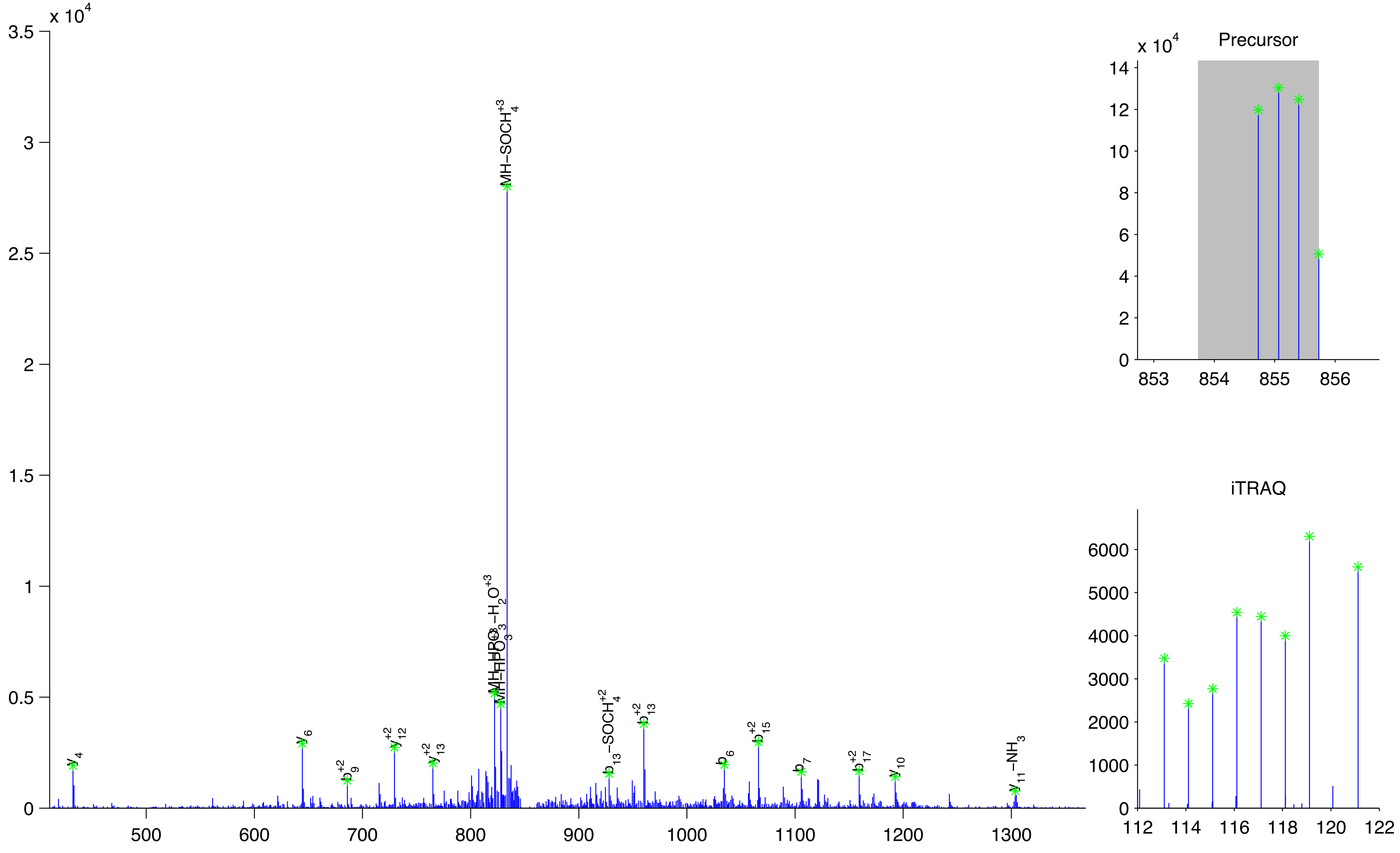

W D S Y E N L S A D G E V L H T Q G P V D G S L y A K

tensin 3 [Homo sapiens]
Charge State: +4
Scan Number: 14384
File Name: 120527_A549_TSAEGF_pY34_el.raw

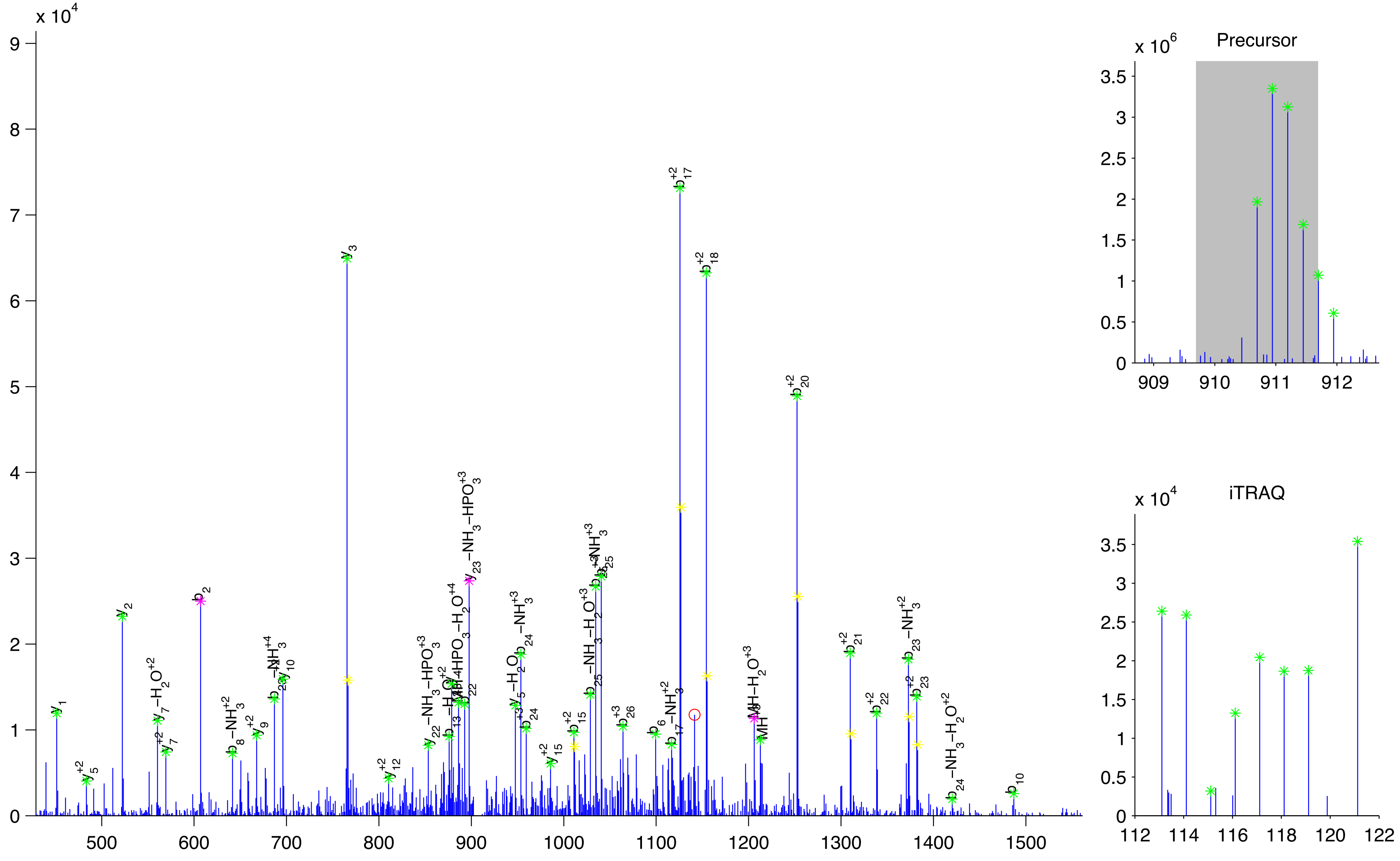

L S L G Q y D N D A G G Q L P F S K

tensin 3 [Homo sapiens]
Charge State: +2
Scan Number: 14426
File Name: 120527_A549_TSAEGF_pY34_el.raw

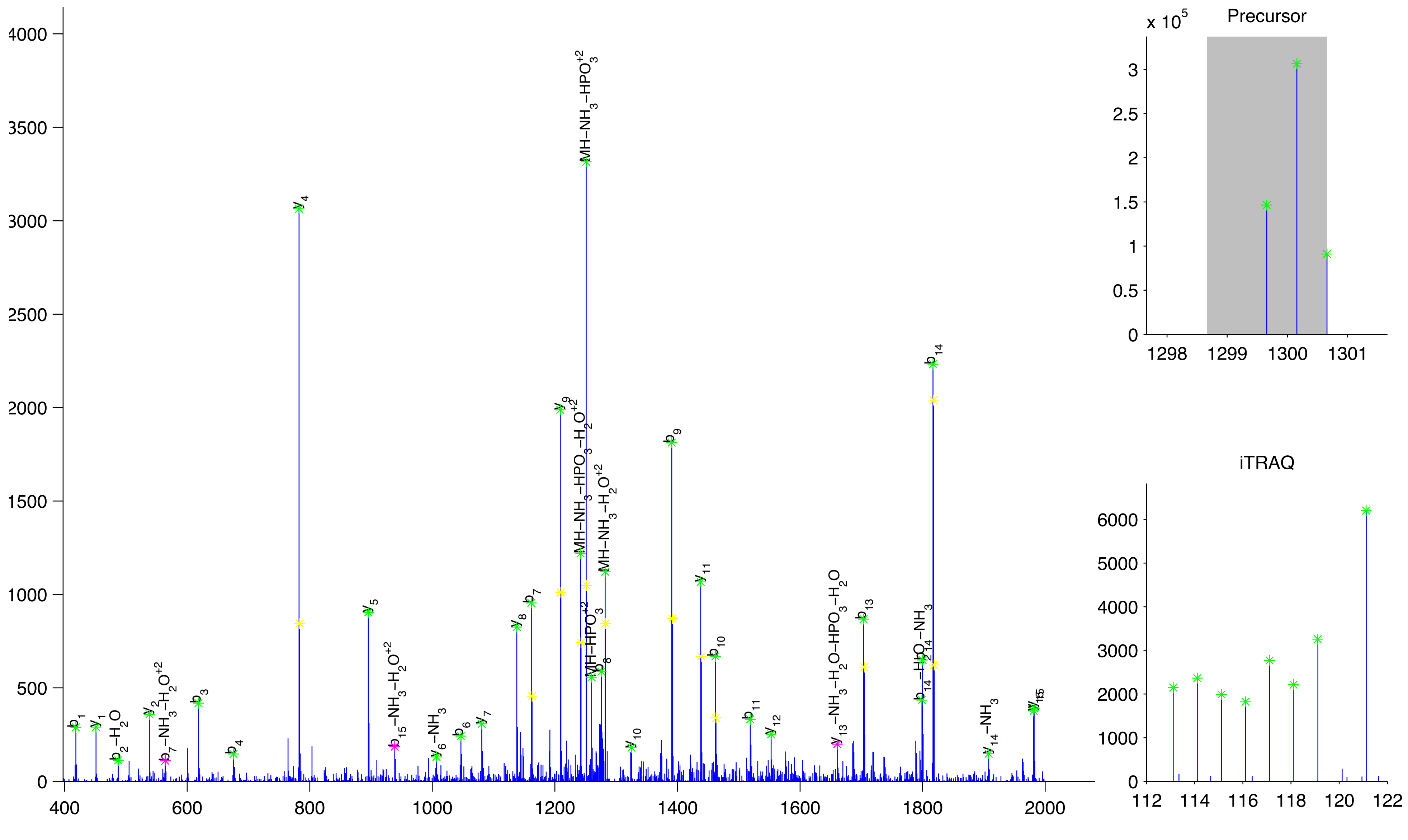

K L S L G Q y D N D A G G Q L P F S K

tensin 3 [Homo sapiens]
Charge State: +3
Scan Number: 24247
File Name: 120518_A549_EGFTSA_pY.raw

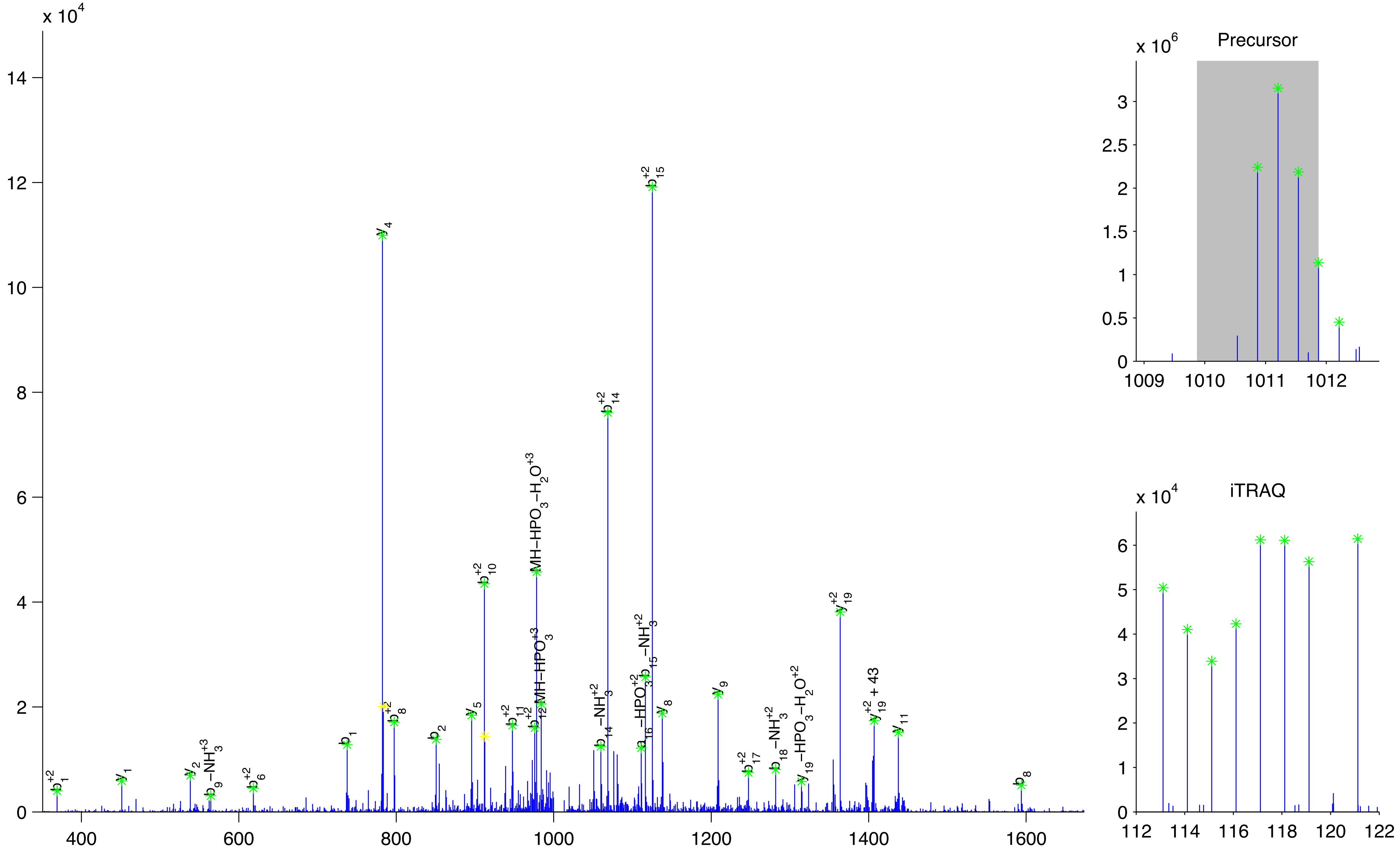

E S M c S T P A F P V S P E T P y V K

tensin 3 [Homo sapiens]
Charge State: +3
Scan Number: 25192
File Name: 120518_A549_EGFTSA_pY.raw

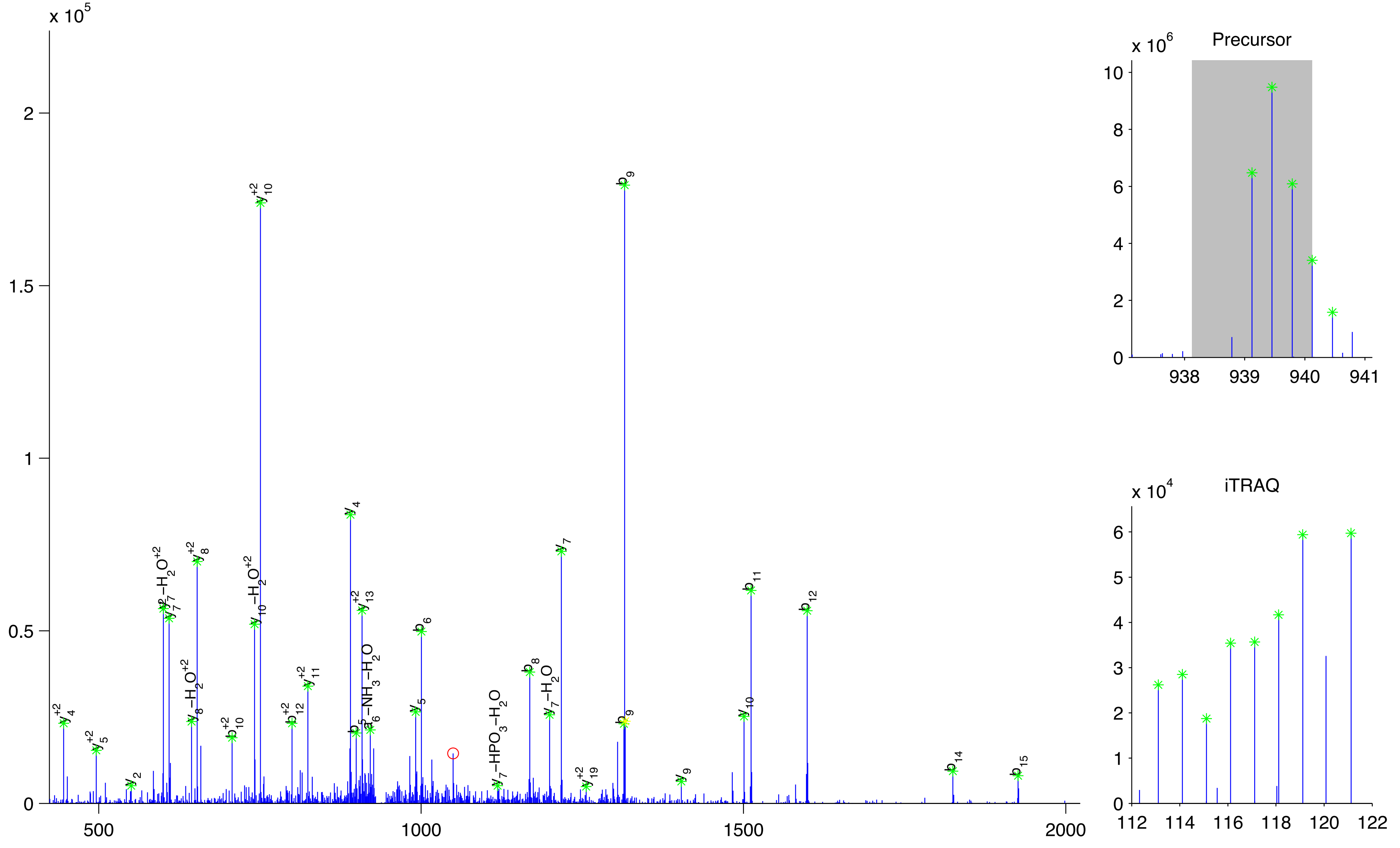

L S L G Q y D N D A G G Q L P F S K

tensin 3 [Homo sapiens]
Charge State: +2
Scan Number: 26979
File Name: 120518_A549_EGFTSA_pY.raw

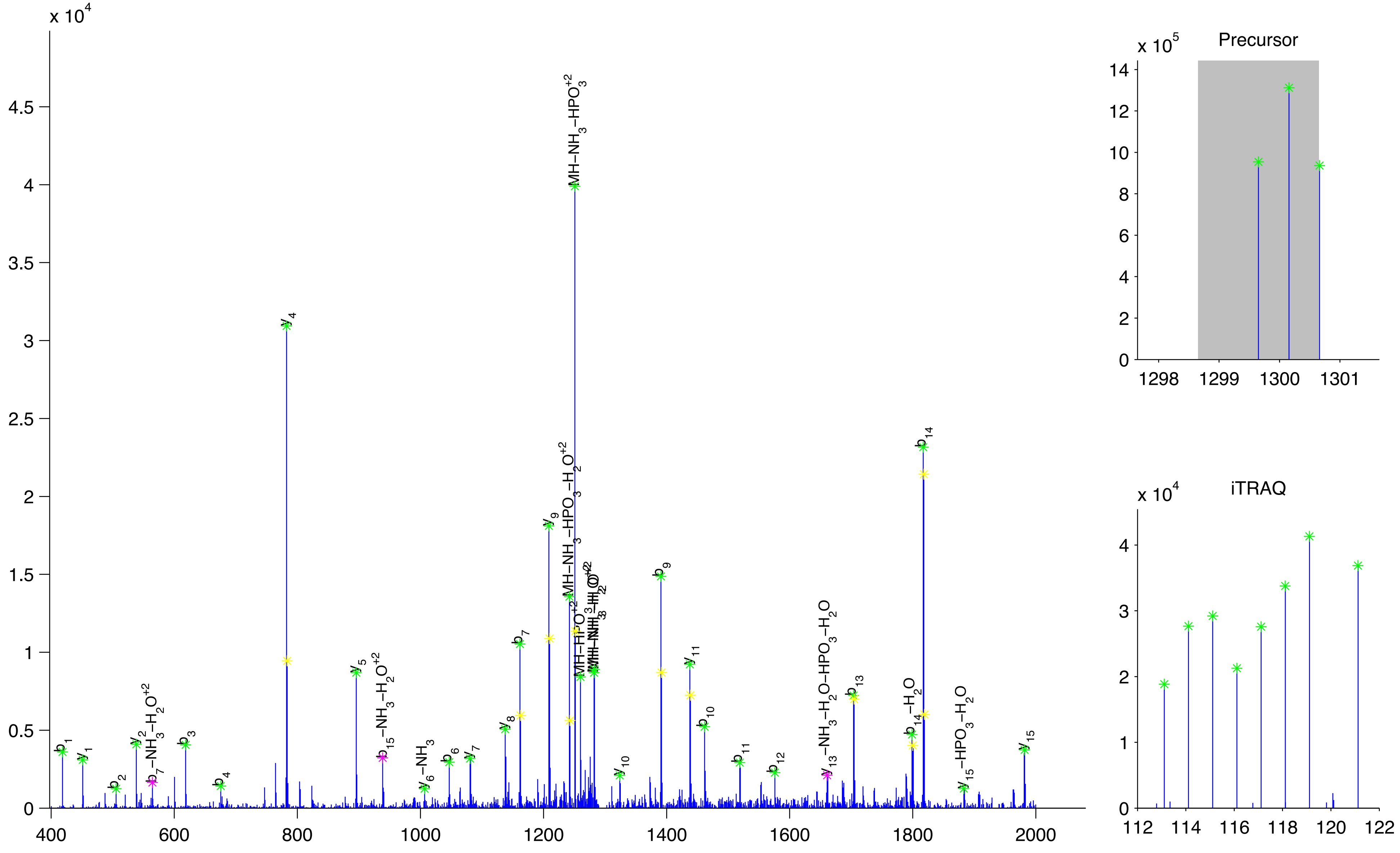

L S L G Q y D N D A G G Q L P F S K

tensin 3 [Homo sapiens]
Charge State: +3
Scan Number: 27055
File Name: 120518_A549_EGFTSA_pY.raw

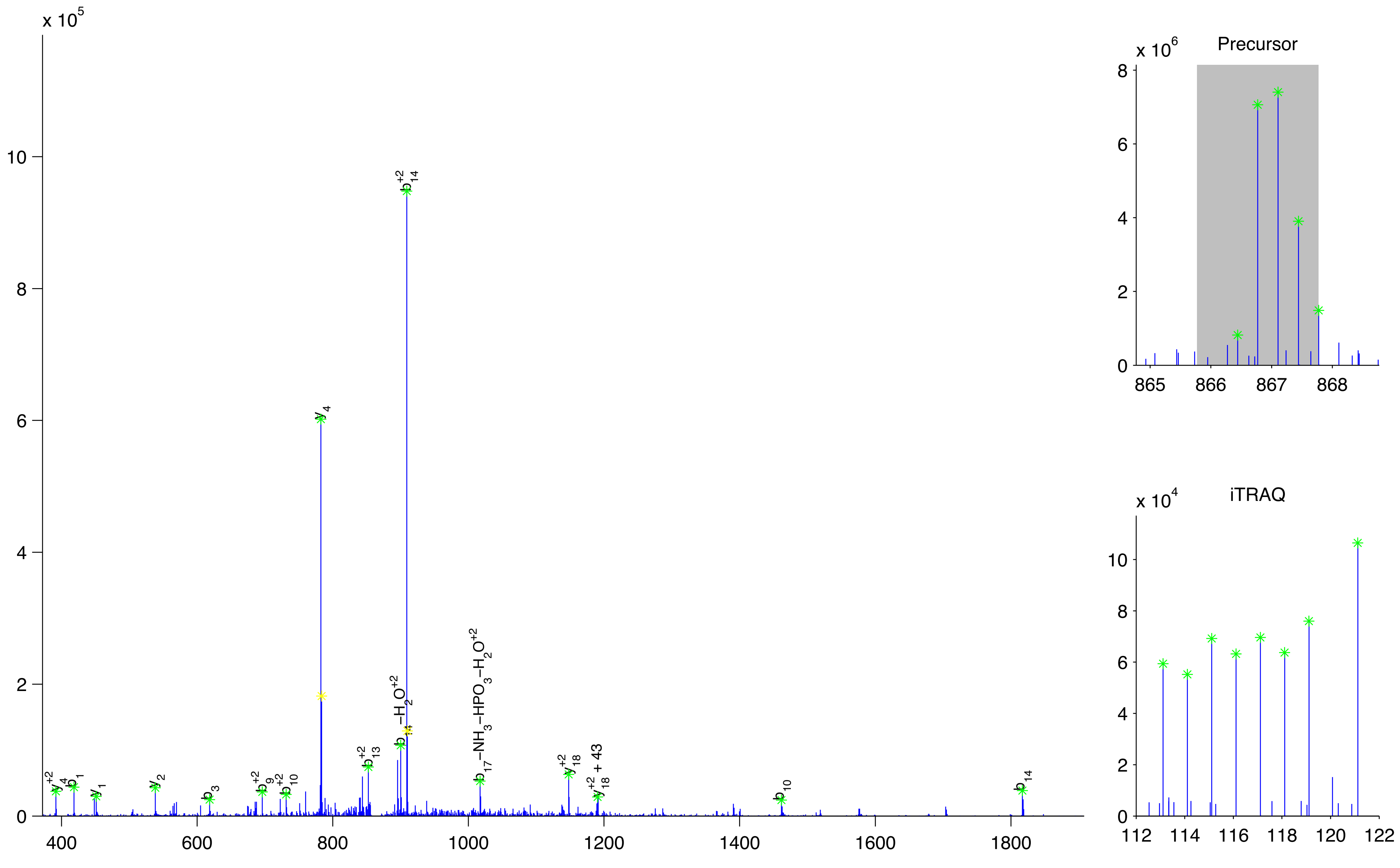

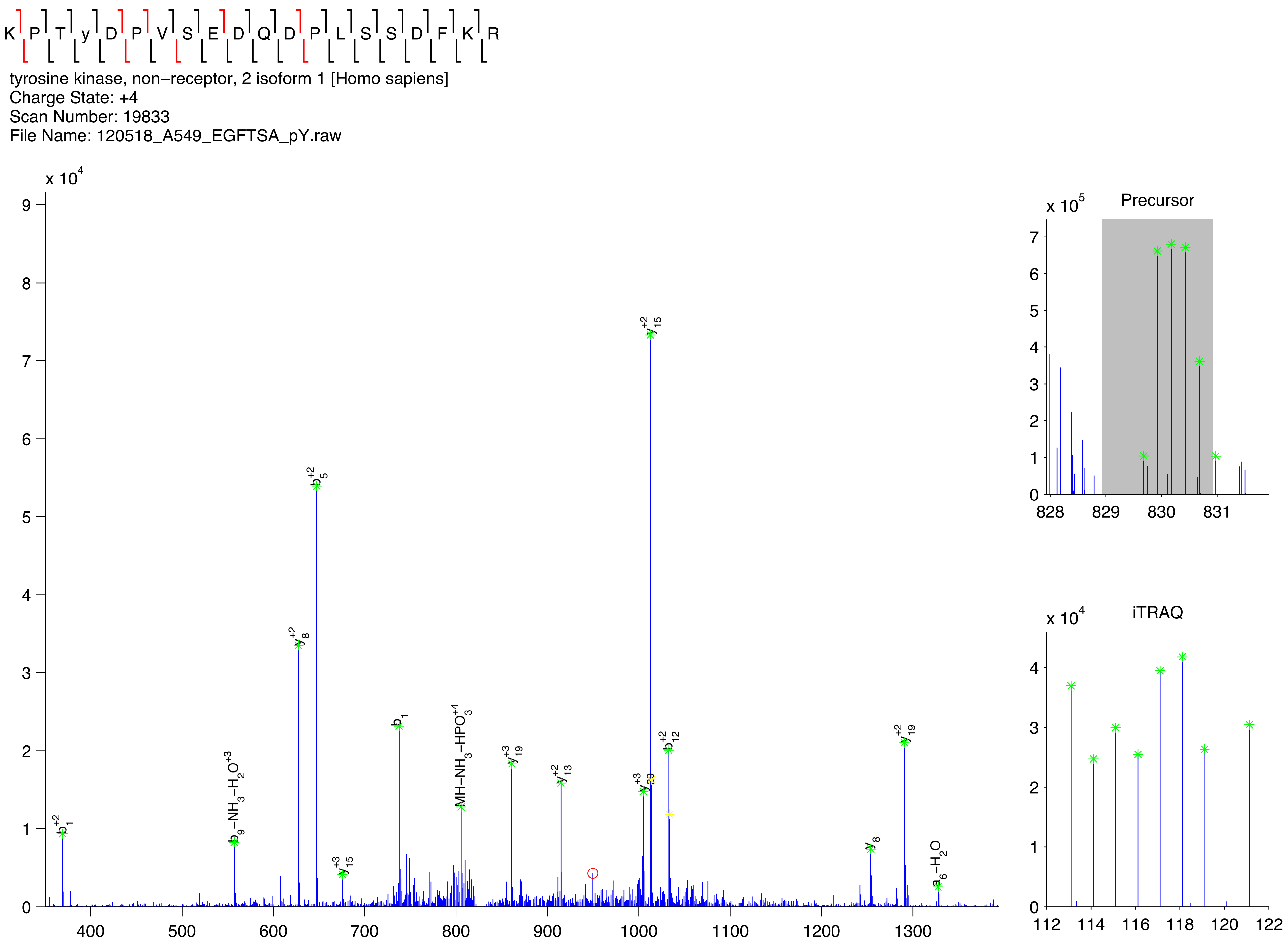
K P T y D P V S E D Q D P L S S D F K R
tyrosine kinase, non–receptor, 2 isoform 1 [Homo sapiens]
Charge State: +4
Scan Number: 19833
File Name: 120518_A549_EGFTSA_pY.raw
x 10^4
b1+2
b9–NH3–H2O+3
y8+2
b5+2
y15+3
b1
MH–NH3–HPO3+4
y19+3
y13+2
y20+3
y15+2
b12+2
y8
y19+2
a6–H2O
Precursor
x 10^5
iTRAQ
x 10^4

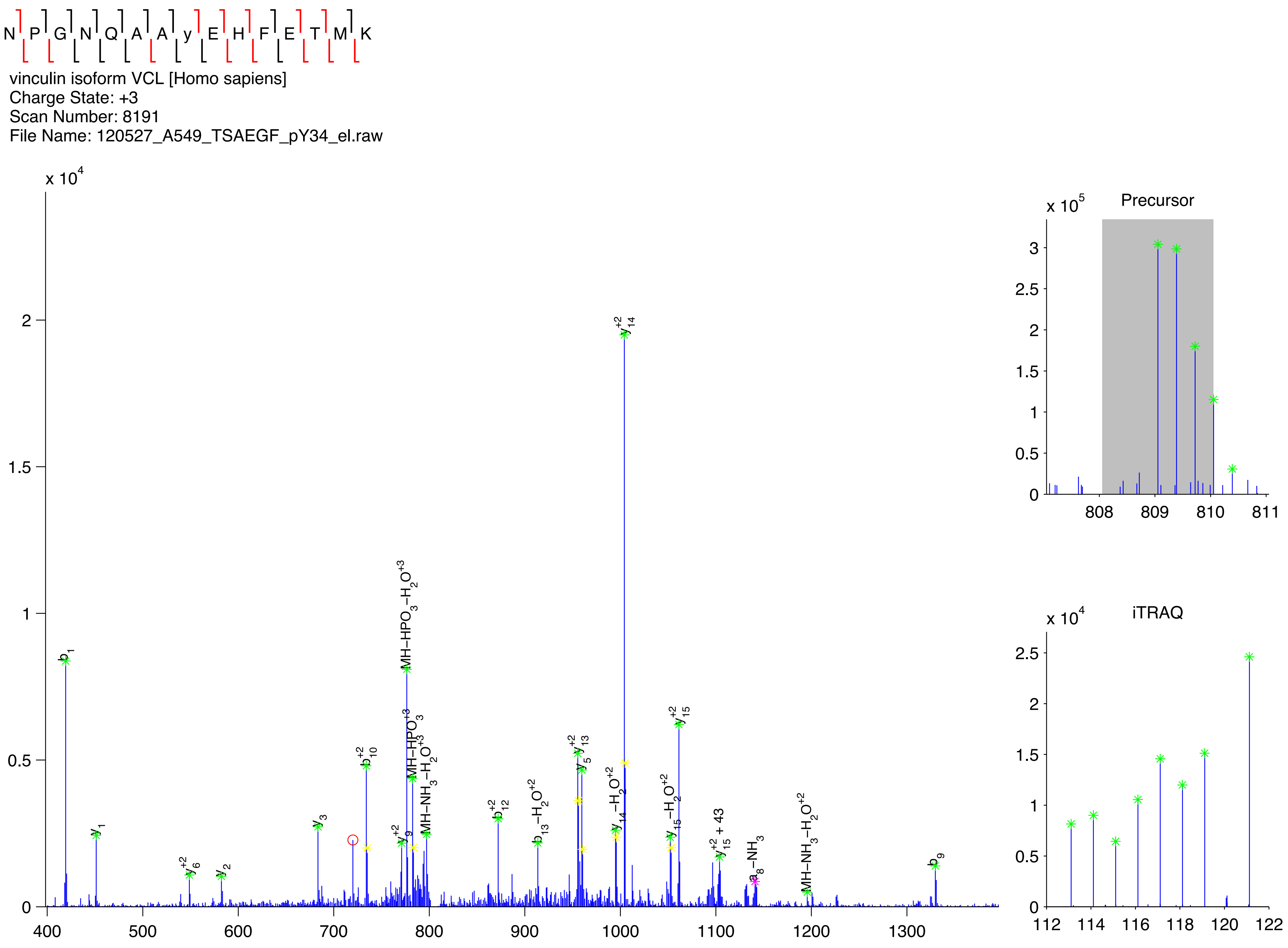
N P G N Q A A y E H F E T M K
vinculin isoform VCL [Homo sapiens]
Charge State: +3
Scan Number: 8191
File Name: 120527_A549_TSAEGF_pY34_el.raw
x 10^4
Precursor
x 10^5
iTRAQ
x 10^4

V I y D F I E K

Wiskott–Aldrich syndrome gene–like protein [Homo sapiens]
Charge State: +3
Scan Number: 17565
File Name: 120527_A549_TSAEGF_pY34_el.raw

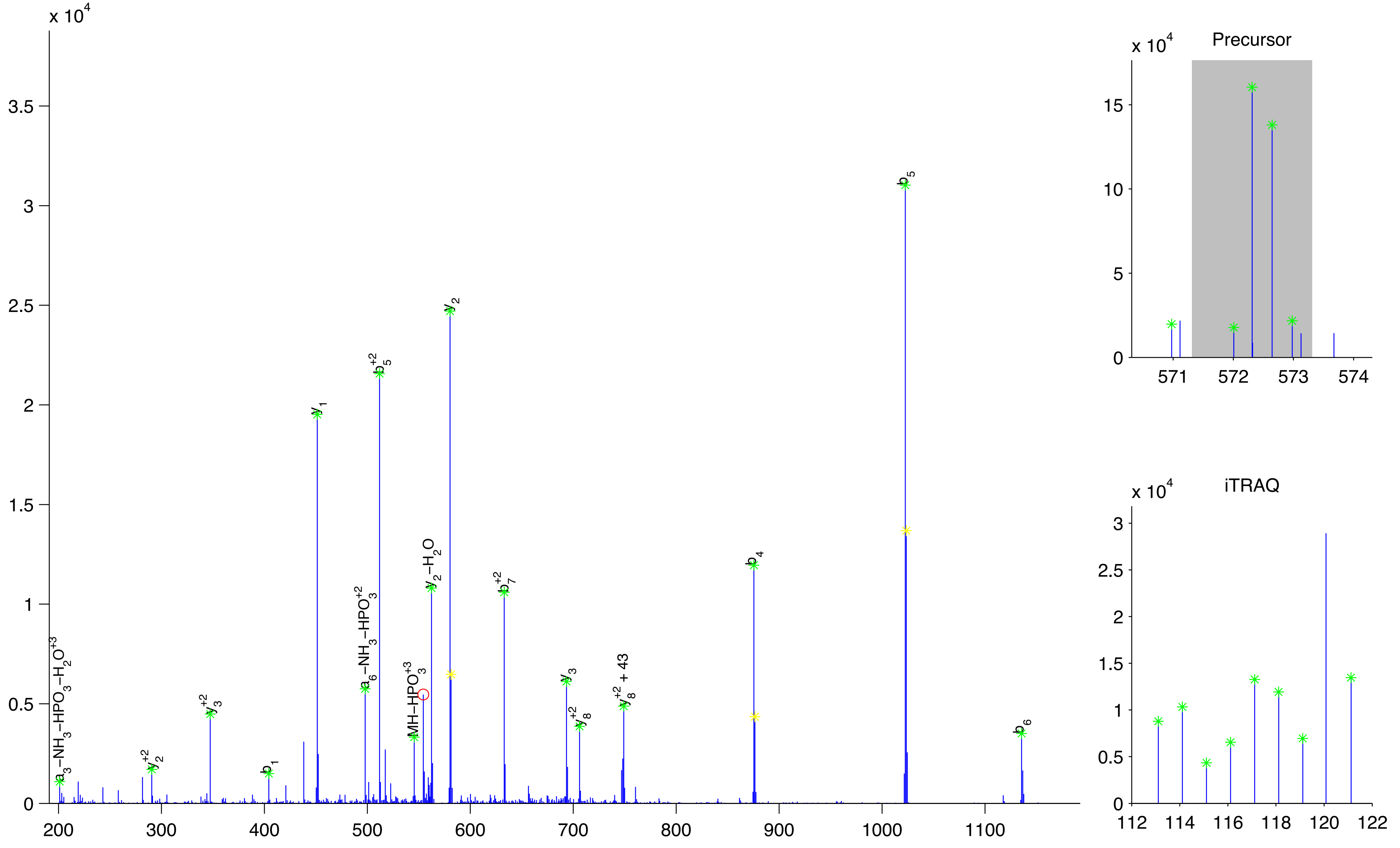

V I y D F I E K

Wiskott–Aldrich syndrome gene–like protein [Homo sapiens]
Charge State: +3
Scan Number: 30228
File Name: 120518_A549_EGFTSA_pY.raw

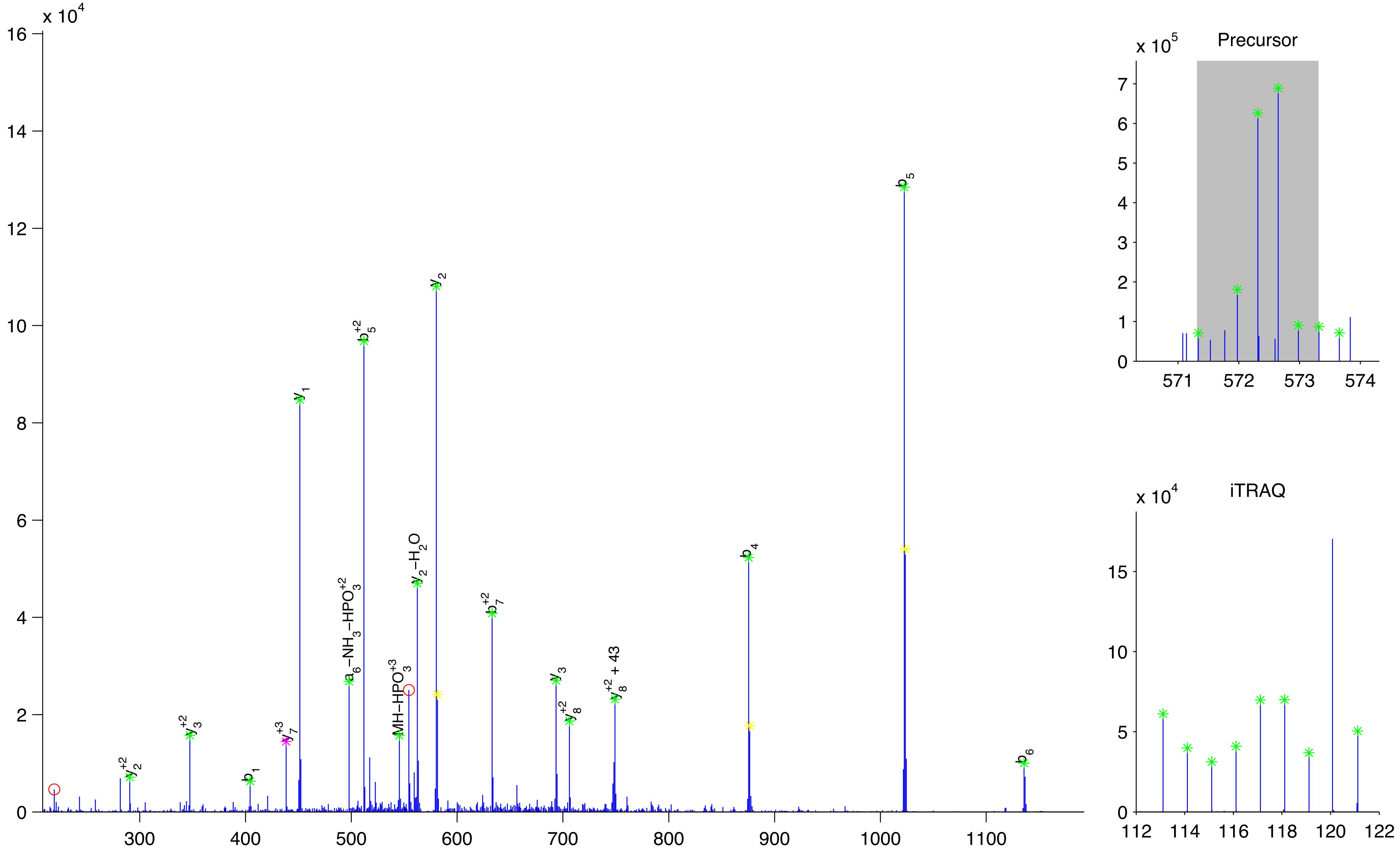

V E N c P D E L y D I M K

Yamaguchi sarcoma viral (v–yes–1) oncogene homolog isoform A [Homo sapiens]
Charge State: +3
Scan Number: 14489
File Name: 120527_A549_TSAEGF_pY34_el.raw

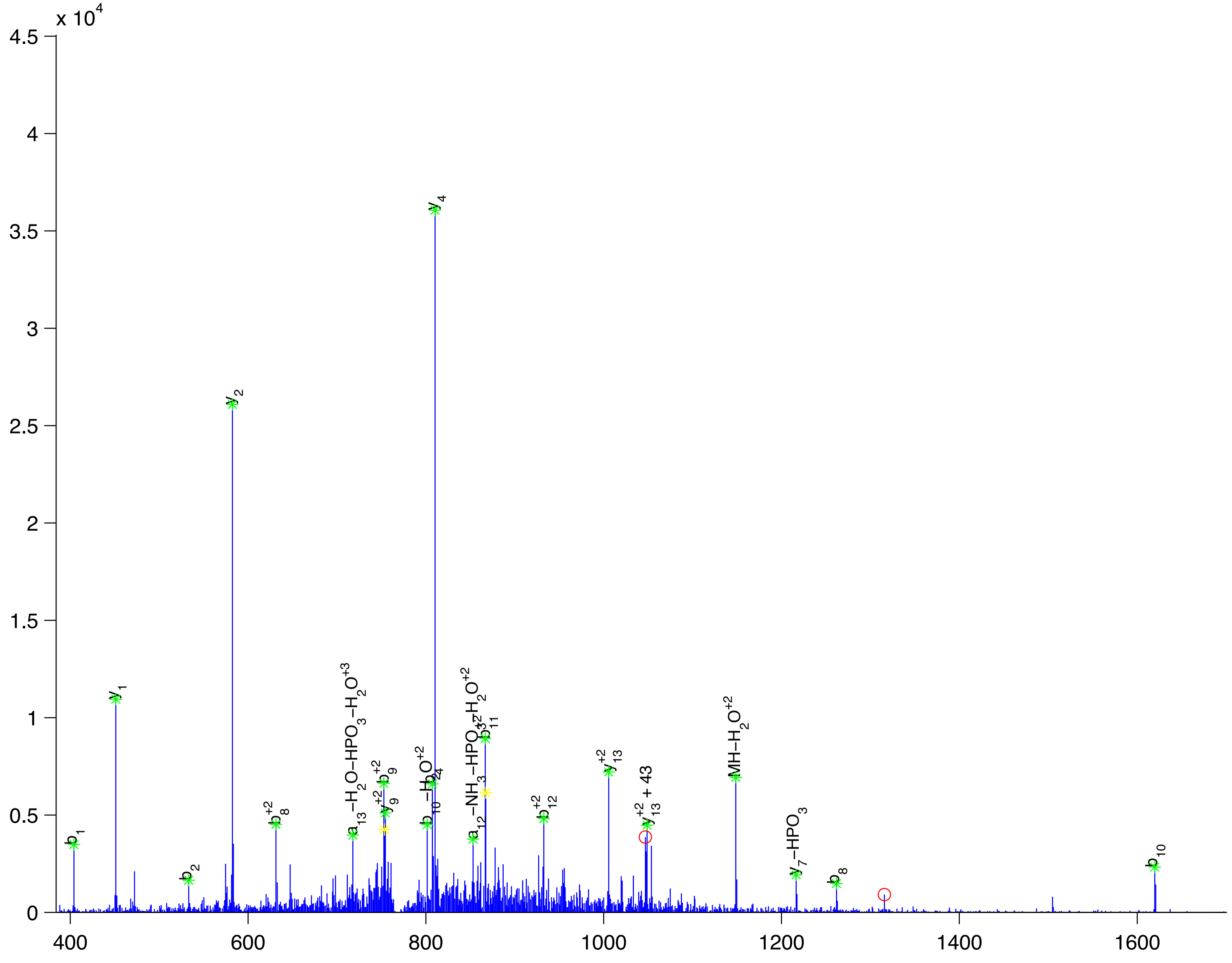

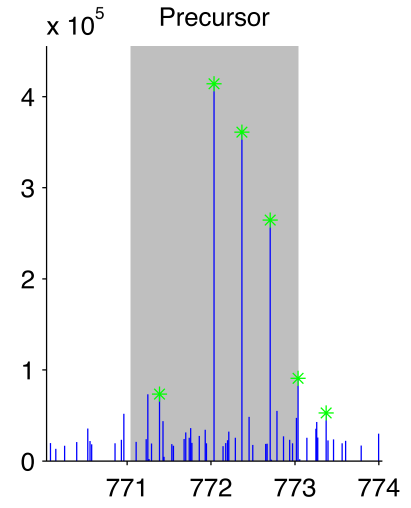

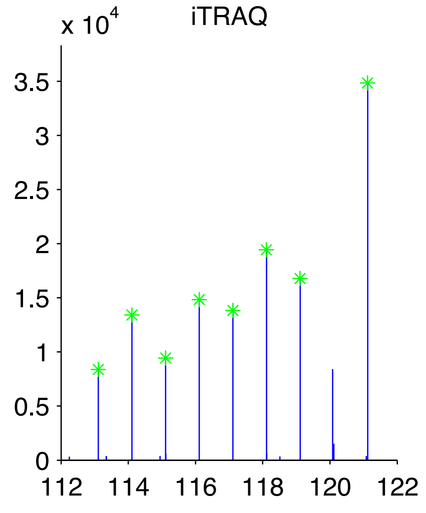

Supplement: S6 Fig — Instead of relying on an FDR analysis where the identity of true positives and true negatives are unknown, we manually validated each MS/MS spectra manually. Each page represents a manually validated MS/MS spectrum. (PDF) [file pone.0126242.s006.pdf]
