# Supplementary figures and images for: Quantitative Profiling of Lysine Acetylation Reveals Dynamic Crosstalk between Receptor Tyrosine Kinases and Lysine Acetylation
Source: PLoS One. 2015 May 15;10(5):e0126242. doi: 10.1371/journal.pone.0126242 (PMC4433260; doi:10.1371/journal.pone.0126242)

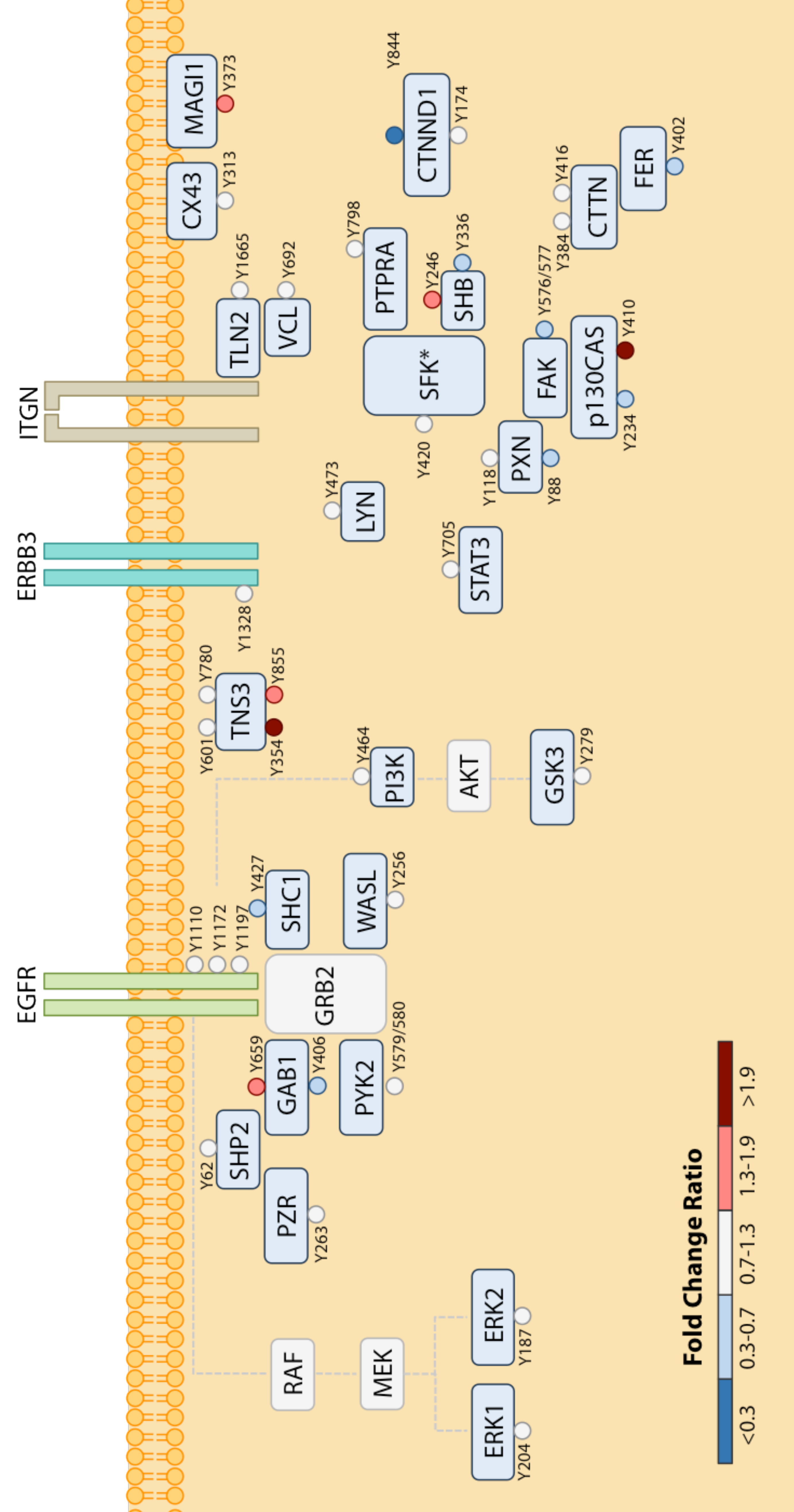

Supplement: S7 Fig — The fold change ratio in response to TSA treatment mapped to a visual representation of proteins involved in kinase signaling, scaffolding, and cell adhesion. Each circle represents a unique phosphorylation site identified on the protein. The sites are color coded to characterize the relative effect of TSA pre-treatment on the EGF response. Proteins are labeled by their gene name. Sites of phosphorylation are labeled. (TIF) [file pone.0126242.s007.tif]
