# Supplementary material for: Quantitative Profiling of Lysine Acetylation Reveals Dynamic Crosstalk between Receptor Tyrosine Kinases and Lysine Acetylation
Source: PLoS One. 2015 May 15;10(5):e0126242. doi: 10.1371/journal.pone.0126242 (PMC4433260; doi:10.1371/journal.pone.0126242)

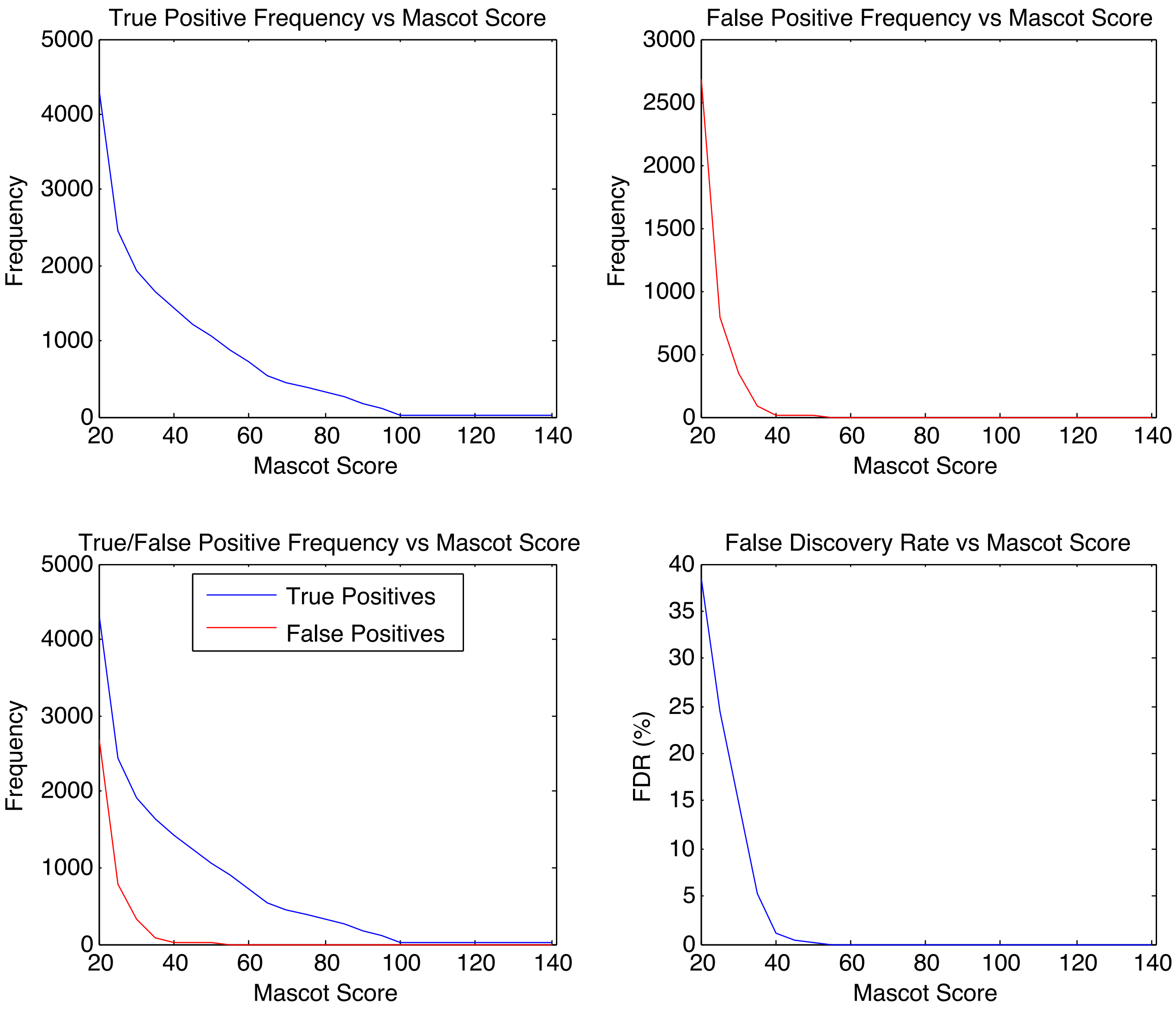
True Positive Frequency vs Mascot Score
Frequency
Mascot Score
5000
4000
3000
2000
1000
0
20
40
60
80
100
120
140
False Positive Frequency vs Mascot Score
Frequency
Mascot Score
3000
2500
2000
1500
1000
500
0
20
40
60
80
100
120
140
True/False Positive Frequency vs Mascot Score
True Positives
False Positives
Frequency
Mascot Score
5000
4000
3000
2000
1000
0
20
40
60
80
100
120
140
False Discovery Rate vs Mascot Score
FDR (%)
Mascot Score
40
35
30
25
20
15
10
5
0
20
40
60
80
100
120
140

G E W k P R

calreticulin precursor [Homo sapiens]
Charge State: +2
Scan Number: 4730
File Name: 111016_HG2_INSC_ACK.raw

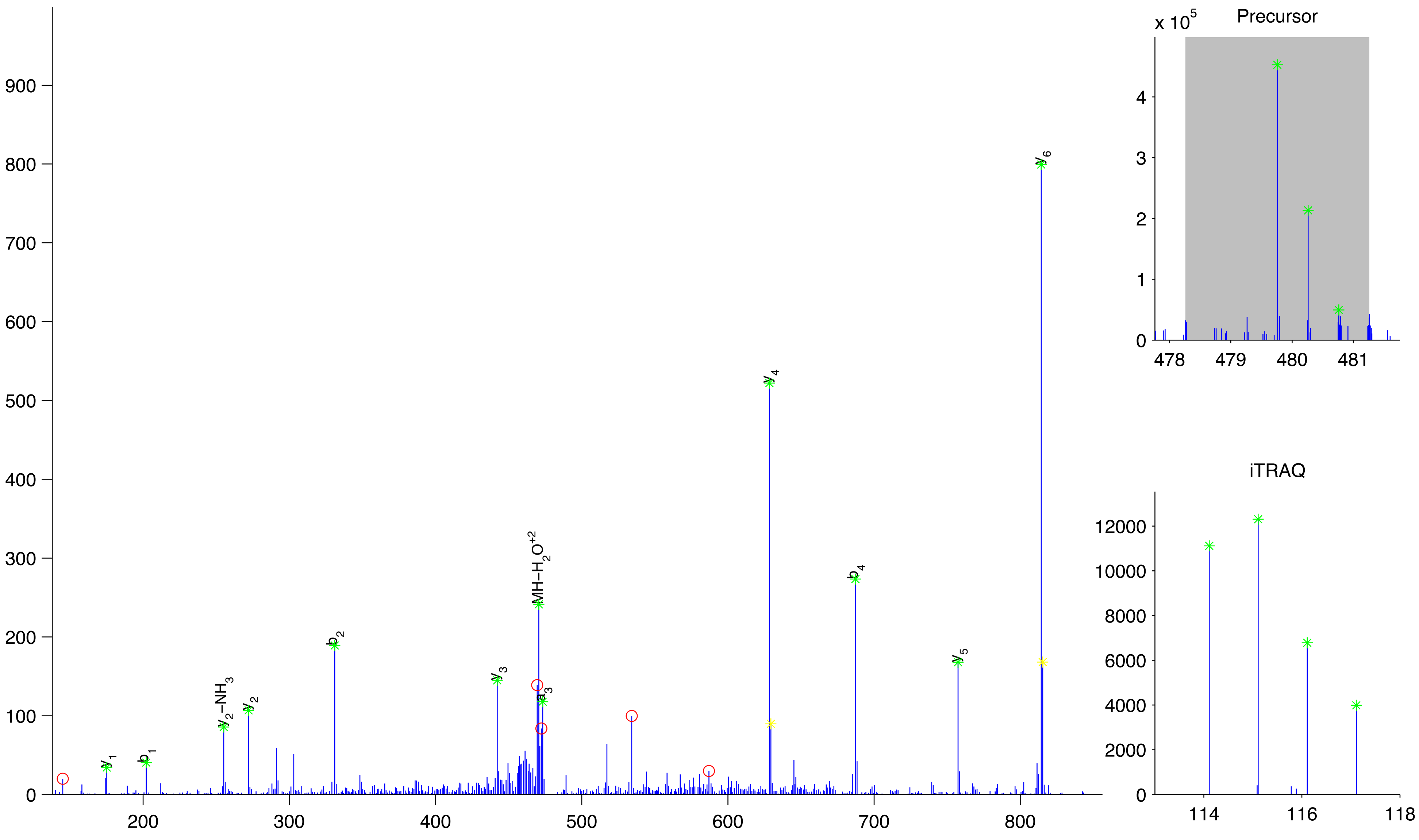

I k E L L k R

centromere protein E [Homo sapiens]
Charge State: +2
Scan Number: 9810
File Name: 111016_HG2_INSC_ACK.raw

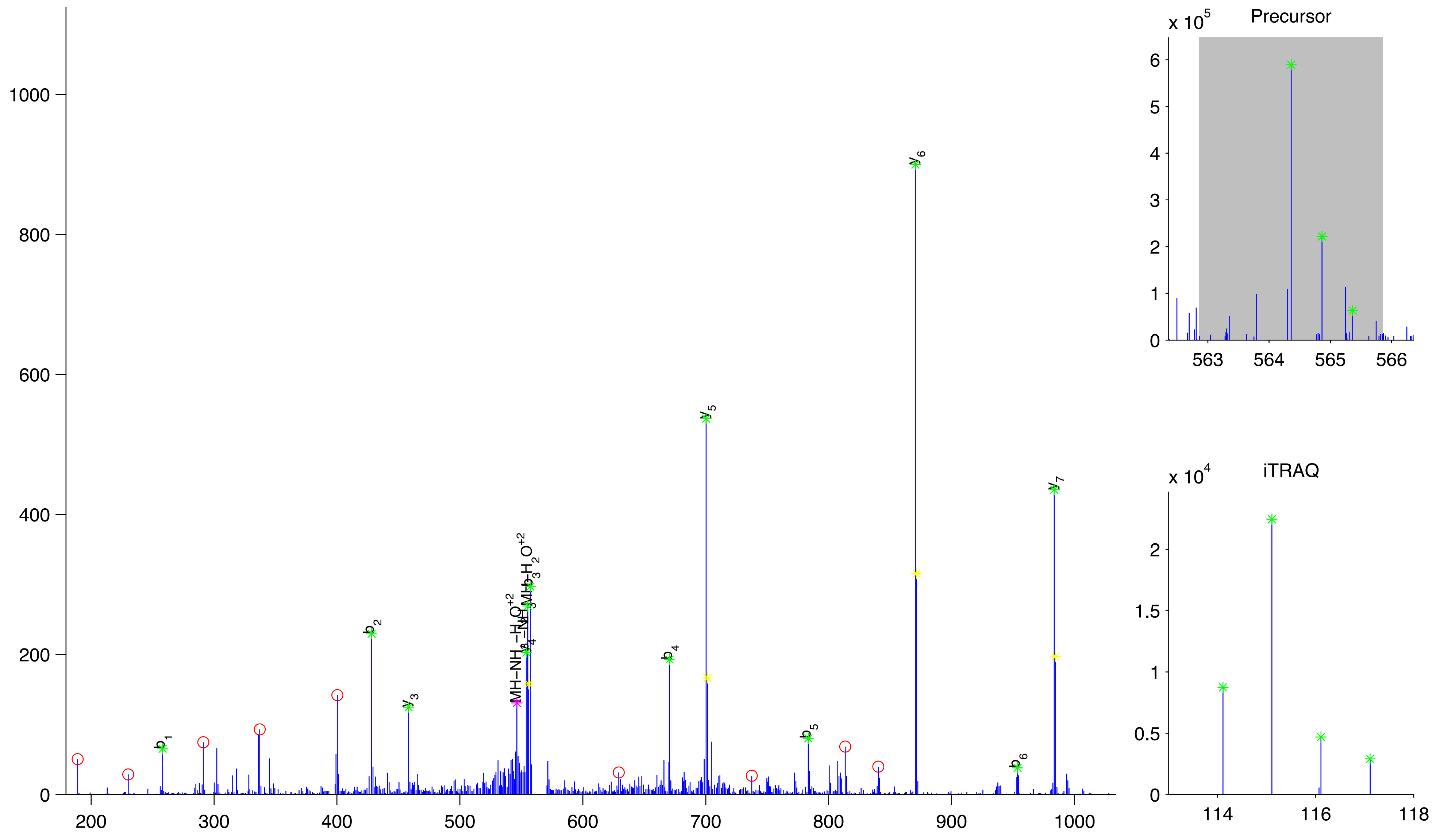

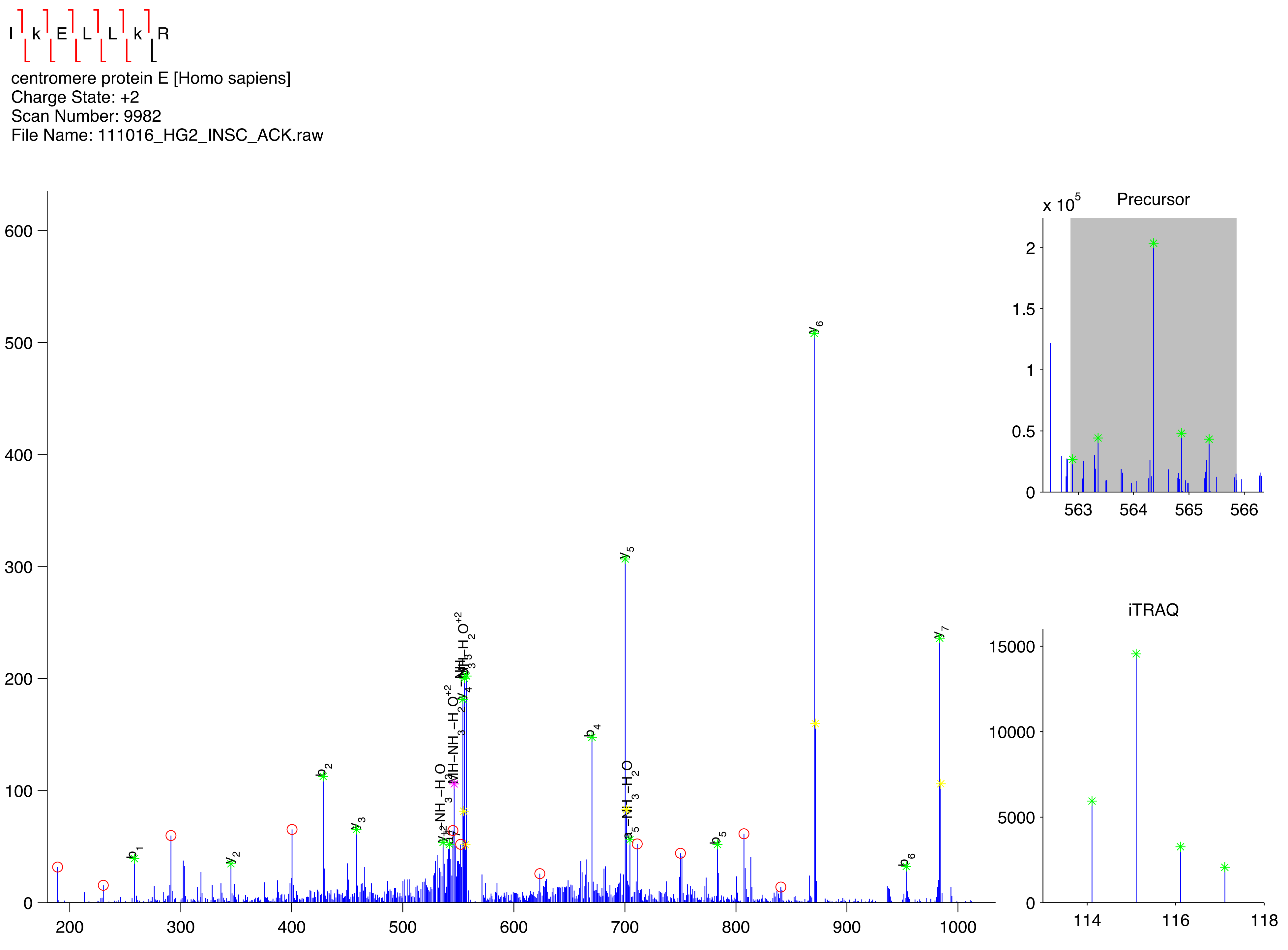
I k E L L k R
centromere protein E [Homo sapiens]
Charge State: +2
Scan Number: 9982
File Name: 111016_HG2_INSC_ACK.raw
Precursor
x 10^5
iTRAQ

k T Q k R I T K

DEAD (Asp–Glu–Ala–Asp) box polypeptide 60–like [Homo sapiens]
Charge State: +2
Scan Number: 13722
File Name: 110924_HG2_INSA_ACK.raw

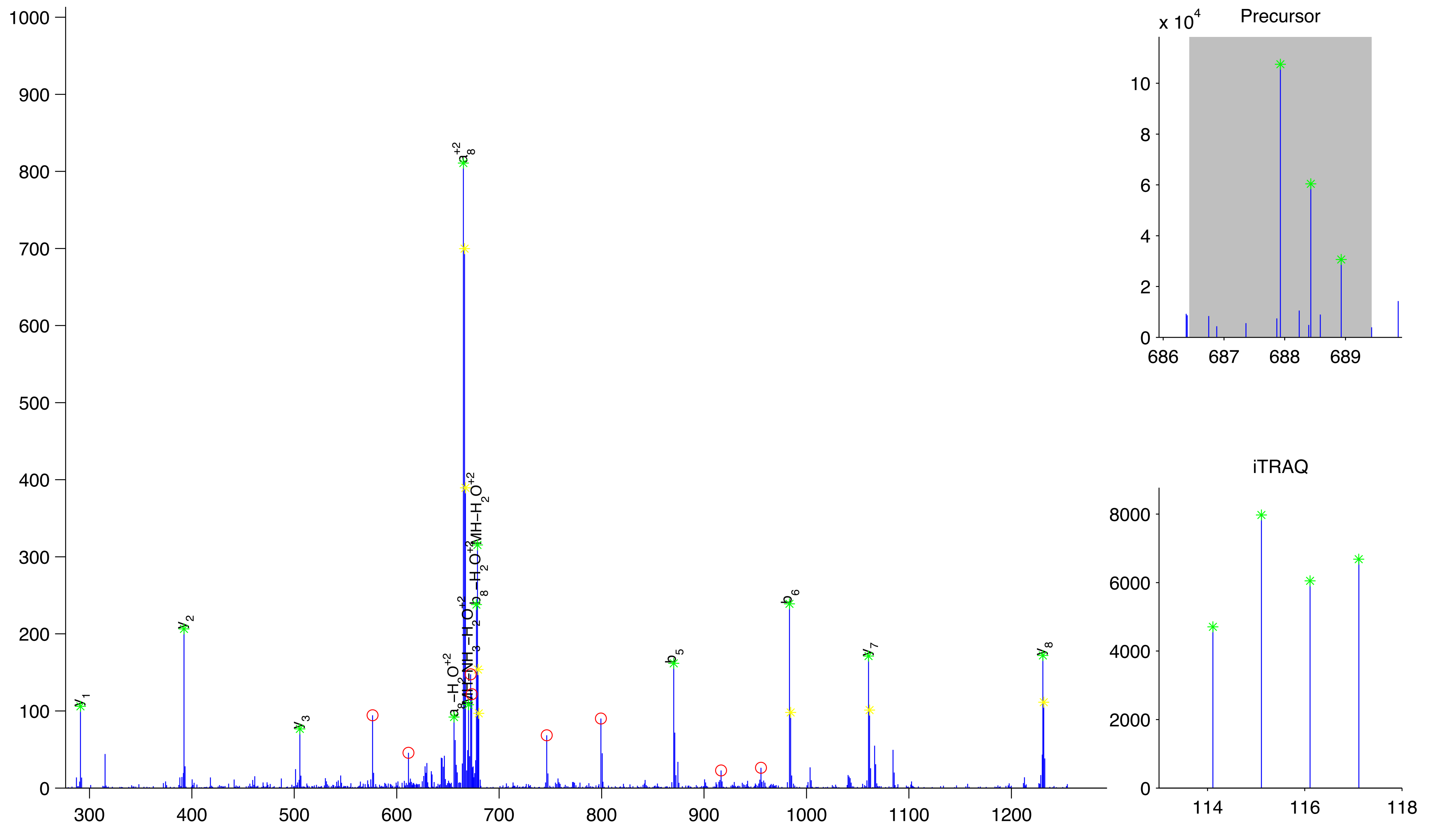

k T S k N k S S L S R

E1A binding protein p300 [Homo sapiens]
Charge State: +2
Scan Number: 12105
File Name: 110924_HG2_INSA_ACK.raw

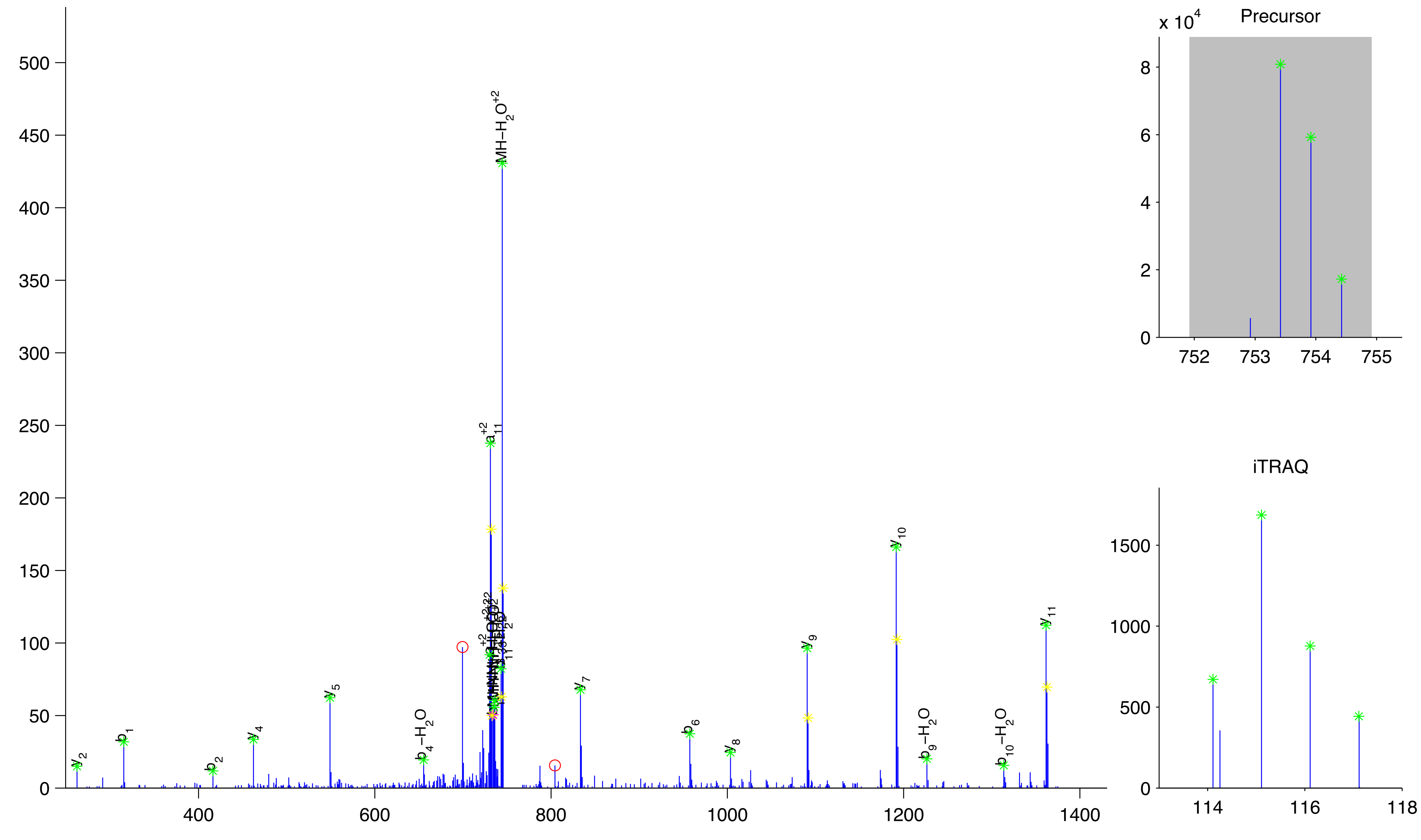

S G k Y D L D F K

enolase 1 [Homo sapiens]
Charge State: +2
Scan Number: 7863
File Name: 111016_HG2_INSC_ACK.raw

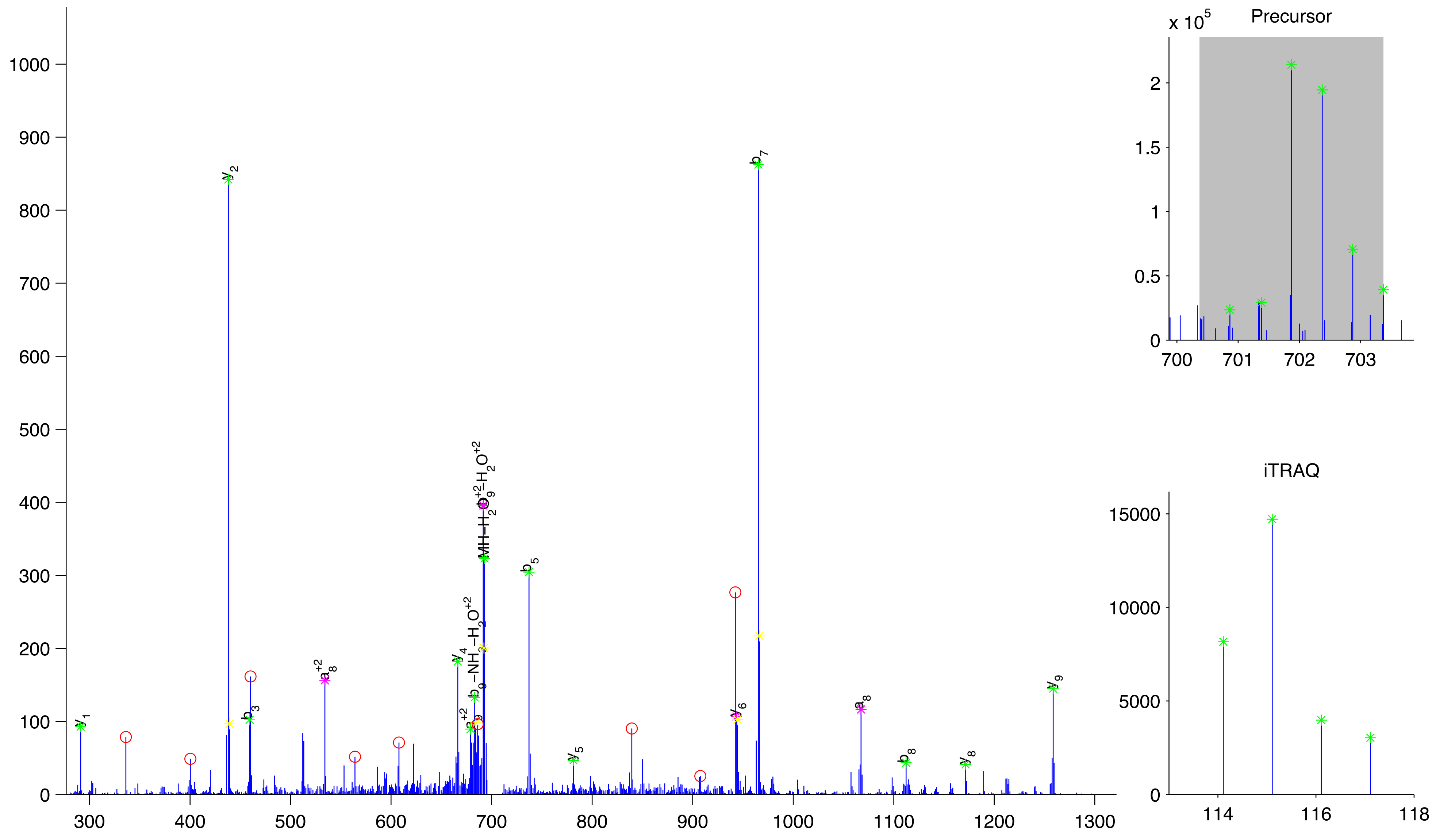

A F N Q G k I F K

eukaryotic translation elongation factor 1 gamma [Homo sapiens]
Charge State: +2
Scan Number: 6945
File Name: 111011_HG2_INSB_ACK.raw

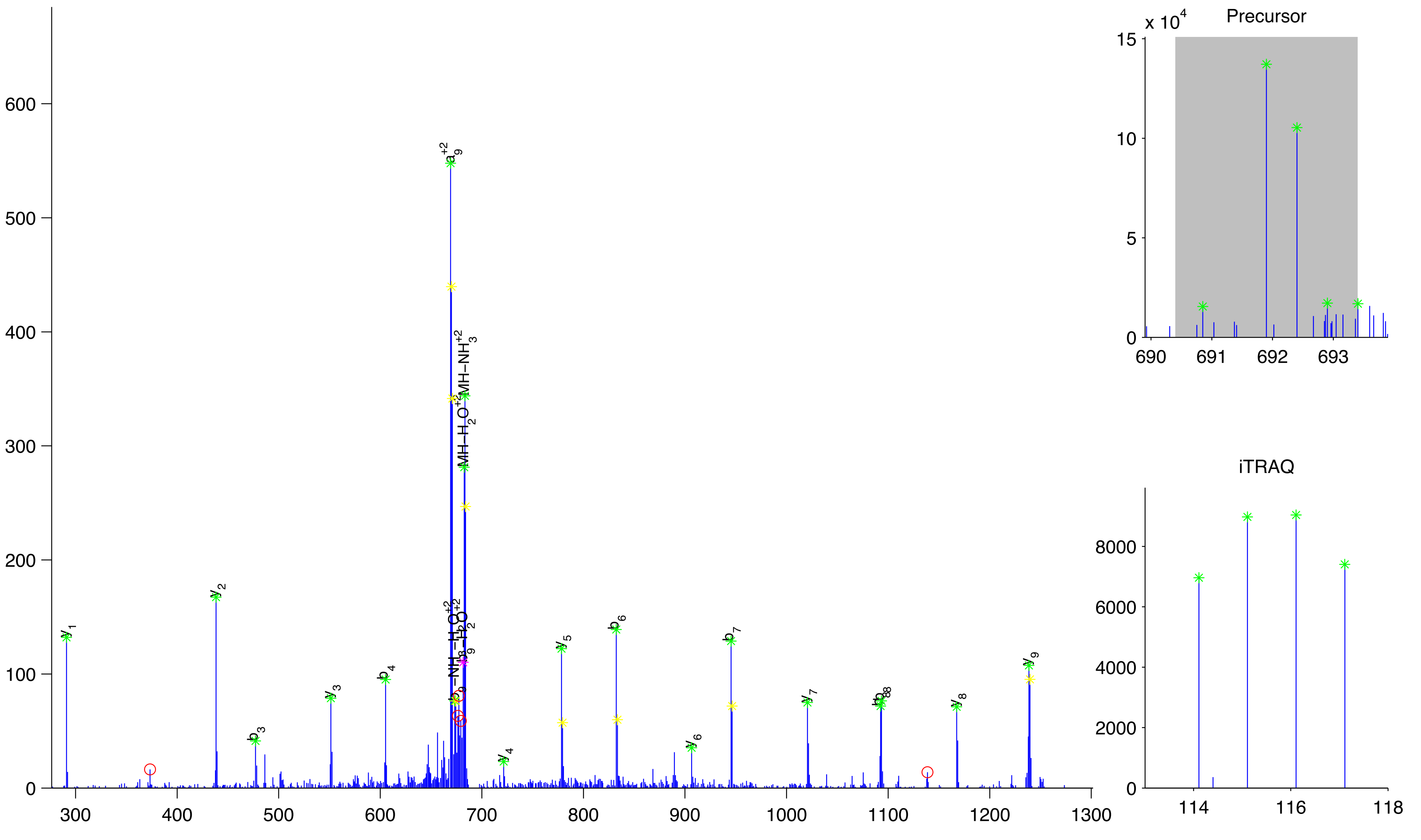

A F N Q G k I F K

eukaryotic translation elongation factor 1 gamma [Homo sapiens]
Charge State: +2
Scan Number: 7521
File Name: 111016_HG2_INSC_ACK.raw

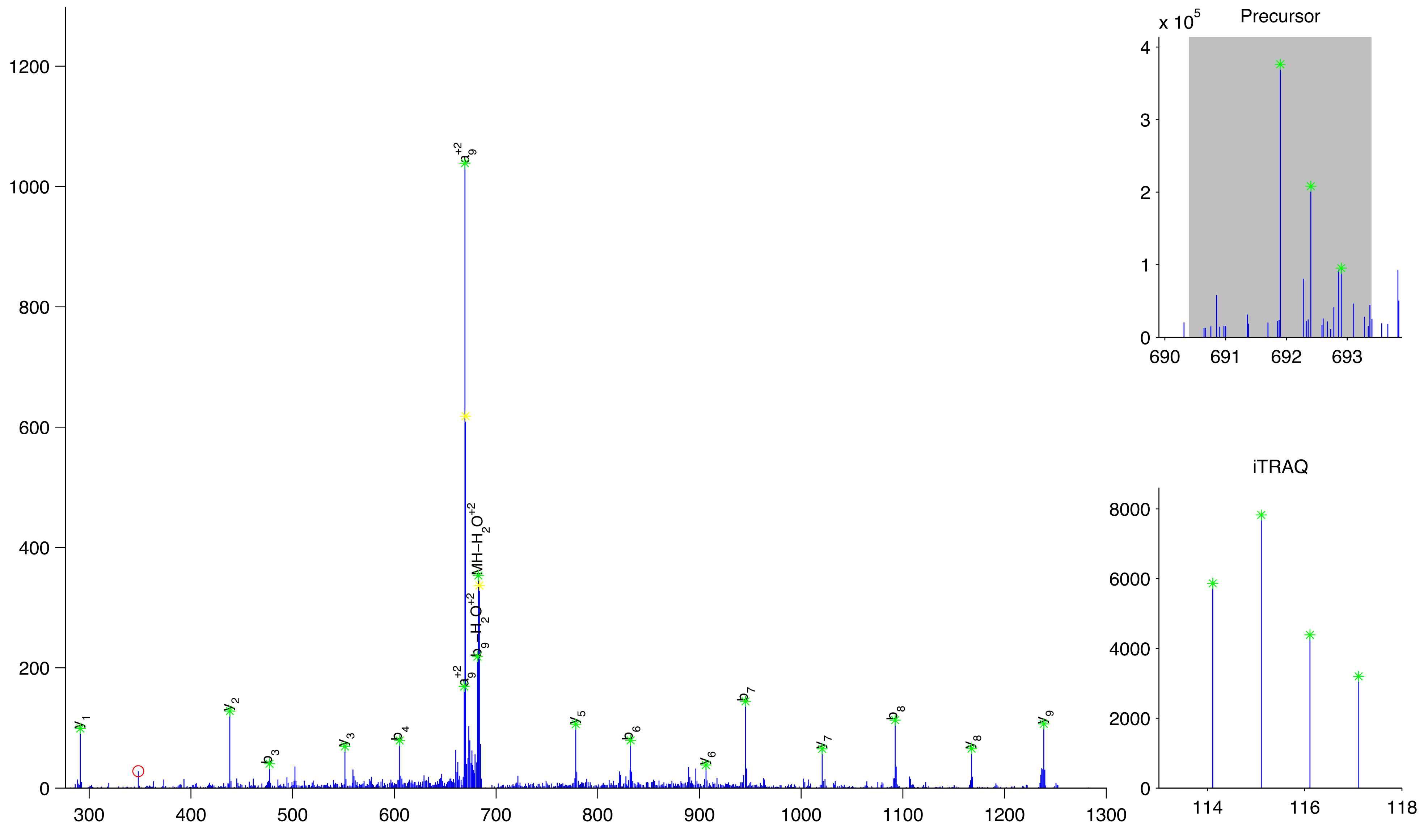

k L W G D R

eukaryotic translation elongation factor 2 [Homo sapiens]
Charge State: +2
Scan Number: 6118
File Name: 111011_HG2_INSB_ACK.raw

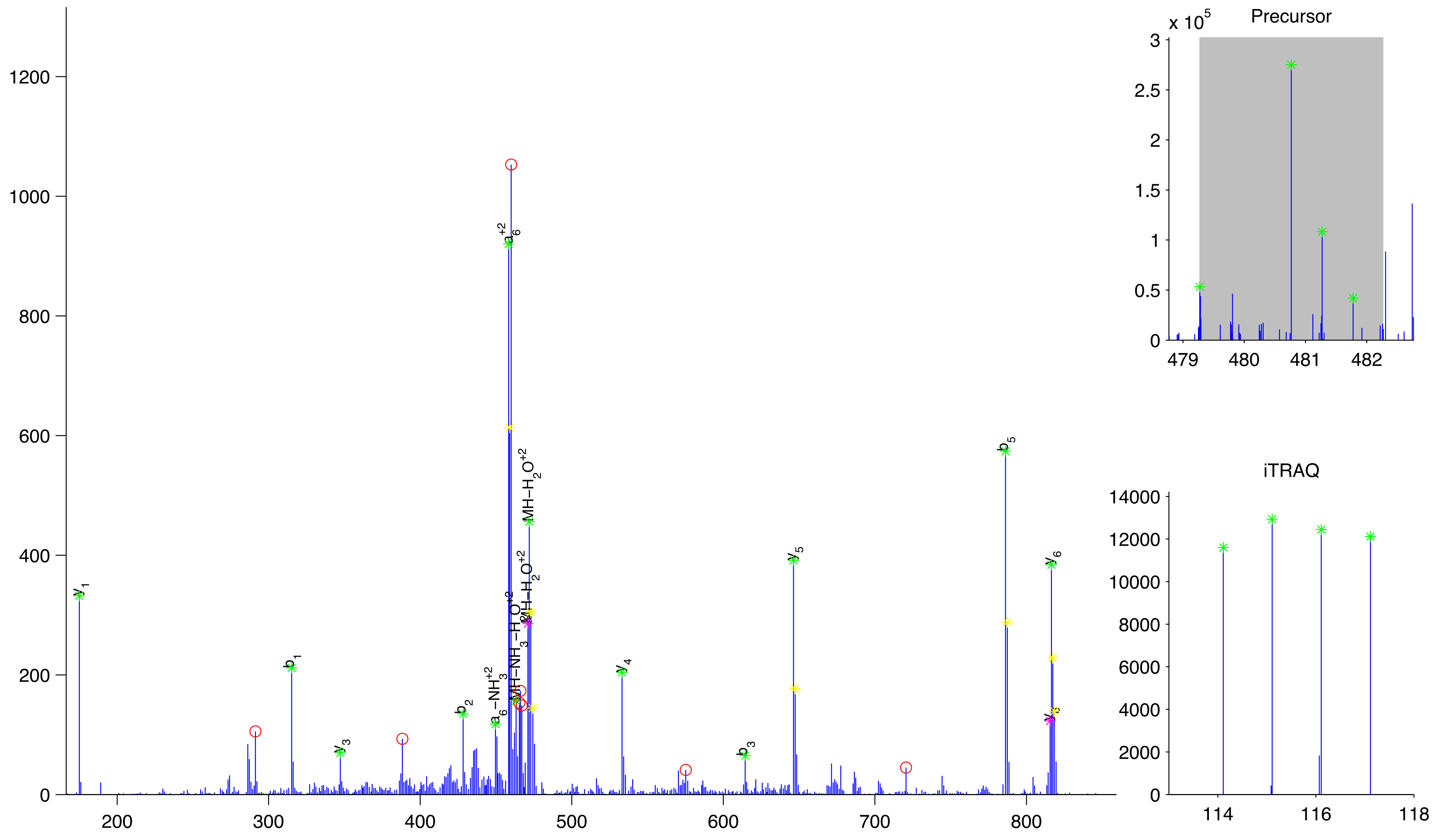

I V E M S T S k T G K

eukaryotic translation initiation factor 5A [Homo sapiens]
Charge State: +2
Scan Number: 5731
File Name: 111011_HG2_INSB_ACK.raw

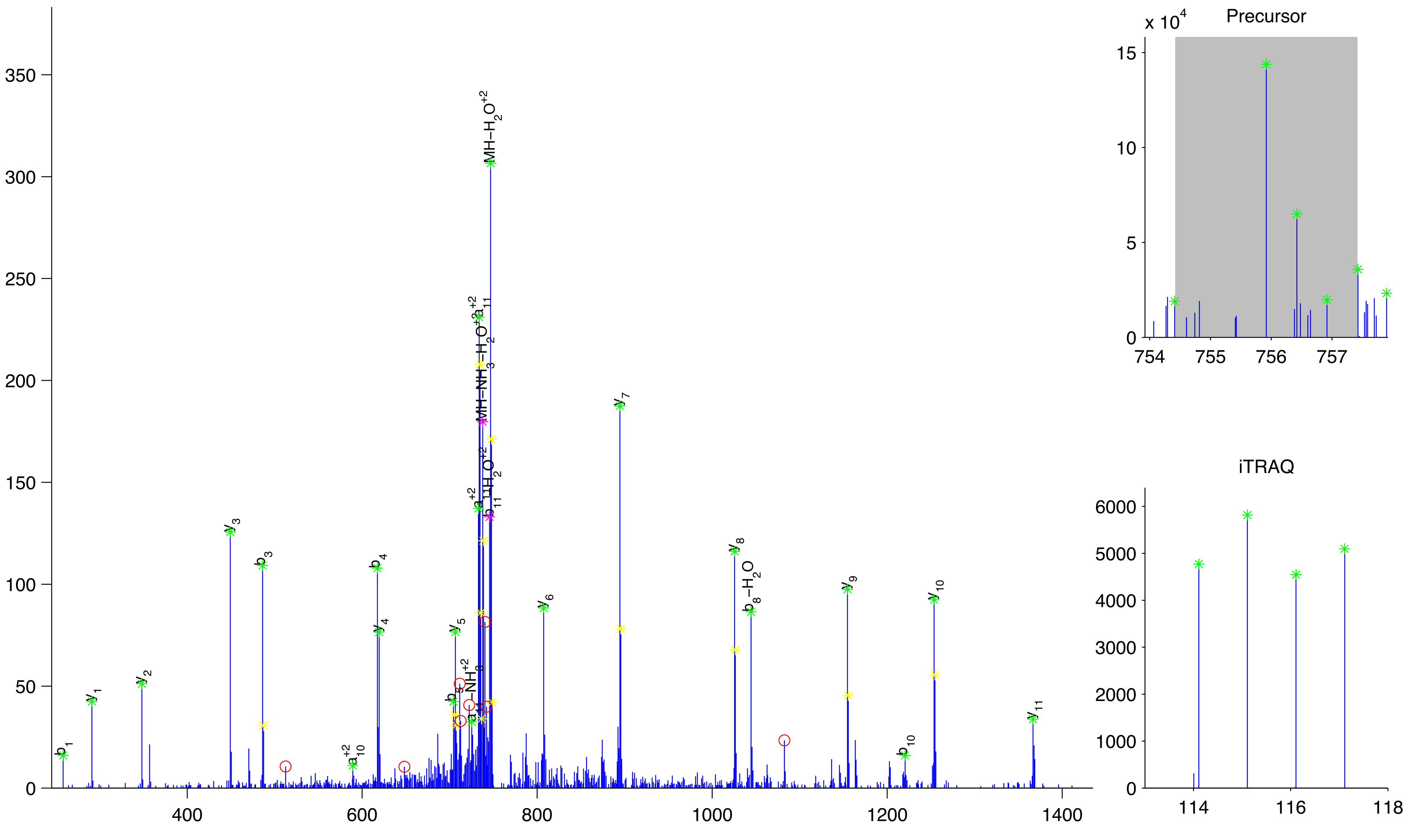

E G V F A k E V R

fatty acid synthase [Homo sapiens]
Charge State: +2
Scan Number: 6139
File Name: 111011_HG2_INSB_ACK.raw

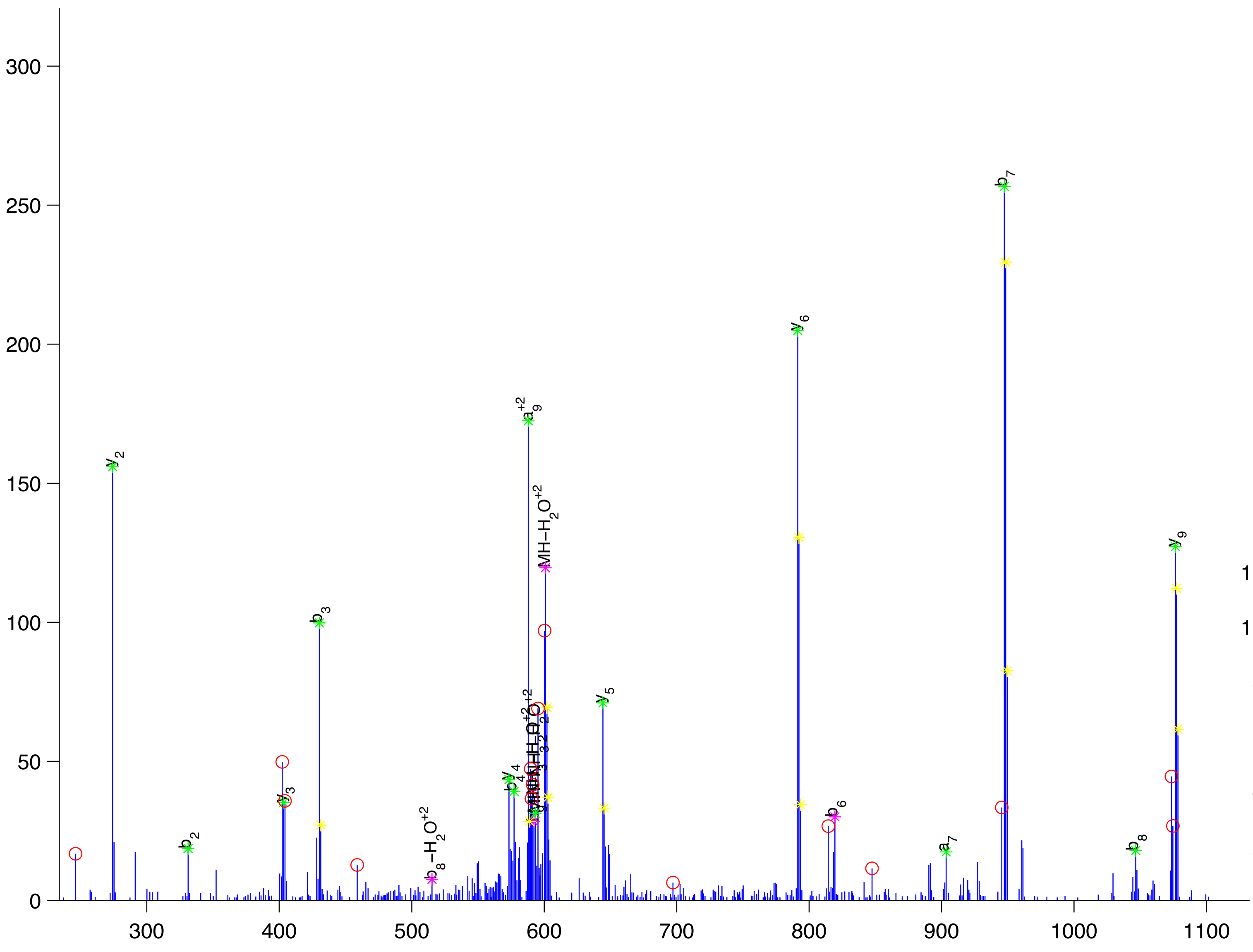

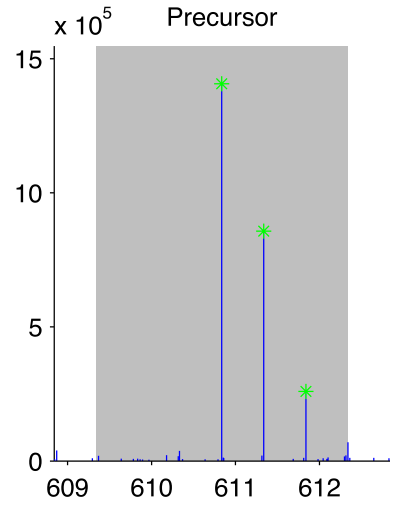

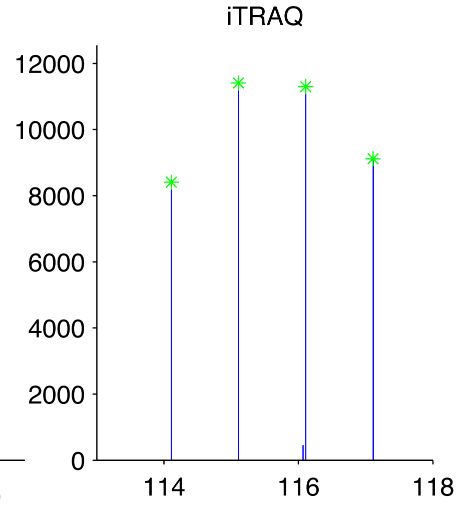

E G V F A k E V R

fatty acid synthase [Homo sapiens]
Charge State: +2
Scan Number: 6300
File Name: 111011_HG2_INSB_ACK.raw

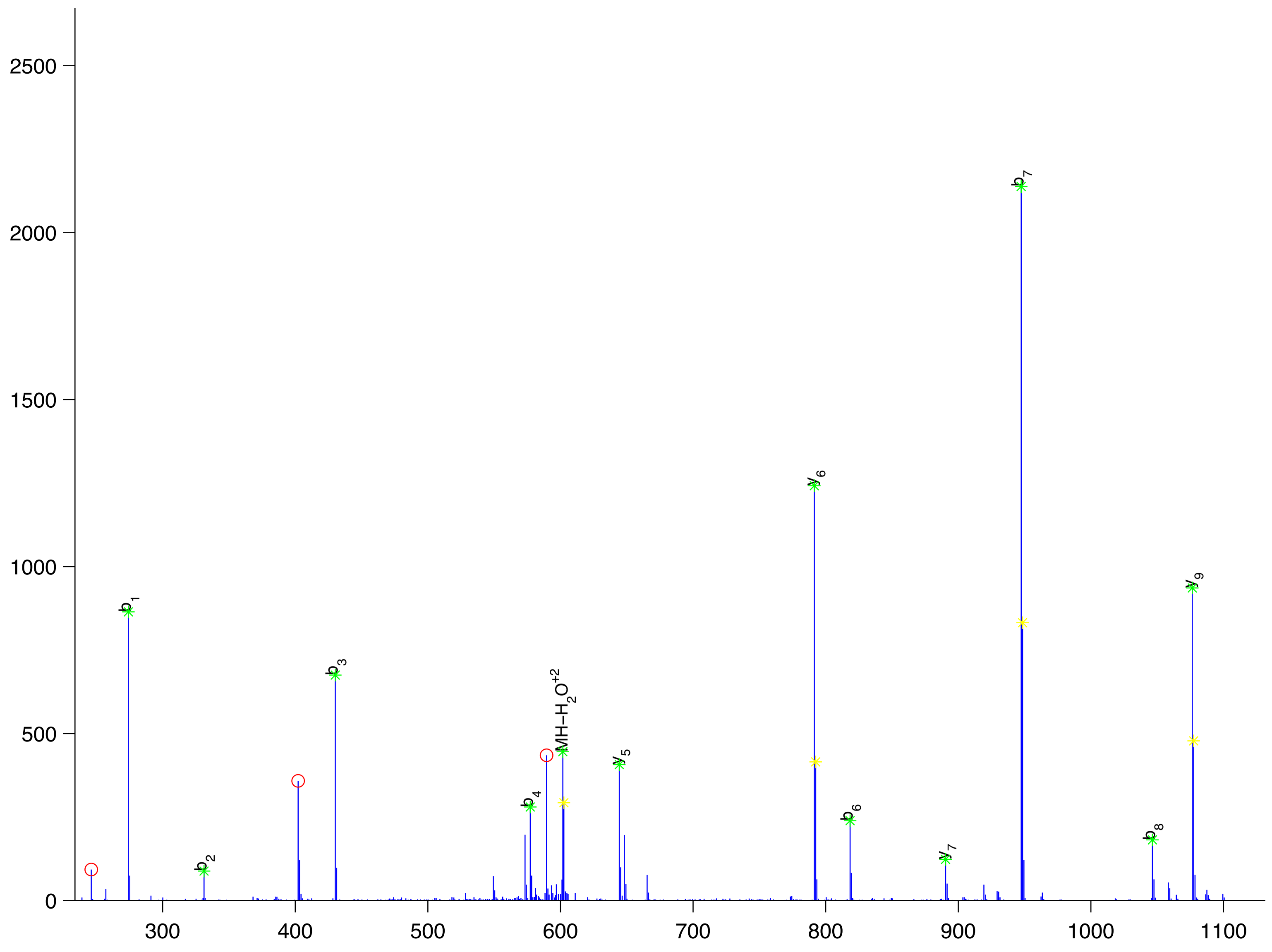

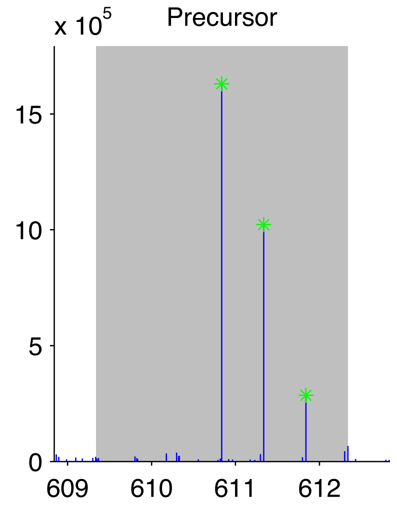

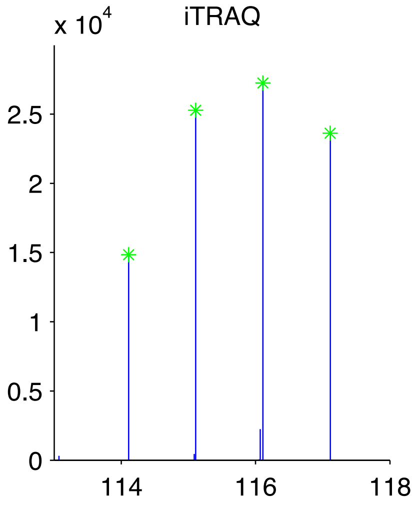

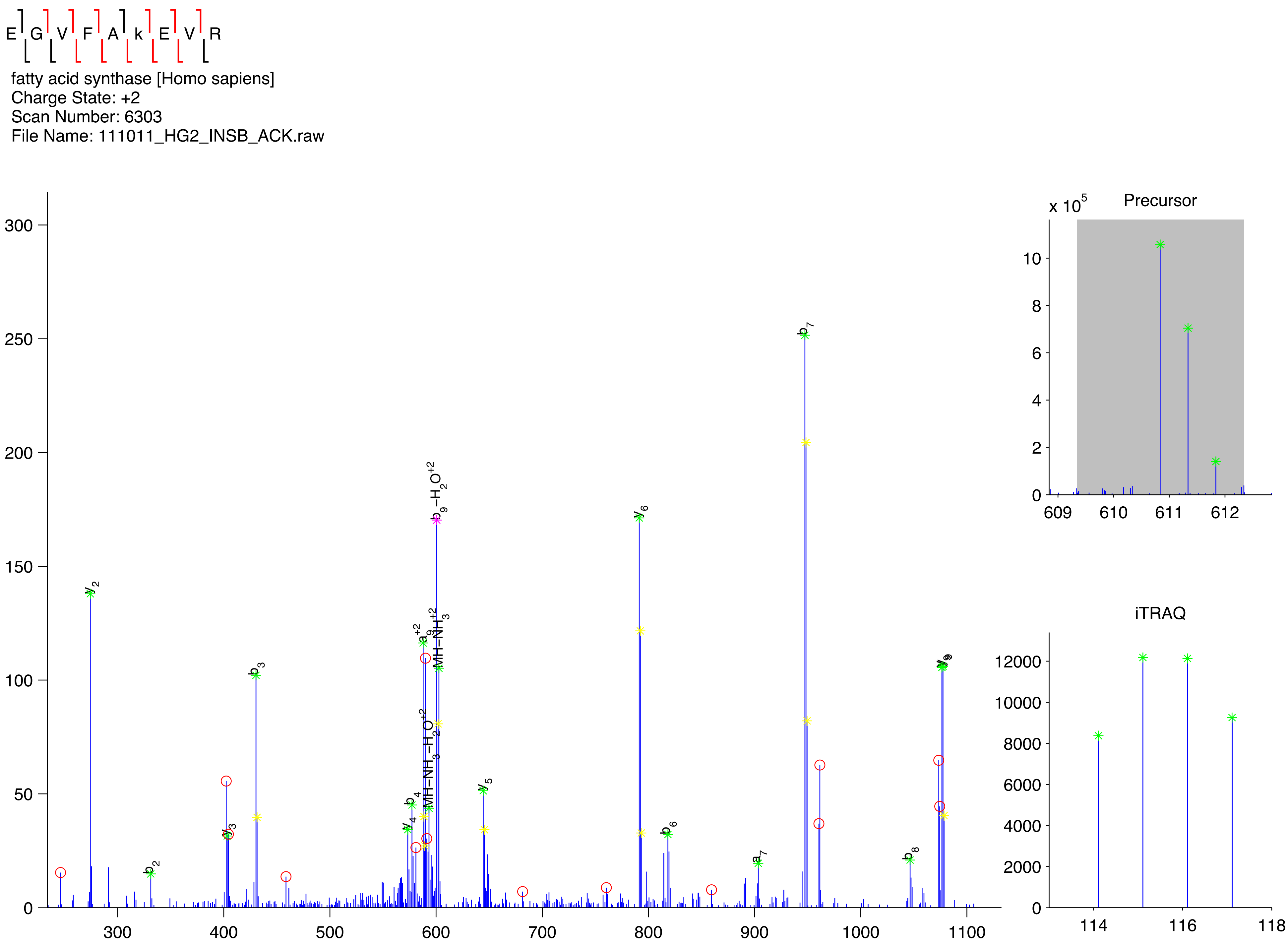

E G V F A k E V R
fatty acid synthase [Homo sapiens]
Charge State: +2
Scan Number: 6303
File Name: 111011_HG2_INSB_ACK.raw
Precursor
x 10^5
iTRAQ

T V D G P S G k L W R

glyceraldehyde−3−phosphate dehydrogenase [Homo sapiens]
Charge State: +2
Scan Number: 6744
File Name: 111011_HG2_INSB_ACK.raw

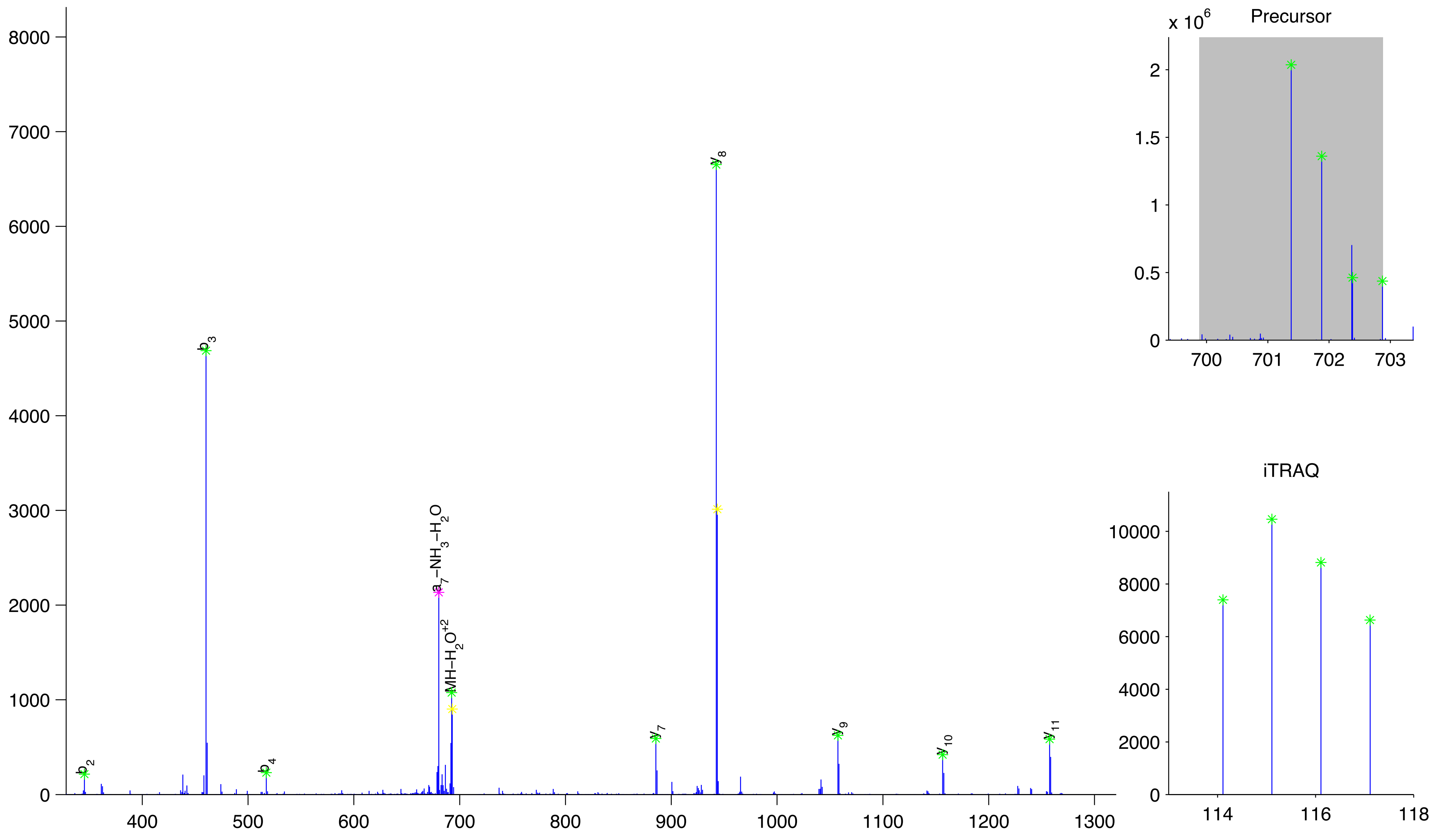

T V D G P S G k L W R

glyceraldehyde−3−phosphate dehydrogenase [Homo sapiens]
Charge State: +2
Scan Number: 6914
File Name: 111011_HG2_INSB_ACK.raw

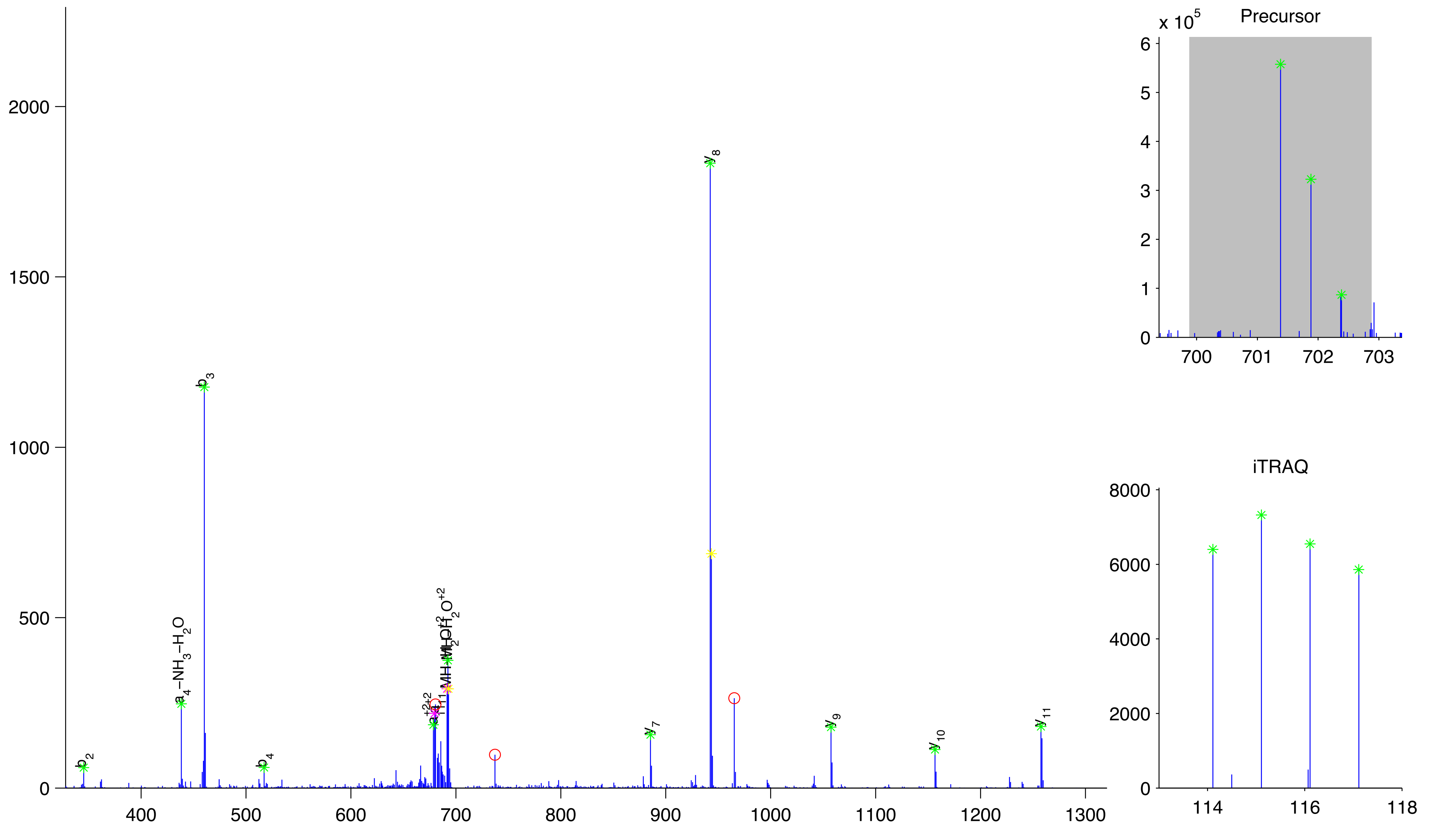

G k T G G k A R

H2A histone family, member X [Homo sapiens]
Charge State: +2
Scan Number: 2903
File Name: 111016_HG2_INSC_ACK.raw

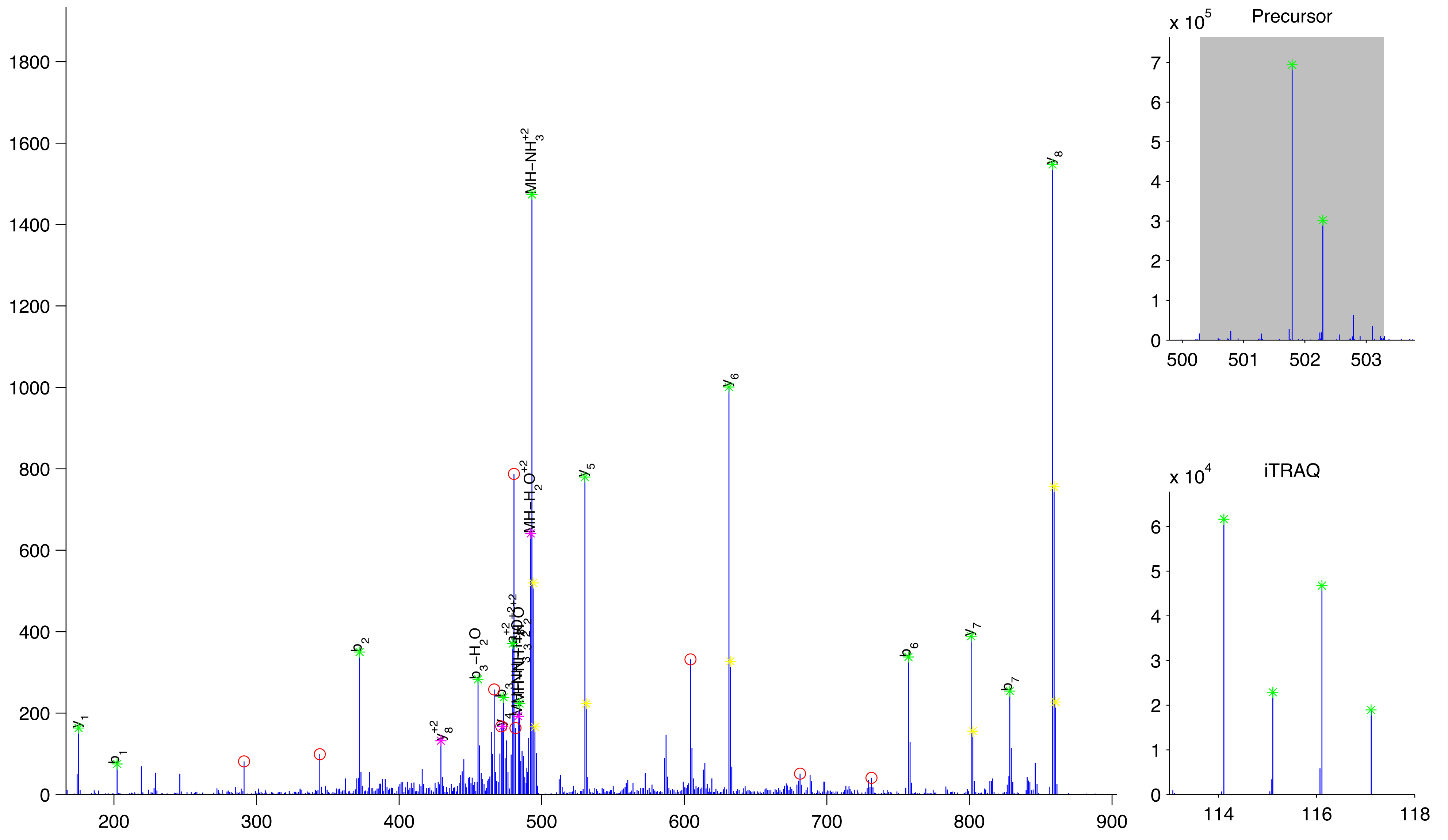

A G G k A G k D S G K

H2A histone family, member Z [Homo sapiens]
Charge State: +2
Scan Number: 3073
File Name: 111016_HG2_INSC_ACK.raw

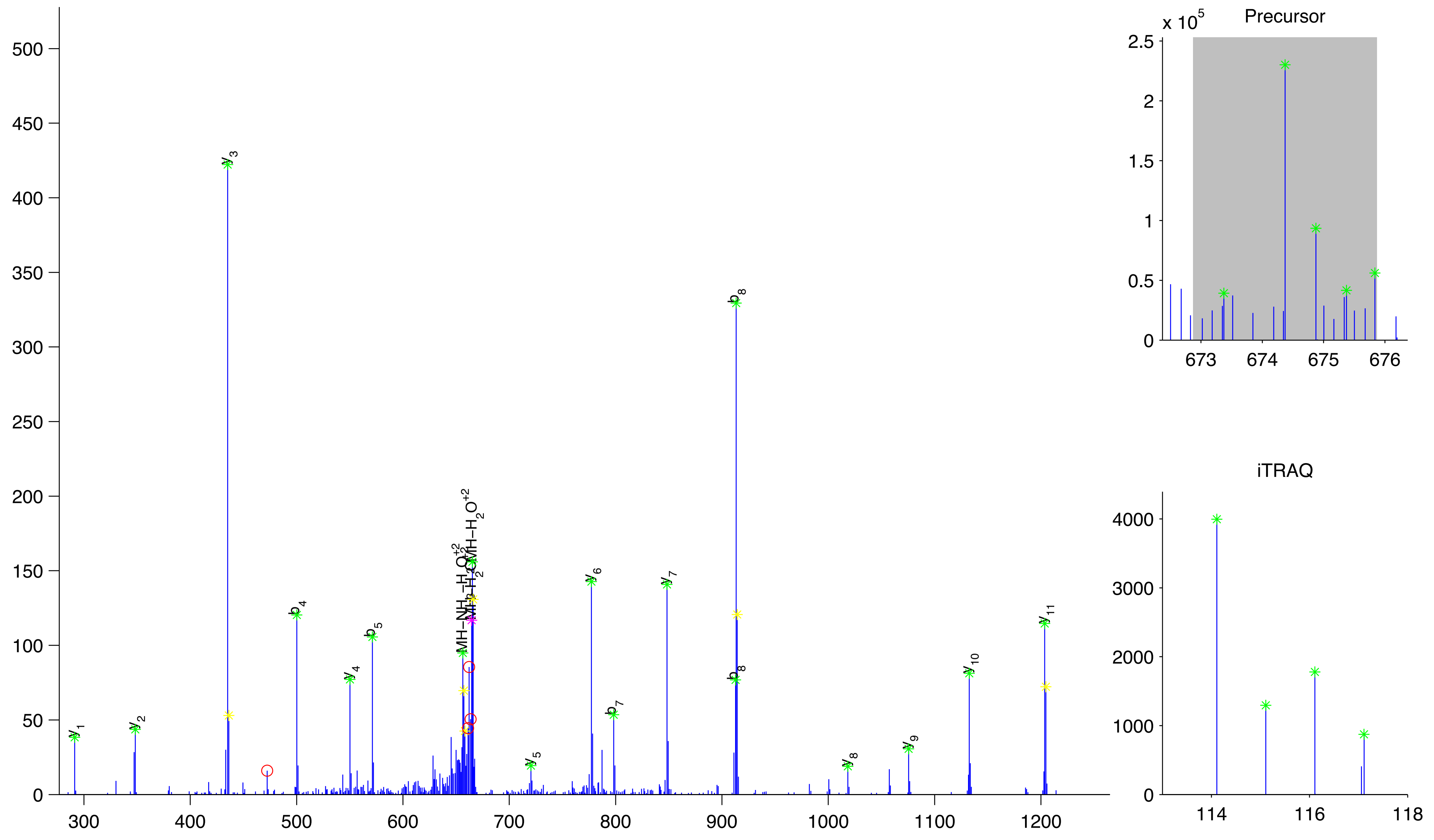

A G G k A G k D S G K

H2A histone family, member Z [Homo sapiens]
Charge State: +2
Scan Number: 4585
File Name: 111011_HG2_INSB_ACK.raw

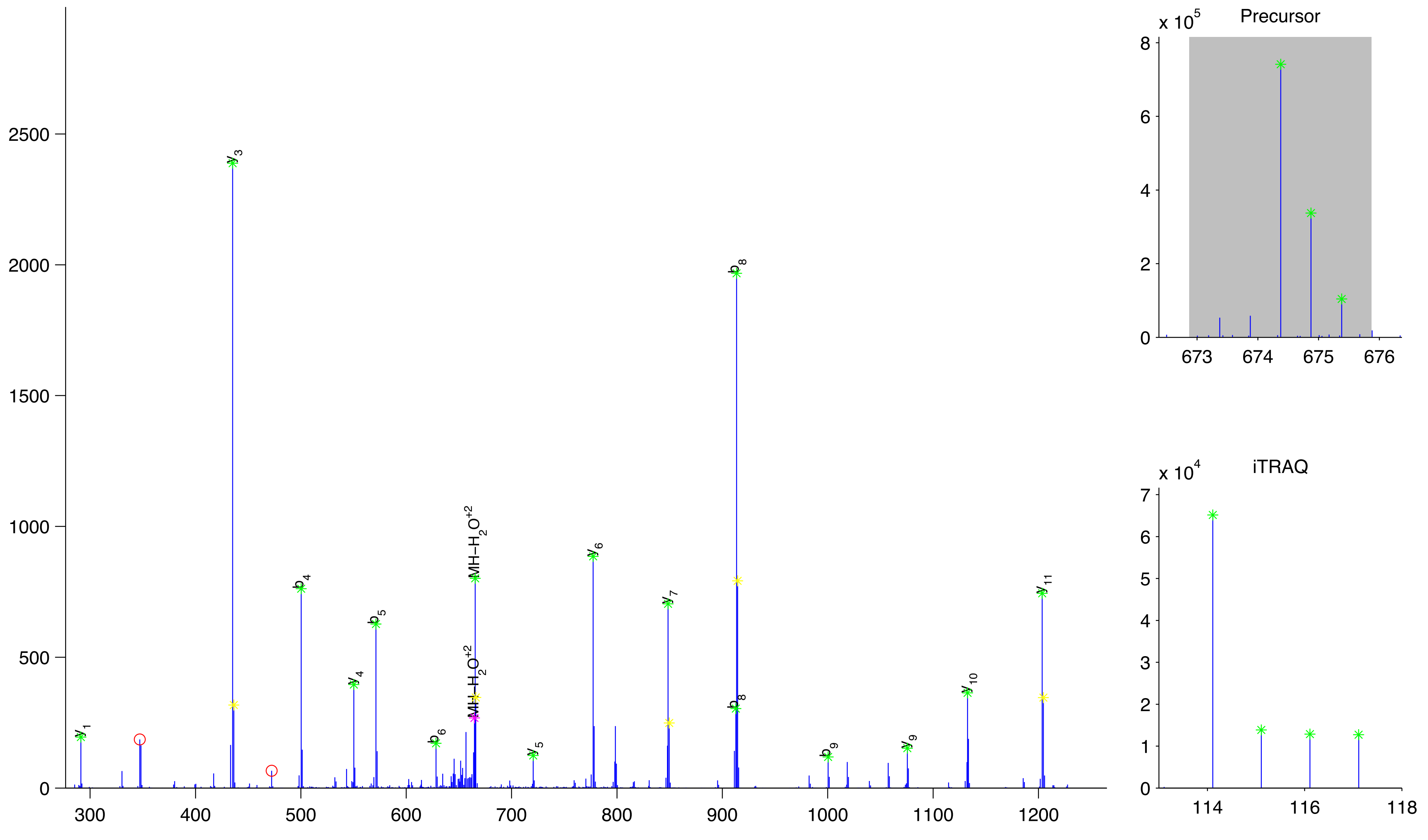

A G G k A G k D S G K

H2A histone family, member Z [Homo sapiens]
Charge State: +2
Scan Number: 11208
File Name: 110924_HG2_INSA_ACK.raw

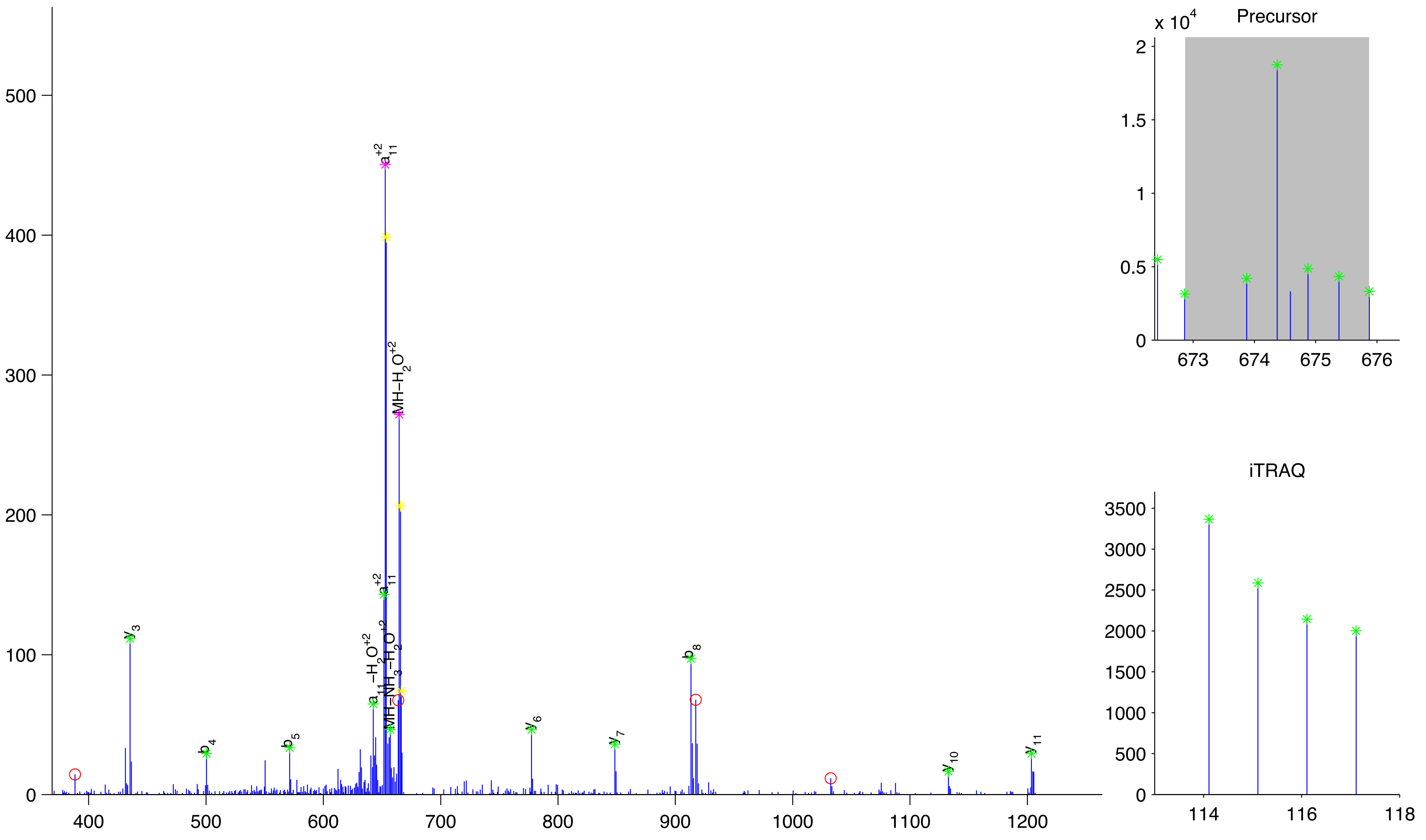

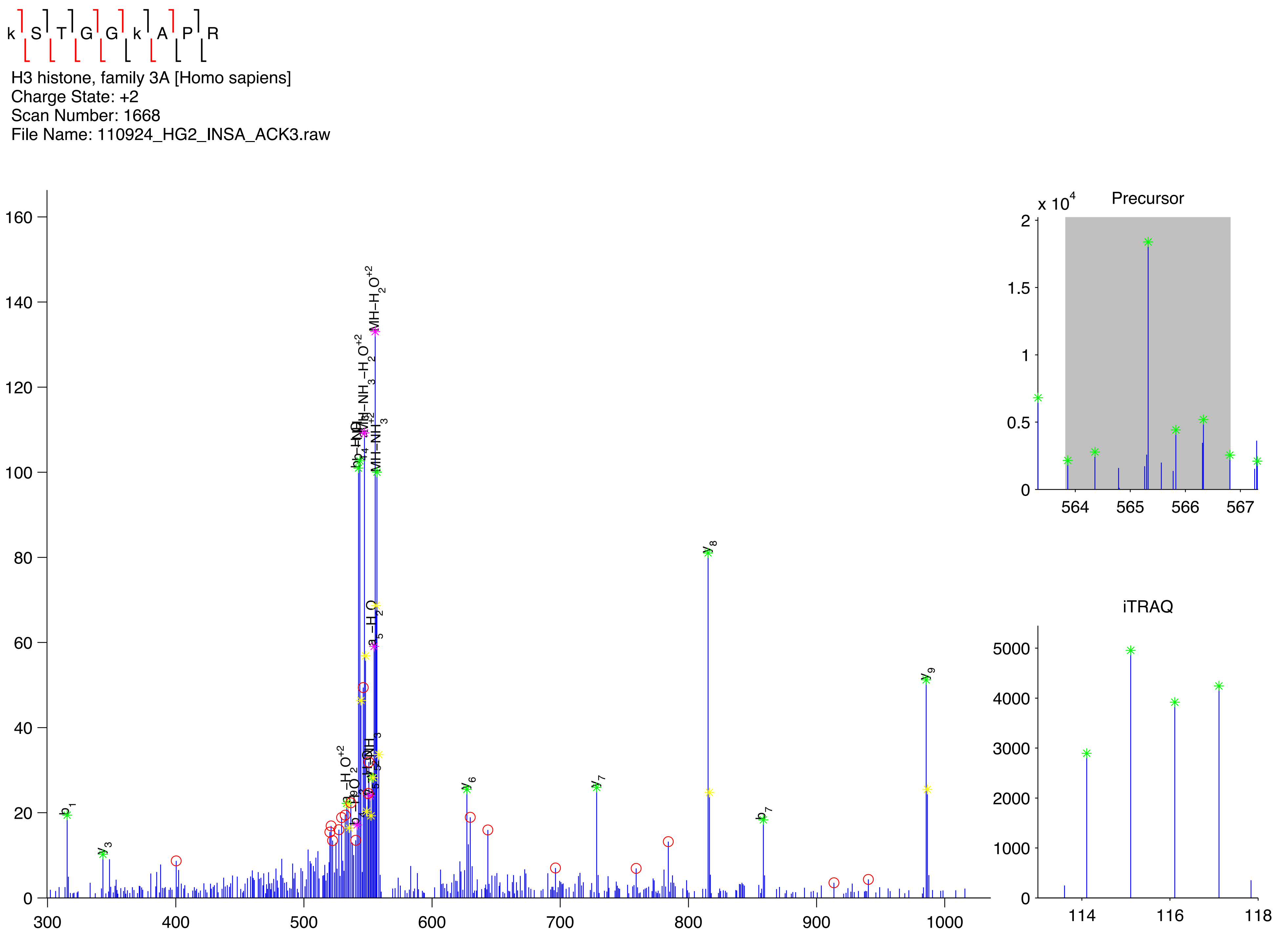

k S T G G k A P R
H3 histone, family 3A [Homo sapiens]
Charge State: +2
Scan Number: 1668
File Name: 110924_HG2_INSA_ACK3.raw
Precursor
x 10^4
iTRAQ
b1
y3
y6
y7
y8
b7
y9
MH-H2O+2
MH-NH3
a5-H2O

k S T G G k A P R

H3 histone, family 3A [Homo sapiens]
Charge State: +2
Scan Number: 3031
File Name: 111016_HG2_INSC_ACK.raw

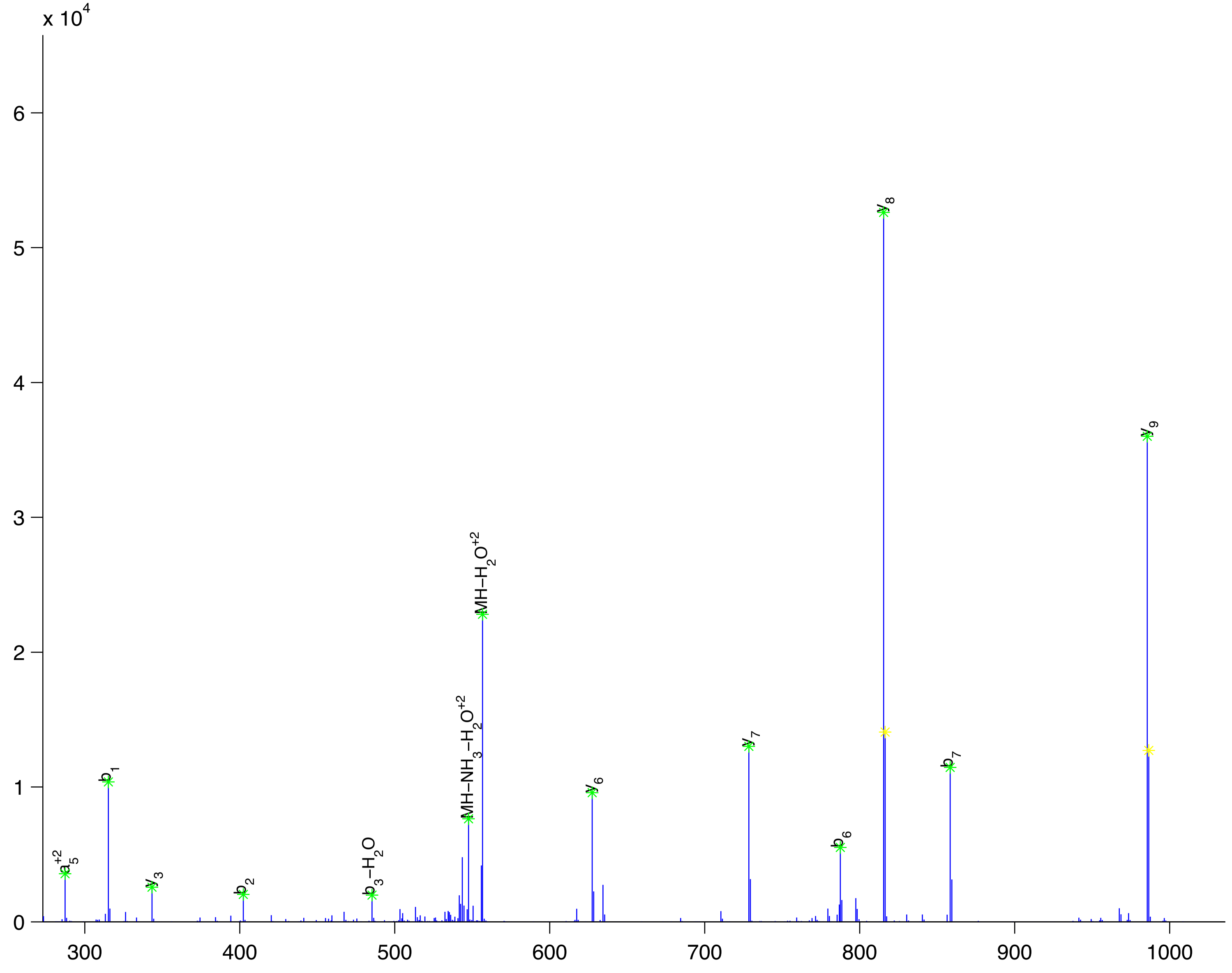

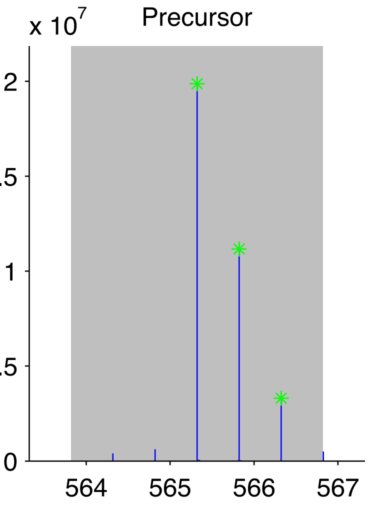

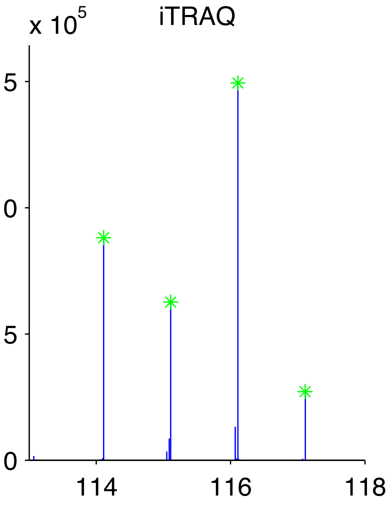

k S T G G k A P R

H3 histone, family 3A [Homo sapiens]
Charge State: +2
Scan Number: 3041
File Name: 111016_HG2_INSC_ACK.raw

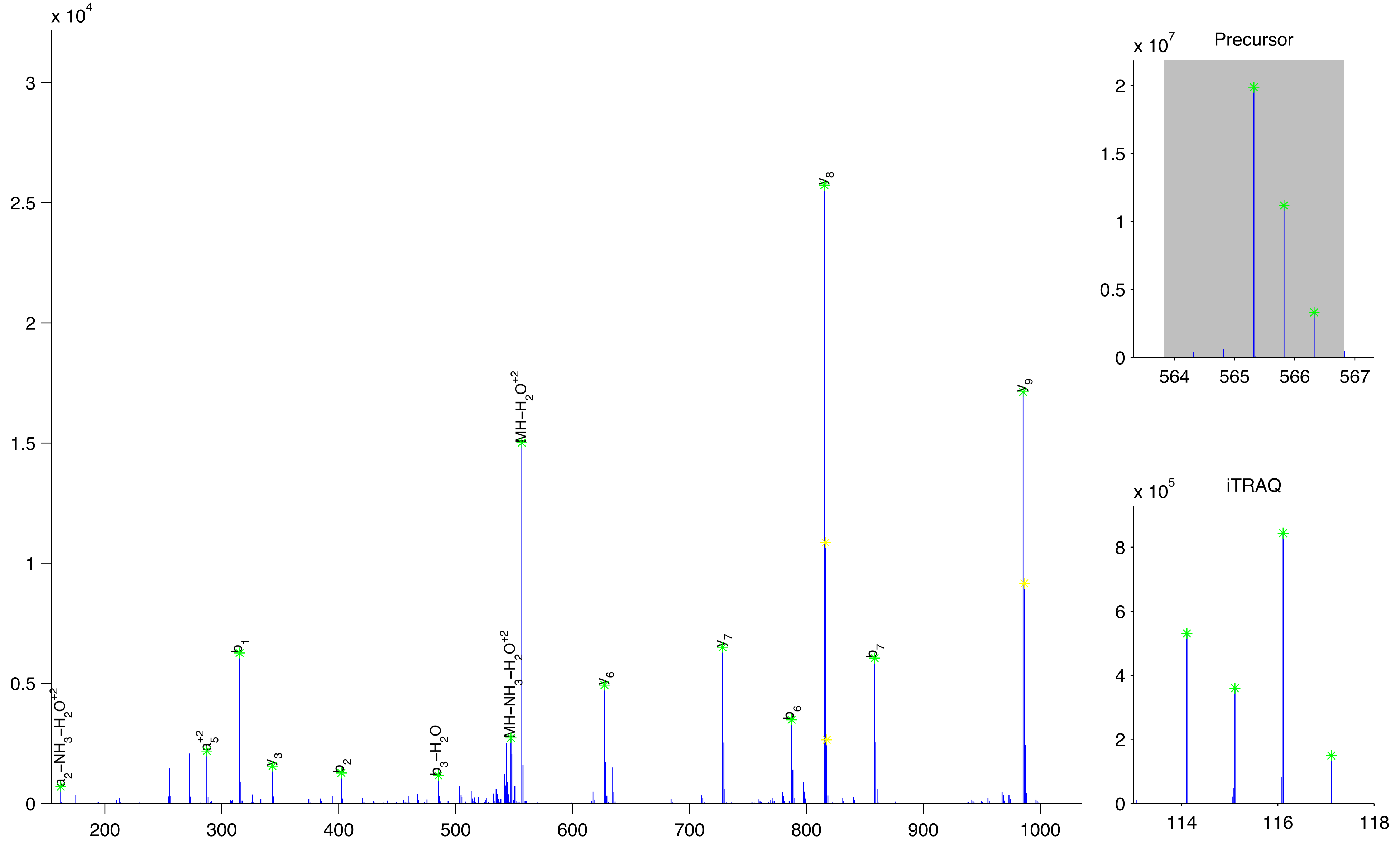

k S T G G k A P R

H3 histone, family 3A [Homo sapiens]
Charge State: +2
Scan Number: 3361
File Name: 110924_HG2_INSA_ACK3.raw

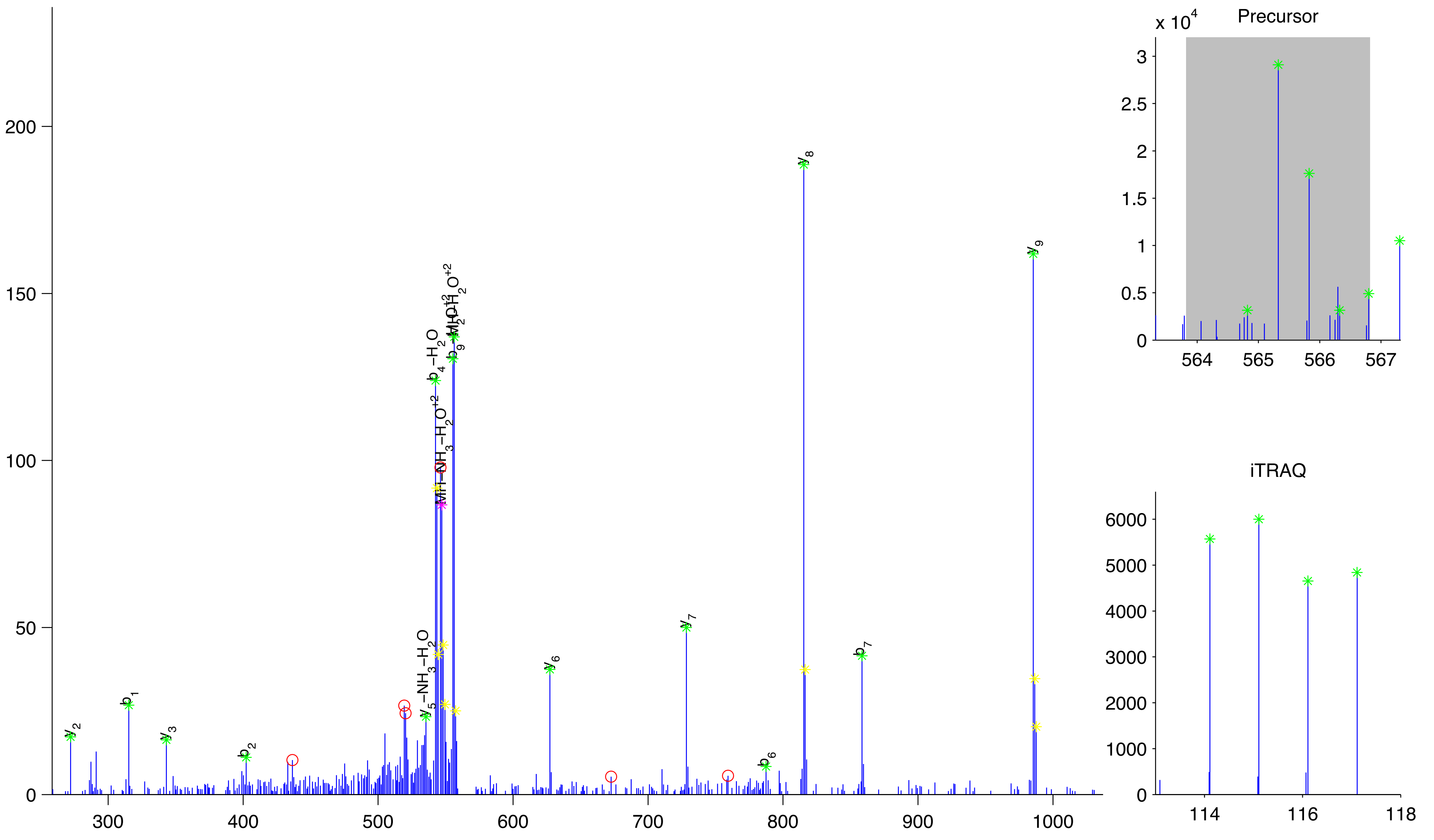

k Q L A T k A A R

H3 histone, family 3A [Homo sapiens]
Charge State: +2
Scan Number: 4014
File Name: 111016_HG2_INSC_ACK.raw

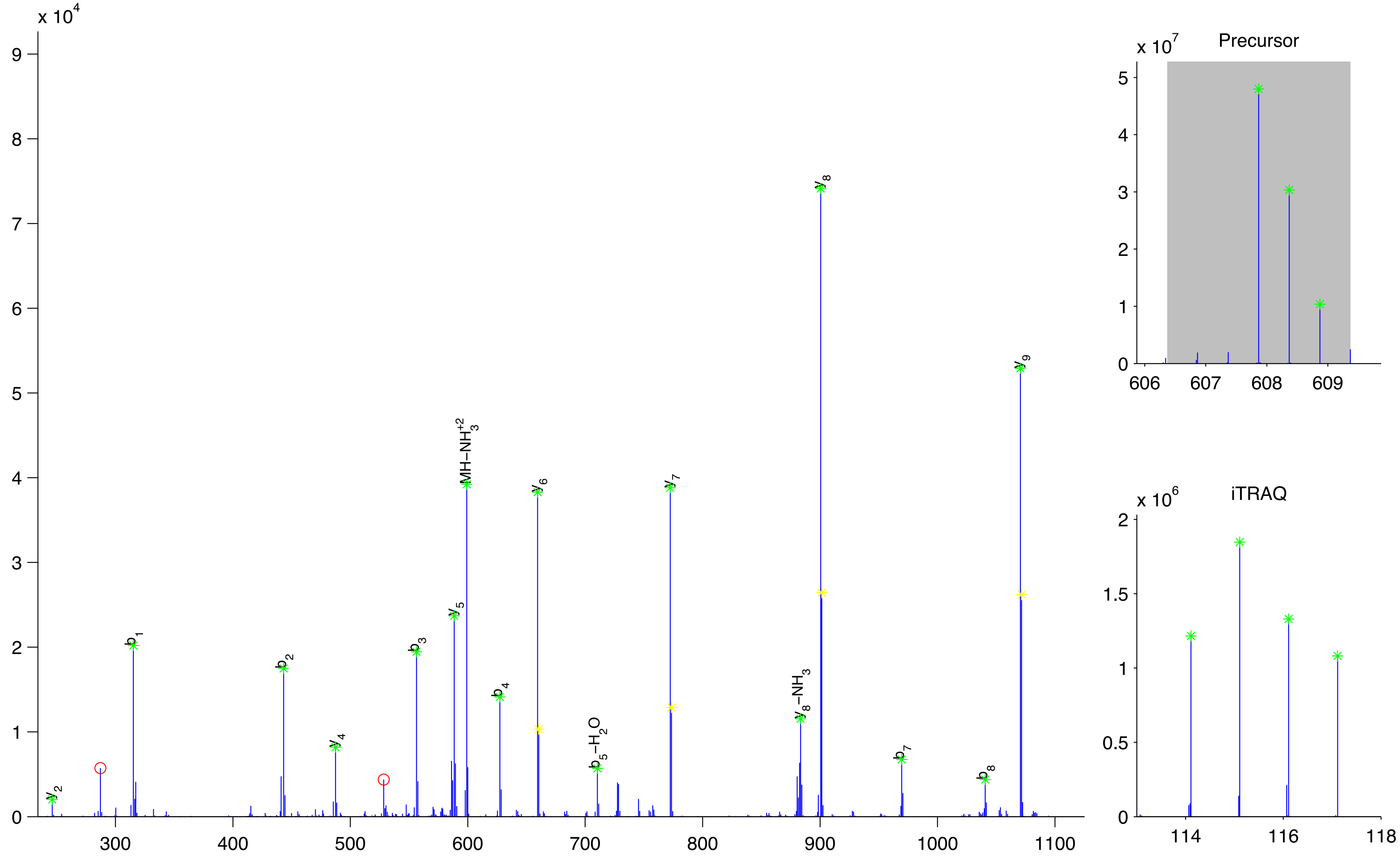

k Q L A T k A A R

H3 histone, family 3A [Homo sapiens]
Charge State: +2
Scan Number: 4020
File Name: 111016_HG2_INSC_ACK.raw

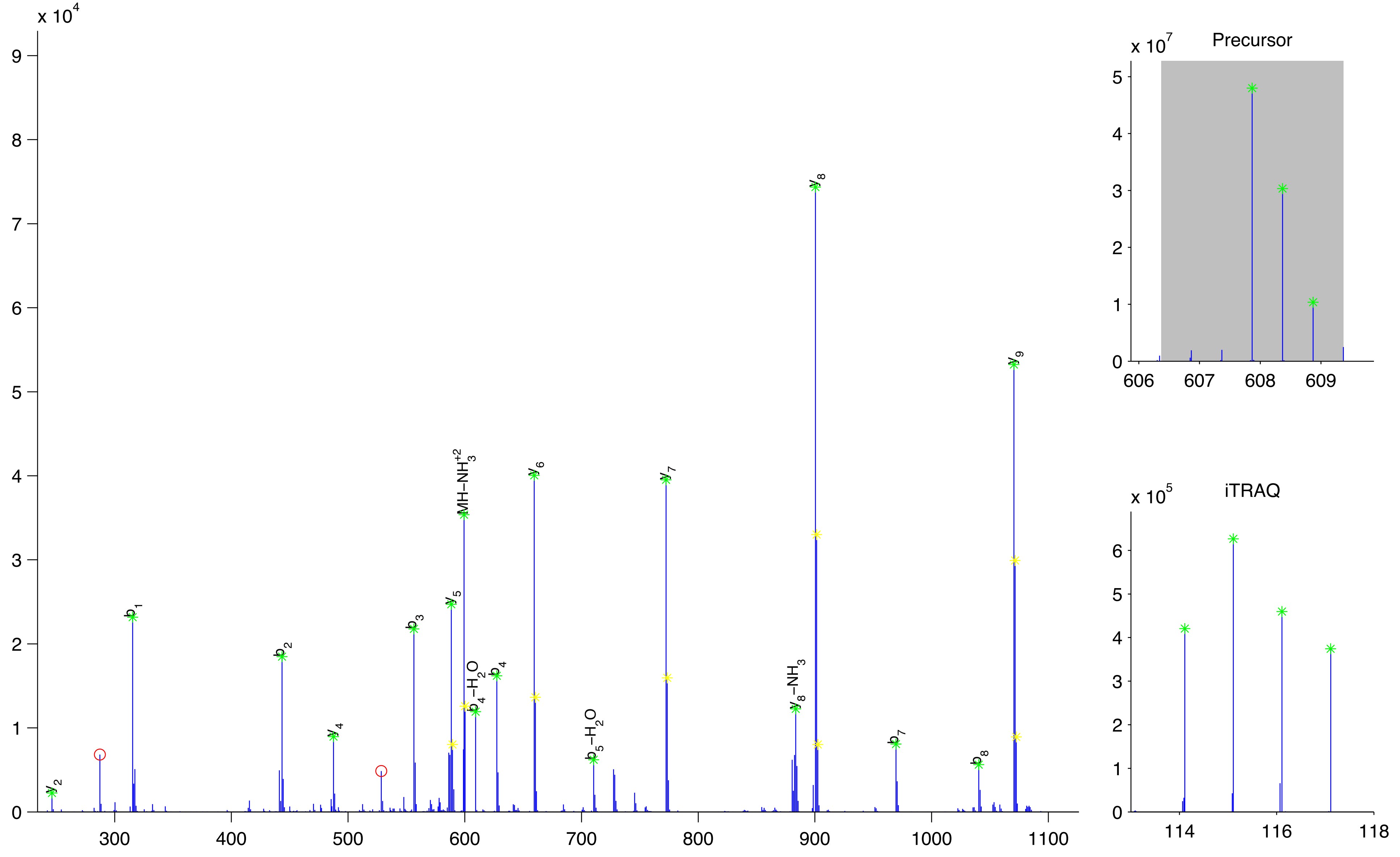

k Q L A T k A A R

H3 histone, family 3A [Homo sapiens]
Charge State: +2
Scan Number: 4182
File Name: 111016_HG2_INSC_ACK.raw

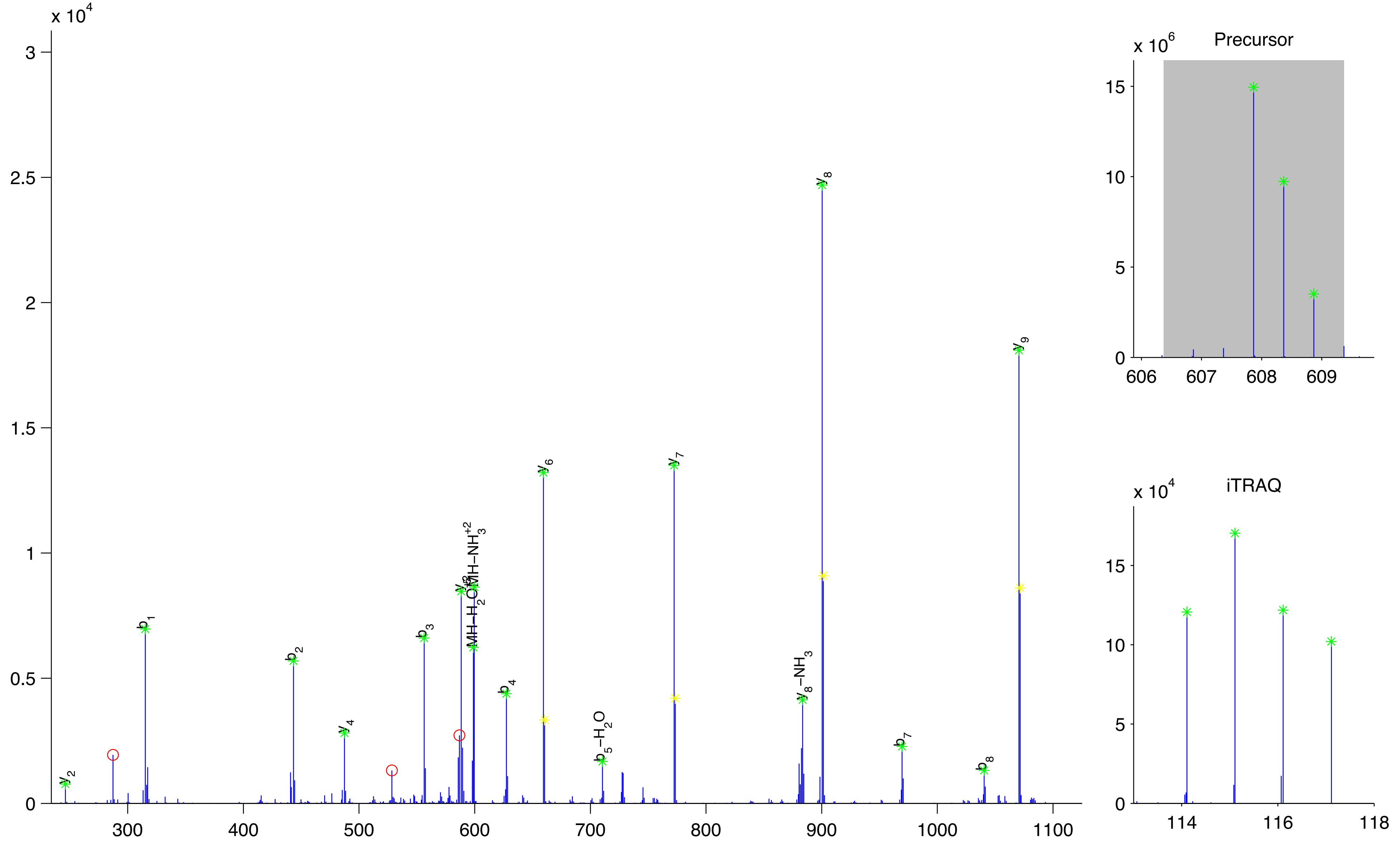

k Q L A T k A A R

H3 histone, family 3A [Homo sapiens]
Charge State: +2
Scan Number: 4190
File Name: 111016_HG2_INSC_ACK.raw

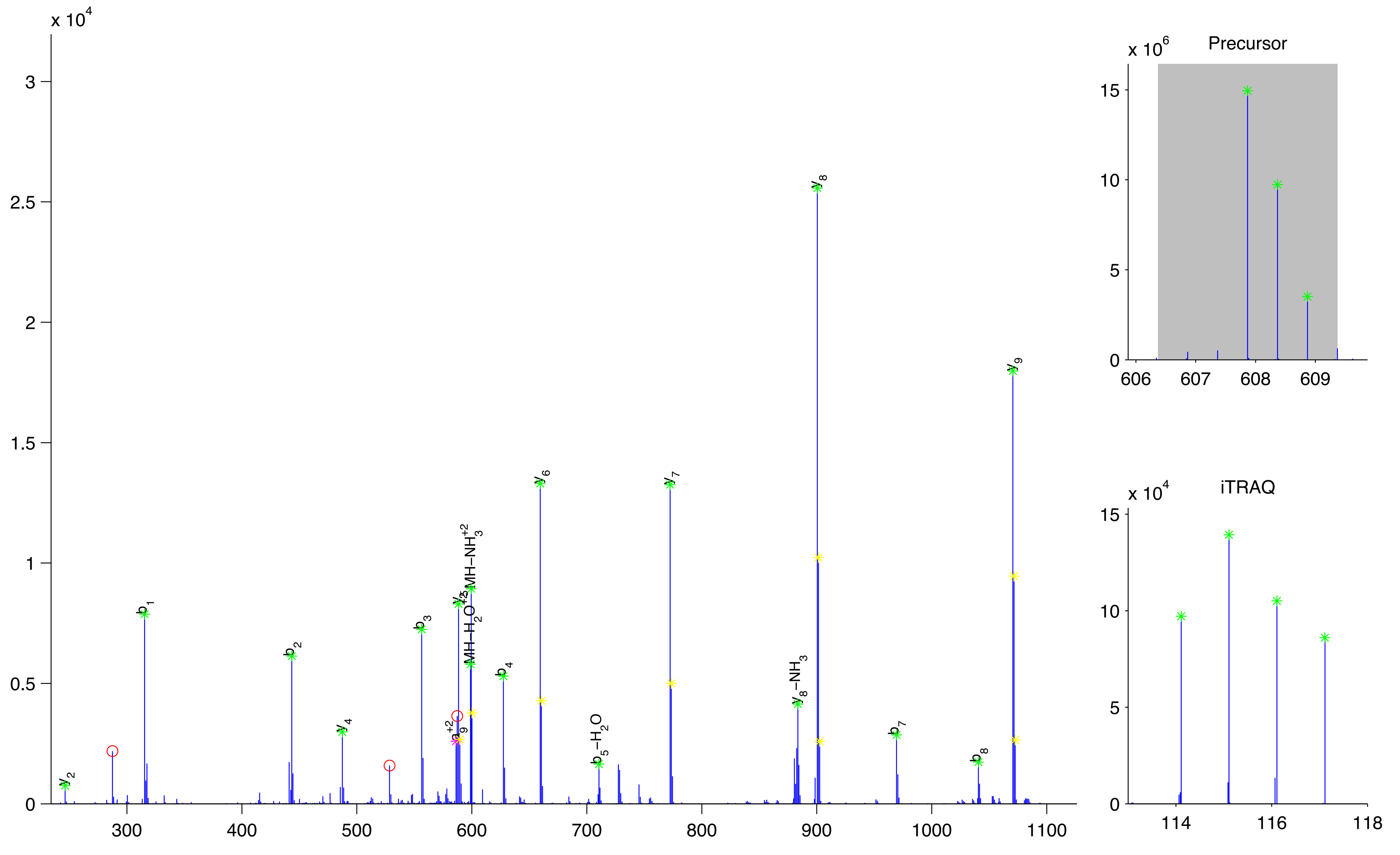

k Q L A T k A A R

H3 histone, family 3A [Homo sapiens]
Charge State: +2
Scan Number: 4350
File Name: 111016_HG2_INSC_ACK.raw

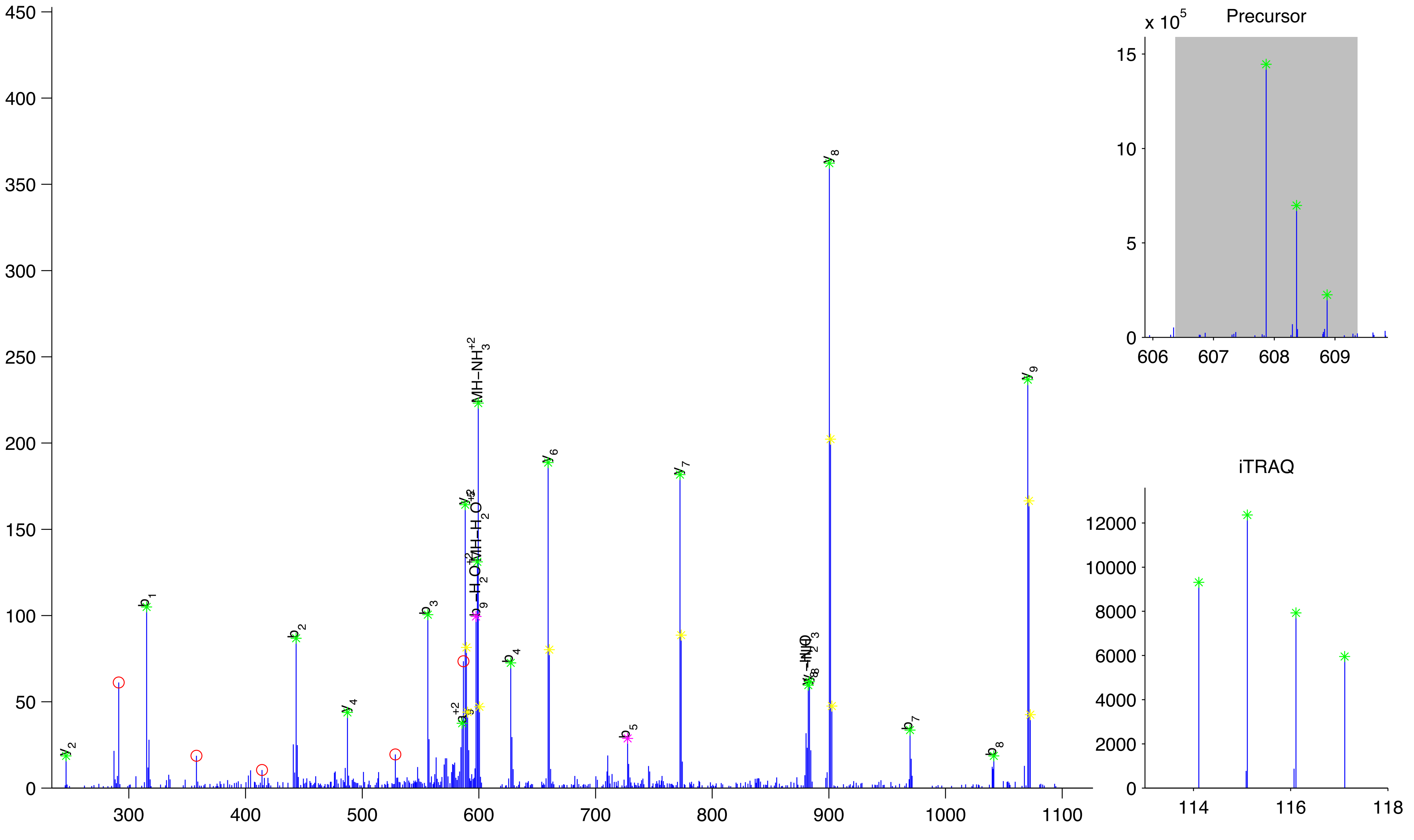

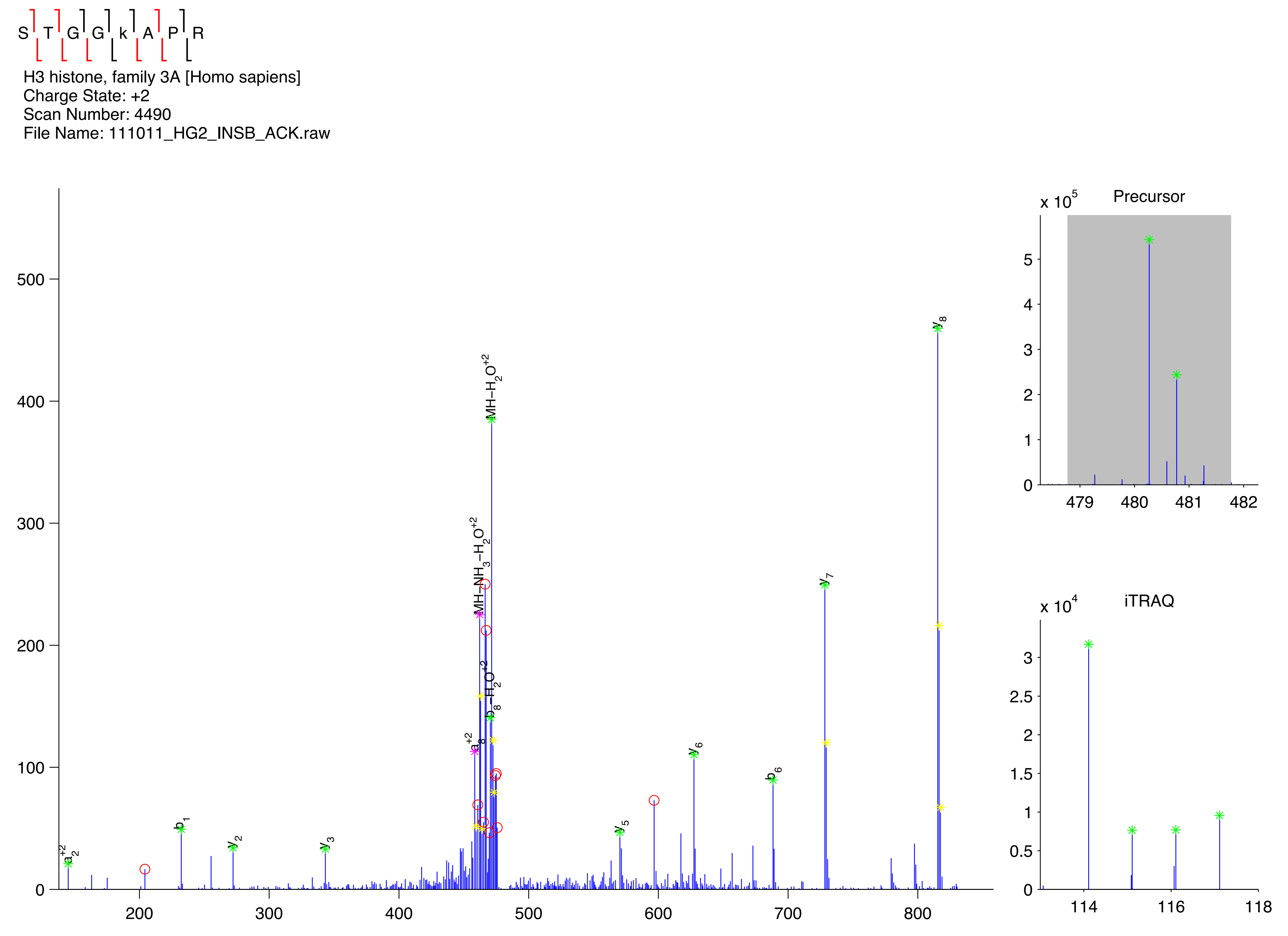

S T G G k A P R
H3 histone, family 3A [Homo sapiens]
Charge State: +2
Scan Number: 4490
File Name: 111011_HG2_INSB_ACK.raw
a_2^{+2}
b_1
y_2
y_3
a_8^{+2}
$MH-NH_3-H_2O^{+2}$
$b_8-H_2O^{+2}$
$MH-H_2O^{+2}$
y_5
y_6
b_6
y_7
y_8
x 10^5
Precursor
x 10^4
iTRAQ

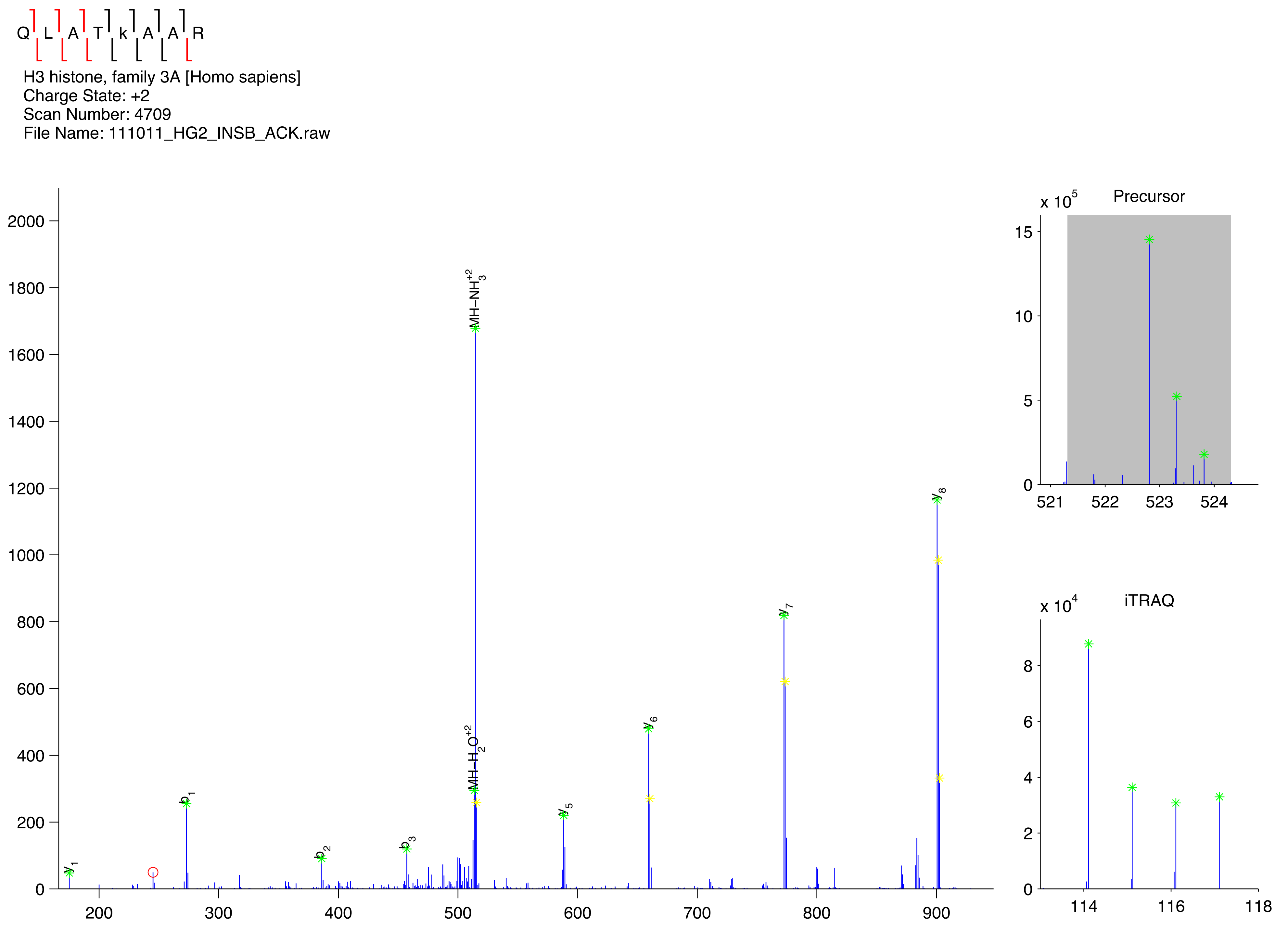
Q L A T k A A R
H3 histone, family 3A [Homo sapiens]
Charge State: +2
Scan Number: 4709
File Name: 111011_HG2_INSB_ACK.raw
MH−NH3+2
MH−H2O+2
y1
b1
b2
b3
y5
y6
y7
y8
x 10^5
Precursor
x 10^4
iTRAQ

k Q L A T K

H3 histone, family 3A [Homo sapiens]
Charge State: +2
Scan Number: 4736
File Name: 111011_HG2_INSB_ACK.raw

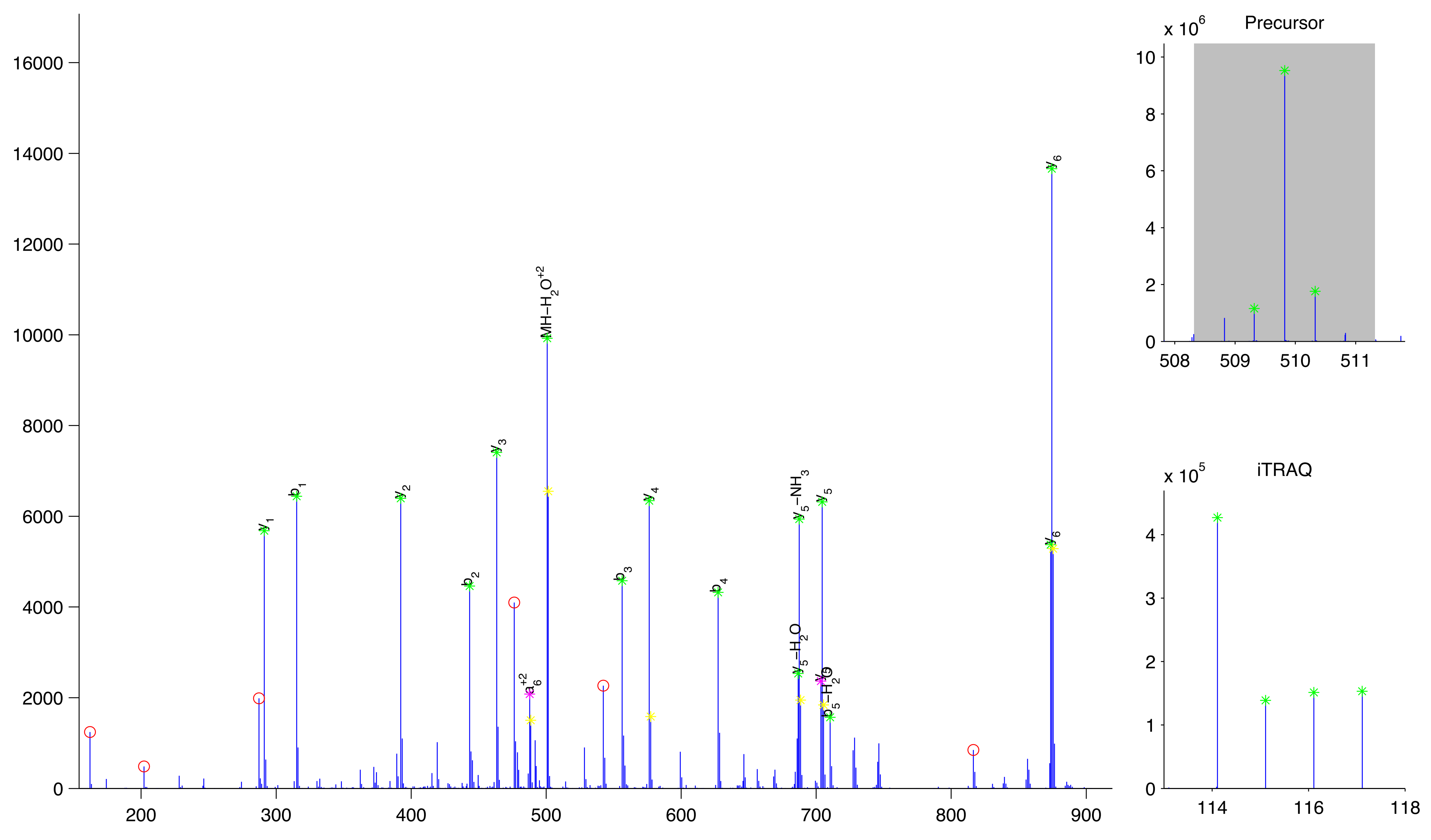

Q L A T k A A R

H3 histone, family 3A [Homo sapiens]
Charge State: +2
Scan Number: 4742
File Name: 111011_HG2_INSB_ACK.raw

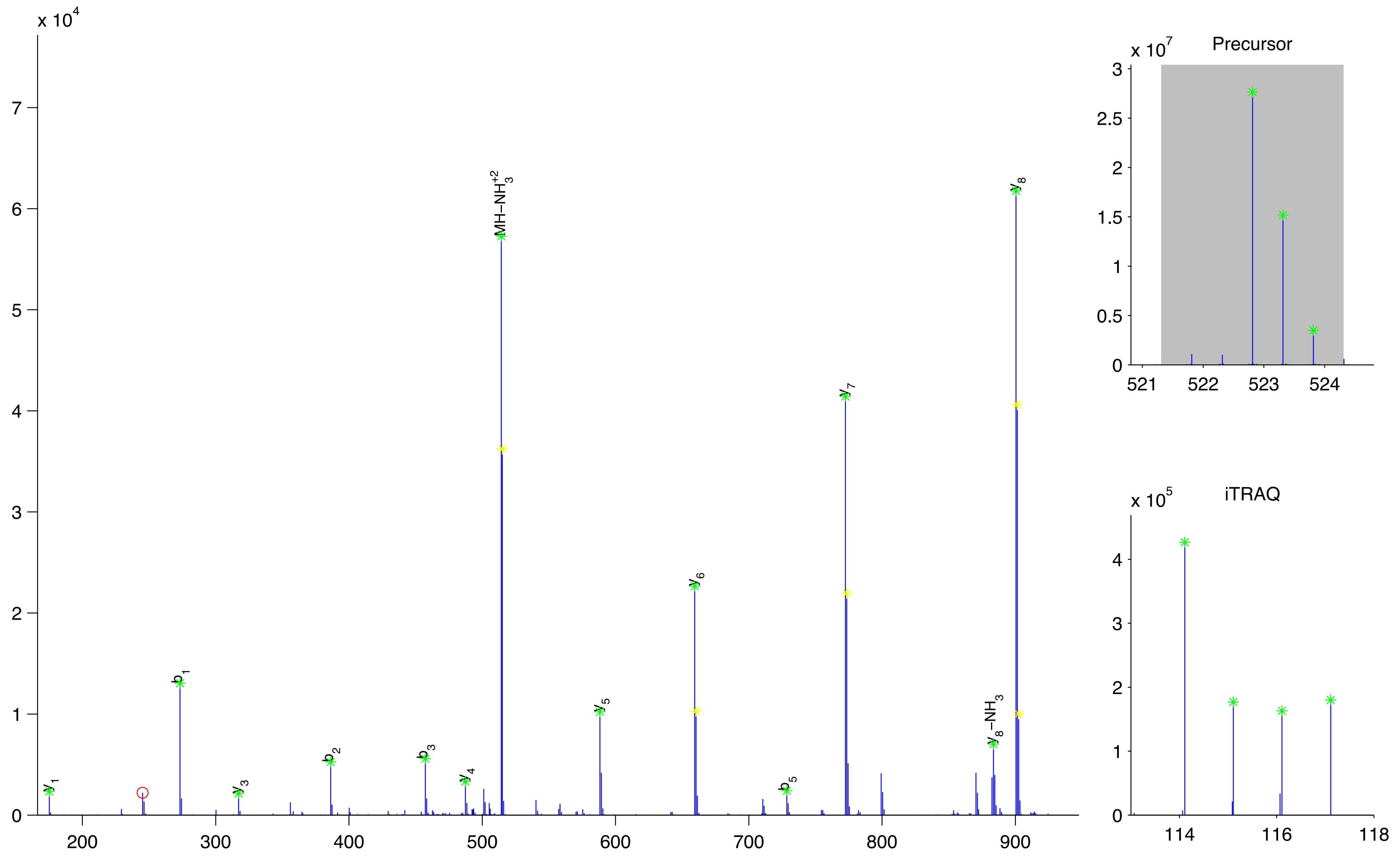

k Q L A T k A A R

H3 histone, family 3A [Homo sapiens]
Charge State: +2
Scan Number: 4980
File Name: 111011_HG2_INSB_ACK.raw

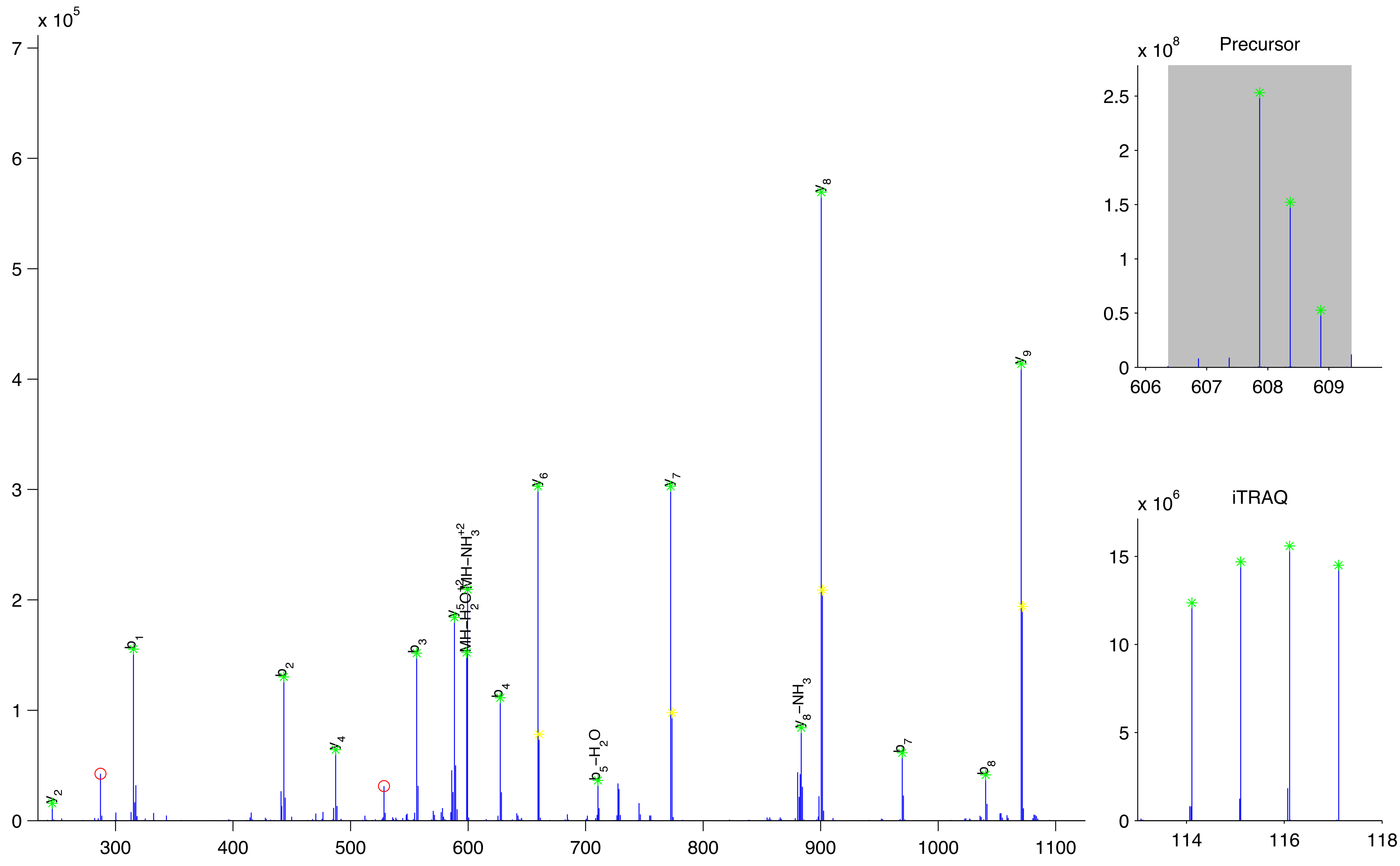

k Q L A T k A A R

H3 histone, family 3A [Homo sapiens]
Charge State: +2
Scan Number: 4982
File Name: 111011_HG2_INSB_ACK.raw

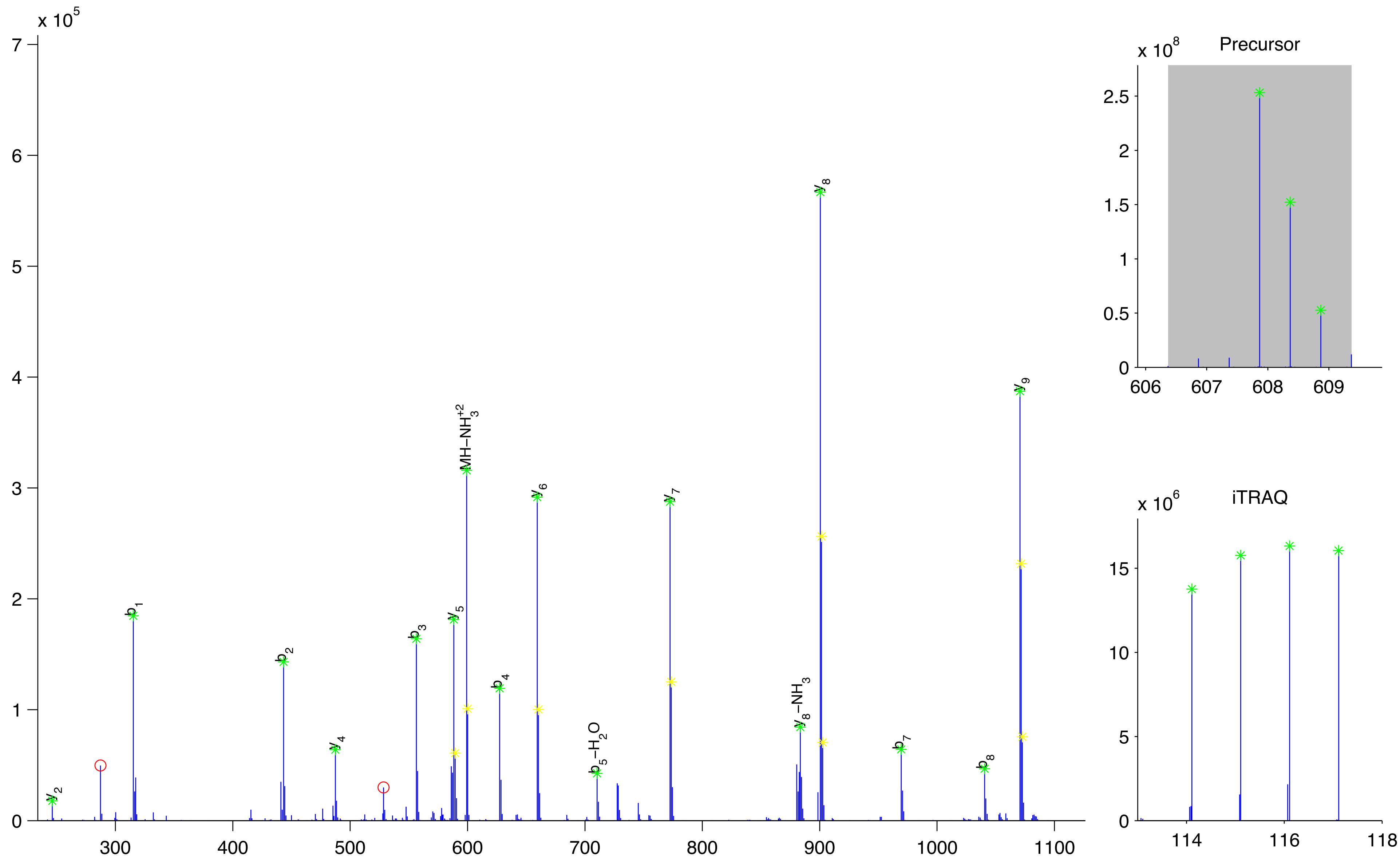

k Q L A T k A A R

H3 histone, family 3A [Homo sapiens]
Charge State: +2
Scan Number: 5171
File Name: 111011_HG2_INSB_ACK.raw

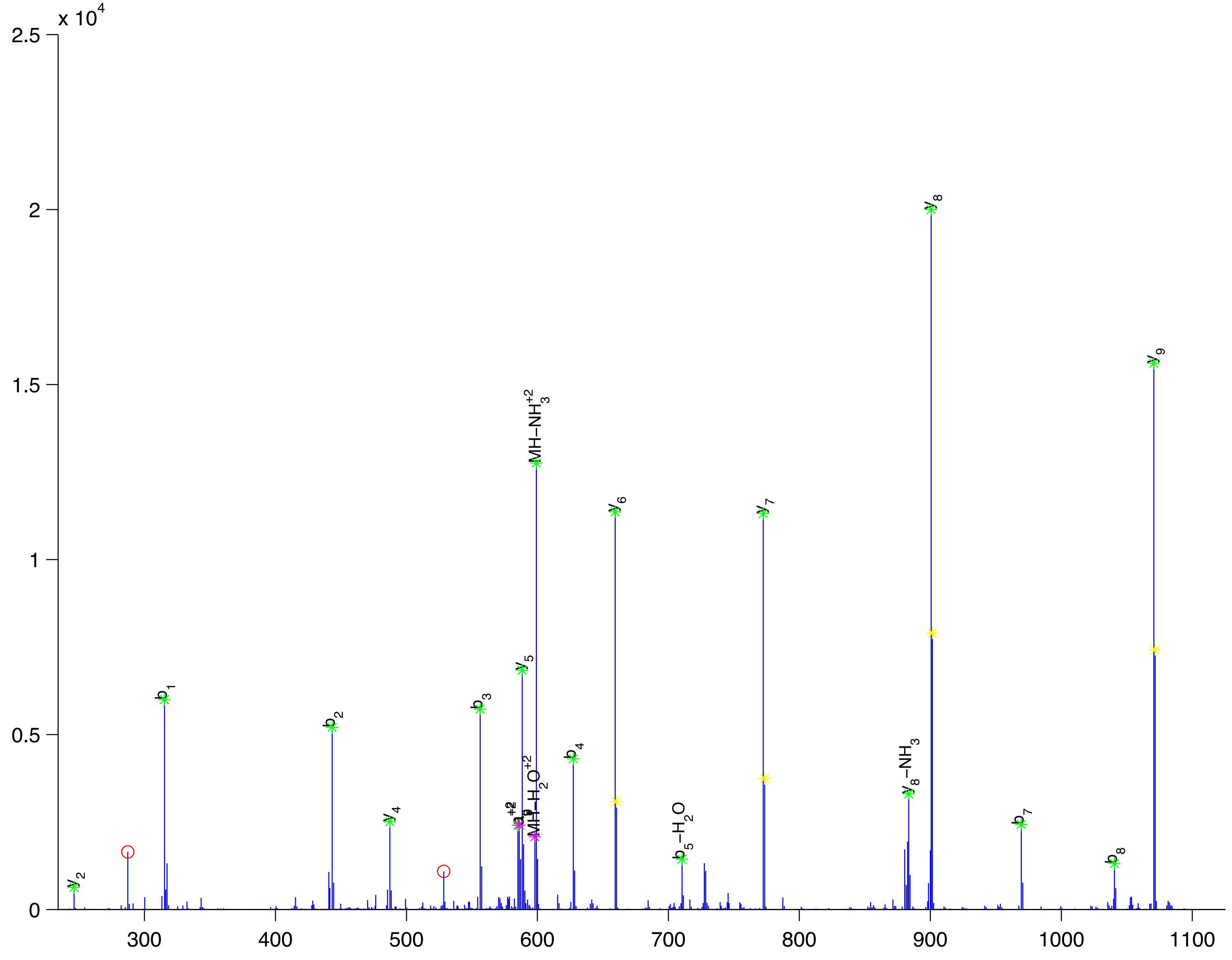

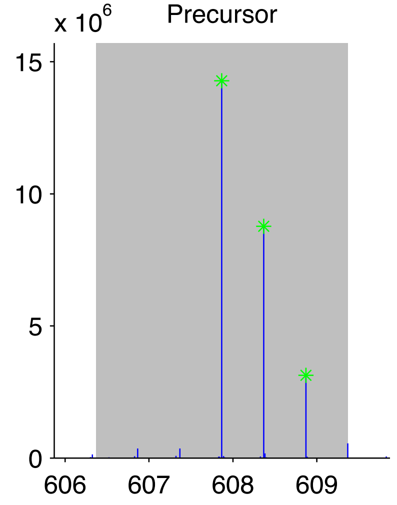

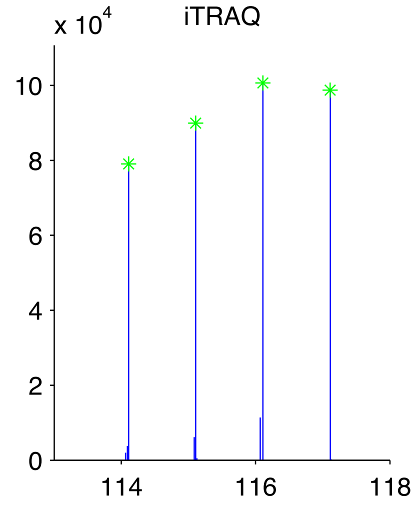

k Q L A T k A A R

H3 histone, family 3A [Homo sapiens]
Charge State: +2
Scan Number: 5179
File Name: 111011_HG2_INSB_ACK.raw

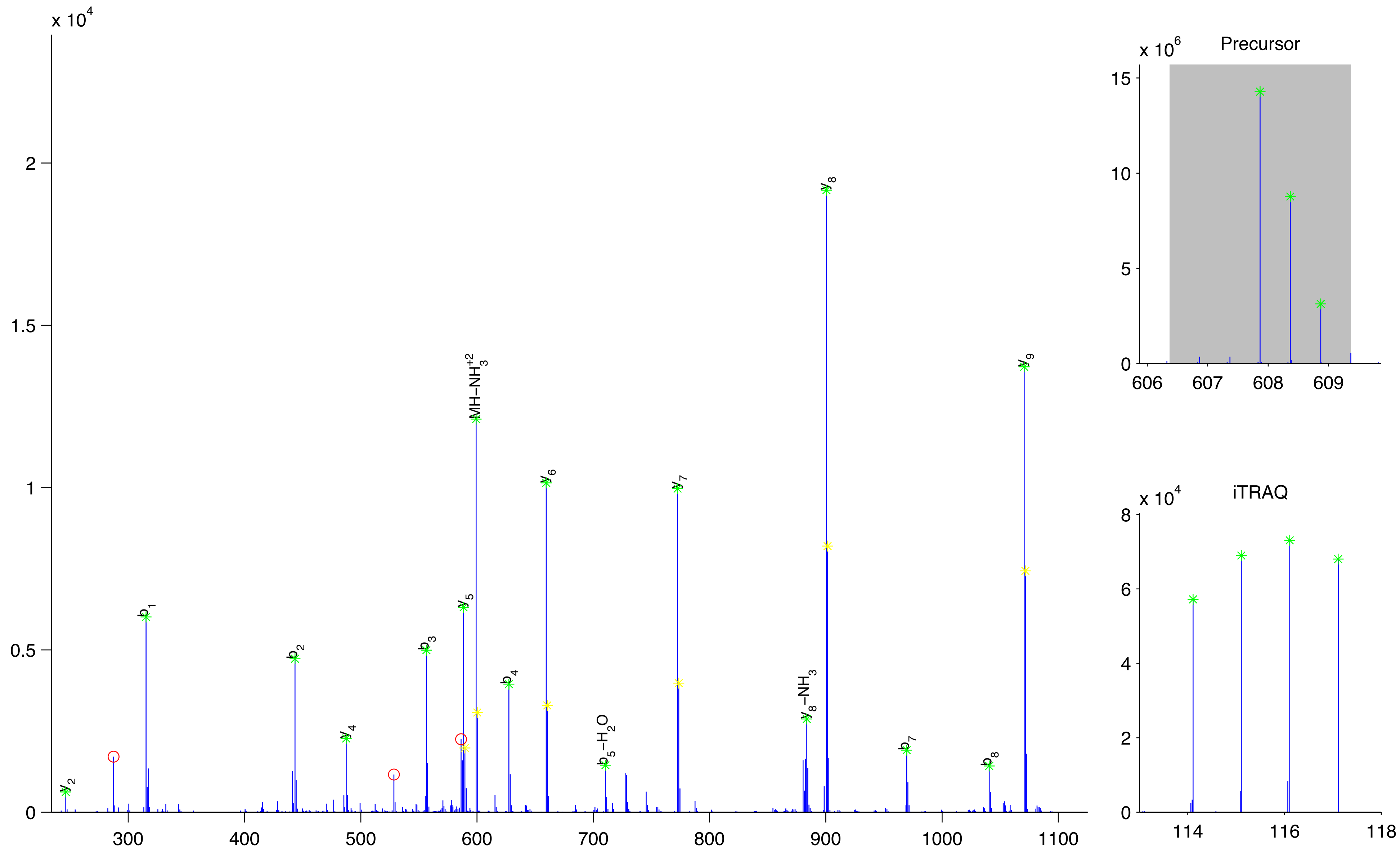

k Q L A T k A A R

H3 histone, family 3A [Homo sapiens]
Charge State: +2
Scan Number: 5339
File Name: 111011_HG2_INSB_ACK.raw

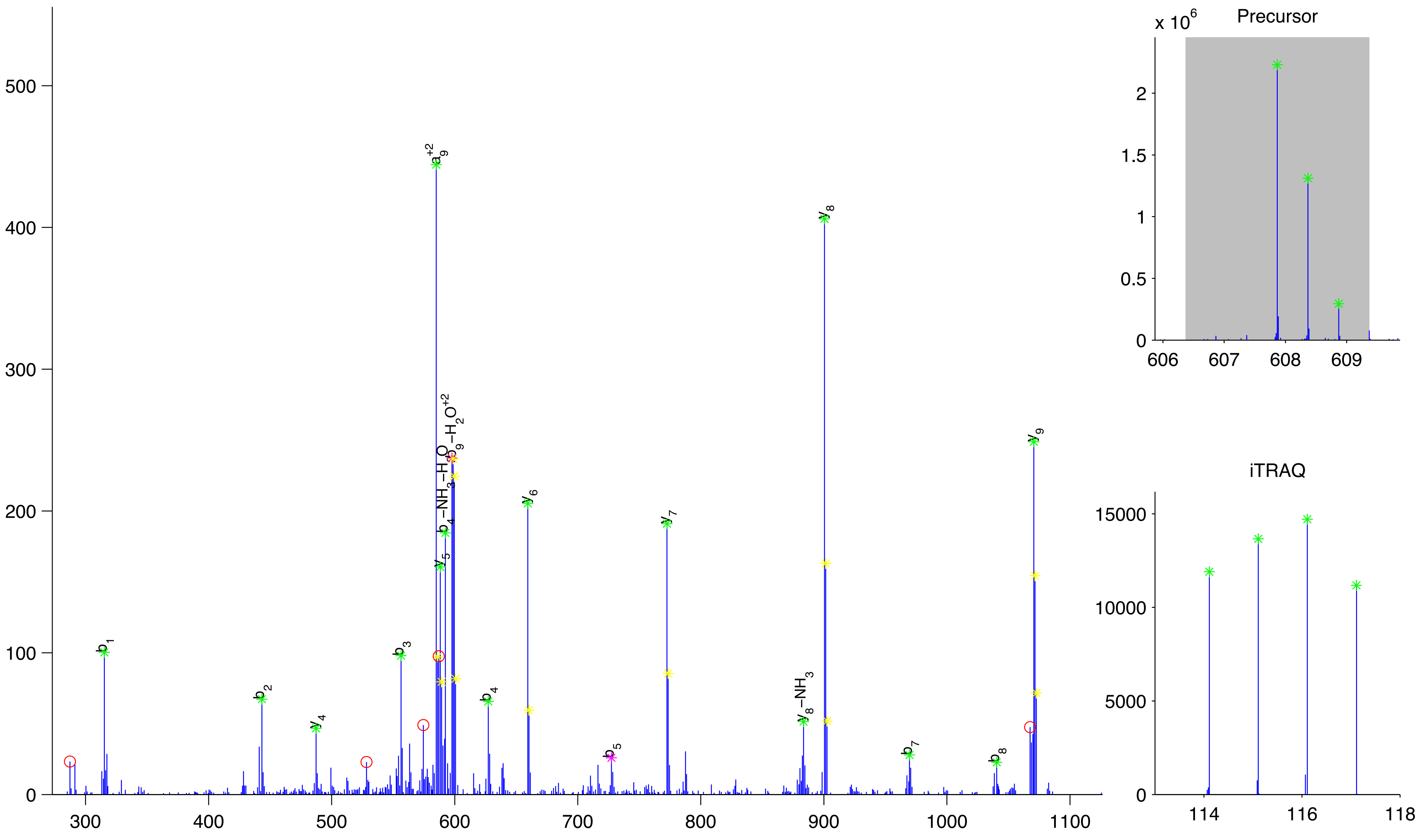

k Q L A T k A A R

H3 histone, family 3A [Homo sapiens]
Charge State: +2
Scan Number: 5507
File Name: 111011_HG2_INSB_ACK.raw

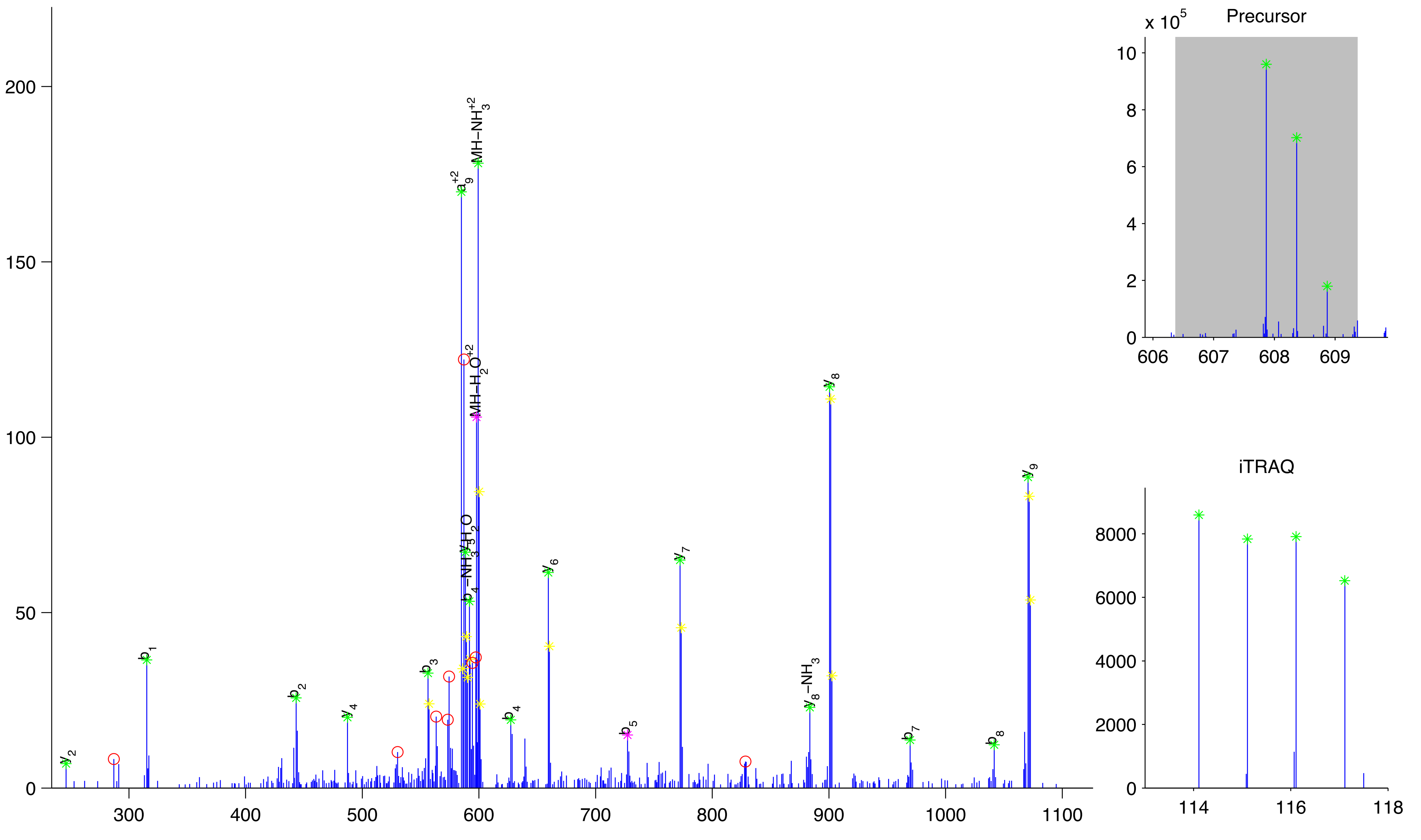

k Q L A T k A A R

H3 histone, family 3A [Homo sapiens]
Charge State: +2
Scan Number: 7189
File Name: 111011_HG2_INSB_ACK.raw

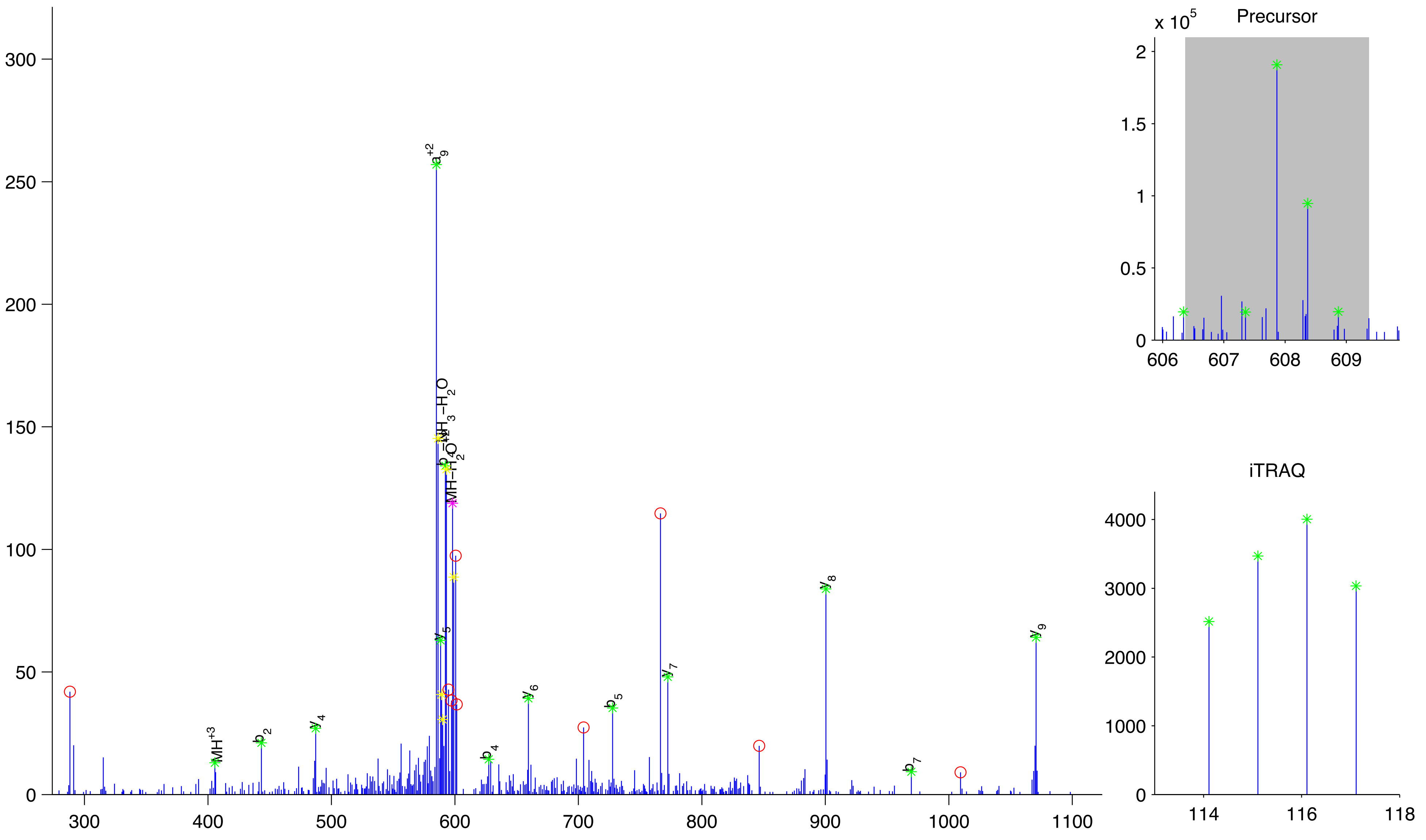

k Q L A T k A A R

H3 histone, family 3A [Homo sapiens]
Charge State: +2
Scan Number: 7359
File Name: 111011_HG2_INSB_ACK.raw

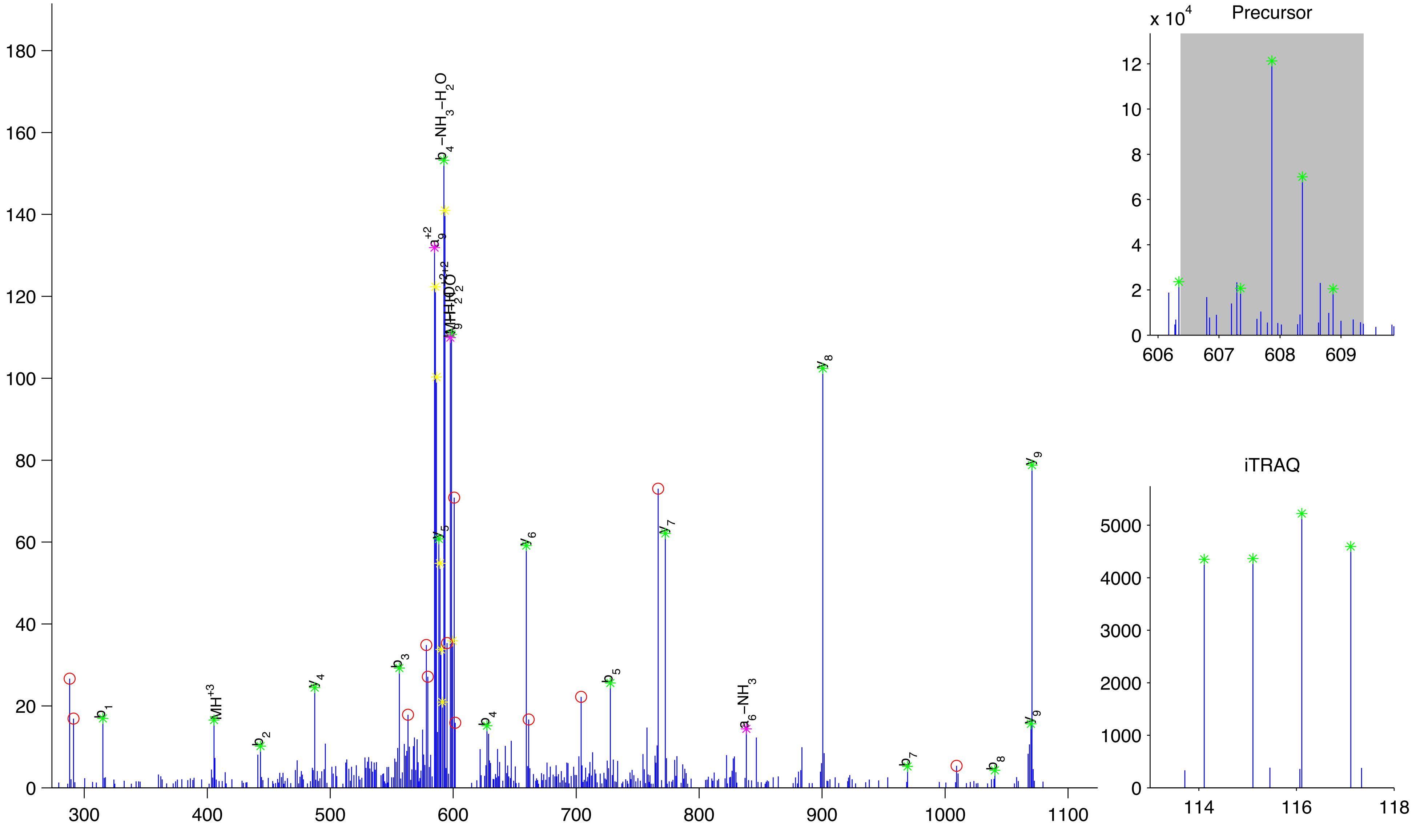

k S T G G k A P R

H3 histone, family 3A [Homo sapiens]
Charge State: +2
Scan Number: 9665
File Name: 110924_HG2_INSA_ACK.raw

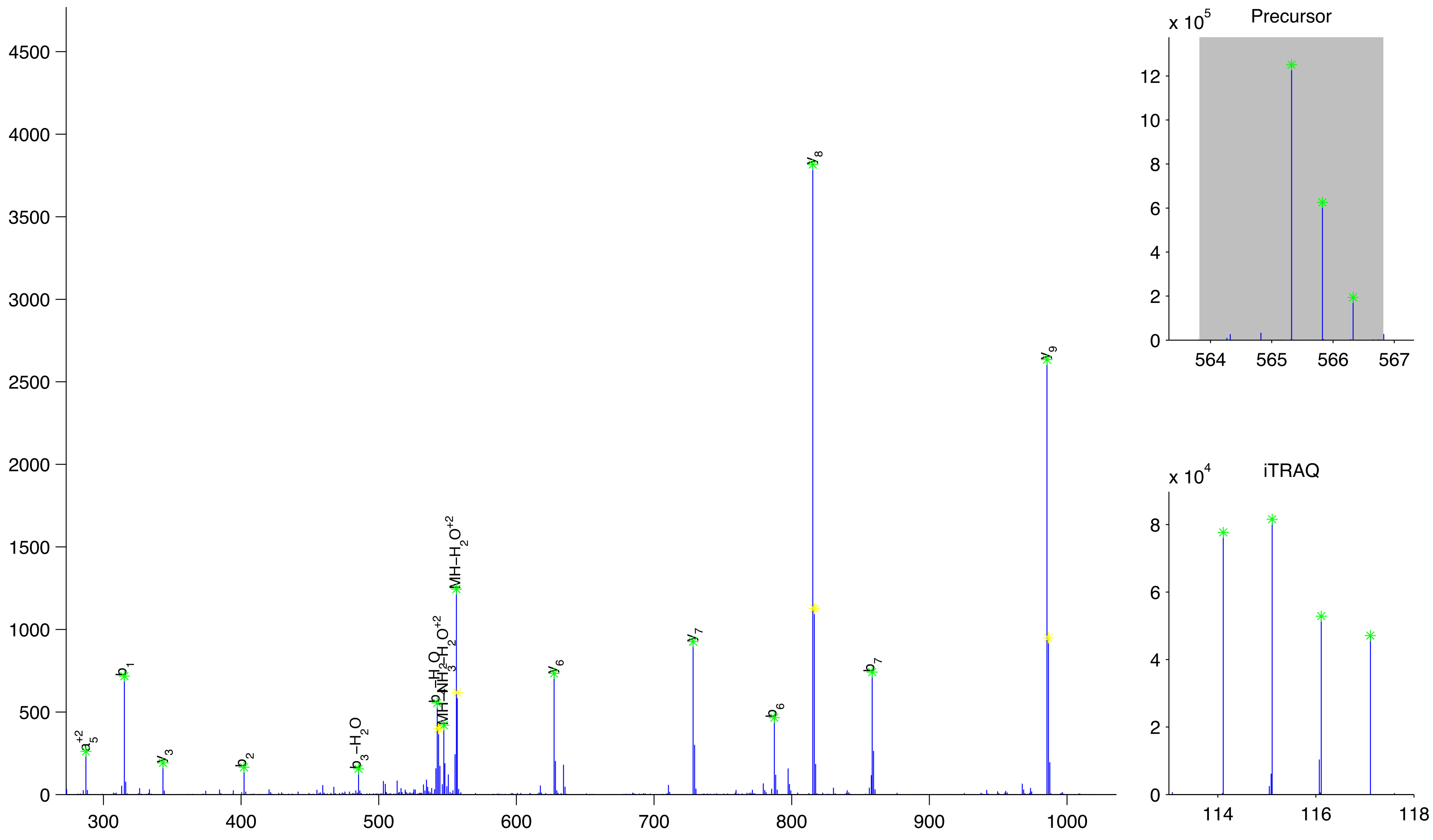

k S T G G k A P R

H3 histone, family 3A [Homo sapiens]
Charge State: +2
Scan Number: 9837
File Name: 110924_HG2_INSA_ACK.raw

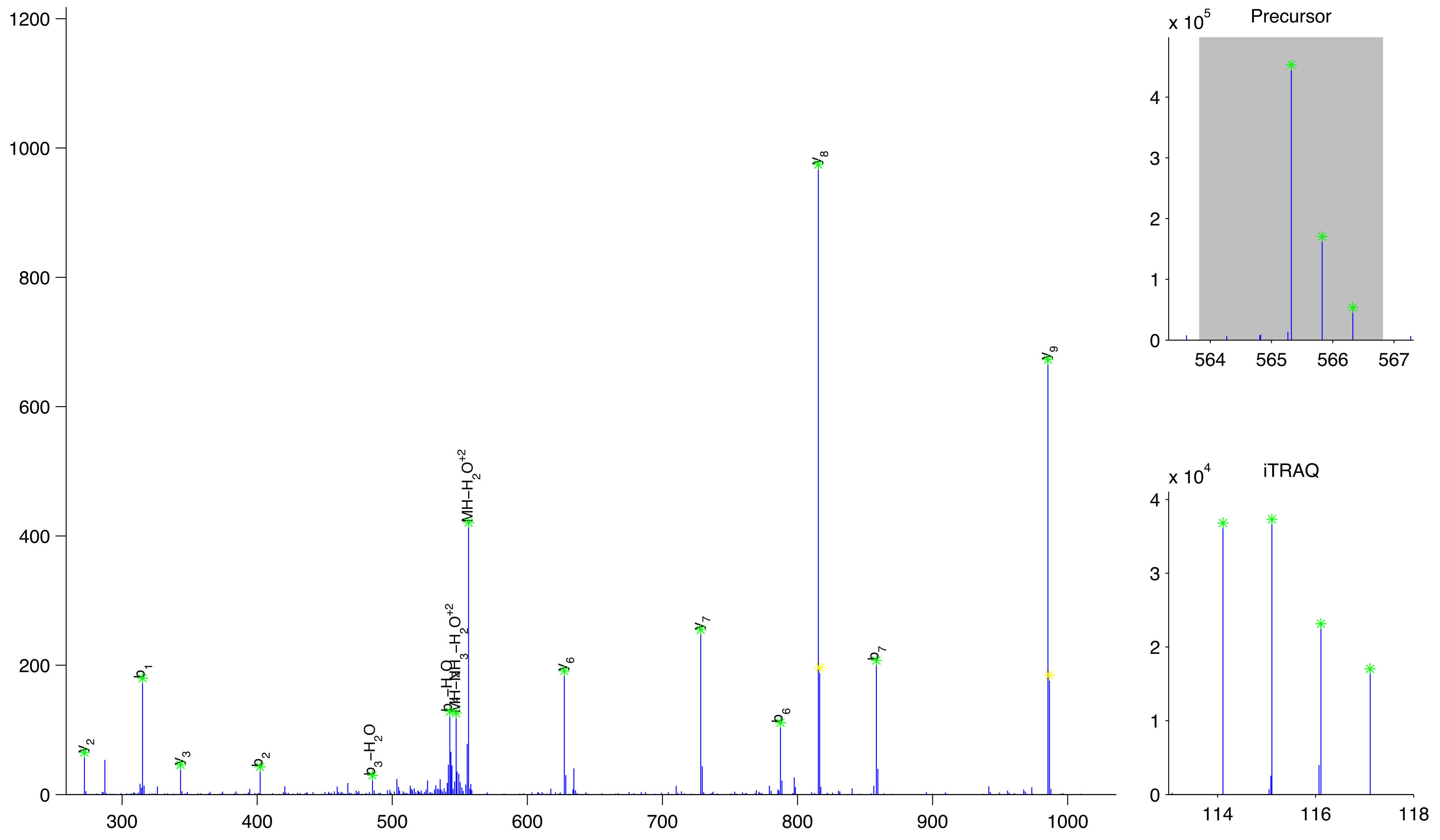

k S T G G k A P R

H3 histone, family 3A [Homo sapiens]
Charge State: +2
Scan Number: 10003
File Name: 110924_HG2_INSA_ACK.raw

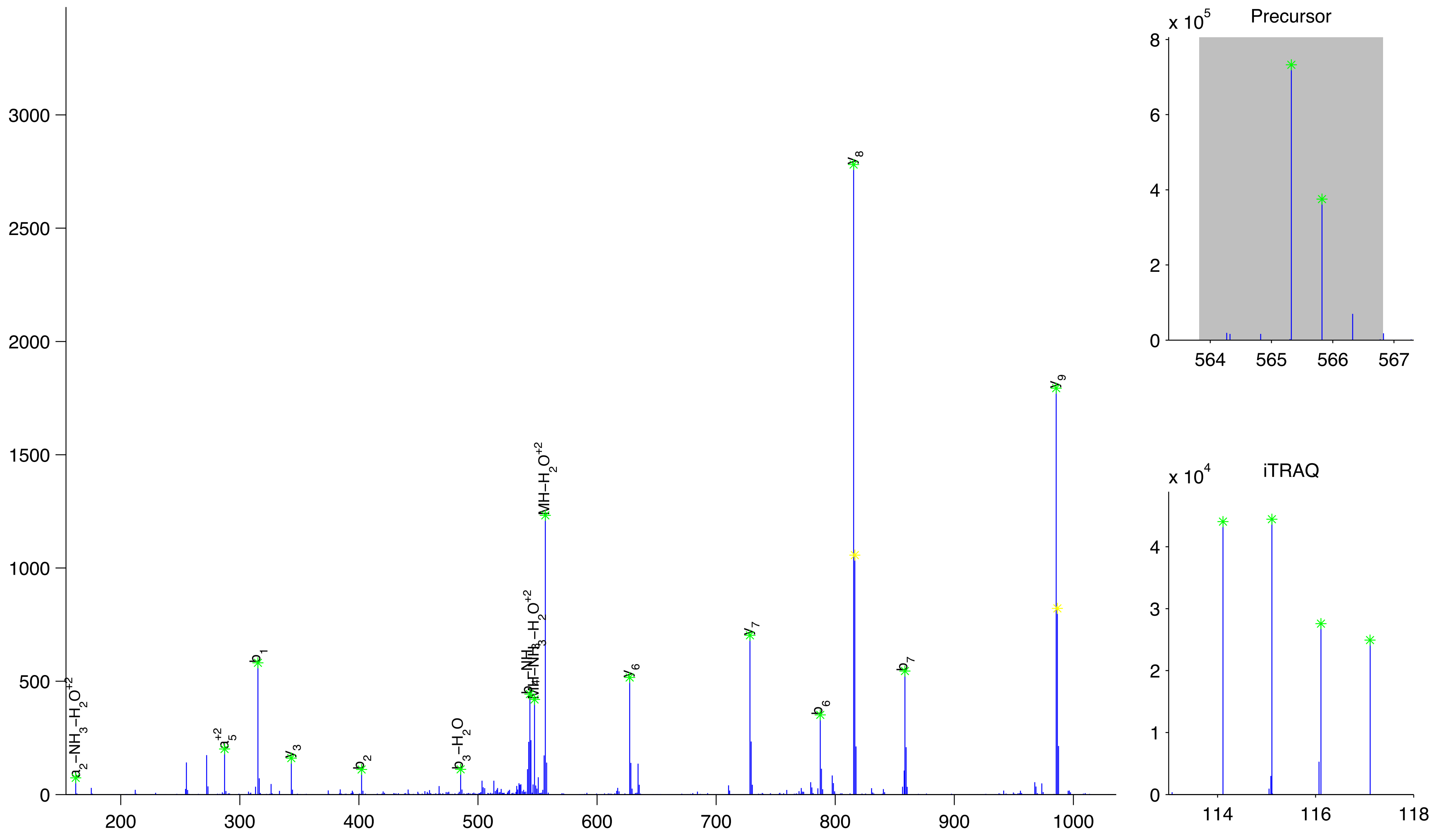

k S T G G k A P R

H3 histone, family 3A [Homo sapiens]
Charge State: +2
Scan Number: 10167
File Name: 110924_HG2_INSA_ACK.raw

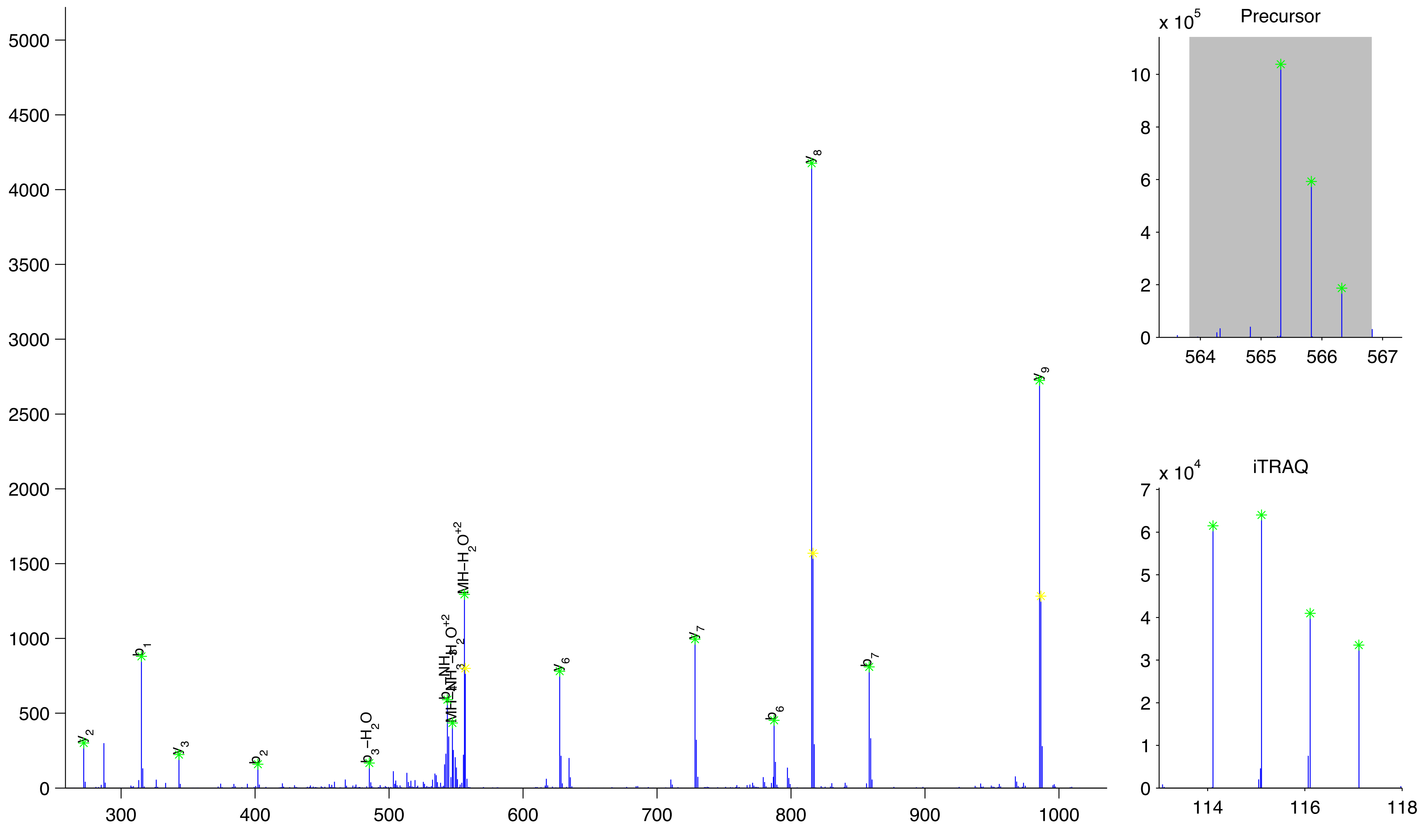

k S T G G k A P R

H3 histone, family 3A [Homo sapiens]
Charge State: +2
Scan Number: 10211
File Name: 110924_HG2_INSA_ACK.raw

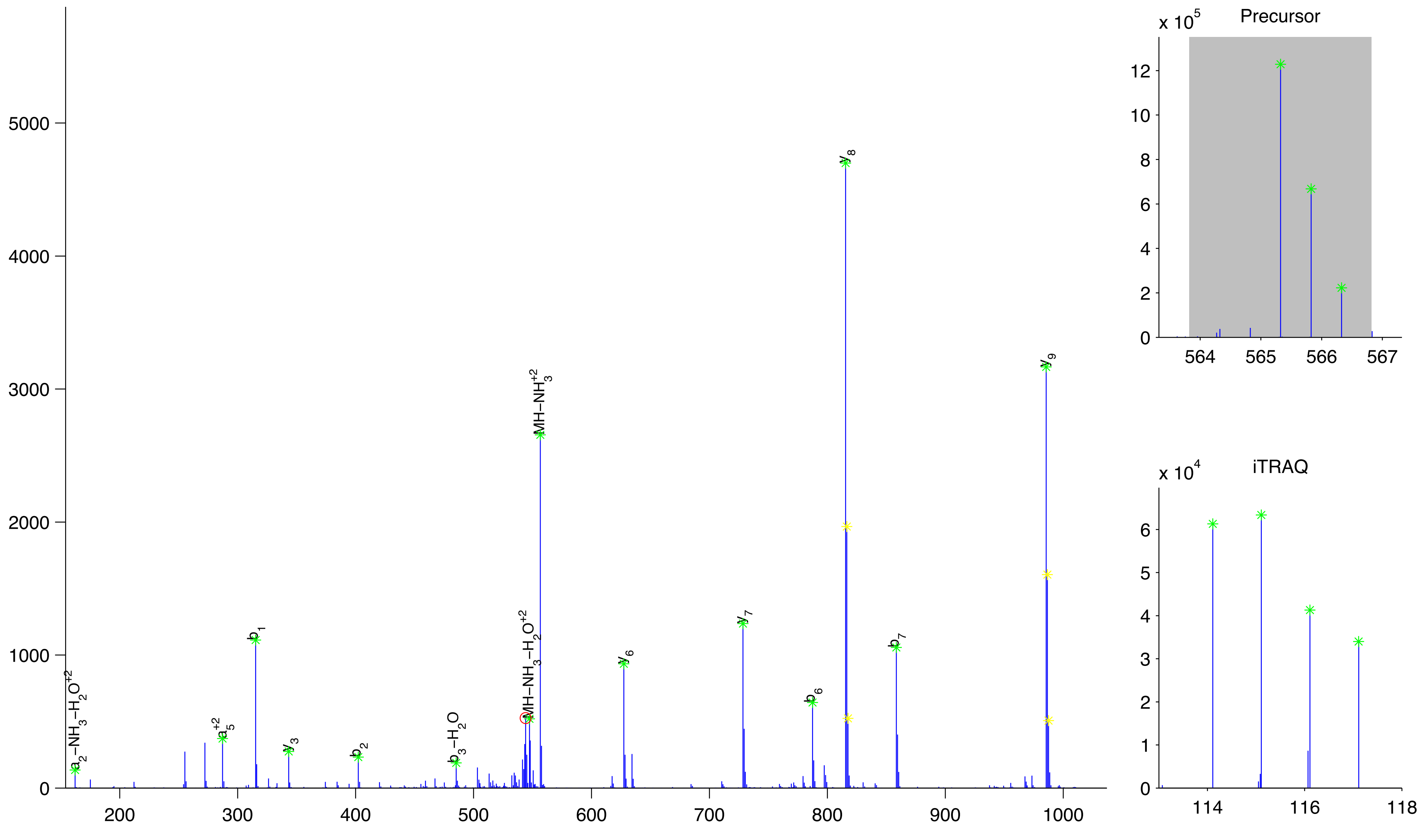

k S T G G k A P R

H3 histone, family 3A [Homo sapiens]
Charge State: +2
Scan Number: 10335
File Name: 110924_HG2_INSA_ACK.raw

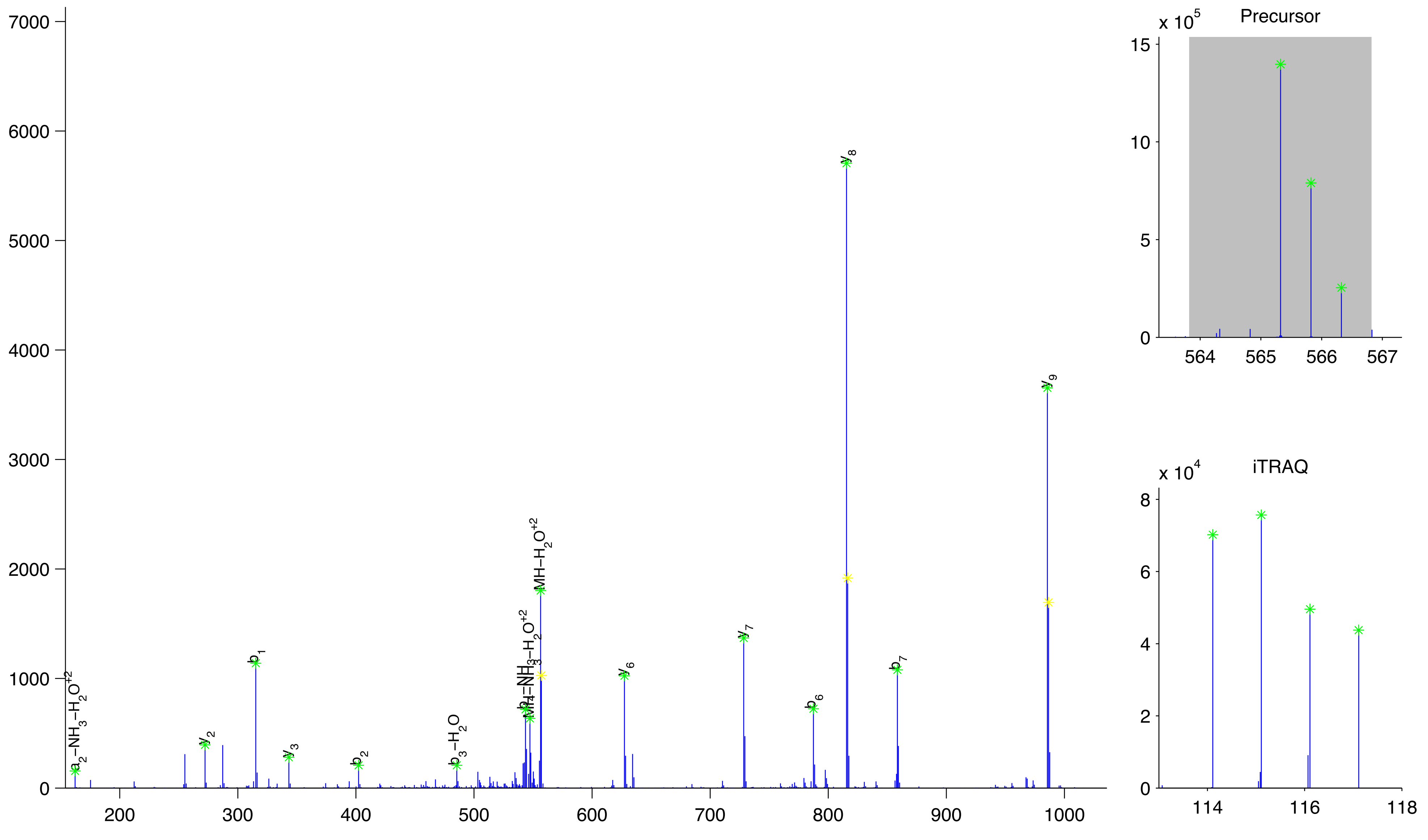

k S T G G k A P R

H3 histone, family 3A [Homo sapiens]
Charge State: +2
Scan Number: 10377
File Name: 110924_HG2_INSA_ACK.raw

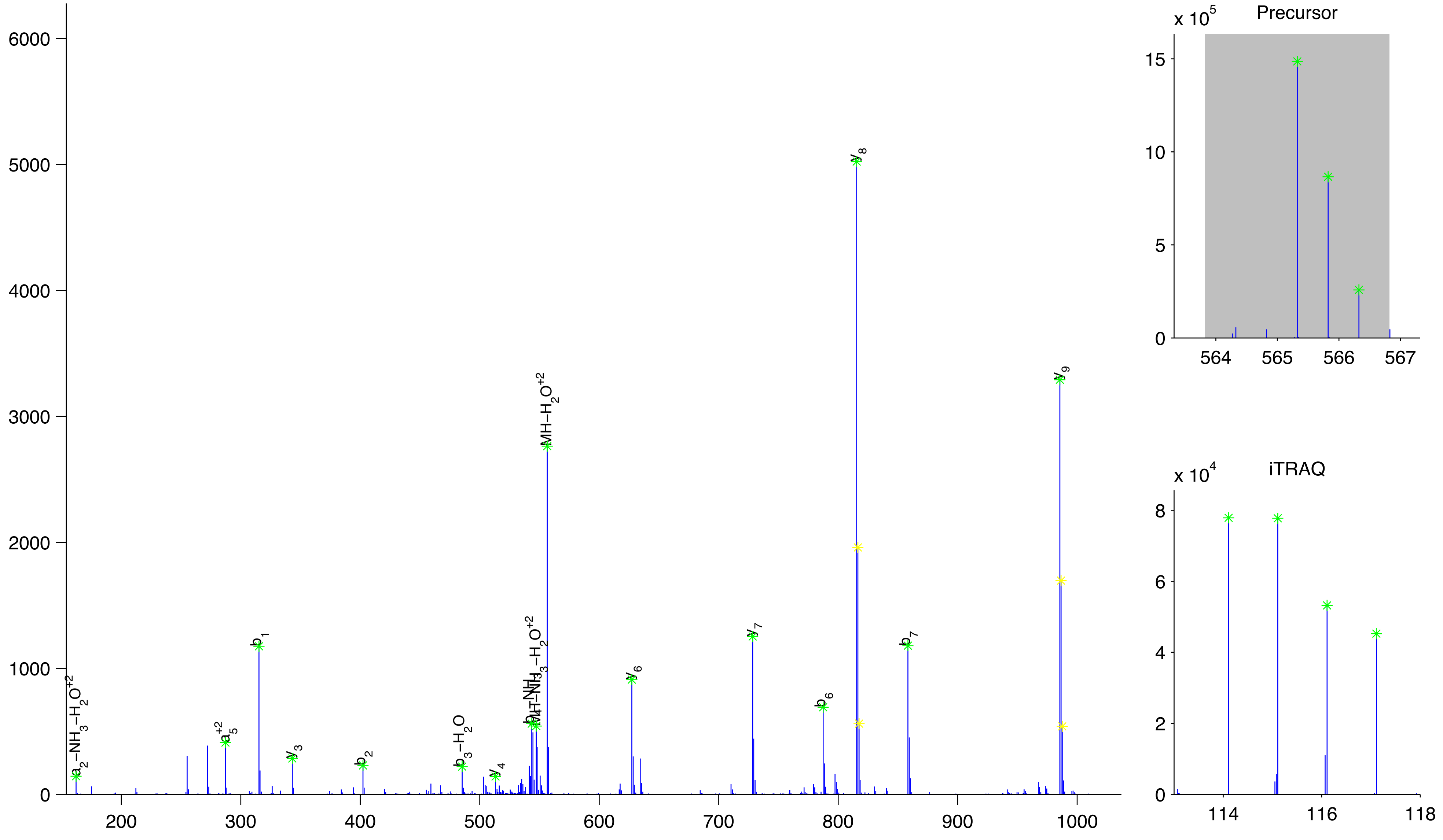

k S T G G k A P R

H3 histone, family 3A [Homo sapiens]
Charge State: +2
Scan Number: 10503
File Name: 110924_HG2_INSA_ACK.raw

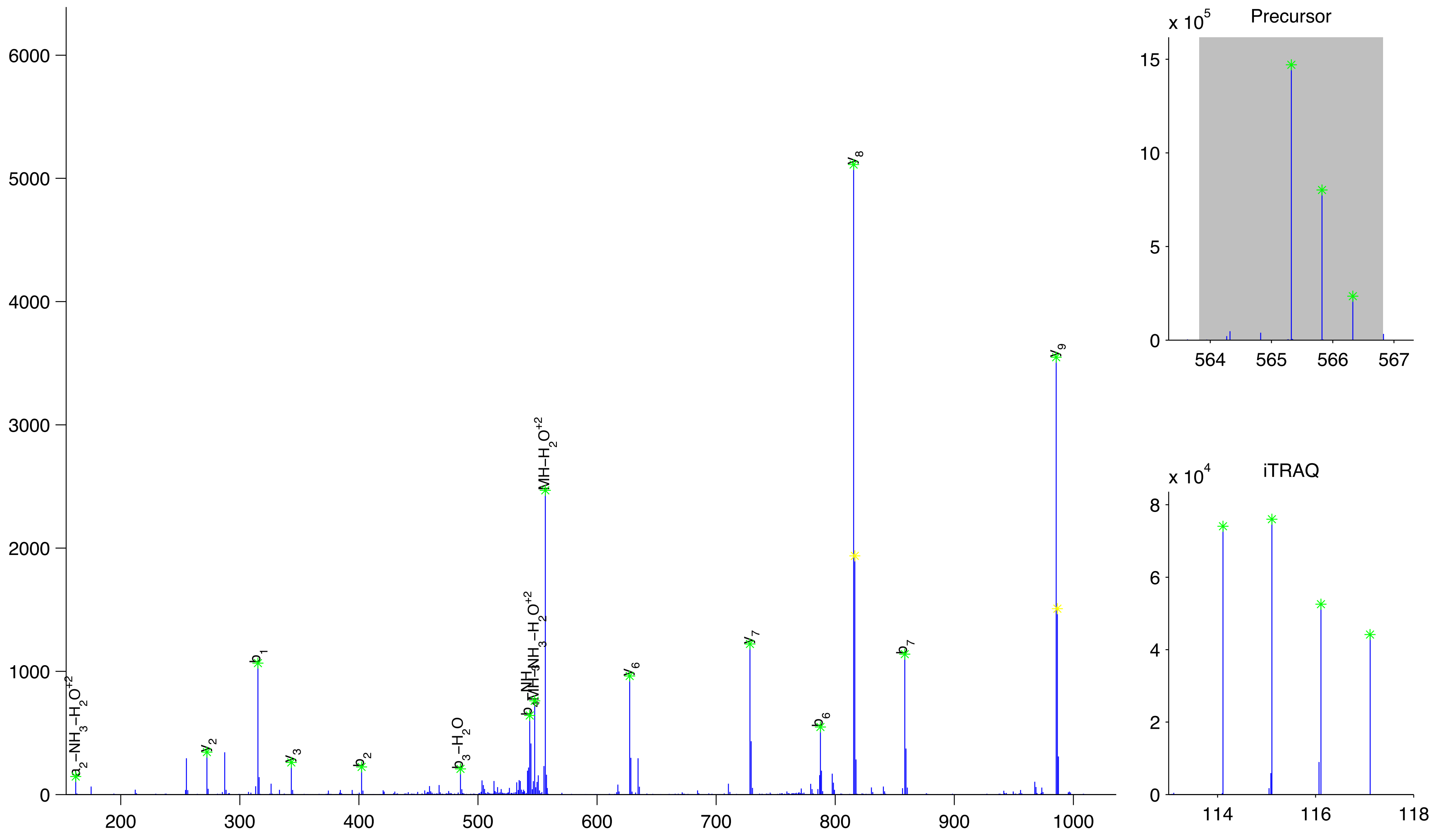

k S T G G k A P R

H3 histone, family 3A [Homo sapiens]
Charge State: +2
Scan Number: 10549
File Name: 110924_HG2_INSA_ACK.raw

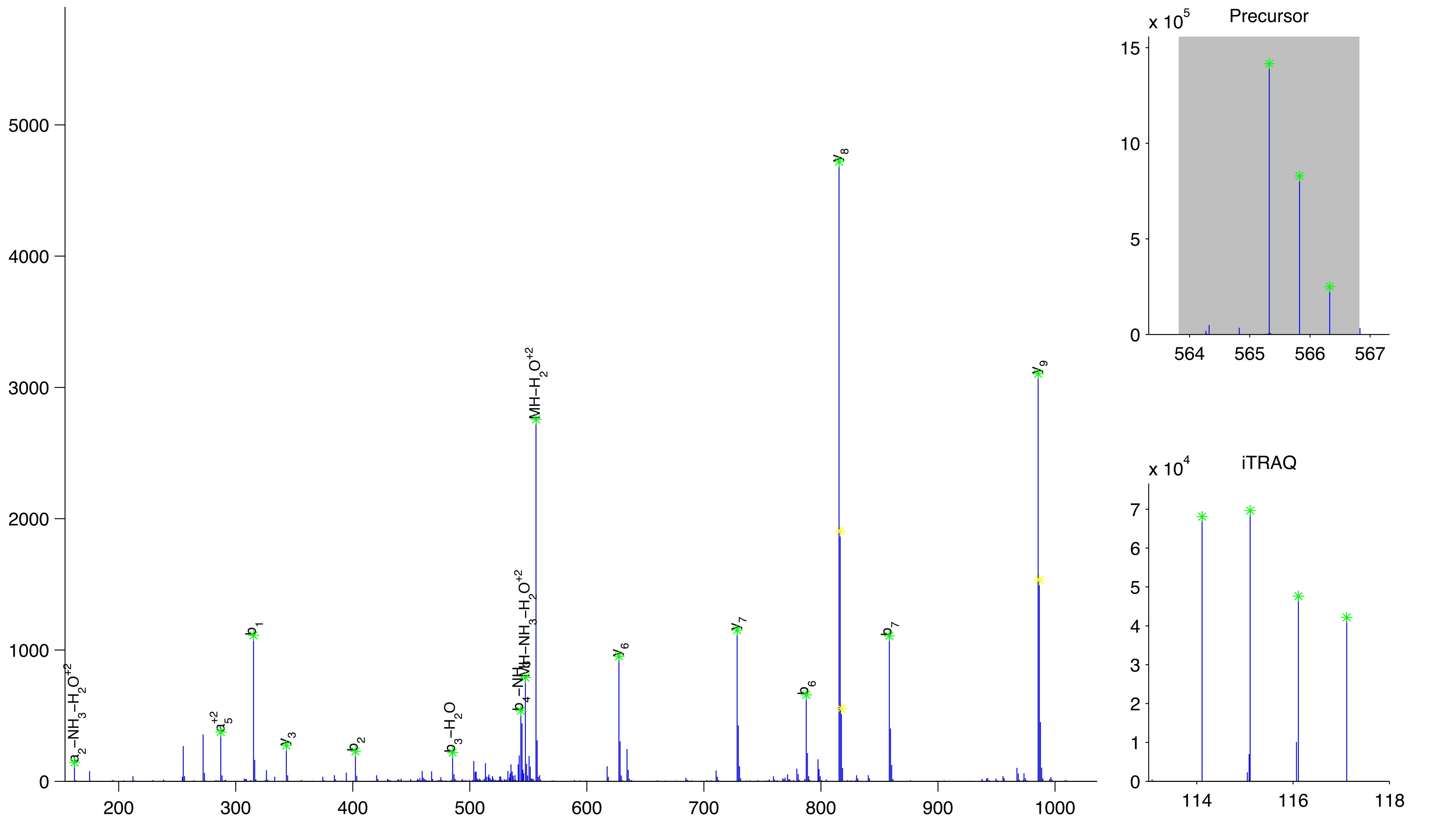

k S T G G k A P R

H3 histone, family 3A [Homo sapiens]
Charge State: +2
Scan Number: 10673
File Name: 110924_HG2_INSA_ACK.raw

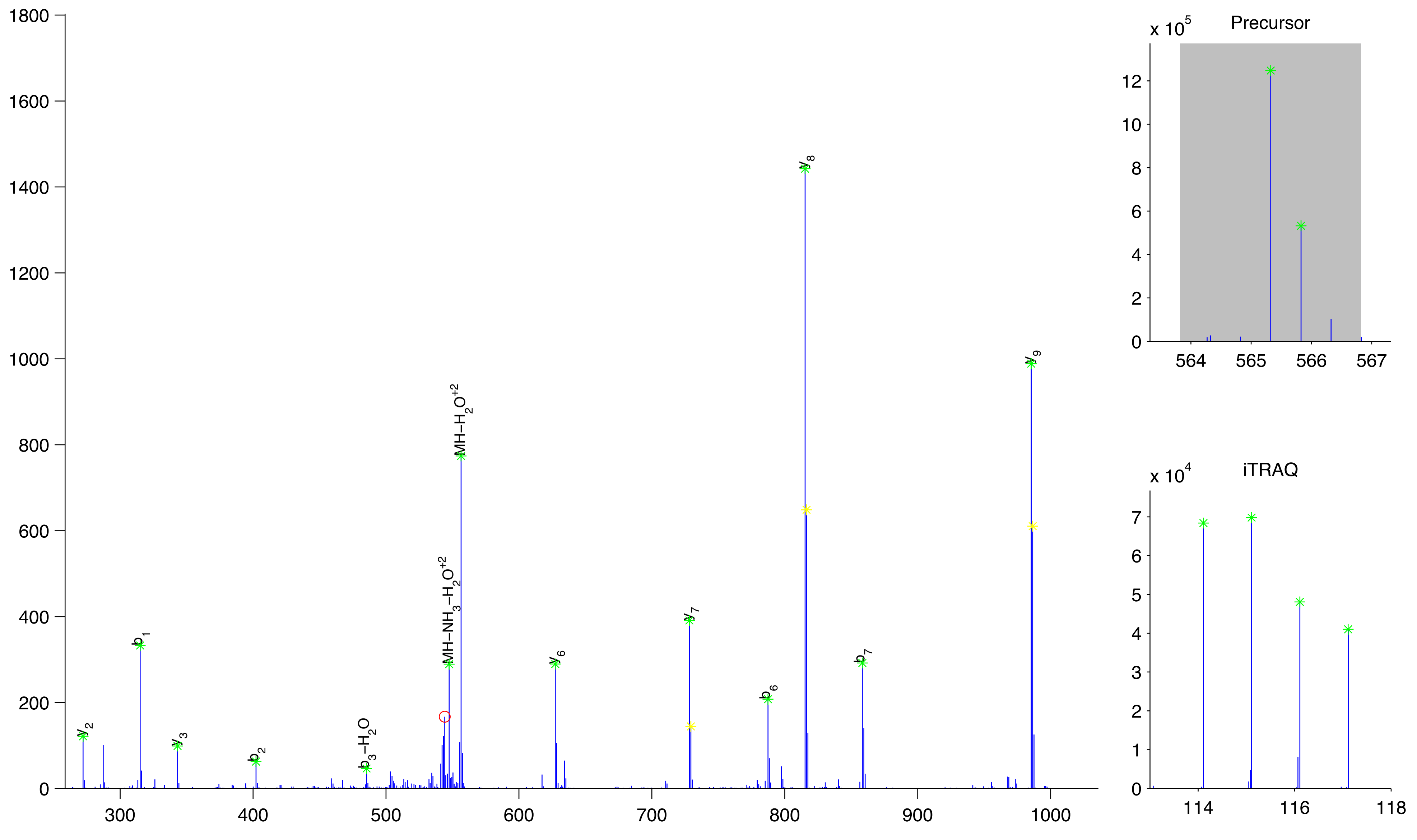

k S T G G k A P R

H3 histone, family 3A [Homo sapiens]
Charge State: +2
Scan Number: 10723
File Name: 110924_HG2_INSA_ACK.raw

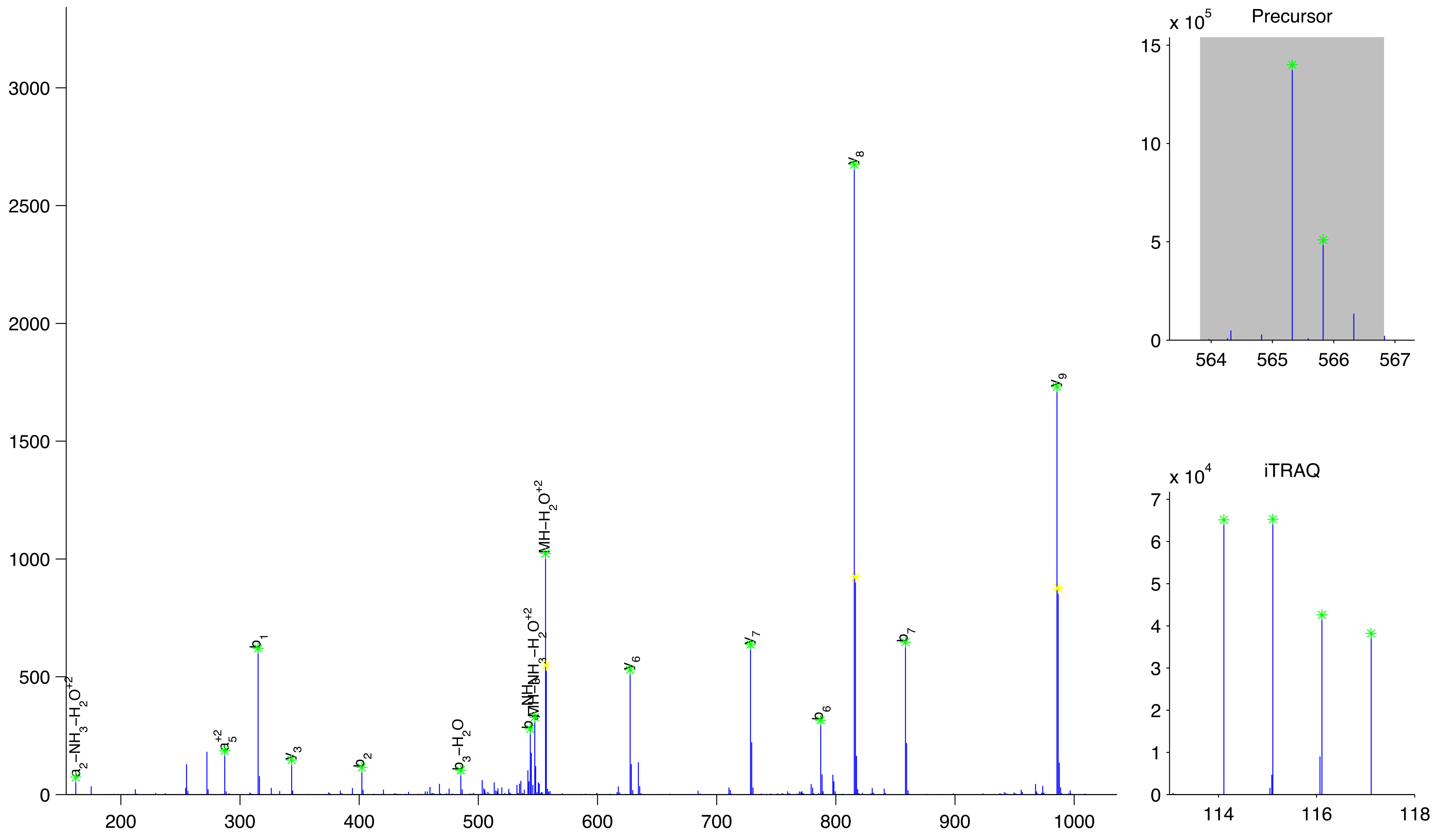

k S T G G k A P R

H3 histone, family 3A [Homo sapiens]
Charge State: +2
Scan Number: 10841
File Name: 110924_HG2_INSA_ACK.raw

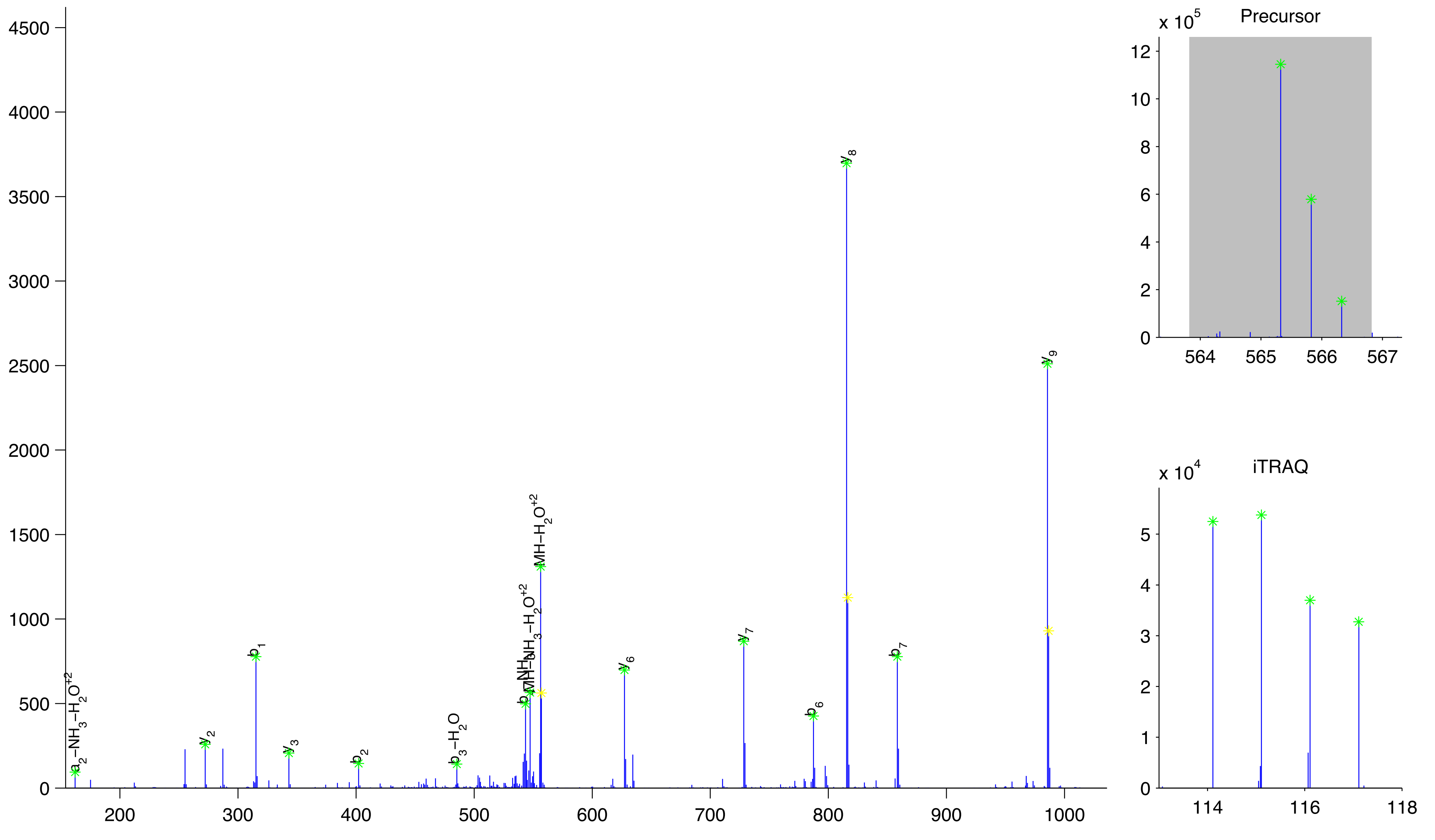

k S T G G k A P R

H3 histone, family 3A [Homo sapiens]
Charge State: +2
Scan Number: 10891
File Name: 110924_HG2_INSA_ACK.raw

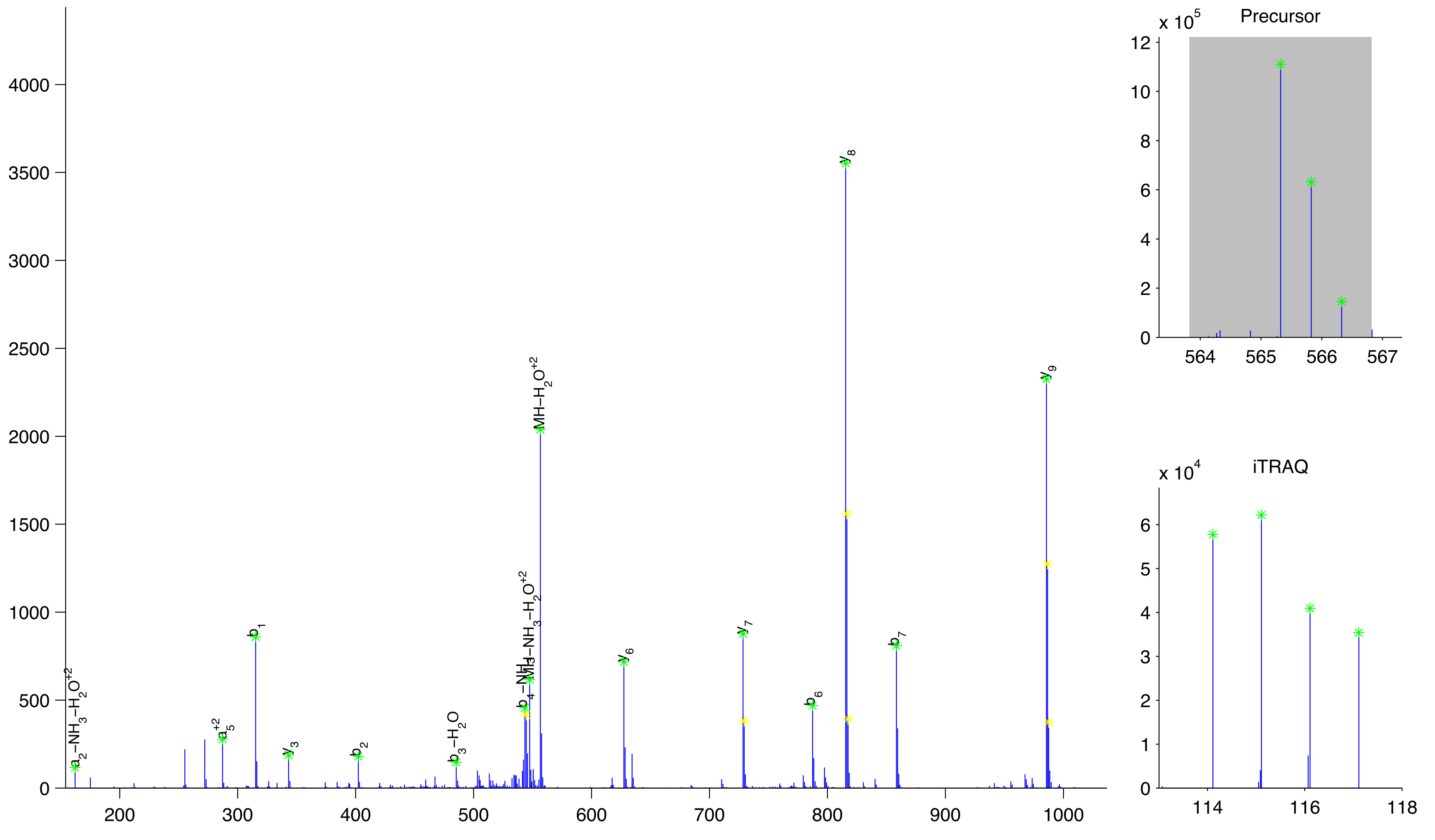

Q L A T k A A R

H3 histone, family 3A [Homo sapiens]
Charge State: +2
Scan Number: 10965
File Name: 110924_HG2_INSA_ACK.raw

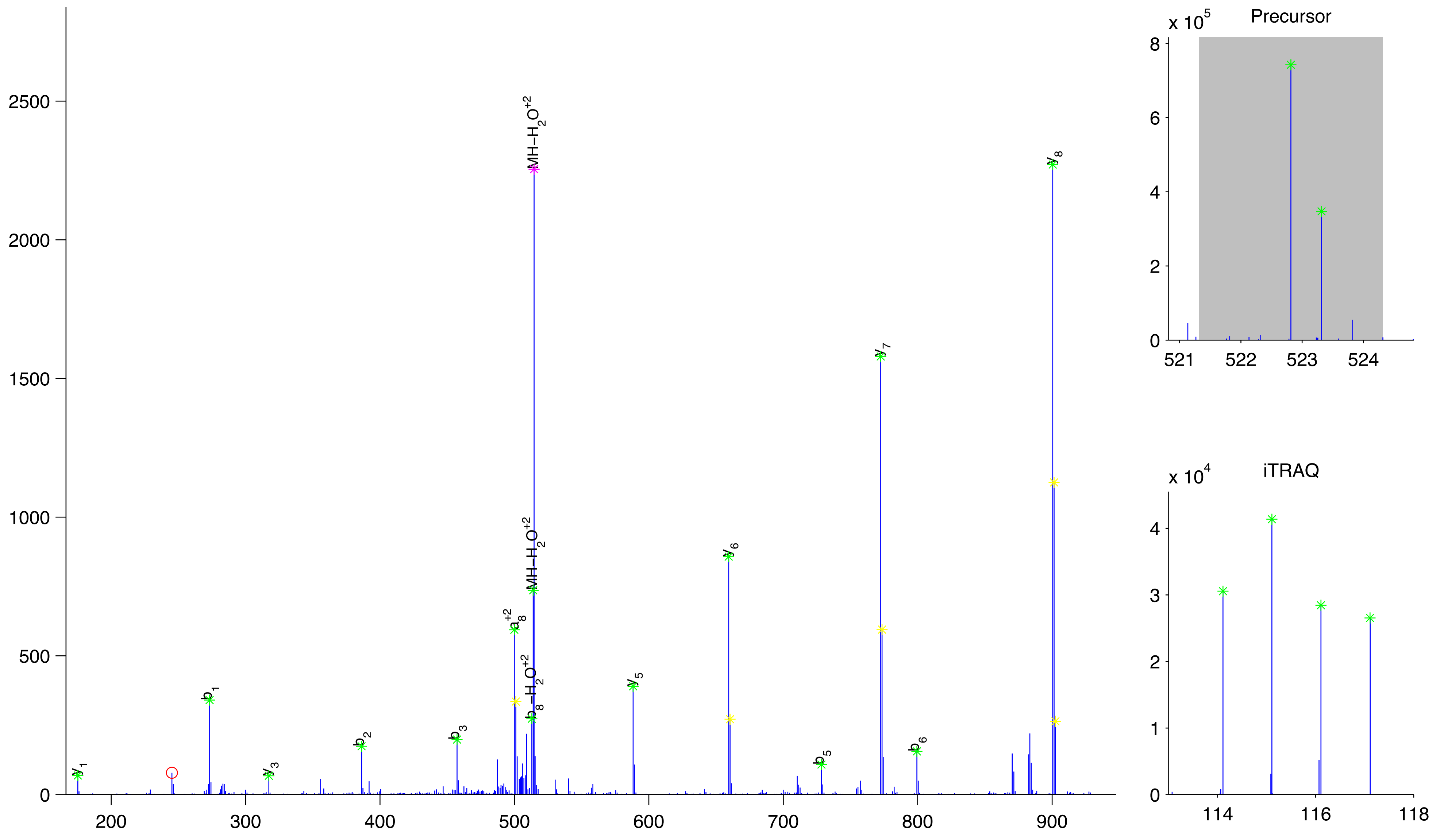

Q L A T k A A R

H3 histone, family 3A [Homo sapiens]
Charge State: +2
Scan Number: 10981
File Name: 110924_HG2_INSA_ACK.raw

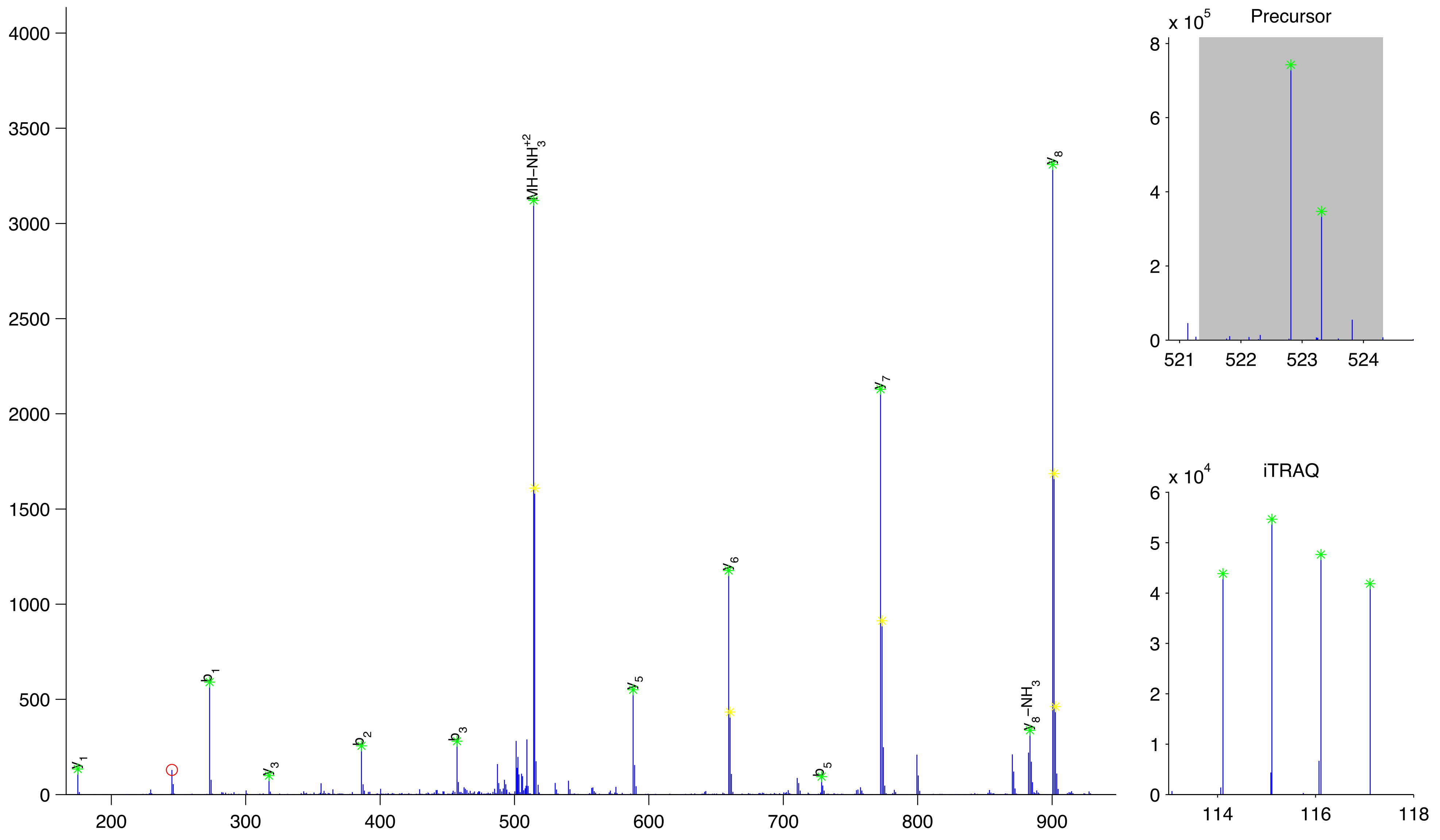

k S T G G k A P R

H3 histone, family 3A [Homo sapiens]
Charge State: +2
Scan Number: 11009
File Name: 110924_HG2_INSA_ACK.raw

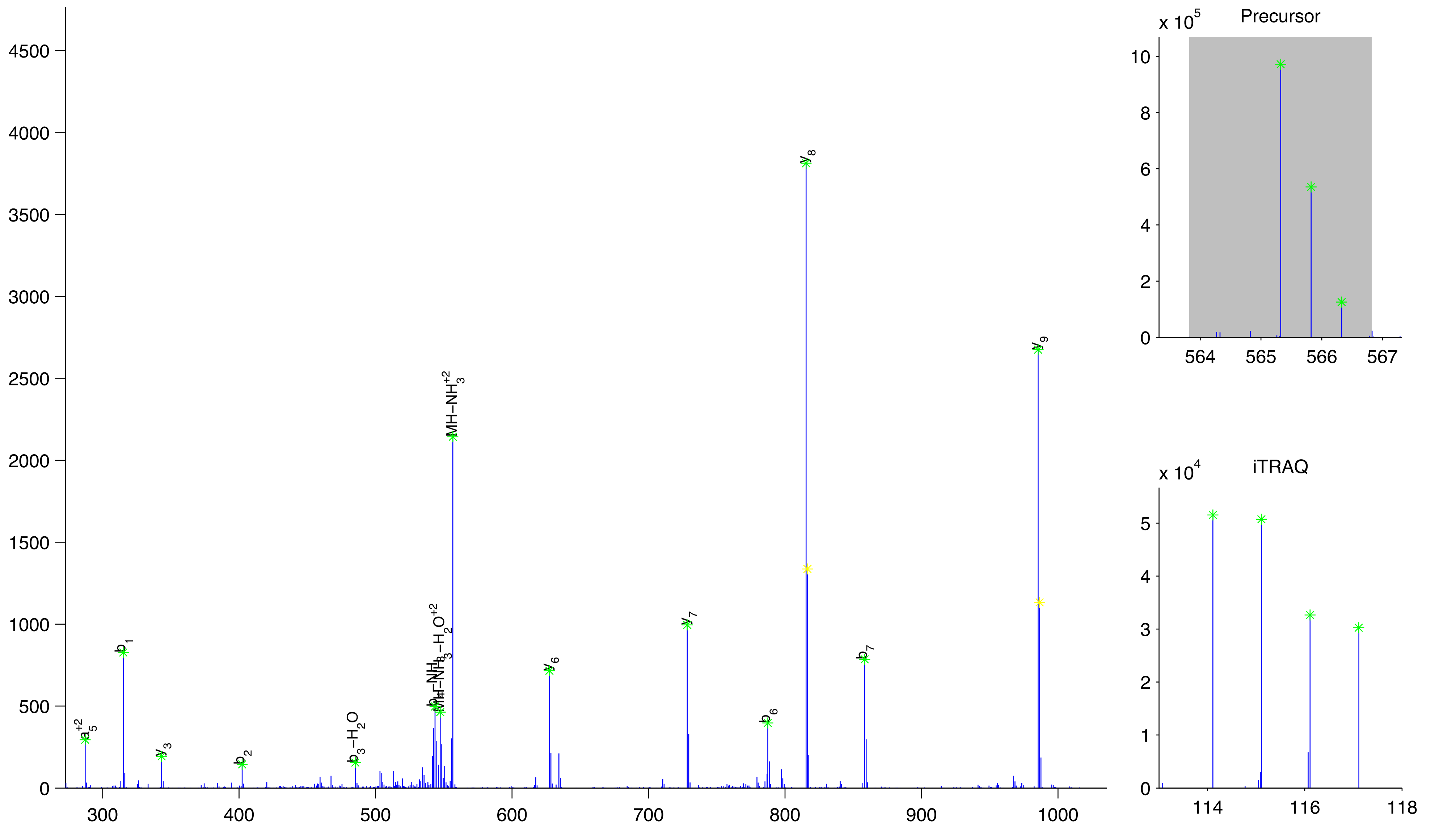

k S T G G k A P R

H3 histone, family 3A [Homo sapiens]
Charge State: +2
Scan Number: 11057
File Name: 110924_HG2_INSA_ACK.raw

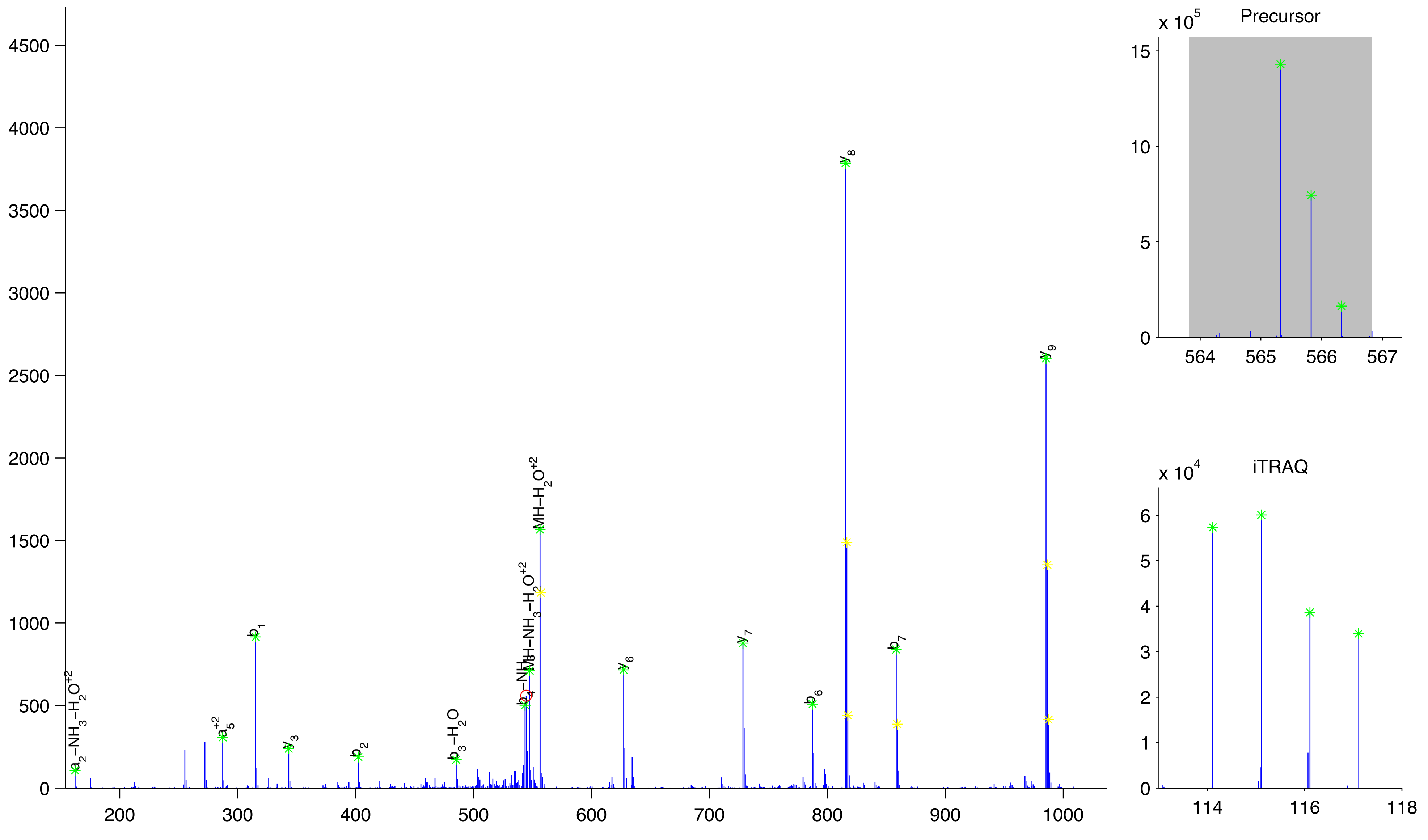

QLATkAAR

H3 histone, family 3A [Homo sapiens]
Charge State: +2
Scan Number: 11133
File Name: 110924_HG2_INSA_ACK.raw

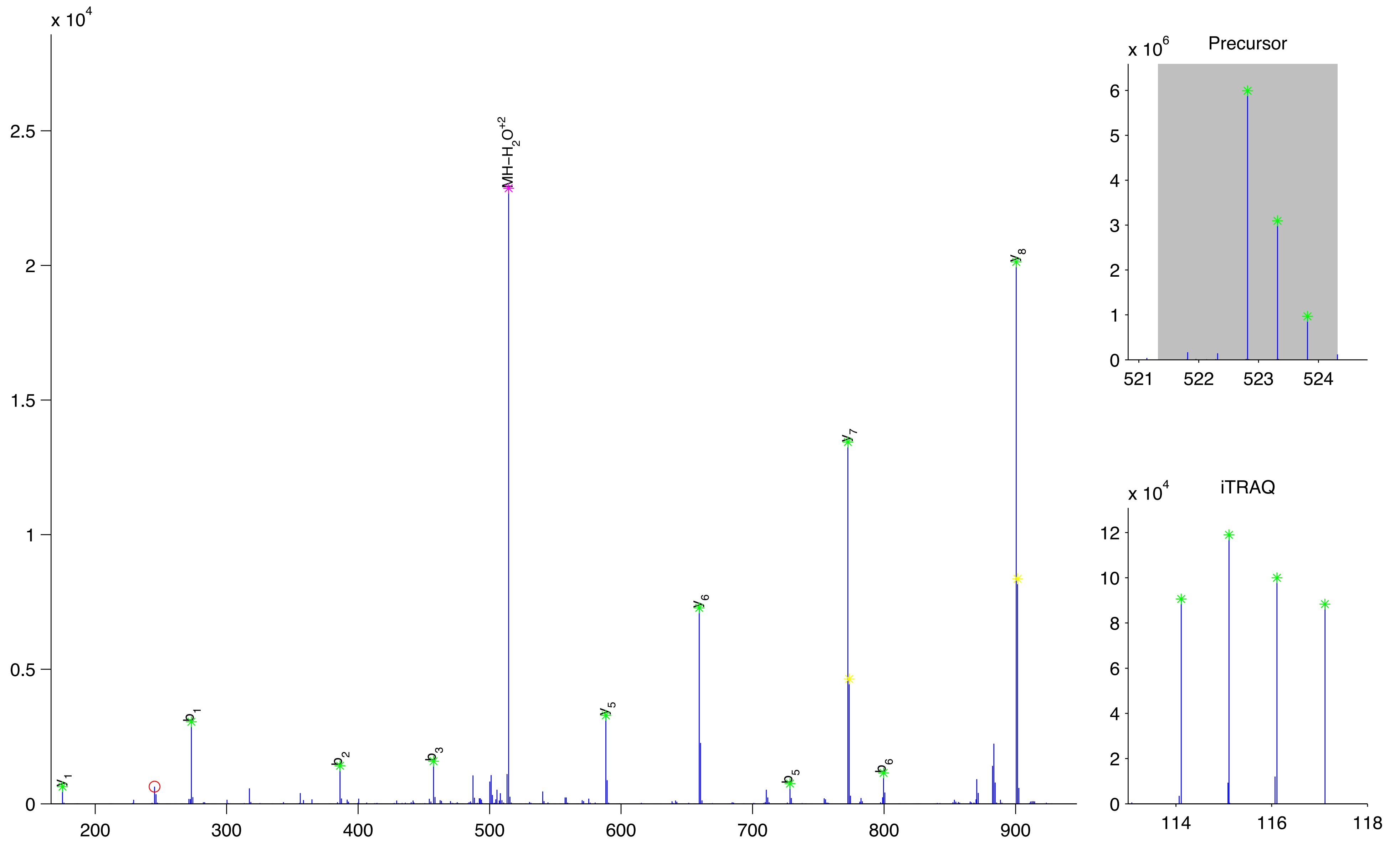

Q L A T k A A R

H3 histone, family 3A [Homo sapiens]
Charge State: +2
Scan Number: 11135
File Name: 110924_HG2_INSA_ACK.raw

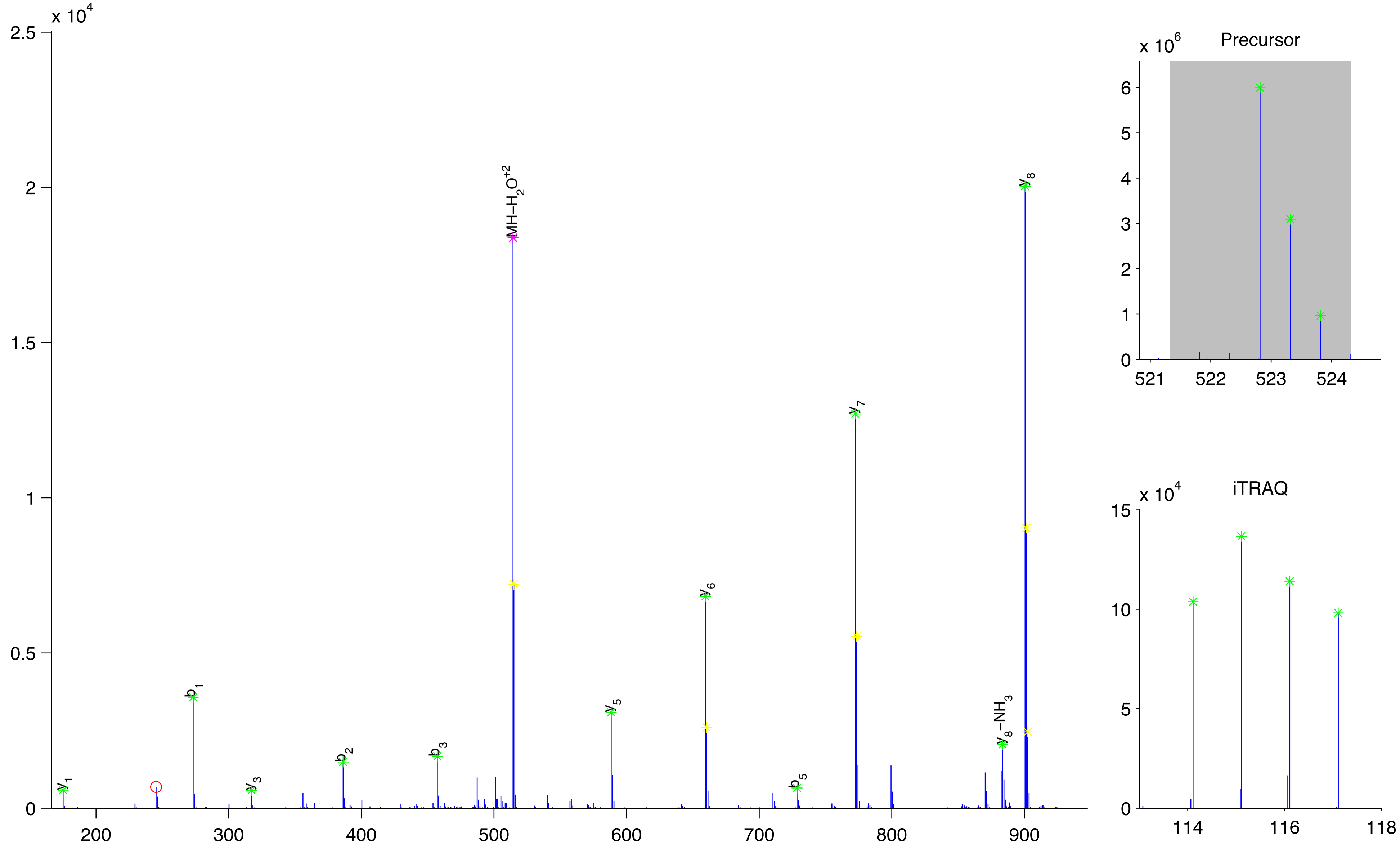

k S T G G k A P R

H3 histone, family 3A [Homo sapiens]
Charge State: +2
Scan Number: 11177
File Name: 110924_HG2_INSA_ACK.raw

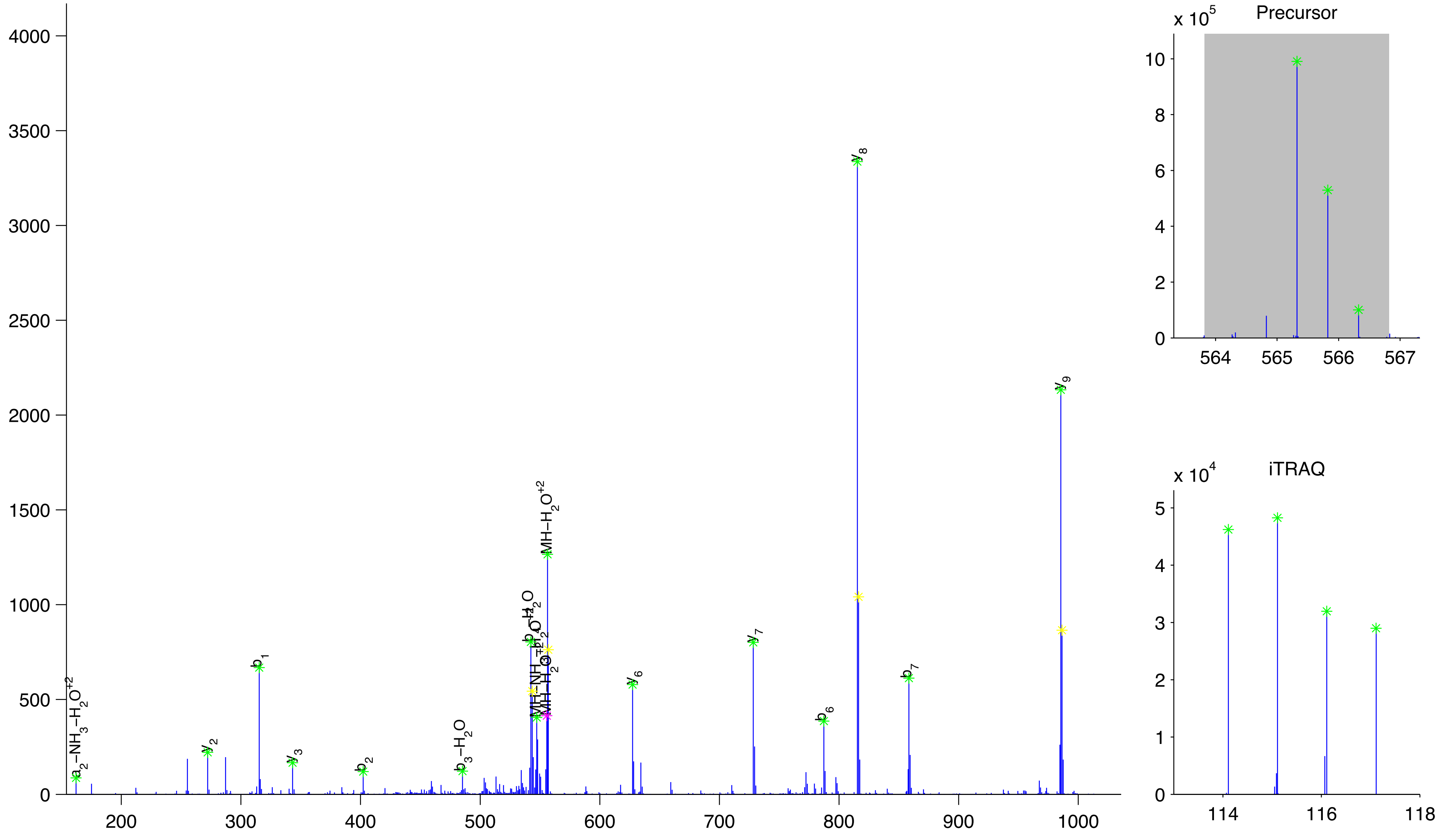

k S T G G k A P R

H3 histone, family 3A [Homo sapiens]
Charge State: +2
Scan Number: 11231
File Name: 110924_HG2_INSA_ACK.raw

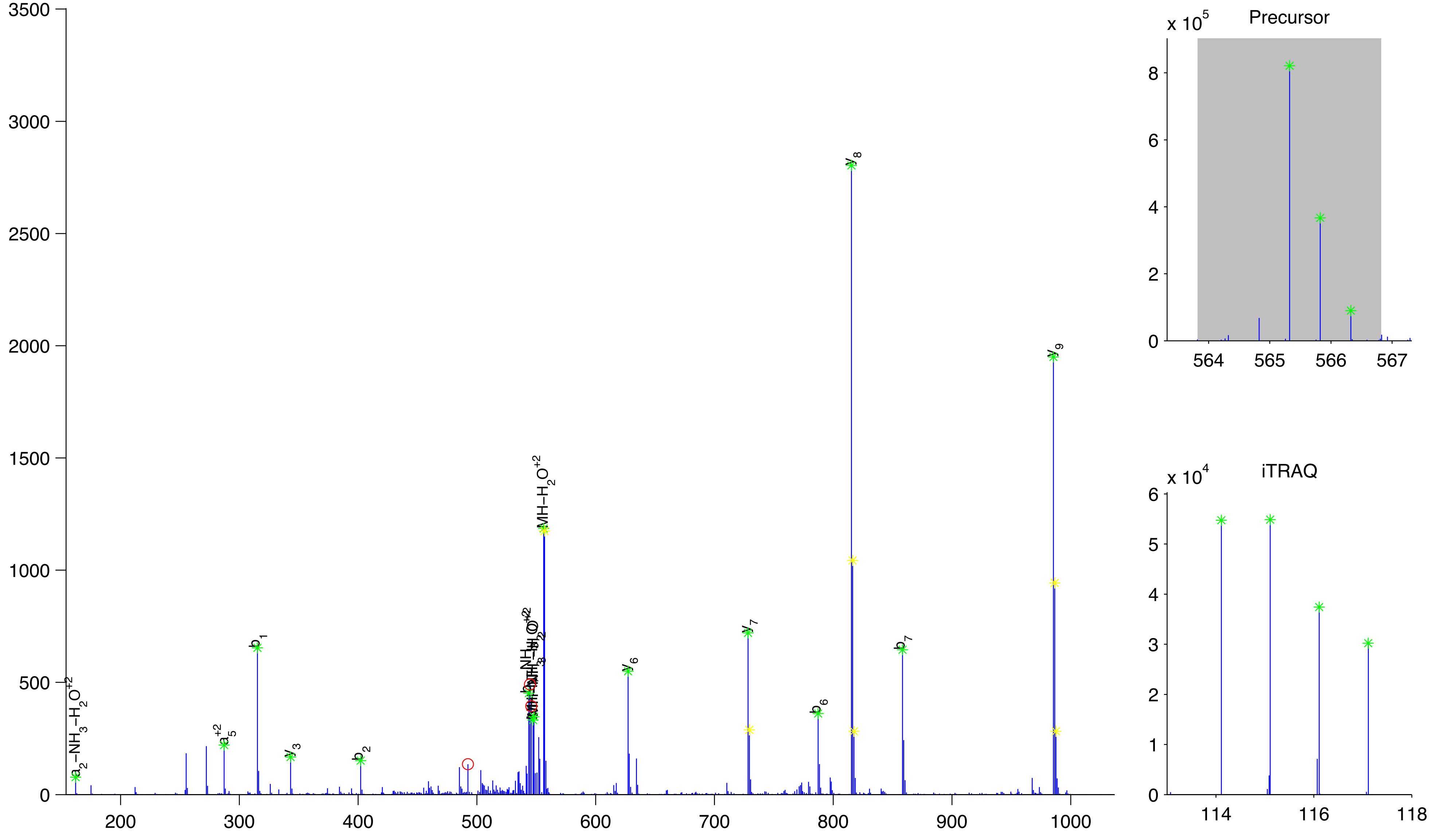

Q L A T k A A R

H3 histone, family 3A [Homo sapiens]
Charge State: +2
Scan Number: 11301
File Name: 110924_HG2_INSA_ACK.raw

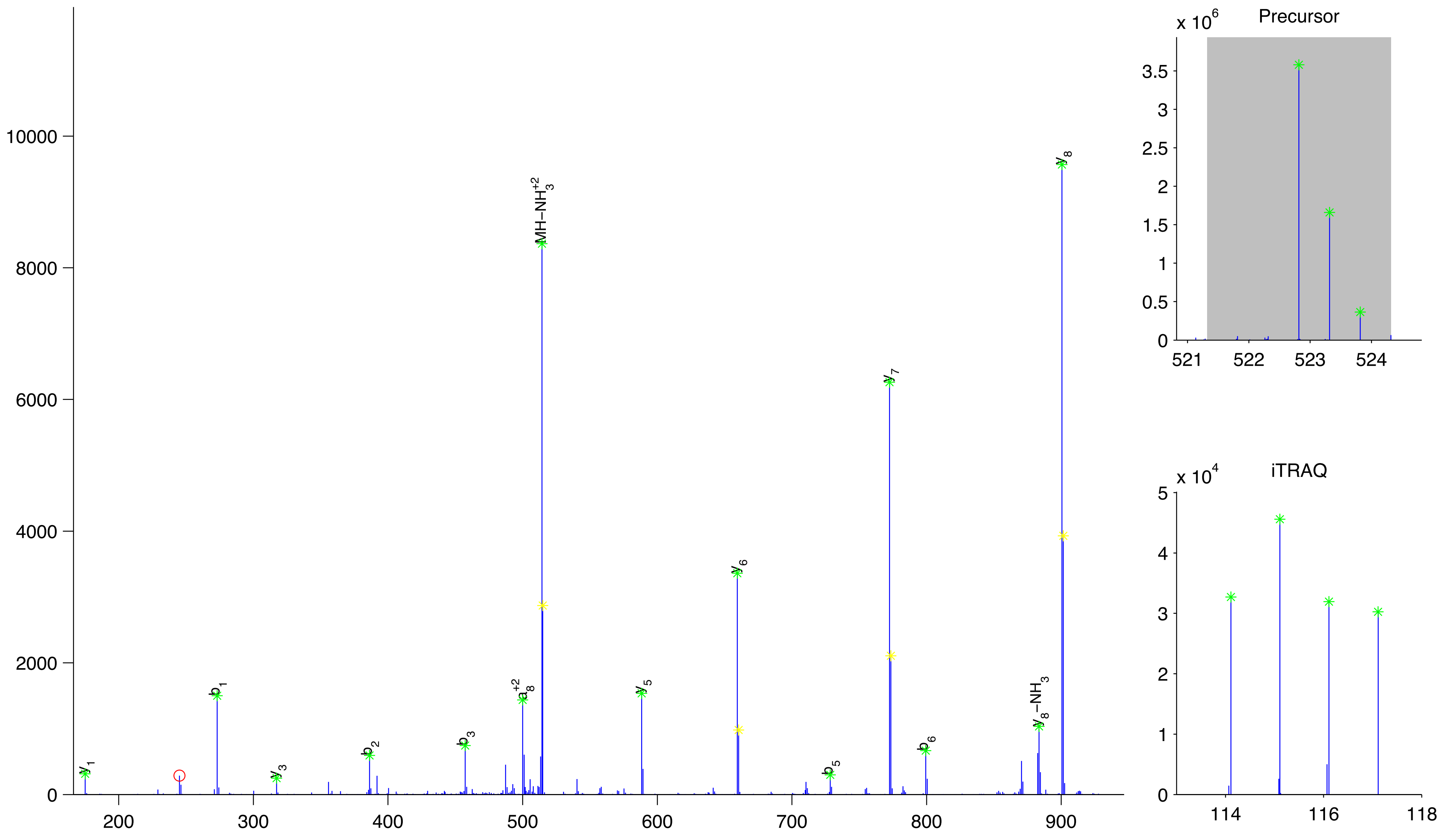

Q L A T k A A R

H3 histone, family 3A [Homo sapiens]
Charge State: +2
Scan Number: 11305
File Name: 110924_HG2_INSA_ACK.raw

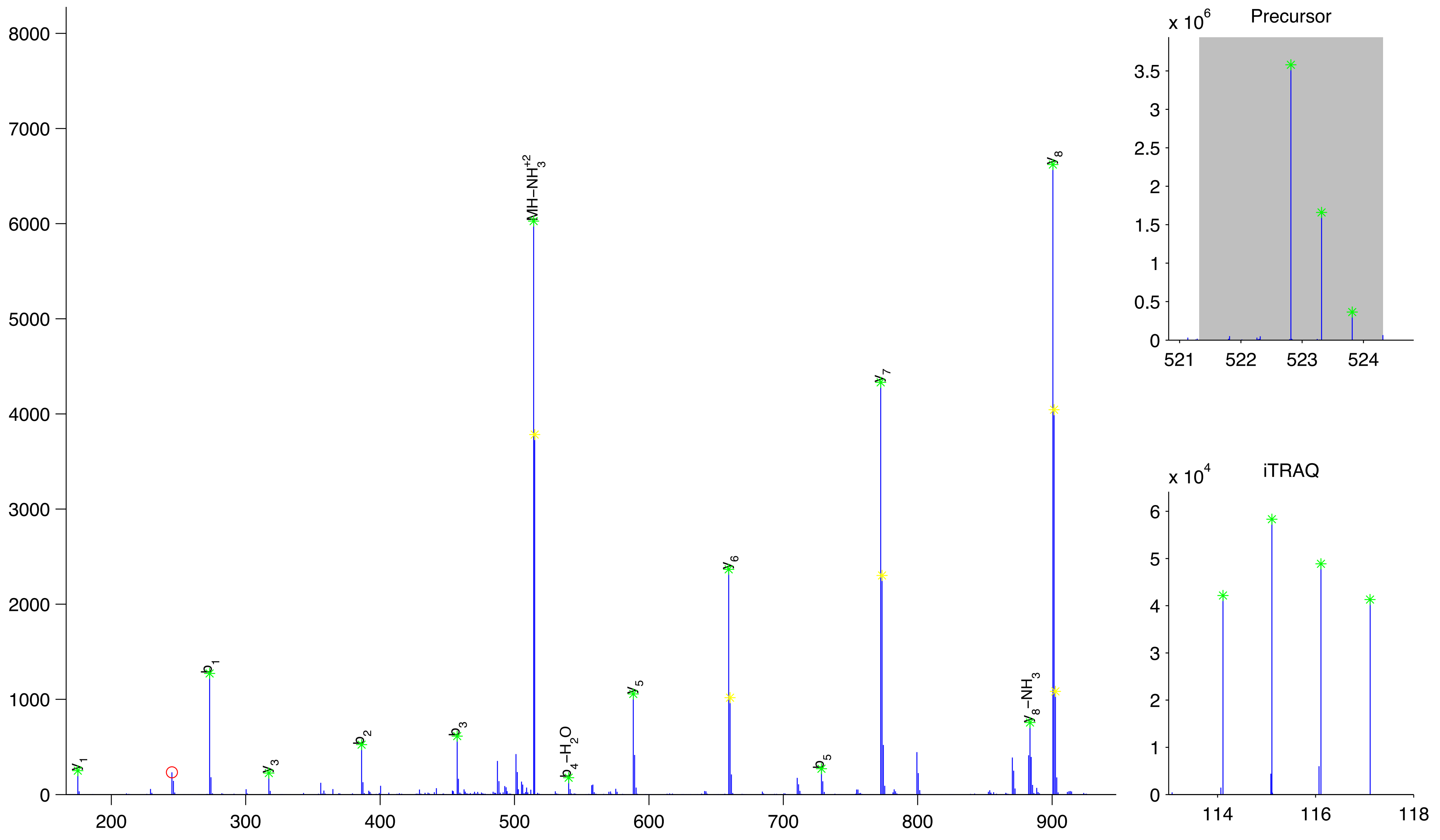

k S T G G k A P R

H3 histone, family 3A [Homo sapiens]
Charge State: +2
Scan Number: 11345
File Name: 110924_HG2_INSA_ACK.raw

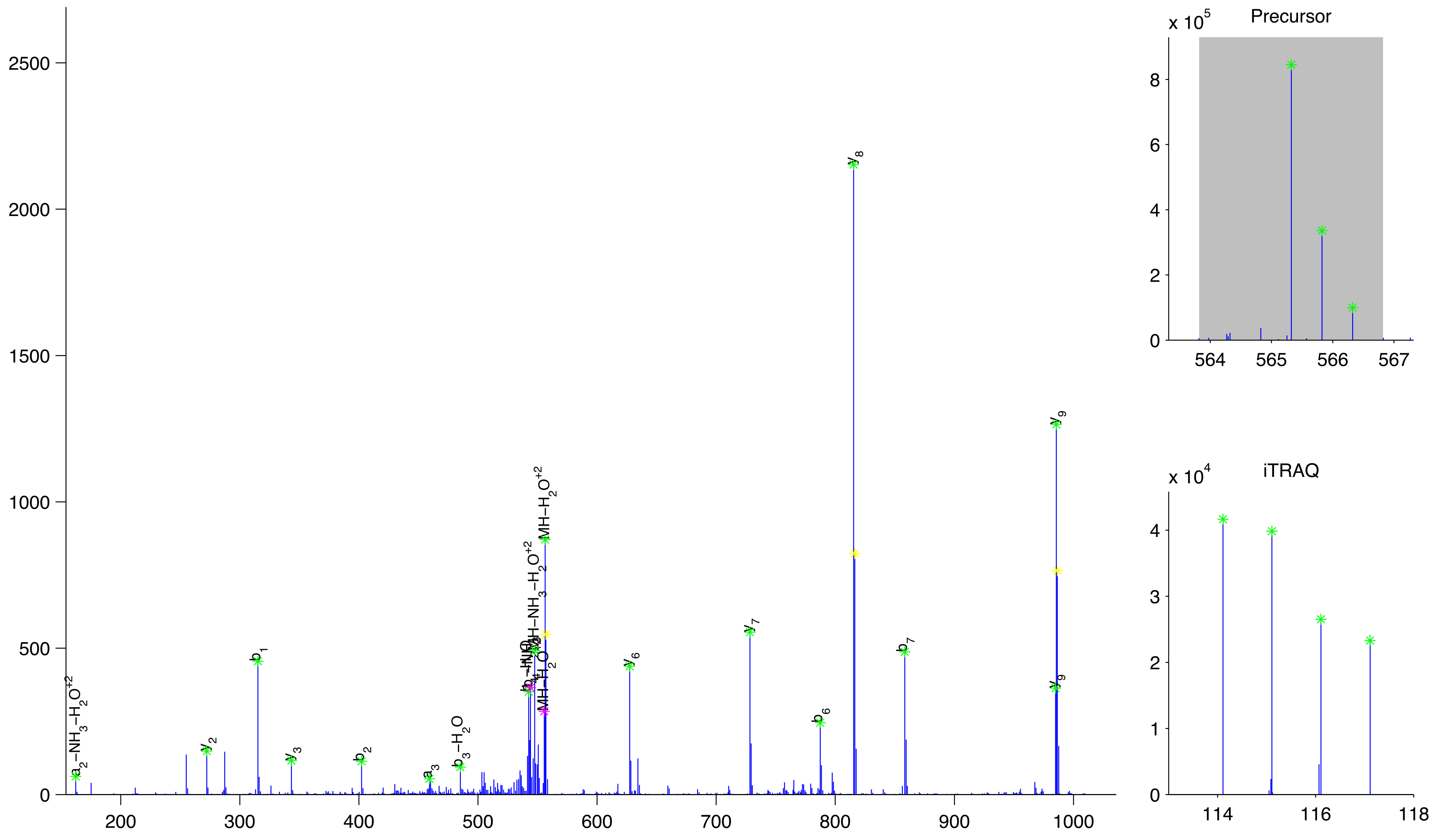

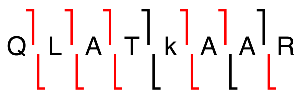

H3 histone, family 3A [Homo sapiens]
Charge State: +2
Scan Number: 11469
File Name: 110924_HG2_INSA_ACK.raw

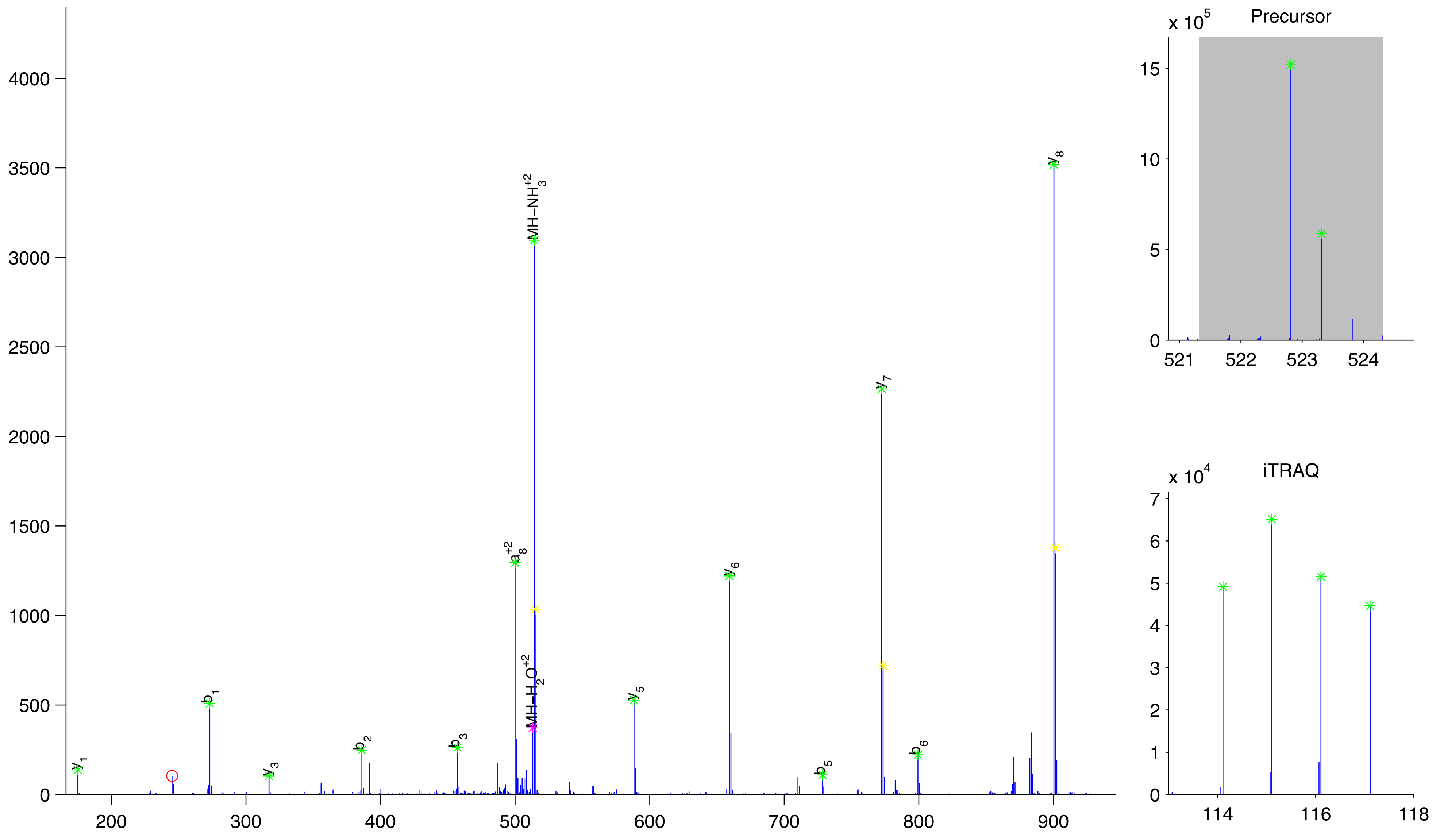

Q L A T k A A R

H3 histone, family 3A [Homo sapiens]
Charge State: +2
Scan Number: 11485
File Name: 110924_HG2_INSA_ACK.raw

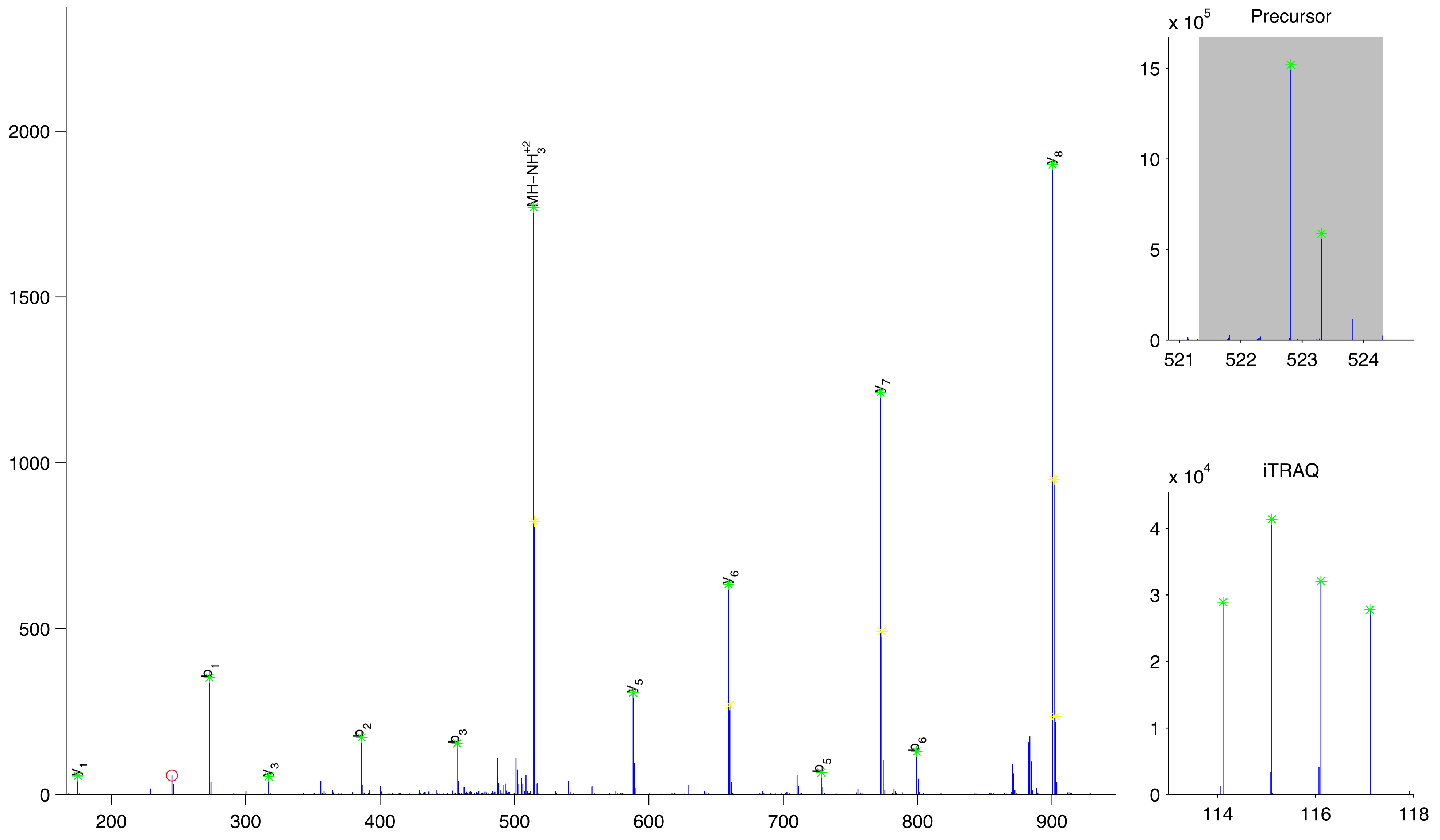

Q L A T k A A R

H3 histone, family 3A [Homo sapiens]
Charge State: +2
Scan Number: 11639
File Name: 110924_HG2_INSA_ACK.raw

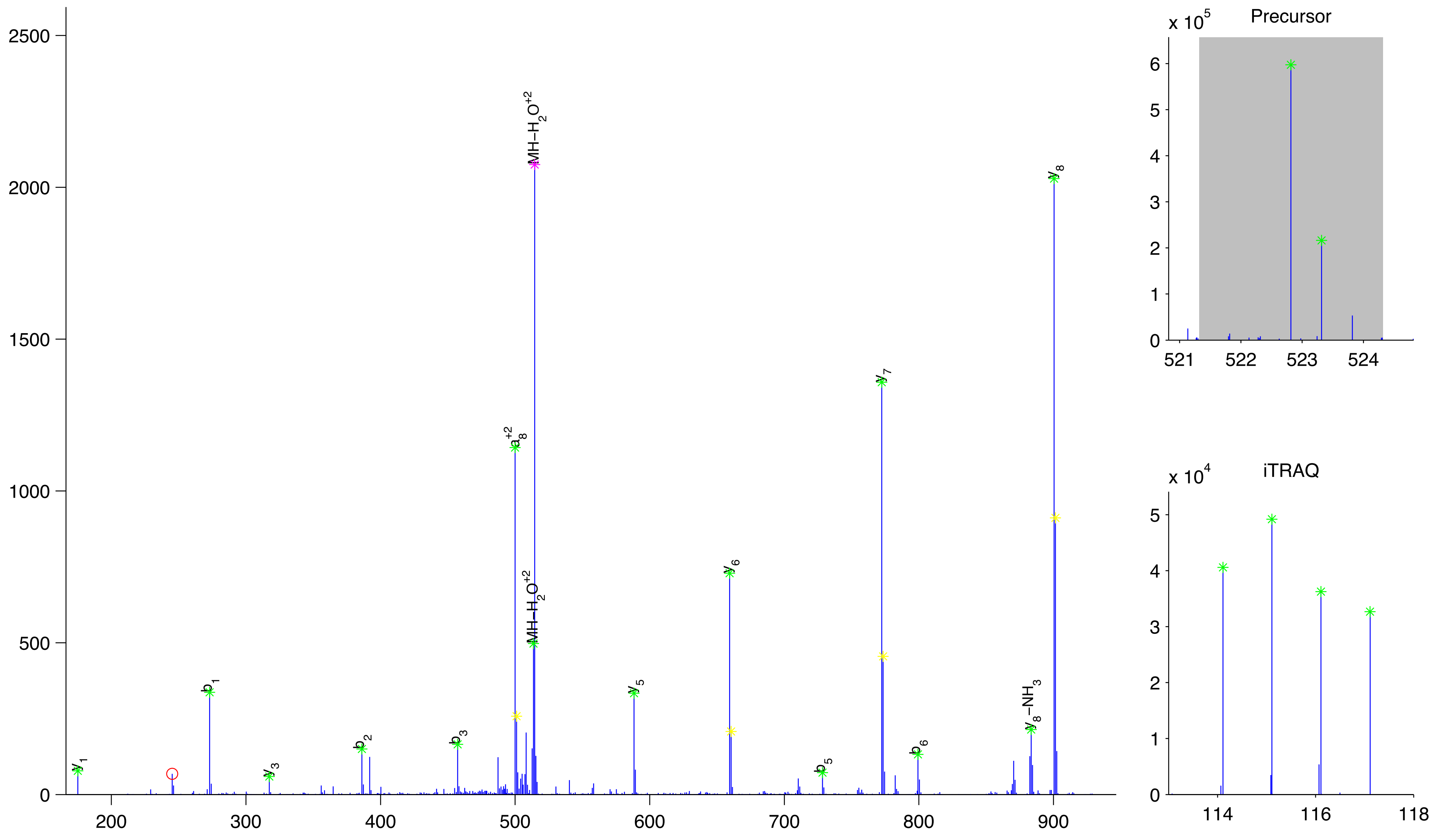

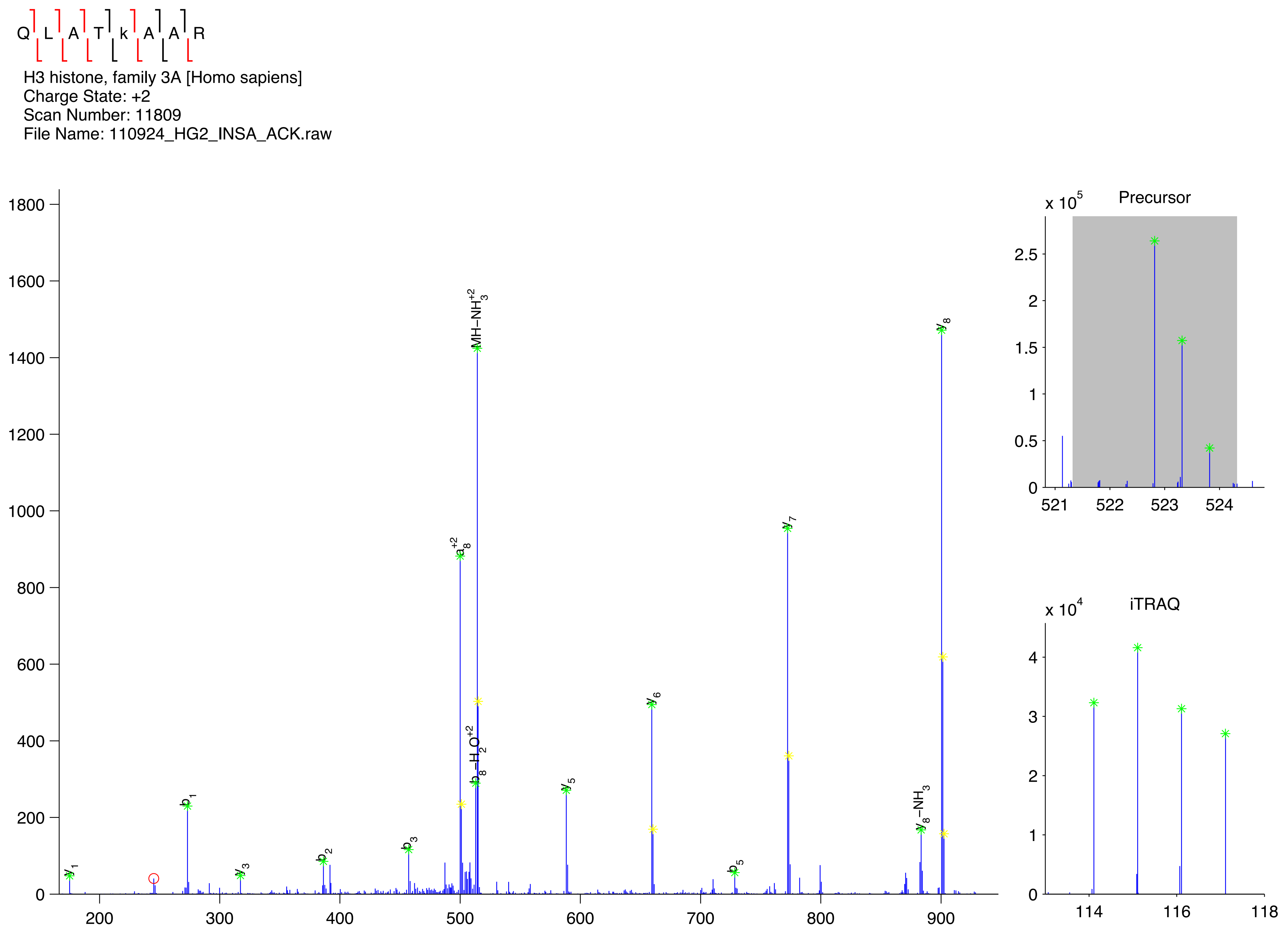
Q L A T k A A R
H3 histone, family 3A [Homo sapiens]
Charge State: +2
Scan Number: 11809
File Name: 110924_HG2_INSA_ACK.raw
y1
b1
y3
b2
b3
a8+2
MH−NH3+2
b8−H2O+2
y5
y6
b5
y7
y8−NH3
y8
Precursor
x 10^5
iTRAQ
x 10^4

Q L A T k A A R

H3 histone, family 3A [Homo sapiens]
Charge State: +2
Scan Number: 11973
File Name: 110924_HG2_INSA_ACK.raw

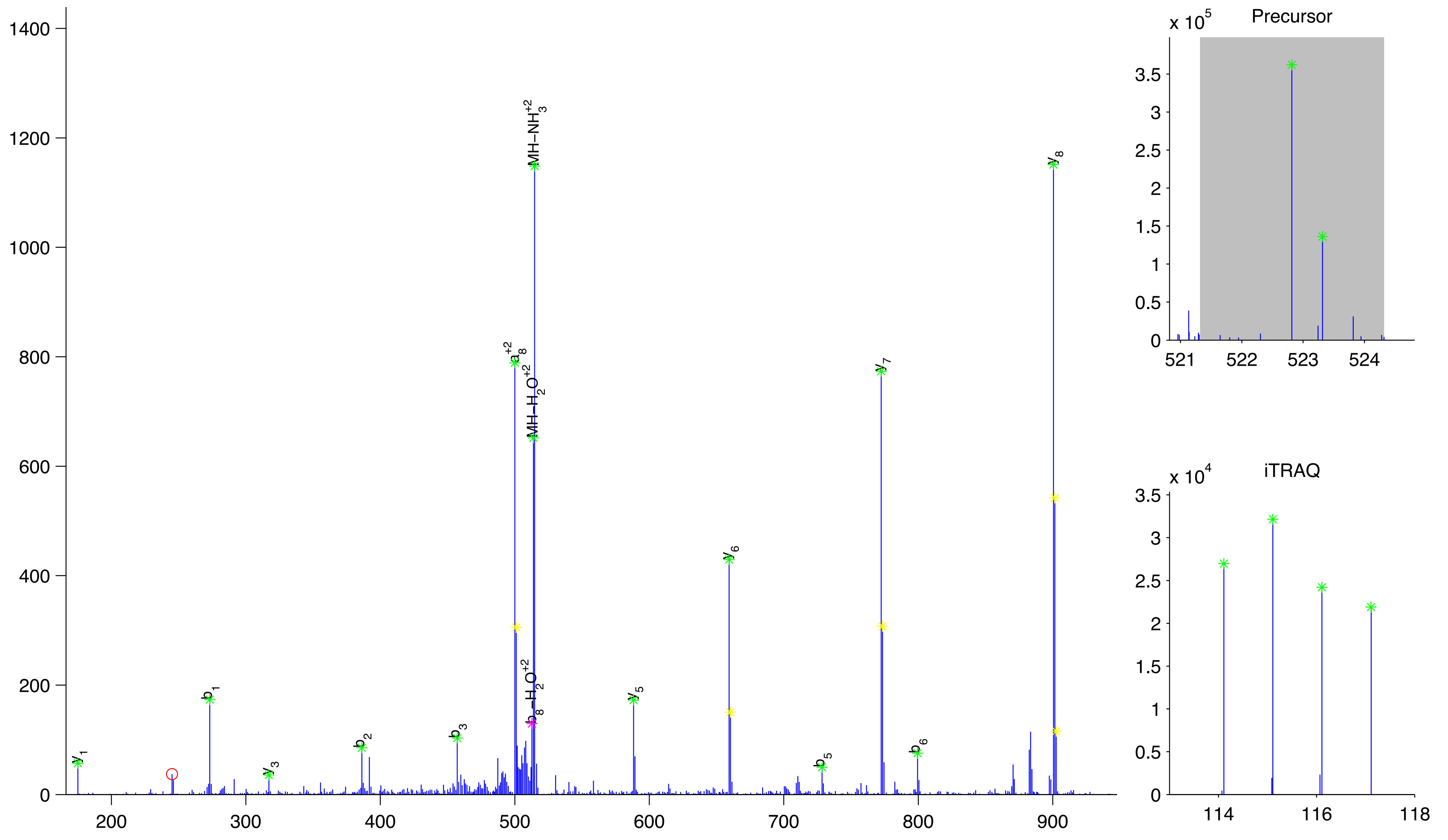

Q L A T k A A R

H3 histone, family 3A [Homo sapiens]
Charge State: +2
Scan Number: 12141
File Name: 110924_HG2_INSA_ACK.raw

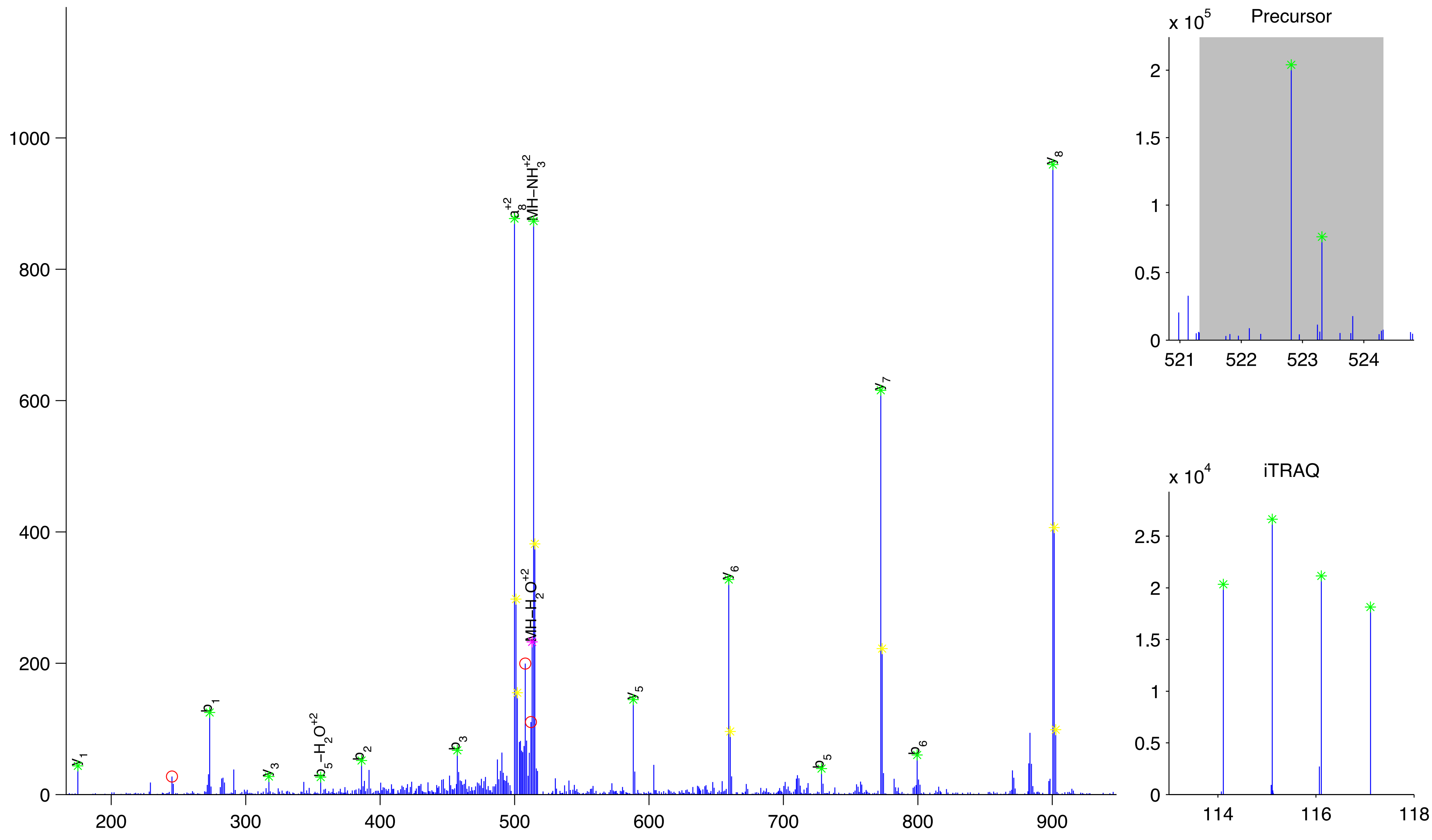

k Q L A T k A A R

H3 histone, family 3A [Homo sapiens]
Charge State: +2
Scan Number: 12309
File Name: 110924_HG2_INSA_ACK.raw

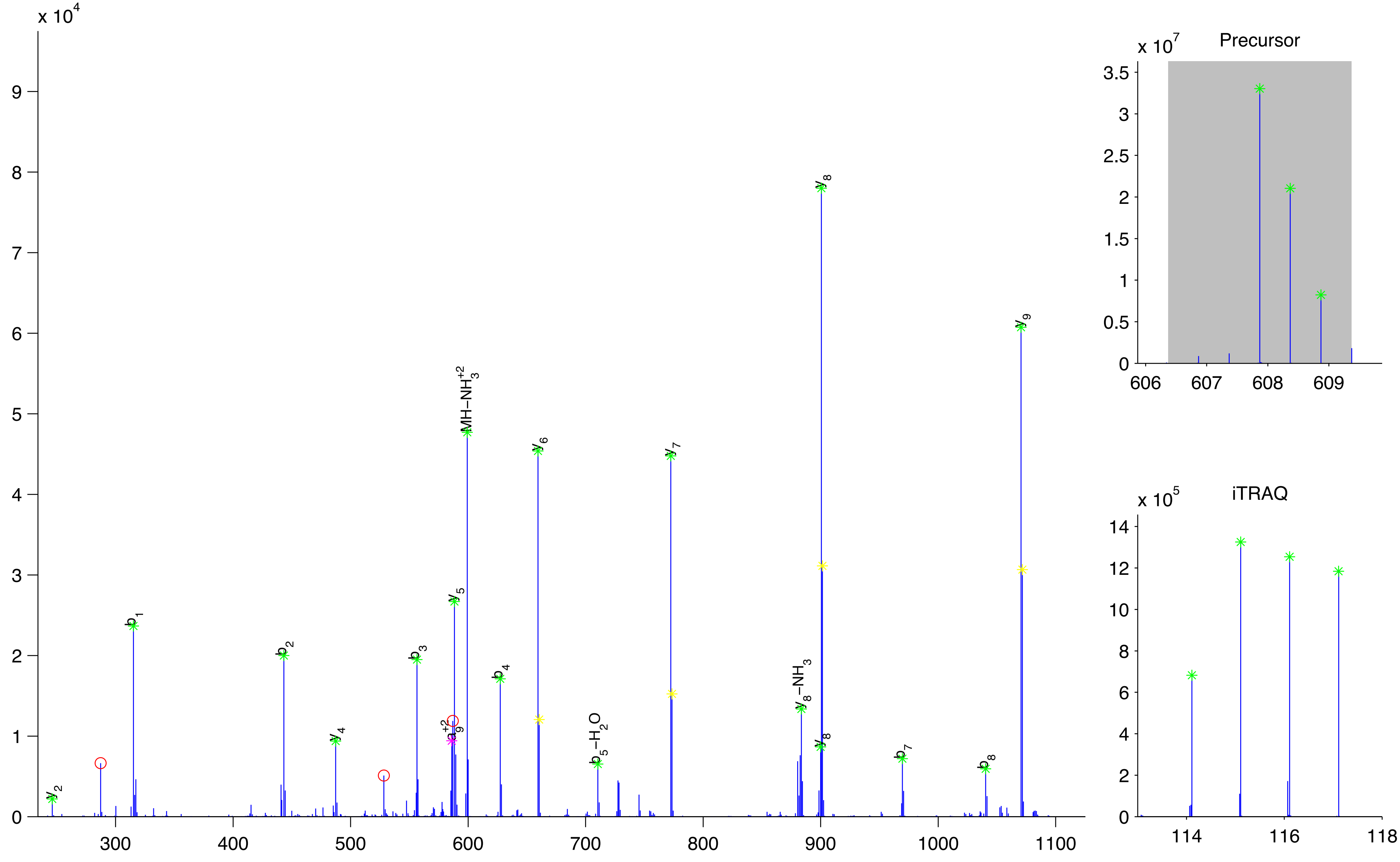

Q L A T k A A R

H3 histone, family 3A [Homo sapiens]
Charge State: +2
Scan Number: 12321
File Name: 110924_HG2_INSA_ACK.raw

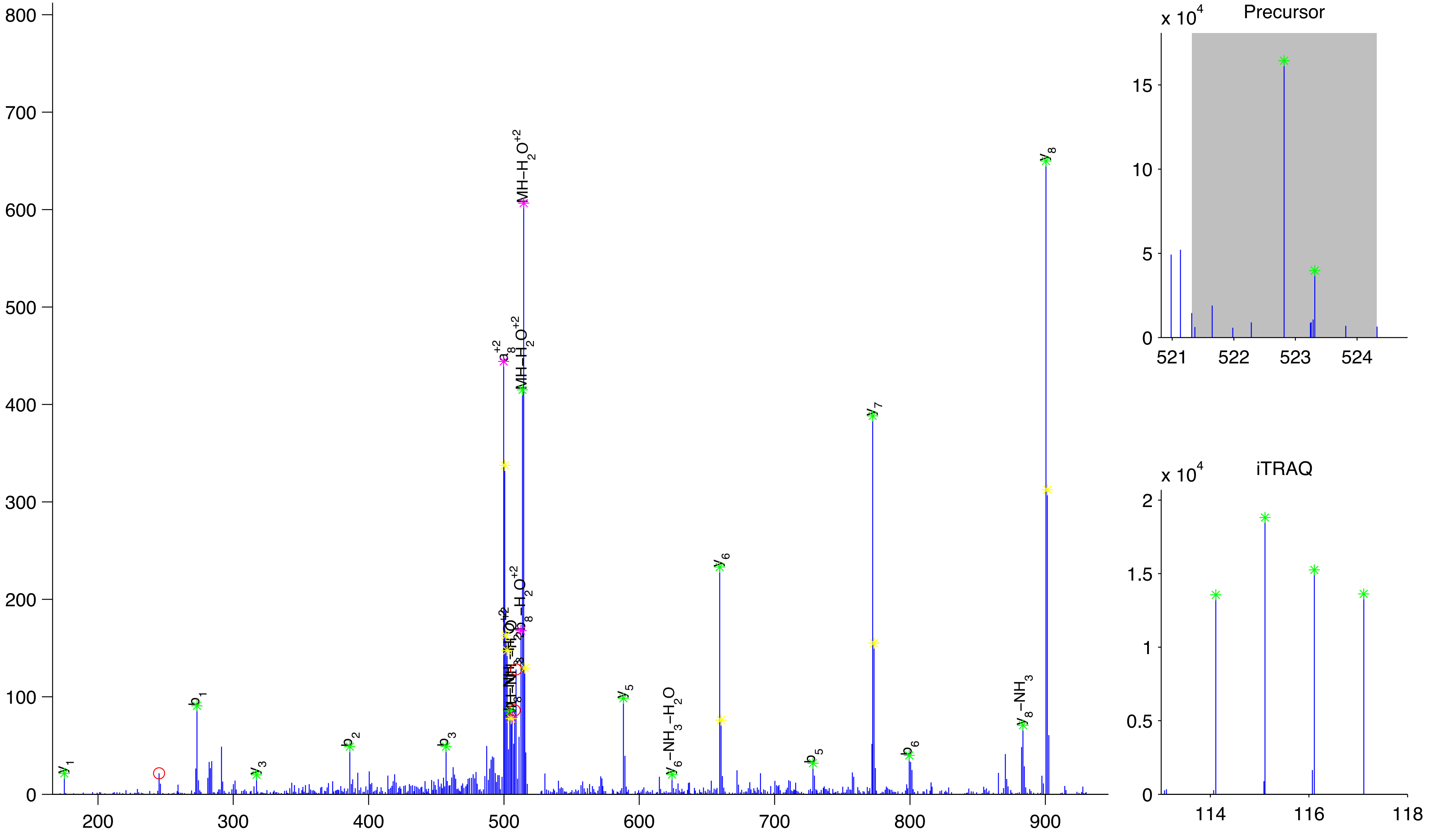

k Q L A T k A A R

H3 histone, family 3A [Homo sapiens]
Charge State: +2
Scan Number: 12393
File Name: 110924_HG2_INSA_ACK.raw

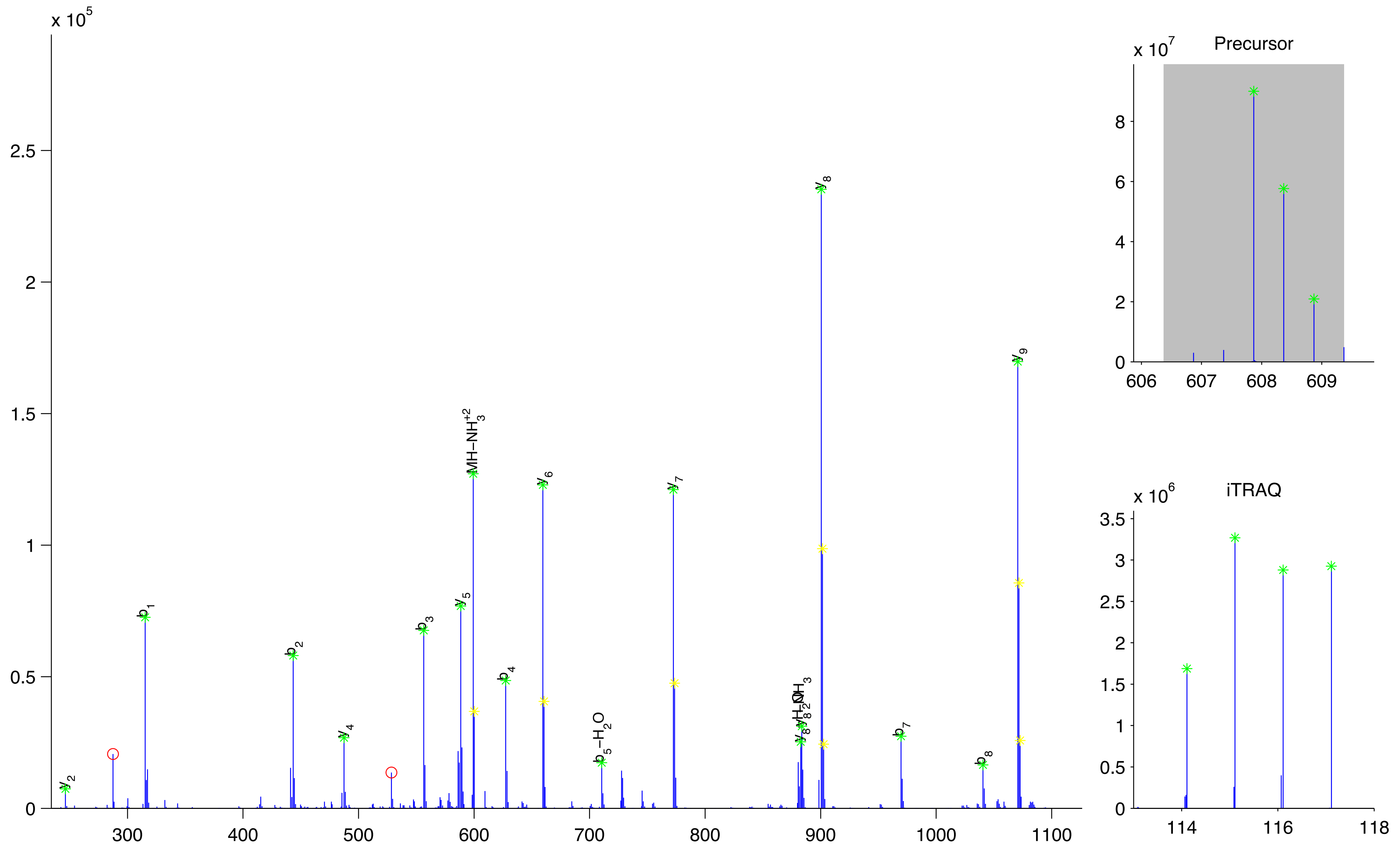

k Q L A T k A A R

H3 histone, family 3A [Homo sapiens]
Charge State: +2
Scan Number: 12498
File Name: 110924_HG2_INSA_ACK.raw

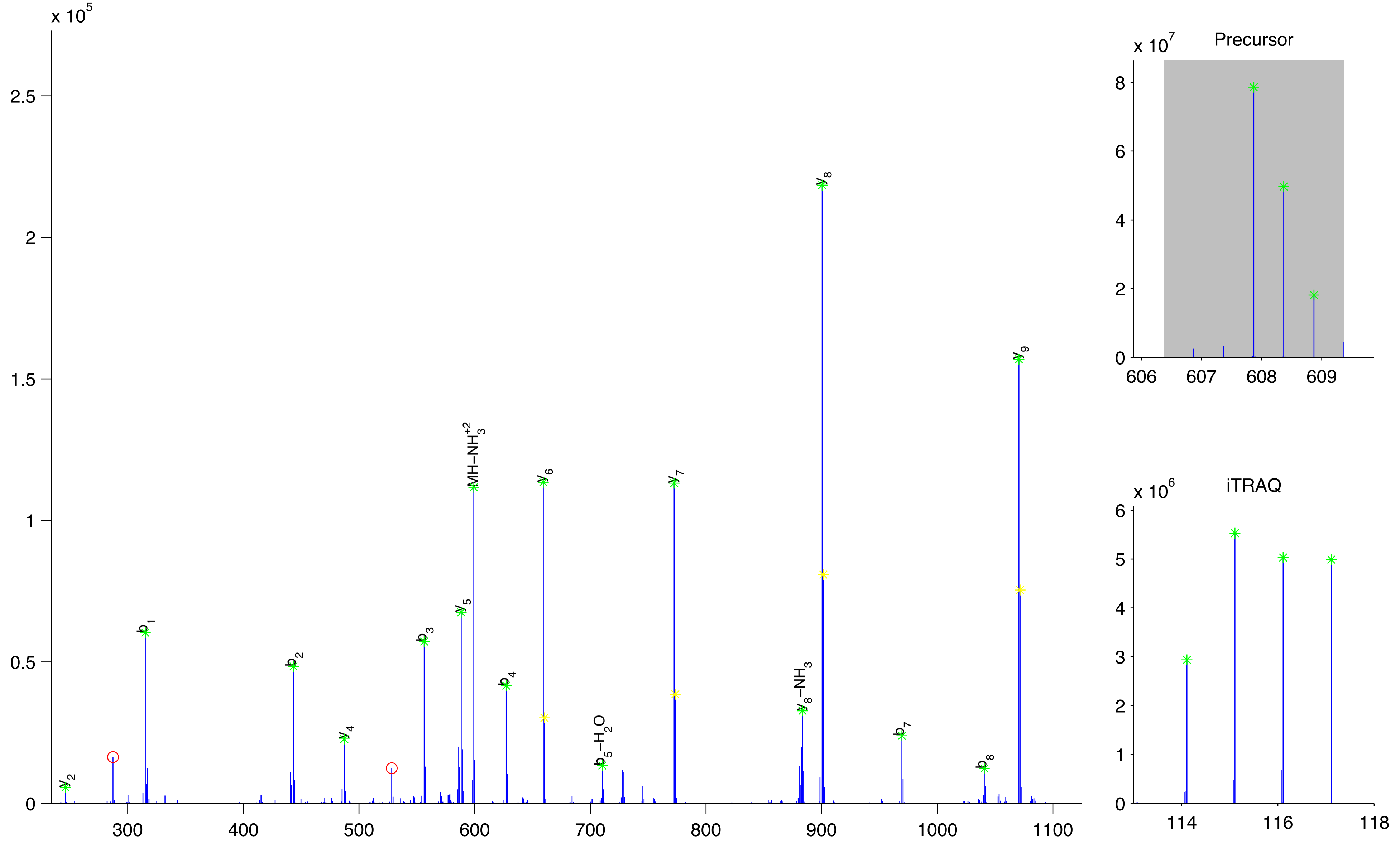

Q L A T k A A R

H3 histone, family 3A [Homo sapiens]
Charge State: +2
Scan Number: 12510
File Name: 110924_HG2_INSA_ACK.raw

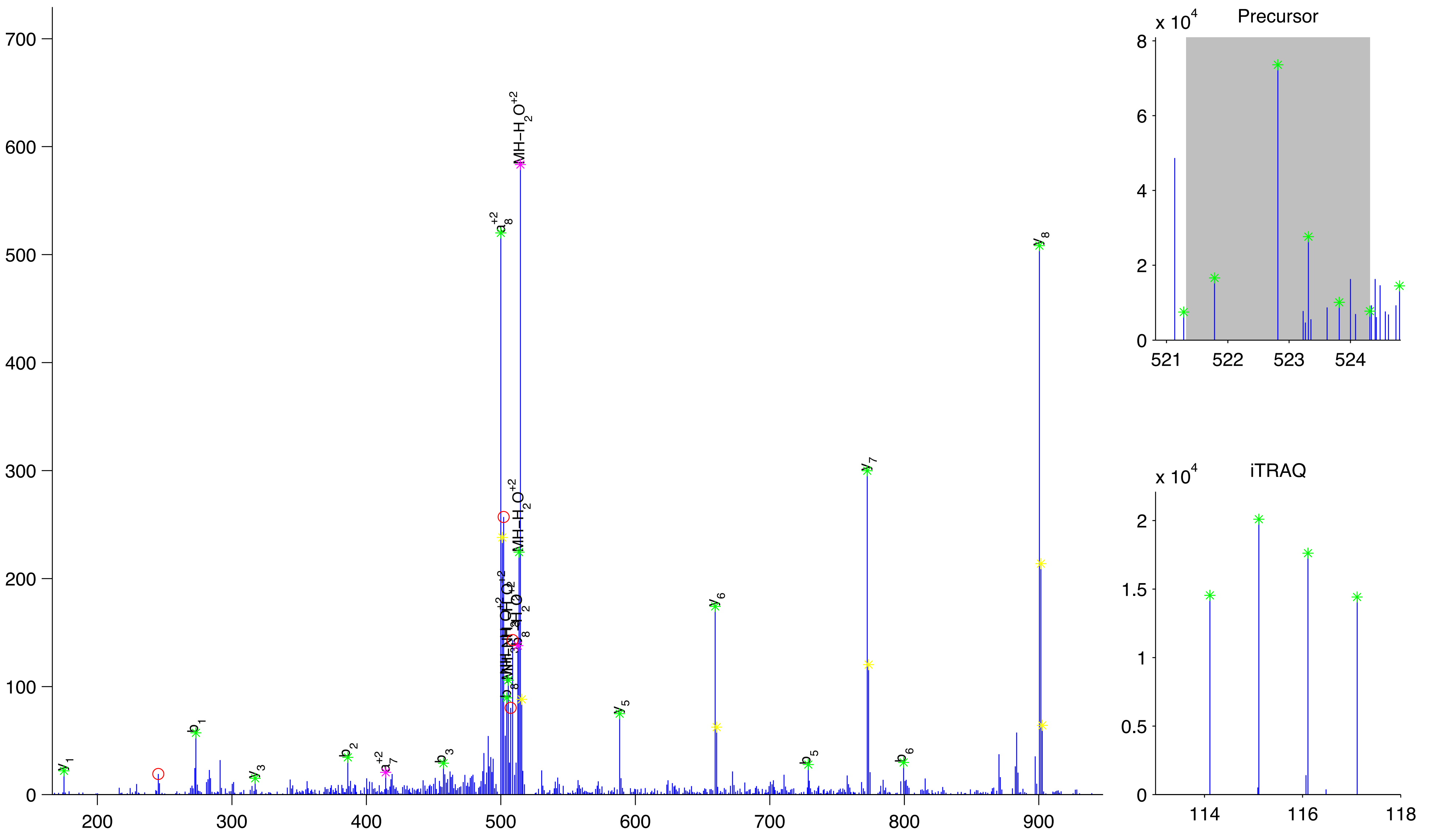

k Q L A T k A A R

H3 histone, family 3A [Homo sapiens]
Charge State: +2
Scan Number: 12582
File Name: 110924_HG2_INSA_ACK.raw

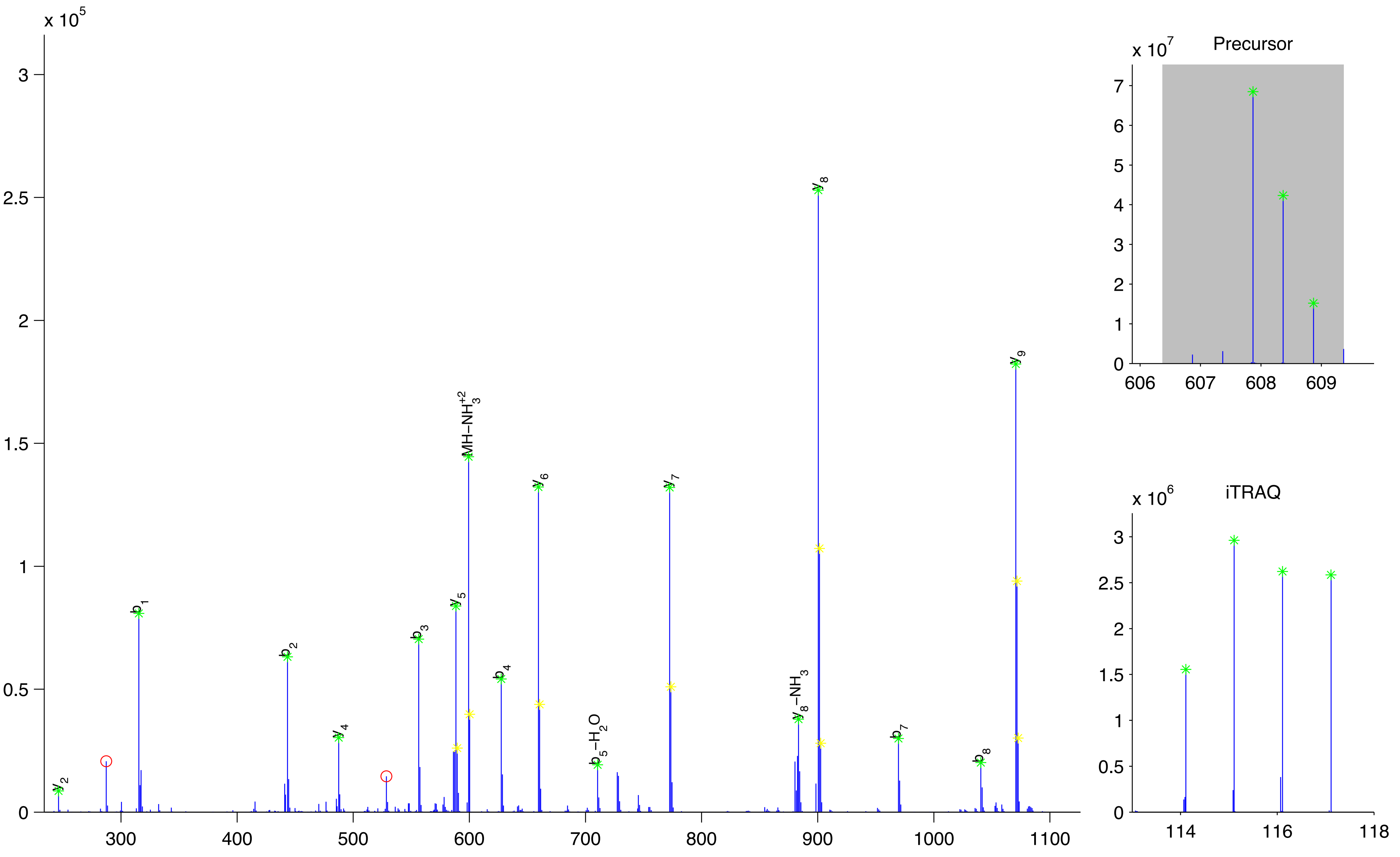

k S T G G k A P R

H3 histone, family 3A [Homo sapiens]
Charge State: +2
Scan Number: 12638
File Name: 110924_HG2_INSA_ACK.raw

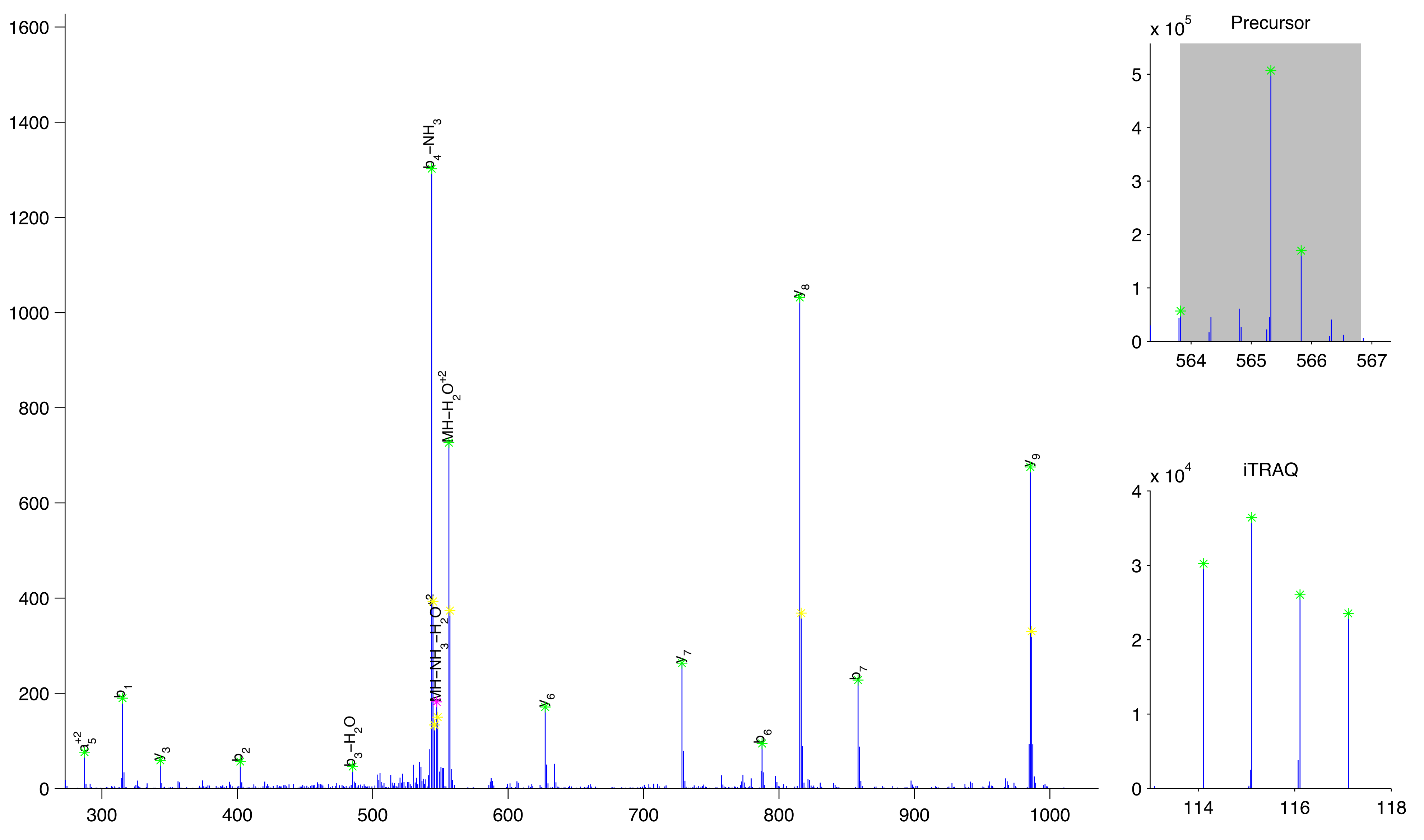

k Q L A T k A A R

H3 histone, family 3A [Homo sapiens]
Charge State: +2
Scan Number: 12687
File Name: 110924_HG2_INSA_ACK.raw

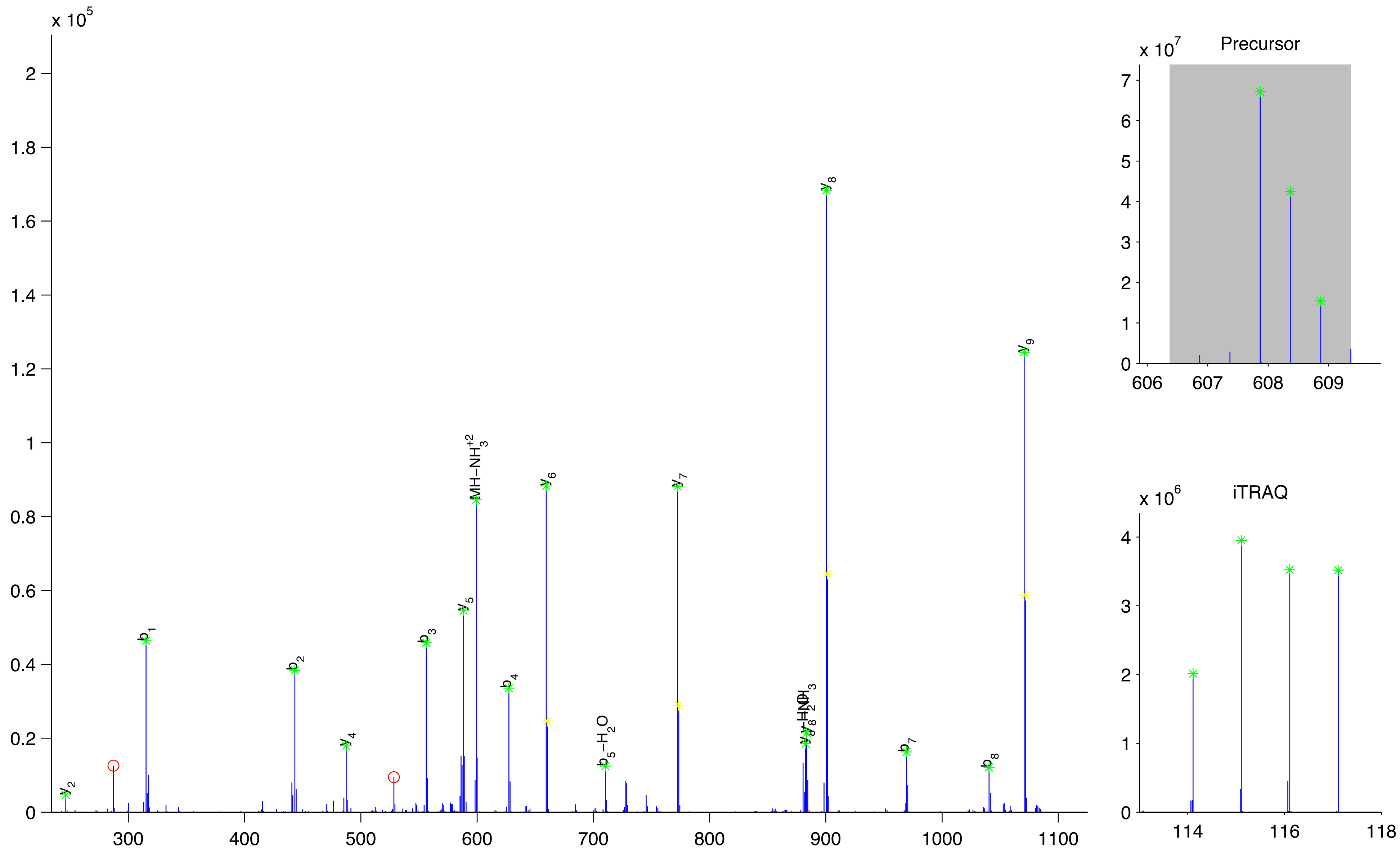

k Q L A T k A A R

H3 histone, family 3A [Homo sapiens]
Charge State: +2
Scan Number: 12771
File Name: 110924_HG2_INSA_ACK.raw

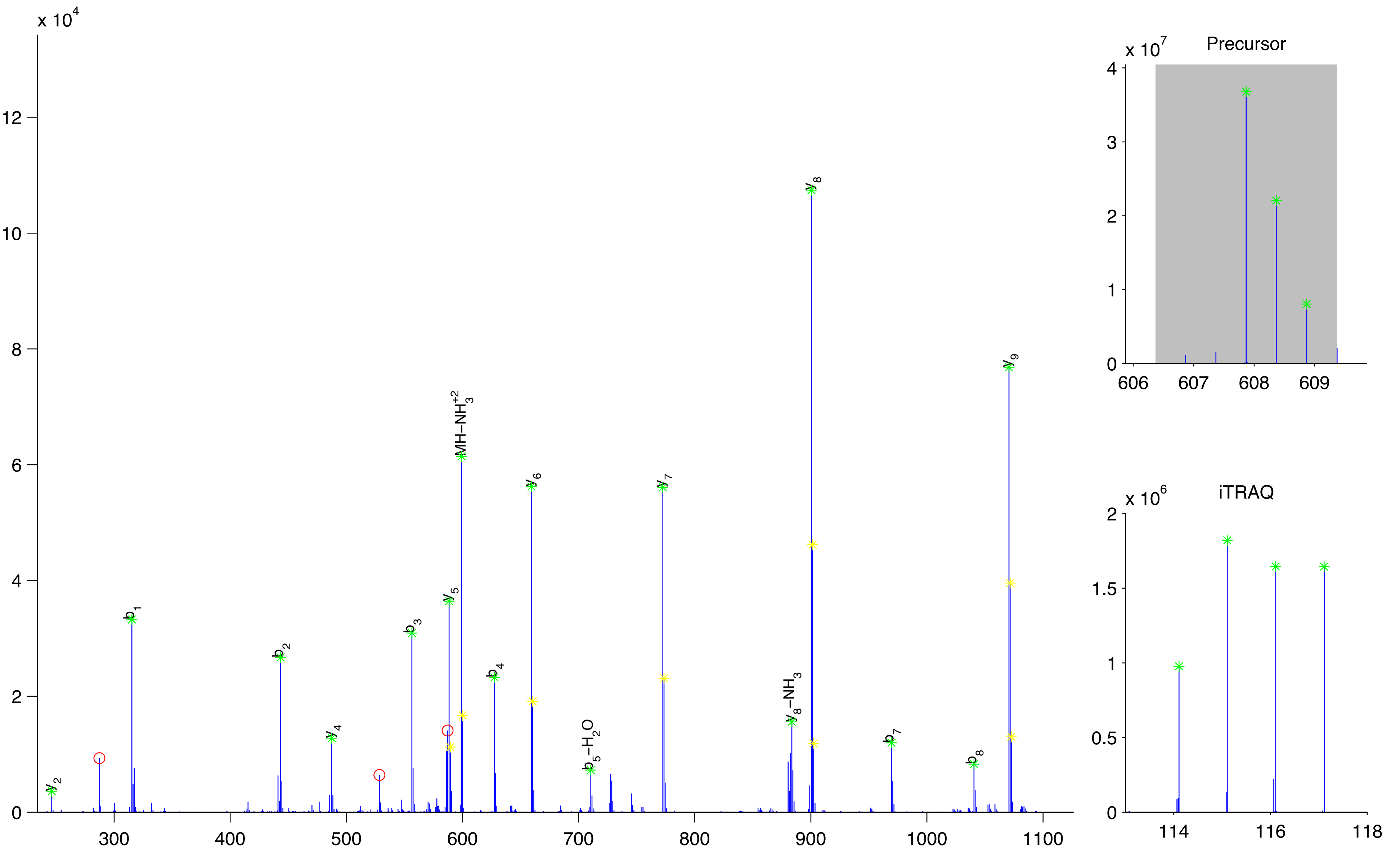

k S T G G k A P R

H3 histone, family 3A [Homo sapiens]
Charge State: +2
Scan Number: 12827
File Name: 110924_HG2_INSA_ACK.raw

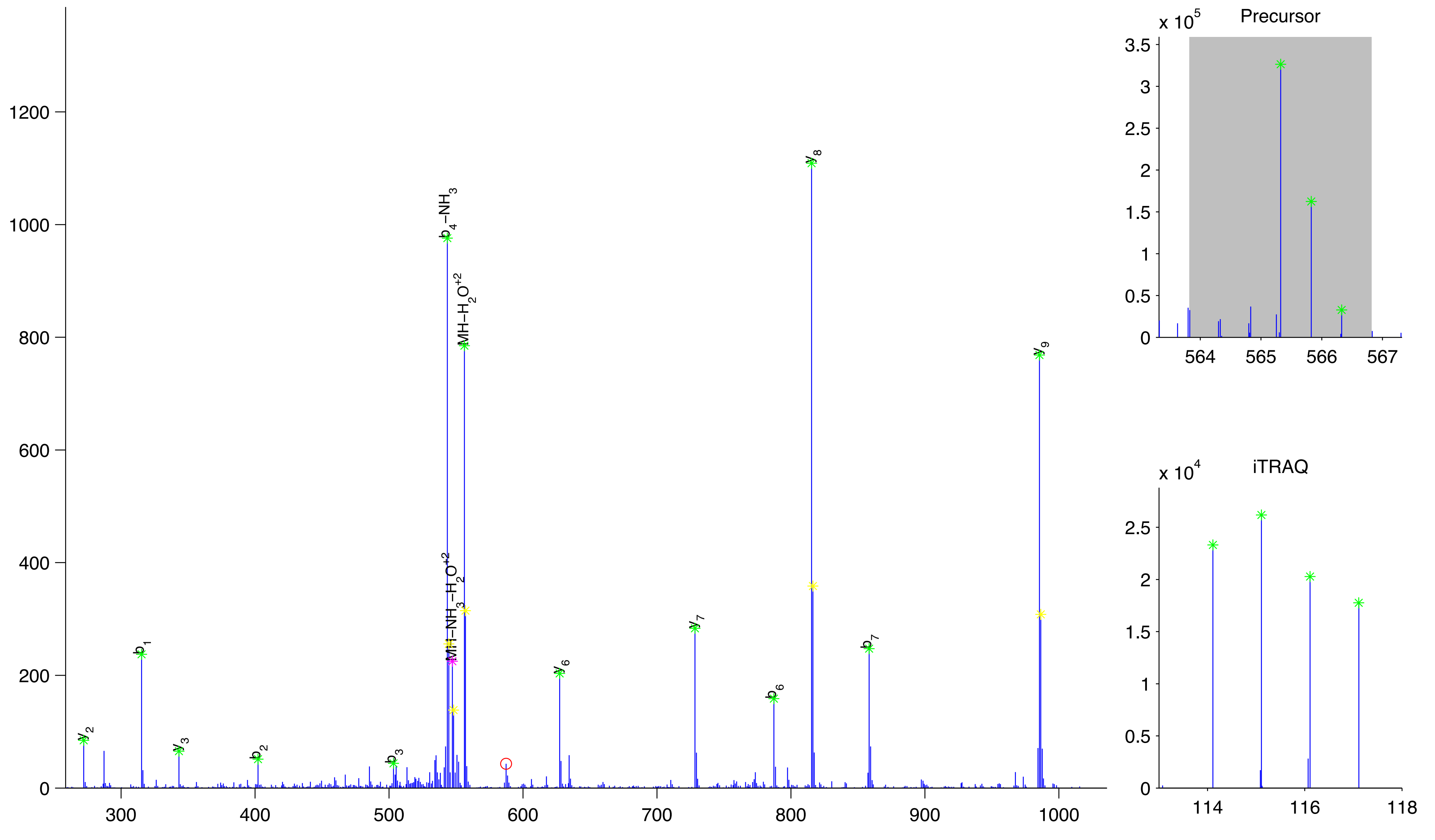

k Q L A T k A A R

H3 histone, family 3A [Homo sapiens]
Charge State: +2
Scan Number: 12876
File Name: 110924_HG2_INSA_ACK.raw

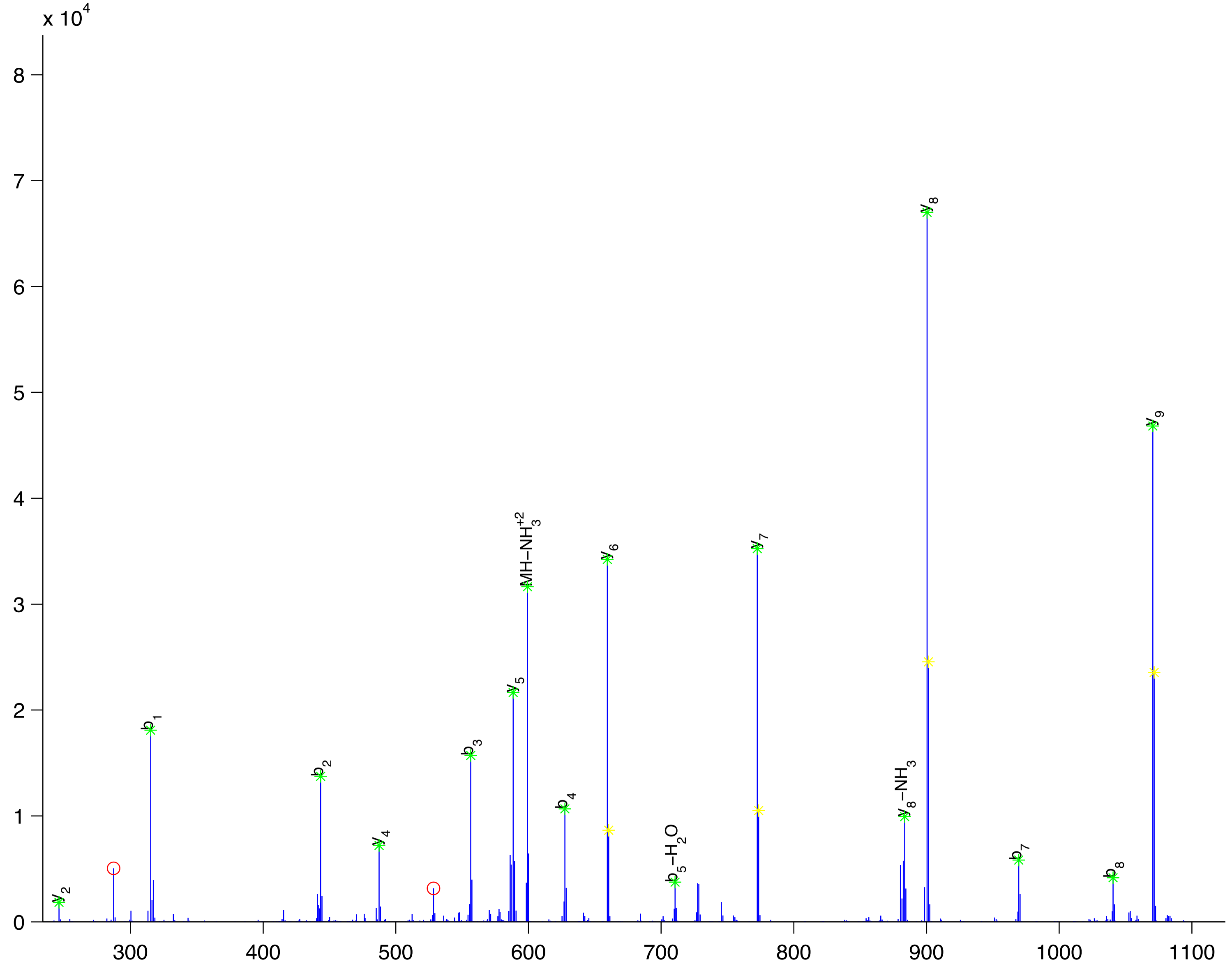

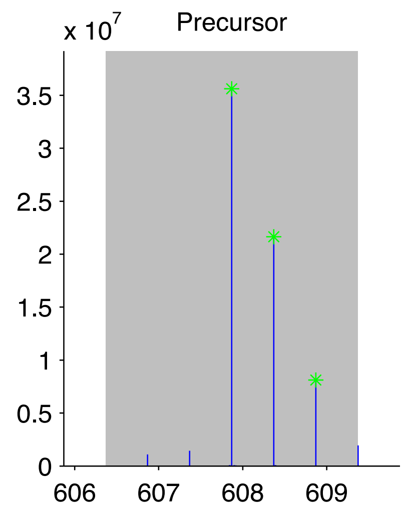

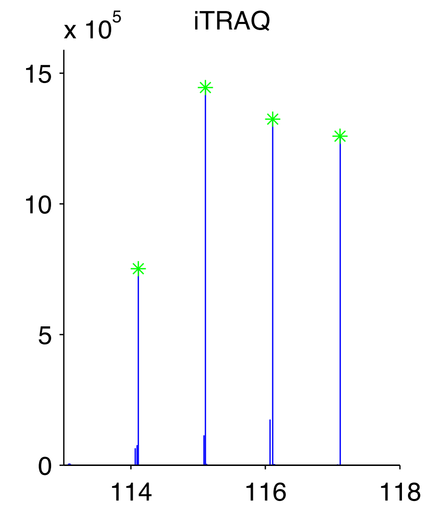

k Q L A T k A A R

H3 histone, family 3A [Homo sapiens]
Charge State: +2
Scan Number: 12960
File Name: 110924_HG2_INSA_ACK.raw

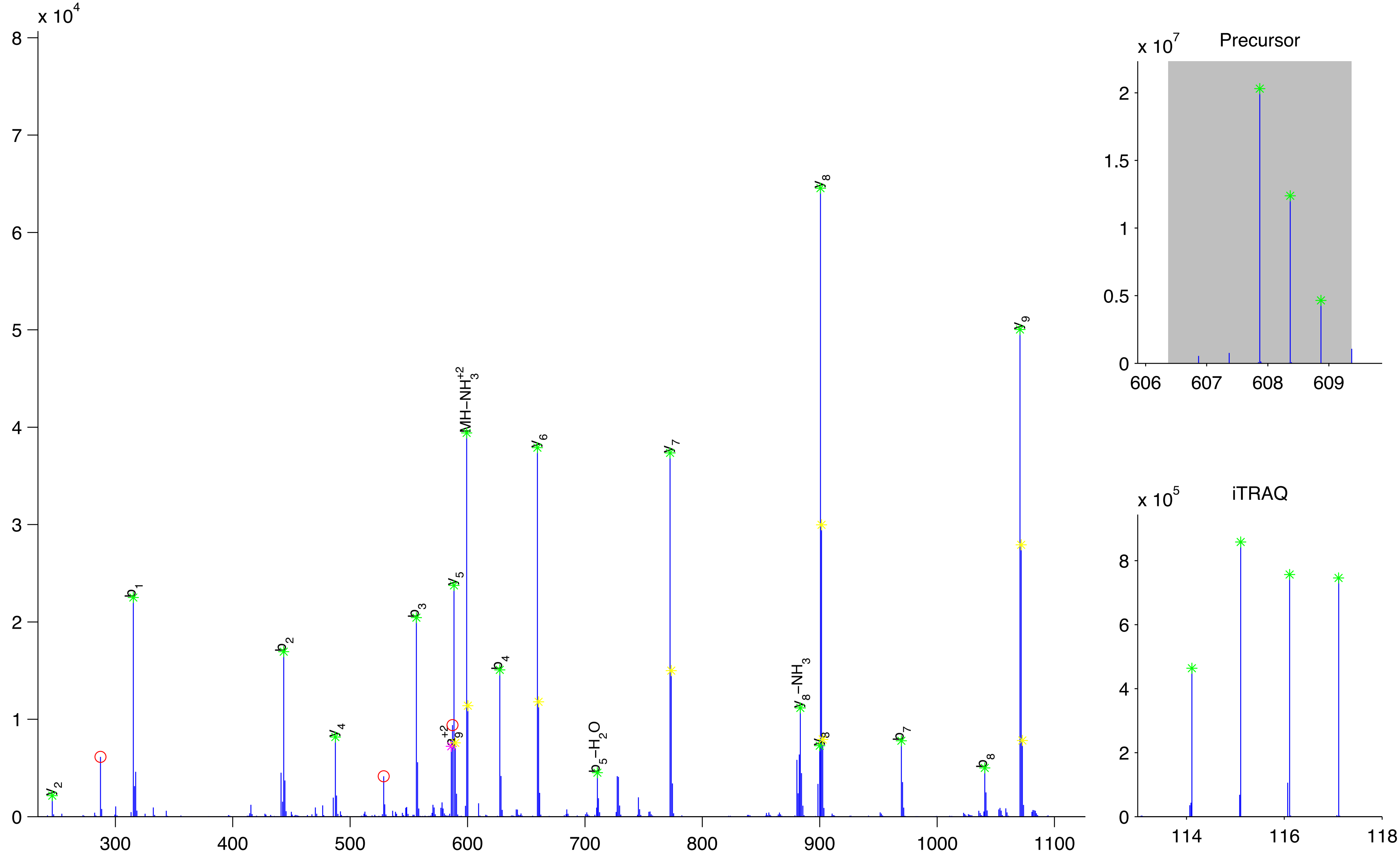

k S T G G k A P R

H3 histone, family 3A [Homo sapiens]
Charge State: +2
Scan Number: 13012
File Name: 110924_HG2_INSA_ACK.raw

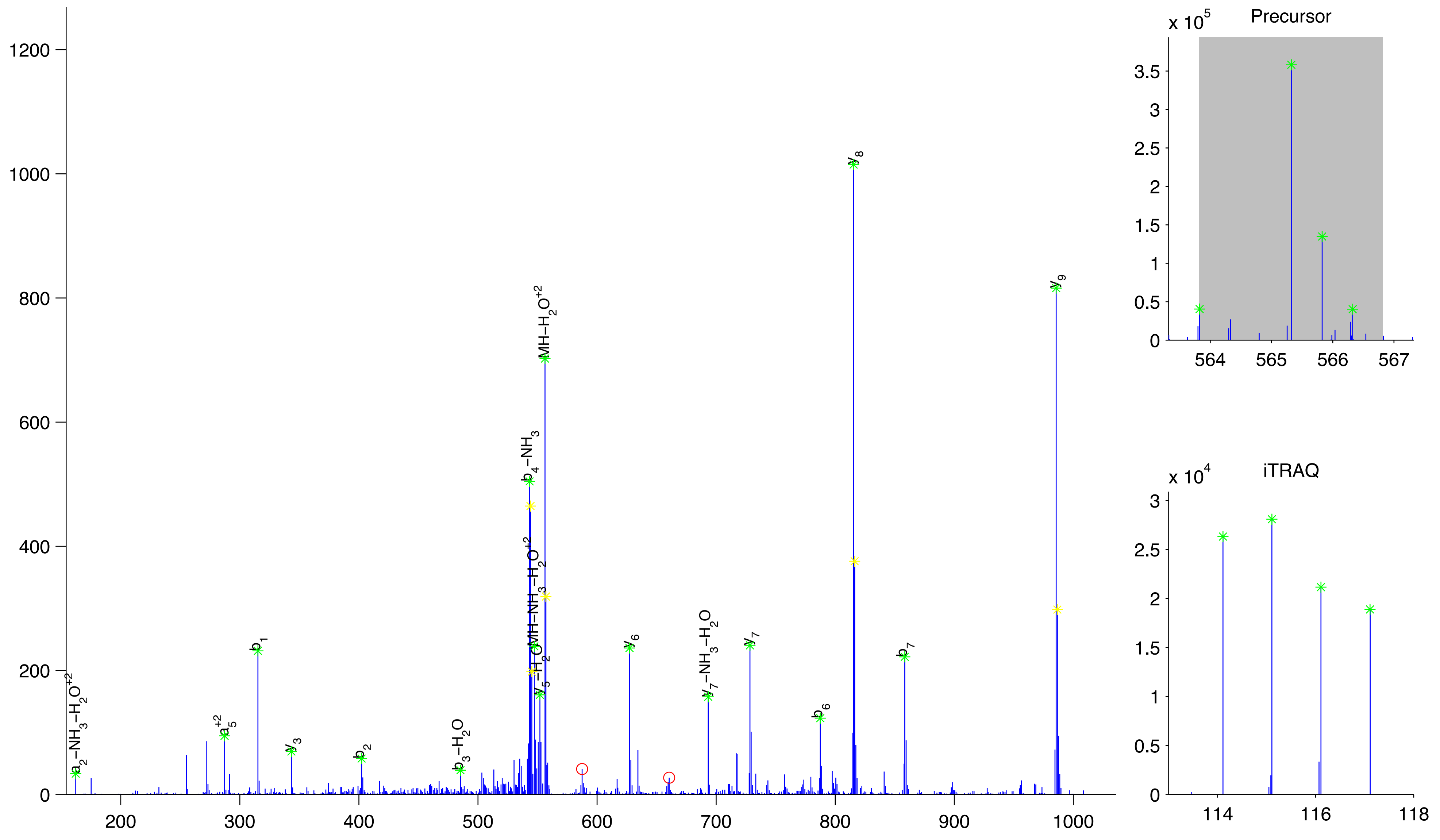

k Q L A T k A A R

H3 histone, family 3A [Homo sapiens]
Charge State: +2
Scan Number: 13065
File Name: 110924_HG2_INSA_ACK.raw

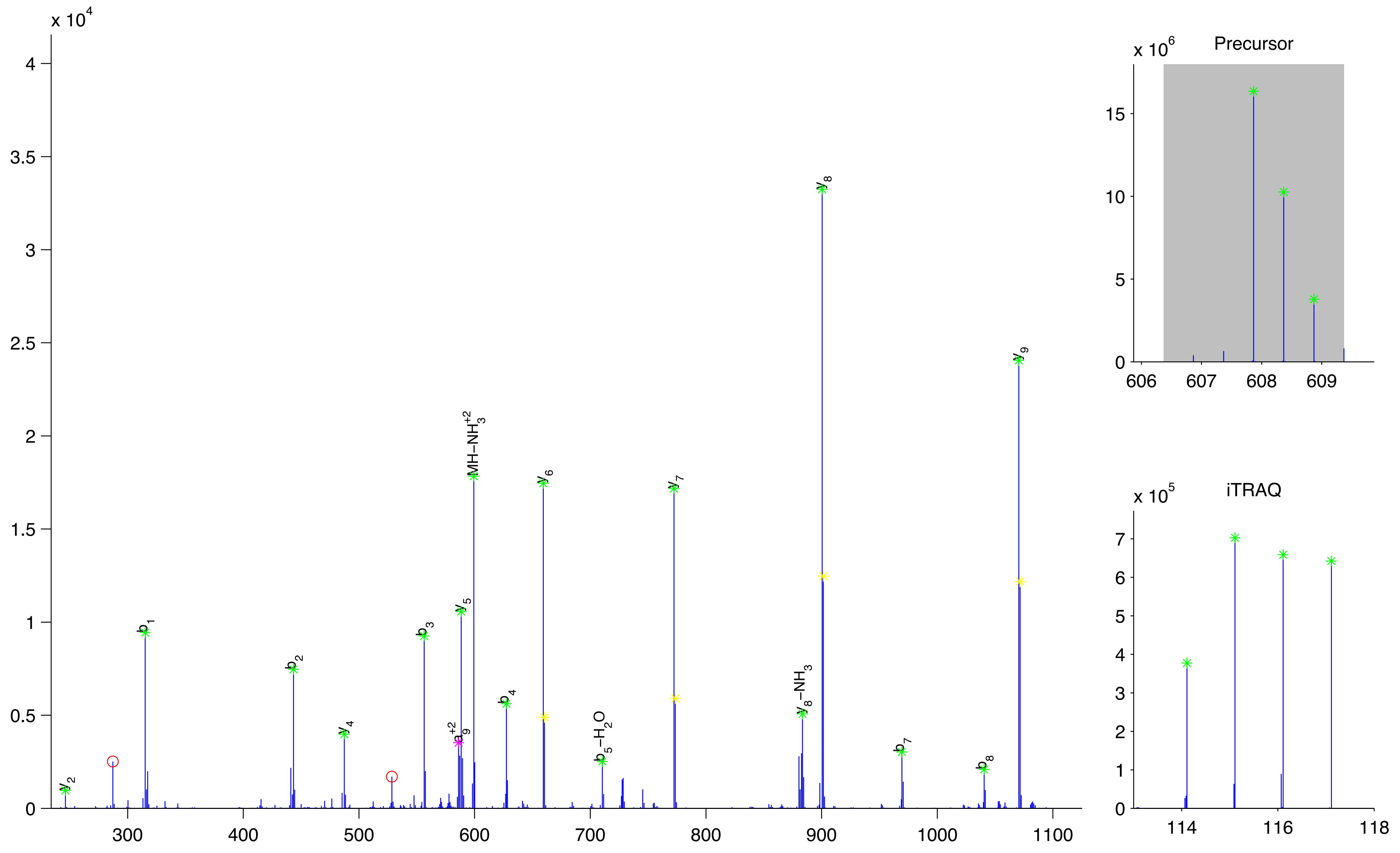

k Q L A T k A A R

H3 histone, family 3A [Homo sapiens]
Charge State: +2
Scan Number: 13151
File Name: 110924_HG2_INSA_ACK.raw

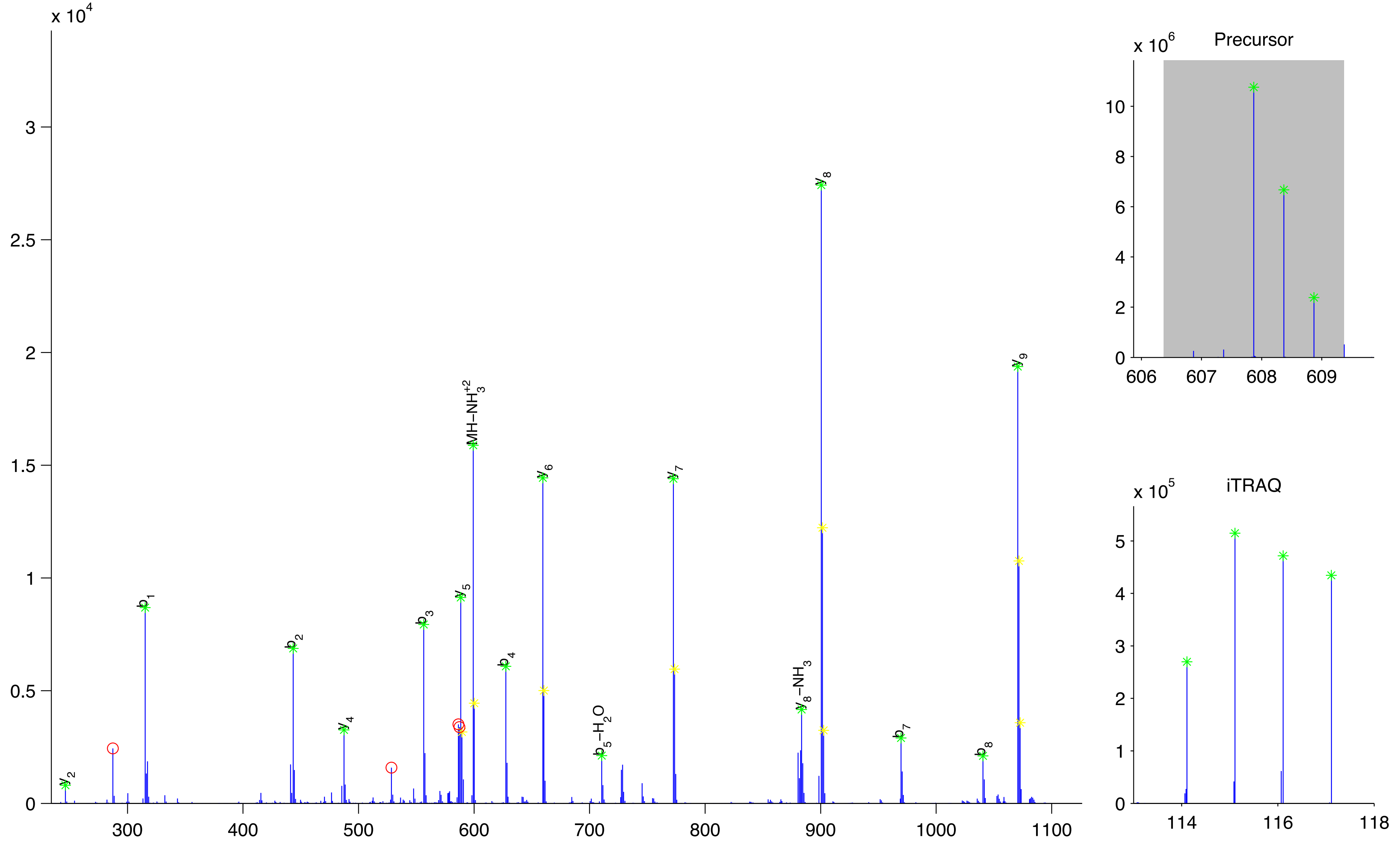

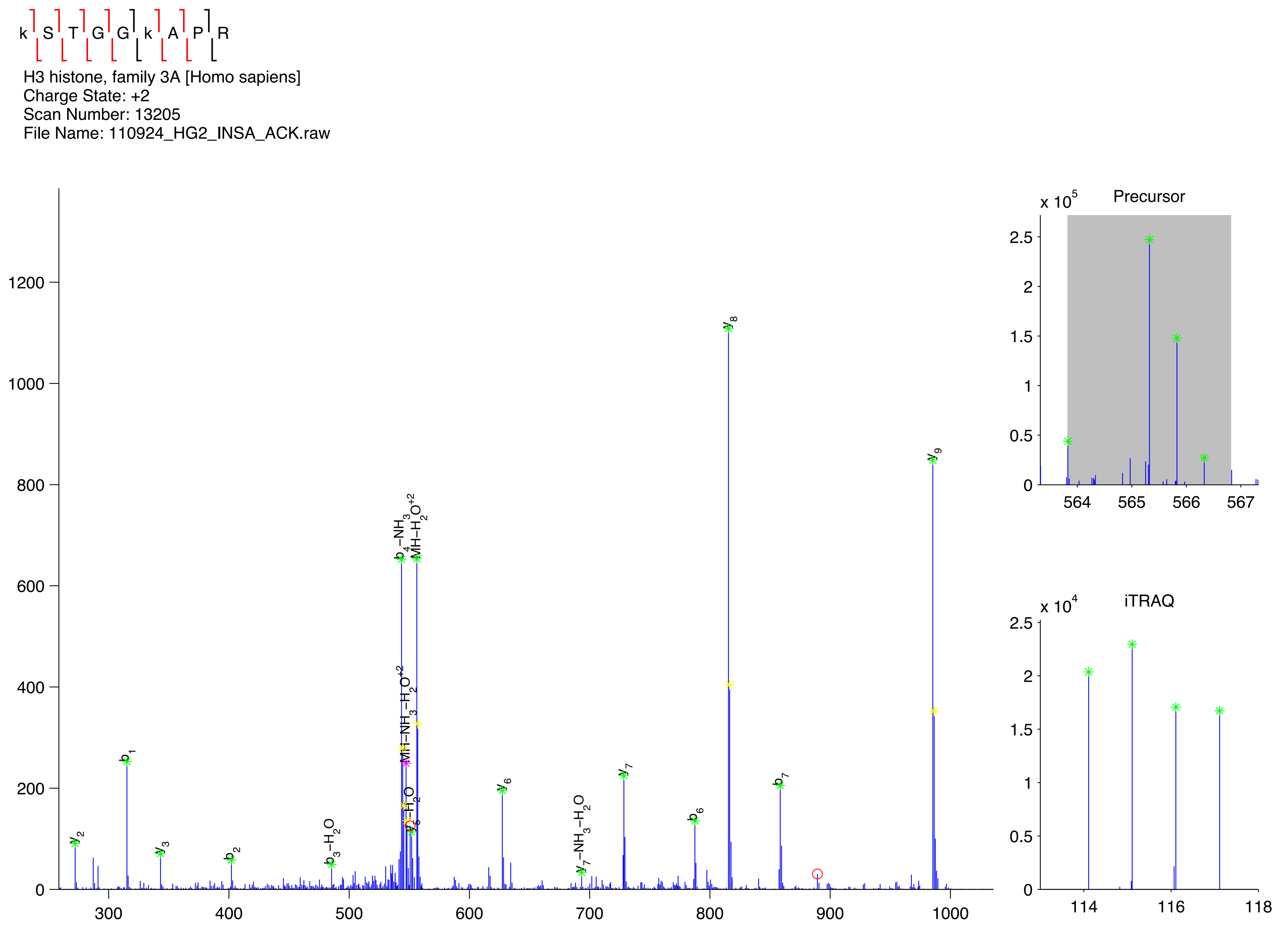

k S T G G k A P R
H3 histone, family 3A [Homo sapiens]
Charge State: +2
Scan Number: 13205
File Name: 110924_HG2_INSA_ACK.raw
y2
b1
y3
b2
b3-H2O
b4-NH3
MH-H2O+2
MH-NH3-H2O+2
b5-H2O
y6
y7-NH3-H2O
y7
b6
y8
b7
y9
Precursor
x 10^5
iTRAQ
x 10^4

k Q L A T k A A R

H3 histone, family 3A [Homo sapiens]
Charge State: +2
Scan Number: 13233
File Name: 110924_HG2_INSA_ACK.raw

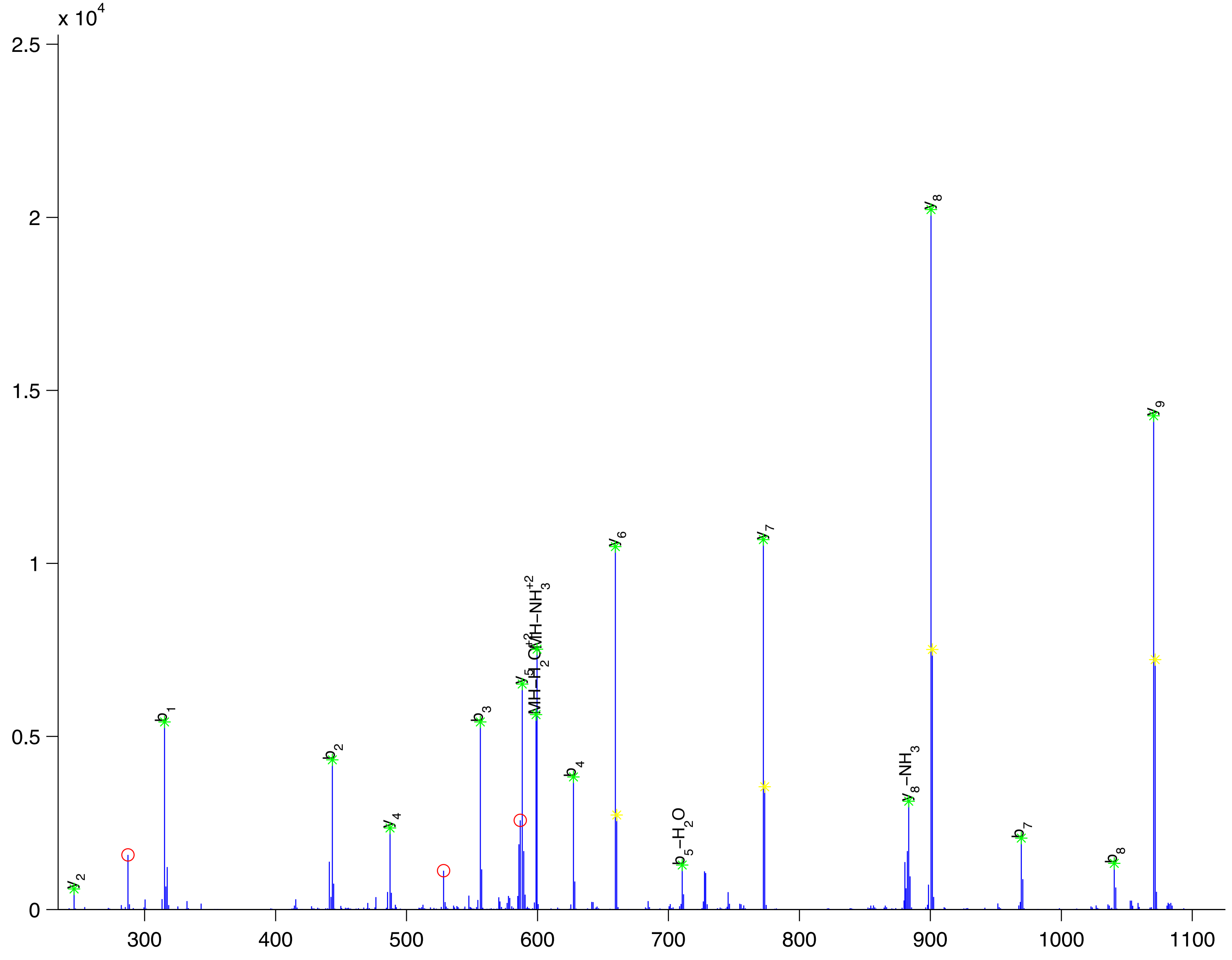

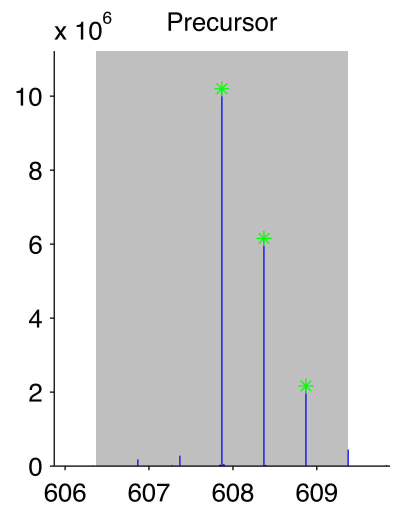

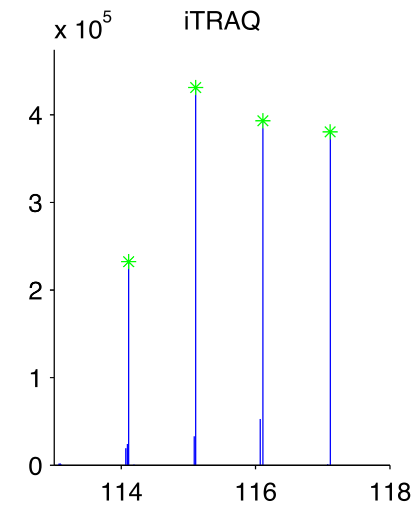

k Q L A T k A A R

H3 histone, family 3A [Homo sapiens]
Charge State: +2
Scan Number: 13319
File Name: 110924_HG2_INSA_ACK.raw

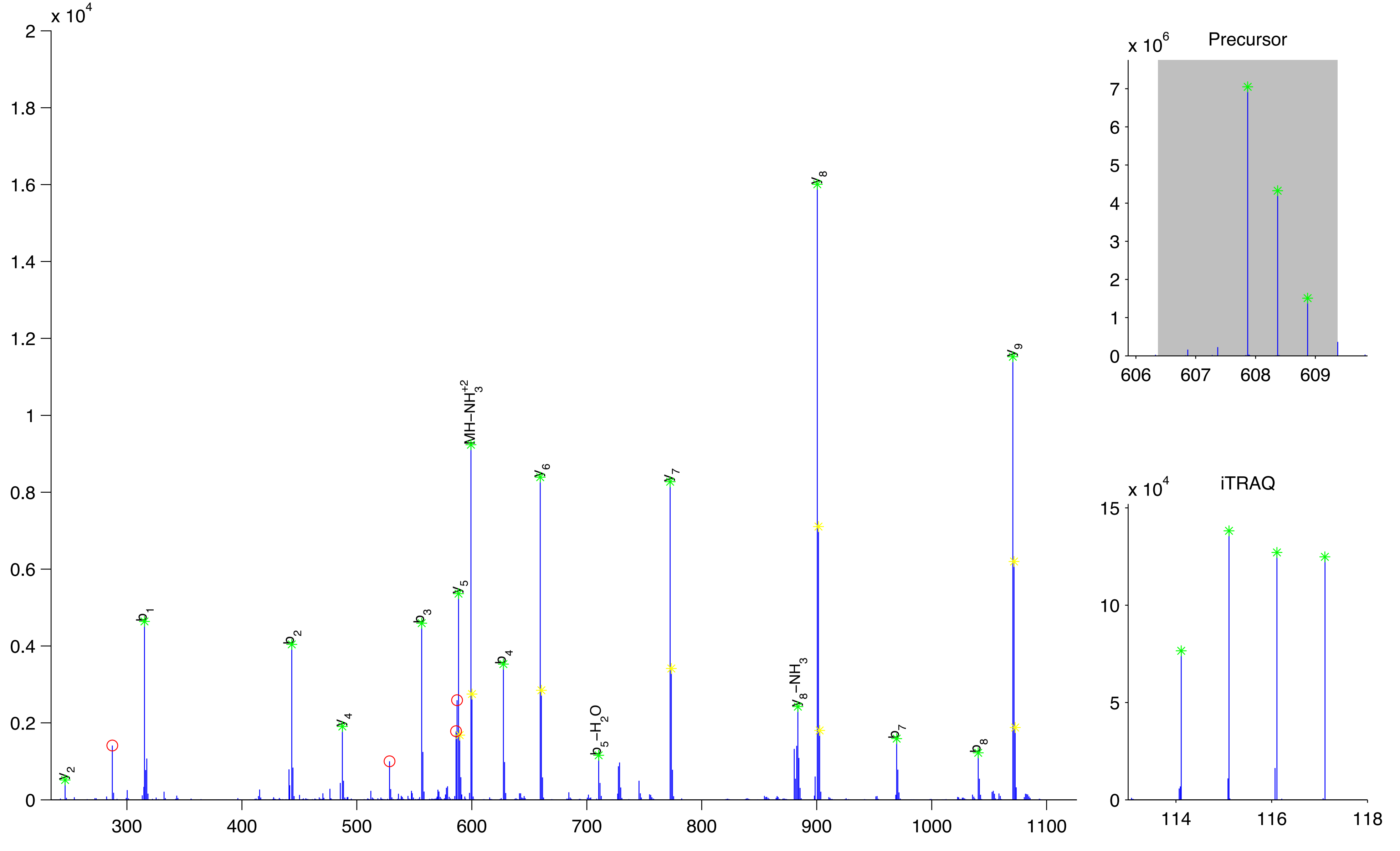

k S T G G k A P R

H3 histone, family 3A [Homo sapiens]
Charge State: +2
Scan Number: 13361
File Name: 110924_HG2_INSA_ACK.raw

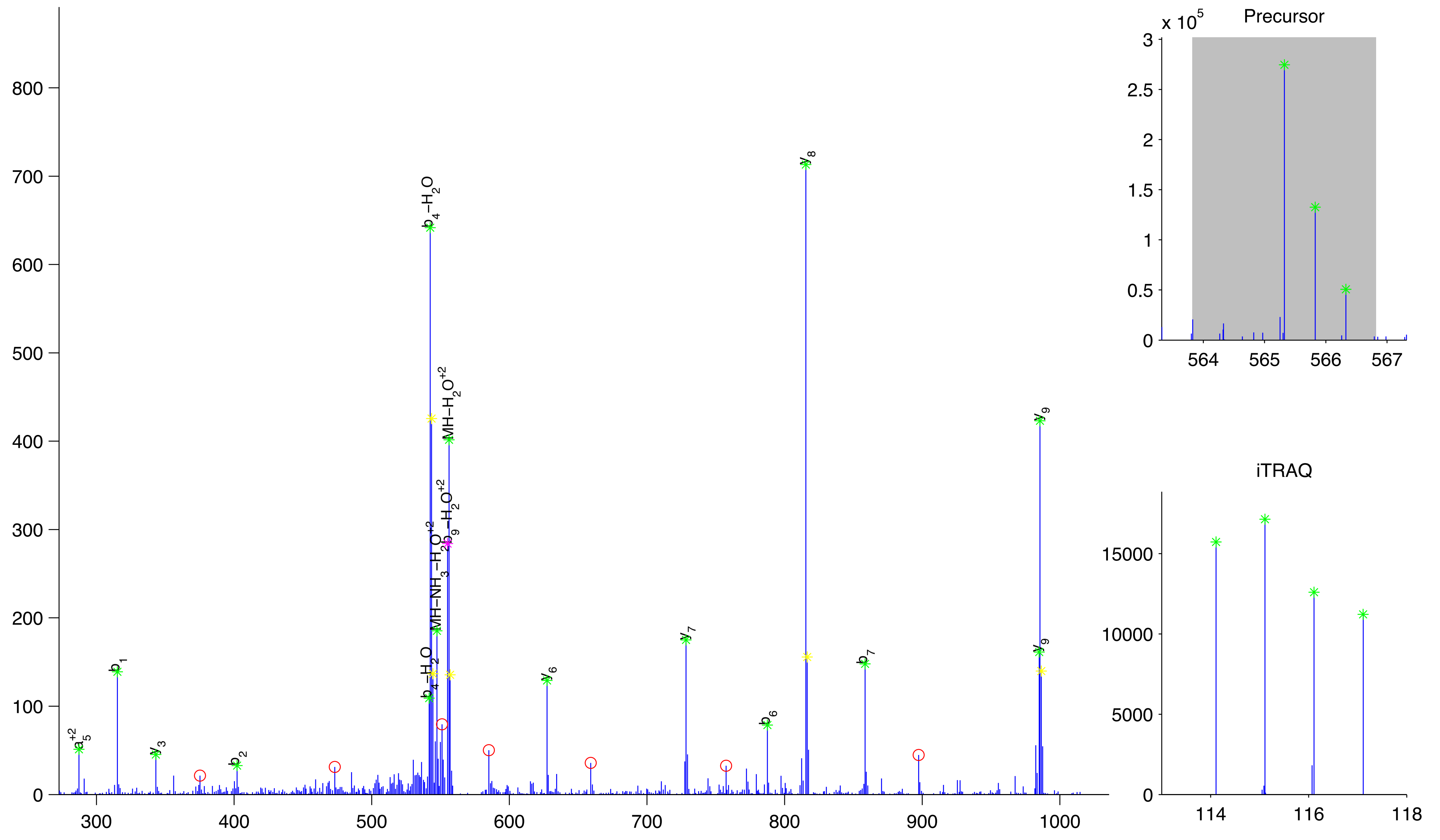

k Q L A T k A A R

H3 histone, family 3A [Homo sapiens]
Charge State: +2
Scan Number: 13401
File Name: 110924_HG2_INSA_ACK.raw

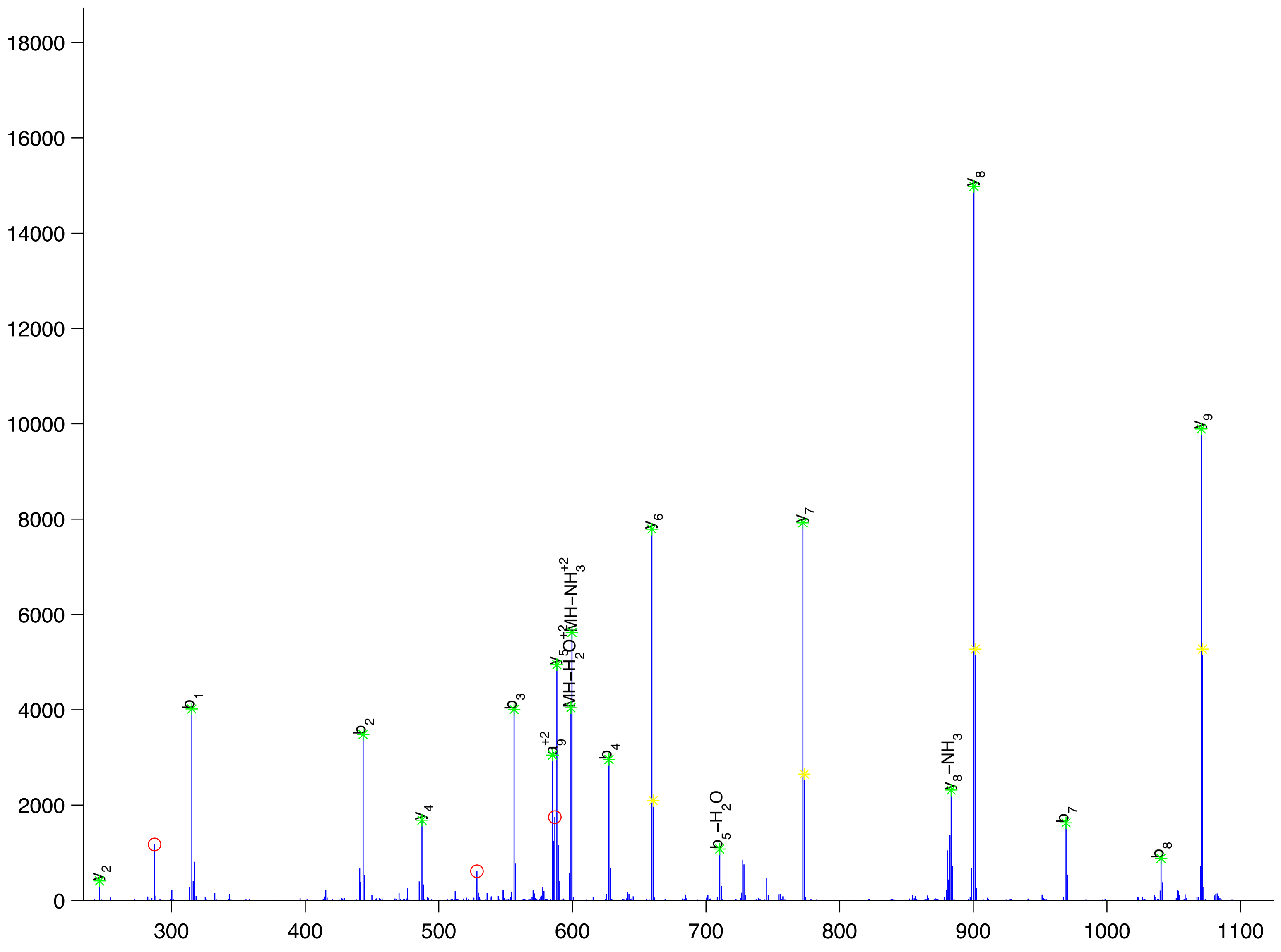

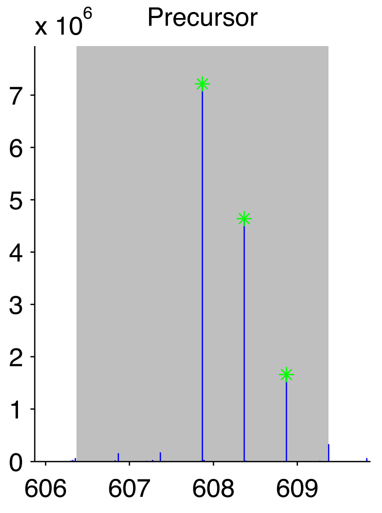

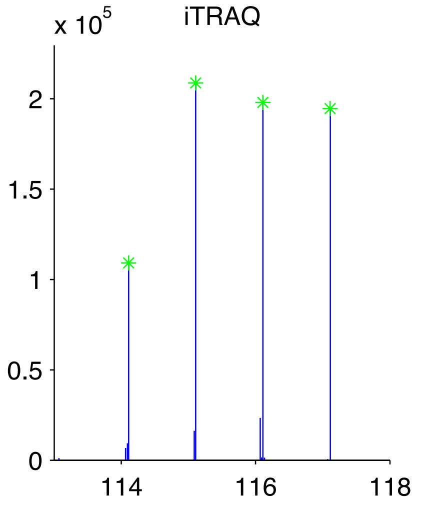

k Q L A T k A A R

H3 histone, family 3A [Homo sapiens]
Charge State: +2
Scan Number: 13489
File Name: 110924_HG2_INSA_ACK.raw

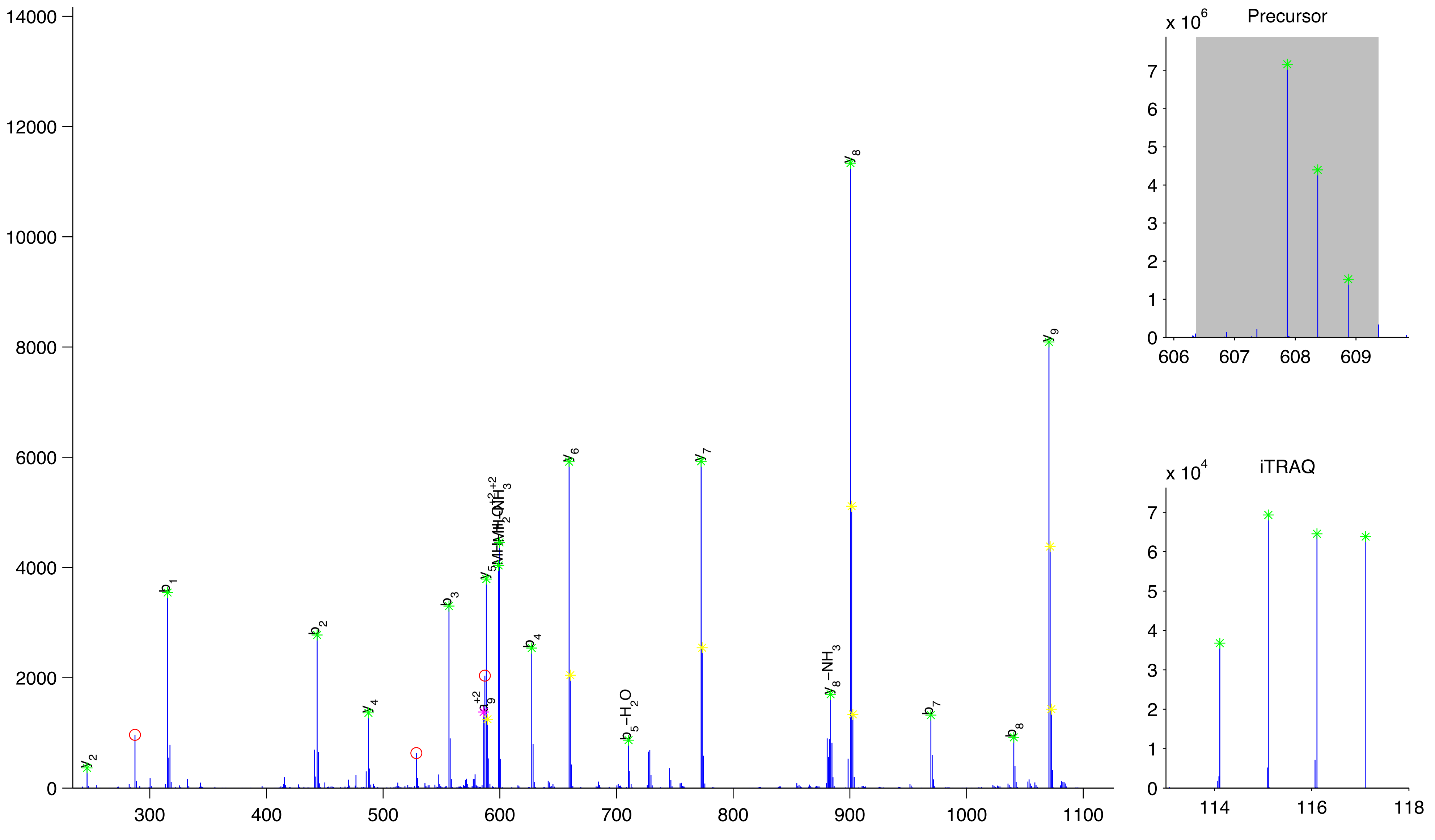

k S T G G k A P R

H3 histone, family 3A [Homo sapiens]
Charge State: +2
Scan Number: 13529
File Name: 110924_HG2_INSA_ACK.raw

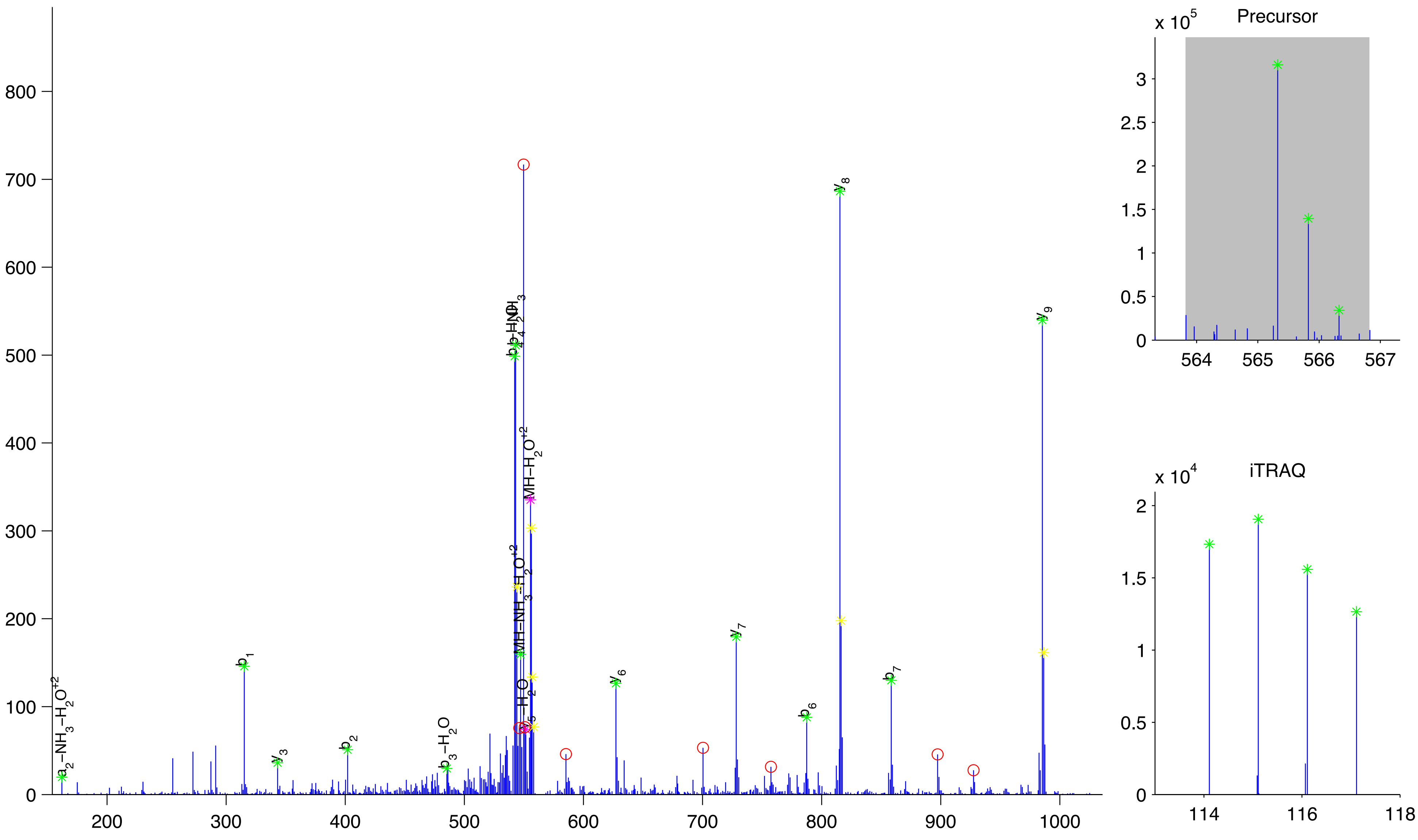

k Q L A T k A A R

H3 histone, family 3A [Homo sapiens]
Charge State: +2
Scan Number: 13569
File Name: 110924_HG2_INSA_ACK.raw

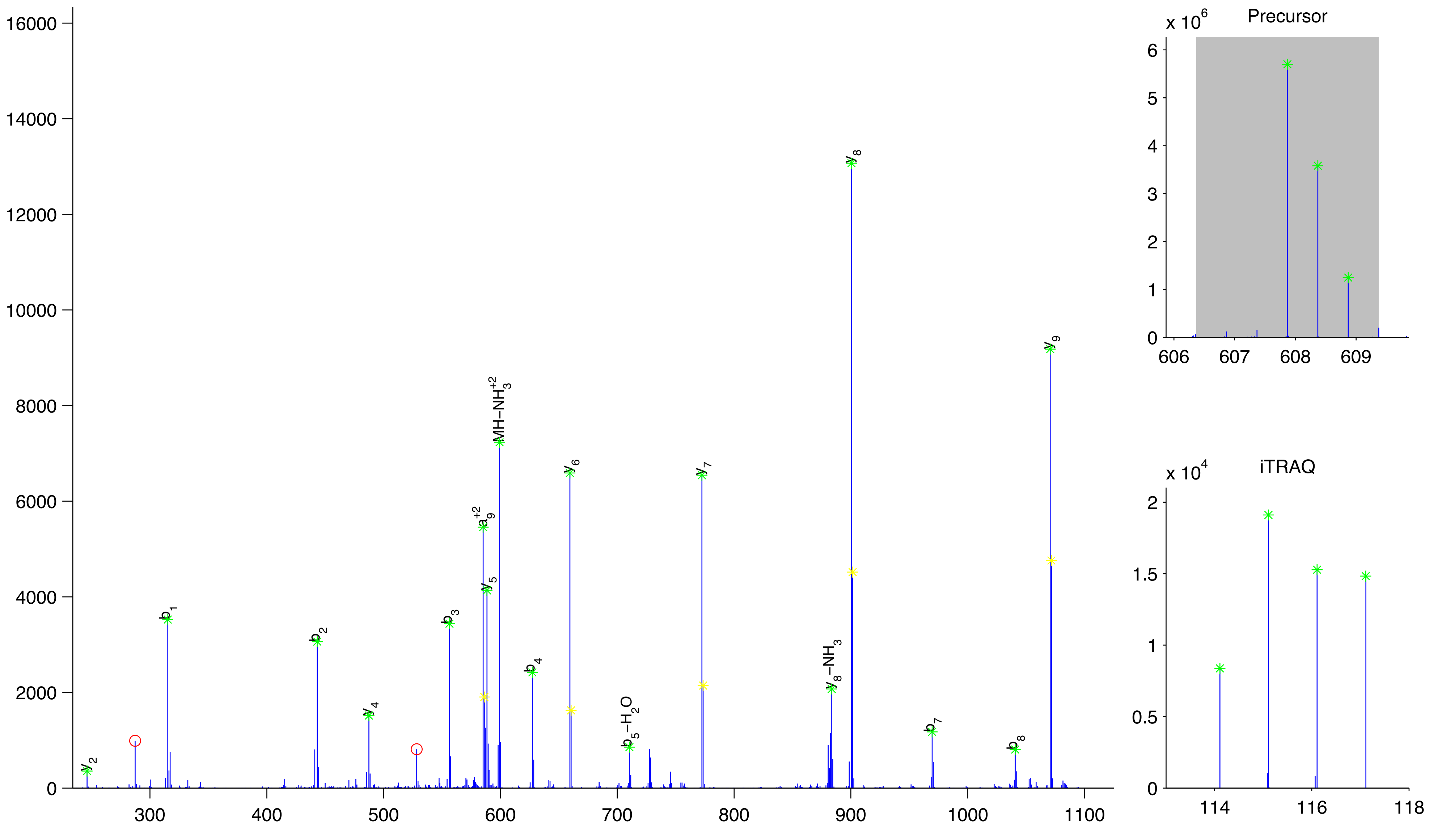

k Q L A T k A A R

H3 histone, family 3A [Homo sapiens]
Charge State: +2
Scan Number: 13657
File Name: 110924_HG2_INSA_ACK.raw

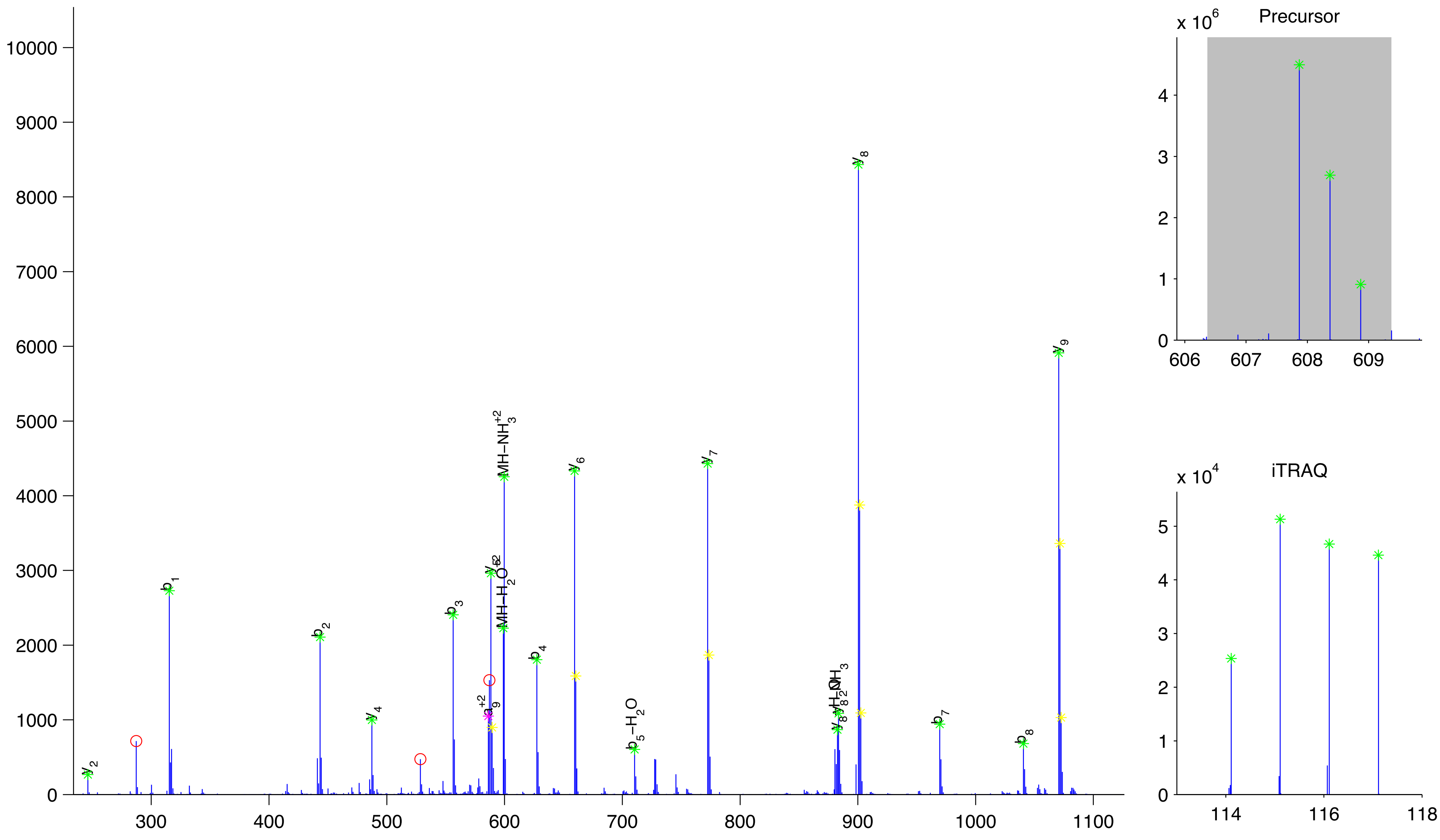

k Q L A T k A A R

H3 histone, family 3A [Homo sapiens]
Charge State: +2
Scan Number: 13737
File Name: 110924_HG2_INSA_ACK.raw

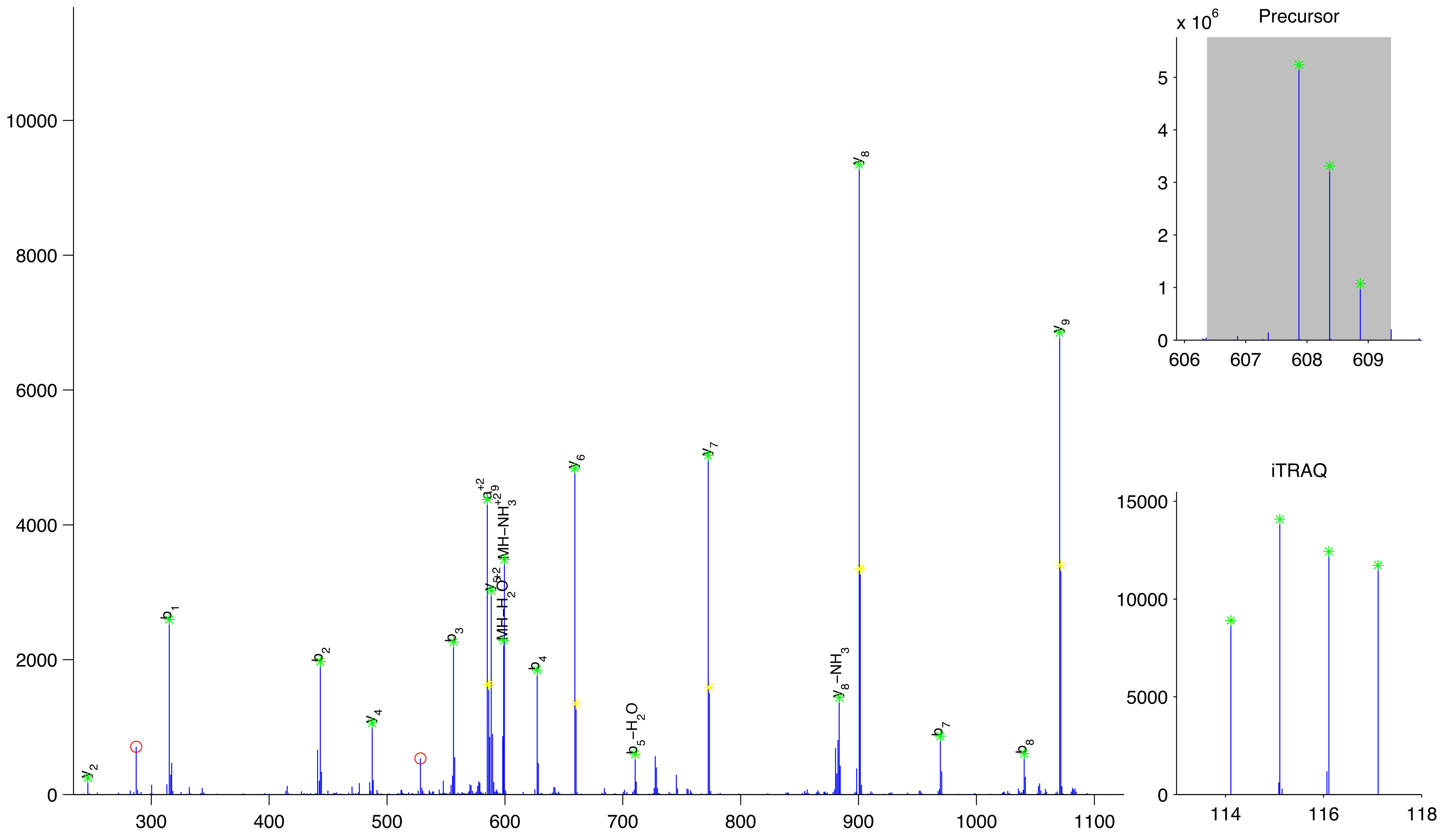

k Q L A T k A A R

H3 histone, family 3A [Homo sapiens]
Charge State: +2
Scan Number: 13825
File Name: 110924_HG2_INSA_ACK.raw

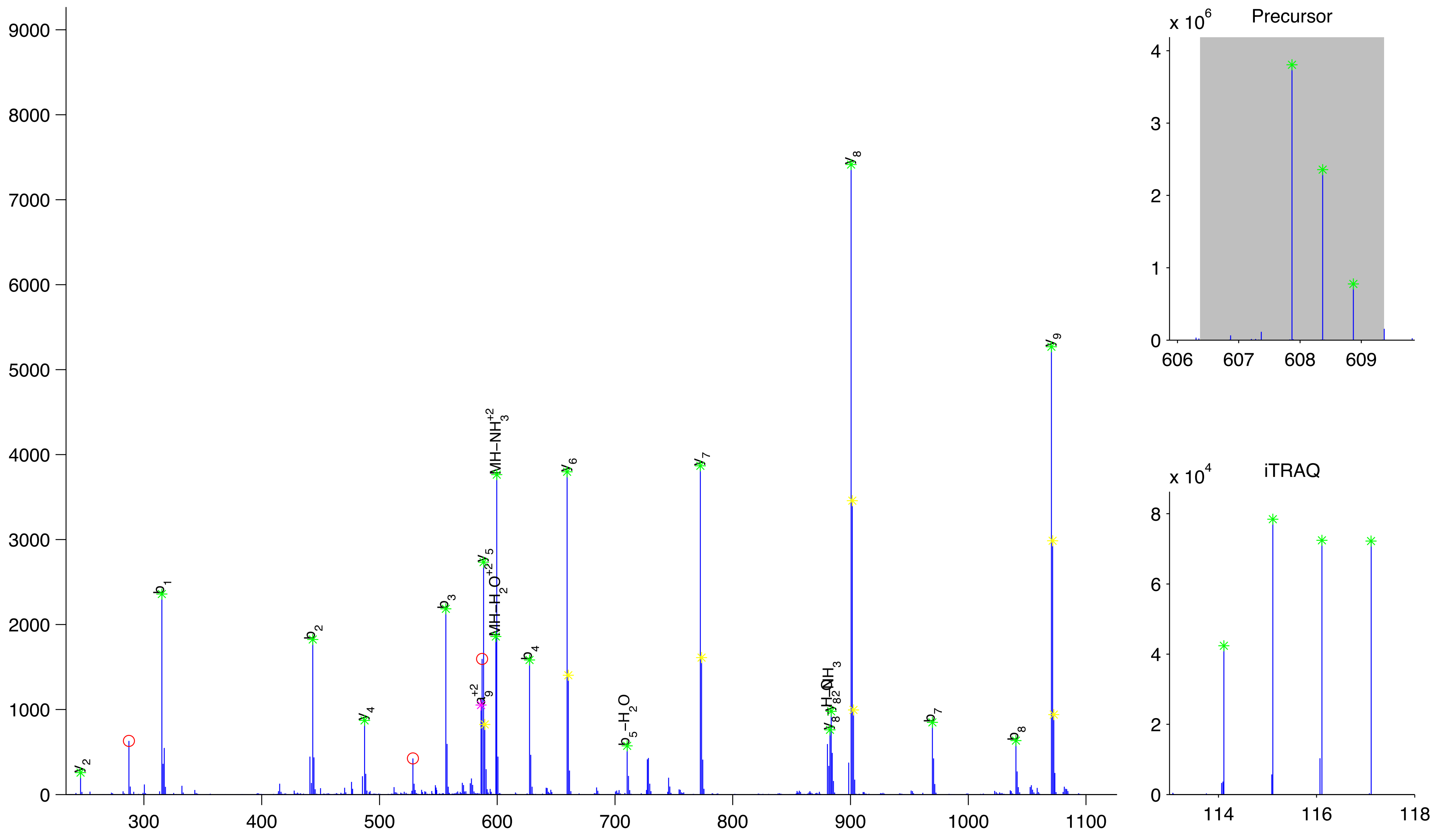

k Q L A T k A A R

H3 histone, family 3A [Homo sapiens]
Charge State: +2
Scan Number: 13905
File Name: 110924_HG2_INSA_ACK.raw

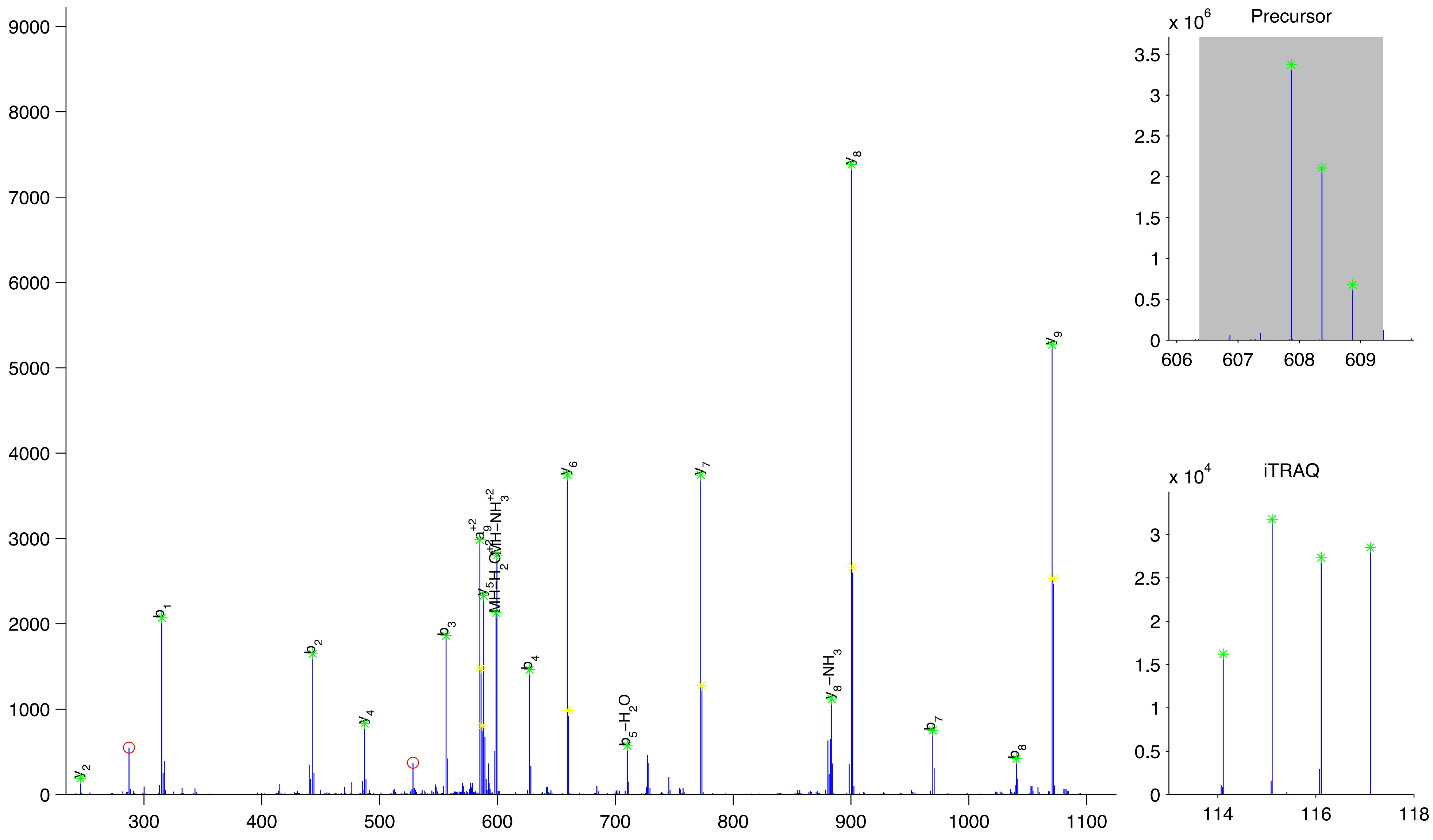

k Q L A T k A A R

H3 histone, family 3A [Homo sapiens]
Charge State: +2
Scan Number: 13993
File Name: 110924_HG2_INSA_ACK.raw

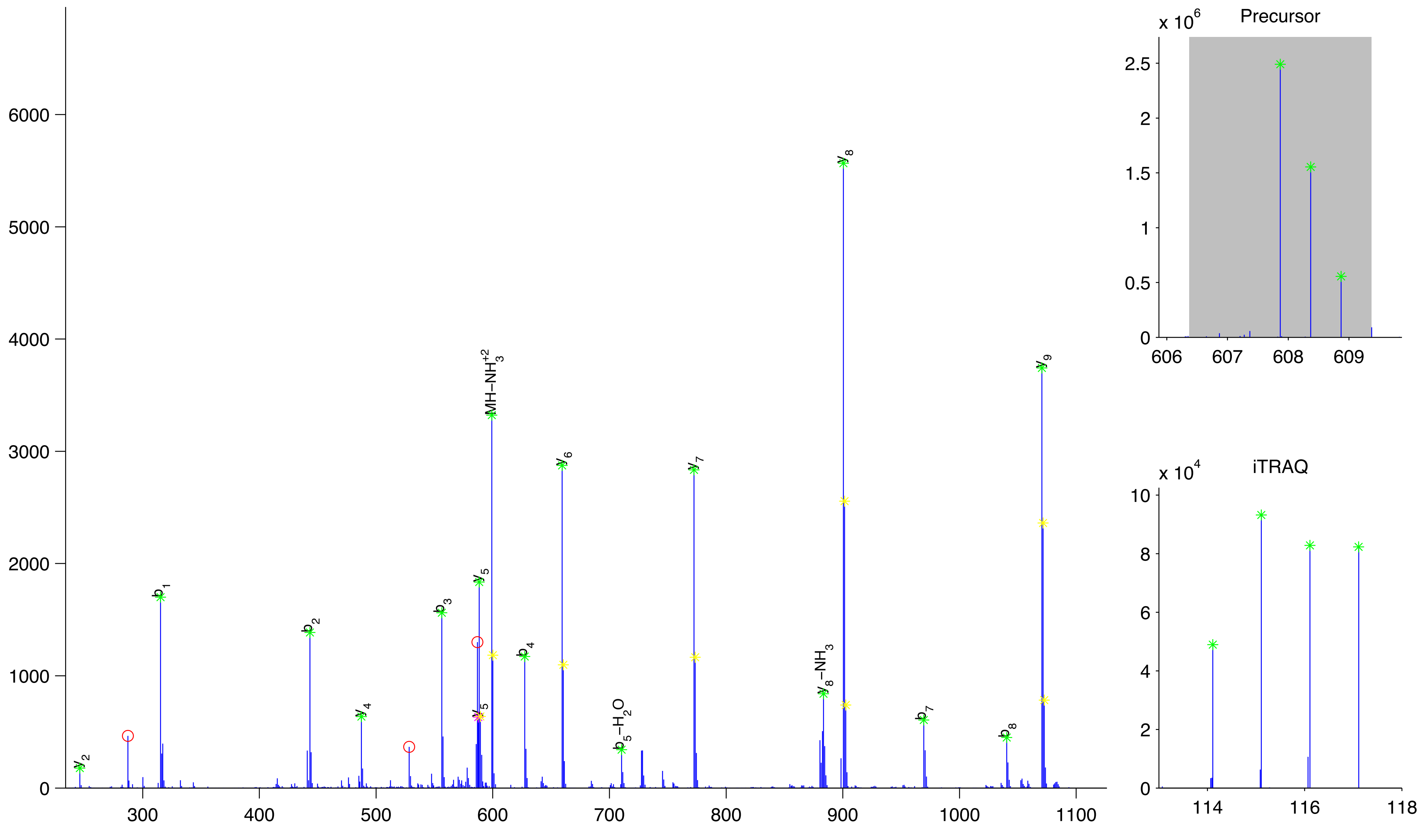

k Q L A T k A A R

H3 histone, family 3A [Homo sapiens]
Charge State: +2
Scan Number: 14073
File Name: 110924_HG2_INSA_ACK.raw

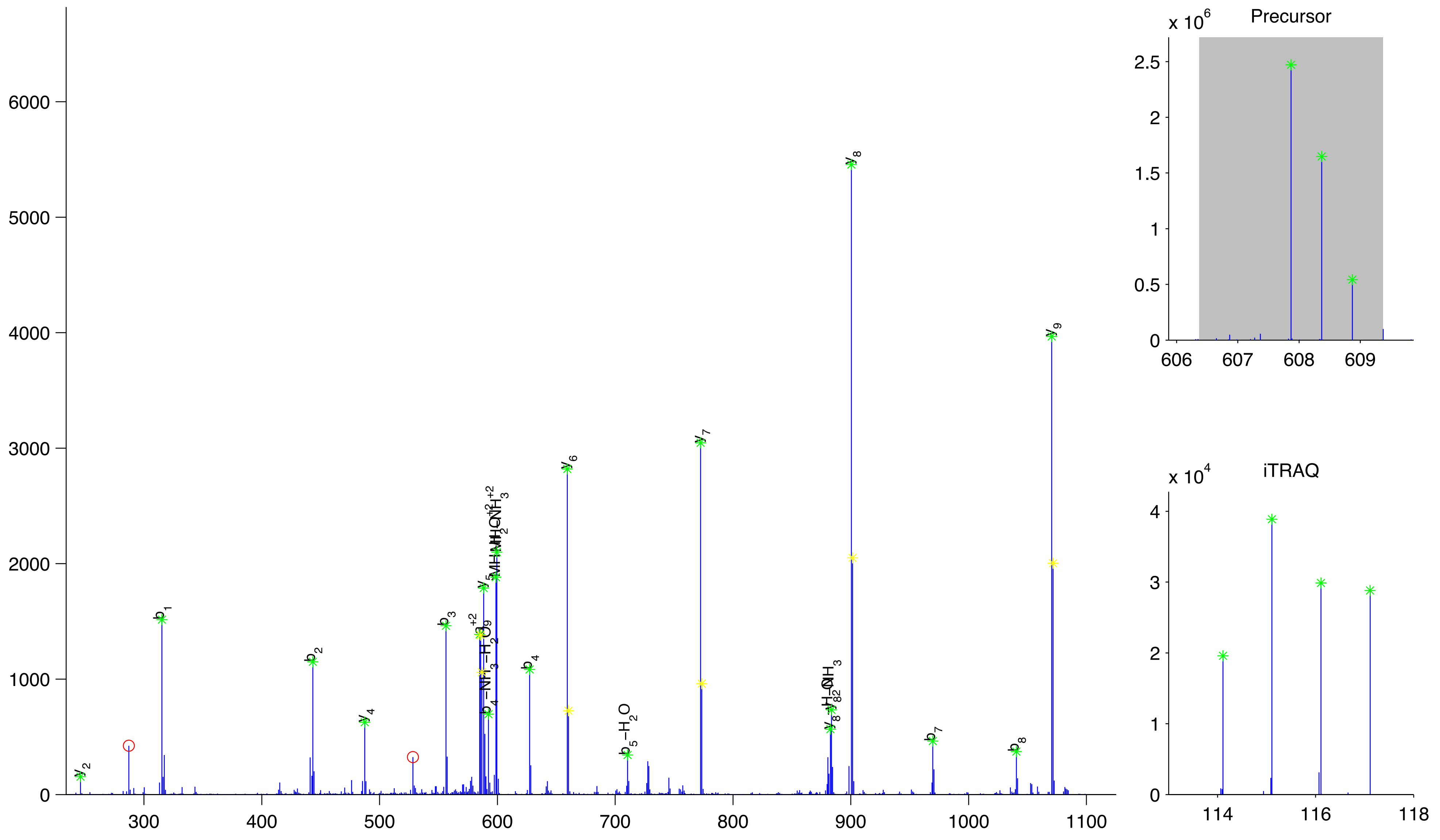

k Q L A T k A A R

H3 histone, family 3A [Homo sapiens]
Charge State: +2
Scan Number: 14241
File Name: 110924_HG2_INSA_ACK.raw

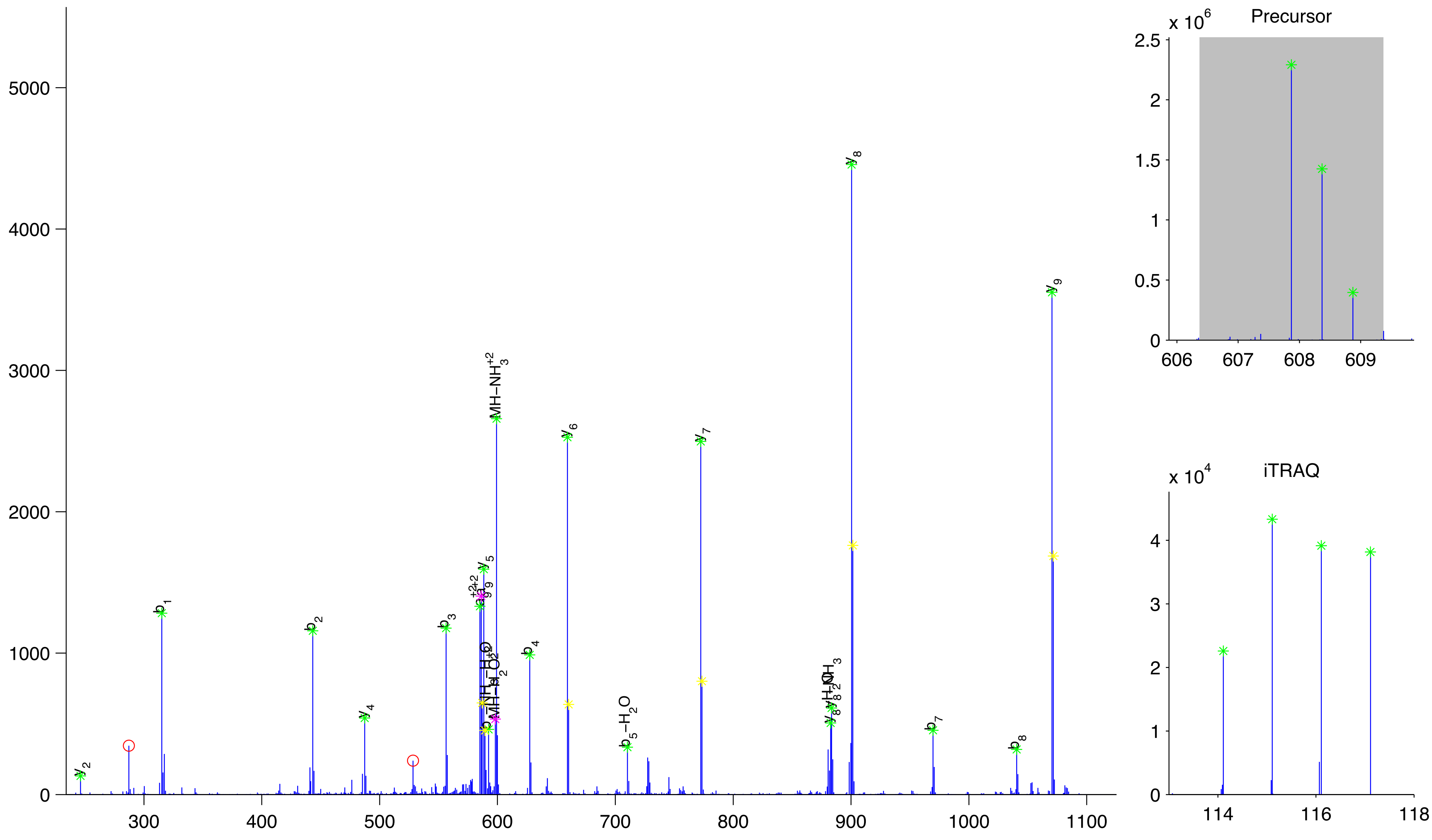

k Q L A T k A A R

H3 histone, family 3A [Homo sapiens]
Charge State: +2
Scan Number: 14409
File Name: 110924_HG2_INSA_ACK.raw

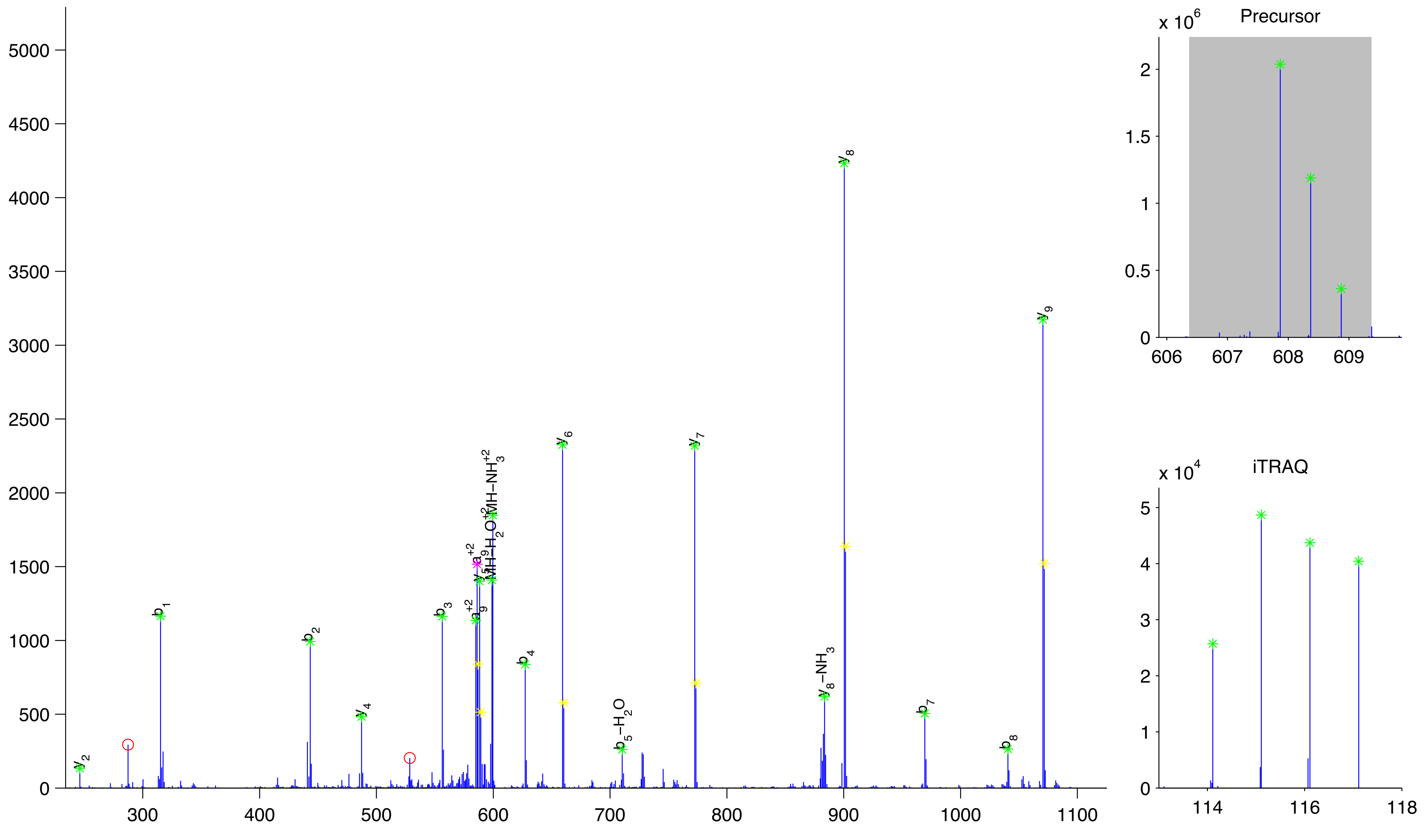

k Q L A T k A A R

H3 histone, family 3A [Homo sapiens]
Charge State: +2
Scan Number: 14577
File Name: 110924_HG2_INSA_ACK.raw

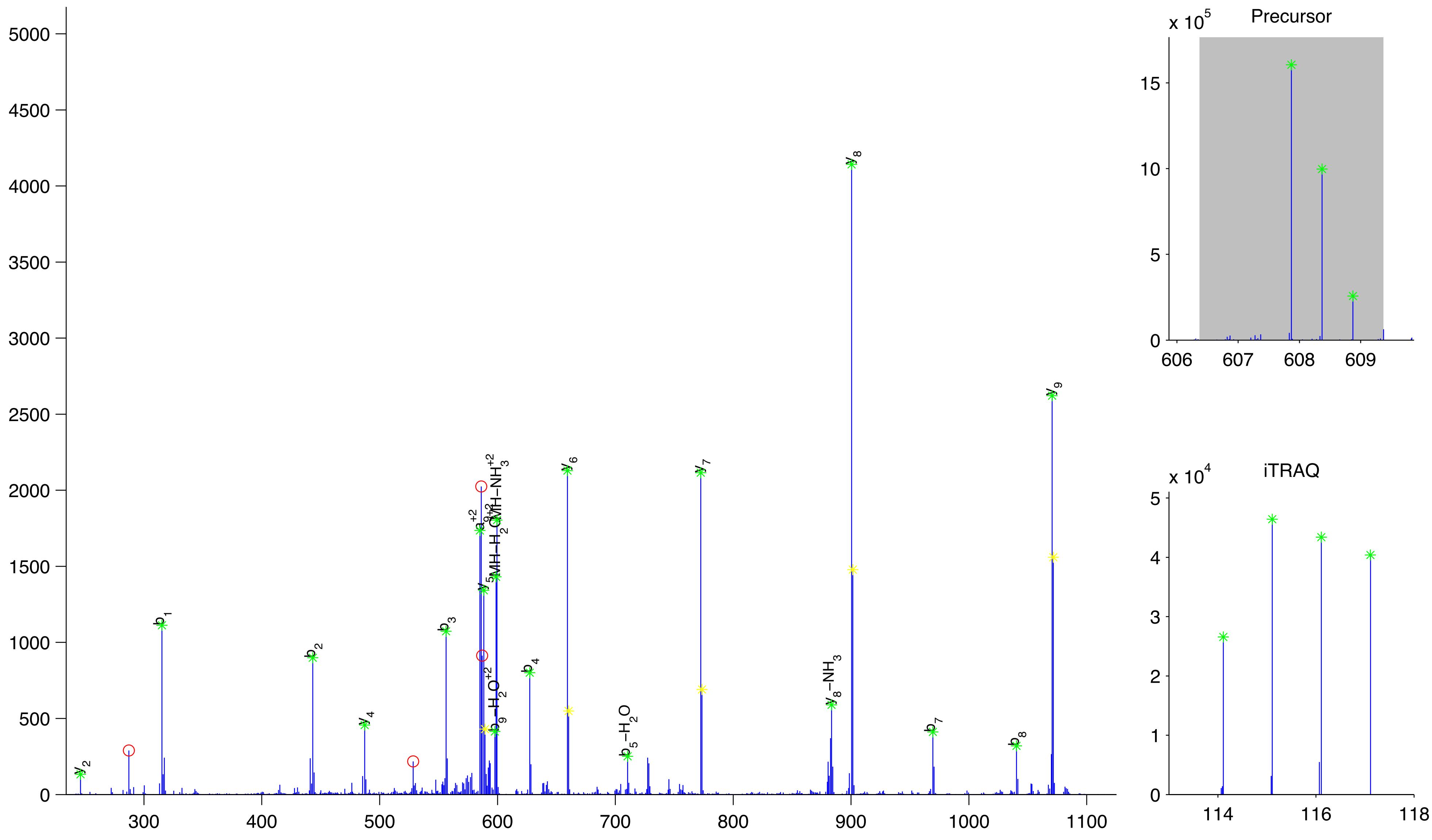

k Q L A T k A A R

H3 histone, family 3A [Homo sapiens]
Charge State: +2
Scan Number: 14745
File Name: 110924_HG2_INSA_ACK.raw

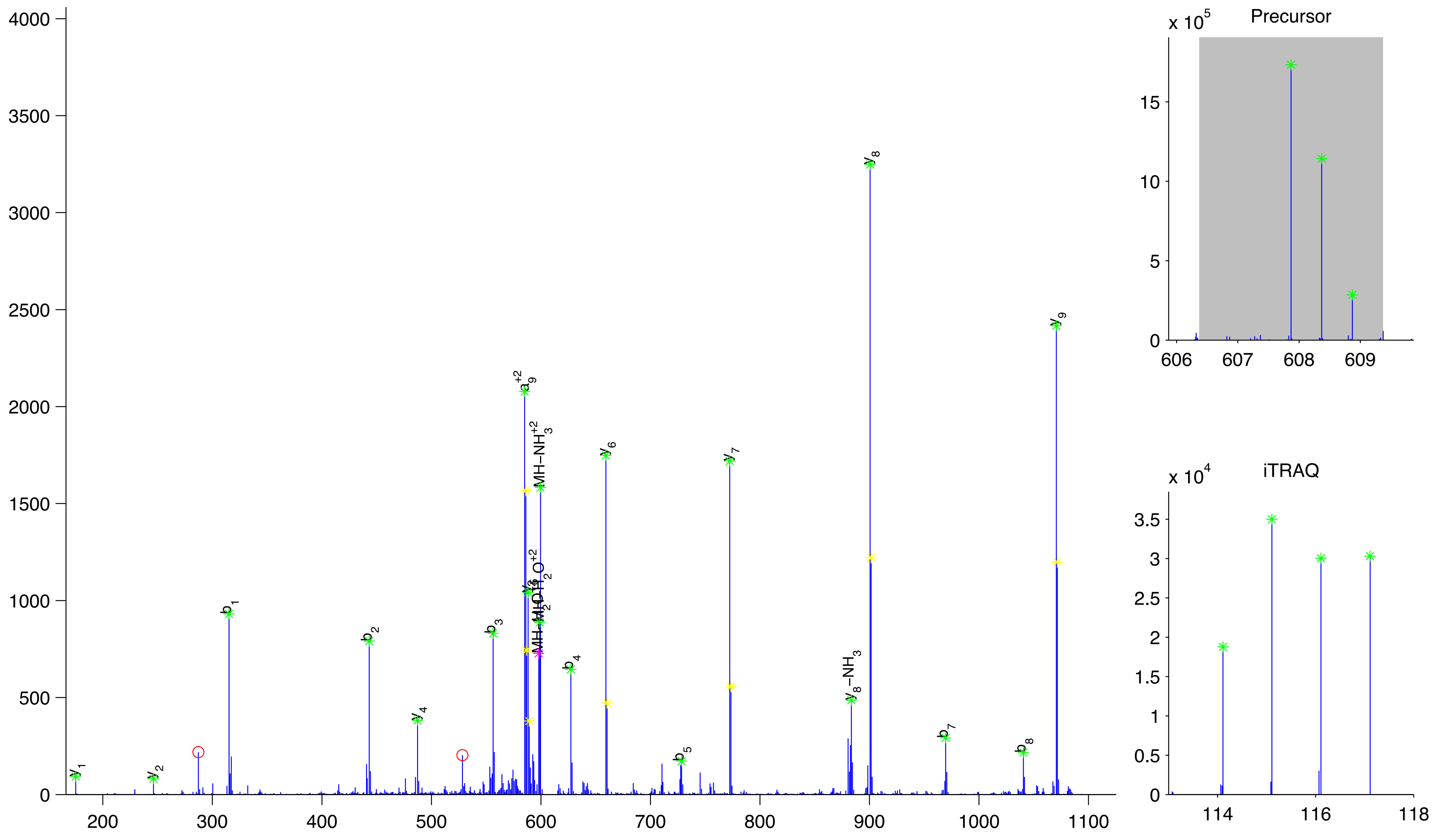

k Q L A T k A A R

H3 histone, family 3A [Homo sapiens]
Charge State: +2
Scan Number: 14913
File Name: 110924_HG2_INSA_ACK.raw

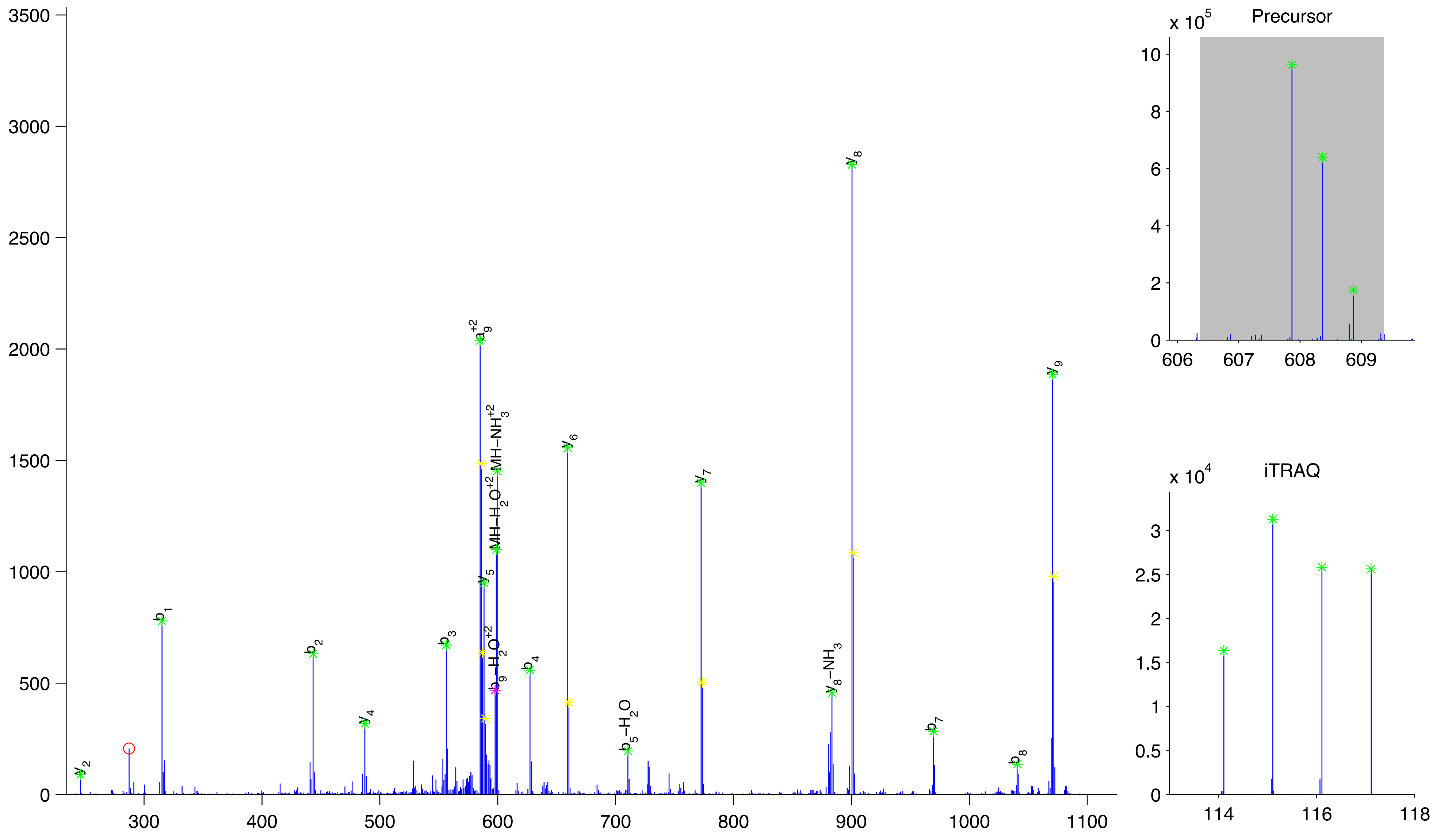

k Q L A T k A A R

H3 histone, family 3A [Homo sapiens]
Charge State: +2
Scan Number: 15081
File Name: 110924_HG2_INSA_ACK.raw

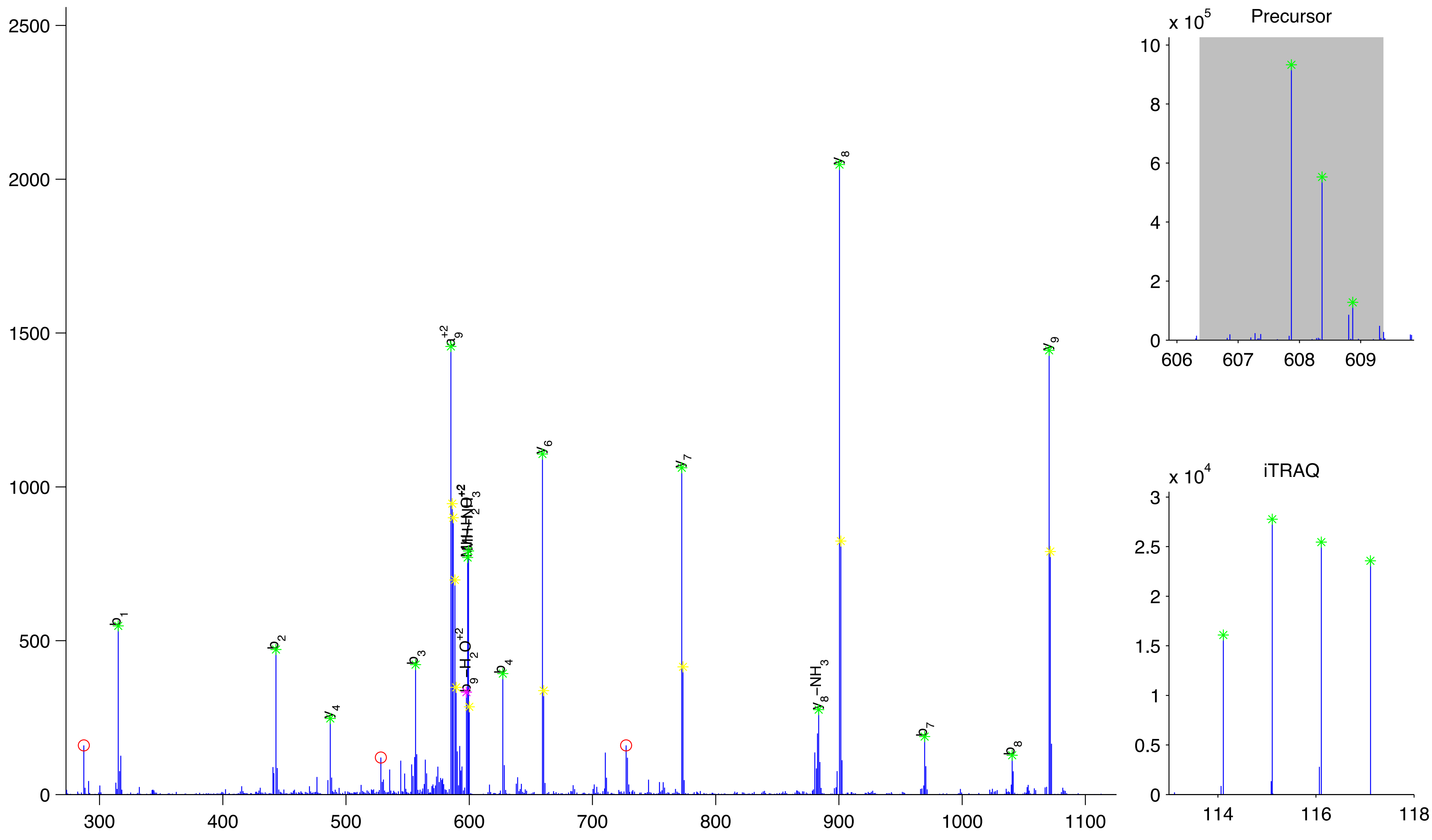

k Q L A T k A A R

H3 histone, family 3A [Homo sapiens]
Charge State: +2
Scan Number: 15251
File Name: 110924_HG2_INSA_ACK.raw

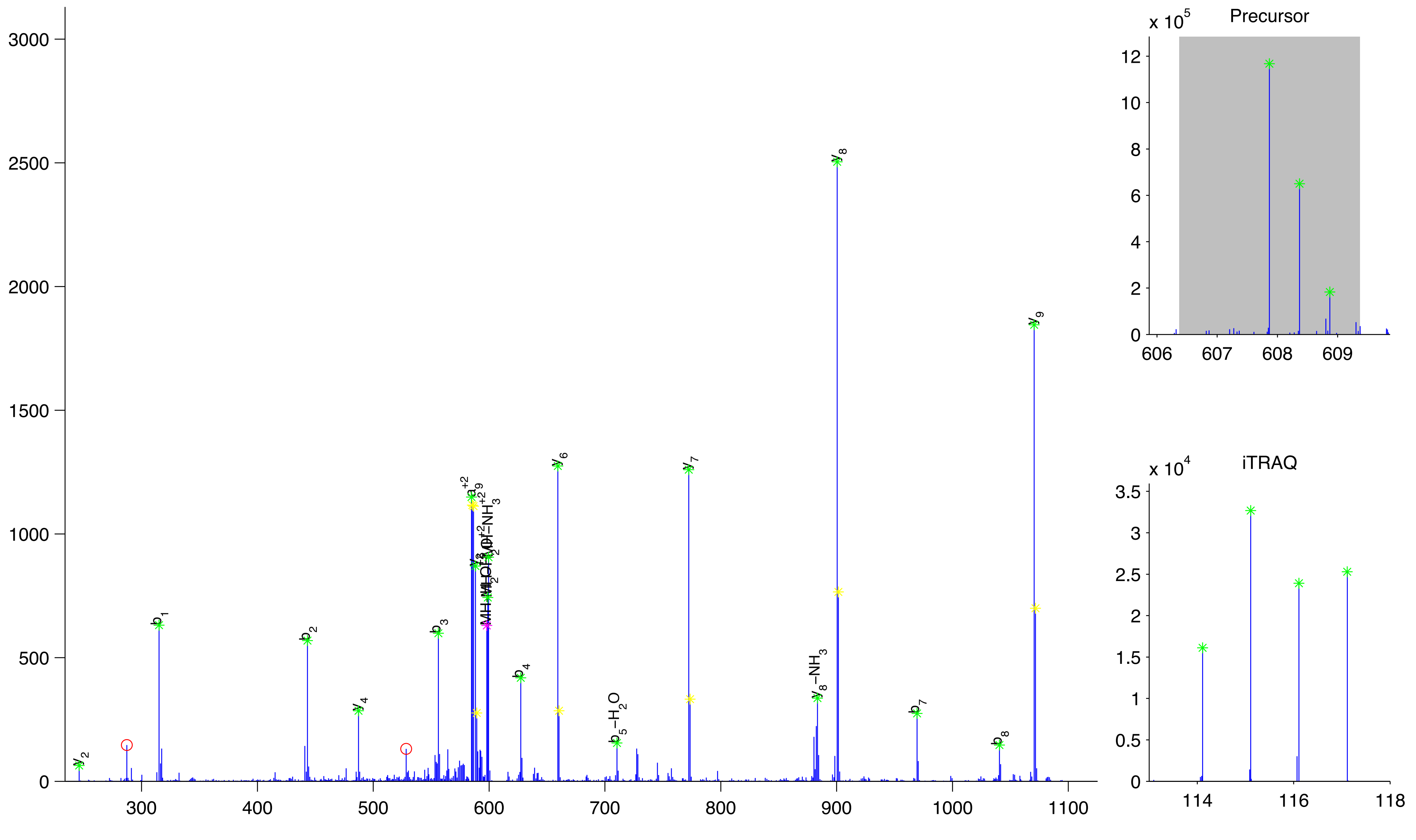

k Q L A T k A A R

H3 histone, family 3A [Homo sapiens]
Charge State: +2
Scan Number: 15419
File Name: 110924_HG2_INSA_ACK.raw

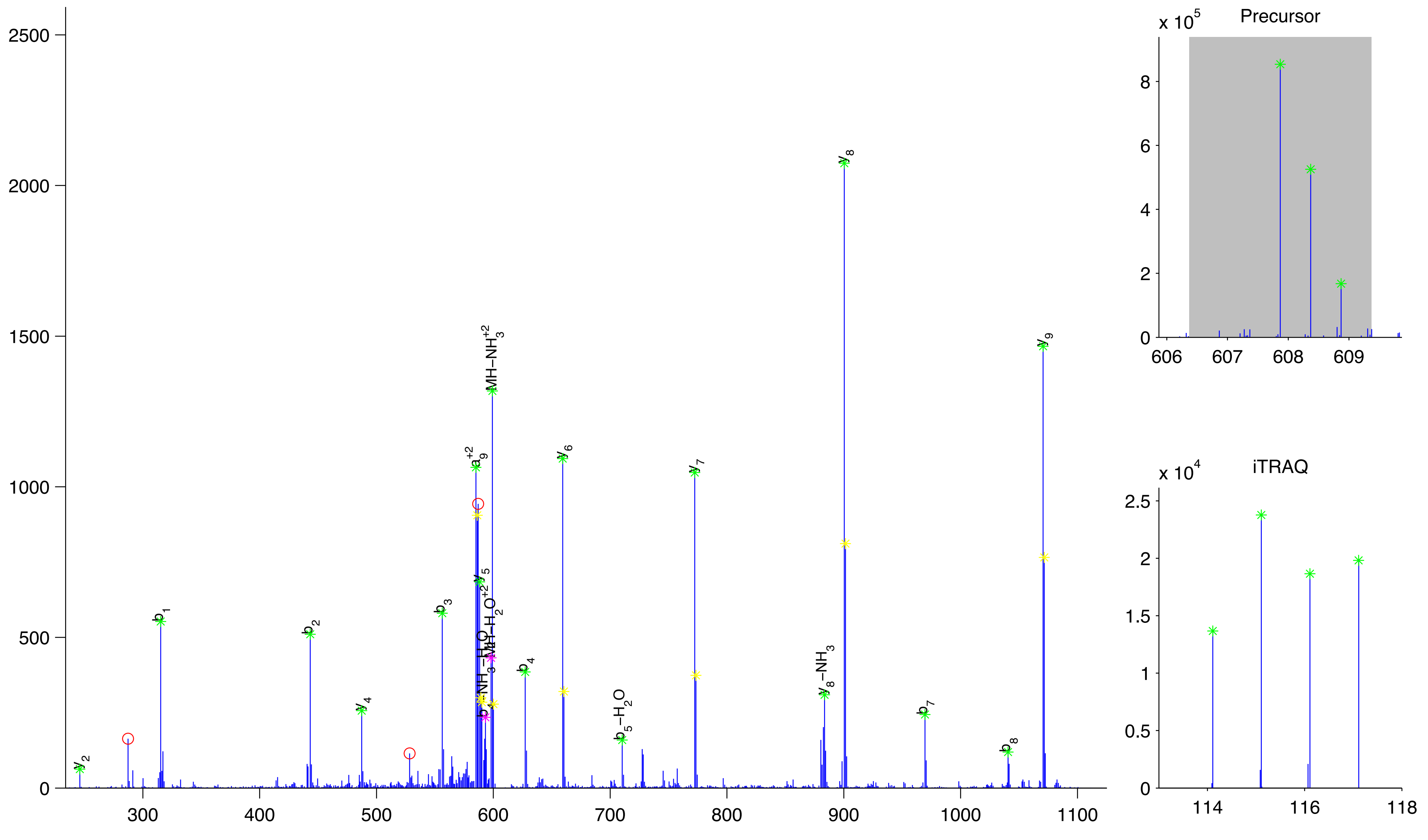

k Q L A T k A A R

H3 histone, family 3A [Homo sapiens]
Charge State: +2
Scan Number: 15587
File Name: 110924_HG2_INSA_ACK.raw

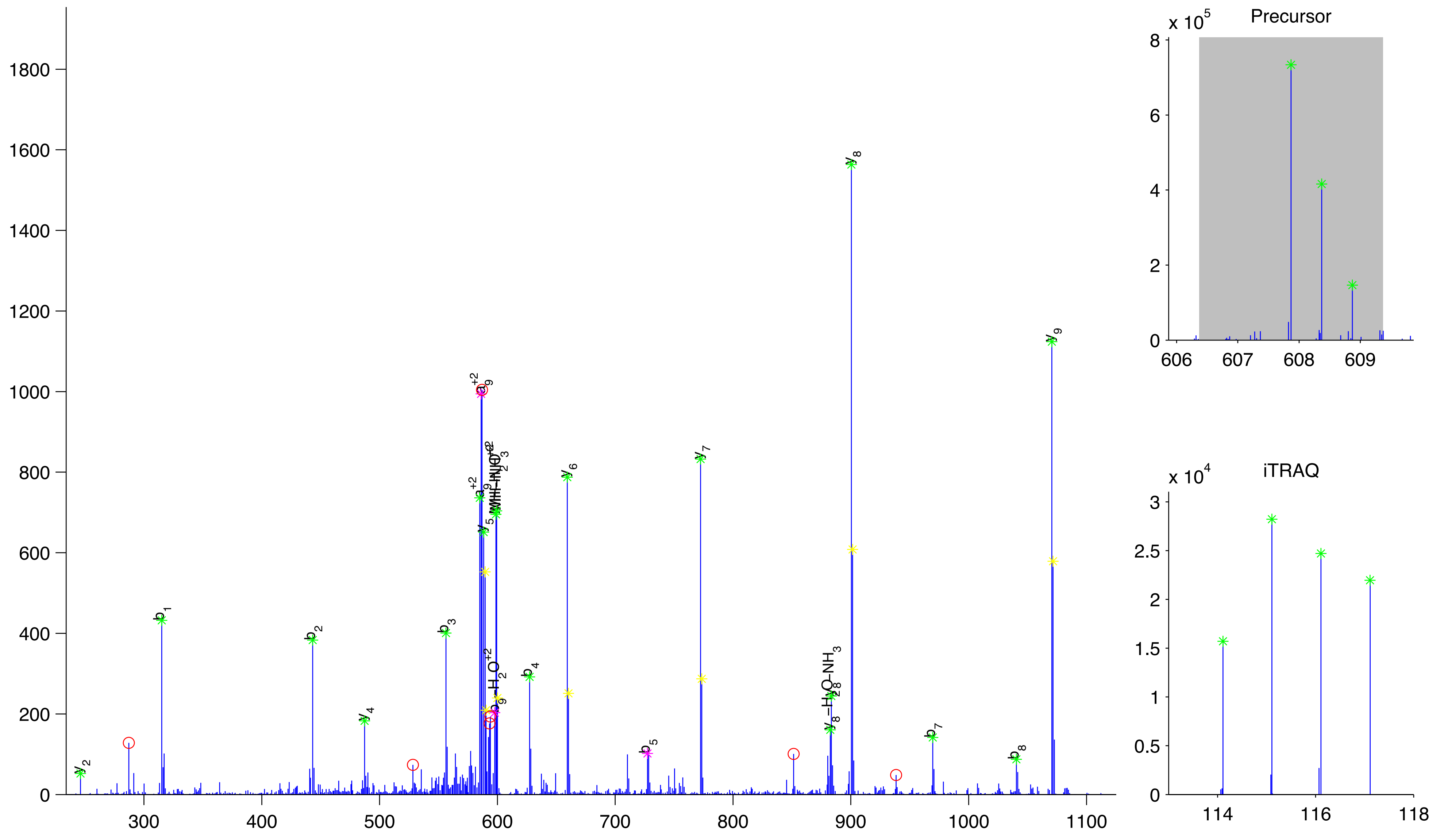

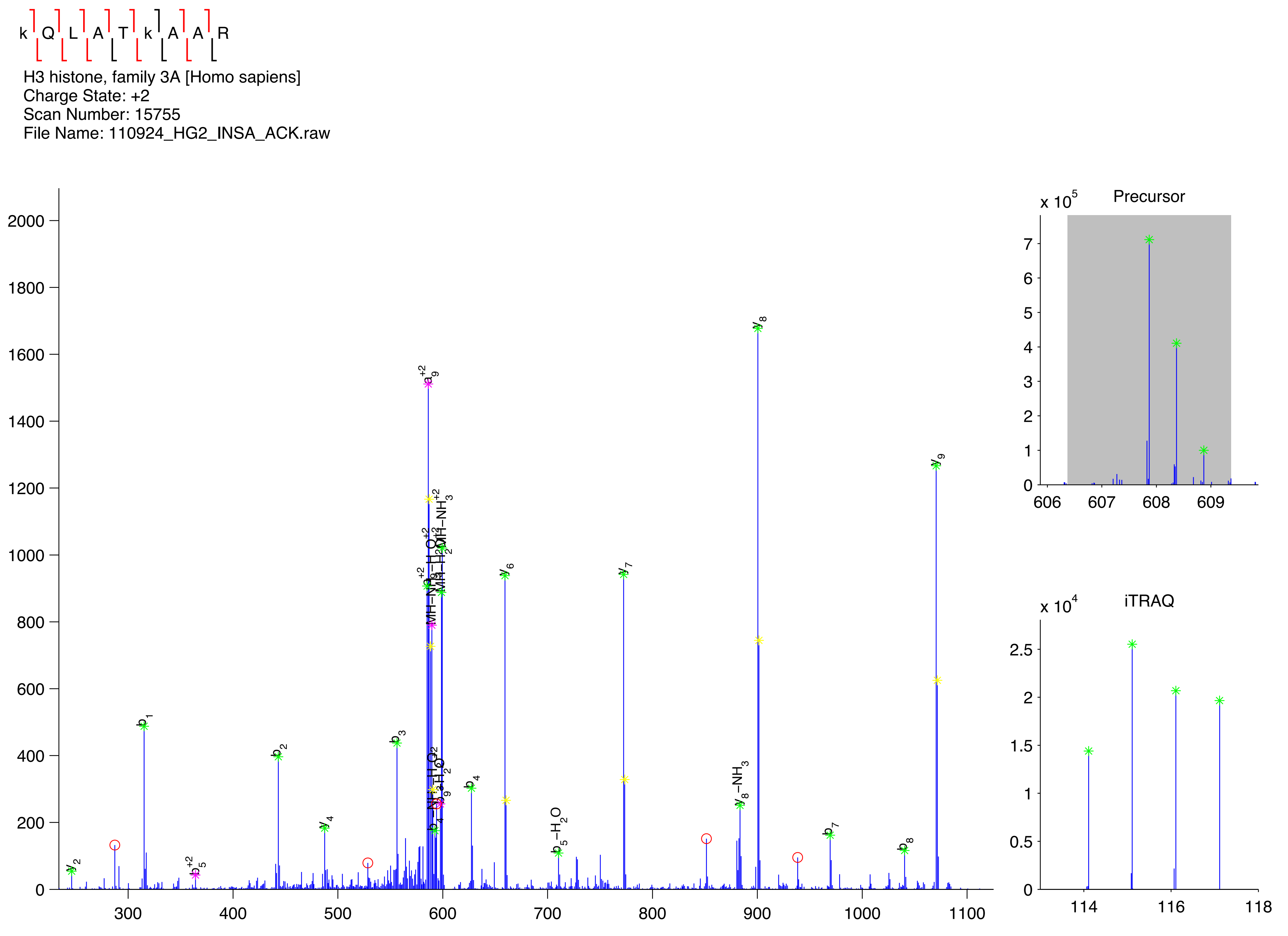
k Q L A T k A A R
H3 histone, family 3A [Homo sapiens]
Charge State: +2
Scan Number: 15755
File Name: 110924_HG2_INSA_ACK.raw
Precursor
x 10^5
iTRAQ
x 10^4

k Q L A T k A A R

H3 histone, family 3A [Homo sapiens]
Charge State: +2
Scan Number: 15923
File Name: 110924_HG2_INSA_ACK.raw

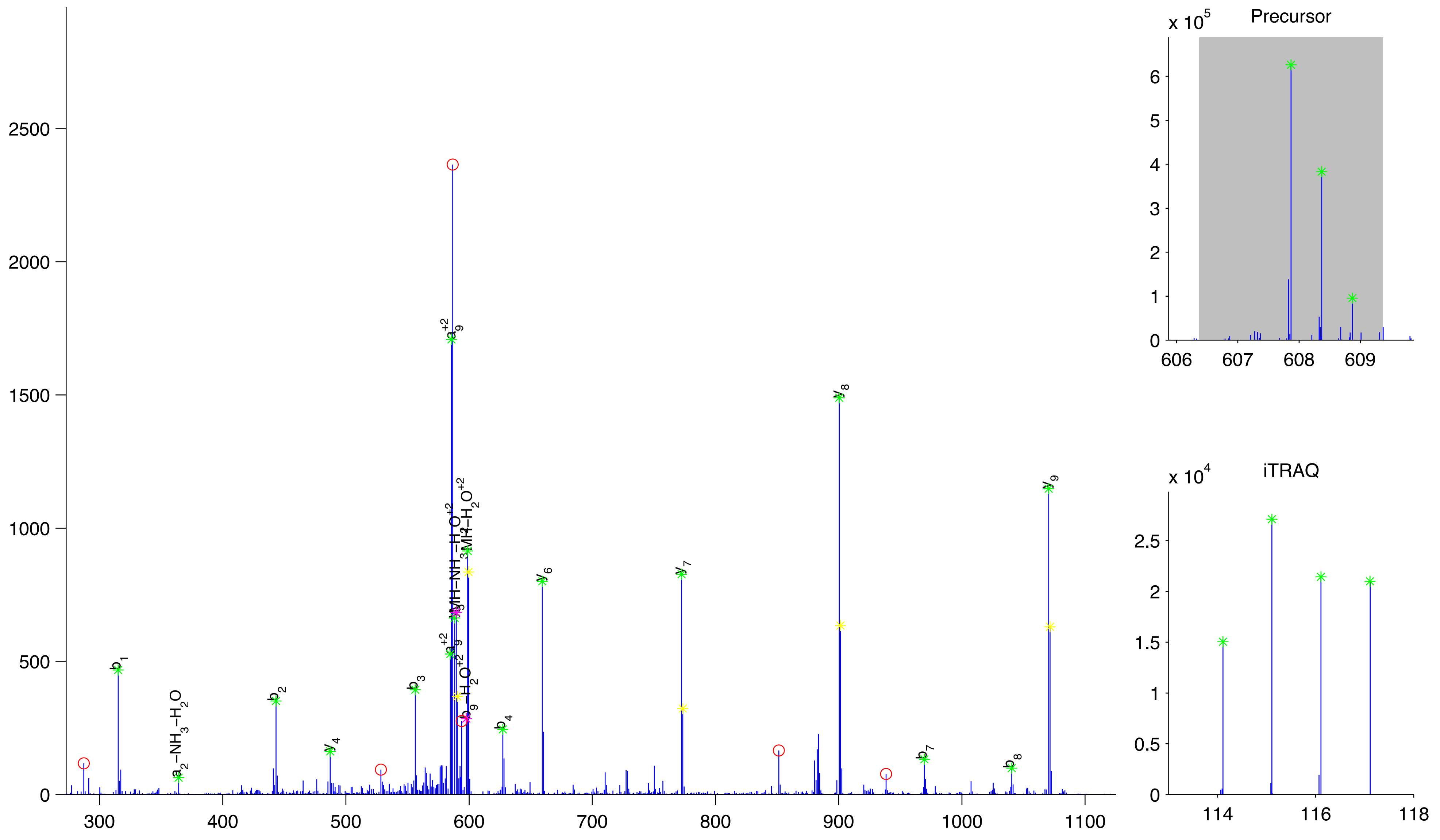

k Q L A T k A A R

H3 histone, family 3A [Homo sapiens]
Charge State: +2
Scan Number: 16091
File Name: 110924_HG2_INSA_ACK.raw

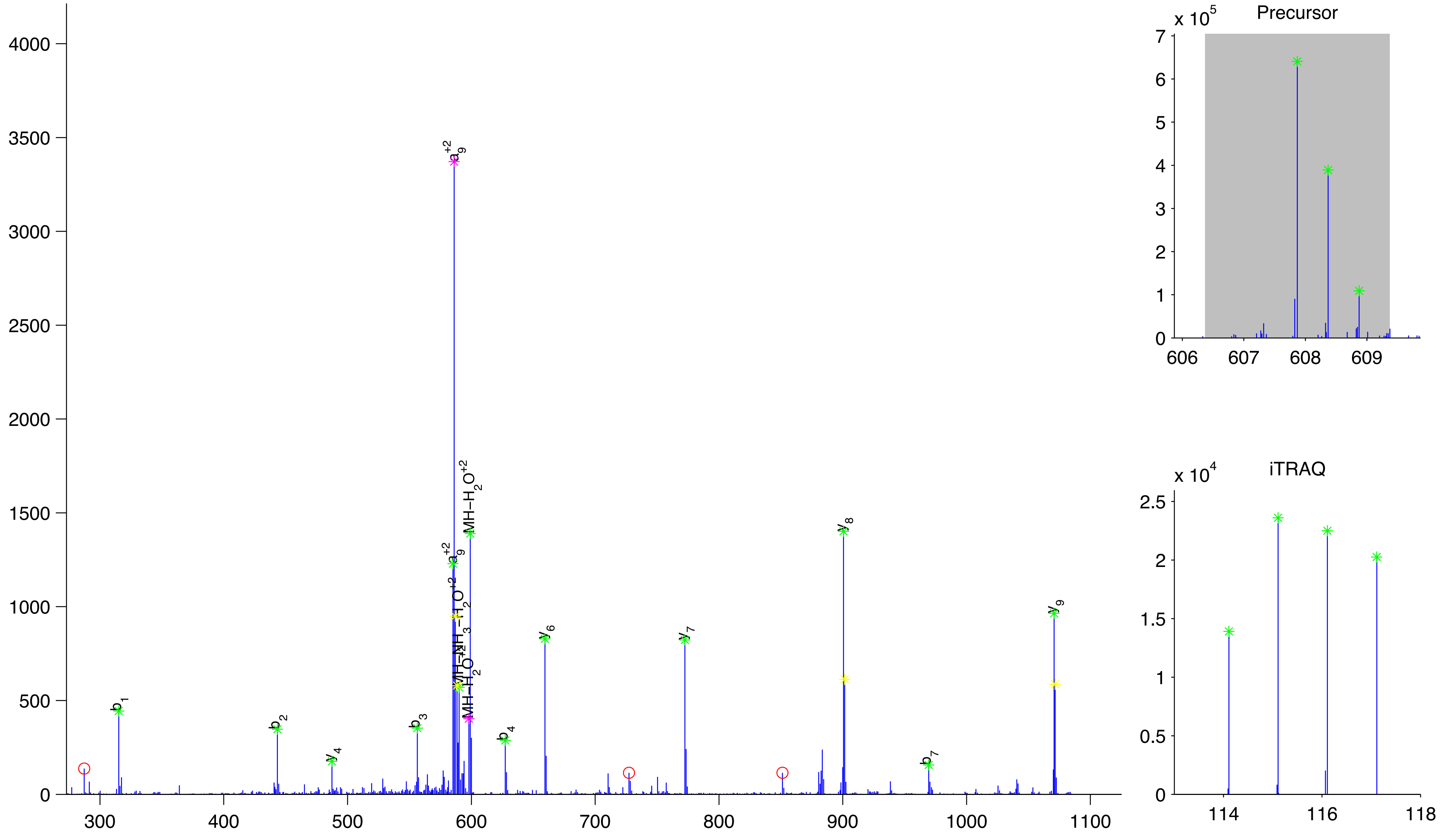

k Q L A T k A A R

H3 histone, family 3A [Homo sapiens]
Charge State: +2
Scan Number: 16259
File Name: 110924_HG2_INSA_ACK.raw

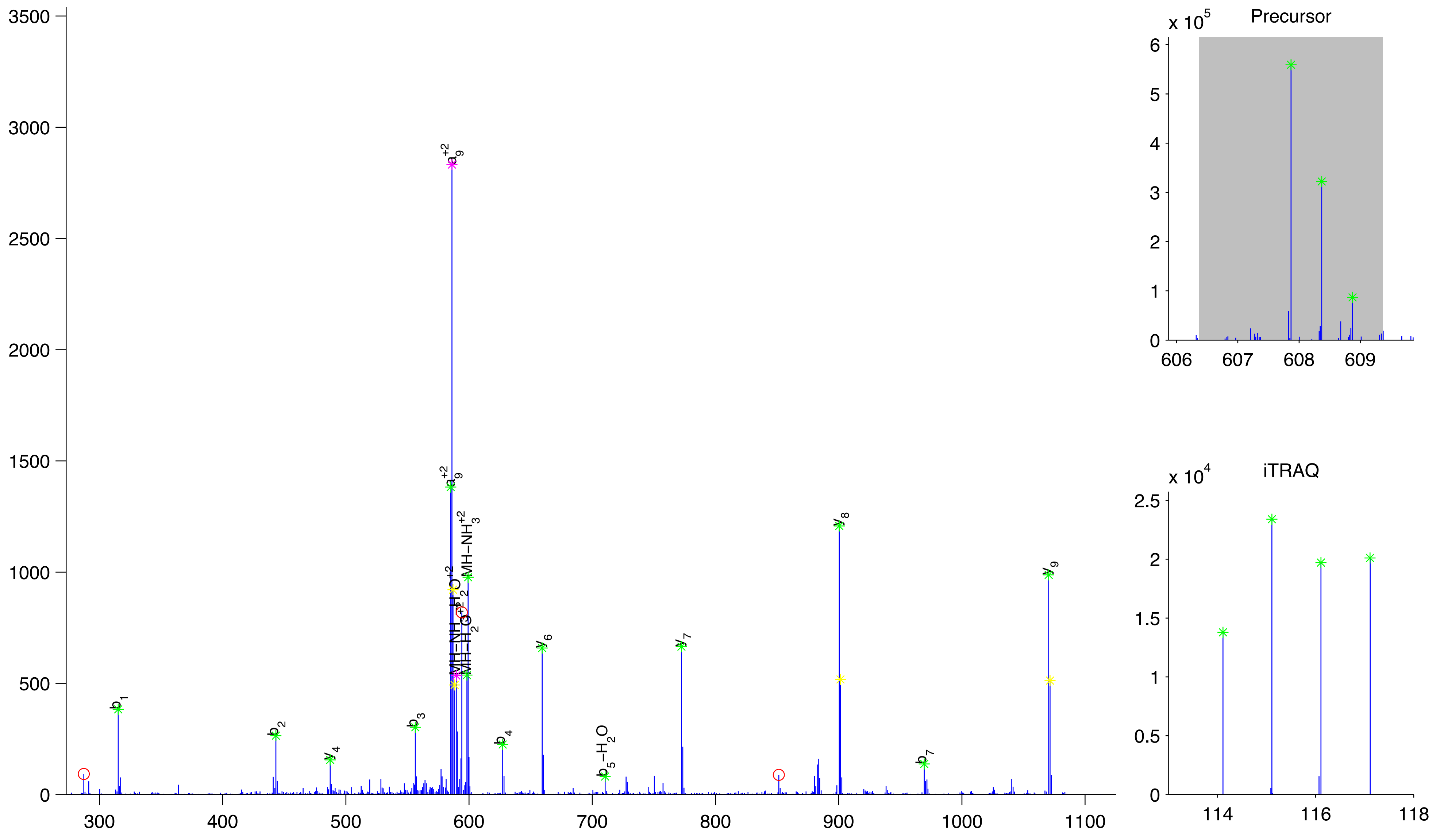

k Q L A T k A A R

H3 histone, family 3A [Homo sapiens]
Charge State: +2
Scan Number: 16427
File Name: 110924_HG2_INSA_ACK.raw

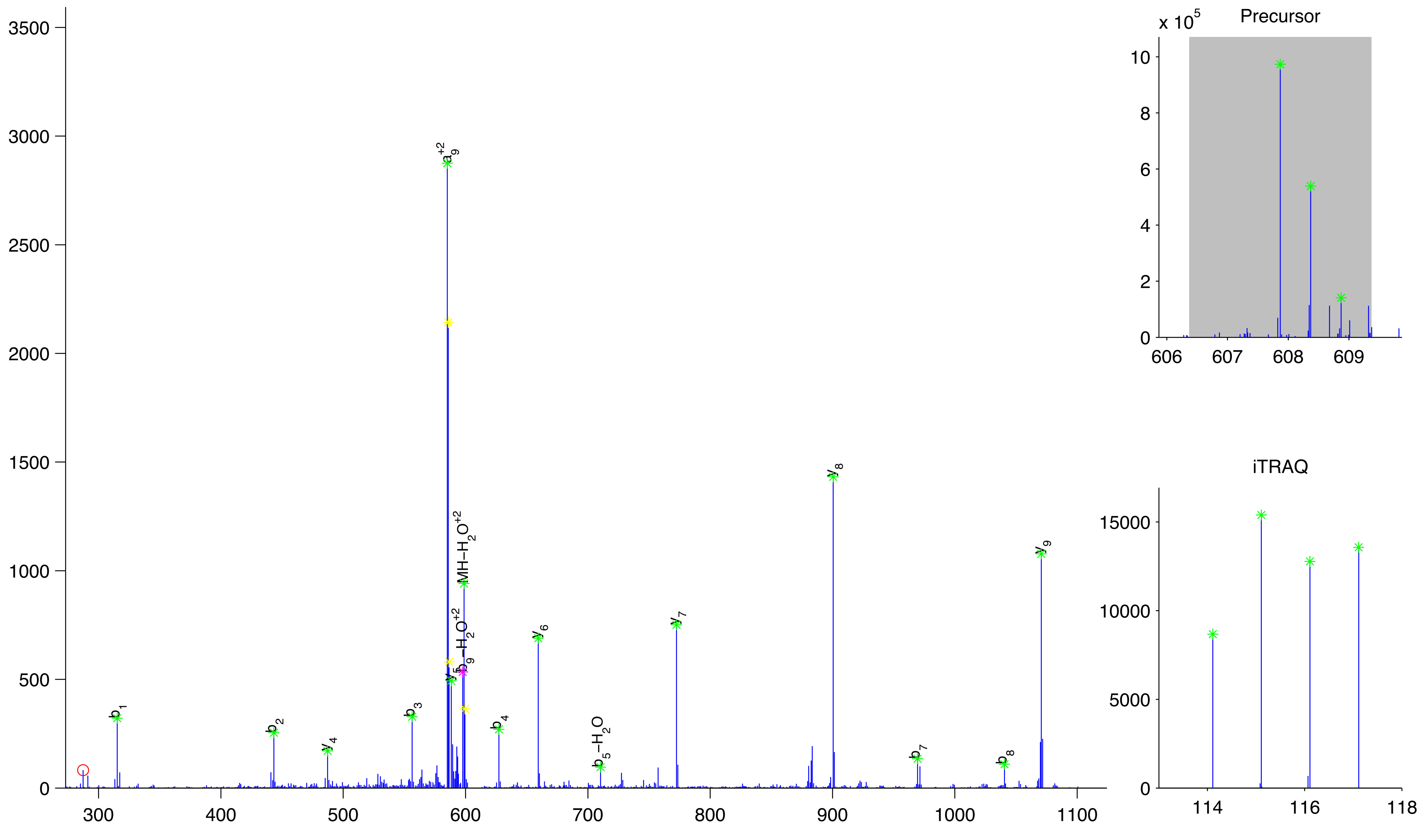

k Q L A T k A A R

H3 histone, family 3A [Homo sapiens]
Charge State: +2
Scan Number: 17099
File Name: 110924_HG2_INSA_ACK.raw

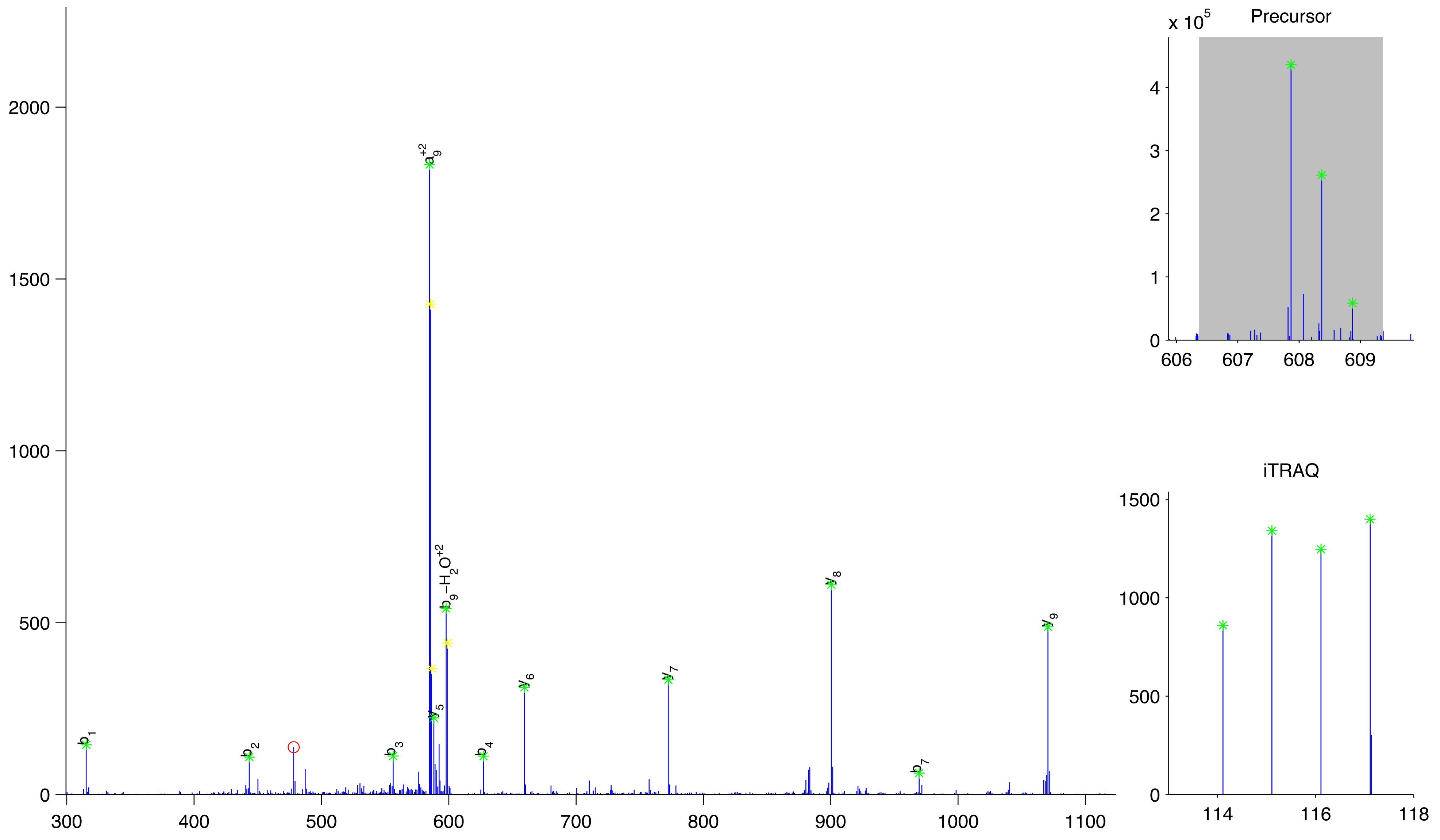

k Q L A T k A A R

H3 histone, family 3A [Homo sapiens]
Charge State: +2
Scan Number: 17267
File Name: 110924_HG2_INSA_ACK.raw

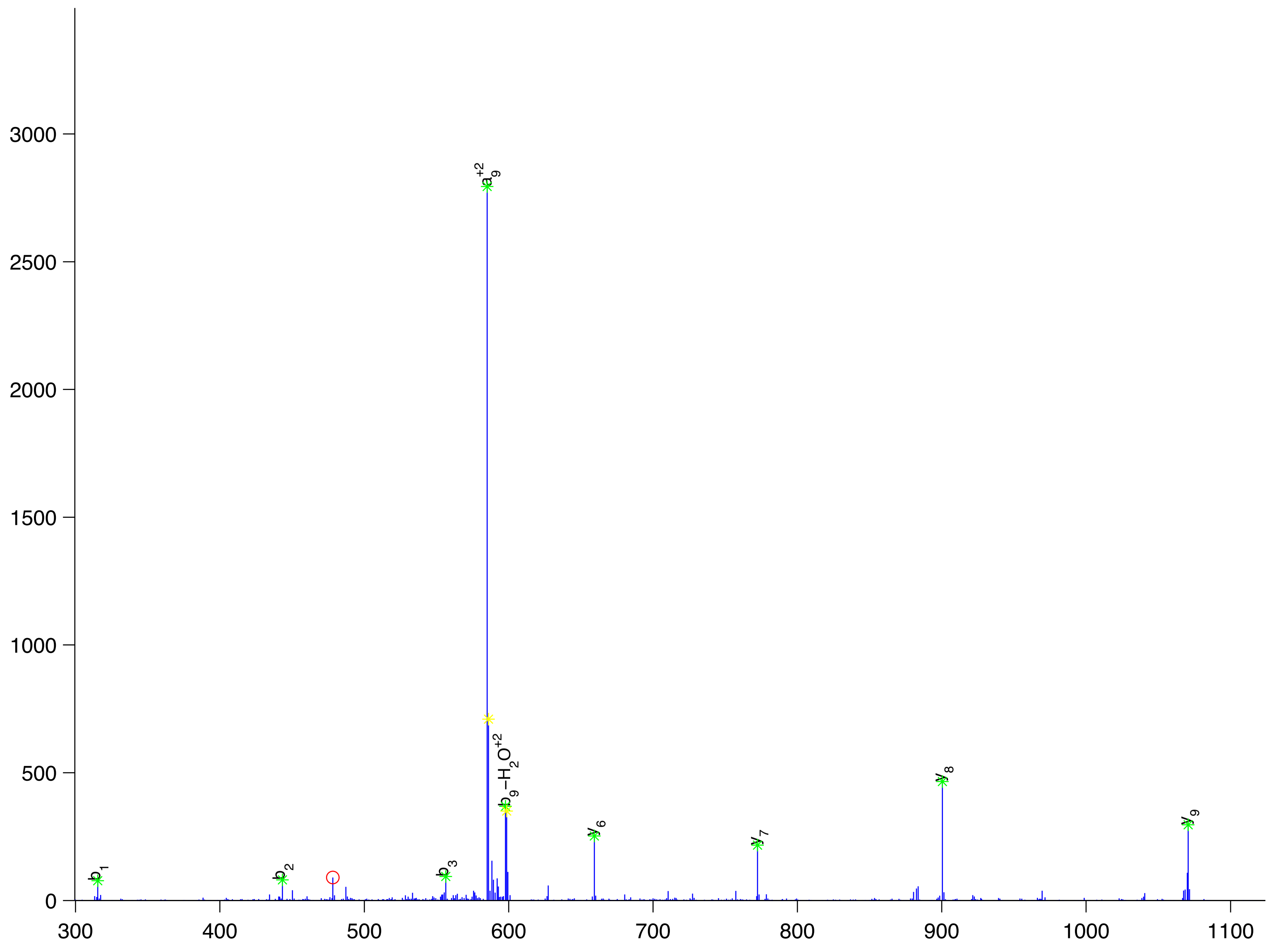

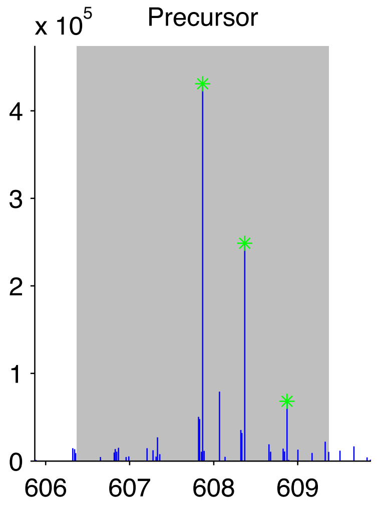

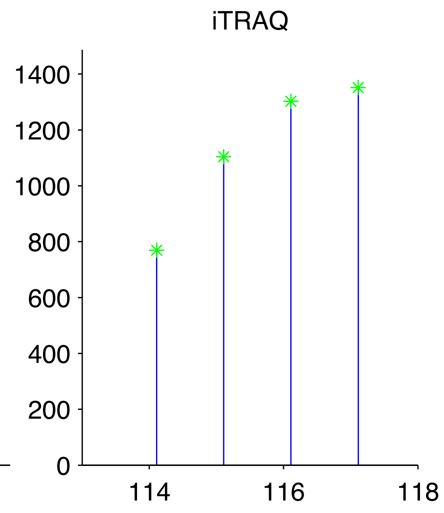

k Q L A T k A A R

H3 histone, family 3A [Homo sapiens]
Charge State: +2
Scan Number: 17437
File Name: 110924_HG2_INSA_ACK.raw

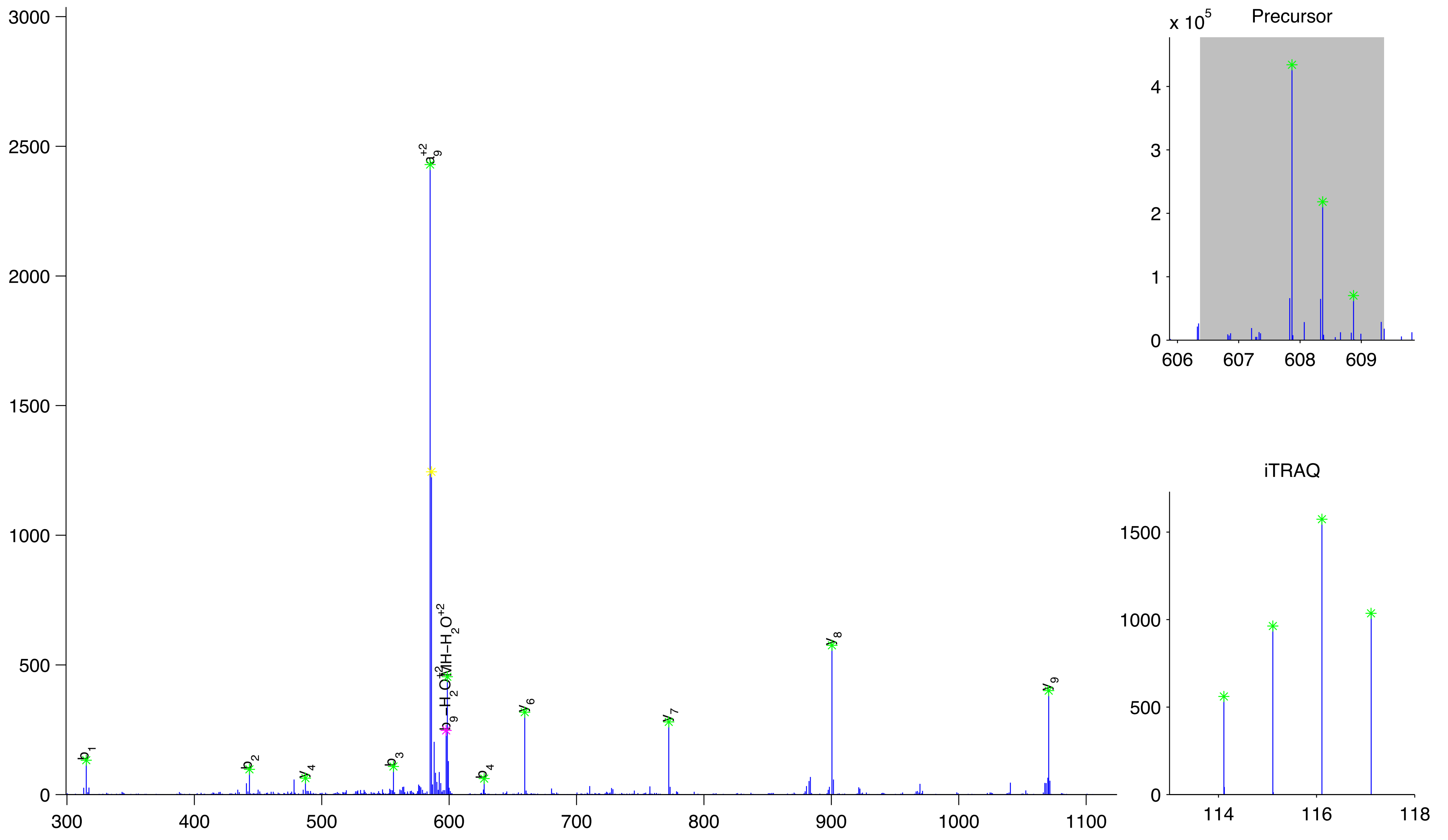

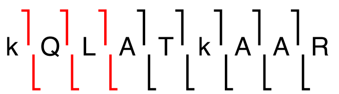

H3 histone, family 3A [Homo sapiens]
Charge State: +2
Scan Number: 18951
File Name: 110924_HG2_INSA_ACK.raw

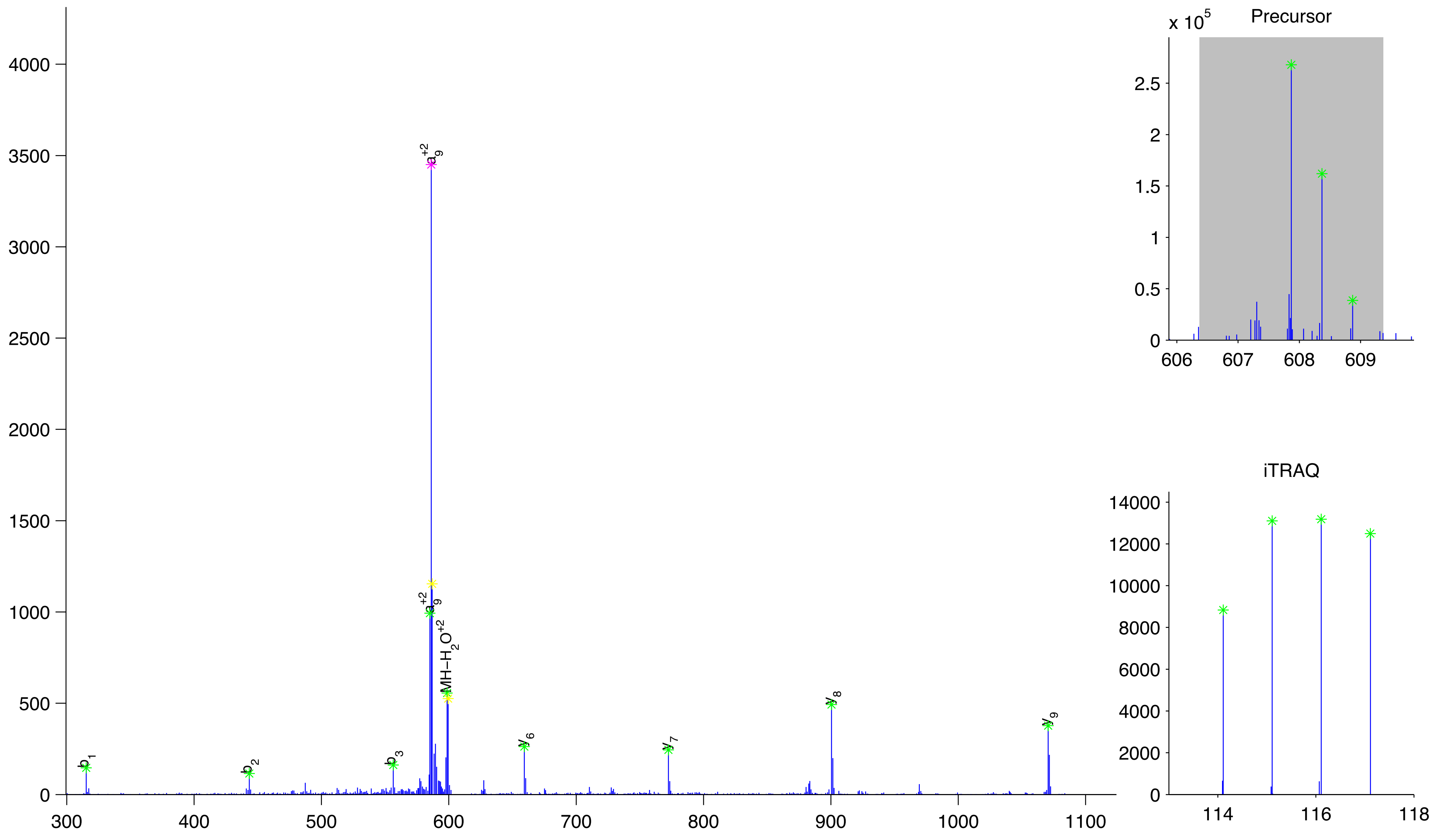

k Q L A T k A A R

H3 histone, family 3A [Homo sapiens]
Charge State: +2
Scan Number: 19119
File Name: 110924_HG2_INSA_ACK.raw

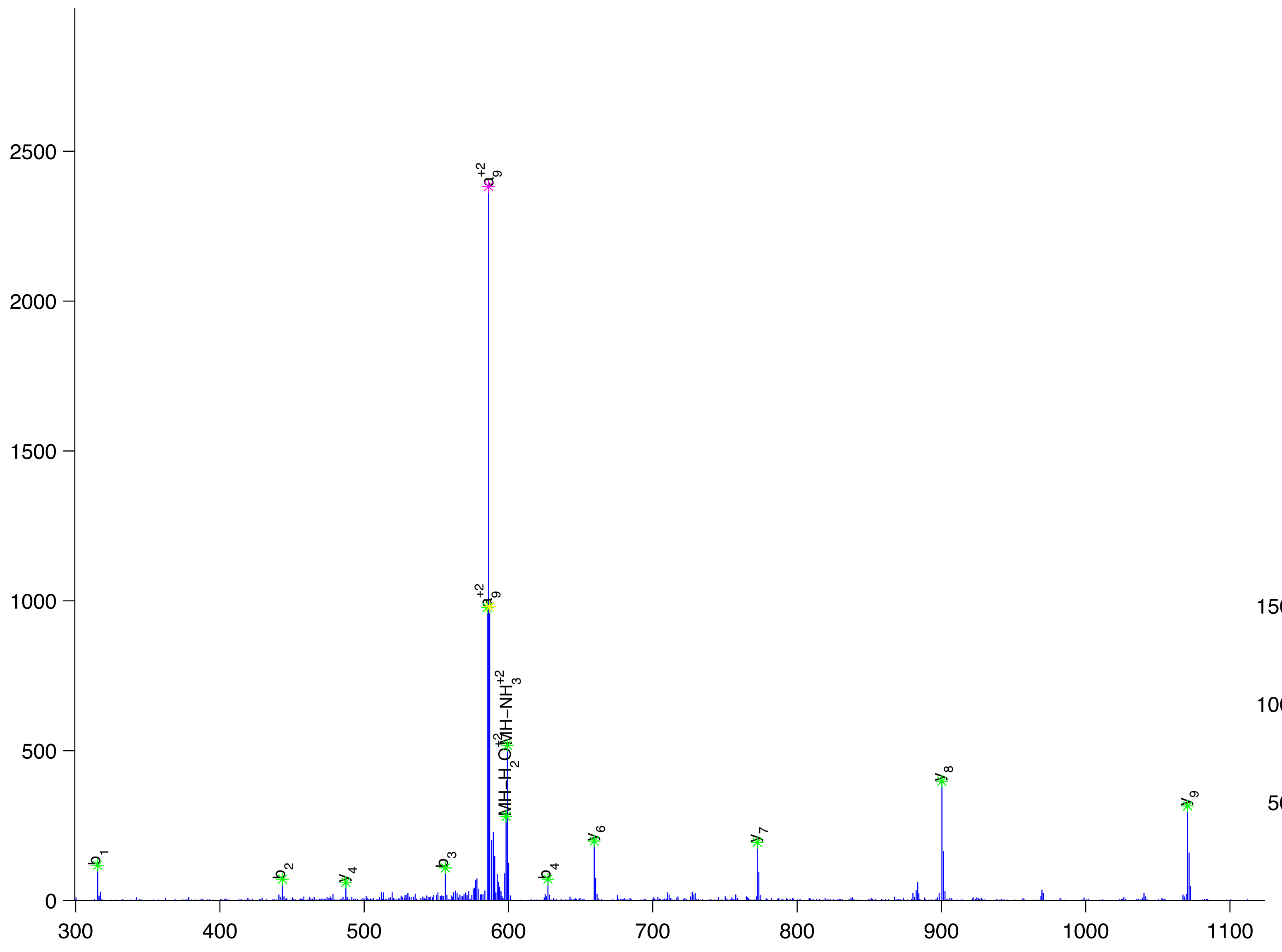

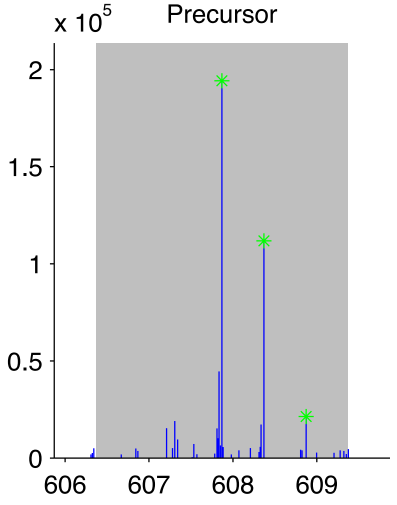

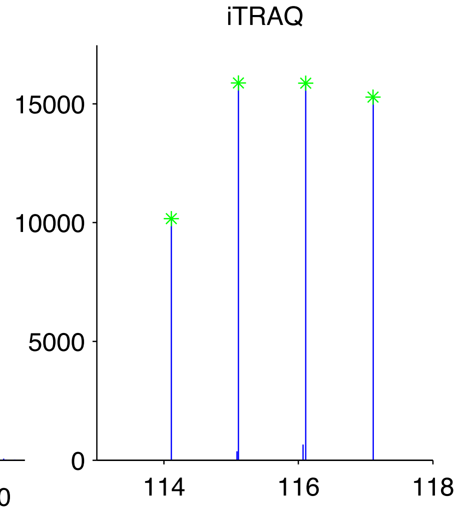

k Q L A T k A A R

H3 histone, family 3A [Homo sapiens]
Charge State: +2
Scan Number: 19287
File Name: 110924_HG2_INSA_ACK.raw

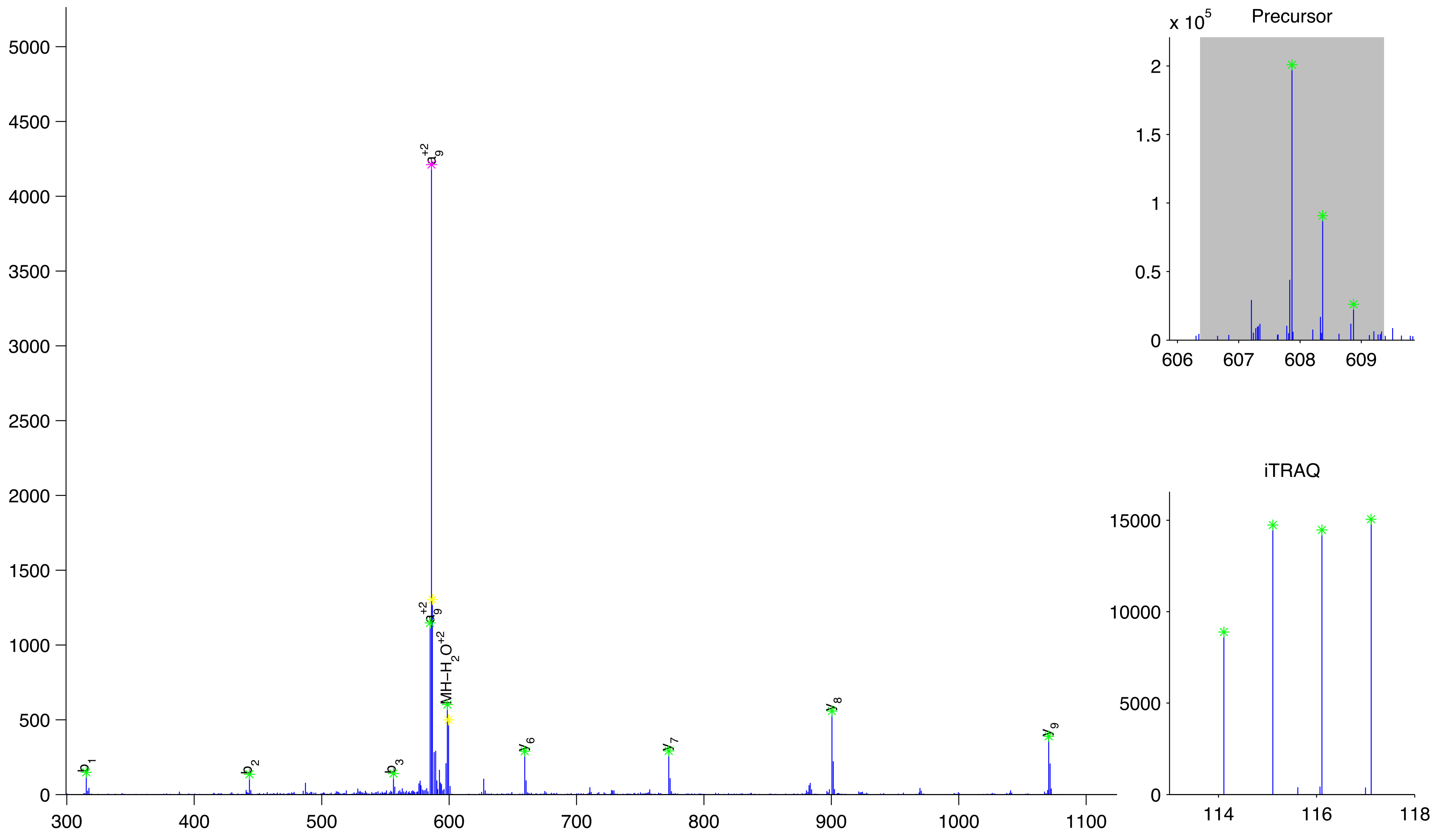

k Q L A T k A A R

H3 histone, family 3A [Homo sapiens]
Charge State: +2
Scan Number: 19455
File Name: 110924_HG2_INSA_ACK.raw

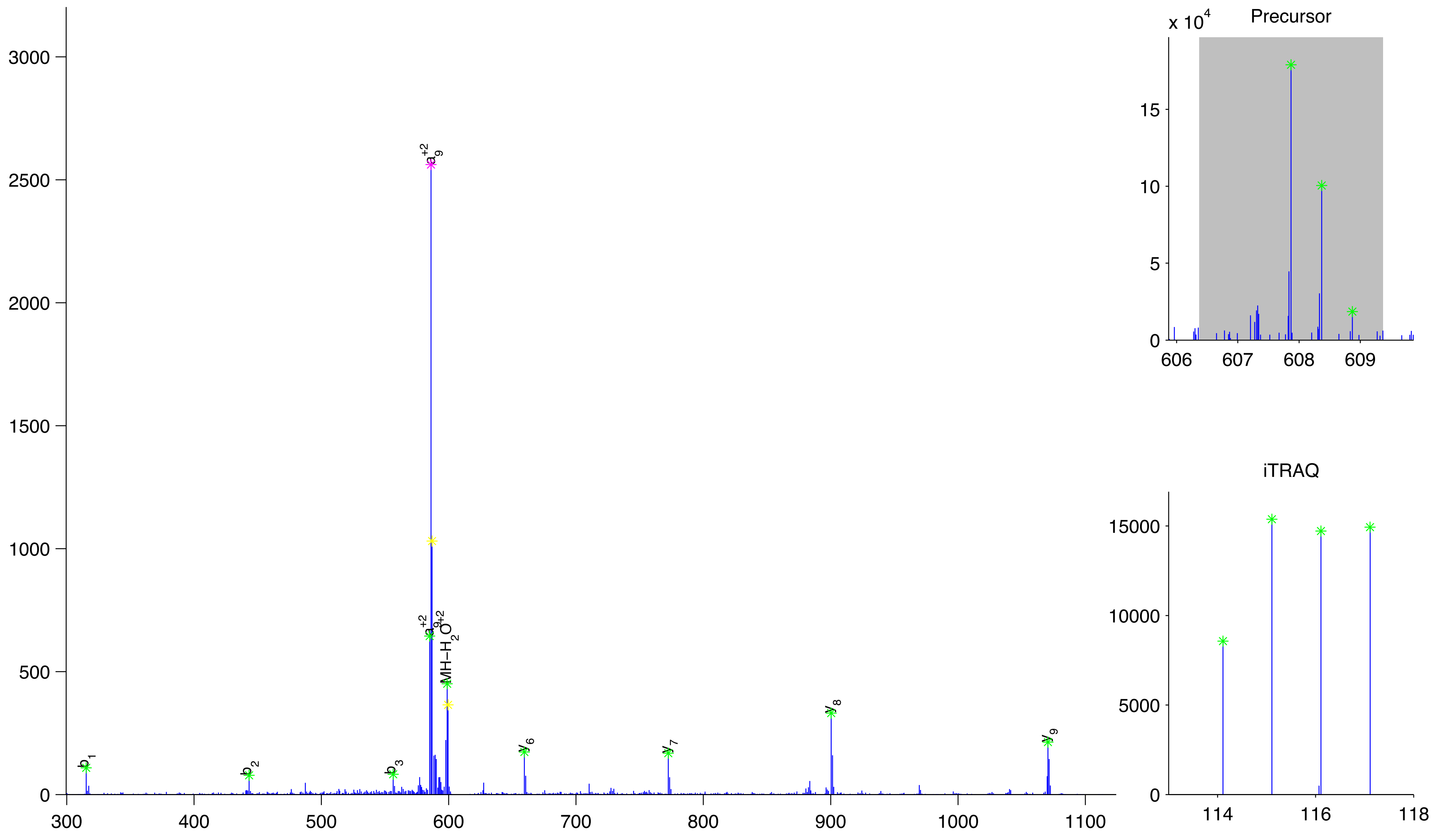

k Q L A T k A A R

H3 histone, family 3A [Homo sapiens]
Charge State: +2
Scan Number: 19621
File Name: 110924_HG2_INSA_ACK.raw

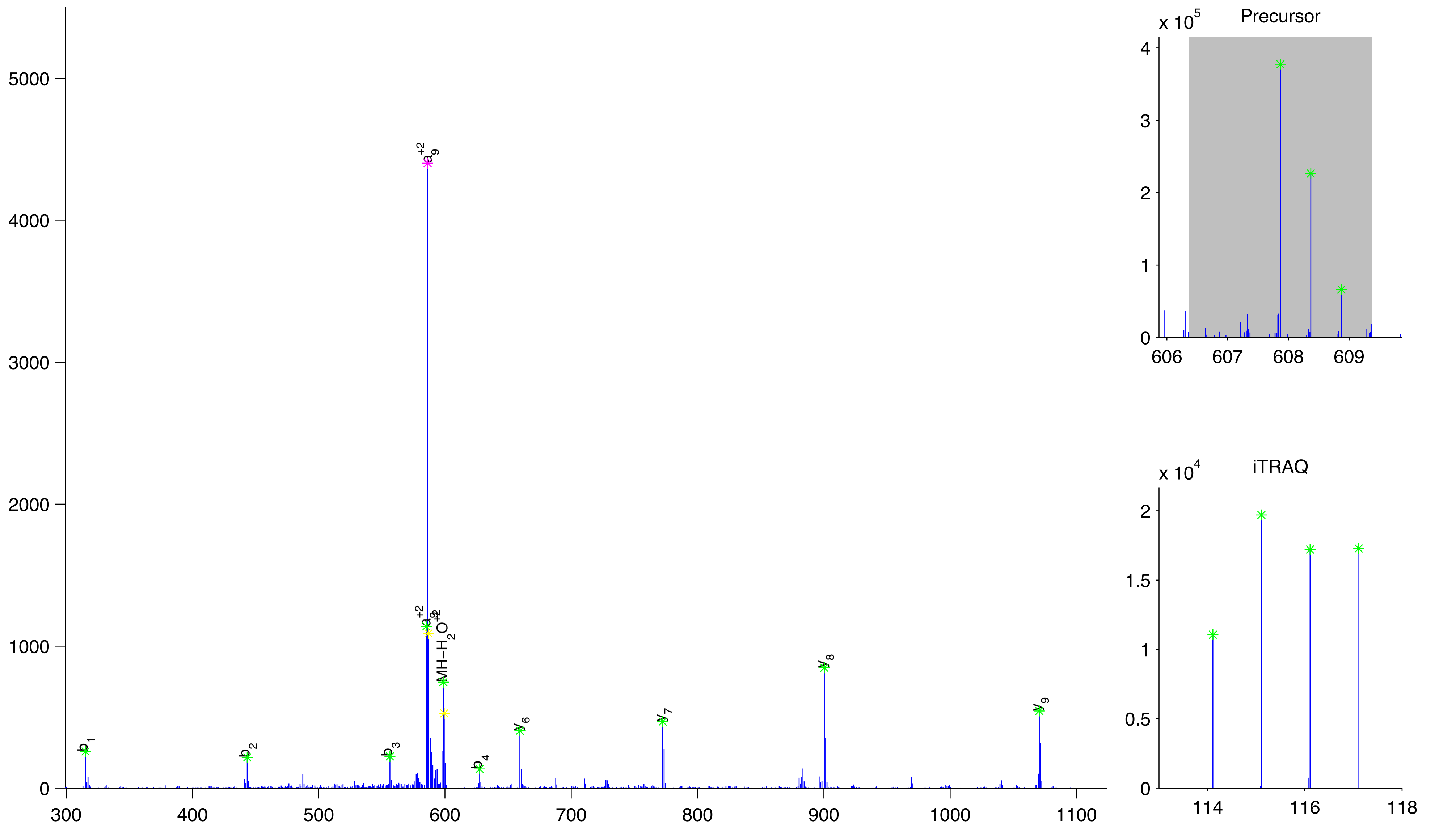

k Q L A T k A A R

H3 histone, family 3A [Homo sapiens]
Charge State: +2
Scan Number: 19789
File Name: 110924_HG2_INSA_ACK.raw

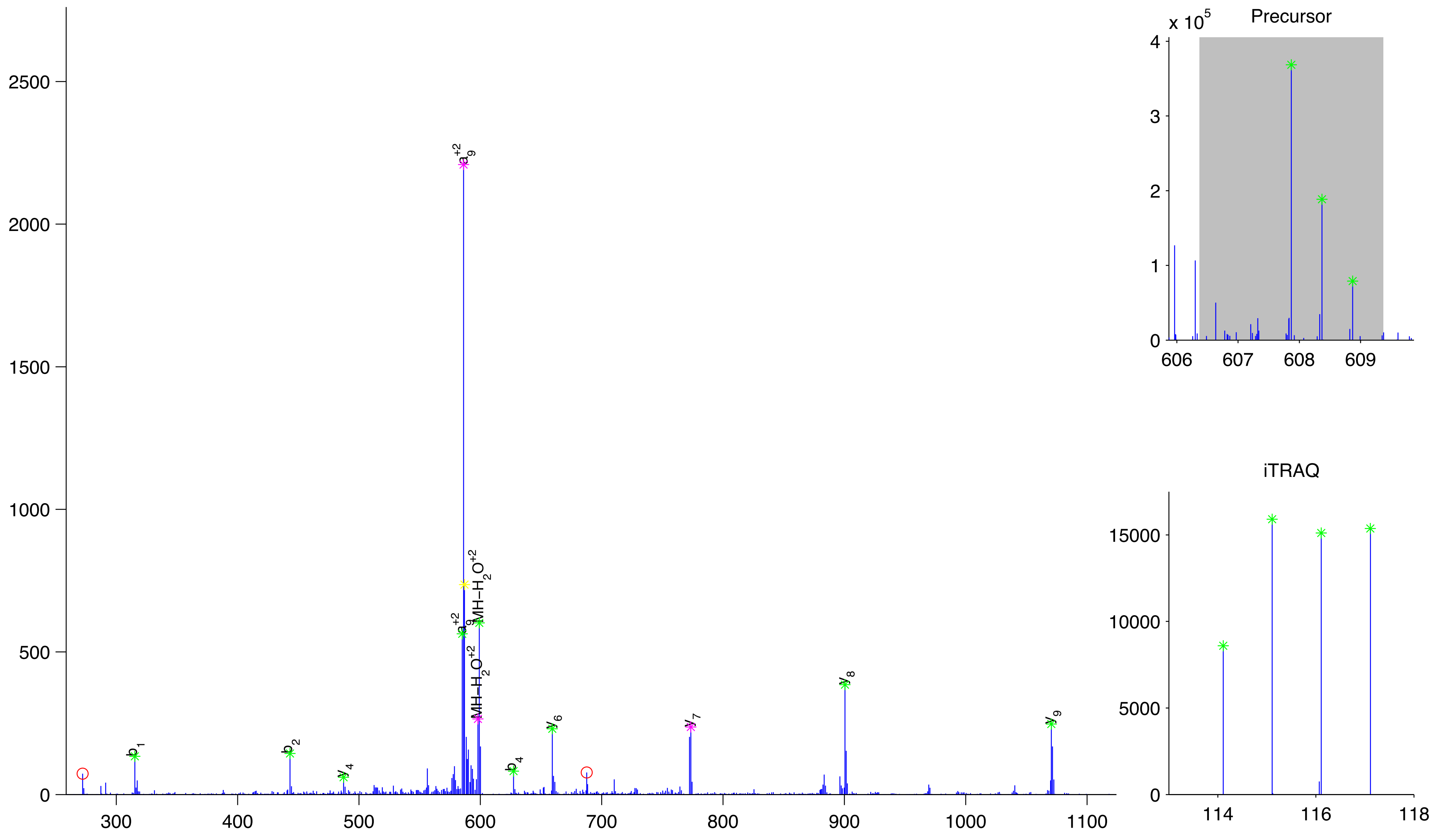

k Q L A T k A A R

H3 histone, family 3A [Homo sapiens]
Charge State: +2
Scan Number: 19961
File Name: 110924_HG2_INSA_ACK.raw

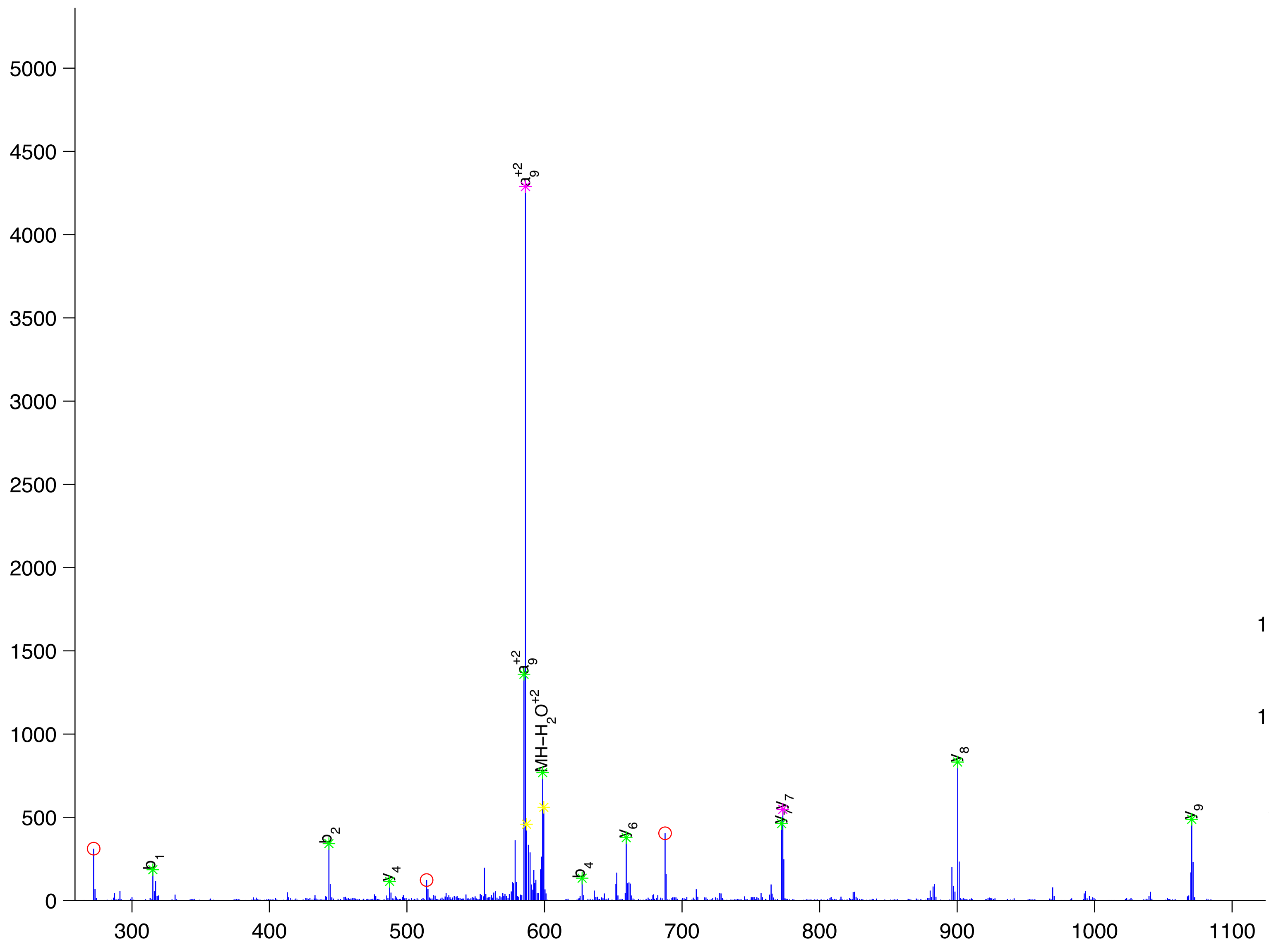

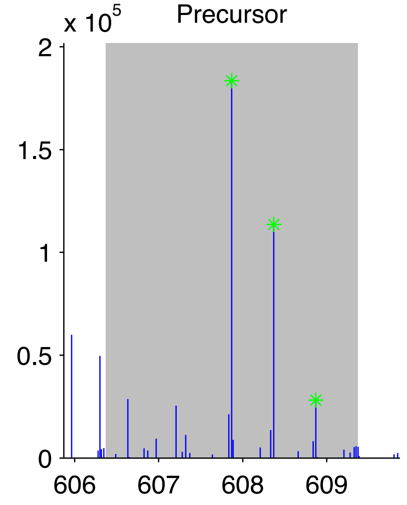

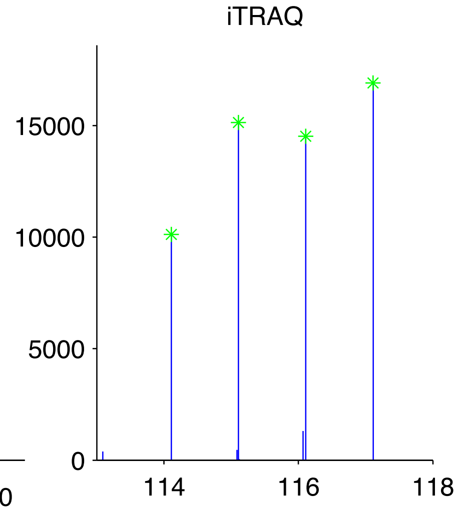

k S T G G k A P R

H3 histone, family 3A [Homo sapiens]
Charge State: +2
Scan Number: 20083
File Name: 110924_HG2_INSA_ACK.raw

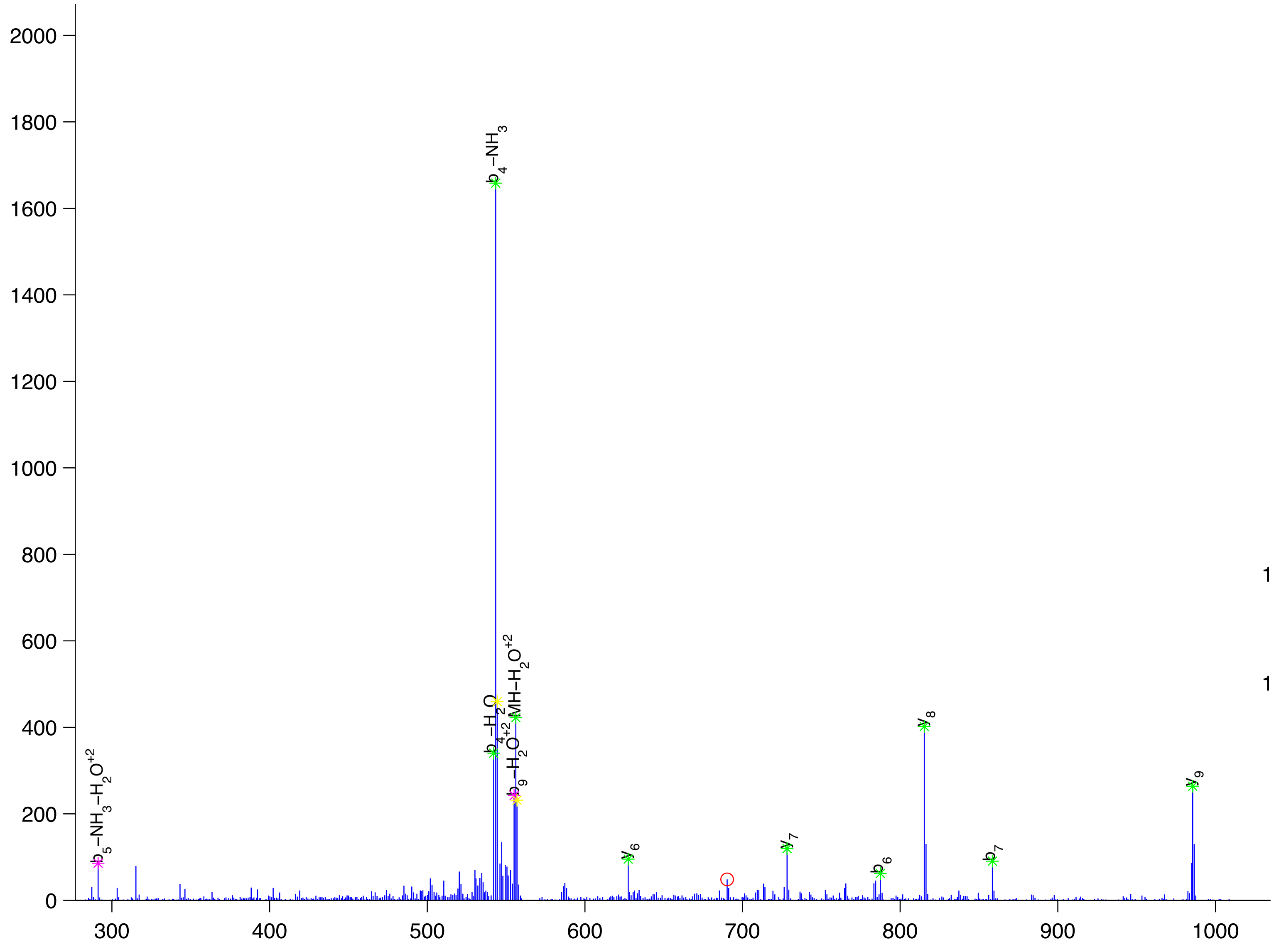

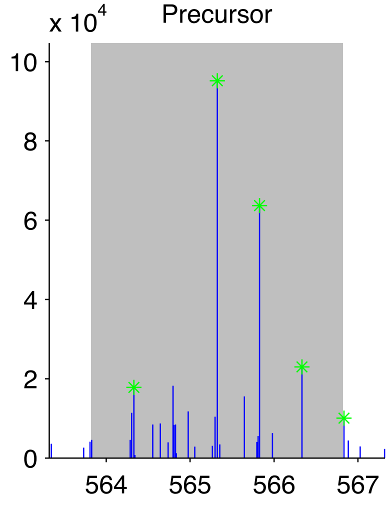

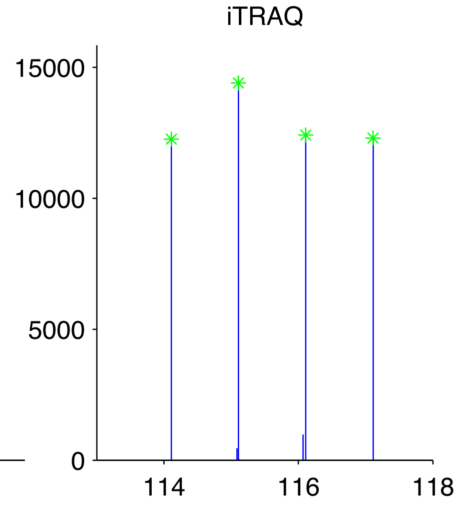

k Q L A T k A A R

H3 histone, family 3A [Homo sapiens]
Charge State: +2
Scan Number: 20129
File Name: 110924_HG2_INSA_ACK.raw

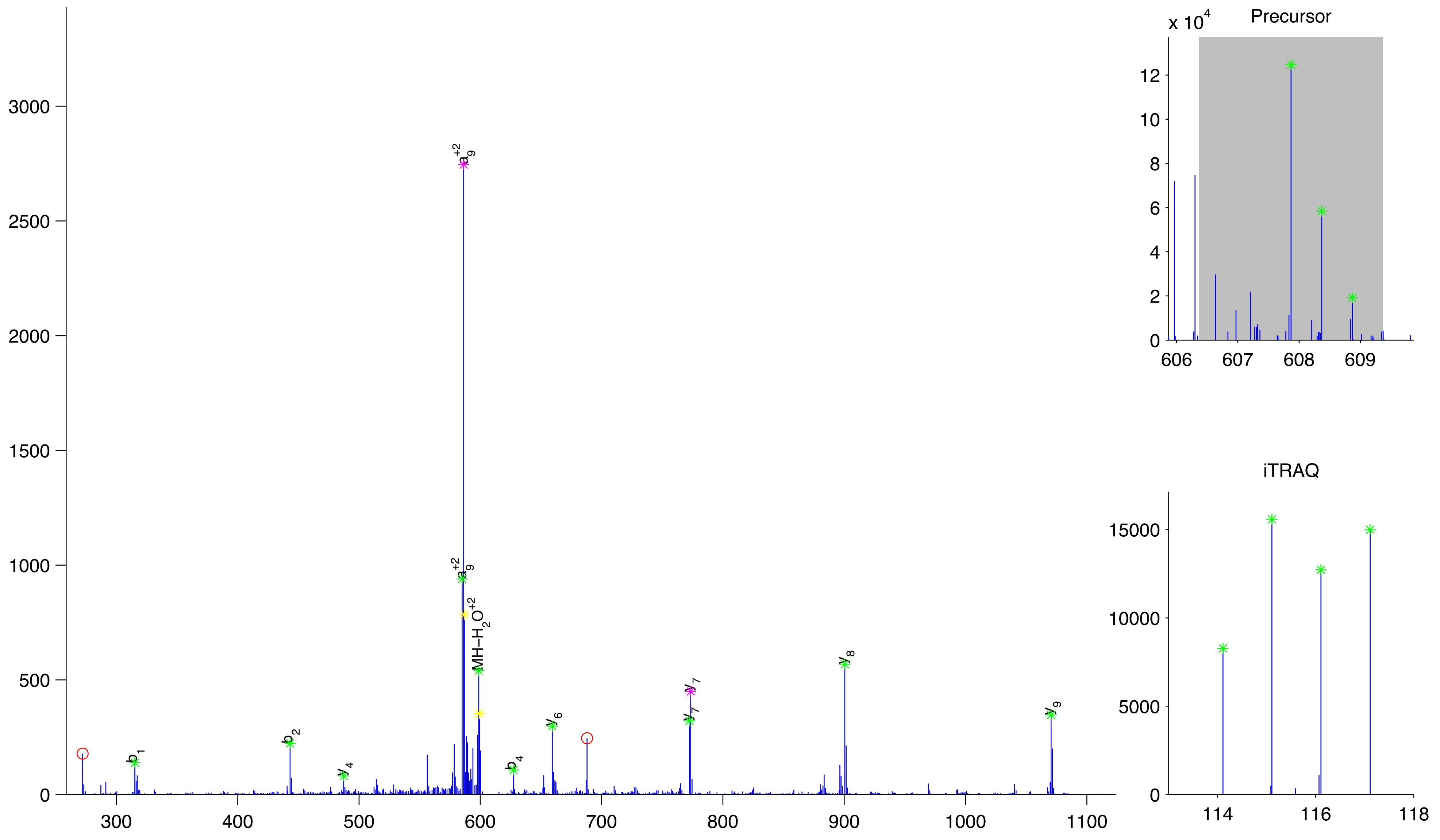

H3 histone, family 3A [Homo sapiens]
Charge State: +2
Scan Number: 20465
File Name: 110924_HG2_INSA_ACK.raw

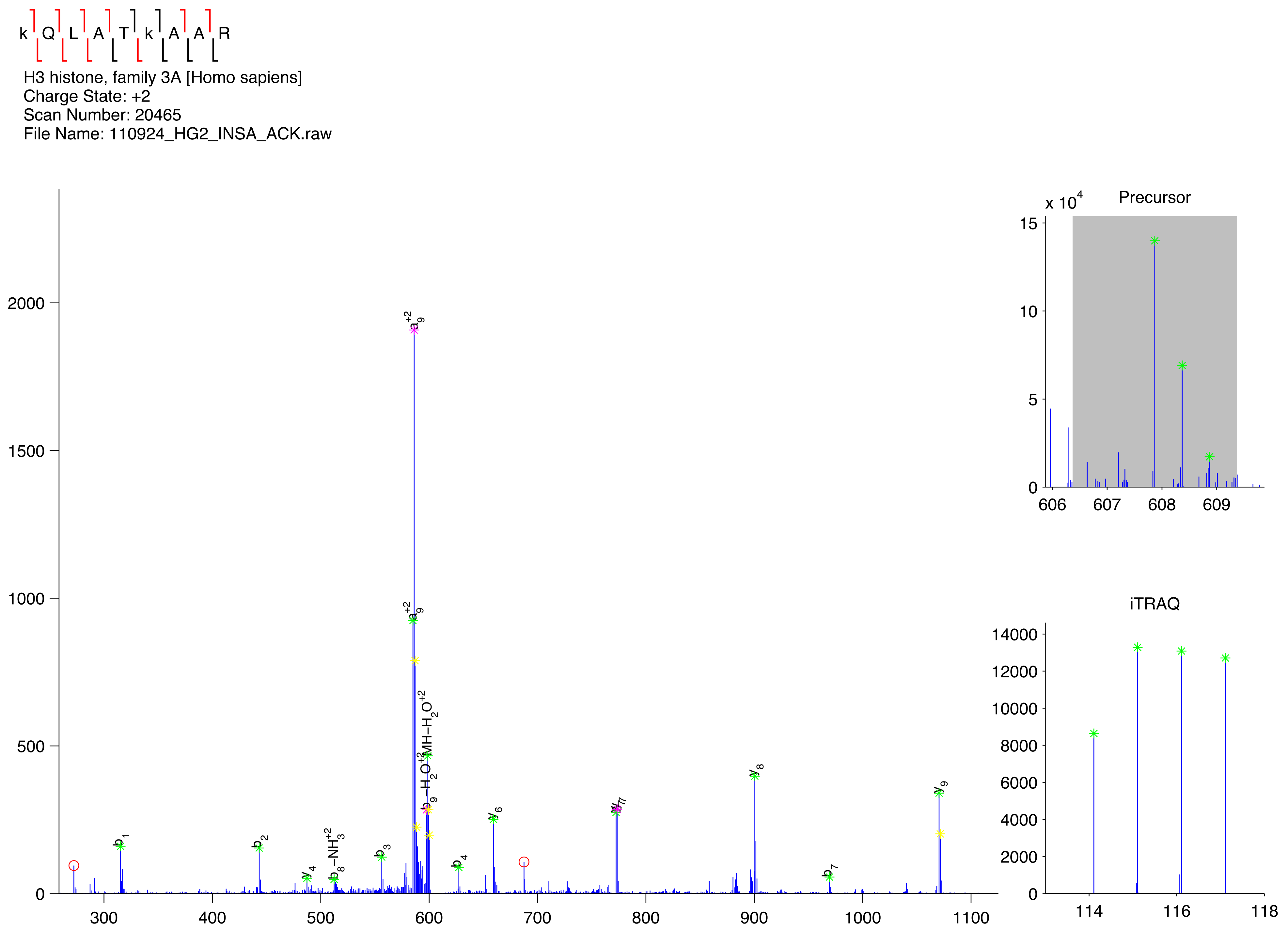

k Q L A T k A A R

H3 histone, family 3A [Homo sapiens]
Charge State: +2
Scan Number: 20633
File Name: 110924_HG2_INSA_ACK.raw

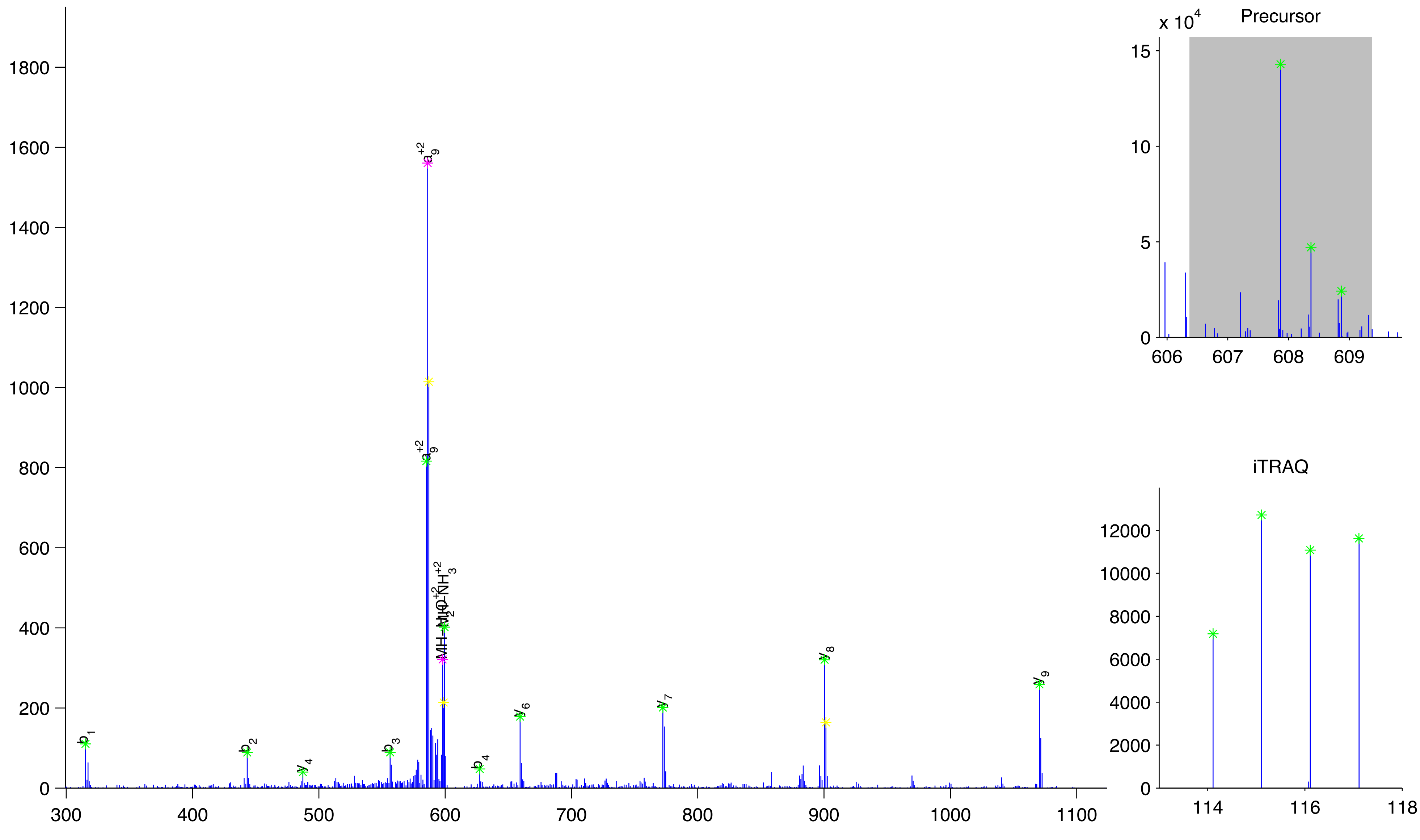

k S T G G k A P R

H3 histone, family 3A [Homo sapiens]
Charge State: +2
Scan Number: 21091
File Name: 110924_HG2_INSA_ACK.raw

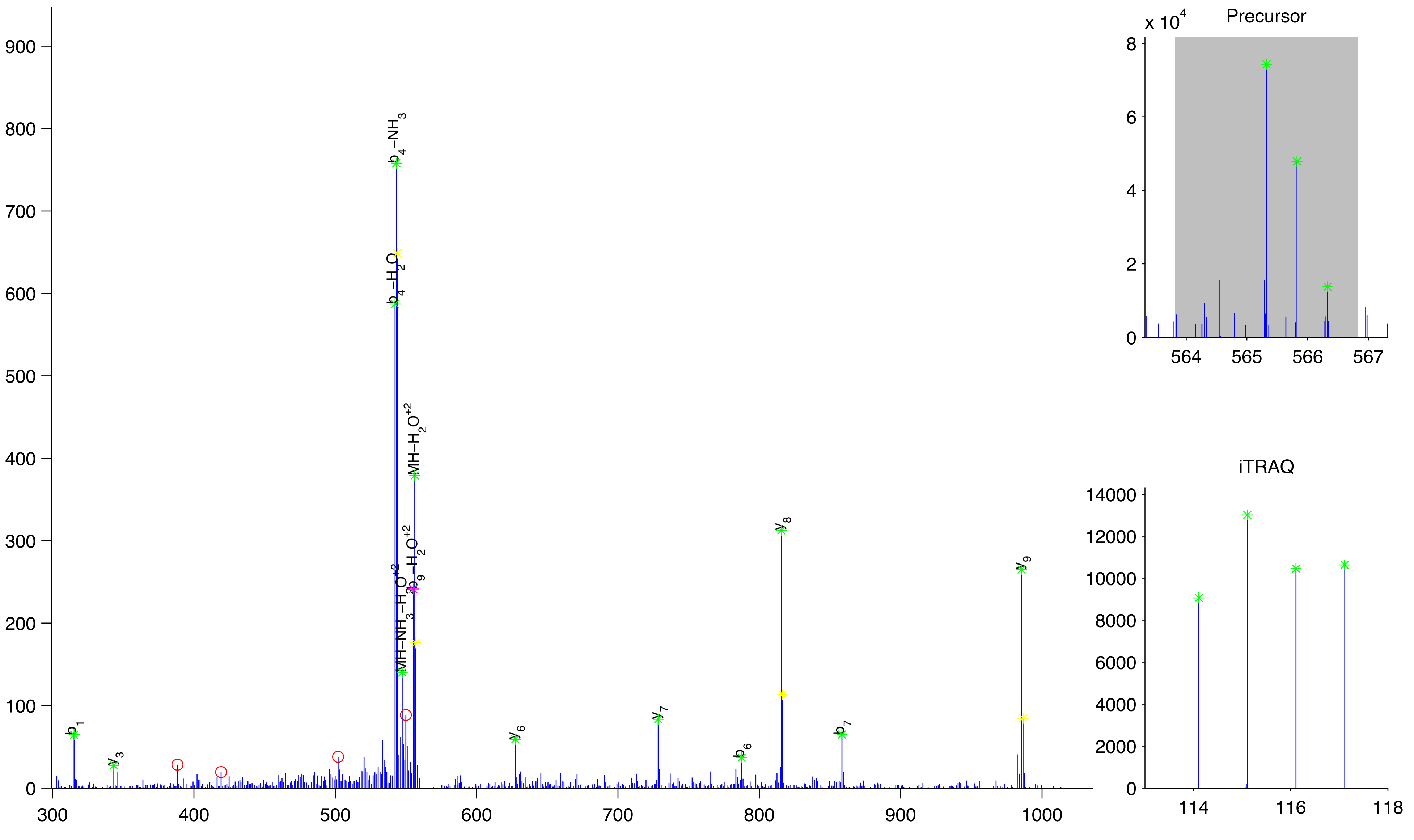

L k E E I S K

heat shock 70kDa protein 9 precursor [Homo sapiens]
Charge State: +2
Scan Number: 16141
File Name: 110924_HG2_INSA_ACK.raw

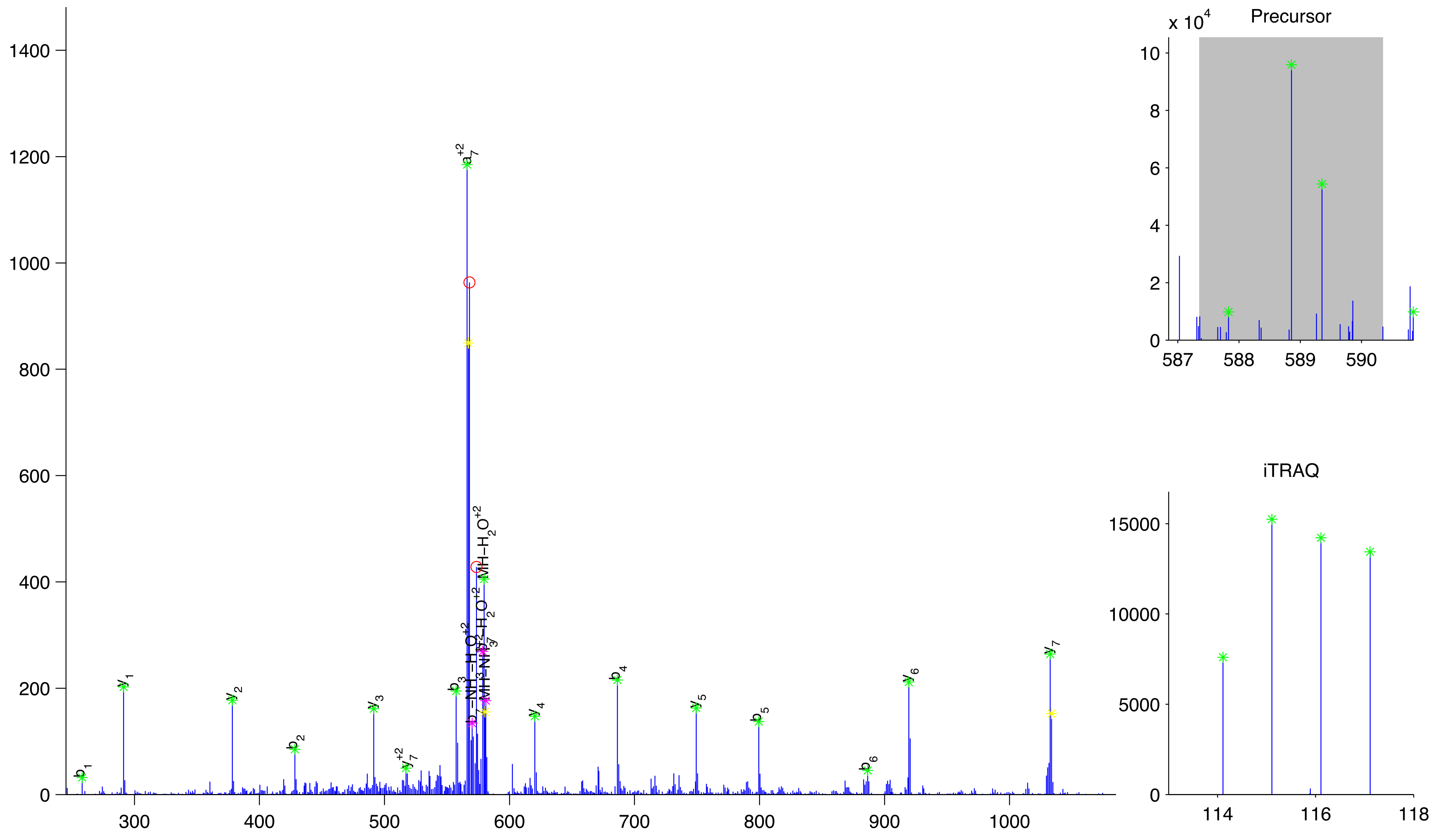

L k E E I S K

heat shock 70kDa protein 9 precursor [Homo sapiens]
Charge State: +2
Scan Number: 16311
File Name: 110924_HG2_INSA_ACK.raw

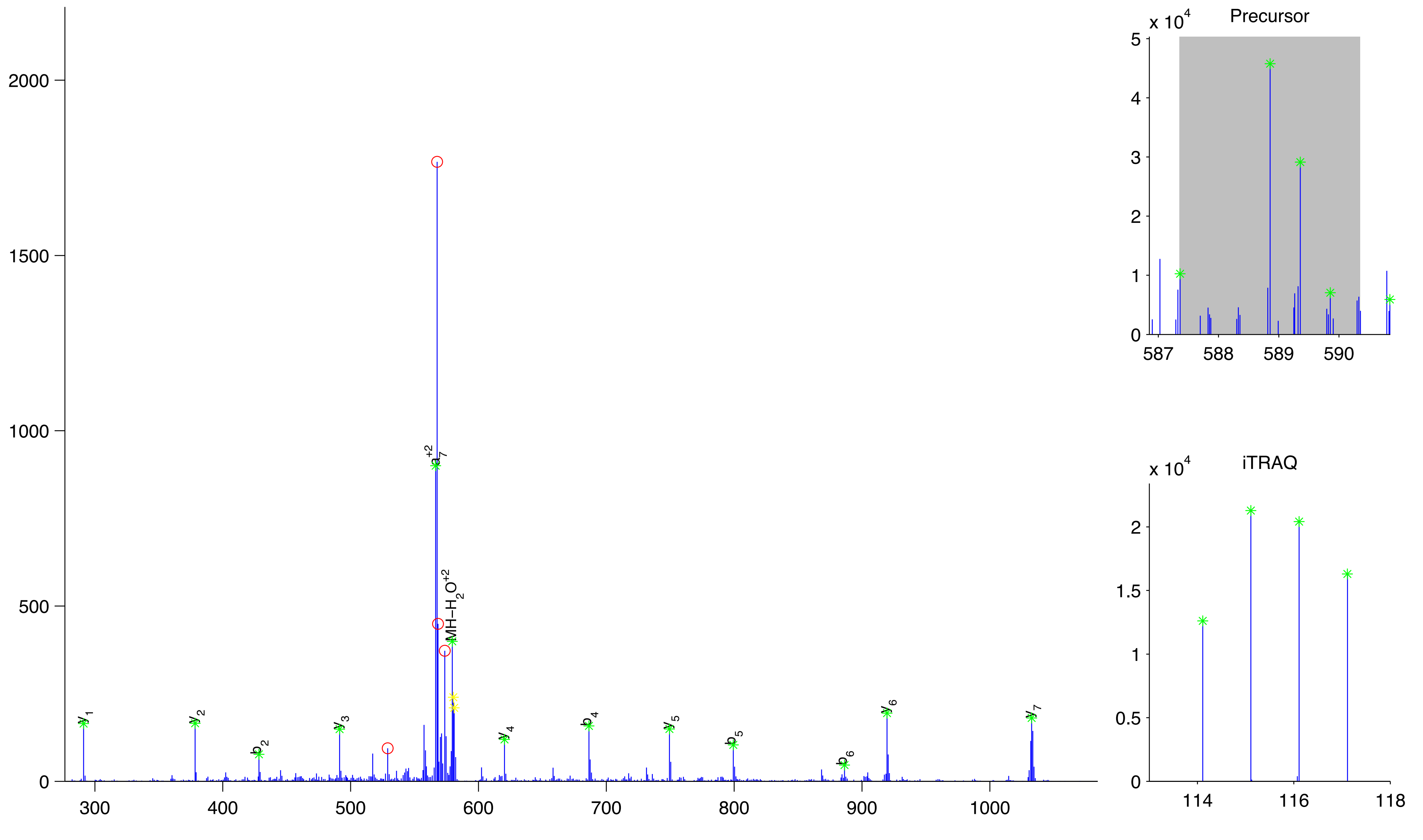

A k F E N L c K

heat shock 90kDa protein 1, beta [Homo sapiens]
Charge State: +2
Scan Number: 6038
File Name: 111016_HG2_INSC_ACK.raw

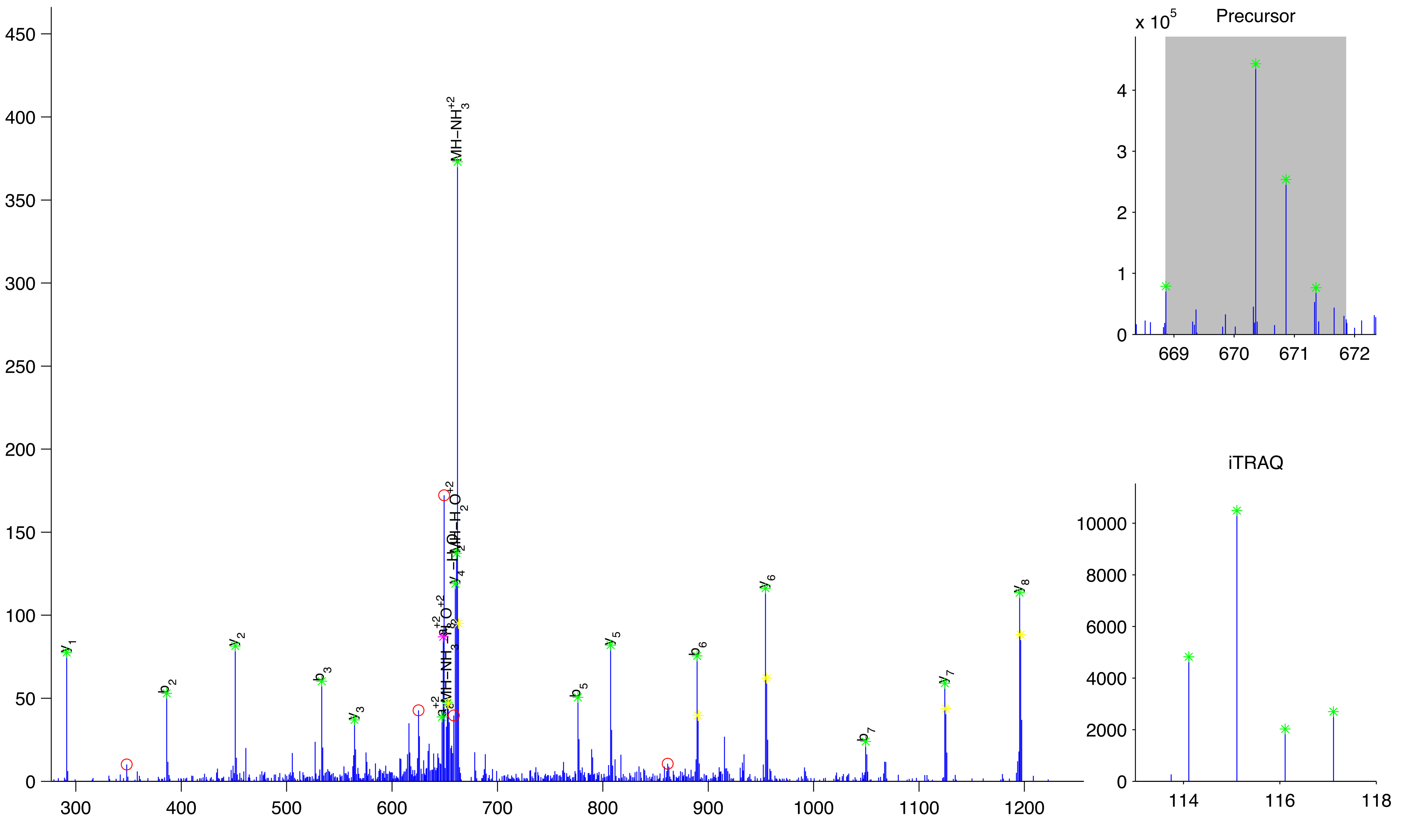

S S Q P L A S k Q E K

high mobility group AT−hook 1 isoform b [Homo sapiens]
Charge State: +2
Scan Number: 4822
File Name: 111011_HG2_INSB_ACK.raw

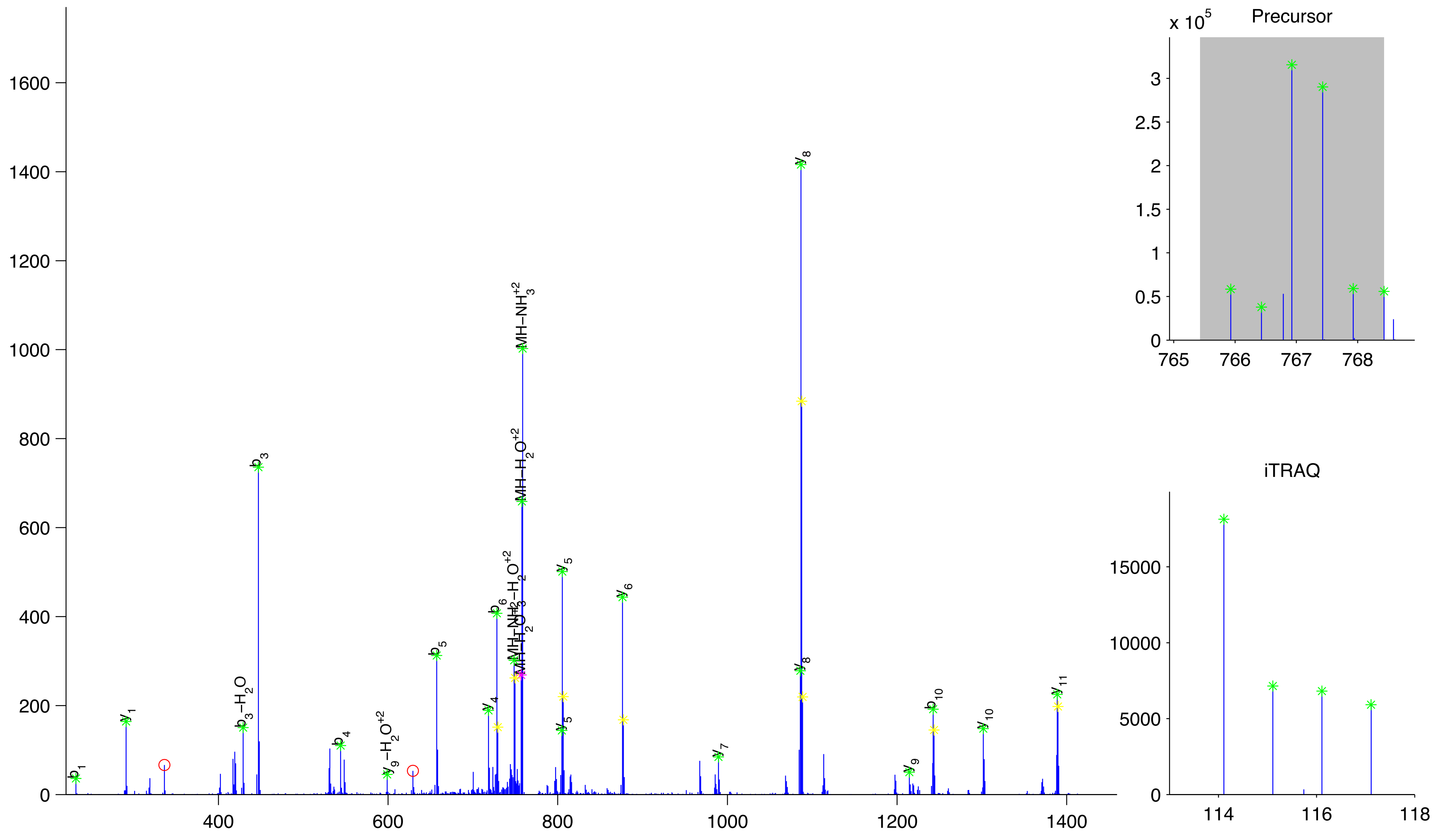

S S Q P L A S k Q E K

high mobility group AT−hook 1 isoform b [Homo sapiens]
Charge State: +2
Scan Number: 11479
File Name: 110924_HG2_INSA_ACK.raw

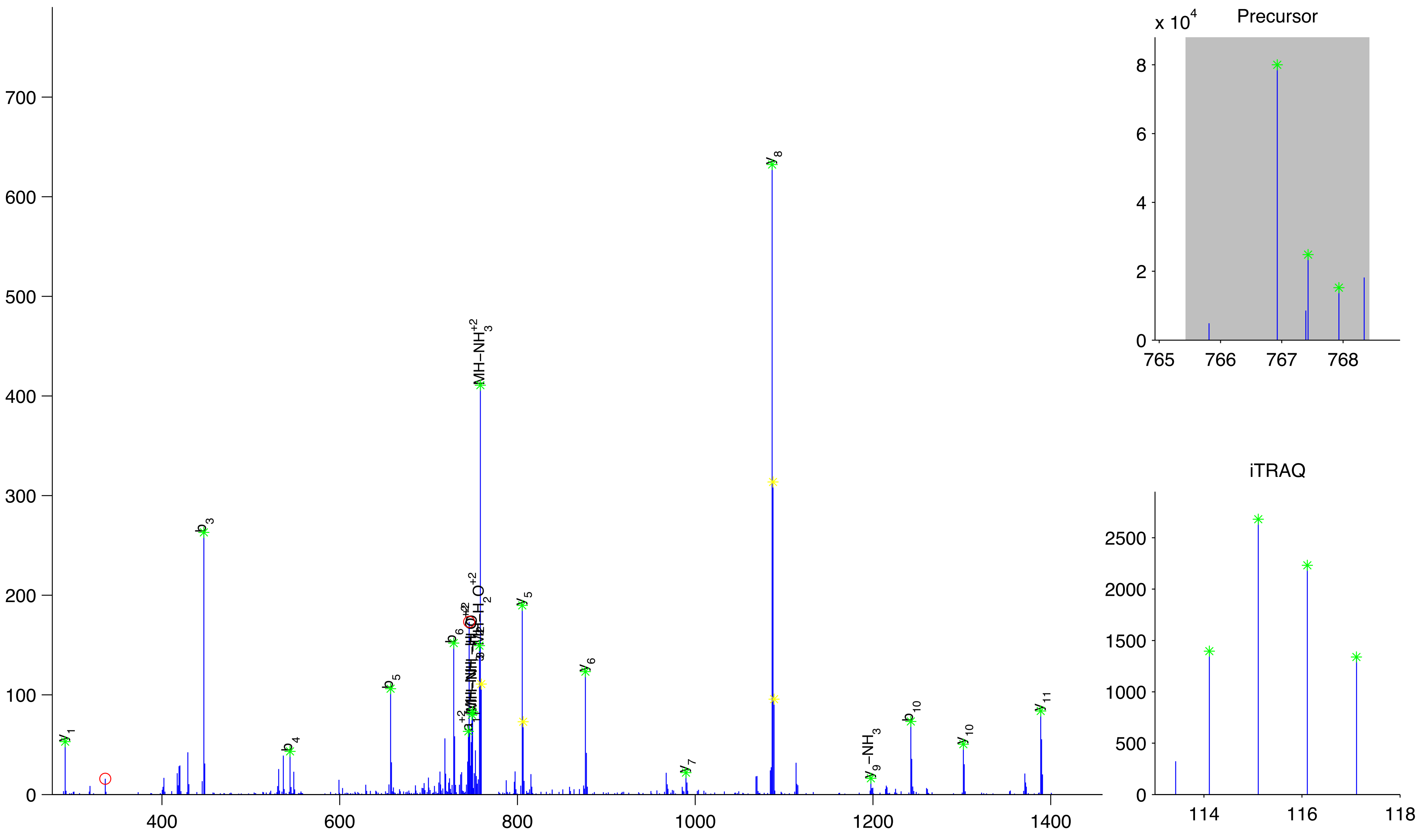

S S Q P L A S k Q E K

high mobility group AT−hook 1 isoform b [Homo sapiens]
Charge State: +2
Scan Number: 11540
File Name: 110924_HG2_INSA_ACK.raw

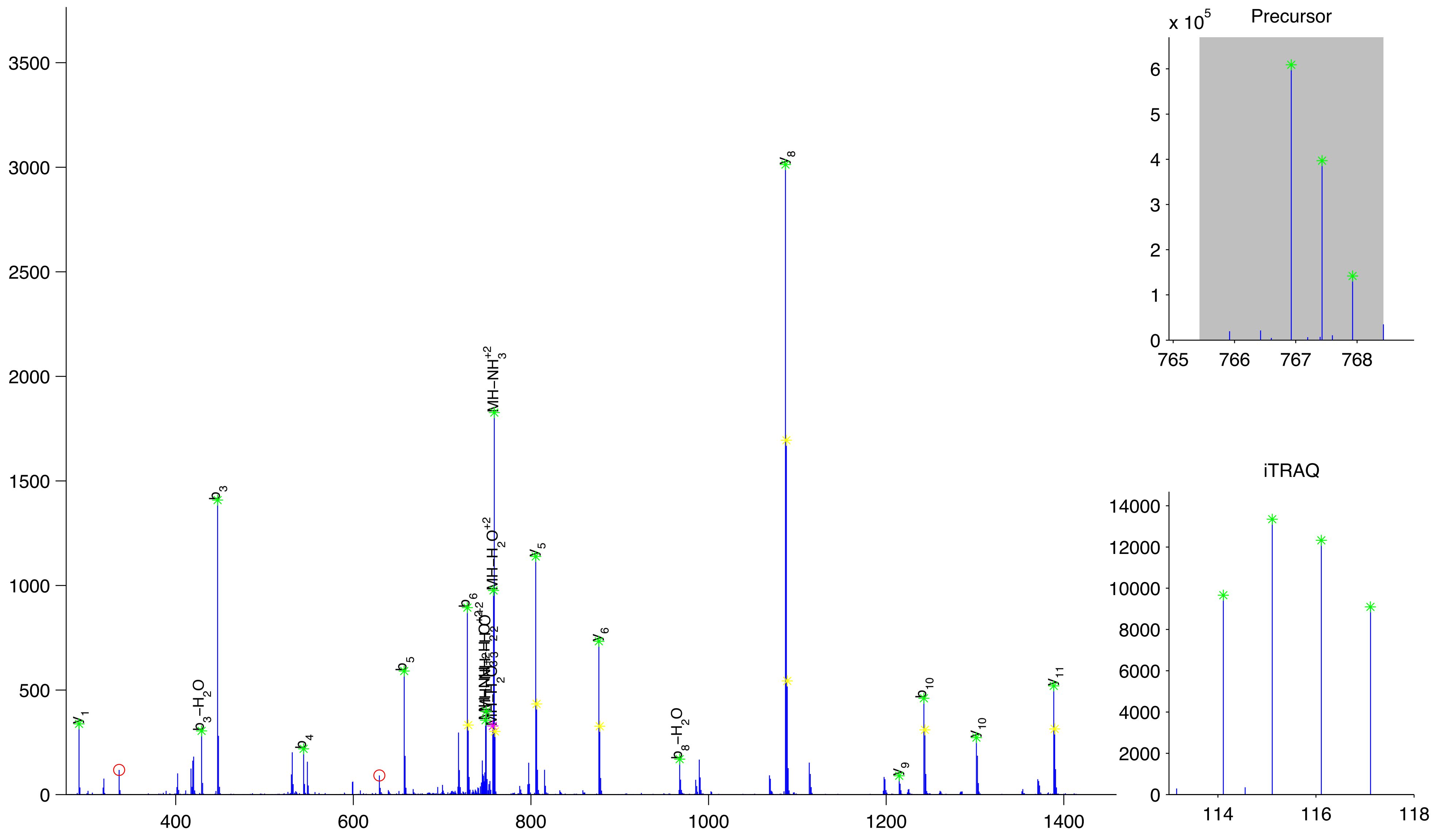

S S Q P L A S k Q E K

high mobility group AT−hook 1 isoform b [Homo sapiens]
Charge State: +3
Scan Number: 11595
File Name: 110924_HG2_INSA_ACK.raw

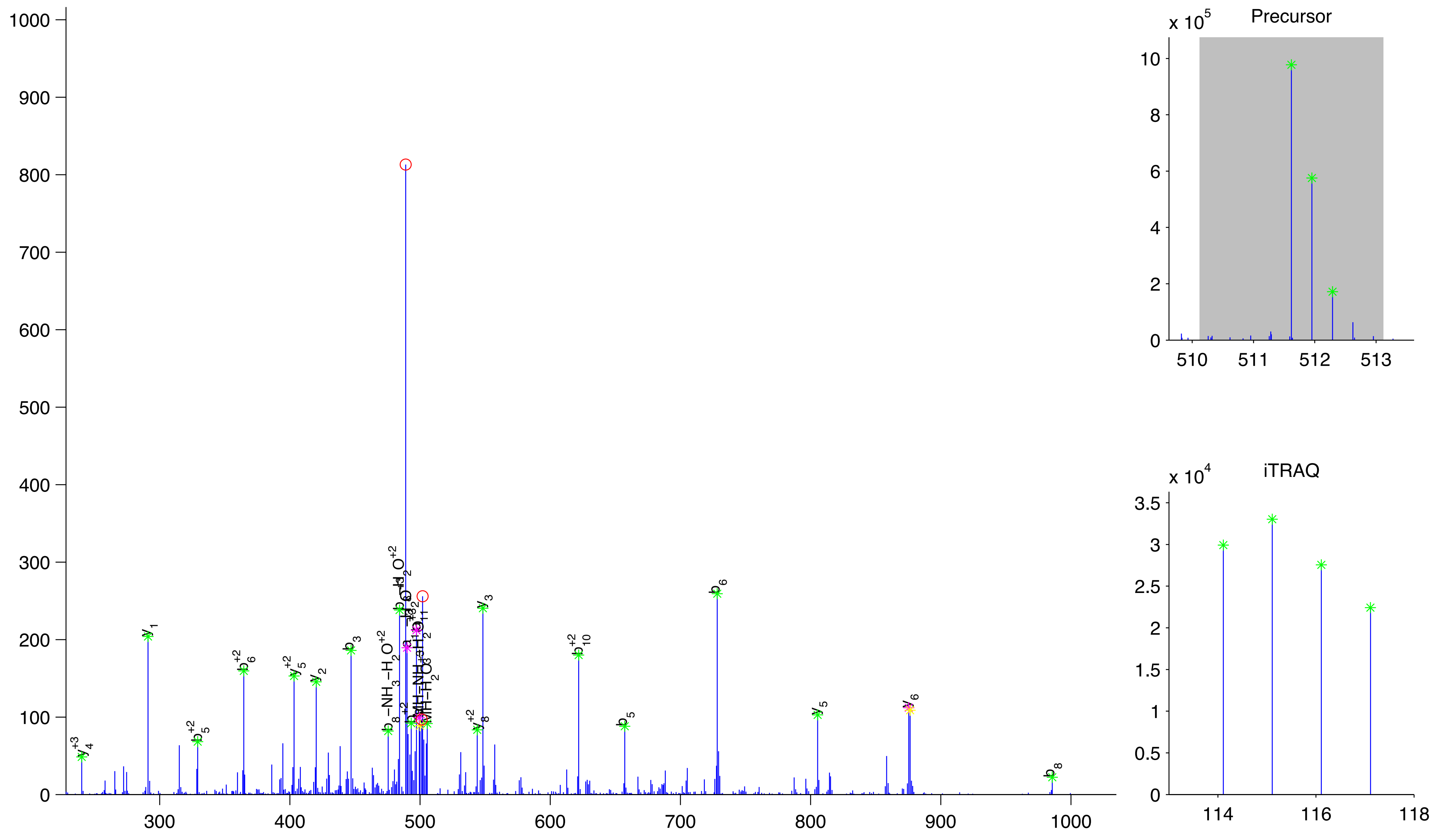

S S Q P L A S k Q E K

high mobility group AT−hook 1 isoform b [Homo sapiens]
Charge State: +2
Scan Number: 11637
File Name: 110924_HG2_INSA_ACK.raw

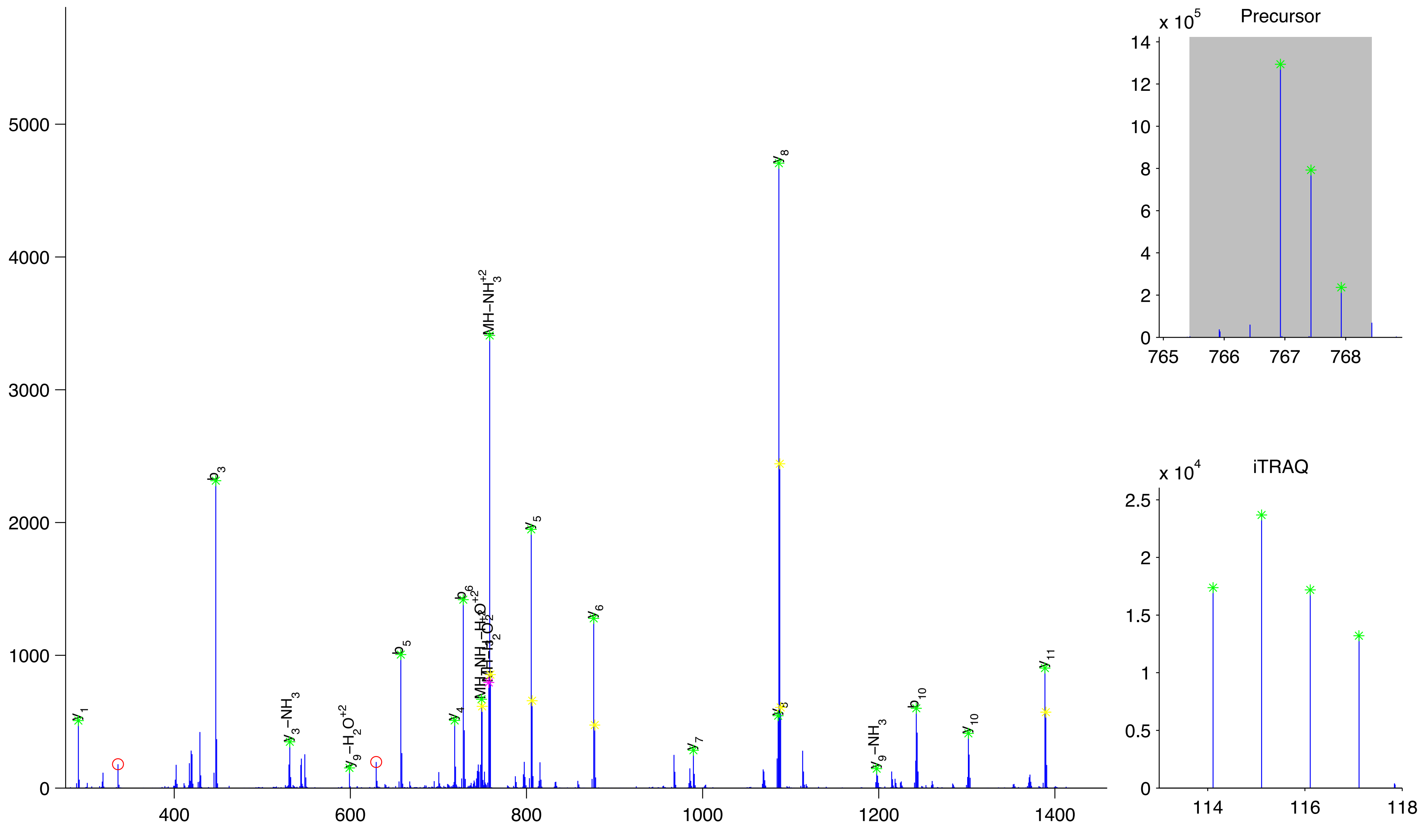

S S Q P L A S k Q E K

high mobility group AT−hook 1 isoform b [Homo sapiens]
Charge State: +2
Scan Number: 11710
File Name: 110924_HG2_INSA_ACK.raw

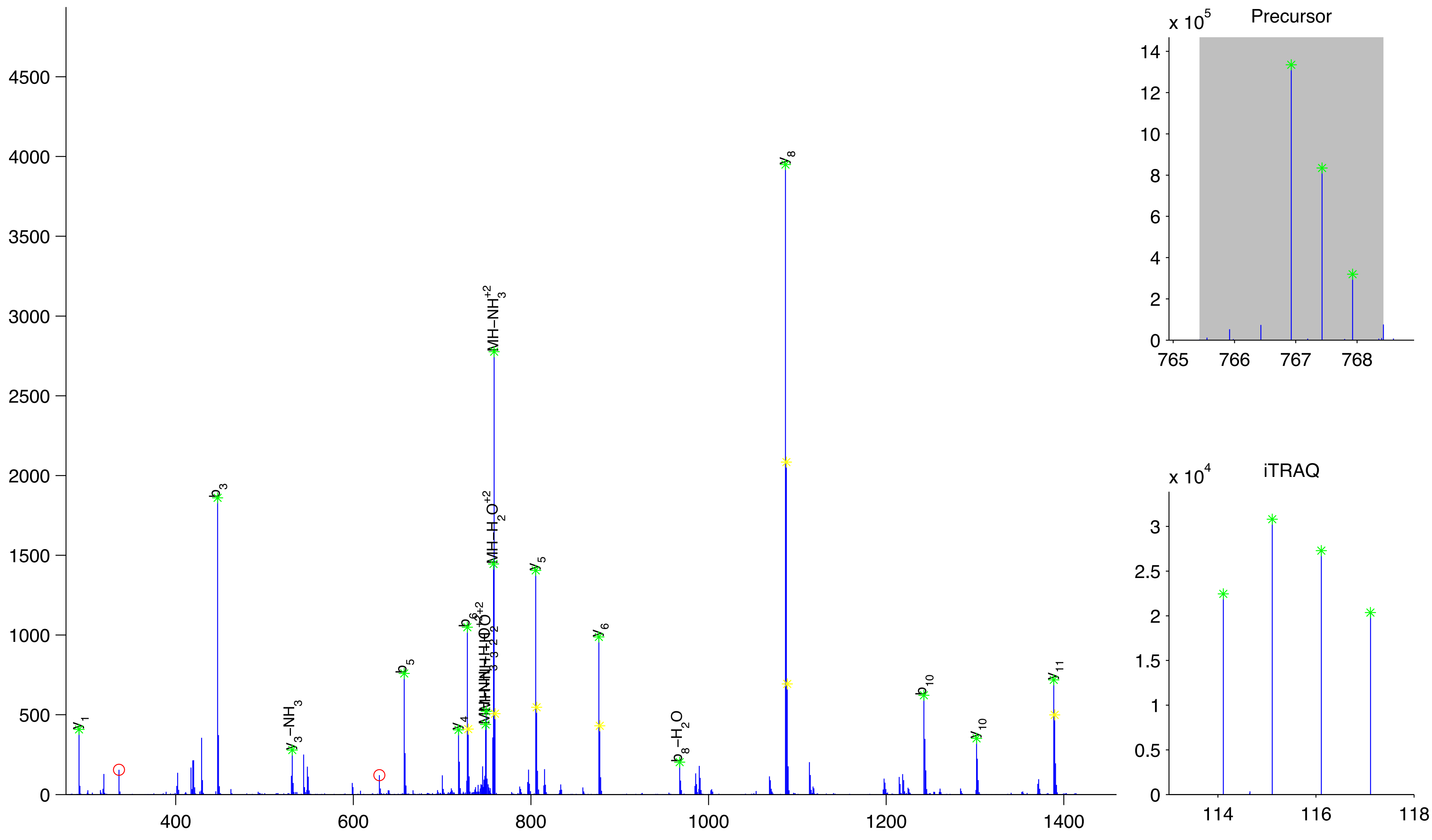

S S Q P L A S k Q E K

high mobility group AT−hook 1 isoform b [Homo sapiens]
Charge State: +2
Scan Number: 11805
File Name: 110924_HG2_INSA_ACK.raw

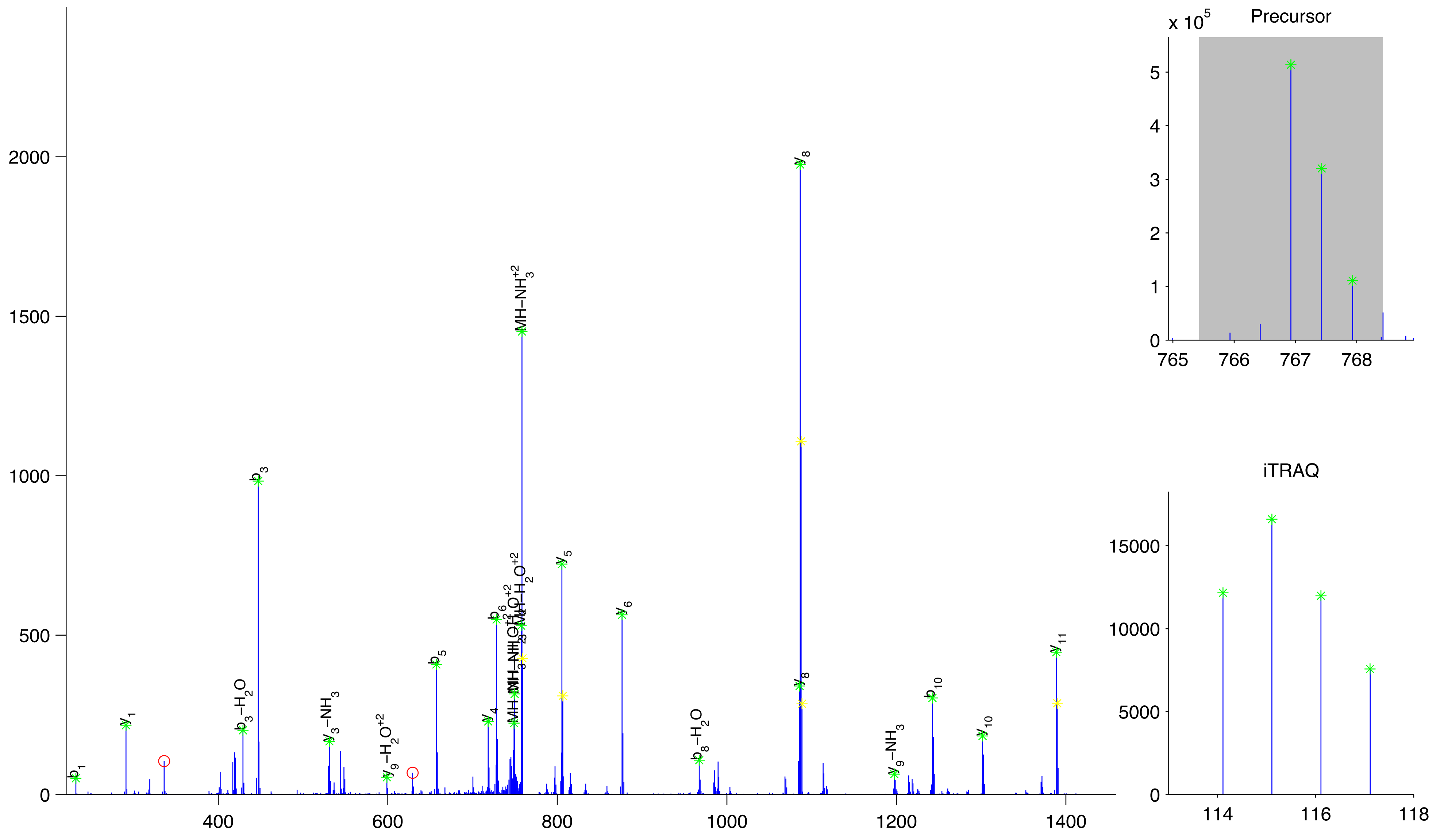

S S Q P L A S k Q E K

high mobility group AT−hook 1 isoform b [Homo sapiens]
Charge State: +2
Scan Number: 11886
File Name: 110924_HG2_INSA_ACK.raw

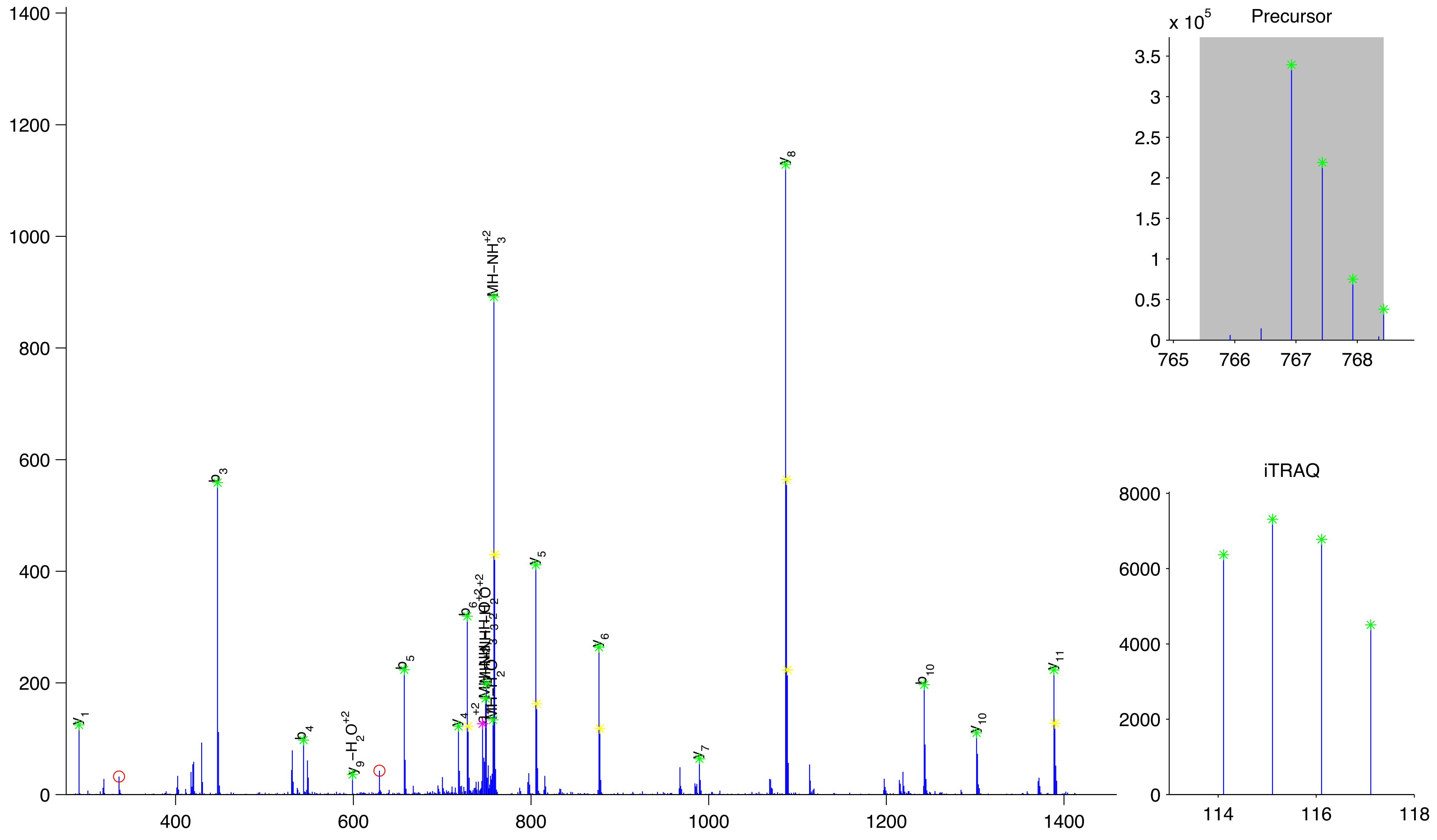

S S Q P L A S k Q E K

high mobility group AT−hook 1 isoform b [Homo sapiens]
Charge State: +2
Scan Number: 11977
File Name: 110924_HG2_INSA_ACK.raw

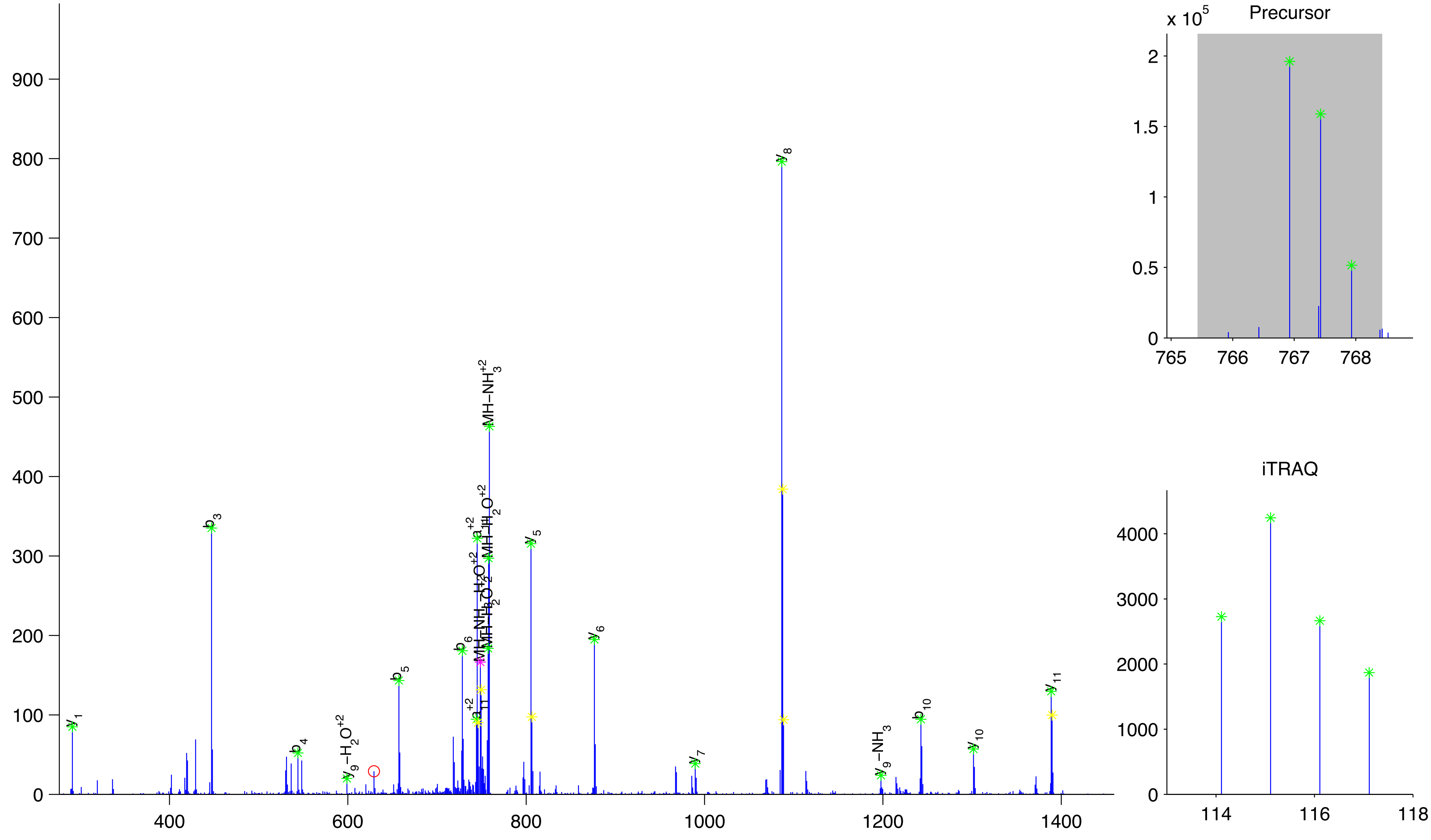

S S Q P L A S k Q E K

high mobility group AT−hook 1 isoform b [Homo sapiens]
Charge State: +2
Scan Number: 12147
File Name: 110924_HG2_INSA_ACK.raw

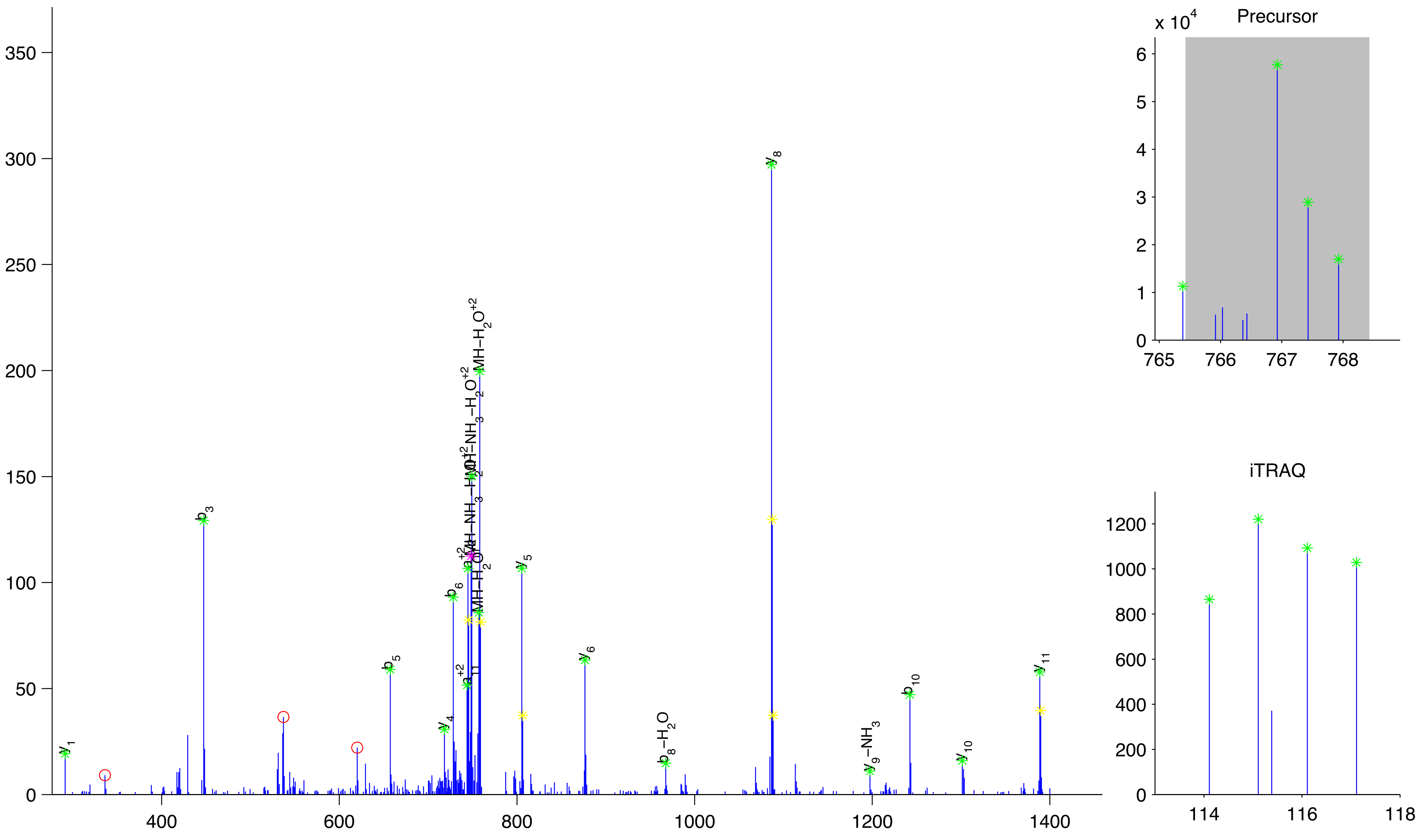

G k Q G G k A R

histone cluster 1, H2ai [Homo sapiens]
Charge State: +2
Scan Number: 2827
File Name: 111016_HG2_INSC_ACK.raw

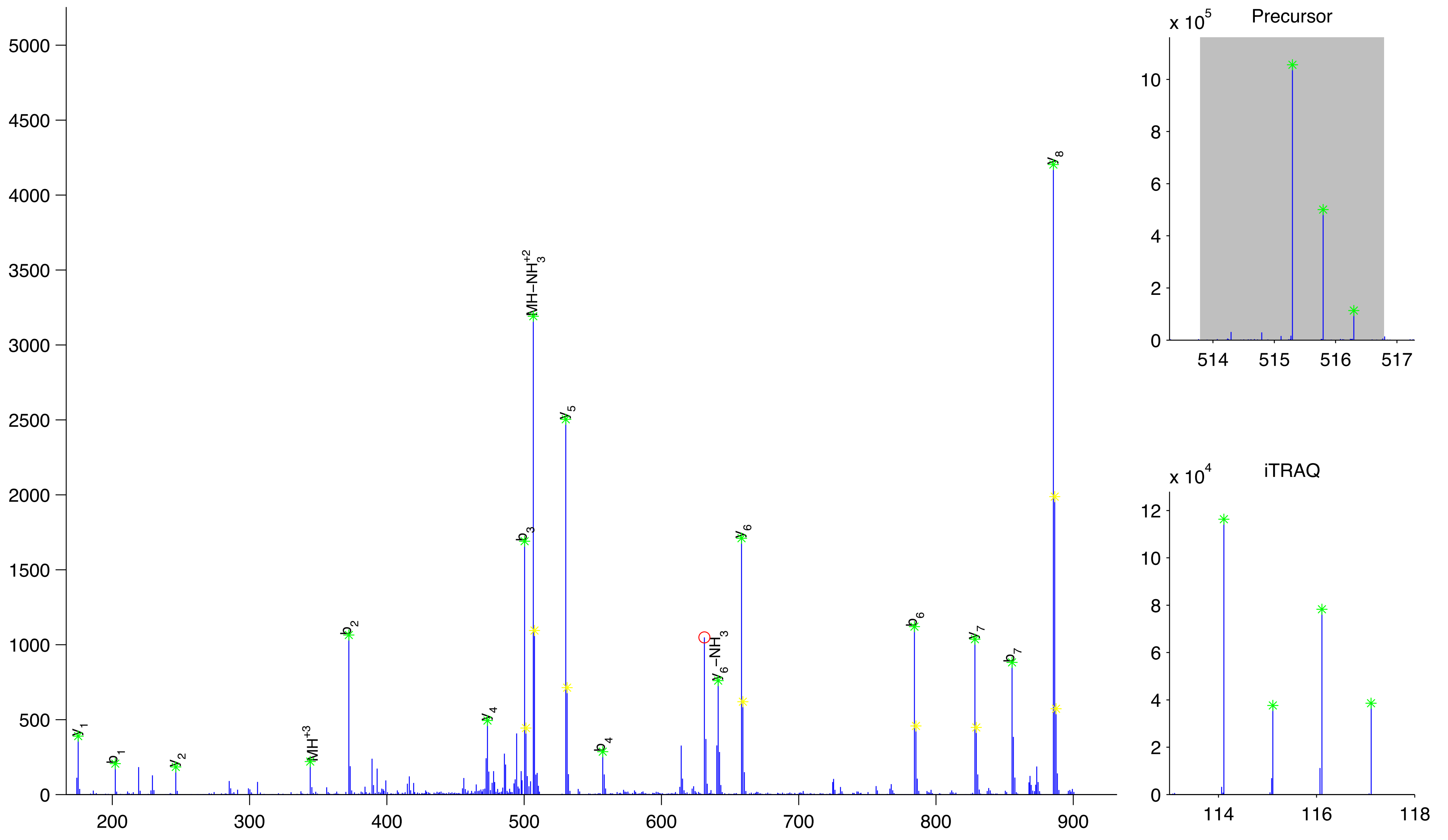

G k Q G G k A R

histone cluster 1, H2ai [Homo sapiens]
Charge State: +2
Scan Number: 8615
File Name: 110924_HG2_INSA_ACK.raw

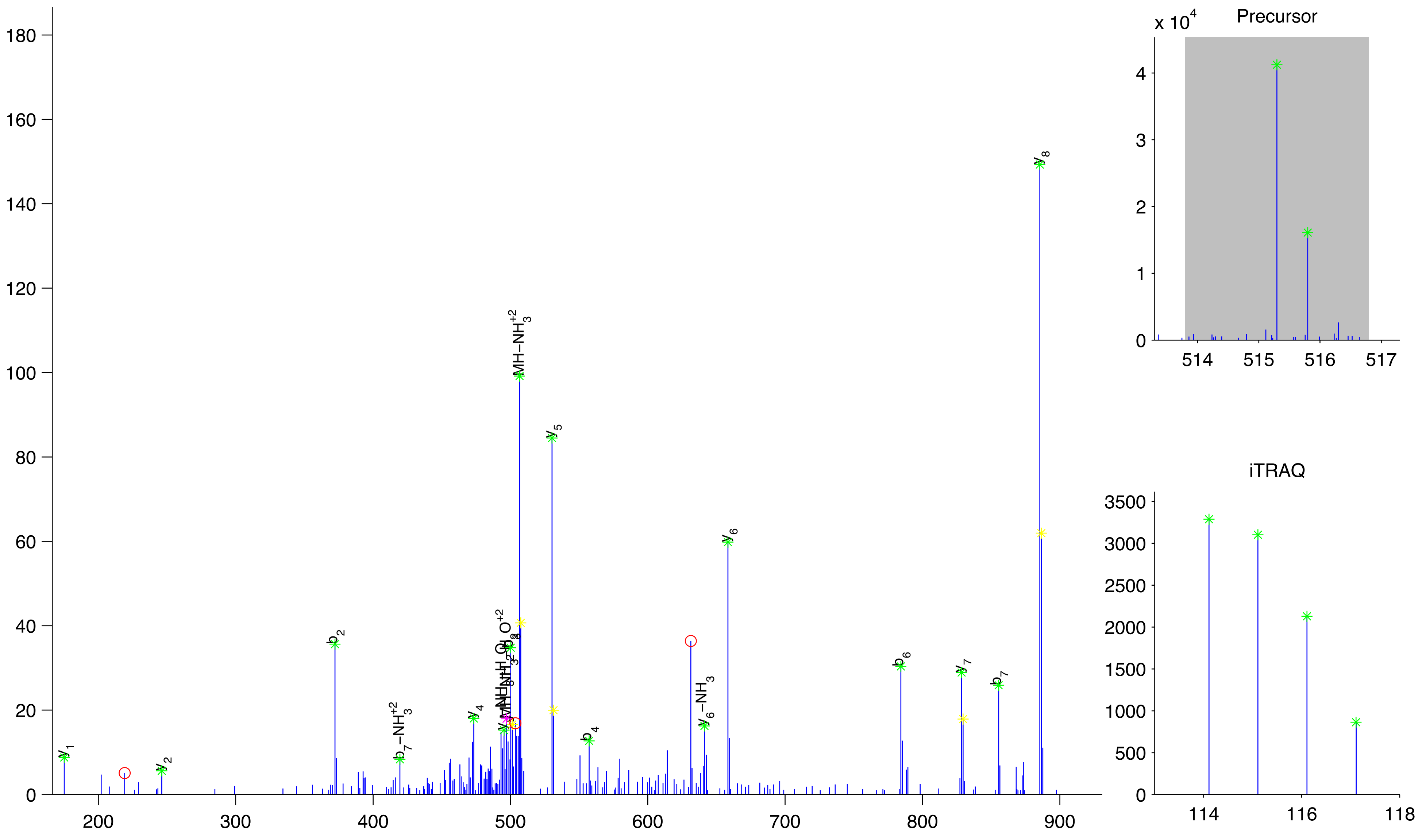

L L L P G E L A K

histone cluster 1, H2ba [Homo sapiens]
Charge State: +2
Scan Number: 10463
File Name: 111016_HG2_INSC_ACK.raw

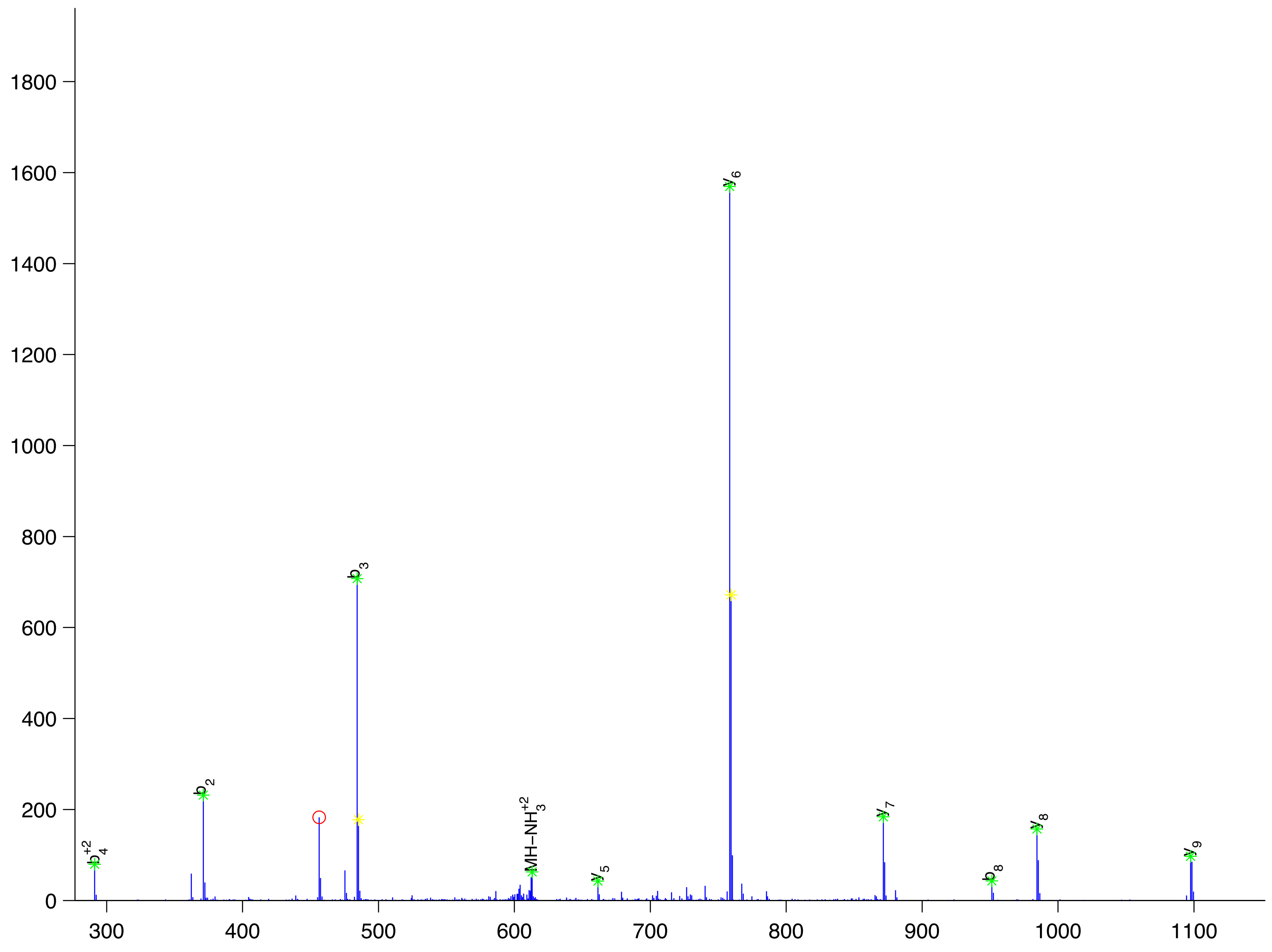

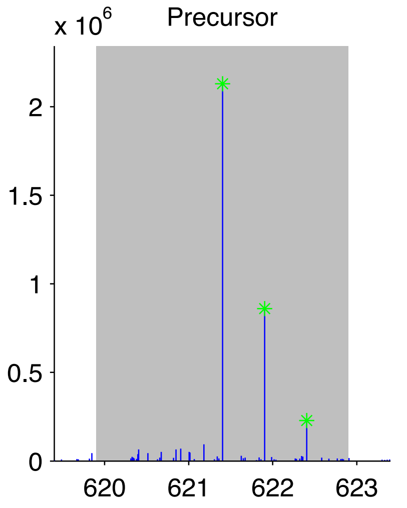

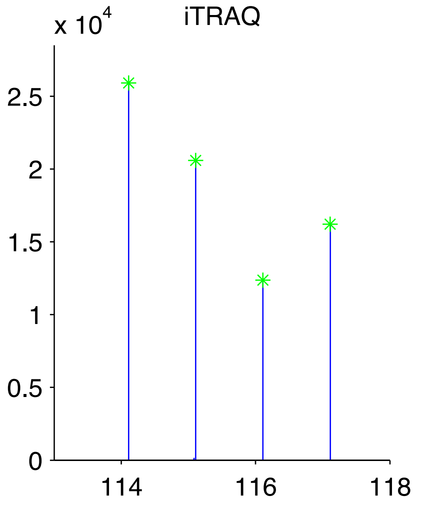

L L L P G E L A K

histone cluster 1, H2ba [Homo sapiens]
Charge State: +2
Scan Number: 10631
File Name: 111016_HG2_INSC_ACK.raw

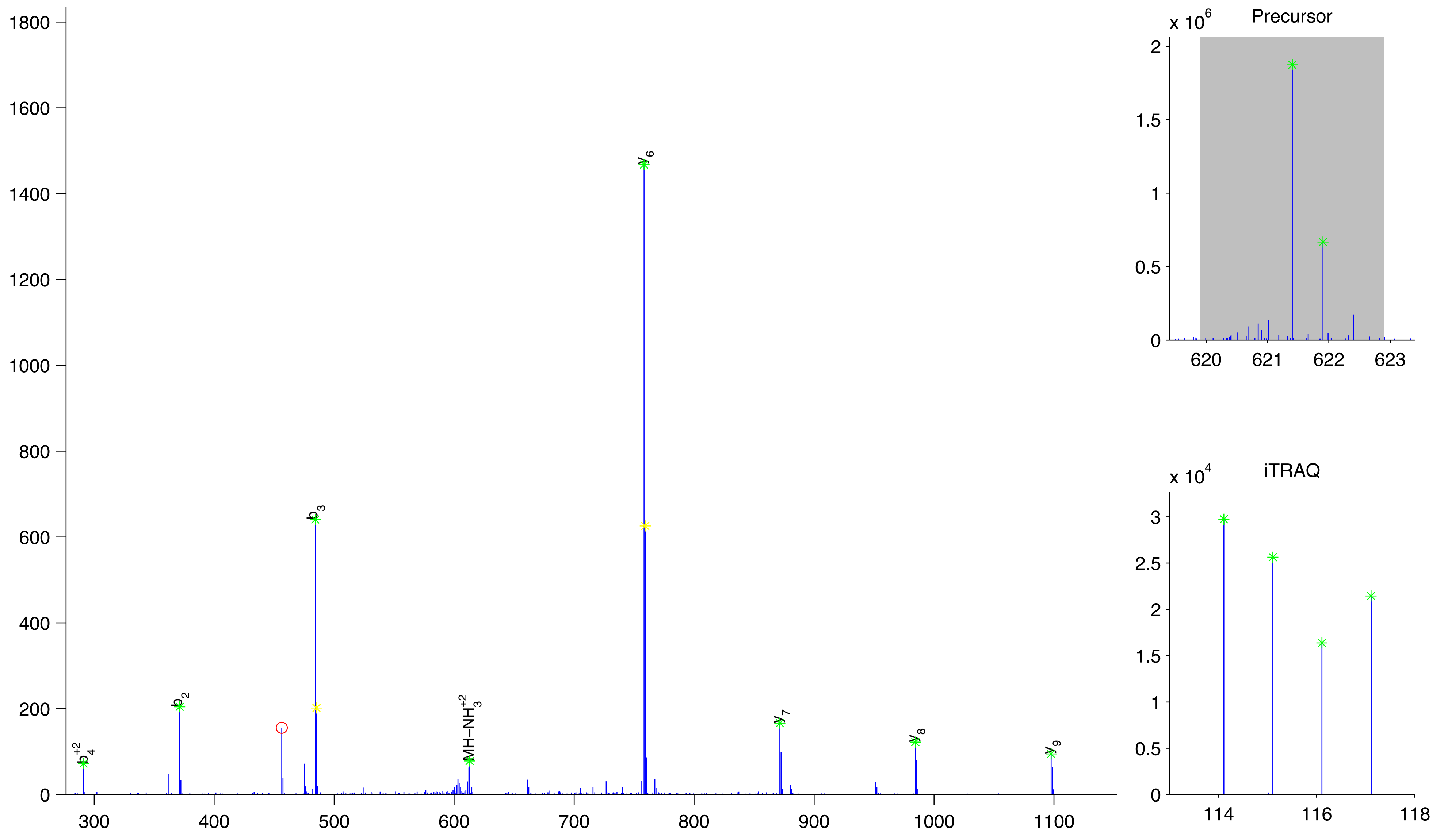

k A V V k T Q K

histone cluster 1, H2ba [Homo sapiens]
Charge State: +2
Scan Number: 12647
File Name: 110924_HG2_INSA_ACK.raw

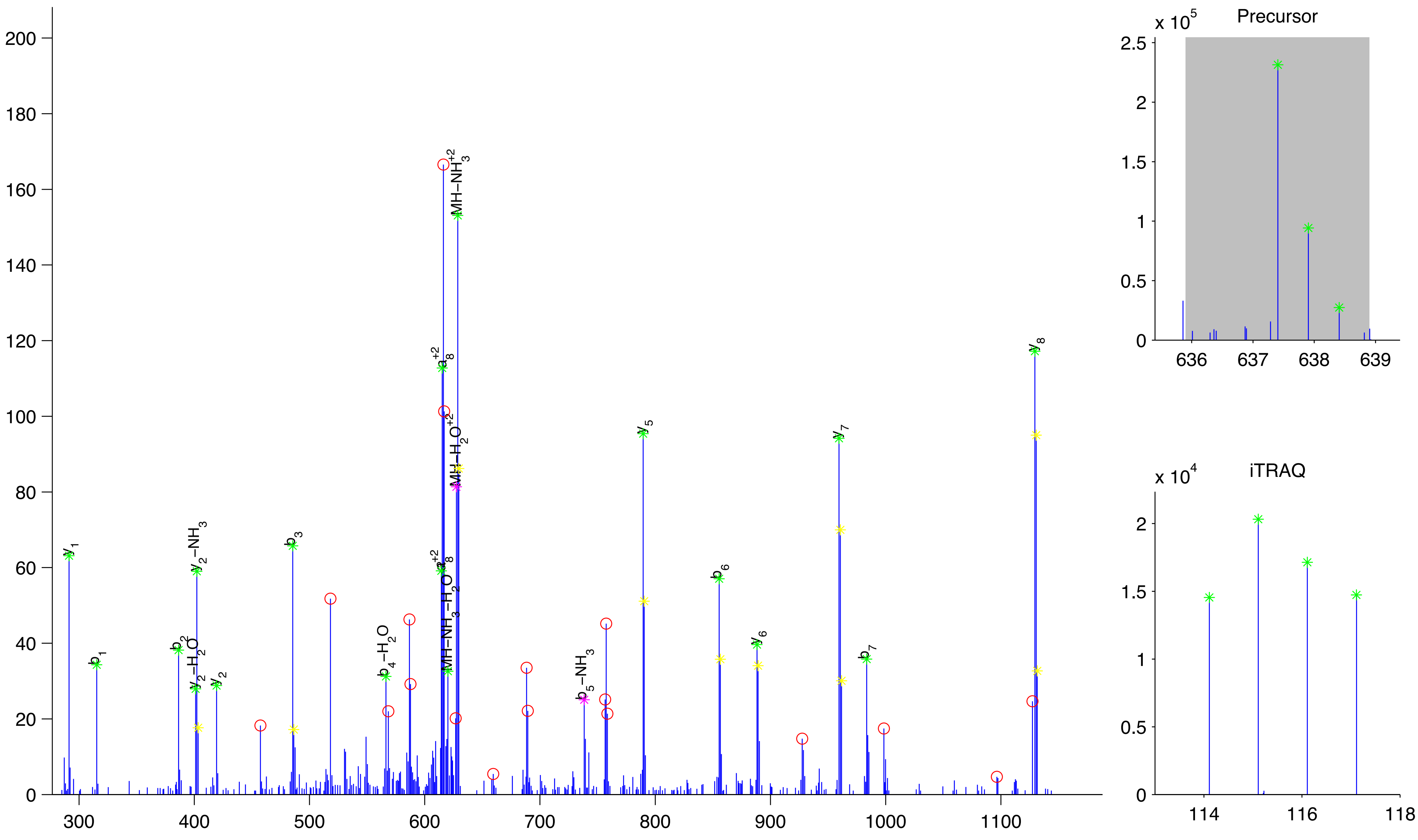

k G S k k A I T K

histone cluster 1, H2bb [Homo sapiens]
Charge State: +2
Scan Number: 12840
File Name: 110924_HG2_INSA_ACK.raw

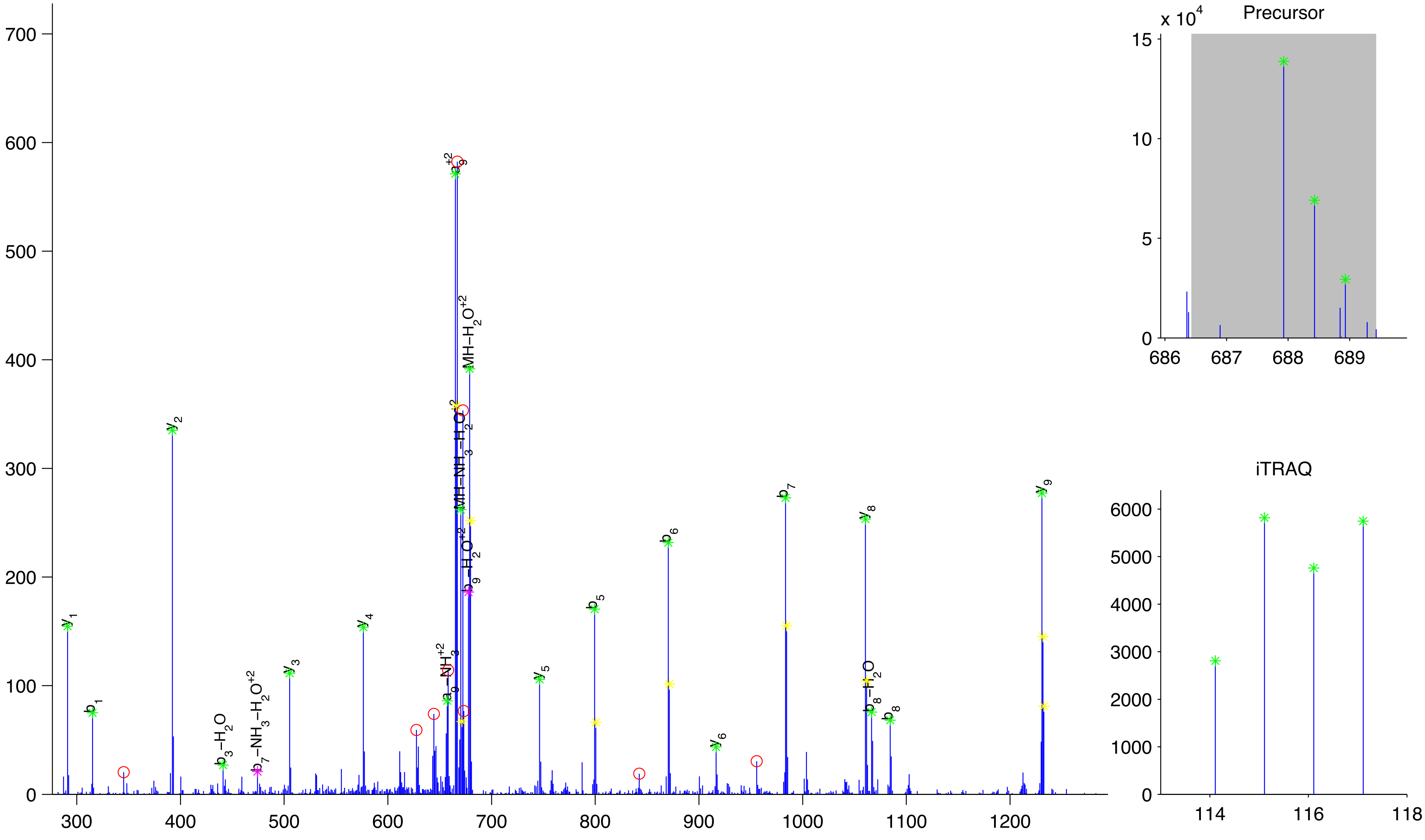

k G S k k A I T K

histone cluster 1, H2bb [Homo sapiens]
Charge State: +2
Scan Number: 13027
File Name: 110924_HG2_INSA_ACK.raw

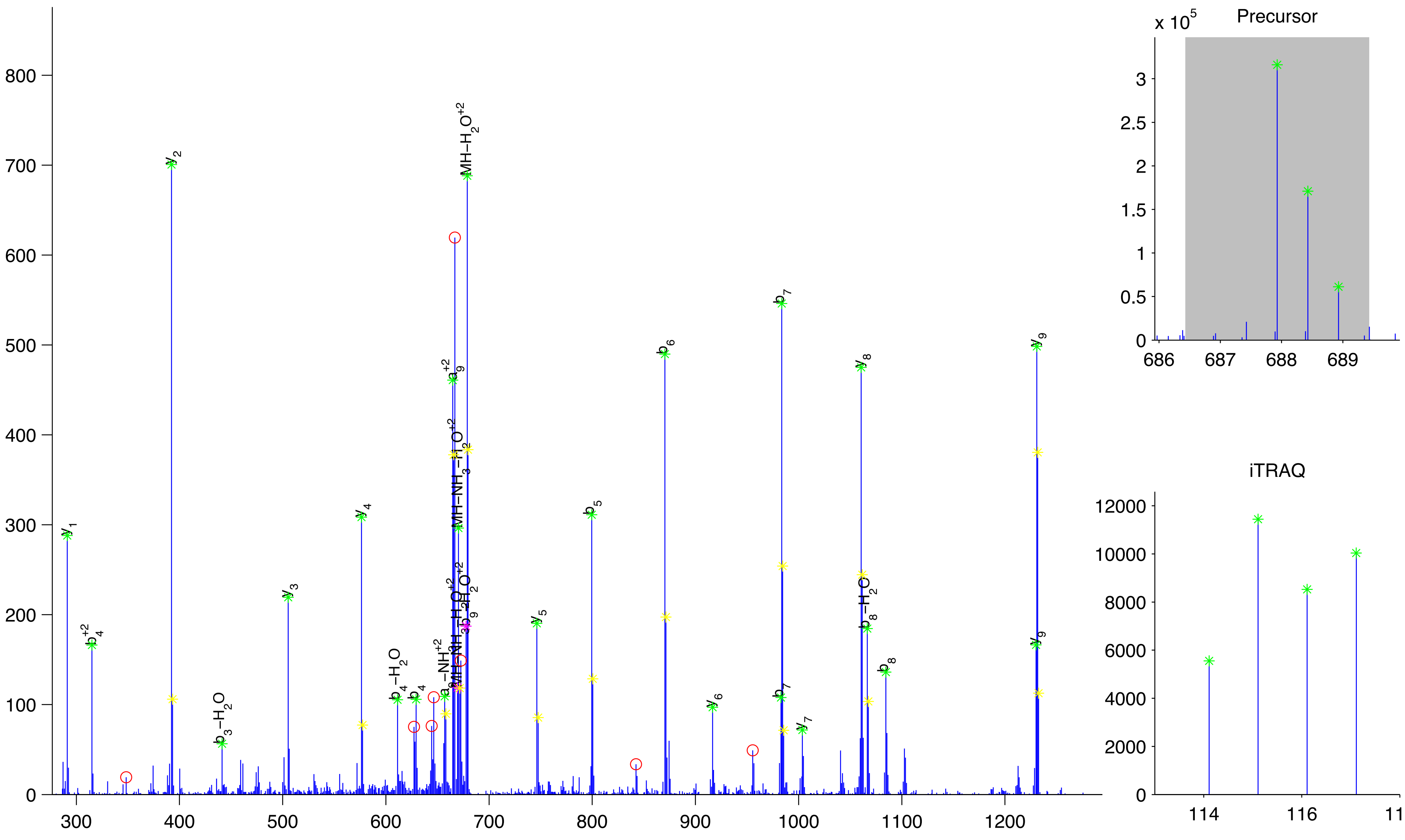

k G S k k A I T K

histone cluster 1, H2bb [Homo sapiens]
Charge State: +2
Scan Number: 13216
File Name: 110924_HG2_INSA_ACK.raw

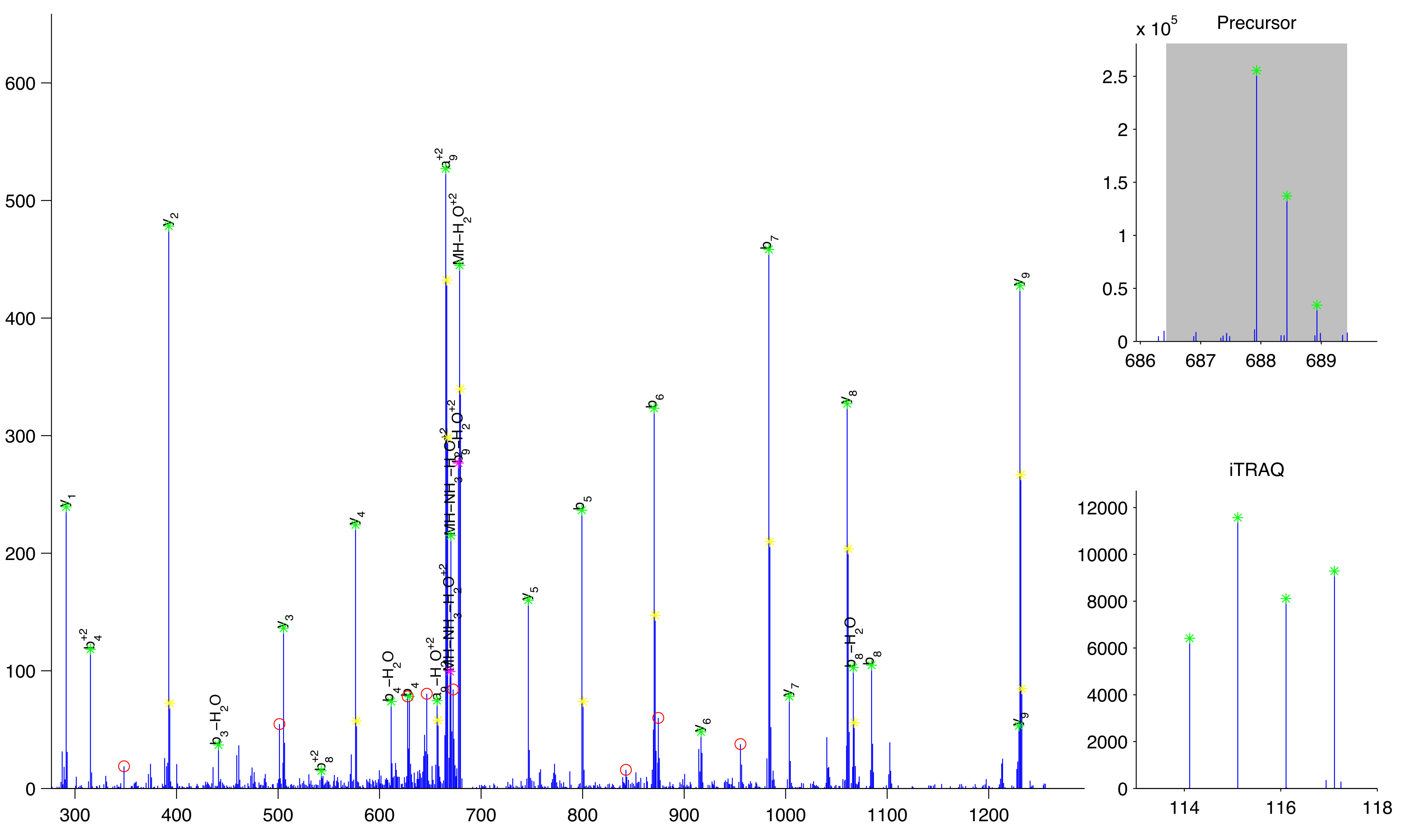

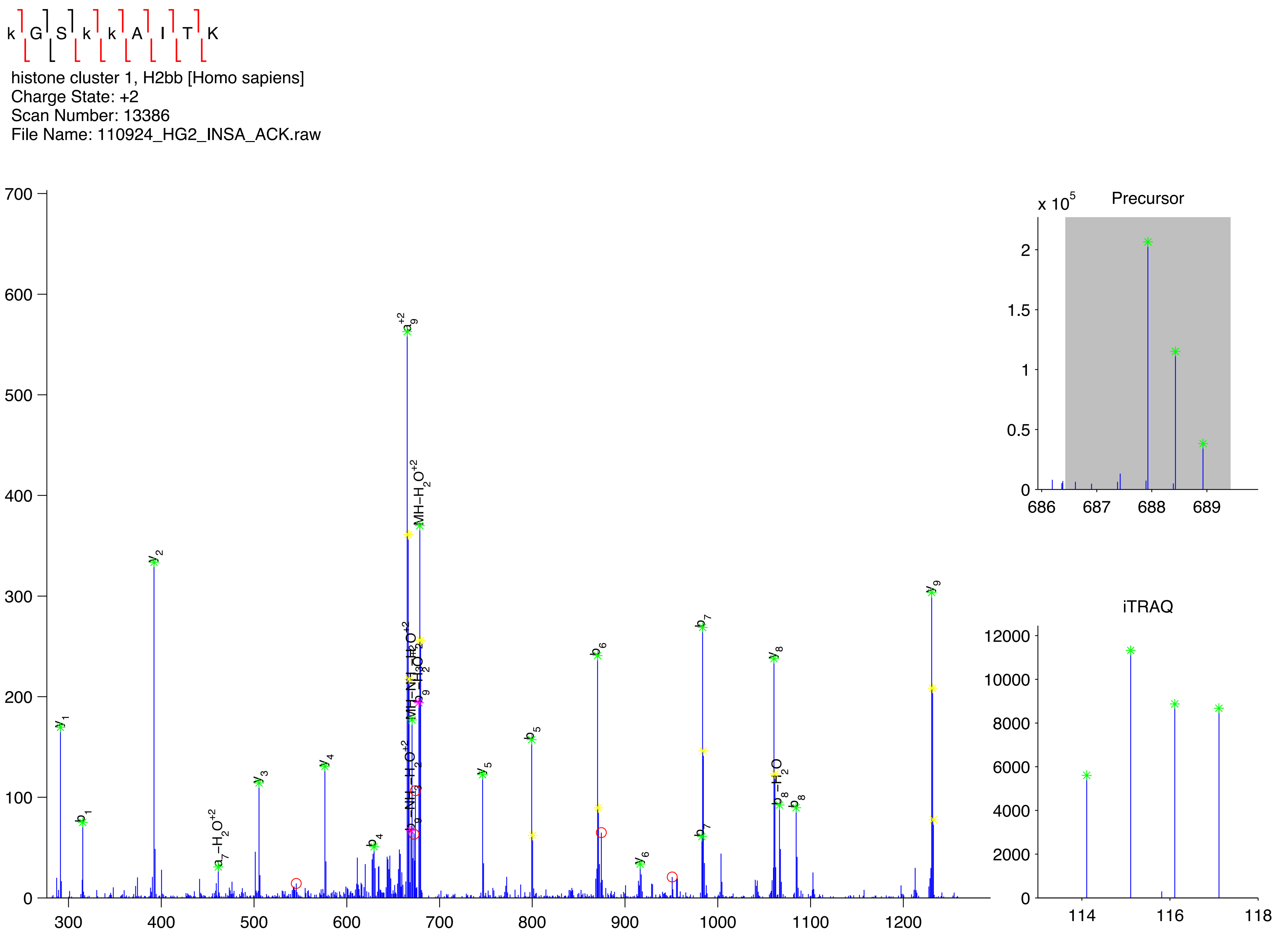
k G S k k A I T K
histone cluster 1, H2bb [Homo sapiens]
Charge State: +2
Scan Number: 13386
File Name: 110924_HG2_INSA_ACK.raw
Precursor
x 10^5
iTRAQ

k G S k k A I T K

histone cluster 1, H2bb [Homo sapiens]
Charge State: +2
Scan Number: 13554
File Name: 110924_HG2_INSA_ACK.raw

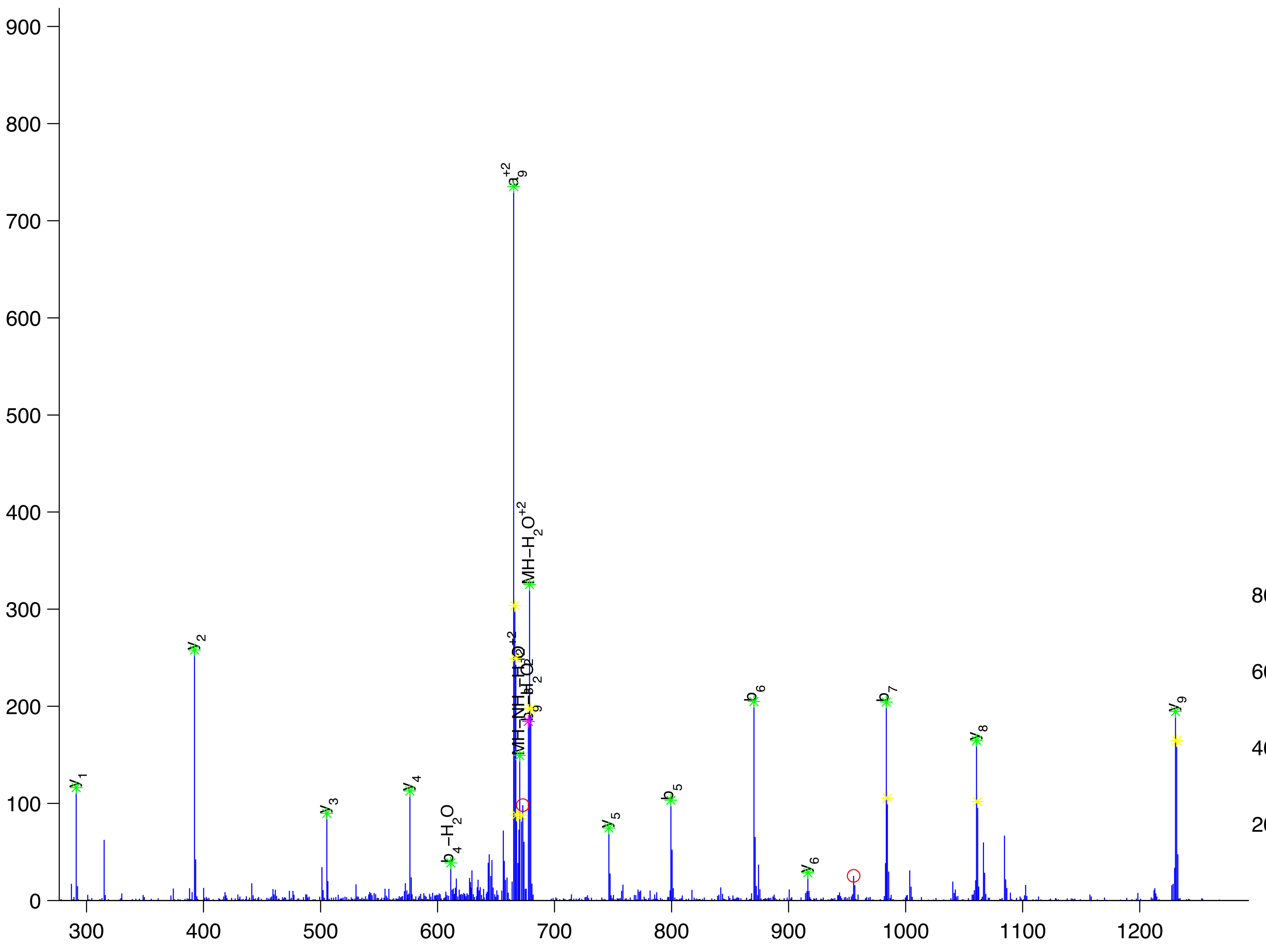

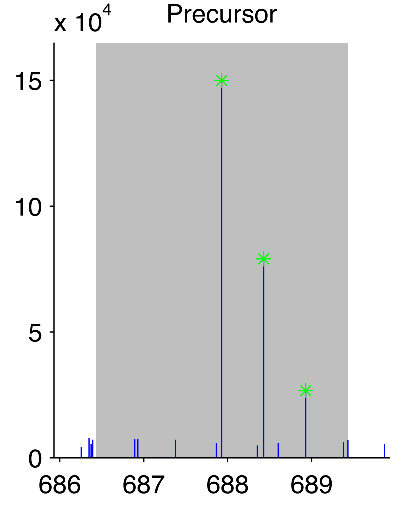

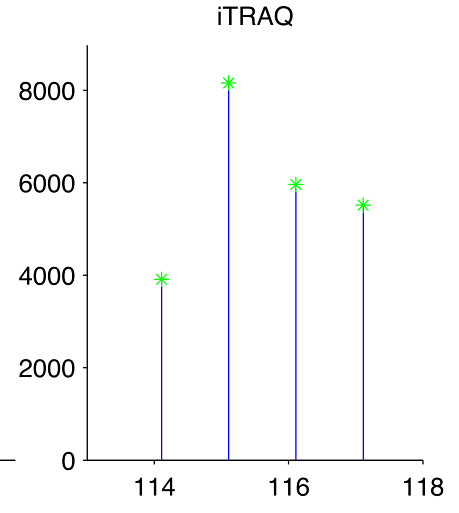

k G S k k A I T K

histone cluster 1, H2bb [Homo sapiens]
Charge State: +2
Scan Number: 13722
File Name: 110924_HG2_INSA_ACK.raw

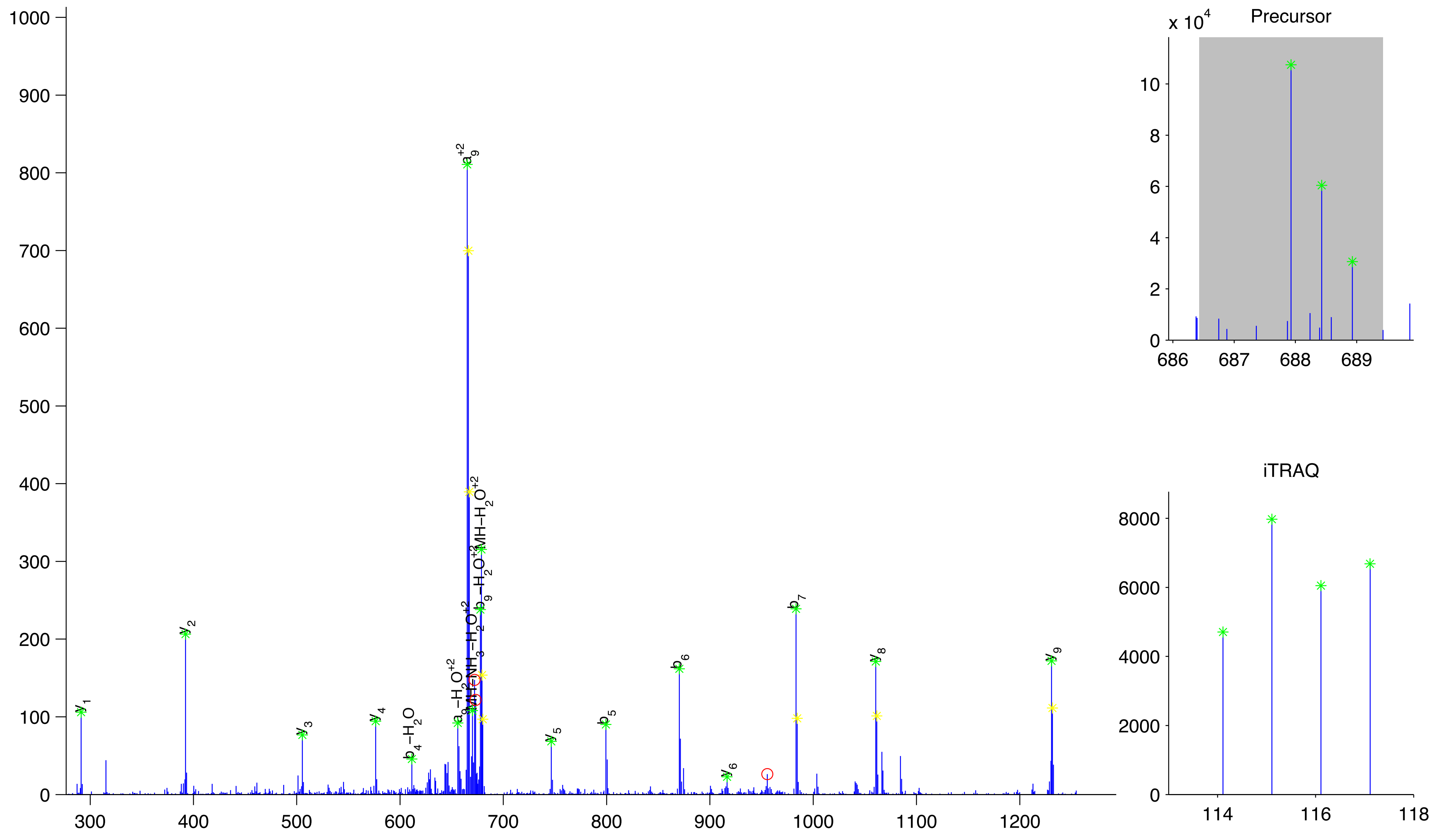

k G S k k A I T K

histone cluster 1, H2bb [Homo sapiens]
Charge State: +2
Scan Number: 13892
File Name: 110924_HG2_INSA_ACK.raw

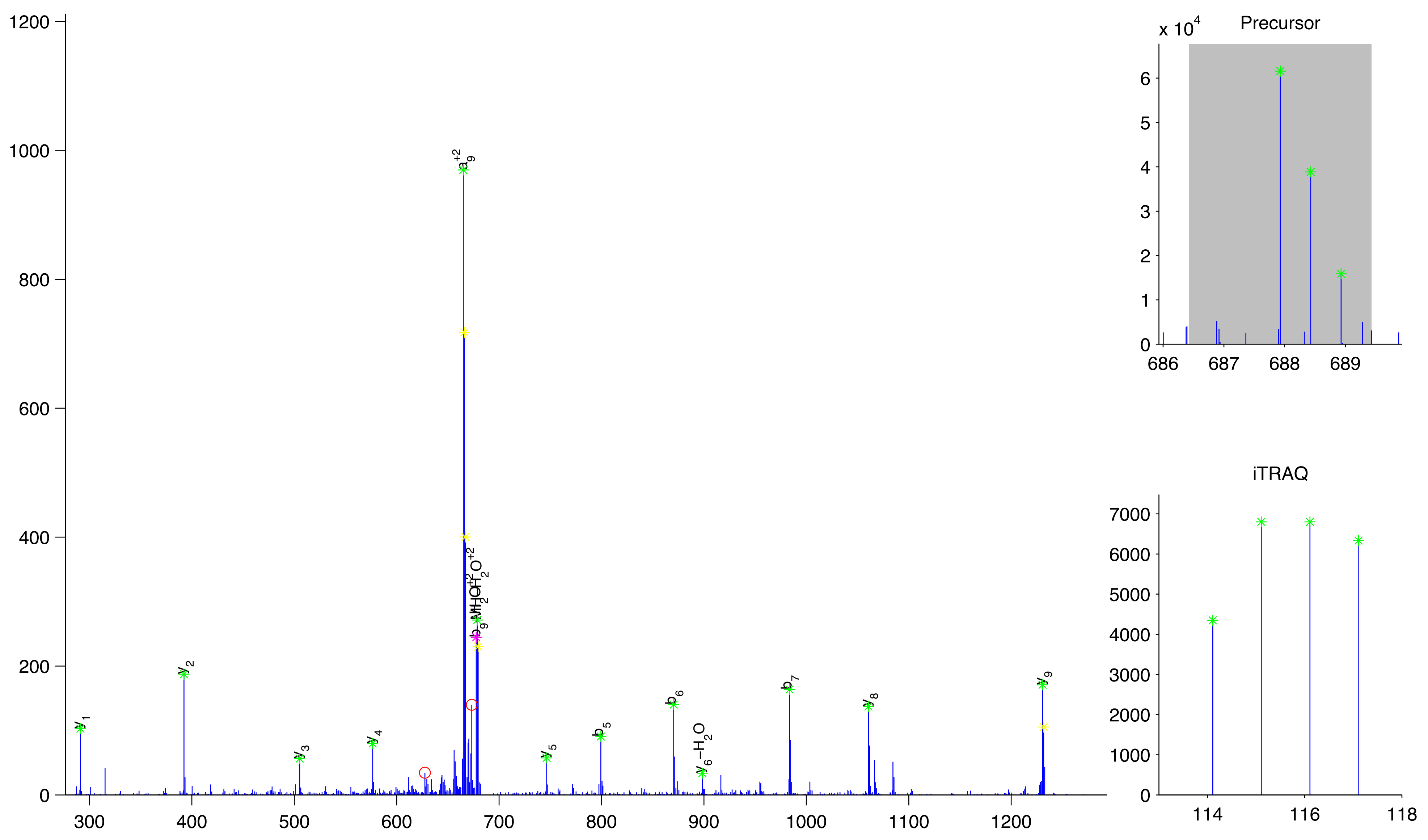

k G S k k A I T K

histone cluster 1, H2bb [Homo sapiens]
Charge State: +2
Scan Number: 14060
File Name: 110924_HG2_INSA_ACK.raw

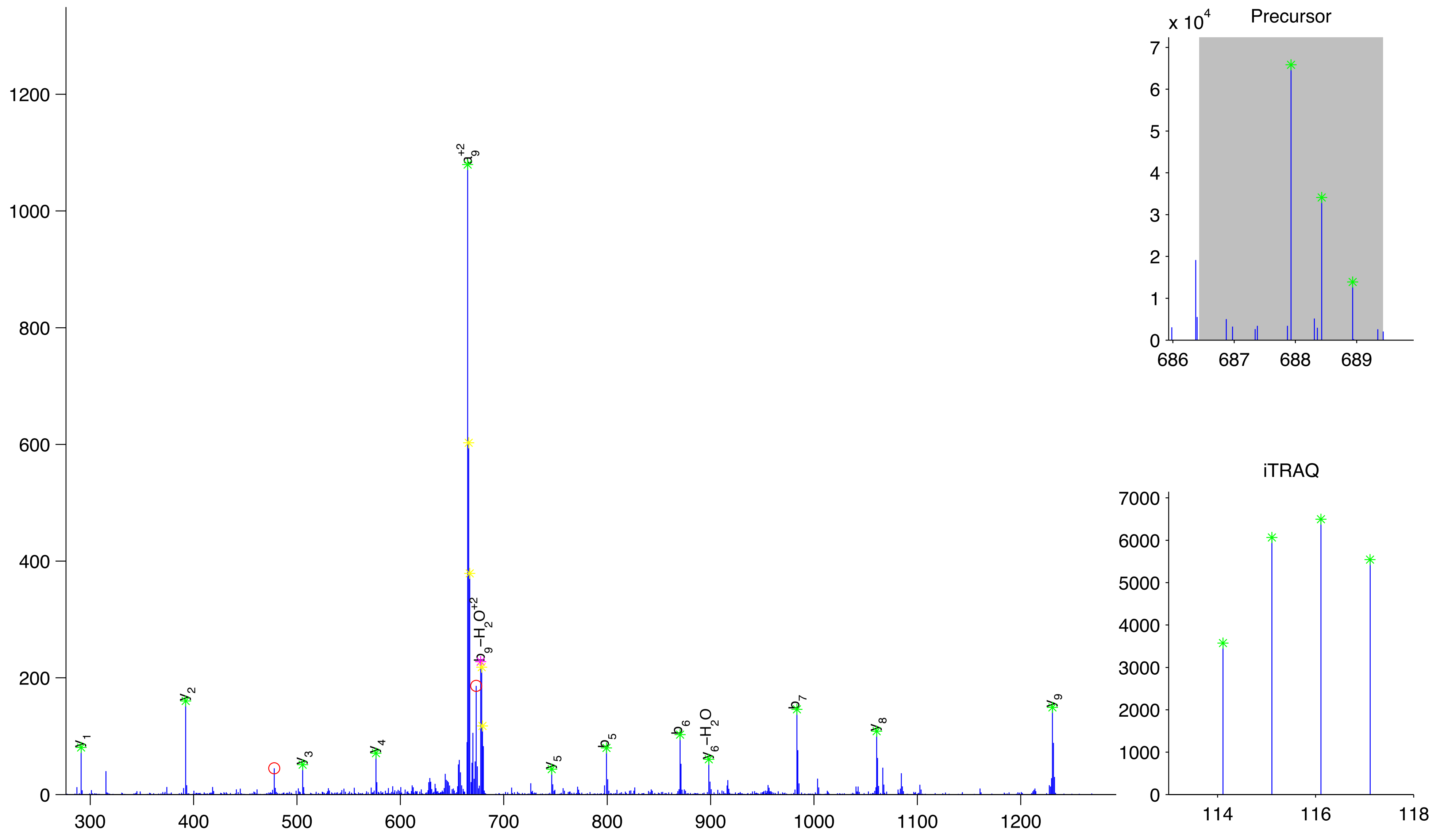

k G S k k A I T K

histone cluster 1, H2bb [Homo sapiens]
Charge State: +2
Scan Number: 14226
File Name: 110924_HG2_INSA_ACK.raw

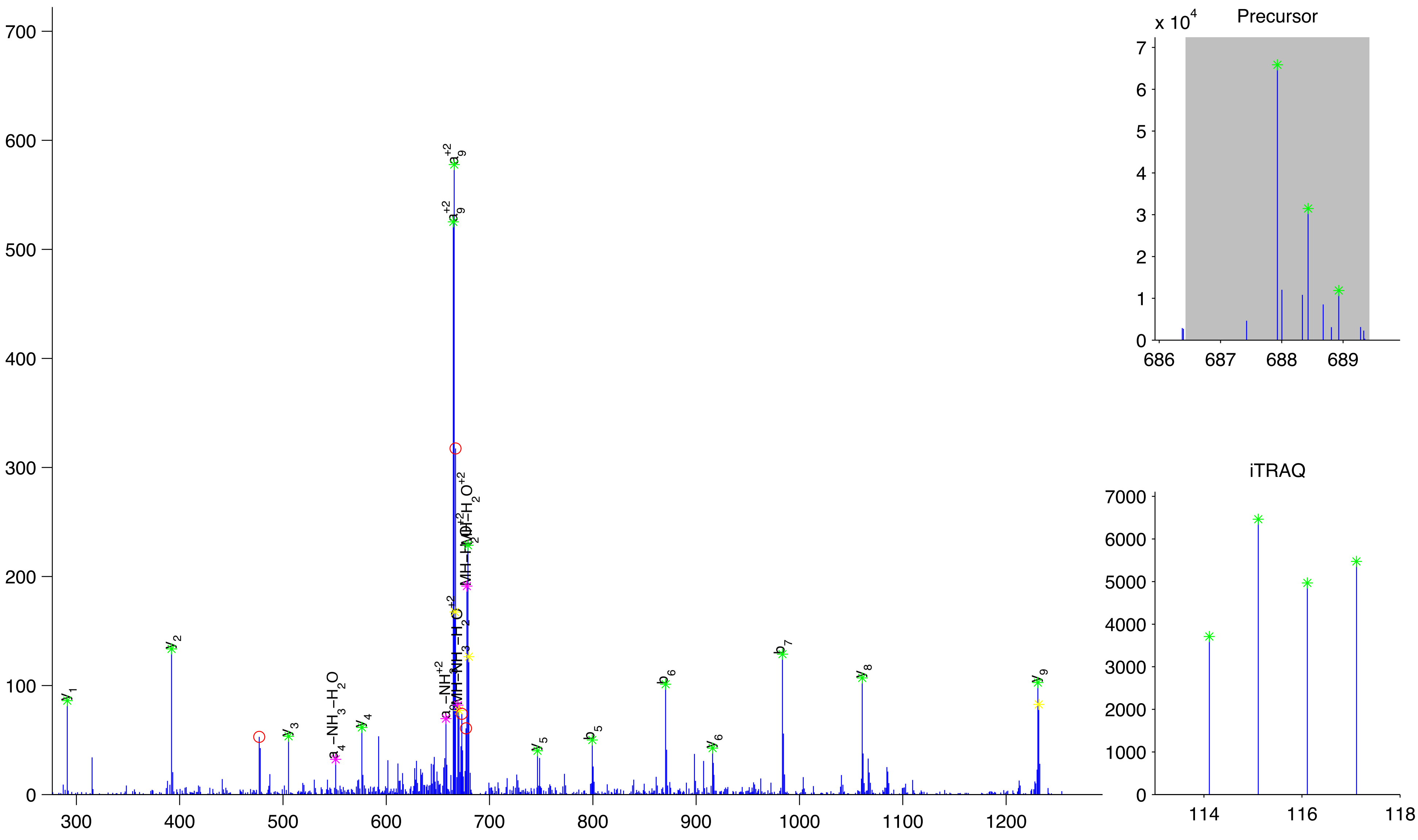

k A V T k A Q K

histone cluster 1, H2bd [Homo sapiens]
Charge State: +2
Scan Number: 11030
File Name: 110924_HG2_INSA_ACK.raw

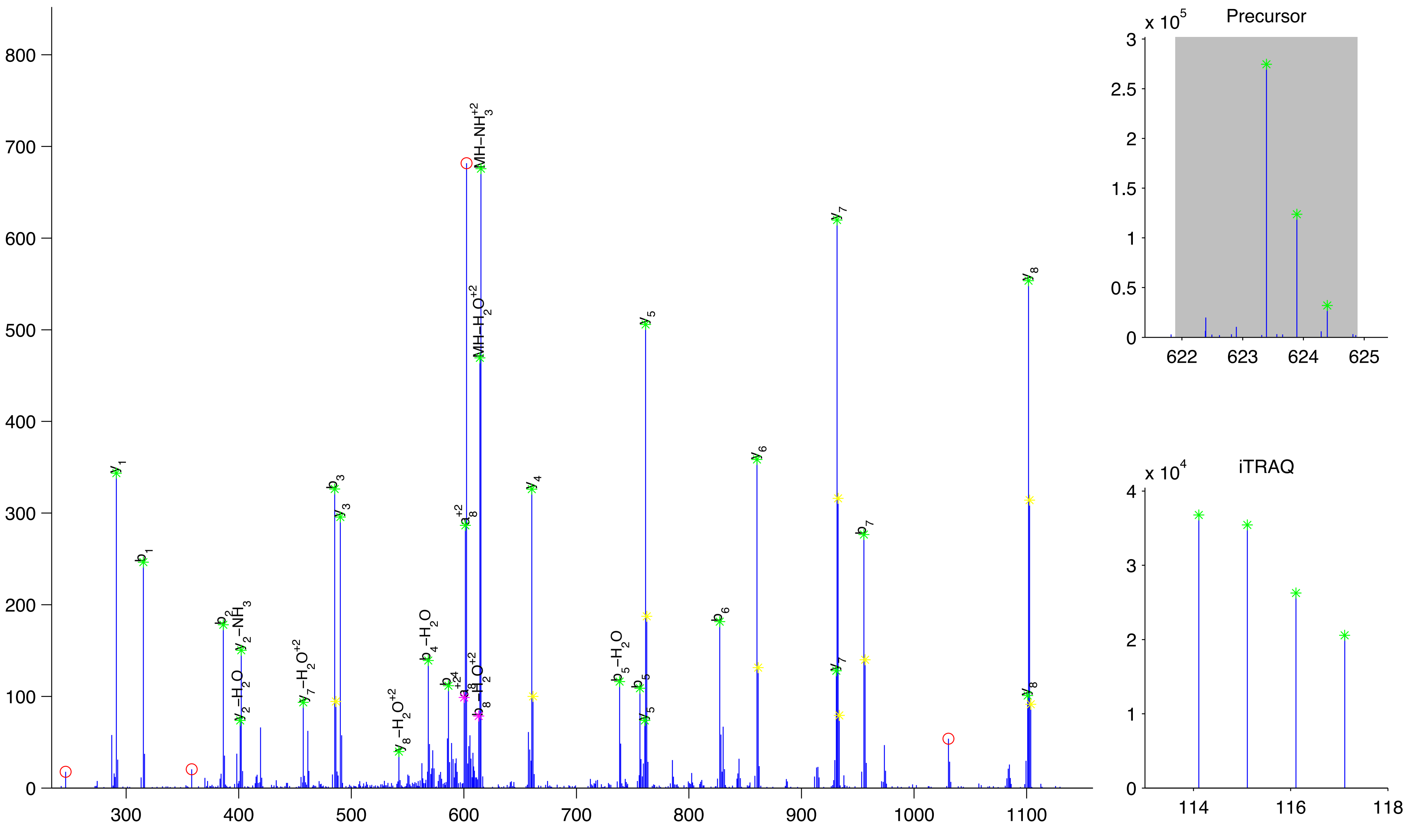

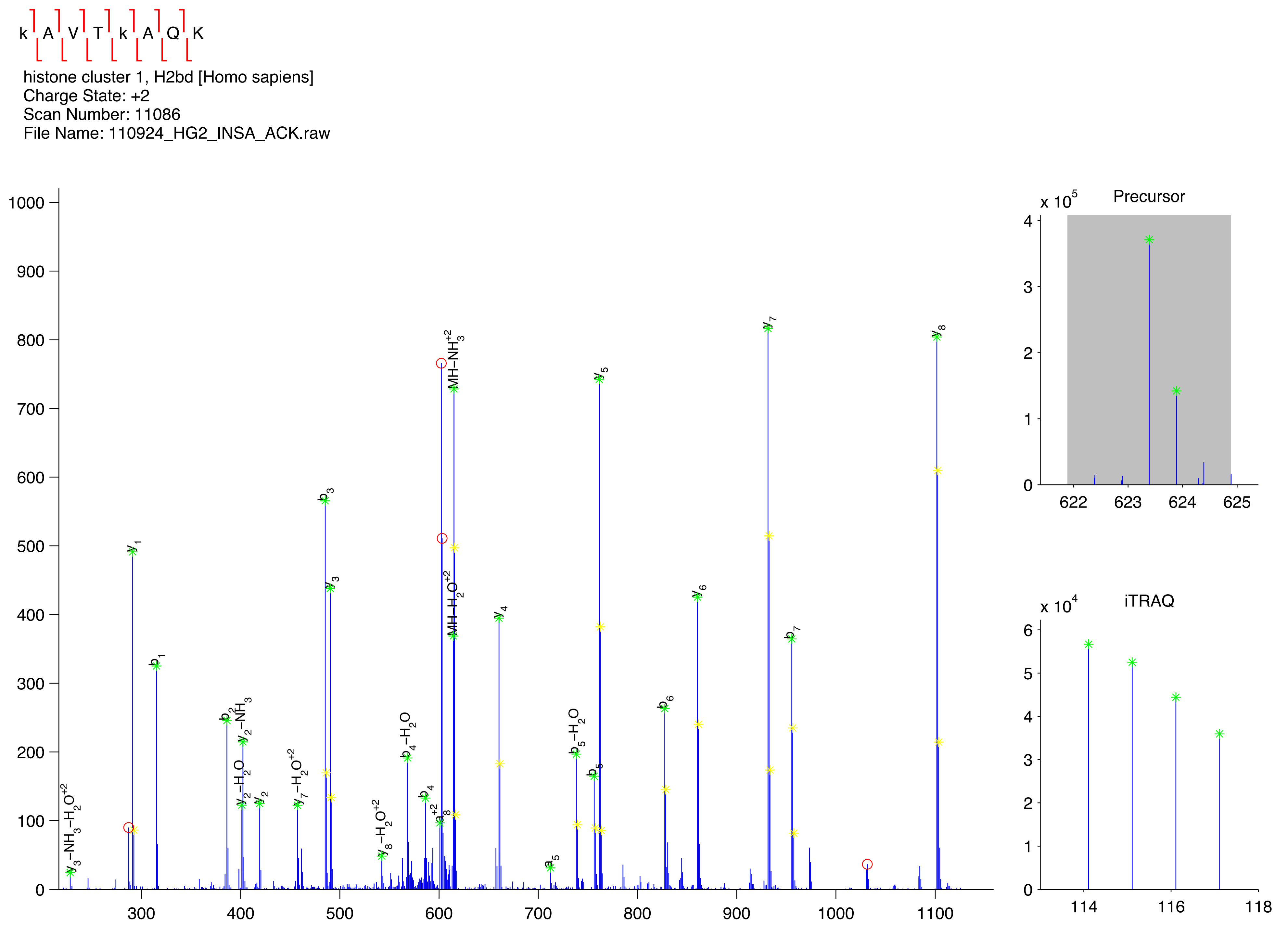
k A V T k A Q K
histone cluster 1, H2bd [Homo sapiens]
Charge State: +2
Scan Number: 11086
File Name: 110924_HG2_INSA_ACK.raw
Precursor
x 10^5
iTRAQ
x 10^4
MH−NH3+2
MH−H2O+2
y1
b1
b2
y2−NH3
y2−H2O
y2
y7−H2O+2
b3
y3
y8−H2O+2
b4−H2O
b4
a8+2
y4
a5
b5−H2O
b5
y5
b6
y6
y7
b7
y8

k A V T k A Q K

histone cluster 1, H2bd [Homo sapiens]
Charge State: +2
Scan Number: 11198
File Name: 110924_HG2_INSA_ACK.raw

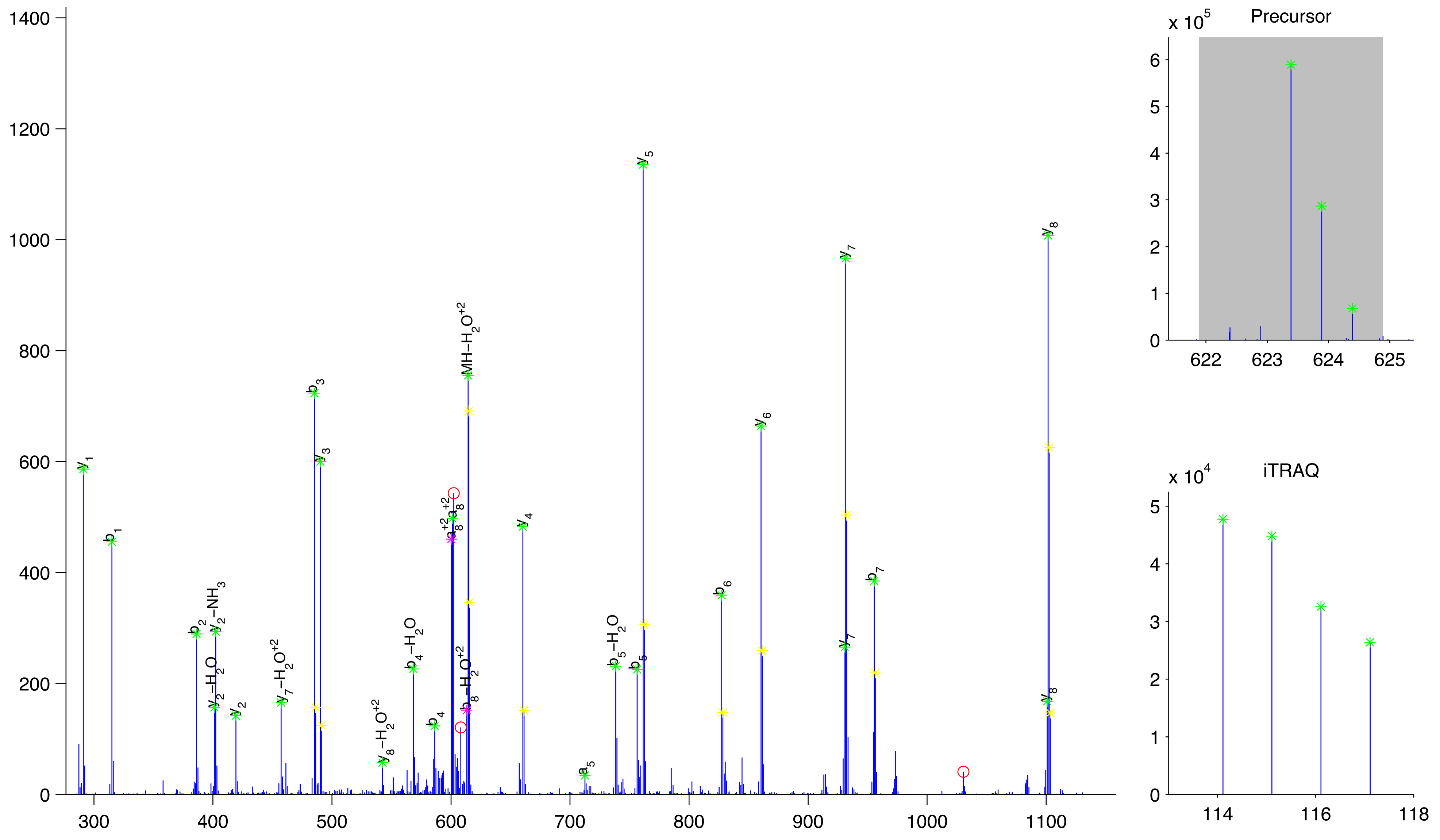

k A V T k A Q K

histone cluster 1, H2bd [Homo sapiens]
Charge State: +2
Scan Number: 11277
File Name: 110924_HG2_INSA_ACK.raw

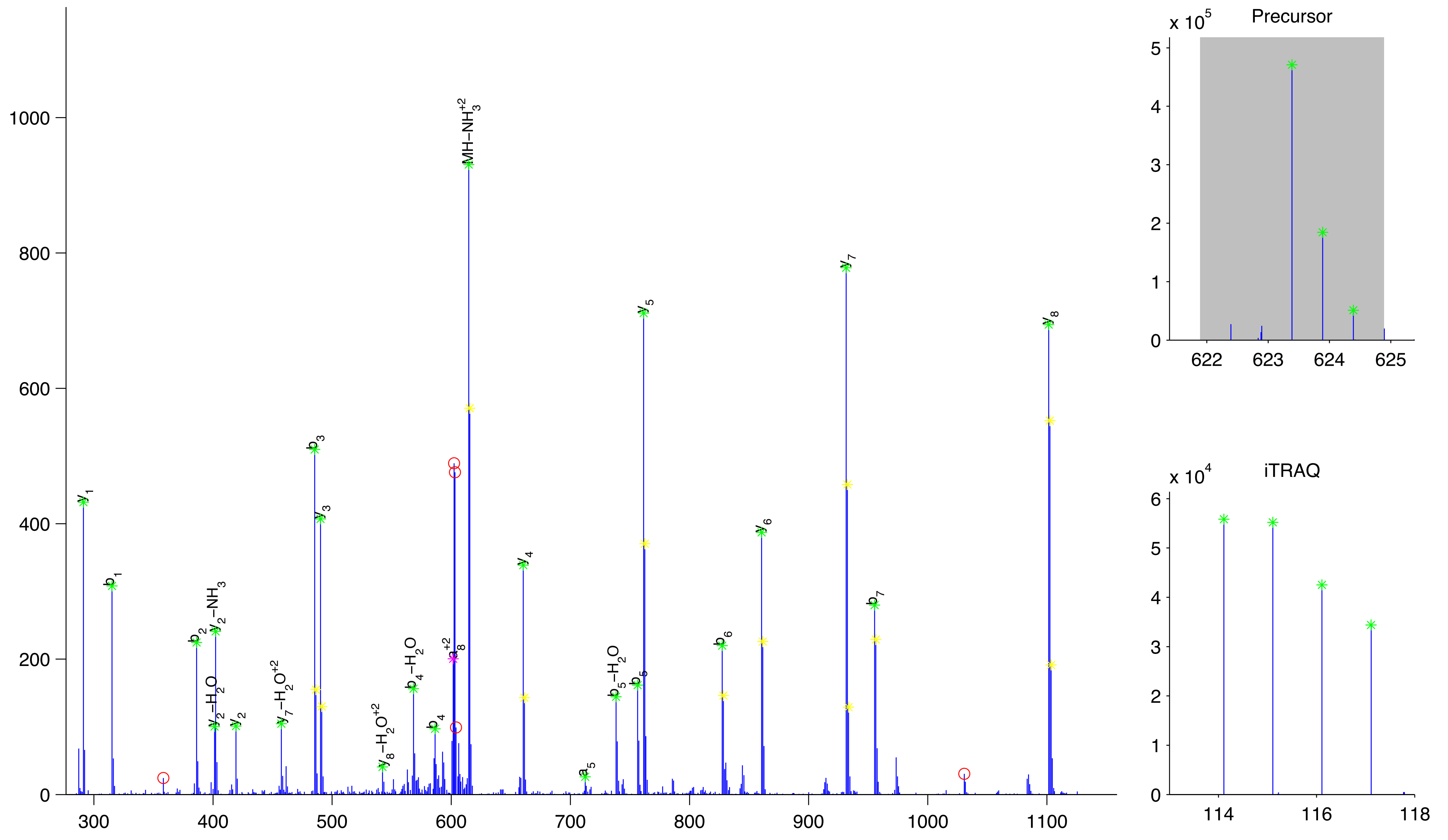

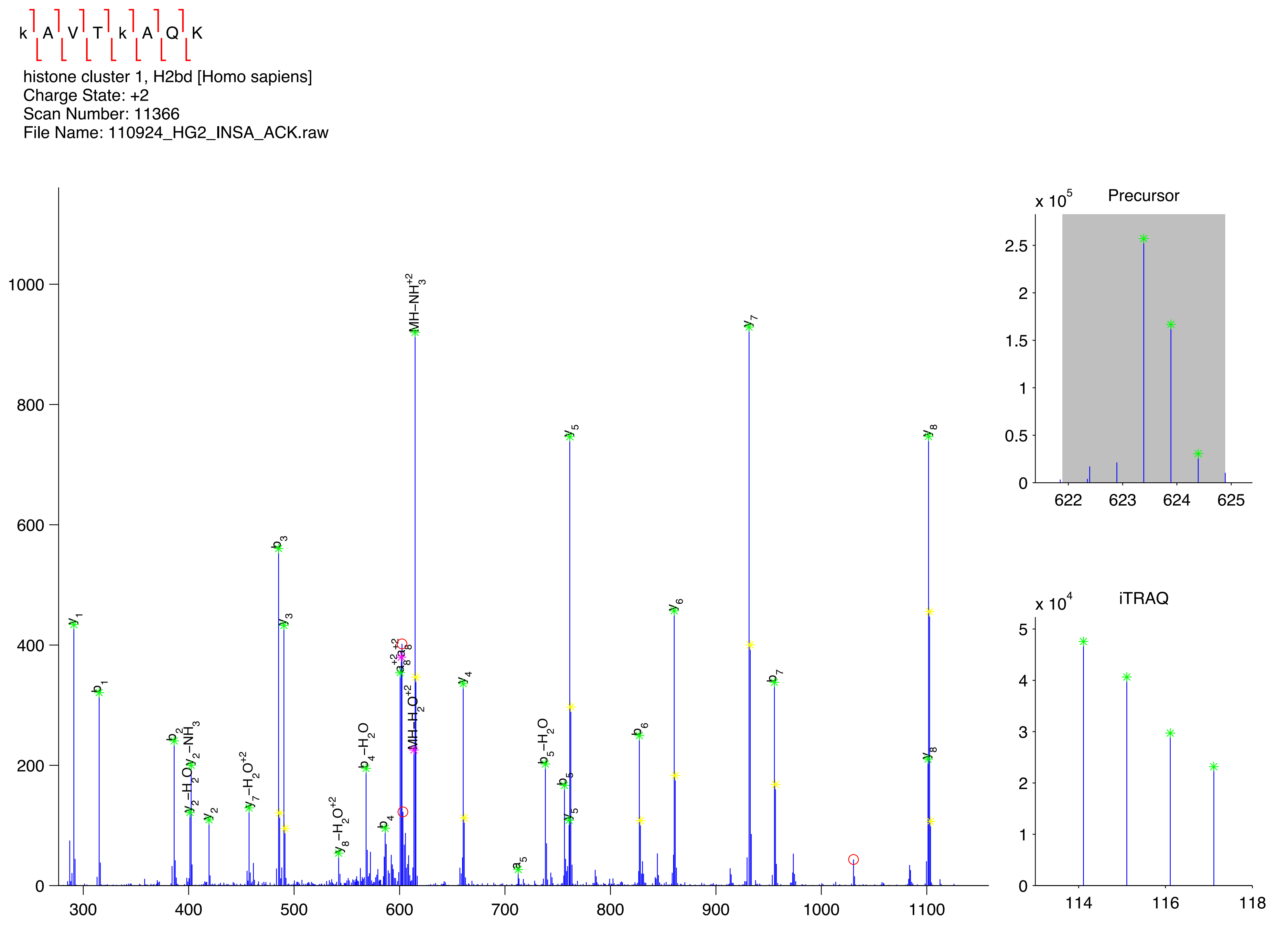

k A V T k A Q K
histone cluster 1, H2bd [Homo sapiens]
Charge State: +2
Scan Number: 11366
File Name: 110924_HG2_INSA_ACK.raw
Precursor
x 10^5
iTRAQ
x 10^4

k G S k k A V T k A Q K

histone cluster 1, H2bd [Homo sapiens]
Charge State: +3
Scan Number: 13237
File Name: 110924_HG2_INSA_ACK.raw

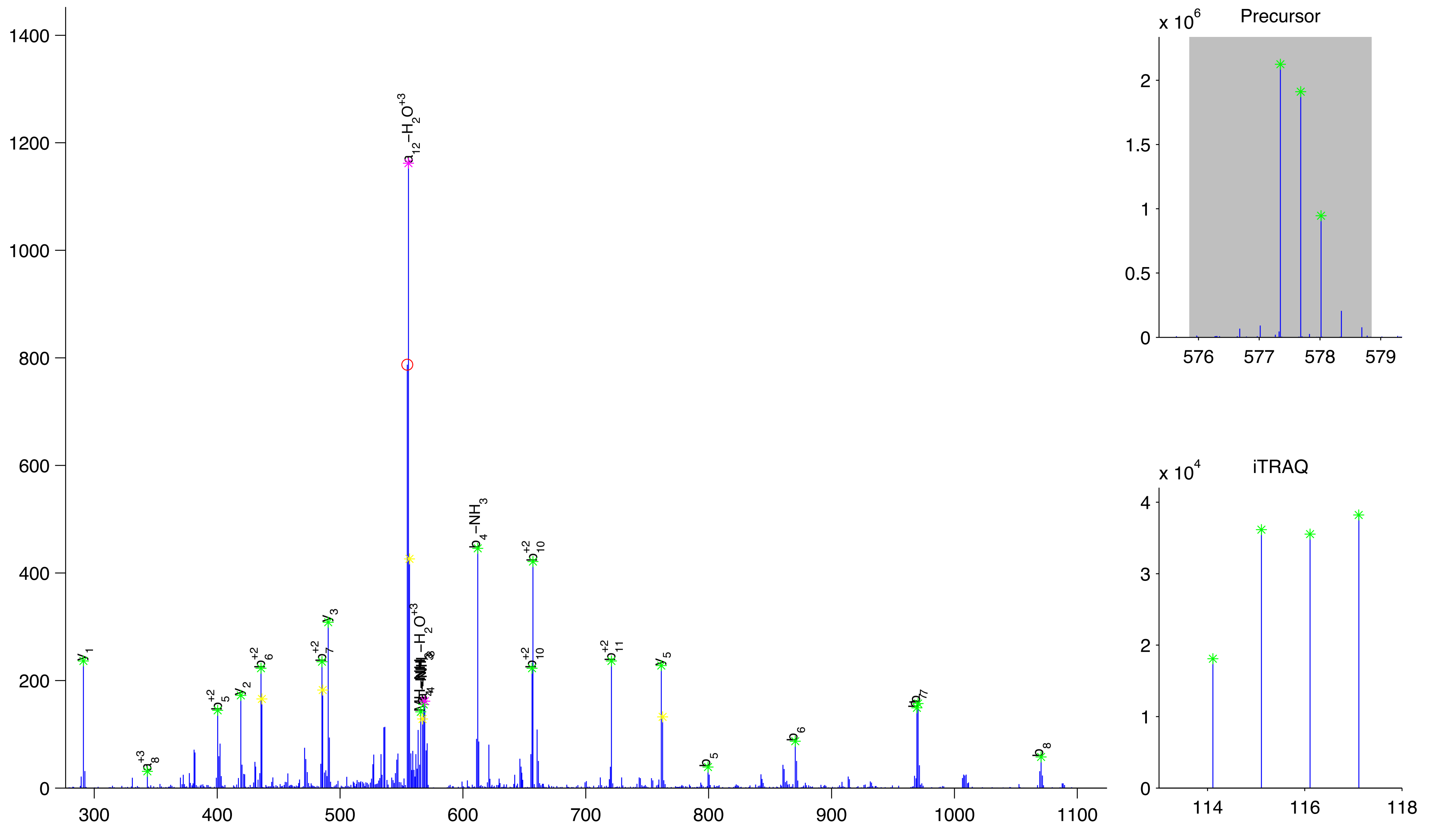

k G S k k A V T k A Q K

histone cluster 1, H2bd [Homo sapiens]
Charge State: +3
Scan Number: 13403
File Name: 110924_HG2_INSA_ACK.raw

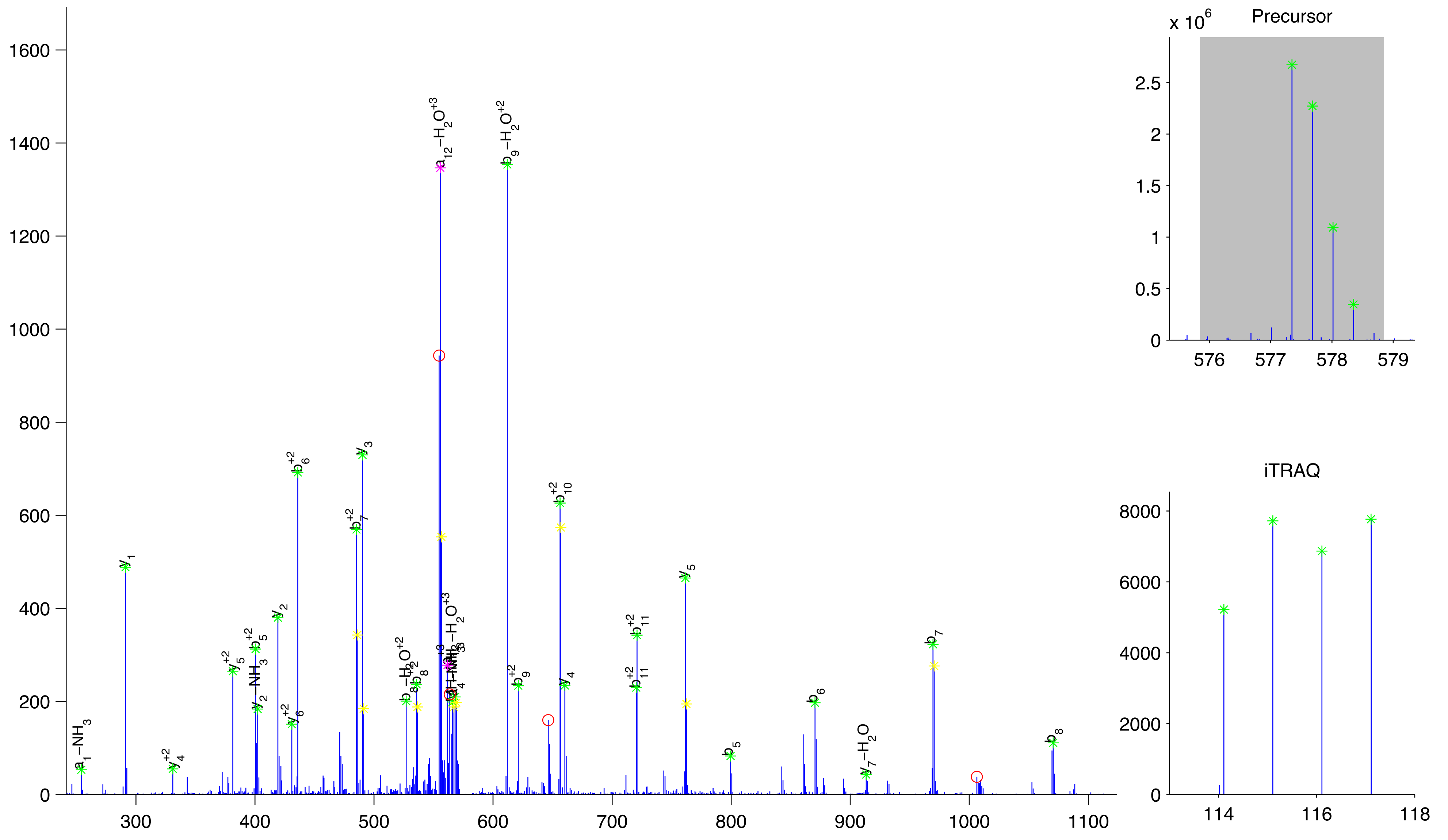

k G S k k A V T k A Q K

histone cluster 1, H2bd [Homo sapiens]
Charge State: +3
Scan Number: 13573
File Name: 110924_HG2_INSA_ACK.raw

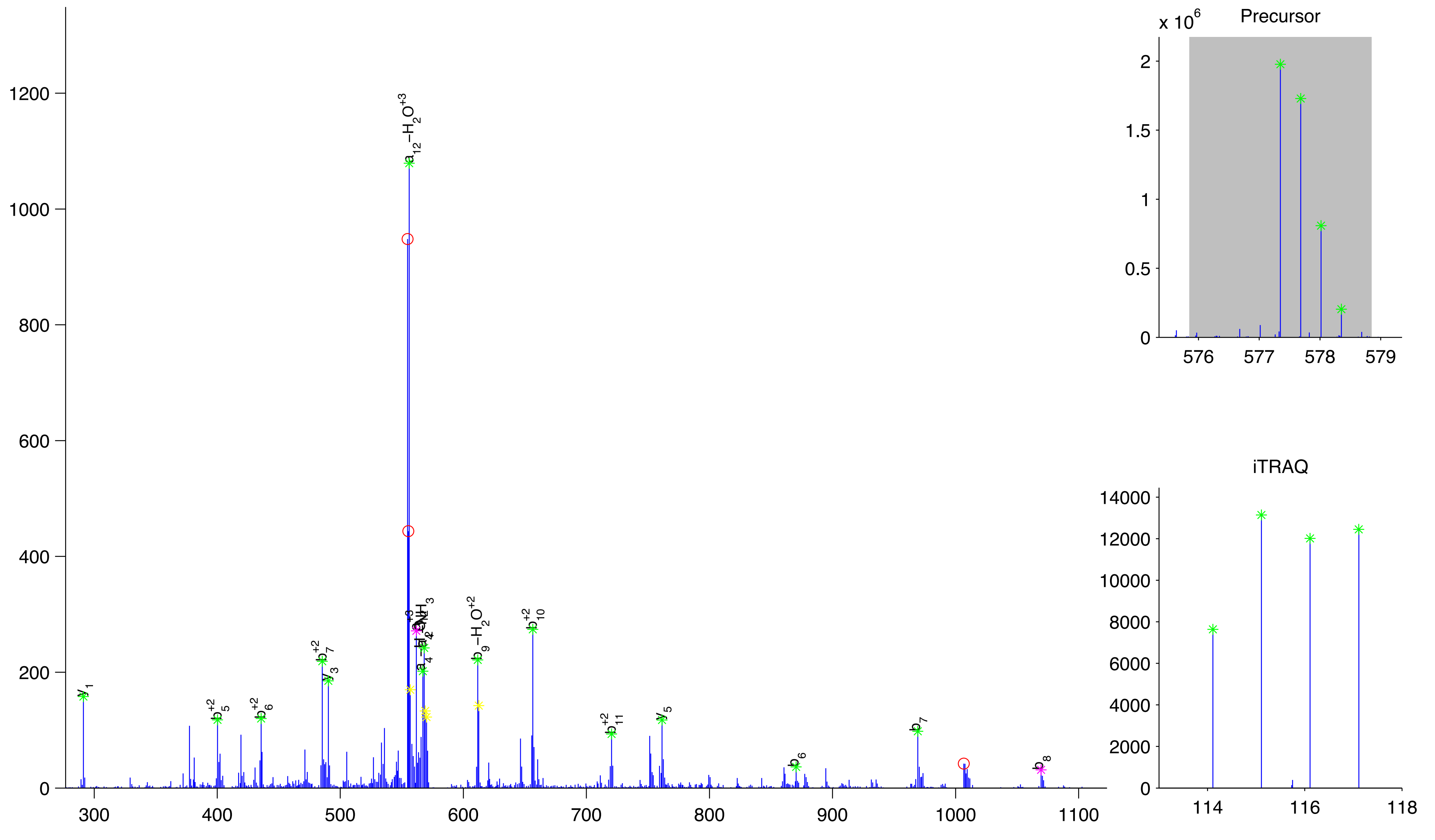

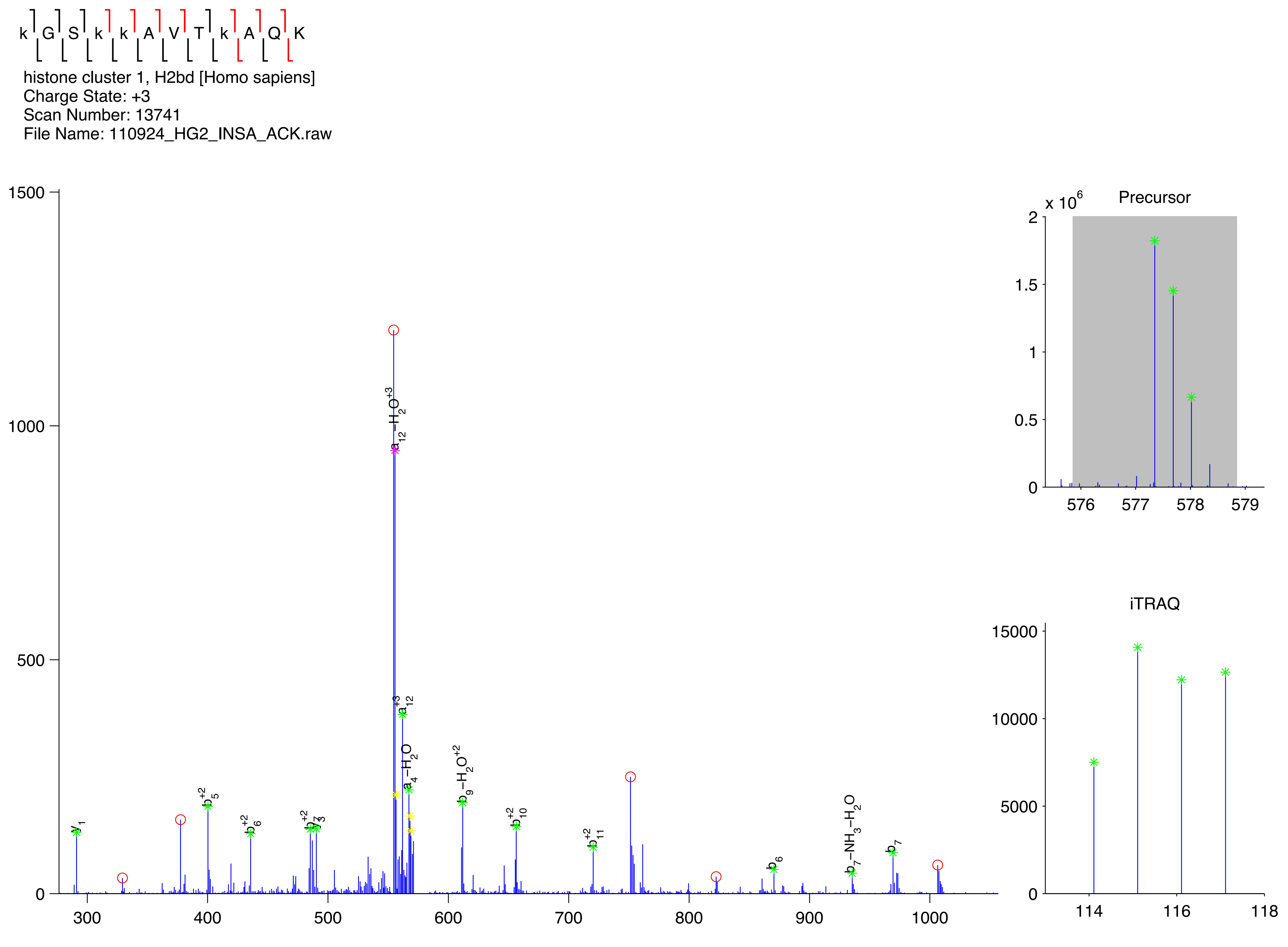

k G S k k A V T k A Q K
histone cluster 1, H2bd [Homo sapiens]
Charge State: +3
Scan Number: 13741
File Name: 110924_HG2_INSA_ACK.raw
1500
1000
500
0
300
400
500
600
700
800
900
1000
y_1
b_5^{+2}
b_6^{+2}
b_7^{+2}
y_3
$a_{12}-H_2O^{+3}$
a_{12}^{+3}
a_4-H_2O
$b_9-H_2O^{+2}$
b_{10}^{+2}
b_{11}^{+2}
b_6
$b_7-NH_3-H_2O$
b_7
x 10^6
Precursor
2
1.5
1
0.5
0
576
577
578
579
iTRAQ
15000
10000
5000
0
114
116
118

k G S k k A V T k A Q K

histone cluster 1, H2bd [Homo sapiens]
Charge State: +2
Scan Number: 14039
File Name: 110924_HG2_INSA_ACK.raw

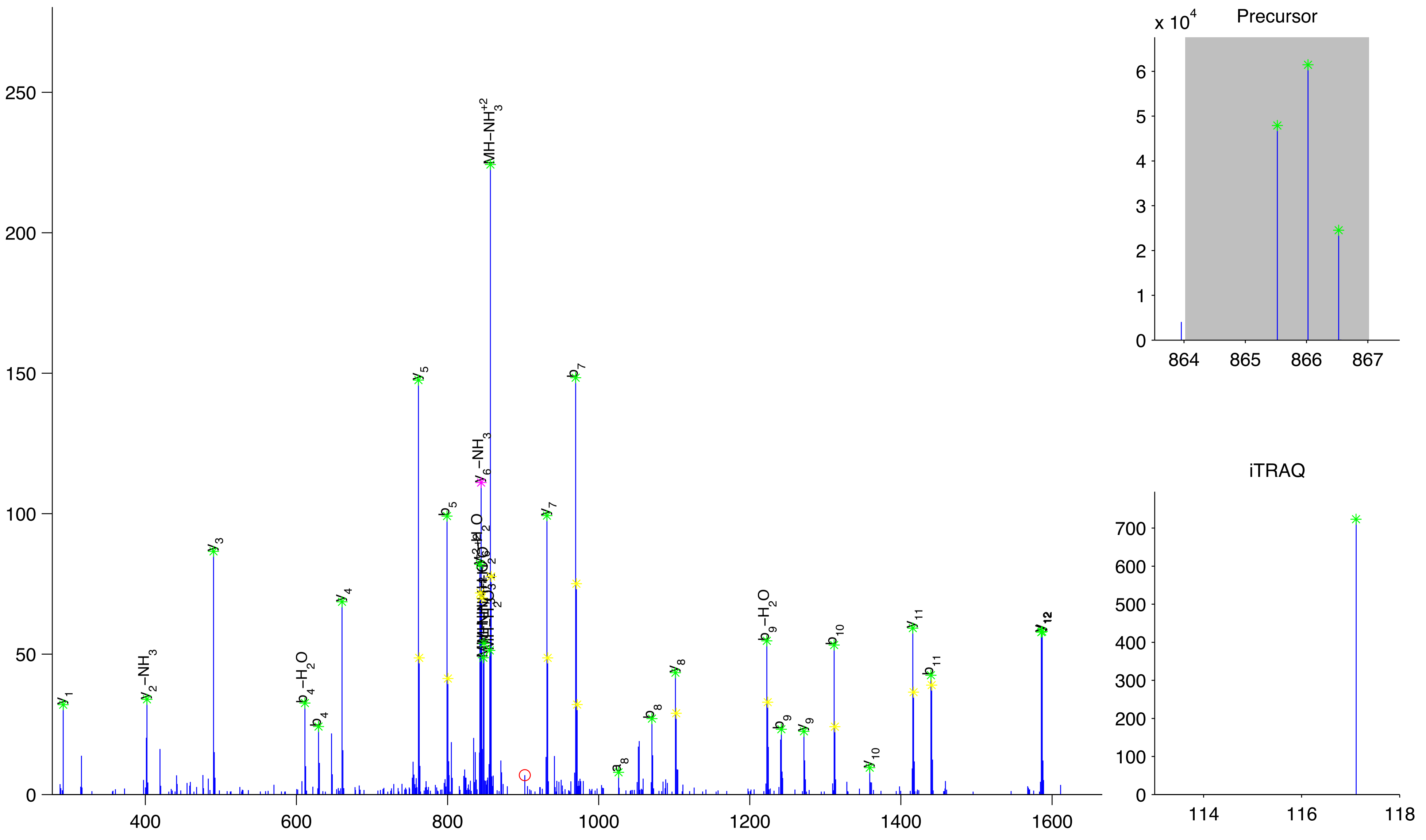

k G S k k A V T k A Q K

histone cluster 1, H2bd [Homo sapiens]
Charge State: +3
Scan Number: 14077
File Name: 110924_HG2_INSA_ACK.raw

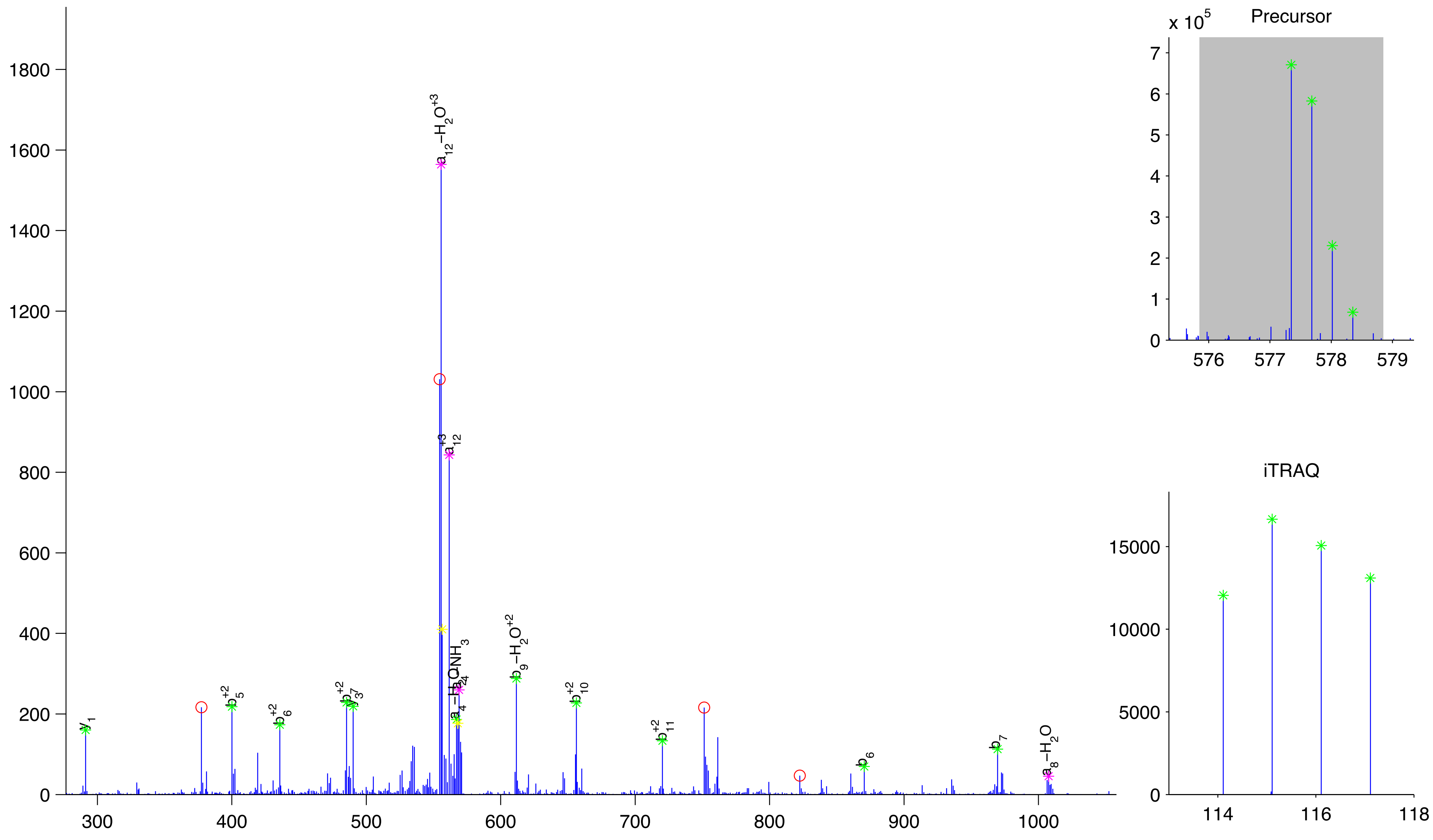

k G S k k A V T k A Q K

histone cluster 1, H2bd [Homo sapiens]
Charge State: +3
Scan Number: 14245
File Name: 110924_HG2_INSA_ACK.raw

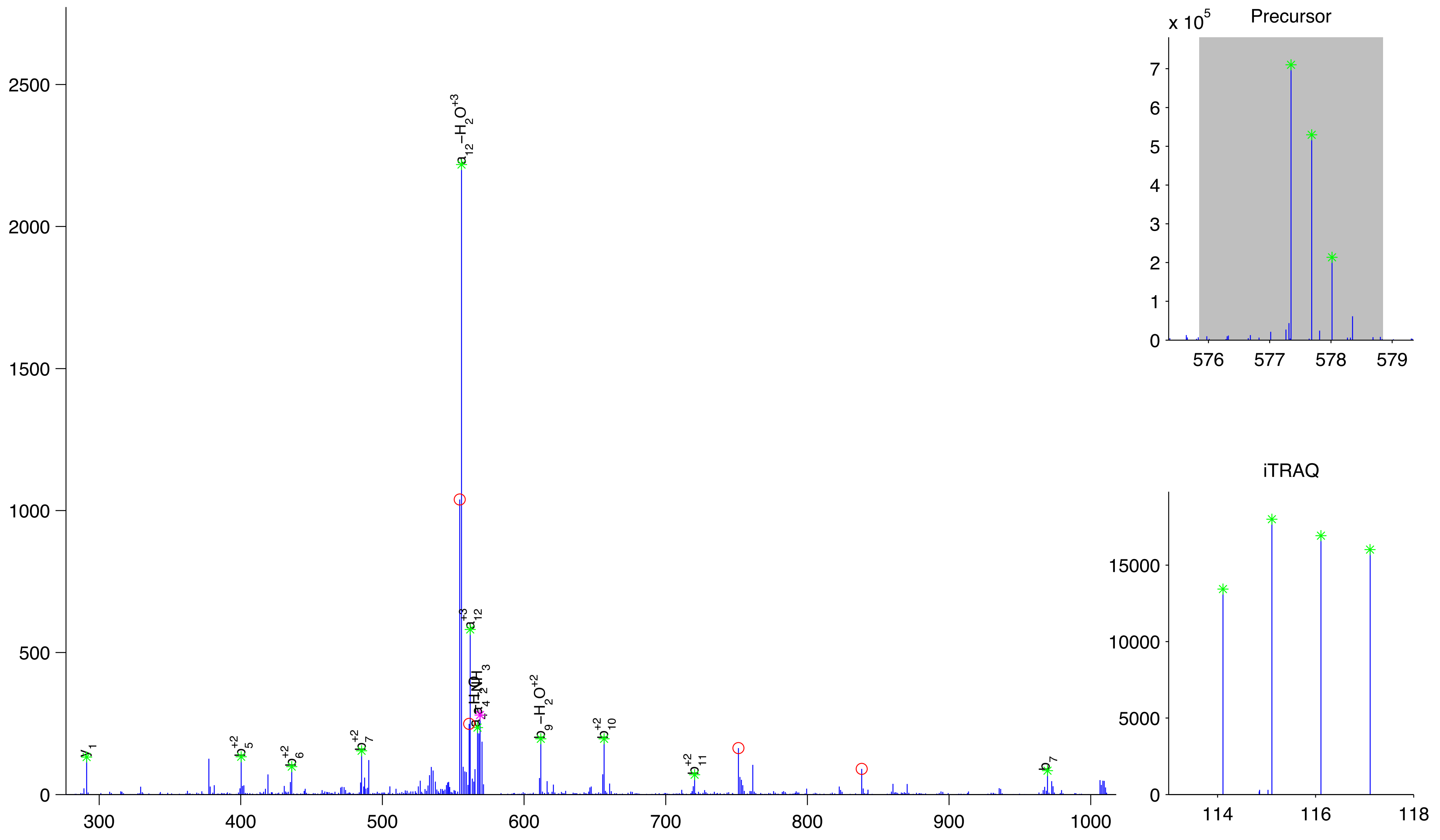

k G S k k A V T k A Q K

histone cluster 1, H2bd [Homo sapiens]
Charge State: +3
Scan Number: 14415
File Name: 110924_HG2_INSA_ACK.raw

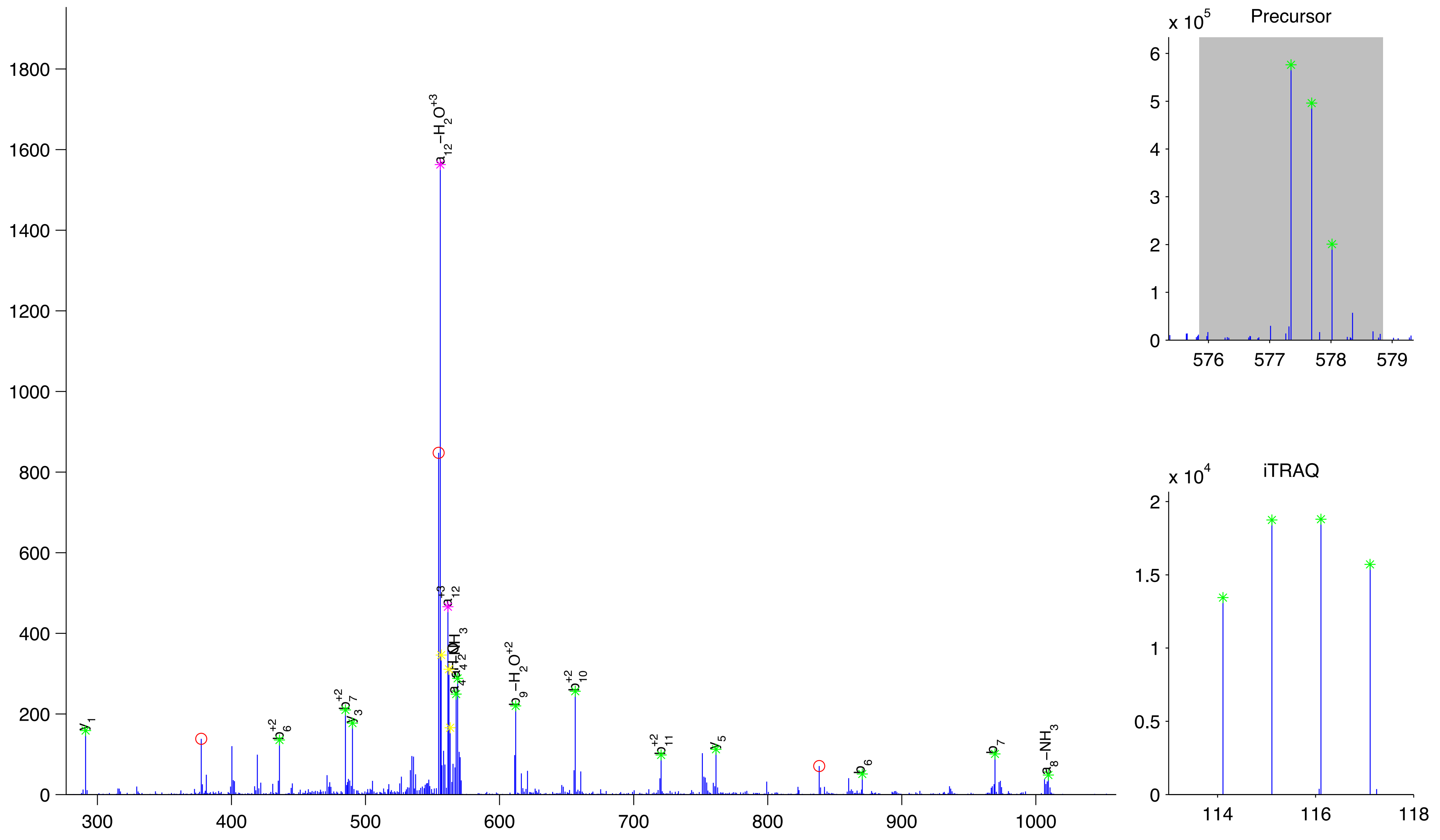

k G S k k A V T k A Q K

histone cluster 1, H2bd [Homo sapiens]
Charge State: +2
Scan Number: 15114
File Name: 110924_HG2_INSA_ACK.raw

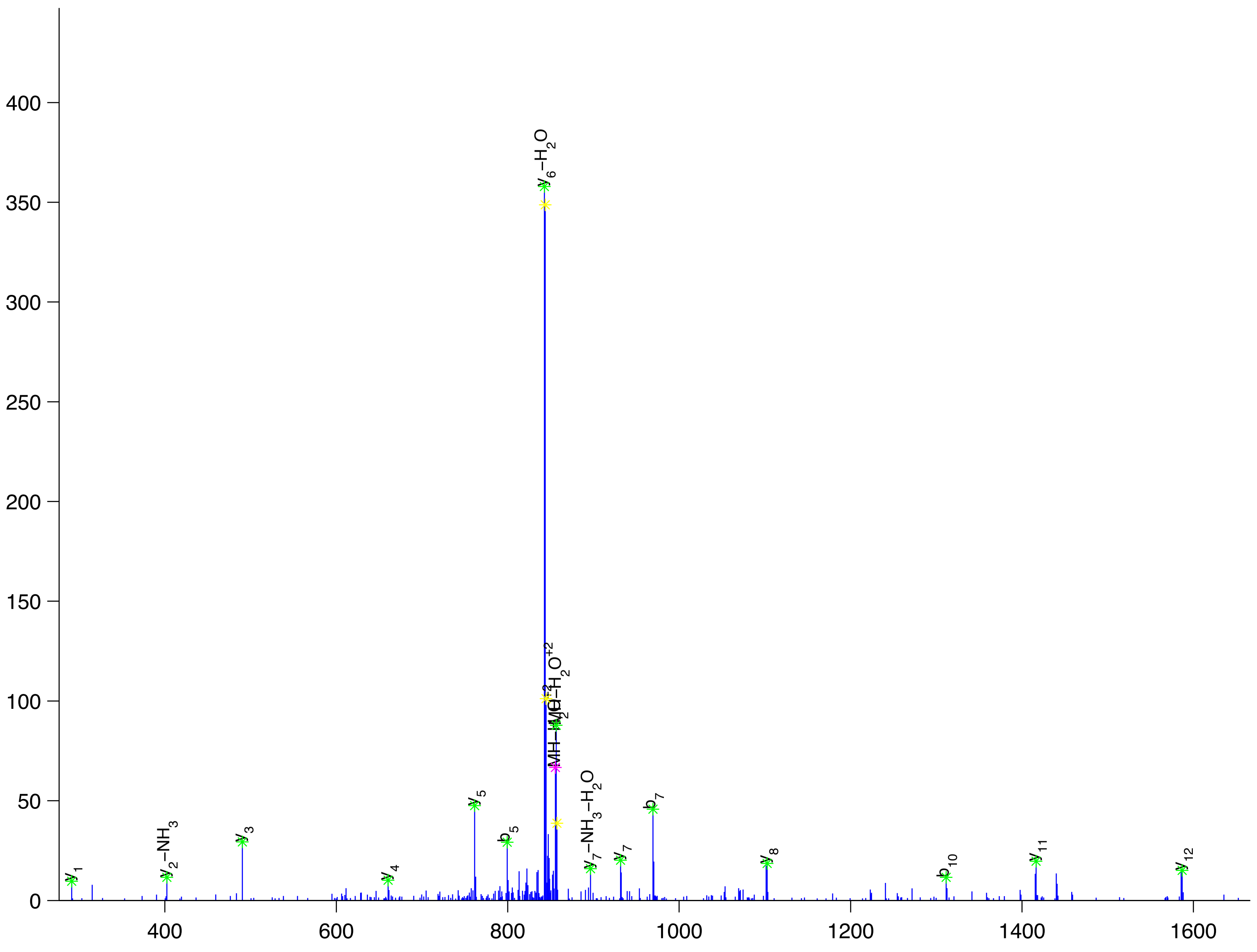

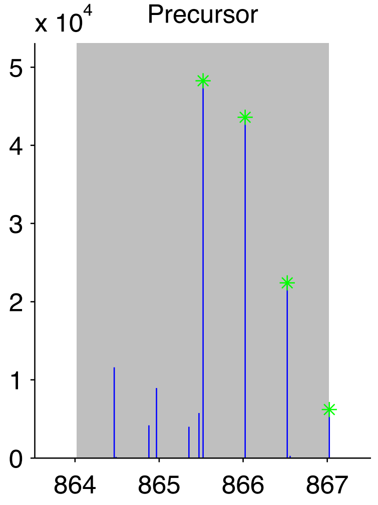

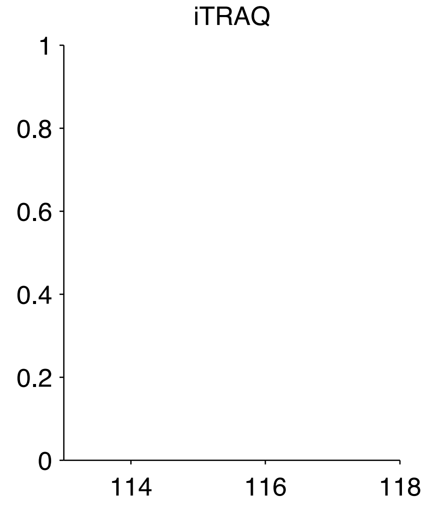

k G S k k A V T k A Q K

histone cluster 1, H2bd [Homo sapiens]
Charge State: +3
Scan Number: 19020
File Name: 110924_HG2_INSA_ACK.raw

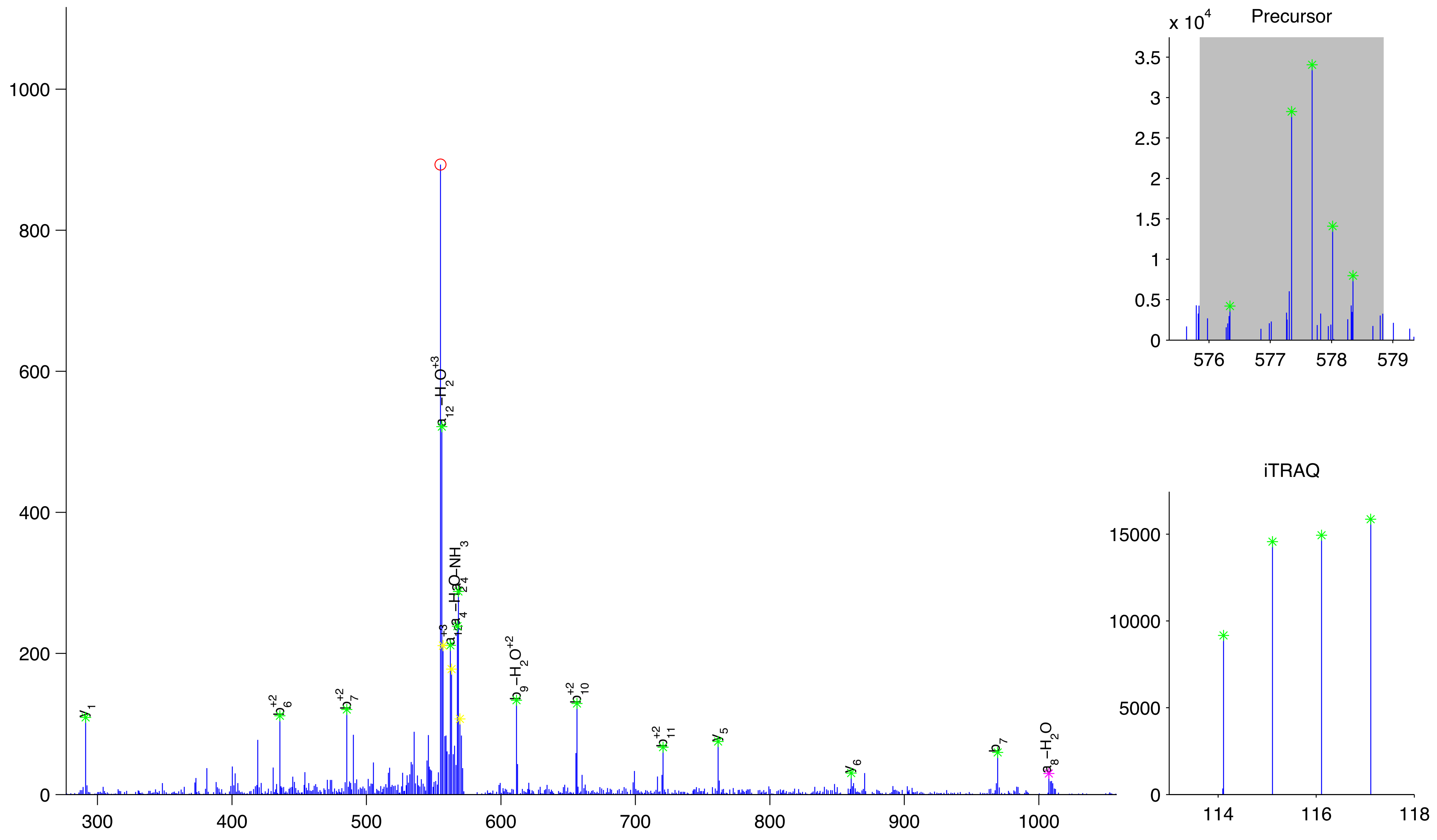

k A V T k A Q K

histone cluster 1, H2bh [Homo sapiens]
Charge State: +2
Scan Number: 4707
File Name: 111011_HG2_INSB_ACK.raw

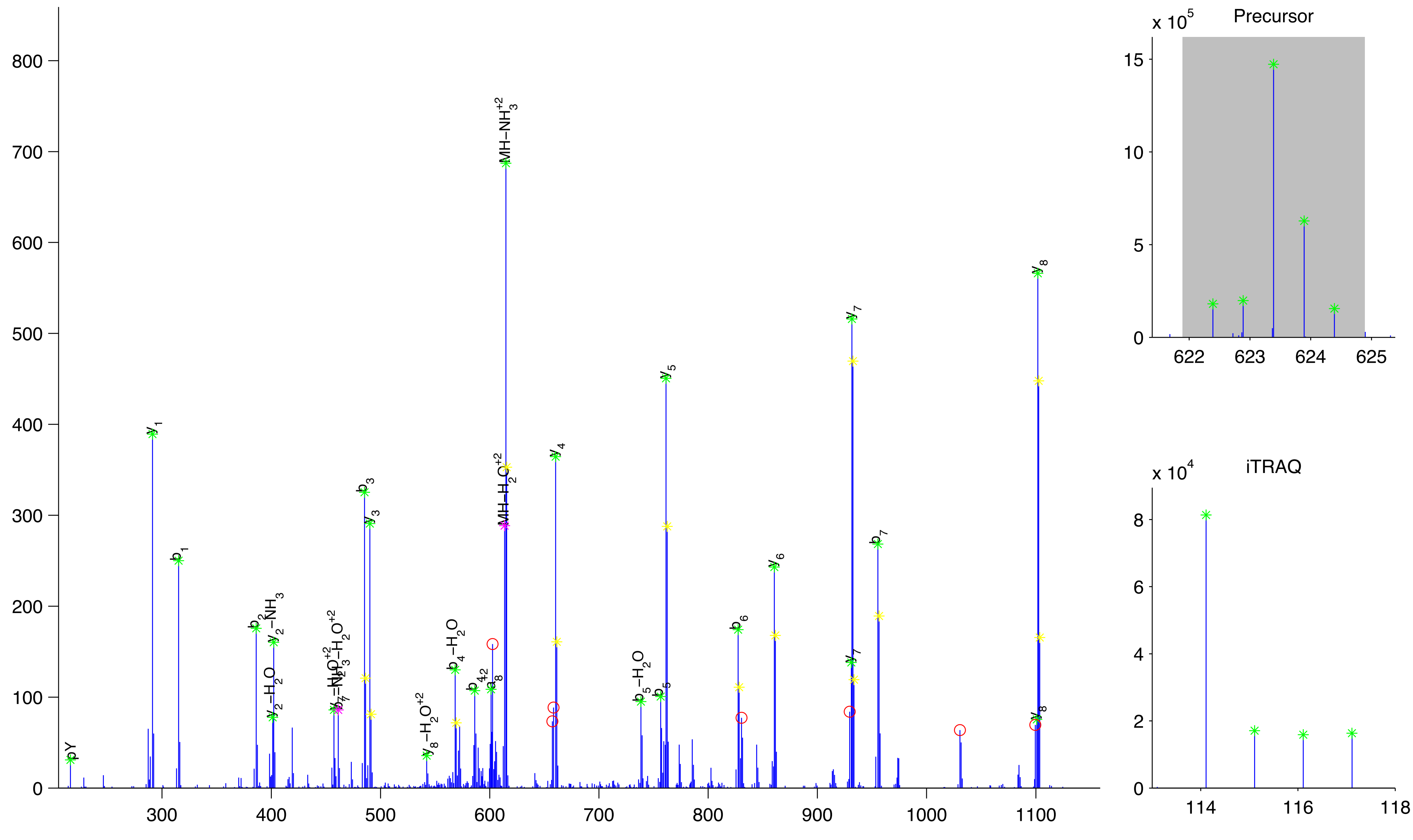

k G S k k A V T K

histone cluster 1, H2bh [Homo sapiens]
Charge State: +2
Scan Number: 4814
File Name: 111011_HG2_INSB_ACK.raw

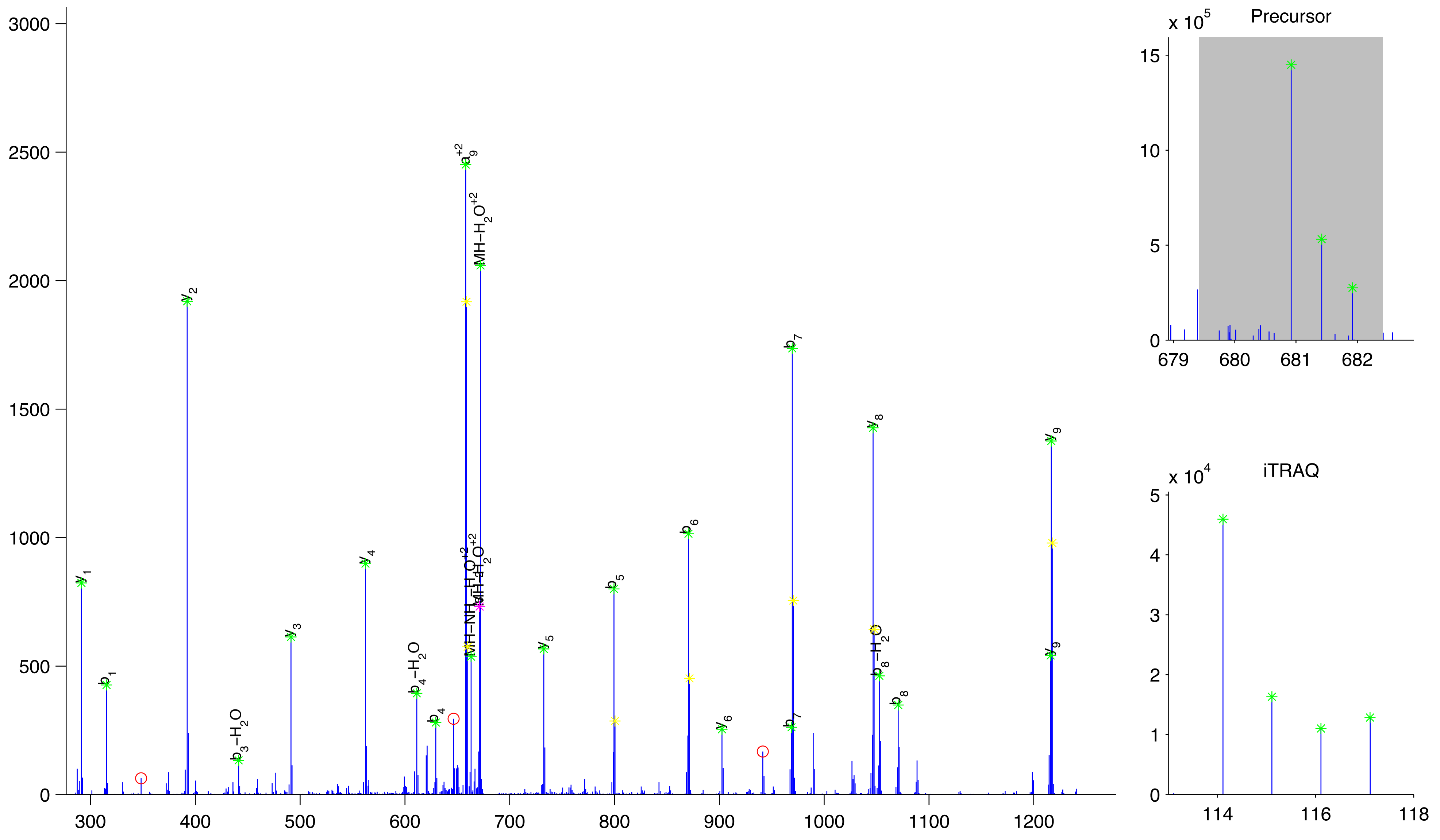

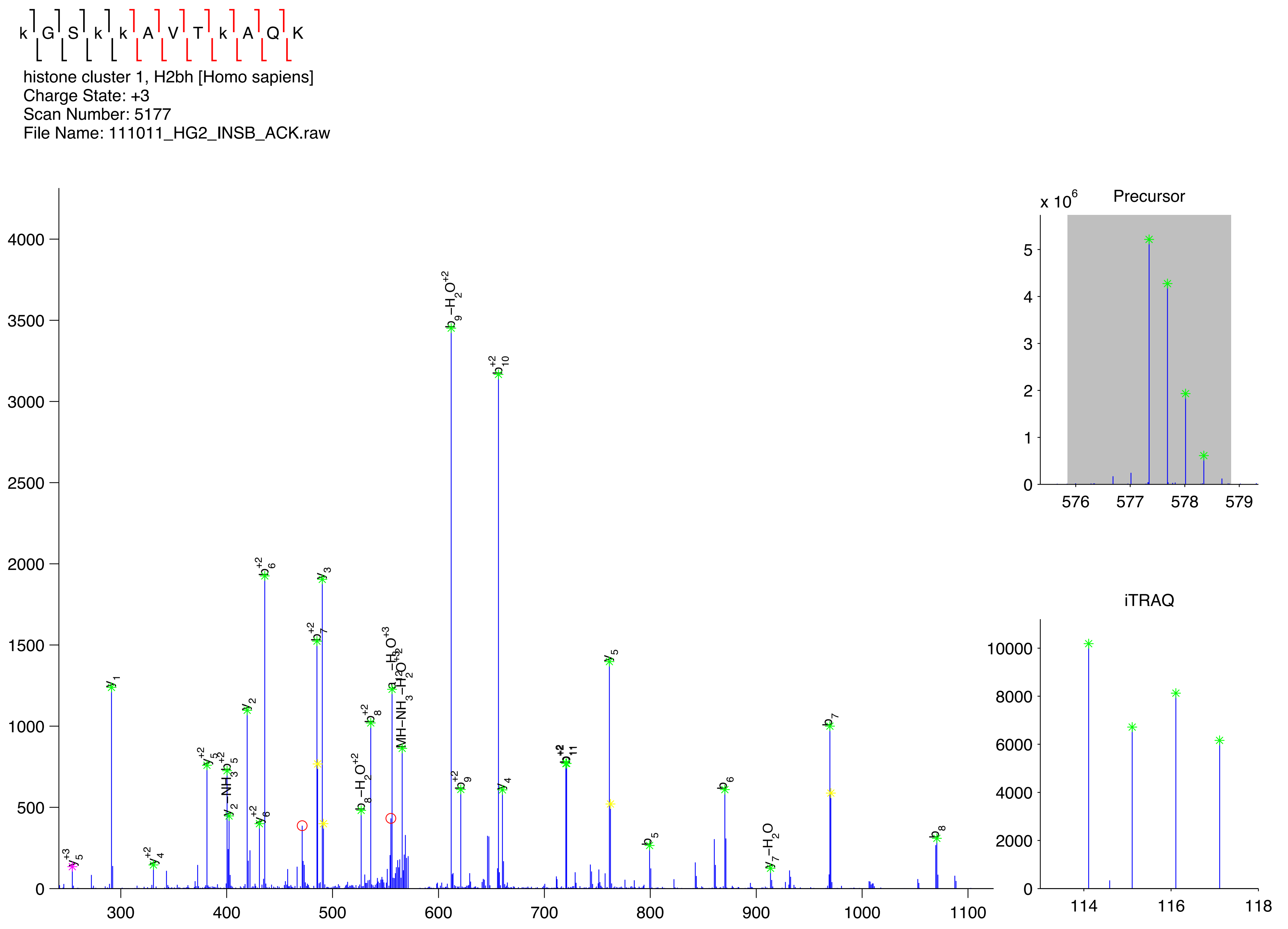
k G S k k A V T k A Q K
histone cluster 1, H2bh [Homo sapiens]
Charge State: +3
Scan Number: 5177
File Name: 111011_HG2_INSB_ACK.raw
Precursor
x 10^6
iTRAQ

k G S k k A V T k A Q K

histone cluster 1, H2bh [Homo sapiens]
Charge State: +3
Scan Number: 5343
File Name: 111011_HG2_INSB_ACK.raw

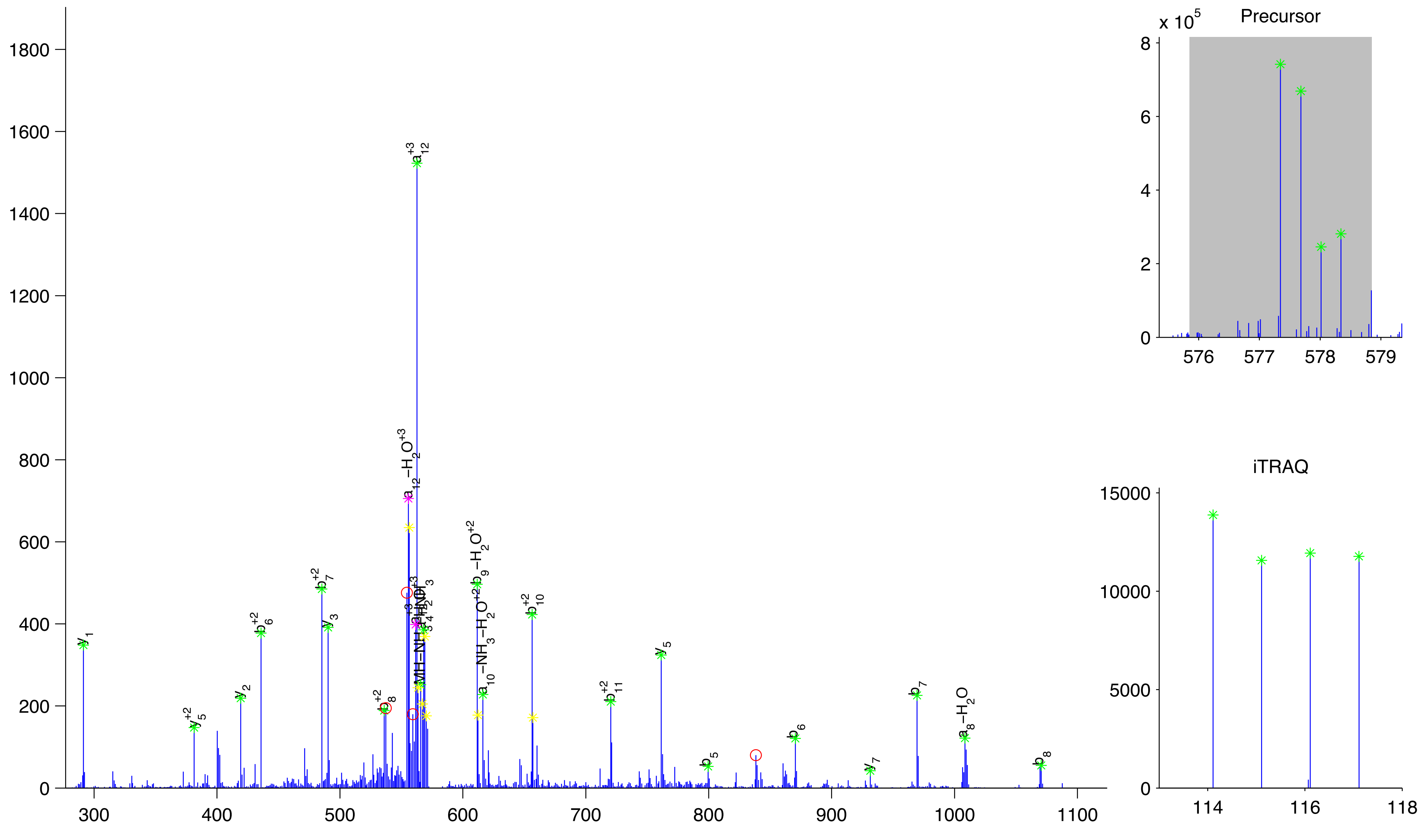

k A V T k A Q K

histone cluster 1, H2bn [Homo sapiens]
Charge State: +2
Scan Number: 3493
File Name: 111016_HG2_INSC_ACK.raw

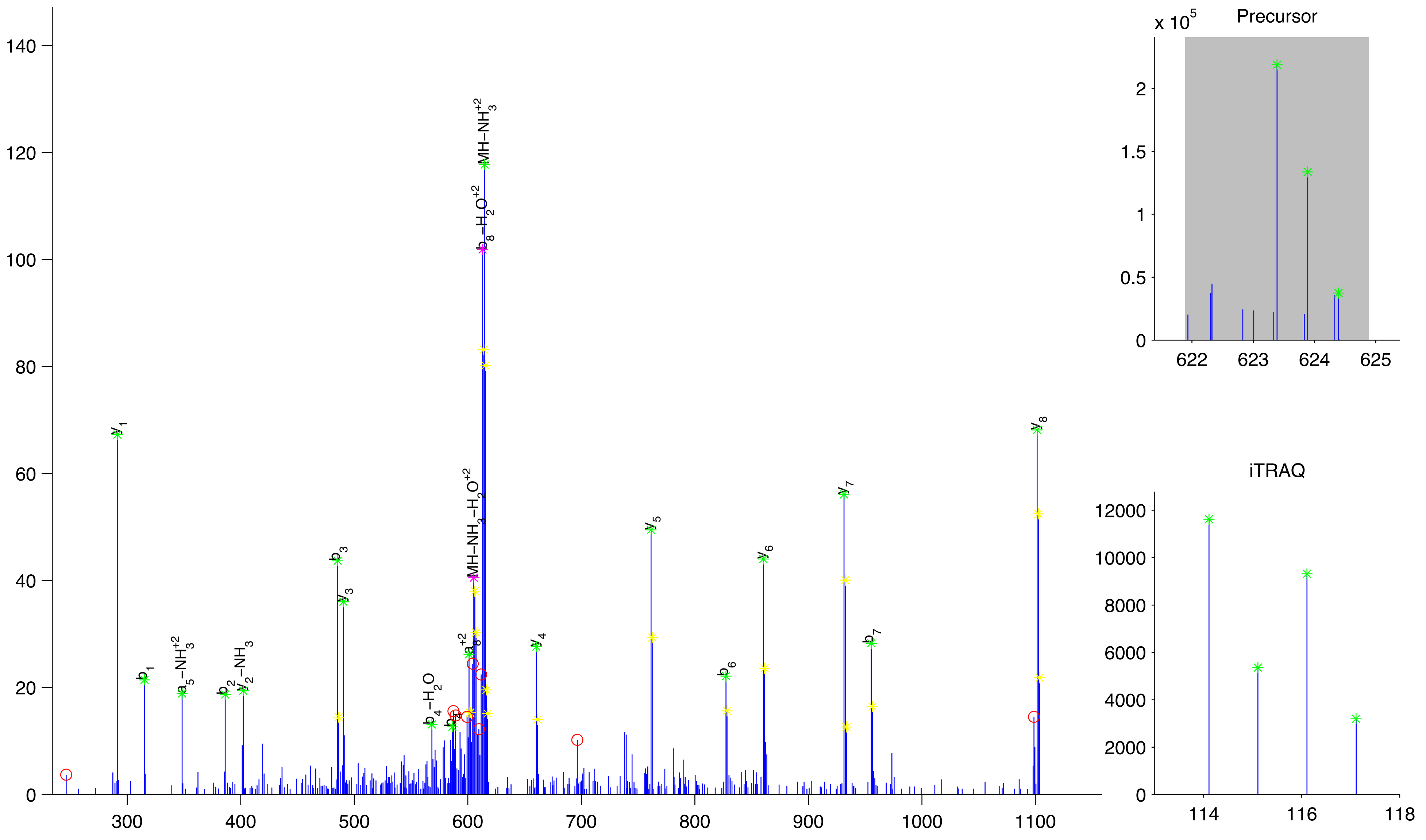

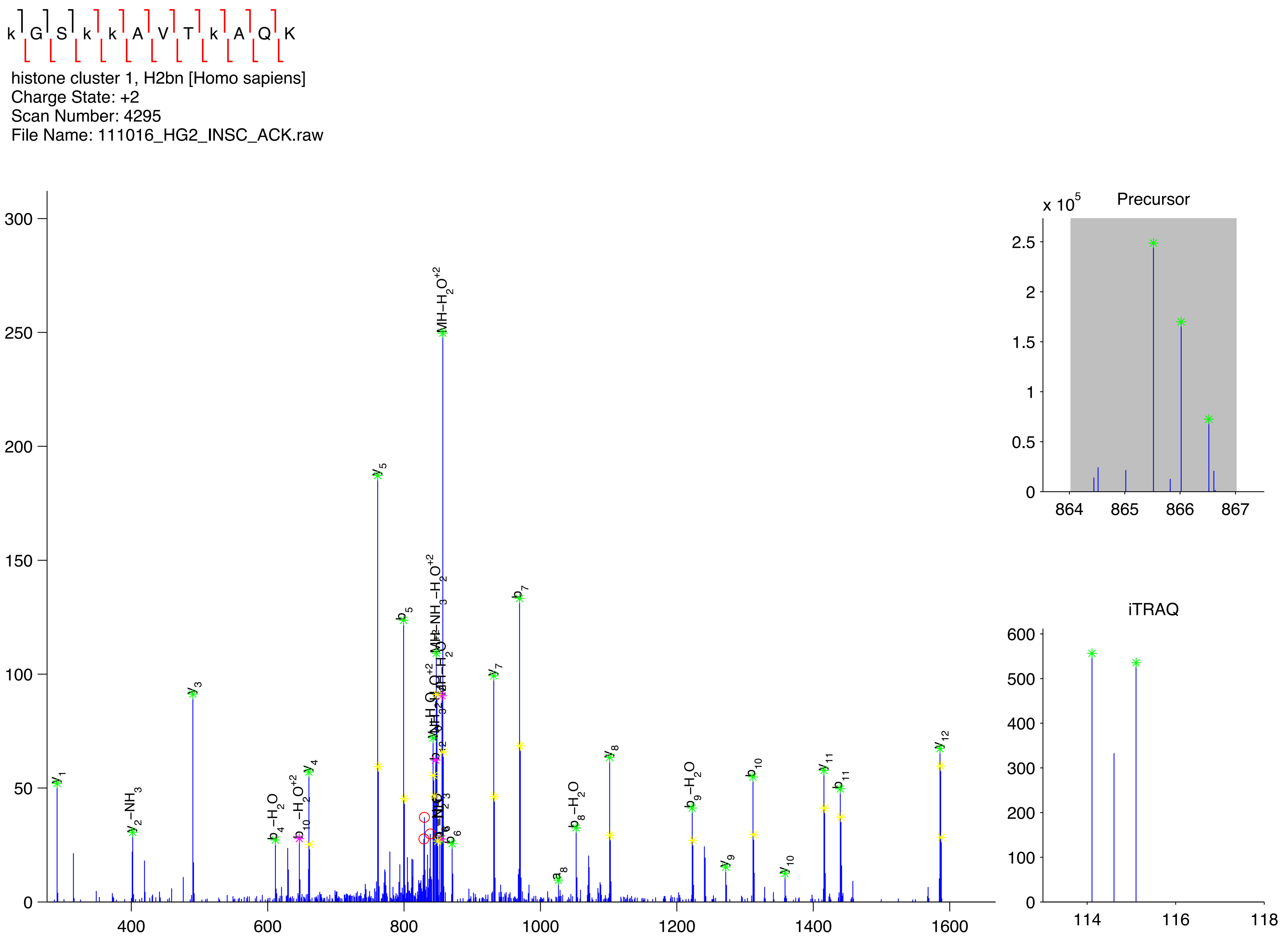
k G S k k A V T k A Q K
histone cluster 1, H2bn [Homo sapiens]
Charge State: +2
Scan Number: 4295
File Name: 111016_HG2_INSC_ACK.raw
y1
y2−NH3
y3
b4−H2O
b10−H2O+2
y4
y5
b5
MH−H2O+2
MH−NH3−H2O+2
y7
b7
a8
b8−H2O
y8
b9−H2O
y9
b10
y10
y11
b11
y12
Precursor
x 10^5
iTRAQ

k G S k k A V T k A Q K

histone cluster 1, H2bn [Homo sapiens]
Charge State: +3
Scan Number: 4308
File Name: 111016_HG2_INSC_ACK.raw

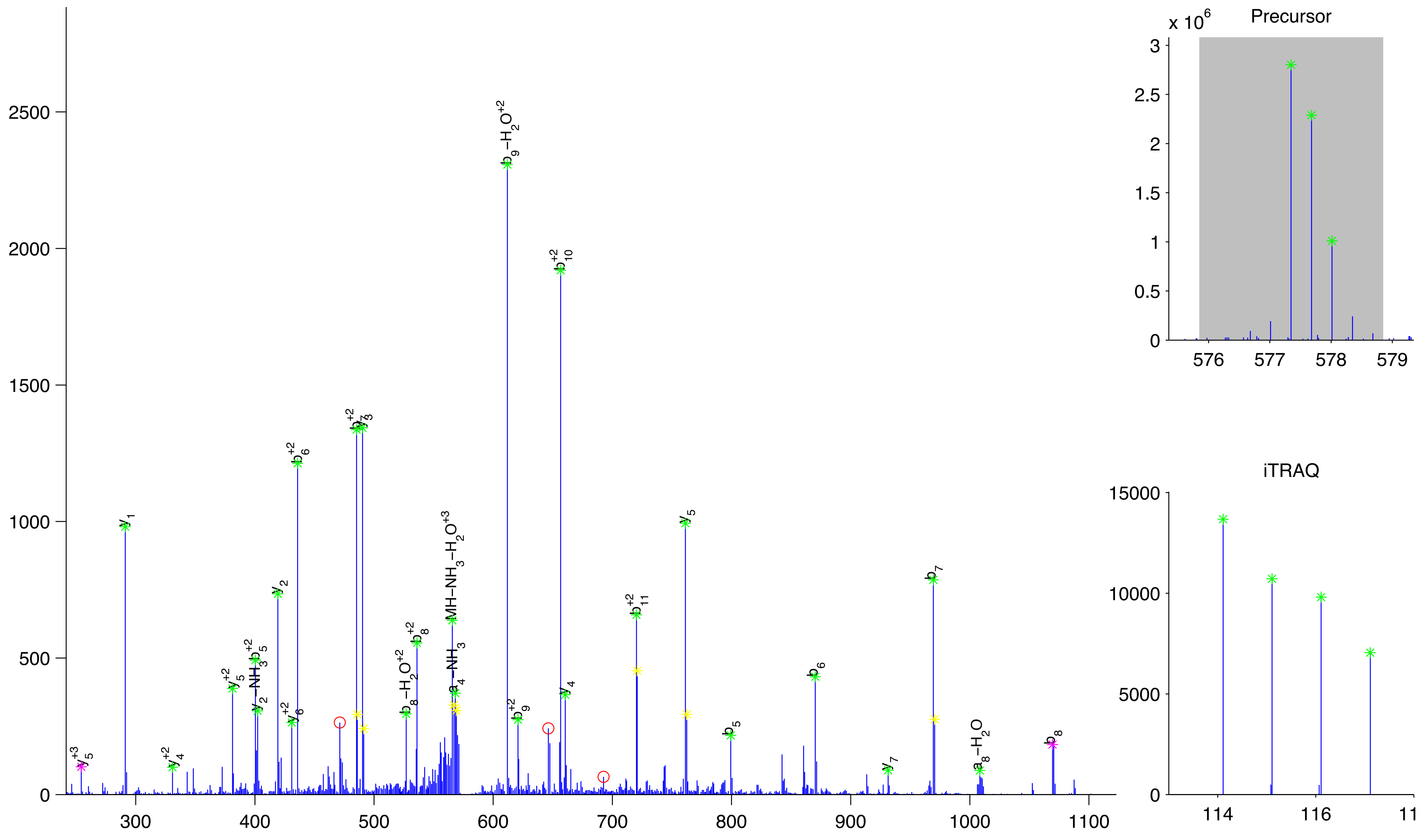

E S Y S V Y V Y K

histone cluster 1, H2bn [Homo sapiens]
Charge State: +2
Scan Number: 6639
File Name: 111016_HG2_INSC_ACK.raw

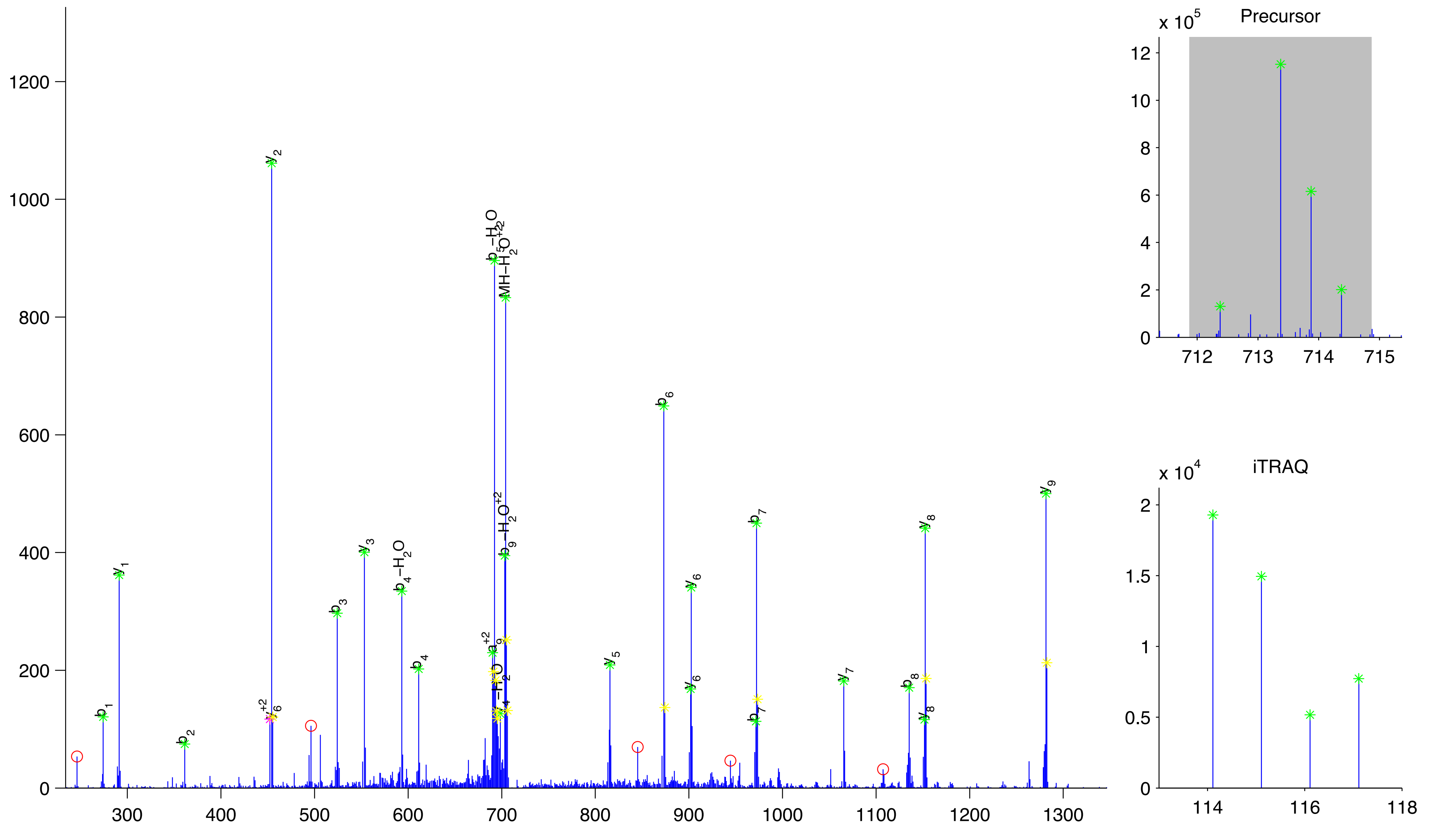

L L L P G E L A K

histone cluster 1, H2bn [Homo sapiens]
Charge State: +2
Scan Number: 10631
File Name: 111016_HG2_INSC_ACK.raw

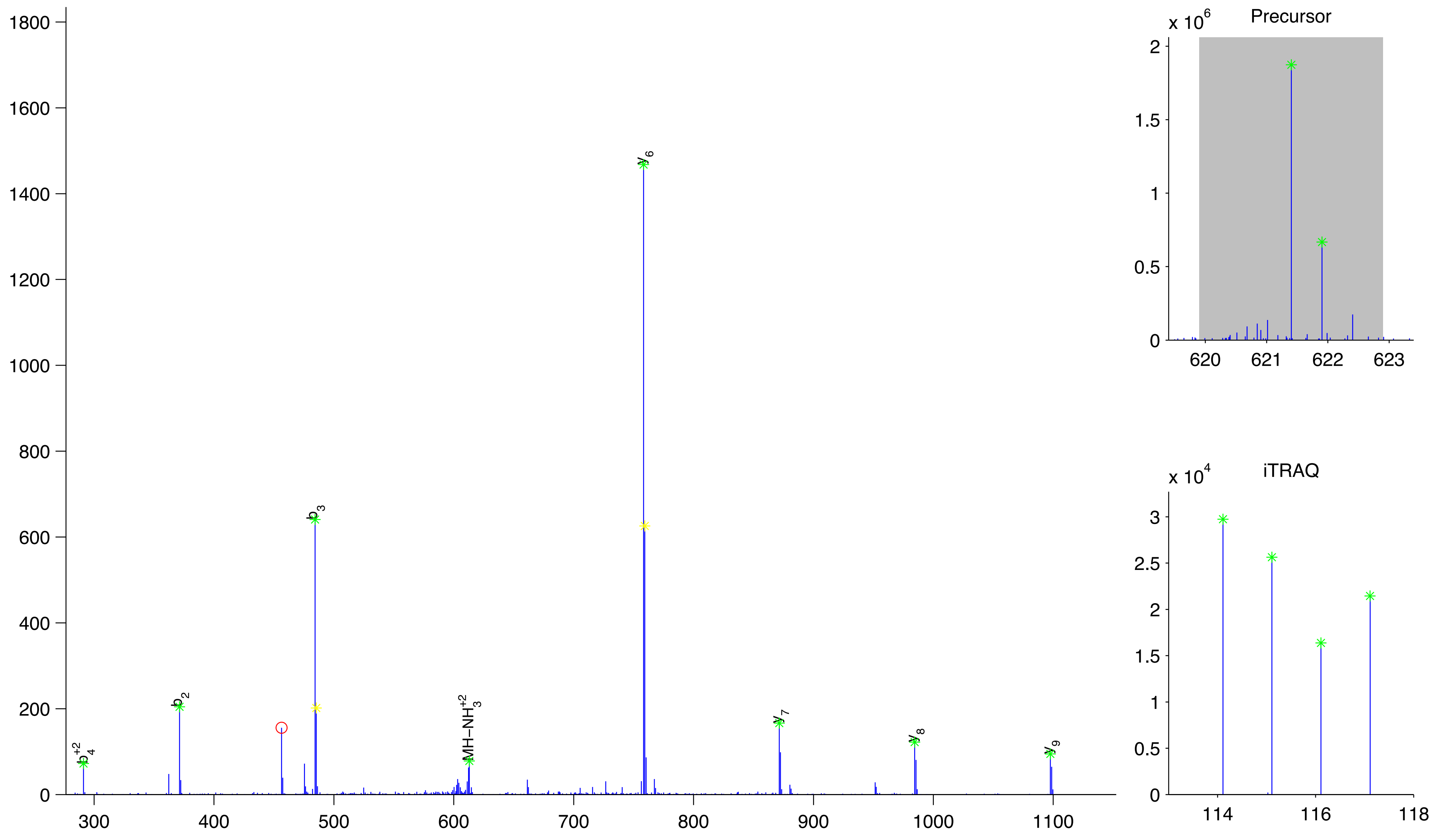

k A V T k A Q K

histone cluster 1, H2bo [Homo sapiens]
Charge State: +2
Scan Number: 3493
File Name: 111016_HG2_INSC_ACK.raw

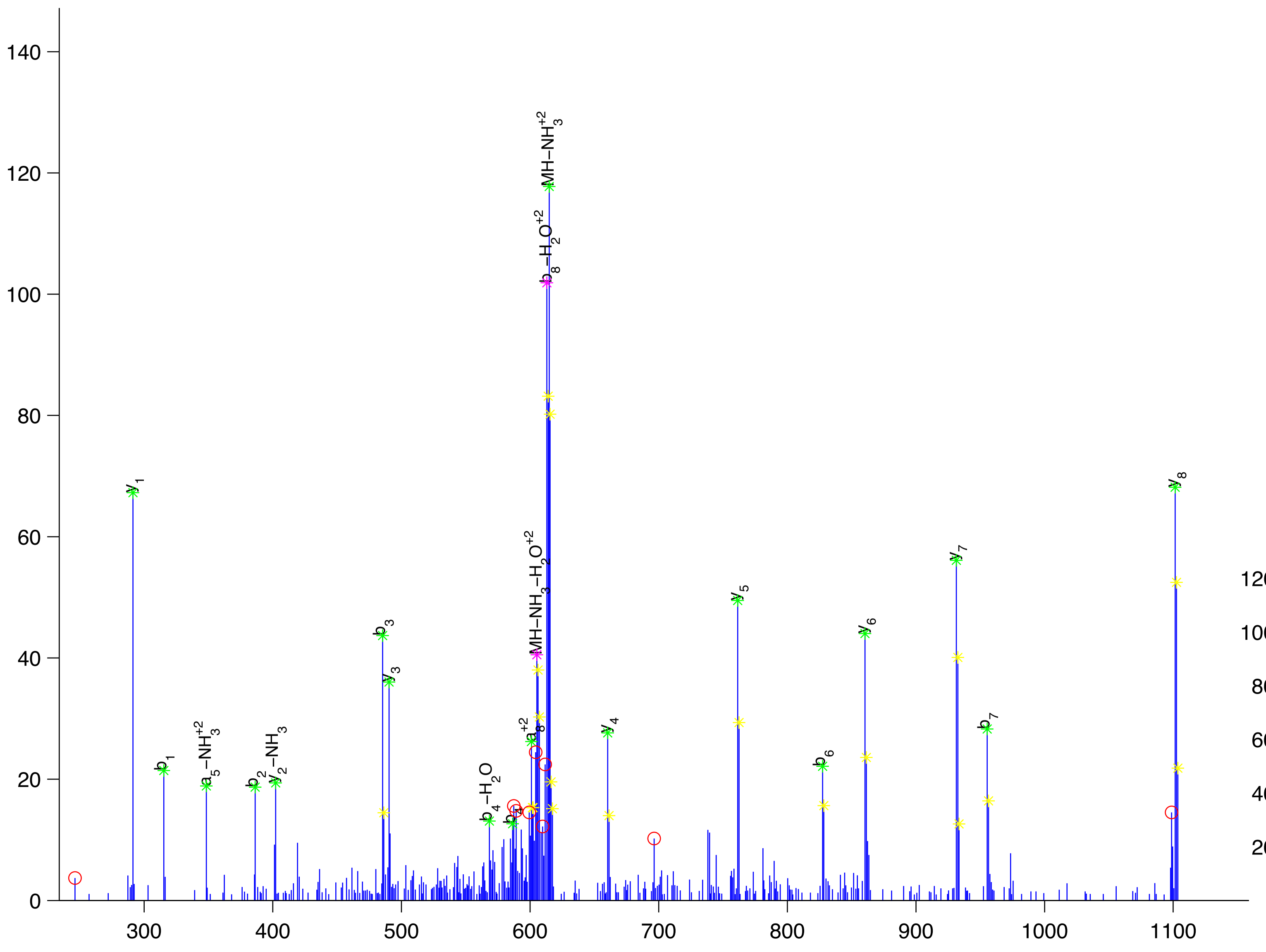

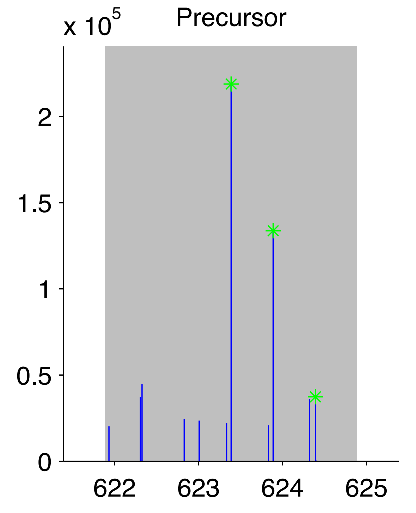

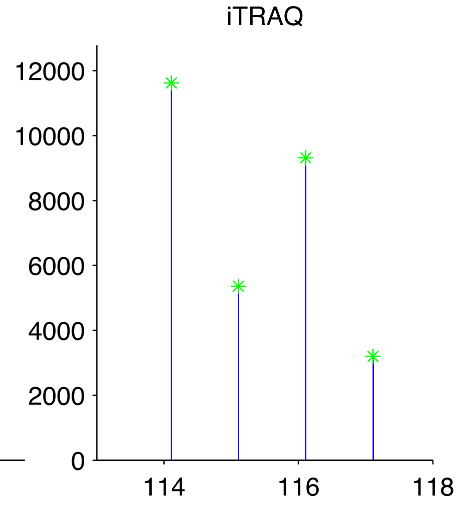

k G S k k A V T k A Q K

histone cluster 1, H2bo [Homo sapiens]
Charge State: +3
Scan Number: 4308
File Name: 111016_HG2_INSC_ACK.raw

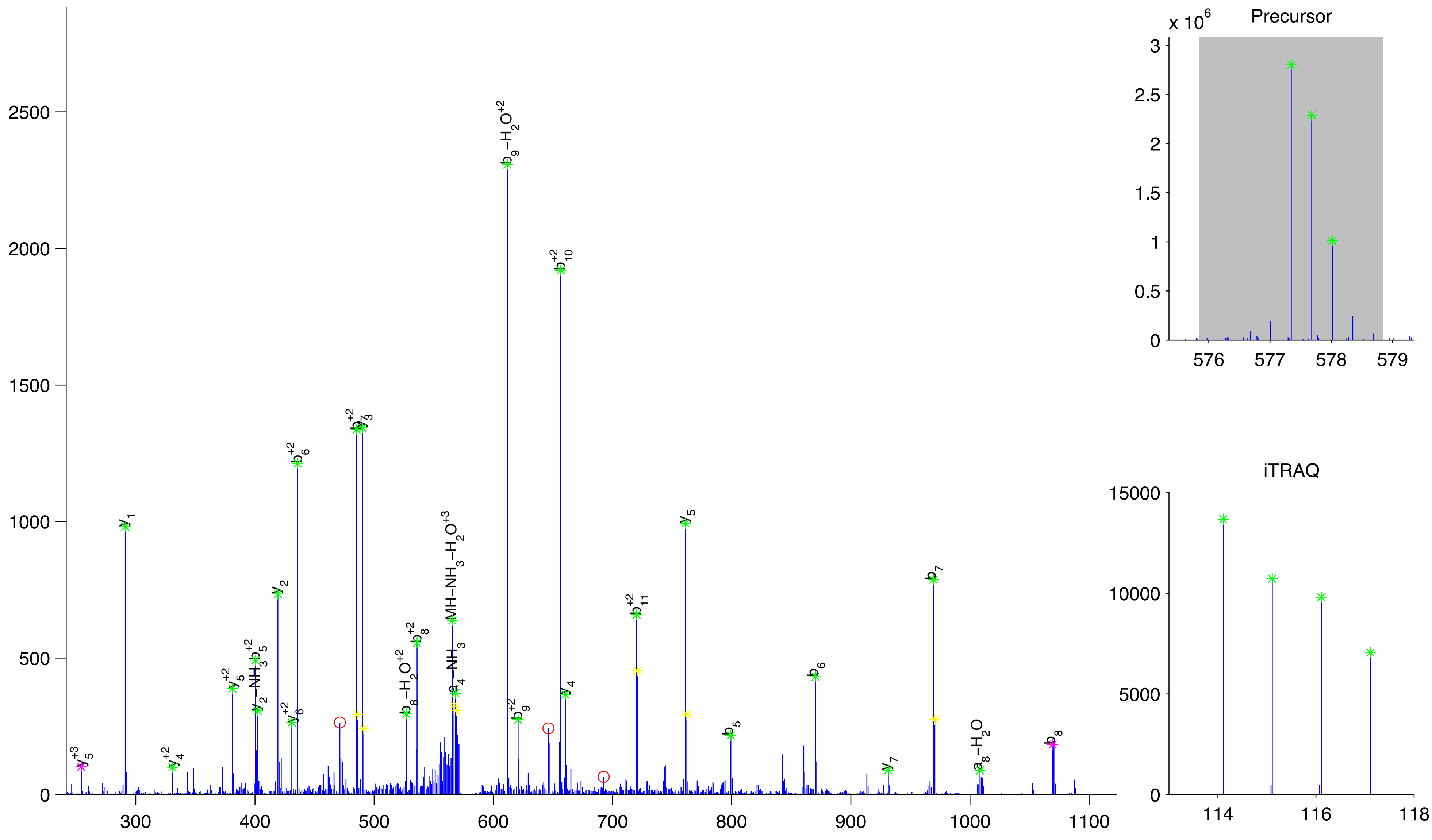

k A V T k A Q K

histone cluster 1, H2bo [Homo sapiens]
Charge State: +2
Scan Number: 4707
File Name: 111011_HG2_INSB_ACK.raw

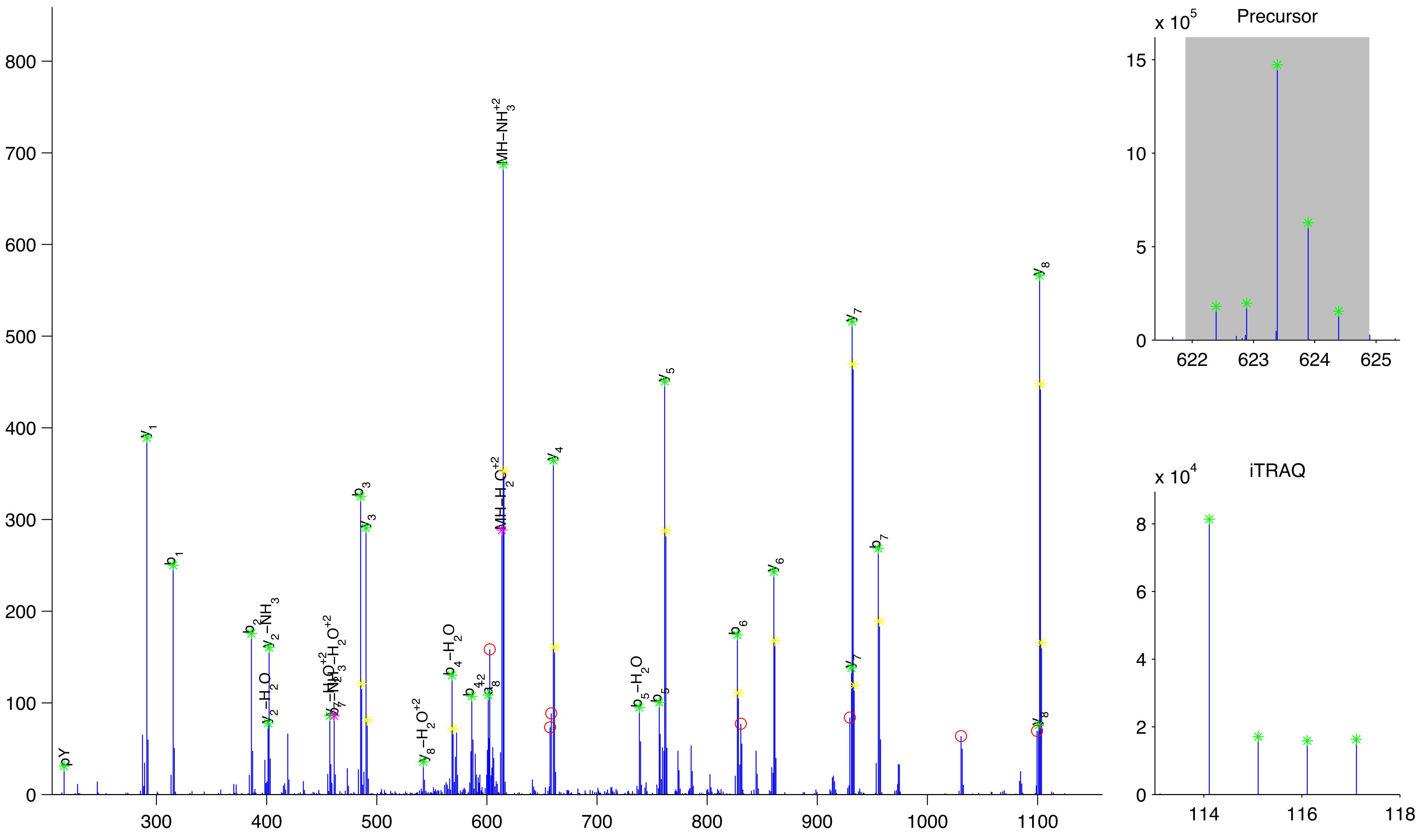

k G S k k A V T K

histone cluster 1, H2bo [Homo sapiens]
Charge State: +2
Scan Number: 4814
File Name: 111011_HG2_INSB_ACK.raw

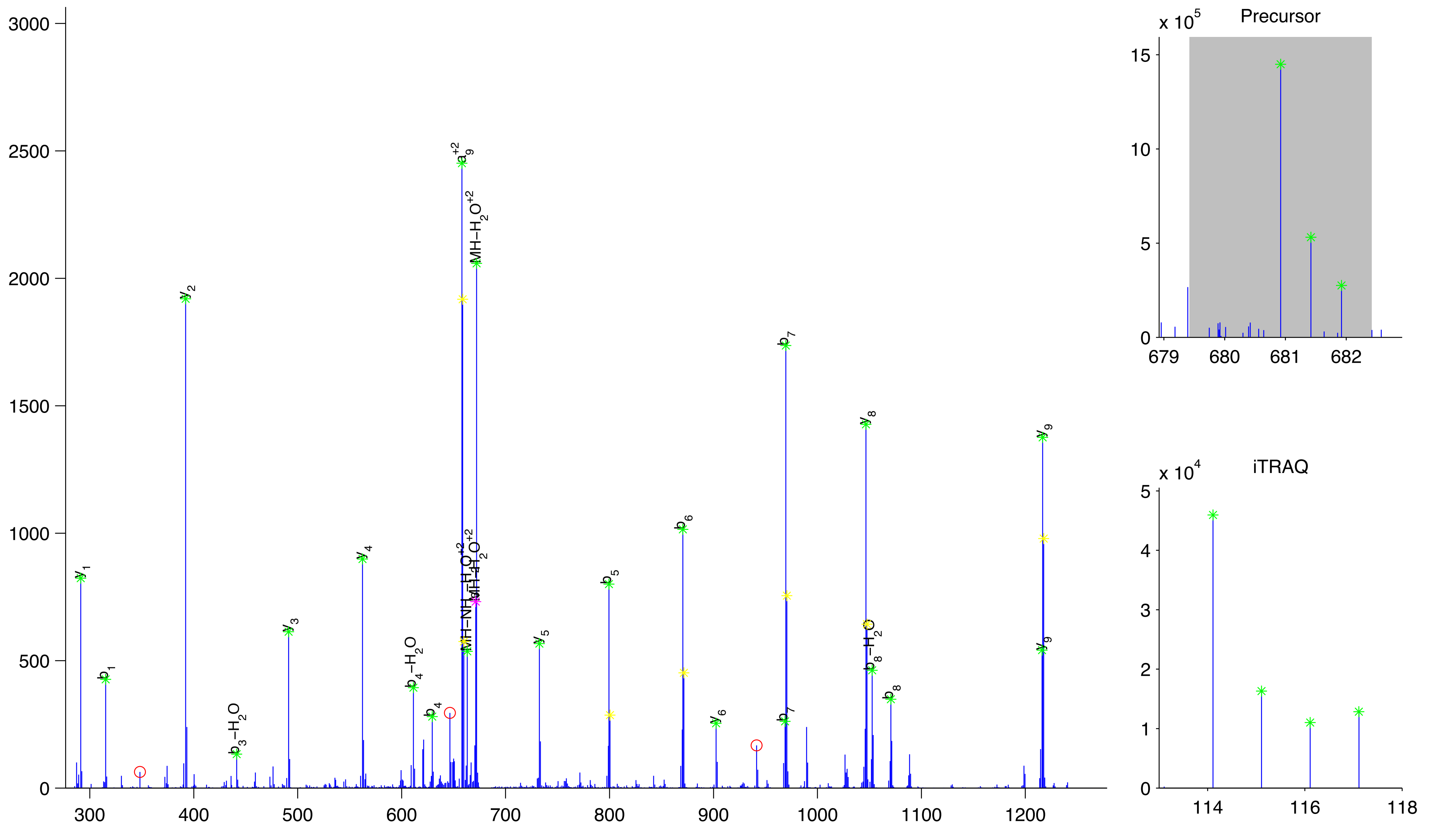

k G S k k A V T k A Q K

histone cluster 1, H2bo [Homo sapiens]
Charge State: +3
Scan Number: 5177
File Name: 111011_HG2_INSB_ACK.raw

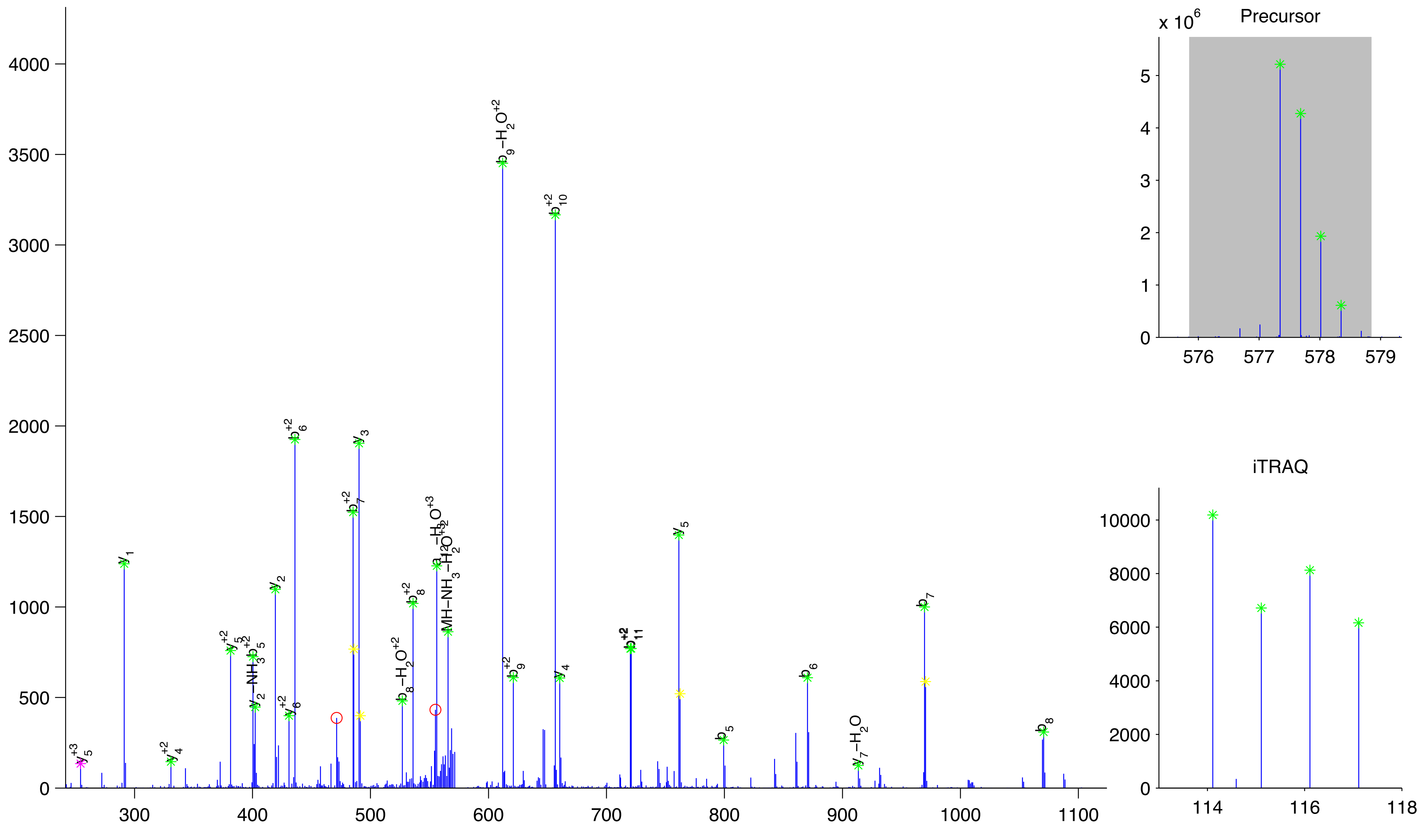

k G S k k A V T k A Q K

histone cluster 1, H2bo [Homo sapiens]
Charge State: +3
Scan Number: 5343
File Name: 111011_HG2_INSB_ACK.raw

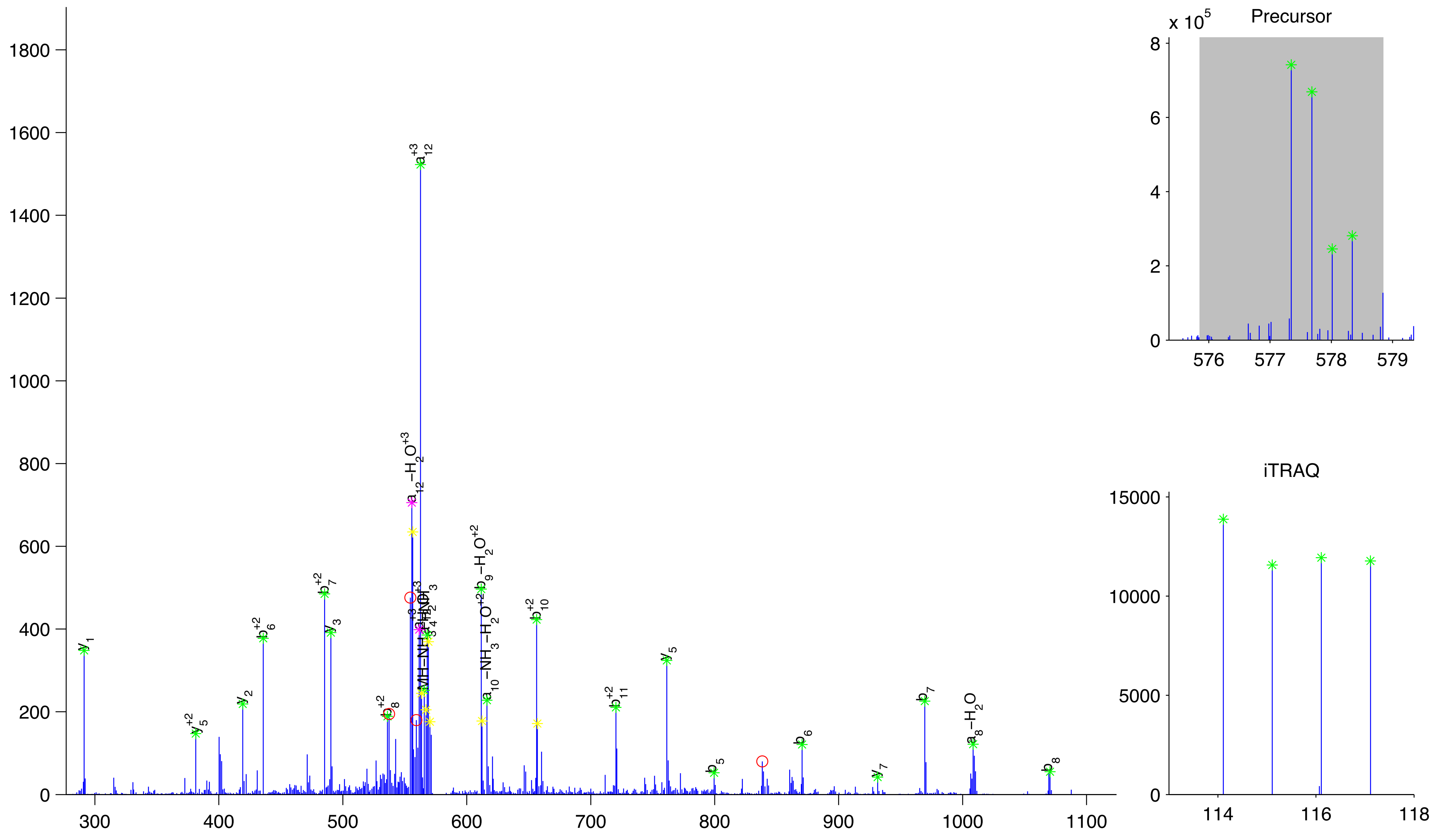

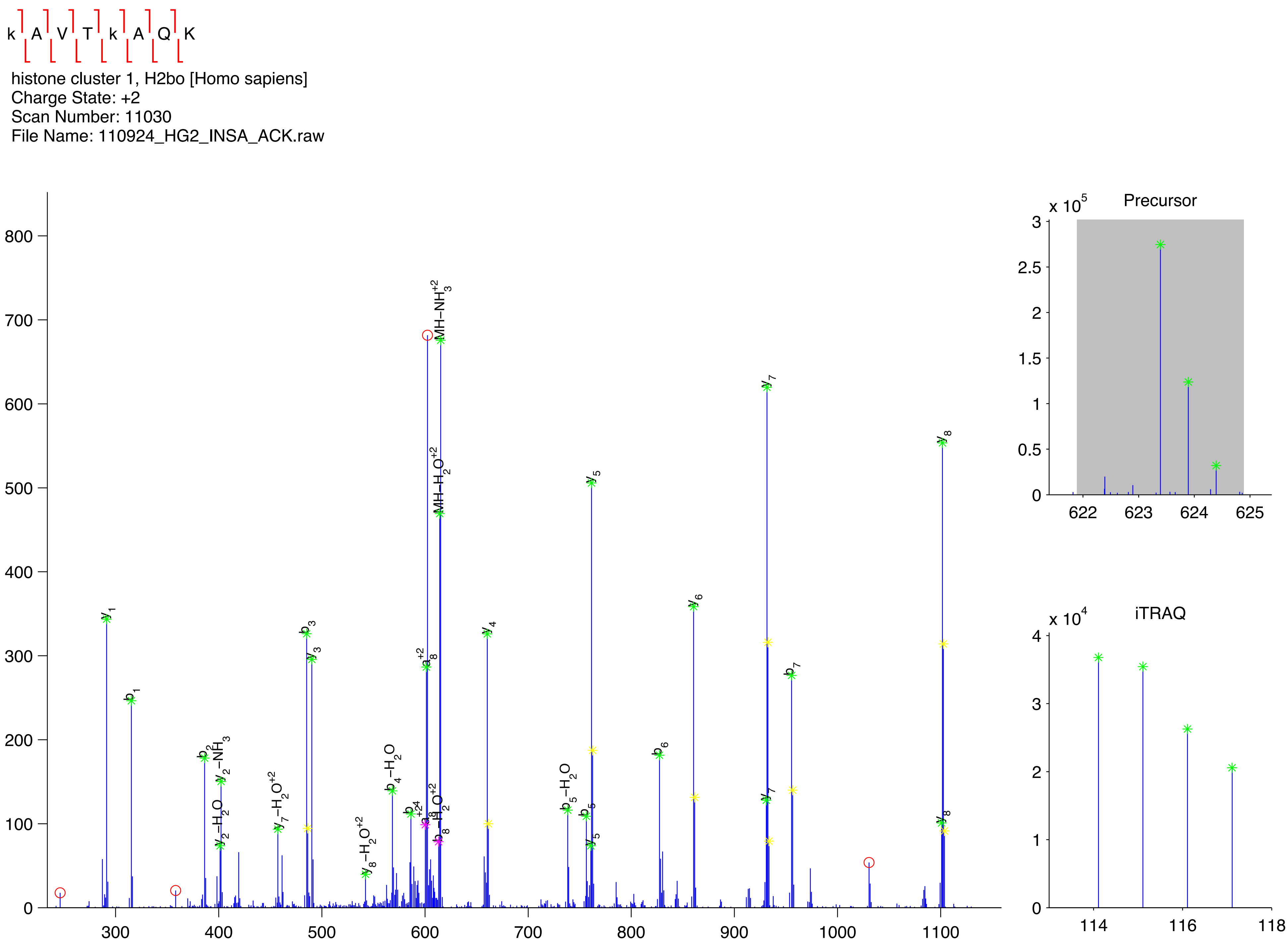
k A V T k A Q K
histone cluster 1, H2bo [Homo sapiens]
Charge State: +2
Scan Number: 11030
File Name: 110924_HG2_INSA_ACK.raw
Precursor
x 10^5
iTRAQ
x 10^4
MH−NH3+2
MH−H2O+2
y1
b1
b2
y2−NH3
y2−H2O
y7−H2O+2
b3
y3
y8−H2O+2
b4−H2O
b+24
a8+2
b8−H2O+2
y4
b5−H2O
b5
y5
b6
y6
y7
b7
y8

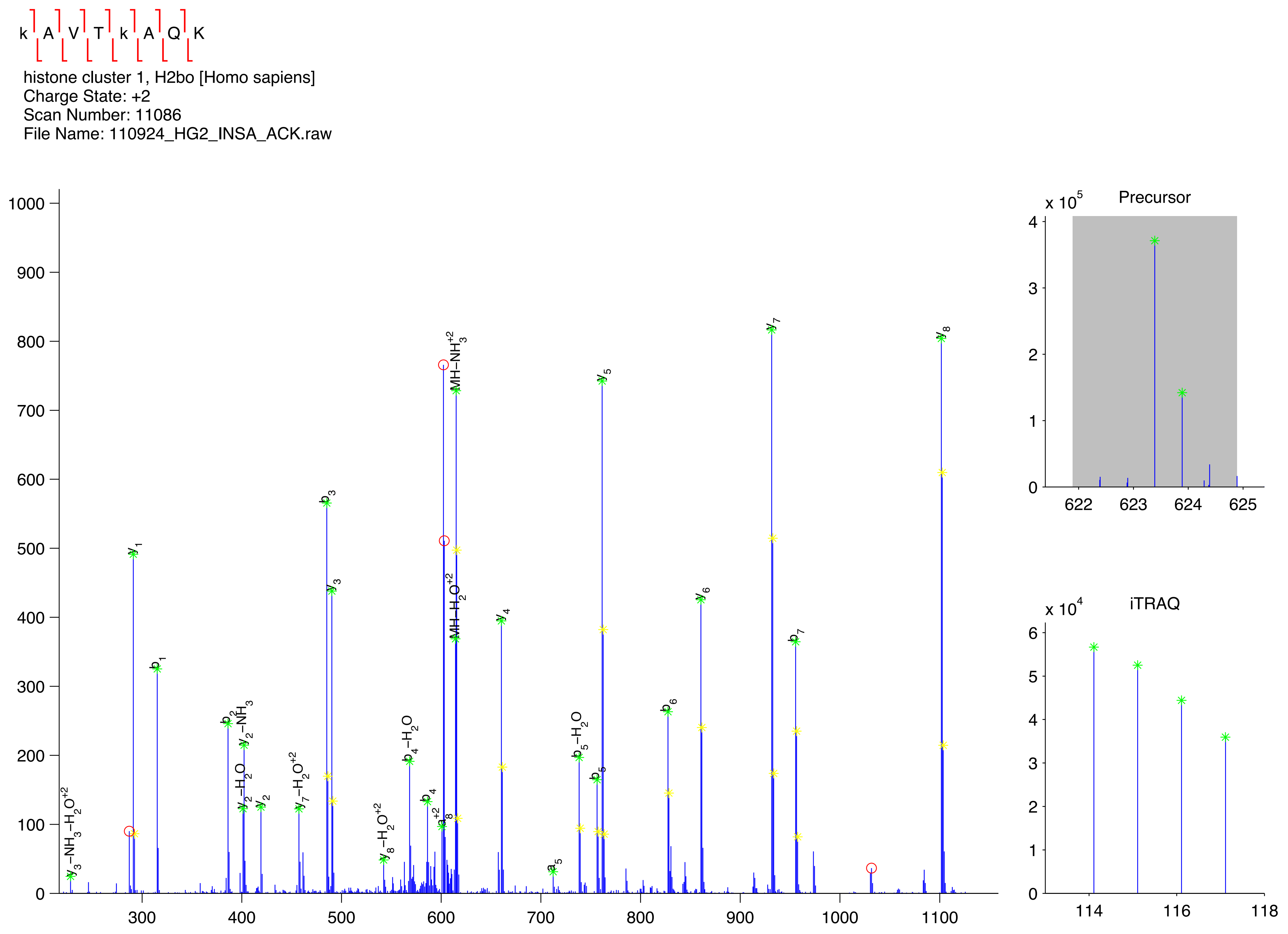

k A V T k A Q K
histone cluster 1, H2bo [Homo sapiens]
Charge State: +2
Scan Number: 11086
File Name: 110924_HG2_INSA_ACK.raw
Precursor
x 10^5
iTRAQ
x 10^4

k A V T k A Q K

histone cluster 1, H2bo [Homo sapiens]
Charge State: +2
Scan Number: 11198
File Name: 110924_HG2_INSA_ACK.raw

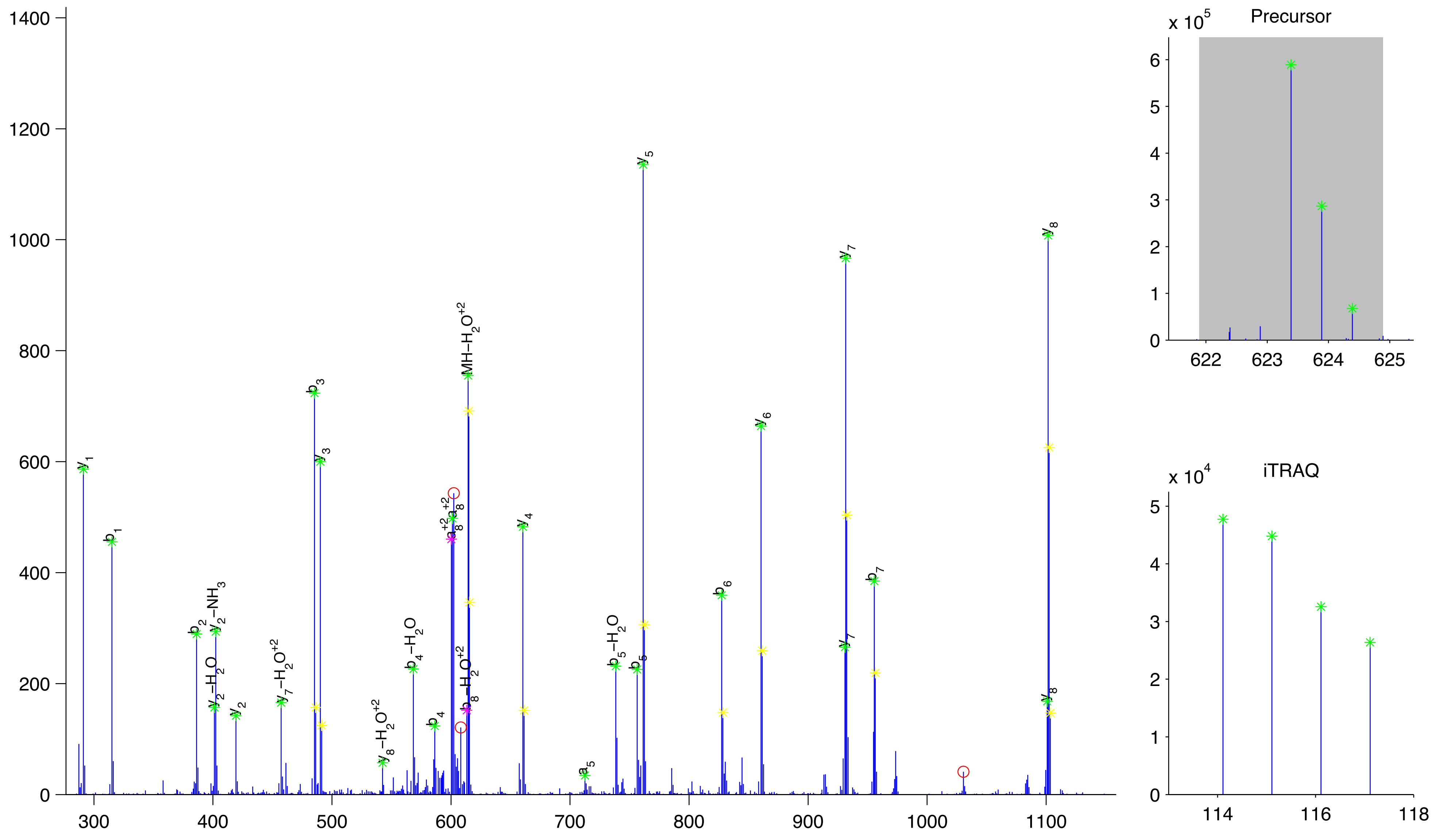

k A V T k A Q K

histone cluster 1, H2bo [Homo sapiens]
Charge State: +2
Scan Number: 11277
File Name: 110924_HG2_INSA_ACK.raw

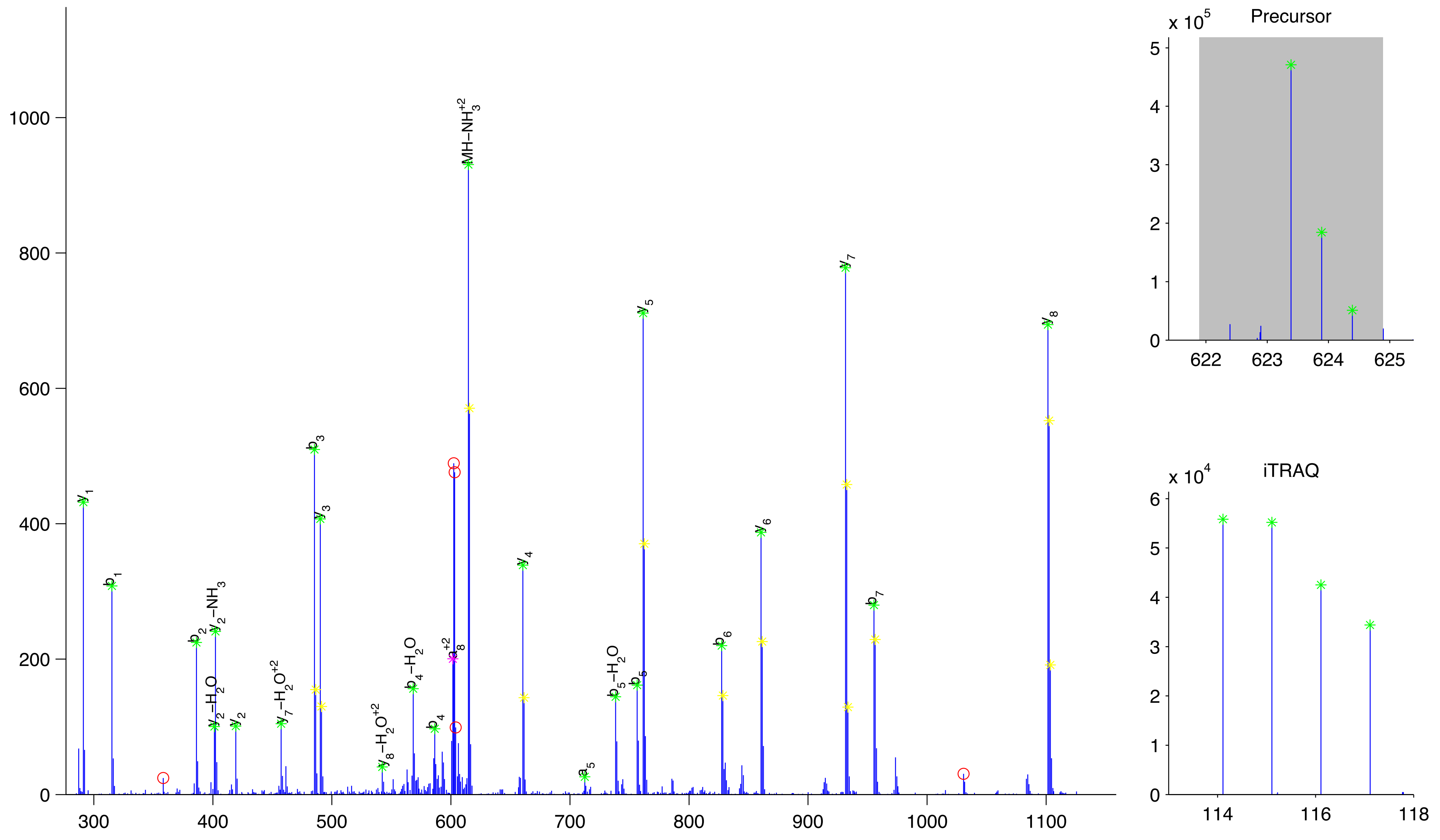

k G S k k A V T k A Q K

histone cluster 1, H2bo [Homo sapiens]
Charge State: +2
Scan Number: 13193
File Name: 110924_HG2_INSA_ACK.raw

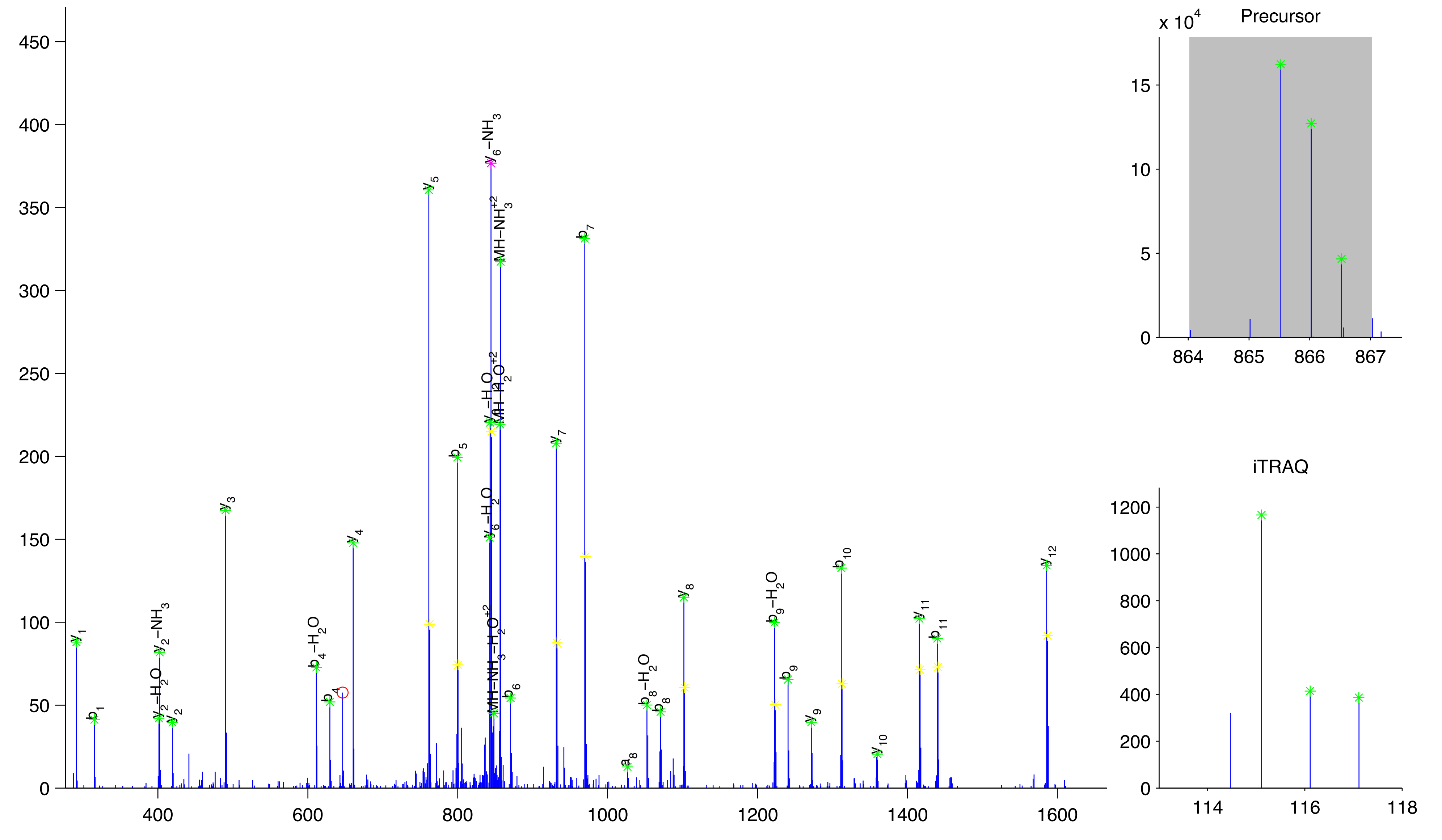

k G S k k A V T k A Q K

histone cluster 1, H2bo [Homo sapiens]
Charge State: +3
Scan Number: 13237
File Name: 110924_HG2_INSA_ACK.raw

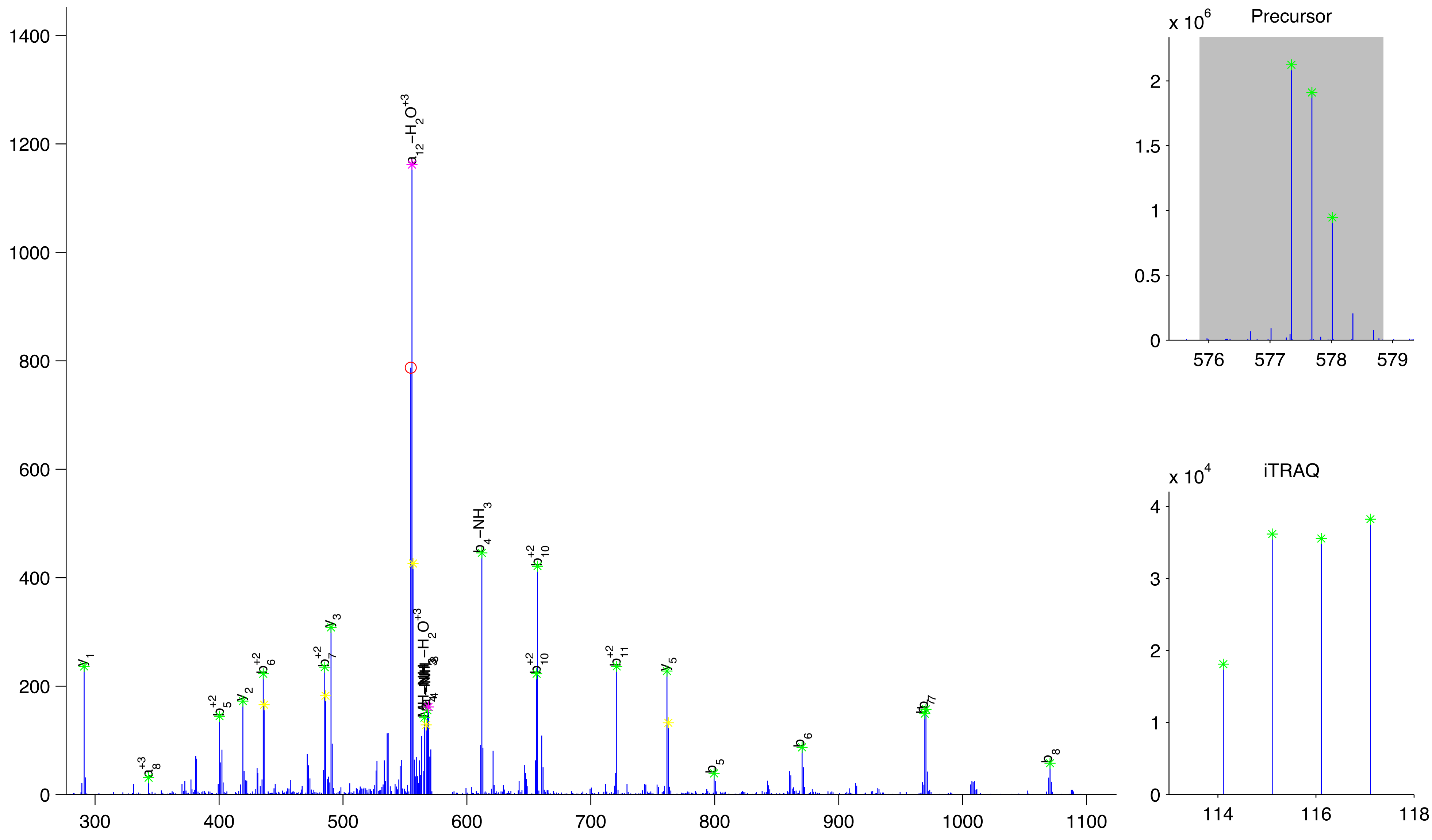

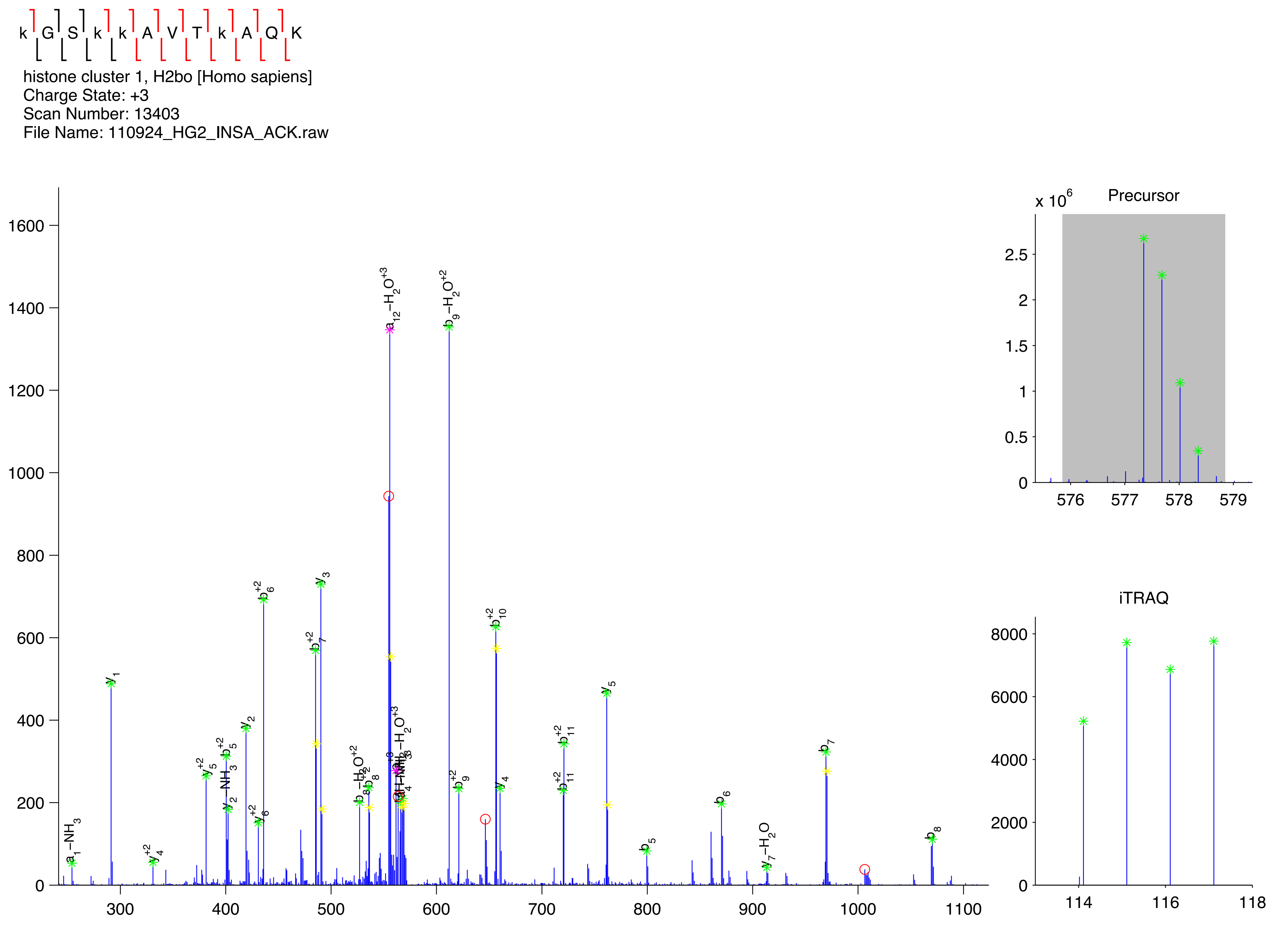

k G S k k A V T k A Q K
histone cluster 1, H2bo [Homo sapiens]
Charge State: +3
Scan Number: 13403
File Name: 110924_HG2_INSA_ACK.raw
Precursor
x 10^6
iTRAQ

k G S k k A V T k A Q K

histone cluster 1, H2bo [Homo sapiens]
Charge State: +2
Scan Number: 13531
File Name: 110924_HG2_INSA_ACK.raw

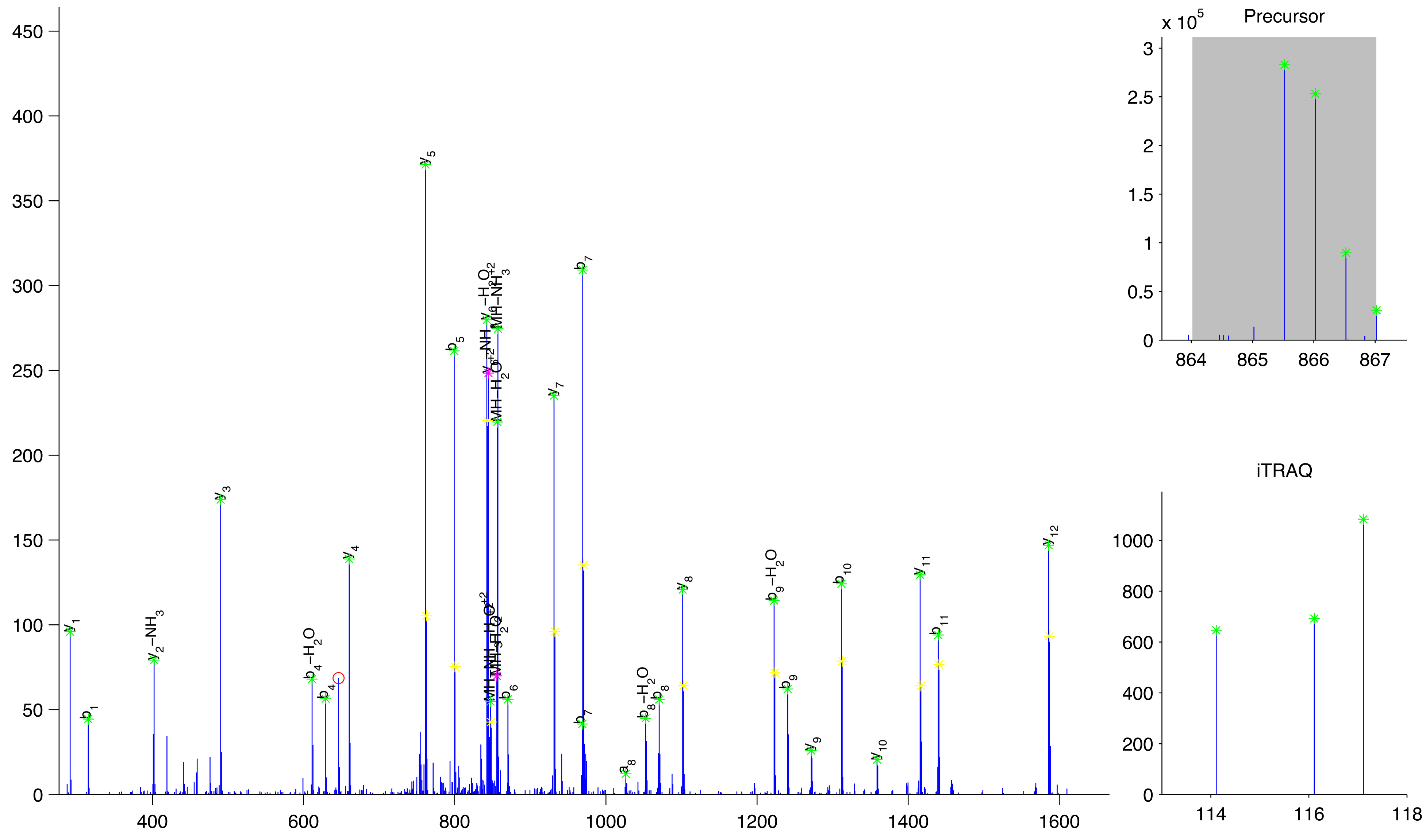

k G S k k A V T k A Q K

histone cluster 1, H2bo [Homo sapiens]
Charge State: +2
Scan Number: 13543
File Name: 110924_HG2_INSA_ACK.raw

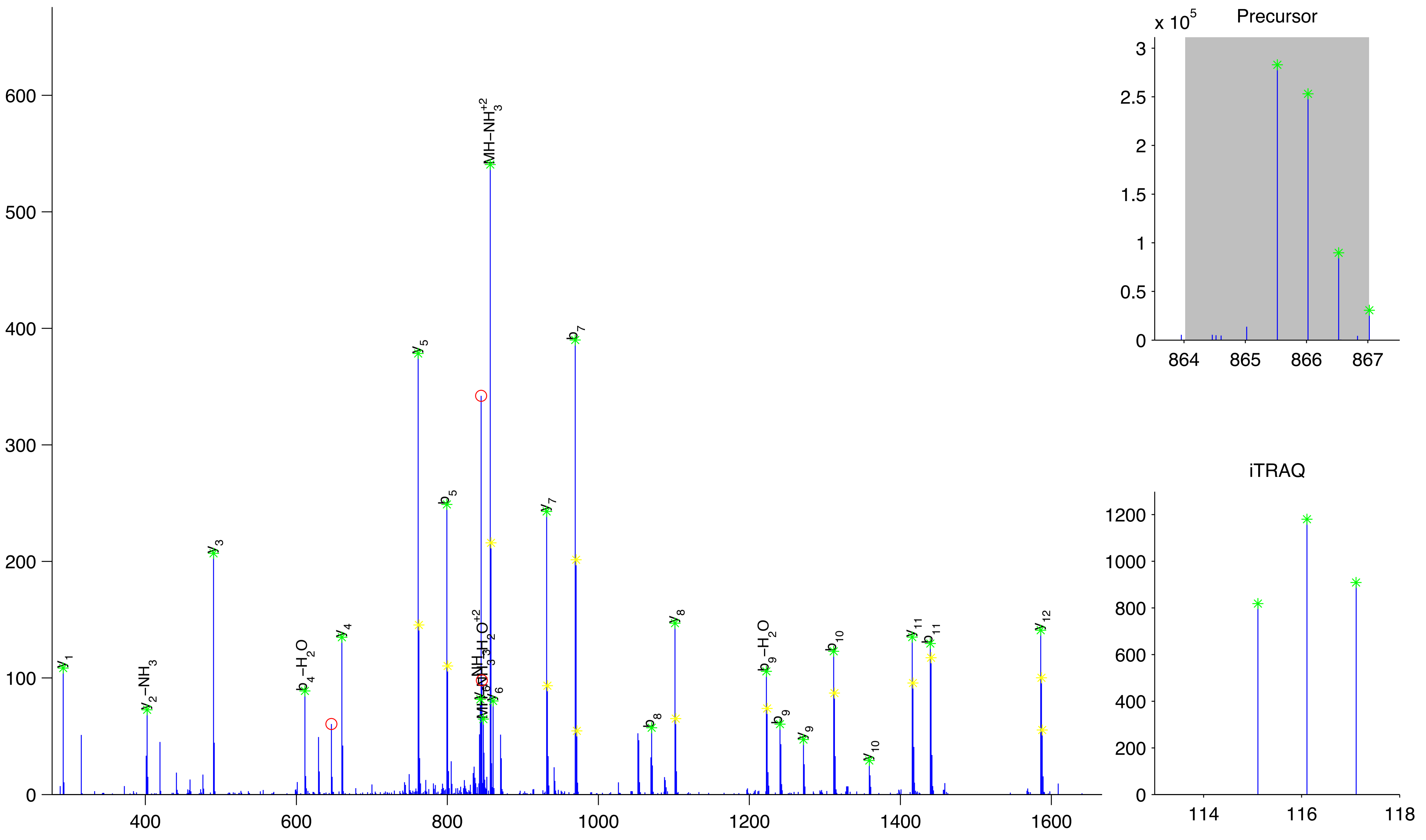

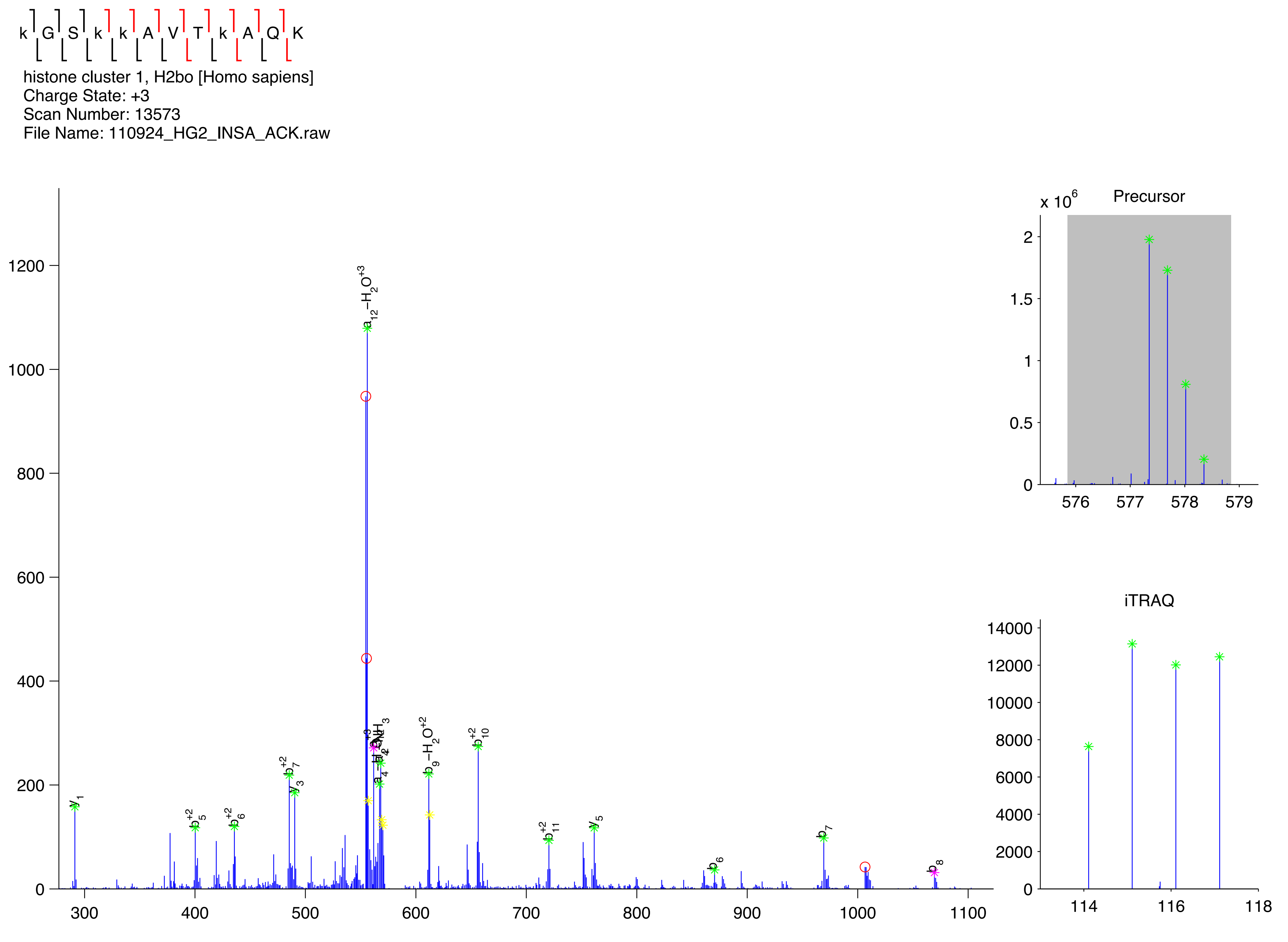
k G S k k A V T k A Q K
histone cluster 1, H2bo [Homo sapiens]
Charge State: +3
Scan Number: 13573
File Name: 110924_HG2_INSA_ACK.raw
Precursor
x 10^6
iTRAQ

k G S k k A V T k A Q K

histone cluster 1, H2bo [Homo sapiens]
Charge State: +3
Scan Number: 13741
File Name: 110924_HG2_INSA_ACK.raw

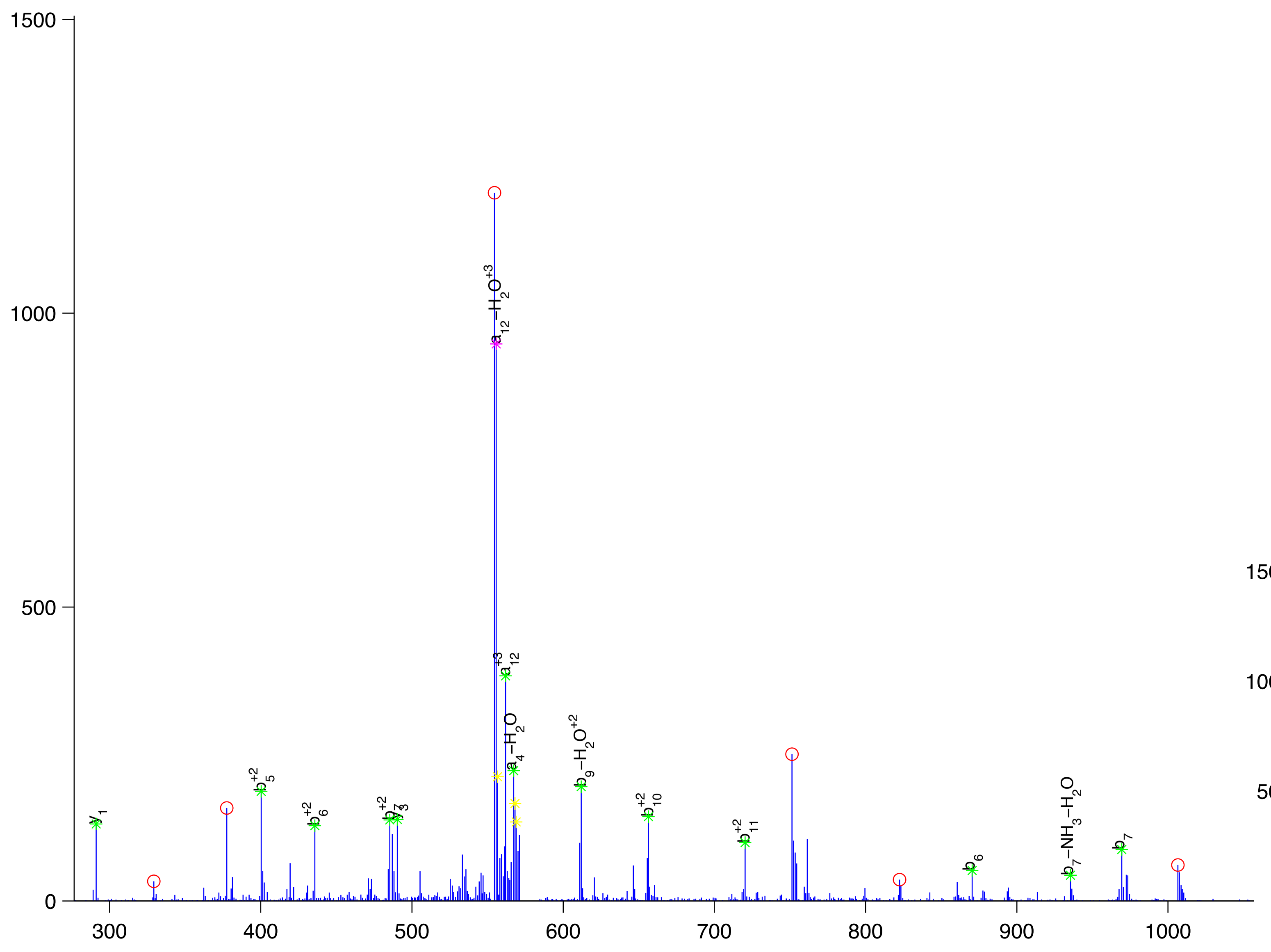

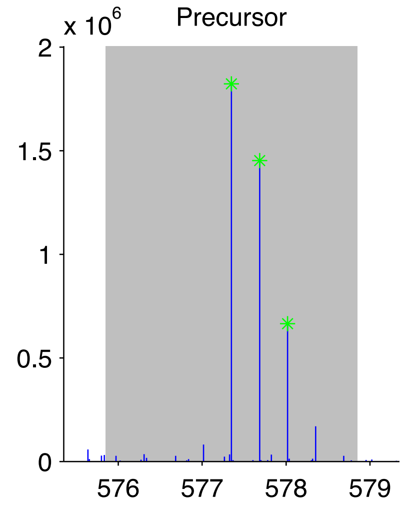

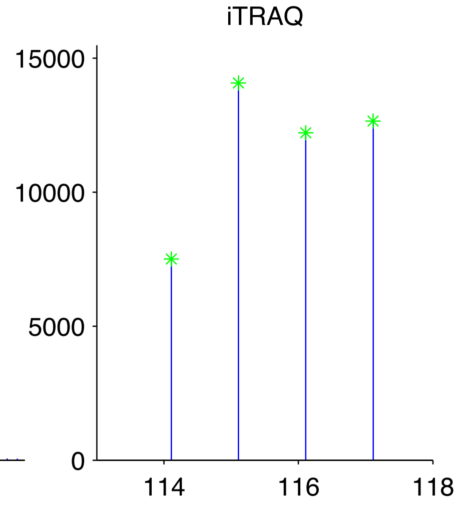

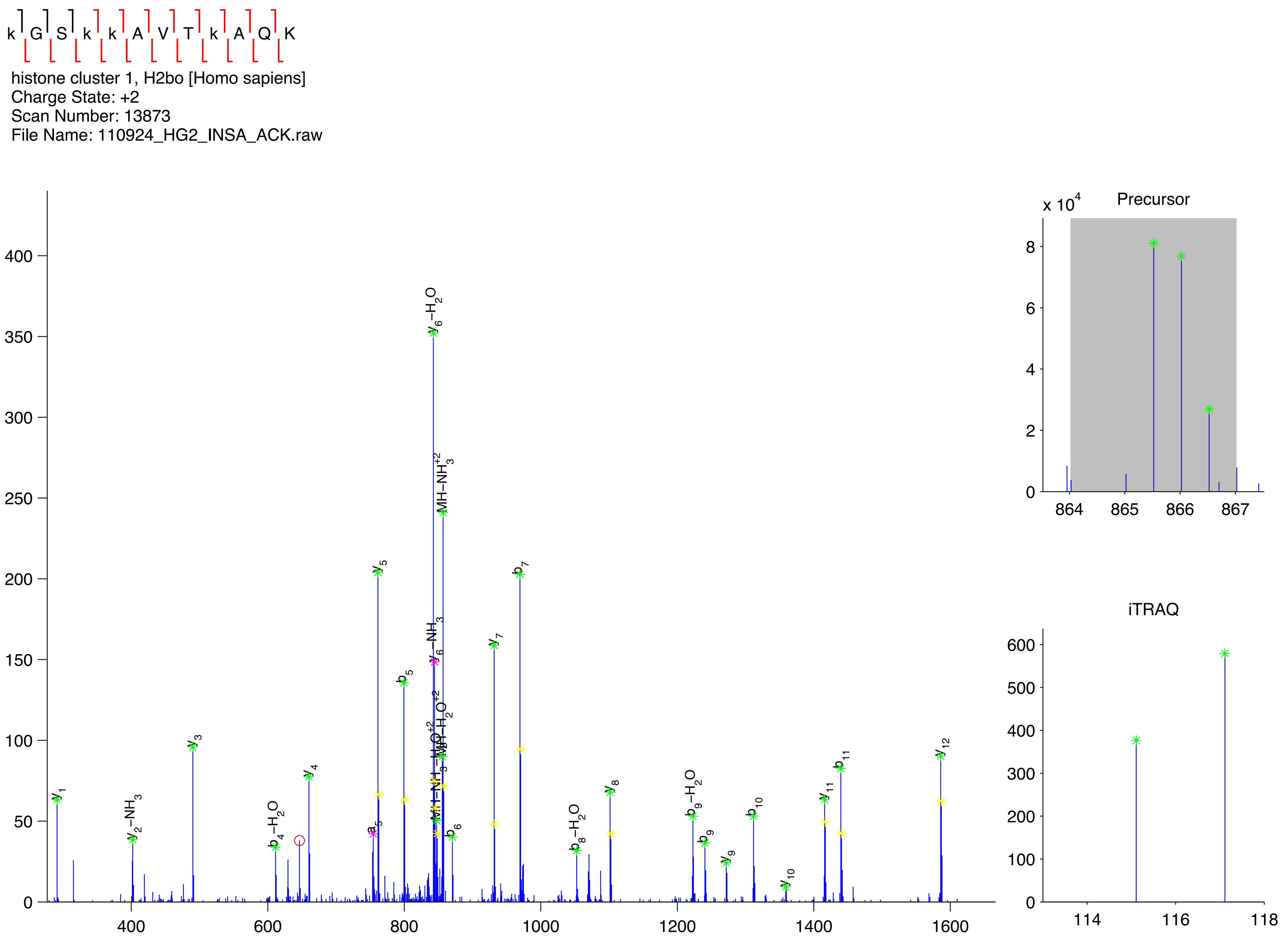
k G S k k A V T k A Q K
histone cluster 1, H2bo [Homo sapiens]
Charge State: +2
Scan Number: 13873
File Name: 110924_HG2_INSA_ACK.raw
Precursor
x 10^4
iTRAQ
y1
y2−NH3
y3
b4−H2O
y4
a5
y5
b5
y6−H2O
y6−NH3
MH−NH3+2
b6
y7
b7
b8−H2O
y8
b9−H2O
b9
y9
b10
y10
y11
b11
y12

k G S k k A V T k A Q K

histone cluster 1, H2bo [Homo sapiens]
Charge State: +2
Scan Number: 14039
File Name: 110924_HG2_INSA_ACK.raw

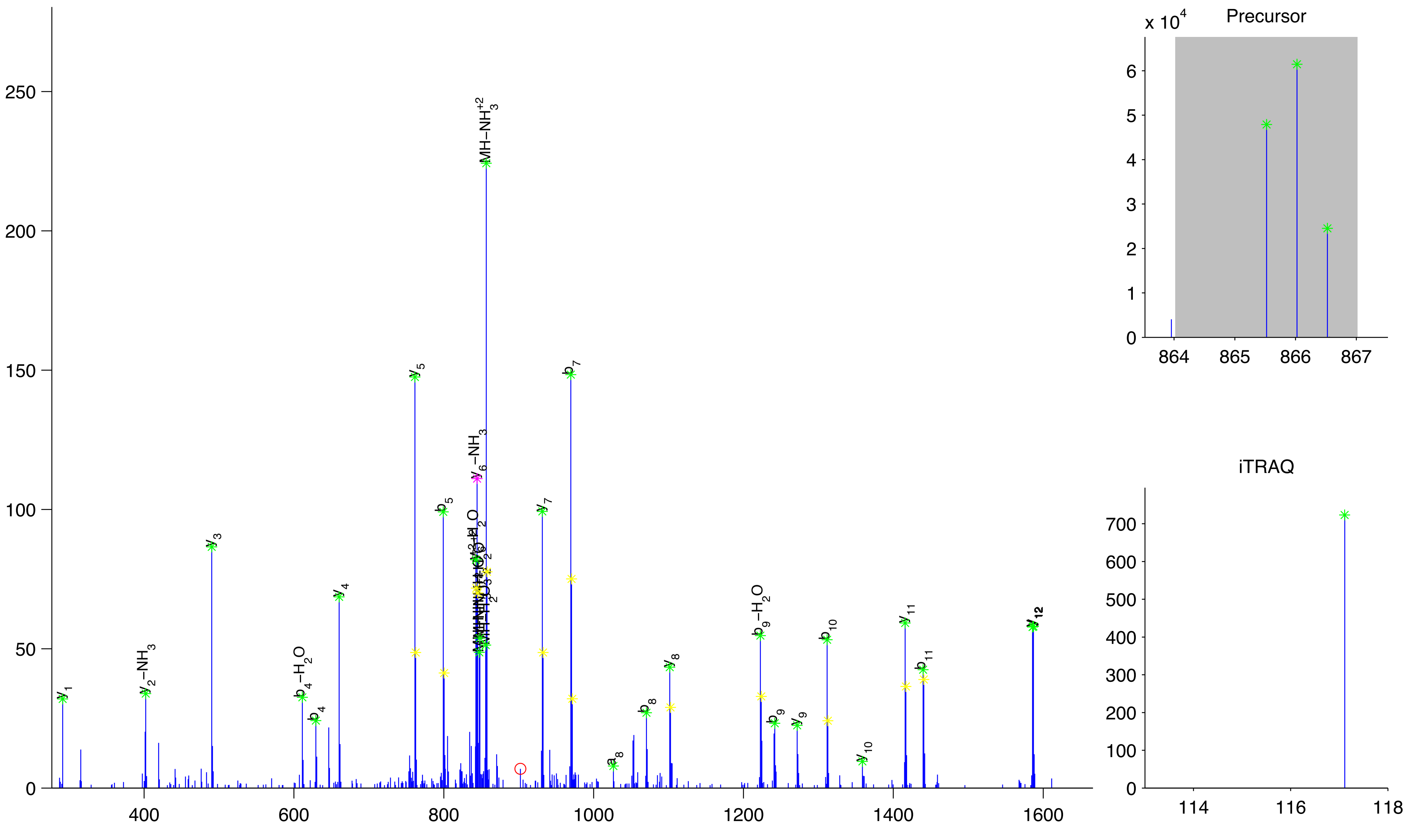

k G S k k A V T k A Q K

histone cluster 1, H2bo [Homo sapiens]
Charge State: +3
Scan Number: 14077
File Name: 110924_HG2_INSA_ACK.raw

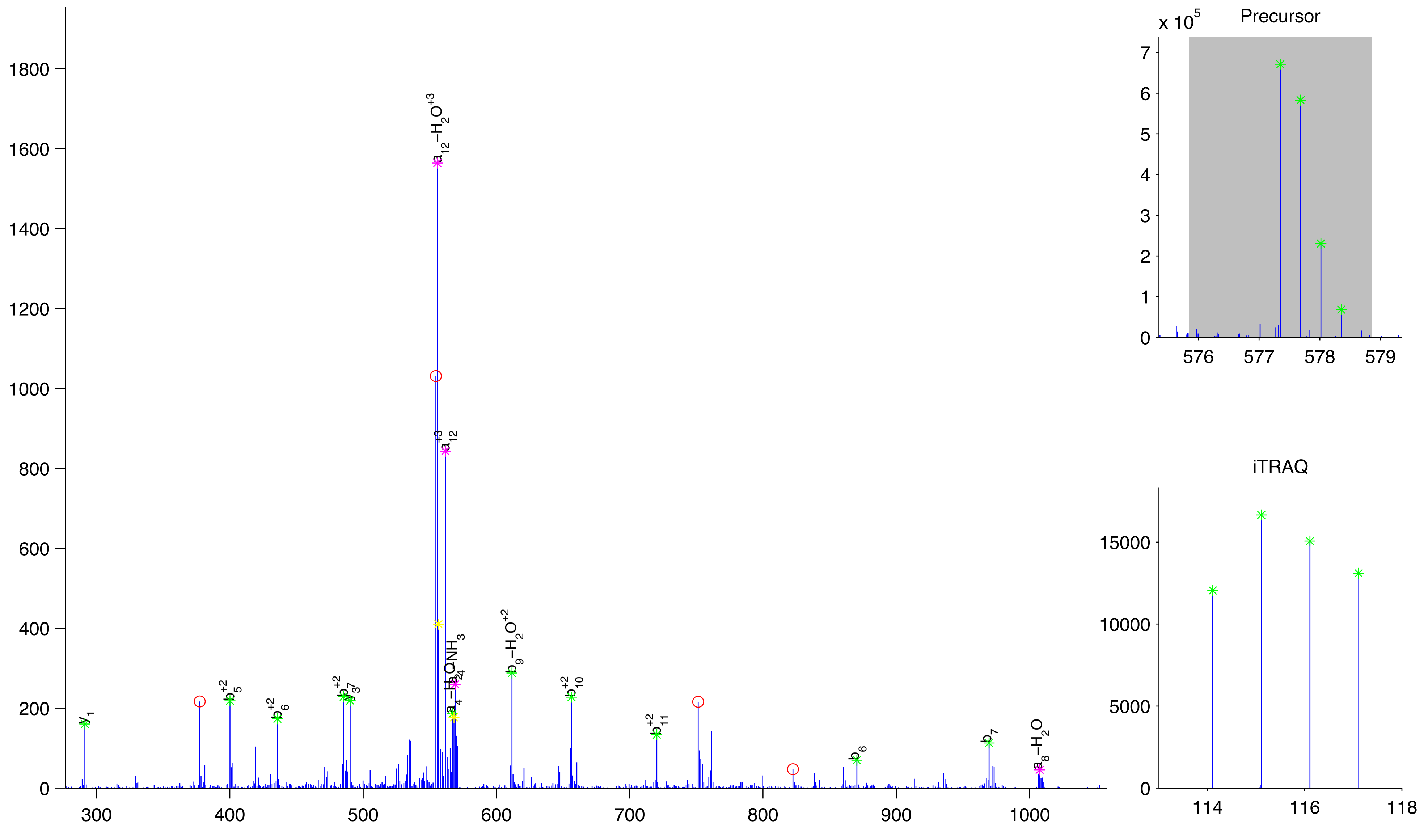

k G S k k A V T k A Q K

histone cluster 1, H2bo [Homo sapiens]
Charge State: +2
Scan Number: 14209
File Name: 110924_HG2_INSA_ACK.raw

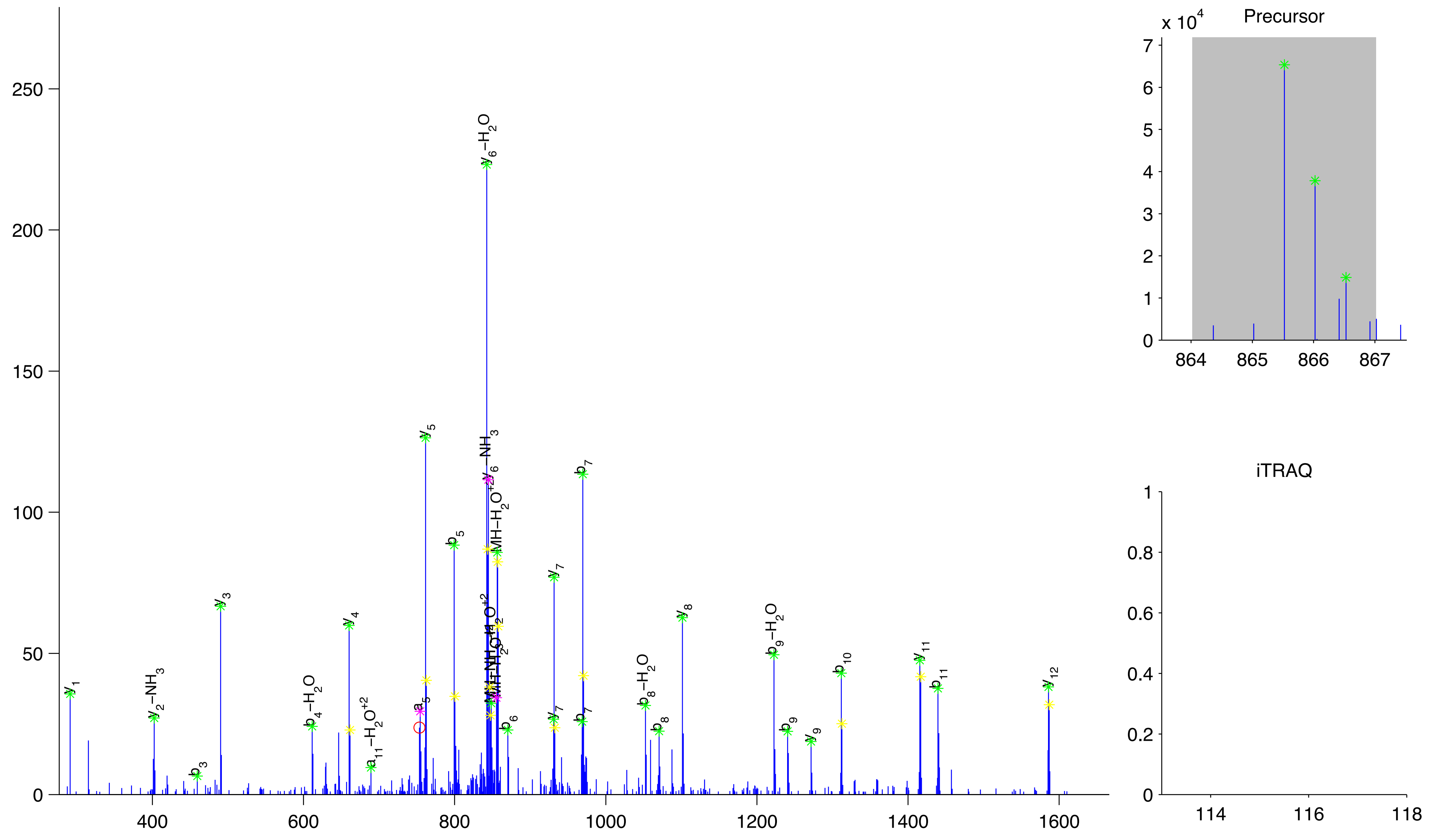

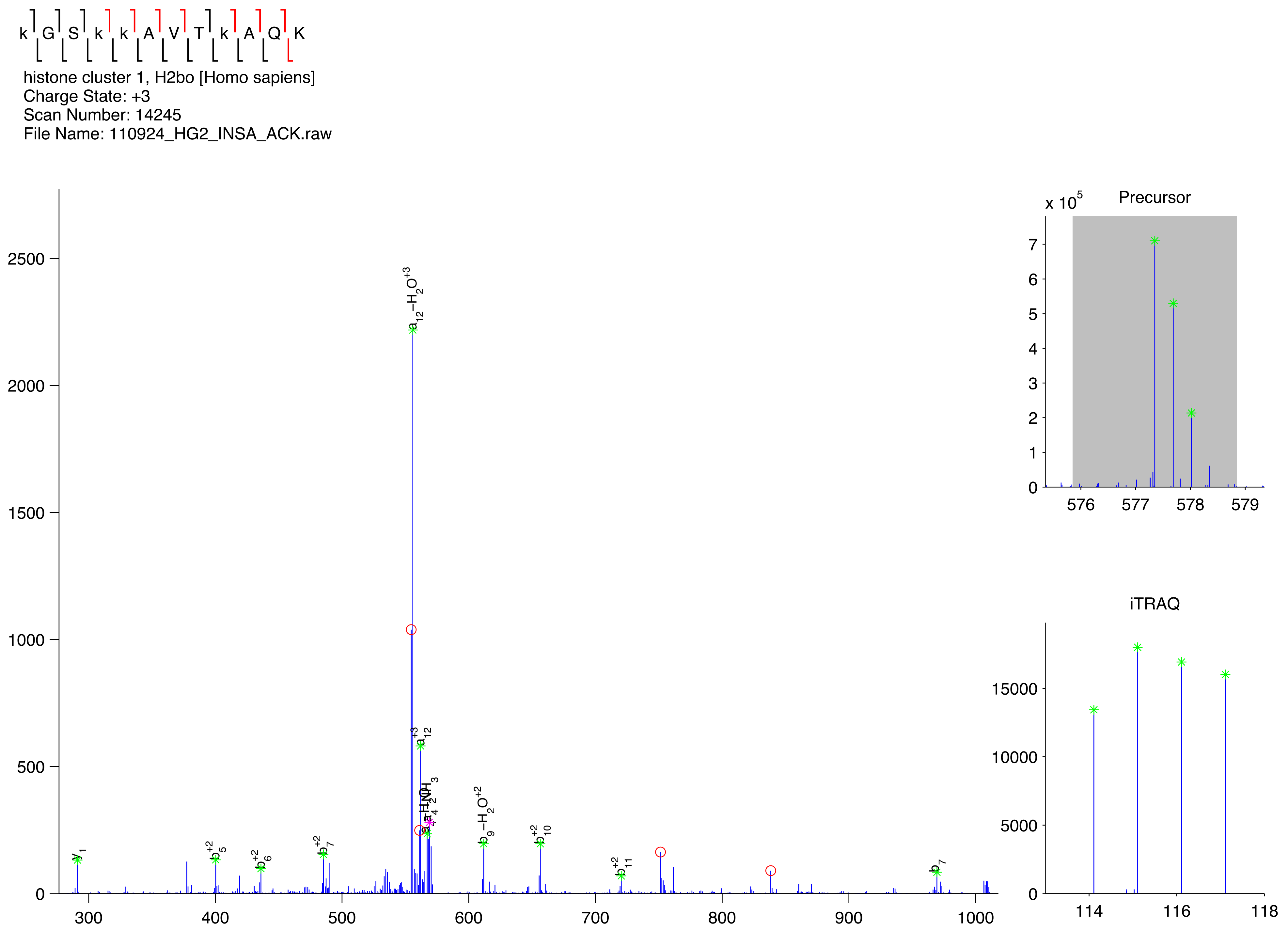
k G S k k A V T k A Q K
histone cluster 1, H2bo [Homo sapiens]
Charge State: +3
Scan Number: 14245
File Name: 110924_HG2_INSA_ACK.raw
Precursor
x 10^5
iTRAQ

k G S k k A V T k A Q K

histone cluster 1, H2bo [Homo sapiens]
Charge State: +2
Scan Number: 14377
File Name: 110924_HG2_INSA_ACK.raw

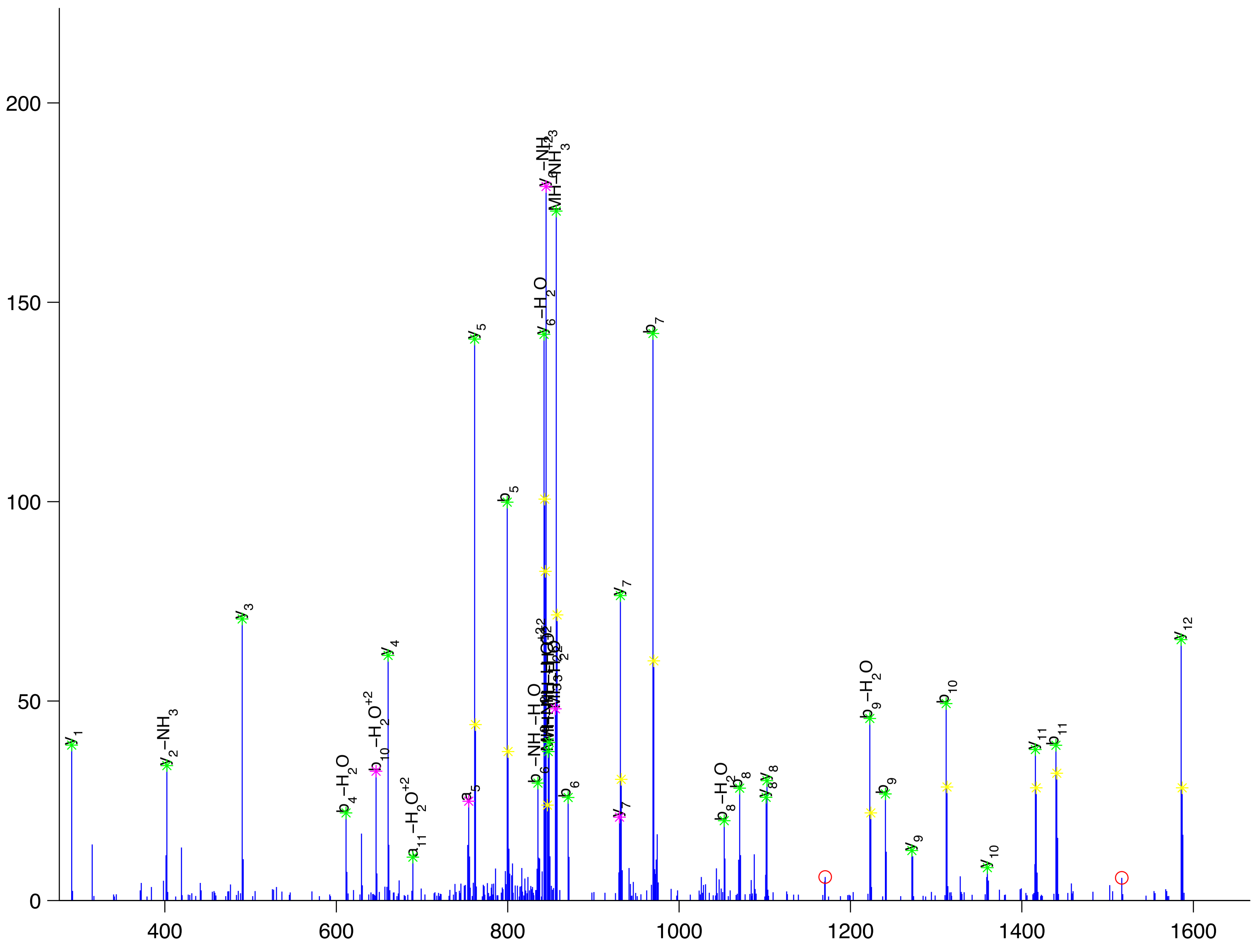

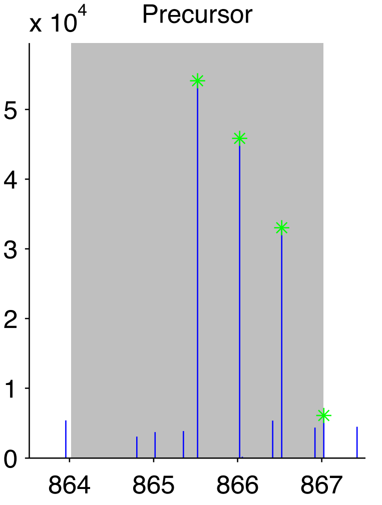

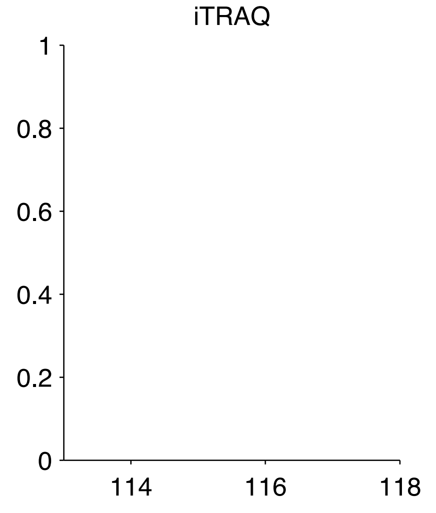

k G S k k A V T k A Q K

histone cluster 1, H2bo [Homo sapiens]
Charge State: +3
Scan Number: 14415
File Name: 110924_HG2_INSA_ACK.raw

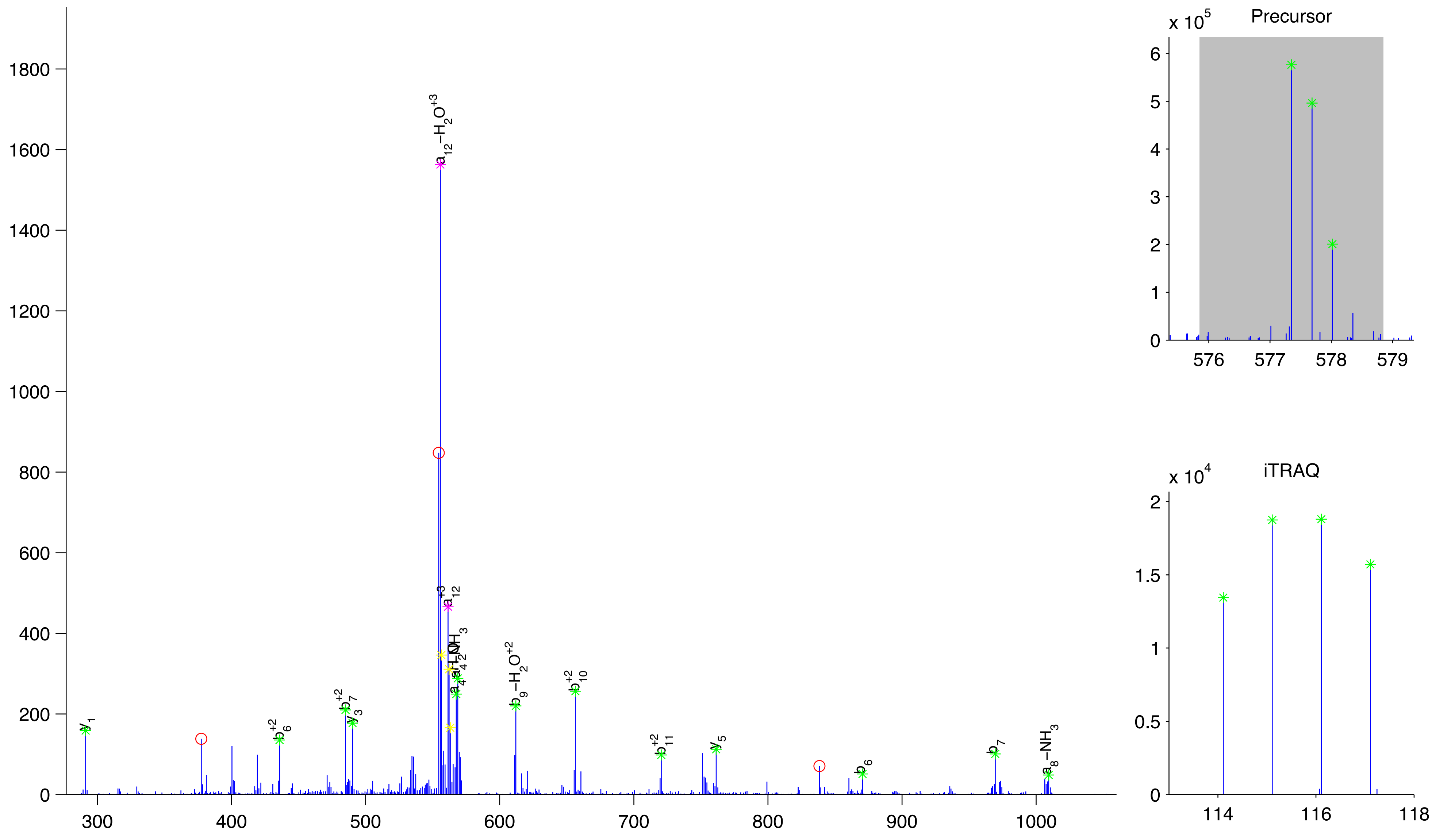

kGSkkAVTkAQK

histone cluster 1, H2bo [Homo sapiens]
Charge State: +2
Scan Number: 14547
File Name: 110924_HG2_INSA_ACK.raw

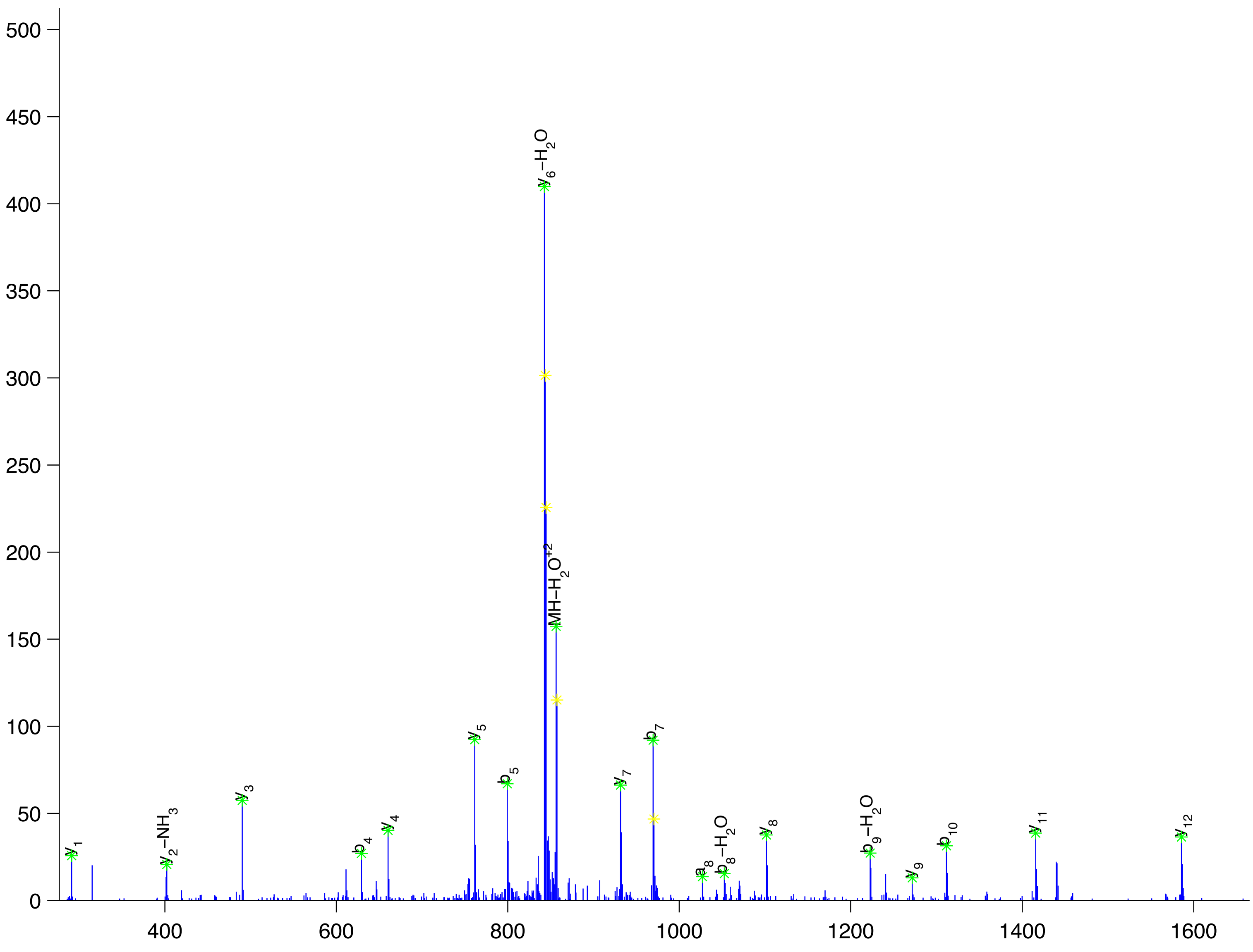

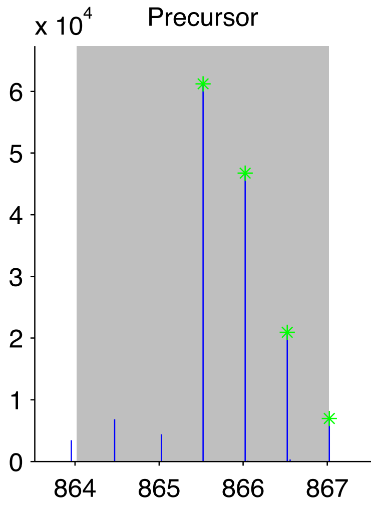

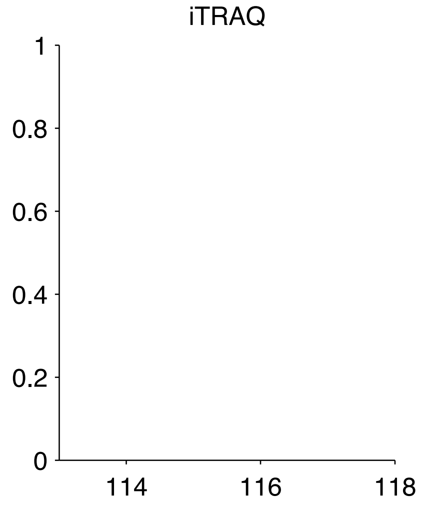

k G S k k A V T k A Q K

histone cluster 1, H2bo [Homo sapiens]
Charge State: +2
Scan Number: 15114
File Name: 110924_HG2_INSA_ACK.raw

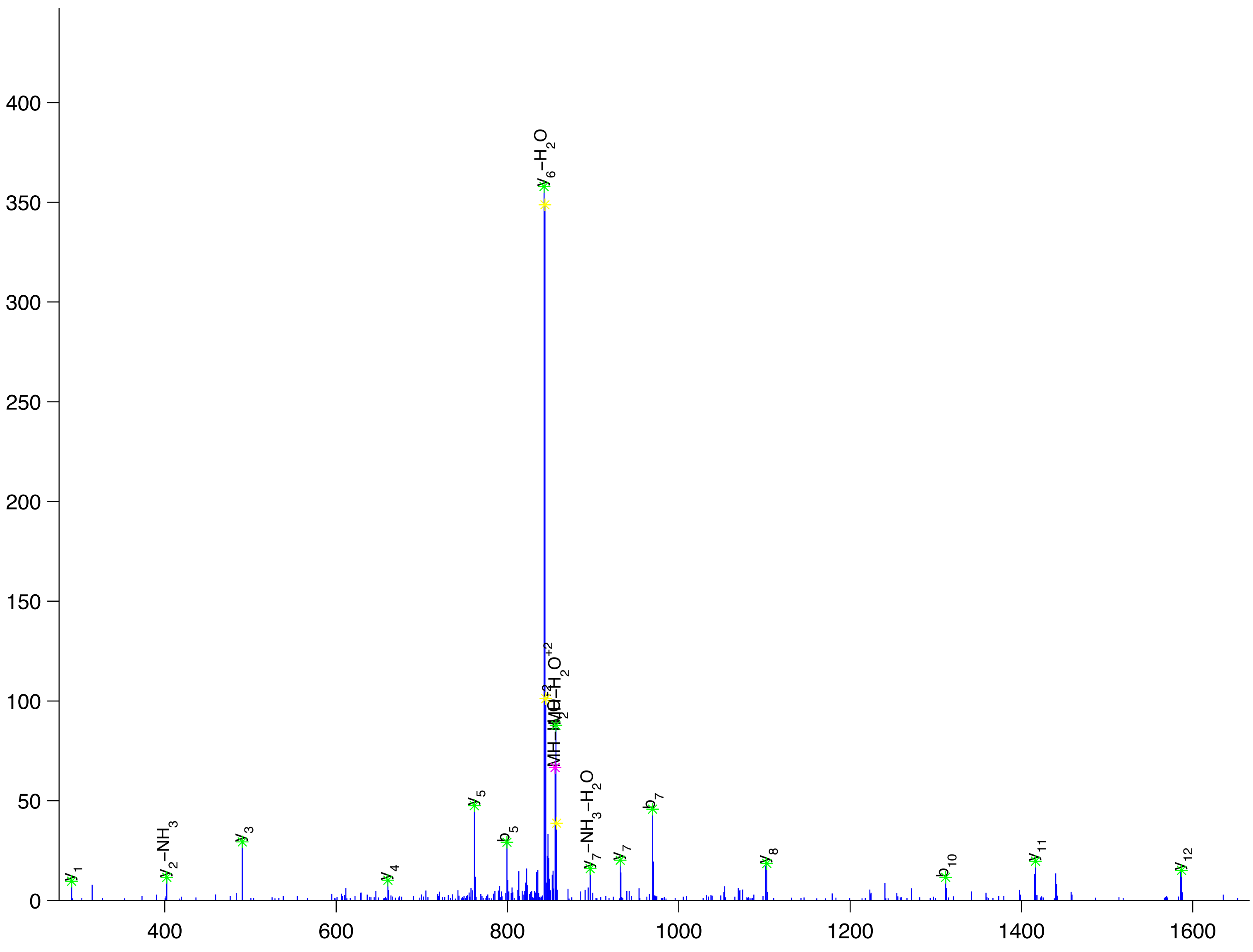

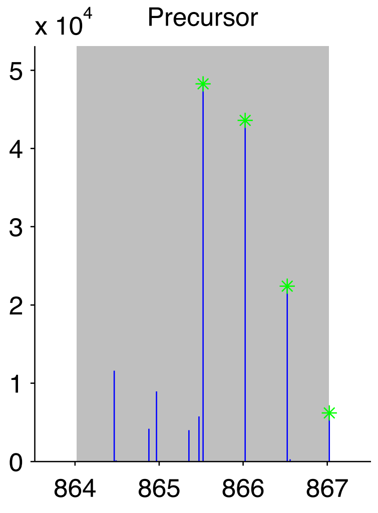

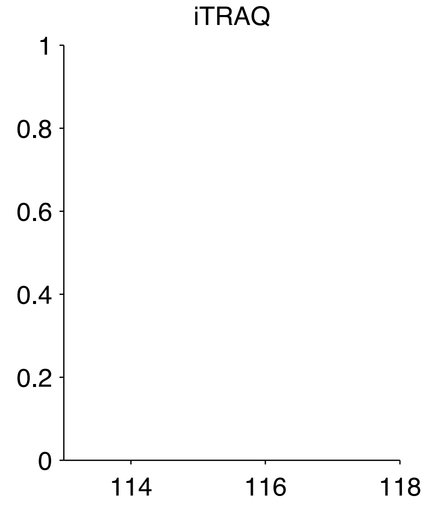

k G S k k A V T k A Q K

histone cluster 1, H2bo [Homo sapiens]
Charge State: +3
Scan Number: 19020
File Name: 110924_HG2_INSA_ACK.raw

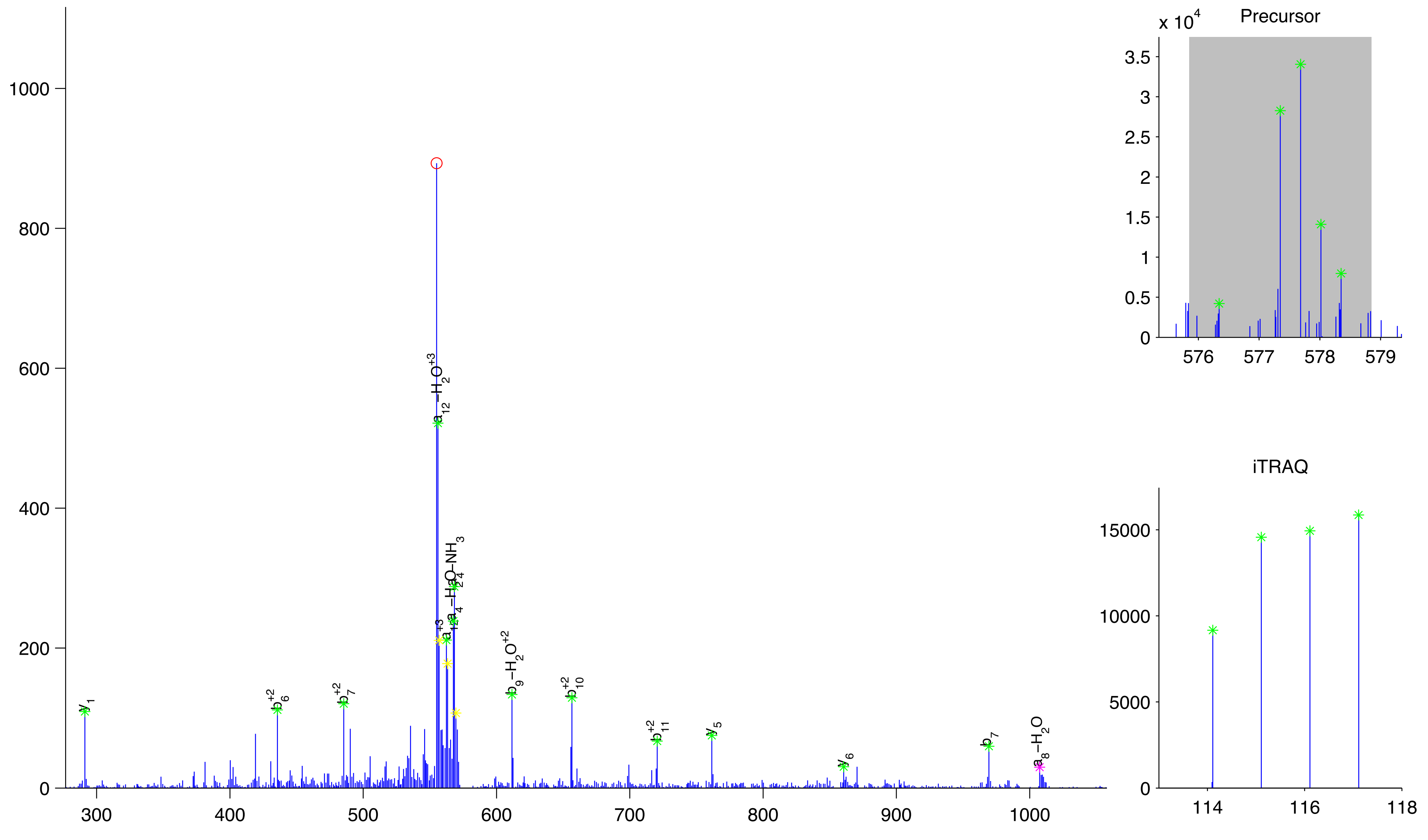

G G k G L G k G G A k R

histone cluster 1, H4a [Homo sapiens]
Charge State: +2
Scan Number: 30
File Name: 110924_HG2_INSA_ACK4.raw

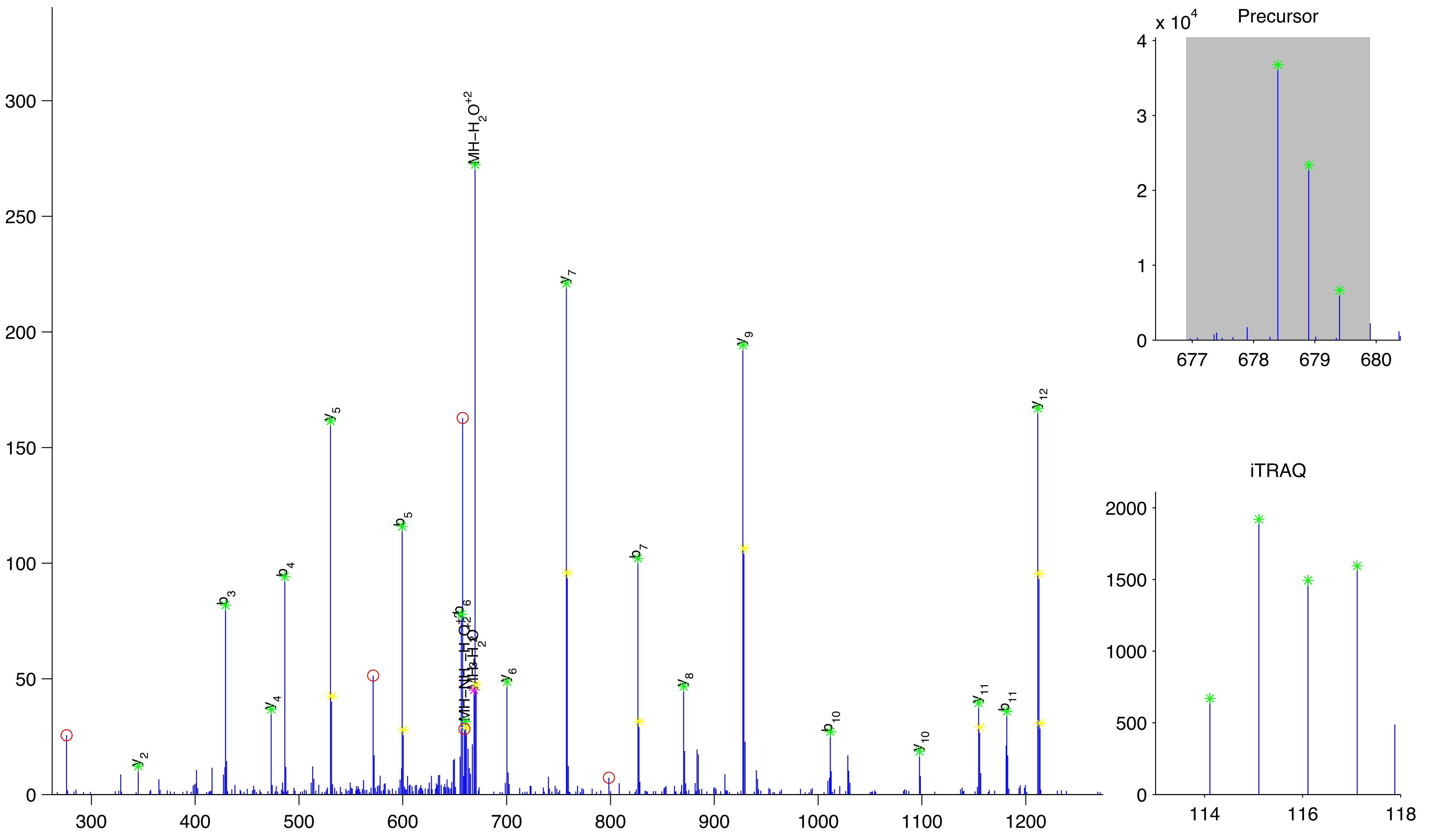

G k G G k G L G k G G A k R

histone cluster 1, H4a [Homo sapiens]
Charge State: +2
Scan Number: 68
File Name: 110924_HG2_INSA_ACK3.raw

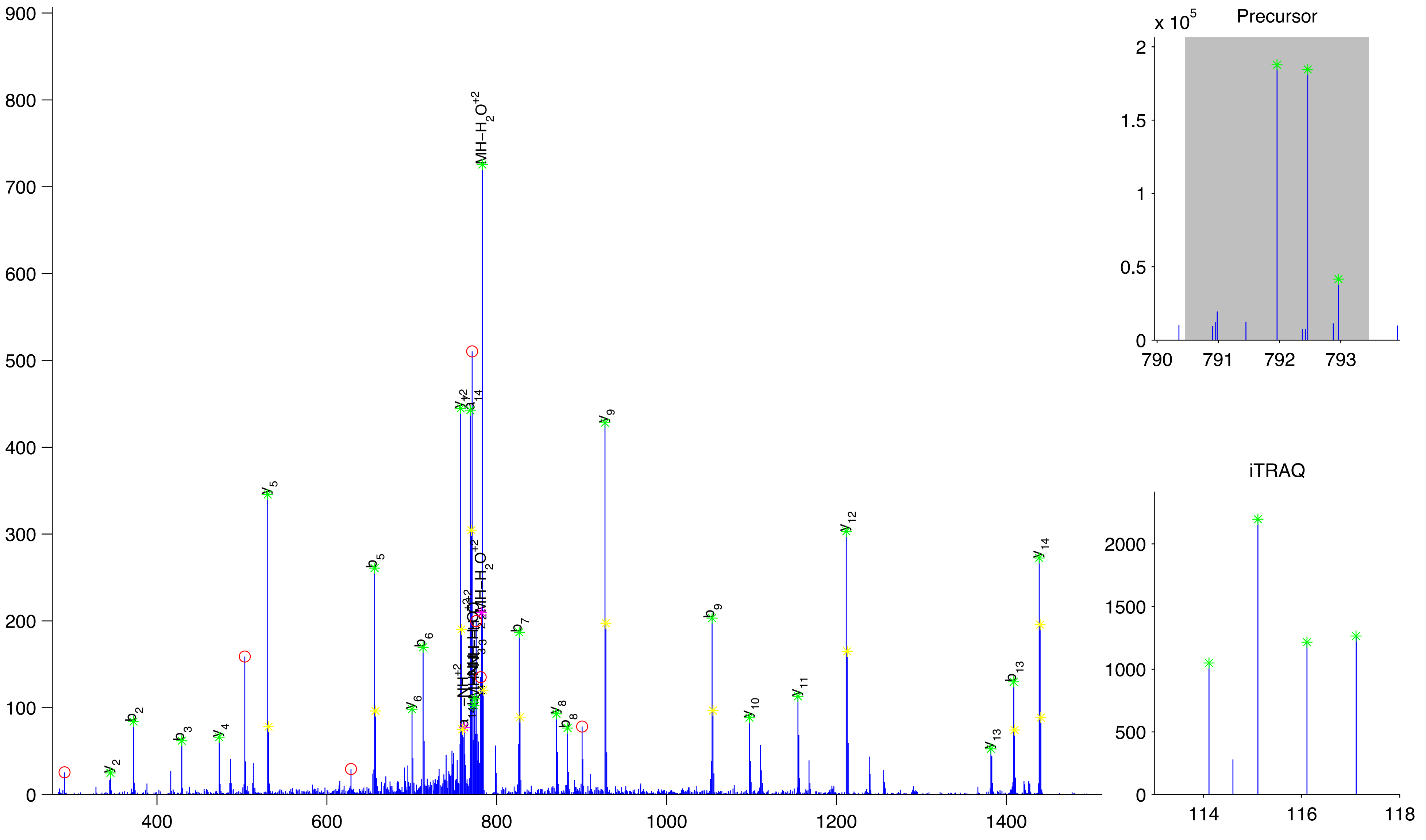

G L G k G G A k R

histone cluster 1, H4a [Homo sapiens]
Charge State: +2
Scan Number: 173
File Name: 110924_HG2_INSA_ACK4.raw

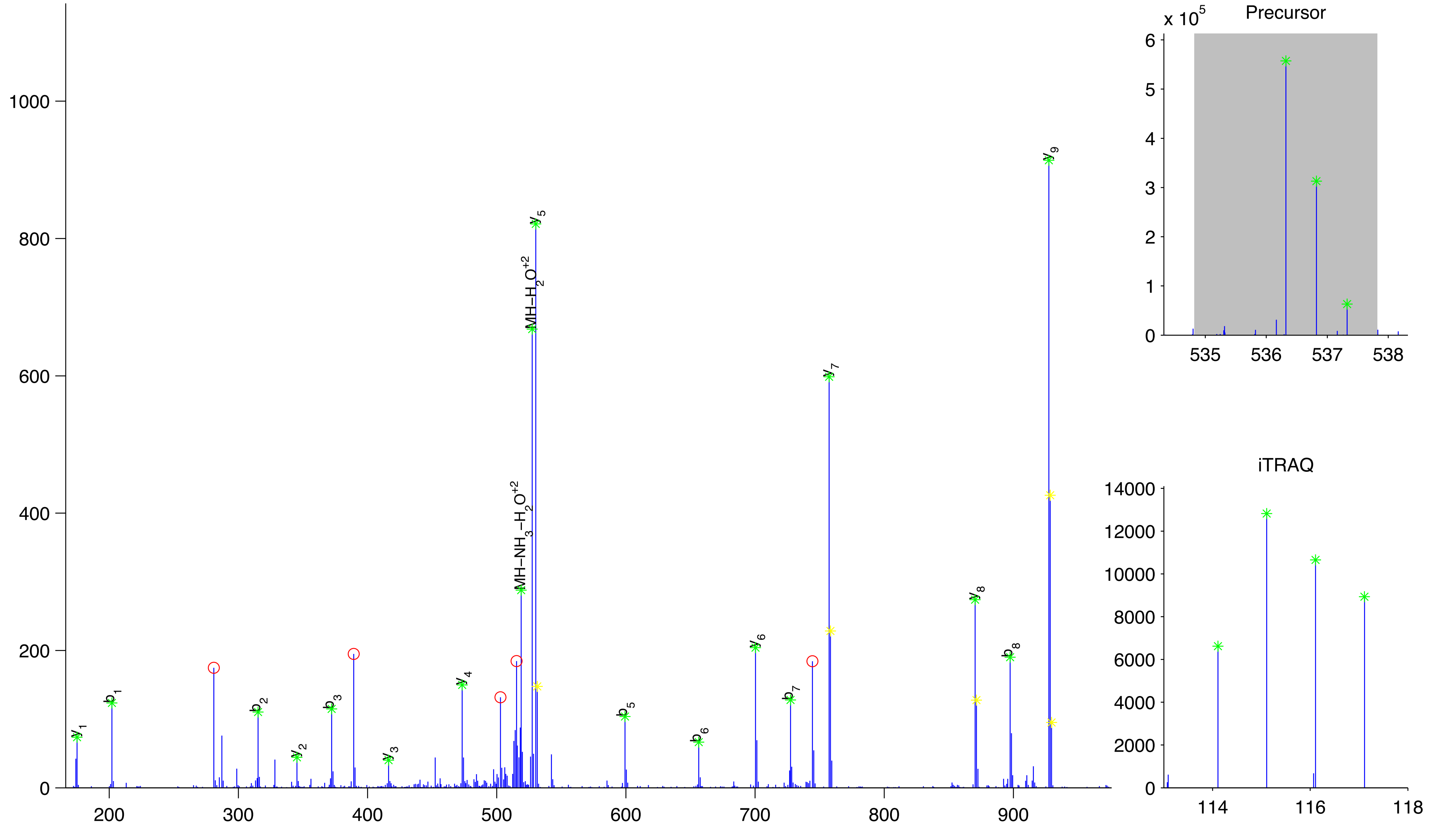

G k G G k G L G k G G A k R

histone cluster 1, H4a [Homo sapiens]
Charge State: +2
Scan Number: 183
File Name: 110924_HG2_INSA_ACK3.raw

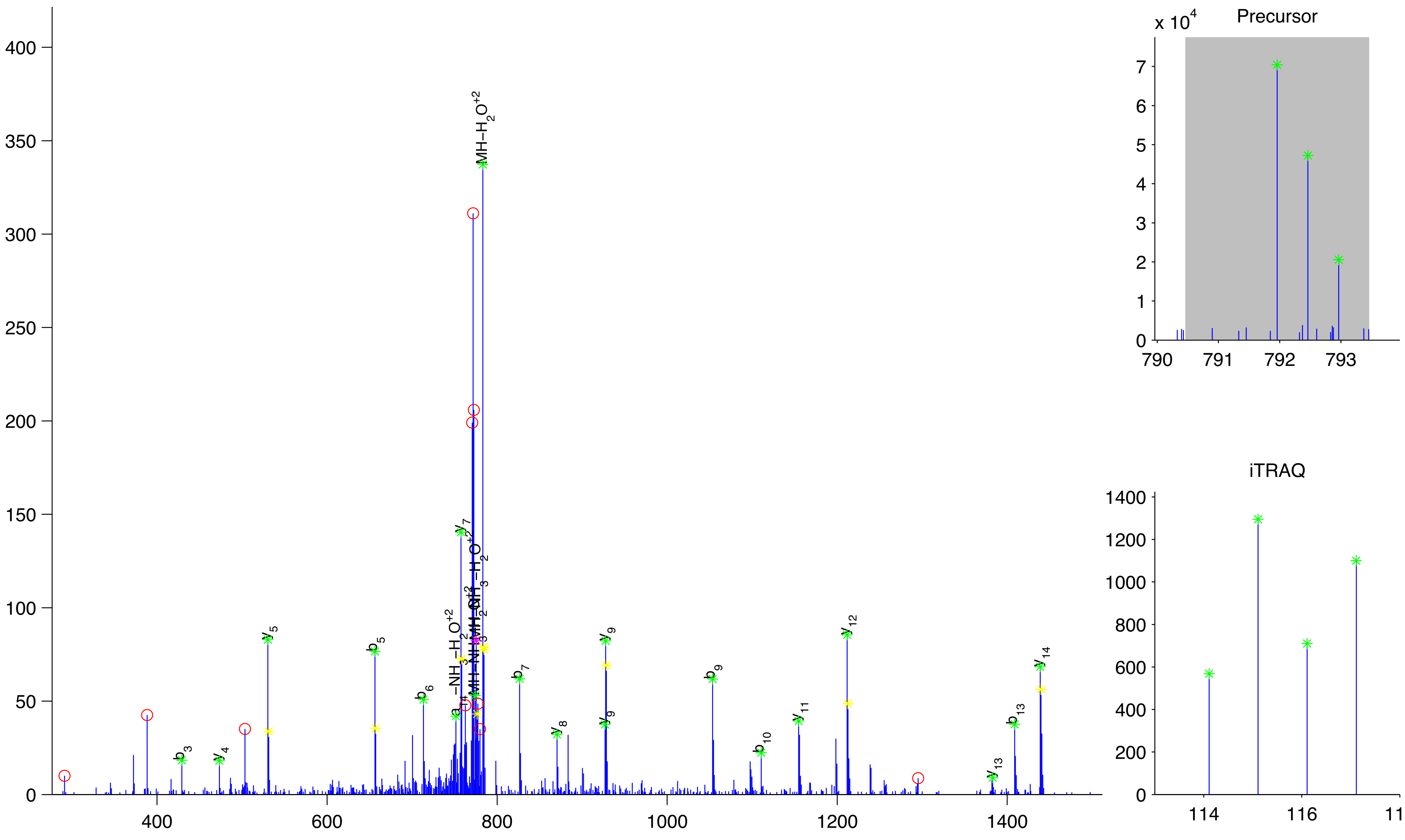

G L G k G G A k R

histone cluster 1, H4a [Homo sapiens]
Charge State: +2
Scan Number: 188
File Name: 110924_HG2_INSA_ACK3.raw

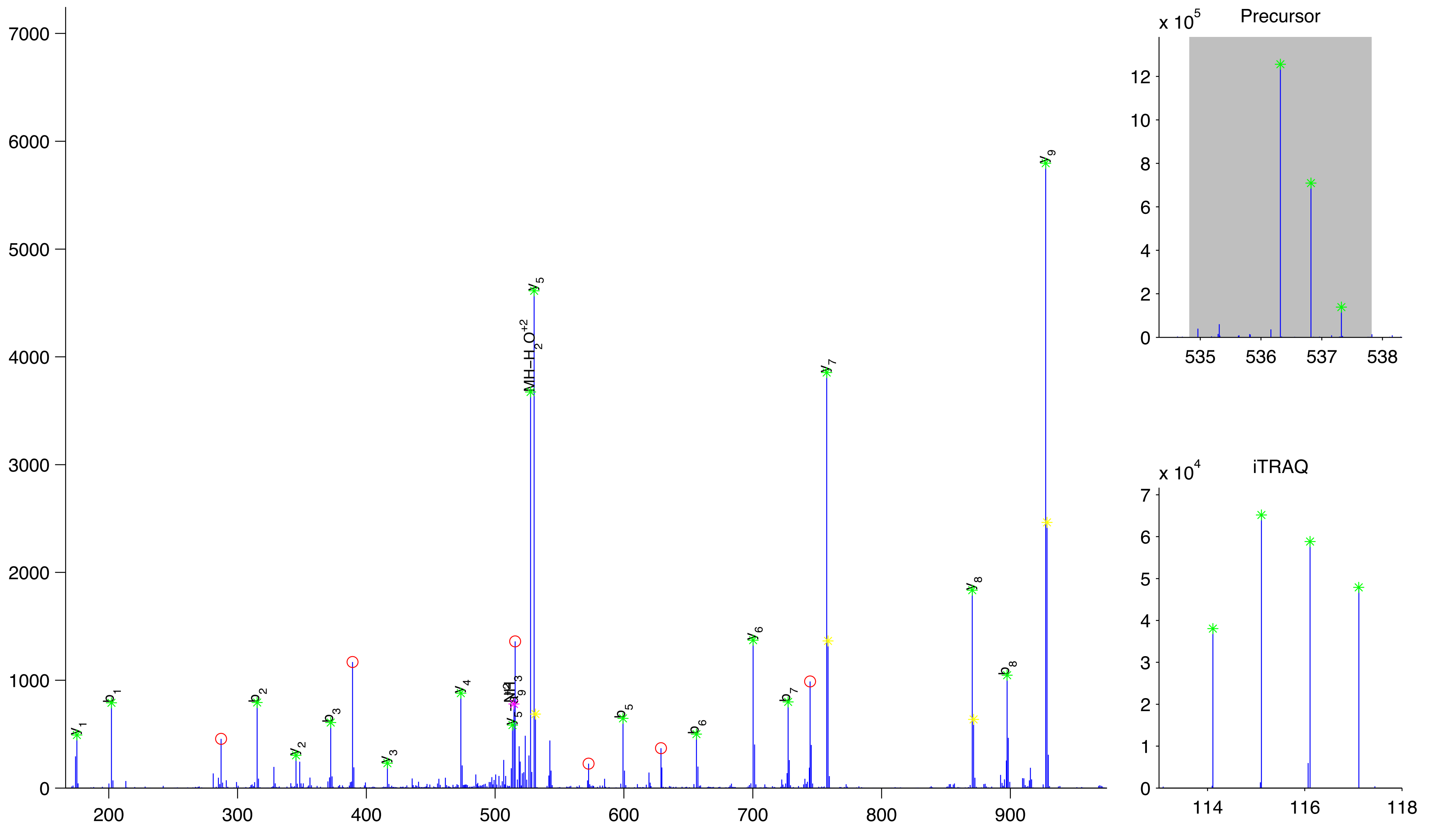

G k G G k G L G k G G A k R

histone cluster 1, H4a [Homo sapiens]
Charge State: +3
Scan Number: 234
File Name: 110924_HG2_INSA_ACK4.raw

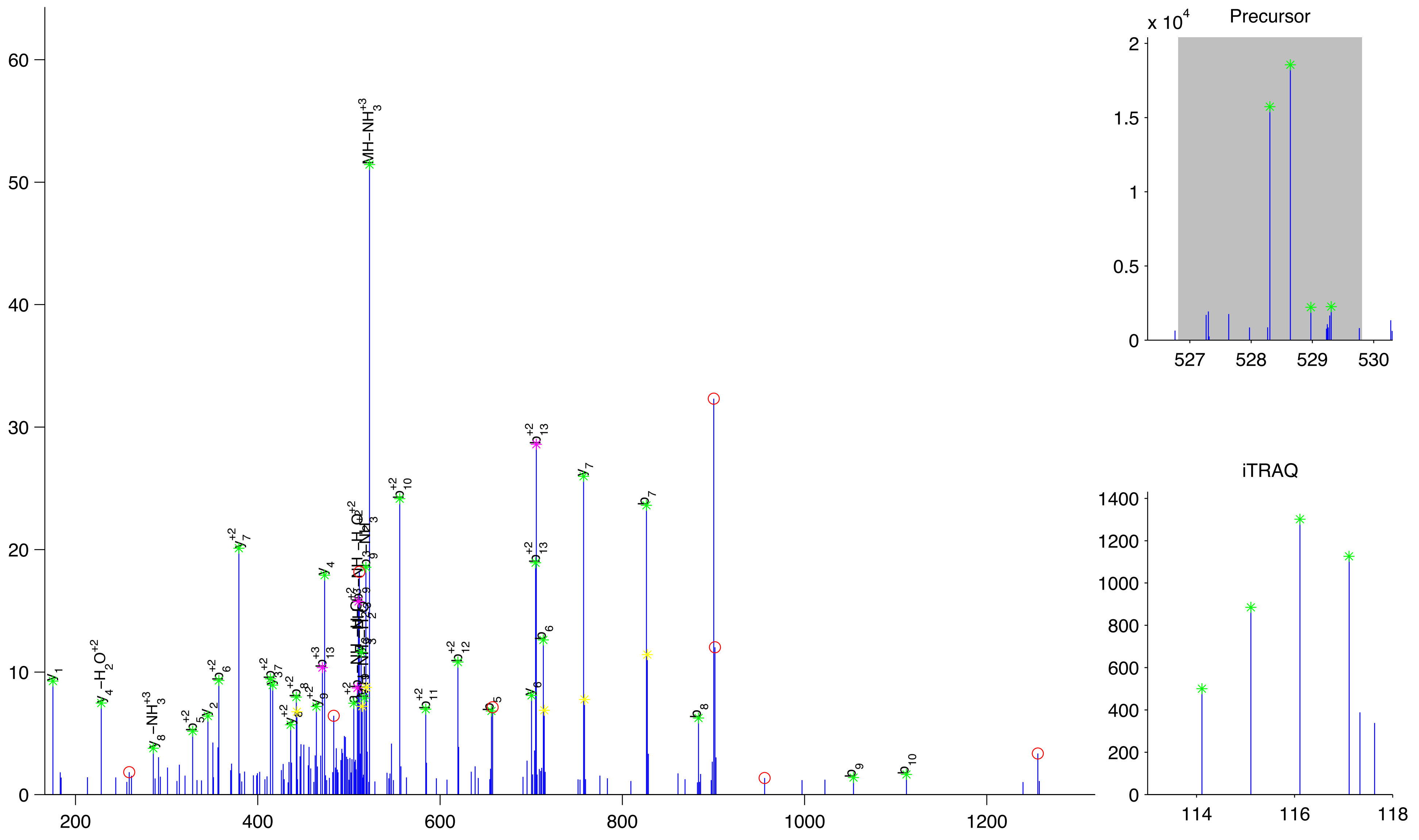

G L G k G G A k R

histone cluster 1, H4a [Homo sapiens]
Charge State: +2
Scan Number: 345
File Name: 110924_HG2_INSA_ACK4.raw

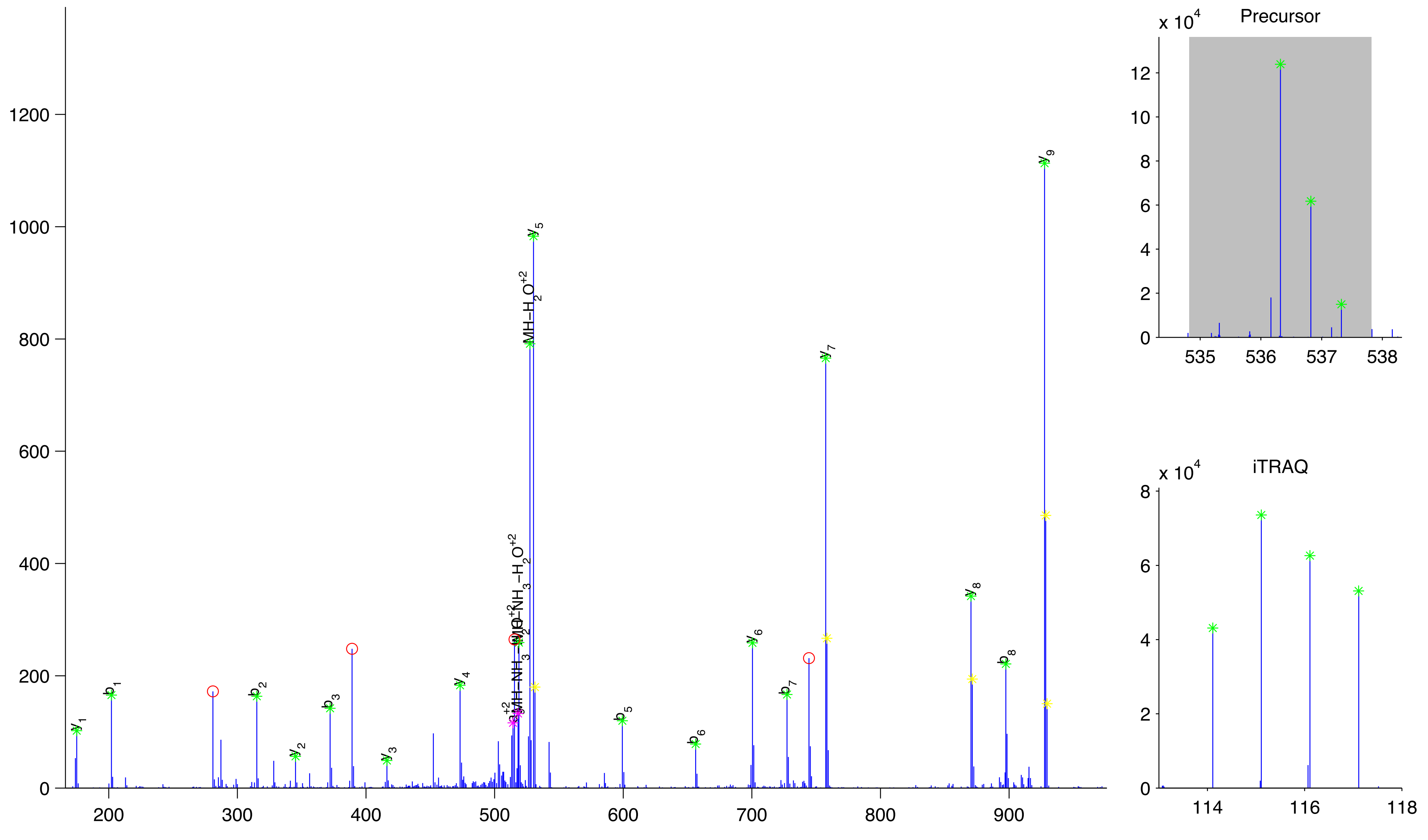

G L G k G G A k R

histone cluster 1, H4a [Homo sapiens]
Charge State: +2
Scan Number: 356
File Name: 110924_HG2_INSA_ACK3.raw

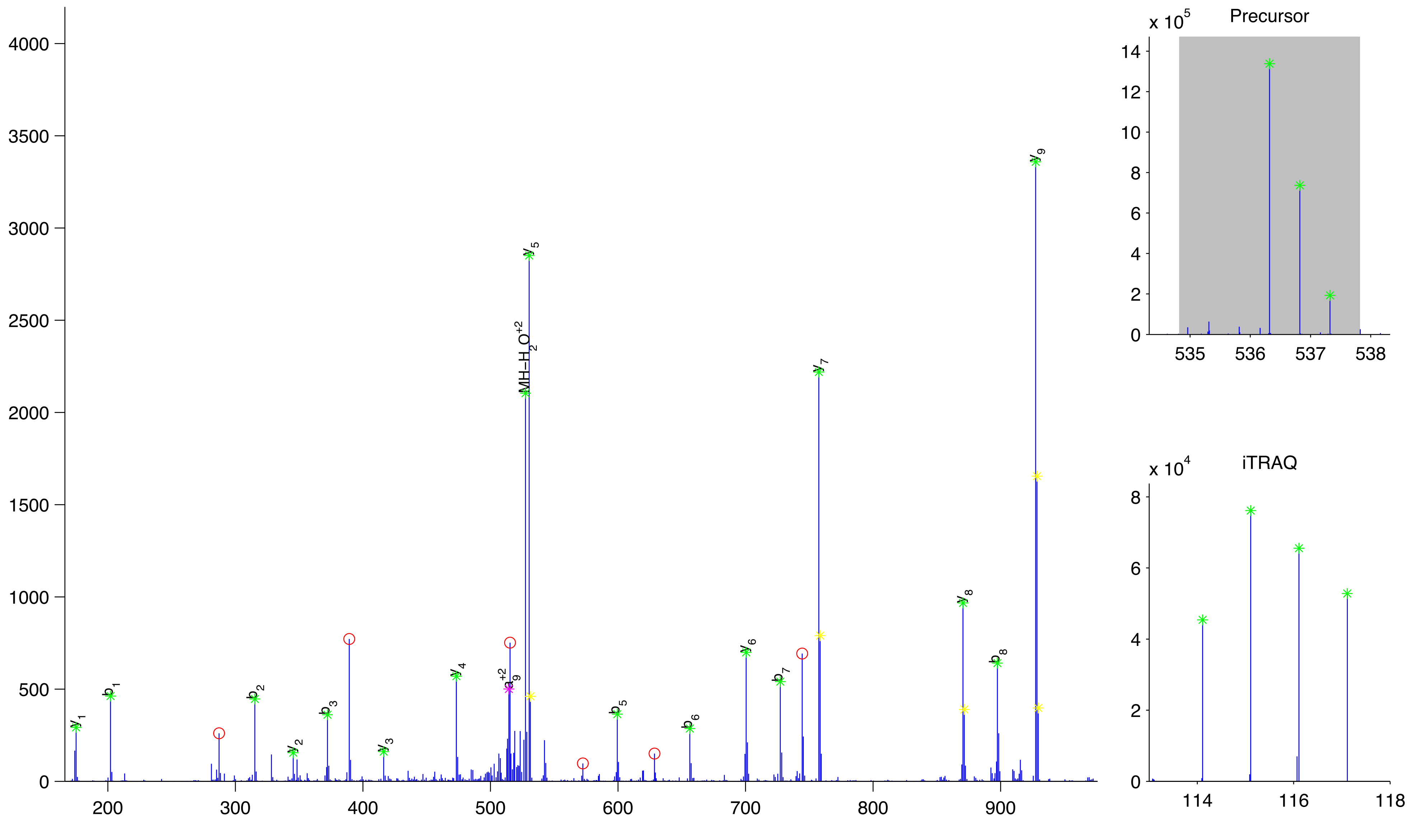

G L G k G G A k R

histone cluster 1, H4a [Homo sapiens]
Charge State: +2
Scan Number: 368
File Name: 110924_HG2_INSA_ACK3.raw

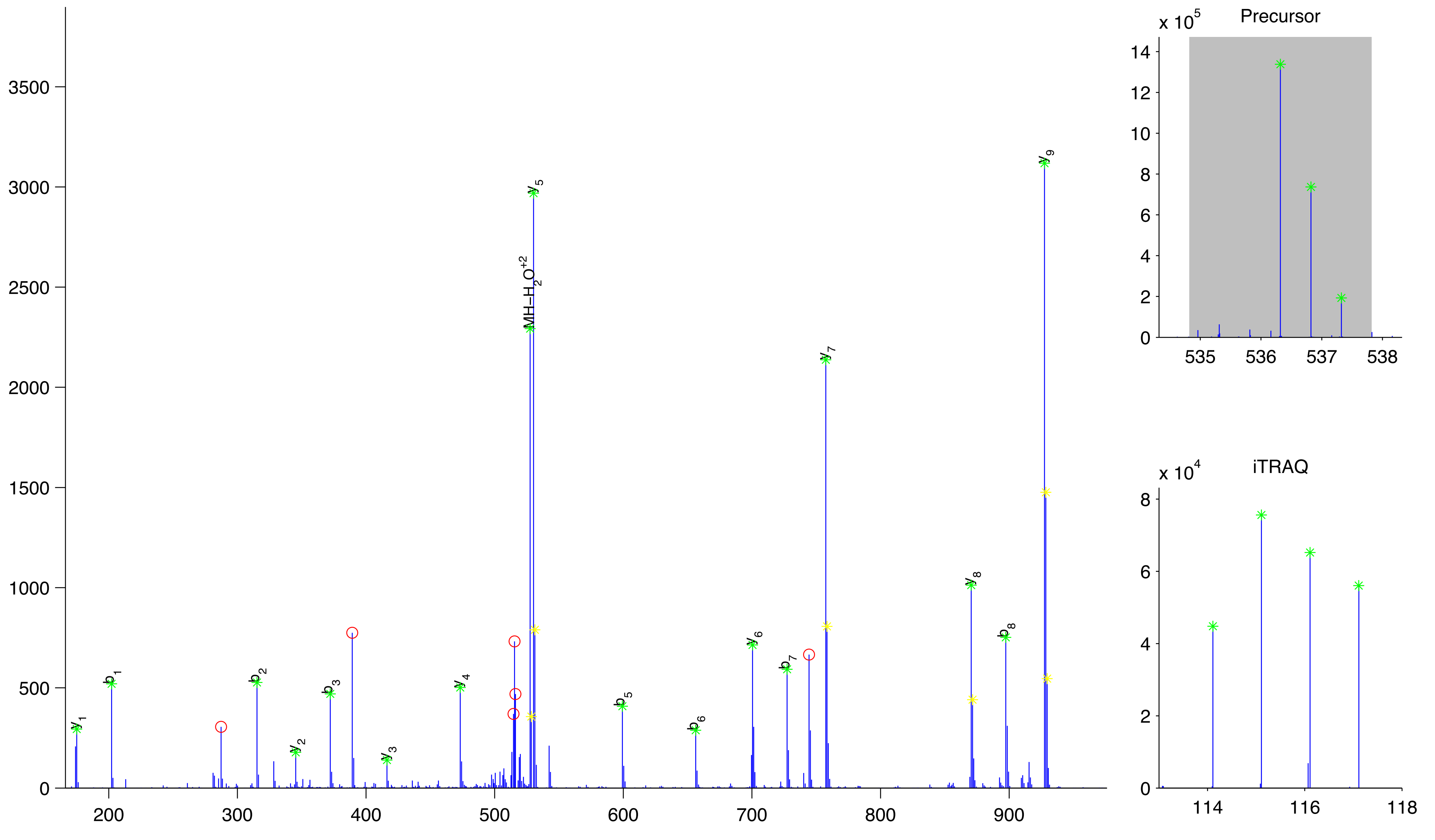

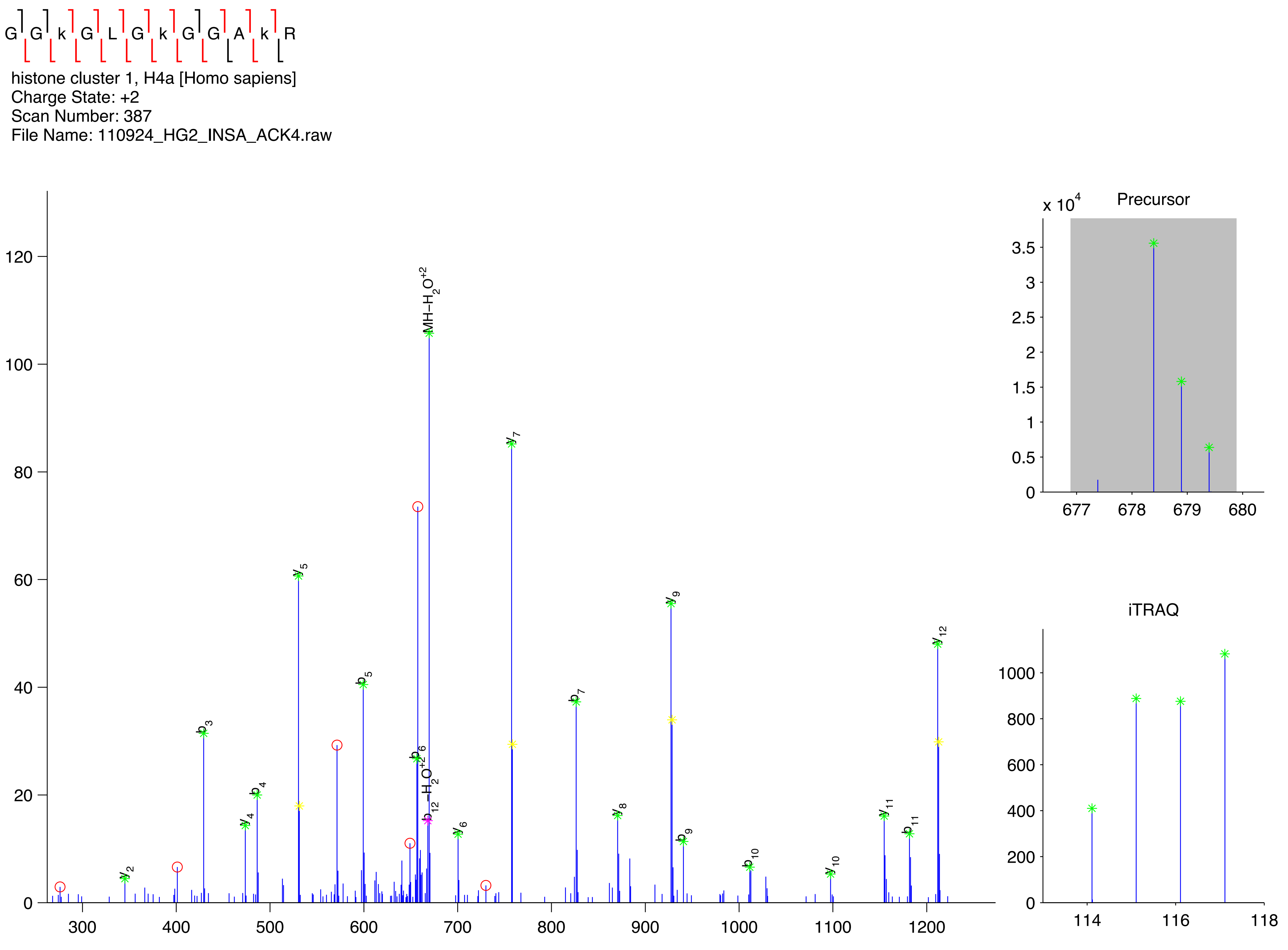
G G k G L G k G G A k R
histone cluster 1, H4a [Homo sapiens]
Charge State: +2
Scan Number: 387
File Name: 110924_HG2_INSA_ACK4.raw
MH-H2O+2
y7
y5
y9
y12
b5
b3
b7
b6+2
b12-H2O+2
b4
y4
y8
y6
y2
b9
y11
b11
b10
y10
x 10^4
Precursor
iTRAQ

G L G k G G A k R

histone cluster 1, H4a [Homo sapiens]
Charge State: +2
Scan Number: 509
File Name: 110924_HG2_INSA_ACK4.raw

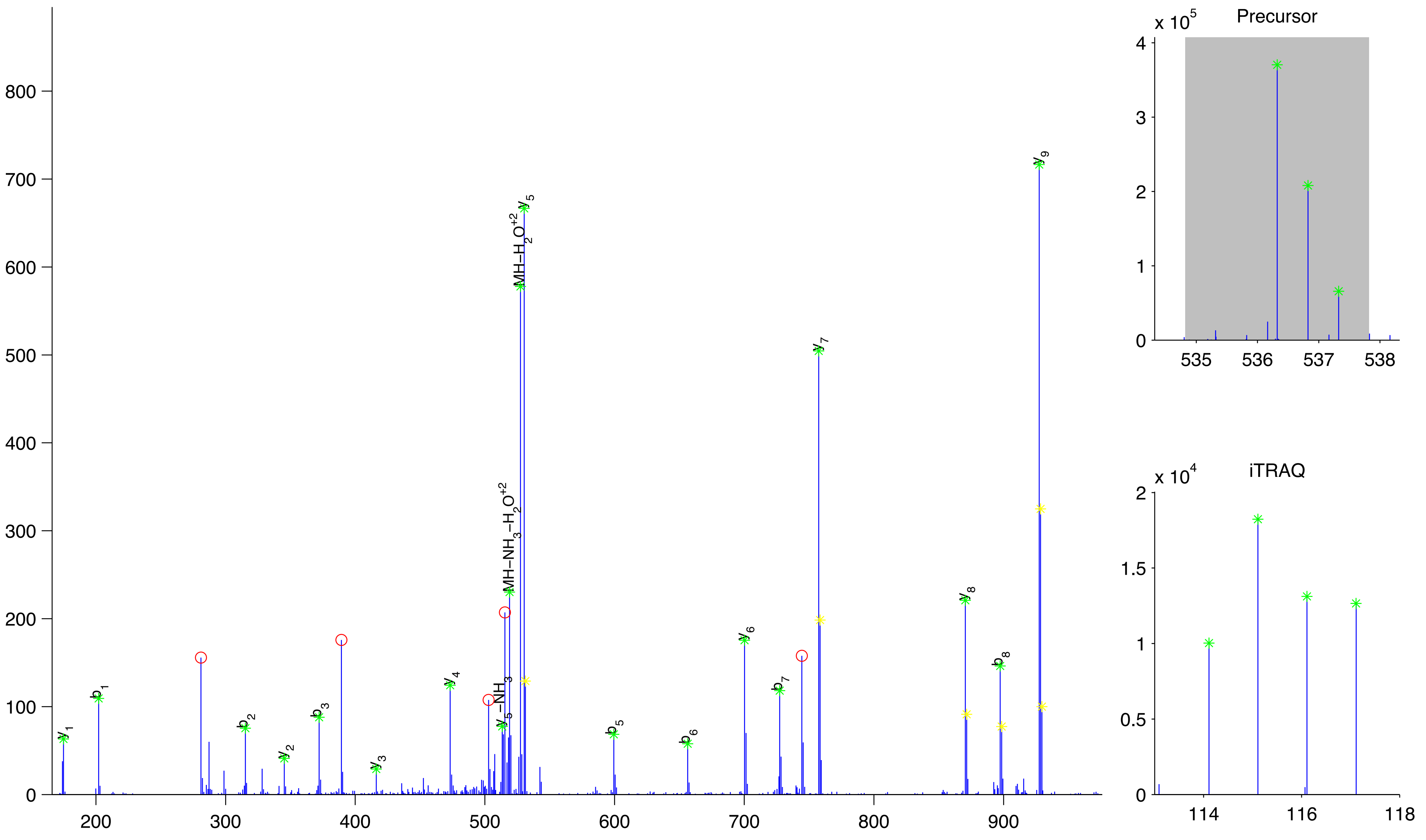

G L G k G G A k R

histone cluster 1, H4a [Homo sapiens]
Charge State: +2
Scan Number: 524
File Name: 110924_HG2_INSA_ACK3.raw

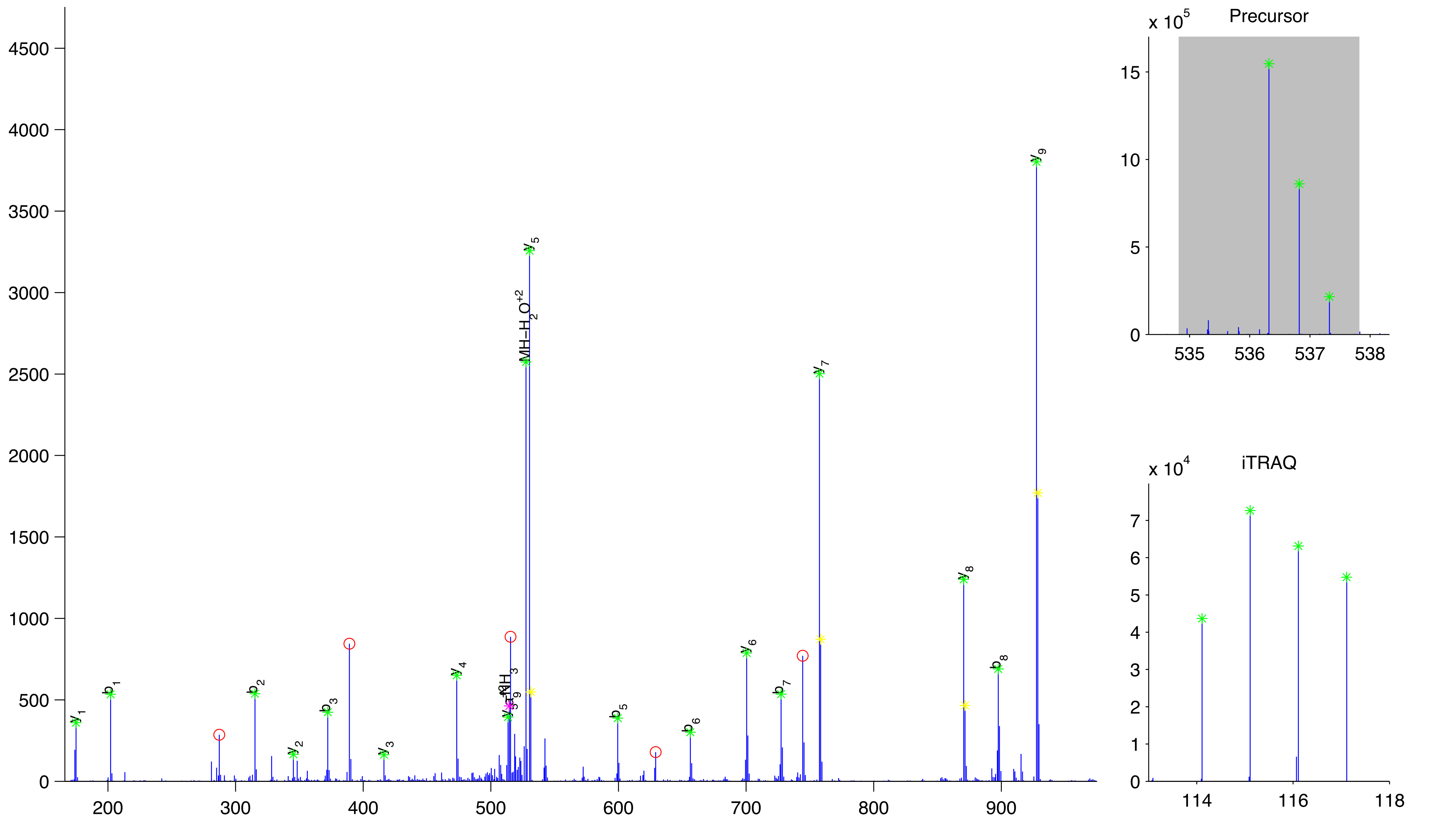

G L G k G G A k R

histone cluster 1, H4a [Homo sapiens]
Charge State: +2
Scan Number: 538
File Name: 110924_HG2_INSA_ACK3.raw

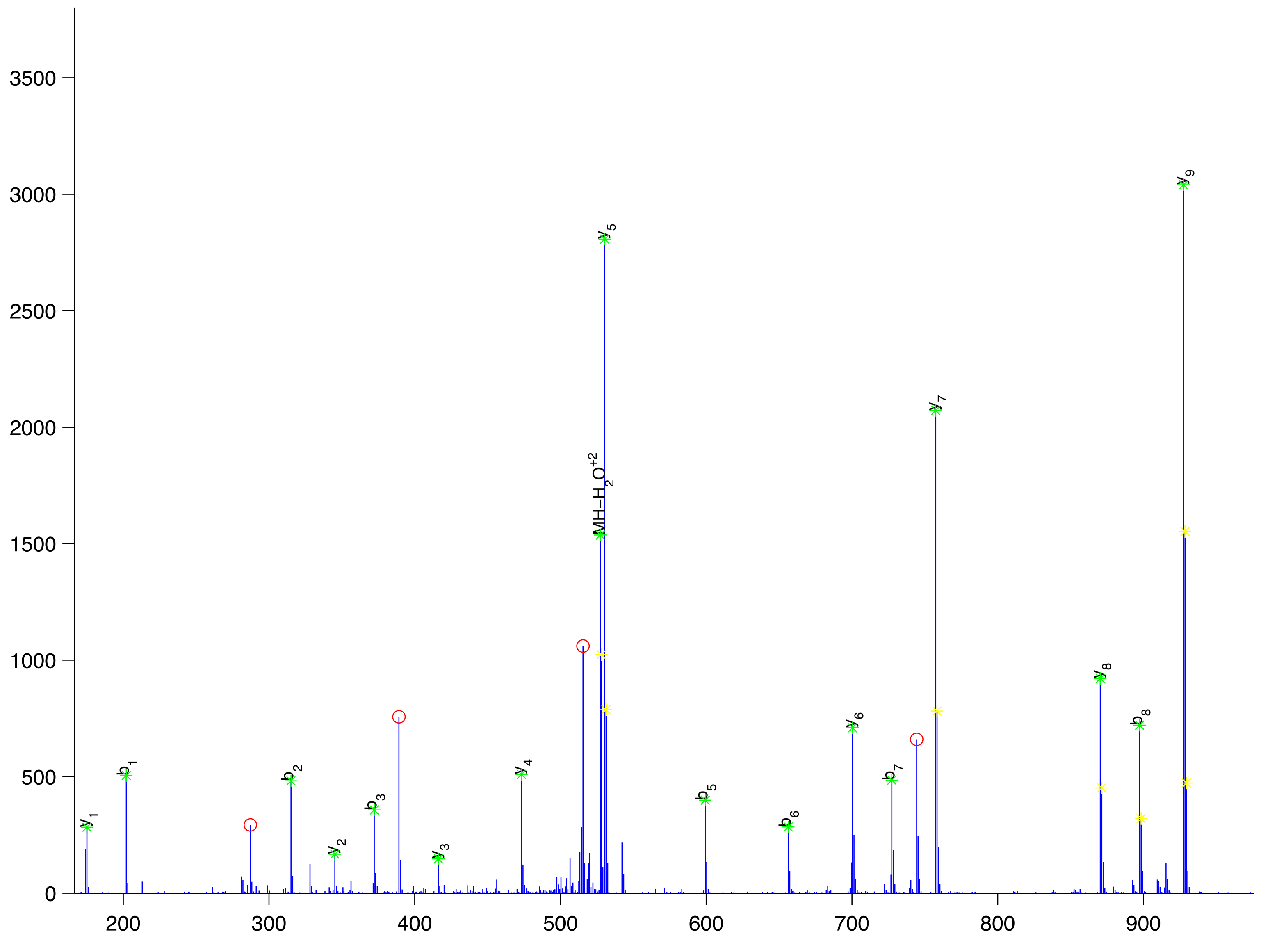

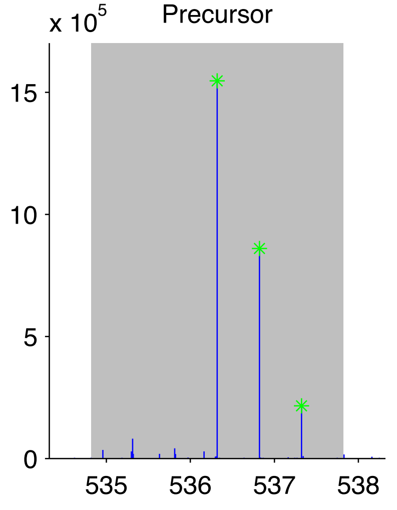

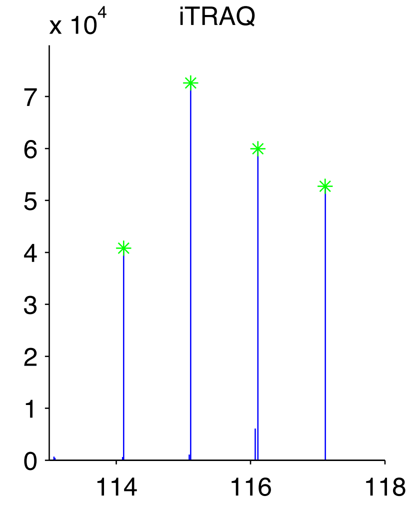

G G k G L G k G G A k R

histone cluster 1, H4a [Homo sapiens]
Charge State: +2
Scan Number: 549
File Name: 110924_HG2_INSA_ACK4.raw

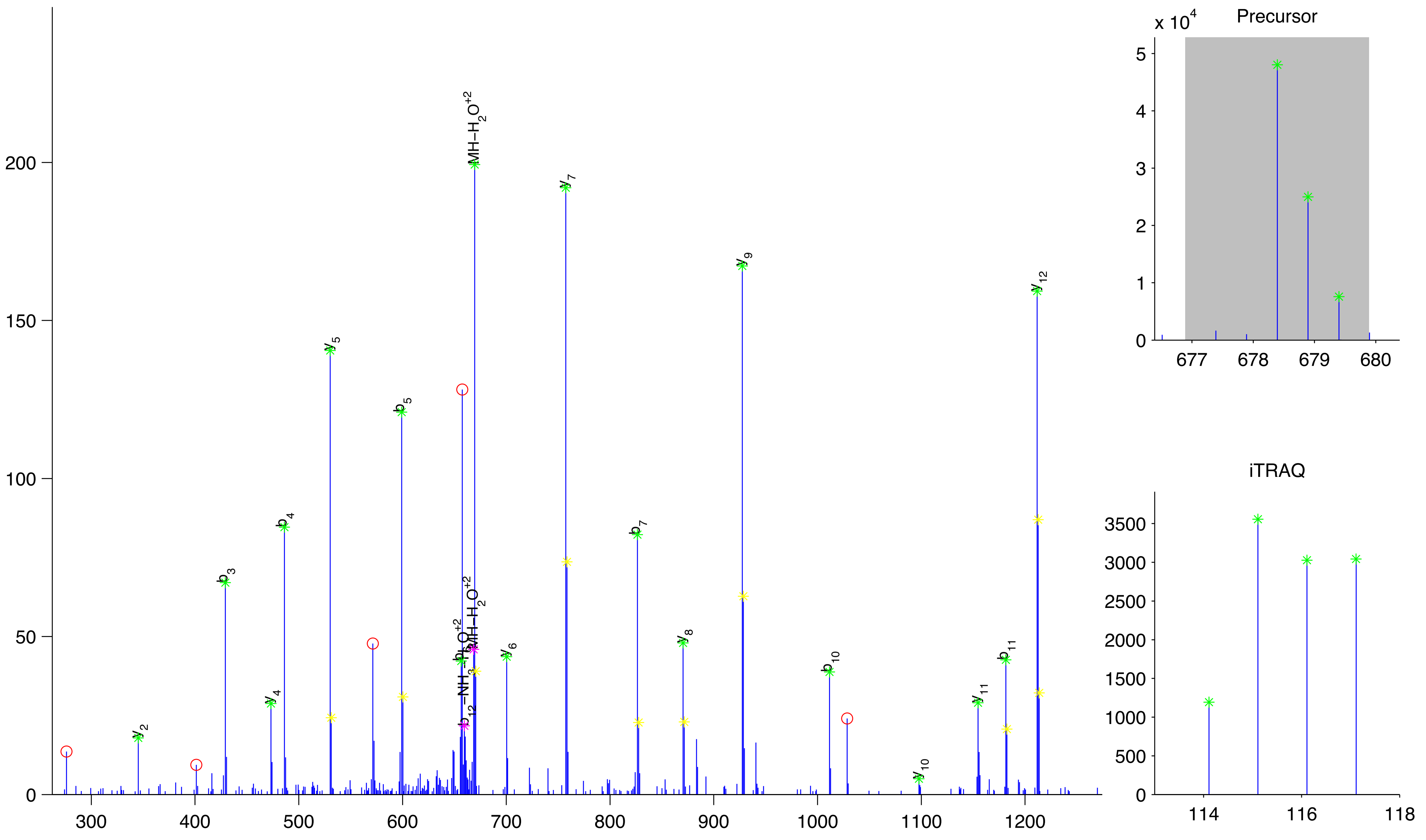

G L G k G G A k R

histone cluster 1, H4a [Homo sapiens]
Charge State: +2
Scan Number: 597
File Name: 110924_HG2_INSA_ACK4.raw

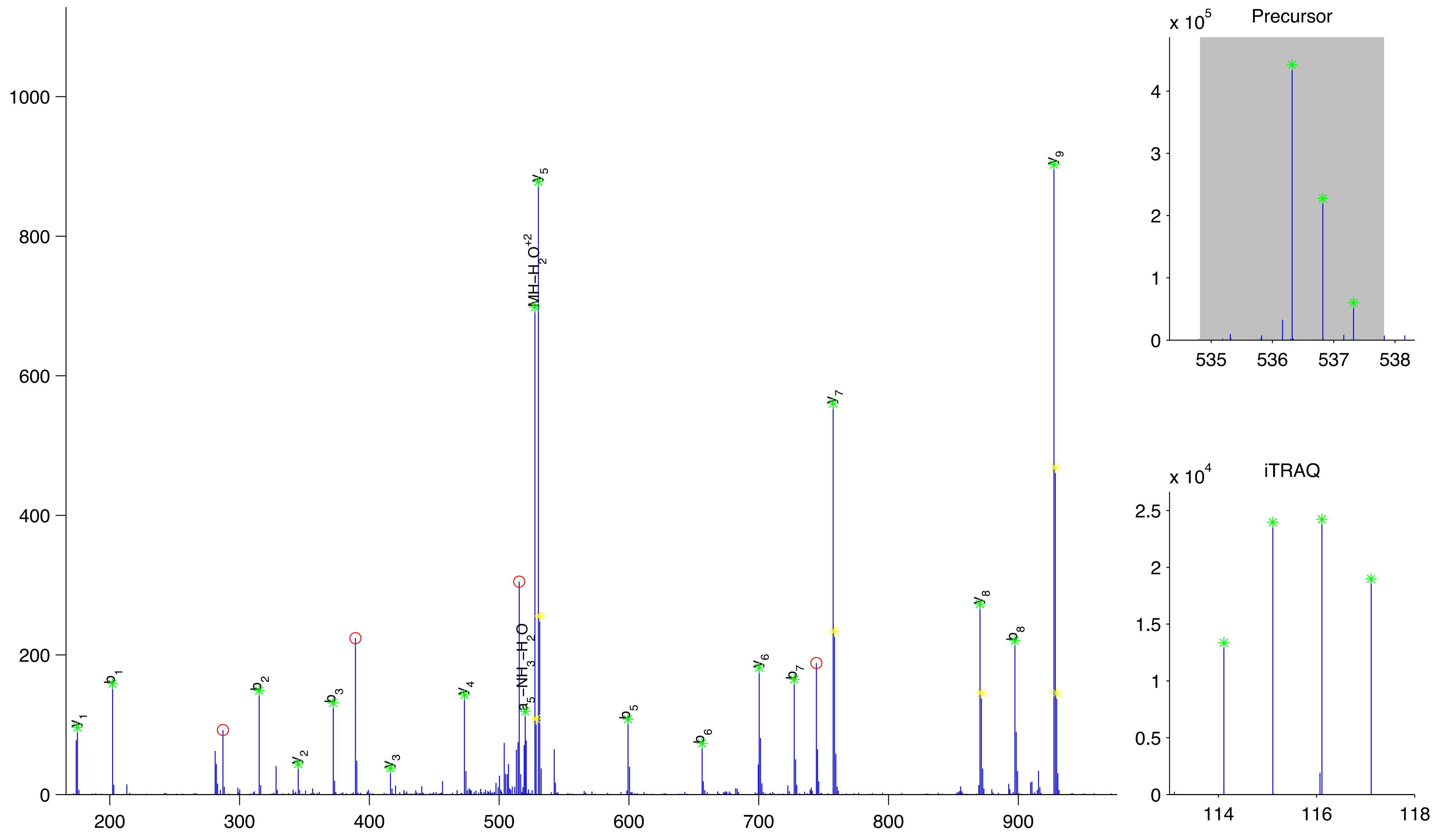

G L G k G G A k R

histone cluster 1, H4a [Homo sapiens]
Charge State: +2
Scan Number: 677
File Name: 110924_HG2_INSA_ACK4.raw

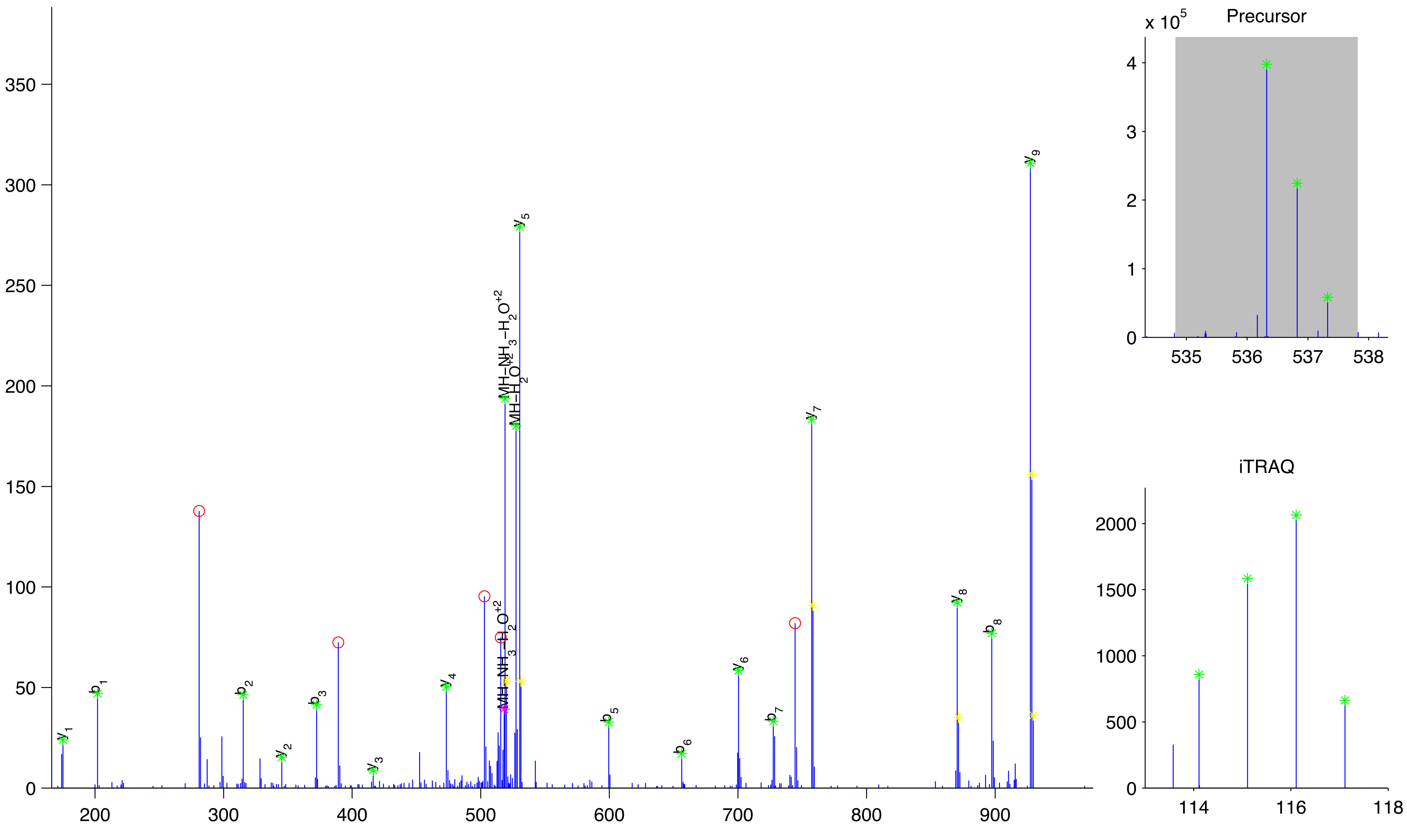

G L G k G G A k R

histone cluster 1, H4a [Homo sapiens]
Charge State: +2
Scan Number: 860
File Name: 110924_HG2_INSA_ACK3.raw

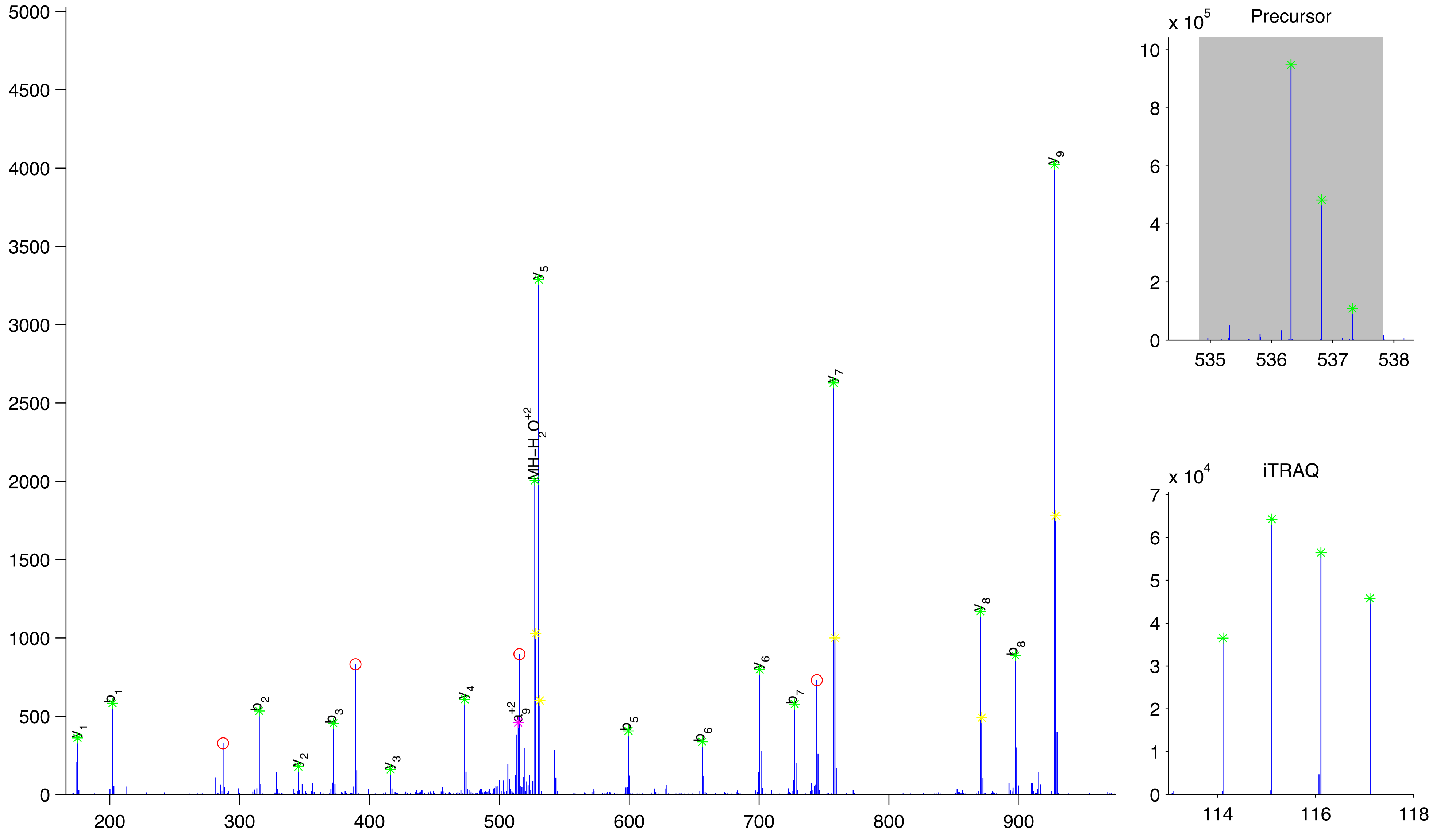

G L G k G G A k R

histone cluster 1, H4a [Homo sapiens]
Charge State: +2
Scan Number: 874
File Name: 110924_HG2_INSA_ACK3.raw

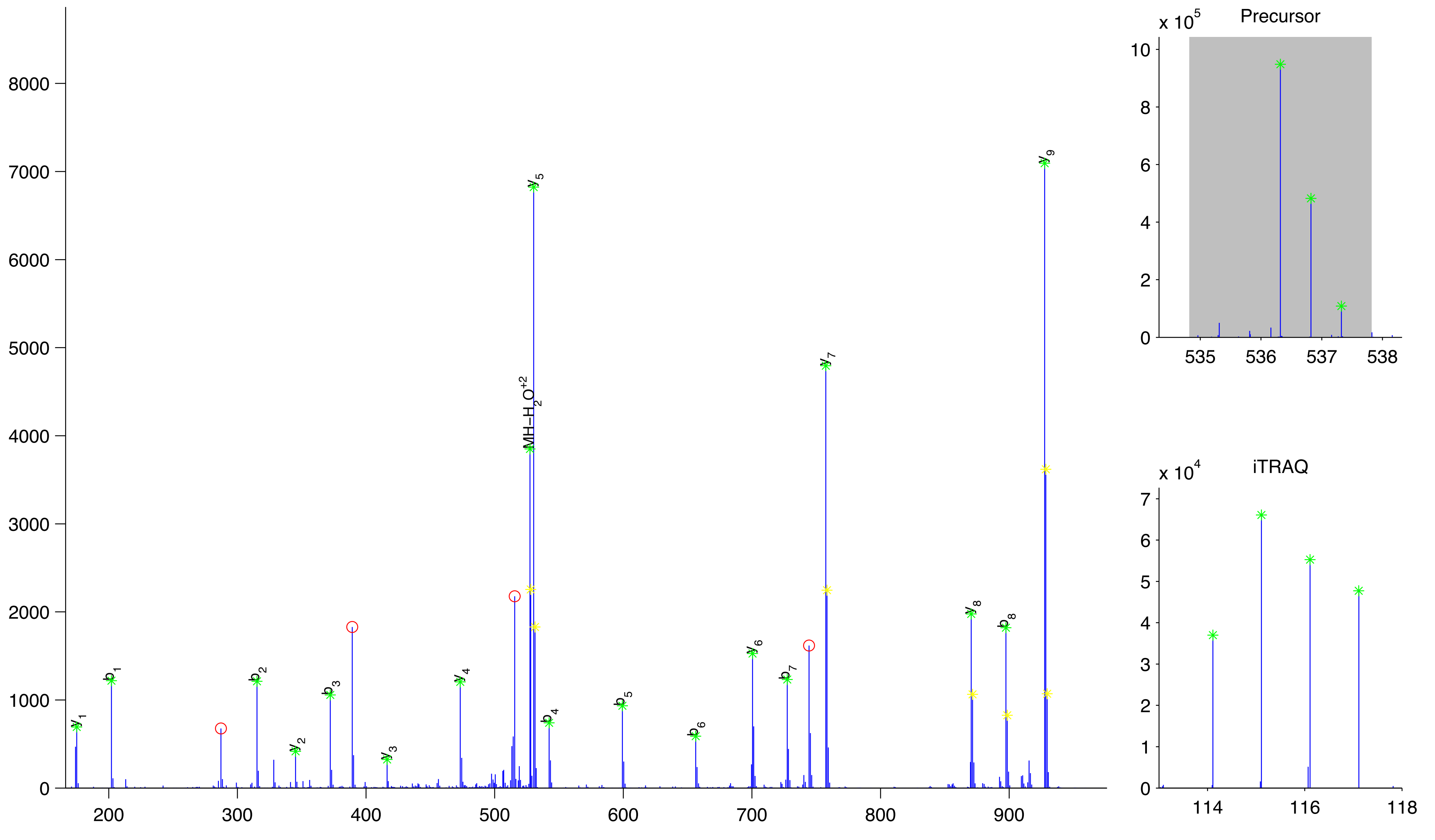

G k G G k G L G k G G A k R

histone cluster 1, H4a [Homo sapiens]
Charge State: +2
Scan Number: 908
File Name: 110924_HG2_INSA_ACK3.raw

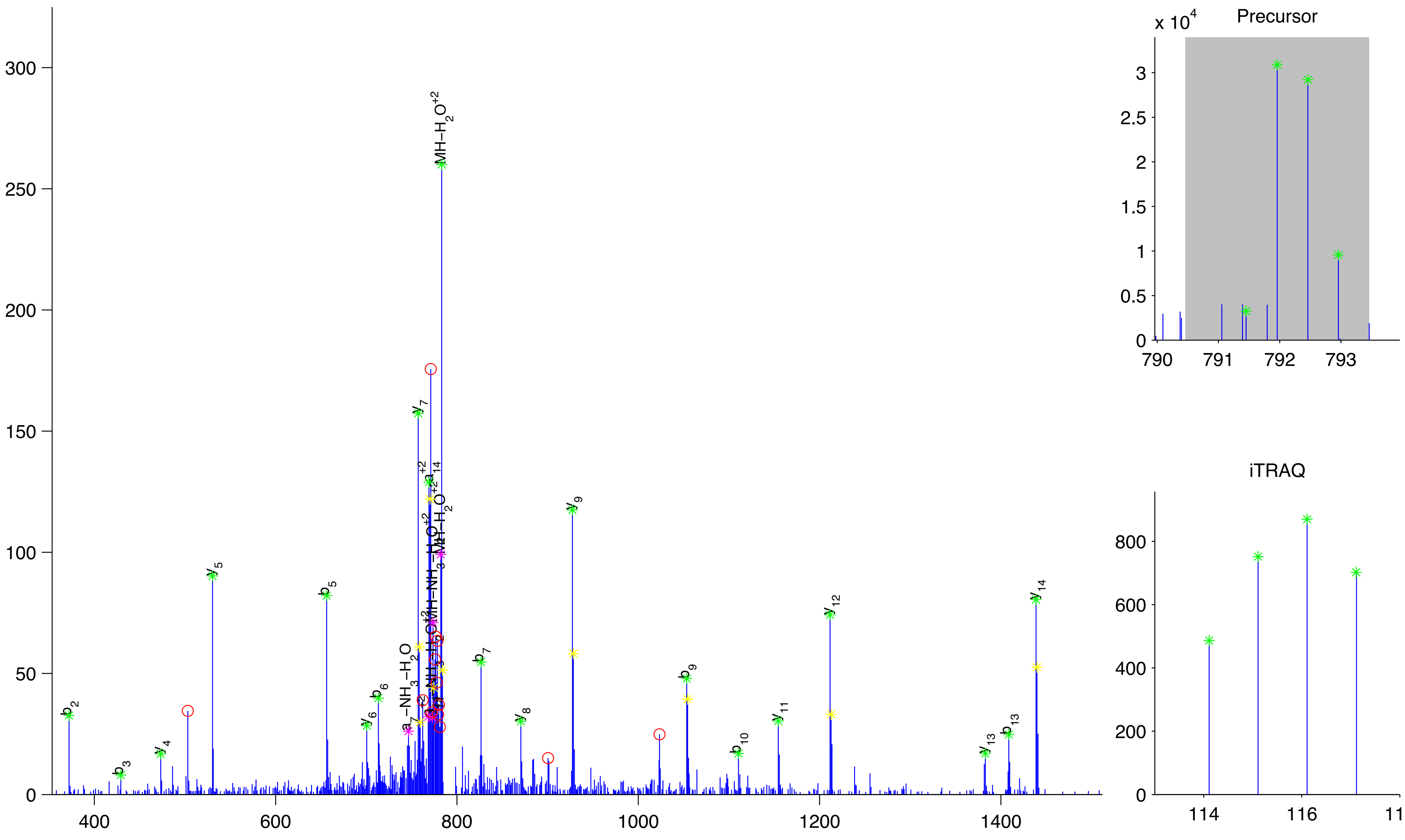

G L G k G G A k R

histone cluster 1, H4a [Homo sapiens]
Charge State: +2
Scan Number: 1013
File Name: 110924_HG2_INSA_ACK4.raw

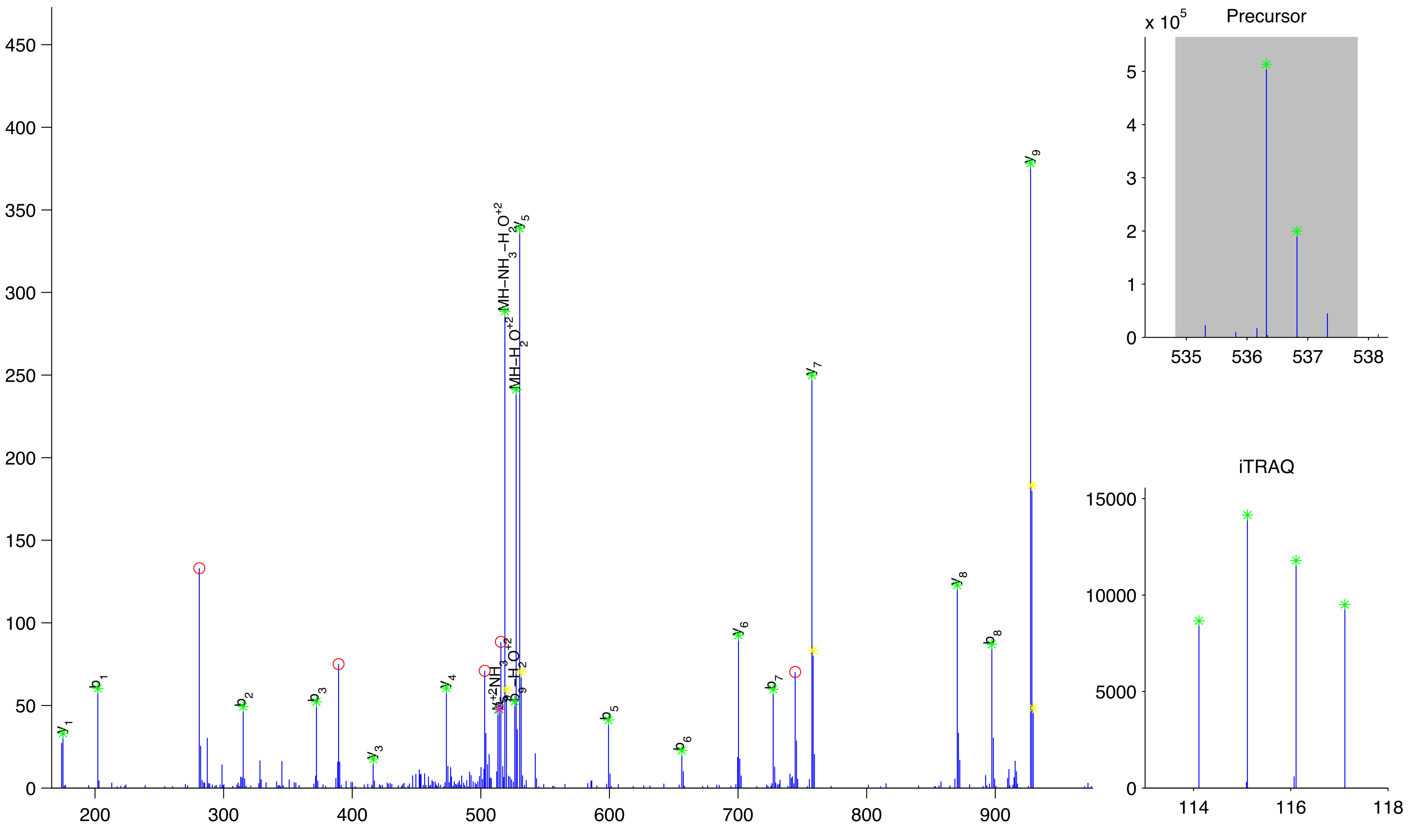

G L G k G G A k R

histone cluster 1, H4a [Homo sapiens]
Charge State: +2
Scan Number: 1028
File Name: 110924_HG2_INSA_ACK3.raw

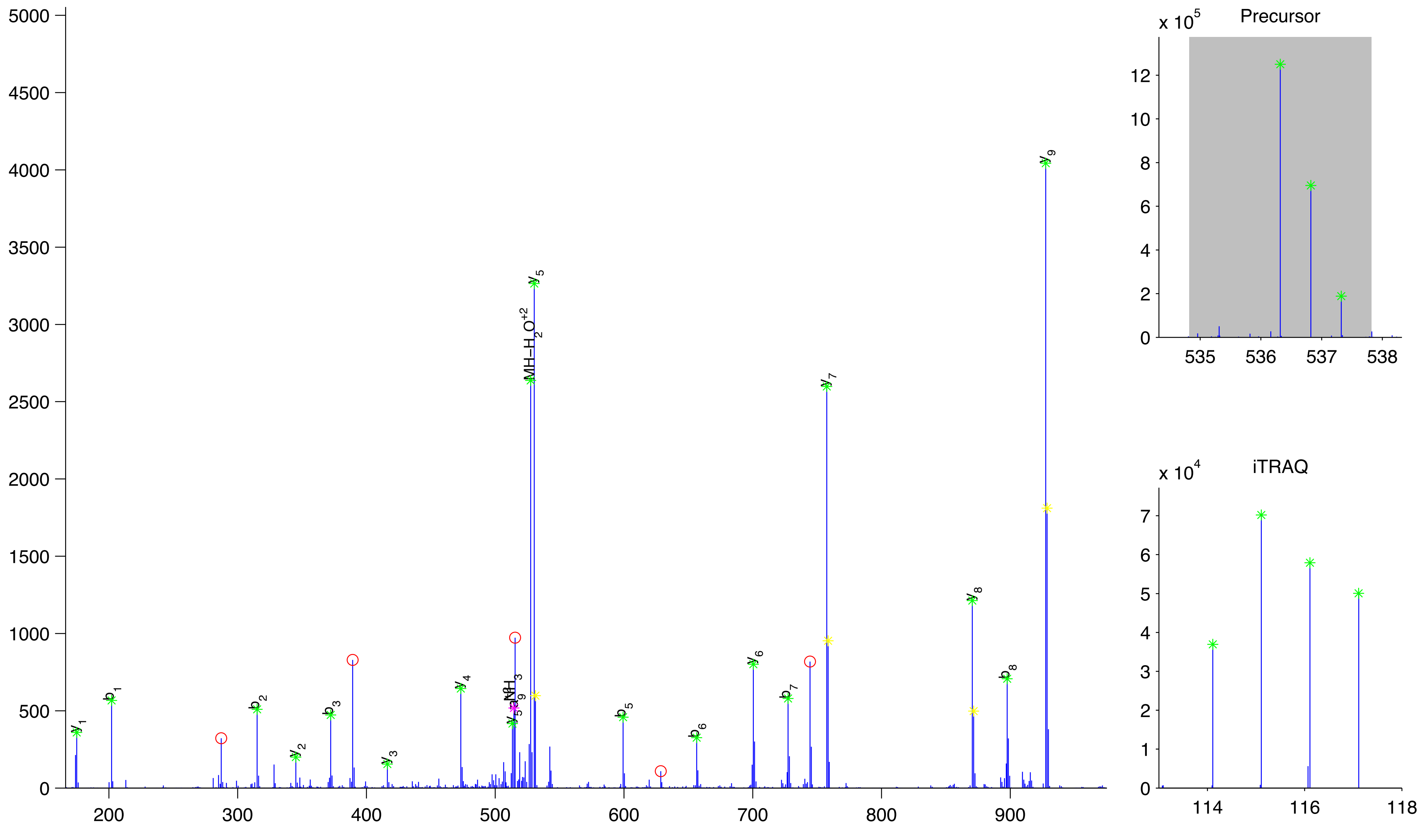

G L G k G G A k R

histone cluster 1, H4a [Homo sapiens]
Charge State: +2
Scan Number: 1149
File Name: 110924_HG2_INSA_ACK4.raw

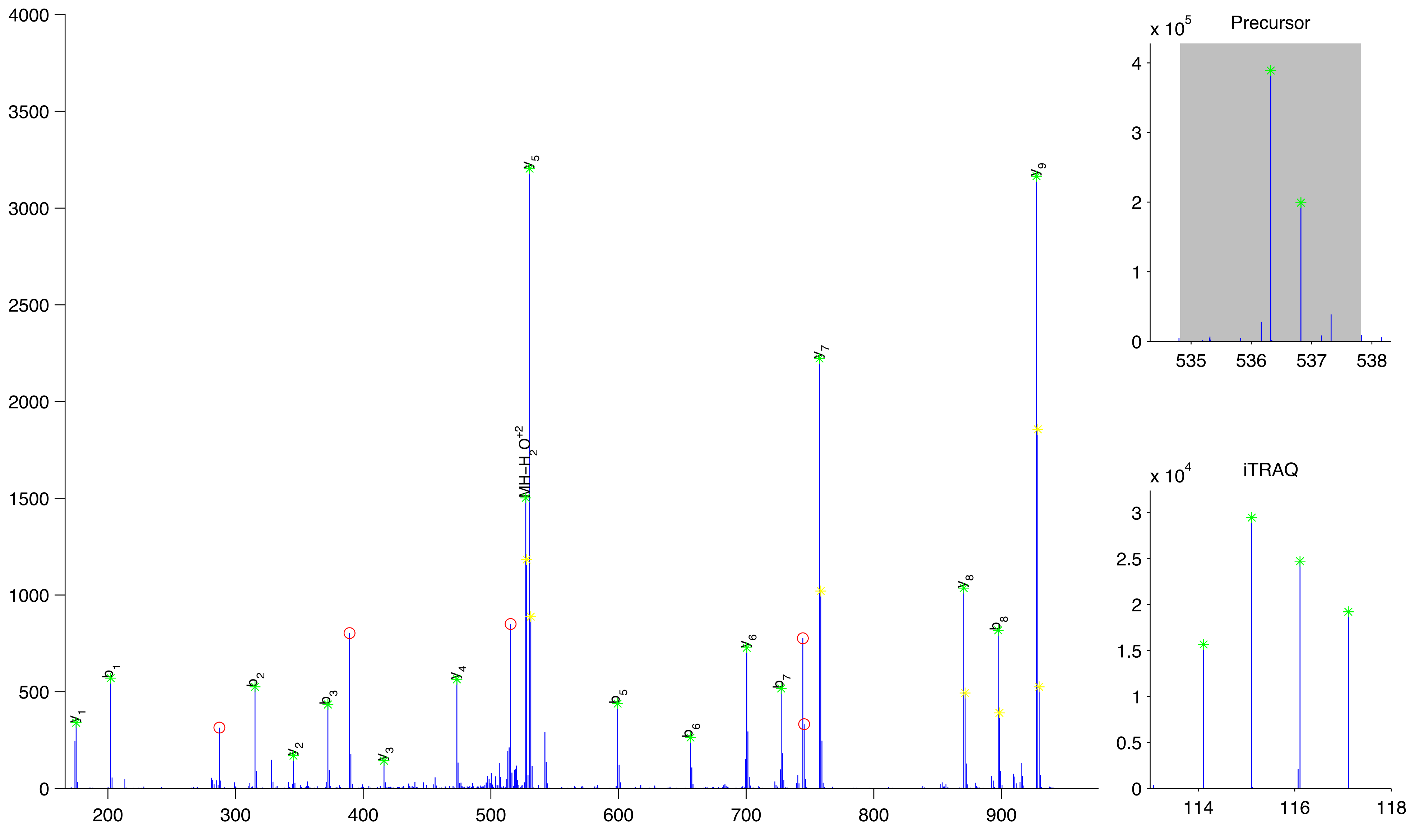

G L G k G G A k R

histone cluster 1, H4a [Homo sapiens]
Charge State: +2
Scan Number: 1181
File Name: 110924_HG2_INSA_ACK4.raw

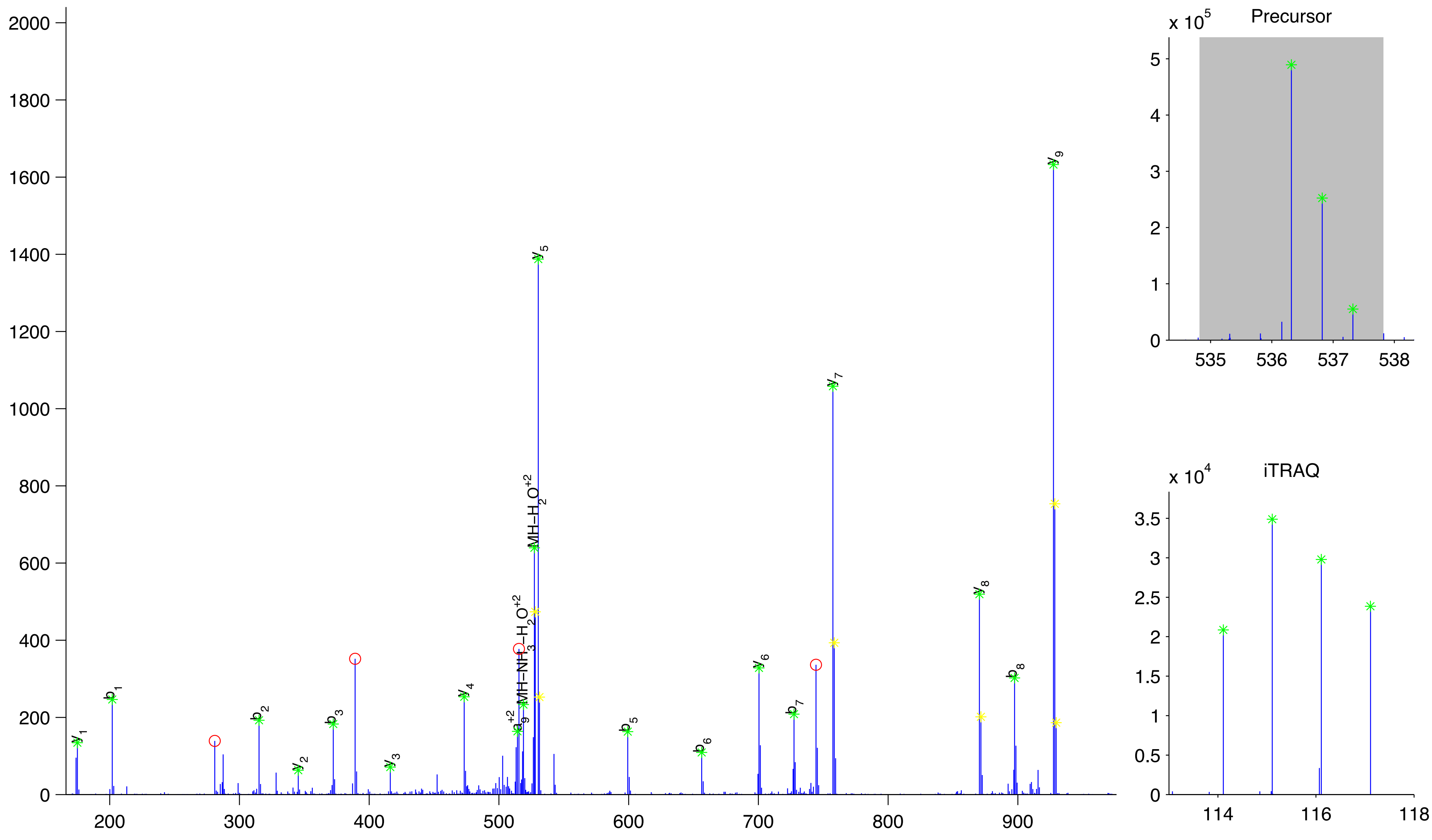

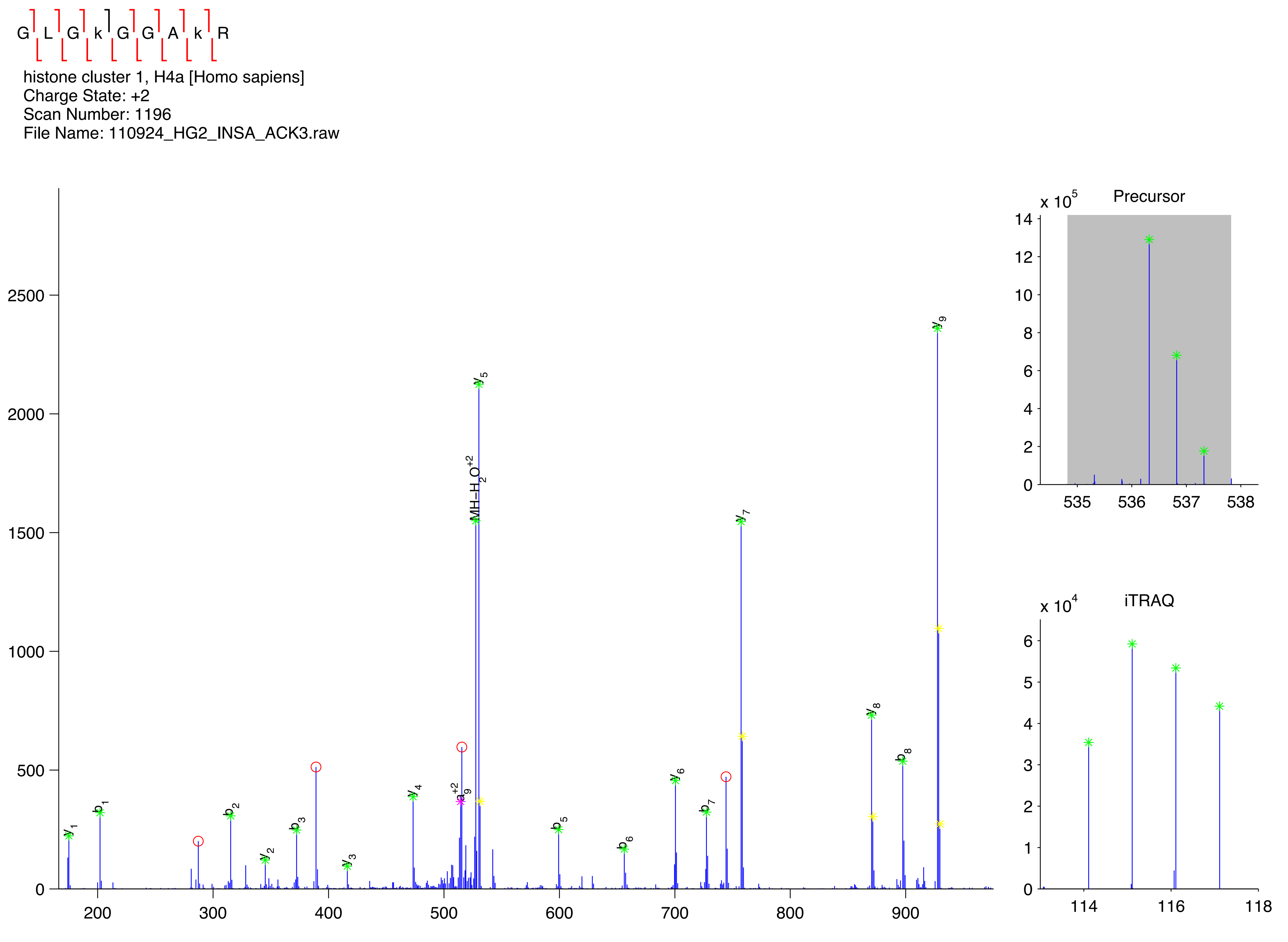
G L G k G G A k R
histone cluster 1, H4a [Homo sapiens]
Charge State: +2
Scan Number: 1196
File Name: 110924_HG2_INSA_ACK3.raw
y1
b1
b2
y2
b3
y3
y4
a9 +2
MH-H2O+2
y5
b5
b6
y6
b7
y7
y8
b8
y9
x 10^5
Precursor
x 10^4
iTRAQ

G G k G L G k G G A k R

histone cluster 1, H4a [Homo sapiens]
Charge State: +2
Scan Number: 1225
File Name: 110924_HG2_INSA_ACK4.raw

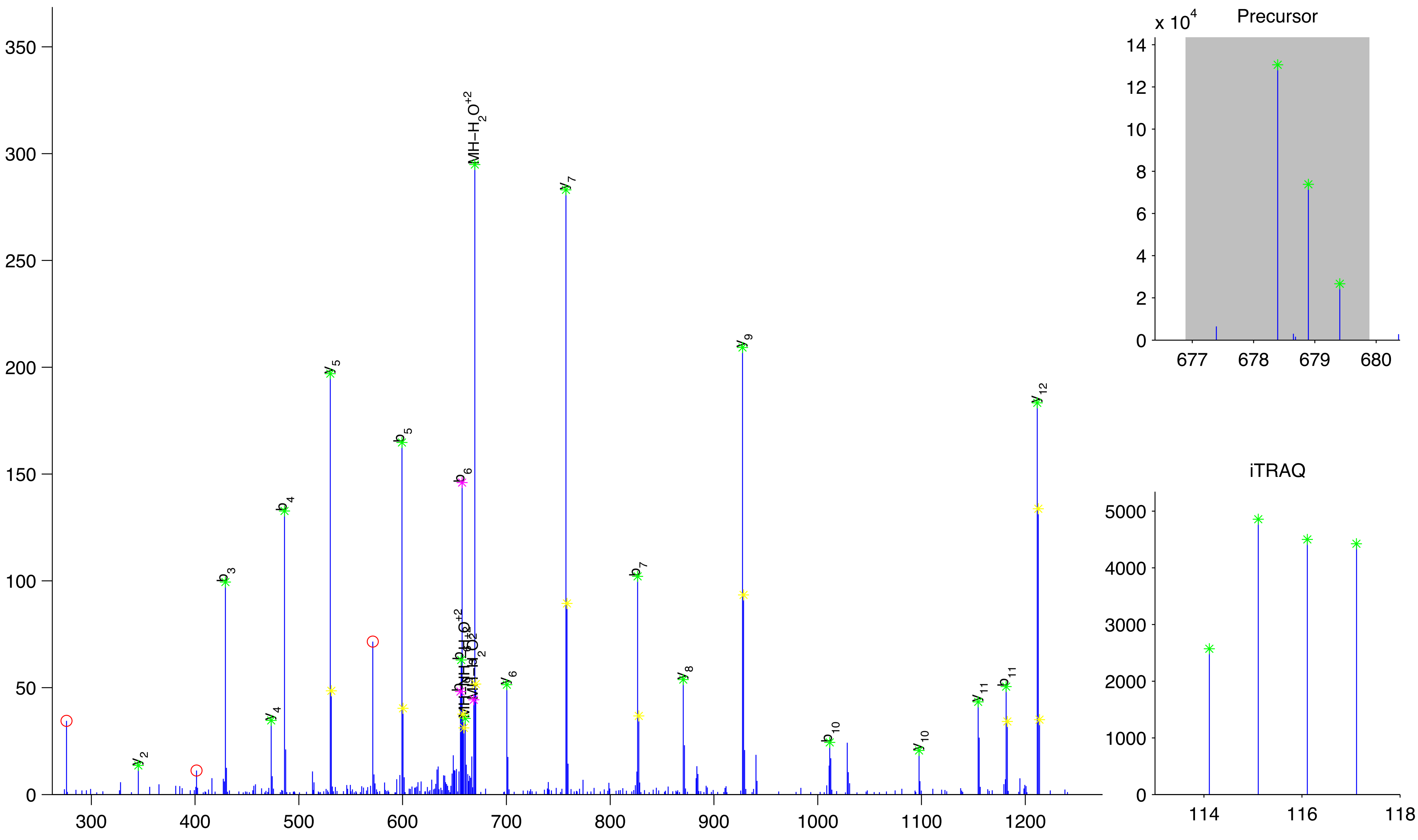

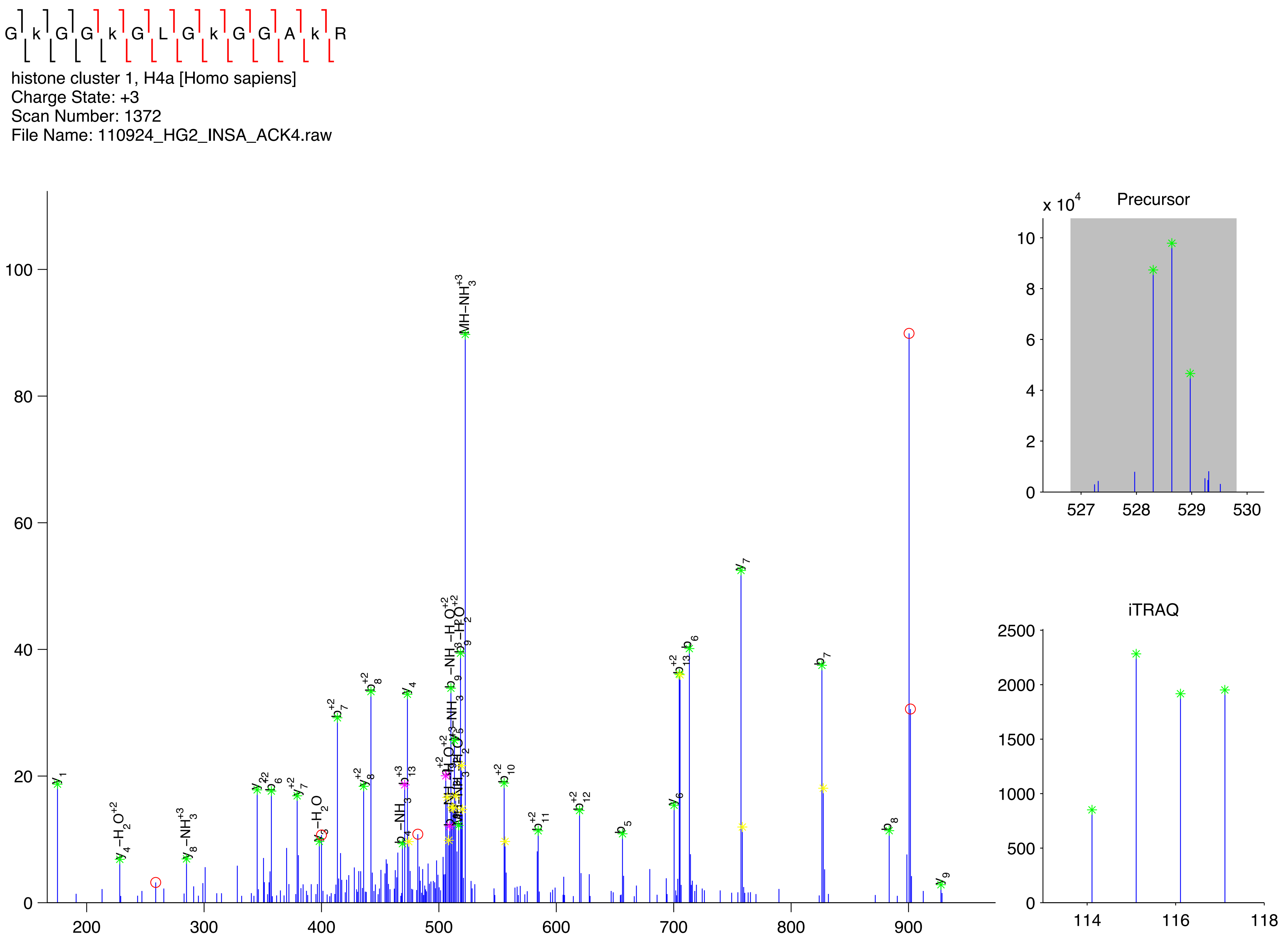
G k G G k G L G k G G A k R
histone cluster 1, H4a [Homo sapiens]
Charge State: +3
Scan Number: 1372
File Name: 110924_HG2_INSA_ACK4.raw
Precursor
x 10^4
iTRAQ
MH−NH3+3

G L G k G G A k R

histone cluster 1, H4a [Homo sapiens]
Charge State: +2
Scan Number: 1403
File Name: 110924_HG2_INSA_ACK3.raw

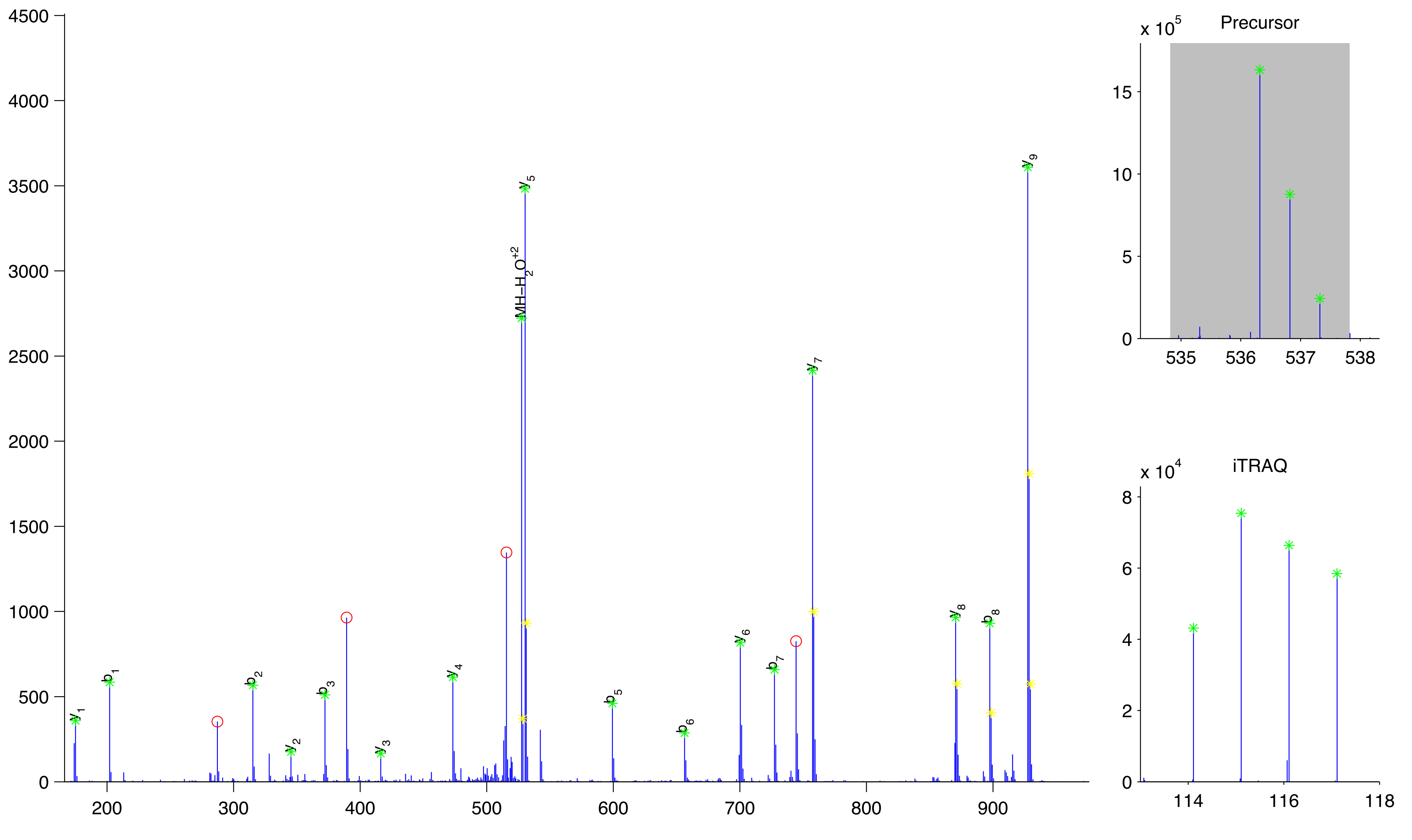

G G k G L G k G G A k R

histone cluster 1, H4a [Homo sapiens]
Charge State: +2
Scan Number: 1435
File Name: 110924_HG2_INSA_ACK4.raw

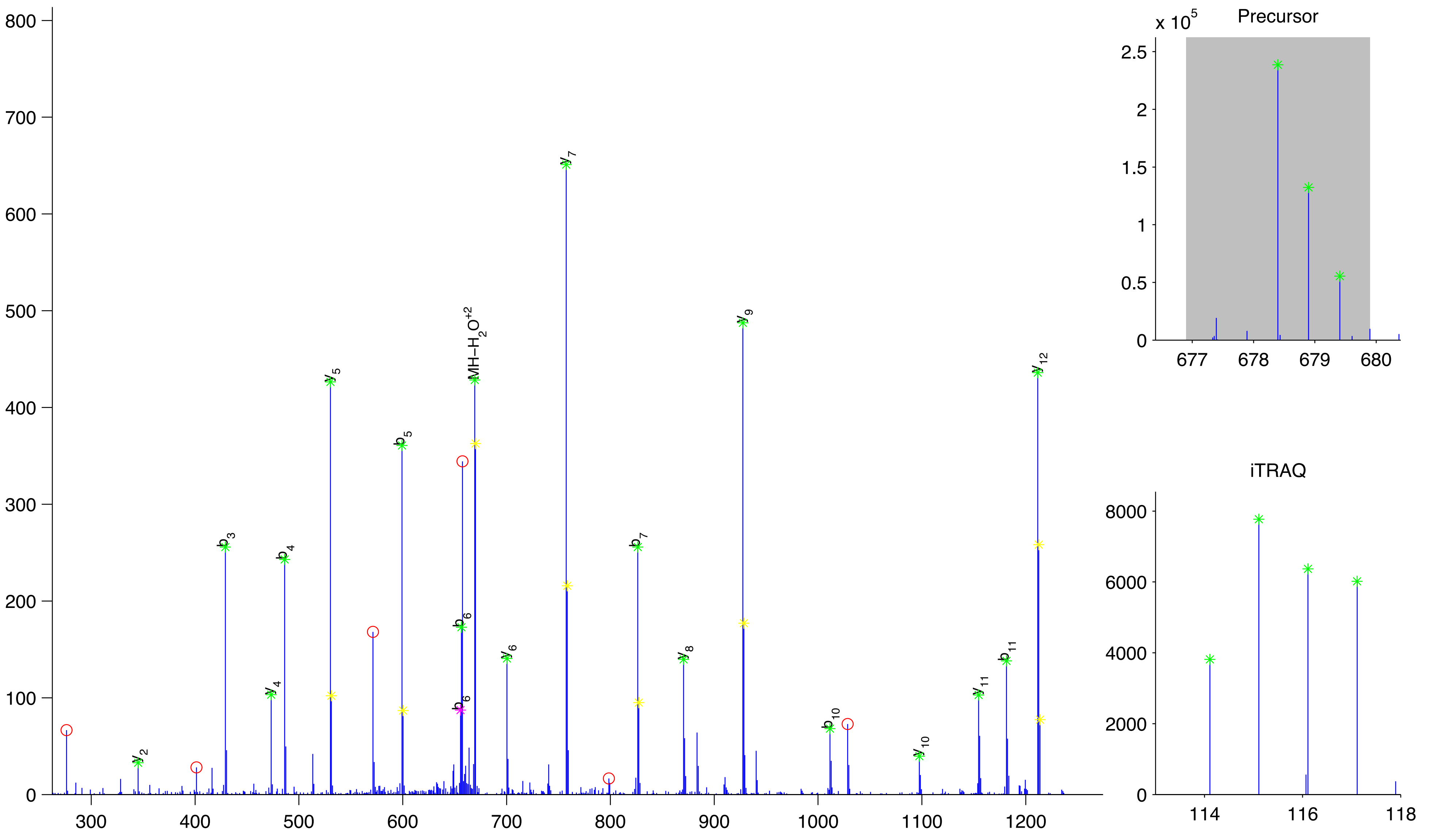

G L G k G G A k R

histone cluster 1, H4a [Homo sapiens]
Charge State: +2
Scan Number: 1508
File Name: 110924_HG2_INSA_ACK4.raw

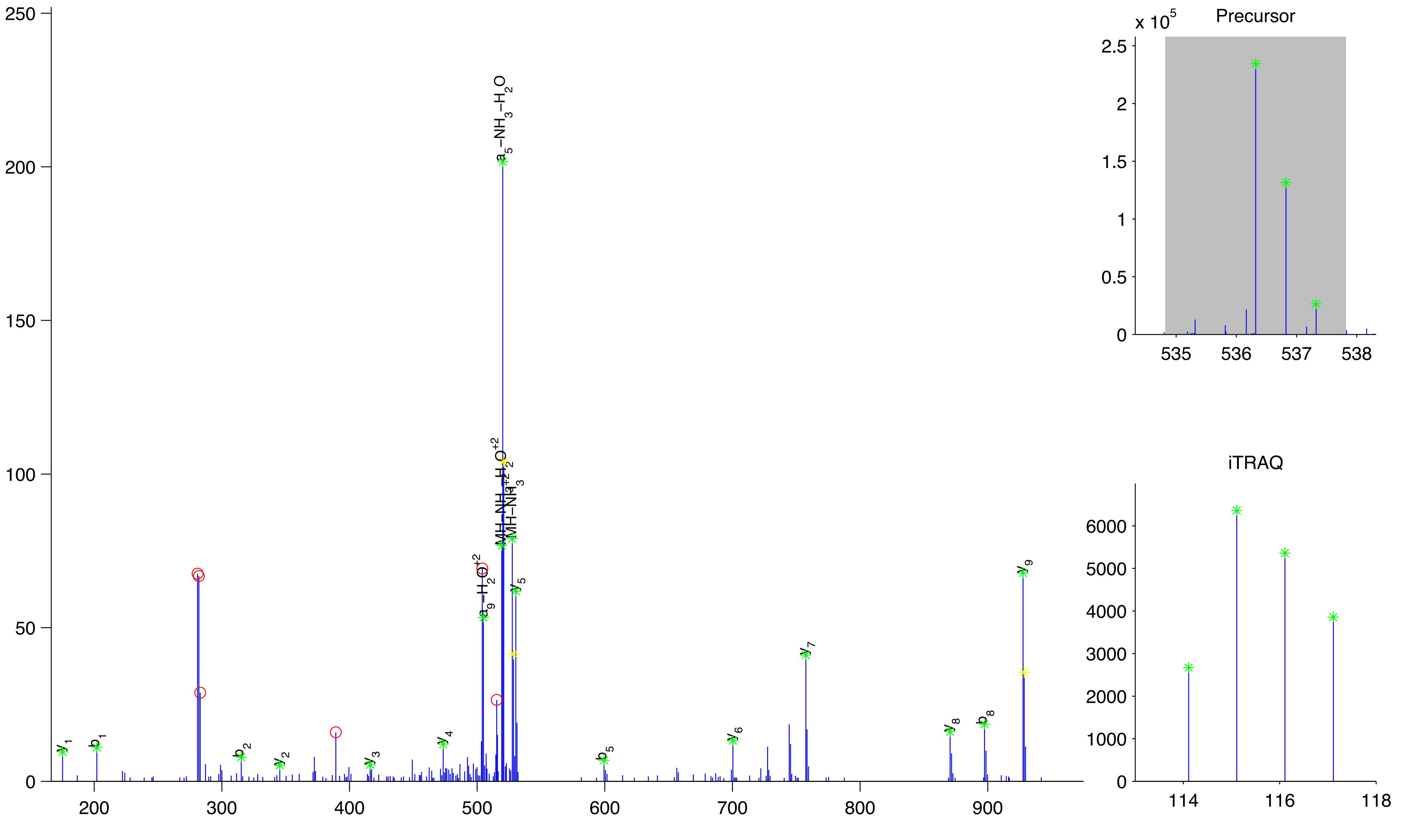

G L G k G G A k R

histone cluster 1, H4a [Homo sapiens]
Charge State: +2
Scan Number: 1532
File Name: 110924_HG2_INSA_ACK3.raw

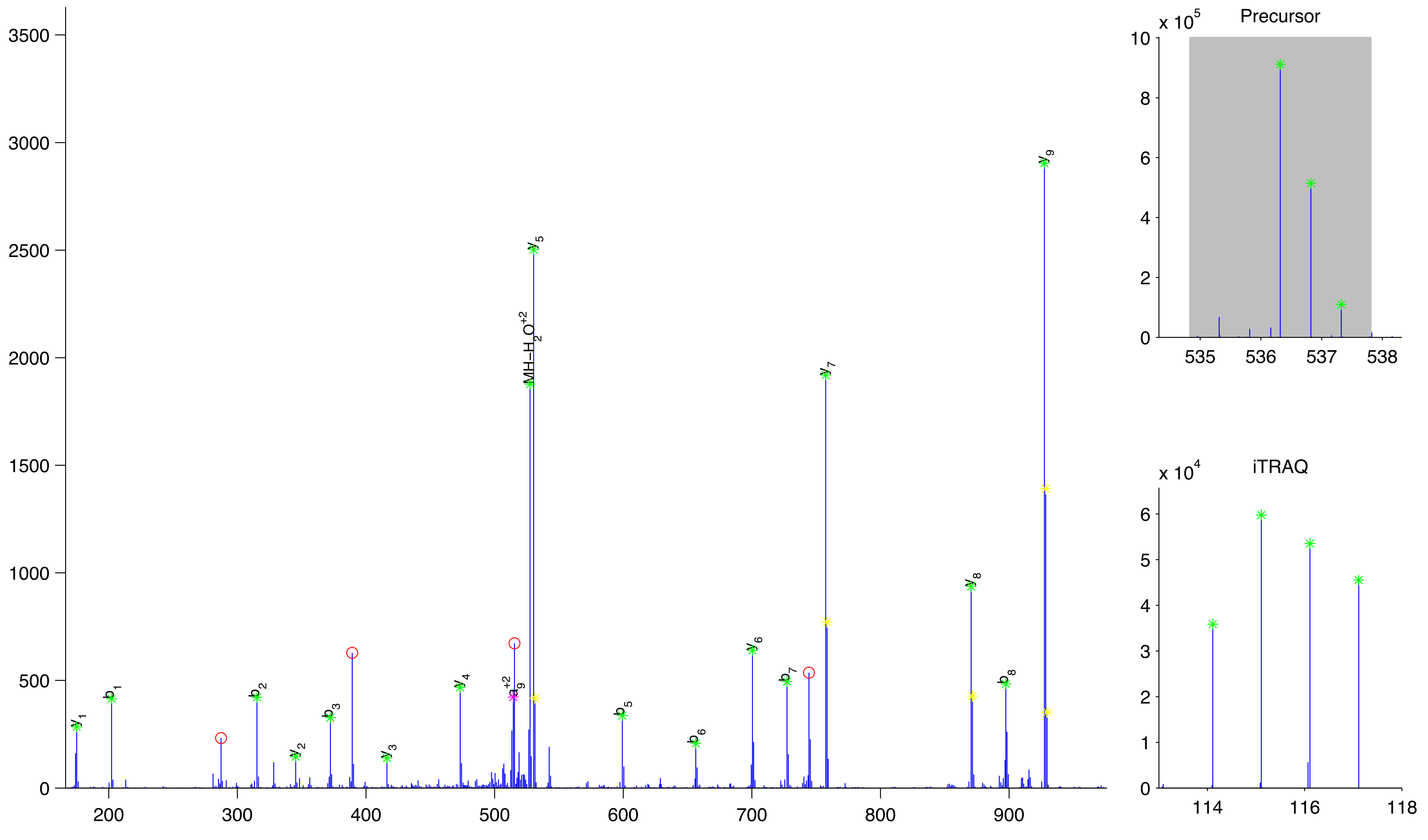

G L G k G G A k R

histone cluster 1, H4a [Homo sapiens]
Charge State: +2
Scan Number: 1569
File Name: 110924_HG2_INSA_ACK3.raw

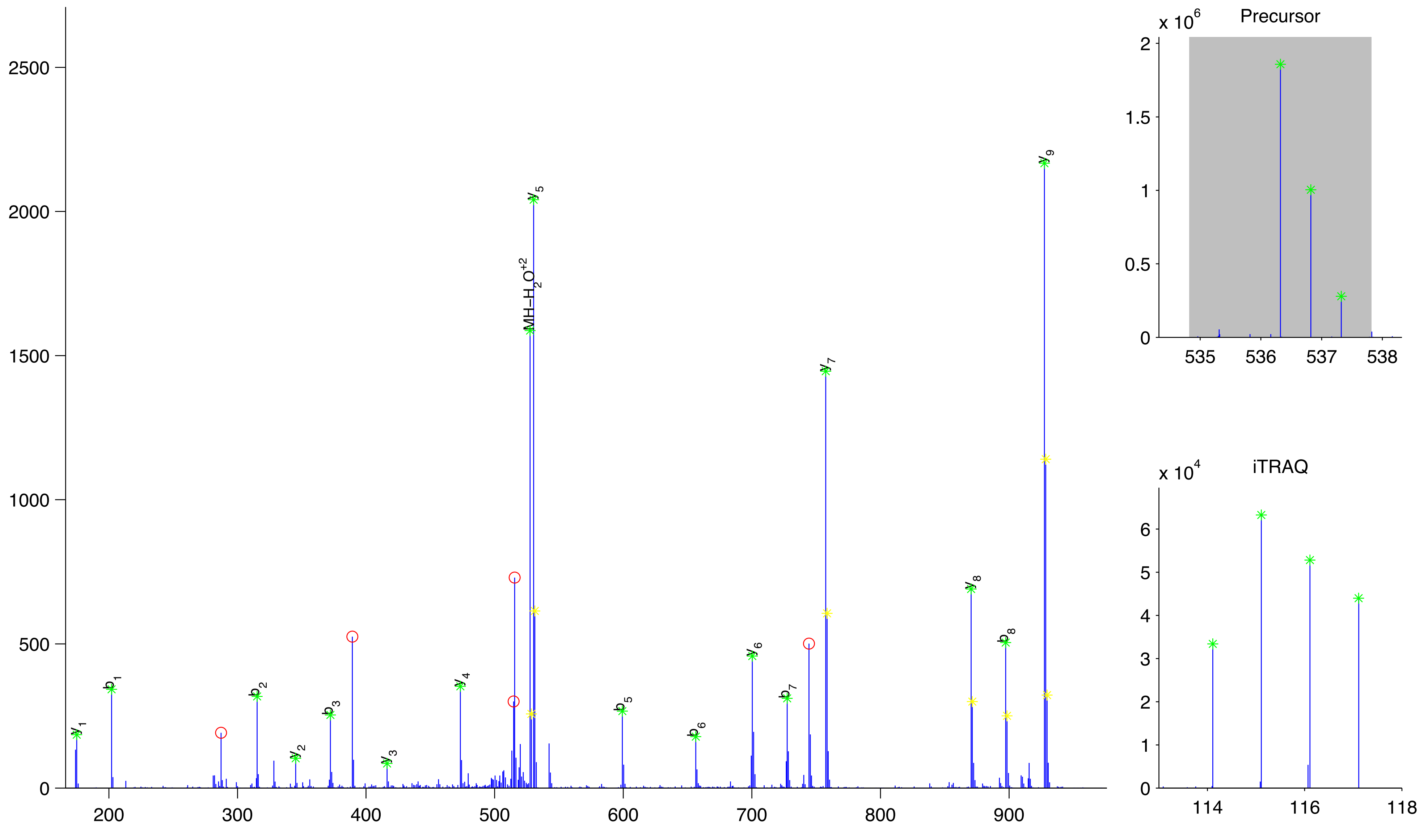

G G k G L G k G G A k R

histone cluster 1, H4a [Homo sapiens]
Charge State: +2
Scan Number: 1599
File Name: 110924_HG2_INSA_ACK4.raw

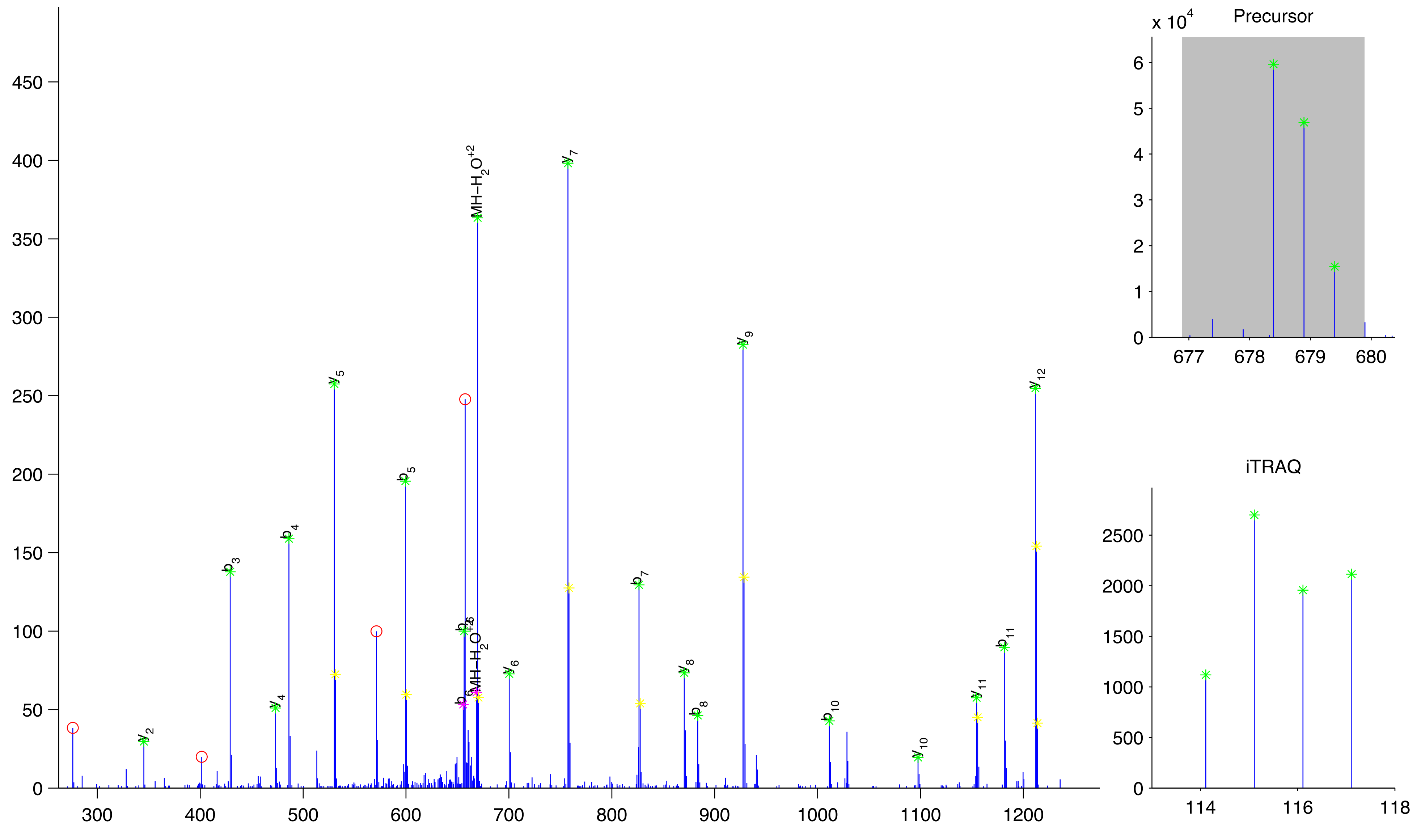

G L G k G G A k R

histone cluster 1, H4a [Homo sapiens]
Charge State: +2
Scan Number: 1700
File Name: 110924_HG2_INSA_ACK3.raw

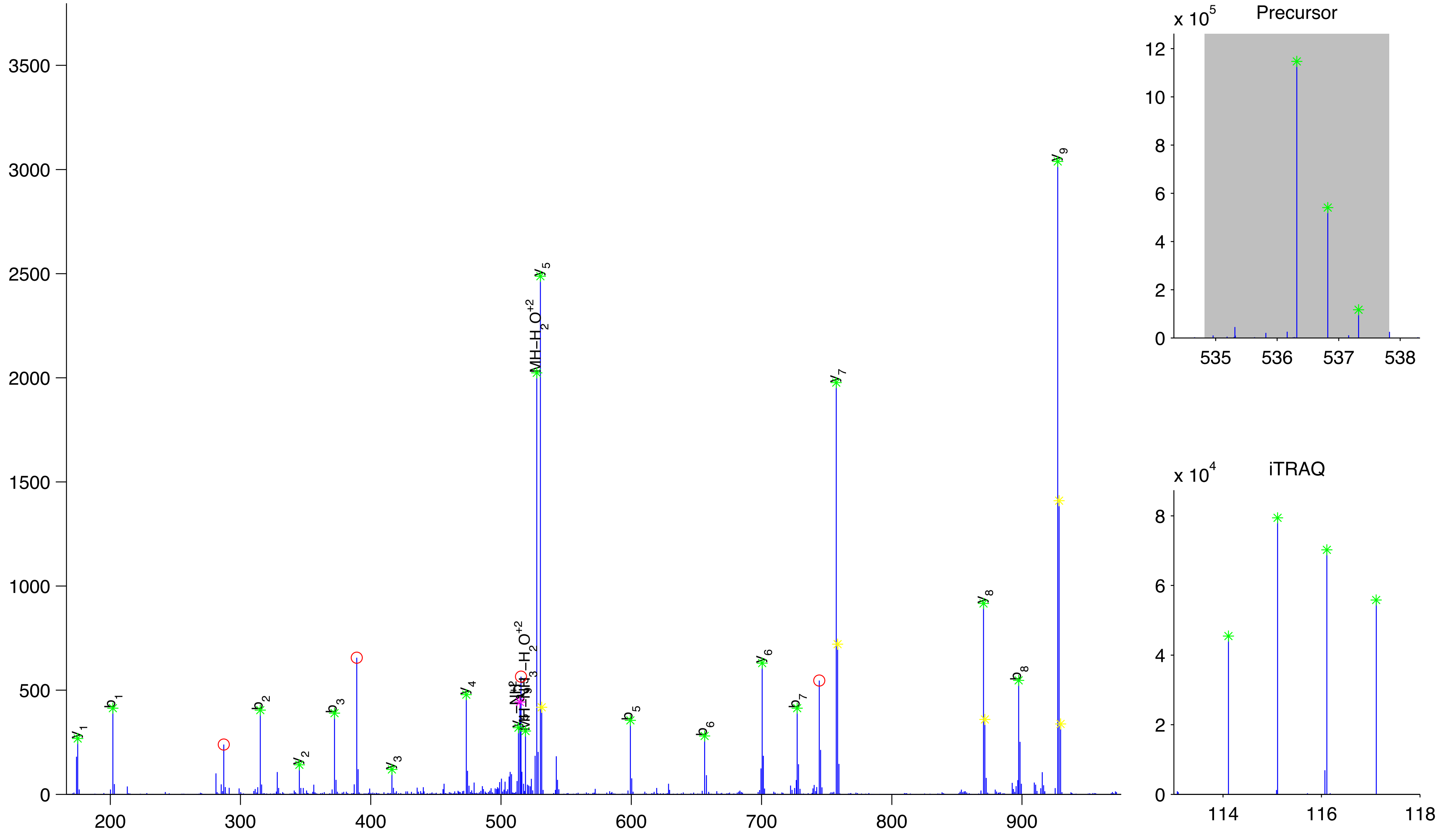

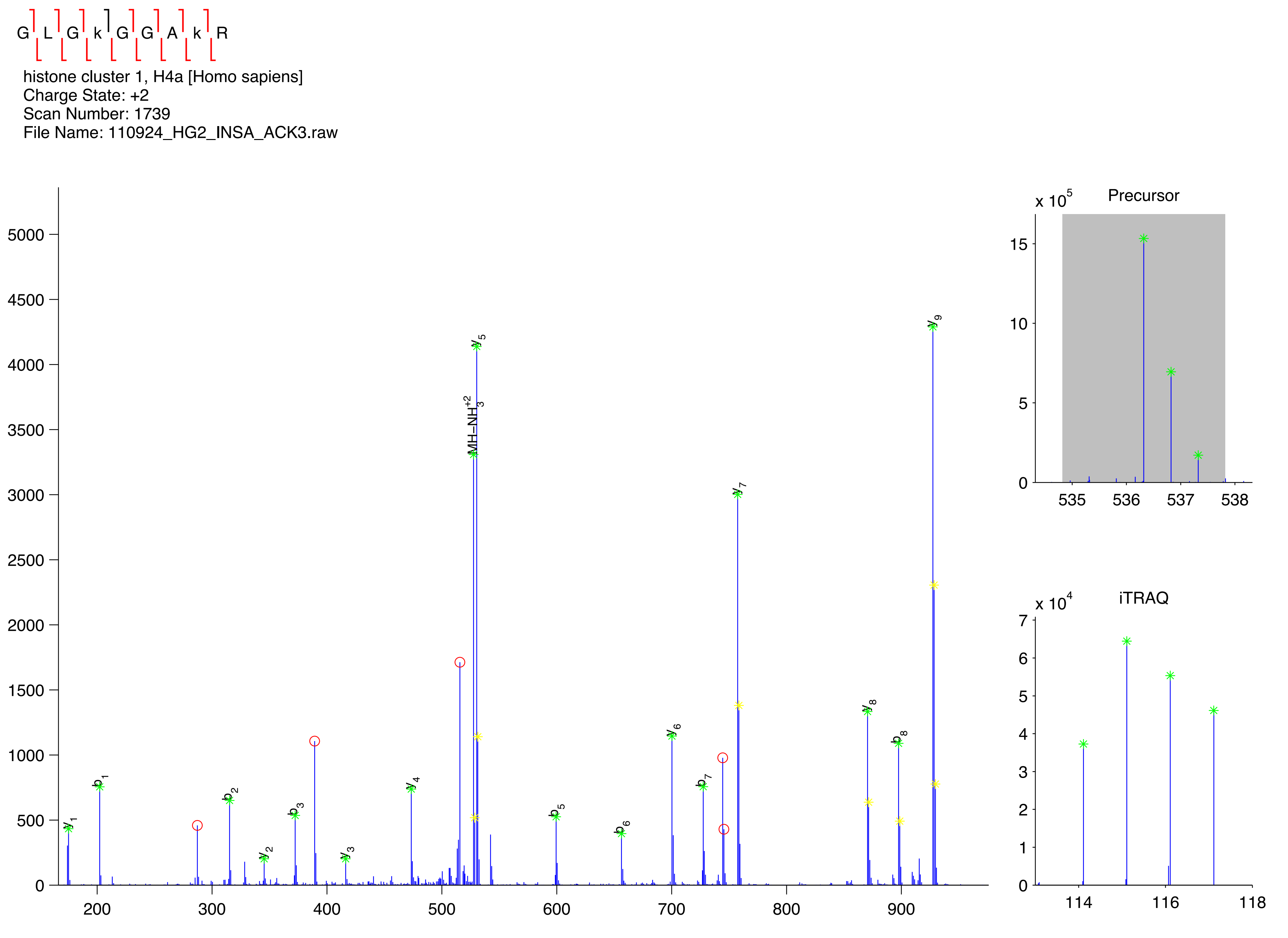

G L G k G G A k R
histone cluster 1, H4a [Homo sapiens]
Charge State: +2
Scan Number: 1739
File Name: 110924_HG2_INSA_ACK3.raw
y1
b1
b2
y2
b3
y3
y4
MH-NH3+2
y5
b5
b6
y6
b7
y7
y8
b8
y9
Precursor
x 10^5
iTRAQ
x 10^4

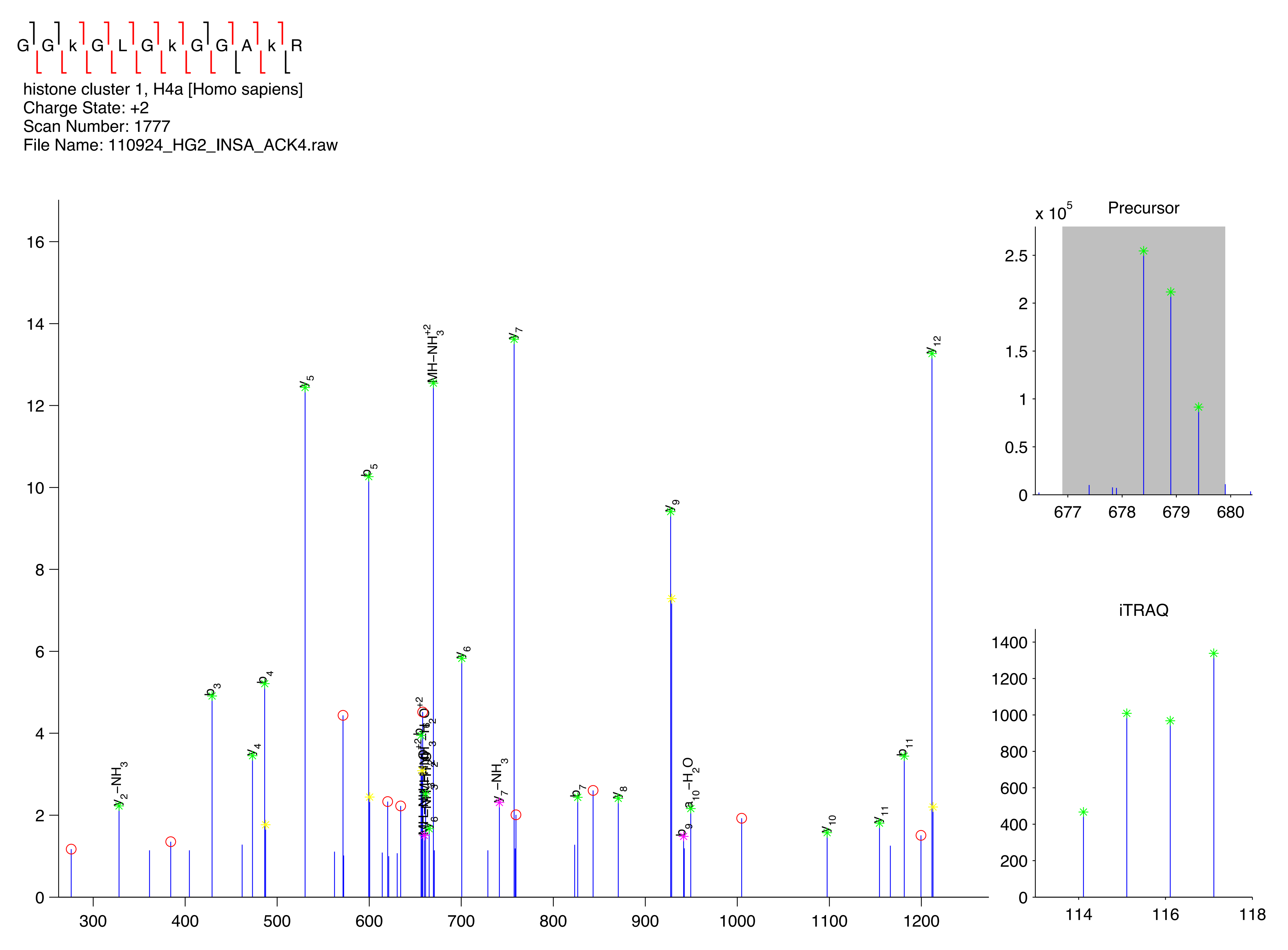

G G k G L G k G G A k R
histone cluster 1, H4a [Homo sapiens]
Charge State: +2
Scan Number: 1777
File Name: 110924_HG2_INSA_ACK4.raw
Precursor
x 10^5
iTRAQ

G L G k G G A k R

histone cluster 1, H4a [Homo sapiens]
Charge State: +2
Scan Number: 1853
File Name: 110924_HG2_INSA_ACK4.raw

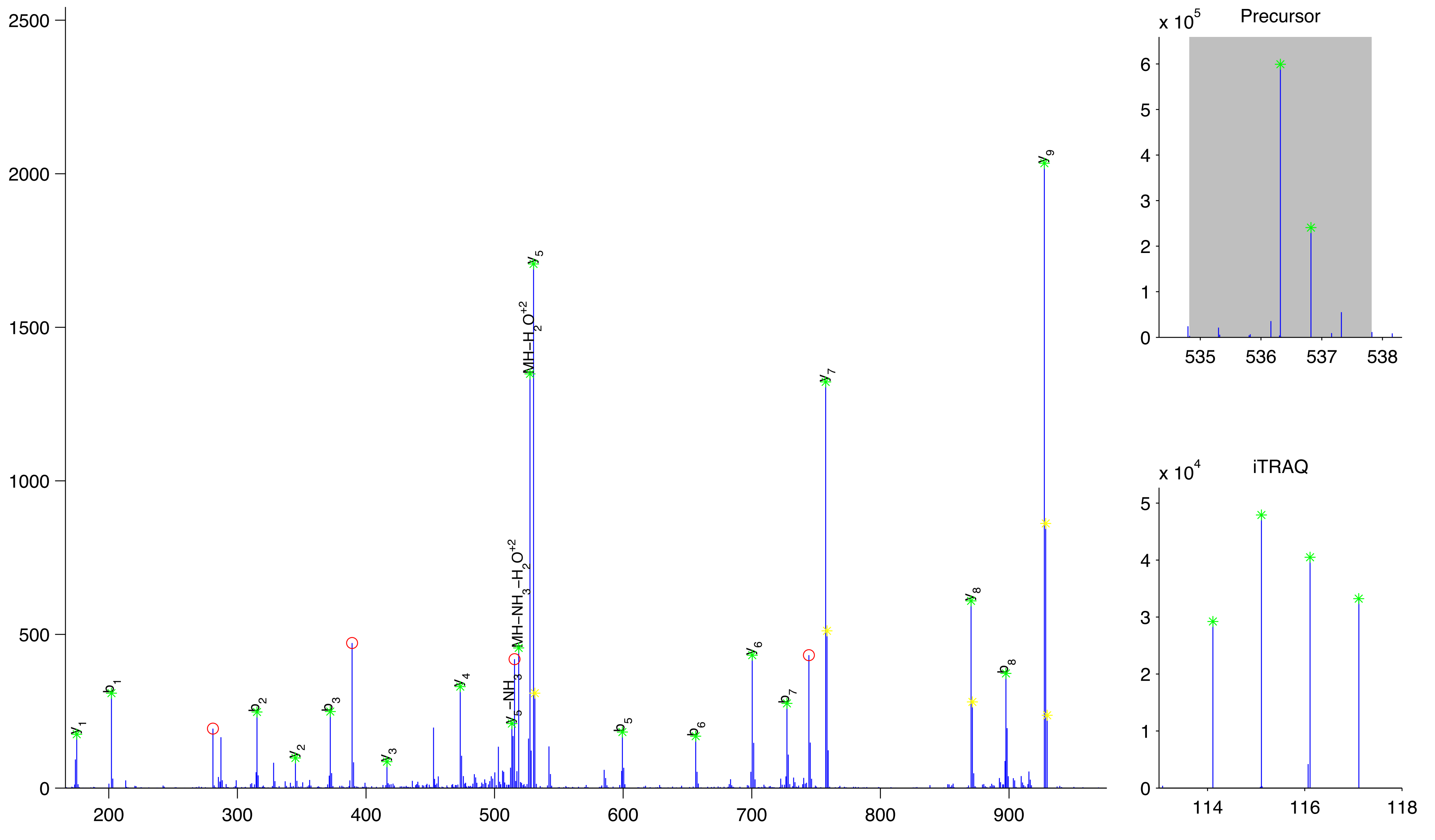

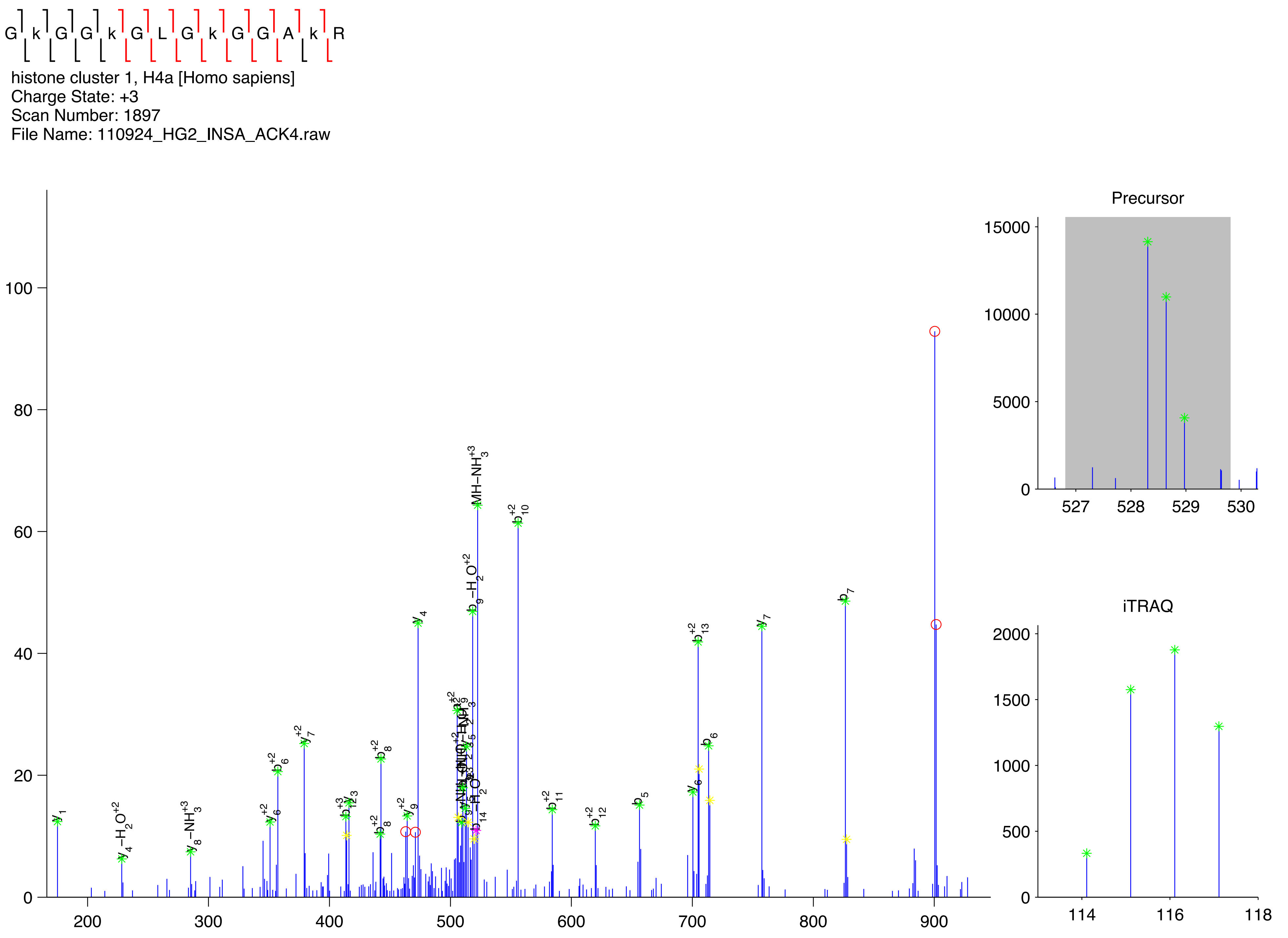

G k G G k G L G k G G A k R
histone cluster 1, H4a [Homo sapiens]
Charge State: +3
Scan Number: 1897
File Name: 110924_HG2_INSA_ACK4.raw
Precursor
iTRAQ

G G k G L G k G G A k R

histone cluster 1, H4a [Homo sapiens]
Charge State: +2
Scan Number: 1935
File Name: 110924_HG2_INSA_ACK4.raw

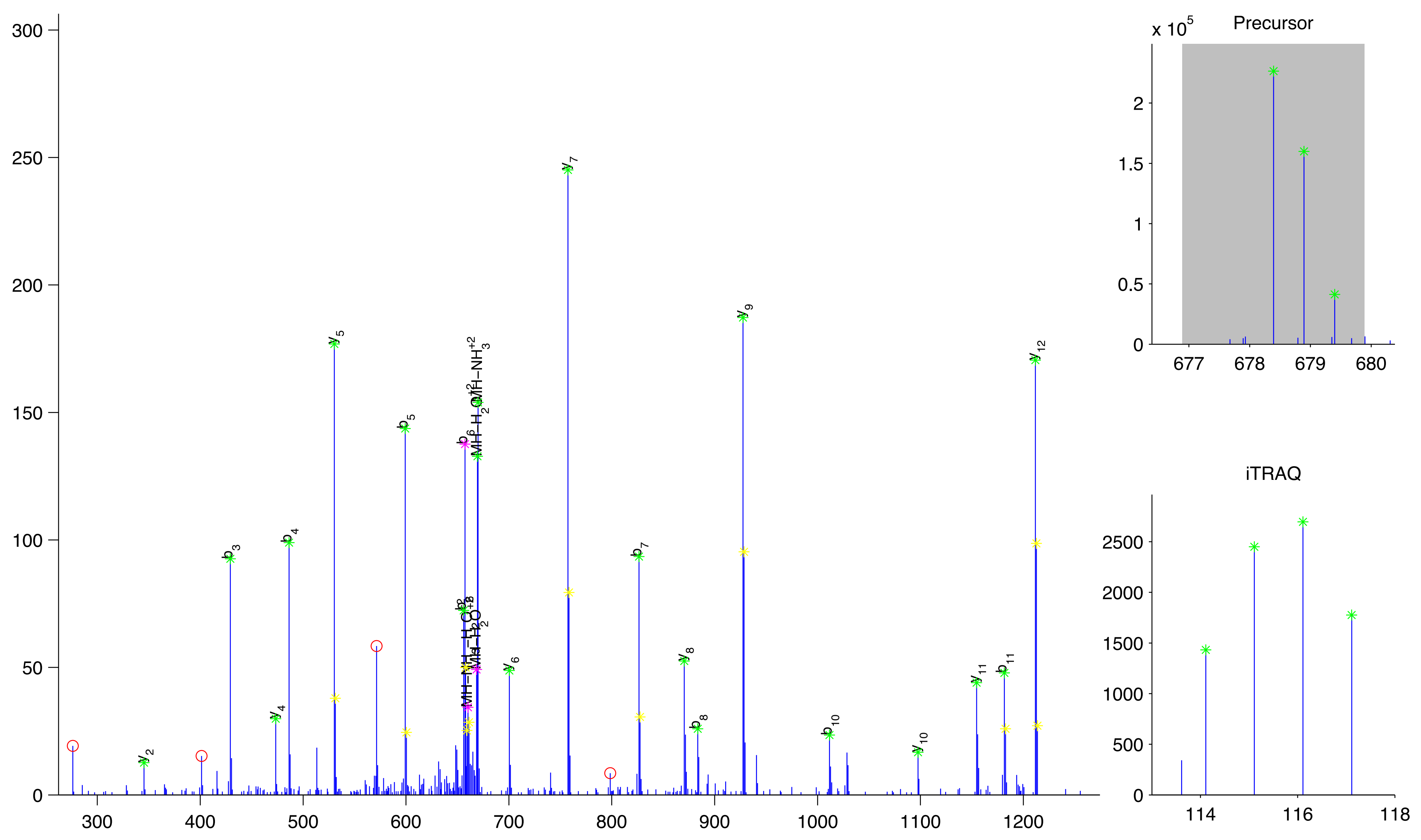

G L G k G G A k R

histone cluster 1, H4a [Homo sapiens]
Charge State: +2
Scan Number: 1935
File Name: 110924_HG2_INSA_ACK3.raw

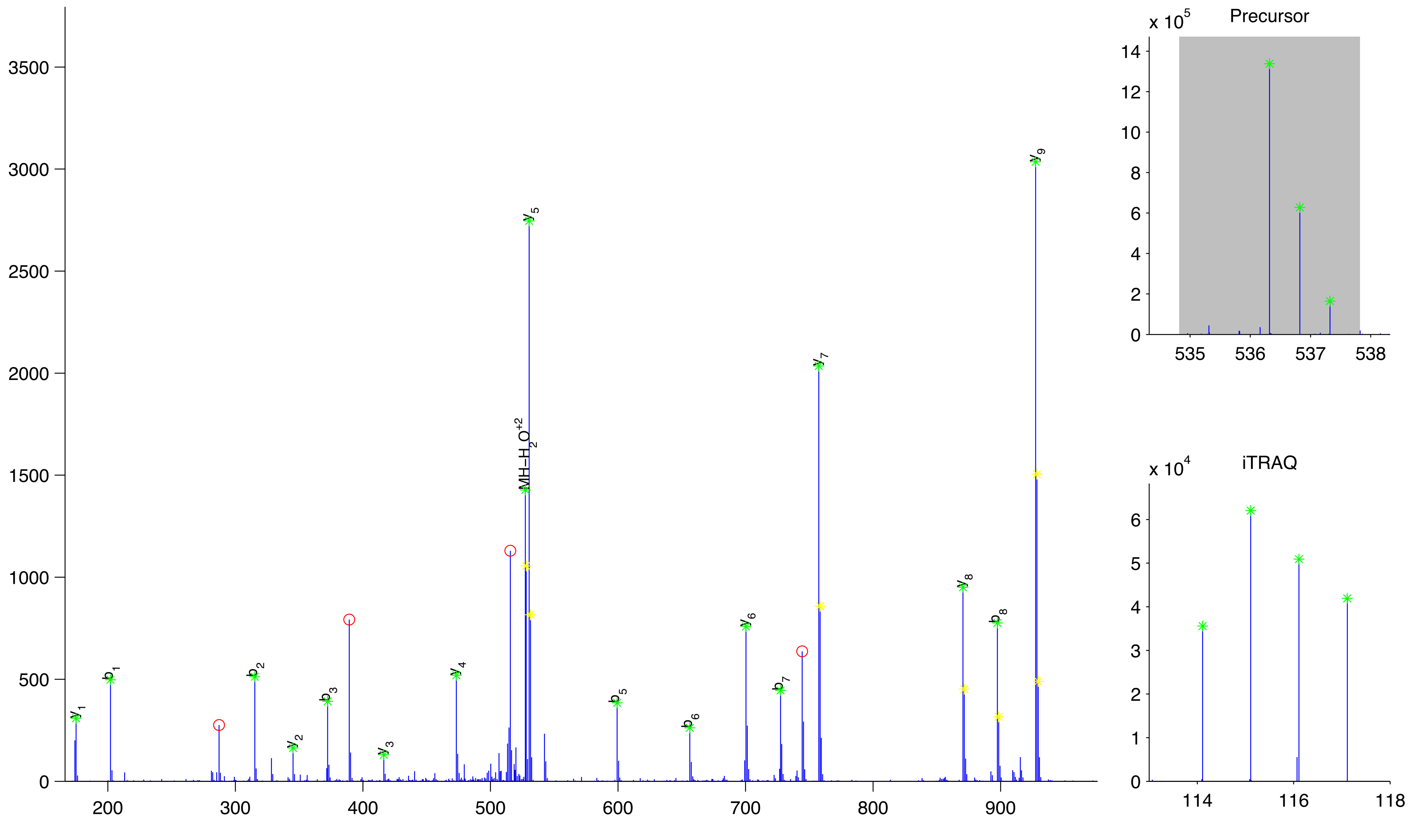

G L G k G G A k R

histone cluster 1, H4a [Homo sapiens]
Charge State: +2
Scan Number: 2038
File Name: 110924_HG2_INSA_ACK3.raw

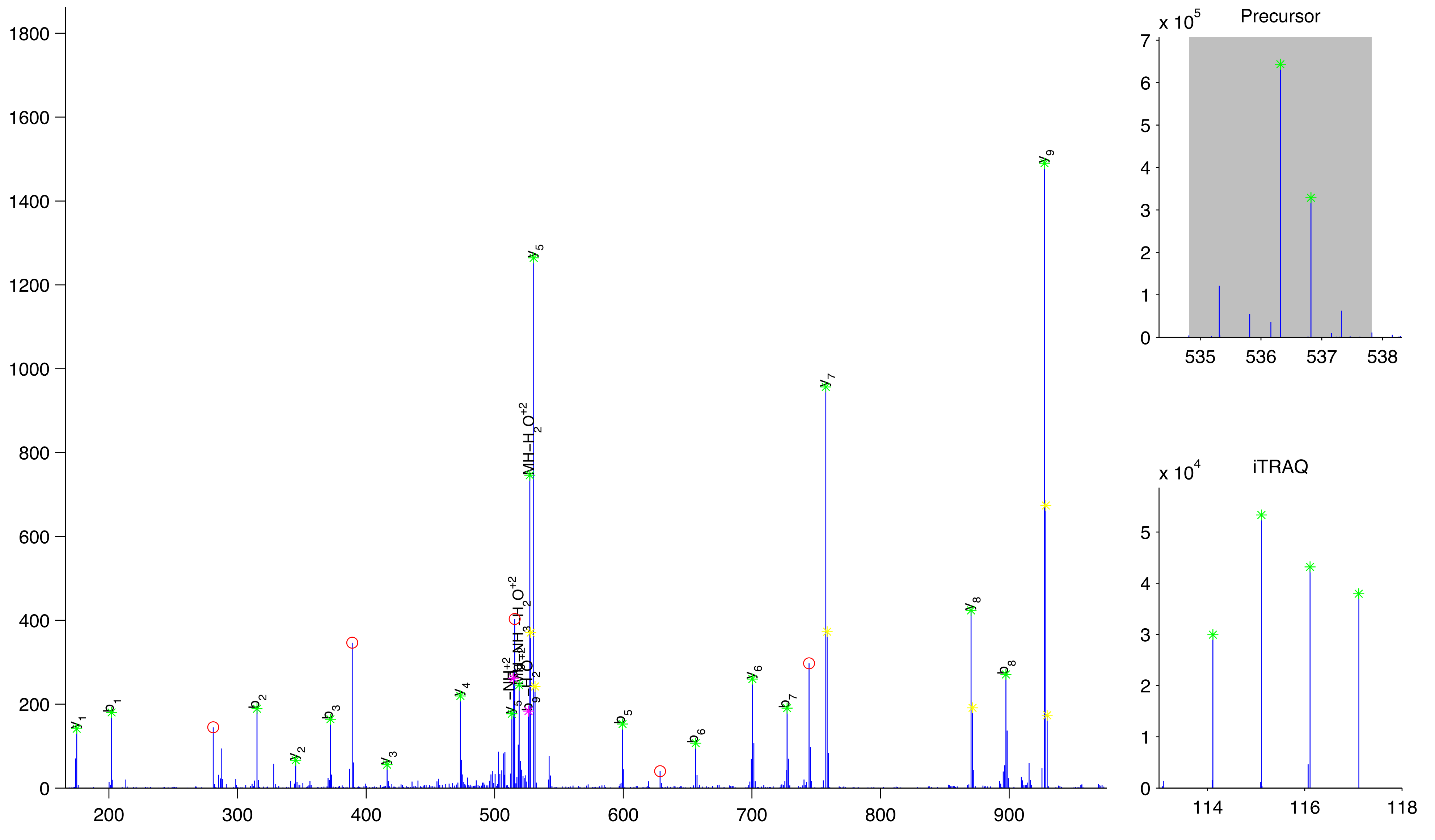

G G k G L G k G G A k R

histone cluster 1, H4a [Homo sapiens]
Charge State: +2
Scan Number: 2117
File Name: 110924_HG2_INSA_ACK4.raw

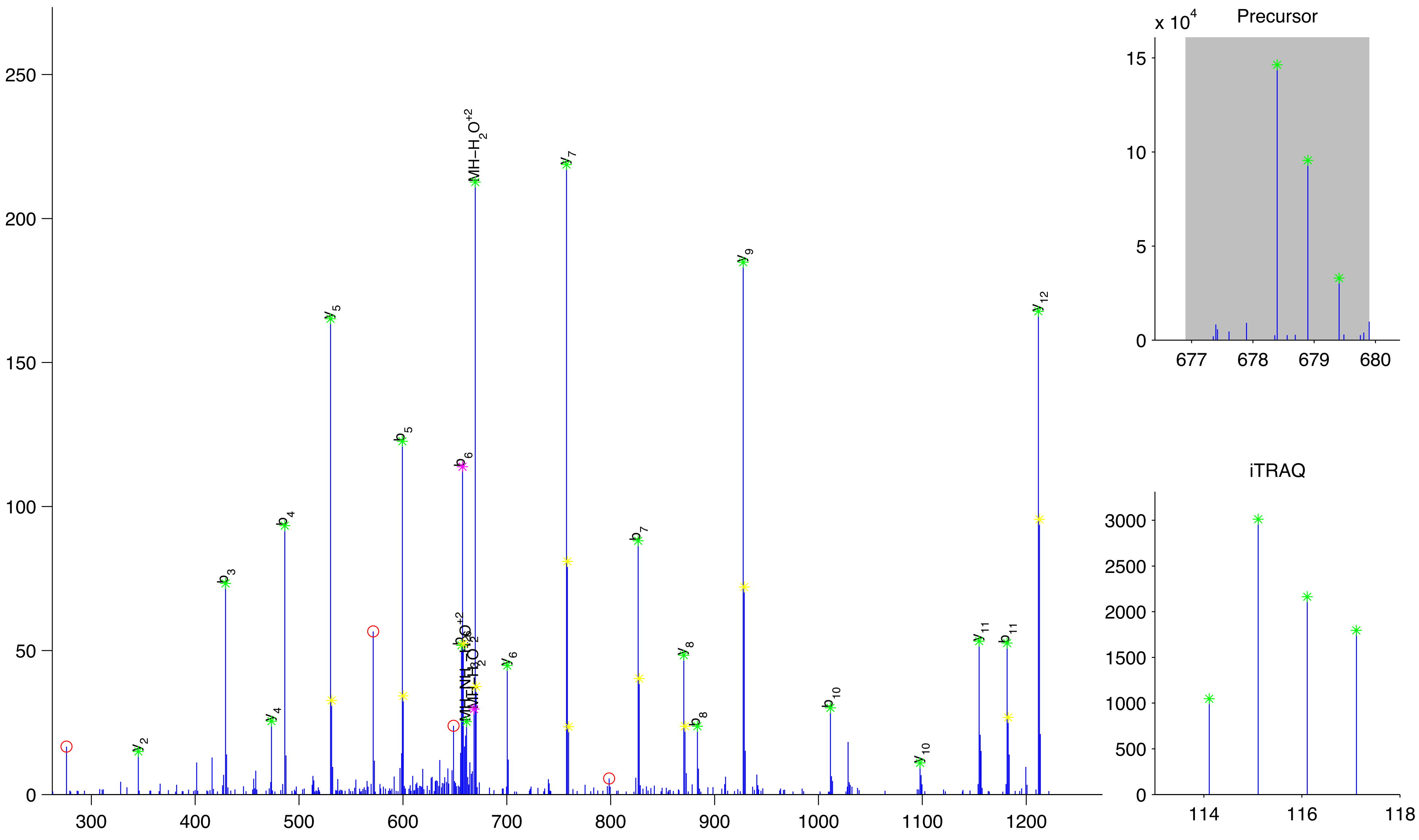

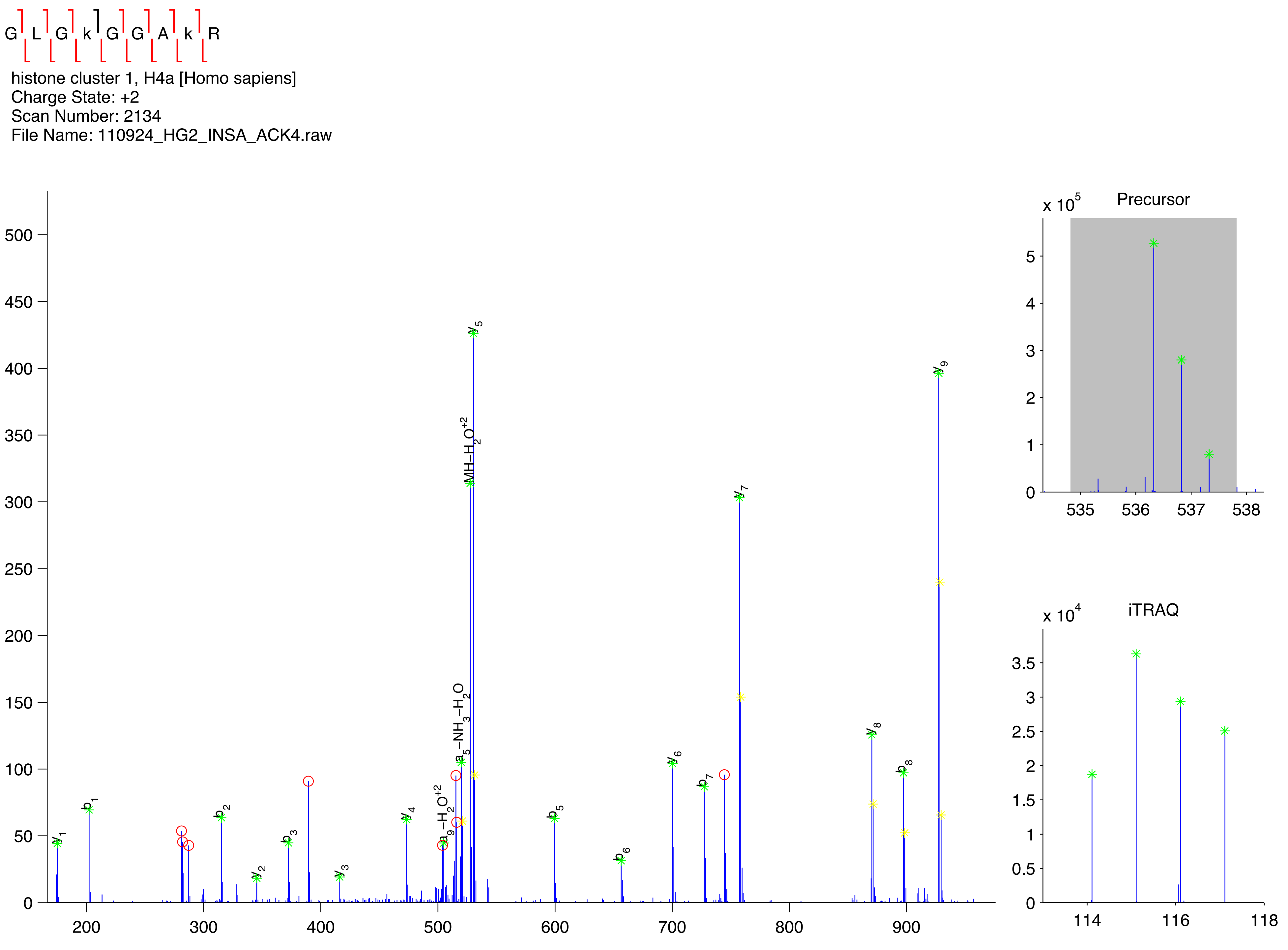
G L G k G G A k R
histone cluster 1, H4a [Homo sapiens]
Charge State: +2
Scan Number: 2134
File Name: 110924_HG2_INSA_ACK4.raw
Precursor
x 10^5
iTRAQ
x 10^4
MH−H2O+2
a5−NH3−H2O
a9−H2O+2

G L G k G G A k R

histone cluster 1, H4a [Homo sapiens]
Charge State: +2
Scan Number: 2187
File Name: 110924_HG2_INSA_ACK4.raw

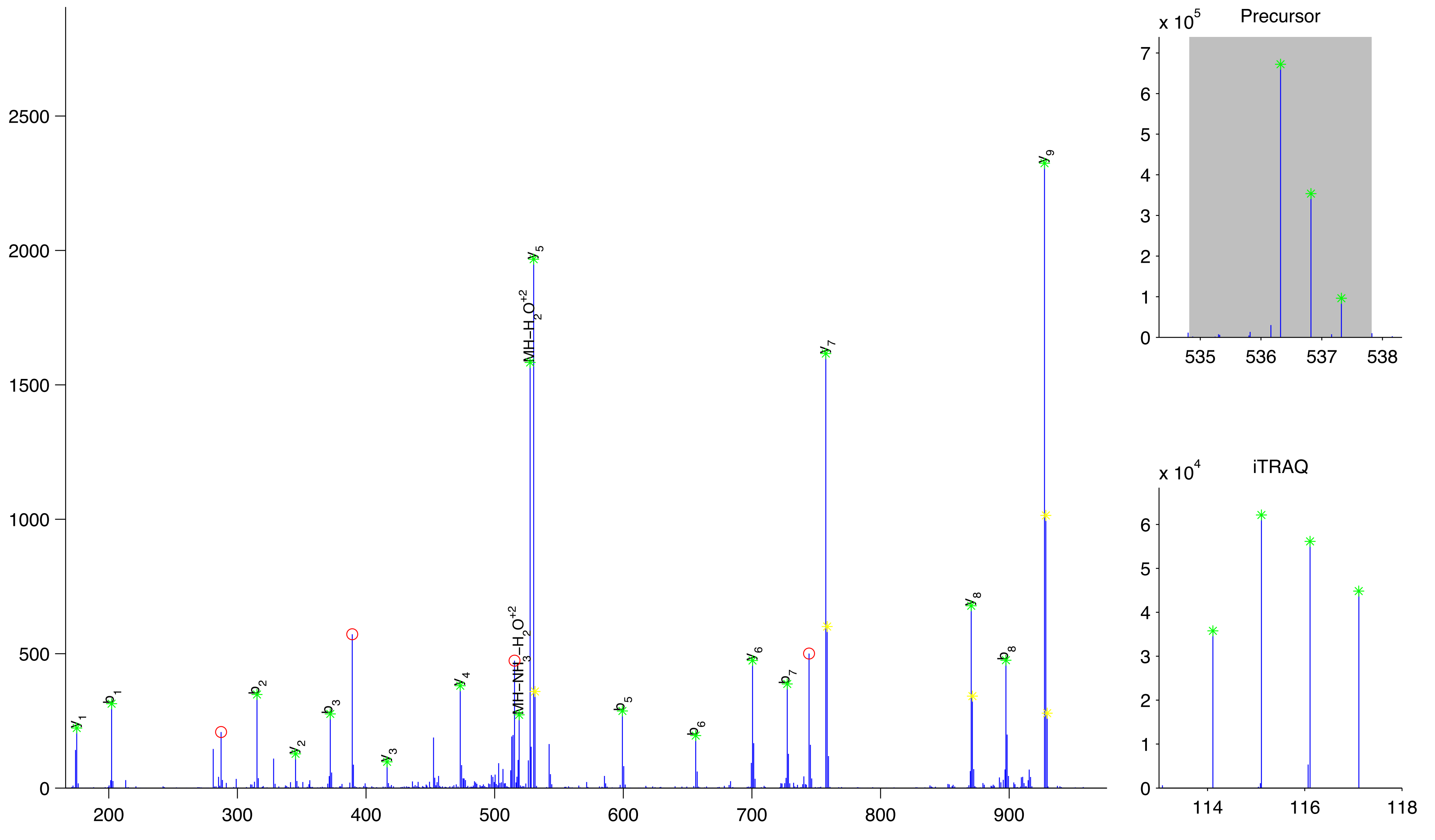

G G k G L G k G G A k R

histone cluster 1, H4a [Homo sapiens]
Charge State: +2
Scan Number: 2281
File Name: 110924_HG2_INSA_ACK4.raw

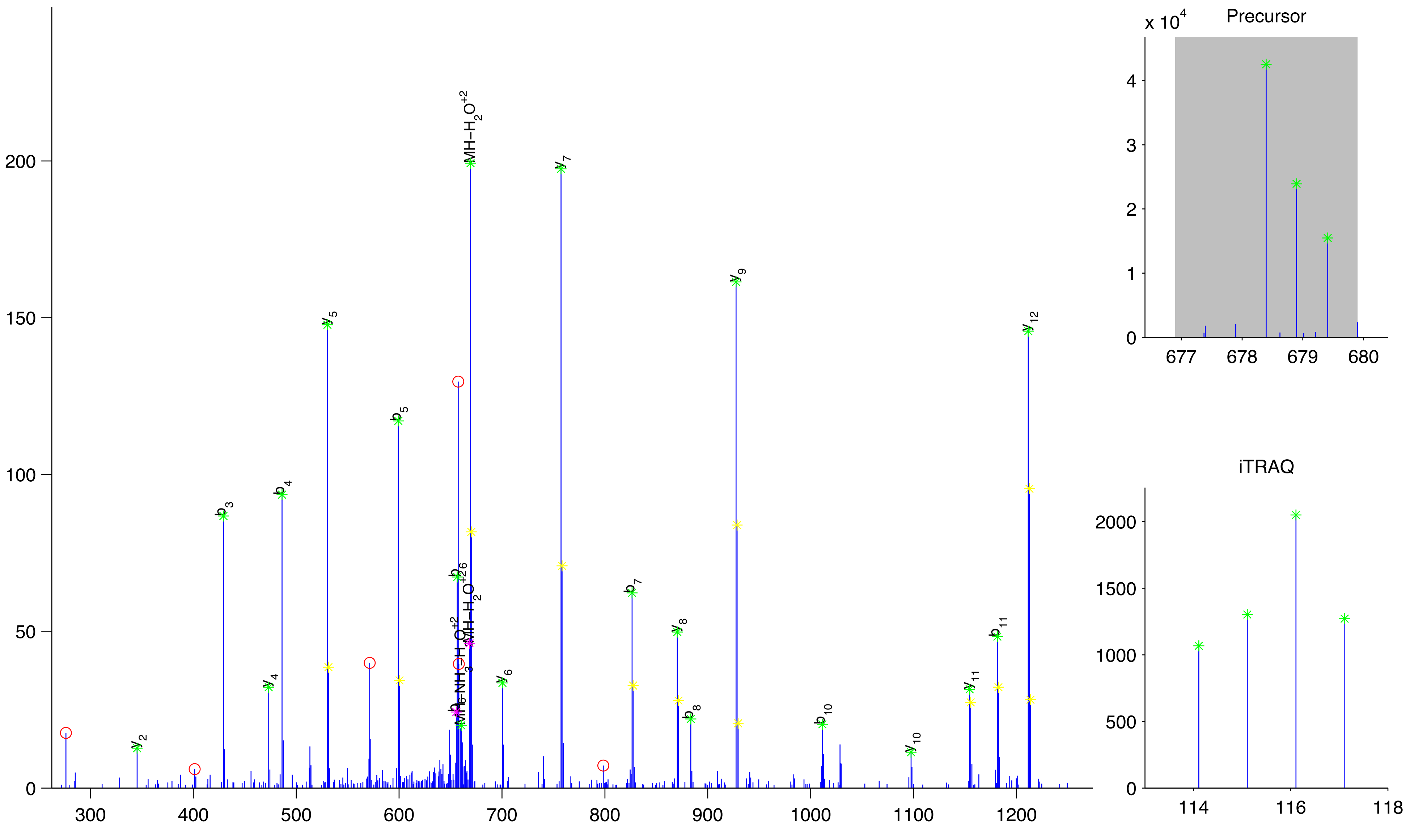

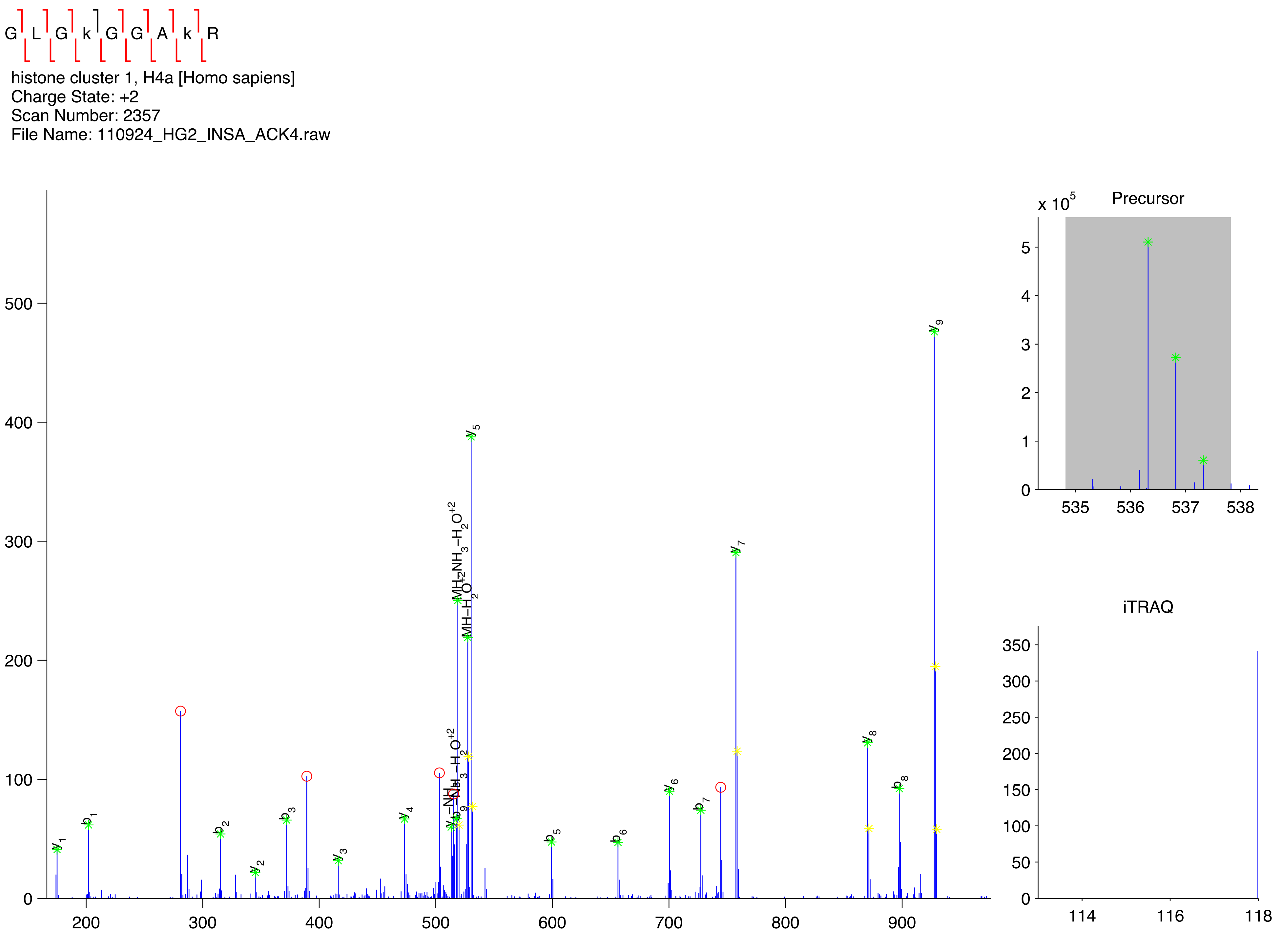

G L G k G G A k R
histone cluster 1, H4a [Homo sapiens]
Charge State: +2
Scan Number: 2357
File Name: 110924_HG2_INSA_ACK4.raw
Precursor
x 10^5
iTRAQ

G L G k G G A k R

histone cluster 1, H4a [Homo sapiens]
Charge State: +2
Scan Number: 2371
File Name: 110924_HG2_INSA_ACK4.raw

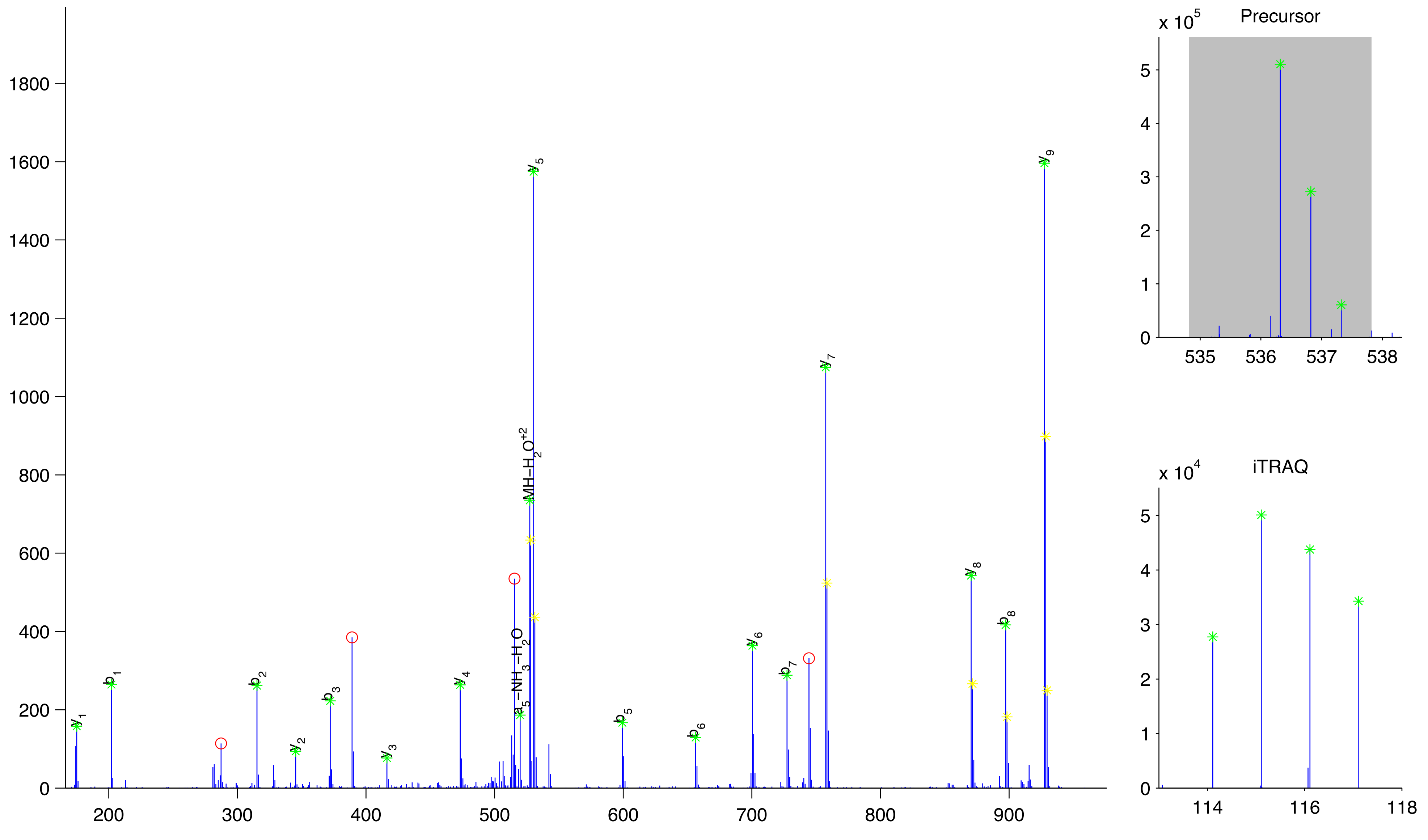

G G k G L G k G G A k R

histone cluster 1, H4a [Homo sapiens]
Charge State: +2
Scan Number: 2449
File Name: 110924_HG2_INSA_ACK4.raw

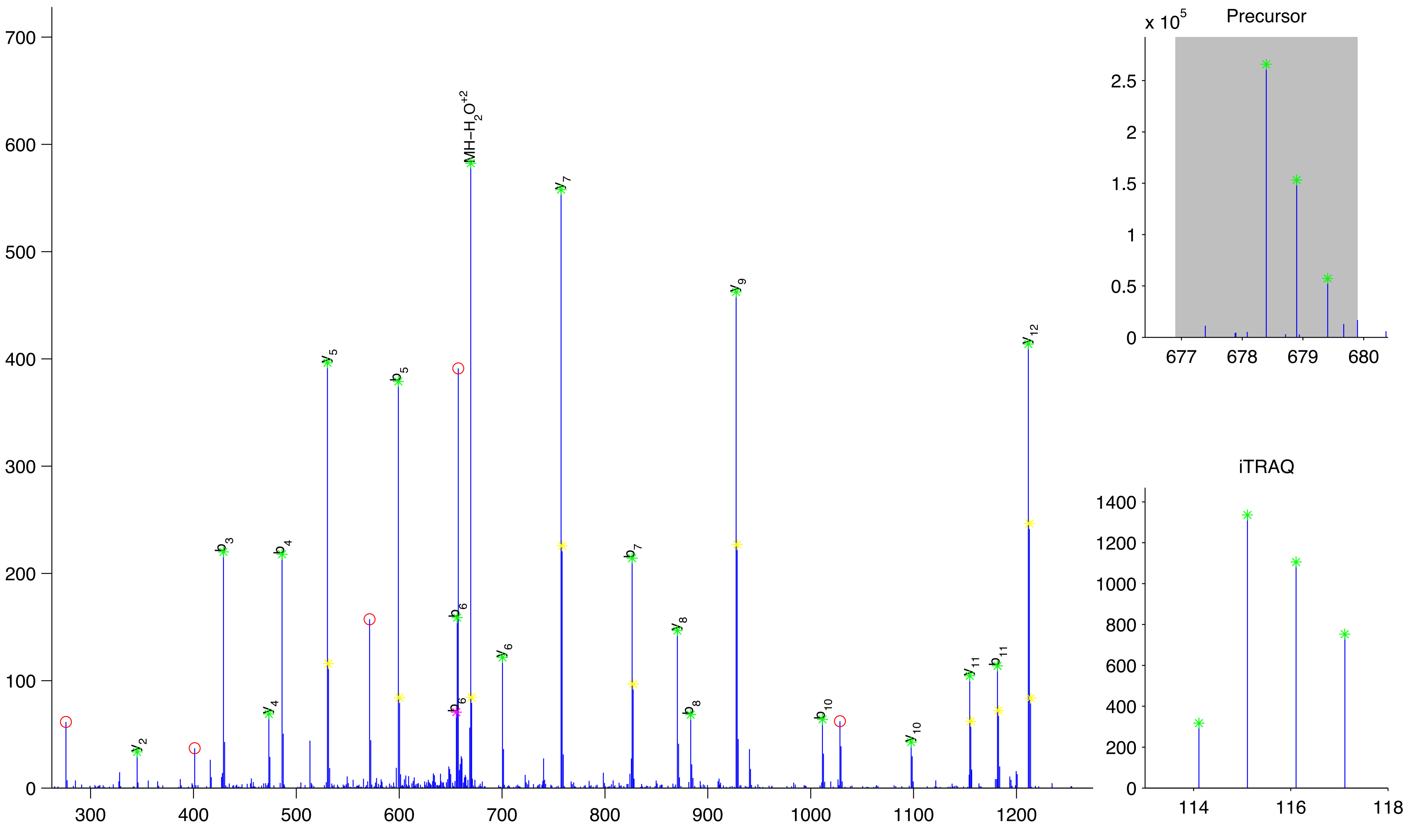

G L G k G G A k R

histone cluster 1, H4a [Homo sapiens]
Charge State: +2
Scan Number: 2466
File Name: 110924_HG2_INSA_ACK3.raw

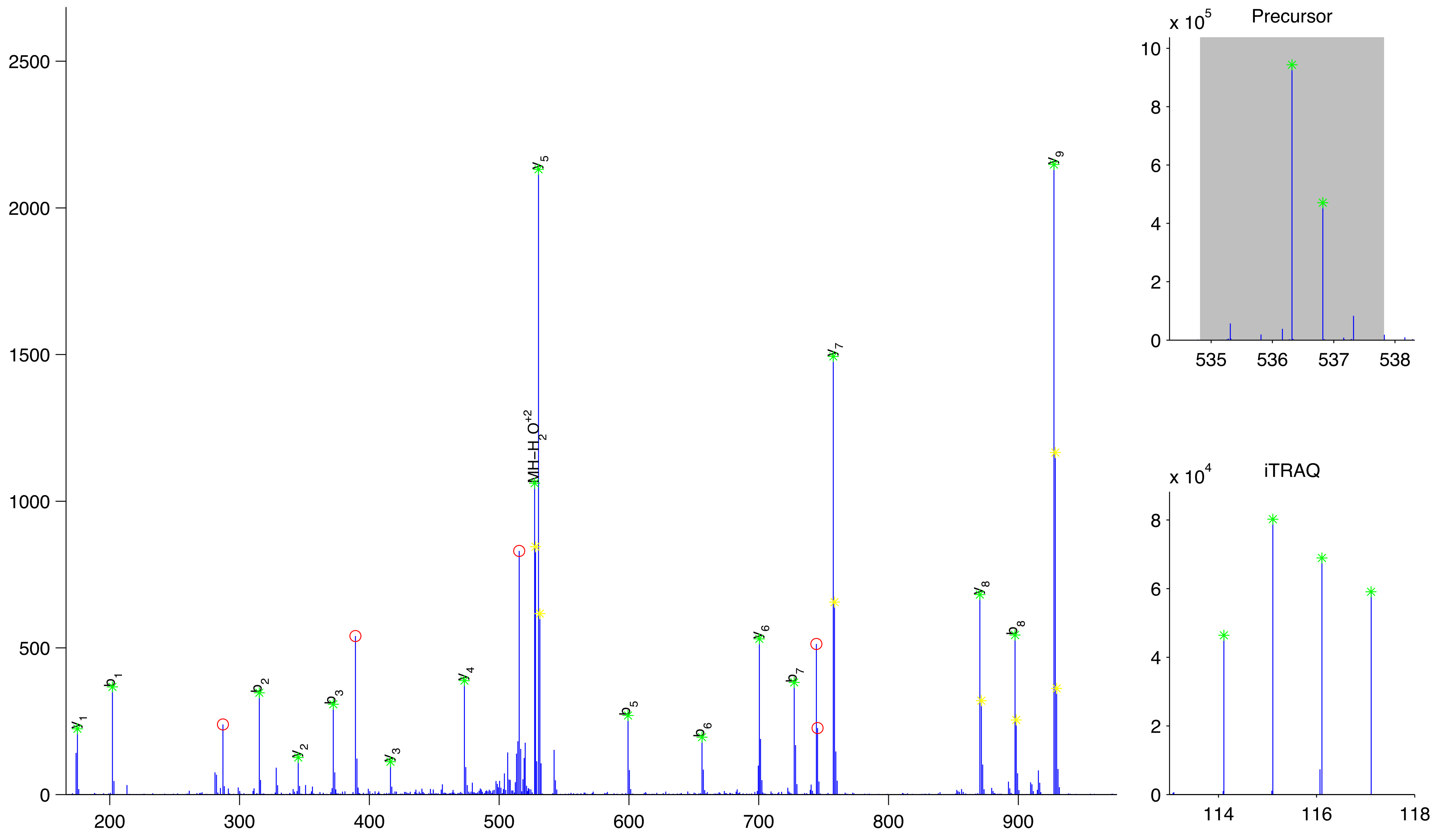

G L G k G G A k R

histone cluster 1, H4a [Homo sapiens]
Charge State: +2
Scan Number: 2525
File Name: 110924_HG2_INSA_ACK4.raw

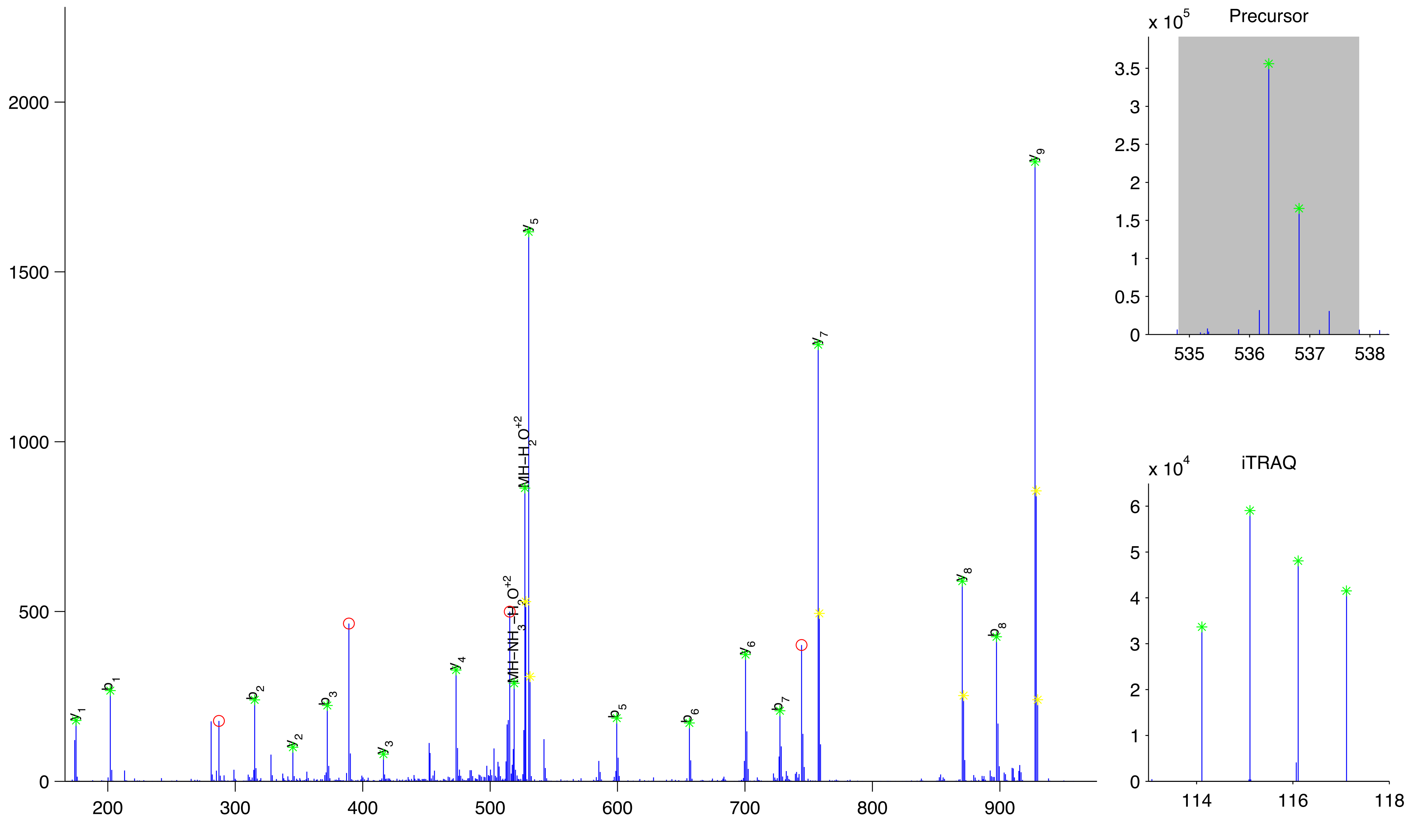

G L G k G G A k R

histone cluster 1, H4a [Homo sapiens]
Charge State: +2
Scan Number: 2540
File Name: 110924_HG2_INSA_ACK3.raw

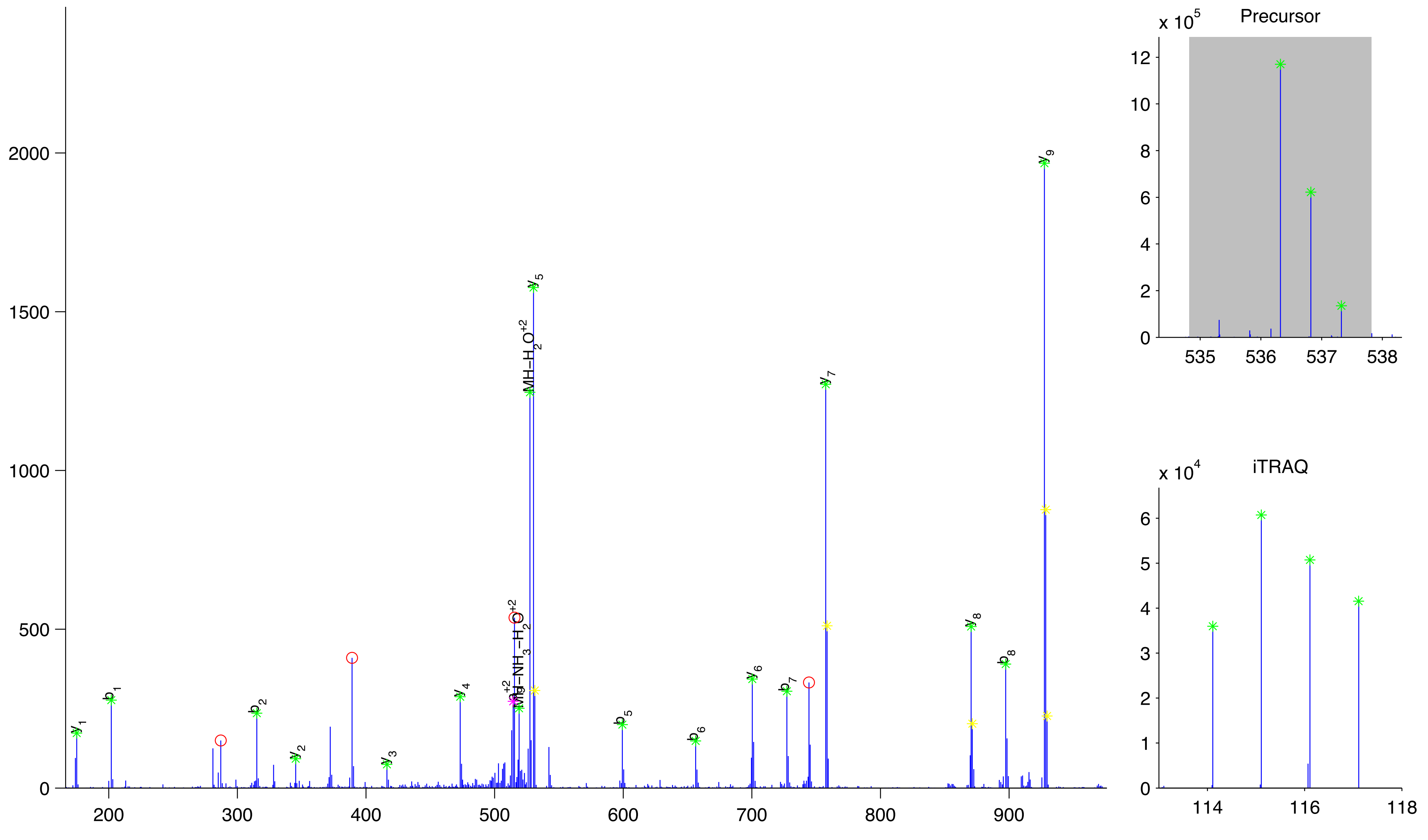

G G k G L G k G G A k R

histone cluster 1, H4a [Homo sapiens]
Charge State: +2
Scan Number: 2607
File Name: 110924_HG2_INSA_ACK4.raw

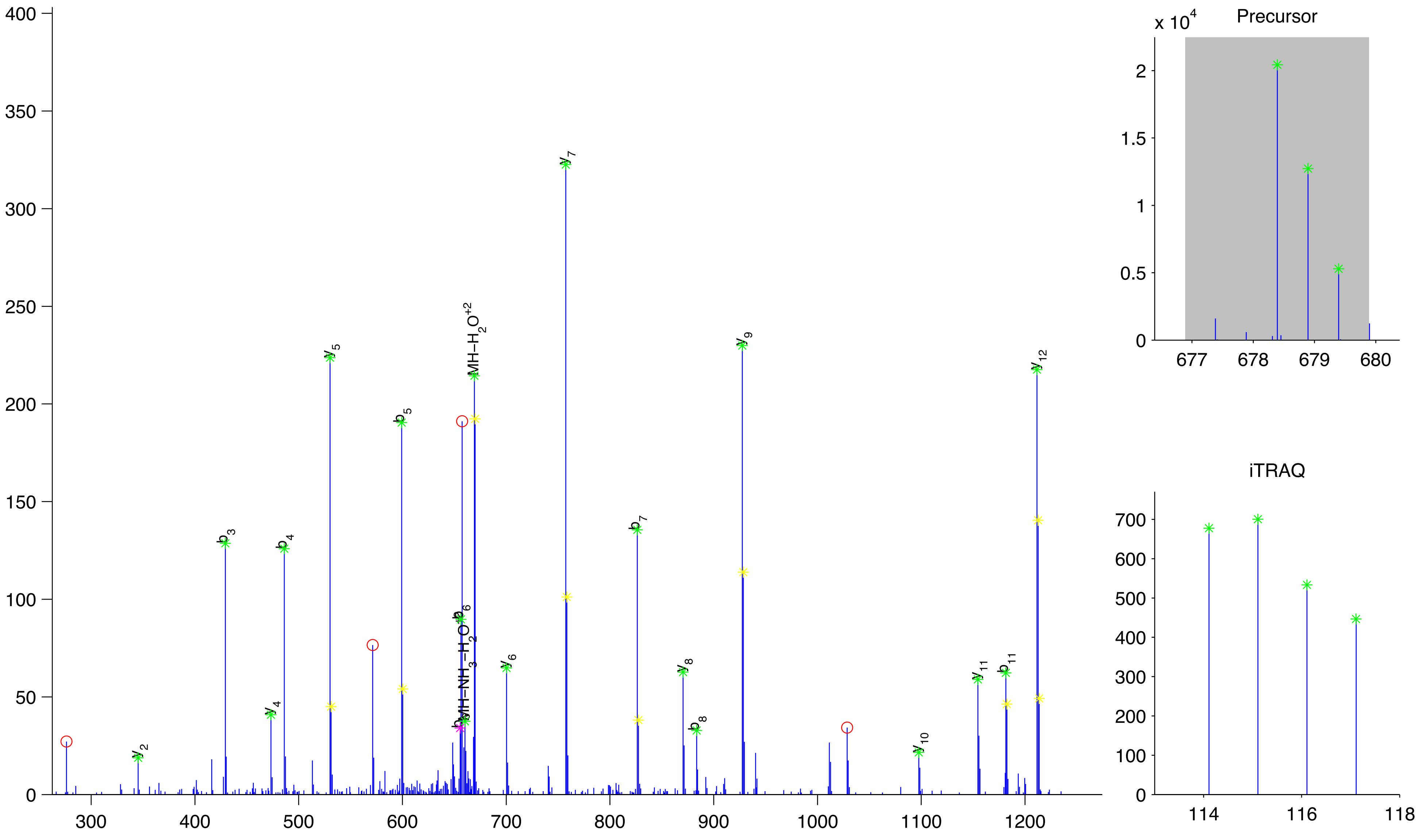

G L G k G G A k R

histone cluster 1, H4a [Homo sapiens]
Charge State: +2
Scan Number: 2691
File Name: 110924_HG2_INSA_ACK4.raw

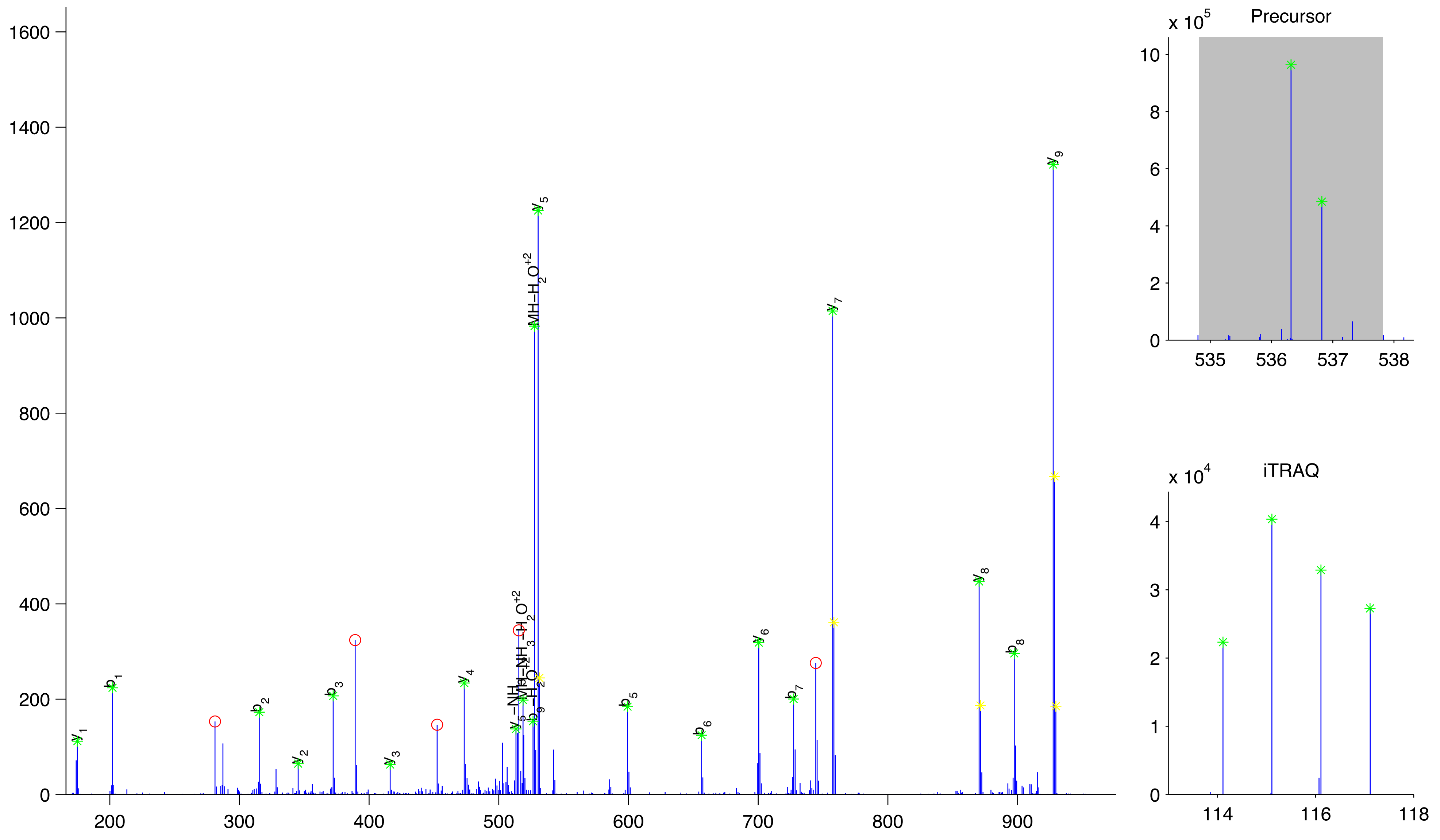

G L G k G G A k R

histone cluster 1, H4a [Homo sapiens]
Charge State: +2
Scan Number: 2762
File Name: 110924_HG2_INSA_ACK4.raw

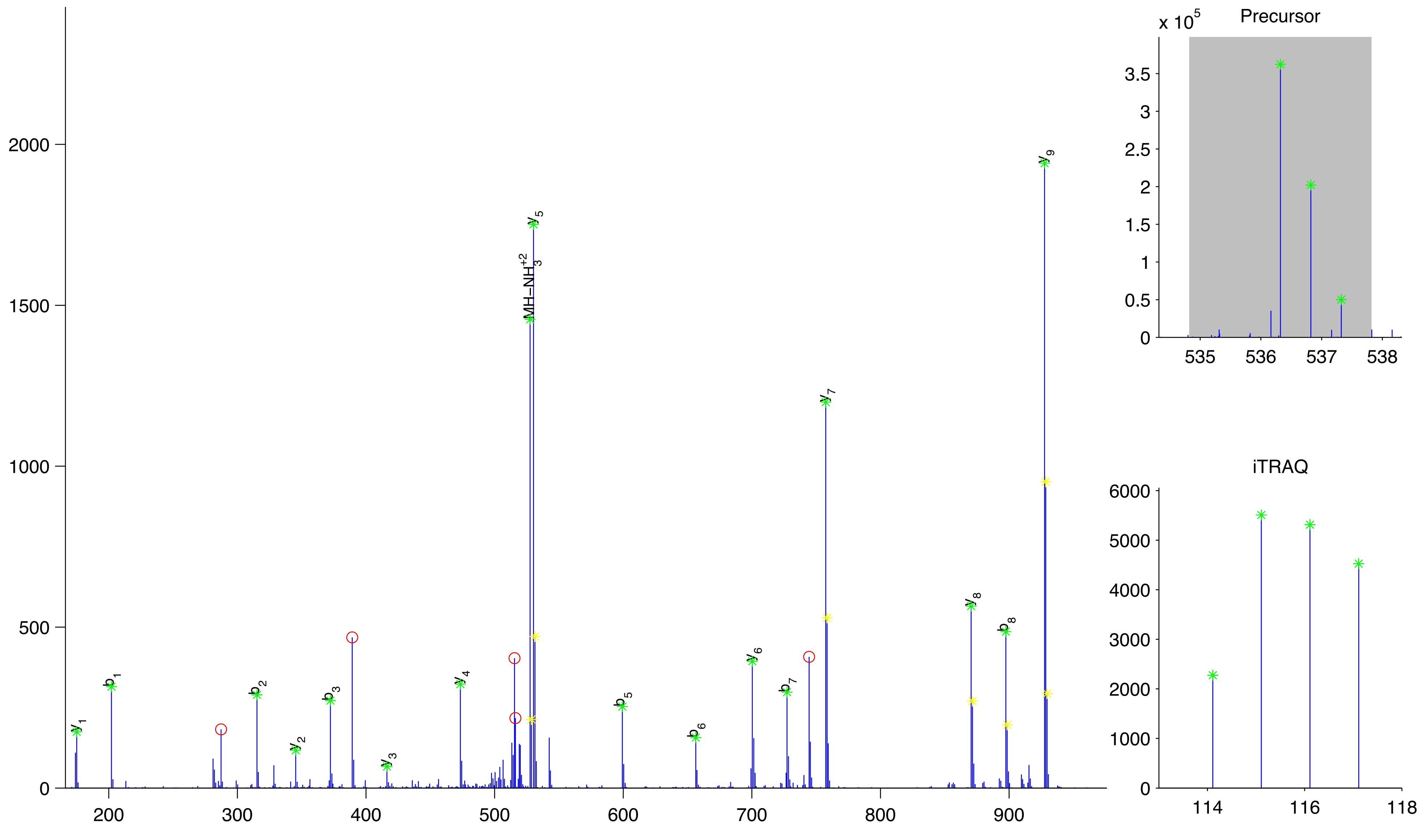

G L G k G G A k R

histone cluster 1, H4a [Homo sapiens]
Charge State: +2
Scan Number: 2859
File Name: 110924_HG2_INSA_ACK4.raw

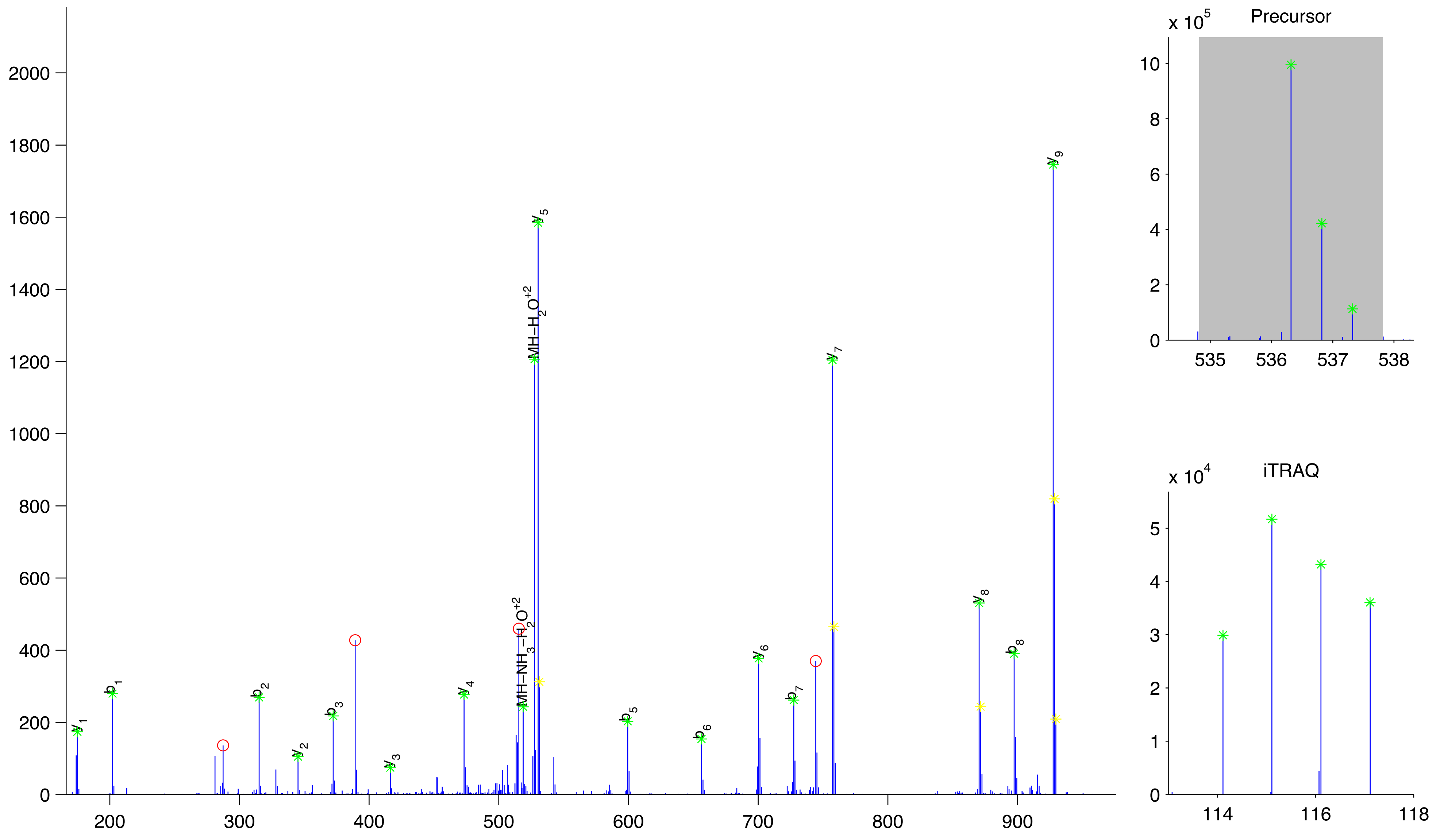

G L G k G G A k R

histone cluster 1, H4a [Homo sapiens]
Charge State: +2
Scan Number: 2876
File Name: 110924_HG2_INSA_ACK3.raw

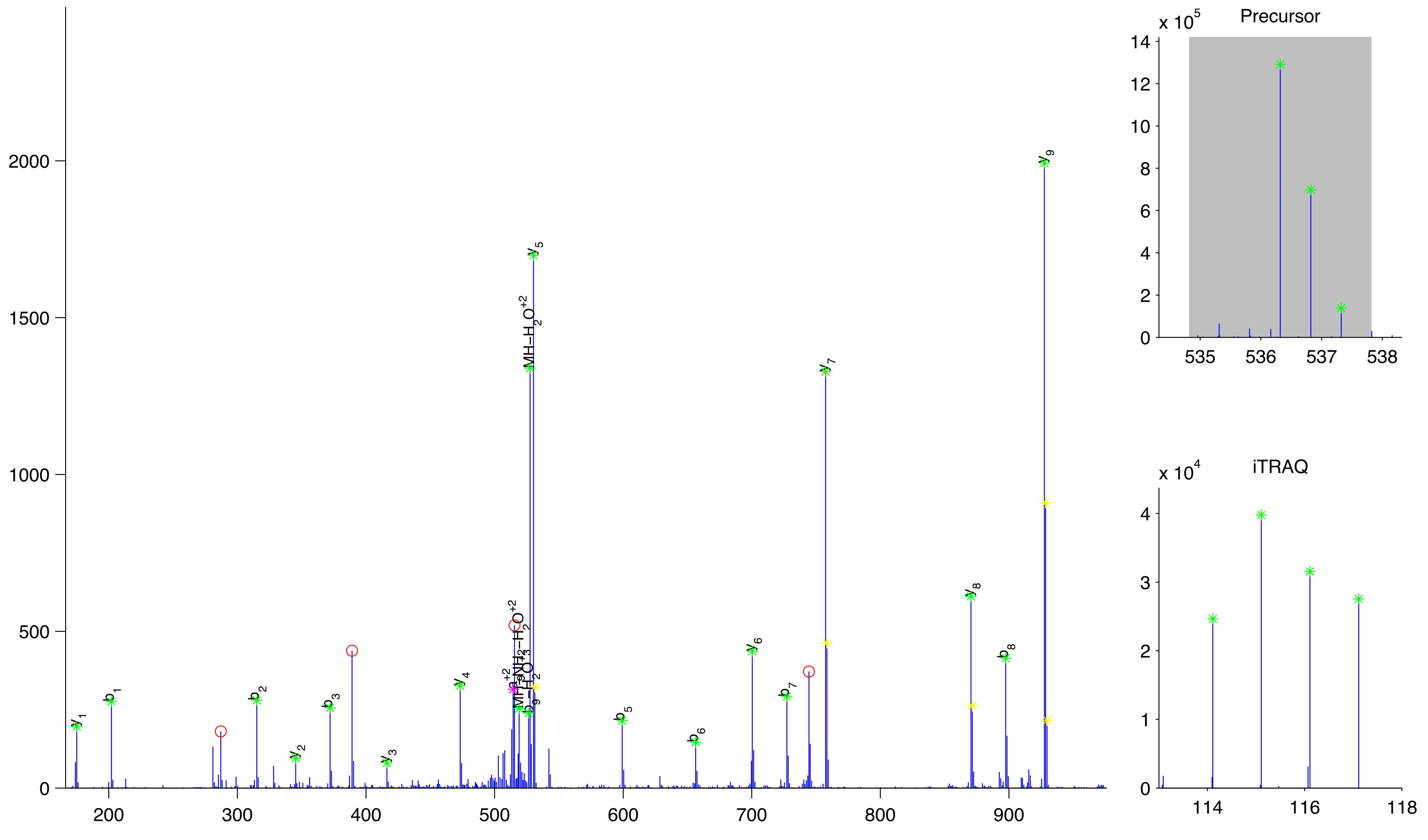

G L G k G G A k R

histone cluster 1, H4a [Homo sapiens]
Charge State: +2
Scan Number: 2968
File Name: 110924_HG2_INSA_ACK3.raw

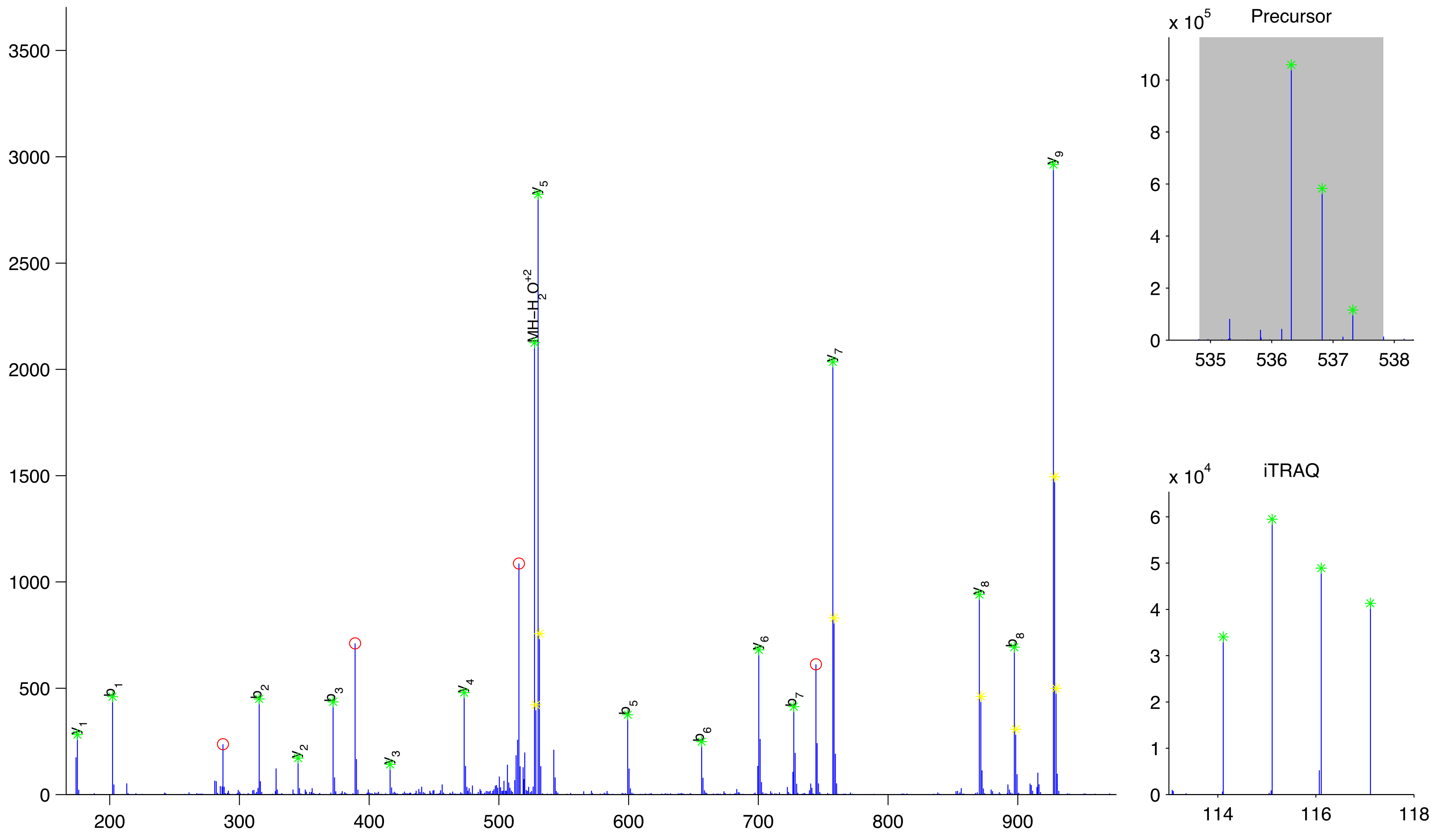

G L G k G G A k R

histone cluster 1, H4a [Homo sapiens]
Charge State: +2
Scan Number: 3044
File Name: 110924_HG2_INSA_ACK3.raw

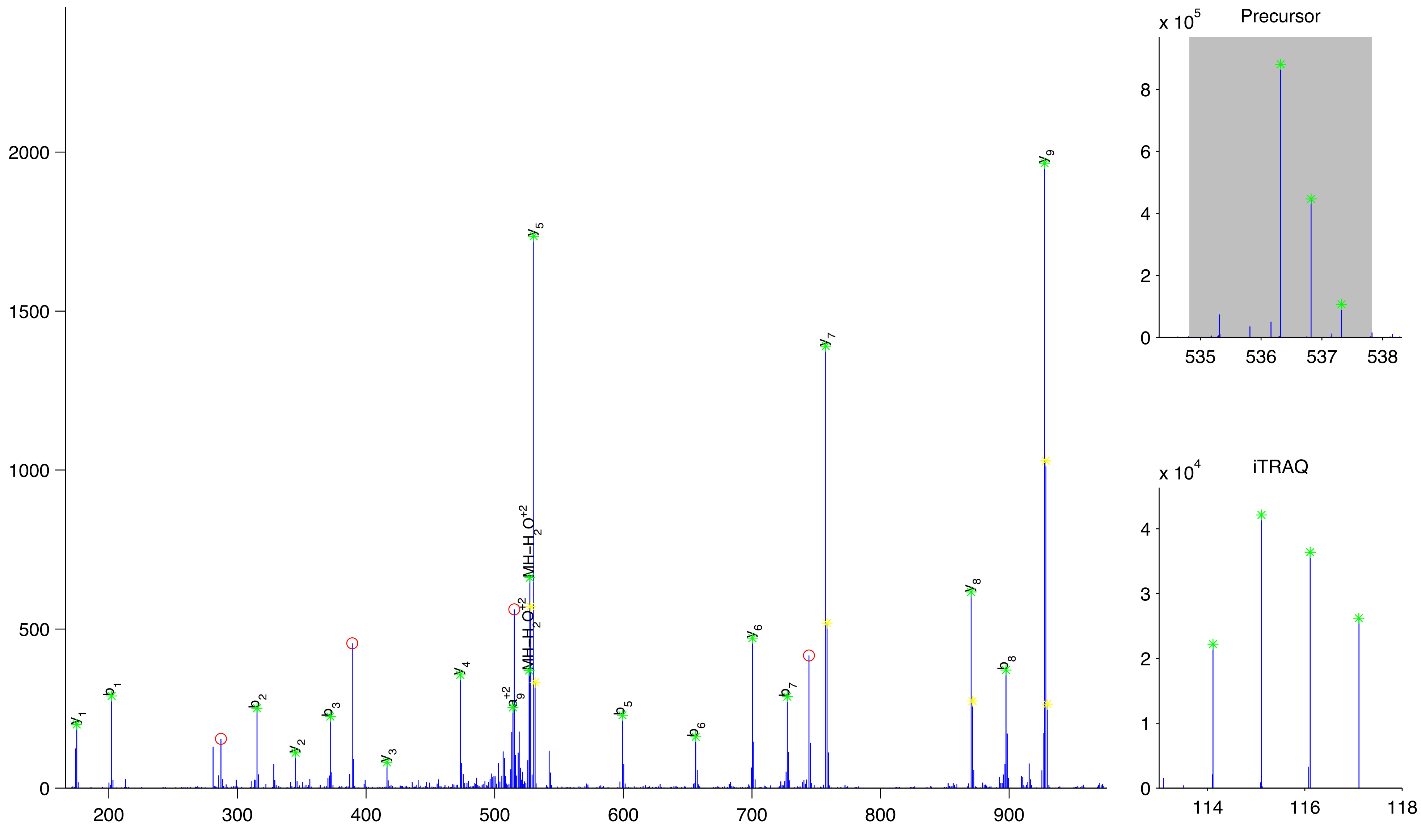

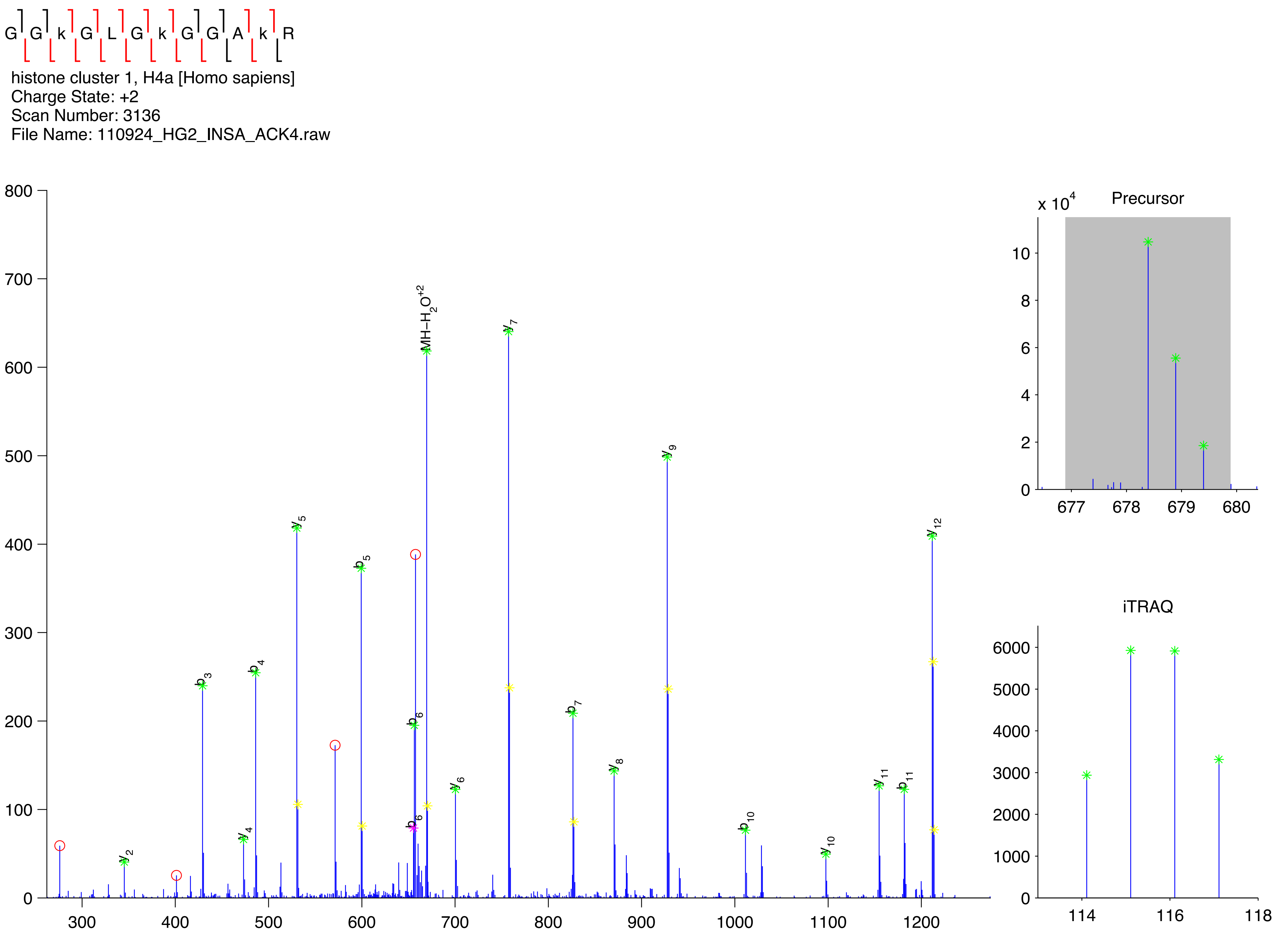
G G k G L G k G G A k R
histone cluster 1, H4a [Homo sapiens]
Charge State: +2
Scan Number: 3136
File Name: 110924_HG2_INSA_ACK4.raw
MH−H2O+2
y7
y9
y5
b5
y12
b3
b4
b6
b7
y8
y6
y4
y2
b10
y10
y11
b11
x 10^4
Precursor
iTRAQ

G L G k G G A k R

histone cluster 1, H4a [Homo sapiens]
Charge State: +2
Scan Number: 3155
File Name: 110924_HG2_INSA_ACK3.raw

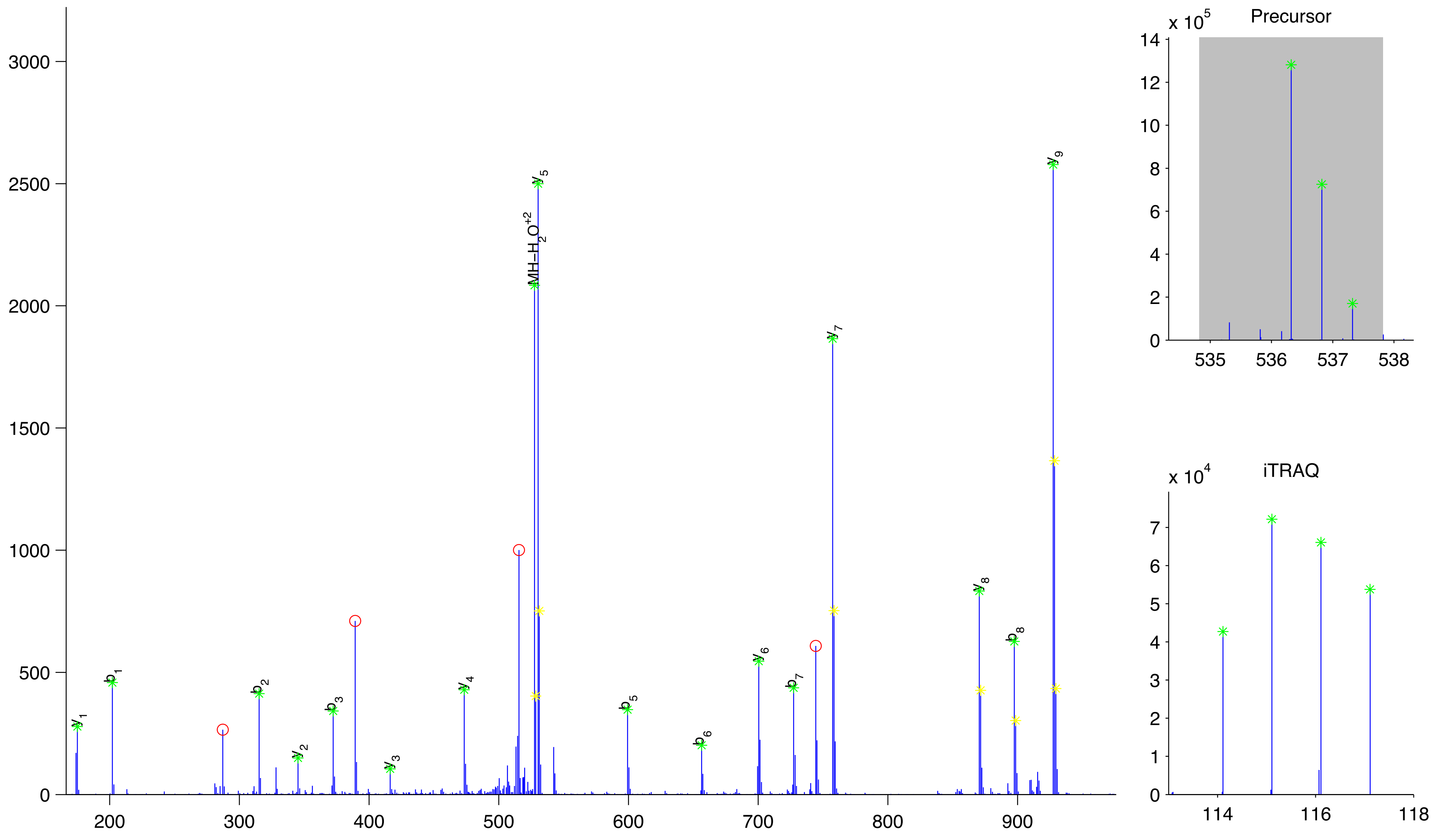

G L G k G G A k R

histone cluster 1, H4a [Homo sapiens]
Charge State: +2
Scan Number: 3201
File Name: 110924_HG2_INSA_ACK4.raw

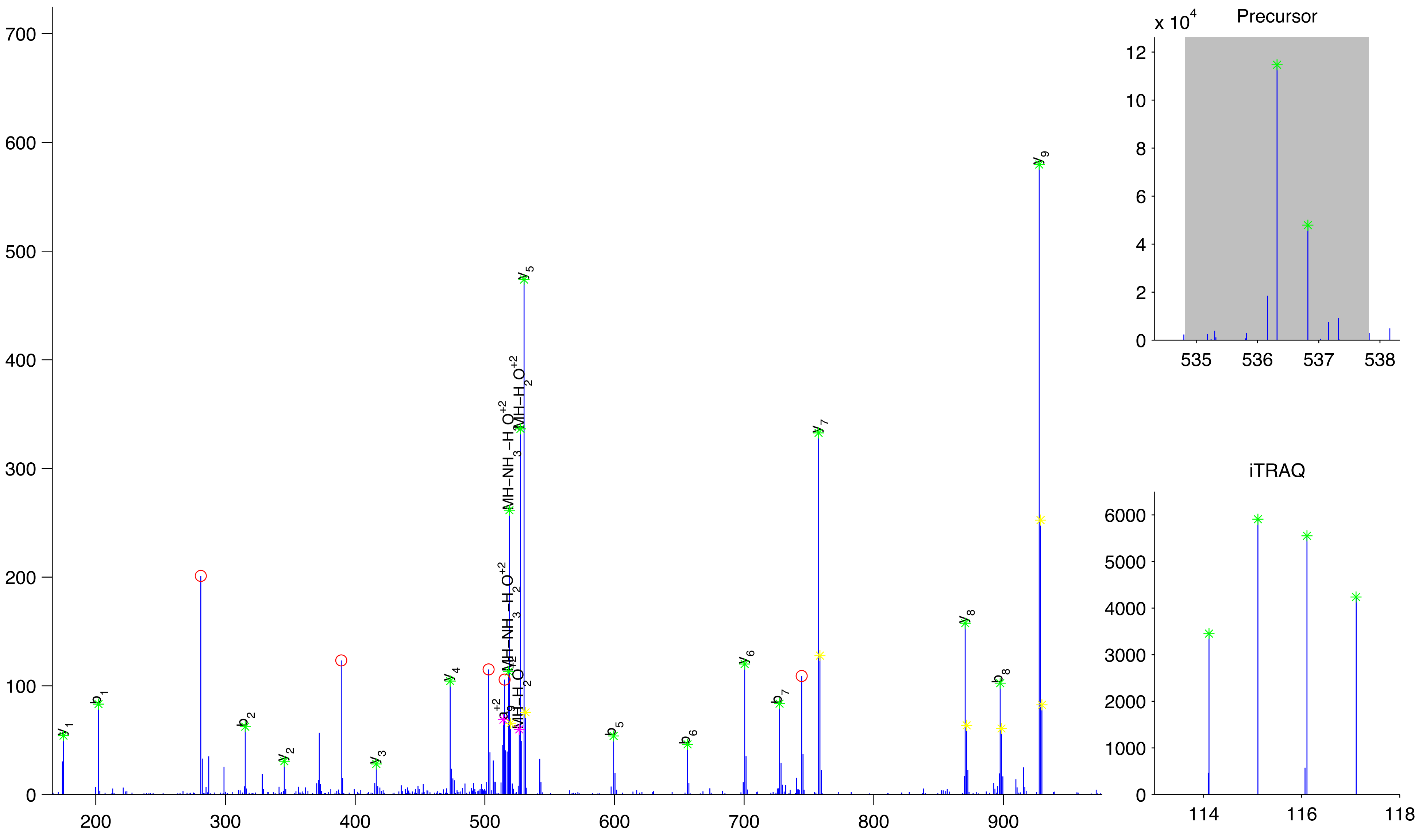

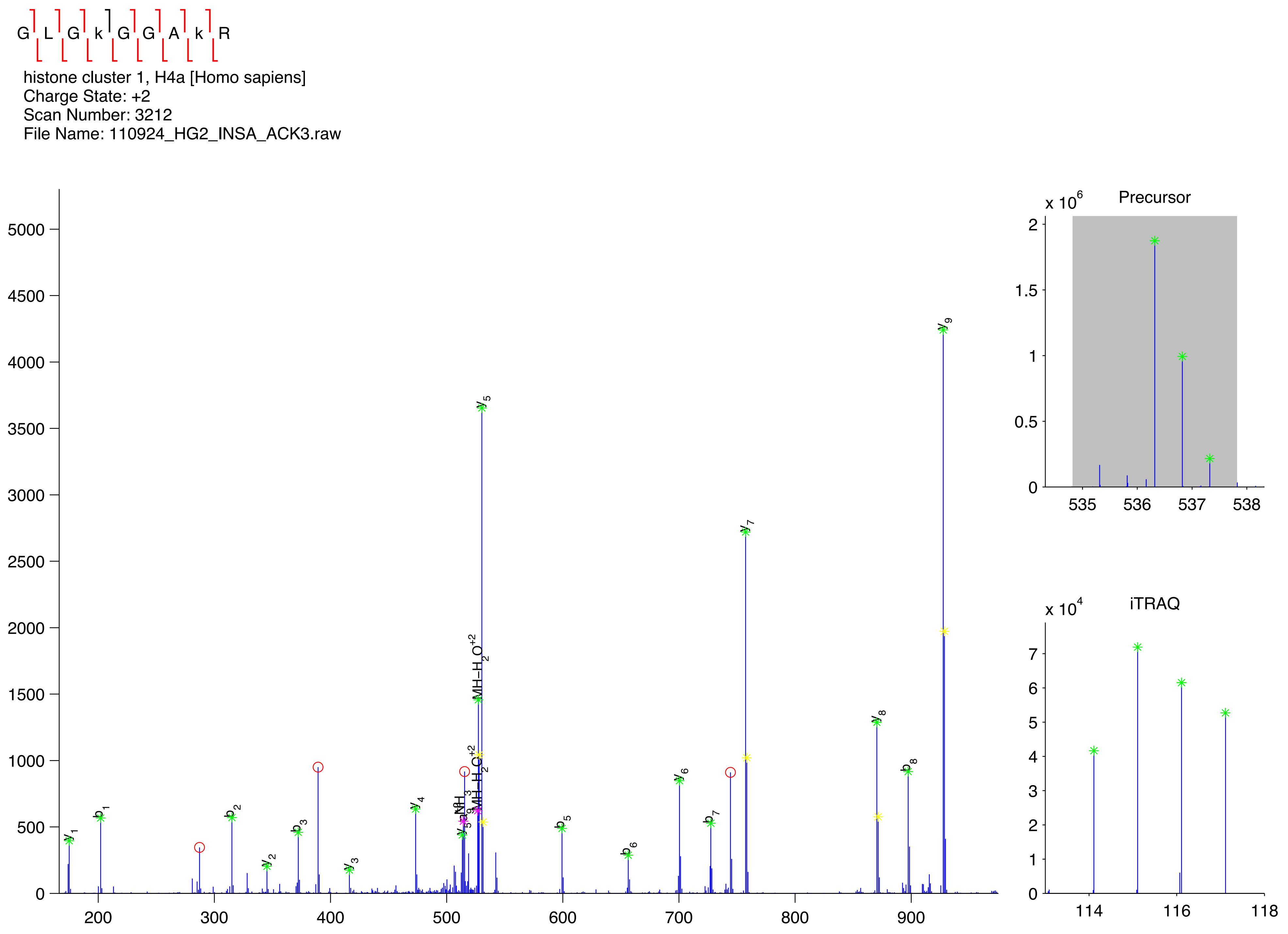

histone cluster 1, H4a [Homo sapiens]
Charge State: +2
Scan Number: 3212
File Name: 110924_HG2_INSA_ACK3.raw

G L G k G G A k R

histone cluster 1, H4a [Homo sapiens]
Charge State: +2
Scan Number: 3319
File Name: 110924_HG2_INSA_ACK3.raw

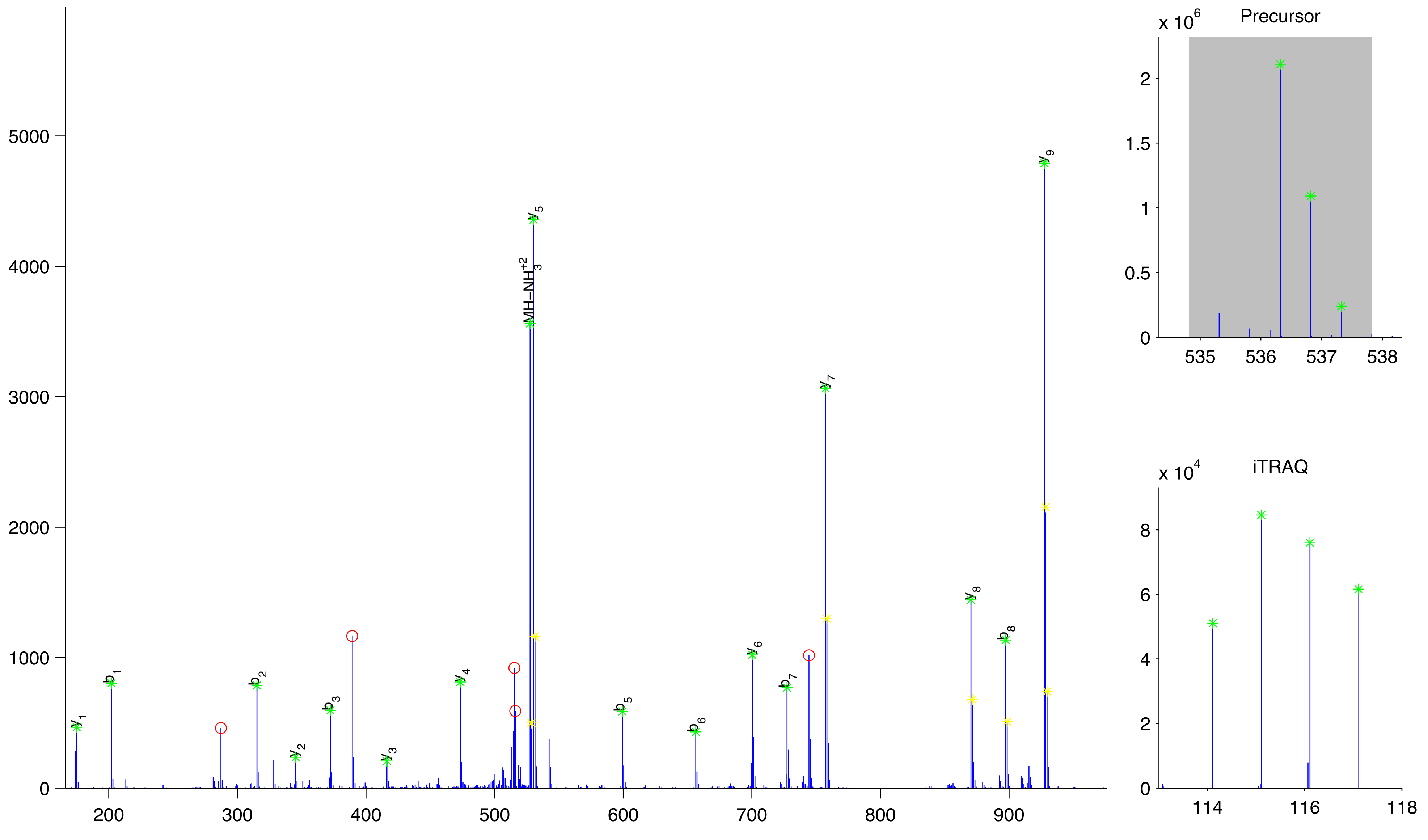

G L G k G G A k R

histone cluster 1, H4a [Homo sapiens]
Charge State: +2
Scan Number: 3365
File Name: 110924_HG2_INSA_ACK4.raw

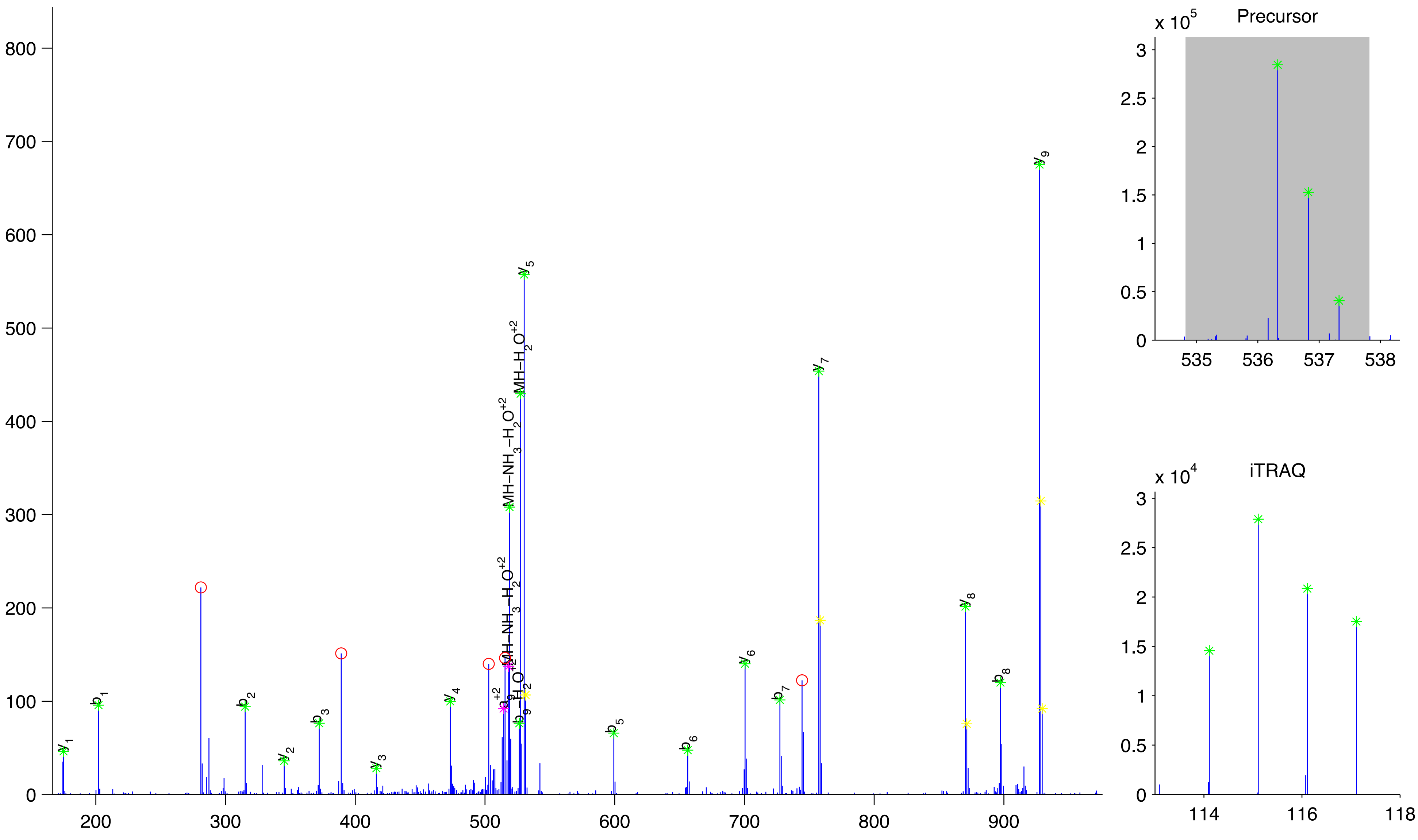

G L G k G G A k R

histone cluster 1, H4a [Homo sapiens]
Charge State: +2
Scan Number: 3382
File Name: 110924_HG2_INSA_ACK3.raw

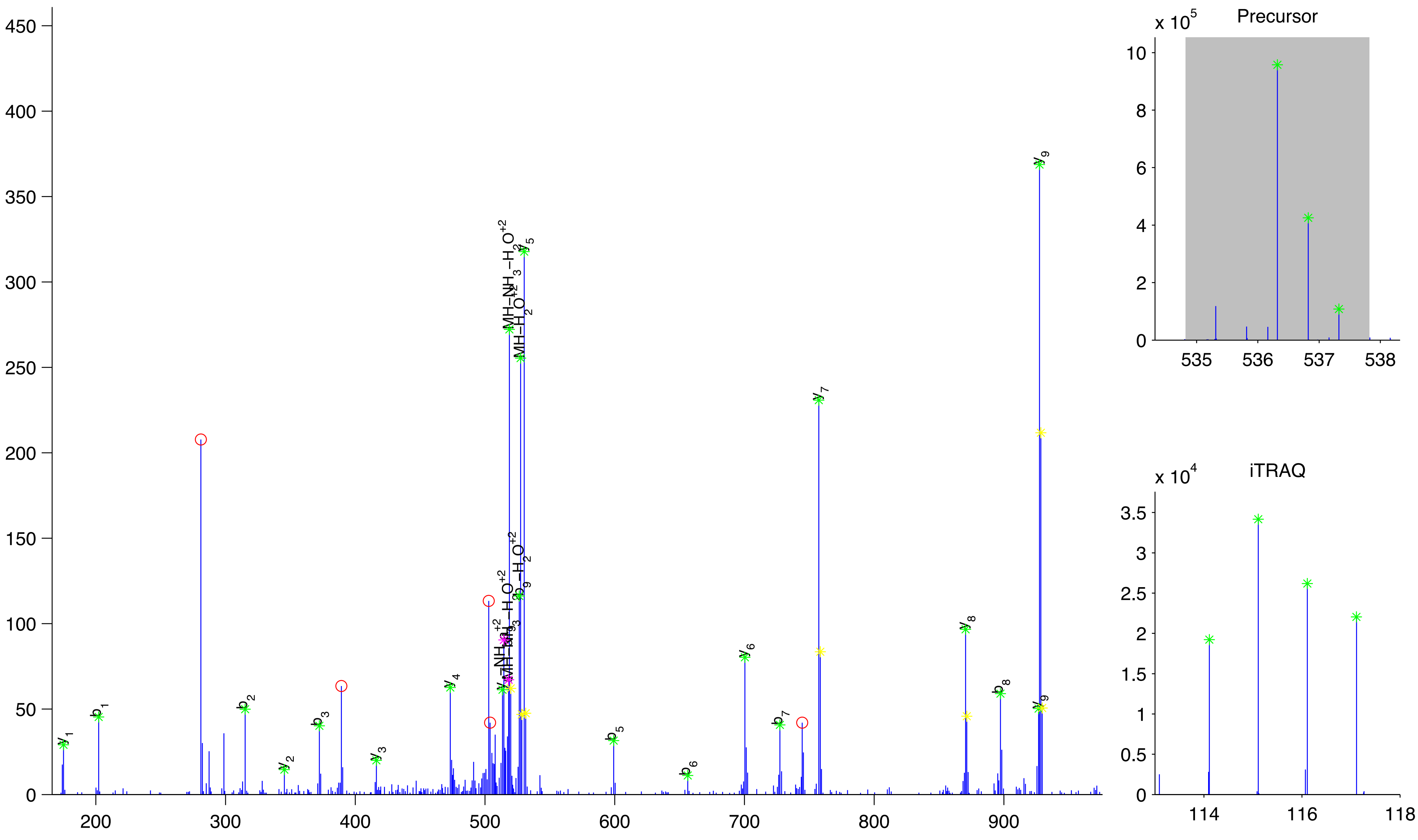

G G k G L G k G G A k R

histone cluster 1, H4a [Homo sapiens]
Charge State: +2
Scan Number: 3569
File Name: 110924_HG2_INSA_ACK3.raw

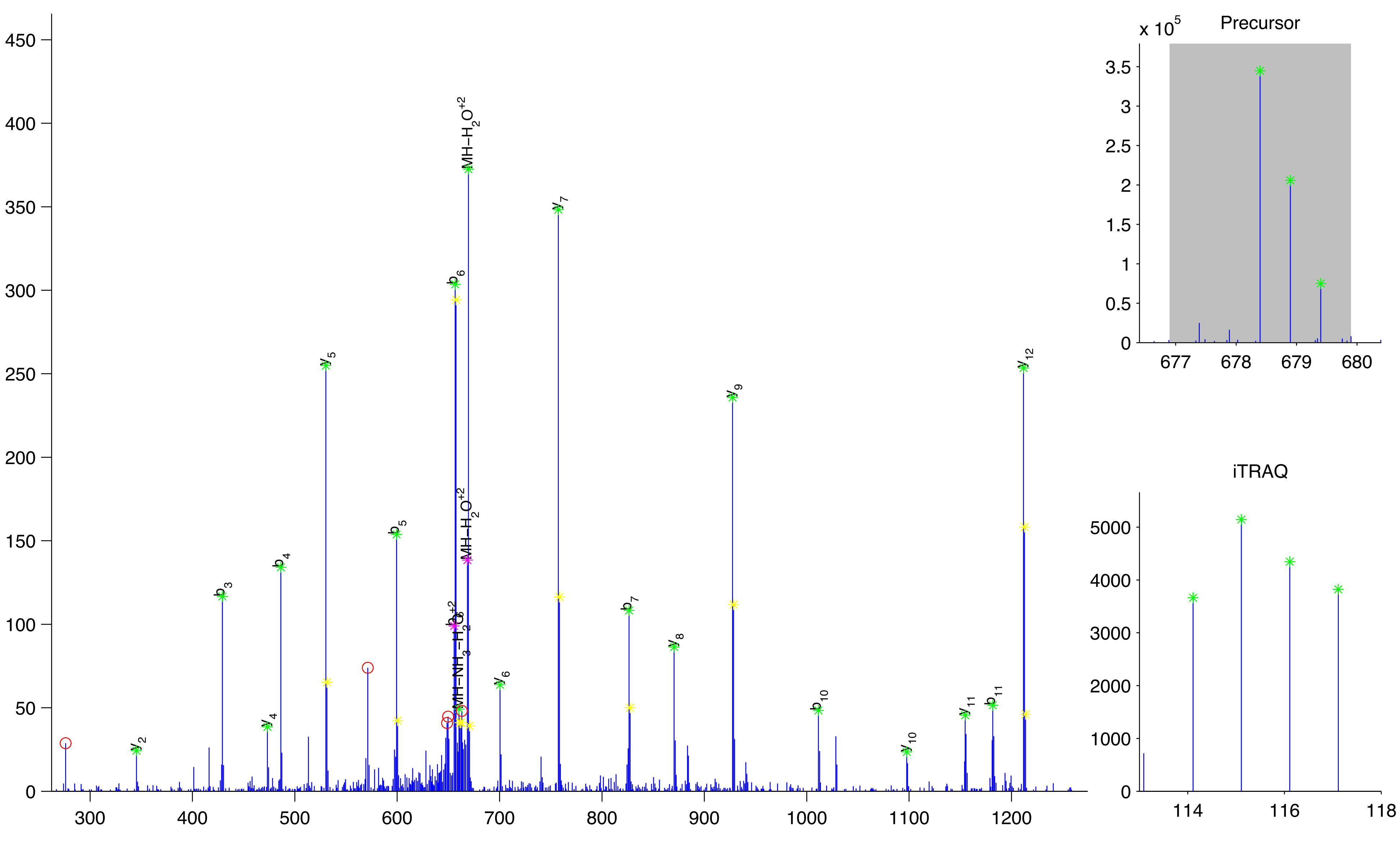

G G k G L G k G G A k R

histone cluster 1, H4a [Homo sapiens]
Charge State: +2
Scan Number: 3636
File Name: 110924_HG2_INSA_ACK4.raw

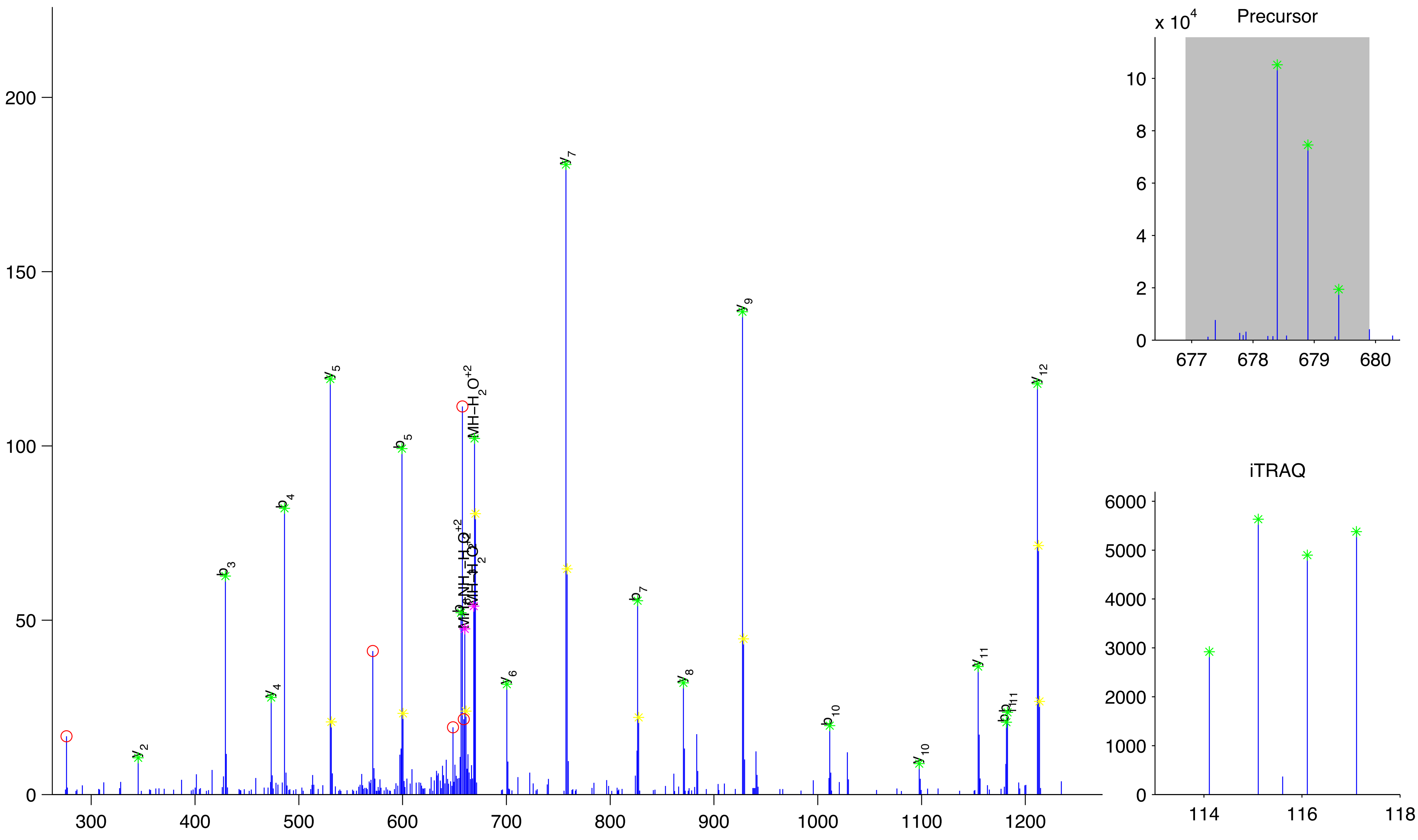

G G k G L G k G G A k R

histone cluster 1, H4a [Homo sapiens]
Charge State: +2
Scan Number: 3694
File Name: 111016_HG2_INSC_ACK.raw

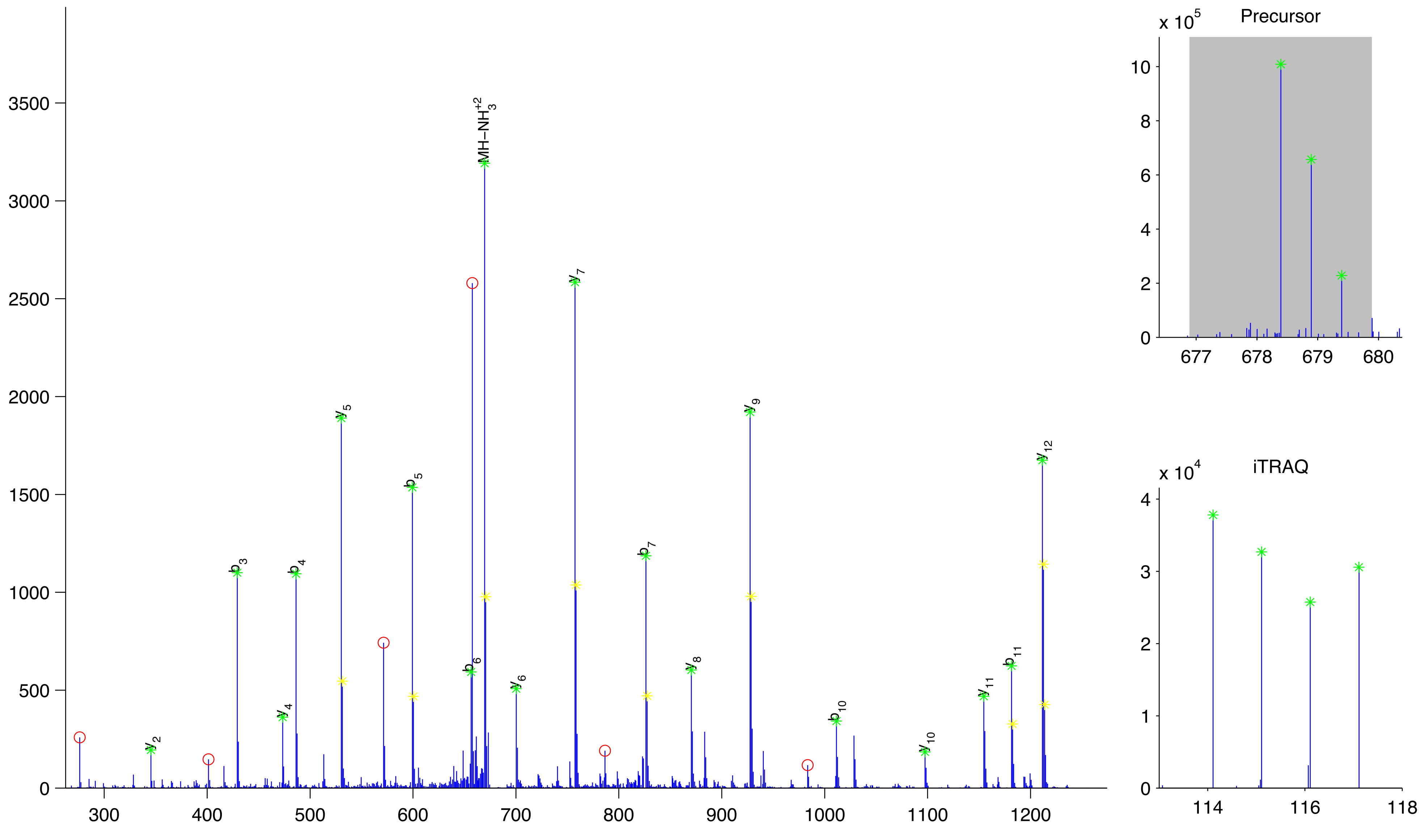

G L G k G G A k R

histone cluster 1, H4a [Homo sapiens]
Charge State: +2
Scan Number: 3703
File Name: 110924_HG2_INSA_ACK4.raw

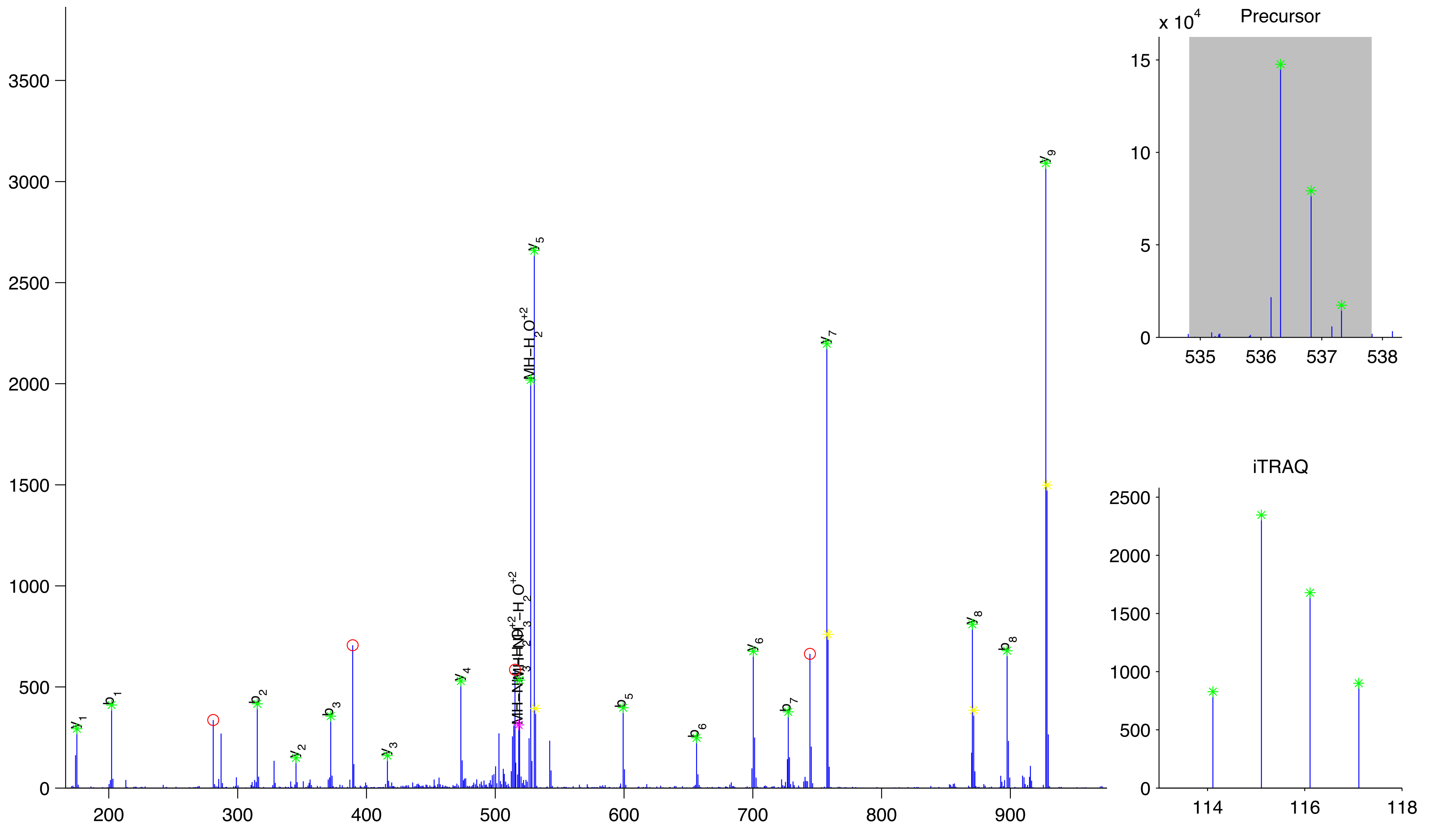

G L G k G G A k R

histone cluster 1, H4a [Homo sapiens]
Charge State: +2
Scan Number: 3720
File Name: 110924_HG2_INSA_ACK3.raw

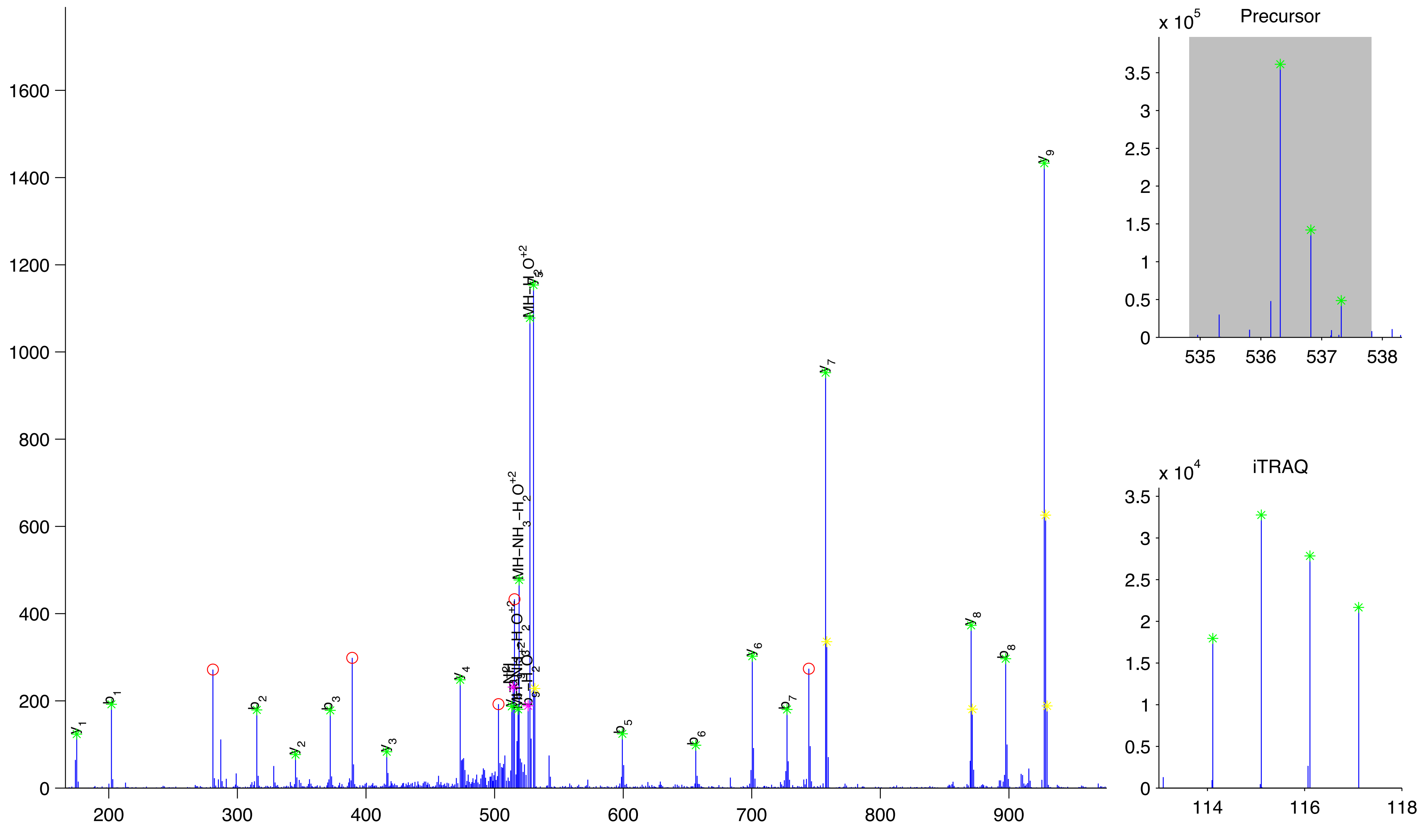

G G k G L G k G G A k R

histone cluster 1, H4a [Homo sapiens]
Charge State: +2
Scan Number: 3825
File Name: 111016_HG2_INSC_ACK.raw

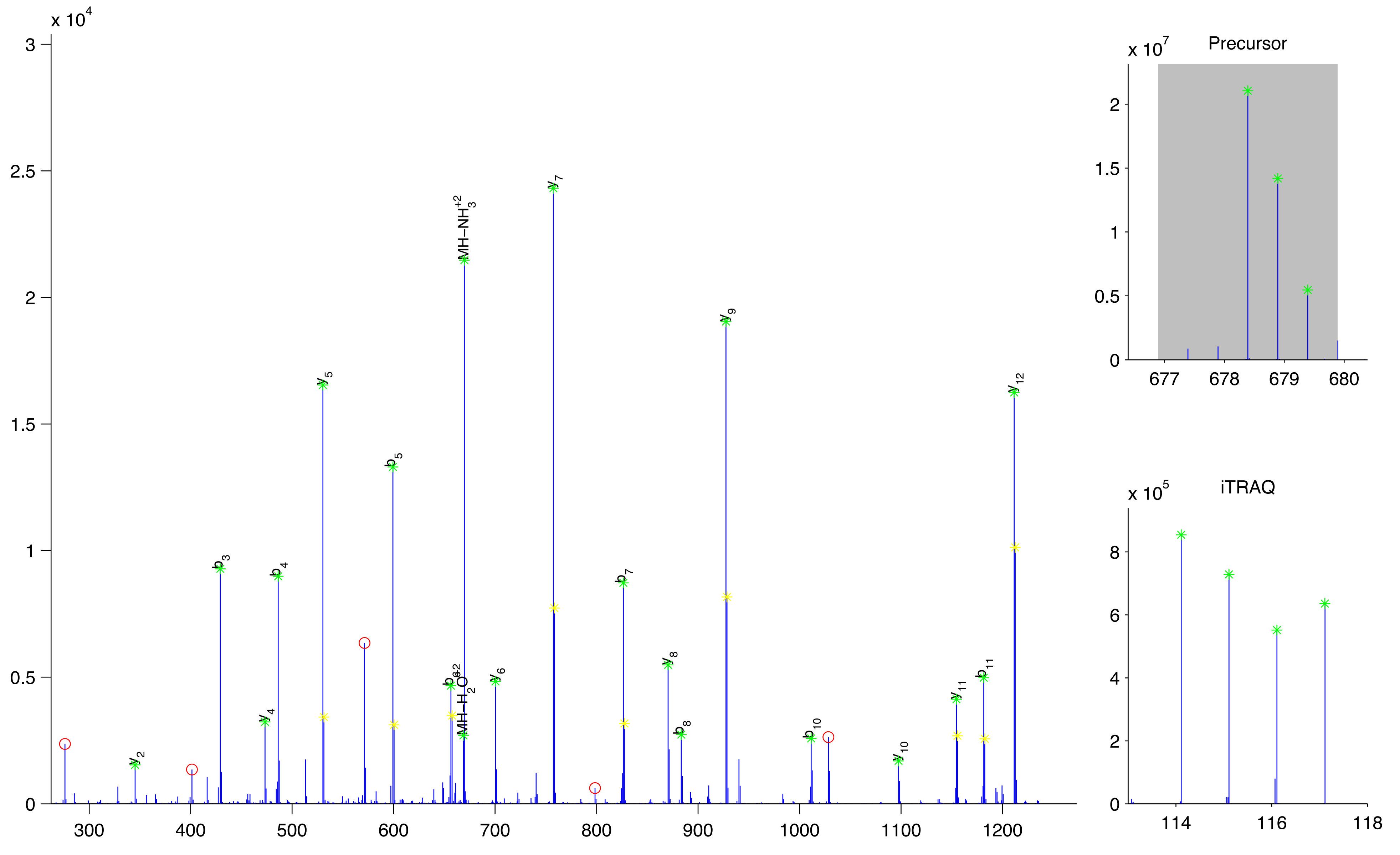

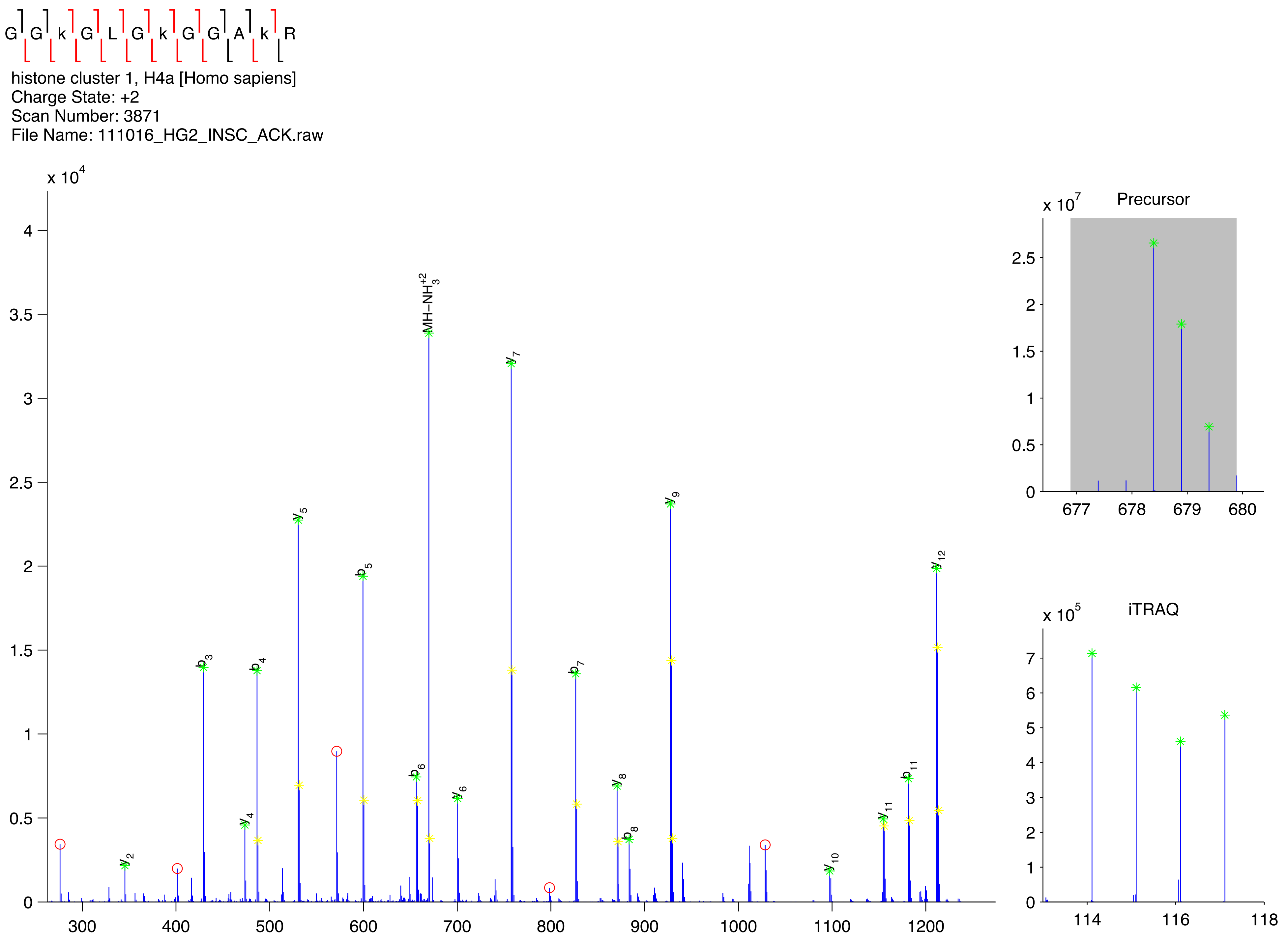

G G k G L G k G G A k R
histone cluster 1, H4a [Homo sapiens]
Charge State: +2
Scan Number: 3871
File Name: 111016_HG2_INSC_ACK.raw
x 10^4
4
3.5
3
2.5
2
1.5
1
0.5
0
300
400
500
600
700
800
900
1000
1100
1200
y2
b3
y4
b4
y5
b5
b6
MH−NH3+2
y6
y7
b7
y8
b8
y9
y10
y11
b11
y12
x 10^7
Precursor
2.5
2
1.5
1
0.5
0
677
678
679
680
x 10^5
iTRAQ
7
6
5
4
3
2
1
0
114
116
118

G G k G L G k G G A k R

histone cluster 1, H4a [Homo sapiens]
Charge State: +2
Scan Number: 3972
File Name: 110924_HG2_INSA_ACK4.raw

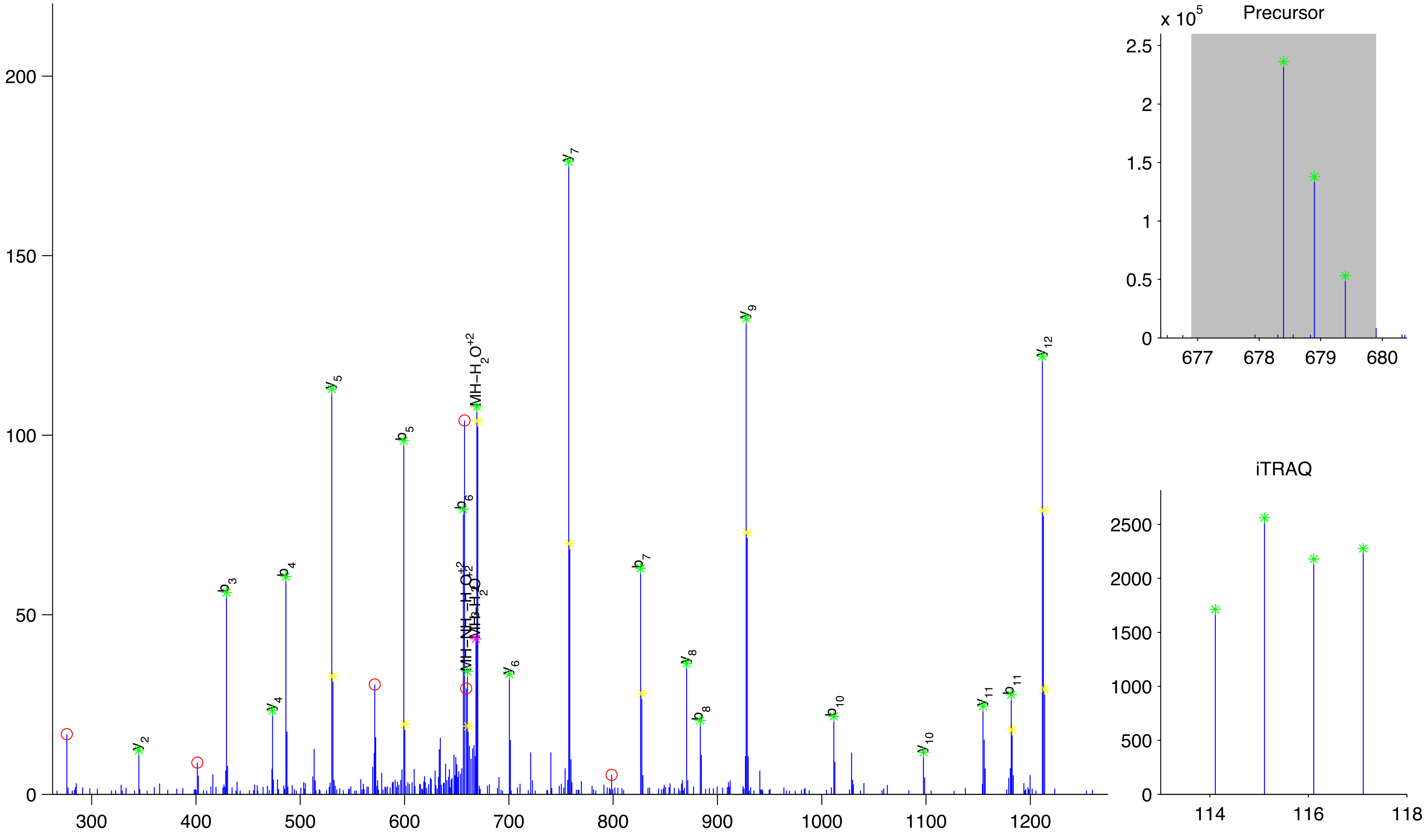

G G k G L G k G G A k R

histone cluster 1, H4a [Homo sapiens]
Charge State: +2
Scan Number: 4016
File Name: 111016_HG2_INSC_ACK.raw

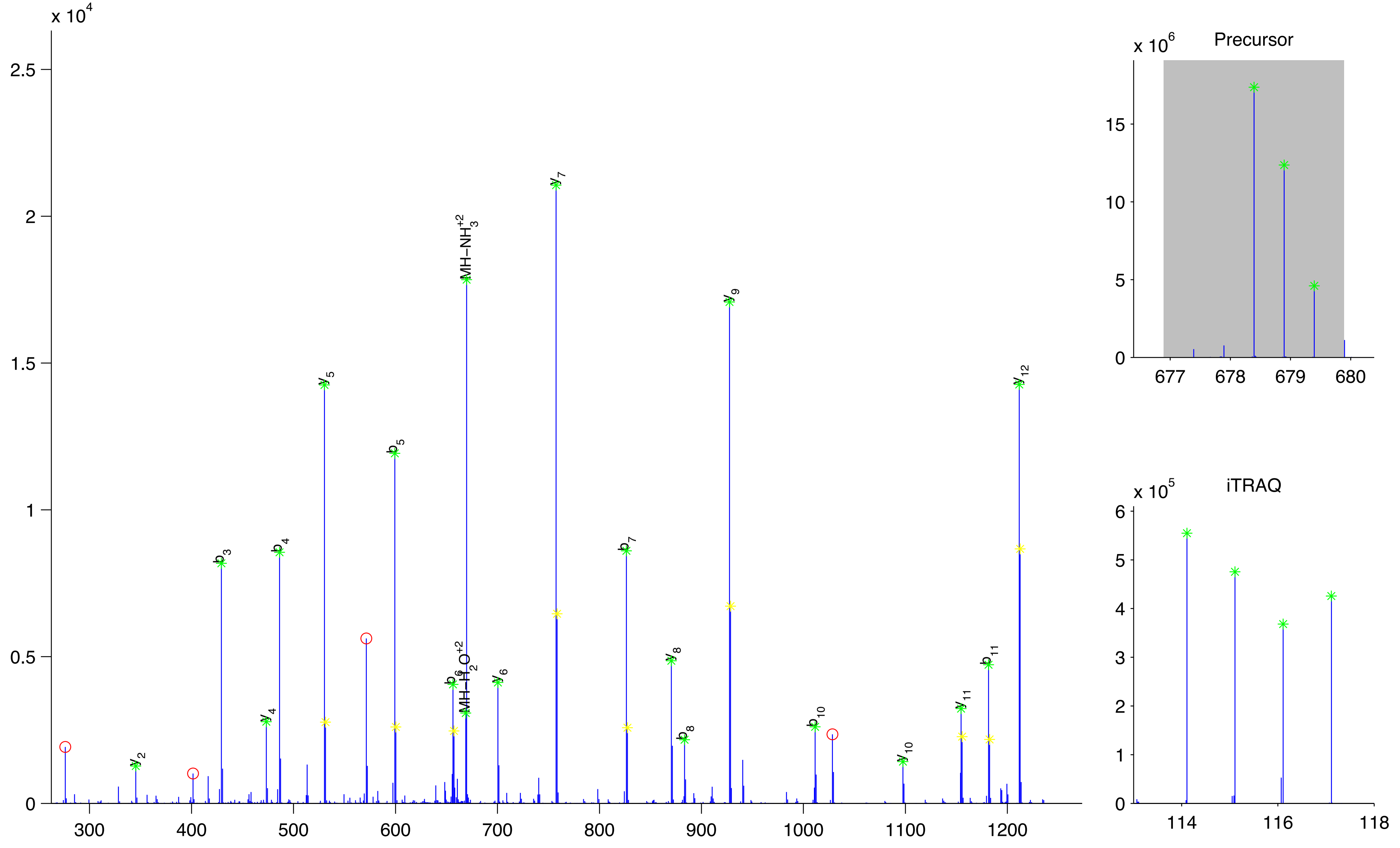

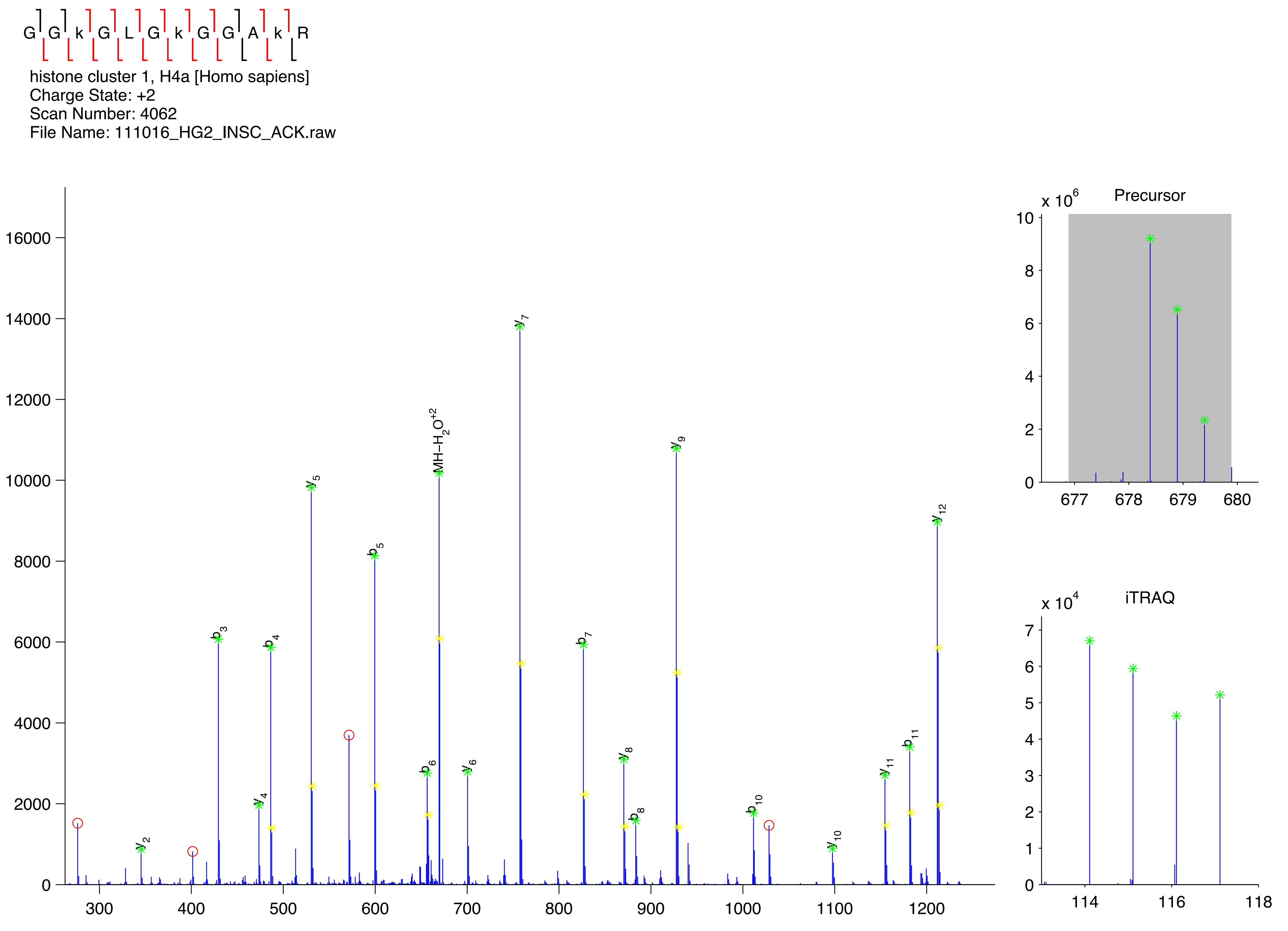

G G k G L G k G G A k R
histone cluster 1, H4a [Homo sapiens]
Charge State: +2
Scan Number: 4062
File Name: 111016_HG2_INSC_ACK.raw
y2
b3
y4
b4
y5
b5
b6
MH−H2O+2
y6
y7
b7
y8
b8
y9
b10
y10
y11
b11
y12
Precursor
x 10^6
iTRAQ
x 10^4

G k G G k G L G k G G A k R

histone cluster 1, H4a [Homo sapiens]
Charge State: +2
Scan Number: 4179
File Name: 111016_HG2_INSC_ACK.raw

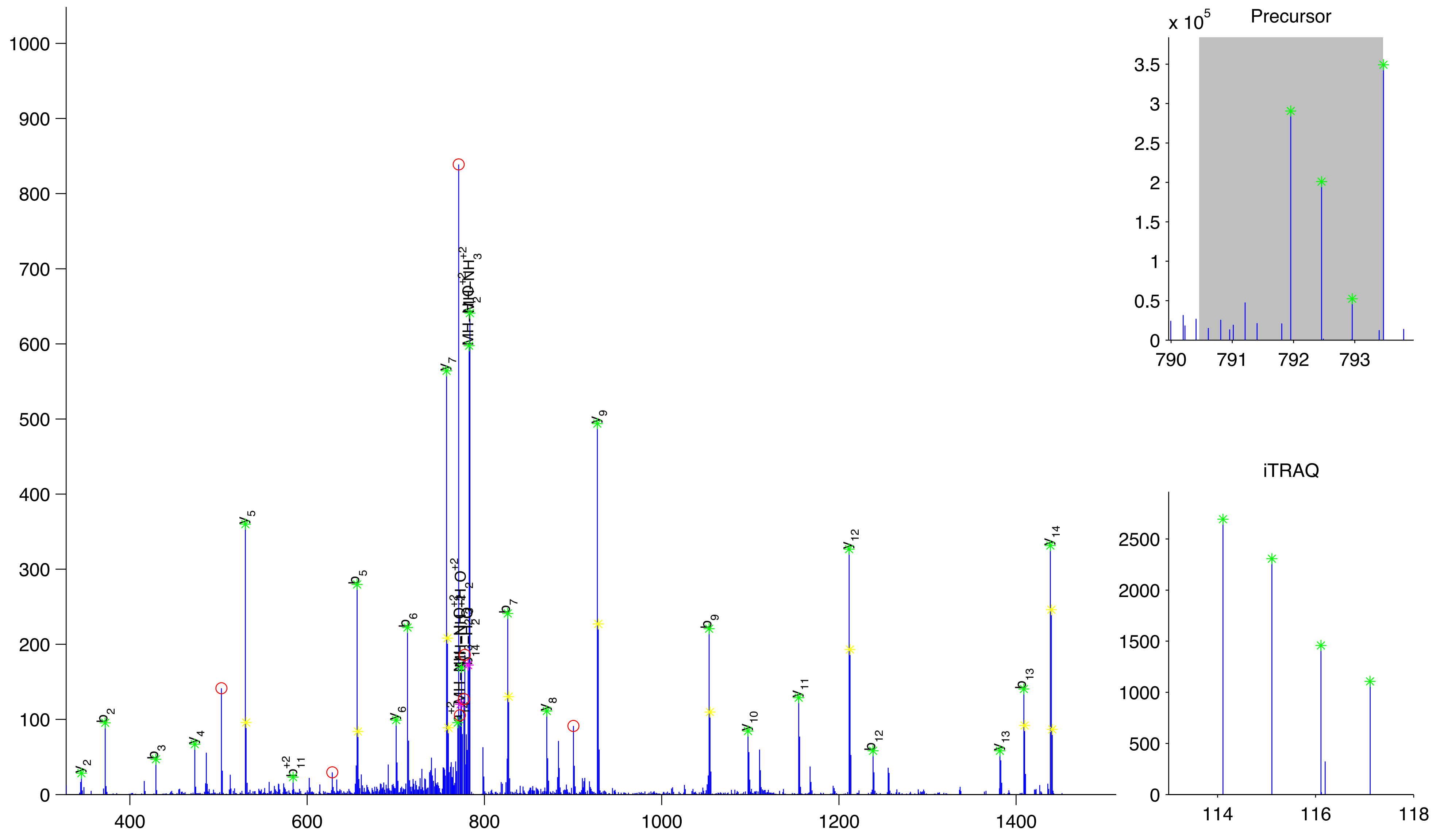

G G k G L G k G G A k R

histone cluster 1, H4a [Homo sapiens]
Charge State: +2
Scan Number: 4184
File Name: 111016_HG2_INSC_ACK.raw

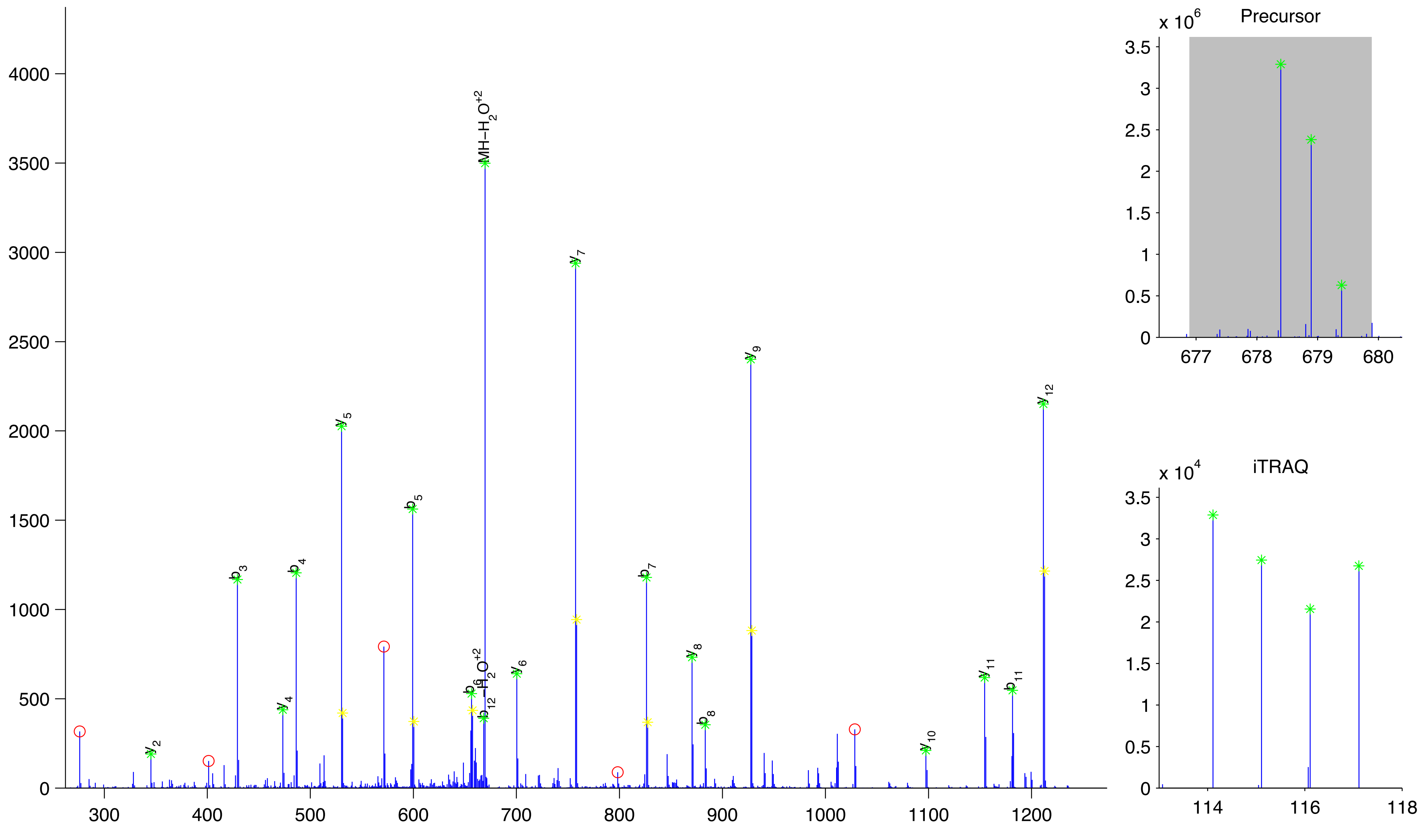

G L G k G G A k R

histone cluster 1, H4a [Homo sapiens]
Charge State: +2
Scan Number: 4222
File Name: 110924_HG2_INSA_ACK3.raw

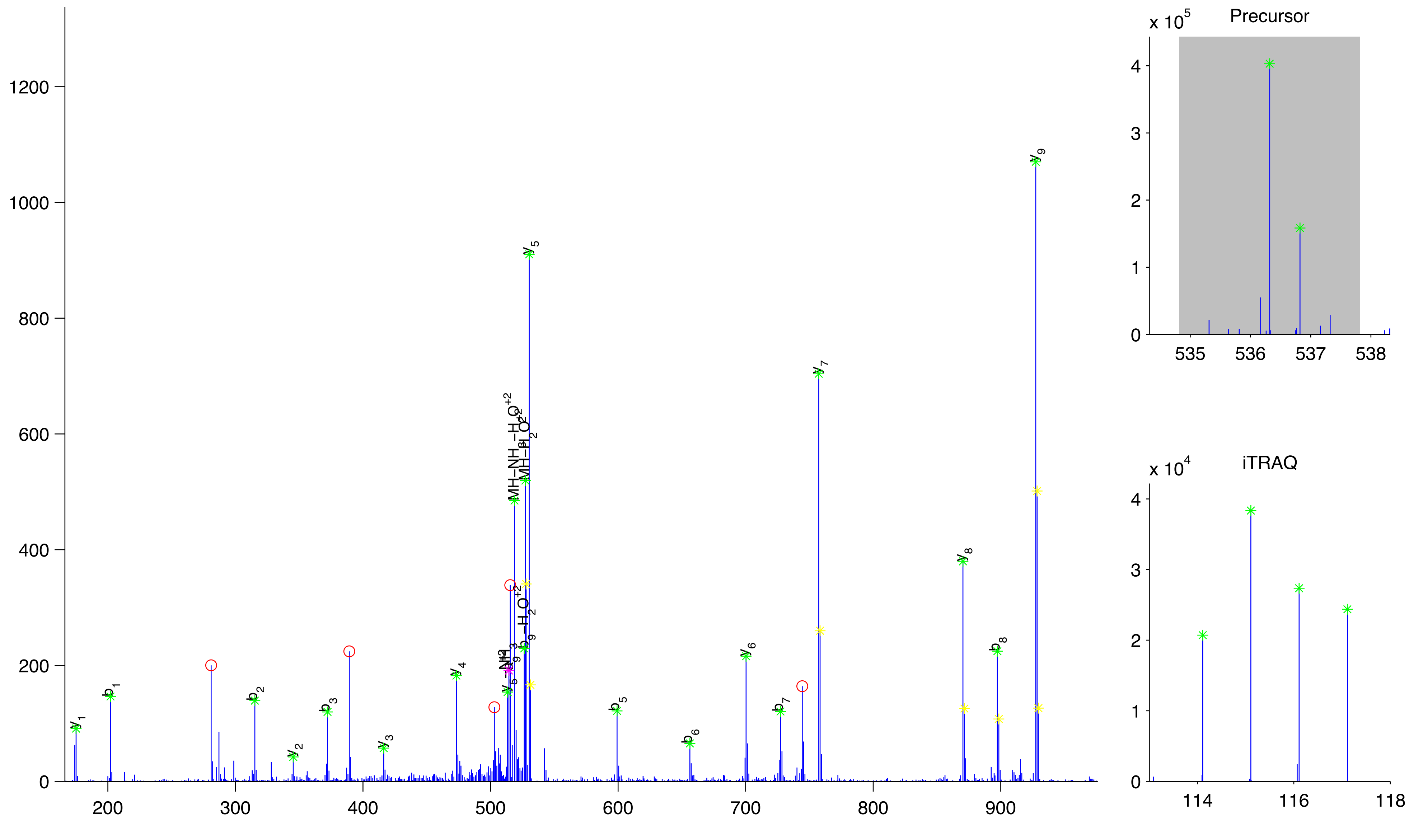

G G k G L G k G G A k R

histone cluster 1, H4a [Homo sapiens]
Charge State: +2
Scan Number: 4242
File Name: 111016_HG2_INSC_ACK.raw

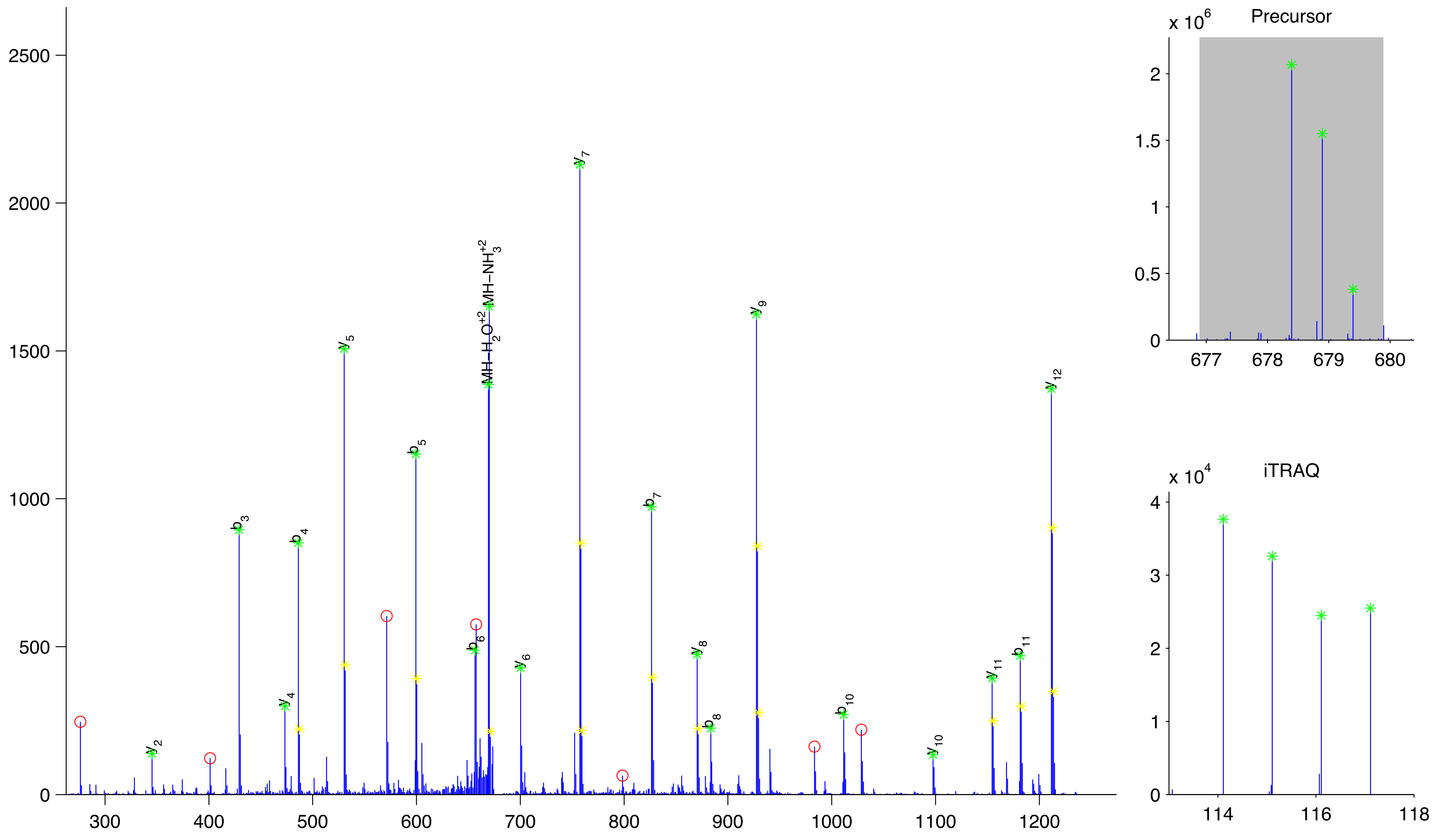

G k G G k G L G k G G A k R

histone cluster 1, H4a [Homo sapiens]
Charge State: +2
Scan Number: 4249
File Name: 111016_HG2_INSC_ACK.raw

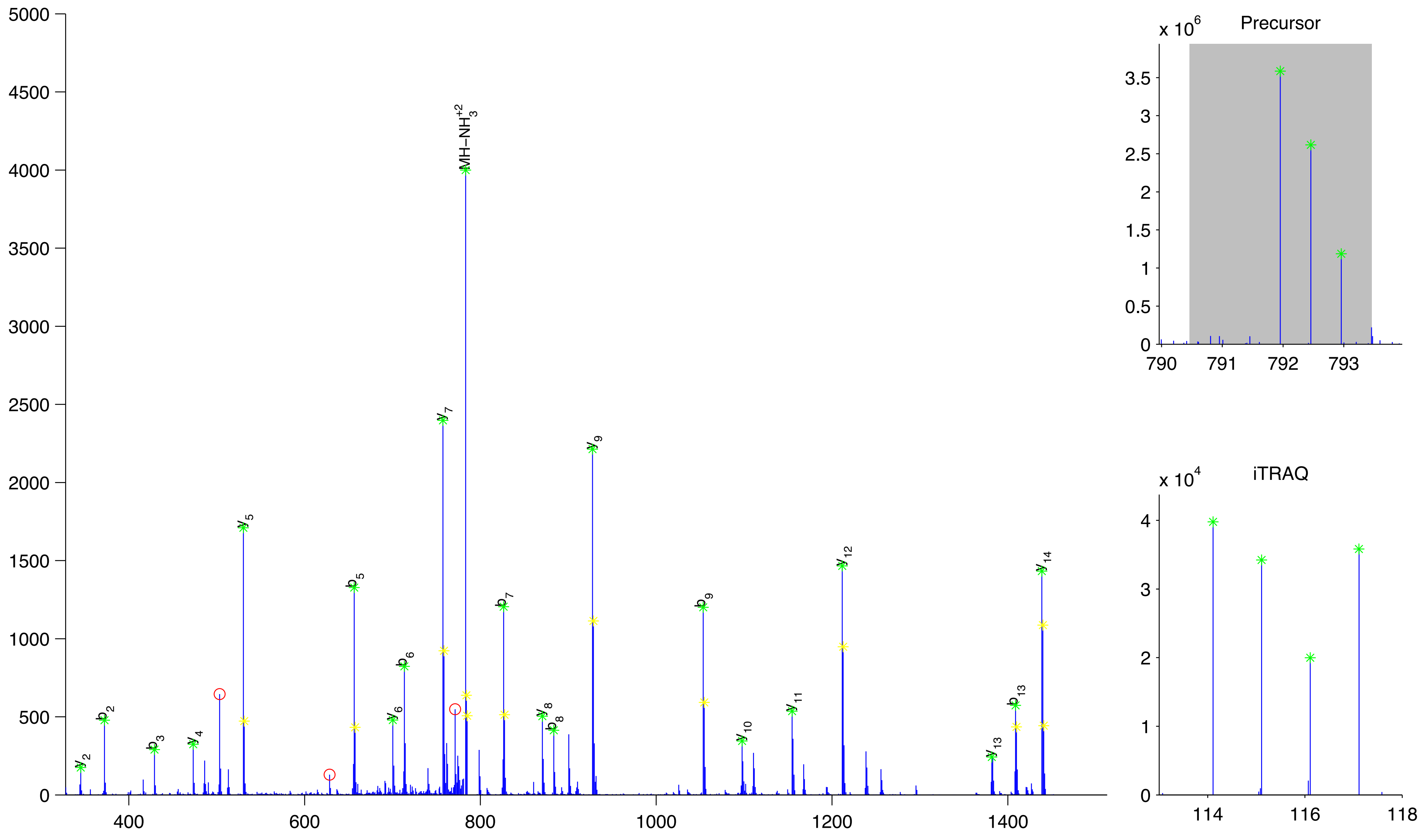

G k G G k G L G k G G A k R

histone cluster 1, H4a [Homo sapiens]
Charge State: +3
Scan Number: 4287
File Name: 111016_HG2_INSC_ACK.raw

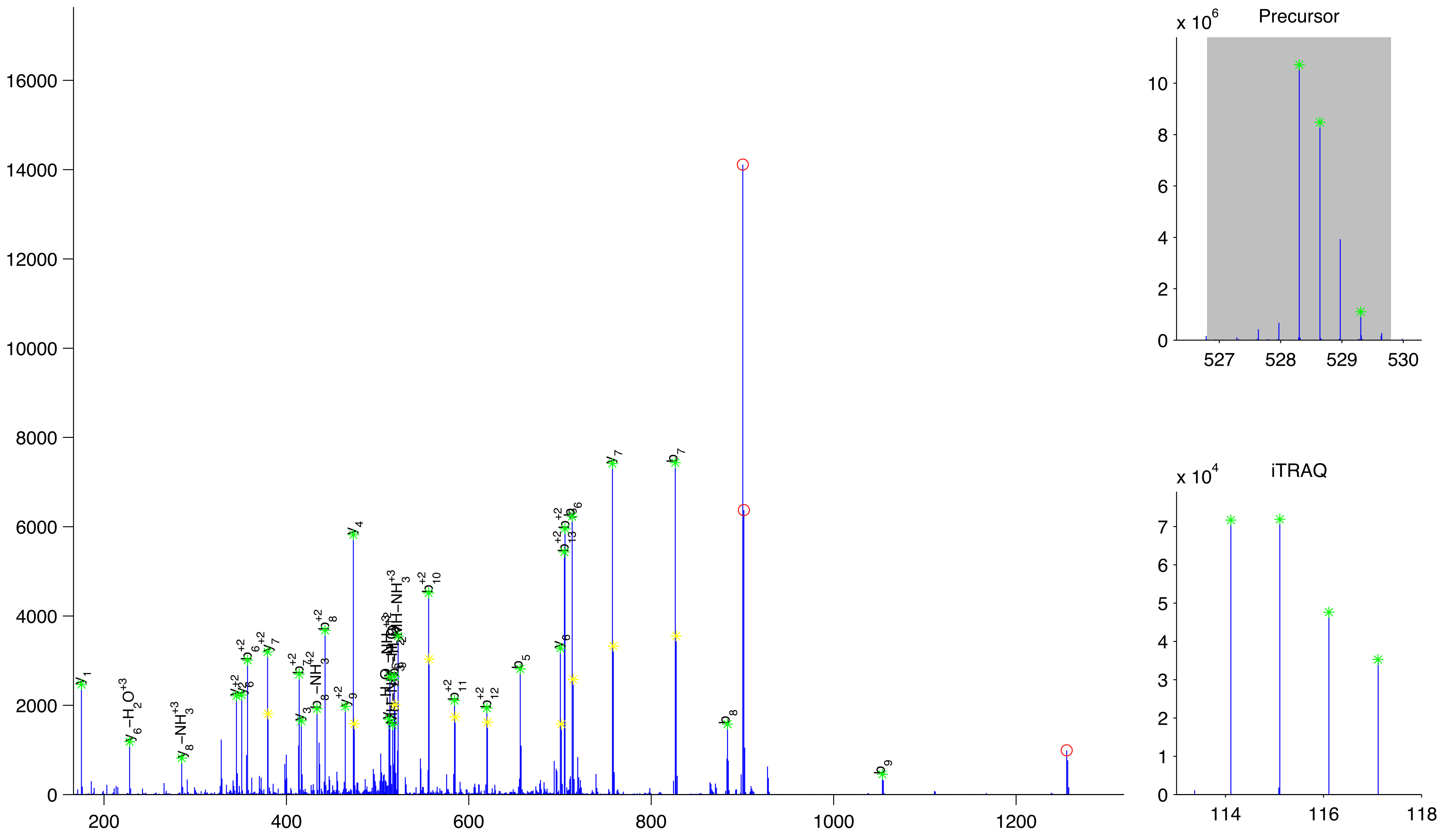

G k G G k G L G k G G A k R

histone cluster 1, H4a [Homo sapiens]
Charge State: +3
Scan Number: 4313
File Name: 111016_HG2_INSC_ACK.raw

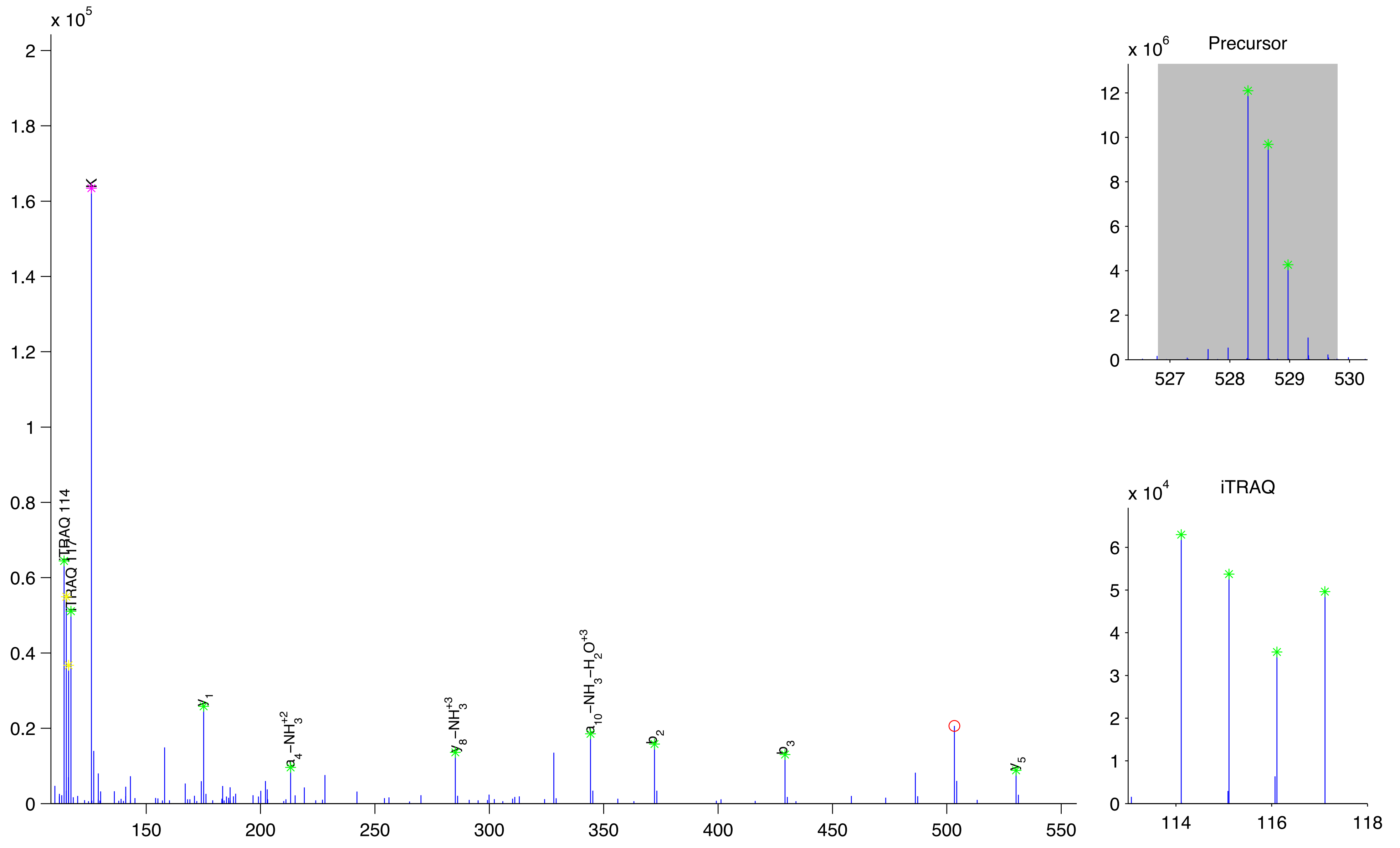

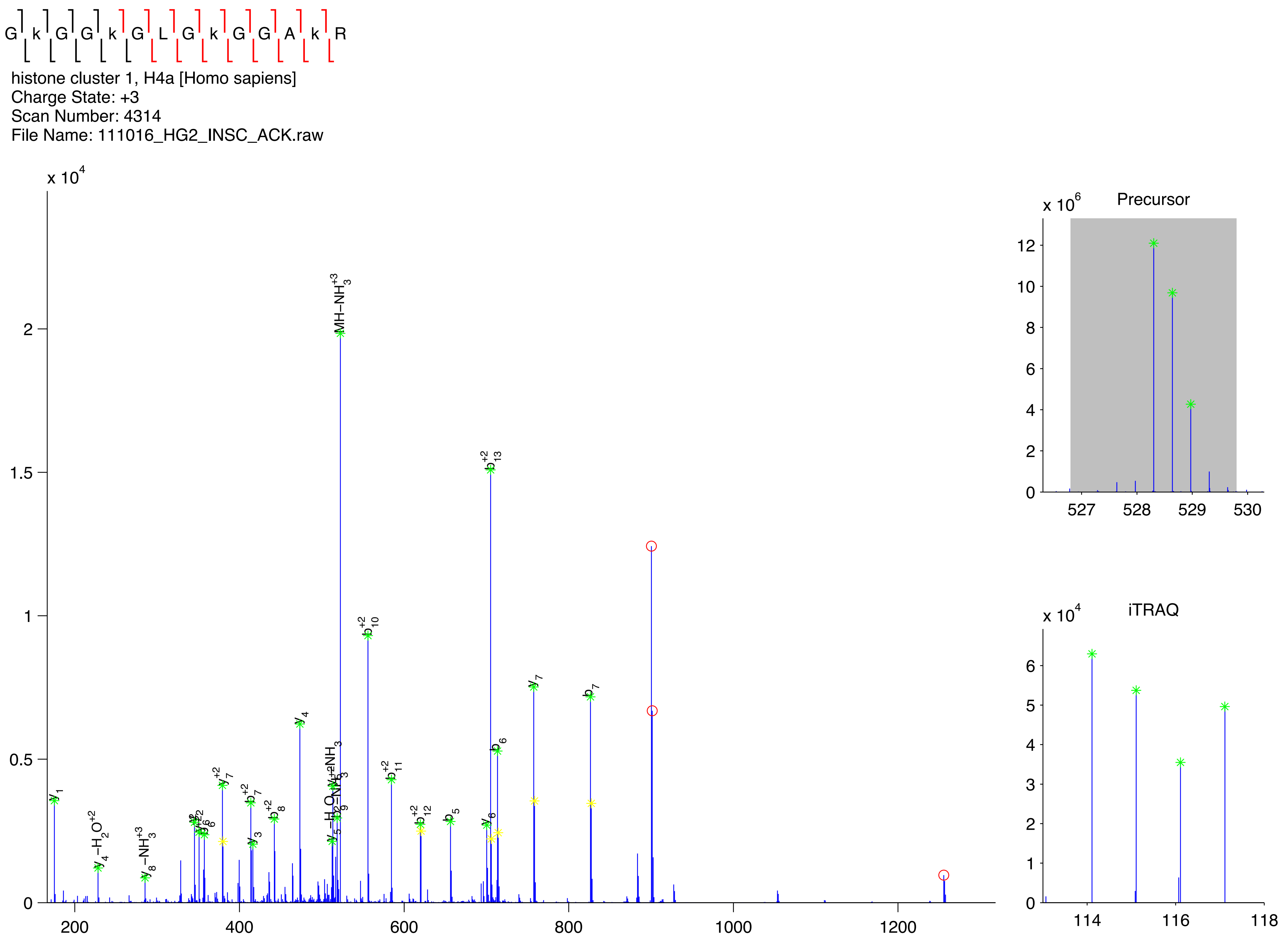

G k G G k G L G k G G A k R
histone cluster 1, H4a [Homo sapiens]
Charge State: +3
Scan Number: 4314
File Name: 111016_HG2_INSC_ACK.raw
x 10^4
MH−NH3+3
b13+2
b10+2
y4
y7
b7
b6
b11+2
y1
y4−H2O+2
y8−NH3+3
y7+2
b7+2
b8+2
y3
b12+2
b5
y6
0 0.5 1 1.5 2
200 400 600 800 1000 1200
x 10^6
Precursor
0 2 4 6 8 10 12
527 528 529 530
x 10^4
iTRAQ
0 1 2 3 4 5 6
114 116 118

G G k G L G k G G A k R

histone cluster 1, H4a [Homo sapiens]
Charge State: +2
Scan Number: 4329
File Name: 110924_HG2_INSA_ACK4.raw

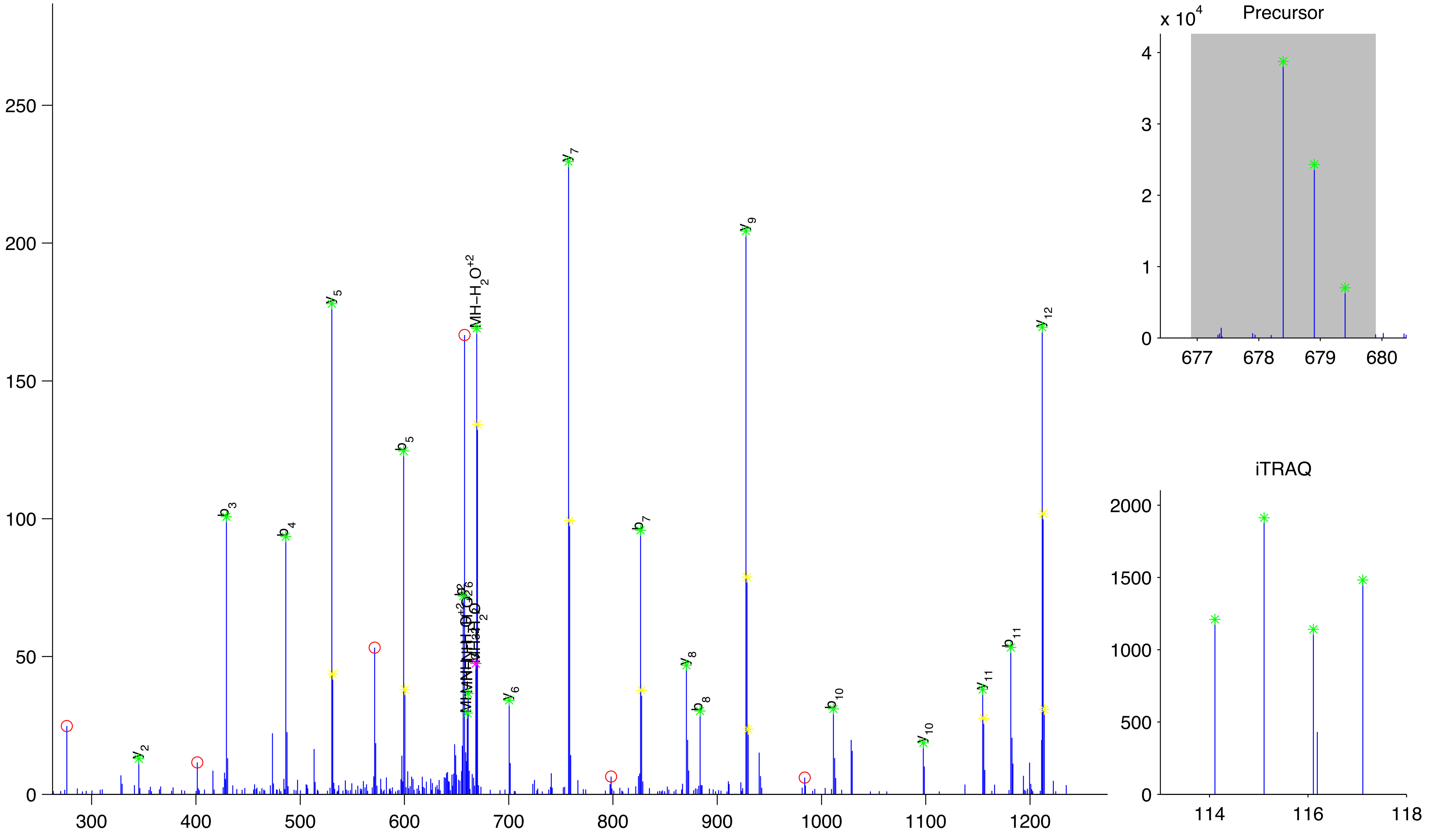

G k G G k G L G k G G A k R

histone cluster 1, H4a [Homo sapiens]
Charge State: +2
Scan Number: 4331
File Name: 111016_HG2_INSC_ACK.raw

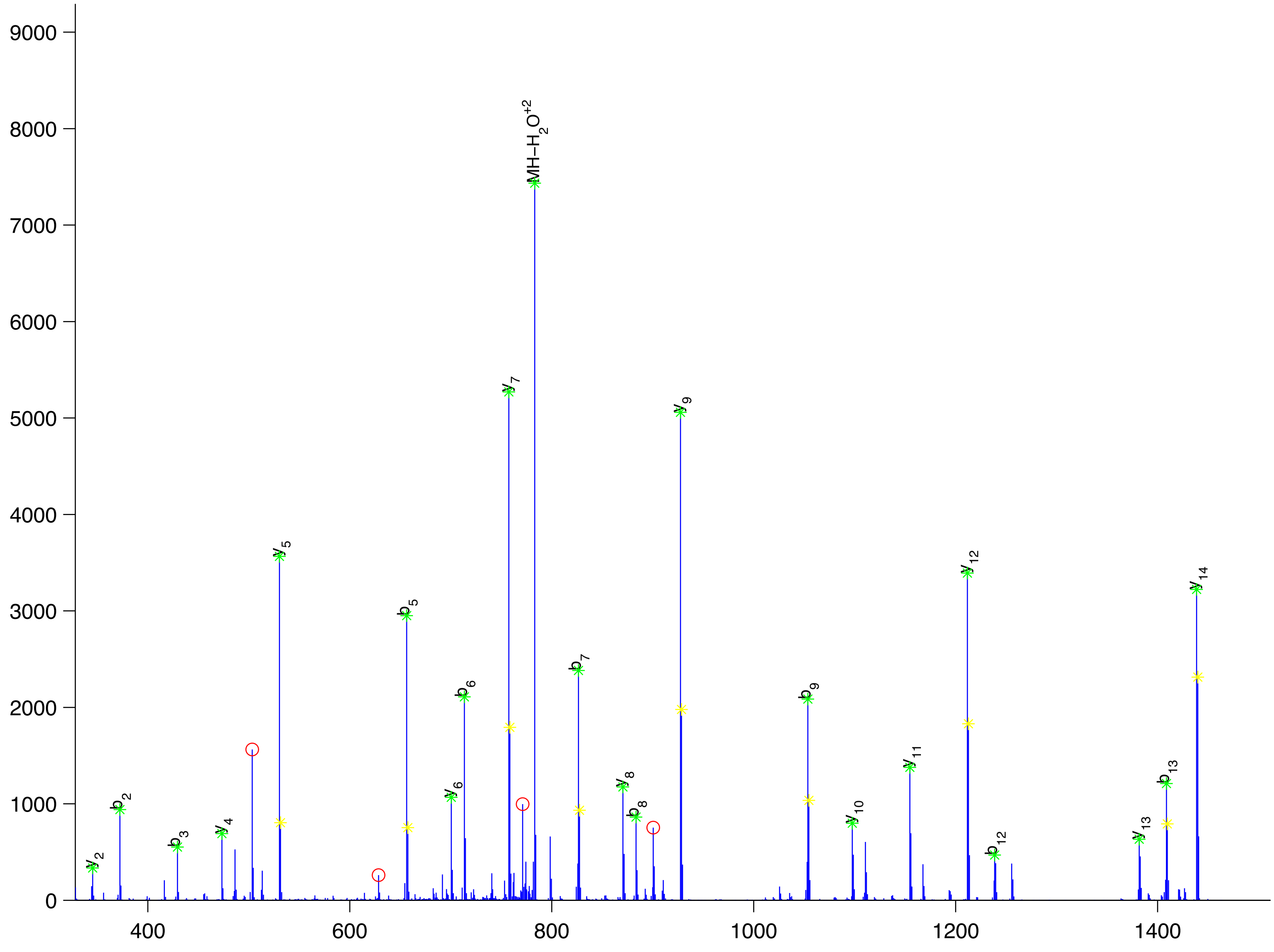

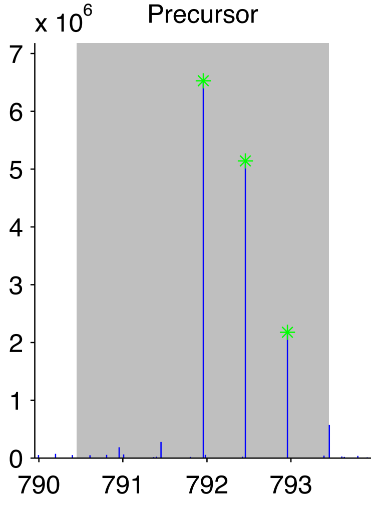

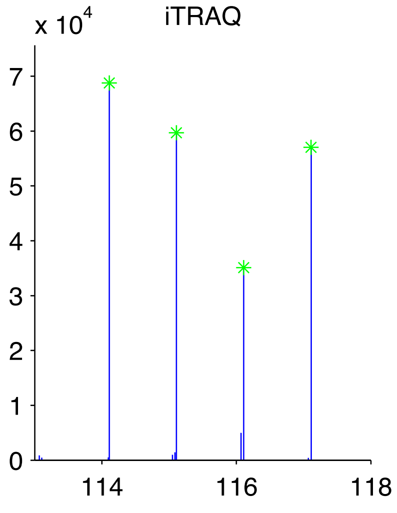

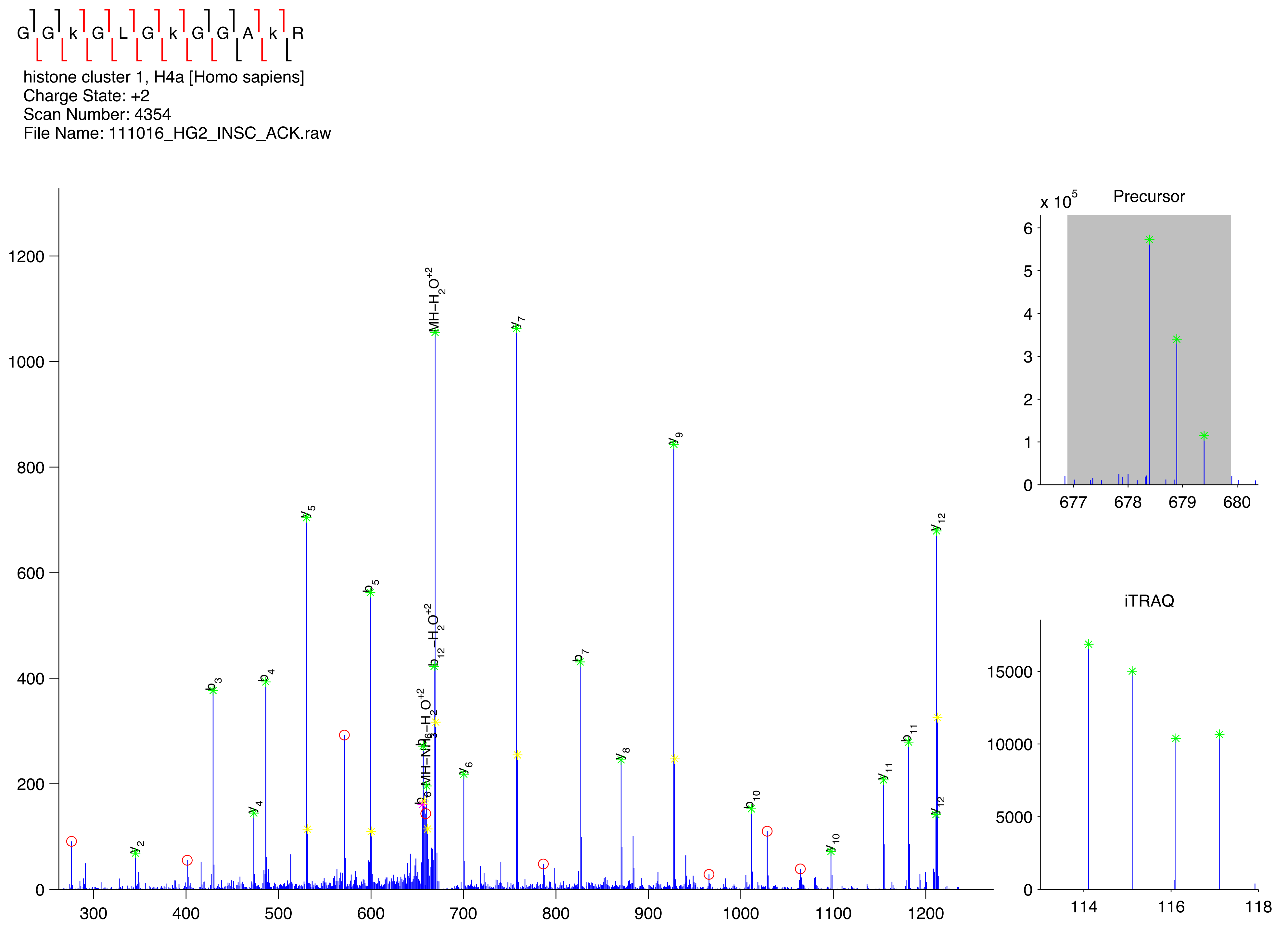

G G k G L G k G G A k R
histone cluster 1, H4a [Homo sapiens]
Charge State: +2
Scan Number: 4354
File Name: 111016_HG2_INSC_ACK.raw
Precursor
x 10^5
iTRAQ

G k G G k G L G k G G A k R

histone cluster 1, H4a [Homo sapiens]
Charge State: +2
Scan Number: 4423
File Name: 111016_HG2_INSC_ACK.raw

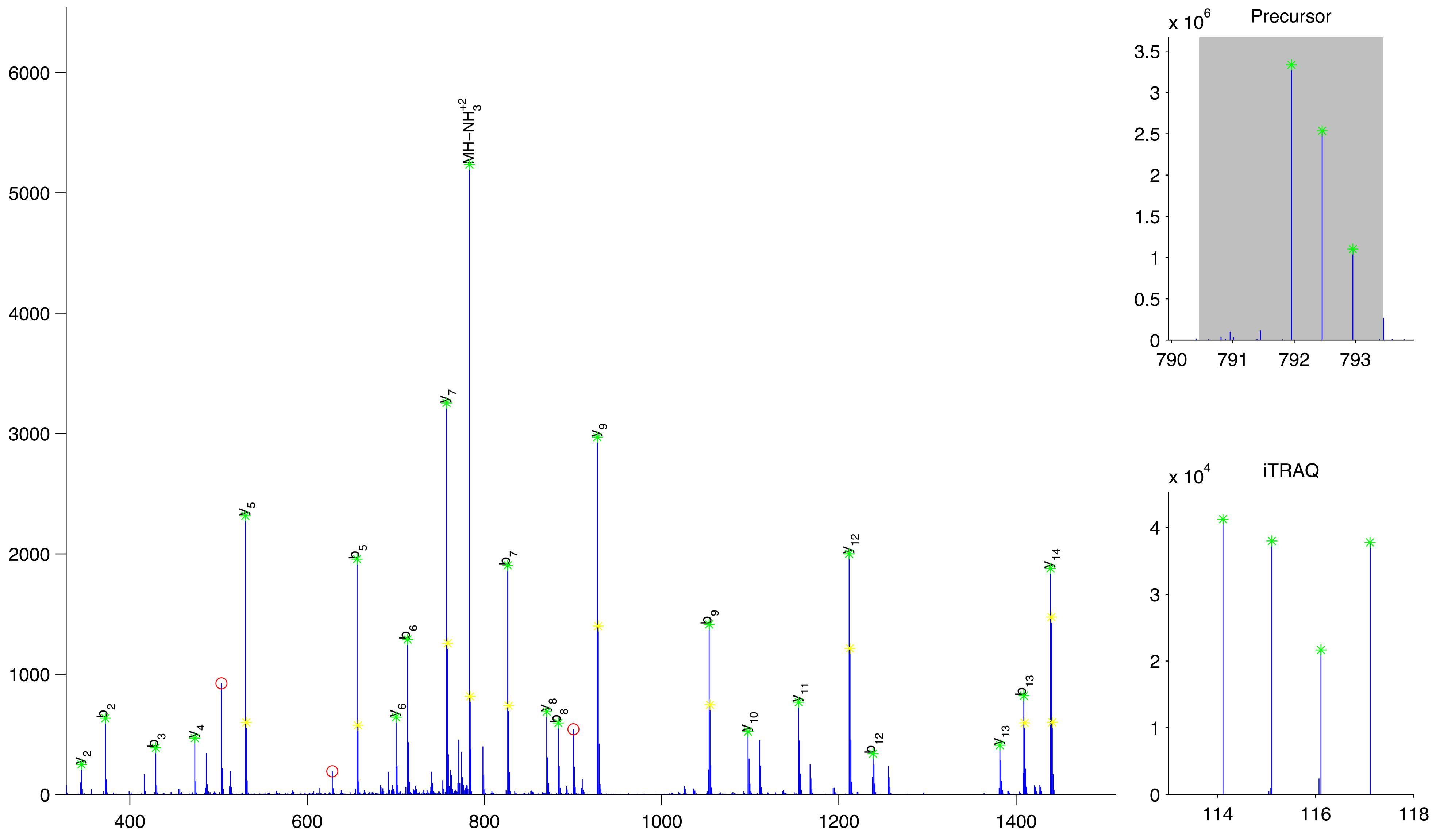

G k G G k G L G k G G A k R

histone cluster 1, H4a [Homo sapiens]
Charge State: +3
Scan Number: 4455
File Name: 111016_HG2_INSC_ACK.raw

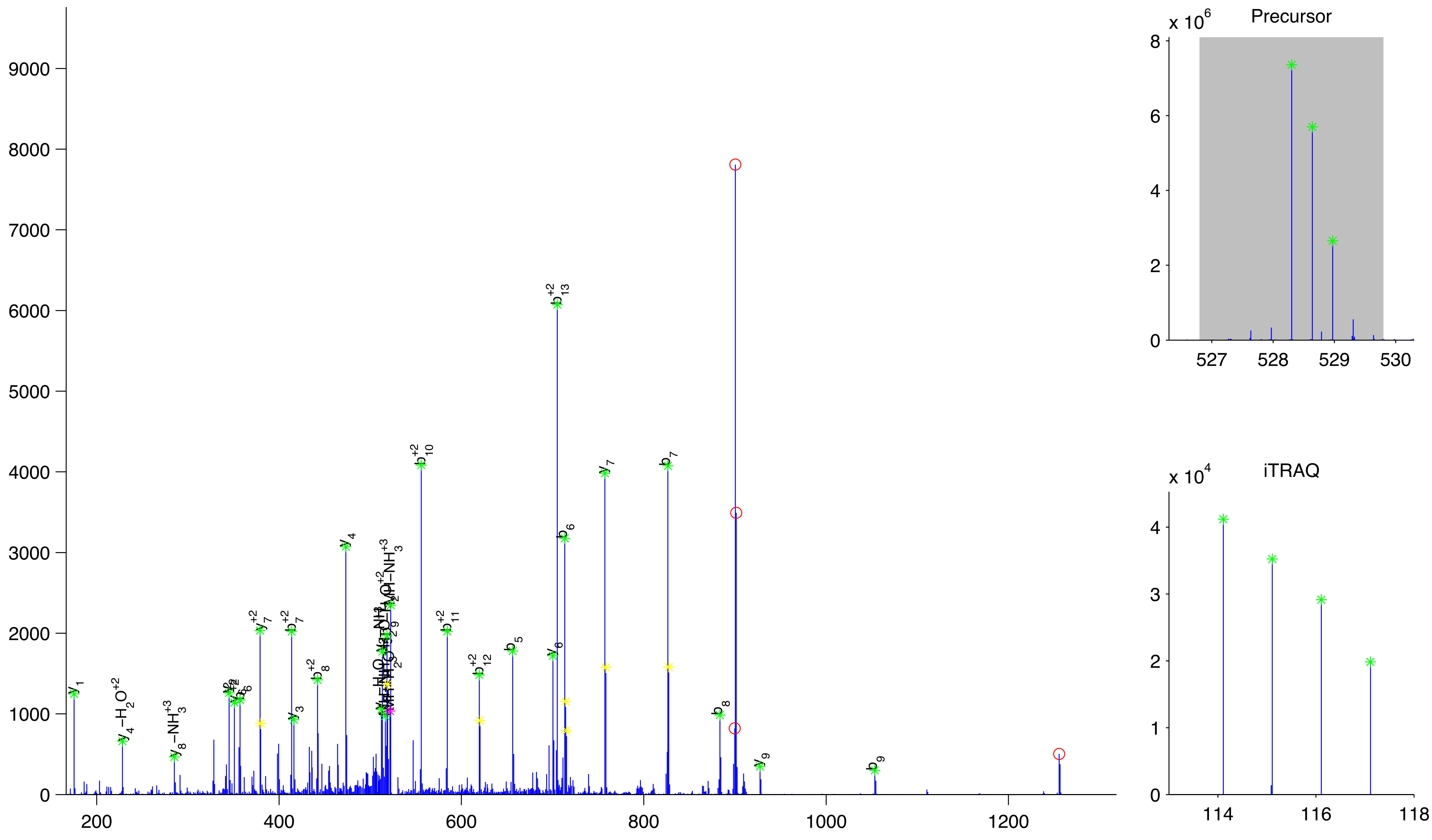

G L G k G G A k R

histone cluster 1, H4a [Homo sapiens]
Charge State: +2
Scan Number: 4490
File Name: 110924_HG2_INSA_ACK3.raw

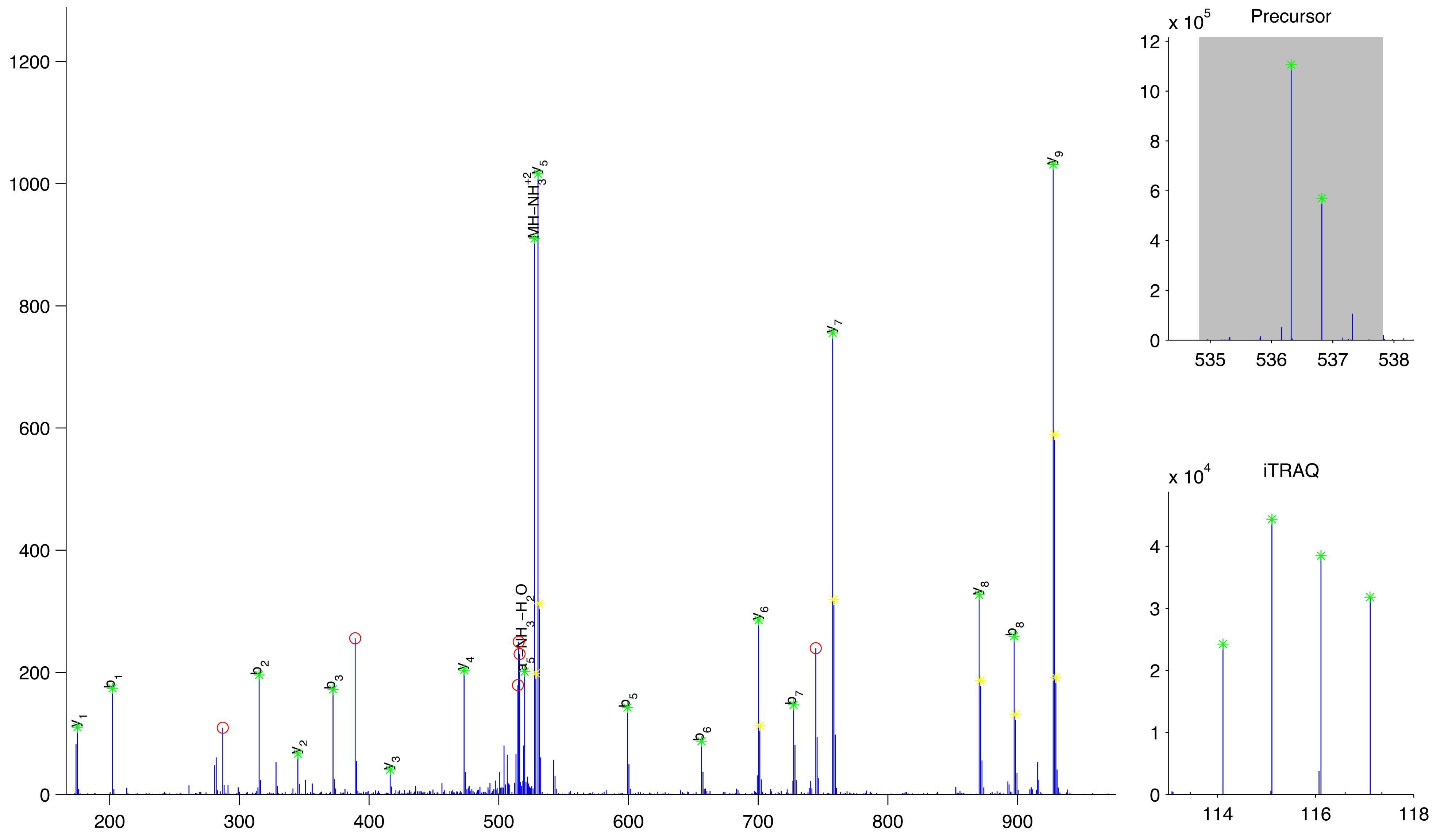

G k G G k G L G k G G A k R

histone cluster 1, H4a [Homo sapiens]
Charge State: +2
Scan Number: 4503
File Name: 111016_HG2_INSC_ACK.raw

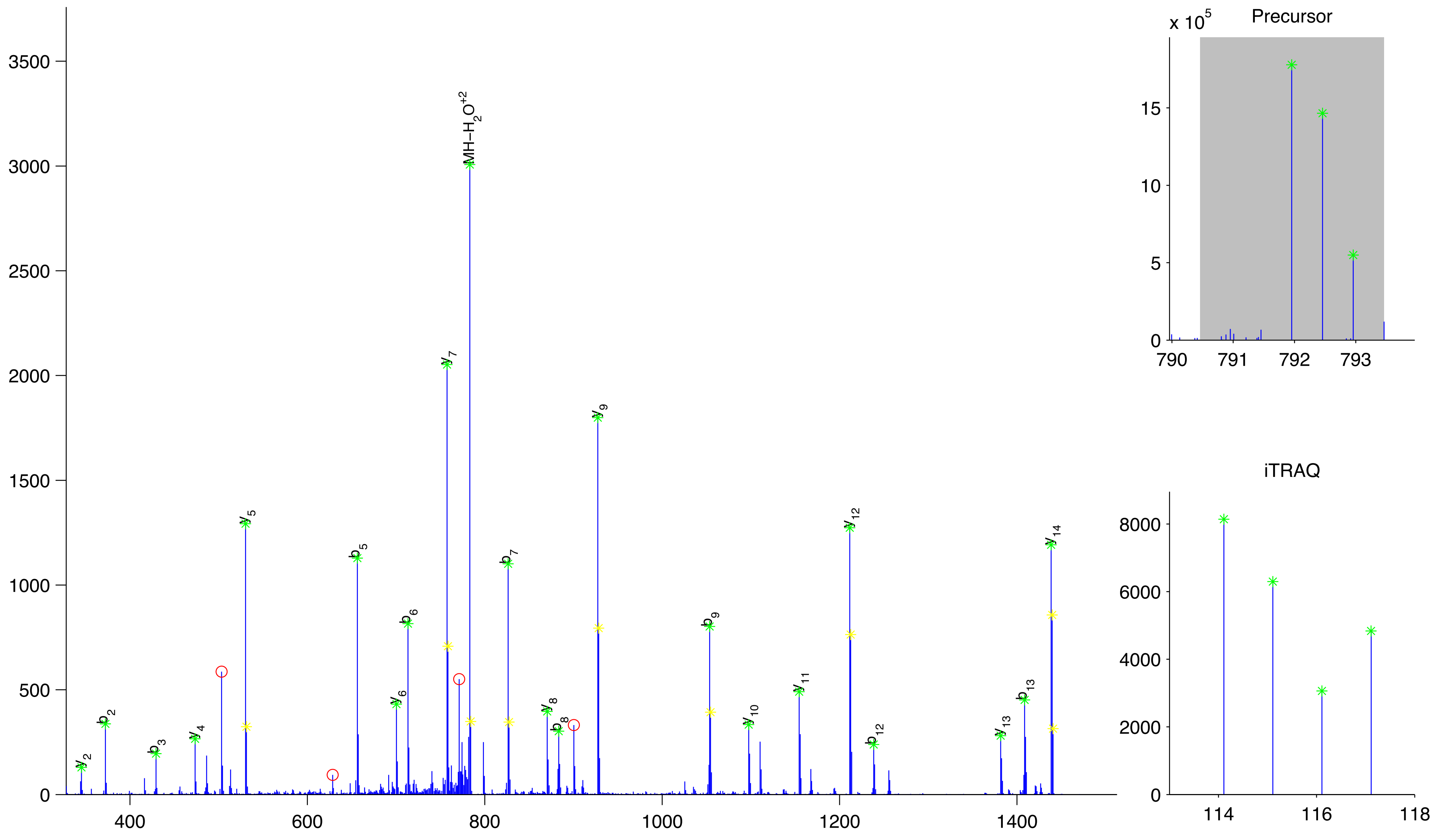

G G k G L G k G G A k R

histone cluster 1, H4a [Homo sapiens]
Charge State: +2
Scan Number: 4522
File Name: 111016_HG2_INSC_ACK.raw

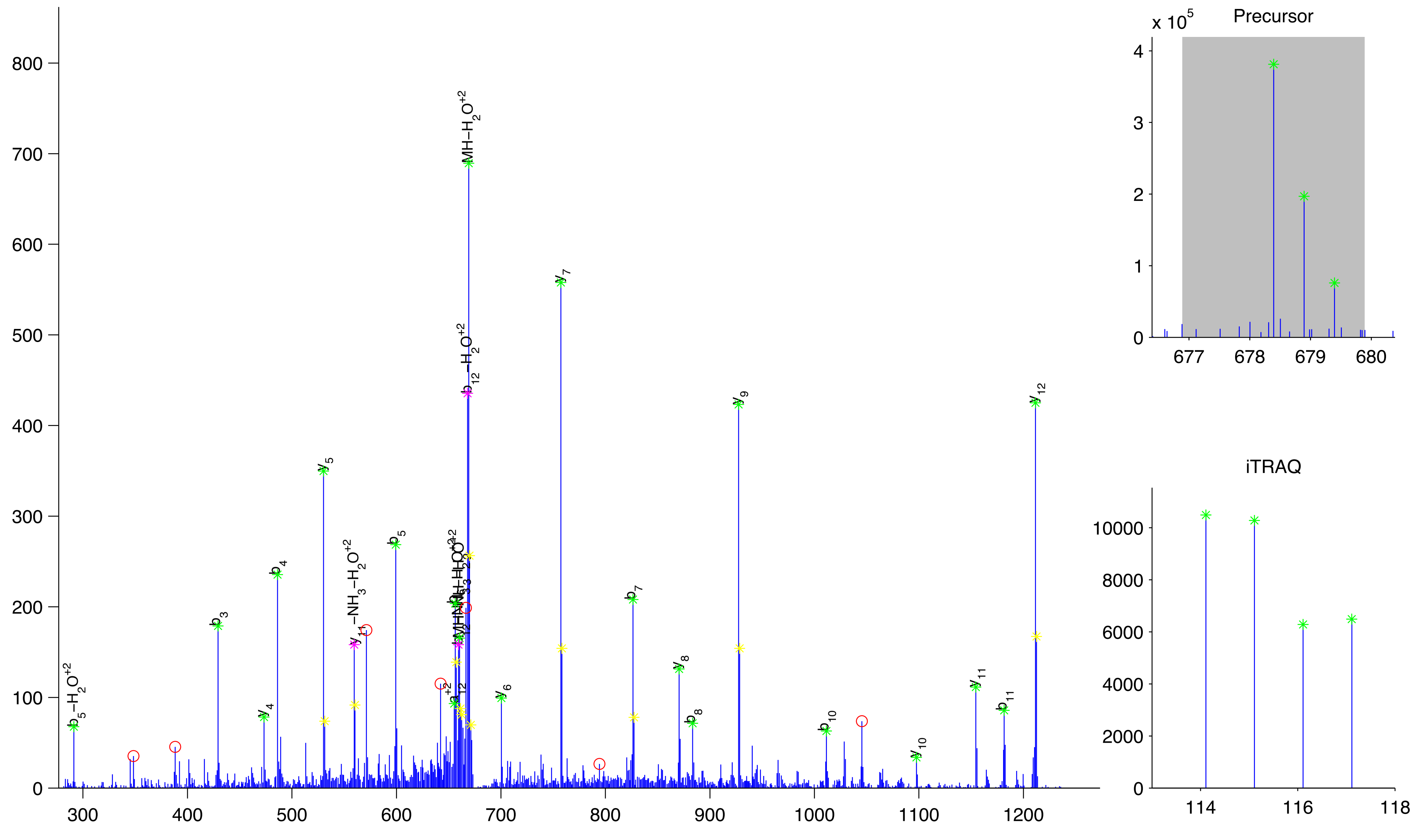

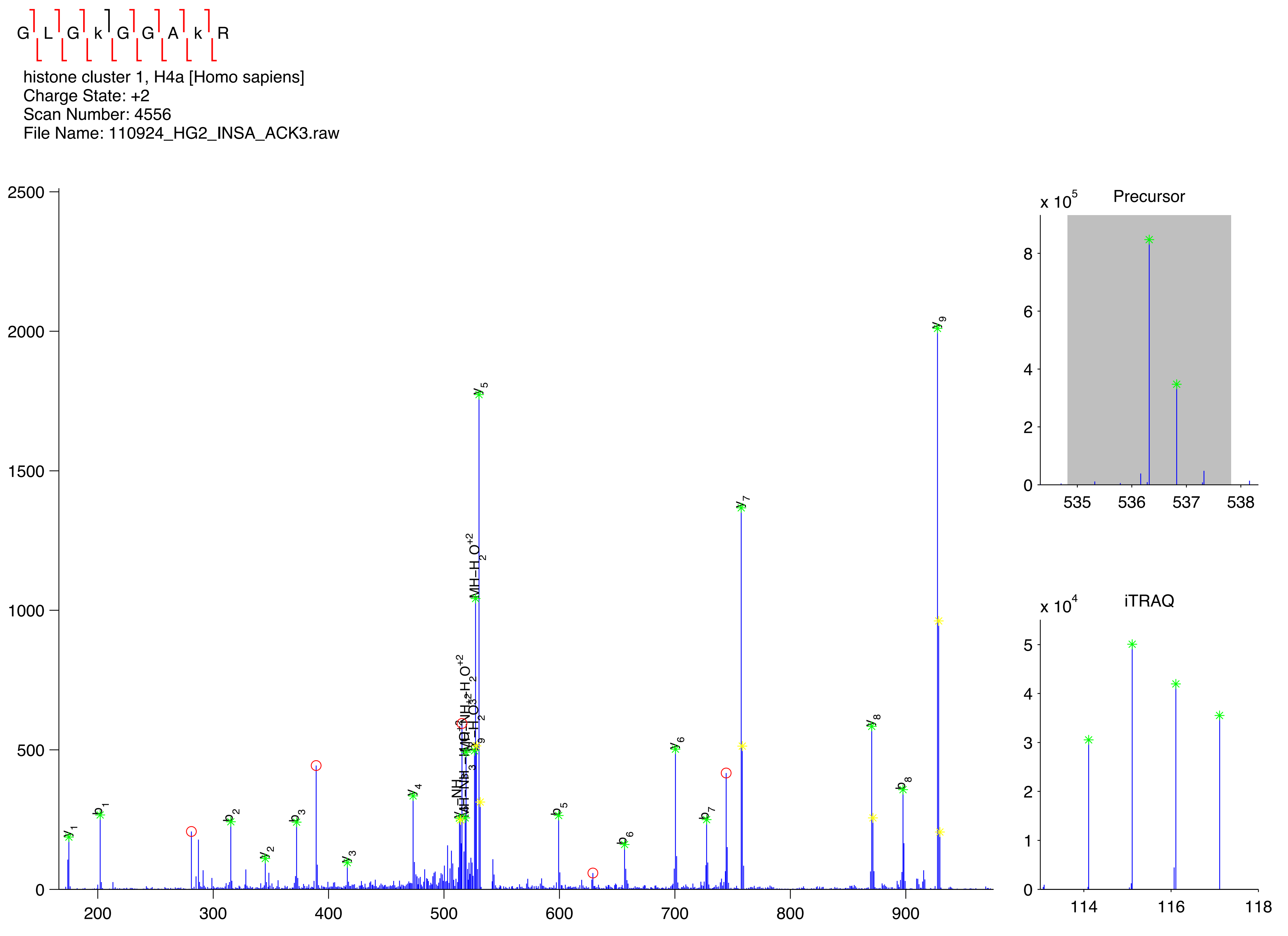

G L G k G G A k R
histone cluster 1, H4a [Homo sapiens]
Charge State: +2
Scan Number: 4556
File Name: 110924_HG2_INSA_ACK3.raw
Precursor
x 10^5
iTRAQ
x 10^4

G L G k G G A K

histone cluster 1, H4a [Homo sapiens]
Charge State: +2
Scan Number: 4671
File Name: 111011_HG2_INSB_ACK.raw

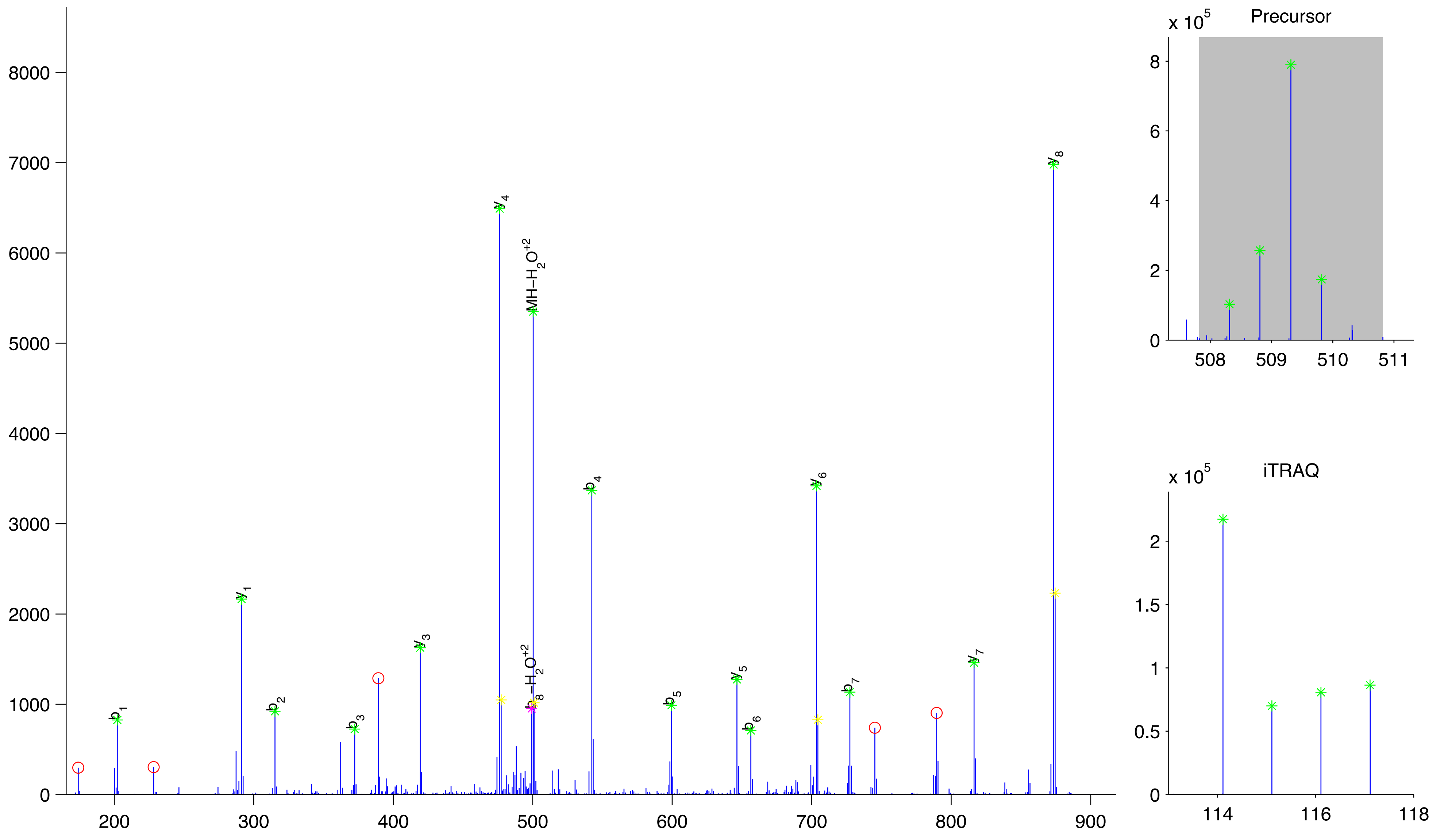

G k G G k G L G k G G A k R

histone cluster 1, H4a [Homo sapiens]
Charge State: +3
Scan Number: 4688
File Name: 110924_HG2_INSA_ACK3.raw

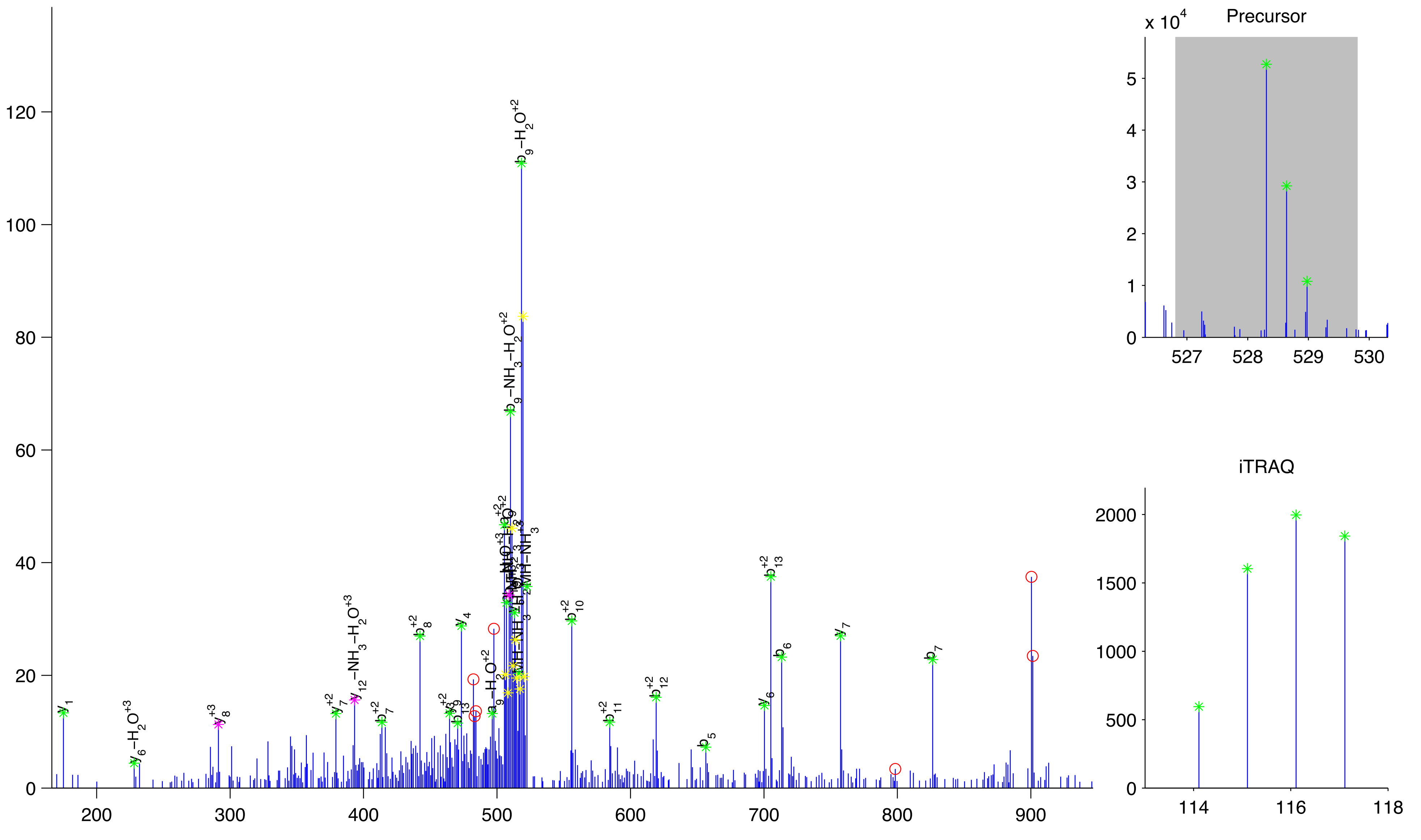

G G k G L G K

histone cluster 1, H4a [Homo sapiens]
Charge State: +2
Scan Number: 4690
File Name: 111011_HG2_INSB_ACK.raw

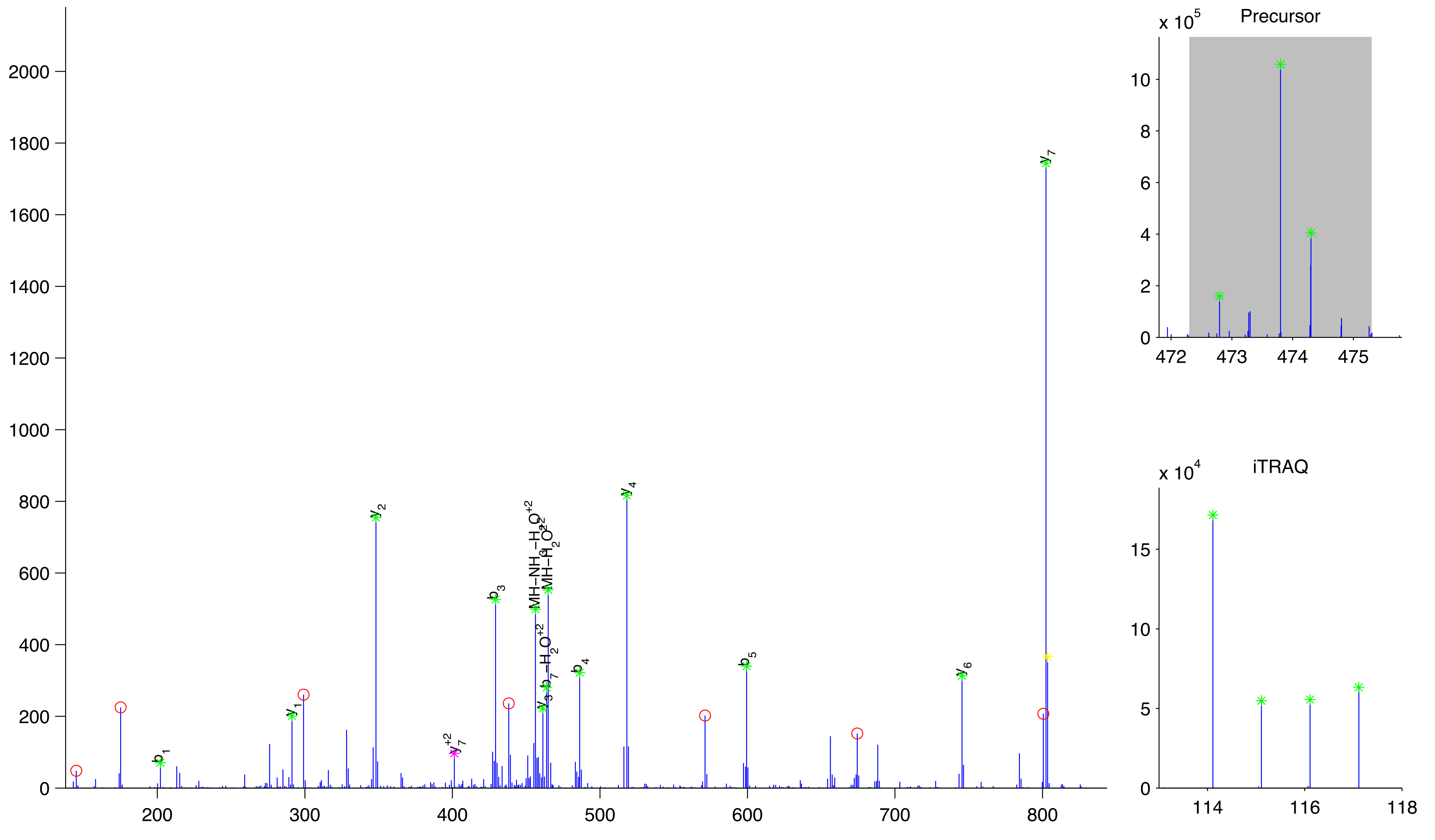

G G k G L G k G G A k R

histone cluster 1, H4a [Homo sapiens]
Charge State: +2
Scan Number: 4713
File Name: 111016_HG2_INSC_ACK.raw

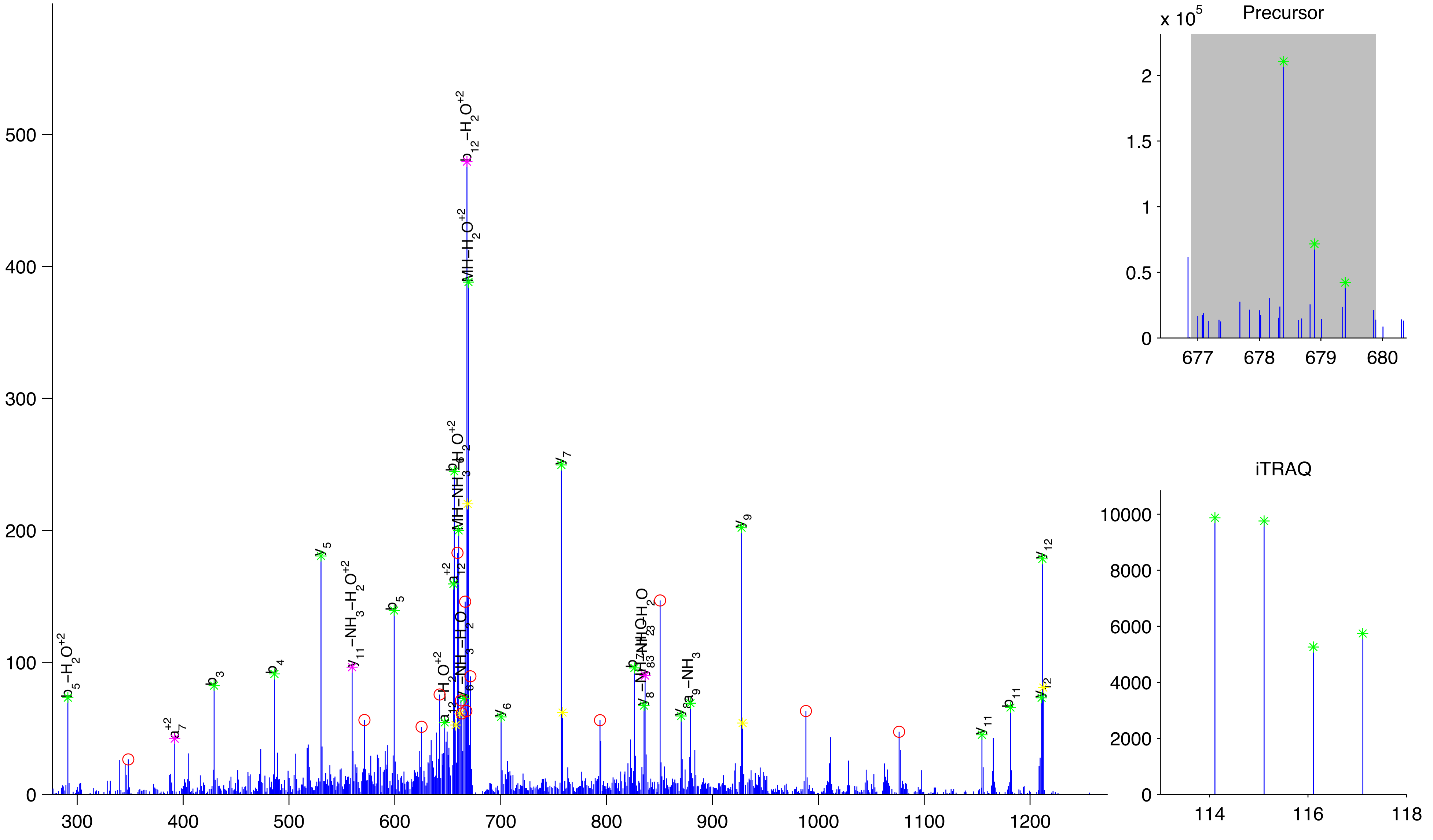

G L G k G G A k R

histone cluster 1, H4a [Homo sapiens]
Charge State: +2
Scan Number: 4726
File Name: 110924_HG2_INSA_ACK3.raw

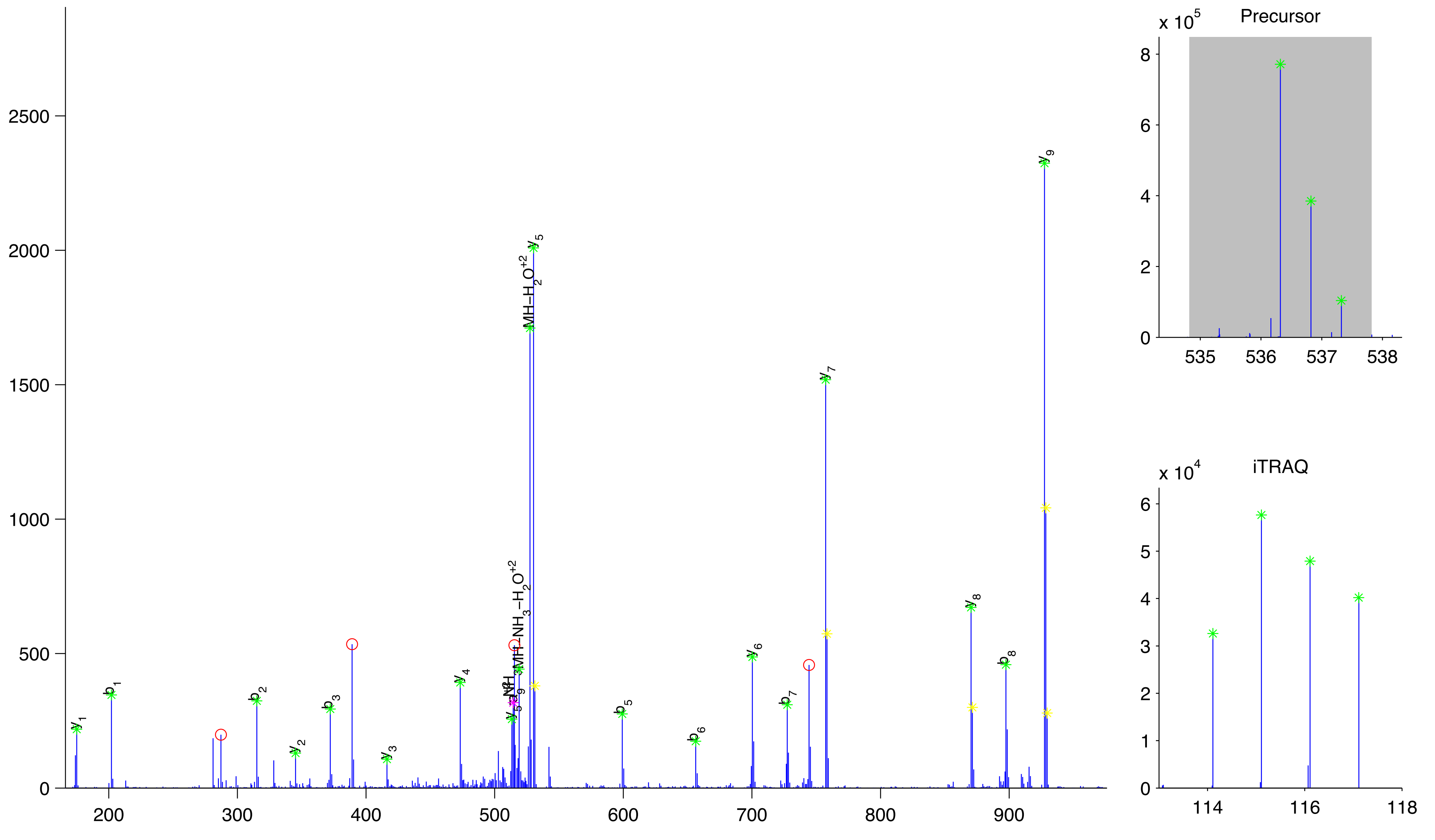

G L G k G G A k R

histone cluster 1, H4a [Homo sapiens]
Charge State: +2
Scan Number: 4728
File Name: 111011_HG2_INSB_ACK.raw

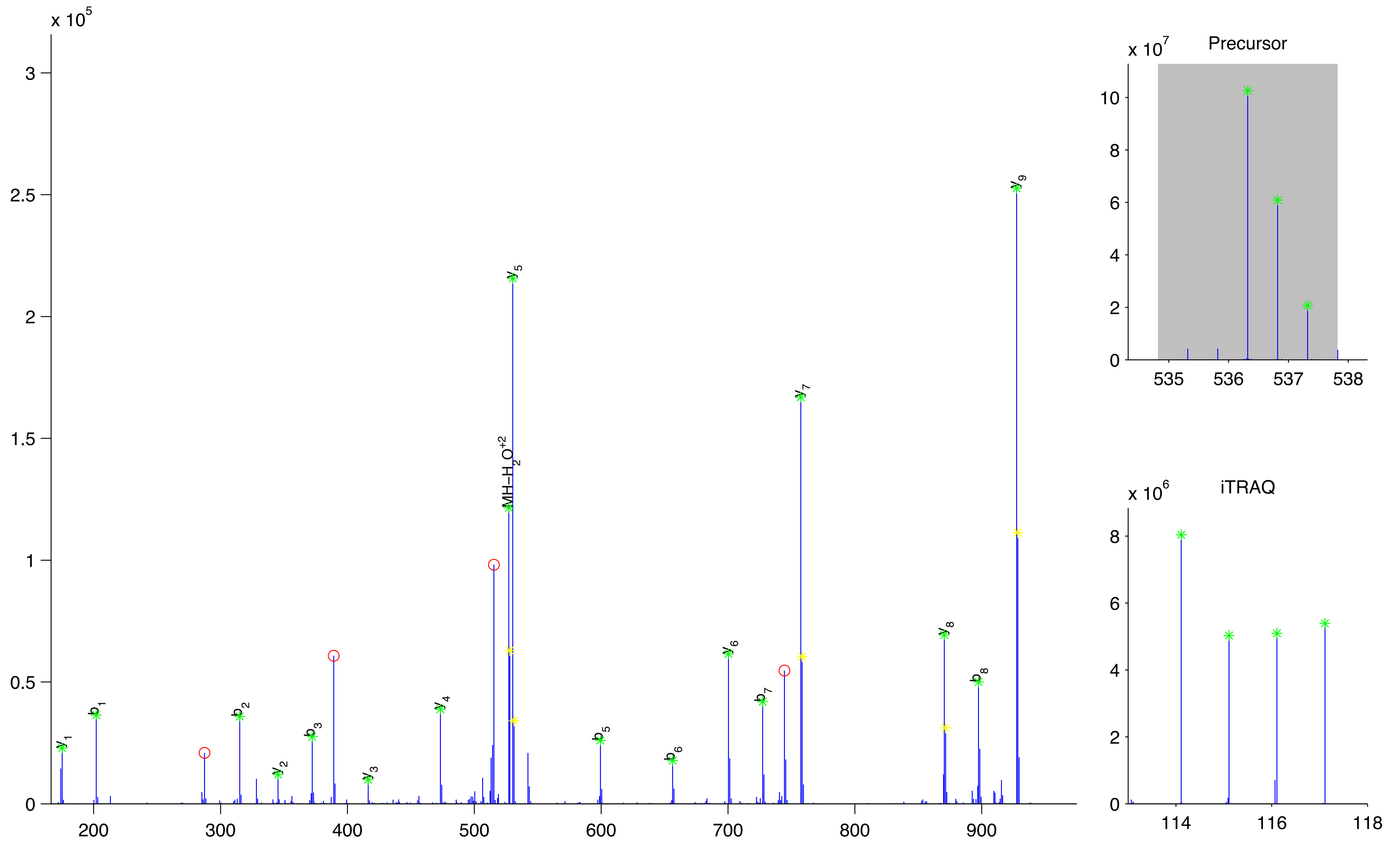

G L G k G G A k R

histone cluster 1, H4a [Homo sapiens]
Charge State: +2
Scan Number: 4791
File Name: 111011_HG2_INSB_ACK.raw

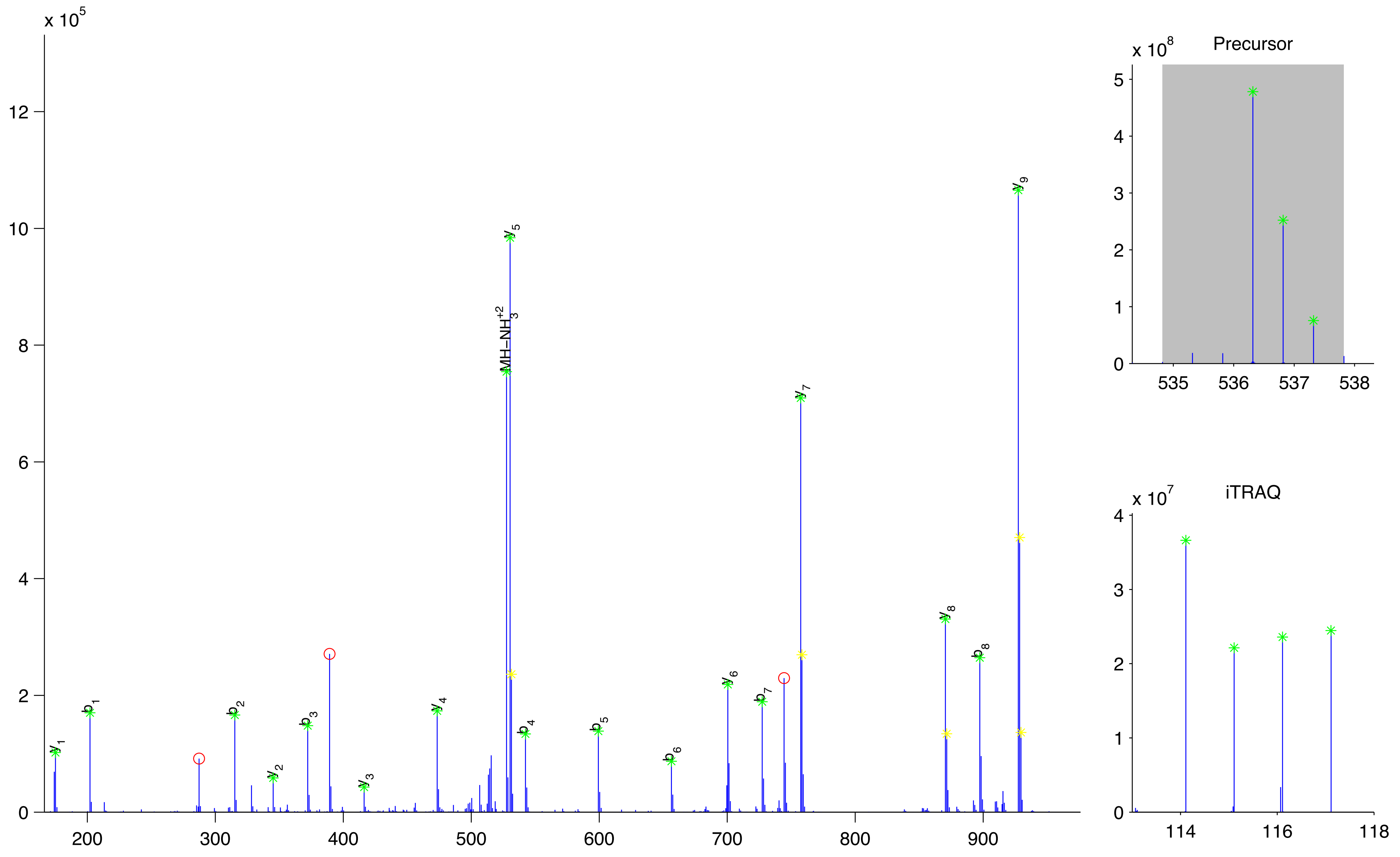

G G k G L G k G G A k R

histone cluster 1, H4a [Homo sapiens]
Charge State: +3
Scan Number: 4856
File Name: 111011_HG2_INSB_ACK.raw

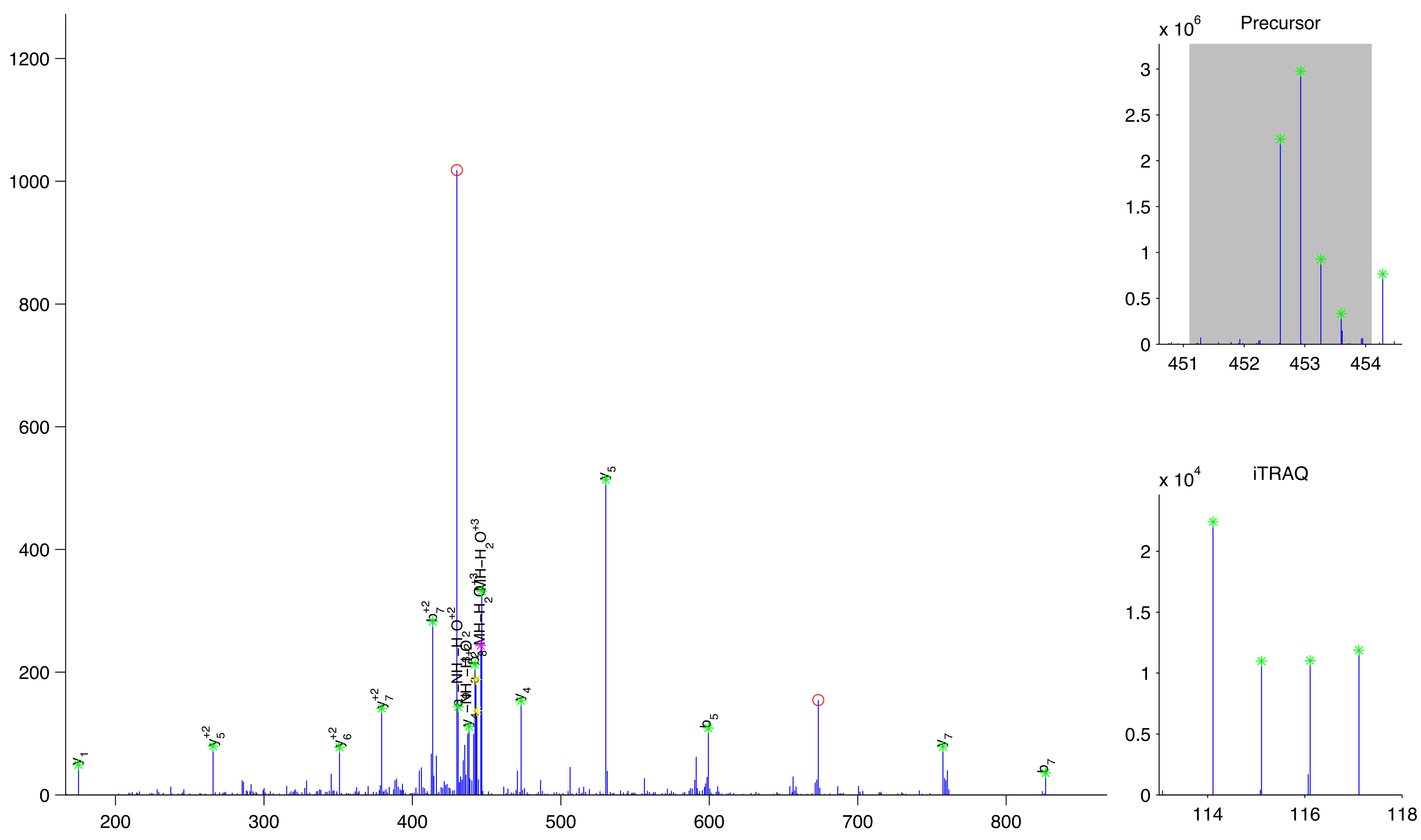

G G k G L G k G G A k R

histone cluster 1, H4a [Homo sapiens]
Charge State: +2
Scan Number: 4902
File Name: 110924_HG2_INSA_ACK4.raw

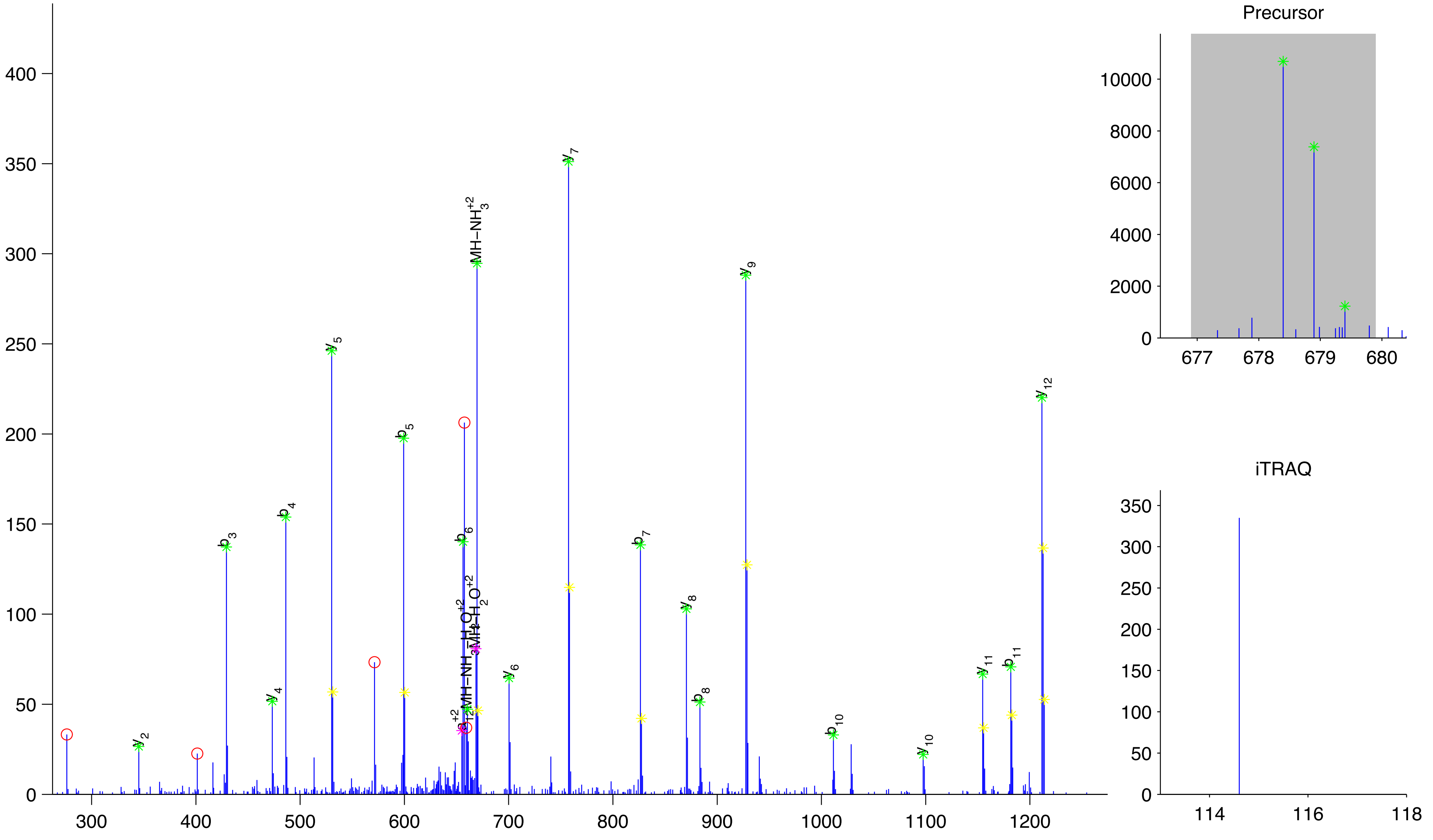

G L G k G G A k R

histone cluster 1, H4a [Homo sapiens]
Charge State: +2
Scan Number: 4917
File Name: 111011_HG2_INSB_ACK.raw

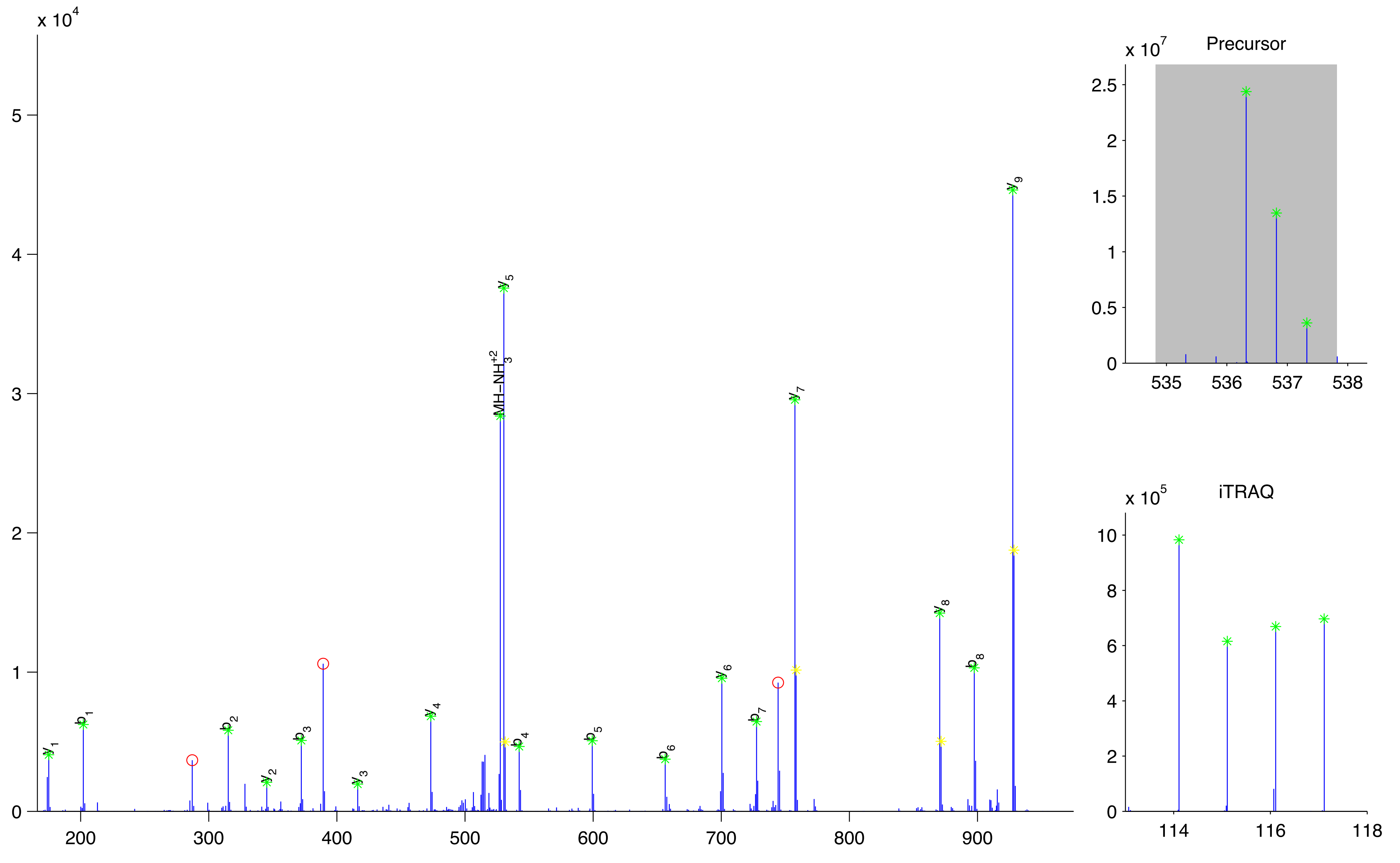

G k G G k G L G k G G A k R

histone cluster 1, H4a [Homo sapiens]
Charge State: +2
Scan Number: 5001
File Name: 111011_HG2_INSB_ACK.raw

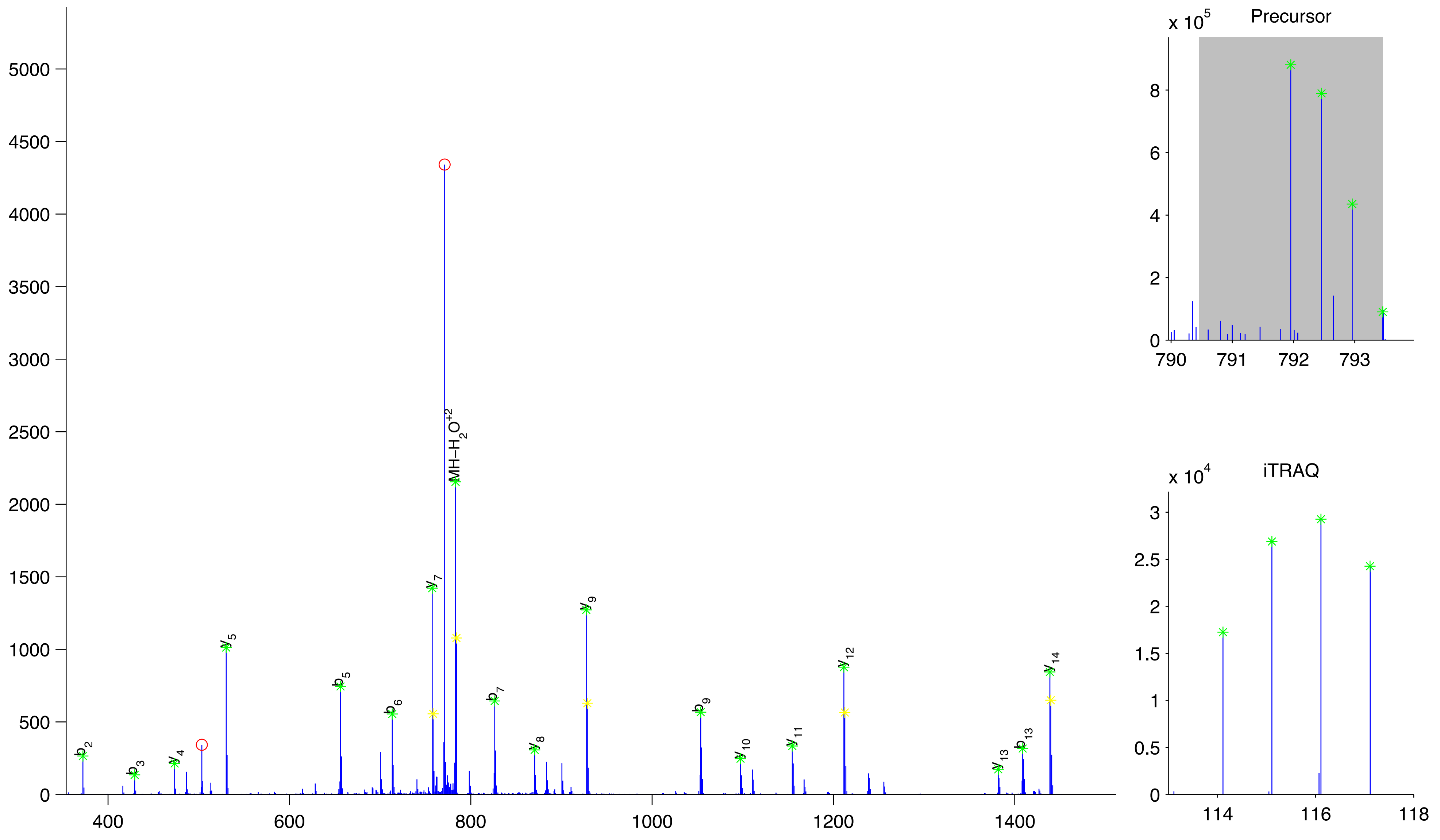

G G k G L G k G G A k R

histone cluster 1, H4a [Homo sapiens]
Charge State: +2
Scan Number: 5022
File Name: 111011_HG2_INSB_ACK.raw

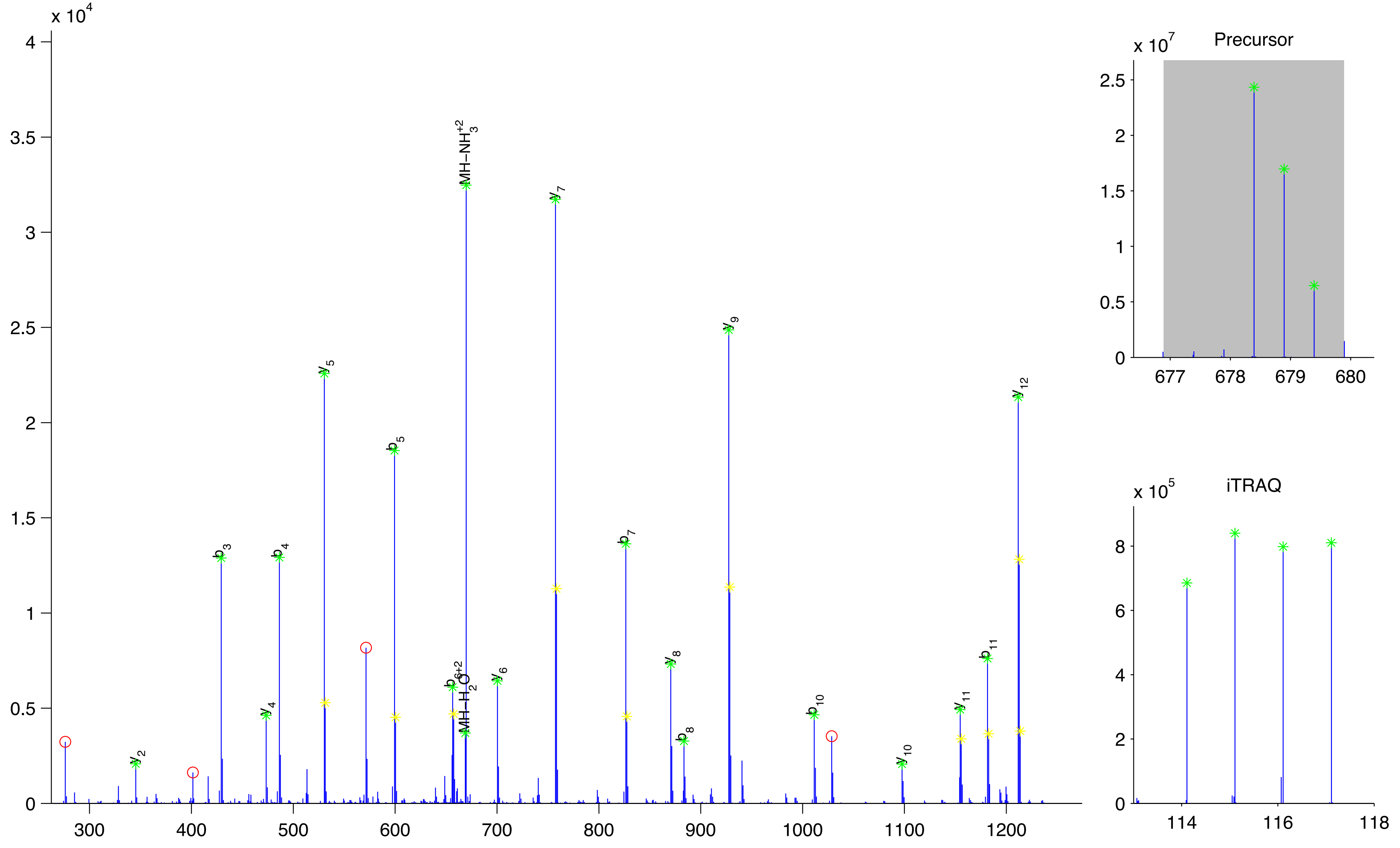

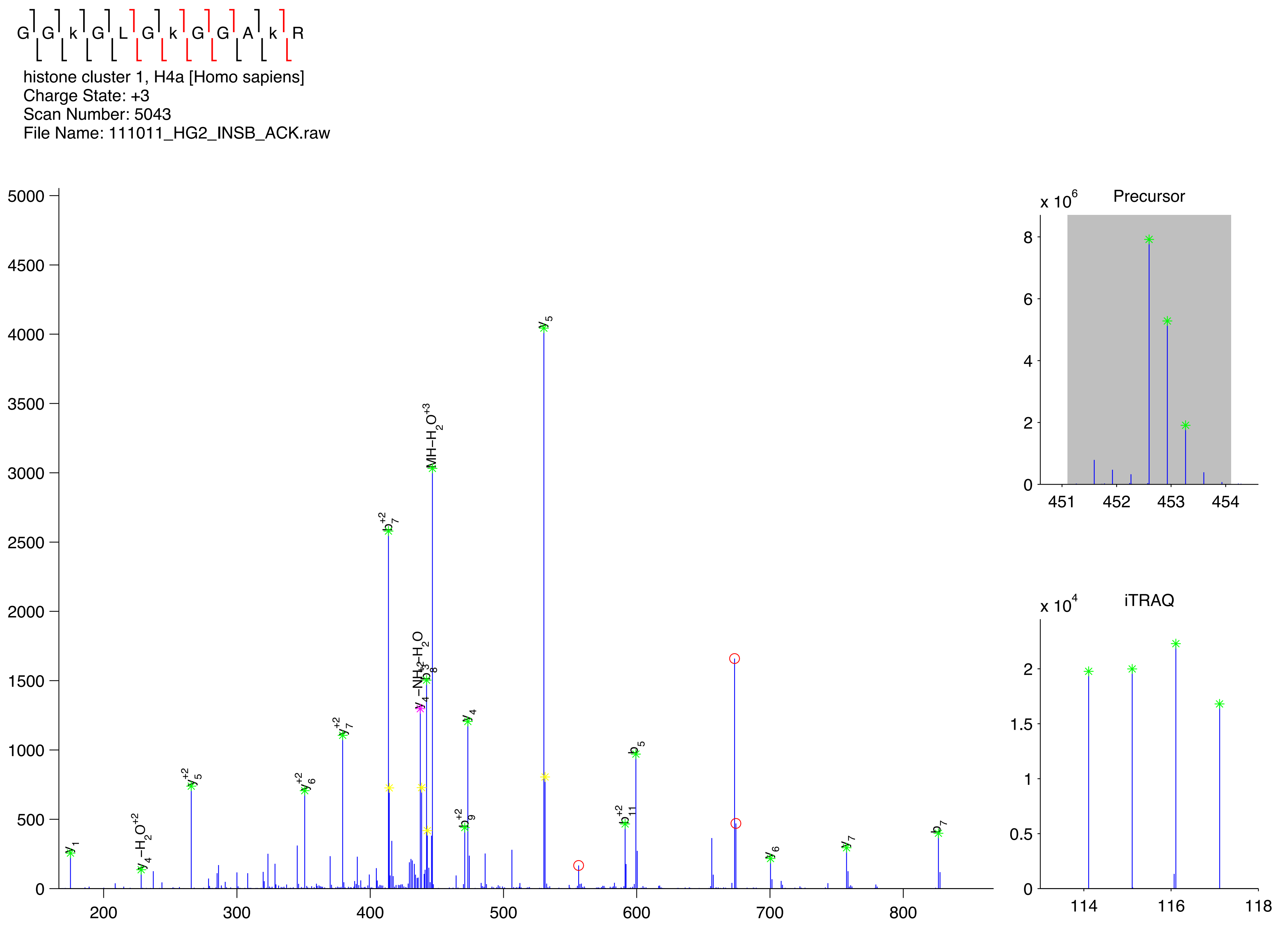
G G k G L G k G G A k R
histone cluster 1, H4a [Homo sapiens]
Charge State: +3
Scan Number: 5043
File Name: 111011_HG2_INSB_ACK.raw
Precursor
x 10^6
iTRAQ
x 10^4

G k G G k G L G k G G A k R

histone cluster 1, H4a [Homo sapiens]
Charge State: +2
Scan Number: 5057
File Name: 111011_HG2_INSB_ACK.raw

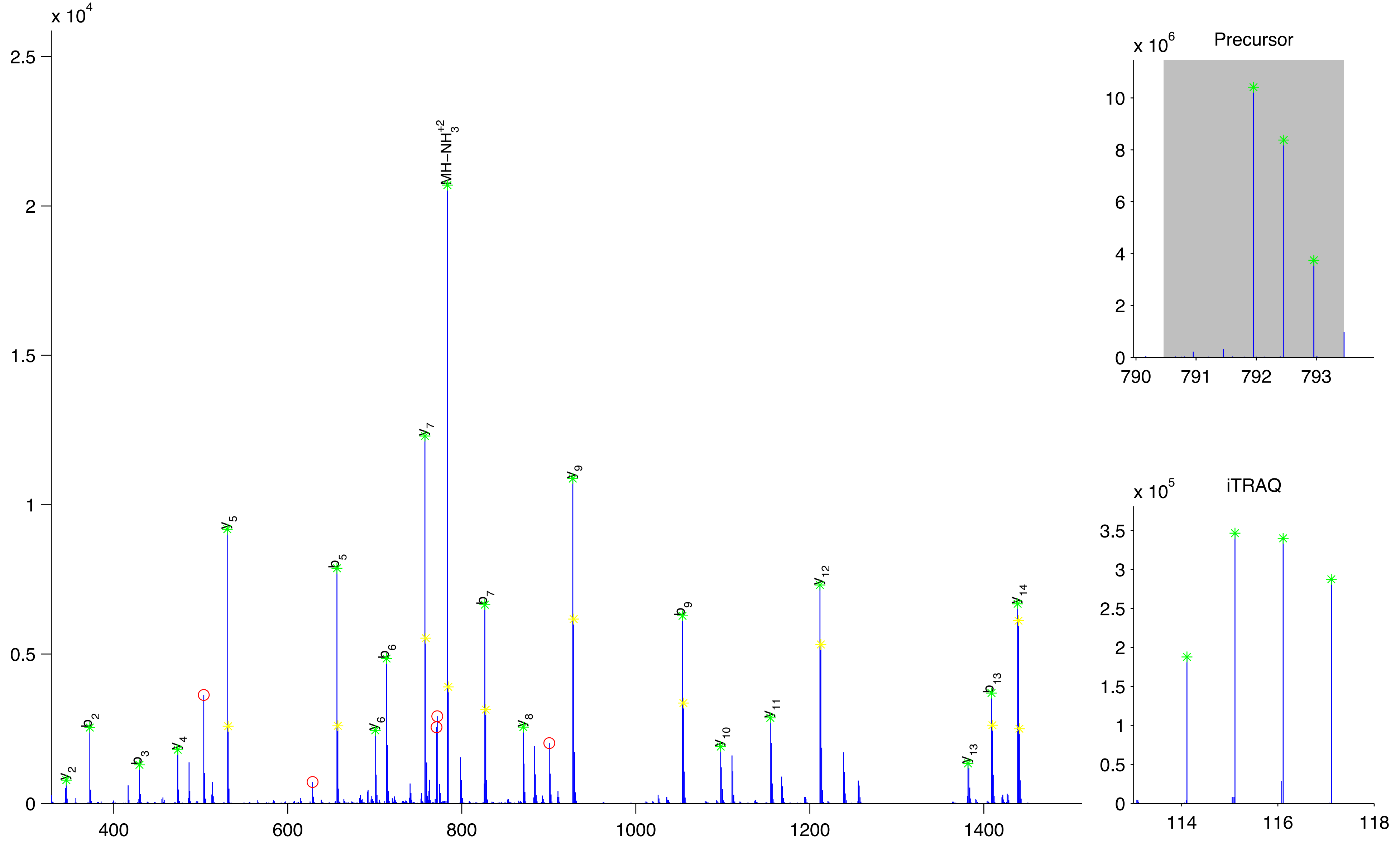

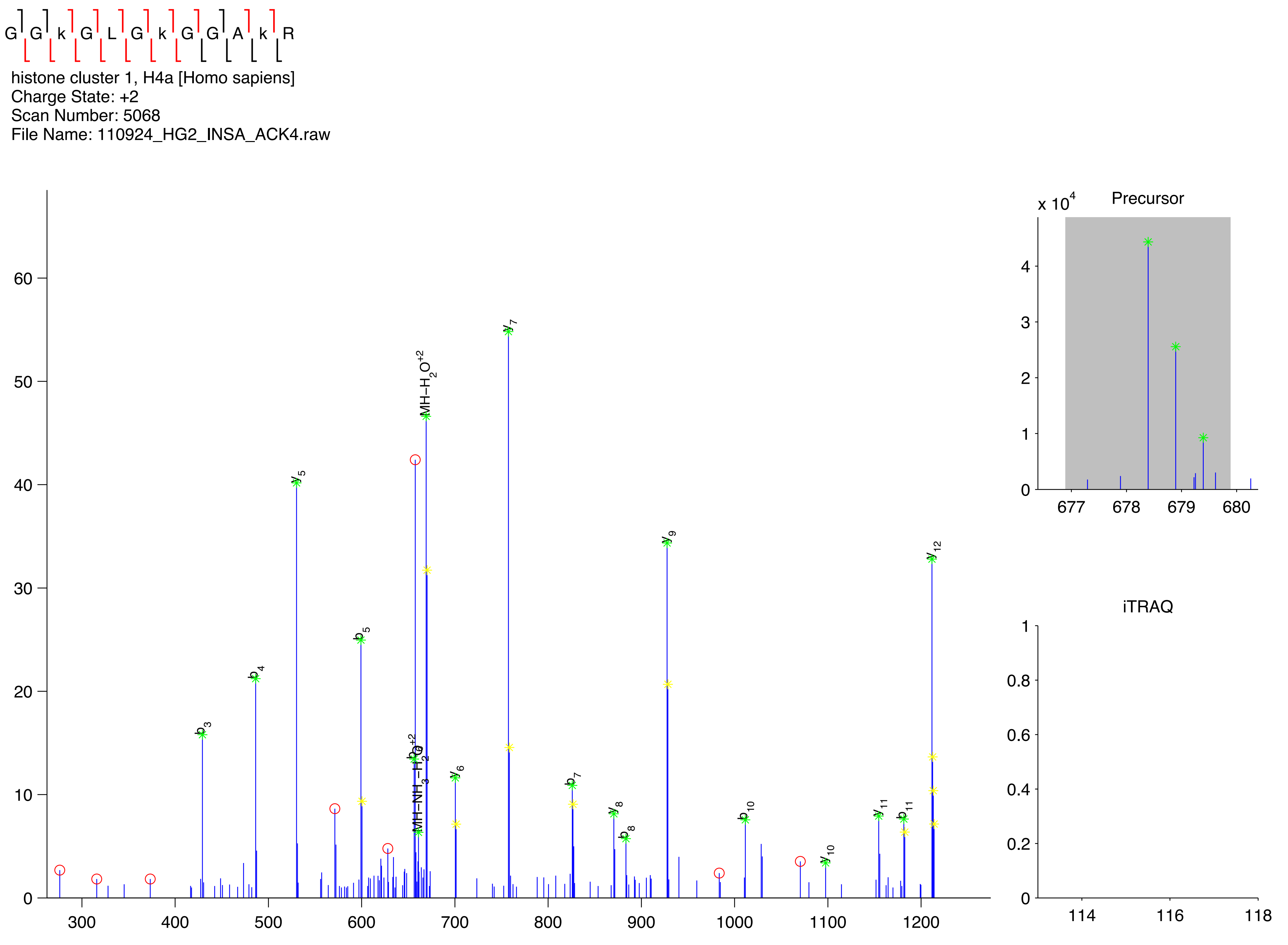
G G k G L G k G G A k R
histone cluster 1, H4a [Homo sapiens]
Charge State: +2
Scan Number: 5068
File Name: 110924_HG2_INSA_ACK4.raw
Precursor
x 10^4
iTRAQ
b3
b4
y5
b5
MH-H2O+2
y6
y7
b7
y8
b8
y9
b10
y10
y11
b11
y12

G L G k G G A k R

histone cluster 1, H4a [Homo sapiens]
Charge State: +2
Scan Number: 5106
File Name: 111011_HG2_INSB_ACK.raw

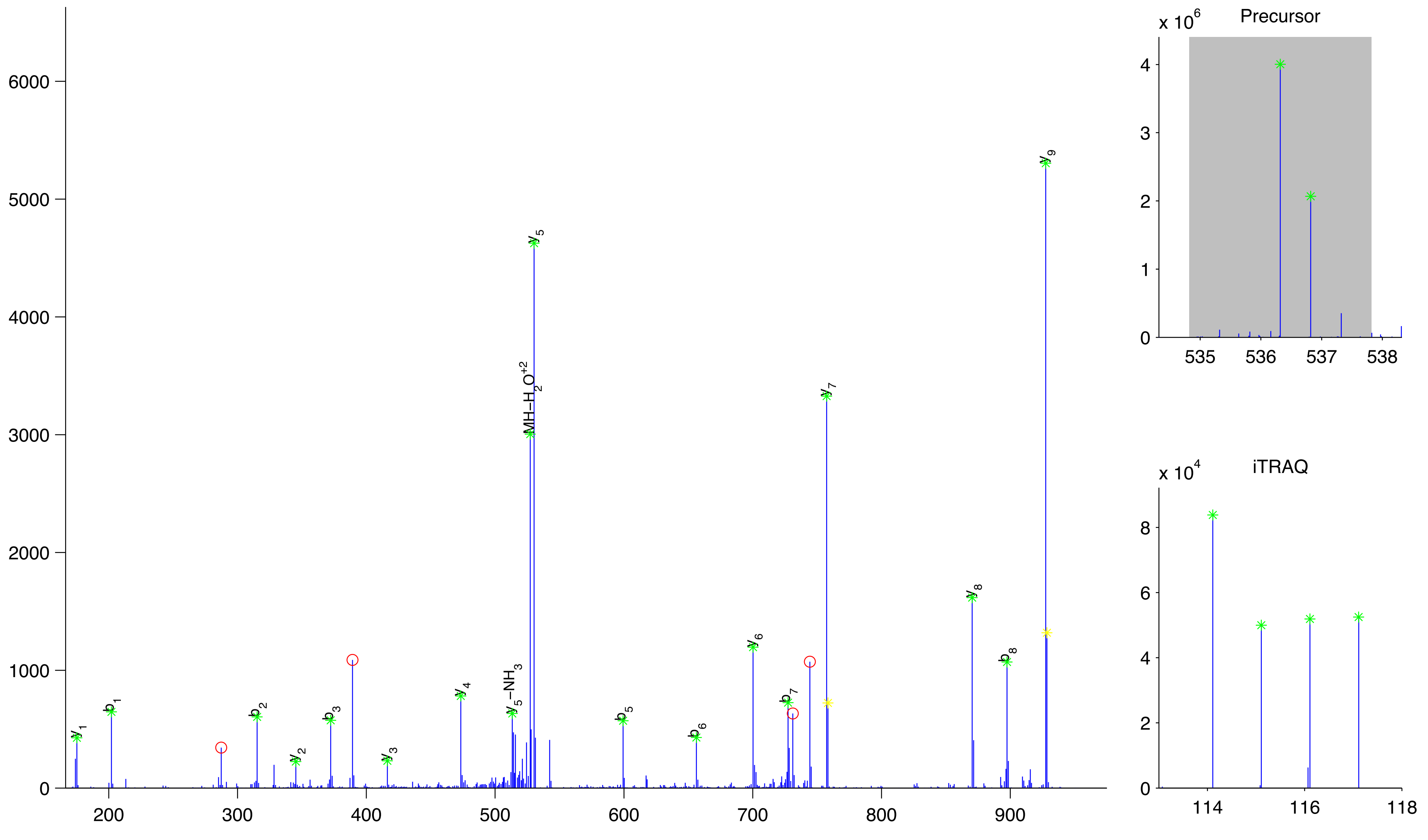

G k G G k G L G k G G A k R

histone cluster 1, H4a [Homo sapiens]
Charge State: +3
Scan Number: 5112
File Name: 111011_HG2_INSB_ACK.raw

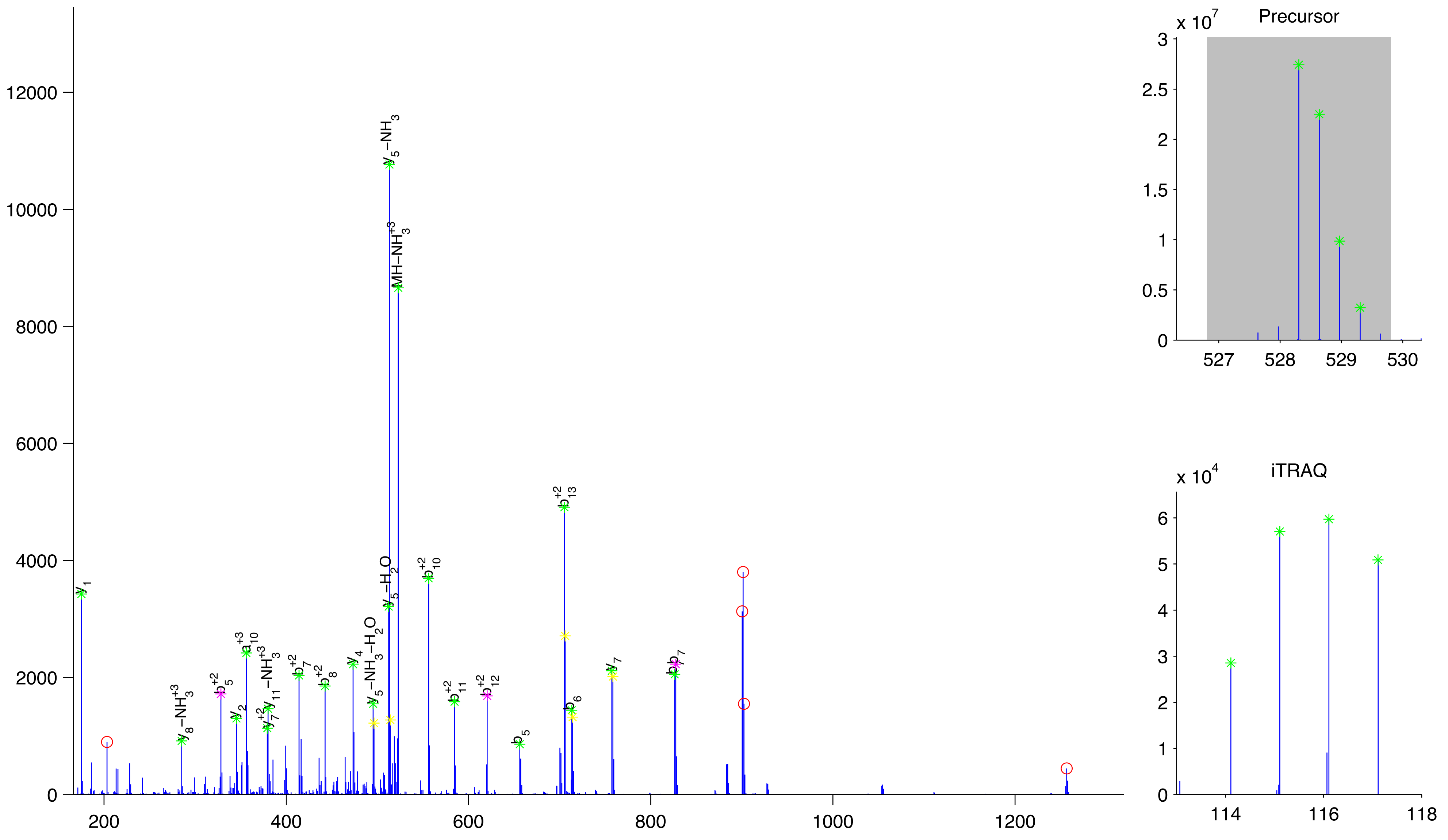

m S G R G k G G k G L G k G G A k R

histone cluster 1, H4a [Homo sapiens]
Charge State: +3
Scan Number: 5127
File Name: 111011_HG2_INSB_ACK.raw

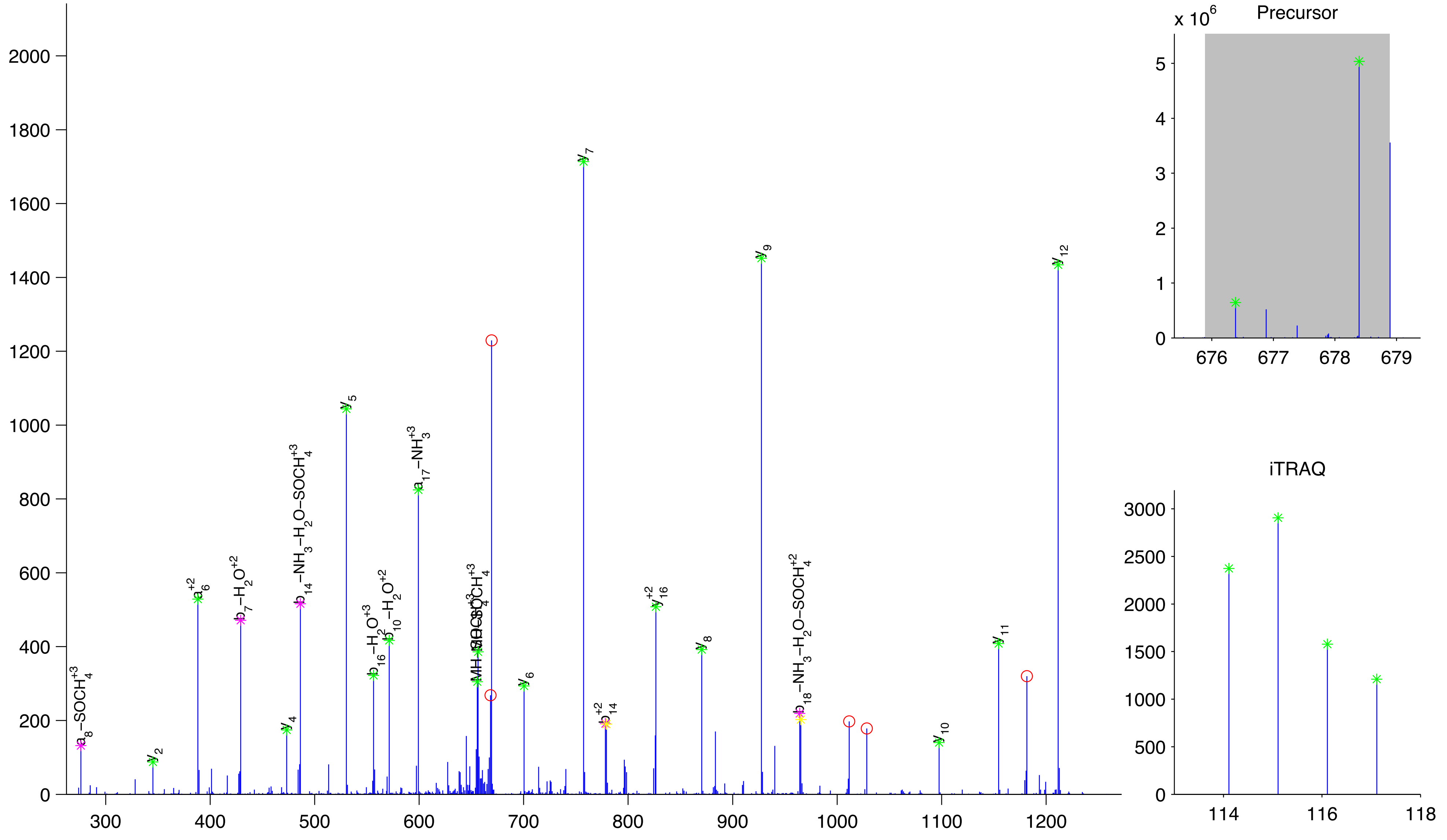

G L G k G G A k R

histone cluster 1, H4a [Homo sapiens]
Charge State: +2
Scan Number: 5135
File Name: 110924_HG2_INSA_ACK3.raw

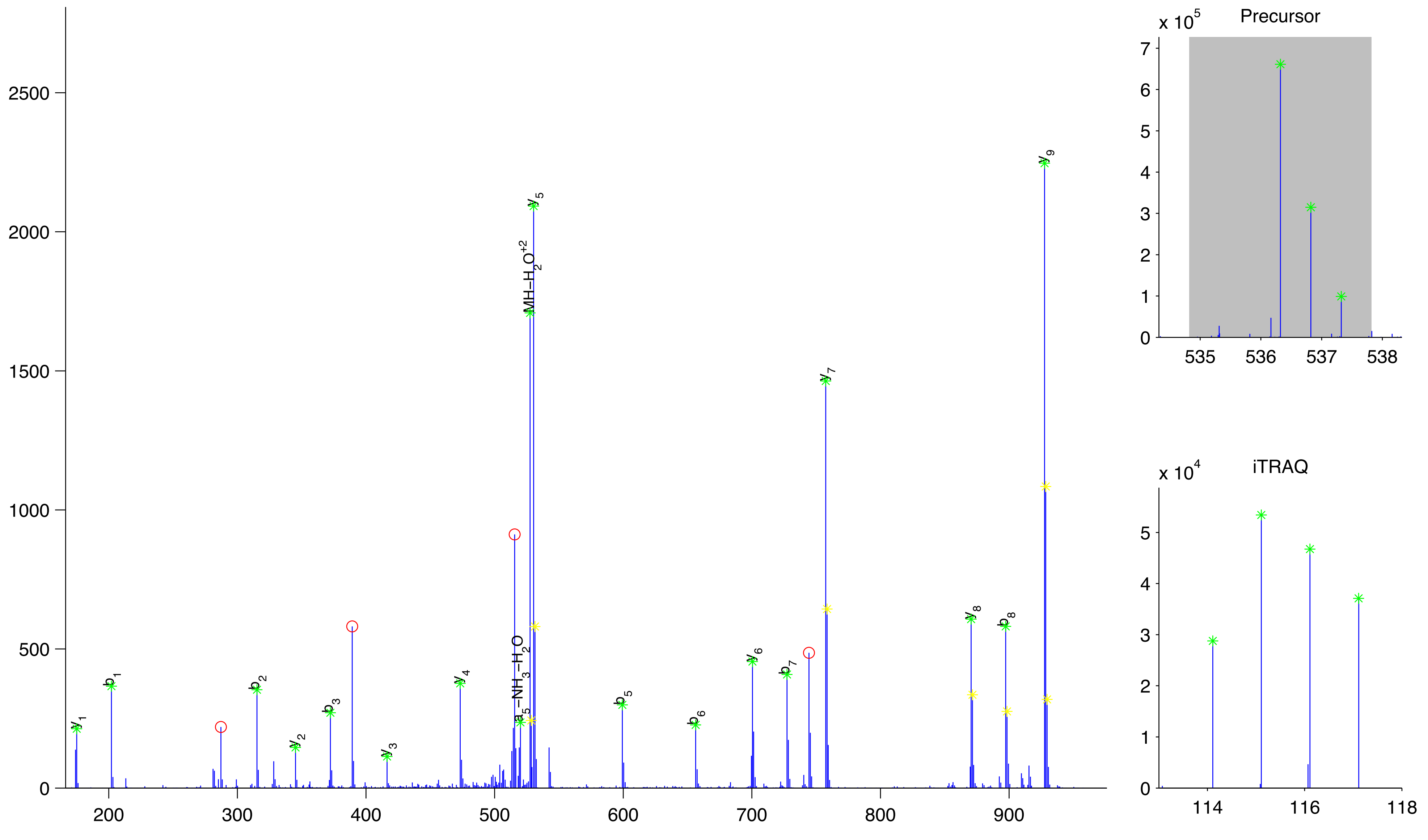

G k G G k G L G k G G A k R

histone cluster 1, H4a [Homo sapiens]
Charge State: +3
Scan Number: 5169
File Name: 111011_HG2_INSB_ACK.raw

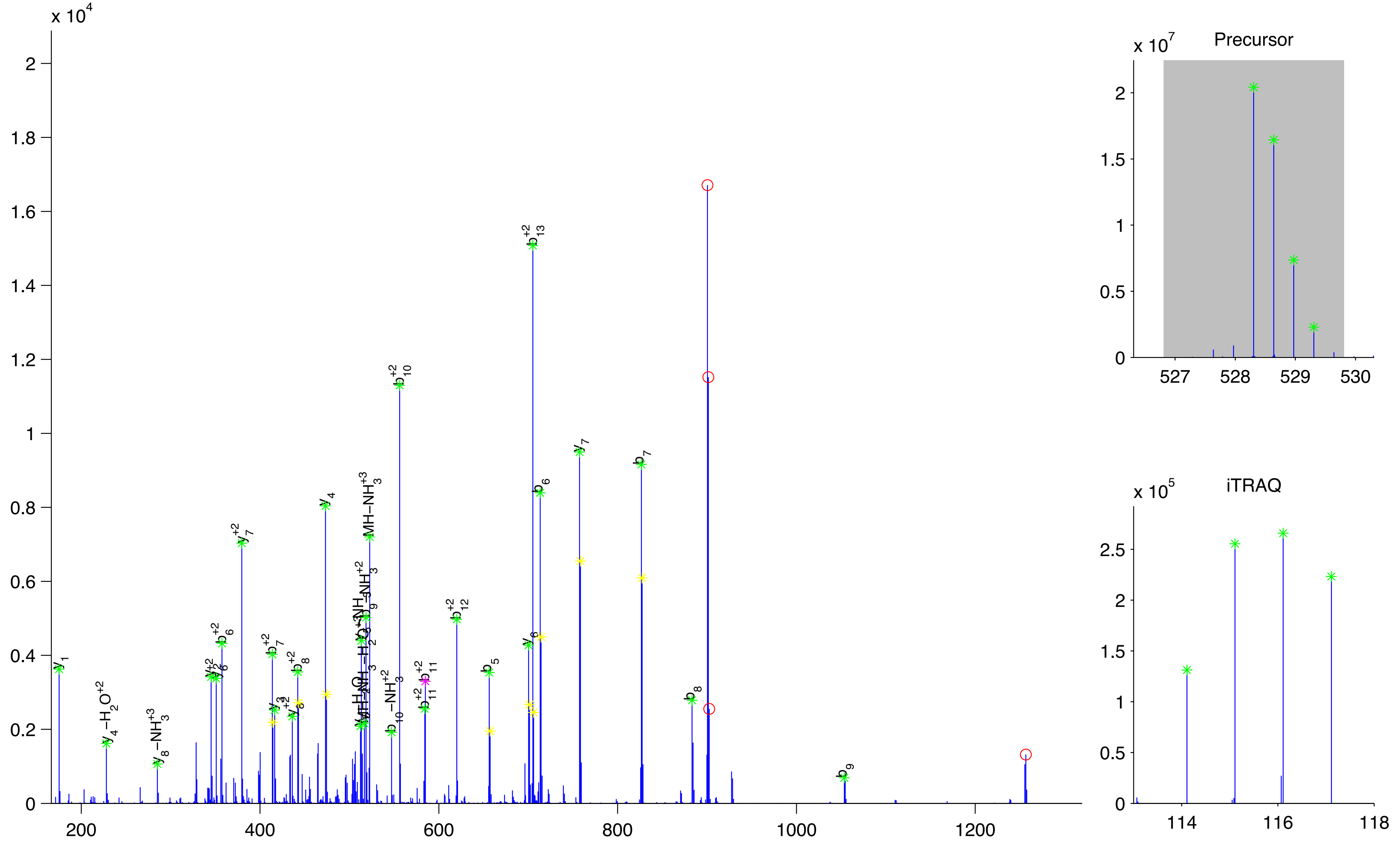

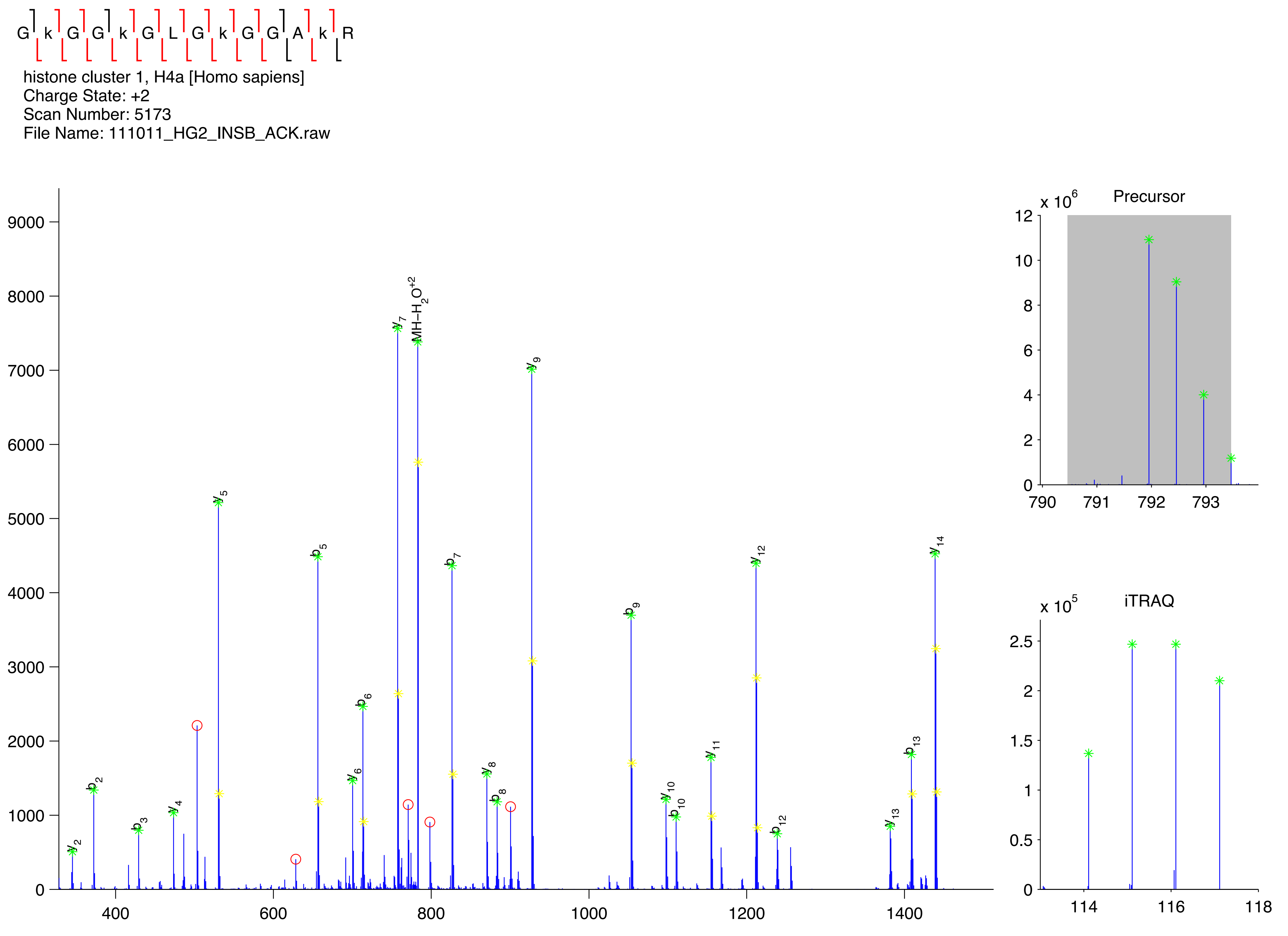
G k G G k G L G k G G A k R
histone cluster 1, H4a [Homo sapiens]
Charge State: +2
Scan Number: 5173
File Name: 111011_HG2_INSB_ACK.raw
Precursor
x 10^6
iTRAQ
x 10^5
MH-H2O+2
y2 b2 b3 y4 y5 b5 y6 b6 y7 b7 y8 b8 y9 b9 y10 b10 y11 y12 b12 y13 b13 y14

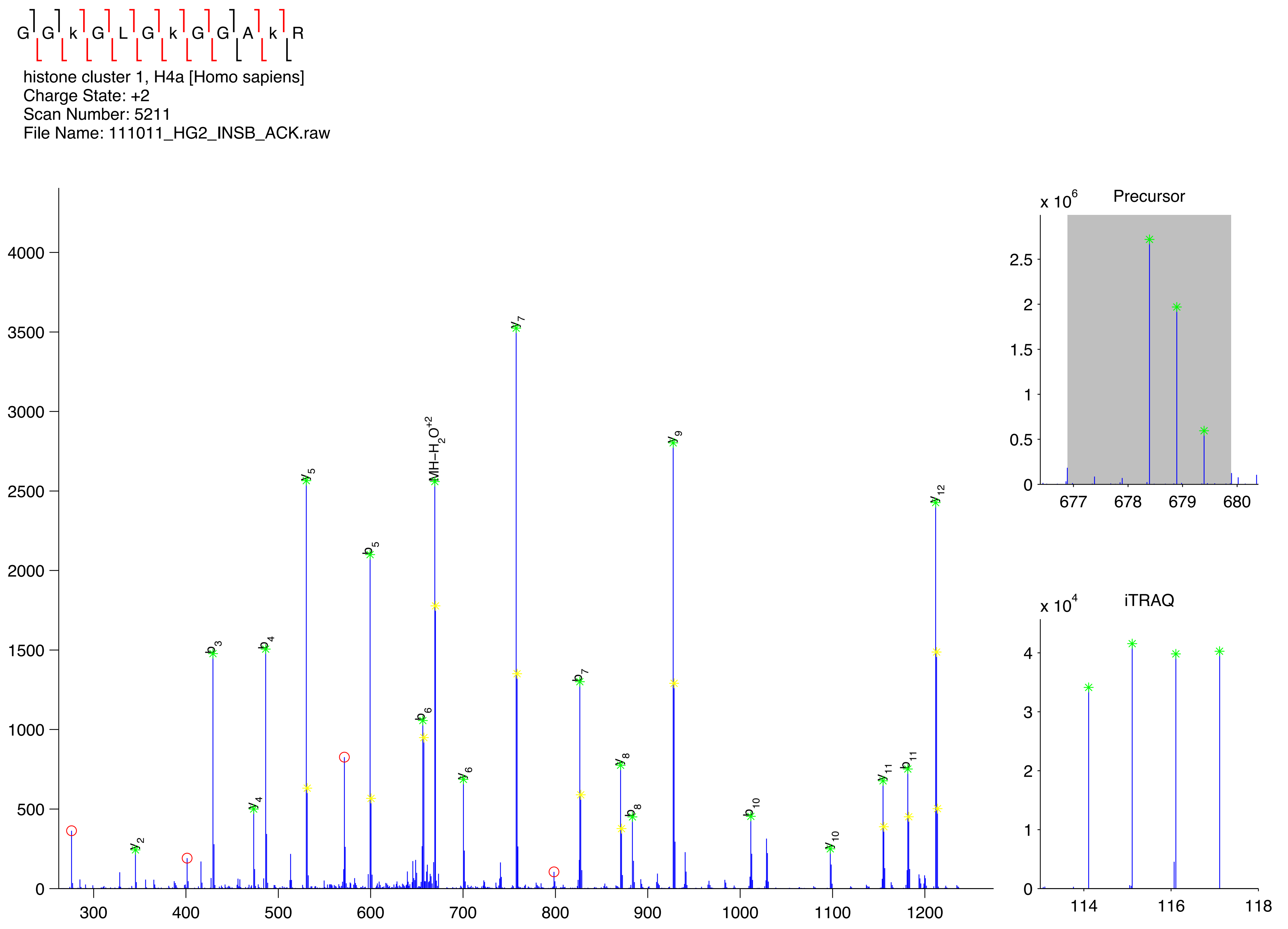
G G k G L G k G G A k R
histone cluster 1, H4a [Homo sapiens]
Charge State: +2
Scan Number: 5211
File Name: 111011_HG2_INSB_ACK.raw
y_2 b_3 y_4 b_4 y_5 b_5 b_6 $MH-H_2O^{+2}$ y_6 y_7 b_7 y_8 b_8 y_9 b_{10} y_{10} y_{11} b_{11} y_{12}
Precursor
x 10^6
iTRAQ
x 10^4

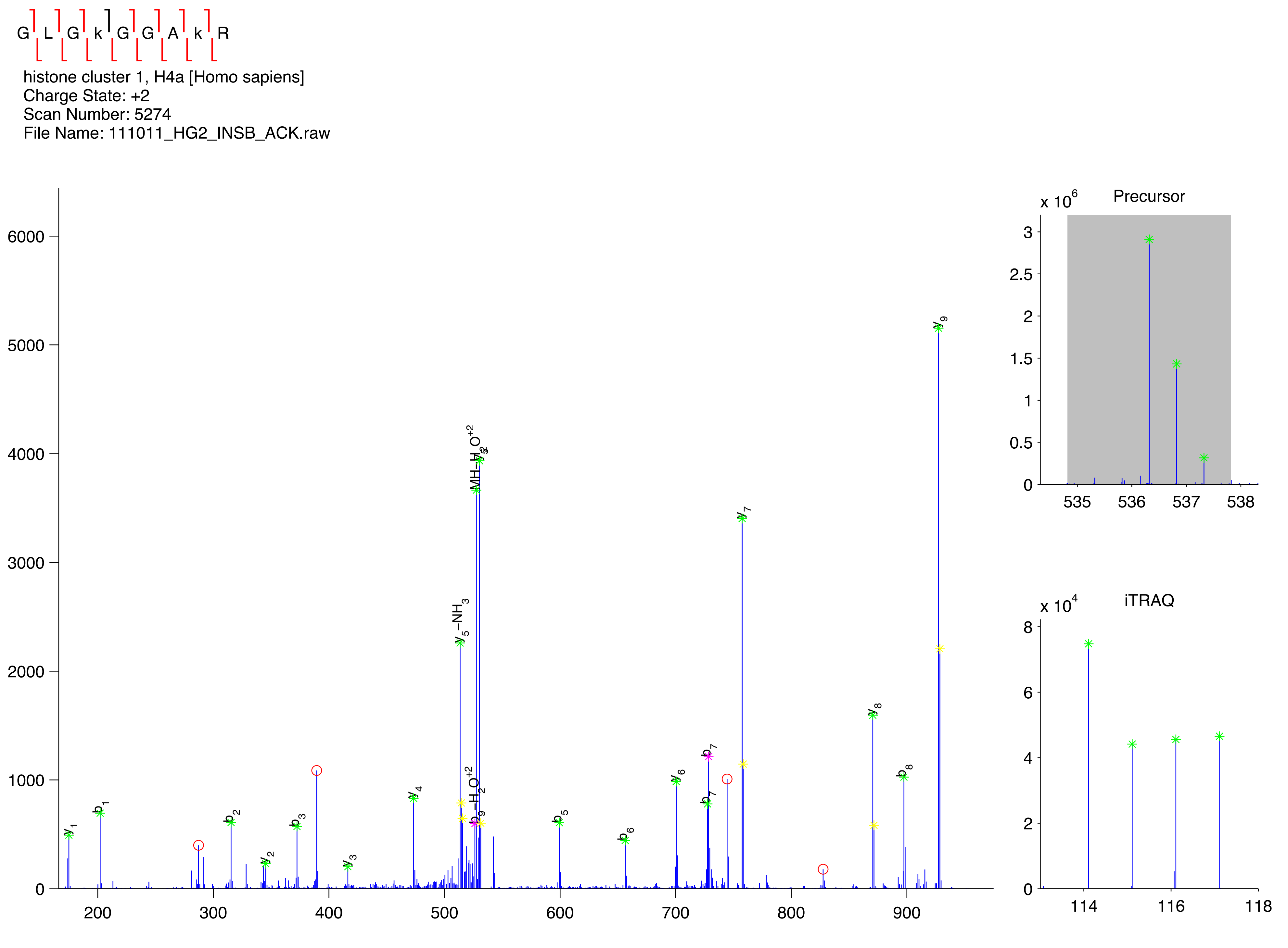
G L G k G G A k R
histone cluster 1, H4a [Homo sapiens]
Charge State: +2
Scan Number: 5274
File Name: 111011_HG2_INSB_ACK.raw
Precursor
x 10^6
iTRAQ
x 10^4
0
1000
2000
3000
4000
5000
6000
200
300
400
500
600
700
800
900
y1
b1
b2
y2
b3
y3
y4
y5-NH3
MH-H2O+2
b9-H2O+2
b5
b6
y6
b7
y7
y8
b8
y9
535
536
537
538
0
0.5
1
1.5
2
2.5
3
114
116
118
0
2
4
6
8

G k G G k G L G k G G A k R

histone cluster 1, H4a [Homo sapiens]
Charge State: +3
Scan Number: 5337
File Name: 111011_HG2_INSB_ACK.raw

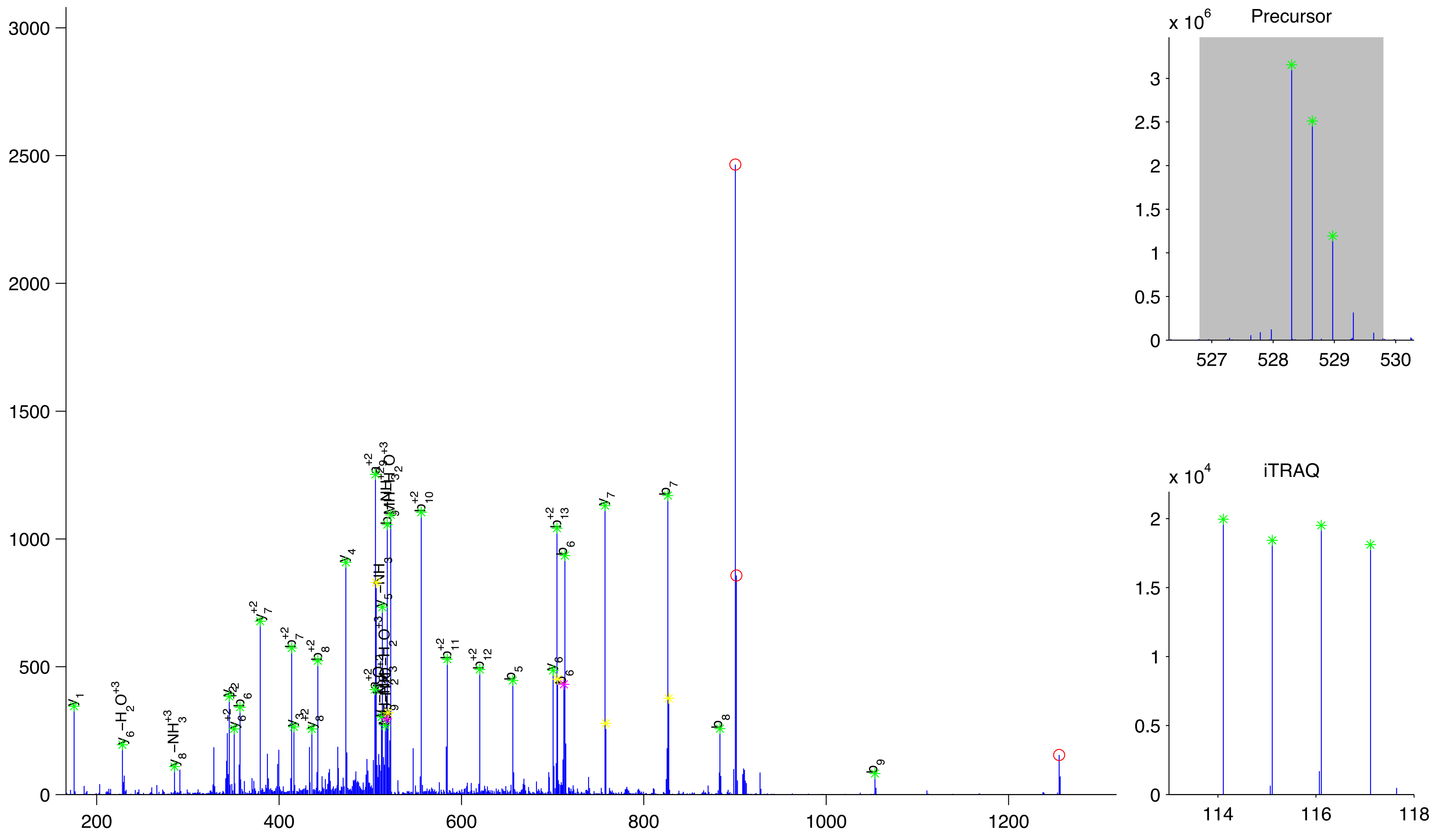

G k G G k G L G k G G A k R

histone cluster 1, H4a [Homo sapiens]
Charge State: +2
Scan Number: 5341
File Name: 111011_HG2_INSB_ACK.raw

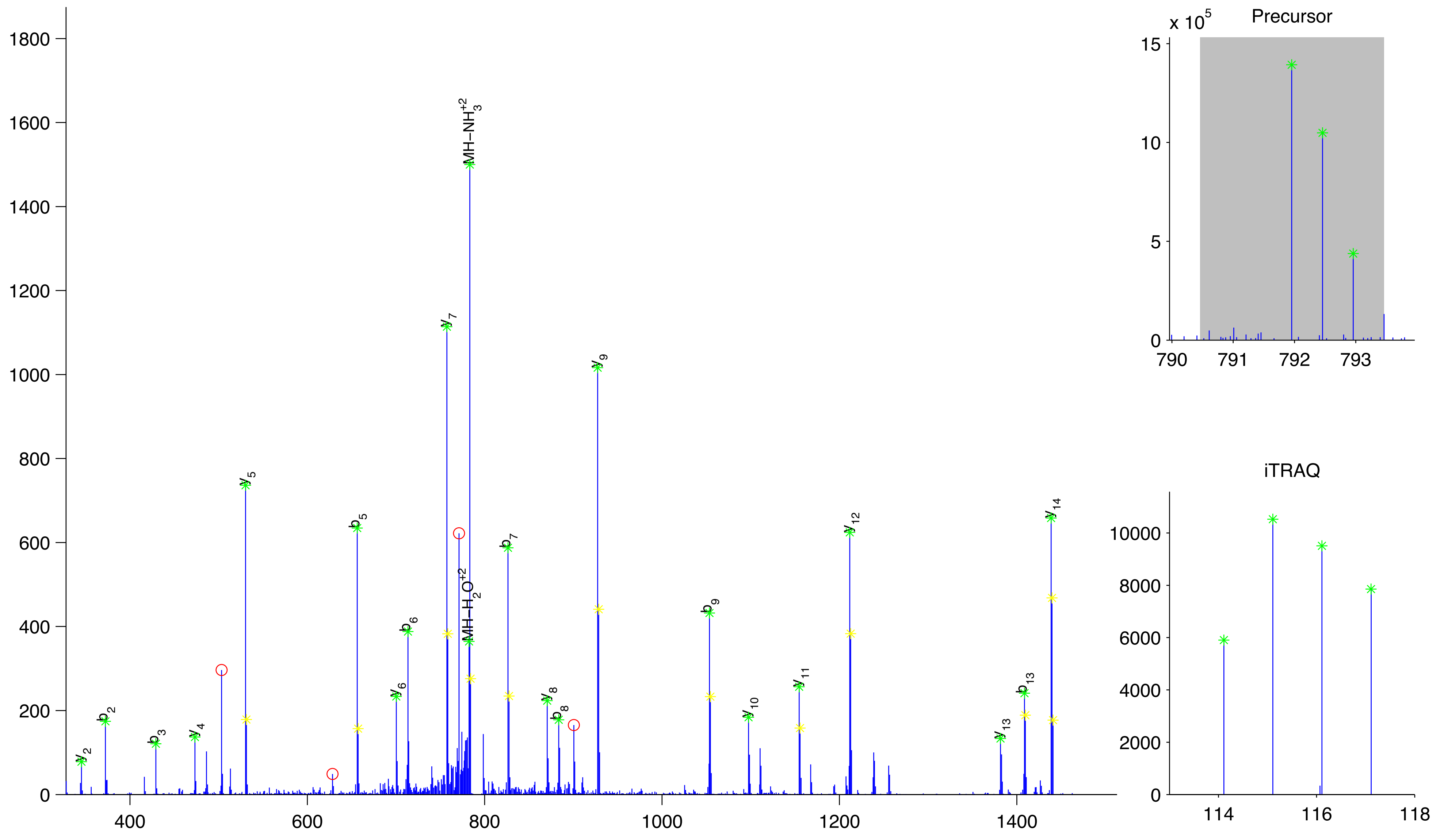

G k G G k G L G k G G A k R

histone cluster 1, H4a [Homo sapiens]
Charge State: +2
Scan Number: 5351
File Name: 111011_HG2_INSB_ACK.raw

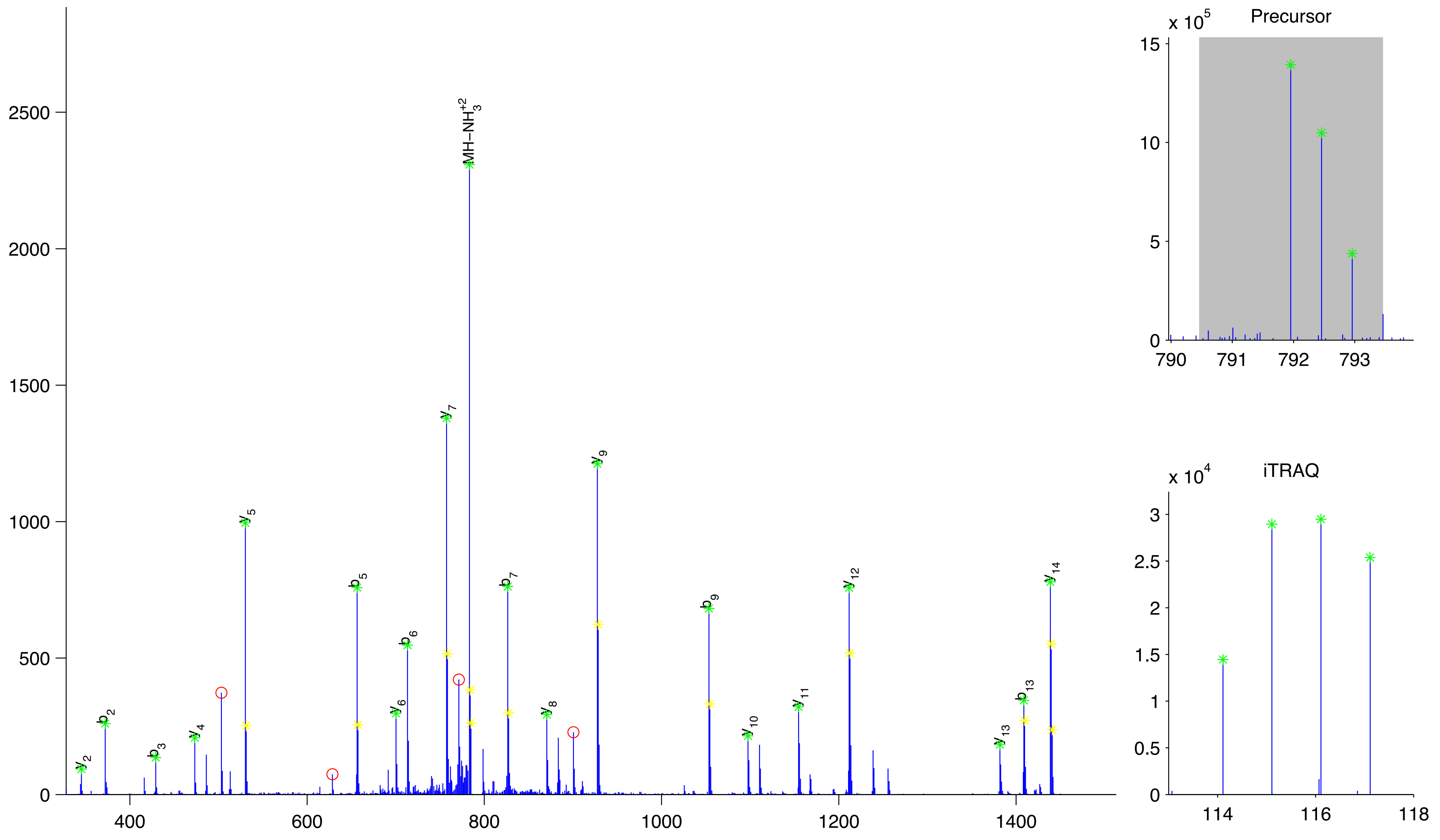

G G k G L G k G G A k R

histone cluster 1, H4a [Homo sapiens]
Charge State: +2
Scan Number: 5381
File Name: 111011_HG2_INSB_ACK.raw

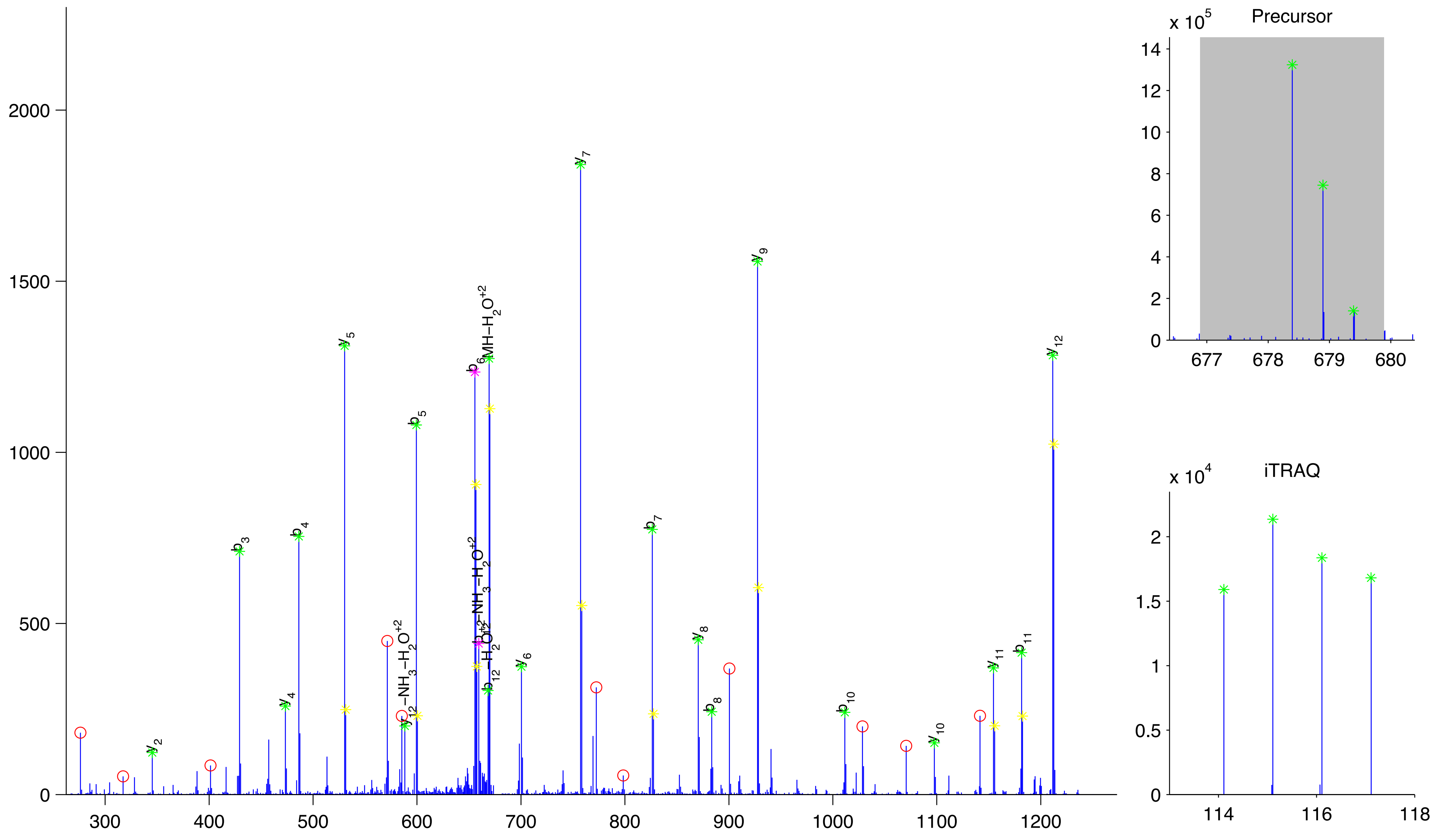

G L G k G G A k R

histone cluster 1, H4a [Homo sapiens]
Charge State: +2
Scan Number: 5444
File Name: 111011_HG2_INSB_ACK.raw

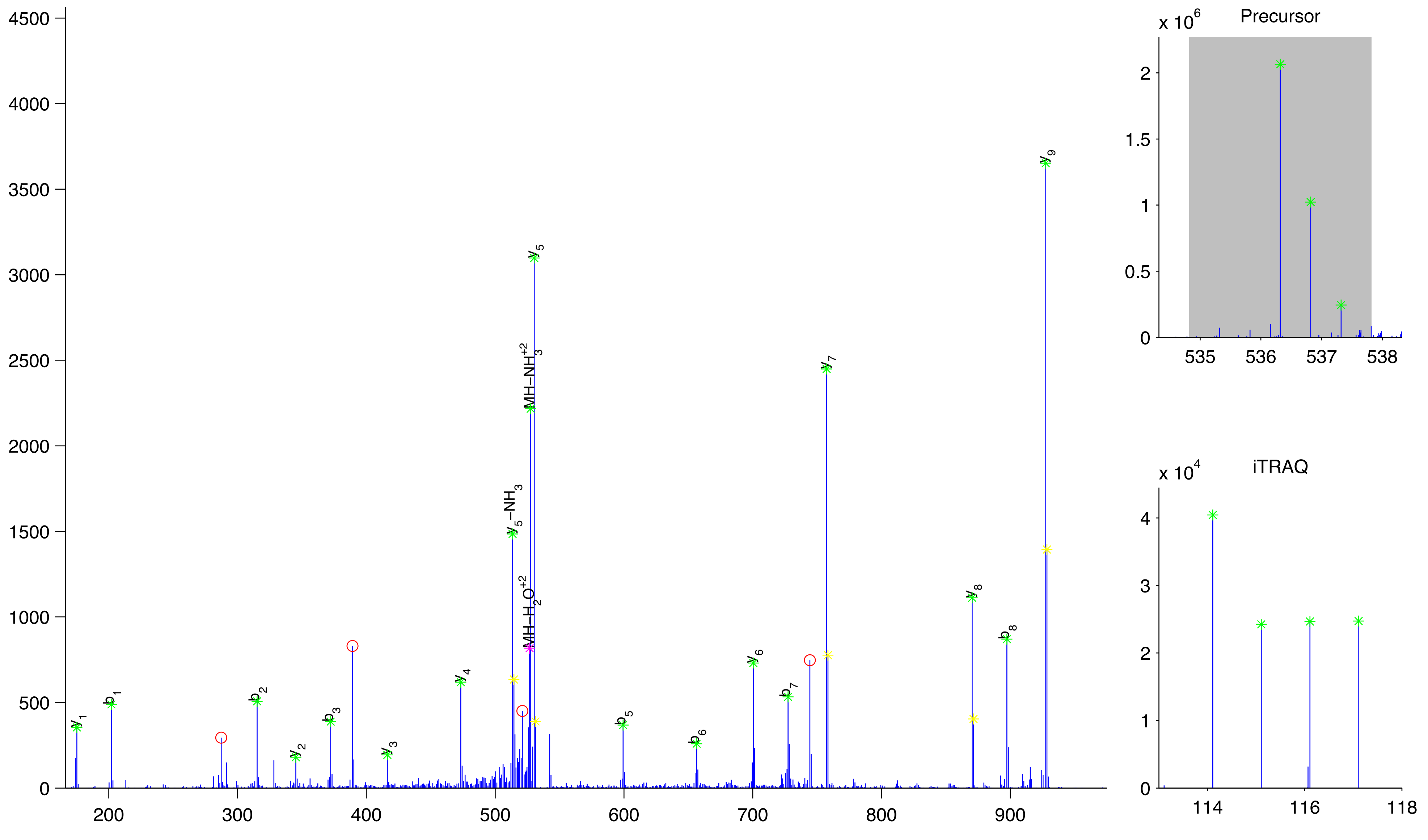

G L G k G G A k R

histone cluster 1, H4a [Homo sapiens]
Charge State: +2
Scan Number: 5610
File Name: 111011_HG2_INSB_ACK.raw

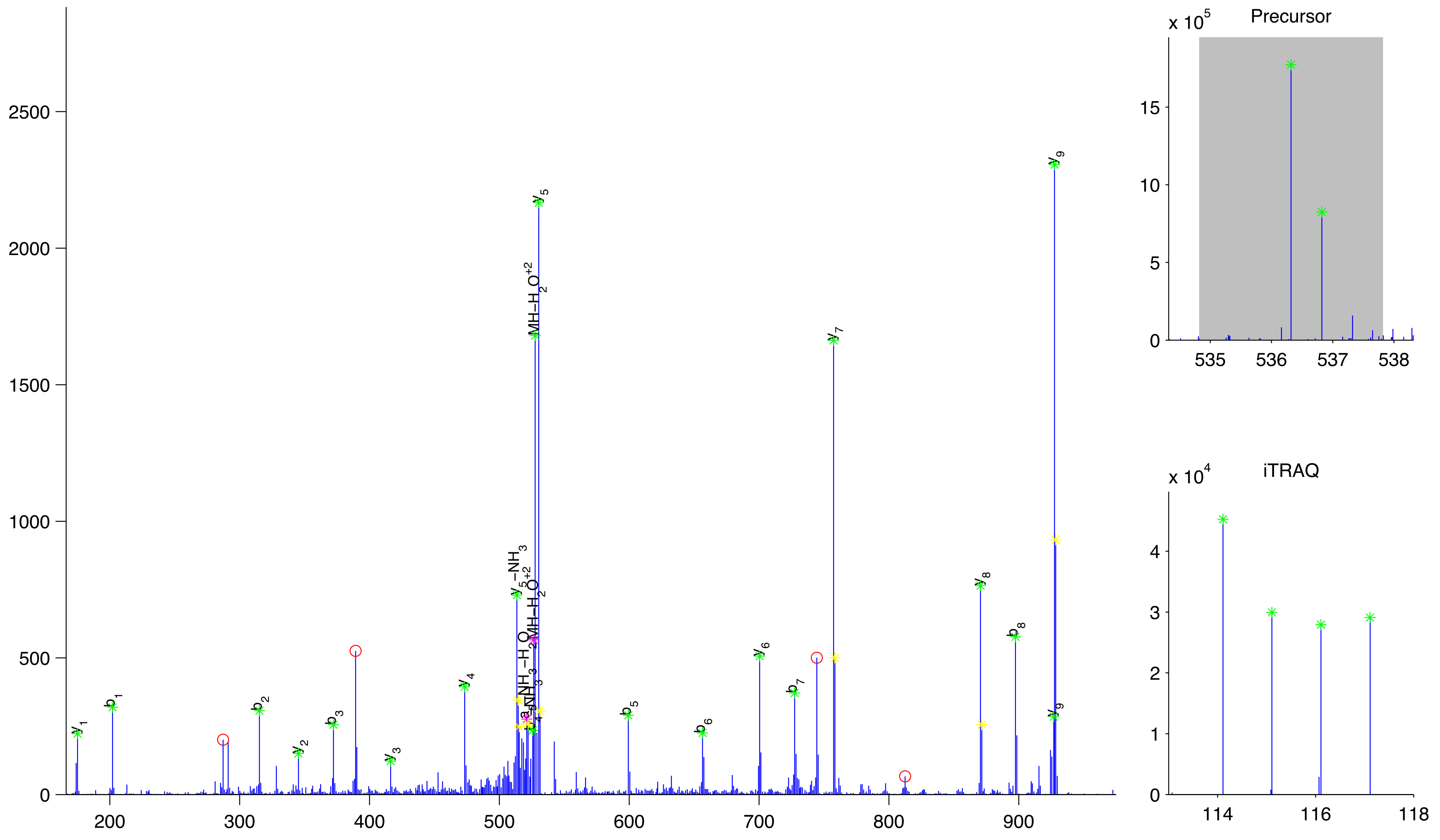

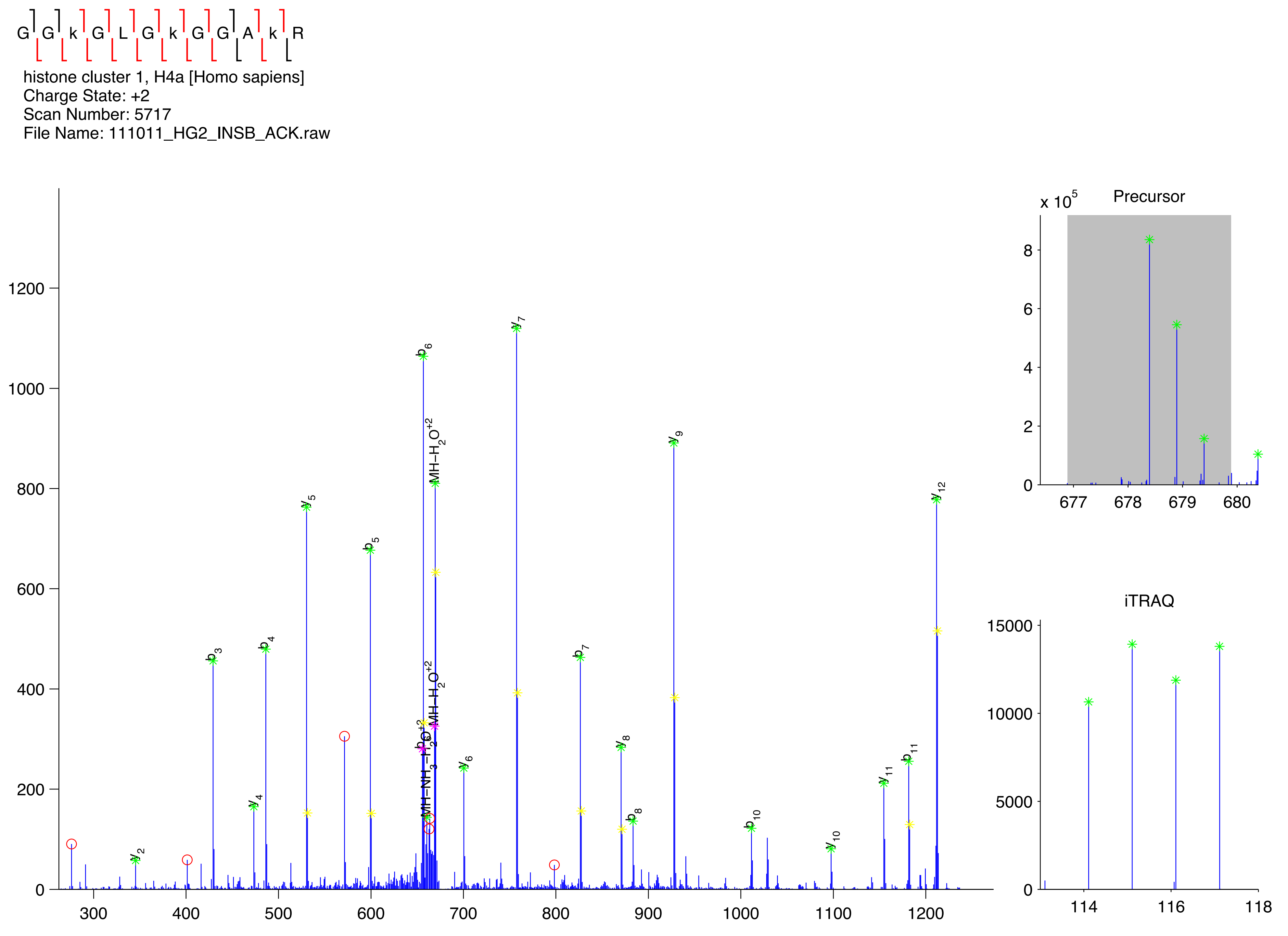
G G k G L G k G G A k R
histone cluster 1, H4a [Homo sapiens]
Charge State: +2
Scan Number: 5717
File Name: 111011_HG2_INSB_ACK.raw
Precursor
x 10^5
iTRAQ
MH-H2O+2
MH-NH3-H2O+2
b6+2

G L G k G G A k R

histone cluster 1, H4a [Homo sapiens]
Charge State: +2
Scan Number: 5736
File Name: 110924_HG2_INSA_ACK3.raw

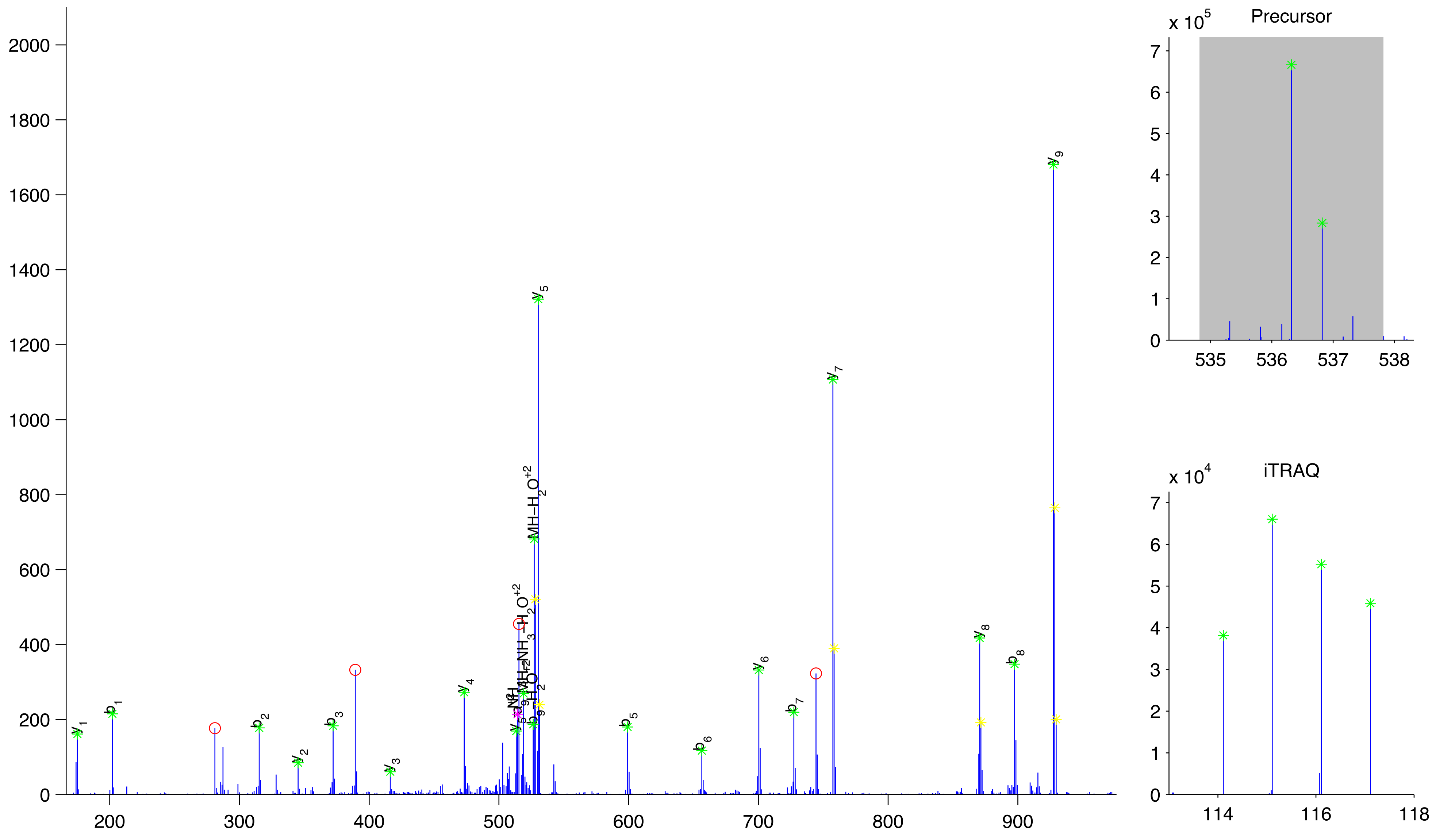

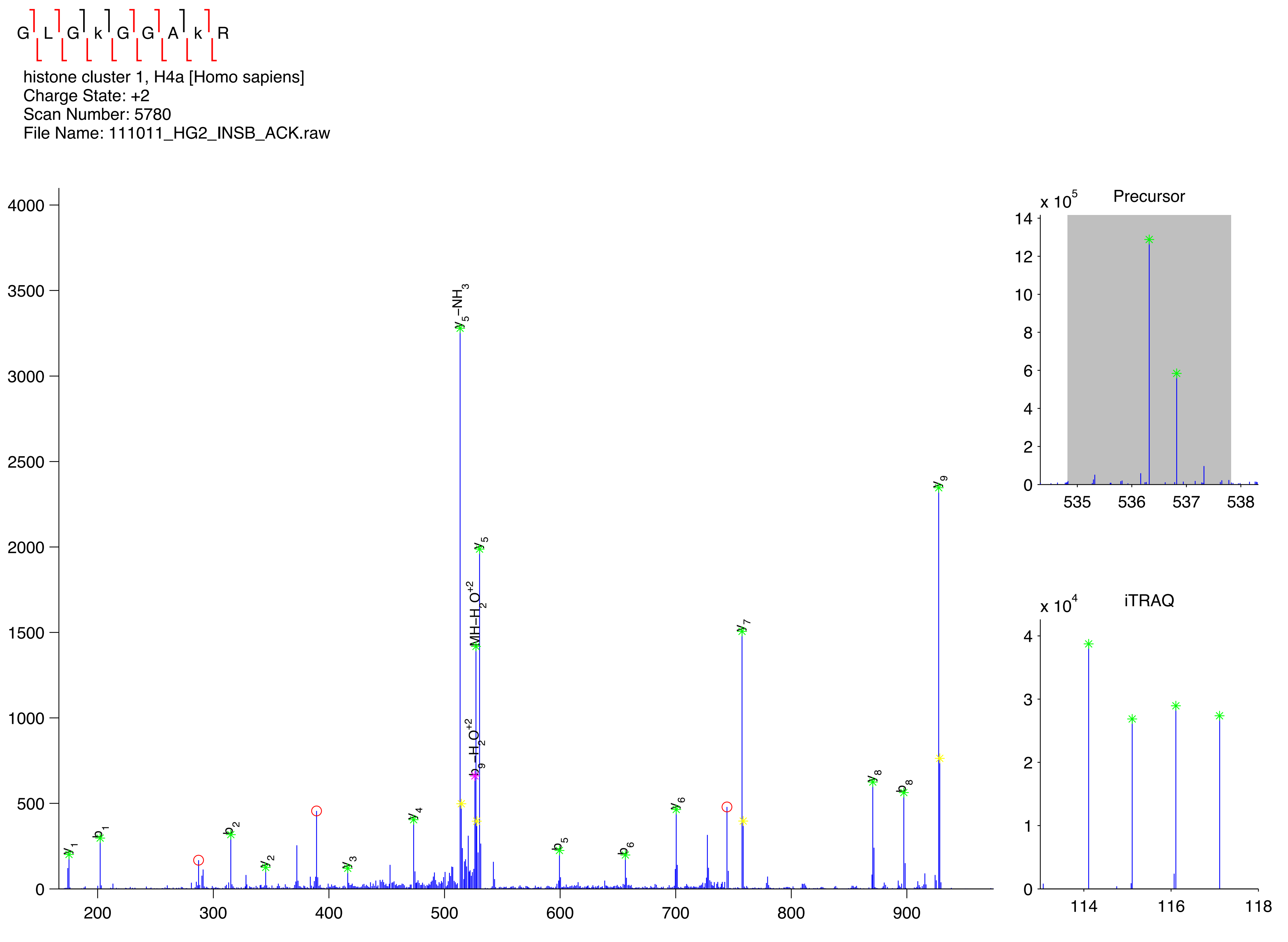

G L G k G G A k R
histone cluster 1, H4a [Homo sapiens]
Charge State: +2
Scan Number: 5780
File Name: 111011_HG2_INSB_ACK.raw
y5–NH3
y5
MH–H2O+2
b9–H2O+2
y1
b1
b2
y2
y3
y4
b5
b6
y6
y7
y8
b8
y9
Precursor
x 10^5
iTRAQ
x 10^4

G L G k G G A k R

histone cluster 1, H4a [Homo sapiens]
Charge State: +2
Scan Number: 5805
File Name: 110924_HG2_INSA_ACK3.raw

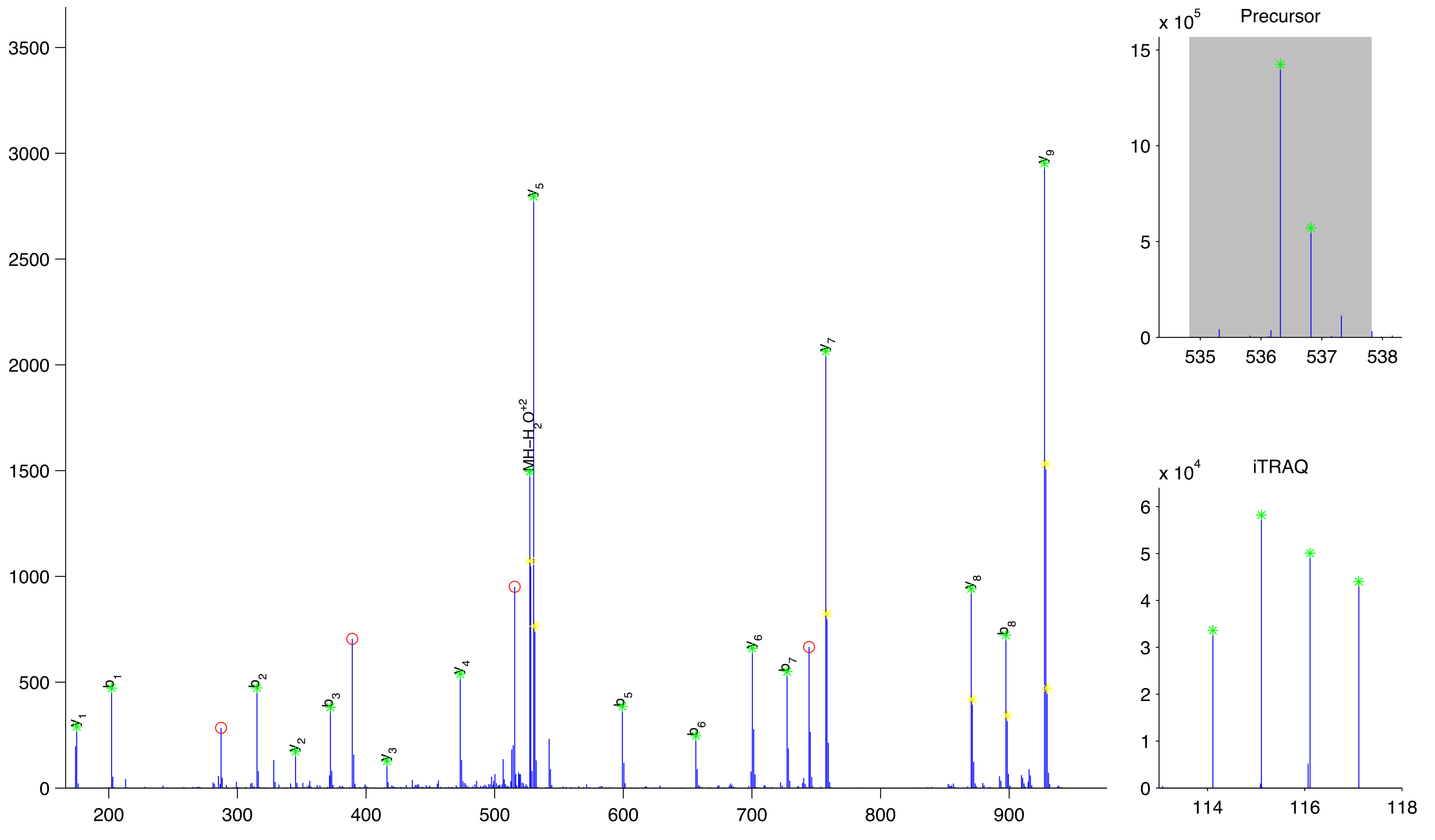

G k G G k G L G k G G A k R

histone cluster 1, H4a [Homo sapiens]
Charge State: +2
Scan Number: 5851
File Name: 111011_HG2_INSB_ACK.raw

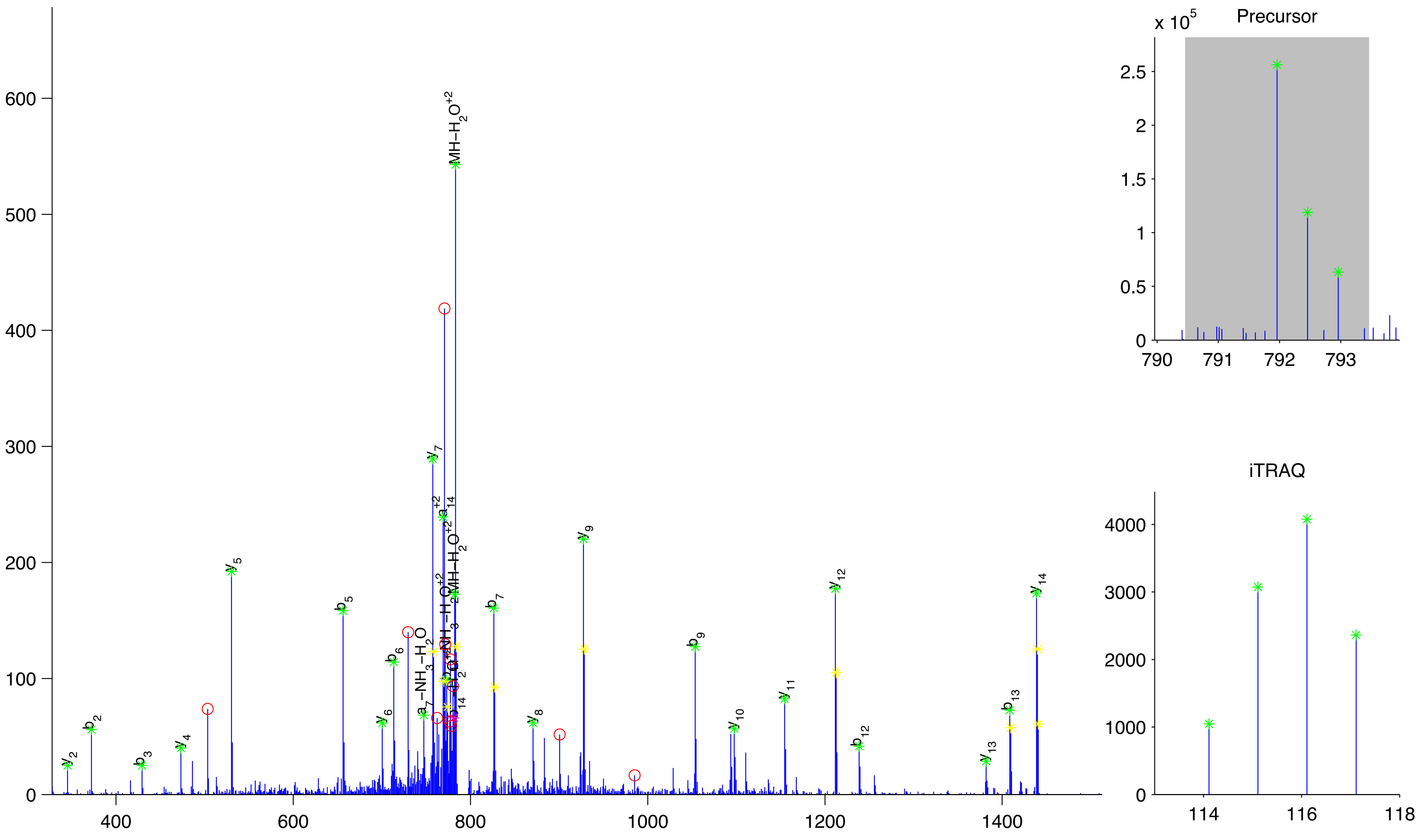

G k G G k G L G k G G A k R

histone cluster 1, H4a [Homo sapiens]
Charge State: +2
Scan Number: 5864
File Name: 110924_HG2_INSA_ACK3.raw

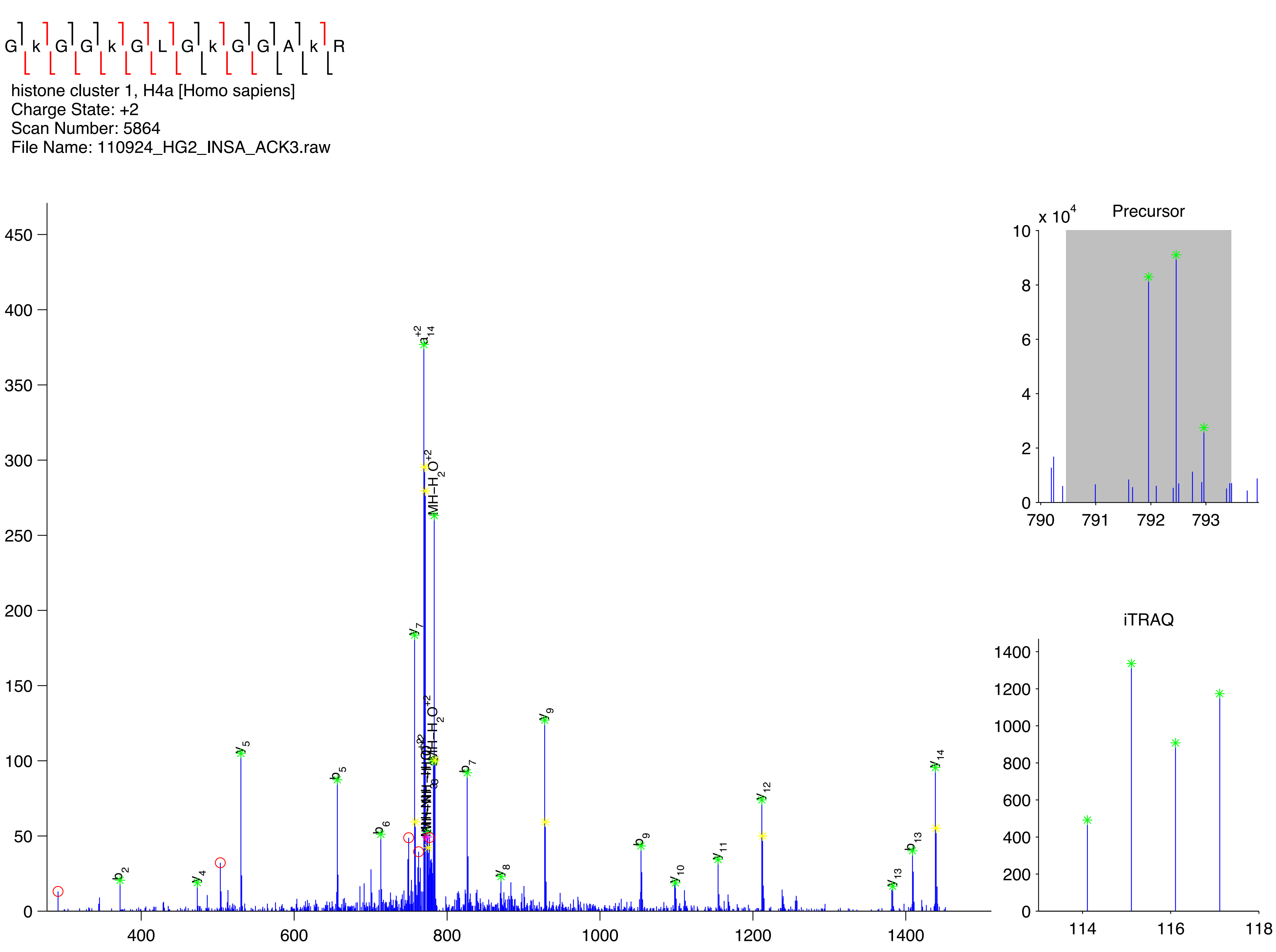

G L G k G G A k R

histone cluster 1, H4a [Homo sapiens]
Charge State: +2
Scan Number: 5876
File Name: 110924_HG2_INSA_ACK4.raw

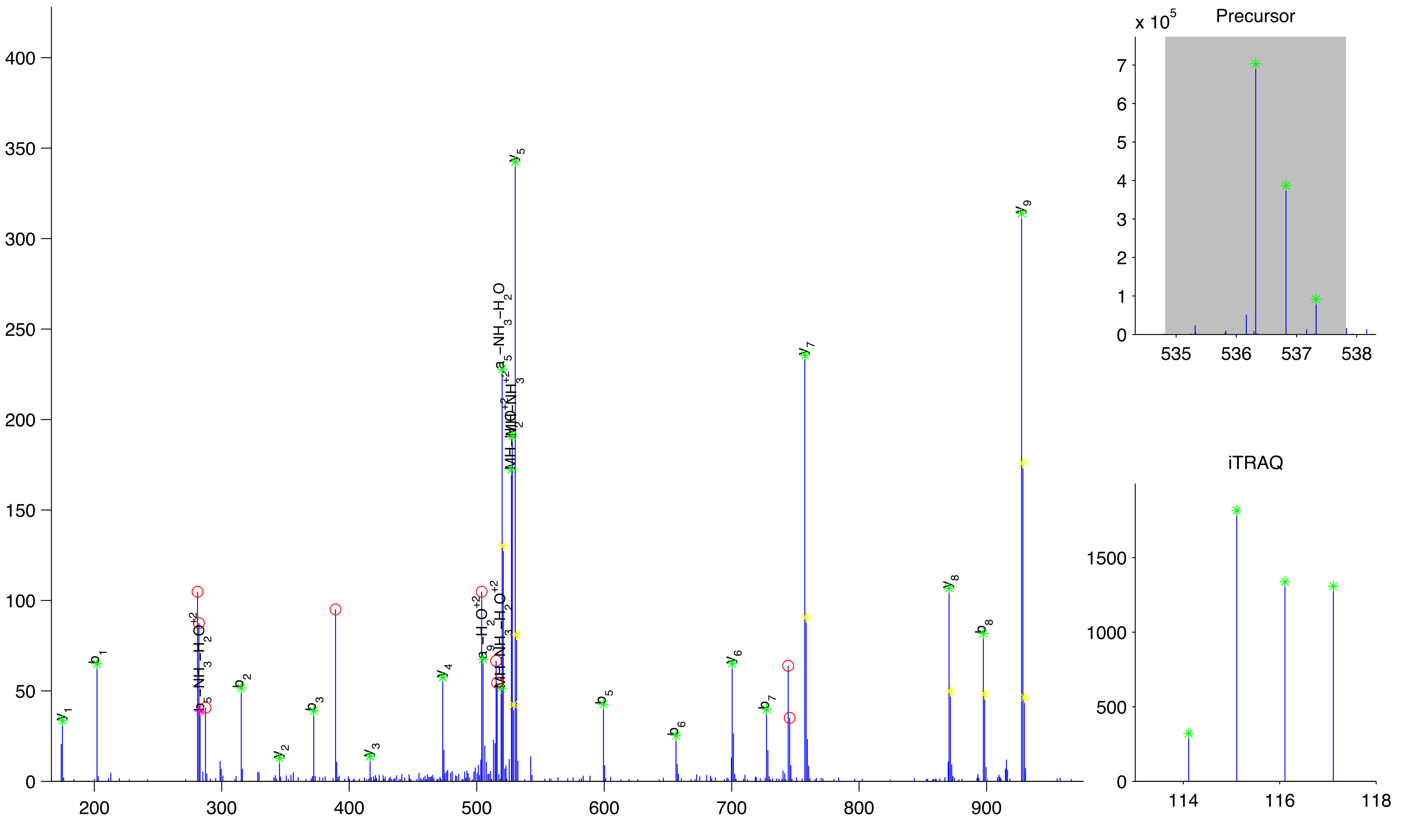

G L G k G G A k R

histone cluster 1, H4a [Homo sapiens]
Charge State: +2
Scan Number: 5883
File Name: 110924_HG2_INSA_ACK4.raw

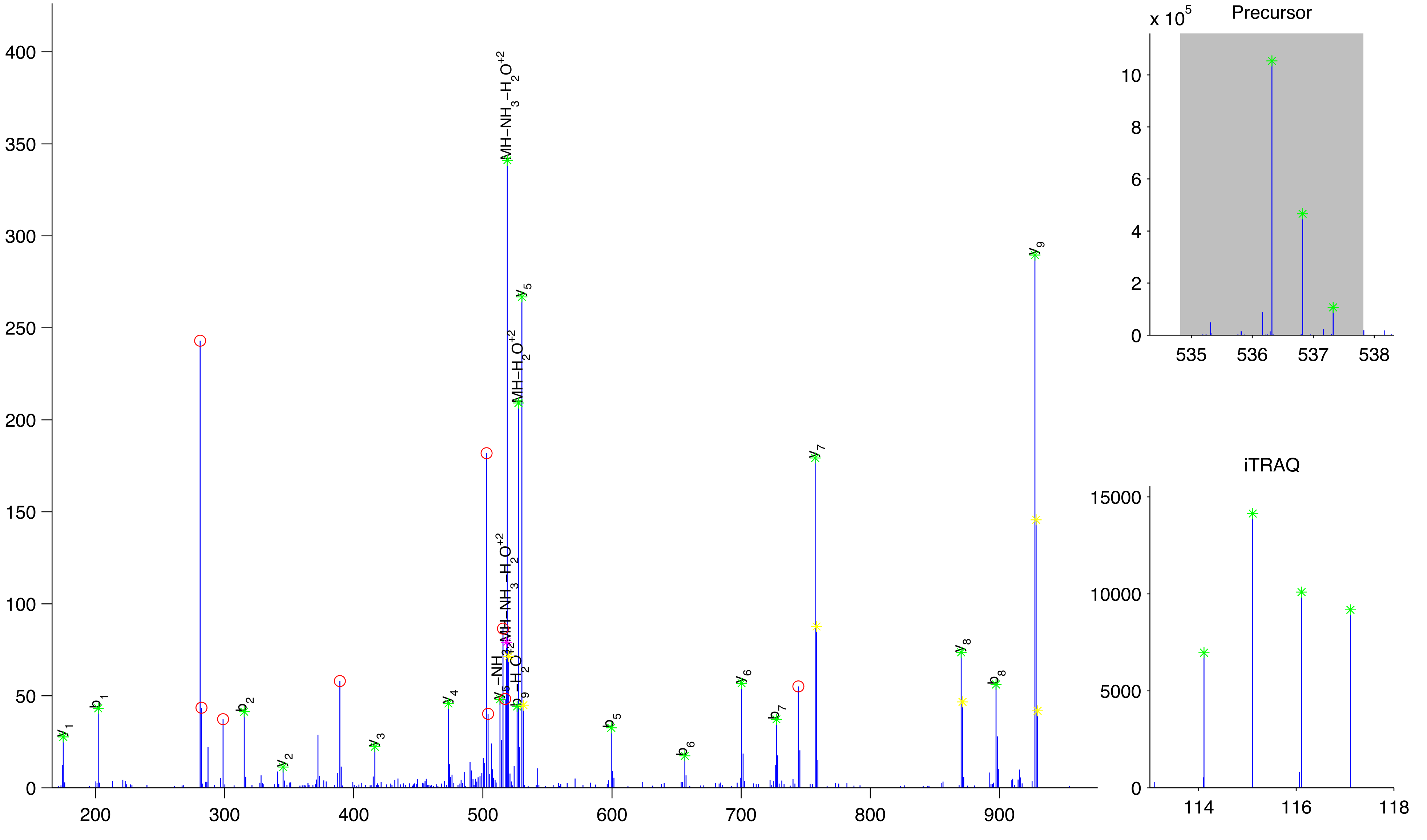

G G k G L G k G G A k R

histone cluster 1, H4a [Homo sapiens]
Charge State: +2
Scan Number: 5887
File Name: 111011_HG2_INSB_ACK.raw

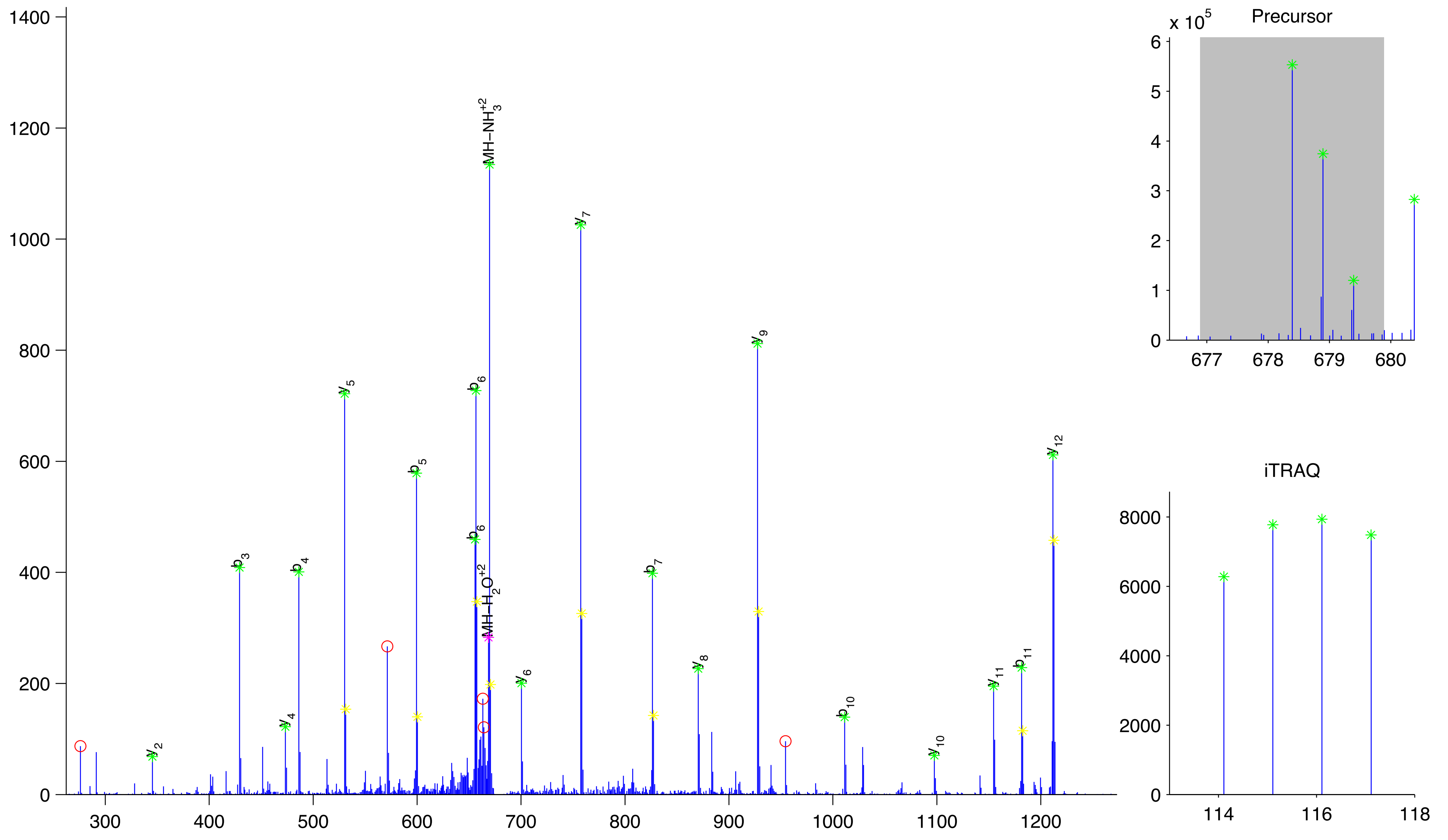

m S G R G k G G k G L G k G G A k R

histone cluster 1, H4a [Homo sapiens]
Charge State: +3
Scan Number: 5897
File Name: 110924_HG2_INSA_ACK2.raw

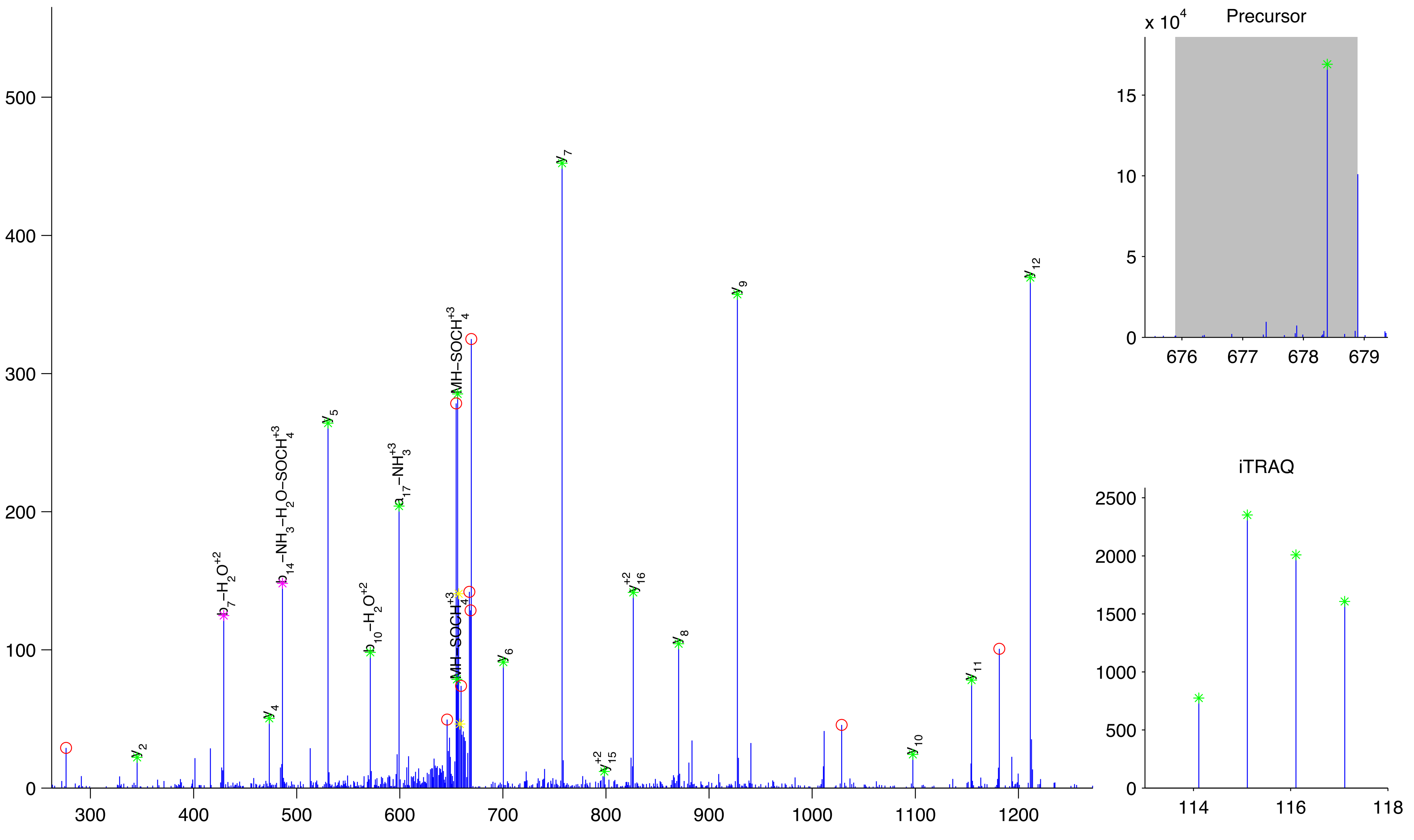

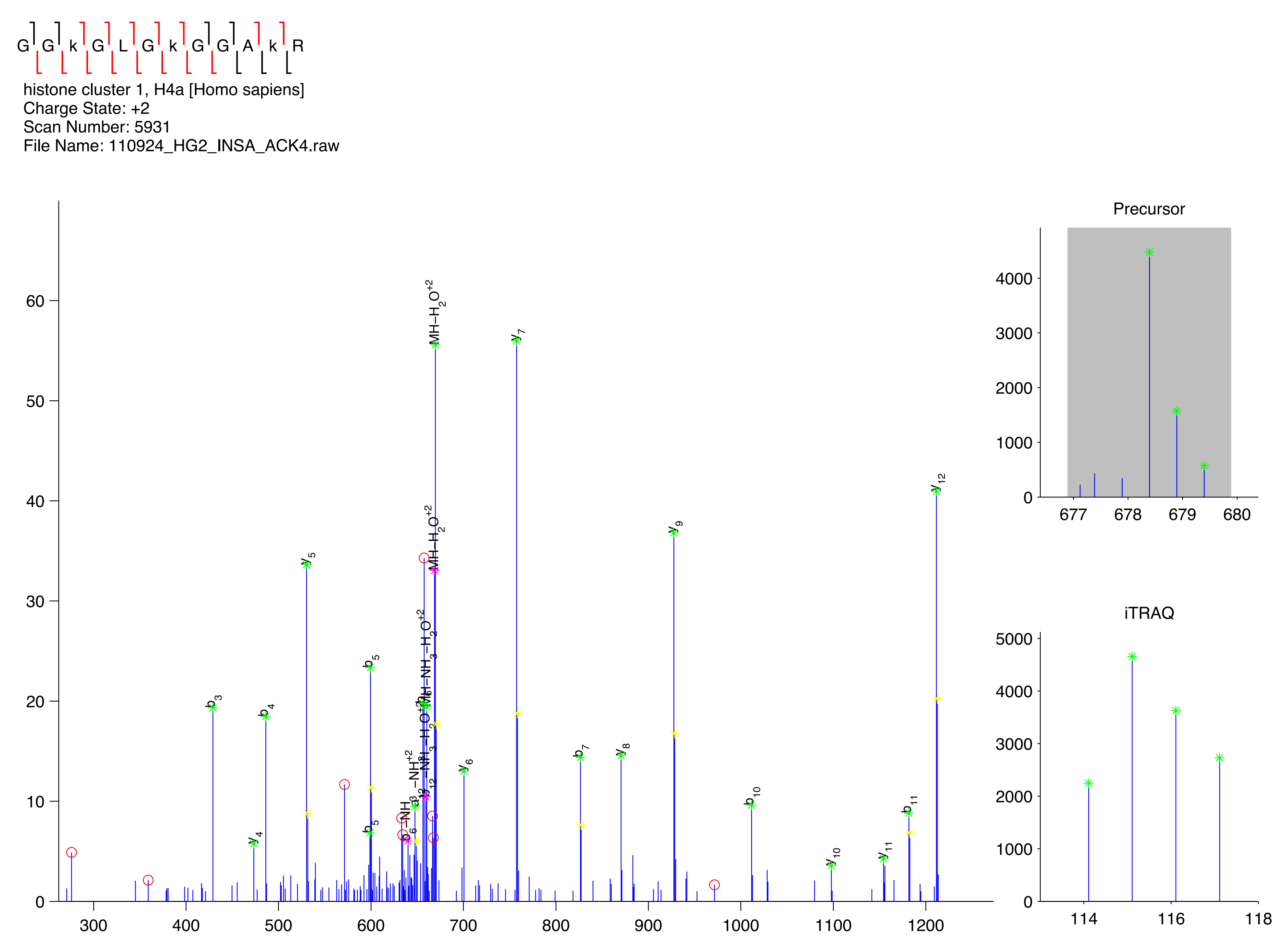

G G k G L G k G G A k R
histone cluster 1, H4a [Homo sapiens]
Charge State: +2
Scan Number: 5931
File Name: 110924_HG2_INSA_ACK4.raw
Precursor
iTRAQ
b3
b4
y4
y5
b5
y6
y7
b7
y8
y9
b10
y10
y11
b11
y12
MH−H2O+2

G L G k G G A k R

histone cluster 1, H4a [Homo sapiens]
Charge State: +2
Scan Number: 5946
File Name: 111011_HG2_INSB_ACK.raw

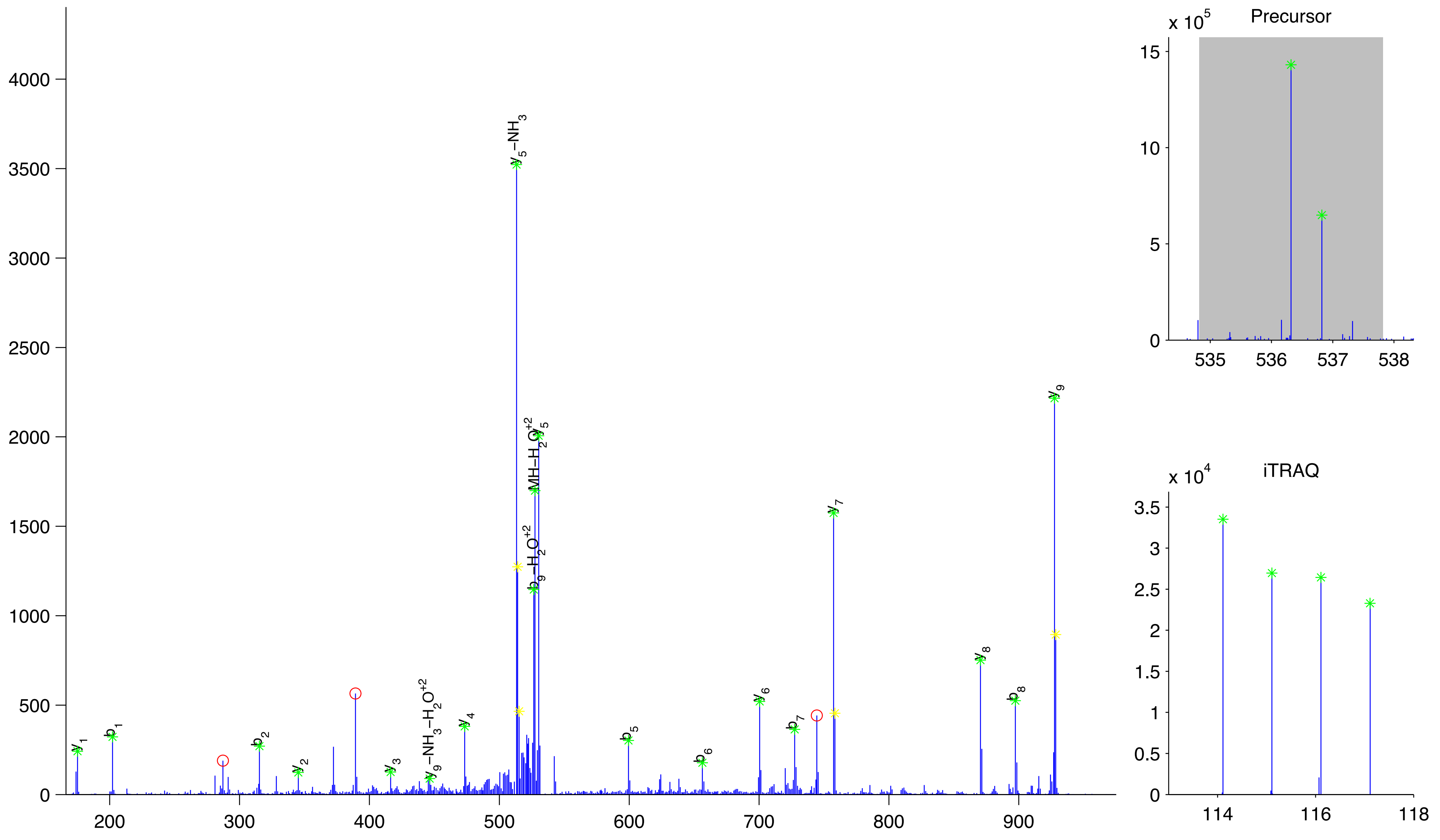

G G k G L G k G G A k R

histone cluster 1, H4a [Homo sapiens]
Charge State: +2
Scan Number: 6051
File Name: 111011_HG2_INSB_ACK.raw

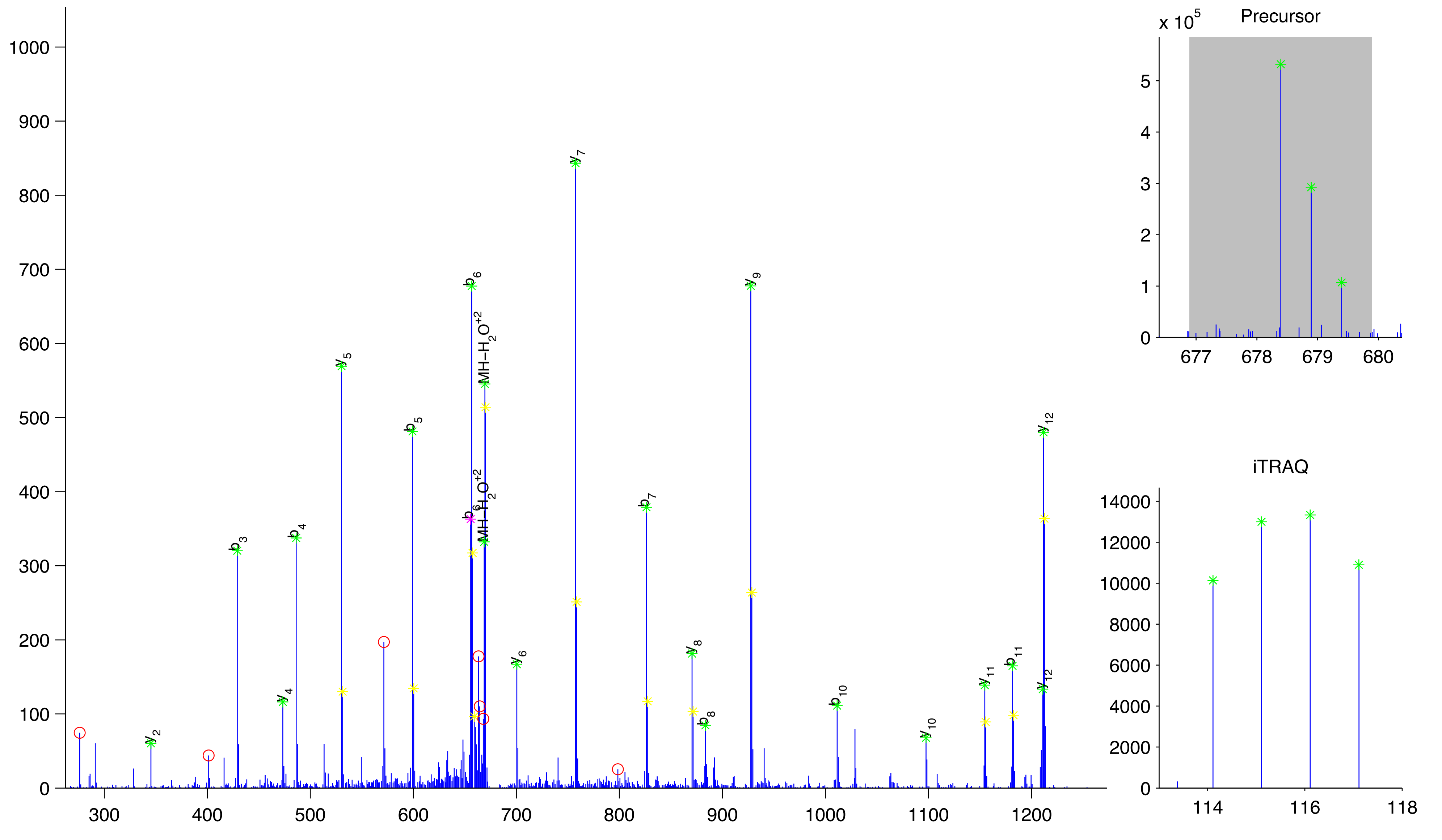

G G k G L G k G G A k R

histone cluster 1, H4a [Homo sapiens]
Charge State: +2
Scan Number: 6114
File Name: 110924_HG2_INSA_ACK3.raw

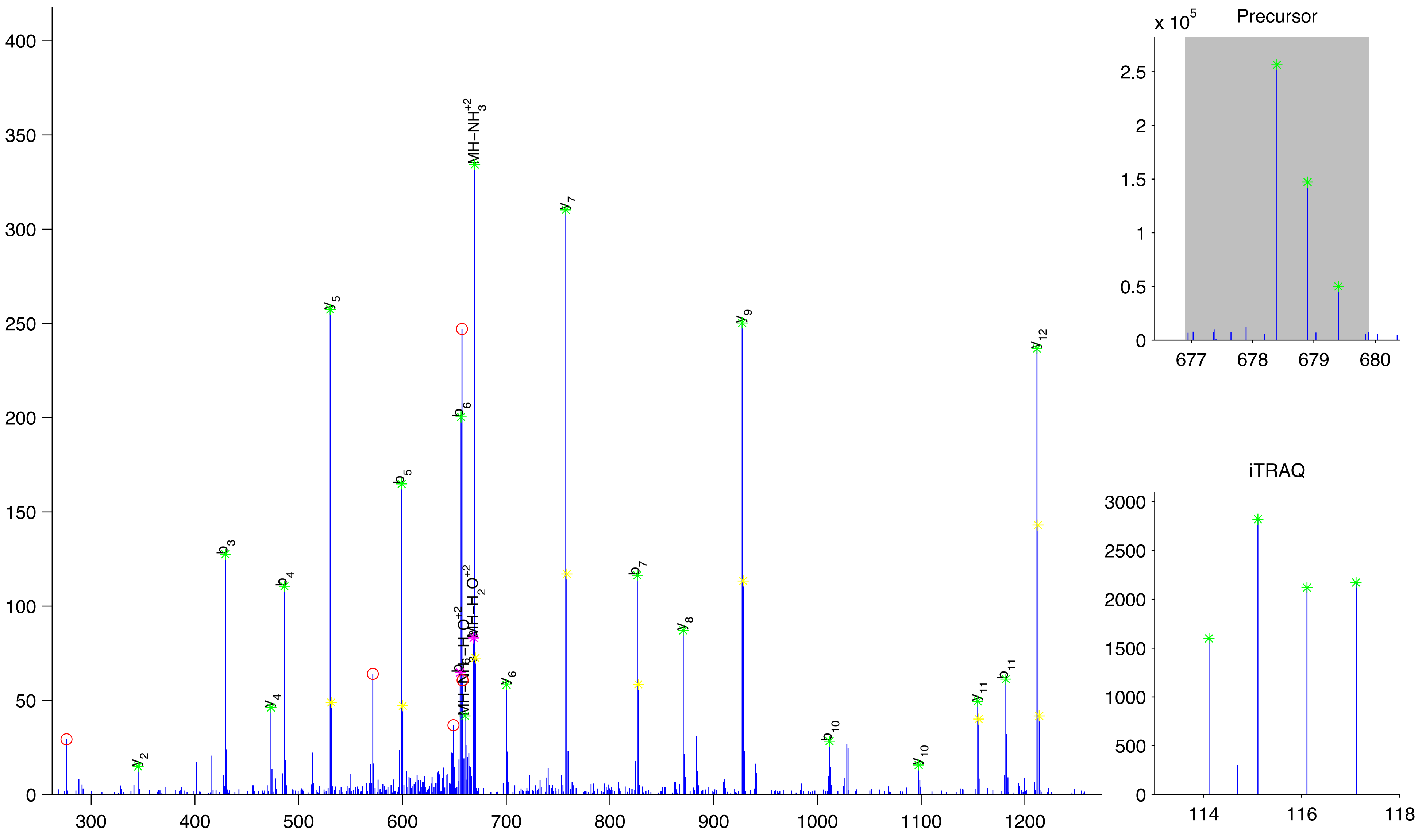

G L G k G G A k R

histone cluster 1, H4a [Homo sapiens]
Charge State: +2
Scan Number: 6114
File Name: 111011_HG2_INSB_ACK.raw

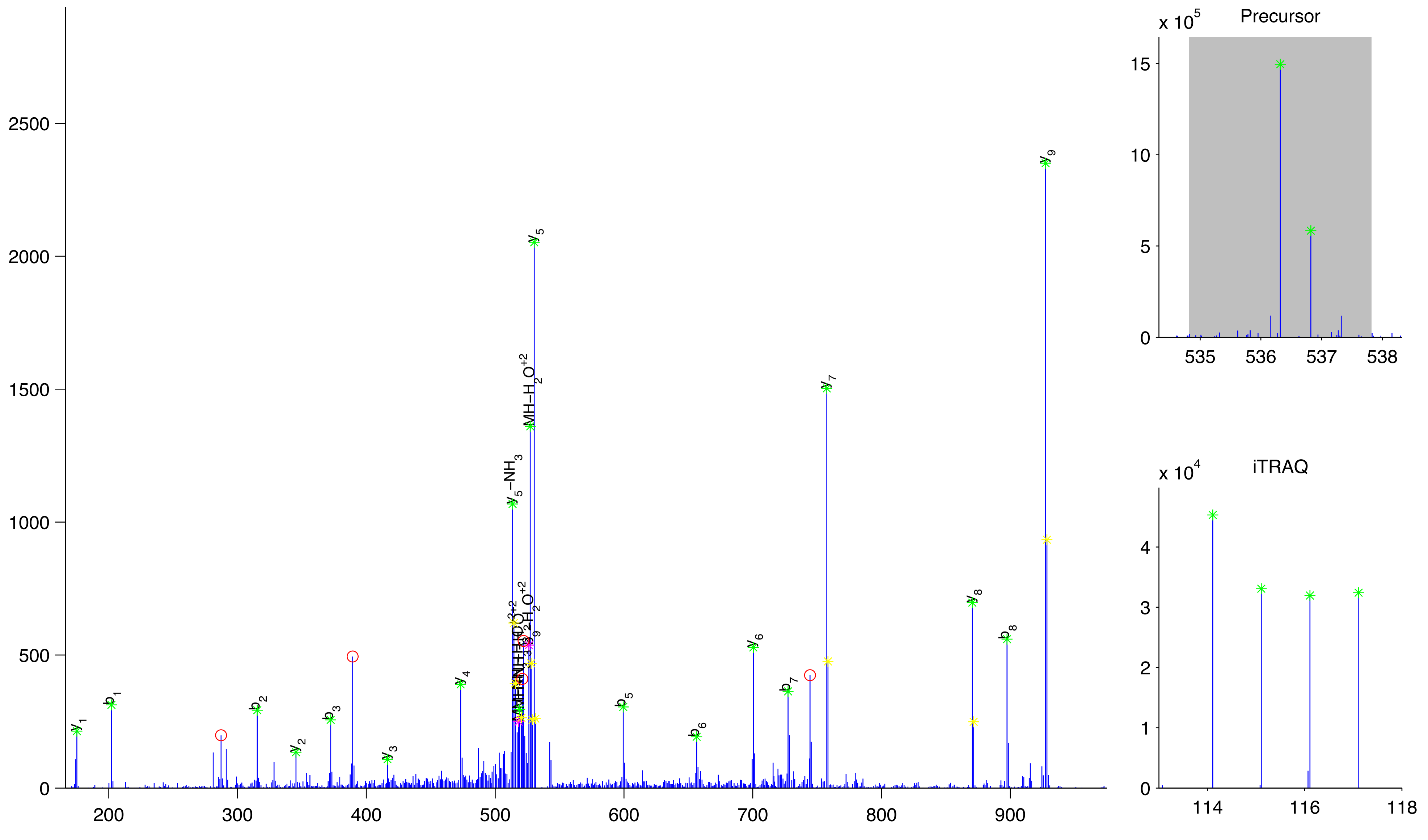

G k G G k G L G k G G A k R

histone cluster 1, H4a [Homo sapiens]
Charge State: +3
Scan Number: 6179
File Name: 111011_HG2_INSB_ACK.raw

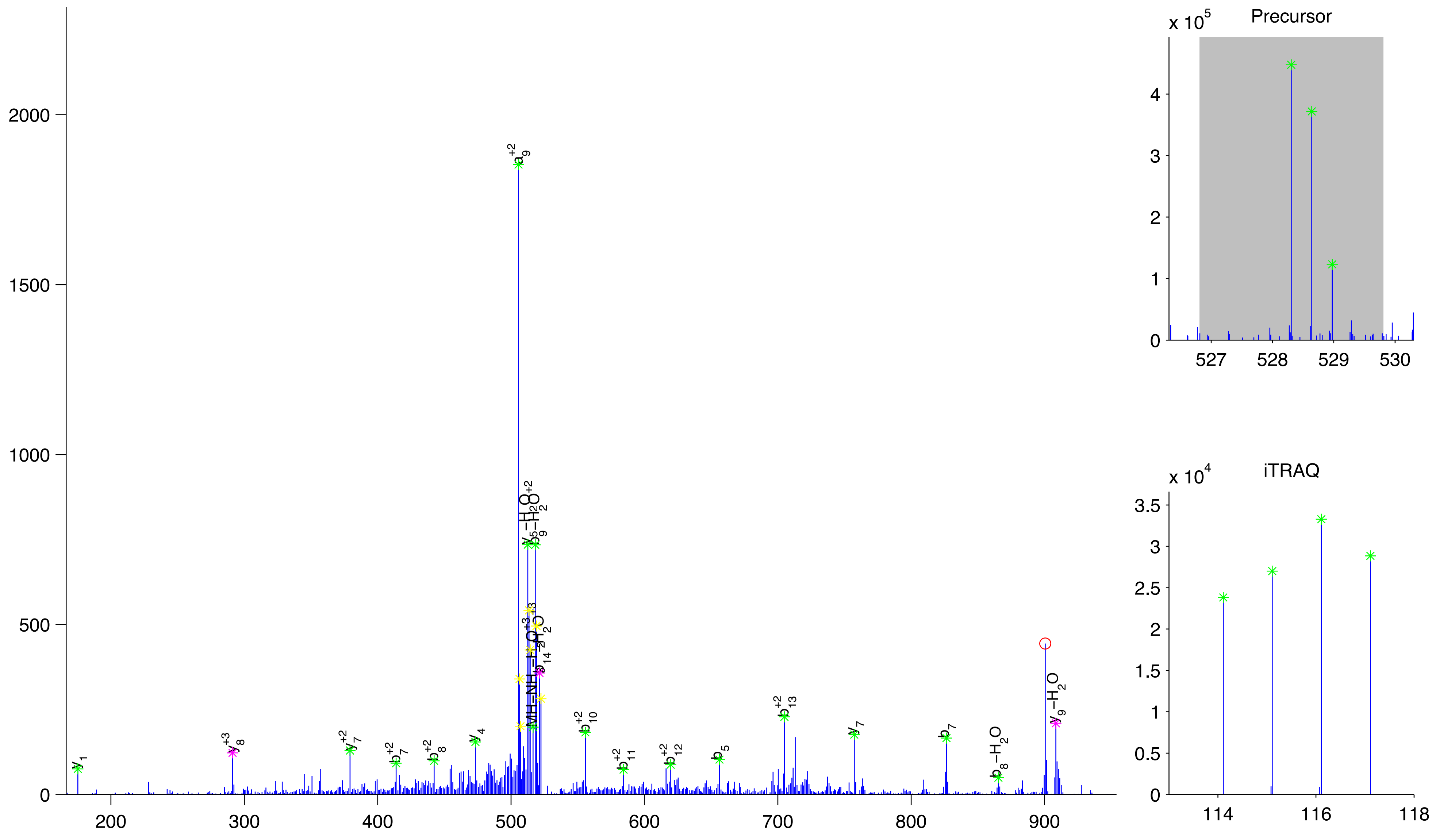

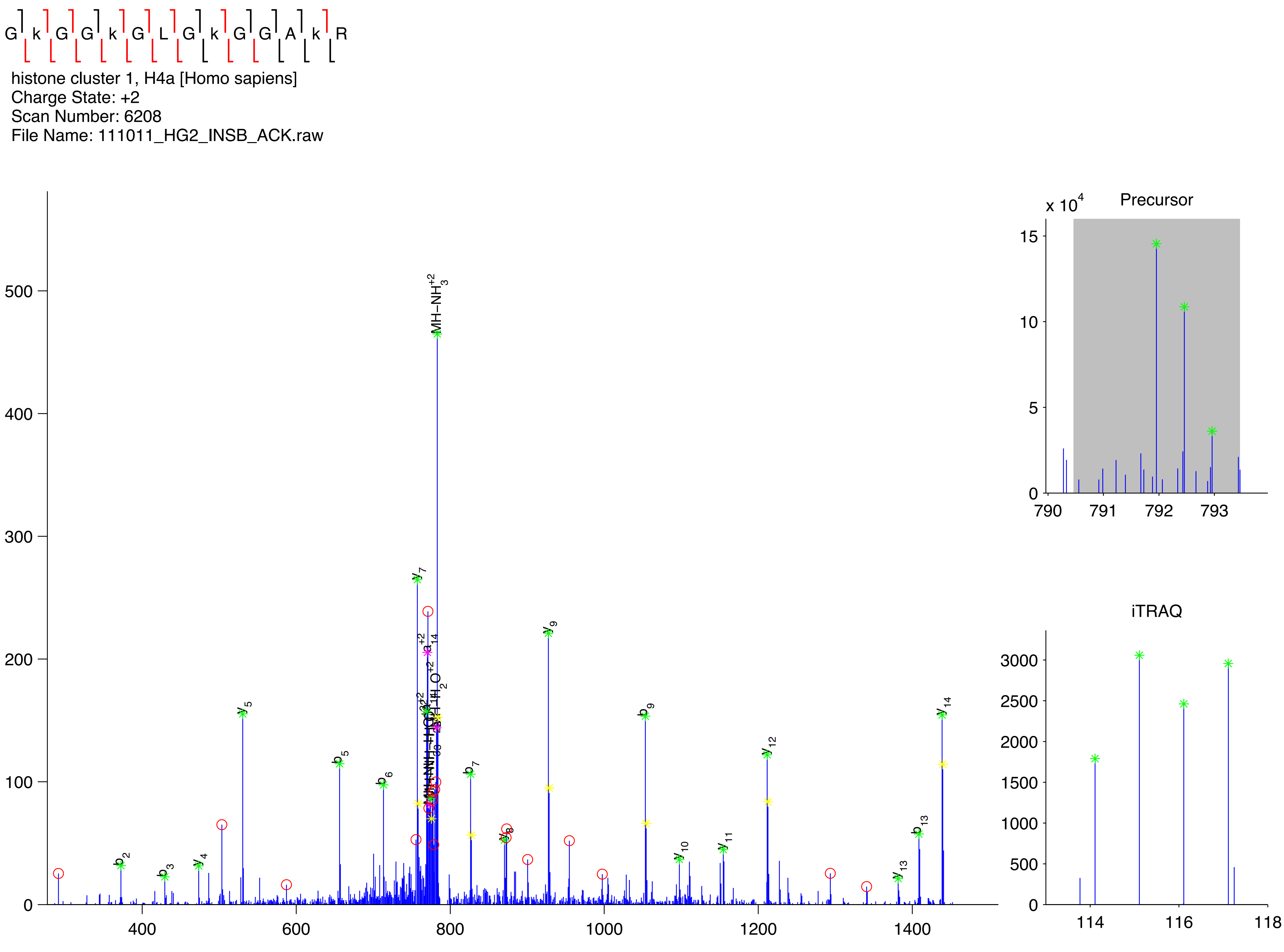

G k G G k G L G k G G A k R
histone cluster 1, H4a [Homo sapiens]
Charge State: +2
Scan Number: 6208
File Name: 111011_HG2_INSB_ACK.raw
Precursor
x 10^4
iTRAQ

G G k G L G k G G A k R

histone cluster 1, H4a [Homo sapiens]
Charge State: +2
Scan Number: 6225
File Name: 111011_HG2_INSB_ACK.raw

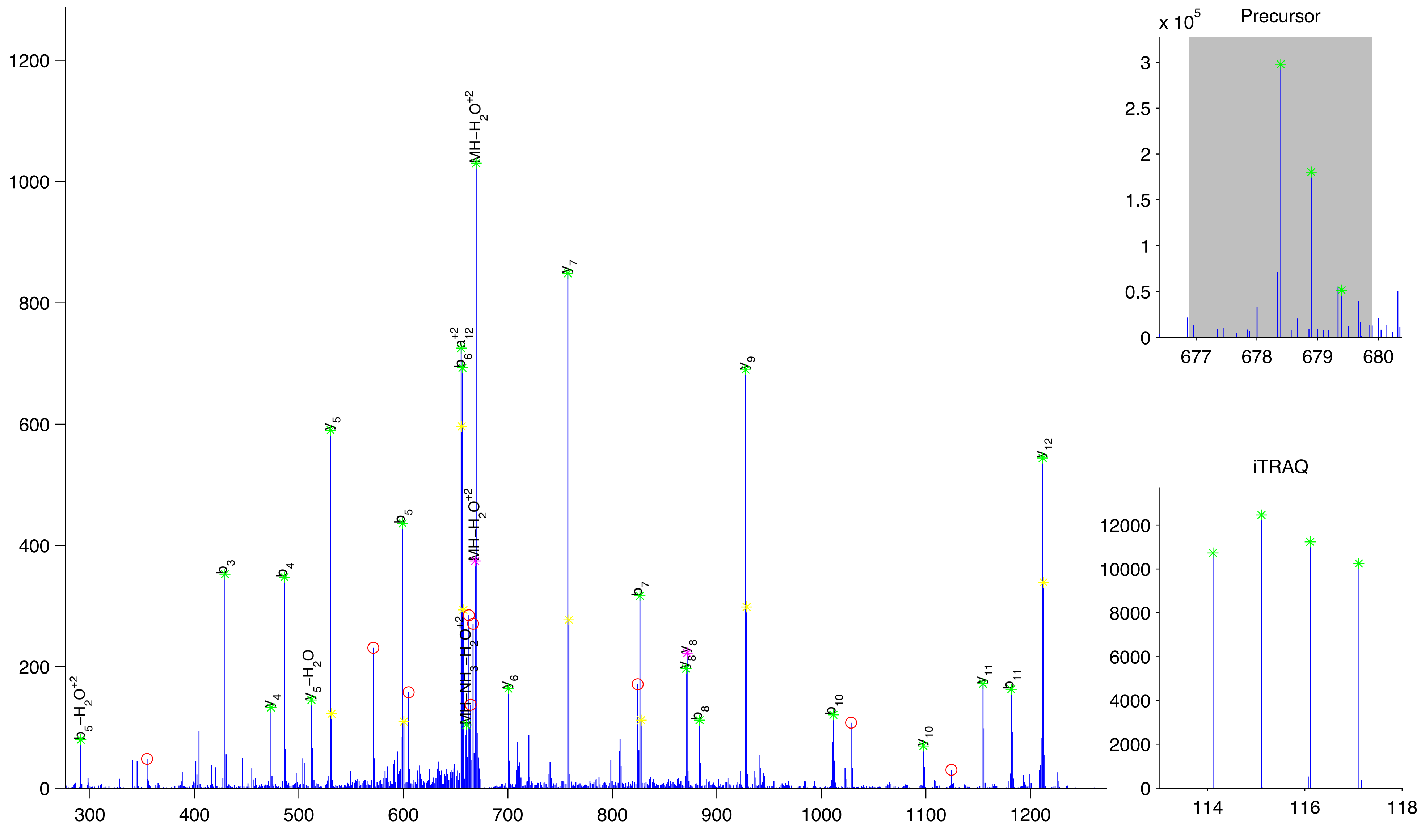

G L G k G G A k R

histone cluster 1, H4a [Homo sapiens]
Charge State: +2
Scan Number: 6284
File Name: 111011_HG2_INSB_ACK.raw

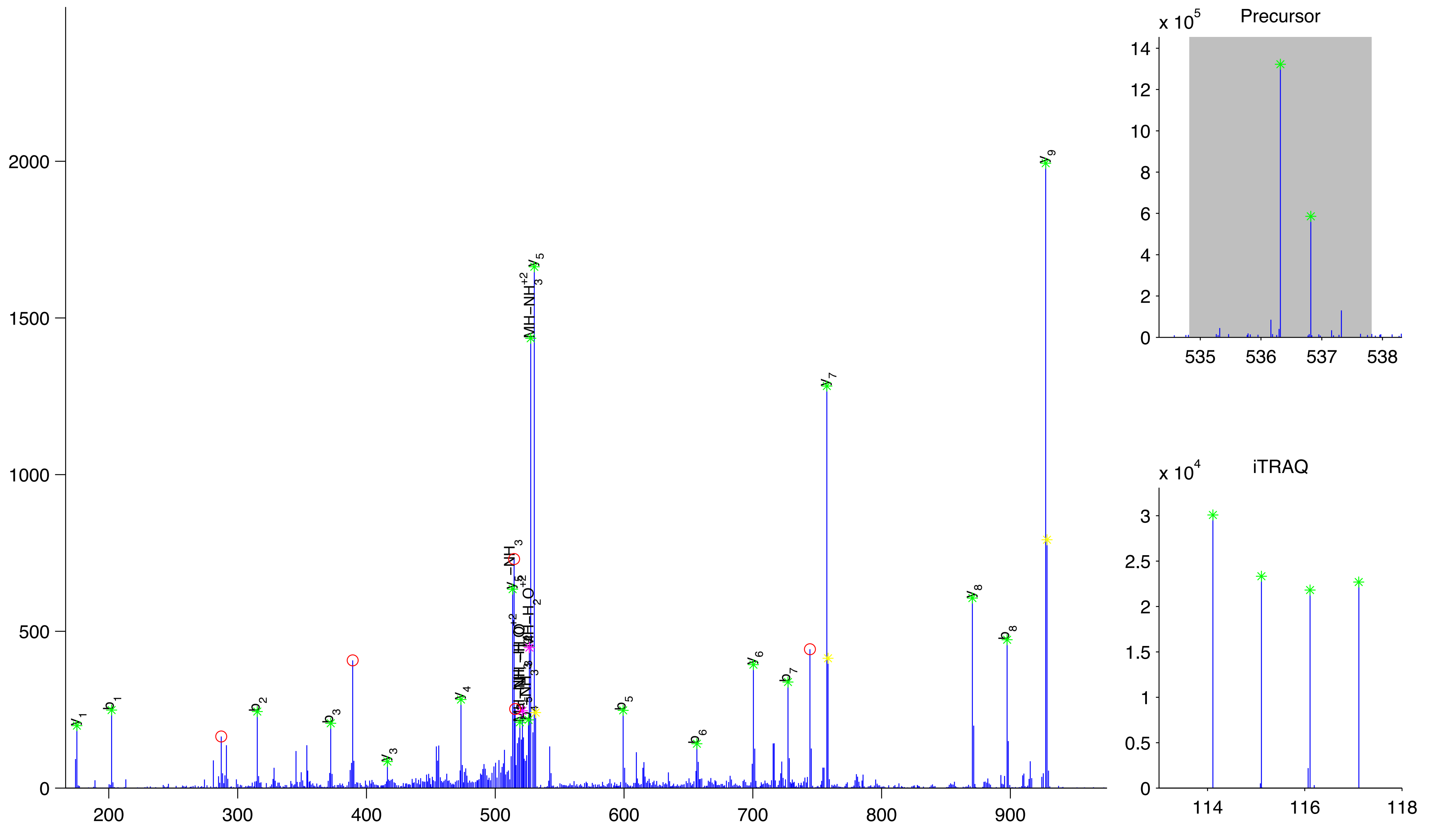

G G k G L G k G G A k R

histone cluster 1, H4a [Homo sapiens]
Charge State: +2
Scan Number: 6387
File Name: 111011_HG2_INSB_ACK.raw

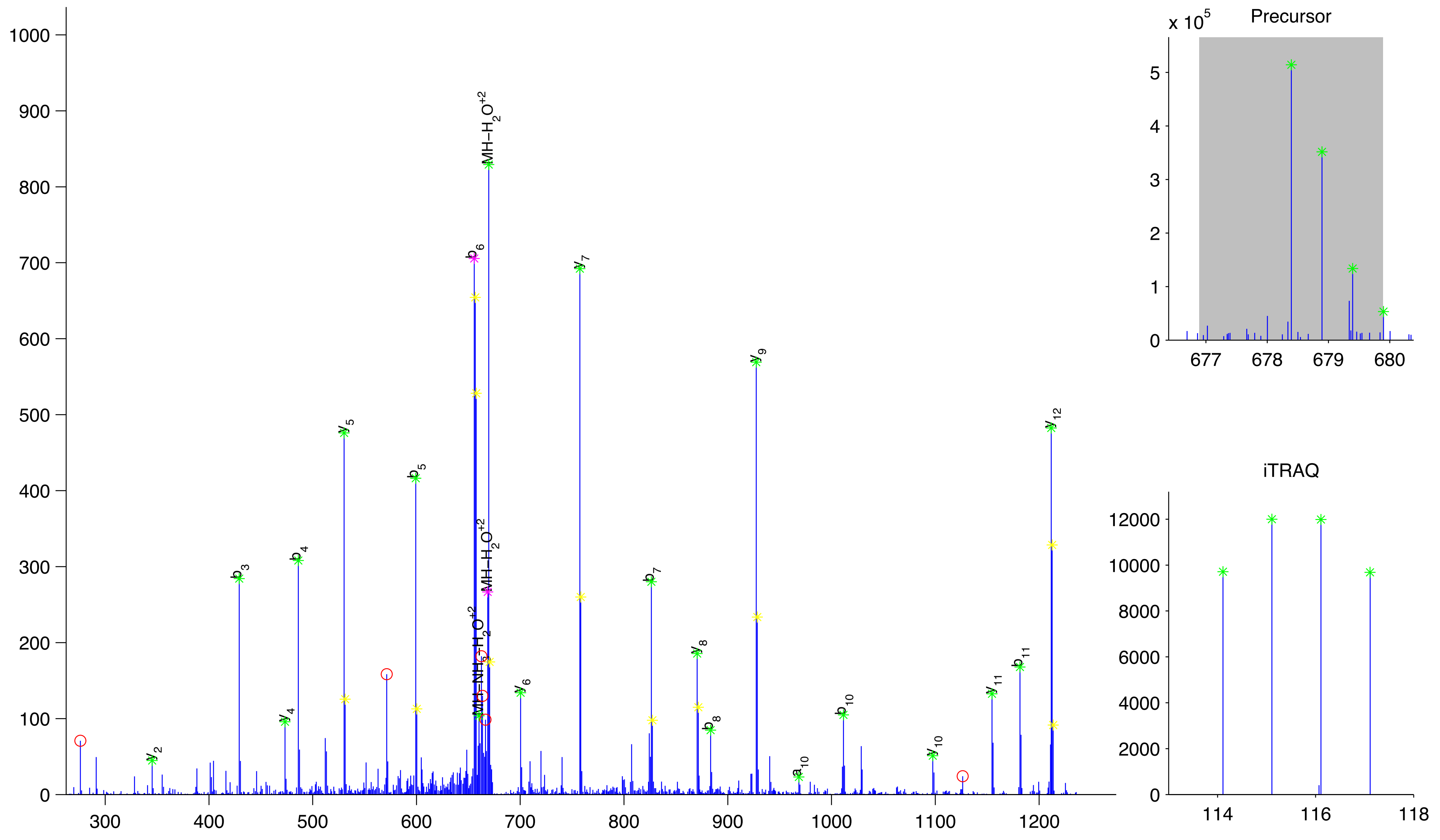

G L G k G G A k R

histone cluster 1, H4a [Homo sapiens]
Charge State: +2
Scan Number: 6404
File Name: 110924_HG2_INSA_ACK3.raw

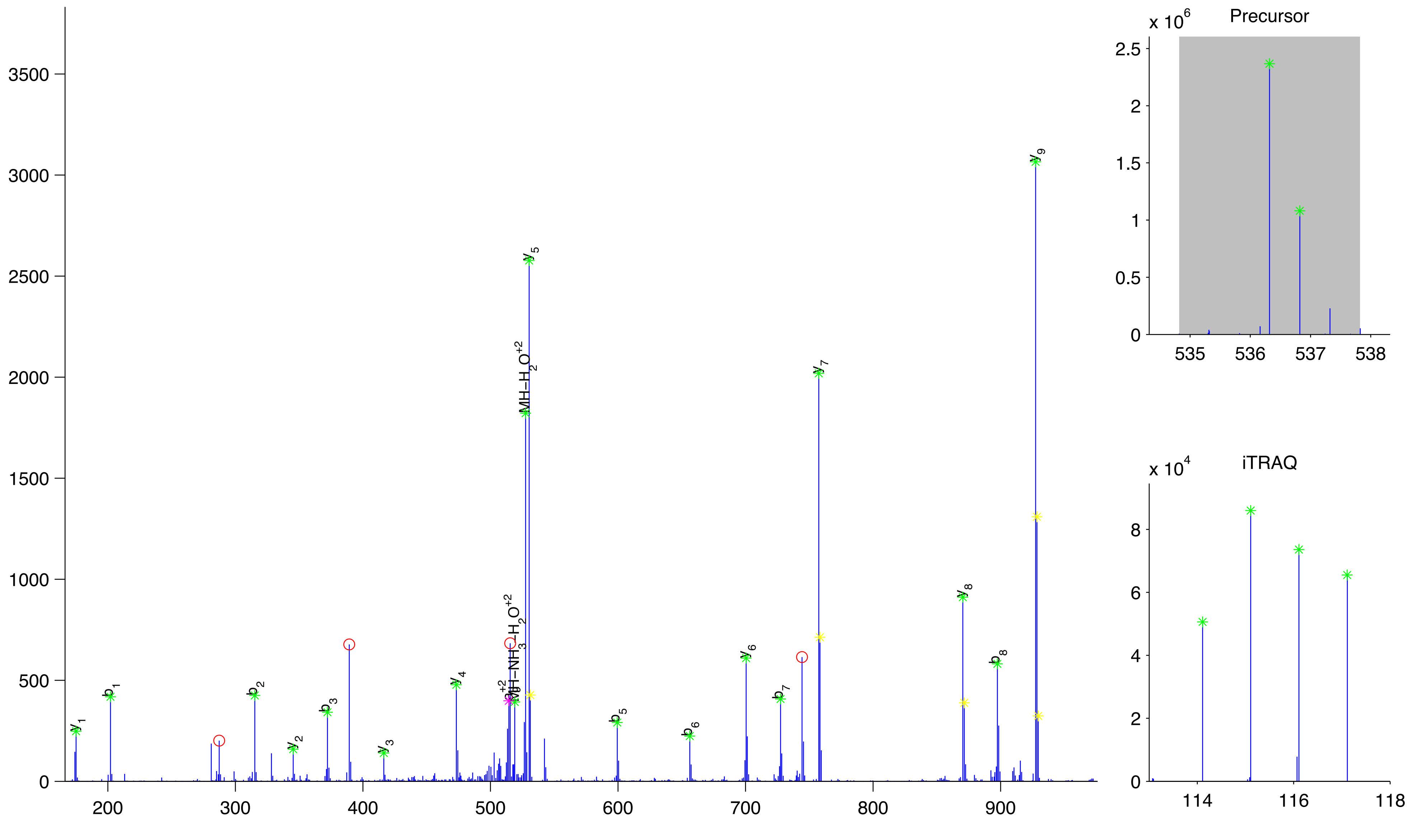

G G k G L G k G G A k R

histone cluster 1, H4a [Homo sapiens]
Charge State: +2
Scan Number: 6446
File Name: 110924_HG2_INSA_ACK3.raw

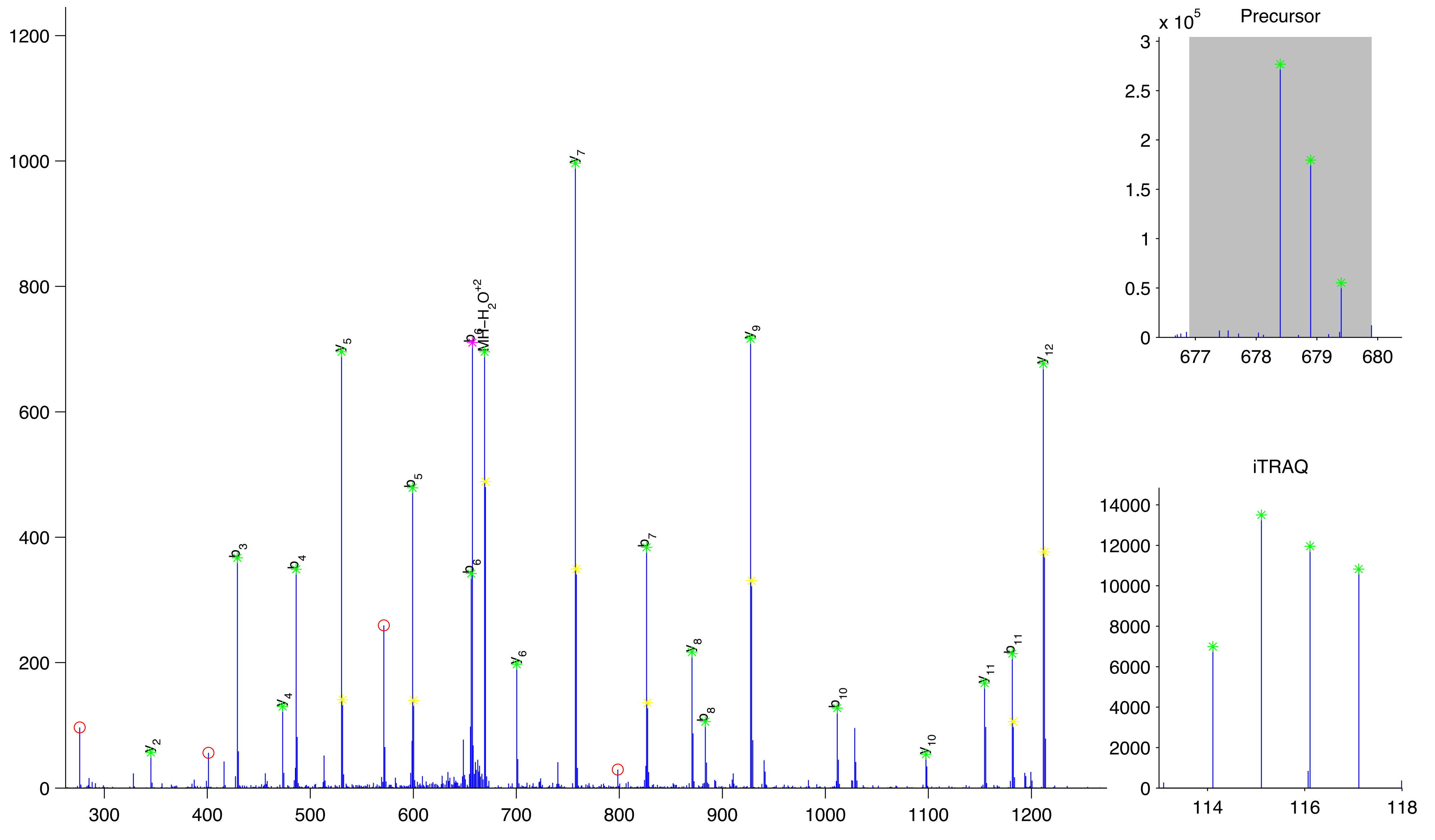

histone cluster 1, H4a [Homo sapiens]
Charge State: +2
Scan Number: 6452
File Name: 111011_HG2_INSB_ACK.raw

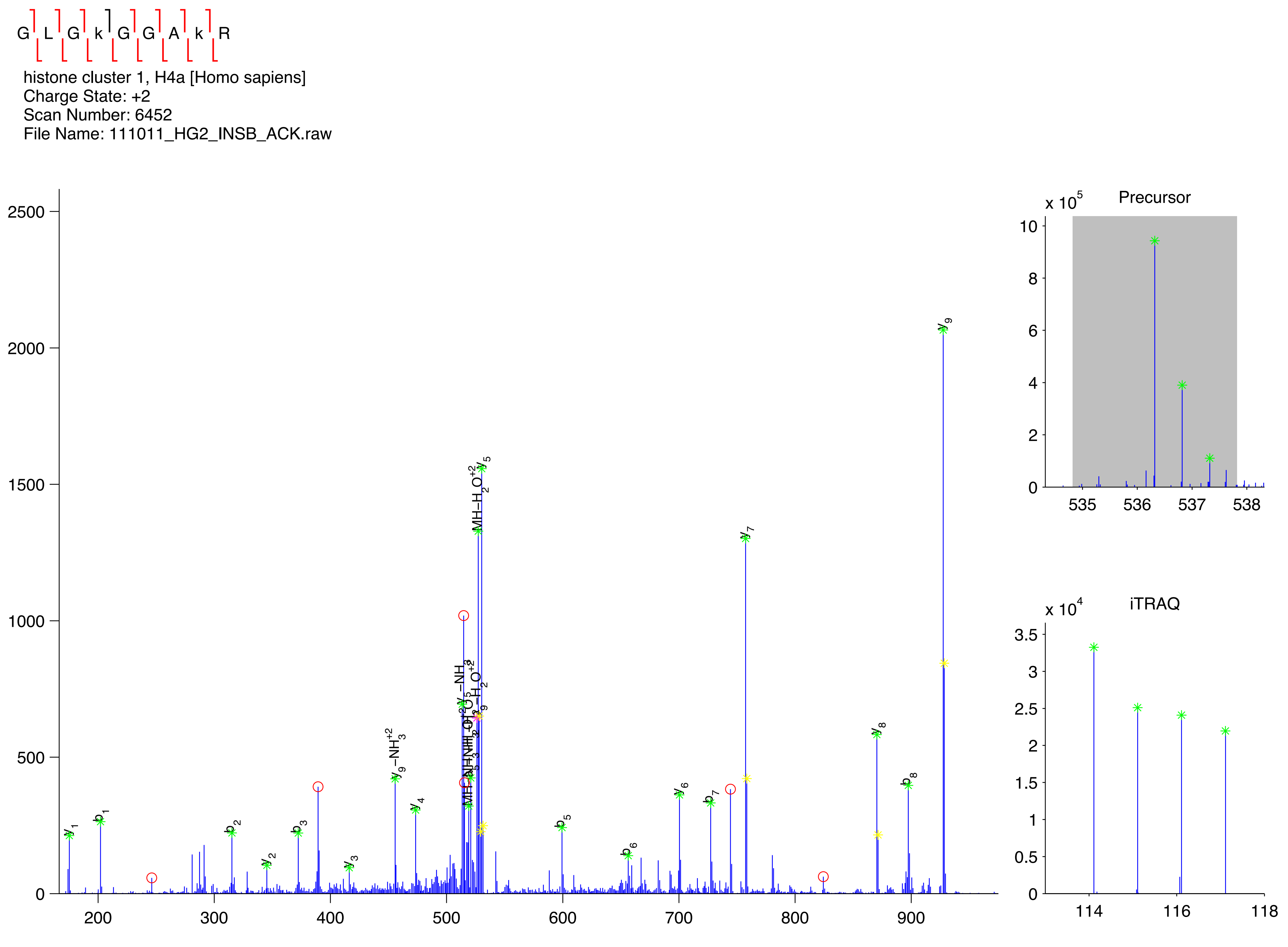

G k G G k G L G k G G A k R

histone cluster 1, H4a [Homo sapiens]
Charge State: +2
Scan Number: 6552
File Name: 111011_HG2_INSB_ACK.raw

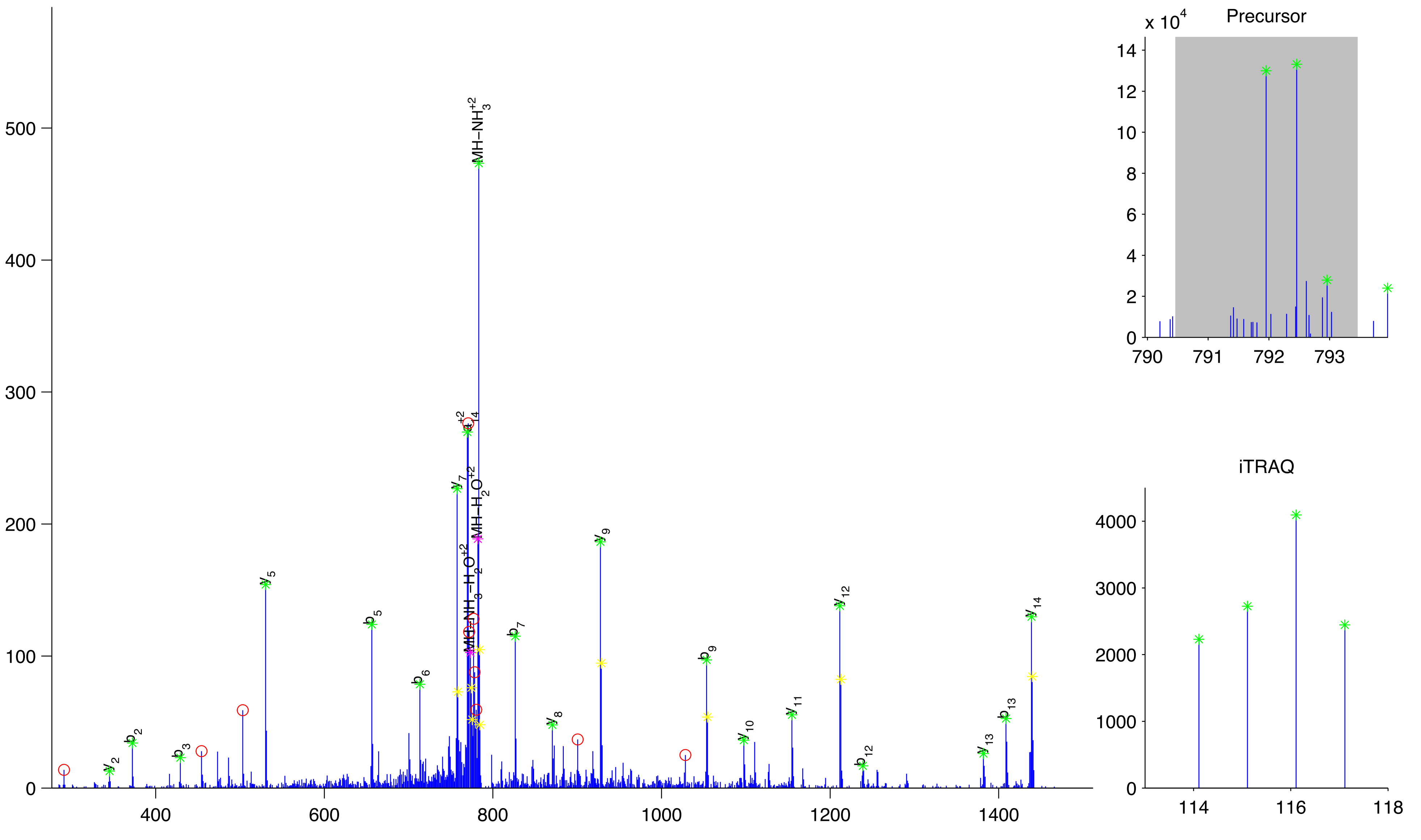

G G k G L G k G G A k R

histone cluster 1, H4a [Homo sapiens]
Charge State: +2
Scan Number: 6557
File Name: 111011_HG2_INSB_ACK.raw

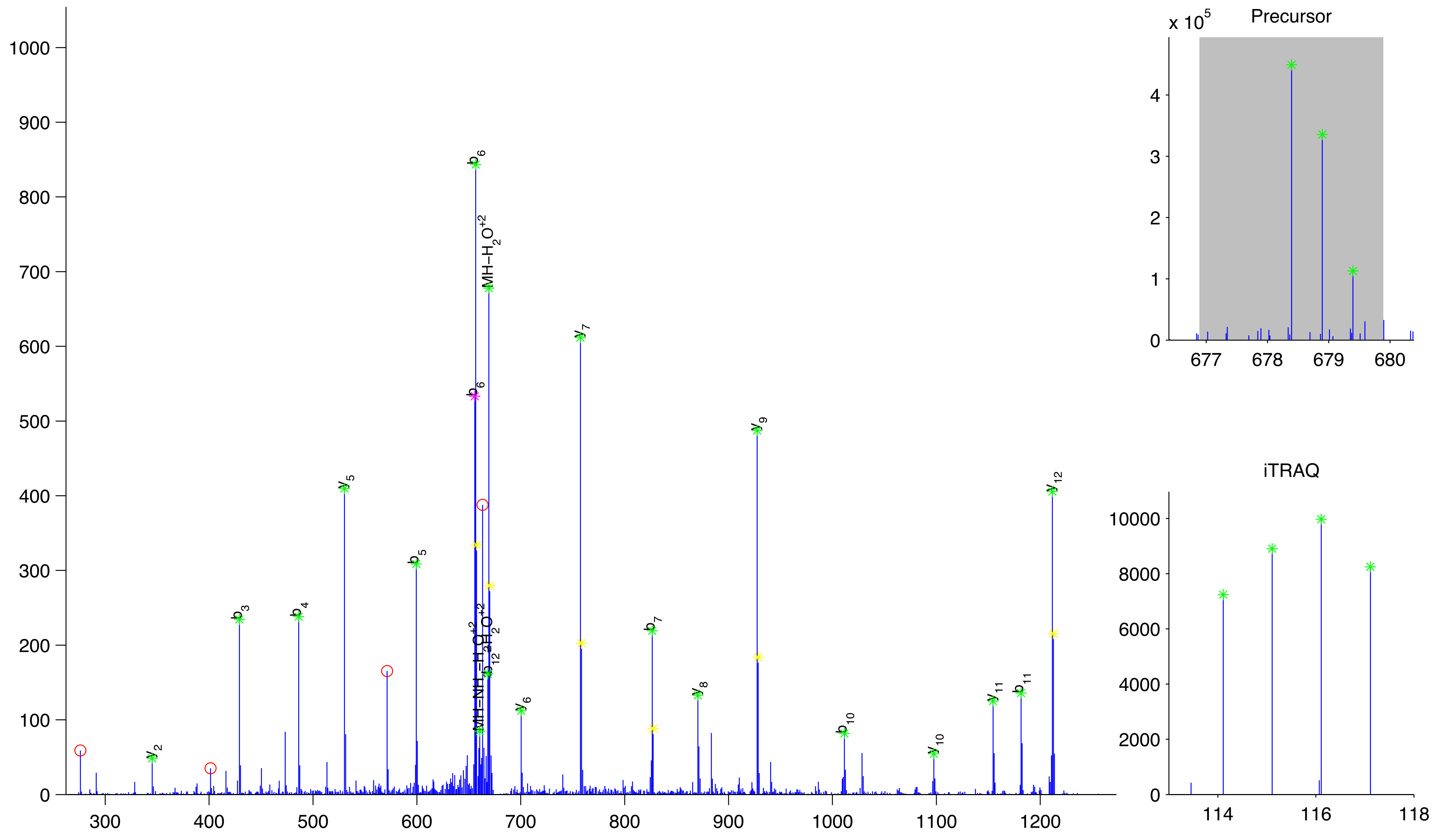

G L G k G G A k R

histone cluster 1, H4a [Homo sapiens]
Charge State: +2
Scan Number: 6620
File Name: 111011_HG2_INSB_ACK.raw

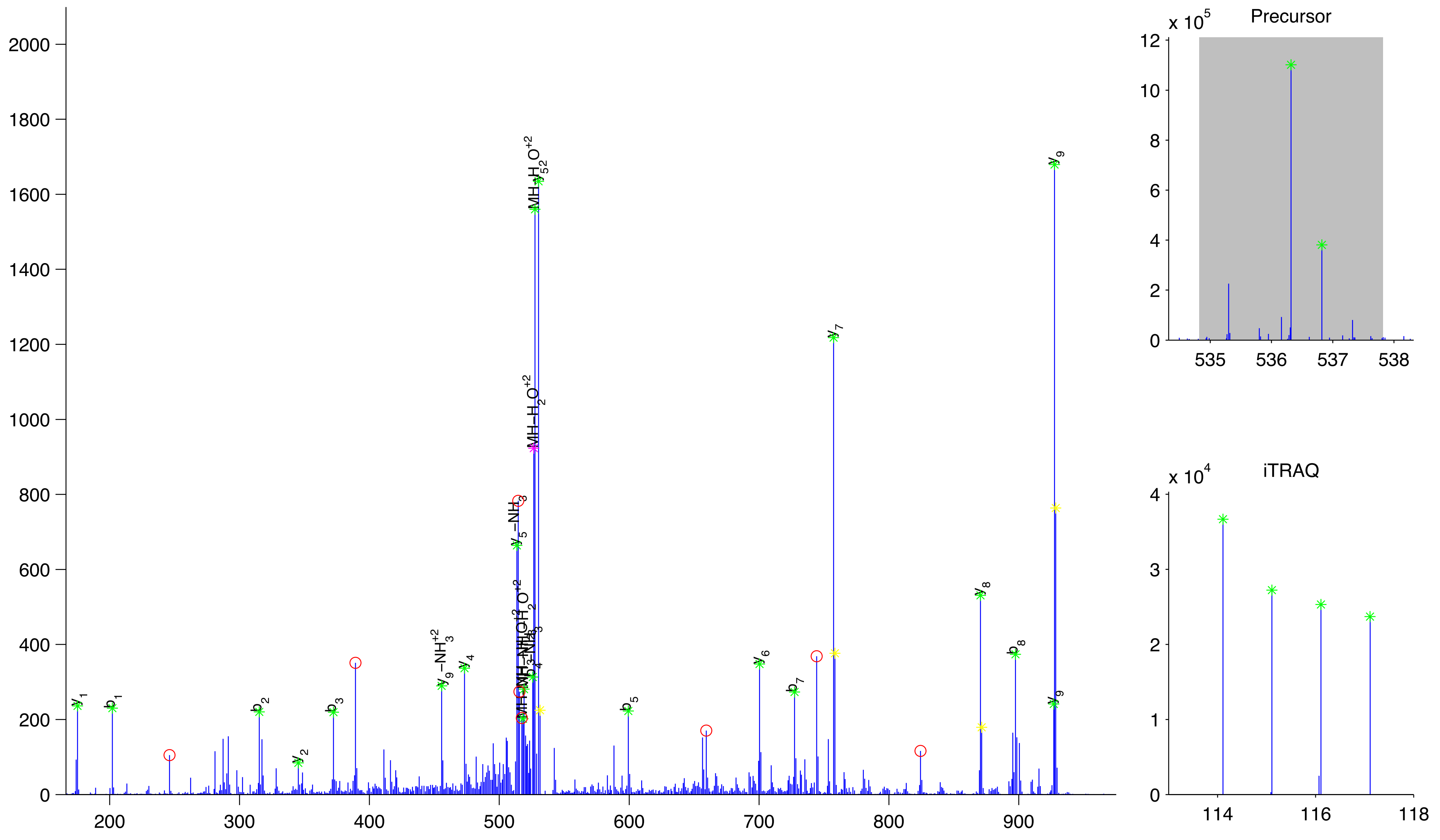

G G k G L G k G G A k R

histone cluster 1, H4a [Homo sapiens]
Charge State: +2
Scan Number: 6725
File Name: 111011_HG2_INSB_ACK.raw

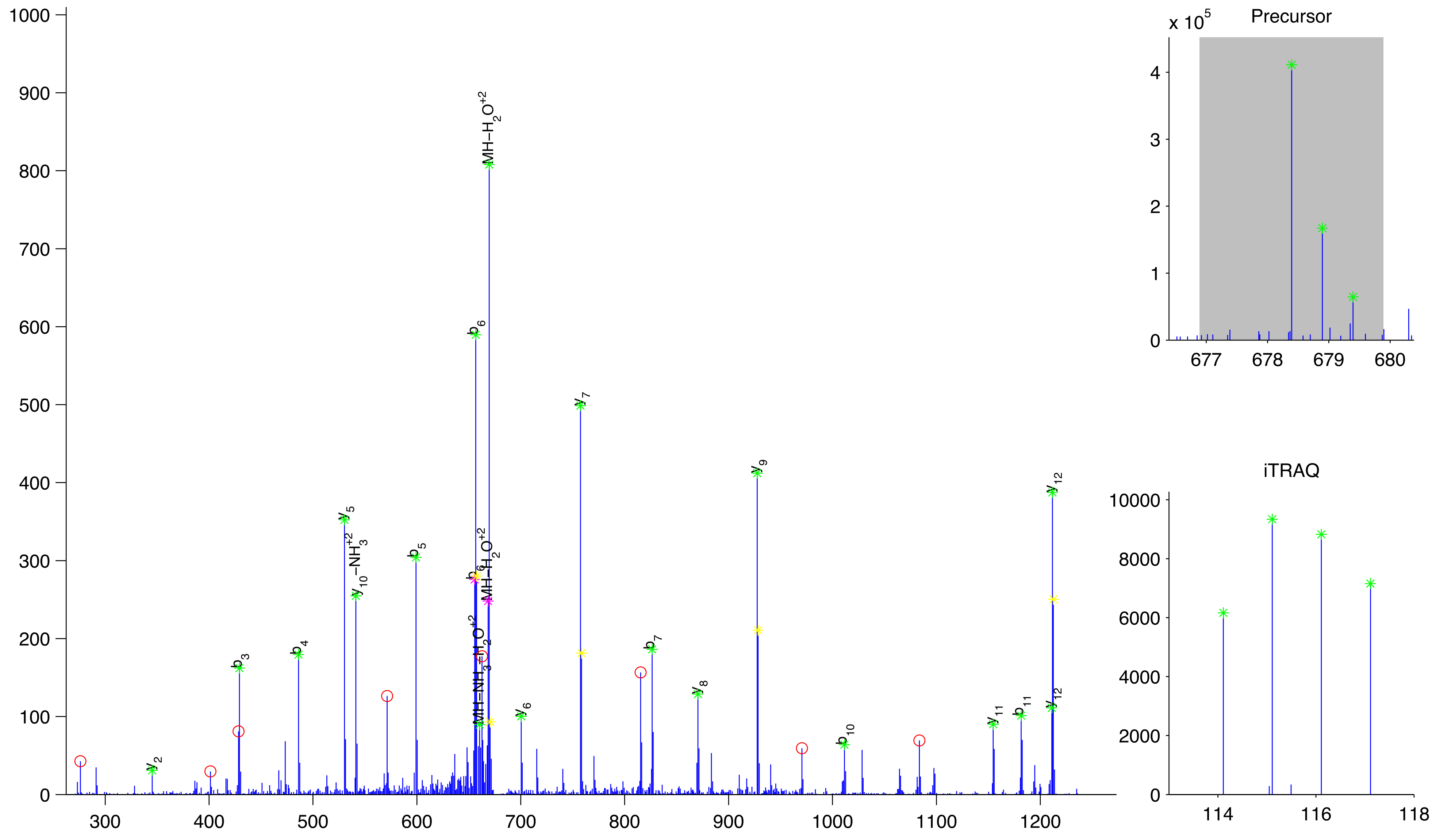

G G k G L G k G G A k R

histone cluster 1, H4a [Homo sapiens]
Charge State: +2
Scan Number: 6788
File Name: 110924_HG2_INSA_ACK3.raw

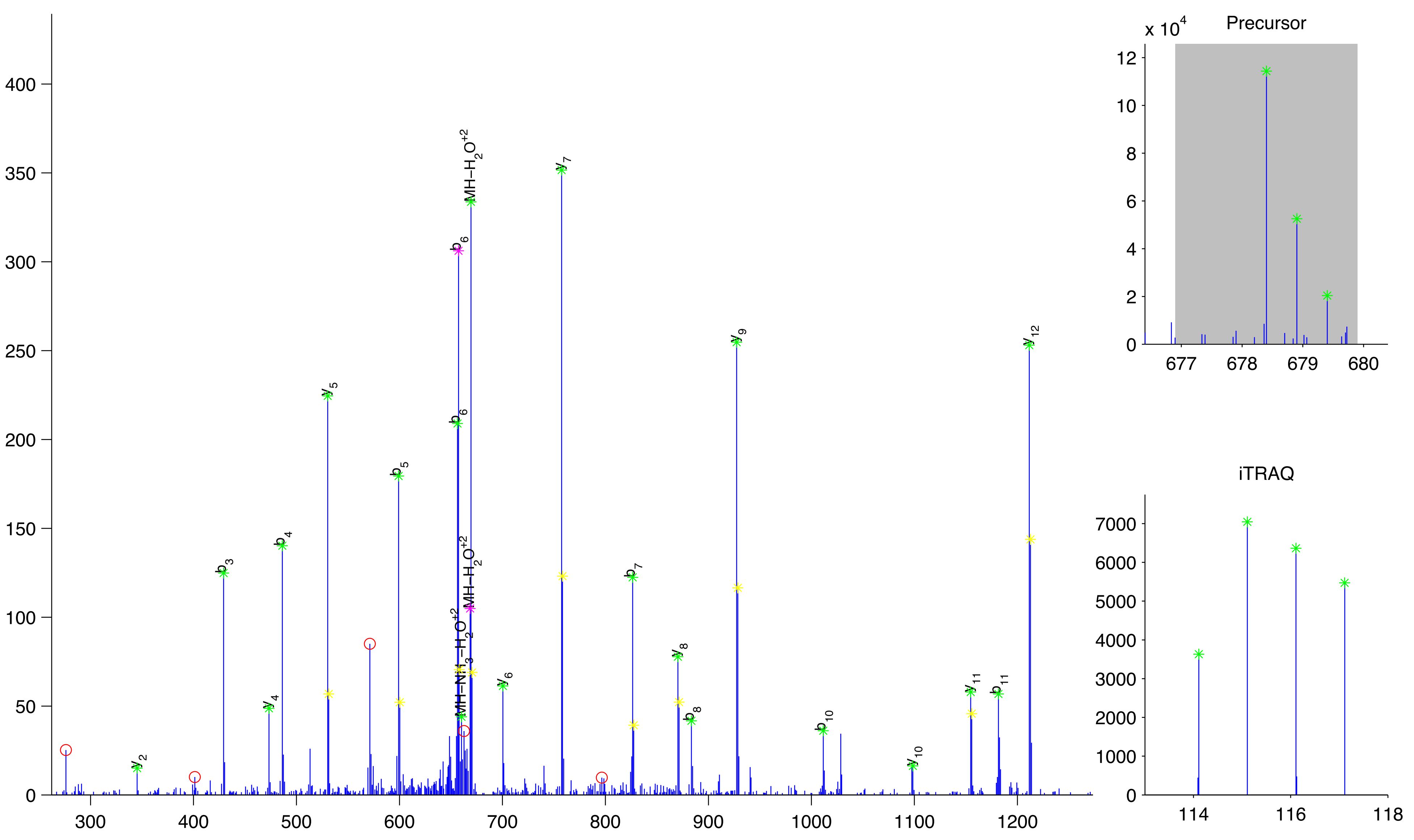

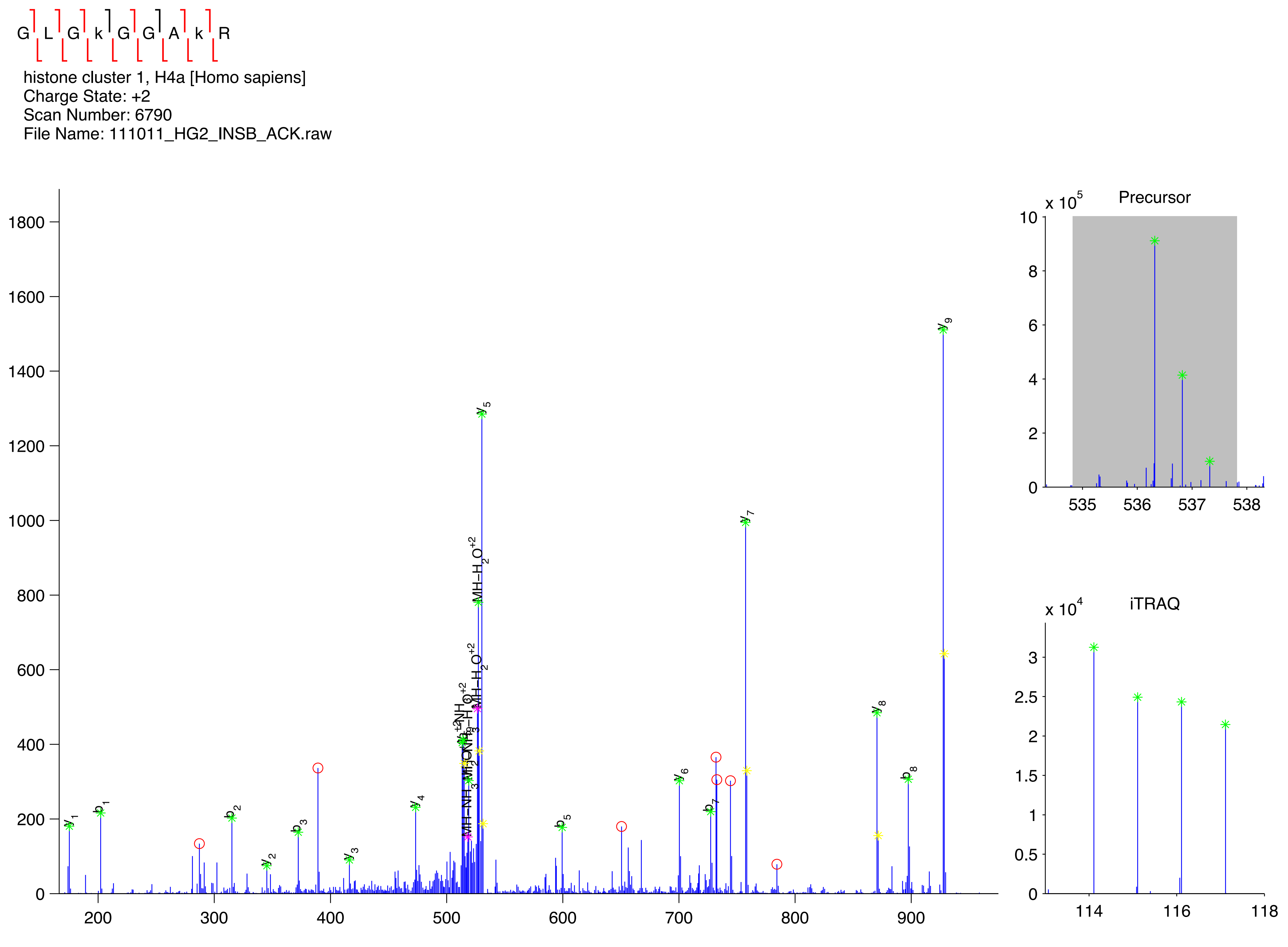

G L G k G G A k R
histone cluster 1, H4a [Homo sapiens]
Charge State: +2
Scan Number: 6790
File Name: 111011_HG2_INSB_ACK.raw
Precursor
iTRAQ

G G k G L G k G G A k R

histone cluster 1, H4a [Homo sapiens]
Charge State: +2
Scan Number: 6891
File Name: 111011_HG2_INSB_ACK.raw

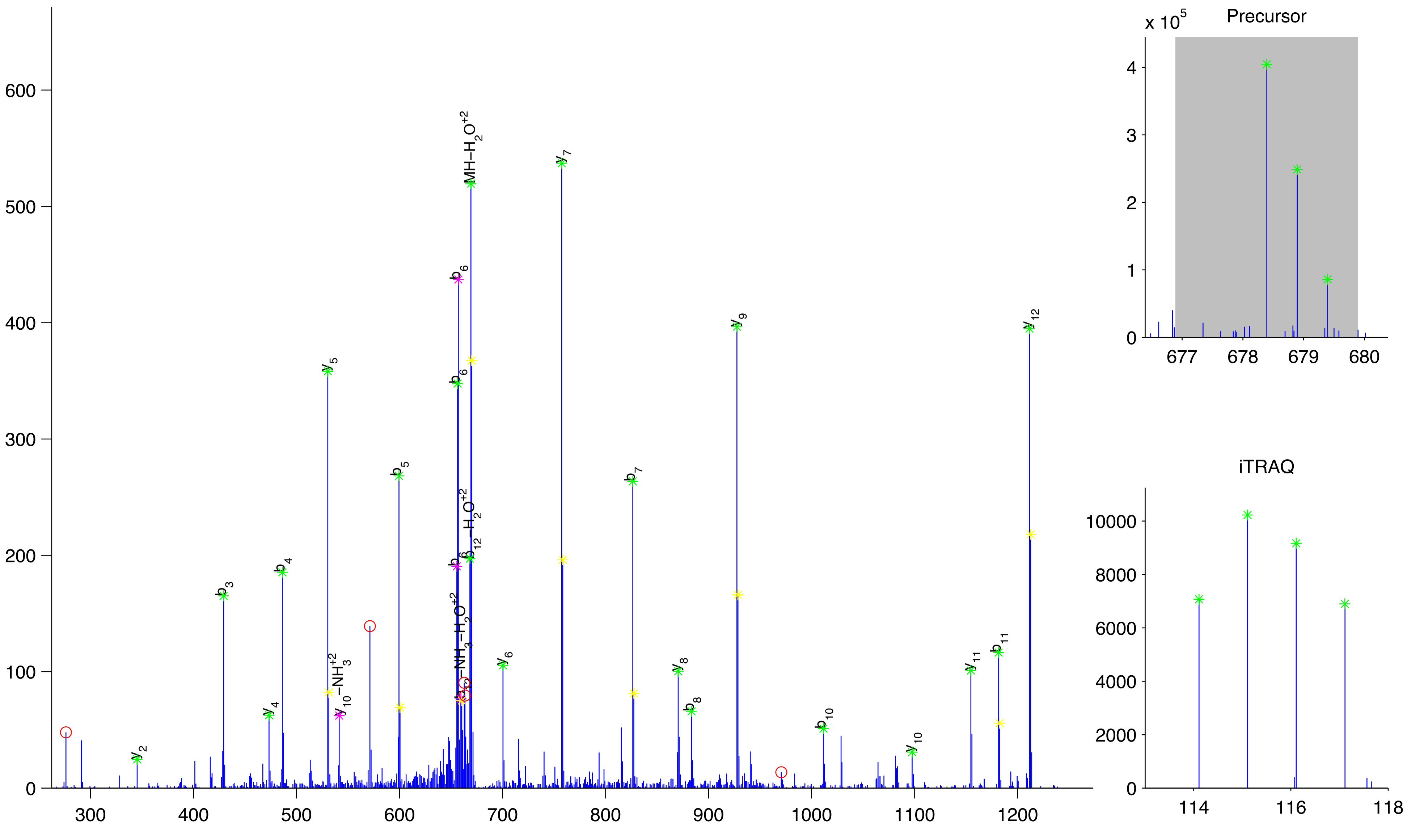

G L G k G G A k R

histone cluster 1, H4a [Homo sapiens]
Charge State: +2
Scan Number: 6910
File Name: 110924_HG2_INSA_ACK3.raw

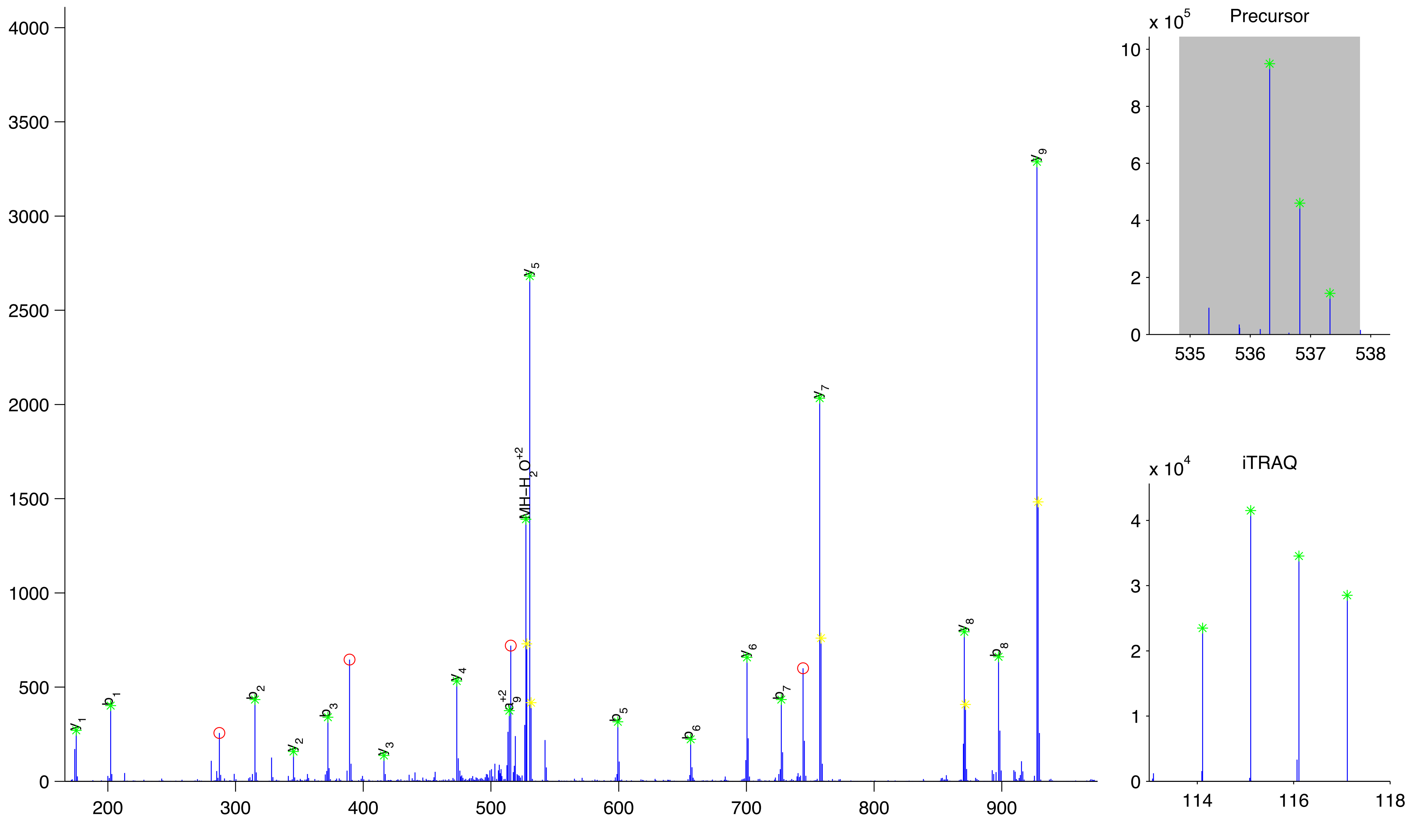

G G k G L G k G G A k R

histone cluster 1, H4a [Homo sapiens]
Charge State: +2
Scan Number: 6956
File Name: 110924_HG2_INSA_ACK3.raw

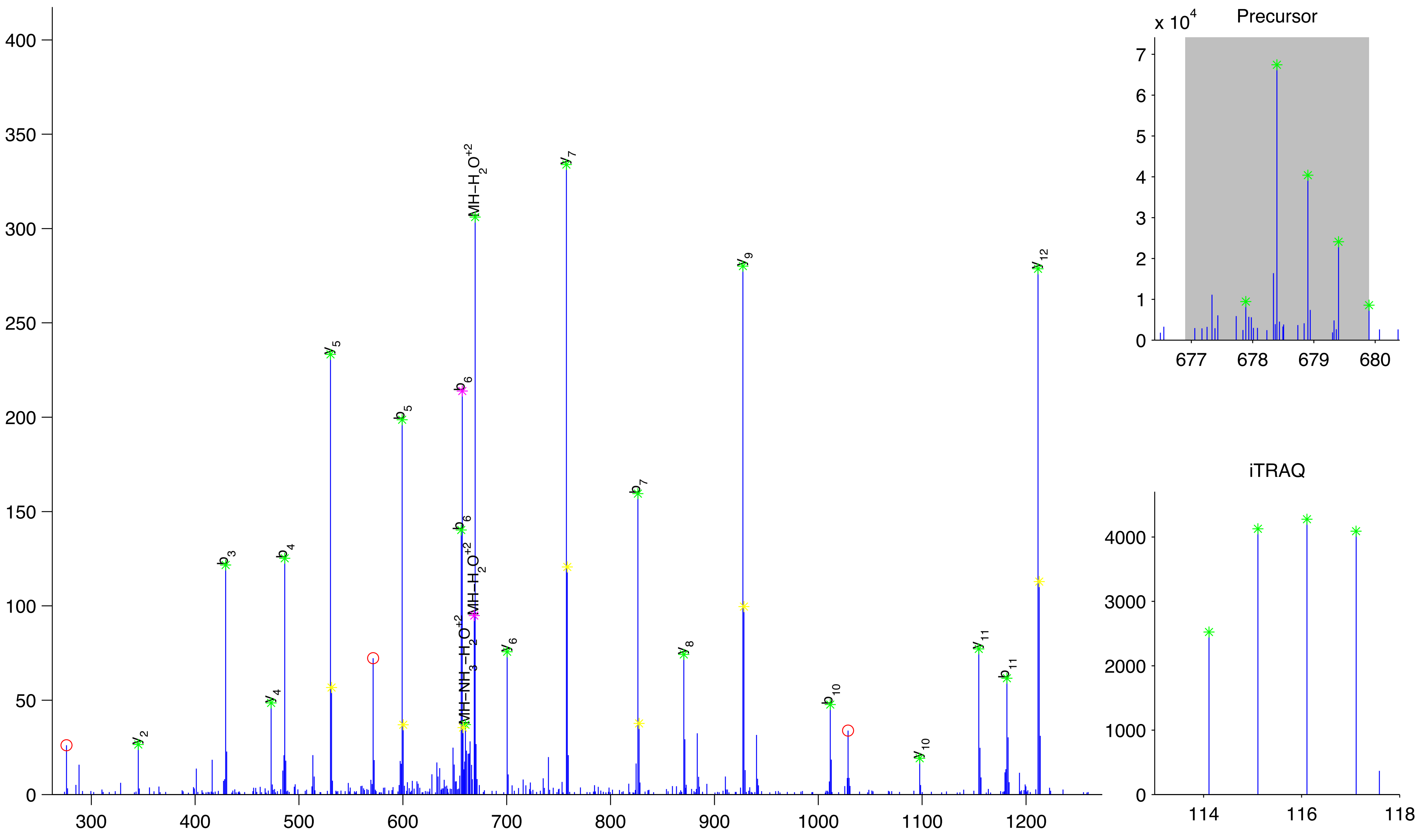

G G k G L G k G G A k R

histone cluster 1, H4a [Homo sapiens]
Charge State: +2
Scan Number: 7061
File Name: 111011_HG2_INSB_ACK.raw

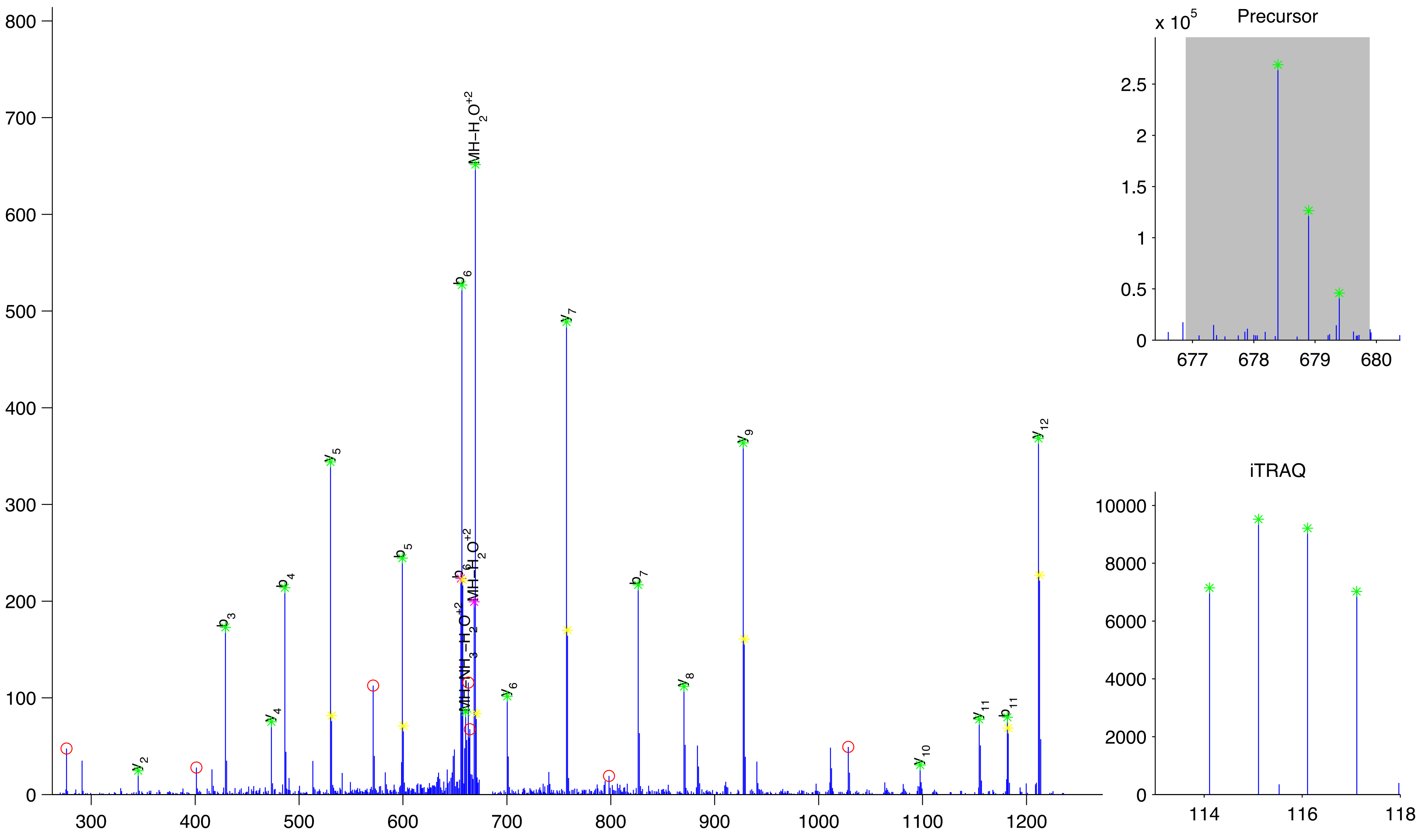

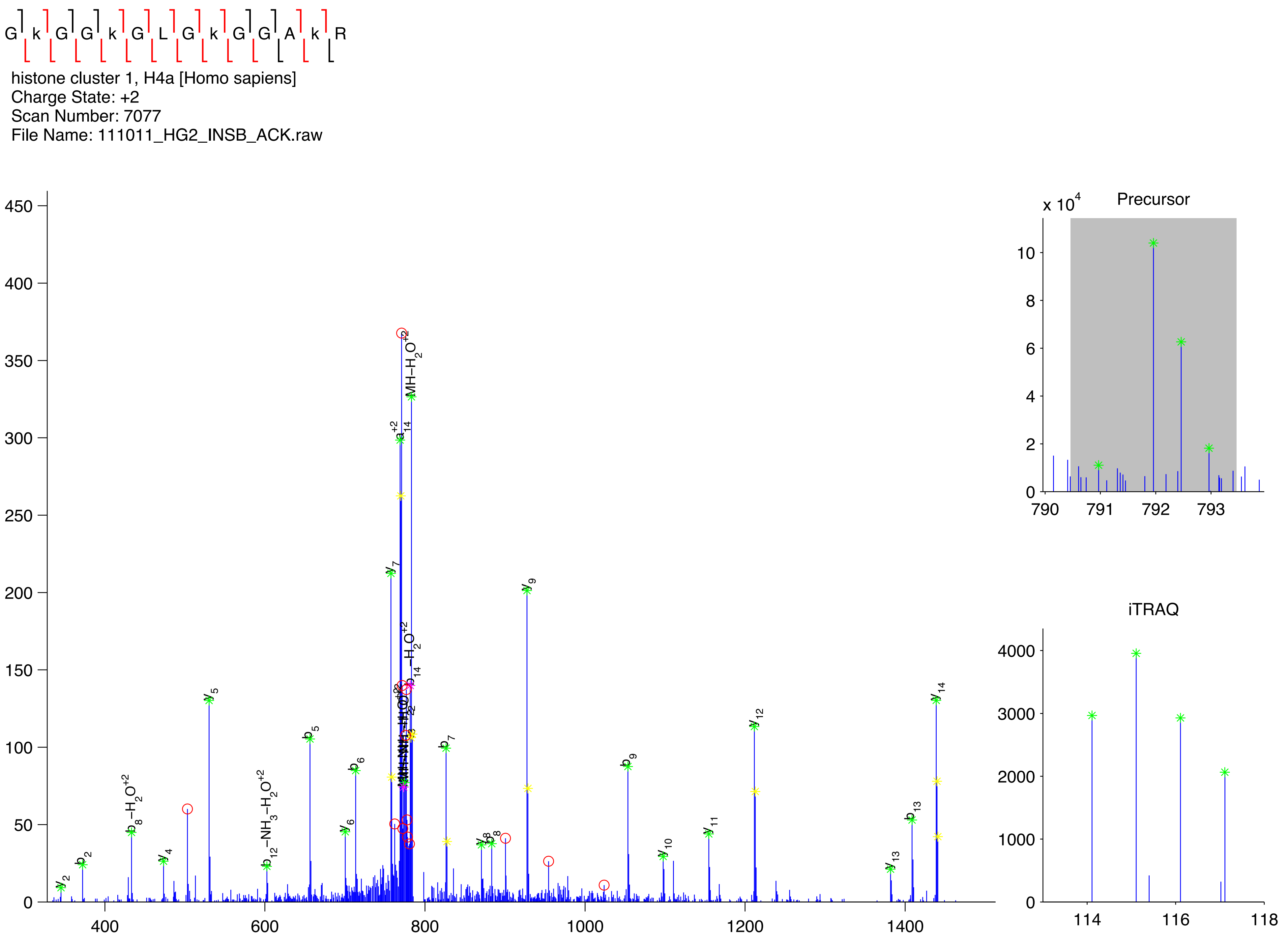
G k G G k G L G k G G A k R
histone cluster 1, H4a [Homo sapiens]
Charge State: +2
Scan Number: 7077
File Name: 111011_HG2_INSB_ACK.raw
Precursor
x 10^4
iTRAQ
y2
b2
b8−H2O+2
y4
y5
b12−NH3−H2O+2
b5
y6
b6
y7
a14+2
MH−H2O+2
b14−H2O+2
b7
y8
b8
y9
b9
y10
y11
y12
y13
b13
y14

G G k G L G k G G A k R

histone cluster 1, H4a [Homo sapiens]
Charge State: +2
Scan Number: 7126
File Name: 110924_HG2_INSA_ACK3.raw

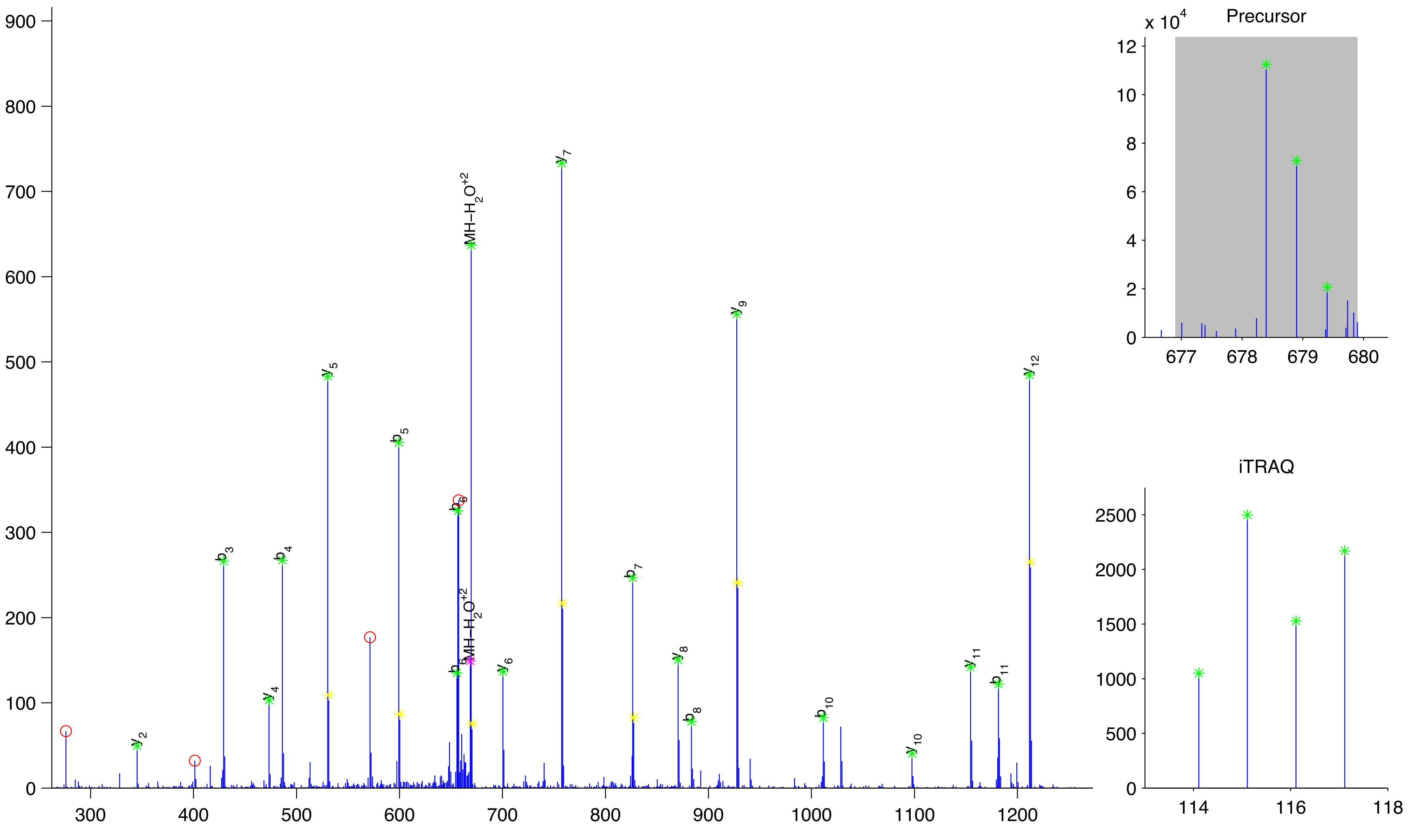

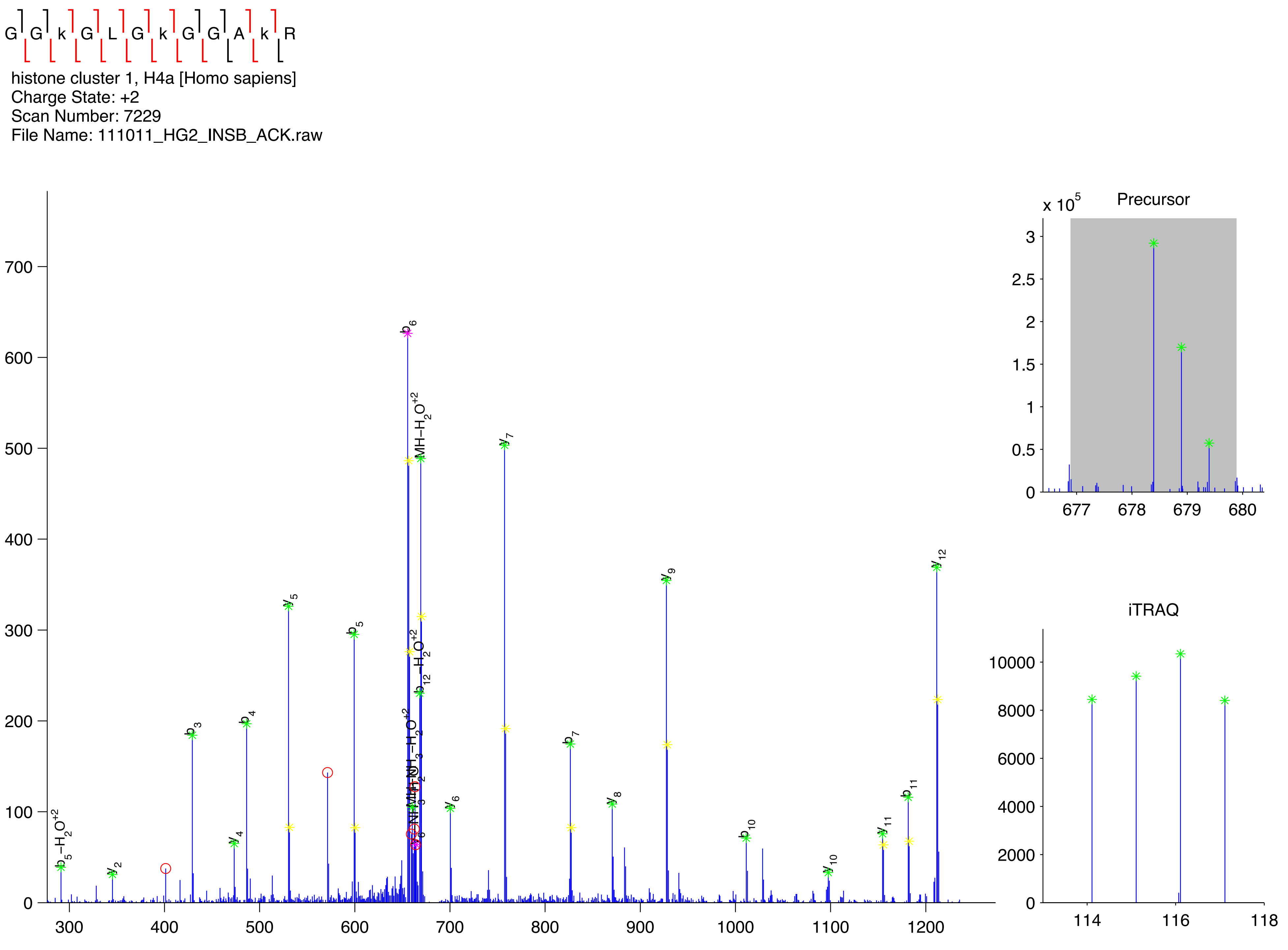

G G k G L G k G G A k R
histone cluster 1, H4a [Homo sapiens]
Charge State: +2
Scan Number: 7229
File Name: 111011_HG2_INSB_ACK.raw
Precursor
x 10^5
iTRAQ

G k G G k G L G k G G A k R

histone cluster 1, H4a [Homo sapiens]
Charge State: +2
Scan Number: 7231
File Name: 111011_HG2_INSB_ACK.raw

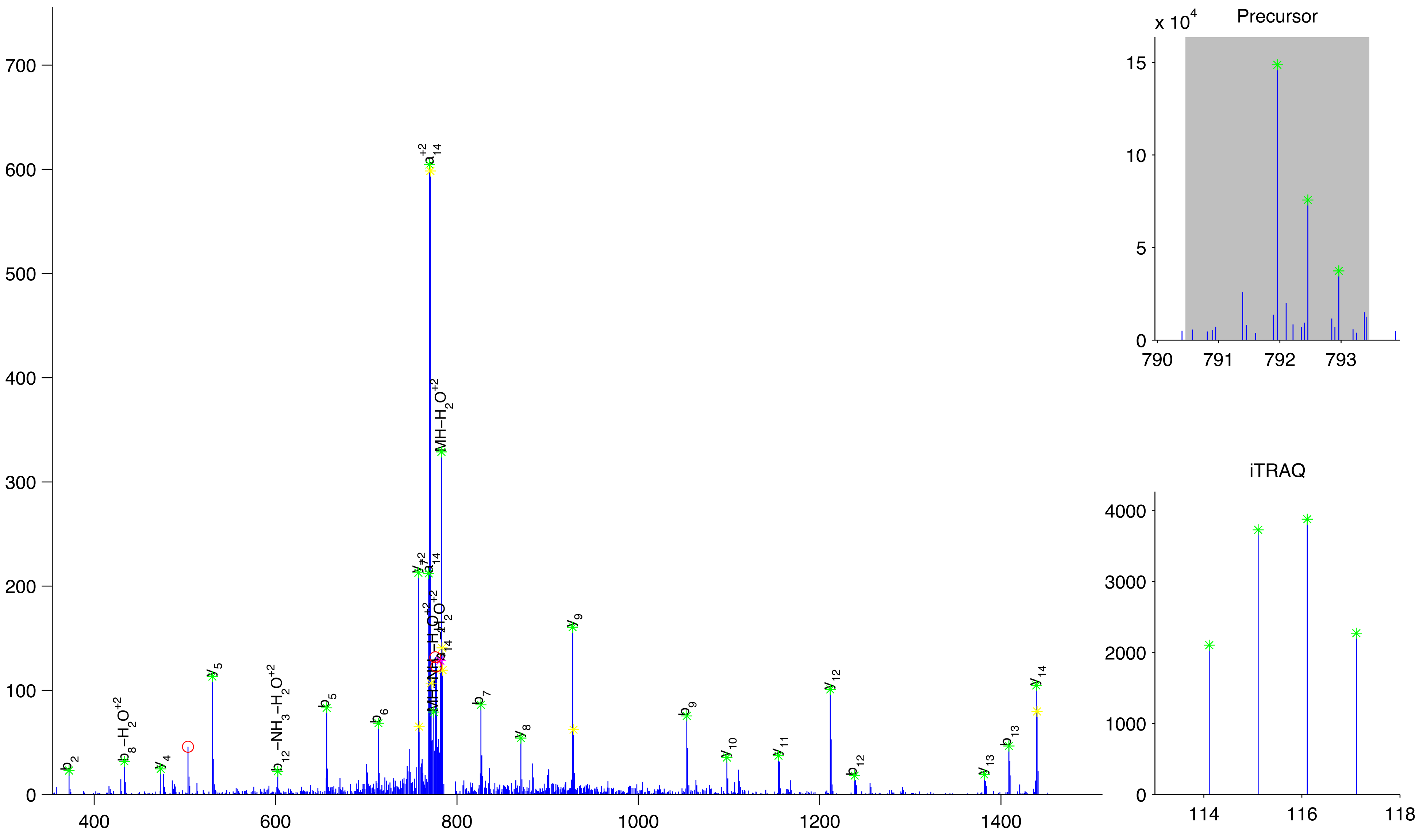

D N I Q G I T k P A I R

histone cluster 1, H4a [Homo sapiens]
Charge State: +2
Scan Number: 7292
File Name: 111016_HG2_INSC_ACK.raw

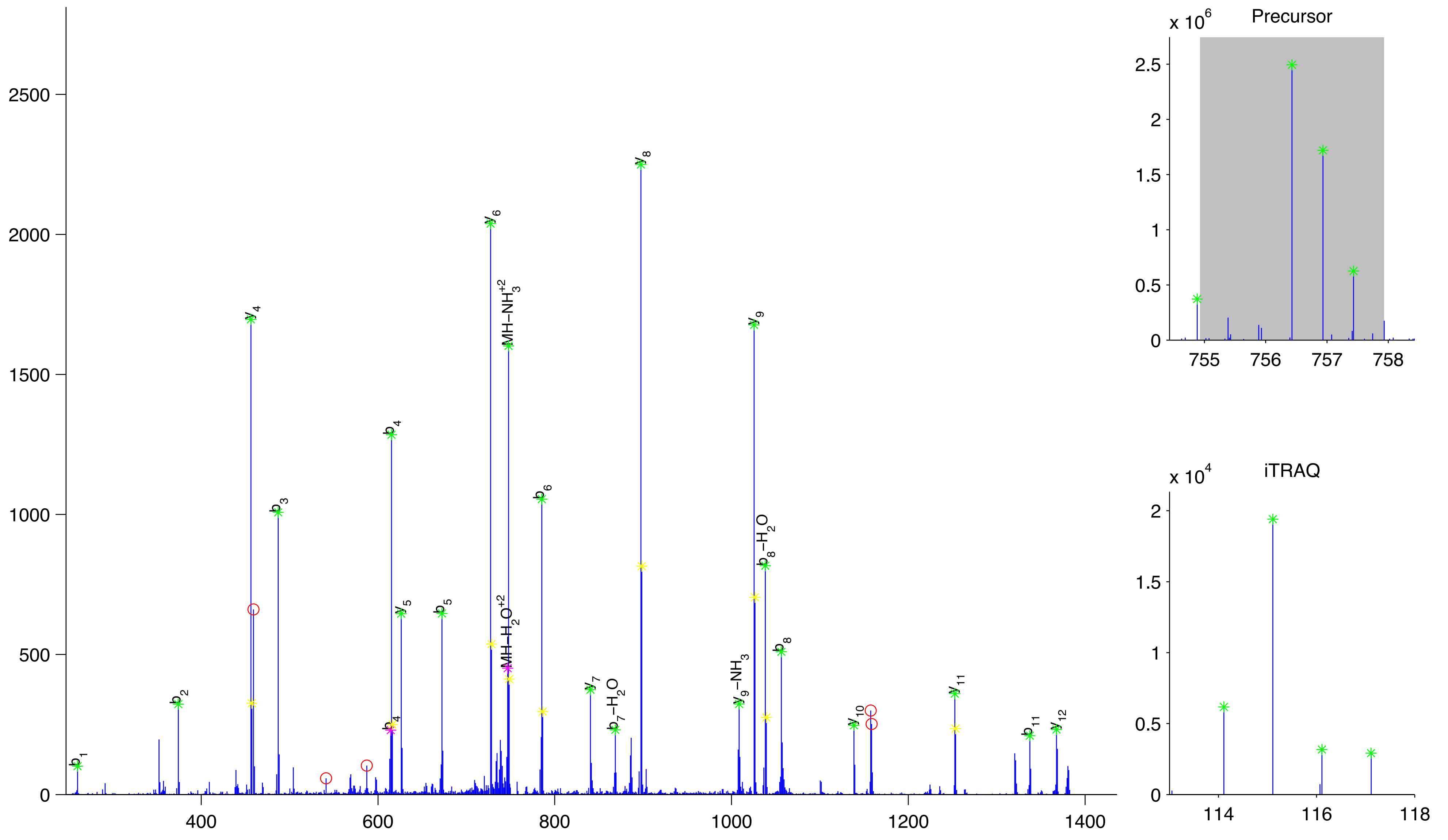

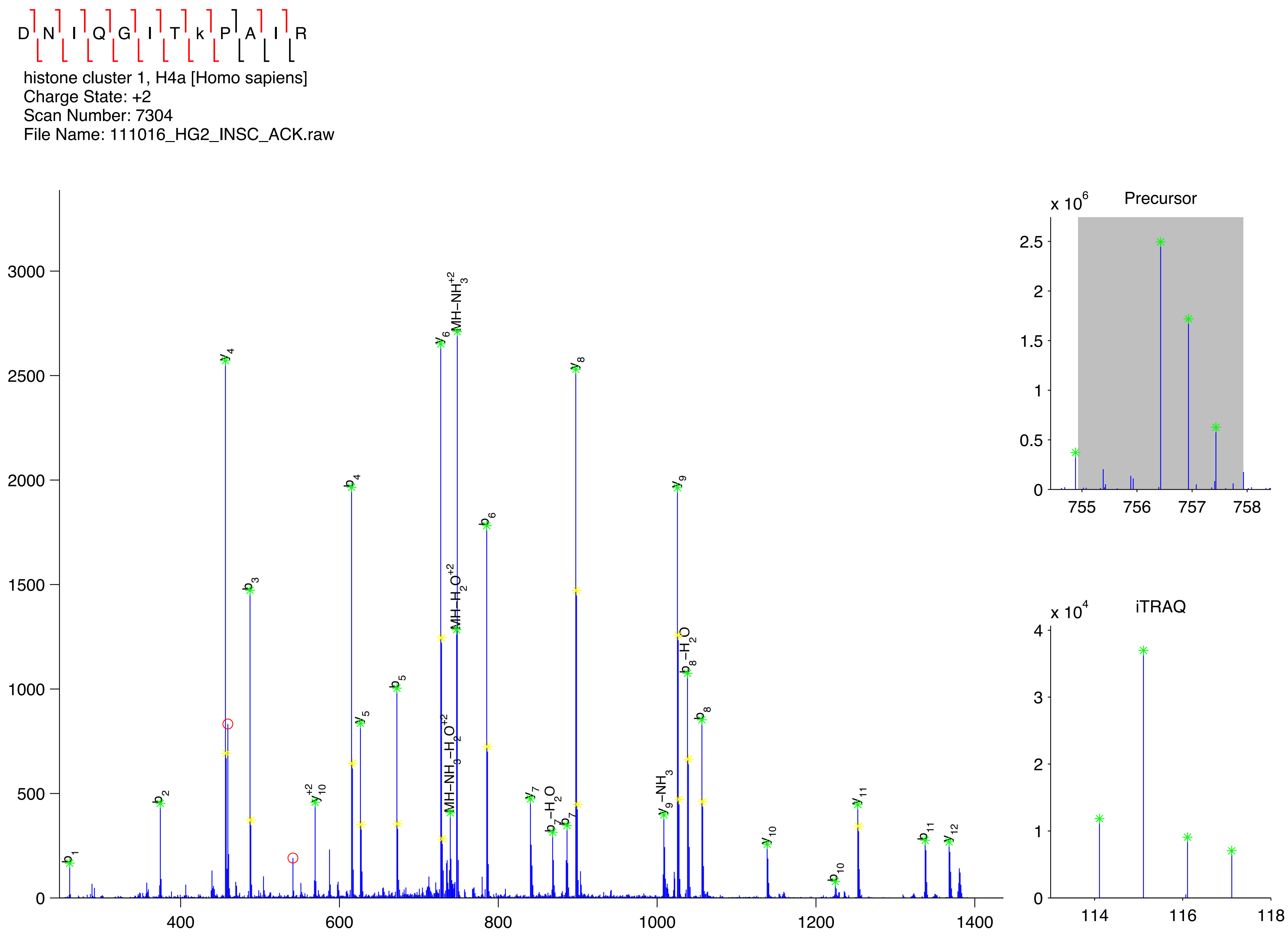
D N I Q G I T k P A I R
histone cluster 1, H4a [Homo sapiens]
Charge State: +2
Scan Number: 7304
File Name: 111016_HG2_INSC_ACK.raw
Precursor
x 10^6
iTRAQ
x 10^4

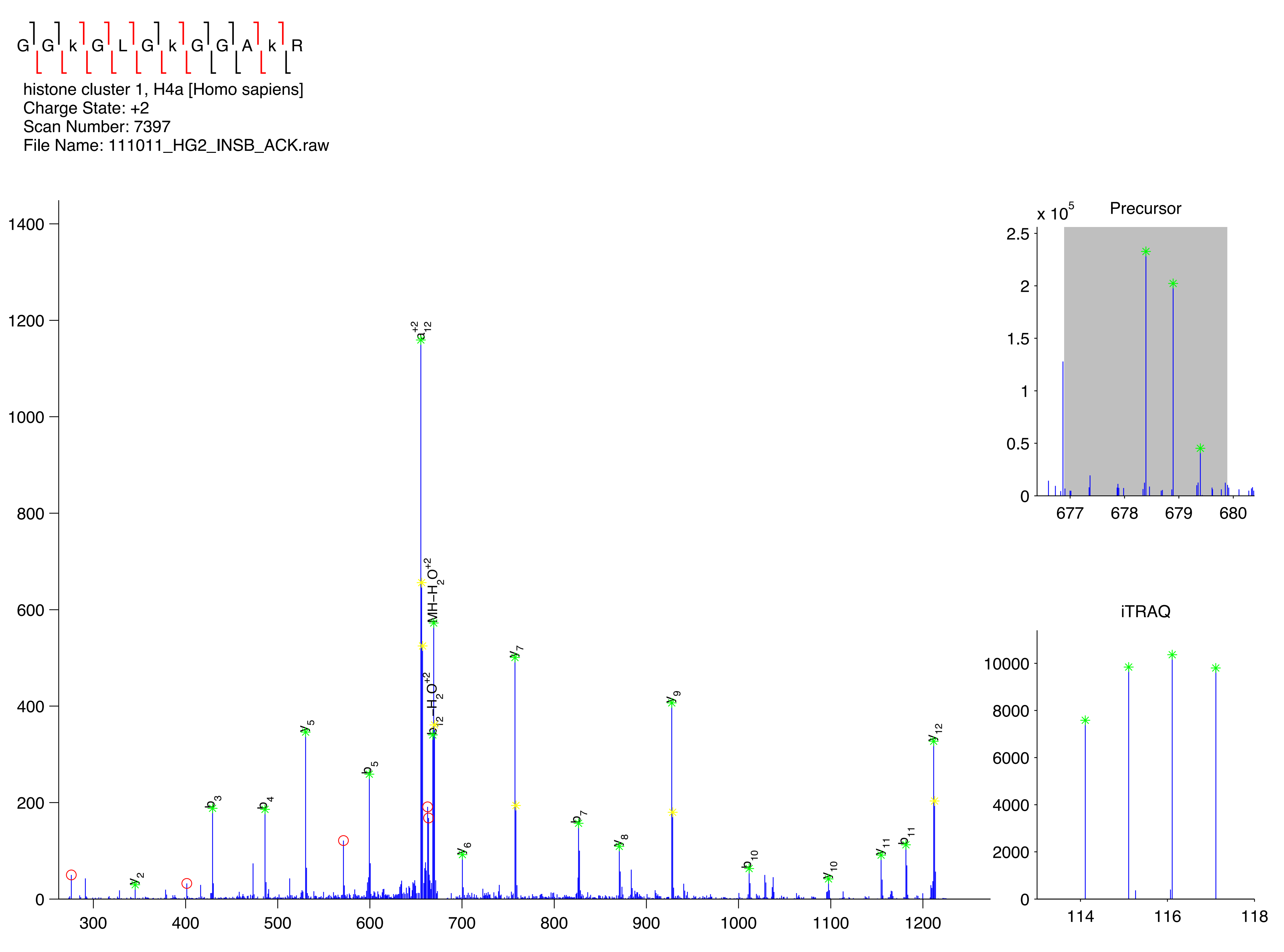
G G k G L G k G G A k R
histone cluster 1, H4a [Homo sapiens]
Charge State: +2
Scan Number: 7397
File Name: 111011_HG2_INSB_ACK.raw
Precursor
x 10^5
iTRAQ
a12+2
MH-H2O+2
b12-H2O+2
y2
b3
b4
y5
b5
y6
y7
b7
y8
y9
b10
y10
y11
b11
y12

G L G k G G A k R

histone cluster 1, H4a [Homo sapiens]
Charge State: +2
Scan Number: 7414
File Name: 110924_HG2_INSA_ACK3.raw

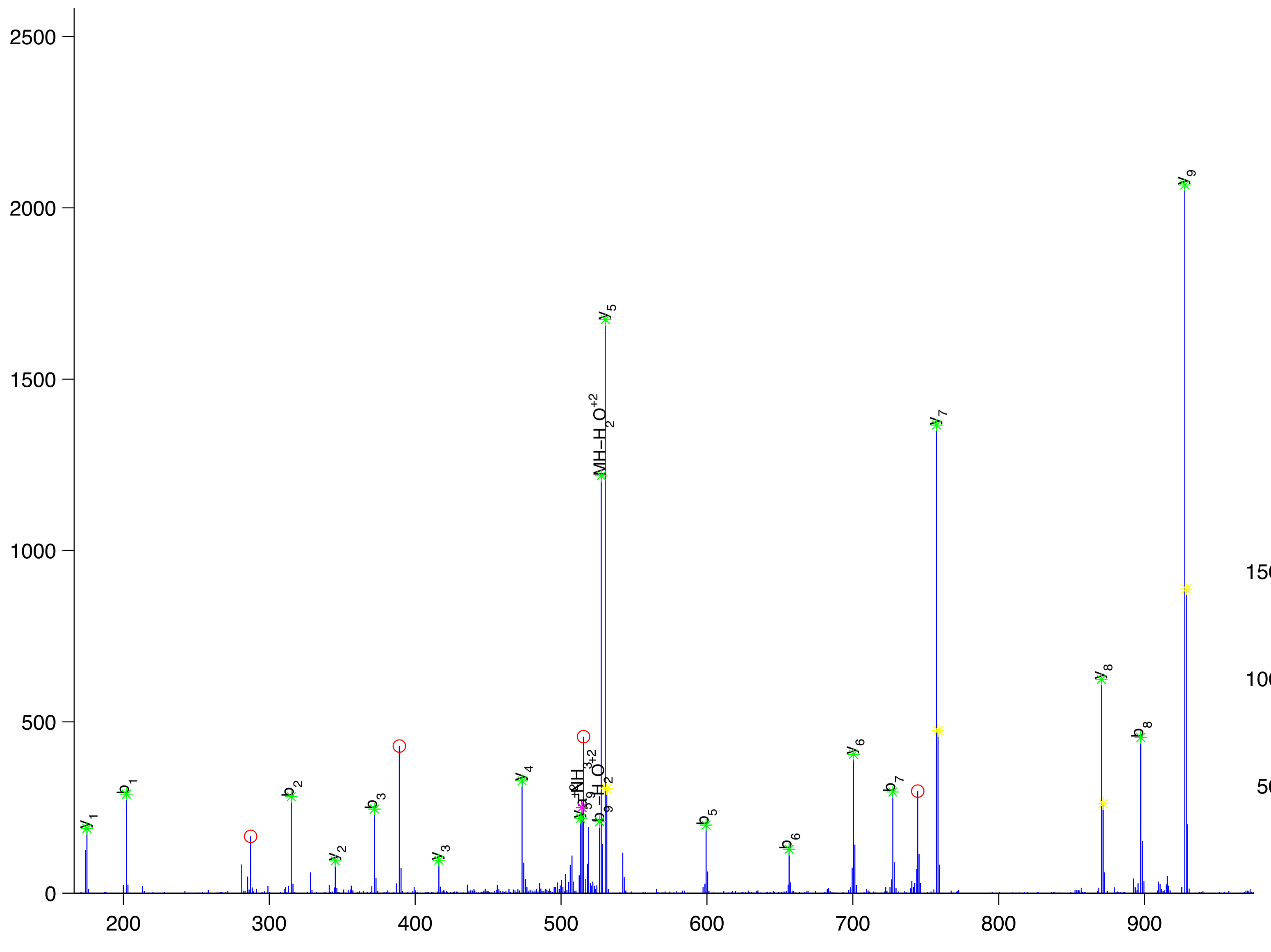

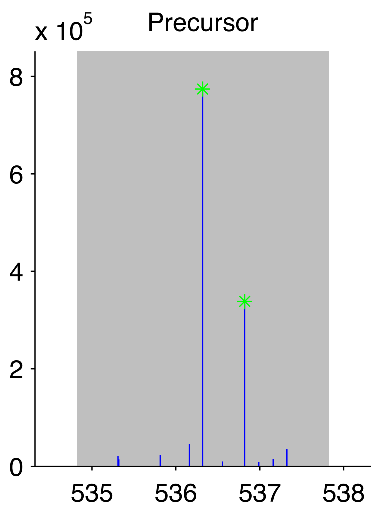

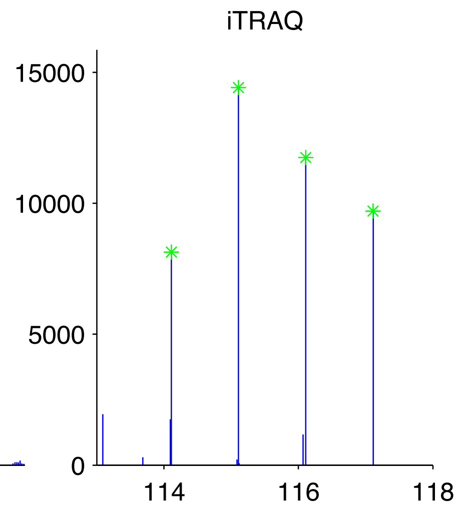

G G k G L G k G G A k R

histone cluster 1, H4a [Homo sapiens]
Charge State: +2
Scan Number: 7458
File Name: 110924_HG2_INSA_ACK3.raw

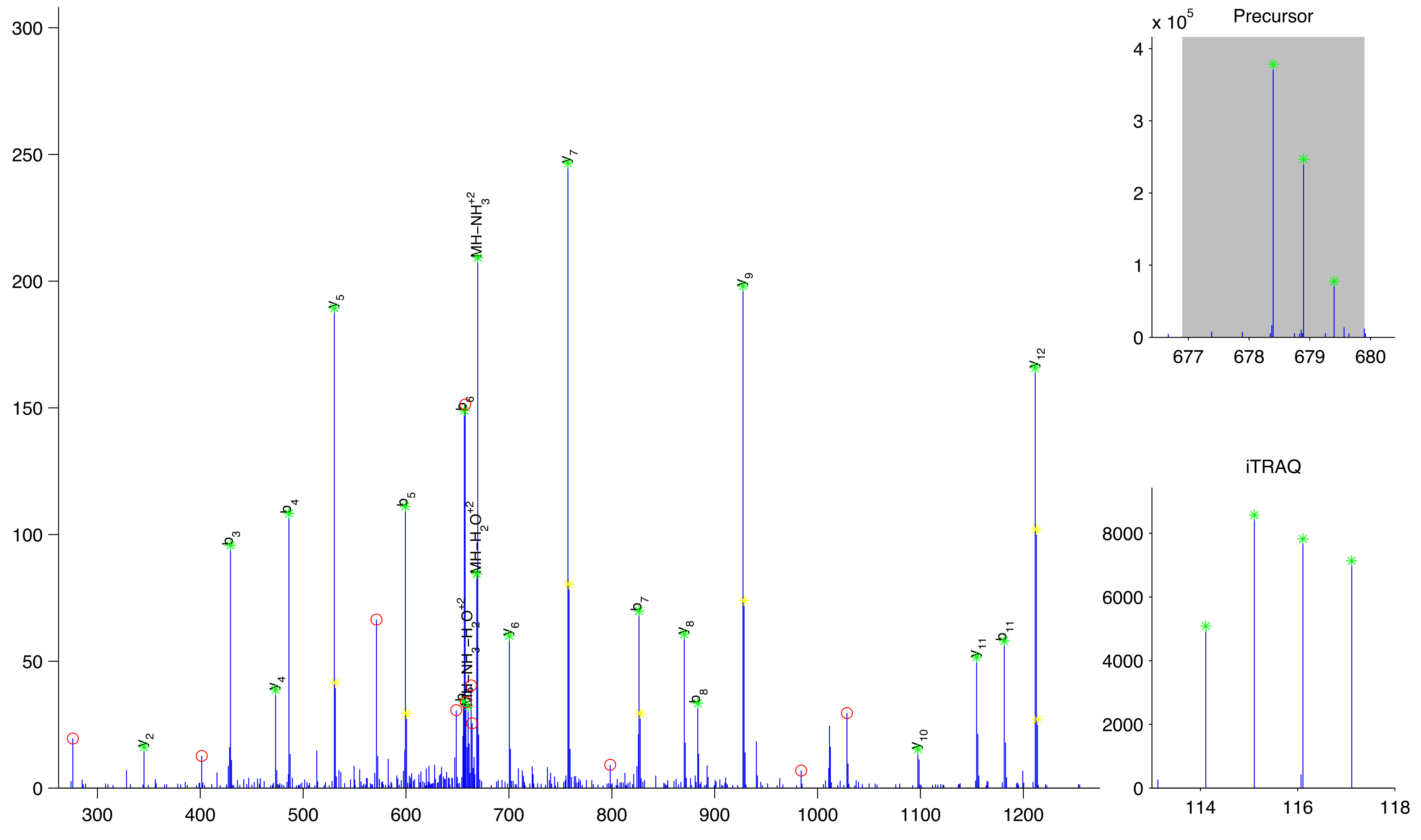

G L G k G G A k R

histone cluster 1, H4a [Homo sapiens]
Charge State: +2
Scan Number: 7458
File Name: 111011_HG2_INSB_ACK.raw

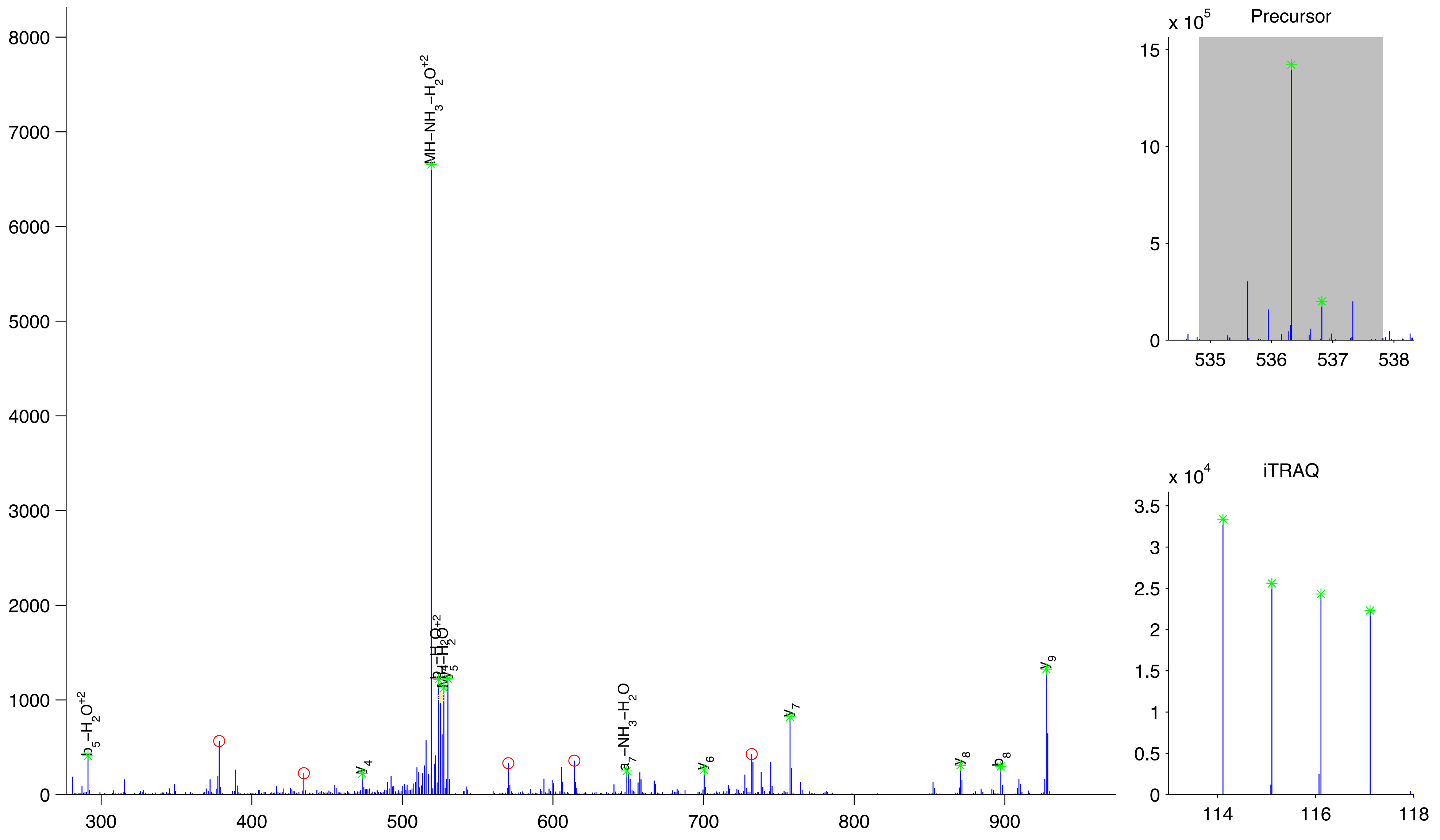

G k G G k G L G k G G A k R

histone cluster 1, H4a [Homo sapiens]
Charge State: +3
Scan Number: 7527
File Name: 111011_HG2_INSB_ACK.raw

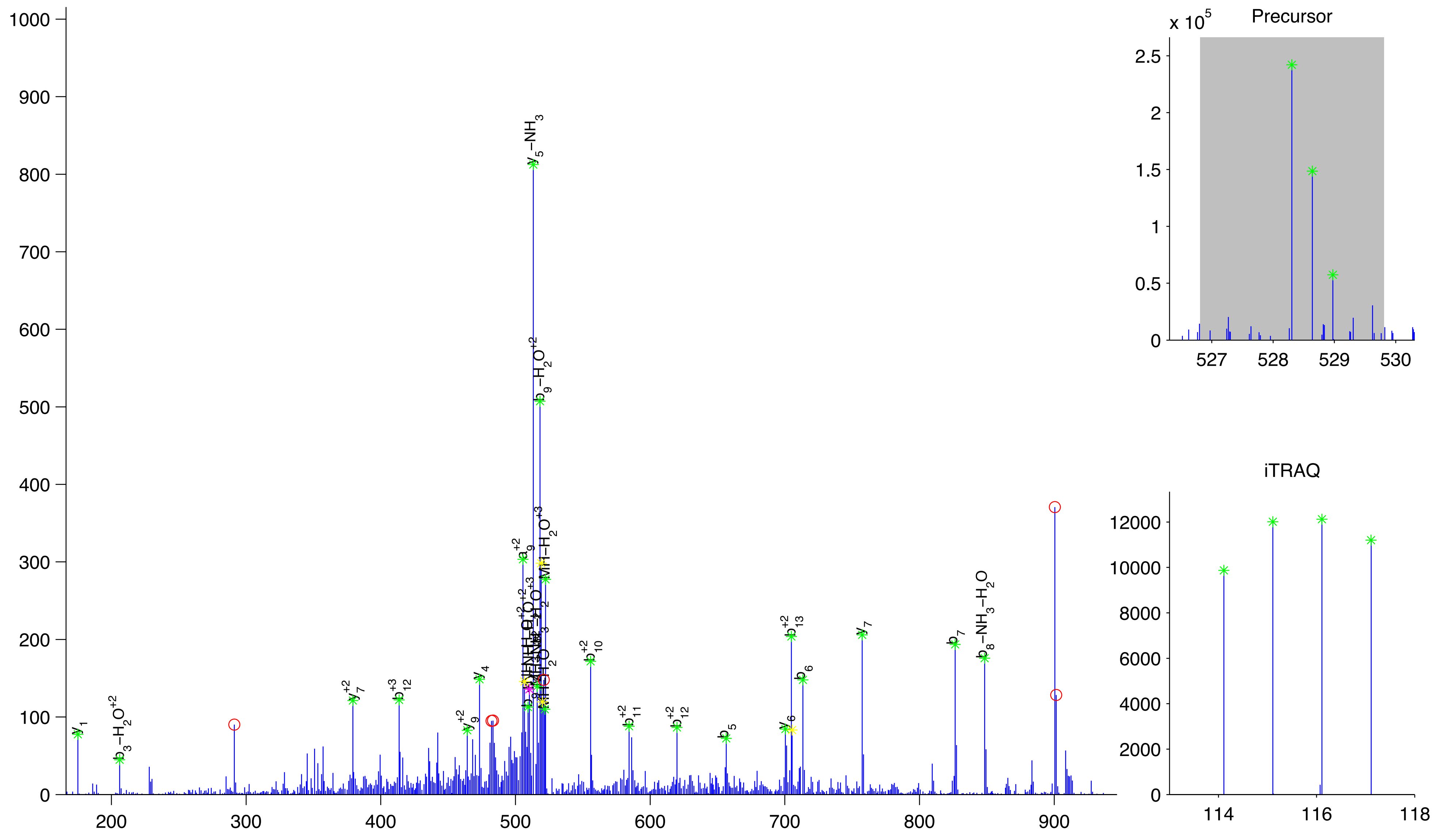

G G k G L G k G G A k R

histone cluster 1, H4a [Homo sapiens]
Charge State: +2
Scan Number: 7565
File Name: 111011_HG2_INSB_ACK.raw

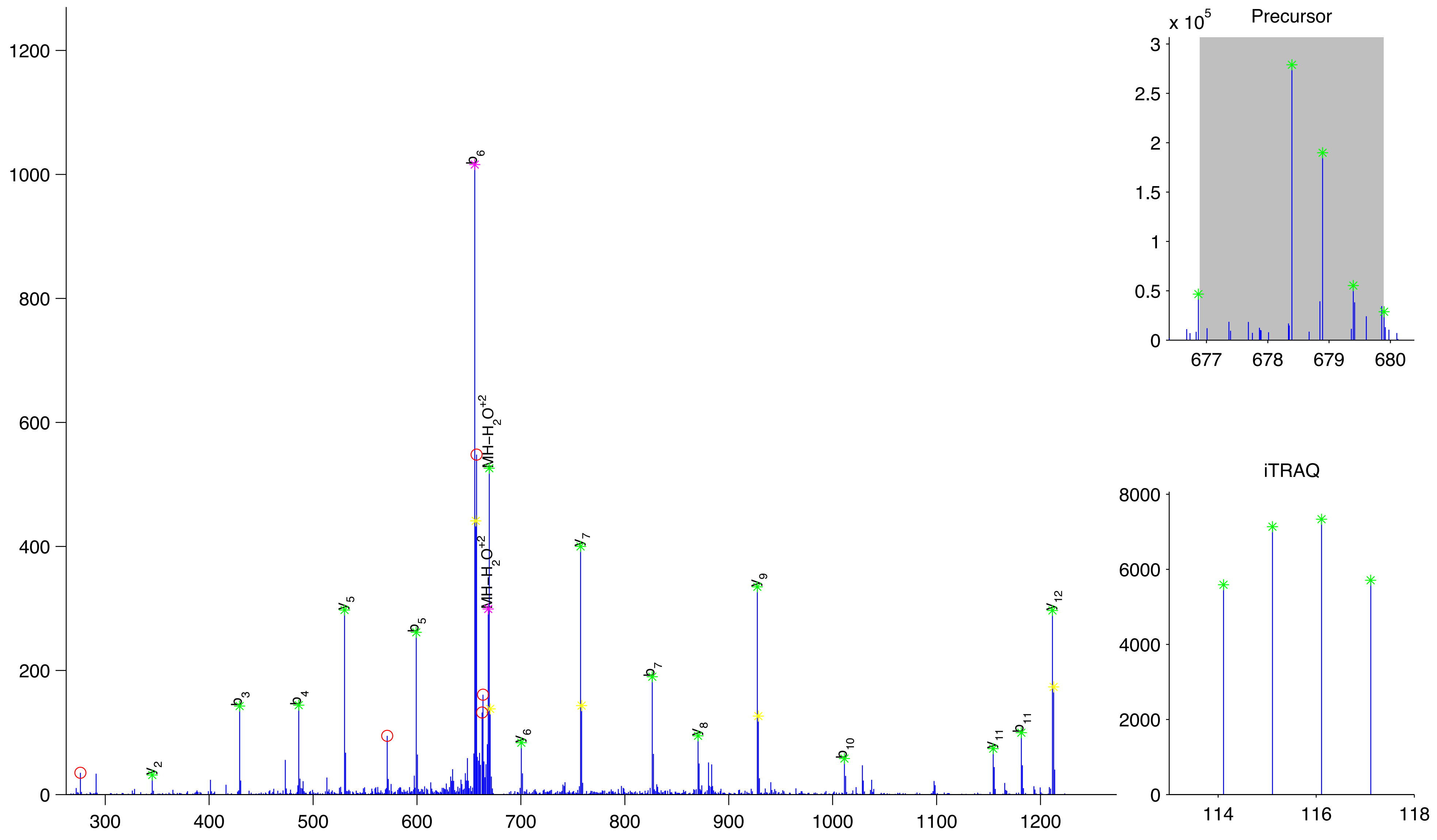

GGkGLGkGGAkR

histone cluster 1, H4a [Homo sapiens]
Charge State: +2
Scan Number: 7626
File Name: 110924_HG2_INSA_ACK3.raw

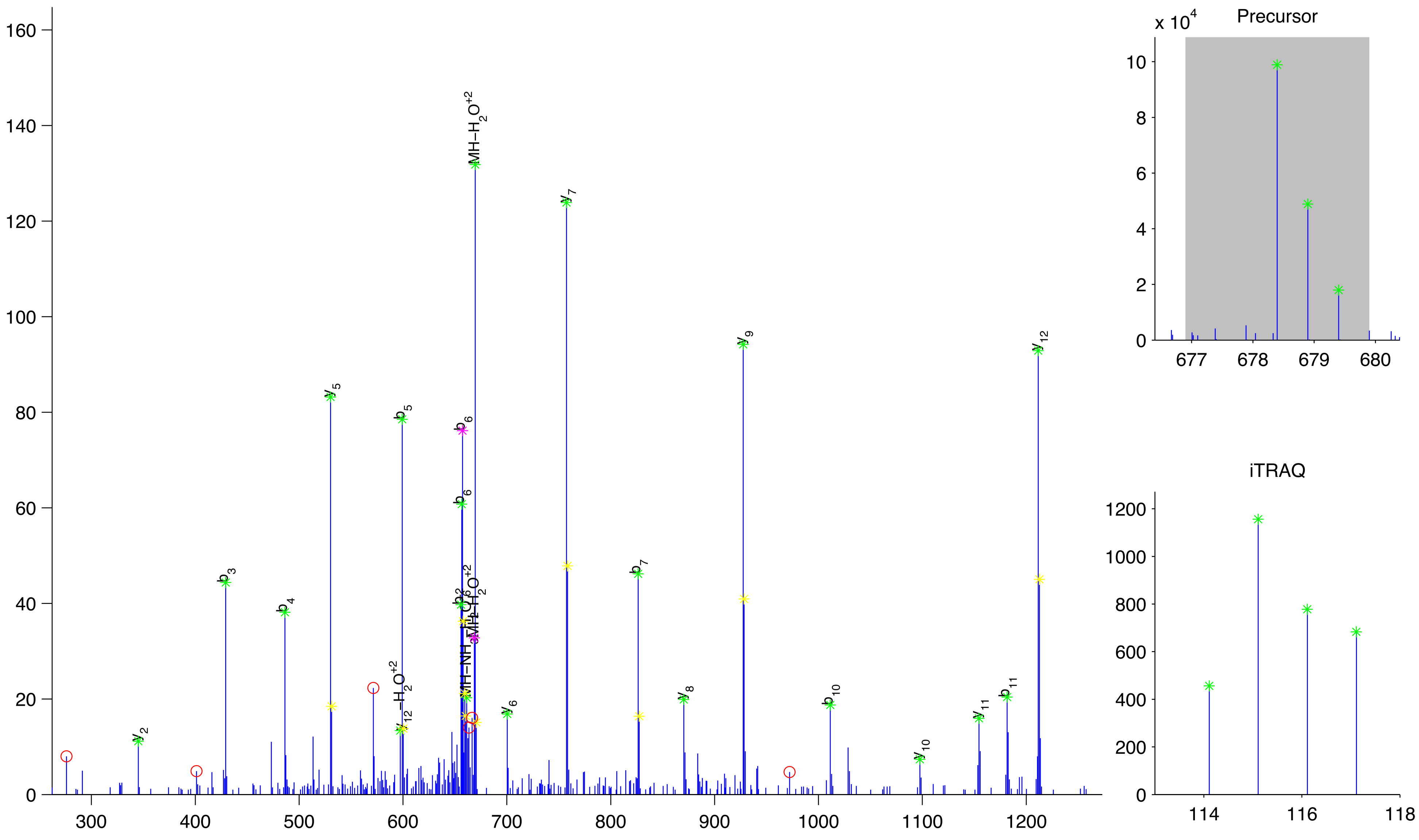

G L G k G G A k R

histone cluster 1, H4a [Homo sapiens]
Charge State: +2
Scan Number: 7626
File Name: 111011_HG2_INSB_ACK.raw

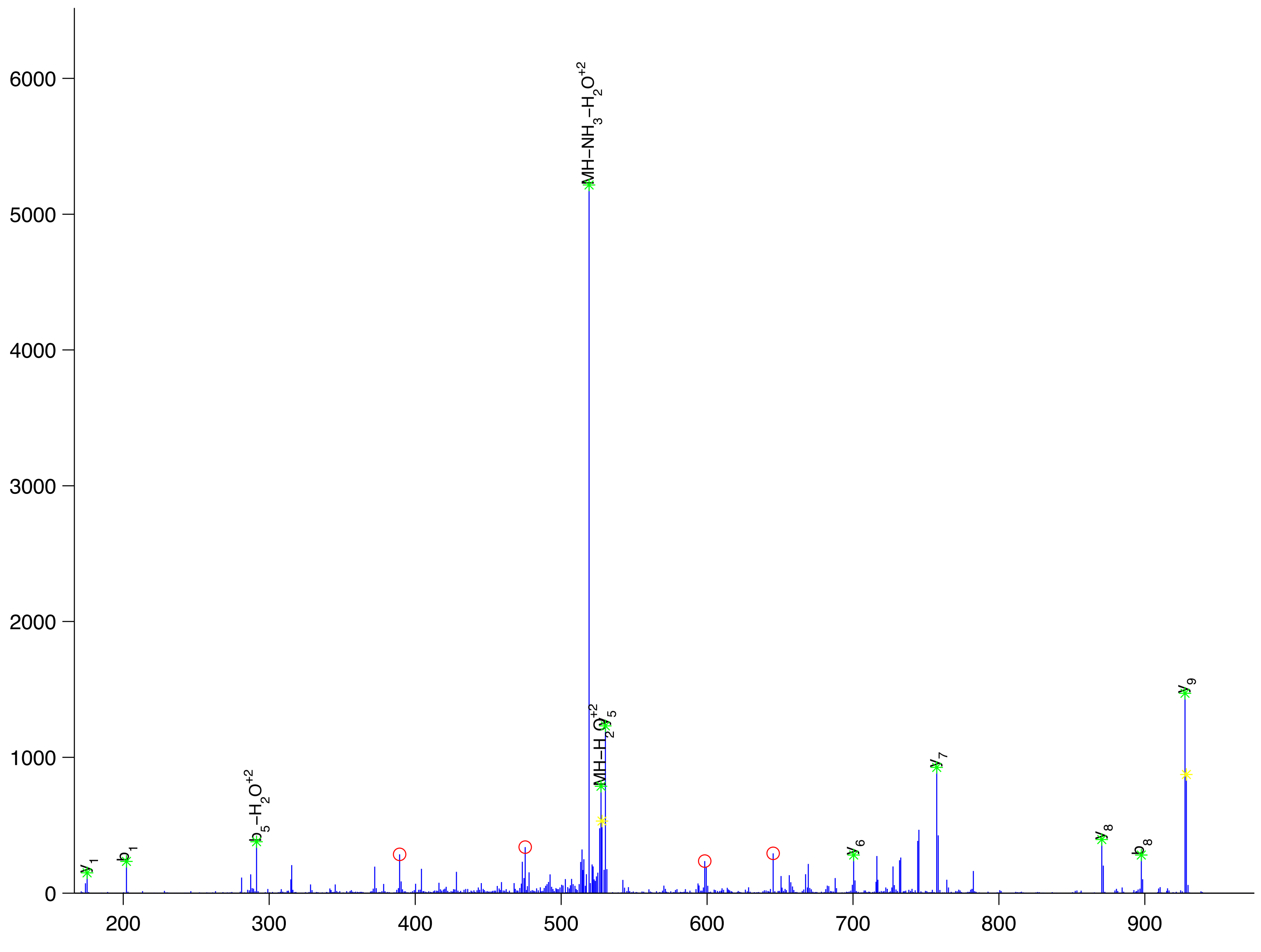

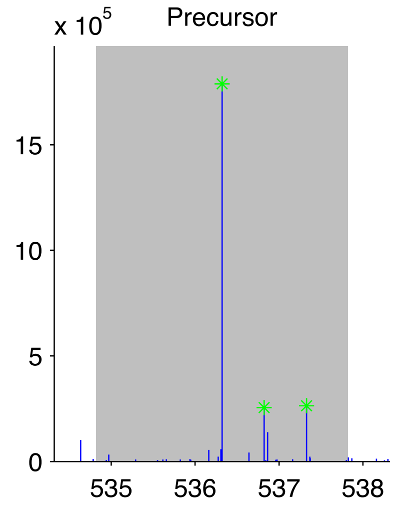

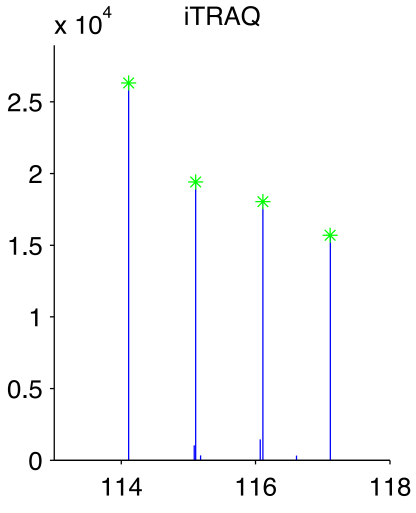

G k G G k G L G k G G A k R

histone cluster 1, H4a [Homo sapiens]
Charge State: +3
Scan Number: 7697
File Name: 111011_HG2_INSB_ACK.raw

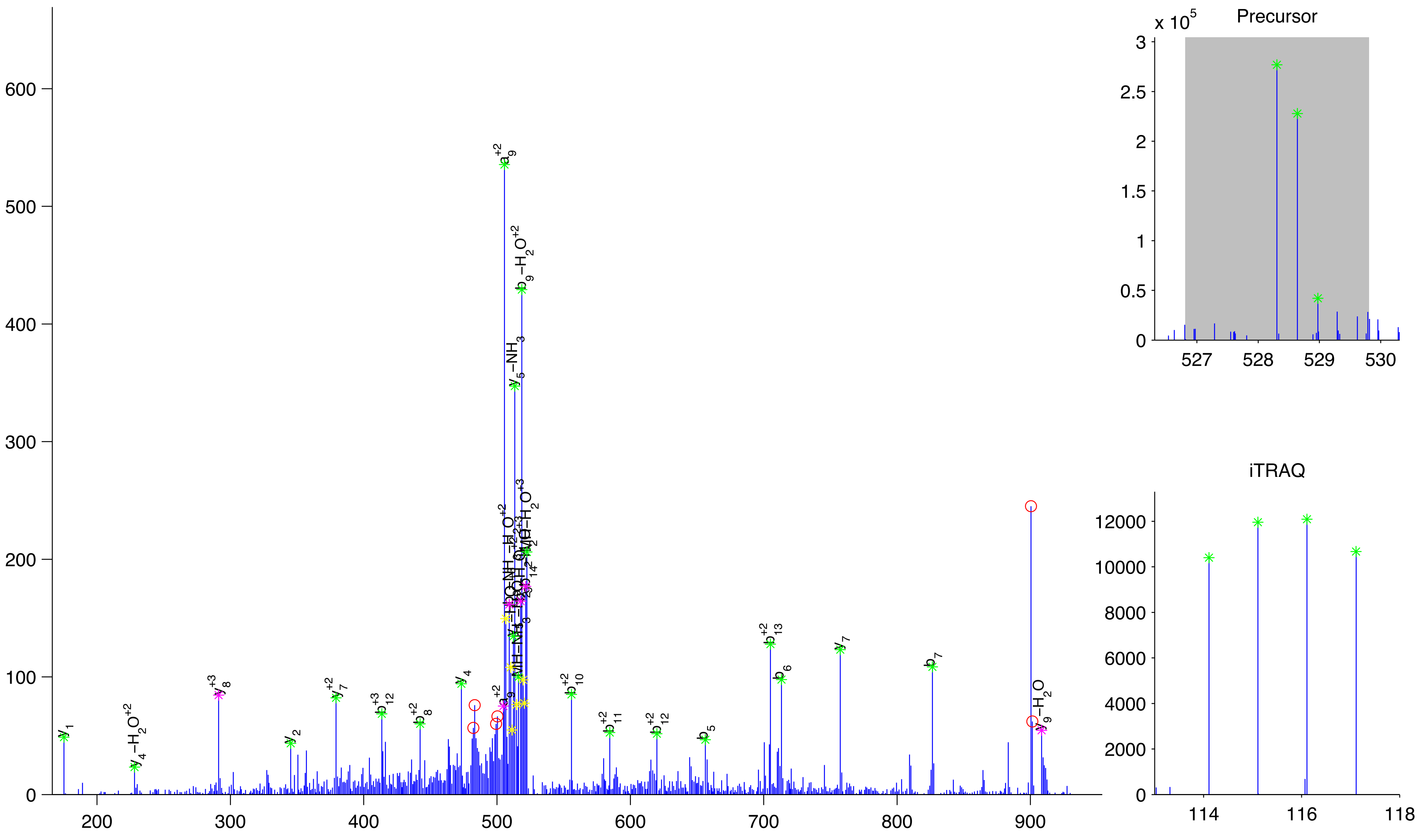

G L G k G G A k R

histone cluster 1, H4a [Homo sapiens]
Charge State: +2
Scan Number: 7748
File Name: 110924_HG2_INSA_ACK3.raw

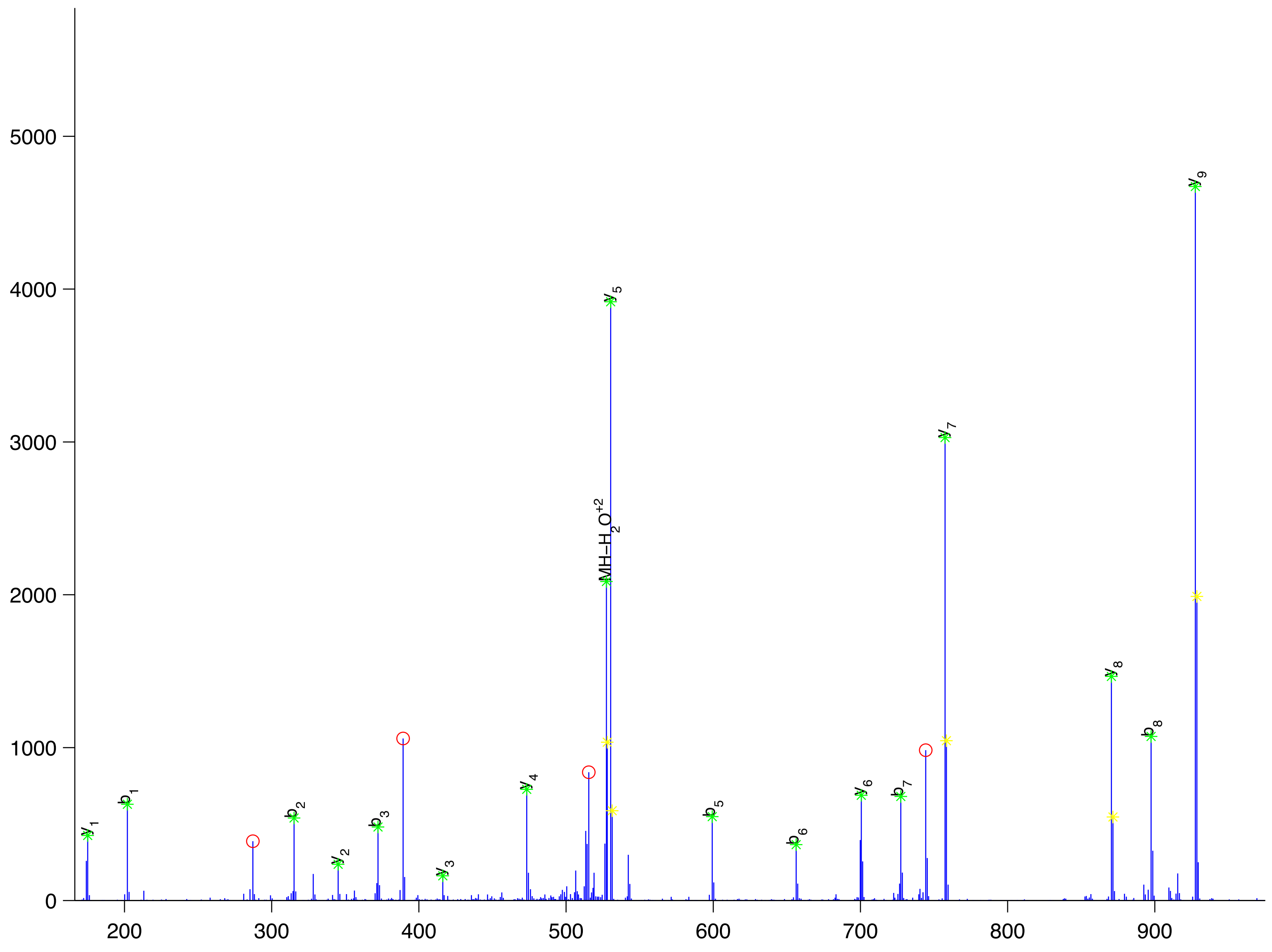

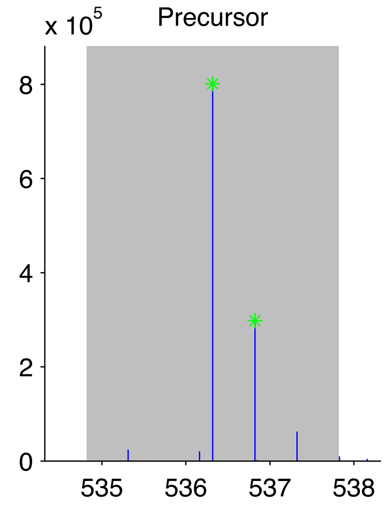

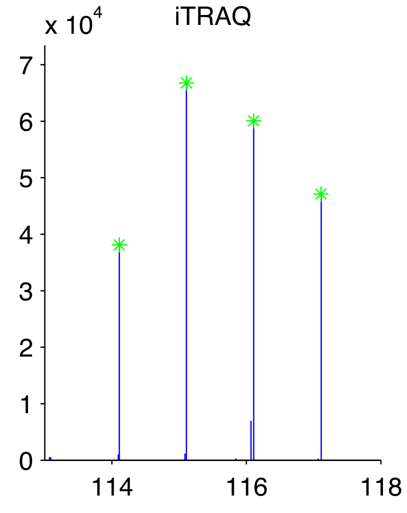

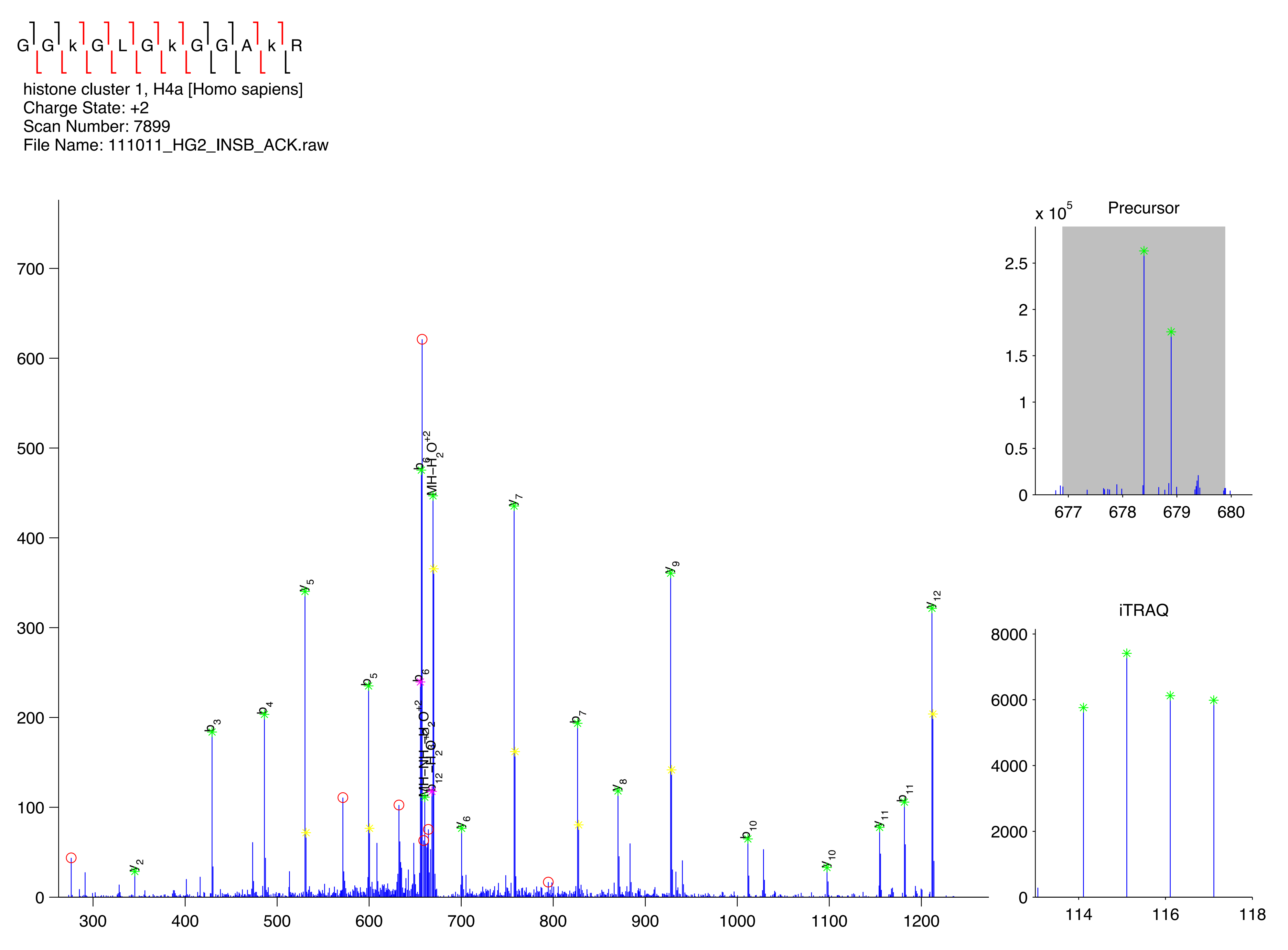

G G k G L G k G G A k R
histone cluster 1, H4a [Homo sapiens]
Charge State: +2
Scan Number: 7899
File Name: 111011_HG2_INSB_ACK.raw
Precursor
x 10^5
iTRAQ

G G k G L G k G G A k R

histone cluster 1, H4a [Homo sapiens]
Charge State: +2
Scan Number: 8071
File Name: 111011_HG2_INSB_ACK.raw

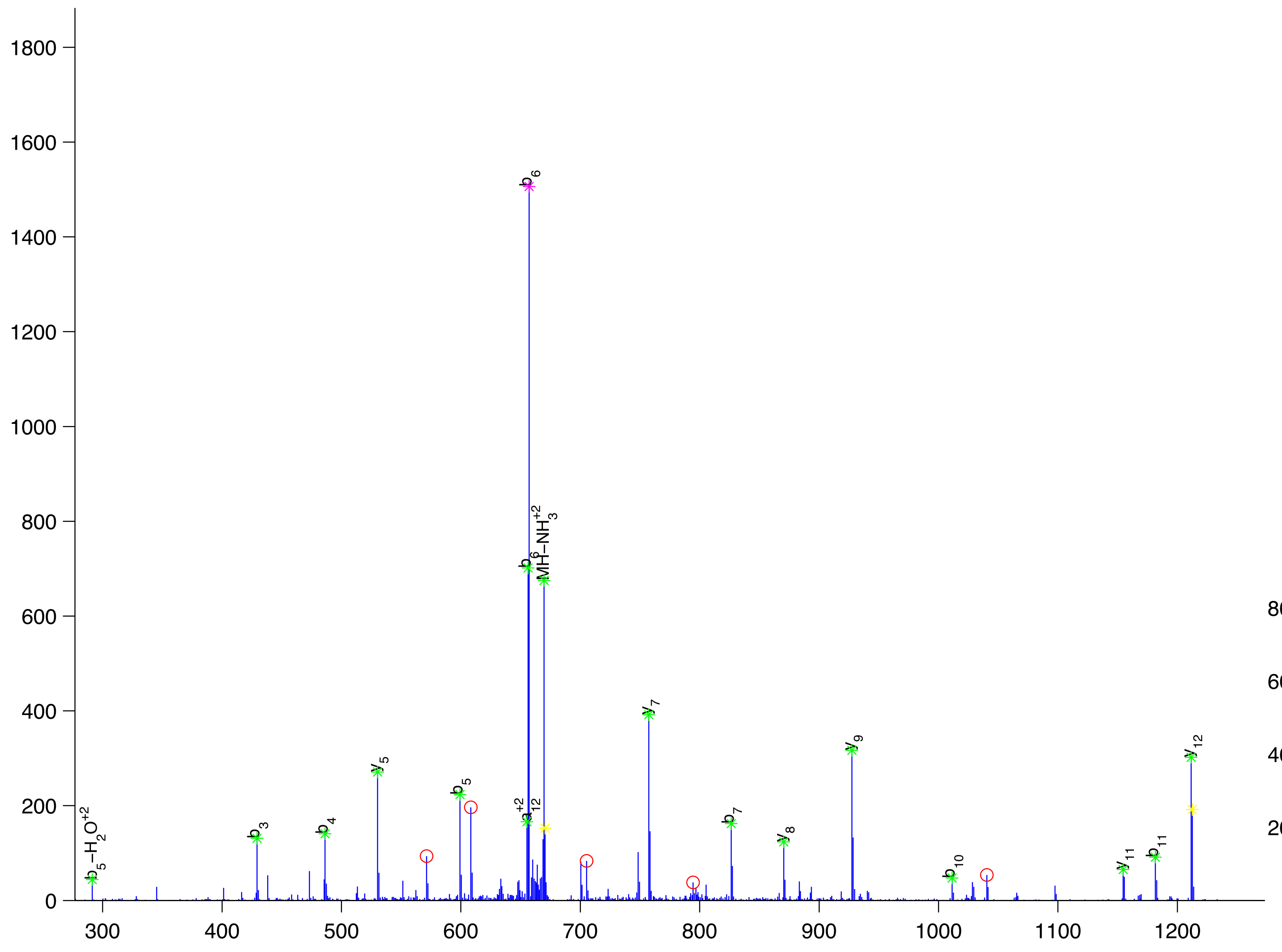

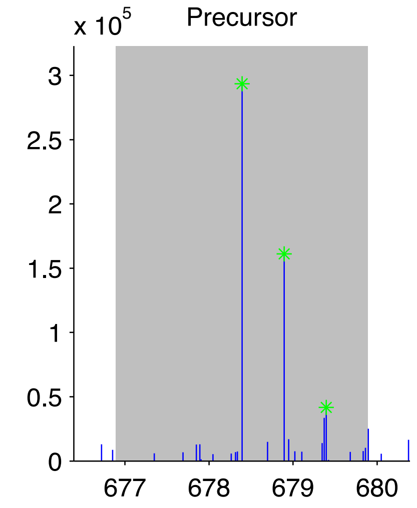

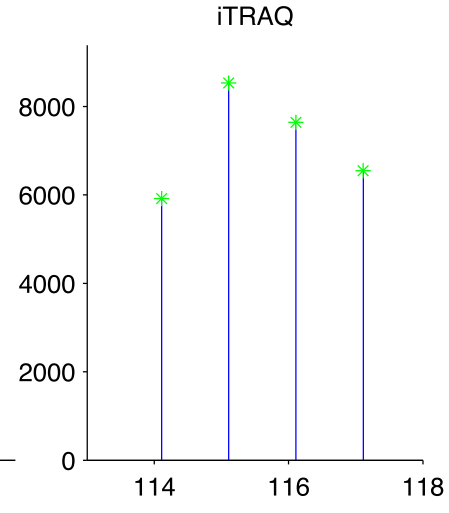

m S G R G k G G k G L G k G G A k R

histone cluster 1, H4a [Homo sapiens]
Charge State: +3
Scan Number: 8102
File Name: 110924_HG2_INSA_ACK2.raw

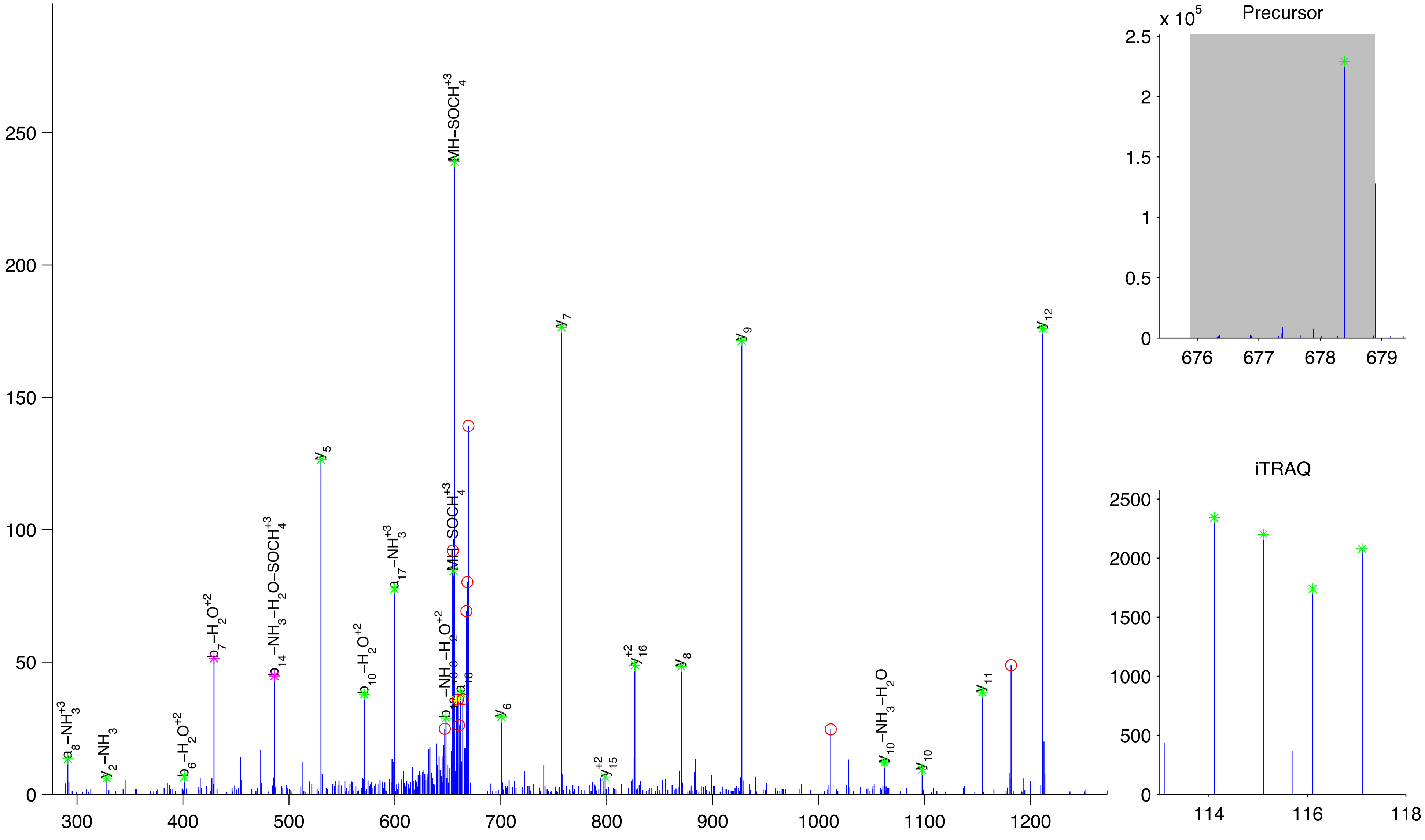

G G k G L G k G G A k R

histone cluster 1, H4a [Homo sapiens]
Charge State: +2
Scan Number: 8245
File Name: 111011_HG2_INSB_ACK.raw

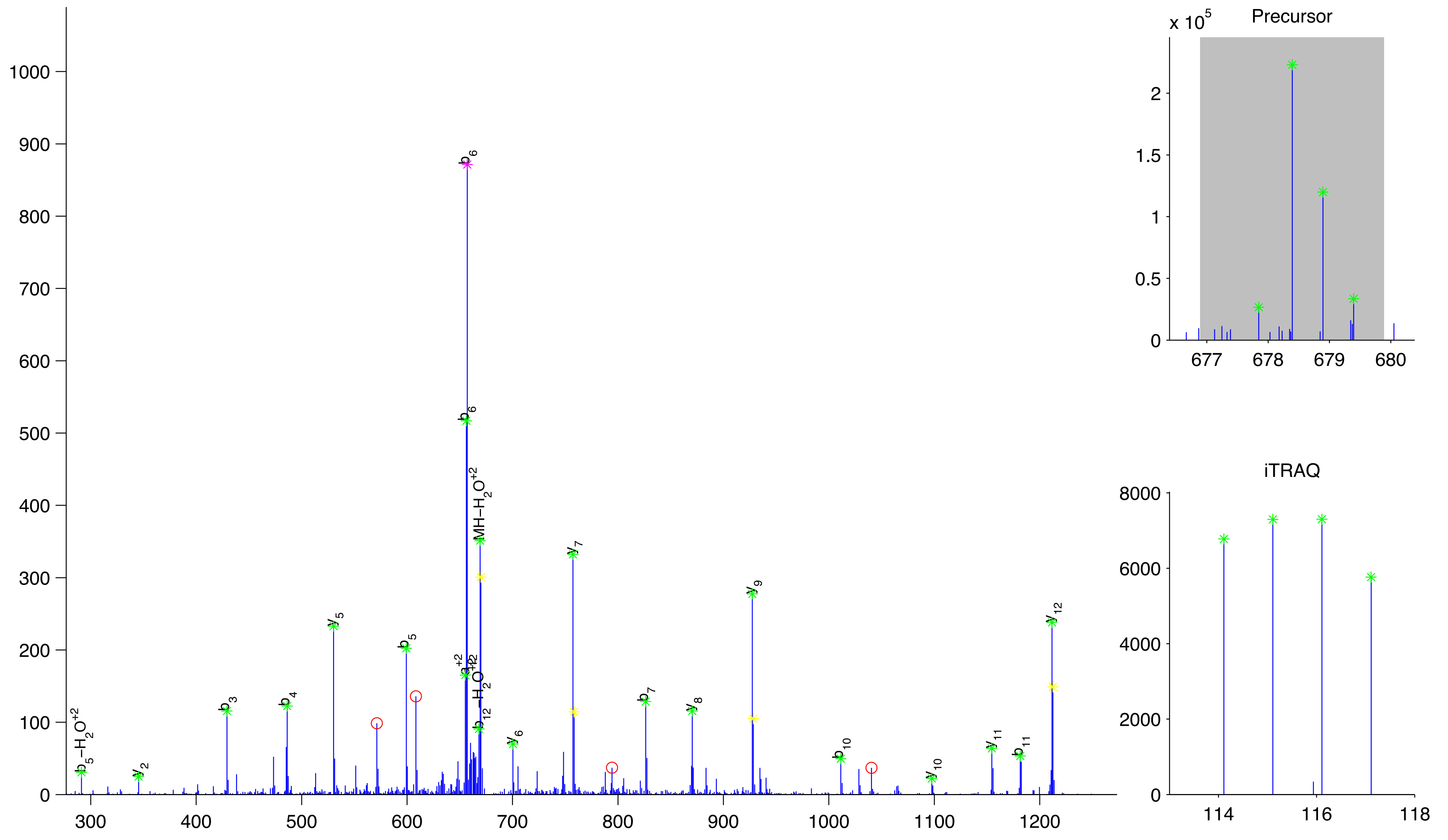

G G k G L G k G G A k R

histone cluster 1, H4a [Homo sapiens]
Charge State: +2
Scan Number: 8413
File Name: 111011_HG2_INSB_ACK.raw

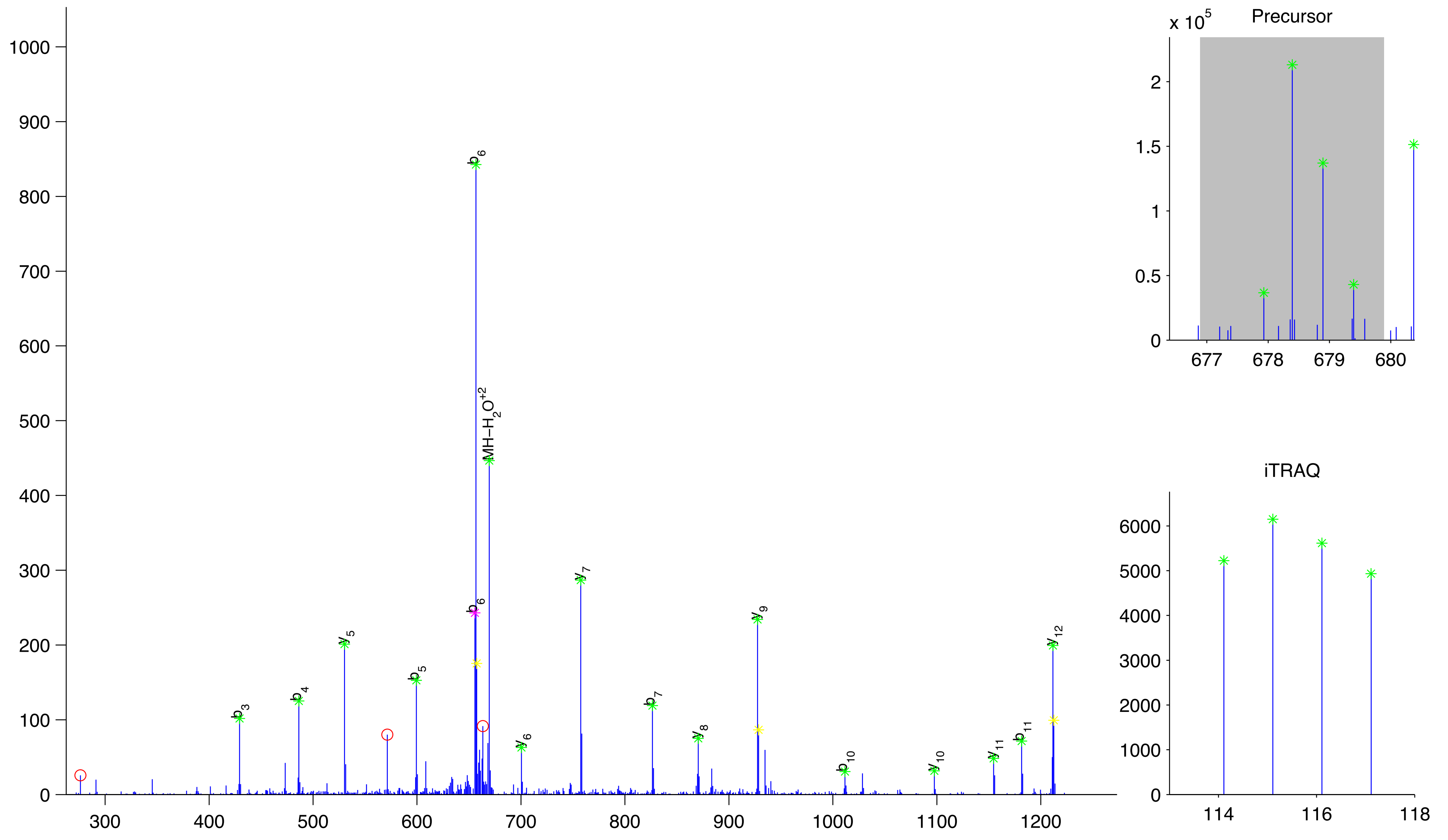

G L G k G G A k R

histone cluster 1, H4a [Homo sapiens]
Charge State: +2
Scan Number: 8420
File Name: 110924_HG2_INSA_ACK3.raw

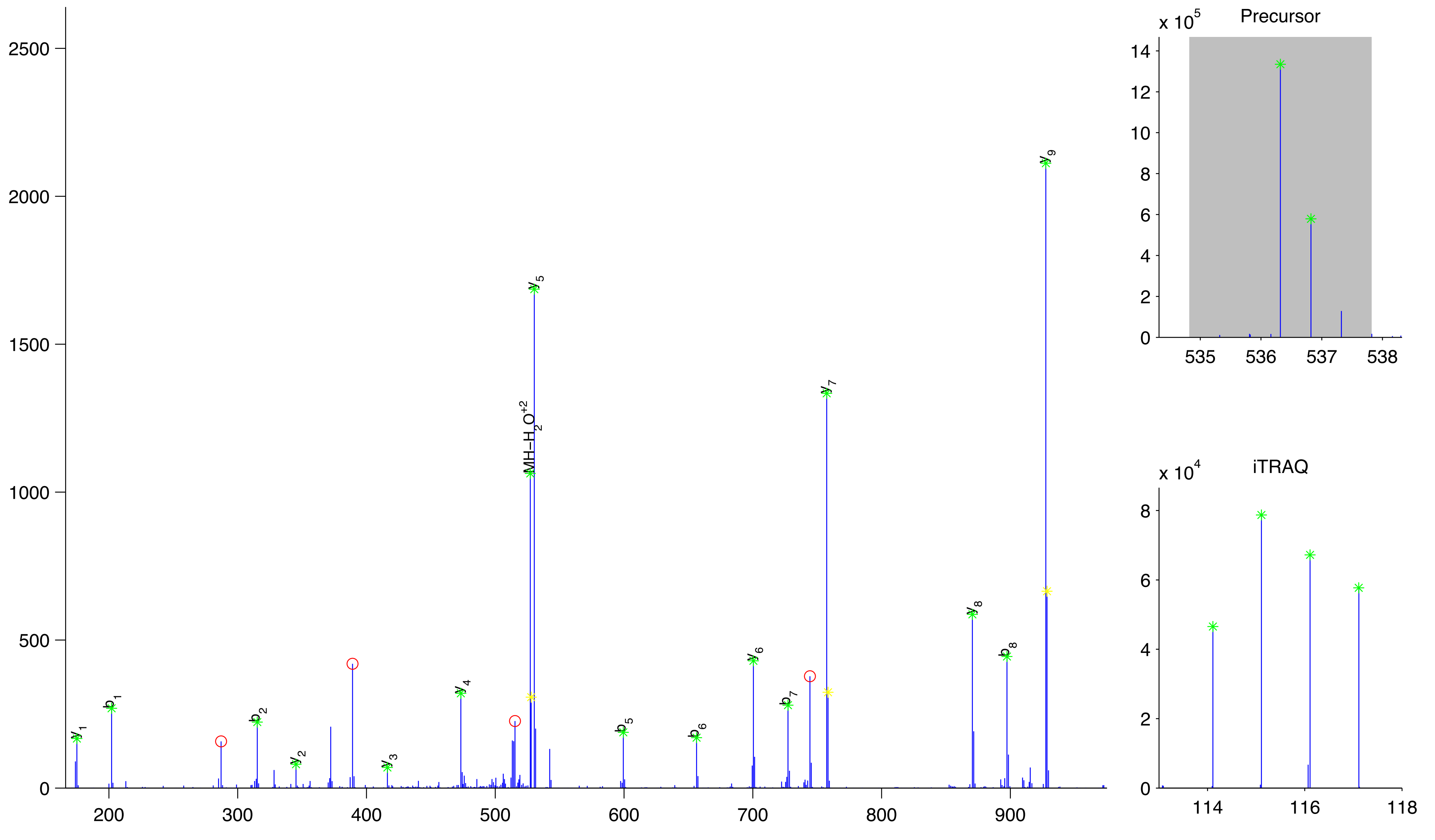

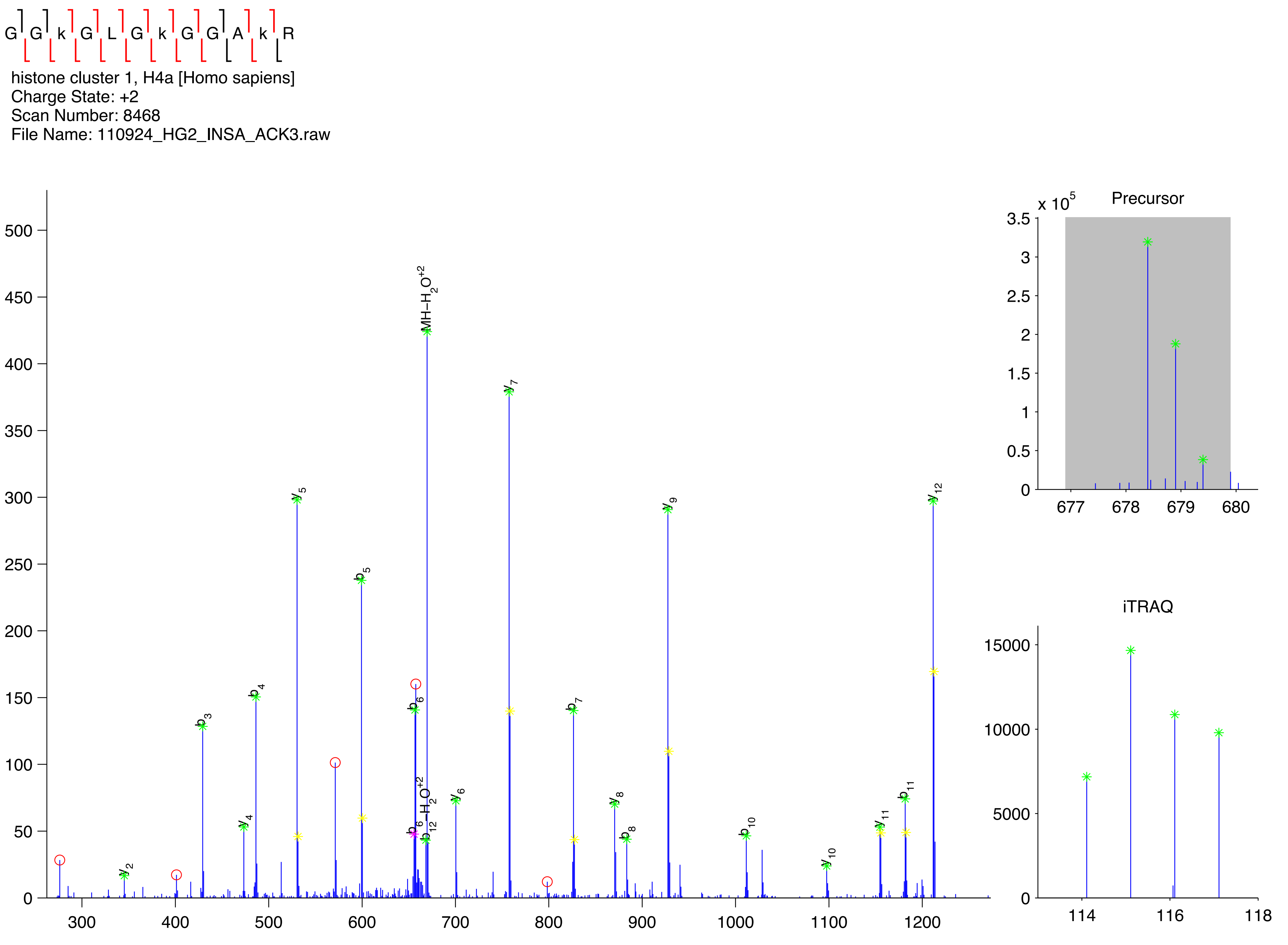

G G k G L G k G G A k R
histone cluster 1, H4a [Homo sapiens]
Charge State: +2
Scan Number: 8468
File Name: 110924_HG2_INSA_ACK3.raw
y2
b3
y4
b4
y5
b5
b6
b6-H2O+2
b12
MH-H2O+2
y6
y7
b7
y8
b8
y9
b10
y10
y11
b11
y12
Precursor
x 10^5
iTRAQ

G L G k G G A k R

histone cluster 1, H4a [Homo sapiens]
Charge State: +2
Scan Number: 8522
File Name: 110924_HG2_INSA_ACK3.raw

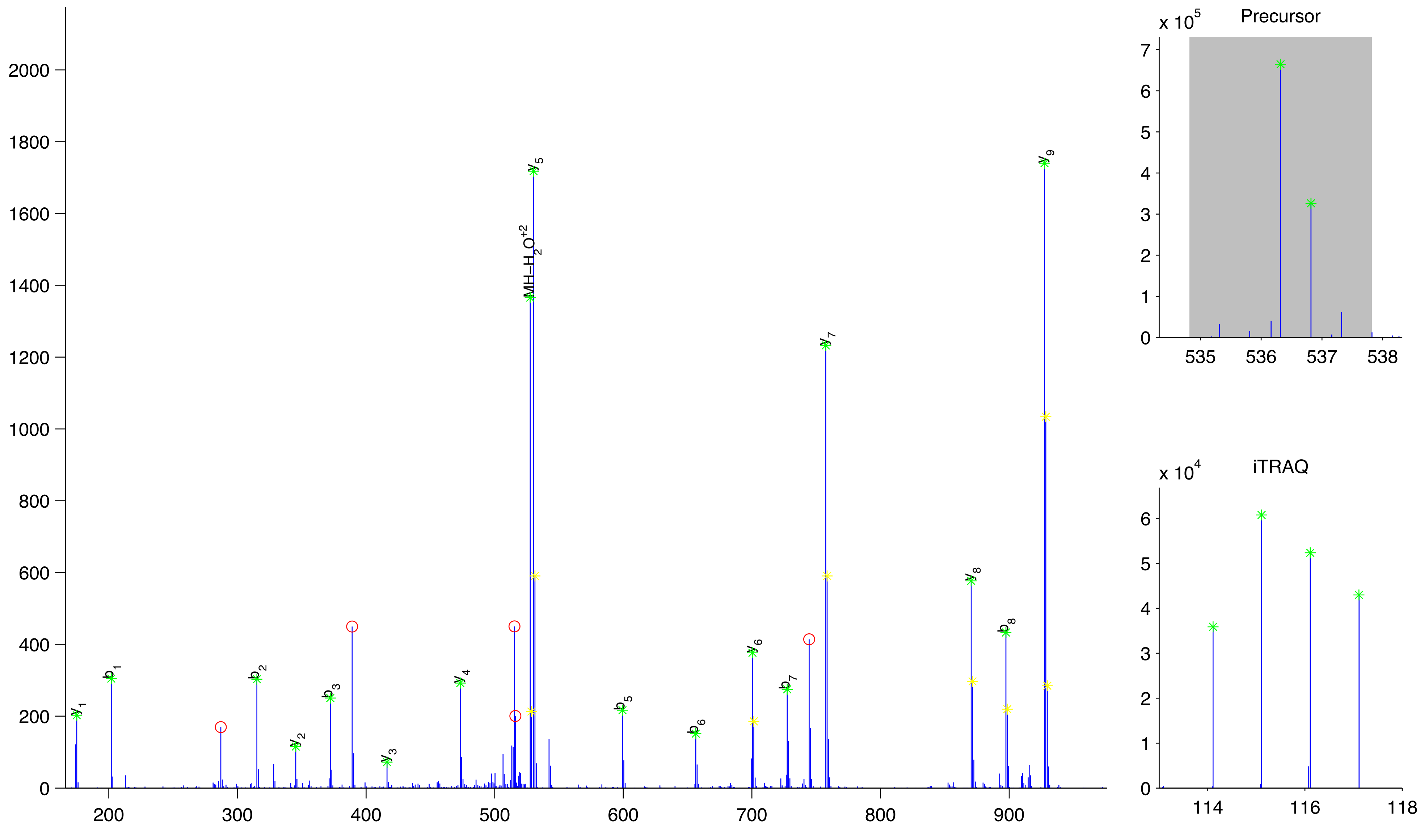

G G k G L G k G G A k R

histone cluster 1, H4a [Homo sapiens]
Charge State: +2
Scan Number: 8753
File Name: 111011_HG2_INSB_ACK.raw

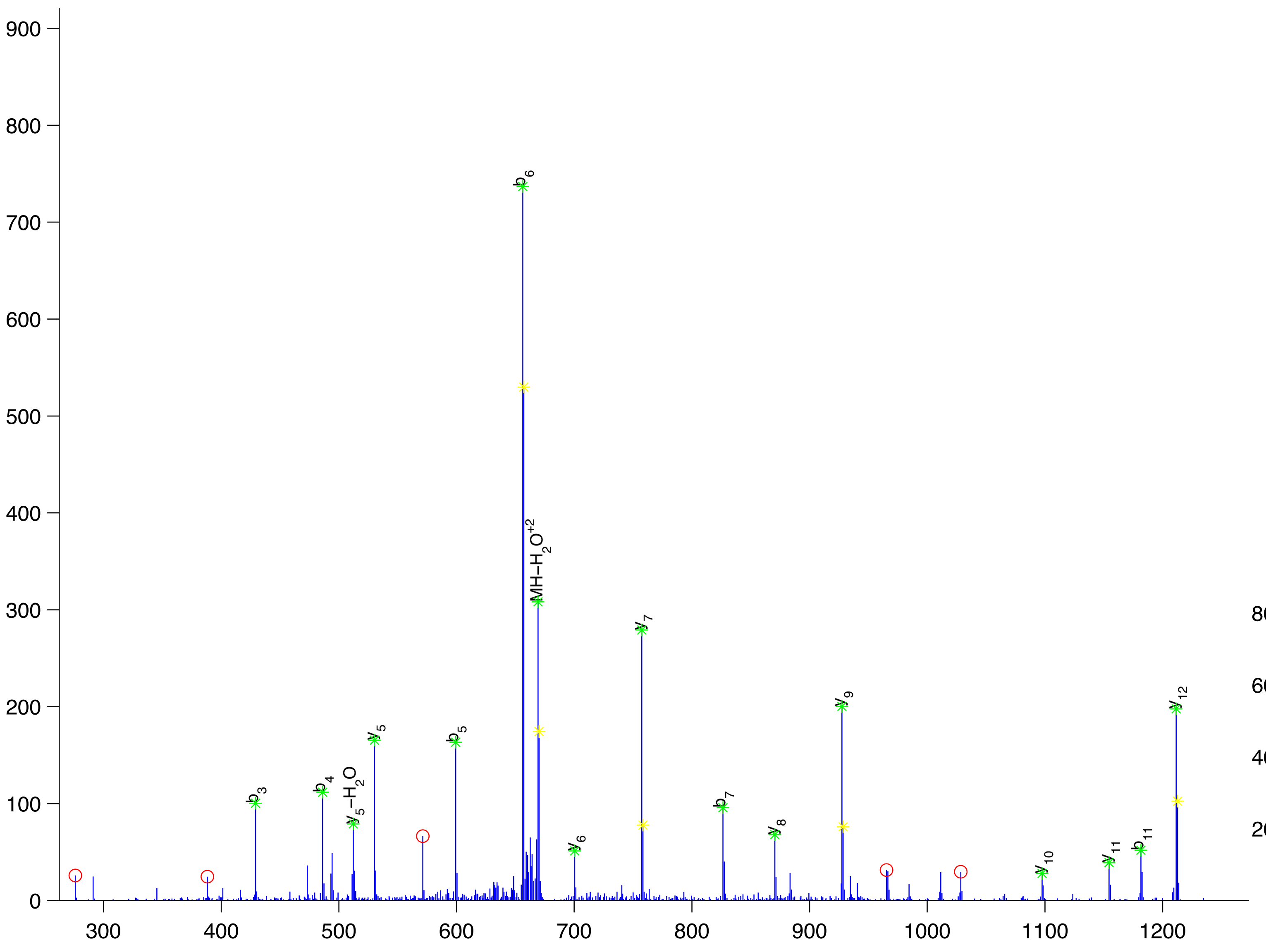

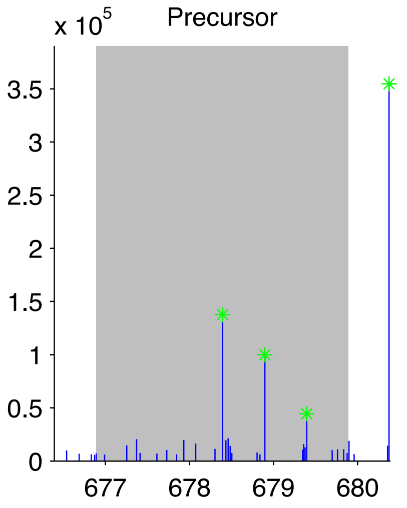

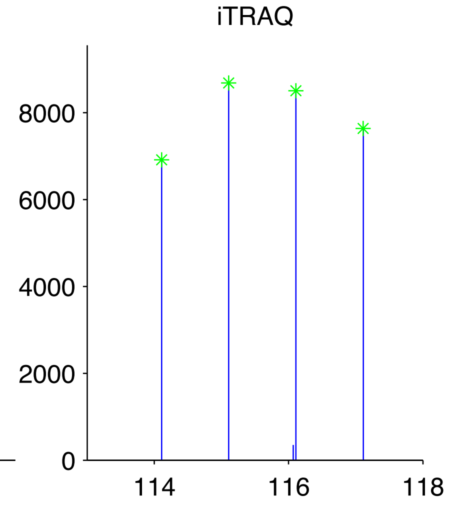

G L G k G G A k R

histone cluster 1, H4a [Homo sapiens]
Charge State: +2
Scan Number: 8777
File Name: 110924_HG2_INSA_ACK3.raw

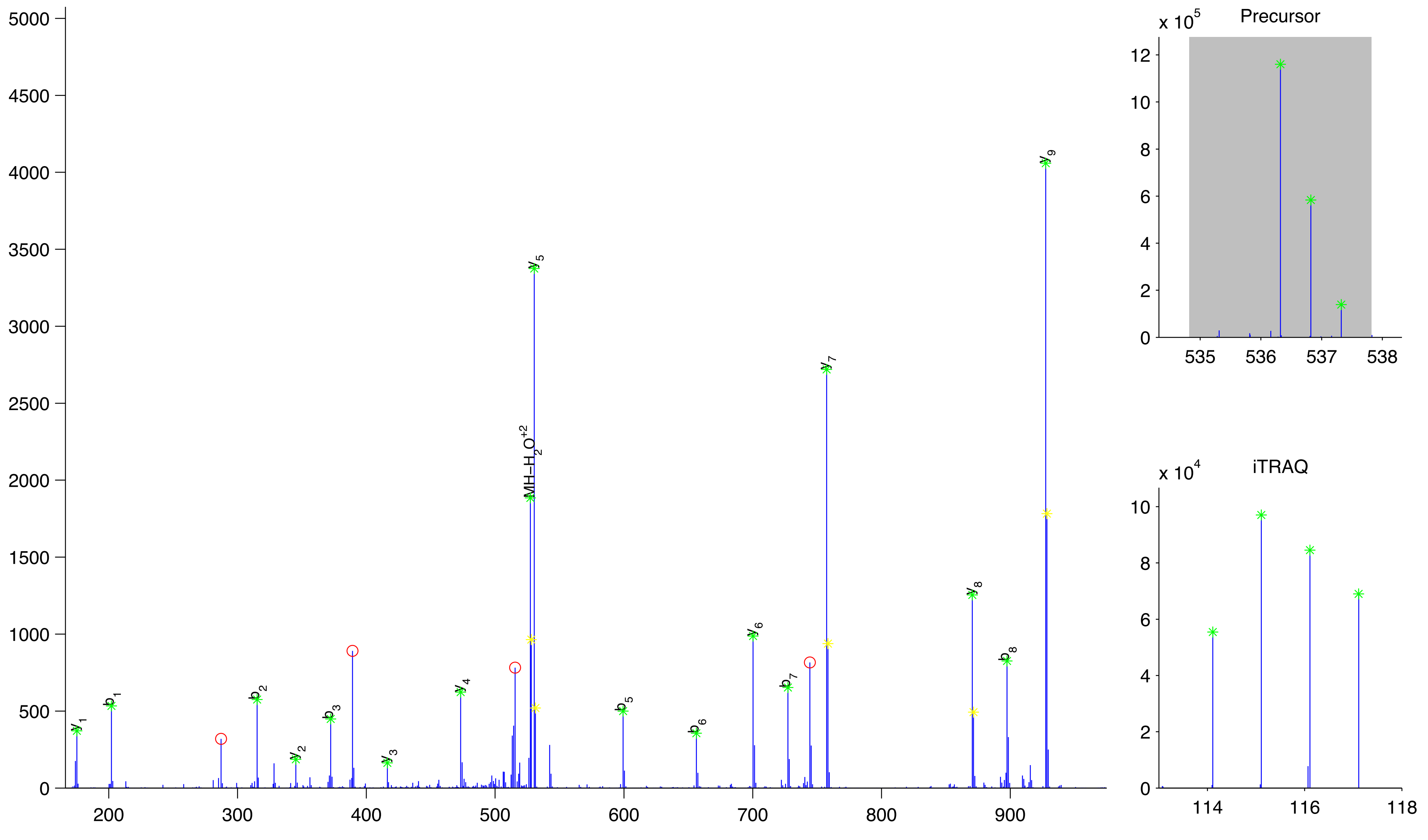

G G k G L G k G G A k R

histone cluster 1, H4a [Homo sapiens]
Charge State: +2
Scan Number: 8966
File Name: 110924_HG2_INSA_ACK3.raw

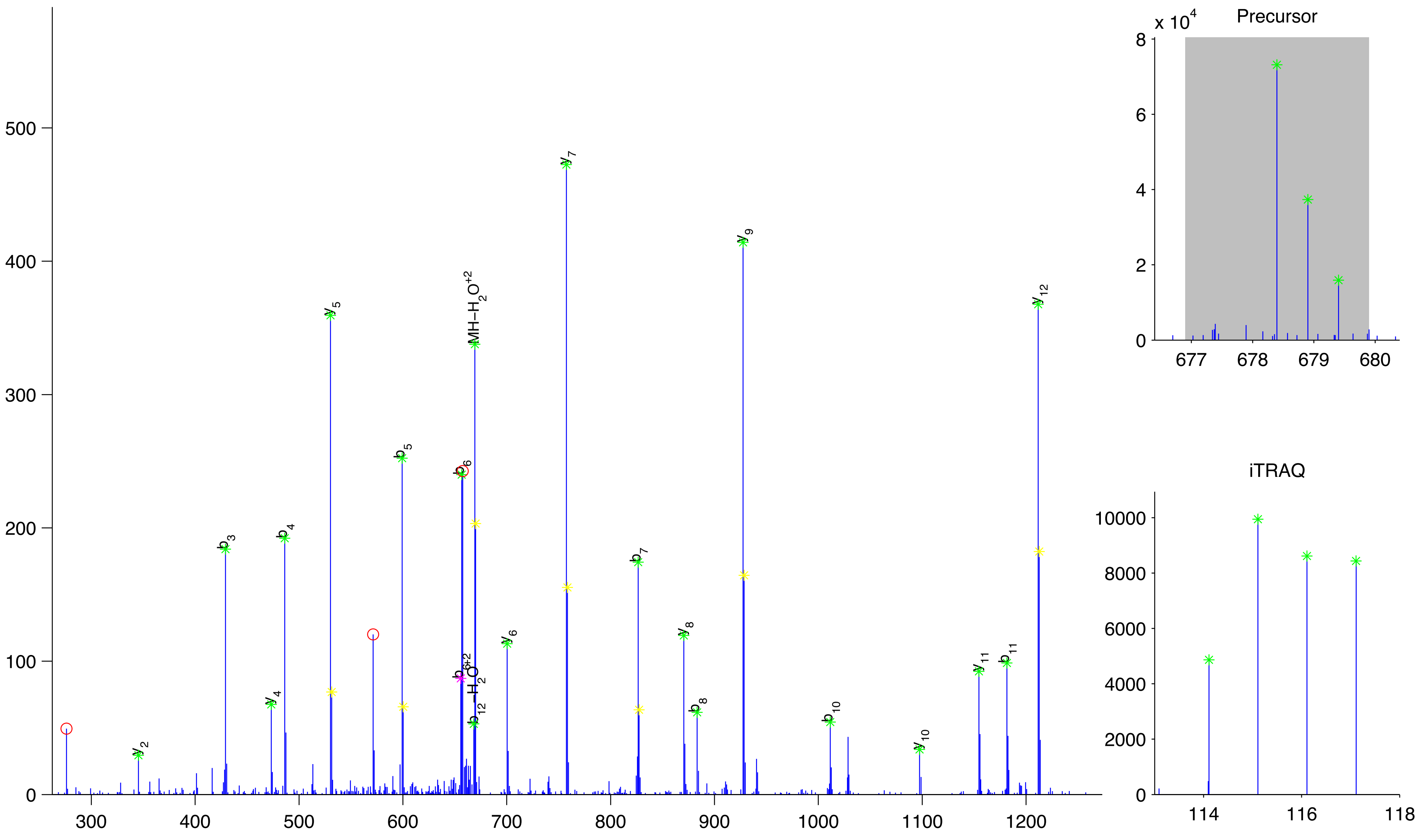

G G k G L G k G G A k R

histone cluster 1, H4a [Homo sapiens]
Charge State: +2
Scan Number: 9007
File Name: 111011_HG2_INSB_ACK.raw

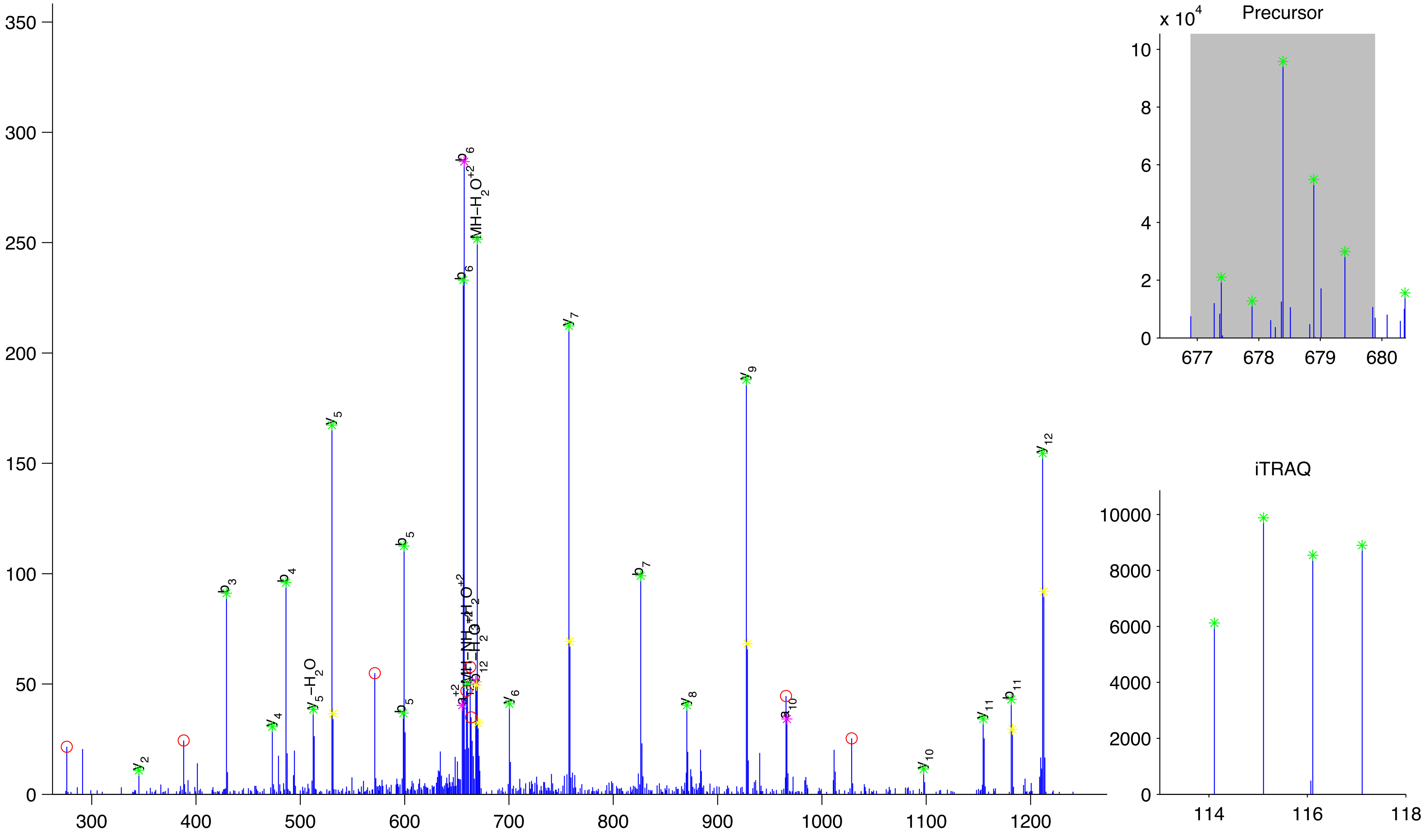

G L G k G G A k R

histone cluster 1, H4a [Homo sapiens]
Charge State: +2
Scan Number: 9113
File Name: 110924_HG2_INSA_ACK3.raw

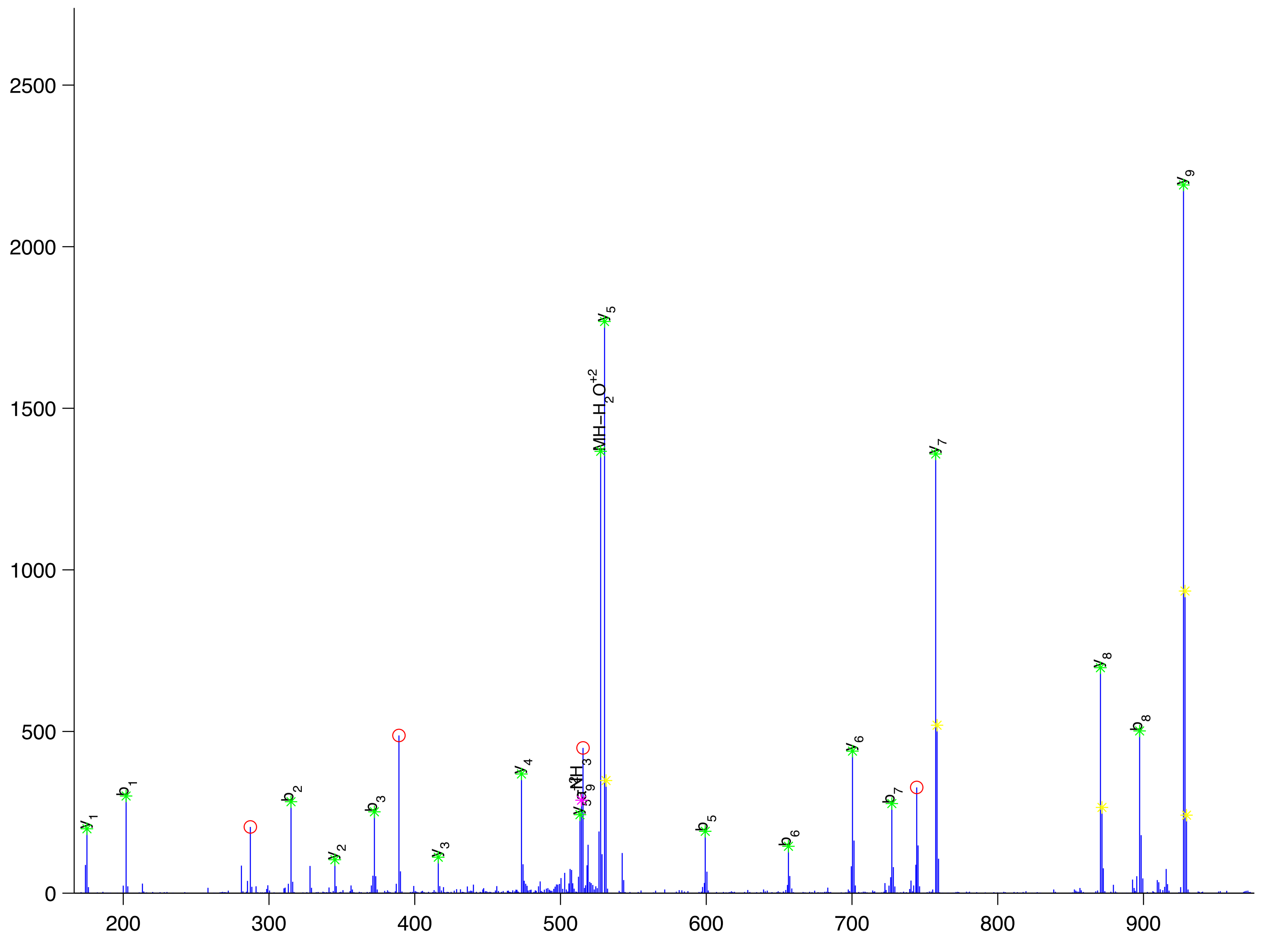

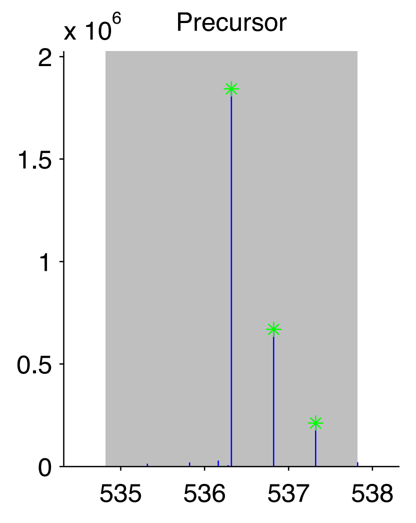

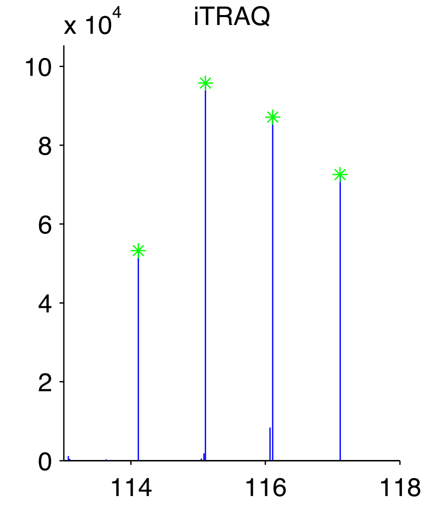

G G k G L G k G G A k R

histone cluster 1, H4a [Homo sapiens]
Charge State: +2
Scan Number: 9140
File Name: 110924_HG2_INSA_ACK3.raw

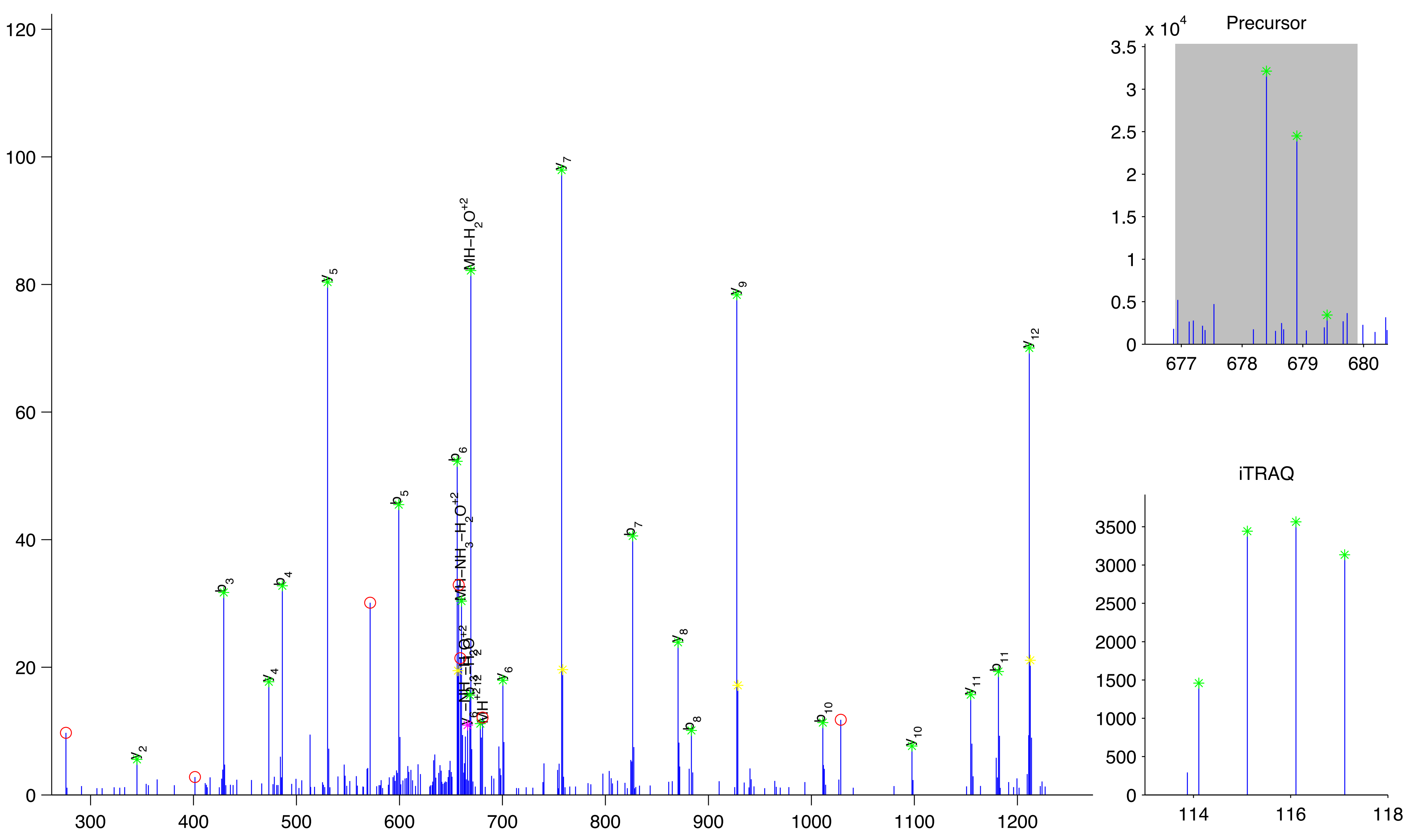

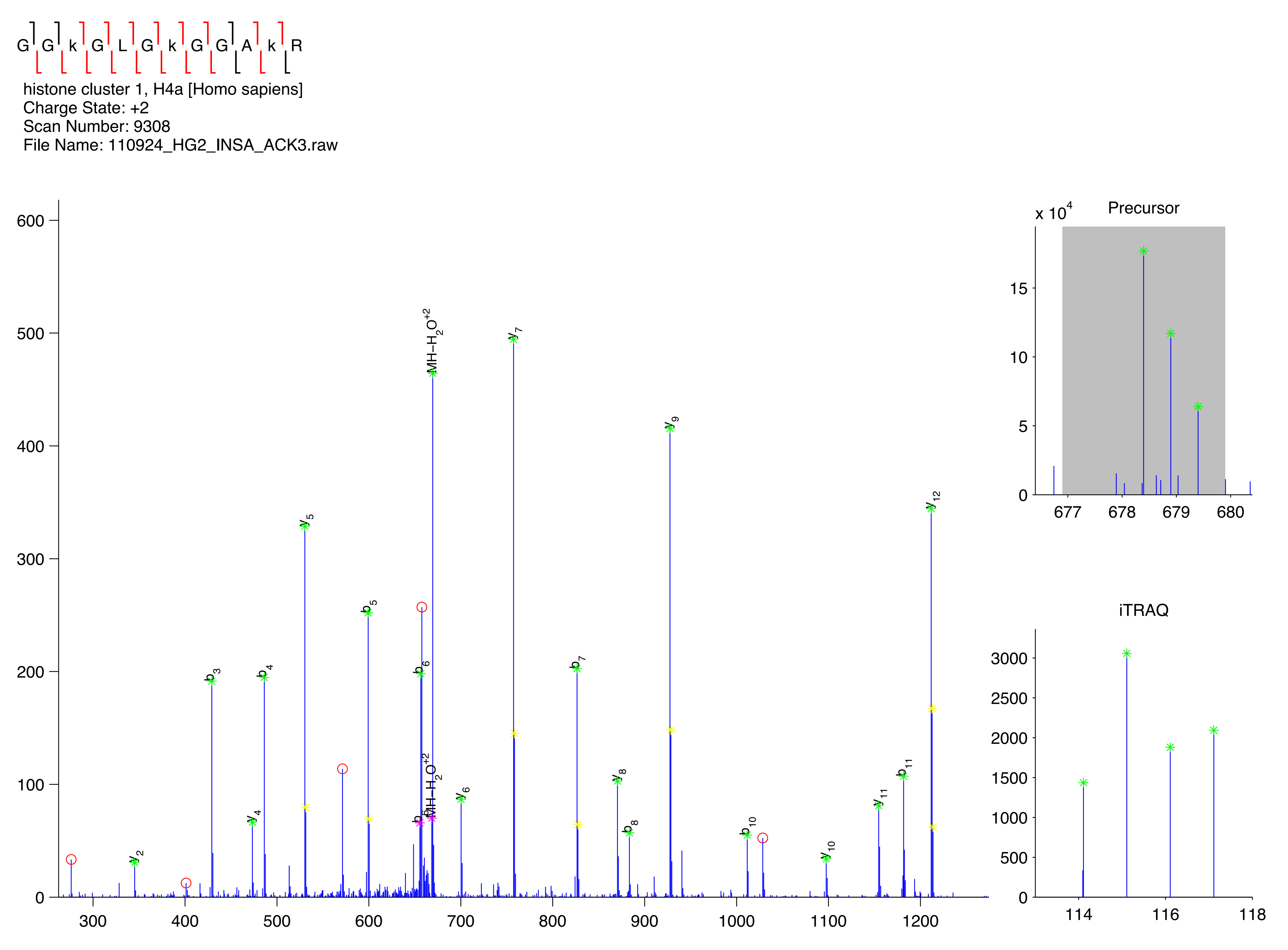

G G k G L G k G G A k R
histone cluster 1, H4a [Homo sapiens]
Charge State: +2
Scan Number: 9308
File Name: 110924_HG2_INSA_ACK3.raw
y_2
b_3
y_4
b_4
y_5
b_5
b_6
$MH-H_2O^{+2}$
y_6
y_7
b_7
y_8
b_8
y_9
b_{10}
y_{10}
y_{11}
b_{11}
y_{12}
x 10^4
Precursor
iTRAQ

G L G k G G A k R

histone cluster 1, H4a [Homo sapiens]
Charge State: +2
Scan Number: 9318
File Name: 111011_HG2_INSB_ACK.raw

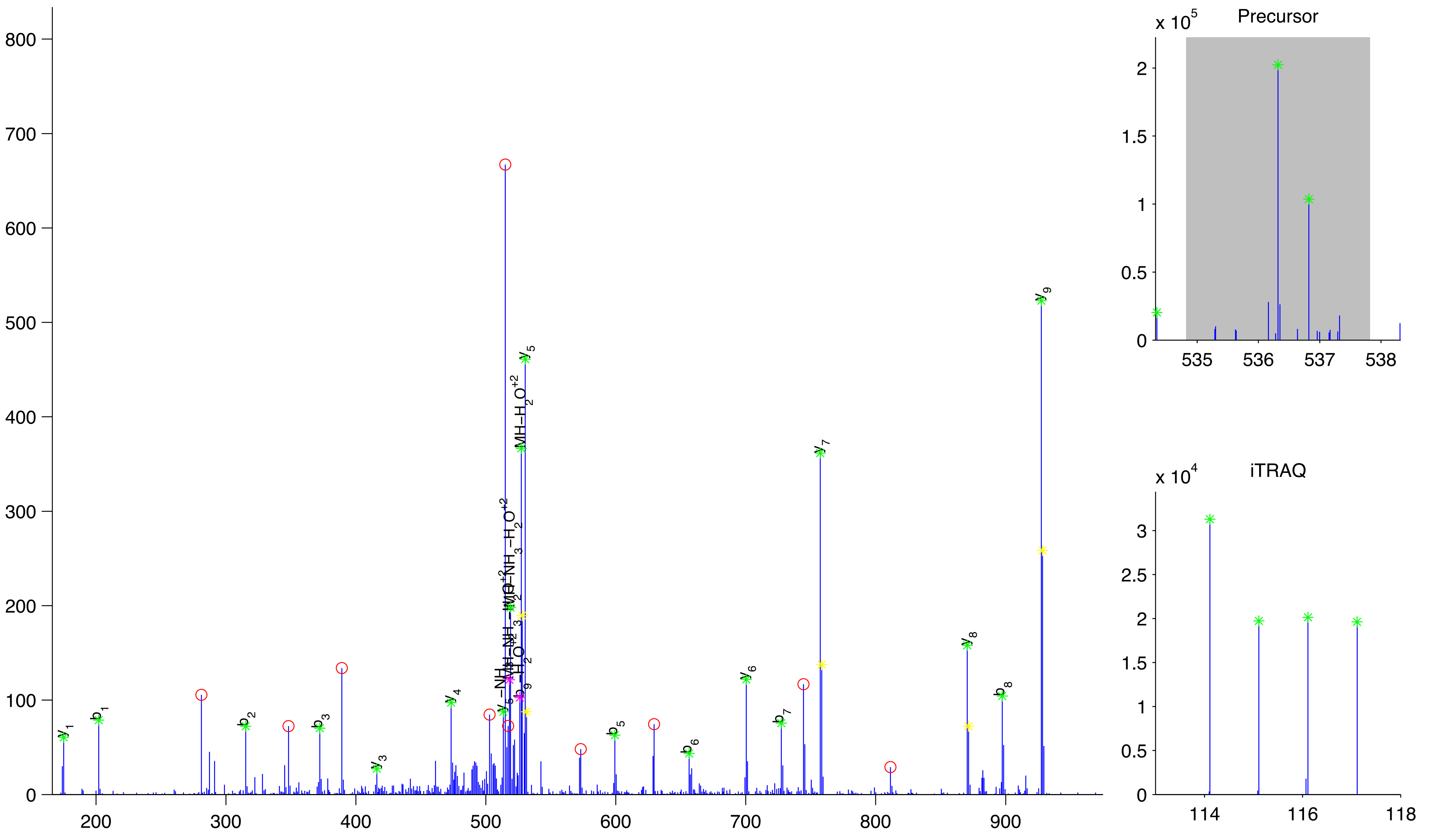

G L G k G G A k R

histone cluster 1, H4a [Homo sapiens]
Charge State: +2
Scan Number: 9449
File Name: 110924_HG2_INSA_ACK3.raw

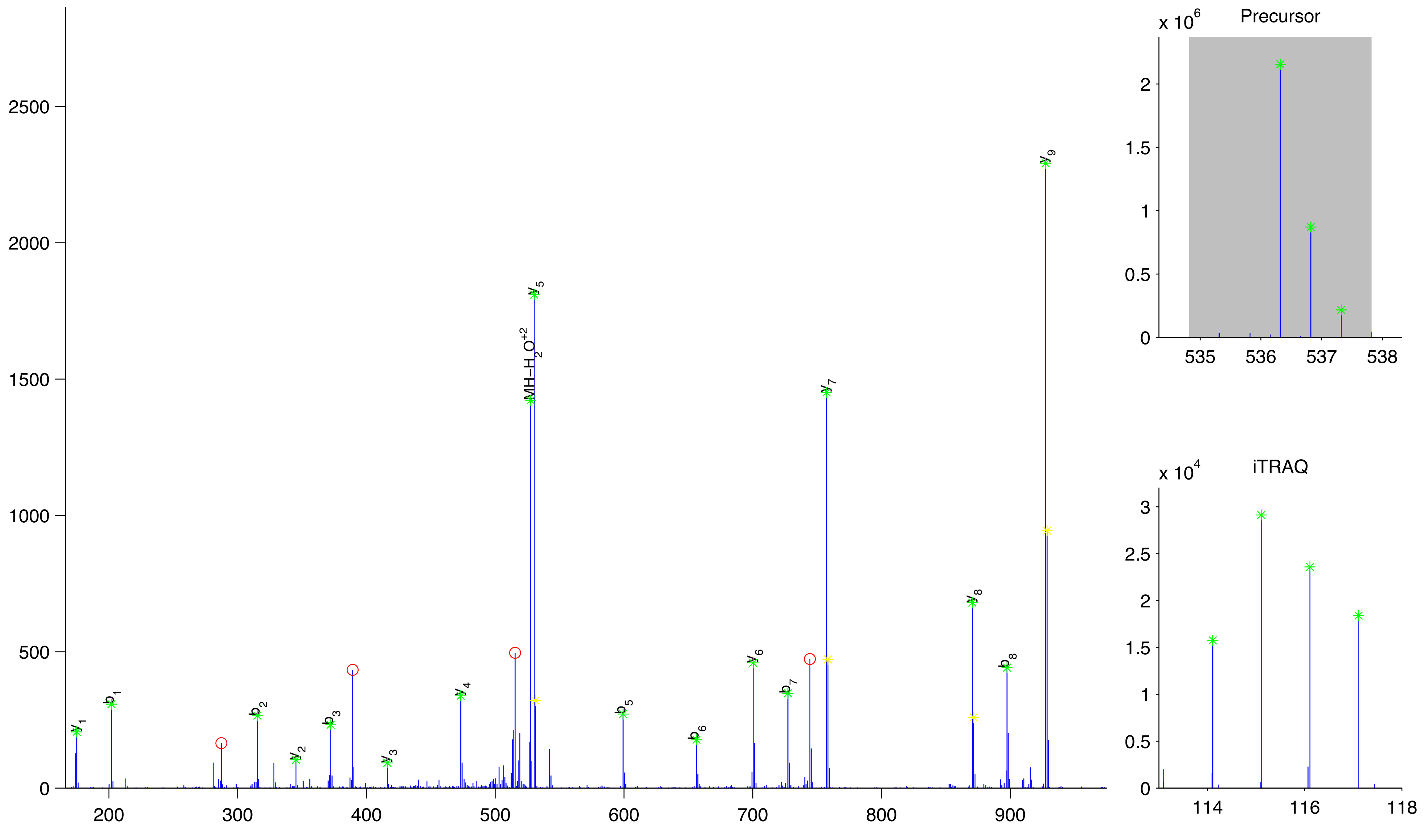

G L G k G G A k R

histone cluster 1, H4a [Homo sapiens]
Charge State: +2
Scan Number: 9617
File Name: 110924_HG2_INSA_ACK3.raw

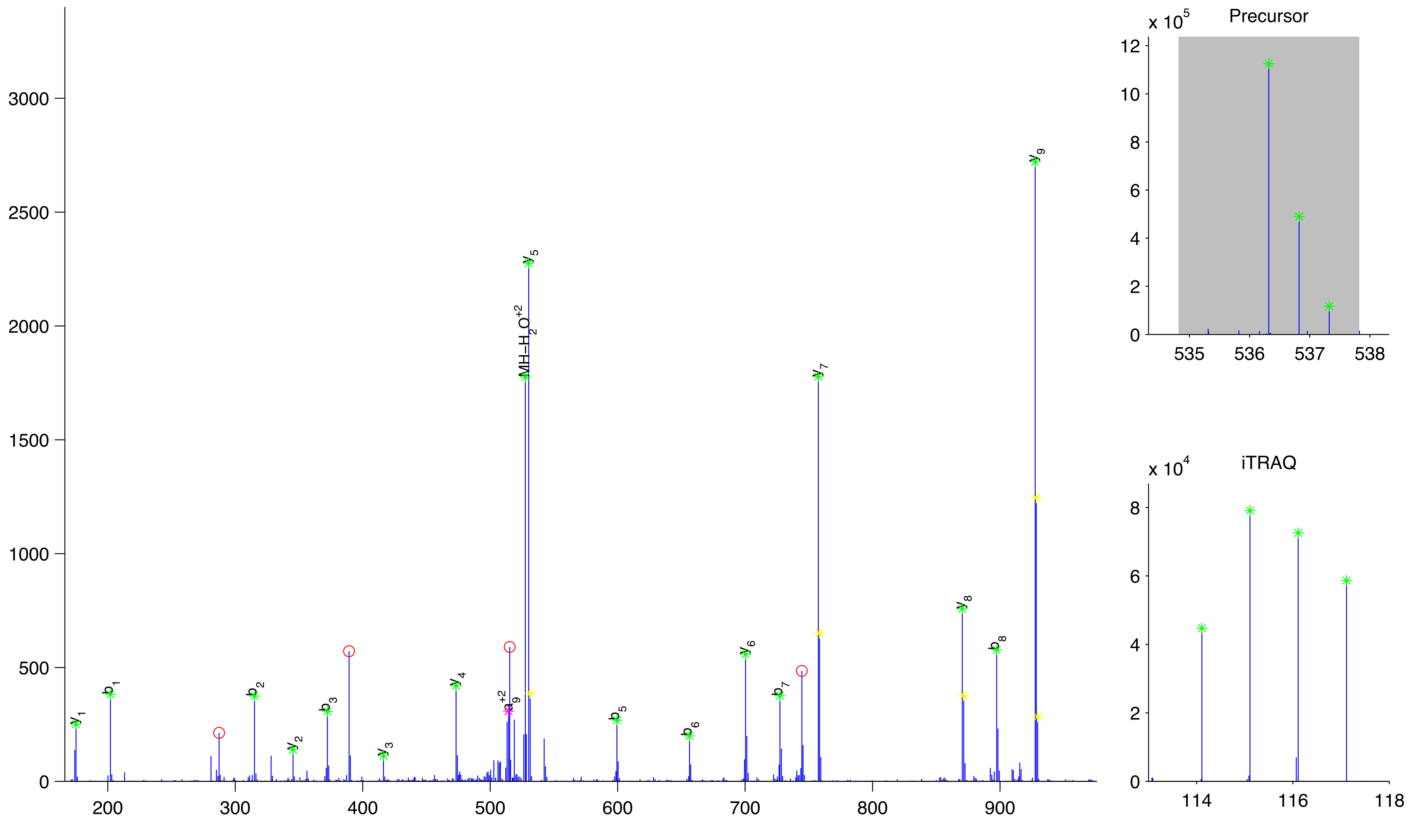

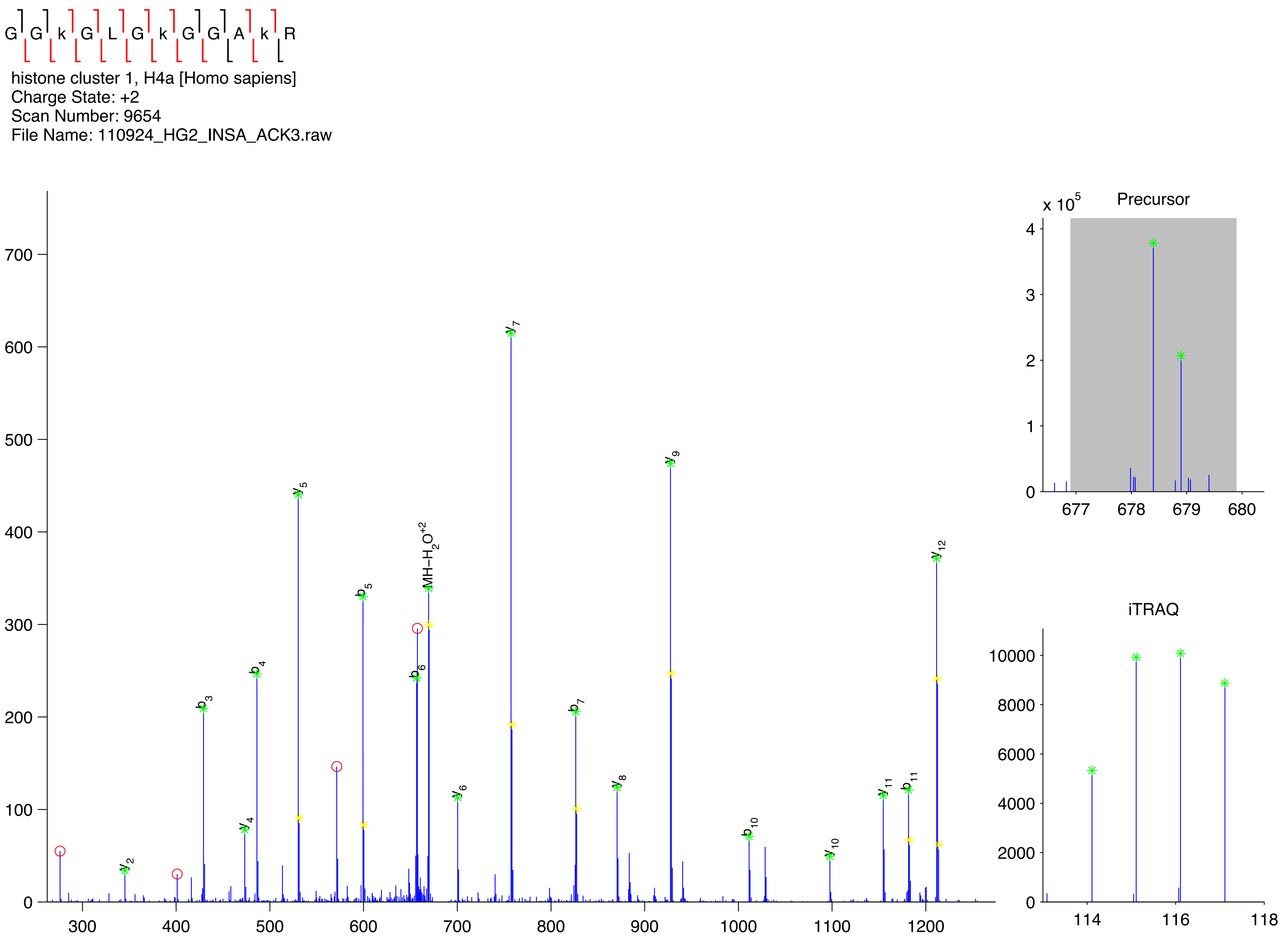
G G k G L G k G G A k R
histone cluster 1, H4a [Homo sapiens]
Charge State: +2
Scan Number: 9654
File Name: 110924_HG2_INSA_ACK3.raw
Precursor
x 10^5
iTRAQ

G G k G L G k G G A k R

histone cluster 1, H4a [Homo sapiens]
Charge State: +2
Scan Number: 9806
File Name: 110924_HG2_INSA_ACK3.raw

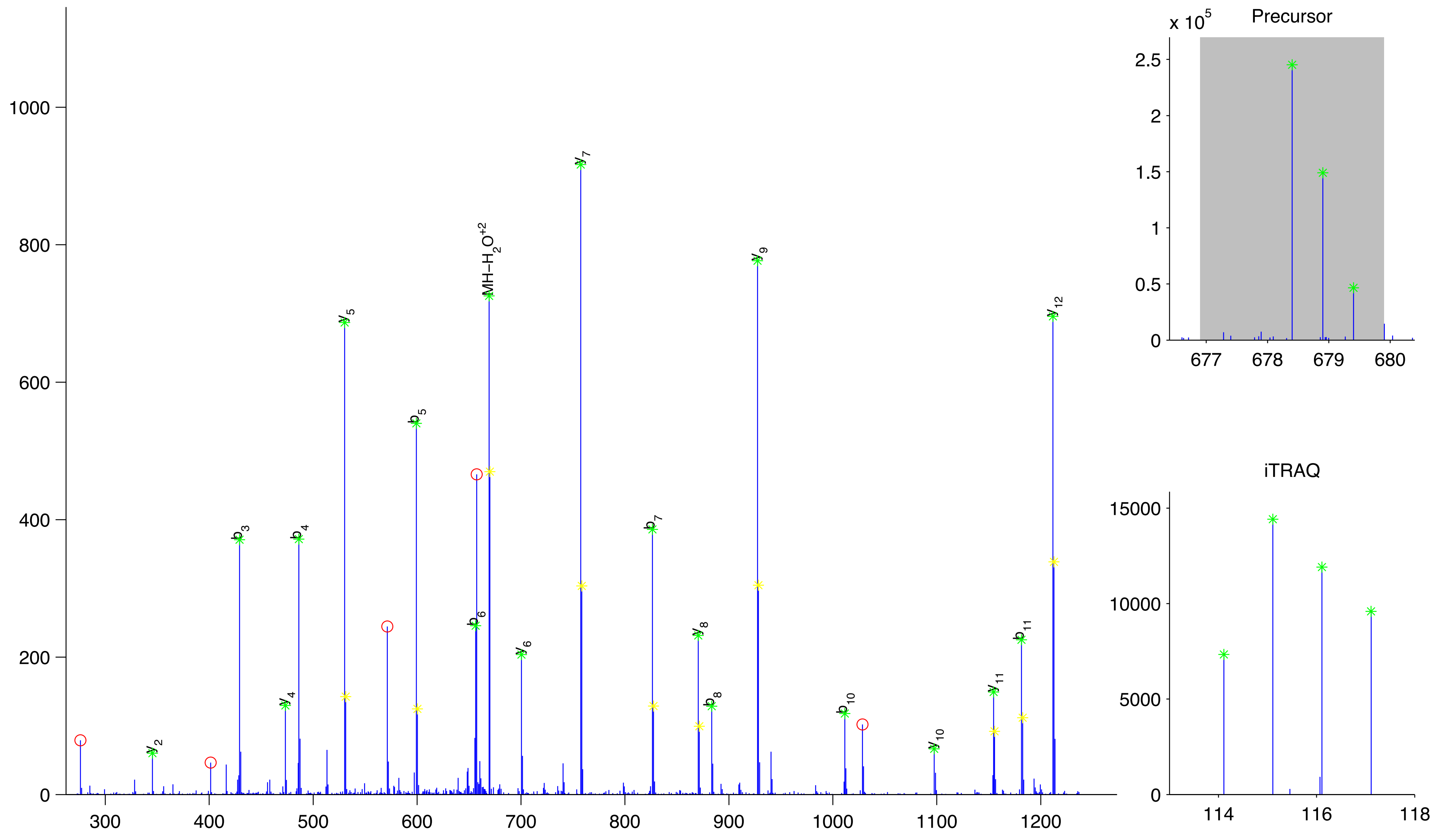

G L G k G G A k R

histone cluster 1, H4a [Homo sapiens]
Charge State: +2
Scan Number: 9953
File Name: 110924_HG2_INSA_ACK3.raw

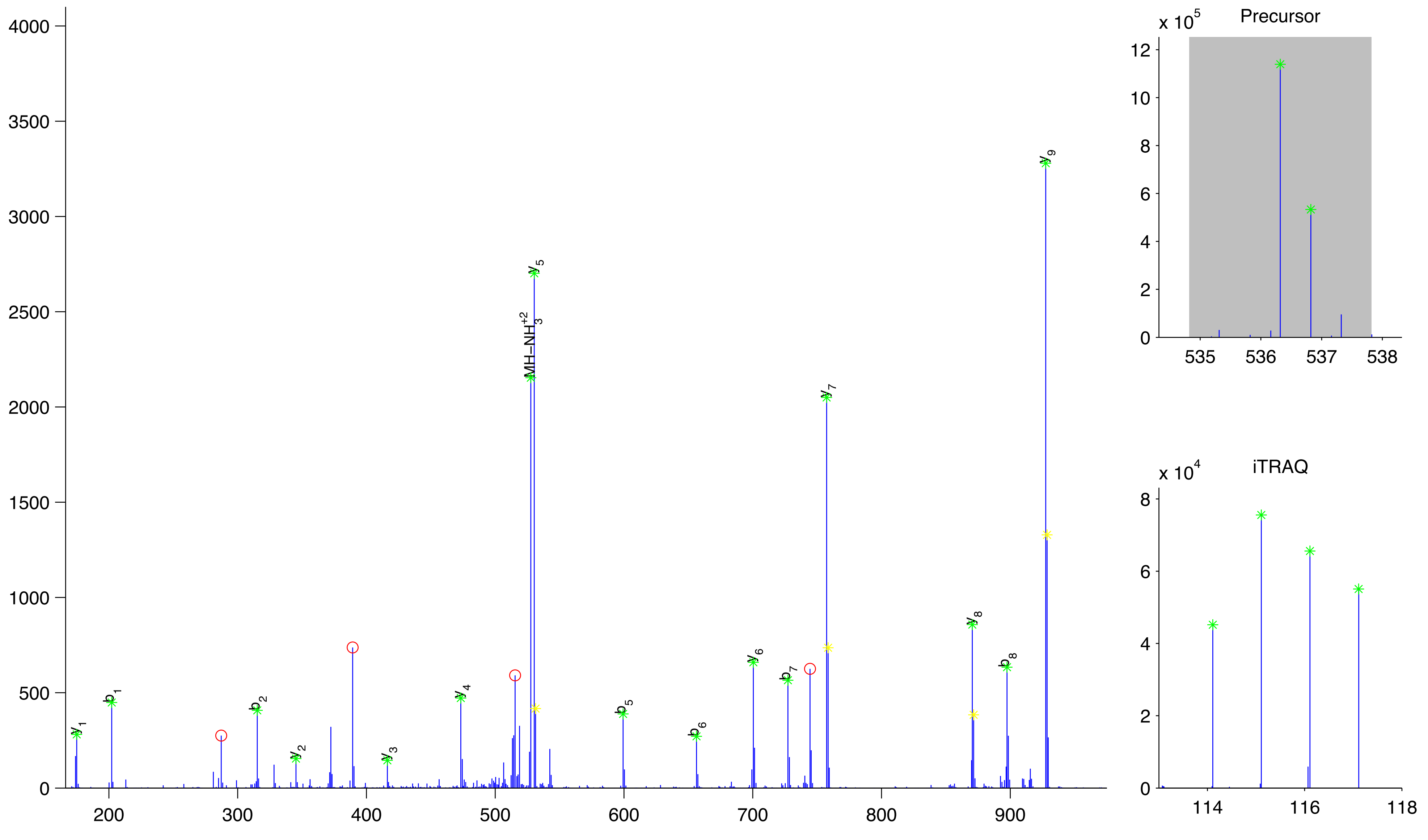

G G k G L G k G G A k R

histone cluster 1, H4a [Homo sapiens]
Charge State: +2
Scan Number: 9978
File Name: 110924_HG2_INSA_ACK3.raw

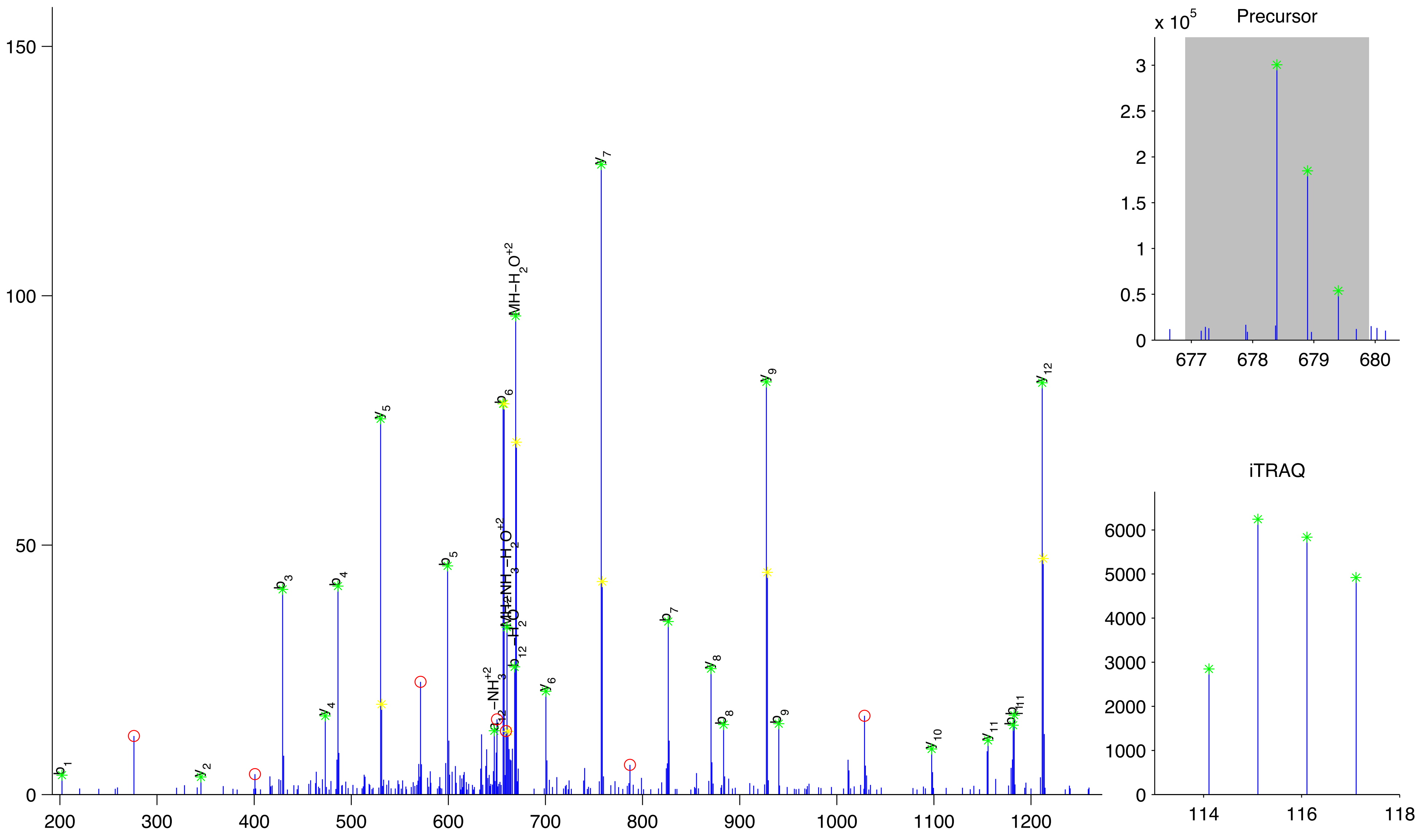

G L G k G G A k R

histone cluster 1, H4a [Homo sapiens]
Charge State: +2
Scan Number: 9992
File Name: 111011_HG2_INSB_ACK.raw

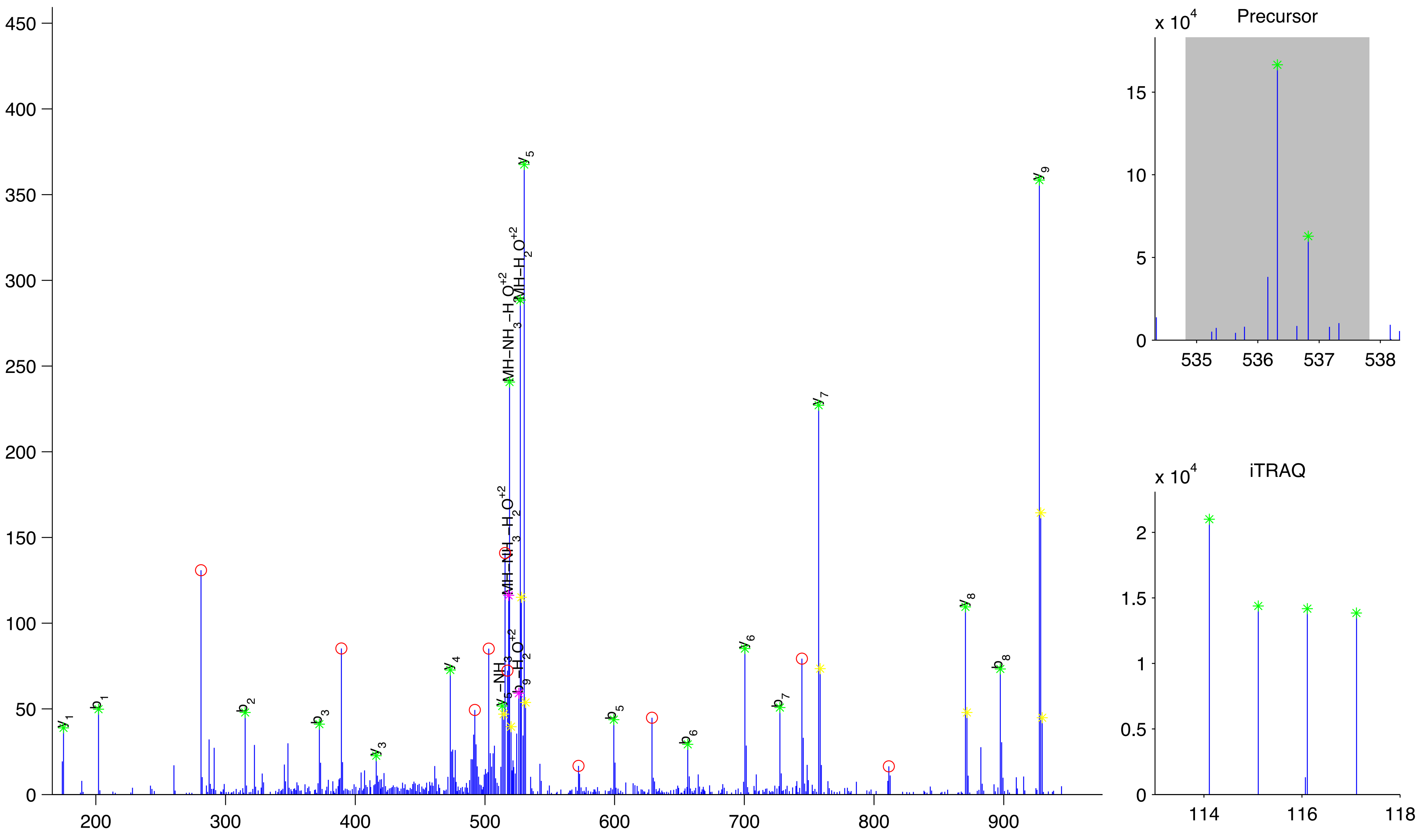

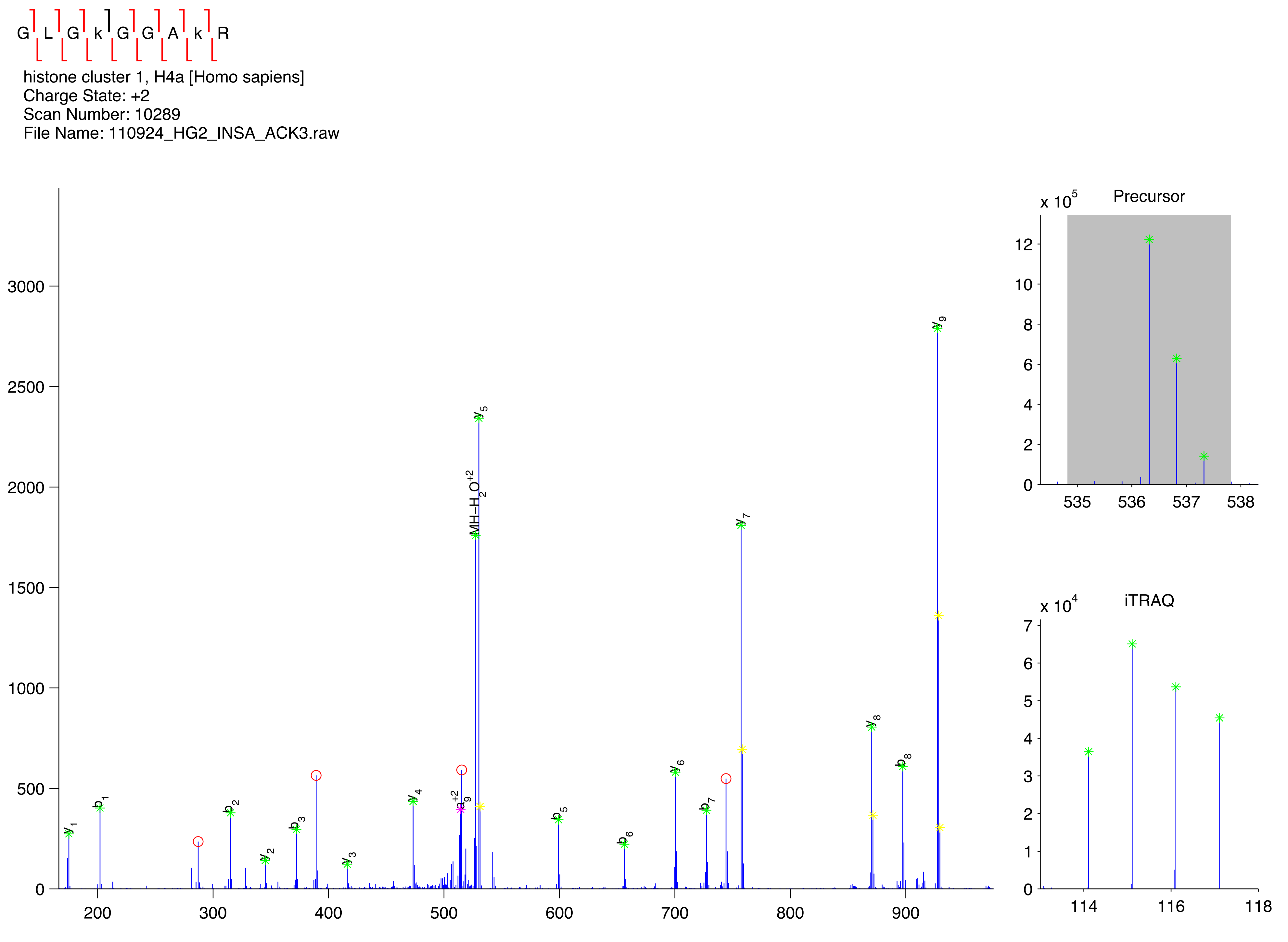
G L G k G G A k R
histone cluster 1, H4a [Homo sapiens]
Charge State: +2
Scan Number: 10289
File Name: 110924_HG2_INSA_ACK3.raw
Precursor
iTRAQ
MH-H2O+2

G G k G L G k G G A k R

histone cluster 1, H4a [Homo sapiens]
Charge State: +2
Scan Number: 10352
File Name: 110924_HG2_INSA_ACK3.raw

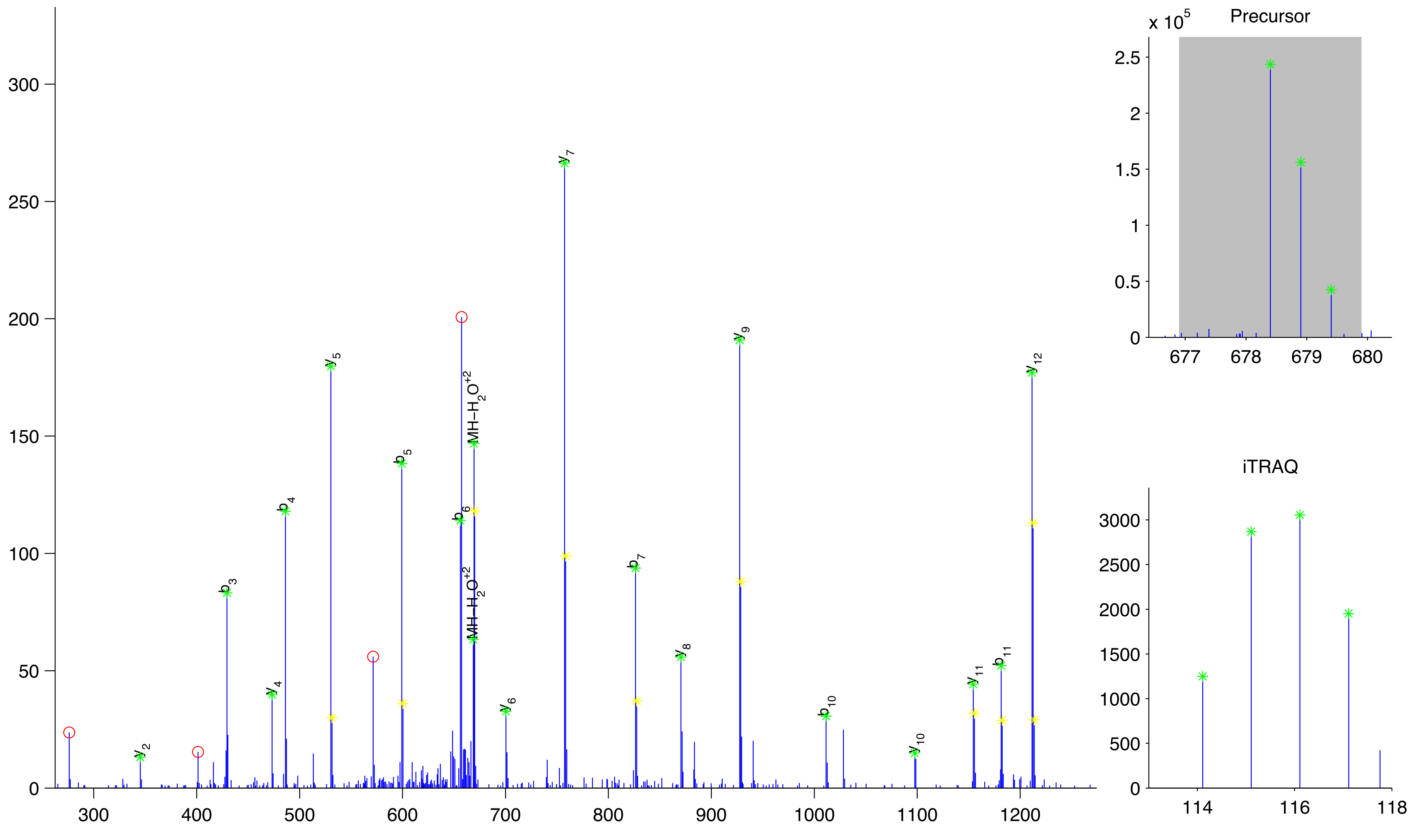

G L G k G G A k R

histone cluster 1, H4a [Homo sapiens]
Charge State: +2
Scan Number: 10625
File Name: 110924_HG2_INSA_ACK3.raw

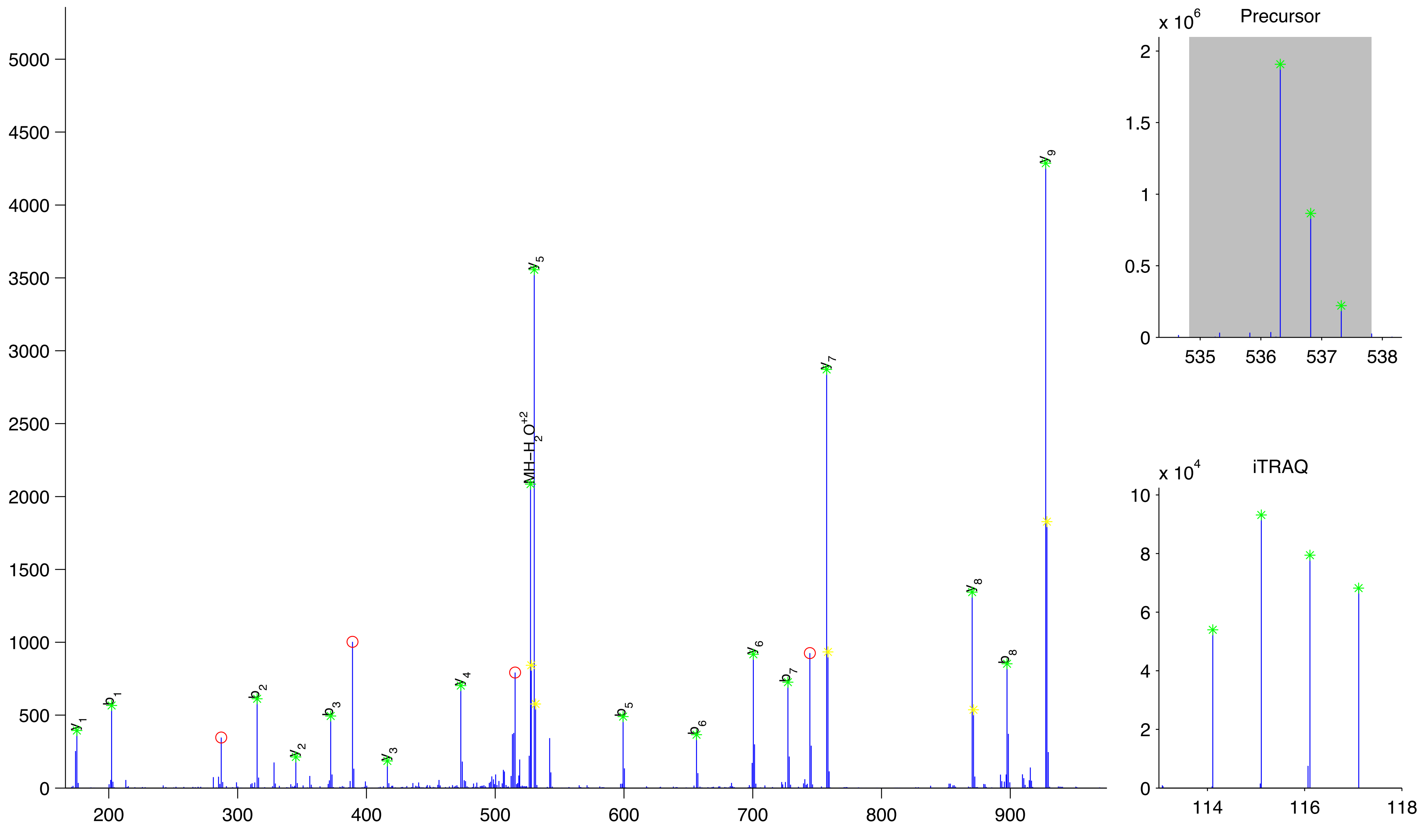

G G k G L G k G G A k R

histone cluster 1, H4a [Homo sapiens]
Charge State: +2
Scan Number: 10709
File Name: 110924_HG2_INSA_ACK3.raw

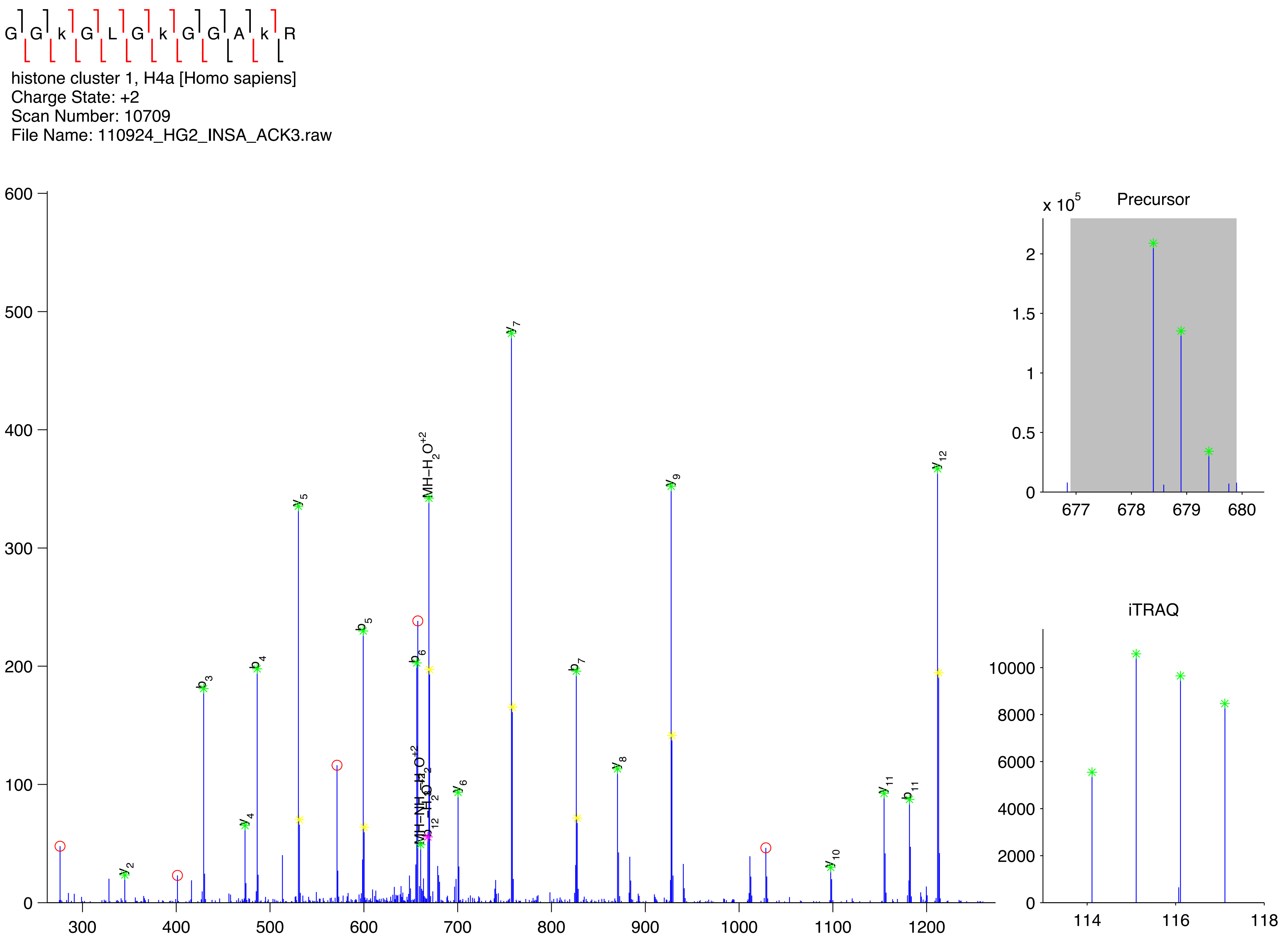

G k G G k G L G k G G A k R

histone cluster 1, H4a [Homo sapiens]
Charge State: +3
Scan Number: 10755
File Name: 110924_HG2_INSA_ACK3.raw

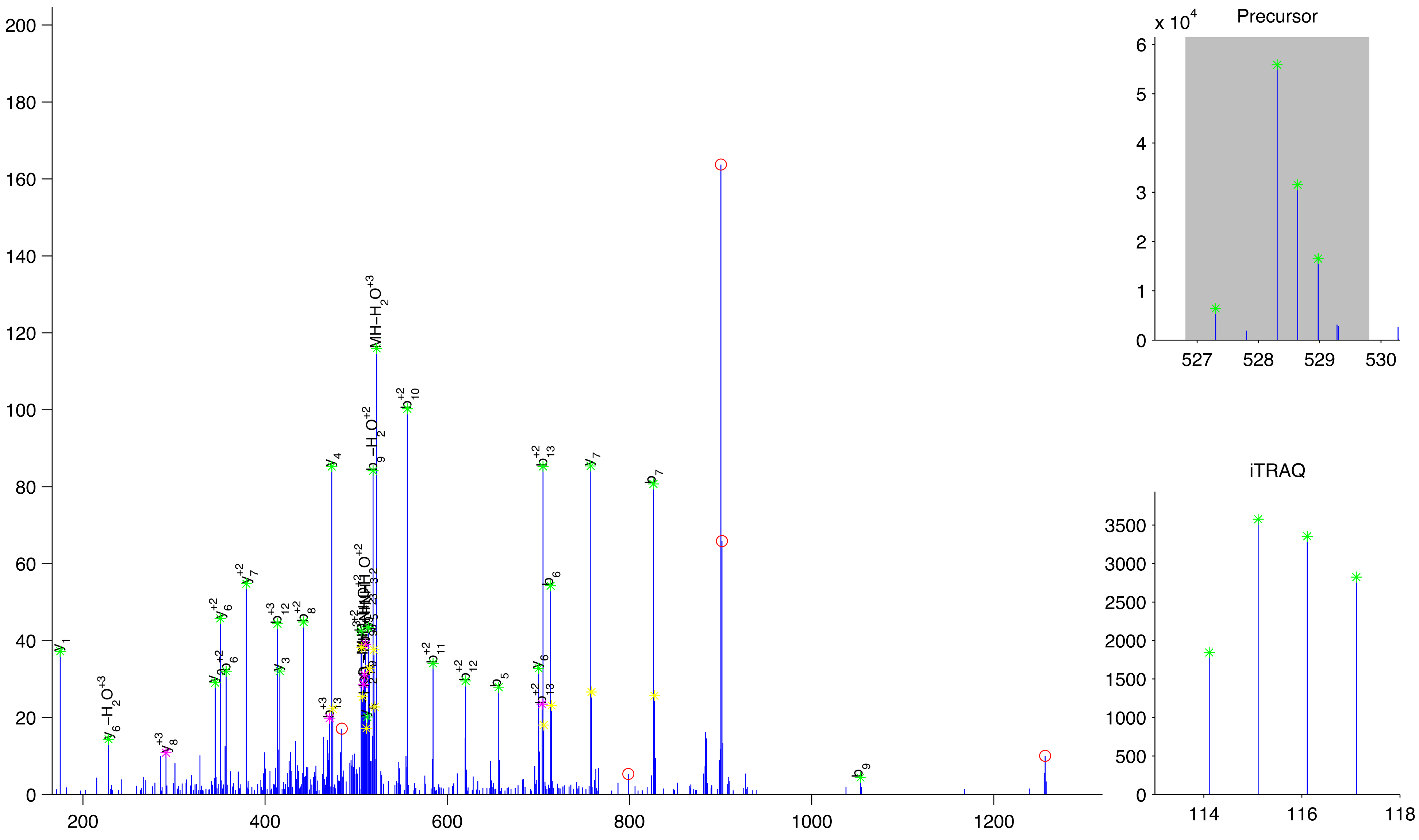

G G k G L G k G G A k R

histone cluster 1, H4a [Homo sapiens]
Charge State: +2
Scan Number: 10773
File Name: 111011_HG2_INSB_ACK.raw

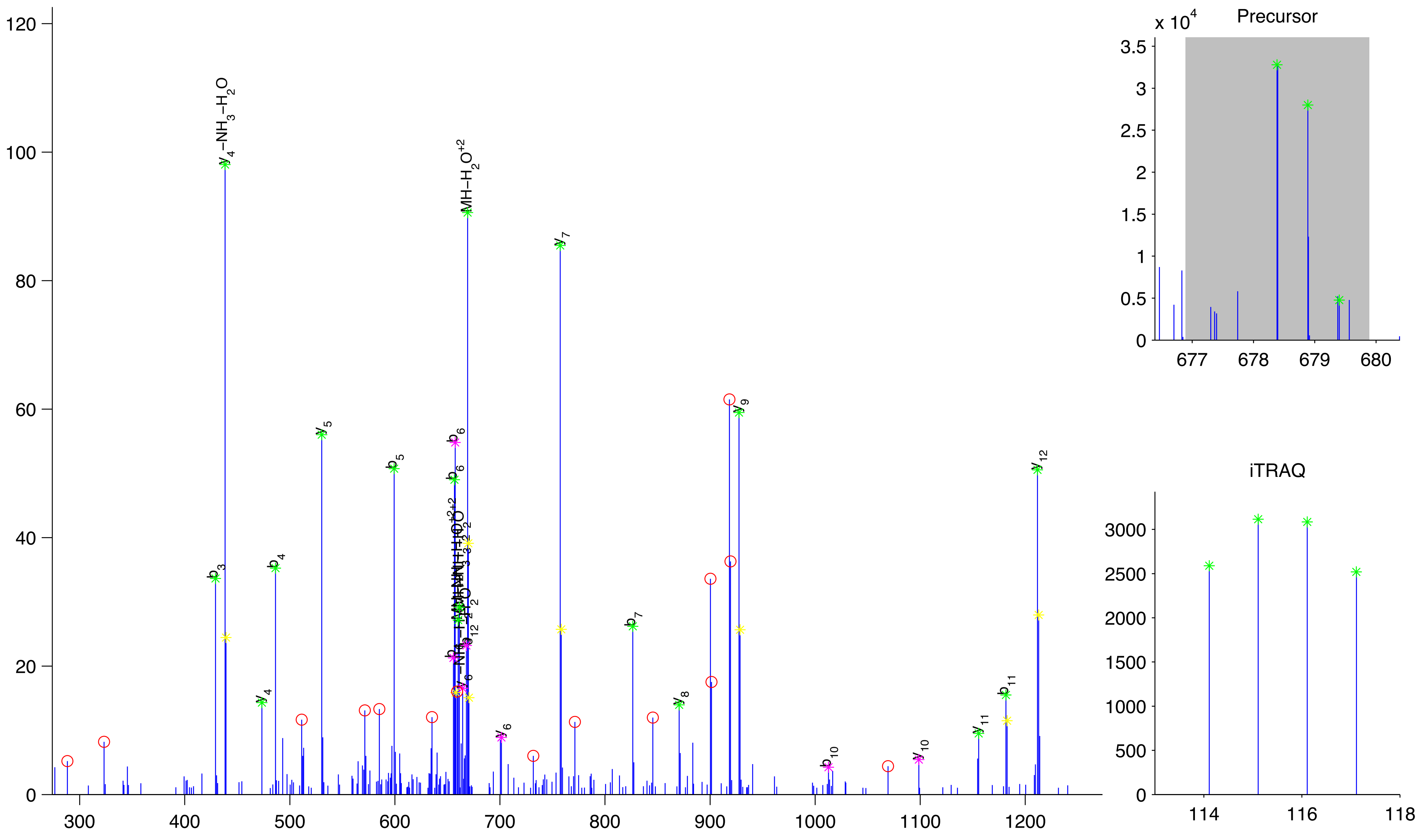

G G k G L G k G G A k R

histone cluster 1, H4a [Homo sapiens]
Charge State: +2
Scan Number: 10879
File Name: 110924_HG2_INSA_ACK3.raw

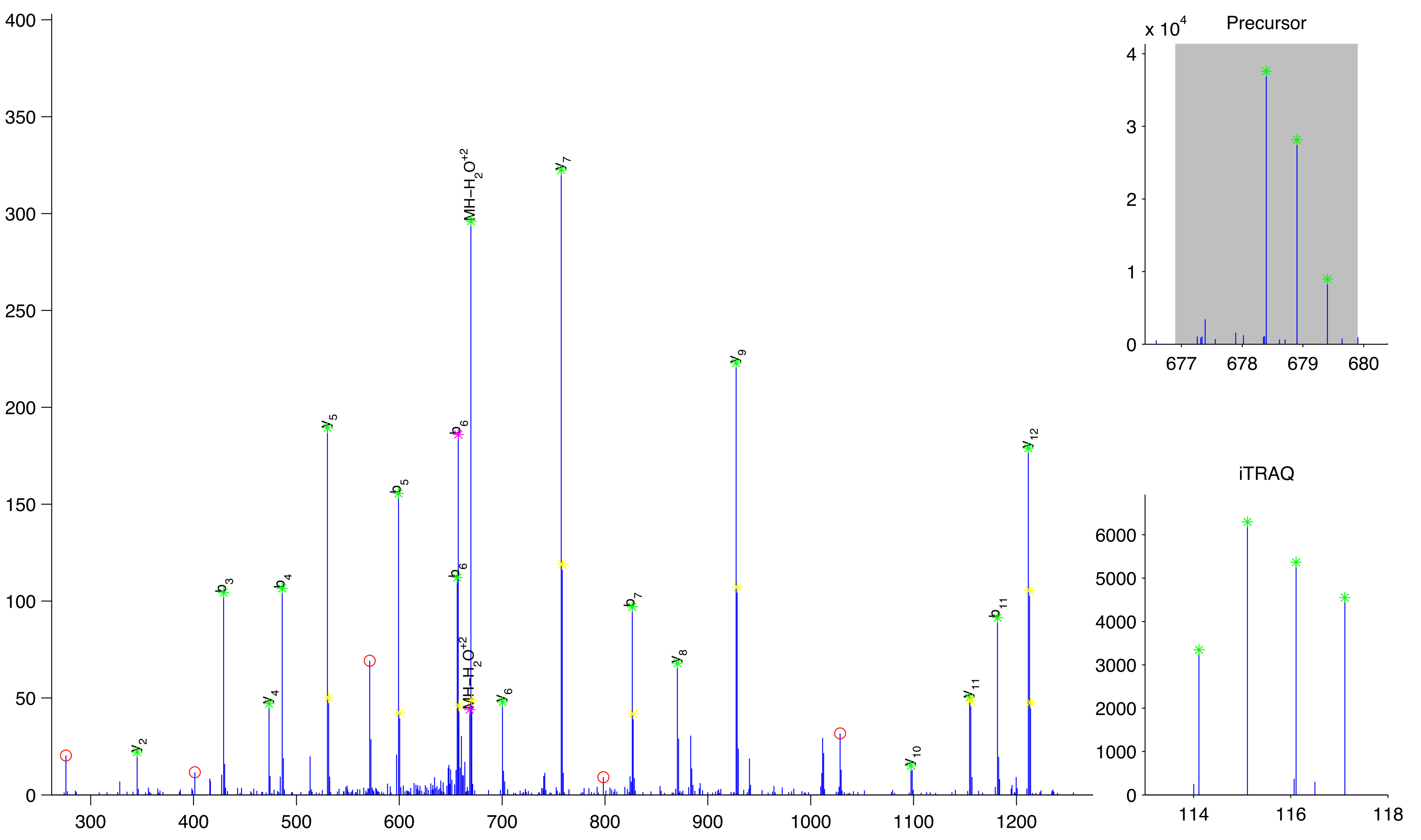

G L G k G G A k R

histone cluster 1, H4a [Homo sapiens]
Charge State: +2
Scan Number: 10961
File Name: 110924_HG2_INSA_ACK3.raw

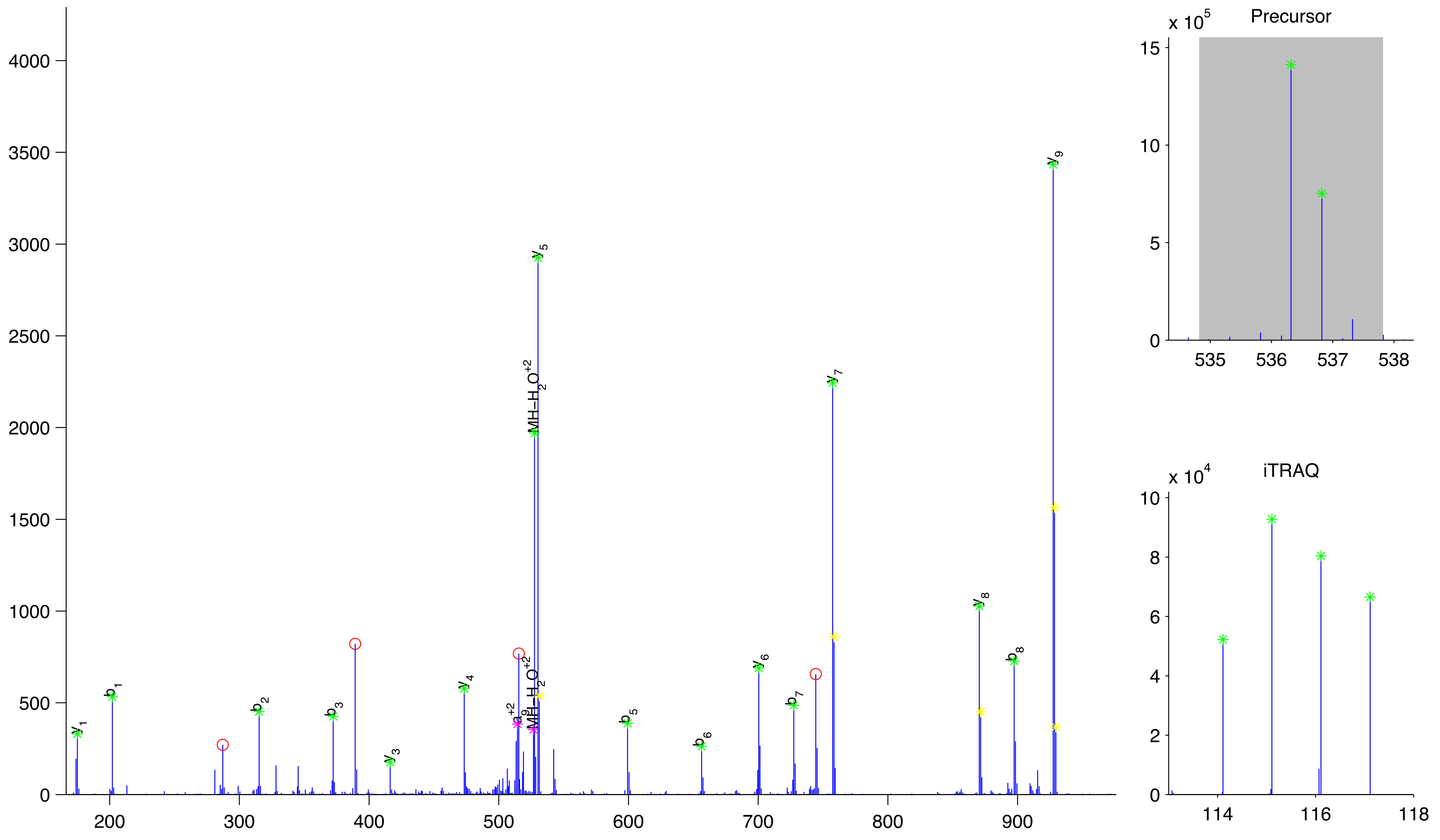

G G k G L G k G G A k R

histone cluster 1, H4a [Homo sapiens]
Charge State: +2
Scan Number: 11045
File Name: 110924_HG2_INSA_ACK3.raw

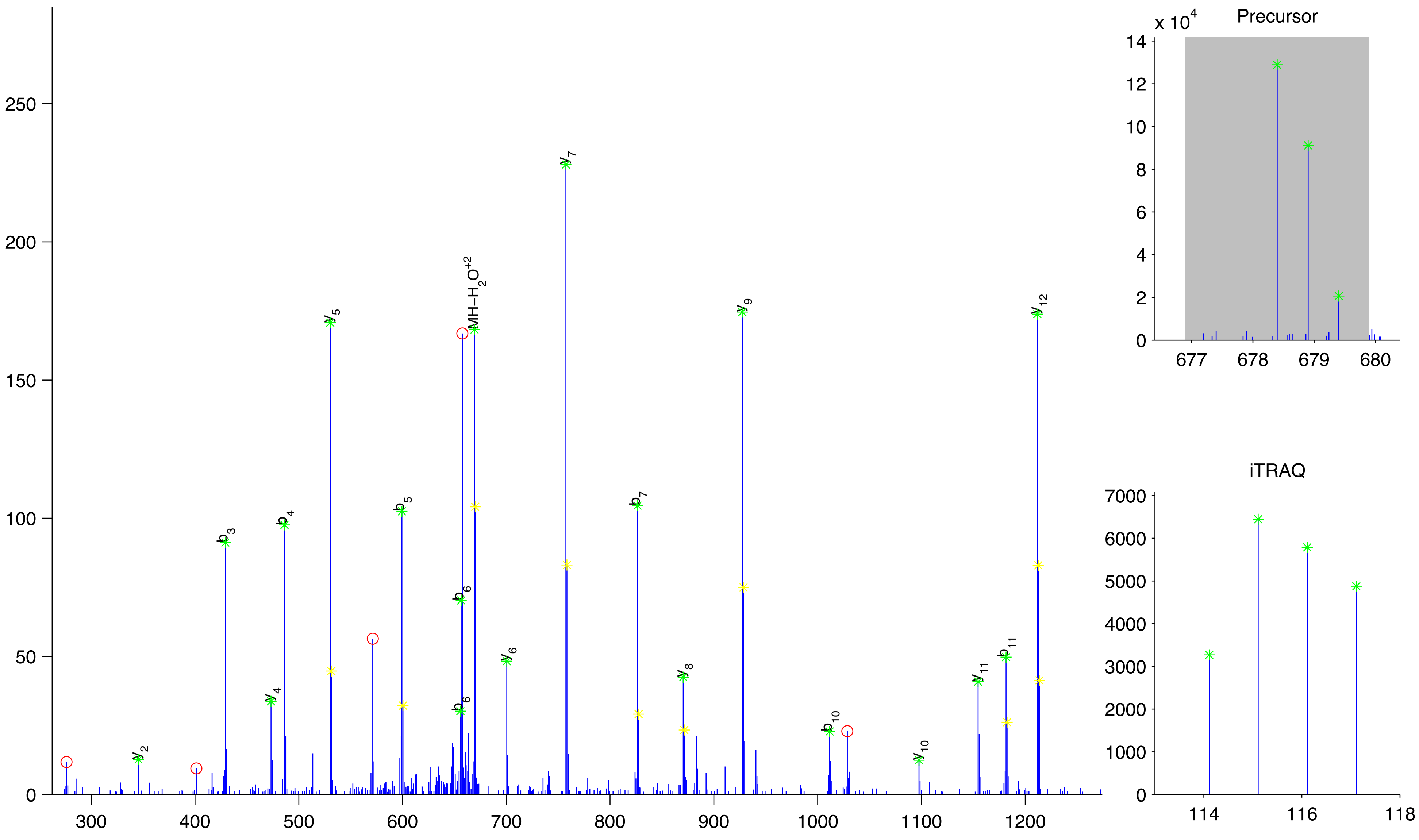

G k G G k G L G k G G A k R

histone cluster 1, H4a [Homo sapiens]
Charge State: +3
Scan Number: 11114
File Name: 110924_HG2_INSA_ACK3.raw

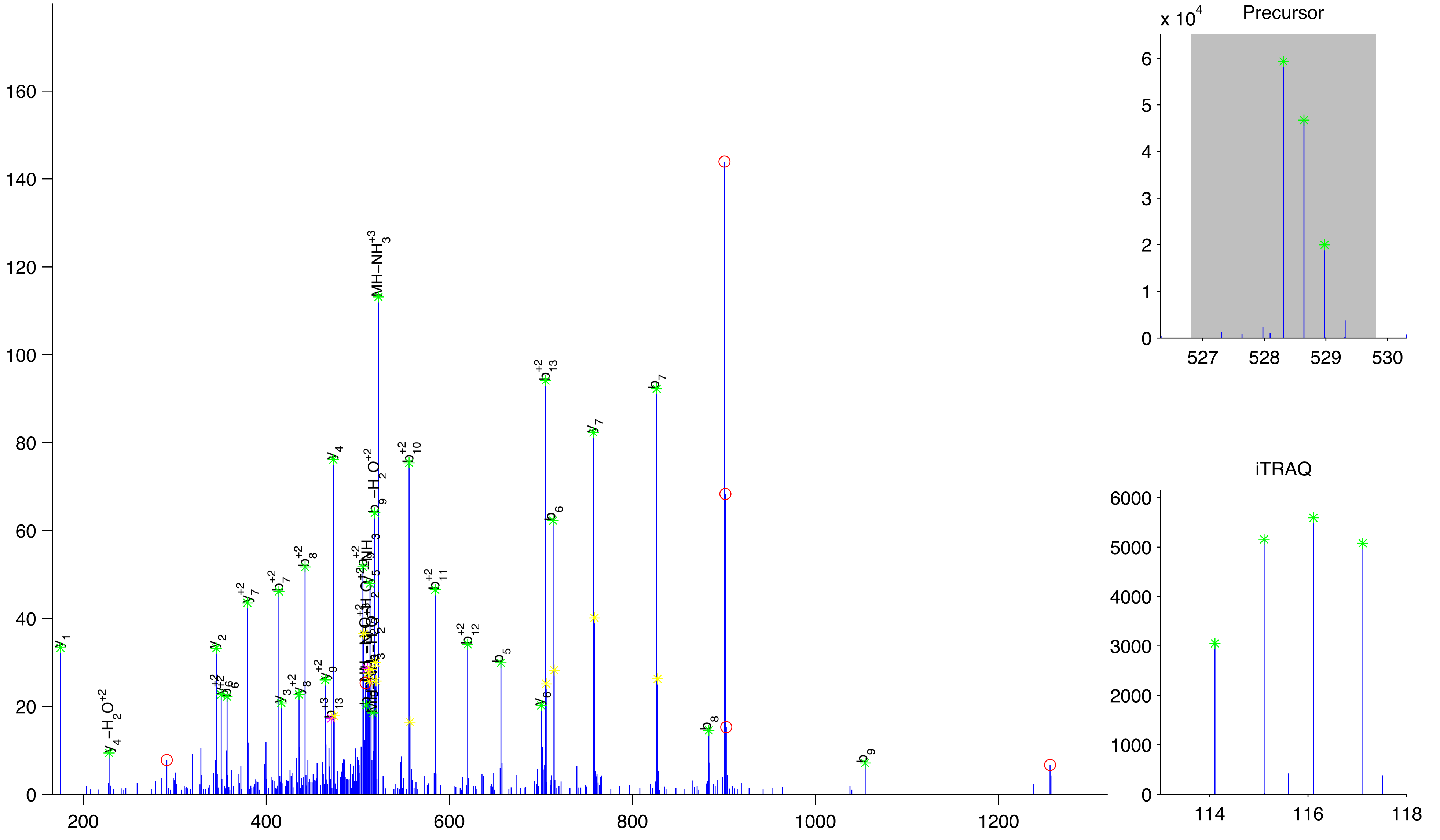

G G k G L G k G G A k R

histone cluster 1, H4a [Homo sapiens]
Charge State: +2
Scan Number: 11118
File Name: 111011_HG2_INSB_ACK.raw

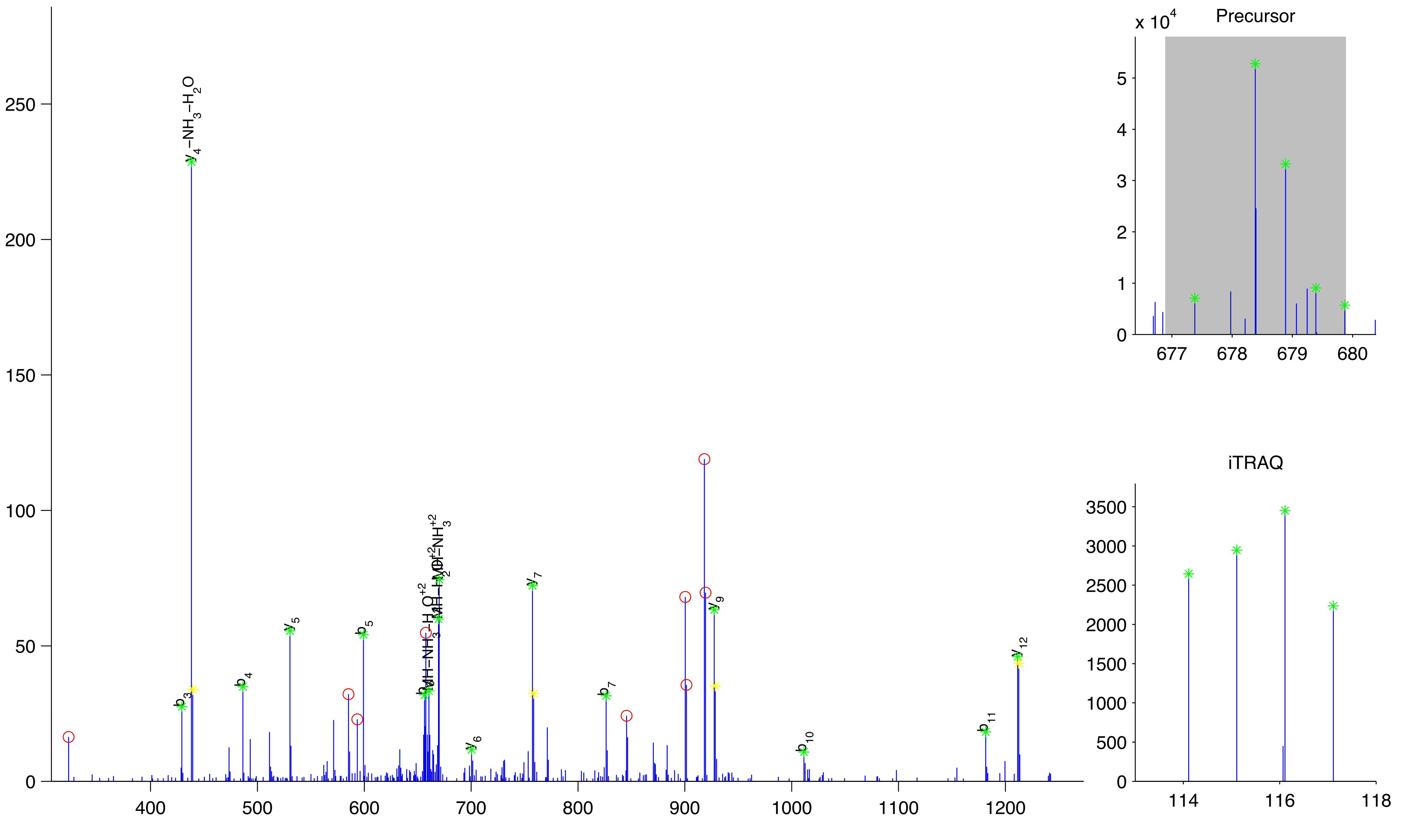

G L G k G G A k R

histone cluster 1, H4a [Homo sapiens]
Charge State: +2
Scan Number: 11129
File Name: 110924_HG2_INSA_ACK3.raw

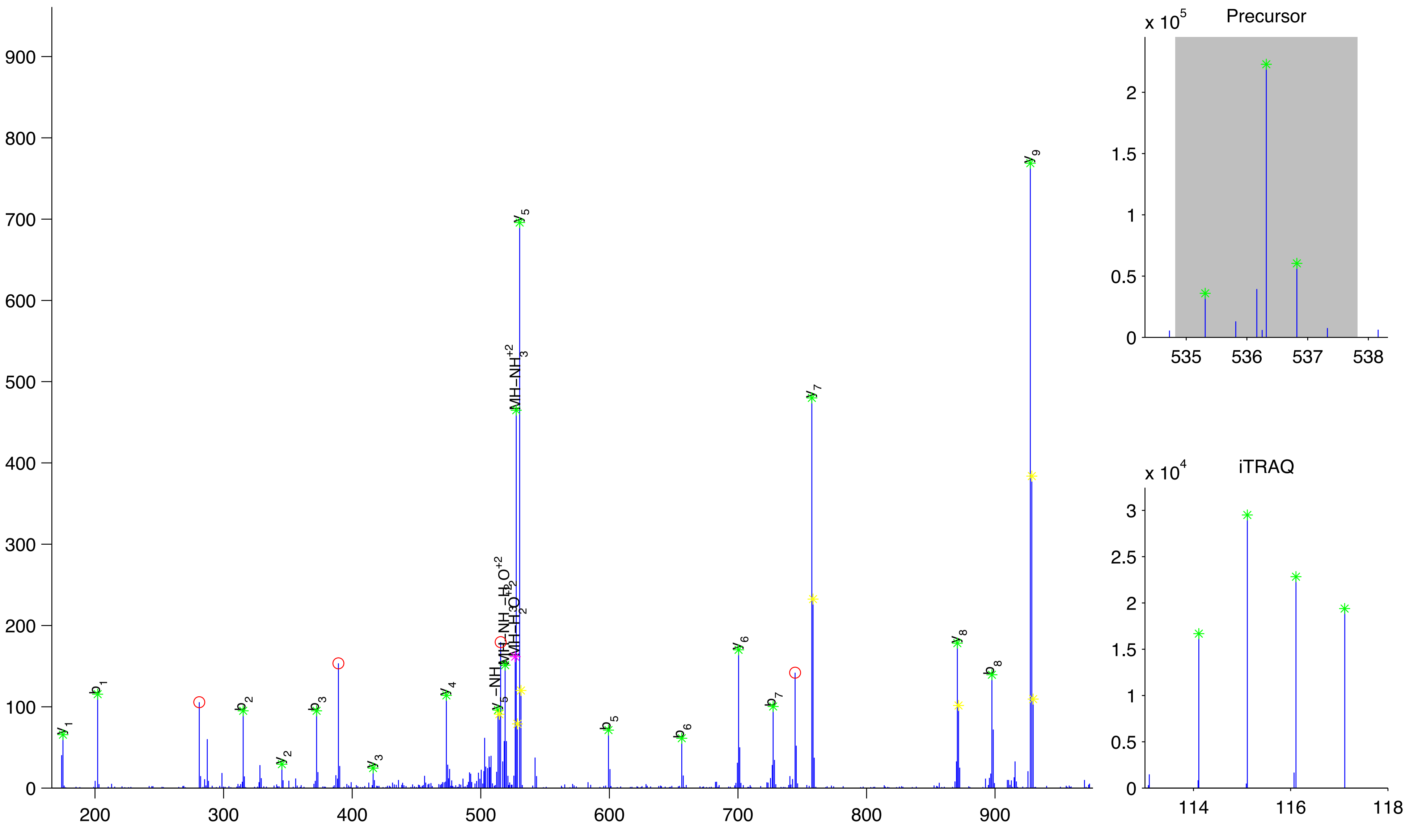

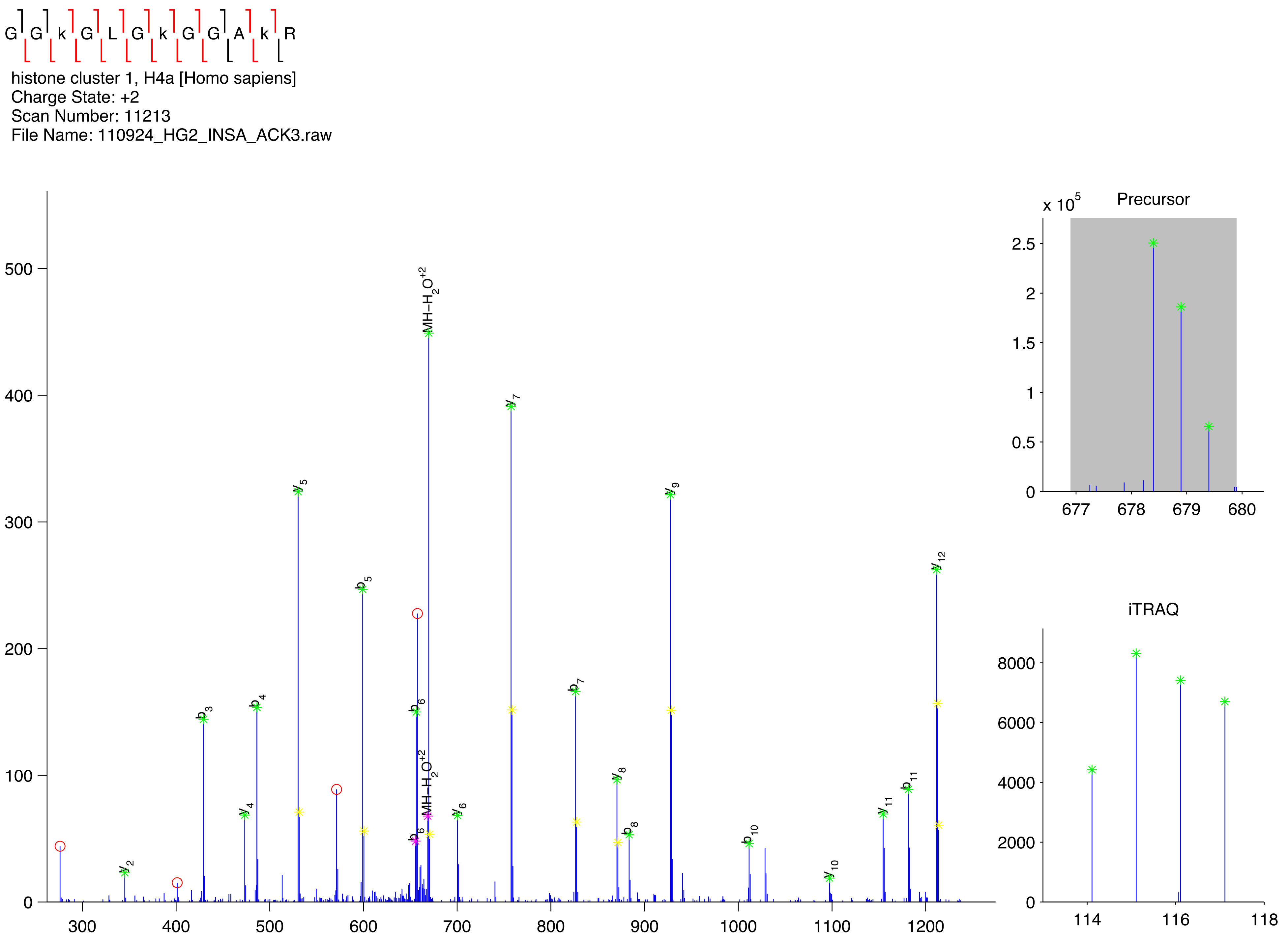
G G k G L G k G G A k R
histone cluster 1, H4a [Homo sapiens]
Charge State: +2
Scan Number: 11213
File Name: 110924_HG2_INSA_ACK3.raw
Precursor
x 10^5
iTRAQ
MH−H2O+2
y2 b3 y4 b4 y5 b5 b6 y6 y7 b7 y8 b8 y9 b10 y10 y11 b11 y12

G k G G k G L G k G G A k R

histone cluster 1, H4a [Homo sapiens]
Charge State: +3
Scan Number: 11282
File Name: 110924_HG2_INSA_ACK3.raw

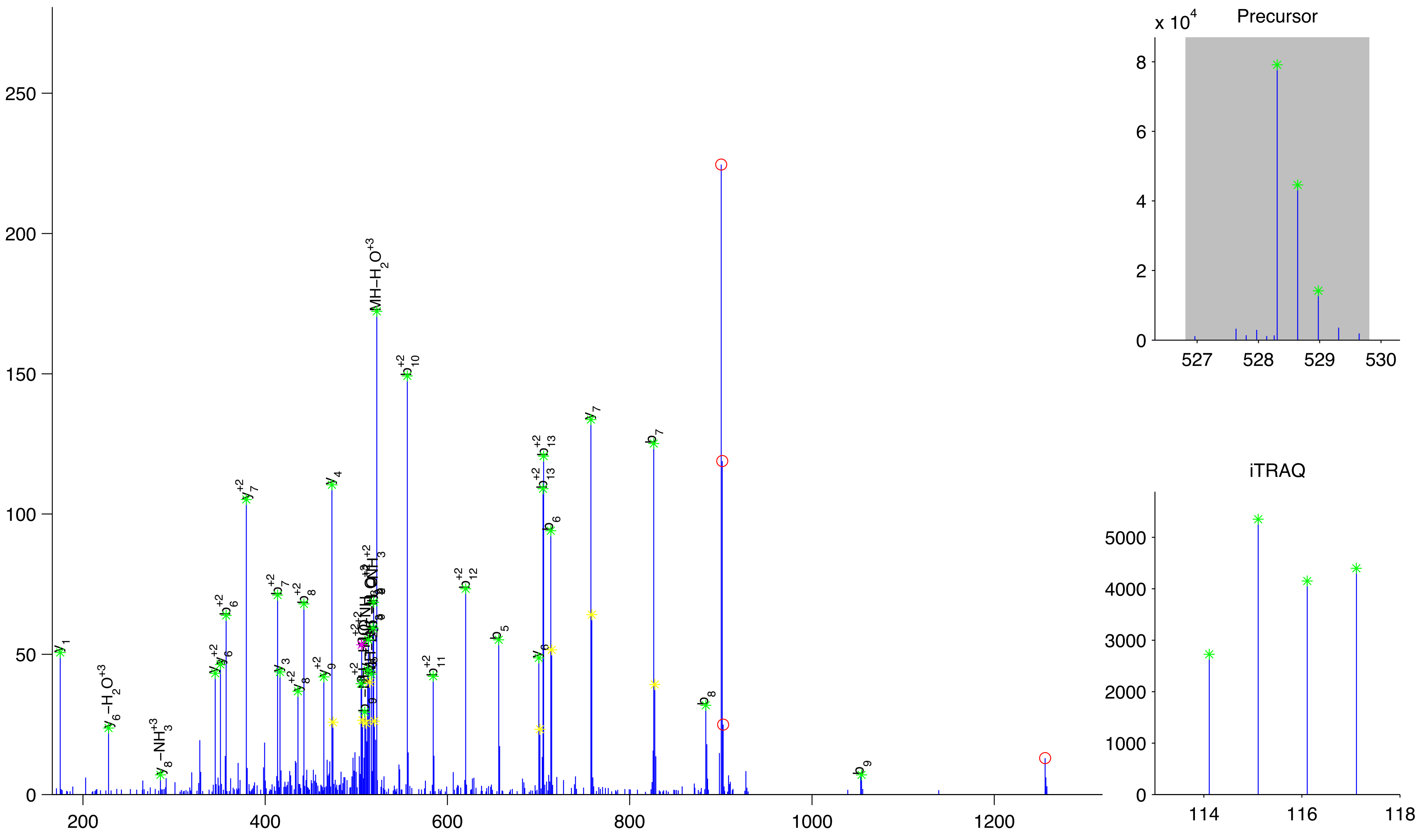

G G k G L G k G G A k R

histone cluster 1, H4a [Homo sapiens]
Charge State: +2
Scan Number: 11292
File Name: 111011_HG2_INSB_ACK.raw

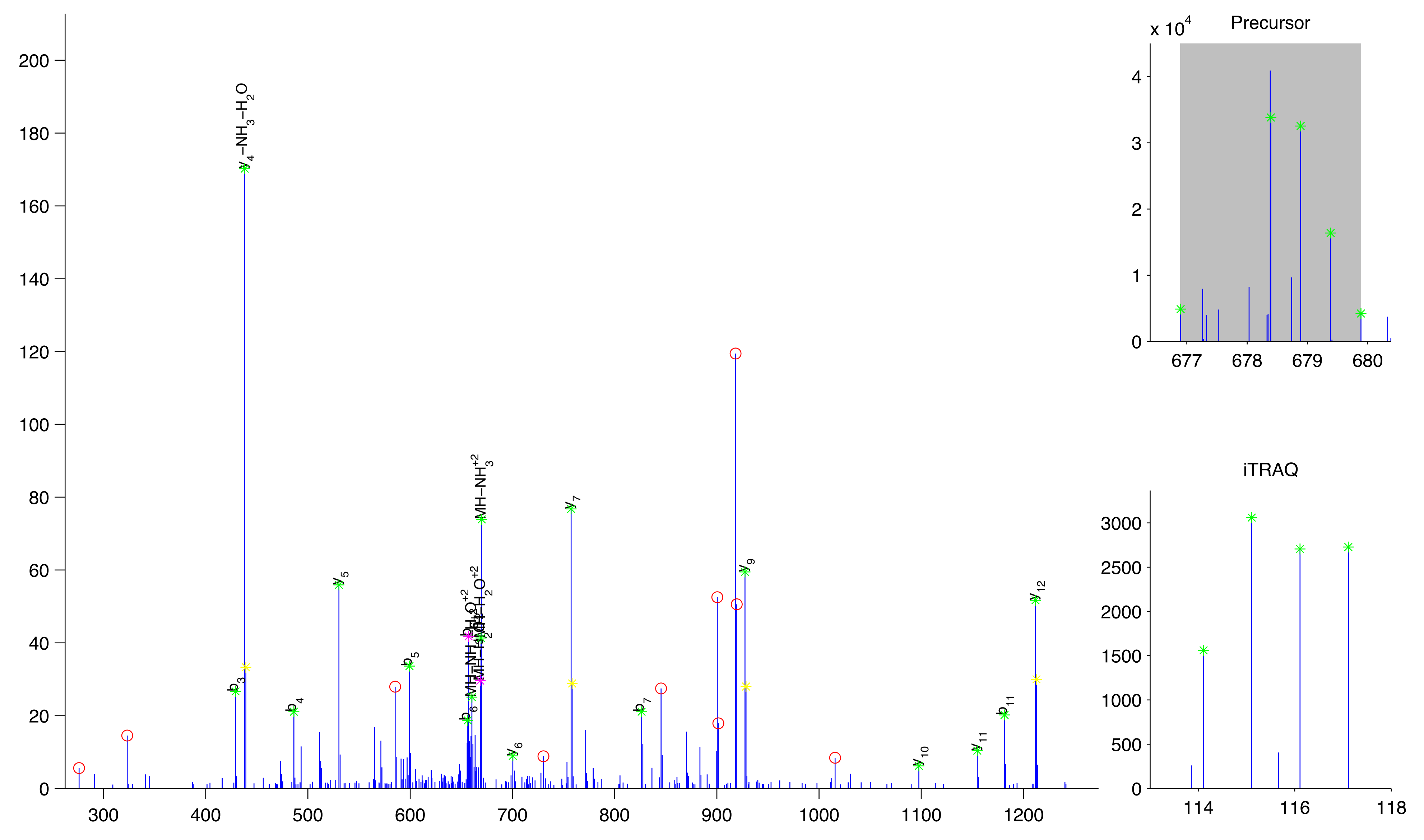

G L G k G G A k R

histone cluster 1, H4a [Homo sapiens]
Charge State: +2
Scan Number: 11297
File Name: 110924_HG2_INSA_ACK3.raw

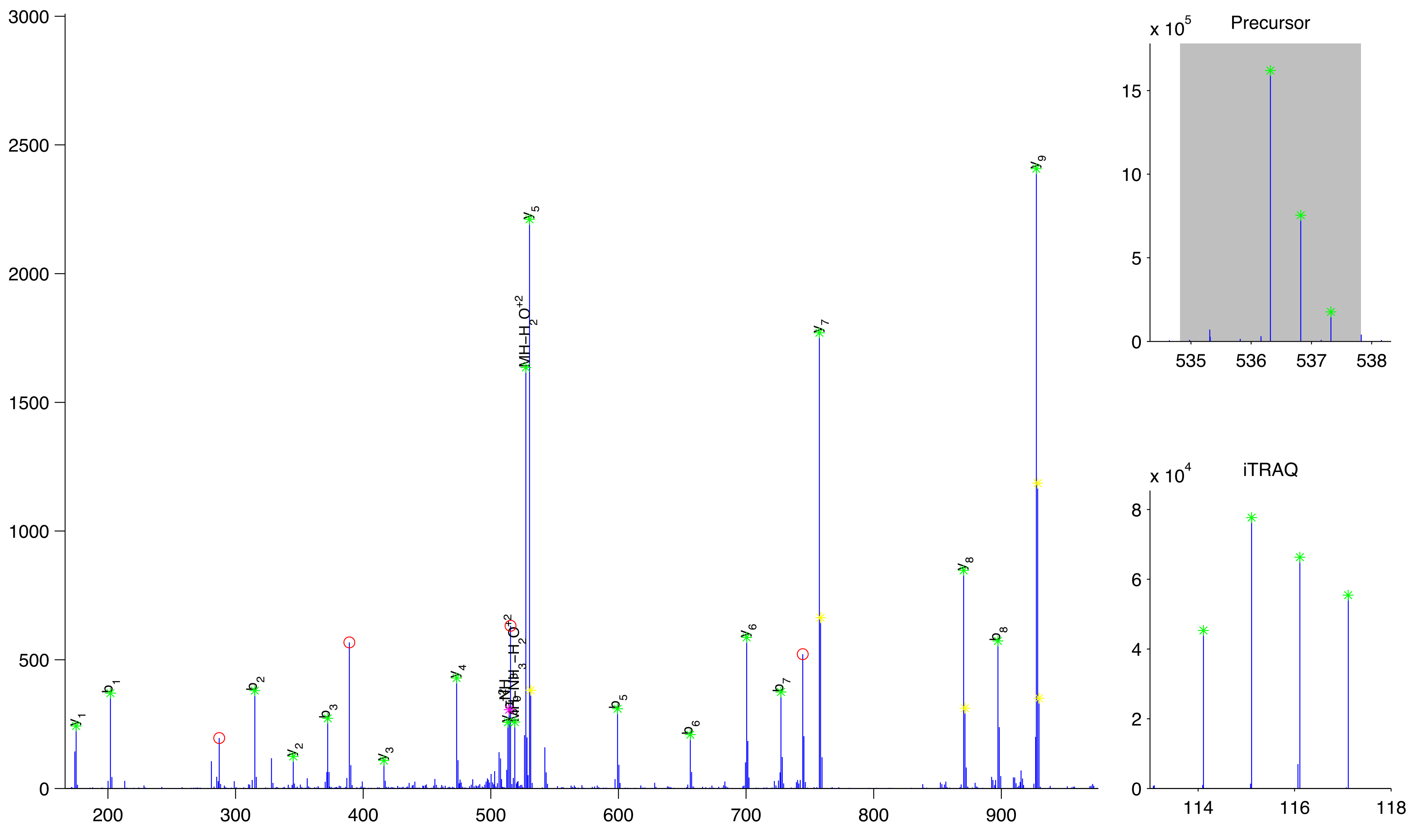

histone cluster 1, H4a [Homo sapiens]
Charge State: +2
Scan Number: 11381
File Name: 110924_HG2_INSA_ACK3.raw

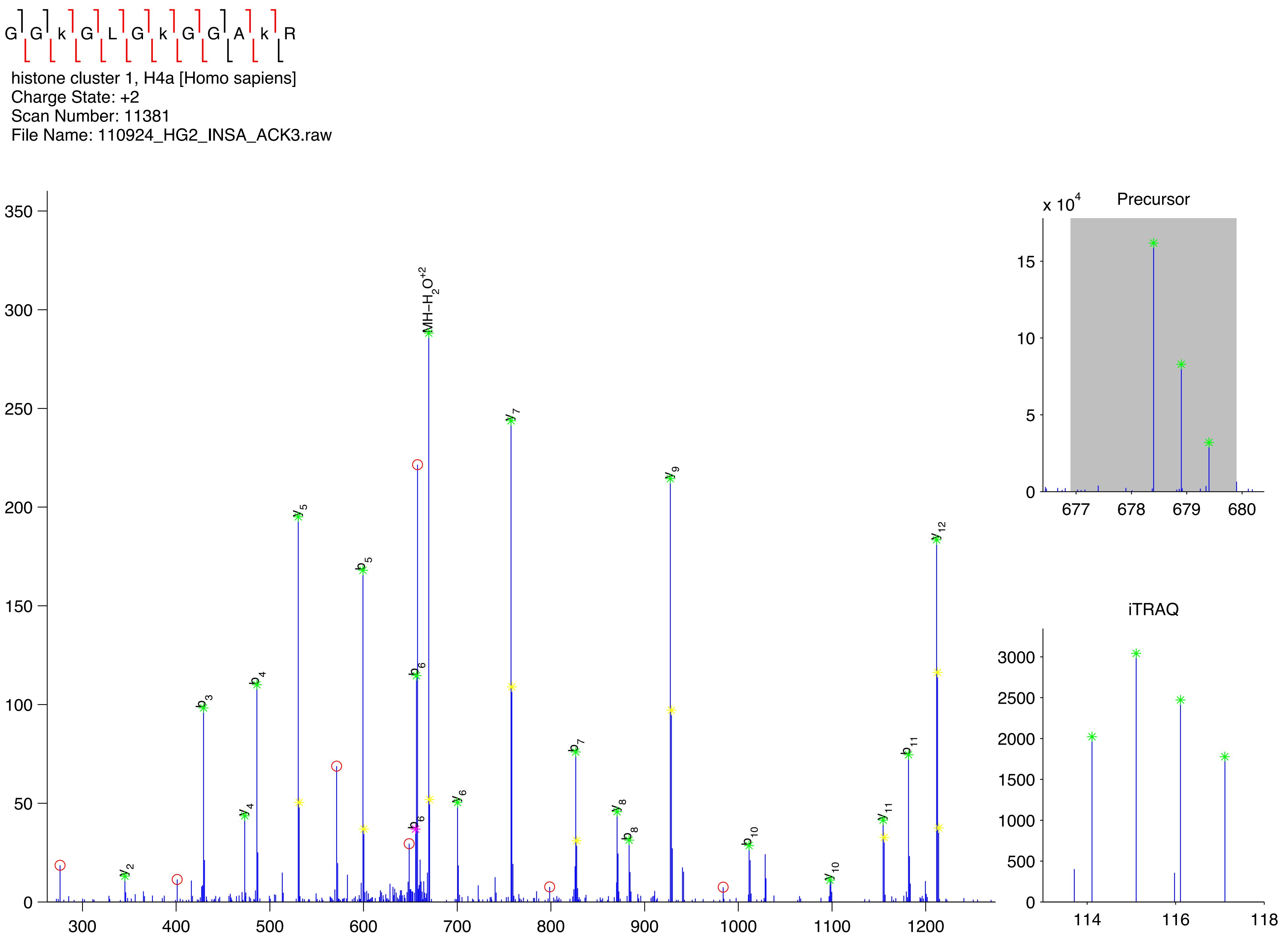

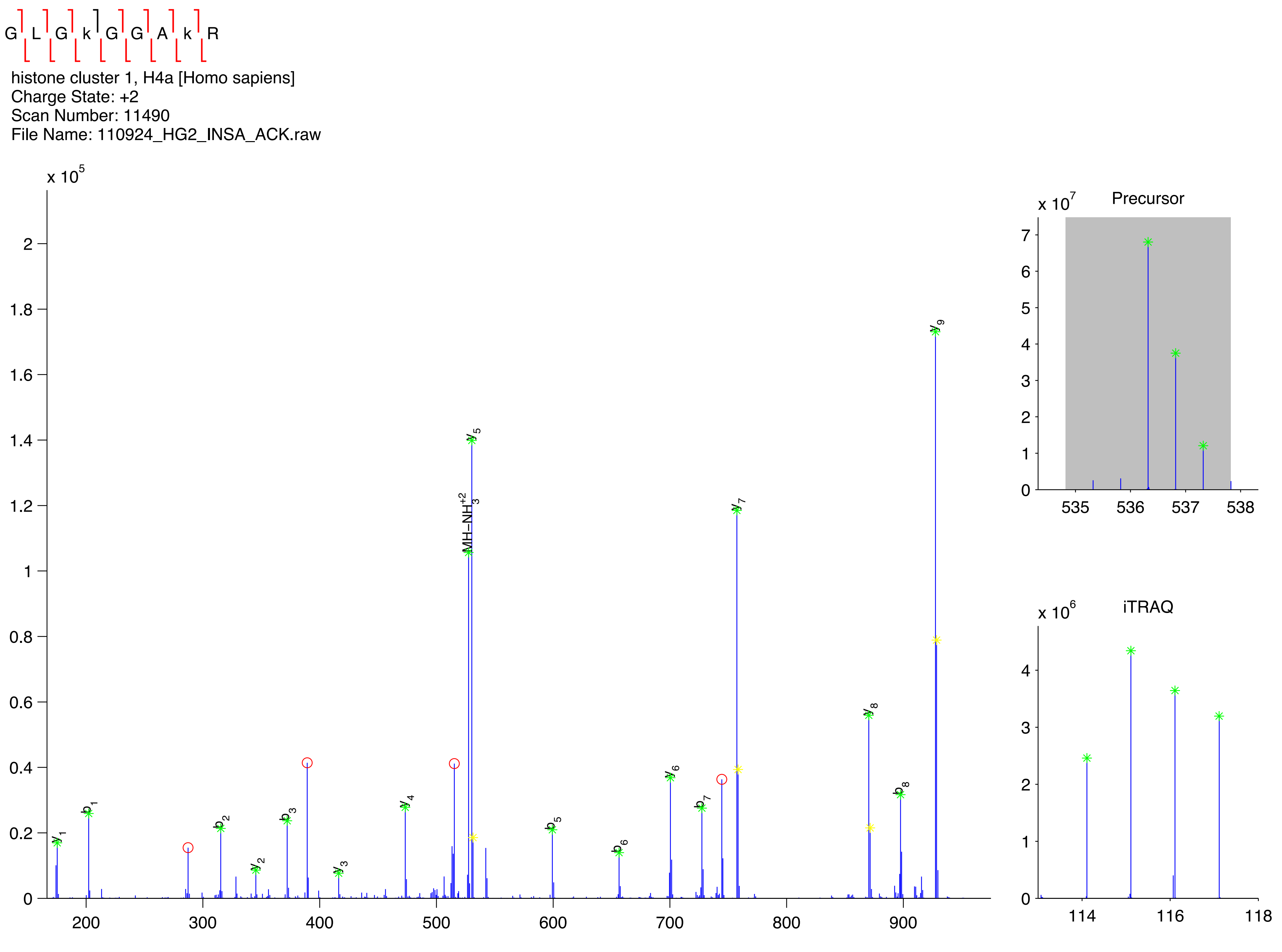
G L G k G G A k R
histone cluster 1, H4a [Homo sapiens]
Charge State: +2
Scan Number: 11490
File Name: 110924_HG2_INSA_ACK.raw
x 10^5
y1 b1 b2 y2 b3 y3 y4 MH-NH3+2 y5 b5 b6 y6 b7 y7 y8 b8 y9
200 300 400 500 600 700 800 900
0 0.2 0.4 0.6 0.8 1 1.2 1.4 1.6 1.8 2
x 10^7
Precursor
535 536 537 538
x 10^6
iTRAQ
114 116 118

G L G k G G A k R

histone cluster 1, H4a [Homo sapiens]
Charge State: +2
Scan Number: 11492
File Name: 110924_HG2_INSA_ACK.raw

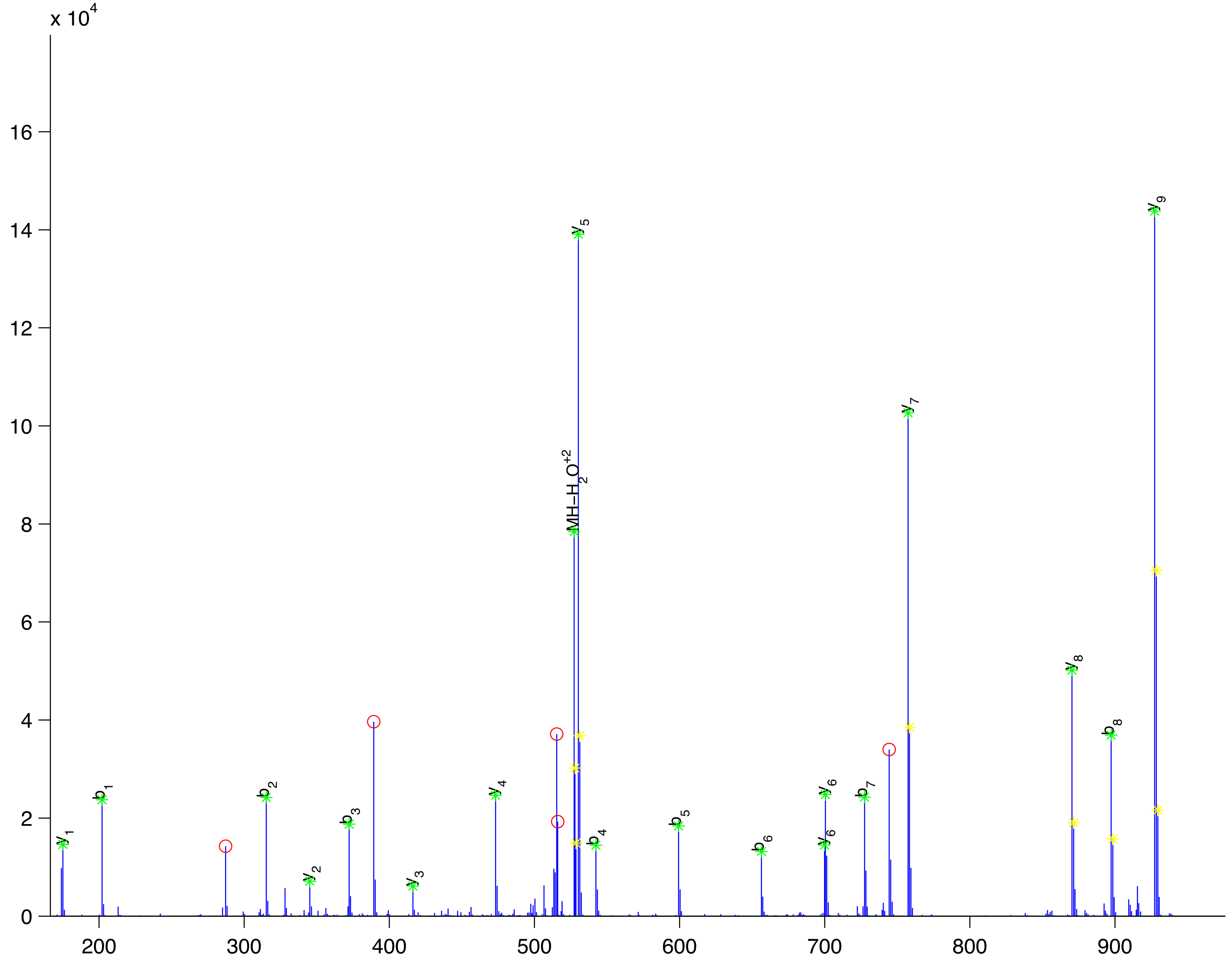

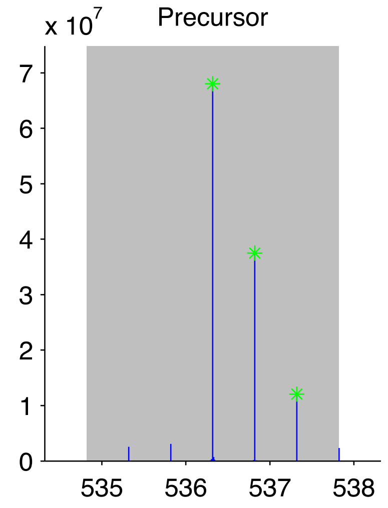

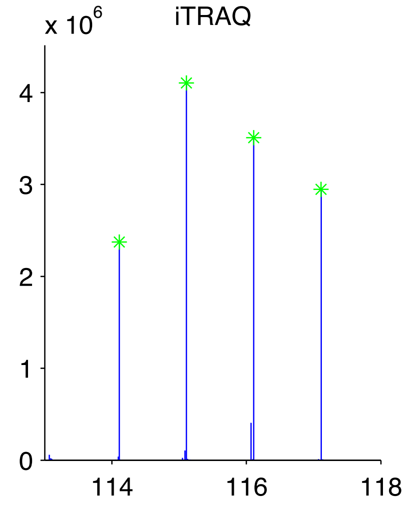

G G k G L G k G G A k R

histone cluster 1, H4a [Homo sapiens]
Charge State: +2
Scan Number: 11634
File Name: 111011_HG2_INSB_ACK.raw

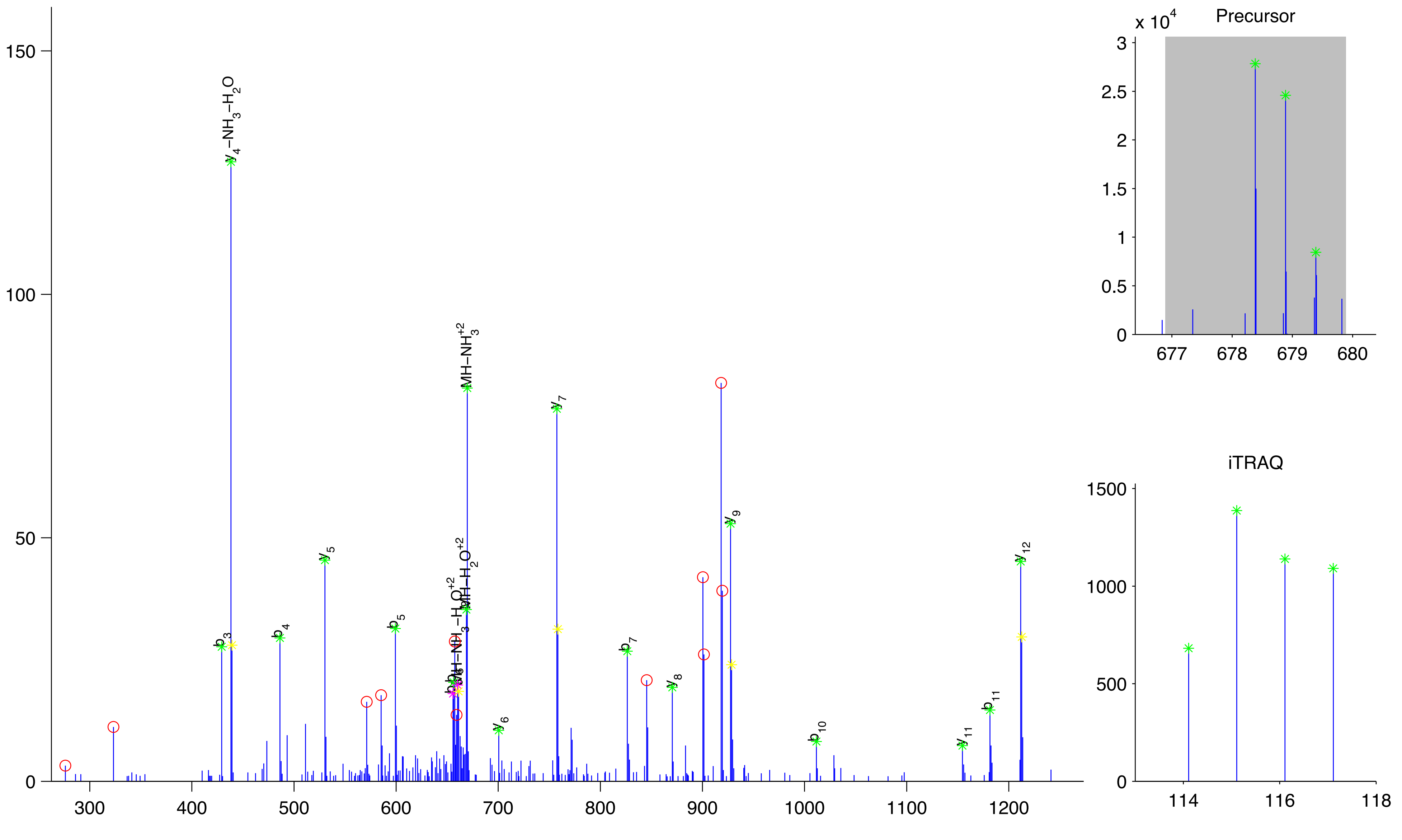

G k G G k G L G k G G A k R

histone cluster 1, H4a [Homo sapiens]
Charge State: +3
Scan Number: 11637
File Name: 110924_HG2_INSA_ACK3.raw

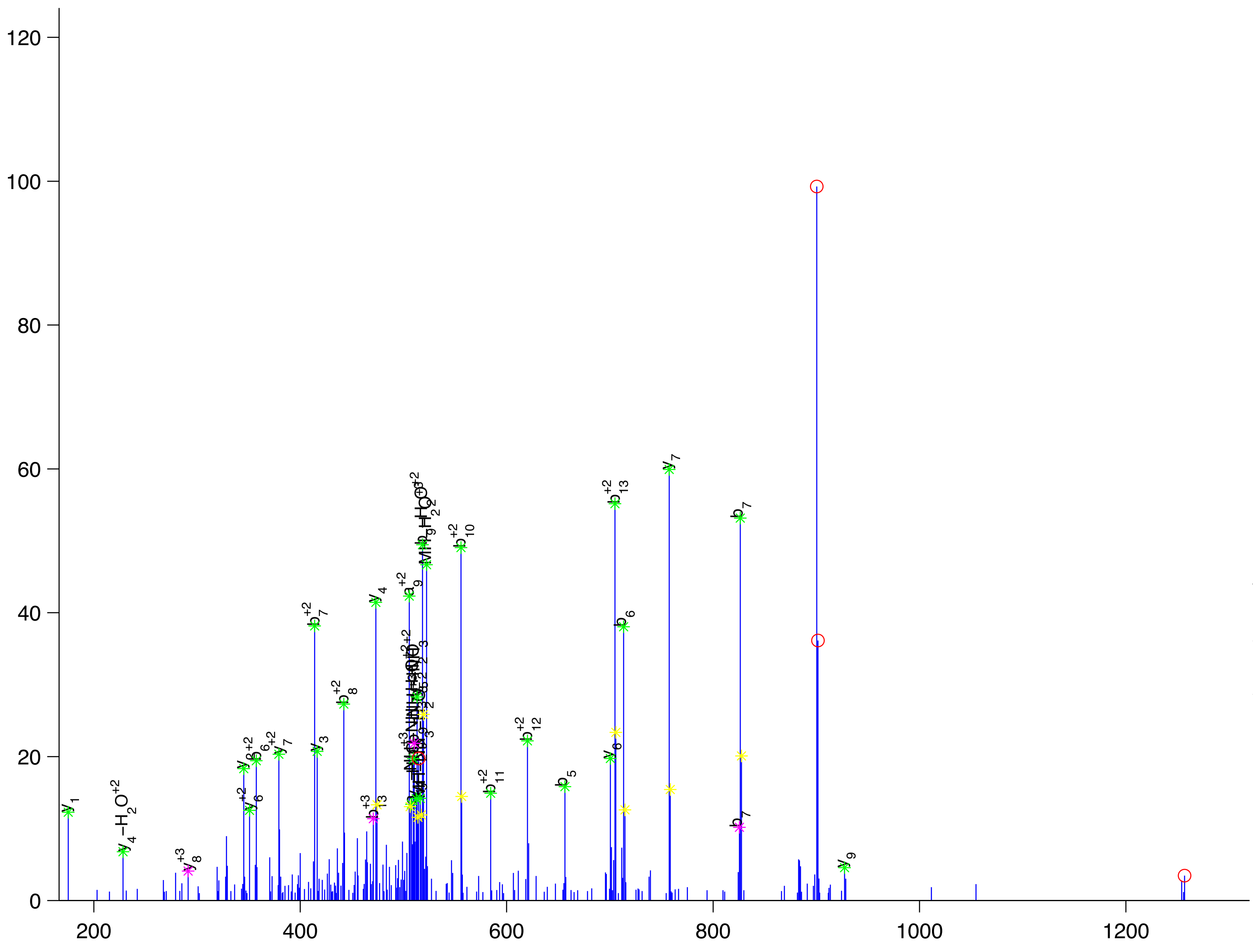

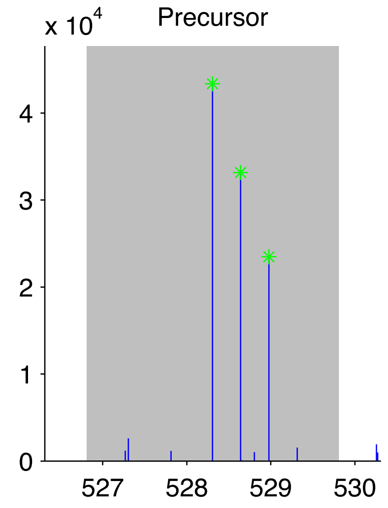

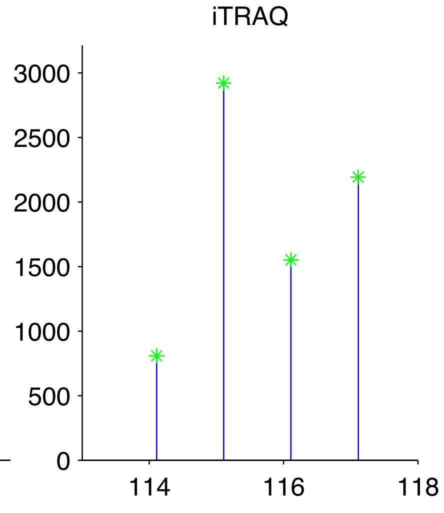

G L G k G G A k R

histone cluster 1, H4a [Homo sapiens]
Charge State: +2
Scan Number: 11660
File Name: 110924_HG2_INSA_ACK.raw

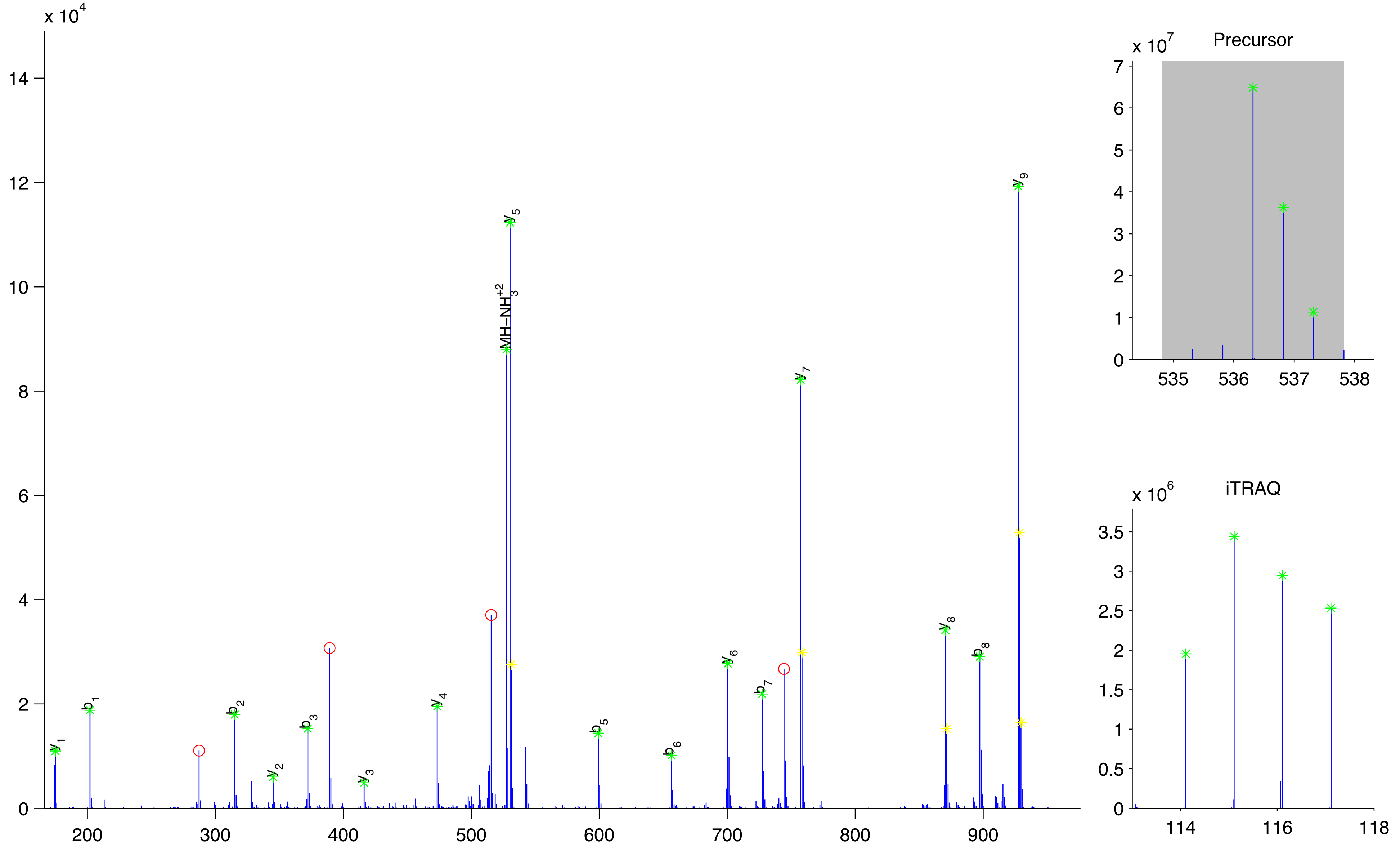

G L G k G G A k R

histone cluster 1, H4a [Homo sapiens]
Charge State: +2
Scan Number: 11704
File Name: 111011_HG2_INSB_ACK.raw

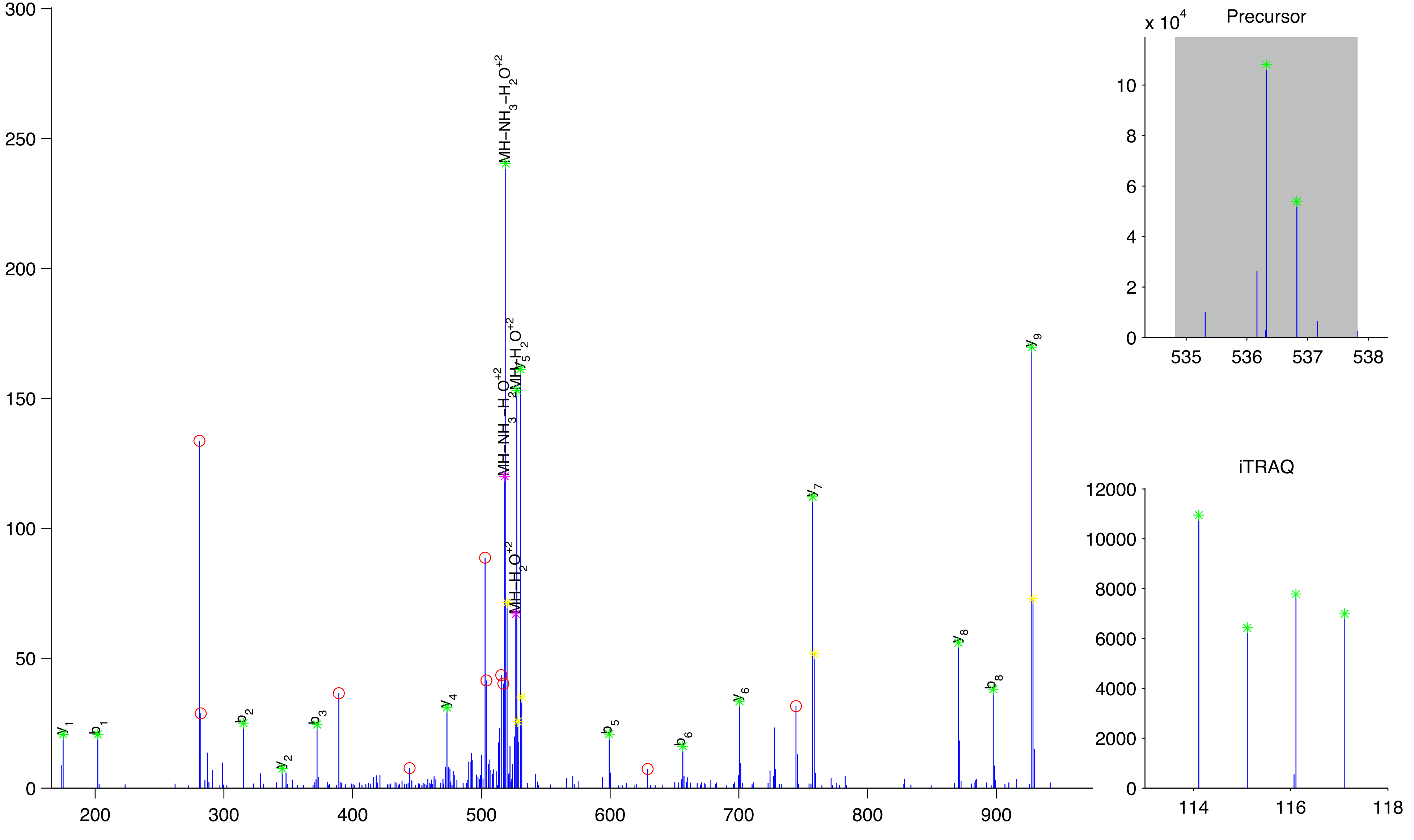

G G k G L G k G G A k R

histone cluster 1, H4a [Homo sapiens]
Charge State: +2
Scan Number: 11717
File Name: 110924_HG2_INSA_ACK3.raw

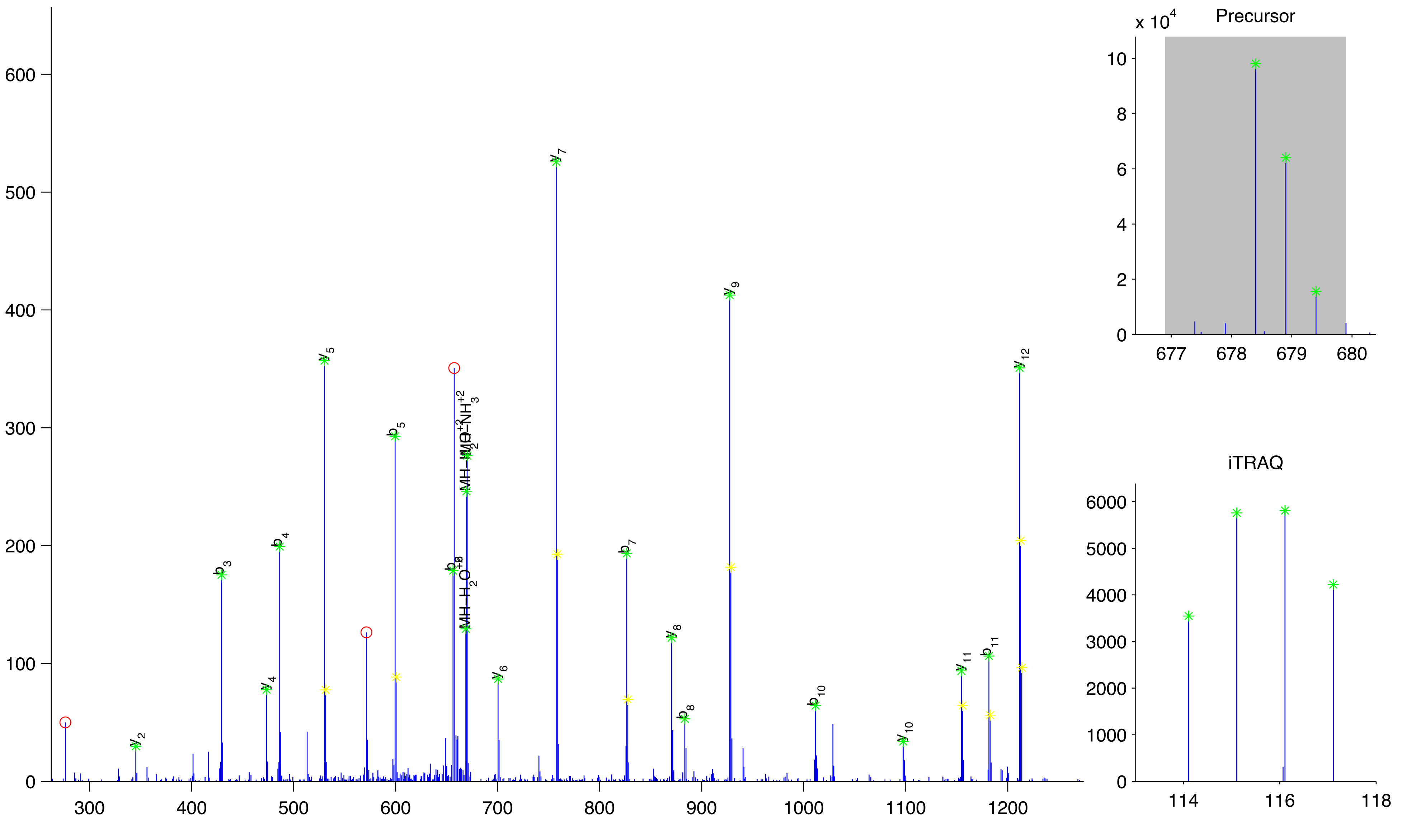

G G k G L G k G G A k R

histone cluster 1, H4a [Homo sapiens]
Charge State: +2
Scan Number: 11802
File Name: 111011_HG2_INSB_ACK.raw

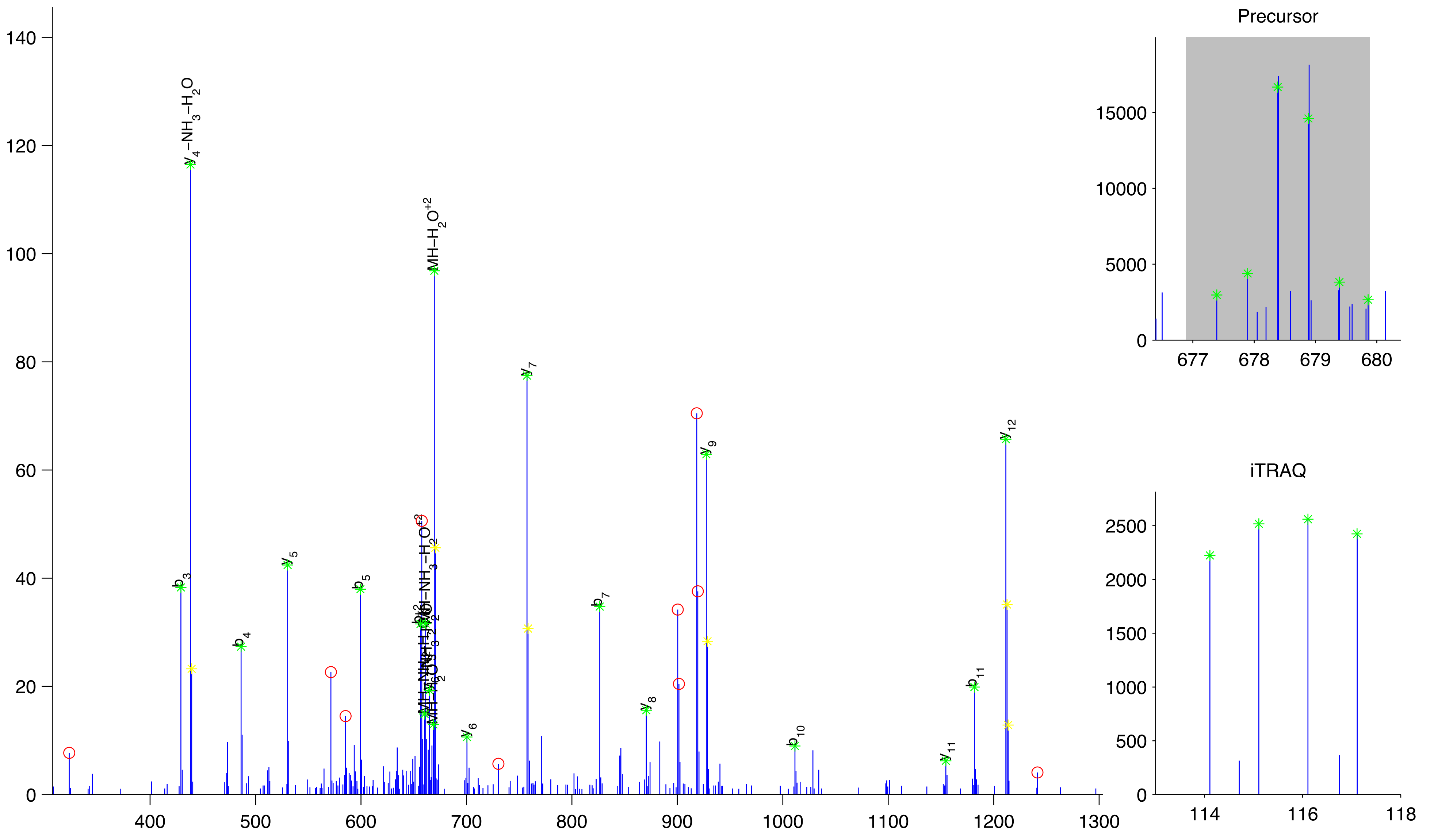

G L G k G G A k R

histone cluster 1, H4a [Homo sapiens]
Charge State: +2
Scan Number: 11969
File Name: 110924_HG2_INSA_ACK3.raw

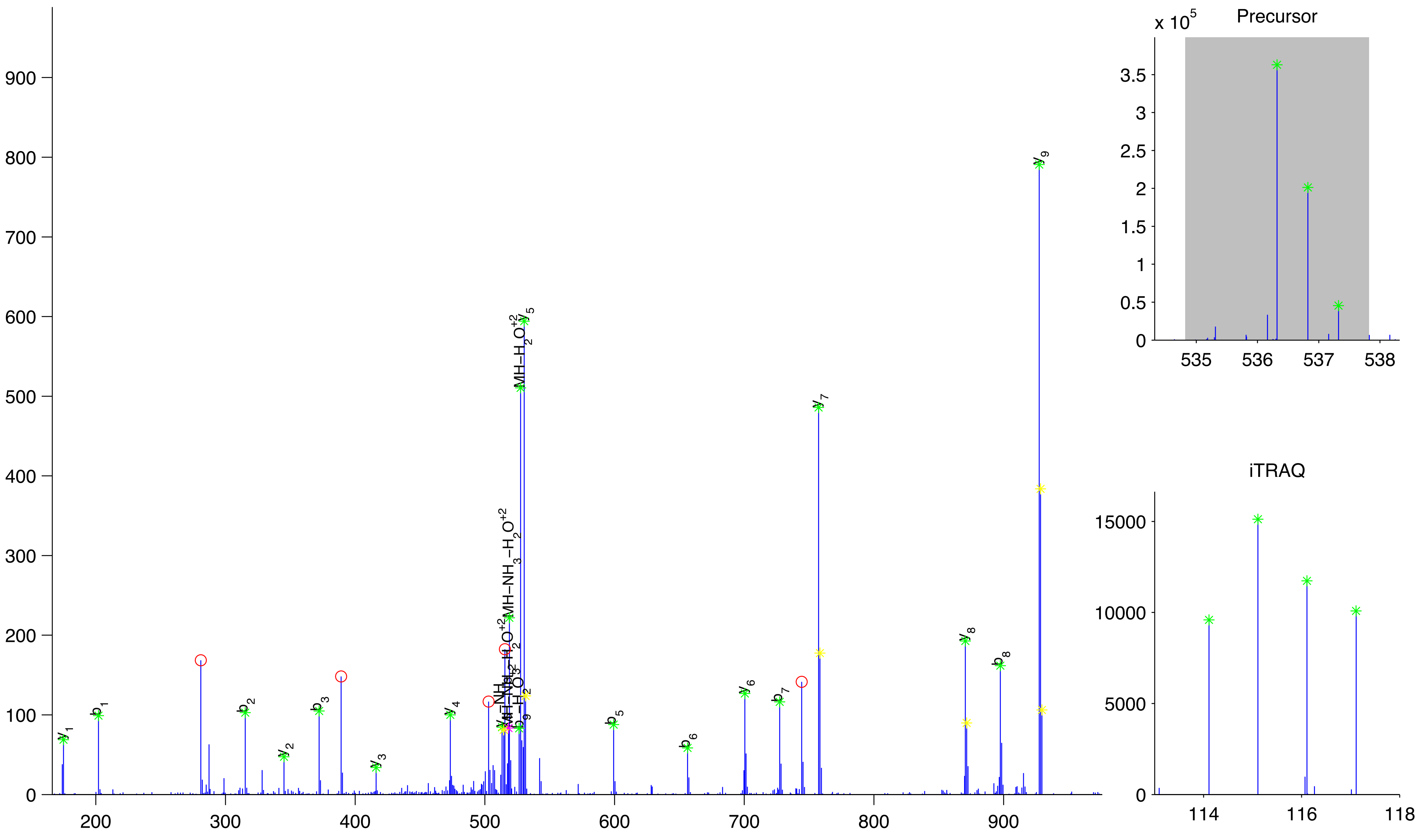

G G k G L G k G G A k R

histone cluster 1, H4a [Homo sapiens]
Charge State: +2
Scan Number: 12227
File Name: 110924_HG2_INSA_ACK.raw

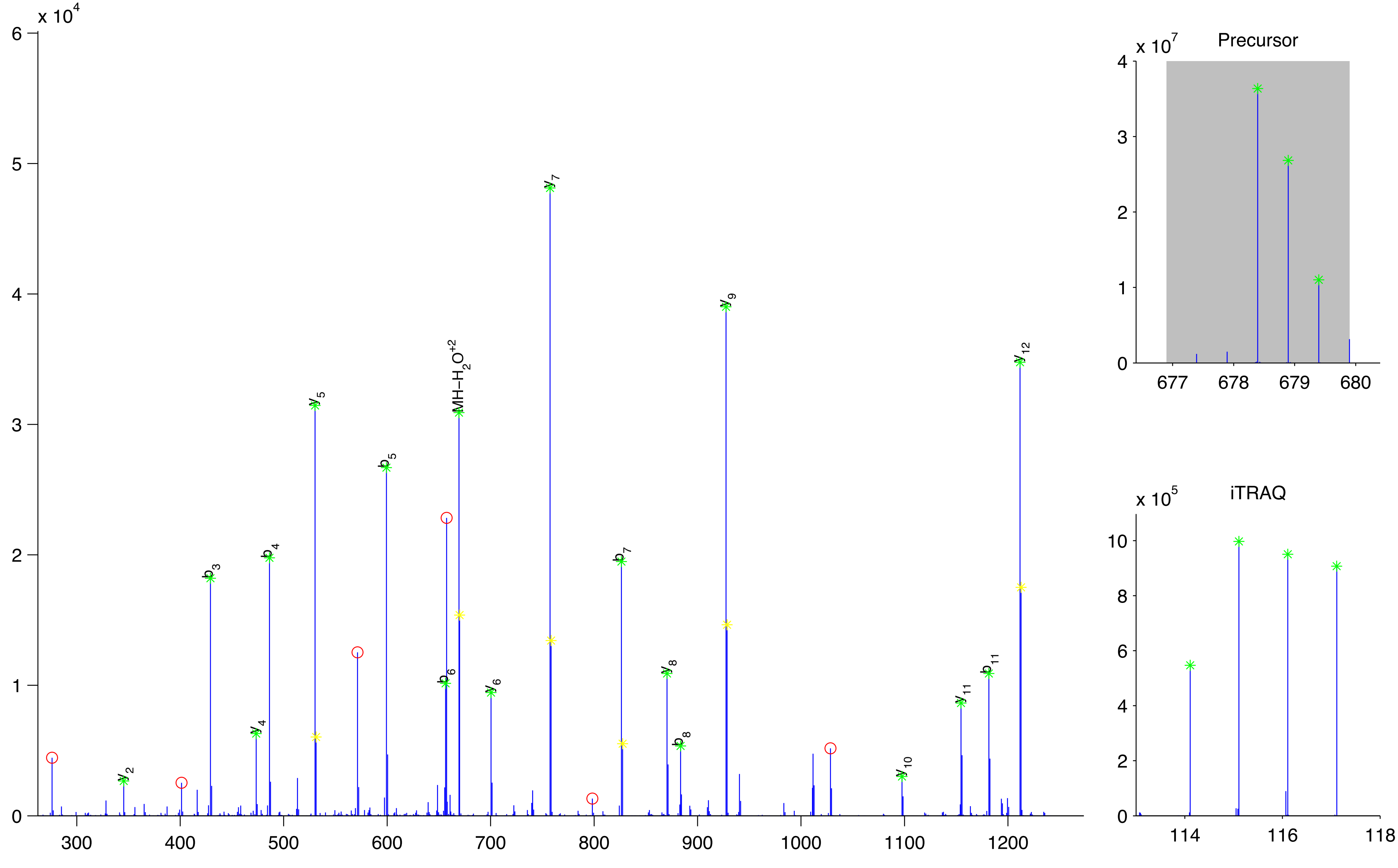

G k G G k G L G k G G A k R

histone cluster 1, H4a [Homo sapiens]
Charge State: +3
Scan Number: 12326
File Name: 110924_HG2_INSA_ACK3.raw

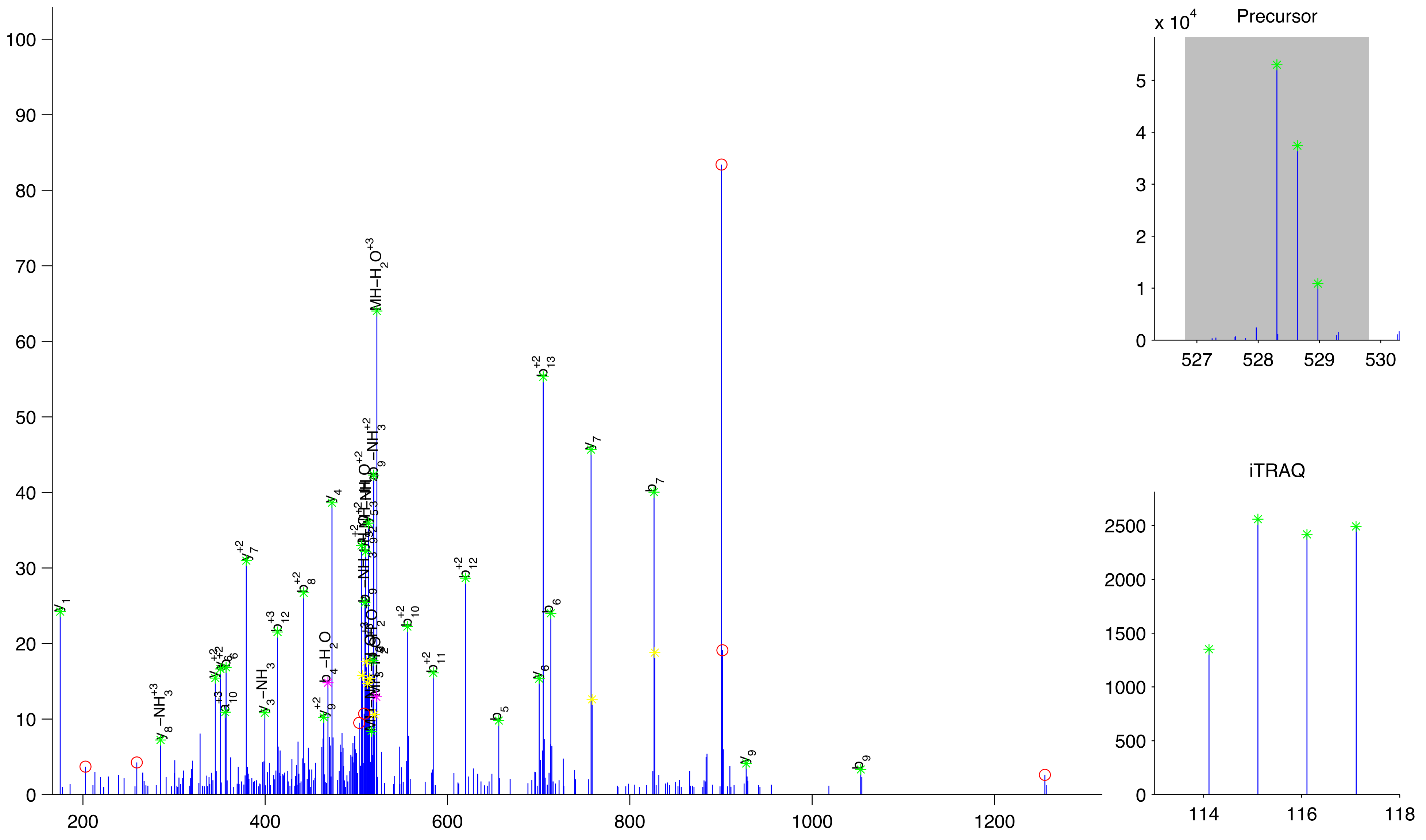

G G k G L G k G G A k R

histone cluster 1, H4a [Homo sapiens]
Charge State: +2
Scan Number: 12414
File Name: 110924_HG2_INSA_ACK.raw

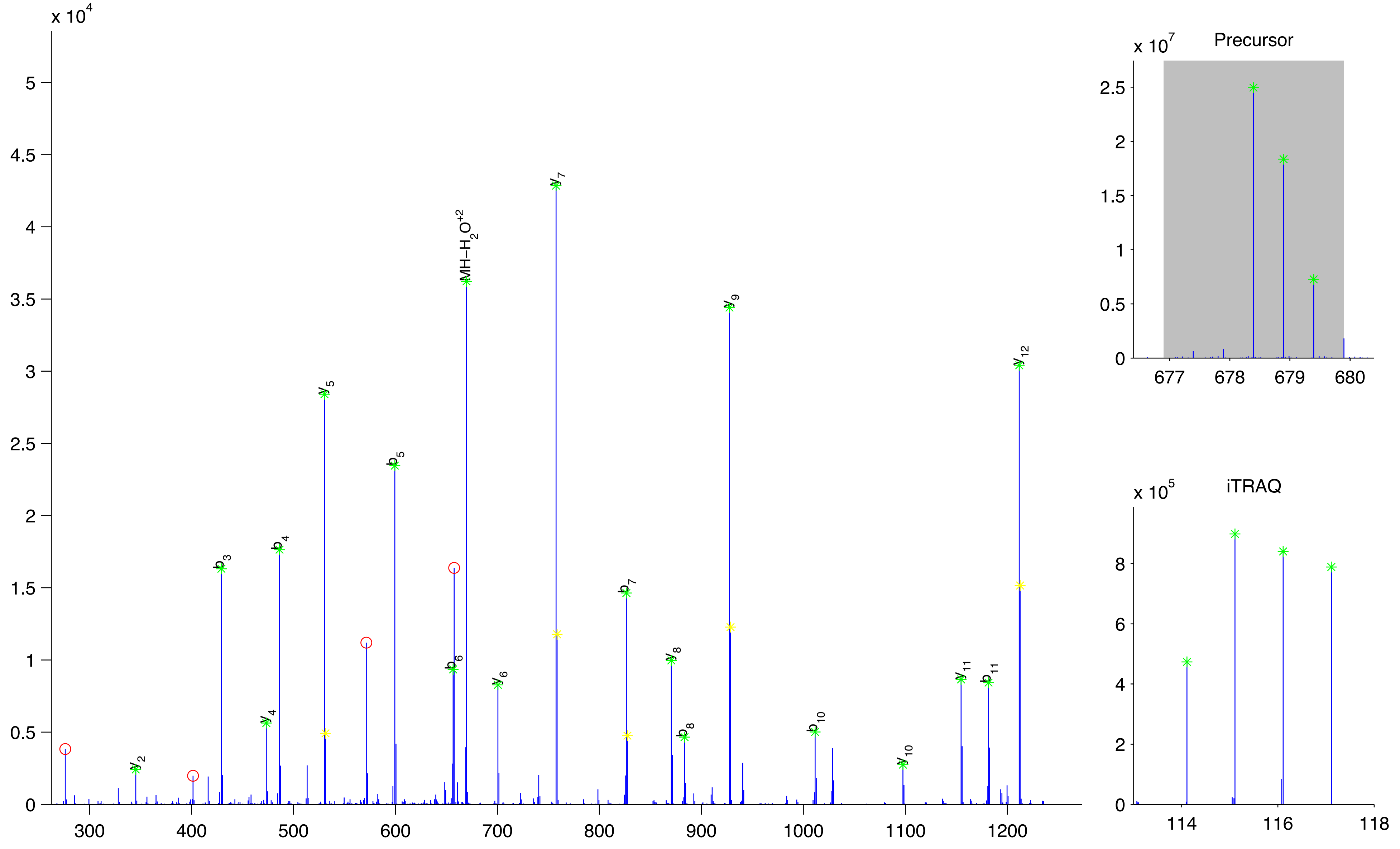

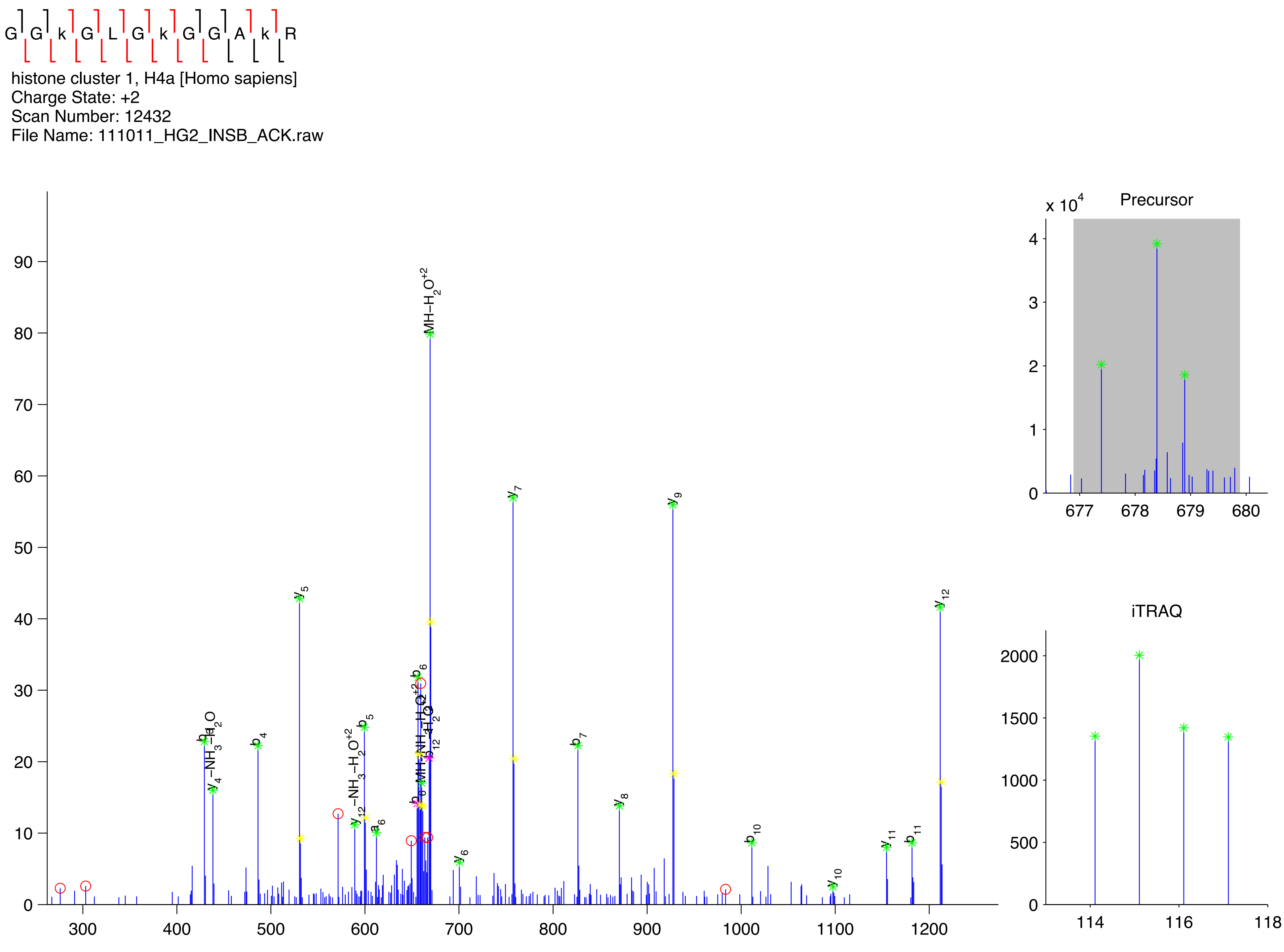
G G k G L G k G G A k R
histone cluster 1, H4a [Homo sapiens]
Charge State: +2
Scan Number: 12432
File Name: 111011_HG2_INSB_ACK.raw
MH−H2O+2
y7
y9
y5
y12
b4
b5
b7
y8
a6
y6
b10
y10
y11
b11
x 10^4
Precursor
iTRAQ

G G k G L G k G G A k R

histone cluster 1, H4a [Homo sapiens]
Charge State: +2
Scan Number: 12557
File Name: 110924_HG2_INSA_ACK3.raw

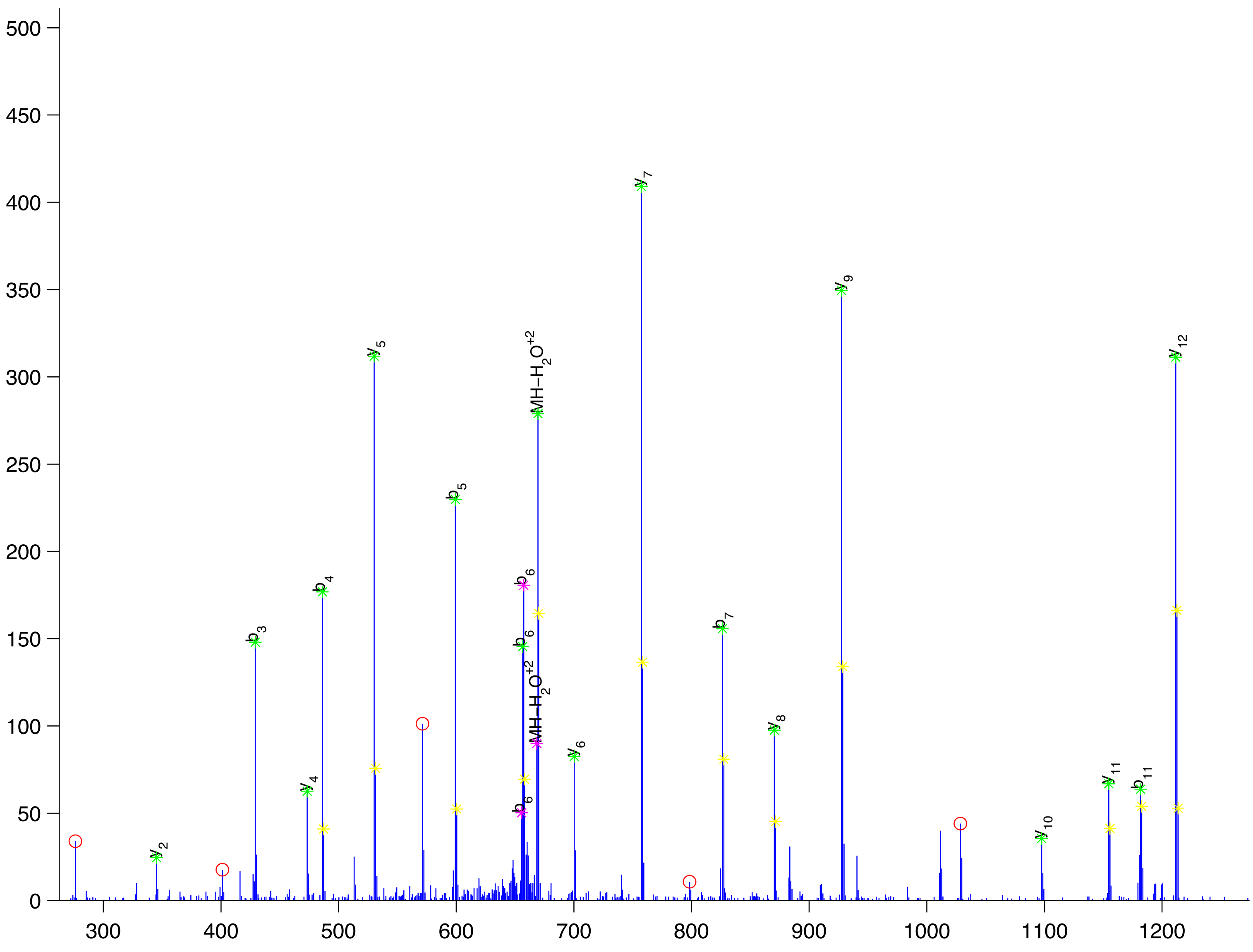

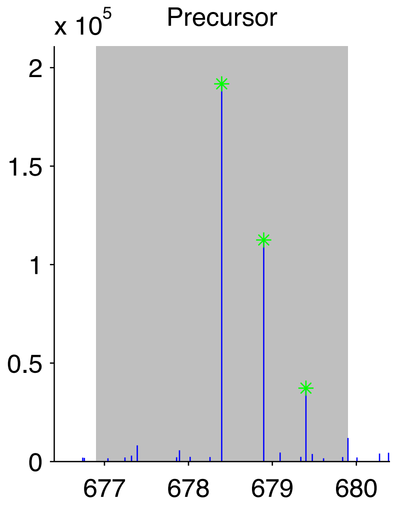

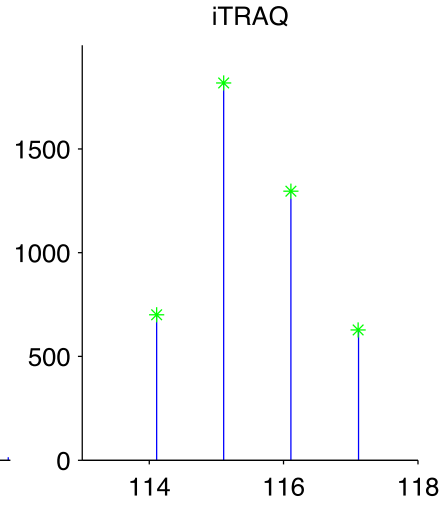

G G k G L G k G G A k R

histone cluster 1, H4a [Homo sapiens]
Charge State: +2
Scan Number: 12603
File Name: 110924_HG2_INSA_ACK.raw

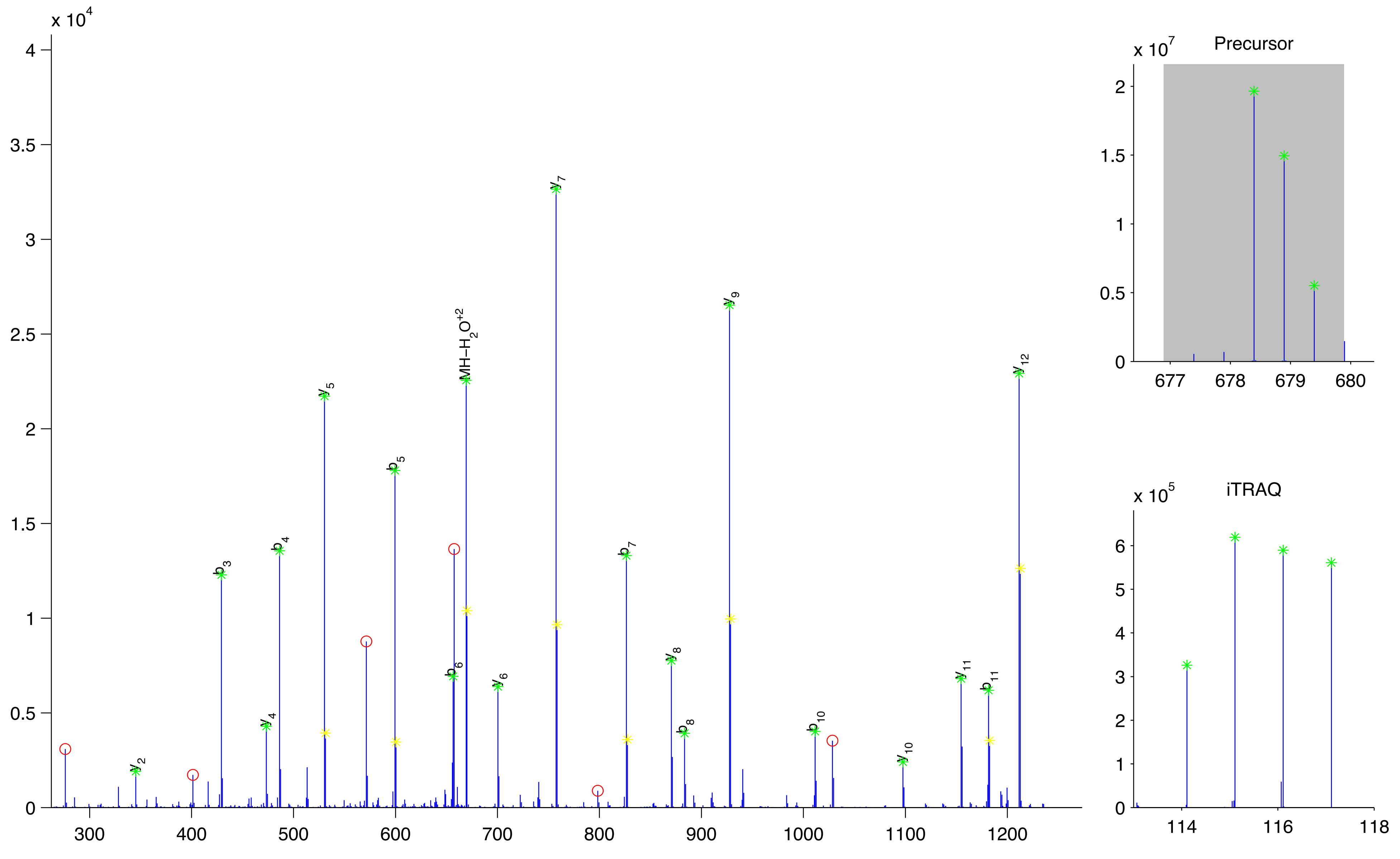

G L G k G G A k R

histone cluster 1, H4a [Homo sapiens]
Charge State: +2
Scan Number: 12641
File Name: 110924_HG2_INSA_ACK3.raw

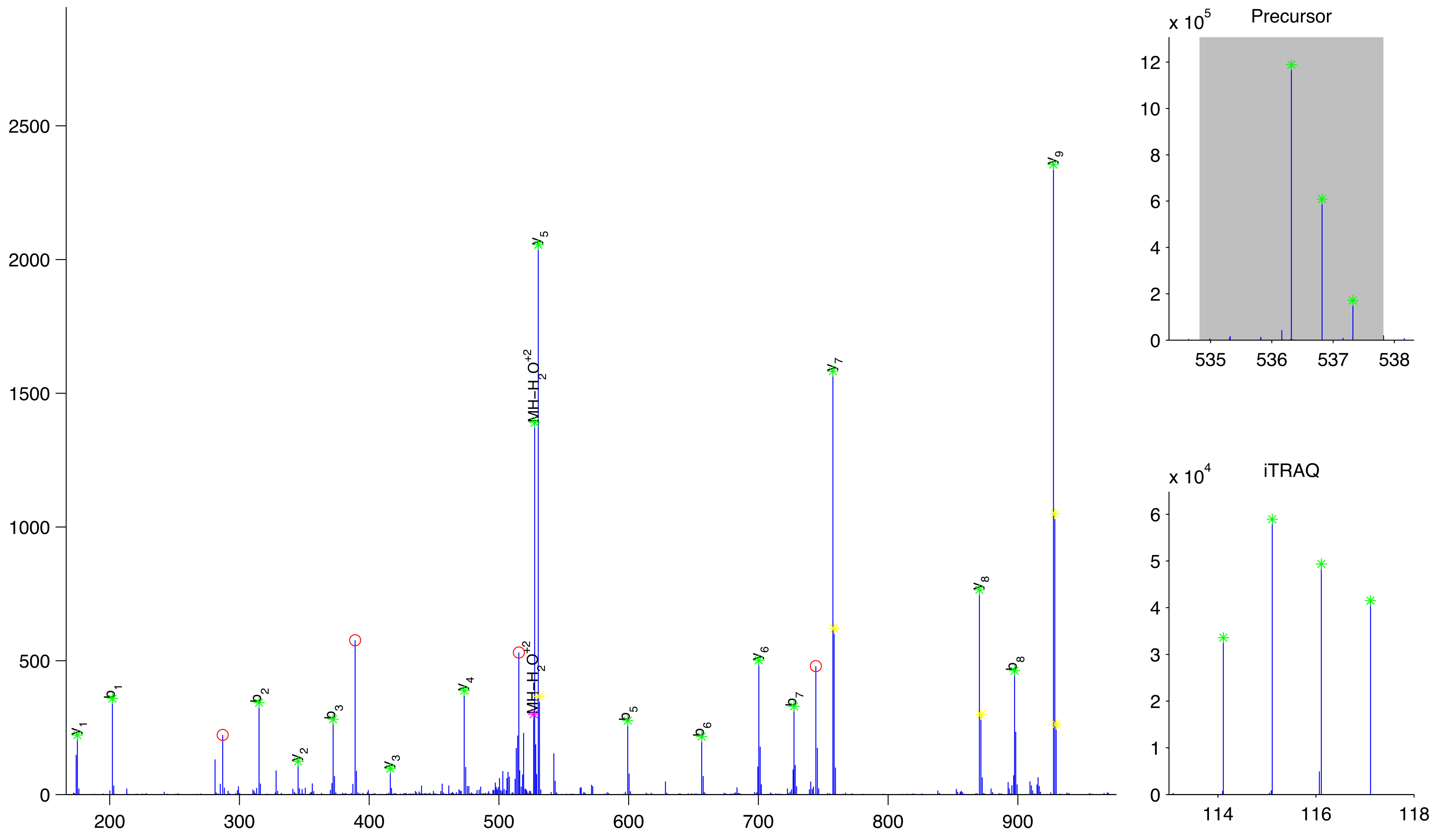

G G k G L G k G G A k R

histone cluster 1, H4a [Homo sapiens]
Charge State: +3
Scan Number: 12653
File Name: 110924_HG2_INSA_ACK.raw

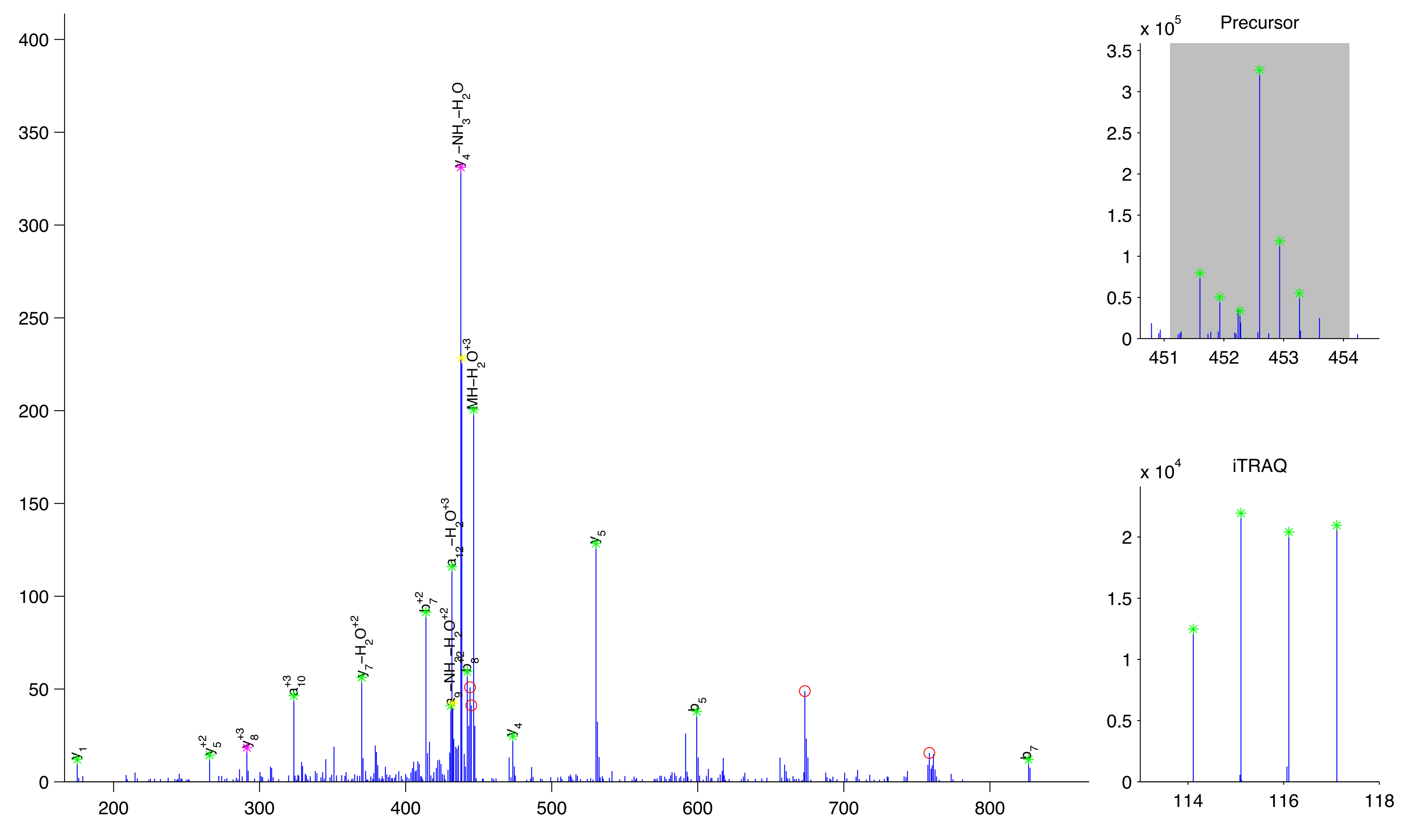

G G k G L G k G G A k R

histone cluster 1, H4a [Homo sapiens]
Charge State: +2
Scan Number: 12661
File Name: 111011_HG2_INSB_ACK.raw

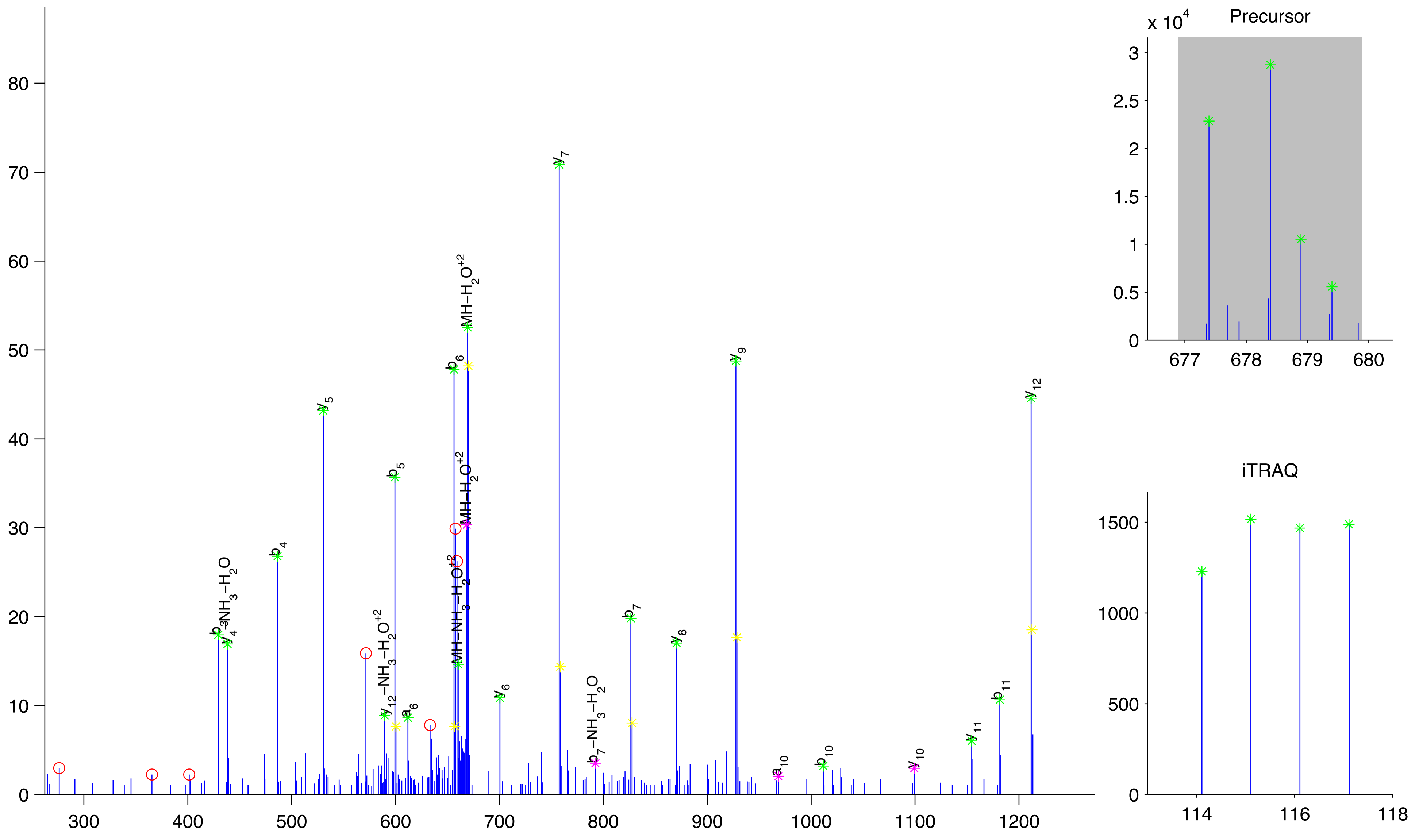

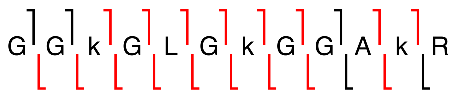

histone cluster 1, H4a [Homo sapiens]
Charge State: +2
Scan Number: 12716
File Name: 110924_HG2_INSA_ACK3.raw

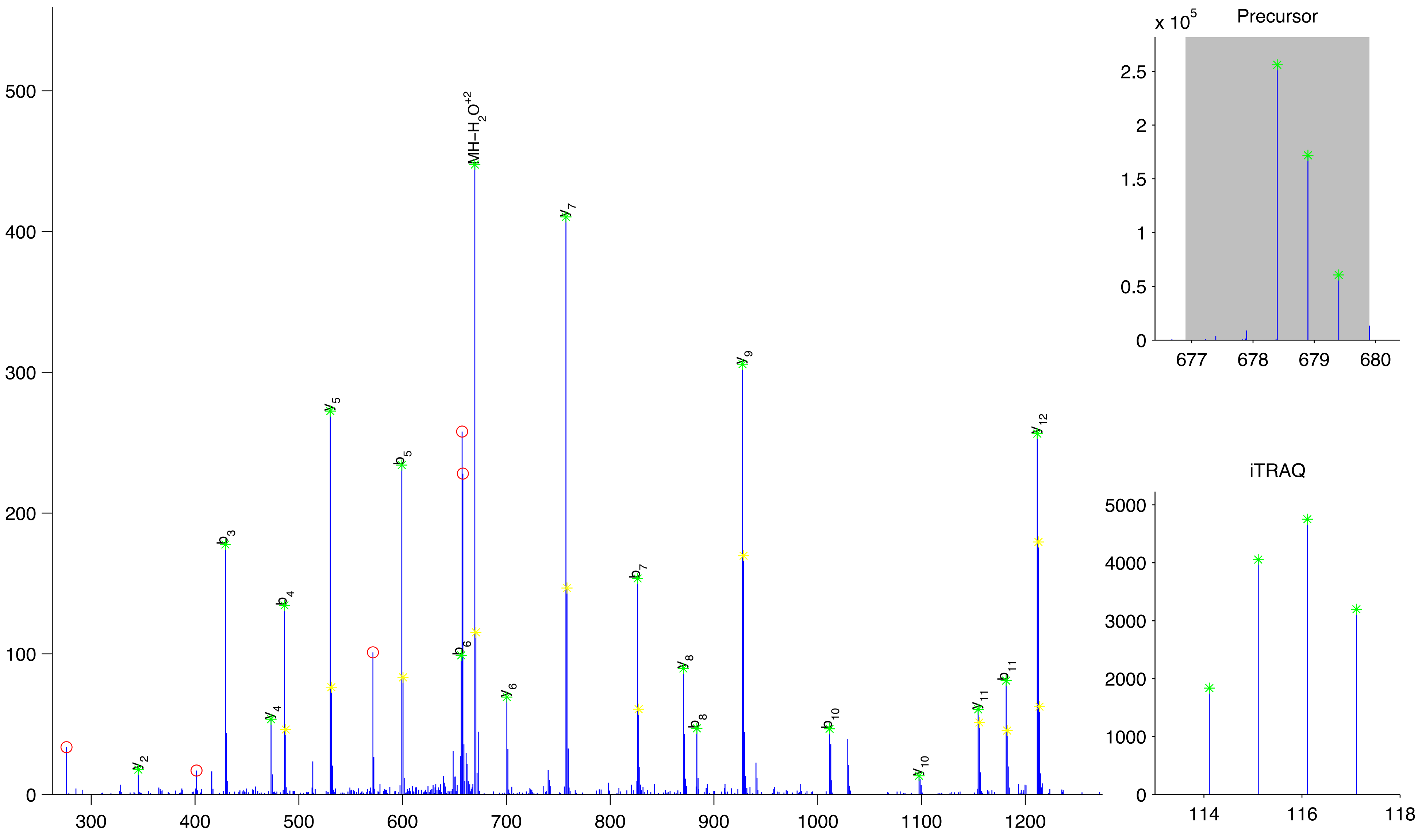

G G k G L G k G G A k R

histone cluster 1, H4a [Homo sapiens]
Charge State: +2
Scan Number: 12725
File Name: 110924_HG2_INSA_ACK3.raw

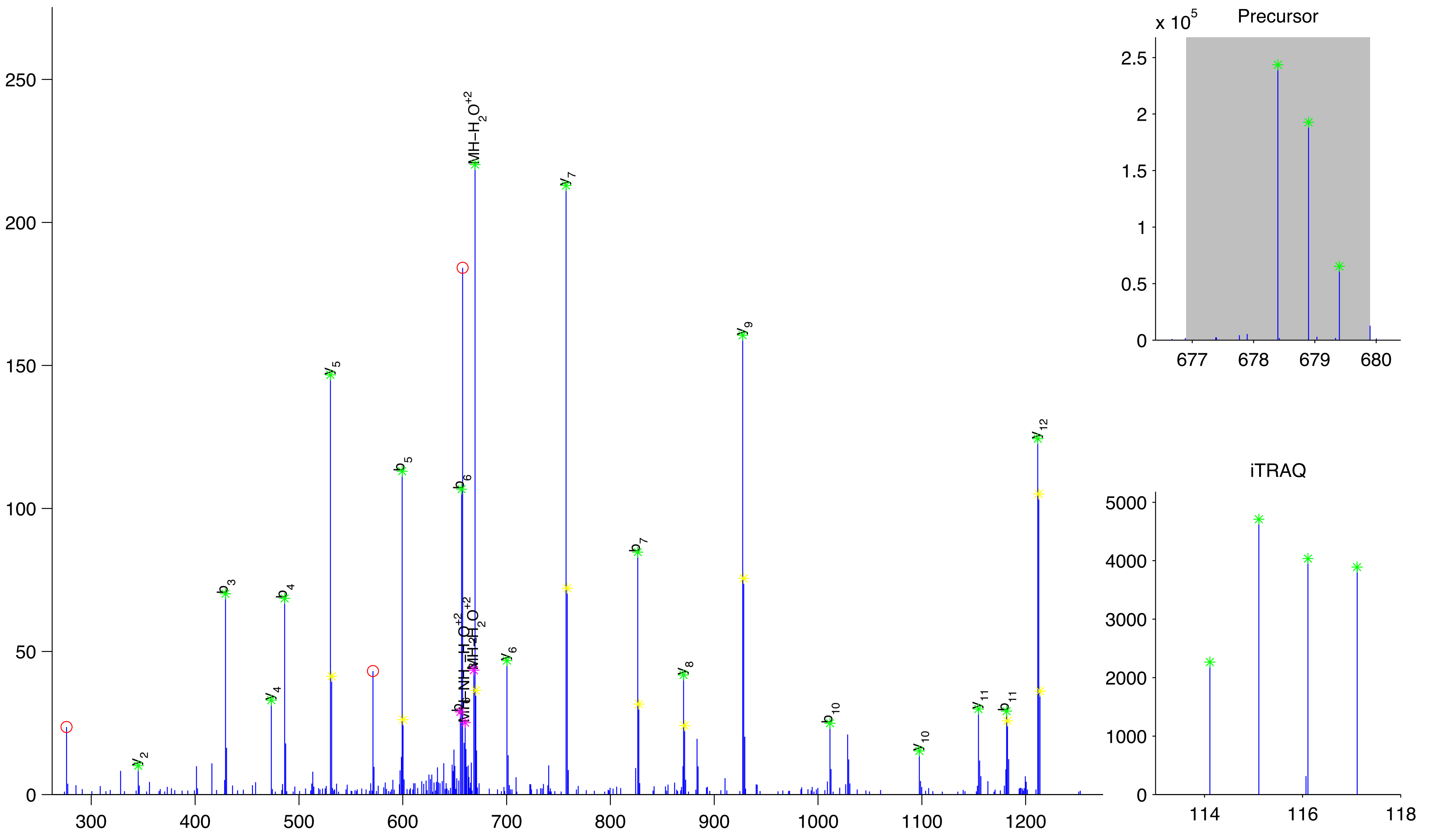

G L G k G G A k R

histone cluster 1, H4a [Homo sapiens]
Charge State: +2
Scan Number: 12754
File Name: 110924_HG2_INSA_ACK3.raw

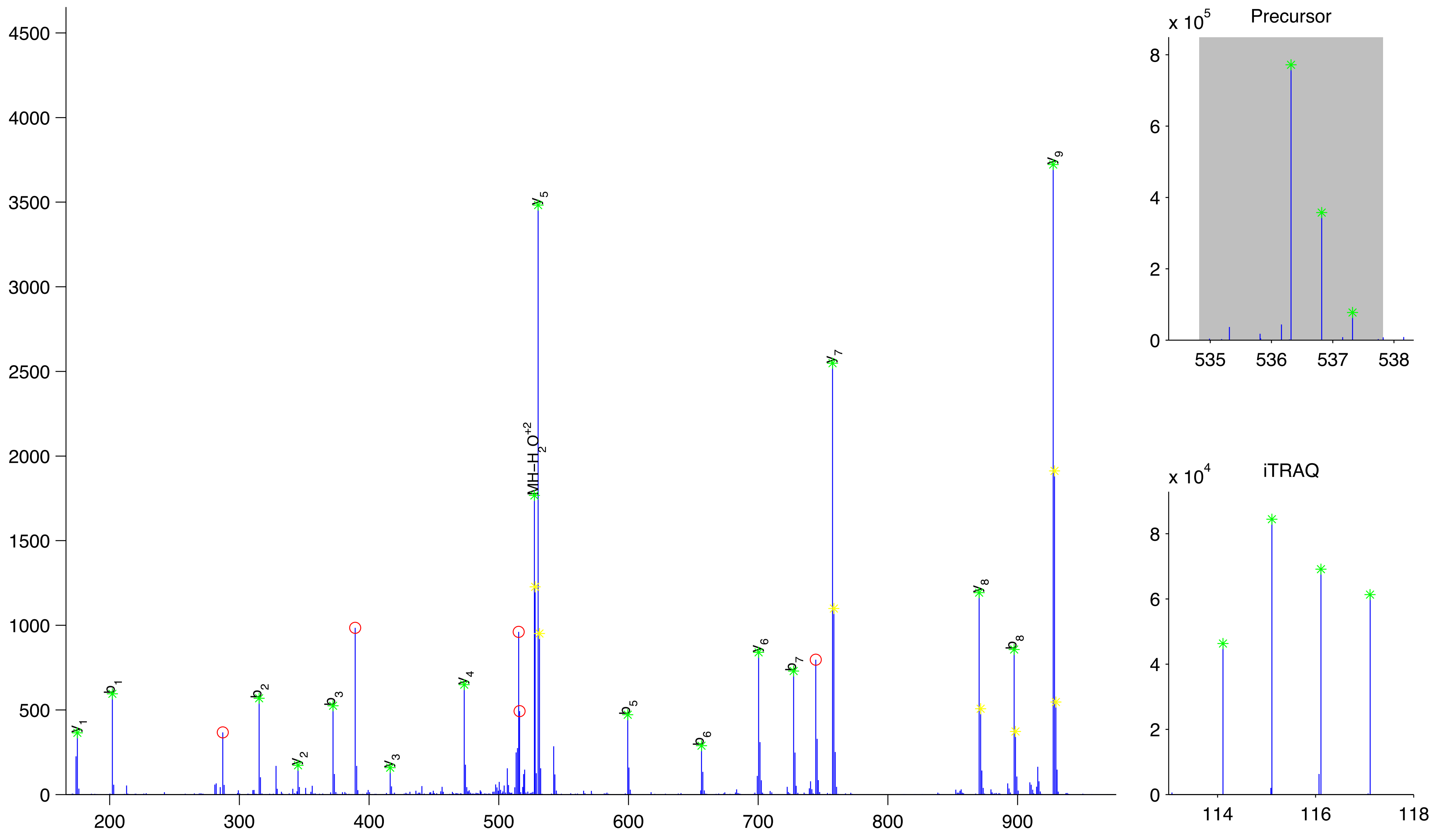

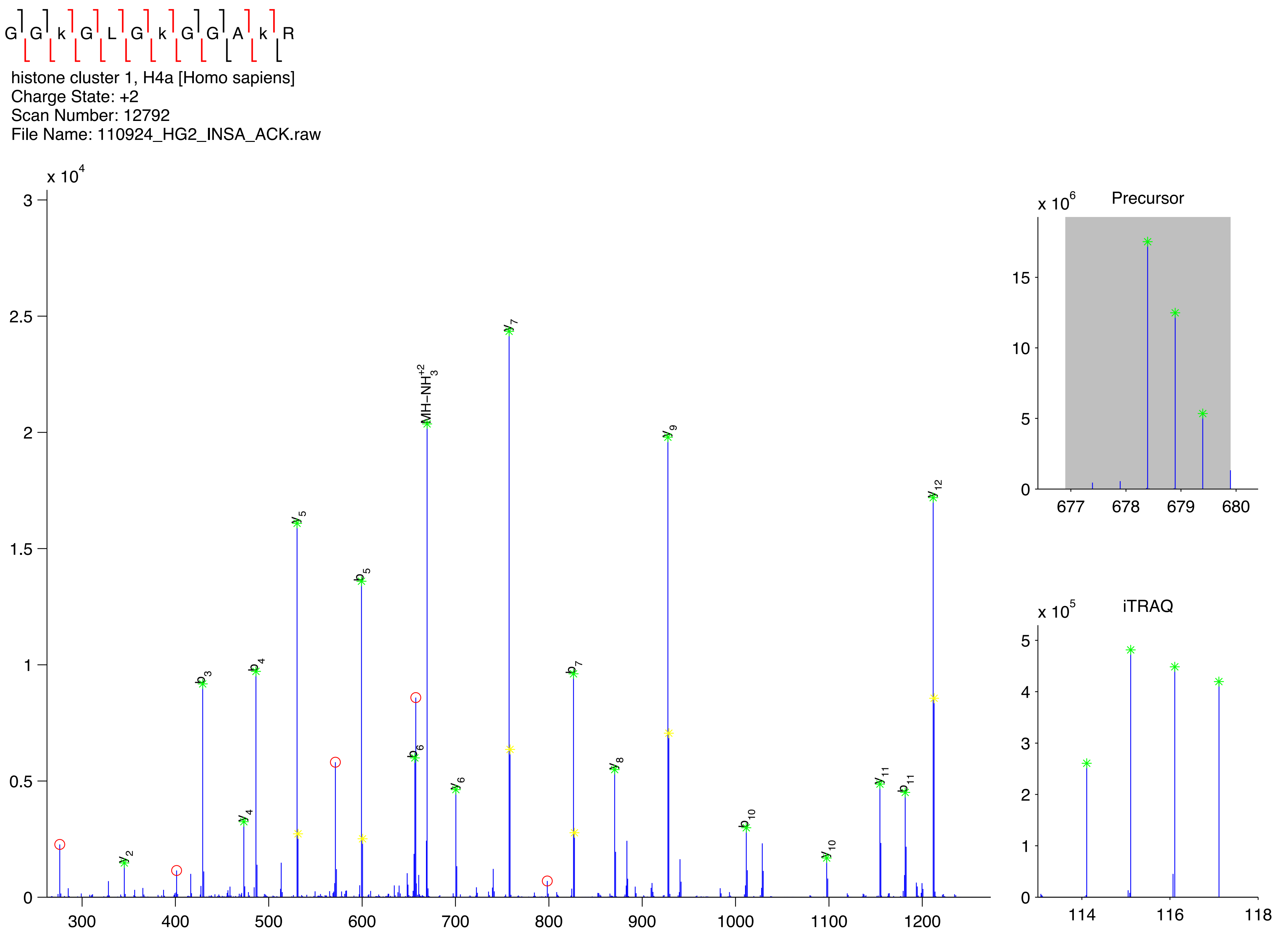

G G k G L G k G G A k R
histone cluster 1, H4a [Homo sapiens]
Charge State: +2
Scan Number: 12792
File Name: 110924_HG2_INSA_ACK.raw
x 10^4
0
0.5
1
1.5
2
2.5
3
300
400
500
600
700
800
900
1000
1100
1200
y2
b3
y4
b4
y5
b5
b6
MH−NH3+2
y6
y7
b7
y8
y9
b10
y10
y11
b11
y12
x 10^6
Precursor
0
5
10
15
677
678
679
680
x 10^5
iTRAQ
0
1
2
3
4
5
114
116
118

G L G k G G A k R

histone cluster 1, H4a [Homo sapiens]
Charge State: +2
Scan Number: 12809
File Name: 110924_HG2_INSA_ACK3.raw

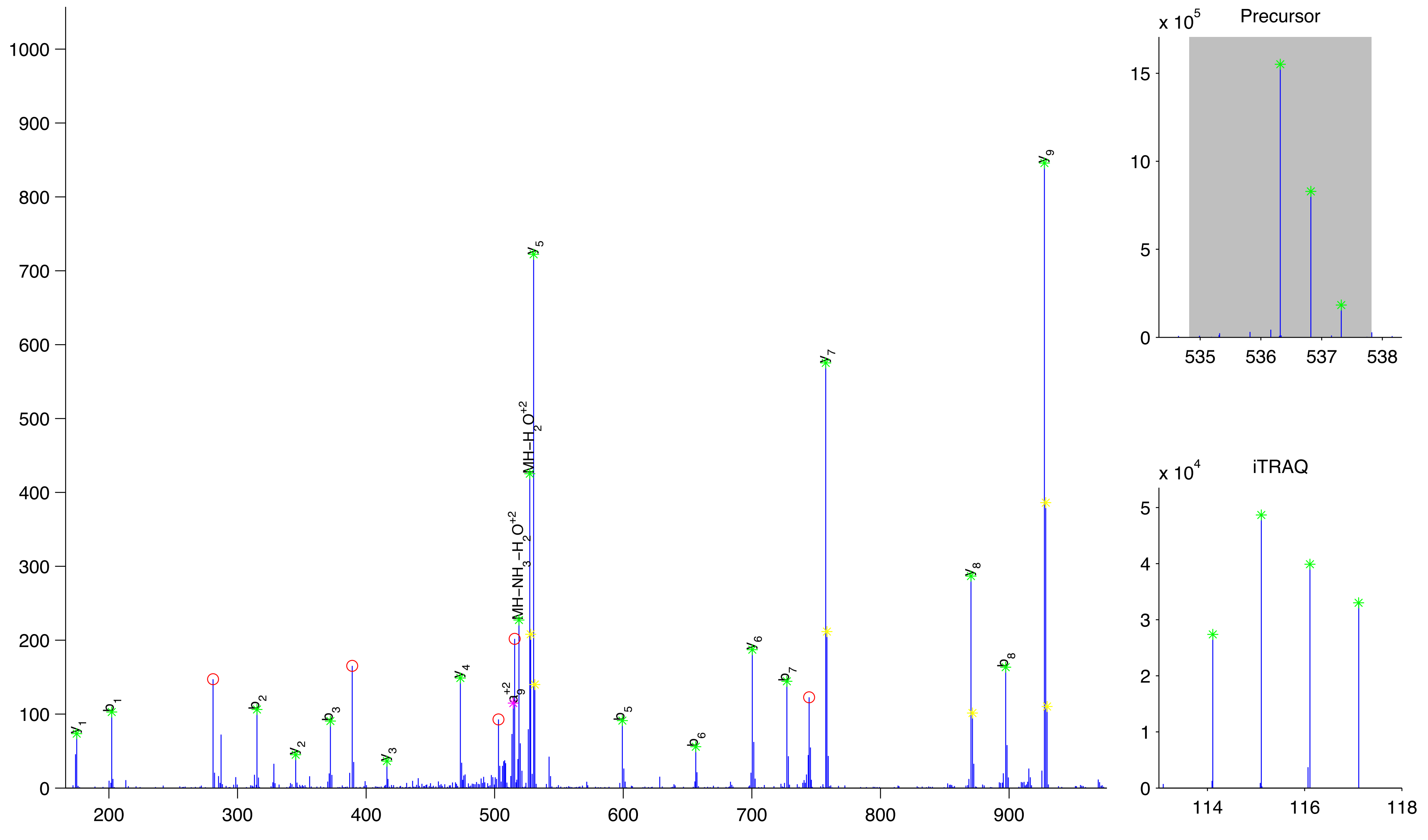

G k G G k G L G k G G A k R

histone cluster 1, H4a [Homo sapiens]
Charge State: +3
Scan Number: 12836
File Name: 110924_HG2_INSA_ACK3.raw

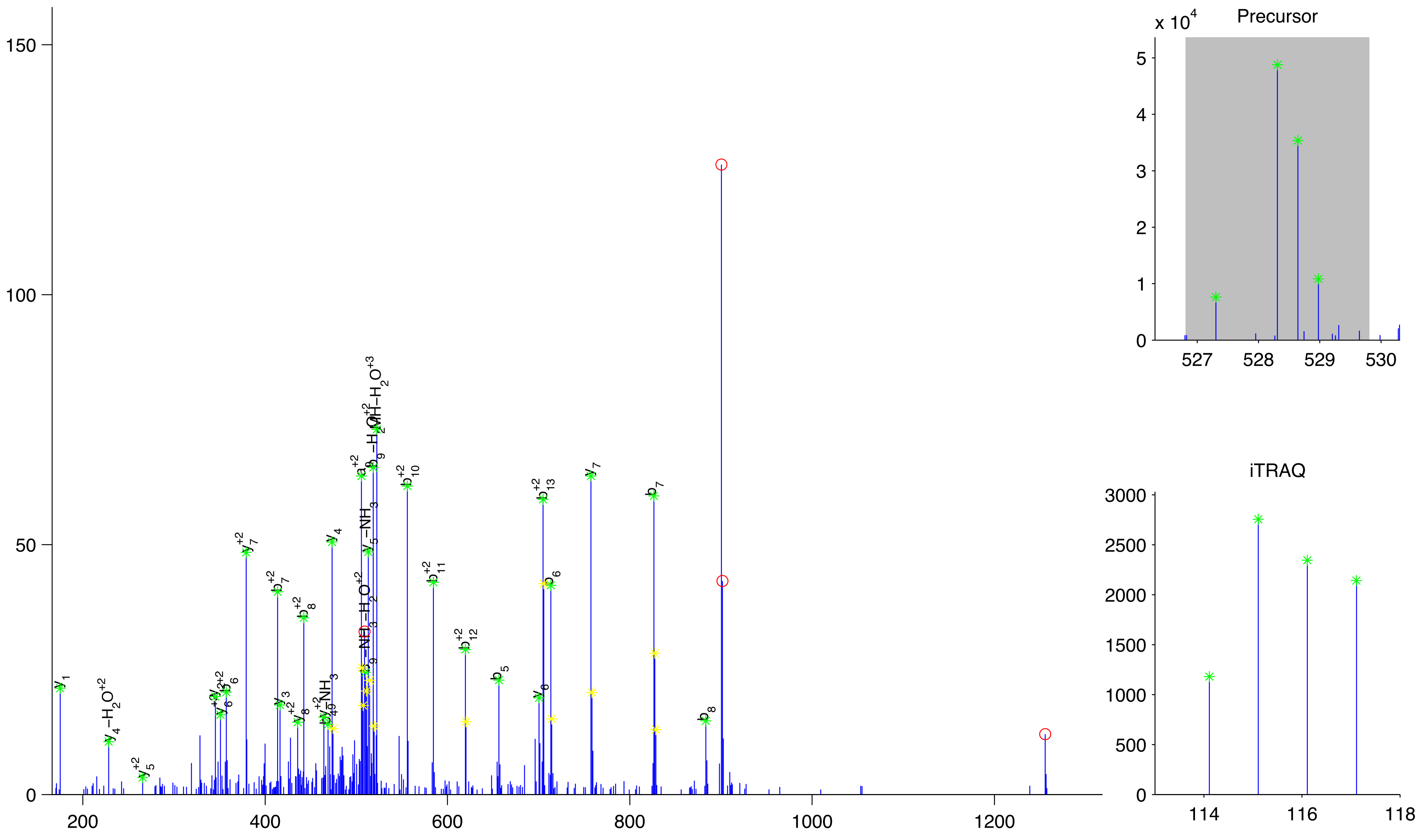

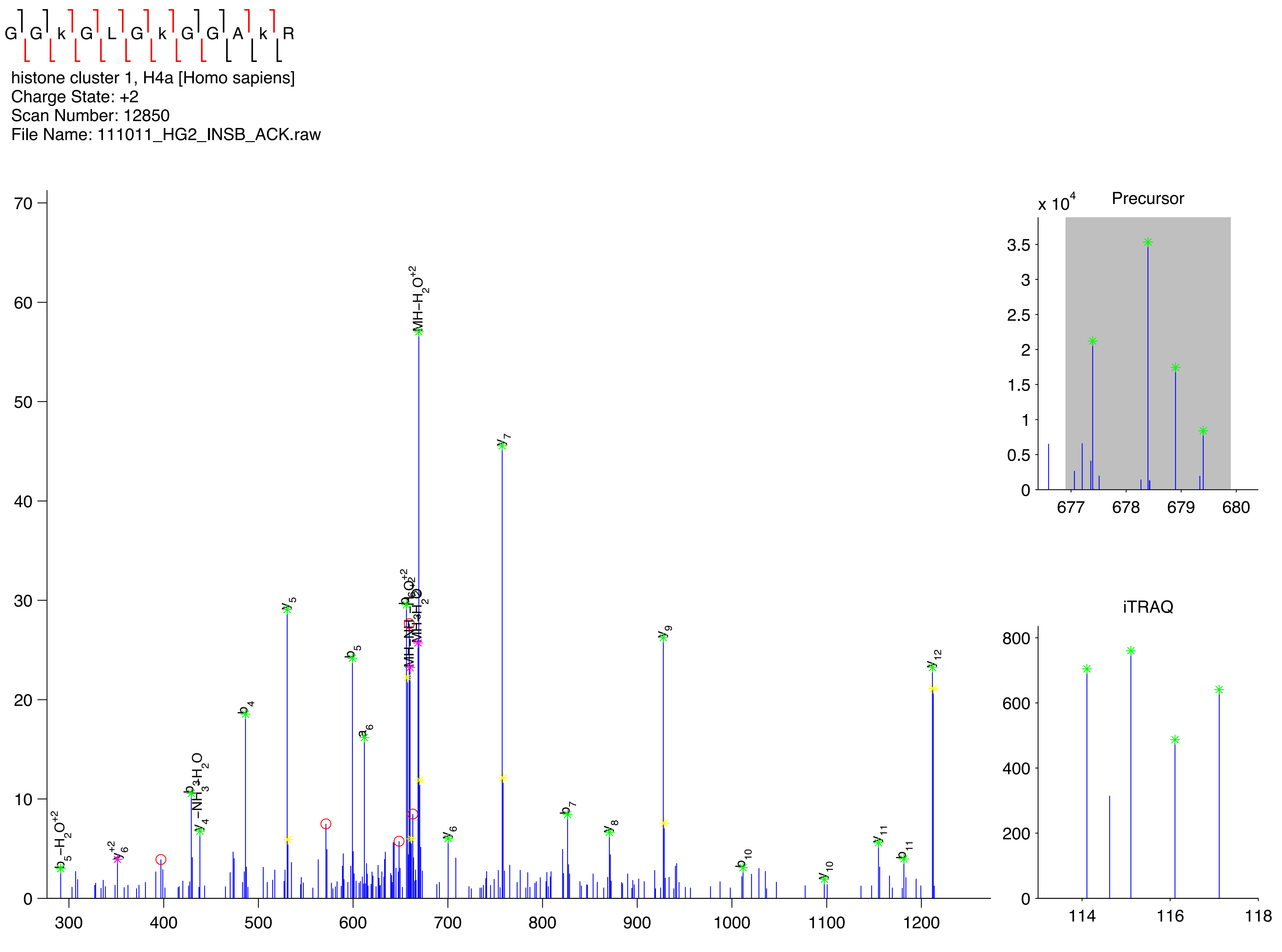
G G k G L G k G G A k R
histone cluster 1, H4a [Homo sapiens]
Charge State: +2
Scan Number: 12850
File Name: 111011_HG2_INSB_ACK.raw
Precursor
x 10^4
iTRAQ

G G k G L G k G G A k R

histone cluster 1, H4a [Homo sapiens]
Charge State: +2
Scan Number: 12878
File Name: 110924_HG2_INSA_ACK.raw

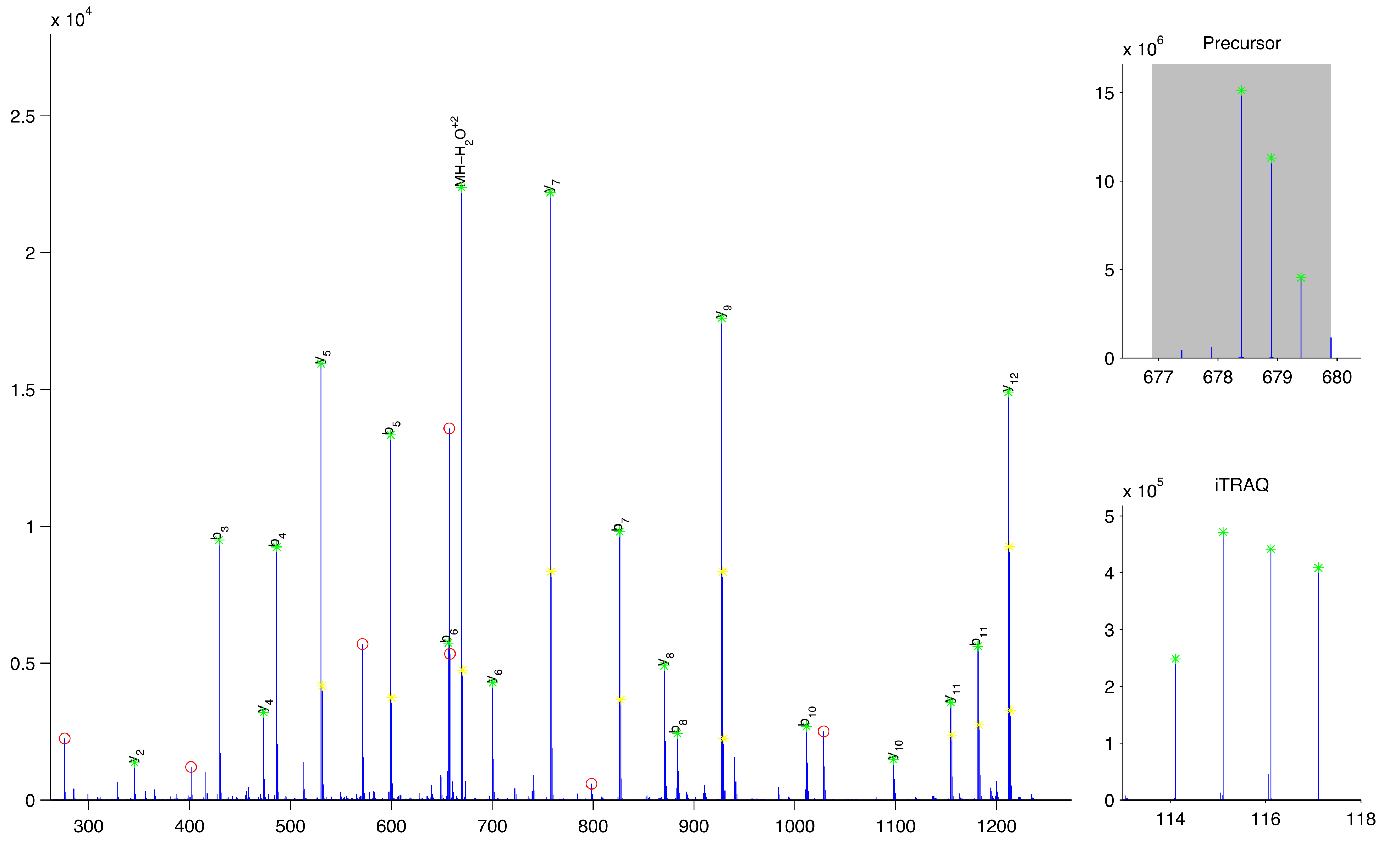

G G k G L G k G G A k R

histone cluster 1, H4a [Homo sapiens]
Charge State: +2
Scan Number: 12981
File Name: 110924_HG2_INSA_ACK.raw

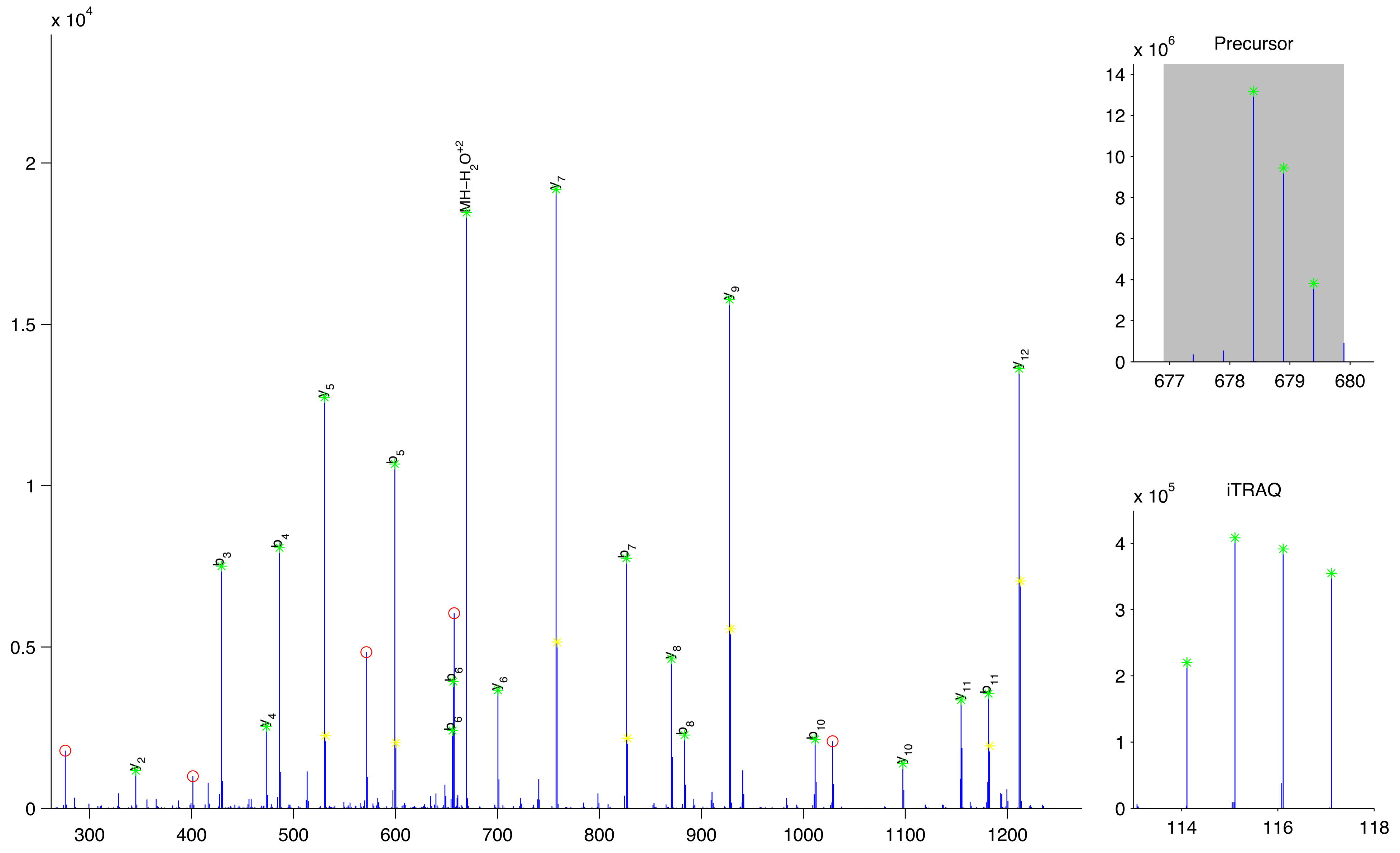

G G k G L G k G G A k R

histone cluster 1, H4a [Homo sapiens]
Charge State: +2
Scan Number: 13010
File Name: 111011_HG2_INSB_ACK.raw

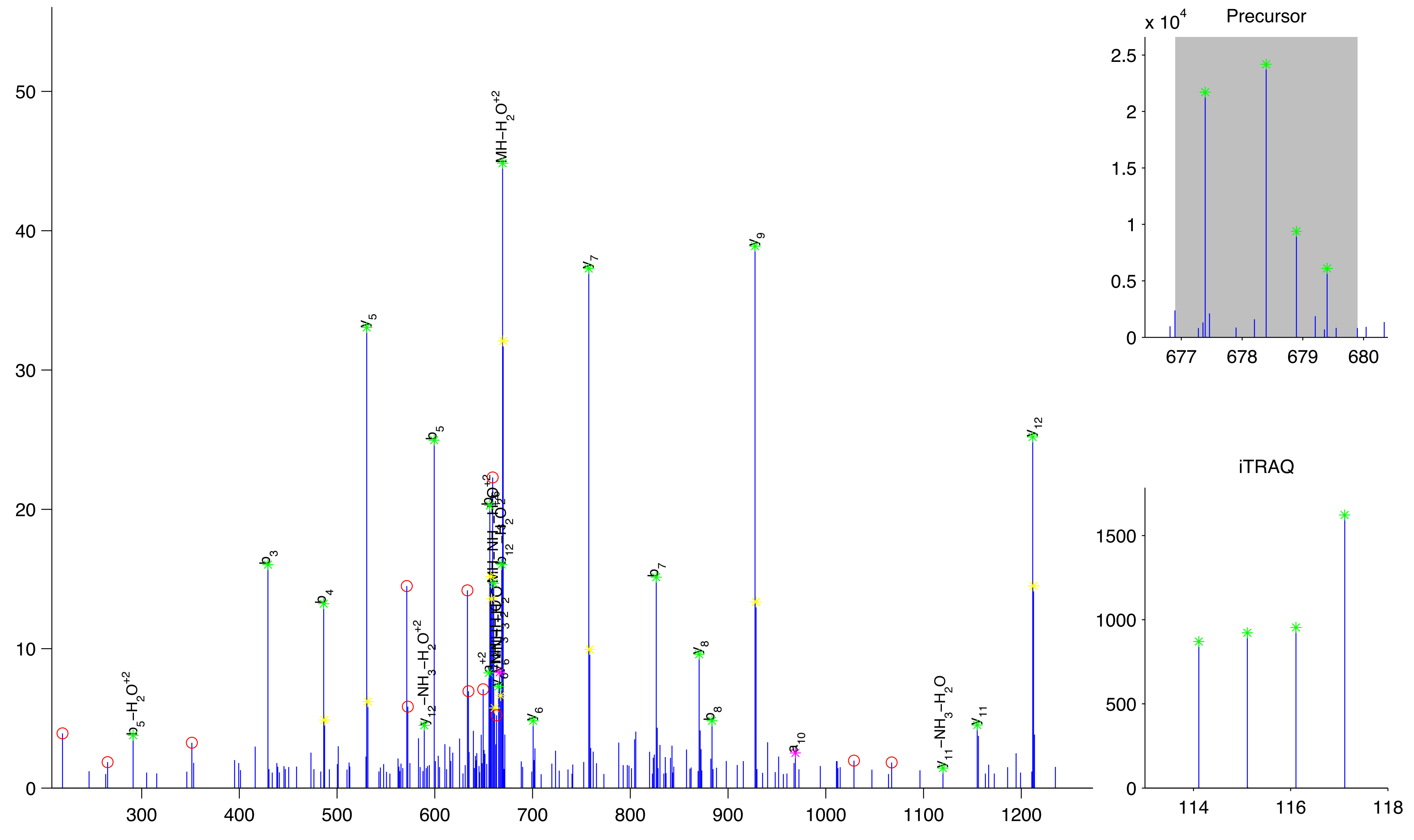

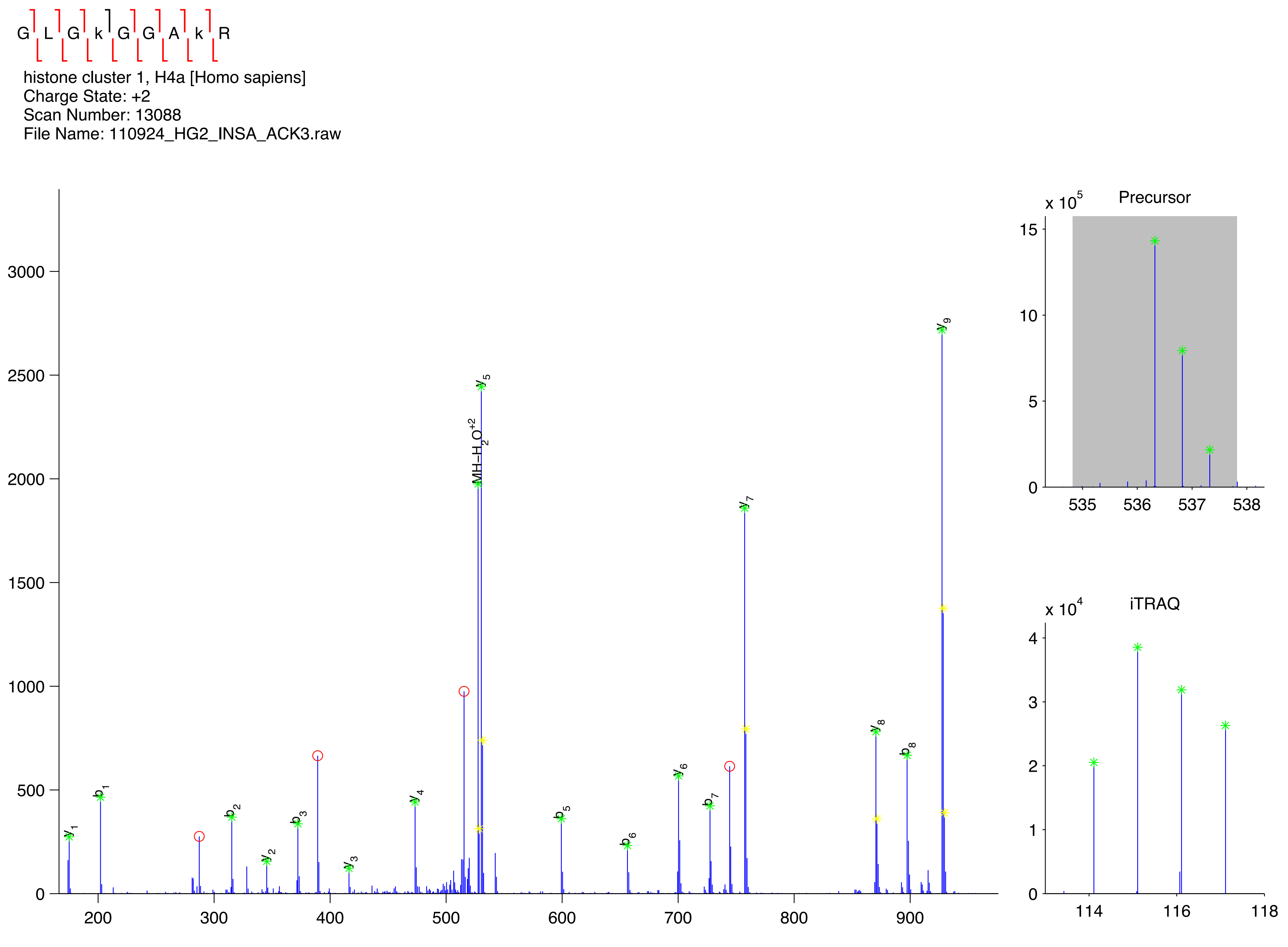

G L G k G G A k R
histone cluster 1, H4a [Homo sapiens]
Charge State: +2
Scan Number: 13088
File Name: 110924_HG2_INSA_ACK3.raw
Precursor
x 10^5
iTRAQ
x 10^4
MH-H2O+2
y1 b1 b2 y2 b3 y3 y4 y5 b5 b6 y6 b7 y7 y8 b8 y9

G G k G L G k G G A k R

histone cluster 1, H4a [Homo sapiens]
Charge State: +2
Scan Number: 13153
File Name: 110924_HG2_INSA_ACK.raw

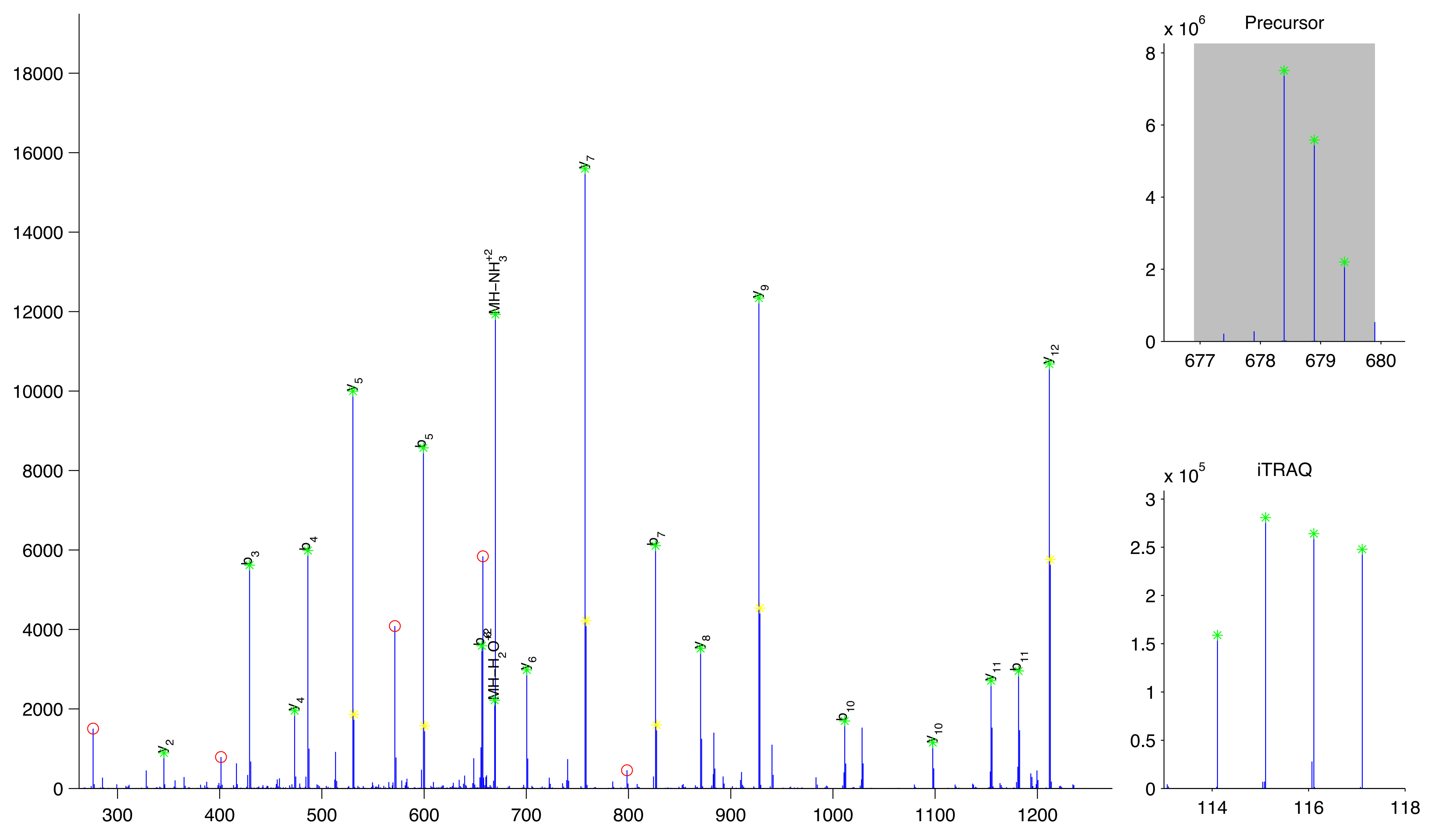

G k G G k G L G k G G A k R

histone cluster 1, H4a [Homo sapiens]
Charge State: +2
Scan Number: 13212
File Name: 110924_HG2_INSA_ACK.raw

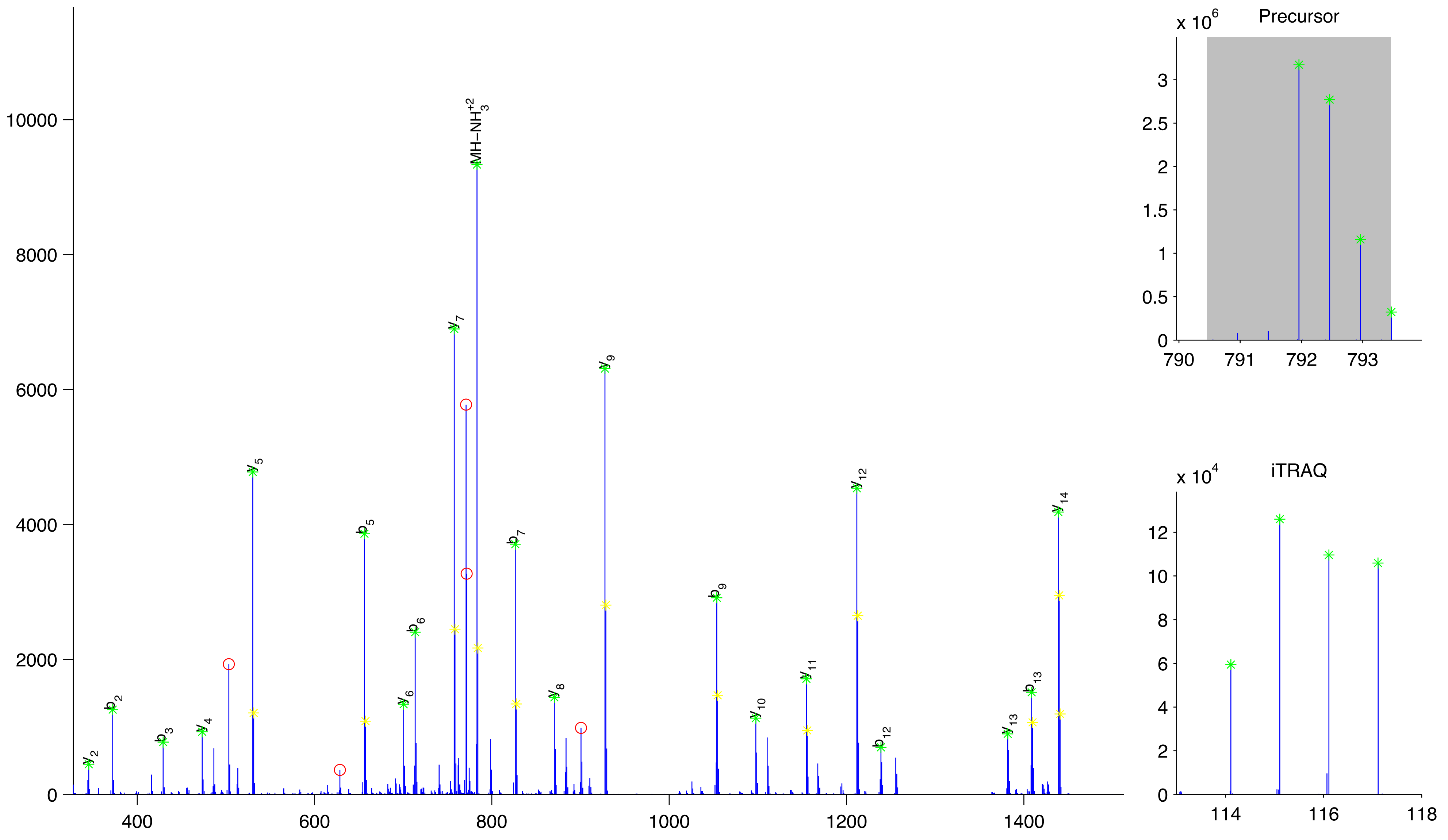

G k G G k G L G k G G A k R

histone cluster 1, H4a [Homo sapiens]
Charge State: +2
Scan Number: 13222
File Name: 110924_HG2_INSA_ACK.raw

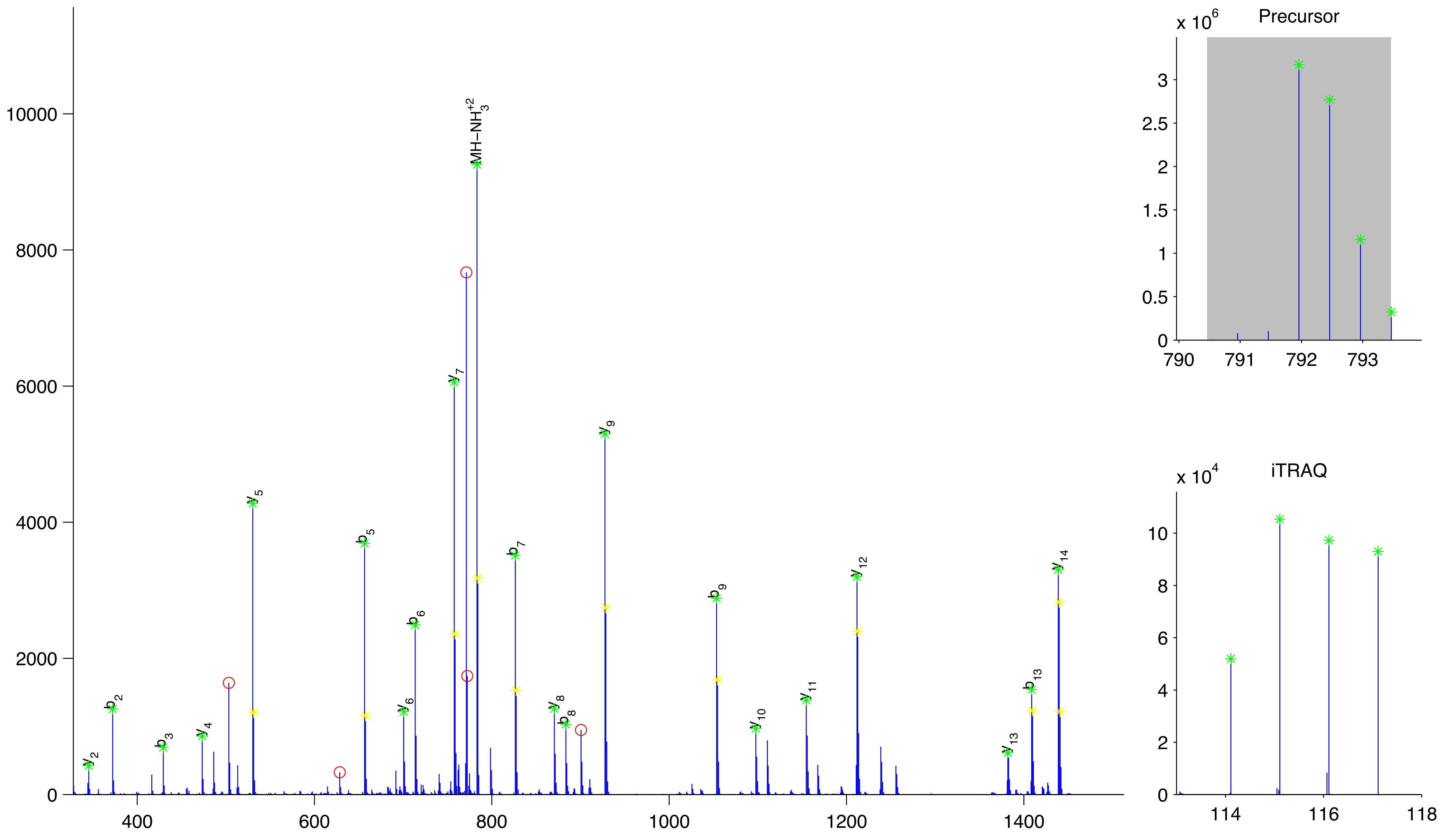

G G k G L G k G G A k R

histone cluster 1, H4a [Homo sapiens]
Charge State: +2
Scan Number: 13229
File Name: 110924_HG2_INSA_ACK3.raw

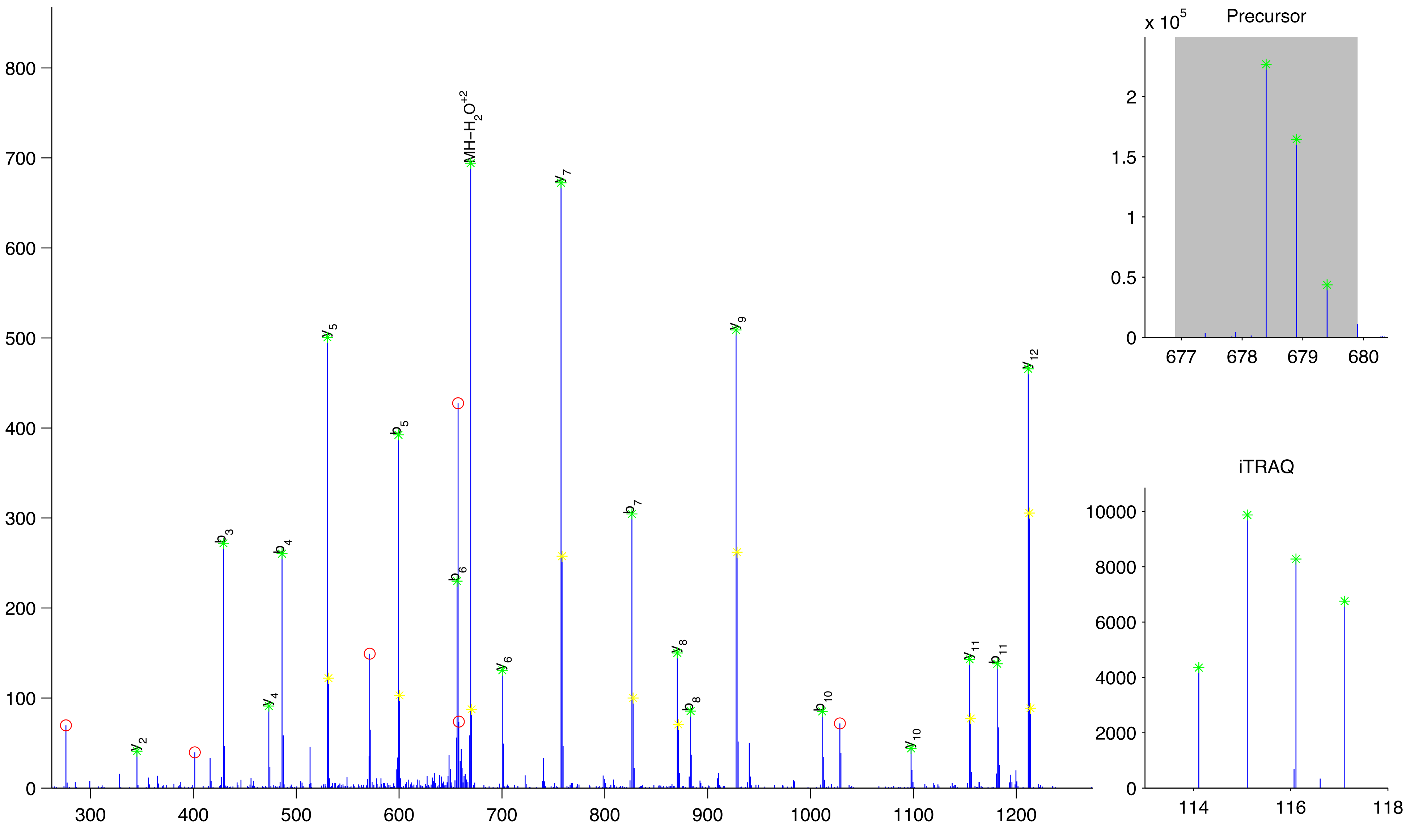

G G k G L G k G G A k R

histone cluster 1, H4a [Homo sapiens]
Charge State: +2
Scan Number: 13235
File Name: 110924_HG2_INSA_ACK.raw

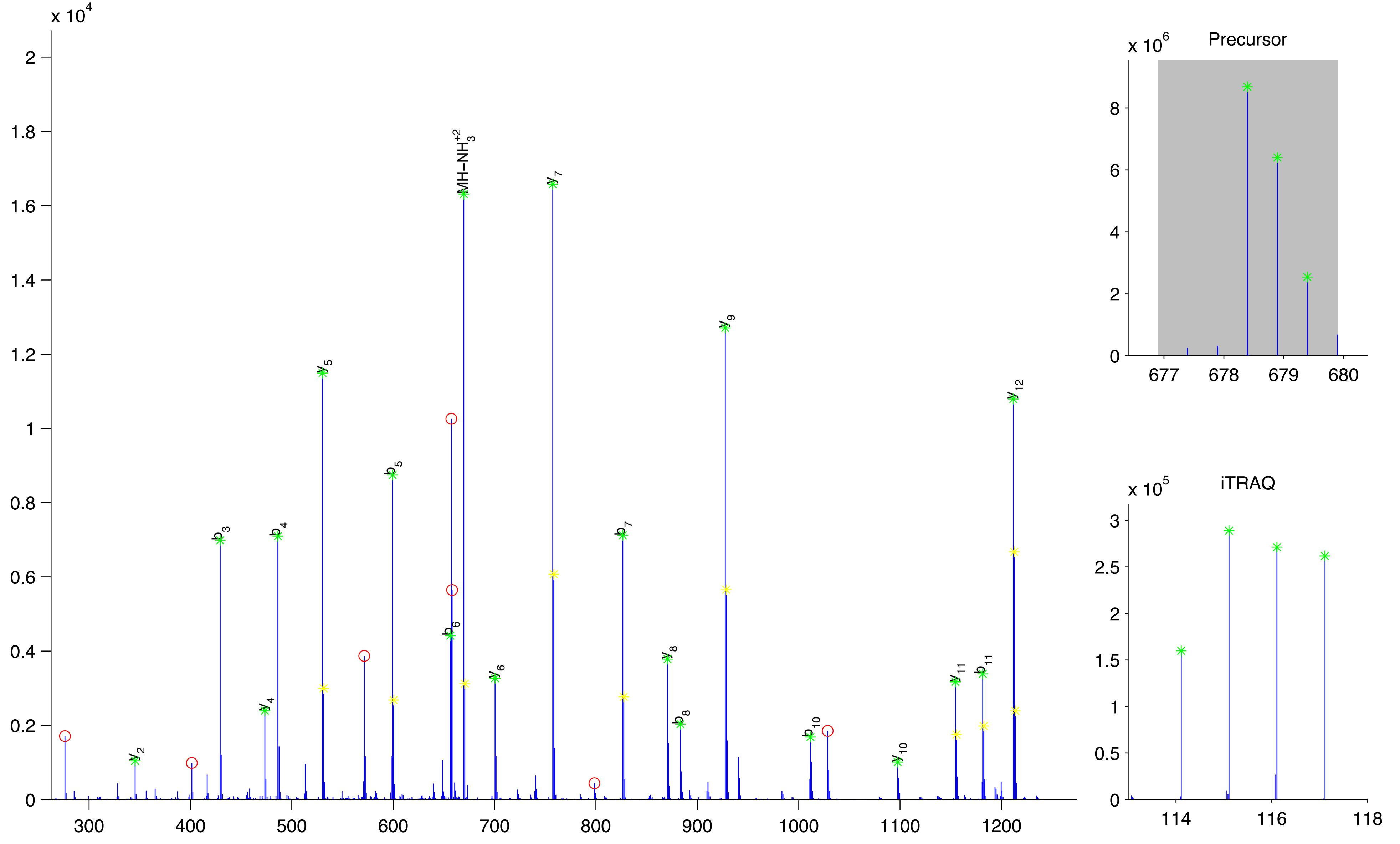

G G k G L G k G G A k R

histone cluster 1, H4a [Homo sapiens]
Charge State: +2
Scan Number: 13243
File Name: 110924_HG2_INSA_ACK3.raw

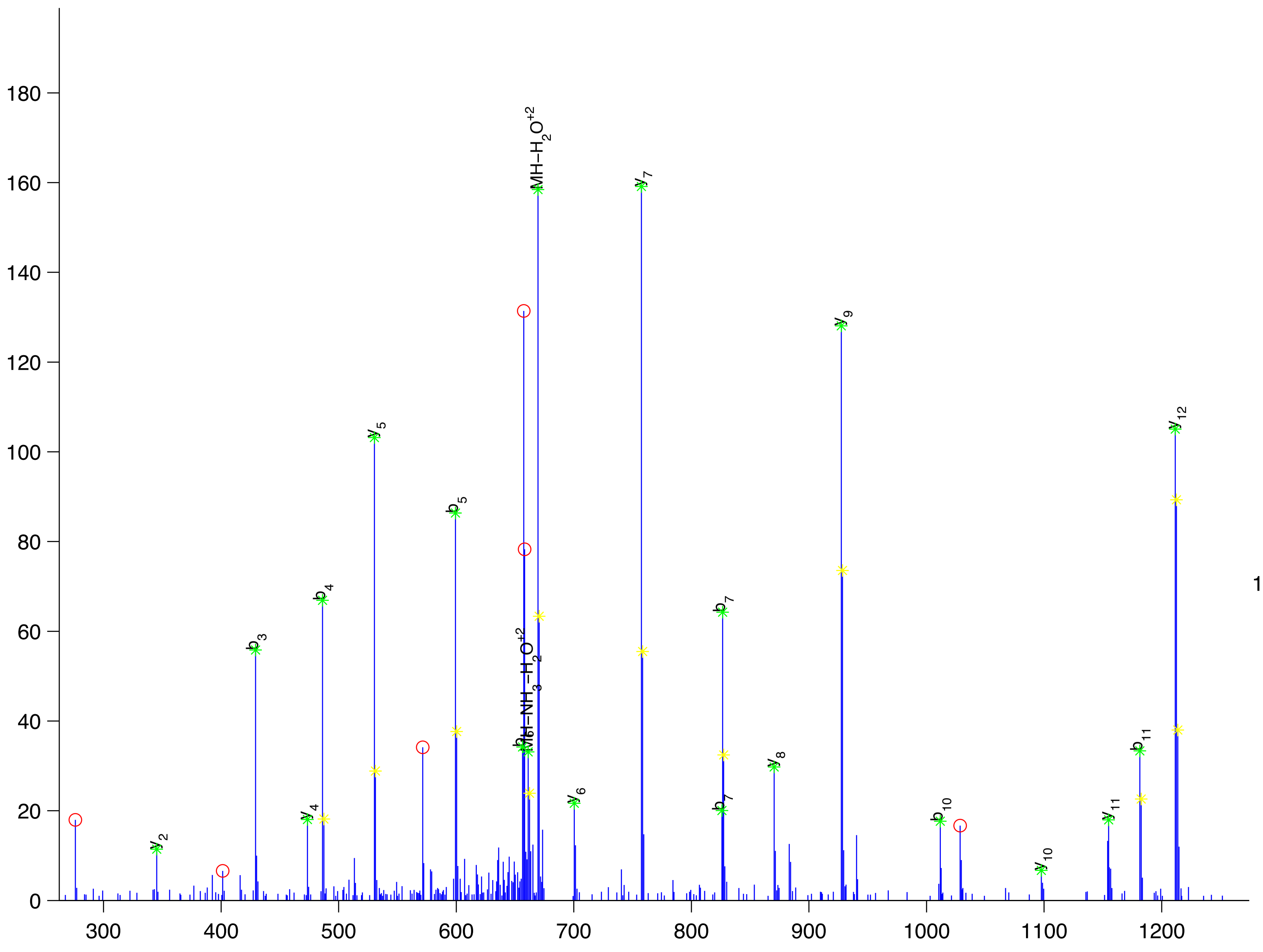

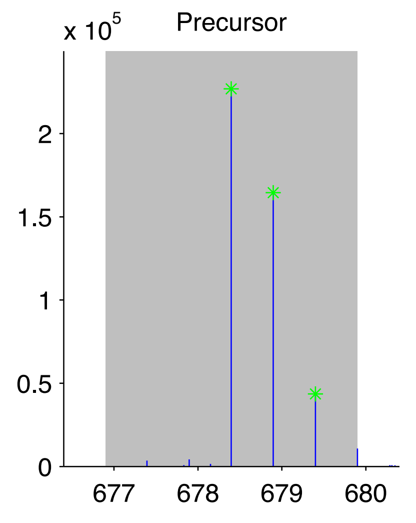

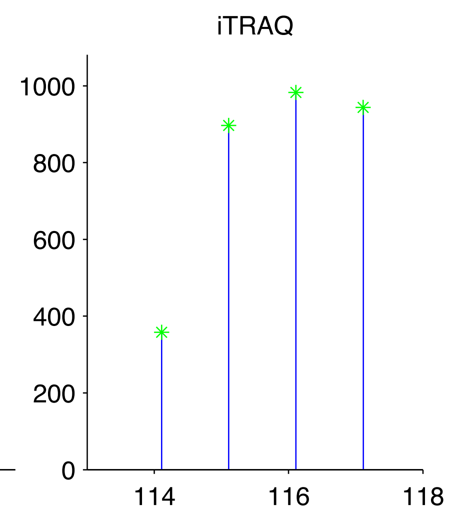

G G k G L G k G G A k R

histone cluster 1, H4a [Homo sapiens]
Charge State: +2
Scan Number: 13321
File Name: 110924_HG2_INSA_ACK.raw

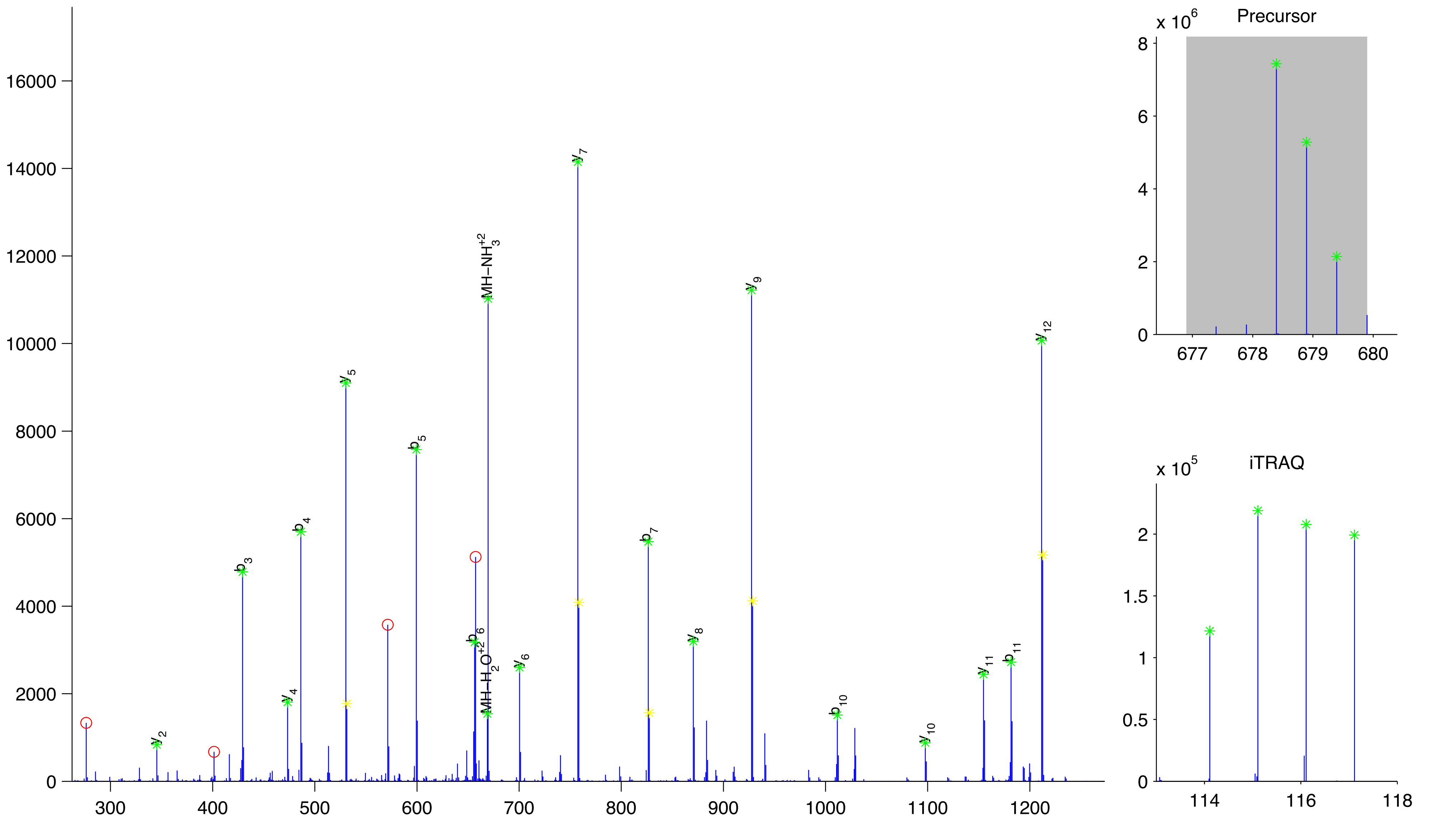

G k G G k G L G k G G A k R

histone cluster 1, H4a [Homo sapiens]
Charge State: +2
Scan Number: 13380
File Name: 110924_HG2_INSA_ACK.raw

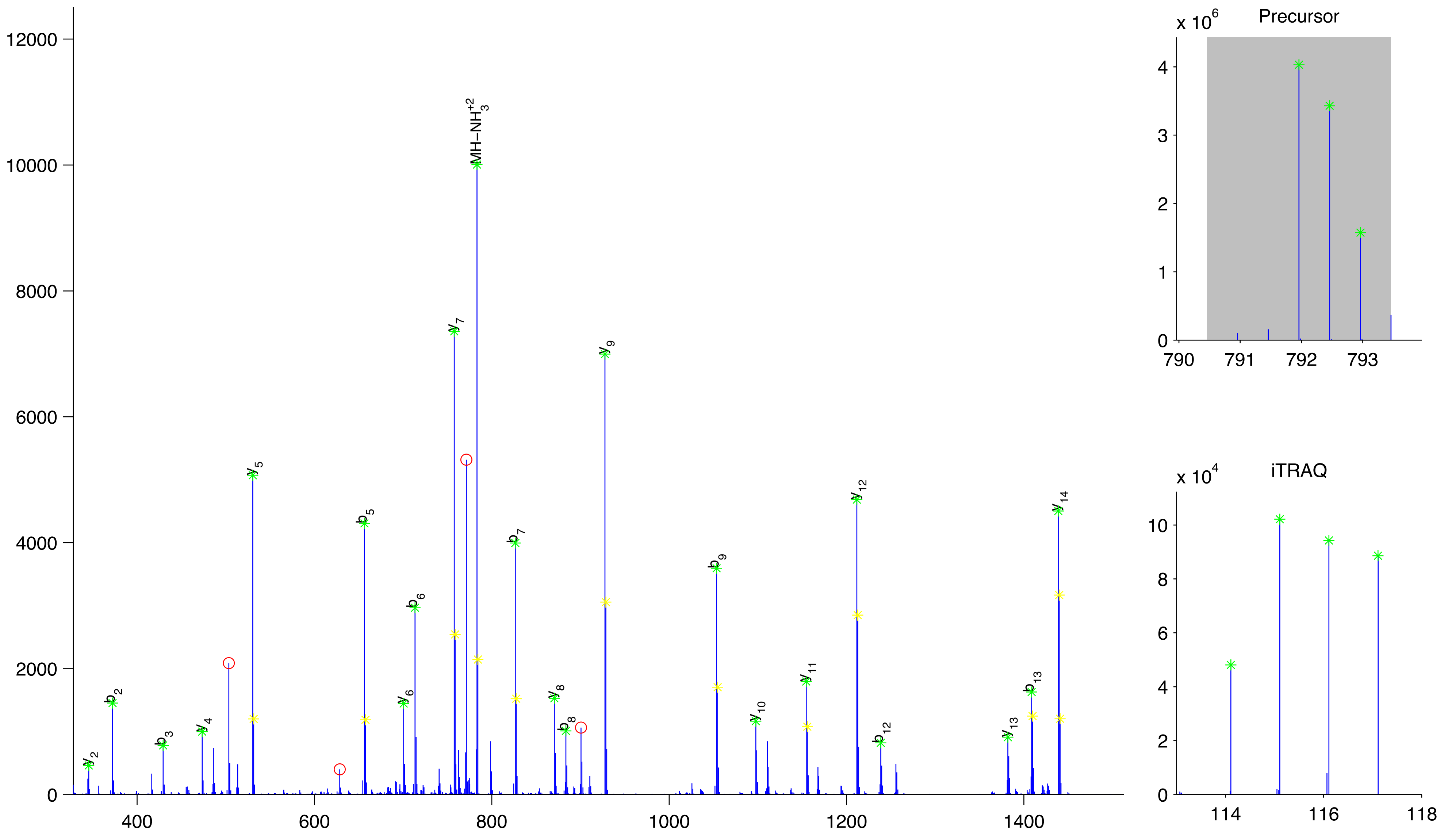

G k G G k G L G k G G A k R

histone cluster 1, H4a [Homo sapiens]
Charge State: +2
Scan Number: 13396
File Name: 110924_HG2_INSA_ACK.raw

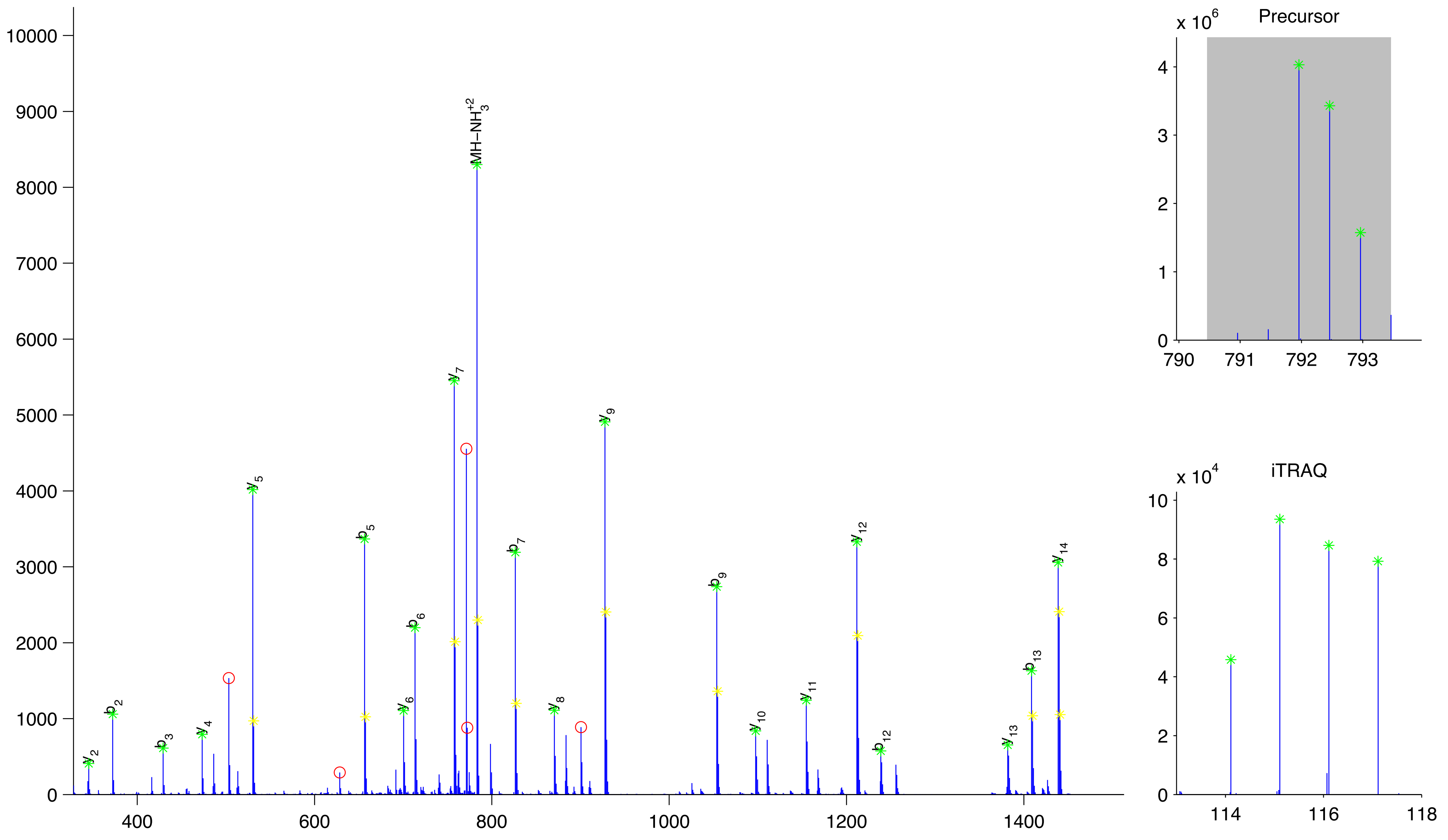

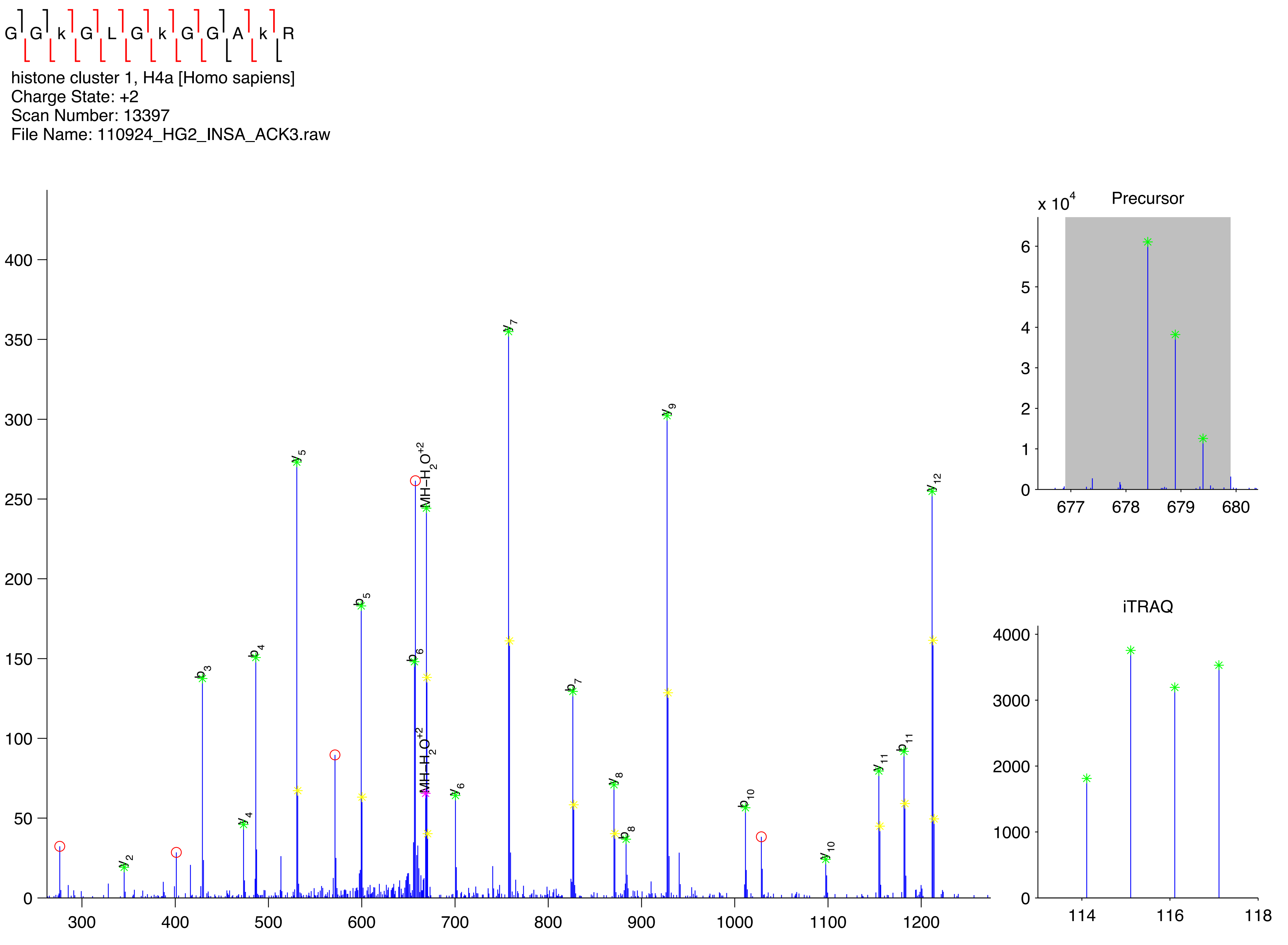
G G k G L G k G G A k R
histone cluster 1, H4a [Homo sapiens]
Charge State: +2
Scan Number: 13397
File Name: 110924_HG2_INSA_ACK3.raw
Precursor
x 10^4
iTRAQ

G G k G L G k G G A k R

histone cluster 1, H4a [Homo sapiens]
Charge State: +2
Scan Number: 13428
File Name: 110924_HG2_INSA_ACK3.raw

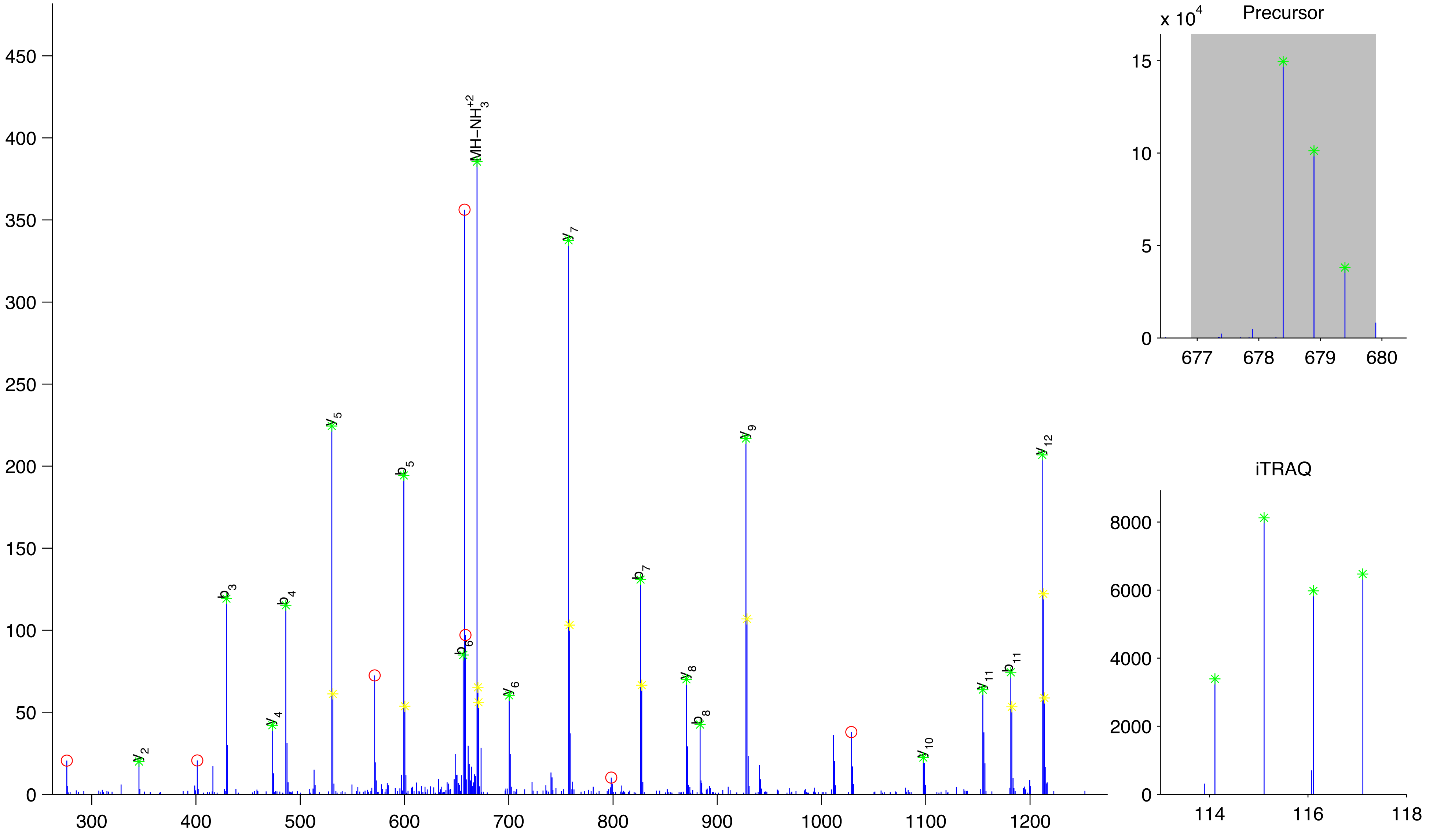

G k G G k G L G k G G A k R

histone cluster 1, H4a [Homo sapiens]
Charge State: +3
Scan Number: 13485
File Name: 110924_HG2_INSA_ACK.raw

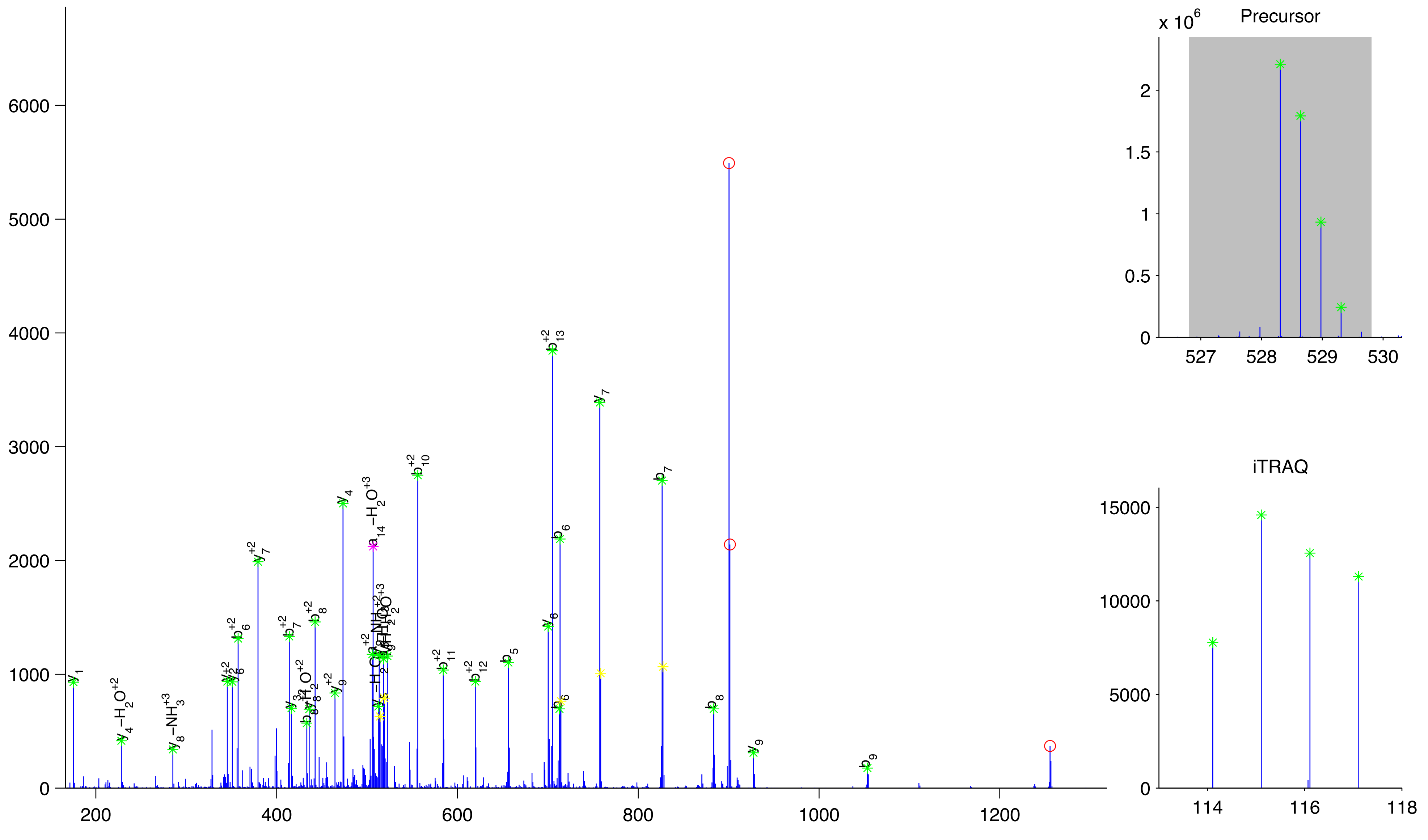

G G k G L G k G G A k R

histone cluster 1, H4a [Homo sapiens]
Charge State: +2
Scan Number: 13487
File Name: 110924_HG2_INSA_ACK.raw

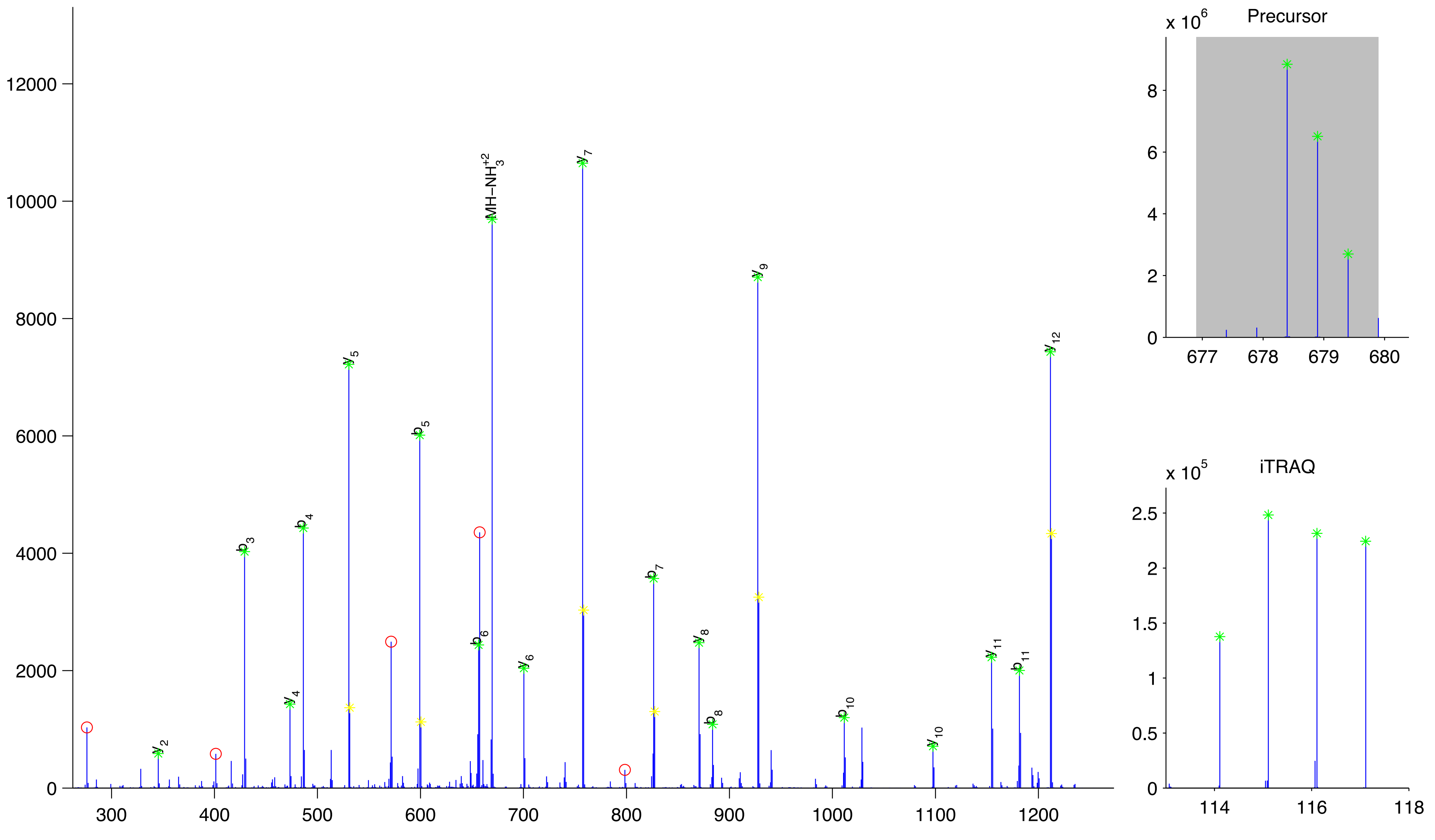

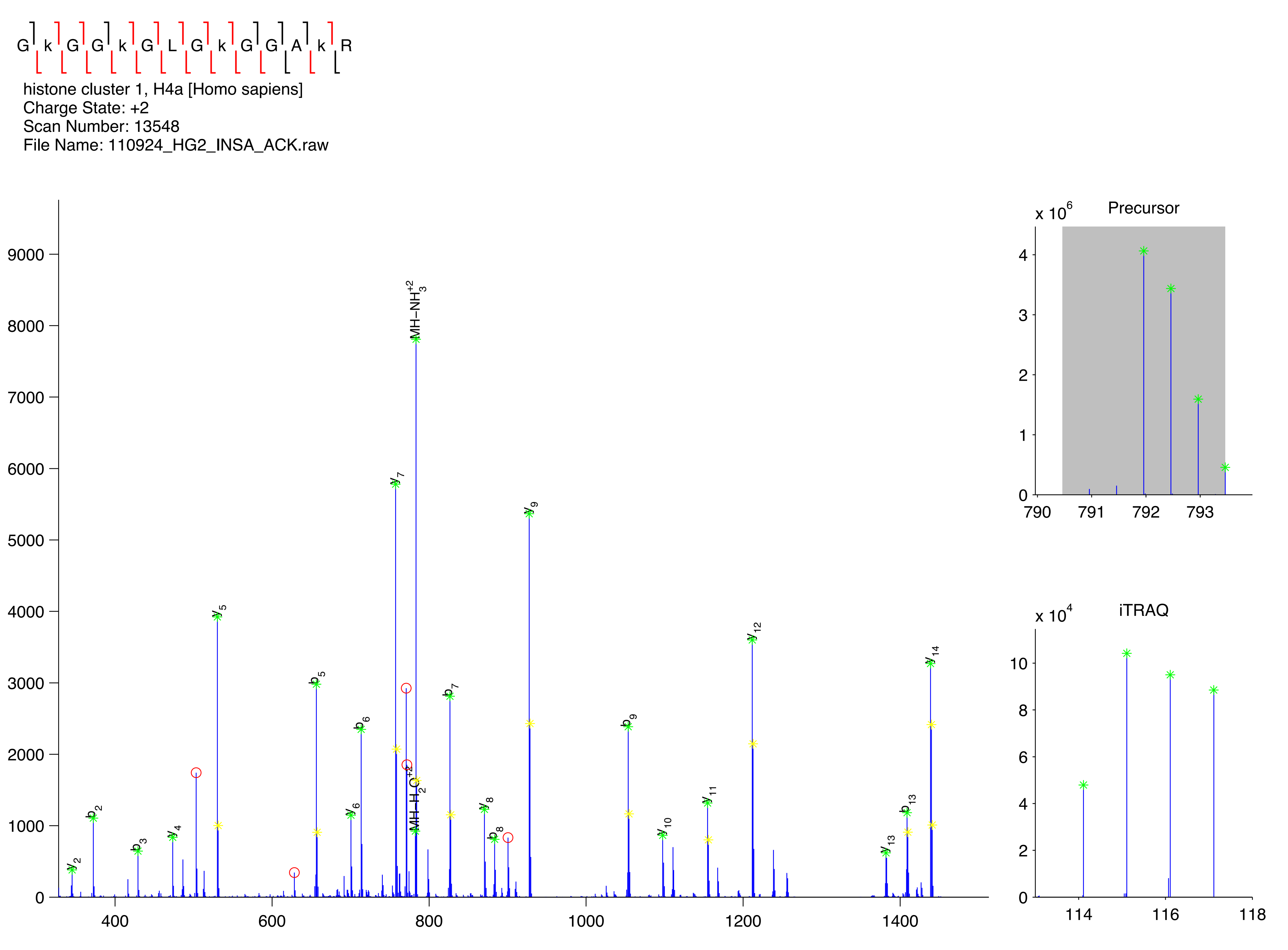

G k G G k G L G k G G A k R
histone cluster 1, H4a [Homo sapiens]
Charge State: +2
Scan Number: 13548
File Name: 110924_HG2_INSA_ACK.raw
y2
b2
b3
y4
y5
b5
y6
b6
y7
MH-H2O+2
MH-NH3+2
b7
y8
b8
y9
b9
y10
y11
y12
y13
b13
y14
0
1000
2000
3000
4000
5000
6000
7000
8000
9000
400
600
800
1000
1200
1400
x 10^6
Precursor
0
1
2
3
4
790
791
792
793
x 10^4
iTRAQ
0
2
4
6
8
10
114
116
118

G k G G k G L G k G G A k R

histone cluster 1, H4a [Homo sapiens]
Charge State: +2
Scan Number: 13556
File Name: 110924_HG2_INSA_ACK.raw

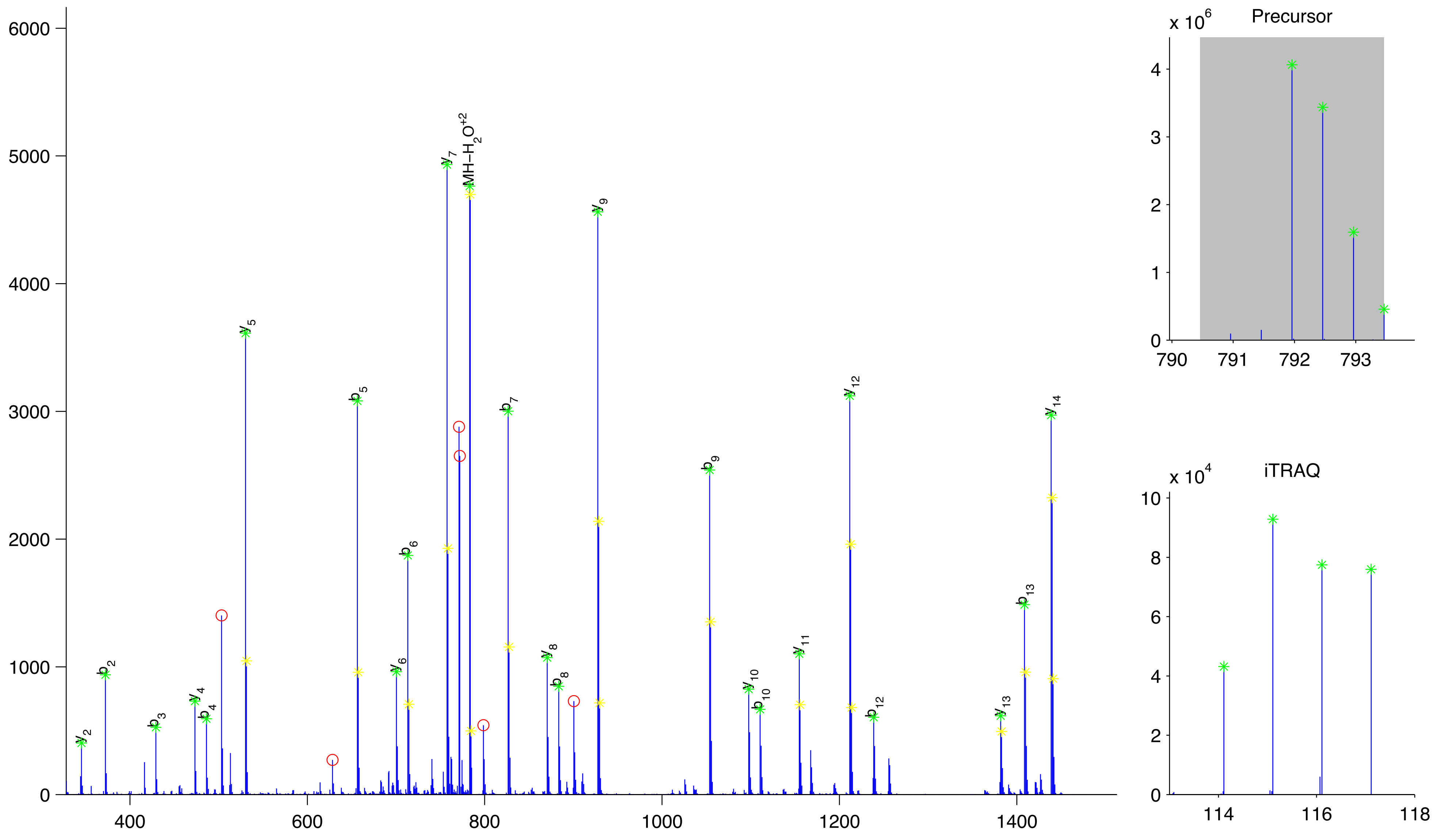

G G k G L G k G G A k R

histone cluster 1, H4a [Homo sapiens]
Charge State: +2
Scan Number: 13655
File Name: 110924_HG2_INSA_ACK.raw

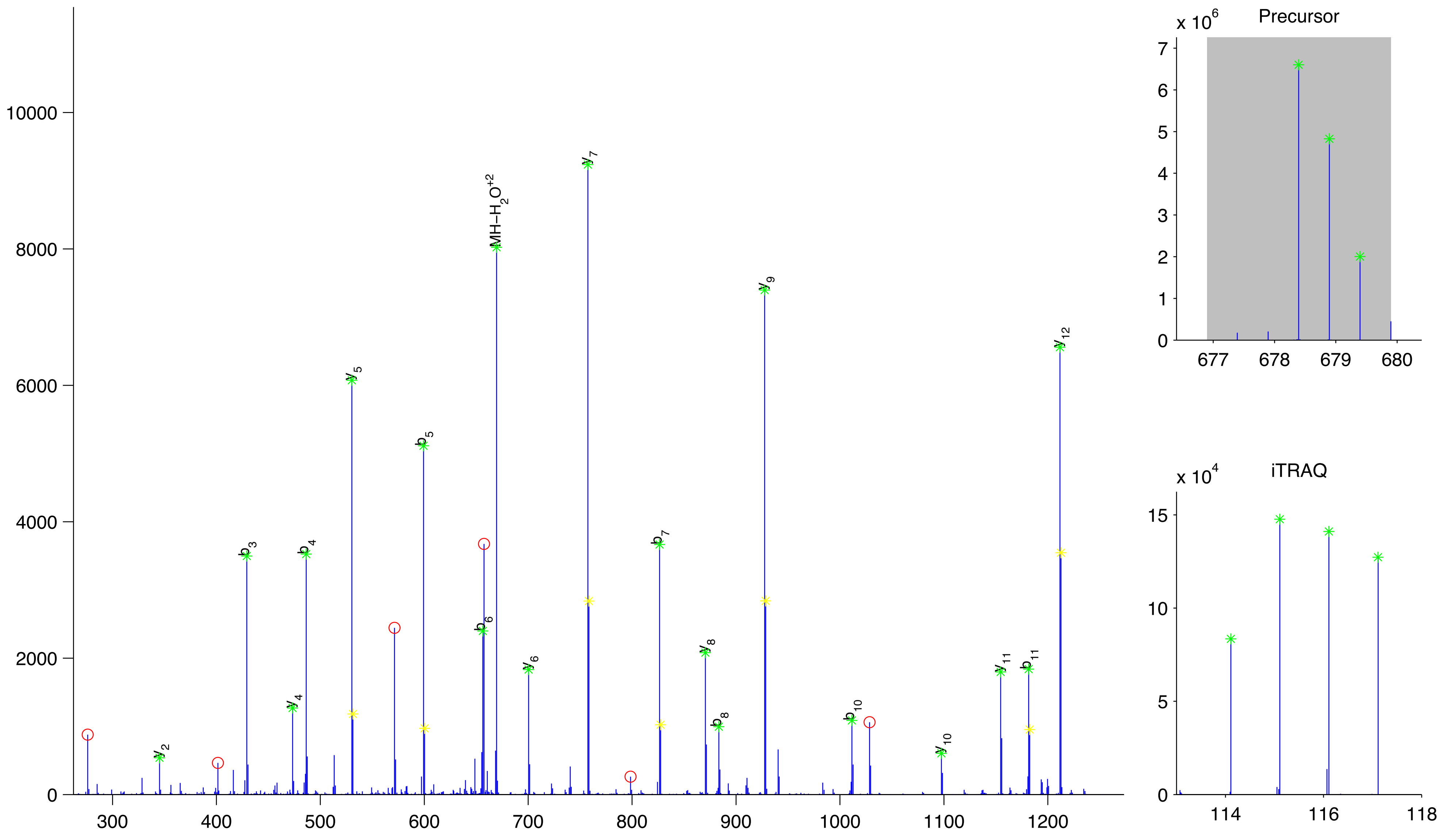

G L G k G G A k R

histone cluster 1, H4a [Homo sapiens]
Charge State: +2
Scan Number: 13707
File Name: 110924_HG2_INSA_ACK3.raw

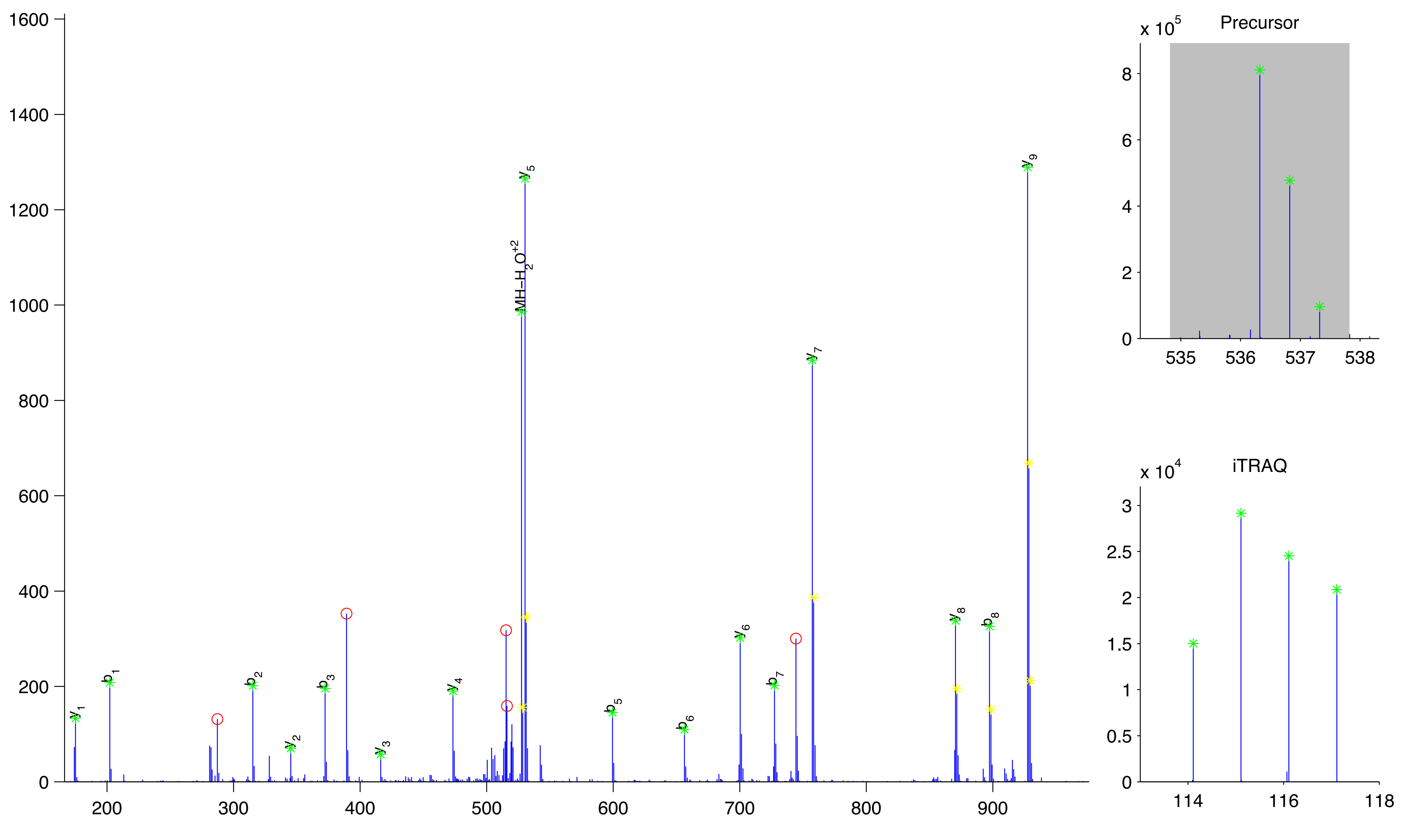

G k G G k G L G k G G A k R

histone cluster 1, H4a [Homo sapiens]
Charge State: +2
Scan Number: 13716
File Name: 110924_HG2_INSA_ACK.raw

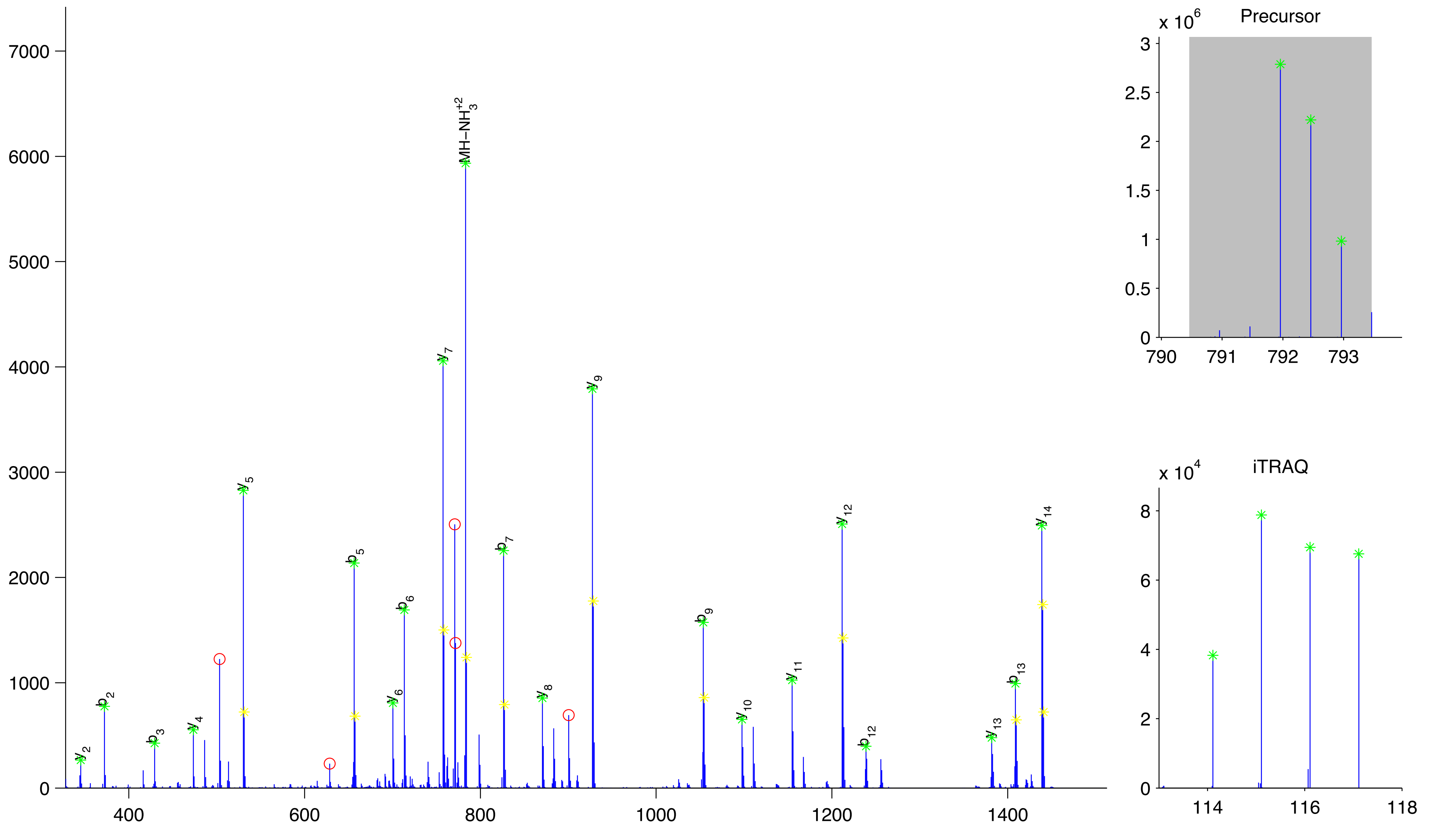

G k G G k G L G k G G A k R

histone cluster 1, H4a [Homo sapiens]
Charge State: +2
Scan Number: 13728
File Name: 110924_HG2_INSA_ACK.raw

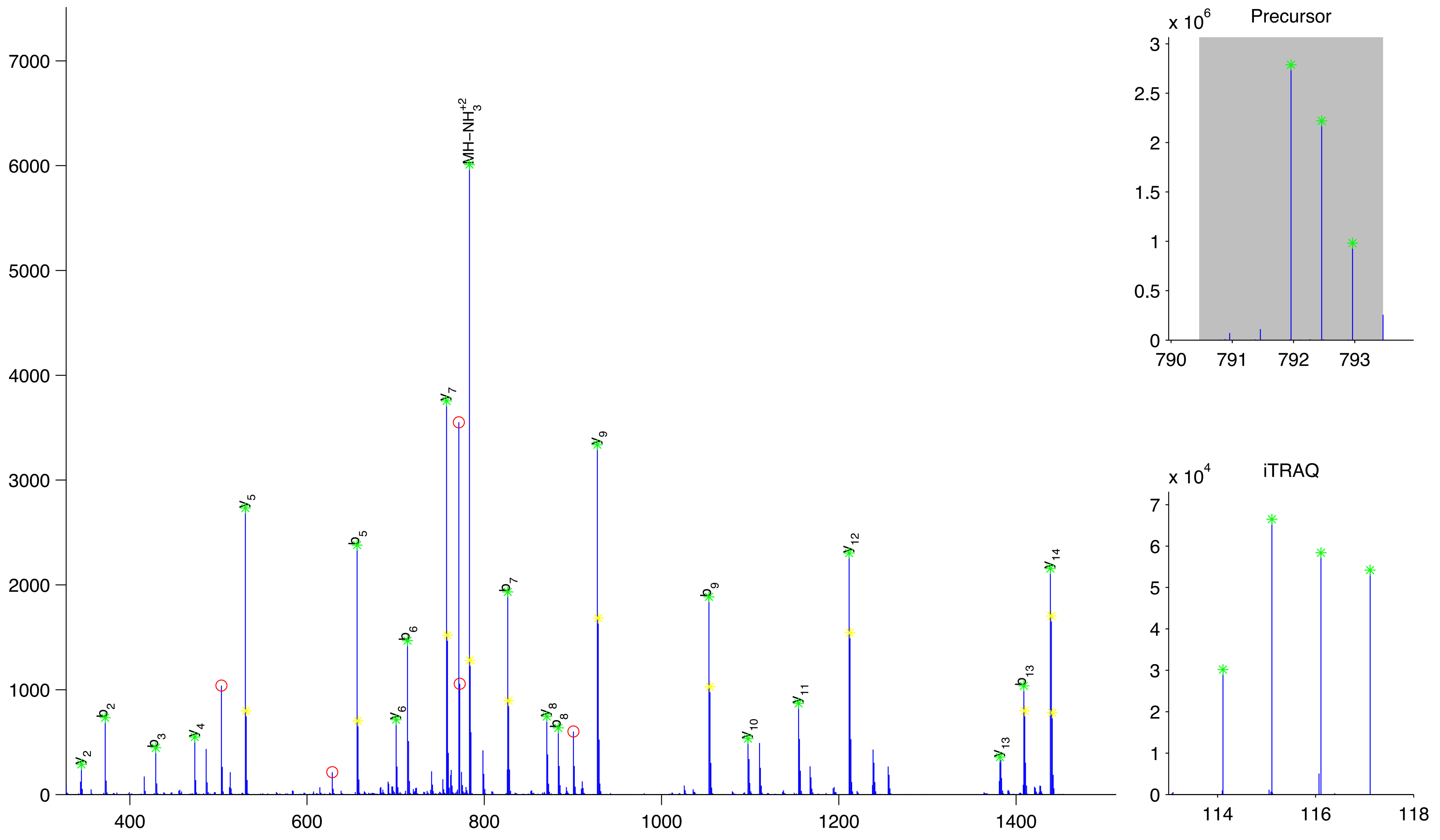

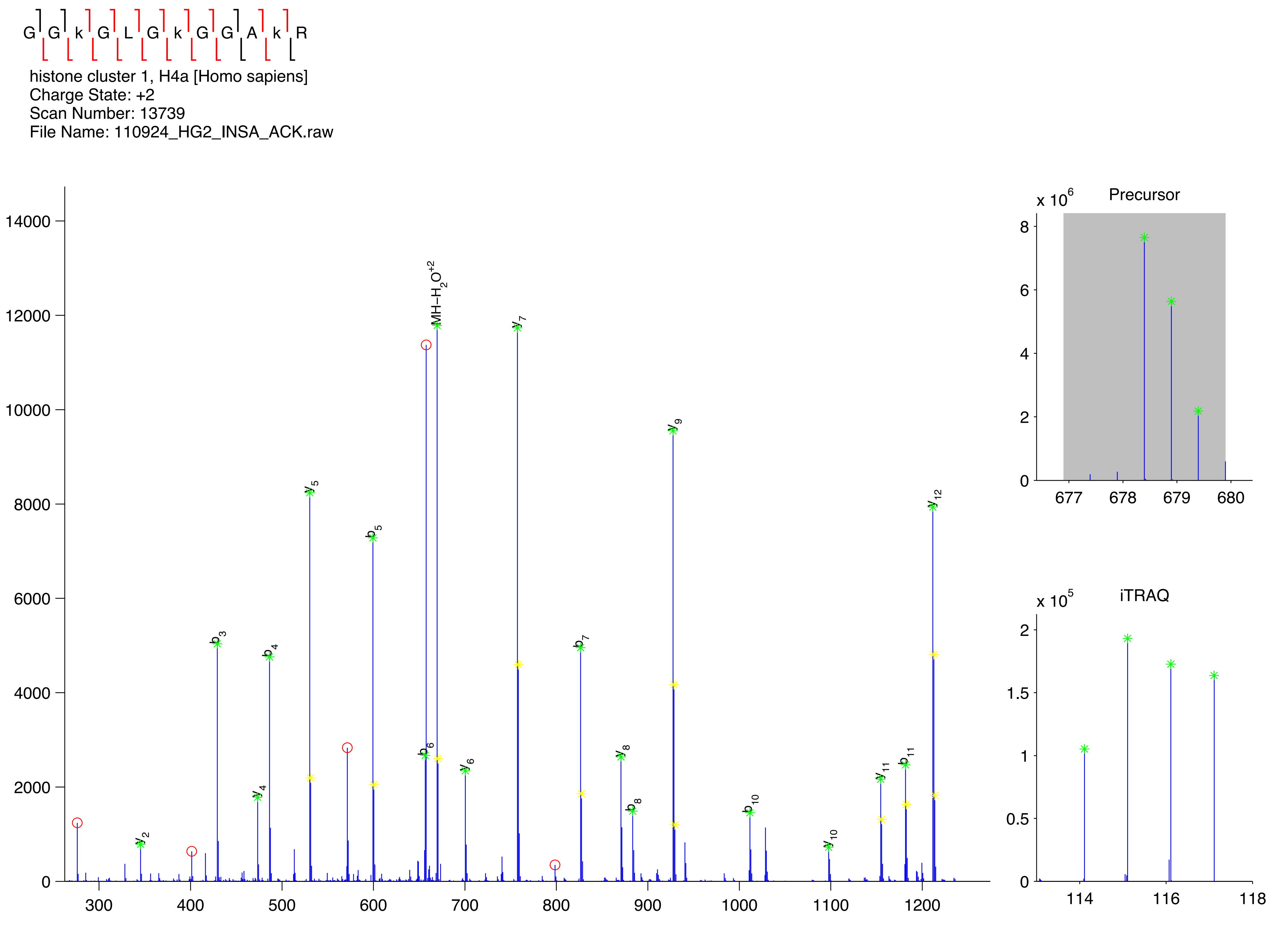
G G k G L G k G G A k R
histone cluster 1, H4a [Homo sapiens]
Charge State: +2
Scan Number: 13739
File Name: 110924_HG2_INSA_ACK.raw
y2
b3
y4
b4
y5
b5
b6
MH−H2O+2
y6
y7
b7
y8
b8
y9
b10
y10
y11
b11
y12
x 10^6
Precursor
677
678
679
680
x 10^5
iTRAQ
114
116
118

G G k G L G k G G A k R

histone cluster 1, H4a [Homo sapiens]
Charge State: +2
Scan Number: 13823
File Name: 110924_HG2_INSA_ACK.raw

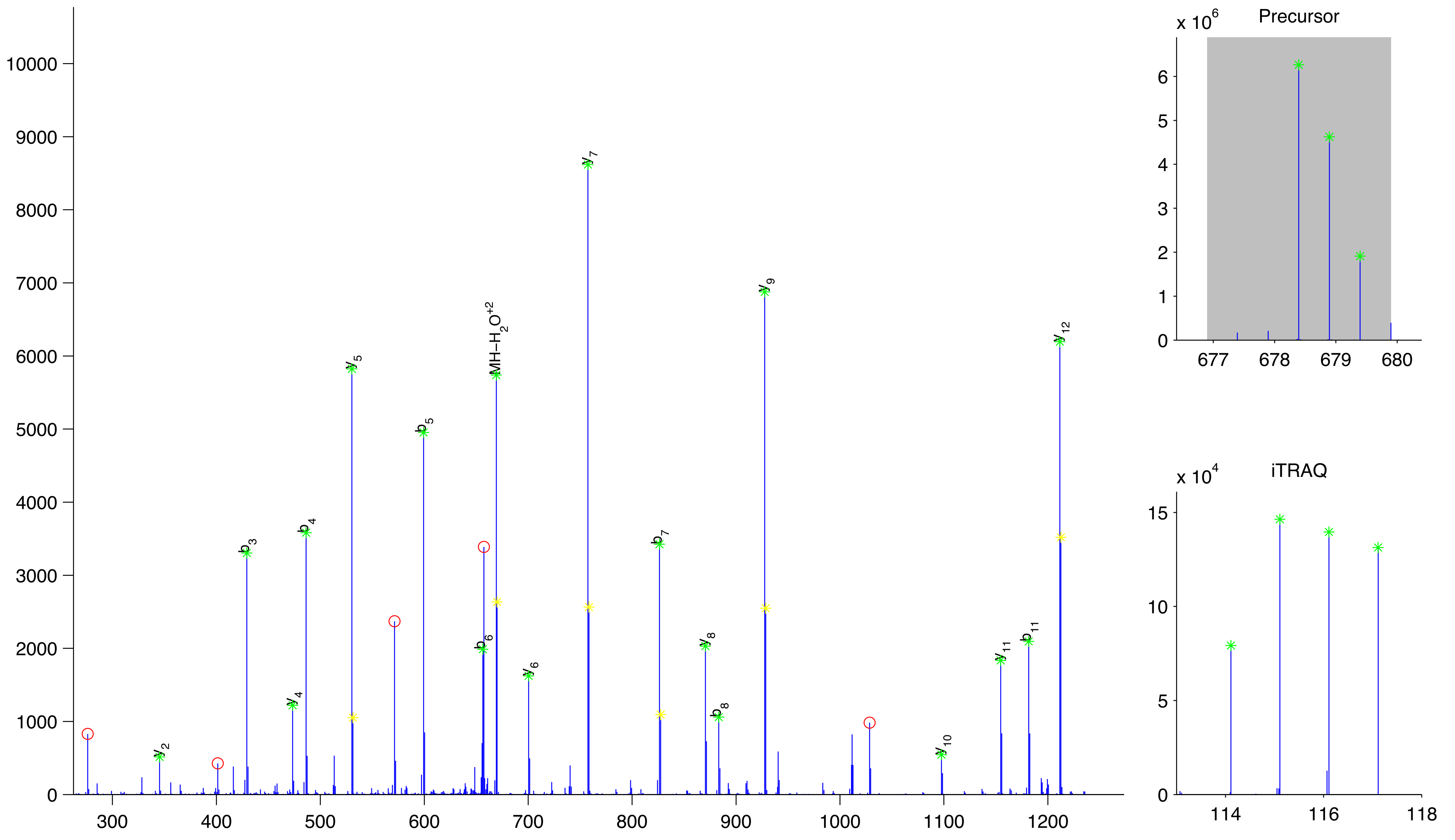

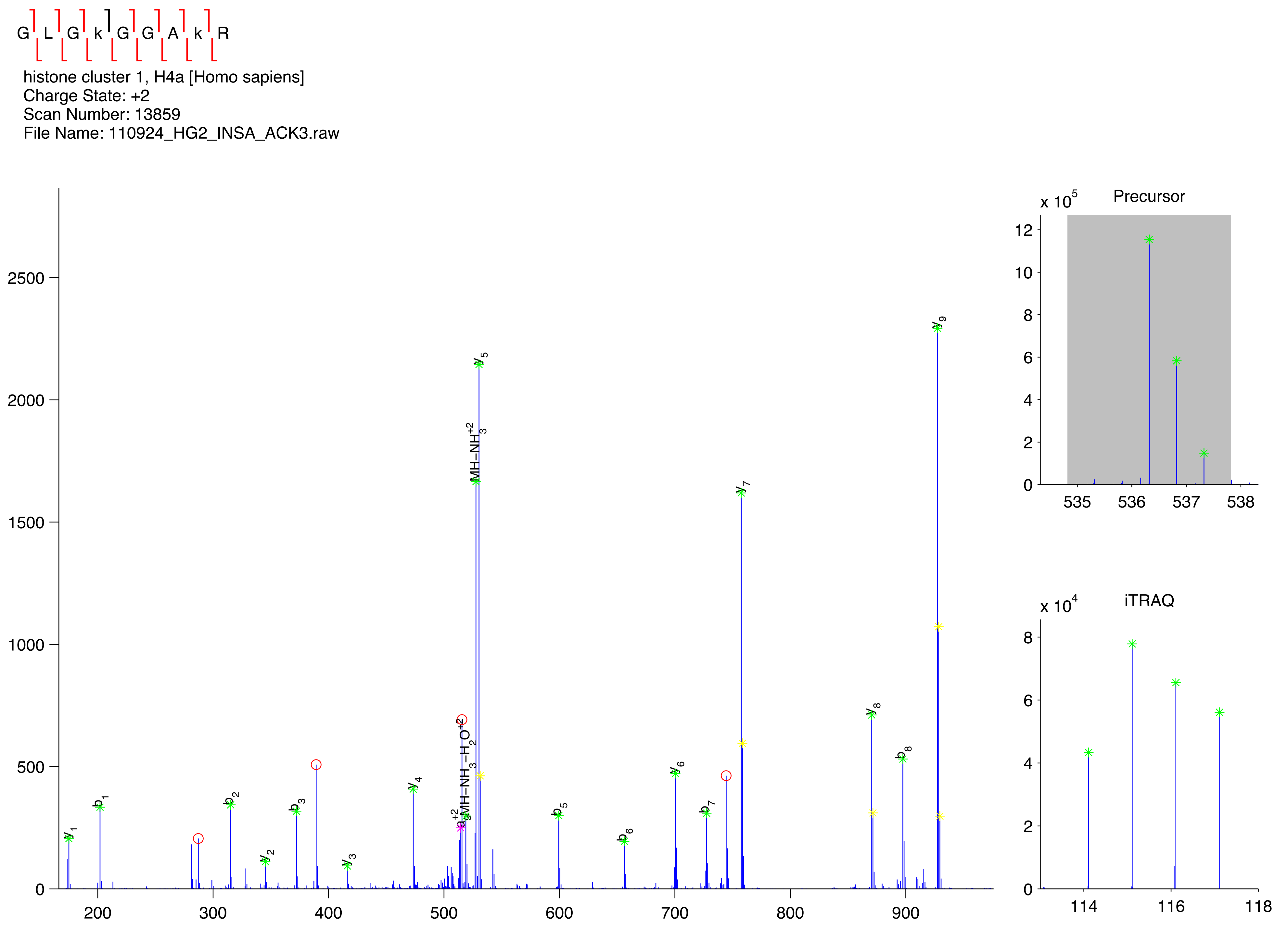
G L G k G G A k R
histone cluster 1, H4a [Homo sapiens]
Charge State: +2
Scan Number: 13859
File Name: 110924_HG2_INSA_ACK3.raw
Precursor
iTRAQ

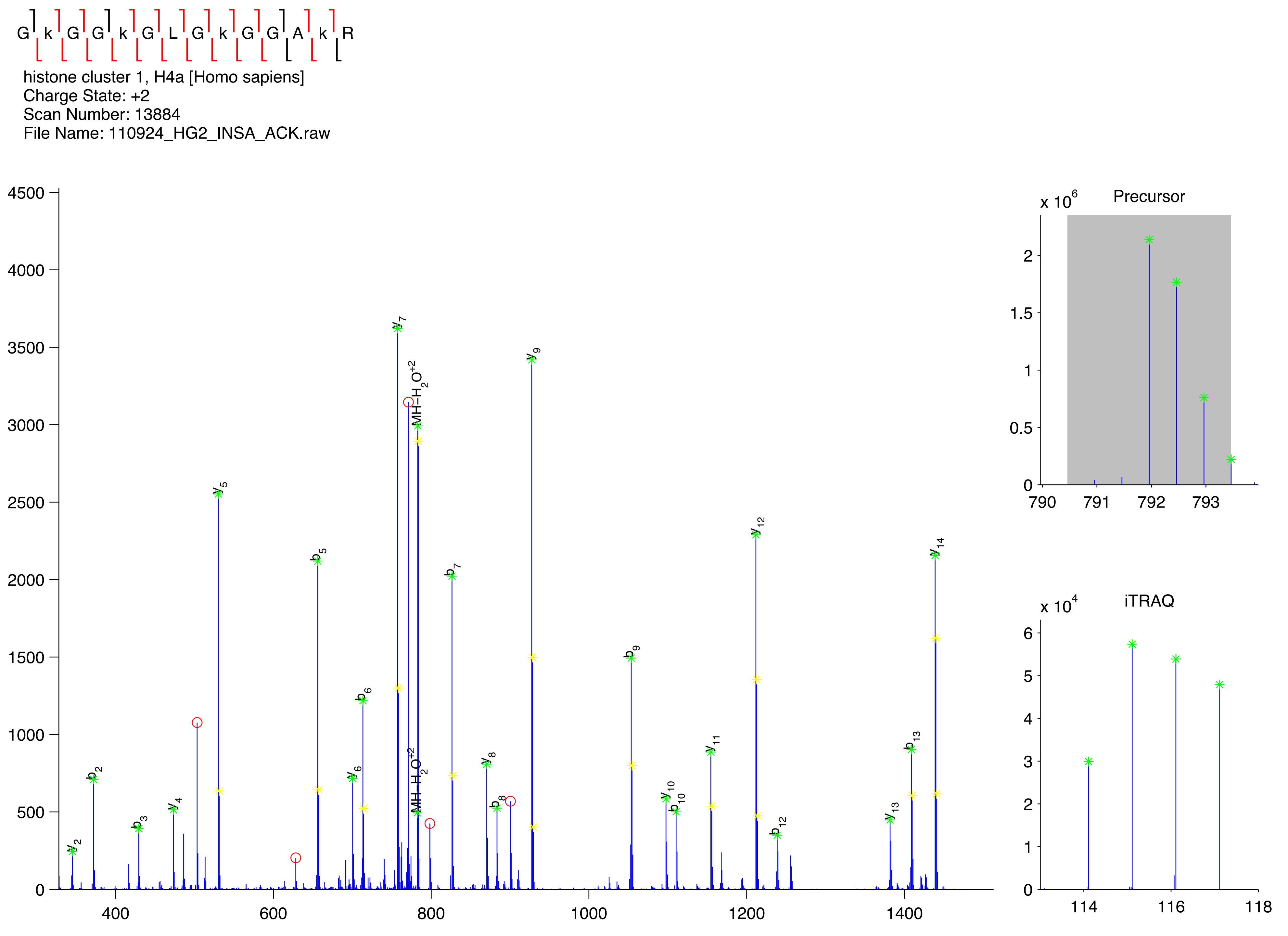
G k G G k G L G k G G A k R
histone cluster 1, H4a [Homo sapiens]
Charge State: +2
Scan Number: 13884
File Name: 110924_HG2_INSA_ACK.raw
y2
b2
b3
y4
y5
b5
y6
b6
y7
MH-H2O+2
b7
y8
b8
y9
b9
y10
b10
y11
y12
b12
y13
b13
y14
x 10^6
Precursor
x 10^4
iTRAQ

G k G G k G L G k G G A k R

histone cluster 1, H4a [Homo sapiens]
Charge State: +2
Scan Number: 13900
File Name: 110924_HG2_INSA_ACK.raw

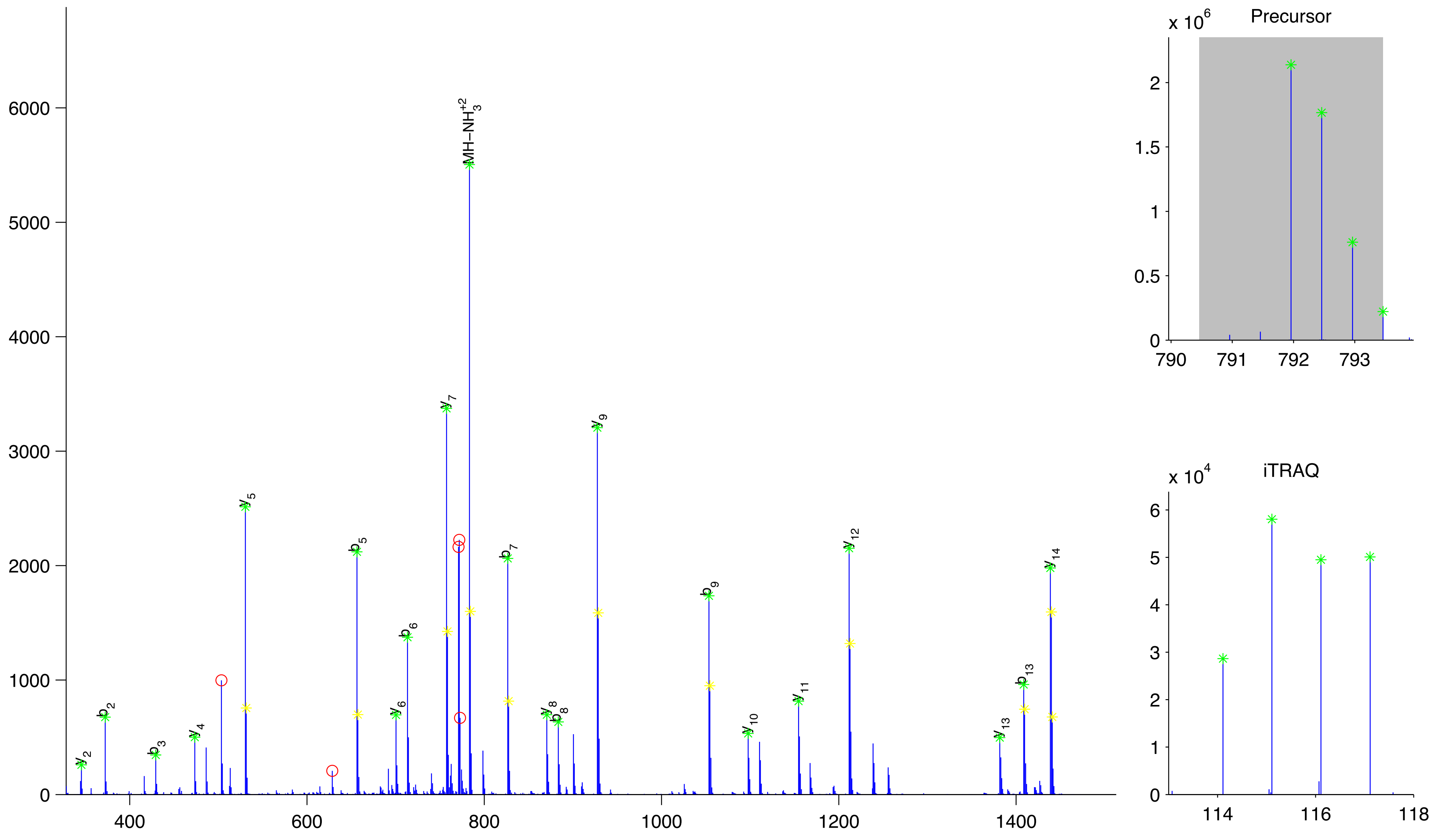

G G k G L G k G G A k R

histone cluster 1, H4a [Homo sapiens]
Charge State: +2
Scan Number: 13907
File Name: 110924_HG2_INSA_ACK.raw

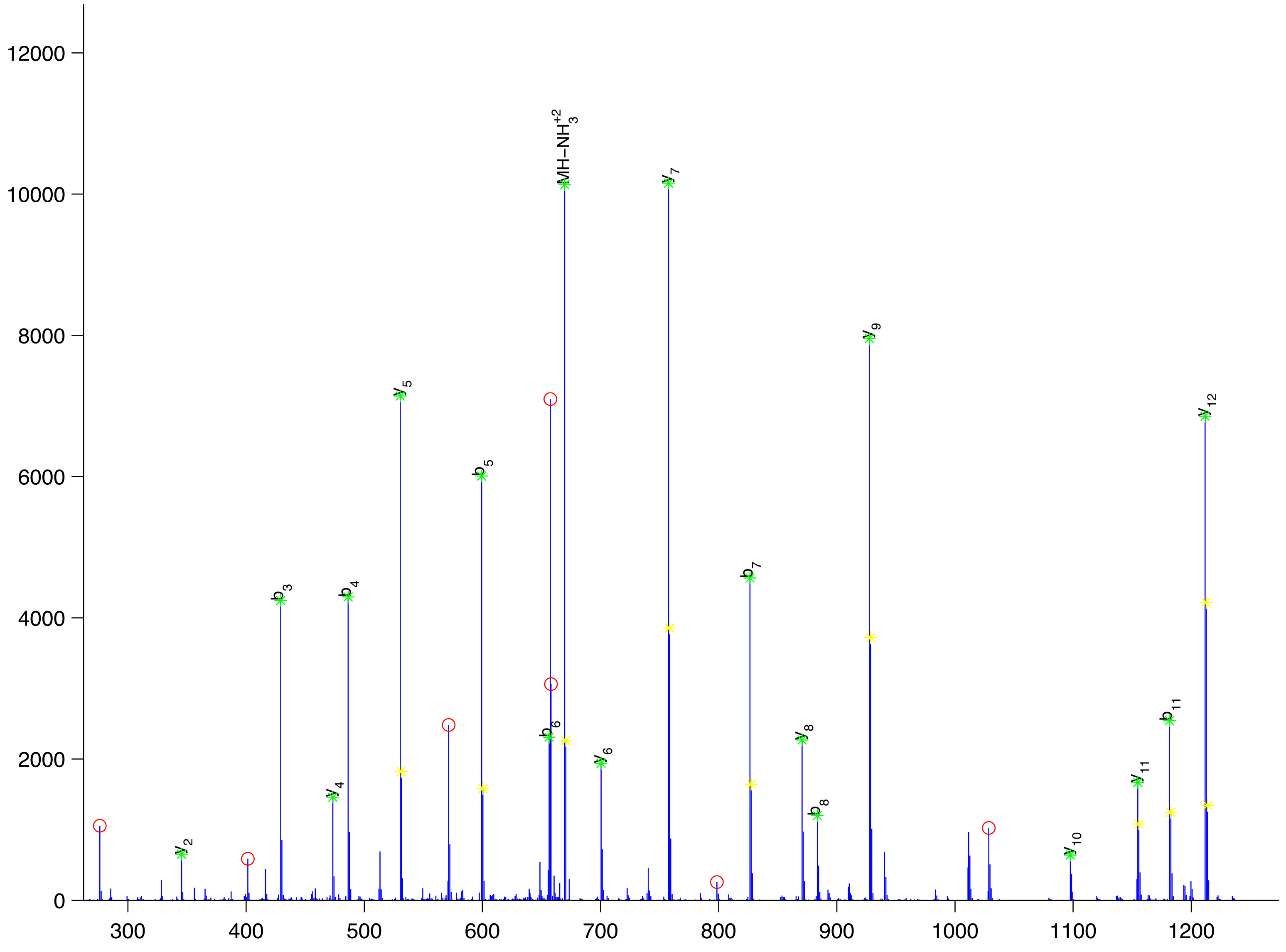

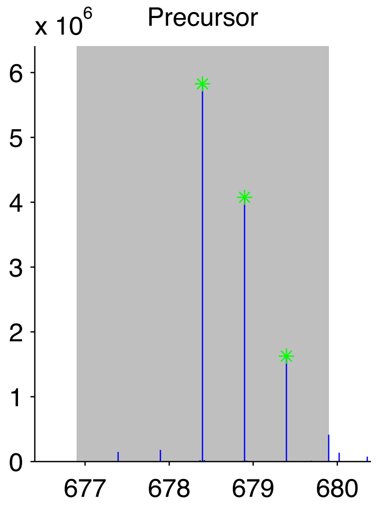

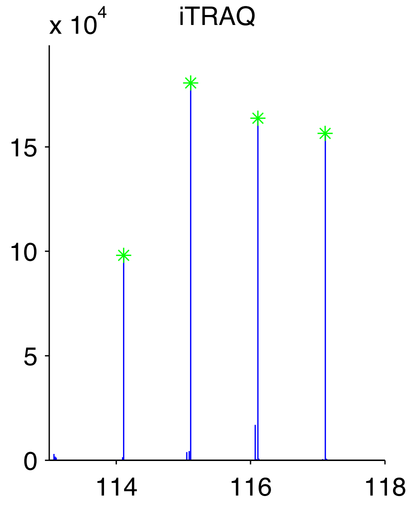

G G k G L G k G G A k R

histone cluster 1, H4a [Homo sapiens]
Charge State: +2
Scan Number: 13991
File Name: 110924_HG2_INSA_ACK.raw

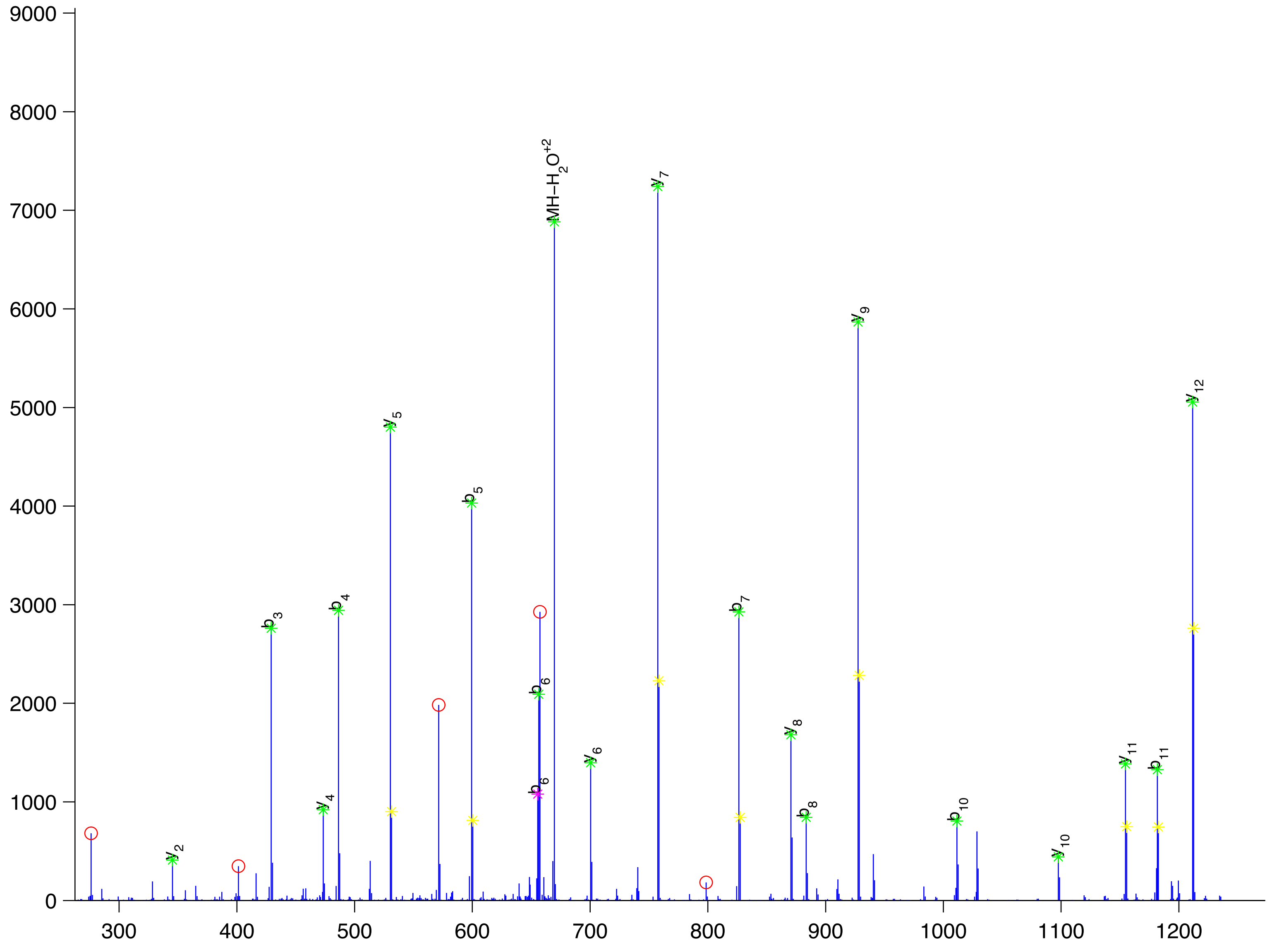

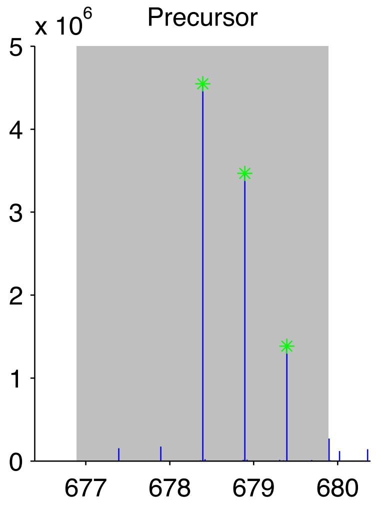

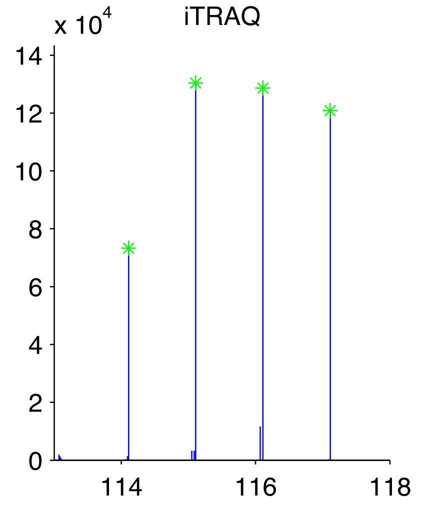

G k G G k G L G k G G A k R

histone cluster 1, H4a [Homo sapiens]
Charge State: +3
Scan Number: 14039
File Name: 110924_HG2_INSA_ACK3.raw

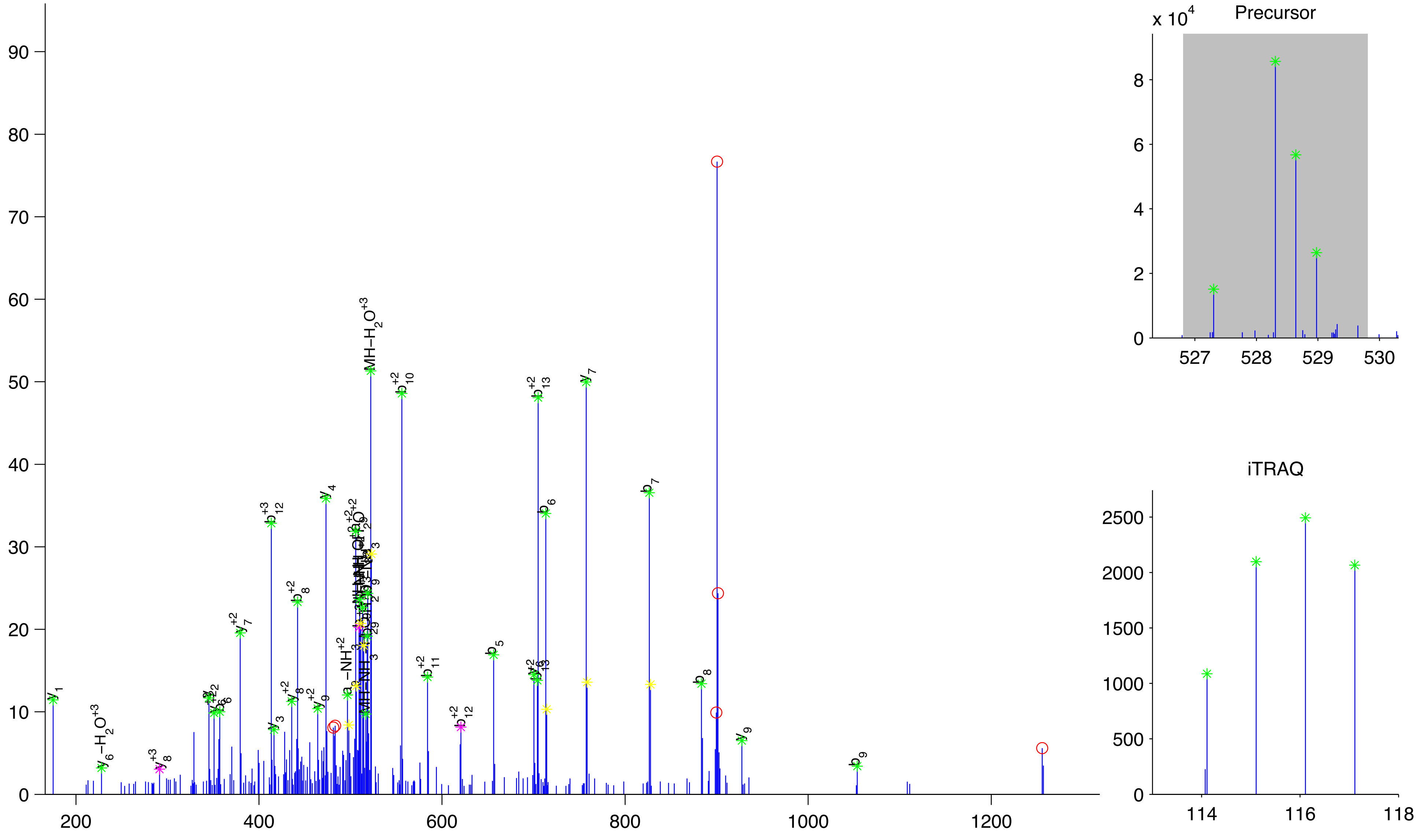

G k G G k G L G k G G A k R

histone cluster 1, H4a [Homo sapiens]
Charge State: +2
Scan Number: 14052
File Name: 110924_HG2_INSA_ACK.raw

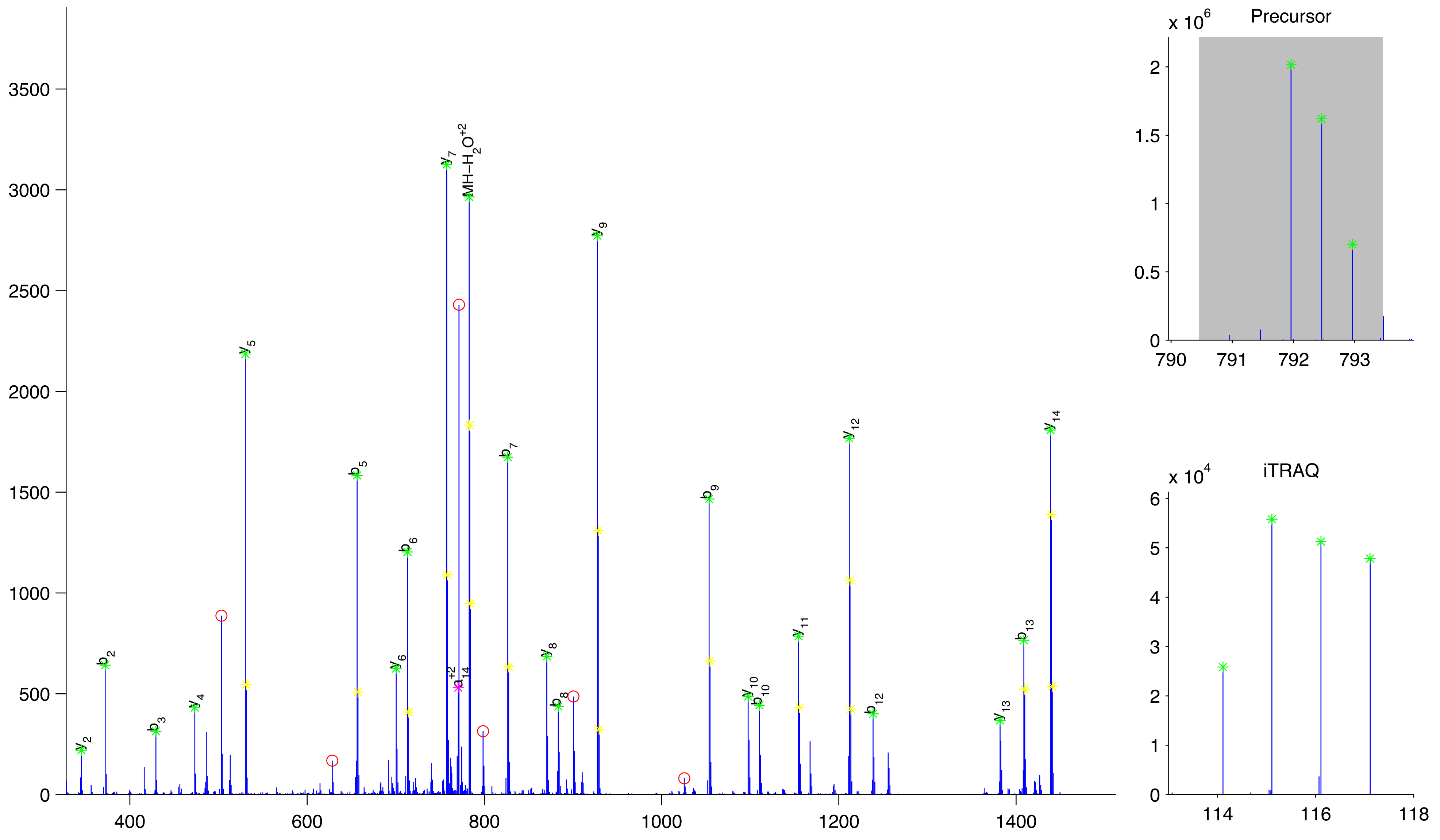

G k G G k G L G k G G A k R

histone cluster 1, H4a [Homo sapiens]
Charge State: +2
Scan Number: 14070
File Name: 110924_HG2_INSA_ACK.raw

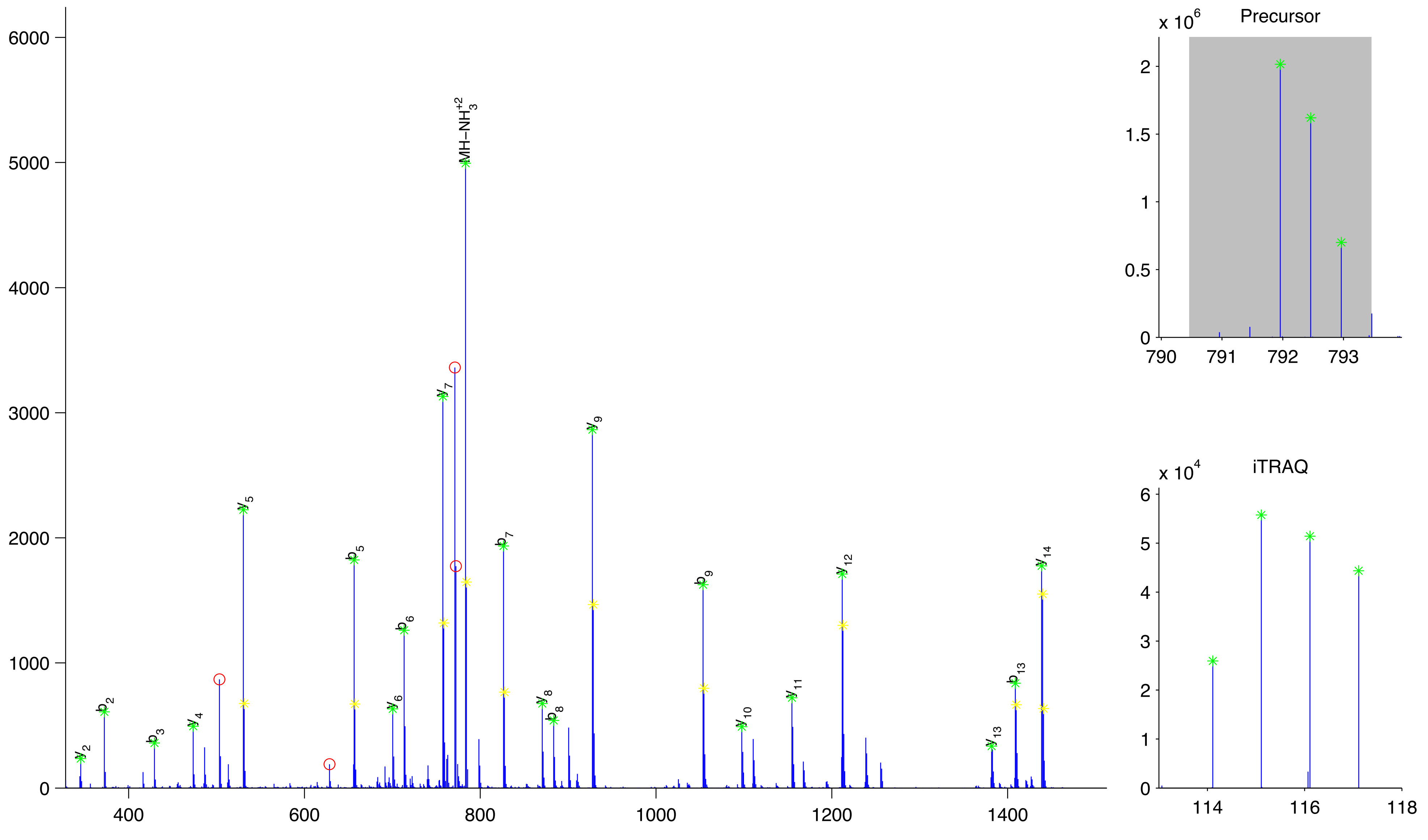

G G k G L G k G G A k R

histone cluster 1, H4a [Homo sapiens]
Charge State: +2
Scan Number: 14159
File Name: 110924_HG2_INSA_ACK.raw

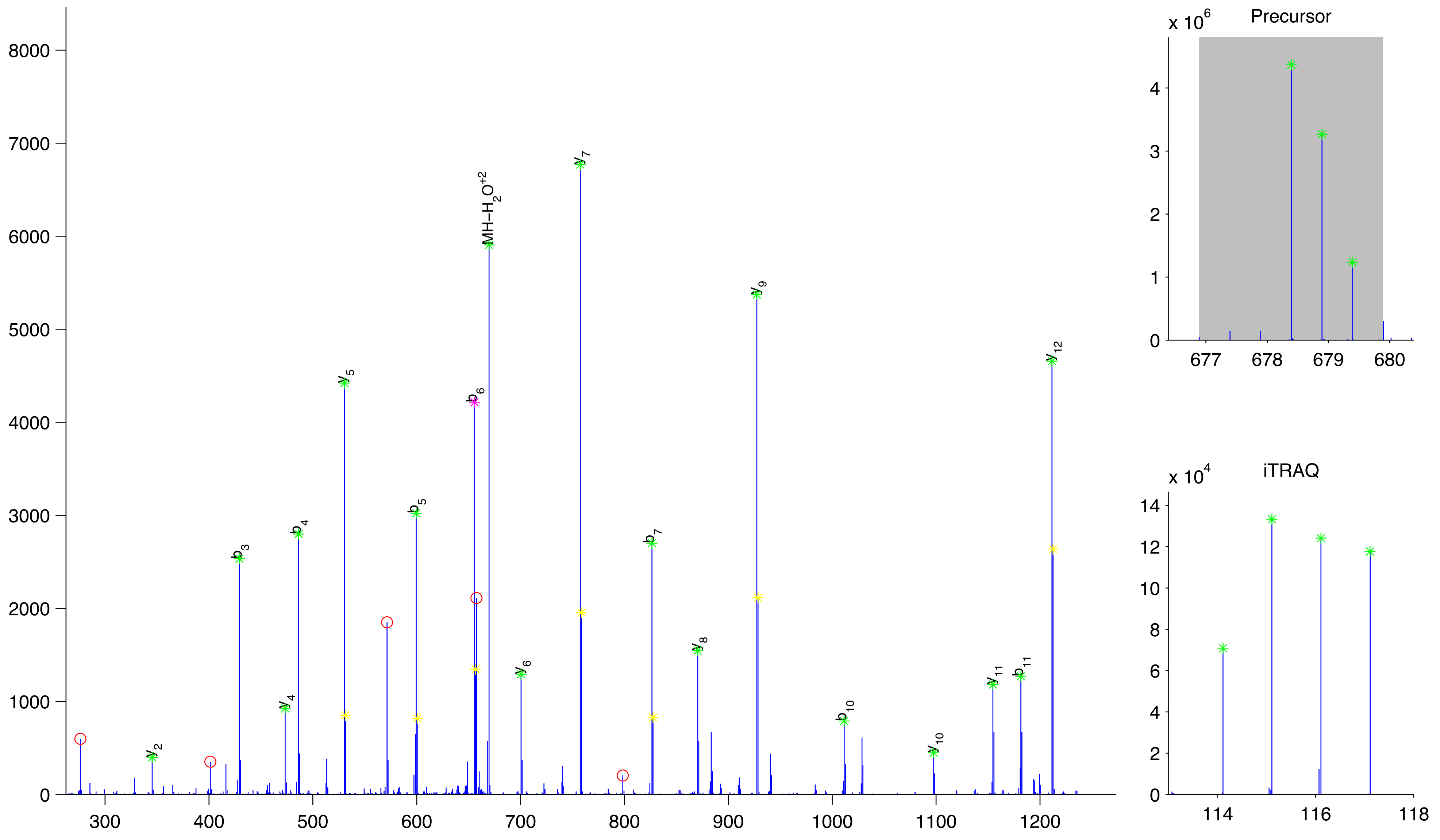

G L G k G G A k R

histone cluster 1, H4a [Homo sapiens]
Charge State: +2
Scan Number: 14195
File Name: 110924_HG2_INSA_ACK3.raw

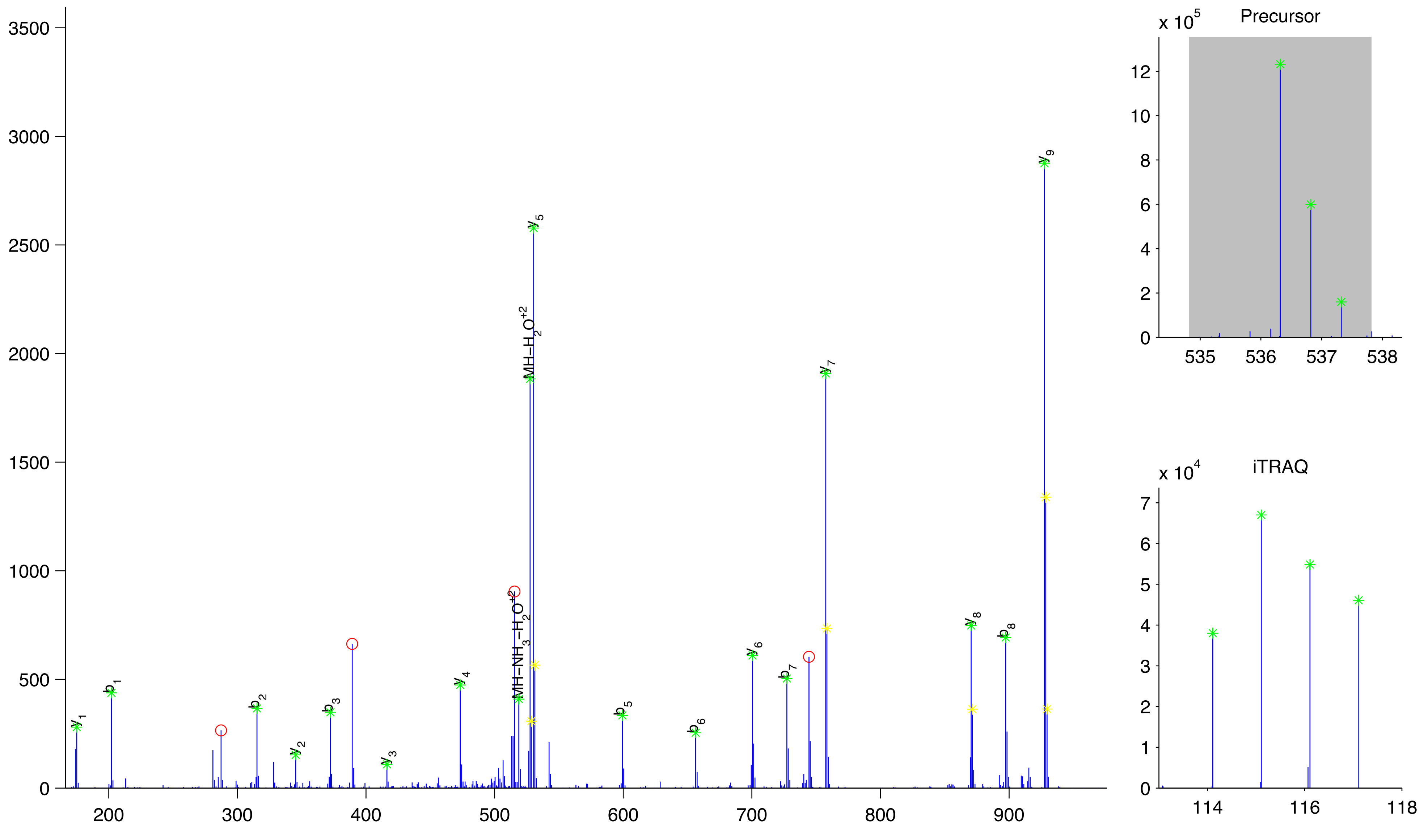

G k G G k G L G k G G A k R

histone cluster 1, H4a [Homo sapiens]
Charge State: +2
Scan Number: 14220
File Name: 110924_HG2_INSA_ACK.raw

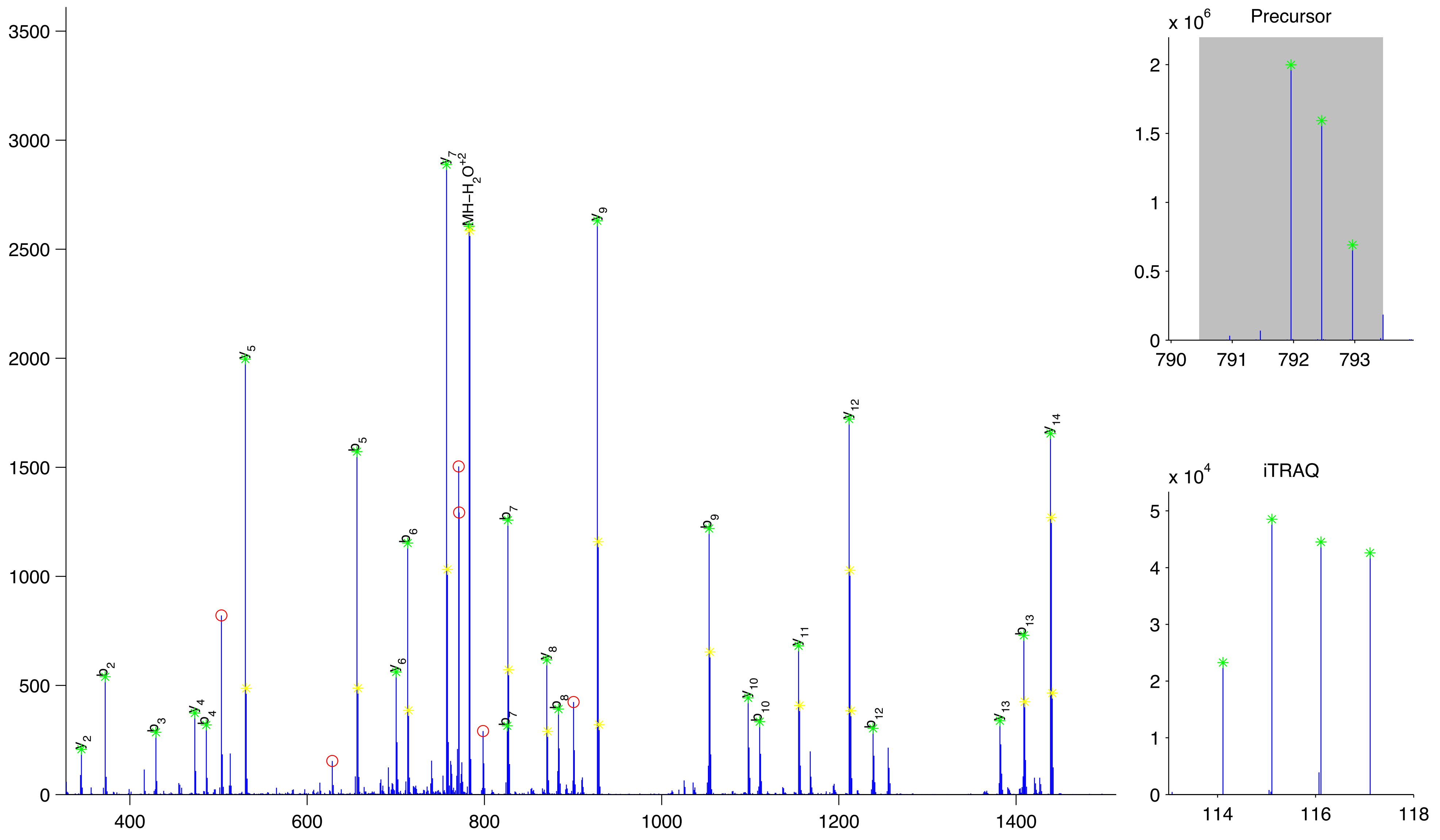

G k G G k G L G k G G A k R

histone cluster 1, H4a [Homo sapiens]
Charge State: +2
Scan Number: 14234
File Name: 110924_HG2_INSA_ACK.raw

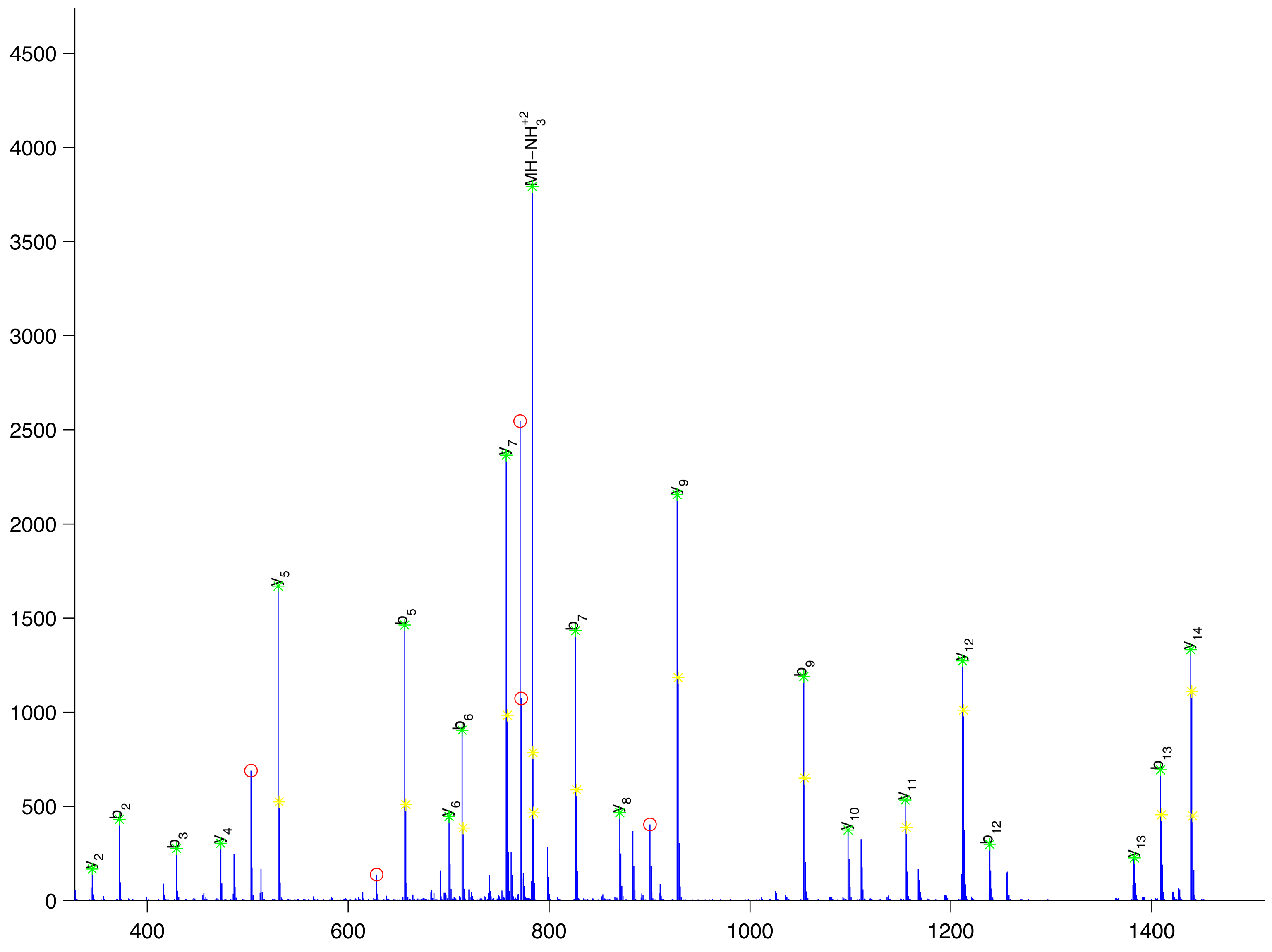

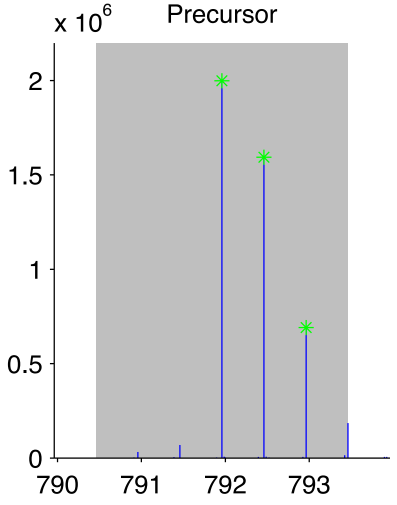

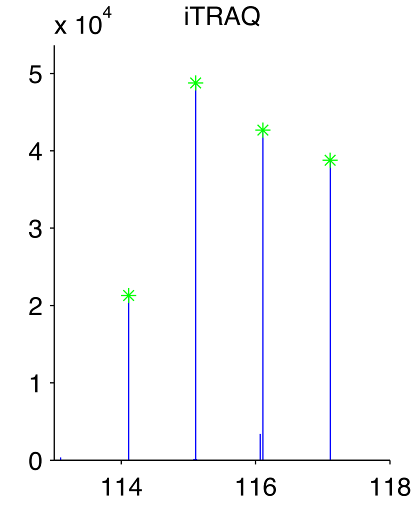

G G k G L G k G G A k R

histone cluster 1, H4a [Homo sapiens]
Charge State: +2
Scan Number: 14237
File Name: 110924_HG2_INSA_ACK3.raw

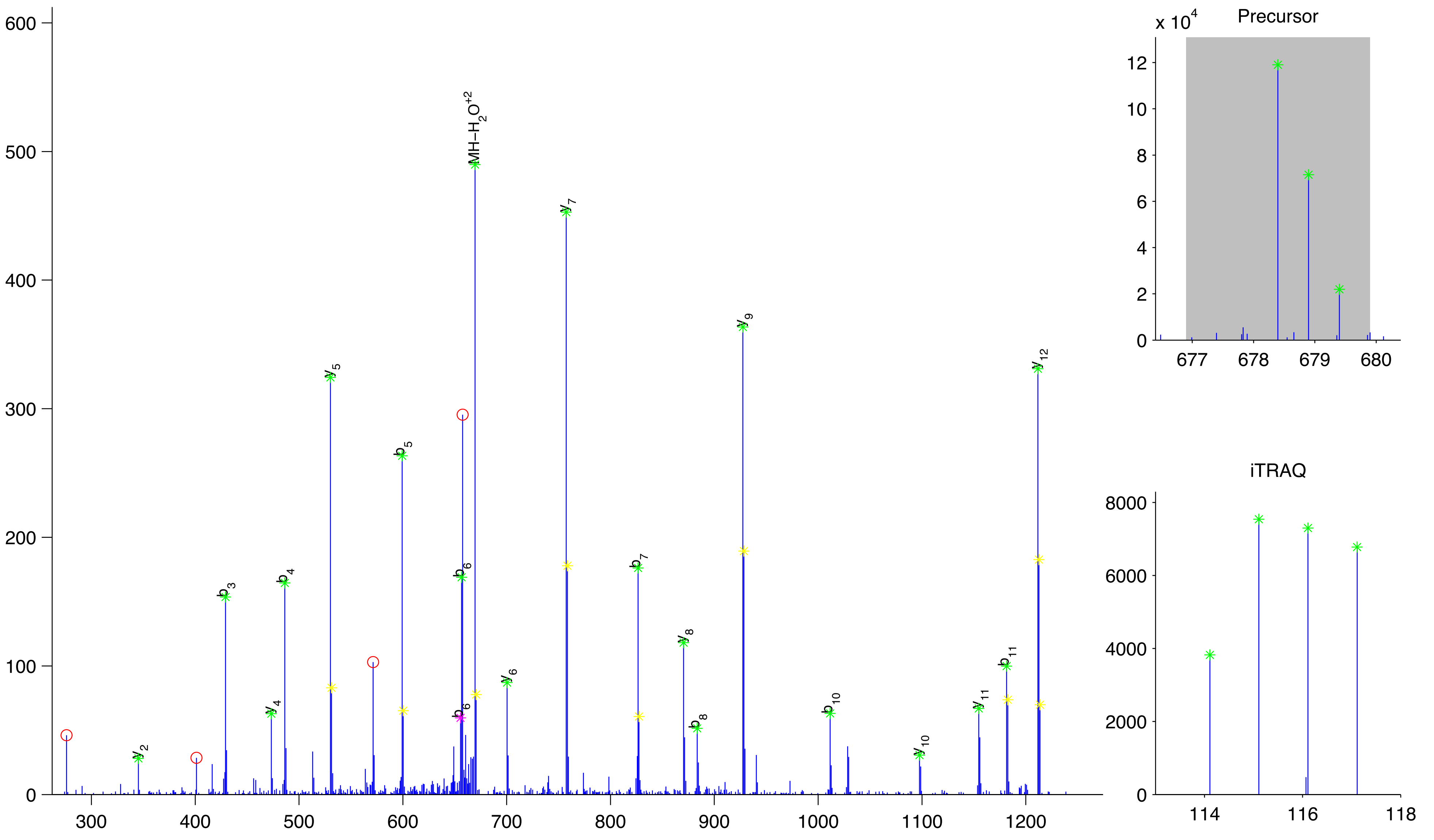

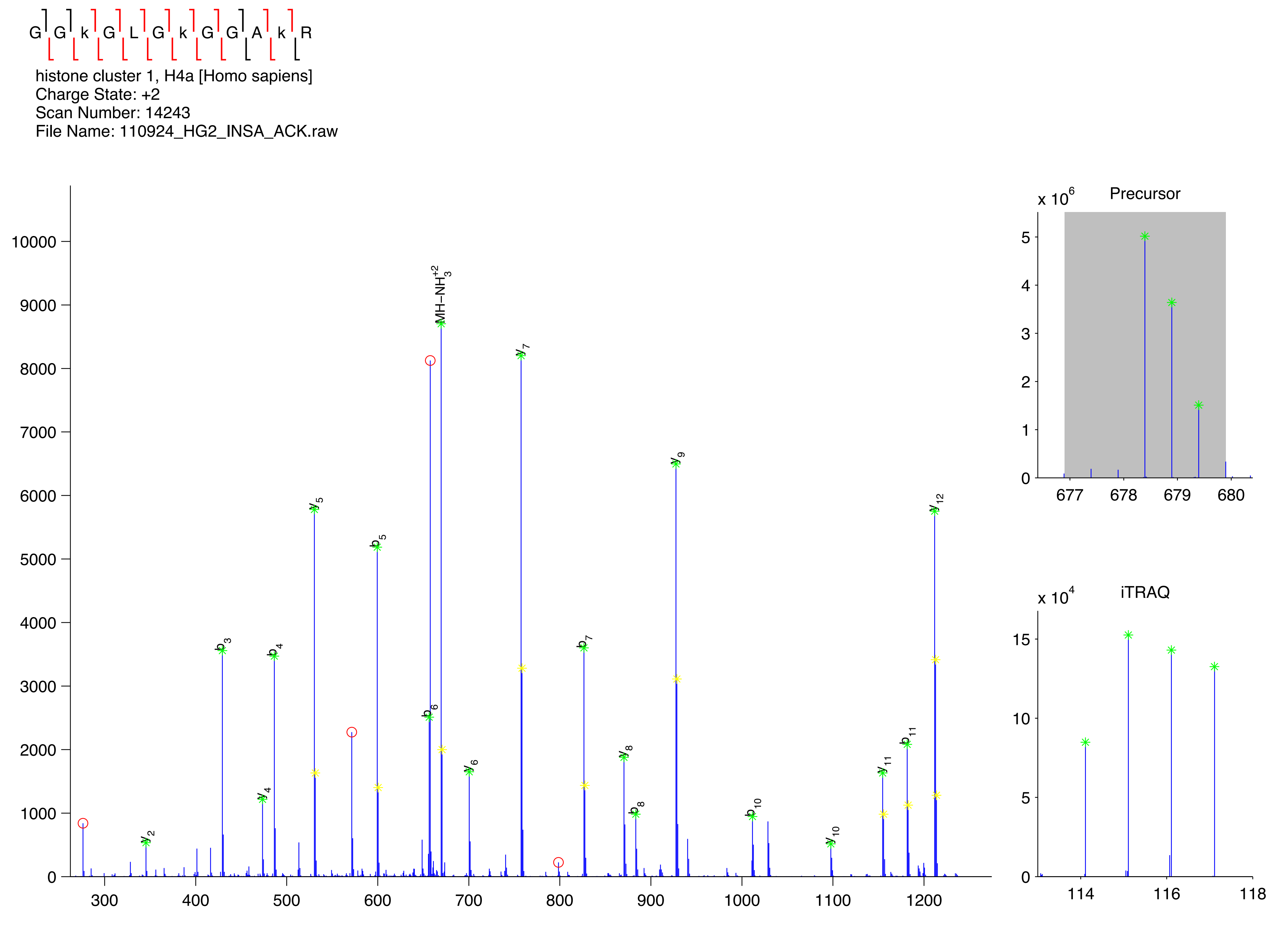
G G k G L G k G G A k R
histone cluster 1, H4a [Homo sapiens]
Charge State: +2
Scan Number: 14243
File Name: 110924_HG2_INSA_ACK.raw
y2
b3
y4
b4
y5
b5
b6
MH−NH3+2
y6
y7
b7
y8
b8
y9
b10
y10
y11
b11
y12
x 10^6
Precursor
x 10^4
iTRAQ

G G k G L G k G G A k R

histone cluster 1, H4a [Homo sapiens]
Charge State: +2
Scan Number: 14329
File Name: 110924_HG2_INSA_ACK.raw

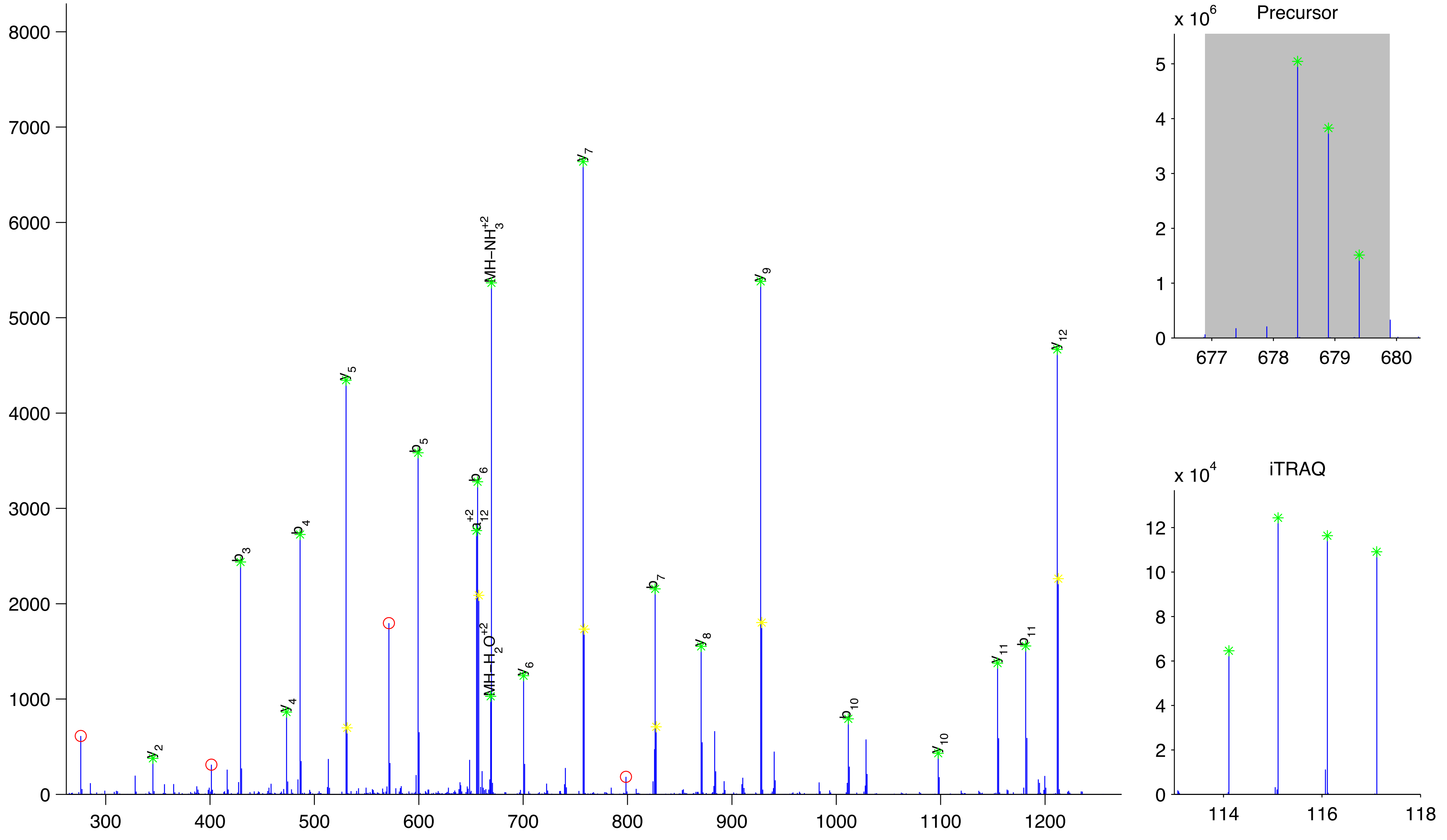

G k G G k G L G k G G A k R

histone cluster 1, H4a [Homo sapiens]
Charge State: +2
Scan Number: 14388
File Name: 110924_HG2_INSA_ACK.raw

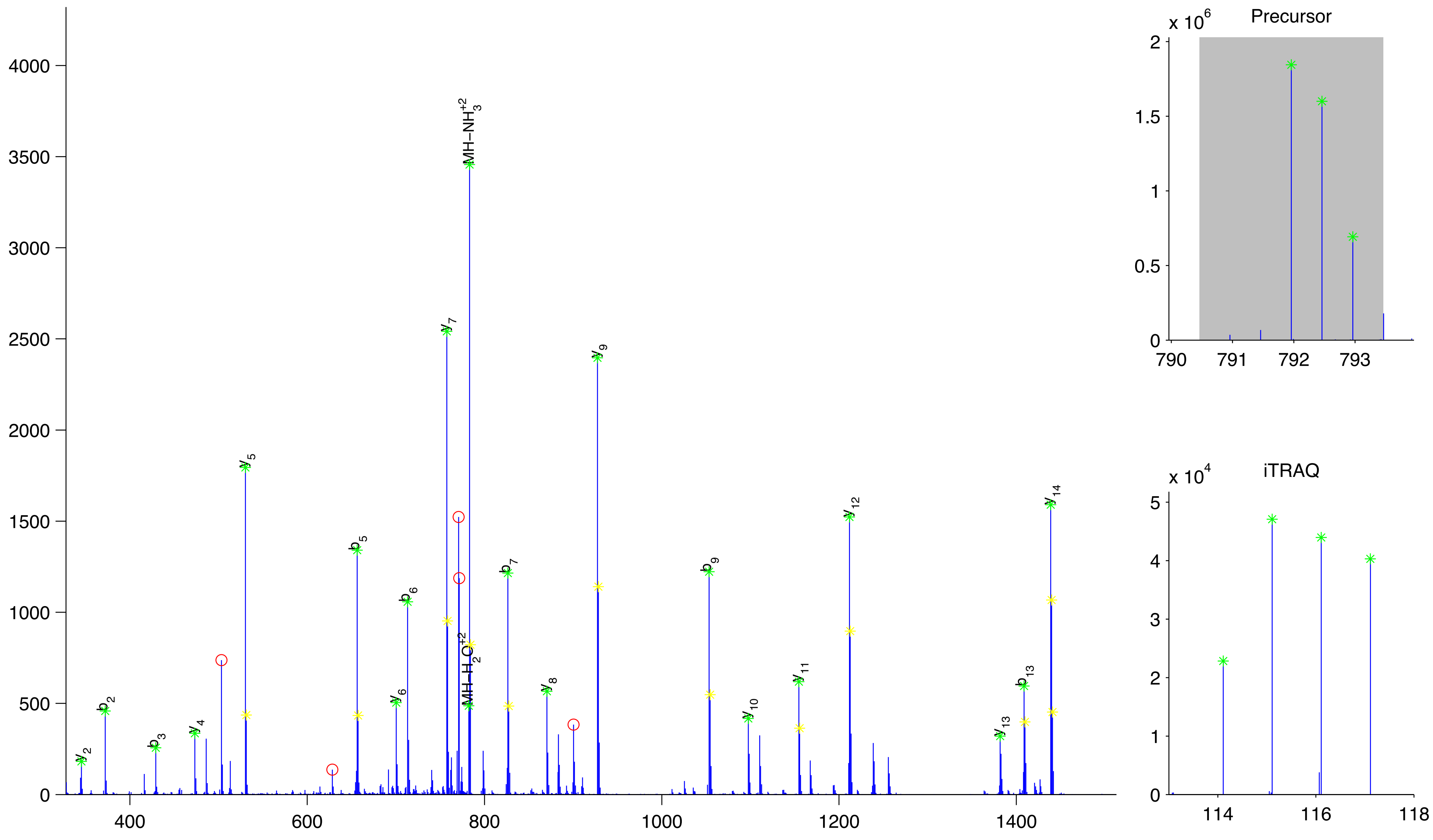

G k G G k G L G k G G A k R

histone cluster 1, H4a [Homo sapiens]
Charge State: +3
Scan Number: 14390
File Name: 110924_HG2_INSA_ACK3.raw

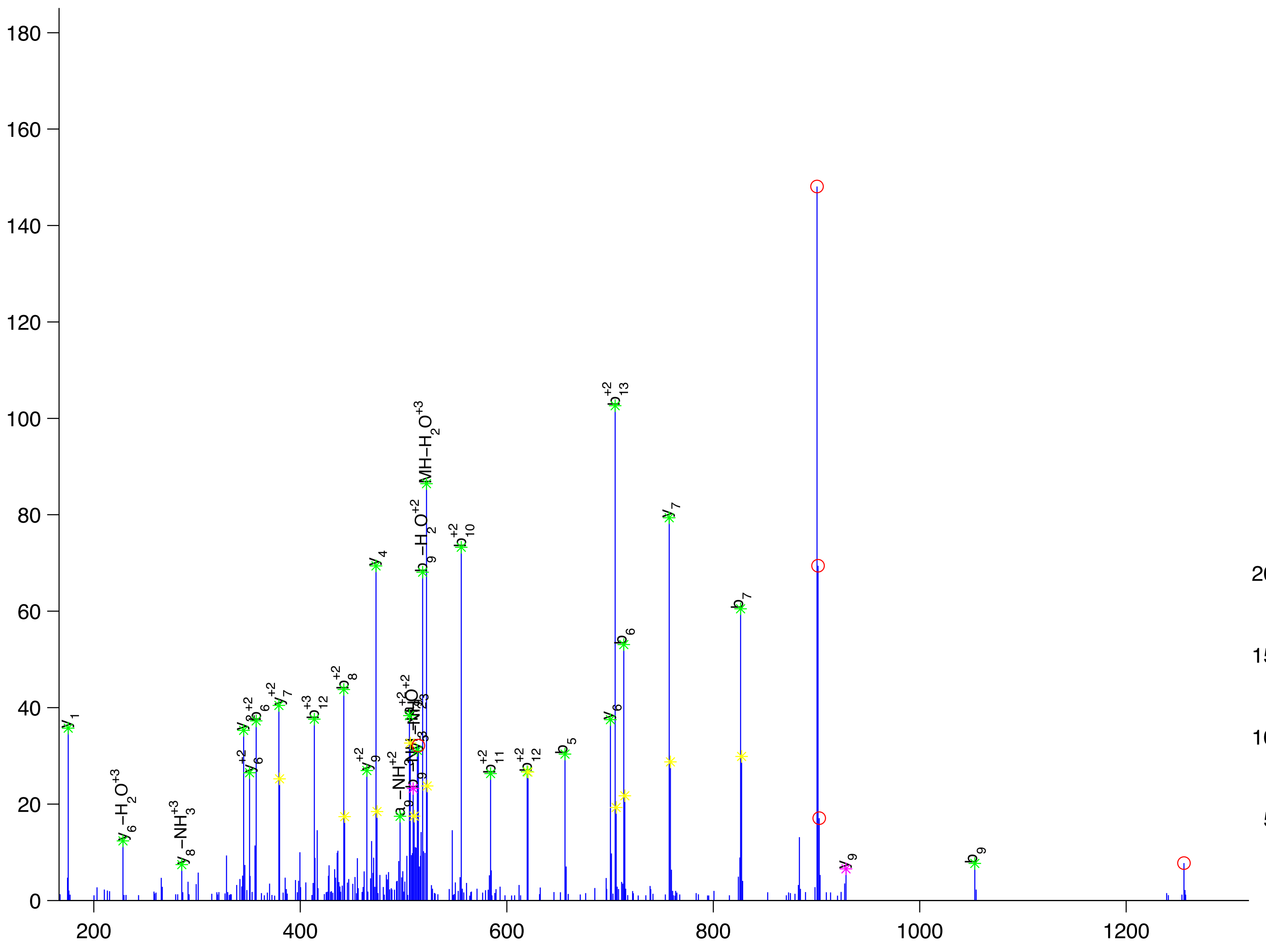

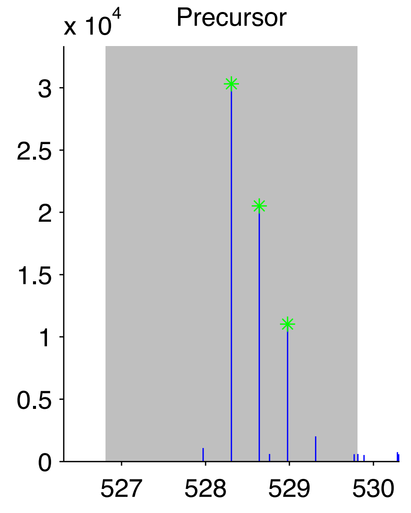

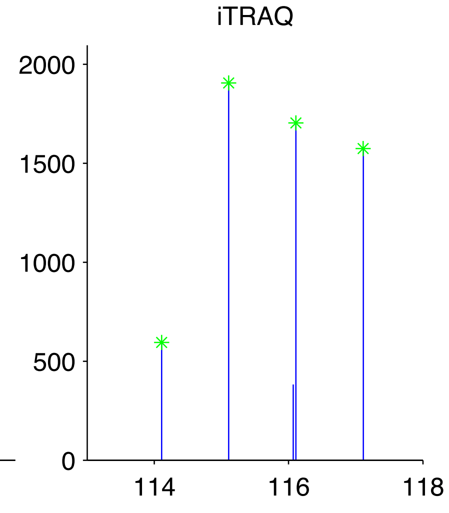

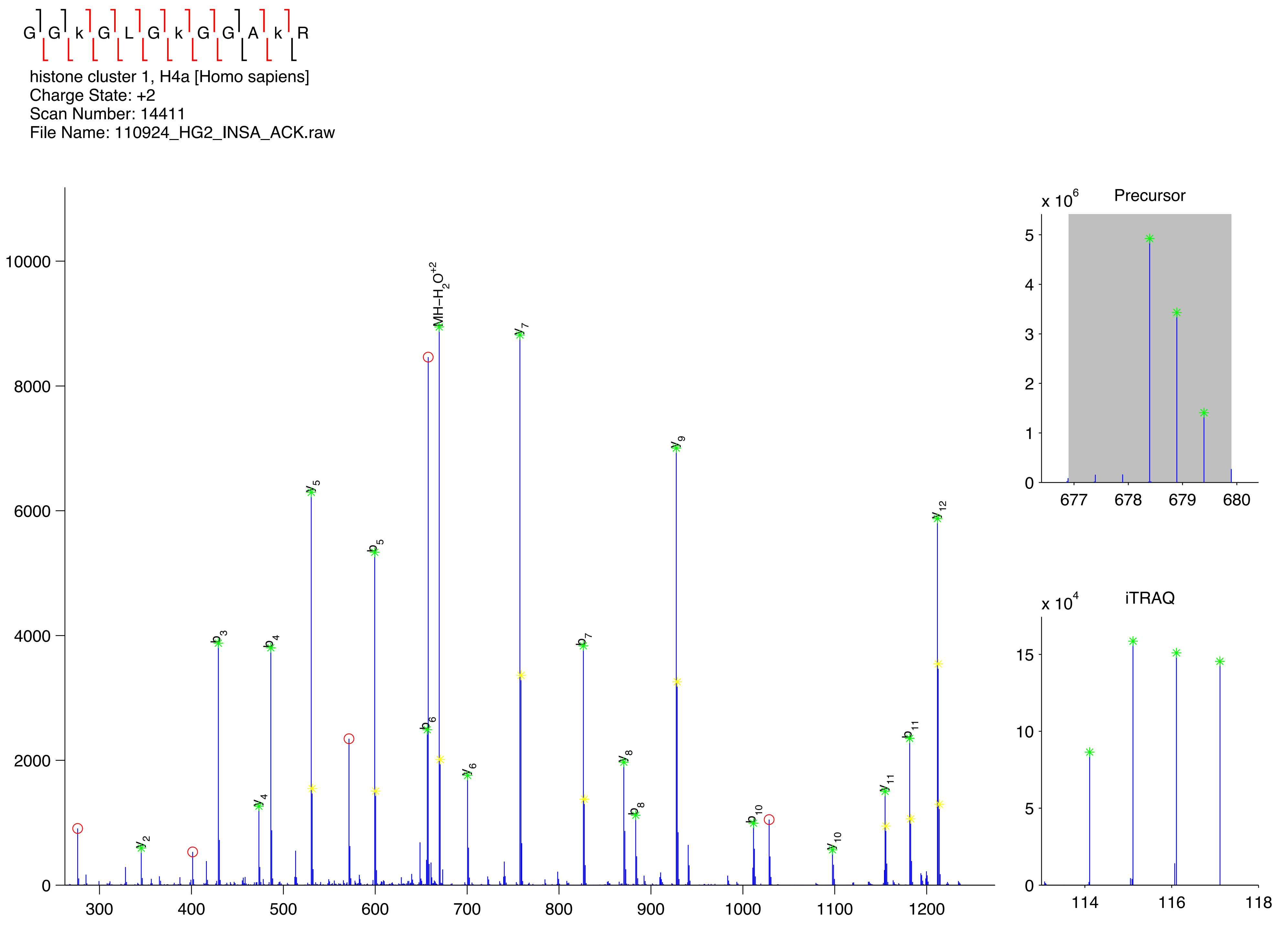
G G k G L G k G G A k R
histone cluster 1, H4a [Homo sapiens]
Charge State: +2
Scan Number: 14411
File Name: 110924_HG2_INSA_ACK.raw
MH−H2O+2
y2 b3 y4 b4 y5 b5 b6 y6 y7 b7 y8 b8 y9 b10 y10 y11 b11 y12
0 2000 4000 6000 8000 10000
300 400 500 600 700 800 900 1000 1100 1200
x 10^6
Precursor
0 1 2 3 4 5
677 678 679 680
x 10^4
iTRAQ
0 5 10 15
114 116 118

G k G G k G L G k G G A k R

histone cluster 1, H4a [Homo sapiens]
Charge State: +2
Scan Number: 14425
File Name: 110924_HG2_INSA_ACK.raw

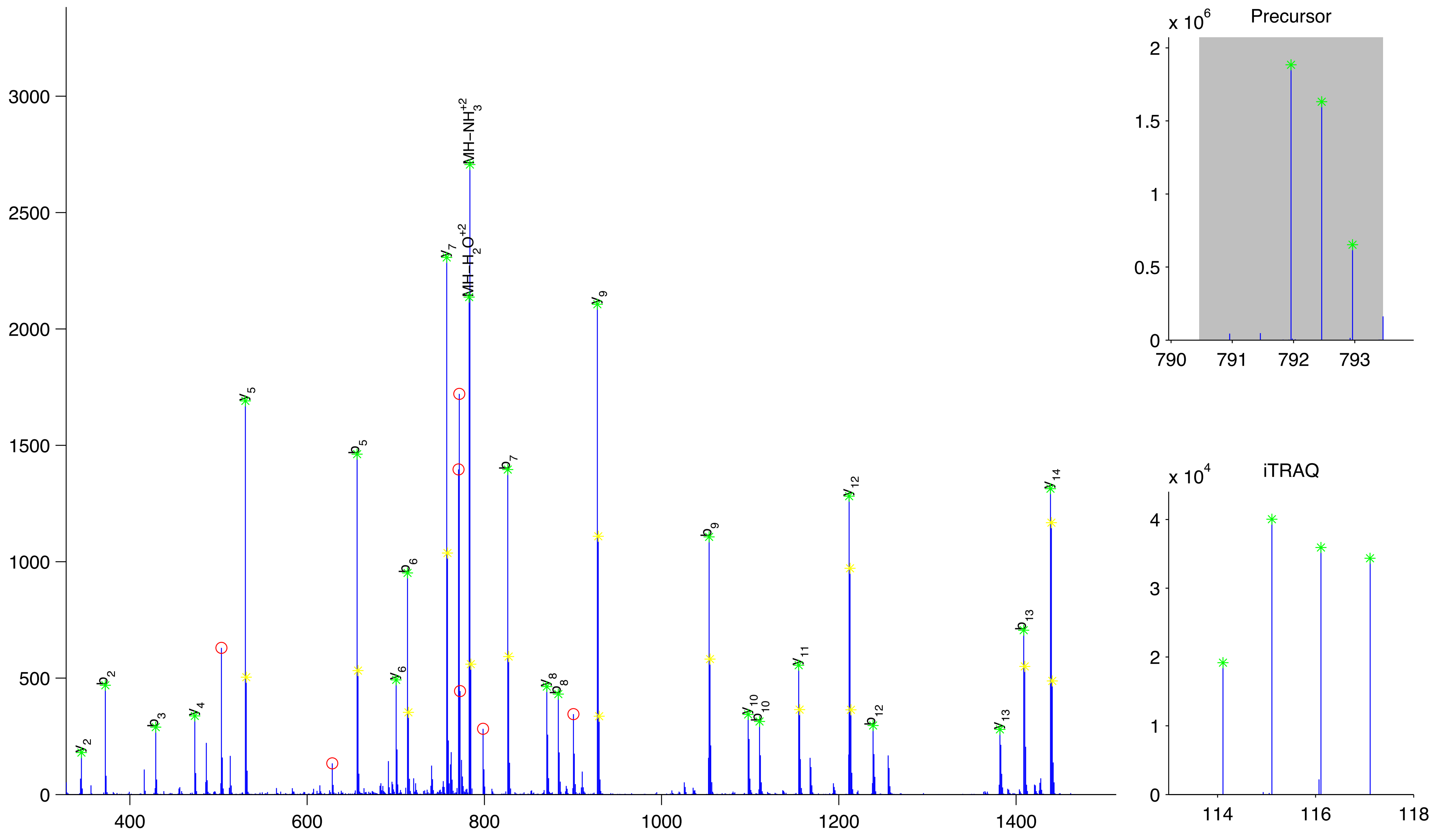

G G k G L G k G G A k R

histone cluster 1, H4a [Homo sapiens]
Charge State: +2
Scan Number: 14426
File Name: 110924_HG2_INSA_ACK3.raw

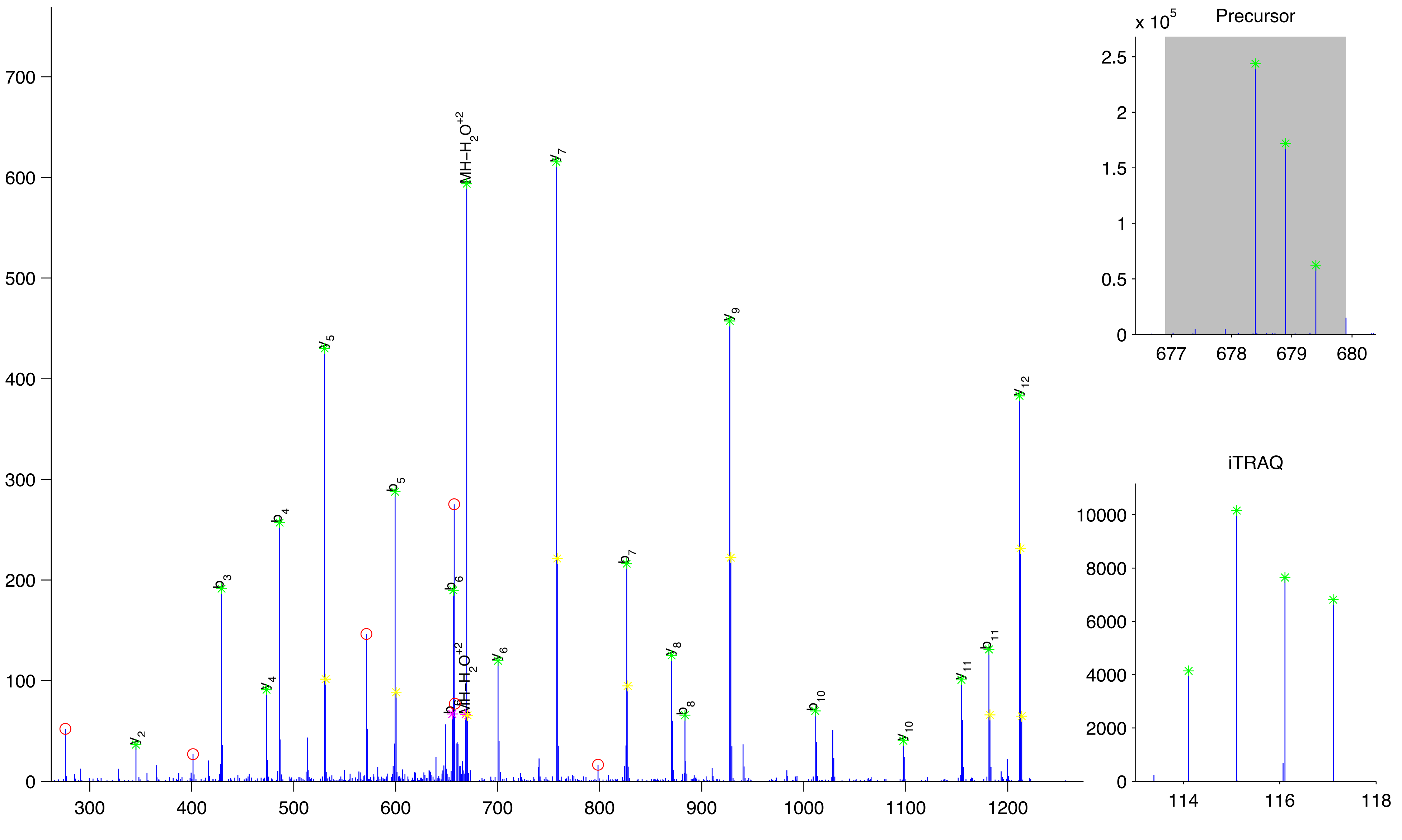

G G k G L G k G G A k R

histone cluster 1, H4a [Homo sapiens]
Charge State: +2
Scan Number: 14499
File Name: 110924_HG2_INSA_ACK.raw

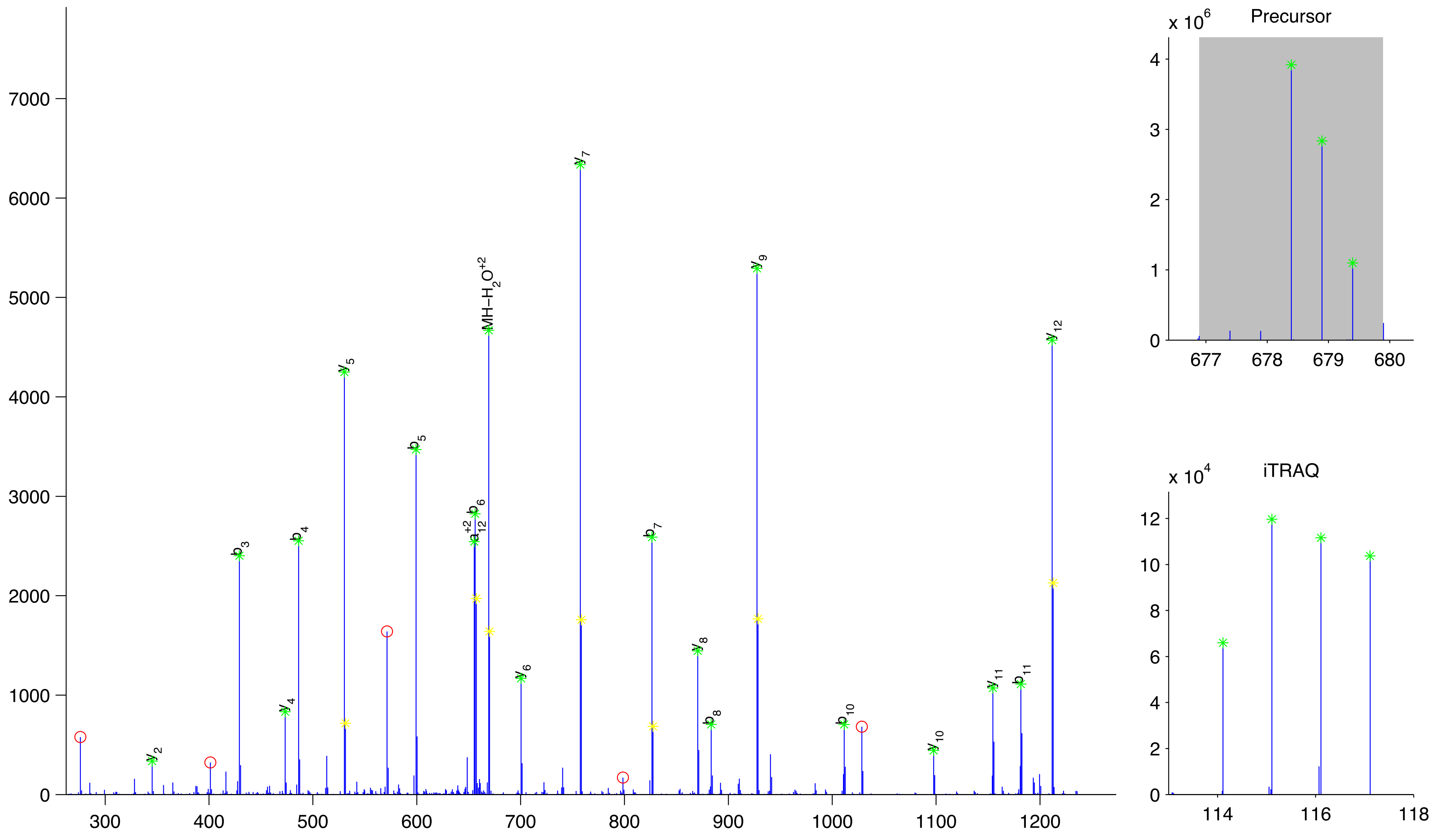

G L G k G G A k R

histone cluster 1, H4a [Homo sapiens]
Charge State: +2
Scan Number: 14531
File Name: 110924_HG2_INSA_ACK3.raw

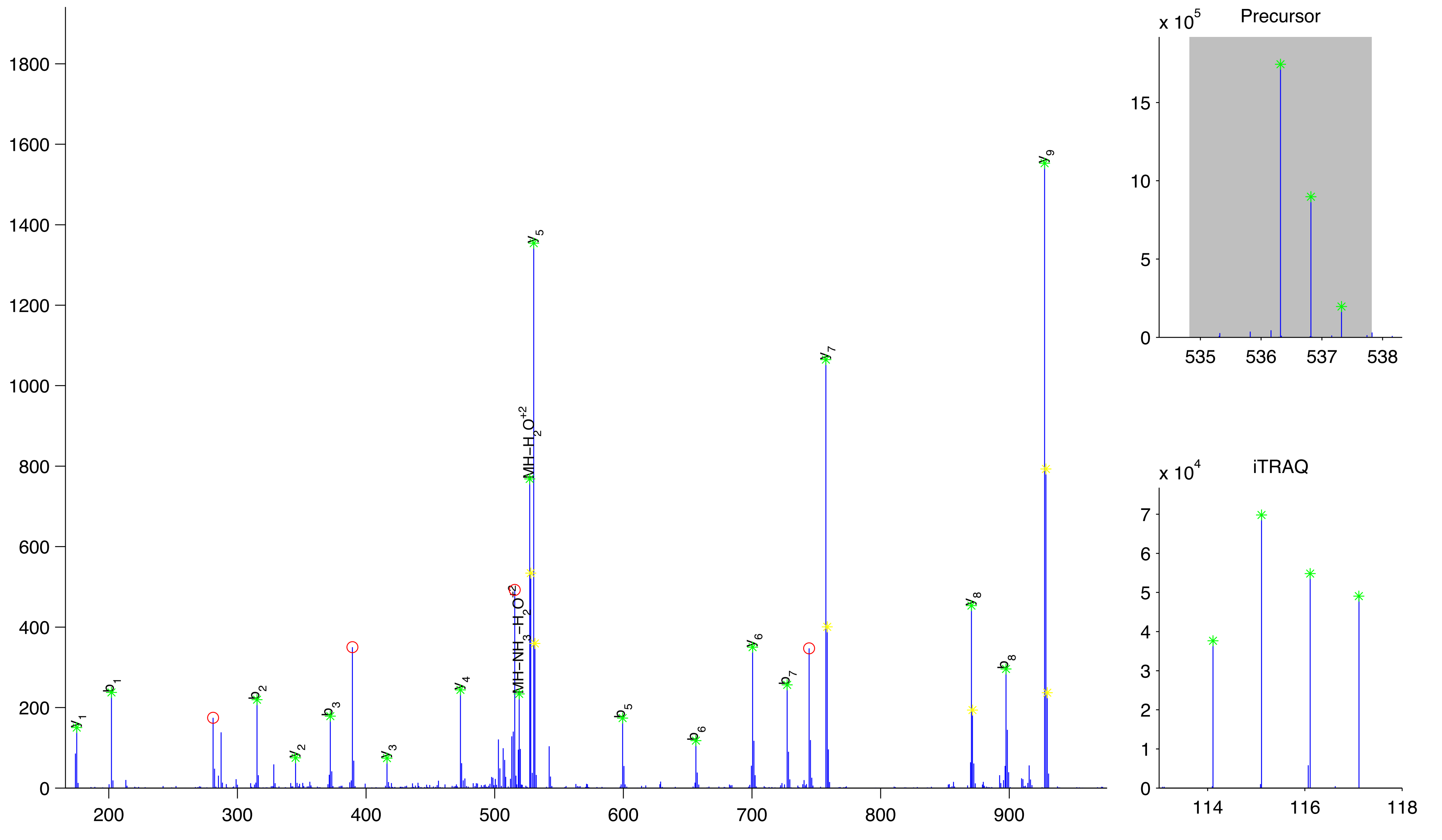

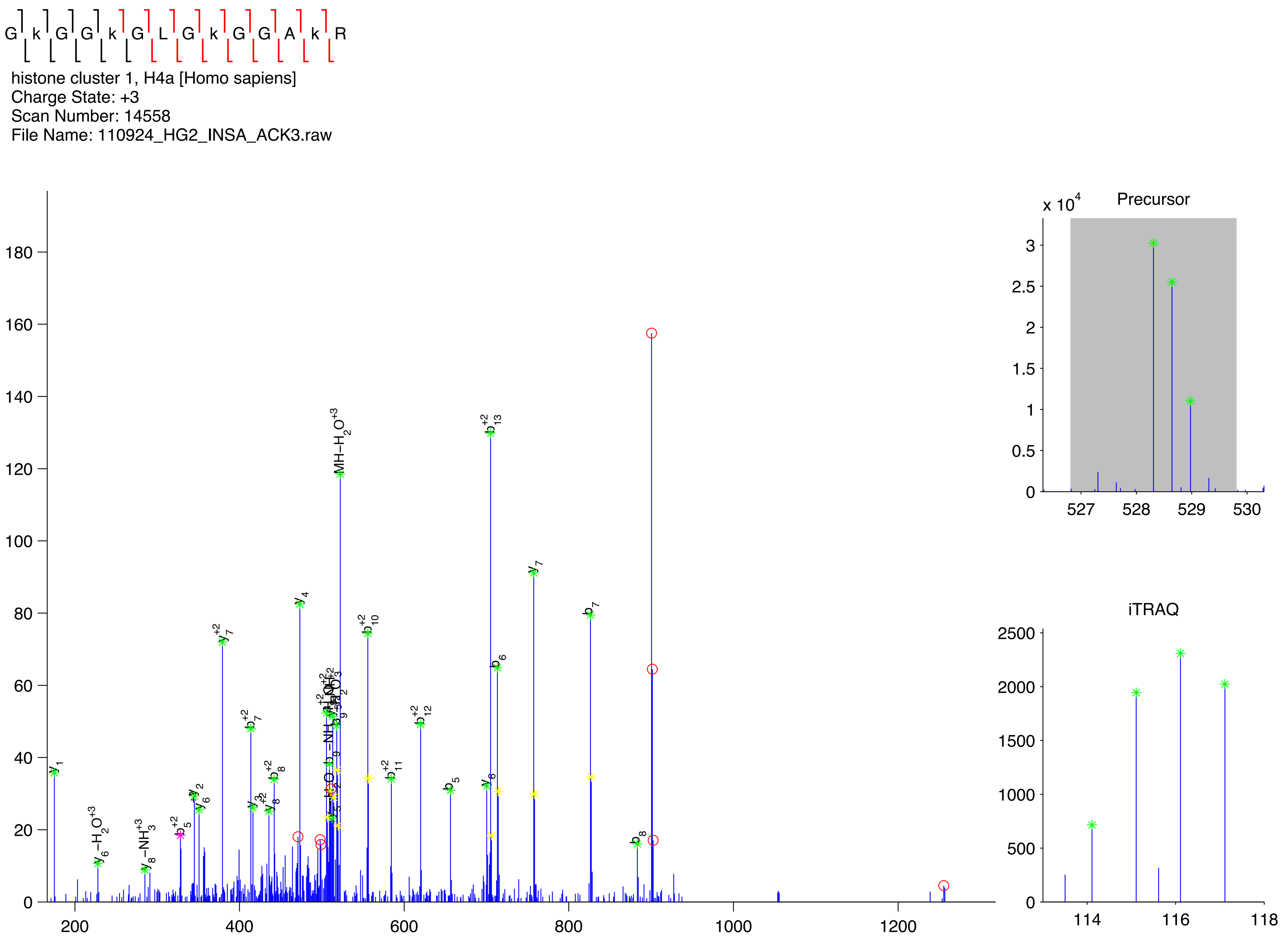
G k G G k G L G k G G A k R
histone cluster 1, H4a [Homo sapiens]
Charge State: +3
Scan Number: 14558
File Name: 110924_HG2_INSA_ACK3.raw
Precursor
x 10^4
iTRAQ
MH−H2O+3
b+2 13
y7
b7
b6
y4
b+2 10
y+2 7
b+2 12
b+2 11
b5
y6
b8
y1
y6−H2O+3
y8−NH3+3
b+2 5
b+2 7
b+2 8
y2
y6
y3
y+2 8
180
160
140
120
100
80
60
40
20
0
200
400
600
800
1000
1200
3
2.5
2
1.5
1
0.5
527
528
529
530
2500
2000
1500
1000
500
114
116
118

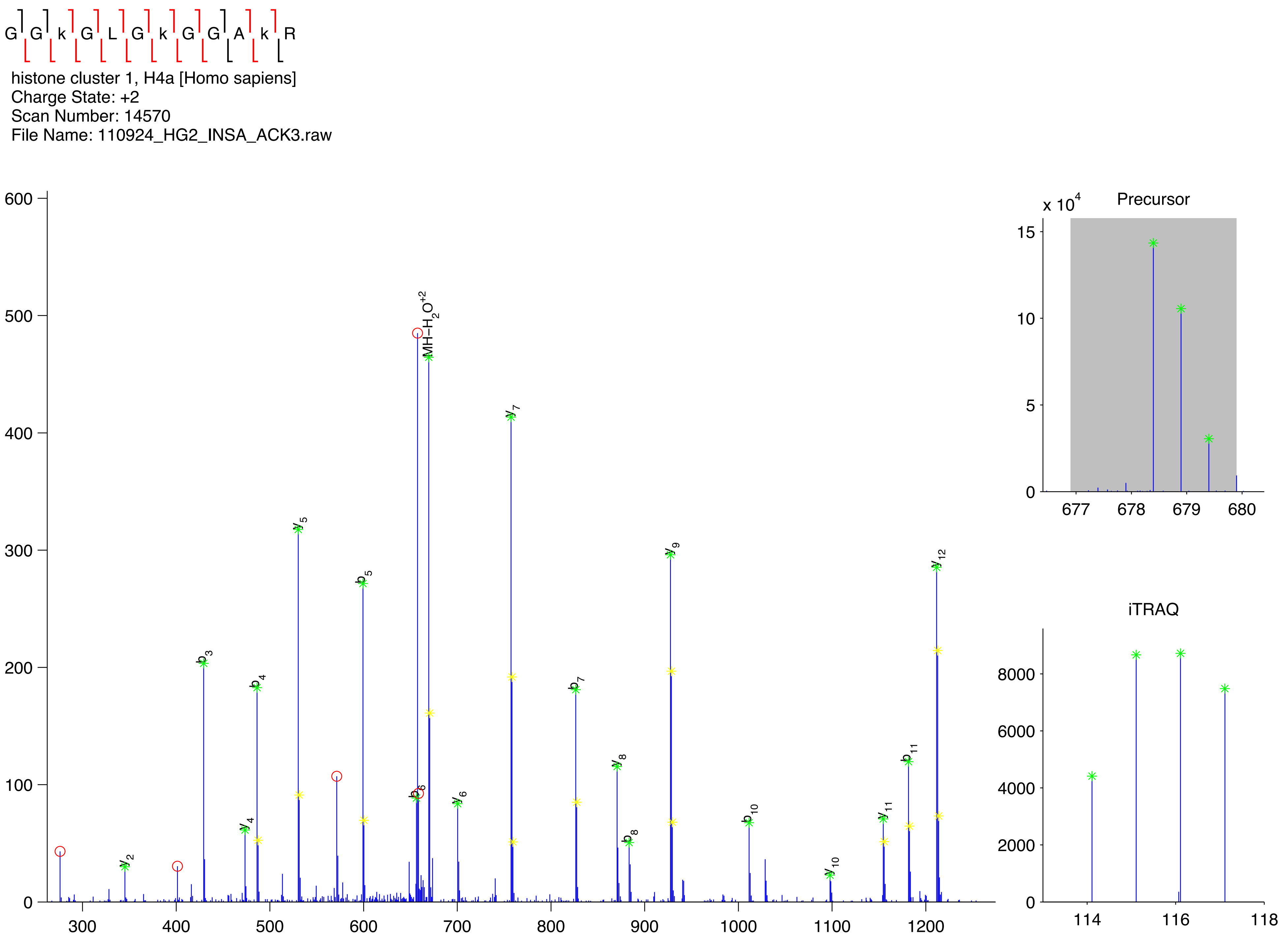

G G k G L G k G G A k R
histone cluster 1, H4a [Homo sapiens]
Charge State: +2
Scan Number: 14570
File Name: 110924_HG2_INSA_ACK3.raw
MH−H2O+2
Precursor
x 10^4
iTRAQ

G G k G L G k G G A k R

histone cluster 1, H4a [Homo sapiens]
Charge State: +2
Scan Number: 14579
File Name: 110924_HG2_INSA_ACK.raw

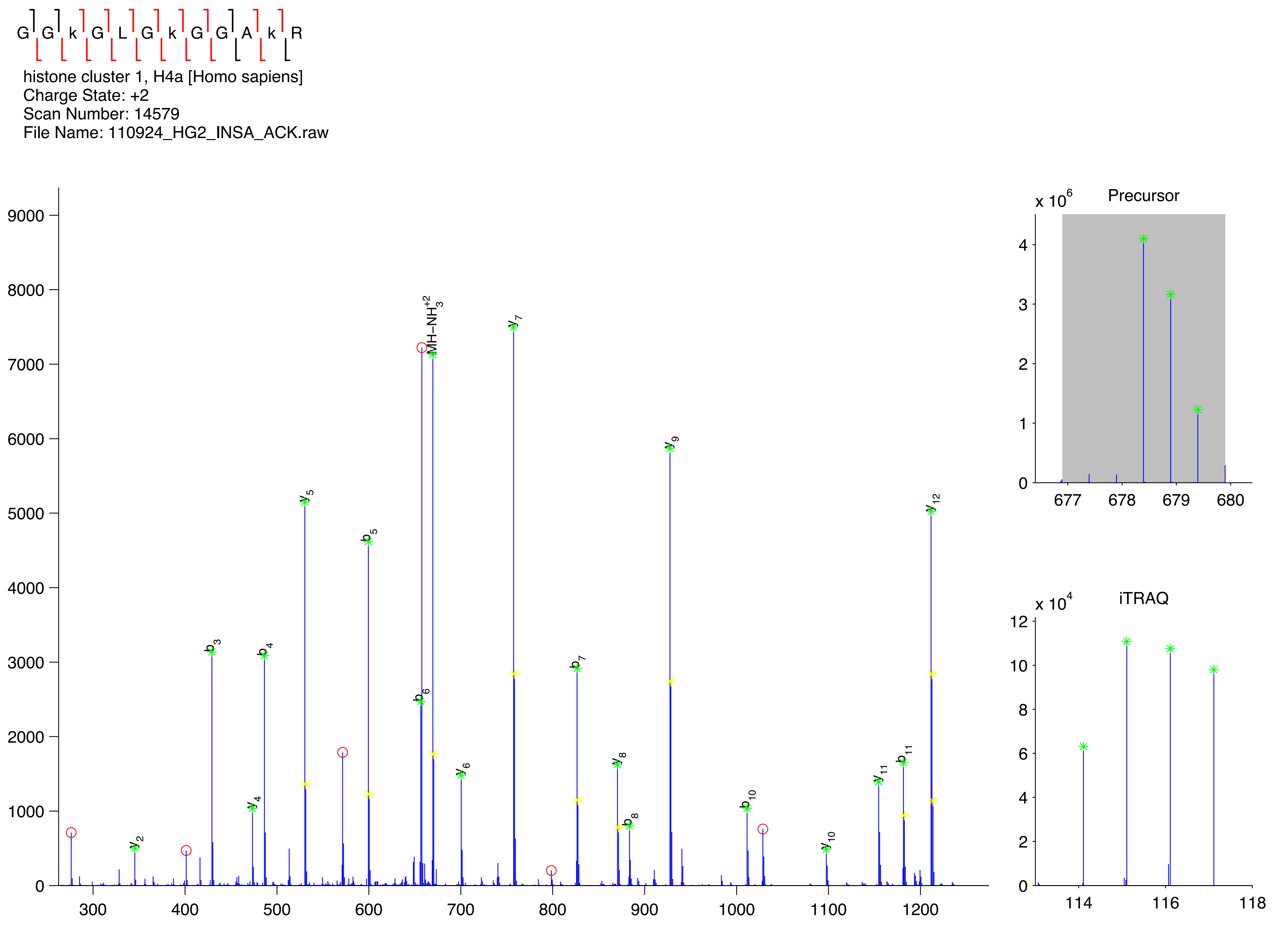

G G k G L G k G G A k R

histone cluster 1, H4a [Homo sapiens]
Charge State: +2
Scan Number: 14594
File Name: 110924_HG2_INSA_ACK3.raw

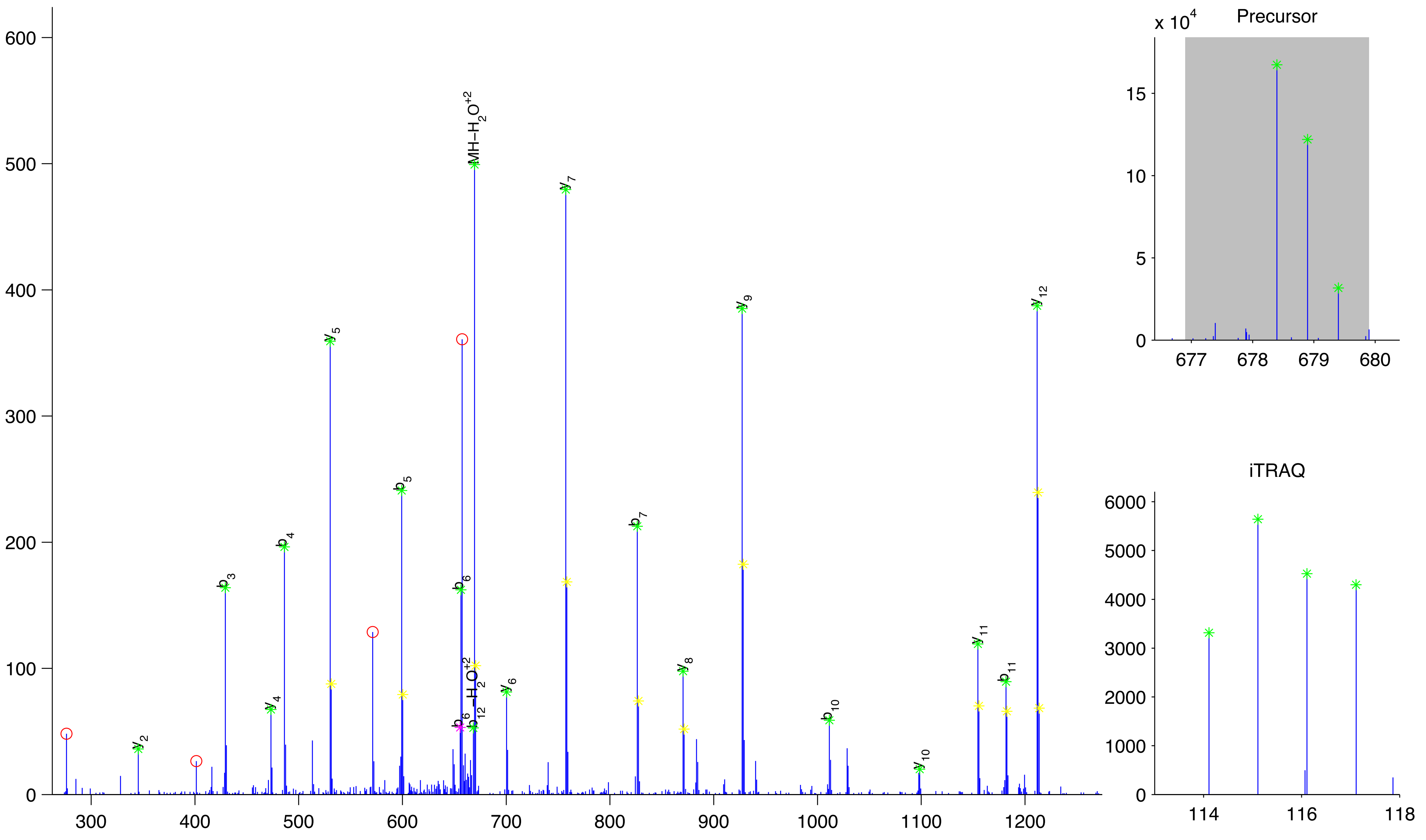

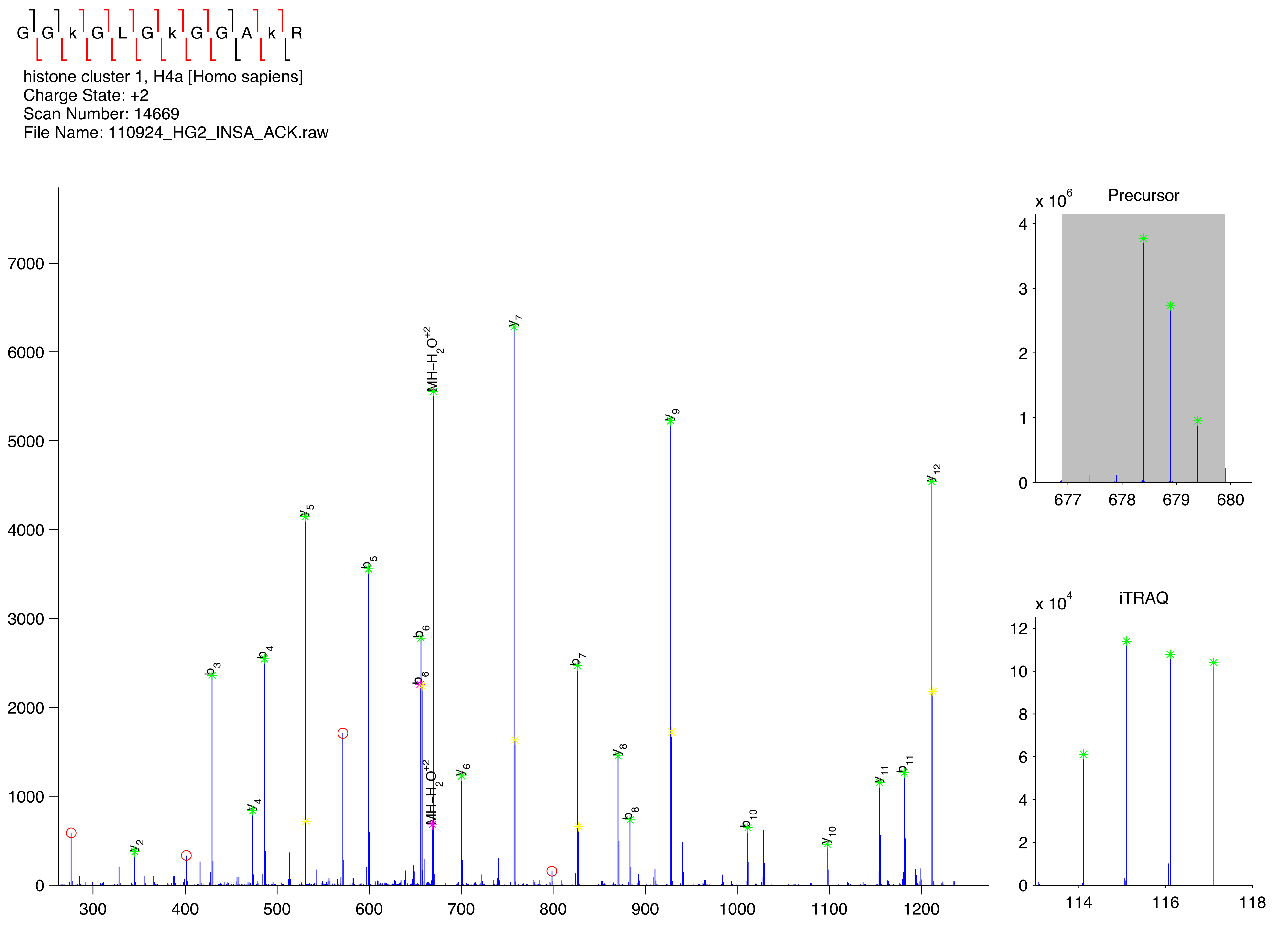
G G k G L G k G G A k R
histone cluster 1, H4a [Homo sapiens]
Charge State: +2
Scan Number: 14669
File Name: 110924_HG2_INSA_ACK.raw
y2
b3
y4
b4
y5
b5
b6
b6
MH-H2O+2
MH-H2O+2
y6
y7
b7
y8
b8
y9
b10
y10
y11
b11
y12
x 10^6
Precursor
x 10^4
iTRAQ

G k G G k G L G k G G A k R

histone cluster 1, H4a [Homo sapiens]
Charge State: +2
Scan Number: 14724
File Name: 110924_HG2_INSA_ACK.raw

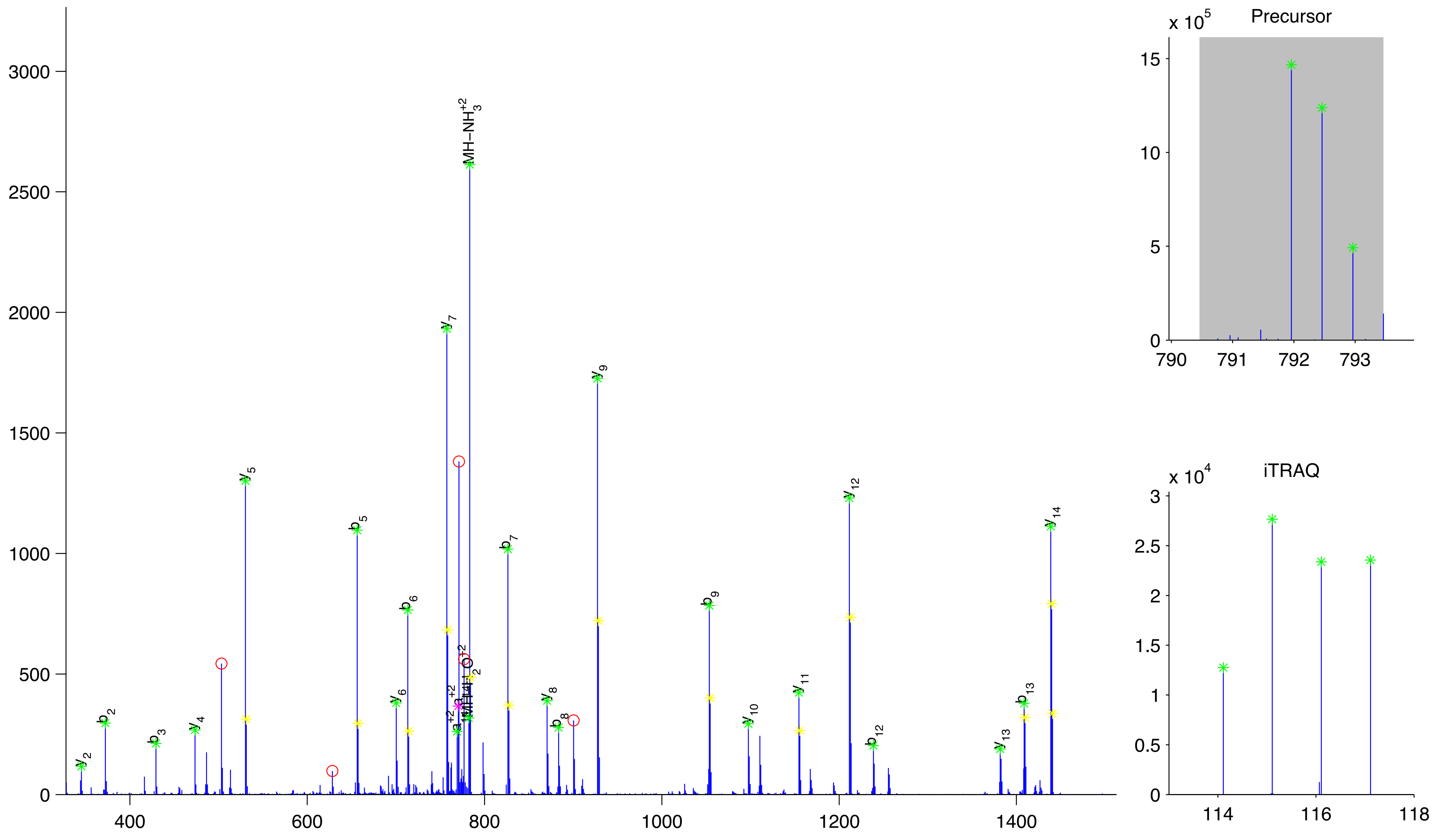

G k G G k G L G k G G A k R

histone cluster 1, H4a [Homo sapiens]
Charge State: +2
Scan Number: 14728
File Name: 110924_HG2_INSA_ACK.raw

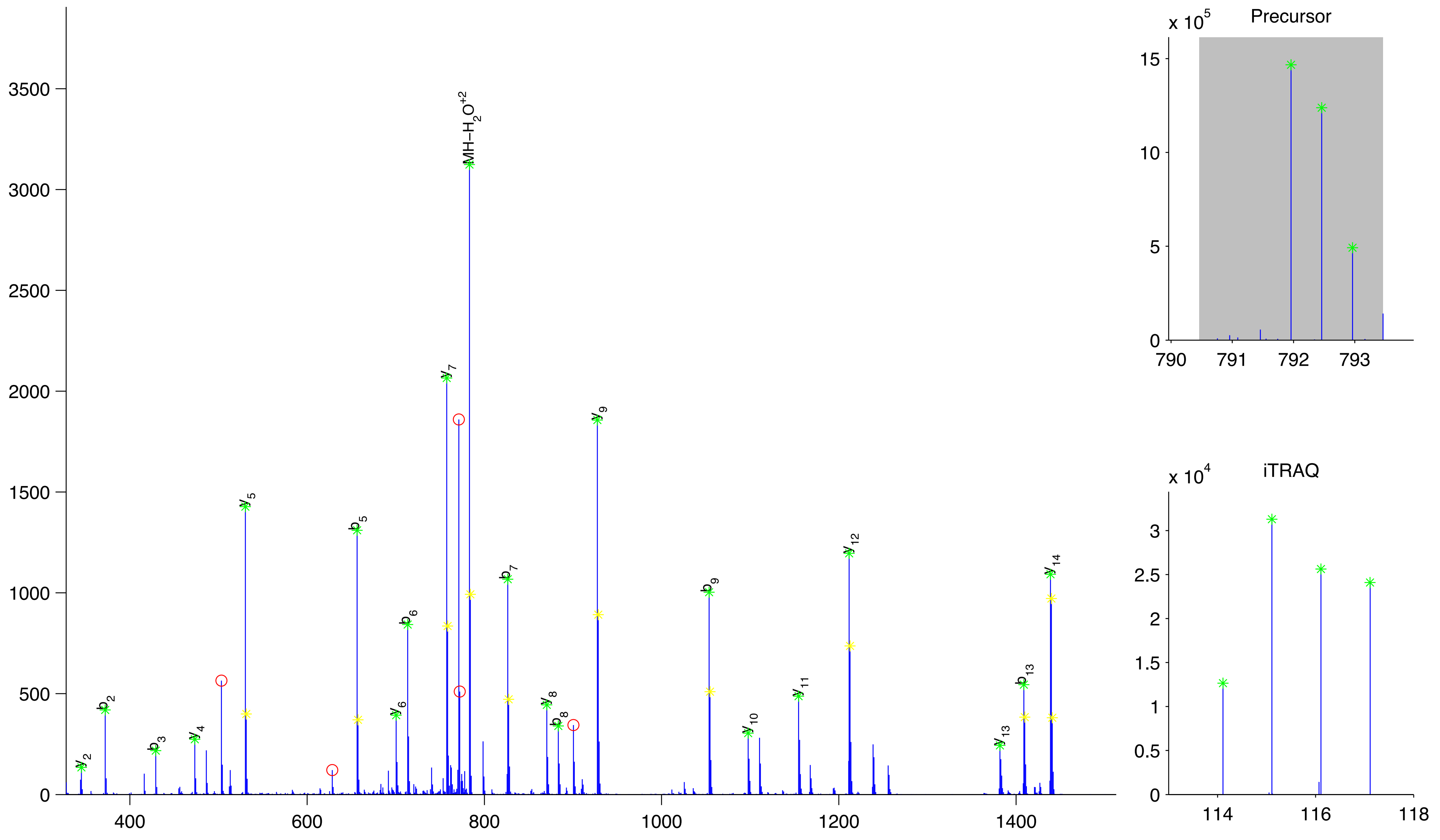

G G k G L G k G G A k R

histone cluster 1, H4a [Homo sapiens]
Charge State: +2
Scan Number: 14747
File Name: 110924_HG2_INSA_ACK.raw

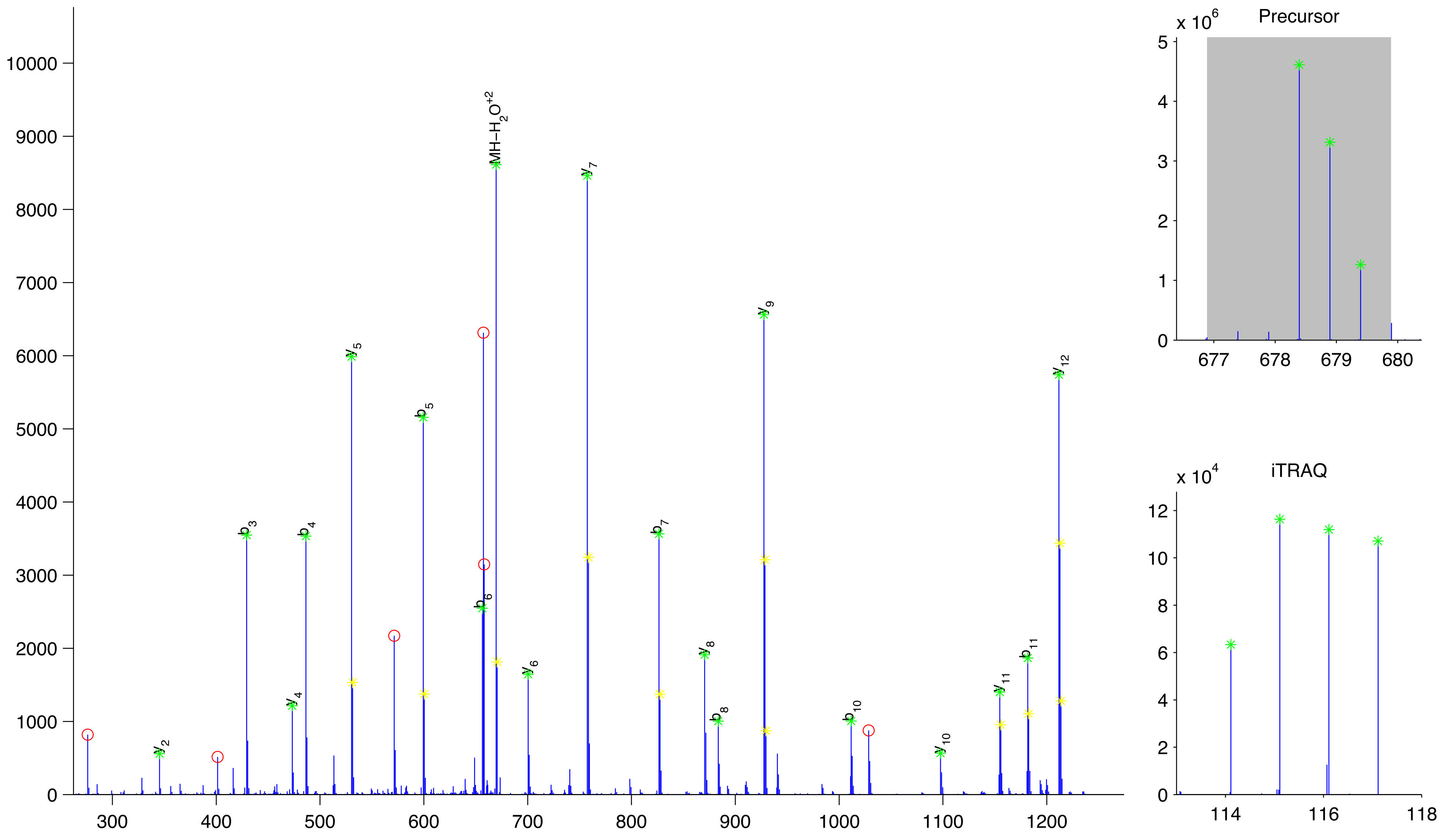

G G k G L G k G G A k R

histone cluster 1, H4a [Homo sapiens]
Charge State: +2
Scan Number: 14837
File Name: 110924_HG2_INSA_ACK.raw

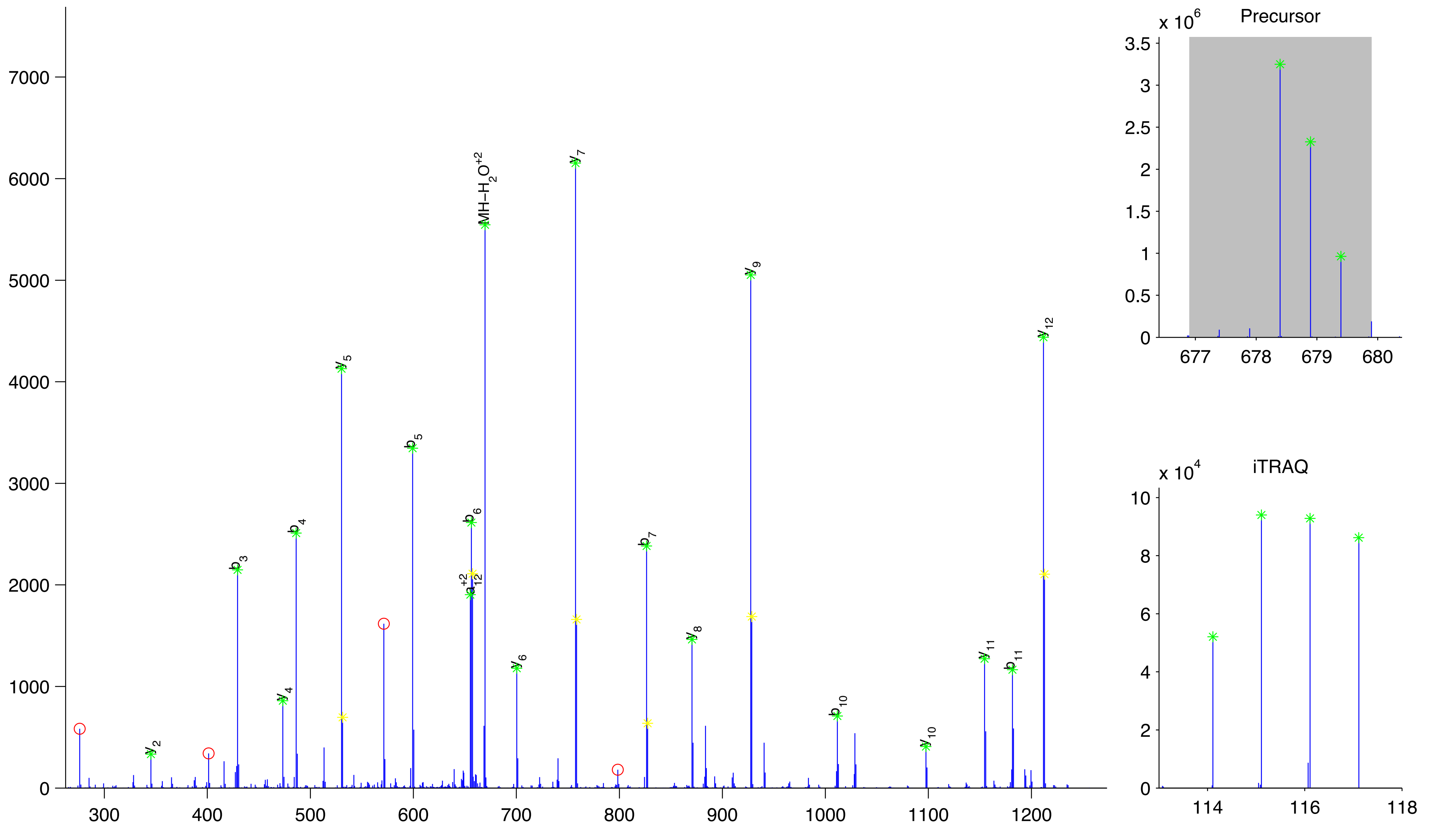

G L G k G G A k R

histone cluster 1, H4a [Homo sapiens]
Charge State: +2
Scan Number: 14867
File Name: 110924_HG2_INSA_ACK3.raw

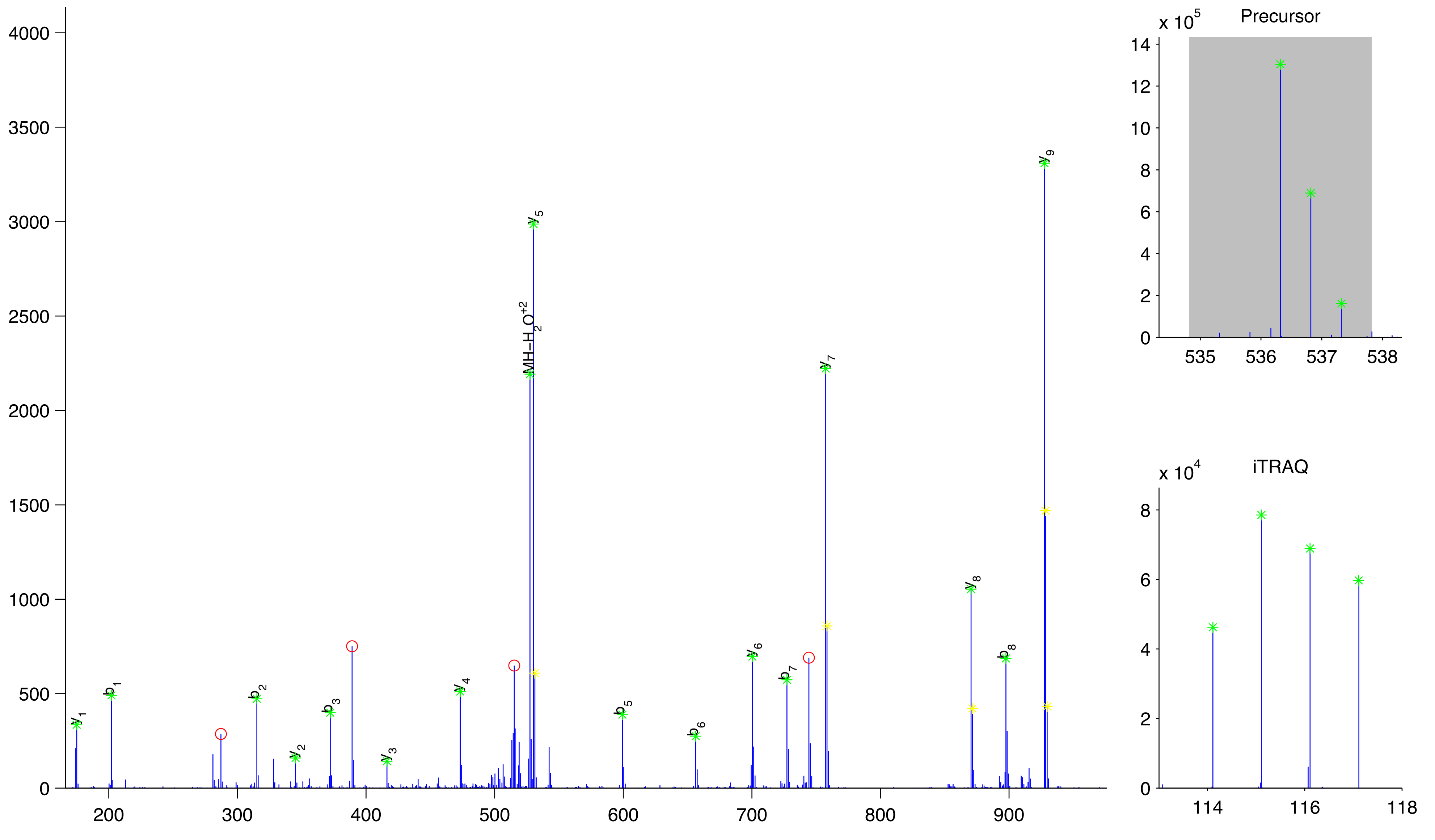

G k G G k G L G k G G A k R

histone cluster 1, H4a [Homo sapiens]
Charge State: +2
Scan Number: 14892
File Name: 110924_HG2_INSA_ACK.raw

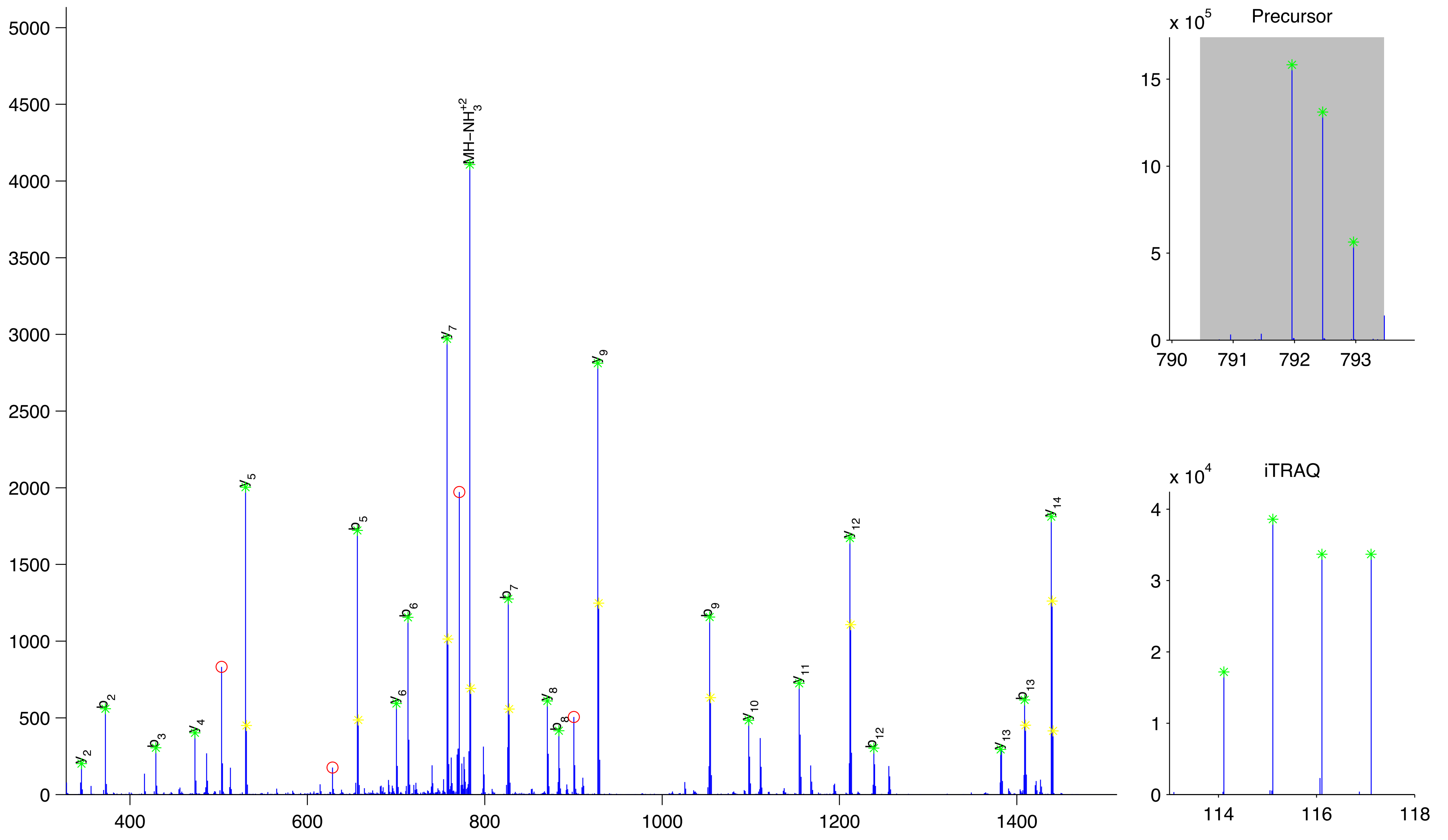

G k G G k G L G k G G A k R

histone cluster 1, H4a [Homo sapiens]
Charge State: +2
Scan Number: 14898
File Name: 110924_HG2_INSA_ACK.raw

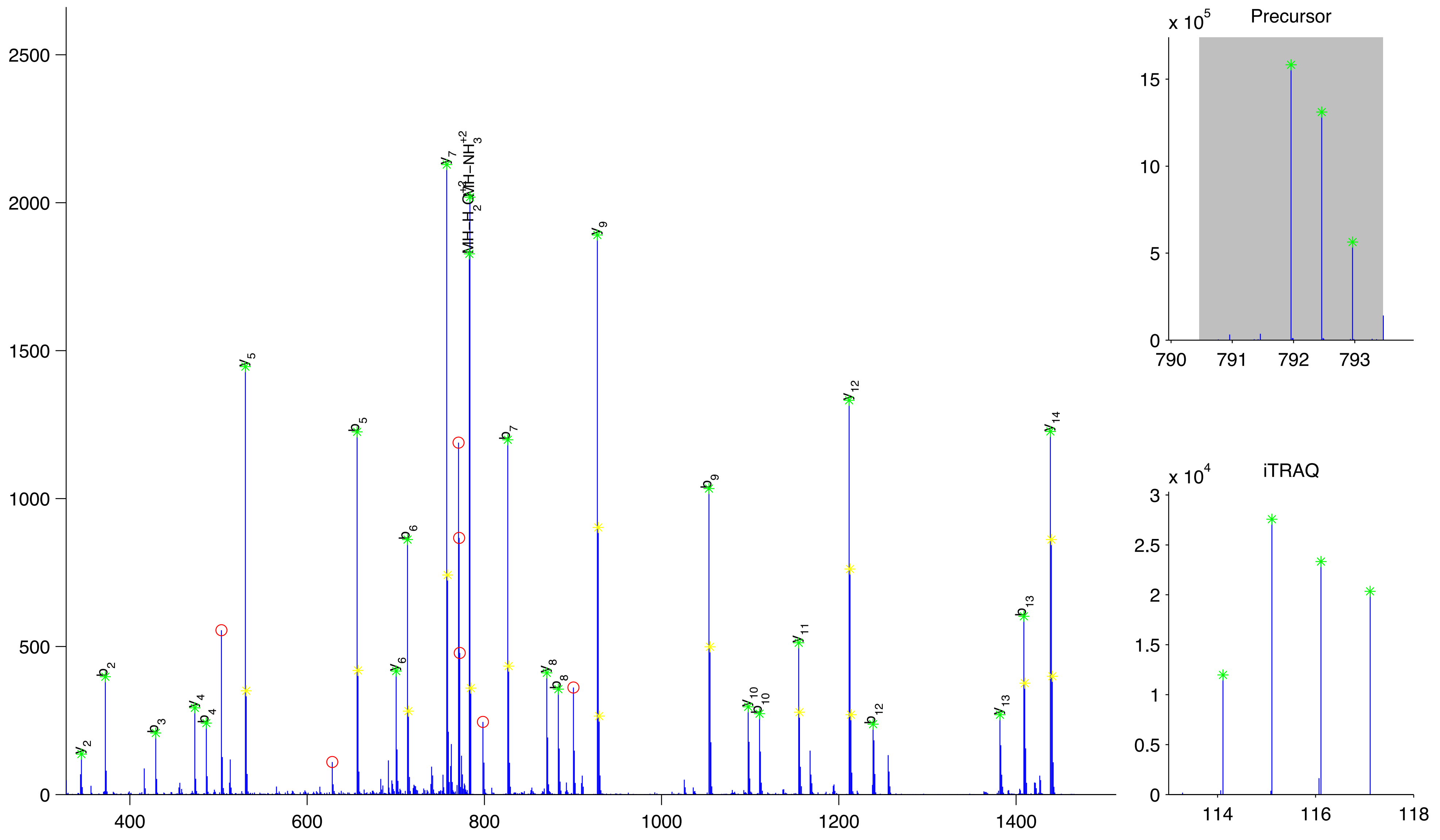

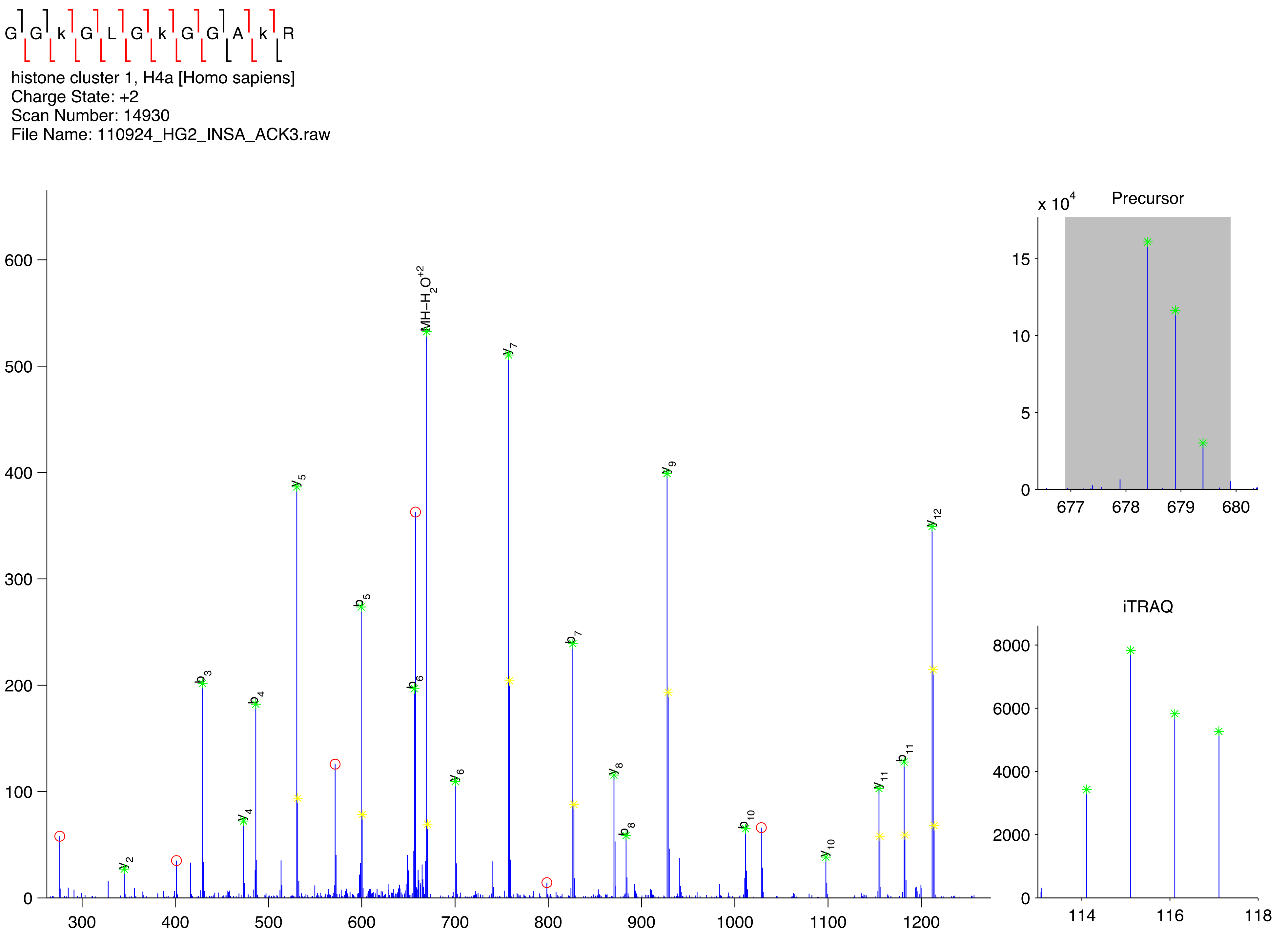
G G k G L G k G G A k R
histone cluster 1, H4a [Homo sapiens]
Charge State: +2
Scan Number: 14930
File Name: 110924_HG2_INSA_ACK3.raw
MH−H2O+2
y5
y7
y9
y12
b3
b4
b5
b6
b7
b8
b10
b11
y2
y4
y6
y8
y10
y11
x 10^4
Precursor
iTRAQ

G L G k G G A k R

histone cluster 1, H4a [Homo sapiens]
Charge State: +2
Scan Number: 15035
File Name: 110924_HG2_INSA_ACK3.raw

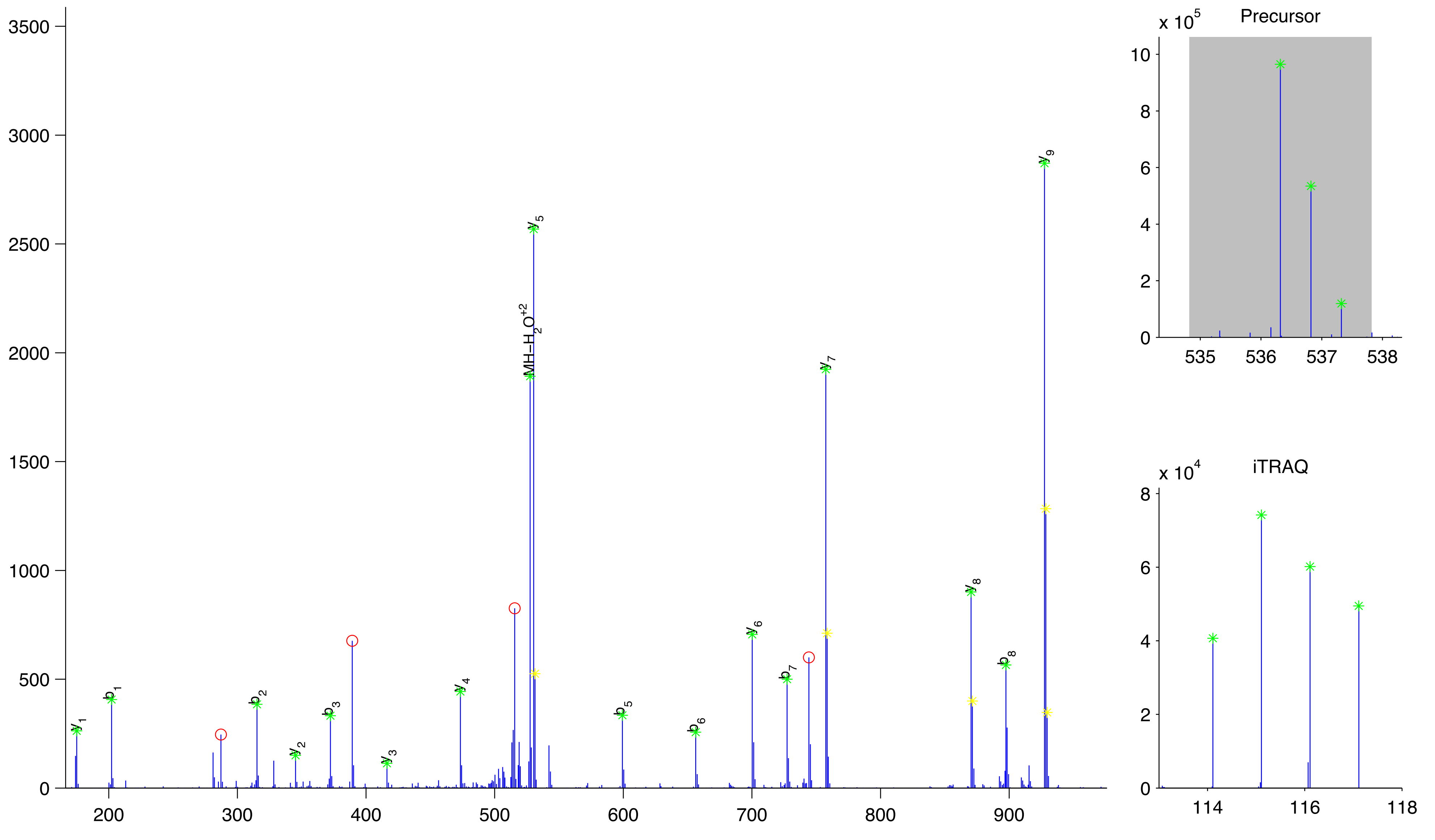

G k G G k G L G k G G A k R

histone cluster 1, H4a [Homo sapiens]
Charge State: +2
Scan Number: 15060
File Name: 110924_HG2_INSA_ACK.raw

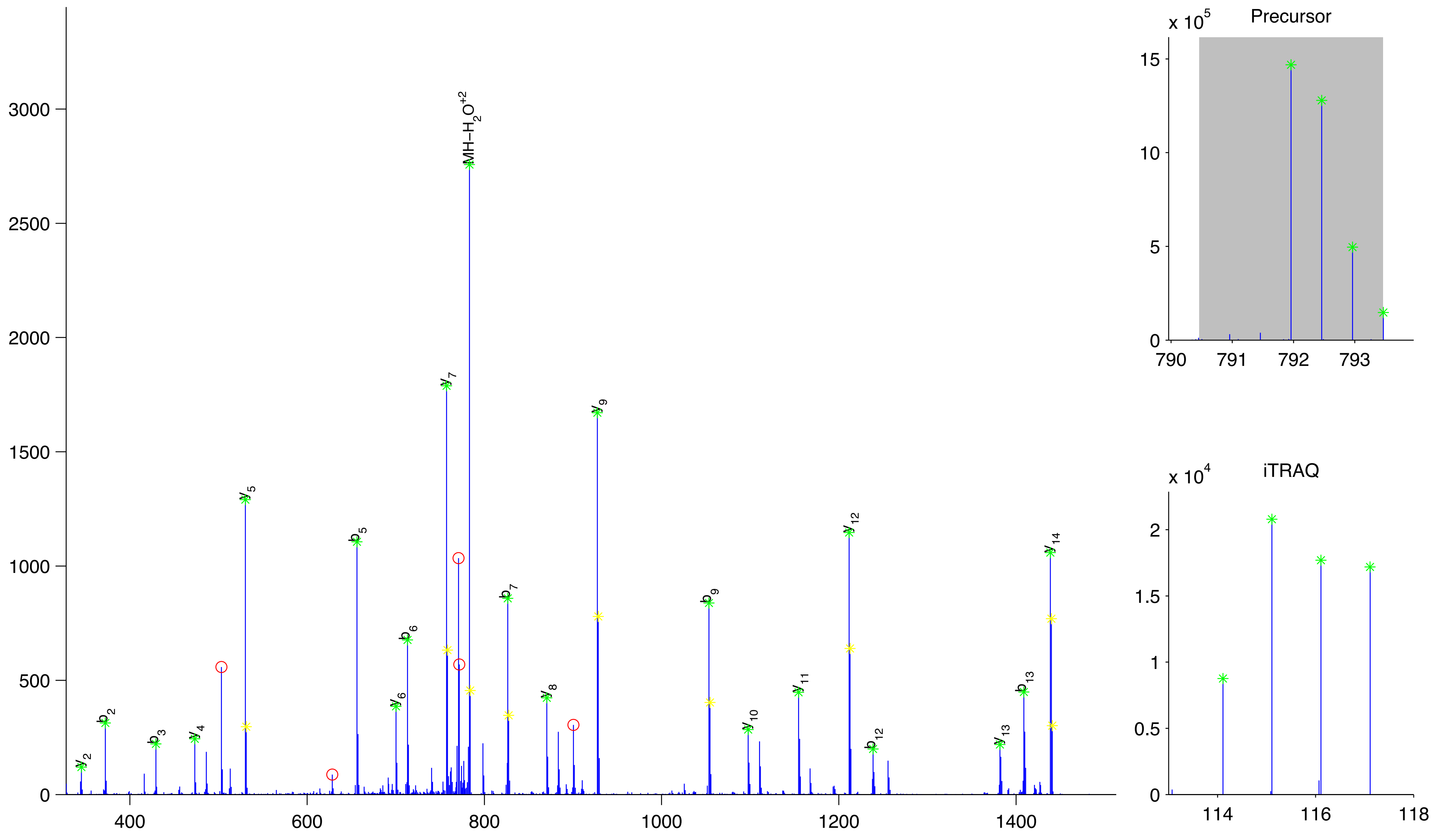

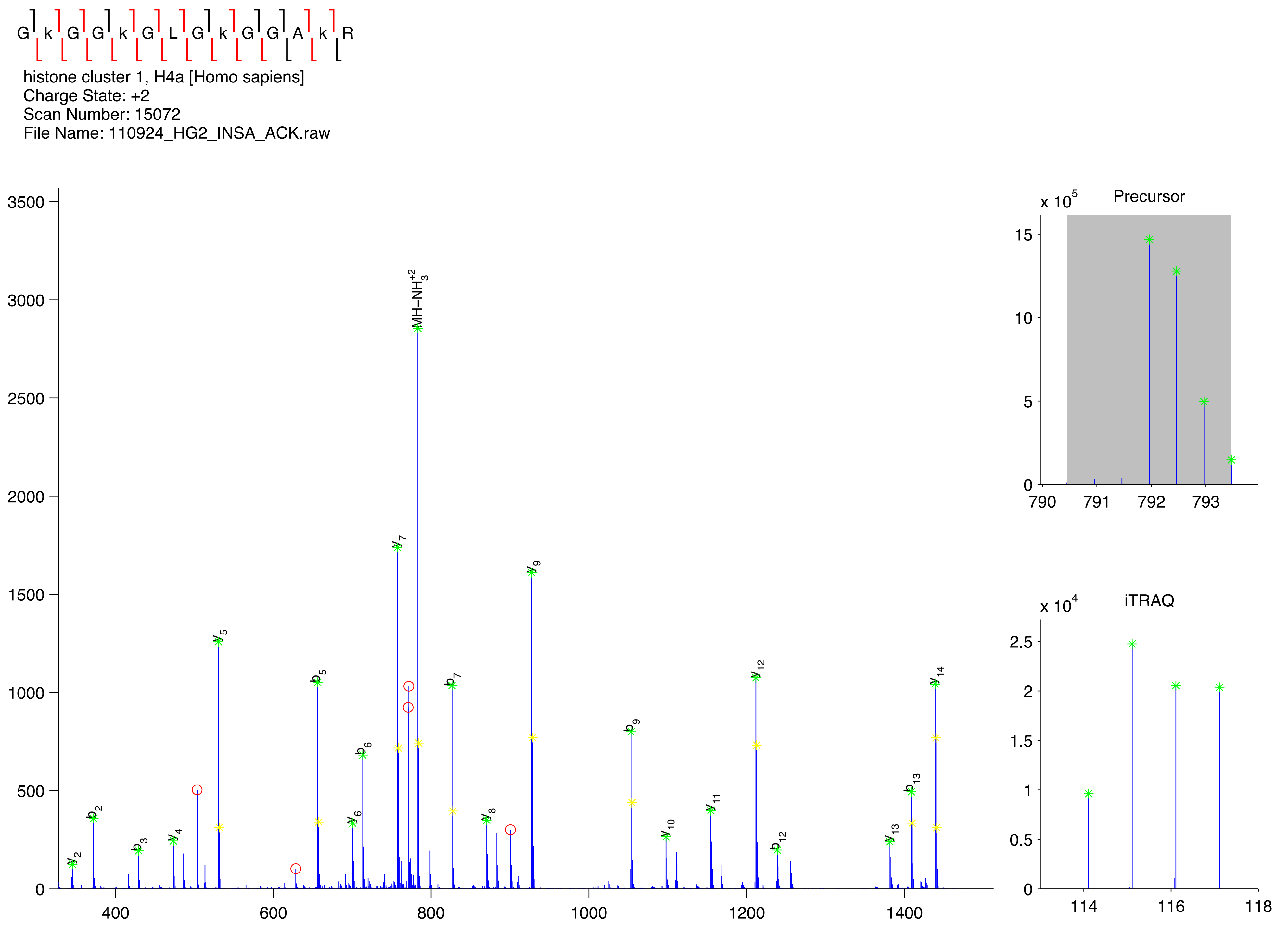
G k G G k G L G k G G A k R
histone cluster 1, H4a [Homo sapiens]
Charge State: +2
Scan Number: 15072
File Name: 110924_HG2_INSA_ACK.raw
y_2 b_2 b_3 y_4 y_5 b_5 y_6 b_6 y_7 $MH-NH_3^{+2}$ b_7 y_8 y_9 b_9 y_{10} y_{11} y_{12} b_{12} y_{13} b_{13} y_{14}
Precursor
x 10^5
iTRAQ
x 10^4

G G k G L G k G G A k R

histone cluster 1, H4a [Homo sapiens]
Charge State: +2
Scan Number: 15091
File Name: 110924_HG2_INSA_ACK3.raw

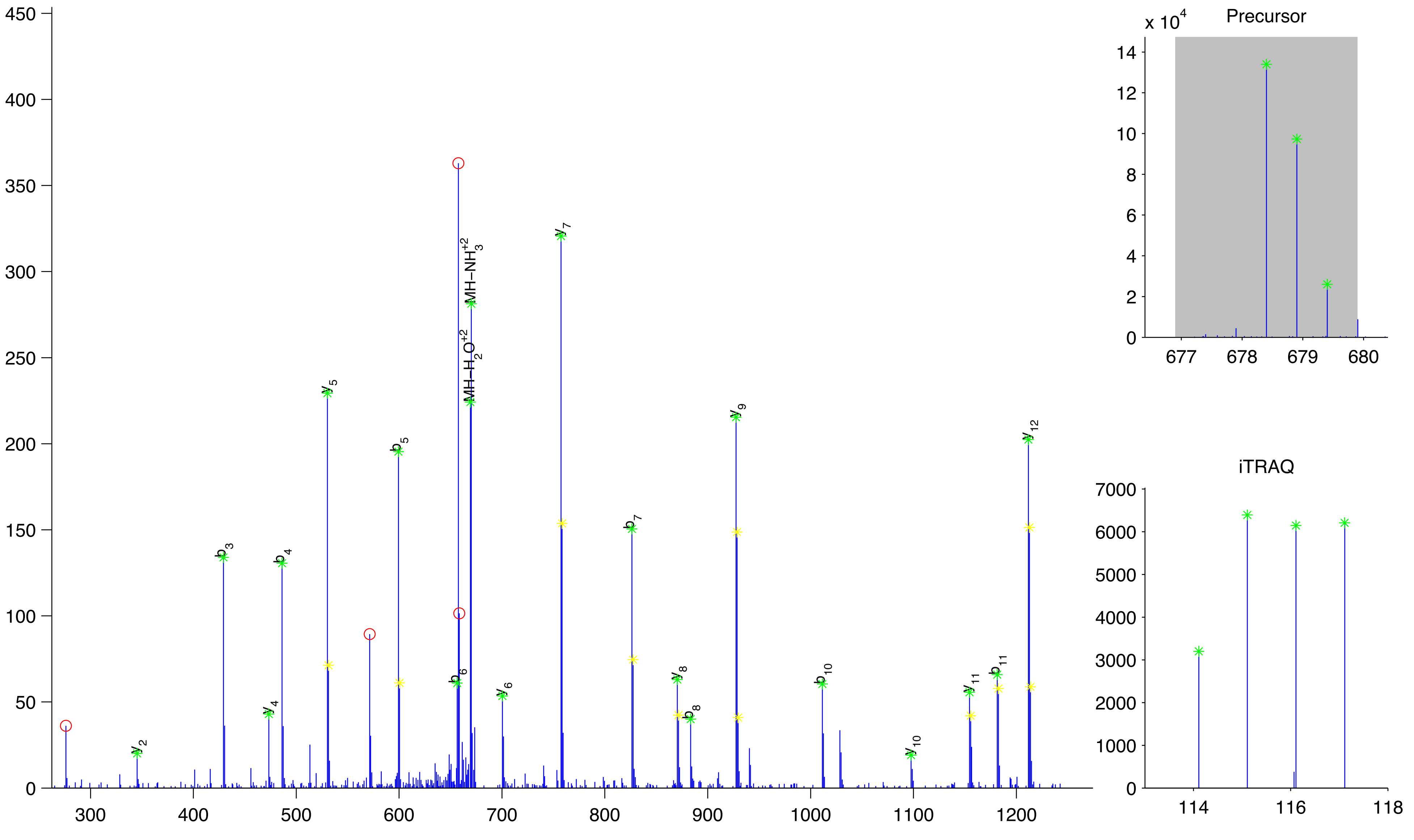

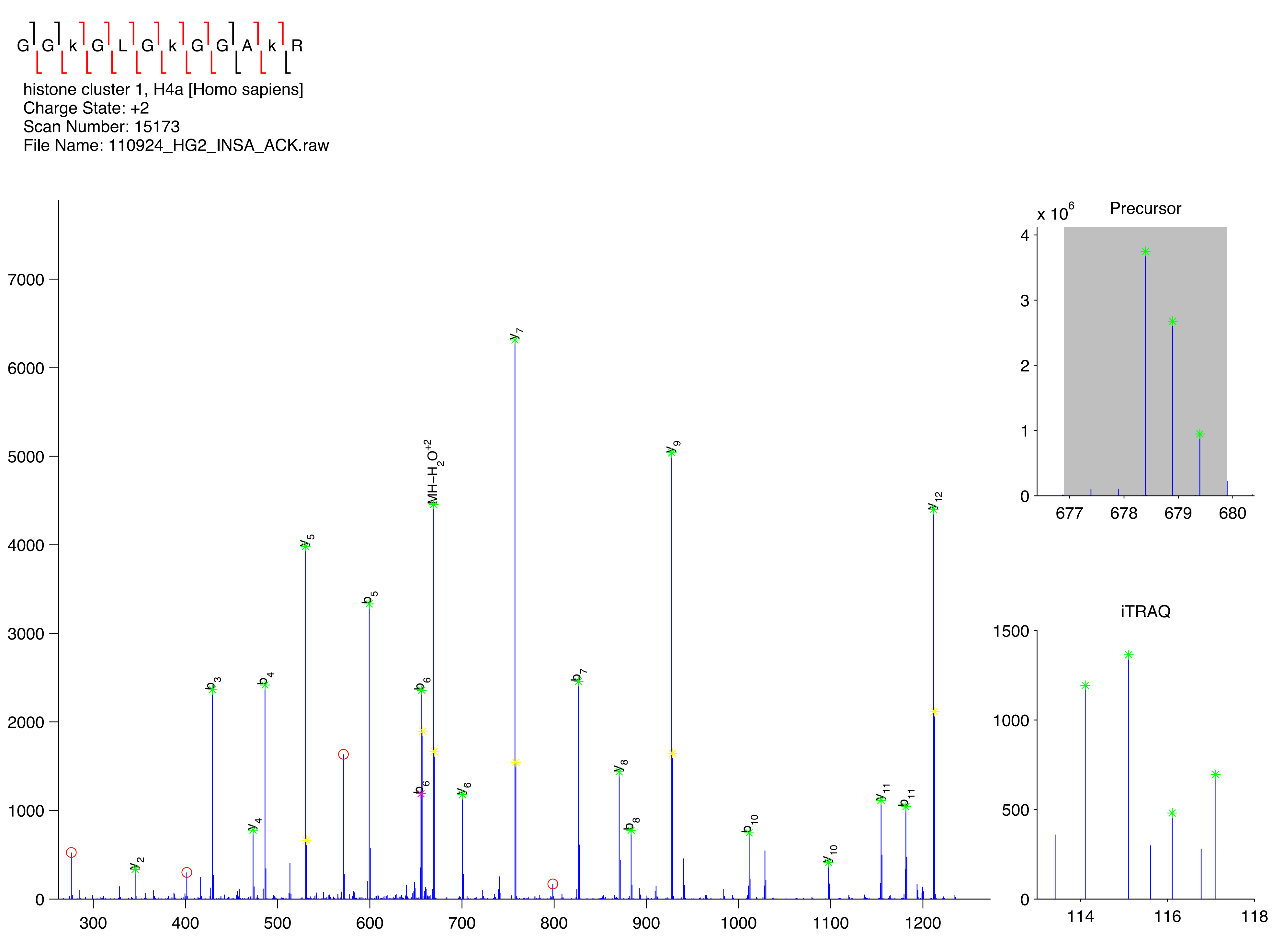

G G k G L G k G G A k R
histone cluster 1, H4a [Homo sapiens]
Charge State: +2
Scan Number: 15173
File Name: 110924_HG2_INSA_ACK.raw
Precursor
x 10^6
iTRAQ
MH−H2O+2
y2 y4 y5 y6 y7 y8 y9 y10 y11 y12
b3 b4 b5 b6 b7 b8 b10 b11

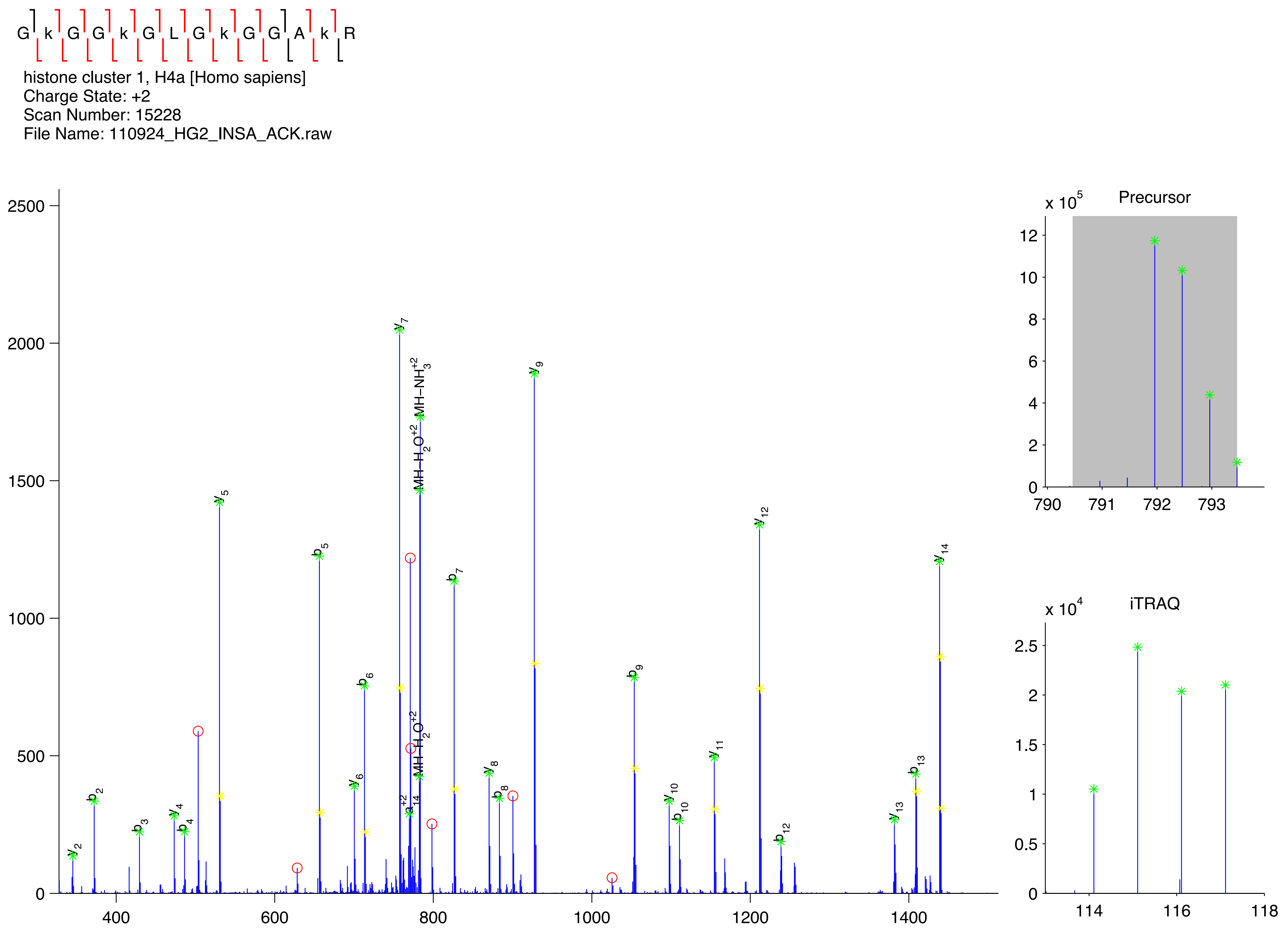
G k G G k G L G k G G A k R
histone cluster 1, H4a [Homo sapiens]
Charge State: +2
Scan Number: 15228
File Name: 110924_HG2_INSA_ACK.raw
Precursor
iTRAQ

G k G G k G L G k G G A k R

histone cluster 1, H4a [Homo sapiens]
Charge State: +2
Scan Number: 15238
File Name: 110924_HG2_INSA_ACK.raw

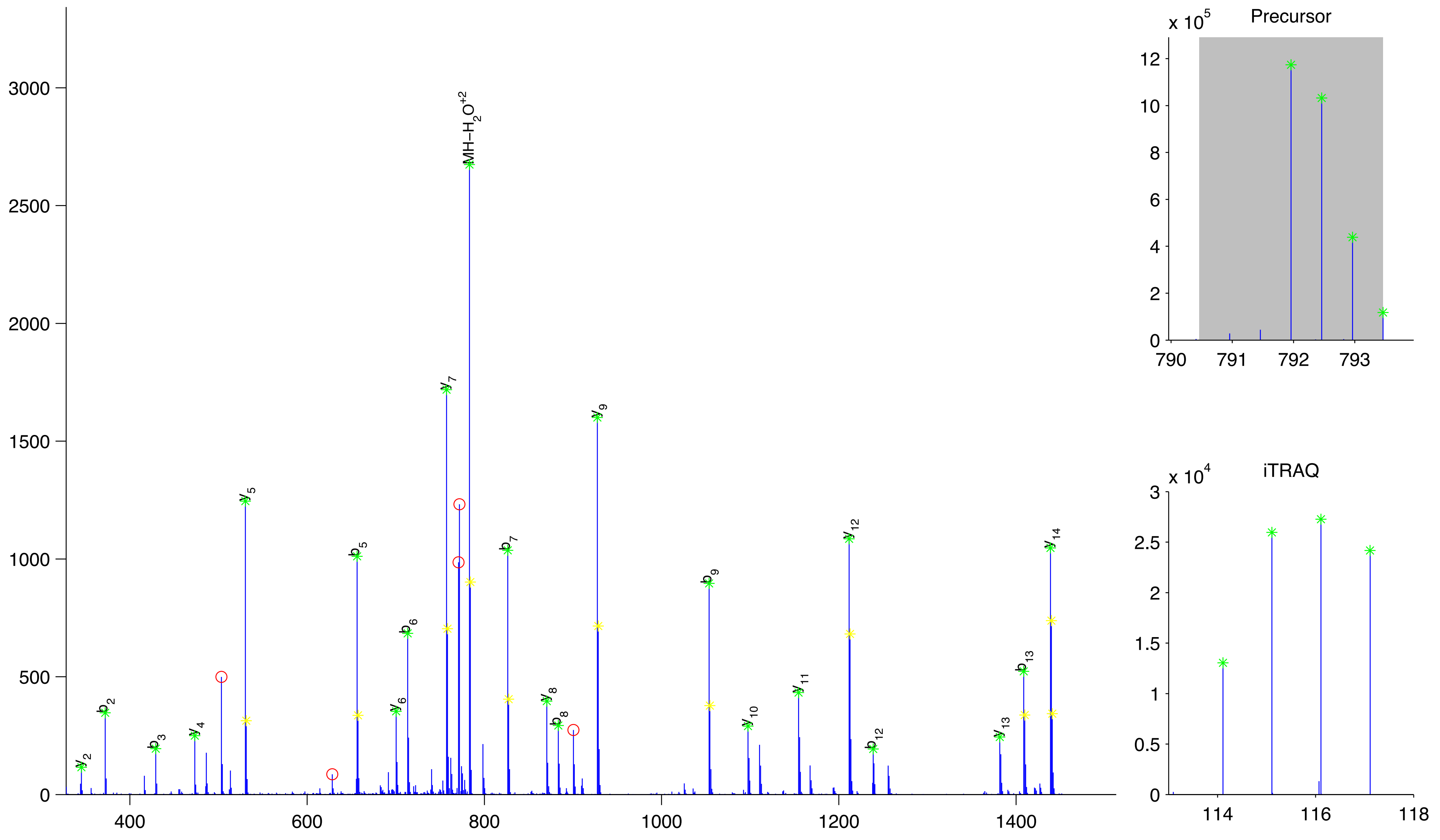

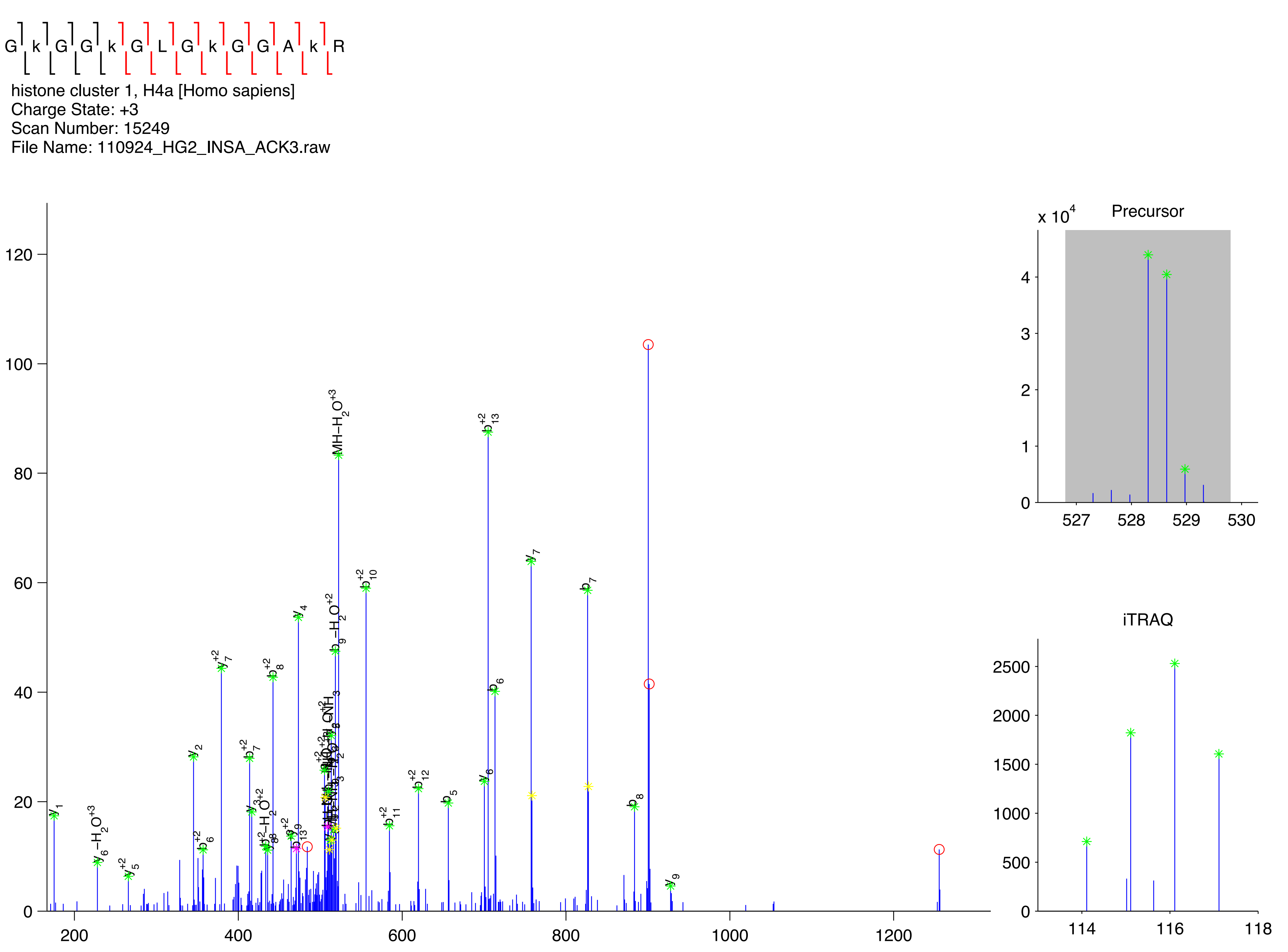

G k G G k G L G k G G A k R
histone cluster 1, H4a [Homo sapiens]
Charge State: +3
Scan Number: 15249
File Name: 110924_HG2_INSA_ACK3.raw
Precursor
x 10^4
iTRAQ

G G k G L G k G G A k R

histone cluster 1, H4a [Homo sapiens]
Charge State: +2
Scan Number: 15339
File Name: 110924_HG2_INSA_ACK.raw

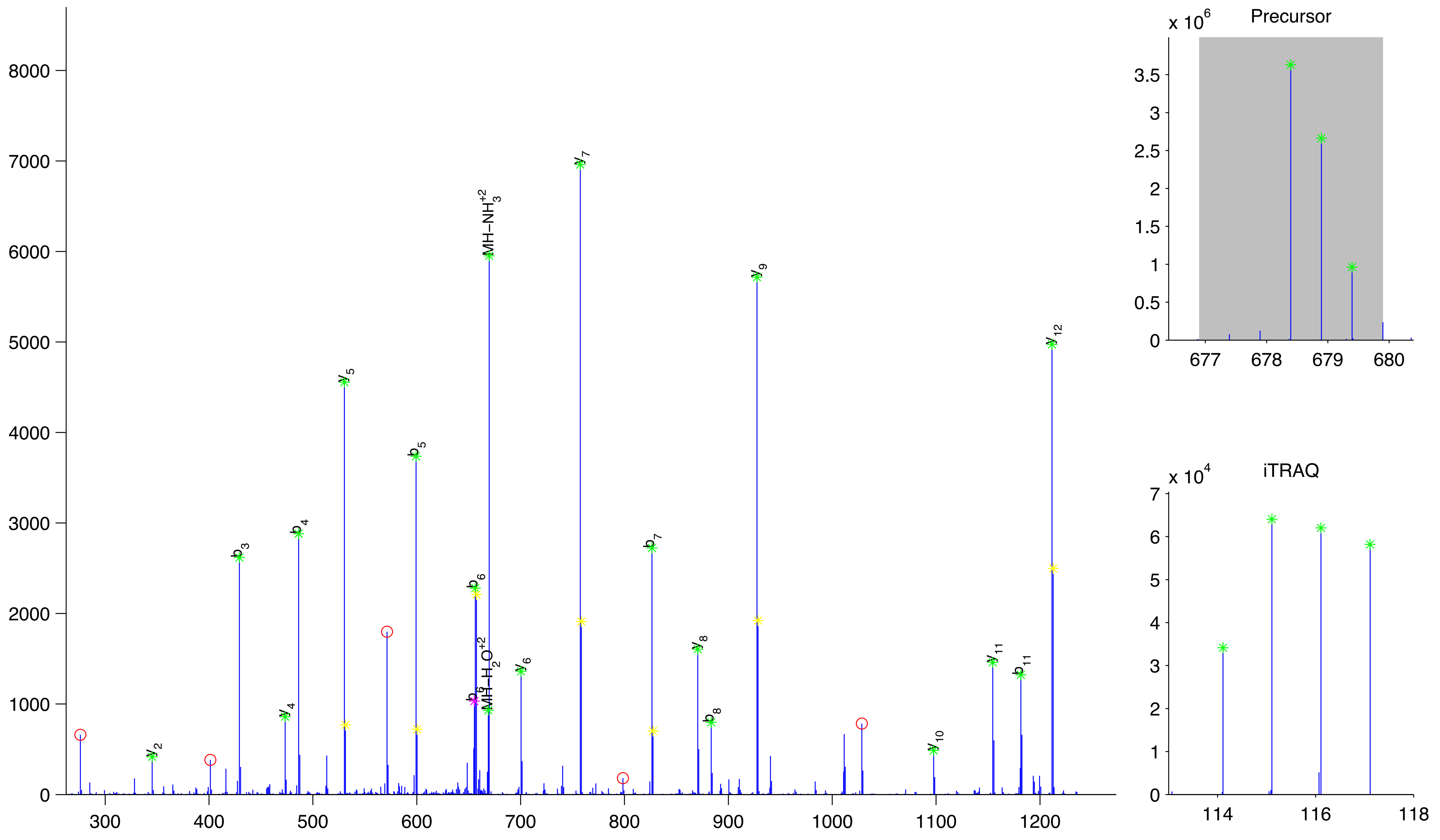

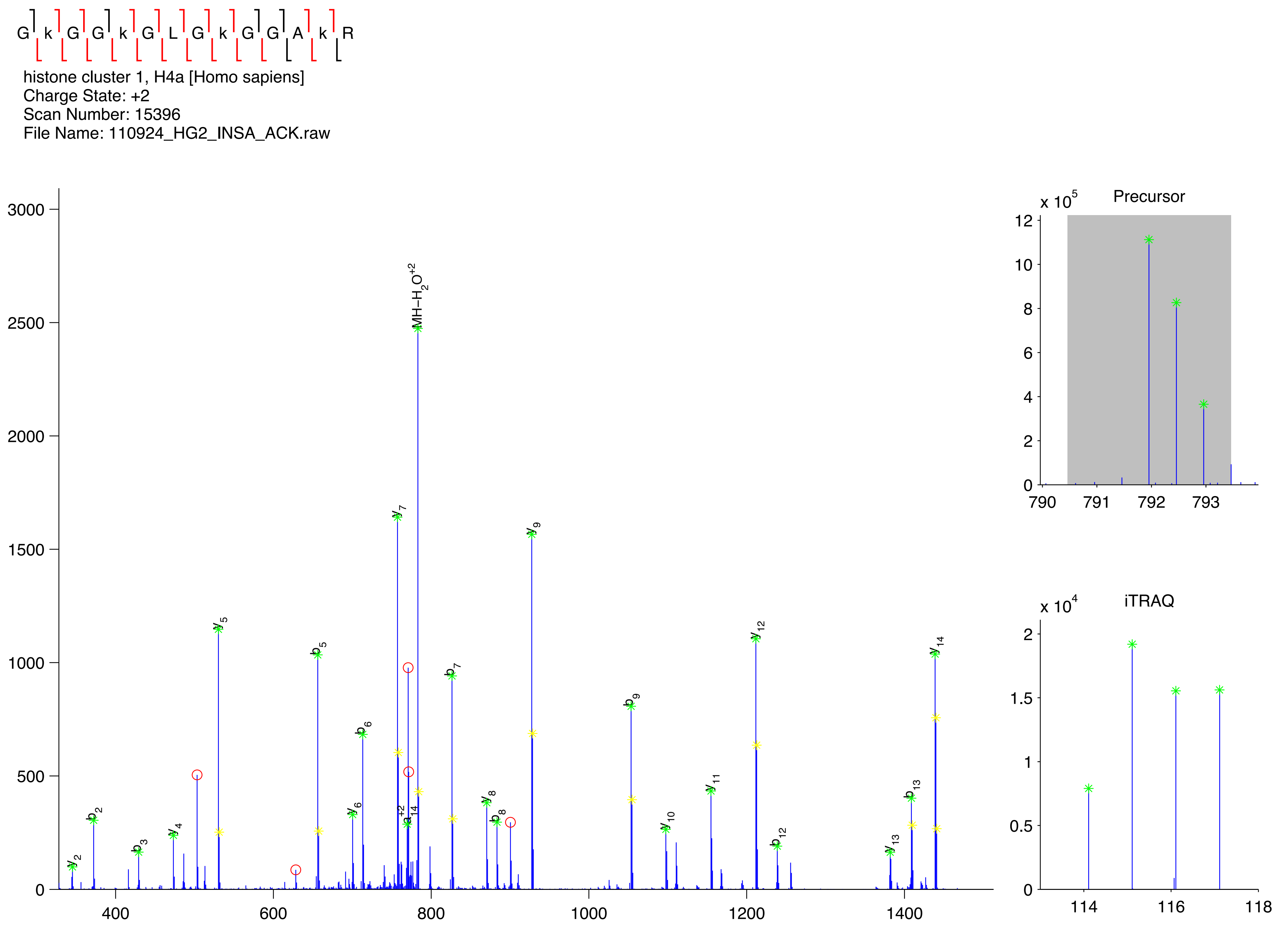
G k G G k G L G k G G A k R
histone cluster 1, H4a [Homo sapiens]
Charge State: +2
Scan Number: 15396
File Name: 110924_HG2_INSA_ACK.raw
MH−H2O+2
y2
b2
b3
y4
y5
b5
y6
b6
y7
a14+2
b7
y8
b8
y9
b9
y10
y11
y12
b12
y13
b13
y14
Precursor
x 10^5
iTRAQ
x 10^4

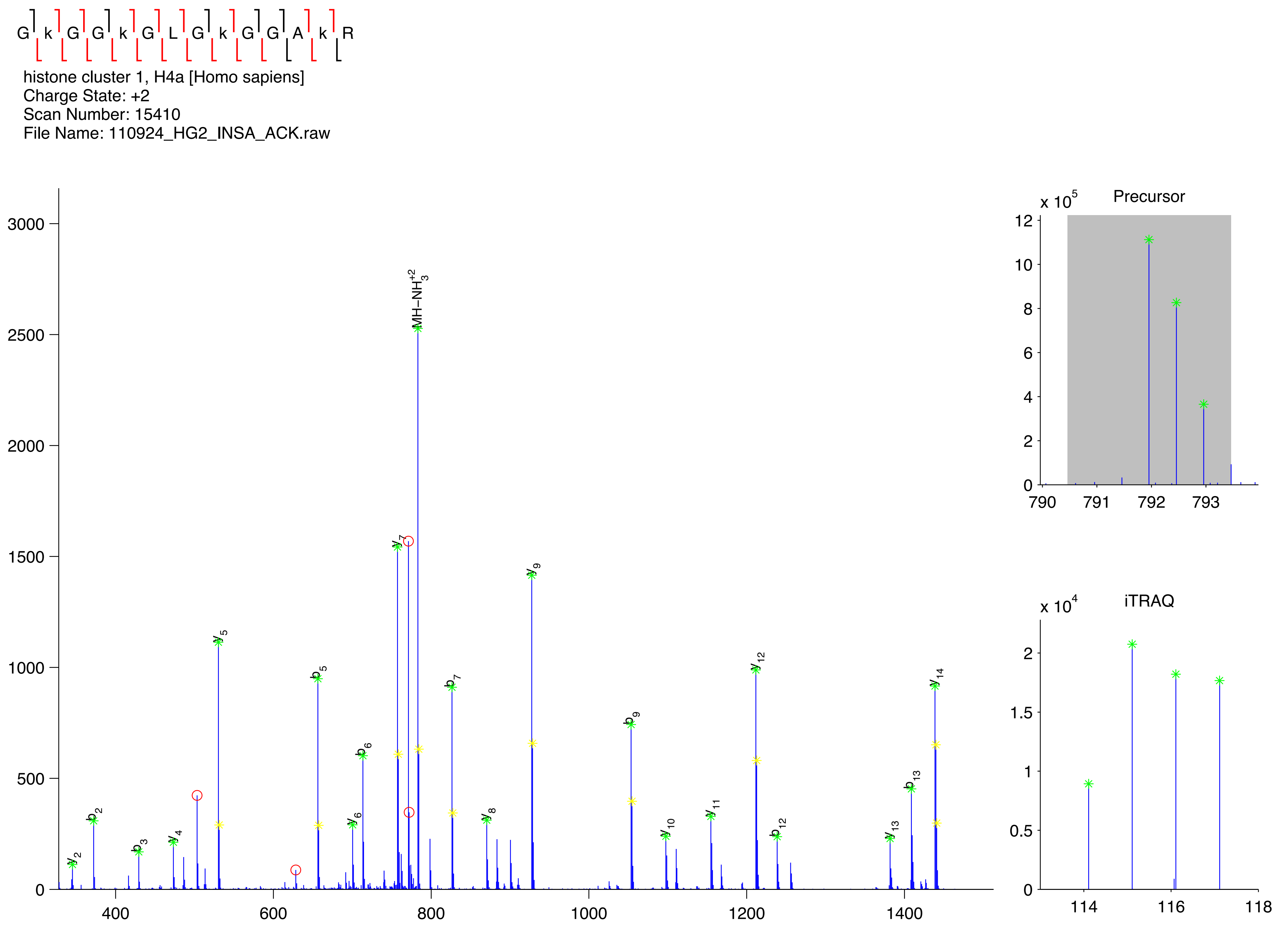
G k G G k G L G k G G A k R
histone cluster 1, H4a [Homo sapiens]
Charge State: +2
Scan Number: 15410
File Name: 110924_HG2_INSA_ACK.raw
MH−NH3+2
y2
b2
b3
y4
y5
b5
y6
b6
y7
b7
y8
y9
b9
y10
y11
y12
b12
y13
b13
y14
Precursor
x 10^5
iTRAQ
x 10^4

G k G G k G L G k G G A k R

histone cluster 1, H4a [Homo sapiens]
Charge State: +2
Scan Number: 15564
File Name: 110924_HG2_INSA_ACK.raw

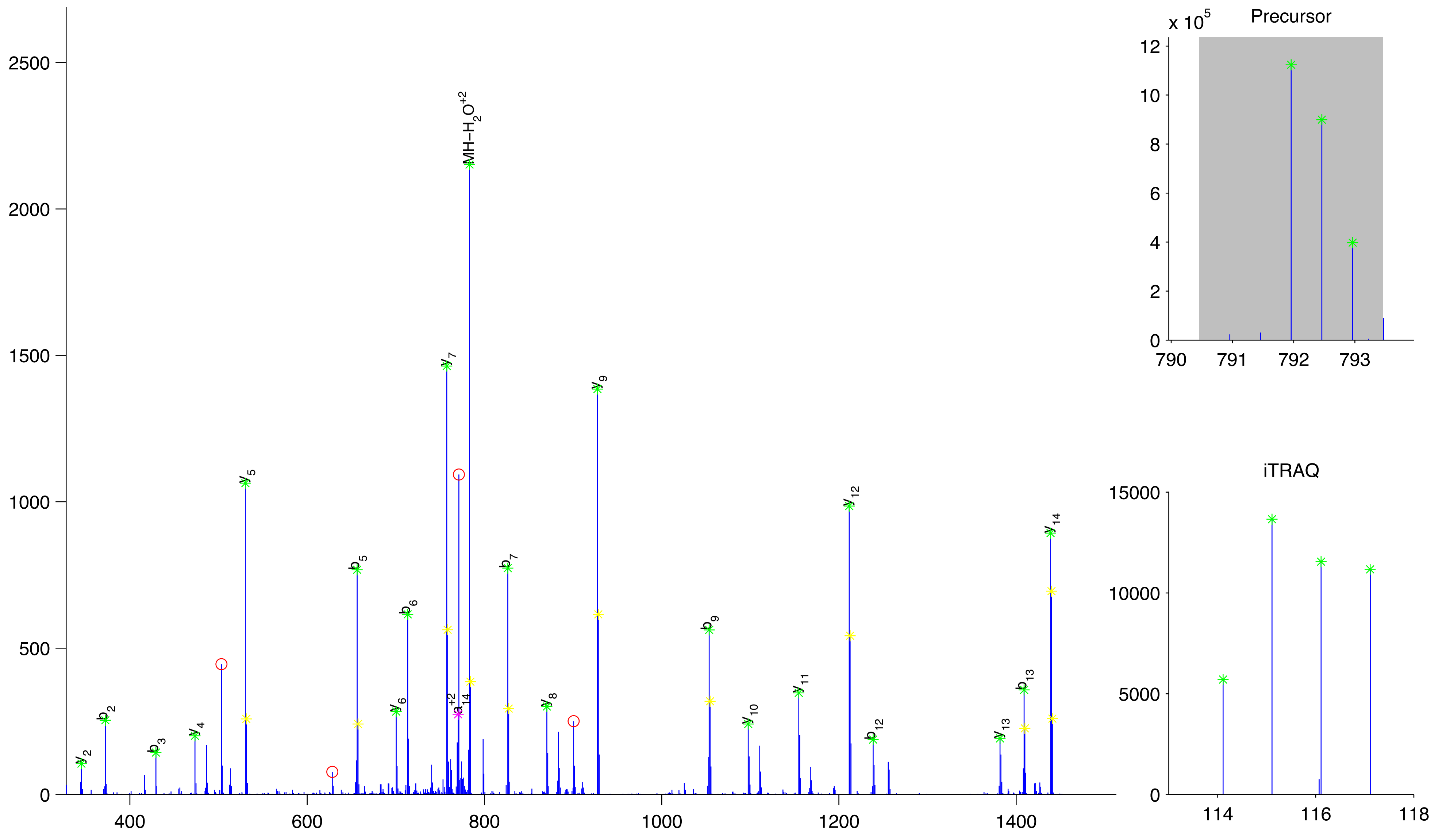

G k G G k G L G k G G A k R

histone cluster 1, H4a [Homo sapiens]
Charge State: +2
Scan Number: 15572
File Name: 110924_HG2_INSA_ACK.raw

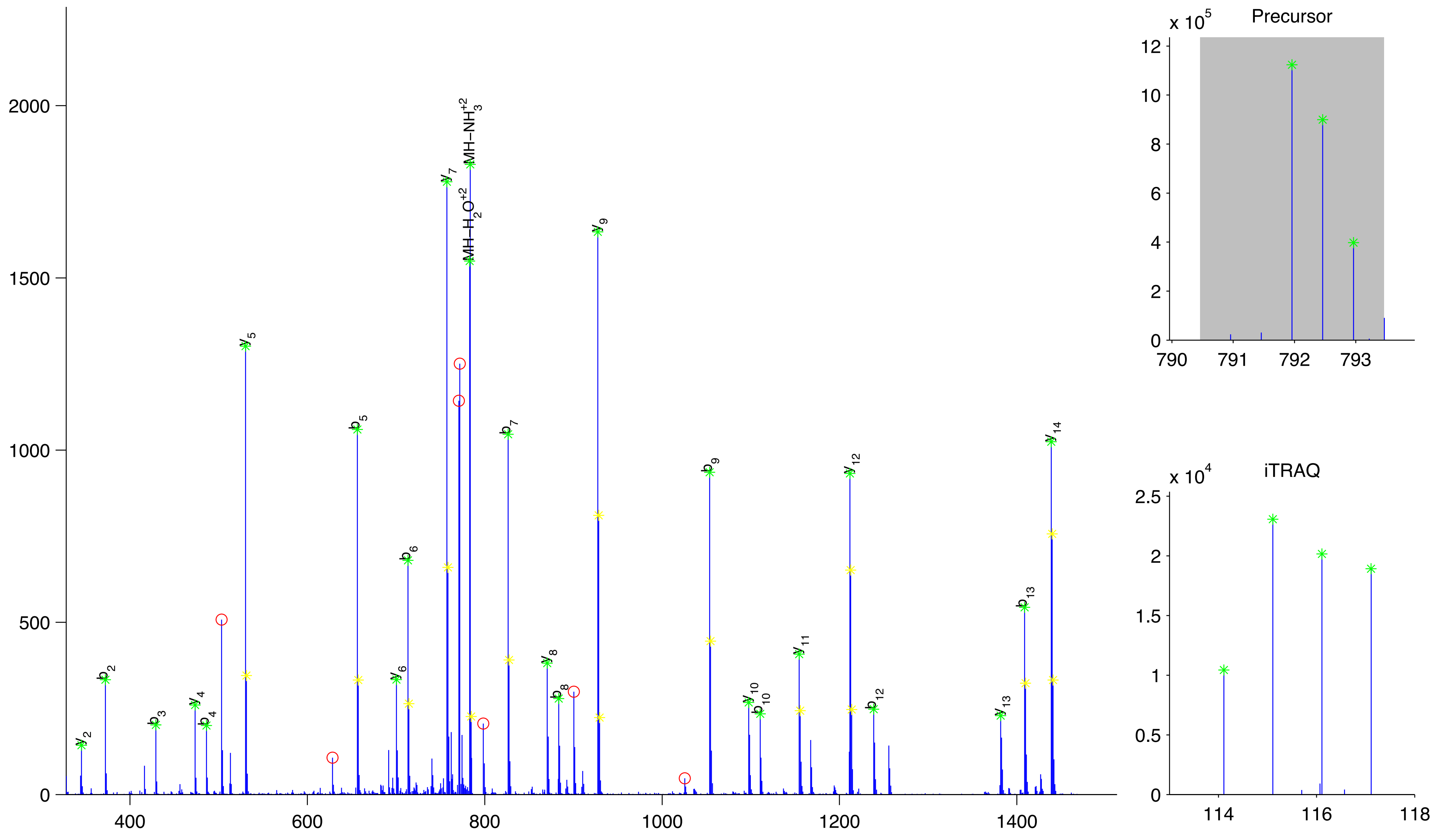

G G k G L G k G G A k R

histone cluster 1, H4a [Homo sapiens]
Charge State: +2
Scan Number: 15675
File Name: 110924_HG2_INSA_ACK.raw

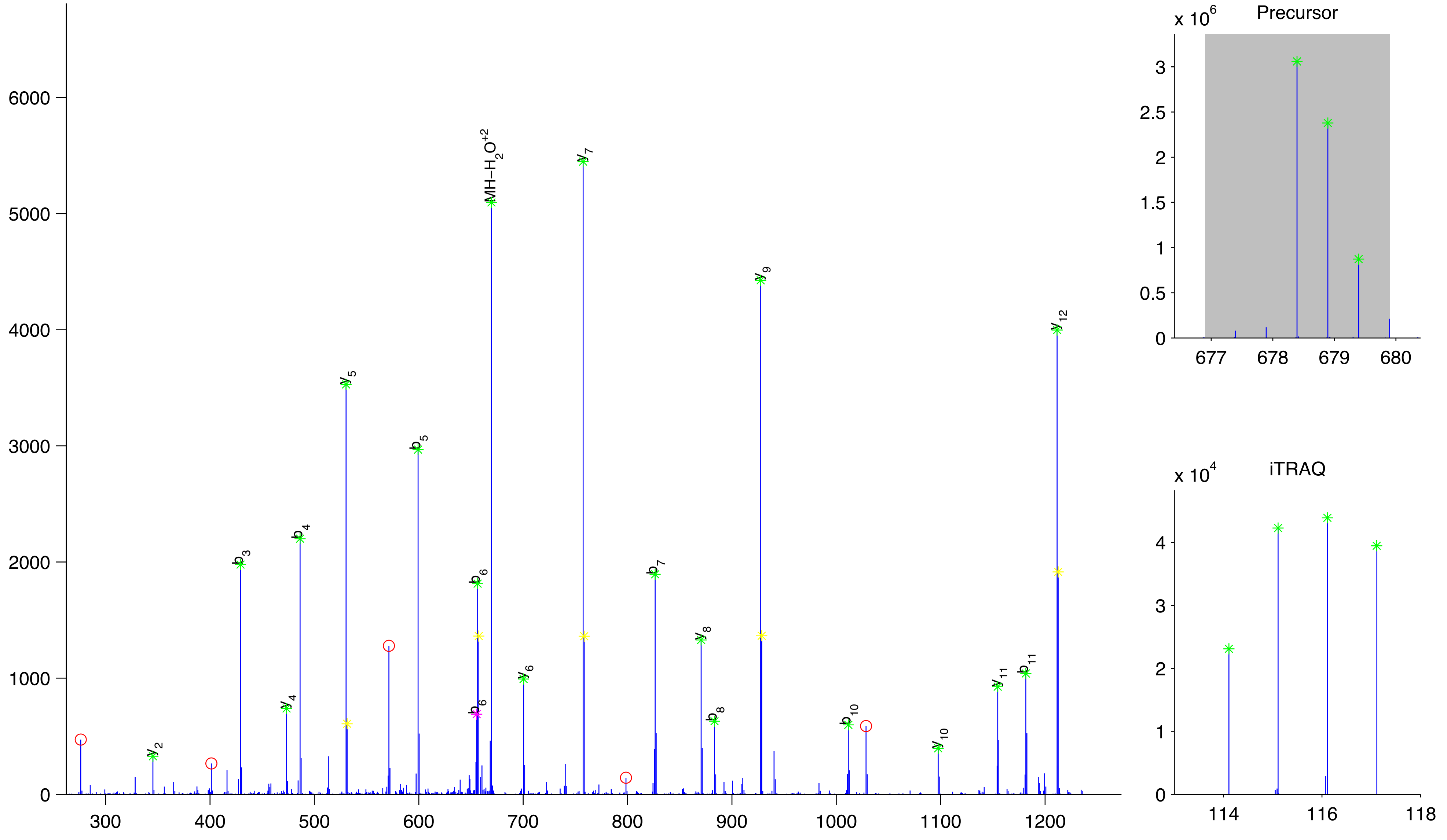

G L G k G G A k R

histone cluster 1, H4a [Homo sapiens]
Charge State: +2
Scan Number: 15707
File Name: 110924_HG2_INSA_ACK3.raw

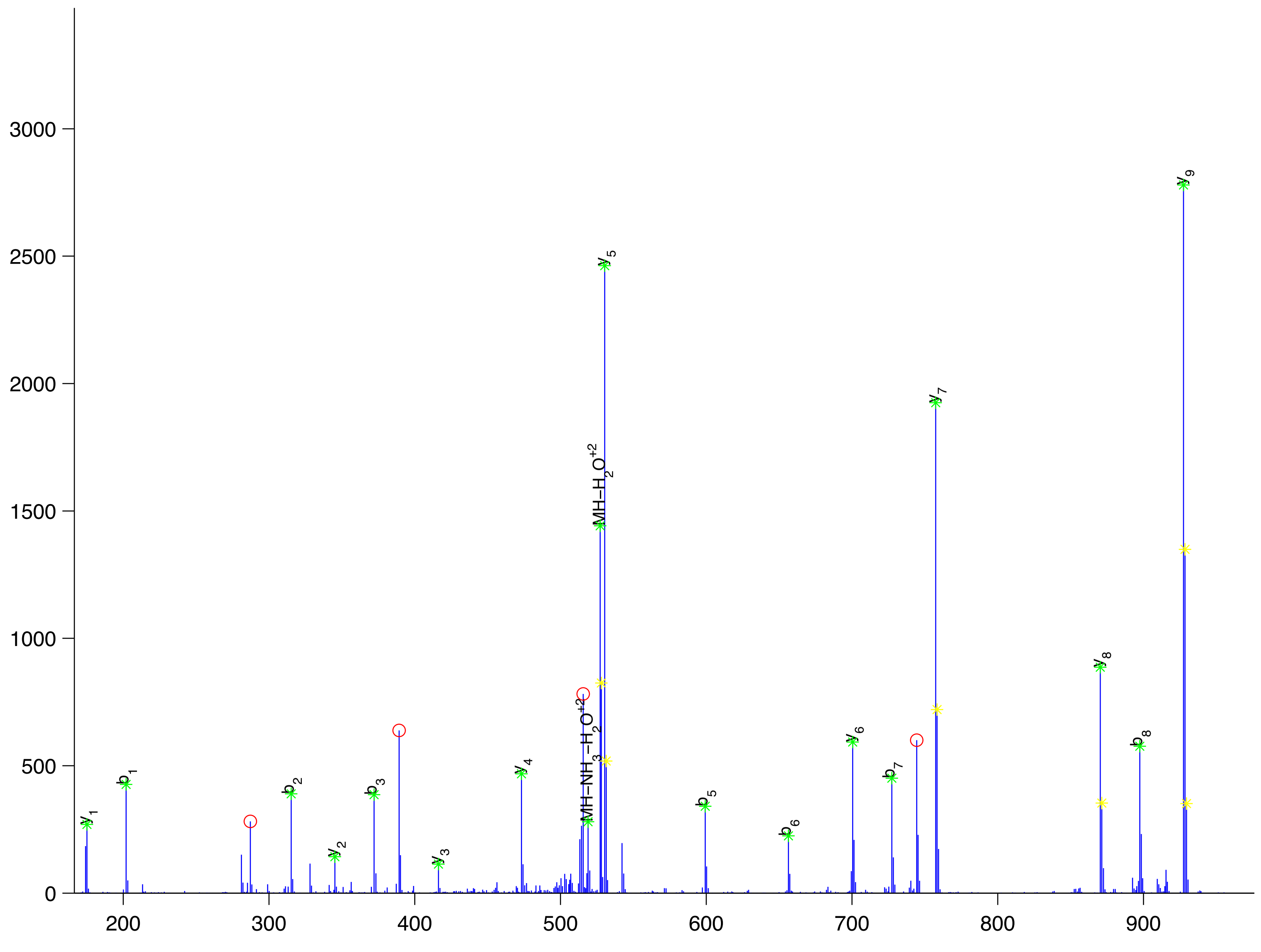

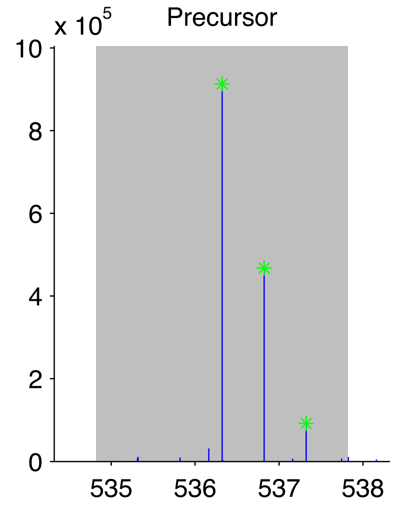

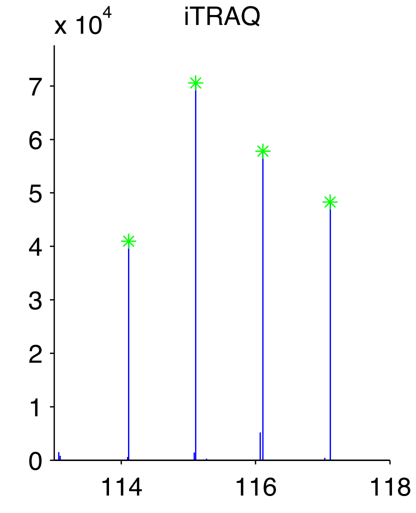

G k G G k G L G k G G A k R

histone cluster 1, H4a [Homo sapiens]
Charge State: +2
Scan Number: 15732
File Name: 110924_HG2_INSA_ACK.raw

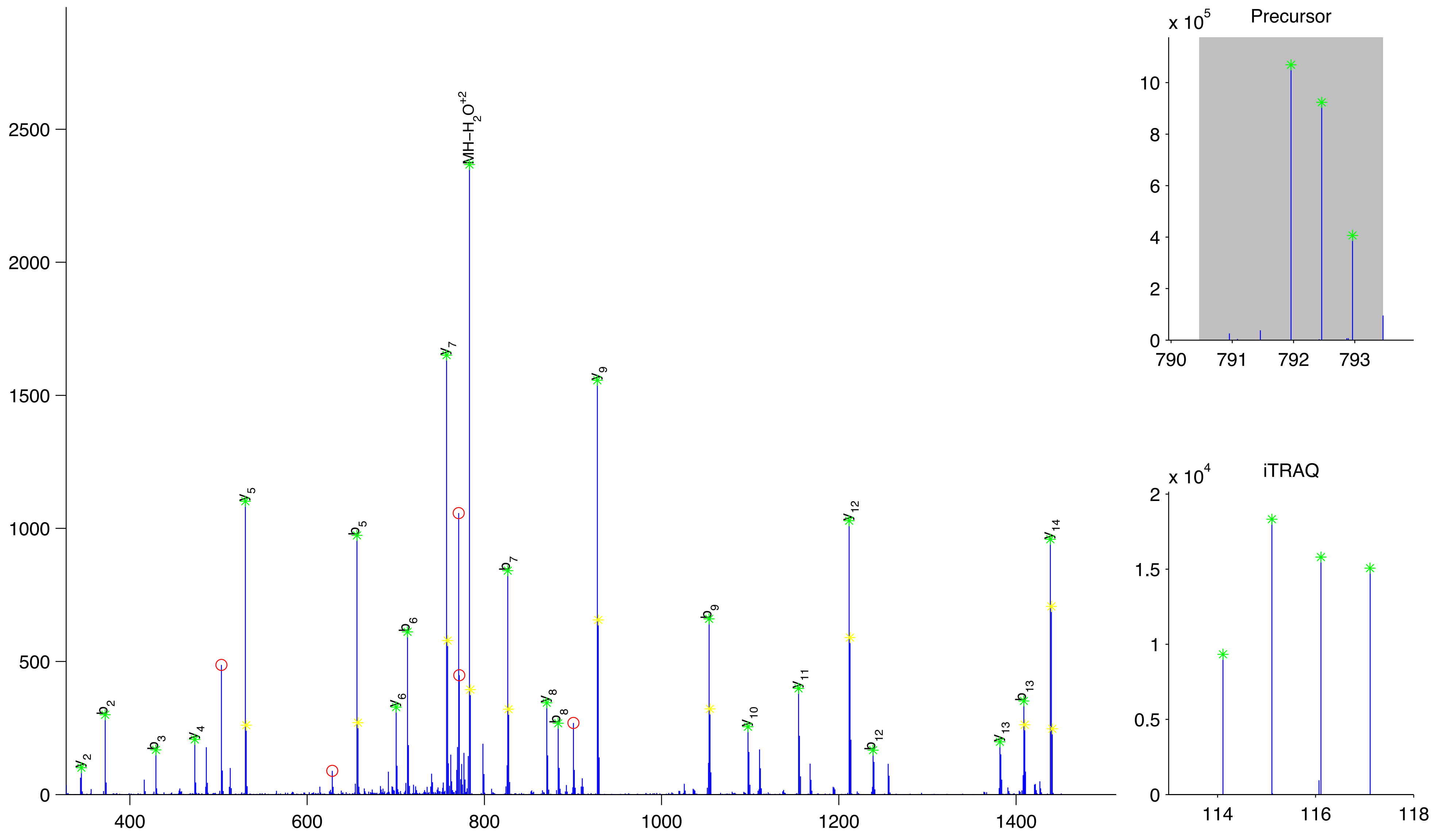

G k G G k G L G k G G A k R

histone cluster 1, H4a [Homo sapiens]
Charge State: +2
Scan Number: 15740
File Name: 110924_HG2_INSA_ACK.raw

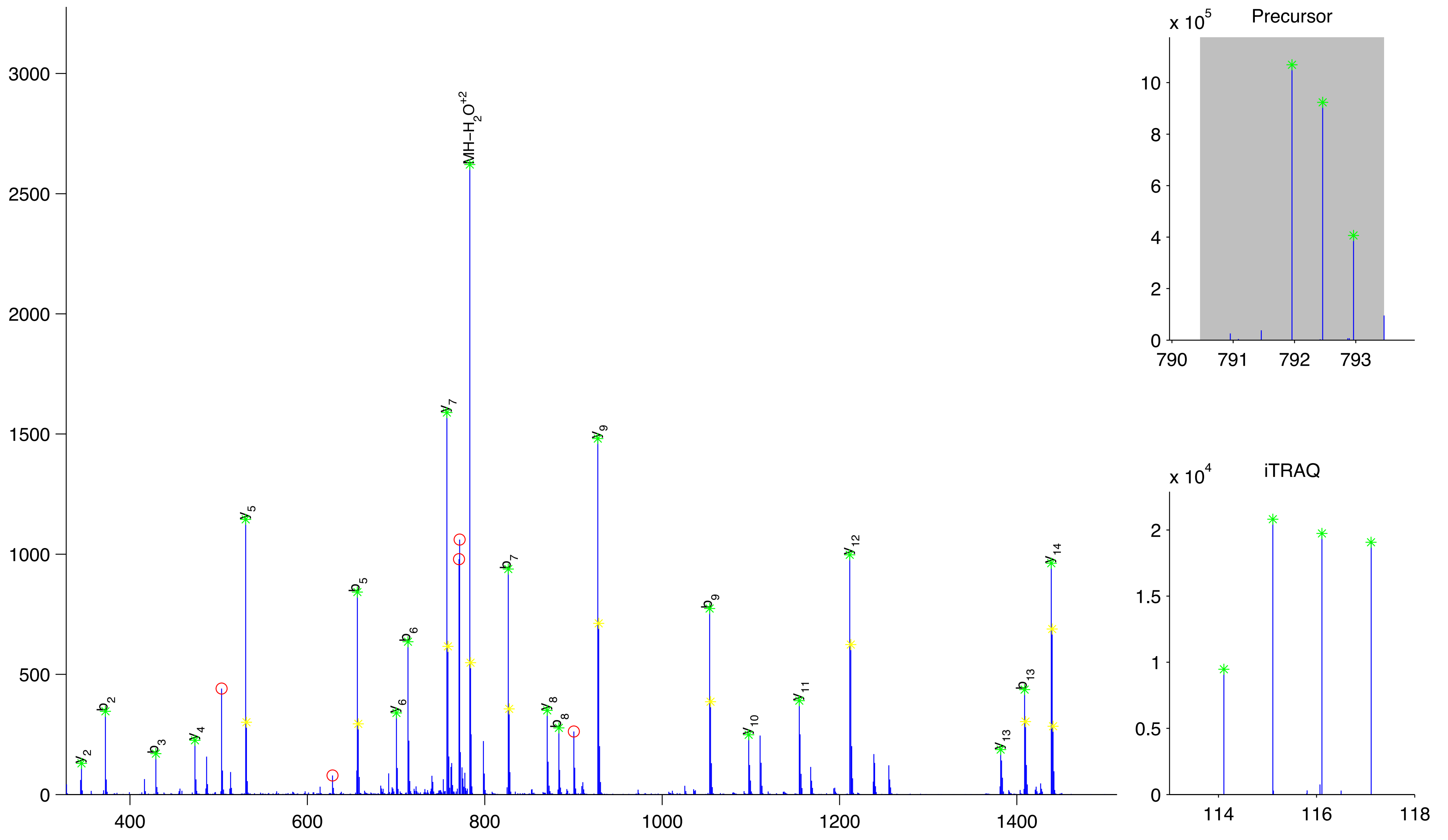

G L G k G G A k R

histone cluster 1, H4a [Homo sapiens]
Charge State: +2
Scan Number: 15877
File Name: 110924_HG2_INSA_ACK3.raw

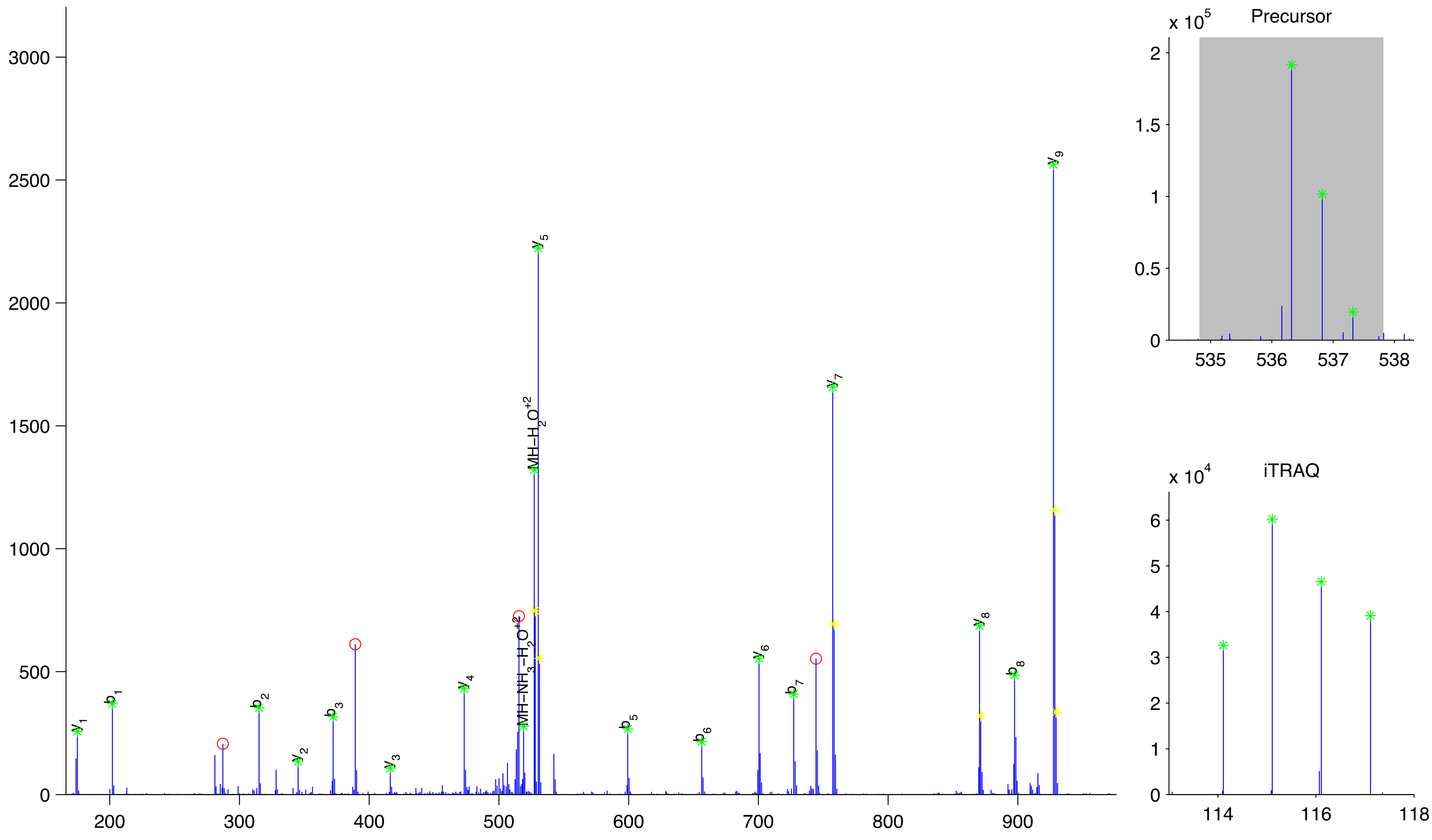

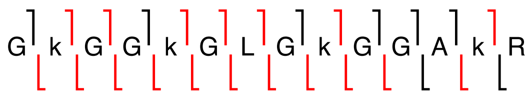

histone cluster 1, H4a [Homo sapiens]
Charge State: +2
Scan Number: 15900
File Name: 110924_HG2_INSA_ACK.raw

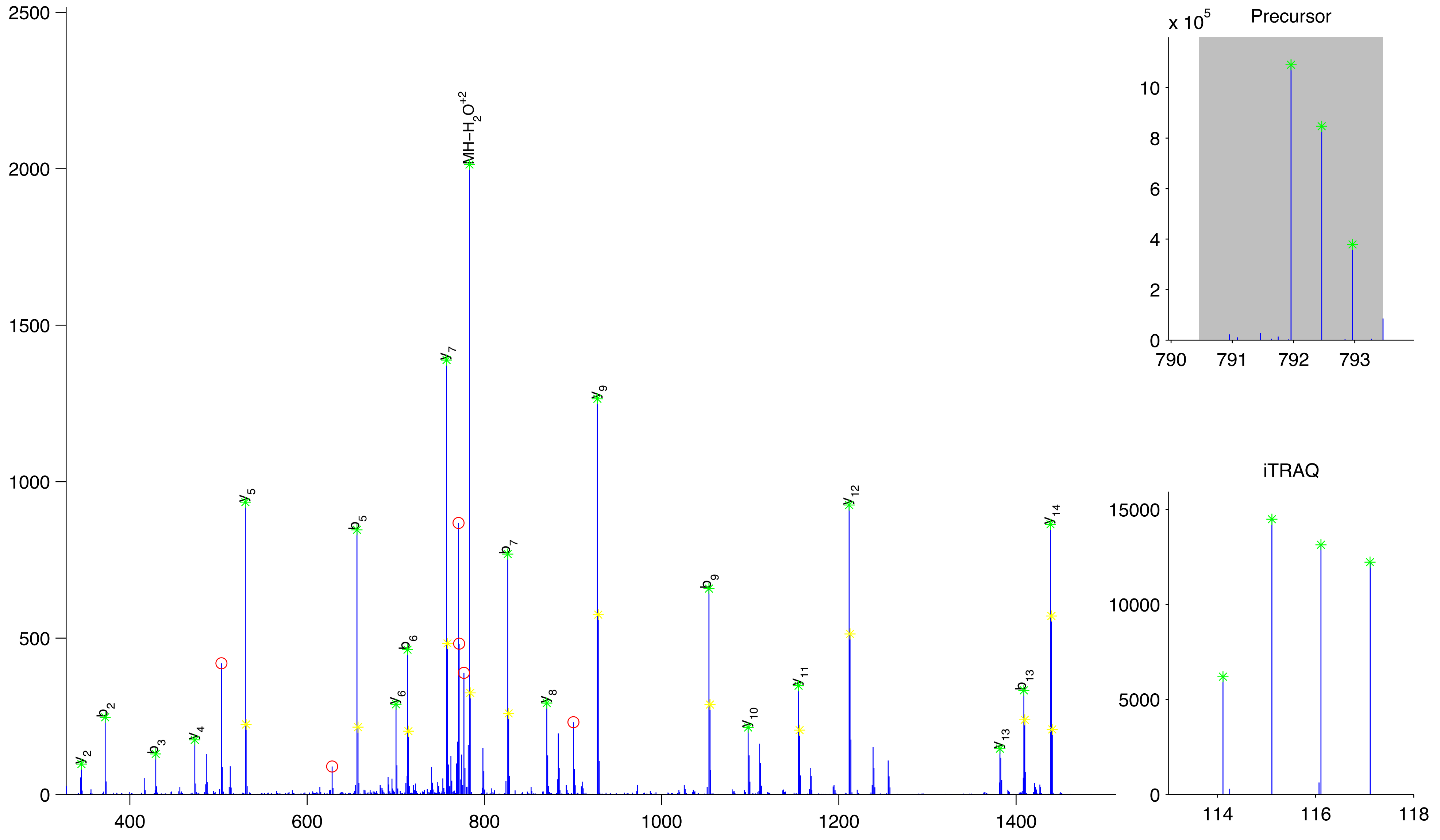

GkGGkGLGkGGAkR

histone cluster 1, H4a [Homo sapiens]
Charge State: +2
Scan Number: 15910
File Name: 110924_HG2_INSA_ACK.raw

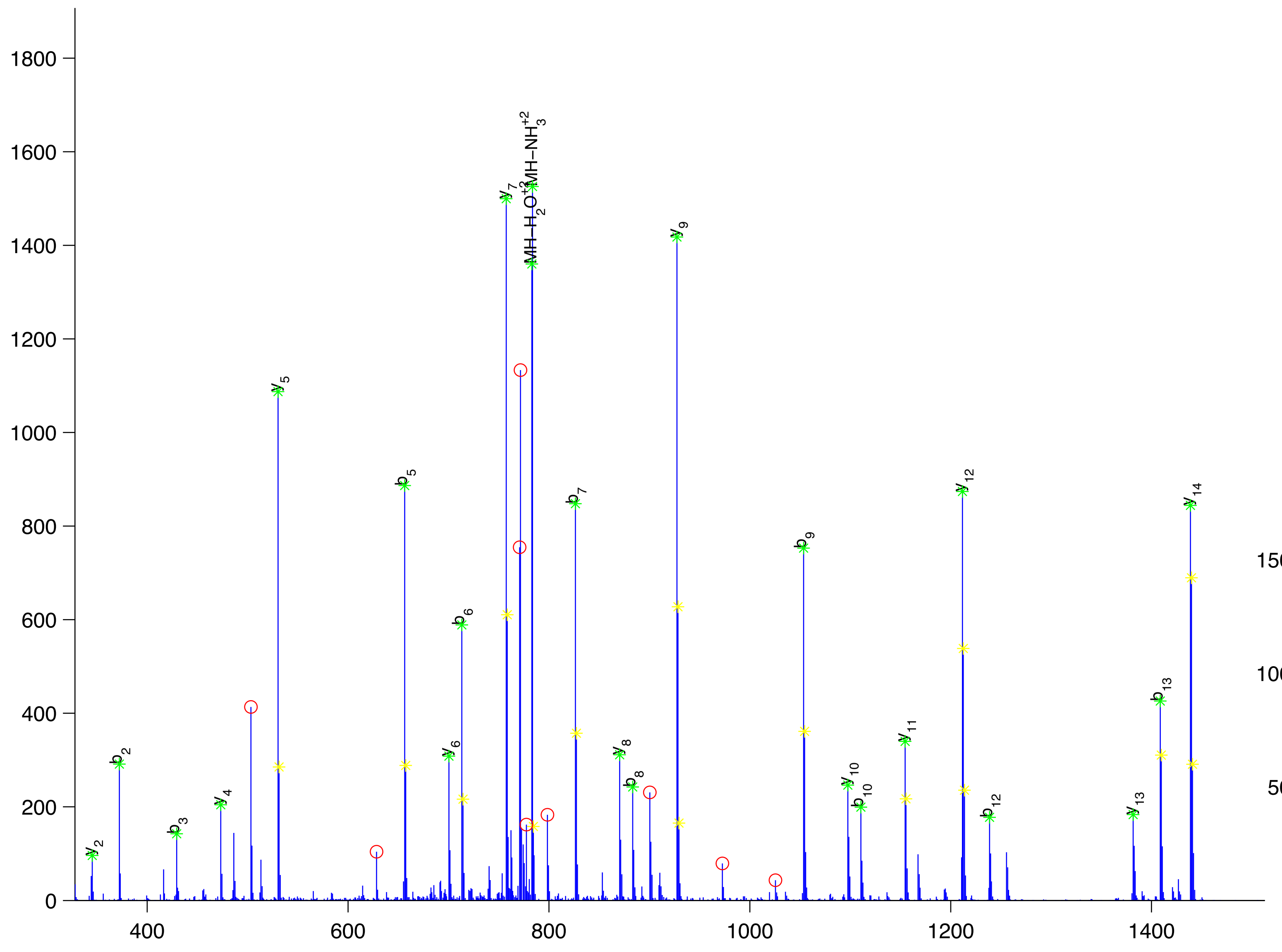

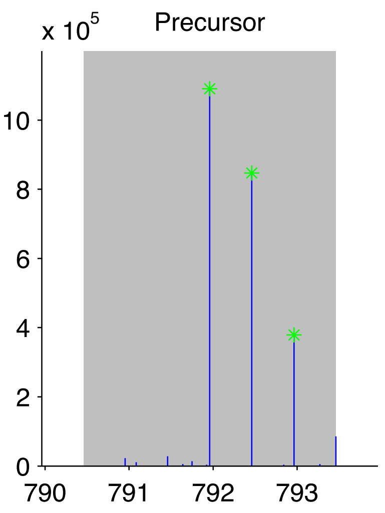

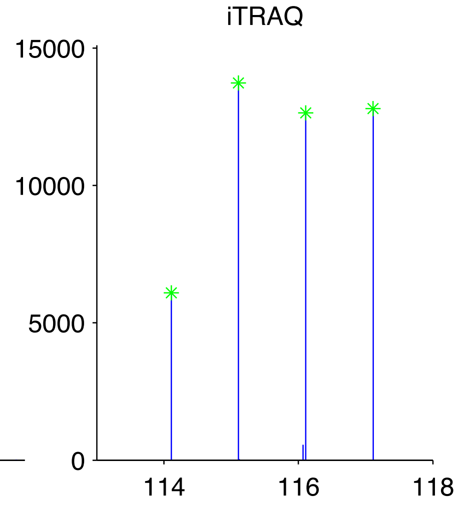

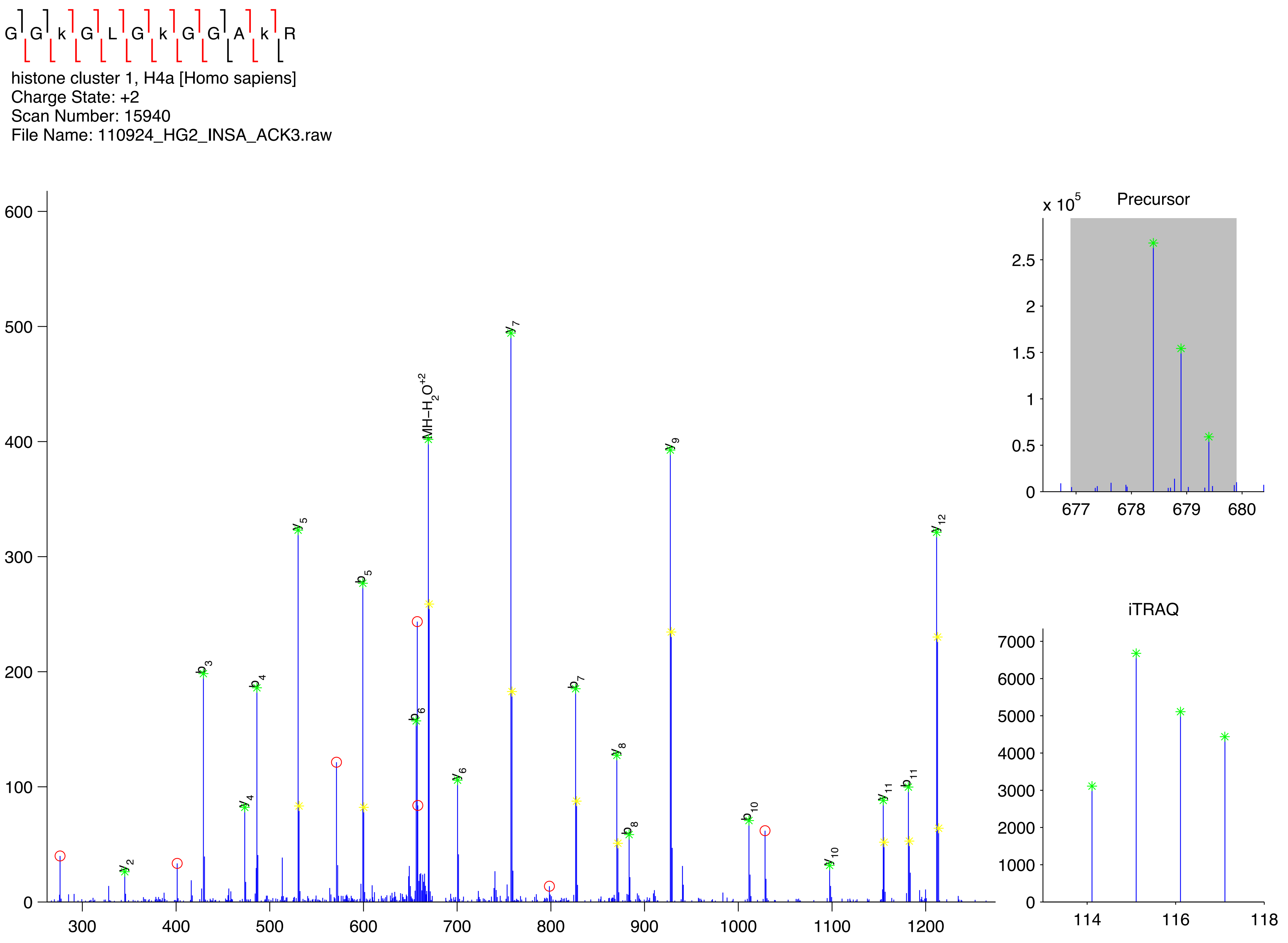
G G k G L G k G G A k R
histone cluster 1, H4a [Homo sapiens]
Charge State: +2
Scan Number: 15940
File Name: 110924_HG2_INSA_ACK3.raw
b3
b4
b5
b6
b7
b8
b10
b11
y2
y4
y5
y6
y7
y8
y9
y10
y11
y12
MH−H2O+2
x 10^5
Precursor
iTRAQ

G G k G L G k G G A k R

histone cluster 1, H4a [Homo sapiens]
Charge State: +2
Scan Number: 16011
File Name: 110924_HG2_INSA_ACK.raw

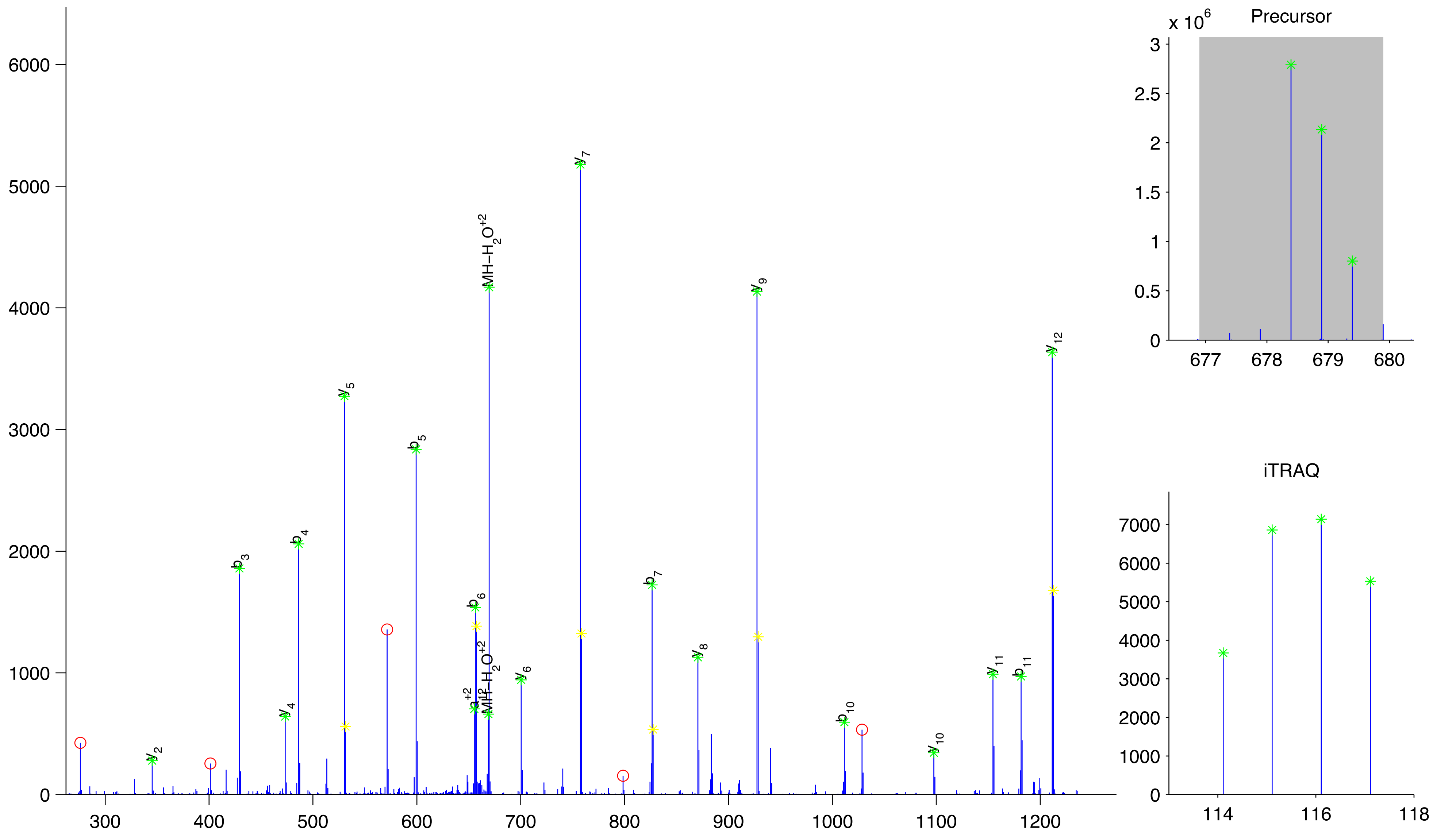

G k G G k G L G k G G A k R

histone cluster 1, H4a [Homo sapiens]
Charge State: +2
Scan Number: 16076
File Name: 110924_HG2_INSA_ACK.raw

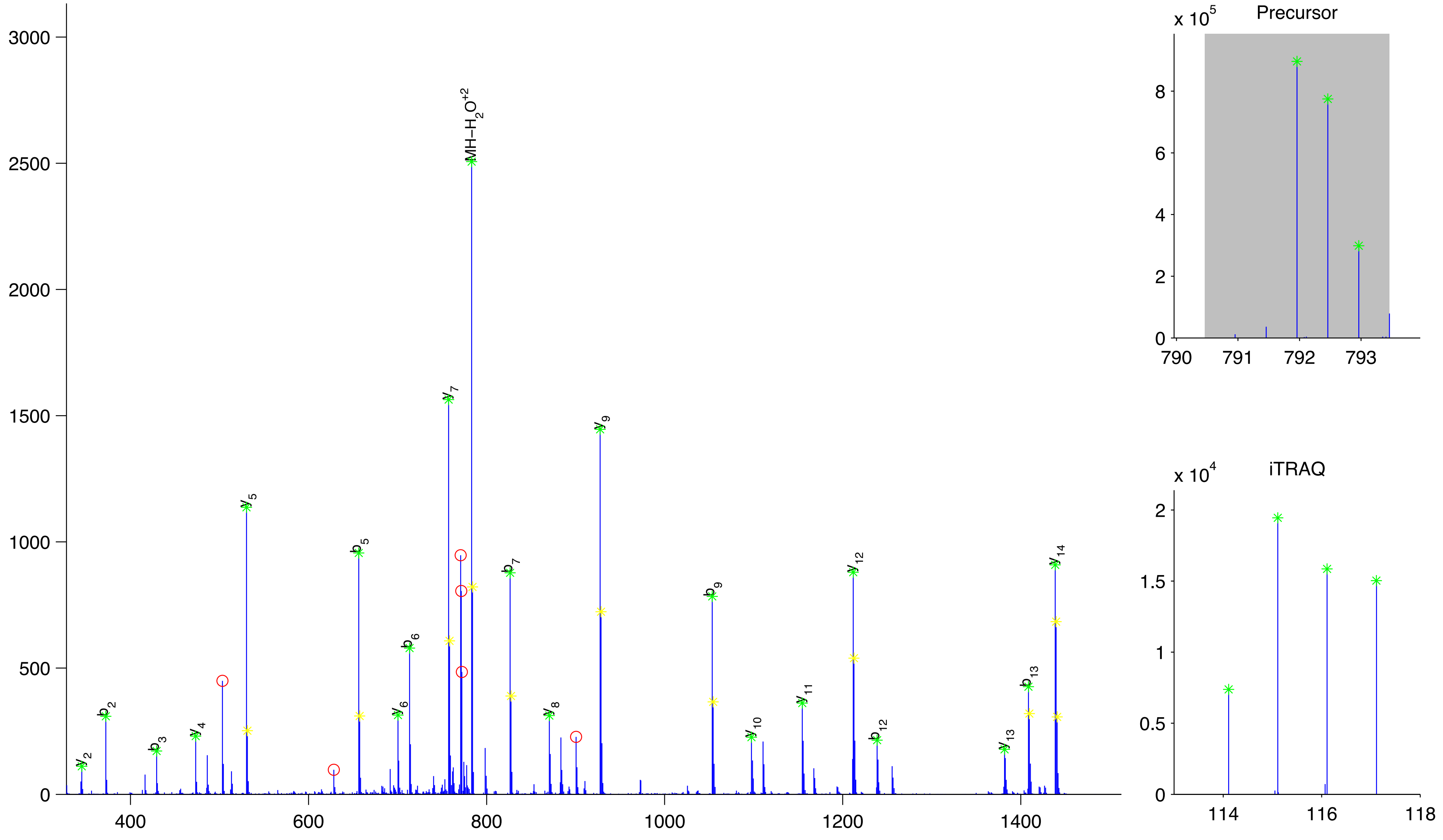

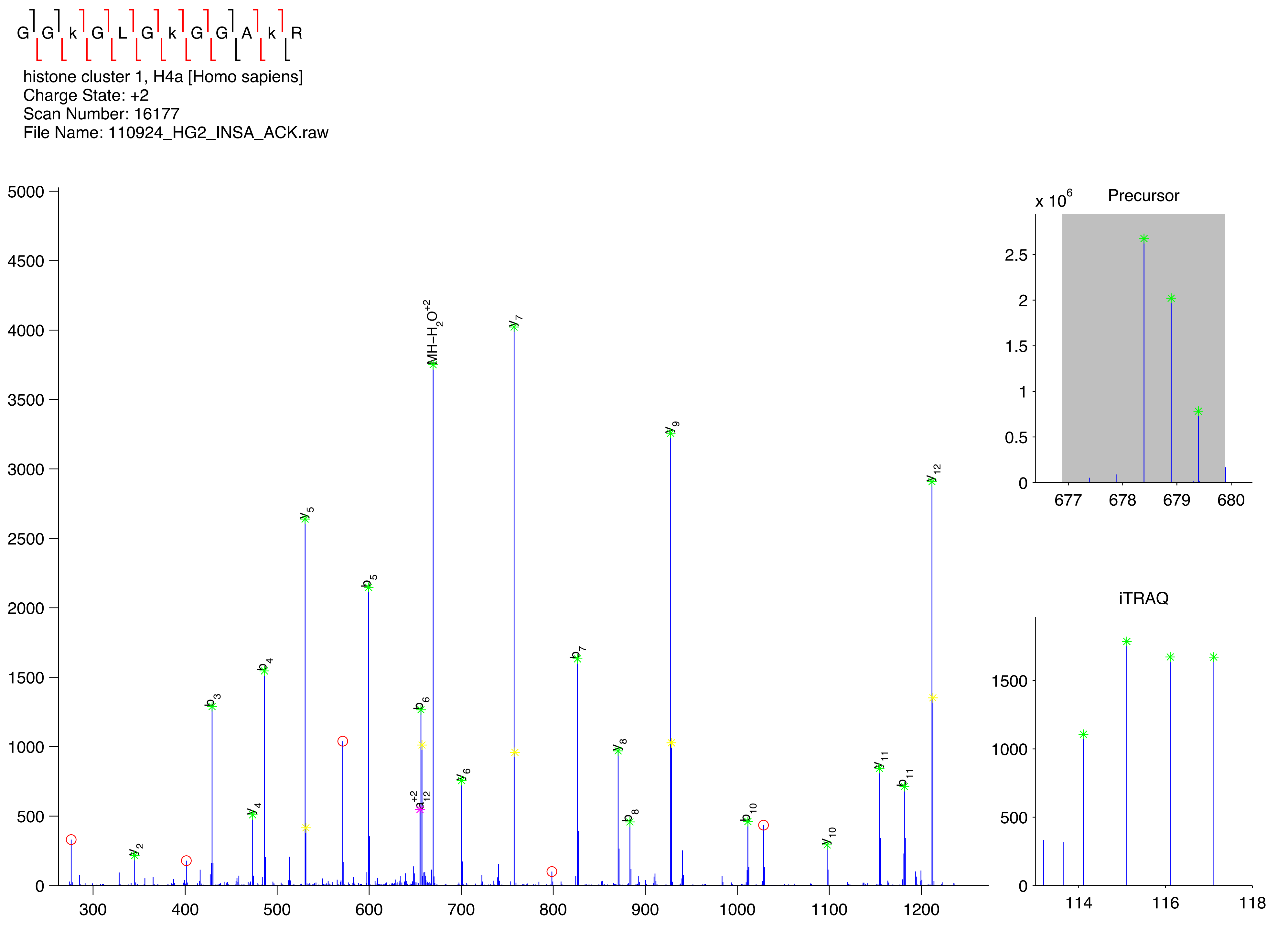

G G k G L G k G G A k R
histone cluster 1, H4a [Homo sapiens]
Charge State: +2
Scan Number: 16177
File Name: 110924_HG2_INSA_ACK.raw
Precursor
x 10^6
iTRAQ

G k G G k G L G k G G A k R

histone cluster 1, H4a [Homo sapiens]
Charge State: +2
Scan Number: 16223
File Name: 110924_HG2_INSA_ACK3.raw

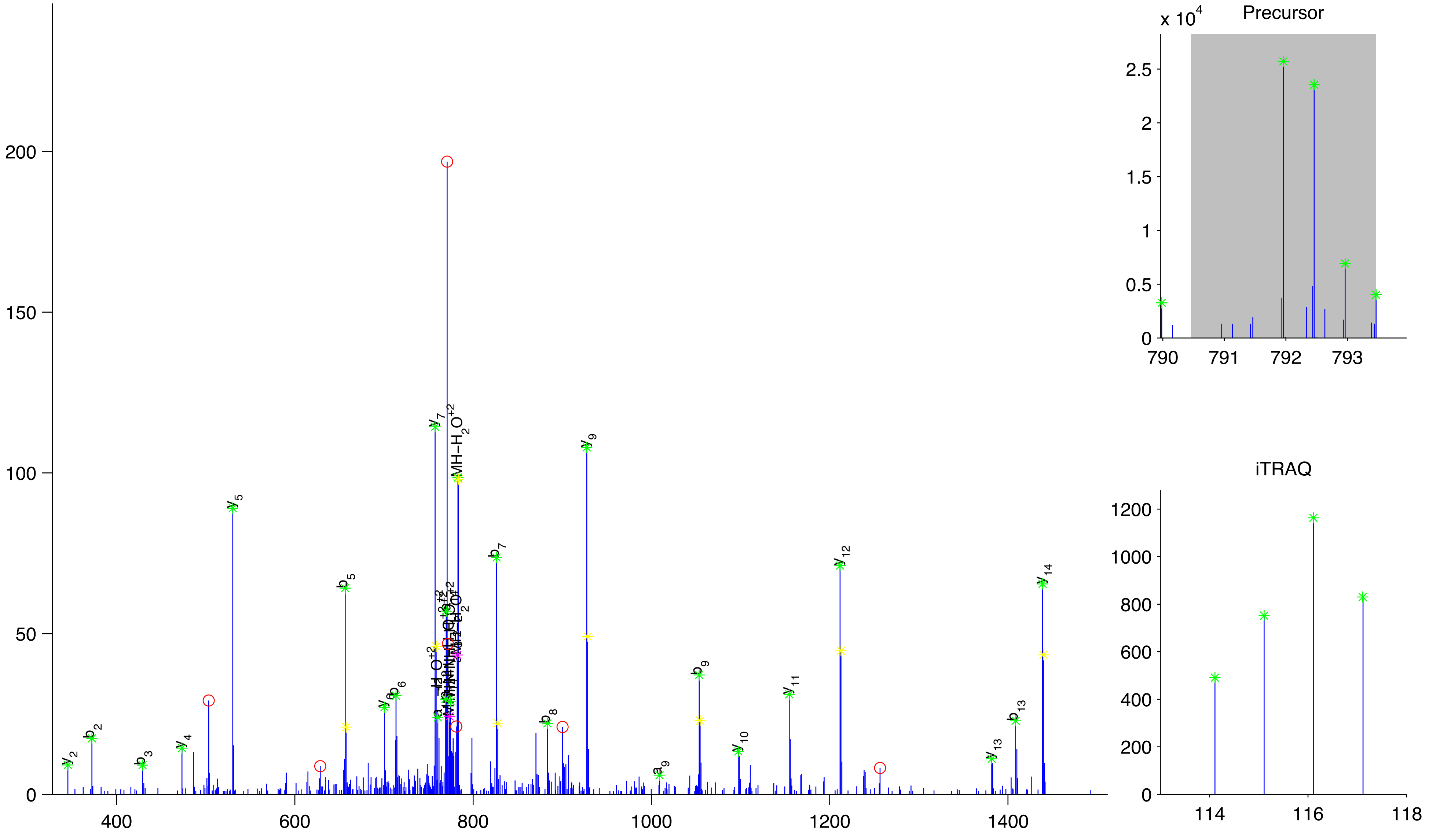

G k G G k G L G k G G A k R

histone cluster 1, H4a [Homo sapiens]
Charge State: +2
Scan Number: 16236
File Name: 110924_HG2_INSA_ACK.raw

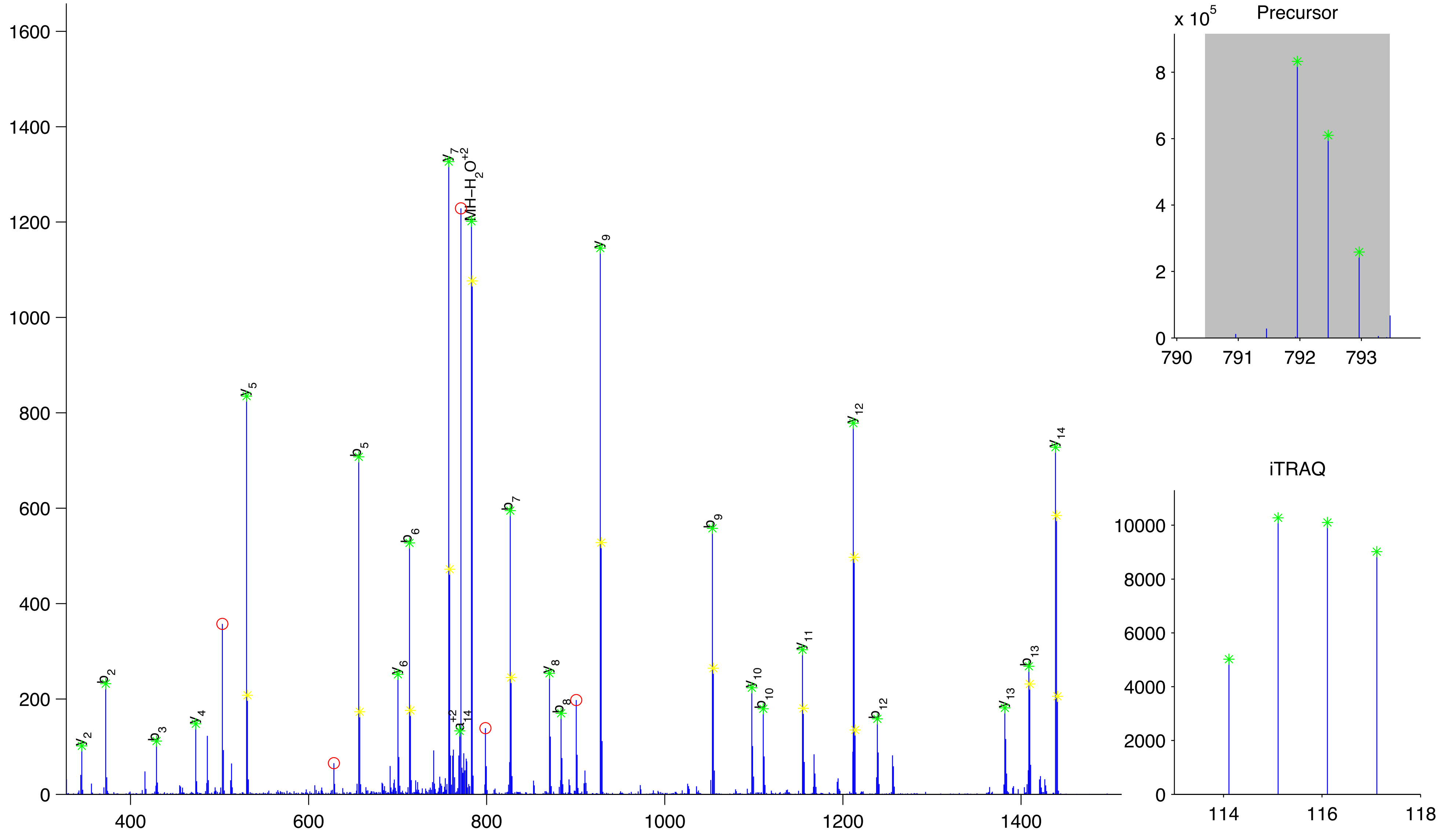

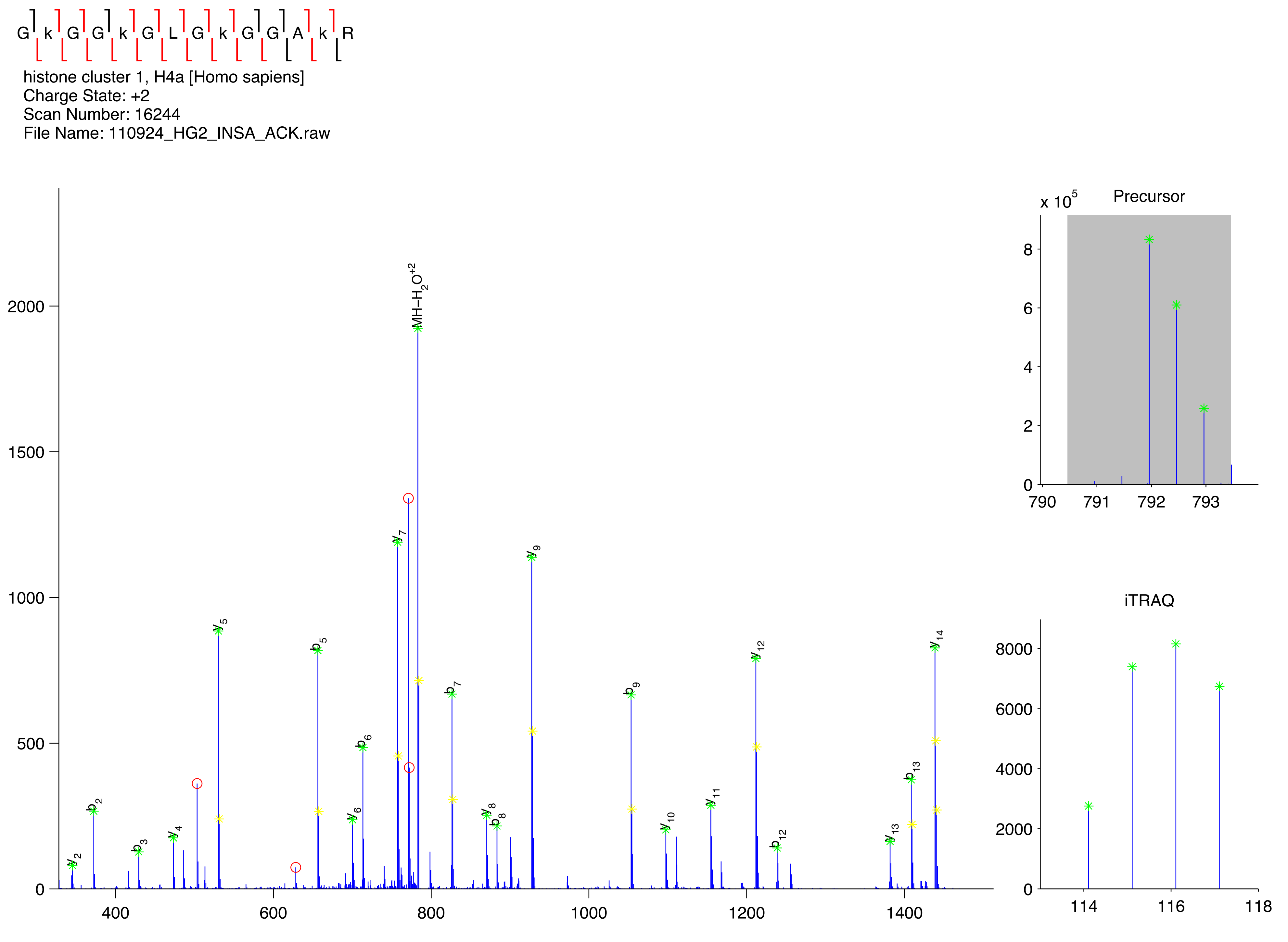

G k G G k G L G k G G A k R
histone cluster 1, H4a [Homo sapiens]
Charge State: +2
Scan Number: 16244
File Name: 110924_HG2_INSA_ACK.raw
MH−H2O+2
y2
b2
b3
y4
y5
b5
y6
b6
y7
b7
y8
b8
y9
b9
y10
y11
y12
b12
y13
b13
y14
0
500
1000
1500
2000
400
600
800
1000
1200
1400
x 10^5
Precursor
0
2
4
6
8
790
791
792
793
iTRAQ
0
2000
4000
6000
8000
114
116
118

G k G G k G L G k G G A k R

histone cluster 1, H4a [Homo sapiens]
Charge State: +3
Scan Number: 16337
File Name: 110924_HG2_INSA_ACK3.raw

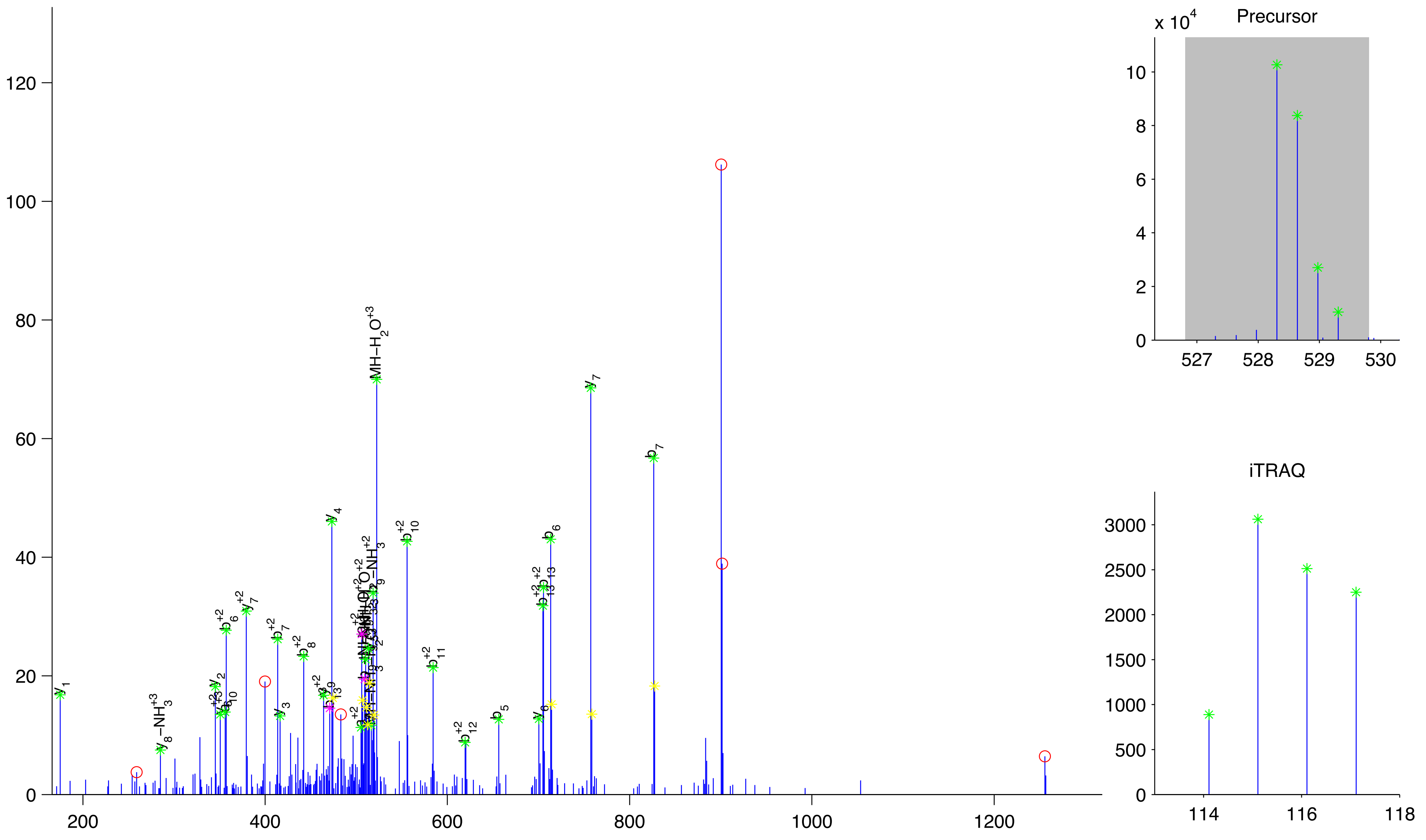

G G k G L G k G G A k R

histone cluster 1, H4a [Homo sapiens]
Charge State: +2
Scan Number: 16347
File Name: 110924_HG2_INSA_ACK.raw

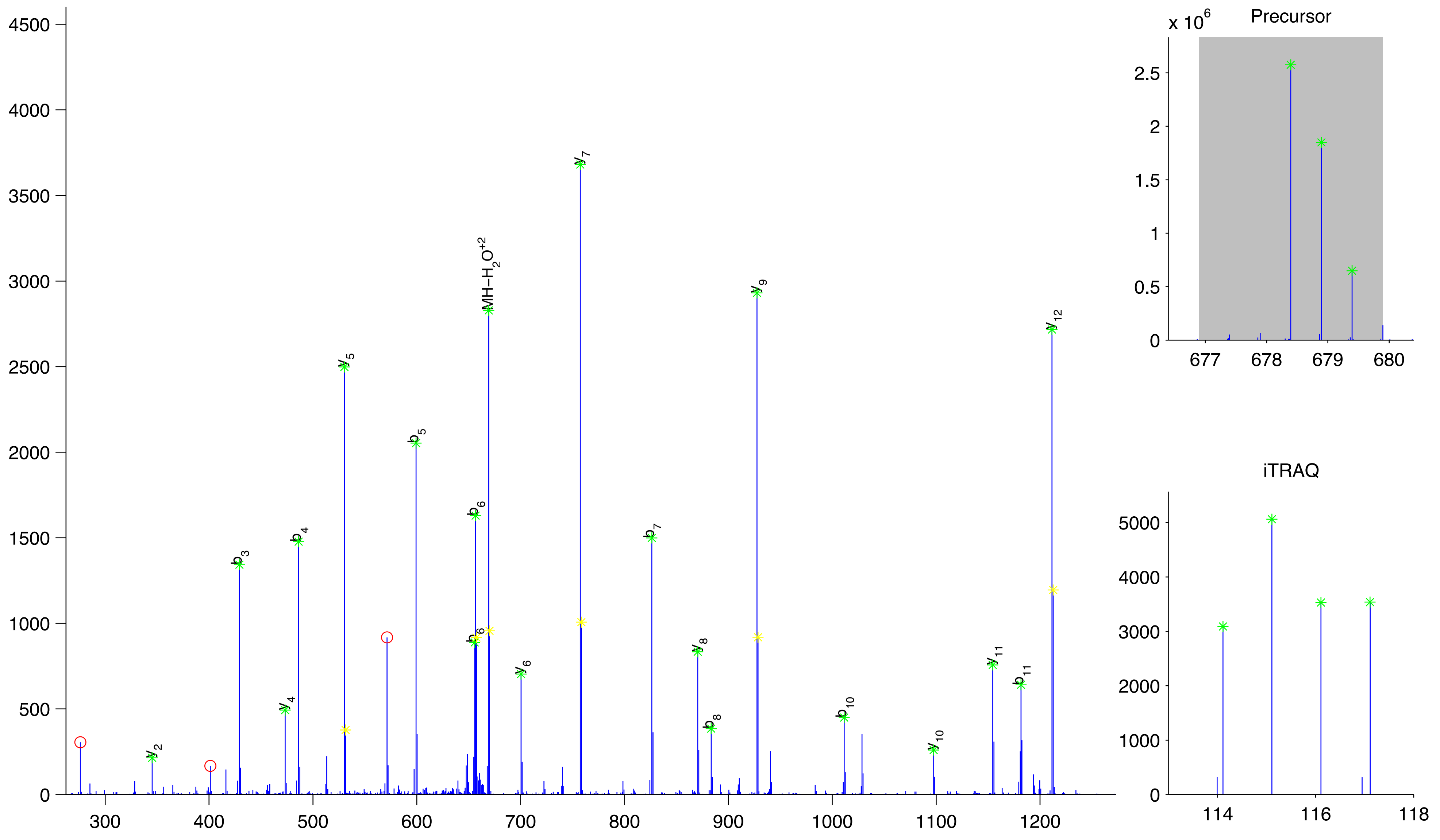

G k G G k G L G k G G A k R

histone cluster 1, H4a [Homo sapiens]
Charge State: +2
Scan Number: 16404
File Name: 110924_HG2_INSA_ACK.raw

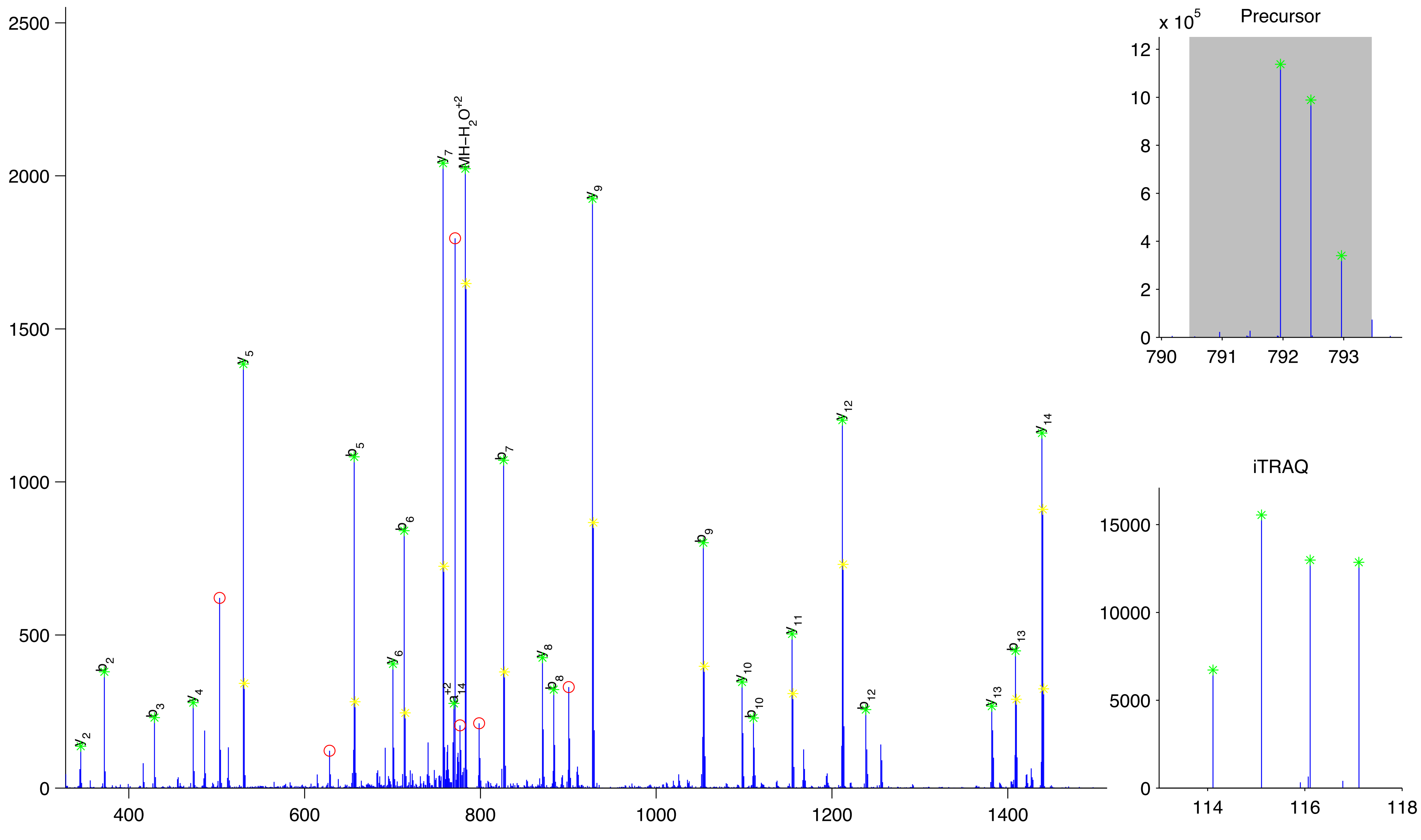

G k G G k G L G k G G A k R

histone cluster 1, H4a [Homo sapiens]
Charge State: +2
Scan Number: 16412
File Name: 110924_HG2_INSA_ACK.raw

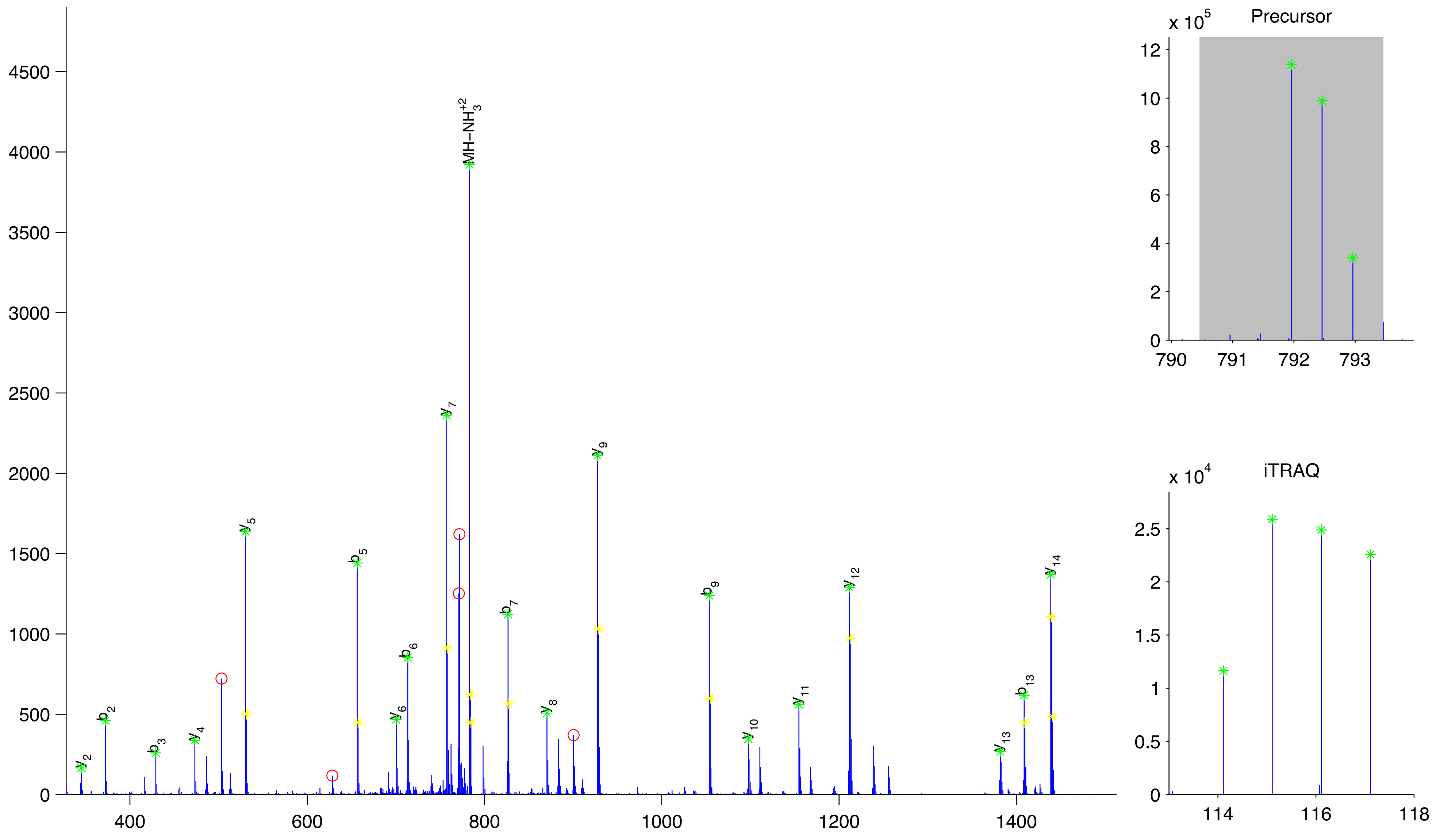

G G k G L G k G G A k R

histone cluster 1, H4a [Homo sapiens]
Charge State: +2
Scan Number: 16463
File Name: 110924_HG2_INSA_ACK3.raw

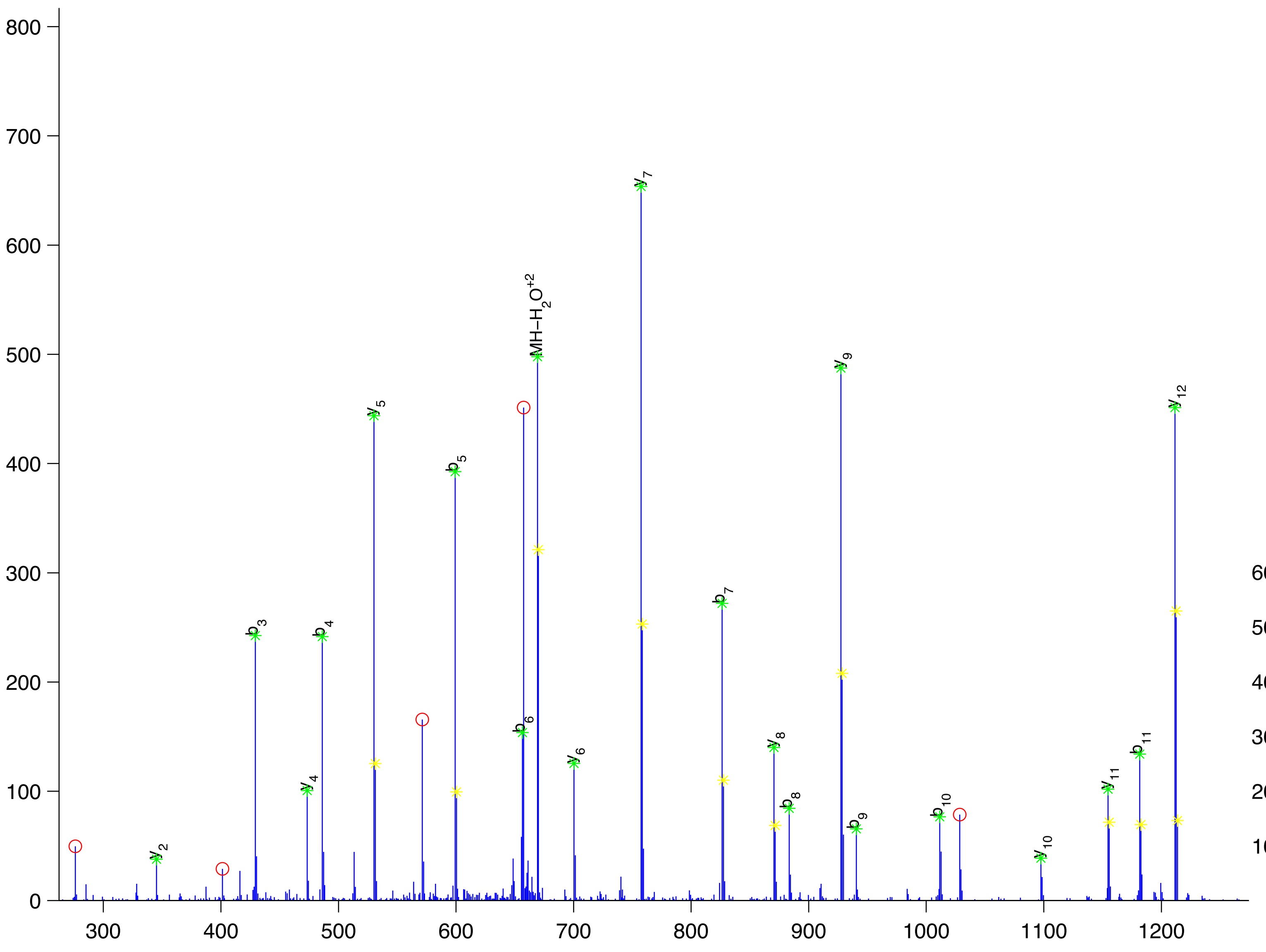

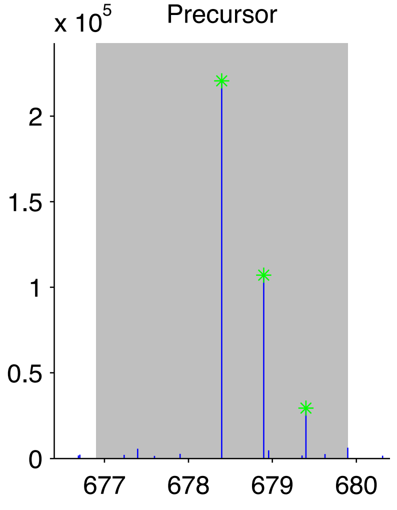

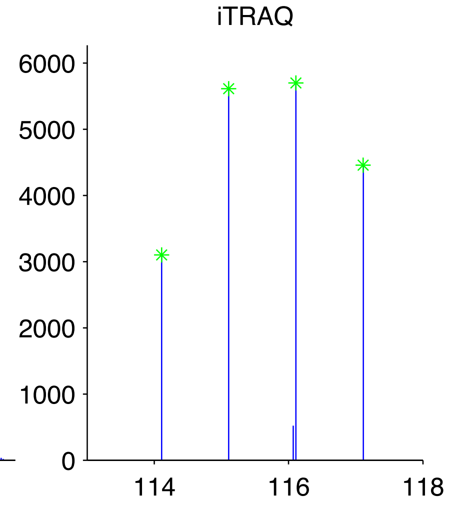

G k G G k G L G k G G A k R

histone cluster 1, H4a [Homo sapiens]
Charge State: +3
Scan Number: 16507
File Name: 110924_HG2_INSA_ACK3.raw

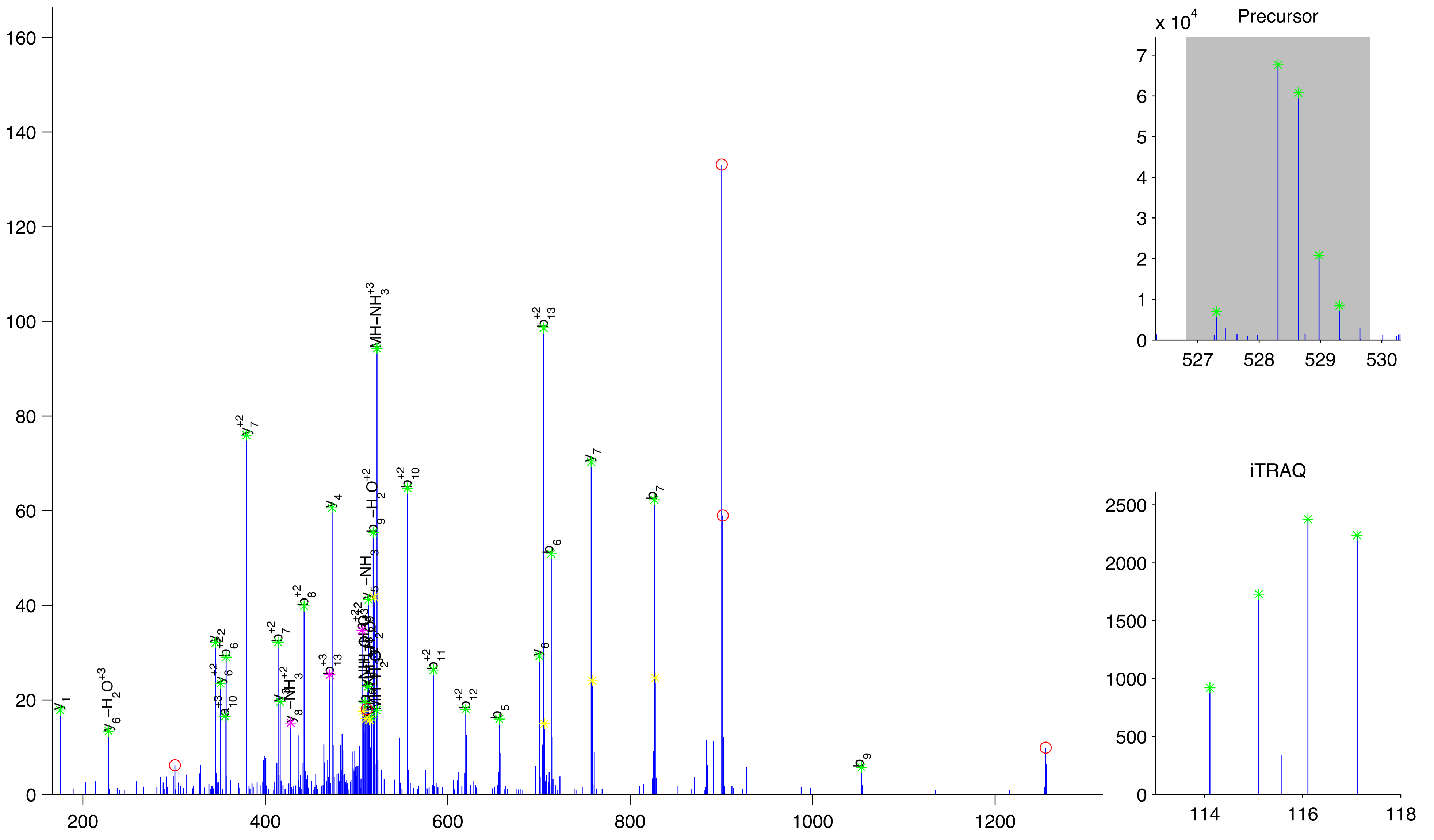

G G k G L G k G G A k R

histone cluster 1, H4a [Homo sapiens]
Charge State: +2
Scan Number: 16517
File Name: 110924_HG2_INSA_ACK.raw

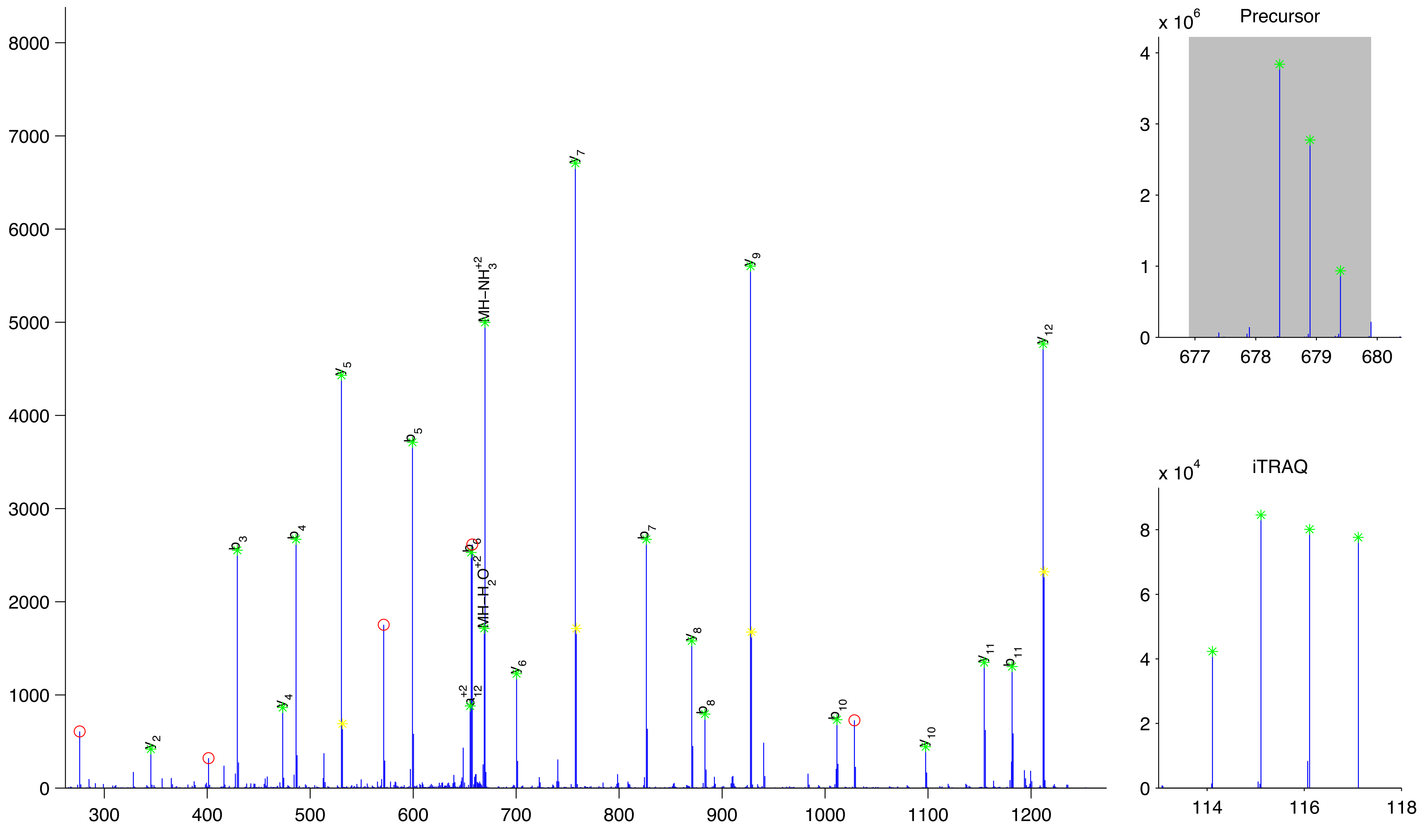

G L G k G G A k R

histone cluster 1, H4a [Homo sapiens]
Charge State: +2
Scan Number: 16547
File Name: 110924_HG2_INSA_ACK3.raw

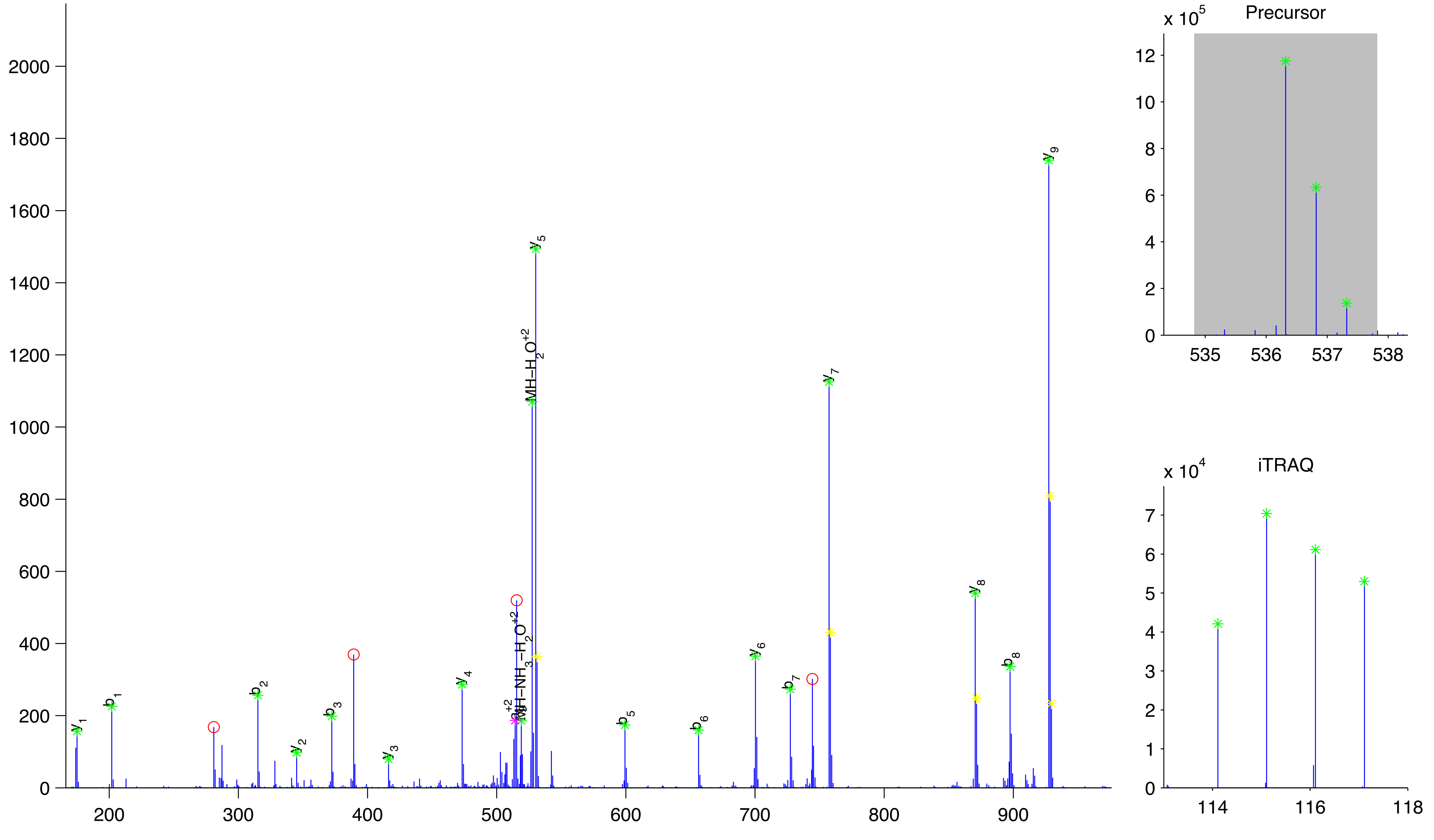

G k G G k G L G k G G A k R

histone cluster 1, H4a [Homo sapiens]
Charge State: +2
Scan Number: 16572
File Name: 110924_HG2_INSA_ACK.raw

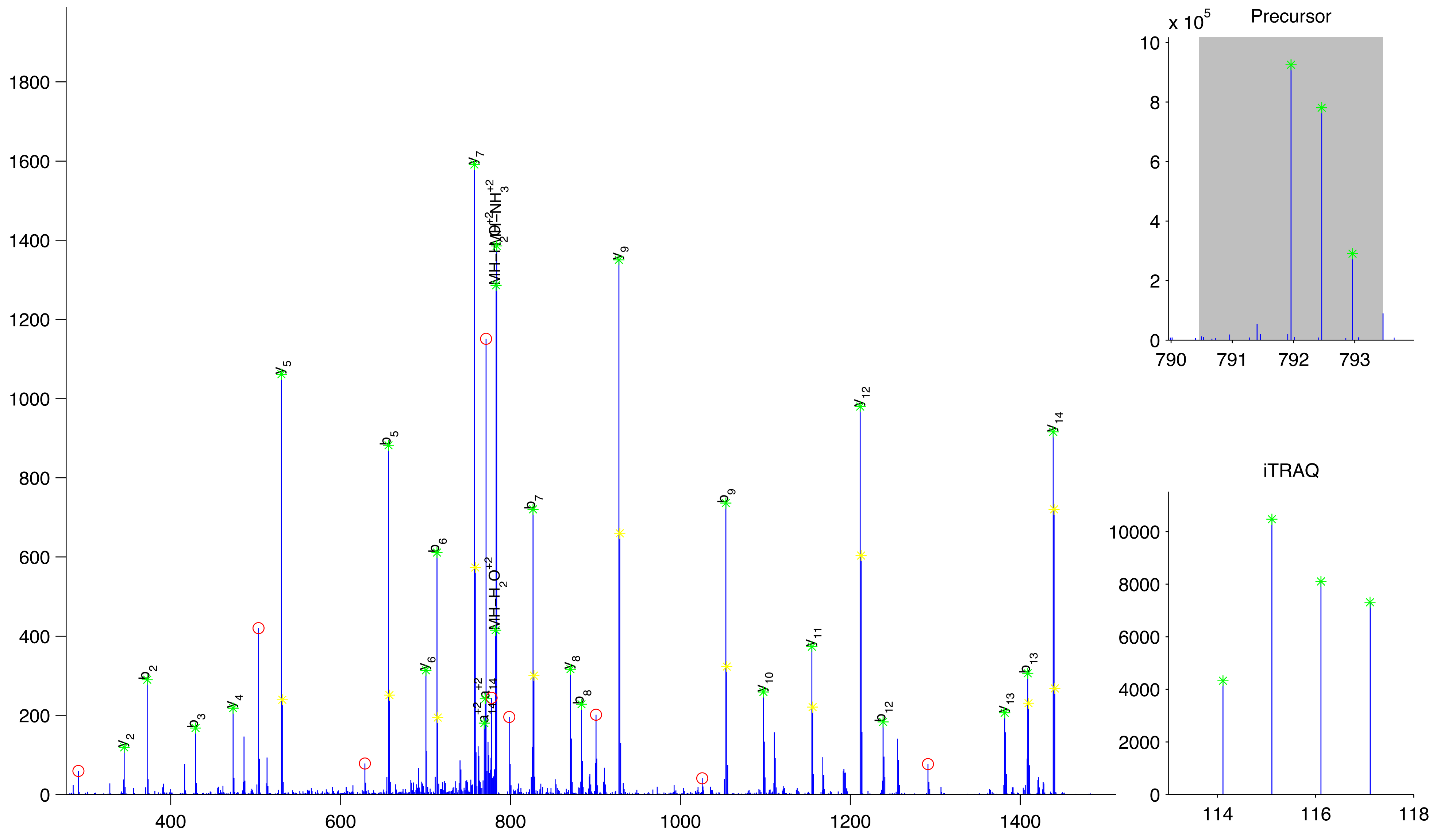

G k G G k G L G k G G A k R

histone cluster 1, H4a [Homo sapiens]
Charge State: +2
Scan Number: 16576
File Name: 110924_HG2_INSA_ACK.raw

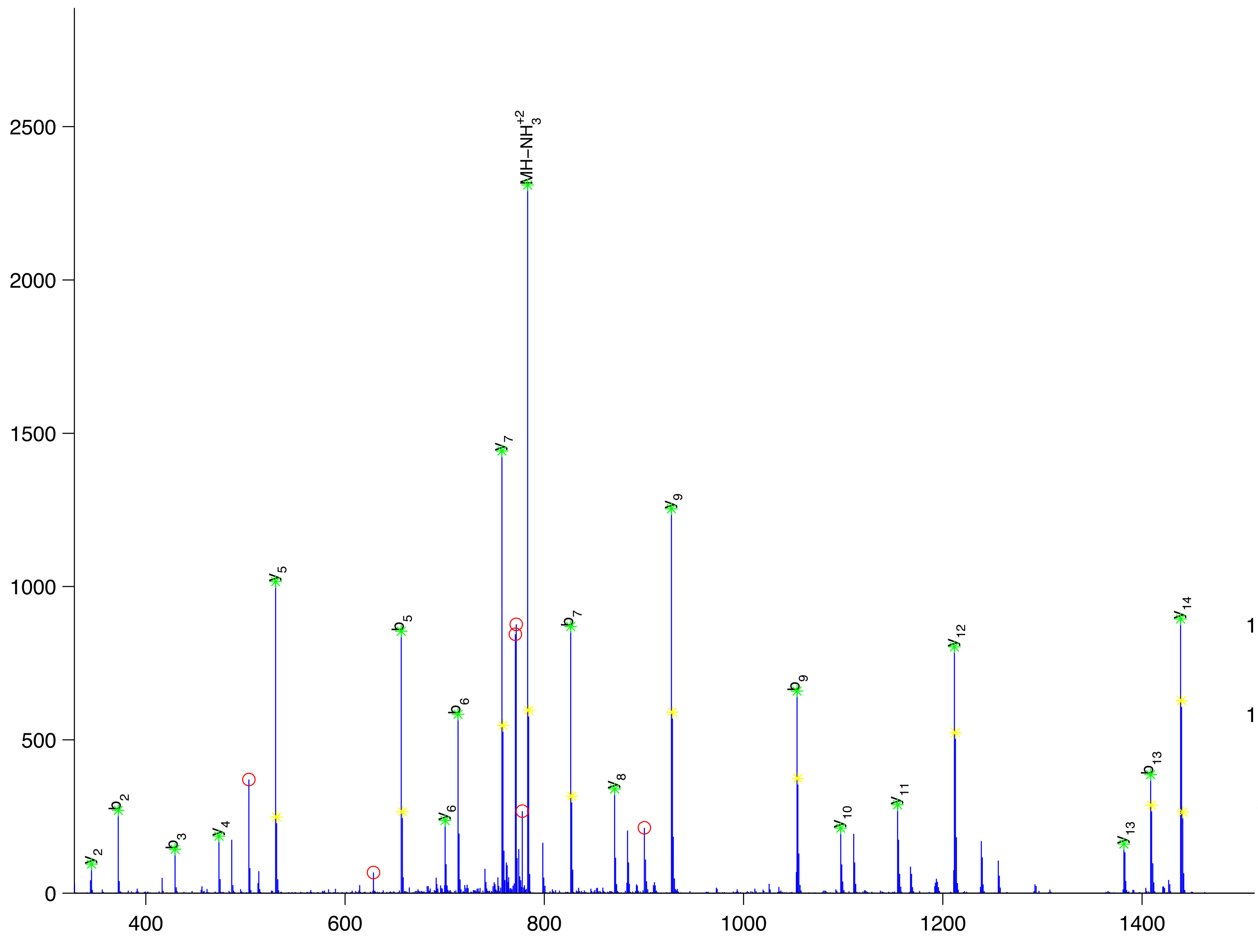

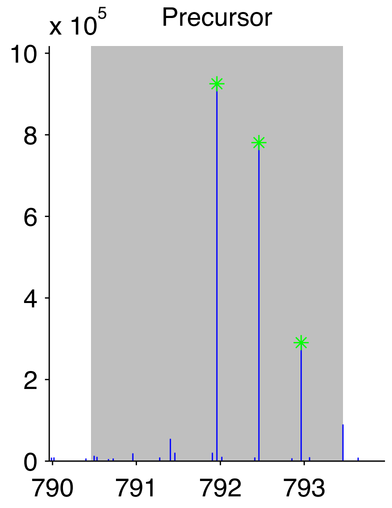

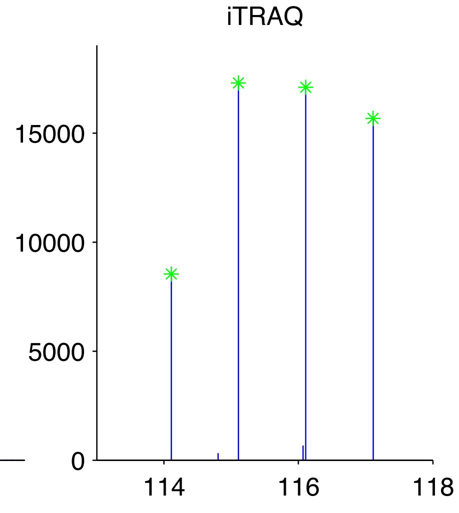

G G k G L G k G G A k R

histone cluster 1, H4a [Homo sapiens]
Charge State: +2
Scan Number: 16631
File Name: 110924_HG2_INSA_ACK3.raw

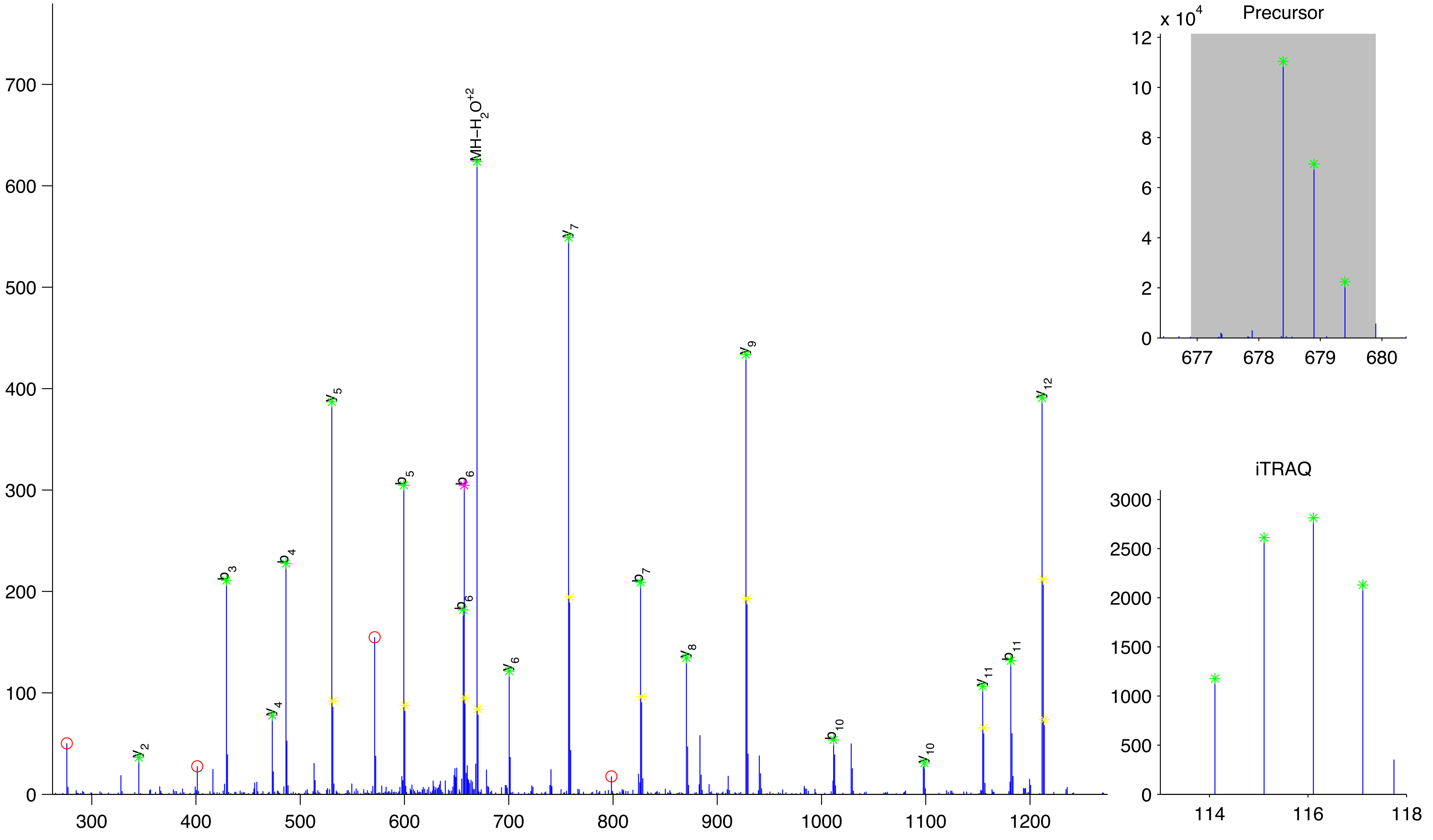

G G k G L G k G G A k R

histone cluster 1, H4a [Homo sapiens]
Charge State: +2
Scan Number: 16689
File Name: 110924_HG2_INSA_ACK.raw

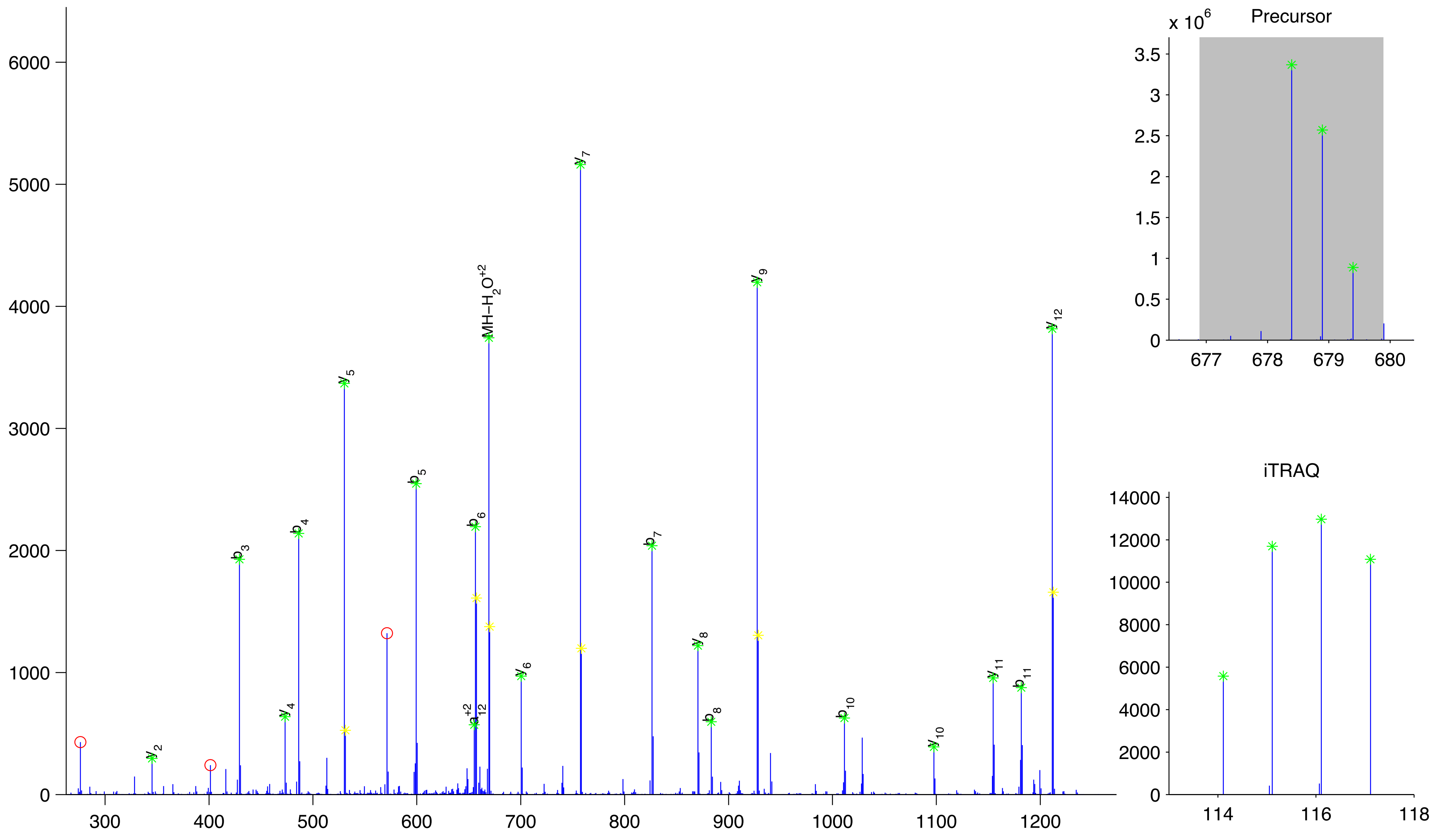

G L G k G G A k R

histone cluster 1, H4a [Homo sapiens]
Charge State: +2
Scan Number: 16717
File Name: 110924_HG2_INSA_ACK3.raw

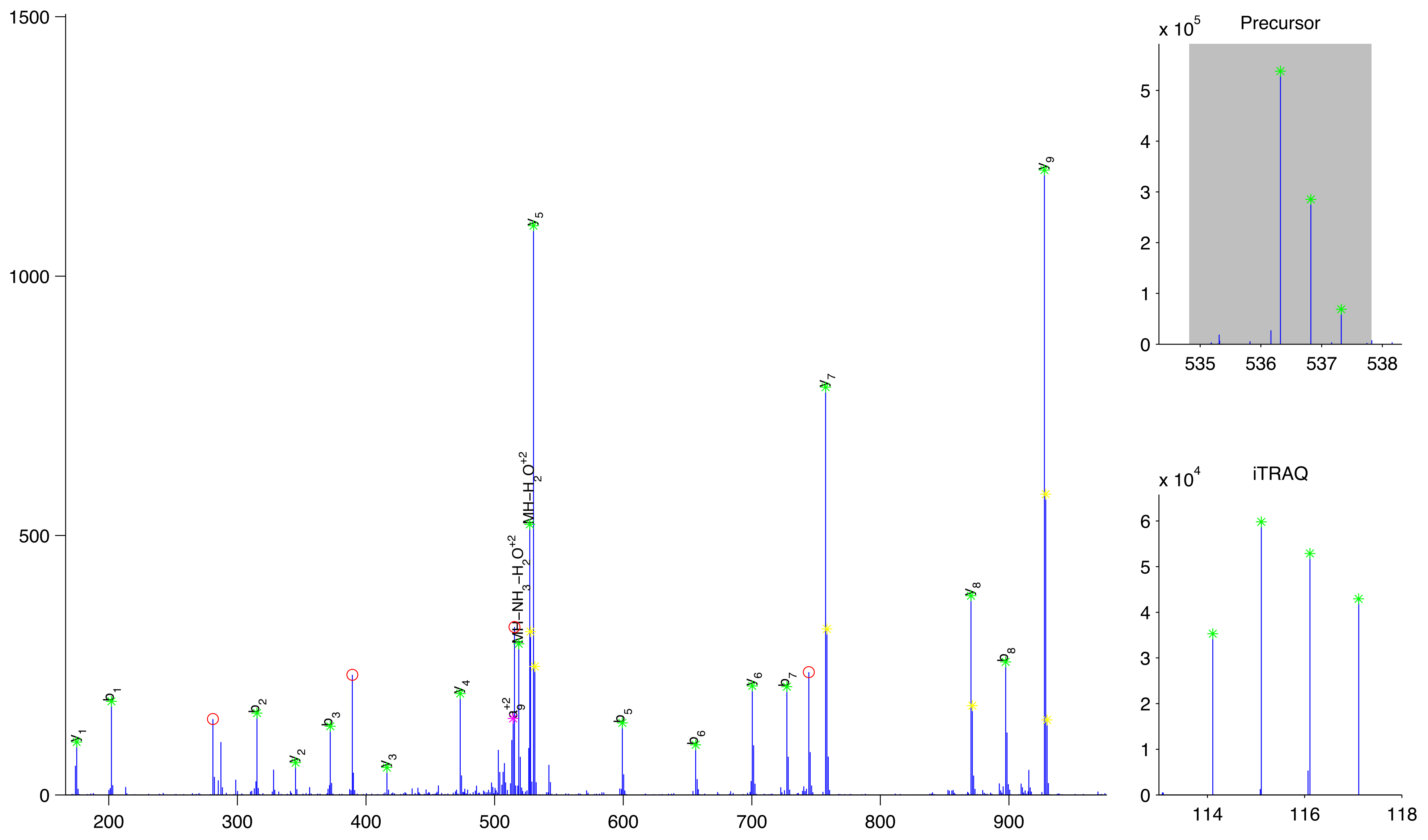

G k G G k G L G k G G A k R

histone cluster 1, H4a [Homo sapiens]
Charge State: +2
Scan Number: 16740
File Name: 110924_HG2_INSA_ACK.raw

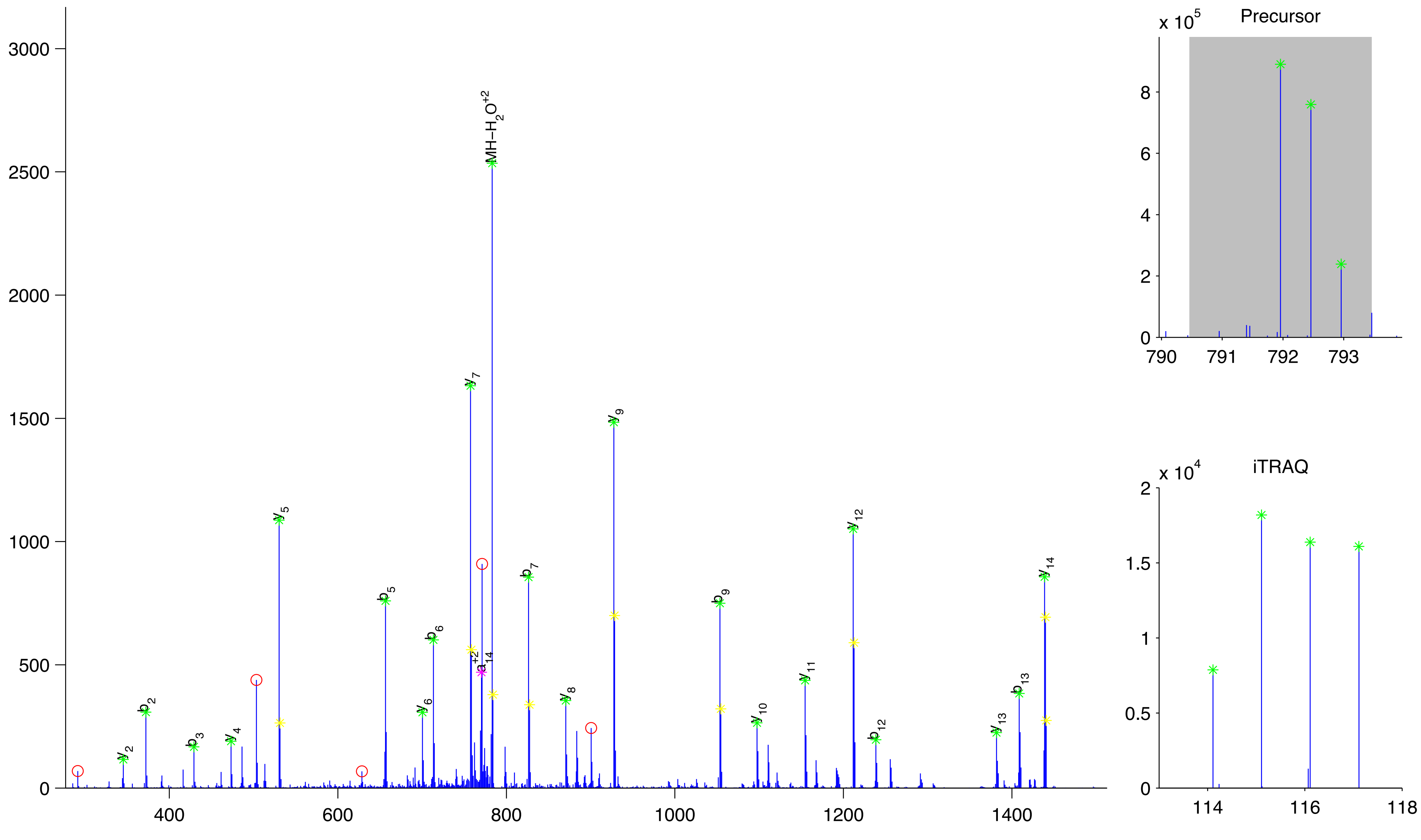

G k G G k G L G k G G A k R

histone cluster 1, H4a [Homo sapiens]
Charge State: +2
Scan Number: 16750
File Name: 110924_HG2_INSA_ACK.raw

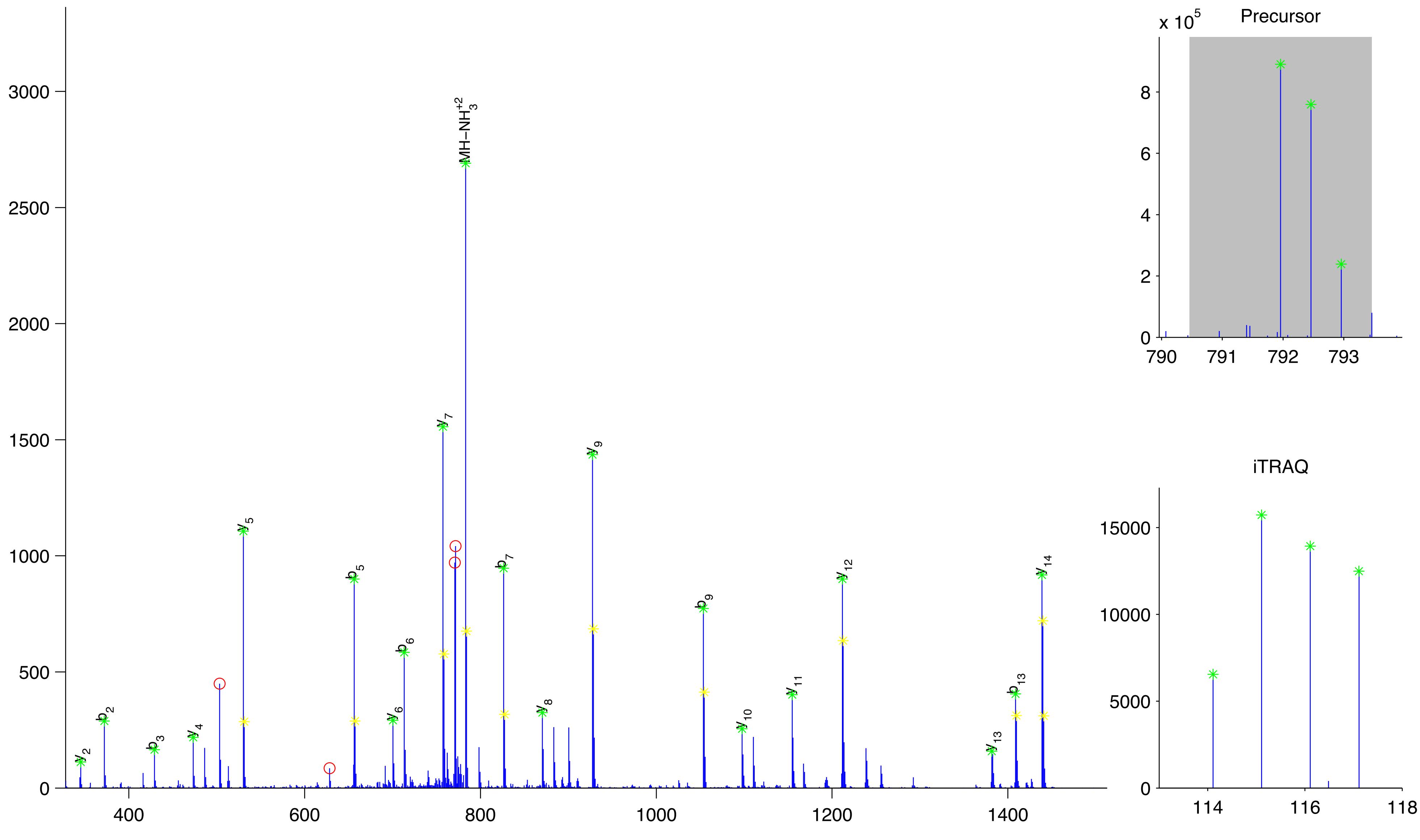

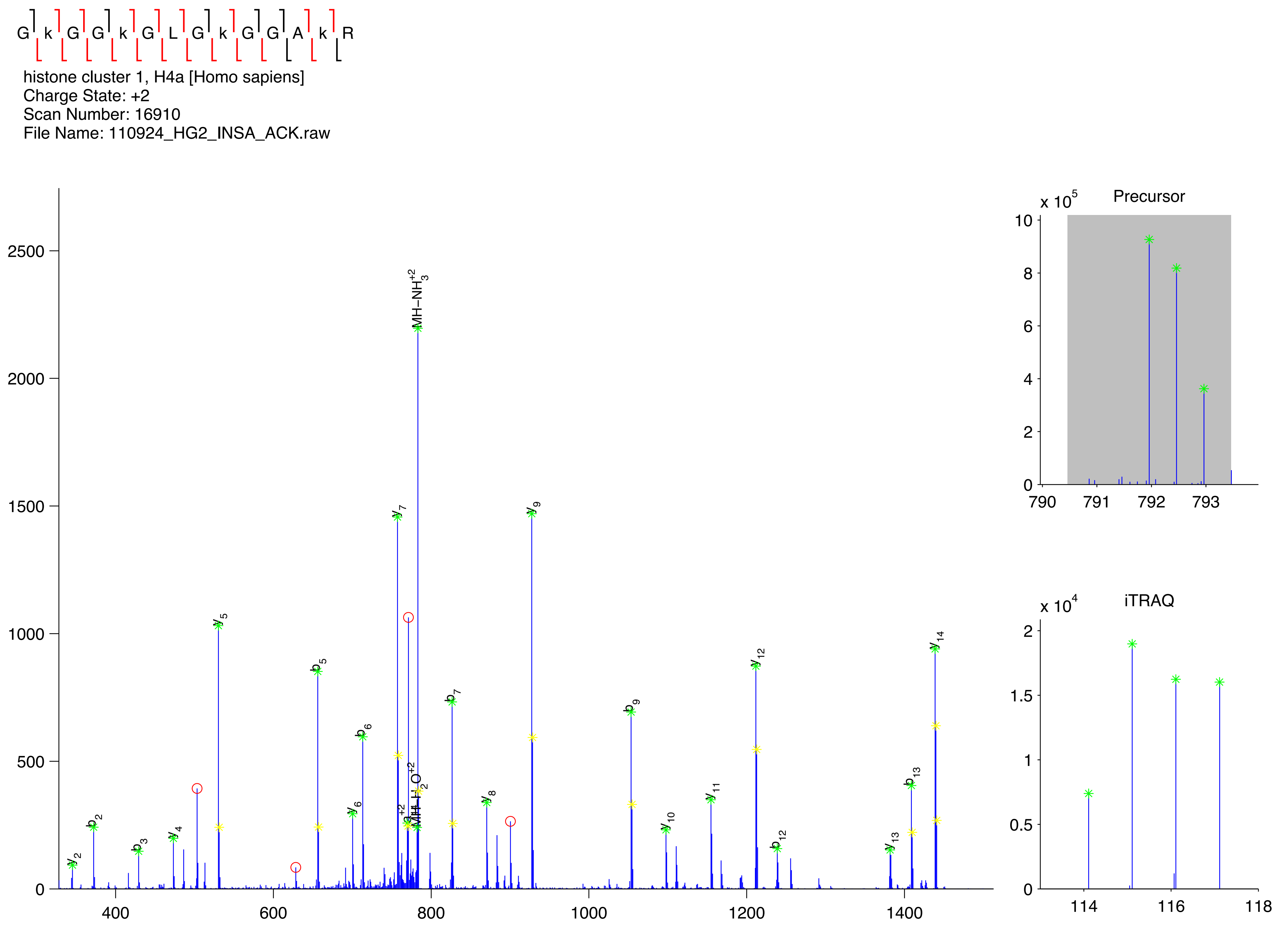
G k G G k G L G k G G A k R
histone cluster 1, H4a [Homo sapiens]
Charge State: +2
Scan Number: 16910
File Name: 110924_HG2_INSA_ACK.raw
Precursor
x 10^5
iTRAQ
x 10^4

G k G G k G L G k G G A k R

histone cluster 1, H4a [Homo sapiens]
Charge State: +2
Scan Number: 16922
File Name: 110924_HG2_INSA_ACK.raw

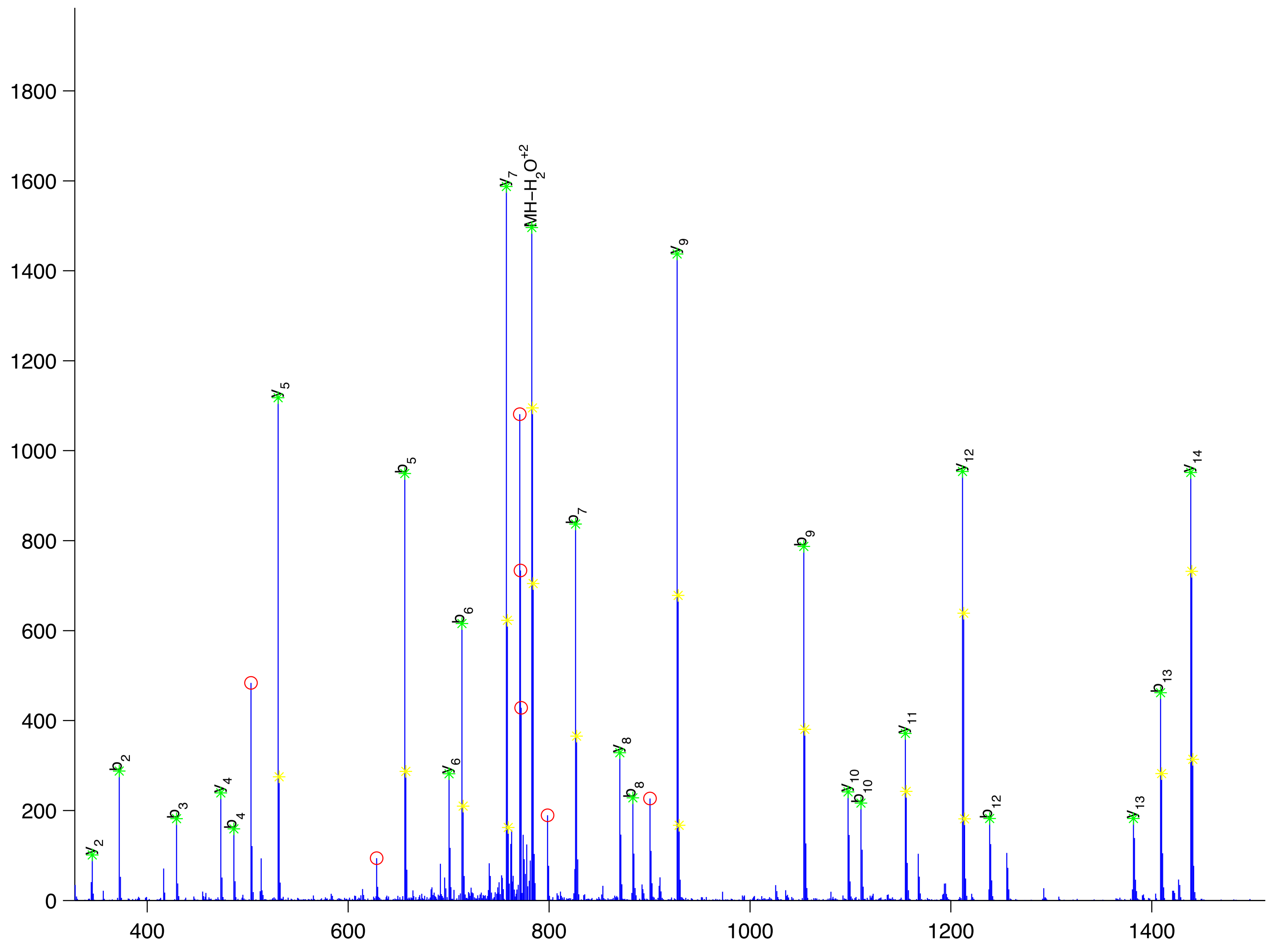

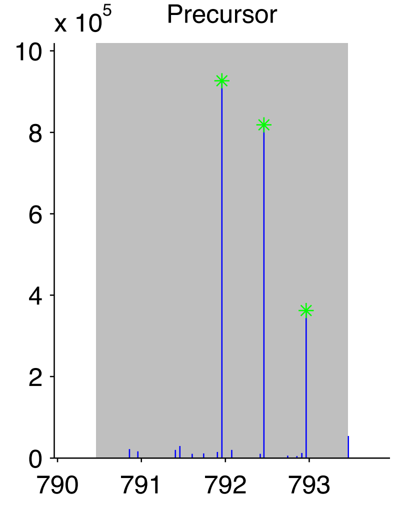

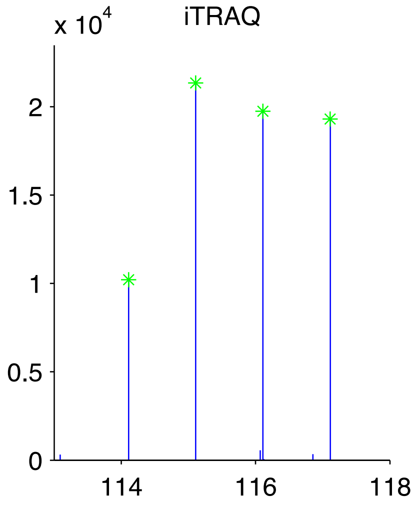

G L G k G G A k R

histone cluster 1, H4a [Homo sapiens]
Charge State: +2
Scan Number: 17053
File Name: 110924_HG2_INSA_ACK3.raw

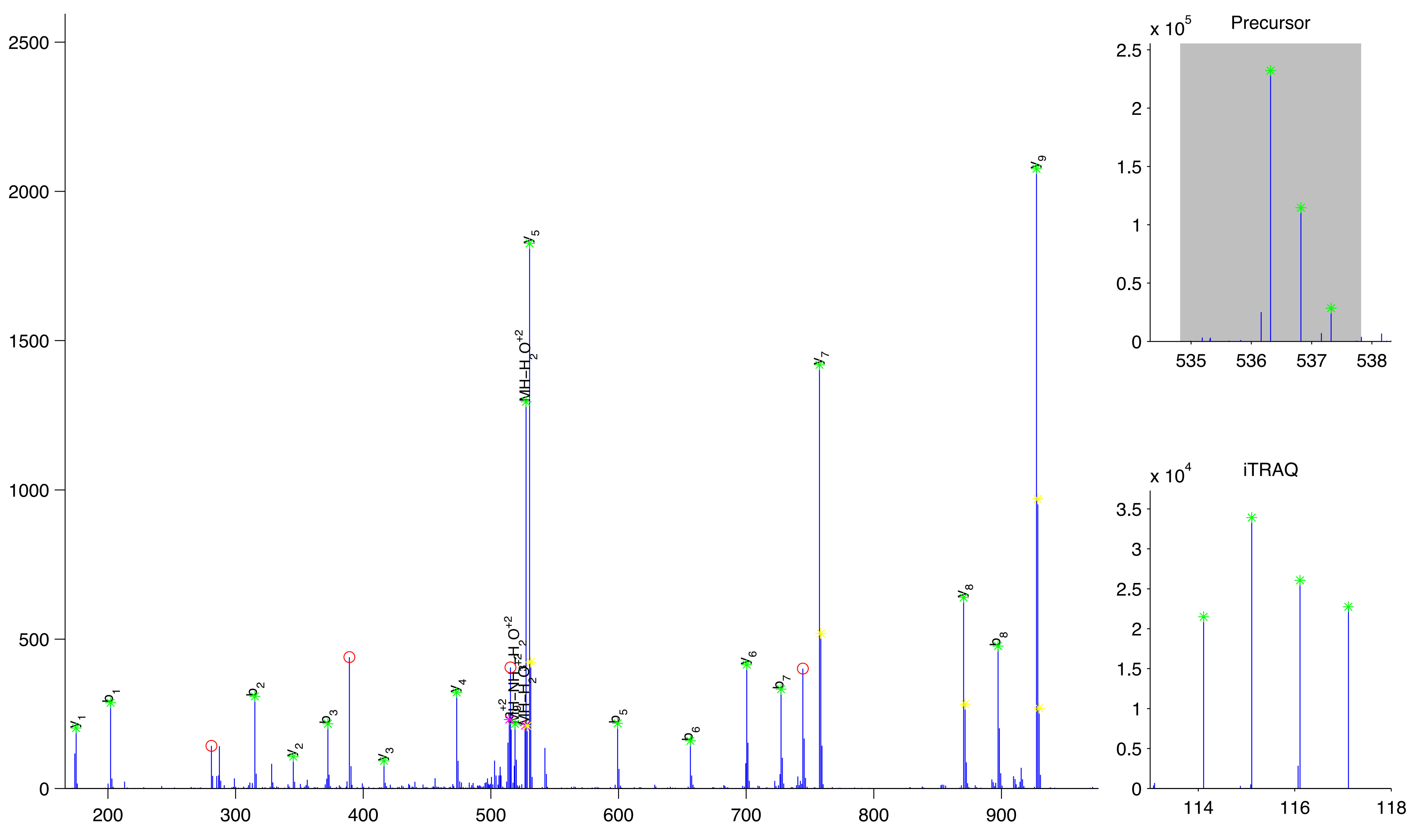

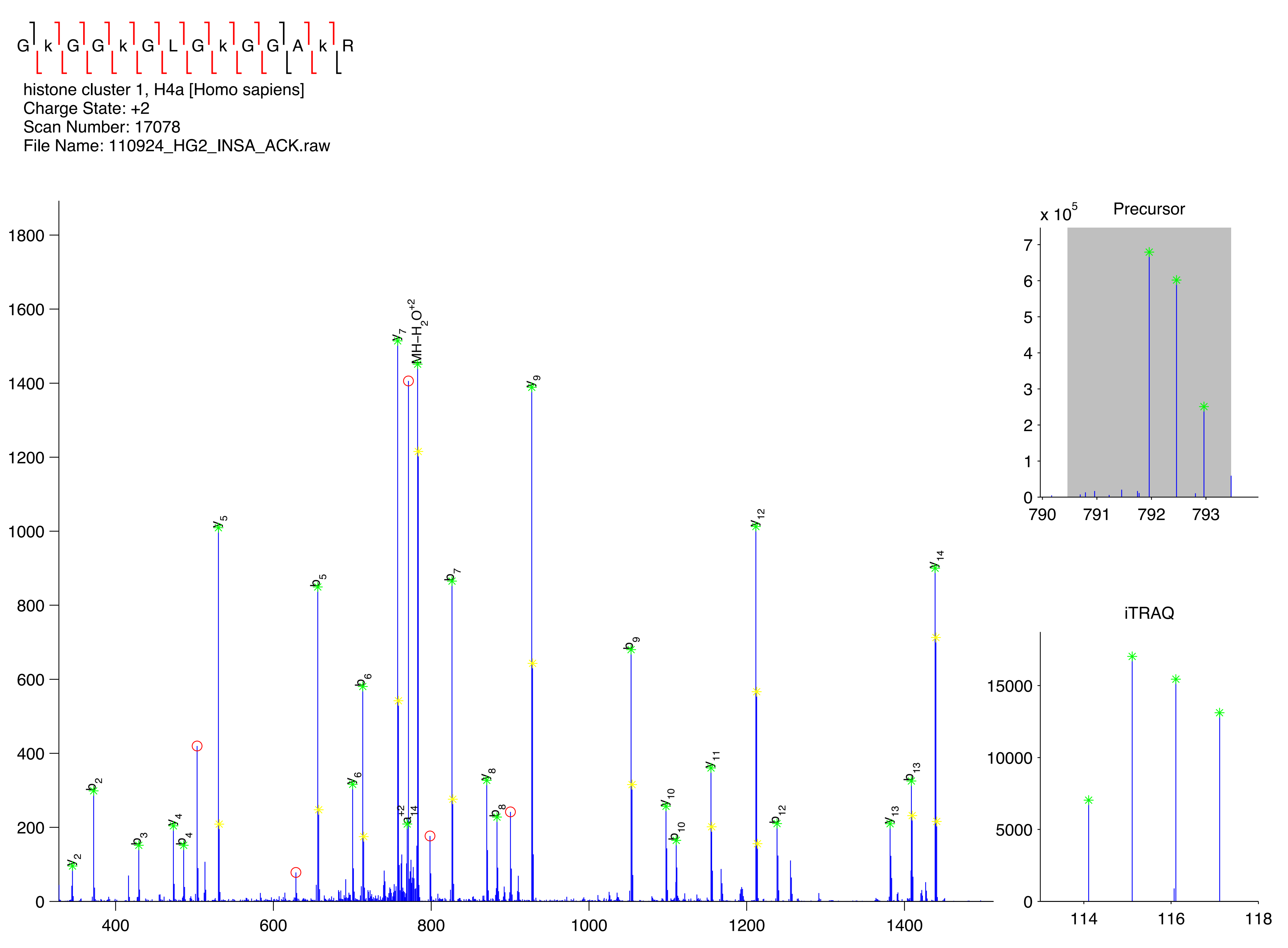
G k G G k G L G k G G A k R
histone cluster 1, H4a [Homo sapiens]
Charge State: +2
Scan Number: 17078
File Name: 110924_HG2_INSA_ACK.raw
y2
b2
b3
y4
b4
y5
b5
y6
b6
y7
a14 +2
MH-H2O+2
b7
y8
b8
y9
b9
y10
b10
y11
y12
b12
y13
b13
y14
x 10^5
Precursor
iTRAQ

G k G G k G L G k G G A k R

histone cluster 1, H4a [Homo sapiens]
Charge State: +2
Scan Number: 17111
File Name: 110924_HG2_INSA_ACK.raw

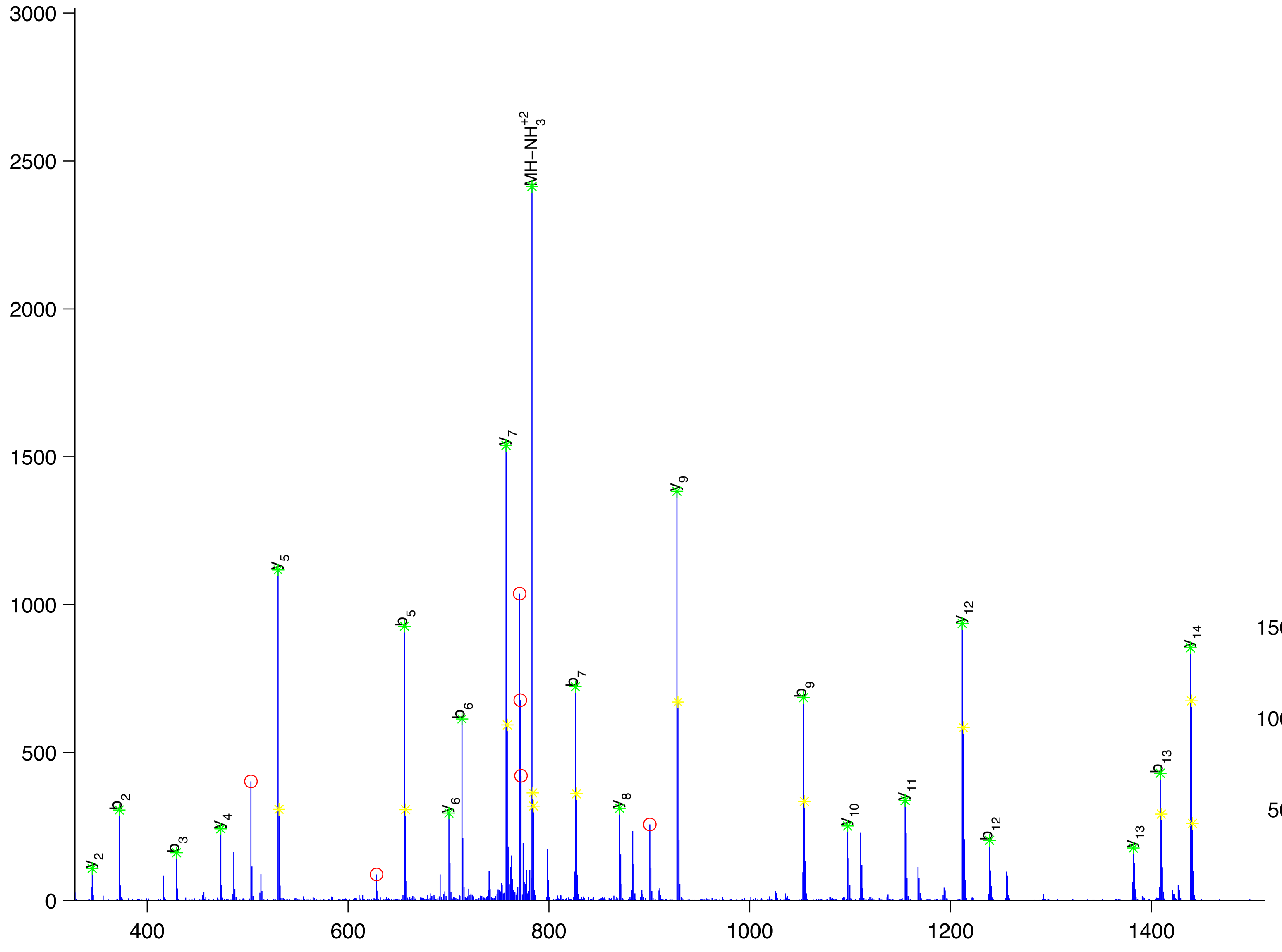

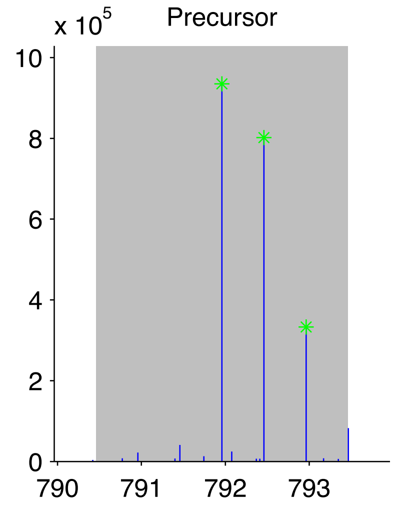

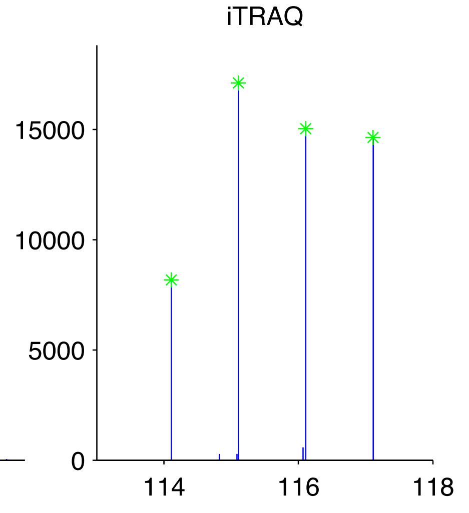

G k G G k G L G k G G A k R

histone cluster 1, H4a [Homo sapiens]
Charge State: +3
Scan Number: 17216
File Name: 110924_HG2_INSA_ACK3.raw

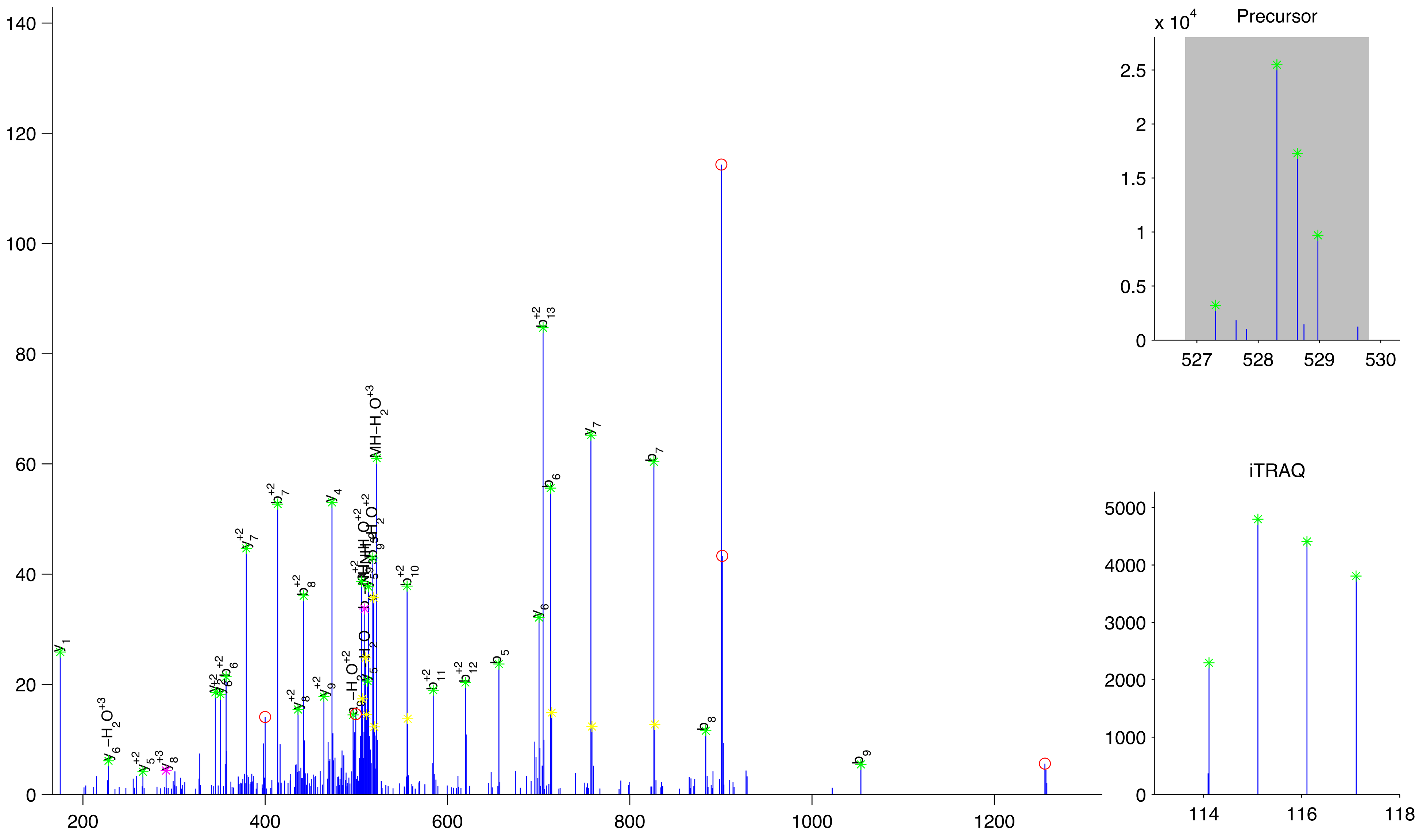

G L G k G G A k R

histone cluster 1, H4a [Homo sapiens]
Charge State: +2
Scan Number: 17219
File Name: 110924_HG2_INSA_ACK3.raw

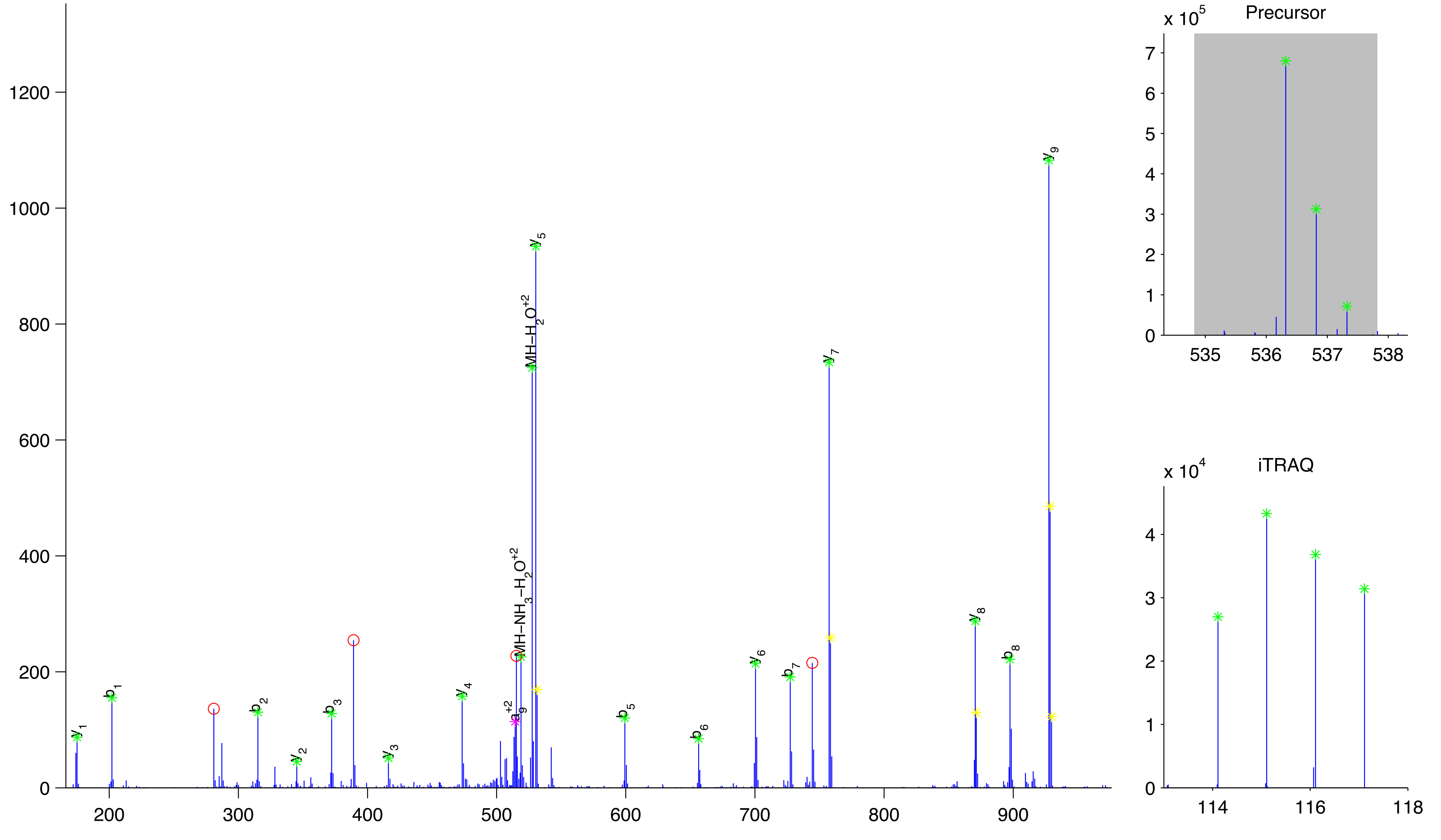

G L G k G G A k R

histone cluster 1, H4a [Homo sapiens]
Charge State: +2
Scan Number: 17231
File Name: 110924_HG2_INSA_ACK3.raw

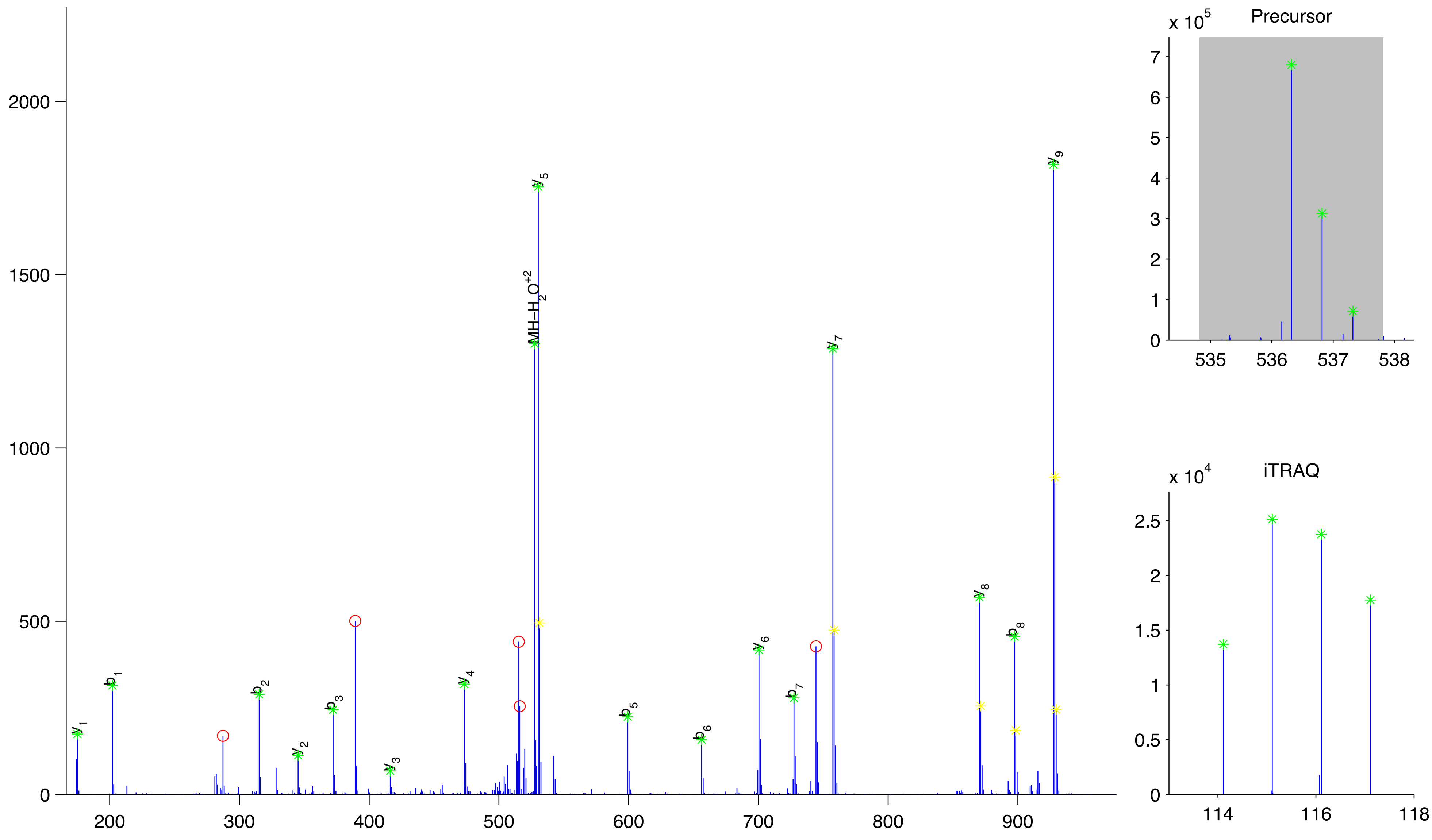

G G k G L G k G G A k R

histone cluster 1, H4a [Homo sapiens]
Charge State: +2
Scan Number: 17265
File Name: 110924_HG2_INSA_ACK.raw

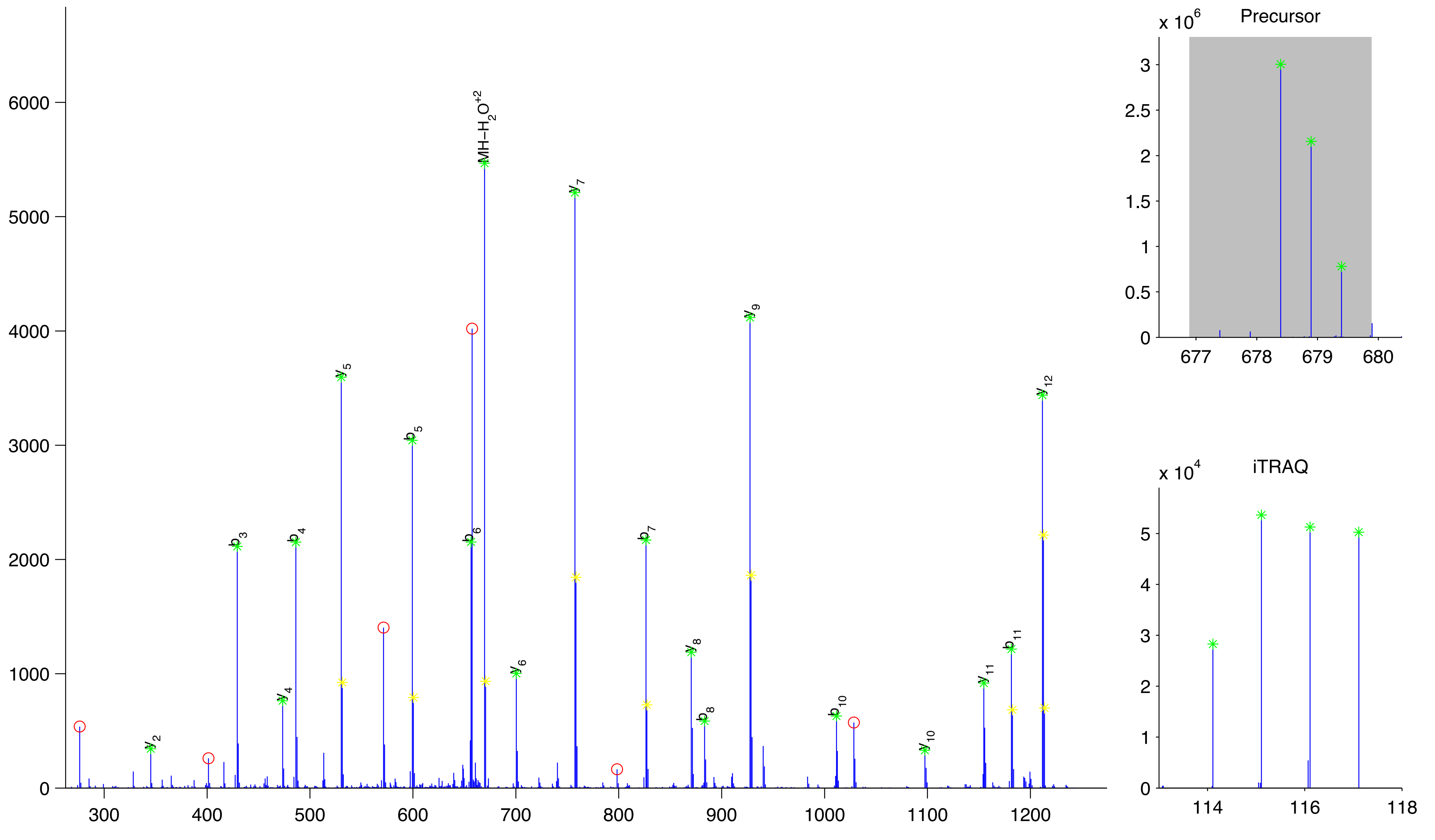

G k G G k G L G k G G A k R

histone cluster 1, H4a [Homo sapiens]
Charge State: +2
Scan Number: 17277
File Name: 110924_HG2_INSA_ACK.raw

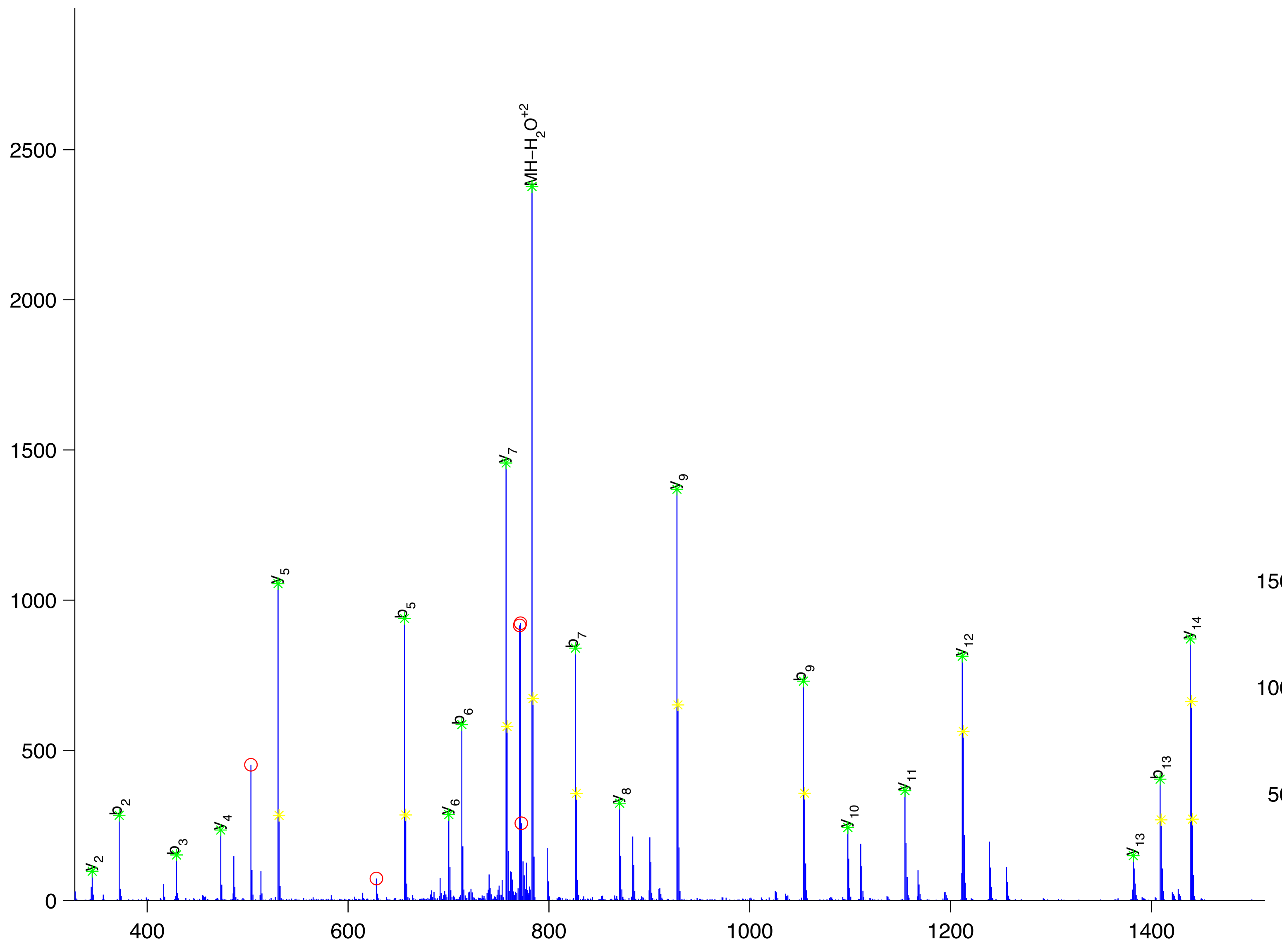

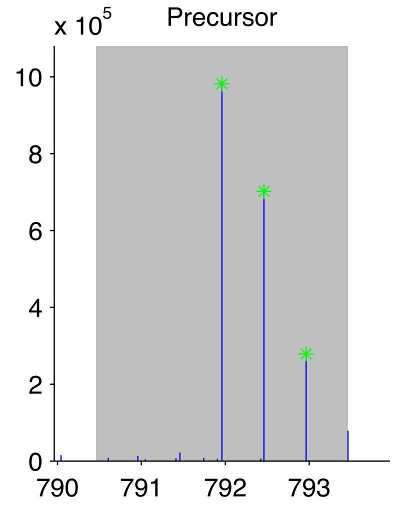

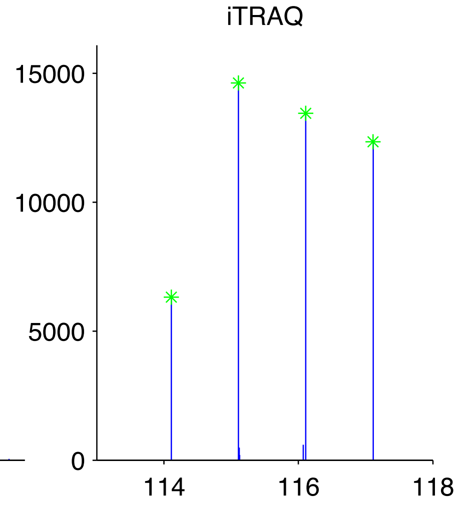

GGkGLGkGGAkR

histone cluster 1, H4a [Homo sapiens]
Charge State: +2
Scan Number: 17305
File Name: 110924_HG2_INSA_ACK3.raw

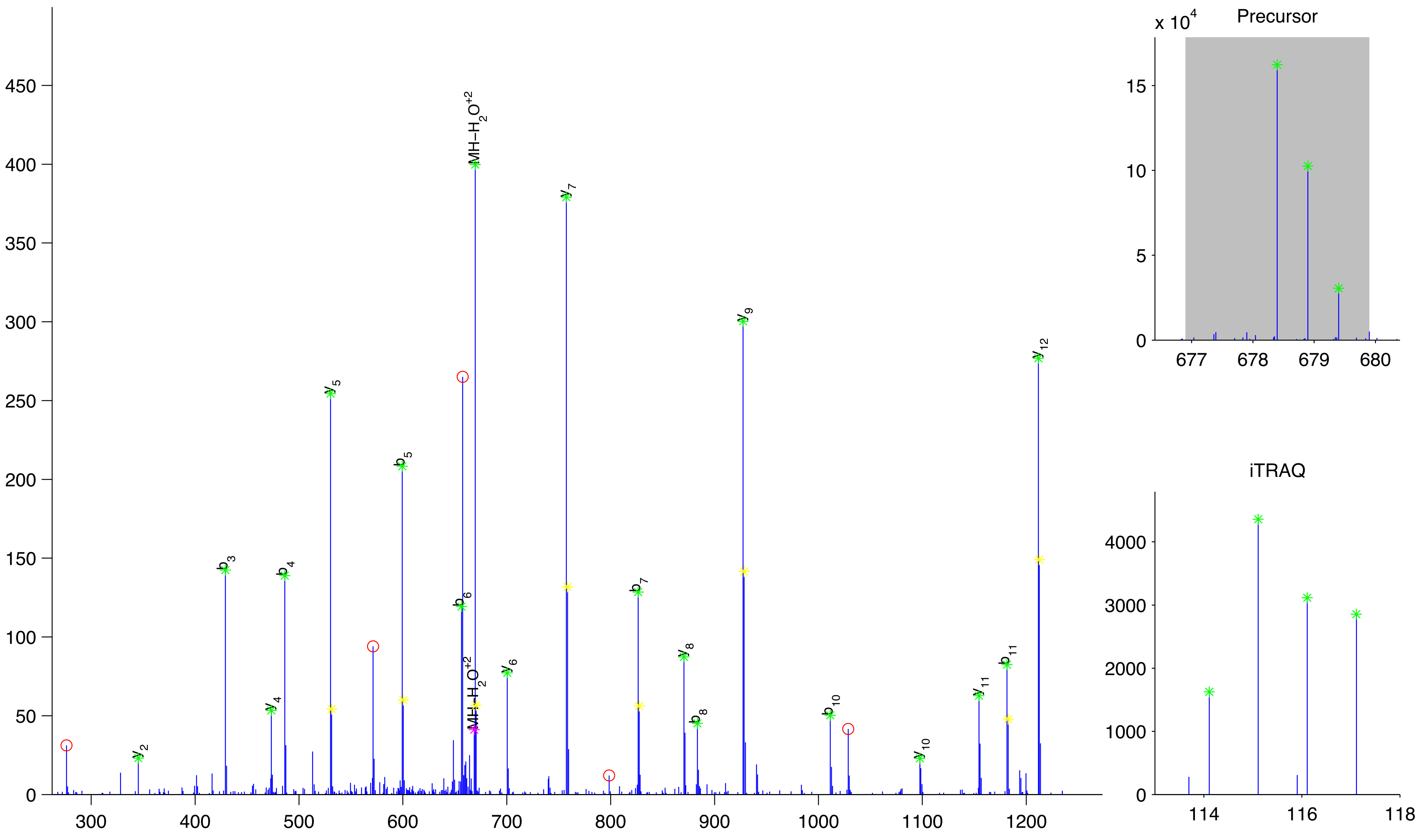

G k G G k G L G k G G A k R

histone cluster 1, H4a [Homo sapiens]
Charge State: +2
Scan Number: 17412
File Name: 110924_HG2_INSA_ACK.raw

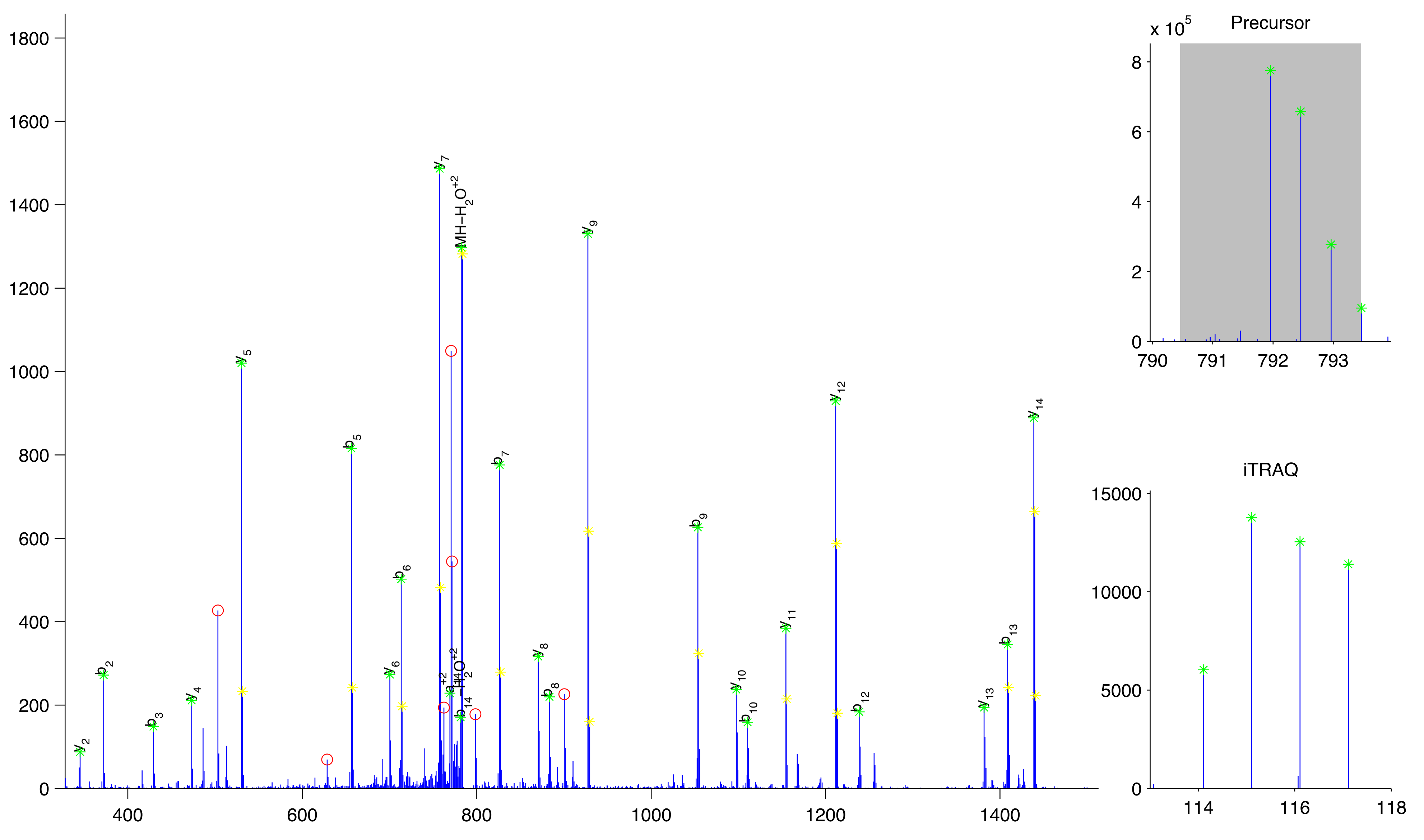

G k G G k G L G k G G A k R

histone cluster 1, H4a [Homo sapiens]
Charge State: +2
Scan Number: 17443
File Name: 110924_HG2_INSA_ACK.raw

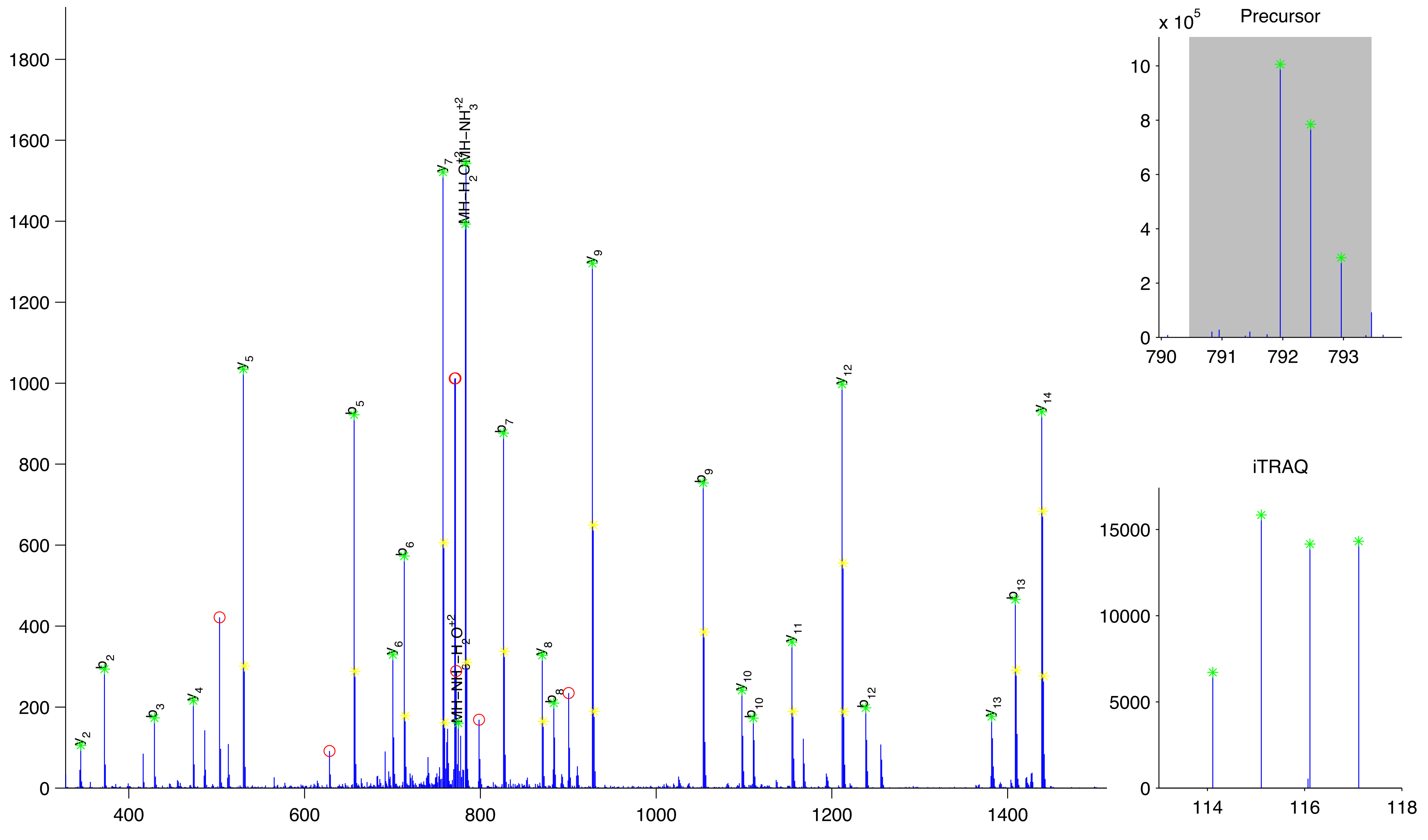

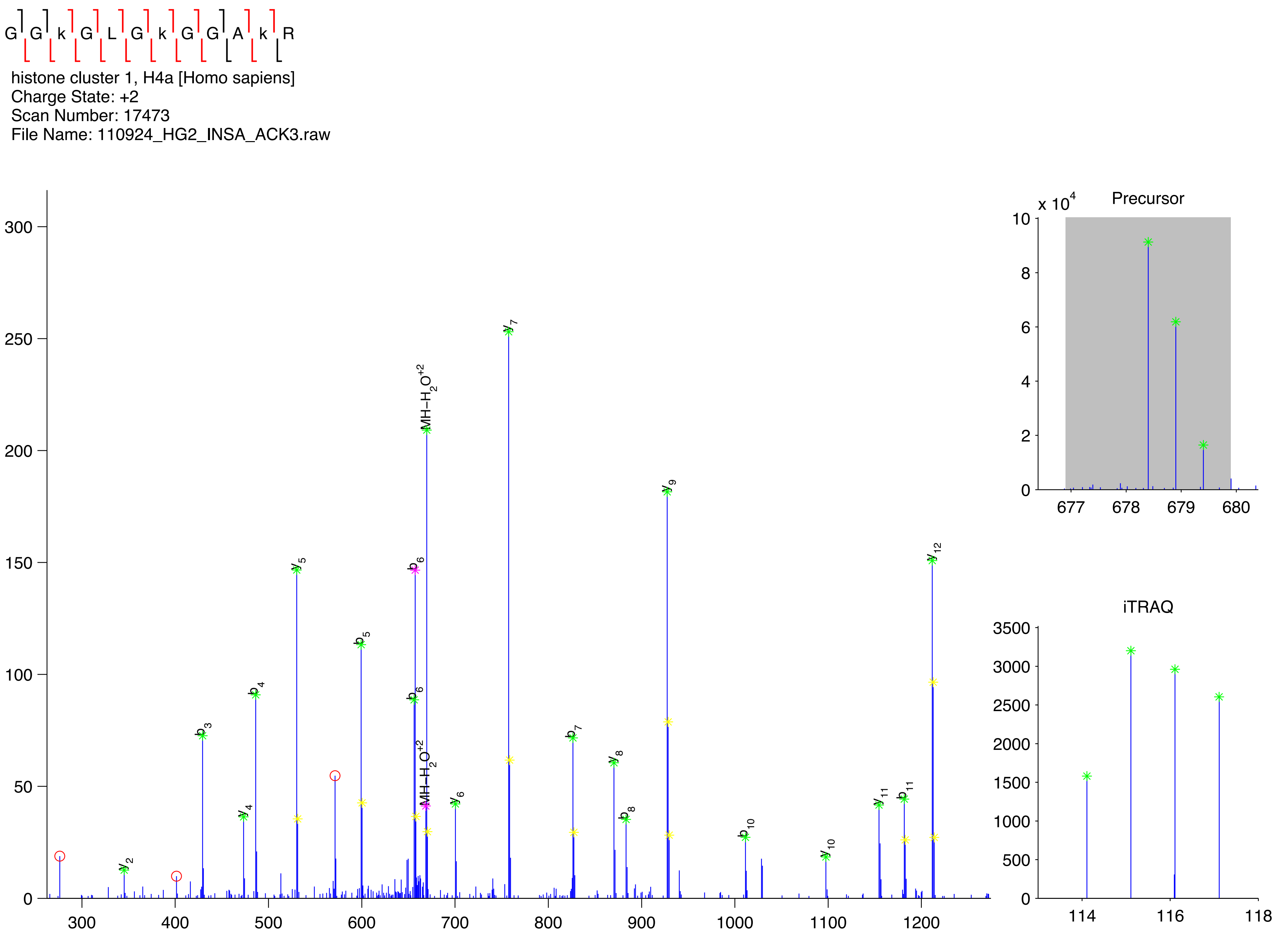
G G k G L G k G G A k R
histone cluster 1, H4a [Homo sapiens]
Charge State: +2
Scan Number: 17473
File Name: 110924_HG2_INSA_ACK3.raw
y2
b3
y4
b4
y5
b5
b6
MH-H2O+2
y6
y7
b7
y8
b8
y9
b10
y10
y11
b11
y12
x 10^4
Precursor
iTRAQ

G L G k G G A k R

histone cluster 1, H4a [Homo sapiens]
Charge State: +2
Scan Number: 17555
File Name: 110924_HG2_INSA_ACK3.raw

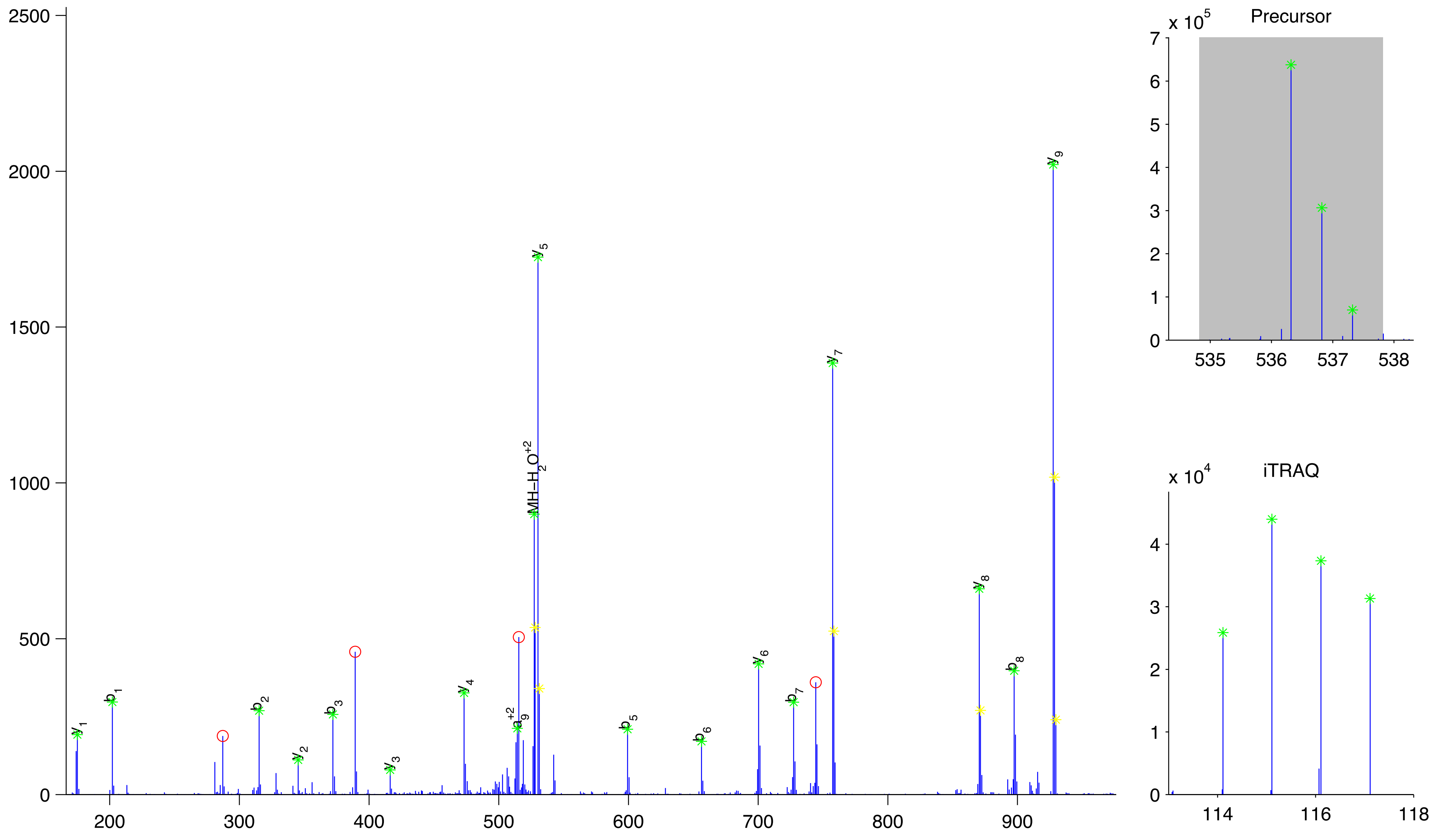

G k G G k G L G k G G A k R

histone cluster 1, H4a [Homo sapiens]
Charge State: +2
Scan Number: 17580
File Name: 110924_HG2_INSA_ACK.raw

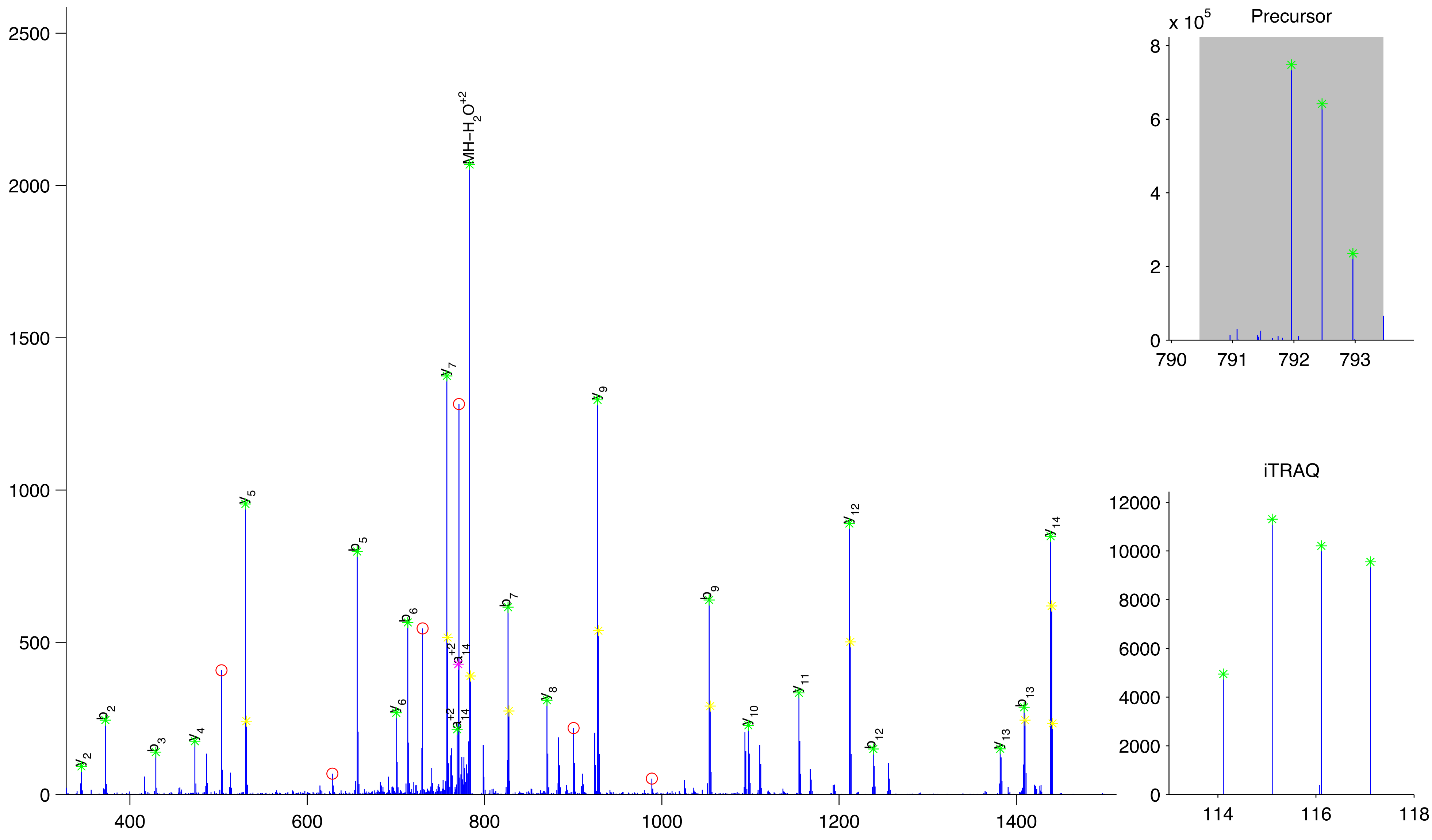

G G k G L G k G G A k R

histone cluster 1, H4a [Homo sapiens]
Charge State: +2
Scan Number: 17601
File Name: 110924_HG2_INSA_ACK.raw

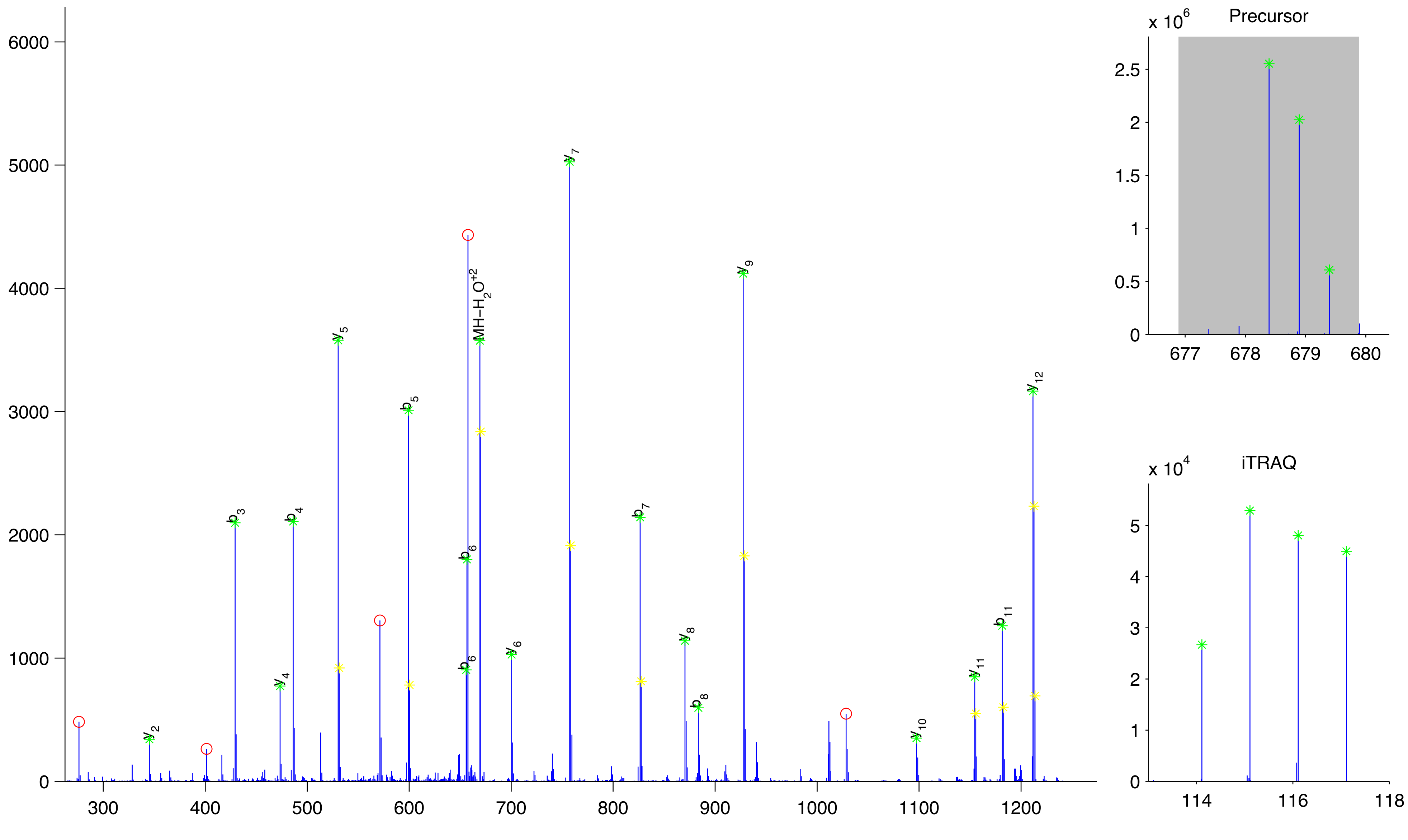

G k G G k G L G k G G A k R

histone cluster 1, H4a [Homo sapiens]
Charge State: +3
Scan Number: 17712
File Name: 110924_HG2_INSA_ACK.raw

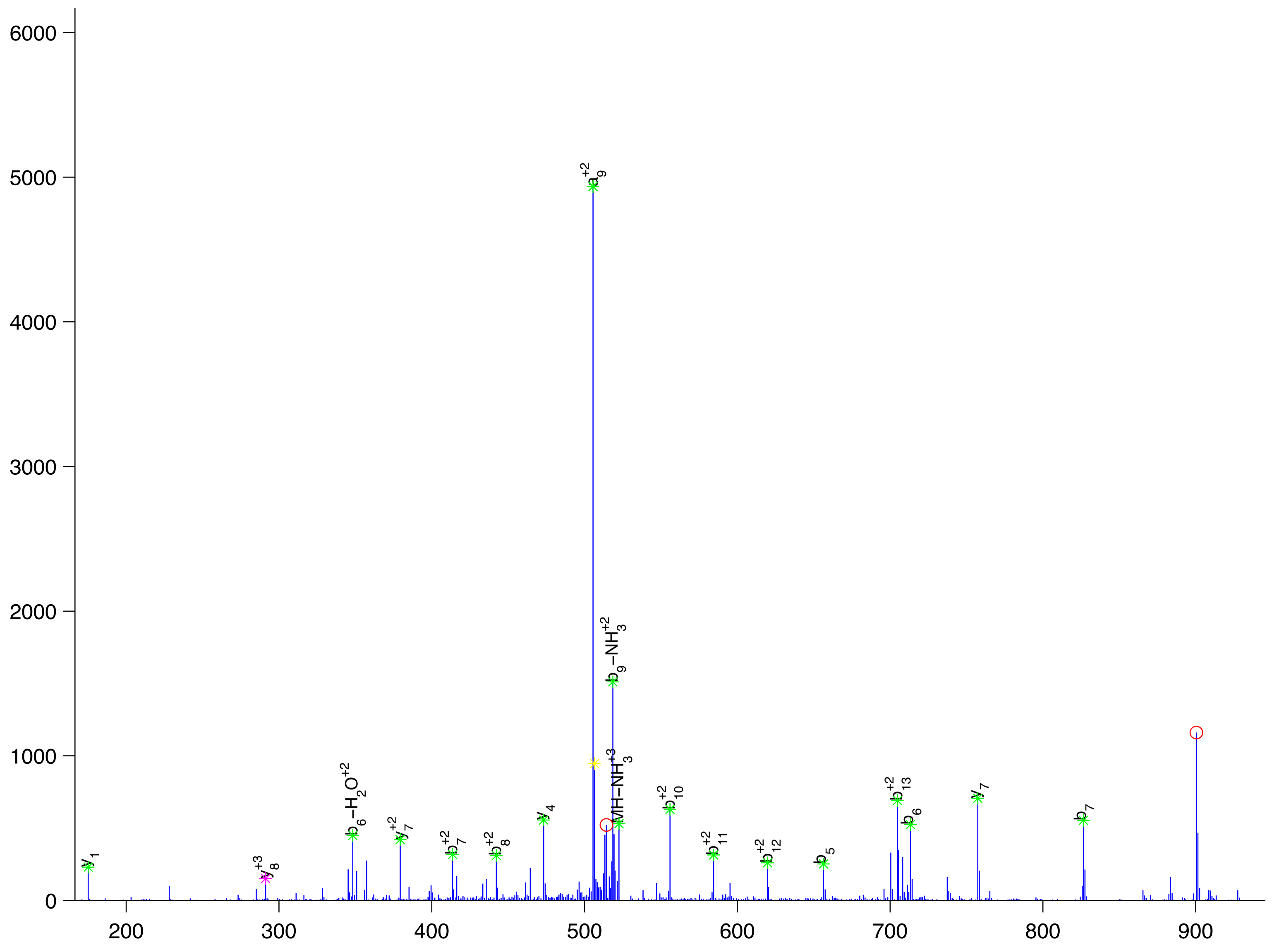

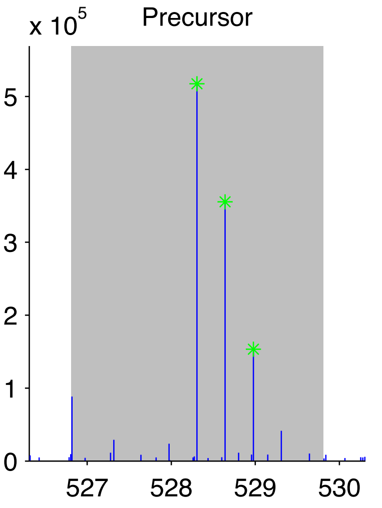

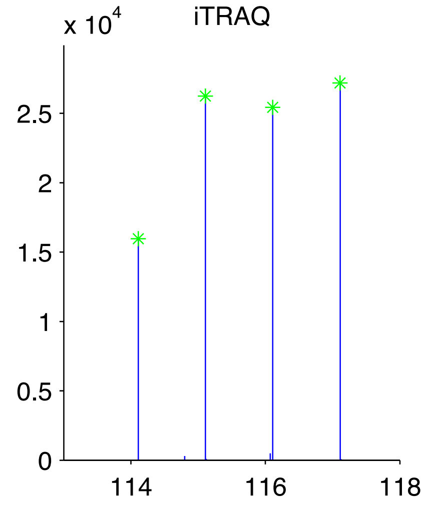

G L G k G G A k R

histone cluster 1, H4a [Homo sapiens]
Charge State: +2
Scan Number: 17725
File Name: 110924_HG2_INSA_ACK3.raw

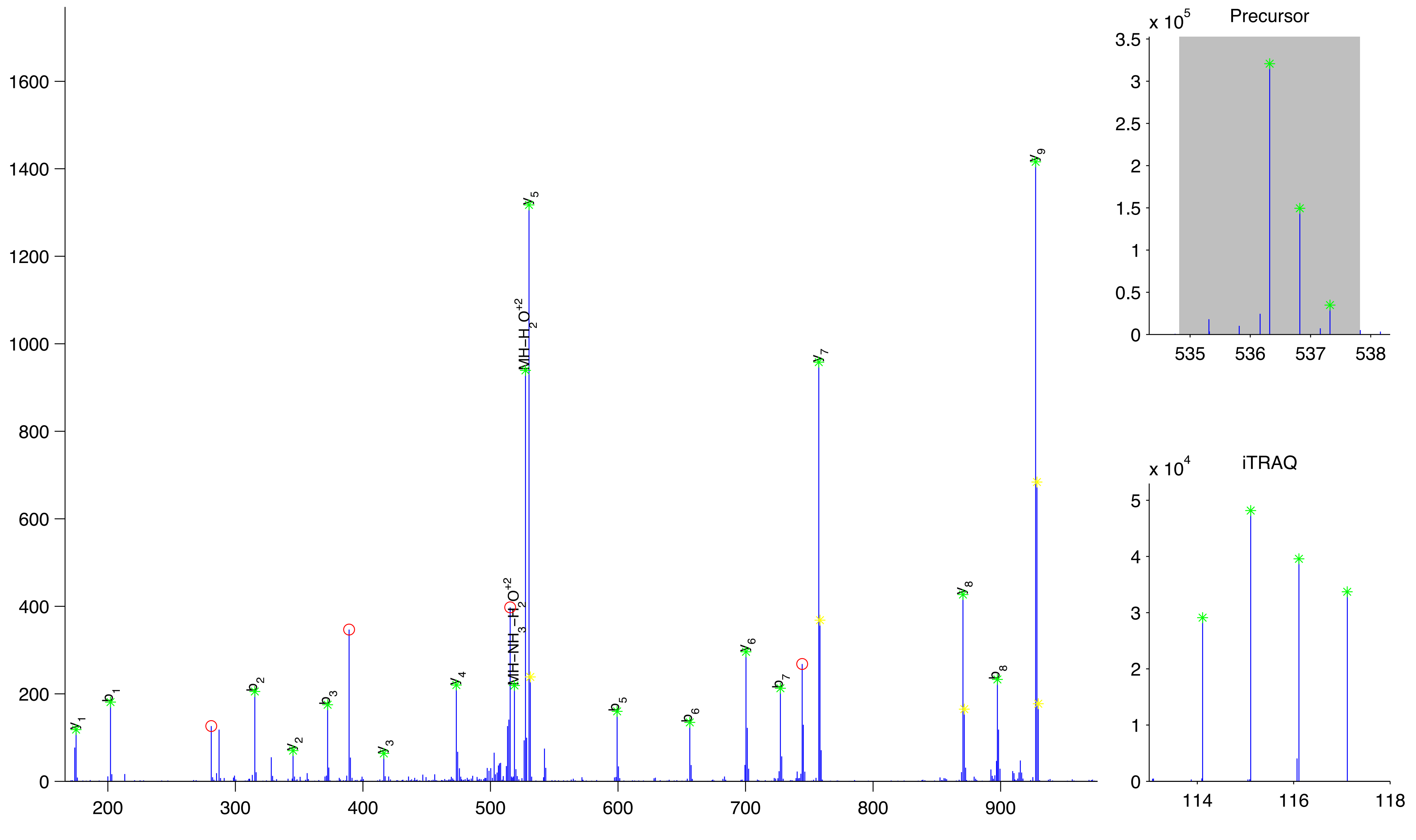

G k G G k G L G k G G A k R

histone cluster 1, H4a [Homo sapiens]
Charge State: +2
Scan Number: 17748
File Name: 110924_HG2_INSA_ACK.raw

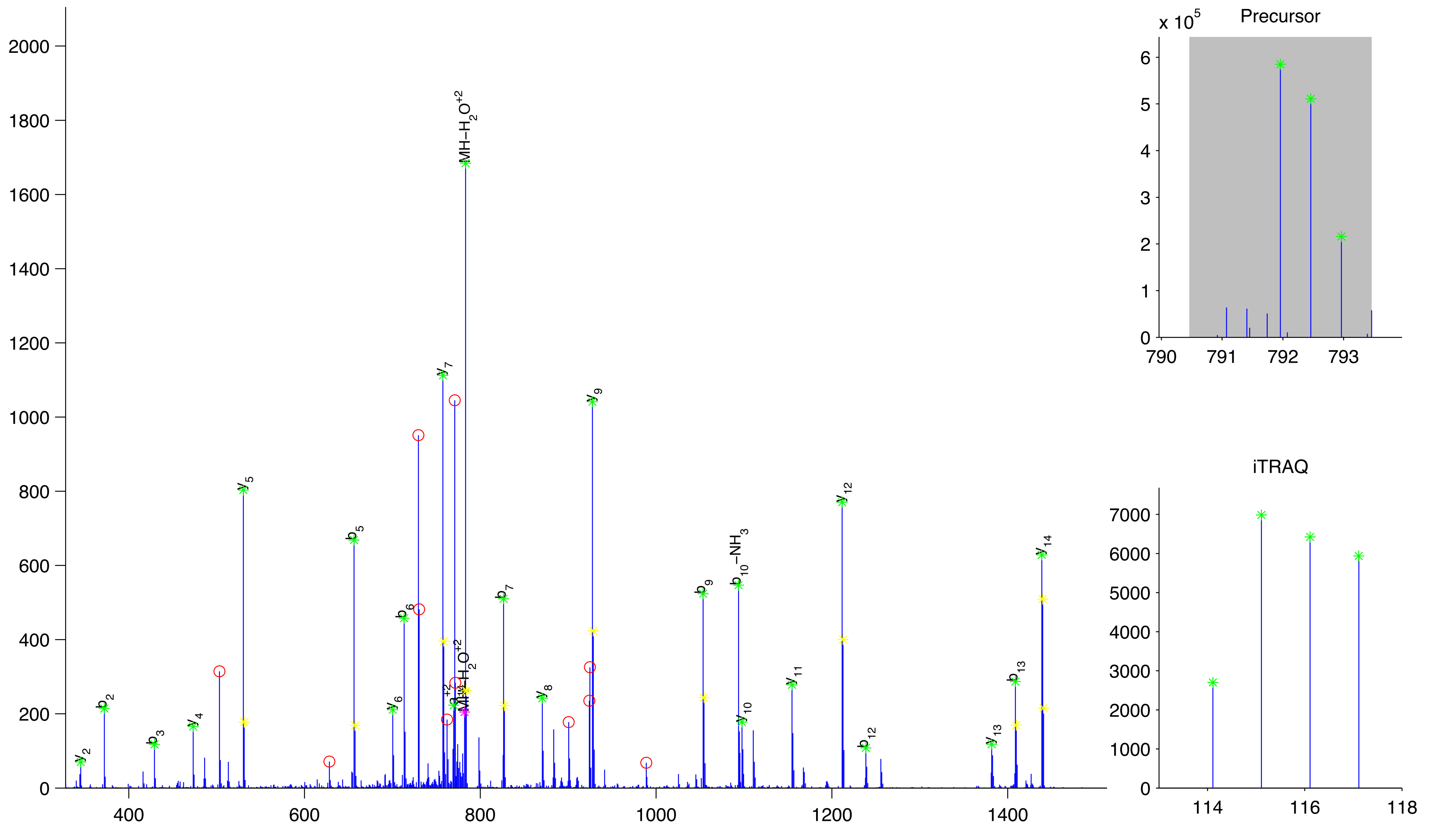

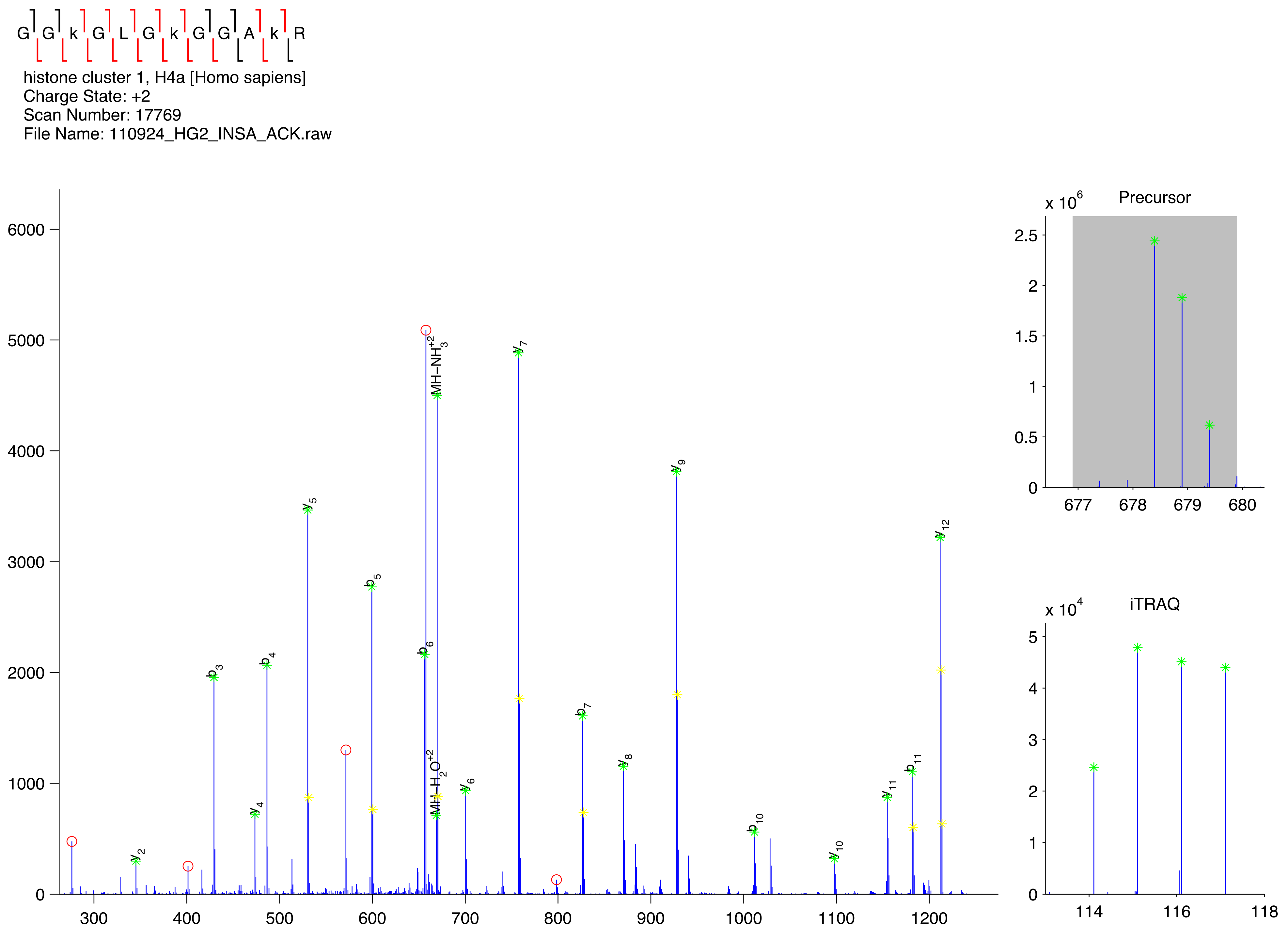
G G k G L G k G G A k R
histone cluster 1, H4a [Homo sapiens]
Charge State: +2
Scan Number: 17769
File Name: 110924_HG2_INSA_ACK.raw
Precursor
x 10^6
iTRAQ
x 10^4
b3
b4
b5
b6
b7
b10
b11
y2
y4
y5
y6
y7
y8
y9
y10
y11
y12
MH−NH3+2
MH−H2O+2

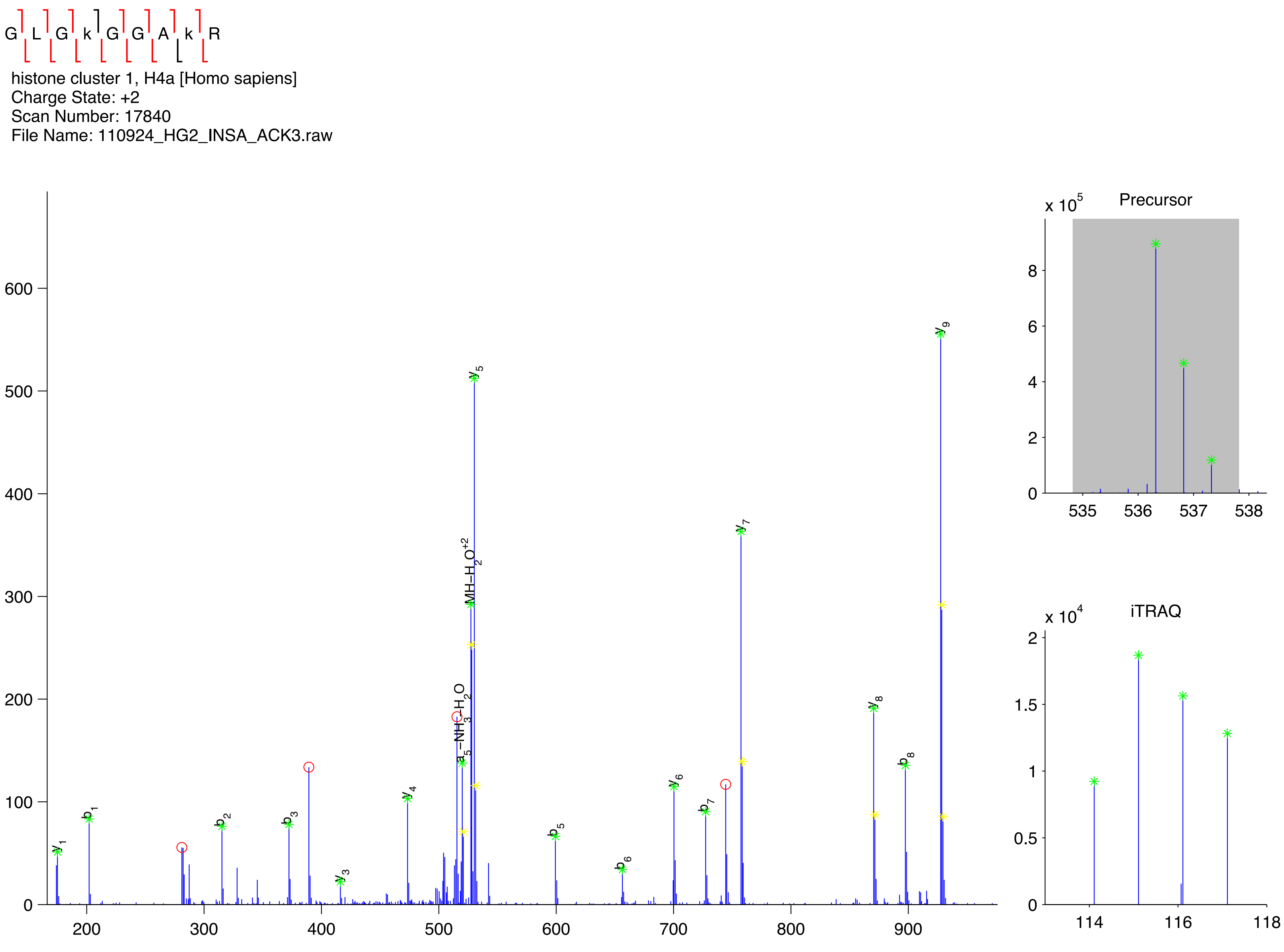
G L G k G G A k R
histone cluster 1, H4a [Homo sapiens]
Charge State: +2
Scan Number: 17840
File Name: 110924_HG2_INSA_ACK3.raw
y1
b1
b2
b3
y3
y4
a5−NH3−H2O
MH−H2O+2
y5
b5
b6
y6
b7
y7
y8
b8
y9
x 10^5
Precursor
x 10^4
iTRAQ

G k G G k G L G k G G A k R

histone cluster 1, H4a [Homo sapiens]
Charge State: +3
Scan Number: 17880
File Name: 110924_HG2_INSA_ACK.raw

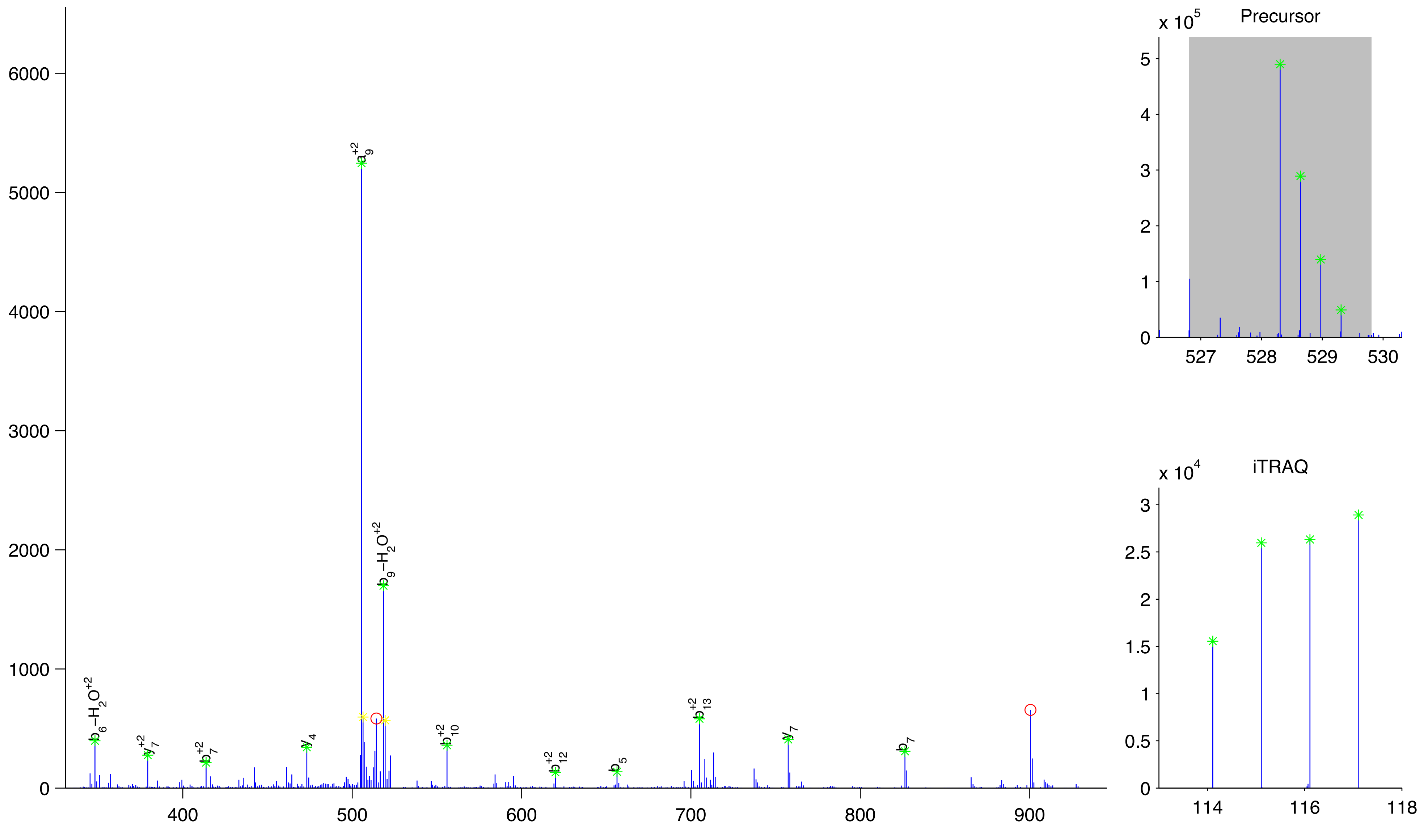

G k G G k G L G k G G A k R

histone cluster 1, H4a [Homo sapiens]
Charge State: +2
Scan Number: 17916
File Name: 110924_HG2_INSA_ACK.raw

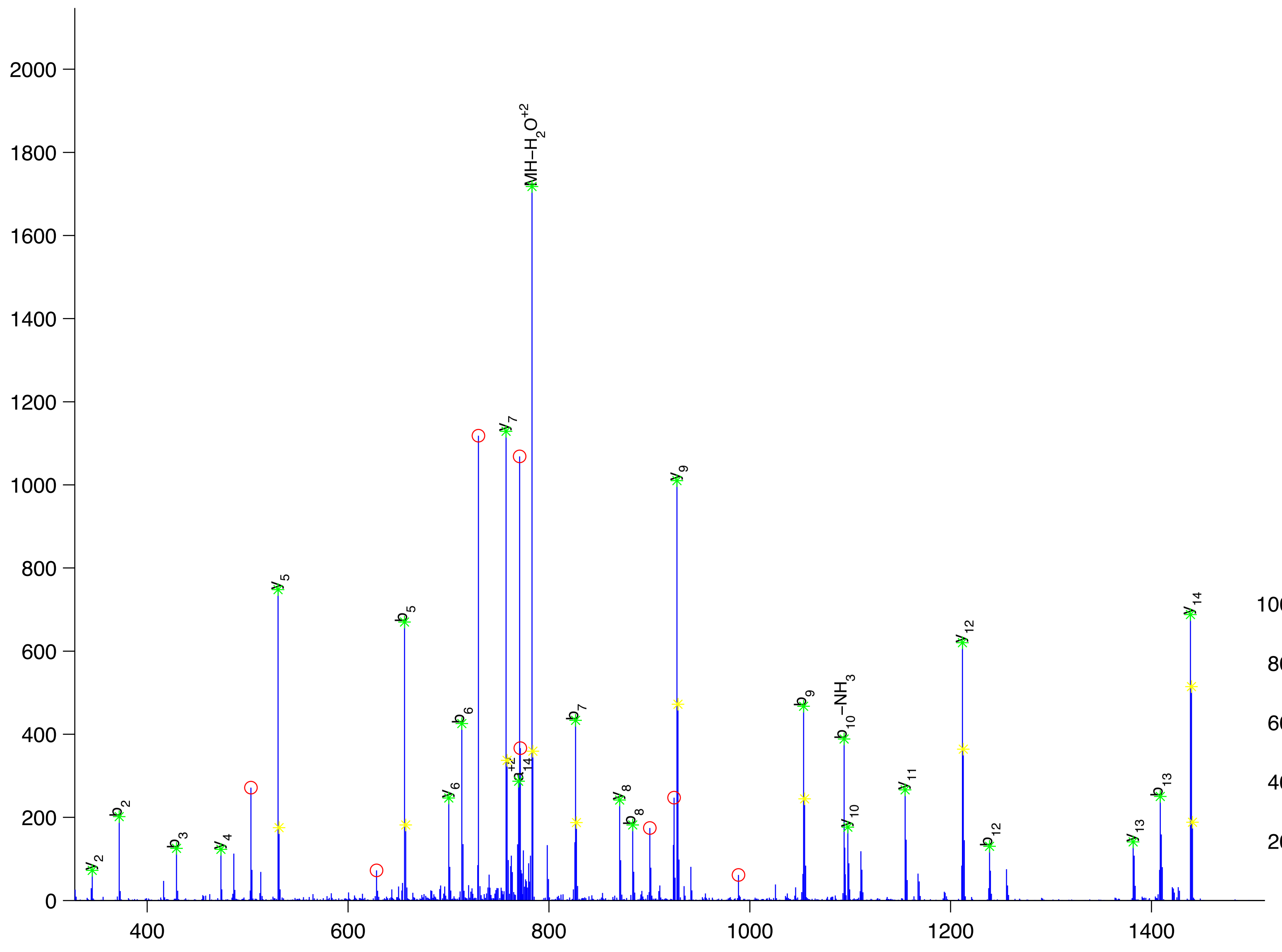

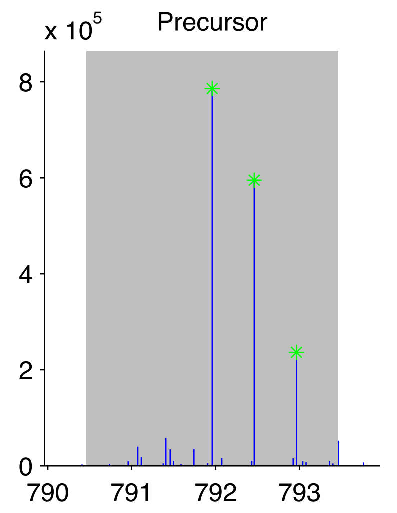

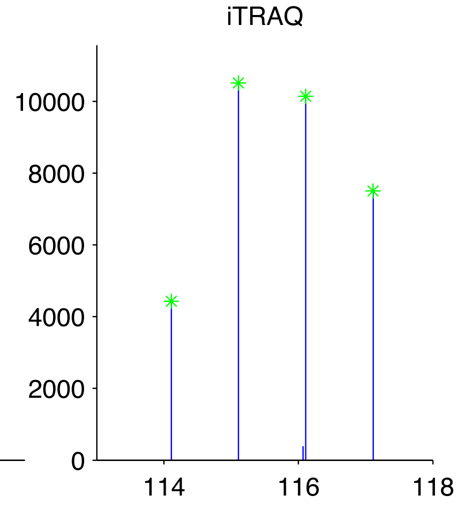

G G k G L G k G G A k R

histone cluster 1, H4a [Homo sapiens]
Charge State: +2
Scan Number: 17937
File Name: 110924_HG2_INSA_ACK.raw

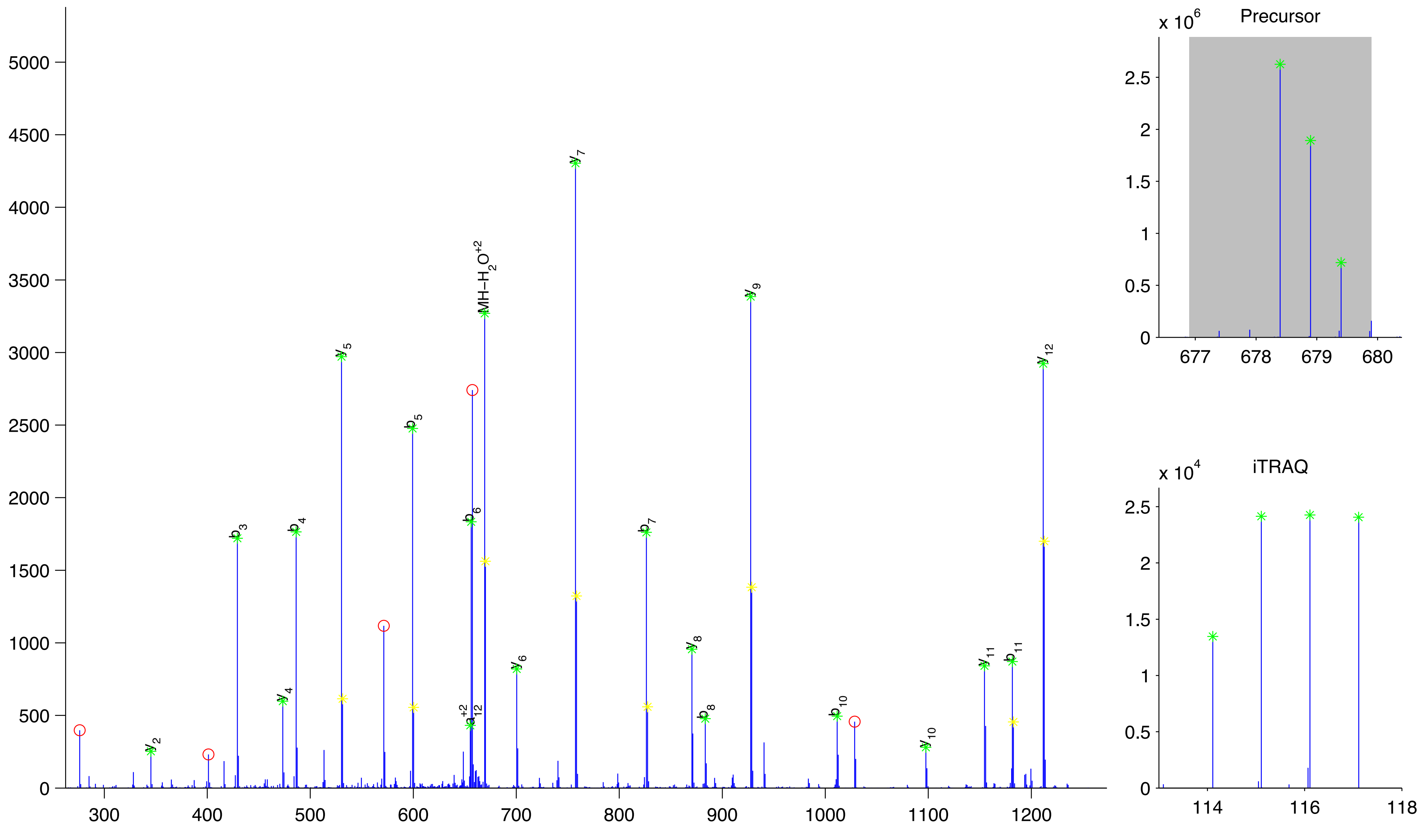

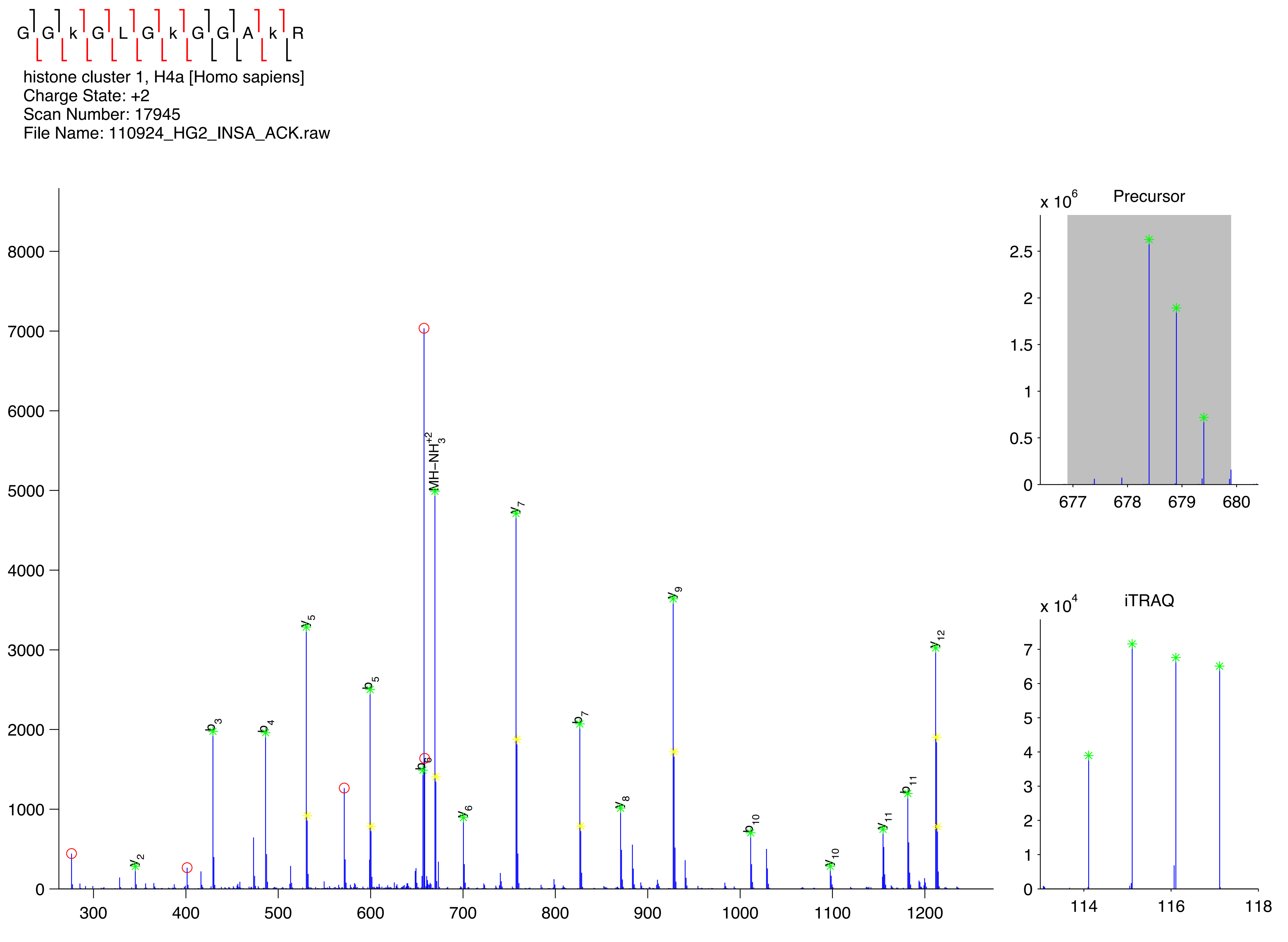
G G k G L G k G G A k R
histone cluster 1, H4a [Homo sapiens]
Charge State: +2
Scan Number: 17945
File Name: 110924_HG2_INSA_ACK.raw
8000
7000
6000
5000
4000
3000
2000
1000
0
300
400
500
600
700
800
900
1000
1100
1200
y2
b3
b4
y5
b5
b6
MH−NH3+2
y6
y7
b7
y8
y9
b10
y10
y11
b11
y12
x 10^6
Precursor
2.5
2
1.5
1
0.5
0
677
678
679
680
x 10^4
iTRAQ
7
6
5
4
3
2
1
0
114
116
118

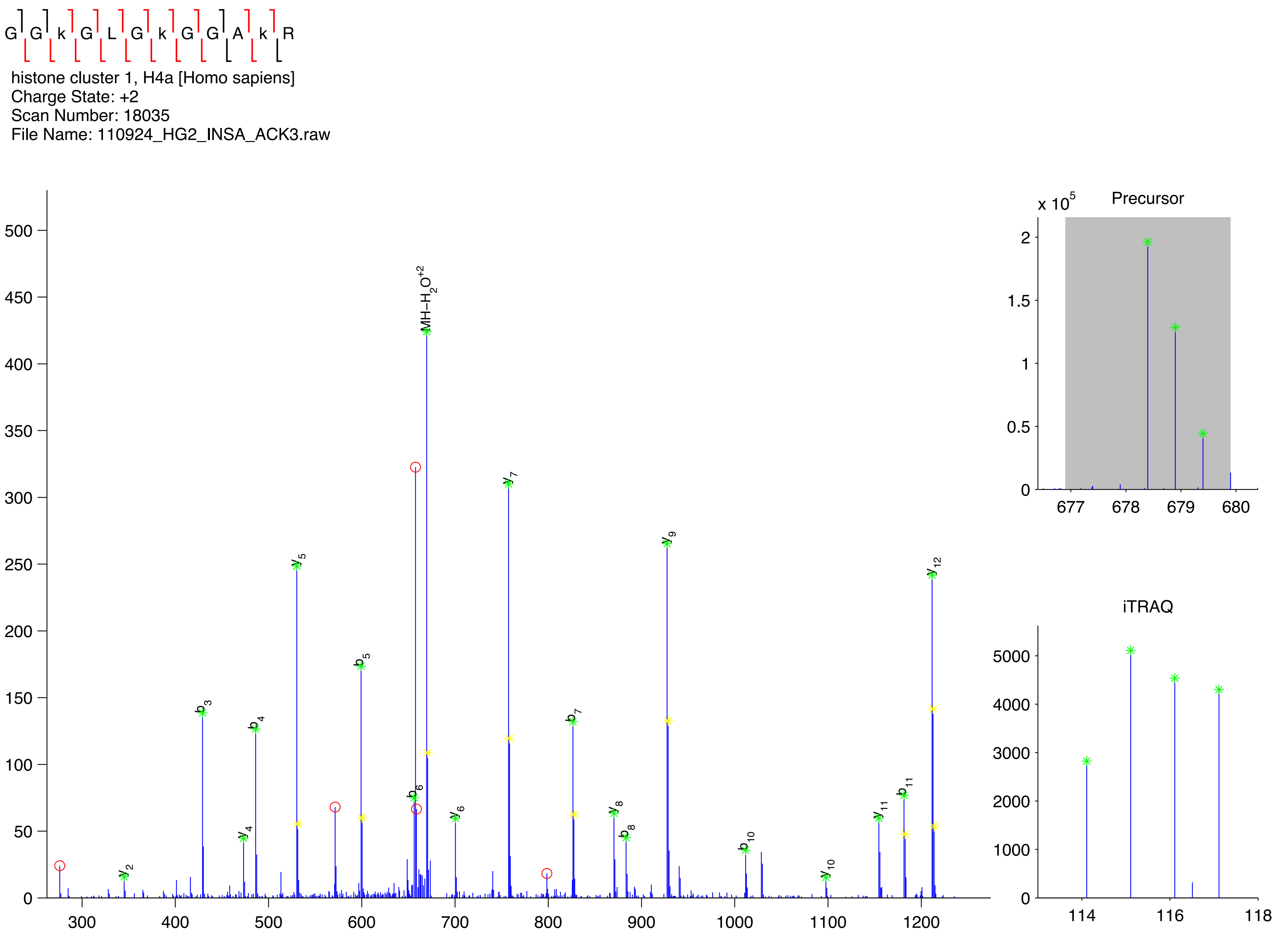

G G k G L G k G G A k R
histone cluster 1, H4a [Homo sapiens]
Charge State: +2
Scan Number: 18035
File Name: 110924_HG2_INSA_ACK3.raw
MH−H2O+2
y2
b3
y4
b4
y5
b5
b6
y6
y7
b7
y8
b8
y9
b10
y10
y11
b11
y12
x 10^5
Precursor
iTRAQ

G L G k G G A k R

histone cluster 1, H4a [Homo sapiens]
Charge State: +2
Scan Number: 18059
File Name: 110924_HG2_INSA_ACK3.raw

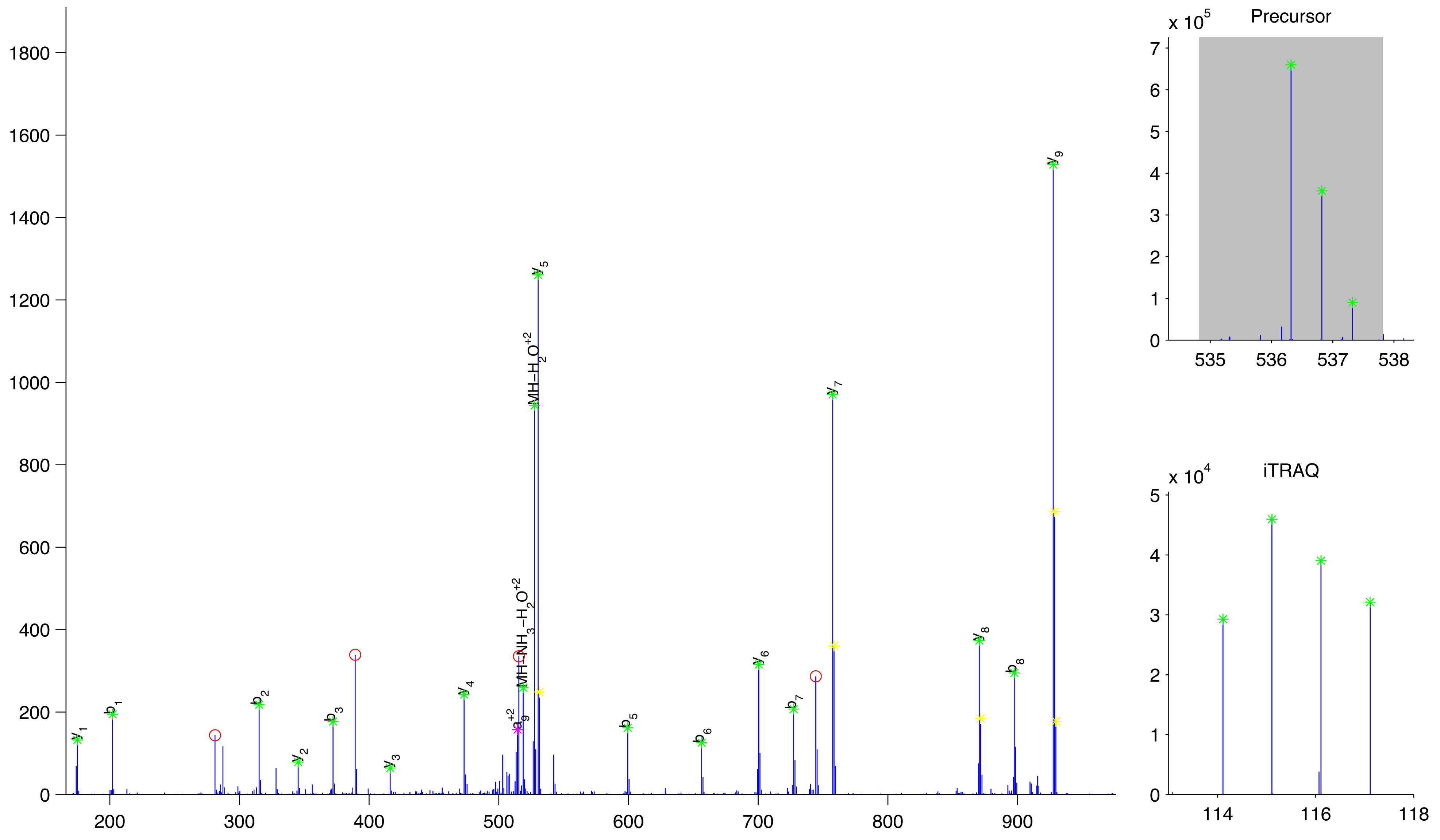

G k G G k G L G k G G A k R

histone cluster 1, H4a [Homo sapiens]
Charge State: +2
Scan Number: 18077
File Name: 110924_HG2_INSA_ACK.raw

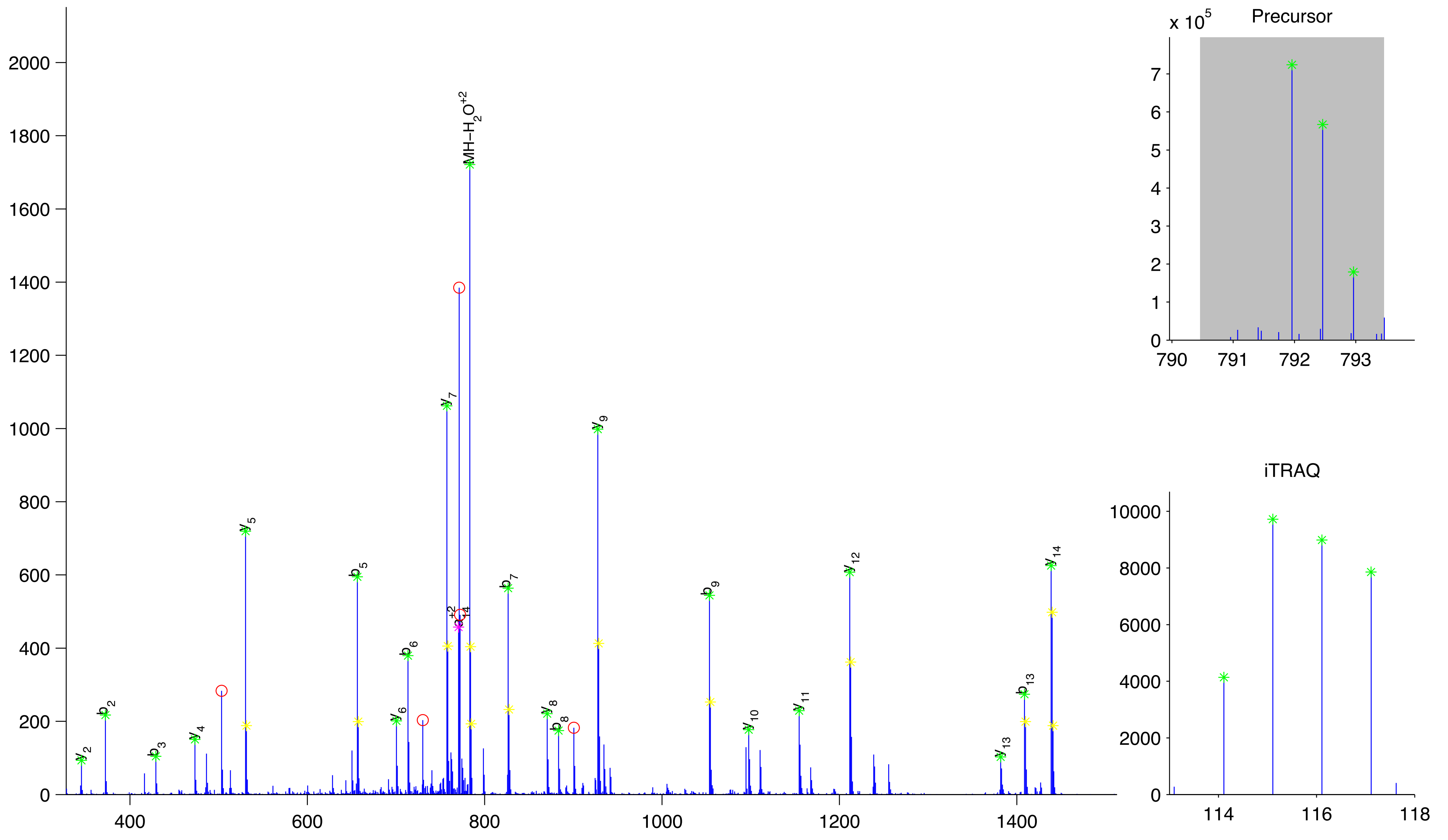

G k G G k G L G k G G A k R

histone cluster 1, H4a [Homo sapiens]
Charge State: +2
Scan Number: 18084
File Name: 110924_HG2_INSA_ACK.raw

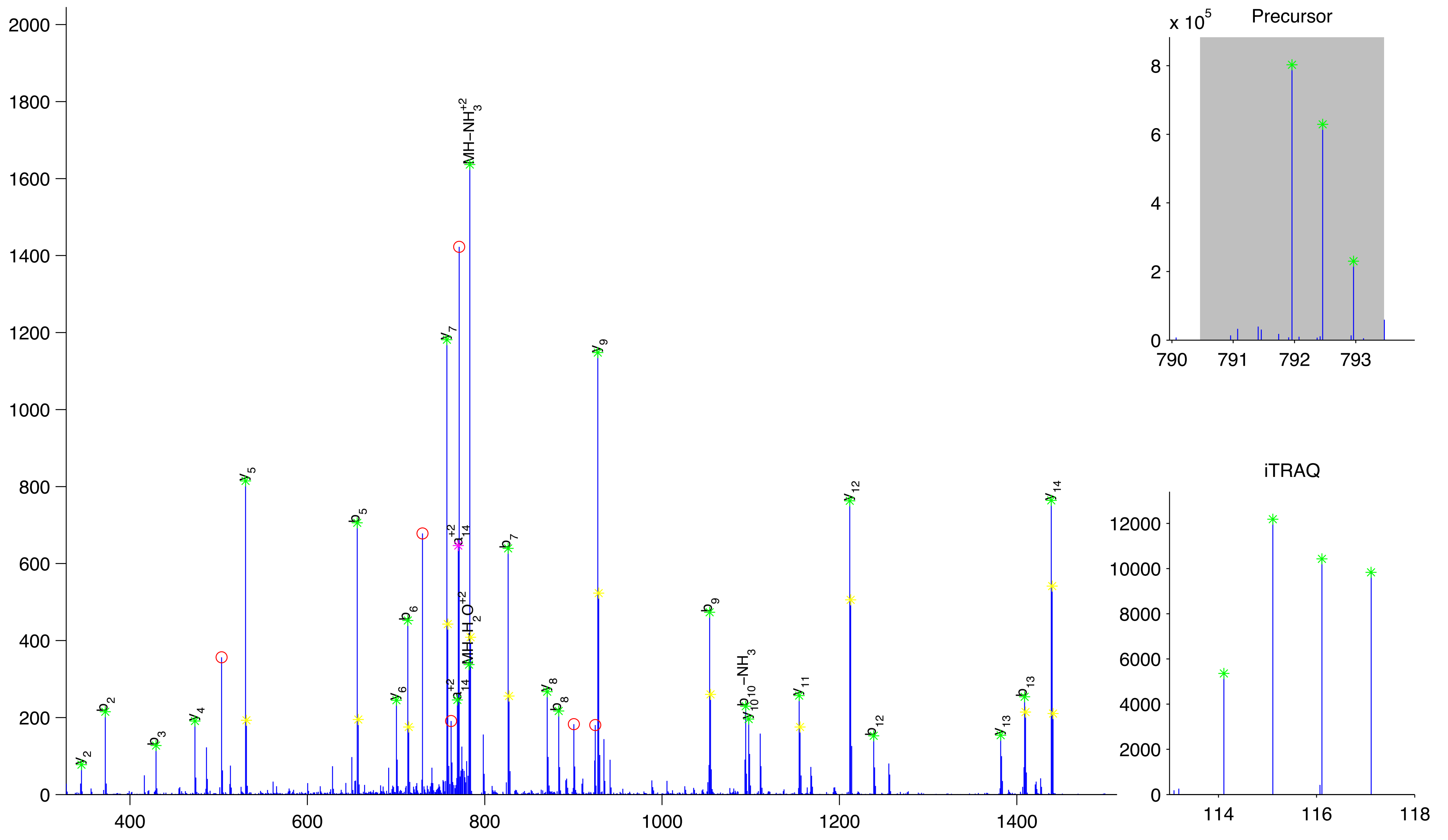

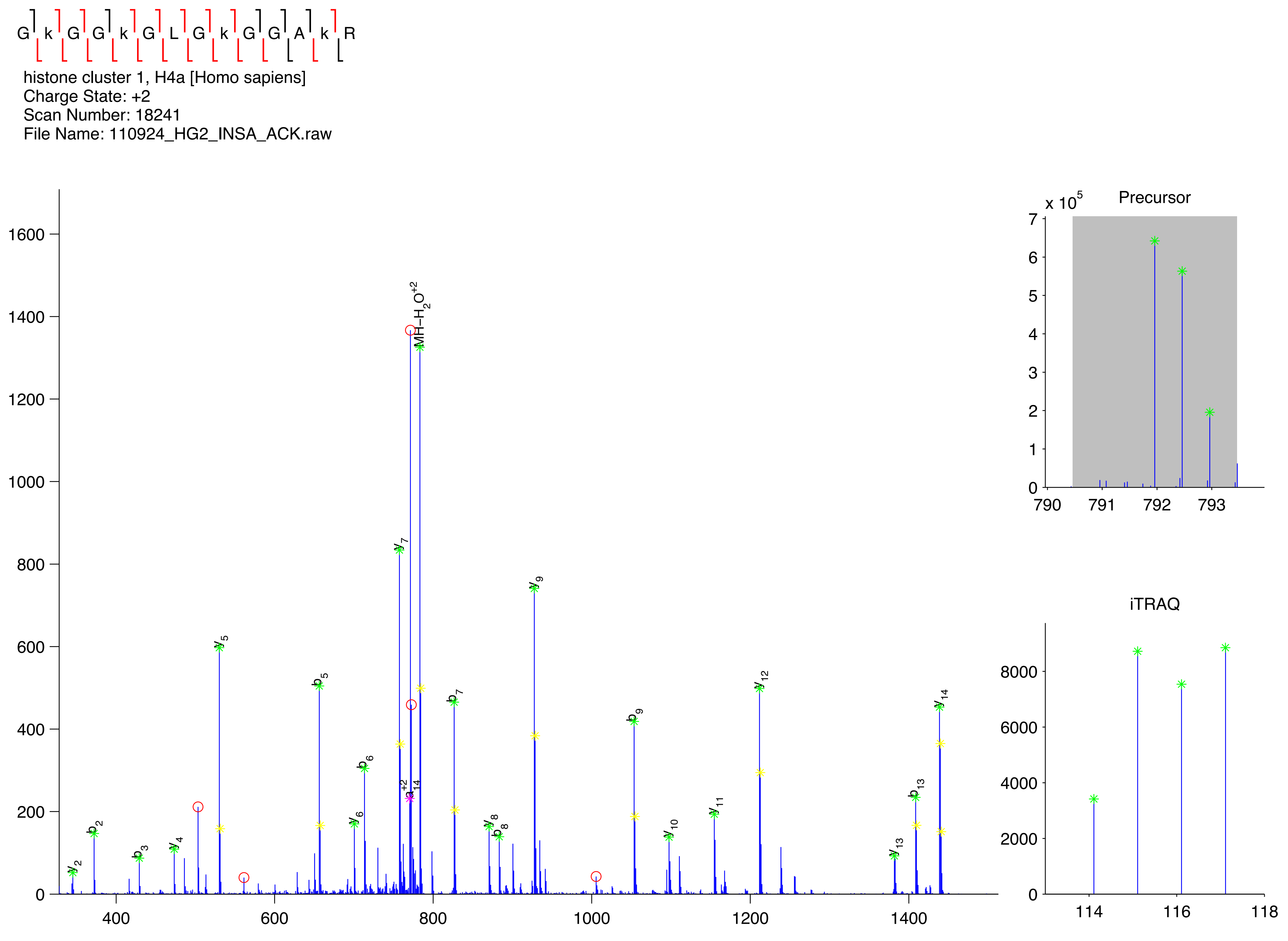

G k G G k G L G k G G A k R
histone cluster 1, H4a [Homo sapiens]
Charge State: +2
Scan Number: 18241
File Name: 110924_HG2_INSA_ACK.raw
MH−H2O+2
Precursor
x 10^5
iTRAQ

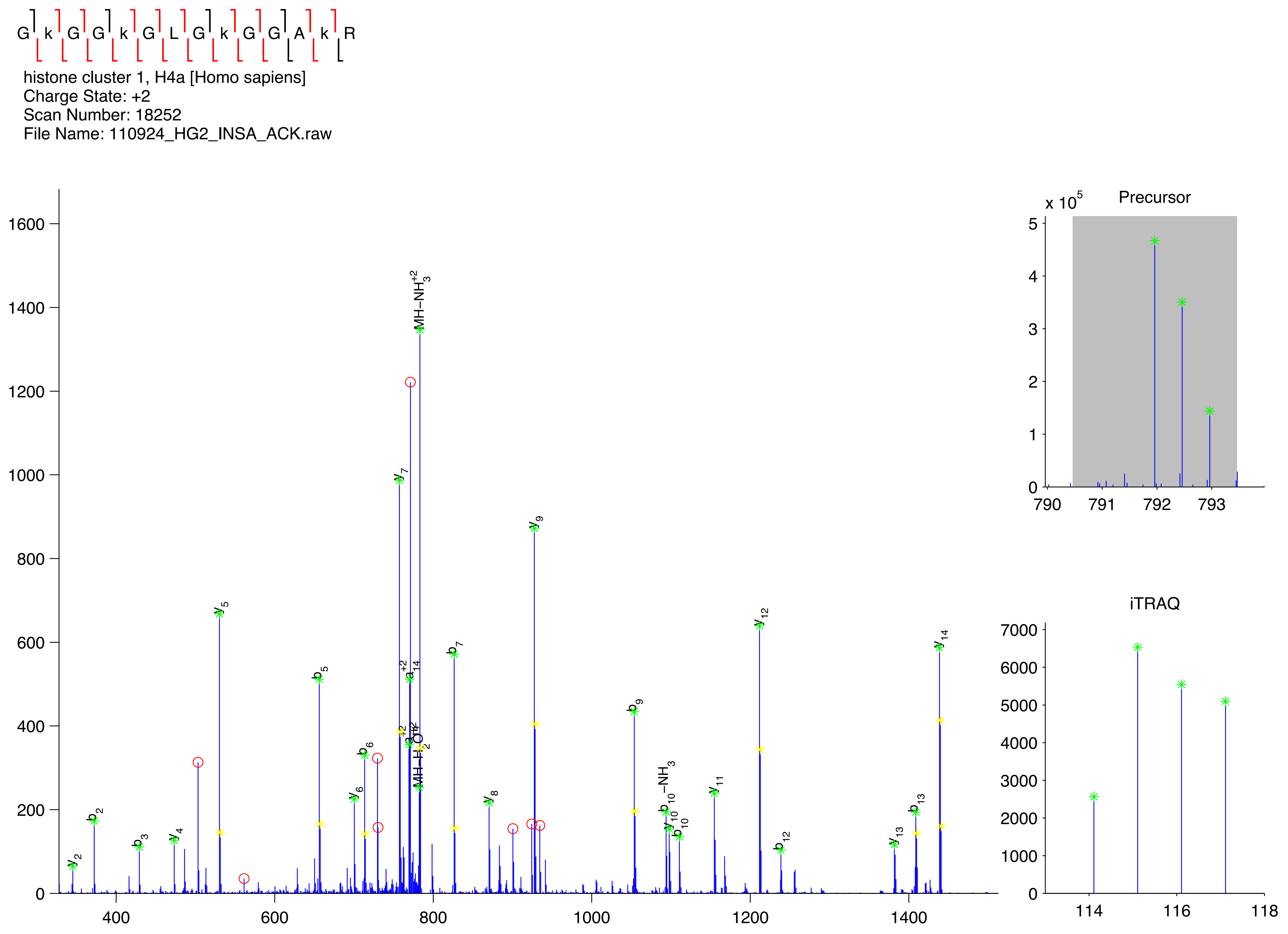
G k G G k G L G k G G A k R
histone cluster 1, H4a [Homo sapiens]
Charge State: +2
Scan Number: 18252
File Name: 110924_HG2_INSA_ACK.raw
Precursor
x 10^5
iTRAQ

G G k G L G k G G A k R

histone cluster 1, H4a [Homo sapiens]
Charge State: +2
Scan Number: 18273
File Name: 110924_HG2_INSA_ACK.raw

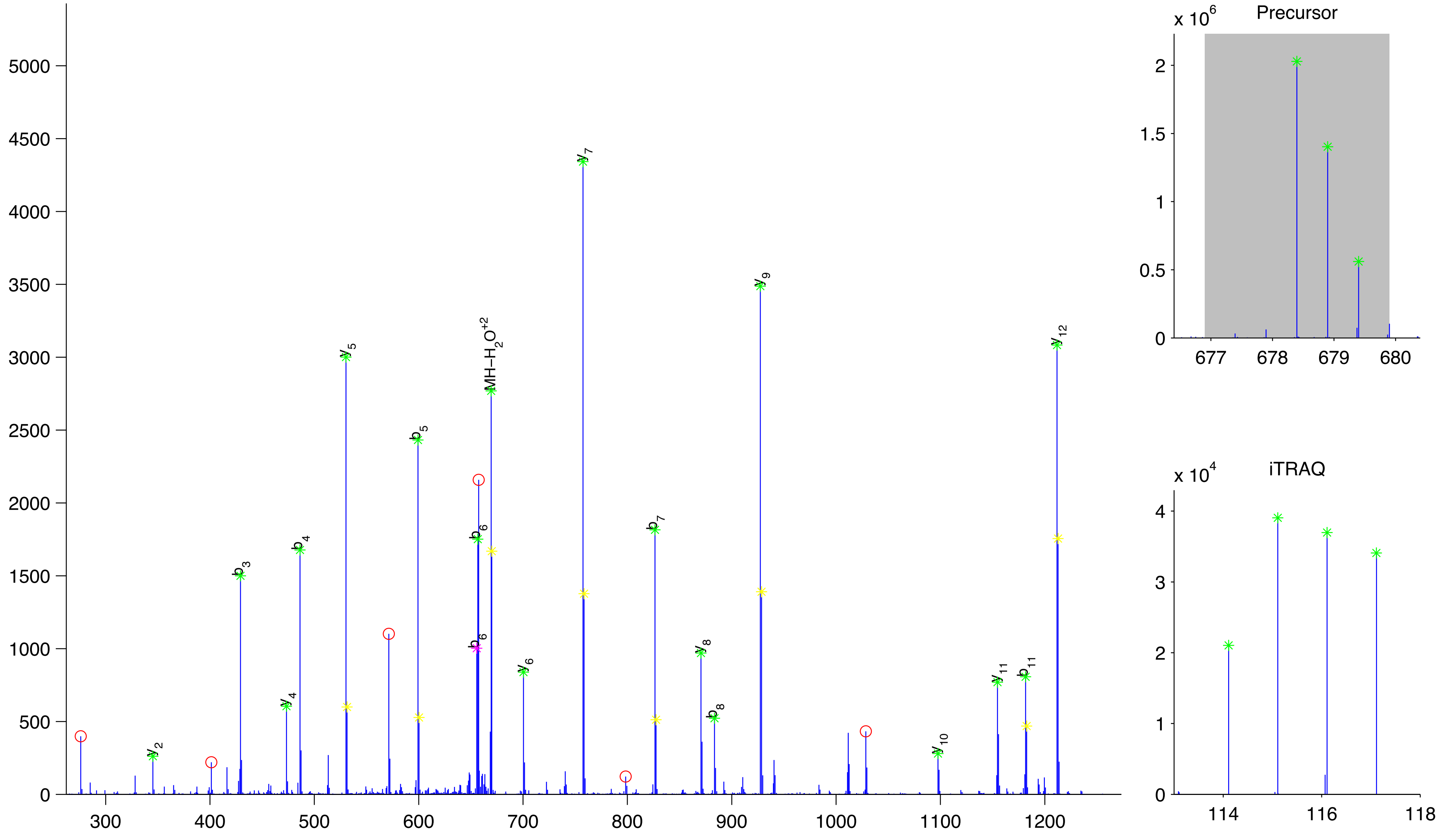

G G k G L G k G G A k R

histone cluster 1, H4a [Homo sapiens]
Charge State: +2
Scan Number: 18283
File Name: 110924_HG2_INSA_ACK.raw

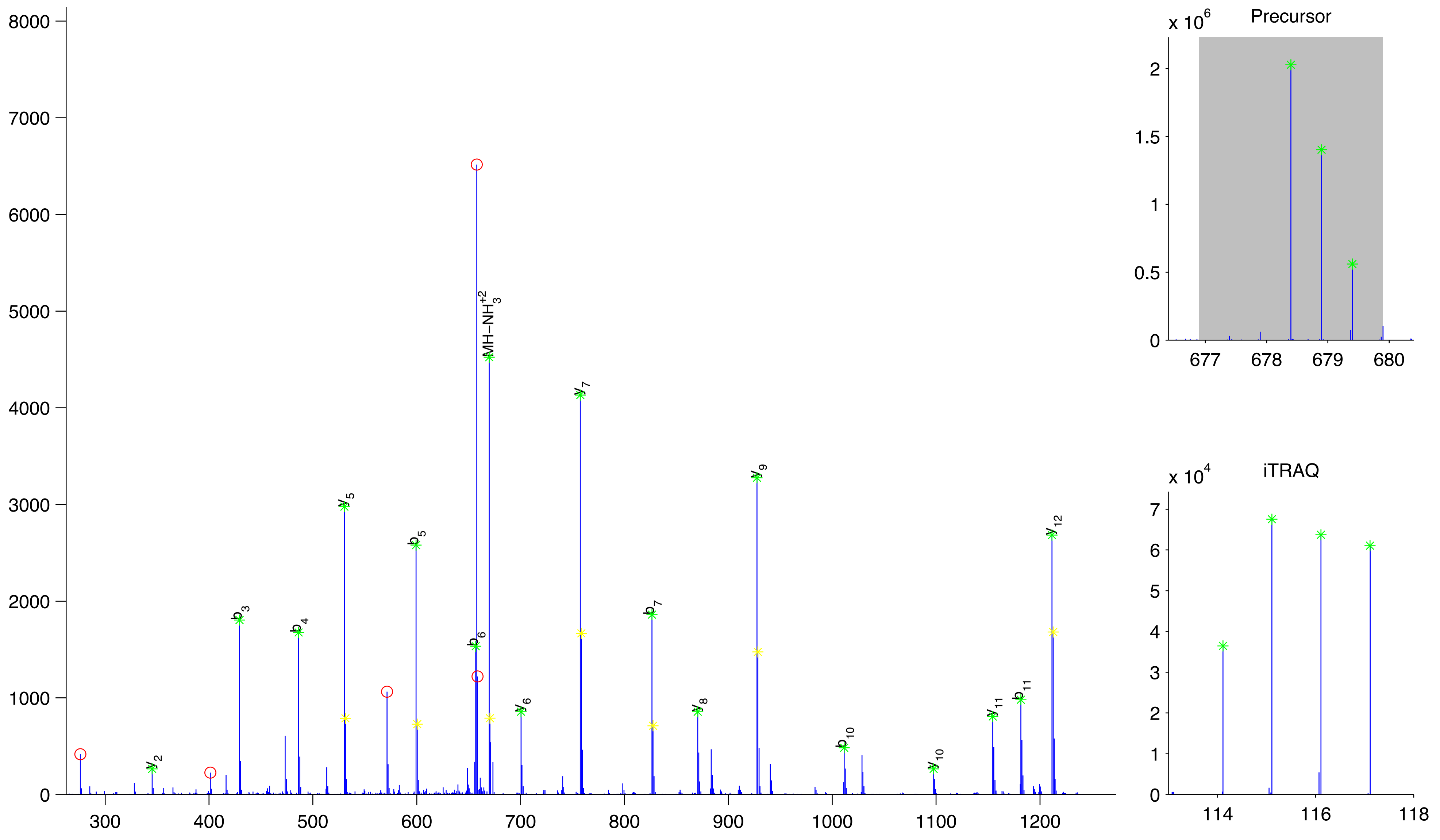

G G k G L G k G G A k R

histone cluster 1, H4a [Homo sapiens]
Charge State: +2
Scan Number: 18340
File Name: 110924_HG2_INSA_ACK3.raw

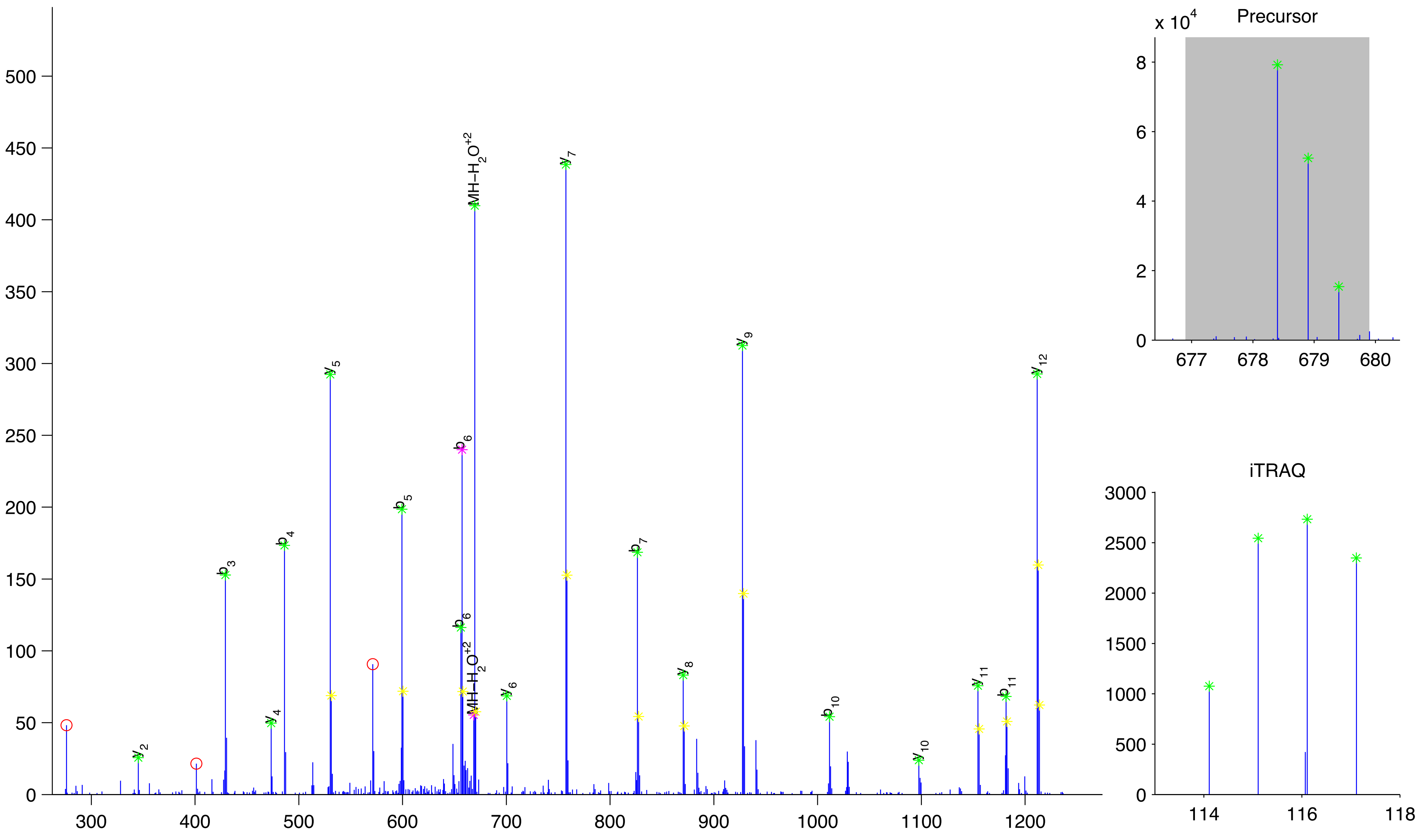

G G k G L G k G G A k R

histone cluster 1, H4a [Homo sapiens]
Charge State: +2
Scan Number: 18411
File Name: 110924_HG2_INSA_ACK3.raw

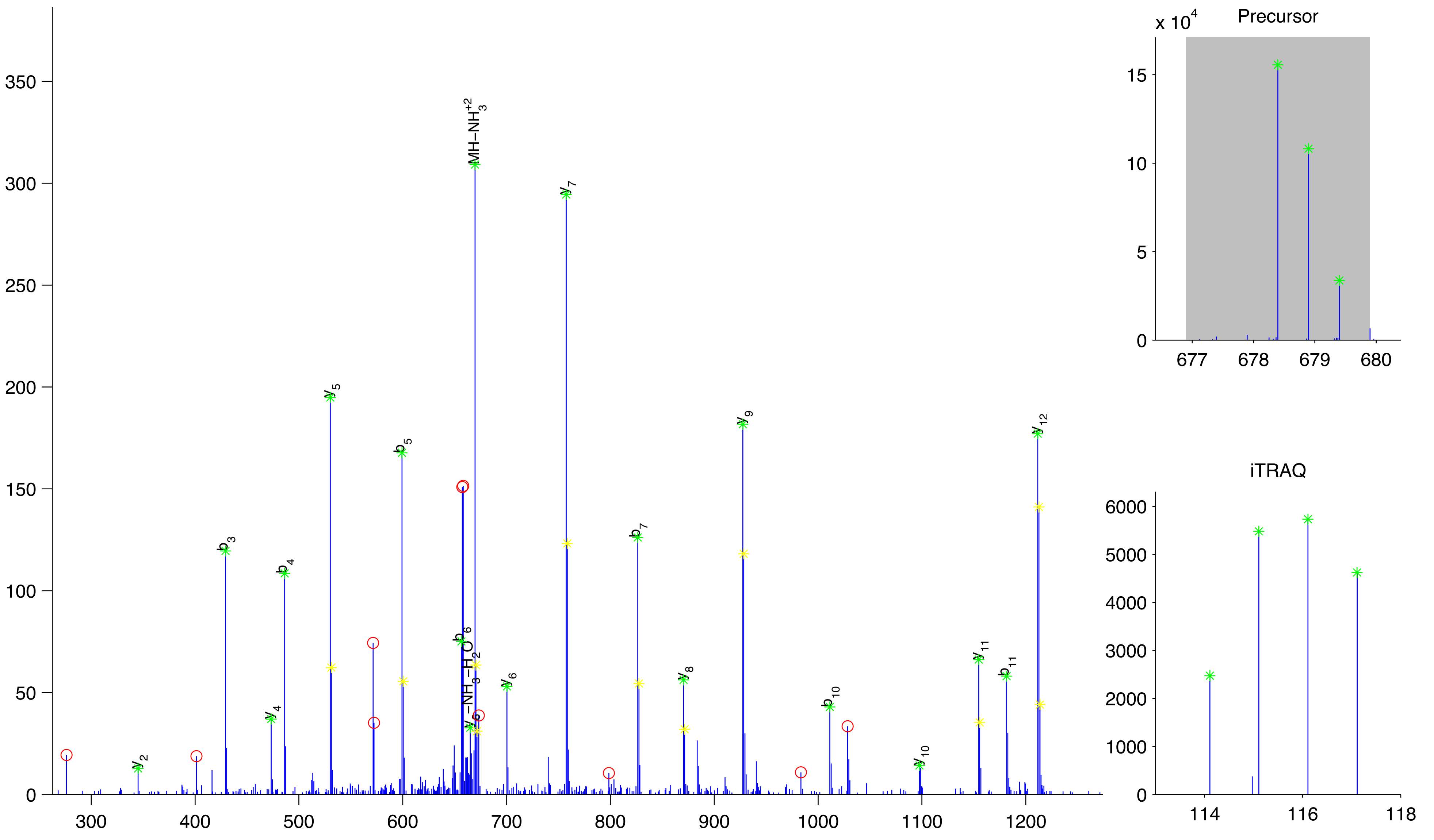

G k G G k G L G k G G A k R

histone cluster 1, H4a [Homo sapiens]
Charge State: +3
Scan Number: 18420
File Name: 110924_HG2_INSA_ACK3.raw

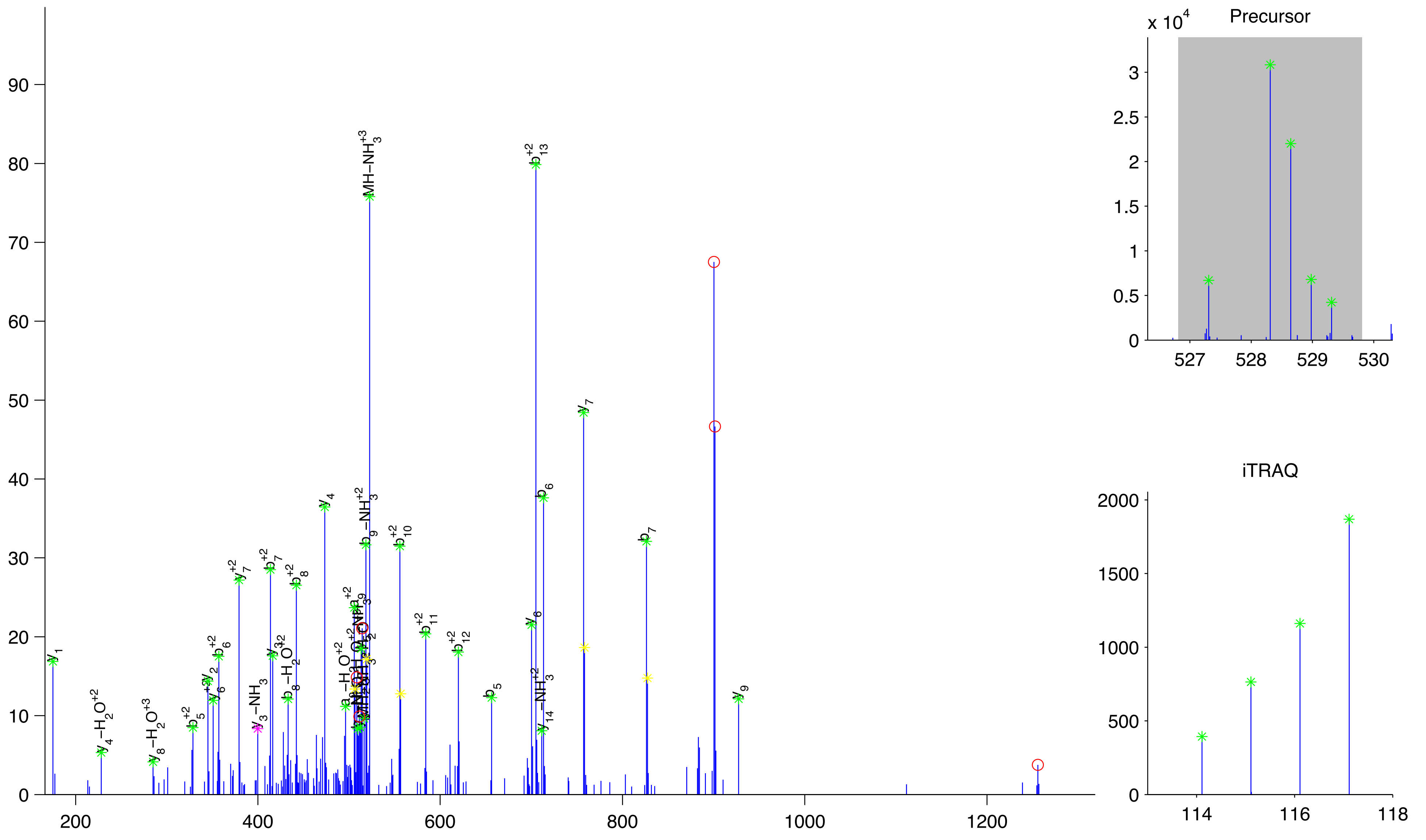

G k G G k G L G k G G A k R

histone cluster 1, H4a [Homo sapiens]
Charge State: +2
Scan Number: 18420
File Name: 110924_HG2_INSA_ACK.raw

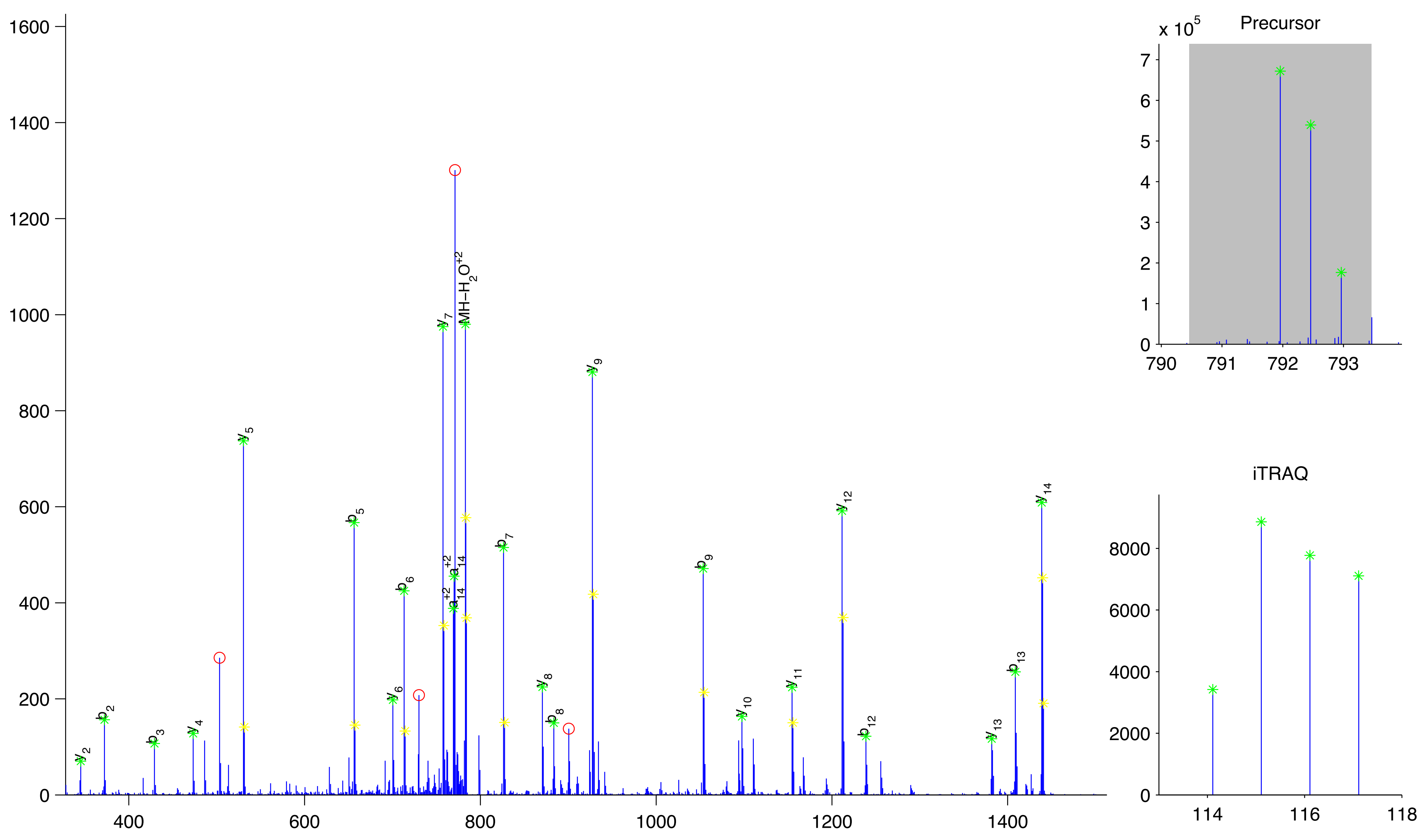

G k G G k G L G k G G A k R

histone cluster 1, H4a [Homo sapiens]
Charge State: +2
Scan Number: 18438
File Name: 110924_HG2_INSA_ACK.raw

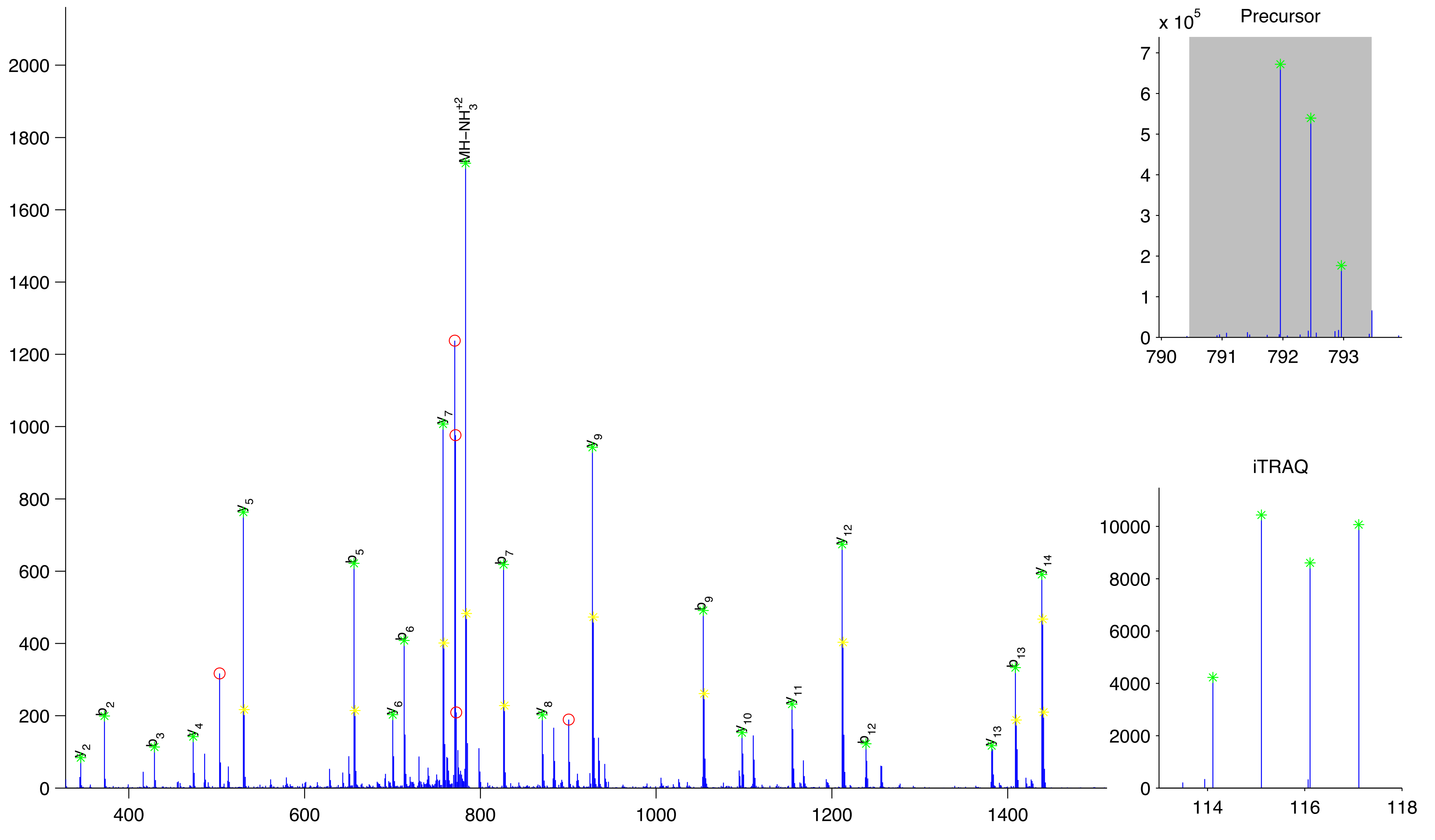

G G k G L G k G G A k R

histone cluster 1, H4a [Homo sapiens]
Charge State: +2
Scan Number: 18441
File Name: 110924_HG2_INSA_ACK.raw

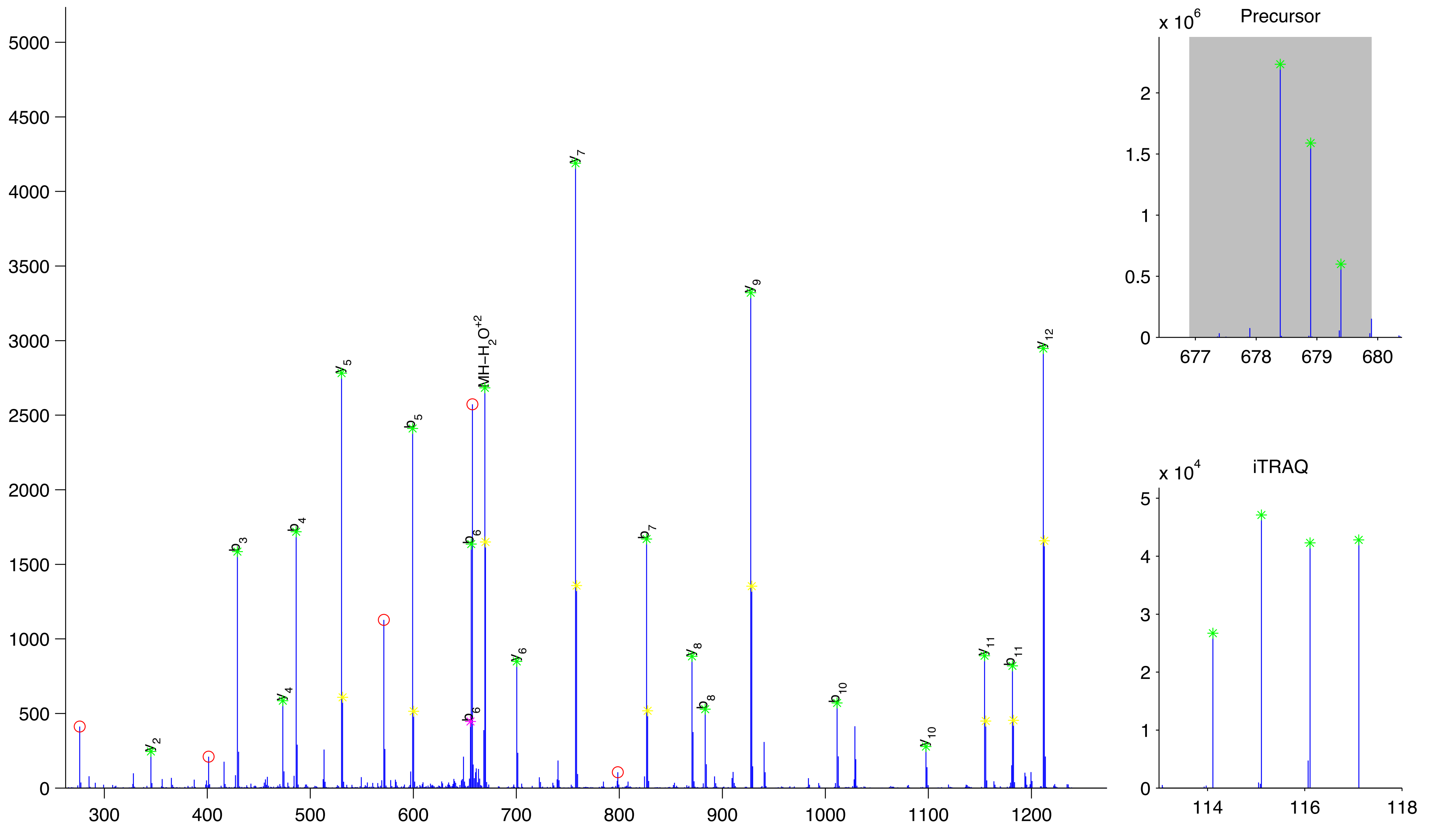

G G k G L G k G G A k R

histone cluster 1, H4a [Homo sapiens]
Charge State: +2
Scan Number: 18449
File Name: 110924_HG2_INSA_ACK.raw

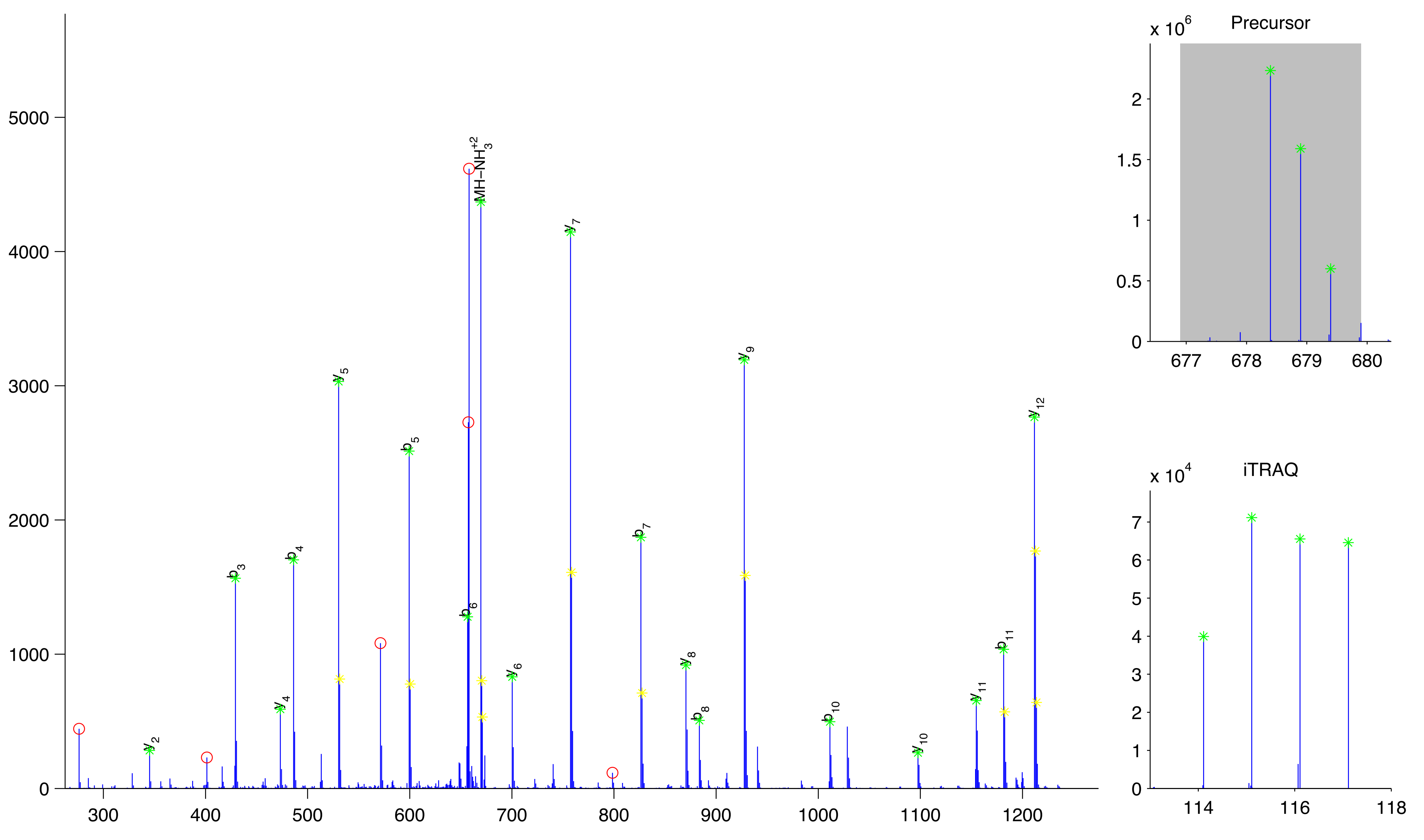

G k G G k G L G k G G A k R

histone cluster 1, H4a [Homo sapiens]
Charge State: +2
Scan Number: 18588
File Name: 110924_HG2_INSA_ACK.raw

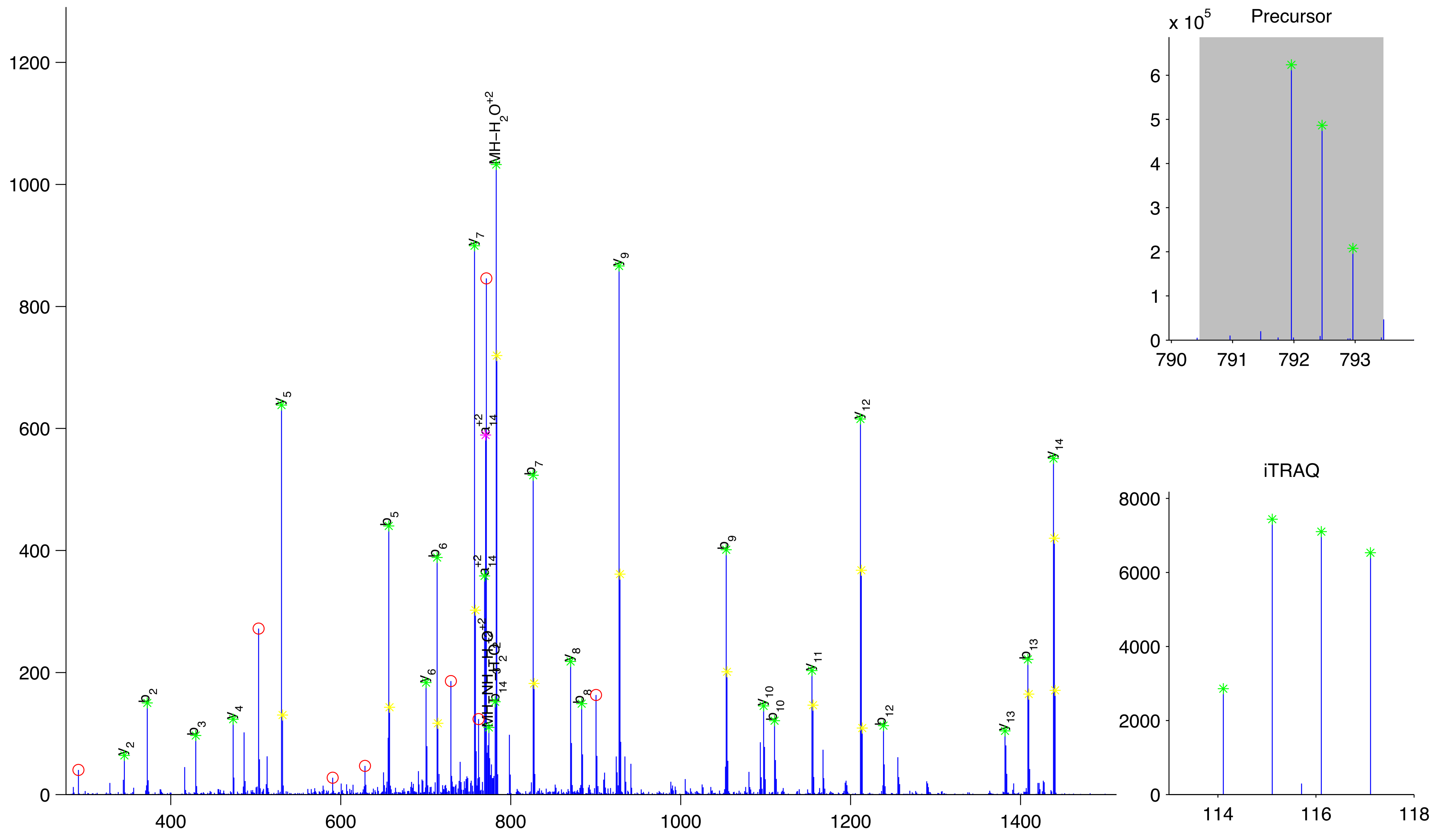

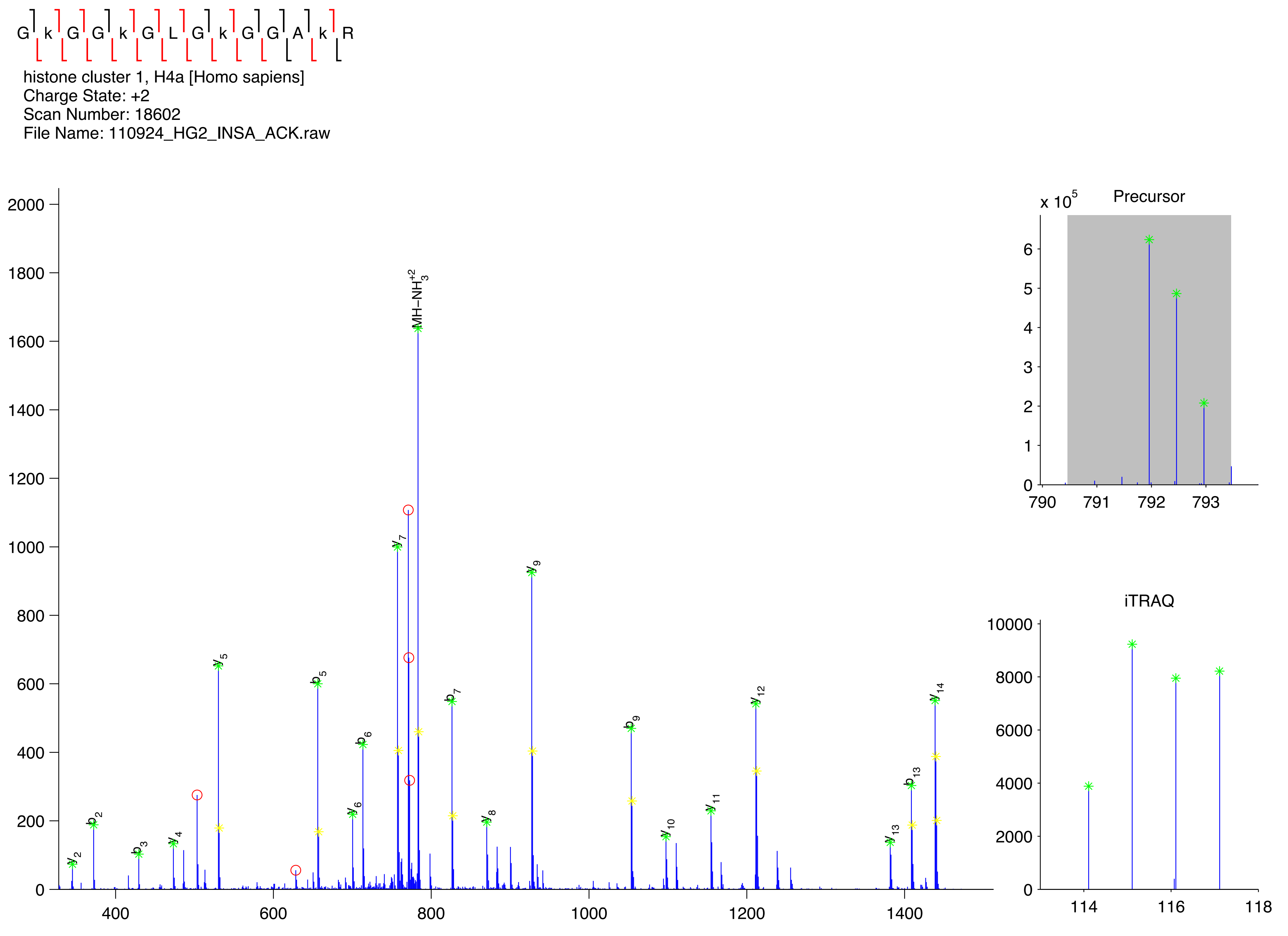
G k G G k G L G k G G A k R
histone cluster 1, H4a [Homo sapiens]
Charge State: +2
Scan Number: 18602
File Name: 110924_HG2_INSA_ACK.raw
MH−NH3+2
y7
y9
y5
b5
b6
y6
b7
y8
b9
y10
y11
y12
y13
b13
y14
y2
b2
b3
y4
Precursor
x 10^5
iTRAQ

G G k G L G k G G A k R

histone cluster 1, H4a [Homo sapiens]
Charge State: +2
Scan Number: 18609
File Name: 110924_HG2_INSA_ACK.raw

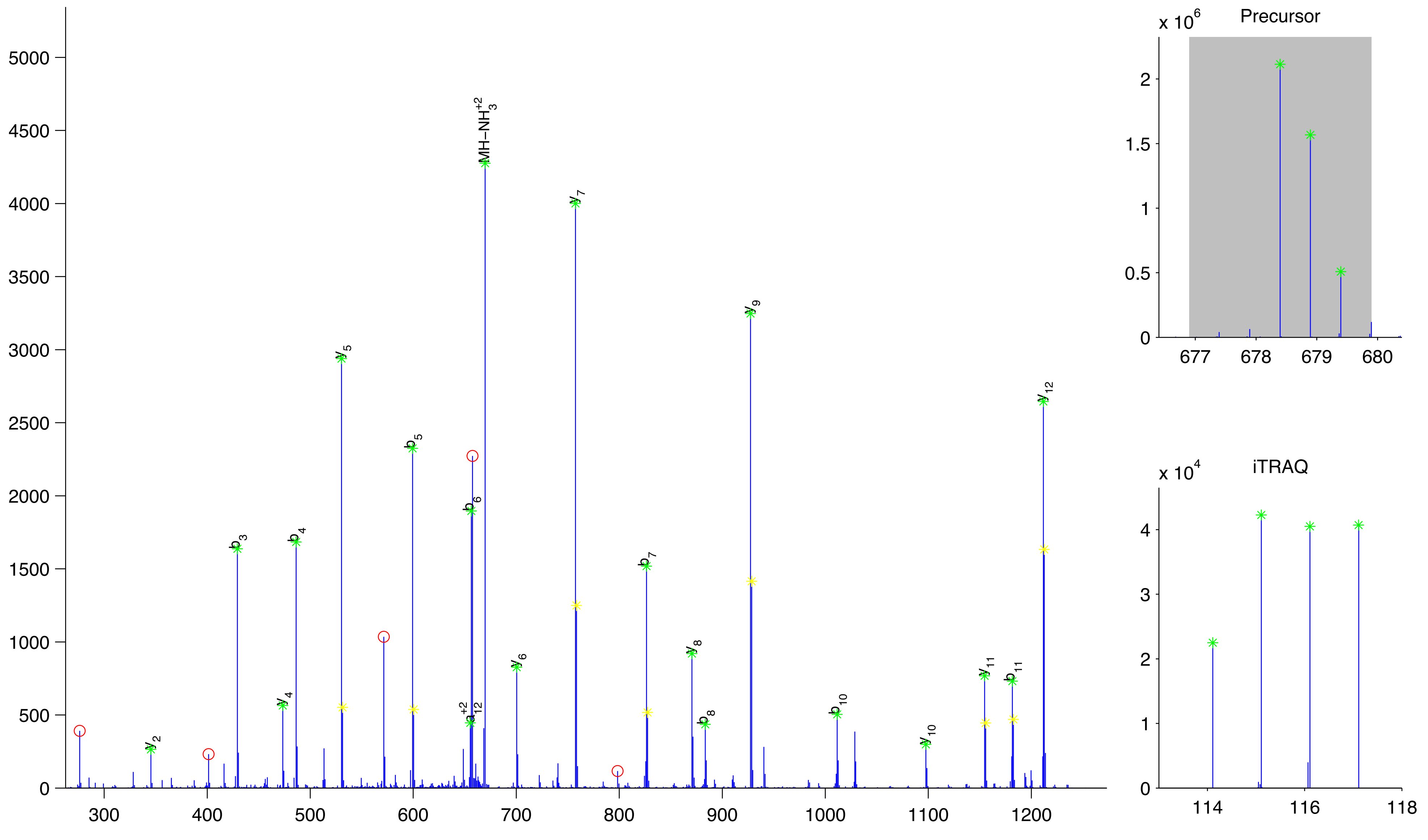

G G k G L G k G G A k R

histone cluster 1, H4a [Homo sapiens]
Charge State: +2
Scan Number: 18617
File Name: 110924_HG2_INSA_ACK.raw

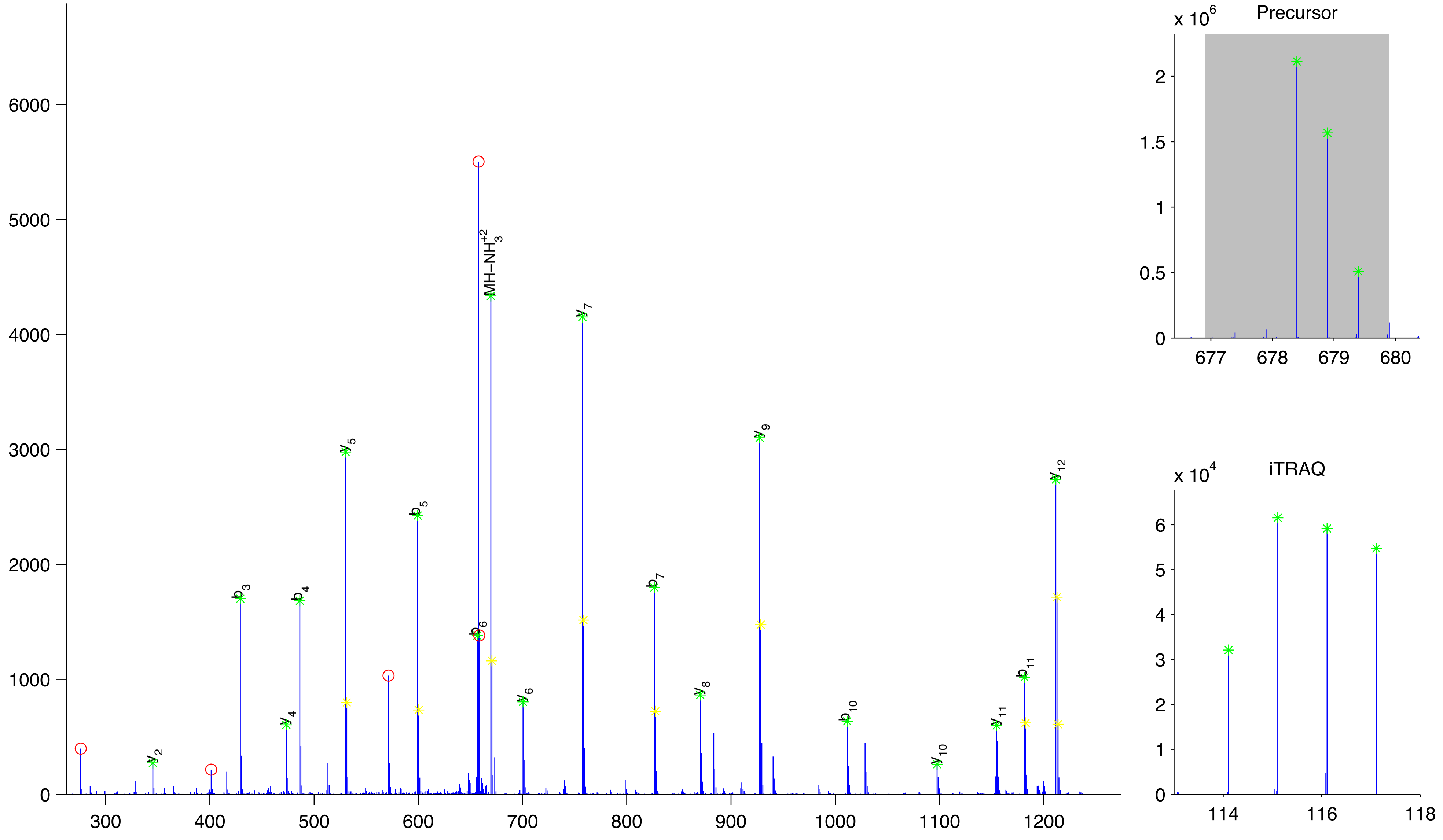

G L G k G G A k R

histone cluster 1, H4a [Homo sapiens]
Charge State: +2
Scan Number: 18697
File Name: 110924_HG2_INSA_ACK3.raw

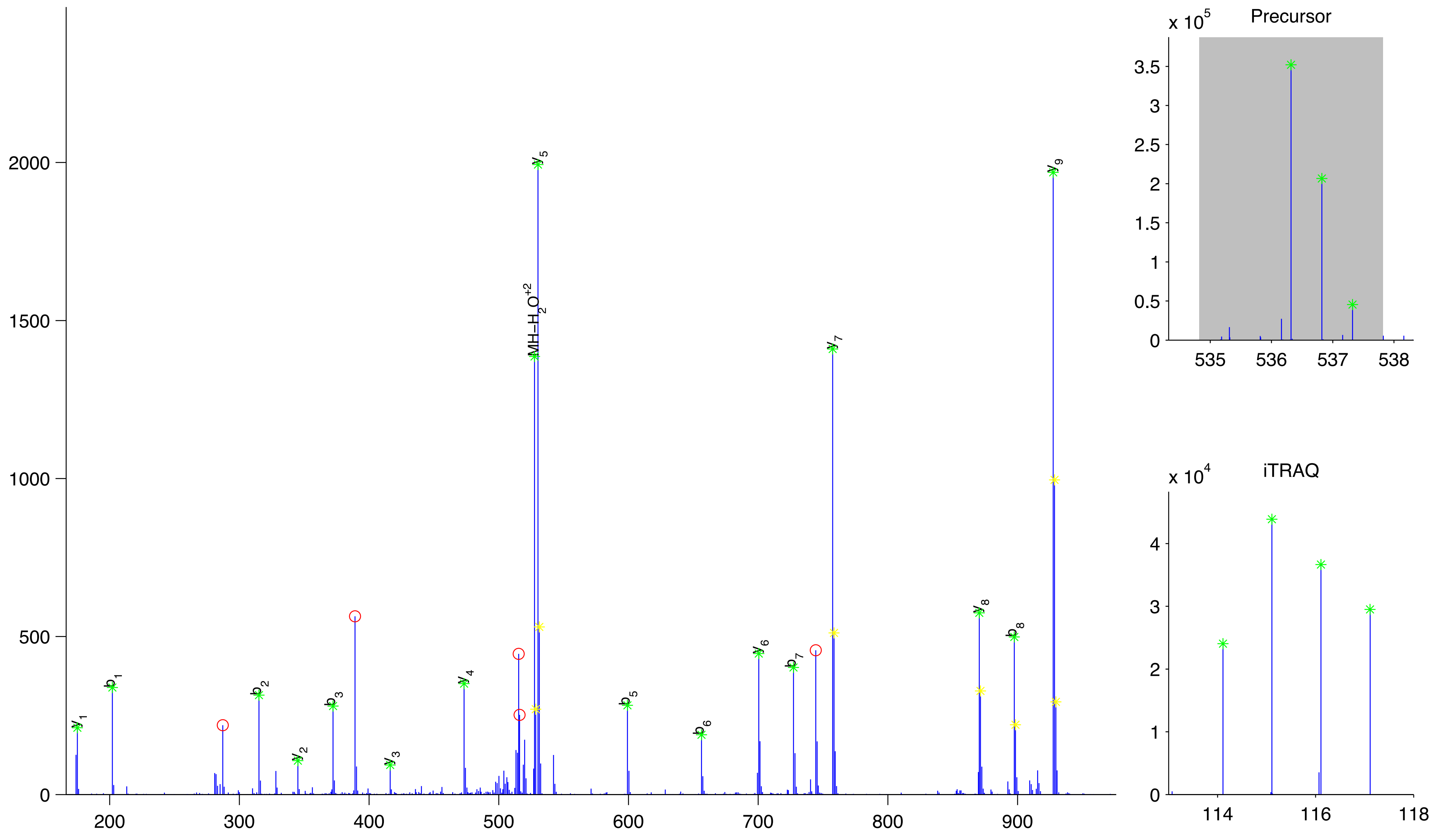

G k G G k G L G k G G A k R

histone cluster 1, H4a [Homo sapiens]
Charge State: +2
Scan Number: 18756
File Name: 110924_HG2_INSA_ACK.raw

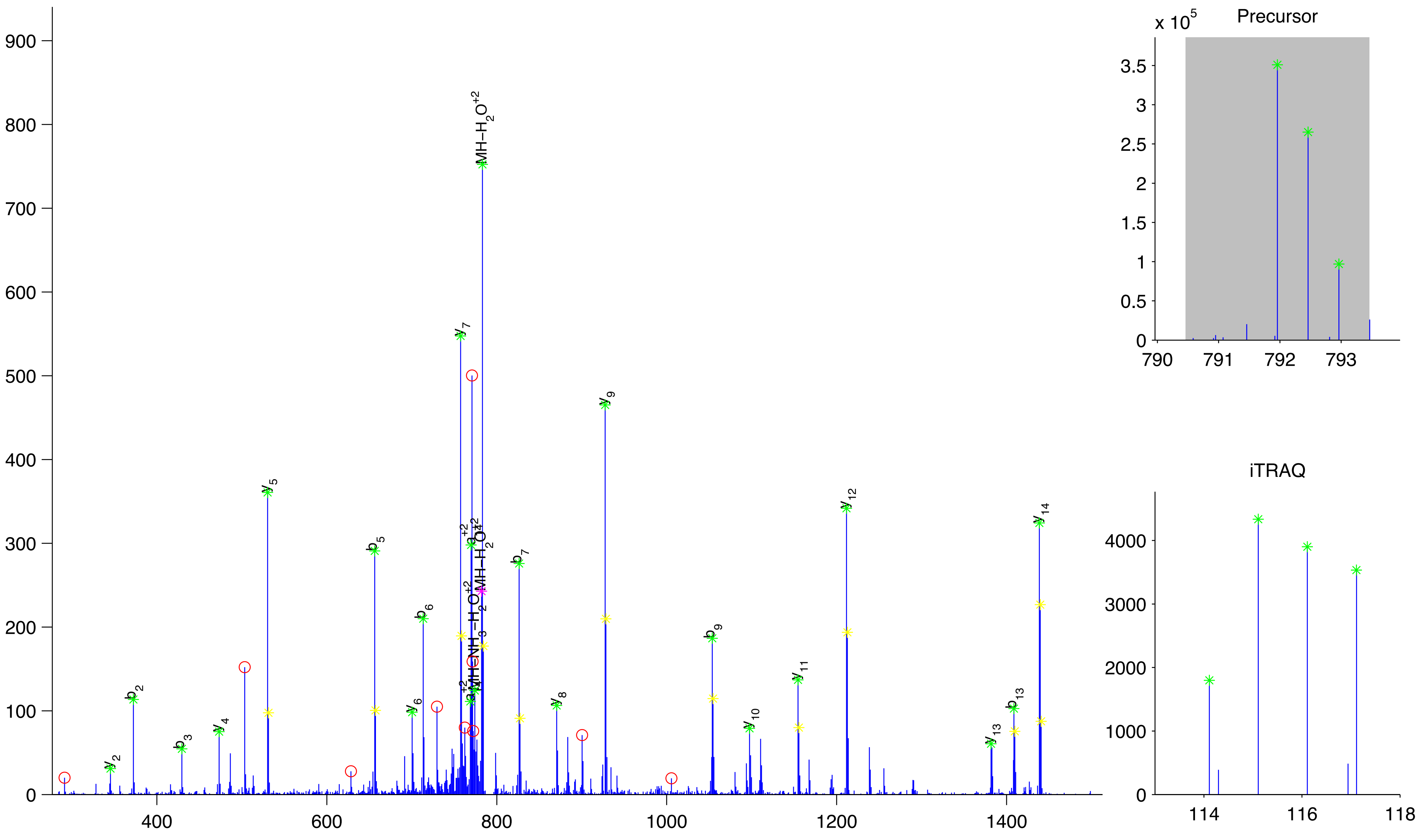

G k G G k G L G k G G A k R

histone cluster 1, H4a [Homo sapiens]
Charge State: +3
Scan Number: 18758
File Name: 110924_HG2_INSA_ACK3.raw

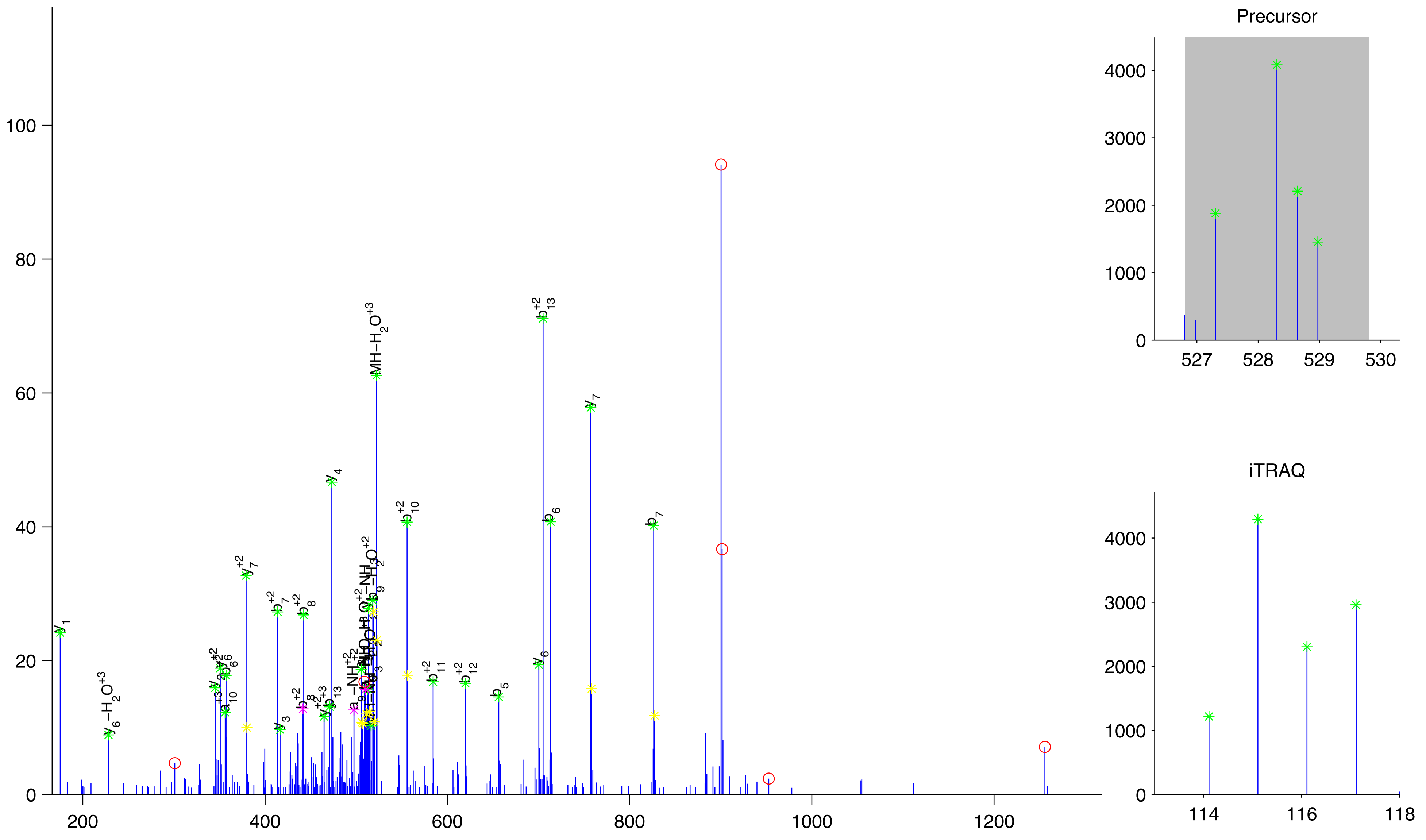

G k G G k G L G k G G A k R

histone cluster 1, H4a [Homo sapiens]
Charge State: +2
Scan Number: 18768
File Name: 110924_HG2_INSA_ACK.raw

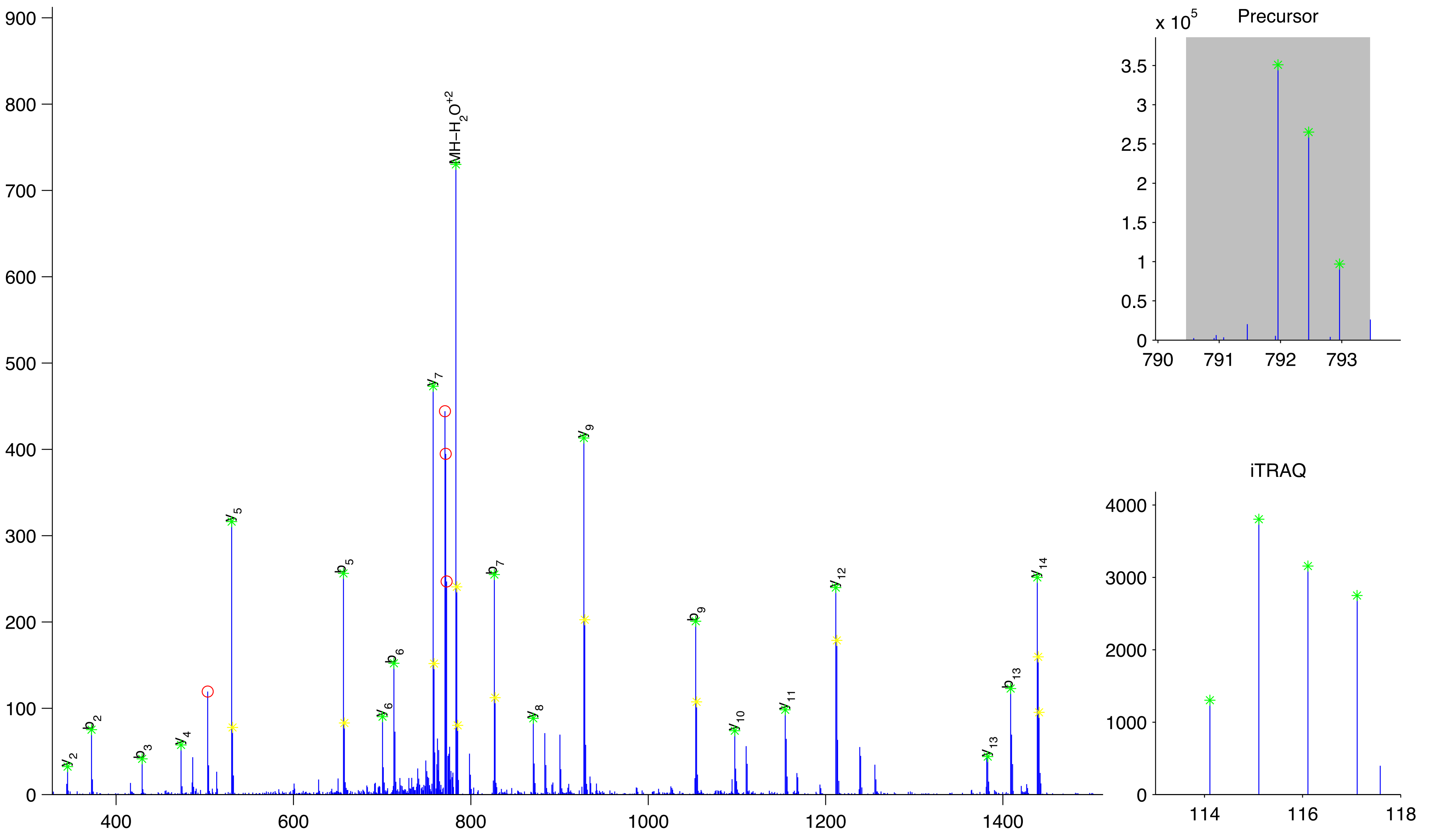

G G k G L G k G G A k R

histone cluster 1, H4a [Homo sapiens]
Charge State: +2
Scan Number: 18777
File Name: 110924_HG2_INSA_ACK.raw

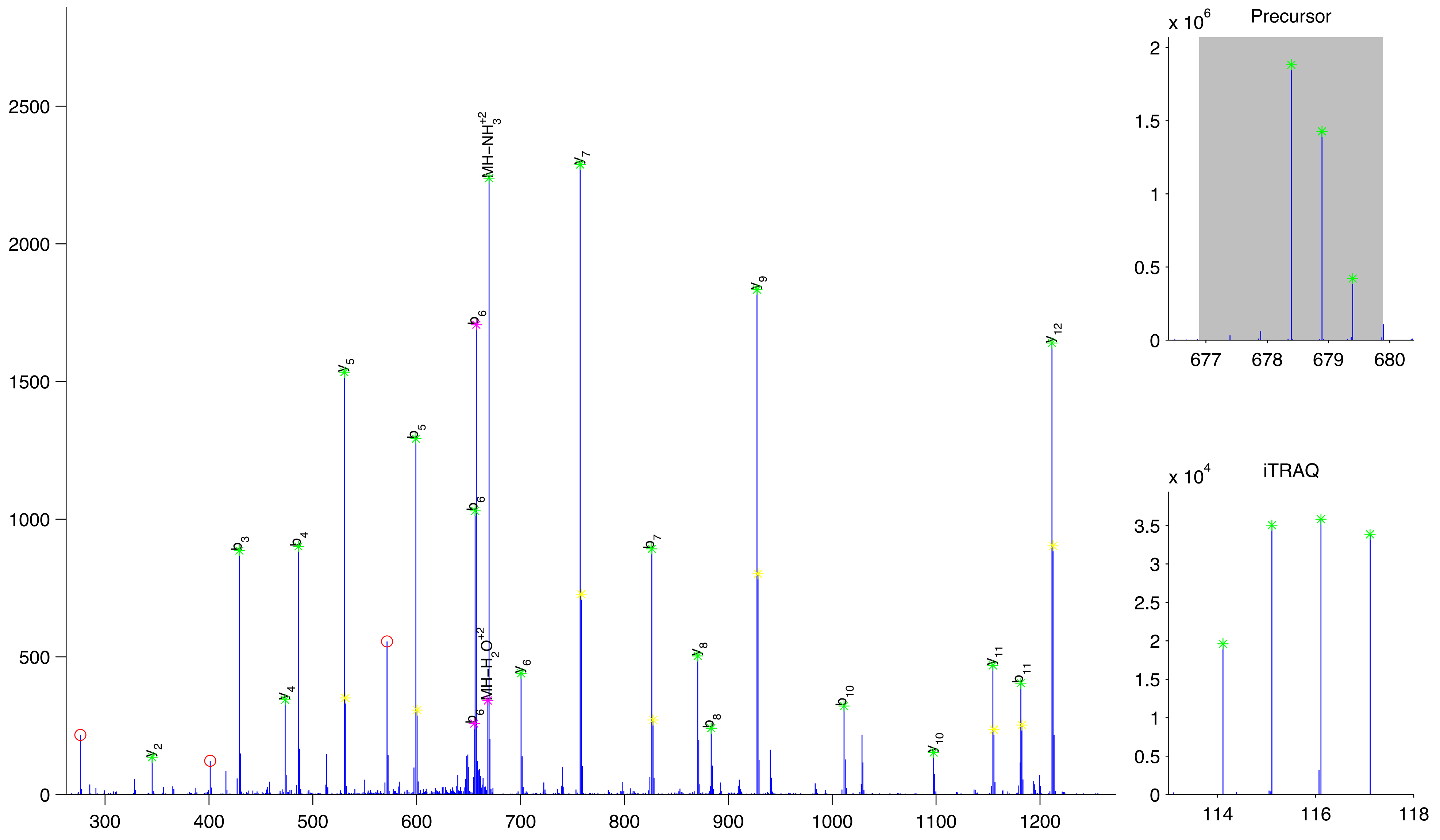

G G k G L G k G G A k R

histone cluster 1, H4a [Homo sapiens]
Charge State: +2
Scan Number: 18787
File Name: 110924_HG2_INSA_ACK.raw

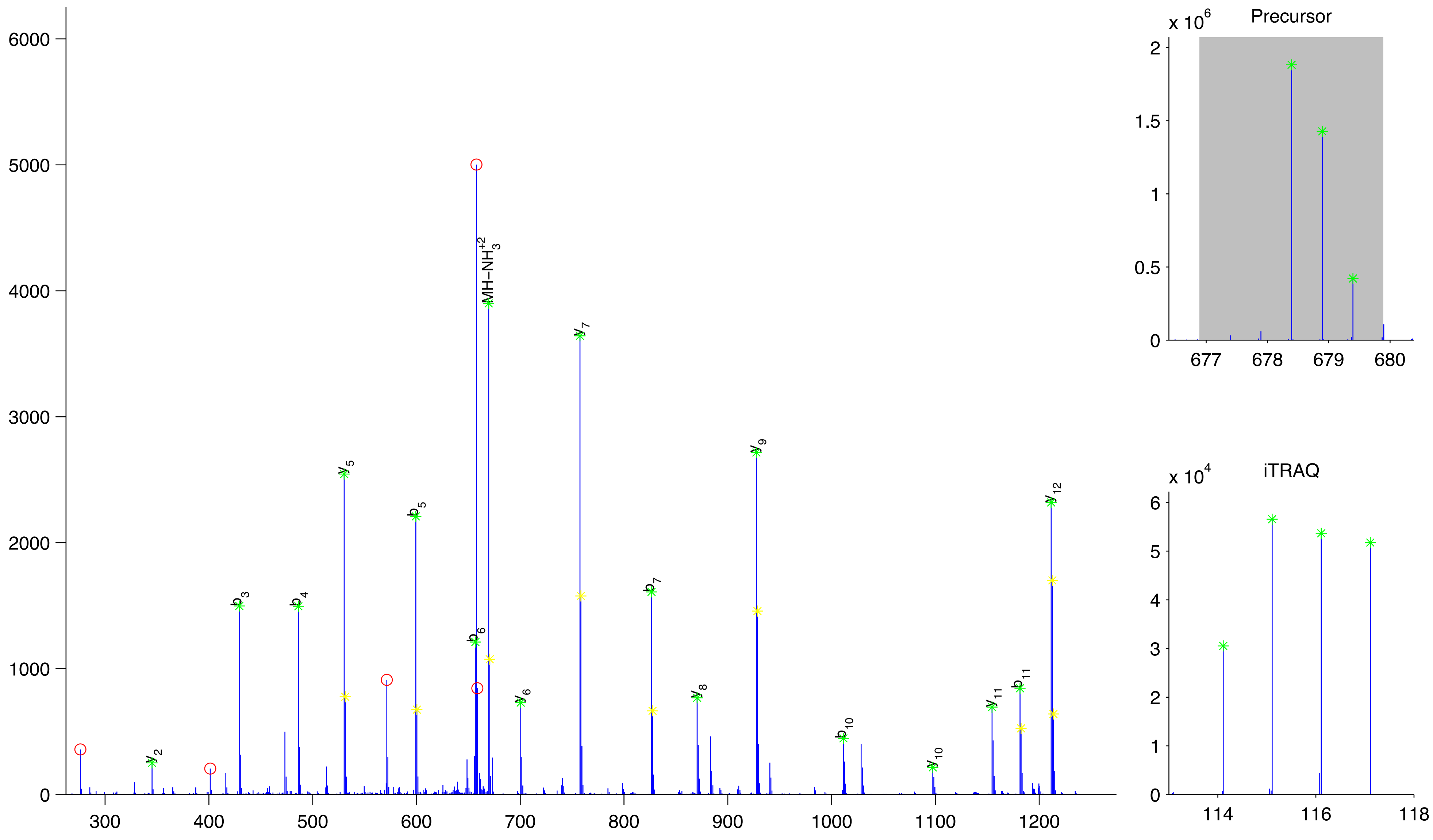

G k G G k G L G k G G A k R

histone cluster 1, H4a [Homo sapiens]
Charge State: +3
Scan Number: 18852
File Name: 110924_HG2_INSA_ACK.raw

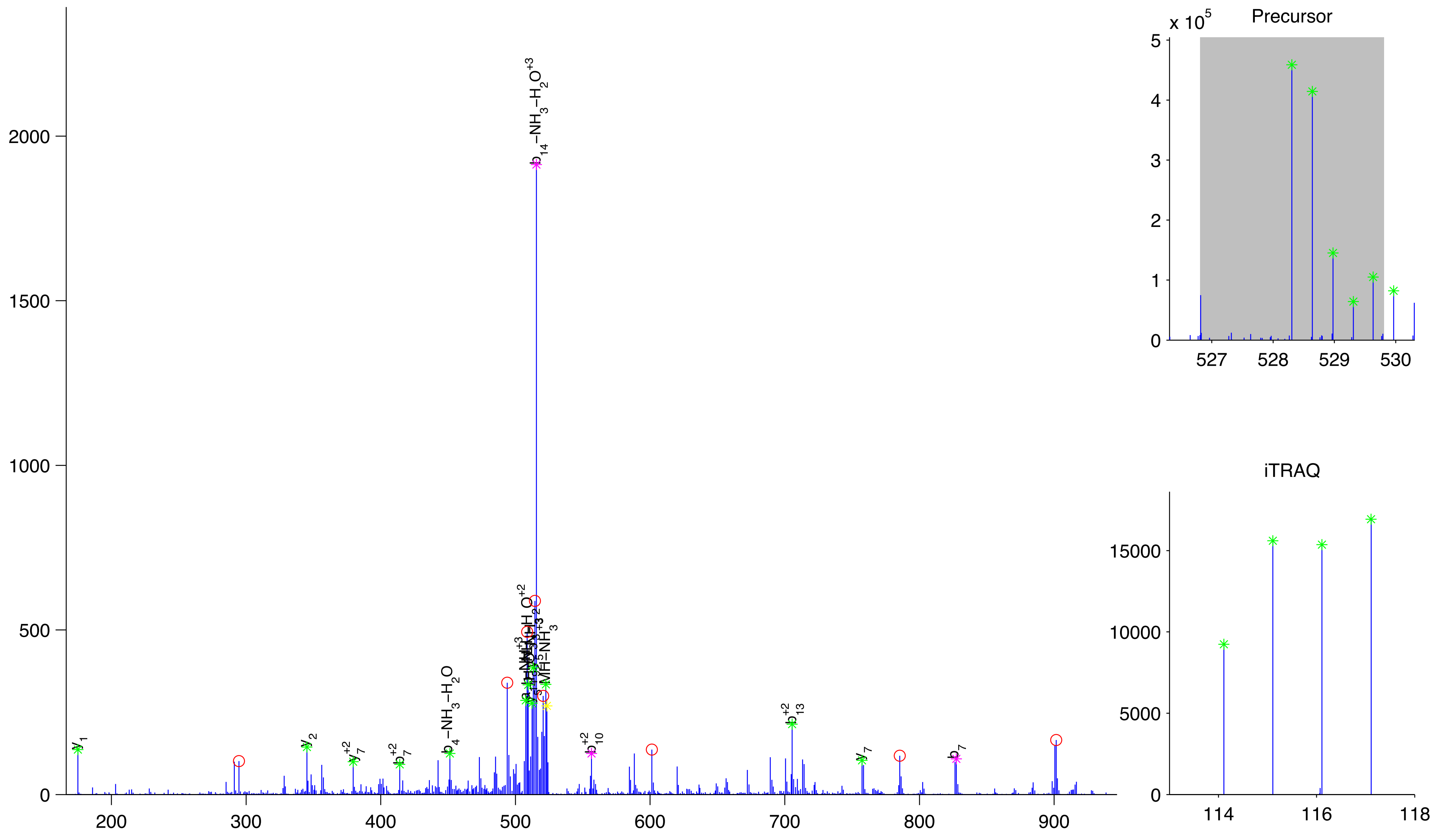

G L G k G G A k R

histone cluster 1, H4a [Homo sapiens]
Charge State: +2
Scan Number: 18880
File Name: 110924_HG2_INSA_ACK3.raw

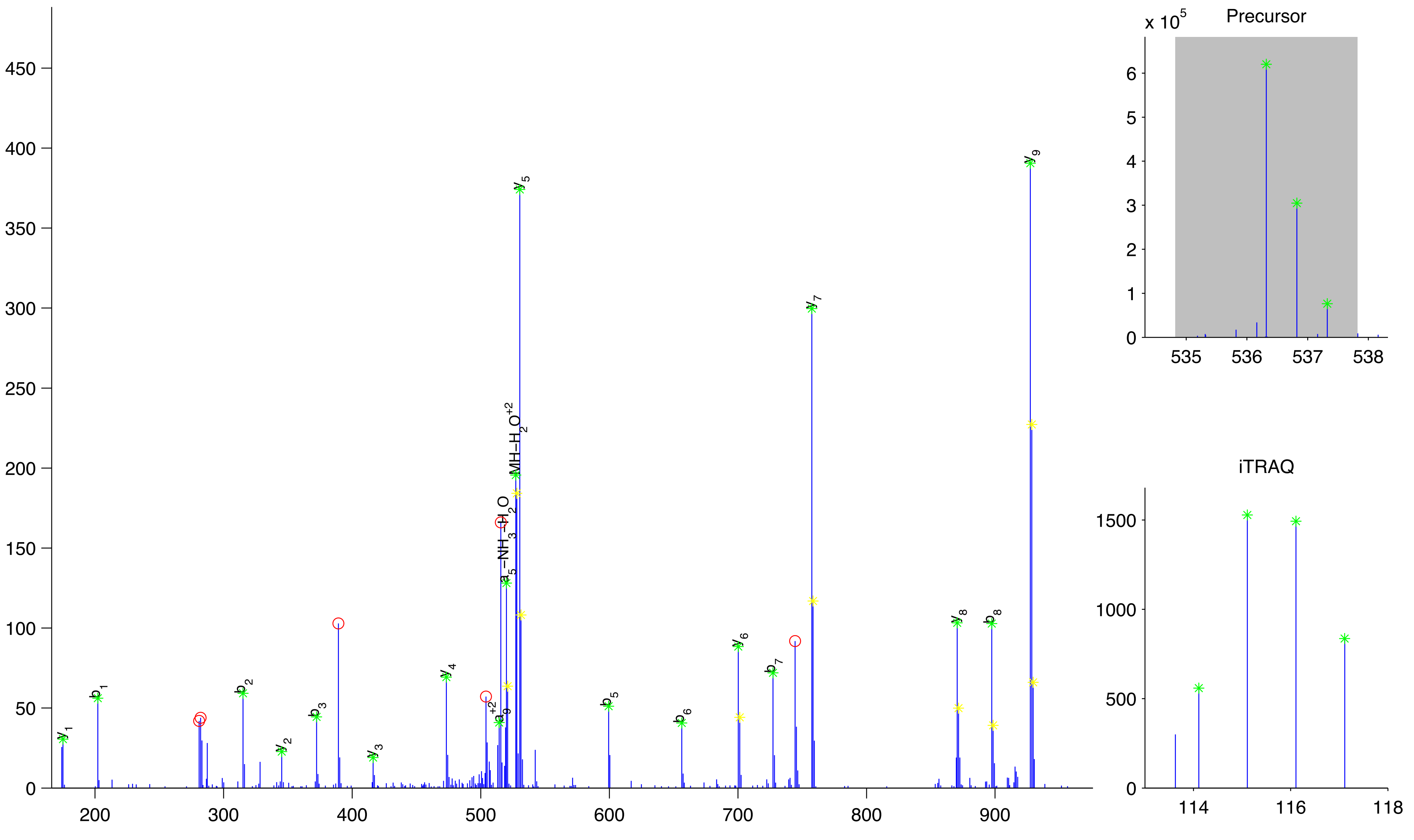

G L G k G G A k R

histone cluster 1, H4a [Homo sapiens]
Charge State: +2
Scan Number: 18899
File Name: 110924_HG2_INSA_ACK3.raw

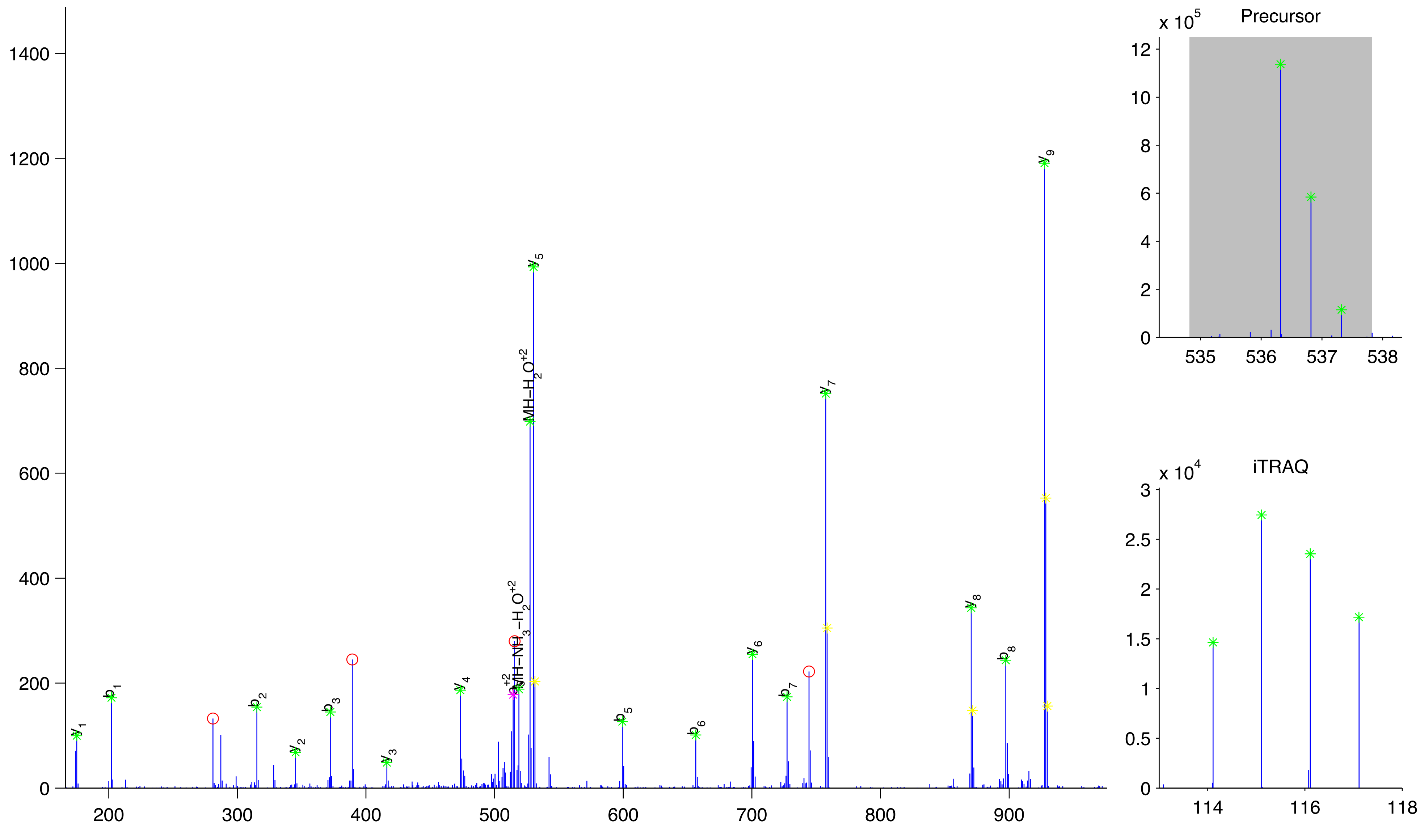

G k G G k G L G k G G A k R

histone cluster 1, H4a [Homo sapiens]
Charge State: +2
Scan Number: 18926
File Name: 110924_HG2_INSA_ACK.raw

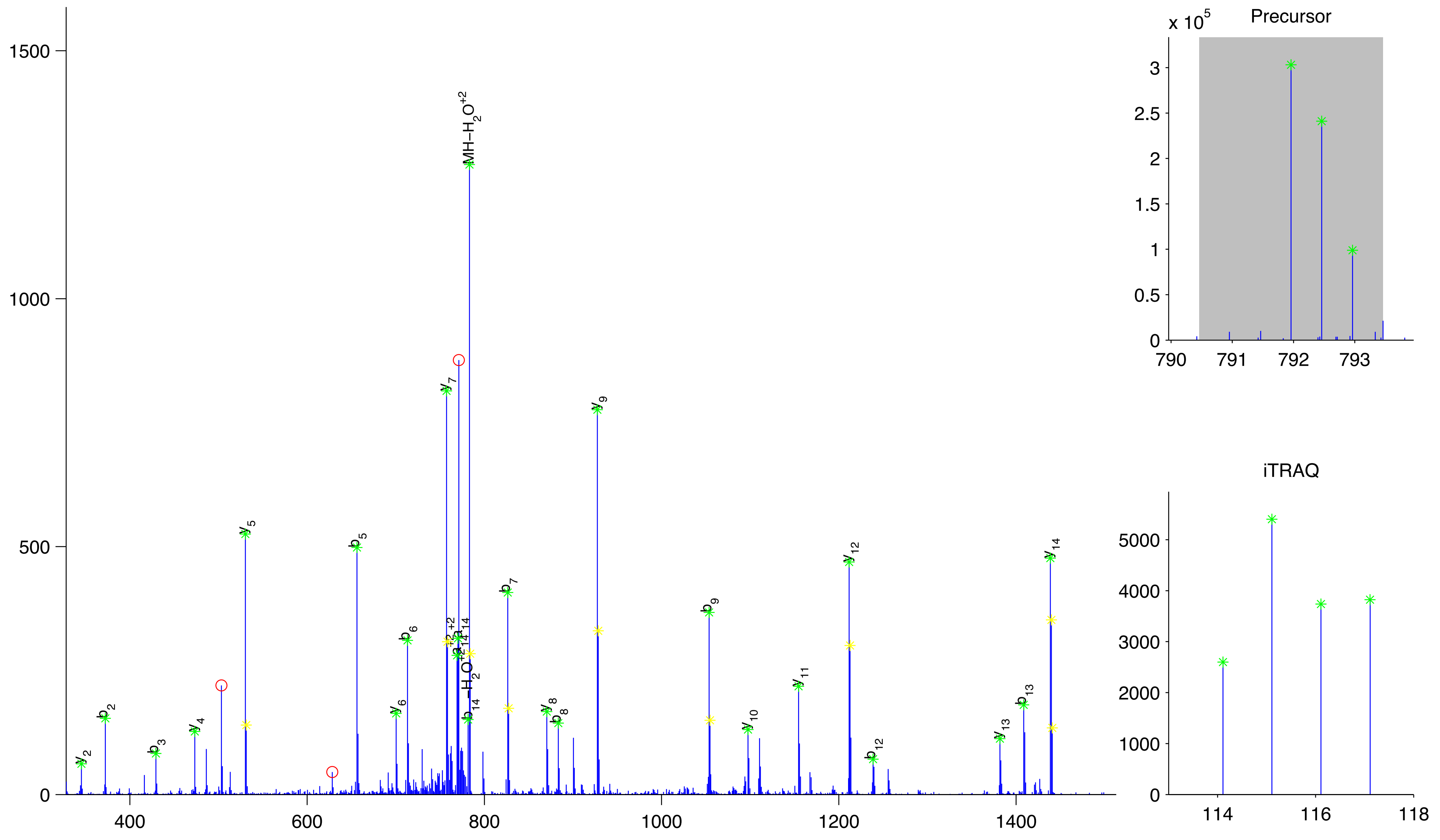

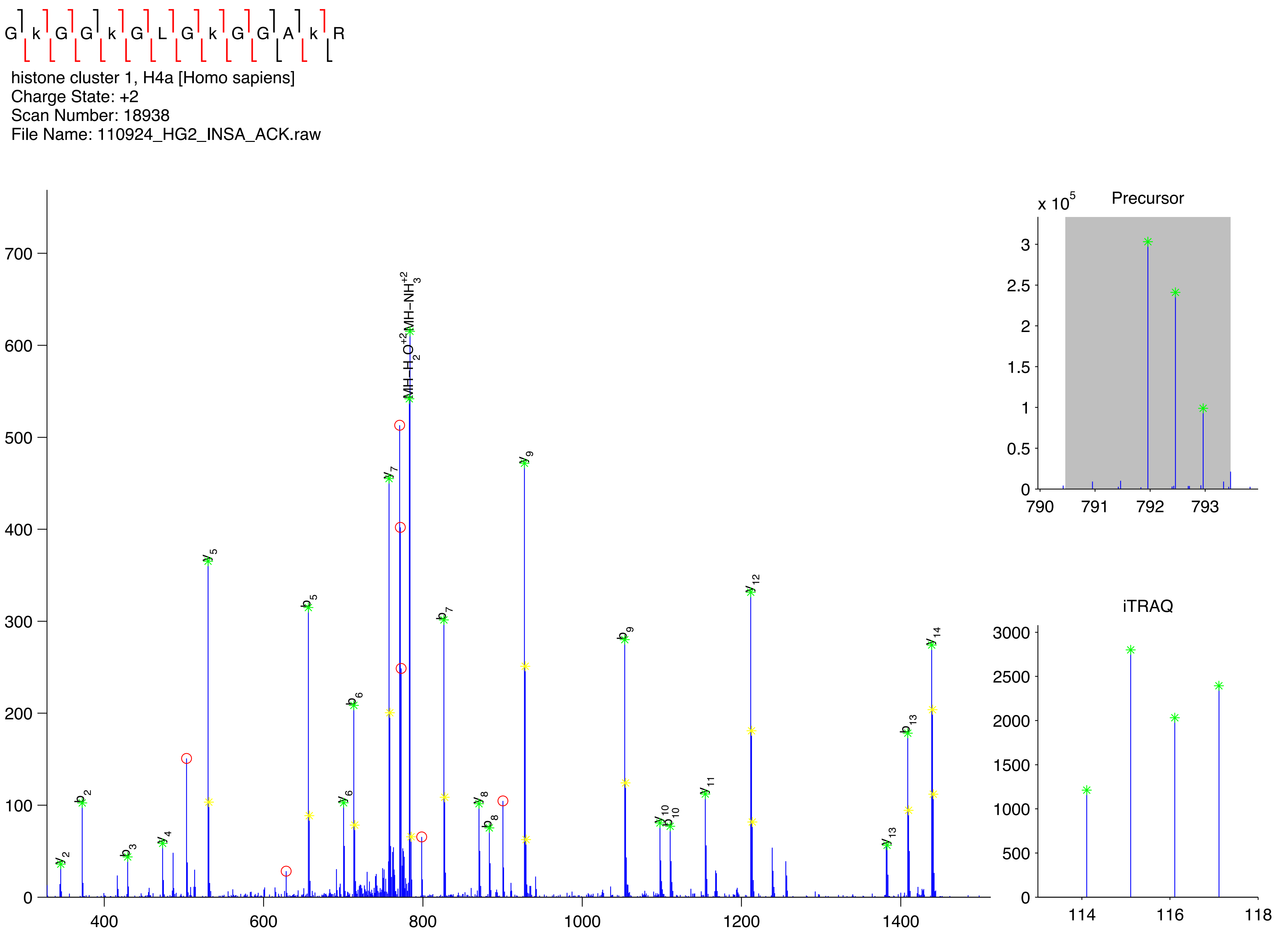
G k G G k G L G k G G A k R
histone cluster 1, H4a [Homo sapiens]
Charge State: +2
Scan Number: 18938
File Name: 110924_HG2_INSA_ACK.raw
Precursor
x 10^5
iTRAQ
MH−H2O+2
MH−NH3+2

G k G G k G L G k G G A k R

histone cluster 1, H4a [Homo sapiens]
Charge State: +2
Scan Number: 19094
File Name: 110924_HG2_INSA_ACK.raw

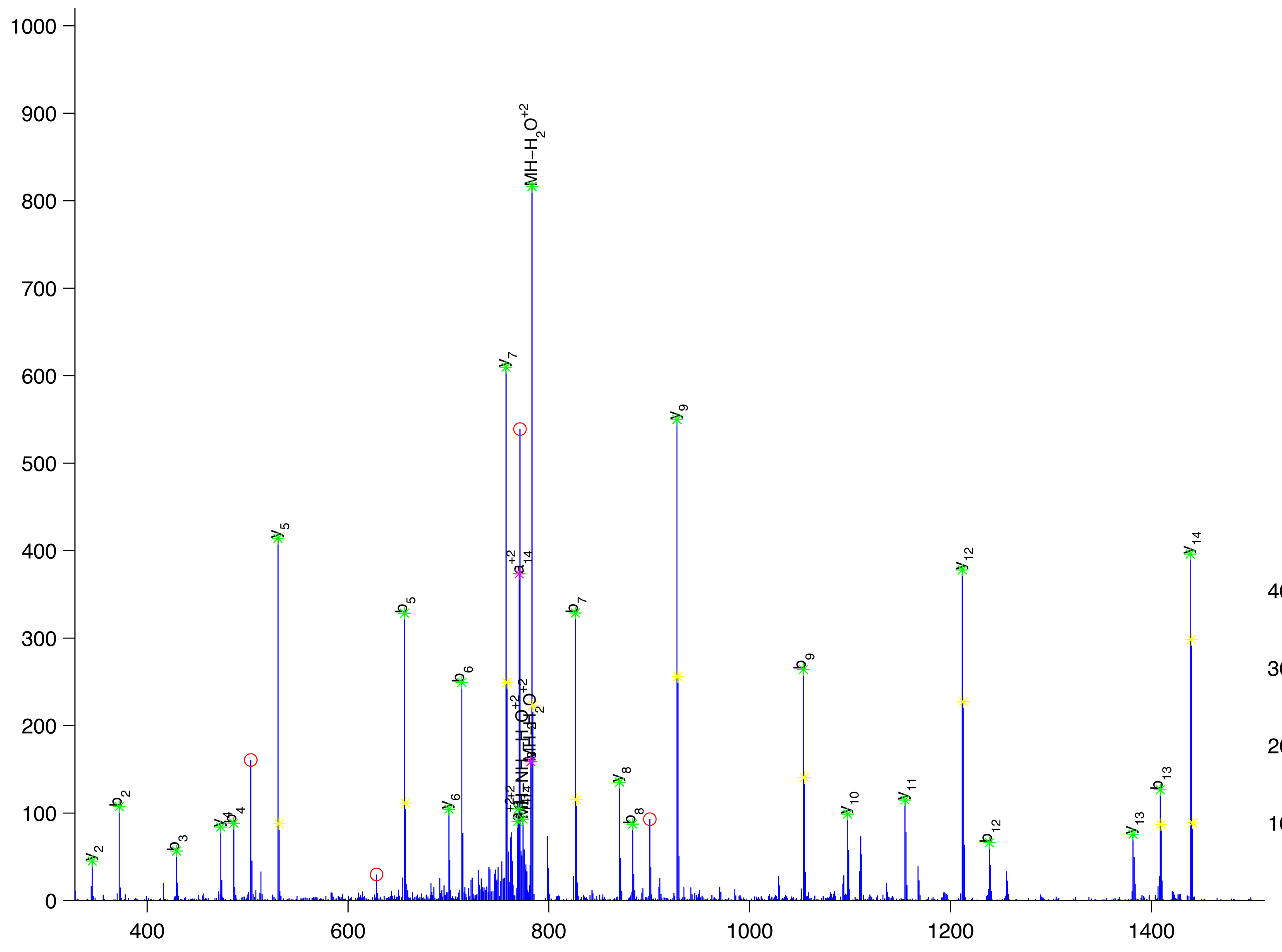

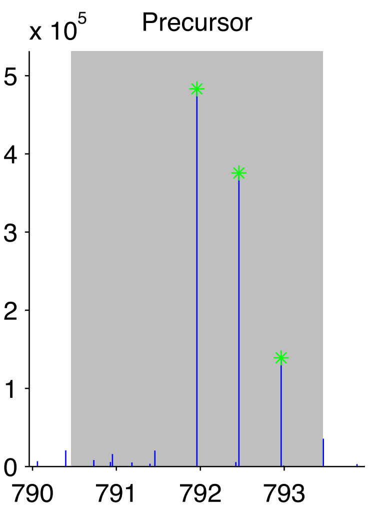

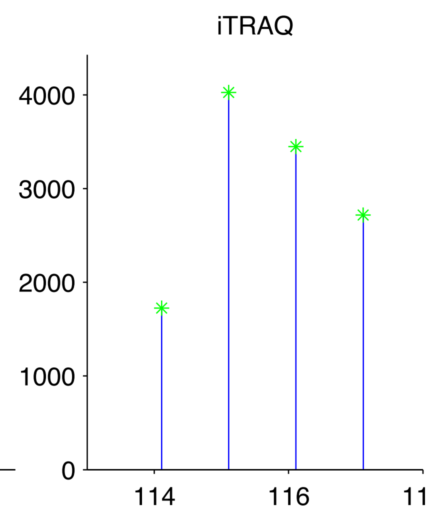

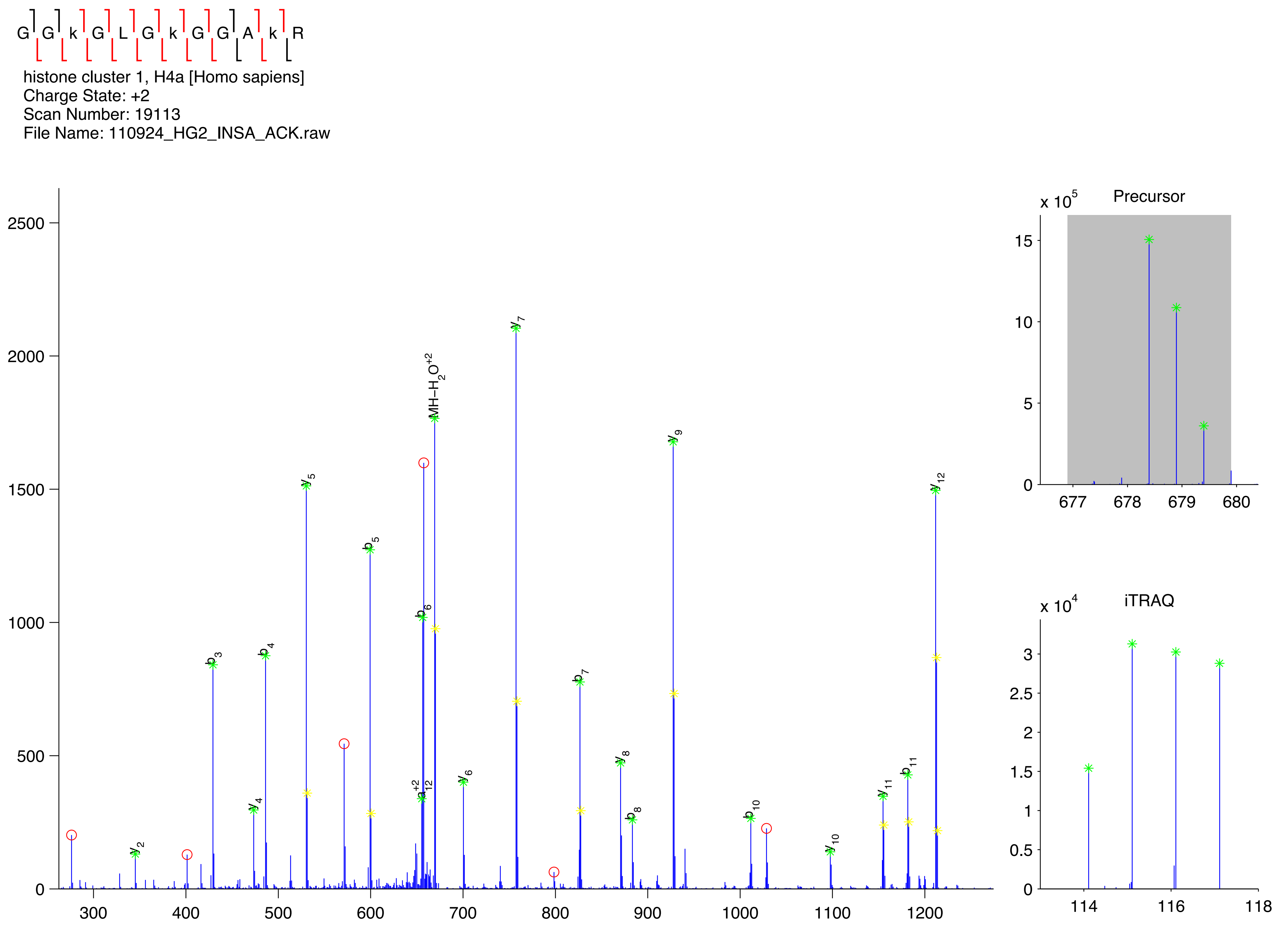
G G k G L G k G G A k R
histone cluster 1, H4a [Homo sapiens]
Charge State: +2
Scan Number: 19113
File Name: 110924_HG2_INSA_ACK.raw
Precursor
x 10^5
iTRAQ
x 10^4
MH−H2O+2

G G k G L G k G G A k R

histone cluster 1, H4a [Homo sapiens]
Charge State: +2
Scan Number: 19123
File Name: 110924_HG2_INSA_ACK.raw

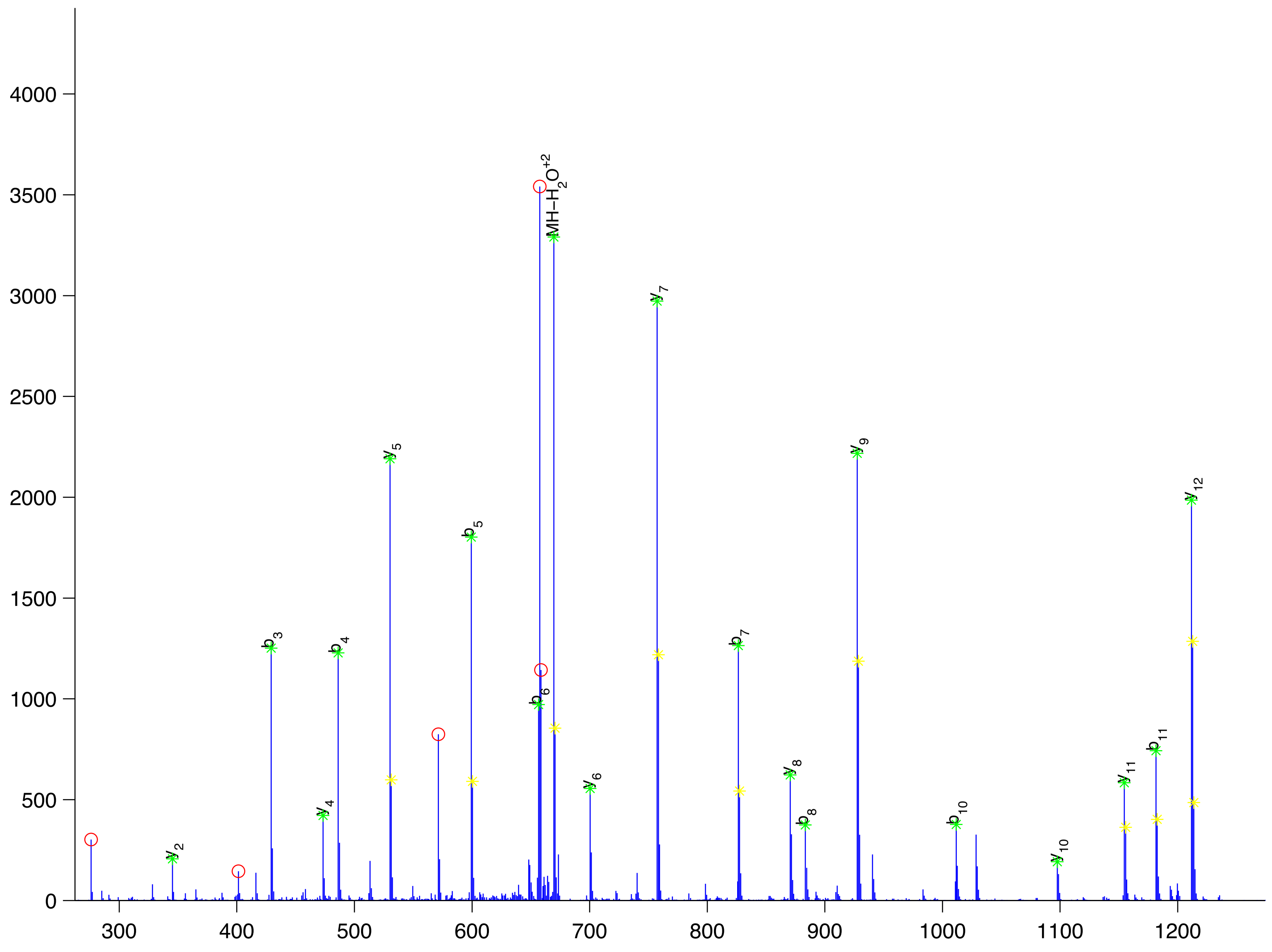

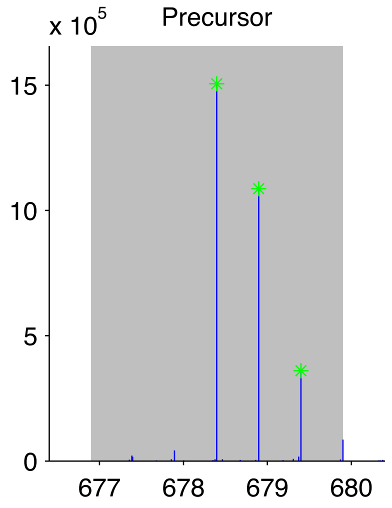

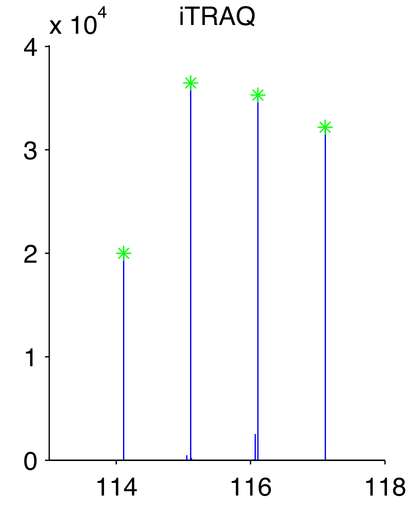

G k G G k G L G k G G A k R

histone cluster 1, H4a [Homo sapiens]
Charge State: +2
Scan Number: 19146
File Name: 110924_HG2_INSA_ACK.raw

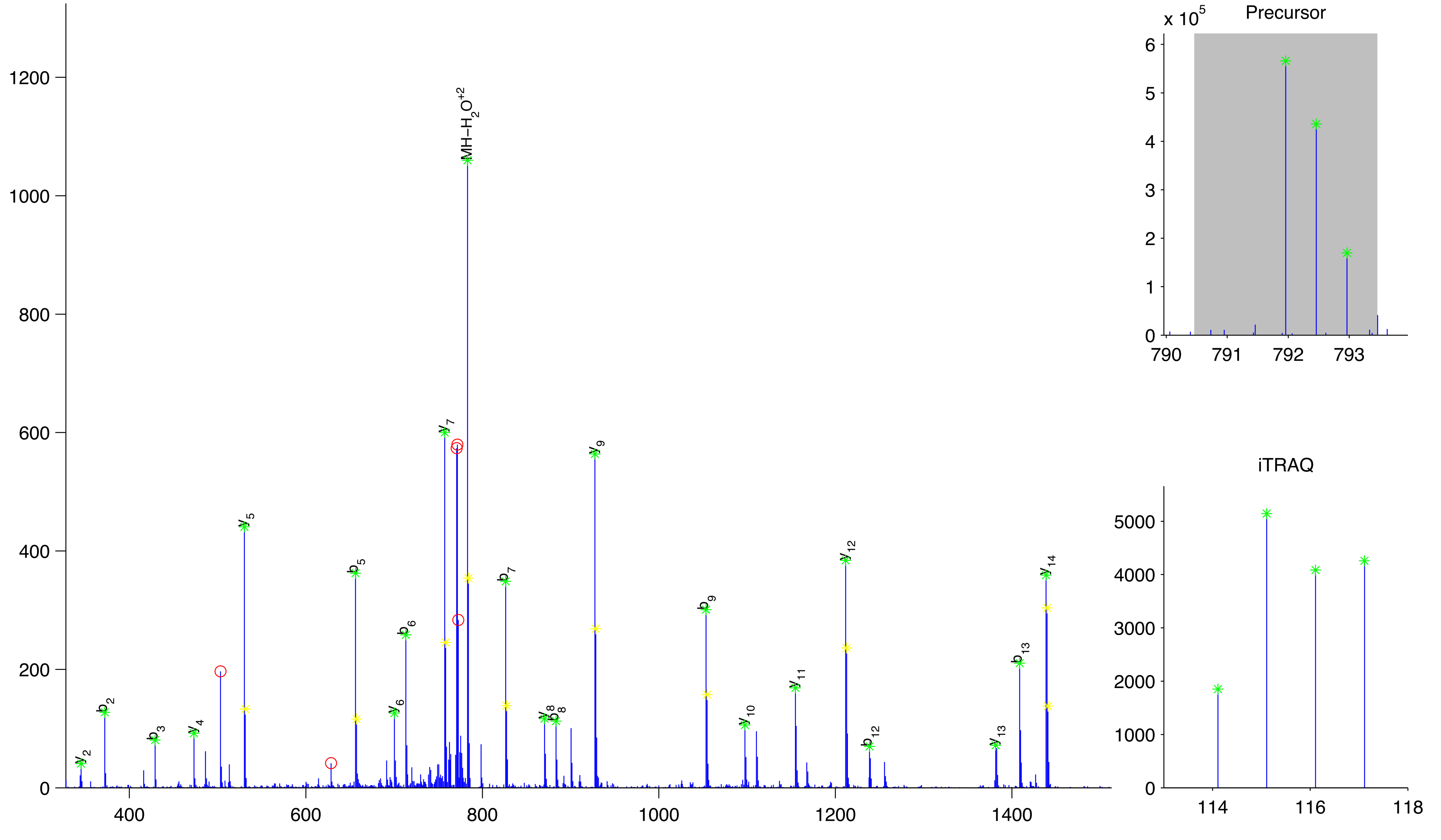

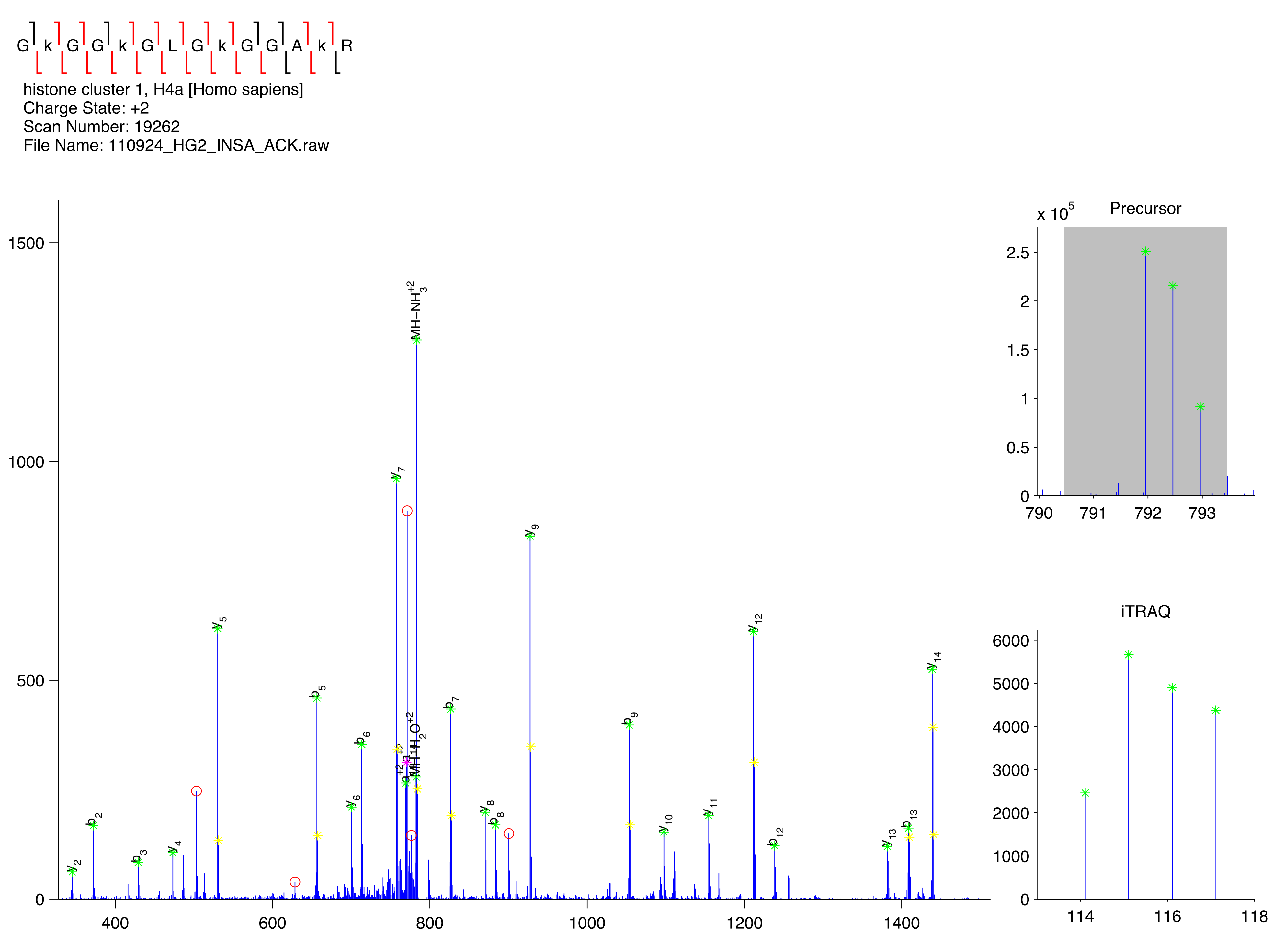

G k G G k G L G k G G A k R
histone cluster 1, H4a [Homo sapiens]
Charge State: +2
Scan Number: 19262
File Name: 110924_HG2_INSA_ACK.raw
Precursor
x 10^5
iTRAQ
MH−NH3+2
MH−H2O+2
y2 b2 b3 y4 y5 b5 y6 b6 y7 b7 y8 b8 y9 b9 y10 y11 y12 b12 y13 b13 y14

G k G G k G L G k G G A k R

histone cluster 1, H4a [Homo sapiens]
Charge State: +2
Scan Number: 19316
File Name: 110924_HG2_INSA_ACK.raw

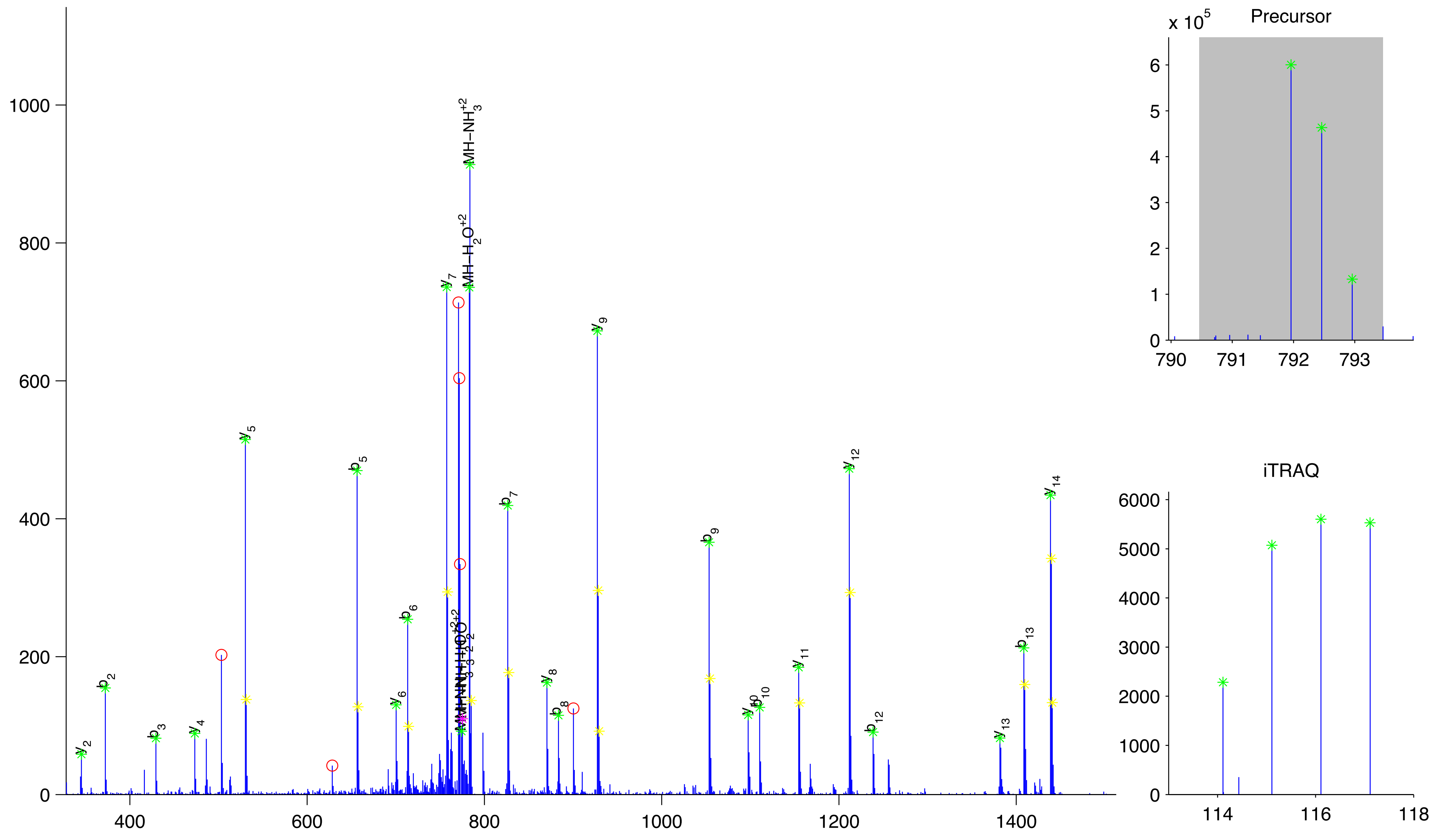

G L G k G G A k R

histone cluster 1, H4a [Homo sapiens]
Charge State: +2
Scan Number: 19403
File Name: 110924_HG2_INSA_ACK3.raw

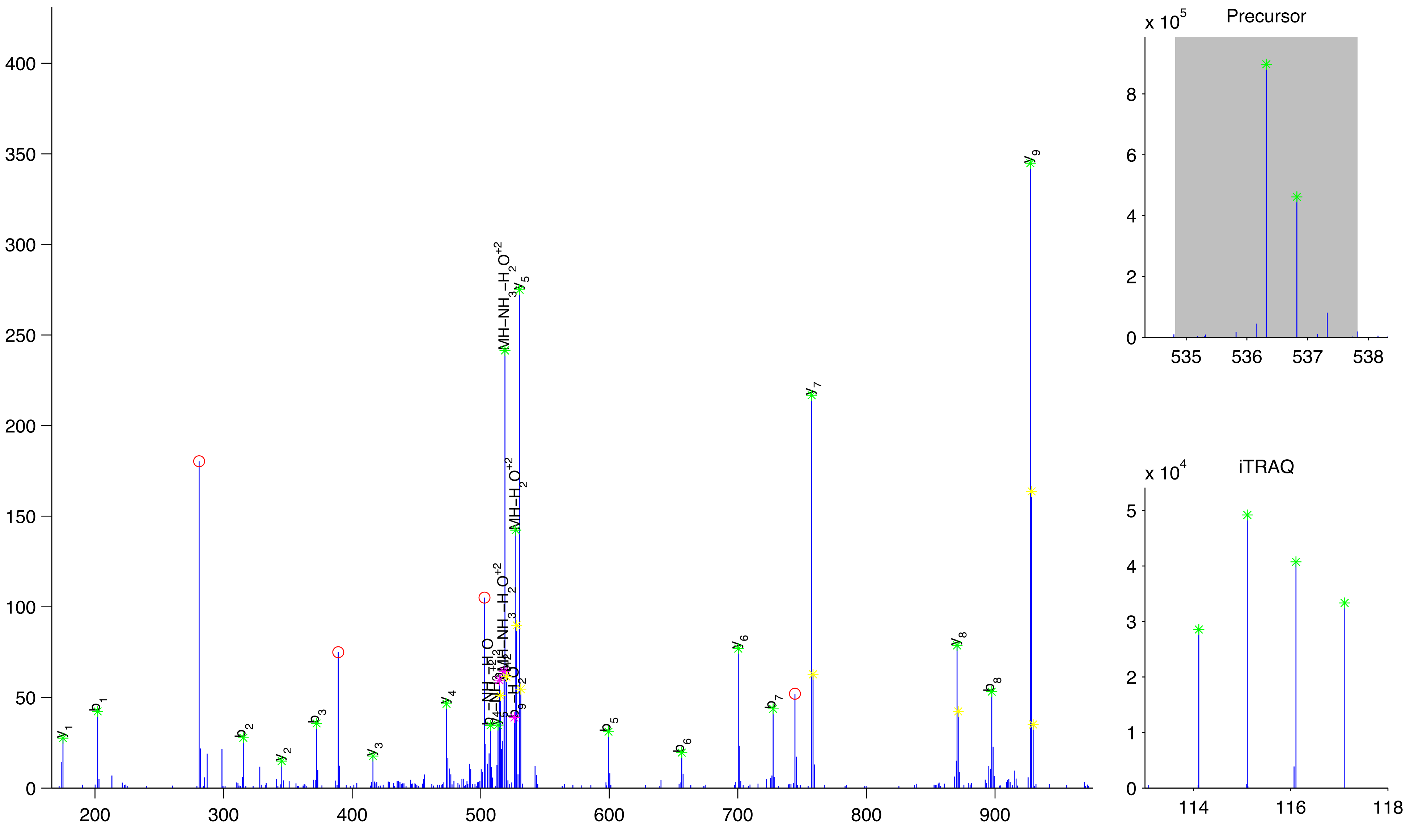

G k G G k G L G k G G A k R

histone cluster 1, H4a [Homo sapiens]
Charge State: +3
Scan Number: 19428
File Name: 110924_HG2_INSA_ACK3.raw

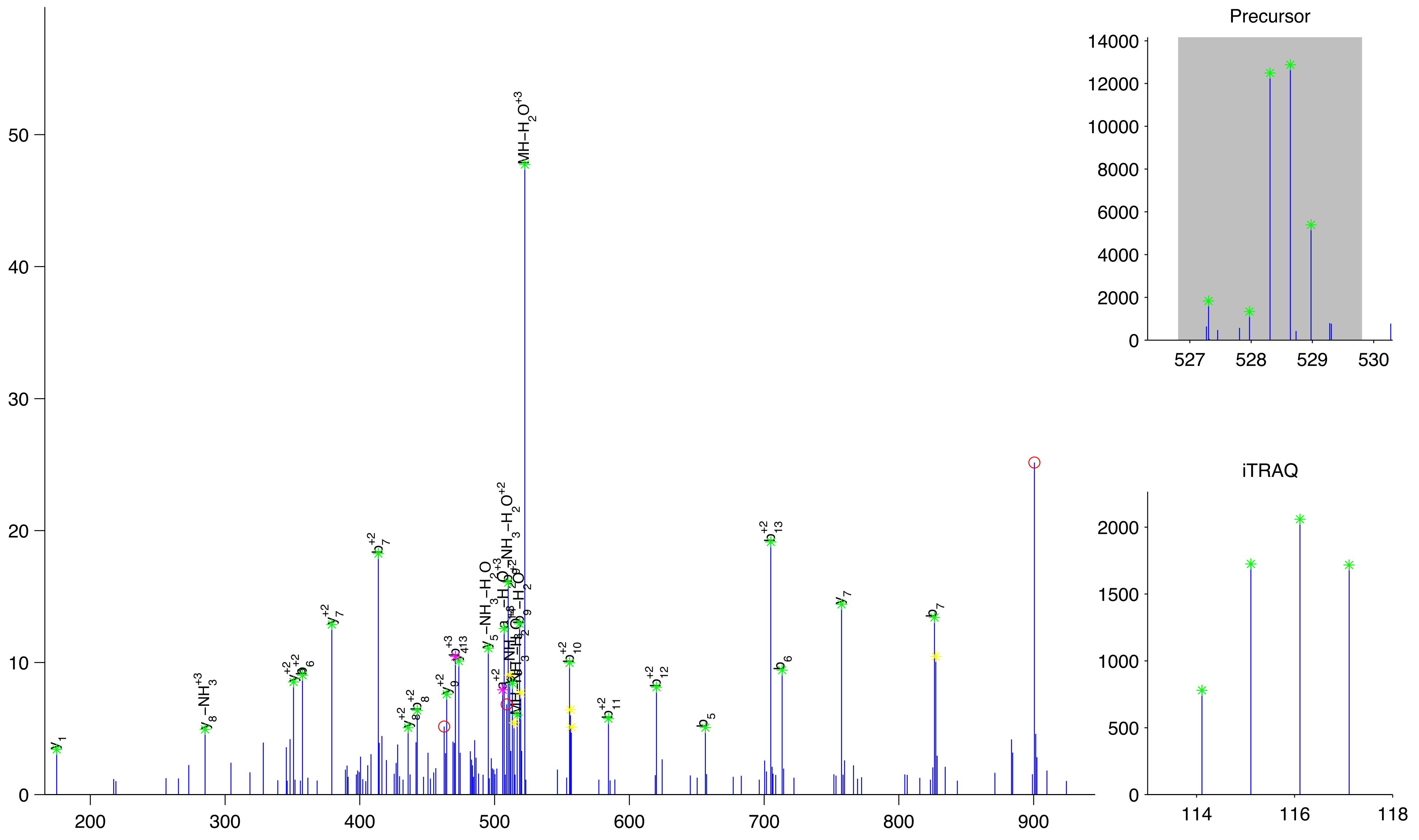

G k G G k G L G k G G A k R

histone cluster 1, H4a [Homo sapiens]
Charge State: +2
Scan Number: 19430
File Name: 110924_HG2_INSA_ACK.raw

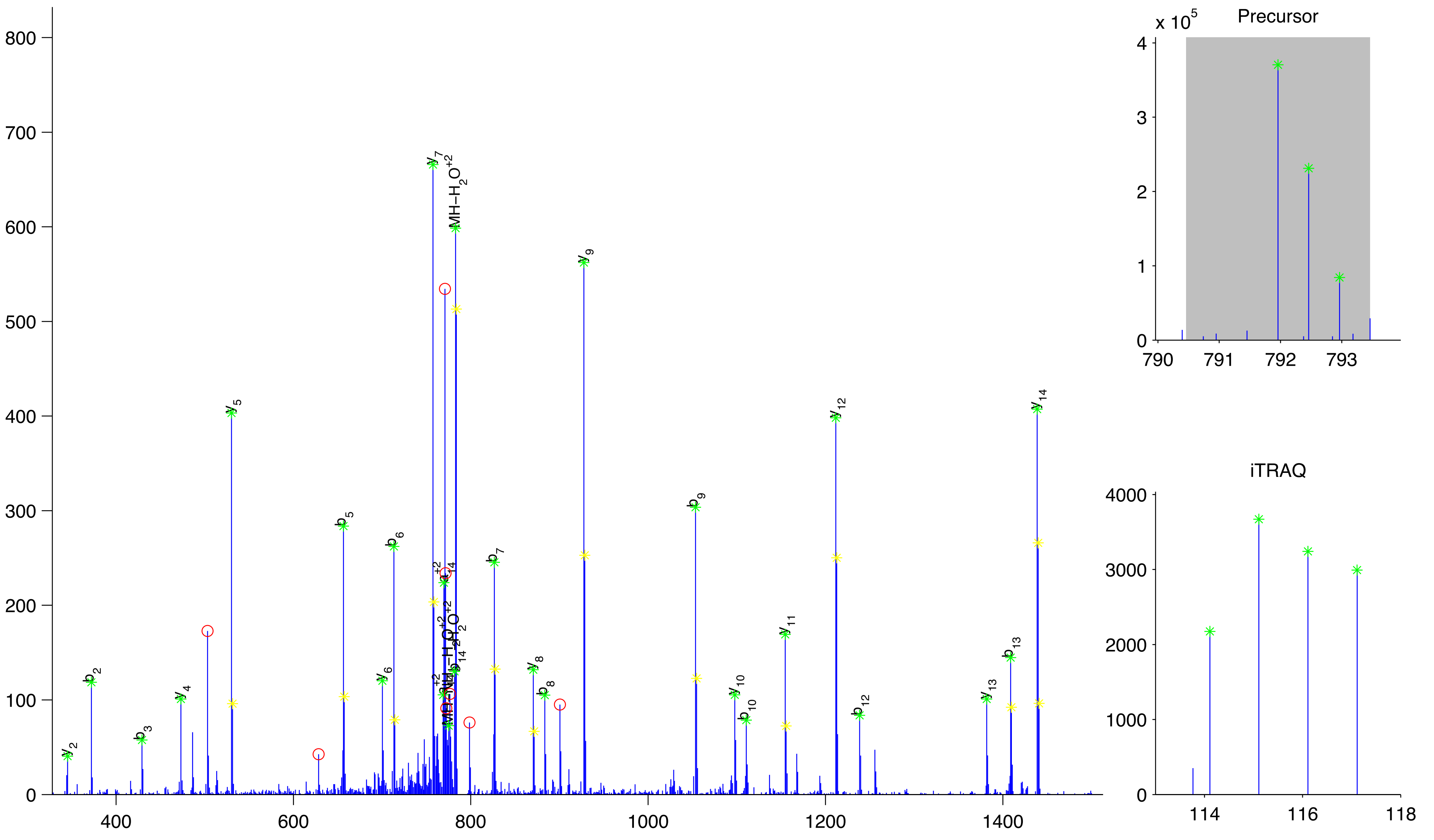

G k G G k G L G k G G A k R

histone cluster 1, H4a [Homo sapiens]
Charge State: +2
Scan Number: 19480
File Name: 110924_HG2_INSA_ACK.raw

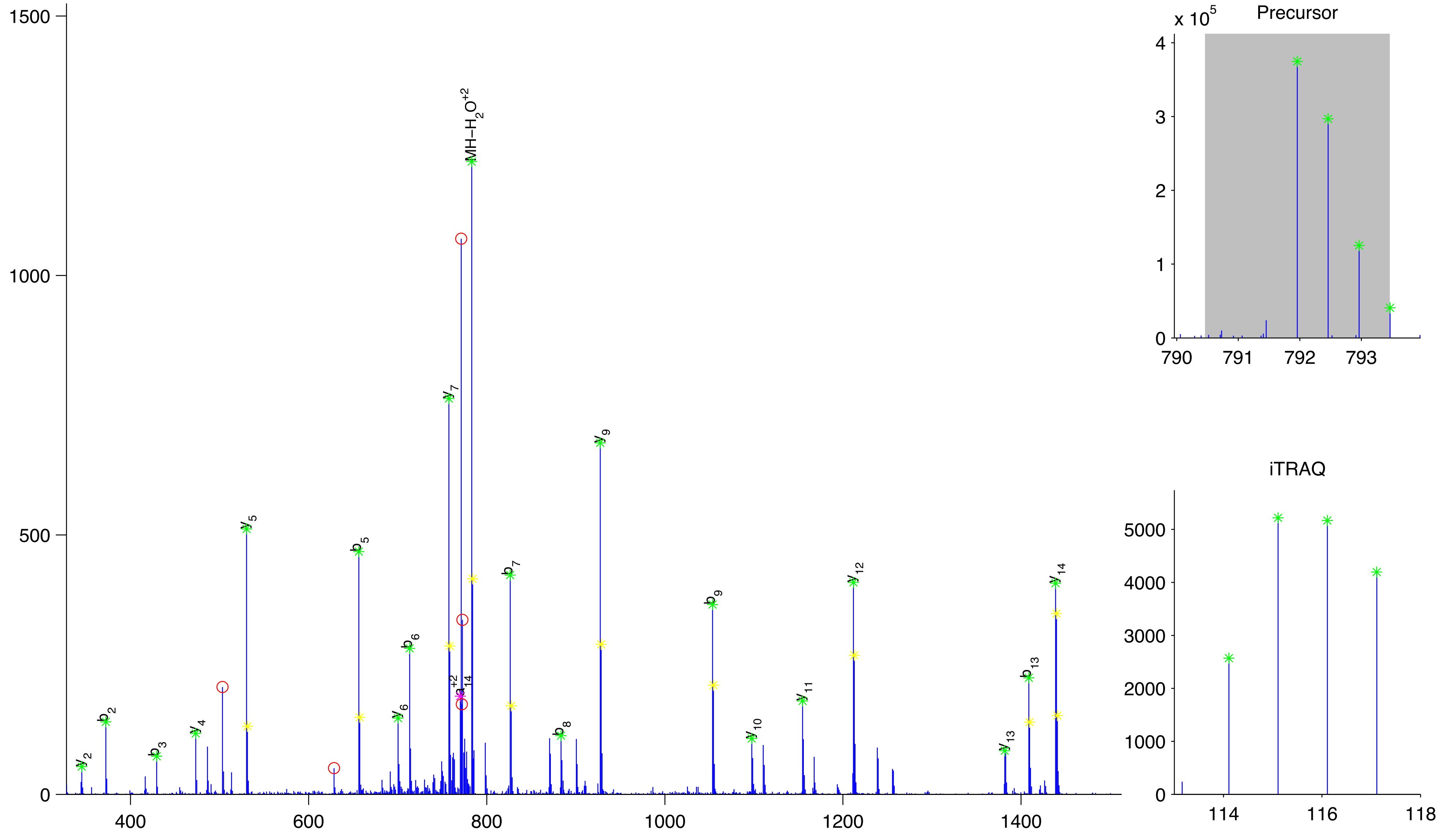

G G k G L G k G G A k R

histone cluster 1, H4a [Homo sapiens]
Charge State: +2
Scan Number: 19617
File Name: 110924_HG2_INSA_ACK.raw

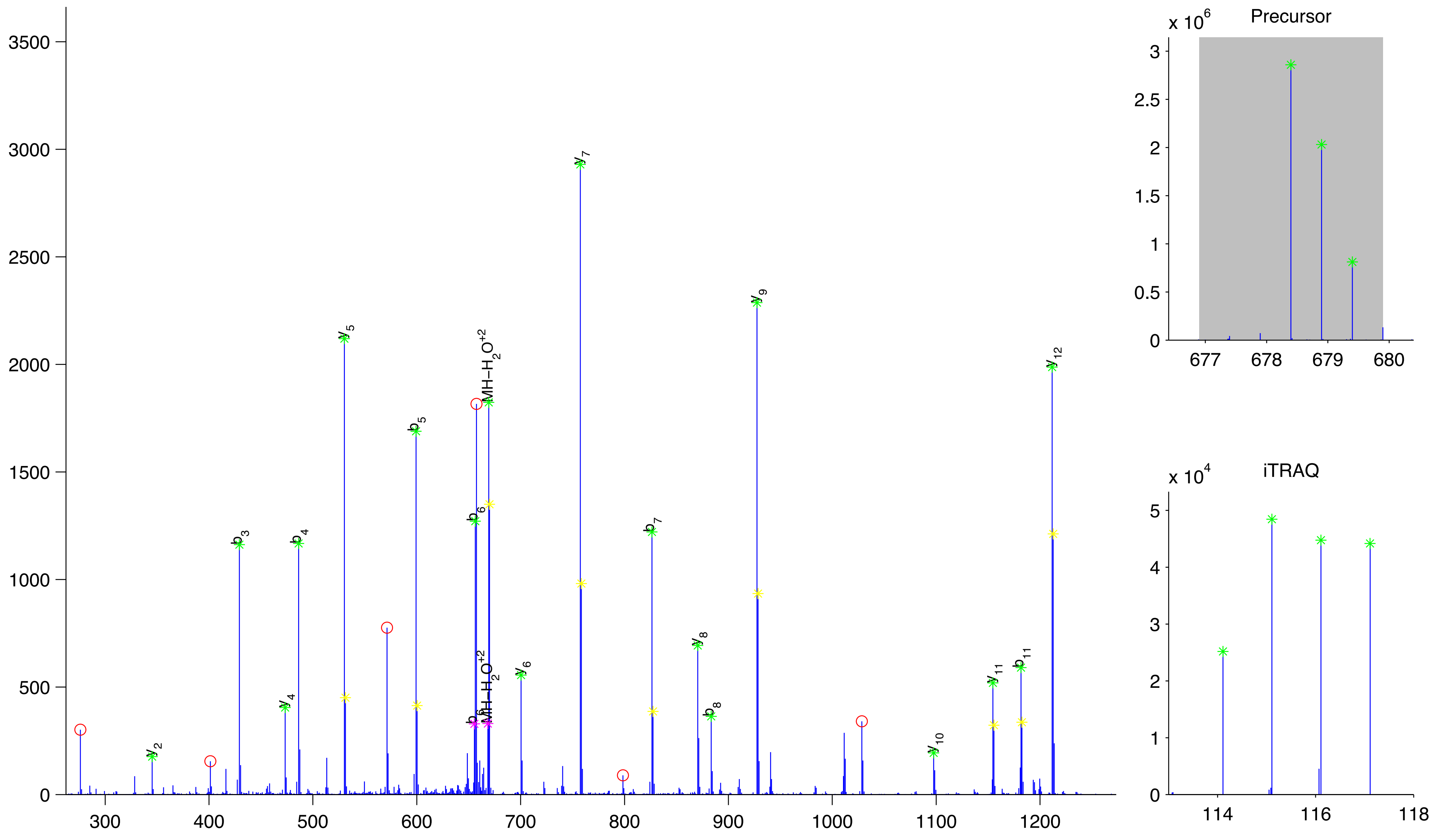

G G k G L G k G G A k R

histone cluster 1, H4a [Homo sapiens]
Charge State: +2
Scan Number: 19629
File Name: 110924_HG2_INSA_ACK.raw

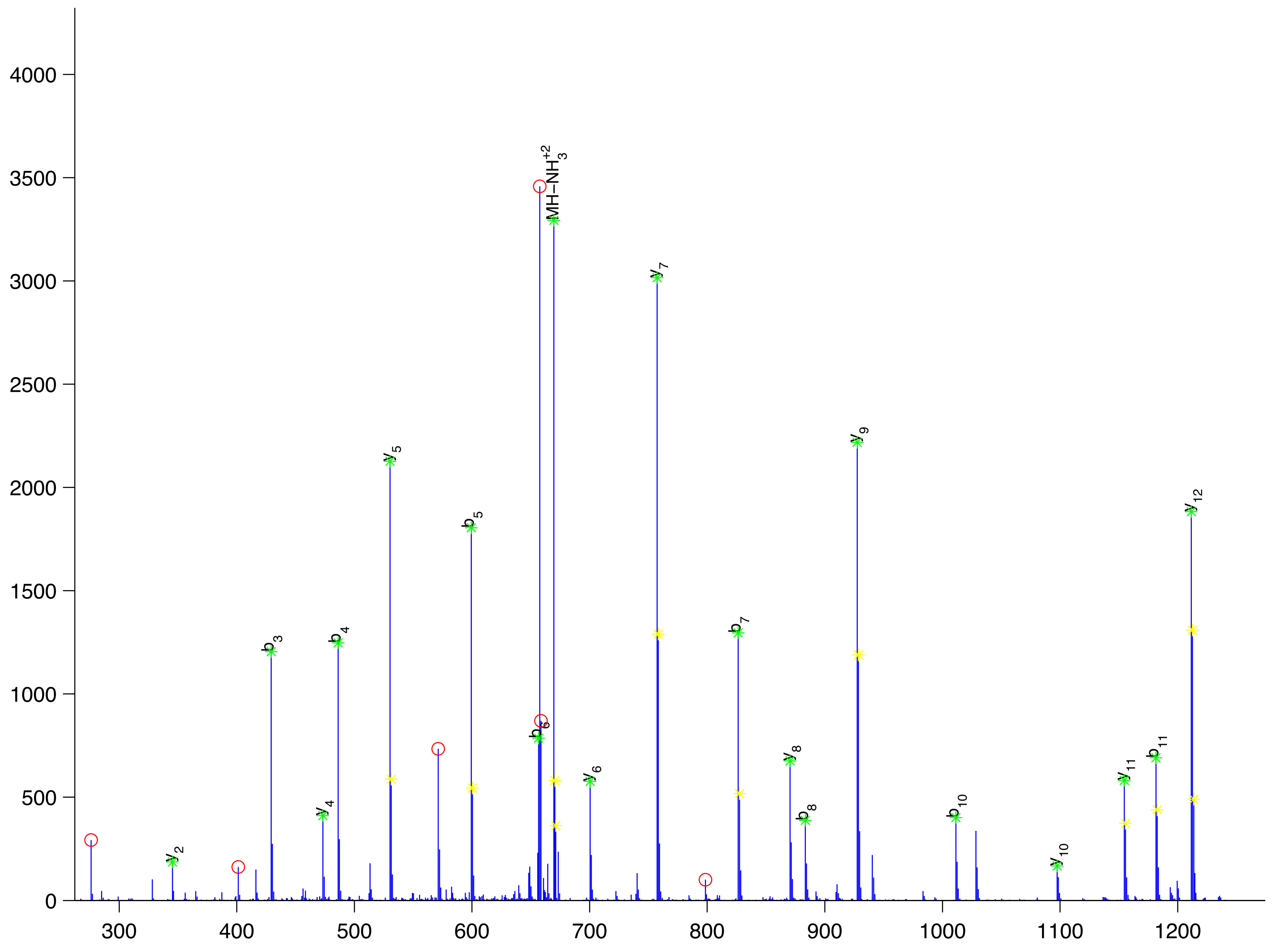

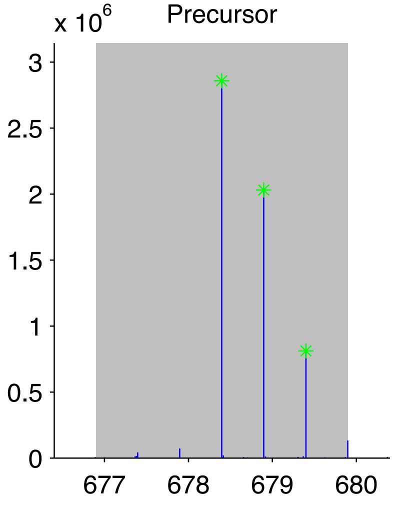

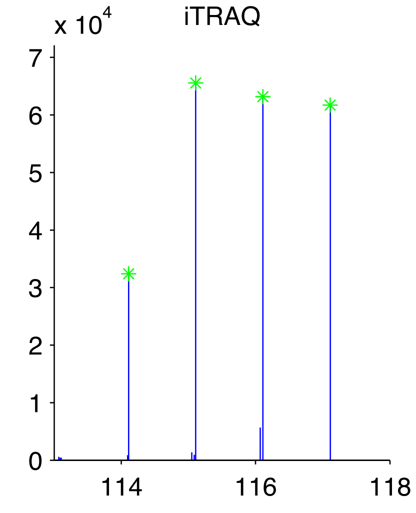

G k G G k G L G k G G A k R

histone cluster 1, H4a [Homo sapiens]
Charge State: +2
Scan Number: 19766
File Name: 110924_HG2_INSA_ACK.raw

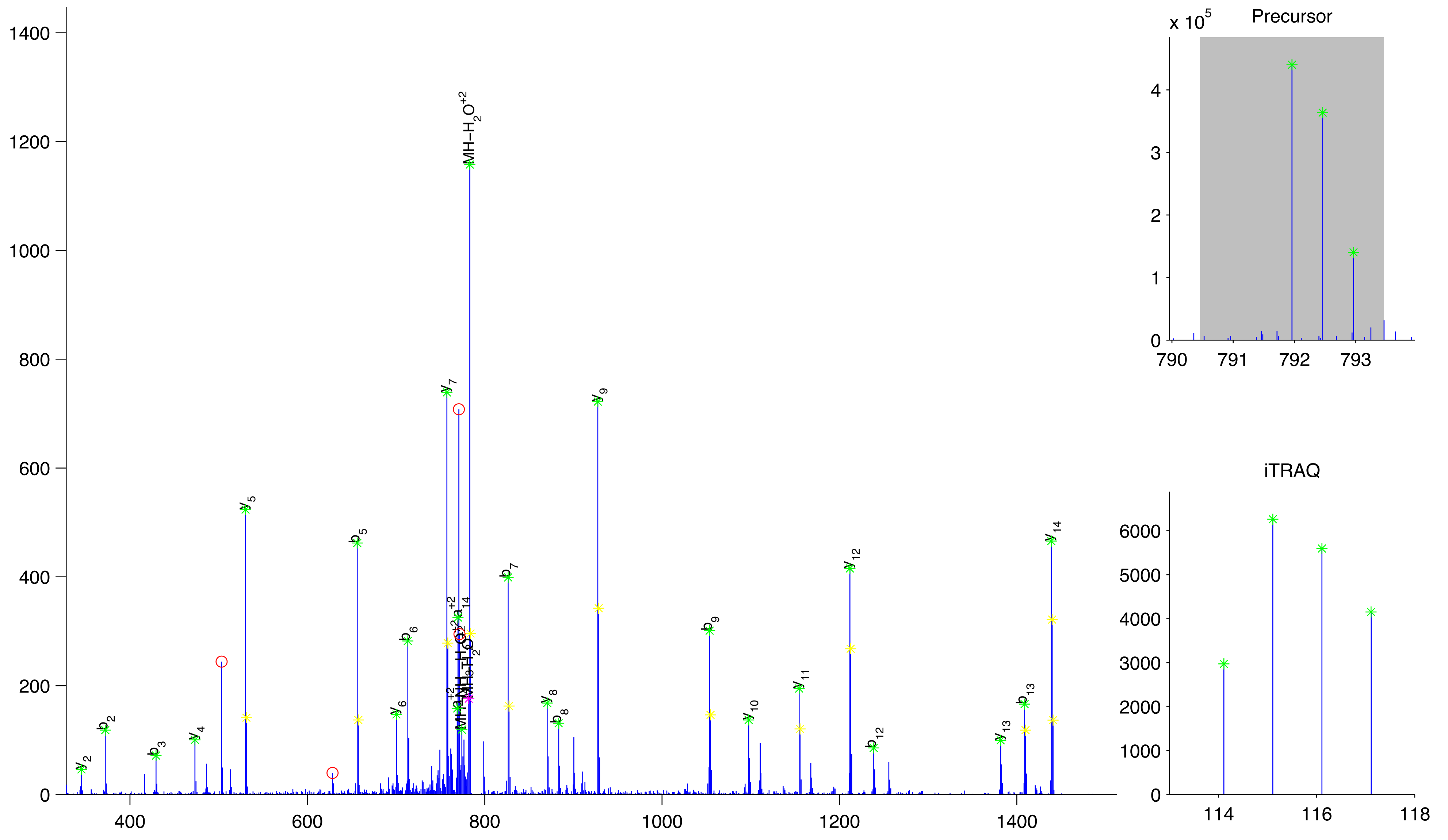

G k G G k G L G k G G A k R

histone cluster 1, H4a [Homo sapiens]
Charge State: +3
Scan Number: 19770
File Name: 110924_HG2_INSA_ACK3.raw

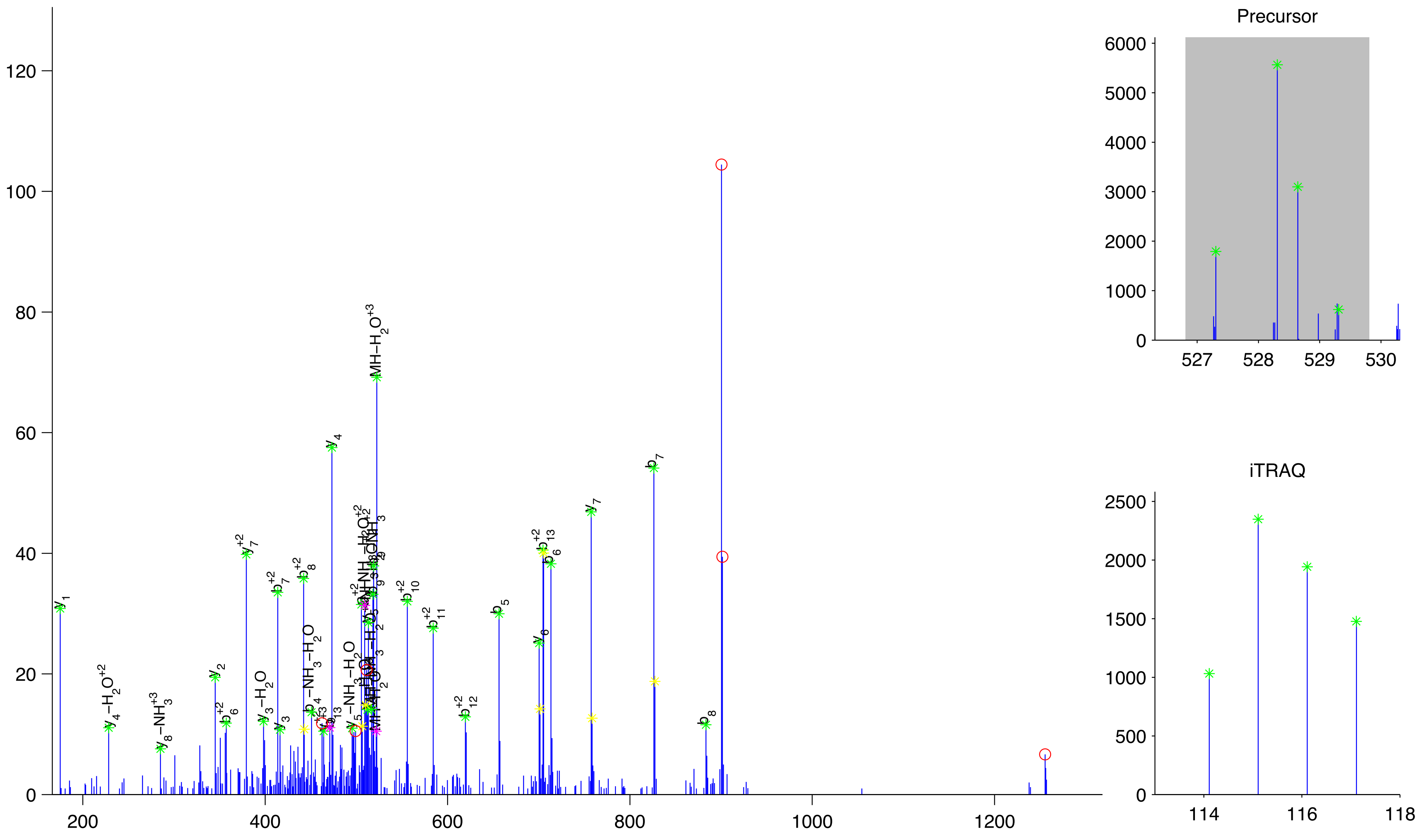

G k G G k G L G k G G A k R

histone cluster 1, H4a [Homo sapiens]
Charge State: +2
Scan Number: 19862
File Name: 110924_HG2_INSA_ACK.raw

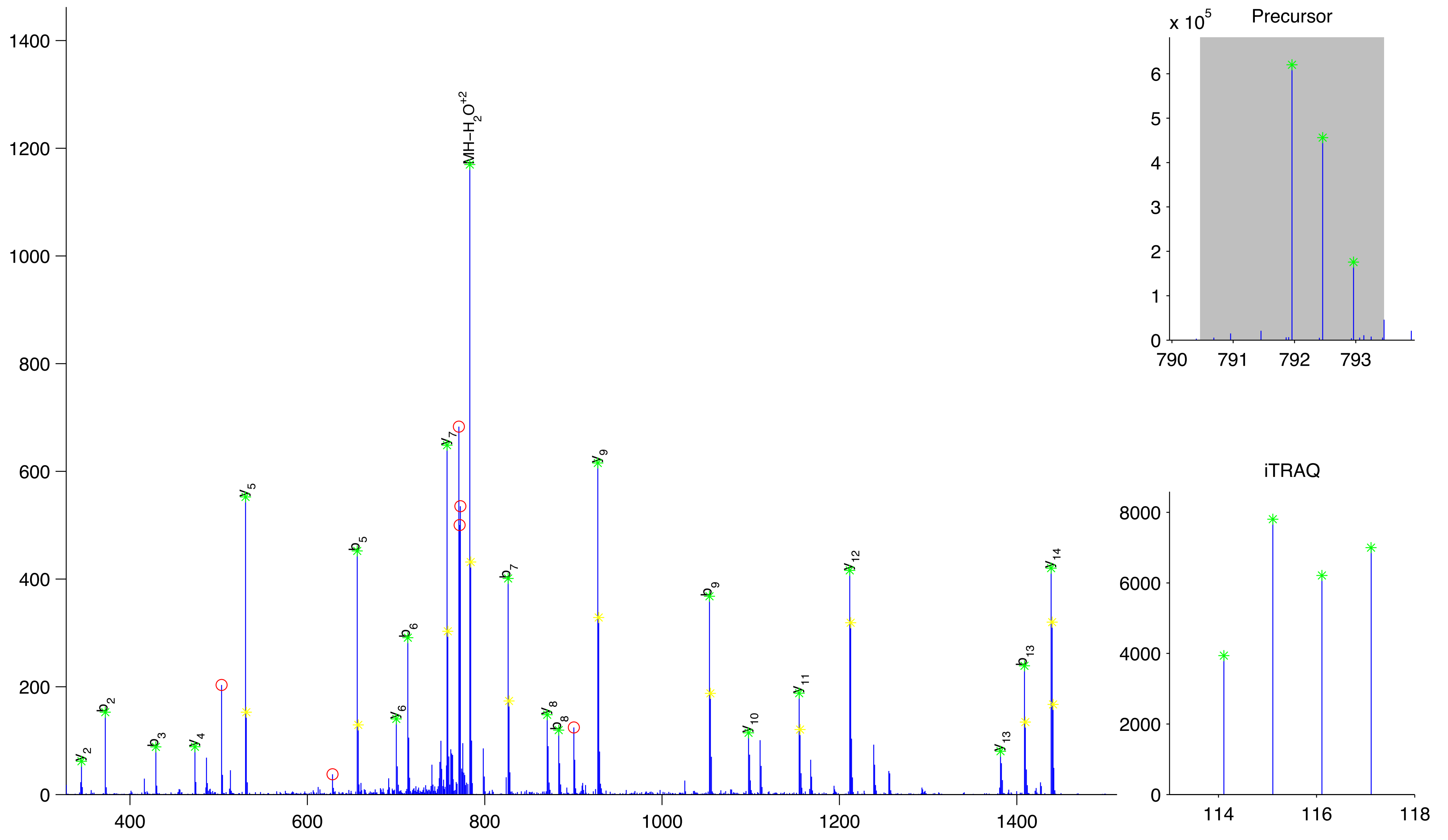

G k G G k G L G k G G A k R

histone cluster 1, H4a [Homo sapiens]
Charge State: +2
Scan Number: 19934
File Name: 110924_HG2_INSA_ACK.raw

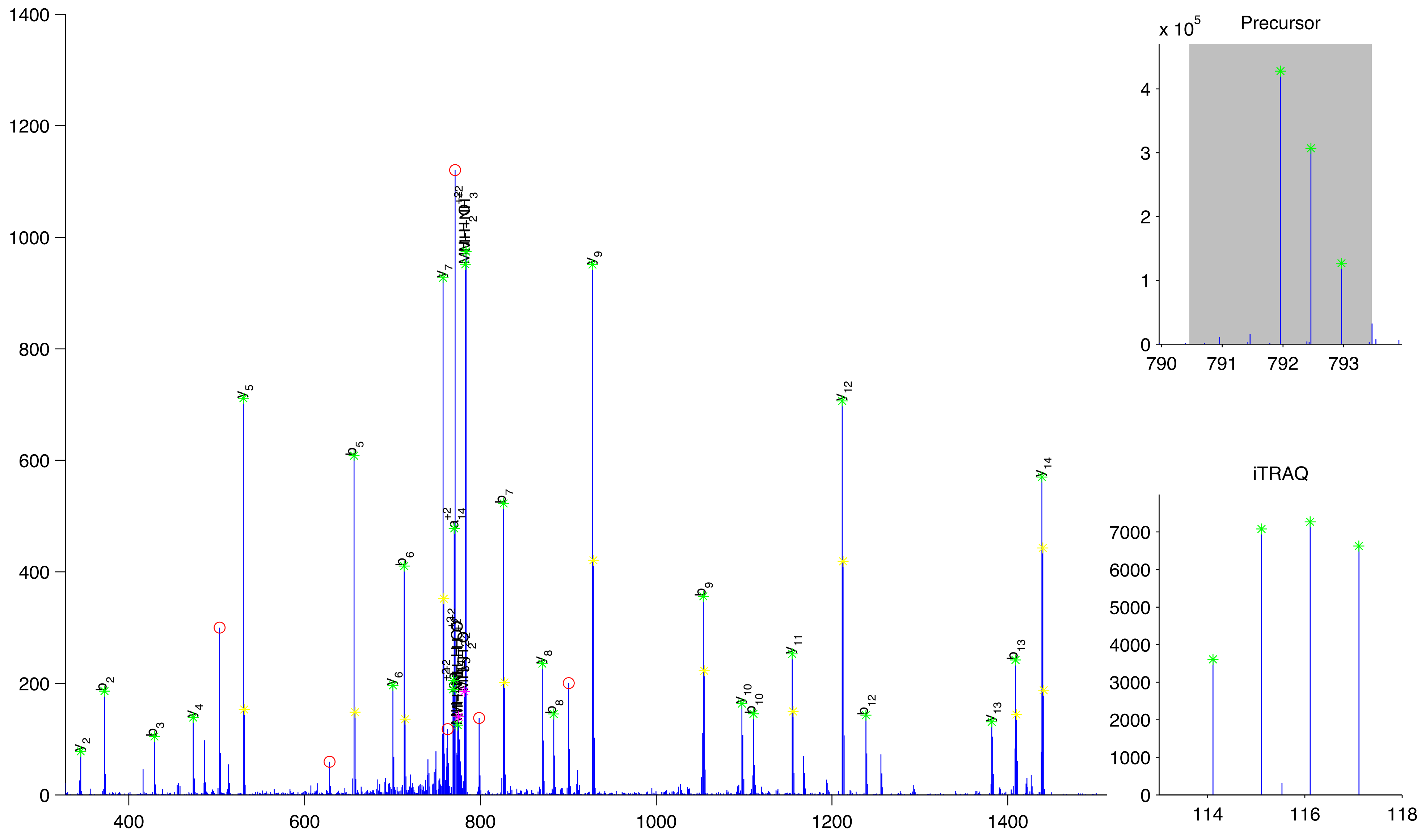

G G k G L G k G G A k R

histone cluster 1, H4a [Homo sapiens]
Charge State: +2
Scan Number: 19950
File Name: 111011_HG2_INSB_ACK.raw

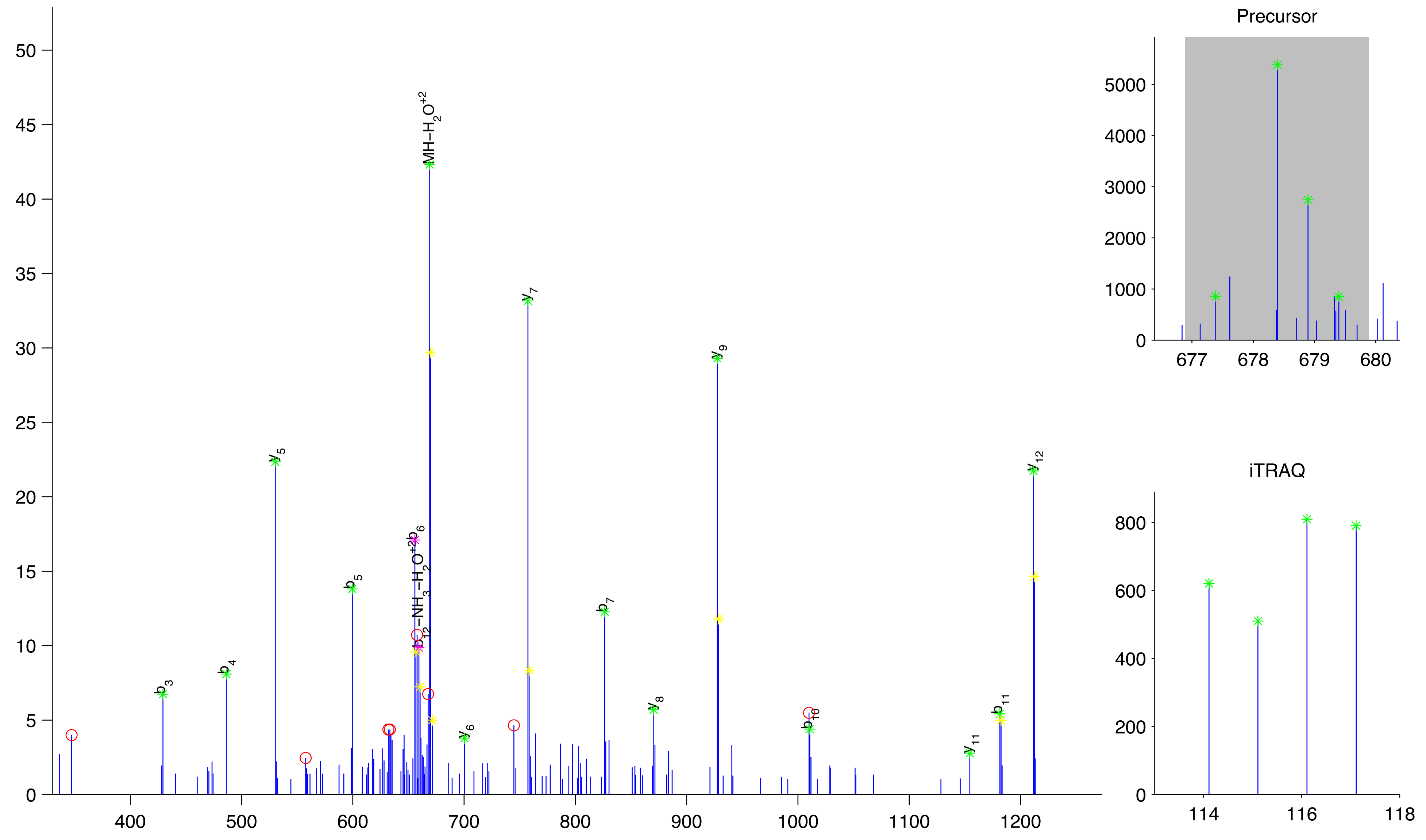

G G k G L G k G G A k R

histone cluster 1, H4a [Homo sapiens]
Charge State: +2
Scan Number: 20033
File Name: 110924_HG2_INSA_ACK3.raw

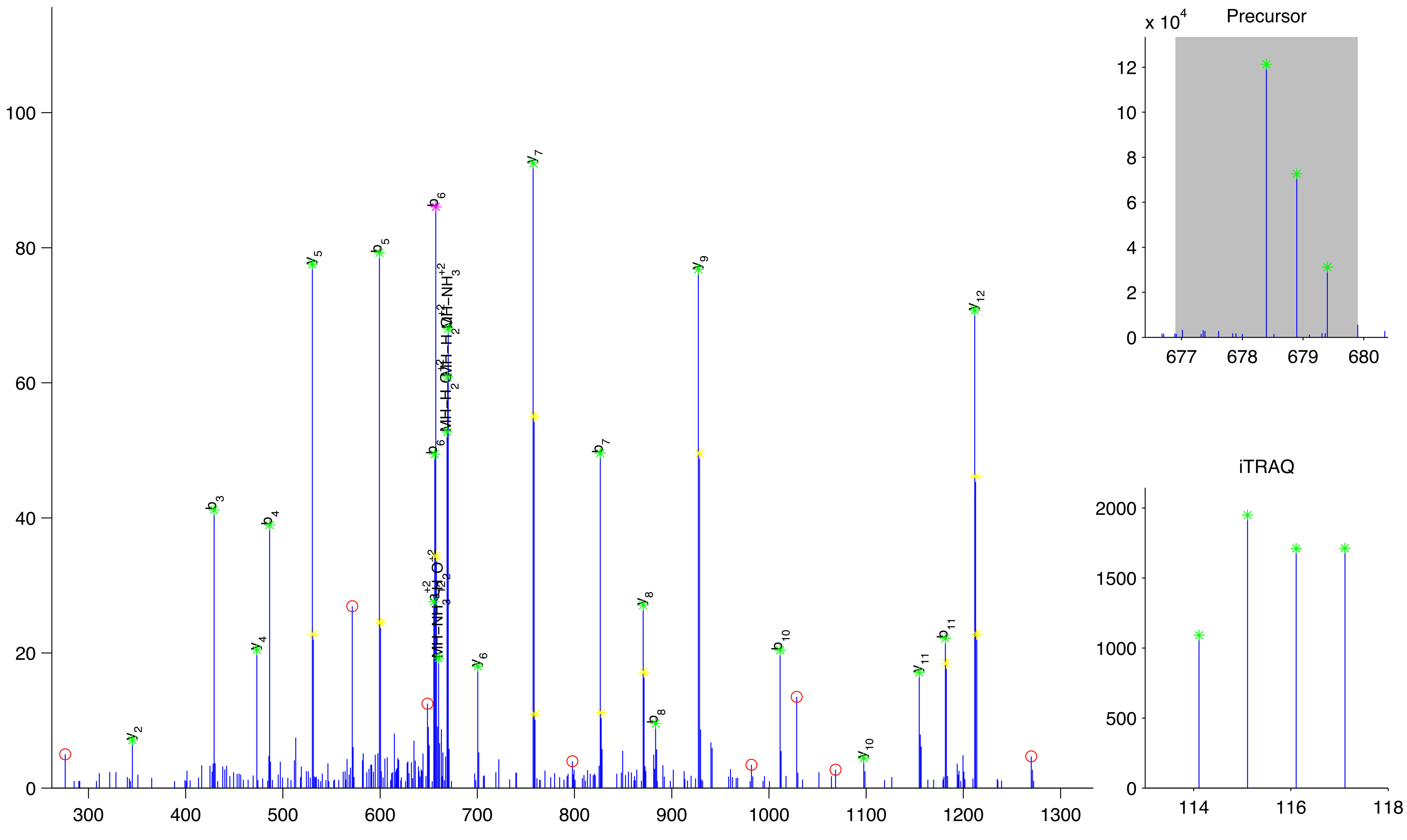

G L G k G G A k R

histone cluster 1, H4a [Homo sapiens]
Charge State: +2
Scan Number: 20075
File Name: 110924_HG2_INSA_ACK3.raw

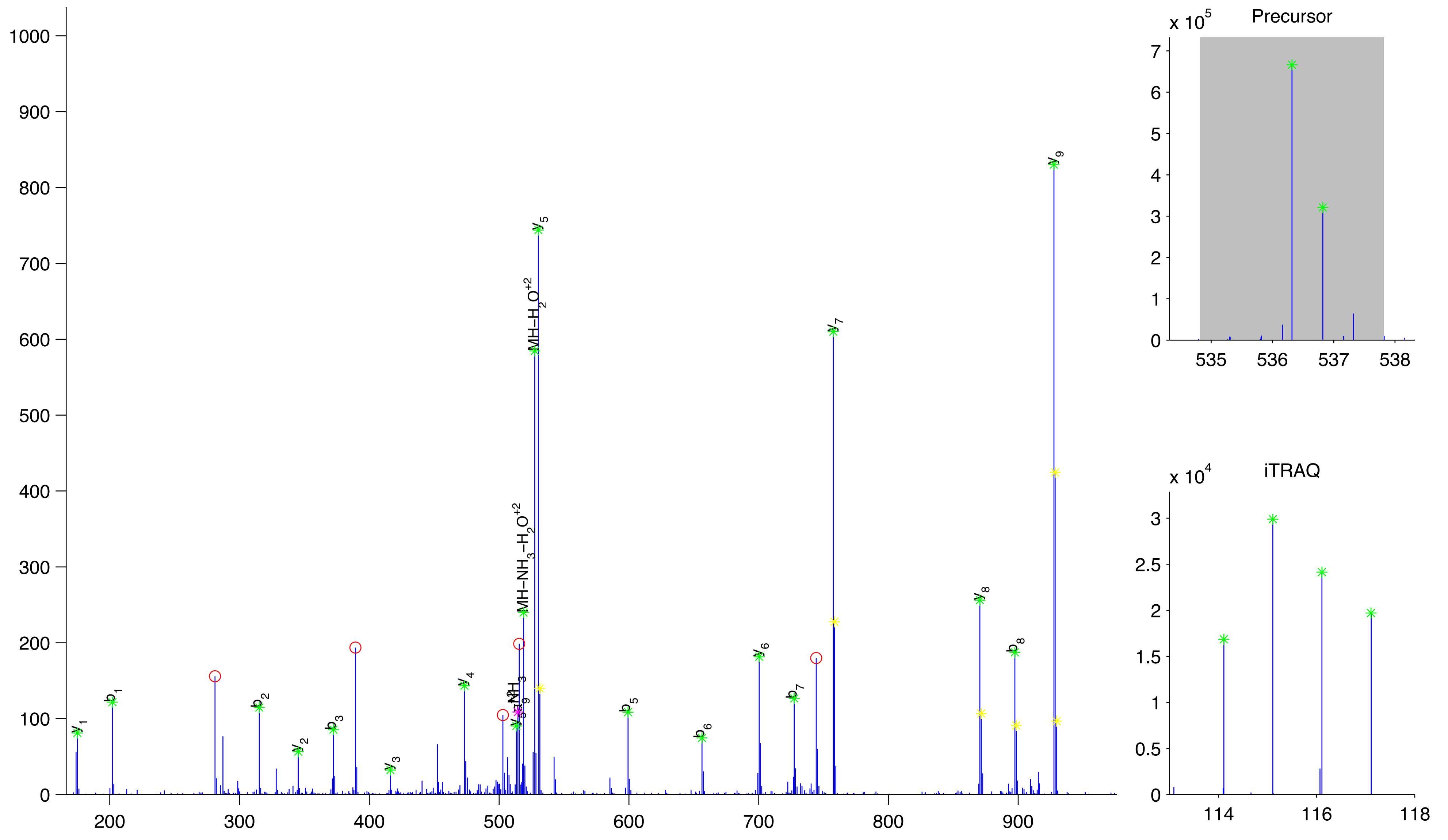

G k G G k G L G k G G A k R

histone cluster 1, H4a [Homo sapiens]
Charge State: +2
Scan Number: 20102
File Name: 110924_HG2_INSA_ACK.raw

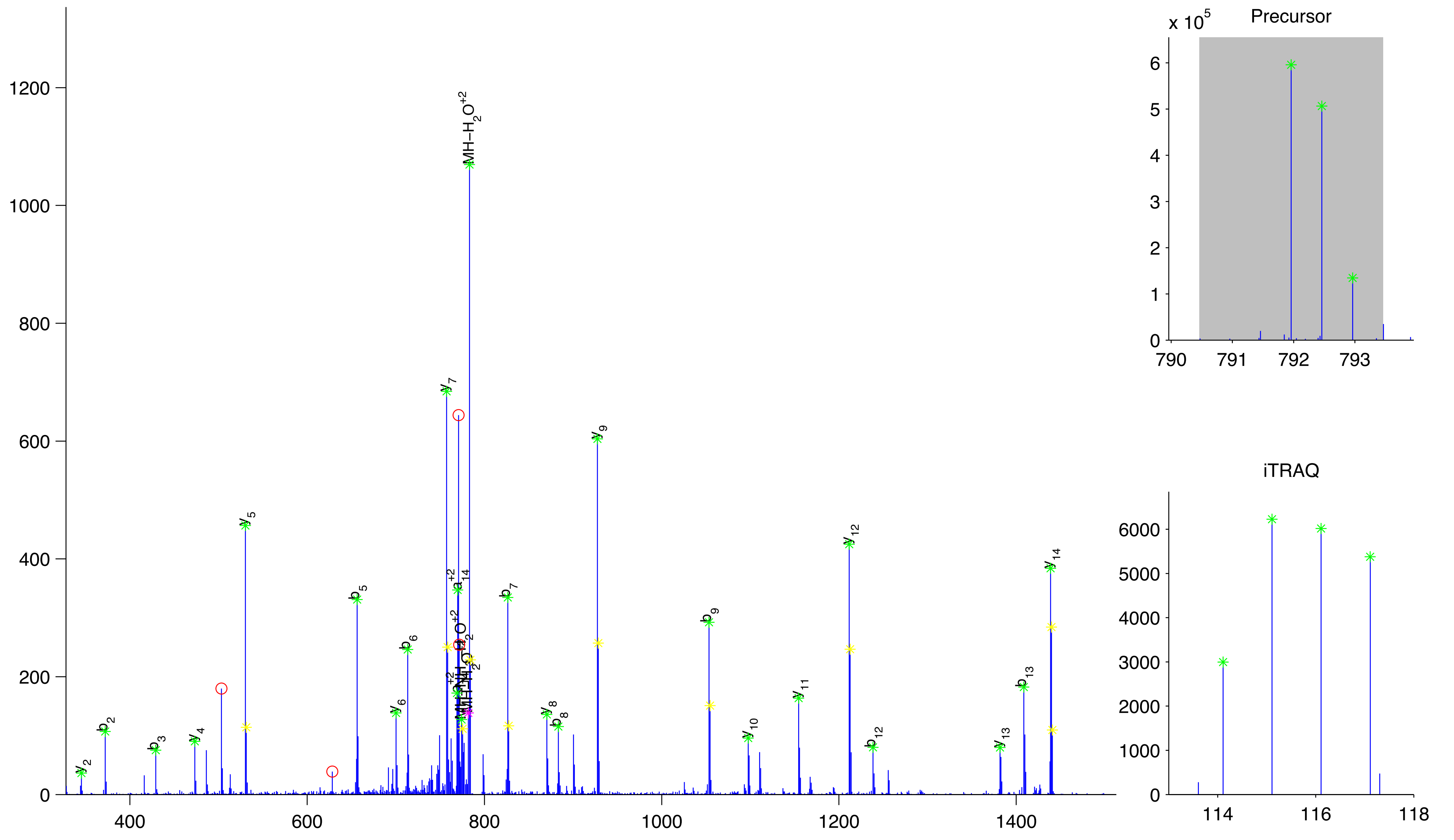

G k G G k G L G k G G A k R

histone cluster 1, H4a [Homo sapiens]
Charge State: +2
Scan Number: 20270
File Name: 110924_HG2_INSA_ACK.raw

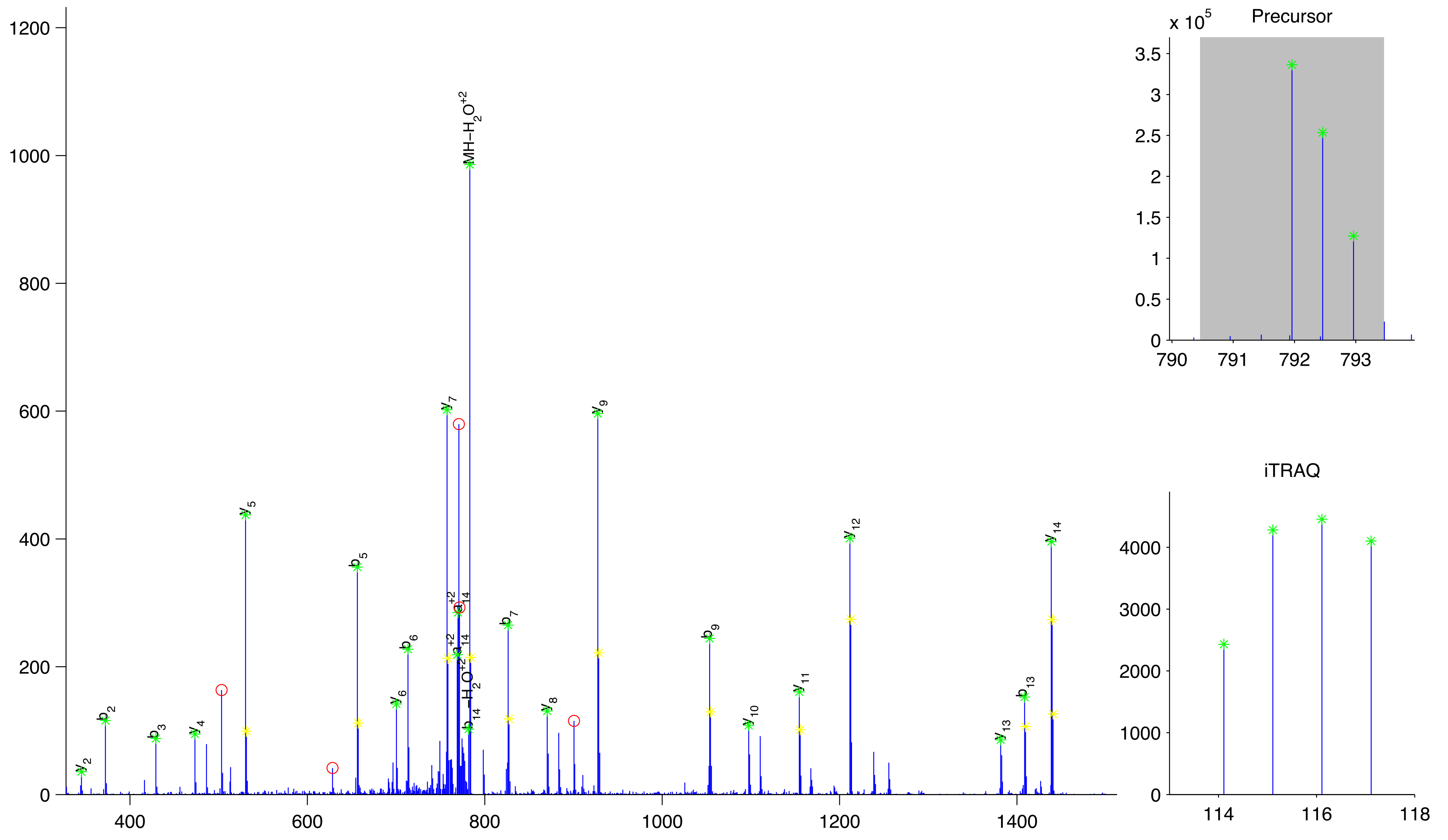

G G k G L G k G G A k R

histone cluster 1, H4a [Homo sapiens]
Charge State: +2
Scan Number: 20289
File Name: 110924_HG2_INSA_ACK.raw

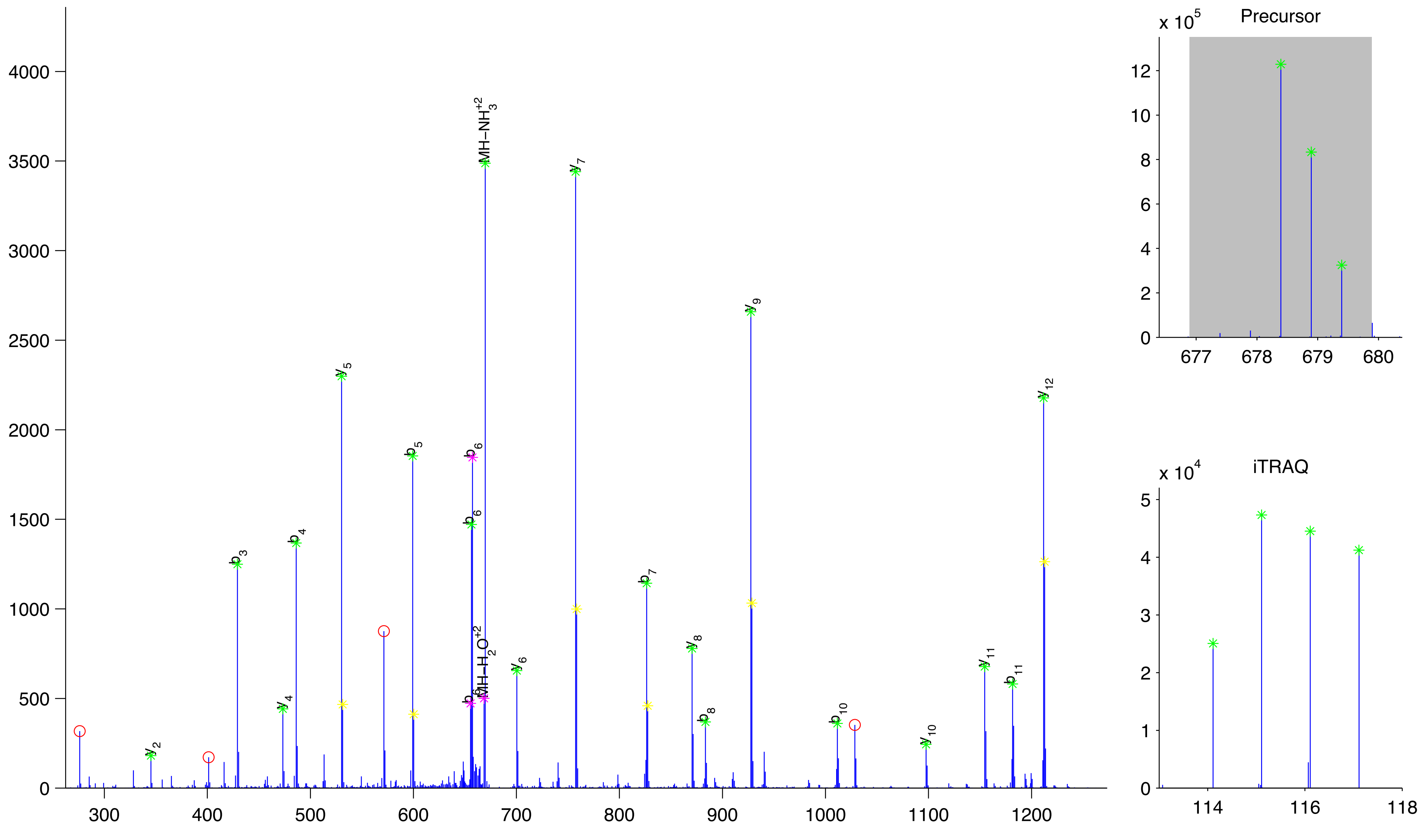

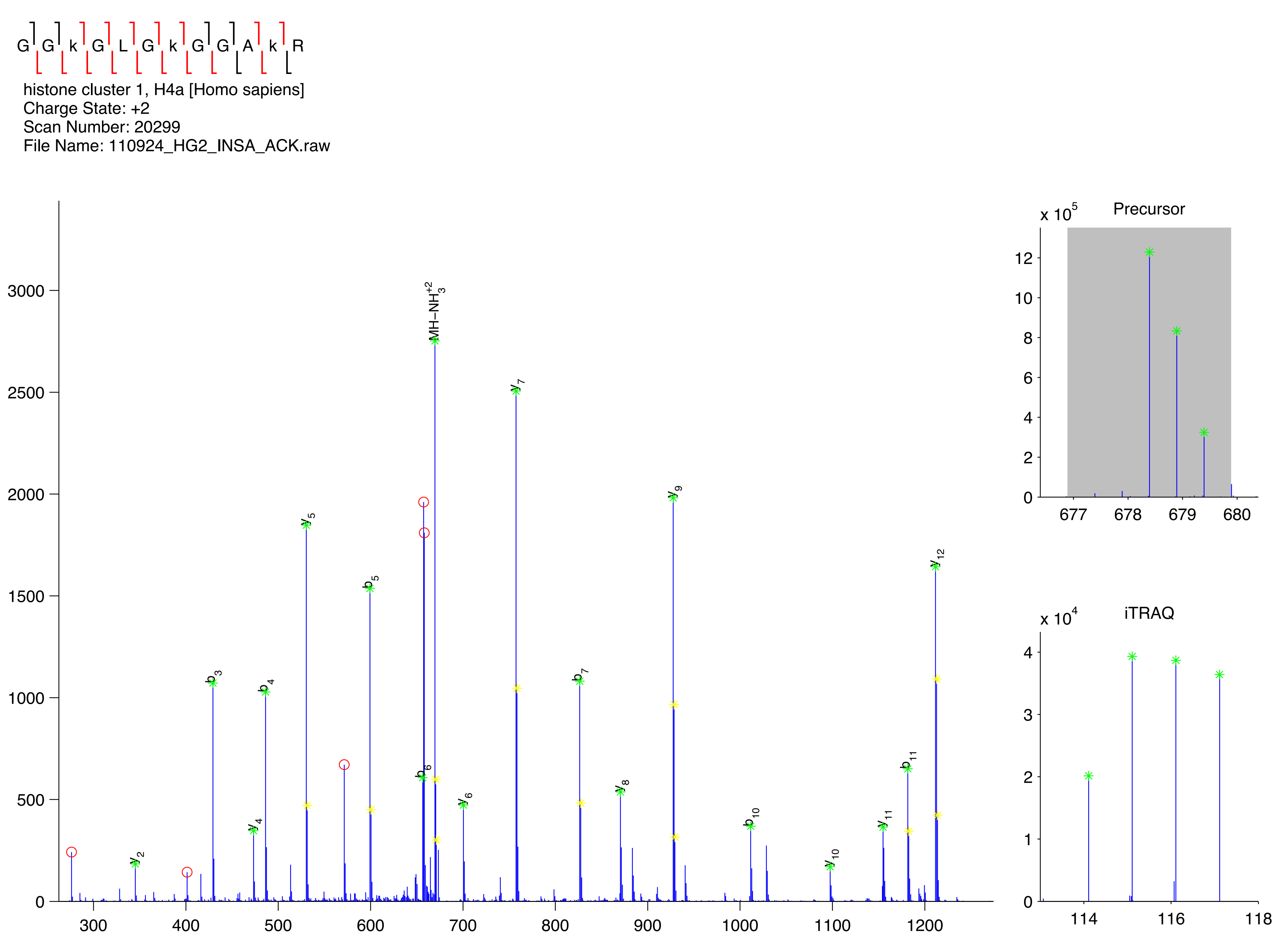

G G k G L G k G G A k R
histone cluster 1, H4a [Homo sapiens]
Charge State: +2
Scan Number: 20299
File Name: 110924_HG2_INSA_ACK.raw
MH−NH3+2
y2
b3
y4
b4
y5
b5
b6
y6
y7
b7
y8
y9
b10
y10
y11
b11
y12
Precursor
x 10^5
iTRAQ
x 10^4

G k G G k G L G k G G A k R

histone cluster 1, H4a [Homo sapiens]
Charge State: +2
Scan Number: 20303
File Name: 110924_HG2_INSA_ACK.raw

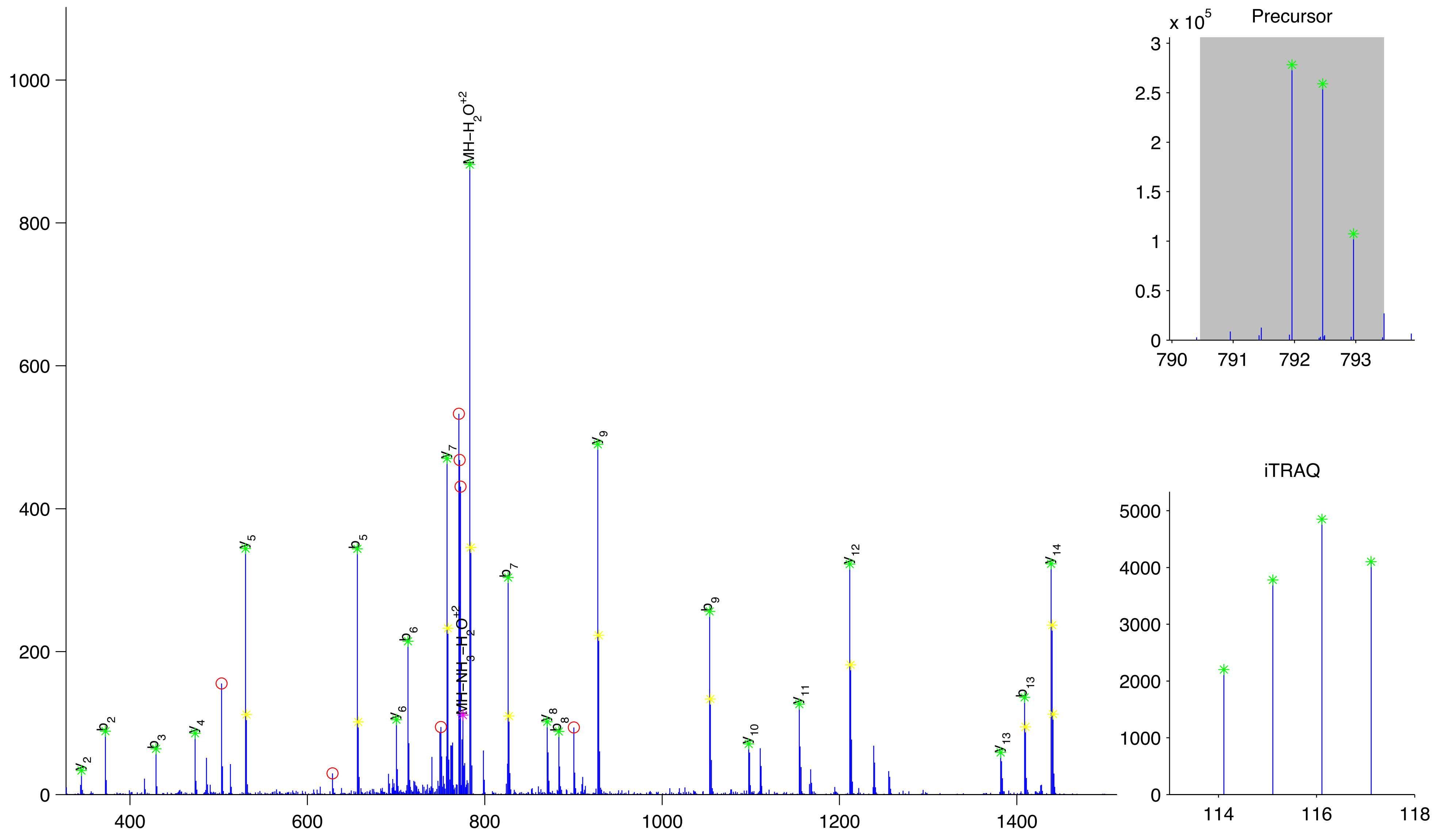

G k G G k G L G k G G A k R

histone cluster 1, H4a [Homo sapiens]
Charge State: +2
Scan Number: 20438
File Name: 110924_HG2_INSA_ACK.raw

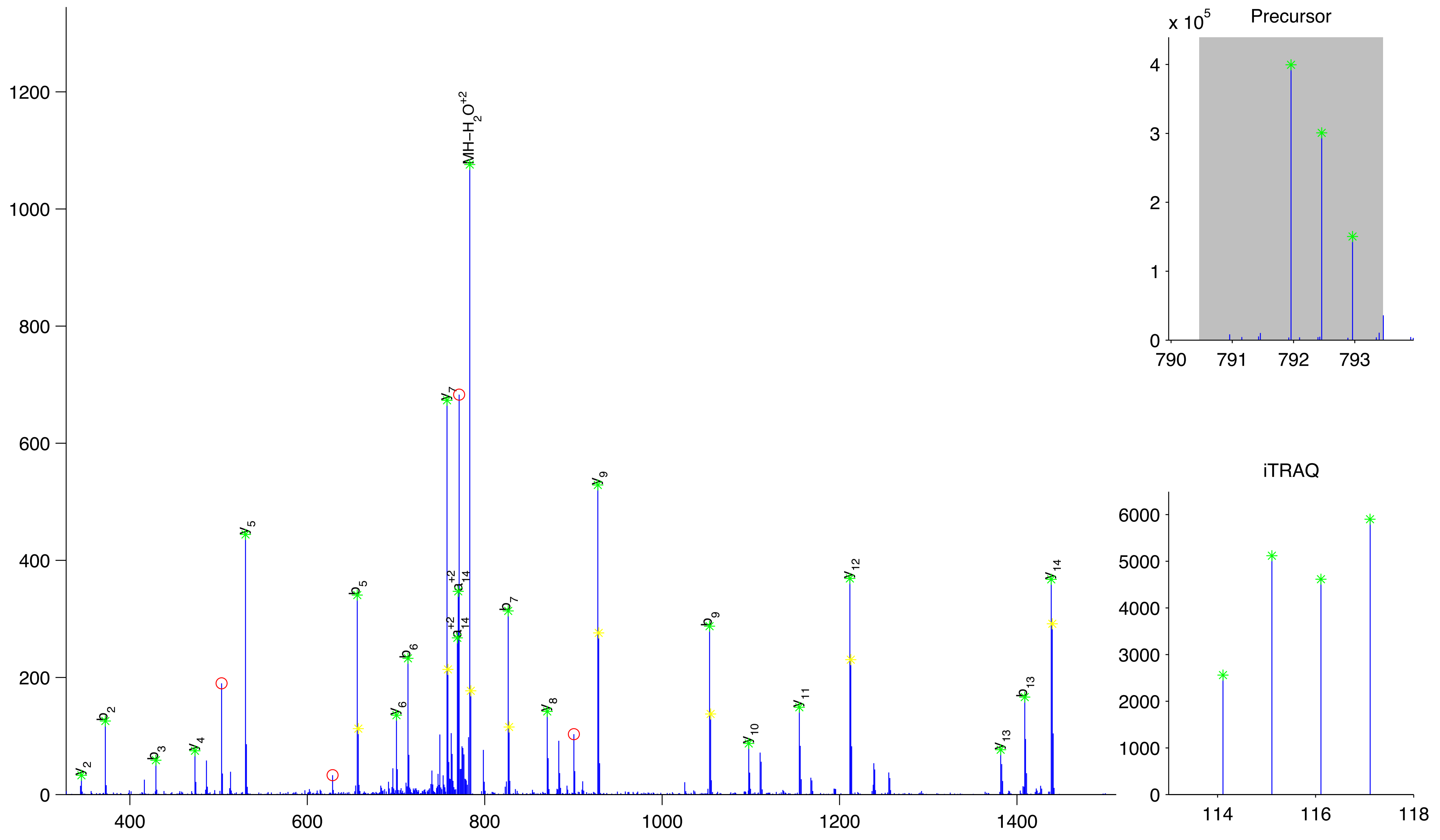

G G k G L G k G G A k R

histone cluster 1, H4a [Homo sapiens]
Charge State: +2
Scan Number: 20457
File Name: 110924_HG2_INSA_ACK.raw

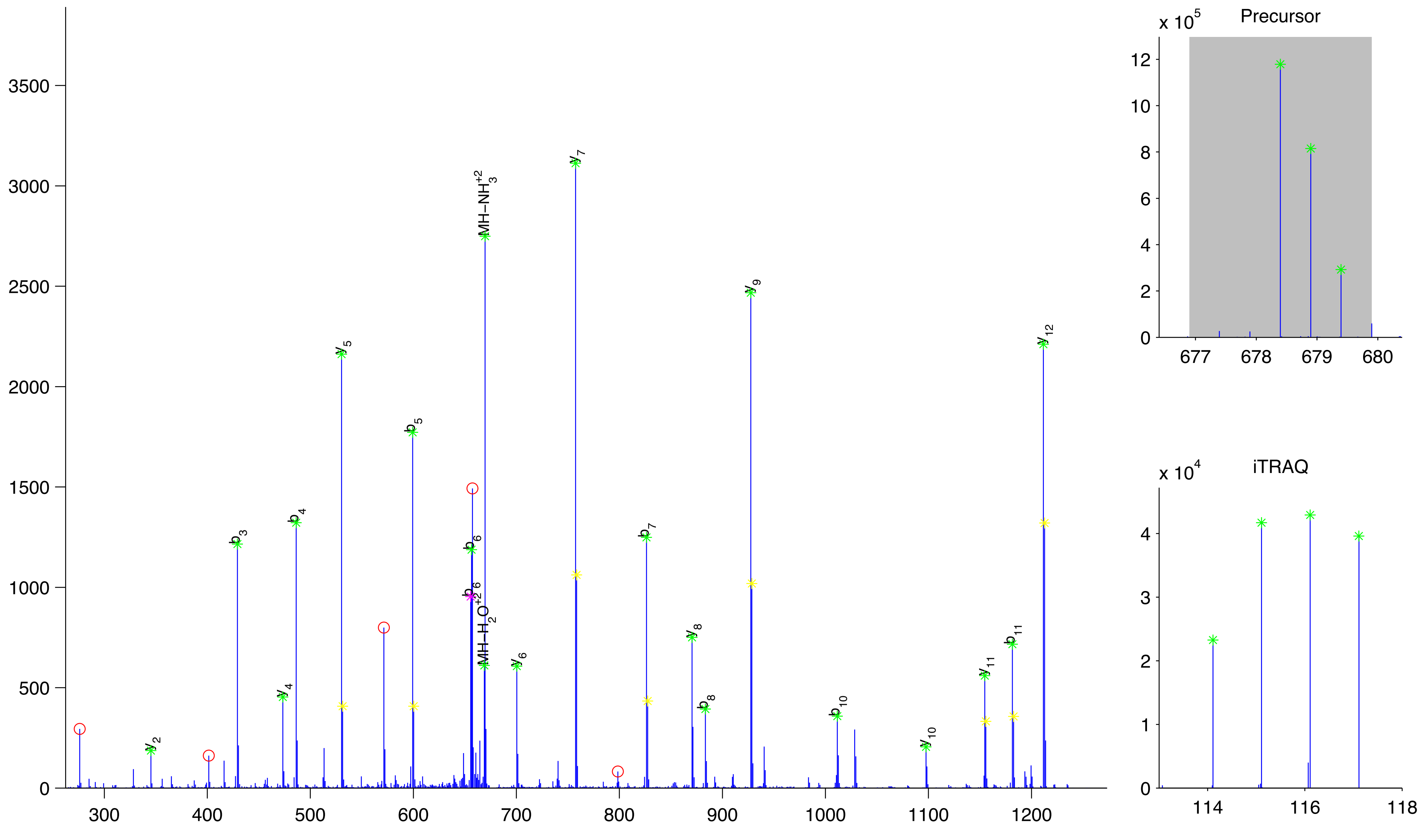

G G k G L G k G G A k R

histone cluster 1, H4a [Homo sapiens]
Charge State: +2
Scan Number: 20467
File Name: 110924_HG2_INSA_ACK.raw

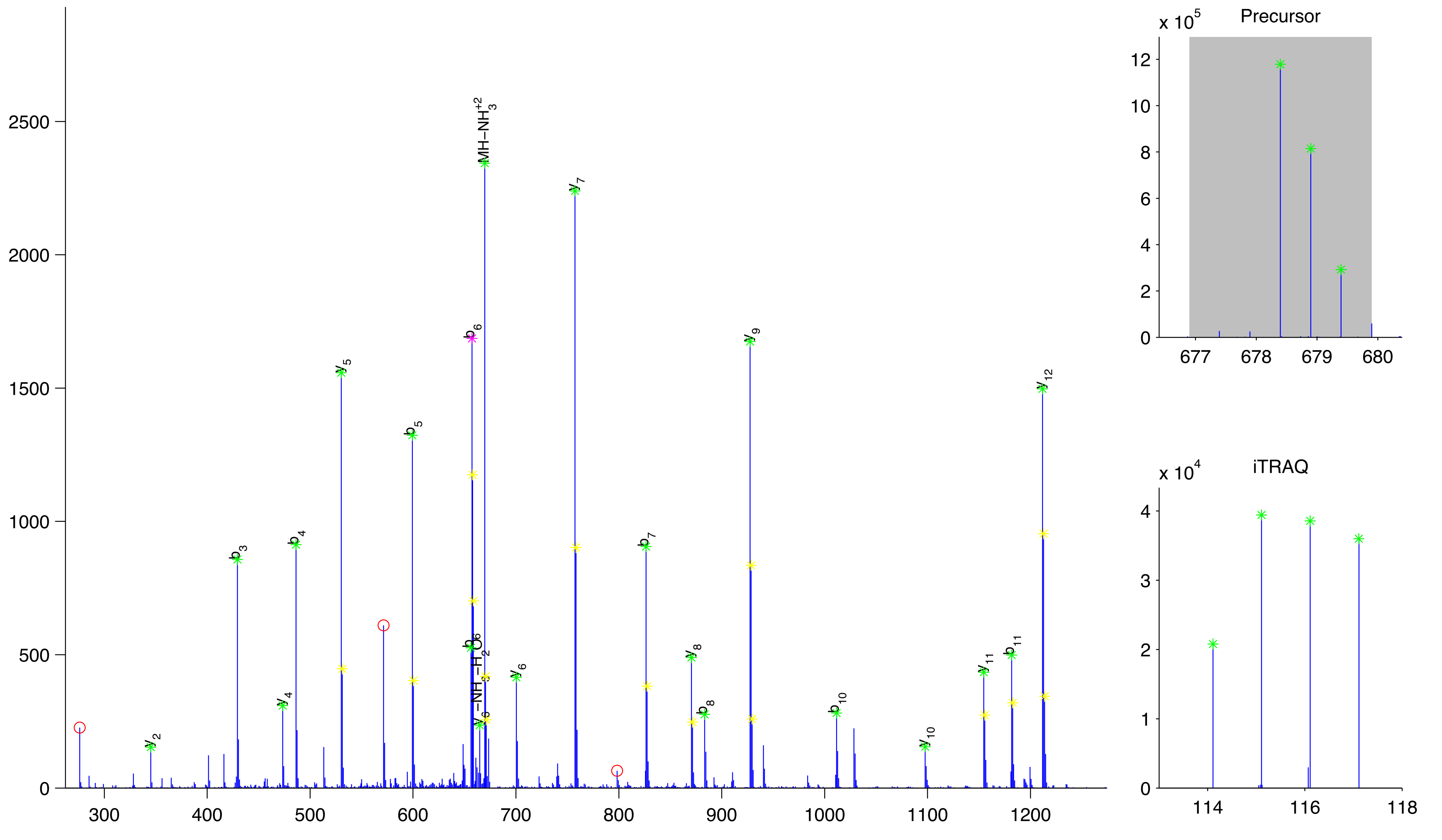

G k G G k G L G k G G A k R

histone cluster 1, H4a [Homo sapiens]
Charge State: +2
Scan Number: 20606
File Name: 110924_HG2_INSA_ACK.raw

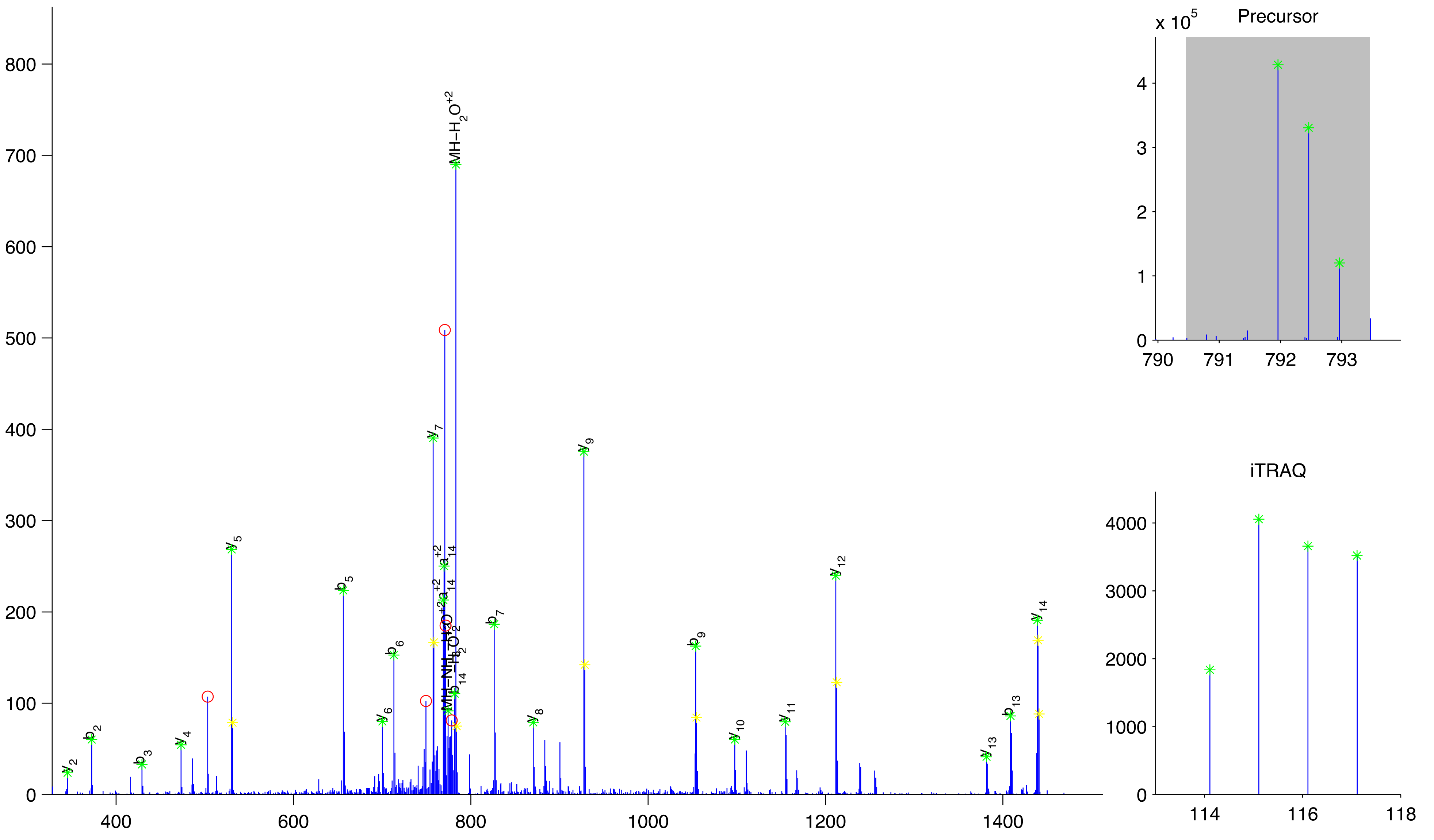

G k G G k G L G k G G A k R

histone cluster 1, H4a [Homo sapiens]
Charge State: +2
Scan Number: 20774
File Name: 110924_HG2_INSA_ACK.raw

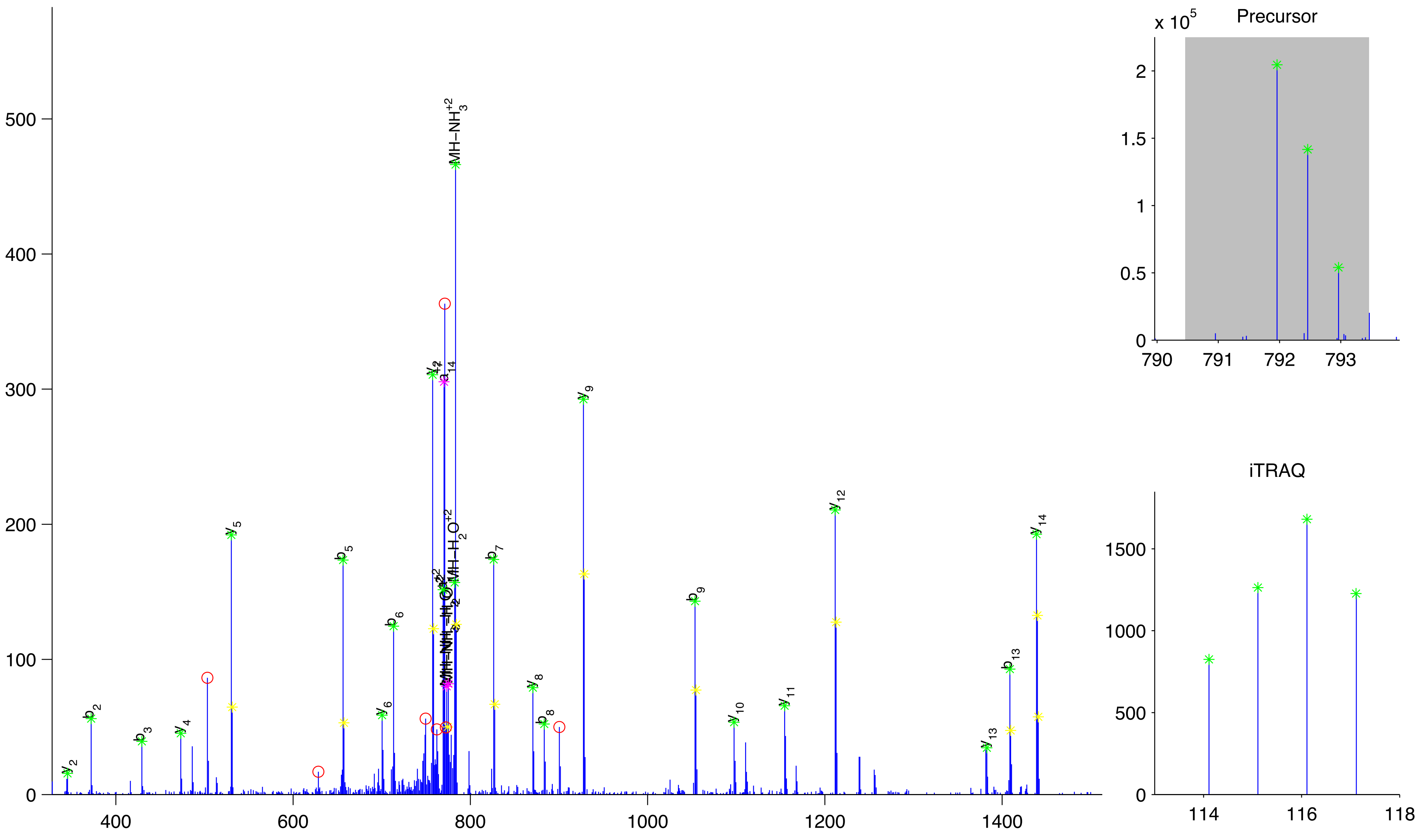

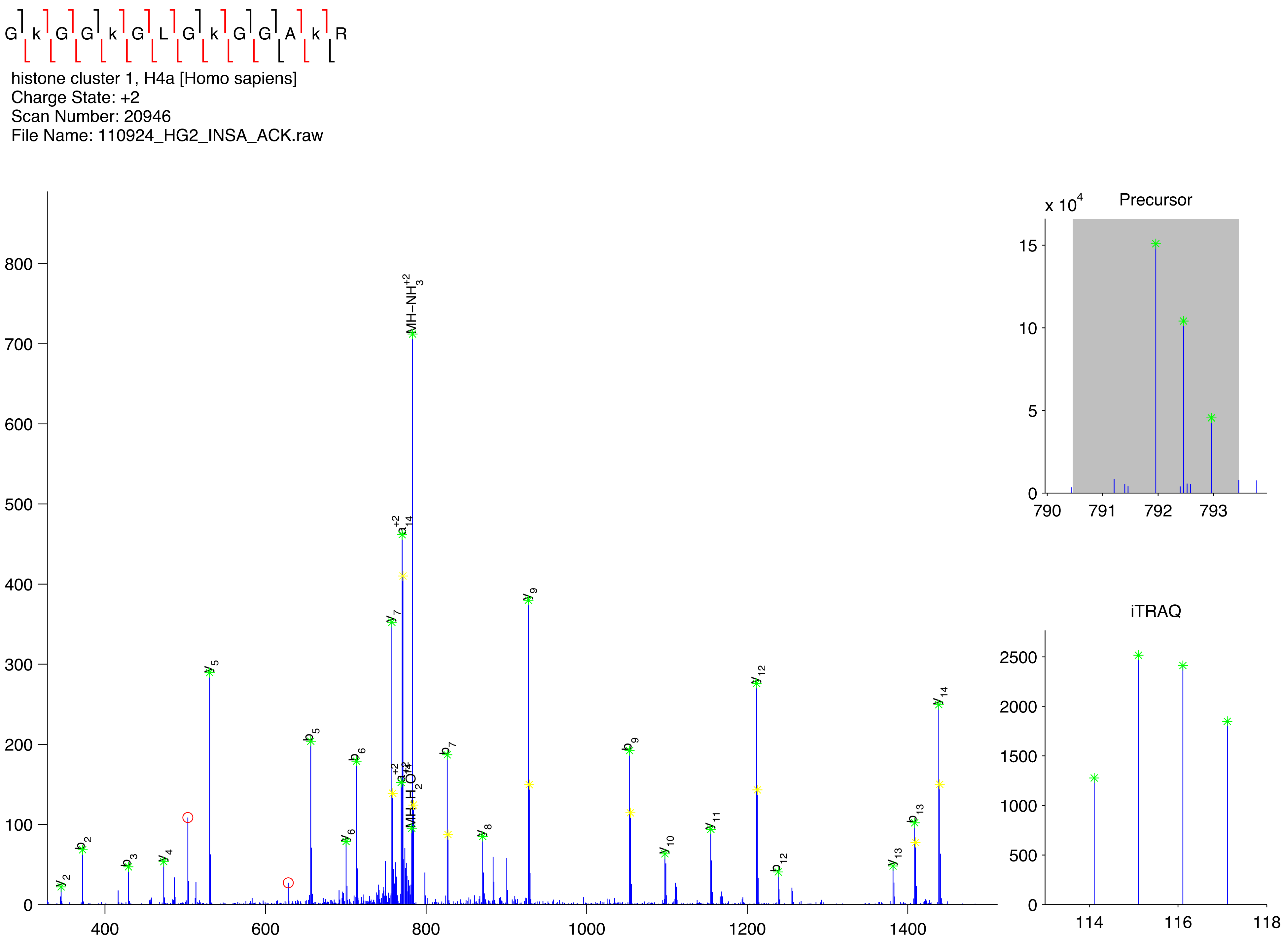
G k G G k G L G k G G A k R
histone cluster 1, H4a [Homo sapiens]
Charge State: +2
Scan Number: 20946
File Name: 110924_HG2_INSA_ACK.raw
Precursor
x 10^4
iTRAQ
MH−NH3+2
MH-H2O+2

G G k G L G k G G A k R

histone cluster 1, H4a [Homo sapiens]
Charge State: +2
Scan Number: 20963
File Name: 110924_HG2_INSA_ACK.raw

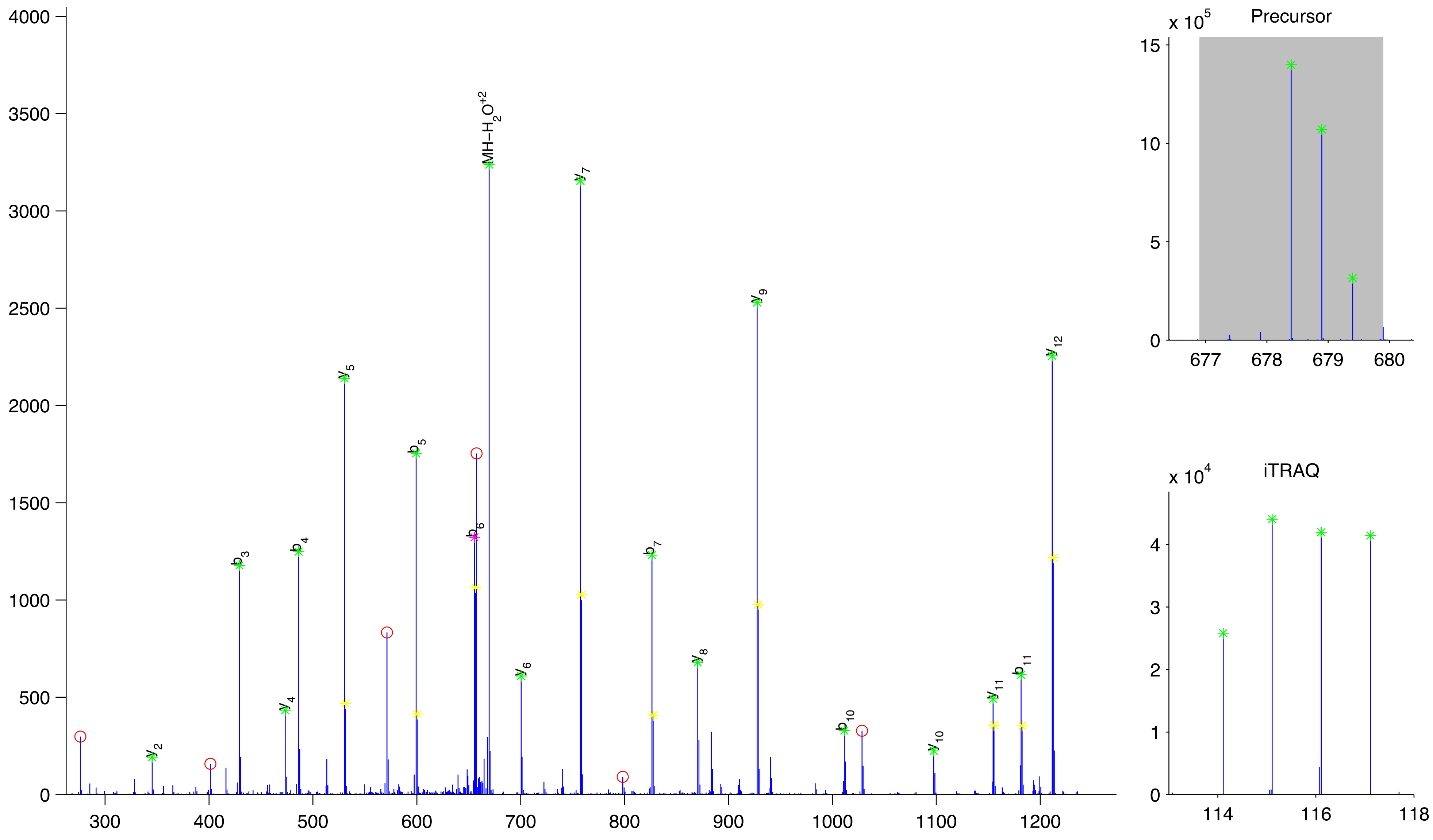

G G k G L G k G G A k R

histone cluster 1, H4a [Homo sapiens]
Charge State: +2
Scan Number: 20977
File Name: 110924_HG2_INSA_ACK.raw

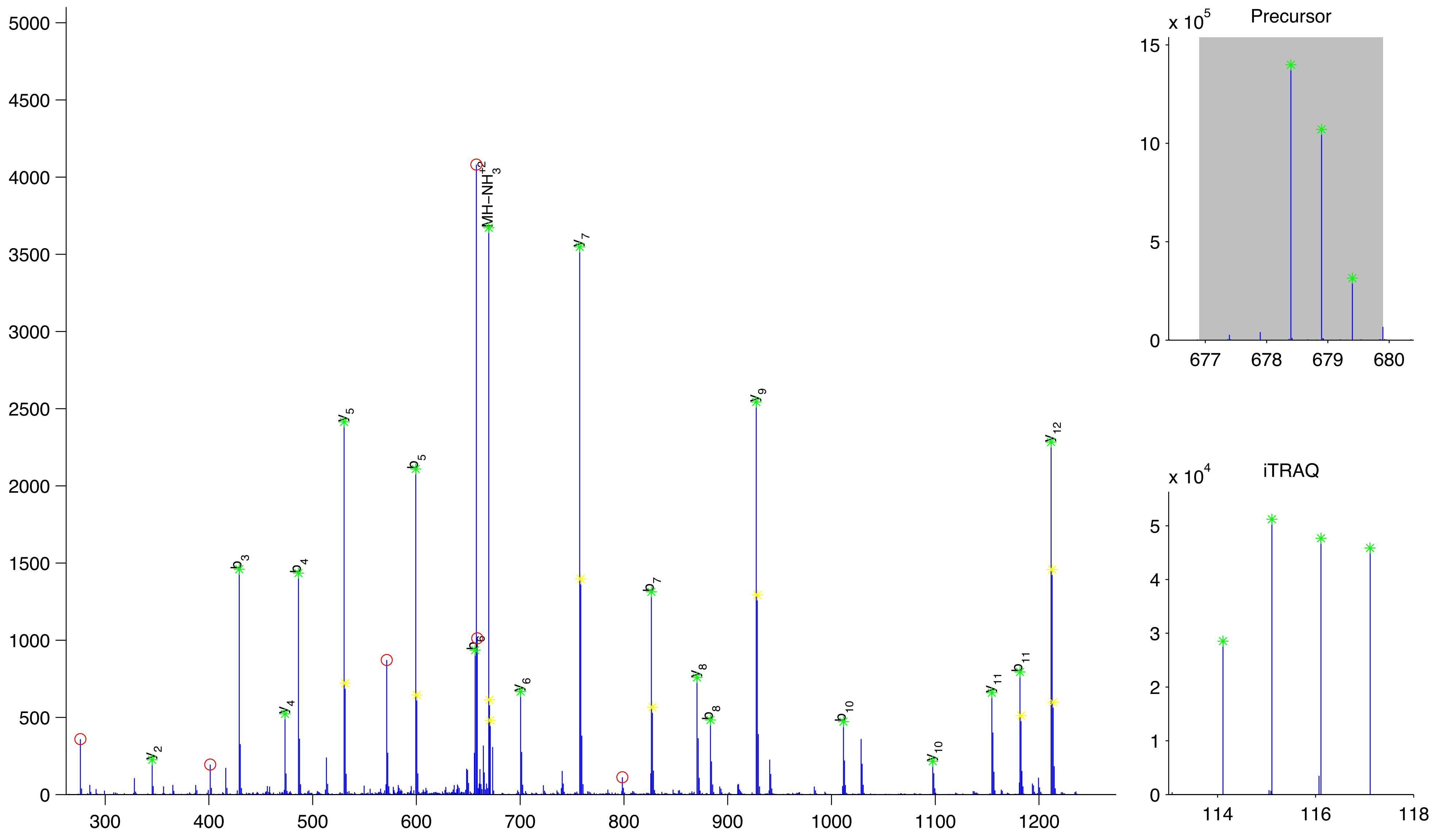

G k G G k G L G k G G A k R

histone cluster 1, H4a [Homo sapiens]
Charge State: +2
Scan Number: 21110
File Name: 110924_HG2_INSA_ACK.raw

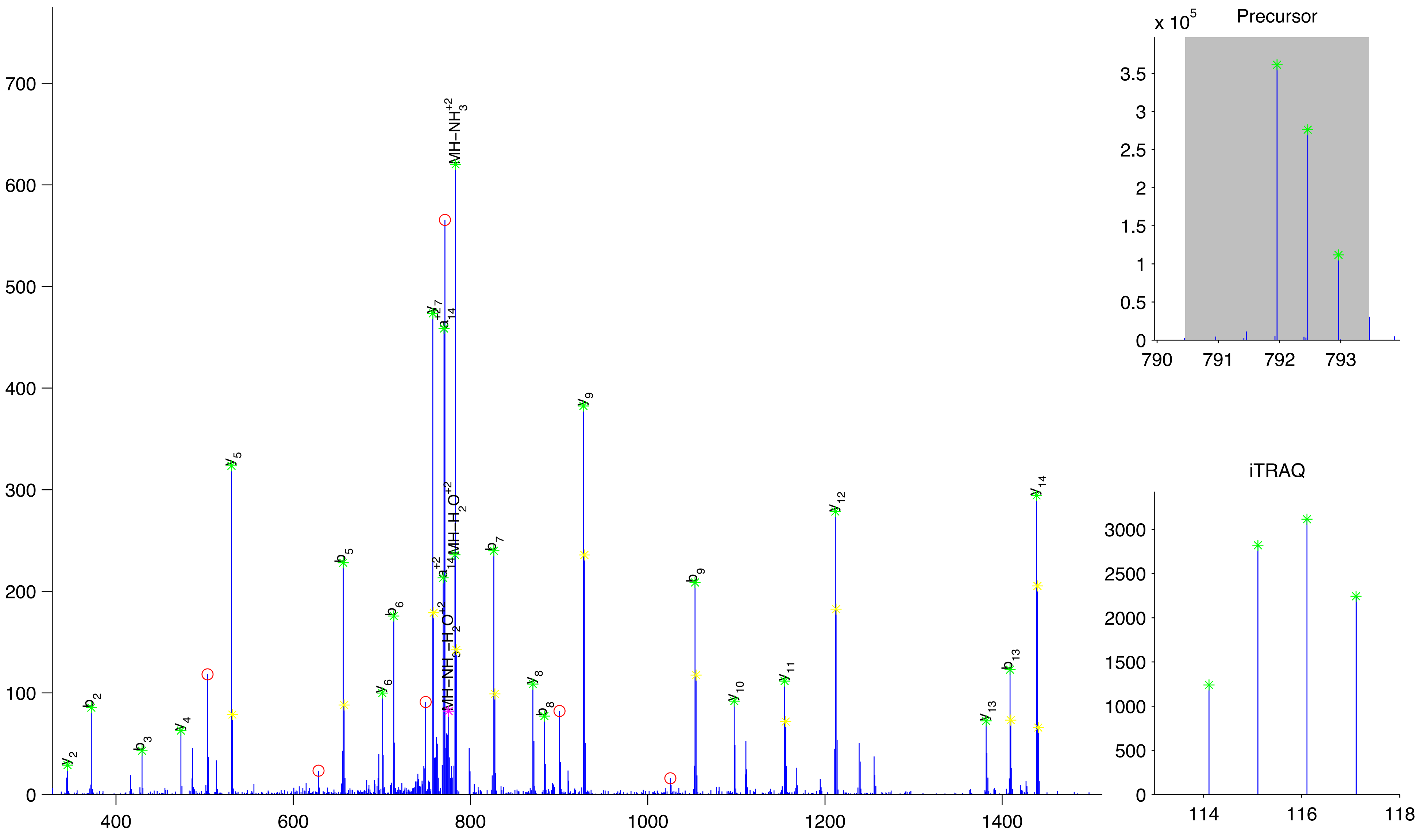

G k G G k G L G k G G A k R

histone cluster 1, H4a [Homo sapiens]
Charge State: +2
Scan Number: 21118
File Name: 110924_HG2_INSA_ACK.raw

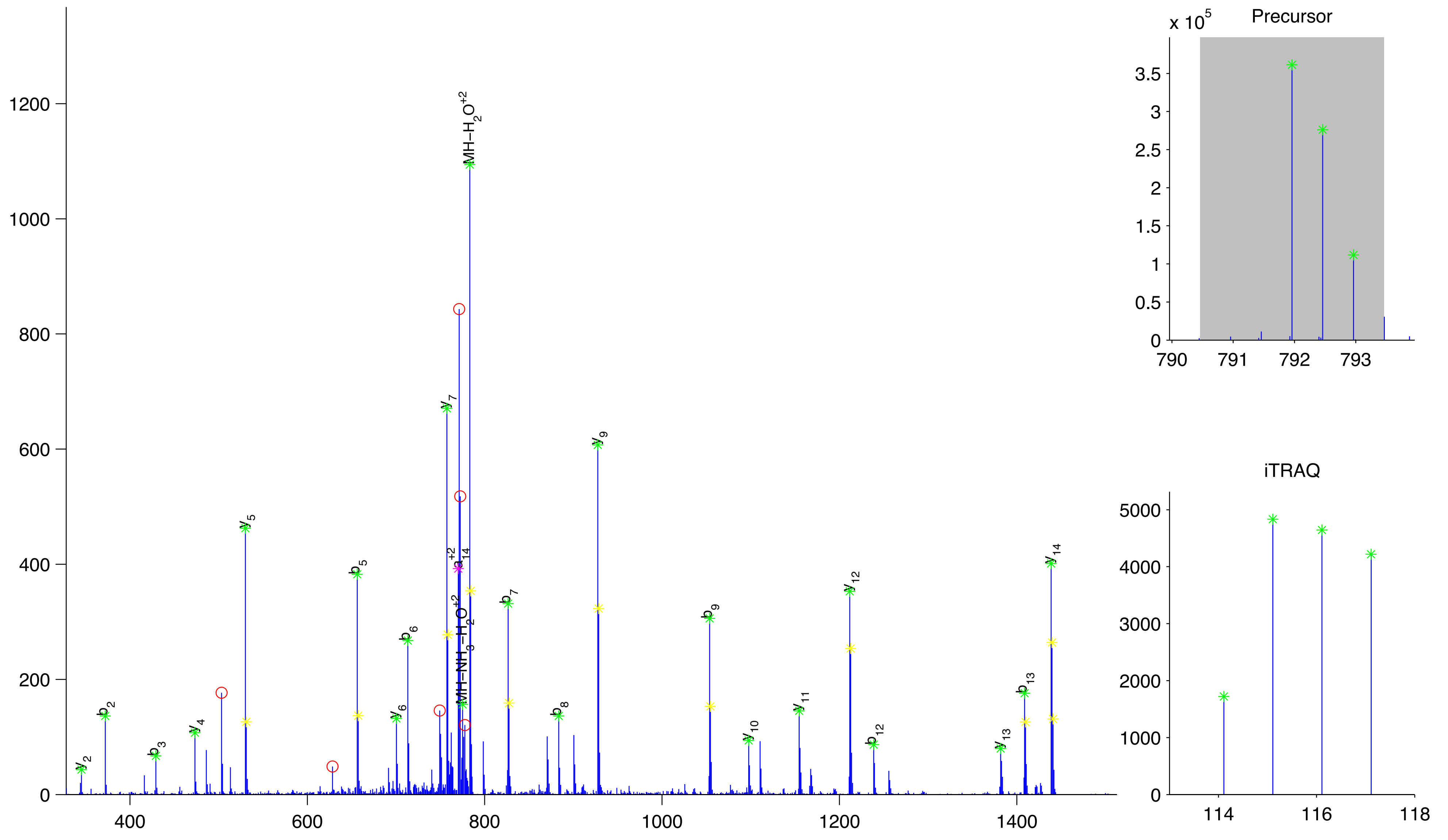

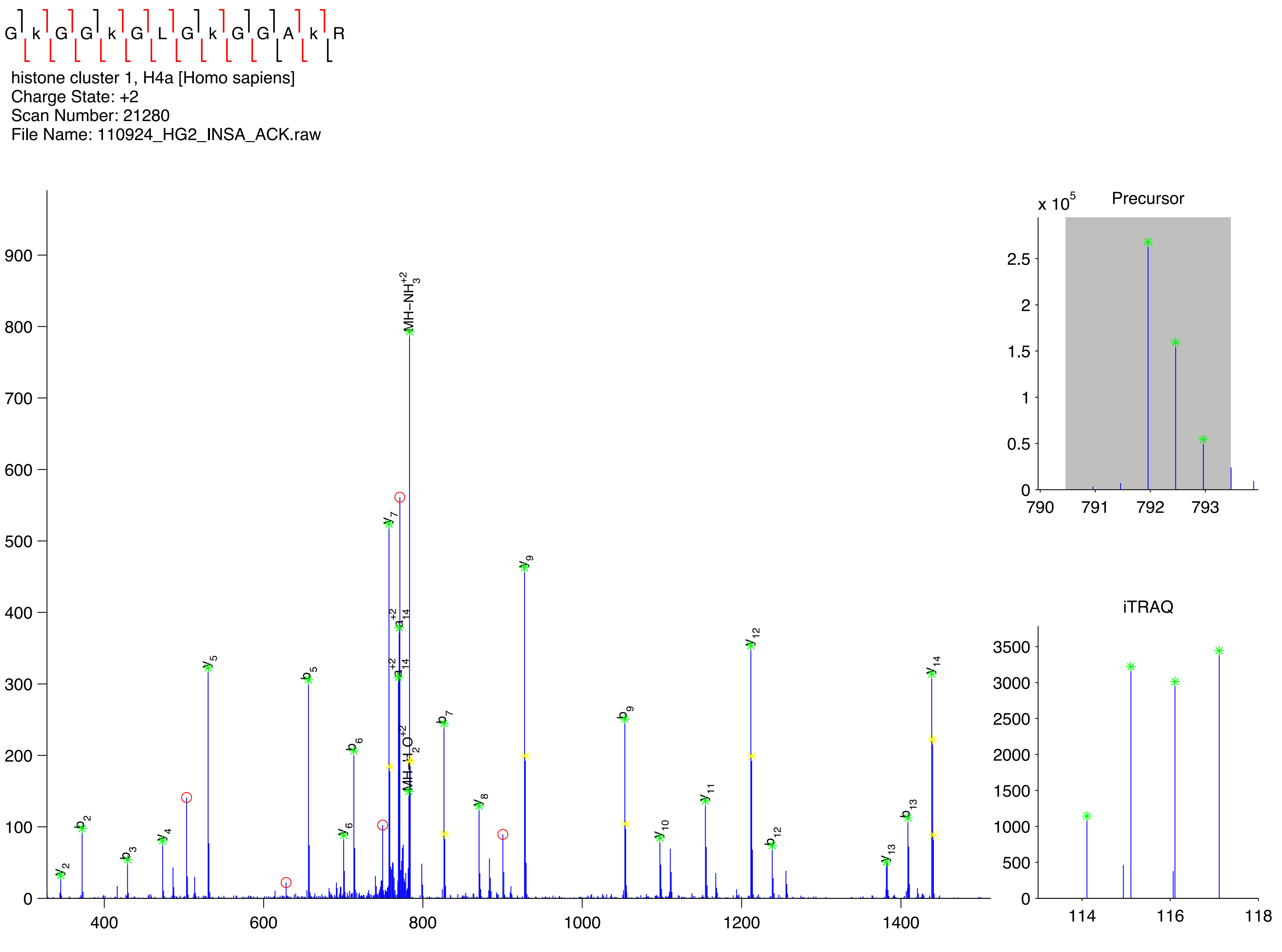

G k G G k G L G k G G A k R
histone cluster 1, H4a [Homo sapiens]
Charge State: +2
Scan Number: 21280
File Name: 110924_HG2_INSA_ACK.raw
Precursor
iTRAQ

G G k G L G k G G A k R

histone cluster 1, H4a [Homo sapiens]
Charge State: +2
Scan Number: 21299
File Name: 110924_HG2_INSA_ACK.raw

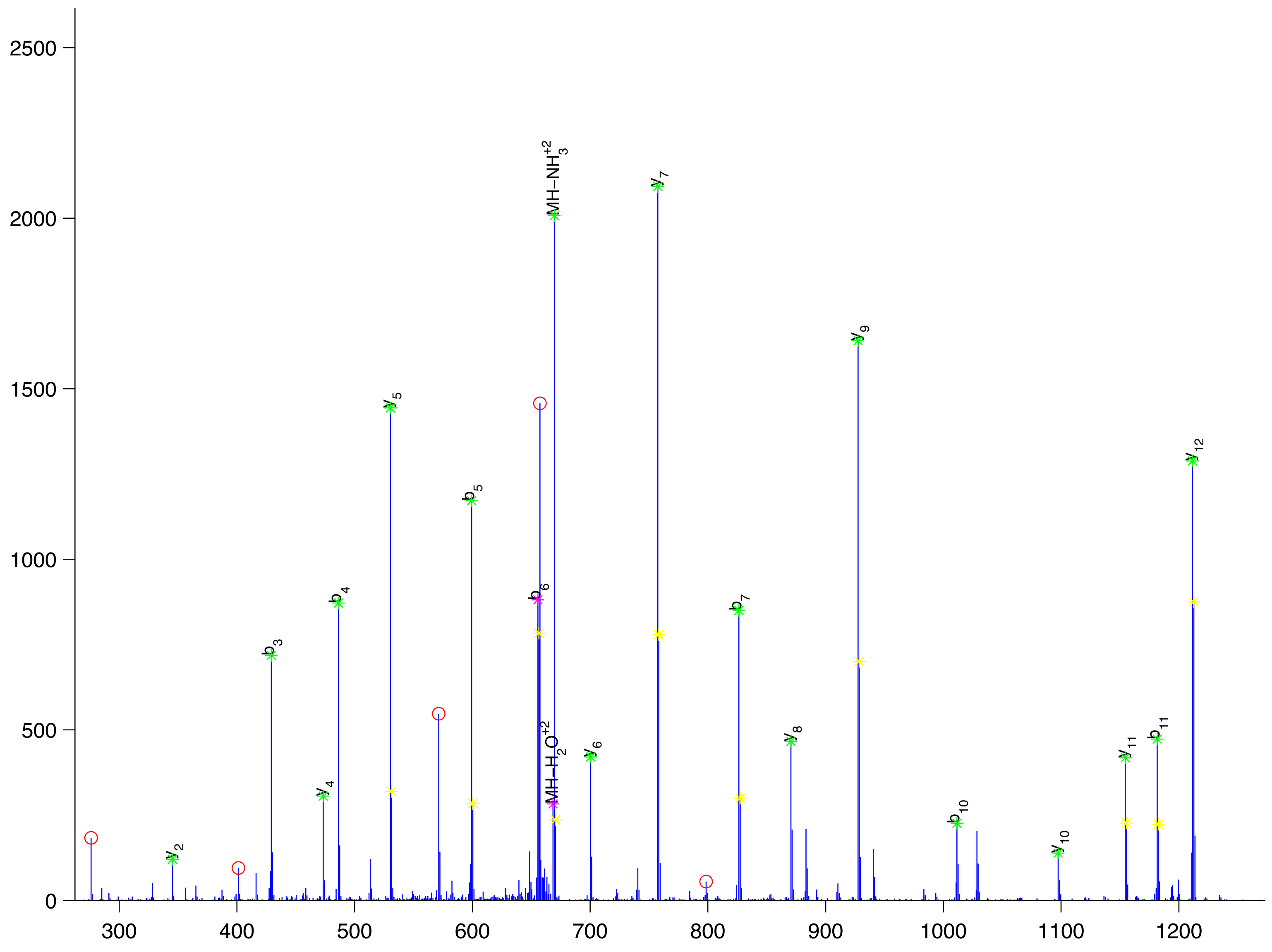

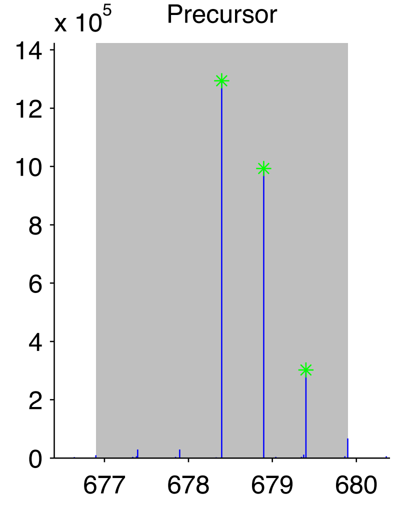

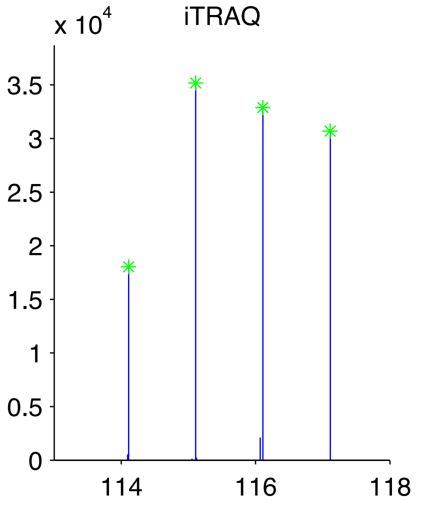

G G k G L G k G G A k R

histone cluster 1, H4a [Homo sapiens]
Charge State: +2
Scan Number: 21315
File Name: 110924_HG2_INSA_ACK.raw

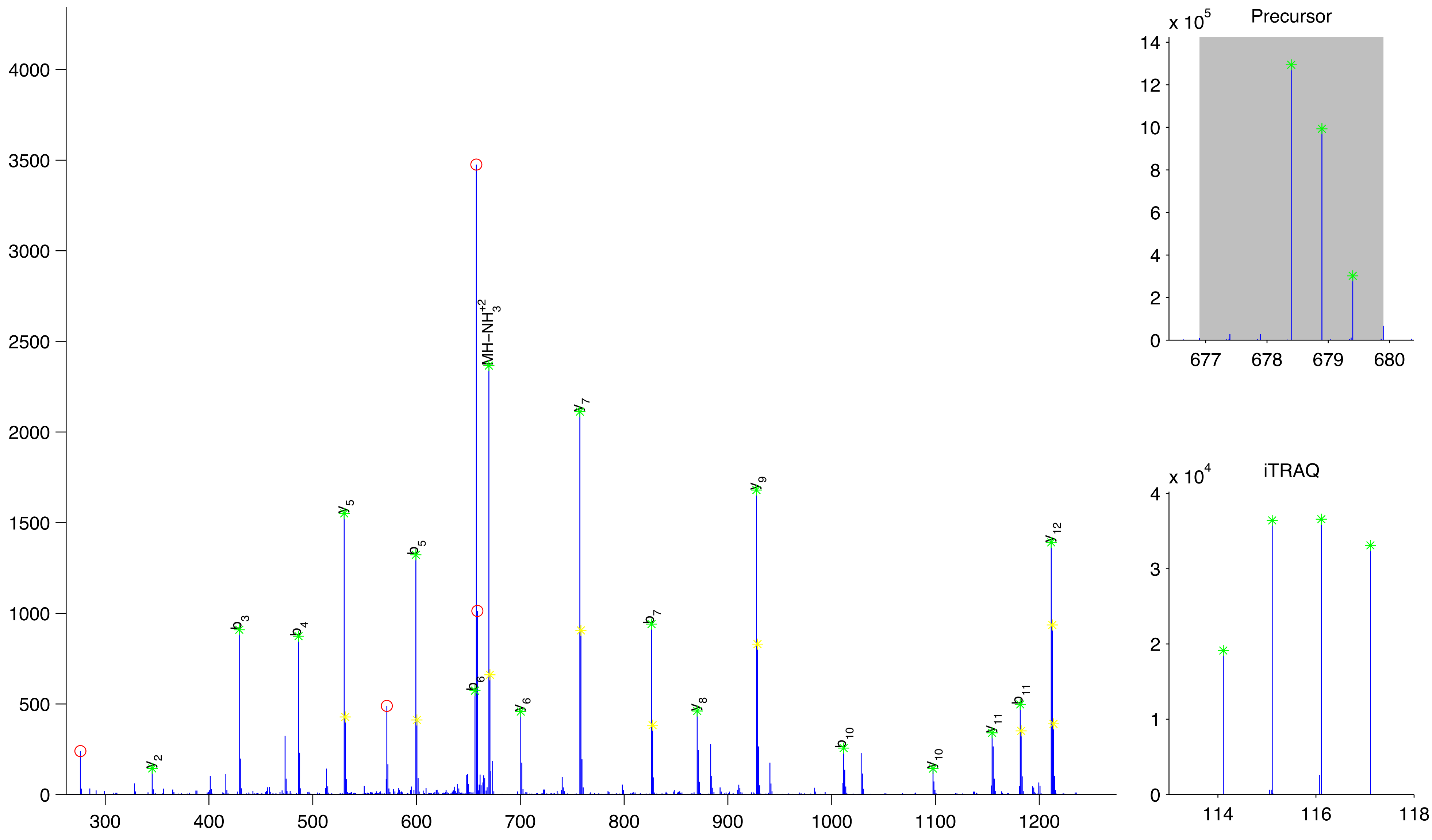

G k G G k G L G k G G A k R

histone cluster 1, H4a [Homo sapiens]
Charge State: +2
Scan Number: 21444
File Name: 110924_HG2_INSA_ACK.raw

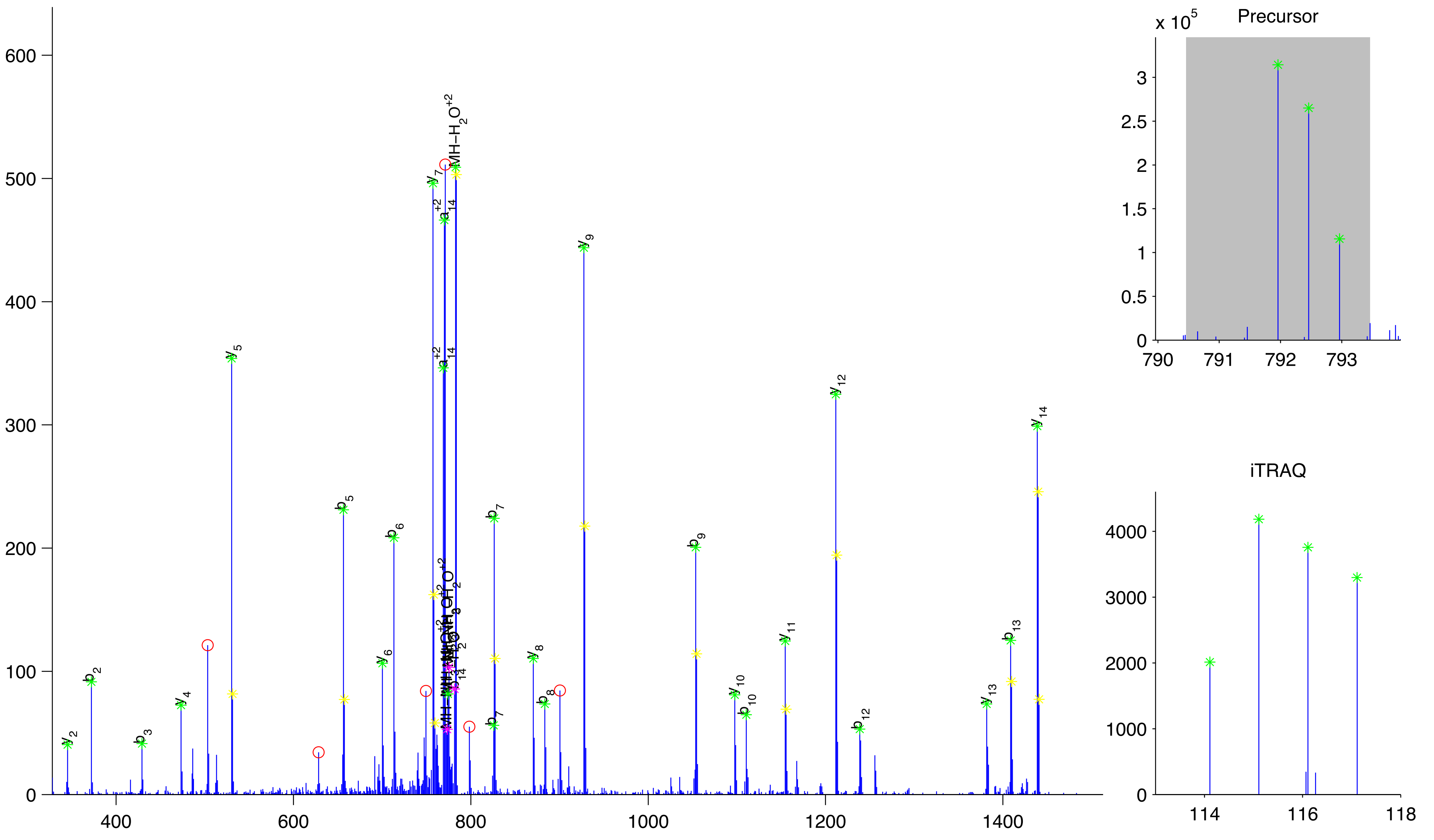

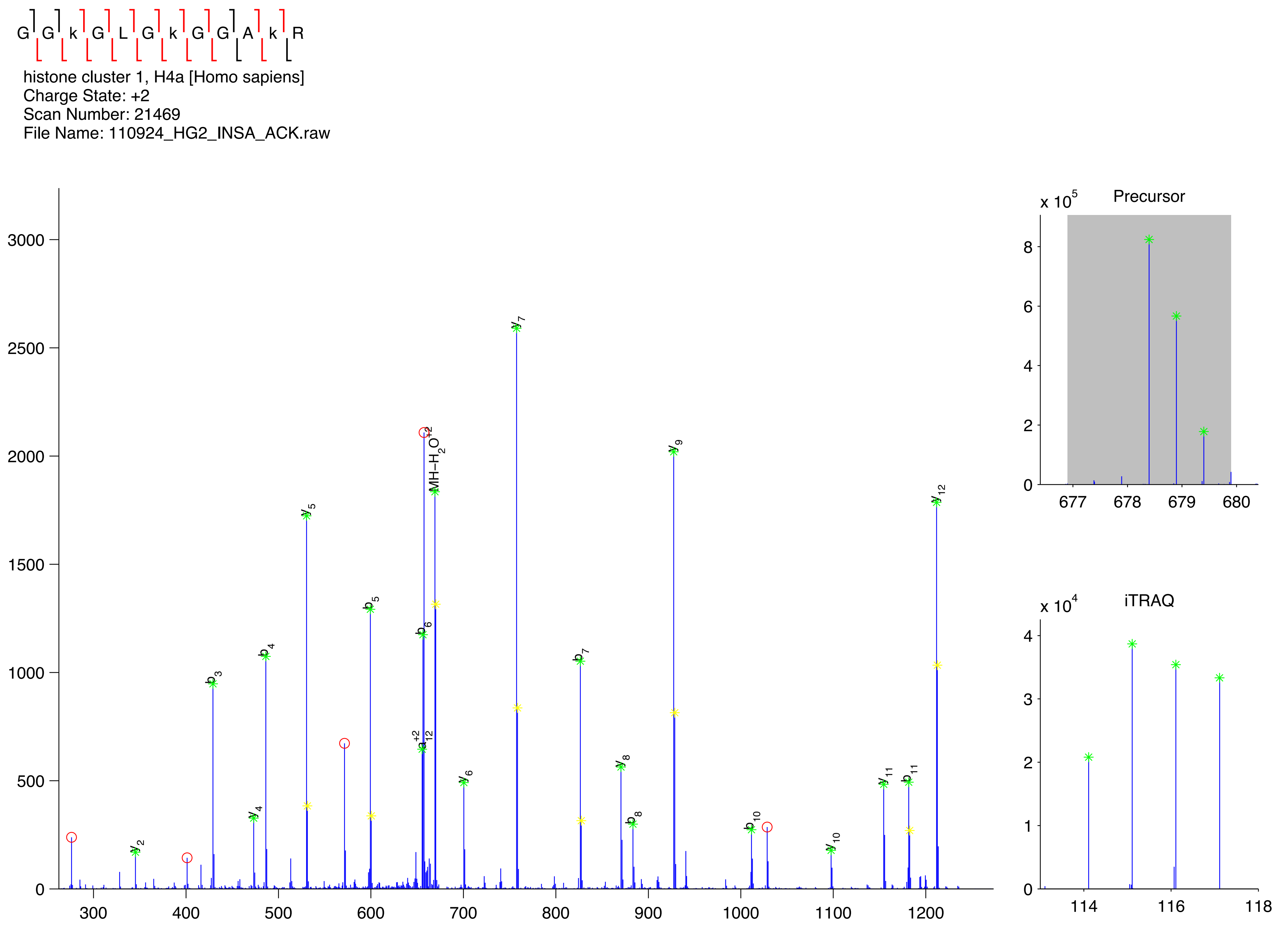
G G k G L G k G G A k R
histone cluster 1, H4a [Homo sapiens]
Charge State: +2
Scan Number: 21469
File Name: 110924_HG2_INSA_ACK.raw
y2
b3
y4
b4
y5
b5
b6
a12 +2
MH−H2O +2
y6
y7
b7
y8
b8
y9
b10
y10
y11
b11
y12
Precursor
x 10^5
iTRAQ
x 10^4

G G k G L G k G G A k R

histone cluster 1, H4a [Homo sapiens]
Charge State: +2
Scan Number: 21483
File Name: 110924_HG2_INSA_ACK.raw

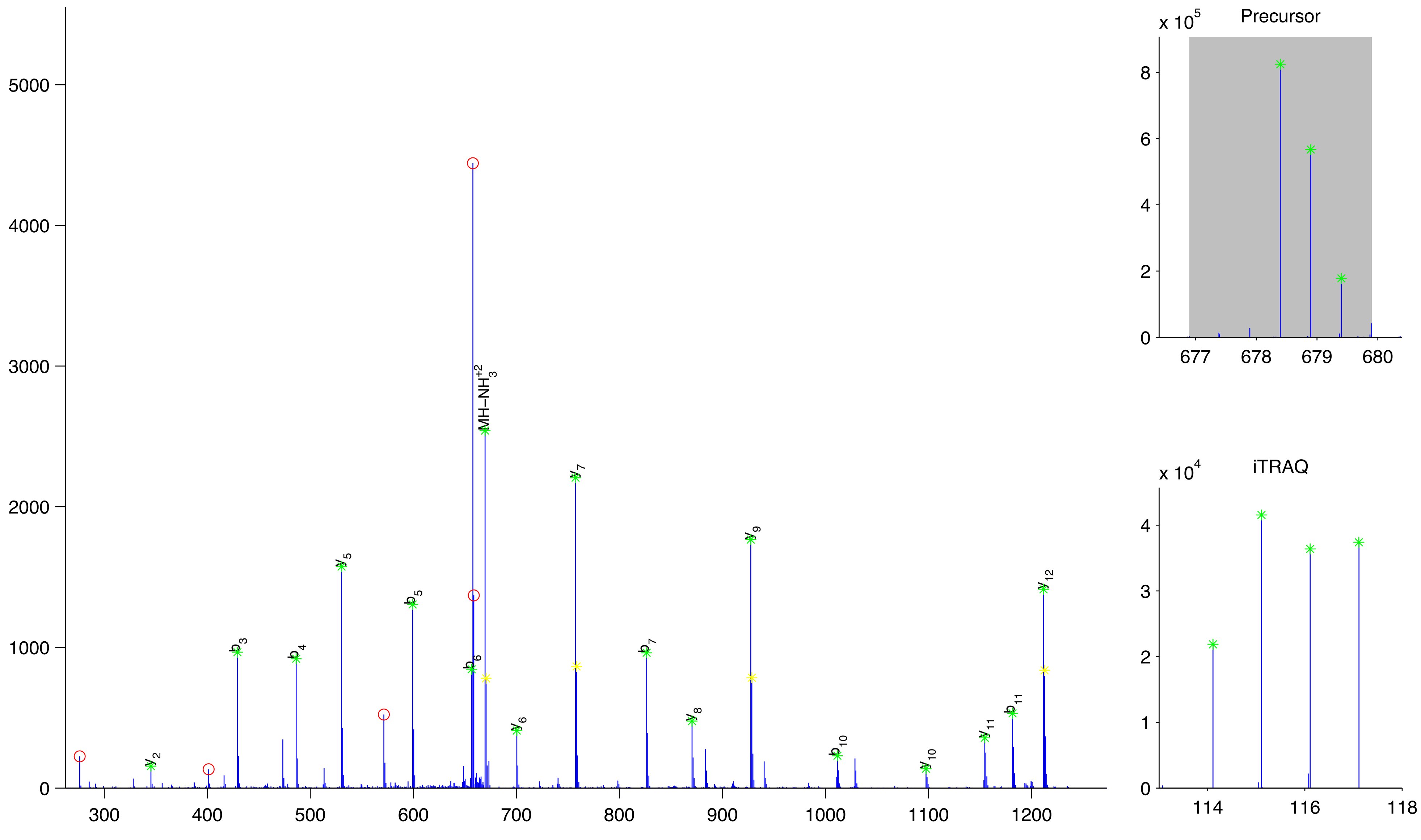

G G k G L G k G G A k R

histone cluster 1, H4a [Homo sapiens]
Charge State: +2
Scan Number: 21637
File Name: 110924_HG2_INSA_ACK.raw

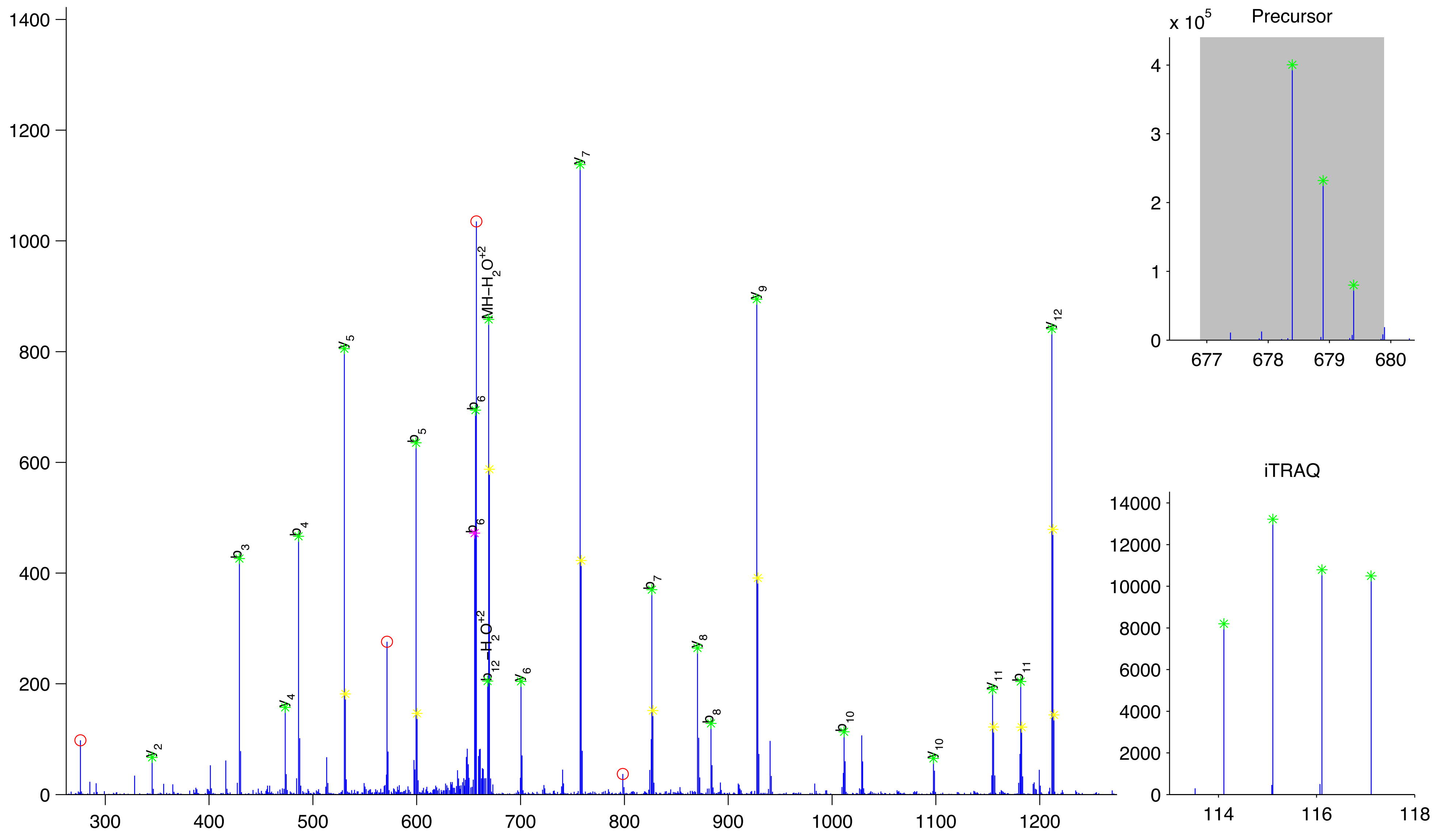

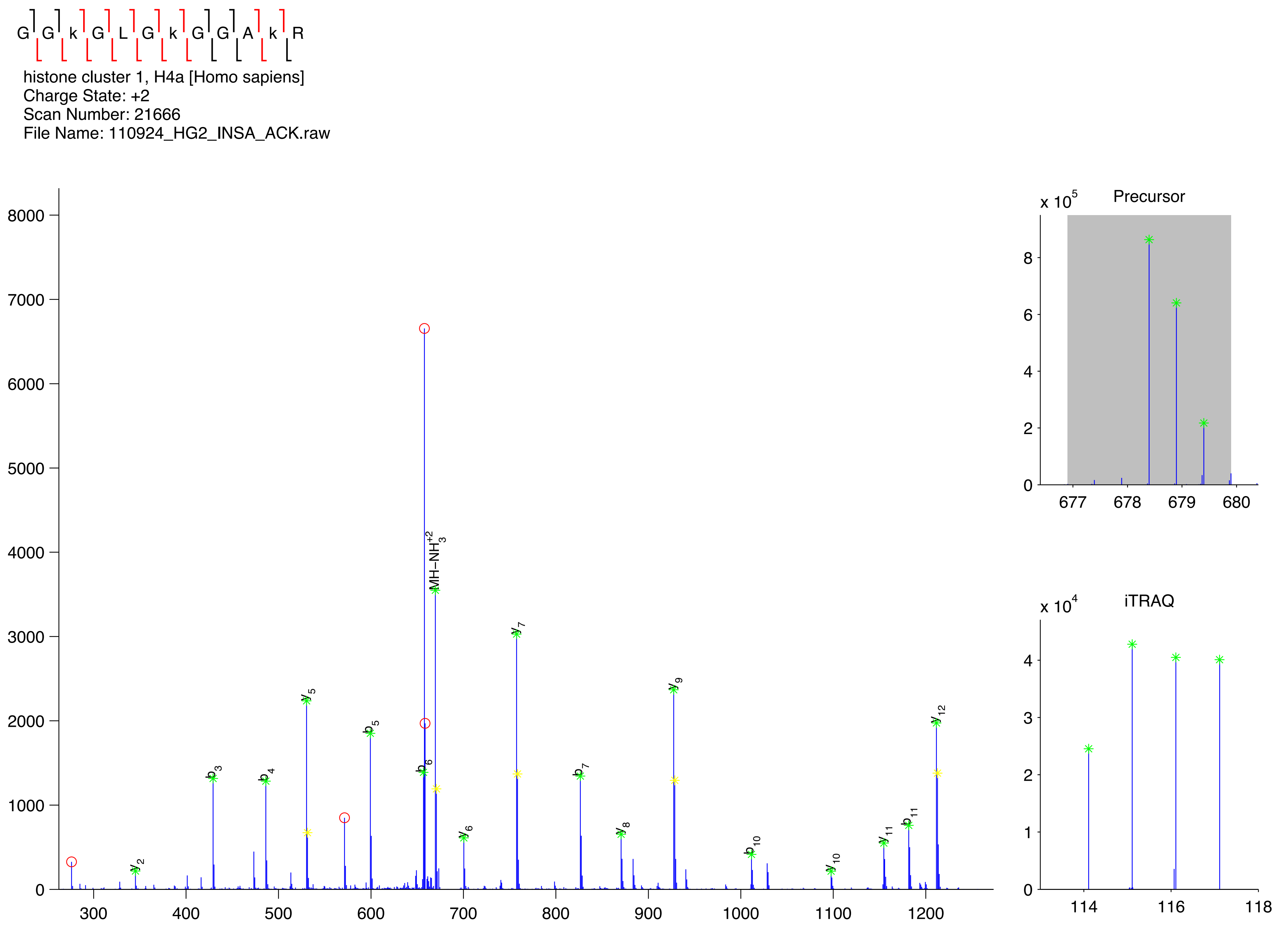
G G k G L G k G G A k R
histone cluster 1, H4a [Homo sapiens]
Charge State: +2
Scan Number: 21666
File Name: 110924_HG2_INSA_ACK.raw
Precursor
x 10^5
iTRAQ
x 10^4
y2
b3
b4
y5
b5
b6
MH−NH3+2
y6
y7
b7
y8
y9
b10
y10
y11
b11
y12

G k G G k G L G k G G A k R

histone cluster 1, H4a [Homo sapiens]
Charge State: +2
Scan Number: 21773
File Name: 110924_HG2_INSA_ACK.raw

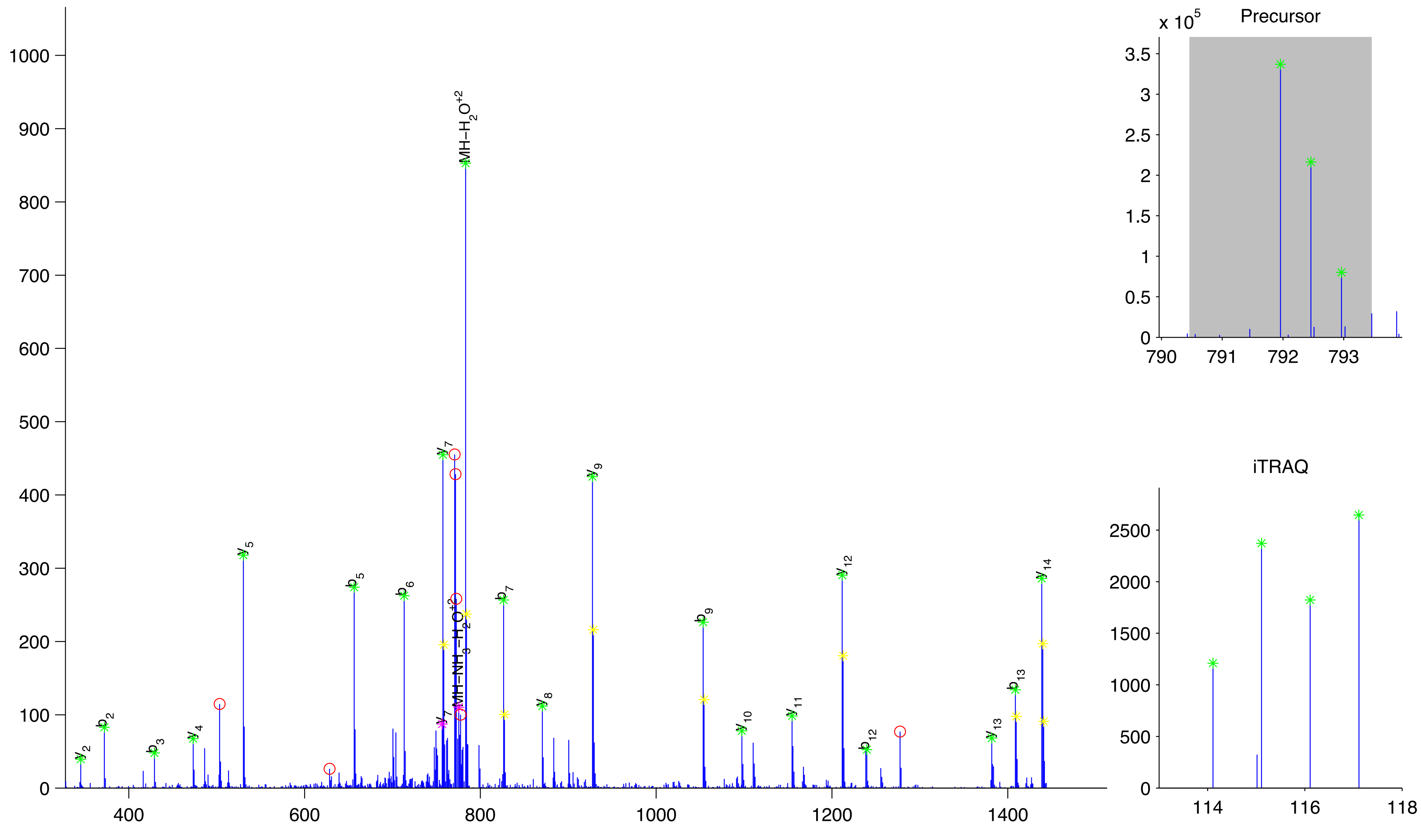

G G k G L G k G G A k R

histone cluster 1, H4a [Homo sapiens]
Charge State: +2
Scan Number: 21832
File Name: 110924_HG2_INSA_ACK.raw

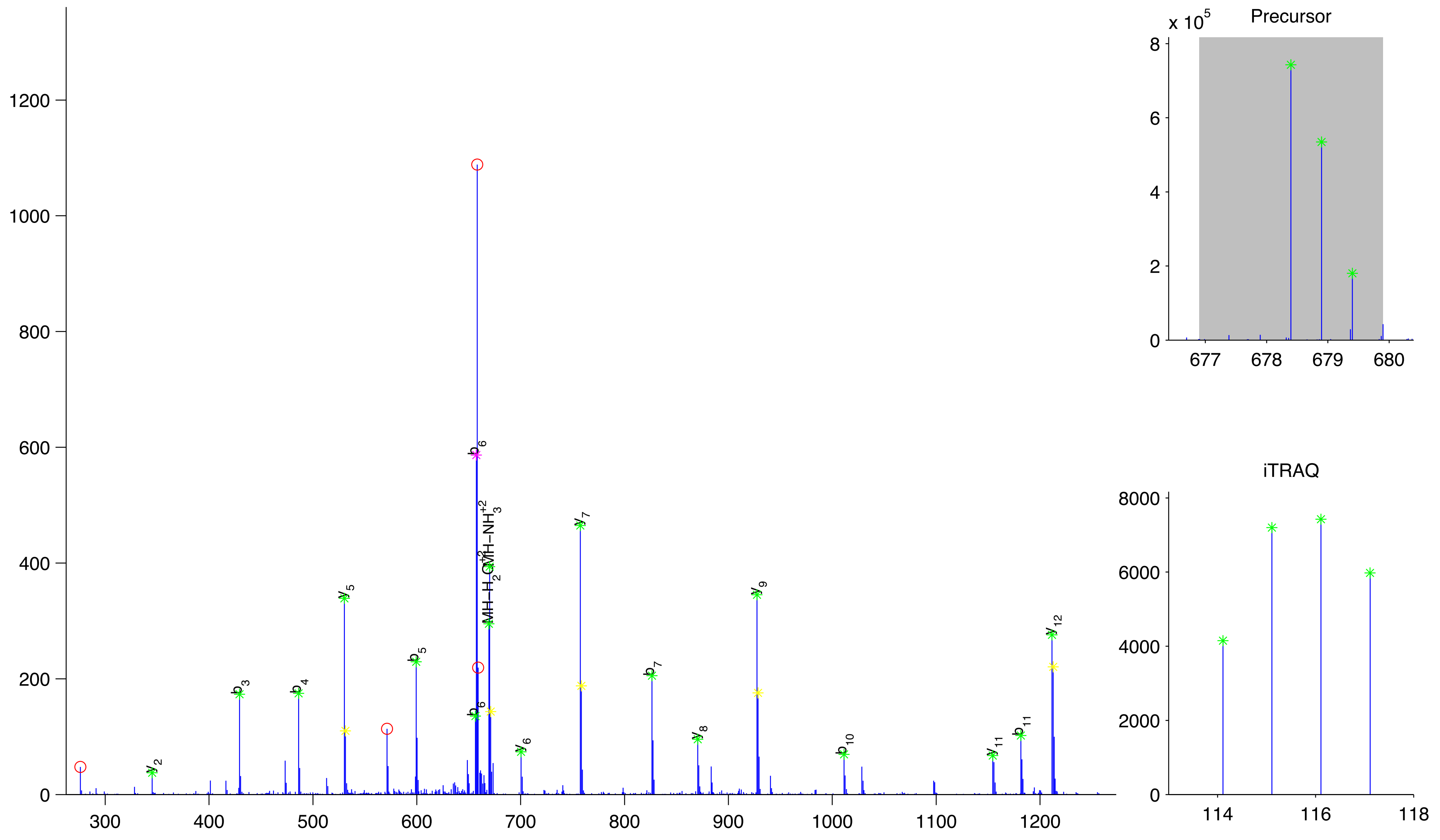

G G k G L G k G G A k R

histone cluster 1, H4a [Homo sapiens]
Charge State: +2
Scan Number: 21971
File Name: 110924_HG2_INSA_ACK.raw

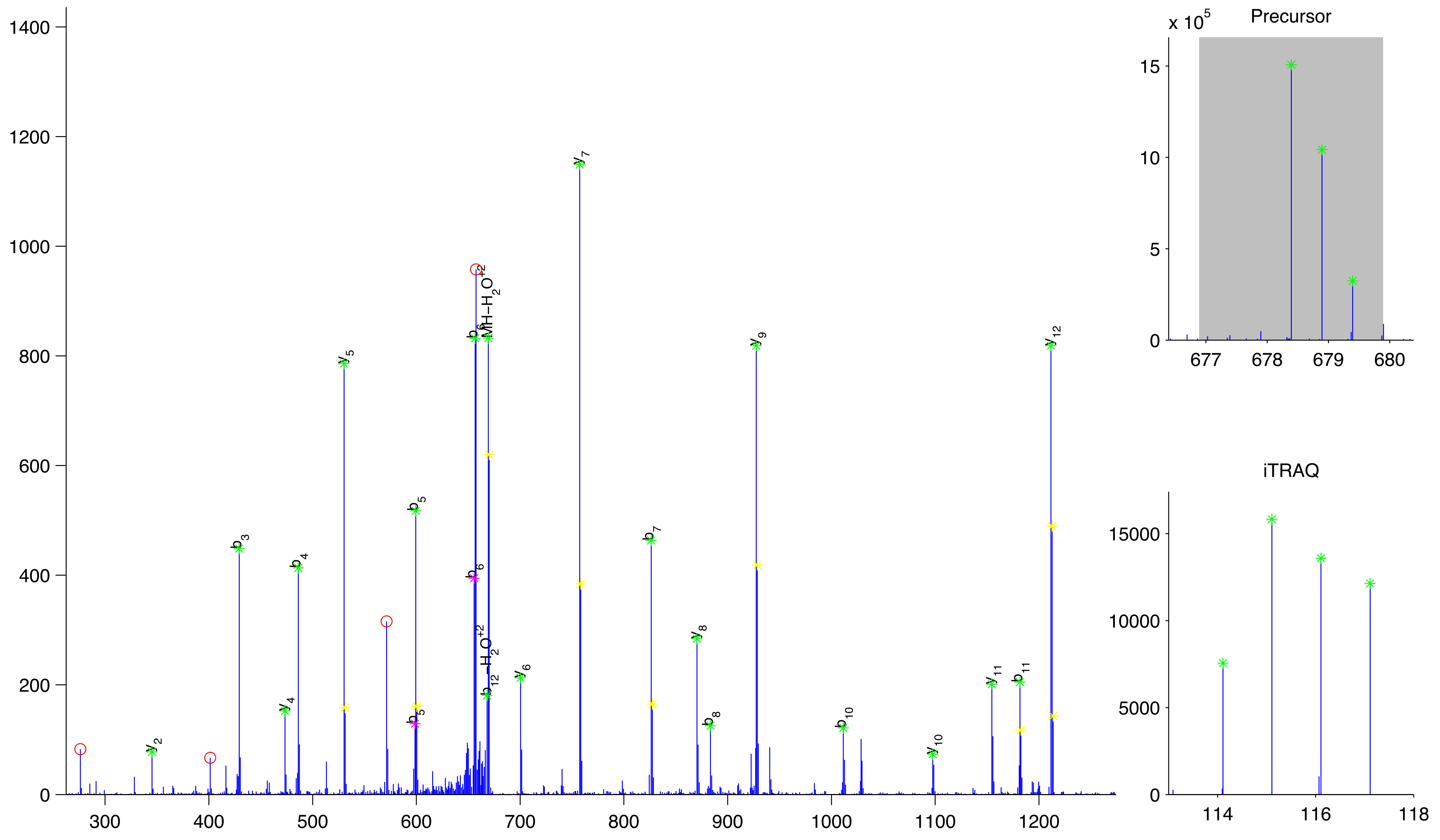

G G k G L G k G G A k R

histone cluster 1, H4a [Homo sapiens]
Charge State: +2
Scan Number: 22004
File Name: 110924_HG2_INSA_ACK.raw

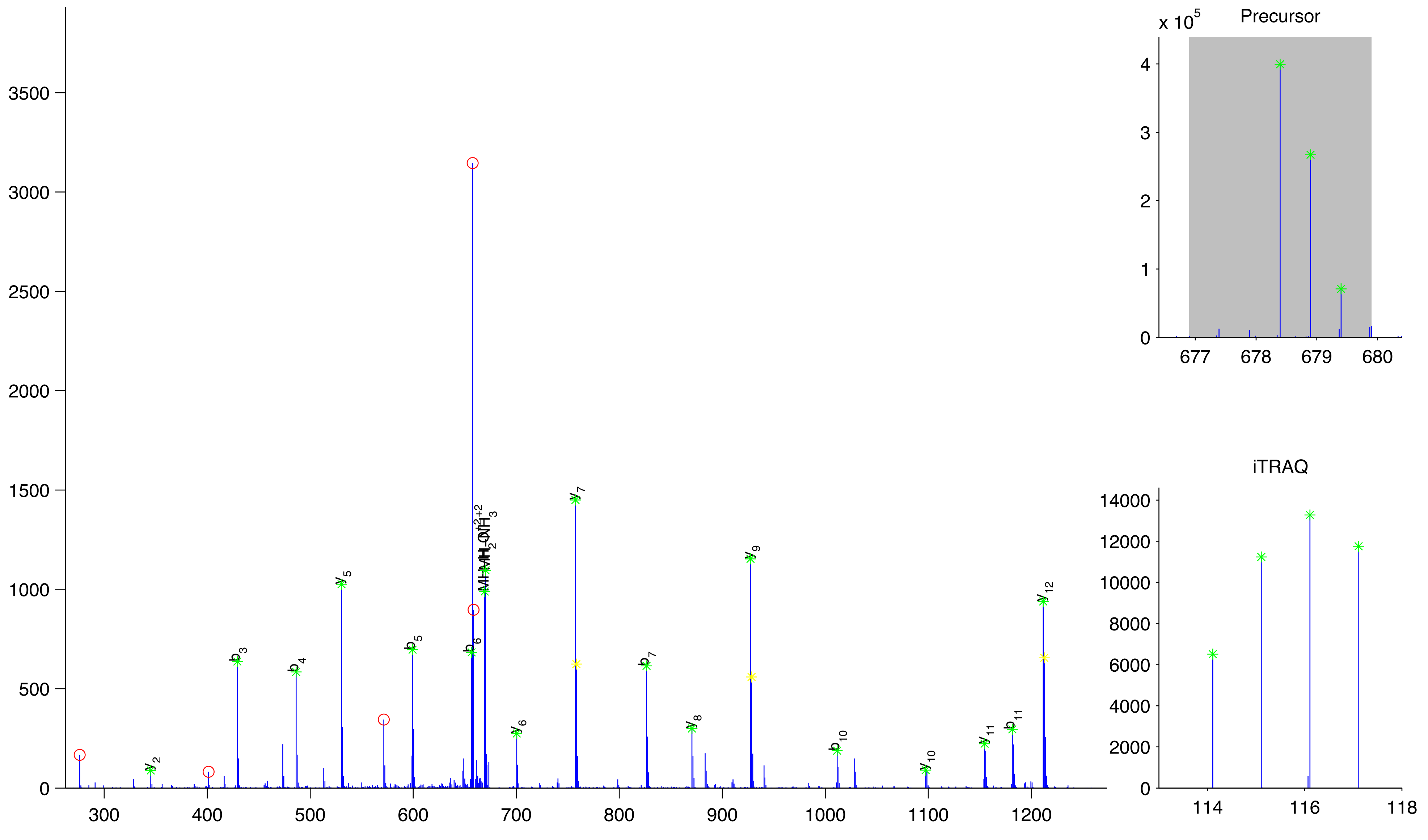

G G k G L G k G G A k R

histone cluster 1, H4a [Homo sapiens]
Charge State: +2
Scan Number: 22475
File Name: 110924_HG2_INSA_ACK.raw

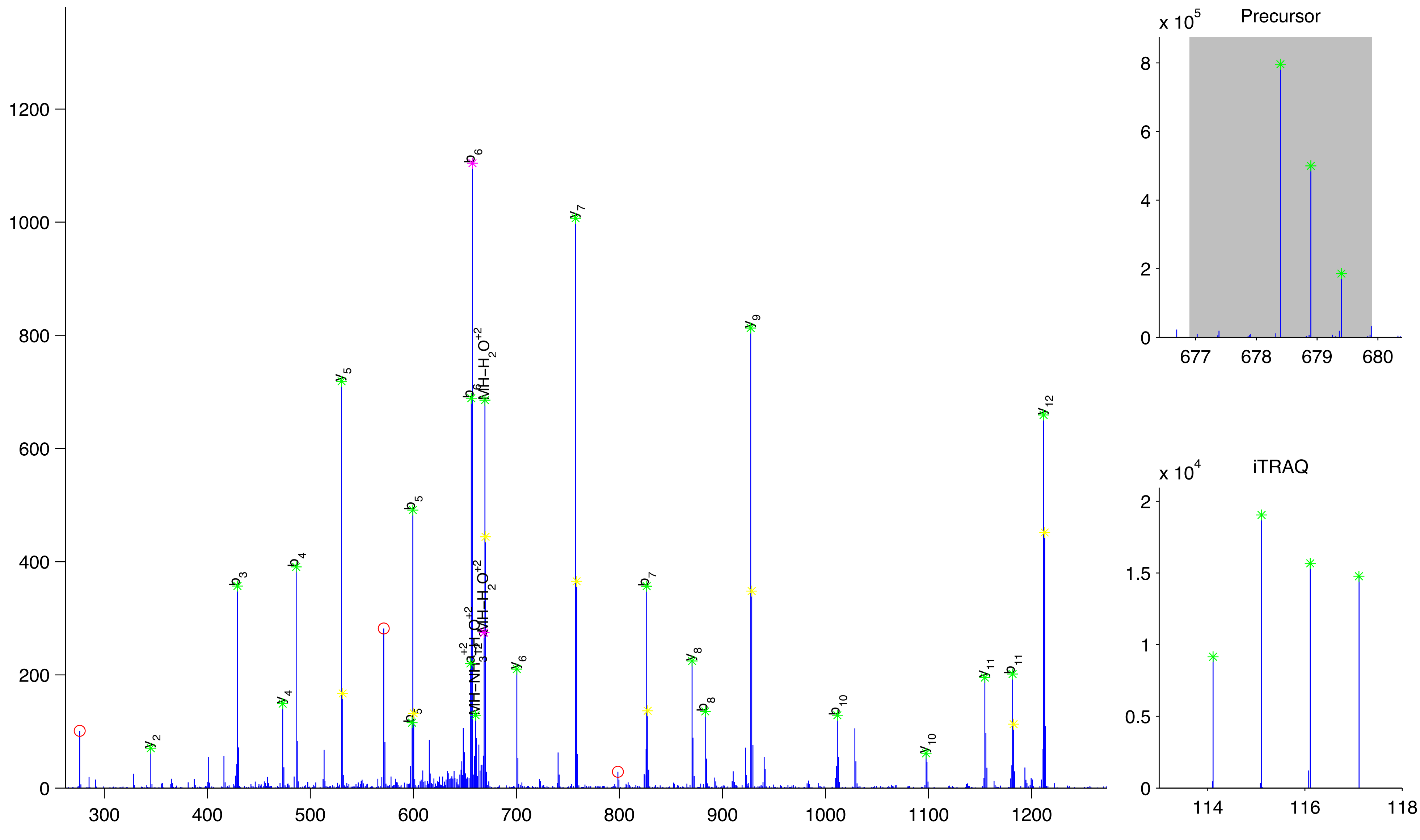

G G k G L G k G G A k R

histone cluster 1, H4a [Homo sapiens]
Charge State: +2
Scan Number: 22762
File Name: 110924_HG2_INSA_ACK.raw

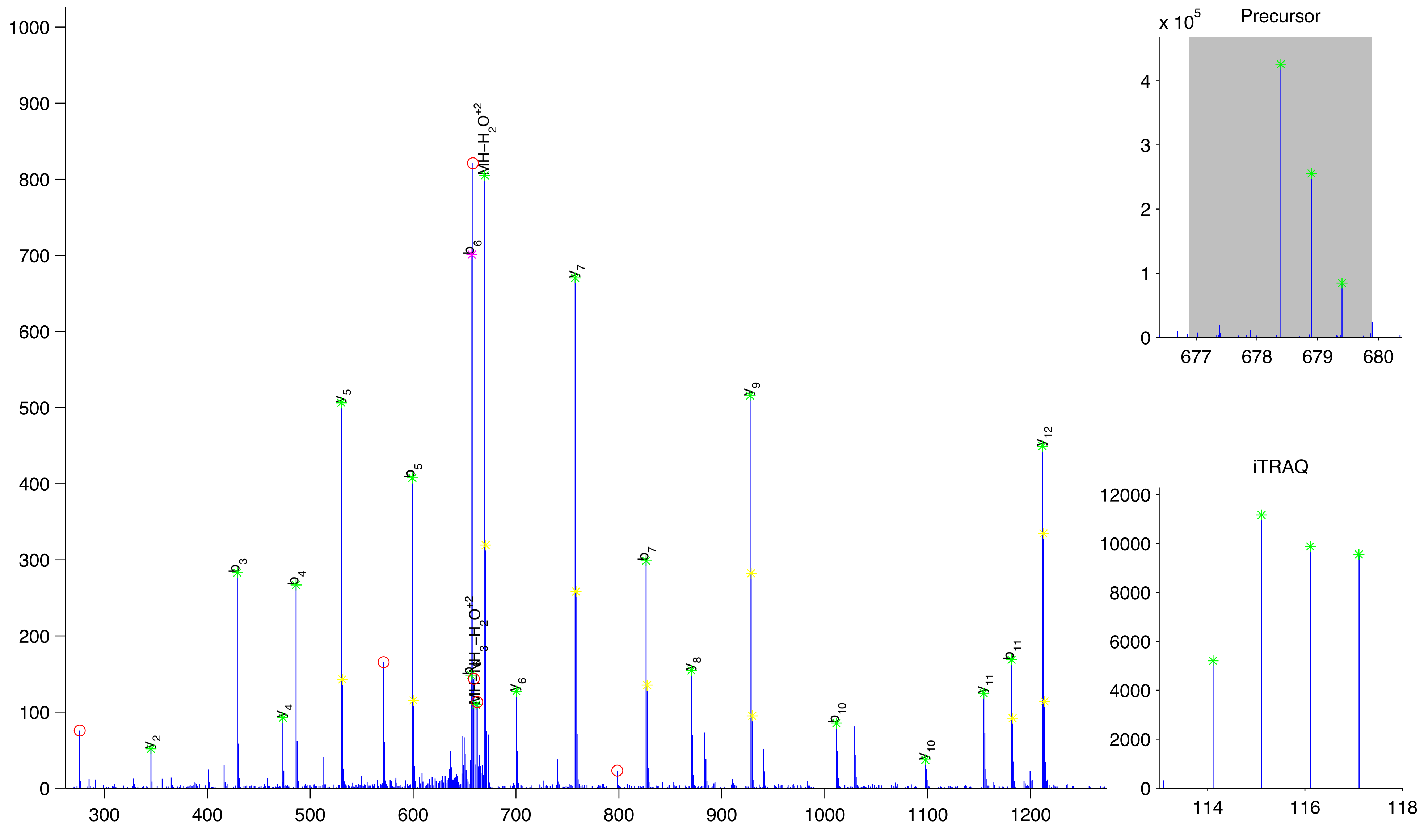

G k G G k G L G k G G A k R

histone cluster 1, H4a [Homo sapiens]
Charge State: +2
Scan Number: 22788
File Name: 110924_HG2_INSA_ACK.raw

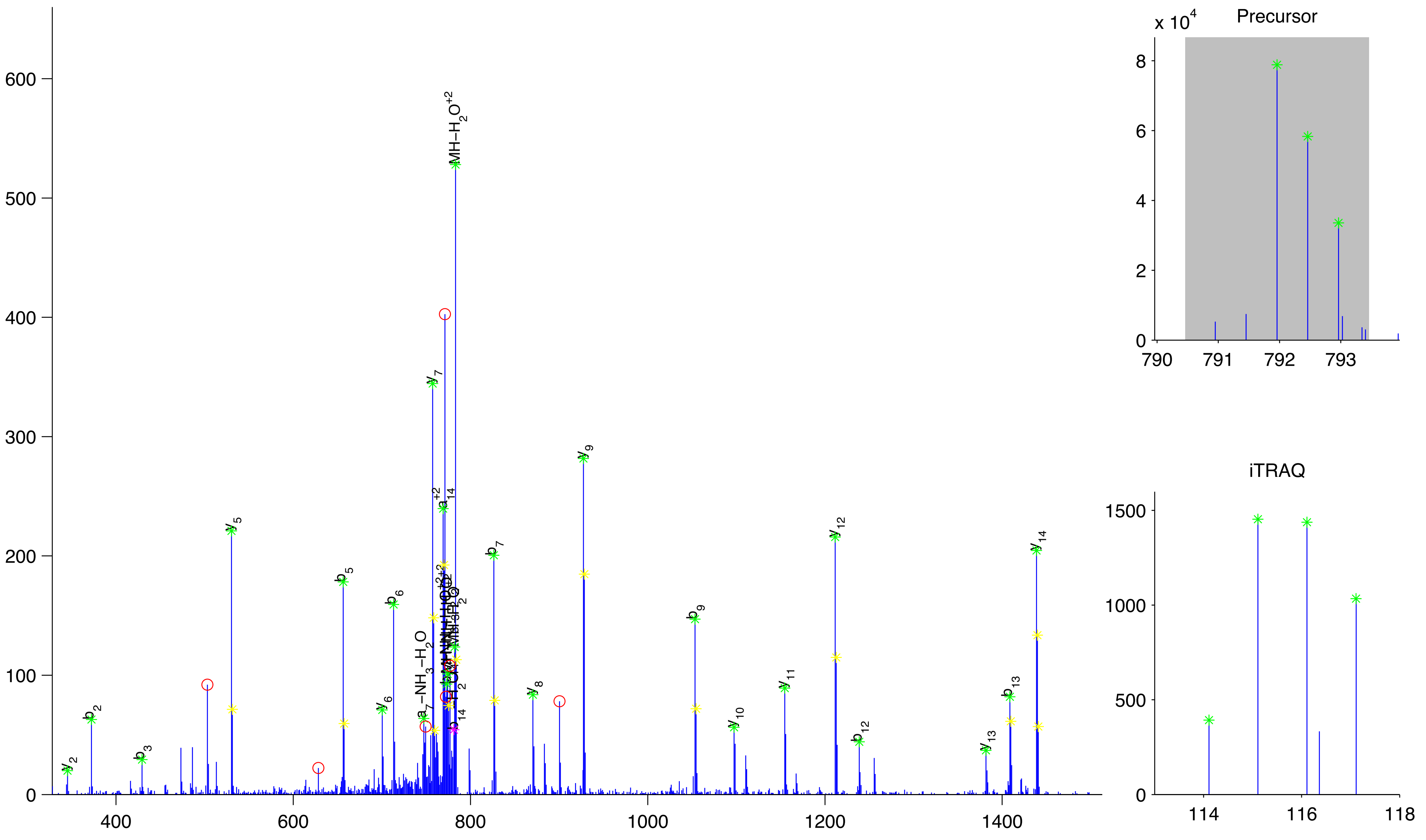

D N I Q G I T k P A I R

histone cluster 1, H4a [Homo sapiens]
Charge State: +2
Scan Number: 22951
File Name: 110924_HG2_INSA_ACK.raw

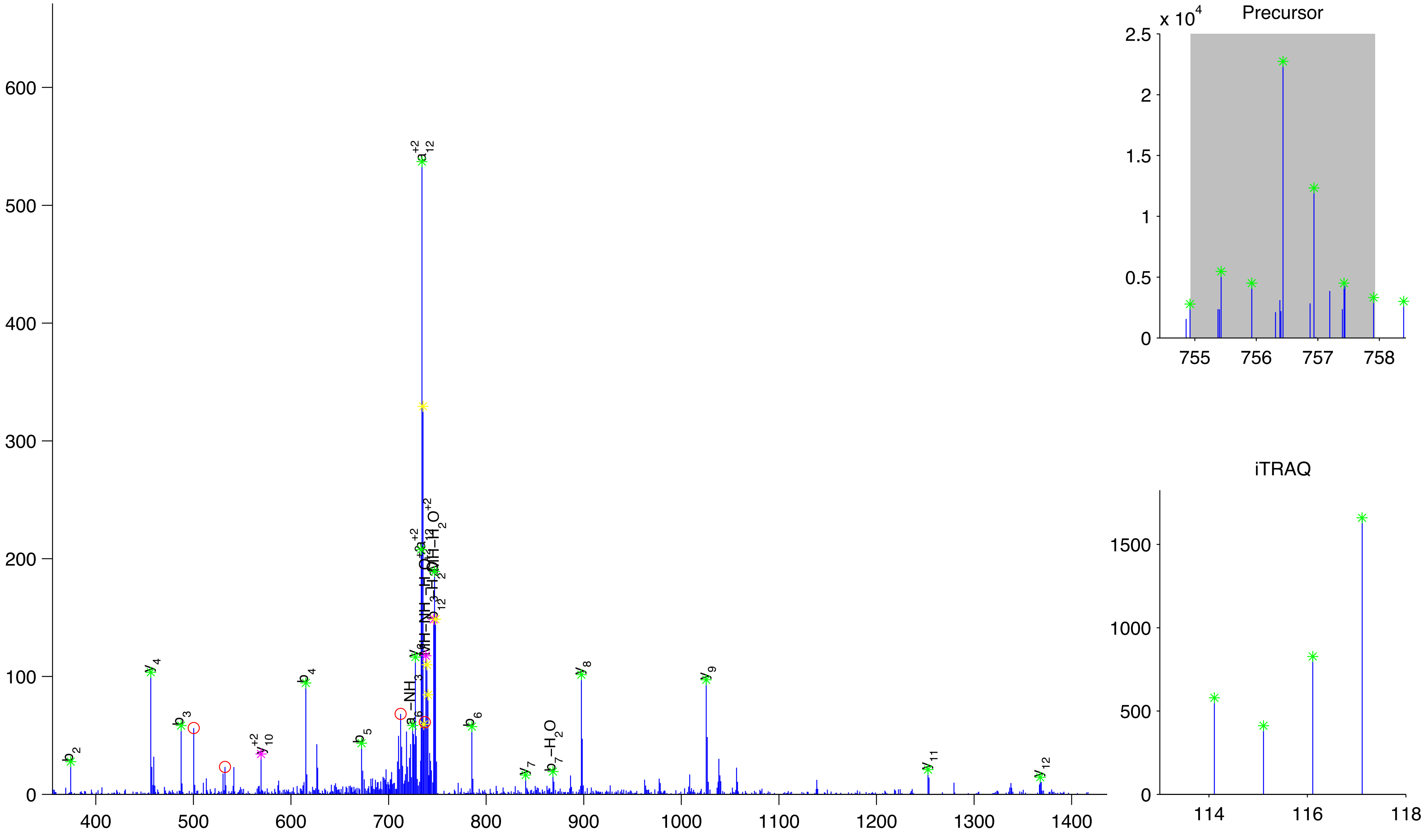

G G k G L G k G G A k R

histone cluster 1, H4a [Homo sapiens]
Charge State: +2
Scan Number: 22953
File Name: 110924_HG2_INSA_ACK.raw

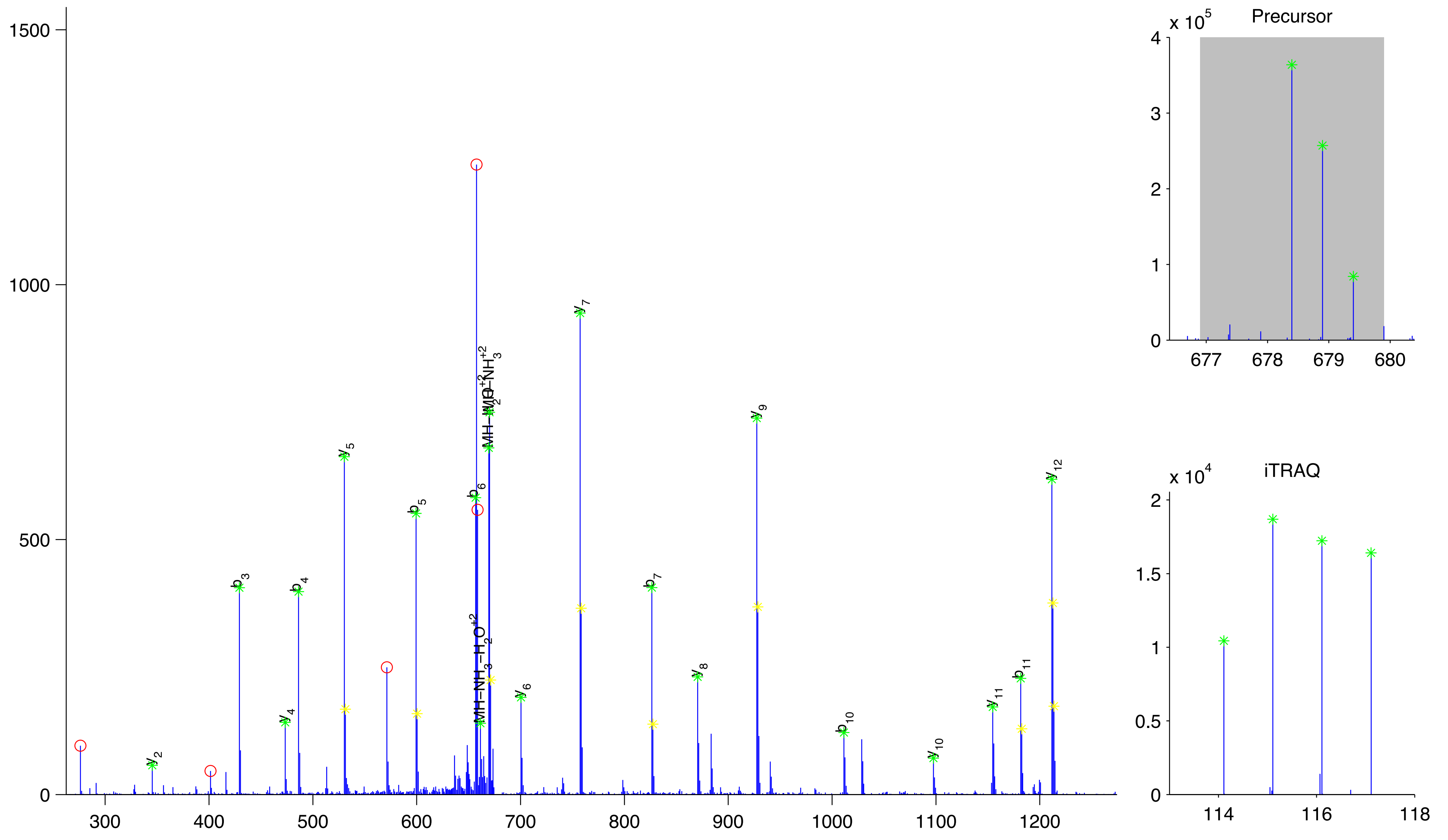

G k G G k G L G k G G A k R

histone cluster 1, H4a [Homo sapiens]
Charge State: +2
Scan Number: 23128
File Name: 110924_HG2_INSA_ACK.raw

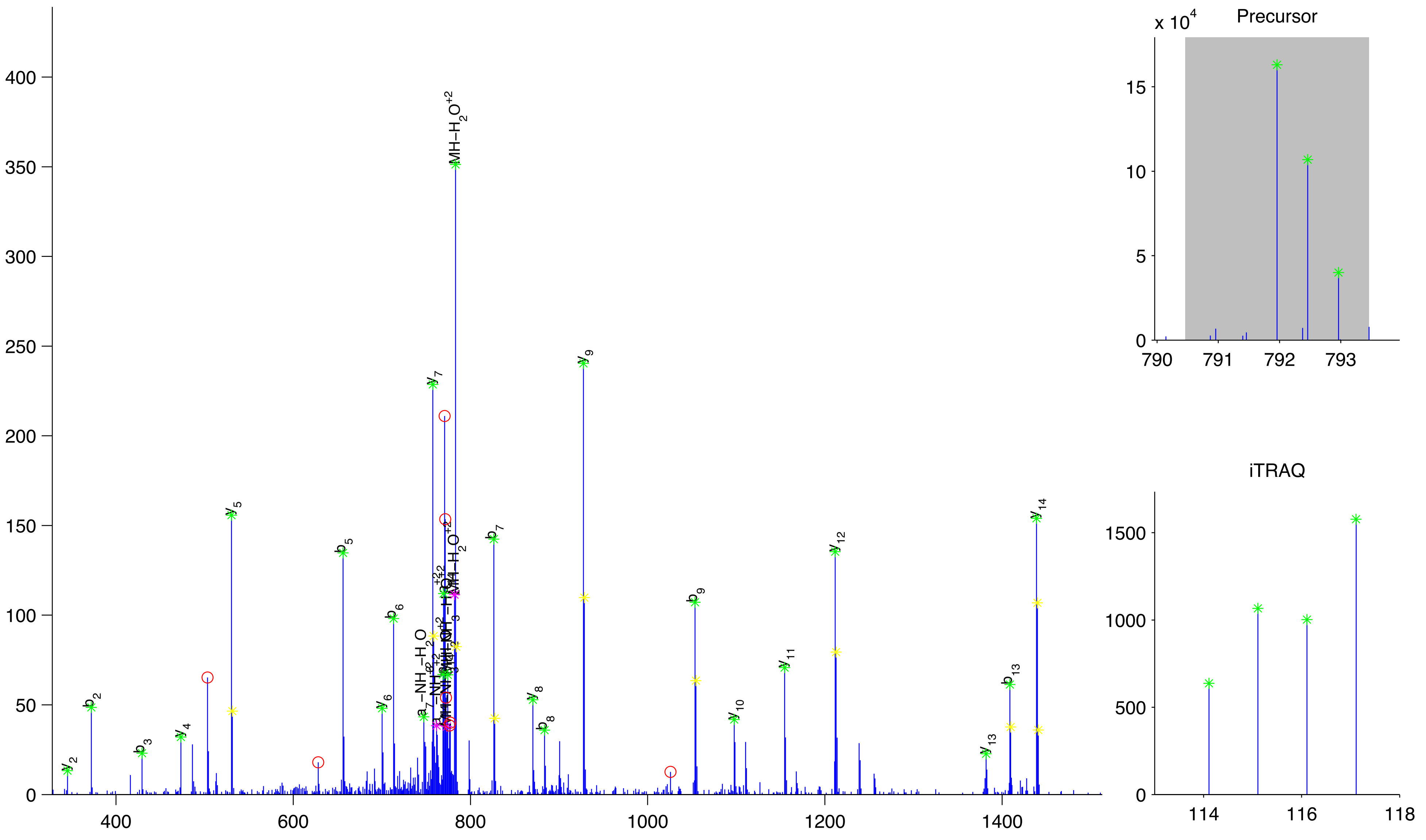

G G k G L G k G G A k R

histone cluster 1, H4a [Homo sapiens]
Charge State: +2
Scan Number: 23136
File Name: 110924_HG2_INSA_ACK.raw

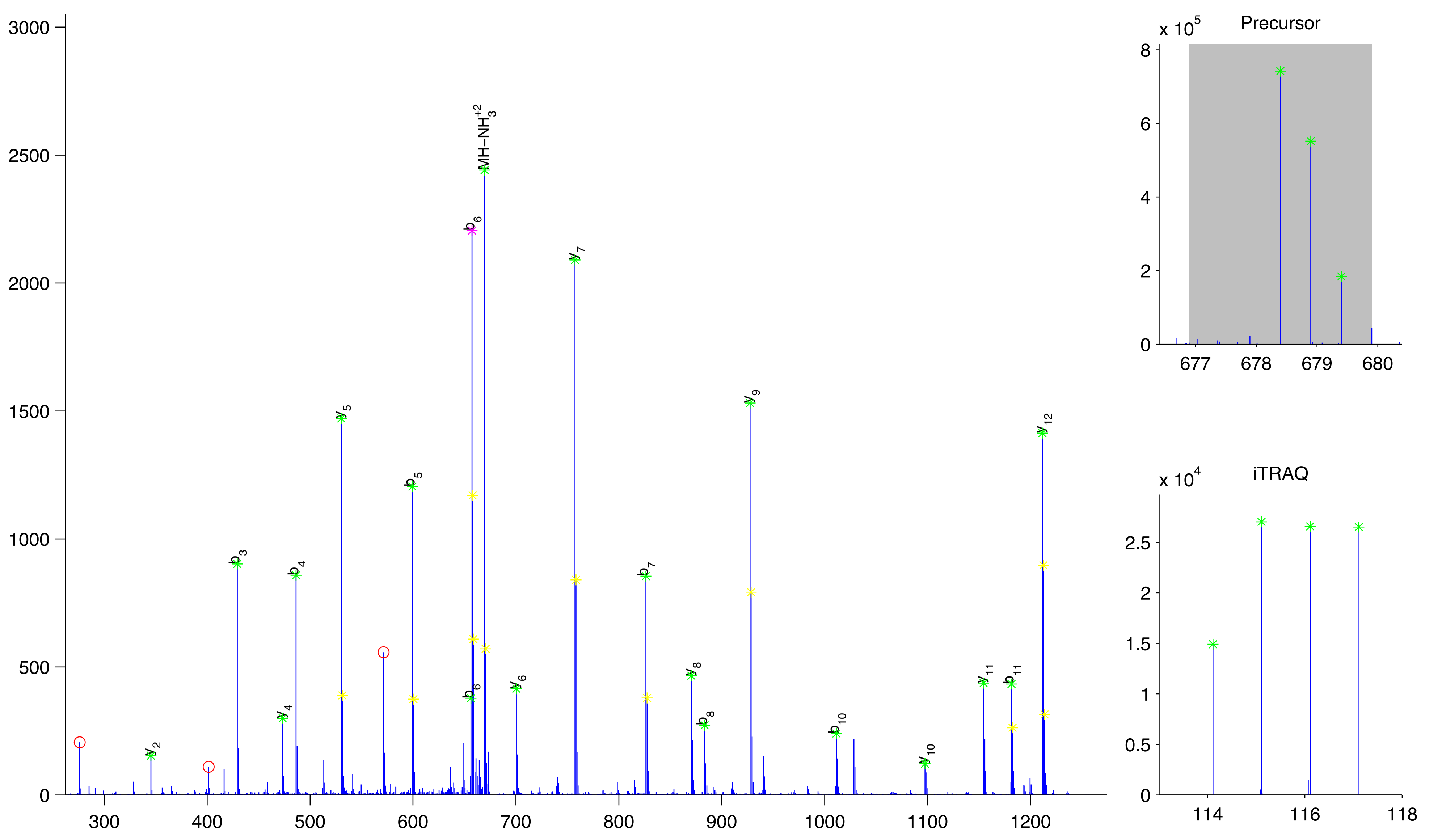

D N I Q G I T k P A I R

histone cluster 1, H4a [Homo sapiens]
Charge State: +2
Scan Number: 23138
File Name: 110924_HG2_INSA_ACK.raw

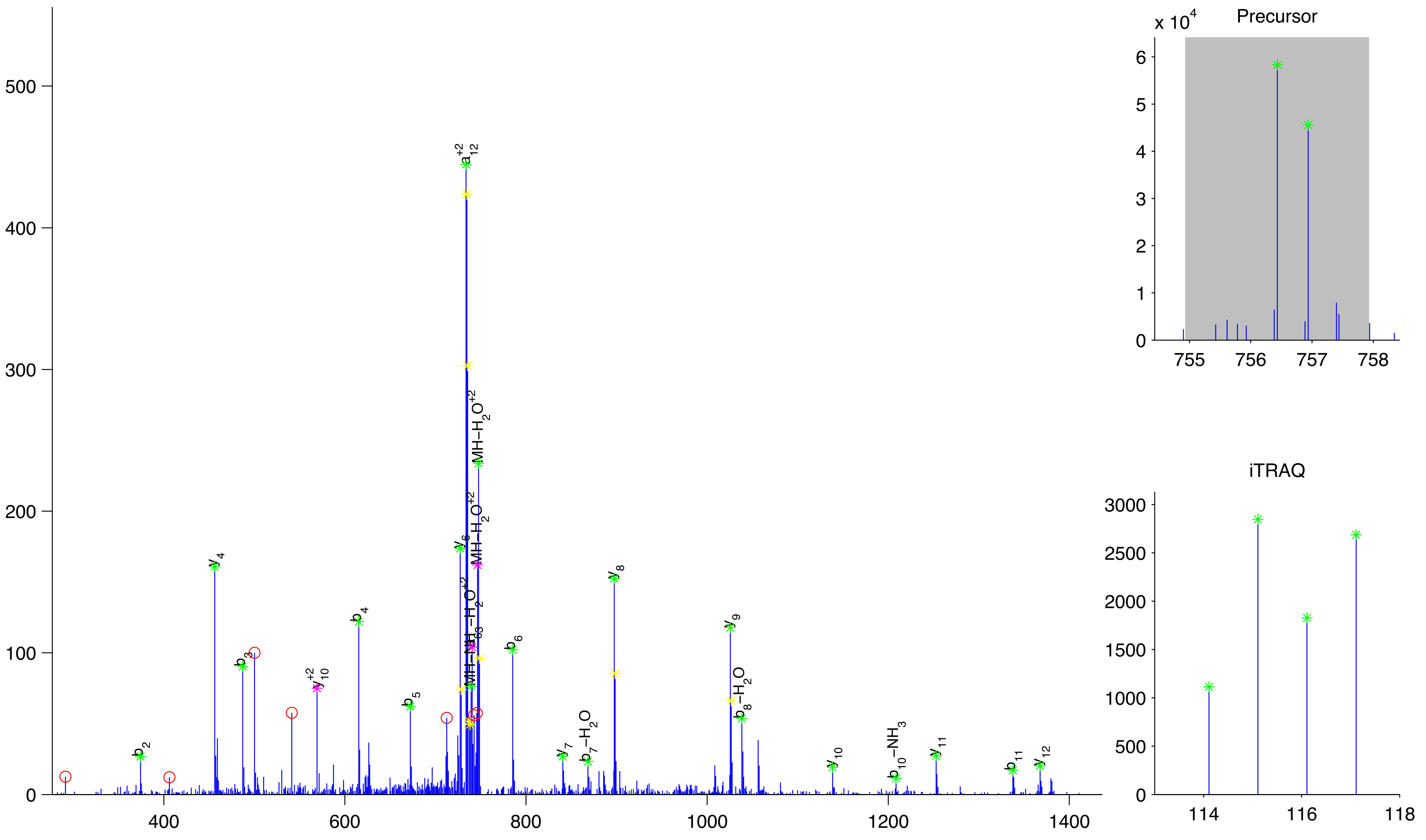

G L G k G G A k R

histone cluster 1, H4a [Homo sapiens]
Charge State: +2
Scan Number: 23273
File Name: 110924_HG2_INSA_ACK3.raw

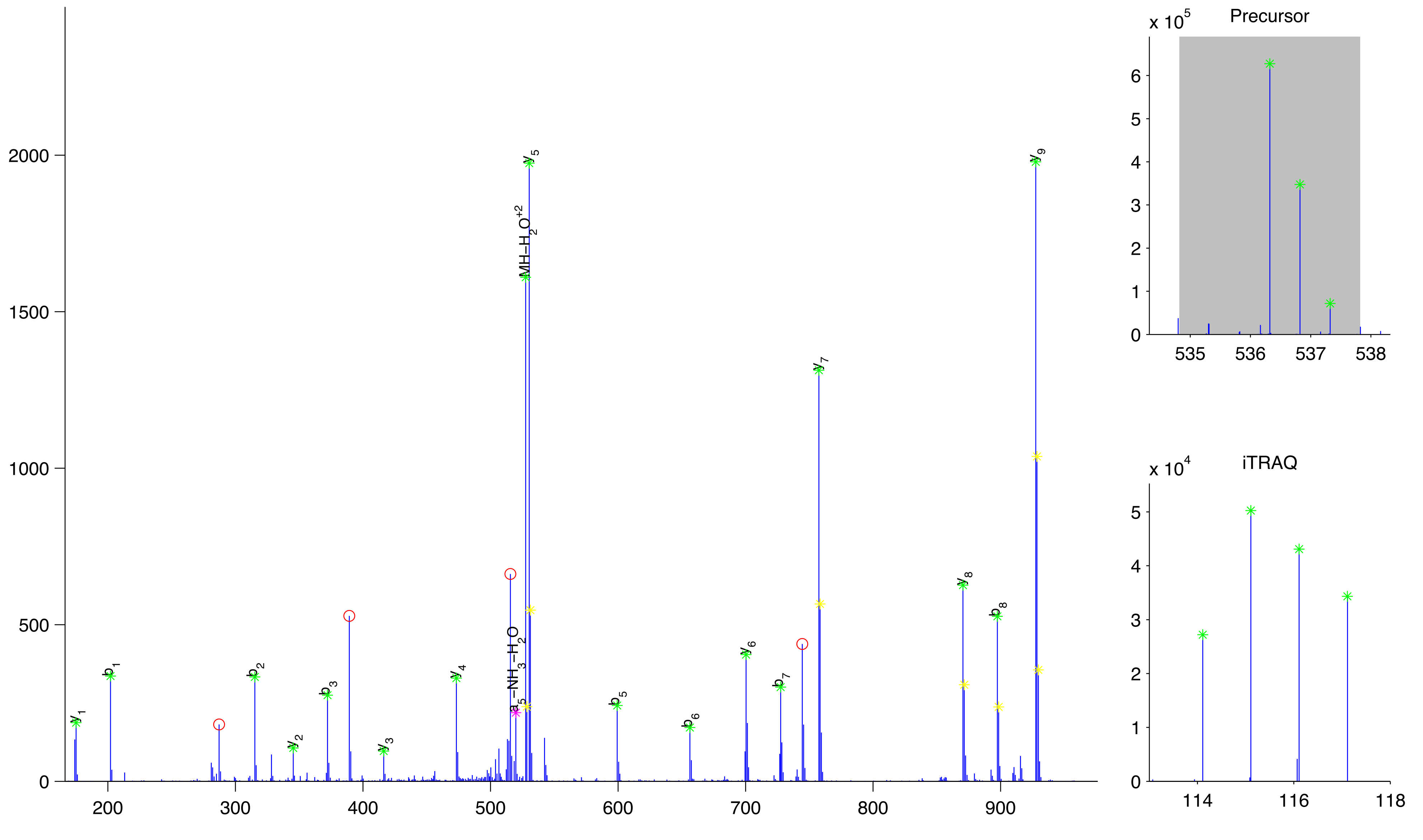

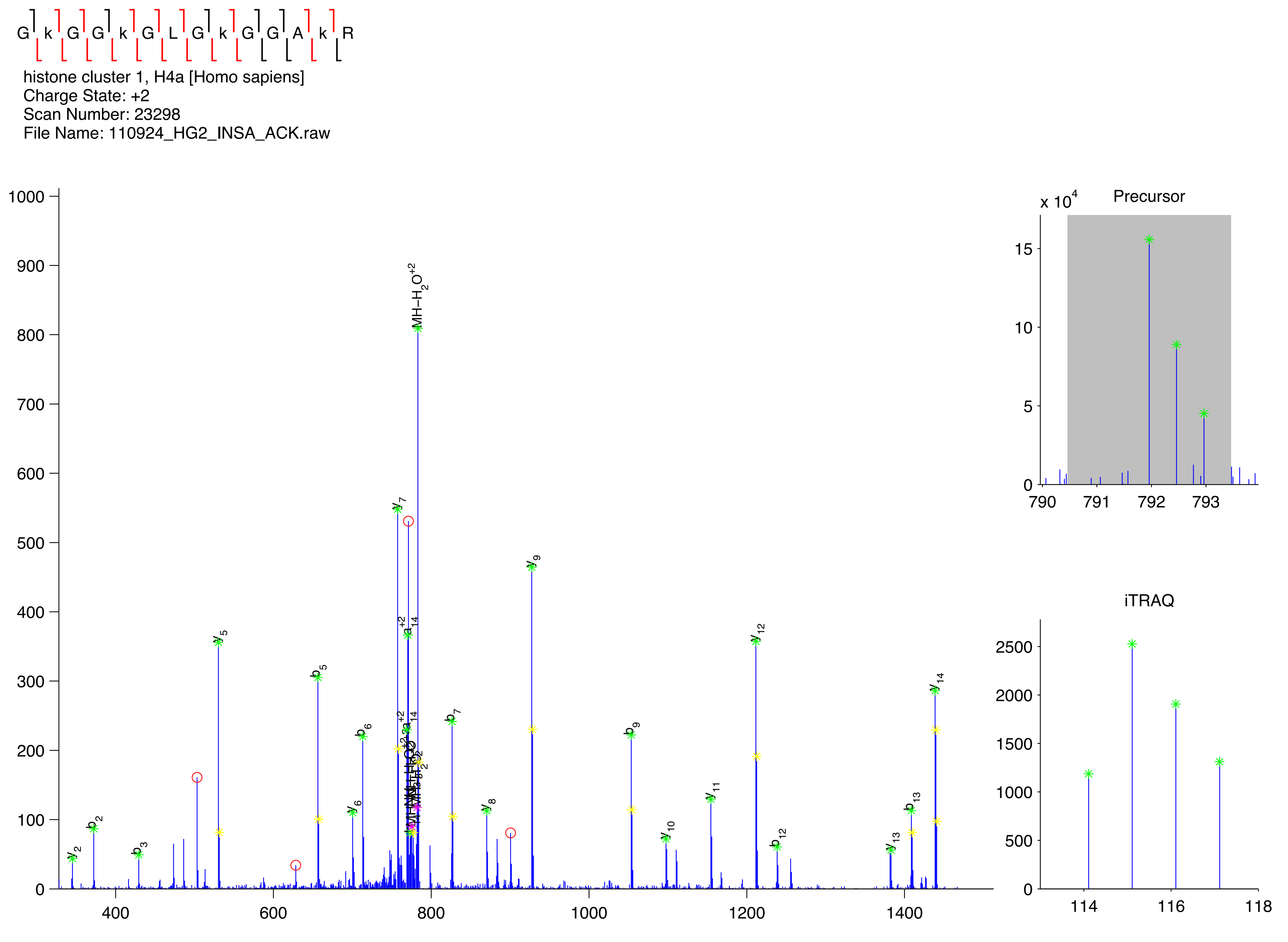
G k G G k G L G k G G A k R
histone cluster 1, H4a [Homo sapiens]
Charge State: +2
Scan Number: 23298
File Name: 110924_HG2_INSA_ACK.raw
y2
b2
b3
y5
b5
y6
b6
y7
MH−H2O+2
b7
y8
y9
b9
y10
y11
y12
b12
y13
b13
y14
Precursor
x 10^4
iTRAQ

G G k G L G k G G A k R

histone cluster 1, H4a [Homo sapiens]
Charge State: +2
Scan Number: 23336
File Name: 110924_HG2_INSA_ACK3.raw

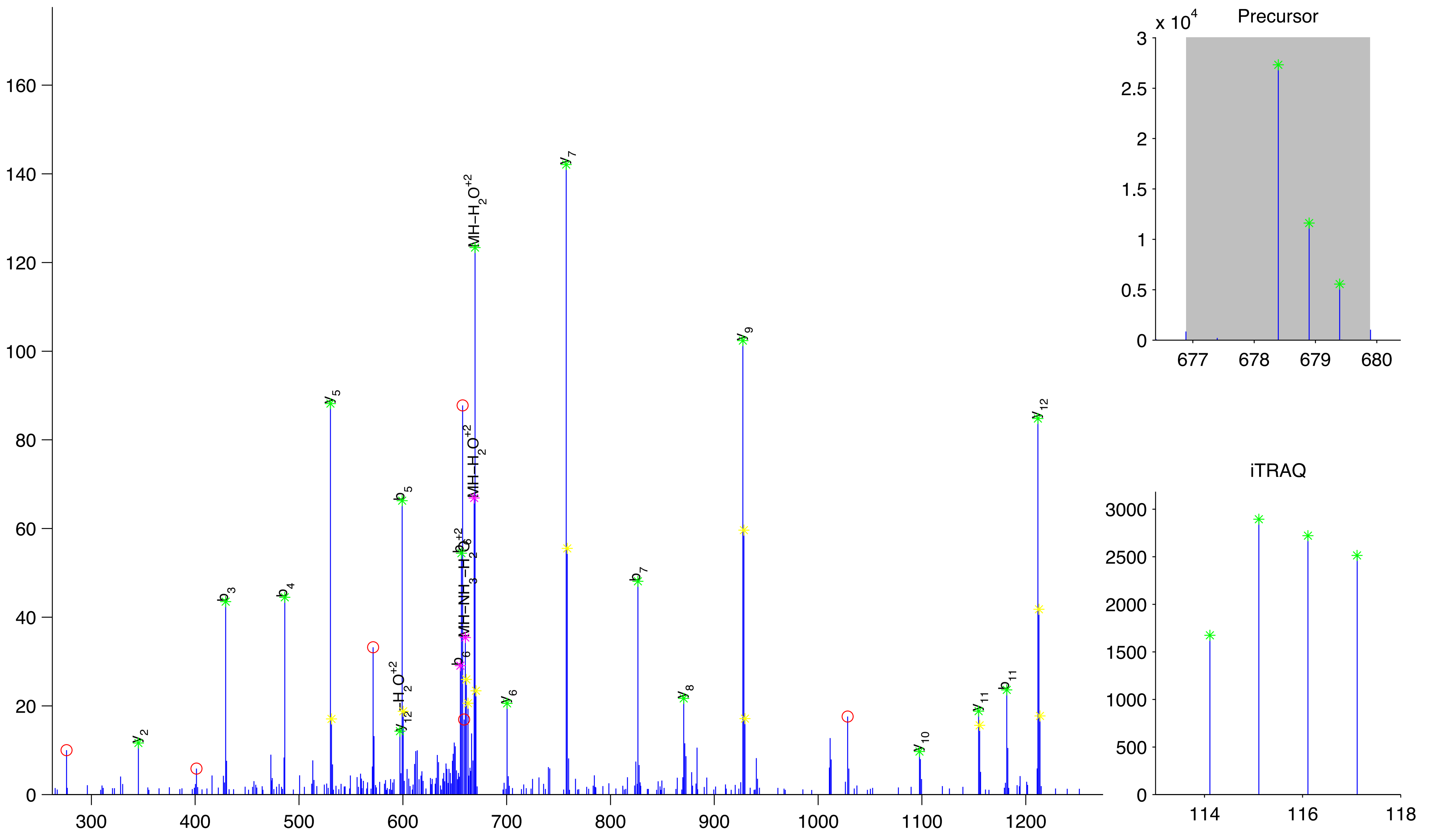

G L G k G G A k R

histone cluster 1, H4a [Homo sapiens]
Charge State: +2
Scan Number: 23624
File Name: 110924_HG2_INSA_ACK3.raw

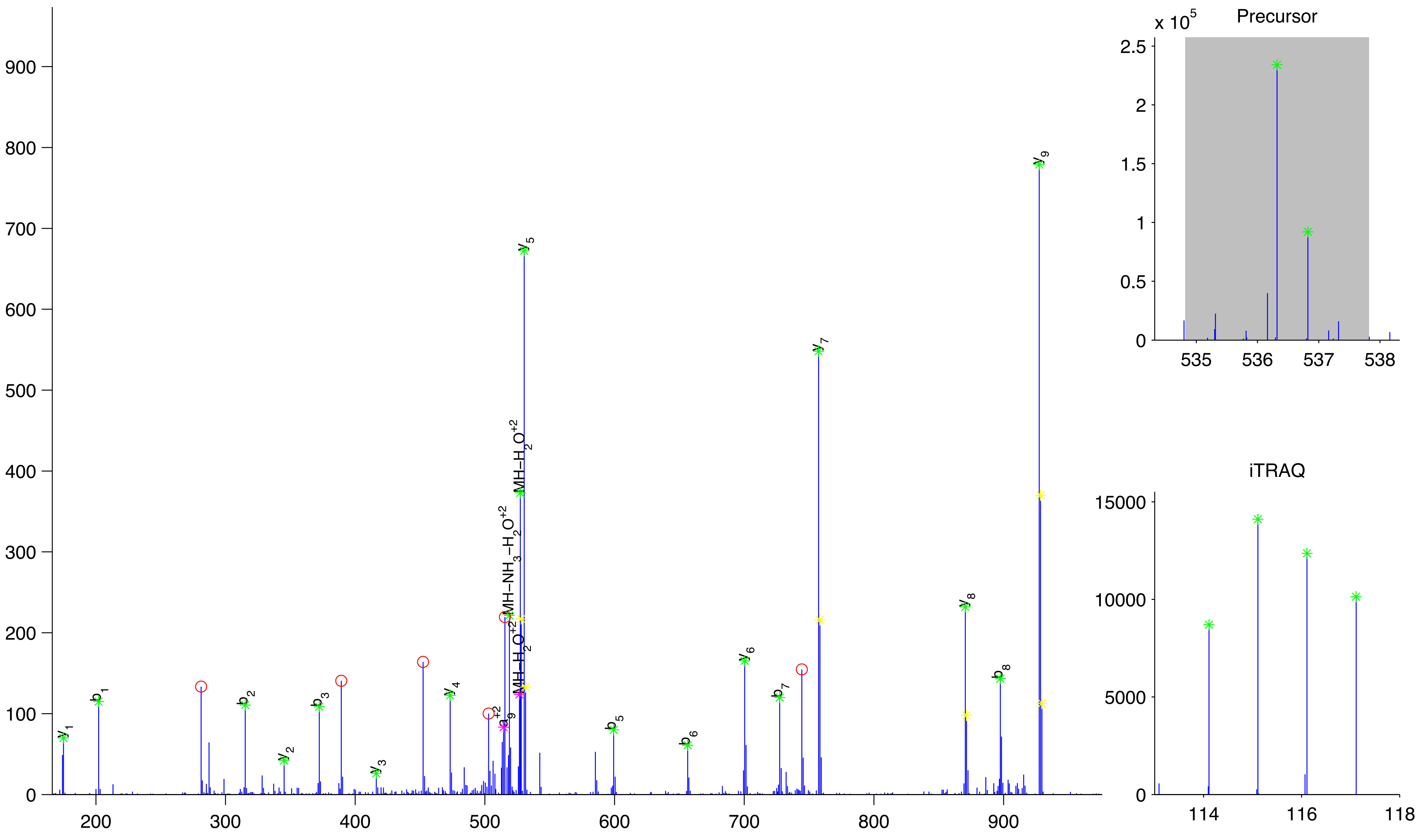

G G k G L G k G G A k R

histone cluster 1, H4a [Homo sapiens]
Charge State: +2
Scan Number: 23821
File Name: 110924_HG2_INSA_ACK.raw

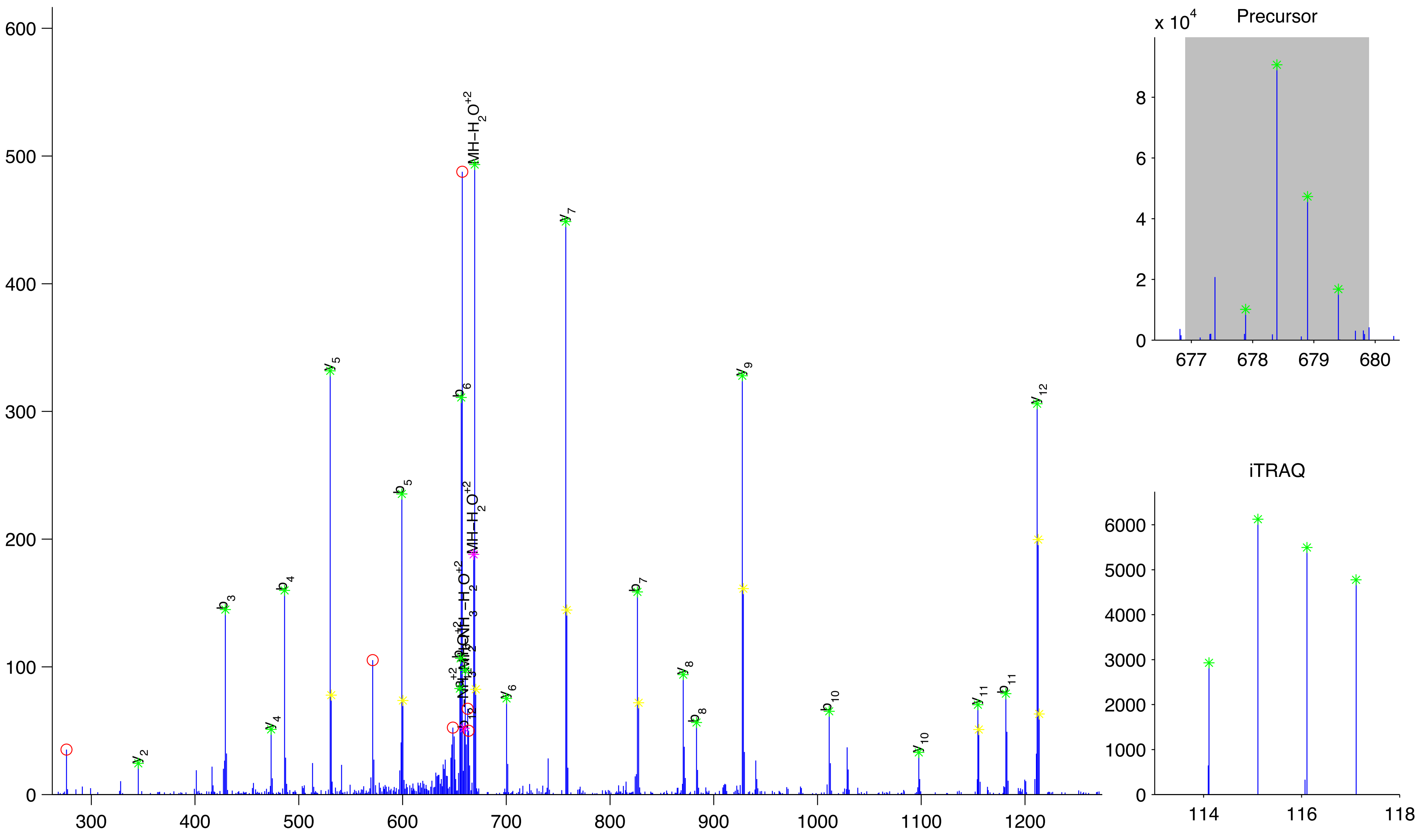

G G k G L G k G G A k R

histone cluster 1, H4a [Homo sapiens]
Charge State: +2
Scan Number: 24178
File Name: 110924_HG2_INSA_ACK3.raw

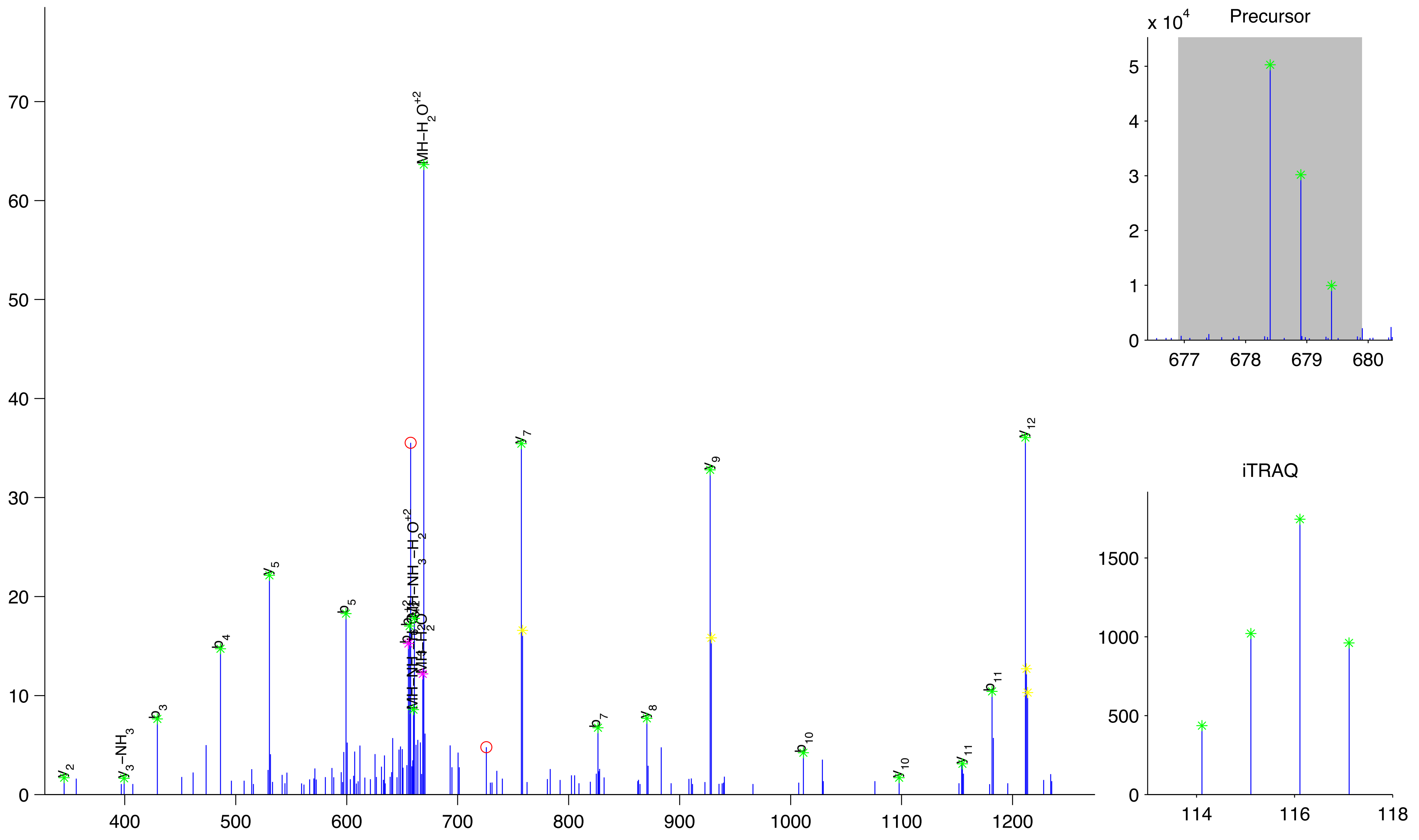

G G k G L G k G G A k R

histone cluster 1, H4a [Homo sapiens]
Charge State: +2
Scan Number: 24680
File Name: 110924_HG2_INSA_ACK3.raw

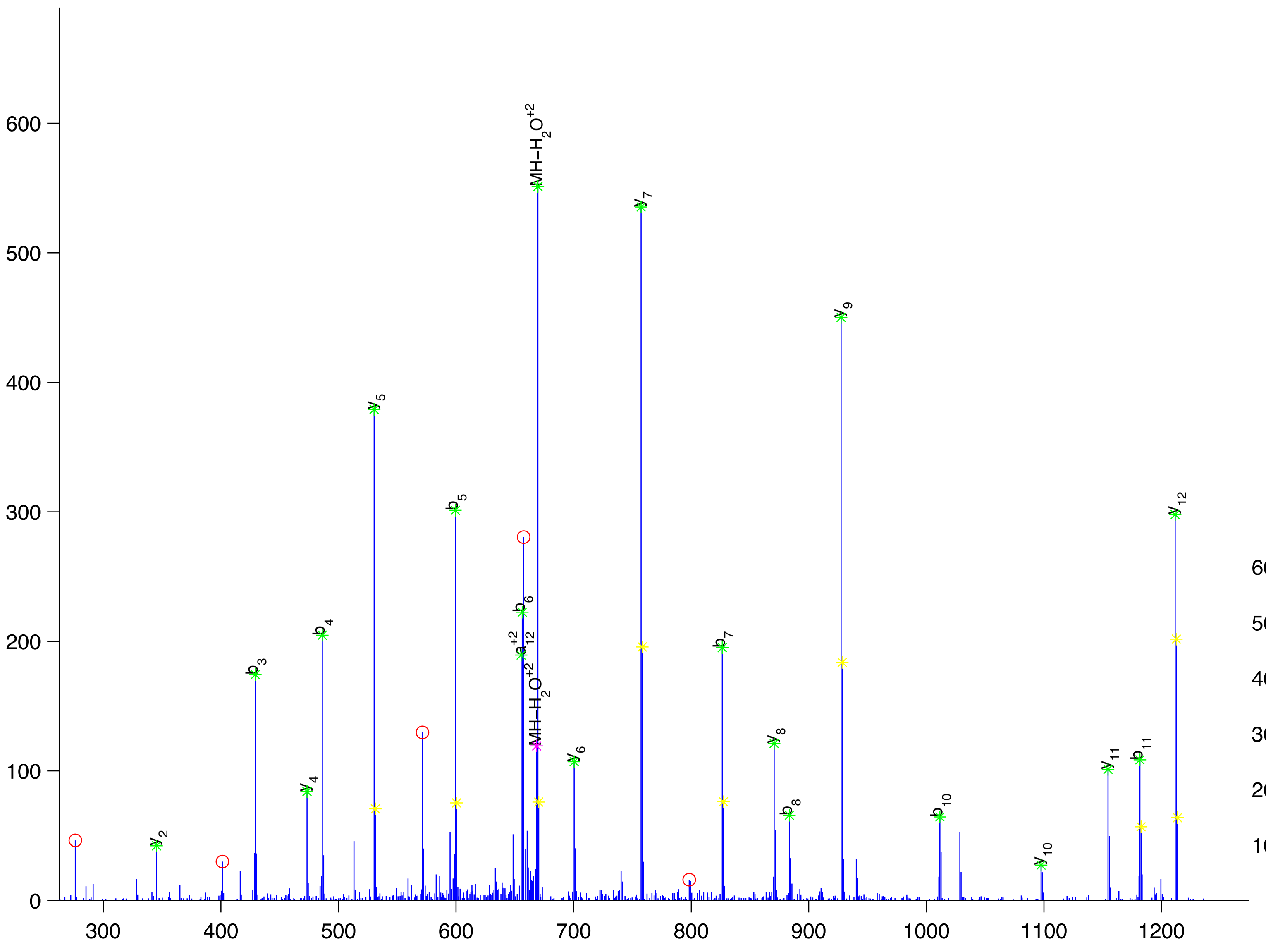

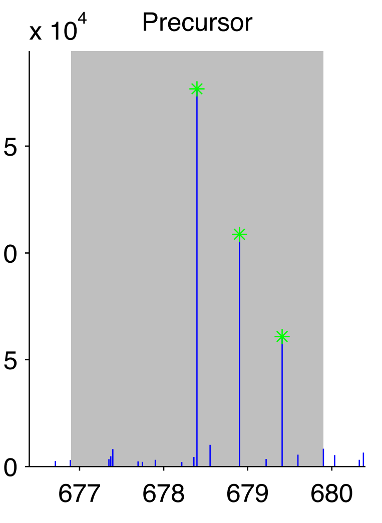

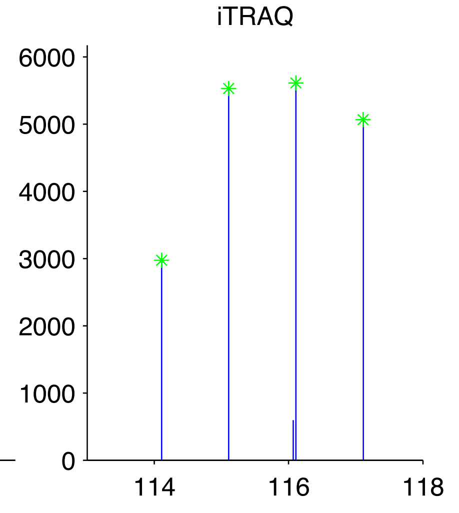

G k G G k G L G k G G A k R

histone cluster 1, H4a [Homo sapiens]
Charge State: +3
Scan Number: 25007
File Name: 110924_HG2_INSA_ACK3.raw

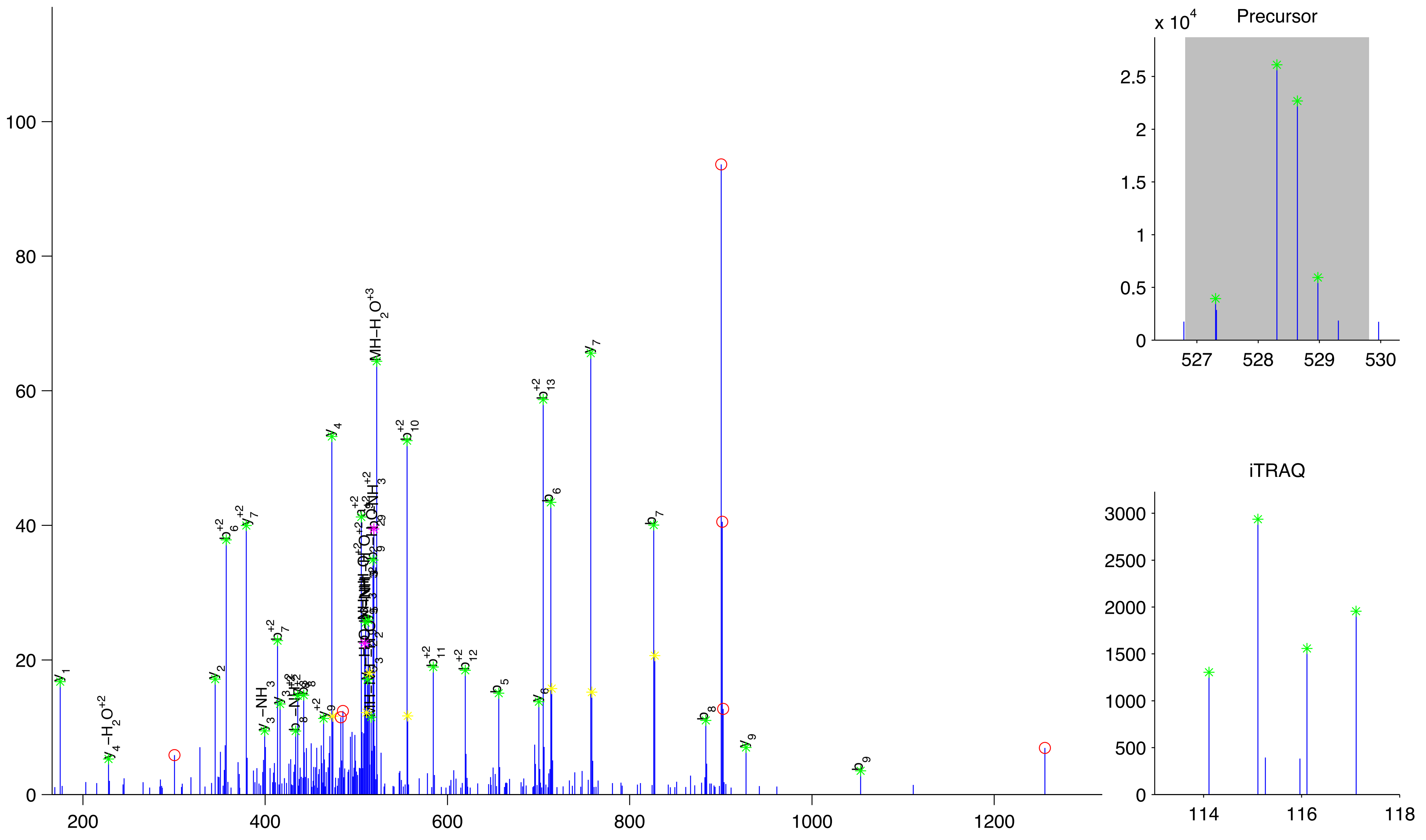

G G k G L G k G G A k R

histone cluster 1, H4a [Homo sapiens]
Charge State: +2
Scan Number: 25180
File Name: 110924_HG2_INSA_ACK3.raw

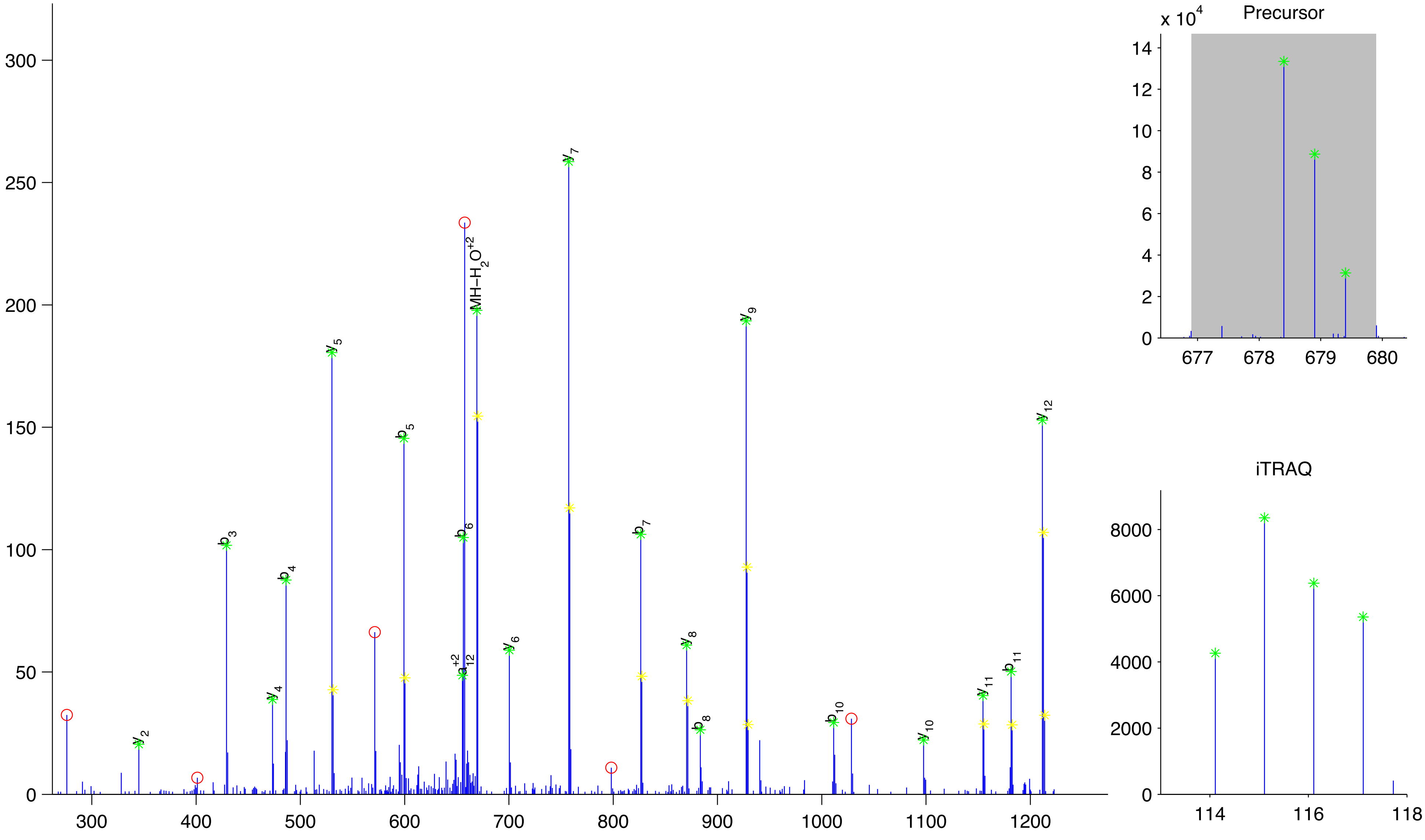

G L G k G G A k R

histone cluster 1, H4a [Homo sapiens]
Charge State: +2
Scan Number: 25640
File Name: 110924_HG2_INSA_ACK3.raw

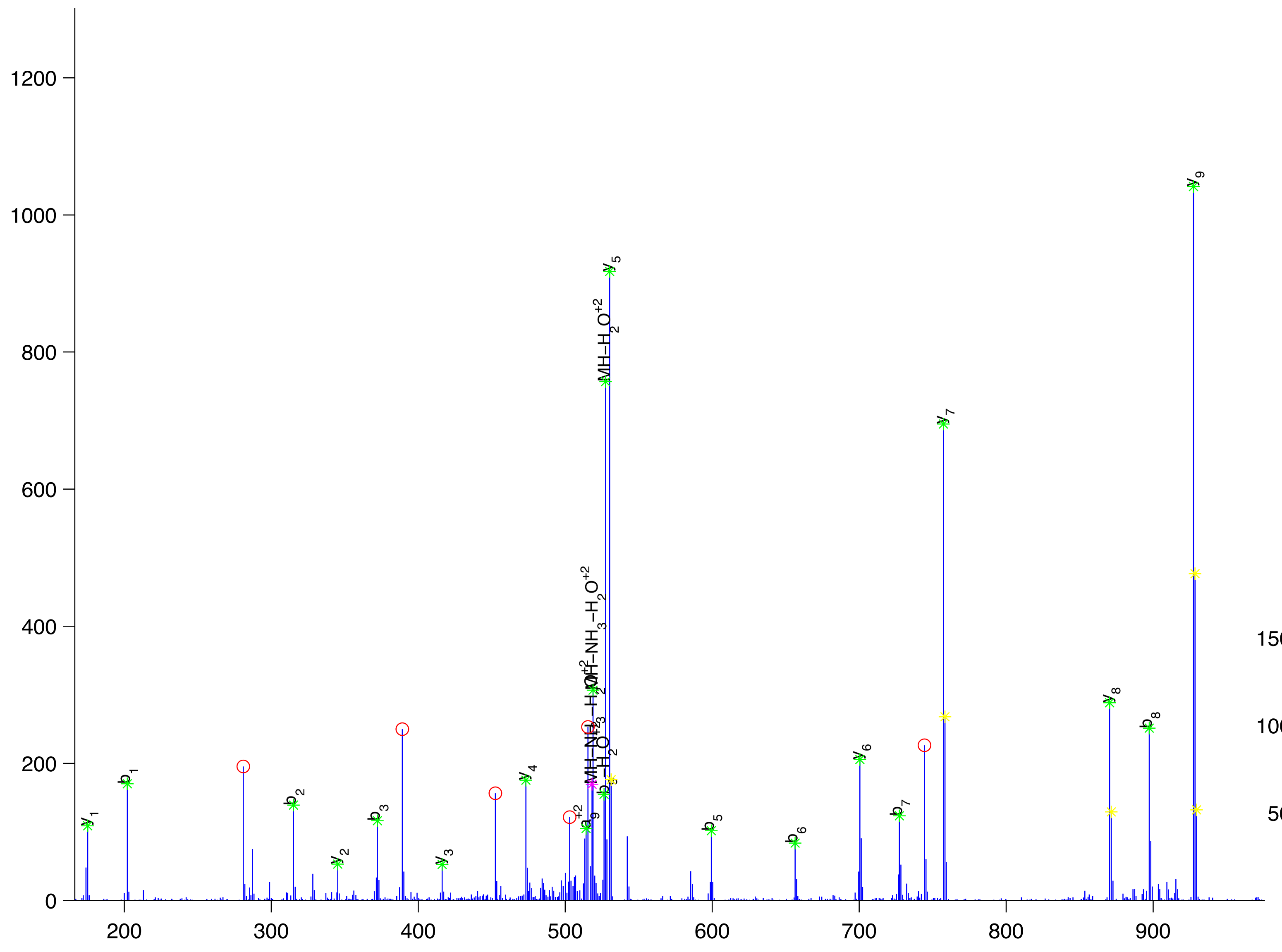

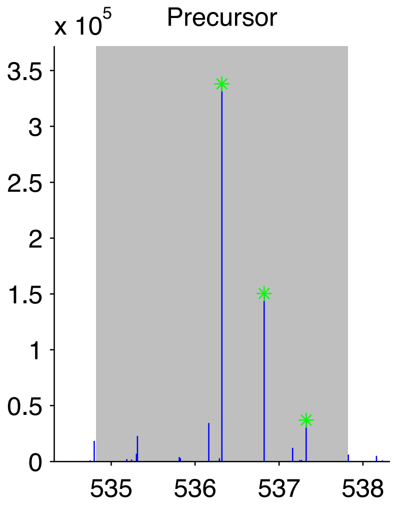

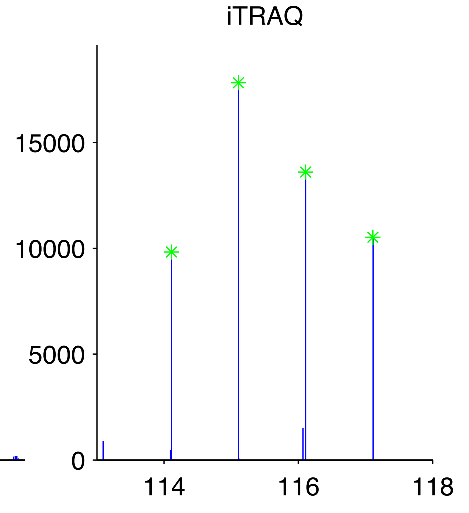

G G k G L G k G G A k R

histone cluster 1, H4a [Homo sapiens]
Charge State: +2
Scan Number: 26018
File Name: 110924_HG2_INSA_ACK3.raw

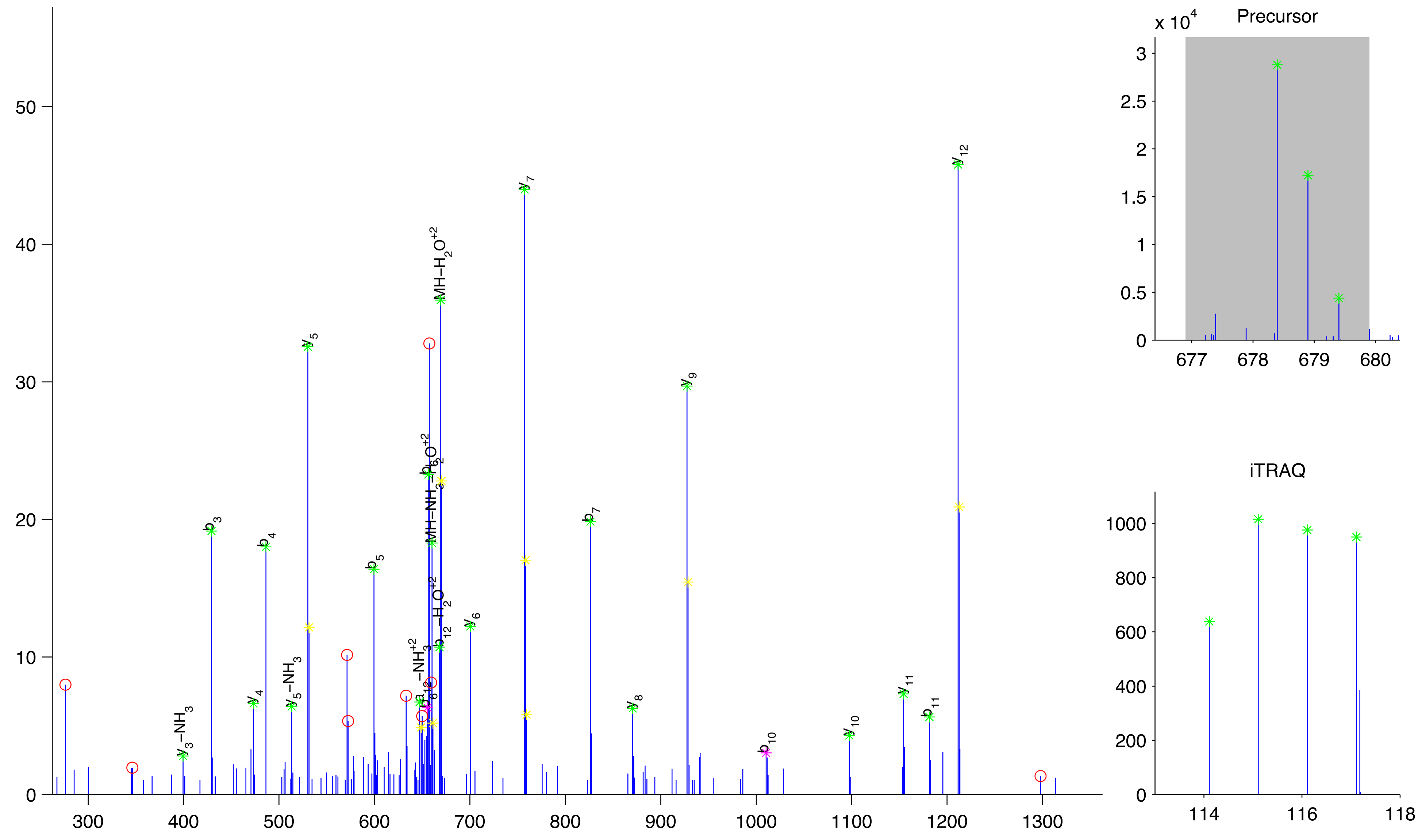

G L G k G G A k R

histone cluster 1, H4a [Homo sapiens]
Charge State: +2
Scan Number: 26312
File Name: 110924_HG2_INSA_ACK3.raw

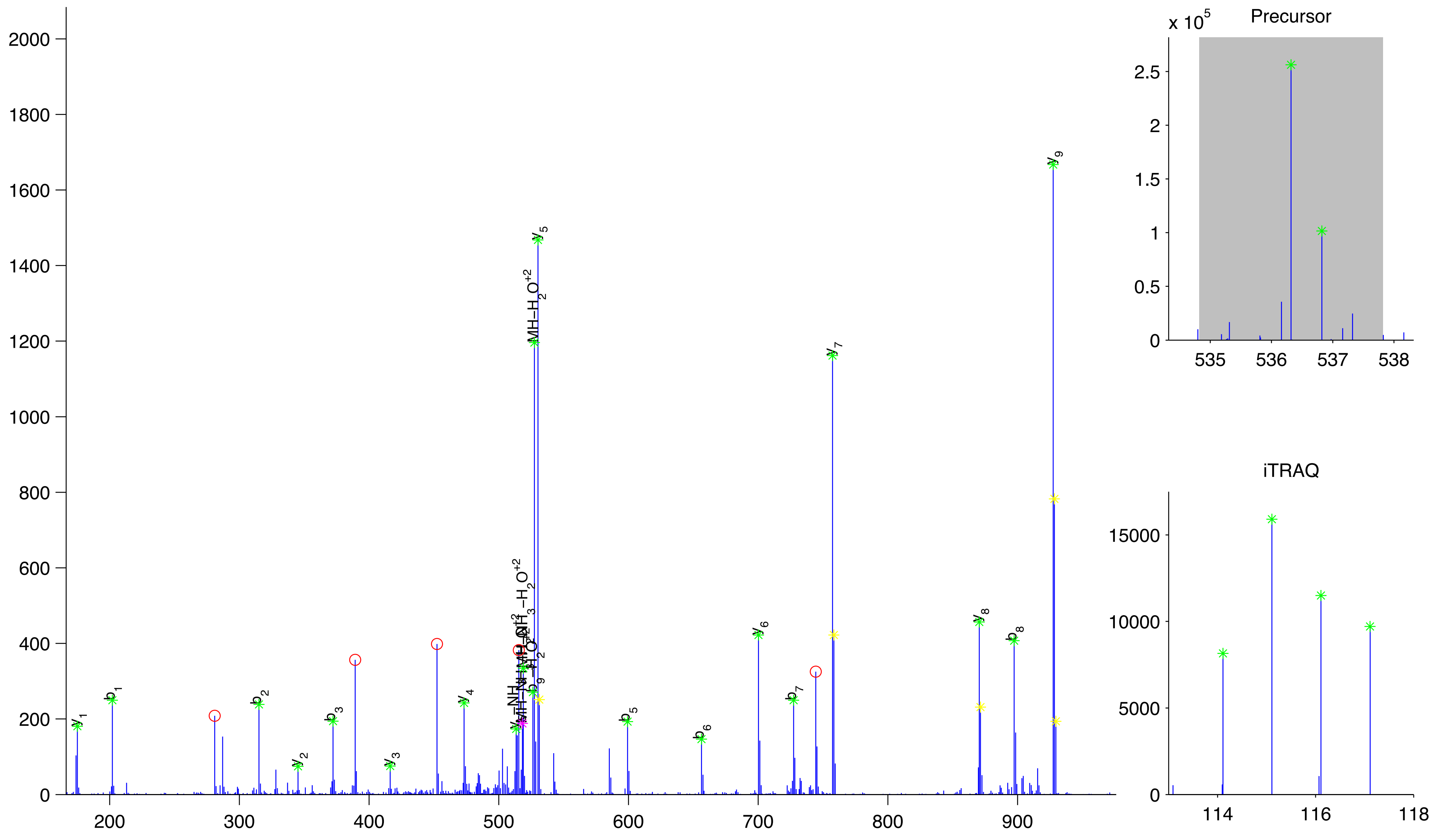

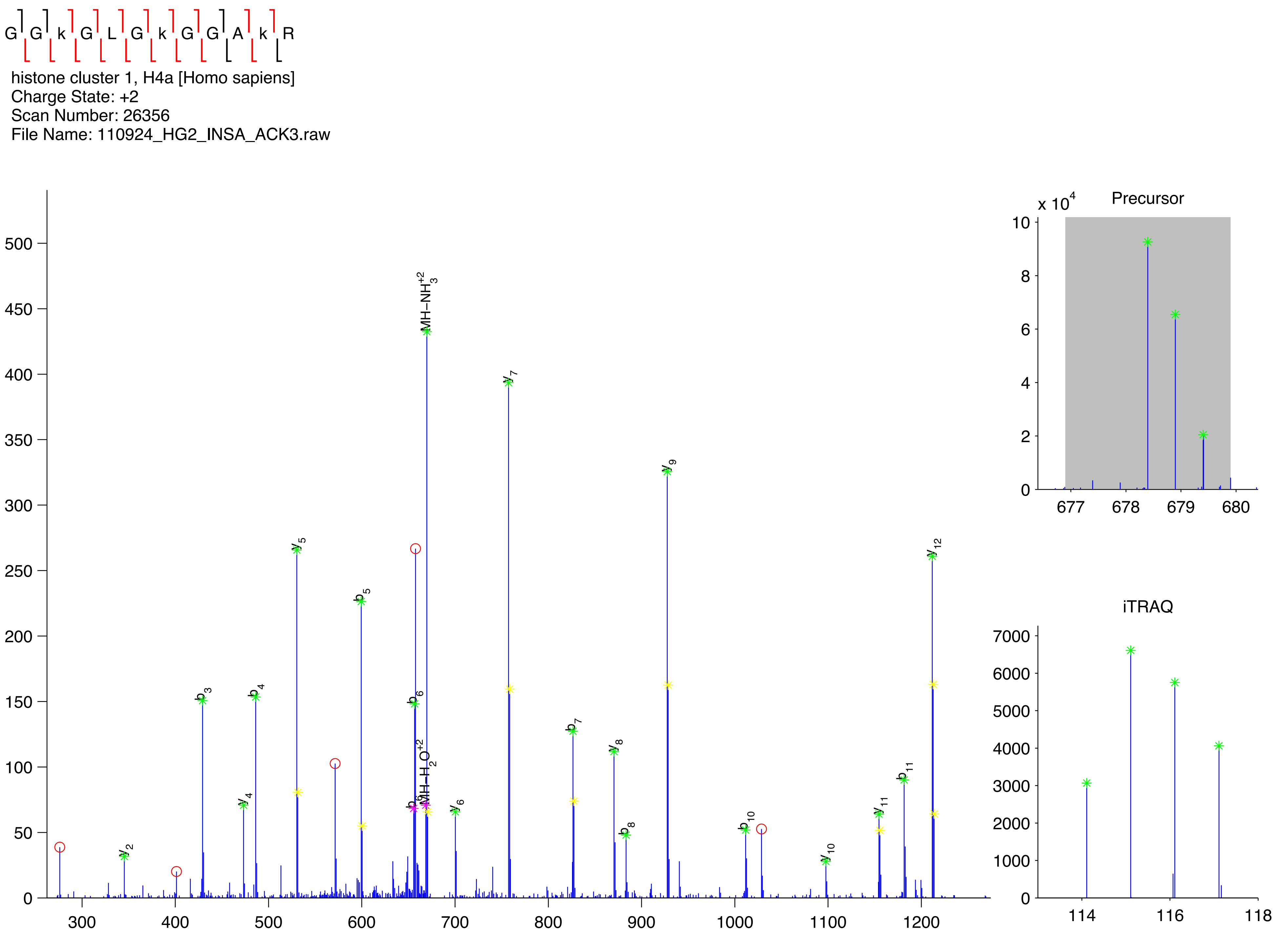
G G k G L G k G G A k R
histone cluster 1, H4a [Homo sapiens]
Charge State: +2
Scan Number: 26356
File Name: 110924_HG2_INSA_ACK3.raw
MH−NH3 +2
MH−H2O +2
y2
b3
y4
b4
y5
b5
b6
y6
y7
b7
y8
b8
y9
b10
y10
y11
b11
y12
x 10^4
Precursor
iTRAQ

G G k G L G k G G A k R

histone cluster 1, H4a [Homo sapiens]
Charge State: +2
Scan Number: 26524
File Name: 110924_HG2_INSA_ACK3.raw

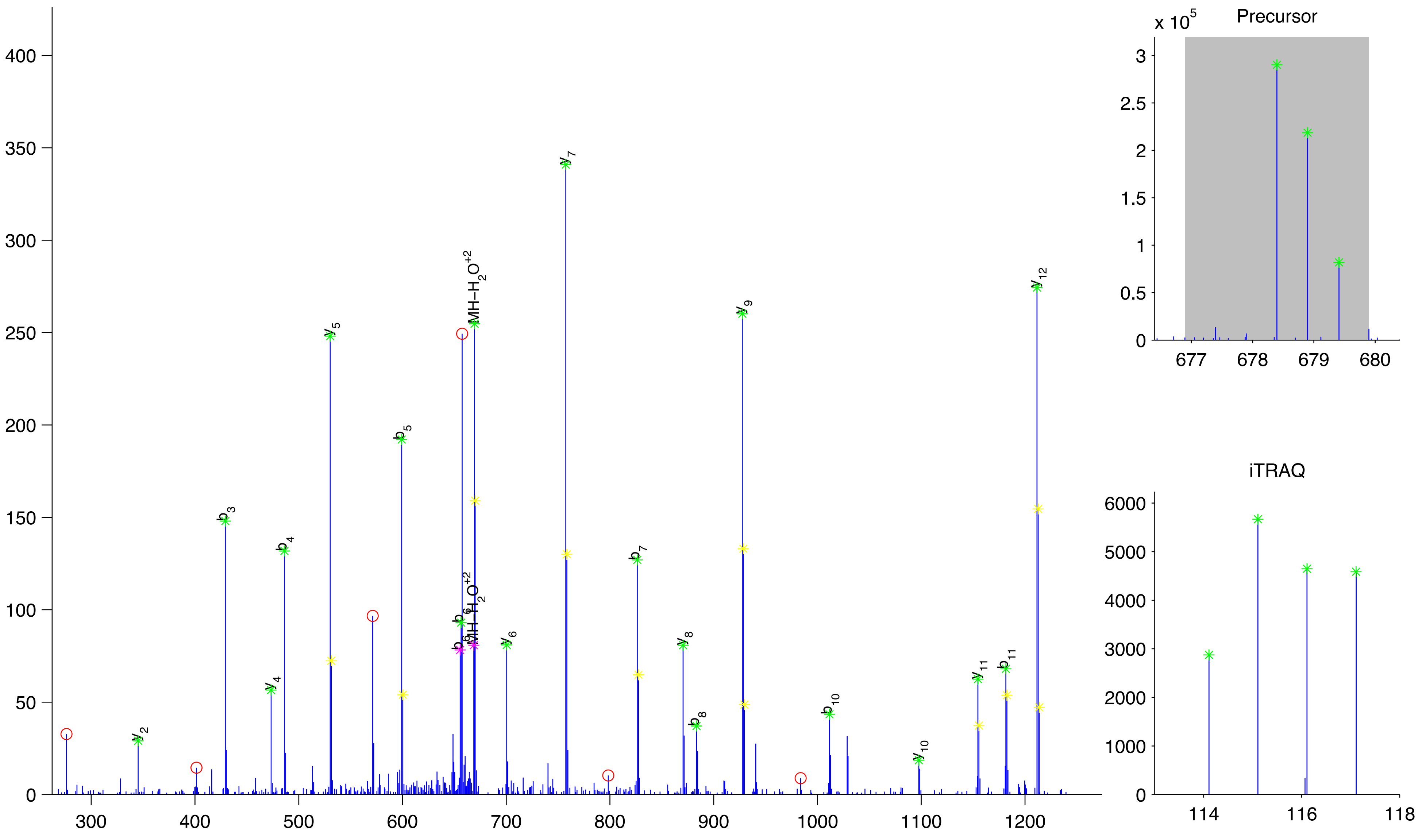

G L G k G G A k R

histone cluster 1, H4a [Homo sapiens]
Charge State: +2
Scan Number: 26816
File Name: 110924_HG2_INSA_ACK3.raw

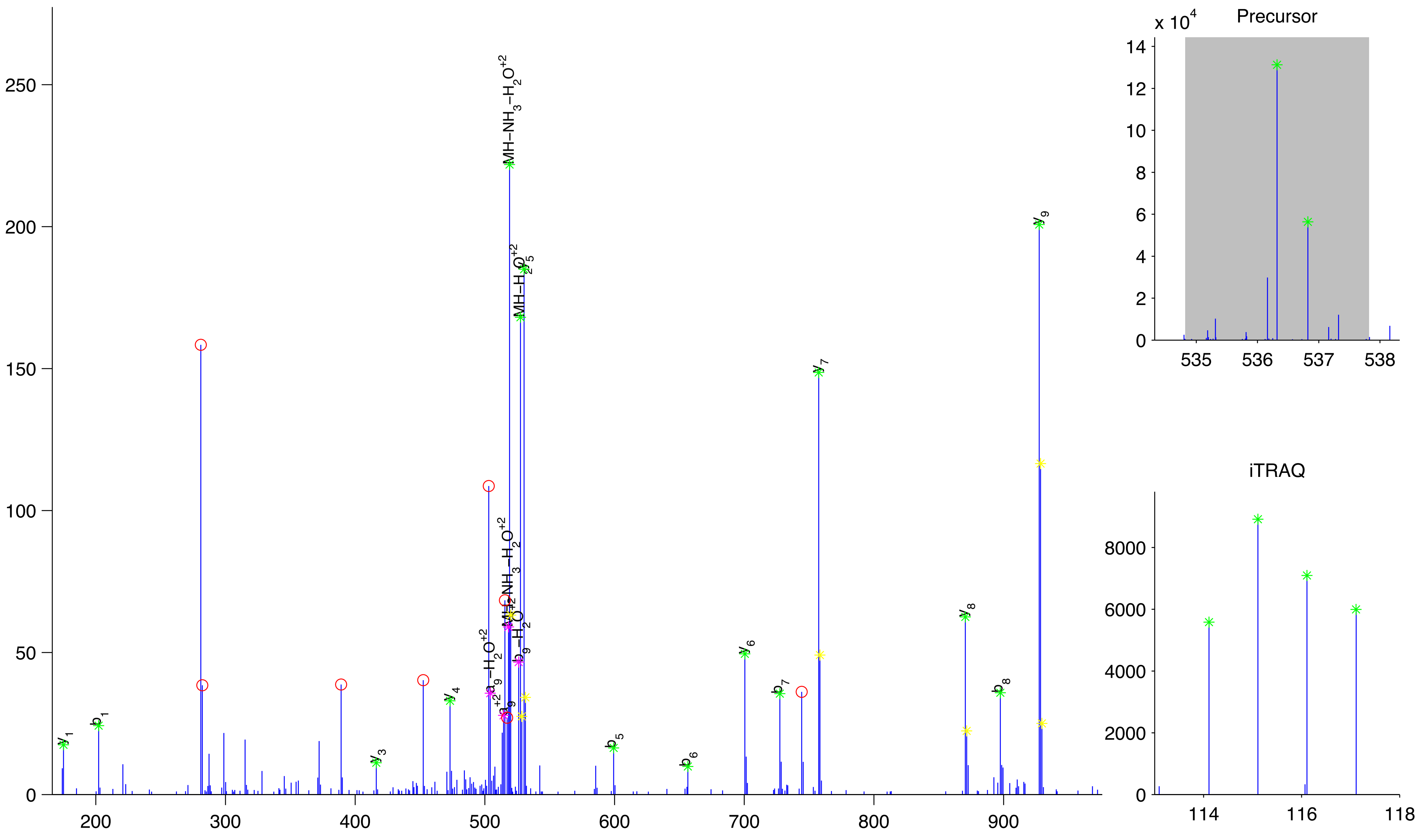

G G k G L G k G G A k R

histone cluster 1, H4a [Homo sapiens]
Charge State: +2
Scan Number: 27215
File Name: 110924_HG2_INSA_ACK3.raw

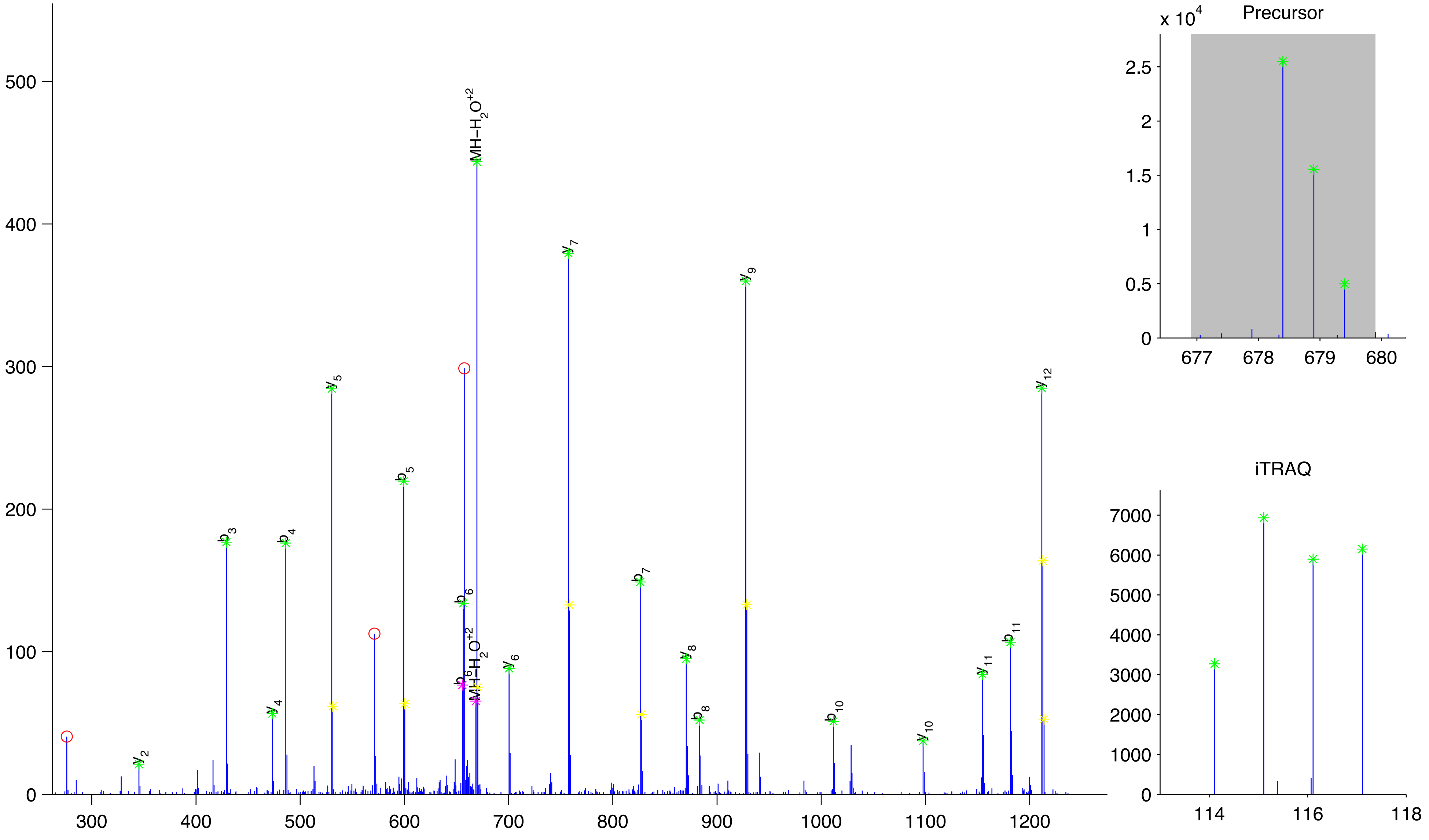

G L G k G G A k R

histone cluster 1, H4a [Homo sapiens]
Charge State: +2
Scan Number: 27479
File Name: 110924_HG2_INSA_ACK3.raw

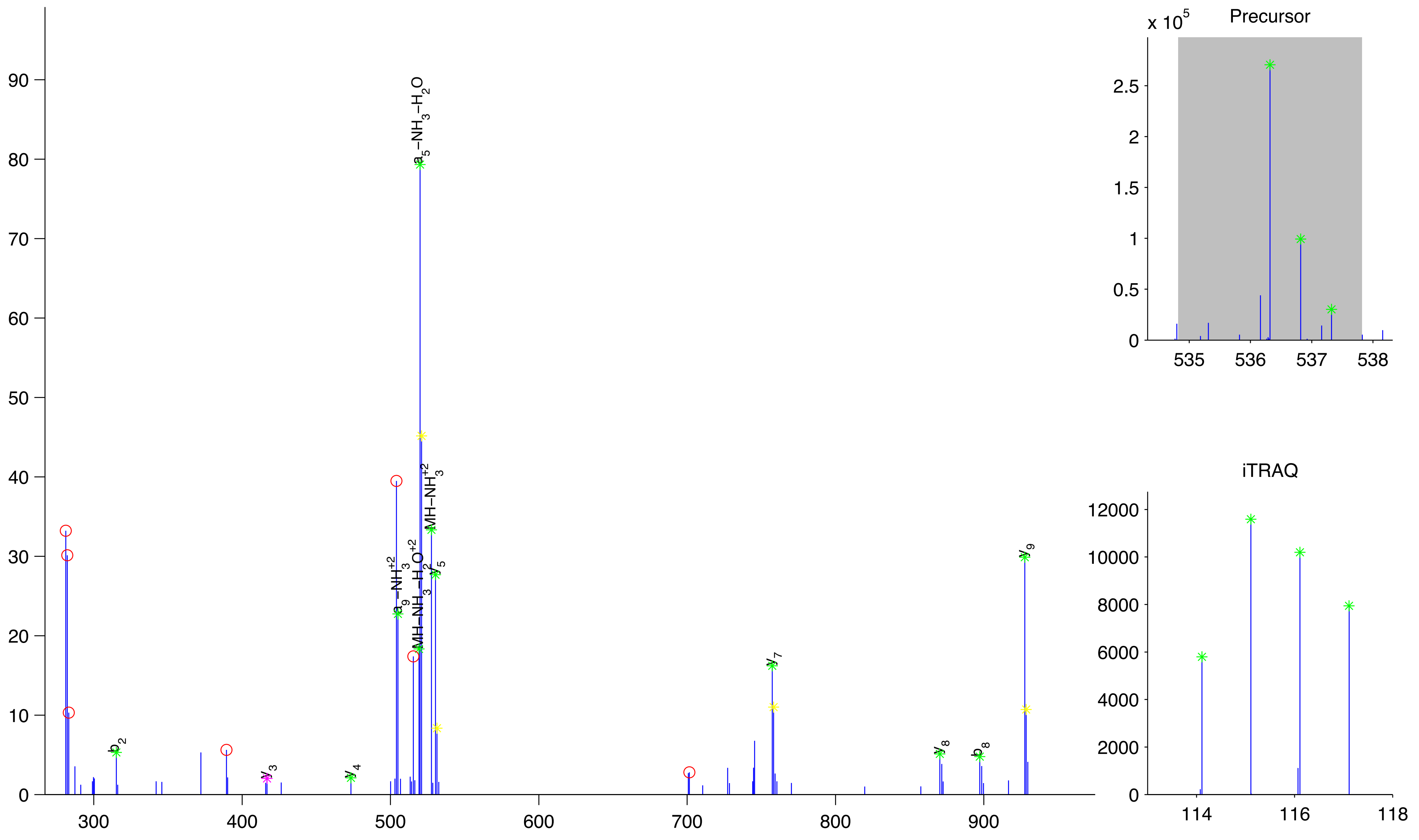

G L G k G G A k R

histone cluster 1, H4a [Homo sapiens]
Charge State: +2
Scan Number: 27656
File Name: 110924_HG2_INSA_ACK3.raw

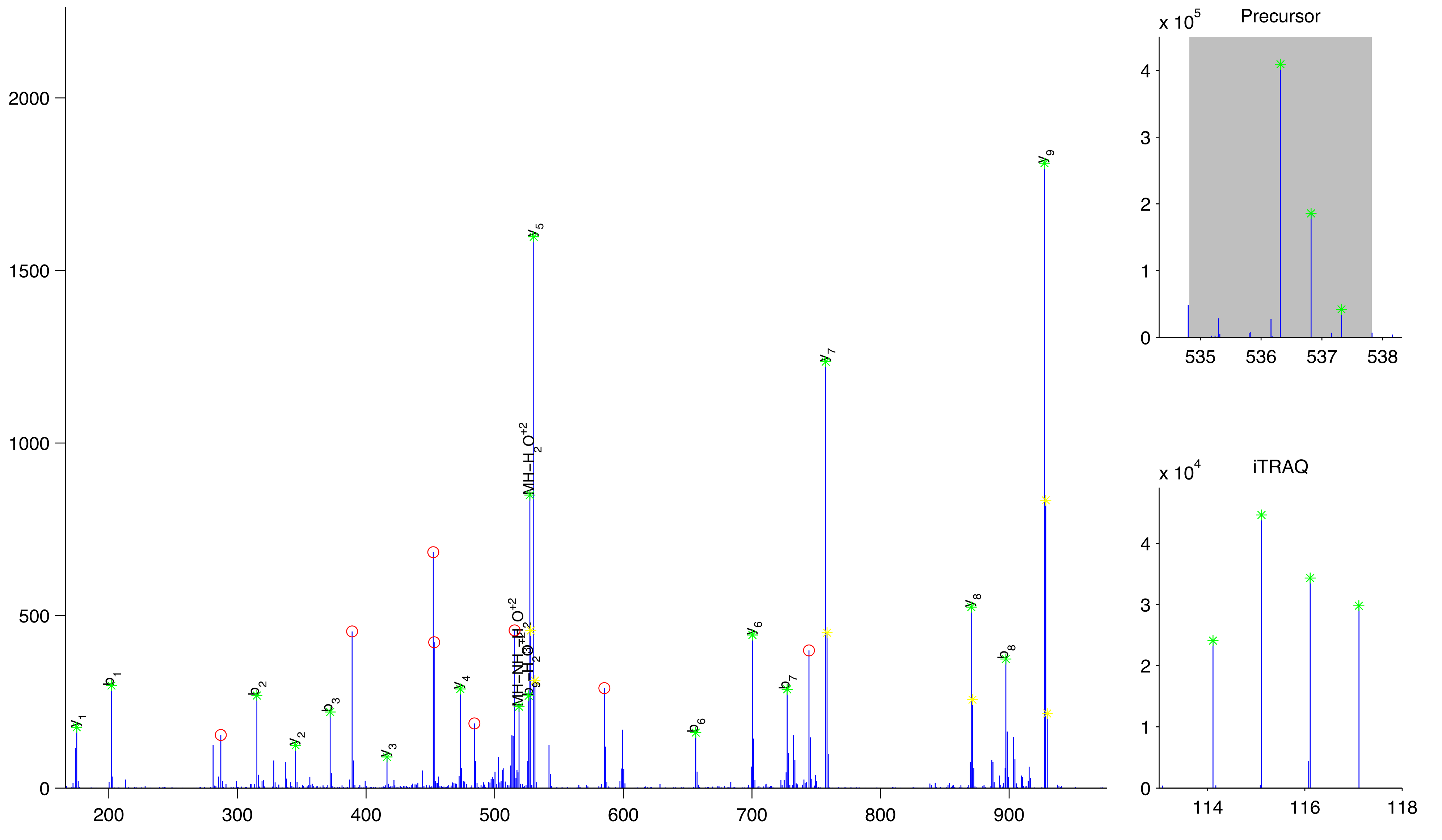

G L G k G G A k R

histone cluster 1, H4a [Homo sapiens]
Charge State: +2
Scan Number: 28126
File Name: 110924_HG2_INSA_ACK3.raw

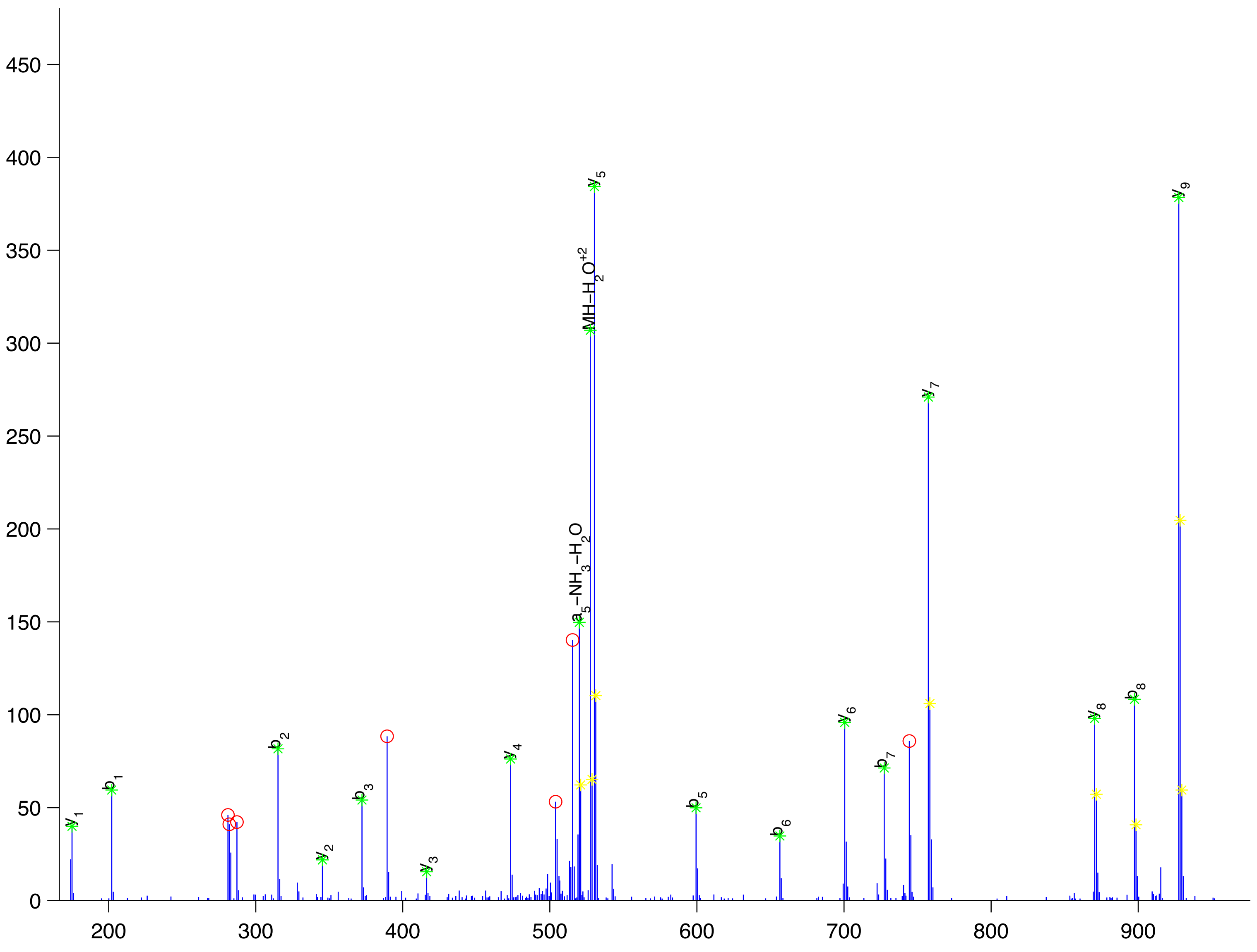

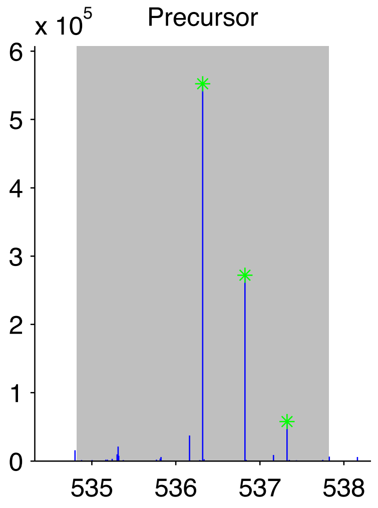

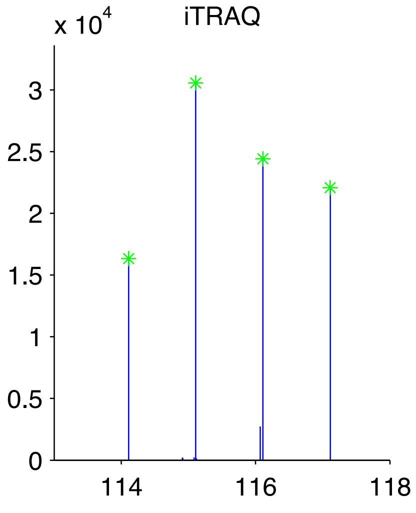

G L G k G G A k R

histone cluster 1, H4a [Homo sapiens]
Charge State: +2
Scan Number: 28997
File Name: 110924_HG2_INSA_ACK3.raw

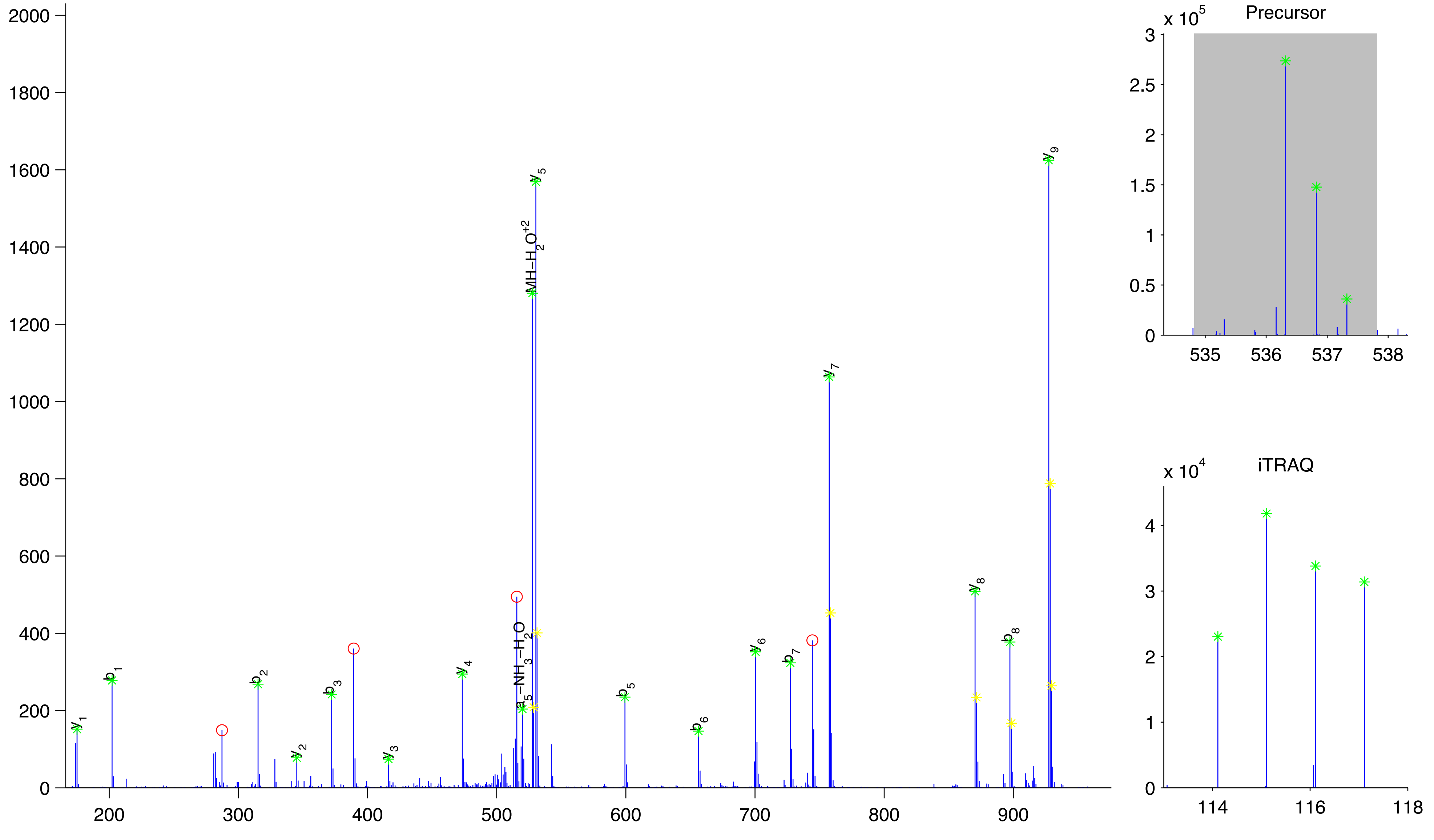

G L G k G G A k R

histone cluster 1, H4a [Homo sapiens]
Charge State: +2
Scan Number: 29184
File Name: 110924_HG2_INSA_ACK3.raw

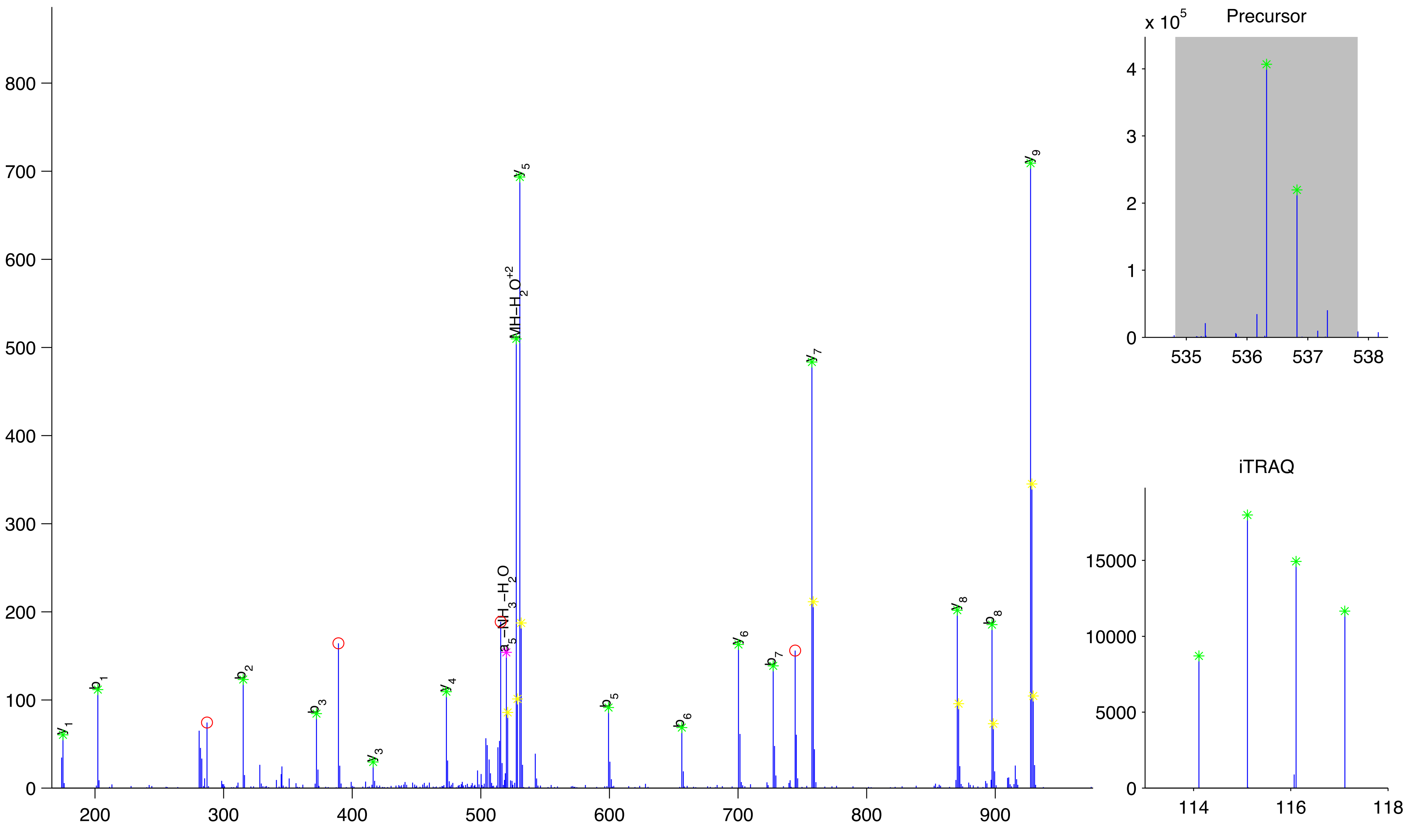

G L G k G G A k R

histone cluster 1, H4a [Homo sapiens]
Charge State: +2
Scan Number: 29504
File Name: 110924_HG2_INSA_ACK3.raw

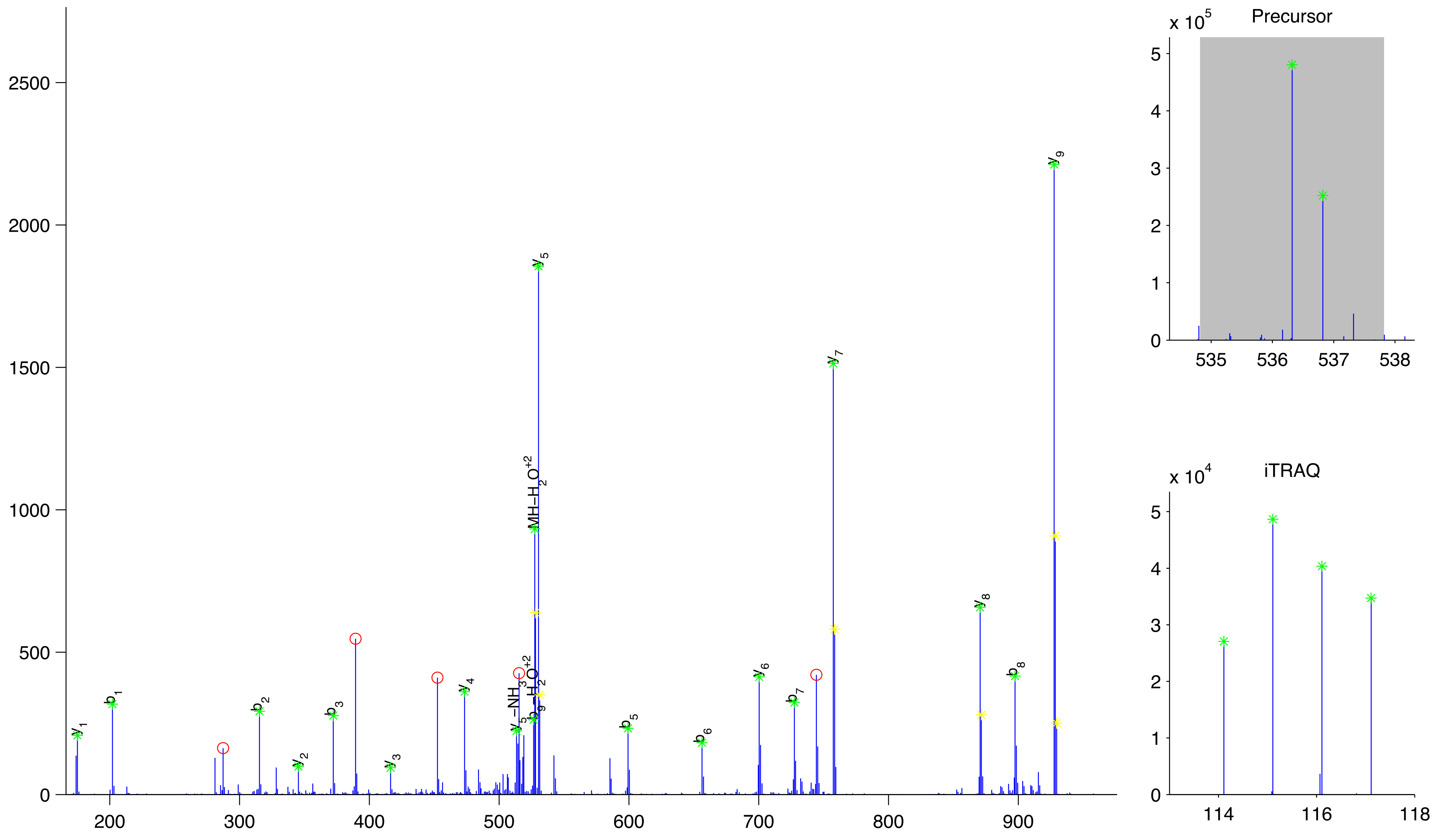

G L G k G G A K

histone cluster 1, H4g [Homo sapiens]
Charge State: +2
Scan Number: 4671
File Name: 111011_HG2_INSB_ACK.raw

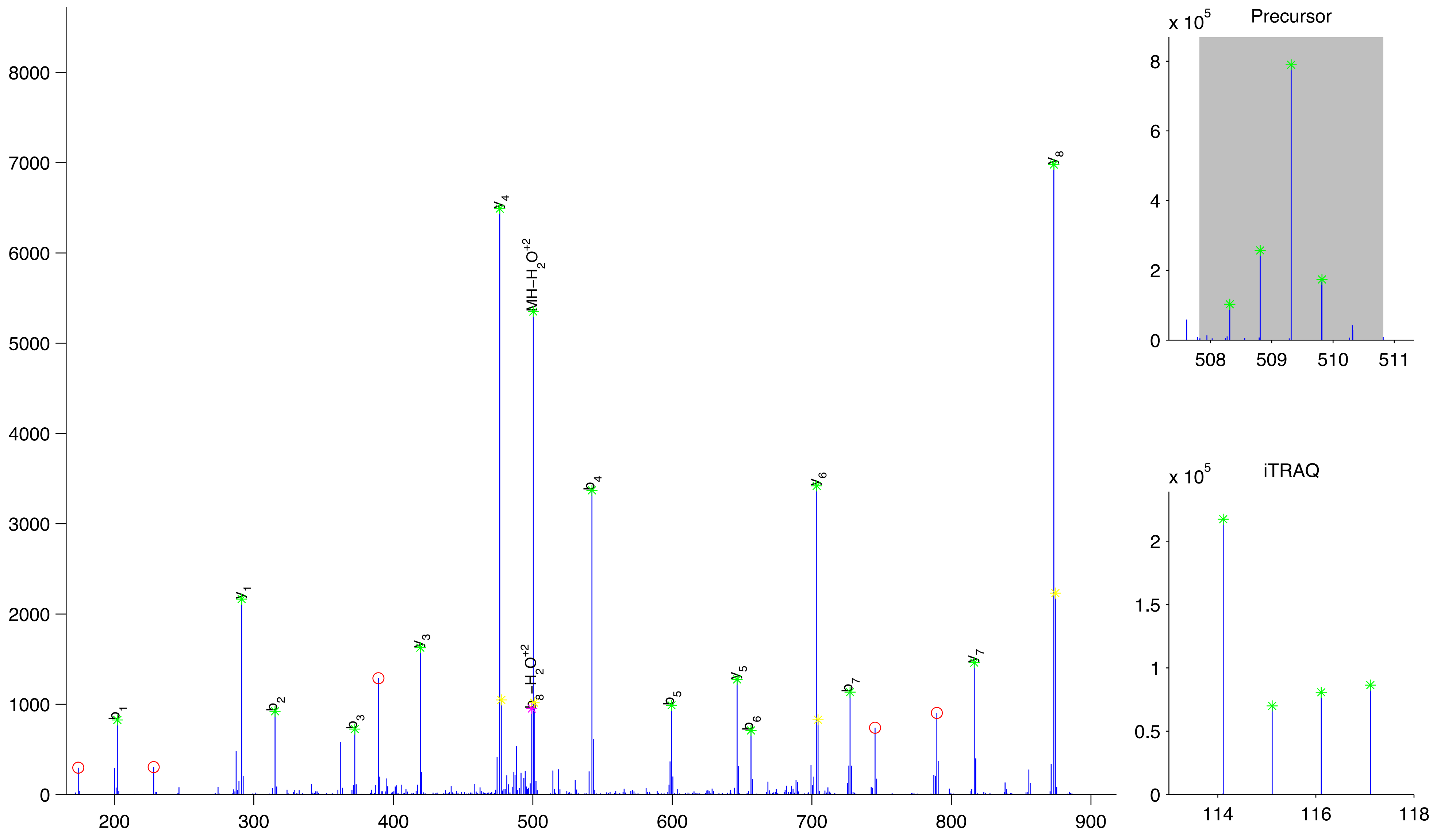

k G S k k A V T K

histone cluster 2, H2bf [Homo sapiens]
Charge State: +2
Scan Number: 4814
File Name: 111011_HG2_INSB_ACK.raw

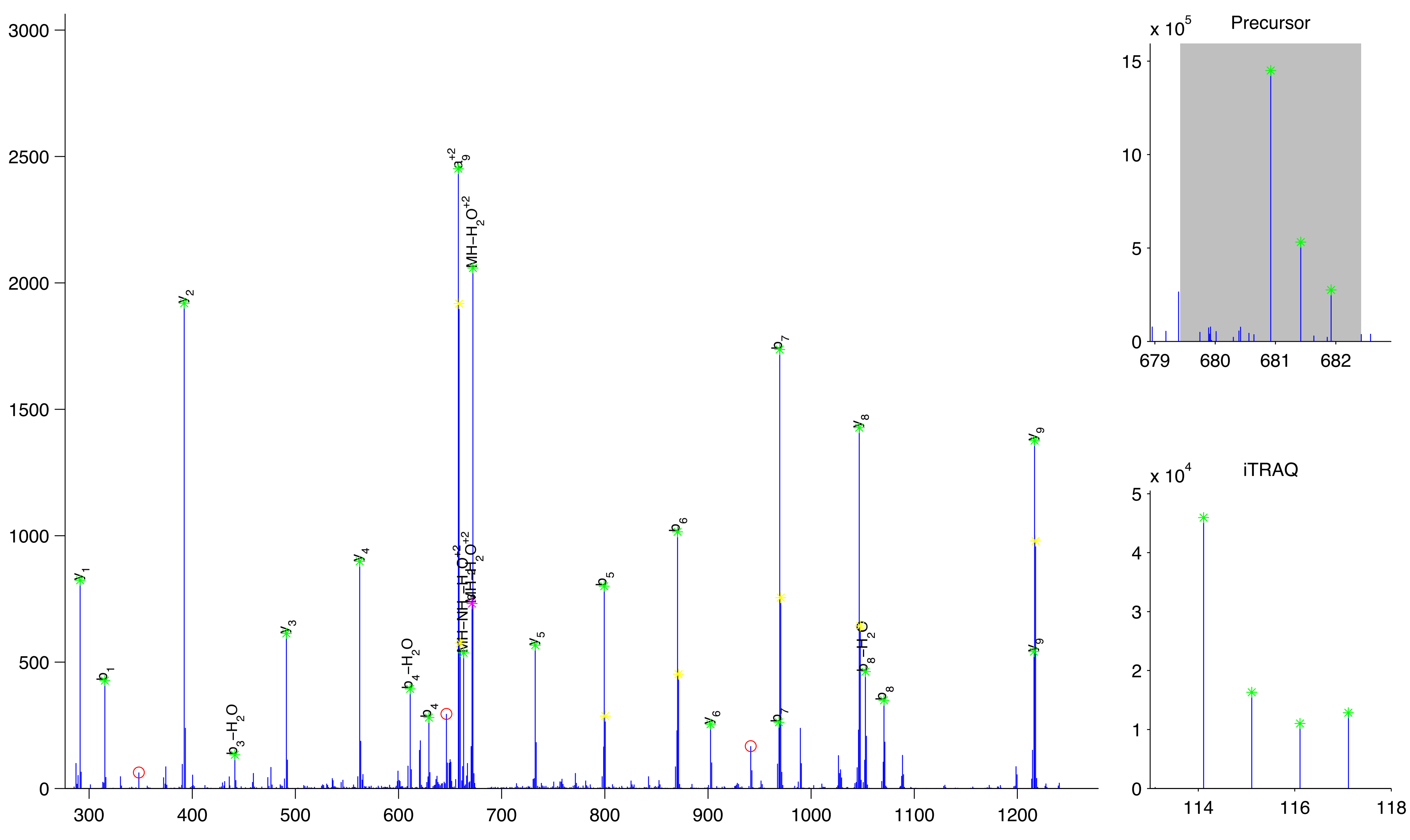

k G S k k A V T k V Q K

histone cluster 2, H2bf [Homo sapiens]
Charge State: +3
Scan Number: 5465
File Name: 111011_HG2_INSB_ACK.raw

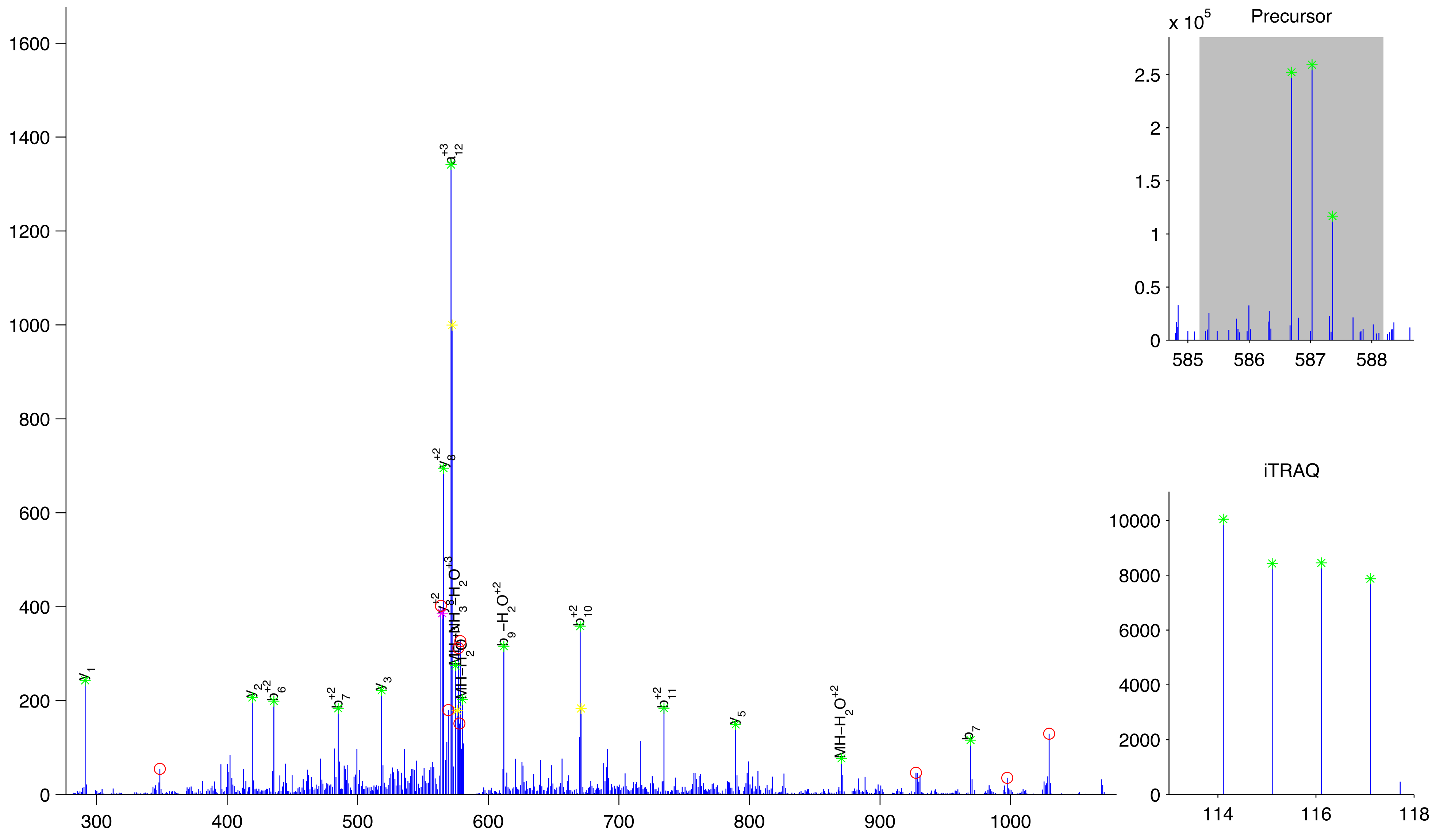

k A V T k V Q K

histone cluster 2, H2bf [Homo sapiens]
Charge State: +2
Scan Number: 12456
File Name: 110924_HG2_INSA_ACK.raw

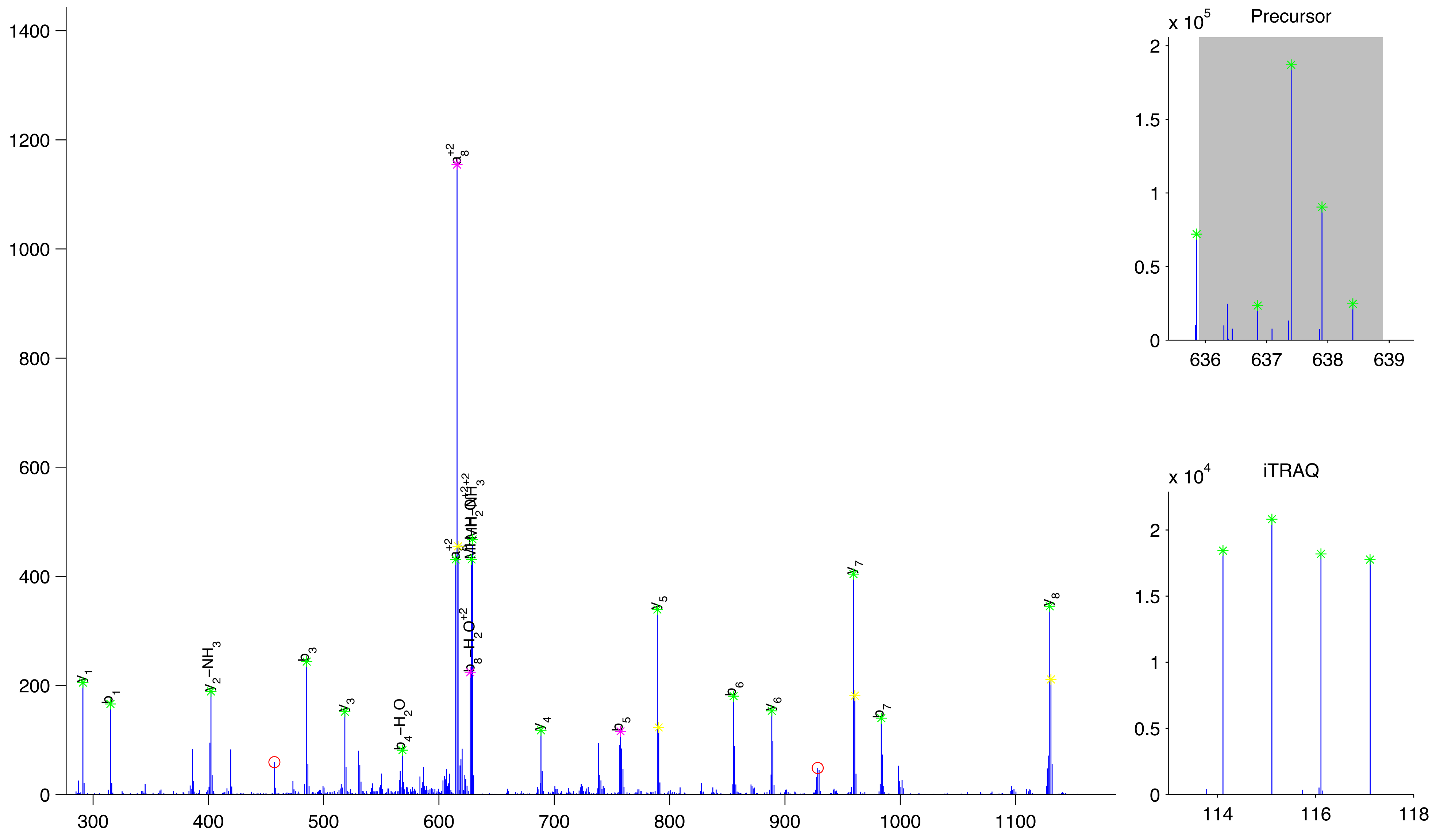

k A V T k V Q K

histone cluster 2, H2bf [Homo sapiens]
Charge State: +2
Scan Number: 12647
File Name: 110924_HG2_INSA_ACK.raw

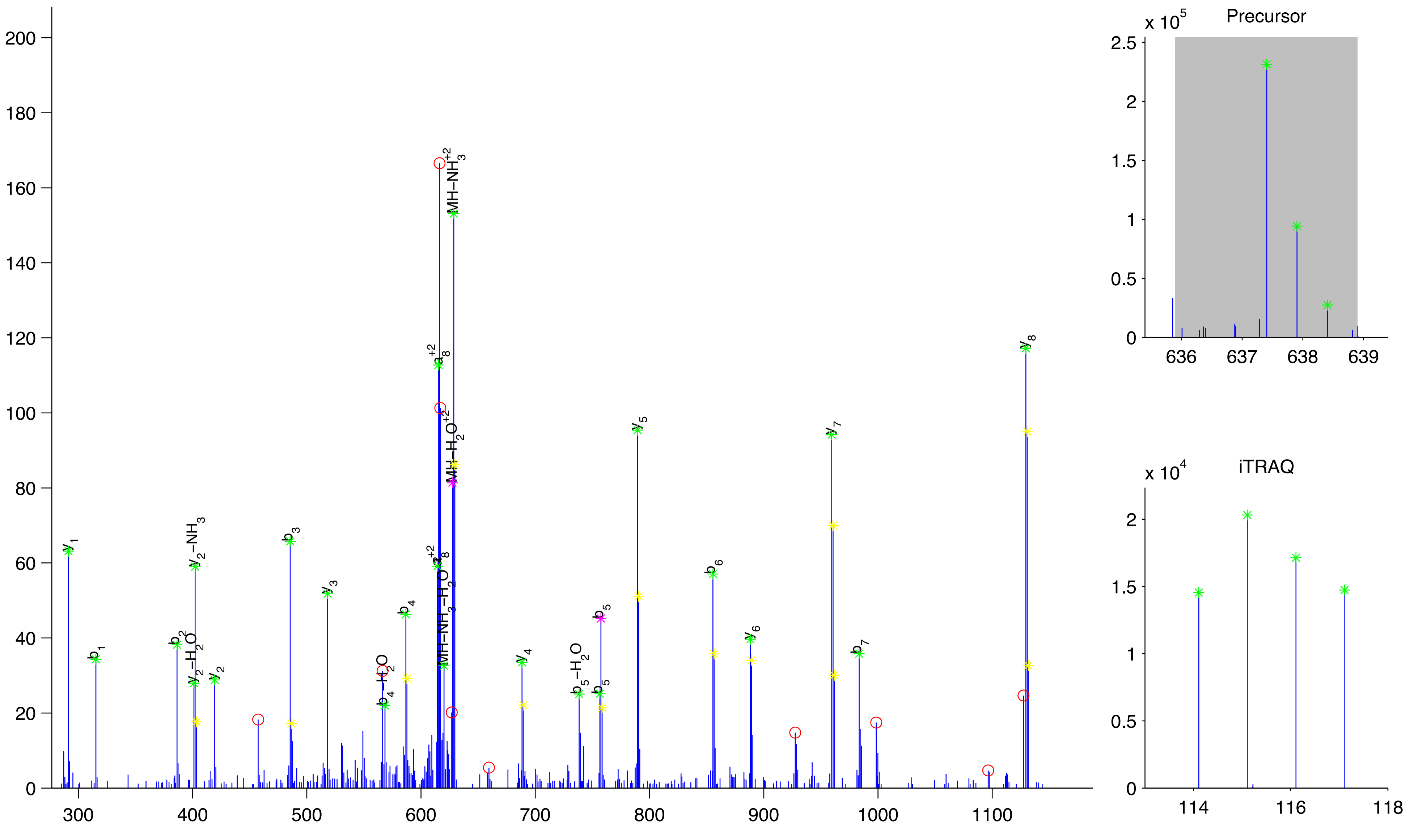

k D G P L D V R

methyltransferase 5 domain containing 1 [Homo sapiens]
Charge State: +2
Scan Number: 5799
File Name: 110924_HG2_INSA_ACK3.raw

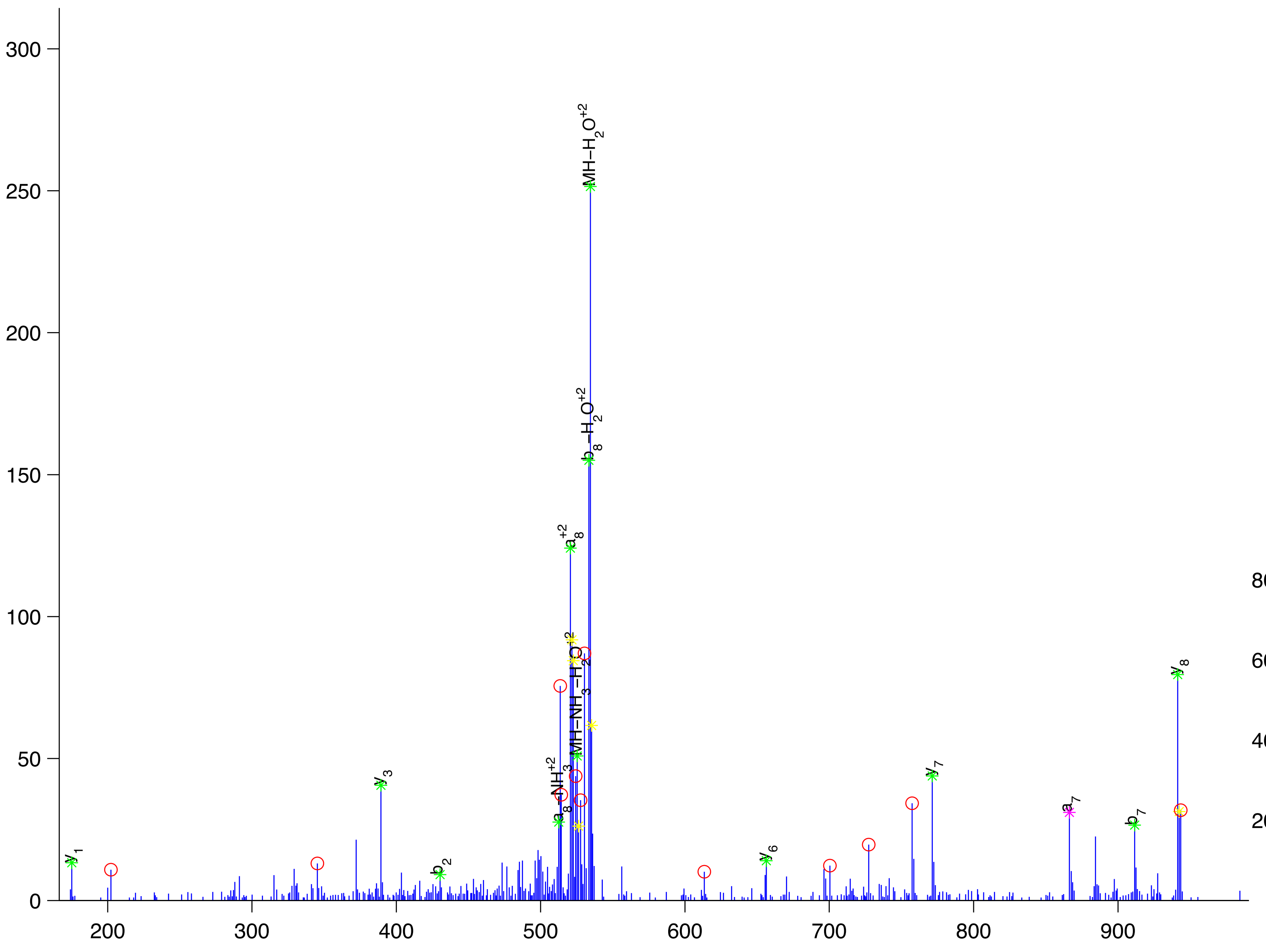

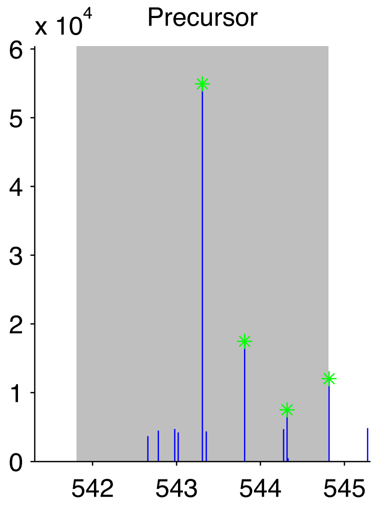

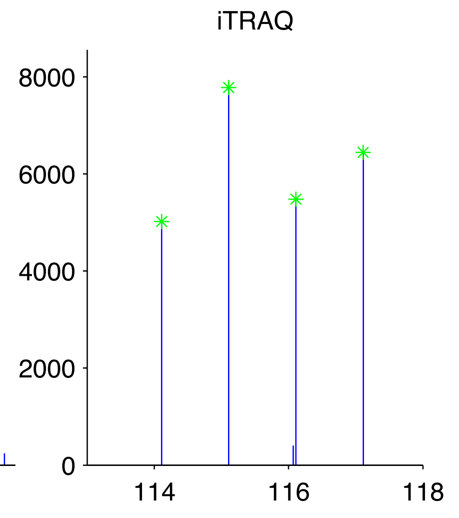

k D G P L D V R

methyltransferase 5 domain containing 1 [Homo sapiens]
Charge State: +2
Scan Number: 7844
File Name: 110924_HG2_INSA_ACK3.raw

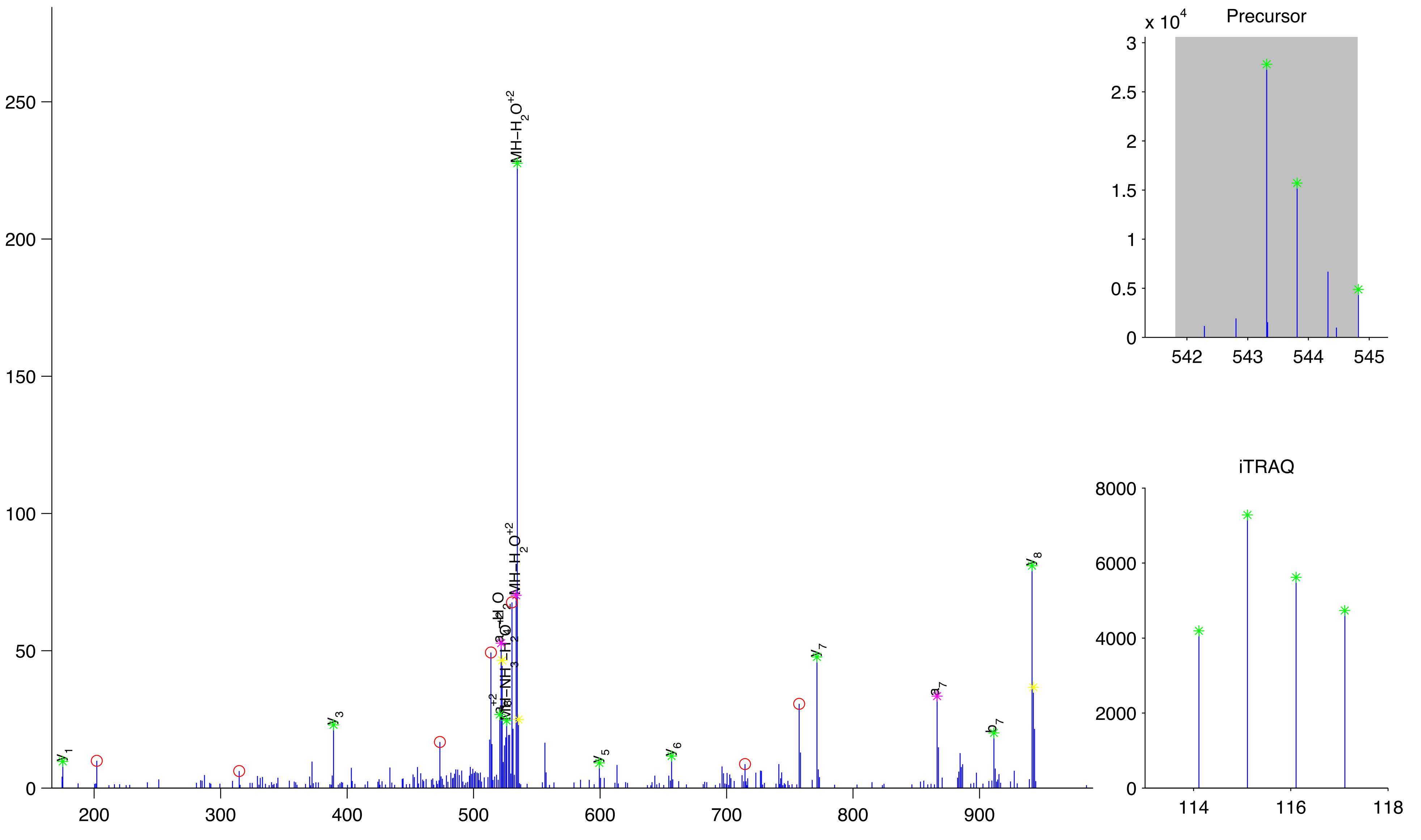

L L S I S G k R

nucleophosmin 1 isoform 1 [Homo sapiens]
Charge State: +2
Scan Number: 6536
File Name: 111011_HG2_INSB_ACK.raw

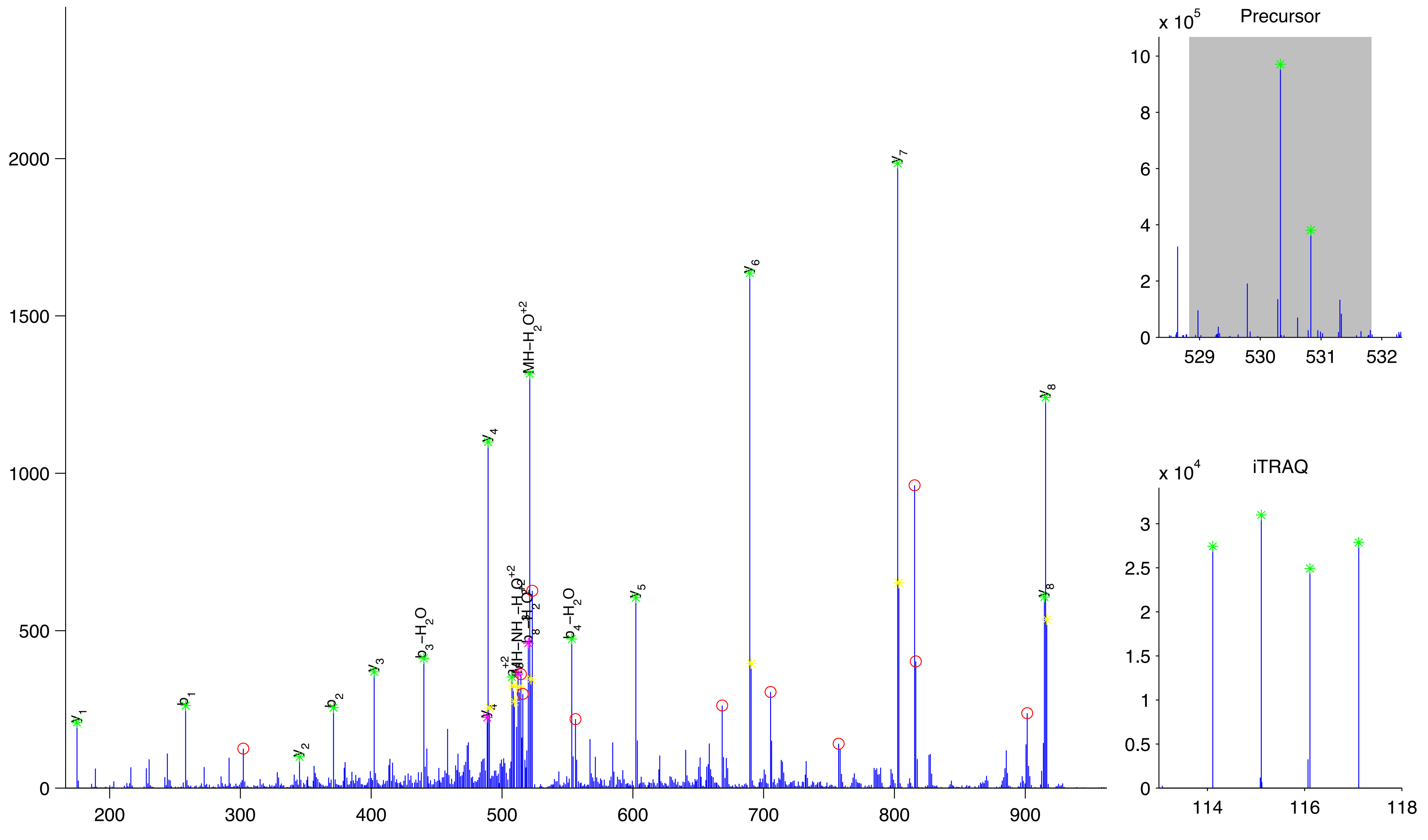

L L S I S G k R

nucleophosmin 1 isoform 1 [Homo sapiens]
Charge State: +2
Scan Number: 6702
File Name: 111011_HG2_INSB_ACK.raw

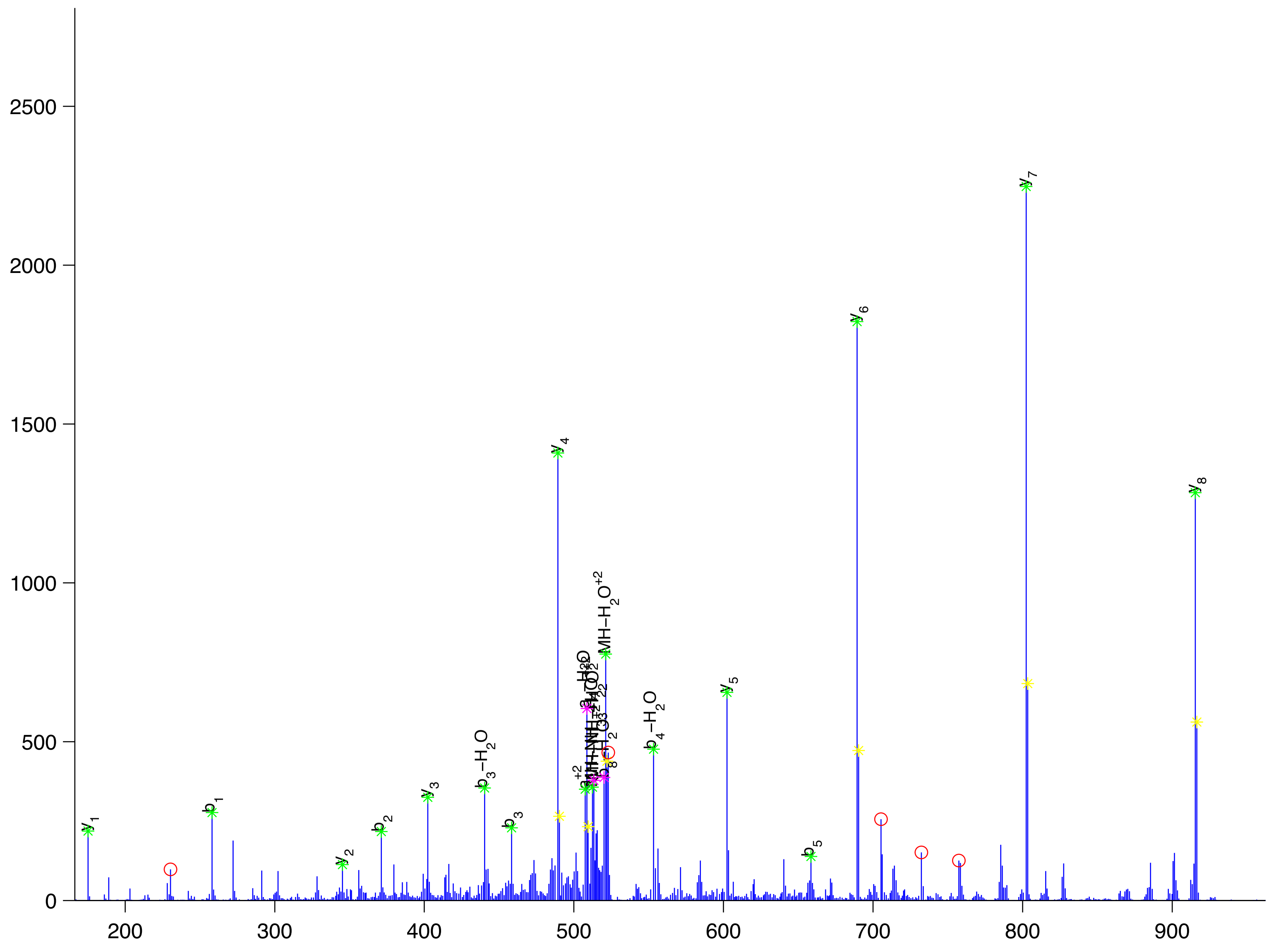

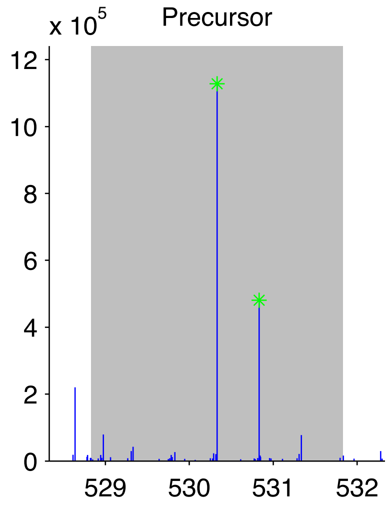

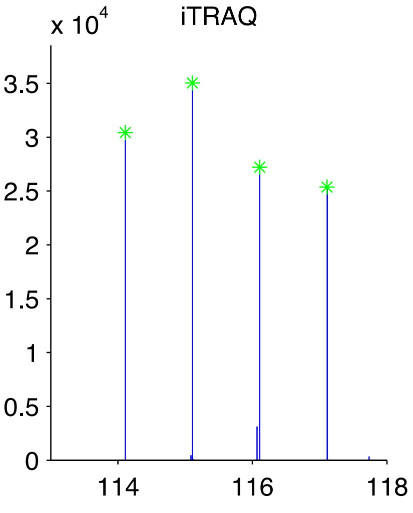

Y k P E S E E L T A E R

prolyl 4–hydroxylase, beta subunit precursor [Homo sapiens]
Charge State: +2
Scan Number: 5681
File Name: 111016_HG2_INSC_ACK.raw

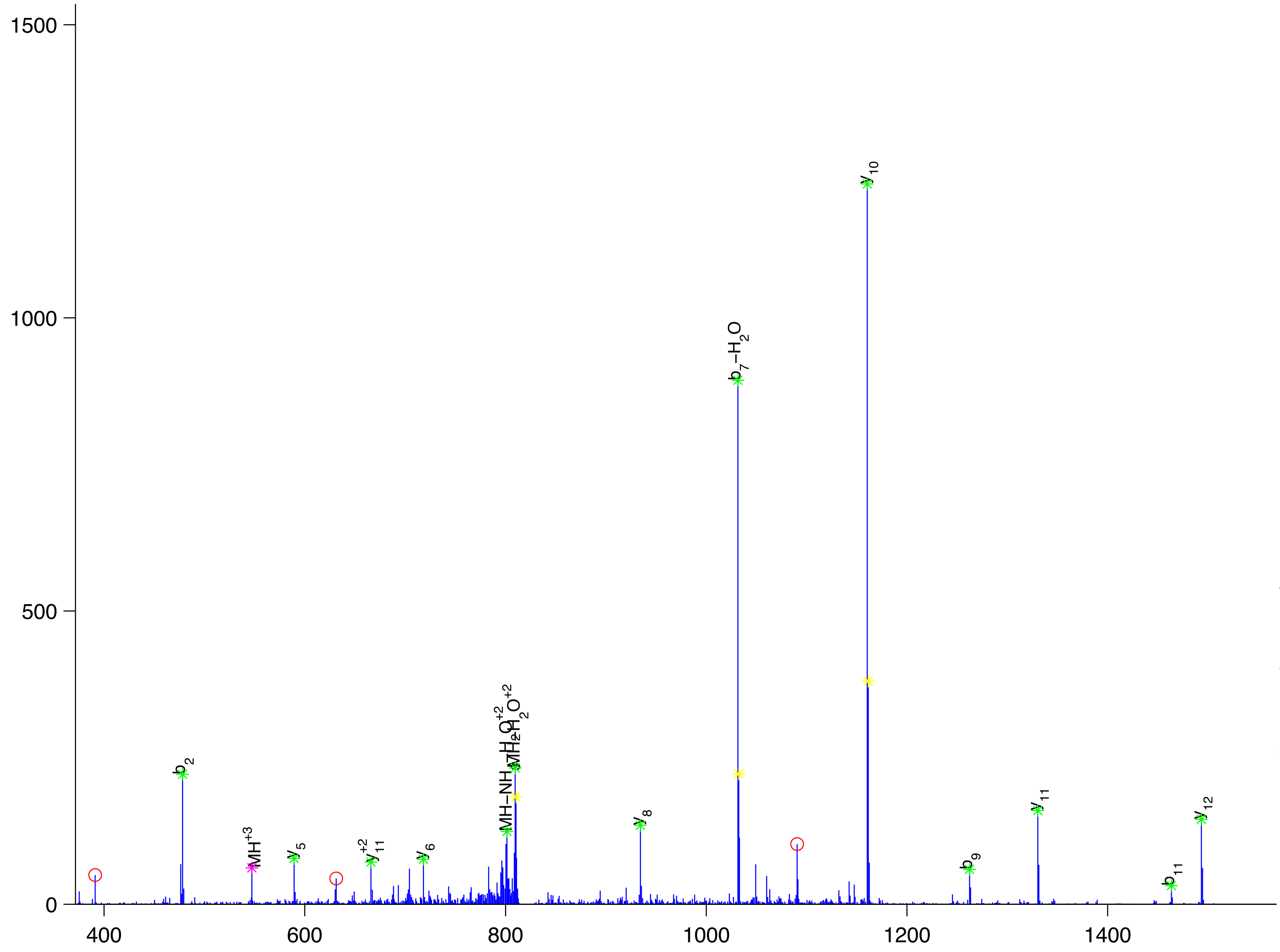

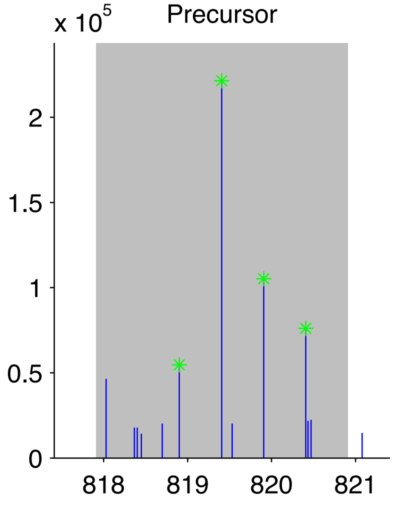

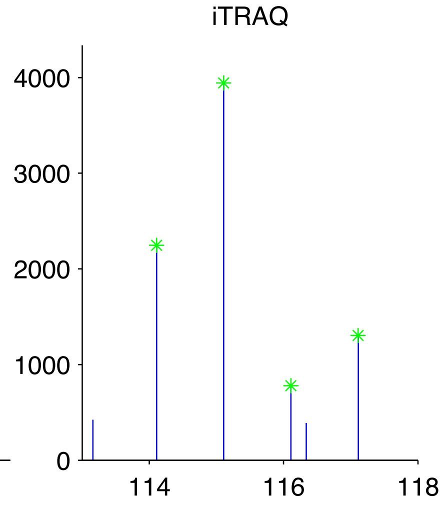

G k D I S T I L D E E R K

remodeling and spacing factor 1 [Homo sapiens]
Charge State: +3
Scan Number: 57
File Name: 110924_HG2_INSA_ACK2.raw

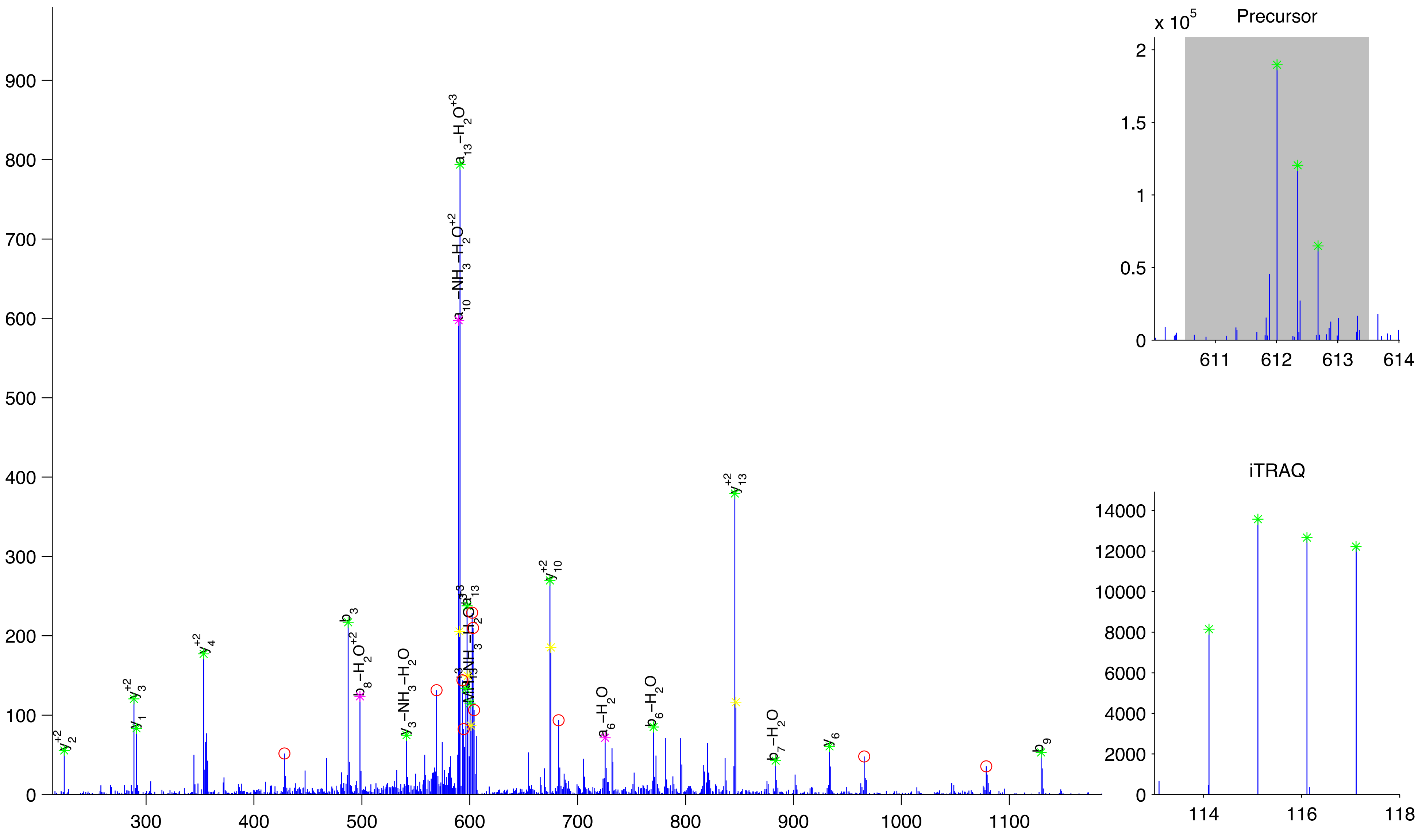

G k D I S T I L D E E R K

remodeling and spacing factor 1 [Homo sapiens]
Charge State: +3
Scan Number: 215
File Name: 110924_HG2_INSA_ACK2.raw

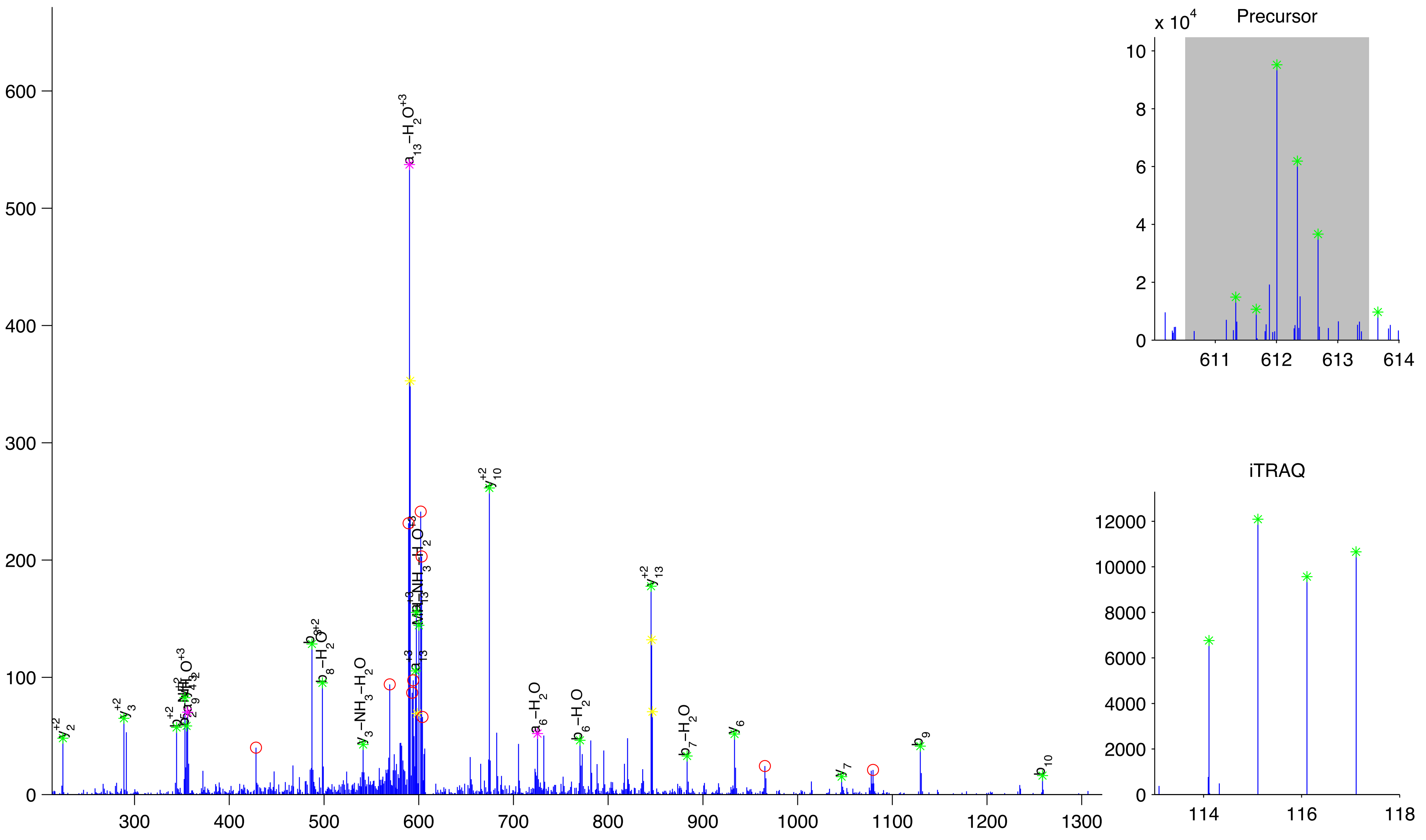

G k D I S T I L D E E R K

remodeling and spacing factor 1 [Homo sapiens]
Charge State: +3
Scan Number: 387
File Name: 110924_HG2_INSA_ACK2.raw

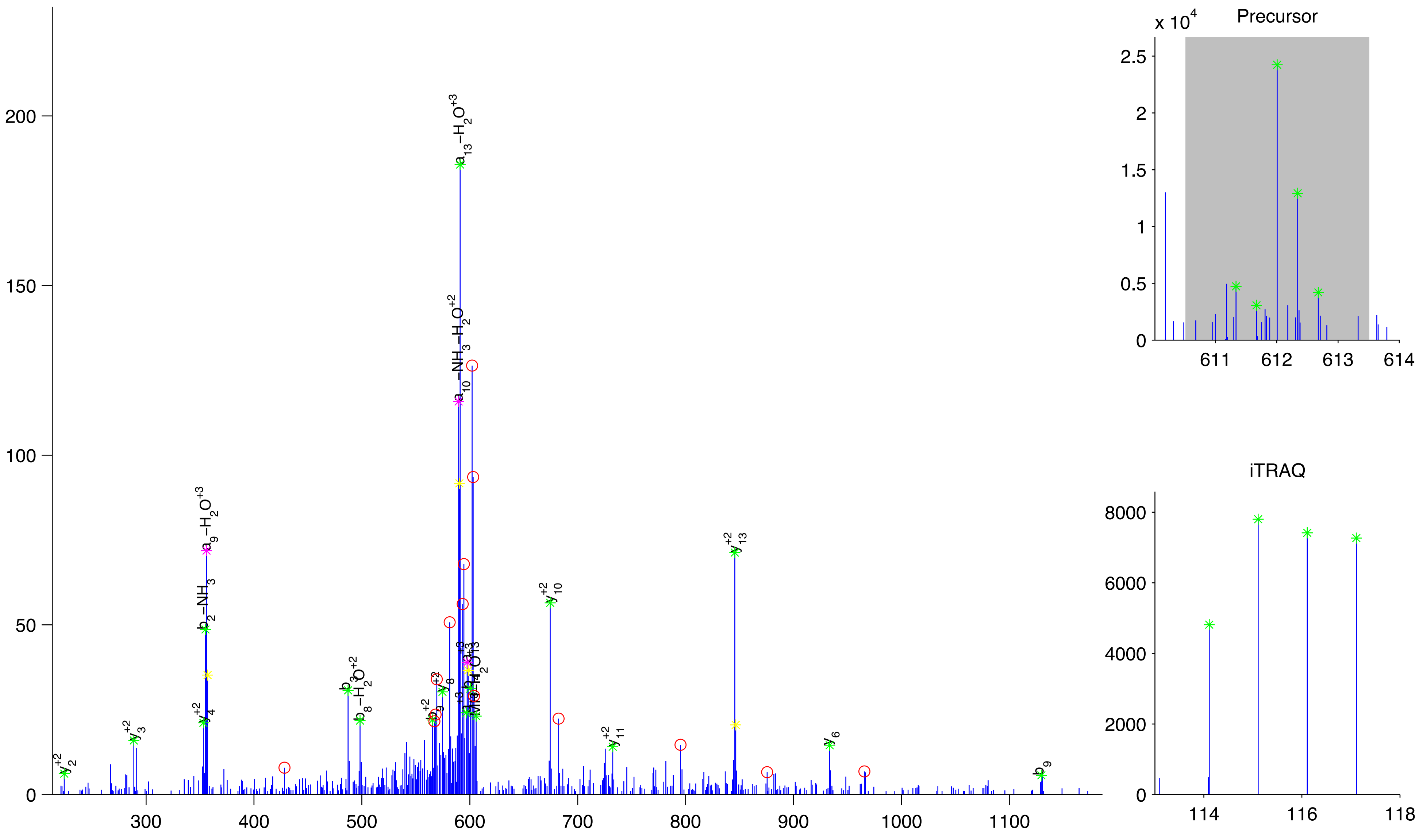

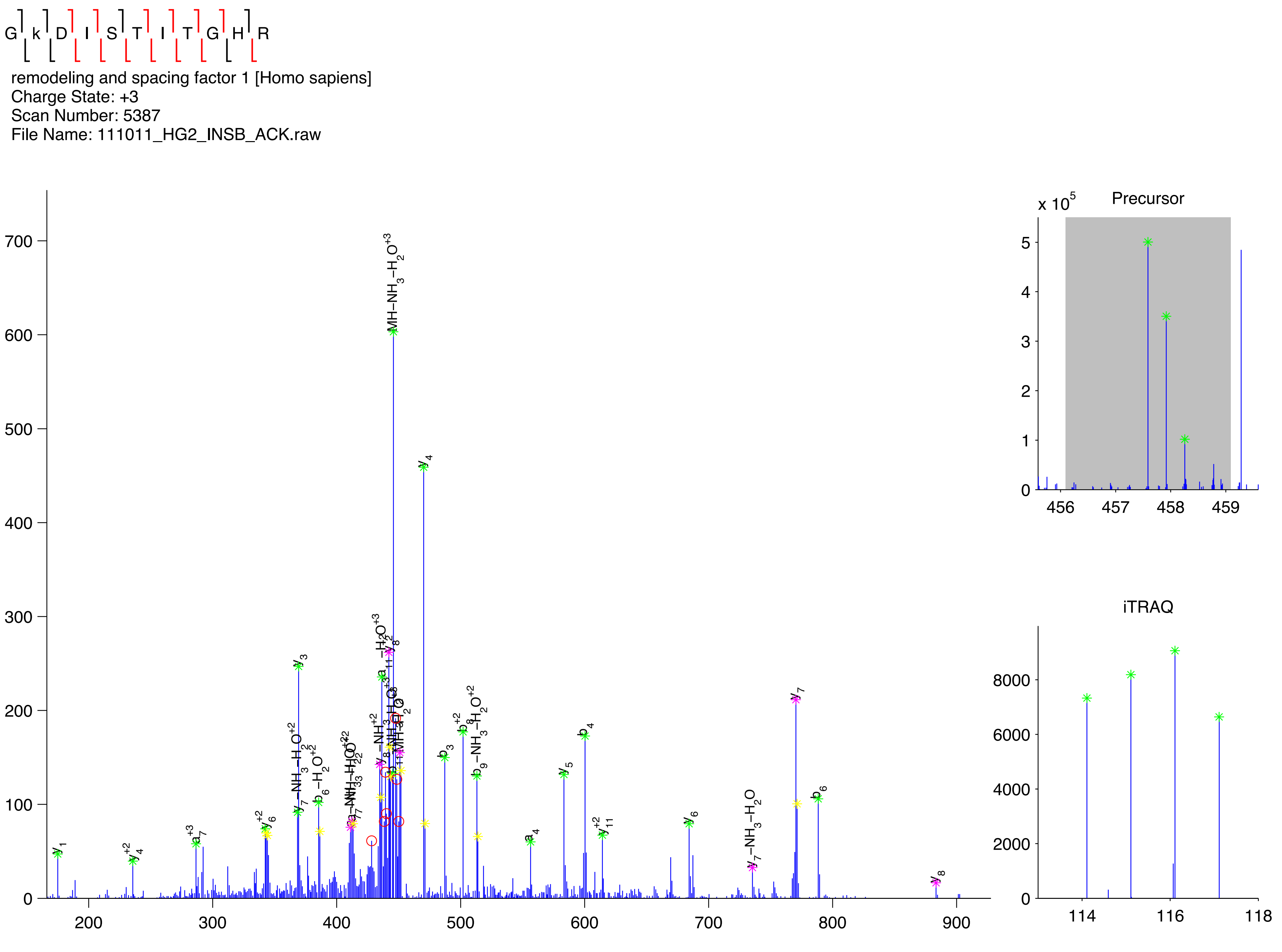
G k D I S T I T G H R
remodeling and spacing factor 1 [Homo sapiens]
Charge State: +3
Scan Number: 5387
File Name: 111011_HG2_INSB_ACK.raw
Precursor
x 10^5
iTRAQ

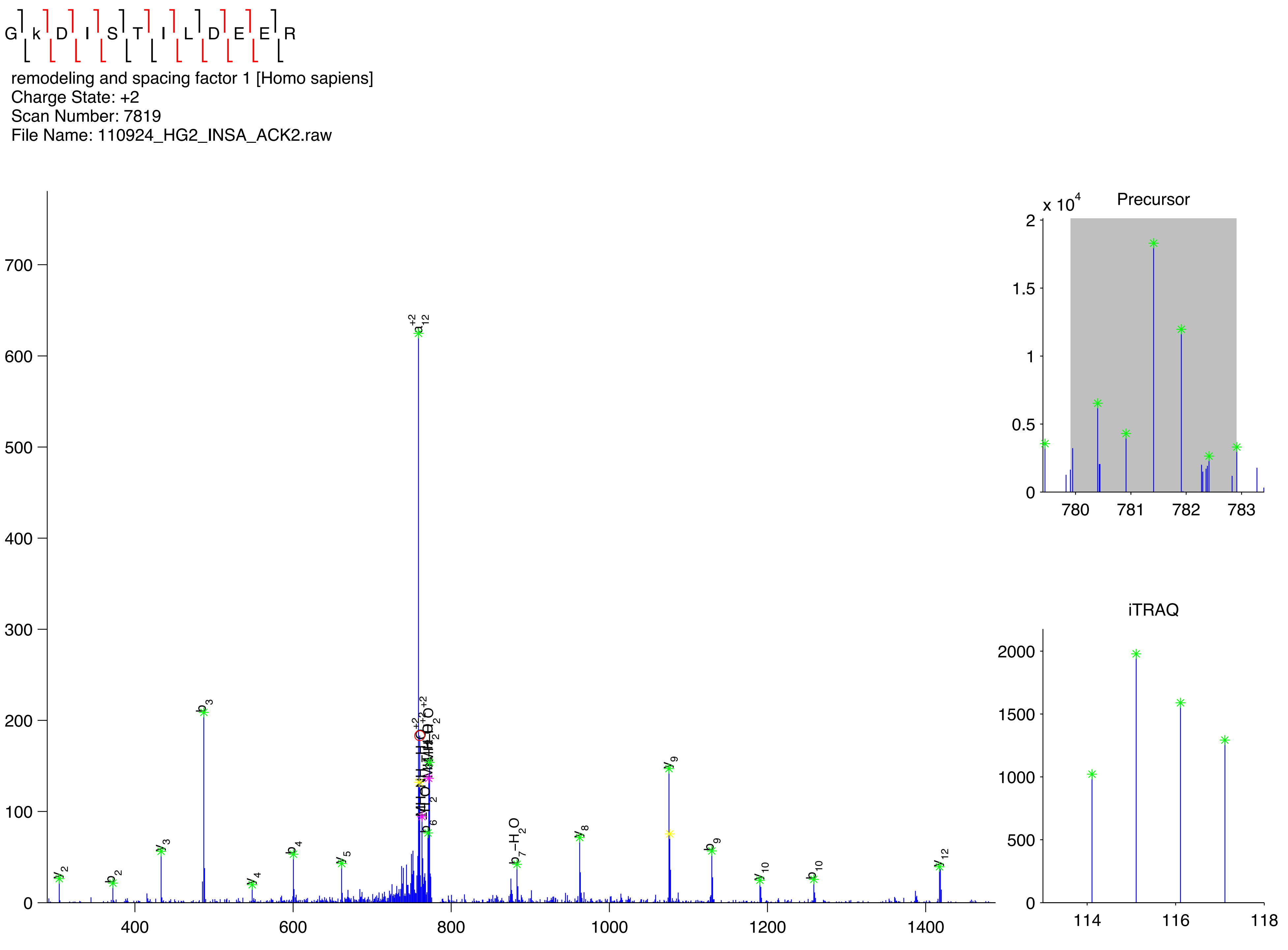
G k D I S T I L D E E R
remodeling and spacing factor 1 [Homo sapiens]
Charge State: +2
Scan Number: 7819
File Name: 110924_HG2_INSA_ACK2.raw
Precursor
iTRAQ

G k D I S T I L D E E R

remodeling and spacing factor 1 [Homo sapiens]
Charge State: +2
Scan Number: 8144
File Name: 111011_HG2_INSB_ACK.raw

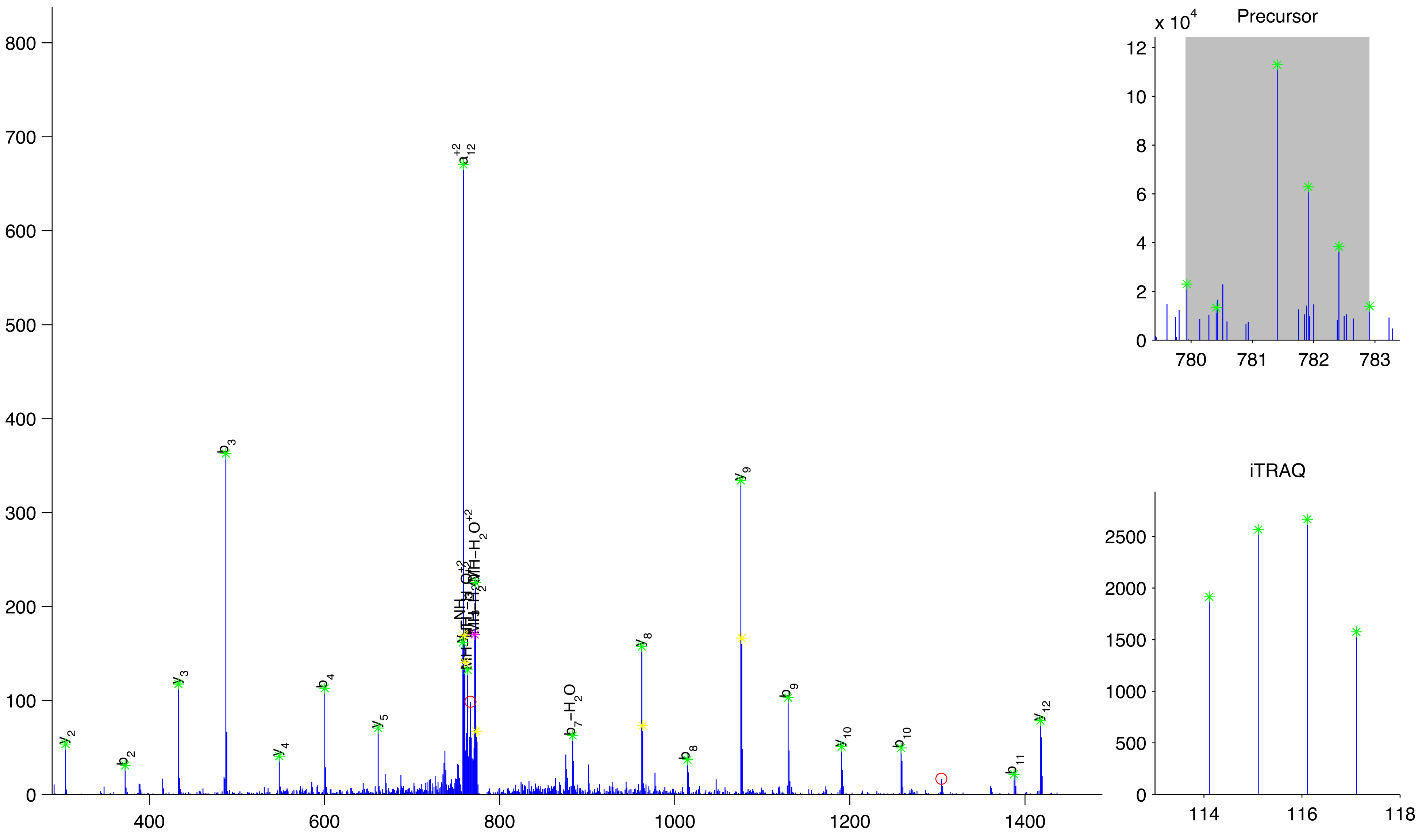

G k D I S T I T G H R

remodeling and spacing factor 1 [Homo sapiens]
Charge State: +3
Scan Number: 14501
File Name: 110924_HG2_INSA_ACK.raw

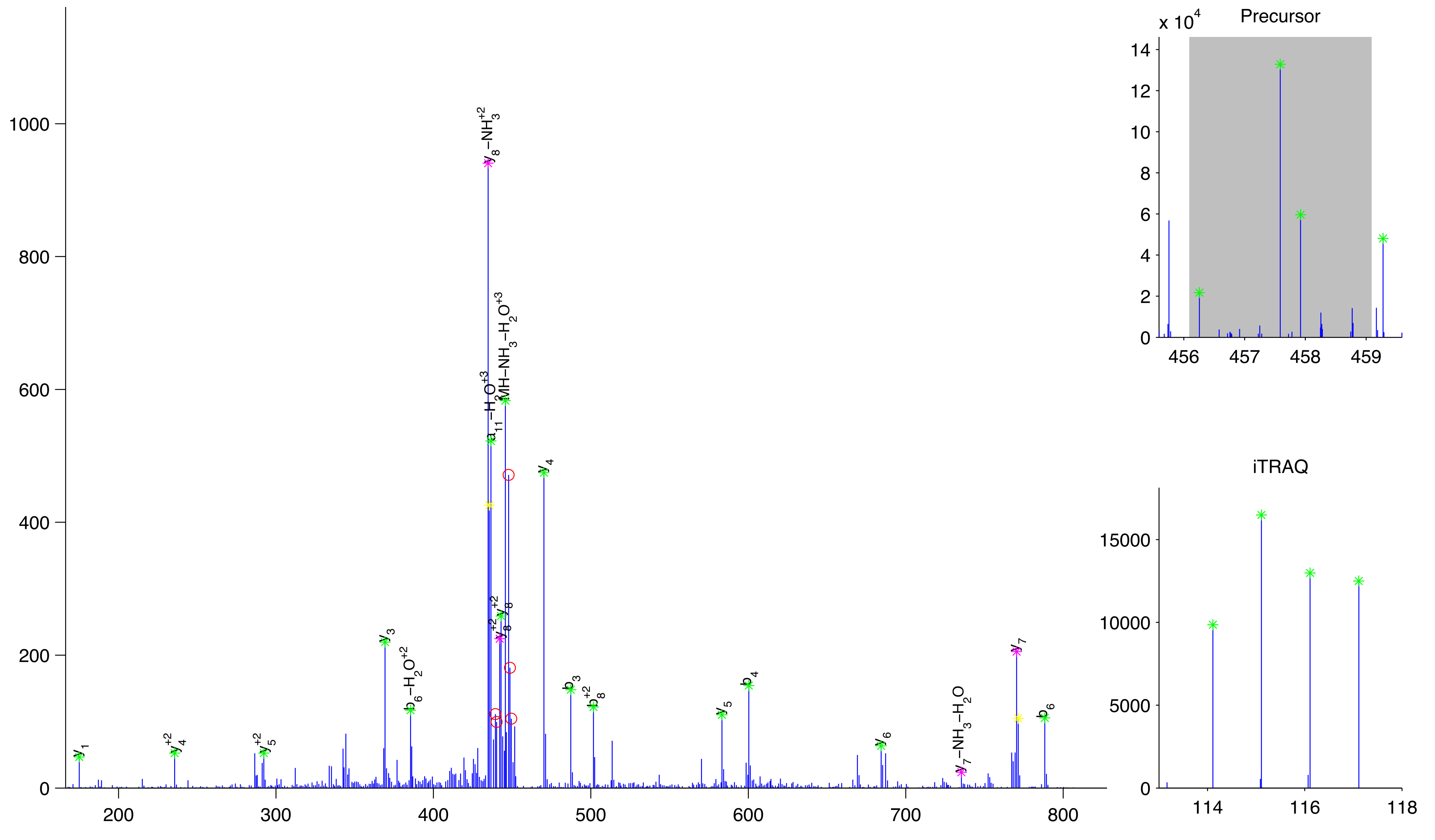

k G k G G A K

SWI/SNF–related matrix–associated actin–dependent regulator of chromatin a2 isoform b [Homo sapiens
Charge State: +2
Scan Number: 4671
File Name: 111011_HG2_INSB_ACK.raw

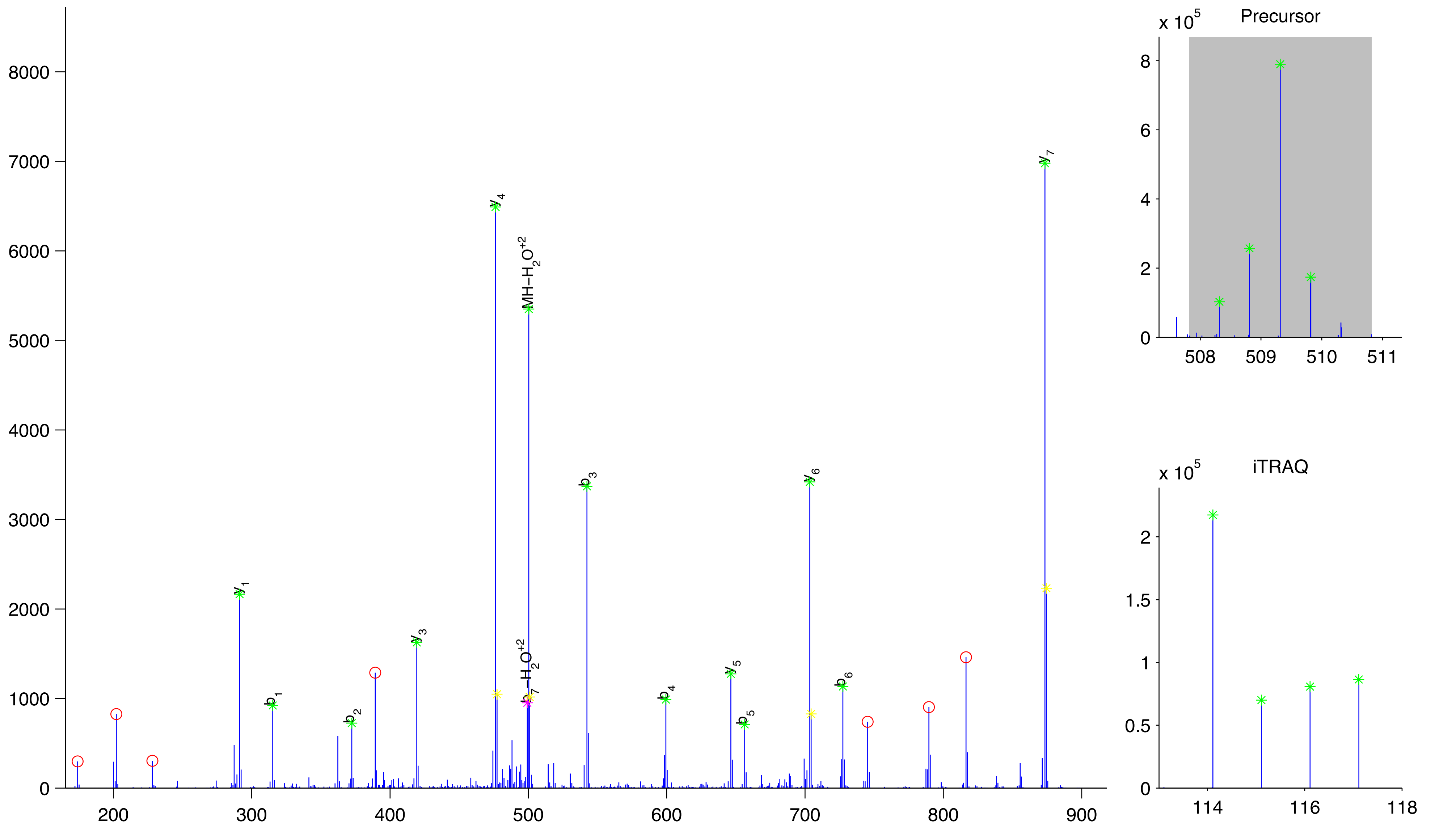

G k T A Q G L S P V D Q R

tripartite motif–containing 33 protein isoform alpha [Homo sapiens]
Charge State: +2
Scan Number: 14138
File Name: 110924_HG2_INSA_ACK.raw

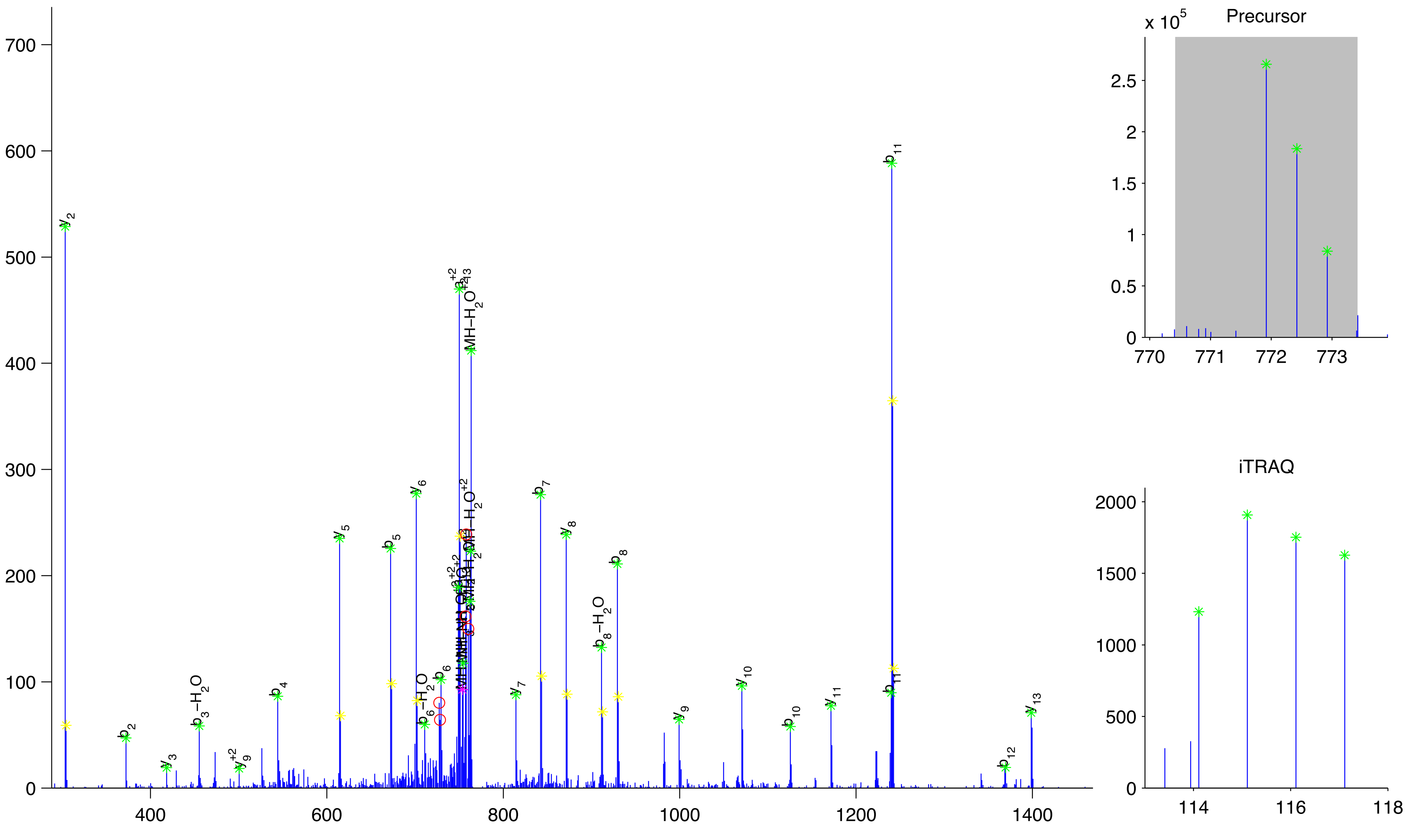

G k T A Q G L S P V D Q R

tripartite motif–containing 33 protein isoform alpha [Homo sapiens]
Charge State: +2
Scan Number: 14304
File Name: 110924_HG2_INSA_ACK.raw

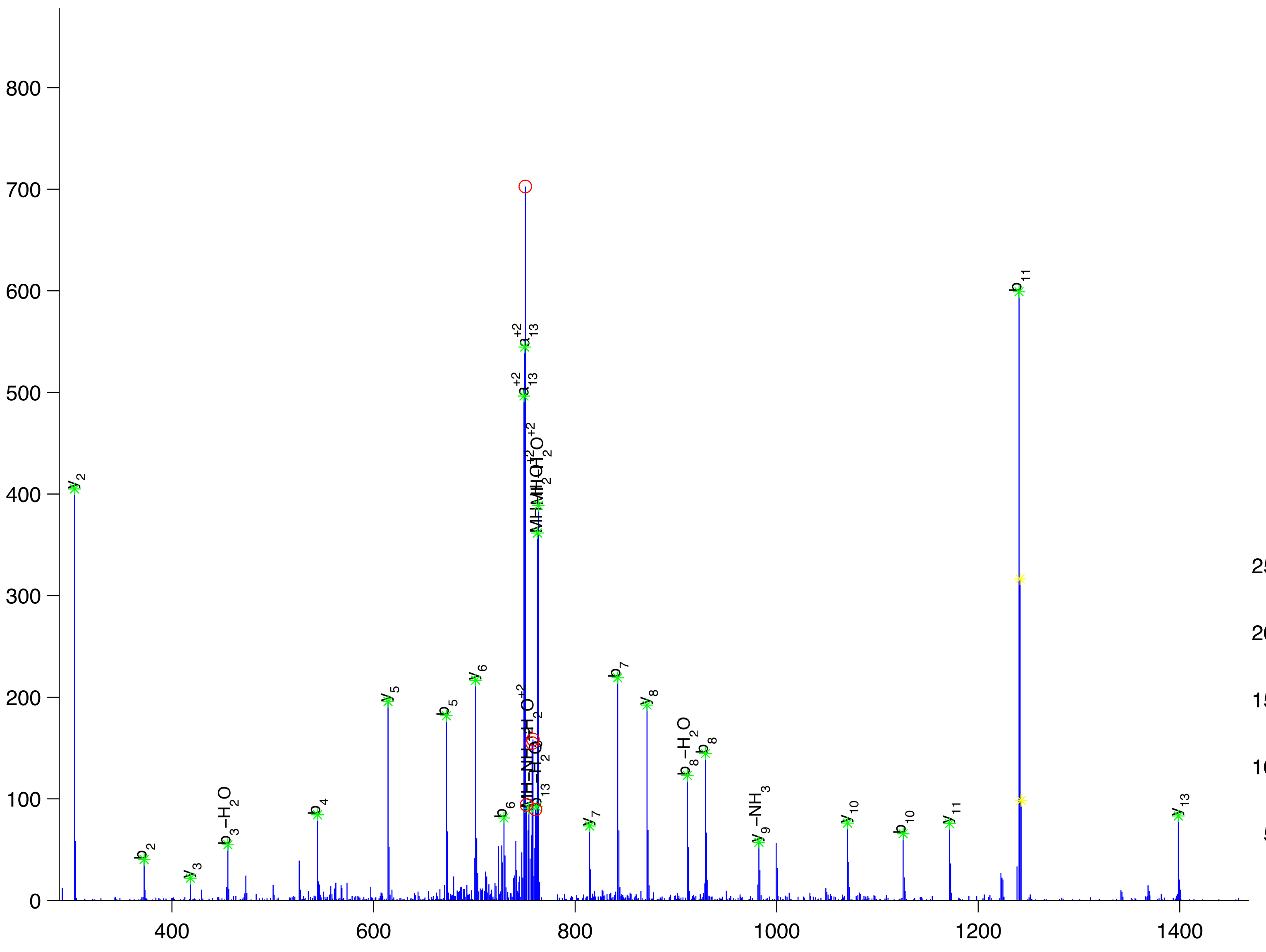

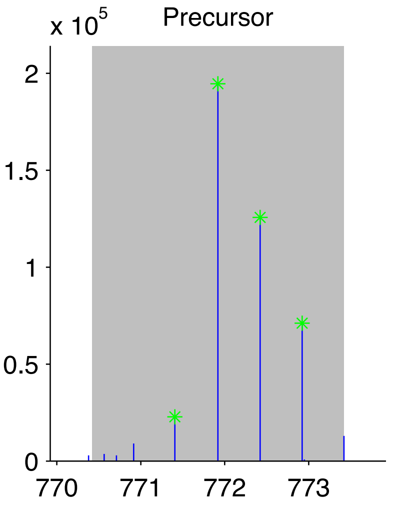

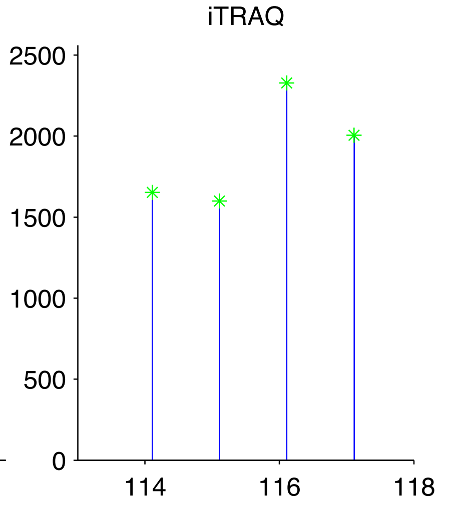

G k T A Q G L S P V D Q R

tripartite motif–containing 33 protein isoform alpha [Homo sapiens]
Charge State: +2
Scan Number: 14472
File Name: 110924_HG2_INSA_ACK.raw

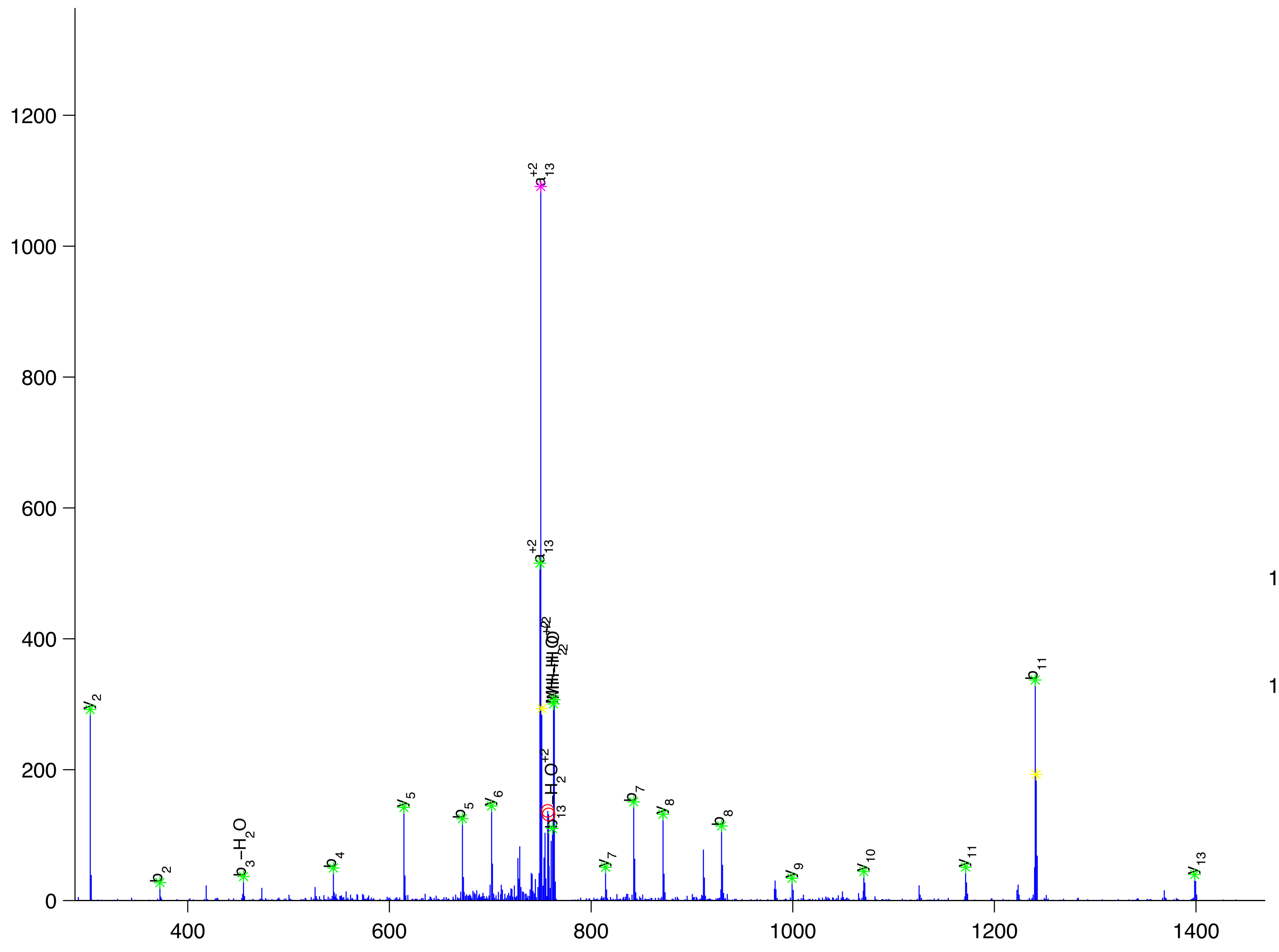

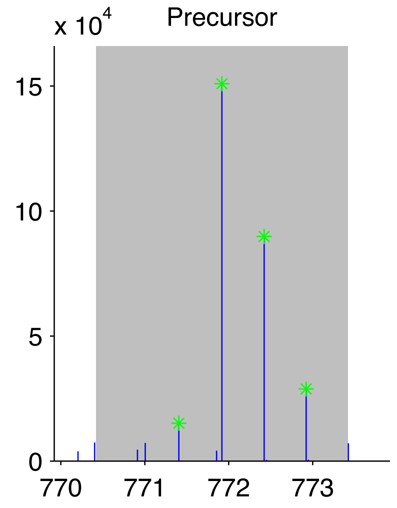

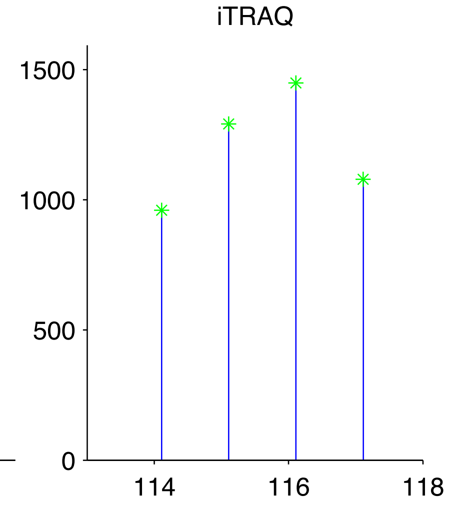

G k T A Q G L S P V D Q R

tripartite motif–containing 33 protein isoform alpha [Homo sapiens]
Charge State: +2
Scan Number: 14644
File Name: 110924_HG2_INSA_ACK.raw

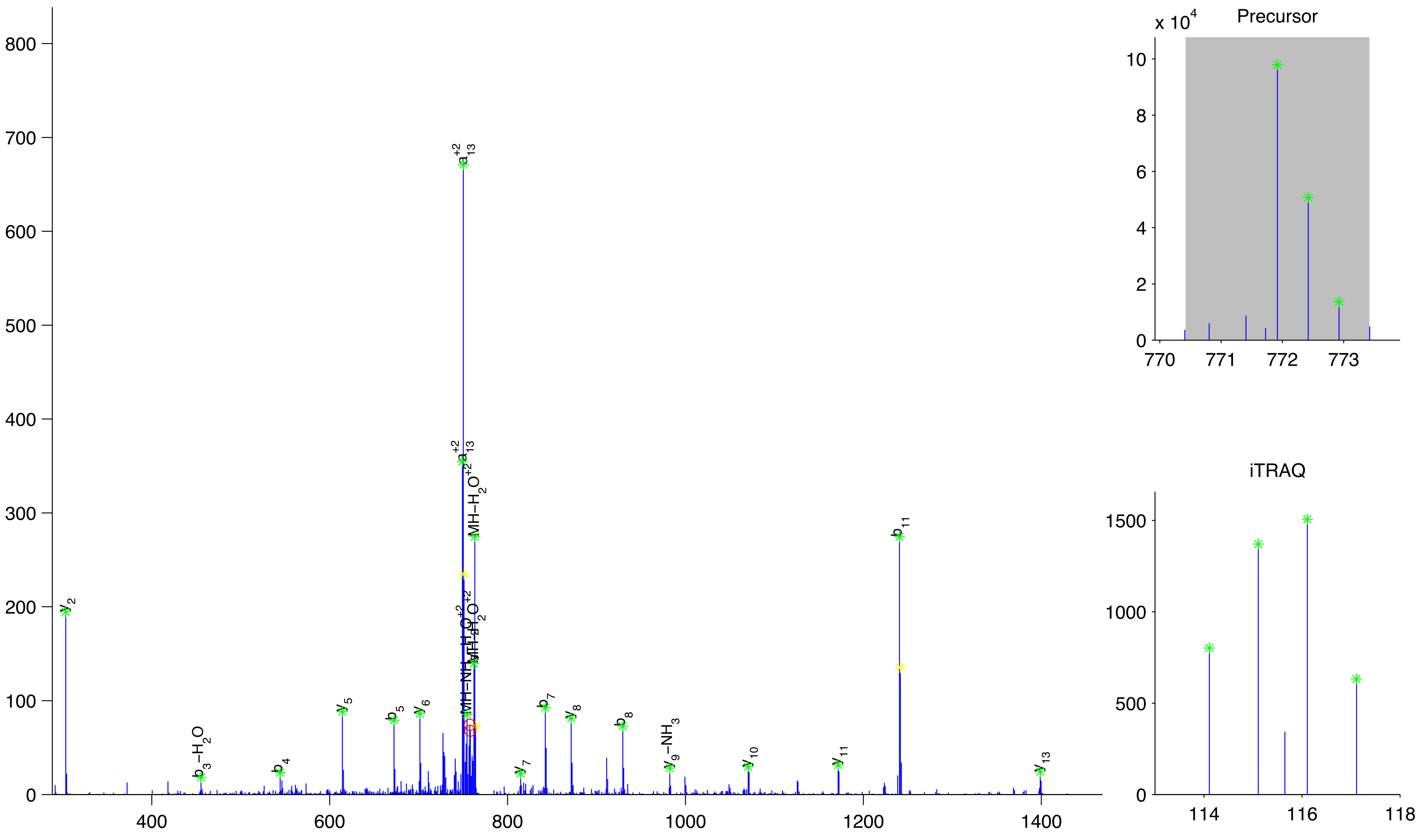

G k T A Q G L S P V D Q R

tripartite motif–containing 33 protein isoform alpha [Homo sapiens]
Charge State: +2
Scan Number: 14824
File Name: 110924_HG2_INSA_ACK.raw

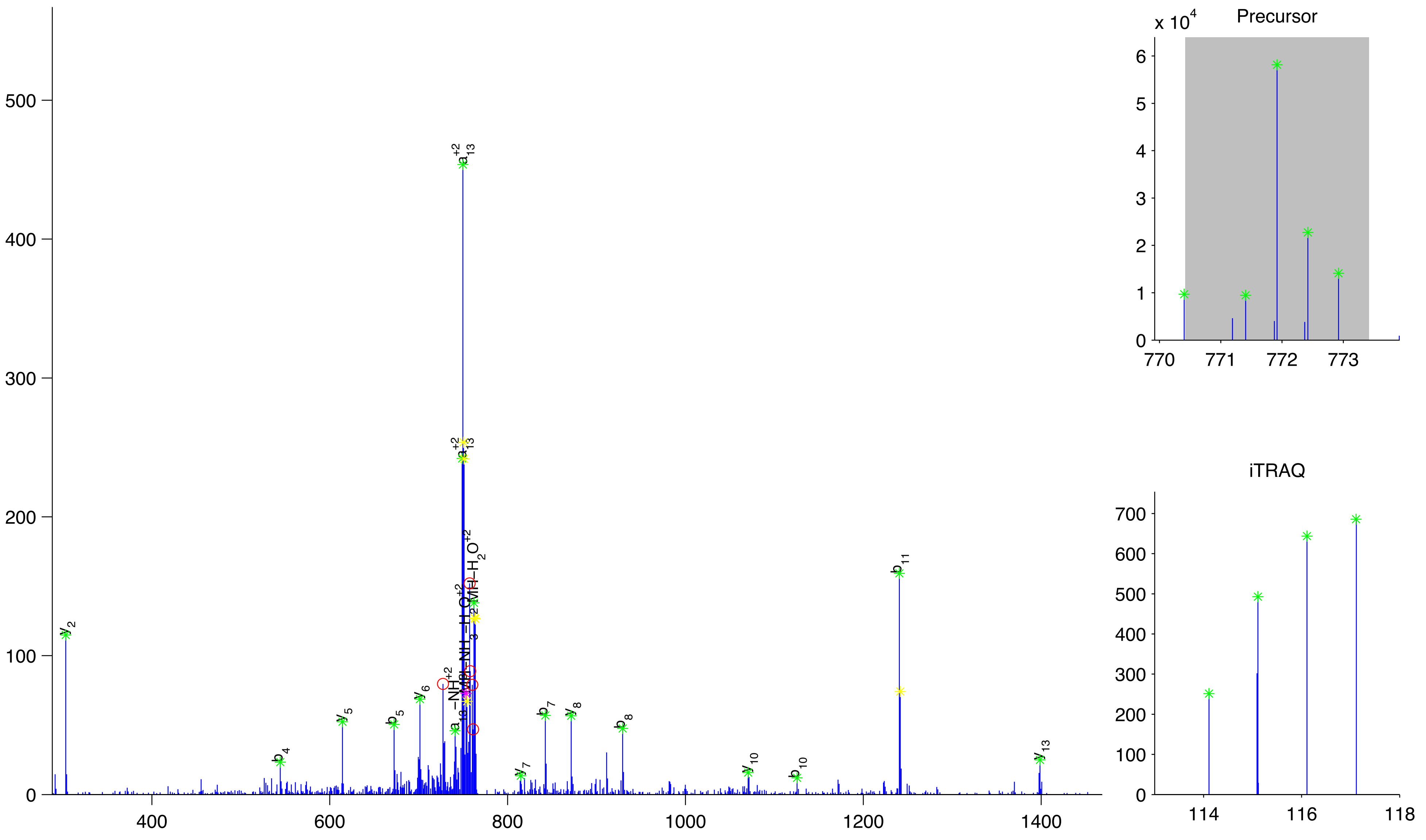

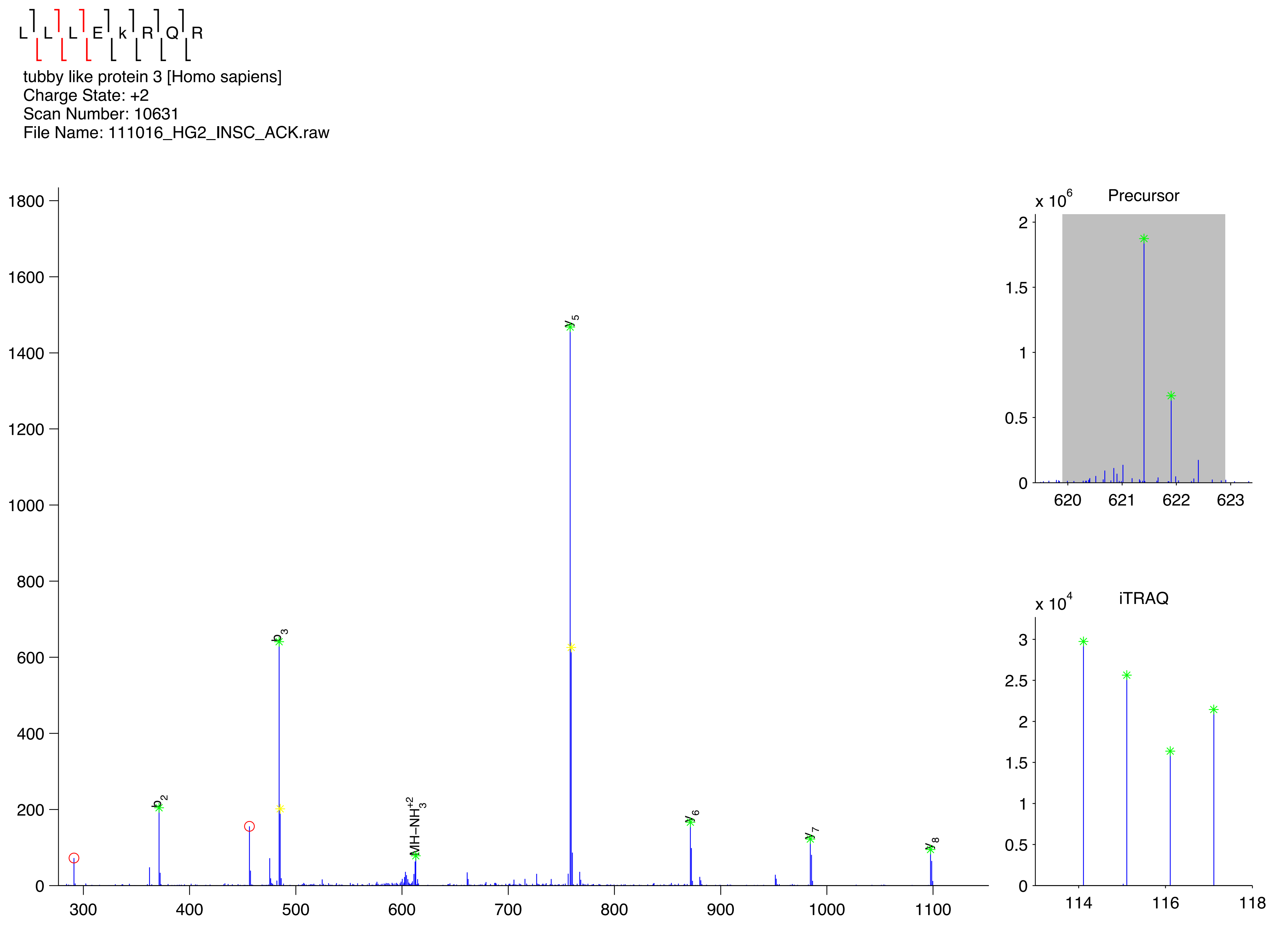
L L L E k R Q R
tubby like protein 3 [Homo sapiens]
Charge State: +2
Scan Number: 10631
File Name: 111016_HG2_INSC_ACK.raw
b2
b3
MH−NH3 +2
y5
y6
y7
y8
Precursor
x 10^6
iTRAQ
x 10^4

F V N V V P T F G k K

ubiquitin–like protein fubi and ribosomal protein S30 precursor [Homo sapiens]
Charge State: +3
Scan Number: 9056
File Name: 111011_HG2_INSB_ACK.raw

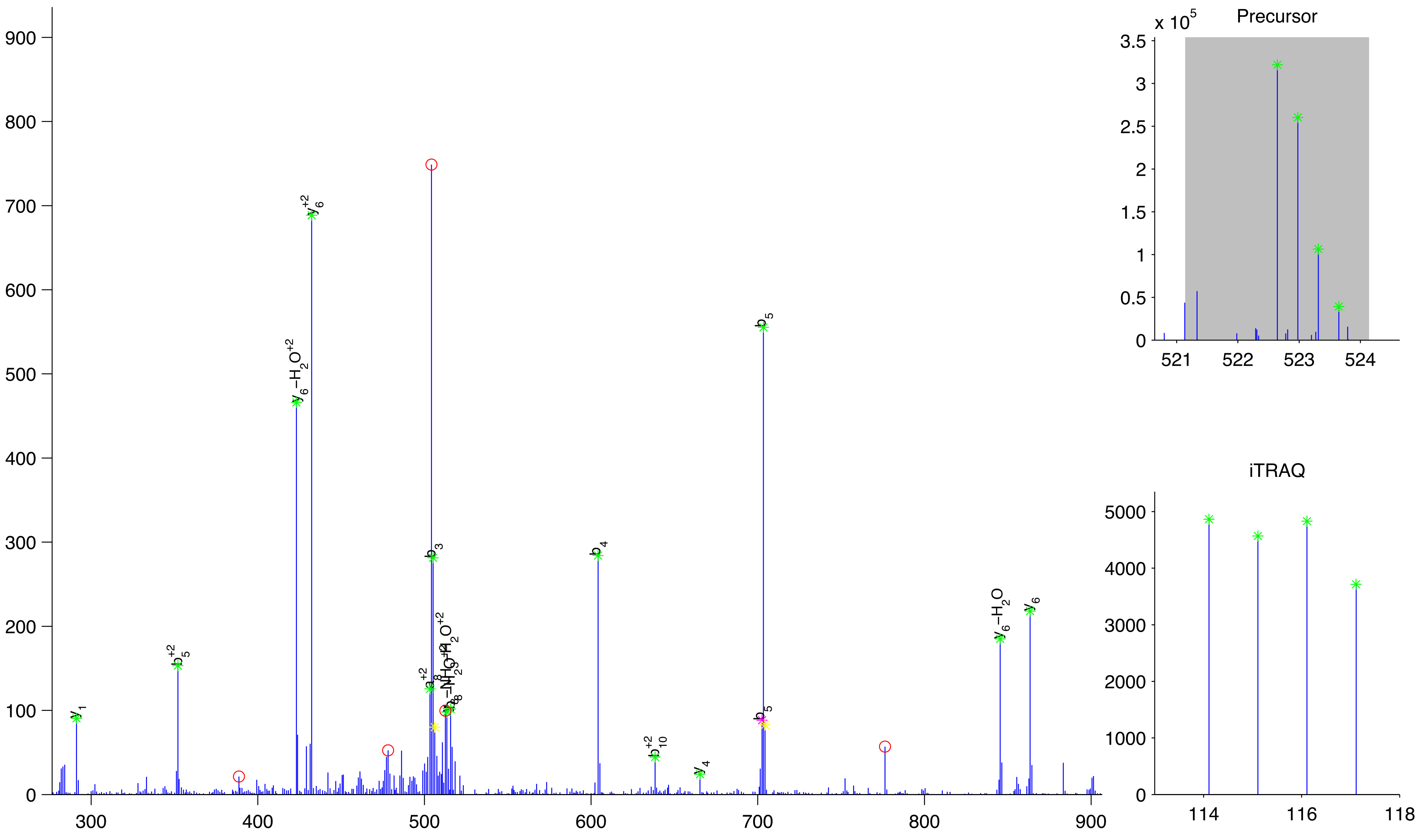

Supplement: S1 Fig — False discovery rate analyses aid in identifying a potential threshold for accepting MS/MS spectra without manual validation. Oftentimes, a particular FDR is selected and a peptide score for which that FDR is met is selected. Here, an FDR analysis was performed as a function of Mascot score. Instead of relying on an FDR analysis where the identity of true positives and true negatives are unknown, we manually validated each MS/MS spectra manually. Each page represents a manually validated MS/MS spectrum. (PDF) [file pone.0126242.s001.pdf]
